# Ochratoxin A Toxicity Research in In Vitro and In Vivo Experimental Settings: A Cross-Software Bibliometric and Science-Mapping Study

**DOI:** 10.3390/toxins18080346

**Published:** 2026-08-11

**Authors:** José Manuel Veiga-del-Baño, José Oliva, Pedro Andreo-Martínez, Miguel Motas, Eva María Mateo, José Miguel Soria, María Ángeles García-Esparza

**Affiliations:** 1 Department of Agricultural Chemistry, Faculty of Chemistry, Regional Campus of International Excellence “Campus Mare Nostrum”, University of Murcia, Campus of Espinardo, 30100 Murcia, Spain; chemavb@um.es (J.M.V.-d.-B.); josoliva@um.es (J.O.); 2 Department of Toxicology, Faculty of Veterinary, Regional Campus of International Excellence “Campus Mare Nostrum”, University of Murcia, Campus of Espinardo, 30100 Murcia, Spain; motas@um.es; 3 Department of Microbiology and Ecology, Faculty of Medicine and Odontology, University of Valencia, 46010 Valencia, Spain; eva.mateo@uv.es; 4 Department of Biomedical Sciences, Universidad Cardenal Herrera-CEU, CEU Universities, 03204 Elche, Spain; 5 Department of Pharmacy, Universidad Cardenal Herrera-CEU, CEU Universities, 03204 Elche, Spain

**Keywords:** bibliometric analysis, toxicity, ochratoxin A (OTA), in vitro, in vivo

## Abstract

Background: Ochratoxin A (OTA) is a widespread mycotoxin with well-established nephrotoxic, immunotoxic, and carcinogenic properties. Despite decades of research, the structure, thematic evolution, and emerging directions of the scientific literature on OTA toxicity remain incompletely mapped. Methods: A bibliometric analysis was conducted using 883 publications indexed in the Web of Science Core Collection (1965–2025). Bibliometrix, VOSviewer, and BibExcel were integrated within a comparative cross-software workflow to assess publication trends, collaboration networks, thematic evolution, and thematic research directions in in vitro and in vivo OTA toxicity studies, while enabling cross-validation of the main bibliometric outputs. Results: After normalisation, authors, journals, author keywords, and Keywords Plus showed strong cross-software agreement, whereas country and institution outputs retained software dependent differences related mainly to country aggregation and affiliation parsing. Publication output increased substantially over the analysed period, particularly during the most recent decade, reflecting the growing visibility of OTA research in food safety and toxicology. The field is highly collaborative and multidisciplinary. Oxidative stress-related terms were prominent in the keyword and thematic analyses and co-occurred with terminology related to apoptosis, DNA damage, and mitochondrial dysfunction. Thematic evolution analyses showed a transition from early studies focused on nephrotoxicity and animal models toward more recent investigations addressing molecular pathways, cellular responses, and microbiota host interactions. Comparatively less prominent or emerging bibliometric themes included the gut immune axis, co-exposure to multiple mycotoxins, and the broader representation of animal-health and productivity-related research. Conclusions: The bibliometric and science mapping analyses indicate that the literature on OTA toxicity has evolved from predominantly organ, and animal model-related research, toward increasing attention to molecular, cellular, intestinal, and microbiota-related topics. These patterns describe changes in the conceptual structure of the literature rather than direct evidence of biological causality. Comparatively, less prominent themes, including human-relevant models, combined exposure scenarios, and microbiome-related research, may warrant further investigation.

## 1. Introduction

Since its discovery and chemical characterization in 1965, ochratoxin A (OTA) has continued to be considered one of the most important and harmful mycotoxins for our health [1]. A secondary metabolite of fungal species such as *Aspergillus* and *Penicillium*, OTA is produced during the storage of cereals and is widely present in various food products due to its stable physicochemical properties, cereal-based products, spices, eggs, coffee, meat, wine, etc. [2,3].

The production of OTA by *Aspergillus* and *Penicillium* species depends on factors such as climatic conditions, substrate water activity, and micronutrient availability [4,5].

OTA biosynthesis is maximised at a water activity of 0.98, irrespective of temperature, with production further enhanced within the optimal temperature range of 25–30 °C. Chemically, OTA is a weak organic acid (pKa = 7.1) with a molecular mass of approximately 404 g/mol. It occurs as colourless to white crystals and exhibits characteristic fluorescence under ultraviolet radiation, emitting green fluorescence under acidic conditions and blue fluorescence under alkaline conditions [6]. OTA is readily soluble in several organic solvents, including alcohols, ketones, and chloroform, under acidic and neutral pH conditions, whereas in alkaline media, it dissolves in aqueous sodium bicarbonate and related alkaline solutions. A key physicochemical property of OTA is its pronounced stability under acidic and thermal conditions, which contributes to its persistence in contaminated food matrices. Consequently, conventional food-processing operations, including cooking and thermal treatments at temperatures up to 200 °C under different moisture regimes, generally have limited effects on OTA degradation or structural integrity [7].

Over the last 60 years, the toxicity of OTA has been thoroughly evaluated, by in vitro and in vivo experiments. Thus, experimental in vitro and in vivo studies have identified multiple toxic effects of OTA, including hepatotoxic [8], nephrotoxic [9], neurotoxic [10,11,12], and genotoxic [13] effects. OTA has also been classified as a possible carcinogen to humans (Group 2B) by the International Agency for Research on Cancer (IARC) [14].

The initial interaction after the ingestion of OTA present in food occurs with the intestinal lining [15]. OTA is absorbed through the gastrointestinal tract into the systemic circulation, where it forms complexes with serum proteins, predominantly albumin with high affinity. The toxin is efficiently transported between tissues because of enterohepatic recirculation [16], a process that involves the reabsorption of OTA from the intestines back into the bloodstream. In addition, OTA is reabsorbed in both the proximal and distal tubular regions of the kidneys [17]. Consequently, OTA accumulates mainly in the bloodstream and in organs such as the kidneys and the liver, which are also the primary sites of its biotransformation [18]. Its strong binding to albumin contributes to a prolonged plasma half-life, which is approximately one month in humans and is longer than that reported for several other species [19,20].

On the other hand, the presence of OTA in the brain appears to suggest that it crosses the blood–brain barrier, where it subsequently settles in different neuronal structures [21,22].

The cellular mechanisms of action of OTA, elucidated through various studies, provide increasingly comprehensive insights into the interactions of this mycotoxin with cellular components and its involvement in the development of pathologies and diseases affecting various tissues and organs. Many investigators have reported that oxidative stress constitutes one of the primary cellular mechanisms of action of OTA [23]. Oxidative stress has been widely recognized as a potential contributing factor in the onset and progression of several diseases [24,25]. The adverse outcomes associated with oxidative stress include DNA damage [25,26,27], protein synthesis inhibition [27] and cell apoptosis [28,29]. In addition, the roles of autophagy and mitophagy in OTA induced toxicity have been explored [30], given that the adaptive function of both processes is to protect organisms against various pathologies.

Over the years, in vitro experiments in different kidney, liver, intestine, brain, and embryonic cell lines and in vivo studies conducted in experimental models, have been conducted, to determine the presence of OTA and to elucidate both the underlying molecular mechanisms and the toxic effects induced by OTA.

This study presents a bibliometric and science mapping analysis of the literature on OTA toxicity in in vitro and in vivo experimental settings. Using the Web of Science Core Collection (WoSCC) as the data source, the analysis combines descriptive bibliometric indicators with thematic mapping, trend topic analysis, bibliographic coupling, and cross-software validation using BibExcel, VOSviewer, and Bibliometrix. Beyond characterizing publication output, influential contributors, and collaboration patterns, the study aims to identify how OTA toxicity in in vivo and in vitro research has evolved conceptually over time. By examining the structure and thematic organisation of the literature, it seeks to identify dominant research themes, emerging areas of investigation, specialised research niches, and comparatively less visible topics that may help formulate questions for future toxicological research. In this context, the objective is not to provide a systematic synthesis of toxicological evidence, but rather to map and interpret the scientific landscape of OTA toxicity in in vivo and in vitro research through quantitative analysis of the published literature. This approach allows the identification of long-term research trends, conceptual transitions, and future research priorities within the field.

Conversely, bibliometrics have gained popularity for creating representative summaries of leading findings across various scientific disciplines [31,32]. Within the scientific domain dedicated to mycotoxin research, the work of Wang et al. (2024) [33] stands out for its rigorous and comprehensive evaluation of current analytical methodologies. Their review synthesizes contemporary detection strategies and highlights the predominance of research on aflatoxins, ochratoxins (including OTA), zearalenone, fumonisins, and trichothecenes, mycotoxins of high toxicological relevance and widespread occurrence. The study further evidences a notable intensification of research activity in recent years, driven by increasing concerns regarding the implications of mycotoxin contamination for food safety, public health, and the agri-food sector [33].

A bibliometric analysis of in vitro and in vivo studies on OTA provides an objective framework for evaluating research output, methodological evolution, and thematic focus within the field. OTA remains one of the most intensively studied mycotoxins due to its documented immunosuppressive, genotoxic, and carcinogenic properties, and its widespread occurrence in food and feed matrices. By quantifying publication trends and identifying research clusters, bibliometric approaches can characterise prominent toxicological terminology, represented biological models, and comparatively less visible topics, thereby helping to formulate priorities for further investigation. Moreover, mapping collaboration networks and identifying leading contributors provides insight into the structure and dynamics of the research landscape. Together, these elements justify the incorporation of bibliometric analysis as a complementary tool for contextualizing current knowledge and guiding the design of new studies on OTA-induced toxicity. In addition to describing the literature, this approach enables the identification of conceptual transitions, emerging bibliometric themes, and comparatively less visible areas that may inform future research questions on OTA toxicity.

## 2. Results and Discussion

### 2.1. Cross-Software Agreement After Data Normalisation

The quantitative comparison showed complete post-normalisation agreement among BibExcel, VOSviewer, and Bibliometrix for authors, journals, Author Keywords, and Keywords Plus (Table 1). The three programs returned 3305 authors, 259 journals, 1779 Author Keywords, and 2086 Keywords Plus. For these four fields, entity-level comparison confirmed identical sets, frequencies, and frequency-based rankings, resulting in pairwise Jaccard coefficients of 1.000, exact frequency agreement of 100%, Spearman coefficients of 1.000, and complete top 20 overlap.

The increase in the BibExcel author count from 3212 to 3305 did not reflect the addition of records or authorship occurrences. The original BibExcel result was derived from the abbreviated AU field, whereas the controlled author vocabulary was constructed through contextual comparison of the complementary AU and AF fields. Direct field-level extraction showed 3212 AU labels and 3669 AF strings before normalisation, compared with 3305 AU labels and 3497 AF strings afterward. In both fields, the total number of author occurrences remained unchanged at 4966. Thus, the increase in the AU based BibExcel output reflected the net effect of variant consolidation and author label disambiguation rather than expansion of the corpus.

Residual discrepancies were restricted to countries and institutions, both of which were derived from affiliation metadata. BibExcel and VOSviewer produced identical post-normalisation country outputs, comprising 73 countries with identical frequencies and rankings. Bibliometrix returned 71 country entries. The 71 Bibliometrix entries were shared with the BibExcel/VOSviewer country set, producing a Jaccard coefficient of 0.973 (Table 1). However, exact frequency agreement and Spearman correlation were not calculated for comparisons involving Bibliometrix because its country production frequencies did not represent the same document-based counting unit as the BibExcel and VOSviewer outputs. The difference was, therefore, reported as a residual software dependent aggregation discrepancy rather than as an error in the normalised WoS file.

Institutional results showed a larger difference. BibExcel and VOSviewer returned identical post-normalisation outputs comprising 987 institutional entities, with identical frequencies and rankings. Bibliometrix returned 808 affiliation entries. Because the Bibliometrix output represented affiliations at a different aggregation level, full list Jaccard similarity, exact frequency agreement, Spearman correlation, and top 20 overlap were not interpreted for comparisons involving this program. The institutional difference was retained as an observable software output discrepancy and was not attributed to an undocumented internal algorithm.

Overall, the validation demonstrated exact cross-software agreement for authors, journals, Author Keywords, and Keywords Plus, as well as exact agreement between BibExcel and VOSviewer for countries and institutions. The remaining differences were confined to the address-derived outputs generated by Bibliometrix. Summary counts, pairwise calculations, metric definitions, and residual discrepancies are provided in Supplementary File S11, while the complete field-level normalisation audits are supplied in Supplementary Files S4, S6, S8, and S10.

### 2.2. Publications Output and Document Characteristics

All descriptive analyses in this section were carried out using BibExcel, our primary environment for characterizing the corpus at a bibliometric level [34]. To ensure metadata integrity, we initially screened the dataset in Bibliometrix, which excels at data conversion and science mapping [35,36]. While VOSviewer is widely known for its visualisation strengths, such as co-citation and co-authorship maps, it was not the main analytical tool for this specific descriptive phase [37].

The final corpus comprised 883 records published between 1965 and 2025. Annual production was initially sporadic, increased progressively from the 2000s onward, and reached its highest value in 2022, with 58 publications. The corpus subsequently included 43 publications in 2023, 38 in 2024, and 48 in 2025. These data indicate sustained long-term growth, although with annual fluctuations.

**Figure 1:**
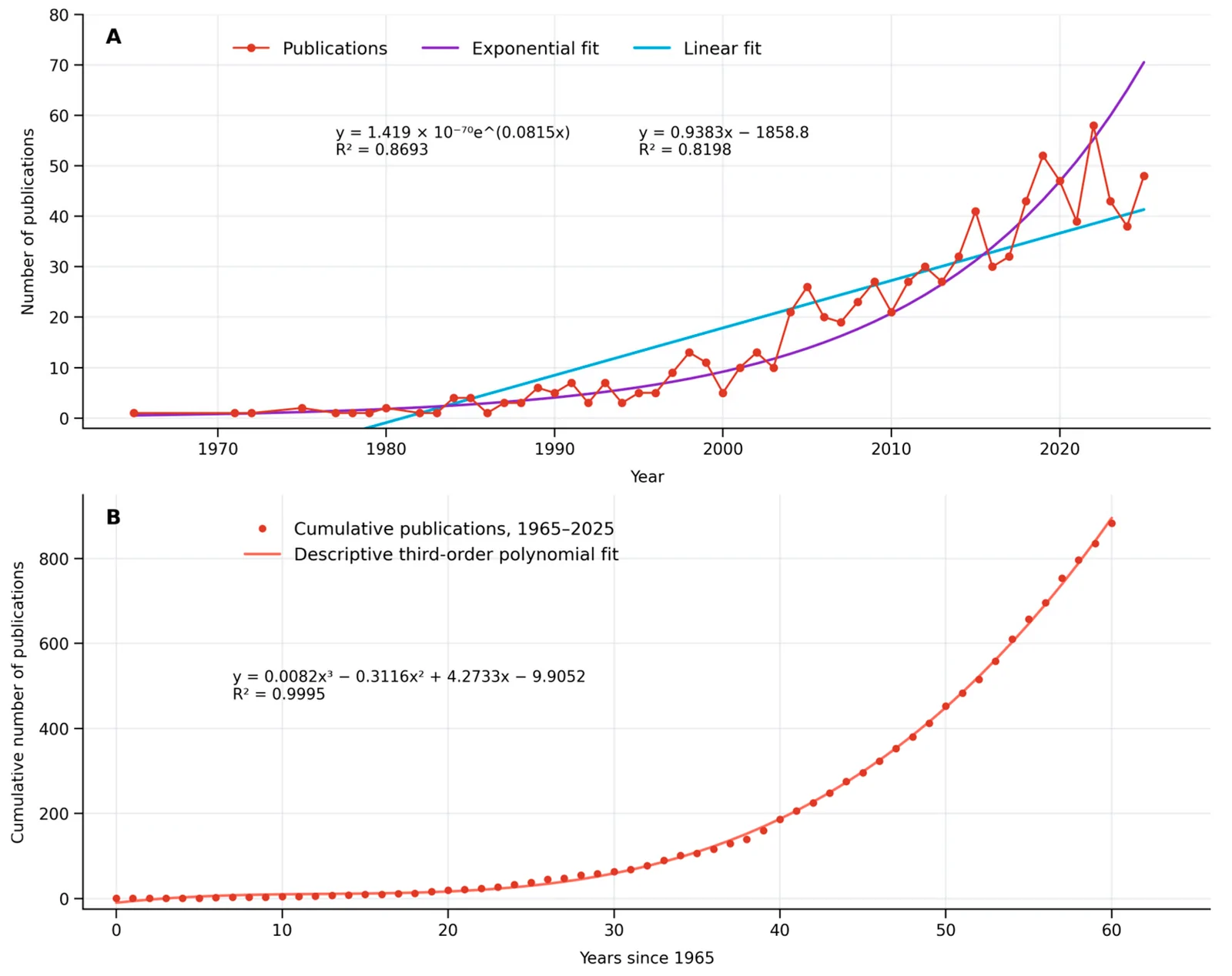
Publication output of the literature on ochratoxin A (OTA) toxicity from 1965 to 2025. (**A**) Annual publication output with linear and exponential fitted models used to compare the observed growth patterns. (**B**) Descriptive third-order polynomial fit to the cumulative publication output observed from 1965 to 2025. The polynomial fit is shown only to summarise the observed cumulative trajectory and was not used for forecasting.

Figure 1A illustrates this growth by comparing linear and exponential models to evaluate whether the publication pattern was consistent with Price’s Law. The exponential model (y = 1.419·10^−70^·e^0.0815x^; R^2^ = 0.8693) outperformed the linear one (R^2^ = 0.8198), indicating that the exponential model provided a better fit than the linear model for the analysed period. This aligns with Price’s classic theories on scientific growth [38], while also reflecting more recent views on the complex, sometimes irregular nature of scientific expansion [39]. Figure 1B provides a descriptive representation of the cumulative publication output observed between 1965 and 2025. The third-order polynomial closely fitted the observed cumulative data (R^2^ = 0.9995), and the correlation between time and cumulative output was r = 0.88 (*p* < 0.0001). These statistics were used solely to summarise the observed cumulative trajectory and were not interpreted as evidence of future growth or of whether the field is approaching a plateau.

In terms of document types, it should be noted that WoS may assign more than one document type label to the same record. Therefore, these values represent field frequencies rather than mutually exclusive document categories. Nevertheless, the predominance of original articles indicates that primary research constituted the main publication type in the analysed corpus, while reviews provided a secondary synthesis layer.

Language wise, the corpus is remarkably uniform. English is the undisputed lingua franca (98.9%), with only a handful of papers in French, German, or Spanish. This dominance underscores the global reach and international visibility of OTA research today.

The initial metadata audit performed in Bibliometrix further supported the overall quality of the dataset. At file import, the fields AU, DT, SO, LA, PY, WC, TI, and TC were classified as excellent. The fields CR (13 missing records; 1.6%), C1 (24 missing records; 2.7%), AB (32 missing records; 3.6%), DI (55 missing records; 6.2%), RP (56 missing records; 6.3%), and ID (59 missing records; 6.7%) were classified as good, whereas DE (127 missing records; 14.4%) was rated as acceptable. With an average document age of 13.2 years, the corpus blends foundational knowledge with a surge of recent activity. This level of data completeness is vital, as bibliometric outcomes are heavily influenced by how data are handled during preprocessing [35,40,41].

The field’s dynamic nature is further highlighted by a Price’s Index of 37.7%. This suggests that while researchers still rely on established foundations, a significant portion of their citations are very recent. Combined with the average document age, this portrays OTA toxicity research not as a brand-new trend but as a maturing area that is constantly renewing itself.

Table 2 summarises the annual publication, authorship, cited-reference, and page indicators for the 52 publication years represented in the corpus. Across the complete period, the 883 records contained 4966 author occurrences, 61,266 cited-reference occurrences, and 11,169 pages. The annual profiles show an increase in the number of authors and cited references per publication during the most recent years. In 2025, the corresponding values were 6.9 author occurrences and 92.9 cited-reference occurrences per publication. The highest annual-cited-reference density was observed in 2020, with 109.6 cited-reference occurrences per publication. These values describe the annual documentary characteristics of the corpus and were not used to calculate changes between non-consecutive publication years [38,39].

At the cited-reference processing level, a distinction must be made between the 61,266 cited reference occurrences recorded across the corpus and the unique cited reference strings identified by each software. BibExcel identified 31,240 unique cited-reference items, VOSviewer identified 32,763, and Bibliometrix identified 32,801. Because cited references were not included in the field-level normalisation procedure and no entity level cross-software validation was performed for this field, similarities or differences in the aggregate counts were not interpreted as evidence of concordance, stricter deduplication, or shared internal processing algorithms. These cited reference outputs are, therefore, reported as software-specific results and should not be considered interchangeable.

Collectively, the longitudinal patterns illustrated in Figure 1, together with the publication, authorship, and cited-reference indicators reported in Table 2, show that the literature on OTA toxicity expanded substantially over the analysed period and became increasingly collaborative and reference-dense. The upward trend in co-authorship indices likely reflects the multidisciplinary convergence characterizing the domain integrating toxicology, food science, and veterinary medicine with analytical chemistry and mechanistic cell biology. Furthermore, the simultaneous increase in reference density and manuscript length points toward a literature that is becoming more integrative, methodologically rigorous, and conceptually mature.

From a comparative software perspective, these findings necessitate two levels of analytical scrutiny. Primary bibliographic attributes, such as publication year (PY), document type (DT), and language (LA), are explicit metadata fields. As such, they yield highly stable outputs that should converge across platforms when the same corpus is analysed. In contrast, cited reference metrics are inherently sensitive to preprocessing decisions, particularly regarding string normalisation and duplicate detection. Therefore, such data should only be compared across environments that utilize equivalent output parameters and deduplication protocols.

### 2.3. Authors, Productivity Patterns, and Intellectual Structure

As shown in Table 1, author normalisation produced identical post-normalisation entity sets, frequencies, and rankings across the three programs. The following analysis therefore focuses on author productivity, impact, and collaboration patterns.

Table 3 presents the leading authors ranked by h-index, as calculated using the Bibliometrix tool. It can be observed that the field is dominated by a small core of highly productive and influential contributors. Huang Kehe ranked first for the normalized dataset with 30 publications since 2015 and an h-index of 20. The following positions were held by Pfohl-Leszkowicz Annie (h-index of 18 with 18 publications), Gan Fang (h-index of 16 with 20 publications), Gekle Michael (h-index of 16 with 20 publications), Mally Angela (h-index of 15 with 17 publications), and Xu Wentao (h-index of 15 with 16 publications). Taken together, these indicators imply that author prominence in this field is not solely a matter of publication count, but also of the long-term citation impact of a relatively small group of key contributors.

**Figure 2:**
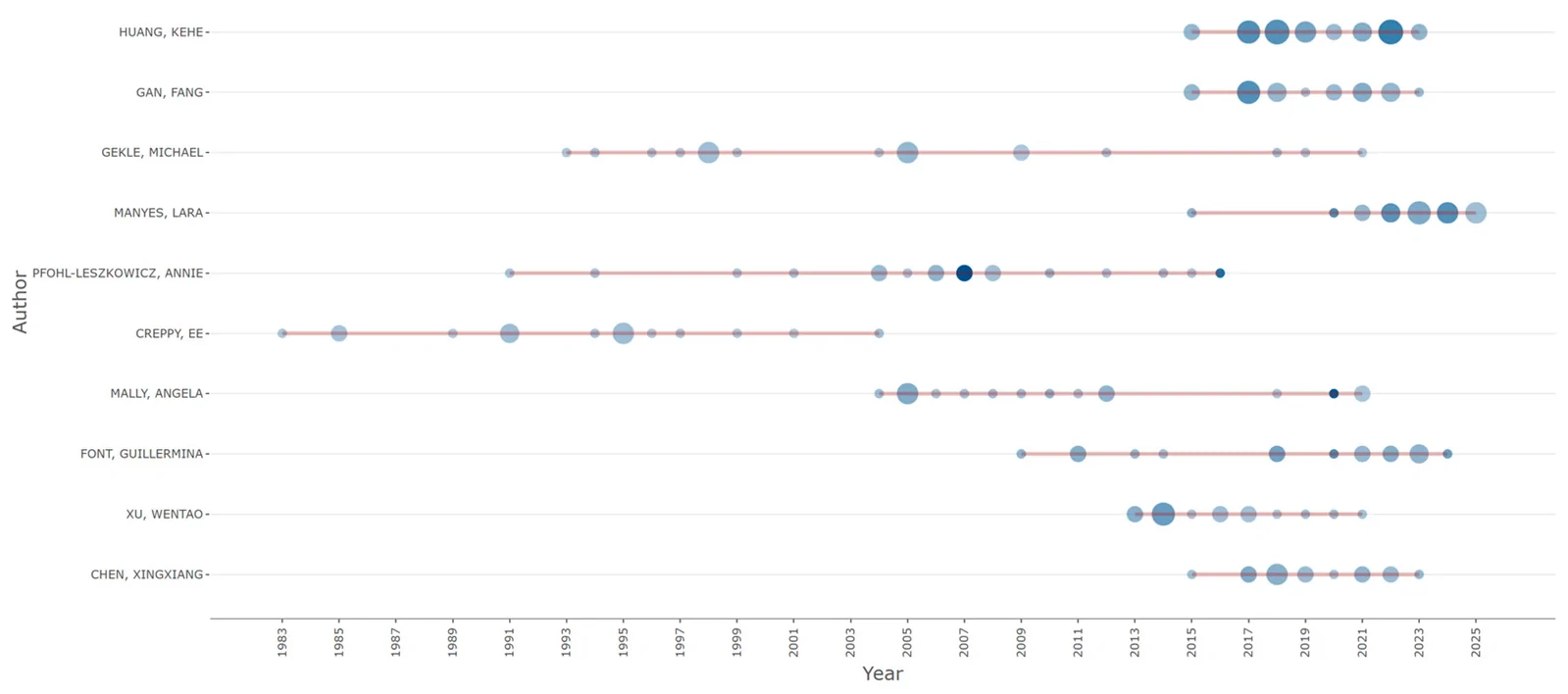
Authors’ production on OTA toxicity over time. Each circle represents an author’s publication output in the corresponding year. Circle size is proportional to the number of articles published by the author in that year, whereas the intensity of the blue colour represents the author’s total citations per year. The red horizontal lines connect publication years and indicate each author’s temporal production trajectory within the analysed corpus.

Figure 2 shows the authors’ production over time, and the temporal production patterns indicate that the field mixes historically established authors with more recent clusters of high output authors. At this historical end of the spectrum, Pfohl-Leszkowicz Annie, Creppy EE, and Gekle Michael provide a foundational layer of research on OTA dating back to the late 20th century. This is in accordance with the well-established role of Pfohl-Leszkowicz in major review papers such as Ochratoxin A: An overview on toxicity and carcinogenicity in animals and humans (2007) and the later review Ochratoxin A: 50 Years of Research (2016), which summarize toxicological and carcinogenic evidence on OTA and have become reference works in the field [42,43].

Looking at the more recent literature, a new wave of researchers, including Huang Kehe, Gan Fang, Chen Xingxiang, and Xu Wentao, has taken centre stage, with their productivity spiking since the mid 2010s. Their work marks a clear departure from traditional toxicology, pivoting instead toward molecular mechanisms. They focus heavily on how things like oxidative stress, apoptosis, and cell signalling pathways actually function. An example of this shift is the 2017 study by Wang and colleagues on ochratoxin A [44]. This paper, co-authored by Chen, Gan, and Huang, provides a detailed look at how OTA triggers intestinal cell injury through mitochondrial pathways. At the same time, the steady influence of authors like Manyes Lara and Font Guillermina comes from their knack for synthesizing complex data, notably in their 2020 review on mycotoxin toxicity in vertebrates [45]. Similarly, Mally Angela’s prominent standing reflects her ongoing leadership in high-level risk assessment, specifically her role in the 2020 EFSA scientific opinion regarding OTA levels in food [46].

Figure 2 also shows a mix of scientific paths that are both diverse and complementary. Some authors have long, steady research careers, while others pop up later on with a more focused but still very noticeable output. Instead of indicating that one generation is replacing another, the timeline suggests a building process, where the foundational toxicological and mechanistic studies laid the groundwork for later explorations into areas like intestinal toxicity, immunotoxicity, oxidative stress, and broader frameworks related to food safety and exposure. In this way, the field seems to have grown through a blend of continuity and fresh perspectives, with seasoned authors continuing to influence the knowledge base while newer voices broaden its themes and methods.

**Figure 3:**
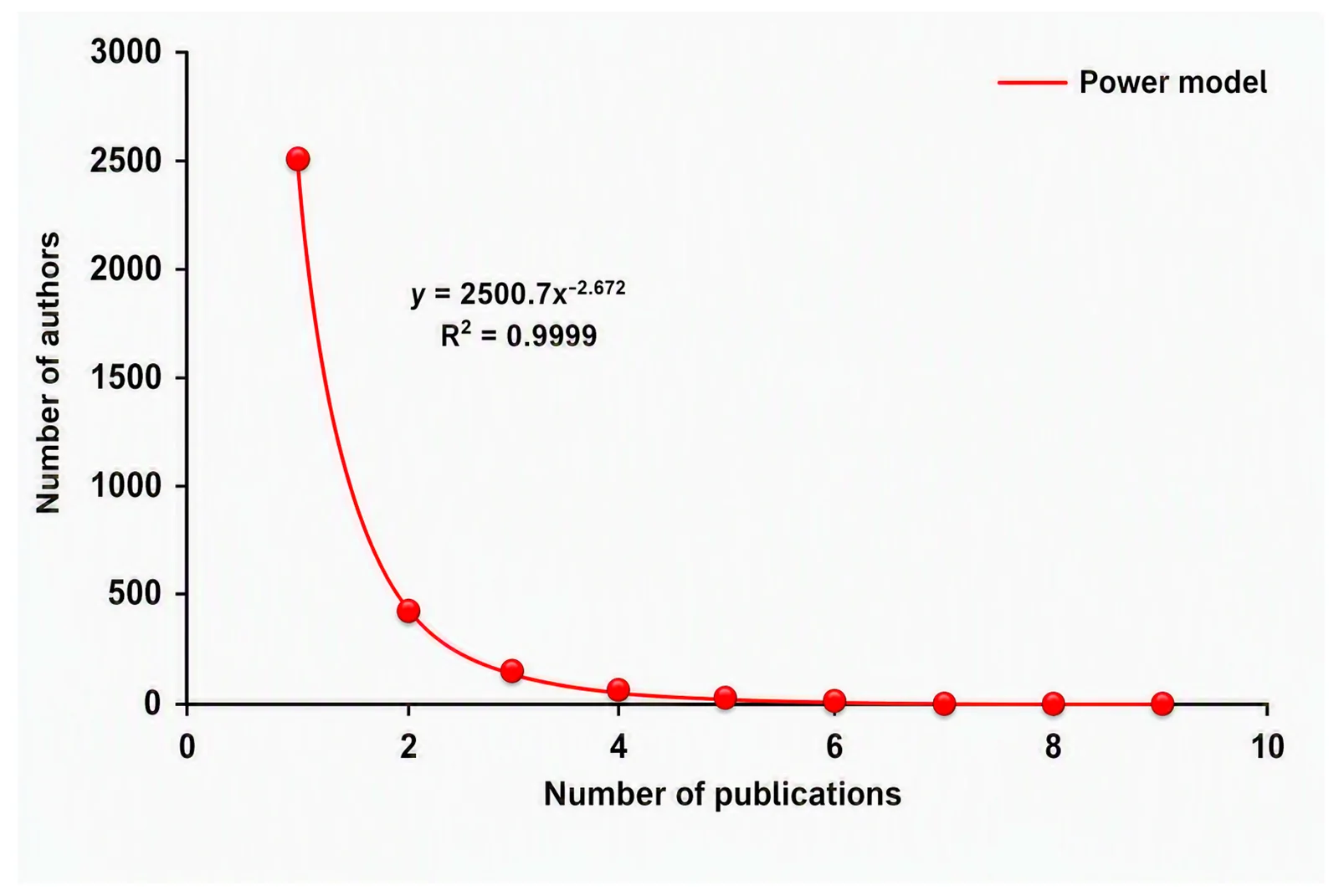
Lotka’s Law for author productivity in the analysed corpus. The figure shows the observed distribution of authors according to their number of publications and the fitted inverse power function for authors with one to nine publications.

Author productivity followed a strongly right-skewed distribution consistent with Lotka’s Law (Figure 3). The fitted inverse power function for authors with one to nine publications was y = 2500.7x^−2.672^, with R^2^ = 0.9999. Most authors contributed one or two publications, whereas progressively fewer authors contributed larger numbers of publications. This pattern is consistent with the concentration of scientific output among a comparatively small group of recurrent contributors described by Lotka’s Law [47].

The collaboration index, calculated as the number of authors involved in multi-authored publications divided by the number of multi authored publications, was 5.72, indicating that collaborative publications in this research area generally involved teams of approximately six authors. This result was close to the co-authors per document value of 5.62 obtained in Bibliometrix.

**Figure 4:**
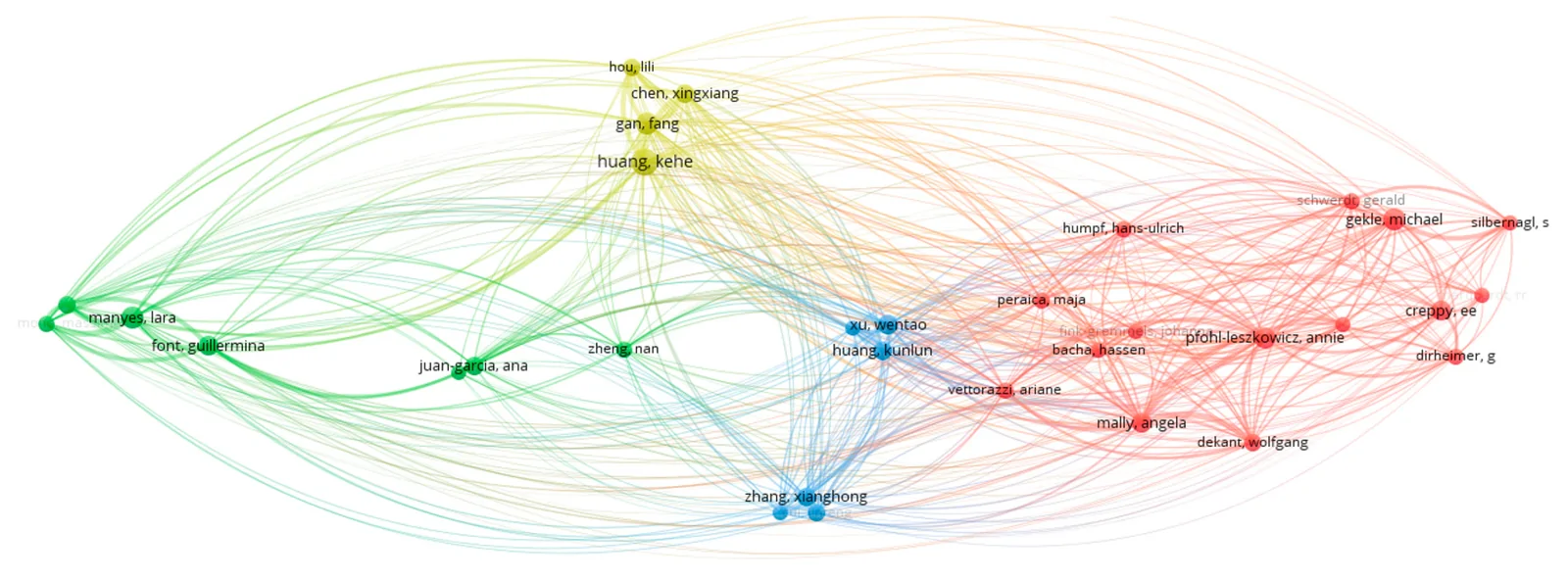
Bibliographic coupling map of authors. This figure presents the network of authors based on bibliographic coupling analysis, where connections are established between authors who share common references in their publications on OTA toxicity. The resulting clusters highlight groups of researchers with similar scientific interests or thematic focus, while the strength of the links reflects the degree of shared citations, indicating closer intellectual relationships within the research field.

Mapping the authors’ bibliographic coupling in VOSviewer (Figure 4, with a 10-document minimum) offers another layer of insight into how the field’s intellectual landscape is built. The resulting network shows a tightly knit group of researchers at its centre. In this core, names like Huang Kehe, Mally Angela, Pfohl-Leszkowicz Annie, and Gan Fang, together with Manyes Lara, Gekle Michael, Chen Xingxiang, and Font Guillermina, stand out as the most influential figures. Because bibliographic coupling is essentially about sharing the same sources, these findings show that the most prominent authors are working from a very similar knowledge base. This points to a research community that is remarkably cohesive, even while maintaining its own internal niches [37,41].

Visually, the coupling map is like a series of interconnected research hubs. On one side, it can be seen a cluster of historically influential figure think Pfohl-Leszkowicz, Mally, Gekle, Creppy, and Dekant. They are tightly linked by a shared foundation in what we would call classical and mechanistic OTA toxicology. In contrast, another major group led by Huang Kehe, Gan Fang, and Chen Xingxiang leans more toward the latest cell based and mechanistic studies. Then, there is a third distinct cluster involving Manyes Lara, Font Guillermina, Juan-Garcia, and Ruiz Maria-Jose, which seems to specialize in food toxicology and synthesizing in vivo/in vitro data. Despite these divisions, the clusters are still very much in conversation with each other. This suggests that the field is not split apart but rather organized into specialized thematic communities that still share common ground.

This author analysis reveals that the field is primarily led by a small, highly influential inner circle, which is surrounded by a larger group of occasional contributors. The consistent results yielded by BibExcel, Bibliometrix, and VOSviewer after normalisation further validate these findings. When we examine h-index rankings, production timelines, Lotka’s distribution, and bibliographic coupling analysis together, it becomes evident that OTA and mycotoxin toxicology is a well-established discipline that continues to evolve. This field is supported by a steady core of experts while being actively propelled by new interdisciplinary perspectives and innovative mechanistic studies.

### 2.4. Journals and WoS Categories

Journal outputs showed complete cross-software agreement before and after normalisation (Table 1). The following results, therefore, focus on journal productivity, impact indicators, and bibliographic-coupling patterns.

As shown in Table 4, performed with the BibExcel tool, the results show that the research field is both specialized and strongly concentrated. Food and Chemical Toxicology ranked first by h-index, with an h-index of 37, 5695 citations, and 72 publications (8.2% of the corpus). Toxins was the most productive journal, with 89 publications (10.1%) and an h-index of 35. Toxicology published 37 papers (4.2%) and reached an h-index of 24, whereas Archives of Toxicology and Toxicology Letters each contributed 35 papers (4.0%), with h-index values of 21 and 20, respectively. Toxicology in vitro contributed 27 papers (3.1%) and reached an h-index of 18. Taken together, these results suggest that the journals attracting the largest share of publications were also those with the greatest citation impact, reinforcing the idea that OTA toxicity research has developed around a recognizable and influential publication core [35,41].

**Figure 5:**
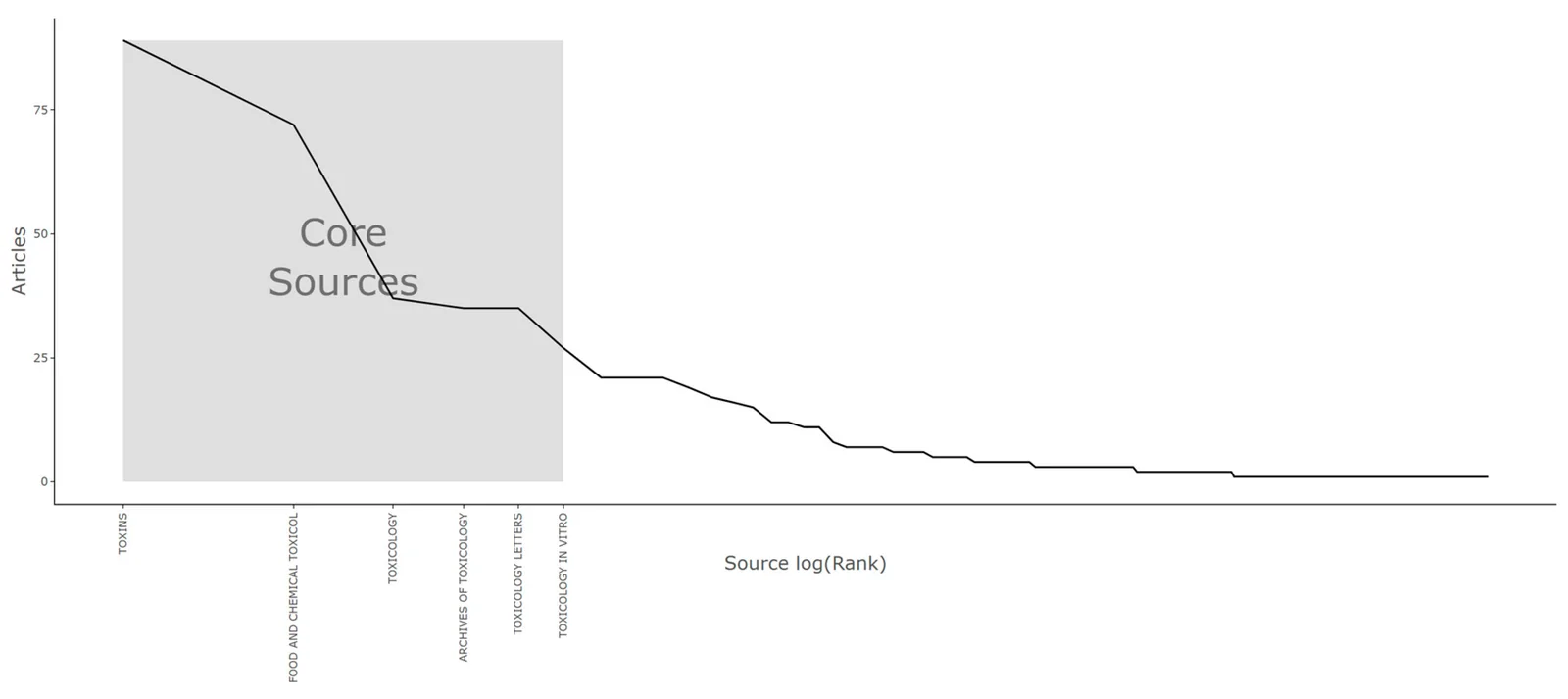
Bradford’s law: journal rank (on a logarithmic scale) versus cumulative articles. Distribution of OTA research articles across journals, highlighting core sources and decreasing productivity zones.

This interpretation is strengthened by the Bradford distribution (see Figure 5 performed with Bibliometrix tool). Zone 1 included only six journals (2.3% of 259 journals), yet these sources already concentrated 295 papers (33.4% of 883 publications). Zone 2 comprised 34 journals (13.1%), whereas the remaining 219 journals (84.6%) were located in Zone 3. Rather than indicating a fragmented literature, this pattern suggests that the field is organized around a small nucleus of journals that shape most of the communication flow, with a much broader peripheral layer contributing more sporadically, exactly as described by Bradford’s law [48].

Notably, the identical IF (5.2) and JCR position (16/106) occupied by both Toxicology and Toxicological Sciences reflects a metric tie rather than a platform anomaly, illustrating the rank compression caused by the current single-decimal rounding convention in the JCR [35].

The WoS/JCR categories add an important interpretative dimension. Most of the leading journals were classified in Toxicology, but several also occupied positions in Food Science & Technology and Pharmacology & Pharmacy. This overlap is especially visible in Toxins and Food and Chemical Toxicology, indicating that the field is not confined to mechanistic toxicology alone, but extends naturally into food safety, exposure, and applied biomedical research. In that sense, the category profile mirrors the interdisciplinary character of ochratoxin toxicity itself [49].

**Figure 6:**
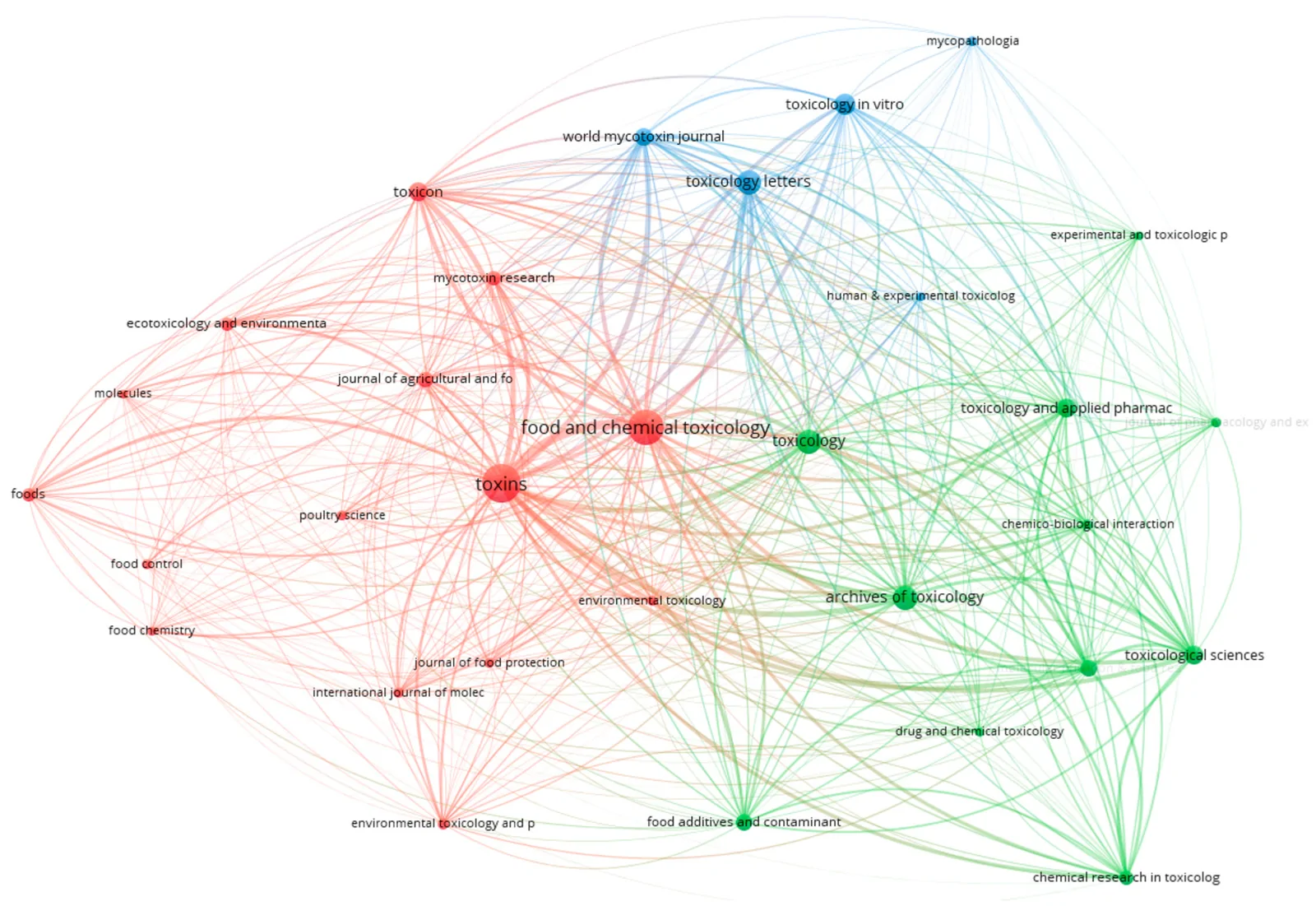
Bibliographic coupling map for journals. Network visualisation of journals based on shared references in OTA research, where link strength indicates the degree of bibliographic coupling and clusters represent related thematic areas.

The bibliographic coupling map provides a complementary representation of the relationships among journals based on shared cited references (Figure 6). The map was built with the VOSviewer tool from journals with a minimum threshold of five publications and shows that the most productive journals are not isolated outlets but part of an interconnected journal landscape based on shared references. This indicates that leading journals not only publish on similar topics but also share a common intellectual foundation, which bibliographic coupling is specifically intended to capture [35,41,50]. Overall, the journal profile of OTA toxicity research can be described as cohesive, specialized, and clearly anchored in toxicology, while still maintaining visible links with adjacent research areas.

### 2.5. Geographical Distribution and Institutional Structure of OTA Toxicity Research

As shown in Table 1, country outputs were identical between BibExcel and VOSviewer after normalisation, whereas Bibliometrix retained a small residual difference in the number and aggregation of country entries. Because its country production frequencies were not directly equivalent to the document-based counts generated by BibExcel and VOSviewer, the concordant BibExcel/VOSviewer output was used for the country productivity and impact analysis presented below.

Table 5 (performed with the BibExcel tool) shows the 20 most productive countries sorted by h-index. As can be observed, Germany and the USA showed the highest h-index values, both with an h-index of 43, followed by China and France with an h-index of 41. China was the most productive country, with 166 articles, whereas France accumulated the highest number of citations among the leading countries, with 7289 citations, followed by the USA, with 6456 citations from 99 articles. This indicates that productivity and impact were not completely aligned. China led in publication volume, whereas Germany, the USA, and France showed the strongest citation performance.

This geographical pattern is consistent with the toxicological and regulatory relevance of OTA. OTA is produced by *Aspergillus* and *Penicillium* species and has been associated with kidney toxicity, genotoxicity, and kidney tumours in experimental systems [9,13,17]. EFSA’s updated risk assessment applied a margin-of-exposure approach because of uncertainties regarding OTA genotoxicity and carcinogenic mode of action, while IARC classified OTA as possibly carcinogenic to humans, Group 2B [14,46]. These concerns help explain the strong contribution of countries with established research traditions in food safety, toxicology, agricultural sciences, and regulatory risk assessment.

It is significant to point out that Germany has emerged as one of the leading contributors to OTA toxicity research, particularly in experimental in vitro and in vivo studies. This position is largely supported by its long standing tradition in toxicology and by the contribution of institutions such as the University of Würzburg, whose research has addressed OTA biotransformation, metabolite formation, and early biomarkers of nephrotoxicity using specialized cellular and animal models [51,52,53]. This body of work helps explain Germany’s high scientific impact in the field, as reflected in its leading position in the country-level analysis.

Germany’s prominence is also linked to its role in the debate on the possible relationship between OTA exposure and Balkan Endemic Nephropathy (BEN). German toxicological studies contributed to clarifying that, although OTA is a potent nephrotoxin, the renal lesions experimentally induced by OTA do not fully reproduce the pathological profile of BEN, and that human exposure levels in endemic regions were below those required to induce nephropathy or carcinogenicity in animal models [54,55]. In addition, German groups contributed to the development of reference methods for OTA detection and biomonitoring in biological samples, strengthening the country’s role in toxicological risk assessment and helping to explain its strong position in mycotoxin research [56].

A similar but more pronounced software-dependent pattern was observed for institutions (Table 1). BibExcel and VOSviewer produced identical post-normalisation institutional entities, frequencies, and rankings, whereas the Bibliometrix affiliation output differed in both the number and aggregation level of entries. Consequently, the concordant BibExcel/VOSviewer output was used for the institutional productivity and impact analysis presented in Table 6.

Table 6 (performed with the BibExcel tool) shows the 20 most productive institutions sorted by h-index. As can be observed, the leading institutions were mostly European and Chinese. The University of Wurzburg ranked first, with an h-index of 22, 30 articles, and 1508 citations; followed by the University of Valencia, with an h-index of 21, 45 articles, and 2718 citations; and Nanjing Agricultural University, also with an h-index of 21 and 35 articles. Other institutions included China Agricultural University, IMROH, the University of Zagreb, the University of Guelph, the University of Navarra, the University of Konstanz, Hebei Medical University, the Chinese Academy of Agricultural Sciences, and CNRS.

It should be noted that no US institution appeared among the 20 leading institutions, despite the USA ranking second at the country level by h-index and recording one of the highest citation totals. This pattern may reflect a comparatively dispersed institutional contribution, whereby publications are distributed across multiple US institutions rather than concentrated in a small number of centres. By contrast, the institutional outputs of Germany, Spain, China, and Croatia were more concentrated among several highly visible organisations.

**Figure 7:**
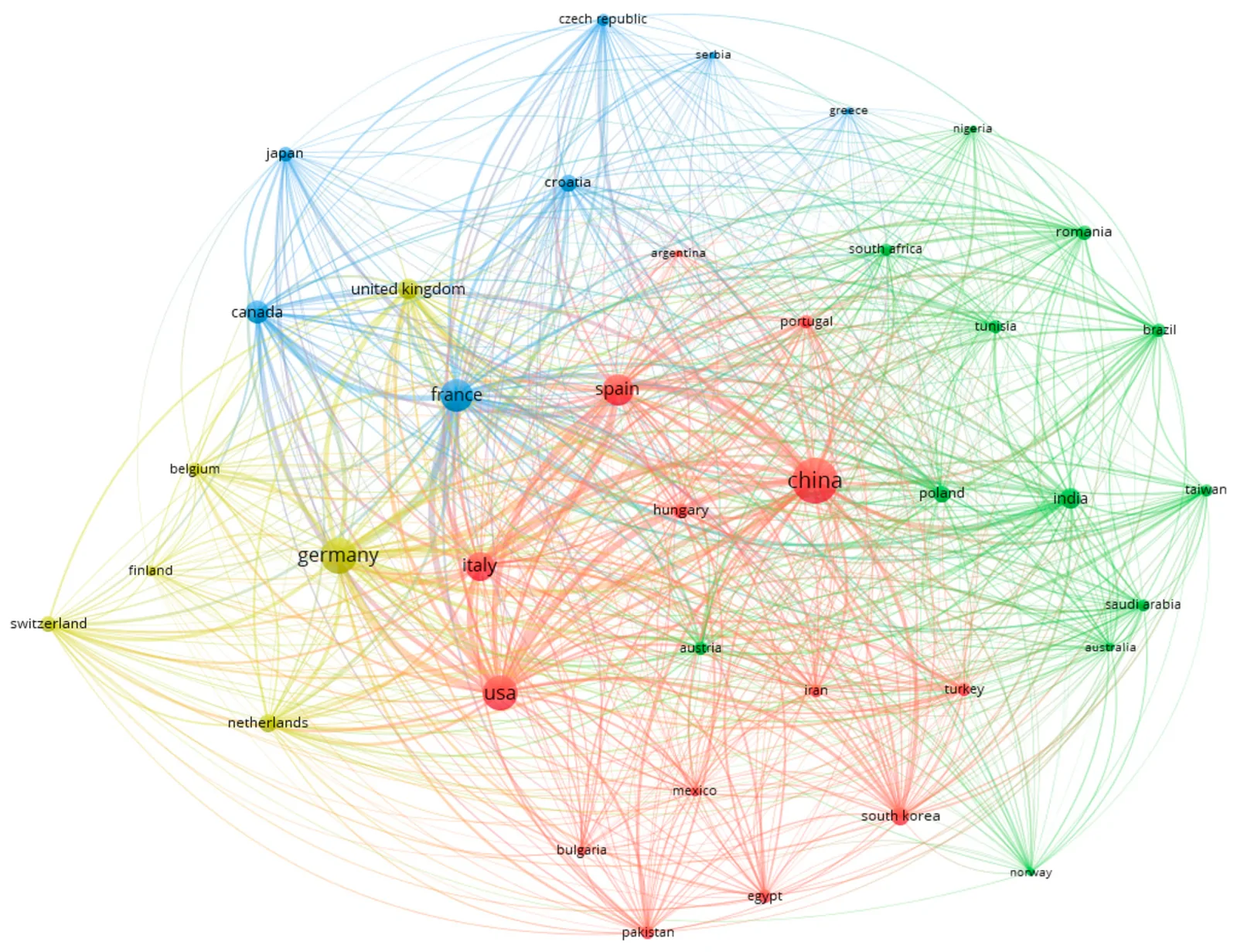
Bibliographic coupling map for countries. Network representation of countries based on shared references in OTA-related publications, where link strength reflects the degree of bibliographic coupling and clusters indicate countries with similar research profiles.

**Figure 8:**
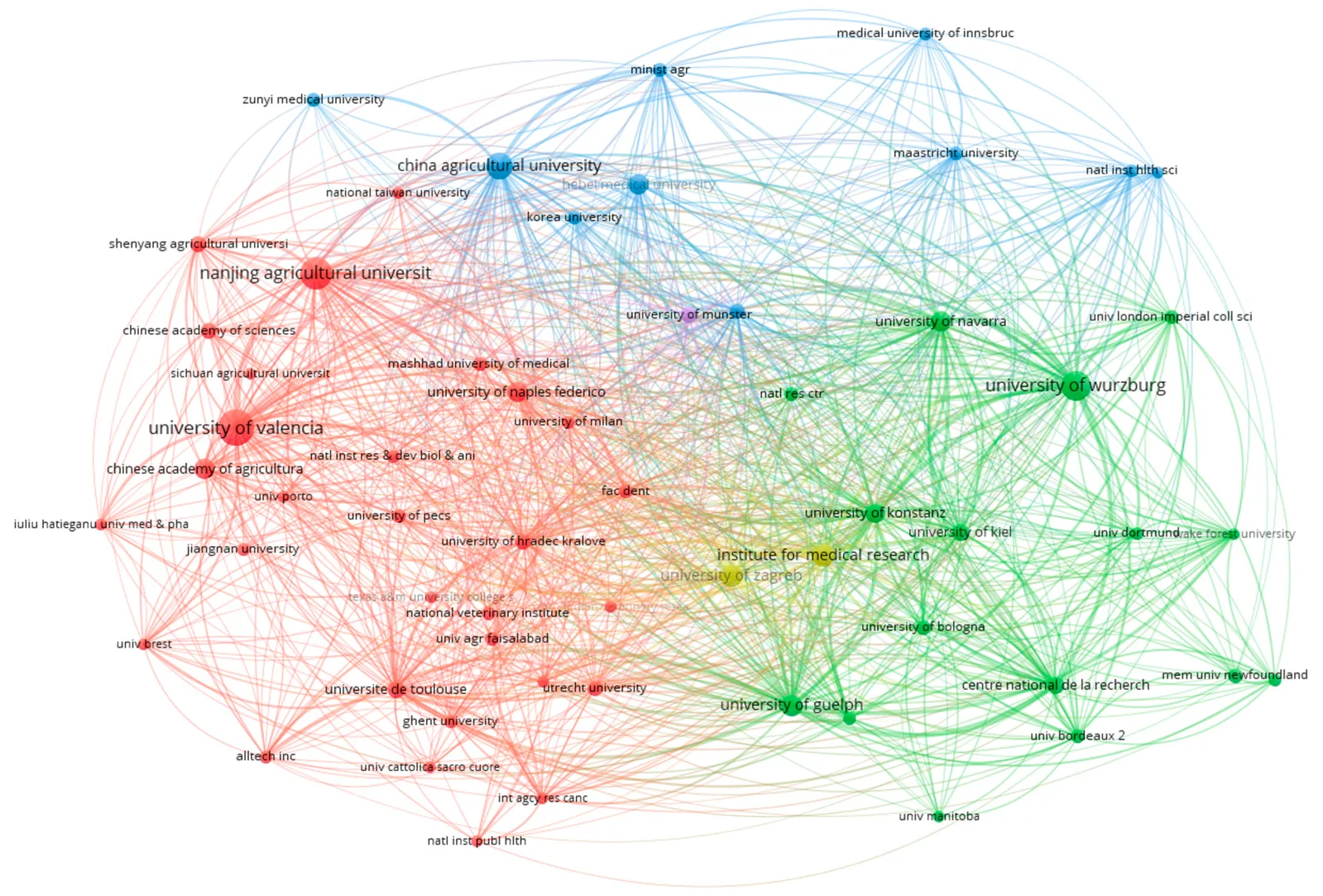
Bibliographic coupling map for institutions. Diagram showing how institutions are interconnected through shared references in OTA research, highlighting groups with similar scientific interests and research focus.

Bibliographic coupling analysis provided an additional representation of the intellectual relationships among countries and institutions. Figure 7, generated using the VOSviewer tool, shows the bibliographic coupling map for a minimum of five documents published by a country, and the analysis identified 39 different countries. Figure 8, also generated using the VOSviewer tool, shows the bibliographic coupling map for a minimum of five documents published by an institution, and the analysis identified 59 different institutions.

While Figure 7 shows broad national intellectual proximity, Figure 8 highlights the institutional hubs around which OTA toxicity research has been organized. The presence of universities, national research centres, toxicology institutes, agricultural institutions, and food safety-related centres in the same network reflects the interdisciplinary nature of the field. As can be observed, OTA toxicity research is not confined to a single disciplinary environment. Rather, it connects experimental toxicology, food safety, veterinary and agricultural sciences, analytical chemistry, and risk assessment. The coupling structure in Figure 8, therefore, suggests that the field is sustained by several specialized institutional clusters that share reference traditions but may focus on different experimental or regulatory dimensions of OTA toxicity.

### 2.6. Keyword Structure and Thematic Evolution of OTA

Author Keywords and Keywords Plus showed complete post-normalisation agreement across the three programs at the entity, frequency, and ranking levels (Table 1). Since the three tools produced concordant keyword outputs after normalisation, Bibliometrix was selected for the keywords analysis because it provides an integrated environment for conceptual structure analysis, trend topic visualisation, and thematic mapping [57]. This decision aligns with the authors’ earlier bibliometric research, where they utilized VOSviewer and Bibliometrix to conduct a keyword analysis of the QuEChERS sample-preparation method. Both tools yielded similar bibliometric results and conclusions, demonstrating that no significant differences were observed between them [58].

**Figure 9:**
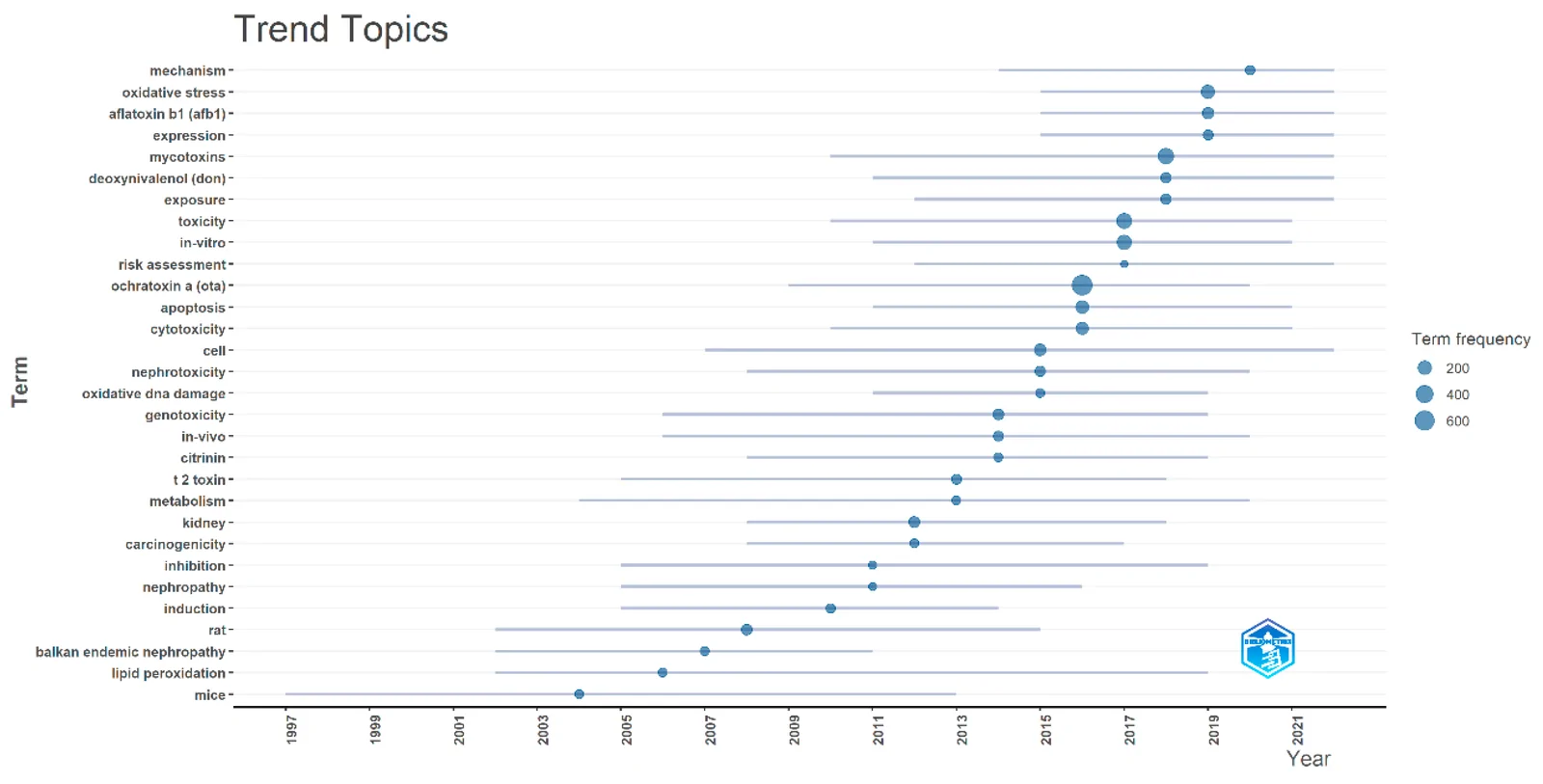
Trend topics analysis. Temporal evolution of key research topics in OTA studies, highlighting emerging themes and shifts in scientific focus over time.

Figure 9 shows the trend topics analysis performed with the three most frequent terms per year and a minimum frequency threshold of 30 across the normalized keyword dataset. The plot reveals a clear temporal shift in the conceptual focus of OTA toxicity research. Earlier years were characterised by terms such as “lipid peroxidation”, “mice”, “rat”, and “Balkan endemic nephropathy”, suggesting an initial emphasis on animal models, oxidative damage, and renal disease-related hypotheses. This is consistent with the historical relevance of OTA as a nephrotoxic mycotoxin and with previous work describing renal transport mechanisms, kidney accumulation, and nephrotoxicity as central elements of OTA toxicology [17].

From the mid-2010s onward, the field became more explicitly mechanistic and mycotoxin oriented, with increasingly prominent terms such as “mycotoxins”, “aflatoxin B1”, “deoxynivalenol”, and “ochratoxin A”. This confirms that OTA toxicity research is embedded within the broader context of mycotoxin toxicology rather than developing as an isolated topic. The same temporal pattern also shows the consolidation of molecular and cellular mechanisms as central research themes. The sustained prominence of “oxidative stress”, together with terms such as “apoptosis”, “cytotoxicity”, “genotoxicity”, “cell”, and “oxidative DNA damage”, indicates that the field has progressively moved from descriptive toxicological observations toward a deeper characterization of the pathways involved in OTA-induced damage [59,60].

The temporal evolution of keywords also reflects changes in experimental approaches. Earlier terms were more closely linked to “in vivo” studies and animal models, whereas “in vitro” became more visible in recent years, consistent with the increasing use of cell-based systems for mechanistic toxicology, toxicity screening, and reduction of animal experimentation. Studies using intestinal cell models, such as differentiated Caco-2 cells, have shown that OTA can induce apoptosis and affect intestinal barrier-related processes, supporting the growing relevance of in vitro approaches in OTA research [61]. Kidney-related terms, including “Balkan endemic nephropathy”, “nephropathy”, and “kidney”, remained recurrent across the period, reinforcing the central role of renal toxicity in OTA research [17,62].

By contrast, terms such as “liver”, “intestine”, “brain”, “gut”, or “microbiota” were not among the three most frequent keywords per year. This should not be interpreted as evidence that these topics are absent from literature but rather as a consequence of the restrictive N = 3 parameter used in the trend topic analysis. In fact, recent studies have shown that OTA exposure can alter the structure and diversity of the gut microbiota in mice, and recent reviews have described the intestine as both a target and a modulator of OTA toxicity [15,63]. Therefore, these topics may become more visible in future analyses using broader keyword thresholds or targeted searches.

The position of this cluster indicates that “OTA”, “toxicity”, and oxidative stress-related terms occupied a highly central position within the keyword co-occurrence structure and thematic organisation of the literature. This finding should be interpreted as a bibliometric observation describing the conceptual structure of OTA toxicity research rather than as direct evidence that oxidative stress is the principal biological mechanism of OTA toxicity. Nevertheless, the prominence of oxidative stress-related terminology in the literature is consistent with the substantial experimental body of work investigating oxidative stress, apoptosis, DNA damage, and related cellular responses associated with OTA exposure [59,60].

**Figure 10:**
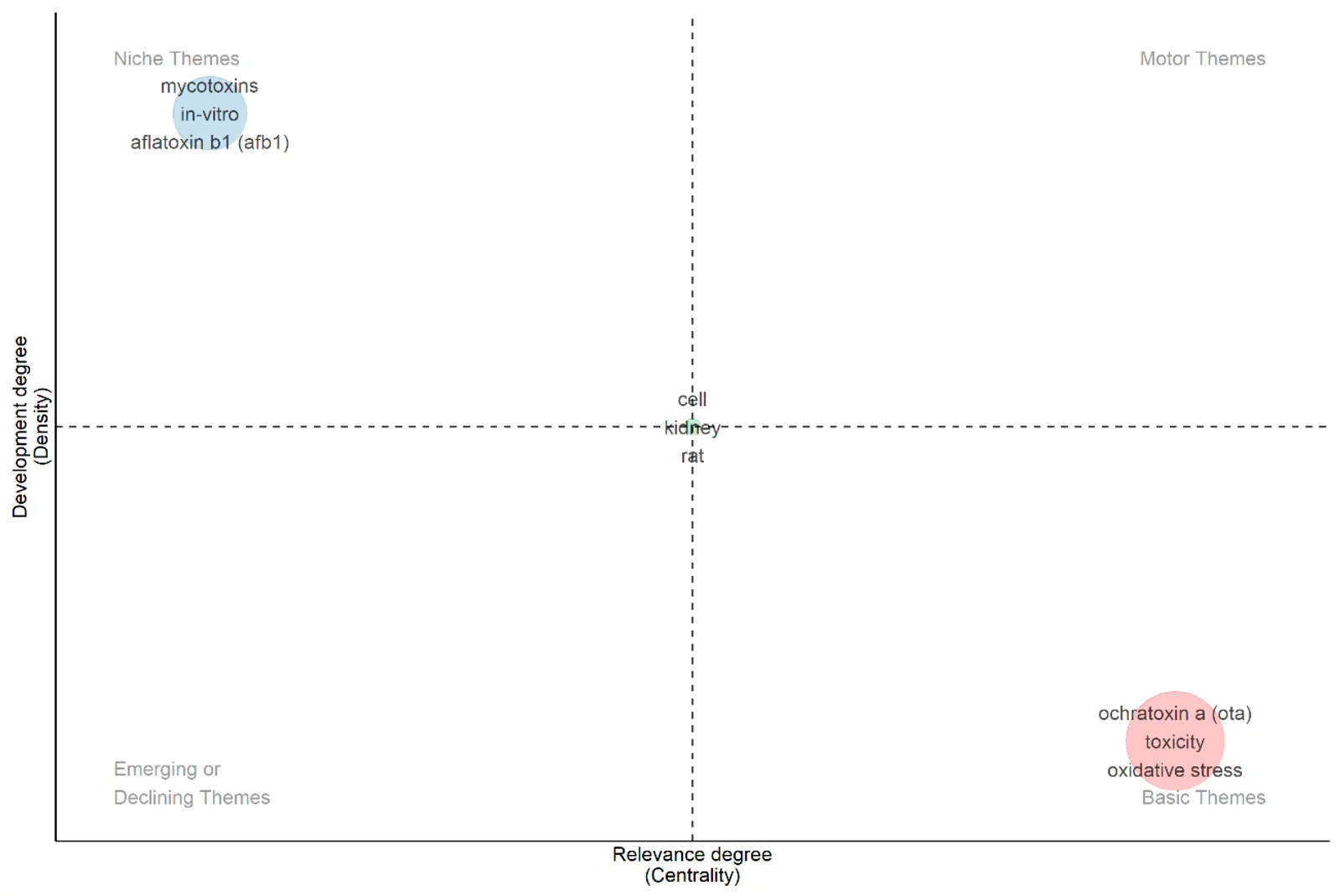
Thematic map. Graphical representation of the main research themes in OTA studies, organized according to their relevance and development, highlighting well-established and emerging topics.

In contrast, the cluster formed by “mycotoxins”, “in-vitro”, and “aflatoxin B1 (AFB1)” appeared as a niche theme, indicating a relatively well developed but less central research line within the overall keyword co-occurrence structure. In thematic maps, niche themes are generally interpreted as internally cohesive topics with lower centrality, meaning that they are specialised but do not act as major conceptual bridges across the field. In this context, AFB1 should not be interpreted as a core OTA specific mechanism but rather as a specialised subtheme linked to comparative mycotoxin toxicology, OTA AFB1 co-exposure, and experimental in vitro approaches. This interpretation is consistent with studies evaluating combined OTA AFB1 toxicity in relation to kidney and liver injury, immune inflammation, oxidative stress, apoptosis, and gut microbiota alterations, as well as with recent evidence showing overlapping toxicological endpoints for AFB1 and OTA in in vitro and in vivo models. Interestingly, no clear motor themes were identified in Figure 10, suggesting that the field does not currently contain a thematic cluster that is both highly developed and highly central. Terms such as “cell”, “kidney”, and “rat” occupied an intermediate position near the centre of the map, reflecting their role as biological and methodological connectors rather than as independent dominant themes. Overall, the thematic map indicates that oxidative stress-related terminology, renal toxicity-related concepts, and experimental model systems occupied prominent or connecting positions within the keyword structure of the analysed corpus [64,65].

### 2.7. Emerging Directions in OTA Research

Recent bibliometric patterns indicate increasing attention to integrative research frameworks linking oxidative stress, genotoxicity, apoptosis, nephrotoxicity, and intestinal effects with gut microbiota interactions, co-exposure to multiple mycotoxins, and advanced experimental models [66,67,68].

The bibliometric patterns observed in this study identify several comparatively less visible topics within the analysed WoSCC corpus. First, despite the increasing use of mechanistic approaches, much of the literature still relies on experimental animal models and immortalised cell lines, suggesting that human-relevant models and translational toxicology frameworks may warrant further bibliometric and experimental attention. Second, while oxidative stress-related terminology occupies a prominent position in the bibliometric structure of the field, the thematic analyses indicate that combined-exposure scenarios involving OTA and other mycotoxins remain comparatively specialised research areas. Third, gut microbiota interactions and gut immune signalling appear as emerging directions with growing scientific relevance but still limited thematic prominence.

Although gut- and microbiota-related terms did not yet appear among the most frequent annual keywords, experimental evidence already indicates that OTA can modify gut microbial communities, and recent reviews emphasize the role of the intestine and microbiota in modulating OTA toxicity [15,63,69,70,71].

Taken together, these bibliometric patterns support further consideration of integrative approaches combining molecular toxicology, microbiome research, co-exposure assessment, and human-relevant experimental platforms.

## 3. Conclusions

This bibliometric and science mapping study provides a quantitative mapping of the literature on OTA toxicity in in vitro and in vivo experimental settings retrieved from the WoSCC. Publication output increased over the analysed period, while bibliographic-coupling, trend-topic, and thematic analyses identified changes in the relational and conceptual organisation of the literature. In particular, the keyword structure showed increasing representation of molecular, cellular, intestinal, microbiota-related, and combined exposure topics alongside the continued prominence of renal toxicity- and oxidative stress-related terminology. These findings describe patterns in the scientific literature and should not be interpreted as direct evidence of biological causality or mechanistic importance.

The cross-software comparison showed exact agreement among BibExcel, VOSviewer, and Bibliometrix for authors, journals, Author Keywords, and Keywords Plus, and exact agreement between BibExcel and VOSviewer for countries and institutions. Residual differences were confined to the address-derived country and affiliation outputs generated by Bibliometrix, demonstrating that these fields were the most sensitive to differences in aggregation and unit definition.

The findings are limited to a query defined WoSCC corpus without record-by-record eligibility screening and, therefore, do not constitute a systematic synthesis of experimental toxicological evidence. Within these limits, comparatively less visible topics, including human-relevant experimental platforms, combined-exposure scenarios, and gut microbiota-related research, may help formulate questions for future experimental investigation.

## 4. Materials and Methods

### 4.1. Study Design

This study was designed as a bibliometric and science mapping analysis of the literature on OTA toxicity in in vitro and in vivo experimental settings. It was not designed as a systematic or scoping review of experimental toxicological evidence and did not include methodological quality assessment, risk of bias evaluation, or evidence grading of individual studies. Its objective was to analyse the structure, evolution, relational patterns, and thematic organisation of the literature through quantitative bibliometric methods.

The analytical workflow combined descriptive bibliometric indicators with selected science mapping approaches. Descriptive analyses were used to characterise publication output, document types, languages, journals, authors, countries, institutions, and keywords. Relational analyses were used to explore bibliographic coupling [50] and keyword-based conceptual structures. This integrated approach was adopted to provide both a quantitative overview of the field and an interpretation of its intellectual and thematic organisation [35,57].

### 4.2. Data Source and Literature Retrieval

The initial search was conducted through the Web of Science (WoS) All Databases interface on 10 February 2026. The Topic Search field (TS), which searches titles, abstracts, author keywords, and Keywords Plus, was queried using the following expression: (toxicity* OR cytotoxicity*) AND (“ochratoxin A” OR “OTA”) AND (“in vitro” OR “in vivo”). The search covered the period from database inception to 10 February 2026. No language or document type filters were applied at the initial retrieval stage. Screenshots of the initial WoS All Databases search, the restriction to the WoSCC, and the corresponding retrieval information are provided in Supplementary File S1. The search strategy combined three conceptual blocks: toxicological framing (toxicity* OR cytotoxicity*), the target compound (“ochratoxin A” OR “OTA”), and the experimental context (“in vitro” OR “in vivo”). The terms “in vitro” and “in vivo” were used without requiring the word “model” to avoid restricting retrieval to publications using the exact expressions “in vitro model” or “in vivo model”. The term cytotoxicity* was included to improve the retrieval of cell-based toxicological studies.

The initial search returned 923 records across the WoS All Databases interface. For the final bibliometric corpus, the source was restricted to WoSCC, resulting in 887 records. Thus, the 36 records retrieved through other WoS databases were not part of the final corpus. WoSCC was selected because it provides a curated and internally consistent bibliographic source with structured metadata suitable for field level normalisation and comparative processing across the bibliometric environments used in this study. Four records published in 2026 were subsequently excluded because 2026 was incomplete at the time of the search. The final corpus therefore comprised 883 records published between 1965 and 2025. The complete original WoS plain-text export containing the 883 records is provided as Supplementary File S2.

Because the final corpus was obtained from a single WoSCC export, no cross-database deduplication was required. Record uniqueness was checked using the WoS accession number (UT). No manual title/abstract eligibility screening or record by record disambiguation of the acronym “OTA” was performed after restricting the dataset to WoSCC. Corpus inclusion was operationally defined by the reproducible search expression, the selected bibliographic source, and the publication-year criterion. Consequently, the dataset should be understood as a query defined bibliometric corpus rather than as a systematically screened collection of experimentally eligible OTA toxicity studies.

The use of a single curated source was a deliberate methodological choice intended to maximise metadata consistency, facilitate field-level normalisation, and ensure comparability across the three bibliometric environments employed in this study. The objective was not to construct the broadest possible multi-database corpus but to analyse a stable and reproducible dataset suitable for cross-software validation. Consequently, the final corpus represents the OTA toxicity literature retrieved from WoSCC under the defined search strategy and should not be interpreted as an exhaustive representation of all of the OTA toxicity literature.

### 4.3. Software Environment and Analytical Integration

Three complementary software environments were used in this study: Bibliometrix/Biblioshiny (bibliometrix package version 5.2.1; Department of Economics and Statistics, University of Naples Federico II, Naples, Italy), run in R 4.5.1 (R Foundation for Statistical Computing, Vienna, Austria) through RStudio Desktop 2026.01.01 (Posit Software, PBC, Boston, MA, USA) [57]; VOSviewer version 1.6.20 (Centre for Science and Technology Studies, Leiden University, Leiden, The Netherlands) [37]; and BibExcel version 2016-02-20 (developed by Olle Persson, Department of Sociology, Umeå University, Umeå, Sweden) [34].

This multi-software strategy was adopted to combine the strengths of an open-source analytical environment, dedicated network visualisation software, and a flexible preprocessing toolbox. The aim was not to force identical outputs across all programs, but to evaluate the stability of the normalized corpus and identify fields that were more sensitive to software-specific parsing.

### 4.4. Data Preprocessing and Field-Level Normalisation

Before analysis, the exported bibliographic records were subjected to field-level normalisation to improve internal consistency and reduce fragmentation caused by spelling variants, initials, abbreviations, diacritics, synonymous terms, journal-title variants, institutional variants, and software-specific parsing rules. Normalisation was applied to author names, source titles, countries, institutions, author keywords, and Keywords Plus.

Author names were harmonized into canonical forms after manual verification. Before normalisation, direct BibExcel extraction identified 3212 unique abbreviated author labels in the WoSCC AU field and 3669 unique full-name strings in the AF field. After normalisation, the corresponding field-level counts were 3305 unique AU labels and 3497 unique AF strings. The changes reflected the combined effect of consolidating variant forms referring to the same author and contextually disambiguating abbreviated author labels during construction of the controlled author vocabulary. The AU and AF fields were used as complementary sources for author identification and were not expected to retain identical raw-string vocabularies because they represent abbreviated and full-name formats, respectively. Importantly, both fields contained 4966 author occurrences before and after normalisation, confirming that the procedure changed author representation and identification without adding or removing authorship occurrences or bibliographic records.

Journal titles were standardized to their full forms. For example, abbreviated or variant journal names were consolidated into full source-title forms such as Food and Chemical Toxicology, Archives of Toxicology, and International Journal of Molecular Sciences.

Countries and institutions were normalized through dictionary-based replacement supported by manual review and comparison of software outputs. Representative country normalisations included “USA” to “United States”, “UK” to “United Kingdom”, “Peoples R China” to “China”, “Turkiye” to “Turkey”, “FED REP GER” to “Germany”, and “Cote Ivoire” to “Cote d’Ivoire”. Representative institutional normalisations included “Univ Valencia” to “University of Valencia”, “Nanjing Agr Univ” to “Nanjing Agricultural University”, “Univ Wurzburg” to “University of Wurzburg”, “Chinese Acad Agr Sci” to “Chinese Academy of Agricultural Sciences”, “Inst Med Res & Occupat Hlth” to “Institute for Medical Research & Occupational Health (IMROH)”, “CNRS” to “Centre National de la Recherche Scientifique (CNRS)”, “Int Agcy Res Canc” to “International Agency for Research on Cancer (IARC)”, and “Texas A&M Univ” to “Texas A&M University College Station”.

Keyword variants were normalized using a controlled equivalence strategy. Representative examples included harmonization of mycotoxin names and acronyms, such as “ochratoxin A”, “OTA”, and “ochratoxin-a”; “aflatoxin B1” and “AFB1”; “deoxynivalenol” and “DON”; “zearalenone” and “ZEN”; and “patulin” and “PAT”. Mechanistic and toxicological terms were also standardized, including variants related to “oxidative stress”, “apoptosis”, “cytotoxicity”, “genotoxicity”, “immunotoxicity”, “oxidative DNA damage”, “NLRP3 inflammasome”, “MAPK/NF-κB”, “PTEN/PI3K/AKT”, “HK-2”, “PBMCs”, “qRT-PCR”, and “western blot”.

The normalised WoS plain-text files and their corresponding audit materials are provided as supplementary files. Supplementary File S3 contains the author-normalised dataset, and Supplementary File S4 contains the complete author-normalisation dictionary and record-level audit. Supplementary File S5 contains the journal-normalised dataset, accompanied by the journal-normalisation audit in Supplementary File S6. Supplementary File S7 contains the countries- and institutions-normalised dataset, with the corresponding normalisation dictionary and audit in Supplementary File S8. Supplementary File S9 contains the keyword-normalised dataset, in which Author Keywords (DE) and Keywords Plus (ID) were normalised jointly but retained as separate fields. The corresponding dictionaries and audit are provided in Supplementary File S10. Supplementary File S11 provides the quantitative cross-software validation based on the final normalised outputs.

Country and institutional outputs were interpreted more cautiously because these entities are derived from address-related information and are therefore more sensitive to software-specific parsing and counting procedures. Methodological studies have shown that bibliometric network construction is not a neutral step and that different counting approaches may affect network structures [72]. Therefore, address derived outputs were validated across software tools and interpreted with attention to software-specific behaviour.

The number of references and cited-references fields were intentionally not normalised because cited-reference counts depend on software-specific preprocessing decisions, particularly reference string parsing and duplicate handling. Therefore, these outputs were compared only within equivalent analytical contexts and were not treated as directly interchangeable metrics.

### 4.5. Bibliometric Indicators and Science Mapping Analyses

Descriptive bibliometric indicators were obtained using BibExcel [34] and included annual publication output, document types, publication languages, leading journals, productive authors, countries, institutions, cited references, recurrent keywords, Lotka’s Law for author productivity, Price’s Law, and h-index values for selected units.

Network visualisation was performed using VOSviewer [37], which supports the construction of bibliometric maps based on citation, bibliographic coupling, co-citation, co-authorship, and term co-occurrence relations. In this study, VOSviewer was used primarily to generate bibliographic-coupling maps [37,50].

Bibliometrix [57] was used as a complementary environment for descriptive outputs, trend-topic analysis, and thematic mapping.

Journal impact factors and journal category positions reported in Table 4 were obtained from the 2025 edition of Journal Citation Reports, accessed on 14 July 2026 [35].

To ensure analytical reproducibility, the software functions, input fields, units of analysis, counting methods, inclusion thresholds, clustering procedures, and normalisation settings used for each bibliometric output are summarised in Table 7. These parameters are reported separately because bibliometric results may vary according to field selection, counting method, inclusion threshold, network-normalisation procedure, and software-specific processing.

### 4.6. Counting Approach and Interpretation of Network Outputs

Bibliographic coupling networks were constructed in VOSviewer using full counting and association strength normalisation. The units of analysis were authors, sources, countries, and organisations. Inclusion thresholds were set at a minimum of 10 documents for authors and five documents for sources, countries, and organisations. For all bibliographic coupling maps, clustering was performed using a resolution of 1.00, a minimum cluster size of one, and the option to merge small clusters. Node size was weighted by the number of documents.

Trend Topics analysis was performed in Bibliometrix using the All Keywords field, which combines Author Keywords and Keywords Plus. The analysis covered the period 1965–2025 and displayed the three most frequent eligible terms per year using a minimum word frequency of 30. The thematic map was generated from the same All Keywords field using 250 terms, a minimum frequency of five, and Louvain clustering. Community repulsion was set to 0.5, with three labels per cluster and a label size of 0.3. No terms were removed and no additional synonyms were applied within Bibliometrix. The general random seed was set to 1234. Parameters not exposed in the Bibliometrix interface were not modified by the authors.

VOSviewer links represent bibliographic coupling relationships based on shared cited references, whereas link strength indicates the strength of these relationships. Because bibliometric maps may vary according to counting procedures, inclusion thresholds, normalisation settings, clustering parameters, and software-specific processing, the resulting networks and thematic structures were interpreted as representations of relational or conceptual proximity rather than as absolute measures of collaboration, scientific influence, or biological importance.

### 4.7. Reproducibility and Cross-Software Validation

Cross-software agreement was assessed for authors, journals, countries, institutions, Author Keywords, and Keywords Plus using the complete entity frequency outputs generated by BibExcel, VOSviewer, and Bibliometrix. For each field and program, the number of unique entries was recorded before and after normalisation. Similarity between entity sets was evaluated using the Jaccard coefficient, calculated as the number of shared entities divided by the number of entities present in the union of both sets [73,74]. Rank order agreement was evaluated using Spearman’s rank correlation coefficient [75,76]. Exact frequency agreement was calculated as the percentage of shared entities assigned an identical frequency by both programs. In addition, overlap between the 20 highest-frequency entities was examined as a transparent comparison of the leading ranked items [77].

Agreement metrics were calculated only when the software outputs represented the same bibliographic unit and the same frequency measure. A Jaccard coefficient of 1.000 indicated identical entity sets, whereas a Spearman coefficient of 1.000 indicated identical rank ordering. Exact frequency agreement of 100% indicated that every shared entity received the same frequency in both programs. Similar unique entry counts were not considered evidence of agreement in the absence of entity-level verification.

For country and institutional outputs, comparability was evaluated separately. BibExcel and VOSviewer generated equivalent document-based country and organisation outputs. However, Bibliometrix produced address derived country production and affiliation outputs that differed in aggregation level. Therefore, full frequency agreement and rank correlation statistics involving Bibliometrix were reported only when the underlying units were directly comparable. Otherwise, the metric was designated as not applicable. Summary counts, pairwise calculations, metric definitions, and residual discrepancies are reported in Supplementary File S11. The complete field-level normalisation dictionaries and audit trails remain available in Supplementary Files S4, S6, S8, and S10.

### 4.8. Use of AI Assisted Tools

Microsoft Copilot [78] was used as an artificial intelligence (AI)-assisted support tool during the metadata normalisation workflow. Its use was limited to helping identify spelling variants; organize normalisation dictionaries; compare outputs across BibExcel [34], VOSviewer [37], and Bibliometrix [57]; and draft audit and methodological descriptions. DeepL Write was used to support language editing and improve the clarity, fluency, and readability of selected sections of the manuscript [79,80]. All AI-assisted outputs and edits were manually reviewed, corrected, and validated by the authors. The AI-assisted tools were not used as autonomous analytical systems, and the authors remain fully responsible for the final dataset, methodological decisions, data interpretation, and conclusions [81,82].

### 4.9. Limitations

This study has several limitations that should be considered when interpreting the results. First, the analysis was based exclusively on records retrieved from the WoSCC. Although this database provides high-quality bibliographic metadata and is widely used in bibliometric studies, it does not include all the scientific literature on OTA toxicity. Therefore, relevant publications indexed only in Scopus, PubMed, Google Scholar, or regional databases may not have been included. In addition, the search strategy was designed to focus specifically on in vitro and in vivo studies related to OTA toxicity. Consequently, studies addressing OTA mainly from analytical, agricultural, regulatory, or food occurrence perspectives may have been underrepresented if they did not match the selected search criteria.

A further limitation concerns the query defined nature of the corpus. The search included the acronym “OTA” and was conducted using the WoS Topic Search field, which considers titles, abstracts, Author Keywords, and Keywords Plus. No subsequent record-by-record title/abstract screening or manual disambiguation of the acronym was performed. Therefore, some records may have been retrieved because ochratoxin A appeared as an indexing term, particularly in Keywords Plus, or because “OTA” occurred in a broader or potentially different context, without OTA toxicity necessarily being the primary focus of the publication. Conversely, relevant studies may not have been retrieved if they did not explicitly use the selected toxicological and experimental descriptors, including “toxicity”, “cytotoxicity”, “in vitro”, or “in vivo”. These characteristics may introduce a degree of thematic noise into the corpus. Accordingly, the results should be interpreted as a bibliometric mapping of the literature retrieved under the specified WoSCC query rather than as a systematically screened or exhaustive collection of all experimental OTA toxicity studies.

A second limitation concerns the quality and completeness of the original metadata exported from WoS. Older records were particularly prone to incomplete indexing. Two records required manual correction because specific bibliographic fields were missing or incomplete. The article “Ochratoxin A and protein utilization in vivo” by Pettersson and Kiessling (1978) [83], did not include an indexed number of cited references in the exported WoS file. Manual verification of the original article identified eight references, which were added to the corresponding annual value. This correction increased the corpus-level total from 61,258 automatically indexed cited-reference occurrences to a final total of 61,266 cited-reference occurrences. In addition, the article “Ochratoxin A, a toxic metabolite produced by *Aspergillus ochraceus* Wilh” [1] lacked indexed page number information and was manually reviewed using the original DOI record. These corrections were limited to incomplete bibliographic metadata and did not modify the structure of the dataset or the main analytical fields.

Regarding the normalisation process, author names, countries, institutions, journals, and keywords required extensive harmonization because bibliometric outputs are highly sensitive to spelling variants, initials, abbreviations, diacritics, institutional name variants, and software-specific parsing rules. Although normalisation was performed through a supervised and auditable workflow combining manual review, dictionary-based replacement, and AI-assisted support, all normalisation decisions were manually reviewed and validated by the authors before being incorporated into the final dataset. Therefore, AI assistance should be understood as part of a human controlled workflow rather than as an autonomous analytical procedure.

Cross-software validation showed exact agreement among BibExcel, VOSviewer, and Bibliometrix for authors, journals, Author Keywords, and Keywords Plus, and exact agreement between BibExcel and VOSviewer for countries and institutions. Residual differences were restricted to the address-derived country and affiliation outputs generated by Bibliometrix. After normalisation, this program returned 71 country entries and 808 affiliations, compared with 73 countries and 987 institutional entities in BibExcel and VOSviewer. Because the country frequencies and institutional units produced by Bibliometrix were not directly equivalent to the document-based and organisation-level outputs of BibExcel and VOSviewer, non-equivalent frequency and ranking metrics were designated as not applicable rather than interpreted as evidence of agreement or disagreement. These differences are reported as observable software output behaviour and are not attributed to undocumented internal algorithms.

The institutional ranking in Table 6 should, therefore, be understood as a robust representation of the most visible research centres according to the concordant BibExcel/VOSviewer output rather than as an absolute institutional census.

Citation-based indicators also have inherent limitations. Metrics such as total citations, h-index, and bibliographic coupling are influenced by publication age, journal visibility, review articles, self-citation, database coverage, and disciplinary citation practices. Older publications have had more time to accumulate citations, whereas recent studies may be underrepresented in impact-based rankings despite their potential scientific relevance. Similarly, bibliographic coupling reflects shared reference structures and intellectual proximity, but it should not be interpreted as direct collaboration among authors, institutions, or countries. For this reason, network maps were used as complementary interpretative tools alongside quantitative indicators such as article counts, citation totals, and h-index values.

Finally, keyword-based analyses depend strongly on the completeness and consistency of author keywords and Keywords Plus. Although the normalisation process produced concordant keyword outputs across the three programs, the interpretation of trend topics and thematic maps is affected by the selected thresholds and parameters. Analyses based on the most frequent annual terms may overlook emerging but still low frequency topics. Therefore, the keyword trends reported here should be interpreted as a representation of the dominant conceptual structure of OTA toxicity research field, not as an exhaustive description of all active or emerging subfields.

Overall, these limitations do not invalidate the findings, but they highlight the importance of transparent preprocessing, careful metadata curation, and cross-software validation in bibliometric research. The combination of manual verification, AI assisted normalisation, and comparative analysis across BibExcel, VOSviewer, and Bibliometrix strengthened the reliability of the final dataset while also making visible the methodological constraints inherent to large scale bibliographic analyses.

## Figures and Tables

**Table 1:** Quantitative cross-software comparison of bibliographic fields before and after normalisation.

Field	Unique Entries Before Normalisation (B/V/M)	Unique Entries After Normalisation (B/V/M)	Post-Normalisation Agreement	Residual Discrepancies and Interpretation
Authors	3212/3509/3509	3305/3305/3305	All pairs: J = 1.000; exact frequency agreement = 100%; Spearman rho = 1.000; top 20 overlap = 20/20	Pre-normalisation differences reflected abbreviated AU labels and software-specific author representations. The controlled vocabulary produced 3305 author entities in all three software outputs. Direct AU and AF field vocabularies remained non-identical, while their 4966 author occurrences were fully preserved.
Journals	259/259/259	259/259/259	All pairs: J = 1.000; exact frequency agreement = 100%; Spearman rho = 1.000; top 20 overlap = 20/20	The SO field required no effective modification.
Countries	76/79/71	73/73/71	B-V: J = 1.000; exact frequency agreement = 100%; Spearman rho = 1.000; top 20 overlap = 20/20. B-M and V-M: J = 0.973; frequency/rank metrics = NA	Bibliometrix returned two fewer independent country entries and used a non-equivalent Country Production frequency measure.
Institutions/affiliations	1005/1005/818	987/987/808	B-V: J = 1.000; exact frequency agreement = 100%; Spearman rho = 1.000; top 20 overlap = 20/20. B-M and V-M: NA	Bibliometrix generated affiliations at a different aggregation level.
Author Keywords (DE)	1890/1890/1890	1779/1779/1779	All pairs: J = 1.000; exact frequency agreement = 100%; Spearman rho = 1.000; top 20 overlap = 20/20	None after normalisation.
Keywords Plus (ID)	2216/2215/2217	2086/2086/2086	All pairs: J = 1.000; exact frequency agreement = 100%; Spearman rho = 1.000; top 20 overlap = 20/20	Pre-normalisation parsing differences disappeared after normalisation.

B: BibExcel; V: VOSviewer; M: Bibliometrix; J: Jaccard similarity coefficient; SO: WoS nomenclature field tag for journal; NA: not applicable because the compared outputs did not represent equivalent frequency or affiliation units. Exact frequency agreement is the percentage of shared entities with identical frequencies. Spearman coefficients were calculated from the frequency-based ranks of shared entities. Top 20 overlap represents the number of shared entities in the first 20 positions. Similar totals alone were not treated as evidence of agreement.

**Table 2:** Annual bibliometric indicators of the OTA toxicity literature. The table reports the number of publications, author occurrences, cited-reference occurrences, and pages for each publication year represented in the corpus, together with the corresponding per-publication ratios. Years without publications are not displayed.

PY	TP	AU	AU/TP	NR	NR/TP	PG	PG/TP
1965	1	5	5.0	4	4.0	2	2.0
1971	1	4	4.0	8	8.0	1	1.0
1972	1	2	2.0	12	12.0	1	1.0
1975	2	9	4.5	49	24.5	18	9.0
1977	1	2	2.0	23	23.0	6	6.0
1978	1	2	2.0	8	8.0	3	3.0
1979	1	2	2.0	21	21.0	13	13.0
1980	2	5	2.5	20	10.0	8	4.0
1982	1	2	2.0	55	55.0	8	8.0
1983	1	5	5.0	19	19.0	9	9.0
1984	4	16	4.0	110	27.5	22	5.5
1985	4	25	6.3	112	28.0	35	8.8
1986	1	9	9.0	48	48.0	9	9.0
1987	3	5	1.7	85	28.3	22	7.3
1988	3	9	3.0	62	20.7	22	7.3
1989	6	23	3.8	158	26.3	38	6.3
1990	5	17	3.4	113	22.6	33	6.6
1991	7	23	3.3	108	15.4	62	8.9
1992	3	9	3.0	136	45.3	21	7.0
1993	7	21	3.0	273	39.0	62	8.9
1994	3	10	3.3	114	38.0	25	8.3
1995	5	18	3.6	181	36.2	63	12.6
1996	5	15	3.0	337	67.4	63	12.6
1997	9	35	3.9	347	38.6	66	7.3
1998	13	38	2.9	689	53.0	112	8.6
1999	11	47	4.3	448	40.7	99	9.0
2000	5	25	5.0	281	56.2	52	10.4
2001	10	48	4.8	726	72.6	125	12.5
2002	13	55	4.2	1014	78.0	170	13.1
2003	10	46	4.6	397	39.7	80	8.0
2004	21	119	5.7	878	41.8	192	9.1
2005	26	121	4.7	1500	57.7	266	10.2
2006	20	109	5.5	1266	63.3	228	11.4
2007	19	99	5.2	1410	74.2	202	10.6
2008	23	123	5.3	1372	59.7	244	10.6
2009	27	154	5.7	1356	50.2	266	9.9
2010	21	104	5.0	1397	66.5	249	11.9
2011	27	157	5.8	1833	67.9	296	11.0
2012	30	181	6.0	1797	59.9	314	10.5
2013	27	148	5.5	2087	77.3	375	13.9
2014	32	204	6.4	1904	59.5	321	10.0
2015	41	241	5.9	3044	74.2	516	12.6
2016	30	157	5.2	2410	80.3	425	14.2
2017	32	191	6.0	2518	78.7	422	13.2
2018	43	282	6.6	2777	64.6	458	10.7
2019	52	276	5.3	3094	59.5	682	13.1
2020	47	308	6.6	5153	109.6	924	19.7
2021	39	241	6.2	2994	76.8	508	13.0
2022	58	351	6.1	5157	88.9	874	15.1
2023	43	283	6.6	3686	85.7	679	15.8
2024	38	254	6.7	3214	84.6	599	15.8
2025	48	331	6.9	4461	92.9	879	18.3
Total	883	4966		61,266		11,169	

PY: publication year; TP: number of publications in the corresponding year; AU: total author occurrences across the publications of that year; AU/TP: mean author occurrences per publication in that year; NR: total cited-reference occurrences across the publications of that year; NR/TP: mean cited-reference occurrences per publication in that year; PG: total number of pages in that year; PG/TP: mean number of pages per publication in that year. Years with no publications are omitted. No rate of change was calculated between non-consecutive publication years.

**Table 3:** Ten most productive authors ranked by h-index.

Author	h_Index	g_Index	m_Index	TC	NP	PY_Start
Huang, Kehe	20	30	1.667	931	30	2015
Pfohl-Leszkowicz, Annie	18	18	0.5	2312	18	1991
Gan, Fang	16	20	1.333	585	20	2015
Gekle, Michael	16	20	0.471	655	20	1993
Mally, Angela	15	17	0.652	1375	17	2004
Xu, Wentao	15	16	1.071	613	16	2013
Creppy, Ee	14	17	0.318	844	17	1983
Huang, Kunlun	14	15	1	582	15	2013
Chen, Xingxiang	13	15	1.083	448	15	2015
Font, Guillermina	13	16	0.722	992	16	2009

TC: time cited; NP: number of publications; PY: publication year.

**Table 4:** The 10 most productive journals, ranked by h-index. Journal impact factor values, JCR categories, and category positions were obtained from the 2025 edition of journal citation reports, accessed on 14 July 2026 [35].

h-Index	Unit	All Citations	All Articles	IF 2025	WoS Category	Position
37	Food and Chemical Toxicology	5695	72	3.2	Food Science & Technology	97/187
					Toxicology	49/106
35	Toxins	4368	89	4.0	Food Science & Technology	72/187
					Toxicology	30/106
24	Toxicology	3005	37	5.2	Pharmacology & Pharmacy	62/356
					Toxicology	16/106
21	Archives of Toxicology	1356	35	10.9	Toxicology	5/106
20	Toxicology Letters	1373	35	3.1	Toxicology	52/106
19	Toxicological Sciences	1384	21	5.2	Toxicology	16/106
18	Toxicon	1044	21	2.8	Pharmacology & Pharmacy	181/356
					Toxicology	60/106
18	Toxicology In Vitro	1004	27	2.7	Toxicology	64/106
17	Toxicology and Applied Pharmacology	867	21	3.6	Pharmacology & Pharmacy	128/356
					Toxicology	35/106
15	Molecular Nutrition & Food Research	1956	16	4.5	Food Science & Technology	61/187

**Table 5:** The 20 most productive countries sorted by h-index. Ranking of the leading countries in OTA research based on h-index, reflecting both scientific productivity and citation impact.

H-Index	Country	Citation Sum Within h-Core	All Citations	All Articles
43	Germany	4380	5671	109
43	USA	5267	6456	99
41	China	3218	5243	166
41	France	6337	7289	85
32	Spain	3704	4318	79
31	Italy	2420	2847	68
23	Canada	2084	2329	42
21	Netherlands	2232	2321	29
20	United Kingdom	1687	1830	35
19	India	1276	1391	33
17	Croatia	1022	1086	24
15	Switzerland	997	1028	21
15	Belgium	1360	1361	16
15	South Korea	637	723	29
14	Japan	881	906	19
13	Austria	1733	1745	16
12	Hungary	398	440	18
12	Poland	723	779	23
12	Portugal	720	723	14
11	Taiwan	429	440	13

**Table 6:** The 20 most productive institutions sorted by h-index. Overview of the leading institutions in OTA research, ranked by h-index to reflect their scientific productivity and impact.

h-Index	Institution	Citation Sum Within h-Core	All Citations	All Articles	Country
22	University of Wurzburg	1408	1508	30	Germany
21	University of Valencia	2554	2718	45	Spain
21	Nanjing Agricultural University	984	1168	35	China
19	China Agricultural University	748	815	25	China
14	Institute for Medical Research & Occupational Health (IMROH)	723	752	18	Croatia
14	University of Zagreb	800	829	18	Croatia
12	University of Guelph	1435	1465	16	Canada
12	University of Navarra	546	563	15	Spain
11	University of Konstanz	945	954	13	Germany
11	Hebei Medical University	348	366	14	China
11	University of Naples Federico II	507	522	15	Italy
10	Chinese Academy of Agricultural Sciences	429	457	14	China
10	Centre National de la Recherche Scientifique (CNRS)	1475	1475	10	France
9	University of Kiel	464	472	10	Germany
8	Universite de Toulouse	1087	1095	10	France
8	Utrecht University	713	713	8	Netherlands
8	University of Hradec Kralove	1355	1359	9	Czech Republic
7	Shenyang Agricultural University	516	516	9	China
7	Chinese Academy of Sciences	296	300	9	China
7	Ministry of Agriculture	317	317	7	China

**Table 7:** Software functions, input fields, counting methods, thresholds, clustering parameters, and normalisation settings used in the bibliometric analyses.

Analysis/Output	Software and Version	Input Field or Unit of Analysis	Function/Procedure	Counting Method	Inclusion Threshold/Timespan	Normalisation and Clustering Settings	Output
Data import and metadata checking	Bibliometrix/Biblioshiny (bibliometrix package version 5.2.1); R 4.5.1; RStudio Desktop 2026.01.01	WoS plain-text file; 883 records	Import, bibliographic-field checking, and descriptive outputs	Record- and field-level frequencies	Complete corpus	Not applicable	Corpus verification
Descriptive bibliometric indicators	BibExcel 2016-02-20	PY, DT, LA, AU/AF, SO, C1/C3/RP, CR, DE, and ID	Field extraction; frequency distributions; annual output; productivity indicators; Lotka’s Law for author productivity; Price’s Law; h-index outputs	Frequency counting of normalised field values	Complete corpus unless otherwise specified	Not applicable	Annual indicators; Price’s Law; Lotka distribution for authors; journal, country, and institution rankings; selected descriptive results
Author impact indicators and Bradford analysis	Bibliometrix/Biblioshiny (bibliometrix package version 5.2.1); R 4.5.1; RStudio Desktop 2026.01.01	Normalised authors and sources	Author local-impact indicators and Bradford’s Law	Record-level publication and citation counts	Complete corpus	Not applicable	Author h-, g-, and m-index table; Bradford plot
Bibliographic coupling by authors	VOSviewer 1.6.20	Authors and cited references	Bibliographic-coupling network	Full counting	Minimum 10 documents per author	Association-strength normalisation; resolution = 1.00; minimum cluster size = 1; merge small clusters enabled; node size weighted by Documents	Figure 4
Bibliographic coupling by sources	VOSviewer 1.6.20	Sources and cited references	Bibliographic-coupling network	Full counting	Minimum 5 documents per source	Association-strength normalisation; resolution = 1.00; minimum cluster size = 1; merge small clusters enabled; node size weighted by Documents	Figure 6
Bibliographic coupling by countries	VOSviewer 1.6.20	Countries and cited references	Bibliographic-coupling network	Full counting	Minimum 5 documents per country	Association-strength normalisation; resolution = 1.00; minimum cluster size = 1; merge small clusters enabled; node size weighted by Documents	Figure 7
Bibliographic coupling by organisations	VOSviewer 1.6.20	Organisations and cited references	Bibliographic-coupling network	Full counting	Minimum 5 documents per organisation	Association-strength normalisation; resolution = 1.00; minimum cluster size = 1; merge small clusters enabled; node size weighted by Documents	Figure 8
Trend Topics analysis	Bibliometrix/Biblioshiny (bibliometrix package version 5.2.1); R 4.5.1; RStudio Desktop 2026.01.01	All Keywords (KW_Merged: DE and ID)	Trend Topics	Keyword occurrences	1965–2025; minimum word frequency = 30; three words per year	No terms removed; no additional synonyms applied within Bibliometrix; random seed = 1234	Figure 9
Thematic map	Bibliometrix/Biblioshiny (bibliometrix package version 5.2.1); R 4.5.1; RStudio Desktop 2026.01.01	All Keywords (KW_Merged: DE and ID)	Co-word analysis and thematic mapping	Keyword co-occurrences	Number of terms = 250; minimum frequency = 5	Louvain clustering; community repulsion = 0.5; three labels per cluster; label size = 0.3; no terms removed; no additional synonyms applied within Biblioshiny; random seed = 1234	Figure 10
Field-level normalisation	Supervised editing of WoS plain-text files, supported by comparison of BibExcel, VOSviewer, and Bibliometrix outputs	AU/AF; SO/J9/JI/CR; C1/C3/RP; DE; ID	Dictionary-based replacement of verified variants followed by manual checking and cross-software comparison	Not applicable	All 883 records; no minimum-frequency threshold	Complete dictionaries or controlled vocabularies and line-by-line audit trails supplied as Supplementary Files	Normalised TXT files and supplementary audits
Cross-software comparison	BibExcel 2016-02-20; VOSviewer 1.6.20; Bibliometrix/Biblioshiny (bibliometrix package version 5.2.1); R 4.5.1; RStudio Desktop 2026.01.01	Authors, journals, countries, institutions, DE, and ID	Comparison of unique-entry counts, field frequencies, and residual discrepancies across software outputs	Software-specific field counting reported separately	Complete normalised outputs for each field	No clustering; field-specific agreement and residual discrepancies reported separately	Cross-software validation results

WoS: Web of Science; DE: WoS nomenclature field tag for Author keywords; ID: WoS nomenclature field tag for Keyword plus; PY: WoS nomenclature field tag for publication year; DT: WoS nomenclature field tag for type of document; LA: WoS nomenclature field tag for language; AU: WoS nomenclature field tag for author; AF: WoS nomenclature field tag for author full names; SO: WoS nomenclature field tag for source title, C1: WoS nomenclature field tag for addresses; C3: WoS nomenclature field tag for affiliations; RP: WoS nomenclature field tag for reprint addresses; CR: WoS nomenclature field tag for cited references; J9: WoS nomenclature field tag for 29-Character journal Abbreviation; JI: WoS nomenclature field tag for ISO journal abbreviation; KW_Merged: Keyword Merged.

## Data Availability

The original contributions presented in this study are included in the article/Supplementary Material. Further inquiries can be directed to the corresponding authors.

## Supplementary Materials

The following supporting information can be downloaded at: https://www.mdpi.com/article/10.3390/toxins18080346/s1, Supplementary File S1: Web of Science Search Strategy, Search History, and Retrieval Information.docx; Supplementary File S2: Original Web of Science Plain-Text Export Containing the 883-Record Corpus.txt; Supplementary File S3: Author-Normalised Web of Science Dataset.txt; Supplementary File S4: Author Normalisation Dictionary and Record-Level Audit.docx; Supplementary File S5: Journal-Normalised Web of Science Dataset.txt; Supplementary File S6: Journal Normalisation Dictionary and Record-Level Audit.docx; Supplementary File S7: Countries- and Institutions-Normalised Web of Science Dataset.txt; Supplementary File S8: Countries and Institutions Normalisation Dictionary and Record-Level Audit.docx; Supplementary File S9: Author Keywords- and Keywords Plus-Normalised Web of Science Dataset.txt; Supplementary File S10: Author Keywords and Keywords Plus Normalisation Dictionaries and Record-Level Audit.docx; Supplementary File S11: Cross-Software Validation Metrics, Pairwise Calculations, and Residual-Discrepancy Audit.xlsx.
