# Supplementary material for: Ochratoxin A Toxicity Research in In Vitro and In Vivo Experimental Settings: A Cross-Software Bibliometric and Science-Mapping Study
Source: Toxins (Basel). 2026 Aug 11;18(8):346. doi: 10.3390/toxins18080346 (PMC13517462; doi:10.3390/toxins18080346)
Supplement: Supplementary file 1 [file toxins-18-00346-s001.zip › Supplementary File S1.pdf]

Supplementary Materials: Ochratoxin A Toxicity Research in In Vitro and In Vivo Experimental Settings: A Cross-Software Bibliometric and Science-Mapping Study

José Manuel Veiga-del-Baño, José Oliva, Pedro Andreo-Martínez, Miguel Motas, Eva María Mateo, José Miguel Soria and María Ángeles García-Esparza

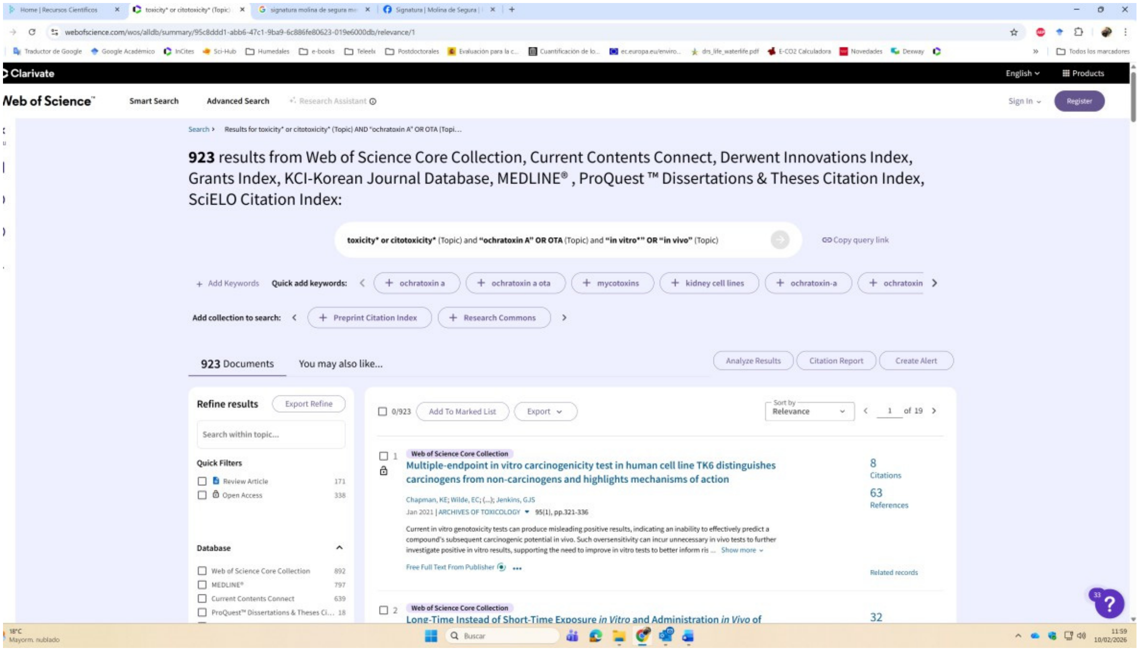

Figure S1. A screenshot of the search performed on 10 February 2026 with the link: https://www.webofscience.com/wos/alldb/summary/95c8ddd1-abb6-47c1-9ba9-6c886fe80623-019e6000db/relevance/1

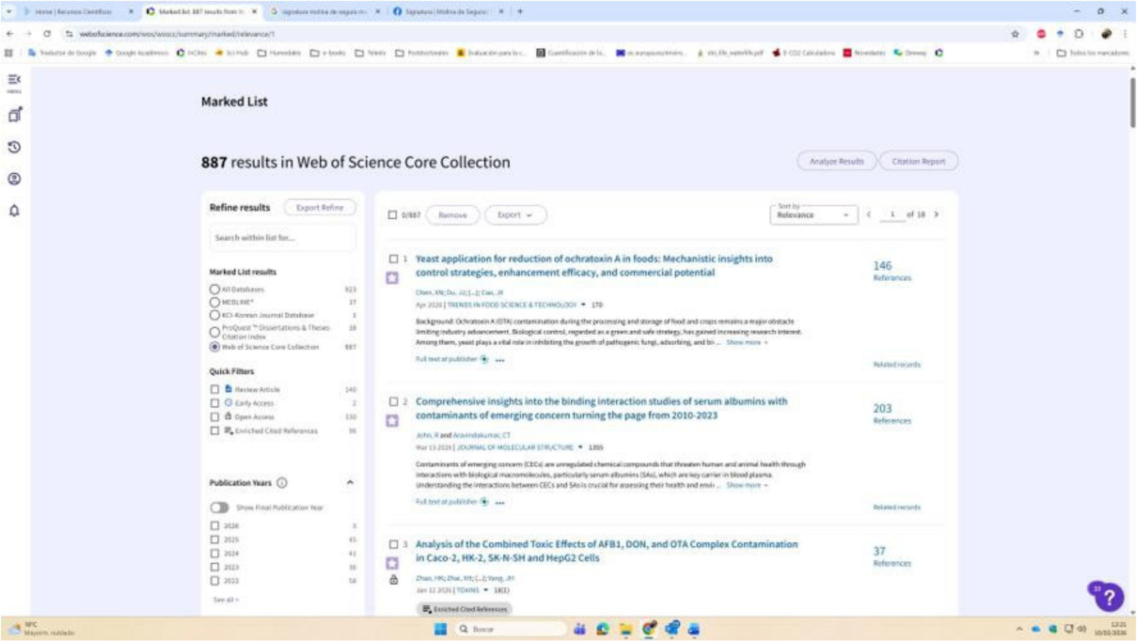

Figure S2. A screenshot of the search conducted on February 10, 2026, with filters set to display only WoSCC results.
