# Supplementary material for: Ochratoxin A Toxicity Research in In Vitro and In Vivo Experimental Settings: A Cross-Software Bibliometric and Science-Mapping Study
Source: Toxins (Basel). 2026 Aug 11;18(8):346. doi: 10.3390/toxins18080346 (PMC13517462; doi:10.3390/toxins18080346)
Supplement: Supplementary file 1 [file toxins-18-00346-s001.zip › Supplementary File S10.pdf]

Keyword normalisation audit – line-by-line and article-by-article

Files compared: WoS 1-883_Original.txt vs WoS_1-883_keywords_Normalized.txt

This Word file lists observed DE/ID keyword changes by article. The accompanying Excel file contains the complete controlled vocabulary, changed dictionary, before/after counts and filterable record-level audit.

Field	Occurrences before	Occurrences after	Changed mapping pairs
DE	4069	4065	1875
ID	7475	7472	2219

Record 1 | WOS:001628370900001

Dietary Quercetin Mitigates DON-Induced Intestinal Injury via Inhibiting MAPK/NF-ÎºB-Mediated Pyroptosis and Tight Junction Disruption in Chicken

Field	Position	Original line	Normalised line	Original value	Normalised value
DE	1	19	19	deoxynivalenol	Deoxynivalenol (DON)
DE	2	19	19	quercetin	Quercetin
DE	3	19	19	pyroptosis	Pyroptosis
DE	4	19	19	intestinal barrier	Intestinal Barrier
DE	5	19	19	chicken	Chicken

Record 2 | WOS:001646793400001

Silent Saboteurs: Decoding Mycotoxins-From Chemistry and Prevalence to Health Risks, Detection, Management and Emerging Frontiers

Field	Position	Original line	Normalised line	Original value	Normalised value
DE	1	154	153	mycotoxins	Mycotoxins
DE	2	154	153	biosynthesis pathways	Biosynthesis Pathway
DE	3	154	153	food safety	Food Safety
DE	4	154	153	fungal metabolites	Fungal Metabolite
DE	5	154	153	machine learning	Machine Learning
DE	6	154	153	risk assessment	Risk Assessment
ID	1	156	155	NEURAL-TUBE DEFECTS	Neural Tube Defect
ID	2	156	155	SPHINGOLIPID METABOLISM	Sphingolipid Metabolism
ID	3	156	155	OCHRATOXIN-A	Ochratoxin A (OTA)
ID	4	156	155	MAIZE GRAIN	Maize Grain
ID	5	156	155	AFLATOXIN EXPOSURE	Aflatoxin Exposure
ID	6	156	155	IN-VIVO	In-Vivo
ID	7	156	155	TOXICITY	Toxicity
ID	8	156	155	PATULIN	Patulin
ID	9	156	155	TRICHOTHECENES	Trichothecene
ID	10	156	155	CONTAMINATION	Contamination

Record 3 | WOS:001648209500001

Dose-Dependent but Non-Interactive Effects of Ochratoxin A and Selenomethionine on Hepatic Lipid Metabolism and Oxidative Stress in Broiler Chickens

Field	Position	Original line	Normalised line	Original value	Normalised value
DE	1	484	482	ochratoxin A	Ochratoxin A (OTA)
DE	2	484	482	selenium	Selenium
DE	3	484	482	broiler chicken	Broiler Chicken
DE	4	484	482	glutathione redox system	Glutathione Redox System
DE	5	484	482	lipid peroxidation	Lipid Peroxidation
DE	6	484	482	fatty acid composition	Fatty Acid Composition
ID	1	486	484	ANIMAL FEED	Animal Feed
ID	2	486	484	VITAMIN-E	Vitamin E
ID	3	486	484	SELENIUM	Selenium

ID	4	486	484	PEROXIDATION	Peroxidation
ID	5	486	484	GLUTATHIONE	Glutathione (GSH)
ID	6	486	484	MYCOTOXINS	Mycotoxins
ID	7	486	484	ADDITIVES	Additive
ID	8	486	484	LIVER	Liver

Record 4 | WOS:001635022200001

Selective Cytotoxicity of Ochratoxin A: Pro-Apoptotic Effects on Healthy Immune Cells Compared to Leukemia Cells

Field	Position	Original line	Normalised line	Original value	Normalised value
DE	1	636	633	ochratoxin A	Ochratoxin A (OTA)
DE	2	636	633	mycotoxin	Mycotoxins
DE	3	636	633	leukemia	Leukemia
DE	4	636	633	immune cells	Immune
DE	5	636	633	hematopoietic toxicity	Hematopoietic Toxicity
DE	6	636	633	hematological disorders	Hematological Disorder
ID	1	638	635	AFLATOXINS	Aflatoxin
ID	2	638	635	BLOOD	Blood
ID	3	638	635	MICE	Mice
ID	4	638	635	DIFFERENTIATION	Differentiation
ID	5	638	635	TOXICITY	Toxicity
ID	6	638	635	DAMAGE	Damage

Record 5 | WOS:001625561500001

Cytotoxic Effects Induced by Combined Exposure of the Patulin, Ochratoxin A, and Acetamiprid to HK-2 and SK-N-SH Cell Lines

Field	Position	Original line	Normalised line	Original value	Normalised value
DE	1	775	771	mycotoxins	Mycotoxins
DE	2	775	771	pesticides	Pesticide
DE	3	775	771	combined toxicity	Combined Toxicity
DE	4	775	771	dose reduction indices (DRI)	Dose Reduction Indice Dri
ID	1	776	772	LIQUID-CHROMATOGRAPHY	Liquid Chromatography
ID	2	776	772	NEONICOTINOID PESTICIDES	Neonicotinoid Pesticide
ID	3	776	772	OXIDATIVE STRESS	Oxidative Stress
ID	4	776	772	RESIDUES	Residue
ID	5	776	772	MYCOTOXINS	Mycotoxins
ID	6	776	772	FOODS	Food
ID	7	776	772	VEGETABLES	Vegetable
ID	8	776	772	PRODUCTS	Product
ID	9	776	772	SAMPLES	Sample
ID	10	776	772	FRUIT	Fruit

Record 6 | WOS:001623564900001

Mycotoxins and the Intestinal Epithelium: From Barrier Injury to Stem Cell Dysfunction

Field	Position	Original line	Normalised line	Original value	Normalised value
DE	1	885	880	mycotoxins	Mycotoxins
DE	2	885	880	intestinal barrier	Intestinal Barrier
DE	3	885	880	oxidative stress	Oxidative Stress
DE	4	885	880	inflammation	Inflammation
DE	5	885	880	intestinal stem cells	Intestinal Stem
ID	1	887	882	FUSARIUM MYCOTOXINS	Fusarium Mycotoxin
ID	2	887	882	DEOXYNIVALENOL VOMITOXIN	Deoxynivalenol (DON)
ID	3	887	882	EXPRESSION	Expression
ID	4	887	882	ZEARALENONE	Zearalenone (ZEA)
ID	5	887	882	IMPACT	Impact
ID	6	887	882	HOMEOSTASIS	Homeostasis
ID	7	887	882	MICROBIOTA	Microbiota

ID	8	887	882	RELEASE	Release
ID	9	887	882	ENERGY	Energy

Record 7 | WOS:001598251800001

Exploration of oxidative stress-mediated genetic toxicology modes of action using a pathway analysis, Connectivity Mapping, and transcriptional benchmark dosing-based framework

Field	Position	Original line	Normalised line	Original value	Normalised value
DE	1	1061	1055	oxidative stress	Oxidative Stress
DE	2	1061	1055	genetic toxicology	Genetic Toxicology
DE	3	1061	1055	Connectivity Mapping (CMap)	Connectivity Mapping Cmap
DE	4	1061	1055	transcriptomic point of departure (tPOD)	Transcriptomic Point Of Departure Tpod
DE	5	1061	1055	new approach methodology (NAM)	New Approach Methodology Nam
DE	6	1061	1055	Next Generation Risk Assessment (NGRA)	Next Generation Risk Assessment Ngra
DE	7	1061	1055	reactive oxygen species (ROS)	Reactive Oxygen Specy ROS
DE	8	1061	1055	mode of action (MoA)	Mode Of Action Moa
ID	1	1065	1059	DNA-DAMAGE	DNA Damage
ID	2	1065	1059	CHROMOSOMAL-ABERRATIONS	Chromosomal Aberration
ID	3	1065	1059	RESPONSE RELATIONSHIPS	Response Relationship
ID	4	1065	1059	MICRONUCLEUS ASSAY	Micronucleus Assay
ID	5	1065	1059	DOSE-RESPONSE	Dose Response
ID	6	1065	1059	OCHRATOXIN-A	Ochratoxin A (OTA)
ID	7	1065	1059	IWGT REPORT	Iwgt Report
ID	8	1065	1059	GENOTOXICITY	Genotoxicity
ID	9	1065	1059	TOXICITY	Toxicity
ID	10	1065	1059	MECHANISMS	Mechanism

Record 8 | WOS:001591221900001

Prevention and detoxification of mycotoxins in food and feed: A review

Field	Position	Original line	Normalised line	Original value	Normalised value
DE	1	1220	1213	Mycotoxin	Mycotoxins
DE	6	1220	1213	Adsorbents	Adsorbent
DE	7	1220	1213	Enzymes	Enzyme
ID	1	1222	1215	LACTIC-ACID BACTERIA	Lactic Acid Bacteria
ID	2	1222	1215	ROSMARINUS-OFFICINALIS L.	Rosmarinus Officinalis L
ID	3	1222	1215	IN-VITRO	In-Vitro
ID	4	1222	1215	AFLATOXIN B-1	Aflatoxin B1 (AFB1)
ID	5	1222	1215	OCHRATOXIN-A	Ochratoxin A (OTA)
ID	6	1222	1215	ESSENTIAL OIL	Essential Oil
ID	7	1222	1215	T-2 TOXIN	T 2 Toxin
ID	8	1222	1215	BIOLOGICAL DETOXIFICATION	Biological Detoxification
ID	9	1222	1215	FUSARIUM-VERTICILLIOIDES	Fusarium Verticillioide
ID	10	1222	1215	TRICHOTHECENE MYCOTOXINS	Trichothecene Mycotoxin

Record 9 | WOS:001603917600001

Astaxanthin Alleviates Ochratoxin A (OTA)-Induced Spleen Dysfunction and Apoptosis in Broiler Chickens by Modulating the PTEN/PI3K/AKT Signaling Pathway

Field	Position	Original line	Normalised line	Original value	Normalised value

DE	1	1568	1560	ochratoxin (OTA)	Ochratoxin OTA
DE	2	1568	1560	apoptosis	Apoptosis
DE	3	1568	1560	astaxanthin	Astaxanthin
DE	4	1568	1560	immunotoxicity	Immunotoxicity
DE	5	1568	1560	oxidative stress injury	Oxidative Stress Injury
DE	6	1568	1560	chicken	Chicken
ID	1	1570	1562	IN-VITRO	In-Vitro
ID	2	1570	1562	CELLS	Cell
ID	3	1570	1562	IMMUNOTOXICITY	Immunotoxicity
ID	4	1570	1562	AUTOPHAGY	Autophagy
ID	5	1570	1562	TOXICITY	Toxicity
ID	6	1570	1562	STRESS	Stress

Record 10 | WOS:001593782400001

Mycotoxin Contamination: Occurrence, Biotransformation, Pathogenic Mechanisms, and Strategies for Nutritional Intervention

Field	Position	Original line	Normalised line	Original value	Normalised value
DE	1	1701	1692	mycotoxins	Mycotoxins
DE	2	1701	1692	biosynthesis	Biosynthesis
DE	3	1701	1692	biotransformation	Biotransformation
DE	4	1701	1692	toxicity	Toxicity
DE	5	1701	1692	detoxification	Detoxification
DE	6	1701	1692	nutritional intervention	Nutritional Intervention
ID	1	1703	1694	BIOSYNTHESIS CLUSTER GENE	Biosynthesis Cluster Gene
ID	2	1703	1694	AFLATOXIN BIOSYNTHESIS	Aflatoxin Biosynthesis
ID	3	1703	1694	OXIDATIVE STRESS	Oxidative Stress
ID	4	1703	1694	VERSICOLORIN-A	Versicolorin A
ID	5	1703	1694	OCHRATOXIN-A	Ochratoxin A (OTA)
ID	6	1703	1694	IN-VITRO	In-Vitro
ID	7	1703	1694	ZEARALENONE	Zearalenone (ZEA)
ID	8	1703	1694	PATHWAY	Pathway
ID	9	1703	1694	ENCODES	Encode
ID	10	1703	1694	B-1	B 1

Record 11 | WOS:001579727300001

Chlorogenic Acid and VX765 Alleviate Deoxynivalenol-Induced Enterohepatic Injury and Lipid Metabolism Disorders by Improving Intestinal Microecology

Field	Position	Original line	Normalised line	Original value	Normalised value
DE	1	1974	1964	deoxynivalenol	Deoxynivalenol (DON)
DE	2	1974	1964	chlorogenic acid	Chlorogenic Acid
DE	3	1974	1964	VX765	Vx765
DE	4	1974	1964	microbiota-gut-liver axis	Microbiota Gut Liver Axis
DE	5	1974	1964	lipid metabolism	Lipid Metabolism
ID	1	1976	1966	GUT MICROBIOTA	Gut Microbiota
ID	2	1976	1966	AXIS	Axis

Record 12 | WOS:001573742900001

Fungal metabolite Ochratoxin A inhibits MrkD1P of multidrug-resistant Klebsiella pneumoniae: Integrated computational and in vitro validation

Field	Position	Original line	Normalised line	Original value	Normalised value
DE	1	2103	2092	Fungal metabolites	Fungal Metabolite
DE	2	2103	2092	Klebsiella pneumoniae	Klebsiella Pneumoniae
DE	3	2103	2092	Fimbrial adhesin protein MrkD1P	Fimbrial Adhesin Protein Mrkd1p
DE	4	2103	2092	Molecular docking	Molecular Docking
DE	6	2103	2092	PCA analysis	Pca Analysis
DE	7	2103	2092	Antibacterial activity	Antibacterial Activity
ID	1	2106	2094	BLOOD-BRAIN-BARRIER	Blood Brain Barrier

ID	2	2106	2094	ANTIMICROBIAL RESISTANCE	Antimicrobial Resistance
ID	3	2106	2094	MOLECULAR-MECHANICS	Molecular Mechanics
ID	4	2106	2094	ESSENTIAL DYNAMICS	Essential Dynamics
ID	5	2106	2094	PREDICTION	Prediction
ID	6	2106	2094	DISCOVERY	Discovery
ID	7	2106	2094	GRAPHICS	Graphics
ID	8	2106	2094	YASARA	Yasara
ID	9	2106	2094	SILICO	Silico

Record 13 | WOS:001581342800001

Biocatalytic Detoxification of Ochratoxins A/B by a Fungal Dye-Decolorizing Peroxidase: Mechanistic Insights and Toxicity Assessment

Field	Position	Original line	Normalised line	Original value	Normalised value
DE	1	2275	2262	ochratoxin A	Ochratoxin A (OTA)
DE	2	2275	2262	ochratoxin B	Ochratoxin B
DE	3	2275	2262	dye-decolorizing peroxidase	Dye Decolorizing Peroxidase
DE	4	2275	2262	detoxification	Detoxification
ID	1	2276	2264	IMPACT	Impact
ID	2	2276	2264	FOOD	Food

Record 14 | WOS:001556476900001

Identification of an M20 Family Metallopeptidase with Amidohydrolase Activity from Alcaligenes faecalis HNGD-HTN06 and Simultaneous Degradation of Ochratoxin A, Aflatoxin B1, and Zearalenone

Field	Position	Original line	Normalised line	Original value	Normalised value
DE	1	2377	2364	Alcaligenes faecalis	Alcaligene Faecalis
DE	2	2377	2364	OchratoxinA	Ochratoxin A (OTA)
DE	3	2377	2364	amidohydrolase	Amidohydrolase
DE	4	2377	2364	multimycotoxin degradation	Multimycotoxin Degradation
DE	5	2377	2364	biodegradation	Biodegradation
ID	1	2379	2366	DEINOCOCCUS-RADIODURANS	Deinococcus Radioduran
ID	2	2379	2366	COPPER	Copper
ID	3	2379	2366	TOXICITY	Toxicity
ID	4	2379	2366	ENZYME	Enzyme
ID	5	2379	2366	BIODEGRADATION	Biodegradation
ID	6	2379	2366	CONTAMINATION	Contamination
ID	7	2379	2366	PATHWAYS	Pathway
ID	8	2379	2366	FOOD	Food

Record 15 | WOS:001590628300001

Roles of microRNAs in multiple organs under mycotoxin stress by bioinformatic analysis microRNA regulation during mycotoxin exposure

Field	Position	Original line	Normalised line	Original value	Normalised value
DE	2	2508	2494	MicroRNAs	Microrna
DE	3	2508	2494	Organ damage	Organ Damage
DE	4	2508	2494	Aflatoxin B1 and ochratoxin A	Aflatoxin B1 And Ochratoxin A
DE	5	2508	2494	Livestock damage	Livestock Damage
ID	1	2510	2496	OCHRATOXIN-A	Ochratoxin A (OTA)
ID	2	2510	2496	FUMONISIN B-1	Fumonisin B1 (FB1)
ID	3	2510	2496	OXIDATIVE STRESS	Oxidative Stress
ID	4	2510	2496	AFLATOXIN B-1	Aflatoxin B1 (AFB1)
ID	5	2510	2496	CELL-PROLIFERATION	Cell Proliferation
ID	6	2510	2496	GUT MICROBIOTA	Gut Microbiota
ID	7	2510	2496	RAT-LIVER	Rat Liver
ID	8	2510	2496	IN-VITRO	In-Vitro
ID	9	2510	2496	EXPRESSION	Expression

ID	10	2510	2496	TOXICITY	Toxicity

Record 16 | WOS:001556296100001

The protective effect of Gentiana lutea flower against mycotoxin toxicity in a gastrointestinal barrier in vitro model

Field	Position	Original line	Normalised line	Original value	Normalised value
DE	1	2738	2723	mycotoxin	Mycotoxins
DE	2	2738	2723	<italic>Gentiana lutea</italic>	Gentiana Lutea
DE	3	2738	2723	qPCR	Qpcr
DE	4	2738	2723	<italic>in vitro</italic> digestion	In Vitro Digestion
DE	5	2738	2723	metabolomics	Metabolomics
DE	6	2738	2723	NMR	Nmr
DE	7	2738	2723	HPLC	Hplc
DE	8	2738	2723	bioactive compounds	Bioactive Compound
ID	1	2740	2725	L.	L
ID	2	2740	2725	IRIDOIDS	Iridoid
ID	3	2740	2725	GENTIOPICROSIDE	Gentiopicroside
ID	4	2740	2725	PHYTOCHEMISTRY	Phytochemistry
ID	5	2740	2725	SECOIRIDOIDS	Secoiridoid
ID	6	2740	2725	ABSORPTION	Absorption
ID	7	2740	2725	XANTHONES	Xanthone
ID	8	2740	2725	ROOTS	Root
ID	9	2740	2725	CELL	Cell

Record 17 | WOS:001546906900003

Mitochondrial dynamics imbalance and energy dyshomeostasis mediated developmental toxicity of ochratoxin a in zebrafish (Danio rerio)

Field	Position	Original line	Normalised line	Original value	Normalised value
DE	1	2883	2867	Ochratoxin A	Ochratoxin A (OTA)
DE	2	2883	2867	Mitochondrial dynamics	Mitochondrial Dynamics
DE	3	2883	2867	Energy metabolism	Energy Metabolism
DE	4	2883	2867	Developmental toxicity	Developmental Toxicity
ID	1	2885	2869	OXIDATIVE STRESS	Oxidative Stress
ID	2	2885	2869	CONTAMINATION	Contamination
ID	3	2885	2869	RESPONSES	Response
ID	4	2885	2869	SAMPLES	Sample
ID	5	2885	2869	WHEAT	Wheat

Record 18 | WOS:001582665400002

L-epicatechin partially alleviates Ochratoxin A-induced oxidative and endoplasmic reticulum stress and improves developmental competence of porcine embryos during in vitro culture

Field	Position	Original line	Normalised line	Original value	Normalised value
DE	1	3023	3006	L-epicatechin	L Epicatechin
DE	2	3023	3006	Porcine embryo	Porcine Embryo
DE	3	3023	3006	Oxidative stress	Oxidative Stress
DE	4	3023	3006	Endoplasmic reticulum stress	Endoplasmic Reticulum Stress
DE	5	3023	3006	Mitochondrial function	Mitochondrial Function
ID	1	3025	3008	ANTIOXIDANTS	Antioxidant
ID	2	3025	3008	ROS	Reactive Oxygen Species (ROS)
ID	3	3025	3008	TOXICITY	Toxicity

Record 19 | WOS:001539646900001

Emerging Mycotoxins in Aquaculture: Current Insights on Toxicity, Biocontrol Strategies, and Occurrence in Aquafeed and Fish

Field	Position	Original line	Normalised line	Original value	Normalised value

DE	1	3133	3115	aquaculture	Aquaculture
DE	2	3133	3115	emerging mycotoxins	Emerging Mycotoxin
DE	3	3133	3115	toxicity	Toxicity
DE	4	3133	3115	occurrence	Occurrence
DE	5	3133	3115	biocontrol strategies	Biocontrol Strategy
ID	1	3135	3116	ENNIATIN B	Enniatin B
ID	2	3135	3116	MITOCHONDRIAL DAMAGE	Mitochondrial Damage
ID	3	3135	3116	DIFFERENT STRAINS	Different Strain
ID	4	3135	3116	CACO-2 CELLS	Caco-2
ID	5	3135	3116	CYTOCHROME-C	Cytochrome C
ID	6	3135	3116	ANIMAL FEED	Animal Feed
ID	7	3135	3116	IN-VITRO	In-Vitro
ID	8	3135	3116	BEAUVERICIN	Beauvericin
ID	9	3135	3116	FUSARIUM	Fusarium
ID	10	3135	3116	DEGRADATION	Degradation

Record 20 | WOS:001529843100001

Decontamination of Ochratoxin A in Food: Emerging Strategies and Safety Perspectives

Field	Position	Original line	Normalised line	Original value	Normalised value
DE	1	3320	3300	food toxicity	Food Toxicity
DE	2	3320	3300	health implications	Health Implication
DE	3	3320	3300	gamma irradiation	Gamma Irradiation
DE	4	3320	3300	cold plasma	Cold Plasma
DE	5	3320	3300	ozone treatment	Ozone Treatment
DE	6	3320	3300	adsorbents	Adsorbent
DE	7	3320	3300	biological decontamination	Biological Decontamination
DE	8	3320	3300	natural preservatives	Natural Preservative
ID	1	3323	3302	A CONTENT	A Content
ID	2	3323	3302	GAMMA-IRRADIATION	Gamma Irradiation
ID	3	3323	3302	IN-VITRO	In-Vitro
ID	4	3323	3302	POTENTIAL APPLICATION	Potential Application
ID	5	3323	3302	GREEN COFFEE	Green Coffee
ID	6	3323	3302	FRUIT JUICES	Fruit Juice
ID	7	3323	3302	COLD-PLASMA	Cold Plasma
ID	8	3323	3302	WHEAT-FLOUR	Wheat Flour
ID	9	3323	3302	REDUCTION	Reduction
ID	10	3323	3302	FATE	Fate

Record 21 | WOS:001527764700005

Through its genoprotective, mitochondrial bioenergetic modulation, and antioxidant effects, Fucoxanthin and its metabolite minimize Ochratoxin A-induced nephrotoxicity in HK-2 human kidney cells

Field	Position	Original line	Normalised line	Original value	Normalised value
DE	3	3541	3519	Ochratoxin A	Ochratoxin A (OTA)
DE	5	3541	3519	Oxidative stress	Oxidative Stress
DE	7	3541	3519	Cellular bioenergetics	Cellular Bioenergetics
ID	1	3543	3521	OXIDATIVE DNA-DAMAGE	Oxidative DNA Damage
ID	2	3543	3521	BROWN-ALGAE	Brown Algae
ID	3	3543	3521	RAT-LIVER	Rat Liver
ID	4	3543	3521	IN-VITRO	In-Vitro
ID	5	3543	3521	TOXICITY	Toxicity
ID	6	3543	3521	STRESS	Stress
ID	7	3543	3521	CYTOTOXICITY	Cytotoxicity
ID	8	3543	3521	GENOTOXICITY	Genotoxicity
ID	9	3543	3521	NEPHROPATHY	Nephropathy

Record 22 | WOS:001539670300001

Oral Exposure to Chlorella sorokiniana Detoxifies Deoxynivalenol, Ochratoxin A, and Fumonisin B1 In Vitro and In Vivo

Field	Position	Original line	Normalised line	Original value	Normalised value
DE	1	3701	3678	deoxynivalenol	Deoxynivalenol (DON)
DE	2	3701	3678	mycotoxin	Mycotoxins
DE	3	3701	3678	<italic>Chlorella sorokiniana</italic>	Chlorella Sorokiniana
DE	4	3701	3678	toxicokinetics	Toxicokinetics

Record 23 | WOS:001513772100001

Schisandrin B mitigates oxidative damage in early porcine embryos exposed to Ochratoxin A via NRF2 pathway activation

Field	Position	Original line	Normalised line	Original value	Normalised value
DE	3	3798	3773	Glutathione (GSH)	Glutathione Gsh
DE	4	3798	3773	In vitro culture (IVC)	In Vitro Culture Ivc
DE	5	3798	3773	Mitochondrial function	Mitochondrial Function
DE	6	3798	3773	Reactive Oxygen Species (ROS)	Reactive Oxygen Specy ROS
ID	1	3800	3775	MITOCHONDRIAL ANTIOXIDANT STATUS	Mitochondrial Antioxidant Status
ID	2	3800	3775	ENDOPLASMIC-RETICULUM STRESS	Endoplasmic Reticulum Stress
ID	3	3800	3775	TOXICITY	Toxicity
ID	4	3800	3775	IMPROVES	Improve
ID	5	3800	3775	TISSUES	Tissue

Record 24 | WOS:001505840900001

Biocontrol Agents and Natural Feed Supplements as a Safe and Cost-Effective Way for Preventing Health Ailments Provoked by Mycotoxins

Field	Position	Original line	Normalised line	Original value	Normalised value
DE	1	3898	3872	food security	Food Security
DE	2	3898	3872	mycotoxins	Mycotoxins
DE	3	3898	3872	foodborne mycotoxicoses	Foodborne Mycotoxicose
DE	4	3898	3872	prevention	Prevention
DE	5	3898	3872	management of the risk	Management Of The Risk
DE	6	3898	3872	natural feed supplements	Natural Feed Supplement
ID	1	3900	3874	SACCHAROMYCES-CEREVISIAE STRAINS	Saccharomyce Cerevisiae Strain
ID	2	3900	3874	AFLATOXIGENIC ASPERGILLUS-FLAVUS	Aflatoxigenic Aspergillus Flavus
ID	3	3900	3874	PENICILLIUM-EXPANSUM GROWTH	Penicillium Expansum Growth
ID	4	3900	3874	RADICAL SCAVENGING ACTIVITY	Radical Scavenging Activity
ID	5	3900	3874	LACTIC-ACID BACTERIA	Lactic Acid Bacteria
ID	6	3900	3874	OCHRATOXIN-A	Ochratoxin A (OTA)
ID	7	3900	3874	TINOSPORA-CORDIFOLIA	Tinospora Cordifolia
ID	8	3900	3874	IN-VITRO	In-Vitro
ID	9	3900	3874	BIOLOGICAL-CONTROL	Biological Control
ID	10	3900	3874	MILK THISTLE	Milk Thistle

Record 25 | WOS:001514636100001

Modulation of the Antioxidant System of Caco-2 Cells in the Presence of Aflatoxin B1, Ochratoxin A, and Ferulic Acid

Field	Position	Original line	Normalised line	Original value	Normalised value

DE	1	4267	4240	ochratoxin A	Ochratoxin A (OTA)
DE	2	4267	4240	aflatoxin B1	Aflatoxin B1 (AFB1)
DE	3	4267	4240	ferulic acid	Ferulic Acid
DE	4	4267	4240	oxidative stress	Oxidative Stress
ID	1	4268	4242	OXIDATIVE STRESS	Oxidative Stress
ID	2	4268	4242	IN-VITRO	In-Vitro
ID	3	4268	4242	LIPID-PEROXIDATION	Lipid Peroxidation
ID	4	4268	4242	TRANSFERASES	Transferase
ID	5	4268	4242	COMBINATION	Combination
ID	6	4268	4242	MYCOTOXINS	Mycotoxins
ID	7	4268	4242	TOXICITY	Toxicity
ID	8	4268	4242	ENZYMES	Enzyme

Record 26 | WOS:001496132800001

Evaluation of Dietary Bioactive Agents Against Aflatoxin B1 and Ochratoxin A-Induced Duodenal Toxicity in Rats

Field	Position	Original line	Normalised line	Original value	Normalised value
DE	1	4418	4391	bioactive compound	Bioactive Compound
DE	2	4418	4391	mycotoxin	Mycotoxins
DE	3	4418	4391	gene expression	Gene Expression
DE	4	4418	4391	apoptosis	Apoptosis
DE	5	4418	4391	biomarker	Biomarker
ID	1	4419	4392	CELLS	Cell

Record 27 | WOS:001488085400001

Bi2WO6-TiO2 carbon nanoparticles controlled postharvest blue mold of table grapes caused by Talaromyces rugulosus and the possible action mechanisms

Field	Position	Original line	Normalised line	Original value	Normalised value
DE	1	4532	4504	Pathogenic fungi	Pathogenic Fungi
DE	2	4532	4504	nano-materials	Nano Material
DE	3	4532	4504	antifungal activity	Antifungal Activity
DE	4	4532	4504	postharvest diseases	Postharvest Disease
DE	5	4532	4504	storage quality	Storage Quality
DE	6	4532	4504	postharvest preservation	Postharvest Preservation

Record 28 | WOS:001486309200001

Immunotoxic effect and mechanisms of Fusarium mycotoxins on human immune cells: A focus on T cells and macrophages

Field	Position	Original line	Normalised line	Original value	Normalised value
DE	2	4640	4611	Fusarium mycotoxins	Fusarium Mycotoxin
DE	3	4640	4611	Deoxynivalenol	Deoxynivalenol (DON)
DE	5	4640	4611	Macrophage alternative activation	Macrophage Alternative Activation
DE	6	4640	4611	Protein processing	Protein Processing
ID	1	4642	4613	DEOXYNIVALENOL DON	Deoxynivalenol DON
ID	2	4642	4613	IN-VITRO	In-Vitro
ID	3	4642	4613	JURKAT	Jurkat
ID	4	4642	4613	CYTOTOXICITY	Cytotoxicity
ID	5	4642	4613	ZEARALENONE	Zearalenone (ZEA)
ID	6	4642	4613	ACTIVATION	Activation
ID	7	4642	4613	EXPRESSION	Expression
ID	8	4642	4613	BIOMARKERS	Biomarker
ID	9	4642	4613	NIVALENOL	Nivalenol

Record 29 | WOS:001484254000002

Quantification and metabolic variations of mycotoxins in raw milk: Implications for dairy cow health and human safety

Field	Position	Original line	Normalised line	Original value	Normalised value

DE	1	4765	4735	lactating dairy cows	Lactating Dairy Cow
DE	2	4765	4735	mycotoxin	Mycotoxins
DE	3	4765	4735	metabolism	Metabolism
DE	4	4765	4735	public health	Public Health
DE	5	4765	4735	food safety	Food Safety
ID	1	4766	4736	AFLATOXIN M-1	Aflatoxin M1 (AFM1)
ID	2	4766	4736	CARRY-OVER	Carry Over
ID	3	4766	4736	MASS-SPECTROMETRY	Mass Spectrometry
ID	4	4766	4736	IN-VITRO	In-Vitro
ID	5	4766	4736	OCHRATOXIN	Ochratoxin A (OTA)
ID	6	4766	4736	ZEARALENONE	Zearalenone (ZEA)
ID	7	4766	4736	PERFORMANCE	Performance
ID	8	4766	4736	FUMONISINS	Fumonisin
ID	9	4766	4736	FEED	Feed
ID	10	4766	4736	B-1	B 1

Record 30 | WOS:001484241000001

Oxidative stress promotes post-translational down-regulation of MRP2 in Caco-2 cells: Involvement of proteasomal degradation and toxicological implications

Field	Position	Original line	Normalised line	Original value	Normalised value
DE	1	4910	4879	MRP2	Mrp2
DE	2	4910	4879	Oxidative stress	Oxidative Stress
DE	4	4910	4879	N-acetylcysteine	N Acetylcysteine
DE	5	4910	4879	Post-translational regulation	Post Translational Regulation
DE	6	4910	4879	Ochratoxin a	Ochratoxin A (OTA)
ID	1	4912	4881	RESISTANCE-ASSOCIATED PROTEIN-2	Resistance Associated Protein 2
ID	2	4912	4881	GLUTATHIONE DEPLETION	Glutathione Depletion
ID	3	4912	4881	N-ACETYLCYSTEINE	N Acetylcysteine
ID	4	4912	4881	DRUG ABSORPTION	Drug Absorption
ID	5	4912	4881	RAT INTESTINE	Rat Intestine
ID	6	4912	4881	OCHRATOXIN-A	Ochratoxin A (OTA)
ID	7	4912	4881	EXPRESSION	Expression
ID	8	4912	4881	LOCALIZATION	Localization
ID	9	4912	4881	TRANSPORTERS	Transporter
ID	10	4912	4881	CYTOTOXICITY	Cytotoxicity

Record 31 | WOS:001471942000001

Ginsenoside Rg1 alleviates ochratoxin A-induced liver inflammation in ducklings: Involvement of intestinal microbiota modulation and the TLR4/NF-ÎºB pathway inhibition

Field	Position	Original line	Normalised line	Original value	Normalised value
DE	2	5058	5026	Ochratoxin A	Ochratoxin A (OTA)
DE	3	5058	5026	Intestinal microbiota	Intestinal Microbiota
DE	4	5058	5026	TLR4/NF-kappa B signaling pathway	Tlr4 Nf Kappa B Signaling Pathway
DE	5	5058	5026	Liver inflammation	Liver Inflammation
ID	1	5060	5028	LIPOPOLYSACCHARIDE	Lipopolysaccharide
ID	2	5060	5028	EXCRETION	Excretion
ID	3	5060	5028	BIOAVAILABILITY	Bioavailability
ID	4	5060	5028	METABOLITES	Metabolite
ID	5	5060	5028	ABSORPTION	Absorption
ID	6	5060	5028	SAPONINS	Saponin
ID	7	5060	5028	BINDING	Binding
ID	8	5060	5028	RAT	Rat

Record 32 | WOS:001471582100001

Mitigating AFB1 and OTA hepatic residues using pumpkin and milk fermented whey as functional ingredients

Field	Position	Original line	Normalised line	Original value	Normalised value
DE	1	5171	5138	Aflatoxin B1	Aflatoxin B1 (AFB1)
DE	2	5171	5138	Ochratoxin A	Ochratoxin A (OTA)
DE	3	5171	5138	QuEChERS	Quecher
DE	5	5171	5138	UHPLC-Q-Orbitrap HRMS	Uhplc Q Orbitrap Hrm
ID	1	5173	5140	AFLATOXIN-B1	Aflatoxin B1 (AFB1)
ID	2	5173	5140	OCHRATOXIN	Ochratoxin A (OTA)
ID	3	5173	5140	LIVER	Liver
ID	4	5173	5140	TOXICITY	Toxicity
ID	5	5173	5140	RAT	Rat

Record 33 | WOS:001456981800001

Ochratoxin A-induced mitochondrial pathway apoptosis and ferroptosis by promoting glycolysis

Field	Position	Original line	Normalised line	Original value	Normalised value
DE	1	5294	5260	Mycotoxin	Mycotoxins
DE	2	5294	5260	Mitochondrial pathway apoptosis	Mitochondrial Pathway Apoptosis
DE	5	5294	5260	Mitochondrial homeostasis imbalance	Mitochondrial Homeostasis Imbalance
ID	1	5296	5262	OXIDATIVE-PHOSPHORYLATION	Oxidative Phosphorylation
ID	2	5296	5262	GLUCOSE-METABOLISM	Glucose Metabolism
ID	3	5296	5262	PYRUVATE CARRIER	Pyruvate Carrier
ID	4	5296	5262	CANCER CELLS	Cancer
ID	5	5296	5262	CYTOTOXICITY	Cytotoxicity

Record 34 | WOS:001463798400001

Combined Cytotoxic Effects of the Fungicide Azoxystrobin and Common Food-Contaminating Mycotoxins

Field	Position	Original line	Normalised line	Original value	Normalised value
DE	1	5436	5401	fungicide	Fungicide
DE	2	5436	5401	mycotoxins	Mycotoxins
DE	3	5436	5401	chemical mixtures	Chemical Mixture
DE	4	5436	5401	interaction	Interaction
DE	5	5436	5401	HepG2 cells	HepG2
ID	1	5437	5402	IN-VITRO	In-Vitro
ID	2	5437	5402	CELL	Cell
ID	3	5437	5402	OCHRATOXIN	Ochratoxin A (OTA)
ID	4	5437	5402	MIXTURES	Mixture

Record 35 | WOS:001475234400001

Mycotoxins in Feed: Hazards, Toxicology, and Plant Extract-Based Remedies

Field	Position	Original line	Normalised line	Original value	Normalised value
DE	1	5577	5541	mycotoxins	Mycotoxins
DE	2	5577	5541	fungal secondary metabolites	Fungal Secondary Metabolite
DE	3	5577	5541	plant extracts	Plant Extract
DE	4	5577	5541	livestock production	Livestock Production
ID	1	5579	5543	ASPERGILLUS-FLAVUS	Aspergillus Flavus
ID	2	5579	5543	AFLATOXIN B-1	Aflatoxin B1 (AFB1)
ID	3	5579	5543	FUMONISIN B-1	Fumonisin B1 (FB1)
ID	4	5579	5543	OCHRATOXIN-A	Ochratoxin A (OTA)
ID	5	5579	5543	MEDICINAL-PLANTS	Medicinal Plant
ID	6	5579	5543	IN-VITRO	In-Vitro
ID	7	5579	5543	METABOLISM	Metabolism
ID	8	5579	5543	GROWTH	Growth
ID	9	5579	5543	DEOXYNIVALENOL	Deoxynivalenol (DON)
ID	10	5579	5543	ANTIOXIDANT	Antioxidant

Record 36 | WOS:001437492200001

Potential role of ochratoxin A in Parkinson's disease: a systematic review of current evidence

Field	Position	Original line	Normalised line	Original value	Normalised value
DE	1	5790	5753	Ochratoxin	Ochratoxin A (OTA)
DE	3	5790	5753	Adverse outcome pathway	Adverse Outcome Pathway
DE	4	5790	5753	Parkinson's disease	Parkinson S Disease
ID	1	5792	5755	RAT-BRAIN	Rat Brain
ID	2	5792	5755	TOXICITY	Toxicity
ID	3	5792	5755	GLUTATHIONE	Glutathione (GSH)
ID	4	5792	5755	INHIBITION	Inhibition
ID	5	5792	5755	EXPOSURE	Exposure
ID	6	5792	5755	CULTURES	Culture

Record 37 | WOS:001427487800001

Neurotoxic implications of gliotoxin and ochratoxin A in SH-SY5Y cells: ROS-induced apoptosis and genotoxicity

Field	Position	Original line	Normalised line	Original value	Normalised value
DE	2	5938	5900	Ochratoxin A	Ochratoxin A (OTA)
DE	3	5938	5900	Mixtures	Mixture
ID	1	5940	5902	GENE-EXPRESSION	Gene Expression
ID	2	5940	5902	DNA-DAMAGE	DNA Damage
ID	3	5940	5902	INDUCTION	Induction
ID	4	5940	5902	CANCER	Cancer
ID	5	5940	5902	DEOXYNIVALENOL	Deoxynivalenol (DON)
ID	6	5940	5902	CYTOTOXICITY	Cytotoxicity
ID	7	5940	5902	LYMPHOCYTES	Lymphocyte
ID	8	5940	5902	METABOLITES	Metabolite
ID	9	5940	5902	PROTEINS	Protein
ID	10	5940	5902	BCL-2	Bcl 2

Record 38 | WOS:001426276300001

Ochratoxin A induces lung cell PANoptosis through activation of the AIM 2 inflammasome

Field	Position	Original line	Normalised line	Original value	Normalised value
DE	1	6060	6021	OTA	Ochratoxin A (OTA)
DE	2	6060	6021	Cell death	Cell Death
DE	3	6060	6021	PANoptosis	Panoptosis
DE	4	6060	6021	ROS	Reactive Oxygen Species (ROS)
ID	1	6061	6023	LIVESTOCK	Livestock
ID	2	6061	6023	TOXICITY	Toxicity
ID	3	6061	6023	DEATH	Death

Record 39 | WOS:001425801400001

Probiotics mitigate reproductive toxicity in male mice induced by co-exposure of ochratoxin A and alcohol

Field	Position	Original line	Normalised line	Original value	Normalised value
DE	1	6159	6120	Ochratoxin A	Ochratoxin A (OTA)
DE	4	6159	6120	Reproductive toxicity	Reproductive Toxicity
DE	5	6159	6120	Sperm quality	Sperm Quality
ID	1	6160	6122	ARISTOLOCHIC ACID	Aristolochic Acid
ID	2	6160	6122	MYCOTOXINS	Mycotoxins

Record 40 | WOS:001416011800001

Metabolic disruption by mycotoxins: focus on metabolic endpoints steatosis, adipogenesis and glucose metabolism in vivo and in vitro

Field	Position	Original line	Normalised line	Original value	Normalised value
DE	2	6265	6226	Glucose tolerance	Glucose Tolerance
DE	3	6265	6226	Insulin tolerance	Insulin Tolerance
DE	4	6265	6226	Lipid accumulation	Lipid Accumulation
DE	5	6265	6226	Metabolic disrupting chemicals	Metabolic Disrupting Chemical
ID	1	6267	6228	11-BETA-HYDROXYSTEROID DEHYDROGENASE TYPE-1	11 Beta Hydroxysteroid Dehydrogenase Type 1
ID	2	6267	6228	EMODIN	Emodin
ID	3	6267	6228	GAMMA	Gamma
ID	4	6267	6228	DEOXYNIVALENOL	Deoxynivalenol (DON)
ID	5	6267	6228	ACCUMULATION	Accumulation
ID	6	6267	6228	HEPATOCYTES	Hepatocyte
ID	7	6267	6228	OBESITY	Obesity

Record 41 | WOS:001403208800001

Curcumin: A Potential Detoxifier Against Chemical and Natural Toxicants

Field	Position	Original line	Normalised line	Original value	Normalised value
DE	1	6390	6350	curcumin	Curcumin
DE	2	6390	6350	detoxification	Detoxification
DE	3	6390	6350	health	Health
DE	4	6390	6350	herbal drugs	Herbal Drug
DE	5	6390	6350	in vivo	In-Vivo
DE	6	6390	6350	toxicity	Toxicity
ID	1	6391	6351	INDUCED LIVER-INJURY	Induced Liver Injury
ID	2	6391	6351	INDUCED TESTICULAR TOXICITY	Induced Testicular Toxicity
ID	3	6391	6351	INDUCED OXIDATIVE STRESS	Induced Oxidative Stress
ID	4	6391	6351	COLISTIN-INDUCED NEPHROTOXICITY	Colistin Induced Nephrotoxicity
ID	5	6391	6351	INDUCED REPRODUCTIVE TOXICITY	Induced Reproductive Toxicity
ID	6	6391	6351	I CLINICAL-TRIAL	I Clinical Trial
ID	7	6391	6351	PROTECTIVE ROLE	Protective Role
ID	8	6391	6351	INDUCED NEUROTOXICITY	Induced Neurotoxicity
ID	9	6391	6351	OCHRATOXIN-A	Ochratoxin A (OTA)
ID	10	6391	6351	DICLOFENAC SODIUM	Diclofenac Sodium

Record 42 | WOS:001416156300001

Ochratoxin A induces immunotoxicity by targeting Annexin A1 mediated neutrophil apoptosis in zebrafish

Field	Position	Original line	Normalised line	Original value	Normalised value
DE	1	6867	6826	ochratoxin A (OTA)	Ochratoxin A (OTA)
DE	2	6867	6826	immunotoxicity	Immunotoxicity
DE	3	6867	6826	apoptosis	Apoptosis
DE	5	6867	6826	aesculetin	Aesculetin
ID	1	6868	6827	TOXICITY	Toxicity

Record 43 | WOS:001393437600001

Chemical Composition, In Vivo, and In Silico Molecular Docking Studies of the Effect of Syzygium aromaticum (Clove) Essential Oil on Ochratoxin A-Induced Acute Neurotoxicity

Field	Position	Original line	Normalised line	Original value	Normalised value
DE	1	6975	6933	ochratoxin A	Ochratoxin A (OTA)

DE	2	6975	6933	<italic>Syzygium aromaticum</italic>	Syzygium Aromaticum
DE	3	6975	6933	GC/MS	Gc Ms
DE	4	6975	6933	neurobehavioral tests	Neurobehavioral Test
DE	5	6975	6933	biochemical parameters	Biochemical Parameter
DE	6	6975	6933	in silico	In Silico
ID	1	6977	6935	OXIDATIVE STRESS	Oxidative Stress
ID	2	6977	6935	EUGENOL	Eugenol
ID	3	6977	6935	TOXICITY	Toxicity
ID	4	6977	6935	ADULT	Adult
ID	5	6977	6935	NEUROGENESIS	Neurogenesis
ID	6	6977	6935	EXPRESSION	Expression
ID	7	6977	6935	NEURONS	Neuron
ID	8	6977	6935	ANXIETY	Anxiety
ID	9	6977	6935	CELLS	Cell
ID	10	6977	6935	MODEL	Model

Record 44 | WOS:001404966700001

Proteomic Analysis of the Murine Liver Response to Oral Exposure to Aflatoxin B1 and Ochratoxin A: The Protective Role to Bioactive Compounds

Field	Position	Original line	Normalised line	Original value	Normalised value
DE	1	7129	7086	proteomics	Proteomics
DE	2	7129	7086	mycotoxin	Mycotoxins
DE	3	7129	7086	bioactive compounds	Bioactive Compound
DE	4	7129	7086	in vivo	In-Vivo
DE	5	7129	7086	LC-MS/MS-QTOF	Lc Ms Ms Qtof
ID	1	7130	7087	METABOLISM	Metabolism
ID	2	7130	7087	MECHANISMS	Mechanism
ID	3	7130	7087	CARCINOMA	Carcinoma
ID	4	7130	7087	TOXICITY	Toxicity
ID	5	7130	7087	ENZYMES	Enzyme
ID	6	7130	7087	RAT	Rat

Record 45 | WOS:001370729500001

Alterations in the proteomes of HepG2 and IHKE cells inflicted by six selected mycotoxins

Field	Position	Original line	Normalised line	Original value	Normalised value
DE	6	7245	7201	Mode of action	Mode Of Action
DE	7	7245	7201	Toxicity pathway	Toxicity Pathway
ID	1	7247	7203	PRIMARY HUMAN HEPATOCYTES	Primary Human Hepatocyte
ID	2	7247	7203	OCHRATOXIN	Ochratoxin A (OTA)
ID	3	7247	7203	CYTOTOXICITY	Cytotoxicity
ID	4	7247	7203	LIVER	Liver
ID	6	7247	7203	REPLICATION	Replication
ID	7	7247	7203	METABOLISM	Metabolism
ID	8	7247	7203	EXPRESSION	Expression
ID	9	7247	7203	INDUCTION	Induction
ID	10	7247	7203	MECHANISM	Mechanism

Record 46 | WOS:001375296600001

Bioavailability study of OTA, ZEN, and AFB1 along with bioactive compounds from tiger nut beverage and its by-products

Field	Position	Original line	Normalised line	Original value	Normalised value
DE	1	7400	7355	Mycotoxin	Mycotoxins
DE	2	7400	7355	Polyphenols	Polyphenol
DE	5	7400	7355	Tiger nut	Tiger Nut
DE	6	7400	7355	By-product	By Product
ID	1	7401	7356	OCHRATOXIN-A	Ochratoxin A (OTA)
ID	2	7401	7356	INTESTINAL BARRIER	Intestinal Barrier
ID	3	7401	7356	PHENOLIC-ACIDS	Phenolic Acid
ID	4	7401	7356	POLYPHENOLS	Polyphenol
ID	5	7401	7356	MYCOTOXINS	Mycotoxins

ID	6	7401	7356	ABSORPTION	Absorption
ID	7	7401	7356	CELLS	Cell
ID	8	7401	7356	OIL	Oil
ID	9	7401	7356	DEOXYNIVALENOL	Deoxynivalenol (DON)
ID	10	7401	7356	EXPRESSION	Expression

Record 47 | WOS:001384323500001

Development of an Easy-To-Use Microfluidic System to Assess Dynamic Exposure to Mycotoxins in 3D Culture Models: Evaluation of Ochratoxin A and Patulin Cytotoxicity

Field	Position	Original line	Normalised line	Original value	Normalised value
DE	1	7555	7509	food contaminants	Food Contaminant
DE	2	7555	7509	mycotoxins	Mycotoxins
DE	3	7555	7509	cytotoxicity	Cytotoxicity
DE	4	7555	7509	in vitro models	In Vitro Model
DE	5	7555	7509	3D culture	3d
DE	6	7555	7509	neuroblastoma spheroid	Neuroblastoma Spheroid
DE	7	7555	7509	microfluidic system	Microfluidic System
ID	1	7557	7511	TOXICITY	Toxicity
ID	2	7557	7511	RAT	Rat

Record 48 | WOS:001356112700001

DNA ligase III mediates deoxynivalenol exposure-induced DNA damage in intestinal epithelial cells by regulating oxidative stress and interaction with PCNA

Field	Position	Original line	Normalised line	Original value	Normalised value
DE	1	7699	7652	LIG3	Lig3
DE	2	7699	7652	DNA damage/repair	DNA Damage Repair
DE	3	7699	7652	PCNA	Pcna
ID	1	7700	7653	ACTIVATED PROTEIN-KINASE	Activated Protein Kinase
ID	2	7700	7653	DOUBLE-STRAND BREAKS	Double Strand Break
ID	3	7700	7653	CYCLE ARREST	Cycle Arrest
ID	4	7700	7653	PATHWAYS	Pathway
ID	5	7700	7653	CONSEQUENCES	Consequence
ID	6	7700	7653	PROGRESSION	Progression
ID	7	7700	7653	GAMMA-H2AX	Gamma H2ax
ID	8	7700	7653	RADIATION	Radiation
ID	9	7700	7653	CANCER	Cancer
ID	10	7700	7653	MODELS	Model

Record 49 | WOS:001366569000001

Chlorophyll-Amended Organoclays for the Detoxification of Ochratoxin A

Field	Position	Original line	Normalised line	Original value	Normalised value
DE	1	7829	7781	mycotoxin	Mycotoxins
DE	2	7829	7781	ochratoxin	Ochratoxin A (OTA)
DE	3	7829	7781	enterosorption	Enterosorption
DE	4	7829	7781	food contamination	Food Contamination
DE	5	7829	7781	thermodynamics	Thermodynamics
DE	6	7829	7781	kinetics	Kinetics
DE	7	7829	7781	isotherms	Isotherm
DE	8	7829	7781	molecular dynamics	Molecular Dynamics
ID	1	7831	7783	AQUEOUS-SOLUTIONS	Aqueous Solution
ID	2	7831	7783	ACTIVATED CARBON	Activated Carbon
ID	3	7831	7783	NOVASIL CLAY	Novasil Clay
ID	4	7831	7783	IN-VITRO	In-Vitro
ID	5	7831	7783	ADSORPTION	Adsorption
ID	6	7831	7783	AFLATOXIN	Aflatoxin

ID	7	7831	7783	EXPOSURE	Exposure
ID	8	7831	7783	CHARMM	Charmm
ID	9	7831	7783	DEGRADATION	Degradation
ID	10	7831	7783	MYCOTOXINS	Mycotoxins

Record 50 | WOS:001351279800001

Protective Effects of Carotenoid-Loaded Nanostructured Lipid Carriers Against Ochratoxin-A-Induced Cytotoxicity

Field	Position	Original line	Normalised line	Original value	Normalised value
DE	1	7973	7924	antioxidant	Antioxidant
DE	2	7973	7924	simulated digestion	Simulated Digestion
DE	3	7973	7924	mycotoxin	Mycotoxins
DE	4	7973	7924	cell line	Cell
ID	1	7974	7925	CELLS	Cell
ID	2	7974	7925	MYCOTOXINS	Mycotoxins
ID	3	7974	7925	BIOACCESSIBILITY	Bioaccessibility
ID	4	7974	7925	ANTIOXIDANTS	Antioxidant
ID	5	7974	7925	MODULATION	Modulation
ID	6	7974	7925	APOPTOSIS	Apoptosis
ID	7	7974	7925	TOXICITY	Toxicity
ID	8	7974	7925	ARREST	Arrest

Record 51 | WOS:001329697100001

Unveiling the hub genes associated with ochratoxin A-induced hepatotoxicity in broiler chickens

Field	Position	Original line	Normalised line	Original value	Normalised value
DE	1	8087	8037	broiler	Broiler
DE	2	8087	8037	gene	Gene
DE	3	8087	8037	liver	Liver
DE	4	8087	8037	ochratoxin A	Ochratoxin A (OTA)
ID	1	8088	8038	LIVER	Liver
ID	2	8088	8038	IDENTIFICATION	Identification
ID	3	8088	8038	APOPTOSIS	Apoptosis

Record 52 | WOS:001314852000005

Investigating the protective properties of Panax ginseng and its constituents against biotoxins and metal toxicity: a mechanistic review

Field	Position	Original line	Normalised line	Original value	Normalised value
DE	2	8188	8137	Cell signaling	Cell Signaling
DE	6	8188	8137	Toxicants	Toxicant
ID	1	8189	8138	KOREAN RED GINSENG	Korean Red Ginseng
ID	2	8189	8138	OXIDATIVE STRESS	Oxidative Stress
ID	3	8189	8138	3-NITROPROPIONIC ACID	3 Nitropropionic Acid
ID	4	8189	8138	CHEMICAL TOXICITIES	Chemical Toxicity
ID	5	8189	8138	D-GALACTOSE	D Galactose
ID	6	8189	8138	MAIN CONSTITUENTS	Main Constituent
ID	7	8189	8138	NATURAL-PRODUCTS	Natural Product
ID	8	8189	8138	AGING MICE	Aging Mice
ID	9	8189	8138	RAT MODEL	Rat Model
ID	10	8189	8138	EXTRACT	Extract

Record 53 | WOS:001323506900001

Phytochemical Characterization of Bilberries and Their Potential as a Functional Ingredient to Mitigate Ochratoxin A Toxicity in Cereal-Based Products

Field	Position	Original line	Normalised line	Original value	Normalised value
DE	1	8408	8356	mycotoxins	Mycotoxins

DE	2	8408	8356	berries	Berry
DE	3	8408	8356	in vitro digestion	In Vitro Digestion
DE	4	8408	8356	mitigation	Mitigation
DE	5	8408	8356	flow cytometry	Flow Cytometry
ID	1	8409	8357	CELL-LINE	Cell
ID	2	8409	8357	BIOMARKERS	Biomarker
ID	3	8409	8357	APOPTOSIS	Apoptosis
ID	4	8409	8357	CACO-2	Caco-2
ID	5	8409	8357	WILD	Wild

Record 54 | WOS:001345720100005

Review: Strategies and technologies in preventing regulated and emerging mycotoxin co-contamination in forage for safeguarding ruminant health

Field	Position	Original line	Normalised line	Original value	Normalised value
DE	1	8540	8487	Animal health	Animal Health
DE	2	8540	8487	Feed safety	Feed Safety
DE	5	8540	8487	Volatile organic compounds	Volatile Organic Compound
ID	1	8541	8488	VOLATILE ORGANIC-COMPOUNDS	Volatile Organic Compound
ID	2	8541	8488	DRY-MATTER INTAKE	Dry Matter Intake
ID	3	8541	8488	AEROBIC STABILITY	Aerobic Stability
ID	4	8541	8488	FUSARIUM-GRAMINEARUM	Fusarium Graminearum
ID	5	8541	8488	MAIZE SILAGE	Maize Silage
ID	6	8541	8488	IN-VITRO	In-Vitro
ID	7	8541	8488	OXYGEN-BARRIER	Oxygen Barrier
ID	8	8541	8488	DAIRY-CATTLE	Dairy Cattle
ID	9	8541	8488	LACTOBACILLUS-BUCHNERI	Lactobacillus Buchneri
ID	10	8541	8488	SECONDARY METABOLITES	Secondary Metabolite

Record 55 | WOS:001296248000001

Effects of isochlorogenic acid A on mitochondrial dynamics imbalance and RLR damage in PAM cells induced by combined mycotoxins

Field	Position	Original line	Normalised line	Original value	Normalised value
DE	1	8805	8751	Isochlorogenic acid A	Isochlorogenic Acid A
DE	2	8805	8751	Combined mycotoxins	Combined Mycotoxin
DE	3	8805	8751	Mitochondrial dynamics	Mitochondrial Dynamics
DE	4	8805	8751	RLR signaling pathway	Rlr Signaling Pathway
ID	1	8807	8753	DEOXYNIVALENOL	Deoxynivalenol (DON)
ID	2	8807	8753	FERROPTOSIS	Ferroptosis
ID	3	8807	8753	DYSFUNCTION	Dysfunction
ID	4	8807	8753	ZEARALENONE	Zearalenone (ZEA)

Record 56 | WOS:001291367000001

Ochratoxin A-induced DNA damage triggers G2 phase arrest via hMLH1-p53-p21 signaling pathway in human gastric epithelium immortalized cells in vitro

Field	Position	Original line	Normalised line	Original value	Normalised value
DE	1	8925	8870	Ochratoxin A	Ochratoxin A (OTA)
DE	2	8925	8870	G 2 phase arrest	G 2 Phase Arrest
DE	3	8925	8870	P53-p21	P53 P21
DE	4	8925	8870	Signaling pathway	Signaling Pathway
DE	5	8925	8870	HMLH1 signaling pathway	Hmlh1 Signaling Pathway
ID	1	8927	8872	MISMATCH REPAIR	Mismatch Repair
ID	2	8927	8872	ROS GENERATION	ROS Generation
ID	3	8927	8872	G(2)/M ARREST	G 2 M Arrest
ID	5	8927	8872	ATM	Atm
ID	6	8927	8872	ASPERGILLUS	Aspergillus
ID	7	8927	8872	ACTIVATION	Activation

ID	8	8927	8872	APOPTOSIS	Apoptosis
ID	9	8927	8872	EXPOSURE	Exposure
ID	10	8927	8872	HMLH1	Hmlh1

Record 57 | WOS:001325349600001

Evaluating the combined and individual cytotoxic effect of beauvericin, enniatin B and ochratoxin a on breast cancer cells, leukemia cells, and fresh peripheral blood mononuclear cells

Field	Position	Original line	Normalised line	Original value	Normalised value
DE	2	9023	8967	PBMC	Pbmc
DE	3	9023	8967	HL-60	Hl 60
DE	4	9023	8967	MDA-MB231	Mda Mb231
DE	6	9023	8967	Mixtures	Mixture
DE	7	9023	8967	Flow cytometry	Flow Cytometry
ID	1	9025	8968	IN-VITRO	In-Vitro
ID	2	9025	8968	HL-60 CELLS	Hl 60
ID	3	9025	8968	INDUCED APOPTOSIS	Induced Apoptosis
ID	4	9025	8968	IMMUNE-RESPONSE	Immune Response
ID	5	9025	8968	MYCOTOXINS	Mycotoxins
ID	6	9025	8968	ZEARALENONE	Zearalenone (ZEA)
ID	7	9025	8968	DEOXYNIVALENOL	Deoxynivalenol (DON)
ID	8	9025	8968	PROLIFERATION	Proliferation
ID	9	9025	8968	ACTIVATION	Activation
ID	10	9025	8968	CITRININ	Citrinin

Record 58 | WOS:001277668700001

The Novel Role of the NLRP3 Inflammasome in Mycotoxin-Induced Toxicological Mechanisms

Field	Position	Original line	Normalised line	Original value	Normalised value
DE	1	9142	9085	mycotoxins	Mycotoxins
DE	2	9142	9085	pyroptosis	Pyroptosis
DE	3	9142	9085	NLRP3 inflammasome	Nlrp3 Inflammasome
DE	4	9142	9085	activation	Activation
DE	5	9142	9085	inhibitor	Inhibitor
ID	1	9143	9086	INTESTINAL INFLAMMATION	Intestinal Inflammation
ID	2	9143	9086	DEOXYNIVALENOL	Deoxynivalenol (DON)
ID	3	9143	9086	ACTIVATION	Activation
ID	4	9143	9086	AFLATOXIN-B1	Aflatoxin B1 (AFB1)
ID	5	9143	9086	OCHRATOXIN	Ochratoxin A (OTA)
ID	6	9143	9086	CASPASES	Caspase
ID	7	9143	9086	IMMUNOTOXICITY	Immunotoxicity
ID	8	9143	9086	DYSFUNCTION	Dysfunction
ID	9	9143	9086	INHIBITION	Inhibition
ID	10	9143	9086	PYROPTOSIS	Pyroptosis

Record 59 | WOS:001263565100001

Reduction of aflatoxins and ochratoxin A by addition of commercial Koji during fermentation of the Korean traditional soybean paste, Doenjang

Field	Position	Original line	Normalised line	Original value	Normalised value
DE	1	9346	9288	Aflatoxins	Aflatoxin
DE	2	9346	9288	Fermented soybean paste	Fermented Soybean Paste
DE	4	9346	9288	Ochratoxin a	Ochratoxin A (OTA)
ID	1	9347	9289	HUMAN HEALTH	Human Health
ID	2	9347	9289	B-1	B 1
ID	3	9347	9289	TOXICITY	Toxicity
ID	4	9347	9289	RISK	Risk
ID	5	9347	9289	FOOD	Food
ID	6	9347	9289	MEJU	Meju

Record 60 | WOS:001257266000001

Zearalenone modulates the function of goat endometrial cells via the mitochondrial quality control system

Field	Position	Original line	Normalised line	Original value	Normalised value
DE	1	9464	9405	cell proliferation	Cell Proliferation
DE	2	9464	9405	endometrial cells	Endometrial
DE	3	9464	9405	goat	Goat
DE	4	9464	9405	mitochondrial quality control system	Mitochondrial Quality Control System
DE	5	9464	9405	zearalenone	Zearalenone (ZEA)
ID	1	9466	9407	MESSENGER-RNA EXPRESSION	Messenger RNA Expression
ID	2	9466	9407	ESTROGEN-RECEPTOR-ALPHA	Estrogen Receptor Alpha
ID	3	9466	9407	PROGESTERONE-RECEPTOR	Progesterone Receptor
ID	4	9466	9407	HYDROGEN-PEROXIDE	Hydrogen Peroxide
ID	5	9466	9407	NUCLEAR ANTIGEN	Nuclear Antigen
ID	6	9466	9407	EARLY-PREGNANCY	Early Pregnancy
ID	7	9466	9407	HUMAN UTERINE	Human Uterine
ID	8	9466	9407	ER-ALPHA	Er Alpha
ID	9	9466	9407	CYCLE	Cycle
ID	10	9466	9407	PROLIFERATION	Proliferation

Record 61 | WOS:001250935500001

Central role of Sigma-1 receptor in ochratoxin A-induced ferroptosis

Field	Position	Original line	Normalised line	Original value	Normalised value
DE	1	9606	9546	Mycotoxin	Mycotoxins
DE	2	9606	9546	Sigma-1 receptor	Sigma 1 Receptor
ID	1	9607	9547	ENDOPLASMIC-RETICULUM STRESS	Endoplasmic Reticulum Stress
ID	2	9607	9547	OXIDATIVE STRESS	Oxidative Stress
ID	3	9607	9547	CELLS	Cell
ID	4	9607	9547	NRF2	Nrf2

Record 62 | WOS:001263872600001

Occurrence of the two major regulated mycotoxins, ochratoxin A and fumonisin B1, in cereal and cereal-based products in Europe and toxicological effects: A review

Field	Position	Original line	Normalised line	Original value	Normalised value
DE	3	9730	9669	Toxicology in vitro	Toxicology In Vitro
DE	4	9730	9669	Combined effects	Combined Effect
DE	5	9730	9669	Food safety	Food Safety
DE	6	9730	9669	Risk assessment	Risk Assessment
ID	1	9732	9671	DRIED BLOOD SPOTS	Dried Blood Spot
ID	2	9732	9671	RISK-ASSESSMENT	Risk Assessment
ID	3	9732	9671	GLUTEN-FREE	Gluten Free
ID	4	9732	9671	IN-VITRO	In-Vitro
ID	5	9732	9671	MULTIPLE MYCOTOXINS	Multiple Mycotoxin
ID	6	9732	9671	EXPOSURE ASSESSMENT	Exposure Assessment
ID	7	9732	9671	BREAKFAST CEREALS	Breakfast Cereal
ID	8	9732	9671	DIETARY EXPOSURE	Dietary Exposure
ID	9	9732	9671	GENE-EXPRESSION	Gene Expression
ID	10	9732	9671	HEALTH-RISK	Health Risk

Record 63 | WOS:001256451400001

High-Coverage UHPLC-MS/MS Analysis of 67 Mycotoxins in Plasma for Male Infertility Exposure Studies

Field	Position	Original line	Normalised line	Original value	Normalised value
DE	1	10030	9968	mycotoxin	Mycotoxins
DE	2	10030	9968	UHPLC-MS/MS	Uhplc Ms Ms
DE	3	10030	9968	emerging mycotoxins	Emerging Mycotoxin
DE	4	10030	9968	male infertility	Male Infertility
DE	5	10030	9968	human biomonitoring	Human Biomonitoring
ID	1	10032	9970	DRIED BLOOD SPOTS	Dried Blood Spot
ID	2	10032	9970	MULTIPLE MYCOTOXINS	Multiple Mycotoxin
ID	3	10032	9970	OCHRATOXIN	Ochratoxin A (OTA)
ID	4	10032	9970	FOOD	Food
ID	5	10032	9970	DEOXYNIVALENOL	Deoxynivalenol (DON)
ID	6	10032	9970	POPULATION	Population
ID	7	10032	9970	PREVALENCE	Prevalence
ID	8	10032	9970	BIOMARKERS	Biomarker
ID	9	10032	9970	AFLATOXIN	Aflatoxin
ID	10	10032	9970	CITRININ	Citrinin

Record 64 | WOS:001234877500001

Investigation of the mechanisms behind ochratoxin A-induced cytotoxicity in human astrocytes and the protective effects of N-acetylcysteine

Field	Position	Original line	Normalised line	Original value	Normalised value
DE	1	10167	10105	apoptosis	Apoptosis
DE	2	10167	10105	astrocytes	Astrocyte
DE	3	10167	10105	mycotoxin	Mycotoxins
DE	4	10167	10105	N-acetylcysteine	N Acetylcysteine
DE	5	10167	10105	ochratoxin A (OTA)	Ochratoxin A (OTA)
DE	6	10167	10105	oxidative stress	Oxidative Stress
ID	1	10169	10107	OXIDATIVE STRESS	Oxidative Stress
ID	2	10169	10107	CELL-DEATH	Cell Death
ID	3	10169	10107	IN-VITRO	In-Vitro
ID	4	10169	10107	TOXICITY	Toxicity
ID	5	10169	10107	DIFFERENTIATION	Differentiation
ID	6	10169	10107	ACTIVATION	Activation
ID	7	10169	10107	MYCOTOXINS	Mycotoxins
ID	8	10169	10107	PATHWAY	Pathway
ID	9	10169	10107	IMPACT	Impact

Record 65 | WOS:001229022000001

The efficiency of mycotoxin binding by sorbents in the in vitro model using a naturally contaminated animal feed

Field	Position	Original line	Normalised line	Original value	Normalised value
DE	1	10282	10219	mycotoxin	Mycotoxins
DE	2	10282	10219	mineral sorbents	Mineral Sorbent
DE	3	10282	10219	mycotoxin binders	Mycotoxin Binder
DE	4	10282	10219	feed additives	Feed Additive
ID	1	10283	10220	AFLATOXIN B-1	Aflatoxin B1 (AFB1)
ID	2	10283	10220	TOXICITY	Toxicity
ID	3	10283	10220	ZEARALENONE	Zearalenone (ZEA)
ID	4	10283	10220	DETOXIFICATION	Detoxification
ID	5	10283	10220	EFFICACY	Efficacy
ID	6	10283	10220	AGENTS	Agent

Record 66 | WOS:001228887800001

In-vivo evaluation of the adverse effects of ochratoxin A on broiler chicken health and adsorption efficacy of indigenous and commercial clay of Balochistan, Pakistan

Field	Position	Original line	Normalised line	Original value	Normalised value
DE	1	10373	10310	Ochratoxin A	Ochratoxin A (OTA)
DE	2	10373	10310	broiler chicken	Broiler Chicken
DE	3	10373	10310	indigenous clay	Indigenous Clay
DE	4	10373	10310	enzymatic activity	Enzymatic Activity

DE	5	10373	10310	average daily gain	Average Daily Gain
ID	1	10375	10312	DIETARY OCHRATOXIN	Ochratoxin A (OTA)
ID	2	10375	10312	AFLATOXIN B-1	Aflatoxin B1 (AFB1)
ID	3	10375	10312	MYCOTOXINS	Mycotoxins
ID	4	10375	10312	PERFORMANCE	Performance
ID	5	10375	10312	ADSORBENTS	Adsorbent

Record 67 | WOS:001222378900001

Role of albumin in the metabolism and excretion of ochratoxin A

Field	Position	Original line	Normalised line	Original value	Normalised value
DE	4	10514	10450	Intravital imaging	Intravital Imaging
ID	1	10515	10451	IN-VITRO	In-Vitro
ID	2	10515	10451	DNA-DAMAGE	DNA Damage
ID	3	10515	10451	TOXICOKINETICS	Toxicokinetics
ID	4	10515	10451	CYTOTOXICITY	Cytotoxicity
ID	5	10515	10451	TOXICITY	Toxicity
ID	6	10515	10451	BINDING	Binding
ID	7	10515	10451	URINE	Urine
ID	8	10515	10451	BILE	Bile
ID	9	10515	10451	RAT	Rat
ID	10	10515	10451	IDENTIFICATION	Identification

Record 68 | WOS:001220328900001

Unveiling the emerging role of curcumin to alleviate ochratoxin A-induced muscle toxicity in grass carp (Ctenopharyngodon idella): in vitro and in vivo studies

Field	Position	Original line	Normalised line	Original value	Normalised value
DE	2	10643	10578	Grass carp	Grass Carp
DE	4	10643	10578	Ochratoxin A	Ochratoxin A (OTA)
DE	5	10643	10578	Protein deposition	Protein Deposition
ID	1	10644	10579	SKELETAL-MUSCLE	Skeletal Muscle
ID	2	10644	10579	PROTEIN-SYNTHESIS	Protein Synthesis
ID	3	10644	10579	OXIDATIVE STRESS	Oxidative Stress
ID	4	10644	10579	EXPRESSION	Expression
ID	5	10644	10579	GROWTH	Growth
ID	6	10644	10579	JUVENILE	Juvenile
ID	7	10644	10579	CELLS	Cell
ID	8	10644	10579	UBIQUITIN	Ubiquitin
ID	9	10644	10579	CARCINOGENICITY	Carcinogenicity
ID	10	10644	10579	PERFORMANCE	Performance

Record 69 | WOS:001215577500001

Protective Effect of Boric Acid Against Ochratoxin A-Induced Toxic Effects in Human Embryonal Kidney Cells (HEK293): A Study on Cytotoxic, Genotoxic, Oxidative, and Apoptotic Effects

Field	Position	Original line	Normalised line	Original value	Normalised value
DE	1	10792	10726	Ochratoxin A	Ochratoxin A (OTA)
DE	2	10792	10726	Boric acid	Boric Acid
DE	3	10792	10726	Protective effect	Protective Effect
ID	1	10793	10727	DNA-DAMAGE	DNA Damage
ID	2	10793	10727	IN-VIVO	In-Vivo
ID	3	10793	10727	BORON	Boron
ID	4	10793	10727	STRESS	Stress
ID	5	10793	10727	MICRONUCLEI	Micronuclei
ID	6	10793	10727	MECHANISMS	Mechanism
ID	7	10793	10727	PREVENTION	Prevention
ID	8	10793	10727	AGENT	Agent
ID	9	10793	10727	BLOOD	Blood
ID	10	10793	10727	RATS	Rat

Record 70 | WOS:001231234500001

Alternariol Monomethyl-Ether Induces Toxicity via Cell Death and Oxidative Stress in Swine Intestinal Epithelial Cells

Field	Position	Original line	Normalised line	Original value	Normalised value
DE	1	10929	10862	alternariol monomethyl-ether	Alternariol Monomethyl Ether
DE	2	10929	10862	intestinal epithelium	Intestinal Epithelium
DE	3	10929	10862	swine	Swine
DE	4	10929	10862	apoptosis	Apoptosis
DE	5	10929	10862	oxidative stress	Oxidative Stress
ID	1	10931	10864	MYCOTOXIN	Mycotoxins
ID	2	10931	10864	TOXINS	Toxin
ID	3	10931	10864	COLON	Colon
ID	4	10931	10864	WHEAT	Wheat
ID	5	10931	10864	ZEARALENONE	Zearalenone (ZEA)
ID	6	10931	10864	MECHANISMS	Mechanism
ID	7	10931	10864	APOPTOSIS	Apoptosis

Record 71 | WOS:001227638700001

Development and application of the physiologically-based toxicokinetic (PBTK) model for ochratoxin A (OTA) in rats and humans

Field	Position	Original line	Normalised line	Original value	Normalised value
DE	1	11051	10983	Ochratoxin A	Ochratoxin A (OTA)
DE	2	11051	10983	human model	Human Model
DE	3	11051	10983	inter-specific extrapolation	Inter Specific Extrapolation
DE	4	11051	10983	dose translation	Dose Translation
ID	1	11053	10985	IN-VIVO	In-Vivo
ID	2	11053	10985	PHARMACOKINETIC MODEL	Pharmacokinetic Model
ID	3	11053	10985	PBPK MODEL	Pbpk Model
ID	4	11053	10985	PLASMA	Plasma
ID	5	11053	10985	TOXICITY	Toxicity
ID	6	11053	10985	METABOLISM	Metabolism
ID	7	11053	10985	TRANSPORT	Transport
ID	8	11053	10985	EXPOSURE	Exposure
ID	9	11053	10985	KINETICS	Kinetics
ID	10	11053	10985	BINDING	Binding

Record 72 | WOS:001209938800001

Comprehensive Insights into Ochratoxin A: Occurrence, Analysis, and Control Strategies

Field	Position	Original line	Normalised line	Original value	Normalised value
DE	1	11213	11144	ochratoxin A	Ochratoxin A (OTA)
DE	2	11213	11144	toxin	Toxin
DE	3	11213	11144	contamination	Contamination
DE	4	11213	11144	health complication	Health Complication
DE	5	11213	11144	control	Control
ID	1	11214	11145	ASPERGILLUS SECTION NIGRI	Aspergillus Section Nigri
ID	2	11214	11145	CYCLODEXTRIN-BASED NANOSPONGES	Cyclodextrin Based Nanosponge
ID	3	11214	11145	GROUND ROASTED COFFEE	Ground Roasted Coffee
ID	4	11214	11145	IN-VITRO CYTOTOXICITY	In Vitro Cytotoxicity
ID	5	11214	11145	LIQUID-CHROMATOGRAPHY	Liquid Chromatography
ID	6	11214	11145	WATER ACTIVITY	Water Activity
ID	7	11214	11145	SACCHAROMYCES-CEREVISIAE	Saccharomyce Cerevisiae
ID	8	11214	11145	PENICILLIUM-VERRUCOSUM	Penicillium Verrucosum

ID	9	11214	11145	NONRIBOSOMAL PEPTIDE	Nonribosomal Peptide
ID	10	11214	11145	SECONDARY METABOLISM	Secondary Metabolism

Record 73 | WOS:001221312500001

In vitro and in vivo assessment of AFB1 and OTA toxic effects and the beneficial role of bioactive compounds. A systematic review

Field	Position	Original line	Normalised line	Original value	Normalised value
DE	2	11606	11536	Functional ingredients	Functional Ingredient
DE	3	11606	11536	Cell lines	Cell
DE	4	11606	11536	Animal models	Animal Model
DE	5	11606	11536	Mechanism of action	Mechanism Of Action
ID	1	11608	11537	OCHRATOXIN	Ochratoxin A (OTA)
ID	2	11608	11537	EXPOSURE	Exposure
ID	3	11608	11537	MYCOTOXINS	Mycotoxins
ID	4	11608	11537	DAMAGE	Damage
ID	5	11608	11537	CELLS	Cell
ID	6	11608	11537	B-1	B 1

Record 74 | WOS:001219077100001

Disruption of intestinal epithelial permeability in the Co-culture system of Caco-2/HT29-MTX cells exposed individually or simultaneously to acrylamide and ochratoxin A

Field	Position	Original line	Normalised line	Original value	Normalised value
DE	1	11776	11704	Acrylamide (AA)	Acrylamide Aa
DE	3	11776	11704	Combined toxicity	Combined Toxicity
DE	4	11776	11704	Caco-2/HT29-MTX cells	Caco-2 Ht29 Mtx
DE	5	11776	11704	Intestinal barrier	Intestinal Barrier
ID	1	11778	11706	GANODERMA-ATRUM POLYSACCHARIDE	Ganoderma Atrum Polysaccharide
ID	2	11778	11706	OXIDATIVE DAMAGE	Oxidative Damage
ID	3	11778	11706	TOXICITY	Toxicity
ID	4	11778	11706	MODELS	Model
ID	5	11778	11706	MUCUS	Mucus

Record 75 | WOS:001185505600042

A high-throughput gut-on-chip platform to study the epithelial responses to enterotoxins

Field	Position	Original line	Normalised line	Original value	Normalised value
ID	1	11911	11837	KAPPA-B ACTIVATION	Kappa B Activation
ID	2	11911	11837	INTESTINAL BARRIER	Intestinal Barrier
ID	3	11911	11837	TIGHT JUNCTIONS	Tight Junction
ID	4	11911	11837	CELL	Cell
ID	5	11911	11837	CYTOTOXICITY	Cytotoxicity
ID	6	11911	11837	PERMEABILITY	Permeability
ID	7	11911	11837	MELITTIN	Melittin
ID	8	11911	11837	DEOXYNIVALENOL	Deoxynivalenol (DON)
ID	9	11911	11837	EXPRESSION	Expression
ID	10	11911	11837	SECRETION	Secretion

Record 76 | WOS:001187481100001

Efficacy of a multicomponent binding agent against combined exposure to zearalenone and ochratoxin A in weaned pigs

Field	Position	Original line	Normalised line	Original value	Normalised value
DE	1	12034	11958	pigs	Pig
DE	2	12034	11958	weaned	Weaned
DE	3	12034	11958	mycotoxins	Mycotoxins
DE	4	12034	11958	zearalenone	Zearalenone (ZEA)

DE	5	12034	11958	ochratoxin A	Ochratoxin A (OTA)
DE	6	12034	11958	adsorbent	Adsorbent
DE	7	12034	11958	mitigation	Mitigation
DE	8	12034	11958	residues	Residue
ID	1	12036	11960	ALPHA-ZEARALENOL	Alpha Zearalenol
ID	2	12036	11960	MYCOTOXIN	Mycotoxins
ID	3	12036	11960	TOXICITY	Toxicity
ID	4	12036	11960	CYTOTOXICITY	Cytotoxicity
ID	5	12036	11960	PERFORMANCE	Performance
ID	6	12036	11960	PARAMETERS	Parameter
ID	7	12036	11960	TISSUE	Tissue
ID	8	12036	11960	CELLS	Cell

Record 77 | WOS:001192691800001

Red Orange and Lemon Extract Ameliorates the Renal Oxidative Stress and Inflammation Induced by Ochratoxin A through the Modulation of Nrf2

Field	Position	Original line	Normalised line	Original value	Normalised value
DE	1	12157	12080	RLE	Rle
DE	2	12157	12080	kidney	Kidney
DE	3	12157	12080	OTA toxicity	OTA Toxicity
DE	5	12157	12080	ROS	Reactive Oxygen Species (ROS)
ID	1	12158	12081	TOXICITY	Toxicity
ID	2	12158	12081	NEPHROPATHY	Nephropathy
ID	3	12158	12081	CELLS	Cell
ID	4	12158	12081	FEED	Feed

Record 78 | WOS:001176181400001

Natural feed additives and bioactive supplements versus chemical additives as a safe and practical approach to combat foodborne mycotoxicoses

Field	Position	Original line	Normalised line	Original value	Normalised value
DE	1	12268	12190	food safety	Food Safety
DE	2	12268	12190	mycotoxins	Mycotoxins
DE	3	12268	12190	foodborne ailments	Foodborne Ailment
DE	4	12268	12190	preventive measures	Preventive Measure
DE	5	12268	12190	risk management	Risk Management
DE	6	12268	12190	feed additives	Feed Additive
DE	7	12268	12190	biological supplements	Biological Supplement
DE	8	12268	12190	herbal additives	Herbal Additive
ID	1	12270	12192	PENICILLIUM-EXPANSUM GROWTH	Penicillium Expansum Growth
ID	2	12270	12192	RADICAL SCAVENGING ACTIVITY	Radical Scavenging Activity
ID	3	12270	12192	OCHRATOXIN-A PRODUCTION	Ochratoxin A Production
ID	4	12270	12192	TINOSPORA-CORDIFOLIA	Tinospora Cordifolia
ID	5	12270	12192	BLUE MOLD	Blue Mold
ID	6	12270	12192	IN-VITRO	In-Vitro
ID	7	12270	12192	AFLATOXIN B-1	Aflatoxin B1 (AFB1)
ID	8	12270	12192	MILK THISTLE	Milk Thistle
ID	9	12270	12192	PATULIN ACCUMULATION	Patulin Accumulation
ID	10	12270	12192	TRICHOSPORON-MYCOTOXINIVORANS	Trichosporon Mycotoxinivoran

Record 79 | WOS:001179683700001

In vitro and in vivo induction of ochratoxin A exposure-related micronucleus formation in rat proximal tubular epithelial cells and expression profiling of chromosomal instability-related genes

Field	Position	Original line	Normalised line	Original value	Normalised value
DE	2	12635	12556	Micronucleus (MN)	Micronucleus Mn

DE	3	12635	12556	Chromosomal instability (CIN)	Chromosomal Instability Cin
DE	5	12635	12556	Renal carcinogen	Renal Carcinogen
ID	1	12637	12558	DNA-DAMAGE	DNA Damage
ID	2	12637	12558	KIDNEY-CELLS	Kidney
ID	3	12637	12558	MICE	Mice
ID	4	12637	12558	CYTOTOXICITY	Cytotoxicity
ID	5	12637	12558	APOPTOSIS	Apoptosis
ID	6	12637	12558	SPINDLE	Spindle
ID	7	12637	12558	3-MONOCHLOROPROPANE-1,2-DIOL	3 Monochloropropane 1 2 Diol
ID	8	12637	12558	CARCINOGENICITY	Carcinogenicity
ID	9	12637	12558	PROLIFERATION	Proliferation
ID	10	12637	12558	GENOTOXICITY	Genotoxicity

Record 80 | WOS:001172173100001

Comparative Study of Spheroids (3D) and Monolayer Cultures (2D) for the In Vitro Assessment of Cytotoxicity Induced by the Mycotoxins Sterigmatocystin, Ochratoxin A and Patulin

Field	Position	Original line	Normalised line	Original value	Normalised value
DE	1	12778	12697	food contaminants	Food Contaminant
DE	2	12778	12697	mycotoxins	Mycotoxins
DE	3	12778	12697	cytotoxicity	Cytotoxicity
DE	4	12778	12697	3D spheroid	3d Spheroid
DE	5	12778	12697	tumour models	Tumour Model
DE	6	12778	12697	mesenchymal stem cells	Mesenchymal Stem
DE	7	12778	12697	endothelial cells	Endothelial
DE	8	12778	12697	in vitro models	In Vitro Model
ID	1	12780	12699	RISK-ASSESSMENT	Risk Assessment
ID	2	12780	12699	FUMONISIN B-1	Fumonisin B1 (FB1)
ID	3	12780	12699	TOXICITY	Toxicity
ID	4	12780	12699	CELLS	Cell
ID	5	12780	12699	NANOPARTICLES	Nanoparticle
ID	6	12780	12699	PRODUCTS	Product
ID	7	12780	12699	EXPOSURE	Exposure
ID	8	12780	12699	COFFEE	Coffee
ID	9	12780	12699	MODEL	Model
ID	10	12780	12699	FOODS	Food

Record 81 | WOS:001175293000001

Involvement of pro-inflammatory mediators and cell cycle disruption in neuronal cells induced by gliotoxin and ochratoxin A after individual and combined exposure

Field	Position	Original line	Normalised line	Original value	Normalised value
DE	2	12910	12828	Ochratoxin A	Ochratoxin A (OTA)
DE	3	12910	12828	SH-SY5Y cells	SH-SY5Y
DE	4	12910	12828	Neuroinflammation and cell cycle	Neuroinflammation And Cell Cycle
ID	1	12911	12829	INDUCED CYTOTOXICITY	Induced Cytotoxicity
ID	2	12911	12829	MYCOTOXINS	Mycotoxins
ID	3	12911	12829	CITRININ	Citrinin
ID	4	12911	12829	FUSARIUM	Fusarium
ID	5	12911	12829	FEED	Feed

Record 82 | WOS:001154746400001

Recent advances in the degradation efficacy and mechanisms of mycotoxins in food by atmospheric cold plasma

Field	Position	Original line	Normalised line	Original value	Normalised value
DE	2	13031	12948	Atmospheric cold plasma	Atmospheric Cold Plasma
DE	3	13031	12948	Degradation mechanism	Degradation Mechanism

DE	4	13031	12948	Food quality	Food Quality
DE	6	13031	12948	Reactive oxygen and nitrogen species	Reactive Oxygen And Nitrogen Specy
ID	1	13033	12950	AFLATOXIN B-1	Aflatoxin B1 (AFB1)
ID	2	13033	12950	DETOXIFICATION	Detoxification
ID	3	13033	12950	INACTIVATION	Inactivation
ID	4	13033	12950	FUNGAL	Fungal
ID	5	13033	12950	IMPACT	Impact

Record 83 | WOS:001201732900001

Comparative Assessment of Thermal Treatment for Mycotoxins Adsorption Potential of Indigenous Clay from Balochistan, Pakistan

Field	Position	Original line	Normalised line	Original value	Normalised value
DE	1	13192	13108	Aflatoxins B1	Aflatoxin B1 (AFB1)
DE	2	13192	13108	Ochratoxin A	Ochratoxin A (OTA)
DE	4	13192	13108	Thermal treatment	Thermal Treatment
ID	1	13194	13110	ASPERGILLUS-FLAVUS	Aspergillus Flavus
ID	2	13194	13110	AFLATOXIN B-1	Aflatoxin B1 (AFB1)
ID	3	13194	13110	OCHRATOXIN	Ochratoxin A (OTA)

Record 84 | WOS:001151094300001

Practical Strategies to Reduce Ochratoxin A in Foods

Field	Position	Original line	Normalised line	Original value	Normalised value
DE	1	13284	13199	ochratoxin A (OTA)	Ochratoxin A (OTA)
DE	2	13284	13199	toxicity	Toxicity
DE	3	13284	13199	reduction	Reduction
DE	4	13284	13199	food processing	Food Processing
DE	5	13284	13199	food additives	Food Additive
ID	1	13285	13200	GREEN COFFEE BEANS	Green Coffee Bean
ID	2	13285	13200	IN-VITRO CYTOTOXICITY	In Vitro Cytotoxicity
ID	3	13285	13200	A CONTENT	A Content
ID	4	13285	13200	BREAKFAST CEREALS	Breakfast Cereal
ID	5	13285	13200	PROTEIN-SYNTHESIS	Protein Synthesis
ID	6	13285	13200	OXIDATIVE STRESS	Oxidative Stress
ID	7	13285	13200	DEGRADATION-PRODUCT	Degradation Product
ID	8	13285	13200	LIPID-PEROXIDATION	Lipid Peroxidation
ID	9	13285	13200	RISK-ASSESSMENT	Risk Assessment
ID	10	13285	13200	HEAT-STABILITY	Heat Stability

Record 85 | WOS:001262485700001

Grape pomace as a novel functional ingredient: Mitigating ochratoxin A bioaccessibility and unraveling cytoprotective mechanisms in vitro

Field	Position	Original line	Normalised line	Original value	Normalised value
DE	4	13517	13431	Flow cytometry	Flow Cytometry
DE	5	13517	13431	Oxidative stress	Oxidative Stress
ID	1	13518	13432	CELL-LINE	Cell
ID	2	13518	13432	MYCOTOXINS	Mycotoxins
ID	3	13518	13432	CACO-2	Caco-2
ID	4	13518	13432	CYTOTOXICITY	Cytotoxicity
ID	5	13518	13432	DAMAGE	Damage
ID	6	13518	13432	FOOD	Food

Record 86 | WOS:001145669600001

Ochratoxin A-enhanced glycolysis induces inflammatory responses in human gastric epithelium cells through mTOR/HIF-1Î± signaling pathway

Field	Position	Original line	Normalised line	Original value	Normalised value

DE	1	13657	13570	Ochratoxin A	Ochratoxin A (OTA)
DE	2	13657	13570	Gastric epithelium cell	Gastric Epithelium
DE	3	13657	13570	Inflammatory responses	Inflammatory Response
DE	5	13657	13570	HIF-1 alpha	Hif 1 Alpha
DE	6	13657	13570	MTOR	Mtor
ID	1	13659	13572	GLUCOSE-METABOLISM	Glucose Metabolism
ID	2	13659	13572	OXIDATIVE STRESS	Oxidative Stress
ID	3	13659	13572	IPEC-J2 CELLS	IPEC-J2
ID	4	13659	13572	TOXICITY	Toxicity
ID	5	13659	13572	CANCER	Cancer
ID	6	13659	13572	HIF-1-ALPHA	Hif 1 Alpha
ID	7	13659	13572	APOPTOSIS	Apoptosis
ID	8	13659	13572	KIDNEY	Kidney

Record 87 | WOS:001119207400001

Elucidating the mechanism of action of mycotoxins through machine learning-driven QSAR models: Focus on lipid peroxidation

Field	Position	Original line	Normalised line	Original value	Normalised value
DE	2	13756	13668	Lipid peroxidation	Lipid Peroxidation
DE	3	13756	13668	QSAR	Qsar
DE	4	13756	13668	Molecular topology	Molecular Topology
DE	5	13756	13668	Machine learning	Machine Learning
ID	1	13758	13669	OXIDATIVE STRESS	Oxidative Stress
ID	2	13758	13669	OCHRATOXIN-A	Ochratoxin A (OTA)
ID	3	13758	13669	IN-VITRO	In-Vitro
ID	4	13758	13669	DEOXYNIVALENOL	Deoxynivalenol (DON)
ID	5	13758	13669	CELLS	Cell
ID	6	13758	13669	TOXIN	Toxin
ID	7	13758	13669	T-2	T 2
ID	8	13758	13669	INVOLVEMENT	Involvement
ID	9	13758	13669	TOXICITY	Toxicity
ID	10	13758	13669	DAMAGE	Damage

Record 88 | WOS:001099952500001

Risks for animal health related to the presence of ochratoxin A (OTA) in feed

Field	Position	Original line	Normalised line	Original value	Normalised value
DE	1	13947	13857	ochratoxin A	Ochratoxin A (OTA)
DE	2	13947	13857	OTA	Feed
DE	3	13947	13857	feed	Exposure
DE	4	13947	13857	exposure	Toxicity
DE	5	13947	13857	toxicity	Animal Health Risk Assessment
DE	6	13947	13857	animal health risk assessment	
ID	1	13949	13858	IN-VITRO CYTOTOXICITY	In Vitro Cytotoxicity
ID	2	13949	13858	ZEALAND WHITE-RABBITS	Zealand White Rabbit
ID	3	13949	13858	OXIDATIVE STRESS	Oxidative Stress
ID	4	13949	13858	BROILER-CHICKENS	Broiler Chicken
ID	5	13949	13858	POULTRY FEEDS	Poultry Feed
ID	6	13949	13858	TISSUE DISTRIBUTION	Tissue Distribution
ID	7	13949	13858	ATLANTIC SALMON	Atlantic Salmon
ID	8	13949	13858	BLOOD-SERUM	Blood Serum
ID	9	13949	13858	RUMEN FLUID	Rumen Fluid
ID	10	13949	13858	GRAIN DIET	Grain Diet

Record 89 | WOS:001072761100001

Farnesoid X Receptor Plays a Key Role in Ochratoxin A-Induced Nephrotoxicity by Targeting Ferroptosis In Vivo and In Vitro

Field	Position	Original line	Normalised line	Original value	Normalised value

DE	1	14192	14100	OTA	Ochratoxin A (OTA)
DE	2	14192	14100	FXR	Fxr
DE	3	14192	14100	ferroptosis	Ferroptosis
DE	4	14192	14100	nephrotoxicity	Nephrotoxicity
DE	5	14192	14100	lipid peroxidation	Lipid Peroxidation
ID	1	14193	14101	ACUTE KIDNEY INJURY	Acute Kidney Injury
ID	2	14193	14101	ACSL4	Acsl4

Record 90 | WOS:001079796800001

Neurotoxicity of ochratoxin A: Molecular mechanisms and neurotherapeutic strategies

Field	Position	Original line	Normalised line	Original value	Normalised value
DE	1	14308	14215	Ochratoxin A	Ochratoxin A (OTA)
DE	4	14308	14215	Experimental models	Experimental Model
DE	5	14308	14215	Human biomonitoring	Human Biomonitoring
ID	1	14310	14217	DOUBLE-STRAND BREAKS	Double Strand Break
ID	2	14310	14217	BLOOD-BRAIN-BARRIER	Blood Brain Barrier
ID	3	14310	14217	HUMAN SERUM-ALBUMIN	Human Serum Albumin
ID	4	14310	14217	OXIDATIVE STRESS	Oxidative Stress
ID	5	14310	14217	HUMAN HEALTH	Human Health
ID	6	14310	14217	ANIMAL FEED	Animal Feed
ID	7	14310	14217	IN-VIVO	In-Vivo
ID	8	14310	14217	RAT	Rat
ID	9	14310	14217	EXPOSURE	Exposure
ID	10	14310	14217	CELLS	Cell

Record 91 | WOS:001078658300001

The coexistence of aflatoxin M1 and ochratoxin A induced intestinal barrier disruption via the regulation of key differentially expressed microRNAs and long non-coding RNAs in BALB/c mice

Field	Position	Original line	Normalised line	Original value	Normalised value
DE	1	14523	14428	Aflatoxin M1	Aflatoxin M1 (AFM1)
DE	2	14523	14428	Ochratoxin A	Ochratoxin A (OTA)
DE	3	14523	14428	Barrier disruption	Barrier Disruption
DE	4	14523	14428	Whole transcriptome	Whole Transcriptome
DE	5	14523	14428	Tight junction proteins	Tight Junction Protein
DE	6	14523	14428	Signaling pathways	Signaling Pathway
ID	1	14525	14430	EPITHELIAL BARRIER	Epithelial Barrier
ID	2	14525	14430	PHOSPHATASE	Phosphatase
ID	3	14525	14430	DOMAIN	Domain
ID	4	14525	14430	CELLS	Cell
ID	5	14525	14430	MILK	Milk

Record 92 | WOS:001077853000001

Ochratoxin A: Overview of Prevention, Removal, and Detoxification Methods

Field	Position	Original line	Normalised line	Original value	Normalised value
DE	1	14639	14543	ochratoxin A	Ochratoxin A (OTA)
DE	2	14639	14543	detoxification	Detoxification
DE	3	14639	14543	biological method	Biological Method
DE	4	14639	14543	degradation	Degradation
ID	1	14640	14544	GAMMA-IRRADIATION	Gamma Irradiation
ID	2	14640	14544	BACILLUS-SUBTILIS	Bacillus Subtilis
ID	3	14640	14544	AQUEOUS-SOLUTIONS	Aqueous Solution
ID	4	14640	14544	COLD-PLASMA	Cold Plasma
ID	5	14640	14544	IN-VITRO	In-Vitro
ID	6	14640	14544	A LEVELS	A Level

ID	7	14640	14544	REDUCTION	Reduction
ID	8	14640	14544	WINE	Wine
ID	9	14640	14544	CONTAMINATION	Contamination
ID	10	14640	14544	ADSORPTION	Adsorption

Record 93 | WOS:001060917800001

Exploring the Toxic Effects of ZEA on IPEC-J2 Cells from the Inflammatory Response and Apoptosis

Field	Position	Original line	Normalised line	Original value	Normalised value
DE	1	14811	14714	zearalenone	Zearalenone (ZEA)
DE	2	14811	14714	IPEC-J2 cells	IPEC-J2
DE	3	14811	14714	oxidative stress	Oxidative Stress
DE	4	14811	14714	apoptosis	Apoptosis
DE	5	14811	14714	inflammatory factors	Inflammatory Factor
ID	1	14813	14716	OXIDATIVE STRESS	Oxidative Stress
ID	2	14813	14716	OCHRATOXIN-A	Ochratoxin A (OTA)
ID	3	14813	14716	ZEARALENONE	Zearalenone (ZEA)
ID	4	14813	14716	MYCOTOXINS	Mycotoxins
ID	5	14813	14716	DEOXYNIVALENOL	Deoxynivalenol (DON)
ID	6	14813	14716	METABOLITES	Metabolite
ID	7	14813	14716	DERIVATIVES	Derivative
ID	8	14813	14716	PATHWAYS	Pathway
ID	9	14813	14716	DAMAGE	Damage
ID	10	14813	14716	DEATH	Death

Record 94 | WOS:001074097100001

The Effect of Mycotoxins and Their Mixtures on Bovine Spermatozoa Characteristics

Field	Position	Original line	Normalised line	Original value	Normalised value
DE	1	14920	14822	mycotoxins	Mycotoxins
DE	2	14920	14822	spermatozoa	Spermatozoa
DE	3	14920	14822	mycotoxin mixtures	Mycotoxin Mixture
DE	4	14920	14822	mycotoxin interactions	Mycotoxin Interaction
DE	5	14920	14822	bovine	Bovine
ID	1	14922	14823	IN-VITRO EXPOSURE	In Vitro Exposure
ID	2	14922	14823	OCHRATOXIN-A	Ochratoxin A (OTA)
ID	3	14922	14823	ALPHA-ZEARALENOL	Alpha Zearalenol
ID	4	14922	14823	SPERM MOTILITY	Sperm Motility
ID	5	14922	14823	BOAR SPERM	Boar Sperm
ID	6	14922	14823	SEMEN QUALITY	Semen Quality
ID	7	14922	14823	DAIRY-COWS	Dairy Cow
ID	8	14922	14823	T-2 TOXIN	T 2 Toxin
ID	9	14922	14823	FERTILITY	Fertility
ID	10	14922	14823	TOXICITY	Toxicity

Record 95 | WOS:001070923600001

Cell cycle and enzymatic activity alterations induced by ROS production in human neuroblastoma cells SH-SY5Y exposed to Fumonisin B1, Ochratoxin A and their combination

Field	Position	Original line	Normalised line	Original value	Normalised value
DE	1	15078	14978	Enzyme's mechanisms	Enzyme S Mechanism
DE	2	15078	14978	Fumonisin B1	Fumonisin B1 (FB1)
DE	3	15078	14978	Ochratoxin a	Ochratoxin A (OTA)
DE	5	15078	14978	ROS	Reactive Oxygen Species (ROS)
ID	1	15079	14980	OXIDATIVE STRESS	Oxidative Stress
ID	2	15079	14980	INDUCED CYTOTOXICITY	Induced Cytotoxicity
ID	3	15079	14980	APOPTOSIS	Apoptosis
ID	4	15079	14980	INVOLVEMENT	Involvement
ID	5	15079	14980	GLUTATHIONE	Glutathione (GSH)
ID	6	15079	14980	MYCOTOXINS	Mycotoxins
ID	7	15079	14980	ENNIATINS	Enniatin

ID	8	15079	14980	B-1	B 1

Record 96 | WOS:001054307000001

An integrated approach for evaluating the interactive effects between azoxystrobin and ochratoxin A: Pathway-based in vivo analyses

Field	Position	Original line	Normalised line	Original value	Normalised value
DE	2	15185	15085	Ochratoxin A	Ochratoxin A (OTA)
DE	3	15185	15085	Combined toxicity	Combined Toxicity
DE	4	15185	15085	Pathway analysis	Pathway Analysis
DE	5	15185	15085	Biological responses	Biological Response
ID	1	15187	15087	OXIDATIVE STRESS	Oxidative Stress
ID	2	15187	15087	DISRUPTION	Disruption
ID	3	15187	15087	MECHANISMS	Mechanism

Record 97 | WOS:001041623900001

The Protective Effect of Pumpkin and Fermented Whey Mixture against AFB1 and OTA Immune Toxicity In Vitro. A Transcriptomic Approach

Field	Position	Original line	Normalised line	Original value	Normalised value
DE	1	15286	15185	bioactive compounds	Bioactive Compound
DE	2	15286	15185	inflammation	Inflammation
DE	3	15286	15185	Jurkat cells	Jurkat
DE	4	15286	15185	mycotoxins	Mycotoxins
DE	5	15286	15185	RNA-sequencing	RNA Sequencing
ID	1	15288	15186	FACTOR-KAPPA-B	Factor Kappa B
ID	2	15288	15186	GENE-EXPRESSION	Gene Expression
ID	3	15288	15186	IN-VITRO	In-Vitro
ID	4	15288	15186	SIGNALING PATHWAY	Signaling Pathway
ID	5	15288	15186	DIGESTION MODEL	Digestion Model
ID	6	15288	15186	AFLATOXIN M1	Aflatoxin M1 (AFM1)
ID	7	15288	15186	OCHRATOXIN	Ochratoxin A (OTA)
ID	8	15288	15186	BIOACCESSIBILITY	Bioaccessibility
ID	9	15288	15186	MYCOTOXINS	Mycotoxins
ID	10	15288	15186	CELLS	Cell

Record 98 | WOS:001055768800001

Mycotoxins in Cereal-Based Products and Their Impacts on the Health of Humans, Livestock Animals and Pets

Field	Position	Original line	Normalised line	Original value	Normalised value
DE	1	15442	15340	cereal grains	Cereal Grain
DE	2	15442	15340	mycotoxins	Mycotoxins
DE	3	15442	15340	health impacts	Health Impact
DE	4	15442	15340	human health	Human Health
DE	5	15442	15340	livestock animals	Livestock Animal
DE	6	15442	15340	pets	Pet
DE	7	15442	15340	management	Management
DE	8	15442	15340	regulation	Regulation
ID	1	15444	15342	BALKAN ENDEMIC NEPHROPATHY	Balkan Endemic Nephropathy
ID	2	15444	15342	GENE-EXPRESSION PROFILES	Gene Expression Profile
ID	3	15444	15342	MONILIFORME CULTURE MATERIAL	Moniliforme Culture Material
ID	4	15444	15342	IN-VITRO CYTOTOXICITY	In Vitro Cytotoxicity
ID	5	15444	15342	FEED RAW-MATERIALS	Feed Raw Material
ID	6	15444	15342	FUMONISIN B-1	Fumonisin B1 (FB1)
ID	7	15444	15342	OCHRATOXIN-A	Ochratoxin A (OTA)
ID	8	15444	15342	FUSARIUM MYCOTOXINS	Fusarium Mycotoxin
ID	9	15444	15342	FOOD SAFETY	Food Safety

ID	10	15444	15342	AFLATOXIN CONTAMINATION	Aflatoxin Contamination

Record 99 | WOS:001036576500001

Pathological Role of Oxidative Stress in Aflatoxin-Induced Toxicity in Different Experimental Models and Protective Effect of Phytochemicals: A Review

Field	Position	Original line	Normalised line	Original value	Normalised value
DE	1	15766	15663	aflatoxins	Aflatoxin
DE	2	15766	15663	oxidative stress	Oxidative Stress
DE	3	15766	15663	phytochemicals	Phytochemical
DE	4	15766	15663	antioxidants	Antioxidant
DE	5	15766	15663	broilers	Broiler
ID	1	15767	15664	THISTLE SILYBUM-MARIANUM	Thistle Silybum Marianum
ID	2	15767	15664	BROILER-CHICKENS	Broiler Chicken
ID	3	15767	15664	OCHRATOXIN-A	Ochratoxin A (OTA)
ID	4	15767	15664	LIVER-INJURY	Liver Injury
ID	5	15767	15664	IN-VIVO	In-Vivo
ID	6	15767	15664	B-1	B 1
ID	7	15767	15664	SILYMARIN	Silymarin
ID	8	15767	15664	CURCUMIN	Curcumin
ID	9	15767	15664	DAMAGE	Damage
ID	10	15767	15664	RESVERATROL	Resveratrol

Record 100 | WOS:001040619100001

MiR-155-5p Elevated by Ochratoxin A Induces Intestinal Fibrosis and Epithelial-to-Mesenchymal Transition through TGF-Î² Regulated Signaling Pathway In Vitro and In Vivo

Field	Position	Original line	Normalised line	Original value	Normalised value
DE	1	15970	15866	ochratoxin A	Ochratoxin A (OTA)
DE	2	15970	15866	miR-155-5p	Mir 155 5p
DE	3	15970	15866	intestinal fibrosis	Intestinal Fibrosis
DE	4	15970	15866	TGF-beta	Tgf Beta
DE	5	15970	15866	EMT	Emt
ID	1	15971	15867	EXPRESSION	Expression
ID	2	15971	15867	MICRORNAS	Microrna
ID	3	15971	15867	TISSUE	Tissue

Record 101 | WOS:001013318000001

Enzymatic degradation of ochratoxin A in the gastrointestinal tract of piglets

Field	Position	Original line	Normalised line	Original value	Normalised value
DE	1	16072	15967	enzymatic hydrolysis	Enzymatic Hydrolysis
DE	2	16072	15967	feed additive	Feed Additive
DE	3	16072	15967	mycotoxins	Mycotoxins
DE	4	16072	15967	ochratoxin A	Ochratoxin A (OTA)
DE	5	16072	15967	OTA amidohydrolase	OTA Amidohydrolase
DE	6	16072	15967	piglet	Piglet
ID	1	16074	15969	PIGS	Pig
ID	2	16074	15969	TOXICITY	Toxicity
ID	3	16074	15969	BIODEGRADATION	Biodegradation
ID	4	16074	15969	HYDROLYSIS	Hydrolysis
ID	5	16074	15969	TISSUES	Tissue

Record 102 | WOS:001011146700001

A Chemically Defined Medium That Supports Mycotoxin Production by Stachybotrys chartarum Enabled Analysis of the Impact of Nitrogen and Carbon Sources on the Biosynthesis of Macrocyclic Trichothecenes and Stachybotrylactam

Field	Position	Original line	Normalised line	Original value	Normalised value
DE	1	16200	16094	Stachybotrys	Stachybotry
DE	2	16200	16094	nitrogen source	Nitrogen Source
DE	3	16200	16094	carbon source	Carbon Source
DE	4	16200	16094	nutrients	Nutrient
DE	5	16200	16094	macrocyclic trichothecenes	Macrocyclic Trichothecene
DE	6	16200	16094	stachybotrylactam	Stachybotrylactam
DE	7	16200	16094	LC-MS	Lc Ms
DE	8	16200	16094	MS	Ms
ID	1	16202	16096	CATABOLITE REPRESSION	Catabolite Repression
ID	2	16202	16096	PULMONARY HEMORRHAGE	Pulmonary Hemorrhage
ID	3	16202	16096	NITRATE ASSIMILATION	Nitrate Assimilation
ID	4	16202	16096	FILAMENTOUS GROWTH	Filamentous Growth
ID	5	16202	16096	BUILDING-MATERIALS	Building Material
ID	6	16202	16096	TOXIGENIC FUNGI	Toxigenic Fungi
ID	7	16202	16096	AMMONIUM-IONS	Ammonium Ion
ID	8	16202	16096	OCHRATOXIN-A	Ochratoxin A (OTA)
ID	9	16202	16096	CELL-CULTURE	Cell
ID	10	16202	16096	SATRATOXIN-G	Satratoxin G

Record 103 | WOS:001013506800001

Citrinin as a potential anti-cancer therapy: A comprehensive review

Field	Position	Original line	Normalised line	Original value	Normalised value
DE	4	16378	16271	Antiplorifactive mechanisms	Antiplorifactive Mechanism
ID	1	16380	16273	PERIPHERAL-BLOOD LYMPHOCYTES	Peripheral Blood Lymphocyte
ID	2	16380	16273	STRESS-INDUCED APOPTOSIS	Stress Induced Apoptosis
ID	3	16380	16273	MYCOTOXINS OCHRATOXIN-A	Ochratoxin A (OTA)
ID	4	16380	16273	NITRIC-OXIDE PRODUCTION	Nitric Oxide Production
ID	5	16380	16273	DEVELOPMENT IN-VITRO	Development In Vitro
ID	6	16380	16273	BREAST-CANCER CELLS	Breast Cancer
ID	7	16380	16273	PENICILLIUM-CITRINUM	Penicillium Citrinum
ID	8	16380	16273	OXIDATIVE STRESS	Oxidative Stress
ID	9	16380	16273	MOLECULAR-MECHANISMS	Molecular Mechanism
ID	10	16380	16273	SIGNALING PATHWAY	Signaling Pathway

Record 104 | WOS:001141751900001

Determination of Ochratoxin-A Detoxification and Antibiotic Resistance Potential of Acinetobacter calcoaceticus

Field	Position	Original line	Normalised line	Original value	Normalised value
DE	2	16590	16481	Ochratoxin A	Ochratoxin A (OTA)
DE	3	16590	16481	Ochratoxin a	Acinetobacter Calcoaceticus
DE	4	16590	16481	Acinetobacter calcoaceticus	Mycotoxins
DE	5	16590	16481	Mycotoxins	Biodegradation
DE	6	16590	16481	Biodegradation	
ID	1	16592	16483	FOOD	Food

Record 105 | WOS:000988737300001

Natural mycotoxin contamination in dog food: A review on toxicity and detoxification methods

Field	Position	Original line	Normalised line	Original value	Normalised value
DE	1	16690	16580	Aflatoxins	Aflatoxin
DE	3	16690	16580	Deoxynivalenol	Deoxynivalenol (DON)
DE	4	16690	16580	Fumonisins	Fumonisin
DE	5	16690	16580	Ochratoxin A	Ochratoxin A (OTA)
DE	6	16690	16580	Zearalenone	Zearalenone (ZEA)
ID	1	16692	16582	LACTIC-ACID BACTERIA	Lactic Acid Bacteria
ID	2	16692	16582	OCHRATOXIN-A	Ochratoxin A (OTA)
ID	3	16692	16582	IN-VITRO	In-Vitro
ID	4	16692	16582	AFLATOXIN B-1	Aflatoxin B1 (AFB1)
ID	5	16692	16582	ANIMAL FEED	Animal Feed
ID	6	16692	16582	BIOLOGICAL DETOXIFICATION	Biological Detoxification
ID	7	16692	16582	GAMMA-IRRADIATION	Gamma Irradiation
ID	8	16692	16582	BEAGLE DOGS	Beagle Dog
ID	9	16692	16582	PET FOOD	Pet Food
ID	10	16692	16582	TRICHOSPORON-MYCOTOXINIVORANS	Trichosporon Mycotoxinivoran

Record 106 | WOS:000983760800001

Excessive ER-phagy contributes to ochratoxin A-induced apoptosis

Field	Position	Original line	Normalised line	Original value	Normalised value
DE	1	16980	16869	Ochratoxin a	Ochratoxin A (OTA)
DE	2	16980	16869	Endoplasmic reticulum stress	Endoplasmic Reticulum Stress
DE	3	16980	16869	ER-Phagy	Er Phagy
DE	5	16980	16869	UFMylation	Ufmylation
ID	1	16982	16871	ENDOPLASMIC-RETICULUM STRESS	Endoplasmic Reticulum Stress
ID	2	16982	16871	OXIDATIVE STRESS	Oxidative Stress
ID	3	16982	16871	QUALITY-CONTROL	Quality Control
ID	4	16982	16871	PROTEIN	Protein
ID	5	16982	16871	UFMYLATION	Ufmylation
ID	6	16982	16871	TEX264	Tex264
ID	7	16982	16871	UFM1	Ufm1

Record 107 | WOS:000977904100001

New perspectives in application of kidney biomarkers in mycotoxin induced nephrotoxicity, with a particular focus on domestic pigs

Field	Position	Original line	Normalised line	Original value	Normalised value
DE	1	17112	17000	nephrotoxicity	Nephrotoxicity
DE	2	17112	17000	KIM-1	Kim 1
DE	3	17112	17000	kidney biomarkers	Kidney Biomarker
DE	4	17112	17000	mycotoxins	Mycotoxins
DE	5	17112	17000	animal toxicity	Animal Toxicity
DE	6	17112	17000	NAG	Nag
DE	7	17112	17000	NGAL biomarkers	Ngal Biomarker
DE	8	17112	17000	mycotoxin induced nephrotoxicity	Mycotoxin Induced Nephrotoxicity
ID	1	17114	17002	FUMONISIN B-1	Fumonisin B1 (FB1)
ID	2	17114	17002	URINARY BIOMARKERS	Urinary Biomarker
ID	3	17114	17002	INJURY MOLECULE-1	Injury Molecule 1
ID	4	17114	17002	IN-VIVO	In-Vivo
ID	5	17114	17002	AFLATOXIN PRODUCTION	Aflatoxin Production
ID	6	17114	17002	MULTIPLE MYCOTOXINS	Multiple Mycotoxin
ID	7	17114	17002	SPHINGOSINE RATIO	Sphingosine Ratio

ID	8	17114	17002	OCHRATOXIN-A	Ochratoxin A (OTA)
ID	9	17114	17002	SPHINGANINE	Sphinganine
ID	10	17114	17002	TOXICITY	Toxicity

Record 108 | WOS:000968395600001

Fermented whey modulated AFB1 and OTA-induced hepatotoxicity and nephrotoxicity in vivo. A relative and absolute quantification about sex differences

Field	Position	Original line	Normalised line	Original value	Normalised value
DE	2	17337	17224	fermented whey	Fermented Whey
DE	3	17337	17224	RT-qPCR	Rt Qpcr
DE	4	17337	17224	droplet digital PCR	Droplet Digital Pcr
DE	5	17337	17224	in vivo	In-Vivo
ID	1	17338	17225	GUIDELINES MINIMUM INFORMATION	Guideline Minimum Information
ID	2	17338	17225	DROPLET DIGITAL PCR	Droplet Digital Pcr
ID	3	17338	17225	UREA CYCLE	Urea Cycle
ID	4	17338	17225	METABOLISM	Metabolism
ID	5	17338	17225	STRESS	Stress
ID	6	17338	17225	CPS1	Cps1
ID	7	17338	17225	SUPPLEMENTATION	Supplementation
ID	8	17338	17225	PUBLICATION	Publication
ID	9	17338	17225	OCHRATOXIN	Ochratoxin A (OTA)
ID	10	17338	17225	AFLATOXINS	Aflatoxin

Record 109 | WOS:000982062800001

Protective Effect of alpha-Tocopherol Against Ochratoxin A in Kidney Cell Line HK-2

Field	Position	Original line	Normalised line	Original value	Normalised value
DE	6	17485	17370	?-Tocopherol	Tocopherol
ID	1	17487	17372	OXIDATIVE STRESS	Oxidative Stress
ID	2	17487	17372	VITAMIN-E	Vitamin E
ID	3	17487	17372	BREAKFAST CEREALS	Breakfast Cereal
ID	4	17487	17372	INFANT CEREALS	Infant Cereal
ID	5	17487	17372	IN-VITRO	In-Vitro
ID	6	17487	17372	MYCOTOXINS	Mycotoxins
ID	7	17487	17372	REDUCTION	Reduction
ID	8	17487	17372	CYTOTOXICITY	Cytotoxicity
ID	9	17487	17372	TOXICITY	Toxicity
ID	10	17487	17372	PRODUCTS	Product

Record 110 | WOS:001094910400007

INITIAL PHASE ESTABLISHMENT OF AN IN VITRO METHOD FOR DEVELOP-MENTAL NEUROTOXICITY TEST USING KI-67 IN HUMAN NEURAL PROGENITOR CELLS

Field	Position	Original line	Normalised line	Original value	Normalised value
DE	1	17608	17492	neurotoxicological test	Neurotoxicological Test
DE	2	17608	17492	5-bromo-2'-deoxyuridine	5 Bromo 2 Deoxyuridine
DE	3	17608	17492	Ki-76	Ki 76
DE	4	17608	17492	developmental neurotoxicity	Developmental Neurotoxicity
DE	5	17608	17492	human neural progenitor cells	Human Neural Progenitor
DE	6	17608	17492	ReNcell CX cells	Rencell Cx
DE	7	17608	17492	aphidicolin	Aphidicolin
DE	8	17608	17492	hydroxyurea	Hydroxyurea
DE	9	17608	17492	cytosine arabinoside	Cytosine Arabinoside
DE	10	17608	17492	5-fluorouracil	5 Fluorouracil
DE	11	17608	17492	ochratoxin A	Ochratoxin A (OTA)
DE	12	17608	17492	sodium gluconate	Sodium Gluconate

DE	13	17608	17492	sodium bicarbonate	Sodium Bicarbonate
DE	14	17608	17492	penicillin G	Penicillin G
DE	15	17608	17492	saccharin	Saccharin
DE	16	17608	17492	propidium iodide	Propidium Iodide
ID	1	17613	17497	STEM-CELLS	Stem
ID	2	17613	17497	CYCLE	Cycle
ID	3	17613	17497	MOUSE	Mouse
ID	4	17613	17497	PROLIFERATION	Proliferation
ID	5	17613	17497	NEUROGENESIS	Neurogenesis
ID	6	17613	17497	REGULATORS	Regulator
ID	7	17613	17497	INHIBITORS	Inhibitor
ID	8	17613	17497	APOPTOSIS	Apoptosis
ID	9	17613	17497	BRDU	Brdu

Record 111 | WOS:000984555200005

Microcapsules loaded with date seed extract and its inhibitory potential to modulate the toxic effects of mycotoxins in mice received mold-contaminated diet

Field	Position	Original line	Normalised line	Original value	Normalised value
DE	1	17731	17614	Date seed	Date Seed
DE	3	17731	17614	Microcapsules	Microcapsule
DE	5	17731	17614	Natural bioactive compounds	Natural Bioactive Compound
ID	1	17733	17616	BIOACTIVE COMPOUNDS	Bioactive Compound
ID	2	17733	17616	ANTIOXIDANT	Antioxidant
ID	3	17733	17616	MICROENCAPSULATION	Microencapsulation
ID	4	17733	17616	PERFORMANCE	Performance
ID	5	17733	17616	EXPRESSION	Expression
ID	6	17733	17616	CAPACITY	Capacity
ID	7	17733	17616	EFFICACY	Efficacy
ID	8	17733	17616	HUMANS	Human
ID	9	17733	17616	FEED	Feed

Record 112 | WOS:000951983500001

Hazard characterisation for significant mycotoxins in food

Field	Position	Original line	Normalised line	Original value	Normalised value
DE	3	17830	17712	Health risk assessment	Health Risk Assessment
DE	4	17830	17712	Hazard characterisation	Hazard Characterisation
DE	5	17830	17712	Total diet study	Total Diet Study
ID	1	17832	17714	EXPOSURE MOE APPROACH	Exposure Moe Approach
ID	2	17832	17714	CYCLOPIAZONIC ACID	Cyclopiazonic Acid
ID	3	17832	17714	ERGOT ALKALOIDS	Ergot Alkaloid
ID	4	17832	17714	HUMAN HEALTH	Human Health
ID	5	17832	17714	T-2 TOXIN	T 2 Toxin
ID	6	17832	17714	SAFETY ASSESSMENT	Safety Assessment
ID	7	17832	17714	DIETARY EXPOSURE	Dietary Exposure
ID	8	17832	17714	CHRONIC TOXICITY	Chronic Toxicity
ID	9	17832	17714	RISK ASSESSMENT	Risk Assessment
ID	10	17832	17714	IN-VIVO	In-Vivo

Record 113 | WOS:000958249600001

The Effect of Mushroom Culture Filtrates on the Inhibition of Mycotoxins Produced by Aspergillus flavus and Aspergillus carbonarius

Field	Position	Original line	Normalised line	Original value	Normalised value
DE	1	18052	17933	aflatoxin B-1	Aflatoxin B1 (AFB1)
DE	2	18052	17933	ochratoxin A	Ochratoxin A (OTA)
DE	3	18052	17933	Schizophyllum commune	Schizophyllum Commune
DE	4	18052	17933	schizophyllan	Schizophyllan

DE	5	18052	17933	biological control	Biological Control
ID	1	18054	17935	AFLATOXIN BIOSYNTHESIS	Aflatoxin Biosynthesis
ID	2	18054	17935	PARASITICUS	Parasiticus
ID	3	18054	17935	FOOD	Food
ID	4	18054	17935	TOXICITY	Toxicity
ID	5	18054	17935	GROWTH	Growth
ID	6	18054	17935	MAIZE	Maize

Record 114 | WOS:000955634100001

Combined Effects of Acrylamide and Ochratoxin A on the Intestinal Barrier in Caco-2 Cells

Field	Position	Original line	Normalised line	Original value	Normalised value
DE	1	18164	18044	acrylamide	Acrylamide (AA)
DE	2	18164	18044	ochratoxin A	Ochratoxin A (OTA)
DE	3	18164	18044	Caco-2 cells	Caco-2
DE	4	18164	18044	tight junction	Tight Junction
DE	5	18164	18044	interactive effects	Interactive Effect
ID	1	18166	18046	MYCOTOXINS	Mycotoxins
ID	2	18166	18046	CEREALS	Cereal
ID	3	18166	18046	CYTOTOXICITY	Cytotoxicity
ID	4	18166	18046	ZEARALENONE	Zearalenone (ZEA)
ID	5	18166	18046	METABOLISM	Metabolism
ID	6	18166	18046	FOODS	Food

Record 115 | WOS:000960233300001

Immunotoxicity of Three Environmental Mycotoxins and Their Risks of Increasing Pathogen Infections

Field	Position	Original line	Normalised line	Original value	Normalised value
DE	1	18277	18156	mycotoxins	Mycotoxins
DE	2	18277	18156	aflatoxin B1	Aflatoxin B1 (AFB1)
DE	3	18277	18156	ochratoxin A	Ochratoxin A (OTA)
DE	4	18277	18156	deoxynivalenol	Deoxynivalenol (DON)
DE	5	18277	18156	bidirectional immunotoxicity	Bidirectional Immunotoxicity
DE	6	18277	18156	pathogen infections	Pathogen Infection
ID	1	18279	18158	DEOXYNIVALENOL DON MYCOTOXIN	Deoxynivalenol DON Mycotoxin
ID	2	18279	18158	IN-VITRO	In-Vitro
ID	3	18279	18158	GUT MICROBIOTA	Gut Microbiota
ID	4	18279	18158	MOLECULAR-MECHANISMS	Molecular Mechanism
ID	5	18279	18158	SIGNALING PATHWAY	Signaling Pathway
ID	6	18279	18158	OCHRATOXIN-A	Ochratoxin A (OTA)
ID	7	18279	18158	AFLATOXIN	Aflatoxin
ID	8	18279	18158	VIVO	Vivo
ID	9	18279	18158	TRICHOTHECENES	Trichothecene
ID	10	18279	18158	NEPHROTOXICITY	Nephrotoxicity

Record 116 | WOS:000944193800011

Dietary Ochratoxin A Contamination Modulates Oxidative Stress, Inflammation Processes and Causes Fibrosis in in vitro and in vivo Lung Models

Field	Position	Original line	Normalised line	Original value	Normalised value
DE	1	18411	18289	ochratoxin A	Ochratoxin A (OTA)
DE	2	18411	18289	lung aging	Lung Aging
DE	3	18411	18289	lung fibrosis	Lung Fibrosis
DE	4	18411	18289	inflammation	Inflammation
DE	5	18411	18289	oxidative stress	Oxidative Stress
ID	1	18412	18291	CELLULAR SENESCENCE	Cellular Senescence
ID	2	18412	18291	DNA-DAMAGE	DNA Damage
ID	3	18412	18291	TOXICITY	Toxicity

Record 117 | WOS:000942033800001

Comparative Assessment of Different Yeast Cell Wall-Based Mycotoxin Adsorbents Using a Model- and Bioassay-Based In Vitro Approach

Field	Position	Original line	Normalised line	Original value	Normalised value
DE	1	18509	18387	adsorption	Adsorption
DE	2	18509	18387	gastrointestinal digestion model	Gastrointestinal Digestion Model
DE	3	18509	18387	isotherm models	Isotherm Model
DE	4	18509	18387	in vitro cell culture	In Vitro Cell
DE	5	18509	18387	liquid chromatography	Liquid Chromatography
DE	6	18509	18387	novel detoxification strategies	Novel Detoxification Strategy
DE	7	18509	18387	remediation	Remediation
DE	8	18509	18387	ruminants	Ruminant
DE	9	18509	18387	sequestration	Sequestration
DE	10	18509	18387	toxicity	Toxicity
ID	1	18512	18390	AFLATOXIN B-1	Aflatoxin B1 (AFB1)
ID	2	18512	18390	ADSORPTION	Adsorption
ID	3	18512	18390	ZEARALENONE	Zearalenone (ZEA)
ID	4	18512	18390	MILK	Milk
ID	5	18512	18390	COMPLEXATION	Complexation
ID	6	18512	18390	PRODUCTS	Product
ID	7	18512	18390	EXPOSURE	Exposure
ID	8	18512	18390	EFFICACY	Efficacy
ID	9	18512	18390	EXTRACT	Extract
ID	10	18512	18390	BARRIER	Barrier

Record 118 | WOS:000929015600001

Potential application of probiotics in mycotoxicosis reduction in mammals and poultry

Field	Position	Original line	Normalised line	Original value	Normalised value
DE	1	18639	18516	Lactic acid bacteria	Lactic Acid Bacteria
DE	2	18639	18516	mycotoxin	Mycotoxins
DE	3	18639	18516	toxicity	Toxicity
DE	4	18639	18516	yeast	Yeast
DE	5	18639	18516	in vivo	In-Vivo
ID	1	18640	18517	LACTIC-ACID BACTERIA	Lactic Acid Bacteria
ID	2	18640	18517	BACILLUS-SUBTILIS ANSB060	Bacillus Subtilis Ansb060
ID	3	18640	18517	FECAL WATER GENOTOXICITY	Fecal Water Genotoxicity
ID	4	18640	18517	HISTOPATHOLOGICAL CHANGES	Histopathological Change
ID	5	18640	18517	SACCHAROMYCES-CEREVISIAE	Saccharomyce Cerevisiae
ID	6	18640	18517	LACTOBACILLUS-CASEI	Lactobacillus Casei
ID	7	18640	18517	SERUM BIOCHEMISTRY	Serum Biochemistry
ID	8	18640	18517	DNA-FRAGMENTATION	DNA Fragmentation
ID	9	18640	18517	OXIDATIVE STRESS	Oxidative Stress
ID	10	18640	18517	BROILER-CHICKENS	Broiler Chicken

Record 119 | WOS:000918850400001

Lonp1 and Sig-1R contribute to the counteraction of ursolic acid against ochratoxin A-induced mitochondrial apoptosis

Field	Position	Original line	Normalised line	Original value	Normalised value
DE	1	18784	18659	Mycotoxin	Mycotoxins
DE	2	18784	18659	Pentacyclic triterpenoid	Pentacyclic Triterpenoid

DE	3	18784	18659	Endoplasmic reticulum stress	Endoplasmic Reticulum Stress
DE	6	18784	18659	Mitochondria-associated ER membranes	Mitochondria Associated Er Membrane

Record 120 | WOS:001000273300006

Protective role of fermented whey and pumpkin extract against aflatoxin B1 and ochratoxin A toxicity in Jurkat T-cells

Field	Position	Original line	Normalised line	Original value	Normalised value
DE	1	18864	18738	aflatoxin B-1	Aflatoxin B1 (AFB1)
DE	2	18864	18738	ochratoxin A	Ochratoxin A (OTA)
DE	3	18864	18738	proteomics	Proteomics
DE	4	18864	18738	Jurkat cells	Jurkat
DE	5	18864	18738	LC/MS/Q-TOF	Lc Ms Q Tof
ID	1	18865	18739	IN-VITRO	In-Vitro
ID	2	18865	18739	REDUCTION	Reduction

Record 121 | WOS:000915148500001

AFB1 and OTA Promote Immune Toxicity in Human LymphoBlastic T Cells at Transcriptomic Level

Field	Position	Original line	Normalised line	Original value	Normalised value
DE	1	18973	18846	mycotoxins	Mycotoxins
DE	2	18973	18846	Jurkat cells	Jurkat
DE	3	18973	18846	RNA-sequencing	RNA Sequencing
DE	4	18973	18846	mechanism of action	Mechanism Of Action
DE	5	18973	18846	immune toxicity	Immune Toxicity
ID	1	18975	18847	METABOLITE OCHRATOXIN ALPHA	Metabolite Ochratoxin Alpha
ID	2	18975	18847	UBIQUITIN LIGASE	Ubiquitin Ligase
ID	3	18975	18847	GENE-EXPRESSION	Gene Expression
ID	4	18975	18847	AFLATOXIN B-1	Aflatoxin B1 (AFB1)
ID	5	18975	18847	MOLECULAR-MECHANISMS	Molecular Mechanism
ID	6	18975	18847	GLUCOSE-METABOLISM	Glucose Metabolism
ID	7	18975	18847	SIGNALING PATHWAY	Signaling Pathway
ID	8	18975	18847	PROTECTIVE ROLE	Protective Role
ID	9	18975	18847	URINE SAMPLES	Urine Sample
ID	10	18975	18847	CYCLE ARREST	Cycle Arrest

Record 122 | WOS:000912325600001

Toxico-pathological effects of ochratoxin A and its diastereoisomer under in ovo conditions and in vitro evaluation of the toxicity of these toxins against the embryo Gallus gallus fibroblast cell line

Field	Position	Original line	Normalised line	Original value	Normalised value
DE	1	19169	19040	ochratoxin A	Ochratoxin A (OTA)
DE	2	19169	19040	2?R-ochratoxin A	2 R Ochratoxin A
DE	3	19169	19040	embryotoxicity	Embryotoxicity
DE	4	19169	19040	Gallus gallus	Gallus Gallus
ID	1	19170	19041	DEGRADATION-PRODUCT	Degradation Product
ID	2	19170	19041	CYTOTOXICITY	Cytotoxicity
ID	3	19170	19041	QUALITY	Quality
ID	4	19170	19041	FEED	Feed

Record 123 | WOS:000900483500001

Modified Mycotoxins, a Still Unresolved Issue

Field	Position	Original line	Normalised line	Original value	Normalised value

DE	1	19282	19152	modified mycotoxins	Modified Mycotoxin
DE	2	19282	19152	metabolites	Metabolite
DE	3	19282	19152	risk assessment	Risk Assessment
DE	4	19282	19152	dose additivity	Dose Additivity
DE	5	19282	19152	toxicity	Toxicity
DE	6	19282	19152	exposure	Exposure
ID	1	19284	19154	NATURALLY CONTAMINATED WHEAT	Naturally Contaminated Wheat
ID	2	19284	19154	MASKED MYCOTOXINS	Masked Mycotoxin
ID	3	19284	19154	FUSARIUM MYCOTOXINS	Fusarium Mycotoxin
ID	4	19284	19154	OCHRATOXIN-A	Ochratoxin A (OTA)
ID	5	19284	19154	HT-2 TOXIN	Ht 2 Toxin
ID	6	19284	19154	T-2 TOXIN	T 2 Toxin
ID	7	19284	19154	IN-VITRO	In-Vitro
ID	8	19284	19154	SUSPENSION-CULTURES	Suspension
ID	9	19284	19154	DEOXYNIVALENOL	Deoxynivalenol (DON)
ID	10	19284	19154	METABOLISM	Metabolism

Record 124 | WOS:000898846200001

Innovative in vitro approaches to toxicological investigations of mycotoxins effects

Field	Position	Original line	Normalised line	Original value	Normalised value
DE	1	19483	19352	Food contaminantsin vitro modelstoxicologymycotoxins	Food Contaminantsin Vitro Modelstoxicologymycotoxin
ID	1	19484	19353	INTESTINAL BARRIER	Intestinal Barrier
ID	2	19484	19353	COCULTURE SYSTEM	Coculture System
ID	3	19484	19353	CELL-CULTURE	Cell
ID	4	19484	19353	MODEL	Model
ID	5	19484	19353	CACO-2	Caco-2
ID	6	19484	19353	BIOACTIVITY	Bioactivity
ID	7	19484	19353	METABOLISM	Metabolism
ID	8	19484	19353	TOXICITY	Toxicity
ID	9	19484	19353	EVALUATE	Evaluate
ID	10	19484	19353	LINE	Line

Record 125 | WOS:000903192900001

Possible Mechanisms of the Interplay between Drugs and Mycotoxins-Is There a Possible Impact?

Field	Position	Original line	Normalised line	Original value	Normalised value
DE	1	19585	19453	food-drug interaction	Food Drug Interaction
DE	2	19585	19453	mycotoxins	Mycotoxins
DE	3	19585	19453	CYP450-enzymes	Cyp450 Enzyme
DE	4	19585	19453	human	Human
DE	5	19585	19453	pharmacotherapeutics	Pharmacotherapeutics
DE	6	19585	19453	drugs	Drug
DE	7	19585	19453	PBPK-modelling	Pbpk Modelling
ID	1	19587	19455	AFLATOXIN B-1 BIOTRANSFORMATION	Aflatoxin B 1 Biotransformation
ID	2	19587	19455	ARYL-HYDROCARBON RECEPTOR	Aryl Hydrocarbon Receptor
ID	3	19587	19455	MESSENGER-RNA EXPRESSION	Messenger RNA Expression
ID	4	19587	19455	IN-VITRO	In-Vitro
ID	5	19587	19455	METABOLIZING-ENZYMES	Metabolizing Enzyme
ID	6	19587	19455	CYTOCHROME-P450 ENZYMES	Cytochrome P450 Enzyme
ID	7	19587	19455	DEEPOXY-DEOXYNIVALENOL	Deepoxy Deoxynivalenol
ID	8	19587	19455	FUSARIUM-MYCOTOXINS	Fusarium Mycotoxin
ID	9	19587	19455	PRIMARY CULTURES	Primary

ID	10	19587	19455	FUMONISIN B-1	Fumonisin B1 (FB1)

Record 126 | WOS:000888695100001

Mycotoxins and cellular senescence: the impact of oxidative stress, hypoxia, and immunosuppression

Field	Position	Original line	Normalised line	Original value	Normalised value
DE	2	19751	19617	Cellular senescence	Cellular Senescence
DE	3	19751	19617	Oxidative stress	Oxidative Stress
DE	4	19751	19617	p53	P53
DE	5	19751	19617	Cell cycle arrest	Cell Cycle Arrest
ID	1	19753	19618	OCHRATOXIN-A	Ochratoxin A (OTA)
ID	2	19753	19618	DOWN-REGULATION	Down Regulation
ID	3	19753	19618	ROS GENERATION	ROS Generation
ID	4	19753	19618	AFLATOXIN B-1	Aflatoxin B1 (AFB1)
ID	5	19753	19618	CYCLE ARREST	Cycle Arrest
ID	6	19753	19618	DNA-DAMAGE	DNA Damage
ID	7	19753	19618	T-2 TOXIN	T 2 Toxin
ID	8	19753	19618	APOPTOSIS	Apoptosis
ID	9	19753	19618	CELLS	Cell
ID	10	19753	19618	CANCER	Cancer

Record 127 | WOS:000892528400007

The efficacy of mycotoxin binders to control mycotoxins in feeds and the potential risk of interactions with nutrient: a review

Field	Position	Original line	Normalised line	Original value	Normalised value
DE	1	19908	19772	efficacy	Efficacy
DE	2	19908	19772	interaction	Interaction
DE	3	19908	19772	mycotoxin	Mycotoxins
DE	4	19908	19772	mycotoxin binder	Mycotoxin Binder
DE	5	19908	19772	review	Review
ID	1	19909	19773	SODIUM-CALCIUM ALUMINOSILICATE	Sodium Calcium Aluminosilicate
ID	2	19909	19773	YEAST-BASED PRODUCTS	Yeast Based Product
ID	3	19909	19773	IN-VITRO AFFINITY	In Vitro Affinity
ID	4	19909	19773	AFLATOXIN B-1	Aflatoxin B1 (AFB1)
ID	5	19909	19773	VITAMIN-A	Vitamin A
ID	6	19909	19773	SEQUESTERING AGENTS	Sequestering Agent
ID	7	19909	19773	ADSORBENT MATERIALS	Adsorbent Material
ID	8	19909	19773	ADSORPTION ABILITY	Adsorption Ability
ID	9	19909	19773	BINDING EFFICACY	Binding Efficacy
ID	10	19909	19773	ACTIVATED CARBON	Activated Carbon

Record 128 | WOS:000886894200008

Protective effect of selenomethionine on kidney injury induced by ochratoxin A in rabbits

Field	Position	Original line	Normalised line	Original value	Normalised value
DE	2	20064	19927	Selenomethionine (SeMet)	Selenomethionine Semet
DE	3	20064	19927	Kidney injury	Kidney Injury
DE	4	20064	19927	Nrf2/HO-1	Nrf2 Ho 1
DE	5	20064	19927	Oxidative stress	Oxidative Stress
DE	6	20064	19927	Inflammatory response	Inflammatory Response
ID	1	20066	19929	SELENOPROTEIN GENE-EXPRESSION	Selenoprotein Gene Expression
ID	2	20066	19929	LIPID-PEROXIDATION	Lipid Peroxidation
ID	3	20066	19929	OXIDATIVE STRESS	Oxidative Stress
ID	4	20066	19929	SELENIUM DEFICIENCY	Selenium Deficiency
ID	5	20066	19929	VITAMIN-E	Vitamin E
ID	6	20066	19929	DIETARY SELENIUM	Dietary Selenium

ID	7	20066	19929	IN-VITRO	In-Vitro
ID	8	20066	19929	TOXICITY	Toxicity
ID	9	20066	19929	APOPTOSIS	Apoptosis
ID	10	20066	19929	MECHANISMS	Mechanism

Record 129 | WOS:000896944500005

In vitro study of ochratoxin A in the expression of genes associated with neuron survival and viability

Field	Position	Original line	Normalised line	Original value	Normalised value
DE	1	20198	20060	Mycotoxin	Mycotoxins
DE	2	20198	20060	Ochratoxin A	Ochratoxin A (OTA)
DE	5	20198	20060	BDNF	Bdnf
ID	1	20199	20061	CELL-DEATH	Cell Death
ID	2	20199	20061	EXPOSURE	Exposure
ID	3	20199	20061	TAU	Tau
ID	5	20199	20061	BIOMARKERS	Biomarker
ID	6	20199	20061	APOPTOSIS	Apoptosis
ID	7	20199	20061	TOXICITY	Toxicity
ID	8	20199	20061	PATHWAYS	Pathway
ID	9	20199	20061	PROTEIN	Protein
ID	10	20199	20061	URINE	Urine

Record 130 | WOS:000974547800001

Low doses of fumonisin B1 exacerbate ochratoxin A-induced renal injury in mice and the protective roles of heat shock protein 70

Field	Position	Original line	Normalised line	Original value	Normalised value
DE	1	20328	20189	Fumonisins B1	Fumonisin B1 (FB1)
DE	2	20328	20189	Ochratoxin A	Ochratoxin A (OTA)
DE	3	20328	20189	Heat shock protein 70	Heat Shock Protein 70
DE	4	20328	20189	Mitochondrial reactive oxygen species	Mitochondrial Reactive Oxygen Specy
DE	5	20328	20189	c-Jun N-Terminal kinase	C Jun N Terminal Kinase
DE	6	20328	20189	Protective effects	Protective Effect
ID	1	20330	20192	MAPK SIGNALING PATHWAY	Mapk Signaling Pathway
ID	2	20330	20192	OXIDATIVE STRESS	Oxidative Stress
ID	3	20330	20192	MULTIPLE MYCOTOXINS	Multiple Mycotoxin
ID	4	20330	20192	INDUCED APOPTOSIS	Induced Apoptosis
ID	5	20330	20192	CELLS	Cell
ID	6	20330	20192	TOXICITY	Toxicity
ID	7	20330	20192	KIDNEY	Kidney
ID	8	20330	20192	JNK	Jnk
ID	9	20330	20192	MECHANISMS	Mechanism
ID	10	20330	20192	INHIBITOR	Inhibitor

Record 131 | WOS:000887299000001

Mycotoxins and Essential Oils-From a Meat Industry Hazard to a Possible Solution: A Brief Review

Field	Position	Original line	Normalised line	Original value	Normalised value
DE	1	20472	20333	meat	Meat
DE	2	20472	20333	meat products	Meat Product
DE	3	20472	20333	essential oils	Essential Oil
DE	4	20472	20333	mycotoxins	Mycotoxins
DE	5	20472	20333	antifungal activity	Antifungal Activity
DE	6	20472	20333	food hazard	Food Hazard
DE	7	20472	20333	consumers' health	Consumer Health
ID	1	20474	20335	LIVER-CANCER RISK	Liver Cancer Risk
ID	2	20474	20335	FUSARIUM MYCOTOXINS	Fusarium Mycotoxin
ID	3	20474	20335	OCHRATOXIN-A	Ochratoxin A (OTA)
ID	4	20474	20335	FUSARENON-X	Fusarenon X

ID	5	20474	20335	CHEMICAL-COMPOSITION	Chemical Composition
ID	6	20474	20335	NATURAL OCCURRENCE	Natural Occurrence
ID	7	20474	20335	THYMUS-VULGARIS	Thymus Vulgaris
ID	8	20474	20335	PROCESSED MEAT	Processed Meat
ID	9	20474	20335	AFLATOXIN B-1	Aflatoxin B1 (AFB1)
ID	10	20474	20335	HEALTH-RISK	Health Risk

Record 132 ǀ WOS:000894684300001

Co-Contamination of Food and Feed with Mycotoxin and Bacteria and Possible Implications for Health

Field	Position	Original line	Normalised line	Original value	Normalised value
DE	1	20683	20543	mycotoxin	Mycotoxins
DE	2	20683	20543	bacteria	Bacteria
DE	3	20683	20543	oxidative stress	Oxidative Stress
DE	4	20683	20543	inflammation	Inflammation
ID	1	20684	20544	OXIDATIVE STRESS	Oxidative Stress
ID	2	20684	20544	OCHRATOXIN	Ochratoxin A (OTA)
ID	3	20684	20544	APOPTOSIS	Apoptosis
ID	4	20684	20544	LIPOPOLYSACCHARIDE	Lipopolysaccharide
ID	5	20684	20544	ZEARALENONE	Zearalenone (ZEA)
ID	6	20684	20544	POULTRY	Poultry
ID	7	20684	20544	CELLS	Cell

Record 133 ǀ WOS:000883142900001

Current Review of Mycotoxin Biodegradation and Bioadsorption: Microorganisms, Mechanisms, and Main Important Applications

Field	Position	Original line	Normalised line	Original value	Normalised value
DE	1	20798	20657	mycotoxins	Mycotoxins
DE	2	20798	20657	aflatoxins	Aflatoxin
DE	3	20798	20657	contamination	Contamination
DE	4	20798	20657	microorganisms	Microorganism
DE	5	20798	20657	biodegradation	Biodegradation
DE	6	20798	20657	enzymes	Enzyme
ID	1	20800	20658	LACTIC-ACID BACTERIA	Lactic Acid Bacteria
ID	2	20800	20658	AFLATOXIN DEGRADATION ENZYME	Aflatoxin Degradation Enzyme
ID	3	20800	20658	IN-VITRO	In-Vitro
ID	4	20800	20658	SACCHAROMYCES-CEREVISIAE	Saccharomyce Cerevisiae
ID	5	20800	20658	OCHRATOXIN-A	Ochratoxin A (OTA)
ID	6	20800	20658	BIOLOGICAL DEGRADATION	Biological Degradation
ID	7	20800	20658	OXIDATIVE STRESS	Oxidative Stress
ID	8	20800	20658	FUMONISIN B-1	Fumonisin B1 (FB1)
ID	9	20800	20658	APPLE JUICE	Apple Juice
ID	10	20800	20658	TRICHOTHECENE MYCOTOXINS	Trichothecene Mycotoxin

Record 134 ǀ WOS:000867775600001

The Effect of Aflatoxin B1 on Tumor-Related Genes and Phenotypic Characters of MCF7 and MCF10A Cells

Field	Position	Original line	Normalised line	Original value	Normalised value
DE	1	21059	20916	aflatoxin B1	Aflatoxin B1 (AFB1)
DE	2	21059	20916	MCF7	Mcf7
DE	3	21059	20916	MCF10A	Mcf10a
DE	4	21059	20916	carcinogen	Carcinogen
DE	5	21059	20916	breast cancer	Breast Cancer
ID	1	21060	20917	BREAST-CANCER	Breast Cancer
ID	2	21060	20917	DNA-DAMAGE	DNA Damage

ID	3	21060	20917	IN-VITRO	In-Vitro
ID	4	21060	20917	C-MYC	C Myc
ID	5	21060	20917	MULTIDRUG-RESISTANCE	Multidrug Resistance
ID	6	21060	20917	BIOLOGICAL RESPONSE	Biological Response
ID	7	21060	20917	CYCLE REGULATION	Cycle Regulation
ID	8	21060	20917	CARCINOMA-CELLS	Carcinoma
ID	9	21060	20917	DOWN-REGULATION	Down Regulation
ID	10	21060	20917	OCHRATOXIN-A	Ochratoxin A (OTA)

Record 135 | WOS:000911765600013

Ochratoxin A induced differentiation nephrotoxicity in renal tubule and glomeruli via autophagy differential regulation

Field	Position	Original line	Normalised line	Original value	Normalised value
DE	1	21233	21089	Ochratoxin A	Ochratoxin A (OTA)
DE	4	21233	21089	Renal tubules	Renal Tubule
ID	1	21234	21090	PROXIMAL TUBULE	Proximal Tubule
ID	2	21234	21090	MESANGIAL CELLS	Mesangial
ID	3	21234	21090	TOXICOKINETICS	Toxicokinetics
ID	4	21234	21090	MECHANISMS	Mechanism
ID	5	21234	21090	EXPRESSION	Expression
ID	6	21234	21090	TOXICITY	Toxicity

Record 136 | WOS:000866918500001

Quercetin: Its Antioxidant Mechanism, Antibacterial Properties and Potential Application in Prevention and Control of Toxipathy

Field	Position	Original line	Normalised line	Original value	Normalised value
DE	1	21338	21192	quercetin	Quercetin
DE	2	21338	21192	antioxidant	Antioxidant
DE	3	21338	21192	toxipathy	Toxipathy
ID	1	21339	21193	INDUCED OXIDATIVE STRESS	Induced Oxidative Stress
ID	2	21339	21193	HUMAN-LIVER-MICROSOMES	Human Liver Microsome
ID	3	21339	21193	NF-KAPPA-B	Nf Kappa B
ID	4	21339	21193	SECONDARY METABOLITE	Secondary Metabolite
ID	5	21339	21193	INDUCED APOPTOSIS	Induced Apoptosis
ID	6	21339	21193	BETA-ZEARALENOL	Beta Zearalenol
ID	7	21339	21193	GENE-EXPRESSION	Gene Expression
ID	8	21339	21193	AFLATOXIN B-1	Aflatoxin B1 (AFB1)
ID	9	21339	21193	UP-REGULATION	Up Regulation
ID	10	21339	21193	OCHRATOXIN-A	Ochratoxin A (OTA)

Record 137 | WOS:000872026500001

Luteolin and Chrysin Could Prevent E. coli Lipopolysaccharide-Ochratoxin A Combination-Caused Inflammation and Oxidative Stress in In Vitro Porcine Intestinal Model

Field	Position	Original line	Normalised line	Original value	Normalised value
DE	1	21553	21406	ochratoxin	Ochratoxin A (OTA)
DE	2	21553	21406	IPEC-J2 cells	IPEC-J2
DE	3	21553	21406	oxidative stress	Oxidative Stress
DE	4	21553	21406	interleukins	Interleukin
DE	5	21553	21406	flavonoids	Flavonoid
DE	6	21553	21406	lipopolysaccharide	Lipopolysaccharide
DE	7	21553	21406	cytotoxicity	Cytotoxicity
ID	1	21555	21408	ESCHERICHIA-COLI	Escherichia Coli
ID	2	21555	21408	IPEC-J2 CELLS	IPEC-J2
ID	3	21555	21408	ENERGY STATUS	Energy Status
ID	4	21555	21408	PIGLETS	Piglet
ID	5	21555	21408	MYCOTOXINS	Mycotoxins
ID	6	21555	21408	QUERCETIN	Quercetin

ID	7	21555	21408	APOPTOSIS	Apoptosis
ID	8	21555	21408	LINE	Line
ID	9	21555	21408	GUT	Gut

Record 138 | WOS:000864053100001

In-vitro alleviation of ochratoxin a mediated cytotoxicity using N-acetyl-L-tryptophan glucoside pretreatment

Field	Position	Original line	Normalised line	Original value	Normalised value
DE	2	21662	21514	Molecular docking	Molecular Docking
DE	4	21662	21514	N-acetyl-L-tryptophan glucoside	N Acetyl L Tryptophan Glucoside
DE	5	21662	21514	HEK-293 cells	Hek 293
DE	6	21662	21514	Oxidative stress	Oxidative Stress
DE	7	21662	21514	Mitochondrial damage	Mitochondrial Damage
ID	1	21665	21516	TOXICITY	Toxicity
ID	2	21665	21516	TOXICOKINETICS	Toxicokinetics
ID	3	21665	21516	APOPTOSIS	Apoptosis
ID	4	21665	21516	ASSAY	Assay
ID	5	21665	21516	DUST	Dust

Record 139 | WOS:000884231200001

Aflatoxin M1 and ochratoxin A induce a competitive endogenous RNA regulatory network of intestinal immunosuppression by whole-transcriptome analysis

Field	Position	Original line	Normalised line	Original value	Normalised value
DE	1	21760	21610	Aflatoxin M1	Aflatoxin M1 (AFM1)
DE	2	21760	21610	Differentially expressed RNA	Differentially Expressed RNA
DE	4	21760	21610	Intestinal toxicity	Intestinal Toxicity
DE	5	21760	21610	Ochratoxin A	Ochratoxin A (OTA)
DE	6	21760	21610	Whole transcriptome	Whole Transcriptome
ID	1	21762	21612	CELLS	Cell
ID	2	21762	21612	M-1	M 1
ID	3	21762	21612	EXPRESSION	Expression
ID	4	21762	21612	CHALLENGES	Challenge
ID	5	21762	21612	ALPHA	Alpha
ID	6	21762	21612	GENE	Gene

Record 140 | WOS:000876186700006

The importance of transcriptomics and proteomics for studying molecular mechanisms of mycotoxin exposure: A review

Field	Position	Original line	Normalised line	Original value	Normalised value
DE	1	21890	21739	Mycotoxin	Mycotoxins
DE	4	21890	21739	Gene expression	Gene Expression
DE	5	21890	21739	LC-MS	Lc Ms
DE	6	21890	21739	MS	Ms
ID	1	21891	21740	AFLATOXIN M1	Aflatoxin M1 (AFM1)
ID	2	21891	21740	ZEARALENONE	Zearalenone (ZEA)
ID	3	21891	21740	DEOXYNIVALENOL	Deoxynivalenol (DON)
ID	4	21891	21740	CYTOTOXICITY	Cytotoxicity
ID	5	21891	21740	REVEALS	Reveal

Record 141 | WOS:000851604100001

Mycotoxins in soybean-based foods fermented with filamentous fungi: Occurrence and preventive strategies

Field	Position	Original line	Normalised line	Original value	Normalised value
DE	1	22016	21865	fermented food	Fermented Food
DE	2	22016	21865	filamentous fungi	Filamentous Fungi
DE	3	22016	21865	mycotoxin	Mycotoxins
DE	4	22016	21865	prevention strategy	Prevention Strategy

DE	5	22016	21865	soybean	Soybean
ID	1	22018	21866	FUSARIUM HEAD BLIGHT	Fusarium Head Blight
ID	2	22018	21866	AFLATOXIN B-1	Aflatoxin B1 (AFB1)
ID	3	22018	21866	NATURAL OCCURRENCE	Natural Occurrence
ID	4	22018	21866	WORLDWIDE OCCURRENCE	Worldwide Occurrence
ID	5	22018	21866	BIOLOGICAL-CONTROL	Biological Control
ID	6	22018	21866	WATER ACTIVITY	Water Activity
ID	7	22018	21866	OCHRATOXIN-A	Ochratoxin A (OTA)
ID	8	22018	21866	ANIMAL FEED	Animal Feed
ID	9	22018	21866	IN-VITRO	In-Vitro
ID	10	22018	21866	DEOXYNIVALENOL	Deoxynivalenol (DON)

Record 142 | WOS:000851281600001

Mechanisms underlying citrinin-induced toxicity via oxidative stress and apoptosis-mediated by mitochondrial-dependent pathway in SH-SY5Y cells

Field	Position	Original line	Normalised line	Original value	Normalised value
DE	2	22214	22061	citrinin	Citrinin
DE	3	22214	22061	neurotoxicity	Neurotoxicity
DE	4	22214	22061	reactive oxygen species	Reactive Oxygen Specy
DE	5	22214	22061	SH-SY5Y cells	SH-SY5Y
ID	1	22216	22062	MYCOTOXIN CITRININ	Mycotoxin Citrinin
ID	2	22216	22062	INDUCED DYSFUNCTION	Induced Dysfunction
ID	3	22216	22062	OCHRATOXIN-A	Ochratoxin A (OTA)
ID	4	22216	22062	HEPG2 CELLS	HepG2
ID	5	22216	22062	DNA-DAMAGE	DNA Damage
ID	6	22216	22062	IN-VITRO	In-Vitro
ID	7	22216	22062	ACTIVATION	Activation
ID	8	22216	22062	INHIBITION	Inhibition
ID	9	22216	22062	INVOLVEMENT	Involvement
ID	10	22216	22062	INDUCTION	Induction

Record 143 | WOS:000862185500005

Co-occurrence of mycotoxins: A review on bioanalytical methods for simultaneous analysis in human biological samples, mixture toxicity and risk assessment strategies

Field	Position	Original line	Normalised line	Original value	Normalised value
DE	1	22358	22203	Bioanalytical methods	Bioanalytical Method
DE	4	22358	22203	Multi-mycotoxins	Multi Mycotoxin
DE	5	22358	22203	Risk assessment	Risk Assessment
ID	1	22360	22205	MASS-SPECTROMETRIC METHOD	Mass Spectrometric Method
ID	2	22360	22205	HIGH-THROUGHPUT METHOD	High Throughput Method
ID	3	22360	22205	IN-VITRO	In-Vitro
ID	4	22360	22205	MULTI-MYCOTOXIN	Multi Mycotoxin
ID	5	22360	22205	FUSARIUM MYCOTOXINS	Fusarium Mycotoxin
ID	6	22360	22205	QUANTITATIVE-DETERMINATION	Quantitative Determination
ID	7	22360	22205	DIETARY EXPOSURE	Dietary Exposure
ID	8	22360	22205	ALPHA-ZEARALENOL	Alpha Zearalenol
ID	9	22360	22205	OCHRATOXIN-A	Ochratoxin A (OTA)
ID	10	22360	22205	BREAST-MILK	Breast Milk

Record 144 | WOS:000857038900001

The Neurotoxic Effect of Ochratoxin-A on the Hippocampal Neurogenic Niche of Adult Mouse Brain

Field	Position	Original line	Normalised line	Original value	Normalised value
DE	1	22548	22392	ochratoxin A	Ochratoxin A (OTA)
DE	2	22548	22392	brain	Brain
DE	3	22548	22392	hippocampus	Hippocampus
DE	4	22548	22392	neurogenic niche	Neurogenic Niche
DE	5	22548	22392	neurotoxicity	Neurotoxicity
DE	6	22548	22392	cell morphology	Cell Morphology
ID	1	22550	22394	NEURAL STEM-CELLS	Neural Stem
ID	2	22550	22394	SUBVENTRICULAR ZONE	Subventricular Zone
ID	3	22550	22394	RAT-BRAIN	Rat Brain
ID	4	22550	22394	TOXICITY	Toxicity
ID	5	22550	22394	ASTROCYTES	Astrocyte
ID	6	22550	22394	FOOD	Food
ID	7	22550	22394	CYTOTOXICITY	Cytotoxicity
ID	8	22550	22394	APOPTOSIS	Apoptosis
ID	9	22550	22394	NEURONS	Neuron
ID	10	22550	22394	BLOOD	Blood

Record 145 | WOS:000859602700001

Ochratoxin A and Citrinin Differentially Modulate Bovine Mammary Epithelial Cell Permeability and Innate Immune Function

Field	Position	Original line	Normalised line	Original value	Normalised value
DE	1	22669	22512	bovine MAC-T cells	Bovine Mac T
DE	2	22669	22512	citrinin	Citrinin
DE	3	22669	22512	epithelial paracellular permeability	Epithelial Paracellular Permeability
DE	4	22669	22512	in vitro cell culture	In Vitro Cell
DE	5	22669	22512	mycotoxins	Mycotoxins
DE	6	22669	22512	ochratoxin A	Ochratoxin A (OTA)
ID	1	22671	22514	GROWTH-FACTOR-ALPHA	Growth Factor Alpha
ID	2	22671	22514	IN-VITRO	In-Vitro
ID	3	22671	22514	INTRAMAMMARY INFECTION	Intramammary Infection
ID	4	22671	22514	PENICILLIUM MYCOTOXINS	Penicillium Mycotoxin
ID	5	22671	22514	BARRIER DYSFUNCTION	Barrier Dysfunction
ID	6	22671	22514	IPEC-J2 CELLS	IPEC-J2
ID	7	22671	22514	EXPRESSION	Expression
ID	8	22671	22514	DEOXYNIVALENOL	Deoxynivalenol (DON)
ID	9	22671	22514	GLAND	Gland
ID	10	22671	22514	MILK	Milk

Record 146 | WOS:000858998000001

Protective activities of ellagic acid and urolithins against kidney toxicity of environmental pollutants: A review

Field	Position	Original line	Normalised line	Original value	Normalised value
DE	1	22845	22687	Antioxidant agents	Antioxidant Agent
DE	2	22845	22687	Kidney injury	Kidney Injury
DE	3	22845	22687	Heavy metals	Heavy Metal
DE	4	22845	22687	Pesticides	Pesticide
DE	6	22845	22687	Air pollutants	Air Pollutant
ID	1	22847	22689	OXIDATIVE STRESS	Oxidative Stress
ID	2	22847	22689	REACTIVE OXYGEN	Reactive Oxygen
ID	3	22847	22689	INDUCED NEPHROTOXICITY	Induced Nephrotoxicity
ID	4	22847	22689	CARBON-TETRACHLORIDE	Carbon Tetrachloride
ID	5	22847	22689	OCHRATOXIN-A	Ochratoxin A (OTA)
ID	6	22847	22689	ORGANOPHOSPHATE INSECTICIDE	Organophosphate Insecticide
ID	7	22847	22689	LIPID-PEROXIDATION	Lipid Peroxidation
ID	8	22847	22689	RENAL-FAILURE	Renal Failure
ID	9	22847	22689	IN-VITRO	In-Vitro

ID	10	22847	22689	ANTIOXIDANT STATUS	Antioxidant Status

Record 147 | WOS:000836768600001

Metabolic profiling as a powerful tool for the analysis of cellular alterations caused by 20 mycotoxins in HepG2 cells

Field	Position	Original line	Normalised line	Original value	Normalised value
DE	3	23133	22974	Mode of action	Mode Of Action
DE	5	23133	22974	Metabolic profiling	Metabolic Profiling
DE	6	23133	22974	HILIC	Hilic
ID	1	23135	22975	T-2 TOXIN	T 2 Toxin
ID	2	23135	22975	QUANTITATIVE-DETERMINATION	Quantitative Determination
ID	3	23135	22975	GLUTATHIONE	Glutathione (GSH)
ID	4	23135	22975	OCHRATOXIN	Ochratoxin A (OTA)
ID	5	23135	22975	RAT	Rat
ID	7	23135	22975	AFLATOXIN-B1	Aflatoxin B1 (AFB1)
ID	8	23135	22975	MECHANISM	Mechanism
ID	9	23135	22975	PROTEINS	Protein
ID	10	23135	22975	EXPOSURE	Exposure

Record 148 | WOS:000836539000001

Combined effects of cadmium and ochratoxin A on intestinal barrier dysfunction in human Caco-2 cells and pig small intestinal epithelial cells

Field	Position	Original line	Normalised line	Original value	Normalised value
DE	2	23242	23081	Ochratoxin A	Ochratoxin A (OTA)
DE	3	23242	23081	Intestinal barrier	Intestinal Barrier
DE	4	23242	23081	Apical junction	Apical Junction
DE	5	23242	23081	Synergistic toxicity	Synergistic Toxicity
ID	1	23244	23083	OXIDATIVE STRESS	Oxidative Stress
ID	2	23244	23083	IN-VITRO	In-Vitro
ID	3	23244	23083	ABSORPTION	Absorption
ID	4	23244	23083	MYCOTOXINS	Mycotoxins
ID	5	23244	23083	TOXICITY	Toxicity
ID	6	23244	23083	CHLORIDE	Chloride
ID	7	23244	23083	IMPACT	Impact
ID	8	23244	23083	KIDNEY	Kidney
ID	9	23244	23083	GUT	Gut

Record 149 | WOS:000827733300003

Antifungal and antimycotoxic activities of 3 essential oils against 3 mycotoxinogenic fungi

Field	Position	Original line	Normalised line	Original value	Normalised value
DE	1	23358	23196	Essential oils	Essential Oil
ID	1	23359	23197	ASPERGILLUS-FLAVUS	Aspergillus Flavus
ID	2	23359	23197	OCHRATOXIN	Ochratoxin A (OTA)
ID	3	23359	23197	GENES	Gene
ID	4	23359	23197	IDENTIFICATION	Identification
ID	5	23359	23197	BIOSYNTHESIS	Biosynthesis
ID	6	23359	23197	EXPRESSION	Expression

Record 150 | WOS:000845596200001

Evaluation of the Safety and Ochratoxin A Degradation Capacity of Pediococcus pentosaceus as a Dietary Probiotic with Molecular Docking Approach and Pharmacokinetic Toxicity Assessment

Field	Position	Original line	Normalised line	Original value	Normalised value
DE	1	23485	23322	Pediococcus sp	Pediococcus Sp
DE	2	23485	23322	probiotics	Probiotics
DE	3	23485	23322	ochratoxin A	Ochratoxin A (OTA)
DE	4	23485	23322	biodetoxification	Biodetoxification

DE	5	23485	23322	toxicity assessment	Toxicity Assessment
ID	1	23487	23324	LACTIC-ACID BACTERIA	Lactic Acid Bacteria
ID	2	23487	23324	IDENTIFICATION	Identification
ID	3	23487	23324	BIODEGRADATION	Biodegradation

Record 151 | WOS:000810935900001

Paper The multiple biotoxicity integrated study in grass carp (Ctenopharyngodon idella) caused by Ochratoxin A: Oxidative damage, apoptosis and immunosuppression

Field	Position	Original line	Normalised line	Original value	Normalised value
DE	1	23610	23446	Ochratoxin A	Ochratoxin A (OTA)
ID	1	23611	23448	ANTIOXIDANT RESPONSES	Antioxidant Response
ID	2	23611	23448	SIGNALING PATHWAY	Signaling Pathway
ID	3	23611	23448	TELEOST FISH	Teleost Fish
ID	4	23611	23448	HEAD-KIDNEY	Head Kidney
ID	5	23611	23448	DNA-DAMAGE	DNA Damage
ID	6	23611	23448	IN-VITRO	In-Vitro
ID	7	23611	23448	STRESS	Stress
ID	8	23611	23448	DIETARY	Dietary
ID	9	23611	23448	IMMUNE	Immune
ID	10	23611	23448	GROWTH	Growth

Record 152 | WOS:000807511200002

Nutritional impact of mycotoxins in food animal production and strategies for mitigation

Field	Position	Original line	Normalised line	Original value	Normalised value
DE	1	23806	23642	Food animals	Food Animal
DE	2	23806	23642	Mitigation strategies	Mitigation Strategy
DE	4	23806	23642	Nutritional impact	Nutritional Impact
DE	5	23806	23642	Risk management	Risk Management
DE	6	23806	23642	Susceptibility differences	Susceptibility Difference
ID	1	23808	23644	YEAST-CELL WALL	Yeast Cell Wall
ID	2	23808	23644	DIETARY-PROTEIN CONCENTRATION	Dietary Protein Concentration
ID	3	23808	23644	AFLATOXIN B-1	Aflatoxin B1 (AFB1)
ID	4	23808	23644	NUTRIENT DIGESTIBILITY	Nutrient Digestibility
ID	5	23808	23644	FUSARIUM MYCOTOXINS	Fusarium Mycotoxin
ID	6	23808	23644	GROWTH-PERFORMANCE	Growth Performance
ID	7	23808	23644	IN-VITRO	In-Vitro
ID	8	23808	23644	TRICHOTHECENE MYCOTOXINS	Trichothecene Mycotoxin
ID	9	23808	23644	INTESTINAL-MUCOSA	Intestinal Mucosa
ID	10	23808	23644	ENZYME-ACTIVITIES	Enzyme Activity

Record 153 | WOS:000818403700001

Genotoxicity of 12 Mycotoxins by the SOS/umu Test: Comparison of Liver and Kidney S9 Fraction

Field	Position	Original line	Normalised line	Original value	Normalised value
DE	1	24072	23907	genotoxicity	Genotoxicity
DE	2	24072	23907	liver S9	Liver S9
DE	3	24072	23907	kidney S9	Kidney S9
DE	4	24072	23907	in vitro	In-Vitro
DE	5	24072	23907	bioactivation	Bioactivation
ID	1	24073	23908	SALMONELLA-TYPHIMURIUM	Salmonella Typhimurium
ID	2	24073	23908	UMU-TEST	Umu Test
ID	3	24073	23908	ESCHERICHIA-COLI	Escherichia Coli

ID	4	24073	23908	AROMATIC-AMINES	Aromatic Amine
ID	5	24073	23908	RAPID DETECTION	Rapid Detection
ID	6	24073	23908	4-NITROQUINOLINE 1-OXIDE	4 Nitroquinoline 1 Oxide
ID	7	24073	23908	FUMONISIN B-1	Fumonisin B1 (FB1)
ID	8	24073	23908	TEST SYSTEM	Test System
ID	9	24073	23908	AMES TEST	Ame Test
ID	10	24073	23908	MUTAGENICITY	Mutagenicity

Record 154 | WOS:000819631900001

Ochratoxin A and Kidney Oxidative Stress: The Role of Nutraceuticals in Veterinary Medicine-A Review

Field	Position	Original line	Normalised line	Original value	Normalised value
DE	1	24204	24038	OTA	Ochratoxin A (OTA)
DE	2	24204	24038	nutraceuticals	Nutraceutical
DE	3	24204	24038	feed supplementation	Feed Supplementation
DE	4	24204	24038	oxidative stress	Oxidative Stress
DE	5	24204	24038	food safety	Food Safety
DE	6	24204	24038	kidney	Kidney
ID	1	24206	24040	INDUCED CELL-DAMAGE	Induced Cell Damage
ID	2	24206	24040	NADPH OXIDASE	Nadph Oxidase
ID	3	24206	24040	METABOLITE OCHRATOXIN	Metabolite Ochratoxin
ID	4	24206	24040	ANTIOXIDANT ACTIVITY	Antioxidant Activity
ID	5	24206	24040	LIPID-PEROXIDATION	Lipid Peroxidation
ID	6	24206	24040	IN-VIVO	In-Vivo
ID	7	24206	24040	FOOD	Food
ID	8	24206	24040	NEPHROPATHY	Nephropathy
ID	9	24206	24040	MYCOTOXIN	Mycotoxins
ID	10	24206	24040	QUERCETIN	Quercetin

Record 155 | WOS:000806405700007

In vitro mycotoxin binding capacities of clays, glucomannan and their combinations

Field	Position	Original line	Normalised line	Original value	Normalised value
DE	1	24377	24210	Mycotoxin	Mycotoxins
DE	5	24377	24210	Plant extract	Plant Extract
DE	6	24377	24210	In vitro	In-Vitro
ID	1	24378	24211	DIETARY ESTERIFIED GLUCOMANNAN	Dietary Esterified Glucomannan
ID	2	24378	24211	CHRONIC AFLATOXIN 50	Chronic Aflatoxin 50
ID	3	24378	24211	BROILER-CHICKENS	Broiler Chicken
ID	4	24378	24211	SODIUM BENTONITE	Sodium Bentonite
ID	5	24378	24211	MYCOTOXIN CONTAMINATION	Mycotoxin Contamination
ID	6	24378	24211	100 PPB	100 Ppb
ID	7	24378	24211	PERFORMANCE	Performance
ID	8	24378	24211	CLINOPTILOLITE	Clinoptilolite
ID	9	24378	24211	ADSORPTION	Adsorption
ID	10	24378	24211	EFFICACY	Efficacy

Record 156 | WOS:000832788700002

Effectiveness of beetroot extract in SH-SY5Y neuronal cell protection against Fumonisin B1, Ochratoxin A and its combination

Field	Position	Original line	Normalised line	Original value	Normalised value
DE	3	24510	24342	FB1	Fumonisin B1 (FB1)
DE	4	24510	24342	OTA	Ochratoxin A (OTA)
DE	5	24510	24342	SH-SY5Y cells	SH-SY5Y
ID	1	24511	24343	OXIDATIVE STRESS	Oxidative Stress
ID	2	24511	24343	RED BEET	Red Beet

ID	3	24511	24343	MYCOTOXINS	Mycotoxins
ID	4	24511	24343	CYTOTOXICITY	Cytotoxicity
ID	5	24511	24343	B-1	B 1
ID	6	24511	24343	15-ACETYL-DEOXYNIVALENOL	15 Acetyl Deoxynivalenol
ID	7	24511	24343	3-ACETYL-DEOXYNIVALENOL	3 Acetyl Deoxynivalenol
ID	8	24511	24343	ALTERNARIOL	Alternariol
ID	9	24511	24343	BEAUVERICIN	Beauvericin
ID	10	24511	24343	INHIBITION	Inhibition

Record 157 | WOS:000793099900001

Ochratoxin a induces hepatic fibrosis through TGF-Î² receptor I/Smad2/3 signaling pathway

Field	Position	Original line	Normalised line	Original value	Normalised value
DE	1	24620	24451	liver fibrosis	Liver Fibrosis
DE	2	24620	24451	Ochratoxin A	Ochratoxin A (OTA)
DE	5	24620	24451	TGF-beta receptor I	Tgf Beta Receptor I
ID	1	24621	24452	EPITHELIAL-MESENCHYMAL TRANSITION	Epithelial Mesenchymal Transition
ID	2	24621	24452	LIVER	Liver
ID	3	24621	24452	EXPRESSION	Expression
ID	4	24621	24452	MECHANISMS	Mechanism
ID	5	24621	24452	TOXICITY	Toxicity
ID	6	24621	24452	CELLS	Cell
ID	7	24621	24452	PREVENTION	Prevention
ID	8	24621	24452	BIOMARKERS	Biomarker
ID	9	24621	24452	INSIGHTS	Insight
ID	10	24621	24452	CADHERIN	Cadherin

Record 158 | WOS:000801766500001

Degradation of Ochratoxin A by a UV-Mutated Aspergillus niger Strain

Field	Position	Original line	Normalised line	Original value	Normalised value
DE	1	24734	24564	Ochratoxin A	Ochratoxin A (OTA)
DE	2	24734	24564	detoxification	Detoxification
DE	3	24734	24564	Aspergillus niger	Aspergillus Niger
DE	4	24734	24564	degradation products	Degradation Product
DE	5	24734	24564	cytotoxicity	Cytotoxicity
ID	1	24736	24566	SECTION NIGRI	Section Nigri
ID	2	24736	24566	HUMAN HEALTH	Human Health
ID	3	24736	24566	IN-VITRO	In-Vitro
ID	4	24736	24566	BIODEGRADATION	Biodegradation
ID	5	24736	24566	FEED	Feed
ID	6	24736	24566	DECONTAMINATION	Decontamination
ID	7	24736	24566	CYTOTOXICITY	Cytotoxicity
ID	8	24736	24566	MUTAGENESIS	Mutagenesis
ID	9	24736	24566	MYCOTOXINS	Mycotoxins
ID	10	24736	24566	TOXICITY	Toxicity

Record 159 | WOS:000802213700006

Pumpkin extract and fermented whey individually and in combination alleviated AFB1-and OTA-induced alterations on neuronal differentiation in vitro

Field	Position	Original line	Normalised line	Original value	Normalised value
DE	2	24860	24689	Bioactive compounds	Bioactive Compound
DE	5	24860	24689	qPCR	Qpcr
ID	1	24861	24690	CELL-CYCLE ARREST	Cell Cycle Arrest
ID	2	24861	24690	DOWN-REGULATION	Down Regulation
ID	3	24861	24690	AFLATOXIN B-1	Aflatoxin B1 (AFB1)
ID	4	24861	24690	DNA-DAMAGE	DNA Damage
ID	5	24861	24690	OCHRATOXIN	Ochratoxin A (OTA)

ID	6	24861	24690	BRAIN	Brain
ID	7	24861	24690	EXPOSURE	Exposure
ID	9	24861	24690	APOPTOSIS	Apoptosis
ID	10	24861	24690	BIOACCESSIBILITY	Bioaccessibility

Record 160 | WOS:000793768800008

Ochratoxin A as an alarming health threat for livestock and human: A review on molecular interactions, mechanism of toxicity, detection, detoxification, and dietary prophylaxis

Field	Position	Original line	Normalised line	Original value	Normalised value
DE	1	25014	24842	Ochratoxin	Ochratoxin A (OTA)
DE	5	25014	24842	Dietary prophylaxis	Dietary Prophylaxis
ID	1	25016	24844	LABEL-FREE APTASENSOR	Label Free Aptasensor
ID	2	25016	24844	IMPEDIMETRIC APTASENSOR	Impedimetric Aptasensor
ID	3	25016	24844	DNA-DAMAGE	DNA Damage
ID	4	25016	24844	ULTRASENSITIVE DETECTION	Ultrasensitive Detection
ID	5	25016	24844	FLUORESCENT APTASENSOR	Fluorescent Aptasensor
ID	6	25016	24844	OXIDATIVE DAMAGE	Oxidative Damage
ID	7	25016	24844	RAPID DETECTION	Rapid Detection
ID	8	25016	24844	PHASE ARREST	Phase Arrest
ID	9	25016	24844	QUANTUM DOTS	Quantum Dot
ID	10	25016	24844	APTAMER	Aptamer

Record 161 | WOS:000786151200001

Cytotoxicity of Mycotoxins and Their Combinations on Different Cell Lines: A Review

Field	Position	Original line	Normalised line	Original value	Normalised value
DE	1	25275	25102	cytotoxicity	Cytotoxicity
DE	2	25275	25102	mycotoxins	Mycotoxins
DE	3	25275	25102	cell line	Cell
DE	4	25275	25102	apoptosis	Apoptosis
DE	5	25275	25102	MTT	Mtt
ID	1	25276	25103	IN-VITRO CYTOTOXICITY	In Vitro Cytotoxicity
ID	2	25276	25103	AFLATOXIN B-1	Aflatoxin B1 (AFB1)
ID	3	25276	25103	FUMONISIN B-1	Fumonisin B1 (FB1)
ID	4	25276	25103	T-2 TOXIN	T 2 Toxin
ID	5	25276	25103	FUSARIUM-MYCOTOXINS	Fusarium Mycotoxin
ID	6	25276	25103	INDUCED APOPTOSIS	Induced Apoptosis
ID	7	25276	25103	OXIDATIVE STRESS	Oxidative Stress
ID	8	25276	25103	ALPHA-ZEARALENOL	Alpha Zearalenol
ID	9	25276	25103	CACO-2 CELLS	Caco-2
ID	10	25276	25103	ENNIATIN-B	Enniatin B

Record 162 | WOS:000784471500001

Mycotoxins in food and feed: toxicity, preventive challenges, and advanced detection techniques for associated diseases

Field	Position	Original line	Normalised line	Original value	Normalised value
DE	1	25447	25273	Fungal metabolites	Fungal Metabolite
DE	2	25447	25273	legislation	Legislation
DE	3	25447	25273	mycotoxin	Mycotoxins
DE	4	25447	25273	signaling pathway	Signaling Pathway
DE	5	25447	25273	toxicity	Toxicity
ID	1	25448	25274	LINKED-IMMUNOSORBENT-ASSAY	Linked Immunosorbent Assay

ID	2	25448	25274	PERFORMANCE LIQUID-CHROMATOGRAPHY	Performance Liquid Chromatography
ID	3	25448	25274	GLUTATHIONE-S-TRANSFERASE	Glutathione S Transferase
ID	4	25448	25274	OCHRATOXIN-A	Ochratoxin A (OTA)
ID	5	25448	25274	T-2 TOXIN	T 2 Toxin
ID	6	25448	25274	AFLATOXIN B-1	Aflatoxin B1 (AFB1)
ID	7	25448	25274	COFFEE BEANS	Coffee Bean
ID	8	25448	25274	FUMONISIN B-1	Fumonisin B1 (FB1)
ID	9	25448	25274	IN-VITRO	In-Vitro
ID	10	25448	25274	BIOLOGICAL DETOXIFICATION	Biological Detoxification

Record 163 | WOS:000793342700006

PCV2 infection aggravates OTA-induced immunotoxicity in vivo and in vitro

Field	Position	Original line	Normalised line	Original value	Normalised value
DE	1	25721	25546	Ochratoxin A	Ochratoxin A (OTA)
DE	2	25721	25546	Porcine circovirus type 2	Porcine Circovirus Type 2
DE	4	25721	25546	TLR4	Tlr4
DE	5	25721	25546	NF-kappa B p65 signaling pathway	Nf Kappa B P65 Signaling Pathway
DE	6	25721	25546	Porcine alveolar macrophages	Porcine Alveolar Macrophage
DE	7	25721	25546	Pigs	Pig
ID	1	25723	25548	PORCINE ALVEOLAR MACROPHAGES	Porcine Alveolar Macrophage
ID	2	25723	25548	RESPIRATORY SYNDROME VIRUS	Respiratory Syndrome Virus
ID	3	25723	25548	CIRCOVIRUS TYPE-2 REPLICATION	Circovirus Type 2 Replication
ID	4	25723	25548	OCHRATOXIN-A	Ochratoxin A (OTA)
ID	5	25723	25548	SIGNALING PATHWAY	Signaling Pathway
ID	6	25723	25548	OXIDATIVE STRESS	Oxidative Stress
ID	7	25723	25548	PK15 CELLS	Pk15
ID	8	25723	25548	EXPRESSION	Expression
ID	9	25723	25548	INFLAMMATION	Inflammation
ID	10	25723	25548	ACTIVATION	Activation

Record 164 | WOS:000805486800007

An update on immunotoxicity and mechanisms of action of six environmental mycotoxins

Field	Position	Original line	Normalised line	Original value	Normalised value
DE	5	25847	25671	Mechanisms	Mechanism
ID	1	25849	25672	IN-VITRO	In-Vitro
ID	2	25849	25672	FUMONISIN B-1	Fumonisin B1 (FB1)
ID	3	25849	25672	AFLATOXIN B-1	Aflatoxin B1 (AFB1)
ID	4	25849	25672	DEOXYNIVALENOL DON	Deoxynivalenol DON
ID	5	25849	25672	OXIDATIVE STRESS	Oxidative Stress
ID	6	25849	25672	PROTECTIVE ROLE	Protective Role
ID	7	25849	25672	OCHRATOXIN-A	Ochratoxin A (OTA)
ID	8	25849	25672	EXPOSURE	Exposure
ID	9	25849	25672	CELLS	Cell
ID	10	25849	25672	T-2	T 2

Record 165 | WOS:000774405900001

Ex Vivo and In Vitro Studies Revealed Underlying Mechanisms of Immature Intestinal Inflammatory Responses Caused by Aflatoxin M1 Together with Ochratoxin A

Field	Position	Original line	Normalised line	Original value	Normalised value
DE	1	25984	25806	aflatoxin M1	Aflatoxin M1 (AFM1)
DE	2	25984	25806	ochratoxin A	Ochratoxin A (OTA)
DE	3	25984	25806	immature intestine	Immature Intestine
DE	4	25984	25806	inflammation	Inflammation
DE	5	25984	25806	RNA-seq	RNA Seq
ID	1	25985	25808	OXIDATIVE STRESS	Oxidative Stress
ID	2	25985	25808	RISK-ASSESSMENT	Risk Assessment
ID	3	25985	25808	BREAST-MILK	Breast Milk
ID	4	25985	25808	BABY FOODS	Baby Food
ID	5	25985	25808	MYCOTOXINS	Mycotoxins
ID	6	25985	25808	EXPOSURE	Exposure
ID	7	25985	25808	CHILDREN	Children
ID	8	25985	25808	AGE	Age
ID	9	25985	25808	ASSOCIATION	Association
ID	10	25985	25808	EPITHELIUM	Epithelium

Record 166 | WOS:000798517400010

Detoxification of ochratoxin A and zearalenone by Pleurotus ostreatus during in vitro gastrointestinal digestion

Field	Position	Original line	Normalised line	Original value	Normalised value
DE	1	26143	25965	Ochratoxin A	Ochratoxin A (OTA)
DE	2	26143	25965	Zearalenone	Zearalenone (ZEA)
DE	3	26143	25965	Pleurotus ostreatus	Pleurotus Ostreatus
DE	5	26143	25965	Mycotoxins adsorption	Mycotoxin Adsorption
DE	6	26143	25965	In vitro gastrointestinal digestion	In Vitro Gastrointestinal Digestion
ID	1	26145	25968	AFLATOXIN B-1	Aflatoxin B1 (AFB1)
ID	2	26145	25968	TRICHOSPORON-MYCOTOXINIVORANS	Trichosporon Mycotoxinivoran
ID	3	26145	25968	CELL-WALL	Cell Wall
ID	4	26145	25968	BINDING	Binding
ID	5	26145	25968	ADSORPTION	Adsorption
ID	6	26145	25968	TOXICITY	Toxicity
ID	7	26145	25968	DEOXYNIVALENOL	Deoxynivalenol (DON)
ID	8	26145	25968	PURIFICATION	Purification
ID	9	26145	25968	DEGRADATION	Degradation
ID	10	26145	25968	ADSORBENTS	Adsorbent

Record 167 | WOS:000760242900001

MicroRNA regulates the toxicological mechanism of four mycotoxins in vivo and in vitro

Field	Position	Original line	Normalised line	Original value	Normalised value
DE	3	26268	26090	MicroRNA	Microrna
DE	4	26268	26090	Mycotoxin	Mycotoxins
ID	1	26269	26091	OCHRATOXIN-A	Ochratoxin A (OTA)
ID	2	26269	26091	GENE-EXPRESSION	Gene Expression
ID	3	26269	26091	CELL-PROLIFERATION	Cell Proliferation
ID	4	26269	26091	RAT-LIVER	Rat Liver
ID	5	26269	26091	APOPTOSIS	Apoptosis
ID	6	26269	26091	EXPOSURE	Exposure
ID	7	26269	26091	DEOXYNIVALENOL	Deoxynivalenol (DON)
ID	8	26269	26091	ZEARALENONE	Zearalenone (ZEA)
ID	9	26269	26091	ACTIVATION	Activation
ID	10	26269	26091	TOXICITY	Toxicity

Record 168 | WOS:000796935500003

Studies on teratogenic effect of ochratoxin A given via mouldy diet in mice in various sensitive periods of the pregnancy and the putative protection of phenylalanine

Field	Position	Original line	Normalised line	Original value	Normalised value
DE	1	26454	26276	Ochratoxin A	Ochratoxin A (OTA)
DE	3	26454	26276	Teratogenic effect	Teratogenic Effect

DE	6	26454	26276	Sensitive periods of pregnancy	Sensitive Period Of Pregnancy
ID	1	26456	26278	NEURAL-TUBE DEFECTS	Neural Tube Defect
ID	2	26456	26278	UNDERESTIMATED HAZARD	Underestimated Hazard
ID	3	26456	26278	PRENATAL DEVELOPMENT	Prenatal Development
ID	4	26456	26278	RISK-ASSESSMENT	Risk Assessment
ID	5	26456	26278	FEED ADDITIVES	Feed Additive
ID	6	26456	26278	MYCO-TOXINS	Myco Toxin
ID	7	26456	26278	EXPOSURE	Exposure
ID	8	26456	26278	MOUSE	Mouse
ID	9	26456	26278	INTERFERENCE	Interference
ID	10	26456	26278	GENOTOXICITY	Genotoxicity

Record 169 | WOS:000762109100001

Spent Coffee Grounds Valorization as Bioactive Phenolic Source Acquired Antifungal, Anti-Mycotoxigenic, and Anti-Cytotoxic Activities

Field	Position	Original line	Normalised line	Original value	Normalised value
DE	1	26564	26385	antifungal	Antifungal
DE	2	26564	26385	anti-mycotoxigenic	Anti Mycotoxigenic
DE	3	26564	26385	aflatoxins	Aflatoxin
DE	4	26564	26385	cytotoxicity	Cytotoxicity
DE	5	26564	26385	degradation	Degradation
DE	6	26564	26385	detoxification	Detoxification
DE	7	26564	26385	phenolic acids	Phenolic Acid
DE	8	26564	26385	flavonoids	Flavonoid
DE	9	26564	26385	spent coffee grounds	Spent Coffee Ground
DE	10	26564	26385	simulated media	Simulated Media
ID	1	26567	26388	ANTIOXIDANT	Antioxidant
ID	2	26567	26388	EXTRACTS	Extract
ID	3	26567	26388	CONSTITUENTS	Constituent
ID	4	26567	26388	POLYPHENOLS	Polyphenol
ID	5	26567	26388	INHIBITION	Inhibition
ID	6	26567	26388	FUNGI	Fungi

Record 170 | WOS:000762022600001

Metabolomic Analysis Reveals the Mechanisms of Hepatotoxicity Induced by Aflatoxin M1 and Ochratoxin A

Field	Position	Original line	Normalised line	Original value	Normalised value
DE	1	26684	26504	aflatoxin M1	Aflatoxin M1 (AFM1)
DE	2	26684	26504	ochratoxin A	Ochratoxin A (OTA)
DE	3	26684	26504	hepatoxicity	Hepatoxicity
DE	4	26684	26504	metabolomics	Metabolomics
DE	5	26684	26504	LysoPCs	Lysopc
ID	1	26685	26505	IN-VITRO	In-Vitro
ID	2	26685	26505	OXIDATIVE STRESS	Oxidative Stress
ID	3	26685	26505	COMBINATION	Combination
ID	4	26685	26505	MYCOTOXINS	Mycotoxins
ID	5	26685	26505	INJURY	Injury
ID	6	26685	26505	CHINA	China
ID	7	26685	26505	MILK	Milk
ID	8	26685	26505	NEPHROTOXICITY	Nephrotoxicity
ID	9	26685	26505	PRODUCTS	Product
ID	10	26685	26505	EXPOSURE	Exposure

Record 171 | WOS:000789897500004

A synergism of in silico and statistical approaches to discover new potential endocrine disruptor mycotoxins

Field	Position	Original line	Normalised line	Original value	Normalised value
DE	1	26806	26625	Endocrine disruptors	Endocrine Disruptor
DE	3	26806	26625	Nuclear receptors	Nuclear Receptor

DE	4	26806	26625	Molecular docking	Molecular Docking
DE	5	26806	26625	Dimension reduction	Dimension Reduction
ID	1	26808	26627	PRIMARY CULTURES	Primary
ID	2	26808	26627	SCORING FUNCTION	Scoring Function
ID	3	26808	26627	ZEARALENONE	Zearalenone (ZEA)
ID	4	26808	26627	OCHRATOXIN	Ochratoxin A (OTA)
ID	5	26808	26627	EXPRESSION	Expression
ID	6	26808	26627	DOCKING	Docking
ID	7	26808	26627	BINDING	Binding
ID	8	26808	26627	PATULIN	Patulin
ID	9	26808	26627	LEVEL	Level
ID	10	26808	26627	PXR	Pxr

Record 172 | WOS:000738826400005

Combined protective effects of icariin and selenomethionine on novel chronic tubulointerstitial nephropathy models in vivo and in vitro

Field	Position	Original line	Normalised line	Original value	Normalised value
DE	3	26916	26734	Chronic tubulointerstitial nephropathy	Chronic Tubulointerstitial Nephropathy
DE	4	26916	26734	Renal fibrosis	Renal Fibrosis
DE	5	26916	26734	Toll-like receptor 4/NF kappa B signalling pathway	Toll Like Receptor 4 Nf Kappa B Signalling Pathway
ID	1	26918	26736	CHRONIC CYCLOSPORINE NEPHROTOXICITY	Chronic Cyclosporine Nephrotoxicity
ID	2	26918	26736	RAT MODEL	Rat Model
ID	3	26918	26736	TGF-BETA	Tgf Beta
ID	4	26918	26736	SIGNALING PATHWAY	Signaling Pathway
ID	5	26918	26736	OCHRATOXIN-A	Ochratoxin A (OTA)
ID	6	26918	26736	KAPPA-B	Kappa B
ID	7	26918	26736	INJURY	Injury
ID	8	26918	26736	INHIBITION	Inhibition
ID	9	26918	26736	EXPRESSION	Expression
ID	10	26918	26736	NEPHRITIS	Nephritis

Record 173 | WOS:000742259000001

Subchronic exposure of individual and combined ochratoxin A and citrinin selectively affects the expression of rat renal organic cation transporters

Field	Position	Original line	Normalised line	Original value	Normalised value
DE	1	27038	26855	In vivo	In-Vivo
DE	3	27038	26855	Mycotoxin	Mycotoxins
DE	6	27038	26855	Slc22 family	Slc22 Family
ID	1	27039	26856	ANION TRANSPORTERS	Anion Transporter
ID	2	27039	26856	TOXICITY	Toxicity
ID	3	27039	26856	OCT2	Oct2
ID	4	27039	26856	LOCALIZATION	Localization
ID	5	27039	26856	RESVERATROL	Resveratrol
ID	6	27039	26856	MECHANISM	Mechanism
ID	7	27039	26856	CELLS	Cell
ID	8	27039	26856	LIVER	Liver
ID	9	27039	26856	OTA	Ochratoxin A (OTA)

Record 174 | WOS:000750801500002

In vitro and in vivo evaluation of AFB1 and OTA-toxicity through immunofluorescence and flow cytometry techniques: A systematic review

Field	Position	Original line	Normalised line	Original value	Normalised value
DE	2	27149	26965	Toxicological mechanism	Toxicological Mechanism
DE	3	27149	26965	Cell line	Cell

DE	4	27149	26965	Animal model	Animal Model
ID	1	27150	26966	CELL-CYCLE ARREST	Cell Cycle Arrest
ID	2	27150	26966	AFLATOXIN B-1	Aflatoxin B1 (AFB1)
ID	3	27150	26966	PROTECTIVE ROLE	Protective Role
ID	4	27150	26966	FUMONISIN B-1	Fumonisin B1 (FB1)
ID	5	27150	26966	INDUCED APOPTOSIS	Induced Apoptosis
ID	6	27150	26966	OXIDATIVE STRESS	Oxidative Stress
ID	7	27150	26966	PHASE ARREST	Phase Arrest
ID	8	27150	26966	OCHRATOXIN	Ochratoxin A (OTA)
ID	9	27150	26966	EXPRESSION	Expression
ID	10	27150	26966	ACTIVATION	Activation

Record 175 ǀ WOS:000767939000005

An overview of mycotoxins, their pathogenic effects, foods where they are found and their diagnostic biomarkers

Field	Position	Original line	Normalised line	Original value	Normalised value
DE	1	27353	27168	mycotoxin biomarkers	Mycotoxin Biomarker
DE	2	27353	27168	fungi	Fungi
DE	3	27353	27168	toxicity	Toxicity
DE	4	27353	27168	carcinogenesis	Carcinogenesis
ID	1	27354	27169	OCHRATOXIN-A	Ochratoxin A (OTA)
ID	2	27354	27169	AFLATOXIN M-1	Aflatoxin M1 (AFM1)
ID	3	27354	27169	URINARY DEOXYNIVALENOL	Urinary Deoxynivalenol
ID	4	27354	27169	FUMONISIN EXPOSURE	Fumonisin Exposure
ID	5	27354	27169	DIETARY FUMONISIN	Dietary Fumonisin
ID	6	27354	27169	GERMAN ADULTS	German Adult
ID	7	27354	27169	HUMAN-MILK	Human Milk
ID	8	27354	27169	SAO-PAULO	Sao Paulo
ID	9	27354	27169	COWS MILK	Cow Milk
ID	10	27354	27169	IN-VITRO	In-Vitro

Record 176 ǀ WOS:000912776700001

Mycotoxin Interactions along the Gastrointestinal Tract: In Vitro Semi-Dynamic Digestion and Static Colonic Fermentation of a Contaminated Meal

Field	Position	Original line	Normalised line	Original value	Normalised value
DE	1	27523	27337	aflatoxin B1	Aflatoxin B1 (AFB1)
DE	2	27523	27337	ochratoxin A	Ochratoxin A (OTA)
DE	3	27523	27337	gut microbiota	Gut Microbiota
DE	4	27523	27337	inflammation	Inflammation
DE	5	27523	27337	in vitro models	In Vitro Model
ID	1	27525	27339	AFLATOXIN B-1	Aflatoxin B1 (AFB1)
ID	2	27525	27339	DAIRY-PRODUCTS	Dairy Product
ID	3	27525	27339	BIOACCESSIBILITY	Bioaccessibility
ID	4	27525	27339	OCHRATOXIN	Ochratoxin A (OTA)
ID	5	27525	27339	DIVERSITY	Diversity
ID	6	27525	27339	COOCCURRENCE	Cooccurrence
ID	7	27525	27339	COMMUNITY	Community
ID	8	27525	27339	EXPOSURE	Exposure
ID	9	27525	27339	CEREALS	Cereal
ID	10	27525	27339	PATULIN	Patulin

Record 177 ǀ WOS:000786544000007

Adsorption efficiency of sodium & calcium bentonite for ochratoxin A in some Egyptian cheeses: an innovative fortification model, in vitro and in vivo experiments

Field	Position	Original line	Normalised line	Original value	Normalised value
DE	1	27648	27461	adsorbent	Adsorbent
DE	2	27648	27461	bentonite	Bentonite
DE	3	27648	27461	ochratoxin A	Ochratoxin A (OTA)
DE	4	27648	27461	cheese	Cheese
DE	5	27648	27461	albino rats	Albino Rat

ID	1	27649	27462	MYCOTOXINS	Mycotoxins
ID	2	27649	27462	AFLATOXIN	Aflatoxin
ID	3	27649	27462	TOXICITY	Toxicity
ID	4	27649	27462	SURFACE	Surface
ID	5	27649	27462	BLUE	Blue

Record 178 | WOS:000746960900001

Development of an Extraction Method of Aflatoxins and Ochratoxin A from Oral, Gastric and Intestinal Phases of Digested Bread by In Vitro Model

Field	Position	Original line	Normalised line	Original value	Normalised value
DE	1	27769	27581	bioaccessibility	Bioaccessibility
DE	2	27769	27581	validation	Validation
DE	3	27769	27581	aflatoxins	Aflatoxin
DE	4	27769	27581	ochratoxin A	Ochratoxin A (OTA)
DE	5	27769	27581	bread	Bread
ID	1	27771	27583	WORLDWIDE OCCURRENCE	Worldwide Occurrence
ID	2	27771	27583	PROBIOTIC BACTERIA	Probiotic Bacteria
ID	3	27771	27583	OXIDATIVE STRESS	Oxidative Stress
ID	4	27771	27583	BIOACCESSIBILITY	Bioaccessibility
ID	5	27771	27583	FOOD	Food
ID	6	27771	27583	B-1	B 1
ID	7	27771	27583	MYCOTOXINS	Mycotoxins
ID	8	27771	27583	CEREALS	Cereal
ID	9	27771	27583	TOXICITY	Toxicity

Record 179 | WOS:000891197500003

Gene expression profiling after ochratoxin A treatment in small intestinal epithelial cells from pigs

Field	Position	Original line	Normalised line	Original value	Normalised value
ID	1	27866	27677	OXIDATIVE STRESS	Oxidative Stress
ID	2	27866	27677	IPEC-J2 CELLS	IPEC-J2
ID	3	27866	27677	BARRIER	Barrier
ID	4	27866	27677	INFLAMMATION	Inflammation
ID	5	27866	27677	DISRUPTION	Disruption
ID	6	27866	27677	TOXICITY	Toxicity
ID	7	27866	27677	IMMUNITY	Immunity
ID	8	27866	27677	HEALTH	Health

Record 180 | WOS:000730029700001

Agro-industrial Waste Products as Mycotoxin Biosorbents: A Review of in Vitro and in Vivo Studies

Field	Position	Original line	Normalised line	Original value	Normalised value
DE	2	27965	27775	biosorbentes	Biosorbente
DE	3	27965	27775	agro-industrial waste	Agro Industrial Waste
ID	1	27966	27776	OF-THE-ART	Of The Art
ID	2	27966	27776	DIETARY-FIBERS	Dietary Fiber
ID	3	27966	27776	GRAPE POMACE	Grape Pomace
ID	4	27966	27776	ZERO CHARGE	Zero Charge
ID	5	27966	27776	OCHRATOXIN	Ochratoxin A (OTA)
ID	6	27966	27776	BIOACCESSIBILITY	Bioaccessibility
ID	7	27966	27776	BIOSORPTION	Biosorption
ID	8	27966	27776	ADSORPTION	Adsorption
ID	9	27966	27776	TOXICITY	Toxicity
ID	10	27966	27776	FOOD	Food

Record 181 | WOS:000740093400002

Ochratoxin A induces NLRP3 inflammasome-mediated pyroptosis via activation of ROS modulated by autophagy in vitro

Field	Position	Original line	Normalised line	Original value	Normalised value

DE	1	28114	27923	Ochratoxin A	Ochratoxin A (OTA)
DE	4	28114	27923	Reactive Oxidative stress	Reactive Oxidative Stress
DE	5	28114	27923	NLRP3 inflammasome	Nlrp3 Inflammasome
ID	1	28116	27925	SIGNALING PATHWAY	Signaling Pathway
ID	2	28116	27925	OXIDATIVE STRESS	Oxidative Stress
ID	3	28116	27925	TOXICITY	Toxicity
ID	4	28116	27925	KIDNEY	Kidney
ID	5	28116	27925	EXPRESSION	Expression
ID	6	28116	27925	CYTOKINES	Cytokine
ID	7	28116	27925	INHIBITION	Inhibition
ID	8	28116	27925	MECHANISMS	Mechanism
ID	9	28116	27925	CASPASES	Caspase
ID	10	28116	27925	EXPOSURE	Exposure

Record 182 | WOS:000737483600001

Urinary Biomarkers of Mycotoxin Induced Nephrotoxicity-Current Status and Expected Future Trends

Field	Position	Original line	Normalised line	Original value	Normalised value
DE	1	28235	28043	nephrotoxicity	Nephrotoxicity
DE	2	28235	28043	mycotoxin	Mycotoxins
DE	3	28235	28043	biomarkers	Biomarker
DE	4	28235	28043	AKI	Aki
DE	5	28235	28043	NAG	Nag
DE	6	28235	28043	KIM-1	Kim 1
DE	7	28235	28043	ochratoxin A	Ochratoxin A (OTA)
DE	8	28235	28043	citrinin	Citrinin
DE	9	28235	28043	NGAL	Ngal
ID	1	28237	28045	ACUTE KIDNEY INJURY	Acute Kidney Injury
ID	2	28237	28045	GELATINASE-ASSOCIATED LIPOCALIN	Gelatinase Associated Lipocalin
ID	3	28237	28045	COMPLEMENTARY FOODS	Complementary Food
ID	4	28237	28045	OXIDATIVE STRESS	Oxidative Stress
ID	5	28237	28045	BLACK ASPERGILLI	Black Aspergilli
ID	6	28237	28045	AFLATOXIN B-1	Aflatoxin B1 (AFB1)
ID	7	28237	28045	CO-EXPOSURES	Co Exposure
ID	8	28237	28045	FUMONISIN B1	Fumonisin B1 (FB1)
ID	9	28237	28045	BREAST-MILK	Breast Milk
ID	10	28237	28045	IN-VIVO	In-Vivo

Record 183 | WOS:000734651000014

Non-embryotoxic dosage of alternariol aggravates ochratoxin A-triggered deleterious effects on embryonic development through ROS-dependent apoptotic processes

Field	Position	Original line	Normalised line	Original value	Normalised value
DE	1	28449	28256	alternariol	Alternariol
DE	2	28449	28256	apoptosis	Apoptosis
DE	3	28449	28256	ochratoxin A	Ochratoxin A (OTA)
DE	4	28449	28256	oxidative stress	Oxidative Stress
DE	5	28449	28256	embryonic development	Embryonic Development
ID	1	28451	28258	INNER CELL MASS	Inner Cell Mass
ID	2	28451	28258	DEVELOPMENT IN-VITRO	Development In Vitro
ID	3	28451	28258	MOUSE BLASTOCYSTS	Mouse Blastocyst
ID	4	28451	28258	OXIDATIVE STRESS	Oxidative Stress
ID	5	28451	28258	STEM-CELLS	Stem
ID	6	28451	28258	DEATH	Death
ID	7	28451	28258	MECHANISMS	Mechanism
ID	8	28451	28258	TOXICITY	Toxicity
ID	9	28451	28258	RESVERATROL	Resveratrol
ID	10	28451	28258	MAINTENANCE	Maintenance

Record 184 | WOS:000721173800002

The individual and combined effects of ochratoxin A with citrinin and their metabolites (ochratoxin B, ochratoxin C, and dihydrocitrinone) on 2D/3D cell cultures, and zebrafish embryo models

Field	Position	Original line	Normalised line	Original value	Normalised value
DE	1	28592	28397	Ochratoxins	Ochratoxin A (OTA)
DE	4	28592	28397	Combined effects	Combined Effect
DE	5	28592	28397	3D cell culture	3d Cell
DE	6	28592	28397	Zebrafish embryo	Zebrafish Embryo
ID	1	28594	28399	IN-VITRO	In-Vitro
ID	2	28594	28399	MYCOTOXIN OCHRATOXIN	Ochratoxin A (OTA)
ID	3	28594	28399	TOXICITY	Toxicity
ID	4	28594	28399	CYTOTOXICITY	Cytotoxicity
ID	5	28594	28399	NEPHROTOXICITY	Nephrotoxicity
ID	6	28594	28399	PROLIFERATION	Proliferation
ID	7	28594	28399	COOCCURRENCE	Cooccurrence
ID	8	28594	28399	INVOLVEMENT	Involvement
ID	9	28594	28399	CEREALS	Cereal
ID	10	28594	28399	ANALOGS	Analog

Record 185 | WOS:000874698700001

Food Mycotoxins: Dietary Interventions Implicated in the Prevention of Mycotoxicosis

Field	Position	Original line	Normalised line	Original value	Normalised value
DE	1	28740	28544	dietary factors	Dietary Factor
DE	2	28740	28544	food safety	Food Safety
DE	3	28740	28544	mycotoxins	Mycotoxins
DE	4	28740	28544	oxidative stress	Oxidative Stress
DE	5	28740	28544	probiotics	Probiotics
ID	1	28741	28545	CALCIUM MONTMORILLONITE CLAY	Calcium Montmorillonite Clay
ID	2	28741	28545	INDUCED OXIDATIVE STRESS	Induced Oxidative Stress
ID	3	28741	28545	AFLATOXIN B-1	Aflatoxin B1 (AFB1)
ID	4	28741	28545	FUMONISIN B-1	Fumonisin B1 (FB1)
ID	5	28741	28545	OCHRATOXIN-A	Ochratoxin A (OTA)
ID	6	28741	28545	IN-VITRO	In-Vitro
ID	7	28741	28545	L-CARNITINE	L Carnitine
ID	8	28741	28545	T-2 TOXIN	T 2 Toxin
ID	9	28741	28545	VITAMIN-E	Vitamin E
ID	10	28741	28545	LYCOPENE PROTECTS	Lycopene Protect

Record 186 | WOS:000724718300001

Human Proximal Tubule Epithelial Cells (HK-2) as a Sensitive In Vitro System for Ochratoxin A Induced Oxidative Stress

Field	Position	Original line	Normalised line	Original value	Normalised value
DE	1	28990	28793	ochratoxin A (OTA)	Ochratoxin A (OTA)
DE	2	28990	28793	oxidative stress	Oxidative Stress
DE	3	28990	28793	renal carcinogen	Renal Carcinogen
DE	4	28990	28793	kidney cell lines	Kidney Cell
DE	5	28990	28793	LLC-PK1	Llc Pk1
ID	1	28992	28795	HUMAN-PAPILLOMAVIRUS TYPE-16	Human Papillomavirus Type 16
ID	2	28992	28795	KIDNEY PK15 CELLS	Kidney Pk15
ID	3	28992	28795	DNA-DAMAGE	DNA Damage
ID	4	28992	28795	PROTEIN-SYNTHESIS	Protein Synthesis
ID	5	28992	28795	APOPTOSIS	Apoptosis
ID	6	28992	28795	RAT	Rat
ID	7	28992	28795	EXPRESSION	Expression
ID	8	28992	28795	TOXICITY	Toxicity
ID	9	28992	28795	CYTOTOXICITY	Cytotoxicity

ID	10	28992	28795	MECHANISM	Mechanism

Record 187 | WOS:000724371000001

Microbiological Decontamination of Mycotoxins: Opportunities and Limitations

Field	Position	Original line	Normalised line	Original value	Normalised value
DE	1	29142	28943	mycotoxins	Mycotoxins
DE	2	29142	28943	decontamination	Decontamination
DE	3	29142	28943	adsorption	Adsorption
DE	4	29142	28943	detoxification	Detoxification
DE	5	29142	28943	microorganisms	Microorganism
DE	6	29142	28943	lactic acid bacteria	Lactic Acid Bacteria
DE	7	29142	28943	yeasts	Yeast
ID	1	29144	28945	LACTIC-ACID BACTERIA	Lactic Acid Bacteria
ID	2	29144	28945	AFLATOXIN B-1 DEGRADATION	Aflatoxin B 1 Degradation
ID	3	29144	28945	IN-VITRO BIODEGRADATION	In Vitro Biodegradation
ID	4	29144	28945	BETA-D-GLUCANS	Beta D Glucan
ID	5	29144	28945	OCHRATOXIN-A	Ochratoxin A (OTA)
ID	6	29144	28945	SACCHAROMYCES-CEREVISIAE	Saccharomyce Cerevisiae
ID	7	29144	28945	FLAVOBACTERIUM-AURANTIACUM	Flavobacterium Aurantiacum
ID	8	29144	28945	PATULIN BIODEGRADATION	Patulin Biodegradation
ID	9	29144	28945	ENTEROBACTER-CLOACAE	Enterobacter Cloacae
ID	10	29144	28945	SURFACE BINDING	Surface Binding

Record 188 | WOS:000723720700001

Anti-Apoptotic Effect of Flavokawain A on Ochratoxin-A-Induced Endothelial Cell Injury by Attenuation of Oxidative Stress via PI3K/AKT-Mediated Nrf2 Signaling Cascade

Field	Position	Original line	Normalised line	Original value	Normalised value
DE	1	29348	29147	flavokawain A	Flavokawain A
DE	2	29348	29147	ochratoxin A	Ochratoxin A (OTA)
DE	3	29348	29147	oxidative stress	Oxidative Stress
DE	4	29348	29147	apoptosis	Apoptosis
ID	1	29349	29148	VASCULAR ENDOTHELIUM	Vascular Endothelium
ID	2	29349	29148	CHALCONE FLAVOKAWAIN	Chalcone Flavokawain
ID	3	29349	29148	KAVA CHALCONE	Kava Chalcone
ID	4	29349	29148	CURCUMIN	Curcumin
ID	5	29349	29148	DYSFUNCTION	Dysfunction
ID	6	29349	29148	MECHANISMS	Mechanism
ID	7	29349	29148	EXPRESSION	Expression
ID	8	29349	29148	PATHWAY	Pathway

Record 189 | WOS:000807169400001

Ochratoxin A Induces Steatosis via PPARÎ³-CD36 Axis

Field	Position	Original line	Normalised line	Original value	Normalised value
DE	1	29472	29270	fatty liver disease	Fatty Liver Disease
DE	2	29472	29270	lipid metabolism	Lipid Metabolism
DE	3	29472	29270	OTA	Ochratoxin A (OTA)
DE	4	29472	29270	PPAR	Ppar
ID	1	29473	29271	LIPID-PEROXIDATION	Lipid Peroxidation
ID	2	29473	29271	PPAR-GAMMA	Ppar Gamma
ID	3	29473	29271	APOPTOSIS	Apoptosis
ID	4	29473	29271	CONTRIBUTES	Contribute

ID	5	29473	29271	EXPRESSION	Expression
ID	6	29473	29271	RECEPTORS	Receptor
ID	7	29473	29271	CITRININ	Citrinin
ID	8	29473	29271	RISK	Risk
ID	9	29473	29271	BETA	Beta
ID	10	29473	29271	CD36	Cd36

Record 190 | WOS:000718125600004

Combination of zinc and selenium alleviates ochratoxin A-induced fibrosis via blocking ROS-dependent autophagy in HK-2 cells

Field	Position	Original line	Normalised line	Original value	Normalised value
DE	1	29596	29393	Ochratoxin A	Ochratoxin A (OTA)
DE	4	29596	29393	Renal fibrosis	Renal Fibrosis
DE	5	29596	29393	ROS	Reactive Oxygen Species (ROS)
ID	1	29597	29395	OXIDATIVE STRESS	Oxidative Stress

Record 191 | WOS:000709727500001

Selenomethionine alleviated Ochratoxin A induced pyroptosis and renal fibrotic factors expressions in MDCK cells

Field	Position	Original line	Normalised line	Original value	Normalised value
DE	1	29716	29513	MDCK cells	MDCK
DE	2	29716	29513	Ochratoxin A	Ochratoxin A (OTA)
DE	3	29716	29513	pyroptosis	Pyroptosis
DE	4	29716	29513	renal fibrotic genes	Renal Fibrotic Gene
DE	5	29716	29513	selenomethionine	Selenomethionine
ID	1	29718	29514	DNA-DAMAGE	DNA Damage
ID	2	29718	29514	INDUCED APOPTOSIS	Induced Apoptosis
ID	3	29718	29514	OXIDATIVE STRESS	Oxidative Stress
ID	4	29718	29514	KIDNEY-CELLS	Kidney
ID	5	29718	29514	IN-VITRO	In-Vitro
ID	6	29718	29514	SELENIUM	Selenium
ID	7	29718	29514	NEPHROTOXICITY	Nephrotoxicity
ID	8	29718	29514	MECHANISMS	Mechanism
ID	9	29718	29514	CYTOTOXICITY	Cytotoxicity
ID	10	29718	29514	TRANSITION	Transition

Record 192 | WOS:000715414400001

Prioritization of Mycotoxins Based on Their Genotoxic Potential with an In Silico-In Vitro Strategy

Field	Position	Original line	Normalised line	Original value	Normalised value
DE	1	29826	29621	mycotoxins	Mycotoxins
DE	2	29826	29621	in silico	In Silico
DE	3	29826	29621	SOS/umu test	Sos Umu Test
DE	4	29826	29621	genotoxicity	Genotoxicity
DE	5	29826	29621	prioritization	Prioritization
ID	1	29827	29622	SALMONELLA-TYPHIMURIUM	Salmonella Typhimurium
ID	2	29827	29622	OCHRATOXIN-A	Ochratoxin A (OTA)
ID	3	29827	29622	MUTAGENICITY TEST	Mutagenicity Test
ID	4	29827	29622	ESCHERICHIA-COLI	Escherichia Coli
ID	5	29827	29622	AMES TEST	Ame Test
ID	6	29827	29622	TESTER STRAINS	Tester Strain
ID	7	29827	29622	SOS/UMU-TEST	Sos Umu Test
ID	8	29827	29622	ACTIVATION	Activation
ID	9	29827	29622	INDUCTION	Induction
ID	10	29827	29622	TOXICITY	Toxicity

Record 193 | WOS:000714429400001

Ochratoxin A-Induced Nephrotoxicity: Up-to-Date Evidence

Field	Position	Original line	Normalised line	Original value	Normalised value
DE	1	29982	29776	ochratoxin A	Ochratoxin A (OTA)
DE	2	29982	29776	nephrotoxicity	Nephrotoxicity
DE	3	29982	29776	molecular interaction	Molecular Interaction
DE	4	29982	29776	prevention	Prevention
ID	1	29983	29777	ENDOPLASMIC-RETICULUM STRESS	Endoplasmic Reticulum Stress
ID	2	29983	29777	OXIDATIVE DNA-DAMAGE	Oxidative DNA Damage
ID	3	29983	29777	PROXIMAL TUBULE	Proximal Tubule
ID	4	29983	29777	KIDNEY-CELLS	Kidney
ID	5	29983	29777	RAT-LIVER	Rat Liver
ID	6	29983	29777	INDUCED APOPTOSIS	Induced Apoptosis
ID	7	29983	29777	HEME OXYGENASE-1	Heme Oxygenase 1
ID	8	29983	29777	GENE-EXPRESSION	Gene Expression
ID	9	29983	29777	NANO PARTICLES	Nano Particle
ID	10	29983	29777	IN-VITRO	In-Vitro

Record 194 ǀ WOS:000726706400001

The role of pumpkin pulp extract carotenoids against mycotoxin damage in the blood brain barrier in vitro

Field	Position	Original line	Normalised line	Original value	Normalised value
DE	1	30172	29965	beauvericin	Beauvericin
DE	2	30172	29965	ECV304	Ecv304
DE	3	30172	29965	metabolomics	Metabolomics
DE	4	30172	29965	ochratoxin A	Ochratoxin A (OTA)
DE	5	30172	29965	zearalenone	Zearalenone (ZEA)
ID	1	30173	29966	BETA-CAROTENE	Beta Carotene
ID	2	30173	29966	BEAUVERICIN	Beauvericin
ID	3	30173	29966	ACID	Acid
ID	4	30173	29966	PERMEABILITY	Permeability
ID	5	30173	29966	ASSOCIATION	Association
ID	6	30173	29966	BRADYKININ	Bradykinin
ID	7	30173	29966	TOCOPHEROL	Tocopherol
ID	8	30173	29966	MODEL	Model

Record 196 ǀ WOS:000701125500001

The Reduction of the Combined Effects of Aflatoxin and Ochratoxin A in Piglet Livers and Kidneys by Dietary Antioxidants

Field	Position	Original line	Normalised line	Original value	Normalised value
DE	1	30345	30136	piglets	Piglet
DE	2	30345	30136	mycotoxins	Mycotoxins
DE	3	30345	30136	CYPs protein expression	Cyp Protein Expression
DE	4	30345	30136	CYPs enzyme activity	Cyp Enzyme Activity
DE	5	30345	30136	feed additives	Feed Additive
DE	6	30345	30136	antioxidant effect	Antioxidant Effect
ID	1	30347	30138	GLUTATHIONE S-TRANSFERASES	Glutathione S Transferase
ID	2	30347	30138	PREGNANE X RECEPTOR	Pregnane X Receptor
ID	3	30347	30138	TRANSCRIPTIONAL REGULATION	Transcriptional Regulation
ID	4	30347	30138	IN-VITRO	In-Vitro
ID	5	30347	30138	CYTOCHROME-P450 ENZYMES	Cytochrome P450 Enzyme
ID	6	30347	30138	GENETIC-POLYMORPHISM	Genetic Polymorphism
ID	7	30347	30138	OXIDATIVE STRESS	Oxidative Stress
ID	8	30347	30138	MYCOTOXINS	Mycotoxins
ID	9	30347	30138	EXPRESSION	Expression
ID	10	30347	30138	B-1	B 1

Record 197 ǀ WOS:000689864300001

Cytotoxicity of Mycotoxins Frequently Present in Aquafeeds to the Fish Cell Line RTGill-W1

Field	Position	Original line	Normalised line	Original value	Normalised value
DE	1	30526	30316	mycotoxins	Mycotoxins
DE	2	30526	30316	cytotoxicity	Cytotoxicity
DE	3	30526	30316	cell viability	Cell Viability
DE	4	30526	30316	RTgill-W1	Rtgill W1
DE	5	30526	30316	fish	Fish
ID	1	30527	30317	IN-VITRO CYTOTOXICITY	In Vitro Cytotoxicity
ID	2	30527	30317	AFLATOXIN B-1	Aflatoxin B1 (AFB1)
ID	3	30527	30317	FUMONISIN B-1	Fumonisin B1 (FB1)
ID	4	30527	30317	TOXICITY	Toxicity
ID	5	30527	30317	DEOXYNIVALENOL	Deoxynivalenol (DON)
ID	6	30527	30317	ZEARALENONE	Zearalenone (ZEA)
ID	7	30527	30317	TROUT	Trout
ID	8	30527	30317	COMBINATIONS	Combination
ID	9	30527	30317	INGREDIENTS	Ingredient
ID	10	30527	30317	AQUACULTURE	Aquaculture

Record 198 | WOS:000688617200001

Curcumin Modulates Nitrosative Stress, Inflammation, and DNA Damage and Protects against Ochratoxin A-Induced Hepatotoxicity and Nephrotoxicity in Rats

Field	Position	Original line	Normalised line	Original value	Normalised value
DE	1	30656	30445	ochratoxin A	Ochratoxin A (OTA)
DE	2	30656	30445	hepatotoxicity	Hepatotoxicity
DE	3	30656	30445	nephrotoxicity	Nephrotoxicity
DE	4	30656	30445	nitrosative stress	Nitrosative Stress
DE	5	30656	30445	inflammation	Inflammation
DE	6	30656	30445	DNA damage	DNA Damage
ID	1	30658	30447	OXIDATIVE STRESS	Oxidative Stress
ID	2	30658	30447	NITRIC-OXIDE	Nitric Oxide
ID	3	30658	30447	IN-VITRO	In-Vitro
ID	4	30658	30447	GENOTOXICITY	Genotoxicity
ID	5	30658	30447	ANTIOXIDANT	Antioxidant
ID	6	30658	30447	BIOMARKER	Biomarker
ID	7	30658	30447	EXPOSURE	Exposure
ID	8	30658	30447	DISEASE	Disease
ID	9	30658	30447	PATHOGENESIS	Pathogenesis
ID	10	30658	30447	PENICILLIUM	Penicillium

Record 199 | WOS:000681272600001

Hepatotoxicity of food-borne mycotoxins: molecular mechanism, anti-hepatotoxic medicines and target prediction

Field	Position	Original line	Normalised line	Original value	Normalised value
DE	1	30801	30589	Anti-hepatotoxic medicines	Anti Hepatotoxic Medicine
DE	2	30801	30589	hepatotoxicity	Hepatotoxicity
DE	3	30801	30589	molecular mechanism	Molecular Mechanism
DE	4	30801	30589	mycotoxins	Mycotoxins
DE	5	30801	30589	target prediction	Target Prediction
ID	1	30803	30591	INDUCED OXIDATIVE STRESS	Induced Oxidative Stress
ID	2	30803	30591	MESSENGER-RNA EXPRESSION	Messenger RNA Expression
ID	3	30803	30591	PEROXIDATIVE HEPATIC DAMAGE	Peroxidative Hepatic Damage
ID	4	30803	30591	NUCLEAR RECEPTORS PXR	Nuclear Receptor Pxr
ID	5	30803	30591	FUMONISIN B-1	Fumonisin B1 (FB1)
ID	6	30803	30591	RAT-LIVER	Rat Liver
ID	7	30803	30591	AFLATOXIN B-1	Aflatoxin B1 (AFB1)
ID	8	30803	30591	OCHRATOXIN-A	Ochratoxin A (OTA)
ID	9	30803	30591	DNA-DAMAGE	DNA Damage

ID	10	30803	30591	IN-VITRO	In-Vitro

Record 200 | WOS:000674669100002

Metabolomics analysis underlay mechanisms in the renal impairment of mice caused by combination of aflatoxin M1 and ochratoxin A

Field	Position	Original line	Normalised line	Original value	Normalised value
DE	1	31064	30851	aflatoxin M1	Aflatoxin M1 (AFM1)
DE	2	31064	30851	ochratoxin A	Ochratoxin A (OTA)
DE	3	31064	30851	metabolomics	Metabolomics
DE	4	31064	30851	nephrotoxicity	Nephrotoxicity
DE	5	31064	30851	LysoPCs	Lysopc
ID	1	31065	30853	ACETYL-L-CARNITINE	Acetyl L Carnitine
ID	2	31065	30853	OXIDATIVE STRESS	Oxidative Stress
ID	3	31065	30853	MASS-SPECTROMETRY	Mass Spectrometry
ID	4	31065	30853	ANIMAL FEED	Animal Feed
ID	5	31065	30853	MYCOTOXINS	Mycotoxins
ID	6	31065	30853	KIDNEY	Kidney
ID	7	31065	30853	LYSOPHOSPHATIDYLCHOLINE	Lysophosphatidylcholine
ID	8	31065	30853	TOXICITY	Toxicity
ID	9	31065	30853	INJURY	Injury
ID	10	31065	30853	RATIO	Ratio

Record 201 | WOS:000675540600002

Ochratoxin A induces ERK1/2 phosphorylation-dependent apoptosis through NF-KB/ERK axis in human proximal tubule HK-2 cell line

Field	Position	Original line	Normalised line	Original value	Normalised value
DE	1	31208	30995	Ochratoxin A	Ochratoxin A (OTA)
DE	2	31208	30995	NF-KB	Nf Kb
DE	3	31208	30995	HK-2 cell line	HK-2 Cell
DE	4	31208	30995	p-ERK1	P Erk1
DE	5	31208	30995	2	Apoptosis
DE	6	31208	30995	Apoptosis	
ID	1	31209	30996	FACTOR-KAPPA-B	Factor Kappa B
ID	2	31209	30996	SIGNALING PATHWAY	Signaling Pathway
ID	3	31209	30996	OXIDATIVE DAMAGE	Oxidative Damage
ID	4	31209	30996	HUMAN HEALTH	Human Health
ID	5	31209	30996	TOXICITY	Toxicity
ID	6	31209	30996	EXPRESSION	Expression
ID	7	31209	30996	MACROPHAGES	Macrophage
ID	8	31209	30996	COMBINATION	Combination
ID	9	31209	30996	STRESS	Stress
ID	10	31209	30996	FEED	Feed

Record 202 | WOS:000656878400004

Mycotoxins in human food: A challenge for research

Field	Position	Original line	Normalised line	Original value	Normalised value
ID	1	31322	31108	FUMONISIN B-1 ALTERS	Fumonisin B 1 Alter
ID	2	31322	31108	IN-VITRO	In-Vitro
ID	3	31322	31108	SPHINGOLIPID METABOLISM	Sphingolipid Metabolism
ID	4	31322	31108	BARRIER FUNCTION	Barrier Function
ID	5	31322	31108	INTESTINAL EPITHELIUM	Intestinal Epithelium
ID	6	31322	31108	MASKED MYCOTOXINS	Masked Mycotoxin
ID	7	31322	31108	ARISTOLOCHIC ACID	Aristolochic Acid
ID	8	31322	31108	ERGOT ALKALOIDS	Ergot Alkaloid
ID	9	31322	31108	OCHRATOXIN-A	Ochratoxin A (OTA)
ID	10	31322	31108	DEOXYNIVALENOL	Deoxynivalenol (DON)

Record 203 | WOS:000661314200027

In vitro blood brain barrier exposure to mycotoxins and carotenoids pumpkin extract alters mitochondrial gene expression and oxidative stress

Field	Position	Original line	Normalised line	Original value	Normalised value
DE	2	31513	31298	Enniatins	Enniatin
DE	3	31513	31298	Ochratoxin A	Ochratoxin A (OTA)
DE	4	31513	31298	Zearalenone	Zearalenone (ZEA)
DE	5	31513	31298	Carotenoids	Carotenoid
ID	1	31514	31299	ALZHEIMERS-DISEASE	Alzheimer Disease
ID	2	31514	31299	PROTEIN	Protein
ID	3	31514	31299	LUTEIN	Lutein
ID	4	31514	31299	INFLAMMATION	Inflammation
ID	5	31514	31299	ZEARALENONE	Zearalenone (ZEA)
ID	6	31514	31299	OCHRATOXIN	Ochratoxin A (OTA)
ID	7	31514	31299	MECHANISMS	Mechanism
ID	8	31514	31299	GENERATION	Generation
ID	9	31514	31299	INJURY	Injury

Record 204 | WOS:000661314200019

Multidimensional analysis of the epigenetic alterations in toxicities induced by mycotoxins

Field	Position	Original line	Normalised line	Original value	Normalised value
DE	1	31623	31407	Mycotoxin	Mycotoxins
DE	2	31623	31407	Epigenetic alterations	Epigenetic Alteration
DE	3	31623	31407	DNA methylation	DNA Methylation
DE	4	31623	31407	Non-coding RNAs	Non Coding RNA
DE	5	31623	31407	Protein post-translational modification	Protein Post Translational Modification
ID	1	31625	31409	DNA METHYLATION	DNA Methylation
ID	2	31625	31409	AFLATOXIN B-1	Aflatoxin B1 (AFB1)
ID	3	31625	31409	PROMOTER HYPERMETHYLATION	Promoter Hypermethylation
ID	4	31625	31409	GENE-EXPRESSION	Gene Expression
ID	5	31625	31409	FUMONISIN B1	Fumonisin B1 (FB1)
ID	6	31625	31409	HEPG2 CELLS	HepG2
ID	7	31625	31409	NEPHROTOXIN OCHRATOXIN	Ochratoxin A (OTA)
ID	8	31625	31409	HISTONE ACETYLATION	Histone Acetylation
ID	9	31625	31409	HEME OXYGENASE-1	Heme Oxygenase 1
ID	10	31625	31409	RAT-KIDNEY	Rat Kidney

Record 205 | WOS:000659169000010

The effect of ochratoxin A on cytotoxicity and glucose metabolism in human esophageal epithelium Het-1A cells

Field	Position	Original line	Normalised line	Original value	Normalised value
DE	1	31804	31587	Ochratoxin A	Ochratoxin A (OTA)
DE	2	31804	31587	Oxidative damage	Oxidative Damage
DE	3	31804	31587	Mitochondrial function	Mitochondrial Function
DE	5	31804	31587	Glucose metabolism	Glucose Metabolism
ID	1	31806	31589	PHASE ARREST	Phase Arrest
ID	2	31806	31589	DNA-DAMAGE	DNA Damage
ID	3	31806	31589	MITOCHONDRIAL DYSFUNCTION	Mitochondrial Dysfunction
ID	4	31806	31589	OXIDATIVE STRESS	Oxidative Stress
ID	5	31806	31589	APOPTOSIS	Apoptosis
ID	6	31806	31589	TOXICITY	Toxicity

Record 206 | WOS:000664290000006

Non-cytotoxic dosage of fumonisin B1 aggravates ochratoxin A-induced nephrocytotoxicity and apoptosis via ROS-dependent JNK/MAPK signaling pathway

Field	Position	Original line	Normalised line	Original value	Normalised value
DE	1	31905	31687	Fumonisin B1	Fumonisin B1 (FB1)
DE	2	31905	31687	Ochratoxin A	Ochratoxin A (OTA)
DE	5	31905	31687	Reactive oxygen species	Reactive Oxygen Specy
DE	6	31905	31687	JNK/MAPK	Jnk Mapk
ID	1	31907	31689	ACTIVATED PROTEIN-KINASE	Activated Protein Kinase
ID	2	31907	31689	OXIDATIVE STRESS	Oxidative Stress
ID	3	31907	31689	IN-VIVO	In-Vivo
ID	4	31907	31689	PK15 CELLS	Pk15
ID	5	31907	31689	B-1	B 1
ID	6	31907	31689	EXPRESSION	Expression
ID	7	31907	31689	NEPHROTOXICITY	Nephrotoxicity
ID	8	31907	31689	NEPHROPATHY	Nephropathy
ID	9	31907	31689	TOXICITY	Toxicity

Record 207 | WOS:000654061000009

Oral subchronic exposure to the mycotoxin ochratoxin A induces key pathological features of Parkinson's disease in mice six months after the end of the treatment

Field	Position	Original line	Normalised line	Original value	Normalised value
DE	1	32029	31810	Parkinson's disease	Parkinson S Disease
DE	2	32029	31810	Alpha-synuclein	Alpha Synuclein
DE	3	32029	31810	Ochratoxin A	Ochratoxin A (OTA)
DE	4	32029	31810	Gut-brain axis	Gut Brain Axis
DE	5	32029	31810	Chaperone-mediated autophagy	Chaperone Mediated Autophagy
DE	6	32029	31810	LAMP-2A	Lamp 2a
ID	1	32031	31812	CHAPERONE-MEDIATED AUTOPHAGY	Chaperone Mediated Autophagy
ID	2	32031	31812	ENVIRONMENTAL TOXINS	Environmental Toxin
ID	3	32031	31812	PESTICIDE EXPOSURE	Pesticide Exposure
ID	4	32031	31812	RAT-BRAIN	Rat Brain
ID	5	32031	31812	ROTENONE	Rotenone
ID	6	32031	31812	SYSTEM	System
ID	7	32031	31812	RISK	Risk
ID	8	32031	31812	NEURODEGENERATION	Neurodegeneration
ID	9	32031	31812	CYTOTOXICITY	Cytotoxicity
ID	10	32031	31812	PROGRESSION	Progression

Record 208 | WOS:000643671100001

Ochratoxin A Induces Oxidative Stress in HepG2 Cells by Impairing the Gene Expression of Antioxidant Enzymes

Field	Position	Original line	Normalised line	Original value	Normalised value
DE	1	32193	31972	ochratoxin A (OTA)	Ochratoxin A (OTA)
DE	2	32193	31972	hepatotoxicity	Hepatotoxicity
DE	4	32193	31972	oxidative stress	Oxidative Stress
DE	5	32193	31972	reactive oxygen species (ROS)	Reactive Oxygen Specy ROS
ID	1	32195	31974	DNA-DAMAGE	DNA Damage
ID	2	32195	31974	MYCOTOXIN OCHRATOXIN	Ochratoxin A (OTA)
ID	3	32195	31974	PROTEIN-SYNTHESIS	Protein Synthesis
ID	4	32195	31974	VINE FRUITS	Vine Fruit
ID	5	32195	31974	IN-VITRO	In-Vitro
ID	6	32195	31974	APOPTOSIS	Apoptosis
ID	7	32195	31974	TOXICITY	Toxicity
ID	8	32195	31974	LIVER	Liver
ID	9	32195	31974	TRANSFORMATION	Transformation
ID	10	32195	31974	ZEARALENONE	Zearalenone (ZEA)

Record 209 | WOS:000637397900008

Exploration of zebrafish larvae as an alternative whole-animal model for nephrotoxicity testing

Field	Position	Original line	Normalised line	Original value	Normalised value
DE	3	32325	32103	Alternative method	Alternative Method
DE	4	32325	32103	Gene expression	Gene Expression

Record 210 | WOS:000634193100001

Rat Tumour Histopathology Associated with Experimental Chronic Dietary Exposure to Ochratoxin A in Prediction of the Mycotoxin's Risk for Human Cancers

Field	Position	Original line	Normalised line	Original value	Normalised value
DE	1	32474	32251	vimentin	Vimentin
DE	2	32474	32251	CD10	Cd10
DE	3	32474	32251	MNF 116	Mnf 116
DE	4	32474	32251	renal cell cancer	Renal Cell Cancer
DE	5	32474	32251	urothelial cancer	Urothelial Cancer
DE	6	32474	32251	testicular cancer	Testicular Cancer
DE	7	32474	32251	immunohistochemistry	Immunohistochemistry

Record 211 | WOS:000634190600001

The Impact of the Nephrotoxin Ochratoxin A on Human Renal Cells Studied by a Novel Co-Culture Model Is Influenced by the Presence of Fibroblasts

Field	Position	Original line	Normalised line	Original value	Normalised value
DE	1	32549	32325	ochratoxin A	Ochratoxin A (OTA)
DE	2	32549	32325	cell culture	Cell
DE	3	32549	32325	energy metabolism	Energy Metabolism
DE	4	32549	32325	apoptosis-necrosis balance	Apoptosis Necrosis Balance
DE	5	32549	32325	mitochondria	Mitochondria

Record 212 | WOS:000637502500004

Screening of Macrofungi Isolates for Aflatoxin B1 and Ochratoxin A Degradation

Field	Position	Original line	Normalised line	Original value	Normalised value
DE	1	32635	32410	food safety	Food Safety
DE	2	32635	32410	macro fungi	Macro Fungi
DE	3	32635	32410	detoxification	Detoxification
DE	4	32635	32410	aflatoxin B-1	Aflatoxin B1 (AFB1)
DE	5	32635	32410	ochratoxin A	Ochratoxin A (OTA)
ID	1	32636	32412	DETOXIFICATION	Detoxification
ID	2	32636	32412	PURIFICATION	Purification
ID	3	32636	32412	MYCOTOXINS	Mycotoxins
ID	4	32636	32412	ENZYME	Enzyme

Record 213 | WOS:000614680800001

Ochratoxin A induces nephrotoxicity in vitro and in vivo via pyroptosis

Field	Position	Original line	Normalised line	Original value	Normalised value
DE	1	32742	32517	Ochratoxin A	Ochratoxin A (OTA)
DE	2	32742	32517	Renal fibrosis	Renal Fibrosis
DE	4	32742	32517	Caspase-1	Caspase 1
DE	5	32742	32517	NLRP3	Nlrp3
DE	6	32742	32517	MDCK cells	MDCK

Record 214 | WOS:000623049400013

Ochratoxin A and its reaction products affected by sugars during heat processing

Field	Position	Original line	Normalised line	Original value	Normalised value
DE	3	32865	32639	Sugars	Sugar
DE	5	32865	32639	Aqueous model system	Aqueous Model System
ID	1	32867	32640	FUMONISIN B-1	Fumonisin B1 (FB1)
ID	2	32867	32640	BREAKFAST CEREALS	Breakfast Cereal
ID	3	32867	32640	GLUCOSE REACTION	Glucose Reaction
ID	4	32867	32640	IN-VITRO	In-Vitro
ID	5	32867	32640	REDUCTION	Reduction
ID	6	32867	32640	FATE	Fate
ID	7	32867	32640	DEGRADATION	Degradation
ID	8	32867	32640	FRUCTOSE	Fructose
ID	9	32867	32640	FOOD	Food
ID	10	32867	32640	SUCROSE	Sucrose

Record 215 | WOS:000615712900001

Editorial overview of the special issue on application of tissue chips in toxicology

Field	Position	Original line	Normalised line	Original value	Normalised value
ID	1	32962	32734	MICROPHYSIOLOGICAL SYSTEMS	Microphysiological System
ID	2	32962	32734	SAFETY ASSESSMENT	Safety Assessment
ID	3	32962	32734	INTESTINE	Intestine
ID	4	32962	32734	LIVER	Liver

Record 216 | WOS:000615712900006

Central role of TRAP1 in the ameliorative effect of oleanolic acid on the mitochondrial-mediated and endoplasmic reticulum stress-excitated apoptosis induced by ochratoxin A

Field	Position	Original line	Normalised line	Original value	Normalised value
DE	1	33063	32834	Mycotoxin	Mycotoxins
DE	2	33063	32834	Oleanolic acid	Oleanolic Acid
DE	3	33063	32834	Protein homeostasis	Protein Homeostasis
DE	4	33063	32834	Tumor necrosis factor receptor-associated protein 1	Tumor Necrosis Factor Receptor Associated Protein 1
DE	5	33063	32834	ER-mitochondria crosstalk	Er Mitochondria Crosstalk
ID	1	33065	32836	PUBLIC-HEALTH PERSPECTIVES	Public Health Perspective
ID	2	33065	32836	OXIDATIVE STRESS	Oxidative Stress
ID	3	33065	32836	ATP SYNTHASE	Atp Synthase
ID	4	33065	32836	URSOLIC ACID	Ursolic Acid
ID	5	33065	32836	NEPHROPATHY	Nephropathy
ID	6	33065	32836	MYCOTOXINS	Mycotoxins
ID	7	33065	32836	HSP60	Hsp60
ID	8	33065	32836	BAX	Bax

Record 217 | WOS:000606891900001

Mycotoxins: Impact on Health and Strategies for Prevention and Detoxification in the Food Chain

Field	Position	Original line	Normalised line	Original value	Normalised value
DE	2	33202	32972	fungi	Fungi
DE	3	33202	32972	health	Health
DE	4	33202	32972	detoxification	Detoxification
DE	5	33202	32972	food safety	Food Safety
ID	1	33203	32973	FUSARIUM HEAD BLIGHT	Fusarium Head Blight
ID	2	33203	32973	CURCUMA-LONGA L.	Curcuma Longa L

ID	3	33203	32973	IN-VITRO	In-Vitro
ID	4	33203	32973	ANIMAL FEED	Animal Feed
ID	5	33203	32973	AFLATOXIN B-1	Aflatoxin B1 (AFB1)
ID	6	33203	32973	ESSENTIAL OIL	Essential Oil
ID	7	33203	32973	OCHRATOXIN-A	Ochratoxin A (OTA)
ID	8	33203	32973	TRICHOTHECENE MYCOTOXINS	Trichothecene Mycotoxin
ID	9	33203	32973	ASPERGILLUS-FLAVUS	Aspergillus Flavus
ID	10	33203	32973	NATURAL OCCURRENCE	Natural Occurrence

Record 218 | WOS:000638197700008

Comet assay: a versatile but complex tool in genotoxicity testing

Field	Position	Original line	Normalised line	Original value	Normalised value
DE	1	33505	33274	comet assay	Comet Assay
DE	2	33505	33274	DNA damage	DNA Damage
DE	3	33505	33274	risk assessment	Risk Assessment
ID	1	33506	33275	DOSE-RESPONSE RELATIONSHIPS	Dose Response Relationship
ID	2	33506	33275	IN-VIVO GENOTOXICITY	In Vivo Genotoxicity
ID	3	33506	33275	OXIDATIVE DNA-DAMAGE	Oxidative DNA Damage
ID	4	33506	33275	OCHRATOXIN-A	Ochratoxin A (OTA)
ID	5	33506	33275	REACTIVE METABOLITE	Reactive Metabolite
ID	6	33506	33275	ALKYLATION DAMAGE	Alkylation Damage
ID	7	33506	33275	STRAND BREAKS	Strand Break
ID	8	33506	33275	FURAN	Furan
ID	9	33506	33275	ADDUCTS	Adduct
ID	10	33506	33275	REPAIR	Repair

Record 219 | WOS:000701996600001

Aspergillus derived mycotoxins in food and the environment: Prevalence, detection, and toxicity

Field	Position	Original line	Normalised line	Original value	Normalised value
DE	1	33662	33430	Aspergillus species	Aspergillus Specy
DE	3	33662	33430	Foodstuffs	Foodstuff
DE	5	33662	33430	OMICS	Omics
DE	6	33662	33430	Aptamers	Aptamer
DE	7	33662	33430	Point of care (PoC)	Point Of Care Poc
ID	1	33664	33432	BIOSYNTHETIC GENE-CLUSTER	Biosynthetic Gene Cluster
ID	2	33664	33432	PERFORMANCE LIQUID-CHROMATOGRAPHY	Performance Liquid Chromatography
ID	3	33664	33432	SECALONIC-ACID-D	Secalonic Acid D
ID	4	33664	33432	CYCLOPIAZONIC ACID	Cyclopiazonic Acid
ID	5	33664	33432	OCHRATOXIN-A	Ochratoxin A (OTA)
ID	6	33664	33432	IN-VITRO	In-Vitro
ID	7	33664	33432	MASS-SPECTROMETRY	Mass Spectrometry
ID	8	33664	33432	NATURAL CONTAMINATION	Natural Contamination
ID	9	33664	33432	PENICILLIUM-EXPANSUM	Penicillium Expansum
ID	10	33664	33432	INDUCED APOPTOSIS	Induced Apoptosis

Record 220 | WOS:000670072600004

Neurotoxicity mechanism of Ochratoxin A

Field	Position	Original line	Normalised line	Original value	Normalised value
DE	1	34021	33788	brain	Brain
DE	2	34021	33788	mycotoxins	Mycotoxins
DE	3	34021	33788	neurotoxicity	Neurotoxicity
DE	4	34021	33788	Ochratoxin	Ochratoxin A (OTA)
ID	1	34022	33789	OXIDATIVE STRESS	Oxidative Stress
ID	2	34022	33789	TRANSCRIPTION FACTORS	Transcription Factor
ID	3	34022	33789	HUMAN KIDNEY	Human Kidney
ID	4	34022	33789	EARLY EVENT	Early Event
ID	5	34022	33789	CELL-DEATH	Cell Death
ID	6	34022	33789	IN-VITRO	In-Vitro
ID	7	34022	33789	TOXICITY	Toxicity
ID	8	34022	33789	CARCINOGENICITY	Carcinogenicity
ID	9	34022	33789	TRANSPORT	Transport
ID	10	34022	33789	REDUCTION	Reduction

Record 221 | WOS:000603090600001

Mycotoxin and Gut Microbiota Interactions

Field	Position	Original line	Normalised line	Original value	Normalised value
DE	1	34156	33922	mycotoxins	Mycotoxins
DE	2	34156	33922	gut microbiota	Gut Microbiota
DE	3	34156	33922	biotransformation	Biotransformation
DE	4	34156	33922	adsorption	Adsorption
DE	5	34156	33922	health	Health
ID	1	34157	33923	DIFFERENT SEQUESTERING AGENTS	Different Sequestering Agent
ID	2	34157	33923	T-2 TOXIN IMPAIRMENT	T 2 Toxin Impairment
ID	3	34157	33923	IN-VITRO METABOLISM	In Vitro Metabolism
ID	4	34157	33923	FUMONISIN B-1	Fumonisin B1 (FB1)
ID	5	34157	33923	SALMONELLA-TYPHIMURIUM	Salmonella Typhimurium
ID	6	34157	33923	AFLATOXIN B-1	Aflatoxin B1 (AFB1)
ID	7	34157	33923	INTESTINAL MICROFLORA	Intestinal Microflora
ID	8	34157	33923	ESCHERICHIA-COLI	Escherichia Coli
ID	9	34157	33923	MASKED MYCOTOXIN	Masked Mycotoxin
ID	10	34157	33923	OCHRATOXIN-A	Ochratoxin A (OTA)

Record 222 | WOS:000597367400001

Ameliorative role of dietary activated carbon against ochratoxin-A induced oxidative damage, suppressed performance and toxicological effects

Field	Position	Original line	Normalised line	Original value	Normalised value
DE	1	34426	34191	Ochratoxin A	Ochratoxin A (OTA)
DE	2	34426	34191	broiler chicken	Broiler Chicken
DE	3	34426	34191	toxicopathology	Toxicopathology
DE	4	34426	34191	oxidative stress	Oxidative Stress
DE	5	34426	34191	activated carbon	Activated Carbon
ID	1	34428	34193	IN-VITRO	In-Vitro
ID	2	34428	34193	T-2 TOXIN	T 2 Toxin
ID	3	34428	34193	CHARCOAL	Charcoal
ID	4	34428	34193	DEOXYNIVALENOL	Deoxynivalenol (DON)
ID	5	34428	34193	AFLATOXIN	Aflatoxin
ID	6	34428	34193	EFFICACY	Efficacy
ID	7	34428	34193	EXPOSURE	Exposure
ID	8	34428	34193	CEREALS	Cereal

Record 223 | WOS:000584401800010

Transcriptomic alterations induced by aflatoxin B1 and ochratoxin A in LMH cell line

Field	Position	Original line	Normalised line	Original value	Normalised value
DE	1	34550	34314	aflatoxin	Aflatoxin
DE	2	34550	34314	ochratoxin	Ochratoxin A (OTA)
DE	3	34550	34314	hepatoxicity	Hepatoxicity
DE	4	34550	34314	chicken liver	Chicken Liver
DE	5	34550	34314	transcriptomics	Transcriptomics
ID	1	34551	34315	GENE-EXPRESSION ANALYSIS	Gene Expression Analysis
ID	2	34551	34315	HEPATOCELLULAR-CARCINOMA	Hepatocellular Carcinoma
ID	3	34551	34315	LIPID-METABOLISM	Lipid Metabolism
ID	4	34551	34315	GRADED-LEVELS	Graded Level
ID	5	34551	34315	BILE-ACID	Bile Acid
ID	6	34551	34315	B-1	B 1
ID	7	34551	34315	DAMAGE	Damage
ID	8	34551	34315	LIVER	Liver
ID	9	34551	34315	MECHANISM	Mechanism
ID	10	34551	34315	CYTOTOXICITY	Cytotoxicity

Record 224 | WOS:000594353000001

Effects of Single and Repeated Oral Doses of Ochratoxin A on the Lipid Peroxidation and Antioxidant Defense Systems in Mouse Kidneys

Field	Position	Original line	Normalised line	Original value	Normalised value
DE	1	34679	34442	ochratoxin-A	Ochratoxin A (OTA)
DE	2	34679	34442	glutathione	Glutathione (GSH)
DE	3	34679	34442	oxidative stress	Oxidative Stress
DE	4	34679	34442	kidney	Kidney
DE	5	34679	34442	gene expression	Gene Expression
ID	1	34680	34444	BALKAN ENDEMIC NEPHROPATHY	Balkan Endemic Nephropathy
ID	2	34680	34444	HEME OXYGENASE-1 GENE	Heme Oxygenase 1 Gene
ID	3	34680	34444	OXIDATIVE STRESS	Oxidative Stress
ID	4	34680	34444	HEPG2 CELLS	HepG2
ID	5	34680	34444	DNA-DAMAGE	DNA Damage
ID	6	34680	34444	HYDROGEN-PEROXIDE	Hydrogen Peroxide
ID	7	34680	34444	RAT-LIVER	Rat Liver
ID	8	34680	34444	GLUTATHIONE	Glutathione (GSH)
ID	9	34680	34444	INDUCTION	Induction
ID	10	34680	34444	EXPRESSION	Expression

Record 225 | WOS:000580526300009

Microphysiological system modeling of ochratoxin A-associated nephrotoxicity

Field	Position	Original line	Normalised line	Original value	Normalised value
DE	1	34824	34587	Ochratoxin A	Ochratoxin A (OTA)
DE	3	34824	34587	Microphysiological systems	Microphysiological System
DE	4	34824	34587	Proximal tubule epithelial cells	Proximal Tubule Epithelial
DE	5	34824	34587	Oxidative stress	Oxidative Stress
DE	6	34824	34587	GSTs	Gst
ID	1	34826	34589	GLUTATHIONE	Glutathione (GSH)
ID	2	34826	34589	EXPOSURE	Exposure
ID	3	34826	34589	TOXICITY	Toxicity
ID	4	34826	34589	NEPHROPATHY	Nephropathy
ID	5	34826	34589	URINE	Urine
ID	6	34826	34589	EXPRESSION	Expression
ID	7	34826	34589	BIOMARKERS	Biomarker
ID	8	34826	34589	INHIBITORS	Inhibitor
ID	9	34826	34589	MECHANISM	Mechanism
ID	10	34826	34589	TRANSPORT	Transport

Record 226 | WOS:000582386000020

Ochratoxins in food and feed: Occurrence and its impact on human health and management strategies

Field	Position	Original line	Normalised line	Original value	Normalised value
DE	1	34971	34733	Ochratoxins	Ochratoxin A (OTA)
DE	2	34971	34733	Aspergillus spp.	Aspergillus Spp
DE	3	34971	34733	Food contamination	Food Contamination
DE	6	34971	34733	Management and control strategies	Management And Control Strategy
ID	1	34973	34735	ASPERGILLUS SECTION NIGRI	Aspergillus Section Nigri
ID	2	34973	34735	PERFORMANCE LIQUID-CHROMATOGRAPHY	Performance Liquid Chromatography
ID	3	34973	34735	A BIOSYNTHESIS GENES	A Biosynthesis Gene
ID	4	34973	34735	WATER ACTIVITY	Water Activity
ID	5	34973	34735	PENICILLIUM-VERRUCOSUM	Penicillium Verrucosum
ID	6	34973	34735	TOXIGENIC FUNGI	Toxigenic Fungi
ID	7	34973	34735	IN-VITRO	In-Vitro
ID	8	34973	34735	ENVIRONMENTAL-CONDITIONS	Environmental Condition
ID	9	34973	34735	IMMUNOAFFINITY CLEANUP	Immunoaffinity Cleanup
ID	10	34973	34735	BREADMAKING PROCESS	Breadmaking Process

Record 227 | WOS:000584874600001

The protective effect of stilbenes resveratrol and pterostilbene individually and combined with mycotoxin citrinin in human adenocarcinoma HT-29 cell line in vitro

Field	Position	Original line	Normalised line	Original value	Normalised value
DE	2	35272	35033	resveratrol	Resveratrol
DE	3	35272	35033	pterostilbene	Pterostilbene
DE	4	35272	35033	viability	Viability
DE	5	35272	35033	superoxide	Superoxide
DE	6	35272	35033	antioxidant effect	Antioxidant Effect
DE	7	35272	35033	HT-29 cell line	Ht 29 Cell
ID	1	35274	35035	VITIS-VINIFERA	Vitis Vinifera
ID	2	35274	35035	OCHRATOXIN-A	Ochratoxin A (OTA)
ID	3	35274	35035	INDUCED DYSFUNCTION	Induced Dysfunction
ID	4	35274	35035	CYTOTOXICITY	Cytotoxicity
ID	5	35274	35035	APOPTOSIS	Apoptosis
ID	6	35274	35035	BIOAVAILABILITY	Bioavailability
ID	7	35274	35035	ULTRASTRUCTURE	Ultrastructure
ID	8	35274	35035	IDENTIFICATION	Identification
ID	9	35274	35035	MITOCHONDRIA	Mitochondria
ID	10	35274	35035	HOMEOSTASIS	Homeostasis

Record 228 | WOS:000563527100012

Cytoprotective effects of curcumin and silymarin on PK-15 cells exposed to ochratoxin A, fumonisin B1 and deoxynivalenol

Field	Position	Original line	Normalised line	Original value	Normalised value
DE	2	35404	35164	Ochratoxin A	Ochratoxin A (OTA)
DE	3	35404	35164	Fumonisin B-1	Fumonisin B1 (FB1)
DE	4	35404	35164	Deoxynivalenol	Deoxynivalenol (DON)
ID	1	35406	35166	OXIDATIVE STRESS	Oxidative Stress
ID	2	35406	35166	AFLATOXIN B-1	Aflatoxin B1 (AFB1)
ID	3	35406	35166	DNA-DAMAGE	DNA Damage
ID	4	35406	35166	IN-VIVO	In-Vivo
ID	5	35406	35166	TOXICITY	Toxicity
ID	6	35406	35166	MYCOTOXINS	Mycotoxins
ID	7	35406	35166	GENOTOXICITY	Genotoxicity
ID	8	35406	35166	MICE	Mice
ID	9	35406	35166	HEPATOTOXICITY	Hepatotoxicity

ID	10	35406	35166	CYTOTOXICITY	Cytotoxicity

Record 229 | WOS:000563527100013

Genotoxic effects of mycotoxins

Field	Position	Original line	Normalised line	Original value	Normalised value
DE	1	35510	35269	Mycotoxin	Mycotoxins
DE	2	35510	35269	Aflatoxins	Aflatoxin
DE	3	35510	35269	Ochratoxin A	Ochratoxin A (OTA)
DE	5	35510	35269	Fumonisins	Fumonisin
DE	6	35510	35269	Zearalenone	Zearalenone (ZEA)
DE	8	35510	35269	Ergot alkaloids	Ergot Alkaloid
DE	10	35510	35269	Health effect	Health Effect
ID	1	35512	35271	INDUCED OXIDATIVE STRESS	Induced Oxidative Stress
ID	2	35512	35271	IN-VITRO GENOTOXICITY	In Vitro Genotoxicity
ID	3	35512	35271	RENAL TUMOR-FORMATION	Renal Tumor Formation
ID	4	35512	35271	FUMONISIN B-1	Fumonisin B1 (FB1)
ID	5	35512	35271	OCHRATOXIN-A	Ochratoxin A (OTA)
ID	6	35512	35271	AFLATOXIN B-1	Aflatoxin B1 (AFB1)
ID	7	35512	35271	DNA-DAMAGE	DNA Damage
ID	8	35512	35271	CHROMOSOME-ABERRATIONS	Chromosome Aberration
ID	9	35512	35271	HEPATOCELLULAR-CARCINOMA	Hepatocellular Carcinoma
ID	10	35512	35271	FUSARIUM MYCOTOXINS	Fusarium Mycotoxin

Record 230 | WOS:000579907000025

Ochratoxin A induces reprogramming of glucose metabolism by switching energy metabolism from oxidative phosphorylation to glycolysis in human gastric epithelium GES-1 cells in vitro

Field	Position	Original line	Normalised line	Original value	Normalised value
DE	1	35704	35462	Ochratoxin A	Ochratoxin A (OTA)
DE	2	35704	35462	Glucose metabolism	Glucose Metabolism
DE	3	35704	35462	Aerobic glycolysis	Aerobic Glycolysis
DE	4	35704	35462	Tricarboxylic acid cycle	Tricarboxylic Acid Cycle
ID	1	35706	35464	PYRUVATE-KINASE M2	Pyruvate Kinase M2
ID	2	35706	35464	NEPHROTOXIN OCHRATOXIN	Ochratoxin A (OTA)
ID	3	35706	35464	PHASE ARREST	Phase Arrest
ID	4	35706	35464	DNA-DAMAGE	DNA Damage
ID	5	35706	35464	CANCER	Cancer
ID	6	35706	35464	TRANSFORMATION	Transformation
ID	7	35706	35464	INFLAMMATION	Inflammation
ID	8	35706	35464	HIF-1-ALPHA	Hif 1 Alpha
ID	9	35706	35464	ACTIVATION	Activation
ID	10	35706	35464	EXPRESSION	Expression

Record 231 | WOS:000566774200008

Dechlorination and demethylation of ochratoxin A enhance blocking activity of PXR activation, suppress PXR expression and reduce cytotoxicity

Field	Position	Original line	Normalised line	Original value	Normalised value
DE	1	35813	35569	Ochratoxin A	Ochratoxin A (OTA)
DE	2	35813	35569	Mycotoxin	Mycotoxins
DE	3	35813	35569	Ochratoxin A derivatives	Ochratoxin A Derivative
DE	4	35813	35569	Pregnane X receptor	Pregnane X Receptor
DE	5	35813	35569	Chemoresistance and metabolic diseases	Chemoresistance And Metabolic Disease

ID	1	35815	35571	PREGNANE-X-RECEPTOR	Pregnane X Receptor
ID	2	35815	35571	CULTURED HUMAN HEPATOCYTES	Cultured Human Hepatocyte
ID	3	35815	35571	XENOBIOTIC RECEPTOR	Xenobiotic Receptor
ID	4	35815	35571	GENE-EXPRESSION	Gene Expression
ID	5	35815	35571	NUCLEAR RECEPTORS	Nuclear Receptor
ID	6	35815	35571	CYP3A4 INDUCTION	Cyp3a4 Induction
ID	7	35815	35571	BINDING	Binding
ID	8	35815	35571	TARGET	Target
ID	9	35815	35571	TRANSPORTERS	Transporter
ID	10	35815	35571	KETOCONAZOLE	Ketoconazole

Record 232 | WOS:000585534200001

The Compromised Intestinal Barrier Induced by Mycotoxins

Field	Position	Original line	Normalised line	Original value	Normalised value
DE	1	35929	35684	mycotoxins	Mycotoxins
DE	2	35929	35684	intestinal barrier	Intestinal Barrier
DE	3	35929	35684	intestinal inflammation	Intestinal Inflammation
DE	4	35929	35684	interactive effects	Interactive Effect
ID	1	35931	35685	TIGHT JUNCTION PERMEABILITY	Tight Junction Permeability
ID	2	35931	35685	IN-VITRO	In-Vitro
ID	3	35931	35685	AFLATOXIN B-1	Aflatoxin B1 (AFB1)
ID	4	35931	35685	EX-VIVO	Ex Vivo
ID	5	35931	35685	GROWTH-PERFORMANCE	Growth Performance
ID	6	35931	35685	MUCOSAL BARRIER	Mucosal Barrier
ID	7	35931	35685	GUT HEALTH	Gut Health
ID	8	35931	35685	CLAUDIN-2 EXPRESSION	Claudin 2 Expression
ID	9	35931	35685	EPITHELIAL BARRIER	Epithelial Barrier
ID	10	35931	35685	JEJUNAL MORPHOLOGY	Jejunal Morphology

Record 233 | WOS:000564335100008

Larval zebrafish as an in vitro model for evaluating toxicological effects of mycotoxins

Field	Position	Original line	Normalised line	Original value	Normalised value
DE	3	36222	35975	Embryos	Embryo
DE	5	36222	35975	Mixtures	Mixture
DE	7	36222	35975	Model system	Model System
DE	8	36222	35975	Embryonic development	Embryonic Development
ID	1	36224	35977	DANIO-RERIO	Danio Rerio
ID	2	36224	35977	DEVELOPMENTAL TOXICITY	Developmental Toxicity
ID	3	36224	35977	OXIDATIVE STRESS	Oxidative Stress
ID	4	36224	35977	ZEARALENONE	Zearalenone (ZEA)
ID	5	36224	35977	OCHRATOXIN	Ochratoxin A (OTA)
ID	6	36224	35977	EXPOSURE	Exposure
ID	7	36224	35977	CITRININ	Citrinin
ID	8	36224	35977	DEOXYNIVALENOL	Deoxynivalenol (DON)
ID	9	36224	35977	ENNIATIN	Enniatin
ID	10	36224	35977	PRODUCTS	Product

Record 234 | WOS:000585500900001

Toxin Degradation by Rumen Microorganisms: A Review

Field	Position	Original line	Normalised line	Original value	Normalised value
DE	1	36375	36128	rumen microorganisms	Rumen Microorganism
DE	2	36375	36128	plant toxins	Plant Toxin

DE	3	36375	36128	probiotic	Probiotic
DE	4	36375	36128	metabolism	Metabolism
DE	5	36375	36128	degradation	Degradation
ID	1	36376	36129	NONPROTEIN AMINO-ACIDS	Nonprotein Amino Acid
ID	2	36376	36129	RAGWORT SENECIO-JACOBAEA	Ragwort Senecio Jacobaea
ID	3	36376	36129	DHP-DEGRADING BACTERIA	Dhp Degrading Bacteria
ID	4	36376	36129	VITRO DIGESTION MODEL	Vitro Digestion Model
ID	5	36376	36129	NEUROTOXIN BETA-ODAP	Neurotoxin Beta Odap
ID	6	36376	36129	INDUCED DNA-DAMAGE	Induced DNA Damage
ID	7	36376	36129	OCHRATOXIN-A	Ochratoxin A (OTA)
ID	8	36376	36129	IN-VITRO	In-Vitro
ID	9	36376	36129	PYRROLIZIDINE ALKALOIDS	Pyrrolizidine Alkaloid
ID	10	36376	36129	LEAFY SPURGE	Leafy Spurge

Record 235 ǀ WOS:000585462600001

Mycotoxins: Biotransformation and Bioavailability Assessment Using Caco-2 Cell Monolayer

Field	Position	Original line	Normalised line	Original value	Normalised value
DE	1	36702	36453	permeability	Permeability
DE	2	36702	36453	bioavailability	Bioavailability
DE	3	36702	36453	intestinal transport	Intestinal Transport
DE	4	36702	36453	metabolism	Metabolism
DE	5	36702	36453	mycotoxins	Mycotoxins
DE	6	36702	36453	biotransformation	Biotransformation
DE	7	36702	36453	cytochrome	Cytochrome
ID	1	36704	36455	MESSENGER-RNA-EXPRESSION	Messenger RNA Expression
ID	2	36704	36455	IN-VITRO METABOLISM	In Vitro Metabolism
ID	3	36704	36455	ALTERNARIOL MONOMETHYL ETHER	Alternariol Monomethyl Ether
ID	4	36704	36455	T-2 TOXIN	T 2 Toxin
ID	5	36704	36455	GENE-EXPRESSION	Gene Expression
ID	6	36704	36455	AFLATOXIN B-1	Aflatoxin B1 (AFB1)
ID	7	36704	36455	FUMONISIN B-1	Fumonisin B1 (FB1)
ID	8	36704	36455	OCHRATOXIN-A	Ochratoxin A (OTA)
ID	9	36704	36455	ENNIATIN B	Enniatin B
ID	10	36704	36455	PHASE-I	Phase I

Record 236 ǀ WOS:000573807900002

Ochratoxin A affects oocyte maturation and subsequent embryo developmental dynamics in the juvenile sheep model

Field	Position	Original line	Normalised line	Original value	Normalised value
DE	1	37025	36775	Ochratoxin A	Ochratoxin A (OTA)
DE	2	37025	36775	Juvenile sheep oocyte	Juvenile Sheep Oocyte
DE	3	37025	36775	In vitro maturation	In Vitro Maturation
DE	4	37025	36775	In vitro fertilization	In Vitro Fertilization
DE	6	37025	36775	oxidative status	Oxidative Status
DE	7	37025	36775	Time-lapse embryo monitoring	Time Lapse Embryo Monitoring
ID	1	37028	36777	IN-VITRO MATURATION	In Vitro Maturation
ID	2	37028	36777	OXIDATIVE STRESS	Oxidative Stress
ID	3	37028	36777	NANOMOLAR CONCENTRATIONS	Nanomolar Concentration
ID	4	37028	36777	SUPPLEMENTATION	Supplementation
ID	5	37028	36777	FERTILIZATION	Fertilization
ID	6	37028	36777	COMPETENCE	Competence

ID	7	37028	36777	MECHANISMS	Mechanism
ID	8	37028	36777	MYCOTOXIN	Mycotoxins
ID	9	37028	36777	APOPTOSIS	Apoptosis
ID	10	37028	36777	FOLLICLE	Follicle

Record 237 | WOS:000545982700021

E ffects of Ochratoxin A exposure on DNA damage in porcine granulosa cells in vitro

Field	Position	Original line	Normalised line	Original value	Normalised value
DE	1	37164	36912	Ochratoxin A	Ochratoxin A (OTA)
DE	2	37164	36912	Porcine granulosa cells	Porcine Granulosa
DE	3	37164	36912	DNA damage	DNA Damage
DE	4	37164	36912	RNA-Seq	RNA Seq
ID	1	37165	36913	BASE-EXCISION-REPAIR	Base Excision Repair
ID	2	37165	36913	KIDNEY PK15 CELLS	Kidney Pk15
ID	3	37165	36913	G2 PHASE ARREST	G2 Phase Arrest
ID	4	37165	36913	STRAND BREAK	Strand Break
ID	5	37165	36913	ALPHA-TOCOPHEROL	Alpha Tocopherol
ID	6	37165	36913	CYTOTOXICITY	Cytotoxicity
ID	7	37165	36913	MYCOTOXIN	Mycotoxins
ID	8	37165	36913	APOPTOSIS	Apoptosis
ID	9	37165	36913	REPLICATION	Replication
ID	10	37165	36913	TOXICITY	Toxicity

Record 238 | WOS:000568157900004

Multiple-endpoint in vitro carcinogenicity test in human cell line TK6 distinguishes carcinogens from non-carcinogens and highlights mechanisms of action

Field	Position	Original line	Normalised line	Original value	Normalised value
DE	1	37292	37038	In vitro	In-Vitro
DE	2	37292	37038	Carcinogenicity testing	Carcinogenicity Testing
DE	4	37292	37038	Multiple endpoints	Multiple Endpoint
ID	1	37293	37039	DISCRIMINATE RODENT CARCINOGENS	Discriminate Rodent Carcinogen
ID	2	37293	37039	GENOTOXICITY TESTS	Genotoxicity Test
ID	3	37293	37039	FOLLOW-UP	Follow Up
ID	4	37293	37039	NONGENOTOXIC CHEMICALS	Nongenotoxic Chemical
ID	5	37293	37039	RECOMMENDED LISTS	Recommended List
ID	6	37293	37039	OCHRATOXIN	Ochratoxin A (OTA)
ID	7	37293	37039	TOXICITY	Toxicity
ID	8	37293	37039	SAFETY	Safety
ID	9	37293	37039	SENSITIVITY	Sensitivity
ID	10	37293	37039	REDUCTION	Reduction

Record 239 | WOS:000567625500001

Protective role of lactic acid bacteria and yeasts as dietary carcinogen-binding agents - a review

Field	Position	Original line	Normalised line	Original value	Normalised value
DE	1	37433	37178	binding agents	Binding Agent
DE	2	37433	37178	cancer	Cancer
DE	3	37433	37178	lactic acid bacteria	Lactic Acid Bacteria
DE	4	37433	37178	yeast	Yeast
ID	1	37434	37179	SODIUM-CALCIUM ALUMINOSILICATE	Sodium Calcium Aluminosilicate
ID	2	37434	37179	POLYCYCLIC AROMATIC-HYDROCARBONS	Polycyclic Aromatic Hydrocarbon
ID	3	37434	37179	CHOLESTEROL OXIDATION-PRODUCTS	Cholesterol Oxidation Product

ID	4	37434	37179	SACCHAROMYCES-CEREVISIAE STRAINS	Saccharomyce Cerevisiae Strain
ID	5	37434	37179	IN-VITRO REMOVAL	In Vitro Removal
ID	6	37434	37179	AFLATOXIN B-1	Aflatoxin B1 (AFB1)
ID	7	37434	37179	HETEROCYCLIC AMINES	Heterocyclic Amine
ID	8	37434	37179	SURFACE BINDING	Surface Binding
ID	9	37434	37179	HEAVY-METALS	Heavy Metal
ID	10	37434	37179	OCHRATOXIN-A	Ochratoxin A (OTA)

Record 240 | WOS:000562731900014

Ochratoxin A induces glomerular injury through activating the ERK/NF-ÎºB signaling pathway

Field	Position	Original line	Normalised line	Original value	Normalised value
DE	1	37719	37463	Ochratoxin A	Ochratoxin A (OTA)
DE	2	37719	37463	Glomerular injury	Glomerular Injury
DE	3	37719	37463	The ERIVNF-kappa B signaling pathway	The Erivnf Kappa B Signaling Pathway
DE	4	37719	37463	Human mesangial cells	Human Mesangial
ID	1	37721	37465	MESANGIAL CELLS	Mesangial
ID	2	37721	37465	IN-VITRO	In-Vitro
ID	3	37721	37465	TOXICITY	Toxicity
ID	4	37721	37465	IMMUNOTOXICITY	Immunotoxicity
ID	5	37721	37465	NEPHROTOXICITY	Nephrotoxicity
ID	6	37721	37465	KIDNEY	Kidney
ID	7	37721	37465	LIVER	Liver

Record 241 | WOS:000585802200008

Synergistic Interaction of Ochratoxin A and Acrylamide Toxins in Human Kidney and Liver Cells

Field	Position	Original line	Normalised line	Original value	Normalised value
DE	1	37824	37567	ochratoxin A	Ochratoxin A (OTA)
DE	2	37824	37567	acrylamide	Acrylamide (AA)
DE	3	37824	37567	combined toxicity	Combined Toxicity
DE	4	37824	37567	CYP	Cyp
DE	5	37824	37567	apoptosis	Apoptosis
ID	1	37825	37568	OXIDATIVE DNA-DAMAGE	Oxidative DNA Damage
ID	2	37825	37568	IN-VITRO	In-Vitro
ID	3	37825	37568	METABOLIC-ACTIVATION	Metabolic Activation
ID	4	37825	37568	STRESS	Stress
ID	5	37825	37568	APOPTOSIS	Apoptosis
ID	6	37825	37568	TOXICITY	Toxicity
ID	7	37825	37568	INDUCTION	Induction
ID	8	37825	37568	CEREALS	Cereal
ID	9	37825	37568	2,3,7,8-TETRACHLORODIBENZO-P-DIOXIN	2 3 7 8 Tetrachlorodibenzo P Dioxin
ID	10	37825	37568	CYTOCHROME-P450	Cytochrome P450

Record 242 | WOS:000560097800001

Ochratoxin A presence in Cabernet Sauvignon wine changes antioxidant activityin vitroand oxidative stress markersin vivo

Field	Position	Original line	Normalised line	Original value	Normalised value
DE	1	37969	37711	Mycotoxin	Mycotoxins
DE	2	37969	37711	oxidative stress	Oxidative Stress
DE	3	37969	37711	phenolic compounds	Phenolic Compound
DE	4	37969	37711	reactive oxygen species	Reactive Oxygen Specy
ID	1	37970	37712	PHENOLIC-COMPOUNDS	Phenolic Compound
ID	2	37970	37712	RED WINE	Red Wine

ID	3	37970	37712	CAENORHABDITIS-ELEGANS	Caenorhabditis Elegan
ID	4	37970	37712	TOXICITY	Toxicity
ID	5	37970	37712	CYTOTOXICITY	Cytotoxicity
ID	6	37970	37712	DAMAGE	Damage
ID	7	37970	37712	GRAPE	Grape
ID	8	37970	37712	ASSAY	Assay

Record 243 | WOS:000539161100006

Mycotoxins, invisible danger of feedstuff with toxic effect on animals

Field	Position	Original line	Normalised line	Original value	Normalised value
DE	2	38082	37823	Forage contamination	Forage Contamination
DE	3	38082	37823	Animals	Animal
DE	4	38082	37823	Aflatoxins	Aflatoxin
DE	5	38082	37823	Zearaelonone trichothecens	Zearaelonone Trichothecen
DE	8	38082	37823	Methods of mycotoxins determination	Method Of Mycotoxin Determination
ID	1	38085	37825	FUSARIUM MYCOTOXINS	Fusarium Mycotoxin
ID	2	38085	37825	NATURAL OCCURRENCE	Natural Occurrence
ID	3	38085	37825	ALPHA-ZEARALENOL	Alpha Zearalenol
ID	4	38085	37825	FUMONISIN B-1	Fumonisin B1 (FB1)
ID	5	38085	37825	OCHRATOXIN-A	Ochratoxin A (OTA)
ID	6	38085	37825	CORN-SILAGE	Corn Silage
ID	7	38085	37825	IN-VITRO	In-Vitro
ID	8	38085	37825	SACCHAROMYCES-CEREVISIAE	Saccharomyce Cerevisiae
ID	9	38085	37825	CELL-PROLIFERATION	Cell Proliferation
ID	10	38085	37825	WEATHER CONDITIONS	Weather Condition

Record 244 | WOS:000547359800001

Subchronic exposure to individual and combined ochratoxin A and citrinin affects the expression of rat renal organic anion transporters

Field	Position	Original line	Normalised line	Original value	Normalised value
DE	3	38267	38006	Oral exposure	Oral Exposure
DE	4	38267	38006	Organic anions	Organic Anion
DE	5	38267	38006	Proximal tubules	Proximal Tubule
ID	1	38269	38008	PROXIMAL-TUBULE	Proximal Tubule
ID	2	38269	38008	NANOMOLAR CONCENTRATIONS	Nanomolar Concentration
ID	3	38269	38008	IN-VIVO	In-Vivo
ID	4	38269	38008	CELLS	Cell
ID	5	38269	38008	FAMILY	Family
ID	6	38269	38008	OAT1	Oat1
ID	7	38269	38008	COMBINATION	Combination
ID	8	38269	38008	RESVERATROL	Resveratrol
ID	9	38269	38008	MECHANISMS	Mechanism
ID	10	38269	38008	TOXICITY	Toxicity

Record 245 | WOS:000545059200001

Ochratoxin A induces epithelial-to-mesenchymal transition and renal fibrosis through TGF-Î²/Smad2/3 and Wnt1/Î²-catenin signaling pathways in vitro and in vivo

Field	Position	Original line	Normalised line	Original value	Normalised value
DE	1	38397	38135	Ochratoxin A	Ochratoxin A (OTA)
DE	2	38397	38135	Epithelial-to-mesenchymal transition	Epithelial To Mesenchymal Transition
DE	4	38397	38135	Smad2/3	Smad2 3
DE	5	38397	38135	beta-Catenin	Beta Catenin

ID	1	38399	38137	TGF-BETA	Tgf Beta
ID	2	38399	38137	FIBROBLASTS	Fibroblast
ID	3	38399	38137	MECHANISMS	Mechanism
ID	4	38399	38137	TISSUE	Tissue
ID	5	38399	38137	CELLS	Cell
ID	6	38399	38137	EXPRESSION	Expression
ID	7	38399	38137	BETA/SMAD	Beta Smad
ID	8	38399	38137	MEMBRANE	Membrane
ID	9	38399	38137	TOXICITY	Toxicity
ID	10	38399	38137	SECRETE	Secrete

Record 246 | WOS:000541110800046

Luteolin alleviates ochratoxin A induced oxidative stress by regulating Nrf2 and HIF-1Î± pathways in NRK-52E rat kidney cells

Field	Position	Original line	Normalised line	Original value	Normalised value
DE	1	38540	38277	Ochratoxin A	Ochratoxin A (OTA)
DE	4	38540	38277	Oxidative stress	Oxidative Stress
DE	6	38540	38277	HIF-1 alpha	Hif 1 Alpha
ID	1	38542	38279	DNA-DAMAGE	DNA Damage
ID	2	38542	38279	INDUCED TOXICITY	Induced Toxicity
ID	3	38542	38279	EXPRESSION	Expression
ID	4	38542	38279	FLAVONOIDS	Flavonoid
ID	5	38542	38279	QUERCETIN	Quercetin
ID	6	38542	38279	APOPTOSIS	Apoptosis
ID	7	38542	38279	HYPOXIA	Hypoxia
ID	8	38542	38279	NEPHROTOXICITY	Nephrotoxicity
ID	9	38542	38279	SUPPRESS	Suppress
ID	10	38542	38279	LYCOPENE	Lycopene

Record 247 | WOS:000556892100001

Reducing Ochratoxin A Content in Grape Pomace by Different Methods

Field	Position	Original line	Normalised line	Original value	Normalised value
DE	1	38657	38393	ochratoxin A	Ochratoxin A (OTA)
DE	2	38657	38393	OTA reduction	OTA Reduction
DE	3	38657	38393	thermal pressure treatment	Thermal Pressure Treatment
DE	4	38657	38393	acid treatment	Acid Treatment
DE	5	38657	38393	enzymatic treatment	Enzymatic Treatment
ID	1	38659	38395	IN-VITRO CYTOTOXICITY	In Vitro Cytotoxicity
ID	2	38659	38395	STABILITY	Stability
ID	3	38659	38395	DEGRADATION	Degradation
ID	4	38659	38395	FOOD	Food
ID	5	38659	38395	DETOXIFICATION	Detoxification
ID	6	38659	38395	TOXICITY	Toxicity
ID	7	38659	38395	OTA	Ochratoxin A (OTA)
ID	8	38659	38395	PH	Ph

Record 248 | WOS:000530851100002

Ochratoxin A causes cell cycle arrest in G1 and G1/S phases through p53 in HK-2 cells

Field	Position	Original line	Normalised line	Original value	Normalised value
DE	5	38755	38490	Ochratoxin A	Ochratoxin A (OTA)
ID	1	38756	38491	OXIDATIVE DNA-DAMAGE	Oxidative DNA Damage
ID	2	38756	38491	DOUBLE-STRAND BREAKS	Double Strand Break
ID	3	38756	38491	KIDNEY	Kidney
ID	4	38756	38491	CARCINOGENICITY	Carcinogenicity
ID	6	38756	38491	GENOTOXICITY	Genotoxicity
ID	7	38756	38491	ACTIVATION	Activation
ID	8	38756	38491	MECHANISMS	Mechanism

ID	9	38756	38491	MUTATIONS	Mutation
ID	10	38756	38491	INDUCTION	Induction

Record 249 | WOS:000541267600001

Cytogenotoxic evaluation of the acetonitrile extract, citrinin and dicitrinin-A fromPenicillium citrinum

Field	Position	Original line	Normalised line	Original value	Normalised value
DE	1	38874	38608	Endophytic fungi	Endophytic Fungi
DE	2	38874	38608	Penicillium citrinum	Penicillium Citrinum
DE	3	38874	38608	mycotoxins	Mycotoxins
DE	4	38874	38608	cytotoxicity	Cytotoxicity
DE	5	38874	38608	genotoxicity	Genotoxicity
DE	6	38874	38608	mutagenicity	Mutagenicity
ID	1	38876	38610	MYCOTOXINS OCHRATOXIN-A	Ochratoxin A (OTA)
ID	2	38876	38610	PENICILLIUM-CITRINUM	Penicillium Citrinum
ID	3	38876	38610	IN-VITRO	In-Vitro
ID	4	38876	38610	ANTIOXIDANT PROPERTIES	Antioxidant Property
ID	5	38876	38610	PHENOLIC-COMPOUNDS	Phenolic Compound
ID	6	38876	38610	MEDICINAL-PLANTS	Medicinal Plant
ID	7	38876	38610	CELLS	Cell
ID	8	38876	38610	APOPTOSIS	Apoptosis
ID	9	38876	38610	DERIVATIVES	Derivative
ID	10	38876	38610	TOXICITY	Toxicity

Record 250 | WOS:000533844900008

Ochratoxin A-induced genotoxic and epigenetic mechanisms lead to Alzheimer disease: its modulation with strategies

Field	Position	Original line	Normalised line	Original value	Normalised value
DE	1	39037	38769	Ochratoxin A	Ochratoxin A (OTA)
DE	4	39037	38769	DNA methylation	DNA Methylation
DE	5	39037	38769	Histone modifications	Histone Modification
DE	8	39037	38769	Alzheimer disease	Alzheimer Disease
ID	1	39039	38771	OXIDATIVE DNA-DAMAGE	Oxidative DNA Damage
ID	2	39039	38771	MAPK SIGNALING PATHWAYS	Mapk Signaling Pathway
ID	3	39039	38771	BLOOD MONONUCLEAR-CELLS	Blood Mononuclear
ID	4	39039	38771	IN-VITRO	In-Vitro
ID	5	39039	38771	MYCOTOXIN OCHRATOXIN	Ochratoxin A (OTA)
ID	6	39039	38771	PHASE ARREST	Phase Arrest
ID	7	39039	38771	KIDNEY-CELLS	Kidney
ID	8	39039	38771	INDUCED CYTOTOXICITY	Induced Cytotoxicity
ID	9	39039	38771	GENE-EXPRESSION	Gene Expression
ID	10	39039	38771	INDUCED NEPHROTOXICITY	Induced Nephrotoxicity

Record 251 | WOS:000542374900003

Milk thistle (Silybum Marianum) as an antidote or a protective agent against natural or chemical toxicities: a review

Field	Position	Original line	Normalised line	Original value	Normalised value
DE	1	39357	39088	Milk thistle	Milk Thistle
DE	2	39357	39088	Silybum marianum	Silybum Marianum
DE	3	39357	39088	silymarin	Silymarin
DE	4	39357	39088	silibinin	Silibinin
DE	5	39357	39088	toxin	Toxin

DE	6	39357	39088	protective	Protective
ID	1	39358	39089	INDUCED OXIDATIVE STRESS	Induced Oxidative Stress
ID	2	39358	39089	DENSITY-LIPOPROTEIN OXIDATION	Density Lipoprotein Oxidation
ID	3	39358	39089	GREEN TEA POLYPHENOLS	Green Tea Polyphenol
ID	4	39358	39089	SATIVA FIXED OIL	Sativa Fixed Oil
ID	5	39358	39089	OCHRATOXIN-A	Ochratoxin A (OTA)
ID	6	39358	39089	INDUCED NEPHROTOXICITY	Induced Nephrotoxicity
ID	7	39358	39089	CARBON-TETRACHLORIDE	Carbon Tetrachloride
ID	8	39358	39089	SILYMARIN PROTECTS	Silymarin Protect
ID	9	39358	39089	LIPID-PEROXIDATION	Lipid Peroxidation
ID	10	39358	39089	CISPLATIN NEPHROTOXICITY	Cisplatin Nephrotoxicity

Record 252 | WOS:000527365800027

Ochratoxin A exposure causes meiotic failure and oocyte deterioration in mice

Field	Position	Original line	Normalised line	Original value	Normalised value
DE	1	39610	39340	Ochratoxin A	Ochratoxin A (OTA)
DE	2	39610	39340	Oocyte development	Oocyte Development
DE	3	39610	39340	Oxidative stress	Oxidative Stress
DE	4	39610	39340	Epigenetic change	Epigenetic Change
ID	1	39611	39341	OXIDATIVE STRESS	Oxidative Stress
ID	2	39611	39341	EMBRYONIC-DEVELOPMENT	Embryonic Development
ID	3	39611	39341	CELL-PROLIFERATION	Cell Proliferation
ID	4	39611	39341	MOUSE OOCYTES	Mouse Oocyte
ID	5	39611	39341	DNA-DAMAGE	DNA Damage
ID	6	39611	39341	IN-VITRO	In-Vitro
ID	7	39611	39341	MATURATION	Maturation
ID	8	39611	39341	APOPTOSIS	Apoptosis
ID	9	39611	39341	FERTILIZATION	Fertilization
ID	10	39611	39341	MITOCHONDRIA	Mitochondria

Record 253 | WOS:000526412300005

Beauvericin and ochratoxin A mycotoxins individually and combined in HepG2 cells alter lipid peroxidation, levels of reactive oxygen species and glutathione

Field	Position	Original line	Normalised line	Original value	Normalised value
DE	1	39747	39475	HepG2 cells	HepG2
DE	2	39747	39475	mycotoxins' mixture	Mycotoxin Mixture
DE	3	39747	39475	ROS	Reactive Oxygen Species (ROS)
DE	4	39747	39475	LPO	Lpo
DE	5	39747	39475	Glutathione	Glutathione (GSH)
ID	1	39748	39477	OXIDATIVE DNA-DAMAGE	Oxidative DNA Damage
ID	2	39748	39477	KIDNEY PK15 CELLS	Kidney Pk15
ID	3	39748	39477	A-INDUCED CYTOTOXICITY	A Induced Cytotoxicity
ID	4	39748	39477	COMPLEMENTARY FOODS	Complementary Food
ID	5	39748	39477	TOXICOLOGICAL INTERACTIONS	Toxicological Interaction
ID	6	39748	39477	T-2 TOXIN	T 2 Toxin
ID	7	39748	39477	APOPTOSIS	Apoptosis
ID	8	39748	39477	STRESS	Stress
ID	9	39748	39477	DEOXYNIVALENOL	Deoxynivalenol (DON)
ID	10	39748	39477	TOXICITY	Toxicity

Record 254 | WOS:000537553700018

Risk assessment of ochratoxin A in food

Field	Position	Original line	Normalised line	Original value	Normalised value
DE	1	39902	39630	Ochratoxin	Ochratoxin A (OTA)
DE	2	39902	39630	hazard characterisation	Hazard Characterisation
DE	3	39902	39630	dietary exposure assessment	Dietary Exposure Assessment
DE	4	39902	39630	margin of exposure approach	Margin Of Exposure Approach
DE	5	39902	39630	risk characterisation	Risk Characterisation
ID	1	39904	39632	PERFORMANCE LIQUID-CHROMATOGRAPHY	Performance Liquid Chromatography
ID	2	39904	39632	OXIDATIVE DNA-DAMAGE	Oxidative DNA Damage
ID	3	39904	39632	CHRONIC INTERSTITIAL NEPHROPATHY	Chronic Interstitial Nephropathy
ID	4	39904	39632	TANDEM MASS-SPECTROMETRY	Tandem Mass Spectrometry
ID	5	39904	39632	DOUBLE-STRAND BREAKS	Double Strand Break
ID	6	39904	39632	EXPERIMENTAL PORCINE NEPHROPATHY	Experimental Porcine Nephropathy
ID	7	39904	39632	IMMUNOAFFINITY COLUMN CLEANUP	Immunoaffinity Column Cleanup
ID	8	39904	39632	MAPK SIGNALING PATHWAYS	Mapk Signaling Pathway
ID	9	39904	39632	BLOOD MONONUCLEAR-CELLS	Blood Mononuclear
ID	10	39904	39632	A-INDUCED CYTOTOXICITY	A Induced Cytotoxicity

Record 255 | WOS:000505957800037

Aptamer assisted ultrafiltration cleanup with high performance liquid chromatography-fluorescence detector for the determination of OTA in green coffee

Field	Position	Original line	Normalised line	Original value	Normalised value
DE	1	40424	40151	Ochratoxin A	Ochratoxin A (OTA)
DE	4	40424	40151	Green coffee	Green Coffee
DE	5	40424	40151	HPLC-FLD	Hplc Fld
ID	1	40425	40152	IN-VITRO SELECTION	In Vitro Selection
ID	2	40425	40152	OCHRATOXIN-A	Ochratoxin A (OTA)
ID	3	40425	40152	MYCOTOXINS	Mycotoxins
ID	4	40425	40152	APTASENSOR	Aptasensor
ID	5	40425	40152	ASSAY	Assay
ID	6	40425	40152	MOLECULES	Molecule
ID	7	40425	40152	FOODS	Food
ID	8	40425	40152	SELEX	Selex
ID	9	40425	40152	GOLD	Gold

Record 256 | WOS:000526746500001

Protective Effect of Hydroxytyrosol Against Oxidative Stress Induced by the Ochratoxin in Kidney Cells: in vitro and in vivo Study

Field	Position	Original line	Normalised line	Original value	Normalised value
DE	1	40538	40264	canine kidney cell line	Canine Kidney Cell
DE	2	40538	40264	ochratoxin	Ochratoxin A (OTA)
DE	3	40538	40264	pig kidney cell line	Pig Kidney Cell
DE	4	40538	40264	rabbit kidney cell line	Rabbit Kidney Cell
DE	5	40538	40264	rats	Rat
ID	1	40540	40266	HEPATIC LIPID-METABOLISM	Hepatic Lipid Metabolism

ID	2	40540	40266	OLIVE OIL	Olive Oil
ID	3	40540	40266	TOXICITY	Toxicity
ID	4	40540	40266	FIBROSIS	Fibrosis
ID	5	40540	40266	PALMITOYLETHANOLAMIDE	Palmitoylethanolamide
ID	6	40540	40266	CARCINOGENICITY	Carcinogenicity
ID	7	40540	40266	NEPHROTOXICITY	Nephrotoxicity
ID	8	40540	40266	GENOTOXICITY	Genotoxicity
ID	9	40540	40266	PREVENTION	Prevention
ID	10	40540	40266	PHENOLS	Phenol

Record 257 | WOS:000524713000001

Development of ELISA and Lateral Flow Immunoassays for Ochratoxins (OTA and OTB) Detection Based on Monoclonal Antibody

Field	Position	Original line	Normalised line	Original value	Normalised value
DE	1	40654	40379	ochratoxins	Ochratoxin A (OTA)
DE	2	40654	40379	monoclonal antibody	Monoclonal Antibody
DE	3	40654	40379	hybridoma technology	Hybridoma Technology
DE	4	40654	40379	ic-ELISA	Ic Elisa
DE	5	40654	40379	colloidal gold strip	Colloidal Gold Strip
DE	6	40654	40379	nanoflowers gold strip	Nanoflower Gold Strip
ID	1	40656	40381	COLLOIDAL GOLD IMMUNOASSAY	Colloidal Gold Immunoassay
ID	2	40656	40381	LINKED-IMMUNOSORBENT-ASSAY	Linked Immunosorbent Assay
ID	3	40656	40381	ELECTROCHEMICAL IMMUNOSENSOR	Electrochemical Immunosensor
ID	4	40656	40381	RAPID DETECTION	Rapid Detection
ID	5	40656	40381	A DETECTION	A Detection
ID	6	40656	40381	IDENTIFICATION	Identification
ID	7	40656	40381	AFLATOXIN	Aflatoxin
ID	8	40656	40381	TOXICITY	Toxicity
ID	9	40656	40381	PROTEINS	Protein
ID	10	40656	40381	AFFINITY	Affinity

Record 258 | WOS:000530248700129

Protective Effect of Astaxanthin on Ochratoxin A-Induced Kidney Injury to Mice by Regulating Oxidative Stress-Related NRF2/KEAP1 Pathway

Field	Position	Original line	Normalised line	Original value	Normalised value
DE	1	40769	40493	astaxanthin	Astaxanthin
DE	2	40769	40493	ochratoxin A	Ochratoxin A (OTA)
DE	3	40769	40493	oxidative damage	Oxidative Damage
DE	4	40769	40493	NRF2	Nrf2
DE	5	40769	40493	KEAP1	Keap1
ID	1	40770	40494	SIGNALING PATHWAY	Signaling Pathway
ID	2	40770	40494	TOXICITY	Toxicity
ID	3	40770	40494	CELLS	Cell
ID	4	40770	40494	GENOTOXICITY	Genotoxicity
ID	5	40770	40494	ANIMALS	Animal
ID	6	40770	40494	SINGLE	Single
ID	7	40770	40494	ACID	Acid

Record 259 | WOS:000517659300003

Toxicity of mycotoxins in vivo on vertebrate organisms: A review

Field	Position	Original line	Normalised line	Original value	Normalised value
DE	1	40883	40606	Mycotoxin	Mycotoxins
DE	2	40883	40606	Molecular biology	Molecular Biology
DE	5	40883	40606	Mechanism of action	Mechanism Of Action
ID	1	40885	40608	NATURALLY CONTAMINATED FEED	Naturally Contaminated Feed

ID	2	40885	40608	OXIDATIVE STRESS	Oxidative Stress
ID	3	40885	40608	AFLATOXIN B-1	Aflatoxin B1 (AFB1)
ID	4	40885	40608	OCHRATOXIN-A	Ochratoxin A (OTA)
ID	5	40885	40608	DEOXYNIVALENOL DON	Deoxynivalenol DON
ID	6	40885	40608	FUMONISIN B-1	Fumonisin B1 (FB1)
ID	7	40885	40608	ZEARALENONE EXPOSURE	Zearalenone Exposure
ID	8	40885	40608	SIGNALING PATHWAY	Signaling Pathway
ID	9	40885	40608	PROTECTIVE ROLE	Protective Role
ID	10	40885	40608	LC-MS/MS	Lc Ms Ms

Record 260 ǀ WOS:000526529800024

Influences of Electron Beam Irradiation on the Physical and Chemical Properties of Zearalenone- and Ochratoxin A-Contaminated Corn and In Vivo Toxicity Assessment

Field	Position	Original line	Normalised line	Original value	Normalised value
DE	1	41133	40855	zearalenone	Zearalenone (ZEA)
DE	2	41133	40855	ochratoxin A	Ochratoxin A (OTA)
DE	3	41133	40855	electron beam irradiation	Electron Beam Irradiation
DE	4	41133	40855	physical and chemical properties	Physical And Chemical Property
DE	5	41133	40855	toxicity in vivo	Toxicity In Vivo
ID	1	41135	40857	PHYSICOCHEMICAL PROPERTIES	Physicochemical Property
ID	2	41135	40857	GAMMA-IRRADIATION	Gamma Irradiation
ID	3	41135	40857	MYCOTOXINS	Mycotoxins
ID	4	41135	40857	METABOLISM	Metabolism
ID	5	41135	40857	AFLATOXINS	Aflatoxin
ID	6	41135	40857	STARCHES	Starche
ID	7	41135	40857	FLOUR	Flour

Record 261 ǀ WOS:000525009500057

In-Vitro Cell Culture for Efficient Assessment of Mycotoxin Exposure, Toxicity and Risk Mitigation

Field	Position	Original line	Normalised line	Original value	Normalised value
DE	1	41254	40975	mycotoxins	Mycotoxins
DE	2	41254	40975	in-vitro cell culture	In Vitro Cell
DE	3	41254	40975	toxicity assessment and mitigation	Toxicity Assessment And Mitigation
ID	1	41255	40976	INTESTINAL EPITHELIAL-CELLS	Intestinal Epithelial
ID	2	41255	40976	MESSENGER-RNA EXPRESSION	Messenger RNA Expression
ID	3	41255	40976	RENAL TUMOR-FORMATION	Renal Tumor Formation
ID	4	41255	40976	OXIDATIVE DNA-DAMAGE	Oxidative DNA Damage
ID	5	41255	40976	YEAST-BASED PRODUCTS	Yeast Based Product
ID	6	41255	40976	OCHRATOXIN-A	Ochratoxin A (OTA)
ID	7	41255	40976	GENE-EXPRESSION	Gene Expression
ID	8	41255	40976	BARRIER FUNCTION	Barrier Function
ID	9	41255	40976	MYCOPHENOLIC-ACID	Mycophenolic Acid
ID	10	41255	40976	TIGHT JUNCTIONS	Tight Junction

Record 262 ǀ WOS:000543783800001

Nontoxic concentration of ochratoxin A decreases the dosage of cyclosporine A to induce chronic nephropathy model via autophagy mediated by toll-like receptor 4

Field	Position	Original line	Normalised line	Original value	Normalised value
ID	1	41598	41317	SIGNALING PATHWAY	Signaling Pathway
ID	2	41598	41317	INDUCED NEPHROTOXICITY	Induced Nephrotoxicity
ID	3	41598	41317	RENAL FIBROSIS	Renal Fibrosis
ID	4	41598	41317	IN-VITRO	In-Vitro
ID	5	41598	41317	STRESS	Stress
ID	6	41598	41317	IMMUNOTOXICITY	Immunotoxicity

Record 263 | WOS:000512092100007

Ochratoxin A induces global DNA hypomethylation and oxidative stress in neuronal cells in vitro

Field	Position	Original line	Normalised line	Original value	Normalised value
DE	1	41703	41421	Ochratoxin A	Ochratoxin A (OTA)
DE	4	41703	41421	Oxidative stress	Oxidative Stress
DE	6	41703	41421	In vitro	In-Vitro
ID	1	41705	41423	METHYLATION STATUS	Methylation Status
ID	2	41705	41423	DAMAGE	Damage
ID	3	41705	41423	TOXICITY	Toxicity
ID	4	41705	41423	CYTOTOXICITY	Cytotoxicity
ID	5	41705	41423	APOPTOSIS	Apoptosis
ID	6	41705	41423	EXPOSURE	Exposure
ID	7	41705	41423	BINDING	Binding
ID	8	41705	41423	MICRONUCLEI	Micronuclei
ID	9	41705	41423	SITE	Site

Record 264 | WOS:000506899900001

Toxicity effects of mycotoxins and autophagy: a mechanistic view

Field	Position	Original line	Normalised line	Original value	Normalised value
DE	2	41803	41520	mycotoxin	Mycotoxins
DE	3	41803	41520	aflatoxin	Aflatoxin
DE	4	41803	41520	patulin	Patulin
DE	5	41803	41520	cytochalasins	Cytochalasin
ID	1	41804	41521	T-2 TOXIN	T 2 Toxin
ID	2	41804	41521	IN-VITRO	In-Vitro
ID	3	41804	41521	BECLIN 1	Beclin 1
ID	4	41804	41521	OXIDATIVE STRESS	Oxidative Stress
ID	5	41804	41521	FUSARIUM TOXINS	Fusarium Toxin
ID	6	41804	41521	FUMONISIN B-1	Fumonisin B1 (FB1)
ID	7	41804	41521	CELL BIOLOGY	Cell Biology
ID	8	41804	41521	ULK1 COMPLEX	Ulk1 Complex
ID	9	41804	41521	APOPTOSIS	Apoptosis
ID	10	41804	41521	FOOD	Food

Record 265 | WOS:000505295600001

Red orange and lemon extract prevents the renal toxicity induced by ochratoxin A in rats

Field	Position	Original line	Normalised line	Original value	Normalised value
DE	1	41963	41679	anthocyanins	Anthocyanin
DE	2	41963	41679	kidney	Kidney
DE	3	41963	41679	ochratoxin-A	Ochratoxin A (OTA)
DE	4	41963	41679	oxidative stress	Oxidative Stress
DE	5	41963	41679	toxicity	Toxicity
ID	1	41964	41680	OXIDATIVE DNA-DAMAGE	Oxidative DNA Damage
ID	2	41964	41680	CYANIDIN 3-O-BETA-D-GLUCOSIDE	Cyanidin 3 O Beta D Glucoside
ID	3	41964	41680	MOLECULAR-MECHANISMS	Molecular Mechanism
ID	4	41964	41680	ALPHA-TOCOPHEROL	Alpha Tocopherol

ID	5	41964	41680	KIDNEY	Kidney
ID	6	41964	41680	STRESS	Stress
ID	7	41964	41680	ANTHOCYANINS	Anthocyanin
ID	8	41964	41680	PROTECTS	Protect
ID	9	41964	41680	PATHWAY	Pathway
ID	10	41964	41680	CELLS	Cell

Record 266 | WOS:000488786200007

Mycotoxin-induced toxicities and diseases

Field	Position	Original line	Normalised line	Original value	Normalised value
ID	1	42080	41794	FUMONISIN B-1 ALTERS	Fumonisin B 1 Alter
ID	2	42080	41794	MODIFIED HALLOYSITE NANOTUBES	Modified Halloysite Nanotube
ID	3	42080	41794	A-INDUCED NEPHROTOXICITY	A Induced Nephrotoxicity
ID	4	42080	41794	OXIDATIVE DNA-DAMAGE	Oxidative DNA Damage
ID	5	42080	41794	OCHRATOXIN-A	Ochratoxin A (OTA)
ID	6	42080	41794	IN-VITRO	In-Vitro
ID	7	42080	41794	AFLATOXIN B-1	Aflatoxin B1 (AFB1)
ID	8	42080	41794	HEPATOCELLULAR-CARCINOMA	Hepatocellular Carcinoma
ID	9	42080	41794	CHROMOSOME-ABERRATIONS	Chromosome Aberration
ID	10	42080	41794	CULTURE MATERIAL	Culture Material

Record 267 | WOS:000680216400001

Mycotoxins Occurrence, Toxicity and Detection Methods

Field	Position	Original line	Normalised line	Original value	Normalised value
DE	2	42370	42084	Occurrence of mycotoxin	Occurrence Of Mycotoxin
DE	3	42370	42084	Toxicities	Toxicity
DE	4	42370	42084	Detection methods	Detection Method
ID	1	42371	42085	PERFORMANCE LIQUID-CHROMATOGRAPHY	Performance Liquid Chromatography
ID	2	42371	42085	LINKED-IMMUNOSORBENT-ASSAY	Linked Immunosorbent Assay
ID	3	42371	42085	OCHRATOXIN-A PRODUCTION	Ochratoxin A Production
ID	4	42371	42085	TOXIN-INDUCED APOPTOSIS	Toxin Induced Apoptosis
ID	5	42371	42085	DRIED VINE FRUITS	Dried Vine Fruit
ID	6	42371	42085	RAW BULK MILK	Raw Bulk Milk
ID	7	42371	42085	OXIDATIVE STRESS	Oxidative Stress
ID	8	42371	42085	T-2 TOXIN	T 2 Toxin
ID	9	42371	42085	AFLATOXIN B-1	Aflatoxin B1 (AFB1)
ID	10	42371	42085	FUMONISIN B-1	Fumonisin B1 (FB1)

Record 268 | WOS:000504757200032

Melatonin ameliorates ochratoxin A-induced oxidative stress and apoptosis in porcine oocytes

Field	Position	Original line	Normalised line	Original value	Normalised value
DE	1	42732	42445	Ochratoxin A	Ochratoxin A (OTA)
DE	4	42732	42445	Oxidative stress	Oxidative Stress
ID	1	42733	42446	PERMEABILITY TRANSITION PORE	Permeability Transition Pore
ID	2	42733	42446	IN-VITRO	In-Vitro

ID	3	42733	42446	AUTOPHAGY	Autophagy
ID	4	42733	42446	PROTECTS	Protect
ID	5	42733	42446	EXPOSURE	Exposure
ID	6	42733	42446	FERTILIZATION	Fertilization
ID	7	42733	42446	MITOCHONDRIA	Mitochondria
ID	8	42733	42446	METABOLISM	Metabolism
ID	9	42733	42446	MATURATION	Maturation

Record 269 | WOS:000514650700067

Modelling Fungal Growth, Mycotoxin Production and Release in Grana Cheese

Field	Position	Original line	Normalised line	Original value	Normalised value
DE	1	42828	42540	ochratoxin A	Ochratoxin A (OTA)
DE	2	42828	42540	sterigmatocystin	Sterigmatocystin
DE	4	42828	42540	Aspergillus versicolor	Aspergillus Versicolor
DE	5	42828	42540	temperature	Temperature
DE	6	42828	42540	ecology	Ecology
DE	7	42828	42540	modelling	Modelling
DE	8	42828	42540	partitioning	Partitioning
ID	1	42830	42542	PENICILLIUM-VERRUCOSUM	Penicillium Verrucosum
ID	2	42830	42542	OCHRATOXIN	Ochratoxin A (OTA)
ID	3	42830	42542	CONTAMINATION	Contamination
ID	4	42830	42542	ASPERGILLUS	Aspergillus
ID	5	42830	42542	SUBSTRATE	Substrate
ID	6	42830	42542	FOOD	Food

Record 270 | WOS:000545943400001

Penicillium spp. mycotoxins found in food and feed and their health effects

Field	Position	Original line	Normalised line	Original value	Normalised value
DE	1	42933	42644	toxicity	Toxicity
DE	2	42933	42644	fungi	Fungi
DE	3	42933	42644	food and feedstuffs	Food And Feedstuff
ID	1	42934	42645	MYCO-TOXIN BOTRYODIPLODIN	Myco Toxin Botryodiplodin
ID	2	42934	42645	CYCLOPIAZONIC ACID	Cyclopiazonic Acid
ID	3	42934	42645	SECONDARY METABOLITES	Secondary Metabolite
ID	4	42934	42645	MYCOPHENOLIC-ACID	Mycophenolic Acid
ID	5	42934	42645	OCHRATOXIN-A	Ochratoxin A (OTA)
ID	6	42934	42645	NATURAL OCCURRENCE	Natural Occurrence
ID	7	42934	42645	ENDOPHYTIC FUNGUS	Endophytic Fungus
ID	8	42934	42645	CACO-2 CELLS	Caco-2
ID	9	42934	42645	IN-VITRO	In-Vitro
ID	10	42934	42645	CYTOTOXIC METABOLITES	Cytotoxic Metabolite

Record 271 | WOS:000514630000034

Comprehensive Evaluation of the Efficiency of Yeast Cell Wall Extract to Adsorb Ochratoxin A and Mitigate Accumulation of the Toxin in Broiler Chickens

Field	Position	Original line	Normalised line	Original value	Normalised value
DE	1	43158	42868	ochratoxin A	Ochratoxin A (OTA)
DE	2	43158	42868	mitigation	Mitigation
DE	3	43158	42868	mycotoxin binding	Mycotoxin Binding
DE	4	43158	42868	yeast cell wall extracts	Yeast Cell Wall Extract
ID	1	43159	42869	IN-VITRO	In-Vitro

ID	2	43159	42869	PENICILLIUM MYCOTOXINS	Penicillium Mycotoxin
ID	3	43159	42869	OXIDATIVE STRESS	Oxidative Stress
ID	4	43159	42869	TOXICITY	Toxicity
ID	5	43159	42869	EXPOSURE	Exposure
ID	6	43159	42869	GROWTH	Growth
ID	7	43159	42869	WATER	Water
ID	8	43159	42869	COMPLEXATION	Complexation
ID	9	43159	42869	SIMULATIONS	Simulation
ID	10	43159	42869	TEMPERATURE	Temperature

Record 272 | WOS:000514630000026

Transcriptome Analysis of Ochratoxin A-Induced Apoptosis in Differentiated Caco-2 Cells

Field	Position	Original line	Normalised line	Original value	Normalised value
DE	1	43268	42977	ochratoxin A	Ochratoxin A (OTA)
DE	2	43268	42977	differentiated Caco-2 cells	Differentiated Caco-2
DE	3	43268	42977	cell apoptosis	Cell Apoptosis
DE	4	43268	42977	transcriptome analysis	Transcriptome Analysis
ID	1	43270	42979	DOUBLE-STRAND BREAKS	Double Strand Break
ID	2	43270	42979	DOWN-REGULATION	Down Regulation
ID	3	43270	42979	MOLECULAR-MECHANISMS	Molecular Mechanism
ID	4	43270	42979	INDUCED GENOTOXICITY	Induced Genotoxicity
ID	5	43270	42979	EXPRESSION ANALYSIS	Expression Analysis
ID	6	43270	42979	INTESTINAL BARRIER	Intestinal Barrier
ID	7	43270	42979	EPITHELIAL-CELLS	Epithelial
ID	8	43270	42979	HUMAN KIDNEY	Human Kidney
ID	9	43270	42979	MYCOTOXINS	Mycotoxins
ID	10	43270	42979	FOOD	Food

Record 273 | WOS:000498748700011

Epithelial-fibroblast cross talk aggravates the impact of the nephrotoxin ochratoxin A

Field	Position	Original line	Normalised line	Original value	Normalised value
DE	1	43427	43135	Epithelial-fibroblast crosstalk	Epithelial Fibroblast Crosstalk
DE	2	43427	43135	CKD	Ckd
DE	3	43427	43135	Ochratoxin A	Ochratoxin A (OTA)
DE	4	43427	43135	Epithelial-to-mesenchymal transition	Epithelial To Mesenchymal Transition
DE	5	43427	43135	miRNA	Mirna
DE	6	43427	43135	COX-2	Cox 2
ID	1	43429	43137	CHRONIC KIDNEY-DISEASE	Chronic Kidney Disease
ID	2	43429	43137	TO-MESENCHYMAL TRANSITION	To Mesenchymal Transition
ID	3	43429	43137	HEME OXYGENASE-1	Heme Oxygenase 1
ID	4	43429	43137	IN-VITRO	In-Vitro
ID	5	43429	43137	ACTIVATION	Activation
ID	6	43429	43137	CELLS	Cell
ID	7	43429	43137	PROSTAGLANDIN-E2	Prostaglandin E2
ID	8	43429	43137	MICRORNAS	Microrna
ID	9	43429	43137	RISK	Risk
ID	10	43429	43137	REABSORPTION	Reabsorption

Record 274 | WOS:000498194100002

Capsaicin, an inhibitor of Ochratoxin A production by Aspergillus section Nigri strains in grapes (Vitis vinifera L.)

Field	Position	Original line	Normalised line	Original value	Normalised value
DE	1	43540	43246	Ochratoxin A	Ochratoxin A (OTA)
DE	2	43540	43246	Aspergillus section Nigri	Aspergillus Section Nigri
DE	3	43540	43246	Aspergillus carbonarius	Aspergillus Carbonarius
DE	4	43540	43246	capsaicin	Capsaicin
DE	5	43540	43246	grapes	Grape
DE	6	43540	43246	risk assessment	Risk Assessment
ID	1	43542	43248	AFLATOXIN B-1 PRODUCTION	Aflatoxin B 1 Production
ID	2	43542	43248	HEALTH-RISK ASSESSMENT	Health Risk Assessment
ID	3	43542	43248	ANTIMICROBIAL PROPERTIES	Antimicrobial Property
ID	4	43542	43248	ANTIBACTERIAL ACTIVITY	Antibacterial Activity
ID	5	43542	43248	ANNUUM EXTRACT	Annuum Extract
ID	6	43542	43248	ESSENTIAL OILS	Essential Oil
ID	7	43542	43248	IN-VITRO	In-Vitro
ID	8	43542	43248	PARASITICUS	Parasiticus
ID	9	43542	43248	GROWTH	Growth
ID	10	43542	43248	WINE	Wine

Record 275 | WOS:000501604700005

Ochratoxin A Sequentially Activates Autophagy and the Ubiquitin-Proteasome System

Field	Position	Original line	Normalised line	Original value	Normalised value
DE	1	43654	43359	ochratoxin A	Ochratoxin A (OTA)
DE	2	43654	43359	ubiquitin-proteasome system	Ubiquitin Proteasome System
DE	3	43654	43359	autophagy	Autophagy
DE	4	43654	43359	proteolytic pathways	Proteolytic Pathway
DE	5	43654	43359	PI3K	Pi3k
DE	6	43654	43359	AKT	Akt
DE	7	43654	43359	MAPK	Mapk
DE	8	43654	43359	ERK1-2	Erk1 2
ID	1	43656	43361	NF-KAPPA-B	Nf Kappa B
ID	2	43656	43361	ENDOPLASMIC-RETICULUM STRESS	Endoplasmic Reticulum Stress
ID	3	43656	43361	OXIDATIVE STRESS	Oxidative Stress
ID	4	43656	43361	KIDNEY-CELLS	Kidney
ID	5	43656	43361	DNA-DAMAGE	DNA Damage
ID	6	43656	43361	PROTEIN-DEGRADATION	Protein Degradation
ID	7	43656	43361	HEPG2 CELLS	HepG2
ID	8	43656	43361	CARCINOGENICITY	Carcinogenicity
ID	9	43656	43361	EXPRESSION	Expression
ID	10	43656	43361	TOXICITY	Toxicity

Record 276 | WOS:000501604700006

Comparative In Vitro Assessment of a Range of Commercial Feed Additives with Multiple Mycotoxin Binding Claims

Field	Position	Original line	Normalised line	Original value	Normalised value
DE	1	43787	43490	mycotoxins	Mycotoxins
DE	2	43787	43490	animal feed	Animal Feed
DE	3	43787	43490	mycotoxin binders	Mycotoxin Binder
DE	4	43787	43490	feed safety	Feed Safety
ID	1	43788	43491	AFLATOXIN B-1 ADSORPTION	Aflatoxin B 1 Adsorption
ID	2	43788	43491	YEAST-BASED PRODUCTS	Yeast Based Product
ID	3	43788	43491	ANIMAL FEED	Animal Feed
ID	4	43788	43491	OCHRATOXIN-A	Ochratoxin A (OTA)
ID	5	43788	43491	ZEARALENONE	Zearalenone (ZEA)
ID	6	43788	43491	TOXICITY	Toxicity
ID	7	43788	43491	BINDERS	Binder

ID	8	43788	43491	AGENTS	Agent
ID	9	43788	43491	FUNGI	Fungi
ID	10	43788	43491	WATER	Water

Record 277 | WOS:000477691300004

Reduction of ochratoxin A in oats and rice by twin-screw extrusion processing with baking soda

Field	Position	Original line	Normalised line	Original value	Normalised value
DE	2	43904	43606	Oats	Oat
DE	5	43904	43606	Twin-screw extrusion	Twin Screw Extrusion
DE	6	43904	43606	Baking soda	Baking Soda
ID	1	43906	43607	IN-VITRO CYTOTOXICITY	In Vitro Cytotoxicity
ID	2	43906	43607	BREAKFAST CEREALS	Breakfast Cereal
ID	3	43906	43607	SODIUM-BICARBONATE	Sodium Bicarbonate
ID	4	43906	43607	NATURAL OCCURRENCE	Natural Occurrence
ID	5	43906	43607	REDUCED TOXICITY	Reduced Toxicity
ID	6	43906	43607	MYCOTOXINS	Mycotoxins
ID	7	43906	43607	MECHANISM	Mechanism
ID	8	43906	43607	MARKET	Market
ID	9	43906	43607	FATE	Fate

Record 278 | WOS:000475915000013

A multi-endpoint approach to the combined toxic effects of patulin and ochratoxin a in human intestinal cells

Field	Position	Original line	Normalised line	Original value	Normalised value
DE	1	44015	43715	Mycotoxin mixtures	Mycotoxin Mixture
DE	2	44015	43715	Gastrointestinal toxicity	Gastrointestinal Toxicity
DE	3	44015	43715	Interaction effects	Interaction Effect
DE	5	44015	43715	Barrier integrity	Barrier Integrity
ID	1	44017	43717	OXIDATIVE DNA-DAMAGE	Oxidative DNA Damage
ID	2	44017	43717	MYCOTOXIN PATULIN	Mycotoxin Patulin
ID	3	44017	43717	COMET ASSAY	Comet Assay
ID	4	44017	43717	BARRIER FUNCTION	Barrier Function
ID	5	44017	43717	TIGHT JUNCTIONS	Tight Junction
ID	6	44017	43717	BINARY-MIXTURES	Binary Mixture
ID	7	44017	43717	RISK-ASSESSMENT	Risk Assessment
ID	8	44017	43717	HUMAN EXPOSURE	Human Exposure
ID	9	44017	43717	BABY FOODS	Baby Food
ID	10	44017	43717	CYTOTOXICITY	Cytotoxicity

Record 279 | WOS:000484647100041

Impact of dietary Trichosporon mycotoxinivorans on ochratoxin A induced immunotoxicity; In vivo study

Field	Position	Original line	Normalised line	Original value	Normalised value
DE	1	44172	43871	Ochratoxin A	Ochratoxin A (OTA)
DE	3	44172	43871	Trichosporon mycotoxinivoran	Trichosporon Mycotoxinivoran
DE	4	44172	43871	In-vivo modification	In Vivo Modification
DE	5	44172	43871	Broiler chicks	Broiler Chick
ID	1	44174	43873	BIOLOGICAL DETOXIFICATION	Biological Detoxification
ID	2	44174	43873	PERFORMANCE	Performance
ID	3	44174	43873	BROILER	Broiler
ID	4	44174	43873	SERUM	Serum
ID	5	44174	43873	FOOD	Food
ID	6	44174	43873	BIODEGRADATION	Biodegradation
ID	7	44174	43873	TOXICITY	Toxicity
ID	8	44174	43873	FEED	Feed

ID	9	44174	43873	ANALOGS	Analog
ID	10	44174	43873	HEALTH	Health

Record 280 | WOS:000486105900030

In vitro effects of single and binary mixtures of regulated mycotoxins and persistent organochloride pesticides on steroid hormone production in MA-10 Leydig cell line

Field	Position	Original line	Normalised line	Original value	Normalised value
DE	2	44281	43979	Endocrine activity	Endocrine Activity
DE	3	44281	43979	MA-10 Leydig cells	Ma 10 Leydig
DE	4	44281	43979	Mixture toxicity	Mixture Toxicity
DE	6	44281	43979	Reproductive toxicity	Reproductive Toxicity
ID	1	44283	43981	MALE REPRODUCTIVE-SYSTEM	Male Reproductive System
ID	2	44283	43981	BLOOD-TESTIS BARRIER	Blood Testis Barrier
ID	3	44283	43981	TESTOSTERONE SECRETION	Testosterone Secretion
ID	4	44283	43981	ENDOCRINE DISRUPTORS	Endocrine Disruptor
ID	5	44283	43981	RECEPTOR ACTIVATION	Receptor Activation
ID	6	44283	43981	DEFINED MIXTURES	Defined Mixture
ID	7	44283	43981	GENE-EXPRESSION	Gene Expression
ID	8	44283	43981	DDT METABOLITE	Ddt Metabolite
ID	9	44283	43981	SEMEN QUALITY	Semen Quality
ID	10	44283	43981	EXPOSURE	Exposure

Record 281 | WOS:000498064500026

Detecting Neurodevelopmental Toxicity of Domoic Acid and Ochratoxin A Using Rat Fetal Neural Stem Cells

Field	Position	Original line	Normalised line	Original value	Normalised value
DE	1	44397	44094	stem cell differentiation	Stem Cell Differentiation
DE	2	44397	44094	cytotoxicity	Cytotoxicity
DE	3	44397	44094	neurotoxicity	Neurotoxicity
DE	4	44397	44094	domoic acid	Domoic Acid
DE	5	44397	44094	ochratoxin A	Ochratoxin A (OTA)
DE	6	44397	44094	oligodendrocytes	Oligodendrocyte
DE	7	44397	44094	astrocytes	Astrocyte
DE	8	44397	44094	neurons	Neuron
ID	1	44399	44096	CEREBELLAR GRANULE NEURONS	Cerebellar Granule Neuron
ID	2	44399	44096	NERVOUS-SYSTEM	Nervous System
ID	3	44399	44096	ASTROCYTES	Astrocyte
ID	4	44399	44096	MECHANISMS	Mechanism
ID	5	44399	44096	DISEASE	Disease
ID	6	44399	44096	OLIGODENDROCYTES	Oligodendrocyte
ID	7	44399	44096	NEUROTOXICITY	Neurotoxicity
ID	8	44399	44096	CYTOTOXICITY	Cytotoxicity
ID	9	44399	44096	SEIZURES	Seizure
ID	10	44399	44096	EXPOSURE	Exposure

Record 282 | WOS:000487934500032

Zinc supplementation alleviates OTA-induced oxidative stress and apoptosis in MDCK cells by up-regulating metallothioneins

Field	Position	Original line	Normalised line	Original value	Normalised value
DE	2	44527	44222	Ochratoxin A	Ochratoxin A (OTA)
DE	4	44527	44222	Metallothioneins	Metallothionein
DE	5	44527	44222	MDCK cells	MDCK
ID	1	44528	44223	OCHRATOXIN-A	Ochratoxin A (OTA)
ID	2	44528	44223	DNA-DAMAGE	DNA Damage
ID	3	44528	44223	RADIATION	Radiation

ID	4	44528	44223	TRANSPORTERS	Transporter
ID	5	44528	44223	SUPEROXIDE	Superoxide
ID	6	44528	44223	DEFICIENCY	Deficiency
ID	7	44528	44223	PATHWAYS	Pathway
ID	8	44528	44223	PROTECTS	Protect
ID	9	44528	44223	THERAPY	Therapy
ID	10	44528	44223	HYPOXIA	Hypoxia

Record 283 | WOS:000505648100002

Roles of microRNAs and prospective view of competing endogenous RNAs in mycotoxicosis

Field	Position	Original line	Normalised line	Original value	Normalised value
DE	3	44647	44341	MicroRNA	Microrna
DE	5	44647	44341	Competing endogenous RNA	Competing Endogenous RNA
ID	1	44649	44342	C VIRUS-RNA	C Virus RNA
ID	2	44649	44342	OCHRATOXIN-A	Ochratoxin A (OTA)
ID	3	44649	44342	UP-REGULATION	Up Regulation
ID	4	44649	44342	HEME OXYGENASE-1	Heme Oxygenase 1
ID	5	44649	44342	DOWN-REGULATION	Down Regulation
ID	6	44649	44342	AFLATOXIN B-1	Aflatoxin B1 (AFB1)
ID	7	44649	44342	HEALTH-RISK	Health Risk
ID	8	44649	44342	HEPG2 CELLS	HepG2
ID	9	44649	44342	CYCLIN G1	Cyclin G1
ID	10	44649	44342	IN-VITRO	In-Vitro

Record 284 | WOS:000487079300009

Antifungal activity of selected essential oils against Fusarium culmorum and F. graminearum and their secondary metabolites in wheat seeds

Field	Position	Original line	Normalised line	Original value	Normalised value
DE	1	44830	44521	Antifungal activity	Antifungal Activity
DE	2	44830	44521	Essential oils	Essential Oil
DE	3	44830	44521	Fusarium spp	Fusarium Spp
DE	5	44830	44521	Zearalenone	Zearalenone (ZEA)
DE	6	44830	44521	Trichothecenes	Trichothecene
DE	7	44830	44521	Wheat grain	Wheat Grain
ID	1	44832	44523	POSTHARVEST PHYTOPATHOGENIC FUNGI	Postharvest Phytopathogenic Fungi
ID	2	44832	44523	PLANT ESSENTIAL OILS	Plant Essential Oil
ID	3	44832	44523	IN-VITRO	In-Vitro
ID	4	44832	44523	DEOXYNIVALENOL PRODUCTION	Deoxynivalenol Production
ID	5	44832	44523	MYCOTOXIN CONTAMINATION	Mycotoxin Contamination
ID	6	44832	44523	NATURAL OCCURRENCE	Natural Occurrence
ID	7	44832	44523	OCHRATOXIN-A	Ochratoxin A (OTA)
ID	8	44832	44523	ERGOSTEROL	Ergosterol
ID	9	44832	44523	GROWTH	Growth
ID	10	44832	44523	L.	L

Record 285 | WOS:000475915000016

Annexin A3 may play an important role in ochratoxin-induced malignant transformation of human gastric epithelium cells

Field	Position	Original line	Normalised line	Original value	Normalised value
DE	1	44978	44668	Ochratoxin A	Ochratoxin A (OTA)
DE	2	44978	44668	Human gastric epithelium cells	Human Gastric Epithelium
DE	3	44978	44668	Malignant transformation	Malignant Transformation
DE	4	44978	44668	2-DE	2 De

DE	5	44978	44668	Matrix-assisted laser desorption ionization	Matrix Assisted Laser Desorption Ionization
DE	6	44978	44668	time-of-flight mass spectrometry	Time Of Flight Mass Spectrometry
ID	1	44981	44671	GENE-EXPRESSION	Gene Expression
ID	2	44981	44671	CANCER	Cancer
ID	3	44981	44671	OVEREXPRESSION	Overexpression
ID	4	44981	44671	PROLIFERATION	Proliferation
ID	5	44981	44671	FOOD	Food
ID	6	44981	44671	RAT	Rat

Record 286 ǀ WOS:000468622000013

Nontoxic concentrations of OTA aggravate DON-induced intestinal barrier dysfunction in IPEC-J2 cells via activation of NF-ÎºB signaling pathway

Field	Position	Original line	Normalised line	Original value	Normalised value
DE	1	45086	44775	Deoxynivalenol	Deoxynivalenol (DON)
DE	2	45086	44775	Ochratoxin A	Ochratoxin A (OTA)
DE	3	45086	44775	Barrier function	Barrier Function
DE	4	45086	44775	Tight junction	Tight Junction
DE	5	45086	44775	Nuclear factor-kappa B	Nuclear Factor Kappa B
ID	1	45088	44777	TIGHT JUNCTION PERMEABILITY	Tight Junction Permeability
ID	2	45088	44777	ALPHA-INDUCED INCREASE	Alpha Induced Increase
ID	3	45088	44777	OCHRATOXIN-A	Ochratoxin A (OTA)
ID	4	45088	44777	TRICHOTHECENE MYCOTOXIN	Trichothecene Mycotoxin
ID	5	45088	44777	IN-VITRO	In-Vitro
ID	6	45088	44777	DEOXYNIVALENOL	Deoxynivalenol (DON)
ID	7	45088	44777	ABSORPTION	Absorption
ID	8	45088	44777	DEGRADATION	Degradation
ID	9	45088	44777	TOXICITY	Toxicity
ID	10	45088	44777	METABOLITES	Metabolite

Record 287 ǀ WOS:000482246600031

Transport of mycotoxins across human gastric NCI-N87 and intestinal Caco-2 cell models

Field	Position	Original line	Normalised line	Original value	Normalised value
DE	1	45202	44890	Aflatoxin B1	Aflatoxin B1 (AFB1)
DE	2	45202	44890	Apparent permeability	Apparent Permeability
DE	3	45202	44890	Deoxynivalenol	Deoxynivalenol (DON)
DE	4	45202	44890	Fumonisin B1	Fumonisin B1 (FB1)
DE	5	45202	44890	Ochratoxin A	Ochratoxin A (OTA)
DE	6	45202	44890	Uptake/efflux ratio	Uptake Efflux Ratio
ID	1	45204	44892	AFLATOXIN B-1	Aflatoxin B1 (AFB1)
ID	2	45204	44892	FUMONISIN B-1	Fumonisin B1 (FB1)
ID	3	45204	44892	OCHRATOXIN-A	Ochratoxin A (OTA)
ID	4	45204	44892	WORLDWIDE OCCURRENCE	Worldwide Occurrence
ID	5	45204	44892	DRUG PERMEABILITY	Drug Permeability
ID	6	45204	44892	ABSORPTION	Absorption
ID	7	45204	44892	DEOXYNIVALENOL	Deoxynivalenol (DON)
ID	8	45204	44892	PREDICTION	Prediction
ID	9	45204	44892	FOOD	Food
ID	10	45204	44892	METABOLISM	Metabolism

Record 288 ǀ WOS:000487968300046

Astaxanthin Protects OTA-Induced Lung Injury in Mice through the Nrf2/NF-ÎºB Pathway

Field	Position	Original line	Normalised line	Original value	Normalised value
DE	1	45347	45035	ochratoxin	Ochratoxin A (OTA)
DE	2	45347	45035	astaxanthin	Astaxanthin
DE	3	45347	45035	oxidative stress	Oxidative Stress

DE	4	45347	45035	inflammation	Inflammation
DE	5	45347	45035	Nrf2 pathway	Nrf2
DE	6	45347	45035	NF-kappa B pathway	Nf Kappa B Pathway
DE	7	45347	45035	mouse	Mouse
DE	8	45347	45035	lung	Lung
ID	1	45349	45037	OCHRATOXIN-A	Ochratoxin A (OTA)
ID	2	45349	45037	OXIDATIVE STRESS	Oxidative Stress
ID	3	45349	45037	IN-VITRO	In-Vitro
ID	4	45349	45037	MYCOTOXIN OCHRATOXIN	Induced Inflammation
ID	5	45349	45037	INDUCED INFLAMMATION	Toxicity
ID	6	45349	45037	TOXICITY	Antioxidant
ID	7	45349	45037	ANTIOXIDANT	Genotoxicity
ID	8	45349	45037	GENOTOXICITY	Carotenoid
ID	9	45349	45037	CAROTENOIDS	Inhibition
ID	10	45349	45037	INHIBITION	

Record 289 | WOS:000487968300014

Heterologous Expression and Characterization of A Novel Ochratoxin A Degrading Enzyme, N-acyl-L-amino Acid Amidohydrolase, from Alcaligenes faecalis

Field	Position	Original line	Normalised line	Original value	Normalised value
DE	1	45467	45153	ochratoxin A	Ochratoxin A (OTA)
DE	2	45467	45153	degradation	Degradation
DE	3	45467	45153	amidohydrolase	Amidohydrolase
DE	4	45467	45153	Alcaligenes faecalis	Alcaligene Faecalis
DE	5	45467	45153	mycotoxin	Mycotoxins
ID	1	45469	45155	DEGRADATION	Degradation
ID	2	45469	45155	TOXICITY	Toxicity
ID	3	45469	45155	BIODEGRADATION	Biodegradation
ID	4	45469	45155	ADSORPTION	Adsorption
ID	5	45469	45155	MYCOTOXIN	Mycotoxins
ID	6	45469	45155	FEED	Feed
ID	7	45469	45155	FOOD	Food

Record 290 | WOS:000483690200021

Accuracy, discriminative properties and reliability of a human ESC-based in vitro toxicity assay to distinguish teratogens responsible for neural tube defects

Field	Position	Original line	Normalised line	Original value	Normalised value
DE	1	45554	45239	Developmental neurotoxicity	Developmental Neurotoxicity
DE	2	45554	45239	Neural tube defect	Neural Tube Defect
DE	3	45554	45239	Human embryonic stem cells	Human Embryonic Stem
DE	4	45554	45239	In vitro test	In Vitro Test
ID	1	45556	45241	EMBRYONIC STEM-CELLS	Embryonic Stem
ID	2	45556	45241	DEVELOPMENTAL NEUROTOXICITY	Developmental Neurotoxicity
ID	3	45556	45241	DIFFERENTIATION	Differentiation
ID	4	45556	45241	VARIABILITY	Variability
ID	5	45556	45241	MODELS	Model
ID	6	45556	45241	DRUGS	Drug
ID	7	45556	45241	HETEROGENEITY	Heterogeneity
ID	8	45556	45241	MIGRATION	Migration
ID	9	45556	45241	LINES	Line
ID	10	45556	45241	ACID	Acid

Record 291 | WOS:000477777500023

Effects of Gamma Irradiation on Ochratoxin A Stability and Cytotoxicity in Methanolic Solutions and Potential Application in Tunisian Millet Samples

Field	Position	Original line	Normalised line	Original value	Normalised value
DE	2	45659	45343	Gamma irradiation	Gamma Irradiation
DE	4	45659	45343	Ochratoxin A	Ochratoxin A (OTA)
ID	1	45660	45344	MYCOTOXIN CONTAMINATION	Mycotoxin Contamination
ID	2	45660	45344	OXIDATIVE STRESS	Oxidative Stress
ID	3	45660	45344	HEPG2 CELLS	HepG2
ID	4	45660	45344	ANIMAL FEED	Animal Feed
ID	5	45660	45344	VITAMIN-E	Vitamin E
ID	6	45660	45344	FOOD	Food
ID	7	45660	45344	DETOXIFICATION	Detoxification
ID	8	45660	45344	NEPHROPATHY	Nephropathy
ID	9	45660	45344	ASPERGILLUS	Aspergillus
ID	10	45660	45344	ZEARALENONE	Zearalenone (ZEA)

Record 292 | WOS:000478525600001

An in vitro attempt at precision toxicology reveals the involvement of DNA methylation alteration in ochratoxin A-induced G0/G1 phase arrest

Field	Position	Original line	Normalised line	Original value	Normalised value
DE	1	45781	45464	Precision toxicology	Precision Toxicology
DE	2	45781	45464	single-cell sequencing	Single Cell Sequencing
DE	3	45781	45464	cell cycle arrest	Cell Cycle Arrest
DE	4	45781	45464	DNA methylation	DNA Methylation
DE	5	45781	45464	ochratoxin A	Ochratoxin A (OTA)
ID	1	45783	45466	GENE-EXPRESSION	Gene Expression
ID	2	45783	45466	METHYLOME LANDSCAPES	Methylome Landscape
ID	3	45783	45466	STEM-CELLS	Stem
ID	4	45783	45466	RNA-SEQ	RNA Seq
ID	5	45783	45466	NOTCH	Notch
ID	6	45783	45466	PROTEIN	Protein
ID	7	45783	45466	TRANSCRIPTION	Transcription
ID	8	45783	45466	PATHWAYS	Pathway
ID	9	45783	45466	CANCER	Cancer
ID	10	45783	45466	DAMAGE	Damage

Record 293 | WOS:000474669600029

Long-Time Instead of Short-Time Exposure in Vitro and Administration in Vivo of Ochratoxin A Is Consistent in Immunosuppression

Field	Position	Original line	Normalised line	Original value	Normalised value
DE	1	45910	45592	ochratoxin A	Ochratoxin A (OTA)
DE	2	45910	45592	long-time exposure	Long Time Exposure
DE	3	45910	45592	immunosuppression	Immunosuppression
DE	4	45910	45592	immunostimulation	Immunostimulation
DE	5	45910	45592	polarization	Polarization
DE	6	45910	45592	autophagy	Autophagy
ID	1	45912	45594	INDUCED OXIDATIVE STRESS	Induced Oxidative Stress
ID	2	45912	45594	IMMUNE TOXICITY	Immune Toxicity
ID	3	45912	45594	AUTOPHAGY	Autophagy
ID	4	45912	45594	MACROPHAGE	Macrophage
ID	5	45912	45594	CELLS	Cell
ID	6	45912	45594	ACTIVATION	Activation
ID	7	45912	45594	IMMUNOTOXICITY	Immunotoxicity
ID	8	45912	45594	PHAGOCYTOSIS	Phagocytosis
ID	9	45912	45594	COMBINATION	Combination
ID	10	45912	45594	MYCOTOXINS	Mycotoxins

Record 294 | WOS:000482110000051

Ochratoxin A-Induced Hepatotoxicity through Phase I and Phase II Reactions Regulated by AhR in Liver Cells

Field	Position	Original line	Normalised line	Original value	Normalised value
DE	1	46025	45706	hepatotoxicity	Hepatotoxicity

DE	2	46025	45706	ochratoxin A	Ochratoxin A (OTA)
DE	3	46025	45706	oxidative stress	Oxidative Stress
DE	4	46025	45706	phase I reaction	Phase I Reaction
DE	5	46025	45706	phase II reaction	Phase Ii Reaction
ID	1	46027	45708	OXIDATIVE DNA-DAMAGE	Oxidative DNA Damage
ID	2	46027	45708	PENICILLIUM-VERRUCOSUM	Penicillium Verrucosum
ID	3	46027	45708	TOXICITY	Toxicity
ID	4	46027	45708	CYTOTOXICITY	Cytotoxicity
ID	5	46027	45708	METABOLISM	Metabolism
ID	6	46027	45708	APOPTOSIS	Apoptosis
ID	7	46027	45708	STRESS	Stress
ID	8	46027	45708	TOXICOKINETICS	Toxicokinetics
ID	9	46027	45708	NEPHROTOXICITY	Nephrotoxicity
ID	10	46027	45708	TRANSCRIPTION	Transcription

Record 295 | WOS:000475328000001

Assorted Methods for Decontamination of Aflatoxin M1 in Milk Using Microbial Adsorbents

Field	Position	Original line	Normalised line	Original value	Normalised value
DE	1	46140	45820	decontamination	Decontamination
DE	2	46140	45820	mycotoxins	Mycotoxins
DE	3	46140	45820	Aflatoxin M1	Aflatoxin M1 (AFM1)
DE	4	46140	45820	milk	Milk
DE	5	46140	45820	binding	Binding
DE	6	46140	45820	stability	Stability
ID	1	46141	45821	LACTIC-ACID BACTERIA	Lactic Acid Bacteria
ID	2	46141	45821	ASPERGILLUS-FLAVUS	Aspergillus Flavus
ID	3	46141	45821	DAIRY STRAINS	Dairy Strain
ID	4	46141	45821	IN-VITRO	In-Vitro
ID	5	46141	45821	SACCHAROMYCES-CEREVISIAE	Saccharomyce Cerevisiae
ID	6	46141	45821	MYCOTOXIN CONTAMINATION	Mycotoxin Contamination
ID	7	46141	45821	INNOVATIVE TECHNOLOGIES	Innovative Technology
ID	8	46141	45821	POSTHARVEST MANAGEMENT	Postharvest Management
ID	9	46141	45821	GAMMA-RADIATION	Gamma Radiation
ID	10	46141	45821	OCHRATOXIN-A	Ochratoxin A (OTA)

Record 296 | WOS:000475328000038

Cytotoxicity, Genotoxicity and Disturbance of Cell Cycle in HepG2 Cells Exposed to OTA and BEA: Single and Combined Actions

Field	Position	Original line	Normalised line	Original value	Normalised value
DE	1	46353	46032	ochratoxin A	Ochratoxin A (OTA)
DE	2	46353	46032	beauvericin	Beauvericin
DE	3	46353	46032	mixtures	Mixture
DE	4	46353	46032	HepG2 cells	HepG2
DE	5	46353	46032	genotoxicity	Genotoxicity
DE	6	46353	46032	cell cycle	Cell Cycle
ID	1	46355	46033	VITRO MICRONUCLEUS ASSAY	Vitro Micronucleus Assay
ID	2	46355	46033	OCHRATOXIN-A	Ochratoxin A (OTA)
ID	3	46355	46033	IN-VITRO	In-Vitro
ID	4	46355	46033	MYCOTOXIN CITRININ	Mycotoxin Citrinin
ID	5	46355	46033	OXIDATIVE STRESS	Oxidative Stress
ID	6	46355	46033	DNA-DAMAGE	DNA Damage
ID	7	46355	46033	BEAUVERICIN	Beauvericin
ID	8	46355	46033	DEOXYNIVALENOL	Deoxynivalenol (DON)
ID	9	46355	46033	TOXICITY	Toxicity
ID	10	46355	46033	APOPTOSIS	Apoptosis

Record 297 | WOS:000475288200030

Long-Term Effects of Ochratoxin A on the Glutathione Redox System and Its Regulation in Chicken

Field	Position	Original line	Normalised line	Original value	Normalised value
DE	1	46465	46142	Ochratoxin A	Ochratoxin A (OTA)
DE	2	46465	46142	glutathione	Glutathione (GSH)
DE	3	46465	46142	glutathione-peroxidase	Glutathione Peroxidase
DE	4	46465	46142	oxidative stress	Oxidative Stress
DE	5	46465	46142	gene expression	Gene Expression
ID	1	46467	46144	GENE-EXPRESSION	Gene Expression
ID	2	46467	46144	OXIDATIVE STRESS	Oxidative Stress
ID	3	46467	46144	LIPID-PEROXIDATION	Lipid Peroxidation
ID	4	46467	46144	ANTIOXIDANT	Antioxidant
ID	5	46467	46144	TOXICITY	Toxicity
ID	6	46467	46144	NEPHROTOXICITY	Nephrotoxicity
ID	7	46467	46144	MECHANISM	Mechanism
ID	8	46467	46144	RESPONSES	Response
ID	9	46467	46144	ENZYMES	Enzyme
ID	10	46467	46144	KIDNEY	Kidney

Record 298 | WOS:000461534700004

Effects of medicinal herb and Brazilian traditional plant extracts on in vitro mycotoxin decontamination

Field	Position	Original line	Normalised line	Original value	Normalised value
DE	1	46583	46259	Plant extracts	Plant Extract
DE	4	46583	46259	AFB(1)	Aflatoxin B1 (AFB1)
ID	1	46584	46260	ASPERGILLUS-FLAVUS	Aspergillus Flavus
ID	2	46584	46260	PASSIFLORA-ALATA	Passiflora Alata
ID	3	46584	46260	AFLATOXIN B-1	Aflatoxin B1 (AFB1)
ID	4	46584	46260	DETOXIFICATION	Detoxification
ID	5	46584	46260	ANTIOXIDANT	Antioxidant
ID	6	46584	46260	TOXICITY	Toxicity
ID	7	46584	46260	L.	L
ID	8	46584	46260	DEGRADATION	Degradation
ID	9	46584	46260	PRODUCTS	Product
ID	10	46584	46260	M-1	M 1

Record 299 | WOS:000465050300026

Ochratoxin A upregulates biomarkers associated with hypoxia and transformation in human kidney cells

Field	Position	Original line	Normalised line	Original value	Normalised value
DE	1	46669	46344	Ochratoxin A	Ochratoxin A (OTA)
DE	3	46669	46344	Hypoxic response	Hypoxic Response
DE	4	46669	46344	HIF1 alpha	Hif1 Alpha
DE	5	46669	46344	VEGF	Vegf
DE	6	46669	46344	TGF beta	Tgf Beta
ID	1	46670	46346	OXIDATIVE STRESS	Oxidative Stress
ID	2	46670	46346	VEGF EXPRESSION	Vegf Expression
ID	3	46670	46346	DNA-DAMAGE	DNA Damage
ID	4	46670	46346	NEPHROPATHY	Nephropathy
ID	5	46670	46346	APOPTOSIS	Apoptosis
ID	6	46670	46346	TOXICITY	Toxicity
ID	7	46670	46346	CANCER	Cancer
ID	8	46670	46346	HSP90	Hsp90

Record 300 | WOS:000475328000057

The In Vivo and In Vitro Toxicokinetics of Citreoviridin Extracted from Penicillium citreonigrum

Field	Position	Original line	Normalised line	Original value	Normalised value

DE	1	46770	46445	citreoviridin	Citreoviridin
DE	2	46770	46445	toxicokinetics	Toxicokinetics
DE	3	46770	46445	bioavailability	Bioavailability
DE	4	46770	46445	swine	Swine
ID	1	46771	46446	TOXIN DEOXYNIVALENOL DON	Toxin Deoxynivalenol DON
ID	2	46771	46446	OCHRATOXIN-A	Ochratoxin A (OTA)
ID	3	46771	46446	MARANHAO STATE	Maranhao State
ID	4	46771	46446	MYCOTOXIN	Mycotoxins
ID	5	46771	46446	BIOAVAILABILITY	Bioavailability
ID	6	46771	46446	TRANSPORT	Transport
ID	7	46771	46446	BERIBERI	Beriberi
ID	8	46771	46446	CACO-2	Caco-2
ID	9	46771	46446	MODEL	Model
ID	10	46771	46446	PIG	Pig

Record 301 | WOS:000463851800006

Biotransformation of plantain pseudostem fibres using local enzyme sources; analysis of their potential as commercial poultry feed

Field	Position	Original line	Normalised line	Original value	Normalised value
DE	1	46875	46549	Monosaccharides	Monosaccharide
DE	2	46875	46549	plantain pseudostem	Plantain Pseudostem
DE	3	46875	46549	mycotoxins	Mycotoxins
DE	4	46875	46549	free radical	Free Radical
DE	5	46875	46549	amino acid	Amino Acid
ID	1	46877	46550	AMINO-ACIDS	Amino Acid
ID	2	46877	46550	IN-VITRO	In-Vitro
ID	3	46877	46550	HYDROLYSIS	Hydrolysis
ID	4	46877	46550	MYCOTOXINS	Mycotoxins
ID	5	46877	46550	CELLULOSE	Cellulose
ID	6	46877	46550	COMPONENTS	Component
ID	7	46877	46550	TOXICITY	Toxicity
ID	8	46877	46550	BROILERS	Broiler
ID	9	46877	46550	GROWTH	Growth
ID	10	46877	46550	DIETS	Diet

Record 302 | WOS:000472672300064

Degrading Ochratoxin A and Zearalenone Mycotoxins Using a Multifunctional Recombinant Enzyme

Field	Position	Original line	Normalised line	Original value	Normalised value
DE	1	46988	46660	fusion enzyme	Fusion Enzyme
DE	2	46988	46660	ochratoxin A	Ochratoxin A (OTA)
DE	3	46988	46660	carboxypeptidase	Carboxypeptidase
DE	4	46988	46660	zearalenone	Zearalenone (ZEA)
DE	5	46988	46660	ZHD101	Zhd101
DE	6	46988	46660	HK293 cell	Hk293
DE	7	46988	46660	LX2 cell	Lx2
ID	1	46990	46662	DEGRADATION	Degradation
ID	2	46990	46662	DETOXIFICATION	Detoxification
ID	3	46990	46662	BIODEGRADATION	Biodegradation
ID	4	46990	46662	CYTOTOXICITY	Cytotoxicity
ID	5	46990	46662	METABOLISM	Metabolism
ID	6	46990	46662	TOXICITY	Toxicity
ID	7	46990	46662	DESIGN	Design
ID	8	46990	46662	CELLS	Cell
ID	9	46990	46662	FEED	Feed
ID	10	46990	46662	FOOD	Food

Record 303 | WOS:000469753500299

Interaction of Mycotoxin Alternariol with Serum Albumin

Field	Position	Original line	Normalised line	Original value	Normalised value

DE	1	47100	46771	alternariol	Alternariol
DE	2	47100	46771	serum albumin	Serum Albumin
DE	3	47100	46771	albumin-ligand interaction	Albumin Ligand Interaction
DE	4	47100	46771	binding site	Binding Site
DE	5	47100	46771	fluorescence spectroscopy	Fluorescence Spectroscopy
ID	1	47102	46773	PERFORMANCE LIQUID-CHROMATOGRAPHY	Performance Liquid Chromatography
ID	2	47102	46773	SOLID-PHASE EXTRACTION	Solid Phase Extraction
ID	3	47102	46773	DRUG-BINDING	Drug Binding
ID	4	47102	46773	STRUCTURAL BASIS	Structural Basis
ID	5	47102	46773	ENERGY-TRANSFER	Energy Transfer
ID	6	47102	46773	OCHRATOXIN-A	Ochratoxin A (OTA)
ID	7	47102	46773	TOXICITY	Toxicity
ID	8	47102	46773	DISPLACEMENT	Displacement
ID	9	47102	46773	SPECTROSCOPY	Spectroscopy
ID	10	47102	46773	MODULATION	Modulation

Record 304 | WOS:000467512200032

Cytotoxicity against fish and mammalian cell lines and endocrine activity of the mycotoxins beauvericin, deoxynivalenol and ochratoxin-A

Field	Position	Original line	Normalised line	Original value	Normalised value
DE	2	47232	46902	In vitro toxicity	In Vitro Toxicity
DE	3	47232	46902	Mammalian and fish cells	Mammalian And Fish
DE	4	47232	46902	Androgen receptor	Androgen Receptor
DE	5	47232	46902	Estrogen receptor	Estrogen Receptor
DE	6	47232	46902	Thyroid receptor	Thyroid Receptor
ID	1	47234	46904	EMERGING FUSARIUM-MYCOTOXINS	Emerging Fusarium Mycotoxin
ID	2	47234	46904	CYPRINUS-CARPIO L.	Cyprinus Carpio L
ID	3	47234	46904	TOXICITY	Toxicity
ID	4	47234	46904	FEED	Feed
ID	5	47234	46904	ZEARALENONE	Zearalenone (ZEA)
ID	6	47234	46904	EXPOSURE	Exposure
ID	7	47234	46904	DAMAGE	Damage
ID	8	47234	46904	FOOD	Food
ID	9	47234	46904	DON	Deoxynivalenol (DON)
ID	10	47234	46904	ACTIVATION	Activation

Record 305 | WOS:000468044200002

Prevention of ochratoxin A-induced oxidative stress-mediated apoptotic processes and impairment of embryonic development in mouse blastocysts by liquiritigenin

Field	Position	Original line	Normalised line	Original value	Normalised value
DE	1	47367	47036	apoptosis	Apoptosis
DE	2	47367	47036	embryonic development	Embryonic Development
DE	3	47367	47036	liquiritigenin	Liquiritigenin
DE	4	47367	47036	ochratoxin A	Ochratoxin A (OTA)
DE	5	47367	47036	oxidative stress	Oxidative Stress
ID	1	47369	47038	IN-VITRO	In-Vitro
ID	2	47369	47038	GLYCYRRHIZAE-RADIX	Glycyrrhizae Radix
ID	3	47369	47038	LICORICE ROOTS	Licorice Root
ID	4	47369	47038	STEM-CELLS	Stem
ID	5	47369	47038	TOXICITY	Toxicity
ID	6	47369	47038	FERTILIZATION	Fertilization
ID	7	47369	47038	MATURATION	Maturation
ID	8	47369	47038	INHIBITION	Inhibition
ID	9	47369	47038	OOCYTES	Oocyte
ID	10	47369	47038	ISOLIQUIRITIGENIN	Isoliquiritigenin

Record 306 | WOS:000467299600035

Assessment of Toxic Effects of Ochratoxin A in Human Embryonic Stem Cells

Field	Position	Original line	Normalised line	Original value	Normalised value
DE	2	47506	47174	human Stem Cells	Human Stem
DE	3	47506	47174	mycotoxins	Mycotoxins
DE	4	47506	47174	cells	Cell
DE	5	47506	47174	cytotoxicity	Cytotoxicity
DE	6	47506	47174	cell culture	
ID	1	47508	47175	IN-VITRO	In-Vitro
ID	2	47508	47175	DEVELOPMENTAL TOXICITY	Developmental Toxicity
ID	3	47508	47175	OXIDATIVE STRESS	Oxidative Stress
ID	4	47508	47175	AFLATOXIN B-1	Aflatoxin B1 (AFB1)
ID	5	47508	47175	APOPTOSIS	Apoptosis
ID	6	47508	47175	EXPOSURE	Exposure
ID	7	47508	47175	KIDNEY	Kidney
ID	8	47508	47175	MOUSE	Mouse
ID	9	47508	47175	SMOKE	Smoke
ID	10	47508	47175	RATS	Rat

Record 307 | WOS:000467645200017

Nephrotoxicity instead of immunotoxicity of OTA is induced through DNMT1-dependent activation of JAK2/STAT3 signaling pathway by targeting SOCS3

Field	Position	Original line	Normalised line	Original value	Normalised value
DE	1	47648	47314	Ochratoxin A	Ochratoxin A (OTA)
DE	3	47648	47314	DNA damage	DNA Damage
DE	4	47648	47314	DNMT1	Dnmt1
DE	5	47648	47314	SOCS3	Socs3
DE	6	47648	47314	JAK2	Jak2
DE	7	47648	47314	STAT3 signaling pathway	Stat3 Signaling Pathway
ID	1	47650	47316	RENAL PROXIMAL TUBULE	Renal Proximal Tubule
ID	2	47650	47316	OCHRATOXIN-A	Ochratoxin A (OTA)
ID	3	47650	47316	DNA METHYLTRANSFERASES	DNA Methyltransferase
ID	4	47650	47316	OXIDATIVE STRESS	Oxidative Stress
ID	5	47650	47316	FUMONISIN B-1	Fumonisin B1 (FB1)
ID	6	47650	47316	PK15 CELLS	Pk15
ID	7	47650	47316	IN-VITRO	In-Vitro
ID	8	47650	47316	APOPTOSIS	Apoptosis
ID	9	47650	47316	KIDNEY	Kidney
ID	10	47650	47316	INFLAMMATION	Inflammation

Record 308 | WOS:000467299600005

Biomarkers for Exposure as a Tool for Efficacy Testing of a Mycotoxin Detoxifier in Broiler Chickens and Pigs

Field	Position	Original line	Normalised line	Original value	Normalised value
DE	1	47769	47433	biomarkers	Biomarker
DE	2	47769	47433	exposure	Exposure
DE	3	47769	47433	efficacy	Efficacy
DE	4	47769	47433	mycotoxin detoxifier	Mycotoxin Detoxifier
DE	5	47769	47433	pig	Pig
DE	6	47769	47433	broiler chicken	Broiler Chicken
ID	1	47771	47434	FUSARIUM TOXIN DEOXYNIVALENOL	Fusarium Toxin Deoxynivalenol
ID	2	47771	47434	IN-VIVO EFFICACY	In Vivo Efficacy
ID	3	47771	47434	SODIUM BENTONITE	Sodium Bentonite
ID	4	47771	47434	OCHRATOXIN-A	Ochratoxin A (OTA)
ID	5	47771	47434	AFLATOXIN B-1	Aflatoxin B1 (AFB1)
ID	6	47771	47434	COMPARATIVE TOXICOKINETICS	Comparative Toxicokinetics
ID	7	47771	47434	OXIDATIVE STRESS	Oxidative Stress

ID	8	47771	47434	METABOLISM	Metabolism
ID	9	47771	47434	ZEARALENONE	Zearalenone (ZEA)
ID	10	47771	47434	POULTRY	Poultry

Record 309 | WOS:000470119800012

Antifungal and antimycotoxigenic effect of Lactobacillus plantarum CRL 778 at different water activity values

Field	Position	Original line	Normalised line	Original value	Normalised value
DE	1	47892	47554	Lactic acid bacteria	Lactic Acid Bacteria
DE	2	47892	47554	Antifungal activity	Antifungal Activity
DE	3	47892	47554	Ochratoxin A	Ochratoxin A (OTA)
DE	4	47892	47554	Water activity	Water Activity
ID	1	47893	47556	OCHRATOXIN-A PRODUCTION	Ochratoxin A Production
ID	2	47893	47556	LACTIC-ACID BACTERIA	Lactic Acid Bacteria
ID	3	47893	47556	ASPERGILLUS-CARBONARIUS	Aspergillus Carbonarius
ID	4	47893	47556	ECOPHYSIOLOGICAL FACTORS	Ecophysiological Factor
ID	5	47893	47556	TEMPERATURE	Temperature
ID	6	47893	47556	GROWTH	Growth
ID	7	47893	47556	STRAINS	Strain
ID	8	47893	47556	CONTAMINATION	Contamination
ID	9	47893	47556	STRATEGIES	Strategy
ID	10	47893	47556	REDUCTION	Reduction

Record 310 | WOS:000462049400006

Oxidative stress as a mechanism of combined OTA and CTN toxicity in rat plasma, liver and kidney

Field	Position	Original line	Normalised line	Original value	Normalised value
DE	2	47988	47650	ochratoxin A	Ochratoxin A (OTA)
DE	3	47988	47650	oxidative stress	Oxidative Stress
DE	4	47988	47650	rats	Rat
DE	5	47988	47650	resveratrol	Resveratrol
ID	1	47989	47651	OCHRATOXIN-A	Ochratoxin A (OTA)
ID	2	47989	47651	LIPID-PEROXIDATION	Lipid Peroxidation
ID	3	47989	47651	PENICILLIUM-VERRUCOSUM	Penicillium Verrucosum
ID	4	47989	47651	MYCOTOXIN CITRININ	Mycotoxin Citrinin
ID	5	47989	47651	PROTECTIVE ROLE	Protective Role
ID	6	47989	47651	DNA-DAMAGE	DNA Damage
ID	7	47989	47651	IN-VITRO	In-Vitro
ID	8	47989	47651	CELLS	Cell
ID	9	47989	47651	APOPTOSIS	Apoptosis
ID	10	47989	47651	CARCINOGENICITY	Carcinogenicity

Record 311 | WOS:000467299600032

Fluorescence Spectroscopic Investigation of Competitive Interactions between Quercetin and Aflatoxin B1 for Binding to Human Serum Albumin

Field	Position	Original line	Normalised line	Original value	Normalised value
DE	1	48111	47772	aflatoxin B-1	Aflatoxin B1 (AFB1)
DE	2	48111	47772	quercetin	Quercetin
DE	3	48111	47772	human serum albumin	Human Serum Albumin
DE	4	48111	47772	competitive interaction	Competitive Interaction
DE	5	48111	47772	fluorescence spectroscopy	Fluorescence Spectroscopy
ID	1	48113	47774	OCHRATOXIN-A	Ochratoxin A (OTA)
ID	2	48113	47774	OXIDATIVE STRESS	Oxidative Stress
ID	3	48113	47774	DRUG INTERACTION	Drug Interaction
ID	4	48113	47774	IN-VITRO	In-Vitro
ID	5	48113	47774	BOVINE	Bovine

ID	6	48113	47774	PREVENTION	Prevention
ID	7	48113	47774	TOXICITY	Toxicity
ID	8	48113	47774	ZEARALENONE	Zearalenone (ZEA)
ID	9	48113	47774	INHIBITION	Inhibition
ID	10	48113	47774	METABOLISM	Metabolism

Record 312 | WOS:000462420300003

Review: Biotechnology of mycotoxins detoxification using microorganisms and enzymes

Field	Position	Original line	Normalised line	Original value	Normalised value
DE	5	48234	47894	Enzymes	Enzyme
ID	1	48235	47895	LACTIC-ACID BACTERIA	Lactic Acid Bacteria
ID	2	48235	47895	LACTOBACILLUS-PLANTARUM MON03	Lactobacillus Plantarum Mon03
ID	3	48235	47895	AFLATOXIN DEGRADATION ENZYME	Aflatoxin Degradation Enzyme
ID	4	48235	47895	LC-MS/MS METHOD	Lc Ms Ms Method
ID	5	48235	47895	OCHRATOXIN-A	Ochratoxin A (OTA)
ID	6	48235	47895	IN-VITRO	In-Vitro
ID	7	48235	47895	SACCHAROMYCES-CEREVISIAE	Saccharomyce Cerevisiae
ID	8	48235	47895	FUSARIUM-MYCOTOXINS	Fusarium Mycotoxin
ID	9	48235	47895	RISK-ASSESSMENT	Risk Assessment
ID	10	48235	47895	OXIDATIVE STRESS	Oxidative Stress

Record 313 | WOS:000460996800027

Ochratoxin A Exposure Impairs Porcine Granulosa Cell Growth via the PI3K/AKT Signaling Pathway

Field	Position	Original line	Normalised line	Original value	Normalised value
DE	1	48513	48171	Ochratoxin A	Ochratoxin A (OTA)
DE	2	48513	48171	Granulosa cells	Granulosa
DE	3	48513	48171	RNA-seq	RNA Seq
DE	4	48513	48171	PI3K/AKT signaling pathway	Pi3k Akt Signaling Pathway
ID	1	48514	48172	FOLLICULAR ATRESIA	Follicular Atresia
ID	2	48514	48172	CYCLE ARREST	Cycle Arrest
ID	3	48514	48172	APOPTOSIS	Apoptosis
ID	4	48514	48172	ZEARALENONE	Zearalenone (ZEA)
ID	5	48514	48172	EXPRESSION	Expression
ID	6	48514	48172	MYCOTOXIN	Mycotoxins
ID	7	48514	48172	TOXICITY	Toxicity
ID	8	48514	48172	PTEN	Pten
ID	9	48514	48172	AKT	Akt
ID	10	48514	48172	PROLIFERATION	Proliferation

Record 314 | WOS:000465449900001

Cytotoxicity of gamma irradiated aflatoxin B1 and ochratoxin A

Field	Position	Original line	Normalised line	Original value	Normalised value
DE	2	48634	48291	food safety	Food Safety
DE	3	48634	48291	gamma irradiation	Gamma Irradiation
DE	4	48634	48291	mycotoxins	Mycotoxins
DE	5	48634	48291	reduction	Reduction
ID	1	48635	48292	IN-VITRO	In-Vitro
ID	2	48635	48292	MYCOTOXIN CONTAMINATION	Mycotoxin Contamination
ID	3	48635	48292	VITAMIN-E	Vitamin E
ID	4	48635	48292	REDUCTION	Reduction
ID	5	48635	48292	INACTIVATION	Inactivation
ID	6	48635	48292	TOXICITY	Toxicity

ID	7	48635	48292	PRODUCTS	Product
ID	8	48635	48292	CELLS	Cell
ID	9	48635	48292	ACID	Acid

Record 315 | WOS:000481430100007

Inhibitory effect of ochratoxin A on DNMT-mediated flocculation of yeast

Field	Position	Original line	Normalised line	Original value	Normalised value
ID	1	48739	48395	DNA METHYLATION	DNA Methylation
ID	2	48739	48395	CANCER	Cancer
ID	3	48739	48395	EPIGENETICS	Epigenetics
ID	4	48739	48395	METHYLTRANSFERASES	Methyltransferase
ID	5	48739	48395	DEOXYNIVALENOL	Deoxynivalenol (DON)
ID	6	48739	48395	EXPOSURE	Exposure

Record 316 | WOS:000460801500074

Modulation of Mucin (MUC2, MUC5AC and MUC5B) mRNA Expression and Protein Production and Secretion in Caco-2/HT29-MTX Co-Cultures Following Exposure to Individual and Combined Aflatoxin M1 and Ochratoxin A

Field	Position	Original line	Normalised line	Original value	Normalised value
DE	1	48825	48480	aflatoxin M1	Aflatoxin M1 (AFM1)
DE	2	48825	48480	ochratoxin A	Ochratoxin A (OTA)
DE	4	48825	48480	HT29-MTX co-cultures	Ht29 Mtx Co
DE	5	48825	48480	mucin	Mucin
DE	6	48825	48480	interactive effects	Interactive Effect
ID	1	48827	48482	HEALTH-RISK ASSESSMENT	Health Risk Assessment
ID	2	48827	48482	INTESTINAL EPITHELIUM	Intestinal Epithelium
ID	3	48827	48482	DIETARY EXPOSURE	Dietary Exposure
ID	4	48827	48482	GENE-EXPRESSION	Gene Expression
ID	5	48827	48482	CACO-2 CELLS	Caco-2
ID	6	48827	48482	MYCOTOXINS	Mycotoxins
ID	7	48827	48482	BARRIER	Barrier
ID	8	48827	48482	MILK	Milk
ID	9	48827	48482	DEOXYNIVALENOL	Deoxynivalenol (DON)
ID	10	48827	48482	FOOD	Food

Record 317 | WOS:000457296600055

Precision toxicology shows that troxerutin alleviates ochratoxin A-induced renal lipotoxicity

Field	Position	Original line	Normalised line	Original value	Normalised value
DE	1	48964	48617	lipidomics	Lipidomics
DE	2	48964	48617	brown adipose activity	Brown Adipose Activity
DE	3	48964	48617	chronic kidney disease	Chronic Kidney Disease
DE	4	48964	48617	energy metabolism	Energy Metabolism
DE	5	48964	48617	RNA-seq	RNA Seq
ID	1	48966	48619	FATTY-ACID OXIDATION	Fatty Acid Oxidation
ID	2	48966	48619	CHRONIC KIDNEY-DISEASE	Chronic Kidney Disease
ID	3	48966	48619	LIPID-METABOLISM	Lipid Metabolism
ID	4	48966	48619	CELL-DEATH	Cell Death
ID	5	48966	48619	GENE-EXPRESSION	Gene Expression
ID	6	48966	48619	DNA-DAMAGE	DNA Damage
ID	7	48966	48619	RNA-SEQ	RNA Seq
ID	8	48966	48619	PROTEIN	Protein
ID	9	48966	48619	CERAMIDE	Ceramide
ID	10	48966	48619	STRESS	Stress

Record 318 | WOS:000566844400029

Potential Mycotoxin Effects on Coffee Consumers' Health

Field	Position	Original line	Normalised line	Original value	Normalised value
ID	1	49139	48790	IN-VITRO CYTOTOXICITY	In Vitro Cytotoxicity
ID	2	49139	48790	OCHRATOXIN-A	Ochratoxin A (OTA)
ID	3	49139	48790	DEGRADATION-PRODUCT	Degradation Product
ID	4	49139	48790	DIGESTION MODEL	Digestion Model
ID	5	49139	48790	GUT MICROBIOTA	Gut Microbiota
ID	6	49139	48790	AFLATOXIN B-1	Aflatoxin B1 (AFB1)
ID	7	49139	48790	BIOACCESSIBILITY	Bioaccessibility
ID	8	49139	48790	METABOLISM	Metabolism
ID	9	49139	48790	TOXICITY	Toxicity
ID	10	49139	48790	RAT	Rat

Record 319 | WOS:000533508400001

Protective activity of melatonin against mycotoxins-induced toxicity: a review

Field	Position	Original line	Normalised line	Original value	Normalised value
DE	2	49234	48884	aflatoxin	Aflatoxin
DE	3	49234	48884	ochratoxin A	Ochratoxin A (OTA)
DE	4	49234	48884	hepatotoxicity	Hepatotoxicity
DE	5	49234	48884	mycotoxin	Mycotoxins
ID	1	49235	48885	INDUCED OXIDATIVE STRESS	Induced Oxidative Stress
ID	2	49235	48885	OCHRATOXIN-A	Ochratoxin A (OTA)
ID	3	49235	48885	RAT-LIVER	Rat Liver
ID	4	49235	48885	LIPID-PEROXIDATION	Lipid Peroxidation
ID	5	49235	48885	BETA-ZEARALENOL	Beta Zearalenol
ID	6	49235	48885	COENZYME Q(10)	Coenzyme Q 10
ID	7	49235	48885	ANIMAL-MODEL	Animal Model
ID	8	49235	48885	DAMAGE	Damage
ID	9	49235	48885	AFLATOXIN-B1	Aflatoxin B1 (AFB1)
ID	10	49235	48885	ACID	Acid

Record 320 | WOS:000467038800009

OCRATOXINS AND THEIR NEPHROTOXIC POTENTIAL

Field	Position	Original line	Normalised line	Original value	Normalised value
DE	1	49344	48993	ochratoxins	Ochratoxin A (OTA)
DE	2	49344	48993	nephrotoxicity	Nephrotoxicity
DE	3	49344	48993	toxicology	Toxicology
DE	4	49344	48993	renal physiology	Renal Physiology
ID	1	49345	48994	OCHRATOXIN-A	Ochratoxin A (OTA)
ID	2	49345	48994	ENDEMIC NEPHROPATHY	Endemic Nephropathy
ID	3	49345	48994	MOLECULAR-MECHANISM	Molecular Mechanism
ID	4	49345	48994	RISK-ASSESSMENT	Risk Assessment
ID	5	49345	48994	KIDNEY-CELLS	Kidney
ID	6	49345	48994	EXPOSURE	Exposure
ID	7	49345	48994	TOXICITY	Toxicity
ID	8	49345	48994	CYTOTOXICITY	Cytotoxicity
ID	9	49345	48994	TRANSPORTERS	Transporter
ID	10	49345	48994	GENOTOXICITY	Genotoxicity

Record 321 | WOS:000576729800009

Mycotoxins in food and feed

Field	Position	Original line	Normalised line	Original value	Normalised value
ID	1	49452	49100	AFLATOXIN B-1	Aflatoxin B1 (AFB1)
ID	2	49452	49100	OCHRATOXIN-A	Ochratoxin A (OTA)

ID	3	49452	49100	SACCHAROMYCES-CEREVISIAE	Saccharomyce Cerevisiae
ID	4	49452	49100	FUSARIUM MYCOTOXINS	Fusarium Mycotoxin
ID	5	49452	49100	UNPROCESSED CEREALS	Unprocessed Cereal
ID	6	49452	49100	SLAVONSKI KULEN	Slavonski Kulen
ID	7	49452	49100	ERGOT ALKALOIDS	Ergot Alkaloid
ID	8	49452	49100	MEAT-PRODUCTS	Meat Product
ID	9	49452	49100	ANIMAL FEED	Animal Feed
ID	10	49452	49100	IN-VITRO	In-Vitro

Record 322 | WOS:000463362400007

DNA damage in rat kidneys and liver upon subchronic exposure to single and combined ochratoxin A and citrinin

Field	Position	Original line	Normalised line	Original value	Normalised value
DE	1	49638	49285	ochratoxin A	Ochratoxin A (OTA)
DE	2	49638	49285	citrinin	Citrinin
DE	3	49638	49285	hOGG1-modiffied comet assay	Hogg1 Modiffied Comet Assay
DE	4	49638	49285	oxidative stress	Oxidative Stress
DE	5	49638	49285	resveratrol	Resveratrol
ID	1	49640	49287	COMET ASSAY	Comet Assay
ID	2	49640	49287	OXIDATIVE DAMAGE	Oxidative Damage
ID	3	49640	49287	MYCOTOXIN CITRININ	Mycotoxin Citrinin
ID	4	49640	49287	IN-VITRO	In-Vitro
ID	5	49640	49287	CELLS	Cell
ID	6	49640	49287	RESVERATROL	Resveratrol
ID	7	49640	49287	CYTOTOXICITY	Cytotoxicity
ID	8	49640	49287	APOPTOSIS	Apoptosis
ID	9	49640	49287	TOXICITY	Toxicity
ID	10	49640	49287	COMBINATION	Combination

Record 323 | WOS:000456754300029

Sex-dependent gene expression after ochratoxin A insult in F344 rat kidney

Field	Position	Original line	Normalised line	Original value	Normalised value
DE	2	49758	49404	Sex differences	Sex Difference
DE	3	49758	49404	Gene expression	Gene Expression
DE	5	49758	49404	Nuclear receptors	Nuclear Receptor
ID	1	49760	49406	PREGNANE X RECEPTOR	Pregnane X Receptor
ID	2	49760	49406	IN-VIVO	In-Vivo
ID	3	49760	49406	DNA METHYLATION	DNA Methylation
ID	4	49760	49406	CARCINOGENICITY	Carcinogenicity
ID	5	49760	49406	NEPHROTOXICITY	Nephrotoxicity
ID	6	49760	49406	GENOTOXICITY	Genotoxicity
ID	7	49760	49406	INDUCTION	Induction
ID	8	49760	49406	CELLS	Cell
ID	9	49760	49406	LIVER	Liver
ID	10	49760	49406	TOXICOKINETICS	Toxicokinetics

Record 324 | WOS:000694027800516

Modulation of intestinal epithelial permeability and mucin mRNA (MUC2, MUC5AC, and MUC5B) expression and protein secretion in Caco-2/HT29-MTX co-cultures exposed to aflatoxin M1, ochratoxin A, and zearalenone individually or collectively.

Field	Position	Original line	Normalised line	Original value	Normalised value
DE	1	49890	49535	Caco-2/HT29-MTX co-culture	Caco-2 Ht29 Mtx Co
DE	2	49890	49535	tight junction	Tight Junction
DE	3	49890	49535	mucin	Mucin

Record 325 | WOS:000507379000008

The in vivo efficacy of highly porous carbon nanocomposites prepared from sugar beet waste for the Ochratoxin A detoxification

Field	Position	Original line	Normalised line	Original value	Normalised value
DE	1	49939	49583	broiler chicken	Broiler Chicken
DE	2	49939	49583	ochratoxin A	Ochratoxin A (OTA)
DE	3	49939	49583	adsorbent	Adsorbent
DE	4	49939	49583	blood biochemistry	Blood Biochemistry
DE	5	49939	49583	ALT	Alt
DE	6	49939	49583	ALP	Alp
ID	1	49940	49585	CHRONIC AFLATOXIN 50	Chronic Aflatoxin 50
ID	2	49940	49585	PATHOLOGICAL-CHANGES	Pathological Change
ID	3	49940	49585	BROILER-CHICKENS	Broiler Chicken
ID	4	49940	49585	DNA-DAMAGE	DNA Damage
ID	5	49940	49585	100 PPB	100 Ppb
ID	6	49940	49585	TOXICITY	Toxicity
ID	7	49940	49585	MYCOTOXINS	Mycotoxins
ID	8	49940	49585	IMMUNITY	Immunity

Record 326 | WOS:000452098600001

Persistence of Epigenomic Effects After Recovery From Repeated Treatment With Two Nephrocarcinogens

Field	Position	Original line	Normalised line	Original value	Normalised value
DE	1	50043	49687	recovery	Recovery
DE	2	50043	49687	persistence	Persistence
DE	3	50043	49687	epigenomics	Epigenomics
DE	4	50043	49687	stress responses	Stress Response
DE	5	50043	49687	ochratoxin A	Ochratoxin A (OTA)
DE	6	50043	49687	potassium bromate	Potassium Bromate
DE	7	50043	49687	nephrotoxicity	Nephrotoxicity
DE	8	50043	49687	metabolomics	Metabolomics
ID	1	50045	49689	OCHRATOXIN-A	Ochratoxin A (OTA)
ID	2	50045	49689	POTASSIUM BROMATE	Potassium Bromate
ID	3	50045	49689	INDUCED CYTOTOXICITY	Induced Cytotoxicity
ID	4	50045	49689	OXIDATIVE STRESS	Oxidative Stress
ID	5	50045	49689	FOOD-ADDITIVES	Food Additive
ID	6	50045	49689	DNA-DAMAGE	DNA Damage
ID	7	50045	49689	IN-VITRO	In-Vitro
ID	8	50045	49689	TOXICITY	Toxicity
ID	9	50045	49689	KIDNEY	Kidney
ID	10	50045	49689	RAT	Rat

Record 327 | WOS:000447547900018

Fumonisin production and toxic capacity in airborne black Aspergilli

Field	Position	Original line	Normalised line	Original value	Normalised value
DE	1	50183	49826	Airborne fungi	Airborne Fungi
DE	2	50183	49826	A. welwitschiae	A Welwitschiae
DE	3	50183	49826	A. tubingensis	A Tubingensis
DE	4	50183	49826	Fumonisins	Fumonisin
DE	6	50183	49826	DNA damage	DNA Damage
ID	1	50185	49828	DNA-DAMAGE	DNA Damage
ID	2	50185	49828	COMET ASSAY	Comet Assay
ID	3	50185	49828	OCHRATOXIN-A	Ochratoxin A (OTA)
ID	4	50185	49828	FUSARIUM VERTICILLIOIDES	Fusarium Verticillioide
ID	5	50185	49828	INFLAMMATORY CYTOKINES	Inflammatory Cytokine
ID	6	50185	49828	B-1	B 1
ID	7	50185	49828	CYTOTOXICITY	Cytotoxicity

ID	8	50185	49828	MYCOTOXIN	Mycotoxins
ID	9	50185	49828	INDOOR	Indoor
ID	10	50185	49828	GENES	Gene

Record 328 | WOS:000452587500077

Activation of AMPK-dependent SIRT-1 by astragalus polysaccharide protects against ochratoxin A-induced immune stress in vitro and in vivo

Field	Position	Original line	Normalised line	Original value	Normalised value
DE	1	50333	49975	Ochratoxin A	Ochratoxin A (OTA)
DE	2	50333	49975	Immune stress	Immune Stress
DE	3	50333	49975	Astragalus polysaccharide	Astragalus Polysaccharide
DE	4	50333	49975	AMPK	Ampk
DE	5	50333	49975	SIRT-1	Sirt 1
ID	1	50334	49976	NF-KAPPA-B	Nf Kappa B
ID	2	50334	49976	INFLAMMATORY RESPONSE	Inflammatory Response
ID	3	50334	49976	SIGNALING PATHWAY	Signaling Pathway
ID	4	50334	49976	RECEPTOR 4	Receptor 4
ID	5	50334	49976	INHIBITION	Inhibition
ID	6	50334	49976	TOXICITY	Toxicity
ID	7	50334	49976	ACETYLATION	Acetylation
ID	8	50334	49976	MACROPHAGES	Macrophage
ID	9	50334	49976	SUPPRESSION	Suppression
ID	10	50334	49976	MODULATION	Modulation

Record 329 | WOS:000449893700013

Ochratoxin A induces cytoprotective autophagy via blocking AKT/mTOR signaling pathway in PK-15 cells

Field	Position	Original line	Normalised line	Original value	Normalised value
DE	1	50442	50083	Ochratoxin A	Ochratoxin A (OTA)
DE	3	50442	50083	AKT/mTOR	Akt Mtor
ID	1	50443	50084	KIDNEY PK15 CELLS	Kidney Pk15
ID	2	50443	50084	CANCER-CELLS	Cancer
ID	3	50443	50084	INDUCED APOPTOSIS	Induced Apoptosis
ID	4	50443	50084	OXIDATIVE STRESS	Oxidative Stress
ID	5	50443	50084	POTENTIAL ROLE	Potential Role
ID	6	50443	50084	IN-VITRO	In-Vitro
ID	7	50443	50084	ACTIVATION	Activation
ID	8	50443	50084	DEATH	Death
ID	9	50443	50084	NEPHROTOXICITY	Nephrotoxicity
ID	10	50443	50084	MTOR	Mtor

Record 330 | WOS:000451238100005

ERK1/2-mediated autophagy is essential for cell survival under Ochratoxin A exposure in IPEC-J2 cells

Field	Position	Original line	Normalised line	Original value	Normalised value
DE	1	50560	50200	Ochratoxin A	Ochratoxin A (OTA)
DE	2	50560	50200	IPEC-J2 cells	IPEC-J2
DE	3	50560	50200	Extracellular signal-regulated protein kinases 1 and 2	Extracellular Signal Regulated Protein Kinase 1 And 2
DE	5	50560	50200	Mitochondrial reactive oxygen species	Mitochondrial Reactive Oxygen Specy
ID	1	50563	50202	INDUCED APOPTOSIS	Induced Apoptosis
ID	2	50563	50202	SIGNALING PATHWAYS	Signaling Pathway
ID	3	50563	50202	OXIDATIVE STRESS	Oxidative Stress
ID	4	50563	50202	MAP KINASES	Map Kinase
ID	5	50563	50202	ERK1/2	Erk1 2
ID	6	50563	50202	ACTIVATION	Activation
ID	7	50563	50202	INHIBITION	Inhibition

ID	8	50563	50202	CROSSTALK	Crosstalk
ID	9	50563	50202	TOXICITY	Toxicity
ID	10	50563	50202	ARREST	Arrest

Record 331 ∣ WOS:000449242800003

Mitigation of cell apoptosis induced by ochratoxin A (OTA) is possibly through organic cation transport 2 (OCT2) knockout

Field	Position	Original line	Normalised line	Original value	Normalised value
DE	1	50660	50298	Ochratoxin A	Ochratoxin A (OTA)
DE	2	50660	50298	OCT2	Oct2
DE	5	50660	50298	Cell apoptosis	Cell Apoptosis
ID	1	50661	50299	OXIDATIVE STRESS	Oxidative Stress
ID	2	50661	50299	DNA-DAMAGE	DNA Damage
ID	3	50661	50299	KIDNEY-CELLS	Kidney
ID	4	50661	50299	IN-VITRO	In-Vitro
ID	5	50661	50299	NEPHROTOXICITY	Nephrotoxicity
ID	6	50661	50299	CARCINOGENICITY	Carcinogenicity
ID	7	50661	50299	CYTOTOXICITY	Cytotoxicity
ID	8	50661	50299	TOXICITY	Toxicity
ID	9	50661	50299	VIVO	Vivo

Record 332 ∣ WOS:000446150300012

OTA induces intestinal epithelial barrier dysfunction and tight junction disruption in IPEC-J2 cells through ROS/Ca2+-mediated MLCK activation

Field	Position	Original line	Normalised line	Original value	Normalised value
DE	1	50759	50396	Ochratoxin A	Ochratoxin A (OTA)
DE	2	50759	50396	Tight junction	Tight Junction
DE	3	50759	50396	Reactive oxygen species	Reactive Oxygen Specy
DE	4	50759	50396	Intracellular calcium level	Intracellular Calcium Level
DE	5	50759	50396	Myosin light chain kinase	Myosin Light Chain Kinase
ID	1	50761	50398	BRAIN ENDOTHELIAL-CELLS	Brain Endothelial
ID	2	50761	50398	OXIDATIVE STRESS	Oxidative Stress
ID	3	50761	50398	OCHRATOXIN-A	Ochratoxin A (OTA)
ID	4	50761	50398	LIQUID-CHROMATOGRAPHY	Liquid Chromatography
ID	5	50761	50398	IN-VITRO	In-Vitro
ID	6	50761	50398	EXPOSURE	Exposure
ID	7	50761	50398	EXPRESSION	Expression
ID	8	50761	50398	MYCOTOXIN	Mycotoxins
ID	9	50761	50398	KINASE	Kinase
ID	10	50761	50398	PHOSPHORYLATION	Phosphorylation

Record 333 ∣ WOS:000440879500026

Mechanism and effects of Zearalenone on mouse T lymphocytes activation in vitro

Field	Position	Original line	Normalised line	Original value	Normalised value
DE	1	50880	50516	Zearalenone	Zearalenone (ZEA)
DE	2	50880	50516	Activation of T lymphocytes	Activation Of T Lymphocyte
DE	4	50880	50516	Nuclear factor of T cells	Nuclear Factor Of T
DE	5	50880	50516	TCR	Tcr
DE	6	50880	50516	Co-stimulatory molecule	Co Stimulatory Molecule
ID	1	50882	50518	KAPPA-B ACTIVATION	Kappa B Activation
ID	2	50882	50518	AKT PROTEIN-KINASE	Akt Protein Kinase
ID	3	50882	50518	CELL-ACTIVATION	Cell Activation
ID	4	50882	50518	IMMUNE-RESPONSE	Immune Response
ID	5	50882	50518	IMMUNOLOGICAL SYNAPSE	Immunological Synapse

ID	6	50882	50518	SIGNALING PATHWAY	Signaling Pathway
ID	7	50882	50518	OCHRATOXIN-A	Ochratoxin A (OTA)
ID	8	50882	50518	MICE	Mice
ID	9	50882	50518	DERIVATIVES	Derivative
ID	10	50882	50518	TOXICITY	Toxicity

Record 334 | WOS:000448628600032

The registration of aptamer-ligand (ochratoxin A) interactions based on ligand fluorescence changes

Field	Position	Original line	Normalised line	Original value	Normalised value
DE	2	50985	50620	Excitation-emission matrix	Excitation Emission Matrix
DE	4	50985	50620	G-quadruplex	G Quadruplex
DE	5	50985	50620	Ochratoxin A	Ochratoxin A (OTA)
DE	6	50985	50620	Resonance energy transfer	Resonance Energy Transfer
ID	1	50987	50622	PERFORMANCE LIQUID-CHROMATOGRAPHY	Performance Liquid Chromatography
ID	2	50987	50622	IMMUNOAFFINITY COLUMN CLEANUP	Immunoaffinity Column Cleanup
ID	3	50987	50622	DNA APTAMER	DNA Aptamer
ID	4	50987	50622	MULTIVARIATE CALIBRATION	Multivariate Calibration
ID	5	50987	50622	INTRINSIC FLUORESCENCE	Intrinsic Fluorescence
ID	6	50987	50622	LABELED APTAMER	Labeled Aptamer
ID	7	50987	50622	SMALL-MOLECULE	Small Molecule
ID	8	50987	50622	IN-VITRO	In-Vitro
ID	9	50987	50622	BINDING	Binding
ID	10	50987	50622	FOOD	Food

Record 335 | WOS:000447114600010

Dietary L-carnitine and vitamin-E; a strategy to combat ochratoxin-A induced immunosuppression

Field	Position	Original line	Normalised line	Original value	Normalised value
DE	1	51088	50722	Ochratoxin A	Ochratoxin A (OTA)
DE	3	51088	50722	L-carnitine	L Carnitine
DE	5	51088	50722	Dietary supplement	Dietary Supplement
ID	1	51089	50724	WHITE LEGHORN COCKERELS	White Leghorn Cockerel
ID	2	51089	50724	IMMUNE-RESPONSE	Immune Response
ID	3	51089	50724	BROILER CHICKS	Broiler Chick
ID	4	51089	50724	ASPERGILLUS-OCHRACEUS	Aspergillus Ochraceus
ID	5	51089	50724	ALPHA-TOCOPHEROL	Alpha Tocopherol
ID	6	51089	50724	BLOOD PARAMETERS	Blood Parameter
ID	7	51089	50724	FEED	Feed
ID	8	51089	50724	PERFORMANCE	Performance
ID	9	51089	50724	MODULATION	Modulation
ID	10	51089	50724	PREVENTION	Prevention

Record 336 | WOS:000453415000007

In Vivo Detoxification of Ochratoxin A by Highly Porous Magnetic Nanocomposites Prepared from Coconut Shell

Field	Position	Original line	Normalised line	Original value	Normalised value
DE	1	51190	50824	Serum alanine transferase	Serum Alanine Transferase
DE	2	51190	50824	alkaline phosphatase	Alkaline Phosphatase
DE	3	51190	50824	Clinical signs	Clinical Sign
DE	4	51190	50824	Gross lesions	Gross Lesion

ID	1	51192	50826	AFLATOXIN ABSORBENTS	Aflatoxin Absorbent
ID	2	51192	50826	PATHOLOGICAL-CHANGES	Pathological Change
ID	3	51192	50826	BROILER-CHICKENS	Broiler Chicken
ID	4	51192	50826	T-2 TOXIN	T 2 Toxin
ID	5	51192	50826	COMBINATIONS	Combination
ID	6	51192	50826	MECHANISM	Mechanism
ID	7	51192	50826	TOXICITY	Toxicity
ID	8	51192	50826	EFFICACY	Efficacy
ID	9	51192	50826	CARBONS	Carbon
ID	10	51192	50826	GROWTH	Growth

Record 337 | WOS:000442067900032

Research progress on the raw and modified montmorillonites as adsorbents for mycotoxins: A review

Field	Position	Original line	Normalised line	Original value	Normalised value
DE	1	51295	50928	Animal feed	Animal Feed
DE	5	51295	50928	Clay mineral	Clay Mineral
ID	1	51296	50929	SODIUM-CALCIUM ALUMINOSILICATE	Sodium Calcium Aluminosilicate
ID	2	51296	50929	VITRO AFLATOXIN BINDING	Vitro Aflatoxin Binding
ID	3	51296	50929	IN-VITRO	In-Vitro
ID	4	51296	50929	BROILER-CHICKENS	Broiler Chicken
ID	5	51296	50929	OCHRATOXIN-A	Ochratoxin A (OTA)
ID	6	51296	50929	NUTRIENT AVAILABILITY	Nutrient Availability
ID	7	51296	50929	PURIFIED ZEARALENONE	Purified Zearalenone
ID	8	51296	50929	CONTAMINATED DIETS	Contaminated Diet
ID	9	51296	50929	COMBINED TOXICITY	Combined Toxicity
ID	10	51296	50929	THERMAL-TREATMENT	Thermal Treatment

Record 338 | WOS:000439674200027

Restorative effect of L-Dopa treatment against Ochratoxin A induced neurotoxicity

Field	Position	Original line	Normalised line	Original value	Normalised value
DE	1	51497	51129	Ochratoxin A	Ochratoxin A (OTA)
DE	2	51497	51129	L-dopa	L Dopa
DE	3	51497	51129	Parkinson's disease	Parkinson S Disease
DE	4	51497	51129	MPTP	Mptp
ID	1	51498	51130	MPTP MOUSE MODEL	Mptp Mouse Model
ID	2	51498	51130	BEHAVIORAL-TESTING REGIMENS	Behavioral Testing Regimen
ID	3	51498	51130	PARKINSONS-DISEASE	Parkinson Disease
ID	4	51498	51130	ANIMAL-MODELS	Animal Model
ID	5	51498	51130	TYROSINE-HYDROXYLASE	Tyrosine Hydroxylase
ID	6	51498	51130	IN-VITRO	In-Vitro
ID	7	51498	51130	DOPAMINERGIC-NEURONS	Dopaminergic Neuron
ID	8	51498	51130	ENDEMIC NEPHROPATHY	Endemic Nephropathy
ID	9	51498	51130	OXIDATIVE DAMAGE	Oxidative Damage
ID	10	51498	51130	MOTOR DEFICITS	Motor Deficit

Record 339 | WOS:000448339000017

Interaction of 2R-ochratoxin A with Serum Albumins: Binding Site, Effects of Site Markers, Thermodynamics, Species Differences of Albumin-binding, and Influence of Albumin on Its Toxicity in MDCK Cells

Field	Position	Original line	Normalised line	Original value	Normalised value
DE	1	51659	51290	2R-ochratoxin A	2r Ochratoxin A
DE	2	51659	51290	ochratoxin A	Ochratoxin A (OTA)
DE	3	51659	51290	serum albumin	Serum Albumin
DE	4	51659	51290	albumin-ligand interaction	Albumin Ligand Interaction
DE	5	51659	51290	species differences	Specy Difference
DE	6	51659	51290	cellular toxicity	Cellular Toxicity
ID	1	51661	51292	OCHRATOXIN-A	Ochratoxin A (OTA)
ID	2	51661	51292	BLIND DOCKING	Blind Docking
ID	3	51661	51292	DEGRADATION	Degradation
ID	4	51661	51292	PRODUCTS	Product
ID	5	51661	51292	DERIVATIVES	Derivative
ID	6	51661	51292	PARAMETERS	Parameter
ID	7	51661	51292	STABILITY	Stability
ID	8	51661	51292	PROTEINS	Protein
ID	9	51661	51292	IONS	Ion

Record 340 | WOS:000442890400004

Protective effects of compound ammonium glycyrrhizin, L-arginine, silymarin and glucurolactone against liver damage induced by ochratoxin A in primary chicken hepatocytes

Field	Position	Original line	Normalised line	Original value	Normalised value
DE	1	51786	51416	ochratoxin A	Ochratoxin A (OTA)
DE	2	51786	51416	hepatotoxicity	Hepatotoxicity
DE	3	51786	51416	hepatoprotective drugs	Hepatoprotective Drug
DE	4	51786	51416	oxidative stress	Oxidative Stress
DE	5	51786	51416	cell apoptosis	Cell Apoptosis
ID	1	51788	51418	OXIDATIVE DNA-DAMAGE	Oxidative DNA Damage
ID	2	51788	51418	KIDNEY PK15 CELLS	Kidney Pk15
ID	3	51788	51418	MEDIATED TOXICITY	Mediated Toxicity
ID	4	51788	51418	EPITHELIAL-CELLS	Epithelial
ID	5	51788	51418	HEP G2	Hep G2
ID	6	51788	51418	APOPTOSIS	Apoptosis
ID	7	51788	51418	CYTOTOXICITY	Cytotoxicity
ID	8	51788	51418	STRESS	Stress
ID	9	51788	51418	HOMEOSTASIS	Homeostasis
ID	10	51788	51418	ZEARALENONE	Zearalenone (ZEA)

Record 341 | WOS:000442714300074

Micronucleus induction and cell cycle alterations produced by deoxynivalenol and its acetylated derivatives in individual and combined exposure on HepG2 cells

Field	Position	Original line	Normalised line	Original value	Normalised value
DE	1	51900	51528	Deoxynivalenol	Deoxynivalenol (DON)
DE	2	51900	51528	3-ADON	3 Adon
DE	3	51900	51528	15-ADON	15 Adon
DE	5	51900	51528	Cell cycle	Cell Cycle
ID	1	51901	51529	IN-VITRO	In-Vitro
ID	2	51901	51529	DEGRADATION-PRODUCTS	Degradation Product
ID	3	51901	51529	INDUCED CYTOTOXICITY	Induced Cytotoxicity
ID	4	51901	51529	DIETARY-INTAKE	Dietary Intake
ID	5	51901	51529	OCHRATOXIN-A	Ochratoxin A (OTA)
ID	6	51901	51529	LIVER HEPG2	Liver HepG2
ID	7	51901	51529	GENOTOXICITY	Genotoxicity
ID	8	51901	51529	ASSAY	Assay
ID	9	51901	51529	MYCOTOXINS	Mycotoxins
ID	10	51901	51529	TRICHOTHECENES	Trichothecene

Record 342 | WOS:000442489000011

Molecular Dynamics Simulations of Mismatched DNA Duplexes Associated with the Major C8-Linked 2â ²-Deoxyguanosine Adduct of the Food Mutagen Ochratoxin A: Influence of Opposing Base, Adduct Ionization State, and Sequence on the Structure of Damaged DNA

Field	Position	Original line	Normalised line	Original value	Normalised value
ID	1	51997	51624	NUCLEOTIDE EXCISION-REPAIR	Nucleotide Excision Repair
ID	2	51997	51624	COMPUTATIONAL CHARACTERIZATION	Computational Characterization
ID	3	51997	51624	CONFORMATIONAL PREFERENCES	Conformational Preference
ID	4	51997	51624	C8-ARYL-GUANINE ADDUCTS	C8 Aryl Guanine Adduct
ID	5	51997	51624	FORCE-FIELD	Force Field
ID	6	51997	51624	RECOGNITION	Recognition
ID	7	51997	51624	POLYMERASE	Polymerase
ID	8	51997	51624	LESIONS	Lesion
ID	9	51997	51624	AMINOFLUORENE	Aminofluorene
ID	10	51997	51624	INSIGHTS	Insight

Record 343 | WOS:000440099900007

Comparison of base-line and chemical-induced transcriptomic responses in HepaRG and RPTEC/TERT1 cells using TempO-Seq

Field	Position	Original line	Normalised line	Original value	Normalised value
DE	1	52137	51763	HepaRG	Heparg
DE	2	52137	51763	RPTEC/TERT1	Rptec Tert1
DE	3	52137	51763	TempO-Seq	Tempo Seq
DE	4	52137	51763	Stress responses	Stress Response
ID	1	52138	51764	OXIDATIVE STRESS	Oxidative Stress
ID	2	52138	51764	LABEL-FREE	Label Free
ID	3	52138	51764	REAL-TIME	Real Time
ID	4	52138	51764	MECHANISMS	Mechanism
ID	5	52138	51764	EXPRESSION	Expression
ID	6	52138	51764	TOXICITY	Toxicity
ID	7	52138	51764	PROTEIN	Protein
ID	8	52138	51764	CYP3A4	Cyp3a4
ID	9	52138	51764	VISUALIZATION	Visualization
ID	10	52138	51764	DELINEATION	Delineation

Record 344 | WOS:000438651800008

Occurrence of ochratoxin A in Astragalus propinquus root and its transfer to decoction

Field	Position	Original line	Normalised line	Original value	Normalised value
DE	1	52268	51893	Astragalus propinquus Schischkin	Astragalus Propinquus Schischkin
DE	2	52268	51893	Herbal food supplement	Herbal Food Supplement
DE	3	52268	51893	Herbal products	Herbal Product
DE	4	52268	51893	HPLC-FLD	Hplc Fld
DE	5	52268	51893	Ochratoxin A	Ochratoxin A (OTA)
DE	6	52268	51893	Traditional Chinese medicine	Traditional Chinese Medicine
ID	1	52270	51895	IN-VITRO	In-Vitro
ID	2	52270	51895	HUMAN BLOOD	Human Blood
ID	3	52270	51895	MYCOTOXINS	Mycotoxins
ID	4	52270	51895	PHARMACOLOGY	Pharmacology
ID	5	52270	51895	PHYTOCHEMISTRY	Phytochemistry
ID	6	52270	51895	CHROMATOGRAPHY	Chromatography
ID	7	52270	51895	MEMBRANACEUS	Membranaceus
ID	8	52270	51895	CYCLOARTANE	Cycloartane
ID	9	52270	51895	TOXICITY	Toxicity
ID	10	52270	51895	SERUM	Serum

Record 345 | WOS:000434754600071

PCV2 infection aggravates ochratoxin A-induced nephrotoxicity via autophagy involving p38 signaling pathway in vivo and in vitro

Field	Position	Original line	Normalised line	Original value	Normalised value
DE	1	52387	52011	Ochratoxin A	Ochratoxin A (OTA)
DE	2	52387	52011	Porcine circovirus type 2	Porcine Circovirus Type 2
DE	5	52387	52011	p38 MAPK signaling pathway	P38 Mapk Signaling Pathway
ID	1	52389	52013	PORCINE CIRCOVIRUS TYPE-2	Porcine Circovirus Type 2
ID	2	52389	52013	OXIDATIVE STRESS	Oxidative Stress
ID	3	52389	52013	NEPHROPATHY SYNDROME	Nephropathy Syndrome
ID	4	52389	52013	EPITHELIAL-CELLS	Epithelial
ID	5	52389	52013	PK-15 CELLS	Pk 15
ID	6	52389	52013	REPLICATION	Replication
ID	7	52389	52013	PIGS	Pig
ID	8	52389	52013	DERMATITIS	Dermatitis
ID	9	52389	52013	APOPTOSIS	Apoptosis
ID	10	52389	52013	MAPK	Mapk

Record 346 | WOS:000442594000032

Toxicity Assay for Citrinin, Zearalenone and Zearalenone-14-Sulfate Using the Nematode Caenorhabditis elegans as Model Organism

Field	Position	Original line	Normalised line	Original value	Normalised value
DE	1	52505	52127	mycotoxin	Mycotoxins
DE	2	52505	52127	toxicity testing	Toxicity Testing
DE	3	52505	52127	food safety	Food Safety
DE	4	52505	52127	Caenorhabditis elegans	Caenorhabditis Elegan
DE	5	52505	52127	metabolization	Metabolization
ID	1	52507	52129	OCHRATOXIN-A	Ochratoxin A (OTA)
ID	2	52507	52129	NATURAL OCCURRENCE	Natural Occurrence
ID	3	52507	52129	OXIDATIVE STRESS	Oxidative Stress
ID	4	52507	52129	IN-VITRO	In-Vitro
ID	5	52507	52129	MYCOTOXINS	Mycotoxins
ID	6	52507	52129	DAMAGE	Damage
ID	8	52507	52129	REGULATIONS	Regulation
ID	9	52507	52129	METABOLITES	Metabolite
ID	10	52507	52129	RESISTANCE	Resistance

Record 347 | WOS:000430882700061

Toxicological interactions between mycotoxins from ubiquitous fungi: Impact on hepatic and intestinal human epithelial cells

Field	Position	Original line	Normalised line	Original value	Normalised value
DE	2	52613	52234	Combination index	Combination Index
DE	5	52613	52234	Caco-2 cells	Caco-2
DE	6	52613	52234	HepG2 cells	HepG2
ID	1	52615	52235	AFLATOXIN B-1	Aflatoxin B1 (AFB1)
ID	2	52615	52235	FUMONISIN B-1	Fumonisin B1 (FB1)
ID	3	52615	52235	OCHRATOXIN-A	Ochratoxin A (OTA)
ID	4	52615	52235	IN-VITRO	In-Vitro
ID	5	52615	52235	ACETYLATED DERIVATIVES	Acetylated Derivative
ID	6	52615	52235	DEOXYNIVALENOL AFFECTS	Deoxynivalenol Affect
ID	7	52615	52235	WORLDWIDE OCCURRENCE	Worldwide Occurrence
ID	8	52615	52235	B TRICHOTHECENES	B Trichothecene
ID	9	52615	52235	NORTHERN REGION	Northern Region
ID	10	52615	52235	CLIMATE-CHANGE	Climate Change

Record 348 | WOS:000432602600016

Synergistic action of the nephrotoxic mycotoxins ochratoxin A and citrinin at nanomolar concentrations in human proximal tubule-derived cells

Field	Position	Original line	Normalised line	Original value	Normalised value
DE	1	52758	52377	Ochratoxin A	Ochratoxin A (OTA)
DE	3	52758	52377	Proximal tubule	Proximal Tubule
DE	4	52758	52377	Epithelial-to-mesenchymal transition	Epithelial To Mesenchymal Transition
DE	5	52758	52377	Tubulo-interstitial nephropathy	Tubulo Interstitial Nephropathy
ID	1	52760	52379	MESENCHYMAL TRANSITION	Mesenchymal Transition
ID	2	52760	52379	ENDEMIC NEPHROPATHY	Endemic Nephropathy
ID	3	52760	52379	ACTIVATION	Activation
ID	4	52760	52379	EXPRESSION	Expression
ID	5	52760	52379	TOXICITY	Toxicity
ID	6	52760	52379	PATHWAYS	Pathway
ID	7	52760	52379	EXPOSURE	Exposure
ID	8	52760	52379	FIBROSIS	Fibrosis
ID	9	52760	52379	ADULTS	Adult
ID	10	52760	52379	LEVEL	Level

Record 349 | WOS:000437960800009

Risk assessment of exposure to aflatoxin B1 and ochratoxin A through consumption of different Pistachio (Pistacia vera L.) cultivars collected from four geographical regions of Iran

Field	Position	Original line	Normalised line	Original value	Normalised value
DE	1	52869	52487	Aflatoxin B1	Aflatoxin B1 (AFB1)
DE	2	52869	52487	Ochratoxin A	Ochratoxin A (OTA)
DE	3	52869	52487	Pistacia vera	Pistacia Vera
DE	4	52869	52487	Pistachio safety	Pistachio Safety
DE	5	52869	52487	Risk assessment	Risk Assessment
ID	1	52871	52489	ASPERGILLUS-FLAVUS	Aspergillus Flavus
ID	2	52871	52489	DIETARY-INTAKE	Dietary Intake
ID	3	52871	52489	DRIED FRUITS	Dried Fruit
ID	4	52871	52489	HEALTH-RISK	Health Risk
ID	5	52871	52489	IN-VITRO	In-Vitro
ID	6	52871	52489	MYCOTOXINS	Mycotoxins
ID	7	52871	52489	NUTS	Nut
ID	8	52871	52489	TOXICITY	Toxicity

Record 350 | WOS:000435525800022

Combination of Selenomethionine and N-Acetylcysteine Alleviates the Joint Toxicities of Aflatoxin B1 and Ochratoxin A by ERK MAPK Signal Pathway in Porcine Alveolar Macrophages

Field	Position	Original line	Normalised line	Original value	Normalised value
DE	1	52984	52601	combination	Combination
DE	2	52984	52601	selenomethionine	Selenomethionine
DE	3	52984	52601	N-acetylcysteine	N Acetylcysteine
DE	4	52984	52601	aflatoxin B1	Aflatoxin B1 (AFB1)
DE	5	52984	52601	ochratoxin A	Ochratoxin A (OTA)
DE	6	52984	52601	mitogen-activated protein kinases	Mitogen Activated Protein Kinase
ID	1	52986	52603	HEMATOPOIETIC STEM-CELLS	Hematopoietic Stem
ID	2	52986	52603	TRAUMATIC BRAIN-INJURY	Traumatic Brain Injury
ID	3	52986	52603	DORSAL-ROOT GANGLION	Dorsal Root Ganglion
ID	4	52986	52603	OXIDATIVE STRESS	Oxidative Stress
ID	5	52986	52603	IMMUNE TOXICITY	Immune Toxicity
ID	6	52986	52603	FUMONISIN B-1	Fumonisin B1 (FB1)
ID	7	52986	52603	PK15 CELLS	Pk15
ID	8	52986	52603	VITAMIN-E	Vitamin E
ID	9	52986	52603	P38 MAPK	P38 Mapk

ID	10	52986	52603	IN-VITRO	In-Vitro

Record 351 | WOS:000433656100042

Is oxidative stress involved in the sex-dependent response to ochratoxin A renal toxicity?

Field	Position	Original line	Normalised line	Original value	Normalised value
DE	2	53104	52720	Sex differences	Sex Difference
DE	3	53104	52720	Glutathione	Glutathione (GSH)
DE	4	53104	52720	Superoxide dismutase	Superoxide Dismutase
DE	5	53104	52720	Comet assay	Comet Assay
ID	1	53106	52722	DNA-DAMAGE	DNA Damage
ID	2	53106	52722	RAT-KIDNEY	Rat Kidney
ID	3	53106	52722	IN-VIVO	In-Vivo
ID	4	53106	52722	SUPEROXIDE-DISMUTASE	Superoxide Dismutase
ID	5	53106	52722	A EXPOSURE	A Exposure
ID	6	53106	52722	EXPRESSION	Expression
ID	7	53106	52722	CELLS	Cell
ID	8	53106	52722	NEPHROTOXICITY	Nephrotoxicity
ID	9	53106	52722	GENDER	Gender
ID	10	53106	52722	INDUCTION	Induction

Record 352 | WOS:000433016800013

Effects of combined treatment with ochratoxin A and citrinin on oxidative damage in kidneys and liver of rats

Field	Position	Original line	Normalised line	Original value	Normalised value
DE	2	53252	52867	DNA damage	DNA Damage
DE	3	53252	52867	Ochratoxin A	Ochratoxin A (OTA)
DE	4	53252	52867	Oxidative stress	Oxidative Stress
ID	1	53253	52868	DNA-DAMAGE	DNA Damage
ID	2	53253	52868	LIPID-PEROXIDATION	Lipid Peroxidation
ID	3	53253	52868	COMET ASSAY	Comet Assay
ID	4	53253	52868	VERO CELLS	Vero
ID	5	53253	52868	STRESS	Stress
ID	6	53253	52868	GLUTATHIONE	Glutathione (GSH)
ID	7	53253	52868	MYCOTOXINS	Mycotoxins
ID	8	53253	52868	PENICILLIUM	Penicillium
ID	9	53253	52868	APOPTOSIS	Apoptosis
ID	10	53253	52868	BIOSYNTHESIS	Biosynthesis

Record 353 | WOS:000432498000011

The importance of accounting for sex in the search of proteomic signatures of mycotoxin exposure

Field	Position	Original line	Normalised line	Original value	Normalised value
DE	3	53368	52982	Sexual-dimorphism	Sexual Dimorphism
DE	6	53368	52982	Endocrine disruptor	Endocrine Disruptor
ID	1	53370	52984	OCHRATOXIN-A	Ochratoxin A (OTA)
ID	2	53370	52984	IN-VIVO	In-Vivo
ID	3	53370	52984	T-2 TOXIN	T 2 Toxin
ID	4	53370	52984	HEPATOCELLULAR-CARCINOMA	Hepatocellular Carcinoma
ID	5	53370	52984	REPRODUCTIVE TOXICITY	Reproductive Toxicity
ID	6	53370	52984	MOLECULAR-MECHANISMS	Molecular Mechanism
ID	7	53370	52984	FUSARIUM MYCOTOXINS	Fusarium Mycotoxin
ID	8	53370	52984	INTESTINAL TOXICITY	Intestinal Toxicity
ID	9	53370	52984	DEOXYNIVALENOL DON	Deoxynivalenol DON
ID	10	53370	52984	OXIDATIVE STRESS	Oxidative Stress

Record 354 | WOS:000428605400010

Toxicological effects of regulated mycotoxins and persistent organochloride pesticides: In vitro cytotoxic assessment of single and defined mixtures on MA-10 murine Leydig cell line

Field	Position	Original line	Normalised line	Original value	Normalised value
DE	1	53574	53187	Mixture toxicity	Mixture Toxicity
DE	3	53574	53187	Pesticides	Pesticide
DE	5	53574	53187	Leydig cells	Leydig
DE	6	53574	53187	MA-10 cell line	Ma 10 Cell
DE	7	53574	53187	Reproductive toxicity	Reproductive Toxicity
ID	1	53576	53189	ENVIRONMENTAL DDT EXPOSURE	Environmental Ddt Exposure
ID	2	53576	53189	ARYL-HYDROCARBON RECEPTOR	Aryl Hydrocarbon Receptor
ID	3	53576	53189	KIDNEY PK15 CELLS	Kidney Pk15
ID	4	53576	53189	OCHRATOXIN-A	Ochratoxin A (OTA)
ID	5	53576	53189	AFLATOXIN B-1	Aflatoxin B1 (AFB1)
ID	6	53576	53189	ALPHA-ZEARALENOL	Alpha Zearalenol
ID	7	53576	53189	FUMONISIN B-1	Fumonisin B1 (FB1)
ID	8	53576	53189	TESTOSTERONE SECRETION	Testosterone Secretion
ID	9	53576	53189	COMBINATIVE TOXICITY	Combinative Toxicity
ID	10	53576	53189	STEROID-PRODUCTION	Steroid Production

Record 355 | WOS:000434061300005

Ochratoxin A, citrinin and deoxynivalenol decrease claudin-2 expression in mouse rectum CMT93-II cells

Field	Position	Original line	Normalised line	Original value	Normalised value
DE	1	53744	53356	tight junction	Tight Junction
DE	2	53744	53356	claudin	Claudin
DE	3	53744	53356	paracellular permeability	Paracellular Permeability
DE	4	53744	53356	ochratoxin A	Ochratoxin A (OTA)
DE	5	53744	53356	citrinin	Citrinin
DE	6	53744	53356	deoxynivalenol	Deoxynivalenol (DON)
ID	1	53746	53358	ACTIVATED PROTEIN-KINASES	Activated Protein Kinase
ID	2	53746	53358	TIGHT JUNCTION	Tight Junction
ID	3	53746	53358	BARRIER FUNCTION	Barrier Function
ID	4	53746	53358	EPITHELIAL-CELLS	Epithelial
ID	5	53746	53358	IN-VITRO	In-Vitro
ID	6	53746	53358	INTESTINAL BARRIER	Intestinal Barrier
ID	7	53746	53358	PARACELLULAR PERMEABILITY	Paracellular Permeability
ID	8	53746	53358	GENE-EXPRESSION	Gene Expression
ID	9	53746	53358	MDCK CELLS	MDCK
ID	10	53746	53358	HUMAN-MILK	Human Milk

Record 356 | WOS:000429758100013

Toxicity assessment of mycotoxins extracted from contaminated commercial dog pelleted feed on canine blood mononuclear cells

Field	Position	Original line	Normalised line	Original value	Normalised value
DE	2	53883	53494	Pelleted dog feed	Pelleted Dog Feed
DE	3	53883	53494	PBMCs	Pbmc
DE	6	53883	53494	Mitochondrial depolarization	Mitochondrial Depolarization
ID	1	53885	53496	IN-VITRO	In-Vitro
ID	2	53885	53496	GLUTATHIONE	Glutathione (GSH)
ID	3	53885	53496	RISK	Risk
ID	4	53885	53496	NEUROTOXICITY	Neurotoxicity
ID	5	53885	53496	AFLATOXICOSIS	Aflatoxicosis

ID	6	53885	53496	MECHANISMS	Mechanism
ID	7	53885	53496	APOPTOSIS	Apoptosis
ID	8	53885	53496	EXPOSURE	Exposure
ID	9	53885	53496	NECROSIS	Necrosis
ID	10	53885	53496	FOODS	Food

Record 357 | WOS:000428799300003

The efficiency of lactic acid bacteria against pathogenic fungi and mycotoxins

Field	Position	Original line	Normalised line	Original value	Normalised value
DE	2	53996	53606	biological methods	Biological Method
DE	4	53996	53606	inhibition	Inhibition
DE	5	53996	53606	LAB	Lab
ID	1	53997	53607	FUSARIUM TOXINS DEOXYNIVALENOL	Fusarium Toxin Deoxynivalenol
ID	2	53997	53607	DIFFERENT GEOGRAPHICAL REGIONS	Different Geographical Region
ID	3	53997	53607	AFLATOXIN B-1 CONTAMINATION	Aflatoxin B 1 Contamination
ID	4	53997	53607	IN-VITRO	In-Vitro
ID	5	53997	53607	OCHRATOXIN-A	Ochratoxin A (OTA)
ID	6	53997	53607	ANIMAL FEED	Animal Feed
ID	7	53997	53607	SACCHAROMYCES-CEREVISIAE	Saccharomyce Cerevisiae
ID	8	53997	53607	FERMENTATIVE BACTERIA	Fermentative Bacteria
ID	9	53997	53607	CONTROL STRATEGIES	Control Strategy
ID	10	53997	53607	NATURAL OCCURRENCE	Natural Occurrence

Record 358 | WOS:000422811300020

Metabolism-dependent cytotoxicity of citrinin and ochratoxin A alone and in combination as assessed adopting integrated discrete multiple organ co-culture (IdMOC)

Field	Position	Original line	Normalised line	Original value	Normalised value
DE	2	54230	53838	Ochratoxin A	Ochratoxin A (OTA)
DE	4	54230	53838	Metabolism-dependent toxicity	Metabolism Dependent Toxicity
DE	5	54230	53838	IdMOC	Idmoc
DE	6	54230	53838	Molecular interactions	Molecular Interaction
ID	1	54232	53840	MYCOTOXIN CITRININ	Mycotoxin Citrinin
ID	2	54232	53840	HUMAN HEPATOCYTES	Human Hepatocyte
ID	3	54232	53840	XENOBIOTIC TOXICITY	Xenobiotic Toxicity
ID	4	54232	53840	SCIENTIFIC CONCEPTS	Scientific Concept
ID	5	54232	53840	HIGH-THROUGHPUT	High Throughput
ID	6	54232	53840	BLOOD-PLASMA	Blood Plasma
ID	7	54232	53840	LIVER	Liver
ID	8	54232	53840	DIHYDROCITRINONE	Dihydrocitrinone
ID	9	54232	53840	RAT	Rat
ID	10	54232	53840	SINGLE	Single

Record 359 | WOS:000425526000033

Linking site-specific loss of histone acetylation to repression of gene expression by the mycotoxin ochratoxin A

Field	Position	Original line	Normalised line	Original value	Normalised value
DE	1	54362	53969	Ochratoxin A	Ochratoxin A (OTA)
DE	2	54362	53969	Histone hypoacetylation	Histone Hypoacetylation

DE	3	54362	53969	Repression of gene expression	Repression Of Gene Expression
DE	4	54362	53969	Renal carcinogenicity	Renal Carcinogenicity
ID	1	54364	53971	OXIDATIVE DNA-DAMAGE	Oxidative DNA Damage
ID	2	54364	53971	EPIGENETIC MECHANISMS	Epigenetic Mechanism
ID	3	54364	53971	PHASE ARREST	Phase Arrest
ID	4	54364	53971	IN-VIVO	In-Vivo
ID	5	54364	53971	CHROMATIN	Chromatin
ID	6	54364	53971	CELLS	Cell
ID	7	54364	53971	TOXICITY	Toxicity
ID	8	54364	53971	MITOSIS	Mitosis
ID	9	54364	53971	KIDNEY	Kidney
ID	10	54364	53971	REPAIR	Repair

Record 360 | WOS:000425568900007

Toxicity reduction of ochratoxin A by lactic acid bacteria

Field	Position	Original line	Normalised line	Original value	Normalised value
DE	1	54487	54093	Lactic acid bacteria	Lactic Acid Bacteria
DE	2	54487	54093	Ochratoxin A	Ochratoxin A (OTA)
DE	3	54487	54093	Biotechnological degradation	Biotechnological Degradation
DE	4	54487	54093	In vitro gastrointestinal digestion	In Vitro Gastrointestinal Digestion
ID	1	54489	54095	DEGRADATION	Degradation
ID	2	54489	54095	WINE	Wine
ID	3	54489	54095	BIOACCESSIBILITY	Bioaccessibility
ID	4	54489	54095	BIODEGRADATION	Biodegradation
ID	5	54489	54095	MYCOTOXINS	Mycotoxins
ID	6	54489	54095	WHEAT	Wheat
ID	7	54489	54095	FOOD	Food

Record 361 | WOS:000425568900035

Ochratoxin A: Toxicity, oxidative stress and metabolism

Field	Position	Original line	Normalised line	Original value	Normalised value
DE	1	54586	54191	OTA	Ochratoxin A (OTA)
DE	3	54586	54191	ROS	Reactive Oxygen Species (ROS)
DE	4	54586	54191	Oxidative damage	Oxidative Damage
DE	5	54586	54191	Metabolic enzymes	Metabolic Enzyme
DE	6	54586	54191	Reactive oxygen species	Reactive Oxygen Specy
ID	1	54588	54193	A-INDUCED GENOTOXICITY	A Induced Genotoxicity
ID	2	54588	54193	KIDNEY-CELL-LINE	Kidney Cell
ID	3	54588	54193	DNA-DAMAGE	DNA Damage
ID	4	54588	54193	IN-VITRO	In-Vitro
ID	5	54588	54193	GENE-EXPRESSION	Gene Expression
ID	6	54588	54193	APOPTOSIS	Apoptosis
ID	7	54588	54193	PATHWAYS	Pathway
ID	8	54588	54193	LIVER	Liver
ID	9	54588	54193	VIVO	Vivo
ID	10	54588	54193	CARCINOGENICITY	Carcinogenicity

Record 362 | WOS:000424317600001

Disruption of liver development and coagulation pathway by ochratoxin A in embryonic zebrafish

Field	Position	Original line	Normalised line	Original value	Normalised value
ID	1	54782	54386	TOXICITY	Toxicity
ID	2	54782	54386	EXPOSURE	Exposure
ID	3	54782	54386	PROLIFERATION	Proliferation

ID	4	54782	54386	MECHANISMS	Mechanism
ID	5	54782	54386	LESSONS	Lesson
ID	6	54782	54386	TISSUES	Tissue
ID	7	54782	54386	BLOOD	Blood
ID	8	54782	54386	SERUM	Serum

Record 363 | WOS:000424317600006

Ochratoxin A exposure decreased sperm motility via the AMPK and PTEN signaling pathways

Field	Position	Original line	Normalised line	Original value	Normalised value
DE	1	54902	54504	Ochratoxin A	Ochratoxin A (OTA)
DE	2	54902	54504	Sperm motility	Sperm Motility
DE	3	54902	54504	Oxidative stress	Oxidative Stress
DE	5	54902	54504	AMPK/PTEN	Ampk Pten
ID	1	54903	54505	SEMEN-QUALITY PARAMETERS	Semen Quality Parameter
ID	2	54903	54505	A-INDUCED APOPTOSIS	A Induced Apoptosis
ID	3	54903	54505	DEOXYNIVALENOL	Deoxynivalenol (DON)
ID	4	54903	54505	ACTIVATION	Activation
ID	5	54903	54505	PROTEIN	Protein
ID	6	54903	54505	VIABILITY	Viability
ID	7	54903	54505	KIDNEY	Kidney
ID	8	54903	54505	CELLS	Cell
ID	9	54903	54505	BCL-2	Bcl 2

Record 364 | WOS:000423566000030

Individual and combined cytotoxicity assessment of zearalenone with ochratoxin A or Î±-zearalenol by full factorial design

Field	Position	Original line	Normalised line	Original value	Normalised value
DE	1	55017	54618	Zearalenone	Zearalenone (ZEA)
DE	2	55017	54618	Ochratoxin A	Ochratoxin A (OTA)
DE	3	55017	54618	alpha-Zearalenol	Alpha Zearalenol
DE	4	55017	54618	Combined effect	Combined Effect
DE	5	55017	54618	Full factorial design	Full Factorial Design
ID	1	55019	54620	IN-VITRO	In-Vitro
ID	2	55019	54620	TOXICOLOGICAL INTERACTIONS	Toxicological Interaction
ID	3	55019	54620	T-2 TOXIN	T 2 Toxin
ID	4	55019	54620	TOXICITY	Toxicity
ID	5	55019	54620	DEOXYNIVALENOL	Deoxynivalenol (DON)
ID	6	55019	54620	MYCOTOXINS	Mycotoxins
ID	7	55019	54620	ENNIATINS	Enniatin
ID	8	55019	54620	HEPG2	HepG2
ID	9	55019	54620	COMBINATIONS	Combination
ID	10	55019	54620	METABOLITES	Metabolite

Record 365 | WOS:000416024300043

Effect of rMnSOD on Sodium Reabsorption in Renal Proximal Tubule in Ochratoxin ATreated Rats

Field	Position	Original line	Normalised line	Original value	Normalised value
DE	1	55136	54736	OCHRATOXIN A	Ochratoxin A (OTA)
DE	2	55136	54736	rMnSOD	Rmnsod
DE	3	55136	54736	NITRIC OXIDE	Nitric Oxide
DE	4	55136	54736	MICROPUNTURE	Micropunture
DE	5	55136	54736	ANTIOXIDANTS	Antioxidant
ID	1	55137	54737	OXIDATIVE DNA-DAMAGE	Oxidative DNA Damage
ID	2	55137	54737	RECOMBINANT MNSOD	Recombinant Mnsod
ID	3	55137	54737	CELL-LINE	Cell
ID	4	55137	54737	VITAMIN-E	Vitamin E
ID	5	55137	54737	IN-VITRO	In-Vitro

ID	6	55137	54737	STRESS	Stress
ID	7	55137	54737	KIDNEY	Kidney
ID	8	55137	54737	MICE	Mice
ID	9	55137	54737	TOXICOKINETICS	Toxicokinetics
ID	10	55137	54737	TOXICITY	Toxicity

Record 366 | WOS:000435675100011

Cinnamon (Cinnamomum zeylanicum) as an antidote or a protective agent against natural or chemical toxicities: a review

Field	Position	Original line	Normalised line	Original value	Normalised value
DE	2	55235	54834	antidote	Antidote
DE	3	55235	54834	cinnamic acid	Cinnamic Acid
DE	4	55235	54834	cinnamaldehyde	Cinnamaldehyde
DE	5	55235	54834	protective	Protective
DE	6	55235	54834	toxicity	Toxicity
ID	1	55236	54835	INDUCED OXIDATIVE STRESS	Induced Oxidative Stress
ID	2	55236	54835	ACRYLAMIDE-INDUCED NEUROTOXICITY	Acrylamide Induced Neurotoxicity
ID	3	55236	54835	SATIVA FIXED OIL	Sativa Fixed Oil
ID	4	55236	54835	NIGELLA-SATIVA	Nigella Sativa
ID	5	55236	54835	LIVER-INJURY	Liver Injury
ID	6	55236	54835	BARK OIL	Bark Oil
ID	7	55236	54835	INDUCED NEPHROTOXICITY	Induced Nephrotoxicity
ID	8	55236	54835	ASPERGILLUS-FLAVUS	Aspergillus Flavus
ID	9	55236	54835	ETHANOL TOXICITY	Ethanol Toxicity
ID	10	55236	54835	AQUEOUS EXTRACT	Aqueous Extract

Record 367 | WOS:000423248100019

Modified mycotoxins: An updated review on their formation, detection, occurrence, and toxic effects

Field	Position	Original line	Normalised line	Original value	Normalised value
DE	1	55374	54972	Mycotoxigenic fungi	Mycotoxigenic Fungi
DE	2	55374	54972	Masked mycotoxin	Masked Mycotoxin
DE	3	55374	54972	Metabolites	Metabolite
DE	4	55374	54972	Food safety	Food Safety
DE	5	55374	54972	Food toxicants	Food Toxicant
DE	6	55374	54972	Exposure assessment	Exposure Assessment
ID	1	55376	54974	IN-VITRO CYTOTOXICITY	In Vitro Cytotoxicity
ID	2	55376	54974	OCHRATOXIN-A	Ochratoxin A (OTA)
ID	3	55376	54974	MASKED MYCOTOXIN	Masked Mycotoxin
ID	4	55376	54974	FUSARIUM MYCOTOXINS	Fusarium Mycotoxin
ID	5	55376	54974	T-2 TOXIN	T 2 Toxin
ID	6	55376	54974	FUMONISIN B-1	Fumonisin B1 (FB1)
ID	7	55376	54974	HT-2 TOXIN	Ht 2 Toxin
ID	8	55376	54974	STRUCTURAL-CHARACTERIZATION	Structural Characterization
ID	9	55376	54974	STRUCTURE ELUCIDATION	Structure Elucidation
ID	10	55376	54974	ZEARALENONE-SULFATE	Zearalenone Sulfate

Record 368 | WOS:000423248100048

In vitro mechanisms of Beauvericin toxicity: A review

Field	Position	Original line	Normalised line	Original value	Normalised value
DE	2	55632	55229	Biological properties	Biological Property
DE	3	55632	55229	Biochemical activities	Biochemical Activity
DE	4	55632	55229	In vitro cytoxicity	In Vitro Cytoxicity

DE	5	55632	55229	Toxicokinetic studies	Toxicokinetic Study
ID	1	55634	55231	EMERGING FUSARIUM-MYCOTOXINS	Emerging Fusarium Mycotoxin
ID	2	55634	55231	KIDNEY PK15 CELLS	Kidney Pk15
ID	3	55634	55231	PAECILOMYCES-TENUIPES BCC-1614	Paecilomyce Tenuipe Bcc 1614
ID	4	55634	55231	TANDEM MASS-SPECTROMETRY	Tandem Mass Spectrometry
ID	5	55634	55231	CHO-K1 CELLS	Cho K1
ID	6	55634	55231	NATURAL OCCURRENCE	Natural Occurrence
ID	7	55634	55231	OCHRATOXIN-A	Ochratoxin A (OTA)
ID	8	55634	55231	SECONDARY METABOLITES	Secondary Metabolite
ID	9	55634	55231	VENTRICULAR MYOCYTES	Ventricular Myocyte
ID	10	55634	55231	ANTIOXIDANT CAPACITY	Antioxidant Capacity

Record 369 | WOS:000418302000021

Modulations of DNMT1 and HDAC1 are involved in the OTA-induced cytotoxicity and apoptosis in vitro

Field	Position	Original line	Normalised line	Original value	Normalised value
DE	1	55794	55389	Ochratoxin A	Ochratoxin A (OTA)
DE	2	55794	55389	DNMT1	Dnmt1
DE	3	55794	55389	HDAC1	Hdac1
ID	1	55795	55390	OCHRATOXIN-A	Ochratoxin A (OTA)
ID	2	55795	55390	OXIDATIVE STRESS	Oxidative Stress
ID	3	55795	55390	HISTONE DEACETYLASE	Histone Deacetylase
ID	4	55795	55390	MAMMALIAN-CELLS	Mammalian
ID	5	55795	55390	TOXICITY	Toxicity
ID	6	55795	55390	CANCER	Cancer
ID	7	55795	55390	METHYLATION	Methylation
ID	8	55795	55390	RATS	Rat
ID	9	55795	55390	CARCINOGENICITY	Carcinogenicity
ID	10	55795	55390	GENOTOXICITY	Genotoxicity

Record 370 | WOS:000417670500029

Ochratoxin A-Induced Apoptosis of IPEC-J2 Cells through ROS-Mediated Mitochondrial Permeability Transition Pore Opening Pathway

Field	Position	Original line	Normalised line	Original value	Normalised value
DE	1	55903	55497	Ochratoxin A	Ochratoxin A (OTA)
DE	2	55903	55497	IPEC-J2 cells	IPEC-J2
DE	3	55903	55497	mitochondrial reactive oxygen species	Mitochondrial Reactive Oxygen Specy
DE	4	55903	55497	mitochondrial permeability transition pore opening	Mitochondrial Permeability Transition Pore Opening
DE	5	55903	55497	apoptosis	Apoptosis
ID	1	55905	55499	OXIDATIVE DNA-DAMAGE	Oxidative DNA Damage
ID	2	55905	55499	CYTOCHROME-C	Cytochrome C
ID	3	55905	55499	MYCOTOXINS DEOXYNIVALENOL	Mycotoxin Deoxynivalenol
ID	4	55905	55499	CYTOTOXICITY	Cytotoxicity
ID	5	55905	55499	RELEASE	Release
ID	6	55905	55499	STRESS	Stress
ID	7	55905	55499	DEATH	Death
ID	8	55905	55499	COSTUNOLIDE	Costunolide
ID	9	55905	55499	DERIVATIVES	Derivative
ID	10	55905	55499	ABSORPTION	Absorption

Record 371 | WOS:000418211700013

A comprehensive review on biological properties of citrinin

Field	Position	Original line	Normalised line	Original value	Normalised value
ID	1	56054	55646	MYCOTOXINS OCHRATOXIN-A	Ochratoxin A (OTA)
ID	2	56054	55646	IN-VITRO PROLIFERATION	In Vitro Proliferation
ID	3	56054	55646	PREGNANT WISTAR RATS	Pregnant Wistar Rat
ID	4	56054	55646	NATURAL-PRODUCTS	Natural Product
ID	5	56054	55646	PENICILLIUM MYCOTOXINS	Penicillium Mycotoxin
ID	6	56054	55646	INDUCED DYSFUNCTION	Induced Dysfunction
ID	7	56054	55646	RENAL-FUNCTION	Renal Function
ID	8	56054	55646	CELLS	Cell
ID	9	56054	55646	APOPTOSIS	Apoptosis
ID	10	56054	55646	TOXICITY	Toxicity

Record 372 | WOS:000416589800031

Differential Gene Expression Analysis of Bovine Macrophages after Exposure to the Penicillium Mycotoxins Citrinin and/or Ochratoxin A

Field	Position	Original line	Normalised line	Original value	Normalised value
DE	1	56279	55870	ochratoxinA	Ochratoxin A (OTA)
DE	2	56279	55870	citrinin	Citrinin
DE	3	56279	55870	mixture toxicity	Mixture Toxicity
DE	4	56279	55870	bovine macrophage	Bovine Macrophage
DE	5	56279	55870	gene expression	Gene Expression
DE	6	56279	55870	microarray	Microarray
ID	1	56281	55872	IN-VITRO	In-Vitro
ID	2	56281	55872	OXIDATIVE STRESS	Oxidative Stress
ID	3	56281	55872	RAT-LIVER	Rat Liver
ID	4	56281	55872	CELLS	Cell
ID	5	56281	55872	APOPTOSIS	Apoptosis
ID	6	56281	55872	PROLIFERATION	Proliferation
ID	7	56281	55872	ACTIVATION	Activation
ID	8	56281	55872	INHIBITION	Inhibition
ID	9	56281	55872	EXCRETION	Excretion
ID	10	56281	55872	CARCINOMA	Carcinoma

Record 373 | WOS:000415911400030

UPLC-MS/MS analysis of ochratoxin A metabolites produced by Caco-2 and HepG2 cells in a co-culture system

Field	Position	Original line	Normalised line	Original value	Normalised value
DE	1	56410	56000	OTA	Ochratoxin A (OTA)
DE	3	56410	56000	Transwell system	Transwell System
DE	4	56410	56000	In vitro cytotoxicity	In Vitro Cytotoxicity
DE	5	56410	56000	MTS assay	Mts Assay
DE	6	56410	56000	TEER measurement	Teer Measurement
ID	1	56412	56002	IN-VITRO	In-Vitro
ID	2	56412	56002	TISSUE DISTRIBUTION	Tissue Distribution
ID	3	56412	56002	A METABOLITES	A Metabolite
ID	4	56412	56002	DNA-ADDUCT	DNA Adduct
ID	5	56412	56002	MYCOTOXINS	Mycotoxins
ID	6	56412	56002	EXPOSURE	Exposure
ID	7	56412	56002	RAT	Rat
ID	8	56412	56002	EXPRESSION	Expression
ID	9	56412	56002	AFLATOXINS	Aflatoxin
ID	10	56412	56002	TOXICITY	Toxicity

Record 374 | WOS:000410635200006

Effects of ochratoxin A on ER stress, MAPK signaling pathway and autophagy of kidney and spleen in pigs

Field	Position	Original line	Normalised line	Original value	Normalised value
DE	1	56544	56133	Ochratoxin A	Ochratoxin A (OTA)
DE	2	56544	56133	ER stress	Er Stress
DE	3	56544	56133	MAPK signaling pathway	Mapk Signaling Pathway
DE	4	56544	56133	autophagy	Autophagy
DE	5	56544	56133	pig	Pig
ID	1	56545	56134	OXIDATIVE STRESS	Oxidative Stress
ID	2	56545	56134	PHASE ARREST	Phase Arrest
ID	3	56545	56134	DNA-DAMAGE	DNA Damage
ID	4	56545	56134	IN-VITRO	In-Vitro
ID	5	56545	56134	APOPTOSIS	Apoptosis
ID	6	56545	56134	EXPRESSION	Expression
ID	7	56545	56134	CITRININ	Citrinin
ID	8	56545	56134	CELLS	Cell
ID	9	56545	56134	ACTIVATION	Activation
ID	10	56545	56134	MYCOTOXINS	Mycotoxins

Record 375 | WOS:000414913800067

Evaluation of Chitosan and Cellulosic Polymers as Binding Adsorbent Materials to Prevent Aflatoxin B1, Fumonisin B1, Ochratoxin, Trichothecene, Deoxynivalenol, and Zearalenone Mycotoxicoses Through an In Vitro Gastrointestinal Model for Poultry

Field	Position	Original line	Normalised line	Original value	Normalised value
DE	1	56662	56250	adsorption	Adsorption
DE	2	56662	56250	cellulosic polymers	Cellulosic Polymer
DE	3	56662	56250	chitosan	Chitosan
DE	4	56662	56250	in vitro	In-Vitro
DE	5	56662	56250	mycotoxins	Mycotoxins
ID	1	56663	56251	CROSS-LINKED CHITOSAN	Cross Linked Chitosan
ID	2	56663	56251	CARBOXYMETHYL CELLULOSE	Carboxymethyl Cellulose
ID	3	56663	56251	PHOSPHATE REMOVAL	Phosphate Removal
ID	4	56663	56251	ANIMAL FEED	Animal Feed
ID	5	56663	56251	ADSORPTION	Adsorption
ID	6	56663	56251	TOXICITY	Toxicity
ID	7	56663	56251	EFFICACY	Efficacy
ID	8	56663	56251	CHITIN	Chitin
ID	9	56663	56251	DETOXIFICATION	Detoxification
ID	10	56663	56251	CONTAMINATION	Contamination

Record 376 | WOS:000408520200003

Worldwide Occurrence of Mycotoxins in Cereals and Cereal-Derived Food Products: Public Health Perspectives of Their Co-occurrence

Field	Position	Original line	Normalised line	Original value	Normalised value
DE	1	56773	56359	mycotoxins	Mycotoxins
DE	2	56773	56359	cereals	Cereal
DE	3	56773	56359	co-occurrence	Co Occurrence
DE	4	56773	56359	combined toxicity	Combined Toxicity
ID	1	56774	56360	KIDNEY PK15 CELLS	Kidney Pk15
ID	2	56774	56360	CORN-BASED FOOD	Corn Based Food
ID	3	56774	56360	SIMULTANEOUS PRENATAL EXPOSURE	Simultaneous Prenatal Exposure
ID	4	56774	56360	OCHRATOXIN-A	Ochratoxin A (OTA)
ID	5	56774	56360	AFLATOXIN B-1	Aflatoxin B1 (AFB1)
ID	6	56774	56360	FUMONISIN B-1	Fumonisin B1 (FB1)
ID	7	56774	56360	FUSARIUM-MYCOTOXINS	Fusarium Mycotoxin
ID	8	56774	56360	NATURAL OCCURRENCE	Natural Occurrence
ID	9	56774	56360	IN-VITRO	In-Vitro
ID	10	56774	56360	RISK-ASSESSMENT	Risk Assessment

Record 377 | WOS:000408520200008

Mutagenicity of Ochratoxin A: Role for a Carbon-Linked C8-Deoxyguanosine Adduct?

Field	Position	Original line	Normalised line	Original value	Normalised value
DE	1	57062	56647	ochratoxin A(OTA)	Ochratoxin A (OTA)
DE	2	57062	56647	DNA damage	DNA Damage
DE	3	57062	56647	DNA addnct	DNA Addnct
DE	4	57062	56647	iDNA structure	Idna Structure
DE	5	57062	56647	mutagenicity	Mutagenicity
DE	6	57062	56647	DNA replication	DNA Replication
DE	7	57062	56647	DNA polymerase	DNA Polymerase
DE	8	57062	56647	translesion synthesis	Translesion Synthesis
ID	1	57064	56649	COVALENT DNA ADDUCTION	Covalent DNA Adduction
ID	2	57064	56649	DOUBLE-STRAND BREAKS	Double Strand Break
ID	3	57064	56649	NUCLEIC-ACID ADDUCTS	Nucleic Acid Adduct
ID	4	57064	56649	GPT DELTA RATS	Gpt Delta Rat
ID	5	57064	56649	C8-ARYL-GUANINE ADDUCTS	C8 Aryl Guanine Adduct
ID	6	57064	56649	PEROXIDASE ACTIVATION	Peroxidase Activation
ID	7	57064	56649	SEQUENCE INFLUENCE	Sequence Influence
ID	8	57064	56649	PHENOXYL RADICALS	Phenoxyl Radical
ID	9	57064	56649	NUCLEOSIDE ADDUCT	Nucleoside Adduct
ID	10	57064	56649	C8 REACTIVITY	C8 Reactivity

Record 378 | WOS:000408074600028

Overexpression and Low Expression of Selenoprotein S Impact Ochratoxin A-Induced Porcine Cytotoxicity and Apoptosis in Vitro

Field	Position	Original line	Normalised line	Original value	Normalised value
DE	1	57186	56770	selenoprotein S	Selenoprotein S
DE	2	57186	56770	ochratoxin A	Ochratoxin A (OTA)
DE	3	57186	56770	cytotoxicity	Cytotoxicity
DE	4	57186	56770	apoptosis	Apoptosis
DE	5	57186	56770	oxidative stress	Oxidative Stress
DE	6	57186	56770	p38 signal pathway	P38 Signal Pathway
DE	7	57186	56770	PK15 cells	Pk15
ID	1	57188	56772	CIRCOVIRUS TYPE-2 REPLICATION	Circovirus Type 2 Replication
ID	2	57188	56772	INHIBITING OXIDATIVE STRESS	Inhibiting Oxidative Stress
ID	3	57188	56772	MAPK SIGNALING PATHWAYS	Mapk Signaling Pathway
ID	4	57188	56772	INFLAMMATORY RESPONSE	Inflammatory Response
ID	5	57188	56772	PCV2 REPLICATION	Pcv2 Replication
ID	6	57188	56772	GENE-EXPRESSION	Gene Expression
ID	7	57188	56772	PK15 CELLS	Pk15
ID	8	57188	56772	INJURY	Injury
ID	9	57188	56772	SPLENOCYTES	Splenocyte
ID	10	57188	56772	ACTIVATION	Activation

Record 379 | WOS:000411538100005

Nrf2 deficiency exacerbates ochratoxin A-induced toxicity in vitro and in vivo

Field	Position	Original line	Normalised line	Original value	Normalised value
DE	2	57296	56879	microRNA	Microrna
DE	3	57296	56879	Nuclear factor E2-related factor 2	Nuclear Factor E2 Related Factor 2
DE	5	57296	56879	Mycotoxin	Mycotoxins
DE	6	57296	56879	Kidney diseases	Kidney Disease

ID	1	57298	56881	EPITHELIAL-MESENCHYMAL TRANSITION	Epithelial Mesenchymal Transition
ID	2	57298	56881	DIABETIC-NEPHROPATHY	Diabetic Nephropathy
ID	3	57298	56881	HEME OXYGENASE-1	Heme Oxygenase 1
ID	4	57298	56881	RENAL FIBROSIS	Renal Fibrosis
ID	5	57298	56881	INDUCED NEPHROTOXICITY	Induced Nephrotoxicity
ID	6	57298	56881	KIDNEY-DISEASE	Kidney Disease
ID	7	57298	56881	CELL-ADHESION	Cell Adhesion
ID	8	57298	56881	CARCINOGENICITY	Carcinogenicity
ID	9	57298	56881	MICE	Mice
ID	10	57298	56881	SULFORAPHANE	Sulforaphane

Record 380 | WOS:000406735700003

Ochratoxin A transport by the human breast cancer resistance protein (BCRP), multidrug resistance protein 2 (MRP2), and organic anion-transporting polypeptides 1A2, 1B1 and 2B1

Field	Position	Original line	Normalised line	Original value	Normalised value
DE	1	57414	56996	Ochratoxin A	Ochratoxin A (OTA)
DE	2	57414	56996	BCRP	Bcrp
DE	3	57414	56996	MRP2	Mrp2
DE	4	57414	56996	OATP	Oatp
ID	1	57415	56997	CACO-2 CELLS	Caco-2
ID	2	57415	56997	EXPRESSION	Expression
ID	3	57415	56997	SECRETION	Secretion
ID	4	57415	56997	MODELS	Model
ID	5	57415	56997	OAT3	Oat3
ID	6	57415	56997	PH	Ph

Record 381 | WOS:000407370700051

Health Risks Associated with Exposure to Filamentous Fungi

Field	Position	Original line	Normalised line	Original value	Normalised value
DE	1	57487	57068	mycotoxicity	Mycotoxicity
DE	2	57487	57068	infections	Infection
ID	1	57488	57069	INVASIVE PULMONARY ASPERGILLOSIS	Invasive Pulmonary Aspergillosis
ID	2	57488	57069	IN-VITRO CYTOTOXICITY	In Vitro Cytotoxicity
ID	3	57488	57069	MYCOTOXIN PRODUCTION	Mycotoxin Production
ID	4	57488	57069	FUSARIUM-MYCOTOXINS	Fusarium Mycotoxin
ID	5	57488	57069	OXIDATIVE STRESS	Oxidative Stress
ID	6	57488	57069	FUMONISIN B-1	Fumonisin B1 (FB1)
ID	7	57488	57069	OCHRATOXIN-A	Ochratoxin A (OTA)
ID	8	57488	57069	CELL-DEATH	Cell Death
ID	9	57488	57069	INFECTIONS	Infection
ID	10	57488	57069	GLIOTOXIN	Gliotoxin

Record 382 | WOS:000404237400013

Evaluation of the automated MicroFlowÂ® and Metaferâ ¢ platforms for high-throughput micronucleus scoring and dose response analysis in human lymphoblastoid TK6 cells

Field	Position	Original line	Normalised line	Original value	Normalised value
DE	3	57692	57272	Dose response	Dose Response
DE	4	57692	57272	Metafer (TM)	Metafer Tm
DE	5	57692	57272	MicroFlow (R)	Microflow R
ID	1	57693	57273	GENETIC TOXICOLOGY	Genetic Toxicology

ID	2	57693	57273	EMPIRICAL-ANALYSIS	Empirical Analysis
ID	3	57693	57273	FLOW-CYTOMETRY	Flow Cytometry
ID	4	57693	57273	IMAGE-ANALYSIS	Image Analysis
ID	5	57693	57273	BMD METRICS	Bmd Metrics
ID	6	57693	57273	ASSAY	Assay
ID	7	57693	57273	VALIDATION	Validation
ID	8	57693	57273	PROTOCOL	Protocol
ID	9	57693	57273	POTENCY	Potency
ID	10	57693	57273	CYTOTOXICITY	Cytotoxicity

Record 383 | WOS:000404198300012

In vitro immune toxicity of ochratoxin A in porcine alveolar macrophages: A role for the ROS-relative TLR4/MyD88 signaling pathway

Field	Position	Original line	Normalised line	Original value	Normalised value
DE	1	57785	57364	Ochratoxin A	Ochratoxin A (OTA)
DE	2	57785	57364	Immune toxicity	Immune Toxicity
DE	3	57785	57364	ROS	Reactive Oxygen Species (ROS)
DE	4	57785	57364	TLR4	Tlr4
DE	5	57785	57364	MyD88	Myd88
ID	1	57786	57366	NECROSIS-FACTOR-ALPHA	Necrosis Factor Alpha
ID	2	57786	57366	TOLL-LIKE RECEPTORS	Toll Like Receptor
ID	3	57786	57366	OXIDATIVE STRESS	Oxidative Stress
ID	4	57786	57366	MYCOTOXIN OCHRATOXIN	Ochratoxin A (OTA)
ID	5	57786	57366	AFLATOXIN B-1	Aflatoxin B1 (AFB1)
ID	6	57786	57366	CELLS	Cell
ID	7	57786	57366	APOPTOSIS	Apoptosis
ID	8	57786	57366	EXPRESSION	Expression
ID	9	57786	57366	TLR4	Tlr4
ID	10	57786	57366	INFLAMMATION	Inflammation

Record 384 | WOS:000401600400001

Adsorption of aflatoxin B1, zearalenone and ochratoxin A by microorganisms isolated from Kefir grains

Field	Position	Original line	Normalised line	Original value	Normalised value
DE	4	57895	57473	Lactobacillus kefiri	Lactobacillus Kefiri
DE	5	57895	57473	Acetobacter syzygii	Acetobacter Syzygii
DE	6	57895	57473	Kazachstania servazzii	Kazachstania Servazzii
ID	1	57897	57475	LACTIC-ACID BACTERIA	Lactic Acid Bacteria
ID	2	57897	57475	SACCHAROMYCES-CEREVISIAE	Saccharomyce Cerevisiae
ID	3	57897	57475	IN-VITRO	In-Vitro
ID	4	57897	57475	BIODEGRADATION	Biodegradation
ID	5	57897	57475	DETOXIFICATION	Detoxification
ID	6	57897	57475	EXPOSURE	Exposure
ID	7	57897	57475	STRAINS	Strain
ID	8	57897	57475	BINDING	Binding
ID	9	57897	57475	MILK	Milk

Record 385 | WOS:000408731500007

Lycopene restores trace element levels in ochratoxin A-treated rats

Field	Position	Original line	Normalised line	Original value	Normalised value
DE	1	58016	57593	copper	Copper
DE	2	58016	57593	kidney	Kidney
DE	3	58016	57593	liver	Liver
DE	4	58016	57593	mycotoxins	Mycotoxins
DE	5	58016	57593	selenium	Selenium
DE	6	58016	57593	testis	Testis

DE	7	58016	57593	zinc	Zinc
ID	1	58017	57594	DNA-DAMAGE	DNA Damage
ID	2	58017	57594	OXIDATIVE STRESS	Oxidative Stress
ID	3	58017	57594	SELENIUM	Selenium
ID	4	58017	57594	ZINC	Zinc
ID	5	58017	57594	CELLS	Cell
ID	6	58017	57594	HEALTH	Health

Record 386 | WOS:000403637300009

Ochratoxin A induced premature senescence in human renal proximal tubular cells

Field	Position	Original line	Normalised line	Original value	Normalised value
DE	1	58120	57696	Ochratoxin A	Ochratoxin A (OTA)
DE	3	58120	57696	Human renal proximal tubular cell	Human Renal Proximal Tubular
ID	1	58122	57697	CELLULAR SENESCENCE	Cellular Senescence
ID	2	58122	57697	DNA-DAMAGE	DNA Damage
ID	3	58122	57697	HUMAN PLASMA	Human Plasma
ID	4	58122	57697	EXPRESSION	Expression
ID	5	58122	57697	KIDNEY	Kidney
ID	6	58122	57697	PHOSPHORYLATION	Phosphorylation
ID	7	58122	57697	EZRIN	Ezrin
ID	8	58122	57697	ACTIVATION	Activation
ID	9	58122	57697	HALLMARKS	Hallmark
ID	10	58122	57697	PROTEIN	Protein

Record 387 | WOS:000394982800001

The intestinal barrier as an emerging target in the toxicological assessment of mycotoxins

Field	Position	Original line	Normalised line	Original value	Normalised value
DE	2	58248	57822	Tight junction proteins	Tight Junction Protein
DE	3	58248	57822	Intestinal permeability	Intestinal Permeability
DE	4	58248	57822	Mucosal inflammation	Mucosal Inflammation
ID	1	58250	57824	NEURAL-TUBE DEFECTS	Neural Tube Defect
ID	2	58250	57824	TIGHT JUNCTION PROTEINS	Tight Junction Protein
ID	3	58250	57824	OCHRATOXIN-A	Ochratoxin A (OTA)
ID	4	58250	57824	IN-VITRO	In-Vitro
ID	5	58250	57824	T-2 TOXIN	T 2 Toxin
ID	6	58250	57824	FUSARIUM MYCOTOXINS	Fusarium Mycotoxin
ID	7	58250	57824	OXIDATIVE STRESS	Oxidative Stress
ID	8	58250	57824	FUMONISIN B-1	Fumonisin B1 (FB1)
ID	9	58250	57824	CACO-2 CELLS	Caco-2
ID	10	58250	57824	SPHINGOLIPID METABOLISM	Sphingolipid Metabolism

Record 388 | WOS:000394558400005

Assessing interactions of binary mixtures of Penicillium mycotoxins (PMs) by using a bovine macrophage cell line (BoMacs)

Field	Position	Original line	Normalised line	Original value	Normalised value
DE	1	58518	58091	Penicillium mycotoxins	Penicillium Mycotoxin
DE	2	58518	58091	Mixture toxicity	Mixture Toxicity
DE	4	58518	58091	Independent action (IA)	Independent Action Ia
DE	5	58518	58091	Concentration addition (CA)	Concentration Addition Ca
ID	1	58520	58093	OCHRATOXIN-A	Ochratoxin A (OTA)
ID	2	58520	58093	EXCRETION	Excretion
ID	3	58520	58093	CITRININ	Citrinin
ID	4	58520	58093	ACID	Acid
ID	5	58520	58093	EXPOSURE	Exposure

ID	6	58520	58093	SHEEP	Sheep
ID	7	58520	58093	MICE	Mice

Record 389 | WOS:000392347800007

Mycotoxins as human carcinogens-the IARC Monographs classification

Field	Position	Original line	Normalised line	Original value	Normalised value
DE	2	58608	58180	Aflatoxins	Aflatoxin
DE	3	58608	58180	Fumonisins	Fumonisin
DE	4	58608	58180	Ochratoxin A	Ochratoxin A (OTA)
DE	5	58608	58180	IARC	Iarc
ID	1	58609	58181	2'-DEOXYGUANOSINE-CARBON 8-BOUND OCHRATOXIN	2 Deoxyguanosine Carbon 8 Bound Ochratoxin
ID	2	58609	58181	RENAL TUMOR-FORMATION	Renal Tumor Formation
ID	3	58609	58181	DNA-DAMAGE	DNA Damage
ID	4	58609	58181	ARISTOLOCHIC ACID	Aristolochic Acid
ID	5	58609	58181	ADDUCT FORMATION	Adduct Formation
ID	6	58609	58181	MALE RATS	Male Rat
ID	7	58609	58181	IN-VIVO	In-Vivo
ID	8	58609	58181	KIDNEY	Kidney
ID	9	58609	58181	GENOTOXICITY	Genotoxicity
ID	10	58609	58181	EXPOSURE	Exposure

Record 390 | WOS:000389167600013

Mycotoxin detoxifiers detoxifiers attenuate deoxynivalenol-induced pro-inflammatory barrier insult in porcine enterocytes as an in vitro evaluation model of feed mycotoxin reduction

Field	Position	Original line	Normalised line	Original value	Normalised value
DE	1	58770	58341	Mycotoxin detoxifiers	Mycotoxin Detoxifier
DE	2	58770	58341	Porcine enterocytes	Porcine Enterocyte
DE	3	58770	58341	Deoxynivalenol	Deoxynivalenol (DON)
ID	1	58771	58342	FUSARIUM-TOXIN DEOXYNIVALENOL	Fusarium Toxin Deoxynivalenol
ID	2	58771	58342	CONTAMINANT DEOXYNIVALENOL	Contaminant Deoxynivalenol
ID	3	58771	58342	BINDING PROTEIN	Binding Protein
ID	4	58771	58342	AFLATOXIN B-1	Aflatoxin B1 (AFB1)
ID	5	58771	58342	OCHRATOXIN-A	Ochratoxin A (OTA)
ID	6	58771	58342	INTESTINE	Intestine
ID	7	58771	58342	DON	Deoxynivalenol (DON)
ID	8	58771	58342	BENTONITE	Bentonite
ID	9	58771	58342	DIETS	Diet
ID	10	58771	58342	ZEARALENONE	Zearalenone (ZEA)

Record 391 | WOS:000392789800020

Chitosan nanoparticles plus quercetin suppress the oxidative stress, modulate DNA fragmentation and gene expression in the kidney of rats fed ochratoxin A-contaminated diet

Field	Position	Original line	Normalised line	Original value	Normalised value
DE	1	58889	58459	Ochratoxin A	Ochratoxin A (OTA)
DE	2	58889	58459	Chitosan nanoparticles	Chitosan Nanoparticle
DE	4	58889	58459	Oxidative stress	Oxidative Stress
ID	1	58891	58461	NF-KAPPA-B	Nf Kappa B
ID	2	58891	58461	IN-VITRO	In-Vitro
ID	3	58891	58461	INDUCED INFLAMMATION	Induced Inflammation
ID	4	58891	58461	VITAMIN-E	Vitamin E
ID	5	58891	58461	ANTIOXIDANT	Antioxidant
ID	6	58891	58461	DAMAGE	Damage
ID	7	58891	58461	CELLS	Cell
ID	8	58891	58461	PREVENTION	Prevention

ID	9	58891	58461	TOXICITY	Toxicity
ID	10	58891	58461	GENOTOXICITY	Genotoxicity

Record 392 | WOS:000402081300011

Mycotoxins co-contamination: Methodological aspects and biological relevance of combined toxicity studies

Field	Position	Original line	Normalised line	Original value	Normalised value
DE	2	59060	58629	antagonism	Antagonism
DE	3	59060	58629	multi-contamination	Multi Contamination
DE	4	59060	58629	mycotoxins	Mycotoxins
DE	5	59060	58629	synergy	Synergy
DE	6	59060	58629	toxicological interactions	Toxicological Interaction
ID	1	59062	58631	KIDNEY PK15 CELLS	Kidney Pk15
ID	2	59062	58631	STATISTICALLY DESIGNED EXPERIMENTS	Statistically Designed Experiment
ID	3	59062	58631	INTESTINAL EPITHELIAL-CELLS	Intestinal Epithelial
ID	4	59062	58631	MESSENGER-RNA EXPRESSION	Messenger RNA Expression
ID	5	59062	58631	IN-VITRO CYTOTOXICITY	In Vitro Cytotoxicity
ID	6	59062	58631	FUSARIUM MYCOTOXINS	Fusarium Mycotoxin
ID	7	59062	58631	AFLATOXIN B-1	Aflatoxin B1 (AFB1)
ID	8	59062	58631	FUMONISIN B-1	Fumonisin B1 (FB1)
ID	9	59062	58631	OCHRATOXIN-A	Ochratoxin A (OTA)
ID	10	59062	58631	CYCLOPIAZONIC ACID	Cyclopiazonic Acid

Record 393 | WOS:000399597700009

Protective role of bentonite against aflatoxin B1- and ochratoxin A-induced immunotoxicity in broilers

Field	Position	Original line	Normalised line	Original value	Normalised value
DE	1	59268	58836	Aflatoxin B1	Aflatoxin B1 (AFB1)
DE	2	59268	58836	bentonite	Bentonite
DE	3	59268	58836	immune system	Immune System
DE	4	59268	58836	ochratoxin A	Ochratoxin A (OTA)
ID	1	59269	58837	SODIUM BENTONITE	Sodium Bentonite
ID	2	59269	58837	IMMUNE-RESPONSE	Immune Response
ID	3	59269	58837	T-2 TOXIN	T 2 Toxin
ID	4	59269	58837	VITAMIN-E	Vitamin E
ID	5	59269	58837	PERFORMANCE	Performance
ID	6	59269	58837	DIETS	Diet
ID	7	59269	58837	EFFICACY	Efficacy
ID	8	59269	58837	SERUM	Serum
ID	9	59269	58837	FEED	Feed
ID	10	59269	58837	IMMUNOSUPPRESSION	Immunosuppression

Record 394 | WOS:000448708300012

Aflatoxin in Rice Crop: Prevalence and Assessment of Daily Exposure

Field	Position	Original line	Normalised line	Original value	Normalised value
DE	3	59389	58956	Worldwide contamination	Worldwide Contamination
ID	1	59390	58957	ASPERGILLUS SPP.	Aspergillus Spp
ID	2	59390	58957	OCHRATOXIN-A	Ochratoxin A (OTA)
ID	3	59390	58957	RETAIL FOODS	Retail Food
ID	4	59390	58957	IN-VITRO	In-Vitro
ID	5	59390	58957	CONTAMINATION	Contamination
ID	6	59390	58957	B-1	B 1
ID	7	59390	58957	ZEARALENONE	Zearalenone (ZEA)
ID	8	59390	58957	MYCOTOXINS	Mycotoxins

ID	9	59390	58957	PRODUCTS	Product
ID	10	59390	58957	SUDAN	Sudan

Record 395 | WOS:000416384800008

Effect of PR toxin on THP1 and Caco-2 cells: an in vitro study

Field	Position	Original line	Normalised line	Original value	Normalised value
DE	1	59507	59073	PR toxin	Pr Toxin
DE	2	59507	59073	mycotoxin	Mycotoxins
DE	3	59507	59073	cytotoxicity	Cytotoxicity
DE	4	59507	59073	Penicillium roqueforti	Penicillium Roqueforti
DE	5	59507	59073	inflammatory gene expression	Inflammatory Gene Expression
DE	6	59507	59073	apoptosis	Apoptosis
ID	1	59509	59075	PENICILLIUM-ROQUEFORTI GROUP	Penicillium Roqueforti Group
ID	2	59509	59075	ASPERGILLUS-FUMIGATUS	Aspergillus Fumigatus
ID	3	59509	59075	MYCOTOXIN PRODUCTION	Mycotoxin Production
ID	4	59509	59075	CYCLOPIAZONIC ACID	Cyclopiazonic Acid
ID	5	59509	59075	MYCOPHENOLIC-ACID	Mycophenolic Acid
ID	6	59509	59075	ACUTE TOXICITY	Acute Toxicity
ID	7	59509	59075	OCHRATOXIN-A	Ochratoxin A (OTA)
ID	8	59509	59075	GENE-CLUSTER	Gene Cluster
ID	9	59509	59075	CYTOTOXICITY	Cytotoxicity
ID	10	59509	59075	METABOLITES	Metabolite

Record 396 | WOS:000413016300007

Efficacy of mixed adsorbent in ameliorating ochratoxicosis in broilers fed ochratoxin A contaminated diets

Field	Position	Original line	Normalised line	Original value	Normalised value
DE	1	59626	59191	Ochratoxin A	Ochratoxin A (OTA)
DE	2	59626	59191	mixed adsorbent	Mixed Adsorbent
DE	3	59626	59191	sodium montmorillonite	Sodium Montmorillonite
DE	4	59626	59191	yeast cell wall	Yeast Cell Wall
ID	1	59627	59193	HISTOPATHOLOGICAL CHANGES	Histopathological Change
ID	2	59627	59193	IN-VITRO	In-Vitro
ID	3	59627	59193	AFLATOXIN	Aflatoxin
ID	4	59627	59193	CHICKENS	Chicken
ID	5	59627	59193	SERUM	Serum
ID	6	59627	59193	PERFORMANCE	Performance
ID	7	59627	59193	BENTONITE	Bentonite
ID	8	59627	59193	TOXICITY	Toxicity
ID	9	59627	59193	RESIDUES	Residue
ID	10	59627	59193	TISSUES	Tissue

Record 397 | WOS:000417403600001

Citrinin induces apoptosis in human HCT116 colon cancer cells through endoplasmic reticulum stress

Field	Position	Original line	Normalised line	Original value	Normalised value
ID	1	59713	59278	CYTOCHROME-C	Cytochrome C
ID	2	59713	59278	MITOCHONDRIAL DYSFUNCTION	Mitochondrial Dysfunction
ID	3	59713	59278	SODIUM 4-PHENYLBUTYRATE	Sodium 4 Phenylbutyrate
ID	4	59713	59278	OXIDATIVE STRESS	Oxidative Stress
ID	5	59713	59278	OCHRATOXIN-A	Ochratoxin A (OTA)
ID	6	59713	59278	MYCOTOXINS	Mycotoxins
ID	7	59713	59278	SIGNALS	Signal

ID	8	59713	59278	DEATH	Death
ID	9	59713	59278	CYTOTOXICITY	Cytotoxicity
ID	10	59713	59278	INHIBITION	Inhibition

Record 398 | WOS:000424458100023

Mycotoxins

Field	Position	Original line	Normalised line	Original value	Normalised value
ID	1	59845	59409	RIBONUCLEIC-ACID POLYMERASE	Ribonucleic Acid Polymerase
ID	2	59845	59409	NEURAL-TUBE DEFECTS	Neural Tube Defect
ID	3	59845	59409	AFLATOXIN B-1	Aflatoxin B1 (AFB1)
ID	4	59845	59409	HEPATOCELLULAR-CARCINOMA	Hepatocellular Carcinoma
ID	5	59845	59409	T-2 TOXIN	T 2 Toxin
ID	6	59845	59409	ASPERGILLUS-FLAVUS	Aspergillus Flavus
ID	7	59845	59409	FUMONISIN B-1	Fumonisin B1 (FB1)
ID	8	59845	59409	OCHRATOXIN-A	Ochratoxin A (OTA)
ID	9	59845	59409	HEPATITIS-B	Hepatitis B
ID	10	59845	59409	IN-VIVO	In-Vivo

Record 399 | WOS:000448708300031

Application of Fungal Metabolites Against Mycotoxins Production

Field	Position	Original line	Normalised line	Original value	Normalised value
DE	1	60123	59686	Fungal metabolites	Fungal Metabolite
DE	2	60123	59686	Mycotoxins biosynthesis	Mycotoxin Biosynthesis
DE	3	60123	59686	Polyketides	Polyketide
DE	4	60123	59686	Terpenes	Terpene
DE	5	60123	59686	Alkaloids	Alkaloid
DE	6	60123	59686	Mycotoxin biocontrol	Mycotoxin Biocontrol
ID	1	60125	59688	POLYKETIDE SYNTHASE GENES	Polyketide Synthase Gene
ID	2	60125	59688	ASPERGILLUS SECTION FLAVI	Aspergillus Section Flavi
ID	3	60125	59688	POTENTIAL BIOCONTROL AGENTS	Potential Biocontrol Agent
ID	4	60125	59688	HUMAN ESOPHAGEAL CANCER	Human Esophageal Cancer
ID	5	60125	59688	OCHRATOXIN-A	Ochratoxin A (OTA)
ID	6	60125	59688	BIOLOGICAL-CONTROL	Biological Control
ID	7	60125	59688	AFLATOXIN BIOSYNTHESIS	Aflatoxin Biosynthesis
ID	8	60125	59688	IN-VITRO	In-Vitro
ID	9	60125	59688	FUNCTIONAL-CHARACTERIZATION	Functional Characterization
ID	10	60125	59688	HEPATOCELLULAR-CARCINOMA	Hepatocellular Carcinoma

Record 400 | WOS:000391769900002

Toxicity study of ochratoxin A using HEK293 and HepG2 cell lines based on microRNA profiling

Field	Position	Original line	Normalised line	Original value	Normalised value
DE	1	60360	59922	miRNA profiling	Mirna Profiling
DE	2	60360	59922	HEK293	Hek293
DE	4	60360	59922	ochratoxin A	Ochratoxin A (OTA)
DE	5	60360	59922	cytotoxicity	Cytotoxicity
ID	1	60361	59923	OXIDATIVE DNA-DAMAGE	Oxidative DNA Damage
ID	2	60361	59923	IN-VITRO	In-Vitro

ID	3	60361	59923	PHASE ARREST	Phase Arrest
ID	4	60361	59923	CANCER-CELLS	Cancer
ID	5	60361	59923	EXPRESSION	Expression
ID	6	60361	59923	APOPTOSIS	Apoptosis
ID	7	60361	59923	VIVO	Vivo
ID	8	60361	59923	NEPHROTOXICITY	Nephrotoxicity
ID	9	60361	59923	CYTOTOXICITY	Cytotoxicity
ID	10	60361	59923	BIOMARKERS	Biomarker

Record 401 | WOS:000393580000007

Limited Link between Oxidative Stress and Ochratoxin A-Induced Renal Injury in an Acute Toxicity Rat Model

Field	Position	Original line	Normalised line	Original value	Normalised value
DE	1	60484	60045	Ochratoxin A	Ochratoxin A (OTA)
DE	2	60484	60045	acute toxicity	Acute Toxicity
DE	3	60484	60045	nephrotoxicity	Nephrotoxicity
DE	4	60484	60045	oxidative stress	Oxidative Stress
DE	5	60484	60045	DNA damage	DNA Damage
ID	1	60486	60047	DOUBLE-STRAND BREAKS	Double Strand Break
ID	2	60486	60047	KIDNEY-CELL-LINE	Kidney Cell
ID	3	60486	60047	DNA-DAMAGE	DNA Damage
ID	4	60486	60047	MOLECULAR-MECHANISMS	Molecular Mechanism
ID	5	60486	60047	IN-VIVO	In-Vivo
ID	6	60486	60047	LIVER	Liver
ID	7	60486	60047	INDUCTION	Induction
ID	8	60486	60047	MICE	Mice
ID	9	60486	60047	EXPRESSION	Expression
ID	10	60486	60047	EXPOSURE	Exposure

Record 402 | WOS:000387780500027

TOXICOPATHOLOGICAL AND SERUM BIOCHEMICAL ALTERATIONS INDUCED BY OCHRATOXIN A IN BROILER CHICKS AND THEIR AMELIORATION BY LOCALLY AVAILABLE BENTONITE CLAY

Field	Position	Original line	Normalised line	Original value	Normalised value
DE	1	60609	60168	Broilers chicks	Broiler Chick
DE	2	60609	60168	Ochratoxin A	Ochratoxin A (OTA)
DE	3	60609	60168	bentonite clay	Bentonite Clay
DE	4	60609	60168	toxicopathology	Toxicopathology
DE	5	60609	60168	serum biochemical parameters	Serum Biochemical Parameter
DE	6	60609	60168	body weight	Body Weight
DE	7	60609	60168	FCR	Fcr
DE	8	60609	60168	organ weights	Organ Weight
ID	1	60611	60170	VITAMIN-E	Vitamin E
ID	2	60611	60170	DIETARY-PROTEIN	Dietary Protein
ID	3	60611	60170	BREEDER HENS	Breeder Hen
ID	4	60611	60170	IN-VIVO	In-Vivo
ID	5	60611	60170	AFLATOXIN	Aflatoxin
ID	6	60611	60170	FEED	Feed
ID	7	60611	60170	TOXICITY	Toxicity
ID	8	60611	60170	HEALTH	Health
ID	9	60611	60170	DEOXYNIVALENOL	Deoxynivalenol (DON)
ID	10	60611	60170	PERFORMANCE	Performance

Record 403 | WOS:000383825300009

Aflatoxin M1 cytotoxicity against human intestinal Caco-2 cells is enhanced in the presence of other mycotoxins

Field	Position	Original line	Normalised line	Original value	Normalised value
DE	1	60714	60272	Mycotoxin	Mycotoxins
DE	2	60714	60272	Co-contamination	Co Contamination

DE	4	60714	60272	Human colon adenocarcinoma cells	Human Colon Adenocarcinoma
ID	1	60716	60273	IN-VITRO	In-Vitro
ID	2	60716	60273	ALPHA-ZEARALENOL	Alpha Zearalenol
ID	3	60716	60273	OCHRATOXIN-A	Ochratoxin A (OTA)
ID	4	60716	60273	FUSARIUM MYCOTOXINS	Fusarium Mycotoxin
ID	5	60716	60273	EPITHELIAL-CELLS	Epithelial
ID	6	60716	60273	RISK-ASSESSMENT	Risk Assessment
ID	7	60716	60273	CHO-K1 CELLS	Cho K1
ID	8	60716	60273	BABY FOODS	Baby Food
ID	9	60716	60273	T-2 TOXIN	T 2 Toxin
ID	10	60716	60273	MILK	Milk

Record 404 | WOS:000387347300005

Different Toxicity Mechanisms for Citrinin and Ochratoxin A Revealed by Transcriptomic Analysis in Yeast

Field	Position	Original line	Normalised line	Original value	Normalised value
DE	1	60840	60395	Ochratoxin A	Ochratoxin A (OTA)
DE	4	60840	60395	Saccharomyces cerevisiae	Saccharomyce Cerevisiae
DE	5	60840	60395	mycotoxins	Mycotoxins
DE	6	60840	60395	oxidative stress	Oxidative Stress
DE	7	60840	60395	dose response	Dose Response
ID	1	60842	60397	OXIDATIVE STRESS	Oxidative Stress
ID	2	60842	60397	GENE-EXPRESSION	Gene Expression
ID	3	60842	60397	MYCOTOXIN CITRININ	Mycotoxin Citrinin
ID	4	60842	60397	LIPID-PEROXIDATION	Lipid Peroxidation
ID	5	60842	60397	IN-VITRO	In-Vitro
ID	7	60842	60397	APOPTOSIS	Apoptosis
ID	8	60842	60397	CELLS	Cell
ID	9	60842	60397	PENICILLIUM	Penicillium
ID	10	60842	60397	PATHWAY	Pathway

Record 405 | WOS:000383733300020

Quantitation of the DNA Adduct of Semicarbazide in Organs of Semicarbazide-Treated Rats by Isotope-Dilution Liquid Chromatography-Tandem Mass Spectrometry: A Comparative Study with the RNA Adduct

Field	Position	Original line	Normalised line	Original value	Normalised value
ID	1	60969	60522	APURINIC/APYRIMIDINIC SITES	Apurinic Apyrimidinic Site
ID	2	60969	60522	FLUORESCENCE DETECTION	Fluorescence Detection
ID	3	60969	60522	OCHRATOXIN-A	Ochratoxin A (OTA)
ID	4	60969	60522	IN-VIVO	In-Vivo
ID	5	60969	60522	PRODUCTS	Product
ID	6	60969	60522	DERIVATIZATION	Derivatization
ID	7	60969	60522	BREAD	Bread
ID	8	60969	60522	FLOUR	Flour
ID	9	60969	60522	AZODICARBONAMIDE	Azodicarbonamide
ID	10	60969	60522	QUANTIFICATION	Quantification

Record 406 | WOS:000387199300010

Ochratoxin A activates neutrophils and kills these cells through necrosis, an effect eliminated through its conversion into ochratoxin Î±

Field	Position	Original line	Normalised line	Original value	Normalised value
DE	1	61057	60609	Ochratoxin A	Ochratoxin A (OTA)
DE	2	61057	60609	Ochratoxin alpha	Ochratoxin Alpha
DE	3	61057	60609	Oxidative burst	Oxidative Burst
DE	4	61057	60609	ATP depletion	Atp Depletion
DE	5	61057	60609	Necrosis Intracellular Ca2+	Necrosis Intracellular Ca2

ID	1	61059	60611	OXIDATIVE DNA-DAMAGE	Oxidative DNA Damage
ID	2	61059	60611	FLOW-CYTOMETRY	Flow Cytometry
ID	3	61059	60611	IN-VITRO	In-Vitro
ID	4	61059	60611	CYTOTOXICITY	Cytotoxicity
ID	5	61059	60611	CALCIUM	Calcium
ID	6	61059	60611	ATP	Atp
ID	7	61059	60611	APOPTOSIS	Apoptosis
ID	8	61059	60611	TOXICITY	Toxicity
ID	9	61059	60611	BLOOD	Blood
ID	10	61059	60611	DEATH	Death

Record 407 ǀ WOS:000380647600001

Cytotoxic Effects of Ochratoxin A in Neuro-2a Cells: Role of Oxidative Stress Evidenced by N-acetylcysteine

Field	Position	Original line	Normalised line	Original value	Normalised value
DE	1	61187	60738	Ochratoxin-A	Ochratoxin A (OTA)
DE	2	61187	60738	Neuro-2a	Neuro 2a
DE	3	61187	60738	N-acetylcysteine	N Acetylcysteine
DE	4	61187	60738	reactive oxygen species	Reactive Oxygen Specy
DE	5	61187	60738	c-Jun N-terminal kinases	C Jun N Terminal Kinase
DE	6	61187	60738	apoptosis	Apoptosis
ID	1	61189	60740	DNA-DAMAGE	DNA Damage
ID	2	61189	60740	TYROSINE-HYDROXYLASE	Tyrosine Hydroxylase
ID	3	61189	60740	SIGNAL-TRANSDUCTION	Signal Transduction
ID	4	61189	60740	ALZHEIMERS-DISEASE	Alzheimer Disease
ID	5	61189	60740	INDUCED TOXICITY	Induced Toxicity
ID	6	61189	60740	BACOPA-MONNIERA	Bacopa Monniera
ID	7	61189	60740	DOWN-REGULATION	Down Regulation
ID	8	61189	60740	HEPG2 CELLS	HepG2
ID	9	61189	60740	BDNF	Bdnf
ID	10	61189	60740	APOPTOSIS	Apoptosis

Record 408 ǀ WOS:000380763800002

Ochratoxin A: 50 Years of Research

Field	Position	Original line	Normalised line	Original value	Normalised value
DE	1	61315	60864	ochratoxin A	Ochratoxin A (OTA)
DE	2	61315	60864	microfungi	Microfungi
DE	3	61315	60864	food	Food
DE	4	61315	60864	feed	Feed
DE	5	61315	60864	toxicity	Toxicity
DE	6	61315	60864	Balkan endemic nephropathy	Balkan Endemic Nephropathy
DE	7	61315	60864	carcinogenicity	Carcinogenicity
DE	8	61315	60864	urothelial cancer	Urothelial Cancer
DE	9	61315	60864	biomarkers	Biomarker
ID	1	61317	60866	PERFORMANCE LIQUID-CHROMATOGRAPHY	Performance Liquid Chromatography
ID	2	61317	60866	THIN-LAYER CHROMATOGRAPHY	Thin Layer Chromatography
ID	3	61317	60866	DNA ADDUCT FORMATION	DNA Adduct Formation
ID	4	61317	60866	2'-DEOXYGUANOSINE-CARBON 8-BOUND OCHRATOXIN	2 Deoxyguanosine Carbon 8 Bound Ochratoxin
ID	5	61317	60866	CHRONIC INTERSTITIAL NEPHROPATHY	Chronic Interstitial Nephropathy
ID	6	61317	60866	BALKAN ENDEMIC NEPHROPATHY	Balkan Endemic Nephropathy

ID	7	61317	60866	LINKED-IMMUNOSORBENT-ASSAY	Linked Immunosorbent Assay
ID	8	61317	60866	TANDEM MASS-SPECTROMETRY	Tandem Mass Spectrometry
ID	9	61317	60866	IN-VITRO CYTOTOXICITY	In Vitro Cytotoxicity
ID	10	61317	60866	HUMAN PROXIMAL TUBULE	Human Proximal Tubule

Record 409 | WOS:000377720700011

Inhibition of CXCL12-mediated chemotaxis of Jurkat cells by direct immunotoxicants

Field	Position	Original line	Normalised line	Original value	Normalised value
DE	1	61766	61313	Direct immunotoxicity	Direct Immunotoxicity
DE	3	61766	61313	CXCL12	Cxcl12
ID	1	61767	61314	CXCR4 CHEMOKINE RECEPTOR	Cxcr4 Chemokine Receptor
ID	2	61767	61314	LEUKOCYTE MIGRATION	Leukocyte Migration
ID	3	61767	61314	MAMMALIAN TARGET	Mammalian Target
ID	4	61767	61314	IMMUNE-RESPONSE	Immune Response
ID	5	61767	61314	UP-REGULATION	Up Regulation
ID	6	61767	61314	KINASE	Kinase
ID	7	61767	61314	EXPRESSION	Expression
ID	8	61767	61314	RAPAMYCIN	Rapamycin
ID	9	61767	61314	IDENTIFICATION	Identification
ID	10	61767	61314	HEMATOPOIESIS	Hematopoiesis

Record 410 | WOS:000376990200009

Cytotoxicity, apoptosis, DNA damage and methylation in mammary and kidney epithelial cell lines exposed to ochratoxin A

Field	Position	Original line	Normalised line	Original value	Normalised value
DE	3	61884	61430	Ochratoxin A	Ochratoxin A (OTA)
DE	4	61884	61430	5-mC	5 Mc
DE	5	61884	61430	8-OHdG	8 Ohdg
ID	1	61885	61431	IN-VITRO	In-Vitro
ID	2	61885	61431	OXIDATIVE STRESS	Oxidative Stress
ID	3	61885	61431	PHASE ARREST	Phase Arrest
ID	4	61885	61431	MYCOTOXINS	Mycotoxins
ID	5	61885	61431	TOXICITY	Toxicity
ID	6	61885	61431	CHROMATIN	Chromatin
ID	7	61885	61431	BINDING	Binding
ID	8	61885	61431	FORM	Form

Record 411 | WOS:000378297500009

Effects of Ochratoxin A on Mouse Oocyte Maturation and Fertilization, and Apoptosis During Fetal Development

Field	Position	Original line	Normalised line	Original value	Normalised value
DE	1	61969	61514	ochratoxin A	Ochratoxin A (OTA)
DE	2	61969	61514	apoptosis	Apoptosis
DE	3	61969	61514	oocyte maturation	Oocyte Maturation
DE	4	61969	61514	embryonic development	Embryonic Development
ID	1	61970	61515	EMBRYONIC STEM-CELLS	Embryonic Stem
ID	2	61970	61515	DEVELOPMENT IN-VITRO	Development In Vitro
ID	3	61970	61515	OXIDATIVE STRESS	Oxidative Stress
ID	4	61970	61515	GINKGOLIDE-B	Ginkgolide B
ID	5	61970	61515	TOXICITY	Toxicity
ID	6	61970	61515	DEATH	Death
ID	7	61970	61515	BLASTOCYSTS	Blastocyst

ID	8	61970	61515	INJURY	Injury
ID	9	61970	61515	EMODIN	Emodin
ID	10	61970	61515	MASS	Mass

Record 412 | WOS:000375813200002

Innovative technologies for the mitigation of mycotoxins in animal feed and ingredients-A review of recent patents

Field	Position	Original line	Normalised line	Original value	Normalised value
DE	1	62083	61627	Mycotoxin	Mycotoxins
DE	4	62083	61627	Bio-transformation	Bio Transformation
DE	5	62083	61627	Enzymes	Enzyme
ID	1	62084	61628	SODIUM CALCIUM ALUMINOSILICATE	Sodium Calcium Aluminosilicate
ID	2	62084	61628	PARACASEI SUBSP TOLERANS	Paracasei Subsp Toleran
ID	3	62084	61628	AFLATOXIN B-1	Aflatoxin B1 (AFB1)
ID	4	62084	61628	IN-VITRO	In-Vitro
ID	5	62084	61628	FUSARIUM-GRAMINEARUM	Fusarium Graminearum
ID	6	62084	61628	ANTIFUNGAL ACTIVITY	Antifungal Activity
ID	7	62084	61628	SURFACE BINDING	Surface Binding
ID	8	62084	61628	DEOXYNIVALENOL	Deoxynivalenol (DON)
ID	9	62084	61628	TOXICITY	Toxicity
ID	10	62084	61628	LACTOBACILLUS	Lactobacillus

Record 413 | WOS:000373624800006

Multiparametric luminescent cell viability assay in toxicology models: A critical evaluation

Field	Position	Original line	Normalised line	Original value	Normalised value
DE	1	62217	61760	Cell cultures	Cell
DE	2	62217	61760	ATP depletion	Atp Depletion
DE	3	62217	61760	Perchloric acid extraction	Perchloric Acid Extraction
DE	4	62217	61760	Luminescence viability assays	Luminescence Viability Assay
DE	5	62217	61760	ATP/protein ratio	Atp Protein Ratio
DE	6	62217	61760	DAPI staining	Dapi Staining
ID	1	62219	61762	IN-VITRO	In-Vitro
ID	2	62219	61762	ATP DEPLETION	Atp Depletion
ID	3	62219	61762	ADENOSINE-TRIPHOSPHATE	Adenosine Triphosphate
ID	4	62219	61762	TRITON X-100	Triton X 100
ID	5	62219	61762	APOPTOSIS	Apoptosis
ID	6	62219	61762	CALCEIN	Calcein
ID	7	62219	61762	PROTEIN	Protein
ID	8	62219	61762	QUANTITATION	Quantitation
ID	9	62219	61762	TOXICITY	Toxicity
ID	10	62219	61762	ACTIVATION	Activation

Record 414 | WOS:000377731900003

Mycotoxins modify the barrier function of Caco-2 cells through differential gene expression of specific claudin isoforms: Protective effect of illite mineral clay

Field	Position	Original line	Normalised line	Original value	Normalised value
DE	2	62337	61879	Caco-2-cells	Caco-2
DE	4	62337	61879	Barrier function	Barrier Function
DE	5	62337	61879	Tight junctions	Tight Junction
ID	1	62339	61880	SUICIDAL ERYTHROCYTE DEATH	Suicidal Erythrocyte Death
ID	2	62339	61880	INTESTINAL BARRIER	Intestinal Barrier
ID	3	62339	61880	TIGHT JUNCTIONS	Tight Junction

ID	4	62339	61880	FUMONISIN B-1	Fumonisin B1 (FB1)
ID	5	62339	61880	OCHRATOXIN-A	Ochratoxin A (OTA)
ID	6	62339	61880	AFLATOXIN B-1	Aflatoxin B1 (AFB1)
ID	7	62339	61880	T-2 TOXIN	T 2 Toxin
ID	8	62339	61880	IN-VIVO	In-Vivo
ID	9	62339	61880	TOXICITY	Toxicity
ID	10	62339	61880	PROTEIN	Protein

Record 415 | WOS:000372000700010

Alleviative effect of myricetin on ochratoxin A-induced oxidative stress in rat renal cortex: histological and biochemical study

Field	Position	Original line	Normalised line	Original value	Normalised value
DE	2	62491	62031	Ochratoxin A	Ochratoxin A (OTA)
DE	3	62491	62031	Oxidative stress	Oxidative Stress
DE	5	62491	62031	Renal cortex	Renal Cortex
ID	1	62492	62032	TRANSFORMING GROWTH FACTOR-BETA(1)	Transforming Growth Factor Beta 1
ID	2	62492	62032	COENZYME Q(10)	Coenzyme Q 10
ID	3	62492	62032	IN-VITRO	In-Vitro
ID	4	62492	62032	APOPTOSIS	Apoptosis
ID	5	62492	62032	TOXICITY	Toxicity
ID	6	62492	62032	CANCER	Cancer
ID	7	62492	62032	DAMAGE	Damage
ID	8	62492	62032	KIDNEY	Kidney
ID	9	62492	62032	LIVER	Liver
ID	10	62492	62032	MELATONIN	Melatonin

Record 416 | WOS:000375861700010

Natural Co-Occurrence of Mycotoxins in Foods and Feeds and Their in vitro Combined Toxicological Effects

Field	Position	Original line	Normalised line	Original value	Normalised value
DE	1	62621	62160	mycotoxins	Mycotoxins
DE	2	62621	62160	foodstuffs	Foodstuff
DE	3	62621	62160	regulations	Regulation
DE	4	62621	62160	co-occurrence	Co Occurrence
DE	5	62621	62160	combined toxicological effects	Combined Toxicological Effect
ID	1	62623	62162	EMERGING FUSARIUM-MYCOTOXINS	Emerging Fusarium Mycotoxin
ID	2	62623	62162	KIDNEY PK15 CELLS	Kidney Pk15
ID	3	62623	62162	A TRICHOTHECENE MYCOTOXINS	A Trichothecene Mycotoxin
ID	4	62623	62162	CEREAL-BASED FOODS	Cereal Based Food
ID	5	62623	62162	OCHRATOXIN-A	Ochratoxin A (OTA)
ID	6	62623	62162	FUMONISIN B-1	Fumonisin B1 (FB1)
ID	7	62623	62162	AFLATOXIN B-1	Aflatoxin B1 (AFB1)
ID	8	62623	62162	BREAKFAST CEREALS	Breakfast Cereal
ID	9	62623	62162	LIQUID-CHROMATOGRAPHY	Liquid Chromatography
ID	10	62623	62162	CYCLOPIAZONIC ACID	Cyclopiazonic Acid

Record 418 | WOS:000372963300003

Ochratoxin A and T-2 Toxin Induce Clonogenicity and Cell Migration in Human Colon Carcinoma and Fetal Lung Fibroblast Cell Lines

Field	Position	Original line	Normalised line	Original value	Normalised value
DE	1	62934	62471	Ochratoxin A	Ochratoxin A (OTA)
DE	2	62934	62471	T2-Toxin	T2 Toxin
DE	3	62934	62471	Aflatoxin B1	Aflatoxin B1 (AFB1)
ID	1	62935	62473	NF-KAPPA-B	Nf Kappa B

ID	2	62935	62473	C-MYC	C Myc
ID	3	62935	62473	INTERCELLULAR COMMUNICATION	Intercellular Communication
ID	4	62935	62473	RAT-LIVER	Rat Liver
ID	5	62935	62473	MYCOTOXINS	Mycotoxins
ID	6	62935	62473	FUSARIUM	Fusarium
ID	7	62935	62473	ACTIVATION	Activation
ID	8	62935	62473	APOPTOSIS	Apoptosis
ID	9	62935	62473	NEPHROPATHY	Nephropathy
ID	10	62935	62473	ZEARALENONE	Zearalenone (ZEA)

Record 419 | WOS:000370832600004

Malignant transformation of human gastric epithelium cells via reactive oxygen species production and Wnt/Î²-catenin pathway activation following 40-week exposure to ochratoxin A

Field	Position	Original line	Normalised line	Original value	Normalised value
DE	1	63060	62597	Ochratoxin A	Ochratoxin A (OTA)
DE	2	63060	62597	Malignant transformation	Malignant Transformation
DE	3	63060	62597	Oxidative stress	Oxidative Stress
DE	4	63060	62597	Wnt/beta-catenin signaling pathway	Wnt Beta Catenin Signaling Pathway
DE	5	63060	62597	Xenografts	Xenograft
ID	1	63062	62599	OXIDATIVE DNA-DAMAGE	Oxidative DNA Damage
ID	2	63062	62599	BETA-CATENIN	Beta Catenin
ID	3	63062	62599	TUMOR PROGRESSION	Tumor Progression
ID	4	63062	62599	PHASE ARREST	Phase Arrest
ID	5	63062	62599	CANCER	Cancer
ID	6	63062	62599	STRESS	Stress
ID	7	63062	62599	TUMORIGENESIS	Tumorigenesis
ID	8	63062	62599	CARCINOGENESIS	Carcinogenesis
ID	9	63062	62599	METASTASIS	Metastasis
ID	10	63062	62599	INHIBITION	Inhibition

Record 420 | WOS:000369179000011

Effects of aflatoxin B1 on oxidative stress markers and apoptosis of spleens in broilers

Field	Position	Original line	Normalised line	Original value	Normalised value
DE	1	63175	62711	Aflatoxin B-1	Aflatoxin B1 (AFB1)
DE	2	63175	62711	oxidative stress	Oxidative Stress
DE	3	63175	62711	apoptosis	Apoptosis
DE	4	63175	62711	flow cytometry	Flow Cytometry
DE	5	63175	62711	spleen	Spleen
ID	1	63176	62712	LYMPHOCYTES IN-VITRO	Lymphocyte In Vitro
ID	2	63176	62712	LIPID-PEROXIDATION	Lipid Peroxidation
ID	3	63176	62712	DNA-DAMAGE	DNA Damage
ID	4	63176	62712	PERITONEAL-MACROPHAGES	Peritoneal Macrophage
ID	5	63176	62712	SPLENIC LYMPHOCYTES	Splenic Lymphocyte
ID	6	63176	62712	PROTECTIVE ROLE	Protective Role
ID	7	63176	62712	OCHRATOXIN-A	Ochratoxin A (OTA)
ID	8	63176	62712	RAT-LIVER	Rat Liver
ID	9	63176	62712	PROTEIN	Protein
ID	10	63176	62712	CELLS	Cell

Record 421 | WOS:000370094500037

Grafting of gallic acid onto chitosan nano particles enhances antioxidant activities in vitro and protects against ochratoxin A toxicity in catfish (Clarias gariepinus)

Field	Position	Original line	Normalised line	Original value	Normalised value
DE	1	63286	62821	Ochratoxin A	Ochratoxin A (OTA)
DE	2	63286	62821	Chitosan nanoparticles	Chitosan Nanoparticle
DE	3	63286	62821	Gallic acid	Gallic Acid
DE	4	63286	62821	Octyl gallate	Octyl Gallate
ID	1	63288	62823	LACCASE-MEDIATED FUNCTIONALIZATION	Laccase Mediated Functionalization
ID	2	63288	62823	INDUCED OXIDATIVE STRESS	Induced Oxidative Stress
ID	3	63288	62823	NANOPARTICLES	Nanoparticle
ID	4	63288	62823	FOOD	Food
ID	5	63288	62823	GENOTOXICITY	Genotoxicity
ID	6	63288	62823	CARCINOGENICITY	Carcinogenicity
ID	7	63288	62823	NEPHROPATHY	Nephropathy
ID	8	63288	62823	PREVENTION	Prevention
ID	9	63288	62823	EXPOSURE	Exposure
ID	10	63288	62823	REMOVAL	Removal

Record 422 | WOS:000388323700013

Mycotoxin Outbreak in Animal Feed

Field	Position	Original line	Normalised line	Original value	Normalised value
ID	1	63417	62951	HEAD BLIGHT RESISTANCE	Head Blight Resistance
ID	2	63417	62951	OCHRATOXIN-A	Ochratoxin A (OTA)
ID	3	63417	62951	BIOLOGICAL DETOXIFICATION	Biological Detoxification
ID	4	63417	62951	FIELD OUTBREAKS	Field Outbreak
ID	5	63417	62951	PLANT-DISEASE	Plant Disease
ID	6	63417	62951	CARRY-OVER	Carry Over
ID	7	63417	62951	IN-VIVO	In-Vivo
ID	8	63417	62951	DEOXYNIVALENOL	Deoxynivalenol (DON)
ID	9	63417	62951	FOOD	Food
ID	10	63417	62951	FUSARIUM	Fusarium

Record 423 | WOS:000368966200007

Ochratoxin A-induced cytotoxicity, genotoxicity and reactive oxygen species in kidney cells: An integrative approach of complementary endpoints

Field	Position	Original line	Normalised line	Original value	Normalised value
DE	1	63648	63181	Ochratoxin A	Ochratoxin A (OTA)
DE	4	63648	63181	Reactive oxygen species	Reactive Oxygen Specy
DE	5	63648	63181	Antioxidants	Antioxidant
DE	6	63648	63181	MnTnHex-2-PyP	Mntnhex 2 Pyp
ID	1	63650	63183	OXIDATIVE DNA-DAMAGE	Oxidative DNA Damage
ID	2	63650	63183	IN-VITRO	In-Vitro
ID	3	63650	63183	PROTECTIVE ROLE	Protective Role
ID	4	63650	63183	PHASE ARREST	Phase Arrest
ID	5	63650	63183	HEPG2 CELLS	HepG2
ID	6	63650	63183	SUPEROXIDE-DISMUTASE	Superoxide Dismutase
ID	7	63650	63183	COMET ASSAY	Comet Assay
ID	8	63650	63183	VITAMIN-E	Vitamin E
ID	9	63650	63183	TOXICITY	Toxicity
ID	10	63650	63183	MECHANISMS	Mechanism

Record 424 | WOS:000371816100005

Individual and combined effects of roquefortine C and mycophenolic acid on human monocytic and intestinal cells

Field	Position	Original line	Normalised line	Original value	Normalised value
DE	1	63797	63328	apoptosis	Apoptosis
DE	2	63797	63328	Caco-2 cells	Caco-2
DE	3	63797	63328	monocytic cells	Monocytic

DE	4	63797	63328	mycophenolic acid	Mycophenolic Acid
DE	5	63797	63328	roquefortine C	Roquefortine C
ID	1	63799	63329	IN-VITRO PROLIFERATION	In Vitro Proliferation
ID	2	63799	63329	OCHRATOXIN-A	Ochratoxin A (OTA)
ID	3	63799	63329	PENICILLIUM MYCOTOXINS	Penicillium Mycotoxin
ID	4	63799	63329	CYCLOPIAZONIC ACID	Cyclopiazonic Acid
ID	5	63799	63329	CYTOTOXICITY	Cytotoxicity
ID	6	63799	63329	METABOLITES	Metabolite
ID	7	63799	63329	CACO-2	Caco-2
ID	8	63799	63329	INHIBITION	Inhibition
ID	9	63799	63329	TOXICITY	Toxicity
ID	10	63799	63329	FUSARIUM	Fusarium

Record 425 | WOS:000376604100010

Evaluation of renal in vitro models used in ochratoxin research

Field	Position	Original line	Normalised line	Original value	Normalised value
DE	1	63892	63421	ochratoxin A	Ochratoxin A (OTA)
DE	2	63892	63421	in vitro vs in vivo	In Vitro Vs In Vivo
DE	3	63892	63421	cell culture quality control	Cell Culture Quality Control
ID	1	63893	63422	PROXIMAL TUBULAR CELLS	Proximal Tubular
ID	2	63893	63422	CITRININ INDUCED NEPHROSIS	Citrinin Induced Nephrosis
ID	3	63893	63422	A-INDUCED GENOTOXICITY	A Induced Genotoxicity
ID	4	63893	63422	DNA ADDUCT FORMATION	DNA Adduct Formation
ID	5	63893	63422	BALKAN ENDEMIC NEPHROPATHY	Balkan Endemic Nephropathy
ID	6	63893	63422	KIDNEY EPITHELIAL-CELLS	Kidney Epithelial
ID	7	63893	63422	DOUBLE-STRAND BREAKS	Double Strand Break
ID	8	63893	63422	NEPHROTOXIN OCHRATOXIN	Ochratoxin A (OTA)
ID	9	63893	63422	ARISTOLOCHIC ACID	Aristolochic Acid
ID	10	63893	63422	MYCOTOXIC NEPHROPATHY	Mycotoxic Nephropathy

Record 426 | WOS:000389199300011

Diet contaminated with ochratoxin A at the highest level allowed by EU recommendation disturbs liver metabolism in weaned piglets

Field	Position	Original line	Normalised line	Original value	Normalised value
DE	1	64196	63723	ochratoxin A	Ochratoxin A (OTA)
DE	2	64196	63723	swine	Swine
DE	3	64196	63723	mycotoxin	Mycotoxins
DE	4	64196	63723	hepatotoxicity	Hepatotoxicity
ID	1	64197	63724	OXIDATIVE STRESS	Oxidative Stress
ID	2	64197	63724	TOXICITY	Toxicity
ID	3	64197	63724	MYCOTOXINS	Mycotoxins
ID	4	64197	63724	NEPHROPATHY	Nephropathy
ID	5	64197	63724	ZEARALENONE	Zearalenone (ZEA)
ID	6	64197	63724	EXPRESSION	Expression
ID	7	64197	63724	KIDNEY	Kidney
ID	8	64197	63724	FEEDS	Feed
ID	9	64197	63724	FOCUS	Focus

Record 427 | WOS:000368260800005

Ochratoxin A as possible factor trigging autism and its male prevalence via epigenetic mechanism

Field	Position	Original line	Normalised line	Original value	Normalised value
DE	2	64310	63836	Male-prevalence	Male Prevalence
DE	6	64310	63836	Ochratoxin A	Ochratoxin A (OTA)
ID	1	64312	63838	SPECTRUM DISORDERS	Spectrum Disorder
ID	2	64312	63838	GENE	Gene
ID	3	64312	63838	PROFILES	Profile

Record 428 ǀ WOS:000387395900007

In Vitro Assays to Estimate the Toxicological Effects of Masked Mycotoxins

Field	Position	Original line	Normalised line	Original value	Normalised value
ID	1	64394	63919	DIGESTION MODEL	Digestion Model
ID	2	64394	63919	OCHRATOXIN-A	Ochratoxin A (OTA)
ID	3	64394	63919	ENTEROHEPATIC CIRCULATION	Enterohepatic Circulation
ID	4	64394	63919	PROTEIN-SYNTHESIS	Protein Synthesis
ID	5	64394	63919	DRUG DISCOVERY	Drug Discovery
ID	6	64394	63919	FUMONISIN B-1	Fumonisin B1 (FB1)
ID	7	64394	63919	WHEAT-GRAIN	Wheat Grain
ID	8	64394	63919	T-2 TOXIN	T 2 Toxin
ID	9	64394	63919	ZEARALENONE	Zearalenone (ZEA)
ID	10	64394	63919	DEOXYNIVALENOL	Deoxynivalenol (DON)

Record 429 ǀ WOS:000384724200015

Probiotic preparation reduces faecal water genotoxicity and cytotoxicity in chickens fed ochratoxin A contaminated feed (in vivo study)

Field	Position	Original line	Normalised line	Original value	Normalised value
DE	1	64567	64091	ochratoxin A	Ochratoxin A (OTA)
DE	2	64567	64091	probiotics	Probiotics
DE	3	64567	64091	chicken	Chicken
DE	4	64567	64091	genotoxicity	Genotoxicity
DE	5	64567	64091	cytotoxicity	Cytotoxicity
ID	1	64568	64092	AFLATOXIN B-1	Aflatoxin B1 (AFB1)
ID	2	64568	64092	SACCHAROMYCES-CEREVISIAE	Saccharomyce Cerevisiae
ID	4	64568	64092	BACTERIA	Bacteria
ID	5	64568	64092	STRAINS	Strain
ID	6	64568	64092	ADDUCTS	Adduct
ID	7	64568	64092	DAMAGE	Damage
ID	8	64568	64092	MODE	Mode

Record 430 ǀ WOS:000366832700013

miR-122 plays an important role in ochratoxin A-induced hepatocyte apoptosis in vitro and in vivo

Field	Position	Original line	Normalised line	Original value	Normalised value
ID	1	64681	64204	HUMAN HEPATOCELLULAR-CARCINOMA	Human Hepatocellular Carcinoma
ID	2	64681	64204	P53 ACTIVITY	P53 Activity
ID	3	64681	64204	CYCLIN G1	Cyclin G1
ID	4	64681	64204	CELLS	Cell
ID	5	64681	64204	MICRORNA-122	Microrna 122
ID	6	64681	64204	EXPRESSION	Expression
ID	7	64681	64204	LIVER	Liver
ID	8	64681	64204	GENE	Gene
ID	9	64681	64204	PROLIFERATION	Proliferation
ID	10	64681	64204	REPLICATION	Replication

Record 431 ǀ WOS:000367635500005

MiR-122 partly mediates the ochratoxin A-induced GC-2 cell apoptosis

Field	Position	Original line	Normalised line	Original value	Normalised value
DE	1	64799	64321	Ochratoxin A	Ochratoxin A (OTA)
DE	2	64799	64321	GC-2 cell	Gc 2
DE	3	64799	64321	miR-122	Mir 122
DE	4	64799	64321	Cell apoptosis	Cell Apoptosis
ID	1	64800	64322	HUMAN HEPATOCELLULAR-CARCINOMA	Human Hepatocellular Carcinoma
ID	2	64800	64322	IN-VITRO	In-Vitro
ID	3	64800	64322	CYCLIN-G	Cyclin G
ID	4	64800	64322	MDCK-C7 CELLS	Mdck C7
ID	5	64800	64322	MICRORNA	Microrna
ID	6	64800	64322	GENE	Gene
ID	7	64800	64322	TARGET	Target
ID	8	64800	64322	RATS	Rat
ID	10	64800	64322	EXPRESSION	Expression

Record 432 | WOS:000367013500020

Interaction of Citrinin with Human Serum Albumin

Field	Position	Original line	Normalised line	Original value	Normalised value
DE	1	64914	64435	citrinin	Citrinin
DE	2	64914	64435	human serum albumin	Human Serum Albumin
DE	3	64914	64435	fluorescence spectroscopy	Fluorescence Spectroscopy
DE	4	64914	64435	ultrafiltration	Ultrafiltration
DE	5	64914	64435	species differences	Specy Difference
ID	1	64916	64437	BINDING-SITE	Binding Site
ID	2	64916	64437	COMPETITIVE INTERACTIONS	Competitive Interaction
ID	3	64916	64437	FLAVONOID AGLYCONES	Flavonoid Aglycone
ID	4	64916	64437	MYCOTOXIN CITRININ	Mycotoxin Citrinin
ID	5	64916	64437	SUBDOMAIN-IIA	Subdomain Iia
ID	6	64916	64437	BLIND DOCKING	Blind Docking
ID	7	64916	64437	OCHRATOXIN-A	Ochratoxin A (OTA)
ID	8	64916	64437	PROTEIN	Protein
ID	9	64916	64437	WARFARIN	Warfarin
ID	10	64916	64437	TEMPERATURE	Temperature

Record 433 | WOS:000362726700025

The Phytoalexin Resveratrol Ameliorates Ochratoxin A Toxicity in Human Embryonic Kidney (HEK293) Cells

Field	Position	Original line	Normalised line	Original value	Normalised value
DE	1	65033	64553	RESVERATROL	Resveratrol
DE	2	65033	64553	OCHRATOXIN A	Ochratoxin A (OTA)
DE	3	65033	64553	OXIDATIVE STRESS	Oxidative Stress
DE	4	65033	64553	LONP1	Lonp1
DE	5	65033	64553	HEK293 CELLS	Hek293
ID	1	65034	64554	OXIDATIVE STRESS	Oxidative Stress
ID	2	65034	64554	PROTECTIVE ROLE	Protective Role
ID	3	65034	64554	LON PROTEASE	Lon Protease
ID	4	65034	64554	METABOLISM	Metabolism
ID	5	65034	64554	SIRT1	Sirt1
ID	6	65034	64554	CARCINOGENICITY	Carcinogenicity
ID	7	65034	64554	ACTIVATION	Activation
ID	8	65034	64554	DAMAGE	Damage

Record 434 | WOS:000363821500008

A food contaminant ochratoxin A suppresses pregnane X receptor (PXR)-mediated CYP3A4 induction in primary cultures of human hepatocytes

Field	Position	Original line	Normalised line	Original value	Normalised value
DE	1	65121	64640	Ochratoxin A	Ochratoxin A (OTA)
DE	2	65121	64640	CYP3A4	Cyp3a4
DE	3	65121	64640	PXR	Pxr
DE	4	65121	64640	miR-148a	Mir 148a
ID	1	65122	64641	MESSENGER-RNA EXPRESSION	Messenger RNA Expression
ID	2	65122	64641	GENE-EXPRESSION	Gene Expression
ID	3	65122	64641	NUCLEAR RECEPTORS	Nuclear Receptor
ID	4	65122	64641	IN-VITRO	In-Vitro
ID	5	65122	64641	RATS	Rat
ID	6	65122	64641	PXR	Pxr
ID	7	65122	64641	NEPHROTOXICITY	Nephrotoxicity
ID	8	65122	64641	TRANSCRIPTION	Transcription
ID	9	65122	64641	ACTIVATION	Activation
ID	10	65122	64641	TOXICITY	Toxicity

Record 435 | WOS:000363259300002

In vitro exposure of Penicillium mycotoxins with or without a modified yeast cell wall extract (mYCW) on bovine macrophages (BoMacs)

Field	Position	Original line	Normalised line	Original value	Normalised value
DE	1	65213	64731	Penicillium mycotoxins	Penicillium Mycotoxin
DE	2	65213	64731	Bovine macrophage	Bovine Macrophage
DE	3	65213	64731	In vitro bioassay	In Vitro Bioassay
DE	5	65213	64731	Yeast cell wall extract	Yeast Cell Wall Extract
ID	1	65215	64733	OCHRATOXIN-A	Ochratoxin A (OTA)
ID	2	65215	64733	AFLATOXIN B-1	Aflatoxin B1 (AFB1)
ID	3	65215	64733	T-2 TOXIN	T 2 Toxin
ID	4	65215	64733	ESTERIFIED-GLUCOMANNAN	Esterified Glucomannan
ID	5	65215	64733	DESOXYPATULINIC ACID	Desoxypatulinic Acid
ID	6	65215	64733	MAIZE SILAGE	Maize Silage
ID	7	65215	64733	CORN-SILAGE	Corn Silage
ID	8	65215	64733	PATULIN	Patulin
ID	9	65215	64733	ZEARALENONE	Zearalenone (ZEA)
ID	10	65215	64733	CONTAMINATION	Contamination

Record 436 | WOS:000360322300014

Ochratoxin A at low concentrations inhibits in vitro growth of canine umbilical cord matrix mesenchymal stem cells through oxidative chromatin and DNA damage

Field	Position	Original line	Normalised line	Original value	Normalised value
DE	2	65324	64841	Umbilical cord matrix mesenchymal stem	Umbilical Cord Matrix Mesenchymal Stem
DE	3	65324	64841	cells	Cell
DE	4	65324	64841	Ochratoxin A	Ochratoxin A (OTA)
DE	6	65324	64841	Oxidative DNA damage	Oxidative DNA Damage
ID	1	65326	64843	NEPHRITOGENIC OCHRATOXIN	Ochratoxin A (OTA)
ID	2	65326	64843	DIFFERENTIATION POTENCY	Differentiation Potency
ID	3	65326	64843	FETAL ADNEXA	Fetal Adnexa
ID	4	65326	64843	TEST EST	Test Est
ID	5	65326	64843	APOPTOSIS	Apoptosis
ID	6	65326	64843	TOXICITY	Toxicity
ID	7	65326	64843	MECHANISMS	Mechanism
ID	8	65326	64843	ANIMALS	Animal
ID	9	65326	64843	STRESS	Stress
ID	10	65326	64843	BLOOD	Blood

Record 437 | WOS:000365417800020

The influence of ochratoxin A on DNA adduct formation by the carcinogen aristolochic acid in rats

Field	Position	Original line	Normalised line	Original value	Normalised value
DE	1	65486	65002	Balkan endemic nephropathy	Balkan Endemic Nephropathy
DE	2	65486	65002	Aristolochic acid nephropathy	Aristolochic Acid Nephropathy
DE	3	65486	65002	Aristolochic acid	Aristolochic Acid
DE	4	65486	65002	Ochratoxin A	Ochratoxin A (OTA)
DE	5	65486	65002	DNA adducts	DNA Adduct
ID	1	65488	65004	BALKAN ENDEMIC NEPHROPATHY	Balkan Endemic Nephropathy
ID	2	65488	65004	CHINESE HERBS NEPHROPATHY	Chinese Herb Nephropathy
ID	3	65488	65004	CYTOCHROMES P450 1A1	Cytochrome P450 1a1
ID	4	65488	65004	P-32 POSTLABELING ANALYSIS	P 32 Postlabeling Analysis
ID	5	65488	65004	METABOLIC-ACTIVATION	Metabolic Activation
ID	6	65488	65004	REDUCTIVE ACTIVATION	Reductive Activation
ID	7	65488	65004	UROTHELIAL CANCER	Urothelial Cancer
ID	8	65488	65004	NAD(P)H/QUINONE OXIDOREDUCTASE	Nad P H Quinone Oxidoreductase
ID	9	65488	65004	ENVIRONMENTAL EXPOSURE	Environmental Exposure
ID	10	65488	65004	OXIDATIVE STRESS	Oxidative Stress

Record 438 | WOS:000357839100054

Cross-linked chitosan polymers as generic adsorbents for simultaneous adsorption of multiple mycotoxins

Field	Position	Original line	Normalised line	Original value	Normalised value
DE	1	65674	65189	Multiple mycotoxins	Multiple Mycotoxin
DE	2	65674	65189	Cross-linked chitosan	Cross Linked Chitosan
DE	4	65674	65189	In vitro	In-Vitro
DE	5	65674	65189	Adsorption isotherm model	Adsorption Isotherm Model
ID	1	65676	65191	IN-VITRO	In-Vitro
ID	2	65676	65191	OCHRATOXIN-A	Ochratoxin A (OTA)
ID	3	65676	65191	AFLATOXIN B-1	Aflatoxin B1 (AFB1)
ID	4	65676	65191	RED WINE	Red Wine
ID	5	65676	65191	ZEARALENONE	Zearalenone (ZEA)
ID	6	65676	65191	REMOVAL	Removal
ID	7	65676	65191	CHITIN	Chitin
ID	8	65676	65191	TOXICITY	Toxicity
ID	9	65676	65191	BINDING	Binding
ID	10	65676	65191	BIOACCESSIBILITY	Bioaccessibility

Record 440 | WOS:000361263500036

Cytotoxicity of the mycotoxins deoxynivalenol and ochratoxin A on Caco-2 cell line in presence of resveratrol

Field	Position	Original line	Normalised line	Original value	Normalised value
DE	2	65842	65355	Mycotoxin co-exposition	Mycotoxin Co Exposition
DE	3	65842	65355	In vitro cytotoxicity	In Vitro Cytotoxicity
DE	4	65842	65355	Oxidative stress	Oxidative Stress
DE	5	65842	65355	ROS production	ROS Production
ID	1	65844	65357	A-INDUCED CYTOTOXICITY	A Induced Cytotoxicity
ID	2	65844	65357	DNA-DAMAGE	DNA Damage
ID	3	65844	65357	IN-VITRO	In-Vitro
ID	4	65844	65357	OXIDATIVE STRESS	Oxidative Stress
ID	5	65844	65357	MICROMOLAR CONCENTRATIONS	Micromolar Concentration
ID	6	65844	65357	EXPOSURE ASSESSMENT	Exposure Assessment

ID	7	65844	65357	HEPG2 CELLS	HepG2
ID	8	65844	65357	APOPTOSIS	Apoptosis
ID	9	65844	65357	TOXICITY	Toxicity
ID	10	65844	65357	TRICHOTHECENES	Trichothecene

Record 441 | WOS:000367407400003

Dietary mycotoxins, co-exposure, and carcinogenesis in humans: Short review

Field	Position	Original line	Normalised line	Original value	Normalised value
DE	3	65958	65470	Mycotoxin co-exposure	Mycotoxin Co Exposure
DE	4	65958	65470	Mycotoxin carcinogenicity	Mycotoxin Carcinogenicity
DE	5	65958	65470	Colorectal cancer	Colorectal Cancer
DE	6	65958	65470	Hepatocellular cancer	Hepatocellular Cancer
ID	1	65960	65472	HEPATITIS-B-VIRUS	Hepatitis B Virus
ID	2	65960	65472	OCHRATOXIN-A	Ochratoxin A (OTA)
ID	3	65960	65472	AFLATOXIN B-1	Aflatoxin B1 (AFB1)
ID	4	65960	65472	DNA-DAMAGE	DNA Damage
ID	5	65960	65472	X-PROTEIN	X Protein
ID	6	65960	65472	ERGOT ALKALOIDS	Ergot Alkaloid
ID	7	65960	65472	FUMONISIN B-1	Fumonisin B1 (FB1)
ID	8	65960	65472	HUMAN COLON	Human Colon
ID	9	65960	65472	HEPATOCELLULAR-CARCINOMA	Hepatocellular Carcinoma
ID	10	65960	65472	NATURAL OCCURRENCE	Natural Occurrence

Record 442 | WOS:000364960400028

Comparative Ochratoxin Toxicity: A Review of the Available Data

Field	Position	Original line	Normalised line	Original value	Normalised value
DE	1	66128	65639	ochratoxin	Ochratoxin A (OTA)
DE	2	66128	65639	biosynthesis	Biosynthesis
DE	3	66128	65639	metabolites	Metabolite
DE	4	66128	65639	detection	Detection
DE	5	66128	65639	comparative toxicity	Comparative Toxicity
ID	1	66129	65641	ESCHERICHIA-COLI STRAINS	Escherichia Coli Strain
ID	2	66129	65641	RENAL TUMOR-FORMATION	Renal Tumor Formation
ID	3	66129	65641	IN-VITRO	In-Vitro
ID	4	66129	65641	ASPERGILLUS-OCHRACEUS	Aspergillus Ochraceus
ID	5	66129	65641	MYCOTOXIN OCHRATOXIN	Ochratoxin A (OTA)
ID	6	66129	65641	PROTEIN-SYNTHESIS	Protein Synthesis
ID	7	66129	65641	DNA-ADDUCT	DNA Adduct
ID	8	66129	65641	CELL-LINE	Cell
ID	9	66129	65641	PENICILLIUM-NORDICUM	Penicillium Nordicum
ID	10	66129	65641	ENDEMIC NEPHROPATHY	Endemic Nephropathy

Record 443 | WOS:000355886400028

Development of an aptasensor for the fast detection of Versicolorin A

Field	Position	Original line	Normalised line	Original value	Normalised value
DE	3	66401	65912	Isothermal titration calorimetry	Isothermal Titration Calorimetry
DE	5	66401	65912	Aptamer-based solid phase extraction column	Aptamer Based Solid Phase Extraction Column
ID	1	66403	65914	ASPERGILLUS-FLAVUS	Aspergillus Flavus

ID	2	66403	65914	WATER ACTIVITY	Water Activity
ID	3	66403	65914	AFLATOXIN BIOSYNTHESIS	Aflatoxin Biosynthesis
ID	4	66403	65914	GENE-CLUSTER	Gene Cluster
ID	5	66403	65914	IN-VITRO	In-Vitro
ID	6	66403	65914	GROWTH	Growth
ID	7	66403	65914	TEMPERATURE	Temperature
ID	8	66403	65914	STRAINS	Strain
ID	9	66403	65914	CHROMATOGRAPHY	Chromatography
ID	10	66403	65914	MUTAGENICITY	Mutagenicity

Record 444 ǀ WOS:000359029000029

Cytotoxic and genotoxic potencies of single and combined spore extracts of airborne OTA-producing and OTA-non-producing Aspergilli in Human lung A549 cells

Field	Position	Original line	Normalised line	Original value	Normalised value
DE	1	66519	66028	A. sclerotiorum	A Sclerotiorum
DE	2	66519	66028	A. pseudoglaucus	A Pseudoglaucus
DE	3	66519	66028	Ochratoxin A	Ochratoxin A (OTA)
DE	4	66519	66028	Spore extracts	Spore Extract
ID	1	66521	66030	ACUTE INHALATION TOXICITY	Acute Inhalation Toxicity
ID	2	66521	66030	OCHRATOXIN-A	Ochratoxin A (OTA)
ID	3	66521	66030	OCCUPATIONAL-EXPOSURE	Occupational Exposure
ID	4	66521	66030	INHALED MYCOTOXINS	Inhaled Mycotoxin
ID	5	66521	66030	T-2 MYCOTOXIN	T 2 Mycotoxin
ID	6	66521	66030	COMET ASSAY	Comet Assay
ID	7	66521	66030	DNA-DAMAGE	DNA Damage
ID	8	66521	66030	AFLATOXIN	Aflatoxin
ID	9	66521	66030	FUNGI	Fungi
ID	10	66521	66030	METABOLITES	Metabolite

Record 445 ǀ WOS:000362622500013

Ochratoxin A induces DNA damage and G2 phase arrest in human esophageal epithelium Het-1A cells in vitro

Field	Position	Original line	Normalised line	Original value	Normalised value
DE	1	66659	66167	Ochratoxin A	Ochratoxin A (OTA)
DE	2	66659	66167	DNA damage	DNA Damage
DE	3	66659	66167	G2 phase arrest	G2 Phase Arrest
DE	5	66659	66167	Human esophageal epithelium cells	Human Esophageal Epithelium
ID	1	66661	66169	CANCER INCIDENCE	Cancer Incidence
ID	2	66661	66169	CYCLE ARREST	Cycle Arrest
ID	3	66661	66169	5 CONTINENTS	5 Continent
ID	4	66661	66169	G1 PHASE	G1 Phase
ID	5	66661	66169	GENOTOXICITY	Genotoxicity
ID	6	66661	66169	INSTABILITY	Instability
ID	7	66661	66169	ZEARALENONE	Zearalenone (ZEA)
ID	8	66661	66169	CHECKPOINTS	Checkpoint
ID	9	66661	66169	MECHANISMS	Mechanism
ID	10	66661	66169	MUTATIONS	Mutation

Record 446 ǀ WOS:000362860200019

Risk analysis of main mycotoxins occurring in food for children: An overview

Field	Position	Original line	Normalised line	Original value	Normalised value
DE	1	66773	66280	Deoxynivalenol	Deoxynivalenol (DON)
DE	2	66773	66280	Aflatoxins	Aflatoxin
DE	3	66773	66280	Ochratoxins	Ochratoxin A (OTA)
DE	5	66773	66280	Fumonisins	Fumonisin
DE	6	66773	66280	Baby food	Baby Food

DE	8	66773	66280	Risk analysis	Risk Analysis
ID	1	66775	66282	IN-VITRO BIOACCESSIBILITY	In Vitro Bioaccessibility
ID	2	66775	66282	AFLATOXIN B-1	Aflatoxin B1 (AFB1)
ID	3	66775	66282	OCHRATOXIN-A	Ochratoxin A (OTA)
ID	4	66775	66282	APPLE JUICE	Apple Juice
ID	5	66775	66282	DIETARY EXPOSURE	Dietary Exposure
ID	6	66775	66282	INFANT FORMULAS	Infant Formula
ID	7	66775	66282	YOUNG-CHILDREN	Young Children
ID	8	66775	66282	BABY FOODS	Baby Food
ID	9	66775	66282	FUSARIUM MYCOTOXINS	Fusarium Mycotoxin
ID	10	66775	66282	MOLECULAR-MECHANISM	Molecular Mechanism

Record 447 | WOS:000362551400018

Biomonitoring using dried blood spots: Detection of ochratoxin A and its degradation product 2'R-ochratoxin A in blood from coffee drinkers

Field	Position	Original line	Normalised line	Original value	Normalised value
DE	2	67017	66523	Dried blood spot	Dried Blood Spot
DE	4	67017	66523	Mycotoxin	Mycotoxins
DE	5	67017	66523	Ochratoxin	Ochratoxin A (OTA)
ID	1	67018	66524	2'-DEOXYGUANOSINE-CARBON 8-BOUND OCHRATOXIN	2 Deoxyguanosine Carbon 8 Bound Ochratoxin
ID	2	67018	66524	IN-VITRO CYTOTOXICITY	In Vitro Cytotoxicity
ID	3	67018	66524	MASS-SPECTROMETRY	Mass Spectrometry
ID	4	67018	66524	EXPOSURE	Exposure
ID	5	67018	66524	PLASMA	Plasma
ID	6	67018	66524	METABOLITE	Metabolite
ID	8	67018	66524	GENOTOXICITY	Genotoxicity
ID	9	67018	66524	MYCOTOXINS	Mycotoxins
ID	10	67018	66524	STABILITY	Stability

Record 448 | WOS:000361078800019

Hepatotoxic effect of ochratoxin A and citrinin, alone and in combination, and protective effect of vitamin E: In vitro study in HepG2 cell

Field	Position	Original line	Normalised line	Original value	Normalised value
DE	1	67125	66630	Mycotoxin	Mycotoxins
DE	3	67125	66630	Ochratoxin A	Ochratoxin A (OTA)
DE	5	67125	66630	Reactive oxygen species	Reactive Oxygen Specy
ID	1	67127	66632	HUMAN HEPATOMA-CELLS	Human Hepatoma
ID	2	67127	66632	MYCOTOXIN CITRININ	Mycotoxin Citrinin
ID	3	67127	66632	DNA-DAMAGE	DNA Damage
ID	4	67127	66632	POTENTIAL ROLE	Potential Role
ID	5	67127	66632	BLOOD-PLASMA	Blood Plasma
ID	6	67127	66632	COMET ASSAY	Comet Assay
ID	7	67127	66632	APOPTOSIS	Apoptosis
ID	8	67127	66632	CYTOTOXICITY	Cytotoxicity
ID	9	67127	66632	COOCCURRENCE	Cooccurrence
ID	10	67127	66632	TOXICITY	Toxicity

Record 449 | WOS:000361762200007

Effects of Dietary Exposure to Zearalenone (ZEN) on Carp (Cyprinus carpio L.)

Field	Position	Original line	Normalised line	Original value	Normalised value
DE	1	67280	66783	feed quality	Feed Quality
DE	2	67280	66783	aquaculture	Aquaculture
DE	3	67280	66783	blood cell populations	Blood Cell Population
DE	4	67280	66783	genotoxicity	Genotoxicity

DE	5	67280	66783	estrogenic potential	Estrogenic Potential
ID	1	67282	66785	ESTROGENIC MYCOTOXIN ZEARALENONE	Estrogenic Mycotoxin Zearalenone
ID	2	67282	66785	LIQUID-CHROMATOGRAPHY	Liquid Chromatography
ID	3	67282	66785	FUSARIUM MYCOTOXINS	Fusarium Mycotoxin
ID	4	67282	66785	RAINBOW-TROUT	Rainbow Trout
ID	5	67282	66785	IN-VITRO	In-Vitro
ID	6	67282	66785	BIOCHEMICAL PARAMETERS	Biochemical Parameter
ID	7	67282	66785	PERIPHERAL-BLOOD	Peripheral Blood
ID	8	67282	66785	OCHRATOXIN-A	Ochratoxin A (OTA)
ID	9	67282	66785	DEOXYNIVALENOL	Deoxynivalenol (DON)
ID	10	67282	66785	METABOLITES	Metabolite

Record 450 | WOS:000360343000001

Cytotoxic effects of oosporein isolated from endophytic fungus Cochliobolus kusanoi

Field	Position	Original line	Normalised line	Original value	Normalised value
DE	1	67424	66926	oosporein	Oosporein
DE	2	67424	66926	toxicity	Toxicity
DE	3	67424	66926	ROS	Reactive Oxygen Species (ROS)
DE	4	67424	66926	oxidative stress	Oxidative Stress
DE	5	67424	66926	realtime-Q-PCR	Realtime Q Pcr
ID	1	67425	66928	OCHRATOXIN-A	Ochratoxin A (OTA)
ID	2	67425	66928	TOXICITY	Toxicity
ID	3	67425	66928	DAMAGE	Damage
ID	4	67425	66928	GLUTATHIONE	Glutathione (GSH)
ID	5	67425	66928	METABOLITES	Metabolite
ID	6	67425	66928	BIOACTIVITY	Bioactivity
ID	7	67425	66928	MYCOTOXINS	Mycotoxins
ID	8	67425	66928	ADDUCTS	Adduct

Record 451 | WOS:000360202700022

Review on Mycotoxin Issues in Ruminants: Occurrence in Forages, Effects of Mycotoxin Ingestion on Health Status and Animal Performance and Practical Strategies to Counteract Their Negative Effects

Field	Position	Original line	Normalised line	Original value	Normalised value
ID	1	67528	67030	TOXIN-CONTAMINATED TRITICALE	Toxin Contaminated Triticale
ID	2	67528	67030	MICROBIAL PROTEIN-SYNTHESIS	Microbial Protein Synthesis
ID	3	67528	67030	IN-VITRO METABOLISM	In Vitro Metabolism
ID	4	67528	67030	CORN-SILAGE	Corn Silage
ID	5	67528	67030	DAIRY-COWS	Dairy Cow
ID	6	67528	67030	PENICILLIUM-ROQUEFORTI	Penicillium Roqueforti
ID	7	67528	67030	AEROBIC STABILITY	Aerobic Stability
ID	8	67528	67030	MAIZE SILAGE	Maize Silage
ID	9	67528	67030	SECONDARY METABOLITES	Secondary Metabolite
ID	10	67528	67030	FUSARIUM MYCOTOXINS	Fusarium Mycotoxin

Record 452 | WOS:000358238100012

Ochratoxin A activates opposing c-MET/PI3K/Akt and MAPK/ERK 1-2 pathways in human proximal tubule HK-2 cells

Field	Position	Original line	Normalised line	Original value	Normalised value

DE	1	67906	67407	Ochratoxin A	Ochratoxin A (OTA)
DE	2	67906	67407	ERK1-2	Erk1 2
DE	4	67906	67407	c-MET	C Met
DE	6	67906	67407	HK-2 cells	HK-2
ID	1	67907	67408	OXIDATIVE DNA-DAMAGE	Oxidative DNA Damage
ID	2	67907	67408	MET TYROSINE KINASE	Met Tyrosine Kinase
ID	3	67907	67408	PROTEIN-KINASE	Protein Kinase
ID	4	67907	67408	IN-VITRO	In-Vitro
ID	5	67907	67408	SIGNALING PATHWAYS	Signaling Pathway
ID	6	67907	67408	ERK ACTIVATION	Erk Activation
ID	7	67907	67408	HUMAN KIDNEY	Human Kidney
ID	8	67907	67408	INDUCED APOPTOSIS	Induced Apoptosis
ID	9	67907	67408	GROWTH-FACTOR	Growth Factor
ID	10	67907	67408	LUNG-CANCER	Lung Cancer

Record 453 | WOS:000357537400012

Effects of Chronic Ochratoxin A Exposure on p53 Heterozygous and p53 Homozygous Mice

Field	Position	Original line	Normalised line	Original value	Normalised value
DE	1	68060	67560	ochratoxin	Ochratoxin A (OTA)
DE	2	68060	67560	p53	P53
DE	3	68060	67560	mouse	Mouse
DE	4	68060	67560	carcinogenicity	Carcinogenicity
DE	5	68060	67560	chronic toxicity	Chronic Toxicity
ID	1	68061	67561	A-INDUCED GENOTOXICITY	A Induced Genotoxicity
ID	2	68061	67561	RENAL TUMOR-FORMATION	Renal Tumor Formation
ID	3	68061	67561	DOUBLE-STRAND BREAKS	Double Strand Break
ID	4	68061	67561	IN-VIVO	In-Vivo
ID	5	68061	67561	CELL-PROLIFERATION	Cell Proliferation
ID	6	68061	67561	ARISTOLOCHIC ACID	Aristolochic Acid
ID	7	68061	67561	RISK-ASSESSMENT	Risk Assessment
ID	8	68061	67561	HEALTH-RISK	Health Risk
ID	9	68061	67561	KIDNEY	Kidney
ID	10	68061	67561	MOUSE	Mouse

Record 454 | WOS:000355330300006

Neurotoxic effects of ochratoxin A on the subventricular zone of adult mouse brain

Field	Position	Original line	Normalised line	Original value	Normalised value
DE	1	68200	67699	Ochratoxin A	Ochratoxin A (OTA)
DE	2	68200	67699	mycotoxin	Mycotoxins
DE	3	68200	67699	subventricular zone	Subventricular Zone
DE	4	68200	67699	cell proliferation	Cell Proliferation
DE	5	68200	67699	cell viability	Cell Viability
DE	6	68200	67699	cell differentiation	Cell Differentiation
ID	1	68202	67701	NEURAL STEM-CELLS	Neural Stem
ID	2	68202	67701	CELLULAR COMPOSITION	Cellular Composition
ID	3	68202	67701	NEUROGENIC REGIONS	Neurogenic Region
ID	4	68202	67701	STRAND BREAKS	Strand Break
ID	5	68202	67701	IFN-GAMMA	Ifn Gamma
ID	6	68202	67701	RAT-BRAIN	Rat Brain
ID	7	68202	67701	IN-VITRO	In-Vitro
ID	8	68202	67701	EXPOSURE	Exposure
ID	9	68202	67701	EXPRESSION	Expression
ID	10	68202	67701	MICROGLIA	Microglia

Record 455 | WOS:000357490500001

Mycotoxins - prevention and decontamination by yeasts

Field	Position	Original line	Normalised line	Original value	Normalised value
DE	2	68370	67868	Mycotoxin	Mycotoxins
ID	1	68371	67869	SACCHAROMYCES-CEREVISIAE STRAINS	Saccharomyce Cerevisiae Strain
ID	2	68371	67869	LACTIC-ACID BACTERIA	Lactic Acid Bacteria
ID	3	68371	67869	AFLATOXIN B-1	Aflatoxin B1 (AFB1)
ID	4	68371	67869	OCHRATOXIN-A	Ochratoxin A (OTA)
ID	5	68371	67869	IN-VITRO	In-Vitro
ID	6	68371	67869	ASPERGILLUS-CARBONARIUS	Aspergillus Carbonarius
ID	7	68371	67869	BIOCONTROL YEAST	Biocontrol Yeast
ID	8	68371	67869	FUMONISIN B-1	Fumonisin B1 (FB1)
ID	9	68371	67869	TRICHOSPORON-MYCOTOXINIVORANS	Trichosporon Mycotoxinivoran
ID	10	68371	67869	AUREOBASIDIUM-PULLULANS	Aureobasidium Pullulan

Record 456 | WOS:000362080300002

Structure elucidation and in vitro cytotoxicity of ochratoxin Î± amide, a new degradation product of ochratoxin A

Field	Position	Original line	Normalised line	Original value	Normalised value
DE	1	68558	68055	Ochratoxin A	Ochratoxin A (OTA)
DE	6	68558	68055	Ochratoxinaamide	Ochratoxin Aamide
DE	7	68558	68055	Thermal stability	Thermal Stability
ID	1	68560	68057	REDUCTION	Reduction
ID	2	68560	68057	BINDING	Binding
ID	3	68560	68057	BARLEY	Barley

Record 457 | WOS:000357242500002

Polyphenols as possible bioprotectors against cytotoxicity and DNA damage induced by ochratoxin A

Field	Position	Original line	Normalised line	Original value	Normalised value
DE	2	68649	68145	Chlorogenic acid	Chlorogenic Acid
DE	3	68649	68145	Caffeic acid	Caffeic Acid
DE	4	68649	68145	Ochratoxin A	Ochratoxin A (OTA)
DE	6	68649	68145	DNA damage	DNA Damage
ID	1	68651	68147	OXIDATIVE STRESS	Oxidative Stress
ID	2	68651	68147	IN-VITRO	In-Vitro
ID	3	68651	68147	VERO CELLS	Vero
ID	4	68651	68147	TOXICITY	Toxicity
ID	5	68651	68147	MICE	Mice
ID	6	68651	68147	APOPTOSIS	Apoptosis
ID	7	68651	68147	GENOTOXICITY	Genotoxicity
ID	8	68651	68147	FLAVONOIDS	Flavonoid
ID	9	68651	68147	PREVENTION	Prevention
ID	10	68651	68147	LUTEOLIN	Luteolin

Record 458 | WOS:000353230400005

In vitro and in vivo metabolism of ochratoxin A: a comparative study using ultra-performance liquid chromatography-quadrupole/time-of-flight hybrid mass spectrometry

Field	Position	Original line	Normalised line	Original value	Normalised value
DE	1	68773	68268	Ochratoxin A	Ochratoxin A (OTA)
DE	2	68773	68268	Metabolites	Metabolite
DE	3	68773	68268	Chickens	Chicken
DE	4	68773	68268	UPLC-Q/TOF-MS	Uplc Q Tof Ms
ID	1	68774	68269	RAT-LIVER MICROSOMES	Rat Liver Microsome

ID	2	68774	68269	(4S)-4-HYDROXYOCHRATOXIN-A	4s 4 Hydroxyochratoxin A
ID	3	68774	68269	(4R)-4-HYDROXYOCHRATOXIN-A	4r 4 Hydroxyochratoxin A
ID	4	68774	68269	BIOTRANSFORMATION	Biotransformation
ID	5	68774	68269	IDENTIFICATION	Identification
ID	6	68774	68269	ZEARALENONE	Zearalenone (ZEA)
ID	7	68774	68269	MYCOTOXINS	Mycotoxins
ID	8	68774	68269	AFLATOXIN	Aflatoxin
ID	9	68774	68269	PATHWAYS	Pathway
ID	10	68774	68269	URINE	Urine

Record 459 | WOS:000351834600019

Assessment and characterisation of yeast-based products intended to mitigate ochratoxin exposure using in vitro and in vivo models

Field	Position	Original line	Normalised line	Original value	Normalised value
DE	1	68882	68376	ochratoxin A	Ochratoxin A (OTA)
DE	2	68882	68376	yeast-based product	Yeast Based Product
DE	3	68882	68376	cell culture	Cell
DE	4	68882	68376	poultry	Poultry
DE	5	68882	68376	detoxification	Detoxification
ID	1	68883	68377	AFLATOXIN B-1	Aflatoxin B1 (AFB1)
ID	2	68883	68377	SACCHAROMYCES-CEREVISIAE	Saccharomyce Cerevisiae
ID	3	68883	68377	ARISTOLOCHIC ACID	Aristolochic Acid
ID	4	68883	68377	BROILER CHICKS	Broiler Chick
ID	5	68883	68377	DNA ADDUCTION	DNA Adduction
ID	6	68883	68377	A TOXICITY	A Toxicity
ID	7	68883	68377	FEED	Feed
ID	8	68883	68377	MYCOTOXIN	Mycotoxins
ID	9	68883	68377	GENOTOXICITY	Genotoxicity
ID	10	68883	68377	PERFORMANCE	Performance

Record 460 | WOS:000353889600047

Selenium Alleviates Porcine Nephrotoxicity of Ochratoxin A by Improving Selenoenzyme Expression In Vitro

Field	Position	Original line	Normalised line	Original value	Normalised value
ID	1	69011	68504	KIDNEY PK15 CELLS	Kidney Pk15
ID	2	69011	68504	OXIDATIVE STRESS	Oxidative Stress
ID	3	69011	68504	N-ACETYLCYSTEINE	N Acetylcysteine
ID	4	69011	68504	T-2 TOXIN	T 2 Toxin
ID	5	69011	68504	ENRICHED PROBIOTICS	Enriched Probiotics
ID	6	69011	68504	LIPID-PEROXIDATION	Lipid Peroxidation
ID	7	69011	68504	ORGANIC SELENIUM	Organic Selenium
ID	8	69011	68504	DNA-DAMAGE	DNA Damage
ID	9	69011	68504	APOPTOSIS	Apoptosis
ID	10	69011	68504	CYTOTOXICITY	Cytotoxicity

Record 461 | WOS:000349553900004

Determination of Aflatoxins and Ochratoxins in Sicilian Sweet Wines by High-Performance Liquid Chromatography with Fluorometric Detection and Immunoaffinity Cleanup

Field	Position	Original line	Normalised line	Original value	Normalised value
DE	1	69125	68617	Sweet dessert wine	Sweet Dessert Wine
DE	2	69125	68617	Ochratoxin A	Ochratoxin A (OTA)
DE	4	69125	68617	Aflatoxins	Aflatoxin
DE	5	69125	68617	IAC	Iac
DE	6	69125	68617	HPLC-FLD analysis	Hplc Fld Analysis
ID	1	69127	68619	DEVELOPING-COUNTRIES	Developing Country

ID	2	69127	68619	RISK-ASSESSMENT	Risk Assessment
ID	3	69127	68619	RED WINES	Red Wine
ID	4	69127	68619	IN-VITRO	In-Vitro
ID	5	69127	68619	GRAPE	Grape
ID	6	69127	68619	MYCOTOXINS	Mycotoxins
ID	7	69127	68619	TOXICITY	Toxicity
ID	8	69127	68619	ANALOGS	Analog
ID	9	69127	68619	CELLS	Cell

Record 462 | WOS:000343780400062

In vitro genotoxicity of mycotoxins ochratoxin A and fumonisin B1 could be prevented by sodium copper chlorophyllin - Implication to their genotoxic mechanism

Field	Position	Original line	Normalised line	Original value	Normalised value
DE	3	69224	68715	Human lymphocytes	Human Lymphocyte
DE	4	69224	68715	DNA damage	DNA Damage
DE	5	69224	68715	Oxidative stress	Oxidative Stress
DE	6	69224	68715	Protective effect	Protective Effect
ID	1	69226	68717	OXIDATIVE DNA-DAMAGE	Oxidative DNA Damage
ID	2	69226	68717	KIDNEY-CELL-LINE	Kidney Cell
ID	3	69226	68717	CYTOGENETIC STATUS	Cytogenetic Status
ID	4	69226	68717	COMET ASSAY	Comet Assay
ID	5	69226	68717	FRAGMENTATION	Fragmentation
ID	6	69226	68717	ANTIOXIDANT	Antioxidant
ID	7	69226	68717	APOPTOSIS	Apoptosis
ID	8	69226	68717	CARCINOGENICITY	Carcinogenicity
ID	9	69226	68717	CYTOTOXICITY	Cytotoxicity
ID	10	69226	68717	LYMPHOCYTES	Lymphocyte

Record 463 | WOS:000351485000004

Ochratoxin A promotes porcine circovirus type 2 replication in vitro and in vivo

Field	Position	Original line	Normalised line	Original value	Normalised value
DE	1	69355	68845	Ochratoxin A	Ochratoxin A (OTA)
DE	2	69355	68845	Porcine circovirus type 2	Porcine Circovirus Type 2
DE	3	69355	68845	Oxidative stress	Oxidative Stress
DE	4	69355	68845	Signaling pathway	Signaling Pathway
DE	5	69355	68845	Free radicals	Free Radical
ID	1	69357	68847	MULTISYSTEMIC WASTING SYNDROME	Multisystemic Wasting Syndrome
ID	2	69357	68847	KIDNEY PK15 CELLS	Kidney Pk15
ID	3	69357	68847	OXIDATIVE STRESS	Oxidative Stress
ID	4	69357	68847	SIGNALING PATHWAY	Signaling Pathway
ID	5	69357	68847	KINASE ACTIVATION	Kinase Activation
ID	6	69357	68847	PHASE ARREST	Phase Arrest
ID	7	69357	68847	DNA-DAMAGE	DNA Damage
ID	8	69357	68847	GLUTATHIONE	Glutathione (GSH)
ID	9	69357	68847	APOPTOSIS	Apoptosis
ID	10	69357	68847	EPIDEMIOLOGY	Epidemiology

Record 464 | WOS:000350023000003

Ochratoxin A and its effects on immunity

Field	Position	Original line	Normalised line	Original value	Normalised value
DE	1	69482	68971	Cytokines	Cytokine
DE	2	69482	68971	immunity	Immunity
DE	3	69482	68971	inflammation	Inflammation
DE	4	69482	68971	ochratoxin A	Ochratoxin A (OTA)

ID	1	69483	68972	TUMOR-NECROSIS-FACTOR	Tumor Necrosis Factor
ID	2	69483	68972	MITOGEN-INDUCED BLASTOGENESIS	Mitogen Induced Blastogenesis
ID	3	69483	68972	INDUCED IN-VITRO	Induced In Vitro
ID	4	69483	68972	TNF-ALPHA	Tnf Alpha
ID	5	69483	68972	OXIDATIVE STRESS	Oxidative Stress
ID	6	69483	68972	NEPHROTOXIN OCHRATOXIN	Ochratoxin A (OTA)
ID	7	69483	68972	HEPATOCYTE APOPTOSIS	Hepatocyte Apoptosis
ID	8	69483	68972	PENICILLIC ACID	Penicillic Acid
ID	9	69483	68972	FOOD	Food
ID	10	69483	68972	ACTIVATION	Activation

Record 465 | WOS:000350094000016

Genotoxicity of Aflatoxin B1 and Ochratoxin A after simultaneous application of the in vivo micronucleus and comet assay

Field	Position	Original line	Normalised line	Original value	Normalised value
DE	1	69654	69142	Aflatoxin B1	Aflatoxin B1 (AFB1)
DE	2	69654	69142	Ochratoxin A	Ochratoxin A (OTA)
DE	5	69654	69142	Comet assay	Comet Assay
DE	6	69654	69142	Combined exposure	Combined Exposure
ID	1	69656	69144	INDUCED DNA-DAMAGE	Induced DNA Damage
ID	2	69656	69144	RAT	Rat
ID	3	69656	69144	TOXICITY	Toxicity
ID	4	69656	69144	KIDNEY	Kidney
ID	5	69656	69144	LIVER	Liver
ID	6	69656	69144	TOXICOKINETICS	Toxicokinetics
ID	7	69656	69144	PLASMA	Plasma
ID	8	69656	69144	CELLS	Cell
ID	9	69656	69144	VITRO	Vitro
ID	10	69656	69144	B-1	B 1

Record 466 | WOS:000362800100005

Mycotoxin mechanisms of action and health impact: 'in vitro' or 'in vivo' tests, that is the question

Field	Position	Original line	Normalised line	Original value	Normalised value
DE	1	69785	69272	in vivo models	In Vivo Model
DE	2	69785	69272	in vitro models	In Vitro Model
DE	3	69785	69272	mycotoxicity	Mycotoxicity
DE	4	69785	69272	gastrointestinal tract	Gastrointestinal Tract
ID	1	69786	69273	INTESTINAL EPITHELIAL-CELLS	Intestinal Epithelial
ID	2	69786	69273	OCHRATOXIN-A	Ochratoxin A (OTA)
ID	3	69786	69273	T-2 TOXIN	T 2 Toxin
ID	4	69786	69273	FUSARIUM-MYCOTOXINS	Fusarium Mycotoxin
ID	5	69786	69273	BARRIER FUNCTION	Barrier Function
ID	6	69786	69273	FUMONISIN B-1	Fumonisin B1 (FB1)
ID	7	69786	69273	DIGESTION MODEL	Digestion Model
ID	8	69786	69273	AFLATOXIN B-1	Aflatoxin B1 (AFB1)
ID	9	69786	69273	TOXICOLOGICAL INTERACTIONS	Toxicological Interaction
ID	10	69786	69273	ACETYLATED DERIVATIVES	Acetylated Derivative

Record 467 | WOS:000351213600013

Toxic Effects of Aflatoxin B1 on Embryonic Development of Zebrafish (Danio rerio): Potential Activity of Piceatannol Encapsulated Chitosan/poly (Lactic Acid) Nanoparticles

Field	Position	Original line	Normalised line	Original value	Normalised value

DE	1	69990	69476	AflatoxinB1	Aflatoxinb1
DE	2	69990	69476	chitosan	Chitosan
DE	3	69990	69476	poly lactic acid	Poly Lactic Acid
DE	4	69990	69476	piceatannol	Piceatannol
DE	5	69990	69476	toxicity	Toxicity
DE	6	69990	69476	zebrafish	Zebrafish
ID	1	69992	69477	B-1-INDUCED DNA-DAMAGE	B 1 Induced DNA Damage
ID	2	69992	69477	IN-VIVO	In-Vivo
ID	3	69992	69477	OCHRATOXIN-A	Ochratoxin A (OTA)
ID	4	69992	69477	MODEL	Model
ID	5	69992	69477	B-1	B 1
ID	6	69992	69477	STILBENE	Stilbene
ID	7	69992	69477	RESVERATROL	Resveratrol
ID	8	69992	69477	PHYTOALEXIN	Phytoalexin
ID	9	69992	69477	EXPOSURE	Exposure
ID	10	69992	69477	RATS	Rat

Record 468 | WOS:000349133200010

Bioaccessibility of ochratoxin A from red wine in an in vitro dynamic gastrointestinal model

Field	Position	Original line	Normalised line	Original value	Normalised value
DE	1	70126	69610	bioaccessibility	Bioaccessibility
DE	2	70126	69610	in vitro digestion	In Vitro Digestion
DE	3	70126	69610	ochratoxin alpha	Ochratoxin Alpha
ID	1	70127	69611	INTESTINAL-ABSORPTION	Intestinal Absorption
ID	2	70127	69611	BINDING EFFICACY	Binding Efficacy
ID	3	70127	69611	ACTIVATED CARBON	Activated Carbon
ID	4	70127	69611	DIGESTION MODEL	Digestion Model
ID	5	70127	69611	PLASMA	Plasma
ID	6	70127	69611	FERMENTATION	Fermentation
ID	7	70127	69611	SECRETION	Secretion
ID	8	70127	69611	REMOVAL	Removal
ID	9	70127	69611	WHITE	White
ID	10	70127	69611	IRON	Iron

Record 469 | WOS:000415214500006

Current Insights into Mycotoxins

Field	Position	Original line	Normalised line	Original value	Normalised value
ID	1	70220	69703	OCHRATOXIN-A	Ochratoxin A (OTA)
ID	2	70220	69703	TRICHOTHECENE MYCOTOXINS	Trichothecene Mycotoxin
ID	3	70220	69703	FUSARIUM-MONILIFORME	Fusarium Moniliforme
ID	4	70220	69703	RAT-LIVER	Rat Liver
ID	5	70220	69703	PATULIN	Patulin
ID	6	70220	69703	ZEARALENONE	Zearalenone (ZEA)
ID	7	70220	69703	TOXICITY	Toxicity
ID	8	70220	69703	CELLS	Cell
ID	9	70220	69703	FUMONISINS	Fumonisin
ID	10	70220	69703	DEOXYNIVALENOL	Deoxynivalenol (DON)

Record 470 | WOS:000360184800009

Dynamic changes of global DNA methylation and hypermethylation of cell adhesion-related genes in rat kidneys in response to ochratoxin A

Field	Position	Original line	Normalised line	Original value	Normalised value
DE	1	70328	69811	ochratoxin A	Ochratoxin A (OTA)
DE	2	70328	69811	DNA methylation	DNA Methylation
DE	3	70328	69811	DNA methyltransferase	DNA Methyltransferase
DE	4	70328	69811	cadherins	Cadherin
ID	1	70329	69812	E-CADHERIN GENE	E Cadherin Gene

ID	2	70329	69812	NF-KAPPA-B	Nf Kappa B
ID	3	70329	69812	CPG METHYLATION	Cpg Methylation
ID	4	70329	69812	ARSENIC EXPOSURE	Arsenic Exposure
ID	5	70329	69812	EXPRESSION	Expression
ID	6	70329	69812	CARCINOGENESIS	Carcinogenesis
ID	7	70329	69812	PROLIFERATION	Proliferation
ID	8	70329	69812	OVEREXPRESSION	Overexpression
ID	9	70329	69812	TOXICITY	Toxicity
ID	10	70329	69812	WNT	Wnt

Record 471 | WOS:000367698600008

POTENTIAL GENOTOXICITY OF ACETAMIPRID AND PROPINEB SINGLY OR IN COMBINATION IN CULTURED HUMAN PERIPHERAL BLOOD LYMPHOCYTES BY USING MN ASSAY

Field	Position	Original line	Normalised line	Original value	Normalised value
DE	3	70448	69930	in vitro	In-Vitro
DE	4	70448	69930	human lymphocyte culture	Human Lymphocyte
ID	1	70449	69931	DITHIOCARBAMATE FUNGICIDE PROPINEB	Dithiocarbamate Fungicide Propineb
ID	2	70449	69931	IN-VITRO	In-Vitro
ID	3	70449	69931	OXIDATIVE STRESS	Oxidative Stress
ID	4	70449	69931	PESTICIDE MIXTURES	Pesticide Mixture
ID	5	70449	69931	OCHRATOXIN-A	Ochratoxin A (OTA)
ID	6	70449	69931	MICRONUCLEUS	Micronucleus
ID	7	70449	69931	GLUTATHIONE	Glutathione (GSH)
ID	8	70449	69931	TOXICITY	Toxicity
ID	9	70449	69931	CYPERMETHRIN	Cypermethrin
ID	10	70449	69931	CELLS	Cell

Record 472 | WOS:000347274600033

Low doses of ochratoxin A induce micronucleus formation and delay DNA repair in human lymphocytes

Field	Position	Original line	Normalised line	Original value	Normalised value
DE	1	70581	70062	Ochratoxin A	Ochratoxin A (OTA)
DE	2	70581	70062	Comet assay	Comet Assay
DE	3	70581	70062	Cytokinesis-blocked micronucleus (CBMN) assay	Cytokinesis Blocked Micronucleus Cbmn Assay
ID	1	70583	70064	BALKAN ENDEMIC NEPHROPATHY	Balkan Endemic Nephropathy
ID	2	70583	70064	SISTER-CHROMATID EXCHANGE	Sister Chromatid Exchange
ID	3	70583	70064	A-INDUCED GENOTOXICITY	A Induced Genotoxicity
ID	4	70583	70064	DOUBLE-STRAND BREAKS	Double Strand Break
ID	5	70583	70064	OXIDATIVE DAMAGE	Oxidative Damage
ID	6	70583	70064	PHASE ARREST	Phase Arrest
ID	7	70583	70064	HEPG2 CELLS	HepG2
ID	8	70583	70064	IN-VITRO	In-Vitro
ID	9	70583	70064	MYCOTOXINS	Mycotoxins
ID	10	70583	70064	FREQUENCY	Frequency

Record 473 | WOS:000349038800005

In vitro effects of ochratoxin A, deoxynivalenol and zearalenone on cell viability and E-ADA activity in broiler chickens lymphocytes

Field	Position	Original line	Normalised line	Original value	Normalised value
DE	2	70722	70202	leukocytes	Leukocyte
DE	3	70722	70202	cytotoxicity	Cytotoxicity
DE	4	70722	70202	poultry	Poultry
DE	5	70722	70202	colorimetric assays	Colorimetric Assay
ID	1	70723	70203	IMMUNE-SYSTEM	Immune System

ID	2	70723	70203	ADENOSINE	Adenosine
ID	3	70723	70203	CYTOTOXICITY	Cytotoxicity
ID	4	70723	70203	IMMUNOSUPPRESSION	Immunosuppression
ID	5	70723	70203	MYCOTOXINS	Mycotoxins
ID	6	70723	70203	EXPRESSION	Expression

Record 474 | WOS:000344696100002

The mycotoxin beauvericin induces apoptotic cell death in H4IIE hepatoma cells accompanied by an inhibition of NF-Î°B-activity and modulation of MAP-kinases

Field	Position	Original line	Normalised line	Original value	Normalised value
DE	3	70831	70310	ERK	Erk
DE	4	70831	70310	JNK	Jnk
DE	5	70831	70310	MAPK	Mapk
DE	6	70831	70310	Mycotoxin	Mycotoxins
DE	7	70831	70310	NF-kappa B	Nf Kappa B
ID	1	70832	70311	KIDNEY PK15 CELLS	Kidney Pk15
ID	2	70832	70311	OCHRATOXIN-A	Ochratoxin A (OTA)
ID	3	70832	70311	CYTOCHROME-C	Cytochrome C
ID	4	70832	70311	CANCER-CELLS	Cancer
ID	5	70832	70311	FUSAPROLIFERIN	Fusaproliferin
ID	6	70832	70311	CYTOTOXICITY	Cytotoxicity
ID	7	70832	70311	INDUCTION	Induction
ID	8	70832	70311	CASPASE-3	Caspase 3
ID	9	70832	70311	FUMONISIN	Fumonisin
ID	10	70832	70311	ENNIATIN	Enniatin

Record 475 | WOS:000344980400016

Ochratoxin A induces rat renal carcinogenicity with limited induction of oxidative stress responses

Field	Position	Original line	Normalised line	Original value	Normalised value
DE	1	70929	70407	Ochratoxin A	Ochratoxin A (OTA)
DE	2	70929	70407	Renal carcinogenicity	Renal Carcinogenicity
DE	3	70929	70407	Oxidative stress	Oxidative Stress
DE	5	70929	70407	Cell proliferation	Cell Proliferation
ID	1	70931	70409	DNA-DAMAGE	DNA Damage
ID	2	70931	70409	IN-VIVO	In-Vivo
ID	3	70931	70409	LIPID-PEROXIDATION	Lipid Peroxidation
ID	4	70931	70409	ANTIOXIDANT ENZYMES	Antioxidant Enzyme
ID	5	70931	70409	LIPOCALIN 2	Lipocalin 2
ID	6	70931	70409	TOXICITY	Toxicity
ID	7	70931	70409	KIDNEY	Kidney
ID	8	70931	70409	CELLS	Cell
ID	9	70931	70409	LIVER	Liver
ID	10	70931	70409	MECHANISMS	Mechanism

Record 476 | WOS:000343730400082

A New Ochratoxin A Biodegradation Strategy Using Cupriavidus basilensis Or16 Strain

Field	Position	Original line	Normalised line	Original value	Normalised value
ID	1	71057	70534	BALKAN ENDEMIC NEPHROPATHY	Balkan Endemic Nephropathy
ID	2	71057	70534	RENAL-CELL CARCINOMA	Renal Cell Carcinoma
ID	3	71057	70534	IN-VITRO	In-Vitro
ID	4	71057	70534	TRICHOSPORON-MYCOTOXINIVORANS	Trichosporon Mycotoxinivoran
ID	5	71057	70534	BIOLOGICAL DETOXIFICATION	Biological Detoxification
ID	6	71057	70534	BROILER CHICKS	Broiler Chick
ID	7	71057	70534	FUNGAL TOXINS	Fungal Toxin
ID	8	71057	70534	HUMAN BLOOD	Human Blood

ID	9	71057	70534	EXPRESSION	Expression
ID	10	71057	70534	FOOD	Food

Record 477 | WOS:000341677200007

Biodegradation of ochratoxin A by Pediococcus parvulus isolated from Douro wines

Field	Position	Original line	Normalised line	Original value	Normalised value
DE	1	71191	70667	Pediococcus parvulus	Pediococcus Parvulus
DE	2	71191	70667	Ochratoxin A	Ochratoxin A (OTA)
ID	1	71192	70668	LACTIC-ACID BACTERIA	Lactic Acid Bacteria
ID	2	71192	70668	IN-VITRO	In-Vitro
ID	3	71192	70668	STRAINS	Strain
ID	4	71192	70668	ABILITY	Ability
ID	5	71192	70668	FOOD	Food
ID	6	71192	70668	LACTOBACILLUS	Lactobacillus
ID	7	71192	70668	DEGRADATION	Degradation
ID	8	71192	70668	FERMENTATION	Fermentation
ID	9	71192	70668	METABOLITES	Metabolite
ID	10	71192	70668	ZEARALENONE	Zearalenone (ZEA)

Record 478 | WOS:000343630800010

Biological detoxification of zearalenone by Aspergillus niger strain FS10

Field	Position	Original line	Normalised line	Original value	Normalised value
DE	1	71330	70805	Zearalenone	Zearalenone (ZEA)
DE	3	71330	70805	Aspergillus niger FS10	Aspergillus Niger Fs10
DE	4	71330	70805	Corn steep liquor	Corn Steep Liquor
ID	1	71332	70807	AFLATOXIN B-1	Aflatoxin B1 (AFB1)
ID	2	71332	70807	OCHRATOXIN-A	Ochratoxin A (OTA)
ID	3	71332	70807	DEGRADATION	Degradation
ID	4	71332	70807	MYCOTOXINS	Mycotoxins
ID	5	71332	70807	CORN	Corn
ID	6	71332	70807	DECONTAMINATION	Decontamination
ID	7	71332	70807	DEOXYNIVALENOL	Deoxynivalenol (DON)
ID	8	71332	70807	PHOSPHOLIPASE	Phospholipase
ID	9	71332	70807	CONTAMINATION	Contamination
ID	10	71332	70807	PURIFICATION	Purification

Record 479 | WOS:000343630800034

Ochratoxin A kinetics: A review of analytical methods and studies in rat model

Field	Position	Original line	Normalised line	Original value	Normalised value
DE	1	71439	70913	Ochratoxin A	Ochratoxin A (OTA)
DE	3	71439	70913	Analytical techniques	Analytical Technique
ID	1	71440	70914	MYCOTOXIN OCHRATOXIN	Ochratoxin A (OTA)
ID	2	71440	70914	IN-VIVO	In-Vivo
ID	3	71440	70914	CHROMATOGRAPHIC METHODS	Chromatographic Method
ID	4	71440	70914	DIETARY CHOLESTYRAMINE	Dietary Cholestyramine
ID	5	71440	70914	LIQUID-CHROMATOGRAPHY	Liquid Chromatography
ID	6	71440	70914	MOLECULAR-MECHANISM	Molecular Mechanism
ID	7	71440	70914	TUMOR-FORMATION	Tumor Formation
ID	8	71440	70914	PLASMA	Plasma
ID	9	71440	70914	KIDNEY	Kidney
ID	10	71440	70914	PHARMACOKINETICS	Pharmacokinetics

Record 480 | WOS:000340827200008

Synergism of ochratoxin B and calcium-channel antagonist verapamil caused mitochondrial dysfunction

Field	Position	Original line	Normalised line	Original value	Normalised value
DE	1	71598	71071	Ca2+ channel blocker	Ca2 Channel Blocker
DE	2	71598	71071	intercellular Ca2+	Intercellular Ca2
DE	3	71598	71071	ochratoxin B	Ochratoxin B
ID	1	71599	71072	ACETYLCHOLINE	Acetylcholine
ID	2	71599	71072	STIMULATION	Stimulation
ID	3	71599	71072	NEPHROPATHY	Nephropathy
ID	4	71599	71072	NIFEDIPINE	Nifedipine

Record 481 | WOS:000340837600026

Development of an in vitro method for the prediction of mycotoxin binding on yeast-based products: case of aflatoxin B1, zearalenone and ochratoxin A

Field	Position	Original line	Normalised line	Original value	Normalised value
DE	3	71687	71159	Ochratoxin	Ochratoxin A (OTA)
DE	4	71687	71159	Zearalenone	Zearalenone (ZEA)
DE	6	71687	71159	In vitro screening method	In Vitro Screening Method
ID	1	71689	71161	SACCHAROMYCES-CEREVISIAE STRAINS	Saccharomyce Cerevisiae Strain
ID	2	71689	71161	LACTIC-ACID BACTERIA	Lactic Acid Bacteria
ID	3	71689	71161	CELL-WALL	Cell Wall
ID	4	71689	71161	ANIMAL FEED	Animal Feed
ID	5	71689	71161	ADSORPTION	Adsorption
ID	6	71689	71161	TOXICITY	Toxicity
ID	7	71689	71161	ABILITY	Ability
ID	8	71689	71161	DETOXIFICATION	Detoxification
ID	9	71689	71161	CONTAMINATION	Contamination
ID	10	71689	71161	COMPLEXATION	Complexation

Record 482 | WOS:000342990200034

Combination of Metagenomics and Culture-Based Methods to Study the Interaction Between Ochratoxin A and Gut Microbiota

Field	Position	Original line	Normalised line	Original value	Normalised value
DE	1	71819	71290	gut microbiota	Gut Microbiota
DE	2	71819	71290	ochratoxin A	Ochratoxin A (OTA)
DE	3	71819	71290	metagenomics	Metagenomics
ID	1	71820	71291	REDUCED DIVERSITY	Reduced Diversity
ID	2	71820	71291	FECAL MICROBIOTA	Fecal Microbiota
ID	3	71820	71291	AFLATOXIN B-1	Aflatoxin B1 (AFB1)
ID	4	71820	71291	DEGRADATION	Degradation
ID	5	71820	71291	IMPACT	Impact
ID	6	71820	71291	DEOXYNIVALENOL	Deoxynivalenol (DON)
ID	7	71820	71291	INHIBITION	Inhibition
ID	8	71820	71291	MYCOTOXINS	Mycotoxins
ID	9	71820	71291	TOXICITY	Toxicity
ID	10	71820	71291	BACTERIA	Bacteria

Record 483 | WOS:000340087200003

Biological detoxification of mycotoxins: a review

Field	Position	Original line	Normalised line	Original value	Normalised value
ID	1	71916	71386	LACTIC-ACID BACTERIA	Lactic Acid Bacteria
ID	2	71916	71386	AFLATOXIN B-1 DEGRADATION	Aflatoxin B 1 Degradation
ID	3	71916	71386	OCHRATOXIN-A REMOVAL	Ochratoxin A Removal
ID	4	71916	71386	BETA-D-GLUCANS	Beta D Glucan

ID	5	71916	71386	IN-VITRO	In-Vitro
ID	6	71916	71386	SACCHAROMYCES-CEREVISIAE	Saccharomyce Cerevisiae
ID	7	71916	71386	CELL-WALL	Cell Wall
ID	8	71916	71386	DAIRY STRAINS	Dairy Strain
ID	9	71916	71386	FLAVOBACTERIUM-AURANTIACUM	Flavobacterium Aurantiacum
ID	10	71916	71386	LACTOBACILLUS-CASEI	Lactobacillus Casei

Record 484 | WOS:000341466700016

Hepatotoxicity and genotoxicity of patulin in mice, and its modulation by green tea polyphenols administration

Field	Position	Original line	Normalised line	Original value	Normalised value
DE	2	72162	71631	Green tea polyphenol	Green Tea Polyphenol
DE	5	72162	71631	Oxidative stress	Oxidative Stress
DE	6	72162	71631	Bone marrow	Bone Marrow
ID	1	72164	71633	MYCOTOXIN PATULIN	Mycotoxin Patulin
ID	2	72164	71633	HEPG2 CELLS	HepG2
ID	3	72164	71633	OXIDATIVE STRESS	Oxidative Stress
ID	4	72164	71633	MAMMALIAN-CELLS	Mammalian
ID	5	72164	71633	PROTECTIVE ROLE	Protective Role
ID	6	72164	71633	DNA-SYNTHESIS	DNA Synthesis
ID	7	72164	71633	OCHRATOXIN-A	Ochratoxin A (OTA)
ID	8	72164	71633	RAT-LIVER	Rat Liver
ID	9	72164	71633	GLUTATHIONE	Glutathione (GSH)
ID	10	72164	71633	CITRININ	Citrinin

Record 485 | WOS:000341466700027

Cytotoxicity induced by ochratoxin A, zearalenone, and Î±-zearalenol: Effects of individual and combined treatment

Field	Position	Original line	Normalised line	Original value	Normalised value
DE	1	72290	71757	Ochratoxin A	Ochratoxin A (OTA)
DE	2	72290	71757	Zearalenone	Zearalenone (ZEA)
DE	3	72290	71757	alpha-Zearalenol	Alpha Zearalenol
ID	1	72292	71759	IN-VITRO CYTOTOXICITY	In Vitro Cytotoxicity
ID	2	72292	71759	OXIDATIVE STRESS	Oxidative Stress
ID	3	72292	71759	T-2 TOXIN	T 2 Toxin
ID	4	72292	71759	FUSARIUM MYCOTOXINS	Fusarium Mycotoxin
ID	5	72292	71759	BETA-ZEARALENOL	Beta Zearalenol
ID	6	72292	71759	FUMONISIN B-1	Fumonisin B1 (FB1)
ID	7	72292	71759	DNA-DAMAGE	DNA Damage
ID	8	72292	71759	COMBINATIONS	Combination
ID	9	72292	71759	TOXICITY	Toxicity
ID	10	72292	71759	CELLS	Cell

Record 486 | WOS:000338616800026

Cytotoxicity and immunotoxicity of cyclopiazonic acid on human cells

Field	Position	Original line	Normalised line	Original value	Normalised value
DE	1	72391	71857	Cyclopiazonic acid	Cyclopiazonic Acid
DE	2	72391	71857	Food contaminant	Food Contaminant
DE	3	72391	71857	Human cells	Human
DE	5	72391	71857	Mycotoxin	Mycotoxins
ID	1	72393	71859	HEMATOPOIETIC STEM-CELLS	Hematopoietic Stem
ID	2	72393	71859	ASPERGILLUS-FLAVUS	Aspergillus Flavus
ID	3	72393	71859	IN-VITRO	In-Vitro
ID	4	72393	71859	BROILER-CHICKENS	Broiler Chicken
ID	5	72393	71859	OXIDATIVE STRESS	Oxidative Stress

ID	6	72393	71859	CACO-2 CELLS	Caco-2
ID	7	72393	71859	OCHRATOXIN-A	Ochratoxin A (OTA)
ID	8	72393	71859	LINE CACO-2	Line Caco-2
ID	9	72393	71859	T-2 TOXIN	T 2 Toxin
ID	10	72393	71859	MYCOTOXIN	Mycotoxins

Record 487 | WOS:000338817900023

Central role of Nix in the autophagic response to ochratoxin A

Field	Position	Original line	Normalised line	Original value	Normalised value
DE	1	72515	71980	Ochratoxin A	Ochratoxin A (OTA)
DE	5	72515	71980	Renal cytotoxicity	Renal Cytotoxicity
ID	1	72516	71981	MITOCHONDRIAL AUTOPHAGY	Mitochondrial Autophagy
ID	2	72516	71981	CYTOCHROME-C	Cytochrome C
ID	3	72516	71981	CELL-DEATH	Cell Death
ID	4	72516	71981	IN-VIVO	In-Vivo
ID	5	72516	71981	MITOPHAGY	Mitophagy
ID	6	72516	71981	NEPHROPATHY	Nephropathy
ID	7	72516	71981	MECHANISMS	Mechanism
ID	8	72516	71981	NEPHROTOXICITY	Nephrotoxicity
ID	9	72516	71981	DYSFUNCTION	Dysfunction
ID	10	72516	71981	EVENTS	Event

Record 488 | WOS:000336730800004

Toxicity of the mycotoxin citrinin and its metabolite dihydrocitrinone and of mixtures of citrinin and ochratoxin A in vitro

Field	Position	Original line	Normalised line	Original value	Normalised value
DE	3	72623	72087	Metabolite u Mixtures	Metabolite U Mixture
ID	1	72624	72088	CHROMOSOME-ABERRATIONS	Chromosome Aberration
ID	2	72624	72088	CELL-CYCLE	Cell Cycle
ID	3	72624	72088	CYTOTOXICITY	Cytotoxicity
ID	4	72624	72088	APOPTOSIS	Apoptosis
ID	5	72624	72088	MICRONUCLEI	Micronuclei
ID	6	72624	72088	INDUCTION	Induction
ID	7	72624	72088	PROLIFERATION	Proliferation
ID	8	72624	72088	GENOTOXICITY	Genotoxicity
ID	9	72624	72088	DISRUPTION	Disruption
ID	10	72624	72088	VIABILITY	Viability

Record 489 | WOS:000362078100003

Modulation of innate and antigen-specific immune functions directed against Listeria monocytogenes by fungal toxins in vitro

Field	Position	Original line	Normalised line	Original value	Normalised value
DE	3	72726	72188	Immune cells	Immune
ID	1	72727	72189	FUMONISIN B-1	Fumonisin B1 (FB1)
ID	2	72727	72189	ASPERGILLUS-FUMIGATUS	Aspergillus Fumigatus
ID	3	72727	72189	OCHRATOXIN-A	Ochratoxin A (OTA)
ID	4	72727	72189	MYCOPHENOLATE-MOFETIL	Mycophenolate Mofetil
ID	5	72727	72189	GLIOTOXIN PRODUCTION	Gliotoxin Production
ID	6	72727	72189	CYTOKINE PRODUCTION	Cytokine Production
ID	7	72727	72189	CELL-LINES	Cell
ID	8	72727	72189	MYCOTOXINS	Mycotoxins
ID	9	72727	72189	APOPTOSIS	Apoptosis
ID	10	72727	72189	CYTOTOXICITY	Cytotoxicity

Record 490 | WOS:000333247100013

Downregulation of Rad51 participates in OTA-induced DNA double-strand breaks in GES-1 cells in vitro

Field	Position	Original line	Normalised line	Original value	Normalised value
DE	2	72837	72298	Ochratoxin A	Ochratoxin A (OTA)
DE	3	72837	72298	homologous recombination repair pathway	Homologous Recombination Repair Pathway
DE	4	72837	72298	DNA double strand breaks	DNA Double Strand Break
DE	5	72837	72298	Cell cycle arrest	Cell Cycle Arrest
DE	6	72837	72298	Human gastric epithelium immortalized cell	Human Gastric Epithelium Immortalized
ID	1	72840	72301	OCHRATOXIN-A	Ochratoxin A (OTA)
ID	2	72840	72301	INDUCED CYTOTOXICITY	Induced Cytotoxicity
ID	3	72840	72301	HOMOLOGOUS RECOMBINATION	Homologous Recombination
ID	4	72840	72301	IONIZING-RADIATION	Ionizing Radiation
ID	5	72840	72301	PHASE ARREST	Phase Arrest
ID	6	72840	72301	DAMAGE	Damage
ID	7	72840	72301	REPAIR	Repair
ID	8	72840	72301	PATHWAYS	Pathway
ID	10	72840	72301	MUTAGENICITY	Mutagenicity

Record 491 | WOS:000335104500012

Protective role of the mitochondrial Lon protease 1 in ochratoxin A-induced cytotoxicity in HEK293 cells

Field	Position	Original line	Normalised line	Original value	Normalised value
DE	1	72951	72410	Ochratoxin A	Ochratoxin A (OTA)
DE	2	72951	72410	Renal cytotoxicity	Renal Cytotoxicity
DE	4	72951	72410	RNA interference	RNA Interference
DE	5	72951	72410	iTRAQ	Itraq
ID	1	72952	72411	PROTEIN-QUALITY CONTROL	Protein Quality Control
ID	2	72952	72411	OXIDATIVE DNA-DAMAGE	Oxidative DNA Damage
ID	3	72952	72411	INTERMEDIATE-FILAMENTS	Intermediate Filament
ID	4	72952	72411	ENDOPLASMIC-RETICULUM	Endoplasmic Reticulum
ID	5	72952	72411	MALATE-DEHYDROGENASE	Malate Dehydrogenase
ID	6	72952	72411	INDUCED APOPTOSIS	Induced Apoptosis
ID	7	72952	72411	DOWN-REGULATION	Down Regulation
ID	8	72952	72411	JNK ACTIVATION	Jnk Activation
ID	9	72952	72411	CANCER CELLS	Cancer
ID	10	72952	72411	GLYOXALASE-I	Glyoxalase I

Record 492 | WOS:000332034900015

Mutant frequency in comparison to oxidative DNA damage induced by ochratoxin A in L5178Y tk+/- (3.7.2C) mouse lymphoma cells

Field	Position	Original line	Normalised line	Original value	Normalised value
DE	1	73110	72568	Comet assay	Comet Assay
DE	2	73110	72568	mouse lymphoma assay	Mouse Lymphoma Assay
DE	3	73110	72568	mutant frequency	Mutant Frequency
DE	4	73110	72568	ochratoxin A	Ochratoxin A (OTA)
DE	5	73110	72568	oxidative DNA damage	Oxidative DNA Damage
ID	1	73112	72570	GENE MUTATION ASSAY	Gene Mutation Assay
ID	2	73112	72570	INTERNATIONAL WORKSHOP	International Workshop
ID	3	73112	72570	MUTAGENICITY TEST	Mutagenicity Test
ID	4	73112	72570	KIDNEY-CELLS	Kidney
ID	5	73112	72570	GENOTOXICITY	Genotoxicity

ID	6	73112	72570	MICRONUCLEI	Micronuclei
ID	7	73112	72570	INDUCTION	Induction
ID	8	73112	72570	RAT	Rat
ID	9	73112	72570	ADDUCTS	Adduct
ID	10	73112	72570	STRESS	Stress

Record 493 | WOS:000335760200003

Analysis of Individual and Combined Effects of Ochratoxin A and Zearalenone on HepG2 and KK-1 Cells with Mathematical Models

Field	Position	Original line	Normalised line	Original value	Normalised value
DE	1	73231	72688	concentration addition	Concentration Addition
DE	2	73231	72688	ochratoxin A	Ochratoxin A (OTA)
DE	3	73231	72688	independent addition	Independent Addition
DE	4	73231	72688	combined effect	Combined Effect
DE	5	73231	72688	risk assessment	Risk Assessment
DE	6	73231	72688	zearalenone	Zearalenone (ZEA)
ID	1	73233	72690	DNA-DAMAGE	DNA Damage
ID	2	73233	72690	OXIDATIVE STRESS	Oxidative Stress
ID	3	73233	72690	PROTEIN-SYNTHESIS	Protein Synthesis
ID	4	73233	72690	RISK-ASSESSMENT	Risk Assessment
ID	5	73233	72690	TOXICITY	Toxicity
ID	6	73233	72690	MECHANISMS	Mechanism
ID	7	73233	72690	MYCOTOXINS	Mycotoxins
ID	8	73233	72690	GENOTOXICITY	Genotoxicity
ID	9	73233	72690	HORMESIS	Hormesis
ID	10	73233	72690	AFLATOXIN-B1	Aflatoxin B1 (AFB1)

Record 494 | WOS:000338818900001

Flavonoid diosmetin increases ATP levels in kidney cells and relieves ATP depleting effect of ochratoxin A

Field	Position	Original line	Normalised line	Original value	Normalised value
DE	2	73357	72813	ATP	Atp
DE	3	73357	72813	Flavonoids	Flavonoid
DE	4	73357	72813	Ochratoxin A	Ochratoxin A (OTA)
DE	5	73357	72813	Kidney cells	Kidney
ID	1	73358	72814	RADICAL SCAVENGING ACTIVITY	Radical Scavenging Activity
ID	2	73358	72814	NF-KAPPA-B	Nf Kappa B
ID	3	73358	72814	OXIDATIVE-PHOSPHORYLATION	Oxidative Phosphorylation
ID	4	73358	72814	DIOSMIN PROTECTS	Diosmin Protect
ID	5	73358	72814	HEPG2 CELLS	HepG2
ID	6	73358	72814	ANTIOXIDANT	Antioxidant
ID	7	73358	72814	ACTIVATION	Activation
ID	8	73358	72814	MITOCHONDRIA	Mitochondria
ID	9	73358	72814	QUERCETIN	Quercetin
ID	10	73358	72814	FRACTION	Fraction

Record 495 | WOS:000332451700009

Natural feed contaminant zearalenone decreases the expressions of important pro- and anti-inflammatory mediators and mitogen-activated protein kinase/NF-kB signalling molecules in pigs

Field	Position	Original line	Normalised line	Original value	Normalised value
DE	1	73480	72934	Zearalenone	Zearalenone (ZEA)
DE	2	73480	72934	Pigs	Pig
DE	3	73480	72934	Immune responses	Immune Response
DE	5	73480	72934	Signalling pathways	Signalling Pathway
ID	1	73481	72935	NF-KAPPA-B	Nf Kappa B
ID	2	73481	72935	ALPHA-ZEARALENOL	Alpha Zearalenol
ID	3	73481	72935	FUMONISIN B-1	Fumonisin B1 (FB1)
ID	4	73481	72935	OCHRATOXIN-A	Ochratoxin A (OTA)

ID	5	73481	72935	MYCOTOXIN ZEARALENONE	Mycotoxin Zearalenone
ID	6	73481	72935	REPRODUCTIVE-ORGANS	Reproductive Organ
ID	7	73481	72935	FUNGAL METABOLITE	Fungal Metabolite
ID	8	73481	72935	OXIDATIVE DAMAGE	Oxidative Damage
ID	9	73481	72935	IMMUNE-RESPONSE	Immune Response
ID	10	73481	72935	BETA-ZEARALENOL	Beta Zearalenol

Record 496 | WOS:000334436600041

Mycotoxins' Activity at Toxic and Sub-Toxic Concentrations: Differential Cytotoxic and Genotoxic Effects of Single and Combined Administration of Sterigmatocystin, Ochratoxin A and Citrinin on the Hepatocellular Cancer Cell Line Hep3B

Field	Position	Original line	Normalised line	Original value	Normalised value
DE	1	73627	73080	sterigmatocystin	Sterigmatocystin
DE	2	73627	73080	ochratoxin A	Ochratoxin A (OTA)
DE	3	73627	73080	citrinin	Citrinin
DE	4	73627	73080	metabolic activity	Metabolic Activity
DE	5	73627	73080	genotoxicity	Genotoxicity
DE	6	73627	73080	cytostaticity	Cytostaticity
DE	7	73627	73080	cytotoxicity	Cytotoxicity
DE	8	73627	73080	picoMolar concentrations	Picomolar Concentration
ID	1	73629	73082	SISTER-CHROMATID EXCHANGES	Sister Chromatid Exchange
ID	2	73629	73082	OXIDATIVE DNA-DAMAGE	Oxidative DNA Damage
ID	3	73629	73082	HAMSTER OVARY CELLS	Hamster Ovary
ID	4	73629	73082	BONE-MARROW-CELLS	Bone Marrow
ID	5	73629	73082	IN-VITRO	In-Vitro
ID	6	73629	73082	PHASE ARREST	Phase Arrest
ID	7	73629	73082	HUMAN HEALTH	Human Health
ID	8	73629	73082	MUTAGENICITY	Mutagenicity
ID	9	73629	73082	INDUCTION	Induction
ID	10	73629	73082	PENICILLIUM	Penicillium

Record 497 | WOS:000335746400013

Deficient Glutathione in the Pathophysiology of Mycotoxin-Related Illness

Field	Position	Original line	Normalised line	Original value	Normalised value
DE	1	73746	73198	oxidative stress	Oxidative Stress
DE	2	73746	73198	genetic polymorphism	Genetic Polymorphism
DE	3	73746	73198	mycotoxin	Mycotoxins
DE	4	73746	73198	glutathione	Glutathione (GSH)
DE	5	73746	73198	Nrf2 modification	Nrf2 Modification
DE	6	73746	73198	aflatoxin	Aflatoxin
DE	7	73746	73198	trichothecene	Trichothecene
DE	8	73746	73198	ochratoxin	Ochratoxin A (OTA)
ID	1	73748	73200	GAMMA-GLUTAMYL-TRANSPEPTIDASE	Gamma Glutamyl Transpeptidase
ID	2	73748	73200	ANTIGEN-PRESENTING CELLS	Antigen Presenting
ID	3	73748	73200	OCHRATOXIN-A	Ochratoxin A (OTA)
ID	4	73748	73200	OXIDATIVE STRESS	Oxidative Stress
ID	5	73748	73200	GENETIC POLYMORPHISMS	Genetic Polymorphism
ID	6	73748	73200	AFLATOXIN B-1	Aflatoxin B1 (AFB1)
ID	7	73748	73200	IN-VIVO	In-Vivo
ID	8	73748	73200	EPIGENETIC MECHANISMS	Epigenetic Mechanism
ID	9	73748	73200	ANTIOXIDANT DEFENSES	Antioxidant Defense
ID	10	73748	73200	MOLECULAR-MECHANISMS	Molecular Mechanism

Record 498 | WOS:000329417100014

Interaction effects of Fusarium enniatins (A, A1, B and B1) combinations on in vitro cytotoxicity of Caco-2 cells

Field	Position	Original line	Normalised line	Original value	Normalised value
DE	1	73895	73346	Enniatins	Enniatin
DE	3	73895	73346	Interactions	Interaction
DE	4	73895	73346	Isobolograms	Isobologram
DE	5	73895	73346	Caco-2 cells	Caco-2
ID	1	73896	73347	CHO-K1 CELLS	Cho K1
ID	2	73896	73347	MYCOTOXINS BEAUVERICIN	Mycotoxin Beauvericin
ID	3	73896	73347	FUMONISIN B-1	Fumonisin B1 (FB1)
ID	4	73896	73347	OCHRATOXIN-A	Ochratoxin A (OTA)
ID	5	73896	73347	DEOXYNIVALENOL	Deoxynivalenol (DON)
ID	6	73896	73347	PROLIFERATION	Proliferation
ID	7	73896	73347	AVENACEUM	Avenaceum
ID	8	73896	73347	TOXICITY	Toxicity

Record 499 | WOS:000334568500006

Cell-based models for mycotoxin screening and toxicity evaluation: an update

Field	Position	Original line	Normalised line	Original value	Normalised value
DE	3	73981	73431	cell culture	Cell
DE	4	73981	73431	cytotoxicity	Cytotoxicity
DE	5	73981	73431	exposure	Exposure
ID	1	73982	73432	T-2 TOXIN	T 2 Toxin
ID	2	73982	73432	FUSARIUM-MYCOTOXINS	Fusarium Mycotoxin
ID	3	73982	73432	OCHRATOXIN-A	Ochratoxin A (OTA)
ID	4	73982	73432	WORLDWIDE OCCURRENCE	Worldwide Occurrence
ID	5	73982	73432	INDUCED APOPTOSIS	Induced Apoptosis
ID	6	73982	73432	ALPHA-TOCOPHEROL	Alpha Tocopherol
ID	7	73982	73432	MAMMALIAN-CELLS	Mammalian
ID	8	73982	73432	DEOXYNIVALENOL	Deoxynivalenol (DON)
ID	9	73982	73432	FOOD	Food
ID	10	73982	73432	CULTURE	Culture

Record 500 | WOS:000339460900017

Ochratoxin A induces cytotoxicity, DNA damage and apoptosis in rat hepatocyte primary cell culture at nanomolar concentration

Field	Position	Original line	Normalised line	Original value	Normalised value
DE	1	74142	73591	cell viability	Cell Viability
DE	2	74142	73591	genotoxicity	Genotoxicity
DE	3	74142	73591	hepatotoxicity	Hepatotoxicity
DE	4	74142	73591	in vitro	In-Vitro
DE	5	74142	73591	ochratoxin A	Ochratoxin A (OTA)
ID	1	74143	73592	ENDOTHELIAL GROWTH-FACTOR	Endothelial Growth Factor
ID	2	74143	73592	STRAND BREAKS	Strand Break
ID	3	74143	73592	LIVER	Liver
ID	4	74143	73592	GENOTOXICITY	Genotoxicity
ID	5	74143	73592	KIDNEY	Kidney
ID	6	74143	73592	EXPOSURE	Exposure
ID	7	74143	73592	EXPRESSION	Expression
ID	8	74143	73592	BIOMARKERS	Biomarker
ID	9	74143	73592	BIOACTIVATION	Bioactivation
ID	10	74143	73592	MYCOTOXINS	Mycotoxins

Record 501 | WOS:000329058600004

Ochratoxin A induces DNA double-strand breaks and large deletion mutations in the carcinogenic target site of gpt delta rats

Field	Position	Original line	Normalised line	Original value	Normalised value
ID	1	74280	73728	HISTONE H2AX PHOSPHORYLATION	Histone H2ax Phosphorylation
ID	2	74280	73728	ION-INDUCED MUTATIONS	Ion Induced Mutation
ID	3	74280	73728	TRANSGENIC MOUSE	Transgenic Mouse
ID	4	74280	73728	HOMOLOGOUS RECOMBINATION	Homologous Recombination
ID	5	74280	73728	BASE SUBSTITUTIONS	Base Substitution
ID	6	74280	73728	TOPOISOMERASE-II	Topoisomerase Ii
ID	7	74280	73728	MOLECULAR NATURE	Molecular Nature
ID	8	74280	73728	MAMMALIAN-CELLS	Mammalian
ID	9	74280	73728	HEAVY-ION	Heavy Ion
ID	10	74280	73728	GENOTOXICITY	Genotoxicity

Record 502 | WOS:000336092900022

A Review of the Evidence that Ochratoxin A Is an Nrf2 Inhibitor: Implications for Nephrotoxicity and Renal Carcinogenicity

Field	Position	Original line	Normalised line	Original value	Normalised value
DE	1	74399	73846	proximal tubule	Proximal Tubule
DE	2	74399	73846	ochratoxin	Ochratoxin A (OTA)
DE	3	74399	73846	oxidative	Oxidative
ID	1	74400	73847	TRANSCRIPTIONAL REGULATION	Transcriptional Regulation
ID	2	74400	73847	MOLECULAR-MECHANISM	Molecular Mechanism
ID	3	74400	73847	PROTEIN EXPRESSION	Protein Expression
ID	4	74400	73847	OXIDATIVE STRESS	Oxidative Stress
ID	5	74400	73847	EPITHELIAL-CELLS	Epithelial
ID	6	74400	73847	HEME OXYGENASE-1	Heme Oxygenase 1
ID	7	74400	73847	IN-VITRO	In-Vitro
ID	8	74400	73847	TOXICITY	Toxicity
ID	9	74400	73847	GENES	Gene
ID	10	74400	73847	MICE	Mice

Record 503 | WOS:000339460900010

Effects of lactic acid bacteria and Saccharomyces cerevisiae on growth of Aspergillus westerdijkiae and ochratoxin A production and toxicity

Field	Position	Original line	Normalised line	Original value	Normalised value
DE	1	74501	73947	yeast	Yeast
DE	2	74501	73947	lactic acid bacteria	Lactic Acid Bacteria
DE	3	74501	73947	antifungal activity	Antifungal Activity
DE	4	74501	73947	antigenotoxicity	Antigenotoxicity
DE	5	74501	73947	anticytotoxicity	Anticytotoxicity
ID	1	74503	73949	ANTIFUNGAL ACTIVITY	Antifungal Activity
ID	2	74503	73949	AFLATOXIN B-1	Aflatoxin B1 (AFB1)
ID	3	74503	73949	IN-VITRO	In-Vitro
ID	4	74503	73949	BIOLOGICAL-CONTROL	Biological Control
ID	5	74503	73949	MUTAGENICITY TEST	Mutagenicity Test
ID	6	74503	73949	GENE-EXPRESSION	Gene Expression
ID	7	74503	73949	SURFACE BINDING	Surface Binding
ID	8	74503	73949	MYCOTOXINS	Mycotoxins
ID	9	74503	73949	FOOD	Food
ID	10	74503	73949	CYTOTOXICITY	Cytotoxicity

Record 504 | WOS:000329960400028

Ochratoxin-induced toxicity, oxidative stress and apoptosis ameliorated by quercetin - Modulation by Nrf2

Field	Position	Original line	Normalised line	Original value	Normalised value

DE	1	74604	74049	Ochratoxin	Ochratoxin A (OTA)
DE	3	74604	74049	Oxidative stress	Oxidative Stress
DE	4	74604	74049	DNA damage	DNA Damage
ID	1	74605	74050	INDUCED CELL-DAMAGE	Induced Cell Damage
ID	2	74605	74050	DNA-DAMAGE	DNA Damage
ID	3	74605	74050	KIDNEY	Kidney
ID	4	74605	74050	GLUTATHIONE	Glutathione (GSH)
ID	5	74605	74050	PROTECTS	Protect
ID	6	74605	74050	CALCIUM	Calcium
ID	7	74605	74050	ACID	Acid
ID	8	74605	74050	ANTIOXIDANT	Antioxidant
ID	9	74605	74050	ACTIVATION	Activation
ID	10	74605	74050	INDUCTION	Induction

Record 505 | WOS:000328628900003

Deleterious Effects of Mycotoxin Combinations Involving Ochratoxin A

Field	Position	Original line	Normalised line	Original value	Normalised value
DE	1	74722	74166	mycotoxin interactions	Mycotoxin Interaction
DE	2	74722	74166	chronic renal diseases	Chronic Renal Disease
DE	3	74722	74166	genotoxicity	Genotoxicity
DE	4	74722	74166	carcinogenicity	Carcinogenicity
ID	1	74724	74167	KIDNEY PK15 CELLS	Kidney Pk15
ID	2	74724	74167	CITRININ-INDUCED DYSFUNCTION	Citrinin Induced Dysfunction
ID	3	74724	74167	FUMONISIN B-1	Fumonisin B1 (FB1)
ID	4	74724	74167	PENICILLIC ACID	Penicillic Acid
ID	5	74724	74167	AFLATOXIN B-1	Aflatoxin B1 (AFB1)
ID	6	74724	74167	DNA-DAMAGE	DNA Damage
ID	7	74724	74167	IN-VIVO	In-Vivo
ID	8	74724	74167	FUSARIUM-MONILIFORME	Fusarium Moniliforme
ID	9	74724	74167	NATURAL COOCCURRENCE	Natural Cooccurrence
ID	10	74724	74167	ENDEMIC NEPHROPATHY	Endemic Nephropathy

Record 506 | WOS:000327686300025

In vitro investigation of individual and combined cytotoxic effects of aflatoxin B1 and other selected mycotoxins on the cell line porcine kidney 15

Field	Position	Original line	Normalised line	Original value	Normalised value
DE	1	74910	74352	Aflatoxin B1	Aflatoxin B1 (AFB1)
DE	2	74910	74352	Zearalenone	Zearalenone (ZEA)
DE	3	74910	74352	Deoxynivalenol	Deoxynivalenol (DON)
DE	4	74910	74352	Porcine kidney 15 cells	Porcine Kidney 15
DE	5	74910	74352	ROS production	ROS Production
ID	1	74912	74354	FUMONISIN B-1	Fumonisin B1 (FB1)
ID	2	74912	74354	OCHRATOXIN-A	Ochratoxin A (OTA)
ID	3	74912	74354	PK15 CELLS	Pk15
ID	4	74912	74354	T-2 TOXIN	T 2 Toxin
ID	5	74912	74354	DEOXYNIVALENOL	Deoxynivalenol (DON)
ID	6	74912	74354	ZEARALENONE	Zearalenone (ZEA)
ID	7	74912	74354	BEAUVERICIN	Beauvericin
ID	8	74912	74354	APOPTOSIS	Apoptosis
ID	9	74912	74354	TOXICITY	Toxicity
ID	10	74912	74354	CARCINOGENICITY	Carcinogenicity

Record 507 | WOS:000326211800003

Multi-mycotoxin determination in baby foods and in vitro combined cytotoxic effects of aflatoxin M1 and ochratoxin A

Field	Position	Original line	Normalised line	Original value	Normalised value
DE	1	75004	74445	aflatoxin M-1	Aflatoxin M1 (AFM1)

DE	2	75004	74445	ochratoxin A	Ochratoxin A (OTA)
DE	3	75004	74445	HPLC-FLD	Hplc Fld
DE	4	75004	74445	mixtures modelling	Mixture Modelling
DE	5	75004	74445	cytotoxicity	Cytotoxicity
ID	1	75005	74447	INFANT FORMULAS	Infant Formula
ID	2	75005	74447	BINARY-MIXTURES	Binary Mixture
ID	3	75005	74447	COMET ASSAY	Comet Assay
ID	4	75005	74447	T-2 TOXIN	T 2 Toxin
ID	5	75005	74447	GENOTOXICITY	Genotoxicity
ID	6	75005	74447	TOXICITY	Toxicity
ID	7	75005	74447	CELLS	Cell
ID	8	75005	74447	B-1	B 1
ID	9	75005	74447	DEOXYNIVALENOL	Deoxynivalenol (DON)
ID	10	75005	74447	BEAUVERICIN	Beauvericin

Record 508 | WOS:000324301900014

Beauvericin-induced cytotoxicity via ROS production and mitochondrial damage in Caco-2 cells

Field	Position	Original line	Normalised line	Original value	Normalised value
DE	2	75122	74563	Oxidative stress	Oxidative Stress
DE	3	75122	74563	Cell cycle	Cell Cycle
DE	5	75122	74563	Mitochondrial membrane potential	Mitochondrial Membrane Potential
DE	6	75122	74563	DNA damage	DNA Damage
ID	1	75124	74565	EMERGING MYCOTOXINS ENNIATINS	Emerging Mycotoxin Enniatin
ID	2	75124	74565	KIDNEY PK15 CELLS	Kidney Pk15
ID	3	75124	74565	OCHRATOXIN-A	Ochratoxin A (OTA)
ID	4	75124	74565	FUSARIUM MYCOTOXINS	Fusarium Mycotoxin
ID	5	75124	74565	OXIDATIVE STRESS	Oxidative Stress
ID	6	75124	74565	CYTOCHROME-C	Cytochrome C
ID	7	75124	74565	CHO-K1 CELLS	Cho K1
ID	8	75124	74565	DNA-DAMAGE	DNA Damage
ID	9	75124	74565	T-2 TOXIN	T 2 Toxin
ID	10	75124	74565	IN-VITRO	In-Vitro

Record 509 | WOS:000324773300006

Oxidative DNA damage is involved in ochratoxin A-induced G2 arrest through ataxia telangiectasia-mutated (ATM) pathways in human gastric epithelium GES-1 cells in vitro

Field	Position	Original line	Normalised line	Original value	Normalised value
DE	1	75241	74681	Ochratoxin A	Ochratoxin A (OTA)
DE	2	75241	74681	G(2) arrest	G 2 Arrest
DE	3	75241	74681	DNA damage	DNA Damage
DE	4	75241	74681	Ataxia telangiectasia mutated	Ataxia Telangiectasia Mutated
DE	5	75241	74681	Oxidative stress	Oxidative Stress
ID	1	75243	74683	DOUBLE-STRAND BREAKS	Double Strand Break
ID	2	75243	74683	CYCLE ARREST	Cycle Arrest
ID	3	75243	74683	ROS GENERATION	ROS Generation
ID	4	75243	74683	PHASE ARREST	Phase Arrest
ID	5	75243	74683	APOPTOSIS	Apoptosis
ID	6	75243	74683	CHECKPOINT	Checkpoint
ID	7	75243	74683	CANCER	Cancer
ID	8	75243	74683	INDUCTION	Induction
ID	10	75243	74683	KIDNEY	Kidney

Record 510 | WOS:000326384200009

Toxicogenomics-Based Identification of Mechanisms for Direct Immunotoxicity

Field	Position	Original line	Normalised line	Original value	Normalised value
DE	1	75368	74807	toxicogenomics	Toxicogenomics
DE	2	75368	74807	direct immunotoxicity	Direct Immunotoxicity
ID	1	75369	74808	UNFOLDED PROTEIN RESPONSE	Unfolded Protein Response
ID	2	75369	74808	NF-KAPPA-B	Nf Kappa B
ID	3	75369	74808	ENDOPLASMIC-RETICULUM STRESS	Endoplasmic Reticulum Stress
ID	4	75369	74808	BLOOD MONONUCLEAR-CELLS	Blood Mononuclear
ID	5	75369	74808	LIVER-X-RECEPTOR	Liver X Receptor
ID	6	75369	74808	PROLIFERATOR-ACTIVATED RECEPTOR	Proliferator Activated Receptor
ID	7	75369	74808	GENE-EXPRESSION PROFILES	Gene Expression Profile
ID	8	75369	74808	IN-VITRO	In-Vitro
ID	9	75369	74808	OCHRATOXIN-A	Ochratoxin A (OTA)
ID	10	75369	74808	HUMAN-LYMPHOCYTES	Human Lymphocyte

Record 511 | WOS:000332372600004

Toxicity of Ochratoxin A and Its Modulation by Antioxidants: A Review

Field	Position	Original line	Normalised line	Original value	Normalised value
DE	1	75582	75019	ochratoxin A	Ochratoxin A (OTA)
DE	2	75582	75019	oxidative stress	Oxidative Stress
DE	3	75582	75019	DNA damage	DNA Damage
DE	4	75582	75019	antioxidants	Antioxidant
ID	1	75583	75020	NITRIC-OXIDE SYNTHASE	Nitric Oxide Synthase
ID	2	75583	75020	OXIDATIVE DNA-DAMAGE	Oxidative DNA Damage
ID	3	75583	75020	BALKAN ENDEMIC NEPHROPATHY	Balkan Endemic Nephropathy
ID	4	75583	75020	2'-DEOXYGUANOSINE-CARBON 8-BOUND OCHRATOXIN	2 Deoxyguanosine Carbon 8 Bound Ochratoxin
ID	5	75583	75020	INDUCED CELL-DAMAGE	Induced Cell Damage
ID	6	75583	75020	ALPHA-TOCOPHEROL	Alpha Tocopherol
ID	7	75583	75020	GREEN TEA	Green Tea
ID	8	75583	75020	SALMONELLA-TYPHIMURIUM	Salmonella Typhimurium
ID	9	75583	75020	HEME OXYGENASE-1	Heme Oxygenase 1
ID	10	75583	75020	RAT-KIDNEY	Rat Kidney

Record 512 | WOS:000322929800012

Identification of proteins related to early changes observed in Human hepatocellular carcinoma cells after treatment with the mycotoxin Zearalenone

Field	Position	Original line	Normalised line	Original value	Normalised value
DE	1	75816	75251	Zearalenone (ZEA)	Zearalenone ZEA
DE	2	75816	75251	Hepatocarcinoma cells (HepG2)	Hepatocarcinoma Cell HepG2
DE	3	75816	75251	Isobaric tagging for relative and absolute quantification (iTRAQ)	Isobaric Tagging For Relative And Absolute Quantification Itraq
ID	1	75818	75253	HEAT-SHOCK	Heat Shock
ID	2	75818	75253	CULTURED-CELLS	Cultured
ID	3	75818	75253	PROTEASOME INHIBITION	Proteasome Inhibition
ID	4	75818	75253	OXIDATIVE STRESS	Oxidative Stress
ID	5	75818	75253	OCHRATOXIN-A	Ochratoxin A (OTA)
ID	6	75818	75253	BONE-MARROW	Bone Marrow
ID	7	75818	75253	DNA-DAMAGE	DNA Damage

ID	8	75818	75253	INDUCTION	Induction
ID	9	75818	75253	NUCLEOPHOSMIN	Nucleophosmin
ID	10	75818	75253	TOXICITY	Toxicity

Record 513 | WOS:000324847800043

Individual and combined effects of mycotoxins from typical indoor moulds

Field	Position	Original line	Normalised line	Original value	Normalised value
DE	1	75946	75379	Indoor mould	Indoor Mould
DE	3	75946	75379	Combined effects	Combined Effect
DE	4	75946	75379	Concentration addition	Concentration Addition
DE	5	75946	75379	Independent action	Independent Action
ID	1	75948	75381	FUNGAL METABOLITE GLIOTOXIN	Fungal Metabolite Gliotoxin
ID	2	75948	75381	IN-VITRO PROLIFERATION	In Vitro Proliferation
ID	3	75948	75381	TETRAHYMENA-PYRIFORMIS	Tetrahymena Pyriformis
ID	4	75948	75381	ASPERGILLUS-FUMIGATUS	Aspergillus Fumigatus
ID	5	75948	75381	PENICILLIUM MYCOTOXINS	Penicillium Mycotoxin
ID	6	75948	75381	MEDIATED APOPTOSIS	Mediated Apoptosis
ID	7	75948	75381	DNA FRAGMENTATION	DNA Fragmentation
ID	8	75948	75381	MAMMALIAN-CELLS	Mammalian
ID	9	75948	75381	RISK-ASSESSMENT	Risk Assessment
ID	10	75948	75381	OCHRATOXIN-A	Ochratoxin A (OTA)

Record 514 | WOS:000328628700005

Producers and Important Dietary Sources of Ochratoxin A and Citrinin

Field	Position	Original line	Normalised line	Original value	Normalised value
DE	1	76089	75521	ochratoxin A	Ochratoxin A (OTA)
DE	2	76089	75521	citrinin	Citrinin
DE	3	76089	75521	producers	Producer
DE	4	76089	75521	microfungi	Microfungi
DE	5	76089	75521	dietary sources	Dietary Source
DE	6	76089	75521	foods	Food
ID	1	76090	75522	DRY-CURED HAM	Dry Cured Ham
ID	2	76090	75522	PERFORMANCE LIQUID-CHROMATOGRAPHY	Performance Liquid Chromatography
ID	3	76090	75522	BALKAN ENDEMIC NEPHROPATHY	Balkan Endemic Nephropathy
ID	4	76090	75522	PREGNANT WISTAR RATS	Pregnant Wistar Rat
ID	5	76090	75522	MYCOTOXIGENIC FUNGI	Mycotoxigenic Fungi
ID	6	76090	75522	NATURAL OCCURRENCE	Natural Occurrence
ID	7	76090	75522	MASS-SPECTROMETRY	Mass Spectrometry
ID	8	76090	75522	IN-VIVO	In-Vivo
ID	9	76090	75522	TOXICITY	Toxicity
ID	10	76090	75522	FOOD	Food

Record 515 | WOS:000328416400017

Fluorescence spectroscopic investigation of competitive interactions between ochratoxin A and 13 drug molecules for binding to human serum albumin

Field	Position	Original line	Normalised line	Original value	Normalised value
DE	1	76244	75675	competitive interaction	Competitive Interaction

DE	2	76244	75675	drug molecules	Drug Molecule
DE	3	76244	75675	fluorescence polarization	Fluorescence Polarization
DE	4	76244	75675	human serum albumin	Human Serum Albumin
DE	5	76244	75675	ochratoxin A	Ochratoxin A (OTA)
ID	1	76246	75677	PERFORMANCE AFFINITY-CHROMATOGRAPHY	Performance Affinity Chromatography
ID	2	76246	75677	BALKAN ENDEMIC NEPHROPATHY	Balkan Endemic Nephropathy
ID	3	76246	75677	PHASE ARREST	Phase Arrest
ID	4	76246	75677	PLASMA	Plasma
ID	5	76246	75677	PROTEIN	Protein
ID	6	76246	75677	UPDATE	Update
ID	7	76246	75677	KIDNEY	Kidney
ID	8	76246	75677	CELLS	Cell
ID	9	76246	75677	RATS	Rat
ID	10	76246	75677	PHENYLBUTAZONE	Phenylbutazone

Record 516 | WOS:000324280000095

A review on ochratoxin A transcriptomic studies

Field	Position	Original line	Normalised line	Original value	Normalised value
DE	1	76357	75787	Mycotoxin	Mycotoxins
DE	2	76357	75787	Ochratoxin A	Ochratoxin A (OTA)
DE	4	76357	75787	Microarrays	Microarray
DE	5	76357	75787	Gene expression	Gene Expression
ID	1	76359	75789	GENE-EXPRESSION ANALYSIS	Gene Expression Analysis
ID	2	76359	75789	A-INDUCED GENOTOXICITY	A Induced Genotoxicity
ID	3	76359	75789	RENAL TUMOR-FORMATION	Renal Tumor Formation
ID	4	76359	75789	INDUCED IN-VITRO	Induced In Vitro
ID	5	76359	75789	RISK-ASSESSMENT	Risk Assessment
ID	6	76359	75789	MOLECULAR-MECHANISM	Molecular Mechanism
ID	7	76359	75789	CELL-PROLIFERATION	Cell Proliferation
ID	8	76359	75789	MALE RATS	Male Rat
ID	9	76359	75789	TOXICITY	Toxicity
ID	10	76359	75789	CARCINOGENICITY	Carcinogenicity

Record 517 | WOS:000320498300035

Individual and combined cytotoxic effects of Fusarium toxins (deoxynivalenol, nivalenol, zearalenone and fumonisins B1) on swine jejunal epithelial cells

Field	Position	Original line	Normalised line	Original value	Normalised value
DE	1	76523	75952	Deoxynivalenol	Deoxynivalenol (DON)
DE	3	76523	75952	Zearalenone	Zearalenone (ZEA)
DE	4	76523	75952	Fumonisin B1	Fumonisin B1 (FB1)
DE	6	76523	75952	Intestinal epithelial cells	Intestinal Epithelial
ID	1	76525	75954	IN-VITRO	In-Vitro
ID	2	76525	75954	MTT BIOASSAY	Mtt Bioassay
ID	3	76525	75954	CELLULAR PROLIFERATION	Cellular Proliferation
ID	4	76525	75954	ALPHA-ZEARALENOL	Alpha Zearalenol
ID	5	76525	75954	ANIMAL HEALTH	Animal Health
ID	6	76525	75954	CACO-2 CELLS	Caco-2
ID	7	76525	75954	LINE IPEC-J2	Line IPEC-J2
ID	8	76525	75954	OCHRATOXIN-A	Ochratoxin A (OTA)
ID	9	76525	75954	MYCOTOXINS	Mycotoxins
ID	10	76525	75954	TOXICITY	Toxicity

Record 518 | WOS:000320770400009

Computational Design of Peptide Ligands for Ochratoxin A

Field	Position	Original line	Normalised line	Original value	Normalised value
DE	1	76668	76096	ochratoxin A	Ochratoxin A (OTA)
DE	2	76668	76096	mycotoxins	Mycotoxins
DE	3	76668	76096	peptide	Peptide
DE	4	76668	76096	computational modelling	Computational Modelling
DE	5	76668	76096	surface plasmon resonance	Surface Plasmon Resonance
DE	6	76668	76096	biosensor	Biosensor
ID	1	76670	76098	PROTEIN-PROTEIN INTERACTIONS	Protein Protein Interaction
ID	2	76670	76098	SERUM-ALBUMIN	Serum Albumin
ID	3	76670	76098	BINDING	Binding
ID	4	76670	76098	CARCINOGENICITY	Carcinogenicity
ID	5	76670	76098	MYCOTOXINS	Mycotoxins
ID	6	76670	76098	RECOGNITION	Recognition
ID	7	76670	76098	PRINCIPLES	Principle
ID	8	76670	76098	BIOSENSORS	Biosensor
ID	9	76670	76098	TOXICITY	Toxicity
ID	10	76670	76098	SURFACE	Surface

Record 519 | WOS:000320571000011

Effect of quercetin on the toxicokinetics of ochratoxin A in rats

Field	Position	Original line	Normalised line	Original value	Normalised value
DE	1	76772	76199	Ochratoxin A	Ochratoxin A (OTA)
DE	2	76772	76199	quercetin	Quercetin
DE	3	76772	76199	toxicokinetic	Toxicokinetic
DE	4	76772	76199	multi-drug resistance associated protein (MRP2)	Multi Drug Resistance Associated Protein Mrp2
ID	1	76774	76201	CANCER RESISTANCE PROTEIN	Cancer Resistance Protein
ID	2	76774	76201	CACO-2 CELLS	Caco-2
ID	3	76774	76201	IN-VITRO	In-Vitro
ID	4	76774	76201	LIPID-PEROXIDATION	Lipid Peroxidation
ID	5	76774	76201	CHRONIC INGESTION	Chronic Ingestion
ID	6	76774	76201	MRP2	Mrp2
ID	7	76774	76201	TRANSPORT	Transport
ID	8	76774	76201	POLYPHENOLS	Polyphenol
ID	9	76774	76201	HYDROLYSIS	Hydrolysis
ID	10	76774	76201	METABOLISM	Metabolism

Record 520 | WOS:000316978700004

Zinc protects HepG2 cells against the oxidative damage and DNA damage induced by ochratoxin A

Field	Position	Original line	Normalised line	Original value	Normalised value
DE	1	76870	76296	Ochratoxin A	Ochratoxin A (OTA)
DE	4	76870	76296	Oxidative damage	Oxidative Damage
DE	5	76870	76296	DNA damage	DNA Damage
ID	1	76871	76297	SALMONELLA-TYPHIMURIUM	Salmonella Typhimurium
ID	2	76871	76297	MONONUCLEAR-CELLS	Mononuclear
ID	3	76871	76297	IN-VITRO	In-Vitro
ID	4	76871	76297	STRESS	Stress
ID	5	76871	76297	TOXICITY	Toxicity
ID	6	76871	76297	METALLOTHIONEIN	Metallothionein
ID	7	76871	76297	SUPPLEMENTATION	Supplementation
ID	8	76871	76297	METABOLISM	Metabolism
ID	9	76871	76297	APOPTOSIS	Apoptosis
ID	10	76871	76297	RAT	Rat

Record 521 | WOS:000315160400007

Role of hMLH1 in sterigmatocystin-induced G2 phase arrest in human esophageal epithelial Het-1A cells in vitro

Field	Position	Original line	Normalised line	Original value	Normalised value
DE	2	76984	76409	G(2) phase arrest	G 2 Phase Arrest
DE	3	76984	76409	DNA damage	DNA Damage
DE	4	76984	76409	Mismatch repair	Mismatch Repair
DE	5	76984	76409	hMLH1	Hmlh1
ID	1	76985	76410	DNA MISMATCH REPAIR	DNA Mismatch Repair
ID	2	76985	76410	SINGLE-STRAND BREAKS	Single Strand Break
ID	3	76985	76410	CYCLE ARREST	Cycle Arrest
ID	4	76985	76410	OCHRATOXIN-A	Ochratoxin A (OTA)
ID	5	76985	76410	G2/M ARREST	G2 M Arrest
ID	6	76985	76410	CHECKPOINT	Checkpoint
ID	7	76985	76410	DAMAGE	Damage
ID	8	76985	76410	DEOXYNIVALENOL	Deoxynivalenol (DON)
ID	9	76985	76410	APOPTOSIS	Apoptosis
ID	10	76985	76410	CANCER	Cancer

Record 522 | WOS:000316229200014

Cross-talk between microRNAs, nuclear factor E2-related factor 2, and heme oxygenase-1 in ochratoxin A-induced toxic effects in renal proximal tubular epithelial cells

Field	Position	Original line	Normalised line	Original value	Normalised value
DE	2	77111	76535	HO-1	Ho 1
DE	3	77111	76535	miR-132	Mir 132
DE	4	77111	76535	miR-200c	Mir 200c
DE	5	77111	76535	Ochratoxin A	Ochratoxin A (OTA)
ID	1	77112	76536	MESENCHYMAL TRANSITION	Mesenchymal Transition
ID	2	77112	76536	OXIDATIVE STRESS	Oxidative Stress
ID	3	77112	76536	TRANSCRIPTION FACTORS	Transcription Factor
ID	4	77112	76536	MOLECULAR-MECHANISMS	Molecular Mechanism
ID	5	77112	76536	EXPRESSION	Expression
ID	6	77112	76536	DAMAGE	Damage
ID	7	77112	76536	ERYTHROPOIETIN	Erythropoietin
ID	8	77112	76536	PROTECTS	Protect
ID	9	77112	76536	SYSTEM	System
ID	10	77112	76536	INJURY	Injury

Record 523 | WOS:000316522300011

Effects of p53 knockout on ochratoxin A-induced genotoxicity in p53-deficient gpt delta mice

Field	Position	Original line	Normalised line	Original value	Normalised value
DE	4	77261	76683	p53	P53
DE	5	77261	76683	Ochratoxin A	Ochratoxin A (OTA)
ID	1	77262	76684	DOUBLE-STRAND BREAKS	Double Strand Break
ID	2	77262	76684	HOMOLOGOUS RECOMBINATION	Homologous Recombination
ID	3	77262	76684	TRANSCRIPTIONAL REGULATION	Transcriptional Regulation
ID	4	77262	76684	DNA-DAMAGE	DNA Damage
ID	5	77262	76684	CELLS	Cell
ID	6	77262	76684	APOPTOSIS	Apoptosis
ID	7	77262	76684	REPAIR	Repair
ID	8	77262	76684	INACTIVATION	Inactivation
ID	9	77262	76684	STABILITY	Stability
ID	10	77262	76684	KIDNEY	Kidney

Record 524 | WOS:000315408000011

Modulation of Intestinal Functions Following Mycotoxin Ingestion: Meta-Analysis of Published Experiments in Animals

Field	Position	Original line	Normalised line	Original value	Normalised value
DE	1	77361	76781	mycotoxin	Mycotoxins
DE	2	77361	76781	gastrointestinal tract	Gastrointestinal Tract
DE	3	77361	76781	nutrients	Nutrient
DE	4	77361	76781	gut permeability	Gut Permeability
DE	5	77361	76781	mucosal immunity	Mucosal Immunity
DE	6	77361	76781	gut microbiota	Gut Microbiota
ID	1	77363	76783	TOXIN-CONTAMINATED WHEAT	Toxin Contaminated Wheat
ID	2	77363	76783	MICROBIAL PROTEIN-SYNTHESIS	Microbial Protein Synthesis
ID	3	77363	76783	AFLATOXIN B-1 EXPOSURE	Aflatoxin B 1 Exposure
ID	4	77363	76783	FUMONISIN B-1	Fumonisin B1 (FB1)
ID	5	77363	76783	NUTRIENT DIGESTIBILITY	Nutrient Digestibility
ID	6	77363	76783	OCHRATOXIN-A	Ochratoxin A (OTA)
ID	7	77363	76783	FUSARIUM MYCOTOXINS	Fusarium Mycotoxin
ID	8	77363	76783	GROWTH-PERFORMANCE	Growth Performance
ID	9	77363	76783	BARRIER FUNCTION	Barrier Function
ID	10	77363	76783	WORLDWIDE OCCURRENCE	Worldwide Occurrence

Record 525 | WOS:000314671300007

Molecular mechanisms underlying ochratoxin A-induced genotoxicity: global gene expression analysis suggests induction of DNA double-strand breaks and cell cycle progression

Field	Position	Original line	Normalised line	Original value	Normalised value
DE	2	77541	76961	DNA damage	DNA Damage
DE	5	77541	76961	Mycotoxin	Mycotoxins
ID	1	77542	76962	HOMOLOGOUS RECOMBINATION	Homologous Recombination
ID	2	77542	76962	IN-VIVO	In-Vivo
ID	3	77542	76962	REPAIR	Repair
ID	5	77542	76962	TOXICITY	Toxicity
ID	6	77542	76962	DAMAGE	Damage
ID	7	77542	76962	KIDNEY	Kidney
ID	8	77542	76962	PATHWAYS	Pathway
ID	9	77542	76962	CARCINOGENICITY	Carcinogenicity
ID	10	77542	76962	APOPTOSIS	Apoptosis

Record 526 | WOS:000316706800031

An iTRAQ-based mitoproteomics approach for profiling the nephrotoxicity mechanisms of ochratoxin A in HEK 293 cells

Field	Position	Original line	Normalised line	Original value	Normalised value
DE	1	77669	77088	Ochratoxin A	Ochratoxin A (OTA)
DE	4	77669	77088	Cell death	Cell Death
DE	5	77669	77088	N-acetyl-L-cysteine	N Acetyl L Cysteine
ID	1	77671	77090	DIFFERENTIALLY EXPRESSED PROTEINS	Differentially Expressed Protein
ID	2	77671	77090	APOPTOSIS-INDUCING FACTOR	Apoptosis Inducing Factor
ID	3	77671	77090	OXIDATIVE DNA-DAMAGE	Oxidative DNA Damage
ID	4	77671	77090	CYTOCHROME-C	Cytochrome C
ID	5	77671	77090	MITOCHONDRIAL PROTEOMICS	Mitochondrial Proteomics

ID	6	77671	77090	PERMEABILITY TRANSITION	Permeability Transition
ID	7	77671	77090	ENDOPLASMIC-RETICULUM	Endoplasmic Reticulum
ID	8	77671	77090	GENE-EXPRESSION	Gene Expression
ID	9	77671	77090	IN-VITRO	In-Vitro
ID	10	77671	77090	MEMBRANE	Membrane

Record 527 | WOS:000318093700001

A Review of the Mechanism of Injury and Treatment Approaches for Illness Resulting from Exposure to Water-Damaged Buildings, Mold, and Mycotoxins

Field	Position	Original line	Normalised line	Original value	Normalised value
ID	1	77803	77221	BLOOD-BRAIN-BARRIER	Blood Brain Barrier
ID	2	77803	77221	MACROCYCLIC TRICHOTHECENE MYCOTOXIN	Macrocyclic Trichothecene Mycotoxin
ID	3	77803	77221	INCREASED OXIDATIVE STRESS	Increased Oxidative Stress
ID	4	77803	77221	ACUTE INHALATION TOXICITY	Acute Inhalation Toxicity
ID	5	77803	77221	CENTRAL-NERVOUS-SYSTEM	Central Nervous System
ID	6	77803	77221	OCHRATOXIN-A	Ochratoxin A (OTA)
ID	7	77803	77221	IN-VITRO	In-Vitro
ID	8	77803	77221	GLUTATHIONE DEFICIENCY	Glutathione Deficiency
ID	9	77803	77221	STACHYBOTRYS-CHARTARUM	Stachybotry Chartarum
ID	10	77803	77221	DNA-DAMAGE	DNA Damage

Record 528 | WOS:000314048800053

Ochratoxin A Inhibits Mouse Embryonic Development by Activating a Mitochondrion-Dependent Apoptotic Signaling Pathway

Field	Position	Original line	Normalised line	Original value	Normalised value
DE	1	78062	77479	ochratoxin A	Ochratoxin A (OTA)
DE	2	78062	77479	blastocyst	Blastocyst
DE	3	78062	77479	apoptosis	Apoptosis
DE	4	78062	77479	development	Development
DE	5	78062	77479	ROS	Reactive Oxygen Species (ROS)
ID	1	78063	77481	INNER CELL MASS	Inner Cell Mass
ID	2	78063	77481	CITRININ INDUCES APOPTOSIS	Citrinin Induce Apoptosis
ID	3	78063	77481	DEVELOPMENT IN-VITRO	Development In Vitro
ID	4	78063	77481	STEM-CELLS	Stem
ID	5	78063	77481	OXIDATIVE STRESS	Oxidative Stress
ID	6	78063	77481	POSTIMPLANTATION DEVELOPMENT	Postimplantation Development
ID	7	78063	77481	CREATIVE AGENT	Creative Agent
ID	8	78063	77481	RETINOIC ACID	Retinoic Acid
ID	9	78063	77481	BLASTOCYSTS	Blastocyst
ID	10	78063	77481	DEATH	Death

Record 529 | WOS:000313124600008

Role of intestinal microflora in xenobiotic-induced toxicity

Field	Position	Original line	Normalised line	Original value	Normalised value
DE	1	78191	77608	In vitro	In-Vitro
DE	2	78191	77608	In vivo	In-Vivo
DE	4	78191	77608	Intestinal microflora	Intestinal Microflora

DE	5	78191	77608	Xenobiotic metabolism	Xenobiotic Metabolism
ID	1	78193	77609	FOOD-BORNE CARCINOGEN	Food Borne Carcinogen
ID	2	78193	77609	GERM-FREE	Germ Free
ID	3	78193	77609	COLON CARCINOGENESIS	Colon Carcinogenesis
ID	4	78193	77609	BETA-GLUCURONIDASE	Beta Glucuronidase
ID	5	78193	77609	PROTECTIVE ROLE	Protective Role
ID	6	78193	77609	ANTIVIRAL DRUG	Antiviral Drug
ID	7	78193	77609	GUT MICROBIOTA	Gut Microbiota
ID	8	78193	77609	OCHRATOXIN-A	Ochratoxin A (OTA)
ID	9	78193	77609	METABOLISM	Metabolism
ID	10	78193	77609	1-NITROPYRENE	1 Nitropyrene

Record 530 | WOS:000315356800004

Amelioration of Ochratoxin A-induced immunotoxic effects by silymarin and vitamin E in White Leghorn cockerels

Field	Position	Original line	Normalised line	Original value	Normalised value
DE	1	78331	77746	Ochratoxin A	Ochratoxin A (OTA)
DE	2	78331	77746	immunology	Immunology
DE	3	78331	77746	silymarin	Silymarin
ID	1	78332	77747	BROILER CHICKS	Broiler Chick
ID	2	78332	77747	IMMUNE-RESPONSE	Immune Response
ID	3	78332	77747	ASPERGILLUS-OCHRACEUS	Aspergillus Ochraceus
ID	4	78332	77747	IN-VITRO	In-Vitro
ID	5	78332	77747	AFLATOXIN	Aflatoxin
ID	6	78332	77747	TOXICITY	Toxicity
ID	7	78332	77747	CELLS	Cell
ID	8	78332	77747	IMMUNOSUPPRESSION	Immunosuppression
ID	9	78332	77747	COMBINATIONS	Combination
ID	10	78332	77747	PERFORMANCE	Performance

Record 531 | WOS:000312386800135

Nivalenol and Deoxynivalenol Affect Rat Intestinal Epithelial Cells: A Concentration Related Study

Field	Position	Original line	Normalised line	Original value	Normalised value
ID	1	78441	77855	NATURAL OCCURRENCE	Natural Occurrence
ID	2	78441	77855	FUSARIUM TOXINS	Fusarium Toxin
ID	3	78441	77855	OCHRATOXIN-A	Ochratoxin A (OTA)
ID	4	78441	77855	MYCOTOXINS	Mycotoxins
ID	5	78441	77855	TRICHOTHECENES	Trichothecene
ID	6	78441	77855	EXPOSURE	Exposure
ID	7	78441	77855	SURVIVAL	Survival
ID	8	78441	77855	PROTEIN	Protein
ID	9	78441	77855	FOOD	Food
ID	10	78441	77855	PHOSPHORYLATION	Phosphorylation

Record 532 | WOS:000312288000010

Lactobacillus reuteri CRL 1098 and Lactobacillus acidophilus CRL 1014 differently reduce in vitro immunotoxic effect induced by Ochratoxin A

Field	Position	Original line	Normalised line	Original value	Normalised value
DE	1	78558	77971	Ochratoxin A	Ochratoxin A (OTA)
DE	3	78558	77971	Cytokines	Cytokine
DE	5	78558	77971	Membrane rafts	Membrane Raft
ID	1	78559	77972	LACTIC-ACID BACTERIA	Lactic Acid Bacteria
ID	2	78559	77972	BLOOD MONONUCLEAR-CELLS	Blood Mononuclear

ID	3	78559	77972	CYTOKINE PRODUCTION	Cytokine Production
ID	4	78559	77972	DEFENSE REACTIONS	Defense Reaction
ID	5	78559	77972	IMMUNE	Immune
ID	6	78559	77972	ACTIVATION	Activation
ID	7	78559	77972	TOXICITY	Toxicity
ID	8	78559	77972	NEPHROPATHY	Nephropathy
ID	9	78559	77972	FOODSTUFFS	Foodstuff
ID	10	78559	77972	AFLATOXIN	Aflatoxin

Record 533 | WOS:000310867400006

Increase in Mitochondrial DNA Copy Number in Response to Ochratoxin A and Methanol-Induced Mitochondrial DNA Damage in Drosophila

Field	Position	Original line	Normalised line	Original value	Normalised value
DE	2	78664	78075	Ochratoxin A	Ochratoxin A (OTA)
DE	3	78664	78075	mtDNA copy number	Mtdna Copy Number
DE	4	78664	78075	mtDNA damage	Mtdna Damage
ID	1	78665	78076	OXIDATIVE STRESS	Oxidative Stress
ID	2	78665	78076	ADDUCT FORMATION	Adduct Formation
ID	3	78665	78076	RAT	Rat
ID	4	78665	78076	CELLS	Cell

Record 534 | WOS:000312288000021

Evaluation of nephrotoxic effects of mycotoxins, citrinin and patulin, on zebrafish (Danio rerio) embryos

Field	Position	Original line	Normalised line	Original value	Normalised value
ID	1	78741	78151	PREGNANT WISTAR RATS	Pregnant Wistar Rat
ID	2	78741	78151	ACUTE-RENAL-FAILURE	Acute Renal Failure
ID	3	78741	78151	OCHRATOXIN-A	Ochratoxin A (OTA)
ID	4	78741	78151	IN-VITRO	In-Vitro
ID	5	78741	78151	PENTOXIFYLLINE	Pentoxifylline
ID	6	78741	78151	TOXICITY	Toxicity
ID	7	78741	78151	PATHWAY	Pathway
ID	8	78741	78151	MODEL	Model
ID	9	78741	78151	VERTEBRATE	Vertebrate
ID	10	78741	78151	APOPTOSIS	Apoptosis

Record 535 | WOS:000308629100003

Human skin penetration of selected model mycotoxins

Field	Position	Original line	Normalised line	Original value	Normalised value
DE	2	78870	78279	Dermal exposure	Dermal Exposure
DE	3	78870	78279	Risk assessment	Risk Assessment
DE	4	78870	78279	Transdermal penetration	Transdermal Penetration
DE	5	78870	78279	Human skin	Human Skin
ID	1	78872	78281	H-3 T-2 TOXIN	H 3 T 2 Toxin
ID	2	78872	78281	WBN/ILA-HT RATS	Wbn Ila Ht Rat
ID	3	78872	78281	TOPICAL APPLICATION	Topical Application
ID	4	78872	78281	OCHRATOXIN-A	Ochratoxin A (OTA)
ID	5	78872	78281	TRICHOTHECENE MYCOTOXINS	Trichothecene Mycotoxin
ID	6	78872	78281	RISK-ASSESSMENT	Risk Assessment
ID	7	78872	78281	MESSENGER-RNA	Messenger RNA
ID	8	78872	78281	FUMONISIN B-1	Fumonisin B1 (FB1)
ID	9	78872	78281	DORSAL SKIN	Dorsal Skin
ID	10	78872	78281	EXPOSURE	Exposure

Record 536 | WOS:000315406500009

Apoptosis Induction by OTA and TNF-Î± in Cultured Primary Rat Hepatocytes and Prevention by Silibinin

Field	Position	Original line	Normalised line	Original value	Normalised value
DE	1	79047	78455	ochratoxin A	Ochratoxin A (OTA)
DE	2	79047	78455	tumor necrosis factor alpha	Tumor Necrosis Factor Alpha
DE	3	79047	78455	UV-C light	Uv C Light
DE	4	79047	78455	hydrogen peroxide	Hydrogen Peroxide
DE	5	79047	78455	rat hepatocytes	Rat Hepatocyte
DE	6	79047	78455	DNA ladder	DNA Ladder
DE	7	79047	78455	caspase-3	Caspase 3
DE	8	79047	78455	apoptosis	Apoptosis
DE	9	79047	78455	malondialdehyde	Malondialdehyde
DE	10	79047	78455	silibinin	Silibinin
ID	1	79050	78458	OCHRATOXIN-A	Ochratoxin A (OTA)
ID	2	79050	78458	OXIDATIVE STRESS	Oxidative Stress
ID	3	79050	78458	LIVER	Liver
ID	4	79050	78458	SILYMARIN	Silymarin
ID	5	79050	78458	ACTIVATION	Activation
ID	6	79050	78458	MECHANISMS	Mechanism
ID	7	79050	78458	TOXICITY	Toxicity
ID	8	79050	78458	PROTECTION	Protection
ID	9	79050	78458	RECEPTOR	Receptor
ID	10	79050	78458	EXPOSURE	Exposure

Record 537 | WOS:000309849700023

Co-exposure of Fusarium mycotoxins: In vitro myelotoxicity assessment on human hematopoietic progenitors

Field	Position	Original line	Normalised line	Original value	Normalised value
DE	2	79155	78562	Co-exposure	Co Exposure
DE	3	79155	78562	Human hematopoietic progenitors	Human Hematopoietic Progenitor
DE	4	79155	78562	Fusariotoxins	Fusariotoxin
DE	5	79155	78562	In vitro	In-Vitro
ID	1	79157	78564	KIDNEY PK15 CELLS	Kidney Pk15
ID	2	79157	78564	GRANULO-MONOCYTIC PROGENITORS	Granulo Monocytic Progenitor
ID	3	79157	78564	OCHRATOXIN-A	Ochratoxin A (OTA)
ID	4	79157	78564	TOXICOLOGICAL INTERACTIONS	Toxicological Interaction
ID	5	79157	78564	MURINE MACROPHAGES	Murine Macrophage
ID	6	79157	78564	ALPHA-ZEARALENOL	Alpha Zearalenol
ID	7	79157	78564	FUMONISIN B-1	Fumonisin B1 (FB1)
ID	8	79157	78564	CYTOCHROME-C	Cytochrome C
ID	9	79157	78564	COMET ASSAY	Comet Assay
ID	10	79157	78564	ENNIATIN B	Enniatin B

Record 538 | WOS:000309897100023

Immune modulatory effects of the foodborne contaminant citrinin in mice

Field	Position	Original line	Normalised line	Original value	Normalised value
DE	2	79261	78667	Immune cells	Immune
DE	5	79261	78667	Cytokines	Cytokine
DE	6	79261	78667	IgM	Igm
ID	1	79262	78668	BLOOD MONONUCLEAR-CELLS	Blood Mononuclear
ID	2	79262	78668	NECROSIS-FACTOR-ALPHA	Necrosis Factor Alpha
ID	3	79262	78668	TOLL-LIKE RECEPTORS	Toll Like Receptor
ID	4	79262	78668	FACTOR-KAPPA-B	Factor Kappa B
ID	5	79262	78668	T-CELLS	T

ID	6	79262	78668	MYCOTOXINS CITRININ	Mycotoxin Citrinin
ID	7	79262	78668	INDUCED DYSFUNCTION	Induced Dysfunction
ID	8	79262	78668	FUNGAL METABOLITE	Fungal Metabolite
ID	9	79262	78668	OCHRATOXIN-A	Ochratoxin A (OTA)
ID	10	79262	78668	APOPTOSIS	Apoptosis

Record 539 | WOS:000309897100058

Fumonisin B1 and the kidney: Modes of action for renal tumor formation by fumonisin B1 in rodents

Field	Position	Original line	Normalised line	Original value	Normalised value
DE	1	79364	78769	Fumonisin B-1	Fumonisin B1 (FB1)
DE	6	79364	78769	Food contaminant	Food Contaminant
ID	1	79366	78771	PROTEIN-KINASE-C	Protein Kinase C
ID	2	79366	78771	NOVO SPHINGOLIPID BIOSYNTHESIS	Novo Sphingolipid Biosynthesis
ID	3	79366	78771	HUMAN ESOPHAGEAL CANCER	Human Esophageal Cancer
ID	4	79366	78771	NECROSIS-FACTOR-ALPHA	Necrosis Factor Alpha
ID	5	79366	78771	CERAMIDE SYNTHASE	Ceramide Synthase
ID	6	79366	78771	RAT-LIVER	Rat Liver
ID	7	79366	78771	IN-VITRO	In-Vitro
ID	8	79366	78771	OCHRATOXIN-A	Ochratoxin A (OTA)
ID	9	79366	78771	DNA-DAMAGE	DNA Damage
ID	10	79366	78771	SPHINGOSINE 1-PHOSPHATE	Sphingosine 1 Phosphate

Record 540 | WOS:000308289000002

The food contaminant and nephrotoxin ochratoxin A enhances Wnt1 inducible signaling protein 1 and tumor necrosis factor-Î± expression in human primary proximal tubule cells

Field	Position	Original line	Normalised line	Original value	Normalised value
DE	1	79560	78964	Gene expression	Gene Expression
DE	2	79560	78964	Mycotoxin	Mycotoxins
DE	3	79560	78964	Ochratoxin A	Ochratoxin A (OTA)
DE	4	79560	78964	Proximal tubule	Proximal Tubule
DE	5	79560	78964	Tubulointerstitial nephritis	Tubulointerstitial Nephritis
ID	1	79562	78966	INDUCED CARDIOMYOCYTE DEATH	Induced Cardiomyocyte Death
ID	2	79562	78966	BALKAN ENDEMIC NEPHROPATHY	Balkan Endemic Nephropathy
ID	3	79562	78966	PRIMARY CULTURE	Primary
ID	4	79562	78966	NANOMOLAR CONCENTRATIONS	Nanomolar Concentration
ID	5	79562	78966	GROWTH-FACTOR	Growth Factor
ID	6	79562	78966	FIBROSIS	Fibrosis
ID	7	79562	78966	ACTIVATION	Activation
ID	8	79562	78966	SURVIVAL	Survival
ID	9	79562	78966	KIDNEY	Kidney
ID	10	79562	78966	GENES	Gene

Record 541 | WOS:000307323900012

Citrinin reduces testosterone secretion by inducing apoptosis in rat Leydig cells

Field	Position	Original line	Normalised line	Original value	Normalised value
DE	4	79673	79075	Leydig cell	Leydig
ID	1	79674	79076	ACUTE REGULATORY PROTEIN	Acute Regulatory Protein

ID	2	79674	79076	MONASCUS FERMENTATION PRODUCTS	Monascus Fermentation Product
ID	3	79674	79076	OCHRATOXIN-A	Ochratoxin A (OTA)
ID	4	79674	79076	IN-VITRO	In-Vitro
ID	5	79674	79076	STEROIDOGENESIS	Steroidogenesis
ID	6	79674	79076	MYCOTOXINS	Mycotoxins
ID	7	79674	79076	CHOLESTEROL	Cholesterol
ID	8	79674	79076	PATHWAY	Pathway
ID	9	79674	79076	PATULIN	Patulin

Record 542 | WOS:000307766500014

Cellular distribution of cell cycle-related molecules in the renal tubules of rats treated with renal carcinogens for 28 days: relationship between cell cycle aberration and carcinogenesis

Field	Position	Original line	Normalised line	Original value	Normalised value
DE	1	79775	79176	Renal carcinogenesis	Renal Carcinogenesis
DE	3	79775	79176	Cell cycle	Cell Cycle
DE	4	79775	79176	Cell proliferation	Cell Proliferation
ID	1	79777	79178	TOPOISOMERASE-II-ALPHA	Topoisomerase Ii Alpha
ID	2	79777	79178	IN-VIVO MUTAGENICITY	In Vivo Mutagenicity
ID	3	79777	79178	OCHRATOXIN-A	Ochratoxin A (OTA)
ID	4	79777	79178	MADDER COLOR	Madder Color
ID	5	79777	79178	TUMORS	Tumor
ID	6	79777	79178	TOXICITY	Toxicity
ID	7	79777	79178	PROTEIN	Protein
ID	8	79777	79178	PROLIFERATION	Proliferation
ID	9	79777	79178	DISSOCIATION	Dissociation
ID	10	79777	79178	NEPHROPATHY	Nephropathy

Record 543 | WOS:000307487100016

Protective Effect of Deferricoprogen Isolated from Monascus purpureus NTU 568 on Citrinin-Induced Apoptosis in HEK-293 Cells

Field	Position	Original line	Normalised line	Original value	Normalised value
DE	2	79898	79297	HEK-293 cells	Hek 293
DE	3	79898	79297	deferricoprogen	Deferricoprogen
DE	4	79898	79297	Monascus purpureus	Monascus Purpureus
DE	5	79898	79297	nephrotoxicity	Nephrotoxicity
ID	1	79900	79298	RED-MOLD-RICE	Red Mold Rice
ID	2	79900	79298	MONACOLIN-K	Monacolin K
ID	3	79900	79298	MYCOTOXIN CITRININ	Mycotoxin Citrinin
ID	4	79900	79298	OCHRATOXIN-A	Ochratoxin A (OTA)
ID	5	79900	79298	DIOSCOREA	Dioscorea
ID	6	79900	79298	RATS	Rat
ID	7	79900	79298	ANTIOXIDANT	Antioxidant
ID	8	79900	79298	ACID	Acid
ID	9	79900	79298	PENICILLIUM	Penicillium
ID	10	79900	79298	ACTIVATION	Activation

Record 544 | WOS:000304513300011

Toxic effects of citrinin on the male reproductive system in mice

Field	Position	Original line	Normalised line	Original value	Normalised value
DE	2	79999	79396	Male mouse	Male Mouse
DE	5	79999	79396	Semen quality	Semen Quality
ID	1	80001	79398	DEVELOPMENT IN-VITRO	Development In Vitro
ID	2	80001	79398	OCHRATOXIN-A	Ochratoxin A (OTA)
ID	3	80001	79398	CELLS	Cell
ID	4	80001	79398	APOPTOSIS	Apoptosis

ID	5	80001	79398	LIVER	Liver
ID	6	80001	79398	NEPHROTOXICITY	Nephrotoxicity
ID	7	80001	79398	MITOCHONDRIA	Mitochondria
ID	8	80001	79398	ACTIVATION	Activation
ID	9	80001	79398	MYCOTOXINS	Mycotoxins
ID	10	80001	79398	RATS	Rat

Record 545 | WOS:000304401100023

Collaborative study for the detection of toxic compounds in shellfish extracts using cell-based assays. Part II: application to shellfish extracts spiked with lipophilic marine toxins

Field	Position	Original line	Normalised line	Original value	Normalised value
DE	1	80112	79508	Cell-based bioassays	Cell Based Bioassay
DE	2	80112	79508	Collaborative study	Collaborative Study
DE	3	80112	79508	Shellfish extracts	Shellfish Extract
DE	5	80112	79508	Lipophilic phycotoxins	Lipophilic Phycotoxin
ID	1	80114	79510	HUMAN NEUROBLASTOMA-CELLS	Human Neuroblastoma
ID	2	80114	79510	OKADAIC ACID	Okadaic Acid
ID	3	80114	79510	CYTOTOXICITY ASSAY	Cytotoxicity Assay
ID	4	80114	79510	EPITHELIAL-CELLS	Epithelial
ID	5	80114	79510	MOUSE BIOASSAY	Mouse Bioassay
ID	6	80114	79510	BREVETOXINS	Brevetoxin
ID	7	80114	79510	MUSSELS	Mussel
ID	8	80114	79510	CIGUATOXINS	Ciguatoxin
ID	9	80114	79510	PHYCOTOXINS	Phycotoxin
ID	10	80114	79510	SAXITOXIN	Saxitoxin

Record 546 | WOS:000304744700011

Ochratoxin A induces oxidative DNA damage and G1 phase arrest in human peripheral blood mononuclear cells in vitro

Field	Position	Original line	Normalised line	Original value	Normalised value
DE	1	80241	79635	Ochratoxin A	Ochratoxin A (OTA)
DE	2	80241	79635	Reactive oxygen species	Reactive Oxygen Specy
DE	3	80241	79635	DNA damage	DNA Damage
DE	4	80241	79635	Cell cycle arrest	Cell Cycle Arrest
DE	5	80241	79635	Human peripheral blood mononuclear cells	Human Peripheral Blood Mononuclear
ID	1	80243	79637	DOUBLE-STRAND BREAKS	Double Strand Break
ID	2	80243	79637	MYCOTOXIN OCHRATOXIN	Ochratoxin A (OTA)
ID	3	80243	79637	ENDOTHELIAL-CELLS	Endothelial
ID	4	80243	79637	HUMAN-LYMPHOCYTES	Human Lymphocyte
ID	5	80243	79637	APOPTOSIS	Apoptosis
ID	6	80243	79637	STRESS	Stress
ID	7	80243	79637	SUPEROXIDE	Superoxide
ID	8	80243	79637	GENERATION	Generation
ID	9	80243	79637	TOXICITY	Toxicity
ID	10	80243	79637	EXPOSURE	Exposure

Record 547 | WOS:000304198400001

Ochratoxin A and Mitotic Disruption: Mode of Action Analysis of Renal Tumor Formation by Ochratoxin A

Field	Position	Original line	Normalised line	Original value	Normalised value
DE	1	80349	79741	ochratoxin A	Ochratoxin A (OTA)
DE	2	80349	79741	carcinogenesis	Carcinogenesis
DE	3	80349	79741	mitosis	Mitosis
DE	4	80349	79741	MoA	Moa
ID	1	80350	79742	OXIDATIVE DNA-DAMAGE	Oxidative DNA Damage

ID	2	80350	79742	HUMAN PROXIMAL TUBULE	Human Proximal Tubule
ID	3	80350	79742	MYCOTOXIN OCHRATOXIN	Ochratoxin A (OTA)
ID	4	80350	79742	IN-VITRO	In-Vitro
ID	5	80350	79742	SALMONELLA-TYPHIMURIUM	Salmonella Typhimurium
ID	6	80350	79742	MESSENGER-RNA	Messenger RNA
ID	7	80350	79742	HISTONE ACETYLTRANSFERASES	Histone Acetyltransferase
ID	8	80350	79742	NEPHROTOXIN OCHRATOXIN	Gender Difference
ID	9	80350	79742	GENDER DIFFERENCE	Urinary Bladder
ID	10	80350	79742	URINARY-BLADDER	

Record 548 | WOS:000304401100022

Collaborative study for the detection of toxic compounds in shellfish extracts using cell-based assays. Part I: screening strategy and pre-validation study with lipophilic marine toxins

Field	Position	Original line	Normalised line	Original value	Normalised value
DE	1	80585	79975	Cell-based assays	Cell Based Assay
DE	2	80585	79975	Collaborative study	Collaborative Study
DE	3	80585	79975	Lipophilic phycotoxins	Lipophilic Phycotoxin
ID	1	80587	79976	EYE IRRITATION TESTS	Eye Irritation Test
ID	2	80587	79976	OKADAIC ACID	Okadaic Acid
ID	3	80587	79976	INTERLABORATORY VALIDATION	Interlaboratory Validation
ID	4	80587	79976	COSMETIC INGREDIENTS	Cosmetic Ingredient
ID	5	80587	79976	CYTOTOXICITY TEST	Cytotoxicity Test
ID	6	80587	79976	AZASPIRACID-1	Azaspiracid 1
ID	7	80587	79976	NEURO-2A	Neuro 2a
ID	8	80587	79976	QUANTIFICATION	Quantification
ID	9	80587	79976	YESSOTOXIN	Yessotoxin
ID	10	80587	79976	SAXITOXIN	Saxitoxin

Record 549 | WOS:000302950000003

Assaying embryotoxicity in the test tube: Current limitations of the embryonic stem cell test (EST) challenging its applicability domain

Field	Position	Original line	Normalised line	Original value	Normalised value
DE	1	80713	80101	Reproductive toxicology	Reproductive Toxicology
DE	2	80713	80101	teratogenicity	Teratogenicity
DE	3	80713	80101	embryotoxicity	Embryotoxicity
DE	4	80713	80101	applicability domain	Applicability Domain
DE	5	80713	80101	in vitro methods	In Vitro Method
DE	6	80713	80101	alternatives to animal testing	Alternative To Animal Testing
DE	7	80713	80101	embryonic stem cell test (EST)	Embryonic Stem Cell Test Est
ID	1	80716	80104	CONGENITAL DIAPHRAGMATIC-HERNIA	Congenital Diaphragmatic Hernia
ID	2	80716	80104	RETINOIC ACID SYNTHESIS	Retinoic Acid Synthesis
ID	3	80716	80104	COENZYME-A REDUCTASE	Coenzyme A Reductase
ID	4	80716	80104	IN-VITRO	In-Vitro
ID	5	80716	80104	DEVELOPMENTAL TOXICITY	Developmental Toxicity
ID	6	80716	80104	BETA-AMINOPROPIONITRILE	Beta Aminopropionitrile
ID	7	80716	80104	OCHRATOXIN-A	Ochratoxin A (OTA)
ID	8	80716	80104	PRENATAL EXPOSURE	Prenatal Exposure
ID	9	80716	80104	D-PENICILLAMINE	D Penicillamine

ID	10	80716	80104	LYSYL OXIDASE	Lysyl Oxidase

Record 550 | WOS:000307982300003

Mutagenicity of Ochratoxin A and Its Hydroquinone Metabolite in the SupF Gene of the Mutation Reporter Plasmid Ps189

Field	Position	Original line	Normalised line	Original value	Normalised value
DE	1	81017	80404	ochratoxin A	Ochratoxin A (OTA)
DE	2	81017	80404	mutagenicity	Mutagenicity
DE	3	81017	80404	DNA adduct	DNA Adduct
DE	4	81017	80404	genotoxicity	Genotoxicity
DE	5	81017	80404	carcinogenesis	Carcinogenesis
ID	1	81018	80405	OXIDATIVE DNA-DAMAGE	Oxidative DNA Damage
ID	2	81018	80405	BALKAN ENDEMIC NEPHROPATHY	Balkan Endemic Nephropathy
ID	3	81018	80405	ADDUCT FORMATION	Adduct Formation
ID	4	81018	80405	CELL-LINES	Cell
ID	5	81018	80405	GENOTOXICITY	Genotoxicity
ID	6	81018	80405	KIDNEY	Kidney
ID	7	81018	80405	COPPER	Copper
ID	8	81018	80405	REACTIVITY	Reactivity
ID	9	81018	80405	EXPOSURE	Exposure
ID	10	81018	80405	BIOACTIVATION	Bioactivation

Record 551 | WOS:000301792400008

Transcriptomic alterations induced by Ochratoxin A in rat and human renal proximal tubular in vitro models and comparison to a rat in vivo model

Field	Position	Original line	Normalised line	Original value	Normalised value
DE	1	81163	80548	OTA	Ochratoxin A (OTA)
DE	4	81163	80548	Renal proximal tubule	Renal Proximal Tubule
ID	1	81164	80550	OXIDATIVE DNA-DAMAGE	Oxidative DNA Damage
ID	2	81164	80550	EPIGENETIC MECHANISMS	Epigenetic Mechanism
ID	3	81164	80550	EPITHELIAL-CELLS	Epithelial
ID	4	81164	80550	GENE-EXPRESSION	Gene Expression
ID	5	81164	80550	A EXPOSURE	A Exposure
ID	6	81164	80550	KIDNEY	Kidney
ID	7	81164	80550	CARCINOGENICITY	Carcinogenicity
ID	8	81164	80550	APOPTOSIS	Apoptosis
ID	9	81164	80550	PHENYLALANINE	Phenylalanine
ID	10	81164	80550	INDUCTION	Induction

Record 552 | WOS:000302917900001

Carcinogens induce loss of the primary cilium in human renal proximal tubular epithelial cells independently of effects on the cell cycle

Field	Position	Original line	Normalised line	Original value	Normalised value
DE	1	81272	80656	deciliation	Deciliation
DE	2	81272	80656	carcinogenesis	Carcinogenesis
DE	3	81272	80656	proximal tubule	Proximal Tubule
DE	4	81272	80656	cilia	Cilia
ID	1	81273	80657	RIGHT AXIS DETERMINATION	Right Axis Determination
ID	2	81273	80657	BARDET-BIEDL-SYNDROME	Bardet Biedl Syndrome
ID	3	81273	80657	BASAL BODY	Basal Body
ID	4	81273	80657	SENSORY ORGANELLE	Sensory Organelle
ID	5	81273	80657	IN-VITRO	In-Vitro
ID	6	81273	80657	DISEASE	Disease
ID	7	81273	80657	MEMBRANE	Membrane
ID	8	81273	80657	MICE	Mice
ID	9	81273	80657	GENE	Gene
ID	10	81273	80657	DISRUPTION	Disruption

Record 553 | WOS:000301277200012

ERK and p38 MAPK signaling pathways are involved in ochratoxin A-induced G2 phase arrest in human gastric epithelium cells

Field	Position	Original line	Normalised line	Original value	Normalised value
DE	1	81395	80778	Ochratoxin A	Ochratoxin A (OTA)
DE	2	81395	80778	G2 arrest	G2 Arrest
DE	3	81395	80778	ERK	Erk
DE	4	81395	80778	p38 MAPK	P38 Mapk
ID	1	81396	80779	CYCLE ARREST	Cycle Arrest
ID	2	81396	80779	APOPTOSIS	Apoptosis
ID	3	81396	80779	TOXICITY	Toxicity
ID	4	81396	80779	ACID	Acid
ID	5	81396	80779	CYTOTOXICITY	Cytotoxicity
ID	6	81396	80779	ZEARALENONE	Zearalenone (ZEA)
ID	7	81396	80779	ACTIVATION	Activation
ID	8	81396	80779	INDUCTION	Induction
ID	9	81396	80779	HEPG2	HepG2

Record 554 | WOS:000302911300002

Assessment on pollution of ochratoxin a in grain in China and its apoptosis effect on vitro-cultured human tubular kidney cells

Field	Position	Original line	Normalised line	Original value	Normalised value
DE	1	81482	80864	Ochratoxin A	Ochratoxin A (OTA)
DE	4	81482	80864	C-Jun NH2 Terminal Kinase	C Jun Nh2 Terminal Kinase
DE	5	81482	80864	Mitogen-Activated Protein Kinase	Mitogen Activated Protein Kinase
ID	1	81484	80866	ENDEMIC NEPHROPATHY	Endemic Nephropathy
ID	2	81484	80866	SIGNAL-TRANSDUCTION	Signal Transduction
ID	3	81484	80866	IN-VITRO	In-Vitro
ID	4	81484	80866	EXPOSURE	Exposure
ID	5	81484	80866	RATS	Rat
ID	6	81484	80866	NEPHROTOXICITY	Nephrotoxicity
ID	7	81484	80866	ACTIVATION	Activation
ID	8	81484	80866	MECHANISMS	Mechanism
ID	9	81484	80866	PATHWAY	Pathway
ID	10	81484	80866	JNK	Jnk

Record 555 | WOS:000300700100002

Inactivation of A. ochraceus Spores and Detoxification of Ochratoxin A in Coffee Beans by Gamma Irradiation

Field	Position	Original line	Normalised line	Original value	Normalised value
DE	1	81577	80958	Aspergillus ochraceus	Aspergillus Ochraceus
DE	2	81577	80958	cytotoxicity	Cytotoxicity
DE	3	81577	80958	gamma radiation	Gamma Radiation
DE	4	81577	80958	green coffee beans	Green Coffee Bean
DE	5	81577	80958	ochratoxin A	Ochratoxin A (OTA)
ID	1	81579	80960	PENICILLIC ACID	Penicillic Acid
ID	2	81579	80960	GREEN	Green
ID	3	81579	80960	GROWTH	Growth
ID	4	81579	80960	OTA	Ochratoxin A (OTA)

Record 556 | WOS:000309118000007

CYTOTOXIC POTENCY OF MYCOTOXINS IN CULTURES OF V79 LUNG FIBROBLAST CELLS

Field	Position	Original line	Normalised line	Original value	Normalised value

ID	1	81671	81051	CHROMOSOMAL GENOTOXICITY	Chromosomal Genotoxicity
ID	2	81671	81051	ENNIATIN-B	Enniatin B
ID	3	81671	81051	IN-VITRO	In-Vitro
ID	4	81671	81051	FUSARIUM	Fusarium
ID	5	81671	81051	DEOXYNIVALENOL	Deoxynivalenol (DON)
ID	6	81671	81051	EXPOSURE	Exposure
ID	7	81671	81051	TOXINS	Toxin

Record 557 | WOS:000209177700107

Transport of aflatoxin M1 in human intestinal Caco-2/TC7 cells

Field	Position	Original line	Normalised line	Original value	Normalised value
DE	1	81746	81125	aflatoxin M-1	Aflatoxin M1 (AFM1)
DE	2	81746	81125	Caco-2/TC7 cells	Caco-2 Tc7
DE	3	81746	81125	transport	Transport
DE	4	81746	81125	tight junctions	Tight Junction
DE	5	81746	81125	intestinal barrier	Intestinal Barrier
ID	1	81748	81127	IN-VITRO MODEL	In Vitro Model
ID	2	81748	81127	OCHRATOXIN-A	Ochratoxin A (OTA)
ID	3	81748	81127	BARRIER FUNCTION	Barrier Function
ID	4	81748	81127	FUMONISIN B-1	Fumonisin B1 (FB1)
ID	5	81748	81127	TIGHT JUNCTIONS	Tight Junction
ID	6	81748	81127	DAIRY-CATTLE	Dairy Cattle
ID	7	81748	81127	T-2 TOXIN	T 2 Toxin
ID	8	81748	81127	MYCOTOXINS	Mycotoxins
ID	9	81748	81127	MILK	Milk
ID	10	81748	81127	ABSORPTION	Absorption

Record 558 | WOS:000300137300023

In vitro myelotoxicity assessment of the emerging mycotoxins Beauvericin, Enniatin b and Moniliformin on human hematopoietic progenitors

Field	Position	Original line	Normalised line	Original value	Normalised value
DE	2	81860	81238	Human hematopoietic progenitors	Human Hematopoietic Progenitor
DE	3	81860	81238	Emerging mycotoxins	Emerging Mycotoxin
DE	5	81860	81238	Enniatin b	Enniatin B
ID	1	81862	81240	GRANULO-MONOCYTIC PROGENITORS	Granulo Monocytic Progenitor
ID	2	81862	81240	KIDNEY PK15 CELLS	Kidney Pk15
ID	3	81862	81240	FUMONISIN B-1	Fumonisin B1 (FB1)
ID	4	81862	81240	OCHRATOXIN-A	Ochratoxin A (OTA)
ID	5	81862	81240	COMPARATIVE CYTOTOXICITY	Comparative Cytotoxicity
ID	6	81862	81240	FUSARIUM-POAE	Fusarium Poae
ID	7	81862	81240	T-2 TOXIN	T 2 Toxin
ID	8	81862	81240	TOXICITY	Toxicity
ID	9	81862	81240	FUSAPROLIFERIN	Fusaproliferin
ID	10	81862	81240	APOPTOSIS	Apoptosis

Record 559 | WOS:000299028700020

Structure-Activity Relationships Imply Different Mechanisms of Action for Ochratoxin A-Mediated Cytotoxicity and Genotoxicity

Field	Position	Original line	Normalised line	Original value	Normalised value
ID	1	81964	81341	OXIDATIVE DNA-DAMAGE	Oxidative DNA Damage
ID	2	81964	81341	FREE-RADICAL GENERATION	Free Radical Generation
ID	3	81964	81341	ADDUCT FORMATION	Adduct Formation
ID	4	81964	81341	HORSERADISH-PEROXIDASE	Horseradish Peroxidase
ID	5	81964	81341	PHENOXYL RADICALS	Phenoxyl Radical

ID	6	81964	81341	RISK-ASSESSMENT	Risk Assessment
ID	7	81964	81341	KIDNEY	Kidney
ID	8	81964	81341	CARCINOGENICITY	Carcinogenicity
ID	9	81964	81341	BIOACTIVATION	Bioactivation
ID	10	81964	81341	INDUCTION	Induction

Record 560 | WOS:000299902300007

In vivo and ex vivo phagocytic potential of macrophages from progeny of breeder hens kept on ochratoxin A (OTA)-contaminated diet

Field	Position	Original line	Normalised line	Original value	Normalised value
DE	1	82104	81480	Ochratoxin A	Ochratoxin A (OTA)
DE	2	82104	81480	breeder hens	Breeder Hen
DE	3	82104	81480	progeny chicks	Progeny Chick
DE	4	82104	81480	macrophages	Macrophage
DE	5	82104	81480	phagocytic potential	Phagocytic Potential
DE	6	82104	81480	nitrite production	Nitrite Production
ID	1	82106	81482	NATURAL OCCURRENCE	Natural Occurrence
ID	2	82106	81482	MYCOTOXINS	Mycotoxins
ID	3	82106	81482	CHICKENS	Chicken
ID	4	82106	81482	BROILER	Broiler
ID	5	82106	81482	FEEDS	Feed
ID	6	82106	81482	NEPHROPATHY	Nephropathy
ID	7	82106	81482	PERFORMANCE	Performance
ID	8	82106	81482	IMPAIRMENT	Impairment
ID	9	82106	81482	FOODSTUFFS	Foodstuff
ID	10	82106	81482	MYCOFLORA	Mycoflora

Record 561 | WOS:000297778200001

Ochratoxin A in feed of food-producing animals: An undesirable mycotoxin with health and performance effects

Field	Position	Original line	Normalised line	Original value	Normalised value
DE	1	82216	81591	Ochratoxin A	Ochratoxin A (OTA)
ID	1	82217	81592	DE-JANEIRO STATE	De Janeiro State
ID	2	82217	81592	T-2 TOXIN	T 2 Toxin
ID	3	82217	81592	ENTEROHEPATIC CIRCULATION	Enterohepatic Circulation
ID	4	82217	81592	IMMUNOAFFINITY CLEANUP	Immunoaffinity Cleanup
ID	5	82217	81592	PATHOLOGICAL-CHANGES	Pathological Change
ID	6	82217	81592	EIMERIA-ACERVULINA	Eimeria Acervulina
ID	7	82217	81592	IMMUNE-RESPONSE	Immune Response
ID	8	82217	81592	IN-VIVO	In-Vivo
ID	9	82217	81592	NEPHROPATHY	Nephropathy
ID	10	82217	81592	TOXICITY	Toxicity

Record 562 | WOS:000298362500059

Effects of zearalenone and its derivatives on porcine immune response

Field	Position	Original line	Normalised line	Original value	Normalised value
DE	1	82415	81789	Zearalenone	Zearalenone (ZEA)
DE	2	82415	81789	alpha-Zearalenol	Alpha Zearalenol
DE	3	82415	81789	beta-Zearalenol	Beta Zearalenol
DE	5	82415	81789	Swine PBMC	Swine Pbmc
ID	1	82417	81791	BLOOD MONONUCLEAR-CELLS	Blood Mononuclear
ID	2	82417	81791	IN-VITRO PROLIFERATION	In Vitro Proliferation
ID	3	82417	81791	FUSARIUM-MYCOTOXINS	Fusarium Mycotoxin

ID	4	82417	81791	VITAMIN-E	Vitamin E
ID	5	82417	81791	DEOXYNIVALENOL VOMITOXIN	Deoxynivalenol (DON)
ID	6	82417	81791	ESTROGENIC MYCOTOXIN	Estrogenic Mycotoxin
ID	7	82417	81791	BROILER BREEDERS	Broiler Breeder
ID	8	82417	81791	CULTURED-CELLS	Cultured
ID	9	82417	81791	OCHRATOXIN-A	Ochratoxin A (OTA)
ID	10	82417	81791	B6C3F1 MOUSE	B6c3f1 Mouse

Record 563 | WOS:000297102800002

Molecular Events Involved in Ochratoxin A Induced Mitochondrial Pathway of Apoptosis, Modulation by Bcl-2 Family Members

Field	Position	Original line	Normalised line	Original value	Normalised value
DE	1	82548	81921	ochratoxin A	Ochratoxin A (OTA)
DE	2	82548	81921	mitochondria	Mitochondria
DE	3	82548	81921	apoptosis	Apoptosis
DE	4	82548	81921	PTPC	Ptpc
DE	5	82548	81921	Bcl-2	Bcl 2
DE	6	82548	81921	ROS	Reactive Oxygen Species (ROS)
ID	1	82549	81923	MEMBRANE PERMEABILIZATION	Membrane Permeabilization
ID	2	82549	81923	OXIDATIVE STRESS	Oxidative Stress
ID	3	82549	81923	PROTECTIVE ROLE	Protective Role
ID	4	82549	81923	NEPHROPATHY	Nephropathy
ID	5	82549	81923	DAMAGE	Damage
ID	6	82549	81923	ZEARALENONE	Zearalenone (ZEA)
ID	7	82549	81923	INDUCTION	Induction
ID	8	82549	81923	EXPOSURE	Exposure
ID	9	82549	81923	TUNISIA	Tunisia
ID	10	82549	81923	KIDNEY	Kidney

Record 564 | WOS:000296681600021

Ochratoxin A reduces aflatoxin B1 induced DNA damage detected by the comet assay in Hep G2 cells

Field	Position	Original line	Normalised line	Original value	Normalised value
DE	1	82663	82036	Aflatoxin B1	Aflatoxin B1 (AFB1)
DE	2	82663	82036	Ochratoxin A	Ochratoxin A (OTA)
DE	3	82663	82036	Combined genotoxicity	Combined Genotoxicity
DE	4	82663	82036	Comet assay	Comet Assay
ID	1	82665	82038	IN-VITRO	In-Vitro
ID	2	82665	82038	COMBINATIVE TOXICITY	Combinative Toxicity
ID	3	82665	82038	FUMONISIN B-1	Fumonisin B1 (FB1)
ID	4	82665	82038	CYTOTOXICITY	Cytotoxicity
ID	5	82665	82038	METABOLISM	Metabolism
ID	6	82665	82038	MYCOTOXINS	Mycotoxins
ID	7	82665	82038	CYTOPROTECTION	Cytoprotection
ID	8	82665	82038	GENOTOXICITY	Genotoxicity
ID	9	82665	82038	INDUCTION	Induction
ID	10	82665	82038	OXIDATION	Oxidation

Record 565 | WOS:000295241700033

Cytotoxic effects of mycotoxin combinations in mammalian kidney cells

Field	Position	Original line	Normalised line	Original value	Normalised value
DE	2	82779	82151	Deoxynivalenol	Deoxynivalenol (DON)
DE	3	82779	82151	T-2 toxin	T 2 Toxin
DE	4	82779	82151	Vero cells	Vero
ID	1	82780	82152	STATISTICALLY DESIGNED EXPERIMENTS	Statistically Designed Experiment

ID	2	82780	82152	OCHRATOXIN-A	Ochratoxin A (OTA)
ID	3	82780	82152	T-2 TOXIN	T 2 Toxin
ID	4	82780	82152	FUSARIUM MYCOTOXINS	Fusarium Mycotoxin
ID	5	82780	82152	FUMONISIN B-1	Fumonisin B1 (FB1)
ID	6	82780	82152	CACO-2 CELLS	Caco-2
ID	7	82780	82152	PK15 CELLS	Pk15
ID	8	82780	82152	DEOXYNIVALENOL	Deoxynivalenol (DON)
ID	9	82780	82152	TRICHOTHECENES	Trichothecene
ID	10	82780	82152	BEAUVERICIN	Beauvericin

Record 566 | WOS:000298538400030

Mycotoxins profiling of the culture material of Fusarium verticillioides (Sacc.) Nirenberg culture (CABI-IMI 392668) isolated from rice in Niger State, Nigeria and its cytotoxic effects on human lymphocytes comparatively to those of some mycotoxin standards

Field	Position	Original line	Normalised line	Original value	Normalised value
DE	2	82867	82238	human lymphocytes	Human Lymphocyte
DE	3	82867	82238	fumonisins	Fumonisin
DE	4	82867	82238	rice	Rice
ID	1	82868	82239	FUMONISIN B-1	Fumonisin B1 (FB1)
ID	2	82868	82239	OCHRATOXIN-A	Ochratoxin A (OTA)
ID	3	82868	82239	ZEARALENONE	Zearalenone (ZEA)
ID	4	82868	82239	APOPTOSIS	Apoptosis
ID	5	82868	82239	TOXICITY	Toxicity
ID	6	82868	82239	CARCINOGENICITY	Carcinogenicity
ID	7	82868	82239	MONILIFORME	Moniliforme
ID	8	82868	82239	EXPOSURE	Exposure
ID	9	82868	82239	FUNGI	Fungi
ID	10	82868	82239	LIVER	Liver

Record 567 | WOS:000295307400004

Toxicological interactions between the mycotoxins beauvericin, deoxynivalenol and T-2 toxin in CHO-K1 cells in vitro

Field	Position	Original line	Normalised line	Original value	Normalised value
DE	2	82969	82339	Deoxynivalenol	Deoxynivalenol (DON)
DE	3	82969	82339	T-2 toxin	T 2 Toxin
DE	4	82969	82339	CHO-K1 cells	Cho K1
DE	5	82969	82339	Interactions	Interaction
ID	1	82970	82340	KIDNEY PK15 CELLS	Kidney Pk15
ID	2	82970	82340	FUSARIUM-MYCOTOXINS	Fusarium Mycotoxin
ID	3	82970	82340	FUMONISIN B-1	Fumonisin B1 (FB1)
ID	4	82970	82340	OCHRATOXIN-A	Ochratoxin A (OTA)
ID	5	82970	82340	CYTOTOXICITY	Cytotoxicity
ID	6	82970	82340	INDUCTION	Induction
ID	7	82970	82340	APOPTOSIS	Apoptosis
ID	8	82970	82340	ENNIATIN	Enniatin
ID	9	82970	82340	TRICHOTHECENES	Trichothecene
ID	10	82970	82340	METABOLITES	Metabolite

Record 568 | WOS:000296746400005

Comparative cytotoxicity of the possibly occurring mycotoxins in wheat on the basis of a mycological analysis

Field	Position	Original line	Normalised line	Original value	Normalised value
DE	3	83062	82430	contamination	Contamination
DE	4	83062	82430	mycotoxins	Mycotoxins
DE	5	83062	82430	cytotoxicity	Cytotoxicity
DE	6	83062	82430	MTT assay	Mtt Assay
ID	1	83063	82431	OCHRATOXIN-A	Ochratoxin A (OTA)
ID	2	83063	82431	ALTERNARIA-ALTERNATA	Alternaria Alternata
ID	3	83063	82431	PHYLOGENETIC ANALYSIS	Phylogenetic Analysis

ID	4	83063	82431	NATURAL OCCURRENCE	Natural Occurrence
ID	5	83063	82431	AIRBORNE MOLDS	Airborne Mold
ID	6	83063	82431	FUMONISIN B-1	Fumonisin B1 (FB1)
ID	7	83063	82431	HEAD BLIGHT	Head Blight
ID	8	83063	82431	CORN-SILAGE	Corn Silage
ID	9	83063	82431	IN-VITRO	In-Vitro
ID	10	83063	82431	FUSARIUM	Fusarium

Record 569 | WOS:000295697800004

Herbal hepatotoxicity by kava: Update on pipermethystine, flavokavain B, and mould hepatotoxins as primarily assumed culprits

Field	Position	Original line	Normalised line	Original value	Normalised value
DE	1	83210	82577	Drug induced liver injury	Drug Induced Liver Injury
DE	2	83210	82577	Herbal hepatotoxicity	Herbal Hepatotoxicity
DE	3	83210	82577	Herbs induced liver injury	Herb Induced Liver Injury
DE	5	83210	82577	Kava hepatotoxicity	Kava Hepatotoxicity
DE	6	83210	82577	Piper methysticum	Piper Methysticum
ID	1	83212	82579	INDUCED LIVER-INJURY	Induced Liver Injury
ID	2	83212	82579	PIPER-METHYSTICUM	Piper Methysticum
ID	3	83212	82579	CAUSALITY ASSESSMENT	Causality Assessment
ID	4	83212	82579	BOTANICAL ROOTS	Botanical Root
ID	5	83212	82579	TOXICITY	Toxicity
ID	6	83212	82579	EXTRACTS	Extract
ID	7	83212	82579	SAFETY	Safety
ID	8	83212	82579	KAVALACTONES	Kavalactone
ID	9	83212	82579	CHEMOTYPES	Chemotype
ID	10	83212	82579	AFLATOXINS	Aflatoxin

Record 570 | WOS:000294029600014

Ochratoxin A at nanomolar concentration perturbs the homeostasis of neural stem cells in highly differentiated but not in immature three-dimensional brain cell cultures

Field	Position	Original line	Normalised line	Original value	Normalised value
DE	1	83321	82687	Ochratoxin A	Ochratoxin A (OTA)
DE	2	83321	82687	Neural stem cell	Neural Stem
DE	3	83321	82687	Transcriptional regulators	Transcriptional Regulator
DE	4	83321	82687	Stem/progenitor cell markers	Stem Progenitor Cell Marker
DE	6	83321	82687	Sonic hedgehog	Sonic Hedgehog
ID	1	83323	82689	ADULT-MOUSE BRAIN	Adult Mouse Brain
ID	2	83323	82689	SELF-RENEWAL	Self Renewal
ID	3	83323	82689	IN-VITRO	In-Vitro
ID	4	83323	82689	PROTEIN EXPRESSION	Protein Expression
ID	5	83323	82689	NERVOUS-SYSTEM	Nervous System
ID	6	83323	82689	VASCULAR NICHE	Vascular Niche
ID	7	83323	82689	SONIC HEDGEHOG	Sonic Hedgehog
ID	8	83323	82689	RAT-BRAIN	Rat Brain
ID	9	83323	82689	CYCLIN D2	Cyclin D2
ID	10	83323	82689	PROLIFERATION	Proliferation

Record 571 | WOS:000293914500008

Perturbation of Mitosis through Inhibition of Histone Acetyltransferases: The Key to Ochratoxin A Toxicity and Carcinogenicity?

Field	Position	Original line	Normalised line	Original value	Normalised value
DE	1	83458	82822	ochratoxin A	Ochratoxin A (OTA)
DE	2	83458	82822	carcinogenesis	Carcinogenesis
DE	3	83458	82822	mitosis	Mitosis

DE	4	83458	82822	histone	Histone
DE	5	83458	82822	histone acetyltransferase	Histone Acetyltransferase
ID	1	83460	82824	OXIDATIVE DNA-DAMAGE	Oxidative DNA Damage
ID	2	83460	82824	CHROMOSOMAL INSTABILITY	Chromosomal Instability
ID	3	83460	82824	THR-3 PHOSPHORYLATION	Thr 3 Phosphorylation
ID	4	83460	82824	EPIGENETIC MECHANISMS	Epigenetic Mechanism
ID	5	83460	82824	CHROMATIN-STRUCTURE	Chromatin Structure
ID	6	83460	82824	KINASE HASPIN	Kinase Haspin
ID	7	83460	82824	ACETYLATION	Acetylation
ID	8	83460	82824	APOPTOSIS	Apoptosis
ID	9	83460	82824	ADDUCT	Adduct
ID	10	83460	82824	KIDNEY	Kidney

Record 572 | WOS:000293914500016

Site-Specific In Vivo Mutagenicity in the Kidney of gpt Delta Rats Given a Carcinogenic Dose of Ochratoxin A

Field	Position	Original line	Normalised line	Original value	Normalised value
DE	1	83595	82958	gpt delta rat	Gpt Delta Rat
DE	2	83595	82958	mutagenicity	Mutagenicity
DE	3	83595	82958	ochratoxin A	Ochratoxin A (OTA)
DE	4	83595	82958	site	Site
DE	5	83595	82958	specificity	Specificity
ID	1	83596	82959	DNA-DAMAGE	DNA Damage
ID	2	83596	82959	TOXICITY	Toxicity
ID	3	83596	82959	GENOTOXICITY	Genotoxicity
ID	4	83596	82959	MUTATION	Mutation
ID	5	83596	82959	COLON	Colon

Record 573 | WOS:000293914500030

Citrinin-Generated Reactive Oxygen Species Cause Cell Cycle Arrest Leading to Apoptosis via the Intrinsic Mitochondrial Pathway in Mouse Skin

Field	Position	Original line	Normalised line	Original value	Normalised value
DE	1	83699	83061	citrinin	Citrinin
DE	2	83699	83061	mouse skin	Mouse Skin
DE	3	83699	83061	ROS	Reactive Oxygen Species (ROS)
DE	4	83699	83061	apoptosis	Apoptosis
DE	5	83699	83061	antioxidants	Antioxidant
ID	1	83700	83062	EPIDEMIC DROPSY PATIENTS	Epidemic Dropsy Patient
ID	2	83700	83062	DNA-DAMAGE	DNA Damage
ID	3	83700	83062	ANTIOXIDANT DEFENSES	Antioxidant Defense
ID	4	83700	83062	MYCOTOXIN CITRININ	Mycotoxin Citrinin
ID	5	83700	83062	MAMMALIAN-CELLS	Mammalian
ID	6	83700	83062	OCHRATOXIN-A	Ochratoxin A (OTA)
ID	7	83700	83062	TOXICITY	Toxicity
ID	8	83700	83062	RAT	Rat
ID	9	83700	83062	INHIBITION	Inhibition
ID	10	83700	83062	INVITRO	Invitro

Record 574 | WOS:000292672700008

Comparative analysis of micronuclei and DNA damage induced by Ochratoxin A in two mammalian cell lines

Field	Position	Original line	Normalised line	Original value	Normalised value
DE	1	83813	83173	Flow cytometry	Flow Cytometry

DE	3	83813	83173	Oxidative DNA damage	Oxidative DNA Damage
DE	4	83813	83173	Ochratoxin A	Ochratoxin A (OTA)
DE	5	83813	83173	Mammalian cells	Mammalian
ID	1	83815	83175	IN-VITRO	In-Vitro
ID	2	83815	83175	KIDNEY-CELLS	Kidney
ID	3	83815	83175	COMET ASSAY	Comet Assay
ID	4	83815	83175	INDUCTION	Induction
ID	5	83815	83175	RAT	Rat
ID	6	83815	83175	GENOTOXICITY	Genotoxicity
ID	7	83815	83175	VALIDATION	Validation
ID	8	83815	83175	FREQUENCY	Frequency
ID	9	83815	83175	APOPTOSIS	Apoptosis
ID	10	83815	83175	TOXICITY	Toxicity

Record 575 | WOS:000286856200015

Enzyme-Linked Aptamer Assays (ELAAs), based on a competition format for a rapid and sensitive detection of Ochratoxin A in wine

Field	Position	Original line	Normalised line	Original value	Normalised value
DE	1	83938	83297	Ochratoxin A	Ochratoxin A (OTA)
DE	2	83938	83297	DNA aptamer	DNA Aptamer
DE	3	83938	83297	SELEX	Selex
DE	4	83938	83297	Competitive Enzyme-Linked Aptamer Assay (ELAA)	Competitive Enzyme Linked Aptamer Assay Elaa
DE	5	83938	83297	Wine analysis	Wine Analysis
ID	1	83940	83299	DNA APTAMERS	DNA Aptamer
ID	2	83940	83299	ELECTROCHEMICAL IMMUNOSENSOR	Electrochemical Immunosensor
ID	3	83940	83299	FOOD ANALYSIS	Food Analysis
ID	4	83940	83299	SELECTION	Selection
ID	5	83940	83299	MOLECULES	Molecule
ID	6	83940	83299	LIGANDS	Ligand
ID	8	83940	83299	DIAGNOSTICS	Diagnostics
ID	9	83940	83299	ANTIBODIES	Antibody

Record 576 | WOS:000288933100012

Aflatoxin B1 and fumonisin B1 affect the oxidative status of bovine peripheral blood mononuclear cells

Field	Position	Original line	Normalised line	Original value	Normalised value
DE	1	84045	83403	Aflatoxin B-1	Aflatoxin B1 (AFB1)
DE	2	84045	83403	Fumonisin B-1	Fumonisin B1 (FB1)
DE	3	84045	83403	Oxidative stress	Oxidative Stress
DE	4	84045	83403	PBMC	Pbmc
DE	5	84045	83403	Dairy cattle	Dairy Cattle
ID	1	84046	83405	LIPID-PEROXIDATION PRODUCTS	Lipid Peroxidation Product
ID	2	84046	83405	LIVER IN-VIVO	Liver In Vivo
ID	3	84046	83405	RAT-LIVER	Rat Liver
ID	4	84046	83405	COENZYME Q(10)	Coenzyme Q 10
ID	5	84046	83405	FREE-RADICALS	Free Radical
ID	6	84046	83405	OCHRATOXIN-A	Ochratoxin A (OTA)
ID	7	84046	83405	DNA-DAMAGE	DNA Damage
ID	8	84046	83405	DAIRY-COWS	Dairy Cow
ID	9	84046	83405	ALPHA-TOCOPHEROL	Alpha Tocopherol
ID	10	84046	83405	L-CARNITINE	L Carnitine

Record 577 | WOS:000289042100014

Lymphocyte blastic transformation test in the evaluation of ochratoxin A influence on the immune system of hens and their progeny

Field	Position	Original line	Normalised line	Original value	Normalised value
DE	1	84176	83534	ochratoxin A	Ochratoxin A (OTA)
DE	2	84176	83534	feed	Feed
DE	3	84176	83534	hens	Hen

DE	4	84176	83534	offspring	Offspring
DE	5	84176	83534	test of blastic transformation of lymphocytes	Test Of Blastic Transformation Of Lymphocyte
ID	1	84178	83536	IN-VITRO PROLIFERATION	In Vitro Proliferation
ID	2	84178	83536	INFECTED CHICKENS	Infected Chicken
ID	3	84178	83536	SPLEEN	Spleen
ID	4	84178	83536	BLOOD	Blood
ID	5	84178	83536	STIMULATION	Stimulation
ID	6	84178	83536	CITRININ	Citrinin
ID	7	84178	83536	EXPOSURE	Exposure
ID	8	84178	83536	PATULIN	Patulin
ID	9	84178	83536	DISEASE	Disease
ID	10	84178	83536	CELLS	Cell

Record 578 | WOS:000288341900004

Zebrafish (Danio rerio) embryos as a model for testing proteratogens

Field	Position	Original line	Normalised line	Original value	Normalised value
DE	2	84247	83604	Metabolic activation	Metabolic Activation
ID	1	84248	83605	BIOACTIVATION IN-VITRO	Bioactivation In Vitro
ID	2	84248	83605	HUMAN CYTOCHROME-P450 ENZYMES	Human Cytochrome P450 Enzyme
ID	3	84248	83605	CLINICAL PHARMACOKINETICS	Clinical Pharmacokinetics
ID	4	84248	83605	GLUTATHIONE CONJUGATION	Glutathione Conjugation
ID	5	84248	83605	HYDROCARBON RECEPTOR	Hydrocarbon Receptor
ID	6	84248	83605	AFLATOXIN B-1	Aflatoxin B1 (AFB1)
ID	7	84248	83605	OCHRATOXIN-A	Ochratoxin A (OTA)
ID	8	84248	83605	P450 1A2	P450 1a2
ID	9	84248	83605	TOXICITY	Toxicity
ID	10	84248	83605	CARBAMAZEPINE	Carbamazepine

Record 579 | WOS:000286679100003

Protective effects of melatonin and Glycyrrhiza glabra extract on ochratoxin A-induced damages on testes in mature rats

Field	Position	Original line	Normalised line	Original value	Normalised value
DE	1	84450	83806	Glycyrrhiza glabra extract	Glycyrrhiza Glabra Extract
DE	2	84450	83806	ochratoxin A	Ochratoxin A (OTA)
DE	3	84450	83806	melatonin	Melatonin
DE	4	84450	83806	testis	Testis
DE	5	84450	83806	antioxidant	Antioxidant
ID	1	84451	83808	OXIDATIVE STRESS	Oxidative Stress
ID	2	84451	83808	TOXICITY	Toxicity
ID	3	84451	83808	LICORICE	Licorice
ID	4	84451	83808	NEPHROTOXICITY	Nephrotoxicity
ID	5	84451	83808	PARAMETERS	Parameter

Record 580 | WOS:000285845400008

Mycotoxin Citrinin Induced Cell Cycle G2/M Arrest and Numerical Chromosomal Aberration Associated with Disruption of Microtubule Formation in Human Cells

Field	Position	Original line	Normalised line	Original value	Normalised value
DE	1	84543	83899	mycotoxin citrinin	Mycotoxin Citrinin
DE	3	84543	83899	M arrest	M Arrest
DE	4	84543	83899	microtubule	Microtubule

DE	5	84543	83899	numerical chromosomal aberration	Numerical Chromosomal Aberration
ID	1	84545	83901	JNK SIGNALING PATHWAYS	Jnk Signaling Pathway
ID	2	84545	83901	BONE-MARROW-CELLS	Bone Marrow
ID	3	84545	83901	NEOPLASTIC TRANSFORMATION	Neoplastic Transformation
ID	4	84545	83901	OCHRATOXIN-A	Ochratoxin A (OTA)
ID	5	84545	83901	P38 KINASE	P38 Kinase
ID	6	84545	83901	ANEUPLOIDY	Aneuploidy
ID	7	84545	83901	CARCINOGENESIS	Carcinogenesis
ID	8	84545	83901	ACTIVATION	Activation
ID	9	84545	83901	APOPTOSIS	Apoptosis
ID	10	84545	83901	CANCER	Cancer

Record 581 | WOS:000285100300026

Metabolic response to low-level toxicant exposure in a novel renal tubule epithelial cell system

Field	Position	Original line	Normalised line	Original value	Normalised value
ID	1	84658	84013	OCHRATOXIN-A	Ochratoxin A (OTA)
ID	2	84658	84013	DNA-DAMAGE	DNA Damage
ID	3	84658	84013	KIDNEY	Kidney
ID	4	84658	84013	CARCINOGENICITY	Carcinogenicity
ID	5	84658	84013	TOXICITY	Toxicity
ID	6	84658	84013	PATTERN	Pattern
ID	7	84658	84013	RATS	Rat
ID	8	84658	84013	PHOTODECOMPOSITION	Photodecomposition
ID	9	84658	84013	CLASSIFICATION	Classification
ID	10	84658	84013	SPECTROSCOPY	Spectroscopy

Record 582 | WOS:000297106700013

Review of mycotoxin reduction in food and feed: from prevention in the field to detoxification by adsorption or transformation

Field	Position	Original line	Normalised line	Original value	Normalised value
DE	1	84783	84137	toxicology	Toxicology
DE	2	84783	84137	microbiology	Microbiology
DE	3	84783	84137	HPLC	Hplc
DE	4	84783	84137	mycotoxins	Mycotoxins
DE	5	84783	84137	animal feed	Animal Feed
DE	6	84783	84137	cereals	Cereal
ID	1	84784	84138	SODIUM-CALCIUM ALUMINOSILICATE	Sodium Calcium Aluminosilicate
ID	2	84784	84138	LACTIC-ACID BACTERIA	Lactic Acid Bacteria
ID	3	84784	84138	FUSARIUM HEAD BLIGHT	Fusarium Head Blight
ID	4	84784	84138	AFLATOXIN B-1 BINDING	Aflatoxin B 1 Binding
ID	5	84784	84138	IN-VITRO	In-Vitro
ID	6	84784	84138	OCHRATOXIN-A	Ochratoxin A (OTA)
ID	7	84784	84138	T-2 TOXIN	T 2 Toxin
ID	8	84784	84138	SACCHAROMYCES-CEREVISIAE	Saccharomyce Cerevisiae
ID	9	84784	84138	ACTIVATED-CHARCOAL	Activated Charcoal
ID	10	84784	84138	FUMONISIN B-1	Fumonisin B1 (FB1)

Record 583 | WOS:000298090700025

Experimentally Induced Toxicity of Ochratoxin A and Endosulfan in Male Wistar Rats: A Hormonal Disorder

Field	Position	Original line	Normalised line	Original value	Normalised value

DE	1	85086	84439	Ochratoxin A	Ochratoxin A (OTA)
DE	2	85086	84439	endosulfan	Endosulfan
DE	3	85086	84439	male rats	Male Rat
DE	4	85086	84439	hormone	Hormone
DE	5	85086	84439	radioimmuno assay	Radioimmuno Assay
ID	1	85087	84440	TROUT ONCORHYNCHUS-MYKISS	Trout Oncorhynchus Mykiss
ID	2	85087	84440	NEONATAL-HYPOTHYROIDISM	Neonatal Hypothyroidism
ID	3	85087	84440	CORTISOL SECRETION	Cortisol Secretion
ID	4	85087	84440	IN-VITRO	In-Vitro
ID	5	85087	84440	CELLS	Cell
ID	6	85087	84440	INTOXICATION	Intoxication
ID	7	85087	84440	INSECTICIDE	Insecticide
ID	8	85087	84440	ANIMALS	Animal
ID	9	85087	84440	PLASMA	Plasma

Record 584 | WOS:000295693800002

Potential of an in vitro toolbox combined with exposure data as a first step for the risk assessment of dietary chemical contaminants

Field	Position	Original line	Normalised line	Original value	Normalised value
DE	1	85202	84554	bioassay	Bioassay
DE	2	85202	84554	receptors	Receptor
DE	3	85202	84554	toxicology	Toxicology
DE	4	85202	84554	cytotoxicity	Cytotoxicity
DE	5	85202	84554	microbial screening	Microbial Screening
DE	6	85202	84554	mycotoxins	Mycotoxins
DE	7	85202	84554	pesticide residues	Pesticide Residue
ID	1	85204	84556	ARYL-HYDROCARBON RECEPTOR	Aryl Hydrocarbon Receptor
ID	2	85204	84556	INTESTINAL CACO-2 CELLS	Intestinal Caco-2
ID	3	85204	84556	TRANS-RETINOIC ACID	Tran Retinoic Acid
ID	4	85204	84556	ESTROGEN-RECEPTOR	Estrogen Receptor
ID	5	85204	84556	OCHRATOXIN-A	Ochratoxin A (OTA)
ID	6	85204	84556	CYP1A1 INDUCTION	Cyp1a1 Induction
ID	7	85204	84556	CYTOCHROME-P450 ISOFORMS	Cytochrome P450 Isoform
ID	8	85204	84556	FUSARIUM-MYCOTOXINS	Fusarium Mycotoxin
ID	9	85204	84556	ENDEMIC NEPHROPATHY	Endemic Nephropathy
ID	10	85204	84556	PESTICIDE-RESIDUES	Pesticide Residue

Record 585 | WOS:000292496700010

Developing mechanism-based and exposure biomarkers for mycotoxins in animals

Field	Position	Original line	Normalised line	Original value	Normalised value
DE	1	85397	84748	aflatoxin	Aflatoxin
DE	3	85397	84748	biomarkers	Biomarker
DE	4	85397	84748	deoxynivalenol	Deoxynivalenol (DON)
DE	5	85397	84748	fumonisin	Fumonisin
DE	7	85397	84748	mycotoxins	Mycotoxins
DE	9	85397	84748	ochratoxin	Ochratoxin A (OTA)
DE	10	85397	84748	trichothecene	Trichothecene
DE	11	85397	84748	zearalenone	Zearalenone (ZEA)
ID	1	85399	84751	ACTIVATED PROTEIN-KINASE	Activated Protein Kinase
ID	2	85399	84751	RIBOTOXIC STRESS-RESPONSE	Ribotoxic Stress Response
ID	3	85399	84751	FREE SPHINGOID BASES	Free Sphingoid Base
ID	4	85399	84751	FUMONISIN B-1	Fumonisin B1 (FB1)

ID	5	85399	84751	OCHRATOXIN-A	Ochratoxin A (OTA)
ID	6	85399	84751	GENE-EXPRESSION	Gene Expression
ID	7	85399	84751	CULTURE MATERIAL	Culture Material
ID	8	85399	84751	IN-VITRO	In-Vitro
ID	9	85399	84751	TRICHOTHECENE DEOXYNIVALENOL	Trichothecene Deoxynivalenol
ID	10	85399	84751	VOMITOXIN DEOXYNIVALENOL	Vomitoxin Deoxynivalenol

Record 586 | WOS:000297106700012

Isotherm modeling of organic activated bentonite and humic acid polymer used as mycotoxin adsorbents

Field	Position	Original line	Normalised line	Original value	Normalised value
DE	1	85612	84963	HPLC	Hplc
DE	2	85612	84963	extraction	Extraction
DE	3	85612	84963	mycotoxins	Mycotoxins
DE	4	85612	84963	ochratoxin A	Ochratoxin A (OTA)
DE	5	85612	84963	zearalenone	Zearalenone (ZEA)
DE	6	85612	84963	animal feed	Animal Feed
ID	1	85613	84965	OCHRATOXIN-A	Ochratoxin A (OTA)
ID	2	85613	84965	ANIMAL FEED	Animal Feed
ID	3	85613	84965	ADSORPTION	Adsorption
ID	4	85613	84965	ZEARALENONE	Zearalenone (ZEA)
ID	5	85613	84965	SUBSTANCES	Substance
ID	6	85613	84965	BIOSORPTION	Biosorption
ID	7	85613	84965	DEOXYNIVALENOL	Deoxynivalenol (DON)
ID	8	85613	84965	PERFORMANCE	Performance
ID	9	85613	84965	STRATEGIES	Strategy
ID	10	85613	84965	TOXICITY	Toxicity

Record 587 | WOS:000290627500016

Aptamer-functionalized magnetic nanoparticle-based bioassay for the detection of ochratoxin a using upconversion nanoparticles as labels

Field	Position	Original line	Normalised line	Original value	Normalised value
ID	1	85714	85065	DOPED SILICA NANOPARTICLES	Doped Silica Nanoparticle
ID	2	85714	85065	IN-VITRO SELECTION	In Vitro Selection
ID	3	85714	85065	HPLC DETERMINATION	Hplc Determination
ID	4	85714	85065	MOLECULES	Molecule
ID	5	85714	85065	NANOCRYSTALS	Nanocrystal
ID	6	85714	85065	TECHNOLOGY	Technology
ID	7	85714	85065	BIOSENSOR	Biosensor
ID	8	85714	85065	ASSAY	Assay
ID	9	85714	85065	BIND	Bind
ID	10	85714	85065	IONS	Ion

Record 588 | WOS:000283743900015

Immunotoxicity of zearalenone in Balb/c mice in a high subchronic dosing study counteracted by Raphanus sativus extract

Field	Position	Original line	Normalised line	Original value	Normalised value
DE	2	85828	85178	isothiocyanates	Isothiocyanate
DE	3	85828	85178	flavonoids	Flavonoid
DE	4	85828	85178	zearalenone	Zearalenone (ZEA)
DE	5	85828	85178	hematology	Hematology
DE	6	85828	85178	cytokines	Cytokine
DE	7	85828	85178	antibody	Antibody
DE	8	85828	85178	spleen	Spleen
DE	9	85828	85178	LPS	Lps
ID	1	85830	85180	SODIUM-CALCIUM ALUMINOSILICATE	Sodium Calcium Aluminosilicate

ID	2	85830	85180	AFLATOXIN-CONTAMINATED DIETS	Aflatoxin Contaminated Diet
ID	3	85830	85180	DRUG-METABOLIZING-ENZYMES	Drug Metabolizing Enzyme
ID	4	85830	85180	OXIDATIVE STRESS	Oxidative Stress
ID	5	85830	85180	OCHRATOXIN-A	Ochratoxin A (OTA)
ID	6	85830	85180	IN-VIVO	In-Vivo
ID	7	85830	85180	ISOTHIOCYANATE	Isothiocyanate
ID	8	85830	85180	GENOTOXICITY	Genotoxicity
ID	9	85830	85180	MYCOTOXINS	Mycotoxins
ID	10	85830	85180	TOXICITY	Toxicity

Record 589 | WOS:000284436000007

Evaluation of a urinary kidney biomarker panel in rat models of acute and subchronic nephrotoxicity

Field	Position	Original line	Normalised line	Original value	Normalised value
DE	4	85945	85294	Kim-1	Kim 1
DE	6	85945	85294	Lipocalin-2	Lipocalin 2
DE	9	85945	85294	beta 2-Microglobulin	Beta 2 Microglobulin
DE	10	85945	85294	Timp-1	Timp 1
ID	1	85947	85296	ENDOTHELIAL GROWTH-FACTOR	Endothelial Growth Factor
ID	2	85947	85296	GELATINASE-ASSOCIATED LIPOCALIN	Gelatinase Associated Lipocalin
ID	3	85947	85296	RENAL-ALLOGRAFT REJECTION	Renal Allograft Rejection
ID	4	85947	85296	INJURY MOLECULE-1	Injury Molecule 1
ID	5	85947	85296	CYSTATIN-C	Cystatin C
ID	6	85947	85296	CYCLOSPORINE NEPHROTOXICITY	Cyclosporine Nephrotoxicity
ID	7	85947	85296	CADMIUM NEPHROTOXICITY	Cadmium Nephrotoxicity
ID	8	85947	85296	OSTEOPONTIN EXPRESSION	Osteopontin Expression
ID	9	85947	85296	TISSUE INHIBITOR	Tissue Inhibitor
ID	10	85947	85296	TUBULAR INJURY	Tubular Injury

Record 590 | WOS:000283810500022

Protein Biomarkers for in Vitro Testing of Embryotoxicity

Field	Position	Original line	Normalised line	Original value	Normalised value
DE	1	86094	85442	Embryonic stem cell test	Embryonic Stem Cell Test
DE	2	86094	85442	developmental toxicology	Developmental Toxicology
DE	3	86094	85442	toxicity biomarker	Toxicity Biomarker
DE	4	86094	85442	Ras pathway	Ras Pathway
DE	5	86094	85442	differential proteomics	Differential Proteomics
DE	6	86094	85442	REACH	Reach
DE	7	86094	85442	chemicals testing	Chemical Testing
ID	1	86096	85444	OCHRATOXIN-A	Ochratoxin A (OTA)
ID	2	86096	85444	RAS	Ras
ID	3	86096	85444	TOXICITY	Toxicity
ID	4	86096	85444	STEM	Stem
ID	5	86096	85444	INHIBITION	Inhibition
ID	6	86096	85444	PROLIFERATION	Proliferation
ID	7	86096	85444	MITOCHONDRIA	Mitochondria
ID	8	86096	85444	VALIDATION	Validation
ID	9	86096	85444	MECHANISM	Mechanism
ID	10	86096	85444	APOPTOSIS	Apoptosis

Record 591 | WOS:000280344000003

Some food-associated mycotoxins as potential risk factors in humans predisposed to chronic intestinal inflammatory diseases

Field	Position	Original line	Normalised line	Original value	Normalised value
DE	1	86217	85564	Bacterial translocation	Bacterial Translocation
DE	2	86217	85564	Chronic intestinal inflammatory disease	Chronic Intestinal Inflammatory Disease
DE	3	86217	85564	Inflammatory bowel disease	Inflammatory Bowel Disease
DE	5	86217	85564	Mycotoxin	Mycotoxins
ID	1	86219	85566	IN-VITRO CYTOTOXICITY	In Vitro Cytotoxicity
ID	2	86219	85566	GUT EPITHELIAL-CELLS	Gut Epithelial
ID	3	86219	85566	PREGNANT WISTAR RATS	Pregnant Wistar Rat
ID	4	86219	85566	CULTURED VERO CELLS	Cultured Vero
ID	5	86219	85566	CELIAC-DISEASE	Celiac Disease
ID	6	86219	85566	OCHRATOXIN-A	Ochratoxin A (OTA)
ID	7	86219	85566	BOWEL-DISEASE	Bowel Disease
ID	8	86219	85566	FUMONISIN B-1	Fumonisin B1 (FB1)
ID	9	86219	85566	CACO-2 CELLS	Caco-2
ID	10	86219	85566	T-2 TOXIN	T 2 Toxin

Record 592 | WOS:000281740900017

Mycotoxins and Their Biotransformation in the Rumen: A Review

Field	Position	Original line	Normalised line	Original value	Normalised value
DE	3	86434	85780	Ruminants	Ruminant
DE	4	86434	85780	Microbes	Microbe
DE	5	86434	85780	Enzymes	Enzyme
ID	1	86435	85781	SODIUM-CALCIUM ALUMINOSILICATE	Sodium Calcium Aluminosilicate
ID	2	86435	85781	OCHRATOXIN-A	Ochratoxin A (OTA)
ID	3	86435	85781	AFLATOXIN B-1	Aflatoxin B1 (AFB1)
ID	4	86435	85781	T-2 TOXIN	T 2 Toxin
ID	5	86435	85781	IN-VIVO	In-Vivo
ID	6	86435	85781	PATHOLOGICAL-CHANGES	Pathological Change
ID	7	86435	85781	COMBINED TOXICITY	Combined Toxicity
ID	8	86435	85781	CARRY-OVER	Carry Over
ID	9	86435	85781	DEOXYNIVALENOL	Deoxynivalenol (DON)
ID	10	86435	85781	PERFORMANCE	Performance

Record 593 | WOS:000282996200006

Free radicals and antioxidants in two oxidative-stress cell models exposed to ochratoxin A and amyloid Î²: unexpected results

Field	Position	Original line	Normalised line	Original value	Normalised value
DE	1	86618	85963	ochratoxin A	Ochratoxin A (OTA)
DE	2	86618	85963	cytotoxicity	Cytotoxicity
DE	3	86618	85963	reactive oxygen species	Reactive Oxygen Specy
DE	4	86618	85963	leontopodic acid	Leontopodic Acid
ID	1	86619	85964	LEONTOPODIC ACID	Leontopodic Acid
ID	2	86619	85964	APOPTOSIS	Apoptosis
ID	3	86619	85964	DAMAGE	Damage
ID	4	86619	85964	LINE	Line
ID	5	86619	85964	NEUROTOXICITY	Neurotoxicity
ID	6	86619	85964	CYTOTOXICITY	Cytotoxicity
ID	7	86619	85964	INVOLVEMENT	Involvement
ID	8	86619	85964	INDUCTION	Induction
ID	9	86619	85964	TOXICITY	Toxicity
ID	10	86619	85964	INFLUX	Influx

Record 594 | WOS:000280991100075

Safety studies on products from whole coffee fruit

Field	Position	Original line	Normalised line	Original value	Normalised value
DE	1	86703	86047	Coffee fruit	Coffee Fruit
DE	2	86703	86047	CoffeeBerry	Coffeeberry
DE	3	86703	86047	Coffea arabica	Coffea Arabica
DE	6	86703	86047	GRAS	Gra
ID	1	86704	86048	MUCILAGE-LAYER	Mucilage Layer
ID	2	86704	86048	OCHRATOXIN-A	Ochratoxin A (OTA)
ID	3	86704	86048	TEST SYSTEM	Test System
ID	4	86704	86048	CHERRY	Cherry
ID	5	86704	86048	ACTIVATION	Activation
ID	6	86704	86048	BACTERIA	Bacteria
ID	7	86704	86048	MUTAGENS	Mutagen

Record 595 | WOS:000280256800007

Beauvericin and ochratoxin A genotoxicity evaluated using the alkaline comet assay: single and combined genotoxic action

Field	Position	Original line	Normalised line	Original value	Normalised value
DE	2	86801	86144	Comet assay	Comet Assay
DE	3	86801	86144	Mycotoxin synergism	Mycotoxin Synergism
ID	1	86802	86145	KIDNEY PK15 CELLS	Kidney Pk15
ID	2	86802	86145	DNA-DAMAGE	DNA Damage
ID	3	86802	86145	APOPTOSIS	Apoptosis
ID	4	86802	86145	FUMONISIN-B-1	Fumonisin B1 (FB1)
ID	5	86802	86145	INDUCTION	Induction
ID	6	86802	86145	LYMPHOCYTES	Lymphocyte
ID	7	86802	86145	MICRONUCLEI	Micronuclei
ID	8	86802	86145	ENNIATIN	Enniatin
ID	9	86802	86145	TOXICITY	Toxicity
ID	10	86802	86145	RAT	Rat

Record 596 | WOS:000282996200009

Mycotoxins in grapes and wine in Europe: occurrence, factors affecting the occurrence and related toxicological effects

Field	Position	Original line	Normalised line	Original value	Normalised value
DE	1	86899	86241	ochratoxin A	Ochratoxin A (OTA)
DE	2	86899	86241	Alternaria toxins	Alternaria Toxin
DE	3	86899	86241	fumonisins	Fumonisin
DE	4	86899	86241	contamination	Contamination
DE	5	86899	86241	toxicity	Toxicity
ID	1	86900	86242	OCHRATOXIN-A PRODUCTION	Ochratoxin A Production
ID	2	86900	86242	OXIDATIVE DNA-DAMAGE	Oxidative DNA Damage
ID	3	86900	86242	BLACK ASPERGILLI	Black Aspergilli
ID	4	86900	86242	IN-VITRO	In-Vitro
ID	5	86900	86242	FUNGI	Fungi
ID	6	86900	86242	ALTERNARIOL	Alternariol
ID	7	86900	86242	CONTAMINATION	Contamination
ID	8	86900	86242	KIDNEY	Kidney
ID	9	86900	86242	FOOD	Food
ID	10	86900	86242	CYTOTOXICITY	Cytotoxicity

Record 597 | WOS:000278803100015

Allium cepa as a biomonitor of ochratoxin A toxicity and genotoxicity

Field	Position	Original line	Normalised line	Original value	Normalised value
DE	2	87038	86379	cell proliferation	Cell Proliferation
DE	3	87038	86379	onion	Onion
DE	5	87038	86379	root chromosomes	Root Chromosome
DE	6	87038	86379	root growth	Root Growth

ID	1	87040	86381	SALMONELLA-TYPHIMURIUM	Salmonella Typhimurium
ID	2	87040	86381	CHROMOSOME-ABERRATION	Chromosome Aberration
ID	3	87040	86381	WASTE-WATER	Waste Water
ID	4	87040	86381	MUTAGENICITY	Mutagenicity
ID	5	87040	86381	MYCOTOXINS	Mycotoxins
ID	6	87040	86381	ASSAYS	Assay
ID	7	87040	86381	CELLS	Cell

Record 598 | WOS:000277103900009

Neuronal models for evaluation of proliferation in vitro using high content screening

Field	Position	Original line	Normalised line	Original value	Normalised value
DE	1	87116	86456	High content screening	High Content Screening
DE	3	87116	86456	Neuronal models	Neuronal Model
ID	1	87117	86457	OCHRATOXIN-A	Ochratoxin A (OTA)
ID	2	87117	86457	DEVELOPMENTAL NEUROTOXICITY	Developmental Neurotoxicity
ID	3	87117	86457	CELL-PROLIFERATION	Cell Proliferation
ID	4	87117	86457	NEURITE OUTGROWTH	Neurite Outgrowth
ID	5	87117	86457	BLASTOMA CELLS	Blastoma
ID	6	87117	86457	DIFFERENTIATION	Differentiation
ID	7	87117	86457	INHIBITION	Inhibition
ID	8	87117	86457	GROWTH	Growth
ID	9	87117	86457	NEUROGENESIS	Neurogenesis
ID	10	87117	86457	TOXICITY	Toxicity

Record 599 | WOS:000208434800018

Antigenotoxic Studies of Different Substances to Reduce the DNA Damage Induced by Aflatoxin B1 and Ochratoxin A

Field	Position	Original line	Normalised line	Original value	Normalised value
DE	1	87220	86559	aflatoxin B-1	Aflatoxin B1 (AFB1)
DE	2	87220	86559	ochratoxin A	Ochratoxin A (OTA)
DE	3	87220	86559	antigenotoxic	Antigenotoxic
DE	4	87220	86559	DNA damage	DNA Damage
ID	1	87221	86560	INDUCED CELL-DAMAGE	Induced Cell Damage
ID	2	87221	86560	IN-VITRO	In-Vitro
ID	3	87221	86560	LIPID-PEROXIDATION	Lipid Peroxidation
ID	4	87221	86560	RISK-ASSESSMENT	Risk Assessment
ID	5	87221	86560	FOOD-ADDITIVES	Food Additive
ID	6	87221	86560	MYCOTOXINS	Mycotoxins
ID	7	87221	86560	DETOXIFICATION	Detoxification
ID	8	87221	86560	TOXICITY	Toxicity
ID	9	87221	86560	MICE	Mice
ID	10	87221	86560	CONTAMINATION	Contamination

Record 600 | WOS:000276175200004

Ochratoxin A induces G2 phase arrest in human gastric epithelium GES-1 cells in vitro

Field	Position	Original line	Normalised line	Original value	Normalised value
DE	1	87382	86719	Ochratoxin A	Ochratoxin A (OTA)
DE	2	87382	86719	Cell cycle	Cell Cycle
DE	3	87382	86719	CyclinB1	Cyclinb1
DE	5	87382	86719	Cdc25C	Cdc25c
ID	1	87383	86720	MITOTIC CHECKPOINT GENES	Mitotic Checkpoint Gene
ID	2	87383	86720	DNA-DAMAGE	DNA Damage
ID	3	87383	86720	APOPTOSIS	Apoptosis
ID	4	87383	86720	INDUCTION	Induction
ID	5	87383	86720	CYCLE	Cycle

ID	6	87383	86720	TOXICITY	Toxicity
ID	7	87383	86720	DEOXYNIVALENOL	Deoxynivalenol (DON)
ID	8	87383	86720	CYTOTOXICITY	Cytotoxicity
ID	9	87383	86720	INSTABILITY	Instability
ID	10	87383	86720	DEPLETION	Depletion

Record 601 | WOS:000274582400001

PREVENTIVE AND THERAPEUTIC METHODS AGAINST THE TOXIC EFFECTS OF MYCOTOXINS - A REVIEW

Field	Position	Original line	Normalised line	Original value	Normalised value
DE	2	87471	86807	mycotoxin detoxification	Mycotoxin Detoxification
DE	3	87471	86807	mycotoxin decontamination	Mycotoxin Decontamination
DE	4	87471	86807	absorbents	Absorbent
DE	5	87471	86807	feed additives	Feed Additive
ID	1	87473	86809	SODIUM-CALCIUM ALUMINOSILICATE	Sodium Calcium Aluminosilicate
ID	2	87473	86809	RELATIVE ORGAN WEIGHT	Relative Organ Weight
ID	3	87473	86809	T-2 TOXIN	T 2 Toxin
ID	4	87473	86809	OCHRATOXIN-A	Ochratoxin A (OTA)
ID	5	87473	86809	AFLATOXIN B-1	Aflatoxin B1 (AFB1)
ID	6	87473	86809	FUSARIUM MYCOTOXINS	Fusarium Mycotoxin
ID	7	87473	86809	ESTERIFIED-GLUCOMANNAN	Esterified Glucomannan
ID	8	87473	86809	ACTIVATED-CHARCOAL	Activated Charcoal
ID	9	87473	86809	IN-VITRO	In-Vitro
ID	10	87473	86809	CONTAMINATED FEED	Contaminated Feed

Record 602 | WOS:000276819800011

Decontamination and detoxification strategies for the Fusarium mycotoxin deoxynivalenol in animal feed and the effectiveness of microbial biodegradation

Field	Position	Original line	Normalised line	Original value	Normalised value
DE	1	87653	86988	clinical study	Clinical Study
DE	2	87653	86988	toxicology	Toxicology
DE	3	87653	86988	animal study	Animal Study
DE	4	87653	86988	mycotoxins	Mycotoxins
DE	5	87653	86988	trichothecenes	Trichothecene
DE	6	87653	86988	animal feed	Animal Feed
DE	7	87653	86988	cereals and grain	Cereal And Grain
ID	1	87655	86990	SODIUM CALCIUM ALUMINOSILICATE	Sodium Calcium Aluminosilicate
ID	2	87655	86990	IN-VITRO	In-Vitro
ID	3	87655	86990	FLAVOBACTERIUM-AURANTIACUM	Flavobacterium Aurantiacum
ID	4	87655	86990	TRICHOTHECENE MYCOTOXINS	Trichothecene Mycotoxin
ID	5	87655	86990	RUMEN MICROORGANISMS	Rumen Microorganism
ID	6	87655	86990	PHYSICAL TREATMENT	Physical Treatment
ID	7	87655	86990	BROILER-CHICKENS	Broiler Chicken
ID	8	87655	86990	FUNGAL TOXINS	Fungal Toxin
ID	9	87655	86990	AFLATOXIN B-1	Aflatoxin B1 (AFB1)
ID	10	87655	86990	OCHRATOXIN-A	Ochratoxin A (OTA)

Record 603 | WOS:000279331700013

Modification of aflatoxin B1 and ochratoxin A toxicokinetics in rats administered a yeast cell wall preparation

Field	Position	Original line	Normalised line	Original value	Normalised value
DE	1	87798	87132	toxicology	Toxicology
DE	2	87798	87132	toxicokinetics	Toxicokinetics
DE	3	87798	87132	mycotoxins	Mycotoxins
DE	4	87798	87132	aflatoxins	Aflatoxin
DE	5	87798	87132	ochratoxin A	Ochratoxin A (OTA)
DE	6	87798	87132	animal feed	Animal Feed
ID	1	87800	87134	BETA-D-GLUCANS	Beta D Glucan
ID	2	87800	87134	FUSARIUM MYCOTOXINS	Fusarium Mycotoxin
ID	3	87800	87134	BILIARY-EXCRETION	Biliary Excretion
ID	4	87800	87134	SERUM CHEMISTRY	Serum Chemistry
ID	5	87800	87134	FEED	Feed
ID	6	87800	87134	MILK	Milk
ID	7	87800	87134	EFFICACY	Efficacy
ID	8	87800	87134	GRAINS	Grain
ID	9	87800	87134	B-1	B 1

Record 604 | WOS:000273528200008

Zearalenone and its Derivatives: Known Toxins in New Aspects

Field	Position	Original line	Normalised line	Original value	Normalised value
ID	1	87910	87243	LIQUID-CHROMATOGRAPHIC DETERMINATION	Liquid Chromatographic Determination
ID	2	87910	87243	OCHRATOXIN-A RESIDUES	Ochratoxin A Residue
ID	3	87910	87243	ALPHA-ZEARALENOL	Alpha Zearalenol
ID	4	87910	87243	NATURAL OCCURRENCE	Natural Occurrence
ID	5	87910	87243	FUSARIUM TOXINS	Fusarium Toxin
ID	6	87910	87243	CEREAL GRAIN	Cereal Grain
ID	7	87910	87243	MYCO-TOXINS	Myco Toxin
ID	8	87910	87243	IN-VITRO	In-Vitro
ID	9	87910	87243	TOXIGENIC FUNGI	Toxigenic Fungi
ID	10	87910	87243	BETA-ZEARALENOL	Beta Zearalenol

Record 605 | WOS:000281731700007

Lifetime, low-dose ochratoxin A dietary study on renal carcinogenesis in male Fischer rats

Field	Position	Original line	Normalised line	Original value	Normalised value
DE	1	88047	87379	toxicology	Toxicology
DE	2	88047	87379	in vivo	In-Vivo
DE	3	88047	87379	ochratoxin A	Ochratoxin A (OTA)
ID	1	88048	87380	EXPOSURE	Exposure
ID	2	88048	87380	OUTCOMES	Outcome
ID	3	88048	87380	CANCER	Cancer

Record 606 | WOS:000273618500012

Structures of Covalent Adducts between DNA and Ochratoxin A: A New Factor in Debate about Genotoxicity and Human Risk Assessment

Field	Position	Original line	Normalised line	Original value	Normalised value
ID	1	88129	87460	2'-DEOXYGUANOSINE-CARBON 8-BOUND OCHRATOXIN	2 Deoxyguanosine Carbon 8 Bound Ochratoxin
ID	2	88129	87460	DOSE-RESPONSE	Dose Response
ID	3	88129	87460	RENAL TUMORS	Renal Tumor
ID	4	88129	87460	RATS	Rat
ID	5	88129	87460	CARCINOGENICITY	Carcinogenicity
ID	6	88129	87460	BIOACTIVATION	Bioactivation
ID	7	88129	87460	TOXICITY	Toxicity
ID	8	88129	87460	EXPOSURE	Exposure
ID	9	88129	87460	ABSENCE	Absence

Record 607 | WOS:000286528700008

Ochratoxin A-induced serum biochemical alterations in New Zealand white rabbits (Oryctolagus cuniculus)

Field	Position	Original line	Normalised line	Original value	Normalised value
DE	1	88241	87570	Ochratoxin-A	Ochratoxin A (OTA)
DE	2	88241	87570	rabbits	Rabbit
DE	3	88241	87570	serum biochemistry	Serum Biochemistry
ID	1	88242	87571	ESCHERICHIA-COLI	Escherichia Coli
ID	2	88242	87571	BROILER-CHICKENS	Broiler Chicken
ID	3	88242	87571	IN-VITRO	In-Vitro
ID	4	88242	87571	TOXICITY	Toxicity
ID	5	88242	87571	MYCOTOXINS	Mycotoxins
ID	6	88242	87571	CITRININ	Citrinin
ID	7	88242	87571	ANIMALS	Animal
ID	8	88242	87571	HUMANS	Human
ID	9	88242	87571	CELLS	Cell

Record 608 | WOS:000274143800002

Toxicology of mycotoxins

Field	Position	Original line	Normalised line	Original value	Normalised value
ID	1	88336	87664	MESSENGER-RNA EXPRESSION	Messenger RNA Expression
ID	2	88336	87664	HEPATITIS-B-VIRUS	Hepatitis B Virus
ID	3	88336	87664	OCHRATOXIN-A	Ochratoxin A (OTA)
ID	4	88336	87664	FUSARIUM MYCOTOXINS	Fusarium Mycotoxin
ID	5	88336	87664	FUMONISIN B-1	Fumonisin B1 (FB1)
ID	6	88336	87664	LIPID-PEROXIDATION	Lipid Peroxidation
ID	7	88336	87664	LIVER-CANCER	Liver Cancer
ID	8	88336	87664	TNF-ALPHA	Tnf Alpha
ID	9	88336	87664	T-2 TOXIN	T 2 Toxin
ID	10	88336	87664	IN-VIVO	In-Vivo

Record 609 | WOS:000272760200016

CYP1A1 and CYP3A4 modulation by dietary flavonoids in human intestinal Caco-2 cells

Field	Position	Original line	Normalised line	Original value	Normalised value
DE	1	88509	87836	Polyphenols	Polyphenol
DE	2	88509	87836	Human intestinal Caco-2 cells	Human Intestinal Caco-2
DE	3	88509	87836	CYP1A1	Cyp1a1
DE	4	88509	87836	CYP3A4	Cyp3a4
DE	5	88509	87836	Food and drug interactions	Food And Drug Interaction
DE	6	88509	87836	EROD assay	Erod Assay
ID	1	88511	87838	ARYL-HYDROCARBON RECEPTOR	Aryl Hydrocarbon Receptor
ID	2	88511	87838	XENOBIOTIC-METABOLIZING ENZYMES	Xenobiotic Metabolizing Enzyme
ID	3	88511	87838	GRAPEFRUIT JUICE	Grapefruit Juice
ID	4	88511	87838	DRUG-INTERACTIONS	Drug Interaction
ID	5	88511	87838	CYTOCHROME-P450 3A4	Cytochrome P450 3a4
ID	6	88511	87838	ENTEROCYTE CYP3A4	Enterocyte Cyp3a4
ID	7	88511	87838	ORAL AVAILABILITY	Oral Availability
ID	8	88511	87838	GENE-EXPRESSION	Gene Expression
ID	9	88511	87838	OCHRATOXIN-A	Ochratoxin A (OTA)
ID	10	88511	87838	INDUCTION	Induction

Record 610 | WOS:000271971100003

Comparative Mechanisms of Zearalenone and Ochratoxin A Toxicities on Cultured HepG2 cells: Is Oxidative Stress a Common Process?

Field	Position	Original line	Normalised line	Original value	Normalised value
DE	1	88641	87967	zearalenone	Zearalenone (ZEA)
DE	2	88641	87967	ochratoxin A	Ochratoxin A (OTA)
DE	3	88641	87967	Hsp responses	Hsp Response
DE	4	88641	87967	oxidative damage	Oxidative Damage
ID	1	88642	87968	CHRONIC INTERSTITIAL NEPHROPATHY	Chronic Interstitial Nephropathy
ID	2	88642	87968	HEAT-SHOCK PROTEINS	Heat Shock Protein
ID	3	88642	87968	VITAMIN-E	Vitamin E
ID	4	88642	87968	MAMMALIAN-CELLS	Mammalian
ID	5	88642	87968	KIDNEY-CELLS	Kidney
ID	6	88642	87968	MOUSE-LIVER	Mouse Liver
ID	7	88642	87968	DNA-DAMAGE	DNA Damage
ID	8	88642	87968	A EXPOSURE	A Exposure
ID	9	88642	87968	VERO CELLS	Vero
ID	10	88642	87968	RAT-LIVER	Rat Liver

Record 611 | WOS:000208434400002

Cyclopiazonic Acid Biosynthesis of Aspergillus flavus and Aspergillus oryzae

Field	Position	Original line	Normalised line	Original value	Normalised value
DE	2	88750	88074	cyclopiazonic acid	Cyclopiazonic Acid
DE	3	88750	88074	gene cluster	Gene Cluster
DE	4	88750	88074	non-ribosomal peptide synthase	Non Ribosomal Peptide Synthase
ID	1	88752	88076	DIMETHYLALLYL TRYPTOPHAN SYNTHASE	Dimethylallyl Tryptophan Synthase
ID	2	88752	88076	NON-AFLATOXIGENIC STRAINS	Non Aflatoxigenic Strain
ID	3	88752	88076	COVALENTLY BOUND FLAVIN	Covalently Bound Flavin
ID	4	88752	88076	IN-VITRO PROLIFERATION	In Vitro Proliferation
ID	5	88752	88076	PENICILLIUM-CYCLOPIUM	Penicillium Cyclopium
ID	6	88752	88076	SECTION FLAVI	Section Flavi
ID	7	88752	88076	T-2 TOXIN	T 2 Toxin
ID	8	88752	88076	SCLEROTIA PRODUCTION	Sclerotia Production
ID	9	88752	88076	GENE-CLUSTER	Gene Cluster
ID	10	88752	88076	OCHRATOXIN-A	Ochratoxin A (OTA)

Record 612 | WOS:000272276600011

Reactive oxygen species induced by beauvericin, patulin and zearalenone in CHO-K1 cells

Field	Position	Original line	Normalised line	Original value	Normalised value
DE	3	88935	88258	Reactive oxygen species	Reactive Oxygen Specy
DE	4	88935	88258	Dichlorodihydrofluorecein diacetate	Dichlorodihydrofluorecein Diacetate
DE	5	88935	88258	TBARS	Tbar
DE	6	88935	88258	CHO-K1 cells	Cho K1
ID	1	88937	88260	OXIDATIVE DNA-DAMAGE	Oxidative DNA Damage
ID	2	88937	88260	FUSARIUM-MYCOTOXINS	Fusarium Mycotoxin
ID	3	88937	88260	MAMMALIAN-CELLS	Mammalian
ID	4	88937	88260	OCHRATOXIN-A	Ochratoxin A (OTA)
ID	5	88937	88260	CYTOTOXICITY	Cytotoxicity

ID	6	88937	88260	INDUCTION	Induction
ID	7	88937	88260	APOPTOSIS	Apoptosis
ID	8	88937	88260	STRESS	Stress
ID	9	88937	88260	CASPASE-3	Caspase 3
ID	10	88937	88260	DEPLETION	Depletion

Record 613 | WOS:000272276600013

Intracellular zinc stores protect the intestinal epithelium from Ochratoxin A toxicity

Field	Position	Original line	Normalised line	Original value	Normalised value
DE	1	89021	88342	Caco-2/TC7	Caco-2 Tc7
DE	2	89021	88342	Mycotoxin	Mycotoxins
DE	4	89021	88342	Zinc transporters	Zinc Transporter
DE	6	89021	88342	Tight junctions	Tight Junction
ID	1	89023	88344	TRANSCRIPTION FACTOR MTF-1	Transcription Factor Mtf 1
ID	2	89023	88344	CACO-2 CELLS	Caco-2
ID	3	89023	88344	EXPRESSION	Expression
ID	4	89023	88344	PERMEABILITY	Permeability
ID	5	89023	88344	TRAFFICKING	Trafficking
ID	6	89023	88344	TRANSPORTER	Transporter
ID	7	89023	88344	BARRIER	Barrier
ID	8	89023	88344	LINE	Line

Record 614 | WOS:000270527000004

Minimum tolerable exposure period and maximum threshold dietary intake of ochratoxin A for causing renal cancer in male Dark Agouti rats

Field	Position	Original line	Normalised line	Original value	Normalised value
DE	3	89108	88428	Renal carcinoma	Renal Carcinoma
DE	4	89108	88428	Dietary ochratoxin A	Ochratoxin A (OTA)
DE	5	89108	88428	Dark Agouti rat	Dark Agouti Rat
ID	1	89110	88430	CARCINOGENICITY	Carcinogenicity
ID	2	89110	88430	TUMORS	Tumor

Record 615 | WOS:000270503100034

Molecular Characterization of Preneoplastic Lesions Provides Insight on the Development of Renal Tumors

Field	Position	Original line	Normalised line	Original value	Normalised value
ID	1	89181	88500	CARCINOGENIC ARISTOLOCHIC ACID	Carcinogenic Aristolochic Acid
ID	2	89181	88500	CELL CARCINOMA	Cell Carcinoma
ID	3	89181	88500	OCHRATOXIN-A	Ochratoxin A (OTA)
ID	4	89181	88500	EKER RAT	Eker Rat
ID	5	89181	88500	METABOLIC-ACTIVATION	Metabolic Activation
ID	6	89181	88500	EXPRESSION PROFILES	Expression Profile
ID	7	89181	88500	MAMMALIAN TARGET	Mammalian Target
ID	8	89181	88500	MTOR PATHWAY	Mtor Pathway
ID	9	89181	88500	RAPAMYCIN	Rapamycin
ID	10	89181	88500	KIDNEY	Kidney

Record 616 | WOS:000269677000009

Low doses of ochratoxin A upregulate the protein expression of organic anion transporters Oat1, Oat2, Oat3 and Oat5 in rat kidney cortex

Field	Position	Original line	Normalised line	Original value	Normalised value
DE	2	89310	88628	Membrane transporters	Membrane Transporter
DE	5	89310	88628	Organic anions	Organic Anion
DE	6	89310	88628	PAH	Pah
DE	7	89310	88628	Proximal tubule	Proximal Tubule

ID	1	89312	88630	IN-VIVO	In-Vivo
ID	2	89312	88630	PROXIMAL-TUBULE	Proximal Tubule
ID	3	89312	88630	NEPHROTOXIN OCHRATOXIN	Ochratoxin A (OTA)
ID	4	89312	88630	PERITUBULAR TRANSPORT	Peritubular Transport
ID	5	89312	88630	PARA-AMINOHIPPURATE	Para Aminohippurate
ID	6	89312	88630	GENDER-DIFFERENCES	Gender Difference
ID	7	89312	88630	OXIDATIVE STRESS	Oxidative Stress
ID	8	89312	88630	MESSENGER-RNA	Messenger RNA
ID	9	89312	88630	A TRANSPORT	A Transport
ID	10	89312	88630	TOXICITY	Toxicity

Record 617 | WOS:000269551500021

The mycotoxin ochratoxin A inhibits DNA topoisomerase II and induces polyploidy in cultured CHO cells

Field	Position	Original line	Normalised line	Original value	Normalised value
DE	1	89444	88761	Ochratoxin A	Ochratoxin A (OTA)
DE	3	89444	88761	DNA damage	DNA Damage
DE	4	89444	88761	Topoisomerase II	Topoisomerase Ii
ID	1	89445	88762	MAMMALIAN-CELLS	Mammalian
ID	2	89445	88762	ENDO-REDUPLICATION	Endo Reduplication
ID	3	89445	88762	KIDNEY-CELLS	Kidney
ID	4	89445	88762	IN-VITRO	In-Vitro
ID	5	89445	88762	DAMAGE	Damage
ID	6	89445	88762	INDUCTION	Induction
ID	7	89445	88762	TETRAPLOIDY	Tetraploidy
ID	8	89445	88762	APOPTOSIS	Apoptosis
ID	9	89445	88762	RAT	Rat
ID	10	89445	88762	LIVER	Liver

Record 618 | WOS:000269551500029

Multiplexed assay panel of cytotoxicity in HK-2 cells for detection of renal proximal tubule injury potential of compounds

Field	Position	Original line	Normalised line	Original value	Normalised value
ID	1	89560	88876	DICHROMATE-INDUCED NEPHROTOXICITY	Dichromate Induced Nephrotoxicity
ID	2	89560	88876	GAMMA-GLUTAMYL-TRANSPEPTIDASE	Gamma Glutamyl Transpeptidase
ID	3	89560	88876	IN-VITRO	In-Vitro
ID	4	89560	88876	CITRININ NEPHROTOXICITY	Citrinin Nephrotoxicity
ID	5	89560	88876	AMPHOTERICIN-B	Amphotericin B
ID	6	89560	88876	OCHRATOXIN-A	Ochratoxin A (OTA)
ID	7	89560	88876	DNA-DAMAGE	DNA Damage
ID	8	89560	88876	CISPLATIN	Cisplatin
ID	9	89560	88876	GENTAMICIN	Gentamicin
ID	10	89560	88876	TOXICITY	Toxicity

Record 619 | WOS:000268006900004

Pro-apoptotic effects of nivalenol and deoxynivalenol trichothecenes in J774A.1 murine macrophages

Field	Position	Original line	Normalised line	Original value	Normalised value
DE	2	89664	88979	Deoxynivalenol	Deoxynivalenol (DON)
DE	4	89664	88979	J774A.1 macrophages	J774a 1 Macrophage
ID	1	89665	88980	MESSENGER-RNA EXPRESSION	Messenger RNA Expression
ID	2	89665	88980	VOMITOXIN DEOXYNIVALENOL	Vomitoxin Deoxynivalenol

ID	3	89665	88980	IN-VITRO	In-Vitro
ID	4	89665	88980	ALPHA-ZEARALENOL	Alpha Zearalenol
ID	5	89665	88980	UP-REGULATION	Up Regulation
ID	6	89665	88980	FUSARIUM TOXINS	Fusarium Toxin
ID	7	89665	88980	FUMONISIN B-1	Fumonisin B1 (FB1)
ID	8	89665	88980	OCHRATOXIN-A	Ochratoxin A (OTA)
ID	9	89665	88980	CELL-LINE	Cell
ID	10	89665	88980	TNF-ALPHA	Tnf Alpha

Record 620 | WOS:000282995500010

Occurrence of ochratoxin A and heavy metals in tissues associated with porcine nephropathy in Serbia

Field	Position	Original line	Normalised line	Original value	Normalised value
DE	1	89768	89082	swine	Swine
DE	2	89768	89082	ochratoxin A	Ochratoxin A (OTA)
DE	3	89768	89082	heavy metals	Heavy Metal
DE	4	89768	89082	residue	Residue
DE	5	89768	89082	nephropathy	Nephropathy
ID	1	89769	89083	A RESIDUES	A Residue
ID	2	89769	89083	PROXIMAL-TUBULE	Proximal Tubule
ID	3	89769	89083	NULL MICE	Null Mice
ID	4	89769	89083	IN-VIVO	In-Vivo
ID	5	89769	89083	CADMIUM	Cadmium
ID	6	89769	89083	KIDNEY	Kidney
ID	7	89769	89083	PIGS	Pig
ID	8	89769	89083	TRANSPORT	Transport
ID	9	89769	89083	TOXICITY	Toxicity
ID	10	89769	89083	MYCOTOXINS	Mycotoxins

Record 621 | WOS:000267227800008

Ochratoxin A-Mediated DNA and Protein Damage: Roles of Nitrosative and Oxidative Stresses

Field	Position	Original line	Normalised line	Original value	Normalised value
DE	1	89901	89214	mycotoxin	Mycotoxins
DE	2	89901	89214	ochratoxin A	Ochratoxin A (OTA)
DE	3	89901	89214	inducible nitric oxide synthase	Inducible Nitric Oxide Synthase
DE	4	89901	89214	nuclear factor-erythroid 2 p45-related factor 2	Nrf2
DE	5	89901	89214	oxidative and nitrosative stress	Oxidative And Nitrosative Stress
DE	6	89901	89214	nephrotoxicity	Nephrotoxicity
DE	7	89901	89214	carcinogenicity	Carcinogenicity
ID	1	89904	89216	2'-DEOXYGUANOSINE-CARBON 8-BOUND OCHRATOXIN	2 Deoxyguanosine Carbon 8 Bound Ochratoxin
ID	2	89904	89216	NITRIC-OXIDE SYNTHASE	Nitric Oxide Synthase
ID	3	89904	89216	IN-VITRO	In-Vitro
ID	4	89904	89216	SUPEROXIDE-DISMUTASE	Superoxide Dismutase
ID	5	89904	89216	MASS-SPECTROMETRY	Mass Spectrometry
ID	6	89904	89216	ARISTOLOCHIC ACID	Aristolochic Acid
ID	7	89904	89216	RAT	Rat
ID	8	89904	89216	KIDNEY	Kidney
ID	9	89904	89216	CELL	Cell
ID	10	89904	89216	EXPRESSION	Expression

Record 622 | WOS:000268796500023

A Review of the Implementation of the Embryonic Stem Cell Test (EST) The report and recommendations of an ECVAM/ReProTect Workshop

Field	Position	Original line	Normalised line	Original value	Normalised value

ID	1	90068	89379	VITRO EMBRYOTOXICITY TESTS	Vitro Embryotoxicity Test
ID	2	90068	89379	IN-VITRO	In-Vitro
ID	3	90068	89379	DEVELOPMENTAL TOXICITY	Developmental Toxicity
ID	4	90068	89379	VALIDATION	Validation
ID	5	90068	89379	LINES	Line

Record 623 | WOS:000265685000003

Oxidative stress and apoptotic changes in primary cultures of rat proximal tubular cells exposed to lead

Field	Position	Original line	Normalised line	Original value	Normalised value
DE	2	90170	89480	Oxidative stress	Oxidative Stress
DE	4	90170	89480	Proximal tubular cells	Proximal Tubular
DE	5	90170	89480	Primary cell culture	Primary Cell
ID	1	90172	89481	REACTIVE OXYGEN	Reactive Oxygen
ID	2	90172	89481	LIPID-PEROXIDATION	Lipid Peroxidation
ID	3	90172	89481	MESO-2,3-DIMERCAPTOSUCCINIC ACID	Meso 2 3 Dimercaptosuccinic Acid
ID	4	90172	89481	OCHRATOXIN-A	Ochratoxin A (OTA)
ID	5	90172	89481	REDOX STATE	Redox State
ID	6	90172	89481	BLOOD LEAD	Blood Lead
ID	7	90172	89481	IN-VITRO	In-Vitro
ID	8	90172	89481	GLUTATHIONE	Glutathione (GSH)
ID	9	90172	89481	EXPRESSION	Expression
ID	10	90172	89481	NEPHROTOXICITY	Nephrotoxicity

Record 624 | WOS:000265474500006

Silibinin protects OTA-mediated TNF-Î± release from perfused rat livers and isolated rat Kupffer cells

Field	Position	Original line	Normalised line	Original value	Normalised value
DE	1	90269	89577	Kupffer cells	Kupffer
DE	3	90269	89577	Ochratoxin A	Ochratoxin A (OTA)
DE	5	90269	89577	TNF-alpha	Tnf Alpha
ID	1	90270	89578	OCHRATOXIN-A	Ochratoxin A (OTA)
ID	2	90270	89578	SILYBUM-MARIANUM	Silybum Marianum
ID	3	90270	89578	FUMONISIN B-1	Fumonisin B1 (FB1)
ID	4	90270	89578	IN-VIVO	In-Vivo
ID	5	90270	89578	MICE	Mice
ID	6	90270	89578	SILYMARIN	Silymarin
ID	7	90270	89578	INHIBITION	Inhibition
ID	8	90270	89578	INJURY	Injury
ID	9	90270	89578	HEPATOCYTES	Hepatocyte
ID	10	90270	89578	APOPTOSIS	Apoptosis

Record 625 | WOS:000265474500007

Mycotoxins and the kidney: Modes of action for renal tumor formation by ochratoxin A in rodents

Field	Position	Original line	Normalised line	Original value	Normalised value
DE	5	90364	89671	Ochratoxin A	Ochratoxin A (OTA)
ID	1	90365	89672	OXIDATIVE DNA-DAMAGE	Oxidative DNA Damage
ID	2	90365	89672	SISTER-CHROMATID EXCHANGE	Sister Chromatid Exchange
ID	3	90365	89672	CELL-PROLIFERATION	Cell Proliferation
ID	4	90365	89672	IN-VITRO	In-Vitro
ID	5	90365	89672	ADDUCT FORMATION	Adduct Formation
ID	6	90365	89672	SALMONELLA-TYPHIMURIUM	Salmonella Typhimurium
ID	7	90365	89672	PORCINE NEPHROPATHY	Porcine Nephropathy

ID	8	90365	89672	ARISTOLOCHIC ACID	Aristolochic Acid
ID	9	90365	89672	MUTAGENICITY TEST	Mutagenicity Test
ID	10	90365	89672	CHRONIC EXPOSURE	Chronic Exposure

Record 626 | WOS:000263803400010

Effects of the mycotoxin fumonisin B1 on cell death in human kidney cells and human lung fibroblasts in primary culture

Field	Position	Original line	Normalised line	Original value	Normalised value
DE	1	90513	89819	mycotoxin	Mycotoxins
DE	2	90513	89819	lung	Lung
DE	3	90513	89819	kidney	Kidney
DE	4	90513	89819	apoptosis	Apoptosis
DE	5	90513	89819	necrosis	Necrosis
DE	6	90513	89819	collagen homeostasis	Collagen Homeostasis
ID	1	90514	89820	HUMAN PROXIMAL TUBULE	Human Proximal Tubule
ID	2	90514	89820	NEURAL-TUBE DEFECTS	Neural Tube Defect
ID	3	90514	89820	SPHINGOLIPID METABOLISM	Sphingolipid Metabolism
ID	4	90514	89820	FUSARIUM-MONILIFORME	Fusarium Moniliforme
ID	5	90514	89820	CERAMIDE SYNTHASE	Ceramide Synthase
ID	6	90514	89820	EPITHELIAL-CELLS	Epithelial
ID	7	90514	89820	OCHRATOXIN-A	Ochratoxin A (OTA)
ID	8	90514	89820	IN-VIVO	In-Vivo
ID	9	90514	89820	APOPTOSIS	Apoptosis
ID	10	90514	89820	TOXICITY	Toxicity

Record 627 | WOS:000266663300004

Ochratoxin A induces apoptosis in neuronal cells

Field	Position	Original line	Normalised line	Original value	Normalised value
DE	1	90627	89932	Ochratoxin A	Ochratoxin A (OTA)
DE	2	90627	89932	Mycotoxin	Mycotoxins
DE	3	90627	89932	Neurons	Neuron
DE	5	90627	89932	Programmed cell death	Programmed Cell Death
DE	7	90627	89932	Alzheimer's disease	Alzheimer S Disease
ID	1	90629	89934	FOOD MYCOTOXIN OCHRATOXIN	Ochratoxin A (OTA)
ID	2	90629	89934	OXIDATIVE DNA-DAMAGE	Oxidative DNA Damage
ID	3	90629	89934	RAT-BRAIN	Rat Brain
ID	4	90629	89934	IN-VITRO	In-Vitro
ID	5	90629	89934	ENDEMIC NEPHROPATHY	Endemic Nephropathy
ID	6	90629	89934	TOXICITY	Toxicity
ID	7	90629	89934	CYTOTOXICITY	Cytotoxicity
ID	8	90629	89934	KIDNEY	Kidney
ID	9	90629	89934	NEPHROTOXICITY	Nephrotoxicity
ID	10	90629	89934	NEUROTOXICITY	Neurotoxicity

Record 628 | WOS:000263382500003

CYP1A1 induction and CYP3A4 inhibition by the fungicide imazalil in the human intestinal Caco-2 cells-Comparison with other conazole pesticides

Field	Position	Original line	Normalised line	Original value	Normalised value
DE	3	90735	90038	Antifungal agents	Antifungal Agent
DE	5	90735	90038	Food contaminant	Food Contaminant
DE	6	90735	90038	Caco-2 cells	Caco-2
ID	1	90737	90040	QUAIL COLINUS-VIRGINIANUS	Quail Colinus Virginianus
ID	2	90737	90040	ANTIFUNGAL AGENTS	Antifungal Agent

ID	3	90737	90040	P-GLYCOPROTEIN	P Glycoprotein
ID	4	90737	90040	IN-VITRO	In-Vitro
ID	5	90737	90040	CYTOCHROME-P450 ISOFORMS	Cytochrome P450 Isoform
ID	6	90737	90040	DRUG-METABOLISM	Drug Metabolism
ID	7	90737	90040	OCHRATOXIN-A	Ochratoxin A (OTA)
ID	8	90737	90040	SERUM-FREE	Serum Free
ID	9	90737	90040	1A1 EXPRESSION	1a1 Expression
ID	10	90737	90040	RAT	Rat

Record 629 | WOS:000262717200017

Effects of live yeast cell supplementation to high concentrate diets on the toxicokinetics of ochratoxin A in sheep

Field	Position	Original line	Normalised line	Original value	Normalised value
DE	1	90870	90172	live yeast cells	Live Yeast
DE	2	90870	90172	ochratoxin A	Ochratoxin A (OTA)
DE	3	90870	90172	toxicokinetics	Toxicokinetics
DE	4	90870	90172	sheep	Sheep
ID	1	90871	90173	NEUTRAL DETERGENT FIBER	Neutral Detergent Fiber
ID	2	90871	90173	SACCHAROMYCES-CEREVISIAE	Saccharomyce Cerevisiae
ID	3	90871	90173	RUMEN FLUID	Rumen Fluid
ID	4	90871	90173	GRAIN DIET	Grain Diet
ID	5	90871	90173	EXCRETION	Excretion
ID	6	90871	90173	MYCOTOXIN	Mycotoxins
ID	7	90871	90173	CULTURE	Culture
ID	8	90871	90173	FERMENTATION	Fermentation
ID	9	90871	90173	METABOLISM	Metabolism
ID	10	90871	90173	HAY	Hay

Record 630 | WOS:000267272900021

The biotransformation of chosen mycotoxins

Field	Position	Original line	Normalised line	Original value	Normalised value
DE	1	90964	90265	biotransformation	Biotransformation
DE	2	90964	90265	aflatoxin B1	Aflatoxin B1 (AFB1)
DE	3	90964	90265	ochratoxin A	Ochratoxin A (OTA)
DE	4	90964	90265	deoxynivalenol	Deoxynivalenol (DON)
DE	5	90964	90265	fumonisins	Fumonisin
DE	6	90964	90265	zearalenon	Zearalenon
ID	1	90966	90267	MULTIDRUG-RESISTANCE PROTEINS	Multidrug Resistance Protein
ID	2	90966	90267	INTESTINAL CACO-2 CELLS	Intestinal Caco-2
ID	3	90966	90267	IN-VITRO MODEL	In Vitro Model
ID	4	90966	90267	ALPHA-ZEARALENOL	Alpha Zearalenol
ID	5	90966	90267	BETA-ZEARALENOL	Beta Zearalenol
ID	6	90966	90267	GRANULOSA-CELLS	Granulosa
ID	7	90966	90267	FUMONISIN B-1	Fumonisin B1 (FB1)
ID	8	90966	90267	OCHRATOXIN-A	Ochratoxin A (OTA)
ID	9	90966	90267	DEOXYNIVALENOL DON	Deoxynivalenol DON
ID	10	90966	90267	RISK-ASSESSMENT	Risk Assessment

Record 631 | WOS:000273001000009

Effects of probiotic bacteria on the bioaccessibility of aflatoxin B1 and ochratoxin A using an in vitro digestion model under fed conditions

Field	Position	Original line	Normalised line	Original value	Normalised value
DE	1	91076	90376	Aflatoxin B-1	Aflatoxin B1 (AFB1)
DE	2	91076	90376	ochratoxin A	Ochratoxin A (OTA)
DE	3	91076	90376	probiotic bacteria	Probiotic Bacteria

DE	4	91076	90376	bioaccessibility	Bioaccessibility
DE	5	91076	90376	in vitro digestion model	In Vitro Digestion Model
DE	6	91076	90376	exposure reduction	Exposure Reduction
ID	1	91078	90378	IMMUNOAFFINITY COLUMN CLEANUP	Immunoaffinity Column Cleanup
ID	2	91078	90378	LACTIC-ACID BACTERIA	Lactic Acid Bacteria
ID	3	91078	90378	DAIRY STRAINS	Dairy Strain
ID	4	91078	90378	BINDING	Binding
ID	5	91078	90378	ABILITY	Ability
ID	6	91078	90378	LACTOBACILLUS	Lactobacillus
ID	7	91078	90378	MYCOTOXINS	Mycotoxins
ID	8	91078	90378	FOOD	Food
ID	9	91078	90378	BIFIDOBACTERIUM	Bifidobacterium
ID	10	91078	90378	ABSORPTION	Absorption

Record 632 | WOS:000292954300008

Microorganisms and their enzymes for detoxifying mycotoxins posing a risk to livestock animals

Field	Position	Original line	Normalised line	Original value	Normalised value
ID	1	91183	90481	T-2 TOXIN	T 2 Toxin
ID	2	91183	90481	OCHRATOXIN-A	Ochratoxin A (OTA)
ID	3	91183	90481	DEOXYNIVALENOL	Deoxynivalenol (DON)
ID	4	91183	90481	DETOXIFICATION	Detoxification
ID	5	91183	90481	DEGRADATION	Degradation
ID	6	91183	90481	TRANSFORMATION	Transformation
ID	7	91183	90481	FUMONISINS	Fumonisin
ID	8	91183	90481	METABOLISM	Metabolism
ID	9	91183	90481	TOXICITY	Toxicity
ID	10	91183	90481	EFFICACY	Efficacy

Record 633 | WOS:000262775900032

Effect of ochratoxin A on cell survival and collagen homeostasis in human mesangial cells in primary culture

Field	Position	Original line	Normalised line	Original value	Normalised value
DE	1	91278	90575	Ochratoxin A	Ochratoxin A (OTA)
ID	1	91279	90576	HUMAN PROXIMAL TUBULE	Human Proximal Tubule
ID	2	91279	90576	BALKAN ENDEMIC NEPHROPATHY	Balkan Endemic Nephropathy
ID	3	91279	90576	CARCINOGENICITY	Carcinogenicity
ID	4	91279	90576	EXPOSURE	Exposure
ID	5	91279	90576	DEATH	Death
ID	6	91279	90576	ACID	Acid

Record 634 | WOS:000264793900023

EFFECTS OF AFLATOXIN B1, OCHRATOXIN A, PATULIN, CITRININ, AND ZEARALENONE ON THE IN VITRO PROLIFERATION OF PIG BLOOD LYMPHOCYTES

Field	Position	Original line	Normalised line	Original value	Normalised value
DE	1	91366	90662	swine	Swine
DE	2	91366	90662	mycotoxins	Mycotoxins
DE	3	91366	90662	lymphocytes	Lymphocyte
DE	4	91366	90662	proliferation assay	Proliferation Assay
ID	1	91367	90663	MITOGEN-INDUCED BLASTOGENESIS	Mitogen Induced Blastogenesis
ID	2	91367	90663	PENICILLIUM-MYCOTOXINS	Penicillium Mycotoxin
ID	3	91367	90663	TOXICITY	Toxicity

Record 635 | WOS:000262790100004

Developmental Toxicity of Ochratoxin A in Rat Embryo Midbrain Micromass Cultures

Field	Position	Original line	Normalised line	Original value	Normalised value
DE	1	91449	90744	Ochratoxin A	Ochratoxin A (OTA)
DE	2	91449	90744	developmental neurotoxicity	Developmental Neurotoxicity
DE	3	91449	90744	in vitro micromass cultures	In Vitro Micromass
DE	4	91449	90744	embryonic midbrain cells	Embryonic Midbrain
DE	5	91449	90744	computer image analysis	Computer Image Analysis
DE	6	91449	90744	immunocytochemistry	Immunocytochemistry
ID	1	91451	90746	VITRO EMBRYOTOXICITY TESTS	Vitro Embryotoxicity Test
ID	2	91451	90746	IN-VITRO	In-Vitro
ID	3	91451	90746	MYCOTOXIN OCHRATOXIN	Ochratoxin A (OTA)
ID	4	91451	90746	DEFENSE REACTIONS	Defense Reaction
ID	5	91451	90746	CELL LINES	Cell
ID	6	91451	90746	NEUROTOXICITY	Neurotoxicity
ID	7	91451	90746	CYTOTOXICITY	Cytotoxicity
ID	8	91451	90746	BRAIN	Brain
ID	9	91451	90746	IMMUNE	Immune
ID	10	91451	90746	ASSAY	Assay

Record 636 | WOS:000261056700090

Safety and Mutagenicity Evaluation of Nanoparticulate Red Mold Rice

Field	Position	Original line	Normalised line	Original value	Normalised value
DE	2	91549	90843	nanoparticulate	Nanoparticulate
DE	3	91549	90843	safety	Safety
DE	4	91549	90843	mutagenicity	Mutagenicity
ID	1	91550	90844	MONACOLIN-K	Monacolin K
ID	2	91550	90844	MONASCUS-PURPUREUS	Monascus Purpureus
ID	3	91550	90844	YEAST-RICE	Yeast Rice
ID	4	91550	90844	CITRININ MYCOTOXICOSIS	Citrinin Mycotoxicosis
ID	5	91550	90844	FORMULATION APPROACH	Formulation Approach
ID	6	91550	90844	CHOLESTEROL LEVELS	Cholesterol Level
ID	7	91550	90844	DIETARY-SUPPLEMENT	Dietary Supplement
ID	8	91550	90844	OCHRATOXIN-A	Ochratoxin A (OTA)
ID	9	91550	90844	TOXICITY	Toxicity
ID	10	91550	90844	LOVASTATIN	Lovastatin

Record 637 | WOS:000207684500010

Oxidative stress induced by ochratoxin A in LLC-PK1 cell line and the chemoprotective effects of carnosic acid

Field	Position	Original line	Normalised line	Original value	Normalised value
DE	1	91668	90961	prevention	Prevention
DE	2	91668	90961	cytotoxicity	Cytotoxicity

Record 638 | WOS:000260367000001

Individual and combined effects of ochratoxin A and citrinin on viability and DNA fragmentation in cultured Vero cells and on chromosome aberrations in mice bone marrow cells

Field	Position	Original line	Normalised line	Original value	Normalised value
DE	1	91749	91041	Ochratoxin A	Ochratoxin A (OTA)

DE	4	91749	91041	DNA fragmentation	DNA Fragmentation
DE	5	91749	91041	Chromosome aberrations	Chromosome Aberration
DE	6	91749	91041	Synergistic effect	Synergistic Effect
ID	1	91751	91043	BALKAN ENDEMIC NEPHROPATHY	Balkan Endemic Nephropathy
ID	2	91751	91043	INDUCED DYSFUNCTION	Induced Dysfunction
ID	3	91751	91043	RENAL-FUNCTION	Renal Function
ID	4	91751	91043	ENZYME-ACTIVITIES	Enzyme Activity
ID	5	91751	91043	KIDNEY-CELLS	Kidney
ID	6	91751	91043	MYCOTOXINS	Mycotoxins
ID	7	91751	91043	DAMAGE	Damage
ID	8	91751	91043	RATS	Rat
ID	9	91751	91043	APOPTOSIS	Apoptosis
ID	10	91751	91043	CANCER	Cancer

Record 639 | WOS:000259798900005

Inula crithmoides extract protects against ochratoxin A-induced oxidative stress, clastogenic and mutagenic alterations in male rats

Field	Position	Original line	Normalised line	Original value	Normalised value
DE	1	91877	91167	ochratoxins A	Ochratoxin A (OTA)
DE	2	91877	91167	Inula crithmoides	Inula Crithmoide
DE	3	91877	91167	oxidative stress	Oxidative Stress
DE	4	91877	91167	cytogenetic	Cytogenetic
ID	1	91878	91168	BALKAN ENDEMIC NEPHROPATHY	Balkan Endemic Nephropathy
ID	2	91878	91168	URINARY-BLADDER	Urinary Bladder
ID	3	91878	91168	DNA-DAMAGE	DNA Damage
ID	4	91878	91168	CELL-LINE	Cell
ID	5	91878	91168	GENOTOXICITY	Genotoxicity
ID	6	91878	91168	TOXICITY	Toxicity
ID	7	91878	91168	KIDNEY	Kidney
ID	8	91878	91168	MICRONUCLEI	Micronuclei
ID	9	91878	91168	ADDUCTS	Adduct
ID	10	91878	91168	SERUM	Serum

Record 640 | WOS:000260145000006

Differential modification of inflammatory enzymes in J774A.1 macrophages by ochratoxin A alone or in combination with lipopolysaccharide

Field	Position	Original line	Normalised line	Original value	Normalised value
DE	1	92011	91300	Ochratoxin A	Ochratoxin A (OTA)
DE	4	92011	91300	Inducible nitric oxide synthase	Inducible Nitric Oxide Synthase
DE	5	92011	91300	Cyclooxygenase-2	Cyclooxygenase 2
DE	6	92011	91300	Nuclear factor-kB	Nuclear Factor Kb
ID	1	92013	91302	NITRIC-OXIDE SYNTHASE	Nitric Oxide Synthase
ID	2	92013	91302	NF-KAPPA-B	Nf Kappa B
ID	3	92013	91302	LIPID-PEROXIDATION	Lipid Peroxidation
ID	4	92013	91302	DEFENSE REACTIONS	Defense Reaction
ID	5	92013	91302	EPITHELIAL-CELLS	Epithelial
ID	6	92013	91302	IN-VITRO	In-Vitro
ID	7	92013	91302	INDUCTION	Induction
ID	8	92013	91302	CYCLOOXYGENASE	Cyclooxygenase
ID	9	92013	91302	EXPRESSION	Expression
ID	10	92013	91302	IMMUNE	Immune

Record 641 | WOS:000258440100010

Effect of ochratoxin A on redox-regulated transcription factors, antioxidant enzymes and glutathione-S-transferase in cultured kidney tubulus cells

Field	Position	Original line	Normalised line	Original value	Normalised value
DE	1	92135	91422	ochratoxin A	Ochratoxin A (OTA)

DE	2	92135	91422	Glutathione-S-transferase	Glutathione S Transferase
DE	4	92135	91422	AP-1	Ap 1
DE	5	92135	91422	kidney tubulus cells	Kidney Tubulus
DE	6	92135	91422	oxidative stress	Oxidative Stress
DE	7	92135	91422	redox signalling	Redox Signalling
DE	8	92135	91422	nephrotoxicity	Nephrotoxicity
ID	1	92137	91424	BALKAN ENDEMIC NEPHROPATHY	Balkan Endemic Nephropathy
ID	2	92137	91424	OXIDATIVE DNA-DAMAGE	Oxidative DNA Damage
ID	3	92137	91424	LINE HEP G2	Line Hep G2
ID	4	92137	91424	IN-VITRO	In-Vitro
ID	5	92137	91424	GENE-EXPRESSION	Gene Expression
ID	6	92137	91424	RAT	Rat
ID	7	92137	91424	TOXICITY	Toxicity
ID	8	92137	91424	CYTOTOXICITY	Cytotoxicity
ID	9	92137	91424	CARCINOGENICITY	Carcinogenicity
ID	10	92137	91424	LIVER	Liver

Record 642 | WOS:000257645600013

Species-specific toxicity of aristolochic acid (AA) in vitro

Field	Position	Original line	Normalised line	Original value	Normalised value
DE	1	92253	91539	aristolochic acid	Aristolochic Acid
DE	2	92253	91539	kidney	Kidney
DE	3	92253	91539	IHKE	Ihke
DE	4	92253	91539	LLC-PK1	Llc Pk1
DE	5	92253	91539	NRK-52E	Nrk 52e
DE	6	92253	91539	primary culture	Primary
DE	7	92253	91539	rat	Rat
DE	8	92253	91539	human	Human
DE	9	92253	91539	pig	Pig
ID	1	92255	91540	DNA-ADDUCTS	DNA Adduct
ID	2	92255	91540	REDUCTIVE ACTIVATION	Reductive Activation
ID	3	92255	91540	EPITHELIAL-CELLS	Epithelial
ID	4	92255	91540	METABOLIC-ACTIVATION	Metabolic Activation
ID	5	92255	91540	OCHRATOXIN-A	Ochratoxin A (OTA)
ID	6	92255	91540	P53 GENE	P53 Gene
ID	7	92255	91540	NEPHROPATHY	Nephropathy
ID	8	92255	91540	EXPRESSION	Expression
ID	9	92255	91540	ENZYMES	Enzyme
ID	10	92255	91540	EXPOSURE	Exposure

Record 643 | WOS:000257721400033

Identification and in vitro cytotoxicity of ochratoxin a degradation products formed during coffee roasting

Field	Position	Original line	Normalised line	Original value	Normalised value
DE	1	92348	91631	ochratoxin A	Ochratoxin A (OTA)
DE	2	92348	91631	coffee roasting	Coffee Roasting
DE	3	92348	91631	degradation	Degradation
DE	4	92348	91631	toxicity	Toxicity
DE	5	92348	91631	cell culture	Cell
DE	6	92348	91631	14-(R)-ochratoxin A	14 R Ochratoxin A
DE	7	92348	91631	cytotoxicity	Cytotoxicity
ID	1	92350	91633	TANDEM MASS-SPECTROMETRY	Tandem Mass Spectrometry
ID	2	92350	91633	NEPHROTOXIN OCHRATOXIN	Ochratoxin A (OTA)
ID	3	92350	91633	STRUCTURAL ELUCIDATION	Structural Elucidation
ID	4	92350	91633	THERMAL-DEGRADATION	Thermal Degradation
ID	5	92350	91633	A CONTENT	A Content

ID	6	92350	91633	CELLS	Cell
ID	7	92350	91633	NEPHROPATHY	Nephropathy
ID	8	92350	91633	REDUCTION	Reduction
ID	9	92350	91633	APOPTOSIS	Apoptosis
ID	10	92350	91633	TOXICITY	Toxicity

Record 644 | WOS:000257577500008

Gene expression changes induced by ochratoxin A in renal and hepatic tissues of male F344 rat after oral repeated administration

Field	Position	Original line	Normalised line	Original value	Normalised value
DE	1	92444	91726	ochratoxin A	Ochratoxin A (OTA)
DE	2	92444	91726	gene expression	Gene Expression
DE	3	92444	91726	repeated oral toxicity	Repeated Oral Toxicity
DE	4	92444	91726	male Fisher 344 rat	Male Fisher 344 Rat
DE	5	92444	91726	kidney and liver OTA accumulation	Kidney And Liver OTA Accumulation
ID	1	92446	91728	OXIDATIVE DNA-DAMAGE	Oxidative DNA Damage
ID	2	92446	91728	BALKAN ENDEMIC NEPHROPATHY	Balkan Endemic Nephropathy
ID	3	92446	91728	OLIGONUCLEOTIDE ARRAY DATA	Oligonucleotide Array Data
ID	4	92446	91728	TOXICITY	Toxicity
ID	5	92446	91728	CELLS	Cell
ID	6	92446	91728	PLASMA	Plasma
ID	7	92446	91728	KIDNEY	Kidney
ID	8	92446	91728	CARCINOGENICITY	Carcinogenicity
ID	9	92446	91728	NEPHROTOXICITY	Nephrotoxicity
ID	10	92446	91728	TUMORIGENESIS	Tumorigenesis

Record 645 | WOS:000256601000019

Structure-activity relationships for the fluorescence of ochratoxin A: Insight for detection of ochratoxin A metabolites

Field	Position	Original line	Normalised line	Original value	Normalised value
DE	1	92555	91836	ochratoxin A	Ochratoxin A (OTA)
DE	2	92555	91836	fluorescence	Fluorescence
DE	3	92555	91836	limit of detection	Limit Of Detection
DE	4	92555	91836	metabolite	Metabolite
ID	1	92556	91837	BALKAN ENDEMIC NEPHROPATHY	Balkan Endemic Nephropathy
ID	2	92556	91837	CONFORMATIONAL-ANALYSIS	Conformational Analysis
ID	3	92556	91837	GENOTOXICITY	Genotoxicity
ID	4	92556	91837	TOXICITY	Toxicity
ID	5	92556	91837	ADDUCTS	Adduct
ID	6	92556	91837	CELLS	Cell
ID	7	92556	91837	ACID	Acid

Record 646 | WOS:000256806600009

In vitro cytogenetic results supporting a DNA nonreactive mechanism for Ochratoxin A, potentially relevant for its carcinogenicity

Field	Position	Original line	Normalised line	Original value	Normalised value
ID	1	92651	91931	PROTEIN-KINASE INHIBITOR	Protein Kinase Inhibitor
ID	2	92651	91931	MYCOTOXIN OCHRATOXIN	Ochratoxin A (OTA)
ID	3	92651	91931	SALMONELLA-TYPHIMURIUM	Salmonella Typhimurium
ID	4	92651	91931	BARRETTS-ESOPHAGUS	Barrett Esophagus
ID	5	92651	91931	MUTAGENICITY TEST	Mutagenicity Test
ID	6	92651	91931	MAMMALIAN-CELLS	Mammalian
ID	7	92651	91931	FLOW-CYTOMETRY	Flow Cytometry

ID	8	92651	91931	IMMUNE-SYSTEM	Immune System
ID	9	92651	91931	GENOTOXICITY	Genotoxicity
ID	10	92651	91931	INDUCTION	Induction

Record 647 | WOS:000256206000004

Modulating effects of fumonisin B1 and ochratoxin A on leukocytes and messenger cytokines of the human immune system

Field	Position	Original line	Normalised line	Original value	Normalised value
DE	1	92775	92054	mycotoxins	Mycotoxins
DE	2	92775	92054	fumonisin B1	Fumonisin B1 (FB1)
DE	3	92775	92054	ochratoxin A	Ochratoxin A (OTA)
DE	4	92775	92054	leukocytes	Leukocyte
DE	5	92775	92054	cytokines	Cytokine
DE	6	92775	92054	oesophageal and breast carcinomas	Oesophageal And Breast Carcinoma
ID	1	92777	92056	NECROSIS-FACTOR-ALPHA	Necrosis Factor Alpha
ID	2	92777	92056	SPHINGOLIPID BIOSYNTHESIS	Sphingolipid Biosynthesis
ID	3	92777	92056	FUSARIUM-MONILIFORME	Fusarium Moniliforme
ID	4	92777	92056	IN-VITRO	In-Vitro
ID	5	92777	92056	CELLS	Cell
ID	6	92777	92056	B-1	B 1
ID	7	92777	92056	CARCINOMA	Carcinoma
ID	8	92777	92056	TOXICITY	Toxicity
ID	9	92777	92056	CARCINOGENESIS	Carcinogenesis
ID	10	92777	92056	INTERLEUKIN-6	Interleukin 6

Record 648 | WOS:000255760800015

Evaluation of putative biomarkers of nephrotoxicity after exposure to ochratoxin a in vivo and in vitro

Field	Position	Original line	Normalised line	Original value	Normalised value
DE	1	92863	92141	ochratoxin A	Ochratoxin A (OTA)
DE	2	92863	92141	kidney	Kidney
DE	3	92863	92141	nephrotoxicity	Nephrotoxicity
DE	4	92863	92141	biomarker	Biomarker
DE	5	92863	92141	in vitro toxicity testing	In Vitro Toxicity Testing
ID	1	92865	92143	GELATINASE-ASSOCIATED LIPOCALIN	Gelatinase Associated Lipocalin
ID	2	92865	92143	KIDNEY INJURY MOLECULE-1	Kidney Injury Molecule 1
ID	3	92865	92143	EARLY URINARY BIOMARKER	Early Urinary Biomarker
ID	4	92865	92143	PROXIMAL TUBULE	Proximal Tubule
ID	5	92865	92143	GENE-EXPRESSION	Gene Expression
ID	6	92865	92143	CELL-LINES	Cell
ID	7	92865	92143	CLUSTERIN	Clusterin
ID	8	92865	92143	IDENTIFICATION	Identification
ID	9	92865	92143	OSTEOPONTIN	Osteopontin
ID	10	92865	92143	TISSUE	Tissue

Record 649 | WOS:000256713600009

Ochratoxin A inhibits the production of tissue factor and plasminogen activator inhibitor-2 by human blood mononuclear cells: Another potential mechanism of immune-suppression

Field	Position	Original line	Normalised line	Original value	Normalised value
DE	1	92981	92258	coagulation	Coagulation
DE	2	92981	92258	fibrinolysis	Fibrinolysis
DE	3	92981	92258	fibrin	Fibrin
DE	4	92981	92258	cell-mediated immune response	Cell Mediated Immune Response

ID	1	92982	92259	IN-VITRO	In-Vitro
ID	2	92982	92259	EXPRESSION	Expression
ID	3	92982	92259	MONOCYTES	Monocyte
ID	4	92982	92259	INDUCTION	Induction
ID	5	92982	92259	INFECTION	Infection
ID	6	92982	92259	FIBRIN	Fibrin

Record 650 | WOS:000256381400006

Medical relevance of mycotoxins

Field	Position	Original line	Normalised line	Original value	Normalised value
DE	1	93066	92342	mycotoxins	Mycotoxins
DE	2	93066	92342	ochratoxin	Ochratoxin A (OTA)
DE	3	93066	92342	trichothecenes	Trichothecene
DE	4	93066	92342	desoxynivalenol	Desoxynivalenol
DE	5	93066	92342	zearalenones	Zearalenone (ZEA)
DE	6	93066	92342	ergotamine	Ergotamine
DE	7	93066	92342	intoxication	Intoxication
ID	1	93068	92344	OCHRATOXIN-A	Ochratoxin A (OTA)

Record 651 | WOS:000254689700022

Detoxification of patulin and ochratoxin A, two abundant mycotoxins, by lactic acid bacteria

Field	Position	Original line	Normalised line	Original value	Normalised value
DE	1	93142	92417	patulin	Patulin
DE	2	93142	92417	ochratoxin A	Ochratoxin A (OTA)
DE	3	93142	92417	detoxification	Detoxification
DE	4	93142	92417	lactic acid bacteria	Lactic Acid Bacteria
DE	5	93142	92417	micronuclei	Micronuclei
ID	1	93144	92419	AFLATOXIN B-1	Aflatoxin B1 (AFB1)
ID	2	93144	92419	IN-VITRO	In-Vitro
ID	3	93144	92419	DAIRY STRAINS	Dairy Strain
ID	4	93144	92419	SALMONELLA-TYPHIMURIUM	Salmonella Typhimurium
ID	5	93144	92419	ANIMAL FEED	Animal Feed
ID	6	93144	92419	LACTOBACILLUS	Lactobacillus
ID	7	93144	92419	BINDING	Binding
ID	8	93144	92419	MUTAGENICITY	Mutagenicity
ID	9	93144	92419	FOOD	Food
ID	10	93144	92419	CELLS	Cell

Record 652 | WOS:000254544300011

Both direct and indirect effects account for the pro-inflammatory activity of enteropathogenic mycotoxins on the human intestinal epithelium:: Stimulation of interleukin-8 secretion, potentiation of interleukin-1Î² effect and increase in the transepithelial passage of commensal bacteria

Field	Position	Original line	Normalised line	Original value	Normalised value
DE	1	93283	92557	enteropathogenic mycotoxin	Enteropathogenic Mycotoxin
DE	2	93283	92557	deoxynivalenol	Deoxynivalenol (DON)
DE	3	93283	92557	ochratoxin a	Ochratoxin A (OTA)
DE	4	93283	92557	patulin	Patulin
DE	6	93283	92557	interleukin 8	Interleukin 8
DE	7	93283	92557	intestinal inflammation	Intestinal Inflammation
ID	1	93285	92559	ESCHERICHIA-COLI STRAIN	Escherichia Coli Strain
ID	2	93285	92559	OCHRATOXIN-A	Ochratoxin A (OTA)
ID	3	93285	92559	PROINFLAMMATORY CYTOKINE	Proinflammatory Cytokine
ID	4	93285	92559	PATULIN MYCOTOXICOSIS	Patulin Mycotoxicosis
ID	5	93285	92559	CROHNS-DISEASE	Crohn Disease
ID	6	93285	92559	CELL-LINES	Cell
ID	7	93285	92559	IN-VITRO	In-Vitro

ID	8	93285	92559	DEOXYNIVALENOL VOMITOXIN	Deoxynivalenol (DON)
ID	9	93285	92559	MOLECULAR-MECHANISMS	Molecular Mechanism
ID	10	93285	92559	BARRIER FUNCTION	Barrier Function

Record 653 | WOS:000257197500001

Risk-benefit considerations of mitigation measures on acrylamide content of foods - A case study on potatoes, cereals and coffee

Field	Position	Original line	Normalised line	Original value	Normalised value
DE	1	93425	92698	risk-benefit	Risk Benefit
DE	2	93425	92698	mitigation	Mitigation
DE	3	93425	92698	probabilistic modelling	Probabilistic Modelling
DE	4	93425	92698	margin of exposure	Margin Of Exposure
DE	5	93425	92698	acrylamide	Acrylamide (AA)
DE	6	93425	92698	potatoes	Potatoe
DE	7	93425	92698	cereals	Cereal
DE	8	93425	92698	coffee	Coffee
ID	1	93427	92700	TYPE-2 DIABETES-MELLITUS	Type 2 Diabete Mellitus
ID	2	93427	92700	CORONARY-HEART-DISEASE	Coronary Heart Disease
ID	3	93427	92700	FREE AMINO-ACIDS	Free Amino Acid
ID	4	93427	92700	POSTPRANDIAL INSULIN-RESPONSE	Postprandial Insulin Response
ID	5	93427	92700	WHOLE-GRAIN INTAKE	Whole Grain Intake
ID	6	93427	92700	BLOOD-PRESSURE	Blood Pressure
ID	7	93427	92700	RYE-BREAD	Rye Bread
ID	8	93427	92700	GLYCEMIC INDEX	Glycemic Index
ID	9	93427	92700	OCHRATOXIN-A	Ochratoxin A (OTA)
ID	10	93427	92700	IN-VITRO	In-Vitro

Record 655 | WOS:000252706500006

Ameliorative effect of artichoke (Cynara scolymus L.) extracts on creatine monohydrate-induced renal dysfunction in male rats

Field	Position	Original line	Normalised line	Original value	Normalised value
DE	1	93836	93107	creatine	Creatine
DE	2	93836	93107	renal function	Renal Function
DE	3	93836	93107	hormones	Hormone
DE	4	93836	93107	artichoke extracts	Artichoke Extract
ID	1	93837	93108	OXIDATIVE DEAMINATION	Oxidative Deamination
ID	2	93837	93108	PROTECTIVE PROPERTIES	Protective Property
ID	3	93837	93108	GLUCOSE-HOMEOSTASIS	Glucose Homeostasis
ID	4	93837	93108	INSULIN-SECRETION	Insulin Secretion
ID	5	93837	93108	ENDOTHELIAL-CELLS	Endothelial
ID	6	93837	93108	OCHRATOXIN-A	Ochratoxin A (OTA)
ID	7	93837	93108	SUPPLEMENTATION	Supplementation
ID	8	93837	93108	SERUM	Serum
ID	9	93837	93108	HEALTH	Health
ID	10	93837	93108	PERFORMANCE	Performance

Record 656 | WOS:000255988000006

Cytotoxicity and oxidative damage in kidney cells exposed to the mycotoxins ochratoxin A and citrinin: Individual and combined effects

Field	Position	Original line	Normalised line	Original value	Normalised value
DE	1	93968	93238	mycotoxins	Mycotoxins
DE	2	93968	93238	ochratoxin A	Ochratoxin A (OTA)
DE	3	93968	93238	citrinin	Citrinin
DE	4	93968	93238	cytotoxicity	Cytotoxicity

DE	5	93968	93238	oxidative damage	Oxidative Damage
DE	6	93968	93238	renal toxicity	Renal Toxicity
DE	7	93968	93238	combined effects	Combined Effect
ID	1	93970	93240	BALKAN ENDEMIC NEPHROPATHY	Balkan Endemic Nephropathy
ID	2	93970	93240	HEAT-SHOCK	Heat Shock
ID	3	93970	93240	IN-VITRO	In-Vitro
ID	4	93970	93240	STRESS	Stress
ID	5	93970	93240	MALONDIALDEHYDE	Malondialdehyde
ID	6	93970	93240	TOXICITY	Toxicity
ID	7	93970	93240	ASSAY	Assay
ID	8	93970	93240	ANTIOXIDANTS	Antioxidant
ID	9	93970	93240	CEREALS	Cereal
ID	10	93970	93240	DOGS	Dog

Record 657 | WOS:000253659900006

Mycotoxins in cattle feeds and carry-over to dairy milk: A review

Field	Position	Original line	Normalised line	Original value	Normalised value
DE	1	94074	93343	mycotoxins	Mycotoxins
DE	2	94074	93343	ruminants	Ruminant
DE	3	94074	93343	dairy cows	Dairy Cow
DE	4	94074	93343	aflatoxins	Aflatoxin
DE	5	94074	93343	ergot alkaloids	Ergot Alkaloid
DE	6	94074	93343	carry over	Carry Over
DE	7	94074	93343	milk	Milk
DE	8	94074	93343	biological barriers	Biological Barrier
ID	1	94076	93345	TOXIN-CONTAMINATED WHEAT	Toxin Contaminated Wheat
ID	2	94076	93345	TROUT SALMO-GAIRDNERI	Trout Salmo Gairdneri
ID	3	94076	93345	BALED GRASS-SILAGE	Baled Grass Silage
ID	4	94076	93345	OCHRATOXIN-A	Ochratoxin A (OTA)
ID	5	94076	93345	IN-VITRO	In-Vitro
ID	6	94076	93345	AFLATOXIN B-1	Aflatoxin B1 (AFB1)
ID	7	94076	93345	SMOOTH-MUSCLE	Smooth Muscle
ID	8	94076	93345	INTAKE LEVEL	Intake Level
ID	9	94076	93345	RUMEN FLUID	Rumen Fluid
ID	10	94076	93345	LOLITREM-B	Lolitrem B

Record 658 | WOS:000268886600003

Combined Toxic Effects of Ochratoxin A and Citrinin, In Vivo and In Vitro

Field	Position	Original line	Normalised line	Original value	Normalised value
ID	1	94204	93472	BALKAN ENDEMIC NEPHROPATHY	Balkan Endemic Nephropathy
ID	2	94204	93472	URINARY-TRACT TUMORS	Urinary Tract Tumor
ID	3	94204	93472	MYCOTOXINS	Mycotoxins
ID	4	94204	93472	TRANSPORT	Transport
ID	5	94204	93472	CEREALS	Cereal
ID	6	94204	93472	CELLS	Cell
ID	7	94204	93472	RAT	Rat
ID	8	94204	93472	CYTOTOXICITY	Cytotoxicity
ID	9	94204	93472	REABSORPTION	Reabsorption
ID	10	94204	93472	NEPHROTOXIN	Nephrotoxin

Record 659 | WOS:000251052700012

Toxigenic fungi and mycotoxins in mature corn silage

Field	Position	Original line	Normalised line	Original value	Normalised value
DE	1	94308	93575	mature corn silage	Mature Corn Silage

DE	2	94308	93575	mycoflora	Mycoflora
DE	3	94308	93575	multimycotoxin	Multimycotoxin
DE	4	94308	93575	HPLC-MS	Hplc Ms
ID	1	94309	93576	MAIZE	Maize
ID	2	94309	93576	BIOSYNTHESIS	Biosynthesis
ID	3	94309	93576	TOXIN	Toxin

Record 660 | WOS:000249598500015

Catechins: natural free-radical scavengers against ochratoxin A-induced cell damage in a pig kidney cell line (LLC-PK1)

Field	Position	Original line	Normalised line	Original value	Normalised value
DE	1	94399	93665	ochratoxin A	Ochratoxin A (OTA)
DE	2	94399	93665	catechins	Catechin
DE	3	94399	93665	cell viability	Cell Viability
DE	4	94399	93665	DNA fragmentation	DNA Fragmentation
DE	5	94399	93665	reactive oxygen species	Reactive Oxygen Specy
DE	6	94399	93665	prevention	Prevention
ID	1	94401	93667	BRIGGS-RAUSCHER REACTION	Brigg Rauscher Reaction
ID	2	94401	93667	GREEN TEA POLYPHENOLS	Green Tea Polyphenol
ID	3	94401	93667	OXIDATIVE DNA-DAMAGE	Oxidative DNA Damage
ID	4	94401	93667	ANTIOXIDANT ACTIVITY	Antioxidant Activity
ID	5	94401	93667	PROTECTIVE ROLE	Protective Role
ID	6	94401	93667	CYTOTOXICITY	Cytotoxicity
ID	7	94401	93667	INHIBITION	Inhibition
ID	8	94401	93667	TOXICITY	Toxicity
ID	9	94401	93667	NRF2	Nrf2
ID	10	94401	93667	NEPHROPATHY	Nephropathy

Record 661 | WOS:000249983300013

New molecular and field evidences for the implication of mycotoxins but not aristolochic acid in human nephropathy and urinary tract tumor

Field	Position	Original line	Normalised line	Original value	Normalised value
DE	1	94507	93772	aristolochic acid	Aristolochic Acid
DE	2	94507	93772	BEN	Ben
DE	3	94507	93772	ochratoxin A	Ochratoxin A (OTA)
DE	4	94507	93772	slimming regimen	Slimming Regimen
DE	5	94507	93772	urinary tract tumor	Urinary Tract Tumor
ID	1	94509	93774	BALKAN ENDEMIC NEPHROPATHY	Balkan Endemic Nephropathy
ID	2	94509	93774	OCHRATOXIN-A	Ochratoxin A (OTA)
ID	3	94509	93774	CHINESE HERBS	Chinese Herb
ID	4	94509	93774	DNA ADDUCTION	DNA Adduction
ID	5	94509	93774	CEREALS	Cereal
ID	6	94509	93774	TOXICITY	Toxicity
ID	7	94509	93774	CITRININ	Citrinin
ID	8	94509	93774	CARCINOGENICITY	Carcinogenicity
ID	9	94509	93774	CYCLOOXYGENASES	Cyclooxygenase
ID	10	94509	93774	GENOTOXICITY	Genotoxicity

Record 662 | WOS:000248014300007

Ochratoxin A-induced mutagenesis in mammalian cells is consistent with the production of oxidative stress

Field	Position	Original line	Normalised line	Original value	Normalised value
ID	1	94631	93895	DNA ADDUCT FORMATION	DNA Adduct Formation
ID	2	94631	93895	SPONTANEOUSLY OCCURRING MUTATIONS	Spontaneously Occurring Mutation

ID	3	94631	93895	SISTER-CHROMATID EXCHANGE	Sister Chromatid Exchange
ID	4	94631	93895	HPRT GENE	Hprt Gene
ID	5	94631	93895	SALMONELLA-TYPHIMURIUM	Salmonella Typhimurium
ID	6	94631	93895	MYCOTOXINS OCHRATOXIN	Ochratoxin A (OTA)
ID	7	94631	93895	MEDIATED TOXICITY	Mediated Toxicity
ID	8	94631	93895	MUTAGENICITY TEST	Mutagenicity Test
ID	9	94631	93895	KIDNEY-CELLS	Kidney
ID	10	94631	93895	RAT-LIVER	Rat Liver

Record 663 | WOS:000247601800018

Adult hippocampal neural stem/progenitor cells in vitro are vulnerable to the mycotoxin ochratoxin-A

Field	Position	Original line	Normalised line	Original value	Normalised value
DE	1	94771	94033	neural stem/progenitor cells	Neural Stem Progenitor
DE	2	94771	94033	hippocampus	Hippocampus
DE	3	94771	94033	ochratoxin-A	Ochratoxin A (OTA)
DE	4	94771	94033	mycotoxin	Mycotoxins
DE	5	94771	94033	oxidative stress	Oxidative Stress
DE	6	94771	94033	DNA repair	DNA Repair
DE	7	94771	94033	8-oxoguanine glycosylase-1 (OGG1)	8 Oxoguanine Glycosylase 1 Ogg1
DE	8	94771	94033	superoxide dismutase (SOD)	Superoxide Dismutase Sod
DE	9	94771	94033	diethyl maleate (DEM)	Diethyl Maleate Dem
ID	1	94774	94036	BASE-EXCISION-REPAIR	Base Excision Repair
ID	2	94774	94036	DNA-DAMAGE	DNA Damage
ID	3	94774	94036	RAT	Rat
ID	4	94774	94036	NEUROGENESIS	Neurogenesis
ID	5	94774	94036	BRAIN	Brain
ID	6	94774	94036	EXPOSURE	Exposure
ID	7	94774	94036	MICE	Mice
ID	8	94774	94036	STEM	Stem
ID	9	94774	94036	DIFFERENTIATION	Differentiation
ID	10	94774	94036	ISCHEMIA	Ischemia

Record 664 | WOS:000246927300041

Ochratoxin A displaces claudins from detergent resistant membrane microdomains

Field	Position	Original line	Normalised line	Original value	Normalised value
DE	1	94886	94147	tight junction	Tight Junction
DE	2	94886	94147	claudin	Claudin
DE	3	94886	94147	detergent resistant membrane microdomain	Detergent Resistant Membrane Microdomain
DE	4	94886	94147	ochratoxin A	Ochratoxin A (OTA)
ID	1	94888	94149	BARRIER	Barrier
ID	2	94888	94149	STRESS	Stress
ID	3	94888	94149	DAMAGE	Damage

Record 665 | WOS:000247178200008

Ochratoxin A:: 13-week oral toxicity and cell proliferation in male F344/N rats

Field	Position	Original line	Normalised line	Original value	Normalised value
DE	1	94973	94233	ochratoxin A	Ochratoxin A (OTA)
DE	2	94973	94233	kidney	Kidney
DE	3	94973	94233	carcinogenicity	Carcinogenicity
DE	4	94973	94233	cell proliferation	Cell Proliferation
DE	5	94973	94233	karyomegaly	Karyomegaly

ID	1	94974	94235	EPIGENETIC MECHANISMS	Epigenetic Mechanism
ID	2	94974	94235	RENAL TOXICITY	Renal Toxicity
ID	3	94974	94235	DNA-DAMAGE	DNA Damage
ID	4	94974	94235	IN-VIVO	In-Vivo
ID	5	94974	94235	KIDNEY	Kidney
ID	6	94974	94235	ALPHA-2U-GLOBULIN	Alpha 2u Globulin
ID	7	94974	94235	NEPHROPATHY	Nephropathy
ID	8	94974	94235	EXPOSURE	Exposure
ID	9	94974	94235	CARCINOGENICITY	Carcinogenicity
ID	10	94974	94235	NEPHROTOXICITY	Nephrotoxicity

Record 666 | WOS:000247092600009

Transepithelial transport of fusariotoxin nivalenol: Mediation of secretion by ABC transporters

Field	Position	Original line	Normalised line	Original value	Normalised value
DE	1	95075	94334	nivalenol	Nivalenol
DE	2	95075	94334	trichothecene	Trichothecene
DE	3	95075	94334	P-glycoprotein	P Glycoprotein
DE	4	95075	94334	multidrug resistance-associated protein 2	Multidrug Resistance Associated Protein 2
DE	5	95075	94334	epithelial cells	Epithelial
DE	6	95075	94334	intestinal transfer	Intestinal Transfer
ID	1	95077	94336	CANCER RESISTANCE PROTEIN	Cancer Resistance Protein
ID	2	95077	94336	CACO-2 CELL MONOLAYERS	Caco-2 Cell Monolayer
ID	3	95077	94336	MULTIDRUG-RESISTANCE	Multidrug Resistance
ID	4	95077	94336	P-GLYCOPROTEIN	P Glycoprotein
ID	5	95077	94336	DRUG EFFLUX	Drug Efflux
ID	6	95077	94336	IN-VITRO	In-Vitro
ID	7	95077	94336	TRICHOTHECENE MYCOTOXINS	Trichothecene Mycotoxin
ID	8	95077	94336	NATURAL OCCURRENCE	Natural Occurrence
ID	9	95077	94336	OCHRATOXIN-A	Ochratoxin A (OTA)
ID	10	95077	94336	LINE CACO-2	Line Caco-2

Record 667 | WOS:000246213800014

Production and characterization of monoclonal antibodies against ochratoxin B

Field	Position	Original line	Normalised line	Original value	Normalised value
DE	1	95177	94435	ochratoxin B	Ochratoxin B
DE	2	95177	94435	monoclonal antibodies	Monoclonal Antibody
DE	3	95177	94435	ELISA	Elisa
ID	1	95178	94436	BALKAN ENDEMIC NEPHROPATHY	Balkan Endemic Nephropathy
ID	2	95178	94436	LINKED-IMMUNOSORBENT-ASSAY	Linked Immunosorbent Assay
ID	3	95178	94436	ACUTE INTRAPERITONEAL TOXICITY	Acute Intraperitoneal Toxicity
ID	4	95178	94436	TROUT SALMO-GAIRDNERI	Trout Salmo Gairdneri
ID	5	95178	94436	IN-VITRO	In-Vitro
ID	6	95178	94436	ETIOLOGY	Etiology
ID	7	95178	94436	MYCOTOXINS	Mycotoxins
ID	8	95178	94436	BLOOD	Blood
ID	9	95178	94436	FEED	Feed
ID	10	95178	94436	PIGS	Pig

Record 668 | WOS:000246875200005

Combined effects of mycotoxin mixtures on human T cell function

Field	Position	Original line	Normalised line	Original value	Normalised value
DE	1	95268	94525	combined effect	Combined Effect
DE	2	95268	94525	concentration addition	Concentration Addition
DE	3	95268	94525	response addition	Response Addition
DE	4	95268	94525	mycotoxin	Mycotoxins
DE	5	95268	94525	mixture toxicity prediction	Mixture Toxicity Prediction
ID	1	95270	94527	EXPERIMENTAL SUBCHRONIC MYCOTOXICOSES	Experimental Subchronic Mycotoxicose
ID	2	95270	94527	IN-VITRO PROLIFERATION	In Vitro Proliferation
ID	3	95270	94527	OCHRATOXIN-A	Ochratoxin A (OTA)
ID	4	95270	94527	PENICILLIUM MYCOTOXINS	Penicillium Mycotoxin
ID	5	95270	94527	PORCINE LYMPHOCYTES	Porcine Lymphocyte
ID	6	95270	94527	ACTING CHEMICALS	Acting Chemical
ID	7	95270	94527	IMMUNE FUNCTIONS	Immune Function
ID	8	95270	94527	CANCER-RISK	Cancer Risk
ID	9	95270	94527	HUMAN BLOOD	Human Blood
ID	10	95270	94527	TOXICITY	Toxicity

Record 669 | WOS:000245707900011

In vitro gene expression data supporting a DNA non-reactive genotoxic mechanism for ochratoxin A

Field	Position	Original line	Normalised line	Original value	Normalised value
DE	1	95372	94628	ochratoxin A	Ochratoxin A (OTA)
DE	2	95372	94628	gene expression	Gene Expression
DE	3	95372	94628	oxidative DNA damage	Oxidative DNA Damage
DE	4	95372	94628	reactive oxygen species	Reactive Oxygen Specy
DE	5	95372	94628	cytotoxicity	Cytotoxicity
DE	6	95372	94628	human kidney cells	Human Kidney
ID	1	95374	94630	OXIDATIVE STRESS	Oxidative Stress
ID	2	95374	94630	CELL-LINE	Cell
ID	3	95374	94630	DAMAGE	Damage
ID	4	95374	94630	RAT	Rat
ID	5	95374	94630	METABOLISM	Metabolism
ID	6	95374	94630	TOXICOLOGY	Toxicology
ID	7	95374	94630	ASSAY	Assay
ID	8	95374	94630	IDENTIFICATION	Identification
ID	9	95374	94630	MICROARRAYS	Microarray
ID	10	95374	94630	TOXICITY	Toxicity

Record 670 | WOS:000244262900005

Reduction in antioxidant defenses may contribute to ochratoxin a toxicity and carcinogenicity

Field	Position	Original line	Normalised line	Original value	Normalised value
DE	1	95497	94752	mycotoxin	Mycotoxins
DE	2	95497	94752	ochratoxin A	Ochratoxin A (OTA)
DE	3	95497	94752	nuclear factor-erythroid 2 p45-related factor 2 (Nrf2)	Nuclear Factor Erythroid 2 P45 Related Factor 2 Nrf2
DE	4	95497	94752	oxidative stress	Oxidative Stress
DE	5	95497	94752	nephrotoxicity	Nephrotoxicity
DE	6	95497	94752	carcinogenicity	Carcinogenicity
ID	1	95499	94754	DNA ADDUCT FORMATION	DNA Adduct Formation
ID	2	95499	94754	OXIDATIVE STRESS	Oxidative Stress
ID	3	95499	94754	RESPONSE ELEMENT	Response Element
ID	4	95499	94754	ENDEMIC NEPHROPATHY	Endemic Nephropathy
ID	5	95499	94754	CULTURED RAT	Cultured Rat
ID	6	95499	94754	GLUTATHIONE	Glutathione (GSH)

ID	7	95499	94754	NRF2	Nrf2
ID	8	95499	94754	METABOLISM	Metabolism
ID	9	95499	94754	ACTIVATION	Activation
ID	10	95499	94754	INDUCTION	Induction

Record 671 | WOS:000244244100003

Lipid peroxidation and glutathione levels in porcine kidney PK15 cells after individual and combined treatment with fumonisin B1, beauvericin and ochratoxin A

Field	Position	Original line	Normalised line	Original value	Normalised value
ID	1	95610	94864	OXIDATIVE STRESS	Oxidative Stress
ID	2	95610	94864	FUSARIUM-MONILIFORME	Fusarium Moniliforme
ID	3	95610	94864	B-1	B 1
ID	4	95610	94864	PROTEIN	Protein
ID	5	95610	94864	CYTOTOXICITY	Cytotoxicity
ID	6	95610	94864	INHIBITION	Inhibition
ID	7	95610	94864	APOPTOSIS	Apoptosis
ID	8	95610	94864	TOXICITY	Toxicity
ID	9	95610	94864	BLOOD	Blood
ID	10	95610	94864	MYCOTOXINS	Mycotoxins

Record 672 | WOS:000244169500009

Effects of repeated ochratoxin exposure on renal cells in vitro

Field	Position	Original line	Normalised line	Original value	Normalised value
DE	1	95706	94959	ochratoxins	Ochratoxin A (OTA)
DE	2	95706	94959	renal cells	Renal
DE	3	95706	94959	repeated exposure	Repeated Exposure
DE	4	95706	94959	nephropathy	Nephropathy
ID	1	95707	94960	BALKAN ENDEMIC NEPHROPATHY	Balkan Endemic Nephropathy
ID	2	95707	94960	TUBULAR EPITHELIAL-CELLS	Tubular Epithelial
ID	3	95707	94960	MESENCHYMAL TRANSITION	Mesenchymal Transition
ID	4	95707	94960	FIBROSIS	Fibrosis
ID	5	95707	94960	RATS	Rat
ID	6	95707	94960	TRANSDIFFERENTIATION	Transdifferentiation
ID	7	95707	94960	MECHANISMS	Mechanism
ID	8	95707	94960	MYCOTOXINS	Mycotoxins
ID	9	95707	94960	ETIOLOGY	Etiology
ID	10	95707	94960	TUMORS	Tumor

Record 673 | WOS:000243327000004

Oxidative DNA damage induced by Ochratoxin A in the HK-2 human kidney cell line: evidence of the relationship with cytotoxicity

Field	Position	Original line	Normalised line	Original value	Normalised value
ID	1	95787	95038	SALMONELLA-TYPHIMURIUM	Salmonella Typhimurium
ID	2	95787	95038	STRAND BREAKS	Strand Break
ID	3	95787	95038	COMET ASSAY	Comet Assay
ID	4	95787	95038	HEP G2	Hep G2
ID	5	95787	95038	RAT	Rat
ID	6	95787	95038	MUTAGENICITY	Mutagenicity
ID	7	95787	95038	INDUCTION	Induction
ID	8	95787	95038	REPAIR	Repair
ID	9	95787	95038	MOUSE	Mouse
ID	10	95787	95038	GENOTOXICITY	Genotoxicity

Record 674 | WOS:000244790500003

Ochratoxin A: An overview on toxicity and carcinogenicity in animals and humans

Field	Position	Original line	Normalised line	Original value	Normalised value
DE	1	95896	95146	carcinogenicity	Carcinogenicity
DE	2	95896	95146	DNA adducts	DNA Adduct
DE	3	95896	95146	genotoxicity	Genotoxicity
DE	4	95896	95146	kidney tumours	Kidney Tumour
DE	5	95896	95146	ochratoxin A	Ochratoxin A (OTA)
ID	1	95897	95148	BALKAN ENDEMIC NEPHROPATHY	Balkan Endemic Nephropathy
ID	2	95897	95148	ORGANIC ANION TRANSPORTER	Organic Anion Transporter
ID	3	95897	95148	URINARY-TRACT TUMORS	Urinary Tract Tumor
ID	4	95897	95148	IMMUNOAFFINITY COLUMN CLEANUP	Immunoaffinity Column Cleanup
ID	5	95897	95148	ARACHIDONIC-ACID METABOLISM	Arachidonic Acid Metabolism
ID	6	95897	95148	PROSTAGLANDIN-H SYNTHASE	Prostaglandin H Synthase
ID	7	95897	95148	SINGLE-STRAND BREAKS	Single Strand Break
ID	8	95897	95148	RENAL-CELL CARCINOMA	Renal Cell Carcinoma
ID	9	95897	95148	DNA ADDUCT FORMATION	DNA Adduct Formation
ID	10	95897	95148	HUMAN SERUM-ALBUMIN	Human Serum Albumin

Record 675 | WOS:000254907600043

Cytotoxicity of feed-borne mycotoxins to animal cell lines in vitro using the MTT assay

Field	Position	Original line	Normalised line	Original value	Normalised value
DE	1	96449	95699	kidney fibroblasts	Kidney Fibroblast
DE	2	96449	95699	mycotoxins	Mycotoxins
DE	3	96449	95699	cytotoxicity	Cytotoxicity
DE	4	96449	95699	MTT assay	Mtt Assay
ID	1	96450	95700	OCHRATOXIN-A	Ochratoxin A (OTA)
ID	2	96450	95700	TOXICITY	Toxicity
ID	3	96450	95700	CULTURE	Culture

Record 676 | WOS:000248276300289

Effect of Saccharomyces cerevisiae and esterified glucomannan on residues of Ochratoxin A in kidney, muscle and blood of laying hens

Field	Position	Original line	Normalised line	Original value	Normalised value
ID	1	96523	95772	PARAMETERS	Parameter
ID	2	96523	95772	FEED	Feed

Record 677 | WOS:000243441300001

Review on the toxicity, occurrence, metabolism, detoxification, regulations and intake of zearalenone:: An oestrogenic mycotoxin

Field	Position	Original line	Normalised line	Original value	Normalised value
DE	1	96589	95837	zearalenone	Zearalenone (ZEA)
DE	2	96589	95837	toxicity	Toxicity
DE	3	96589	95837	occurrence	Occurrence
DE	4	96589	95837	food	Food
DE	5	96589	95837	metabolism	Metabolism
DE	6	96589	95837	detoxification	Detoxification
ID	1	96590	95838	LIQUID-CHROMATOGRAPHIC DETERMINATION	Liquid Chromatographic Determination
ID	2	96590	95838	DNA ADDUCT FORMATION	DNA Adduct Formation

ID	3	96590	95838	NEW-ZEALAND MAIZE	New Zealand Maize
ID	4	96590	95838	NATURAL OCCURRENCE	Natural Occurrence
ID	5	96590	95838	FUSARIUM-MYCOTOXINS	Fusarium Mycotoxin
ID	6	96590	95838	FUMONISIN B-1	Fumonisin B1 (FB1)
ID	7	96590	95838	OCHRATOXIN-A	Ochratoxin A (OTA)
ID	8	96590	95838	IN-VITRO	In-Vitro
ID	9	96590	95838	BIOLOGICAL DETOXIFICATION	Biological Detoxification
ID	10	96590	95838	DEOXYNIVALENOL VOMITOXIN	Deoxynivalenol (DON)

Record 678 | WOS:000242136100016

Interactive effects of fumonisin B1 and Î±-zearalenol on proliferation and cytokine expression in Jurkat T cells

Field	Position	Original line	Normalised line	Original value	Normalised value
DE	1	96838	96085	fumonisin B1	Fumonisin B1 (FB1)
DE	2	96838	96085	alpha-zearalenol	Alpha Zearalenol
DE	3	96838	96085	Jurkat T cells	Jurkat T
DE	4	96838	96085	immunotoxicity	Immunotoxicity
ID	1	96839	96086	NECROSIS-FACTOR-ALPHA	Necrosis Factor Alpha
ID	2	96839	96086	IN-VITRO	In-Vitro
ID	3	96839	96086	EPITHELIAL-CELLS	Epithelial
ID	4	96839	96086	FUSARIUM-MONILIFORME	Fusarium Moniliforme
ID	5	96839	96086	OCHRATOXIN-A	Ochratoxin A (OTA)
ID	6	96839	96086	B-1 ALTERS	B 1 Alter
ID	7	96839	96086	SPHINGOLIPID METABOLISM	Sphingolipid Metabolism
ID	8	96839	96086	BARRIER FUNCTION	Barrier Function
ID	9	96839	96086	MYCOTOXINS	Mycotoxins
ID	10	96839	96086	CYTOTOXICITY	Cytotoxicity

Record 679 | WOS:000243461200014

Ochratoxin A-induced cytotoxicity in liver (HepG2) cells:: Impact of serum concentration, dietary antioxidants and glutathione-modulating compounds

Field	Position	Original line	Normalised line	Original value	Normalised value
ID	1	96949	96195	VITAMIN-E	Vitamin E
ID	2	96949	96195	MITOCHONDRIAL RESPIRATION	Mitochondrial Respiration
ID	3	96949	96195	BUTHIONINE SULFOXIMINE	Buthionine Sulfoximine
ID	4	96949	96195	LIPID-PEROXIDATION	Lipid Peroxidation
ID	5	96949	96195	GENE-EXPRESSION	Gene Expression
ID	6	96949	96195	APOPTOSIS	Apoptosis
ID	7	96949	96195	DAMAGE	Damage
ID	8	96949	96195	RAT	Rat
ID	9	96949	96195	ACTIVATION	Activation
ID	10	96949	96195	INHIBITION	Inhibition

Record 680 | WOS:000241622800019

Critical period and minimum single oral dose of ochratoxin A for inducing developmental toxicity in pregnant Wistar rats

Field	Position	Original line	Normalised line	Original value	Normalised value
DE	1	97059	96304	ochratoxin A	Ochratoxin A (OTA)
DE	2	97059	96304	pregnancy	Pregnancy
DE	3	97059	96304	malformations	Malformation
DE	4	97059	96304	skeletal defects	Skeletal Defect
DE	5	97059	96304	fetuses	Fetuse

DE	6	97059	96304	visceral anomalies	Visceral Anomaly
DE	7	97059	96304	Wistar rats	Wistar Rat
DE	8	97059	96304	teratology	Teratology
ID	1	97061	96306	DOMESTIC-ANIMALS	Domestic Animal
ID	2	97061	96306	EXPOSURE	Exposure
ID	3	97061	96306	TOXICOKINETICS	Toxicokinetics
ID	4	97061	96306	RABBITS	Rabbit
ID	5	97061	96306	MICE	Mice

Record 681 | WOS:000239496200009

A study of zearalenone cytotoxicity on human peripheral blood mononuclear cells

Field	Position	Original line	Normalised line	Original value	Normalised value
DE	1	97150	96394	zearalenone	Zearalenone (ZEA)
DE	2	97150	96394	mycotoxins	Mycotoxins
DE	3	97150	96394	immunocytotoxicity	Immunocytotoxicity
DE	4	97150	96394	necrosis	Necrosis
DE	5	97150	96394	apoptosis	Apoptosis
DE	6	97150	96394	human peripheral blood mononuclear cells	Human Peripheral Blood Mononuclear
ID	1	97152	96396	IN-VITRO	In-Vitro
ID	2	97152	96396	NATURAL OCCURRENCE	Natural Occurrence
ID	3	97152	96396	FUSARIUM MYCOTOXINS	Fusarium Mycotoxin
ID	4	97152	96396	OCHRATOXIN-A	Ochratoxin A (OTA)
ID	5	97152	96396	VITAMIN-E	Vitamin E
ID	6	97152	96396	LYMPHOCYTES	Lymphocyte
ID	7	97152	96396	DEOXYNIVALENOL	Deoxynivalenol (DON)
ID	8	97152	96396	INHIBITION	Inhibition
ID	9	97152	96396	INDUCTION	Induction
ID	10	97152	96396	APOPTOSIS	Apoptosis

Record 682 | WOS:000239973600004

Expression of COX-2 and hsp72 in peritoneal macrophages after an acute ochratoxin A treatment in mice

Field	Position	Original line	Normalised line	Original value	Normalised value
DE	1	97252	96496	ochratoxin A	Ochratoxin A (OTA)
DE	2	97252	96496	cyclooxygenase-2	Cyclooxygenase 2
DE	3	97252	96496	heat shock protein 72	Heat Shock Protein 72
DE	4	97252	96496	peritoneal macrophage	Peritoneal Macrophage
DE	5	97252	96496	lipid peroxidation	Lipid Peroxidation
DE	6	97252	96496	myeloperoxidase activity	Myeloperoxidase Activity
ID	1	97254	96498	NITRIC-OXIDE SYNTHASE	Nitric Oxide Synthase
ID	2	97254	96498	LIPID-PEROXIDATION	Lipid Peroxidation
ID	3	97254	96498	DEFENSE REACTIONS	Defense Reaction
ID	4	97254	96498	BROILER CHICKS	Broiler Chick
ID	5	97254	96498	CELLS	Cell
ID	6	97254	96498	CYCLOOXYGENASE-2	Cyclooxygenase 2
ID	7	97254	96498	SHOCK	Shock
ID	8	97254	96498	CYTOTOXICITY	Cytotoxicity
ID	9	97254	96498	INFLAMMATION	Inflammation
ID	10	97254	96498	INDUCTION	Induction

Record 683 | WOS:000240051000015

Early cytotoxic effects of ochratoxin A in rat liver: A morphological, biochemical and molecular study

Field	Position	Original line	Normalised line	Original value	Normalised value
DE	1	97383	96625	ochratoxin A	Ochratoxin A (OTA)
DE	2	97383	96625	collagen turnover	Collagen Turnover
DE	3	97383	96625	oxidative stress	Oxidative Stress

DE	4	97383	96625	cadherins	Cadherin
DE	5	97383	96625	connexins	Connexin
DE	6	97383	96625	HSP70	Hsp70
ID	1	97385	96627	BALKAN ENDEMIC NEPHROPATHY	Balkan Endemic Nephropathy
ID	2	97385	96627	OXIDATIVE DNA-DAMAGE	Oxidative DNA Damage
ID	3	97385	96627	GAP-JUNCTIONS	Gap Junction
ID	4	97385	96627	CELL-CULTURE	Cell
ID	5	97385	96627	E-CADHERIN	E Cadherin
ID	6	97385	96627	STRESS	Stress
ID	7	97385	96627	KIDNEY	Kidney
ID	8	97385	96627	MELATONIN	Melatonin
ID	9	97385	96627	CONNEXIN-32	Connexin 32
ID	10	97385	96627	BIOMARKERS	Biomarker

Record 684 | WOS:000240051000002

Adverse effects of T-2 toxin on chicken lymphocytes blastogenesis and its protection with Vitamin E

Field	Position	Original line	Normalised line	Original value	Normalised value
DE	1	97496	96736	T-2 toxin	T 2 Toxin
DE	2	97496	96736	lymphocyte blastogenesis	Lymphocyte Blastogenesis
ID	1	97497	96737	MITOGEN-INDUCED BLASTOGENESIS	Mitogen Induced Blastogenesis
ID	2	97497	96737	LIPID-PEROXIDATION	Lipid Peroxidation
ID	3	97497	96737	IN-VITRO	In-Vitro
ID	4	97497	96737	CELL-PROLIFERATION	Cell Proliferation
ID	5	97497	96737	ALPHA-TOCOPHEROL	Alpha Tocopherol
ID	6	97497	96737	OCHRATOXIN-A	Ochratoxin A (OTA)
ID	7	97497	96737	BALB/C MICE	Balb C Mice
ID	8	97497	96737	MYCOTOXINS	Mycotoxins
ID	9	97497	96737	ACTIVATION	Activation
ID	10	97497	96737	TOXICITY	Toxicity

Record 685 | WOS:000239031400009

Glutathione S-transferase polymorphisms and ochratoxin A toxicity in primary human urothelial cells

Field	Position	Original line	Normalised line	Original value	Normalised value
DE	1	97594	96833	ochratoxin A	Ochratoxin A (OTA)
DE	2	97594	96833	glutathione conjugation	Glutathione Conjugation
DE	3	97594	96833	urinary bladder	Urinary Bladder
DE	4	97594	96833	metabolism	Metabolism
DE	5	97594	96833	genotoxicity	Genotoxicity
ID	1	97596	96835	MYCOTOXIN OCHRATOXIN	Ochratoxin A (OTA)
ID	2	97596	96835	OXIDATIVE STRESS	Oxidative Stress
ID	3	97596	96835	PH HOMEOSTASIS	Ph Homeostasis
ID	4	97596	96835	RISK-FACTORS	Risk Factor
ID	5	97596	96835	COMET ASSAY	Comet Assay
ID	6	97596	96835	DNA-DAMAGE	DNA Damage
ID	7	97596	96835	VASA-RECTA	Vasa Recta
ID	8	97596	96835	IN-VITRO	In-Vitro
ID	9	97596	96835	GENOTOXICITY	Genotoxicity
ID	10	97596	96835	METABOLISM	Metabolism

Record 686 | WOS:000238650400009

Ochratoxin A: Apoptosis and aberrant exit from mitosis due to perturbation of microtubule dynamics?

Field	Position	Original line	Normalised line	Original value	Normalised value
DE	1	97695	96933	ochratoxin A	Ochratoxin A (OTA)
DE	2	97695	96933	carcinogenicity	Carcinogenicity
DE	3	97695	96933	apoptosis	Apoptosis

DE	4	97695	96933	mitosis	Mitosis
DE	5	97695	96933	microtubule	Microtubule
ID	1	97696	96934	KIDNEY EPITHELIAL-CELLS	Kidney Epithelial
ID	2	97696	96934	N-TERMINAL KINASE	N Terminal Kinase
ID	3	97696	96934	CHRONIC INTERSTITIAL NEPHROPATHY	Chronic Interstitial Nephropathy
ID	4	97696	96934	PROXIMAL TUBULAR CELLS	Proximal Tubular
ID	5	97696	96934	FOCAL ADHESION KINASE	Focal Adhesion Kinase
ID	6	97696	96934	KAPPA-B	Kappa B
ID	7	97696	96934	INTERCELLULAR COMMUNICATION	Intercellular Communication
ID	8	97696	96934	NEPHRITOGENIC OCHRATOXIN	Ochratoxin A (OTA)
ID	9	97696	96934	NEPHROTOXIN OCHRATOXIN	DNA Adduct
ID	10	97696	96934	DNA-ADDUCTS	

Record 687 | WOS:000237912300008

Deoxynivalenol transport across human intestinal Caco-2 cells and its effects on cellular metabolism at realistic intestinal concentrations

Field	Position	Original line	Normalised line	Original value	Normalised value
DE	1	97806	97042	deoxynivalenol (DON)	Deoxynivalenol DON
DE	2	97806	97042	Caco-2 cells	Caco-2
DE	3	97806	97042	intestinal absorption	Intestinal Absorption
DE	4	97806	97042	MAPKs	Mapk
DE	5	97806	97042	inflammation	Inflammation
ID	1	97808	97043	RIBOTOXIC STRESS-RESPONSE	Ribotoxic Stress Response
ID	2	97808	97043	ACTIVATED PROTEIN-KINASES	Activated Protein Kinase
ID	3	97808	97043	SERUM-FREE	Serum Free
ID	4	97808	97043	TRICHOTHECENE MYCOTOXINS	Trichothecene Mycotoxin
ID	5	97808	97043	MULTIDRUG-RESISTANCE	Multidrug Resistance
ID	6	97808	97043	GROWTH-PERFORMANCE	Growth Performance
ID	7	97808	97043	EPITHELIAL-CELLS	Epithelial
ID	8	97808	97043	TIGHT JUNCTION	Tight Junction
ID	9	97808	97043	OCHRATOXIN-A	Ochratoxin A (OTA)
ID	10	97808	97043	ABSORPTION	Absorption

Record 688 | WOS:000238344300009

Balkan endemic nephropathy: Role of ochratoxins A through biomarkers

Field	Position	Original line	Normalised line	Original value	Normalised value
DE	1	97918	97152	Balkan endemic nephropathy	Balkan Endemic Nephropathy
DE	2	97918	97152	blood	Blood
DE	3	97918	97152	food	Food
DE	4	97918	97152	mycotoxins	Mycotoxins
DE	5	97918	97152	ochratoxin A	Ochratoxin A (OTA)
ID	1	97919	97153	URINARY SYSTEM TUMORS	Urinary System Tumor
ID	2	97919	97153	TRACT TUMORS	Tract Tumor
ID	3	97919	97153	IN-VIVO	In-Vivo
ID	4	97919	97153	PLASMA	Plasma
ID	5	97919	97153	BINDING	Binding
ID	6	97919	97153	TRANSPORT	Transport
ID	7	97919	97153	BULGARIA	Bulgaria
ID	8	97919	97153	CEREALS	Cereal
ID	9	97919	97153	AREA	Area

ID	10	97919	97153	CELL	Cell

Record 689 | WOS:000238344300010

Evidence of a new dechlorinated ochratoxin A derivative formed in opossum kidney cell cultures after pretreatment by modulators of glutathione pathways: Correlation with DNA-adduct formation

Field	Position	Original line	Normalised line	Original value	Normalised value
DE	1	98022	97255	biotransformation	Biotransformation
DE	2	98022	97255	DNA-adduct	DNA Adduct
DE	3	98022	97255	mass spectrometry	Mass Spectrometry
DE	4	98022	97255	ochratoxin A	Ochratoxin A (OTA)
DE	5	98022	97255	opossum kidney cells	Opossum Kidney
ID	1	98024	97257	C8-DEOXYGUANOSINE NUCLEOSIDE ADDUCT	C8 Deoxyguanosine Nucleoside Adduct
ID	2	98024	97257	COVALENT PROTEIN ADDUCTS	Covalent Protein Adduct
ID	3	98024	97257	LIPID-PEROXIDATION	Lipid Peroxidation
ID	4	98024	97257	C8 REACTIVITY	C8 Reactivity
ID	5	98024	97257	RATS	Rat
ID	6	98024	97257	IDENTIFICATION	Identification
ID	7	98024	97257	GENOTOXICITY	Genotoxicity
ID	8	98024	97257	METABOLITES	Metabolite
ID	9	98024	97257	TOXICITY	Toxicity
ID	10	98024	97257	(4S)-4-HYDROXYOCHRATOXIN-A	4s 4 Hydroxyochratoxin A

Record 690 | WOS:000238344300011

Microbiologicals for deactivating mycotoxins

Field	Position	Original line	Normalised line	Original value	Normalised value
DE	1	98165	97397	BBSH	Bbsh
DE	2	98165	97397	biotransformation	Biotransformation
DE	3	98165	97397	mycotoxins	Mycotoxins
DE	4	98165	97397	trichosporon mycotoxinivorans	Trichosporon Mycotoxinivoran
DE	5	98165	97397	trichothecenes	Trichothecene
ID	1	98167	97399	SODIUM CALCIUM ALUMINOSILICATE	Sodium Calcium Aluminosilicate
ID	2	98167	97399	TOXICITY	Toxicity
ID	3	98167	97399	DEOXYNIVALENOL	Deoxynivalenol (DON)
ID	4	98167	97399	OCHRATOXIN	Ochratoxin A (OTA)
ID	5	98167	97399	AFLATOXIN	Aflatoxin
ID	6	98167	97399	EFFICACY	Efficacy
ID	7	98167	97399	REDUCE	Reduce
ID	8	98167	97399	DEGRADATION	Degradation
ID	9	98167	97399	CORN	Corn

Record 691 | WOS:000236846900001

Ochratoxin A secretion by ATP-dependent membrane transporters in Caco-2 cells

Field	Position	Original line	Normalised line	Original value	Normalised value
DE	1	98250	97481	ochratoxin A	Ochratoxin A (OTA)
DE	2	98250	97481	transport	Transport
DE	4	98250	97481	MRP2	Mrp2
DE	5	98250	97481	BCRP	Bcrp
ID	1	98251	97482	CANCER RESISTANCE PROTEIN	Cancer Resistance Protein
ID	2	98251	97482	NORMAL HUMAN TISSUES	Normal Human Tissue
ID	3	98251	97482	CANINE KIDNEY-CELLS	Canine Kidney
ID	4	98251	97482	NEPHRON IN-VIVO	Nephron In Vivo

ID	5	98251	97482	MULTIDRUG-RESISTANCE	Multidrug Resistance
ID	6	98251	97482	P-GLYCOPROTEIN	P Glycoprotein
ID	7	98251	97482	(ABCG2)-MEDIATED TRANSPORT	Abcg2 Mediated Transport
ID	8	98251	97482	MRP2 GENE	Mrp2 Gene
ID	9	98251	97482	EXPRESSION	Expression
ID	10	98251	97482	GF120918	Gf120918

Record 692 | WOS:000236987100010

In vitro investigation of individual and combined cytotoxic effects of ochratoxin A and other selected mycotoxins on renal cells

Field	Position	Original line	Normalised line	Original value	Normalised value
DE	1	98348	97578	mycotoxins	Mycotoxins
DE	2	98348	97578	renal cells	Renal
DE	3	98348	97578	combination toxicity	Combination Toxicity
ID	1	98349	97579	BALKAN ENDEMIC NEPHROPATHY	Balkan Endemic Nephropathy
ID	2	98349	97579	STATISTICALLY DESIGNED EXPERIMENTS	Statistically Designed Experiment
ID	3	98349	97579	ACUTE INTRAPERITONEAL TOXICITY	Acute Intraperitoneal Toxicity
ID	4	98349	97579	TROUT SALMO-GAIRDNERI	Trout Salmo Gairdneri
ID	5	98349	97579	CHEMICAL-MIXTURES	Chemical Mixture
ID	6	98349	97579	PATULIN	Patulin
ID	7	98349	97579	NEPHROTOXICITY	Nephrotoxicity
ID	8	98349	97579	ETIOLOGY	Etiology
ID	9	98349	97579	CITRININ	Citrinin
ID	10	98349	97579	LLC-PK1	Llc Pk1

Record 693 | WOS:000236605600004

Ochratoxin A: Its cancer risk and potential for exposure

Field	Position	Original line	Normalised line	Original value	Normalised value
ID	1	98432	97661	URINARY-TRACT TUMORS	Urinary Tract Tumor
ID	2	98432	97661	LIPID-PEROXIDATION	Lipid Peroxidation
ID	3	98432	97661	HUMAN-BLOOD	Human Blood
ID	4	98432	97661	IN-VITRO	In-Vitro
ID	5	98432	97661	DIETARY CHOLESTYRAMINE	Dietary Cholestyramine
ID	6	98432	97661	PENICILLIUM-VERRUCOSUM	Penicillium Verrucosum
ID	7	98432	97661	ASPERGILLUS-ALUTACEUS	Aspergillus Alutaceus
ID	8	98432	97661	ENDEMIC NEPHROPATHY	Endemic Nephropathy
ID	9	98432	97661	NATURAL TOXINS	Natural Toxin
ID	10	98432	97661	WATER ACTIVITY	Water Activity

Record 694 | WOS:000234793300002

Toxicokinetics and toxicodynamics of ochratoxin A, an update

Field	Position	Original line	Normalised line	Original value	Normalised value
DE	1	98674	97902	ochratoxin A	Ochratoxin A (OTA)
DE	2	98674	97902	toxicokinetics	Toxicokinetics
DE	3	98674	97902	toxicodynamics	Toxicodynamics
DE	4	98674	97902	combined effects of mycotoxins	Combined Effect Of Mycotoxin

ID	1	98676	97904	ORGANIC ANION TRANSPORTER	Organic Anion Transporter
ID	2	98676	97904	TRANSFER-RNA SYNTHETASE	Transfer RNA Synthetase
ID	3	98676	97904	DNA ADDUCT FORMATION	DNA Adduct Formation
ID	4	98676	97904	MYCOTOXIN OCHRATOXIN	Ochratoxin A (OTA)
ID	5	98676	97904	IN-VIVO	In-Vivo
ID	6	98676	97904	ASPERGILLUS-OCHRACEUS	Aspergillus Ochraceus
ID	7	98676	97904	PROTEIN-SYNTHESIS	Protein Synthesis
ID	8	98676	97904	HUMAN KIDNEY	Human Kidney
ID	9	98676	97904	CELL-LINES	Cell
ID	10	98676	97904	HUMAN-MILK	Human Milk

Record 695 | WOS:000234140400042

The impact of plasma protein binding on the renal transport of organic anions

Field	Position	Original line	Normalised line	Original value	Normalised value
ID	1	98967	98194	ASPERGILLUS OCHRACEUS WILH	Aspergillus Ochraceus Wilh
ID	2	98967	98194	HUMAN SERUM-ALBUMIN	Human Serum Albumin
ID	3	98967	98194	PERFUSED-RAT-LIVER	Perfused Rat Liver
ID	4	98967	98194	OCHRATOXIN-A	Ochratoxin A (OTA)
ID	5	98967	98194	PERITUBULAR TRANSPORT	Peritubular Transport
ID	6	98967	98194	MOLECULAR-CLONING	Molecular Cloning
ID	7	98967	98194	PROXIMAL TUBULES	Proximal Tubule
ID	8	98967	98194	ESTRONE SULFATE	Estrone Sulfate
ID	9	98967	98194	PHENOL RED	Phenol Red
ID	10	98967	98194	KIDNEY	Kidney

Record 696 | WOS:000235953600006

Mycoflora, cytotoxicity, and DNA interaction of polyherbal products from Malaysia

Field	Position	Original line	Normalised line	Original value	Normalised value
DE	1	99064	98290	aflatoxins	Aflatoxin
DE	2	99064	98290	cytotoxicity	Cytotoxicity
DE	3	99064	98290	DNA interaction	DNA Interaction
DE	4	99064	98290	herbal products	Herbal Product
DE	5	99064	98290	mycoflora	Mycoflora
DE	6	99064	98290	ochratoxin A	Ochratoxin A (OTA)
DE	7	99064	98290	polyherbal	Polyherbal
ID	1	99066	98292	MEDICINAL-PLANT	Medicinal Plant
ID	2	99066	98292	IN-VITRO	In-Vitro
ID	3	99066	98292	HERBAL REMEDIES	Herbal Remedy
ID	4	99066	98292	HUMAN LUNG	Human Lung
ID	5	99066	98292	MYCOTOXINS	Mycotoxins
ID	6	99066	98292	CONTAMINATION	Contamination
ID	7	99066	98292	EXTRACTS	Extract
ID	8	99066	98292	ANTITUMOR	Antitumor
ID	9	99066	98292	TOXICITY	Toxicity
ID	10	99066	98292	CRITERIA	Criteria

Record 697 | WOS:000233991000012

A toxicogenomics approach to identify new plausible epigenetic mechanisms of ochratoxin a carcinogenicity in rat

Field	Position	Original line	Normalised line	Original value	Normalised value
DE	1	99174	98399	ochratoxin A	Ochratoxin A (OTA)
DE	2	99174	98399	nephrocarcinogenicity	Nephrocarcinogenicity

ID	1	99175	98400	PROTEIN-KINASE-C	Protein Kinase C
ID	2	99175	98400	GLUTATHIONE-S-TRANSFERASE	Glutathione S Transferase
ID	3	99175	98400	GENE-EXPRESSION PROFILE	Gene Expression Profile
ID	4	99175	98400	COVALENT DNA ADDUCTION	Covalent DNA Adduction
ID	5	99175	98400	PROXIMAL TUBULAR CELLS	Proximal Tubular
ID	6	99175	98400	OXIDATIVE STRESS	Oxidative Stress
ID	7	99175	98400	KIDNEY CORTEX	Kidney Cortex
ID	8	99175	98400	IN-VIVO	In-Vivo
ID	9	99175	98400	CULTURED RAT	Cultured Rat
ID	10	99175	98400	RENAL-CELLS	Renal

Record 698 | WOS:000234194400007

An in vitro approach to detect metabolite toxicity due to CYP3A4-dependent bioactivation of xenobiotics

Field	Position	Original line	Normalised line	Original value	Normalised value
DE	1	99315	98539	metabolism-mediated toxicity	Metabolism Mediated Toxicity
DE	2	99315	98539	adverse drug reactions (ADRs)	Adverse Drug Reaction Adr
DE	3	99315	98539	reactive metabolites	Reactive Metabolite
DE	4	99315	98539	in vitro screening	In Vitro Screening
DE	5	99315	98539	CYP3A4	Cyp3a4
DE	6	99315	98539	cytotoxicity	Cytotoxicity
ID	1	99317	98541	IDIOSYNCRATIC DRUG-REACTIONS	Idiosyncratic Drug Reaction
ID	2	99317	98541	HUMAN HEPATOCYTE CULTURES	Human Hepatocyte
ID	3	99317	98541	HUMAN-LIVER-MICROSOMES	Human Liver Microsome
ID	4	99317	98541	CHINESE-HAMSTER CELLS	Chinese Hamster
ID	5	99317	98541	HEPG2 CELLS	HepG2
ID	6	99317	98541	MEDIATED CYTOTOXICITY	Mediated Cytotoxicity
ID	7	99317	98541	STABLE EXPRESSION	Stable Expression
ID	8	99317	98541	CYTOCHROME-P450	Cytochrome P450
ID	9	99317	98541	INDUCTION	Induction
ID	10	99317	98541	CARBAMAZEPINE	Carbamazepine

Record 699 | WOS:000234094800009

Ochratoxin A induces oxidative DNA damage in liver and kidney after oral dosing to rats

Field	Position	Original line	Normalised line	Original value	Normalised value
DE	1	99426	98648	comet-assay	Comet Assay
DE	2	99426	98648	in vivo	In-Vivo
DE	3	99426	98648	ochratoxin A	Ochratoxin A (OTA)
DE	4	99426	98648	oxidative DNA damage	Oxidative DNA Damage
DE	5	99426	98648	protein oxidation	Protein Oxidation
DE	6	99426	98648	rat	Rat
ID	1	99428	98650	CHRONIC INTERSTITIAL NEPHROPATHY	Chronic Interstitial Nephropathy
ID	2	99428	98650	LIPID-PEROXIDATION	Lipid Peroxidation
ID	3	99428	98650	IN-VITRO	In-Vitro
ID	4	99428	98650	NEPHROTOXICITY	Nephrotoxicity
ID	5	99428	98650	INDUCTION	Induction
ID	6	99428	98650	APOPTOSIS	Apoptosis
ID	7	99428	98650	EXPOSURE	Exposure
ID	8	99428	98650	STRESS	Stress
ID	9	99428	98650	CELLS	Cell
ID	10	99428	98650	MICE	Mice

Record 700 | WOS:000233125300003

Teratogenic effects in rabbits of simultaneous exposure to ochratoxin A and aflatoxin B1 with special reference to microscopic effects

Field	Position	Original line	Normalised line	Original value	Normalised value
DE	1	99549	98770	aflatoxin B-1	Aflatoxin B1 (AFB1)
DE	2	99549	98770	combination	Combination
DE	3	99549	98770	ochratoxin A	Ochratoxin A (OTA)
DE	4	99549	98770	pathomorphology	Pathomorphology
DE	5	99549	98770	rabbit	Rabbit
DE	6	99549	98770	teratogenicity	Teratogenicity
ID	1	99551	98772	SIMULTANEOUS PRENATAL EXPOSURE	Simultaneous Prenatal Exposure
ID	2	99551	98772	DEVELOPMENTAL TOXICITY	Developmental Toxicity
ID	3	99551	98772	FETAL	Fetal
ID	4	99551	98772	RATS	Rat
ID	5	99551	98772	B-1	B 1
ID	6	99551	98772	AFLATOXICOSIS	Aflatoxicosis
ID	7	99551	98772	PREVENTION	Prevention
ID	8	99551	98772	HAMSTERS	Hamster
ID	9	99551	98772	CITRININ	Citrinin
ID	10	99551	98772	EMBRYO	Embryo

Record 701 | WOS:000232435300026

The effects of mycotoxins, fungal food contaminants, on the intestinal epithelial cell-derived innate immune response

Field	Position	Original line	Normalised line	Original value	Normalised value
DE	1	99677	98897	intestine	Intestine
DE	2	99677	98897	epithelial cells	Epithelial
DE	3	99677	98897	immune system	Immune System
DE	4	99677	98897	food contaminant	Food Contaminant
DE	5	99677	98897	mycotoxin	Mycotoxins
DE	6	99677	98897	barrier function	Barrier Function
ID	1	99679	98899	OCHRATOXIN-A	Ochratoxin A (OTA)
ID	2	99679	98899	FUMONISIN B-1	Fumonisin B1 (FB1)
ID	3	99679	98899	MESSENGER-RNA	Messenger RNA
ID	4	99679	98899	INFLAMMATORY CYTOKINES	Inflammatory Cytokine
ID	5	99679	98899	BARRIER FUNCTION	Barrier Function
ID	6	99679	98899	T-2 TOXIN	T 2 Toxin
ID	7	99679	98899	IN-VITRO	In-Vitro
ID	8	99679	98899	EXPRESSION	Expression
ID	9	99679	98899	DEOXYNIVALENOL	Deoxynivalenol (DON)
ID	10	99679	98899	PROTEIN	Protein

Record 702 | WOS:000233069800005

Investigation of the teratogenic potential of ochratoxin A and B using the FETAX system

Field	Position	Original line	Normalised line	Original value	Normalised value
DE	1	99804	99023	ochratoxins	Ochratoxin A (OTA)
DE	2	99804	99023	FETAX	Fetax
DE	3	99804	99023	teratogenesis	Teratogenesis
DE	4	99804	99023	mycotoxins	Mycotoxins
ID	1	99805	99024	XENOPUS-TROPICALIS	Xenopus Tropicalis
ID	2	99805	99024	TRANSPORT	Transport
ID	3	99805	99024	MOUSE	Mouse
ID	4	99805	99024	PATHOGENESIS	Pathogenesis
ID	5	99805	99024	VALIDATION	Validation
ID	6	99805	99024	LAEVIS	Laevis
ID	7	99805	99024	PIGS	Pig

Record 703 | WOS:000232409600014

Fate of ochratoxin A during cooking of naturally contaminated polished rice

Field	Position	Original line	Normalised line	Original value	Normalised value
ID	1	99911	99128	AFLATOXIN B-1	Aflatoxin B1 (AFB1)
ID	2	99911	99128	FUMONISIN B-1	Fumonisin B1 (FB1)
ID	3	99911	99128	IN-VIVO	In-Vivo
ID	4	99911	99128	BARLEY	Barley
ID	5	99911	99128	WHEAT	Wheat
ID	6	99911	99128	MYCOTOXINS	Mycotoxins
ID	7	99911	99128	EXPRESSION	Expression
ID	8	99911	99128	DETOXIFICATION	Detoxification
ID	9	99911	99128	TOXICITY	Toxicity
ID	10	99911	99128	GROWTH	Growth

Record 704 | WOS:000232524600005

Alterations induced in vitro by ochratoxin A in rat lymphoid cells

Field	Position	Original line	Normalised line	Original value	Normalised value
DE	1	99989	99205	apoptosis	Apoptosis
DE	2	99989	99205	immunotoxicity	Immunotoxicity
DE	3	99989	99205	in vitro	In-Vitro
DE	4	99989	99205	ochratoxin A	Ochratoxin A (OTA)
DE	5	99989	99205	phenylalanine	Phenylalanine
DE	6	99989	99205	piroxicam	Piroxicam
ID	1	99991	99207	LIPID-PEROXIDATION	Lipid Peroxidation
ID	2	99991	99207	IMMUNE	Immune
ID	3	99991	99207	INHIBITION	Inhibition
ID	4	99991	99207	APOPTOSIS	Apoptosis
ID	5	99991	99207	ASSAY	Assay
ID	6	99991	99207	MICE	Mice
ID	7	99991	99207	IMMUNOSUPPRESSION	Immunosuppression
ID	8	99991	99207	NEPHROTOXICITY	Nephrotoxicity
ID	9	99991	99207	PROLIFERATION	Proliferation
ID	10	99991	99207	GENOTOXICITY	Genotoxicity

Record 705 | WOS:000231364400010

Cyanidin-3-O-Î²-glucopyranoside, a natural free-radical scavenger against aflatoxin B1-and ochratoxin A-induced cell damage in a human hepatoma cell line (Hep G2) and a human colonic adenocarcinoma cell line (CaCo-2)

Field	Position	Original line	Normalised line	Original value	Normalised value
DE	1	100095	99310	mycotoxins	Mycotoxins
DE	2	100095	99310	cyanidin-3-O-beta-glucopyranoside	Cyanidin 3 O Beta Glucopyranoside
DE	3	100095	99310	Hep G2 cells	Hep G2
DE	4	100095	99310	CaCo-2 cells	Caco-2
ID	1	100097	99311	BRIGGS-RAUSCHER REACTION	Brigg Rauscher Reaction
ID	2	100097	99311	LIPID-PEROXIDATION	Lipid Peroxidation
ID	3	100097	99311	ANTIOXIDANT ACTIVITY	Antioxidant Activity
ID	4	100097	99311	OXIDATIVE DAMAGE	Oxidative Damage
ID	5	100097	99311	RATS	Rat
ID	6	100097	99311	ANTHOCYANINS	Anthocyanin
ID	7	100097	99311	TOXICITY	Toxicity
ID	8	100097	99311	B-1	B 1
ID	9	100097	99311	INHIBITION	Inhibition
ID	10	100097	99311	METABOLISM	Metabolism

Record 706 | WOS:000231235000007

Functional, biochemical, and pathological effects of repeated oral administration of ochratoxin A to rats

Field	Position	Original line	Normalised line	Original value	Normalised value
ID	1	100205	99417	SPRAGUE-DAWLEY RATS	Sprague Dawley Rat
ID	2	100205	99417	CELL-PROLIFERATION	Cell Proliferation
ID	3	100205	99417	LIPID-PEROXIDATION	Lipid Peroxidation
ID	4	100205	99417	RENAL CARCINOGEN	Renal Carcinogen
ID	5	100205	99417	IN-VITRO	In-Vitro
ID	6	100205	99417	FERRIC NITRILOTRIACETATE	Ferric Nitrilotriacetate
ID	7	100205	99417	POTASSIUM BROMATE	Potassium Bromate
ID	8	100205	99417	DNA-DAMAGE	DNA Damage
ID	9	100205	99417	KIDNEY	Kidney
ID	10	100205	99417	NEPHROTOXICITY	Nephrotoxicity

Record 707 | WOS:000231235000008

Ochratoxin A causes DNA damage and cytogenetic effects but no DNA adducts in rats

Field	Position	Original line	Normalised line	Original value	Normalised value
ID	1	100338	99549	MYCOTOXIN OCHRATOXIN	Ochratoxin A (OTA)
ID	2	100338	99549	ESCHERICHIA-COLI	Escherichia Coli
ID	3	100338	99549	FPG PROTEIN	Fpg Protein
ID	4	100338	99549	CELL-LINES	Cell
ID	5	100338	99549	SALMONELLA-TYPHIMURIUM	Salmonella Typhimurium
ID	6	100338	99549	SUBSTRATE-SPECIFICITY	Substrate Specificity
ID	7	100338	99549	ARISTOLOCHIC-ACID	Aristolochic Acid
ID	8	100338	99549	MUTAGENICITY TEST	Mutagenicity Test
ID	9	100338	99549	EPITHELIAL-CELLS	Epithelial
ID	10	100338	99549	URINARY-BLADDER	Urinary Bladder

Record 708 | WOS:000230120200005

Biotransformation and nephrotoxicity of ochratoxin B in rats

Field	Position	Original line	Normalised line	Original value	Normalised value
DE	1	100459	99669	ochratoxins	Ochratoxin A (OTA)
DE	2	100459	99669	nephrotoxicity	Nephrotoxicity
DE	3	100459	99669	cytotoxicity	Cytotoxicity
DE	4	100459	99669	biotransformation	Biotransformation
ID	1	100460	99670	IN-VITRO	In-Vitro
ID	2	100460	99670	MUTAGENICITY	Mutagenicity
ID	3	100460	99670	METABOLISM	Metabolism
ID	4	100460	99670	TOXICOKINETICS	Toxicokinetics
ID	5	100460	99670	CYTOTOXICITY	Cytotoxicity
ID	6	100460	99670	REACTIVITY	Reactivity
ID	7	100460	99670	BACTERIAL	Bacterial
ID	8	100460	99670	ANALOGS	Analog
ID	9	100460	99670	SYSTEM	System

Record 709 | WOS:000228398000034

Cisplatin-induced apoptosis is enhanced by hypoxia and by inhibition of mitochondria in renal collecting duct cells

Field	Position	Original line	Normalised line	Original value	Normalised value
DE	1	100549	99758	cisplatin	Cisplatin
DE	2	100549	99758	hypoxia	Hypoxia
DE	3	100549	99758	collecting duct	Collecting Duct
DE	4	100549	99758	apoptosis	Apoptosis
DE	5	100549	99758	mitochondria	Mitochondria
ID	1	100550	99759	NEPHRON IN-VIVO	Nephron In Vivo
ID	2	100550	99759	EXTRACELLULAR ACIDIFICATION	Extracellular Acidification

ID	3	100550	99759	PREFERENTIAL BINDING	Preferential Binding
ID	4	100550	99759	PROXIMAL TUBULE	Proximal Tubule
ID	5	100550	99759	OCHRATOXIN-A	Ochratoxin A (OTA)
ID	6	100550	99759	CYTOCHROME-C	Cytochrome C
ID	7	100550	99759	DEATH	Death
ID	9	100550	99759	PH	Ph
ID	10	100550	99759	PERMEABILITY	Permeability

Record 710 | WOS:000229004100015

Comparison of in vitro cytotoxicity of Fusarium mycotoxins, deoxynivalenol, T-2 toxin and zearalenone on selected human epithelial cell lines

Field	Position	Original line	Normalised line	Original value	Normalised value
DE	1	100641	99849	cytotoxicity	Cytotoxicity
DE	2	100641	99849	deoxynivalenol	Deoxynivalenol (DON)
DE	4	100641	99849	human cell lines	Human Cell
DE	5	100641	99849	T-2 toxin	T 2 Toxin
DE	6	100641	99849	zearalenone	Zearalenone (ZEA)
ID	1	100643	99851	OCHRATOXIN-A	Ochratoxin A (OTA)
ID	2	100643	99851	APOPTOSIS	Apoptosis
ID	3	100643	99851	TRICHOTHECENES	Trichothecene
ID	4	100643	99851	TOXICITY	Toxicity
ID	5	100643	99851	PROLIFERATION	Proliferation
ID	6	100643	99851	METABOLITES	Metabolite
ID	7	100643	99851	DERIVATIVES	Derivative
ID	8	100643	99851	ABSORPTION	Absorption
ID	9	100643	99851	INDUCTION	Induction
ID	10	100643	99851	NIVALENOL	Nivalenol

Record 711 | WOS:000227733000027

Proximal tubular toxicity of ochratoxin A is amplified by simultaneous inhibition of the extracellular signal-regulated kinases 1/2

Field	Position	Original line	Normalised line	Original value	Normalised value
ID	1	100733	99940	KIDNEY EPITHELIAL-CELLS	Kidney Epithelial
ID	2	100733	99940	NEPHROTOXIN OCHRATOXIN	Ochratoxin A (OTA)
ID	3	100733	99940	MESENCHYMAL TRANSITION	Mesenchymal Transition
ID	4	100733	99940	PROTEIN-KINASES	Protein Kinase
ID	5	100733	99940	MAP KINASE	Map Kinase
ID	6	100733	99940	TGF-BETA	Tgf Beta
ID	7	100733	99940	ACTIVATION	Activation
ID	8	100733	99940	NEPHROPATHY	Nephropathy
ID	9	100733	99940	APOPTOSIS	Apoptosis
ID	10	100733	99940	JNK	Jnk

Record 712 | WOS:000227112000002

Interference of arachidonic acid and its metabolites with TNF-Î± release by ochratoxin A from rat liver

Field	Position	Original line	Normalised line	Original value	Normalised value
DE	1	100824	100029	ochratoxin A	Ochratoxin A (OTA)
DE	2	100824	100029	TNF-alpha	Tnf Alpha
DE	3	100824	100029	arachidonic acid	Arachidonic Acid
DE	4	100824	100029	liver	Liver
DE	5	100824	100029	cytokines	Cytokine
ID	1	100825	100030	TUMOR-NECROSIS-FACTOR	Tumor Necrosis Factor
ID	2	100825	100030	FACTOR-KAPPA-B	Factor Kappa B
ID	3	100825	100030	GENE-EXPRESSION	Gene Expression
ID	4	100825	100030	INDUCED APOPTOSIS	Induced Apoptosis
ID	5	100825	100030	INHIBITOR	Inhibitor
ID	6	100825	100030	MICE	Mice

ID	7	100825	100030	ACTIVATION	Activation
ID	8	100825	100030	RECEPTOR	Receptor
ID	9	100825	100030	CELLS	Cell
ID	10	100825	100030	SUPPRESSION	Suppression

Record 713 | WOS:000227242200003

Ochratoxin A at nanomolar concentrations: A signal modulator in renal cells

Field	Position	Original line	Normalised line	Original value	Normalised value
DE	1	100948	100152	cellular signalling networks	Cellular Signalling Network
DE	2	100948	100152	ochratoxin A	Ochratoxin A (OTA)
DE	3	100948	100152	renal cells	Renal
DE	4	100948	100152	review	Review
ID	1	100949	100153	BALKAN ENDEMIC NEPHROPATHY	Balkan Endemic Nephropathy
ID	2	100949	100153	CANINE KIDNEY-CELLS	Canine Kidney
ID	3	100949	100153	ASPERGILLUS OCHRACEUS WILH	Aspergillus Ochraceus Wilh
ID	4	100949	100153	GLOMERULAR-FILTRATION-RATE	Glomerular Filtration Rate
ID	5	100949	100153	PROXIMAL TUBULAR CELLS	Proximal Tubular
ID	6	100949	100153	IN-VIVO	In-Vivo
ID	7	100949	100153	RAT-KIDNEY	Rat Kidney
ID	8	100949	100153	EPITHELIAL-CELLS	Epithelial
ID	9	100949	100153	PH HOMEOSTASIS	Ph Homeostasis
ID	10	100949	100153	HUMAN BLOOD	Human Blood

Record 714 | WOS:000234123000008

Ochratoxin A: Comparative pharmacokinetics and toxicological implications (experimental and domestic animals and humans)

Field	Position	Original line	Normalised line	Original value	Normalised value
DE	1	101113	100316	ochratoxin A	Ochratoxin A (OTA)
DE	2	101113	100316	toxicokinetics	Toxicokinetics
DE	3	101113	100316	modeling	Modeling
DE	4	101113	100316	risk assessment	Risk Assessment
ID	1	101114	100317	ENTEROHEPATIC CIRCULATION	Enterohepatic Circulation
ID	2	101114	100317	TRANSPORT	Transport
ID	3	101114	100317	TOXICOKINETICS	Toxicokinetics
ID	4	101114	100317	BINDING	Binding
ID	5	101114	100317	RAT	Rat
ID	6	101114	100317	METABOLISM	Metabolism
ID	7	101114	100317	SECRETION	Secretion
ID	8	101114	100317	TOXICITY	Toxicity
ID	9	101114	100317	ALBUMIN	Albumin
ID	10	101114	100317	PROTEIN	Protein

Record 715 | WOS:000234123000011

DNA adduct formation by ochratoxin A: Review of the available evidence

Field	Position	Original line	Normalised line	Original value	Normalised value
DE	1	101210	100412	ochratoxin A	Ochratoxin A (OTA)
DE	2	101210	100412	carcinogenicity	Carcinogenicity
DE	3	101210	100412	DNA-adducts	DNA Adduct
ID	1	101211	100413	BLADDER EPITHELIAL-CELLS	Bladder Epithelial
ID	2	101211	100413	SINGLE-STRAND BREAKS	Single Strand Break
ID	3	101211	100413	SPRAGUE-DAWLEY RATS	Sprague Dawley Rat

ID	4	101211	100413	URINARY-BLADDER	Urinary Bladder
ID	5	101211	100413	SALMONELLA-TYPHIMURIUM	Salmonella Typhimurium
ID	6	101211	100413	MUTAGENICITY TEST	Mutagenicity Test
ID	7	101211	100413	IN-VITRO	In-Vitro
ID	8	101211	100413	MYCOTOXINS	Mycotoxins
ID	9	101211	100413	GENOTOXICITY	Genotoxicity
ID	10	101211	100413	METABOLITES	Metabolite

Record 716 | WOS:000226837900002

Ochratoxin A: The continuing enigma

Field	Position	Original line	Normalised line	Original value	Normalised value
DE	1	101318	100519	kidney	Kidney
DE	2	101318	100519	mechanism	Mechanism
DE	3	101318	100519	nephropathy	Nephropathy
DE	4	101318	100519	ochratoxin A	Ochratoxin A (OTA)
ID	1	101319	100520	ORGANIC ANION TRANSPORTER	Organic Anion Transporter
ID	2	101319	100520	DNA ADDUCT FORMATION	DNA Adduct Formation
ID	3	101319	100520	MYCOTOXIN OCHRATOXIN	Ochratoxin A (OTA)
ID	4	101319	100520	IN-VITRO	In-Vitro
ID	5	101319	100520	PORCINE NEPHROPATHY	Porcine Nephropathy
ID	6	101319	100520	MOLECULAR-CLONING	Molecular Cloning
ID	7	101319	100520	OXIDATIVE STRESS	Oxidative Stress
ID	8	101319	100520	IMMUNE-RESPONSE	Immune Response
ID	9	101319	100520	CULTURED RAT	Cultured Rat
ID	10	101319	100520	ENTEROHEPATIC CIRCULATION	Enterohepatic Circulation

Record 717 | WOS:000234123000012

Further arguments in favour of direct covalent binding of Ochratoxin A (OTA) after metabolic biotransformation

Field	Position	Original line	Normalised line	Original value	Normalised value
DE	1	101592	100792	ochratoxin A	Ochratoxin A (OTA)
DE	2	101592	100792	genotoxicity	Genotoxicity
DE	3	101592	100792	DNA adduct	DNA Adduct
DE	4	101592	100792	OTA metabolites	OTA Metabolite
ID	1	101593	100793	BALKAN ENDEMIC NEPHROPATHY	Balkan Endemic Nephropathy
ID	2	101593	100793	DNA ADDUCT FORMATION	DNA Adduct Formation
ID	3	101593	100793	HUMAN CYTOCHROME-P450	Human Cytochrome P450
ID	4	101593	100793	ARISTOLOCHIC ACID	Aristolochic Acid
ID	5	101593	100793	LIVER-MICROSOMES	Liver Microsome
ID	6	101593	100793	IN-VITRO	In-Vitro
ID	7	101593	100793	RAT	Rat
ID	8	101593	100793	SEX	Sex
ID	9	101593	100793	GENOTOXICITY	Genotoxicity
ID	10	101593	100793	FORMS	Form

Record 718 | WOS:000232477800006

Effect of mycotoxins on some activities of isolated human neutrophils

Field	Position	Original line	Normalised line	Original value	Normalised value
DE	1	101708	100906	mycotoxins	Mycotoxins
DE	2	101708	100906	neutrophils	Neutrophil
DE	3	101708	100906	hypochlorous acid	Hypochlorous Acid

DE	4	101708	100906	nitric oxide	Nitric Oxide
DE	5	101708	100906	chemotaxis	Chemotaxis
ID	1	101709	100907	NITRIC-OXIDE SYNTHASE	Nitric Oxide Synthase
ID	2	101709	100907	OCHRATOXIN-A	Ochratoxin A (OTA)
ID	3	101709	100907	MACROPHAGE MODEL	Macrophage Model
ID	4	101709	100907	FUMONISIN B-1	Fumonisin B1 (FB1)
ID	5	101709	100907	WHOLE-BLOOD	Whole Blood
ID	6	101709	100907	CELL-LINE	Cell
ID	7	101709	100907	IN-VITRO	In-Vitro
ID	8	101709	100907	MICE	Mice
ID	9	101709	100907	IMMUNOSUPPRESSION	Immunosuppression
ID	10	101709	100907	DEOXYNIVALENOL	Deoxynivalenol (DON)

Record 719 | WOS:000226875200005

Ochratoxin A-induced DNA damage in human fibroblast:: protective effect of cyanidin 3-O-Î²-D-glucoside

Field	Position	Original line	Normalised line	Original value	Normalised value
DE	1	101804	101001	ochratoxin A	Ochratoxin A (OTA)
DE	2	101804	101001	reactive oxygen species	Reactive Oxygen Specy
DE	3	101804	101001	DNA damage	DNA Damage
DE	4	101804	101001	cyanidin-3-O-beta-D-glucoside	Cyanidin 3 O Beta D Glucoside
ID	1	101806	101003	LIPID-PEROXIDATION	Lipid Peroxidation
ID	2	101806	101003	OXIDATIVE DAMAGE	Oxidative Damage
ID	3	101806	101003	RISK-ASSESSMENT	Risk Assessment
ID	4	101806	101003	IN-VITRO	In-Vitro
ID	5	101806	101003	ADDUCTS	Adduct
ID	6	101806	101003	CELLS	Cell
ID	7	101806	101003	MYCOTOXINS	Mycotoxins
ID	8	101806	101003	RAT	Rat
ID	9	101806	101003	GENOTOXICITY	Genotoxicity
ID	10	101806	101003	HEPATOCYTES	Hepatocyte

Record 720 | WOS:000226469400004

Exposure to nephrotoxic Ochratoxin A enhances collagen secretion in human renal proximal tubular cells

Field	Position	Original line	Normalised line	Original value	Normalised value
DE	1	101914	101110	collagen secretion	Collagen Secretion
DE	2	101914	101110	fibrosis	Fibrosis
DE	3	101914	101110	Ochratoxin A	Ochratoxin A (OTA)
DE	4	101914	101110	renal disease	Renal Disease
DE	5	101914	101110	renal proximal tubule	Renal Proximal Tubule
ID	1	101916	101112	BALKAN ENDEMIC NEPHROPATHY	Balkan Endemic Nephropathy
ID	2	101916	101112	IN-VITRO	In-Vitro
ID	3	101916	101112	DISEASE	Disease
ID	4	101916	101112	KIDNEY	Kidney
ID	5	101916	101112	PROGRESSION	Progression
ID	6	101916	101112	MECHANISMS	Mechanism
ID	7	101916	101112	FIBROSIS	Fibrosis
ID	8	101916	101112	SAMPLES	Sample
ID	9	101916	101112	RATS	Rat
ID	10	101916	101112	VIVO	Vivo

Record 721 | WOS:000226446300003

Applicability of an in vitro digestion model in assessing the bioaccessibility of mycotoxins from food

Field	Position	Original line	Normalised line	Original value	Normalised value
DE	1	102011	101206	aflatoxin B-1	Aflatoxin B1 (AFB1)
DE	2	102011	101206	ochratoxin A	Ochratoxin A (OTA)
DE	3	102011	101206	bioavailability	Bioavailability

DE	4	102011	101206	bioaccessibility	Bioaccessibility
DE	5	102011	101206	in vitro digestion	In Vitro Digestion
DE	6	102011	101206	caco-2 cells	Caco-2
DE	7	102011	101206	chlorophyllin	Chlorophyllin
DE	8	102011	101206	HSCAS	Hsca
DE	9	102011	101206	cholestyramine	Cholestyramine
DE	10	102011	101206	activated charcoal	Activated Charcoal
DE	11	102011	101206	risk assessment	Risk Assessment
ID	1	102014	101209	OCHRATOXIN-A	Ochratoxin A (OTA)
ID	2	102014	101209	DIETARY CHOLESTYRAMINE	Dietary Cholestyramine
ID	3	102014	101209	DRUG ABSORPTION	Drug Absorption
ID	4	102014	101209	CACO-2 CELLS	Caco-2
ID	5	102014	101209	AFLATOXIN	Aflatoxin
ID	6	102014	101209	CALCIUM	Calcium
ID	7	102014	101209	IRON	Iron
ID	8	102014	101209	BIOAVAILABILITY	Bioavailability
ID	9	102014	101209	TRANSPORT	Transport
ID	10	102014	101209	SOIL	Soil

Record 722 | WOS:000234123000002

Ochratoxin A: Previous risk assessments and issues arising

Field	Position	Original line	Normalised line	Original value	Normalised value
DE	1	102101	101295	ochratoxin A	Ochratoxin A (OTA)
DE	2	102101	101295	toxicity	Toxicity
DE	3	102101	101295	risk assessment	Risk Assessment

Record 723 | WOS:000231491200006

Unusual astrocyte reactivity caused by the food mycotoxin ochratoxin A in aggregating rat brain cell cultures

Field	Position	Original line	Normalised line	Original value	Normalised value
DE	1	102163	101356	gliosis	Gliosis
DE	2	102163	101356	glial fibrillary acidic protein	Glial Fibrillary Acidic Protein
DE	3	102163	101356	brain inflammation	Brain Inflammation
ID	1	102164	101357	FIBRILLARY ACIDIC PROTEIN	Fibrillary Acidic Protein
ID	2	102164	101357	NITRIC-OXIDE SYNTHASE	Nitric Oxide Synthase
ID	3	102164	101357	PROLIFERATOR-ACTIVATED RECEPTORS	Proliferator Activated Receptor
ID	4	102164	101357	BROWN ADIPOSE-TISSUE	Brown Adipose Tissue
ID	5	102164	101357	GAMMA PPAR-GAMMA	Gamma Ppar Gamma
ID	6	102164	101357	HEME OXYGENASE-1	Heme Oxygenase 1
ID	7	102164	101357	CYCLOPENTENONE PROSTAGLANDINS	Cyclopentenone Prostaglandin
ID	8	102164	101357	PHENYLALANINE-HYDROXYLASE	Phenylalanine Hydroxylase
ID	9	102164	101357	NEPHRITOGENIC OCHRATOXIN	Ochratoxin A (OTA)
ID	10	102164	101357	IN-VITRO	In-Vitro

Record 724 | WOS:000225312300008

Effects of deoxynivalenol on general performance and electrophysiological properties of intestinal mucosa of broiler chickens

Field	Position	Original line	Normalised line	Original value	Normalised value
DE	1	102298	101490	chicken	Chicken
DE	2	102298	101490	deoxynivalenol	Deoxynivalenol (DON)
DE	3	102298	101490	microbial feed additive	Microbial Feed Additive

DE	4	102298	101490	glucose absorption	Glucose Absorption
DE	5	102298	101490	electrophysiological parameter	Electrophysiological Parameter
ID	1	102300	101492	WHITE LEGHORN	White Leghorn
ID	2	102300	101492	FUSARIUM MYCOTOXINS	Fusarium Mycotoxin
ID	3	102300	101492	CONTAMINATED WHEAT	Contaminated Wheat
ID	4	102300	101492	HEXOSE-TRANSPORT	Hexose Transport
ID	5	102300	101492	CULTURE MATERIAL	Culture Material
ID	6	102300	101492	FEEDING BLENDS	Feeding Blend
ID	7	102300	101492	OCHRATOXIN-A	Ochratoxin A (OTA)
ID	8	102300	101492	T-2 TOXIN	T 2 Toxin
ID	9	102300	101492	VOMITOXIN	Deoxynivalenol (DON)
ID	10	102300	101492	DIETS	Diet

Record 725 | WOS:000226727000007

Combined effects of selected Penicillium mycotoxins on in vitro proliferation of porcine lymphocytes

Field	Position	Original line	Normalised line	Original value	Normalised value
DE	1	102430	101621	citrinin	Citrinin
DE	2	102430	101621	cyclopiazonic acid	Cyclopiazonic Acid
DE	3	102430	101621	ochratoxin A	Ochratoxin A (OTA)
DE	4	102430	101621	patulin	Patulin
DE	5	102430	101621	penicillic acid	Penicillic Acid
DE	6	102430	101621	roquefortine C	Roquefortine C
DE	7	102430	101621	interaction	Interaction
ID	1	102432	101623	OCHRATOXIN	Ochratoxin A (OTA)

Record 726 | WOS:000226361400006

Molecular aspects of the transport and toxicity of ochratoxin A

Field	Position	Original line	Normalised line	Original value	Normalised value
ID	1	102506	101696	HUMAN SERUM-ALBUMIN	Human Serum Albumin
ID	2	102506	101696	C8-DEOXYGUANOSINE NUCLEOSIDE ADDUCT	C8 Deoxyguanosine Nucleoside Adduct
ID	3	102506	101696	PROXIMAL TUBULE	Proximal Tubule
ID	4	102506	101696	PROTEIN-BINDING	Protein Binding
ID	5	102506	101696	C8 REACTIVITY	C8 Reactivity
ID	6	102506	101696	DNA ADDUCTION	DNA Adduction
ID	7	102506	101696	IN-VIVO	In-Vivo
ID	8	102506	101696	RAT	Rat
ID	9	102506	101696	DAMAGE	Damage
ID	10	102506	101696	CELLS	Cell

Record 727 | WOS:000224725300008

Experimental mycotoxicosis in chickens induced by ochratoxin A and penicillic acid and intervention with natural plant extracts

Field	Position	Original line	Normalised line	Original value	Normalised value
DE	1	102620	101808	mycotoxic nephropathy	Mycotoxic Nephropathy
DE	2	102620	101808	mycotoxins	Mycotoxins
DE	3	102620	101808	ochratoxicosis	Ochratoxicosis
DE	4	102620	101808	ochratoxin A	Ochratoxin A (OTA)
DE	5	102620	101808	penicillic acid	Penicillic Acid
ID	1	102622	101810	BROILER CHICKS	Broiler Chick
ID	2	102622	101810	PORCINE NEPHROPATHY	Porcine Nephropathy
ID	3	102622	101810	IN-VITRO	In-Vitro
ID	4	102622	101810	TOXICITY	Toxicity
ID	5	102622	101810	RAT	Rat

ID	7	102622	101810	MICE	Mice
ID	8	102622	101810	PREVENTION	Prevention
ID	9	102622	101810	ASPARTAME	Aspartame
ID	10	102622	101810	BULGARIA	Bulgaria

Record 728 | WOS:000226417300009

The effects of the Penicillium mycotoxins citrinin, cyclopiazonic acid, ochratoxin A, patulin, penicillic acid, and roquefortine C on in vitro proliferation of porcine lymphocytes

Field	Position	Original line	Normalised line	Original value	Normalised value
DE	1	102733	101920	immunotoxicity	Immunotoxicity
DE	2	102733	101920	pigs	Pig
DE	3	102733	101920	food waste	Food Waste
ID	1	102734	101921	MITOGEN-INDUCED BLASTOGENESIS	Mitogen Induced Blastogenesis
ID	2	102734	101921	ABSORPTION	Absorption
ID	3	102734	101921	EXCRETION	Excretion
ID	4	102734	101921	TOXICITY	Toxicity
ID	5	102734	101921	EXPOSURE	Exposure
ID	6	102734	101921	PHARMACOKINETICS	Pharmacokinetics
ID	7	102734	101921	PIGS	Pig
ID	8	102734	101921	MICE	Mice

Record 729 | WOS:000223666000030

Fungal microflora and ochratoxin a risk in French vineyards

Field	Position	Original line	Normalised line	Original value	Normalised value
DE	1	102818	102004	Ochratoxin A	Ochratoxin A (OTA)
DE	2	102818	102004	Aspergillus carbonarius	Aspergillus Carbonarius
DE	3	102818	102004	French vineyards	French Vineyard
DE	4	102818	102004	fungal microflora	Fungal Microflora
DE	5	102818	102004	grapes	Grape
DE	6	102818	102004	musts	Must
ID	1	102820	102006	A PRODUCERS	A Producer
ID	2	102820	102006	WINE GRAPES	Wine Grape
ID	3	102820	102006	ASPERGILLUS	Aspergillus
ID	4	102820	102006	CLEANUP	Cleanup
ID	5	102820	102006	TABLE	Table

Record 730 | WOS:000223753400030

Alkalinization of urinary pH accelerates renal excretion of ochratoxin A in pigs

Field	Position	Original line	Normalised line	Original value	Normalised value
DE	1	102912	102097	bioavailability	Bioavailability
DE	2	102912	102097	excretion	Excretion
DE	3	102912	102097	mycotoxins	Mycotoxins
DE	4	102912	102097	ochratoxin A	Ochratoxin A (OTA)
DE	5	102912	102097	ochratoxin B	Ochratoxin B
ID	1	102913	102098	SODIUM-BICARBONATE	Sodium Bicarbonate
ID	2	102913	102098	IN-VIVO	In-Vivo
ID	3	102913	102098	REABSORPTION	Reabsorption
ID	4	102913	102098	NEPHROTOXIN	Nephrotoxin
ID	5	102913	102098	METABOLITES	Metabolite
ID	6	102913	102098	ABSORPTION	Absorption

Record 731 | WOS:000224015700013

Synergistic effects of fumonisin B1 and ochratoxin A: are in vitro cytotoxicity data predictive of in vivo acute toxicity?

Field	Position	Original line	Normalised line	Original value	Normalised value

DE	1	102997	102181	fumonisin B1	Fumonisin B1 (FB1)
DE	2	102997	102181	ochratoxin A	Ochratoxin A (OTA)
DE	3	102997	102181	neutral red uptake test	Neutral Red Uptake Test
DE	4	102997	102181	Vero cells	Vero
DE	5	102997	102181	Caco-2 cells	Caco-2
DE	6	102997	102181	C6 glioma cells	C6 Glioma
ID	1	102999	102183	STIMULATED LIPID-PEROXIDATION	Stimulated Lipid Peroxidation
ID	2	102999	102183	BALKAN ENDEMIC NEPHROPATHY	Balkan Endemic Nephropathy
ID	3	102999	102183	C6 GLIOMA-CELLS	C6 Glioma
ID	4	102999	102183	FUSARIUM-MONILIFORME	Fusarium Moniliforme
ID	5	102999	102183	PROTEIN-SYNTHESIS	Protein Synthesis
ID	6	102999	102183	CULTURE MATERIAL	Culture Material
ID	7	102999	102183	MICE	Mice
ID	8	102999	102183	PHENYLALANINE	Phenylalanine
ID	9	102999	102183	MYCOTOXINS	Mycotoxins
ID	10	102999	102183	PREVENTION	Prevention

Record 732 | WOS:000224048300016

Evidence for covalent DNA adduction by ochratoxin A following chronic exposure to rat and subacute exposure to pig

Field	Position	Original line	Normalised line	Original value	Normalised value
ID	1	103120	102303	C8-DEOXYGUANOSINE NUCLEOSIDE ADDUCT	C8 Deoxyguanosine Nucleoside Adduct
ID	2	103120	102303	C8 REACTIVITY	C8 Reactivity
ID	3	103120	102303	GENOTOXICITY	Genotoxicity
ID	4	103120	102303	METABOLITES	Metabolite
ID	5	103120	102303	MYCOTOXINS	Mycotoxins
ID	6	103120	102303	ABSENCE	Absence
ID	7	103120	102303	TUMORS	Tumor
ID	8	103120	102303	ACID	Acid

Record 733 | WOS:000225210900008

Levels of mycotoxins and sample cytotoxicity of selected organic and conventional grain-based products purchased from Finnish and Italian markets

Field	Position	Original line	Normalised line	Original value	Normalised value
DE	1	103238	102420	cytotoxicity	Cytotoxicity
DE	2	103238	102420	grain-based products	Grain Based Product
DE	3	103238	102420	mycotoxins	Mycotoxins
DE	4	103238	102420	organic agriculture	Organic Agriculture
ID	1	103239	102421	FUSARIUM MYCOTOXINS	Fusarium Mycotoxin
ID	2	103239	102421	OCHRATOXIN-A	Ochratoxin A (OTA)
ID	3	103239	102421	CHEMICAL-MIXTURES	Chemical Mixture
ID	4	103239	102421	T-2 TOXIN	T 2 Toxin
ID	5	103239	102421	WHEAT	Wheat
ID	6	103239	102421	FUSAPROLIFERIN	Fusaproliferin
ID	7	103239	102421	BEAUVERICIN	Beauvericin
ID	8	103239	102421	ZEARALENONE	Zearalenone (ZEA)
ID	9	103239	102421	TOXICOLOGY	Toxicology
ID	10	103239	102421	CADMIUM	Cadmium

Record 734 | WOS:000223297700006

Effects of rosmarinic acid against aflatoxin B1 and ochratoxin-A-induced cell damage in a human hepatoma cell line (Hep G2)

Field	Position	Original line	Normalised line	Original value	Normalised value
DE	1	103324	102505	aflatoxin B-1	Aflatoxin B1 (AFB1)
DE	2	103324	102505	ochratoxin A	Ochratoxin A (OTA)
DE	3	103324	102505	rosmarinic acid	Rosmarinic Acid

DE	4	103324	102505	Hep G2 cells	Hep G2
DE	5	103324	102505	cytotoxicity	Cytotoxicity
ID	1	103325	102507	LIPID-PEROXIDATION	Lipid Peroxidation
ID	2	103325	102507	OXIDATIVE DAMAGE	Oxidative Damage
ID	3	103325	102507	MECHANISM	Mechanism
ID	4	103325	102507	GENOTOXICITY	Genotoxicity
ID	5	103325	102507	INHIBITION	Inhibition
ID	6	103325	102507	TOXICITY	Toxicity
ID	7	103325	102507	ASSAY	Assay
ID	8	103325	102507	RATS	Rat

Record 735 | WOS:000221551200016

Ochratoxin A induces apoptosis in human lymphocytes through down regulation of Bcl-xL

Field	Position	Original line	Normalised line	Original value	Normalised value
DE	1	103405	102586	ochratoxin A	Ochratoxin A (OTA)
DE	2	103405	102586	Bcl-x(L)	Bcl X L
DE	3	103405	102586	mitochondria	Mitochondria
DE	4	103405	102586	lymphocyte	Lymphocyte
DE	5	103405	102586	apoptosis	Apoptosis
ID	1	103406	102587	INDUCED CASPASE ACTIVATION	Induced Caspase Activation
ID	2	103406	102587	BCL-X-L	Bcl X L
ID	3	103406	102587	CELL-DEATH	Cell Death
ID	4	103406	102587	CYTOCHROME-C	Cytochrome C
ID	5	103406	102587	DEFENSE REACTIONS	Defense Reaction
ID	6	103406	102587	IMMUNE-RESPONSE	Immune Response
ID	8	103406	102587	MITOCHONDRIA	Mitochondria
ID	9	103406	102587	PREVENTION	Prevention
ID	10	103406	102587	FORM	Form

Record 736 | WOS:000221555600005

Metabolism-mediated cytotoxicity of ochratoxin A

Field	Position	Original line	Normalised line	Original value	Normalised value
DE	1	103517	102697	ochratoxin A	Ochratoxin A (OTA)
DE	2	103517	102697	cytochrome P450	Cytochrome P450
DE	3	103517	102697	metabolism	Metabolism
DE	4	103517	102697	cell lines	Cell
DE	5	103517	102697	cytotoxicity	Cytotoxicity
ID	1	103518	102698	PERFORMANCE LIQUID-CHROMATOGRAPHY	Performance Liquid Chromatography
ID	2	103518	102698	RAT-LIVER	Rat Liver
ID	3	103518	102698	GLUTATHIONE	Glutathione (GSH)
ID	4	103518	102698	CYTOCHROME-P450	Cytochrome P450
ID	5	103518	102698	HEPATOCYTES	Hepatocyte
ID	6	103518	102698	TRANSPORT	Transport
ID	7	103518	102698	TOXICITY	Toxicity
ID	8	103518	102698	ENZYMES	Enzyme
ID	9	103518	102698	CELLS	Cell
ID	10	103518	102698	MICROSOMES	Microsome

Record 737 | WOS:000220837300016

Immunotoxic effects of Ochratoxin A in wistar rats after oral administration

Field	Position	Original line	Normalised line	Original value	Normalised value
DE	1	103615	102793	Ochratoxin A	Ochratoxin A (OTA)
DE	2	103615	102793	Wistar rats	Wistar Rat
DE	3	103615	102793	28 day-study	28 Day Study
DE	4	103615	102793	NK cell activity	Nk Cell Activity
DE	5	103615	102793	immunotoxicity	Immunotoxicity
DE	6	103615	102793	micronuclei	Micronuclei

ID	1	103617	102795	SALMONELLA-TYPHIMURIUM	Salmonella Typhimurium
ID	2	103617	102795	URINARY-BLADDER	Urinary Bladder
ID	3	103617	102795	IN-VITRO	In-Vitro
ID	4	103617	102795	DNA-REPAIR	DNA Repair
ID	5	103617	102795	MUTAGENICITY	Mutagenicity
ID	6	103617	102795	GENOTOXICITY	Genotoxicity
ID	7	103617	102795	IMMUNE	Immune
ID	8	103617	102795	CELLS	Cell
ID	9	103617	102795	MICE	Mice
ID	10	103617	102795	ASSAY	Assay

Record 738 | WOS:000221514400030

Synergistic interaction in simultaneous exposure to Streptomyces californicus and Stachybotrys chartarum

Field	Position	Original line	Normalised line	Original value	Normalised value
DE	1	103730	102907	bacteria	Bacteria
DE	2	103730	102907	fungi	Fungi
DE	3	103730	102907	inflammation	Inflammation
DE	4	103730	102907	interaction	Interaction
DE	5	103730	102907	mycotoxins	Mycotoxins
DE	6	103730	102907	toxicity	Toxicity
ID	1	103731	102908	ACUTE INHALATION TOXICITY	Acute Inhalation Toxicity
ID	2	103731	102908	WATER-DAMAGED BUILDINGS	Water Damaged Building
ID	3	103731	102908	INDOOR AIR	Indoor Air
ID	4	103731	102908	TRICHOTHECENE VOMITOXIN	Trichothecene Vomitoxin
ID	5	103731	102908	INFLAMMATORY RESPONSES	Inflammatory Response
ID	6	103731	102908	T-2 MYCOTOXIN	T 2 Mycotoxin
ID	7	103731	102908	CYTOTOXICITY	Cytotoxicity
ID	8	103731	102908	METABOLITES	Metabolite
ID	9	103731	102908	MODULATION	Modulation
ID	10	103731	102908	MEDIATORS	Mediator

Record 739 | WOS:000220235700005

Inhibitory effects of ochratoxin A on nerve growth factor-induced neurite extension through downregulation of p38 map kinase and AP-1 activation in cultured pheochromocytoma cells

Field	Position	Original line	Normalised line	Original value	Normalised value
ID	1	103855	103031	PC12 CELLS	Pc12
ID	2	103855	103031	PC-12 CELLS	Pc 12
ID	3	103855	103031	PROTEIN-KINASE	Protein Kinase
ID	4	103855	103031	NEURONAL DIFFERENTIATION	Neuronal Differentiation
ID	5	103855	103031	TRANSCRIPTION FACTORS	Transcription Factor
ID	6	103855	103031	CALCIUM HOMEOSTASIS	Calcium Homeostasis
ID	7	103855	103031	ENDEMIC NEPHROPATHY	Endemic Nephropathy
ID	8	103855	103031	GENE-EXPRESSION	Gene Expression
ID	9	103855	103031	CHICK-EMBRYOS	Chick Embryo
ID	10	103855	103031	C-JUN	C Jun

Record 740 | WOS:000189087700013

Ochratoxin A: Lack of formation of covalent DNA adducts

Field	Position	Original line	Normalised line	Original value	Normalised value
ID	1	103955	103130	PEROXIDASE ACTIVATION	Peroxidase Activation

ID	2	103955	103130	MUTAGENICITY	Mutagenicity
ID	3	103955	103130	RATS	Rat
ID	4	103955	103130	METABOLISM	Metabolism
ID	5	103955	103130	REACTIVITY	Reactivity
ID	6	103955	103130	INDUCTION	Induction
ID	7	103955	103130	BACTERIAL	Bacterial
ID	8	103955	103130	TOXICITY	Toxicity
ID	9	103955	103130	EXPOSURE	Exposure
ID	10	103955	103130	STRESS	Stress

Record 741 | WOS:000188530700003

Ochratoxin A and zearalenone: a comparative study on genotoxic effects and cell death induced in bovine lymphocytes

Field	Position	Original line	Normalised line	Original value	Normalised value
DE	1	104041	103215	mycotoxins	Mycotoxins
DE	2	104041	103215	chromosome aberrations	Chromosome Aberration
DE	3	104041	103215	sister chromatid exchanges	Sister Chromatid Exchange
DE	4	104041	103215	mitotic index	Mitotic Index
DE	5	104041	103215	apoptosis	Apoptosis
DE	6	104041	103215	cell cultures	Cell
ID	1	104043	103217	BALKAN ENDEMIC NEPHROPATHY	Balkan Endemic Nephropathy
ID	2	104043	103217	IN-VITRO	In-Vitro
ID	3	104043	103217	MYCOTOXIN ZEARALENONE	Mycotoxin Zearalenone
ID	4	104043	103217	HUMAN-BLOOD	Human Blood
ID	5	104043	103217	VITAMIN-E	Vitamin E
ID	6	104043	103217	INDUCTION	Induction
ID	7	104043	103217	MUTAGENICITY	Mutagenicity
ID	8	104043	103217	APOPTOSIS	Apoptosis
ID	9	104043	103217	CULTURES	Culture
ID	10	104043	103217	KIDNEY	Kidney

Record 742 | WOS:000189121300007

New insights into the mechanisms involved in renal proximal tubular damage induced in vitro by ochratoxin A

Field	Position	Original line	Normalised line	Original value	Normalised value
DE	1	104155	103327	ochratoxin A	Ochratoxin A (OTA)
DE	2	104155	103327	LLC-PK1	Llc Pk1
DE	3	104155	103327	nephrotoxicity	Nephrotoxicity
DE	4	104155	103327	in vitro	In-Vitro
ID	1	104156	103328	TOXICITY	Toxicity
ID	2	104156	103328	NEPHROTOXICITY	Nephrotoxicity
ID	3	104156	103328	CELLS	Cell

Record 743 | WOS:000189041600006

Inhibition of mitochondria and extracellular acidification enhance ochratoxin A-induced apoptosis in renal collecting duct-derived MDCK-C7 cells

Field	Position	Original line	Normalised line	Original value	Normalised value
DE	1	104244	103415	apoptosis	Apoptosis
DE	2	104244	103415	ochratoxin A	Ochratoxin A (OTA)
DE	3	104244	103415	mitochondria	Mitochondria
DE	4	104244	103415	collecting duct	Collecting Duct
DE	5	104244	103415	acidosis	Acidosis
ID	1	104245	103416	CANINE KIDNEY-CELLS	Canine Kidney
ID	2	104245	103416	NEPHRON IN-VIVO	Nephron In Vivo
ID	3	104245	103416	EPITHELIAL-CELLS	Epithelial
ID	4	104245	103416	NEPHRITOGENIC OCHRATOXIN	Ochratoxin A (OTA)
ID	5	104245	103416	PH HOMEOSTASIS	Ph Homeostasis
ID	6	104245	103416	CYTOCHROME-C	Cytochrome C

ID	7	104245	103416	ACTIVATION	Activation
ID	8	104245	103416	TUBULE	Tubule
ID	9	104245	103416	DEATH	Death
ID	10	104245	103416	REABSORPTION	Reabsorption

Record 744 | WOS:000231756500075

Natural antioxidants and mycotoxins: Theoretical considerations and practical applications

Field	Position	Original line	Normalised line	Original value	Normalised value
ID	1	104336	103505	STIMULATED LIPID-PEROXIDATION	Stimulated Lipid Peroxidation
ID	2	104336	103505	PRIMARY RAT HEPATOCYTES	Primary Rat Hepatocyte
ID	3	104336	103505	DNA ADDUCT FORMATION	DNA Adduct Formation
ID	4	104336	103505	LIVER IN-VIVO	Liver In Vivo
ID	5	104336	103505	T-2 TOXIN	T 2 Toxin
ID	6	104336	103505	FUMONISIN B-1	Fumonisin B1 (FB1)
ID	7	104336	103505	OCHRATOXIN-A	Ochratoxin A (OTA)
ID	8	104336	103505	VITAMIN-E	Vitamin E
ID	9	104336	103505	ALPHA-TOCOPHEROL	Alpha Tocopherol
ID	10	104336	103505	TOXICITY	Toxicity

Record 745 | WOS:000186953000004

Apoptosis and oxidative stress induced by ochratoxin A in rat kidney

Field	Position	Original line	Normalised line	Original value	Normalised value
DE	1	104434	103602	ochratoxin A	Ochratoxin A (OTA)
DE	2	104434	103602	apoptosis	Apoptosis
DE	3	104434	103602	oxidative stress	Oxidative Stress
DE	4	104434	103602	proximal tubule cells	Proximal Tubule
DE	5	104434	103602	rat kidney	Rat Kidney
ID	1	104436	103604	PROXIMAL-TUBULE	Proximal Tubule
ID	2	104436	103604	NEPHROTOXIN OCHRATOXIN	Ochratoxin A (OTA)
ID	3	104436	103604	LIPID-PEROXIDATION	Lipid Peroxidation
ID	4	104436	103604	HUMAN-BLOOD	Human Blood
ID	5	104436	103604	IN-VIVO	In-Vivo
ID	6	104436	103604	TRANSPORT	Transport
ID	7	104436	103604	TOXICITY	Toxicity
ID	8	104436	103604	CELLS	Cell
ID	9	104436	103604	PATHOGENESIS	Pathogenesis
ID	10	104436	103604	NEPHROPATHY	Nephropathy

Record 746 | WOS:000186282400042

Effects of chronic ingestion of ochratoxin A on blood levels and excretion of the mycotoxin in sheep

Field	Position	Original line	Normalised line	Original value	Normalised value
DE	1	104559	103725	ochratoxin A	Ochratoxin A (OTA)
DE	2	104559	103725	ochratoxin alpha	Ochratoxin Alpha
DE	3	104559	103725	mycotoxin	Mycotoxins
DE	4	104559	103725	sheep	Sheep
DE	5	104559	103725	ruminants	Ruminant
DE	6	104559	103725	metabolism	Metabolism
DE	7	104559	103725	excretion	Excretion
ID	1	104561	103727	RUMEN FLUID	Rumen Fluid
ID	2	104561	103727	RAT	Rat
ID	3	104561	103727	METABOLITES	Metabolite
ID	4	104561	103727	ABSORPTION	Absorption
ID	5	104561	103727	TOXICITY	Toxicity
ID	6	104561	103727	ANALOGS	Analog
ID	7	104561	103727	CELLS	Cell
ID	8	104561	103727	MILK	Milk

ID	9	104561	103727	DIET	Diet

Record 747 | WOS:000184259400007

Effects of the mycotoxin ochratoxin A and some of its metabolites on human kidney cell lines

Field	Position	Original line	Normalised line	Original value	Normalised value
DE	1	104650	103815	ochratoxin A, C	Ochratoxin A C
DE	2	104650	103815	kidney cell lines	Kidney Cell
DE	3	104650	103815	proliferation	Proliferation
DE	4	104650	103815	cell wall integrity	Cell Wall Integrity
DE	5	104650	103815	complement-regulating surface proteins	Complement Regulating Surface Protein
DE	6	104650	103815	CD46, CD55, CD59	Cd46 Cd55 Cd59
ID	1	104652	103817	PORCINE NEPHROPATHY	Porcine Nephropathy
ID	2	104652	103817	DEFENSE REACTIONS	Defense Reaction
ID	3	104652	103817	IN-VITRO	In-Vitro
ID	4	104652	103817	MEMBRANE	Membrane
ID	5	104652	103817	PROTEINS	Protein
ID	6	104652	103817	IMMUNE	Immune
ID	7	104652	103817	PIGS	Pig
ID	8	104652	103817	CYTOTOXICITY	Cytotoxicity
ID	9	104652	103817	EXPRESSION	Expression
ID	10	104652	103817	APOPTOSIS	Apoptosis

Record 748 | WOS:000184243200002

An oxygen-bonded C8-deoxyguanosine nucleoside adduct of pentachlorophenol by peroxidase activation: Evidence for ambident C8 reactivity by phenoxyl radicals

Field	Position	Original line	Normalised line	Original value	Normalised value
ID	1	104750	103914	OCHRATOXIN-A	Ochratoxin A (OTA)
ID	2	104750	103914	AQUEOUS-SOLUTION	Aqueous Solution
ID	3	104750	103914	DNA-ADDUCTS	DNA Adduct
ID	4	104750	103914	POTENTIALS	Potential
ID	5	104750	103914	OXIDATION	Oxidation
ID	6	104750	103914	RATS	Rat

Record 749 | WOS:000184363500006

Effect of Ochratoxin A on human haematopoietic progenitors proliferation and differentiation: an in vitro study

Field	Position	Original line	Normalised line	Original value	Normalised value
DE	1	104819	103982	haematopoietic progenitors	Haematopoietic Progenitor
DE	2	104819	103982	human	Human
DE	3	104819	103982	ochratoxin A	Ochratoxin A (OTA)
DE	4	104819	103982	myelotoxicity	Myelotoxicity
ID	1	104820	103983	CLINICAL-CHEMISTRY	Clinical Chemistry
ID	2	104820	103983	DIFFERENT ORIGINS	Different Origin
ID	3	104820	103983	BONE-MARROW	Bone Marrow
ID	4	104820	103983	TOXICITY	Toxicity
ID	5	104820	103983	MICE	Mice
ID	6	104820	103983	PROTEIN	Protein
ID	7	104820	103983	BLOOD	Blood
ID	8	104820	103983	CELLS	Cell
ID	9	104820	103983	PHENYLALANINE	Phenylalanine
ID	10	104820	103983	INHIBITION	Inhibition

Record 750 | WOS:000185415100022

Evaluation of two mycotoxin binders to reduce toxicity of broiler diets containing ochratoxin A and T-2 toxin contaminated grain

Field	Position	Original line	Normalised line	Original value	Normalised value
DE	1	104921	104083	mycotoxins	Mycotoxins
DE	2	104921	104083	ochratoxin A	Ochratoxin A (OTA)
DE	3	104921	104083	T-2 toxin	T 2 Toxin
DE	4	104921	104083	broilers	Broiler
DE	5	104921	104083	adsorbents	Adsorbent
DE	6	104921	104083	evaluation	Evaluation
ID	1	104922	104084	SODIUM-CALCIUM ALUMINOSILICATE	Sodium Calcium Aluminosilicate
ID	2	104922	104084	AFLATOXIN	Aflatoxin
ID	3	104922	104084	CHICKENS	Chicken
ID	4	104922	104084	EFFICACY	Efficacy
ID	5	104922	104084	PATHOLOGY	Pathology
ID	6	104922	104084	POULTRY	Poultry
ID	7	104922	104084	KIDNEY	Kidney
ID	8	104922	104084	BARLEY	Barley

Record 751 | WOS:000183040200012

A new approach to studying ochratoxin A (OTA)-induced nephrotoxicity: Expression profiling in vivo and in vitro employing cDNA microarrays

Field	Position	Original line	Normalised line	Original value	Normalised value
DE	1	105024	104185	toxicogenomics	Toxicogenomics
DE	2	105024	104185	microarray	Microarray
DE	3	105024	104185	expression profiling	Expression Profiling
DE	4	105024	104185	Ochratoxin A	Ochratoxin A (OTA)
DE	5	105024	104185	nephrotoxicity	Nephrotoxicity
DE	6	105024	104185	kidney	Kidney
DE	7	105024	104185	proximal tubule	Proximal Tubule
DE	8	105024	104185	cell culture	Cell
ID	1	105026	104187	STEM-CELL FACTOR	Stem Cell Factor
ID	2	105026	104187	TRANSCRIPTIONAL ACTIVATION	Transcriptional Activation
ID	3	105026	104187	MESSENGER-RNA	Messenger RNA
ID	4	105026	104187	C-KIT	C Kit
ID	6	105026	104187	PROTEIN	Protein
ID	7	105026	104187	CARCINOMA	Carcinoma
ID	8	105026	104187	GENES	Gene
ID	9	105026	104187	RAT	Rat
ID	10	105026	104187	ISCHEMIA	Ischemia

Record 752 | WOS:000183875100009

Effects of the mycotoxin ochratoxin A in a bacterial and a mammalian in vitro mutagenicity test system

Field	Position	Original line	Normalised line	Original value	Normalised value
DE	1	105138	104298	mycotoxin	Mycotoxins
DE	2	105138	104298	ochratoxin A	Ochratoxin A (OTA)
DE	3	105138	104298	mutagenicity	Mutagenicity
DE	4	105138	104298	Salmonella typhimurium	Salmonella Typhimurium
DE	5	105138	104298	HPRT assay	Hprt Assay
DE	6	105138	104298	metabolic activation	Metabolic Activation
ID	1	105140	104300	BALKAN ENDEMIC NEPHROPATHY	Balkan Endemic Nephropathy
ID	2	105140	104300	SALMONELLA-TYPHIMURIUM	Salmonella Typhimurium
ID	4	105140	104300	RAT	Rat
ID	5	105140	104300	GENOTOXICITY	Genotoxicity
ID	6	105140	104300	CARCINOGENICITY	Carcinogenicity
ID	7	105140	104300	METABOLISM	Metabolism
ID	8	105140	104300	APOPTOSIS	Apoptosis
ID	9	105140	104300	ADDUCTS	Adduct
ID	10	105140	104300	CELLS	Cell

Record 753 | WOS:000182307900052

Interaction of ochratoxin A with human intestinal Caco-2 cells: possible implication of a multidrug resistance-associated protein (MRP2)

Field	Position	Original line	Normalised line	Original value	Normalised value
DE	1	105235	104394	ochratoxin A	Ochratoxin A (OTA)
DE	2	105235	104394	Caco-2 cells	Caco-2
DE	3	105235	104394	transepithelial transport	Transepithelial Transport
DE	4	105235	104394	multidrug resistance-associated protein	Multidrug Resistance Associated Protein
ID	1	105237	104396	CANINE KIDNEY-CELLS	Canine Kidney
ID	2	105237	104396	SERUM-FREE	Serum Free
ID	3	105237	104396	SECRETION	Secretion
ID	4	105237	104396	TRANSPORTER	Transporter
ID	5	105237	104396	EFFLUX	Efflux
ID	6	105237	104396	LOCALIZATION	Localization
ID	7	105237	104396	SPECIFICITY	Specificity
ID	8	105237	104396	ABSORPTION	Absorption
ID	9	105237	104396	EXPRESSION	Expression
ID	10	105237	104396	MONOLAYERS	Monolayer

Record 755 | WOS:000180037400011

Photochemically catalyzed reaction of ochratoxin A with D- and L-cysteine

Field	Position	Original line	Normalised line	Original value	Normalised value
ID	1	105379	104536	HUMAN SERUM-ALBUMIN	Human Serum Albumin
ID	2	105379	104536	ELECTROCHEMICAL OXIDATION	Electrochemical Oxidation
ID	4	105379	104536	BINDING	Binding
ID	5	105379	104536	4-CHLOROPHENOL	4 Chlorophenol
ID	6	105379	104536	GENOTOXICITY	Genotoxicity
ID	7	105379	104536	GLUTATHIONE	Glutathione (GSH)
ID	8	105379	104536	METABOLISM	Metabolism
ID	9	105379	104536	ADDUCTS	Adduct
ID	10	105379	104536	ANALOGS	Analog

Record 756 | WOS:000179850500004

Prevention and control of mycotoxins in the poultry production chain:: a European view

Field	Position	Original line	Normalised line	Original value	Normalised value
DE	1	105469	104625	mycotoxin	Mycotoxins
DE	2	105469	104625	poultry	Poultry
DE	3	105469	104625	prevention	Prevention
ID	1	105470	104626	MONILIFORME CULTURE MATERIAL	Moniliforme Culture Material
ID	2	105470	104626	SODIUM CALCIUM ALUMINOSILICATE	Sodium Calcium Aluminosilicate
ID	3	105470	104626	FUMONISIN B-1 PRESENT	Fumonisin B 1 Present
ID	4	105470	104626	BROILER CHICKS	Broiler Chick
ID	5	105470	104626	T-2 TOXIN	T 2 Toxin
ID	6	105470	104626	FUSARIUM-FUJIKUROI	Fusarium Fujikuroi
ID	7	105470	104626	TURKEY POULTS	Turkey Poult
ID	8	105470	104626	ACUTE TOXICITY	Acute Toxicity
ID	9	105470	104626	OCHRATOXIN-A	Ochratoxin A (OTA)
ID	10	105470	104626	DEOXYNIVALENOL VOMITOXIN	Deoxynivalenol (DON)

Record 757 | WOS:000178857500004

RETRACTED: Antioxidant nutrients and mycotoxins (Retracted Article)

Field	Position	Original line	Normalised line	Original value	Normalised value

DE	1	105650	104805	antioxidants	Antioxidant
DE	2	105650	104805	mycotoxins	Mycotoxins
DE	3	105650	104805	detoxification	Detoxification
DE	4	105650	104805	free radicals	Free Radical
DE	5	105650	104805	lipid peroxidation	Lipid Peroxidation
ID	1	105652	104806	AFLATOXIN B-1-DNA BINDING	Aflatoxin B 1 DNA Binding
ID	2	105652	104806	OCHRATOXIN-PRODUCED GENOTOXICITY	Ochratoxin Produced Genotoxicity
ID	3	105652	104806	INDUCED DNA-DAMAGE	Induced DNA Damage
ID	4	105652	104806	BONE-MARROW-CELLS	Bone Marrow
ID	5	105652	104806	LIPID-PEROXIDATION	Lipid Peroxidation
ID	6	105652	104806	RAT-LIVER	Rat Liver
ID	7	105652	104806	VITAMIN-E	Vitamin E
ID	8	105652	104806	T-2 TOXIN	T 2 Toxin
ID	9	105652	104806	ALPHA-TOCOPHEROL	Alpha Tocopherol
ID	10	105652	104806	IN-VIVO	In-Vivo

Record 758 | WOS:000177393700006

Genotoxic effects of ochratoxin A in human-derived hepatoma (HepG2) cells

Field	Position	Original line	Normalised line	Original value	Normalised value
DE	1	105869	105022	ochratoxin A	Ochratoxin A (OTA)
DE	3	105869	105022	micronouclei	Micronouclei
DE	4	105869	105022	comet assay	Comet Assay
DE	5	105869	105022	Ames test	Ame Test
DE	6	105869	105022	HepG2-S9	HepG2 S9
ID	1	105870	105023	SALMONELLA-TYPHIMURIUM	Salmonella Typhimurium
ID	2	105870	105023	METABOLIC-ACTIVATION	Metabolic Activation
ID	3	105870	105023	IN-VITRO	In-Vitro
ID	4	105870	105023	MYCOTOXINS	Mycotoxins
ID	5	105870	105023	MUTAGENICITY	Mutagenicity
ID	6	105870	105023	INDUCTION	Induction
ID	7	105870	105023	ASSAY	Assay

Record 759 | WOS:000177631900009

Species- and sex-specific variations in binding of ochratoxin A by renal proteins in vitro

Field	Position	Original line	Normalised line	Original value	Normalised value
DE	1	105961	105113	ochratoxin A	Ochratoxin A (OTA)
DE	2	105961	105113	renal protein binding	Renal Protein Binding
DE	3	105961	105113	renal toxicity	Renal Toxicity
DE	4	105961	105113	organic anion transporter	Organic Anion Transporter
ID	1	105963	105115	ORGANIC ANION TRANSPORTER	Organic Anion Transporter
ID	2	105963	105115	MEDIATING BASOLATERAL UPTAKE	Mediating Basolateral Uptake
ID	3	105963	105115	A-CONTAMINATED BARLEY	A Contaminated Barley
ID	4	105963	105115	FUNCTIONAL-CHARACTERIZATION	Functional Characterization
ID	5	105963	105115	PERITUBULAR TRANSPORT	Peritubular Transport
ID	6	105963	105115	FEEDING EXPERIMENTS	Feeding Experiment
ID	7	105963	105115	PROXIMAL TUBULES	Proximal Tubule
ID	8	105963	105115	BACON PIGS	Bacon Pig
ID	9	105963	105115	RAT-KIDNEY	Rat Kidney
ID	10	105963	105115	CLONING	Cloning

Record 760 | WOS:000176910000001

Aristolochic acid as a probable human cancer hazard in herbal remedies: a review

Field	Position	Original line	Normalised line	Original value	Normalised value
ID	1	106057	105208	INTERSTITIAL RENAL FIBROSIS	Interstitial Renal Fibrosis
ID	2	106057	105208	DNA ADDUCT FORMATION	DNA Adduct Formation
ID	3	106057	105208	NATURALLY-OCCURRING SUBSTANCES	Naturally Occurring Substance
ID	4	106057	105208	BALKAN ENDEMIC NEPHROPATHY	Balkan Endemic Nephropathy
ID	5	106057	105208	P-32 POSTLABELING ANALYSIS	P 32 Postlabeling Analysis
ID	6	106057	105208	VALVULAR HEART-DISEASE	Valvular Heart Disease
ID	7	106057	105208	CHINESE HERBS	Chinese Herb
ID	8	106057	105208	SALMONELLA-TYPHIMURIUM	Salmonella Typhimurium
ID	9	106057	105208	REDUCTIVE ACTIVATION	Reductive Activation
ID	10	106057	105208	SLIMMING REGIMEN	Slimming Regimen

Record 761 | WOS:000175922300023

Esterified-glucomannan in broiler chicken diets-contaminated with aflatoxin, ochratoxin and T-2 toxin:: Evaluation of its binding ability (in vitro) and efficacy as immunomodulator

Field	Position	Original line	Normalised line	Original value	Normalised value
DE	1	106237	105387	esterified-glucomannan	Esterified Glucomannan
DE	2	106237	105387	aflatoxin	Aflatoxin
DE	3	106237	105387	ochratoxin	Ochratoxin A (OTA)
DE	4	106237	105387	T-2 toxin	T 2 Toxin
DE	5	106237	105387	broilers	Broiler

Record 762 | WOS:000176518700006

Role of human organic anion transporter 4 in the transport of ochratoxin A

Field	Position	Original line	Normalised line	Original value	Normalised value
DE	1	106321	105470	ochratoxin A	Ochratoxin A (OTA)
DE	2	106321	105470	organic anion transporter	Organic Anion Transporter
DE	3	106321	105470	proximal tubule	Proximal Tubule
DE	4	106321	105470	transport	Transport
DE	5	106321	105470	cell line	Cell
ID	1	106323	105472	RENAL PROXIMAL TUBULES	Renal Proximal Tubule
ID	2	106323	105472	NEPHRON IN-VIVO	Nephron In Vivo
ID	3	106323	105472	FUNCTIONAL-CHARACTERIZATION	Functional Characterization
ID	4	106323	105472	MOLECULAR-CLONING	Molecular Cloning
ID	5	106323	105472	PERITUBULAR TRANSPORT	Peritubular Transport
ID	6	106323	105472	EXPRESSION CLONING	Expression Cloning
ID	7	106323	105472	CATION TRANSPORTER	Cation Transporter
ID	8	106323	105472	RAT-KIDNEY	Rat Kidney
ID	9	106323	105472	CELLS	Cell
ID	10	106323	105472	NEPHROTOXICITY	Nephrotoxicity

Record 763 | WOS:000176216800005

Expression of Hsp70 in kidney cells exposed to ochratoxin A

Field	Position	Original line	Normalised line	Original value	Normalised value
DE	1	106430	105578	ochratoxin A	Ochratoxin A (OTA)
DE	2	106430	105578	endemic nephropathy	Endemic Nephropathy
DE	3	106430	105578	nephrotoxicity	Nephrotoxicity
DE	4	106430	105578	heat shock protein 70	Heat Shock Protein 70
DE	5	106430	105578	cell viability	Cell Viability
DE	6	106430	105578	lactate dehydrogenase release	Lactate Dehydrogenase Release
ID	1	106432	105580	HEAT-SHOCK PROTEINS	Heat Shock Protein
ID	2	106432	105580	MEMBRANE-BOUND ENZYMES	Membrane Bound Enzyme
ID	3	106432	105580	SINGLE-STRAND BREAKS	Single Strand Break
ID	4	106432	105580	ENDEMIC NEPHROPATHY	Endemic Nephropathy
ID	5	106432	105580	MOLECULAR CHAPERONES	Molecular Chaperone
ID	6	106432	105580	LIPID-PEROXIDATION	Lipid Peroxidation
ID	7	106432	105580	OXIDATIVE STRESS	Oxidative Stress
ID	8	106432	105580	MESSENGER-RNAS	Messenger RNA
ID	9	106432	105580	RAT-BRAIN	Rat Brain
ID	10	106432	105580	IN-VIVO	In-Vivo

Record 764 | WOS:000175152300005

Effect of T-2 toxin on in vivo lipid peroxidation and vitamin E status in mice

Field	Position	Original line	Normalised line	Original value	Normalised value
DE	1	106535	105682	T-2 toxin	T 2 Toxin
DE	2	106535	105682	lipid peroxidation	Lipid Peroxidation
DE	3	106535	105682	MDA	Mda
DE	4	106535	105682	vitamin E	Vitamin E
ID	1	106536	105683	OCHRATOXIN-A	Ochratoxin A (OTA)
ID	2	106536	105683	RAT-LIVER	Rat Liver
ID	3	106536	105683	ANTIOXIDANTS	Antioxidant
ID	4	106536	105683	RESISTANCE	Resistance
ID	5	106536	105683	T-2-TOXIN	T 2 Toxin
ID	6	106536	105683	EXPOSURE	Exposure
ID	7	106536	105683	PLASMA	Plasma

Record 765 | WOS:000173055800005

Determination of the epigenetic effects of ochratoxin in a human kidney and a rat liver epithelial cell line

Field	Position	Original line	Normalised line	Original value	Normalised value
DE	1	106620	105766	gap junctional intercellular communication	Gap Junctional Intercellular Communication
DE	2	106620	105766	ochratoxin A	Ochratoxin A (OTA)
DE	3	106620	105766	epigenetic toxicity	Epigenetic Toxicity
DE	4	106620	105766	tumor promotion	Tumor Promotion
DE	5	106620	105766	mitogen activated protein kinase	Mitogen Activated Protein Kinase
ID	1	106622	105768	JUNCTIONAL INTERCELLULAR COMMUNICATION	Junctional Intercellular Communication
ID	2	106622	105768	ACTIVATED PROTEIN-KINASE	Activated Protein Kinase
ID	3	106622	105768	POLYCYCLIC AROMATIC-HYDROCARBONS	Polycyclic Aromatic Hydrocarbon
ID	4	106622	105768	BALKAN ENDEMIC NEPHROPATHY	Balkan Endemic Nephropathy

ID	5	106622	105768	TUMOR PROMOTERS	Tumor Promoter
ID	6	106622	105768	DOWN-REGULATION	Down Regulation
ID	7	106622	105768	GROWTH-FACTOR	Growth Factor
ID	8	106622	105768	IN-VITRO	In-Vitro
ID	9	106622	105768	GAP	Gap
ID	10	106622	105768	PHOSPHORYLATION	Phosphorylation

Record 766 | WOS:000176962600010

Toxins of filamentous fungi

Field	Position	Original line	Normalised line	Original value	Normalised value
ID	1	106753	105897	SODIUM-CALCIUM ALUMINOSILICATE	Sodium Calcium Aluminosilicate
ID	2	106753	105897	AFLATOXIN B-1 BIOTRANSFORMATION	Aflatoxin B 1 Biotransformation
ID	3	106753	105897	FUJIKUROI MATING POPULATION	Fujikuroi Mating Population
ID	4	106753	105897	PATHWAY GENE CLUSTERS	Pathway Gene Cluster
ID	5	106753	105897	HEPATITIS-B-VIRUS	Hepatitis B Virus
ID	6	106753	105897	ASPERGILLUS-PARASITICUS	Aspergillus Parasiticus
ID	7	106753	105897	OCHRATOXIN-A	Ochratoxin A (OTA)
ID	8	106753	105897	FUSARIUM-MONILIFORME	Fusarium Moniliforme
ID	9	106753	105897	FUMONISIN B-1	Fumonisin B1 (FB1)
ID	10	106753	105897	MOLECULAR CHARACTERIZATION	Molecular Characterization

Record 767 | WOS:000174243300013

Detection of ochratoxin A-induced DNA damage in MDCK cells by alkaline single cell gel electrophoresis (comet assay)

Field	Position	Original line	Normalised line	Original value	Normalised value
DE	1	107019	106162	mycotoxin	Mycotoxins
DE	2	107019	106162	ochratoxin A	Ochratoxin A (OTA)
DE	3	107019	106162	DNA damage	DNA Damage
DE	4	107019	106162	DNA repair	DNA Repair
DE	5	107019	106162	MDCK cells	MDCK
DE	6	107019	106162	alkaline single cell gel electrophoresis	Alkaline Single Cell Gel Electrophoresis
ID	1	107021	106164	BALKAN ENDEMIC NEPHROPATHY	Balkan Endemic Nephropathy
ID	2	107021	106164	MYCOTOXIN OCHRATOXIN	Ochratoxin A (OTA)
ID	3	107021	106164	URINARY-BLADDER	Urinary Bladder
ID	4	107021	106164	STRAND BREAKS	Strand Break
ID	5	107021	106164	IN-VITRO	In-Vitro
ID	6	107021	106164	GENOTOXICITY	Genotoxicity
ID	7	107021	106164	LIVER	Liver
ID	8	107021	106164	INDUCTION	Induction
ID	9	107021	106164	APOPTOSIS	Apoptosis
ID	10	107021	106164	CITRININ	Citrinin

Record 768 | WOS:000172508300013

Improvement of megakaryocytic progenitor culture for toxicological investigations

Field	Position	Original line	Normalised line	Original value	Normalised value
DE	1	107112	106253	human CFU-MK	Human Cfu Mk
DE	2	107112	106253	megakaryocytopoiesis	Megakaryocytopoiesis
DE	3	107112	106253	in vitro toxicity	In Vitro Toxicity
DE	4	107112	106253	hematotoxicity	Hematotoxicity
DE	5	107112	106253	methodology	Methodology
ID	1	107114	106255	IN-VITRO TOXICITY	In Vitro Toxicity

ID	2	107114	106255	T-2 TOXIN	T 2 Toxin
ID	3	107114	106255	HEMATOPOIETIC PROGENITORS	Hematopoietic Progenitor
ID	4	107114	106255	RAT PROGENITORS	Rat Progenitor
ID	5	107114	106255	HAEMATOTOXICITY	Haematotoxicity
ID	6	107114	106255	MYCOTOXINS	Mycotoxins
ID	7	107114	106255	TESTS	Test

Record 769 | WOS:000173171400003

Horseradish peroxidase mediates DNA and deoxyguanosine 3â ²-monophosphate adduct formation in the presence of ochratoxin A

Field	Position	Original line	Normalised line	Original value	Normalised value
DE	1	107193	106333	ochratoxin A	Ochratoxin A (OTA)
DE	2	107193	106333	DNA adducts	DNA Adduct
DE	3	107193	106333	dGMP adducts	Dgmp Adduct
DE	4	107193	106333	horseradish peroxidase	Horseradish Peroxidase
DE	5	107193	106333	kidney microsomes	Kidney Microsome
DE	6	107193	106333	prostaglandin H synthase	Prostaglandin H Synthase
DE	7	107193	106333	cumene hydroperoxide	Cumene Hydroperoxide
ID	1	107195	106335	PROSTAGLANDIN-H SYNTHASE	Prostaglandin H Synthase
ID	2	107195	106335	URINARY-BLADDER	Urinary Bladder
ID	3	107195	106335	ARACHIDONATE 12-LIPOXYGENASE	Arachidonate 12 Lipoxygenase
ID	4	107195	106335	BENZIDINE BINDING	Benzidine Binding
ID	5	107195	106335	ACTIVATION	Activation
ID	6	107195	106335	GENOTOXICITY	Genotoxicity
ID	7	107195	106335	OXIDATION	Oxidation
ID	8	107195	106335	LIVER	Liver
ID	9	107195	106335	LIPOXYGENASES	Lipoxygenase
ID	10	107195	106335	MUTAGENICITY	Mutagenicity

Record 770 | WOS:000171478500001

Toxicity, metabolism, and impact of mycotoxins on humans and animals

Field	Position	Original line	Normalised line	Original value	Normalised value
DE	1	107313	106452	mycotoxins	Mycotoxins
DE	2	107313	106452	aflatoxins	Aflatoxin
DE	3	107313	106452	ruminants	Ruminant
DE	4	107313	106452	non-ruminants	Non Ruminant
ID	1	107314	106453	IN-VITRO TOXICITY	In Vitro Toxicity
ID	2	107314	106453	PROTEIN-KINASE-C	Protein Kinase C
ID	3	107314	106453	T-2 TOXIN	T 2 Toxin
ID	4	107314	106453	AFLATOXIN B-1	Aflatoxin B1 (AFB1)
ID	5	107314	106453	OCHRATOXIN-A	Ochratoxin A (OTA)
ID	6	107314	106453	TRICHOTHECENE MYCOTOXINS	Trichothecene Mycotoxin
ID	7	107314	106453	CYCLOPIAZONIC ACID	Cyclopiazonic Acid
ID	8	107314	106453	FUMONISIN B-1	Fumonisin B1 (FB1)
ID	9	107314	106453	ASPERGILLUS-FLAVUS	Aspergillus Flavus
ID	10	107314	106453	HEPATIC-METABOLISM	Hepatic Metabolism

Record 771 | WOS:000171571300006

The mycotoxin ochratoxin A alters intestinal barrier and absorption functions but has no effect on chloride secretion

Field	Position	Original line	Normalised line	Original value	Normalised value
DE	1	107635	106773	absorption	Absorption
DE	2	107635	106773	intestinal epithelium	Intestinal Epithelium
DE	3	107635	106773	xenobiotic	Xenobiotic

DE	4	107635	106773	transepithelial resistance	Transepithelial Resistance
DE	5	107635	106773	ochratoxin A	Ochratoxin A (OTA)
DE	6	107635	106773	microdomains	Microdomain
ID	1	107637	106775	HUMAN-IMMUNODEFICIENCY-VIRUS	Human Immunodeficiency Virus
ID	2	107637	106775	CANINE KIDNEY-CELLS	Canine Kidney
ID	3	107637	106775	PROXIMAL TUBULE CELLS	Proximal Tubule
ID	4	107637	106775	TIGHT JUNCTIONS	Tight Junction
ID	5	107637	106775	VERO-CELLS	Vero
ID	6	107637	106775	RAT	Rat
ID	7	107637	106775	TRANSPORT	Transport
ID	8	107637	106775	GLUCOSE	Glucose
ID	9	107637	106775	GALACTOSYLCERAMIDE	Galactosylceramide
ID	10	107637	106775	DIFFERENTIATION	Differentiation

Record 772 | WOS:000171310400014

Species-, sex-, and cell type-specific effects of ochratoxin A and B

Field	Position	Original line	Normalised line	Original value	Normalised value
DE	1	107744	106881	ochratoxin A	Ochratoxin A (OTA)
DE	2	107744	106881	nephropathy	Nephropathy
DE	3	107744	106881	renal cancer	Renal Cancer
DE	4	107744	106881	uptake	Uptake
DE	5	107744	106881	cytostasis	Cytostasis
DE	6	107744	106881	toxicity	Toxicity
ID	1	107745	106882	BALKAN ENDEMIC NEPHROPATHY	Balkan Endemic Nephropathy
ID	2	107745	106882	BLADDER EPITHELIAL-CELLS	Bladder Epithelial
ID	3	107745	106882	PROXIMAL TUBULE CELLS	Proximal Tubule
ID	4	107745	106882	HUMAN-BLOOD	Human Blood
ID	5	107745	106882	PRIMARY CULTURE	Primary
ID	6	107745	106882	KIDNEY	Kidney
ID	7	107745	106882	APOPTOSIS	Apoptosis
ID	8	107745	106882	RAT	Rat
ID	9	107745	106882	GROWTH	Growth
ID	10	107745	106882	INDUCTION	Induction

Record 773 | WOS:000171274400005

Characterization of ochratoxin A transport by human organic anion transporters

Field	Position	Original line	Normalised line	Original value	Normalised value
DE	1	107879	107014	ochratoxin A	Ochratoxin A (OTA)
DE	2	107879	107014	organic anion transporter	Organic Anion Transporter
DE	3	107879	107014	proximal tubule	Proximal Tubule
DE	4	107879	107014	transport	Transport
DE	5	107879	107014	cell line	Cell
ID	1	107881	107016	NEPHRON IN-VIVO	Nephron In Vivo
ID	2	107881	107016	MOLECULAR-CLONING	Molecular Cloning
ID	3	107881	107016	A NEPHROTOXICITY	A Nephrotoxicity
ID	4	107881	107016	PROXIMAL TUBULE	Proximal Tubule
ID	5	107881	107016	KIDNEY	Kidney
ID	6	107881	107016	REABSORPTION	Reabsorption
ID	7	107881	107016	EXPRESSION	Expression
ID	8	107881	107016	CELLS	Cell
ID	9	107881	107016	SECRETION	Secretion
ID	10	107881	107016	CITRININ	Citrinin

Record 774 | WOS:000170039000017

Species- and sex-specific renal cytotoxicity of Ochratoxin A and B in vitro

Field	Position	Original line	Normalised line	Original value	Normalised value
DE	1	107967	107101	ochratoxin	Ochratoxin A (OTA)
DE	2	107967	107101	cytotoxicity	Cytotoxicity
DE	3	107967	107101	in vitro	In-Vitro
ID	1	107968	107102	BALKAN ENDEMIC NEPHROPATHY	Balkan Endemic Nephropathy
ID	2	107968	107102	TOXICITY	Toxicity
ID	3	107968	107102	KIDNEY	Kidney
ID	4	107968	107102	GROWTH	Growth
ID	5	107968	107102	PIGS	Pig
ID	6	107968	107102	RAT	Rat

Record 775 | WOS:000169038800006

Aspartame prevents the karyomegaly induced by ochratoxin A in rat kidney

Field	Position	Original line	Normalised line	Original value	Normalised value
DE	1	108068	107201	ochratoxin A	Ochratoxin A (OTA)
DE	2	108068	107201	nephrotoxicity	Nephrotoxicity
DE	3	108068	107201	karyomegaly	Karyomegaly
DE	4	108068	107201	prevention	Prevention
DE	5	108068	107201	aspartame	Aspartame
ID	1	108069	107202	LIPID-PEROXIDATION	Lipid Peroxidation
ID	2	108069	107202	MICE	Mice
ID	3	108069	107202	GENOTOXICITY	Genotoxicity
ID	4	108069	107202	MECHANISM	Mechanism
ID	5	108069	107202	TUNISIA	Tunisia
ID	6	108069	107202	BINDING	Binding
ID	7	108069	107202	URINARY	Urinary
ID	8	108069	107202	TUMORS	Tumor
ID	9	108069	107202	CELLS	Cell

Record 776 | WOS:000167652300006

Age-related differences in the toxicity of ochratoxin A in female rats

Field	Position	Original line	Normalised line	Original value	Normalised value
DE	1	108167	107299	ochratoxin A	Ochratoxin A (OTA)
DE	2	108167	107299	ageing	Ageing
DE	3	108167	107299	rat	Rat
DE	4	108167	107299	toxicity	Toxicity
DE	5	108167	107299	immunotoxicity	Immunotoxicity
DE	6	108167	107299	nephrotoxicity	Nephrotoxicity
DE	7	108167	107299	neurotoxicity	Neurotoxicity
ID	1	108169	107301	IN-VIVO	In-Vivo
ID	2	108169	107301	MYCOTOXIN OCHRATOXIN	Ochratoxin A (OTA)
ID	3	108169	107301	PH HOMEOSTASIS	Ph Homeostasis
ID	4	108169	107301	CELLS	Cell
ID	5	108169	107301	MICE	Mice
ID	6	108169	107301	QUANTIFICATION	Quantification
ID	7	108169	107301	IMMUNOTOXICITY	Immunotoxicity
ID	8	108169	107301	NEUROTOXICITY	Neurotoxicity
ID	9	108169	107301	REABSORPTION	Reabsorption
ID	10	108169	107301	INDUCTION	Induction

Record 777 | WOS:000166500400005

Metabolism of ochratoxin A: Absence of formation of genotoxic derivatives by human and rat enzymes

Field	Position	Original line	Normalised line	Original value	Normalised value
ID	1	108279	107410	PROSTAGLANDIN-H SYNTHASE	Prostaglandin H Synthase

ID	2	108279	107410	DNA ADDUCT FORMATION	DNA Adduct Formation
ID	3	108279	107410	HUMAN CYTOCHROME-P450	Human Cytochrome P450
ID	4	108279	107410	LIPID-PEROXIDATION	Lipid Peroxidation
ID	5	108279	107410	STRAND BREAKS	Strand Break
ID	6	108279	107410	MICROSOMAL CYTOCHROME-P-450	Microsomal Cytochrome P 450
ID	7	108279	107410	RENAL CARCINOGEN	Renal Carcinogen
ID	8	108279	107410	LIVER-MICROSOMES	Liver Microsome
ID	9	108279	107410	SEX-DIFFERENCES	Sex Difference
ID	10	108279	107410	MUTAGENICITY	Mutagenicity

Record 778 | WOS:000165378500005

Effects of ochratoxin A on cytotoxicity and cell differentiation in cultured rat embryonic cells

Field	Position	Original line	Normalised line	Original value	Normalised value
ID	1	108408	107538	PEROXISOME PROLIFERATOR CIPROFIBRATE	Peroxisome Proliferator Ciprofibrate
ID	2	108408	107538	IN-VITRO	In-Vitro
ID	3	108408	107538	TOXICITY	Toxicity
ID	4	108408	107538	PATHOGENESIS	Pathogenesis
ID	5	108408	107538	MIDBRAIN	Midbrain
ID	6	108408	107538	SERA	Sera

Record 779 | WOS:000089269500007

Studies of ochratoxin A-induced inhibition of phenylalanine hydroxylase and its reversal by phenylalanine

Field	Position	Original line	Normalised line	Original value	Normalised value
DE	1	108497	107626	ochratoxin A	Ochratoxin A (OTA)
DE	2	108497	107626	phenylalanine hydroxylase	Phenylalanine Hydroxylase
DE	3	108497	107626	experimental	Experimental
DE	4	108497	107626	endemic nephropathy	Endemic Nephropathy
ID	1	108499	107628	MEMBRANE-BOUND ENZYMES	Membrane Bound Enzyme
ID	2	108499	107628	NEPHRON IN-VIVO	Nephron In Vivo
ID	3	108499	107628	INDUCED PHOSPHORYLATION	Induced Phosphorylation
ID	4	108499	107628	RAT HEPATOCYTES	Rat Hepatocyte
ID	5	108499	107628	MICE	Mice
ID	6	108499	107628	PREVENTION	Prevention
ID	7	108499	107628	ASPARTAME	Aspartame
ID	8	108499	107628	BINDING	Binding
ID	9	108499	107628	4-MONOOXYGENASE	4 Monooxygenase
ID	10	108499	107628	REABSORPTION	Reabsorption

Record 780 | WOS:000087816200003

In vitro DNA and dGMP adducts formation caused by ochratoxin A

Field	Position	Original line	Normalised line	Original value	Normalised value
DE	1	108593	107721	ochratoxin A	Ochratoxin A (OTA)
DE	2	108593	107721	mycotoxin	Mycotoxins
DE	3	108593	107721	DNA adducts	DNA Adduct
DE	4	108593	107721	guanine adducts	Guanine Adduct
DE	5	108593	107721	prostaglandin H synthases	Prostaglandin H Synthase
DE	6	108593	107721	lipoxygenases	Lipoxygenase
ID	1	108595	107723	BALKAN ENDEMIC NEPHROPATHY	Balkan Endemic Nephropathy
ID	2	108595	107723	URINARY-BLADDER	Urinary Bladder
ID	3	108595	107723	GENOTOXICITY	Genotoxicity

ID	4	108595	107723	INDUCTION	Induction
ID	5	108595	107723	KIDNEY	Kidney
ID	6	108595	107723	MOUSE	Mouse
ID	7	108595	107723	MYCOTOXIN	Mycotoxins
ID	8	108595	107723	TISSUES	Tissue
ID	9	108595	107723	TUMORS	Tumor
ID	10	108595	107723	CELLS	Cell

Record 781 | WOS:000087555100005

Ochratoxin A from a toxicological perspective

Field	Position	Original line	Normalised line	Original value	Normalised value
ID	1	108706	107833	ANIMAL FEEDING STUFFS	Animal Feeding Stuff
ID	2	108706	107833	BLADDER EPITHELIAL-CELLS	Bladder Epithelial
ID	3	108706	107833	NEPHRON IN-VIVO	Nephron In Vivo
ID	4	108706	107833	HUMAN BLOOD	Human Blood
ID	5	108706	107833	PH HOMEOSTASIS	Ph Homeostasis
ID	6	108706	107833	VASA-RECTA	Vasa Recta
ID	7	108706	107833	HUMAN SERA	Human Sera
ID	8	108706	107833	MYCOTOXINS	Mycotoxins
ID	9	108706	107833	CONTAMINATION	Contamination
ID	10	108706	107833	INGREDIENTS	Ingredient

Record 782 | WOS:000085306200013

Induction of unscheduled DNA synthesis in primary human urothelial cells by the mycotoxin ochratoxin A

Field	Position	Original line	Normalised line	Original value	Normalised value
DE	1	108854	107979	ochratoxin A	Ochratoxin A (OTA)
DE	2	108854	107979	DNA repair	DNA Repair
DE	3	108854	107979	human urothelium	Human Urothelium
DE	4	108854	107979	primary culture	Primary
ID	1	108855	107980	STIMULATED LIPID-PEROXIDATION	Stimulated Lipid Peroxidation
ID	2	108855	107980	BLADDER EPITHELIAL-CELLS	Bladder Epithelial
ID	3	108855	107980	URINARY-BLADDER	Urinary Bladder
ID	4	108855	107980	HUMAN-MILK	Human Milk
ID	5	108855	107980	GENOTOXICITY	Genotoxicity
ID	6	108855	107980	CULTURES	Culture
ID	7	108855	107980	RAT	Rat
ID	8	108855	107980	REPAIR	Repair
ID	9	108855	107980	MOUSE	Mouse
ID	10	108855	107980	CARCINOGENICITY	Carcinogenicity

Record 783 | WOS:000084146300015

Cholestyramine protection against ochratoxin A toxicity: Role of ochratoxin A sorption by the resin and bile enterohepatic circulation

Field	Position	Original line	Normalised line	Original value	Normalised value
ID	1	108965	108088	DIETARY CHOLESTYRAMINE	Dietary Cholestyramine
ID	2	108965	108088	MICE	Mice
ID	3	108965	108088	MYCOTOXIN	Mycotoxins
ID	4	108965	108088	NEPHROPATHY	Nephropathy
ID	5	108965	108088	BINDING	Binding
ID	6	108965	108088	RATS	Rat

Record 784 | WOS:000165381000007

Increased cytotoxicity of food-borne mycotoxins toward human cell lines in vitro via enhanced cytochrome p450 expression using the MTT bioassay

Field	Position	Original line	Normalised line	Original value	Normalised value
DE	1	109054	108176	cell culture	Cell
DE	2	109054	108176	food-borne mycotoxins	Food Borne Mycotoxin
DE	3	109054	108176	human cytochrome P450s	Human Cytochrome P450
DE	4	109054	108176	metabolic activity	Metabolic Activity
DE	5	109054	108176	MTT assay	Mtt Assay
DE	6	109054	108176	transformed human cell lines	Transformed Human Cell
ID	1	109056	108178	OCHRATOXIN-A	Ochratoxin A (OTA)
ID	2	109056	108178	METABOLISM	Metabolism
ID	3	109056	108178	ACTIVATION	Activation
ID	4	109056	108178	AFLATOXIN	Aflatoxin
ID	5	109056	108178	VIRUS	Virus

Record 785 | WOS:000083648200009

Genotoxicity of ochratoxin A by Salmonella mutagenicity test after bioactivation by mouse kidney microsomes

Field	Position	Original line	Normalised line	Original value	Normalised value
DE	1	109134	108255	ochratoxin A	Ochratoxin A (OTA)
DE	2	109134	108255	Salmonella mutagenicity microtest	Salmonella Mutagenicity Microtest
DE	3	109134	108255	kidney microsomes	Kidney Microsome
DE	4	109134	108255	arachidonic acid	Arachidonic Acid
ID	1	109136	108257	BALKAN ENDEMIC NEPHROPATHY	Balkan Endemic Nephropathy
ID	2	109136	108257	SINGLE-STRAND BREAKS	Single Strand Break
ID	3	109136	108257	BACTERIAL MUTAGENICITY	Bacterial Mutagenicity
ID	4	109136	108257	URINARY-BLADDER	Urinary Bladder
ID	5	109136	108257	CELL-LINES	Cell
ID	6	109136	108257	INDUCTION	Induction
ID	7	109136	108257	TYPHIMURIUM	Typhimurium
ID	8	109136	108257	MYCOTOXINS	Mycotoxins
ID	9	109136	108257	CHEMICALS	Chemical
ID	10	109136	108257	ASSAY	Assay

Record 786 | WOS:000082038700010

Ochratoxin binding to phenylalanyl-tRNA synthetase: Computational approach to the mechanism of ochratoxicosis and its antagonism

Field	Position	Original line	Normalised line	Original value	Normalised value
ID	1	109247	108366	TRANSFER-RNA-SYNTHETASE	Transfer RNA Synthetase
ID	2	109247	108366	SERUM-ALBUMIN	Serum Albumin
ID	3	109247	108366	SUBSTRATE-SPECIFICITY	Substrate Specificity
ID	4	109247	108366	MOLECULAR MECHANICS	Molecular Mechanics
ID	5	109247	108366	CRYSTAL-STRUCTURE	Crystal Structure
ID	6	109247	108366	ESCHERICHIA-COLI	Escherichia Coli
ID	7	109247	108366	FORCE-FIELD	Force Field
ID	8	109247	108366	IN-VITRO	In-Vitro
ID	9	109247	108366	PROTEIN	Protein
ID	10	109247	108366	SIMULATION	Simulation

Record 787 | WOS:000081907800002

Mutagenicity of commercial Monascus fermentation products and the role of citrinin contamination

Field	Position	Original line	Normalised line	Original value	Normalised value
DE	2	109342	108460	colorant	Colorant
DE	3	109342	108460	citrinin	Citrinin
DE	4	109342	108460	HPLC	Hplc

DE	5	109342	108460	mutagenicity	Mutagenicity
DE	6	109342	108460	S9-mix	S9 Mix
DE	7	109342	108460	hepatocyte	Hepatocyte
ID	1	109343	108461	PERFORMANCE LIQUID-CHROMATOGRAPHY	Performance Liquid Chromatography
ID	2	109343	108461	RED PIGMENTS	Red Pigment
ID	3	109343	108461	INDUCED DYSFUNCTION	Induced Dysfunction
ID	4	109343	108461	FUNGUS MONASCUS	Fungus Monascus
ID	5	109343	108461	OCHRATOXIN-A	Ochratoxin A (OTA)
ID	6	109343	108461	CULTURE	Culture
ID	7	109343	108461	MITOCHONDRIA	Mitochondria
ID	8	109343	108461	METABOLITES	Metabolite
ID	9	109343	108461	MECHANISM	Mechanism
ID	10	109343	108461	PURPUREUS	Purpureus

Record 788 | WOS:000081759900001

Ochratoxin A-binding proteins in rat organs and plasma and in different cell lines of the kidney

Field	Position	Original line	Normalised line	Original value	Normalised value
DE	1	109437	108554	ochratoxin A	Ochratoxin A (OTA)
DE	2	109437	108554	kidney	Kidney
DE	3	109437	108554	protein binding	Protein Binding
DE	4	109437	108554	renal cell lines	Renal Cell
DE	5	109437	108554	aspartame	Aspartame
ID	1	109438	108555	NEPHRON IN-VIVO	Nephron In Vivo
ID	2	109438	108555	EPITHELIAL-CELLS	Epithelial
ID	3	109438	108555	PROXIMAL TUBULE	Proximal Tubule
ID	4	109438	108555	PHENYLALANINE	Phenylalanine
ID	5	109438	108555	REABSORPTION	Reabsorption
ID	6	109438	108555	INHIBITION	Inhibition
ID	7	109438	108555	REDUCTION	Reduction
ID	8	109438	108555	CULTURES	Culture
ID	9	109438	108555	SUBTYPES	Subtype
ID	10	109438	108555	VITRO	Vitro

Record 789 | WOS:000080471300017

Transport of ochratoxin A by renal multispecific organic anion transporter 1

Field	Position	Original line	Normalised line	Original value	Normalised value
ID	1	109543	108659	EXPRESSION CLONING	Expression Cloning
ID	2	109543	108659	A NEPHROTOXICITY	A Nephrotoxicity
ID	3	109543	108659	IN-VIVO	In-Vivo
ID	4	109543	108659	REABSORPTION	Reabsorption
ID	5	109543	108659	NEPHROPATHY	Nephropathy
ID	6	109543	108659	CITRININ	Citrinin
ID	7	109543	108659	KIDNEY	Kidney
ID	8	109543	108659	CELLS	Cell

Record 790 | WOS:000080762800002

Roles of cyclooxygenase and lipoxygenases in ochratoxin A genotoxicity in human epithelial lung cells

Field	Position	Original line	Normalised line	Original value	Normalised value
DE	1	109623	108738	prostaglandin-synthase	Prostaglandin Synthase
DE	2	109623	108738	lipoxygenase	Lipoxygenase
DE	3	109623	108738	ochratoxin A	Ochratoxin A (OTA)
DE	4	109623	108738	genotoxicity	Genotoxicity
DE	5	109623	108738	ochratoxin A metabolites	Ochratoxin A Metabolite
DE	6	109623	108738	DNA adduct	DNA Adduct
DE	7	109623	108738	pig seminal vesicle	Pig Seminal Vesicle
DE	8	109623	108738	bronchial epithelial cells	Bronchial Epithelial

ID	1	109626	108741	PROSTAGLANDIN-H SYNTHASE	Prostaglandin H Synthase
ID	2	109626	108741	BALKAN ENDEMIC NEPHROPATHY	Balkan Endemic Nephropathy
ID	3	109626	108741	DNA ADDUCT FORMATION	DNA Adduct Formation
ID	4	109626	108741	LIPID-PEROXIDATION	Lipid Peroxidation
ID	5	109626	108741	ARACHIDONIC-ACID	Arachidonic Acid
ID	6	109626	108741	CYTOCHROME-P450	Cytochrome P450
ID	7	109626	108741	INDUCTION	Induction
ID	8	109626	108741	METABOLITES	Metabolite
ID	9	109626	108741	TISSUES	Tissue
ID	10	109626	108741	RATS	Rat

Record 791 | WOS:000080209100001

Inactivation of ochratoxin in ruminal fluid with variation of pH-value and fermentation parameters in an in vitro system

Field	Position	Original line	Normalised line	Original value	Normalised value
DE	1	109743	108857	dairy cows	Dairy Cow
DE	2	109743	108857	ochratoxin A	Ochratoxin A (OTA)
DE	3	109743	108857	ochratoxin alpha	Ochratoxin Alpha
DE	4	109743	108857	pH-value	Ph Value
DE	5	109743	108857	rumen	Rumen
ID	1	109744	108858	RUMEN PROTOZOA	Rumen Protozoa
ID	2	109744	108858	HPLC	Hplc

Record 792 | WOS:000083320300013

Cytotoxic and genotoxic effects of zearalenone:: Prevention by vitamin E

Field	Position	Original line	Normalised line	Original value	Normalised value
ID	1	109823	108936	DNA ADDUCT FORMATION	DNA Adduct Formation
ID	2	109823	108936	NATURAL OCCURRENCE	Natural Occurrence
ID	3	109823	108936	MOUSE-TISSUES	Mouse Tissue
ID	4	109823	108936	OCHRATOXIN-A	Ochratoxin A (OTA)
ID	5	109823	108936	CELLS	Cell
ID	6	109823	108936	MYCOTOXINS	Mycotoxins
ID	7	109823	108936	REPRODUCTION	Reproduction
ID	8	109823	108936	NIVALENOL	Nivalenol
ID	9	109823	108936	TOXIN	Toxin
ID	10	109823	108936	ACID	Acid

Record 793 | WOS:000087386900028

Biotransformation and lack of mutagenicity of ochratoxin A using combinations of mammalian biotransformation enzymes

Field	Position	Original line	Normalised line	Original value	Normalised value
ID	1	109909	109021	RISK ASSESSMENT	Risk Assessment
ID	2	109909	109021	I-COMPOUNDS	I Compound
ID	3	109909	109021	RATS	Rat
ID	4	109909	109021	NEPHROTOXICITY	Nephrotoxicity
ID	5	109909	109021	INDUCTION	Induction
ID	6	109909	109021	TOXICITY	Toxicity
ID	7	109909	109021	EXPOSURE	Exposure
ID	8	109909	109021	CARCINOGENICITY	Carcinogenicity
ID	9	109909	109021	HEPATOCYTES	Hepatocyte
ID	10	109909	109021	MECHANISMS	Mechanism

Record 794 | WOS:000078111000012

Apical uptake of radiolabelled ochratoxin A into Madin-Darby canine kidney cells

Field	Position	Original line	Normalised line	Original value	Normalised value
DE	1	109988	109099	ochratoxin A	Ochratoxin A (OTA)
DE	2	109988	109099	MDCK cells	MDCK
DE	3	109988	109099	collecting duct	Collecting Duct
DE	4	109988	109099	kidney	Kidney
DE	5	109988	109099	dipeptide transport	Dipeptide Transport
DE	6	109988	109099	aspartame	Aspartame
DE	7	109988	109099	organic anion transport	Organic Anion Transport
DE	8	109988	109099	BCECF	Bcecf
DE	9	109988	109099	phorbol ester	Phorbol Ester
DE	10	109988	109099	EIPA	Eipa
ID	1	109990	109101	BRUSH-BORDER MEMBRANE	Brush Border Membrane
ID	2	109990	109101	BETA-LACTAM ANTIBIOTICS	Beta Lactam Antibiotics
ID	3	109990	109101	PROTEIN-KINASE-C	Protein Kinase C
ID	4	109990	109101	PROXIMAL TUBULE	Proximal Tubule
ID	5	109990	109101	IN-VIVO	In-Vivo
ID	6	109990	109101	EXPRESSION CLONING	Expression Cloning
ID	7	109990	109101	FUNCTIONAL-CHARACTERIZATION	Functional Characterization
ID	8	109990	109101	PEPTIDE TRANSPORTERS	Peptide Transporter
ID	9	109990	109101	PH GRADIENT	Ph Gradient
ID	10	109990	109101	RAT-KIDNEY	Rat Kidney

Record 795 | WOS:000077419100015

Apoptosis in the kidney

Field	Position	Original line	Normalised line	Original value	Normalised value
DE	1	110091	109200	review	Review
DE	2	110091	109200	apoptosis	Apoptosis
DE	3	110091	109200	kidney	Kidney
DE	4	110091	109200	epithelial cells	Epithelial
DE	5	110091	109200	cell death	Cell Death
ID	1	110092	109201	EPIDERMAL GROWTH-FACTOR	Epidermal Growth Factor
ID	2	110092	109201	TUMOR-NECROSIS-FACTOR	Tumor Necrosis Factor
ID	3	110092	109201	TUBULAR EPITHELIAL-CELLS	Tubular Epithelial
ID	4	110092	109201	RENAL-ALLOGRAFT REJECTION	Renal Allograft Rejection
ID	5	110092	109201	IN-VIVO PATTERNS	In Vivo Pattern
ID	6	110092	109201	GRANZYME-B	Granzyme B
ID	7	110092	109201	URETERAL OBSTRUCTION	Ureteral Obstruction
ID	8	110092	109201	LUPUS NEPHRITIS	Lupus Nephritis
ID	9	110092	109201	OCHRATOXIN-A	Ochratoxin A (OTA)
ID	10	110092	109201	RAT-KIDNEY	Rat Kidney

Record 796 | WOS:000076860300002

Measurement of antibacterial activities of T-2 toxin, deoxynivalenol, ochratoxin A, aflatoxin B1 and fumonisin B1 using microtitration tray-based turbidimetric techniques

Field	Position	Original line	Normalised line	Original value	Normalised value
ID	1	110312	109419	EXPOSURE	Exposure
ID	2	110312	109419	RESISTANCE	Resistance

Record 797 | WOS:000076443800002

Cytotoxicity of nephrotoxic fungal toxins to kidney-derived LLC-PK1 and OK cell lines

Field	Position	Original line	Normalised line	Original value	Normalised value
DE	1	110369	109475	citrinin	Citrinin
DE	2	110369	109475	cytotoxicity	Cytotoxicity
DE	3	110369	109475	kidney cell lines	Kidney Cell
DE	4	110369	109475	ochratoxins	Ochratoxin A (OTA)
ID	1	110370	109476	CITRININ-INDUCED DYSFUNCTION	Citrinin Induced Dysfunction
ID	2	110370	109476	OCHRATOXIN-A	Ochratoxin A (OTA)
ID	3	110370	109476	ENZYME-ACTIVITIES	Enzyme Activity
ID	4	110370	109476	TISSUE-CULTURE	Tissue
ID	5	110370	109476	CYTO-TOXICITY	Cyto Toxicity
ID	6	110370	109476	IN-VITRO	In-Vitro
ID	7	110370	109476	MYCOTOXINS	Mycotoxins
ID	8	110370	109476	PROLIFERATION	Proliferation
ID	9	110370	109476	HOECHST-33258	Hoechst 33258
ID	10	110370	109476	BIOSYNTHESIS	Biosynthesis

Record 798 | WOS:000076413600003

Exposure to ochratoxin A impairs organic anion transport in proximal-tubule-derived opossum kidney cells

Field	Position	Original line	Normalised line	Original value	Normalised value
ID	1	110456	109561	PROTEIN-SYNTHESIS	Protein Synthesis
ID	2	110456	109561	IN-VIVO	In-Vivo
ID	3	110456	109561	OK	Ok
ID	4	110456	109561	NEPHROPATHY	Nephropathy
ID	5	110456	109561	LINE	Line
ID	6	110456	109561	SYNTHETASE	Synthetase
ID	7	110456	109561	INHIBITION	Inhibition
ID	8	110456	109561	REDUCTION	Reduction
ID	9	110456	109561	CULTURE	Culture
ID	10	110456	109561	BINDING	Binding

Record 799 | WOS:000074665700021

Detailed mapping of ochratoxin a reabsorption along the rat nephron in vivo: The nephrotoxin can be reabsorbed in all nephron segments by different mechanisms

Field	Position	Original line	Normalised line	Original value	Normalised value
ID	1	110549	109653	FUNCTIONAL-CHARACTERIZATION	Functional Characterization
ID	2	110549	109653	PROXIMAL TUBULE	Proximal Tubule
ID	3	110549	109653	IN-VIVO	In-Vivo
ID	4	110549	109653	KIDNEY	Kidney

Record 800 | WOS:000075078200010

Haematotoxicity of mycotoxins

Field	Position	Original line	Normalised line	Original value	Normalised value
DE	1	110628	109731	haematotoxicity	Haematotoxicity
DE	2	110628	109731	mycotoxin	Mycotoxins
DE	3	110628	109731	review	Review
DE	4	110628	109731	myelotoxicity	Myelotoxicity
DE	5	110628	109731	hematologic disorders	Hematologic Disorder
DE	6	110628	109731	hematopoiesis	Hematopoiesis
ID	1	110630	109733	GRANULO-MONOCYTIC PROGENITORS	Granulo Monocytic Progenitor
ID	2	110630	109733	BONE-MARROW CELLS	Bone Marrow
ID	3	110630	109733	IN-VITRO MODEL	In Vitro Model
ID	4	110630	109733	T-2 TOXIN	T 2 Toxin
ID	5	110630	109733	HEMATOPOIETIC PROGENITORS	Hematopoietic Progenitor

ID	6	110630	109733	BIOCHEMICAL PARAMETERS	Biochemical Parameter
ID	7	110630	109733	COAGULATION DEFECTS	Coagulation Defect
ID	8	110630	109733	BLOOD-COAGULATION	Blood Coagulation
ID	9	110630	109733	ACUTE TOXICITY	Acute Toxicity
ID	10	110630	109733	OCHRATOXIN-A	Ochratoxin A (OTA)

Record 801 | WOS:000073042900016

Activated carbons: In vitro affinity for ochratoxin A and deoxynivalenol and relation of adsorption ability to physicochemical parameters

Field	Position	Original line	Normalised line	Original value	Normalised value
ID	1	110767	109869	PERFORMANCE LIQUID-CHROMATOGRAPHY	Performance Liquid Chromatography
ID	2	110767	109869	SODIUM-CALCIUM ALUMINOSILICATE	Sodium Calcium Aluminosilicate
ID	3	110767	109869	AFLATOXIN	Aflatoxin
ID	4	110767	109869	EFFICACY	Efficacy
ID	5	110767	109869	TOXICITY	Toxicity
ID	6	110767	109869	MILK	Milk

Record 802 | WOS:000072874100001

Ochratoxin A in food and feed: occurrence, legislation and mode of action

Field	Position	Original line	Normalised line	Original value	Normalised value
DE	1	110838	109939	Ochratoxin A	Ochratoxin A (OTA)
DE	2	110838	109939	mycotoxins	Mycotoxins
DE	3	110838	109939	occurrence	Occurrence
DE	4	110838	109939	legislation	Legislation
DE	5	110838	109939	mode of action	Mode Of Action
ID	1	110839	109940	STIMULATED LIPID-PEROXIDATION	Stimulated Lipid Peroxidation
ID	2	110839	109940	BALKAN ENDEMIC NEPHROPATHY	Balkan Endemic Nephropathy
ID	3	110839	109940	HUMAN-MILK	Human Milk
ID	4	110839	109940	TOXICITY	Toxicity
ID	5	110839	109940	ANALOGS	Analog
ID	6	110839	109940	CARCINOGENESIS	Carcinogenesis
ID	7	110839	109940	FOODSTUFFS	Foodstuff
ID	8	110839	109940	MECHANISM	Mechanism
ID	9	110839	109940	ENZYMES	Enzyme
ID	10	110839	109940	BLOOD	Blood

Record 803 | WOS:000079327200007

The effects of four mycotoxins on the mitogen stimulated proliferation of bovine peripheral blood mononuclear cells in vitro

Field	Position	Original line	Normalised line	Original value	Normalised value
DE	1	110955	110055	bovine peripheral blood mononuclear cells	Bovine Peripheral Blood Mononuclear
DE	2	110955	110055	cytotoxicity	Cytotoxicity
DE	3	110955	110055	deoxynivalenol	Deoxynivalenol (DON)
DE	4	110955	110055	fumonisin B-1	Fumonisin B1 (FB1)
DE	5	110955	110055	H-3-thymidine uptake	H 3 Thymidine Uptake
DE	6	110955	110055	MTT and LDH bioassays	Mtt And Ldh Bioassay
DE	7	110955	110055	ochratoxin A	Ochratoxin A (OTA)
DE	8	110955	110055	Trypan blue exclusion	Trypan Blue Exclusion
DE	9	110955	110055	T-2 toxin	T 2 Toxin
ID	1	110958	110058	RAPID COLORIMETRIC BIOASSAY	Rapid Colorimetric Bioassay
ID	2	110958	110058	BROILER CHICKS	Broiler Chick

ID	3	110958	110058	OCHRATOXIN-A	Ochratoxin A (OTA)
ID	4	110958	110058	T-2 TOXIN	T 2 Toxin
ID	5	110958	110058	VOMITOXIN DEOXYNIVALENOL	Vomitoxin Deoxynivalenol
ID	6	110958	110058	DIETARY EXPOSURE	Dietary Exposure
ID	7	110958	110058	IMMUNE-RESPONSE	Immune Response
ID	8	110958	110058	FUMONISIN B-1	Fumonisin B1 (FB1)
ID	9	110958	110058	B6C3F1 MOUSE	B6c3f1 Mouse
ID	10	110958	110058	MICE	Mice

Record 804 | WOS:000081940900027

Mycotoxins: Mechanisms of action at the molecular level

Field	Position	Original line	Normalised line	Original value	Normalised value
DE	1	111064	110163	aflatoxin	Aflatoxin
DE	2	111064	110163	ochratoxin	Ochratoxin A (OTA)
DE	3	111064	110163	fumonisin	Fumonisin
DE	4	111064	110163	genotoxicity	Genotoxicity
DE	5	111064	110163	carcinogenicity	Carcinogenicity
DE	6	111064	110163	biotransformation	Biotransformation
ID	1	111066	110165	HUMAN HEPATOCELLULAR CARCINOMAS	Human Hepatocellular Carcinoma
ID	2	111066	110165	NOVO SPHINGOLIPID BIOSYNTHESIS	Novo Sphingolipid Biosynthesis
ID	3	111066	110165	BALKAN ENDEMIC NEPHROPATHY	Balkan Endemic Nephropathy
ID	4	111066	110165	TUMOR-SUPPRESSOR GENE	Tumor Suppressor Gene
ID	5	111066	110165	DNA ADDUCT FORMATION	DNA Adduct Formation
ID	6	111066	110165	OCHRATOXIN-A	Ochratoxin A (OTA)
ID	7	111066	110165	P53 GENE	P53 Gene
ID	8	111066	110165	AFLATOXIN B-1	Aflatoxin B1 (AFB1)
ID	9	111066	110165	FUMONISIN B-1	Fumonisin B1 (FB1)
ID	10	111066	110165	FUSARIUM-MONILIFORME	Fusarium Moniliforme

Record 805 | WOS:000075950800023

Tubulotoxic mechanisms of ochratoxin a

Field	Position	Original line	Normalised line	Original value	Normalised value
DE	1	111188	110286	ochratoxin A	Ochratoxin A (OTA)
DE	2	111188	110286	tubulotoxicity	Tubulotoxicity
DE	3	111188	110286	mycotoxin	Mycotoxins
DE	4	111188	110286	toxokinetics	Toxokinetics
DE	5	111188	110286	toxodynamics	Toxodynamics
ID	1	111189	110287	CANINE KIDNEY-CELLS	Canine Kidney
ID	2	111189	110287	IN-VIVO	In-Vivo
ID	3	111189	110287	PROXIMAL TUBULE	Proximal Tubule
ID	4	111189	110287	PH HOMEOSTASIS	Ph Homeostasis
ID	5	111189	110287	HUMAN BLOOD	Human Blood
ID	6	111189	110287	VASA-RECTA	Vasa Recta
ID	7	111189	110287	NEPHROPATHY	Nephropathy
ID	8	111189	110287	CONDUCTANCE	Conductance
ID	9	111189	110287	FOODSTUFFS	Foodstuff
ID	10	111189	110287	INCREASES	Increase

Record 806 | WOS:000080521300009

Ochratoxins: Molecular strategies for developing an antidote

Field	Position	Original line	Normalised line	Original value	Normalised value

DE	1	111268	110365	receptor-mediated toxicity	Receptor Mediated Toxicity
DE	2	111268	110365	molecular-dynamical simulations	Molecular Dynamical Simulation
DE	3	111268	110365	mechanisms of ochratoxin toxicity	Mechanism Of Ochratoxin Toxicity
DE	4	111268	110365	phenylalanine-t-RNA synthetase	Phenylalanine T RNA Synthetase
DE	5	111268	110365	serum albumin	Serum Albumin
ID	1	111270	110367	TRANSFER-RNA-SYNTHETASE	Transfer RNA Synthetase
ID	2	111270	110367	PROTEINS	Protein
ID	3	111270	110367	ALBUMIN	Albumin
ID	4	111270	110367	CELLS	Cell
ID	5	111270	110367	RATS	Rat

Record 807 ∣ WOS:000071510600005

Acute toxicity of the mycotoxin roridin E on liver and kidney of rats

Field	Position	Original line	Normalised line	Original value	Normalised value
DE	1	111335	110431	mycotoxin roridin E	Mycotoxin Roridin E
DE	2	111335	110431	trichothecenes	Trichothecene
DE	3	111335	110431	linoleic acid	Linoleic Acid
DE	4	111335	110431	free radicals	Free Radical
DE	5	111335	110431	antioxidants	Antioxidant
DE	6	111335	110431	acute toxicity	Acute Toxicity
ID	1	111337	110433	OCHRATOXIN-A	Ochratoxin A (OTA)

Record 808 ∣ WOS:A1997YD92300001

The neurotoxic effects of ochratoxin-A are reduced by protein binding but are not affected by I-phenylalanine

Field	Position	Original line	Normalised line	Original value	Normalised value
ID	1	111402	110497	EMBRYONIC CHICK BRAIN	Embryonic Chick Brain
ID	2	111402	110497	CELL-CULTURES	Cell
ID	3	111402	110497	TOXICITY	Toxicity
ID	4	111402	110497	INVITRO	Invitro
ID	5	111402	110497	MICE	Mice
ID	6	111402	110497	FETAL	Fetal
ID	7	111402	110497	RATS	Rat

Record 809 ∣ WOS:A1997XM61400017

Effects of inorganic adsorbents and cyclopiazonic acid in broiler chickens

Field	Position	Original line	Normalised line	Original value	Normalised value
DE	1	111495	110588	mycotoxin	Mycotoxins
DE	2	111495	110588	cyclopiazonic acid	Cyclopiazonic Acid
DE	3	111495	110588	toxicity	Toxicity
DE	4	111495	110588	inorganic adsorbents	Inorganic Adsorbent
DE	5	111495	110588	chicken	Chicken
ID	1	111496	110589	SODIUM-CALCIUM ALUMINOSILICATE	Sodium Calcium Aluminosilicate
ID	2	111496	110589	ASPERGILLUS-FLAVUS	Aspergillus Flavus
ID	3	111496	110589	GROWING BARROWS	Growing Barrow
ID	4	111496	110589	OCHRATOXIN-A	Ochratoxin A (OTA)
ID	5	111496	110589	AFLATOXIN	Aflatoxin
ID	6	111496	110589	TOXICITY	Toxicity
ID	7	111496	110589	RATS	Rat
ID	8	111496	110589	MYCOTOXIN	Mycotoxins
ID	9	111496	110589	DIETS	Diet
ID	10	111496	110589	SWINE	Swine

Record 810 | WOS:A1997XN68000014

Lipid peroxidation as one mode of action in ochratoxin A toxicity in rats and chicks

Field	Position	Original line	Normalised line	Original value	Normalised value
DE	1	111601	110693	ochratoxin A	Ochratoxin A (OTA)
DE	2	111601	110693	lipid peroxidation	Lipid Peroxidation
DE	3	111601	110693	malondialdehyde	Malondialdehyde
DE	4	111601	110693	rat	Rat
DE	5	111601	110693	chick	Chick
ID	1	111602	110694	ANALOGS	Analog
ID	2	111602	110694	LIVER	Liver

Record 811 | WOS:A1997XA91600004

Induction of micronuclei with ochratoxin A in ovine seminal vesicle cell cultures

Field	Position	Original line	Normalised line	Original value	Normalised value
DE	1	111673	110764	ochratoxin A	Ochratoxin A (OTA)
DE	2	111673	110764	genotoxicity	Genotoxicity
DE	3	111673	110764	micronuclei	Micronuclei
DE	4	111673	110764	ovine seminal vesicle cells	Ovine Seminal Vesicle
DE	5	111673	110764	clastogen	Clastogen
ID	1	111675	110766	ANEUPLOIDY-INDUCING AGENTS	Aneuploidy Inducing Agent
ID	2	111675	110766	BALKAN ENDEMIC NEPHROPATHY	Balkan Endemic Nephropathy
ID	3	111675	110766	PROSTAGLANDIN-H SYNTHASE	Prostaglandin H Synthase
ID	4	111675	110766	SYRIAN-HAMSTER EMBRYO	Syrian Hamster Embryo
ID	5	111675	110766	SINGLE-STRAND BREAKS	Single Strand Break
ID	6	111675	110766	IN-VITRO	In-Vitro
ID	7	111675	110766	MICE	Mice
ID	8	111675	110766	GENOTOXICITY	Genotoxicity
ID	9	111675	110766	METABOLISM	Metabolism
ID	10	111675	110766	MOUSE	Mouse

Record 812 | WOS:A1997WT39200011

Reduction of ochratoxin A toxicity by heat-induced epimerization. In vitro effects of ochratoxins on embryonic chick meningeal and other cell cultures

Field	Position	Original line	Normalised line	Original value	Normalised value
DE	1	111796	110886	ochratoxins	Ochratoxin A (OTA)
DE	2	111796	110886	ochratoxin-A epimer	Ochratoxin A Epimer
DE	3	111796	110886	culture	Culture
DE	4	111796	110886	brain	Brain
DE	5	111796	110886	retina	Retina
DE	6	111796	110886	chick	Chick
DE	7	111796	110886	fibroblast	Fibroblast
ID	1	111798	110888	BRAIN	Brain

Record 813 | WOS:A1997WW09700003

Reduction of the ochratoxin A-induced cytotoxicity in Vero cells by aspartame

Field	Position	Original line	Normalised line	Original value	Normalised value
DE	1	111868	110957	ochratoxin A	Ochratoxin A (OTA)
DE	2	111868	110957	aspartame (A(19))	Aspartame A 19
DE	3	111868	110957	Vero cells	Vero
DE	4	111868	110957	cytotoxicity	Cytotoxicity
DE	5	111868	110957	binding to plasma proteins	Binding To Plasma Protein

ID	1	111870	110959	STIMULATED LIPID-PEROXIDATION	Stimulated Lipid Peroxidation
ID	2	111870	110959	ENDEMIC NEPHROPATHY	Endemic Nephropathy
ID	3	111870	110959	PROTEIN-SYNTHESIS	Protein Synthesis
ID	4	111870	110959	RATS	Rat
ID	5	111870	110959	MICE	Mice
ID	6	111870	110959	PHENYLALANINE	Phenylalanine
ID	7	111870	110959	IMMUNOSUPPRESSION	Immunosuppression
ID	8	111870	110959	METABOLITES	Metabolite
ID	9	111870	110959	INHIBITION	Inhibition
ID	10	111870	110959	PREVENTION	Prevention

Record 814 | WOS:A1997XZ55900019

Effects of the naturally occurring food mycotoxin ochratoxin A on brain cells in culture

Field	Position	Original line	Normalised line	Original value	Normalised value
DE	1	111969	111056	ochratoxin A	Ochratoxin A (OTA)
DE	2	111969	111056	brain cell	Brain
DE	3	111969	111056	differentiation	Differentiation
DE	4	111969	111056	reactive oxygen species	Reactive Oxygen Specy
DE	5	111969	111056	protein synthesis	Protein Synthesis
ID	1	111971	111058	RAT-BRAIN	Rat Brain
ID	2	111971	111058	AGGREGATE CULTURES	Aggregate
ID	3	111971	111058	LIPID-PEROXIDATION	Lipid Peroxidation
ID	4	111971	111058	TRIMETHYLTIN TMT	Trimethyltin Tmt
ID	5	111971	111058	KIDNEY-CELLS	Kidney
ID	6	111971	111058	MICROGLIA	Microglia
ID	7	111971	111058	TOXICITY	Toxicity
ID	8	111971	111058	MICE	Mice
ID	9	111971	111058	DIFFERENTIATION	Differentiation
ID	10	111971	111058	NEUROTOXICITY	Neurotoxicity

Record 815 | WOS:A1997WC04100026

Reabsorption of the nephrotoxin ochratoxin A along the rat nephron in vivo

Field	Position	Original line	Normalised line	Original value	Normalised value
ID	1	112070	111156	PROXIMAL TUBULE	Proximal Tubule
ID	2	112070	111156	KIDNEY	Kidney
ID	3	112070	111156	INVIVO	Invivo

Record 816 | WOS:A1996WB42500013

Influence of vitamins E and C on the toxic effects of ochratoxin A and T-2 toxin in chicks

Field	Position	Original line	Normalised line	Original value	Normalised value
DE	1	112136	111221	ochratoxin A	Ochratoxin A (OTA)
DE	2	112136	111221	T-2 toxin	T 2 Toxin
DE	3	112136	111221	lipid peroxidation	Lipid Peroxidation
DE	4	112136	111221	antioxidants	Antioxidant
DE	5	112136	111221	malondialdehyde	Malondialdehyde
ID	1	112138	111223	STIMULATED LIPID-PEROXIDATION	Stimulated Lipid Peroxidation
ID	2	112138	111223	PERFORMANCE	Performance
ID	3	112138	111223	MALONDIALDEHYDE	Malondialdehyde
ID	4	112138	111223	AFLATOXIN	Aflatoxin
ID	5	112138	111223	ANALOGS	Analog
ID	6	112138	111223	LIVER	Liver
ID	7	112138	111223	HENS	Hen

Record 817 | WOS:A1996UZ36400005

Effects of mycotoxins on cytokine production and proliferation in EL-4 thymoma cells

Field	Position	Original line	Normalised line	Original value	Normalised value
ID	1	112218	111302	OCHRATOXIN-A	Ochratoxin A (OTA)
ID	2	112218	111302	VOMITOXIN DEOXYNIVALENOL	Vomitoxin Deoxynivalenol
ID	3	112218	111302	INTERLEUKIN-2 PRODUCTION	Interleukin 2 Production
ID	4	112218	111302	PROTEIN-SYNTHESIS	Protein Synthesis
ID	5	112218	111302	T-2 TOXIN	T 2 Toxin
ID	6	112218	111302	CYCLOPIAZONIC ACID	Cyclopiazonic Acid
ID	7	112218	111302	IMMUNE-RESPONSE	Immune Response
ID	8	112218	111302	IL-6 SECRETION	Il 6 Secretion
ID	9	112218	111302	T-CELLS	T
ID	10	112218	111302	PATULIN	Patulin

Record 818 | WOS:A1996UT94000012

Prevention of toxic effects of mycotoxins by means of nonnutritive adsorbent compounds

Field	Position	Original line	Normalised line	Original value	Normalised value
DE	1	112326	111409	mycotoxins	Mycotoxins
DE	2	112326	111409	aflatoxins	Aflatoxin
DE	3	112326	111409	detoxification	Detoxification
DE	4	112326	111409	adsorption	Adsorption
DE	5	112326	111409	silicates	Silicate
ID	1	112327	111410	SODIUM-CALCIUM ALUMINOSILICATE	Sodium Calcium Aluminosilicate
ID	2	112327	111410	AFLATOXIN-CONTAMINATED DIETS	Aflatoxin Contaminated Diet
ID	3	112327	111410	ACTIVATED-CHARCOAL	Activated Charcoal
ID	4	112327	111410	T-2 TOXICOSIS	T 2 Toxicosis
ID	5	112327	111410	GROWING-PIGS	Growing Pig
ID	6	112327	111410	BROILER-CHICKENS	Broiler Chicken
ID	7	112327	111410	OCHRATOXIN-A	Ochratoxin A (OTA)
ID	8	112327	111410	EFFICACY	Efficacy
ID	9	112327	111410	ZEOLITE	Zeolite
ID	10	112327	111410	BENTONITE	Bentonite

Record 819 | WOS:A1996VJ28200003

Aspartame as a preventive agent of chronic toxic effects of ochratoxin A in experimental animals

Field	Position	Original line	Normalised line	Original value	Normalised value
ID	1	112496	111578	PROTEIN-SYNTHESIS	Protein Synthesis
ID	2	112496	111578	HEPATOMA-CELLS	Hepatoma
ID	3	112496	111578	MICE	Mice
ID	4	112496	111578	PHENYLALANINE	Phenylalanine
ID	5	112496	111578	RATS	Rat
ID	6	112496	111578	GENOTOXICITY	Genotoxicity
ID	7	112496	111578	LIVER	Liver
ID	8	112496	111578	IMMUNOSUPPRESSION	Immunosuppression
ID	9	112496	111578	METABOLITES	Metabolite
ID	10	112496	111578	SYNTHETASE	Synthetase

Record 820 | WOS:A1996VW20400002

Renal toxicodynamics of ochratoxin A: A pathophysiological approach

Field	Position	Original line	Normalised line	Original value	Normalised value
DE	1	112604	111685	ochratoxin A	Ochratoxin A (OTA)
DE	2	112604	111685	nephrotoxicity	Nephrotoxicity
DE	3	112604	111685	kidney pathophysiology	Kidney Pathophysiology

DE	4	112604	111685	hemodynamics	Hemodynamics
DE	5	112604	111685	proximal tubule	Proximal Tubule
ID	1	112606	111687	CANINE KIDNEY-CELLS	Canine Kidney
ID	2	112606	111687	BALKAN ENDEMIC NEPHROPATHY	Balkan Endemic Nephropathy
ID	3	112606	111687	ANGIOTENSIN-II	Angiotensin Ii
ID	4	112606	111687	PROXIMAL TUBULE	Proximal Tubule
ID	5	112606	111687	IN-VIVO	In-Vivo
ID	6	112606	111687	RAT	Rat
ID	7	112606	111687	PROTEIN	Protein
ID	8	112606	111687	PH	Ph
ID	9	112606	111687	OSCILLATIONS	Oscillation
ID	10	112606	111687	INHIBITION	Inhibition

Record 821 | WOS:A1995TU51400124

Prevention of nephrotoxicity of ochratoxin A, a food contaminant

Field	Position	Original line	Normalised line	Original value	Normalised value
DE	1	112729	111809	ochratoxin A	Ochratoxin A (OTA)
DE	2	112729	111809	aspartame	Aspartame
DE	3	112729	111809	nephrotoxicity	Nephrotoxicity
DE	4	112729	111809	genotoxicity	Genotoxicity
DE	5	112729	111809	phenylalanine	Phenylalanine
ID	1	112730	111810	PROTEIN-SYNTHESIS	Protein Synthesis
ID	2	112730	111810	PIG PERFORMANCE	Pig Performance
ID	3	112730	111810	HEPATOMA-CELLS	Hepatoma
ID	4	112730	111810	LIVER	Liver
ID	5	112730	111810	PHENYLALANINE	Phenylalanine
ID	6	112730	111810	INHIBITION	Inhibition
ID	8	112730	111810	MICE	Mice
ID	9	112730	111810	METABOLITES	Metabolite
ID	10	112730	111810	SYNTHETASE	Synthetase

Record 822 | WOS:A1995TN07400005

Activation markers and cell proliferation as indicators of toxicity: A flow cytometric approach

Field	Position	Original line	Normalised line	Original value	Normalised value
DE	1	112829	111908	activation markers	Activation Marker
DE	2	112829	111908	cyclosporin A	Cyclosporin A
DE	3	112829	111908	flow cytometry	Flow Cytometry
DE	4	112829	111908	human lymphocytes	Human Lymphocyte
DE	5	112829	111908	in vitro cytotoxicity	In Vitro Cytotoxicity
DE	6	112829	111908	ochratoxin A	Ochratoxin A (OTA)
ID	1	112831	111910	INVIVO BROMODEOXYURIDINE	Invivo Bromodeoxyuridine
ID	2	112831	111910	MERCURIC COMPOUNDS	Mercuric Compound
ID	3	112831	111910	HUMAN-LYMPHOCYTES	Human Lymphocyte
ID	4	112831	111910	MESSENGER-RNA	Messenger RNA
ID	5	112831	111910	DNA-SYNTHESIS	DNA Synthesis
ID	6	112831	111910	INVITRO	Invitro
ID	7	112831	111910	KINETICS	Kinetics
ID	8	112831	111910	CYCLOSPORINE	Cyclosporine
ID	9	112831	111910	CYCLE	Cycle
ID	10	112831	111910	SUPPRESSION	Suppression

Record 824 | WOS:A1995QJ43400010

SISTER-CHROMATID EXCHANGE FREQUENCY IN CULTURED ISOLATED PORCINE URINARY-BLADDER EPITHELIAL-CELLS (PUBEC) TREATED WITH OCHRATOXIN-A AND OCHRATOXIN-ALPHA

Field	Position	Original line	Normalised line	Original value	Normalised value

DE	1	112951	112027	MYCOTOXIN	Mycotoxins
DE	2	112951	112027	OCHRATOXIN-A	Ochratoxin A (OTA)
DE	3	112951	112027	GENOTOXIC EFFECTS	Genotoxic Effect
DE	4	112951	112027	PUBEC	Pubec
DE	5	112951	112027	SCE FREQUENCY	Sce Frequency
ID	1	112952	112028	SINGLE-STRAND BREAKS	Single Strand Break
ID	2	112952	112028	CHINESE-HAMSTER CELLS	Chinese Hamster
ID	3	112952	112028	LIPID-PEROXIDATION	Lipid Peroxidation
ID	4	112952	112028	PROTEIN-SYNTHESIS	Protein Synthesis
ID	5	112952	112028	HEPATOMA-CELLS	Hepatoma
ID	6	112952	112028	MYCOTOXINS	Mycotoxins
ID	7	112952	112028	PHENYLALANINE	Phenylalanine
ID	8	112952	112028	RATS	Rat
ID	9	112952	112028	MUTAGENICITY	Mutagenicity
ID	10	112952	112028	GENOTOXICITY	Genotoxicity

Record 825 | WOS:A1995QC67100017

EFFECT OF PIROXICAM ON THE NEPHROTOXICITY INDUCED BY OCHRATOXIN-A IN RATS

Field	Position	Original line	Normalised line	Original value	Normalised value
DE	1	113049	112124	OCHRATOXIN A	Ochratoxin A (OTA)
DE	2	113049	112124	PIROXICAM	Piroxicam
DE	3	113049	112124	PREVENTION OF NEPHROTOXICITY	Prevention Of Nephrotoxicity
ID	1	113050	112125	MICE	Mice
ID	2	113050	112125	PHENYLALANINE	Phenylalanine
ID	3	113050	112125	LIVER	Liver
ID	4	113050	112125	CYTOCHROME-P-450	Cytochrome P 450
ID	5	113050	112125	METABOLISM	Metabolism
ID	6	113050	112125	MYCOTOXIN	Mycotoxins
ID	7	113050	112125	URINARY	Urinary
ID	8	113050	112125	TUMORS	Tumor
ID	9	113050	112125	KIDNEY	Kidney

Record 826 | WOS:A1994NN32900003

EFFECT OF SUPEROXIDE-DISMUTASE AND CATALASE ON THE NEPHROTOXICITY INDUCED BY SUBCHRONICAL ADMINISTRATION OF OCHRATOXIN-A IN RATS

Field	Position	Original line	Normalised line	Original value	Normalised value
DE	1	113153	112227	OCHRATOXIN A	Ochratoxin A (OTA)
DE	2	113153	112227	NEPHROTOXICITY	Nephrotoxicity
DE	3	113153	112227	SUPEROXIDE DISMUTASE	Superoxide Dismutase
DE	4	113153	112227	CATALASE	Catalase
ID	1	113154	112228	LIPID-PEROXIDATION	Lipid Peroxidation
ID	2	113154	112228	MICE	Mice
ID	3	113154	112228	PHENYLALANINE	Phenylalanine
ID	4	113154	112228	MYCOTOXIN	Mycotoxins
ID	5	113154	112228	MOUSE	Mouse
ID	6	113154	112228	LIVER	Liver

Record 827 | WOS:A1994MM85700005

THE ROLE OF THE PROXIMAL TUBULE IN OCHRATOXIN-A NEPHROTOXICITY IN-VIVO - TOXODYNAMIC AND TOXOKINETIC ASPECTS

Field	Position	Original line	Normalised line	Original value	Normalised value
DE	1	113241	112314	OCHRATOXIN A	Ochratoxin A (OTA)
DE	2	113241	112314	PROXIMAL TUBULE	Proximal Tubule
DE	3	113241	112314	NEPHROTOXICITY	Nephrotoxicity
DE	4	113241	112314	P-AMINOHIPPURATE	P Aminohippurate
DE	5	113241	112314	AMINO ACIDS	Amino Acid

DE	6	113241	112314	FREE-FLOW MICROPUNCTURE	Free Flow Micropuncture
ID	1	113243	112316	AMINO-ACIDS	Amino Acid
ID	2	113243	112316	RAT	Rat
ID	3	113243	112316	SECRETION	Secretion
ID	4	113243	112316	TRANSPORT	Transport
ID	5	113243	112316	KIDNEY	Kidney
ID	6	113243	112316	PIGS	Pig

Record 828 | WOS:A1994NV49900013

EFFECT OF MYCOTOXINS ON IN-VITRO MOVEMENT OF TRACHEAL CILIA FROM ONE-DAY-OLD CHICKS

Field	Position	Original line	Normalised line	Original value	Normalised value
ID	1	113319	112391	COTTON TEXTILE WORKERS	Cotton Textile Worker
ID	2	113319	112391	BORDETELLA-BRONCHISEPTICA	Bordetella Bronchiseptica
ID	3	113319	112391	PSEUDOMONAS-AERUGINOSA	Pseudomona Aeruginosa
ID	4	113319	112391	MUCOCILIARY CLEARANCE	Mucociliary Clearance
ID	5	113319	112391	RESPIRATORY CILIA	Respiratory Cilia
ID	6	113319	112391	EXPOSURE	Exposure
ID	7	113319	112391	DUST	Dust
ID	8	113319	112391	BYSSINOSIS	Byssinosis
ID	9	113319	112391	INDUSTRY	Industry

Record 829 | WOS:A1993MU47600008

EVALUATION OF THE DEVELOPMENTAL TOXICITY OF CITRININ USING HYDRA-ATTENUATA AND POSTIMPLANTATION RAT WHOLE-EMBRYO CULTURE

Field	Position	Original line	Normalised line	Original value	Normalised value
DE	1	113410	112481	CITRININ	Citrinin
DE	2	113410	112481	DEVELOPMENTAL TOXICITY	Developmental Toxicity
DE	3	113410	112481	HYDRA ATTENUATA	Hydra Attenuata
DE	4	113410	112481	WHOLE EMBRYO CULTURE	Whole Embryo
ID	1	113411	112482	YOLK-SAC	Yolk Sac
ID	2	113411	112482	OCHRATOXIN-A	Ochratoxin A (OTA)
ID	3	113411	112482	INVITRO	Invitro
ID	4	113411	112482	SYSTEM	System
ID	5	113411	112482	CYCLOPHOSPHAMIDE	Cyclophosphamide
ID	6	113411	112482	QUANTITATION	Quantitation
ID	7	113411	112482	MYCOTOXINS	Mycotoxins
ID	8	113411	112482	METABOLISM	Metabolism
ID	9	113411	112482	MORPHOLOGY	Morphology
ID	10	113411	112482	TRANSPORT	Transport

Record 830 | WOS:A1993MM43500024

POSSIBLE ROLE OF AN IRON-OXYGEN COMPLEX IN 4(S)-4-HYDROXYOCHRATOXIN-A FORMATION BY RAT-LIVER MICROSOMES

Field	Position	Original line	Normalised line	Original value	Normalised value
ID	1	113513	112583	STIMULATED LIPID-PEROXIDATION	Stimulated Lipid Peroxidation
ID	2	113513	112583	OCHRATOXIN-A	Ochratoxin A (OTA)
ID	3	113513	112583	CYTOCHROME-P-450	Cytochrome P 450
ID	4	113513	112583	METABOLISM	Metabolism
ID	5	113513	112583	MECHANISMS	Mechanism
ID	6	113513	112583	REACTIVITY	Reactivity
ID	7	113513	112583	INITIATION	Initiation
ID	8	113513	112583	TOXICITY	Toxicity
ID	9	113513	112583	RADICALS	Radical

Record 831 ǀ WOS:A1993MQ49500006

OCHRATOXIN-A IMPAIRS POSTPROXIMAL NEPHRON FUNCTION IN-VIVO AND BLOCKS PLASMA-MEMBRANE ANION CONDUCTANCE IN MADIN-DARBY CANINE KIDNEY-CELLS IN-VITRO

Field	Position	Original line	Normalised line	Original value	Normalised value
DE	1	113601	112670	OCHRATOXIN A	Ochratoxin A (OTA)
DE	2	113601	112670	ANION CONDUCTANCE	Anion Conductance
DE	3	113601	112670	CHLORIDE CHANNEL	Chloride Channel
DE	4	113601	112670	DISTAL NEPHRON FUNCTION	Distal Nephron Function
DE	5	113601	112670	DISTAL NEPHRON TOXICITY	Distal Nephron Toxicity
ID	1	113603	112672	BALKAN ENDEMIC NEPHROPATHY	Balkan Endemic Nephropathy
ID	2	113603	112672	PROTEIN-SYNTHESIS	Protein Synthesis
ID	3	113603	112672	ALDOSTERONE	Aldosterone
ID	4	113603	112672	TRANSPORT	Transport
ID	5	113603	112672	DOMES	Dome
ID	6	113603	112672	MODEL	Model
ID	7	113603	112672	PIGS	Pig
ID	8	113603	112672	RAT	Rat

Record 832 ǀ WOS:A1993LR82100028

EVALUATION AND APPLICATION OF A BIOLUMINESCENT BACTERIAL GENOTOXICITY TEST

Field	Position	Original line	Normalised line	Original value	Normalised value
ID	1	113679	112747	METABOLIC-ACTIVATION	Metabolic Activation
ID	2	113679	112747	MUTAGENICITY	Mutagenicity
ID	3	113679	112747	CARCINOGENICITY	Carcinogenicity
ID	4	113679	112747	AFLATOXIN-B1	Aflatoxin B1 (AFB1)
ID	5	113679	112747	MYCOTOXINS	Mycotoxins
ID	6	113679	112747	BINDING	Binding
ID	7	113679	112747	LIVER	Liver
ID	8	113679	112747	MICE	Mice

Record 833 ǀ WOS:A1993LN86000012

COMPARISON OF IN-VITRO AND IN-VIVO BIOLOGICAL-ACTIVITY OF MYCOTOXINS

Field	Position	Original line	Normalised line	Original value	Normalised value
ID	1	113752	112819	OCHRATOXIN-A	Ochratoxin A (OTA)
ID	2	113752	112819	T-2 TOXIN	T 2 Toxin
ID	3	113752	112819	CYTOTOXICITY	Cytotoxicity
ID	4	113752	112819	CELLS	Cell
ID	5	113752	112819	RATS	Rat

Record 834 ǀ WOS:A1993LJ01500043

EXTRACELLULAR ACIDOSIS AMELIORATES METABOLIC-INHIBITOR-INDUCED AND POTENTIATES OXIDANT-INDUCED CELL-DEATH IN RENAL PROXIMAL TUBULES

Field	Position	Original line	Normalised line	Original value	Normalised value
ID	1	113837	112903	ASCITES TUMOR-CELLS	Ascite Tumor
ID	2	113837	112903	INTRACELLULAR ACIDOSIS	Intracellular Acidosis
ID	3	113837	112903	GLUTATHIONE-REDUCTASE	Glutathione Reductase
ID	4	113837	112903	RAT HEPATOCYTES	Rat Hepatocyte

ID	5	113837	112903	INDUCED INJURY	Induced Injury
ID	6	113837	112903	ACIDOTIC PH	Acidotic Ph
ID	7	113837	112903	TOXICITY	Toxicity
ID	8	113837	112903	PROTECTION	Protection
ID	9	113837	112903	MECHANISM	Mechanism
ID	10	113837	112903	TRANSPORT	Transport

Record 835 | WOS:A1993KQ30600025

IMMUNOTOXICITY OF MYCOTOXINS

Field	Position	Original line	Normalised line	Original value	Normalised value
DE	1	113912	112977	IMMUNOTOXICITY	Immunotoxicity
DE	2	113912	112977	MYCOTOXINS	Mycotoxins
ID	1	113913	112978	EXPERIMENTAL T-2 TOXICOSIS	Experimental T 2 Toxicosis
ID	2	113913	112978	IMMUNE-SYSTEM	Immune System
ID	3	113913	112978	HUMAN-LYMPHOCYTES	Human Lymphocyte
ID	4	113913	112978	CHICKENS	Chicken
ID	5	113913	112978	TOXIN	Toxin
ID	6	113913	112978	AFLATOXIN	Aflatoxin
ID	7	113913	112978	SWINE	Swine
ID	8	113913	112978	TOXICITY	Toxicity
ID	9	113913	112978	MICE	Mice
ID	10	113913	112978	RESISTANCE	Resistance

Record 836 | WOS:A1992JU40900011

HYDROLYSIS OF OCHRATOXIN-A BY THE MICROBIAL ACTIVITY OF DIGESTA IN THE GASTROINTESTINAL-TRACT OF RATS

Field	Position	Original line	Normalised line	Original value	Normalised value
ID	1	114008	113072	TOXICITY	Toxicity
ID	2	114008	113072	MICE	Mice

Record 837 | WOS:A1992KT90400023

ALTERATIONS IN ATP-DEPENDENT CALCIUM-UPTAKE BY RAT RENAL-CORTEX MICROSOMES FOLLOWING OCHRATOXIN A ADMINISTRATION INVIVO OR ADDITION INVITRO

Field	Position	Original line	Normalised line	Original value	Normalised value
ID	1	114078	113141	ENDOPLASMIC-RETICULUM	Endoplasmic Reticulum
ID	2	114078	113141	LIPID-PEROXIDATION	Lipid Peroxidation
ID	3	114078	113141	HEPATIC MICROSOMES	Hepatic Microsome
ID	4	114078	113141	LIVER	Liver
ID	5	114078	113141	PUMP	Pump
ID	6	114078	113141	CYTOCHROME-P-450	Cytochrome P 450
ID	7	114078	113141	TOXICITY	Toxicity
ID	8	114078	113141	GLUTATHIONE	Glutathione (GSH)
ID	9	114078	113141	INHIBITION	Inhibition
ID	10	114078	113141	BLOOD	Blood

Record 838 | WOS:A1992HP59700018

INVITRO EFFECTS OF THE NEPHROTOXINS OCHRATOXIN-A AND CITRININ UPON BIOCHEMICAL FUNCTION OF PORCINE KIDNEY

Field	Position	Original line	Normalised line	Original value	Normalised value
ID	1	114176	113238	EPITHELIAL-CELL SYSTEMS	Epithelial Cell System
ID	2	114176	113238	MYCOTOXIC NEPHROPATHY	Mycotoxic Nephropathy
ID	3	114176	113238	RENAL-FUNCTION	Renal Function

ID	4	114176	113238	PIGS	Pig
ID	5	114176	113238	MICE	Mice
ID	6	114176	113238	RAT	Rat
ID	7	114176	113238	COMBINATION	Combination
ID	8	114176	113238	INDUCTION	Induction
ID	9	114176	113238	RESIDUES	Residue
ID	10	114176	113238	TISSUES	Tissue

Record 839 | WOS:A1991GJ33400005

ROLE OF CYTOCHROME-P-450 IN OCHRATOXIN-A STIMULATED LIPID-PEROXIDATION

Field	Position	Original line	Normalised line	Original value	Normalised value
DE	1	114277	113338	LIPID PEROXIDATION COBALT PROTOPORPHYRIN-IX	Lipid Peroxidation Cobalt Protoporphyrin Ix
DE	2	114277	113338	CYTOCHROME-P-450	Cytochrome P 450
DE	3	114277	113338	OCHRATOXIN-A	Ochratoxin A (OTA)
ID	1	114279	113340	COBALT-PROTOPORPHYRIN	Cobalt Protoporphyrin
ID	2	114279	113340	LIVER-MICROSOMES	Liver Microsome
ID	3	114279	113340	DRUG-METABOLISM	Drug Metabolism
ID	4	114279	113340	REDUCTASE	Reductase
ID	5	114279	113340	NADPH	Nadph
ID	6	114279	113340	MODEL	Model
ID	7	114279	113340	RAT	Rat

Record 840 | WOS:A1991GJ33400006

INVITRO INHIBITION OF RAT PLATELET-AGGREGATION BY OCHRATOXIN-A

Field	Position	Original line	Normalised line	Original value	Normalised value
DE	1	114350	113410	OCHRATOXIN-A	Ochratoxin A (OTA)
DE	2	114350	113410	PLATELET AGGREGATION	Platelet Aggregation
DE	3	114350	113410	SEROTONIN SECRETION	Serotonin Secretion
DE	4	114350	113410	ARACHIDONIC ACID RELEASE	Arachidonic Acid Release
DE	5	114350	113410	METABOLISM	Metabolism

Record 841 | WOS:A1991GA29400009

CHARACTERIZATION OF PIG-LIVER PURIFIED CYTOCHROME-P-450 ISOENZYMES FOR OCHRATOXIN-A METABOLISM STUDIES

Field	Position	Original line	Normalised line	Original value	Normalised value
DE	1	114424	113483	PURIFIED CYTOCHROMES P-450	Purified Cytochrome P 450
DE	2	114424	113483	PIG LIVER	Pig Liver
DE	3	114424	113483	OCHRATOXIN-A METABOLISM	Ochratoxin A Metabolism
ID	1	114425	113484	EXTENSIVE METABOLIZERS	Extensive Metabolizer
ID	2	114425	113484	RAT HEPATOCYTES	Rat Hepatocyte
ID	3	114425	113484	MICROSOMES	Microsome
ID	4	114425	113484	DEBRISOQUINE	Debrisoquine
ID	5	114425	113484	NEPHROPATHY	Nephropathy
ID	6	114425	113484	SYNTHETASE	Synthetase
ID	7	114425	113484	INVIVO	Invivo
ID	8	114425	113484	POOR	Poor
ID	9	114425	113484	MICE	Mice

Record 843 | WOS:A1991FR33200006

INVIVO ABSORPTION OF C-14 GLUCOSE AND C-14 GLYCINE BY THE RAT INTESTINE DURING OCHRATOXIN A TOXICOSIS

Field	Position	Original line	Normalised line	Original value	Normalised value
ID	1	114552	113609	CARBOHYDRATE-METABOLISM	Carbohydrate Metabolism
ID	2	114552	113609	TOXICITY	Toxicity

Record 846 | WOS:A1990EP18900002

EFFECTS OF OCHRATOXIN-A AND OCHRATOXIN-B ON PRECHONDROGENIC MESENCHYMAL CELLS FROM CHICK-EMBRYO LIMB BUDS

Field	Position	Original line	Normalised line	Original value	Normalised value
DE	1	114708	113762	OCHRATOXIN	Ochratoxin A (OTA)
DE	2	114708	113762	EMBRYOTOXICITY	Embryotoxicity
DE	3	114708	113762	LIMB AND CULTURE	Limb And
DE	4	114708	113762	PROTEIN SYNTHESIS	Protein Synthesis
ID	1	114709	113763	ACUTE INTRAPERITONEAL TOXICITY	Acute Intraperitoneal Toxicity
ID	2	114709	113763	TROUT SALMO-GAIRDNERI	Trout Salmo Gairdneri
ID	3	114709	113763	METABOLISM	Metabolism
ID	4	114709	113763	RATS	Rat
ID	5	114709	113763	INHIBITION	Inhibition
ID	6	114709	113763	INVITRO	Invitro
