# Supplementary material for: Ochratoxin A Toxicity Research in In Vitro and In Vivo Experimental Settings: A Cross-Software Bibliometric and Science-Mapping Study
Source: Toxins (Basel). 2026 Aug 11;18(8):346. doi: 10.3390/toxins18080346 (PMC13517462; doi:10.3390/toxins18080346)
Supplement: Supplementary file 1 [file toxins-18-00346-s001.zip › Supplementary File S4.pdf]

Author normalisation audit – line-by-line and article-by-article

Files compared: WoS 1-883_Original.txt vs WoS_1-883_Authors_Normalized.txt

This Word file lists observed AU/AF changes by article. The accompanying Excel file contains the complete controlled vocabulary, changed dictionary, before/after counts and filterable record-level audit.

Field	Occurrences before	Occurrences after	Changed mapping pairs	Unique labels before	Unique labels after
AU	4966	4966	3036	3.212	3.305
AF	4966	4966	1335	3.669	3.497

Record 1 | WOS:001628370900001

Dietary Quercetin Mitigates DON-Induced Intestinal Injury via Inhibiting MAPK/NF-κB-Mediated Pyroptosis and Tight Junction Disruption in Chicken

Field	Position	Original line	Normalised line	Original value	Normalised value
AU	1	4	4	Guan, CW	Guan, Chunwei
AU	2	5	5	Mei, XY	Mei, Xueyi
AU	3	6	6	Sun, SL	Sun, Shilong
AU	4	7	7	Ding, YY	Ding, Yuyang
AU	5	8	8	Cai, JZ	Cai, Jingzeng

Record 2 | WOS:001646793400001

Silent Saboteurs: Decoding Mycotoxins-From Chemistry and Prevalence to Health Risks, Detection, Management and Emerging Frontiers

Field	Position	Original line	Normalised line	Original value	Normalised value
AU	1	125	125	Thambugala, KM	Thambugala, Kasun_M
AU	2	126	126	Dayananda, D	Dayananda, Dilakshini
AU	3	127	127	Tennakoon, S	Tennakoon, Samawansha
AU	4	128	128	Harischandra, H	Harischandra, Hiruni
AU	5	129	129	Jayatunga, P	Jayatunga, Pamoda
AU	6	130	130	de Silva, N	de_Silva, Nissanka
AU	7	131	131	Dhanusha, A	Dhanusha, Asanthi
AU	8	132	132	Madusanka, S	Madusanka, Sahan
AU	9	133	133	Daranagama, DA	Daranagama, Dinushani_A
AU	10	134	134	Gonapaladeniya, M	Gonapaladeniya, Madhusha
AU	11	135	135	Haituk, S	Haituk, Sukanya
AU	12	136	136	Cheewangkoon, R	Cheewangkoon, Ratchadawan
AF	1	137	137	Thambugala, Kasun M.	Thambugala, Kasun_M
AF	6	142	142	de Silva, Nissanka	de_Silva, Nissanka
AF	9	145	145	Daranagama, Dinushani A.	Daranagama, Dinushani_A

Record 3 | WOS:001648209500001

Dose-Dependent but Non-Interactive Effects of Ochratoxin A and Selenomethionine on Hepatic Lipid Metabolism and Oxidative Stress in Broiler Chickens

Field	Position	Original line	Normalised line	Original value	Normalised value
AU	1	460	460	Kulcsár, S	Kulcsar, Szabina
AU	2	461	461	Balogh, K	Balogh, Krisztian
AU	3	462	462	Zándoki, E	Zandoki, Erika
AU	4	463	463	Agyarko, E	Agyarko, Edward
AU	5	464	464	Ali, O	Ali, Omeralfaroug
AU	6	465	465	Kövesi, B	Kovesi, Benjamin
AU	7	466	466	Freiler-Nagy, A	Freiler-Nagy, Agnes
AU	8	467	467	Szabo, A	Szabo, Andras
AU	9	468	468	Mézes, M	Mezes, Miklos

Record 4 | WOS:001635022200001

Selective Cytotoxicity of Ochratoxin A: Pro-Apoptotic Effects on Healthy Immune Cells Compared to Leukemia Cells

Field	Position	Original line	Normalised line	Original value	Normalised value
AU	1	607	607	Wieckowska, M	Wieckowska, Magdalena
AU	2	608	608	Janik-Karpinska, E	Janik-Karpinska, Edyta
AU	3	609	609	Cichon, N	Cichon, Natalia
AU	4	610	610	Synowiec, E	Synowiec, Ewelina
AU	5	611	611	Szelenberger, R	Szelenberger, Rafal
AU	6	612	612	Stela, M	Stela, Maksymilian
AU	7	613	613	Podogrocki, M	Podogrocki, Marcin
AU	8	614	614	Gorniak, L	Gorniak, Leslaw
AU	9	615	615	Poplawski, T	Poplawski, Tomasz
AU	10	616	616	Sliwinski, T	Sliwinski, Tomasz
AU	11	617	617	Krzowski, L	Krzowski, Lukasz
AU	12	618	618	Bijak, M	Bijak, Michal

Record 5 | WOS:001625561500001

Cytotoxic Effects Induced by Combined Exposure of the Patulin, Ochratoxin A, and Acetamiprid to HK-2 and SK-N-SH Cell Lines

Field	Position	Original line	Normalised line	Original value	Normalised value
AU	1	758	758	Zhu, ZY	Zhu, Zuoyin
AU	2	759	759	Zhao, HK	Zhao, Hanke
AU	3	760	760	Yang, XL	Yang, Xianli
AU	4	761	761	Nie, DX	Nie, Dongxia
AU	5	762	762	Yang, JH	Yang, Junhua
AU	6	763	763	Han, Z	Han, Zheng

Record 6 | WOS:001623564900001

Mycotoxins and the Intestinal Epithelium: From Barrier Injury to Stem Cell Dysfunction

Field	Position	Original line	Normalised line	Original value	Normalised value
AU	1	866	866	Huo, WY	Huo, Wenying
AU	2	867	867	Qiao, YY	Qiao, Yingying
AU	3	868	868	He, XR	He, Xiangru

AU	4	869	869	Wang, CL	Wang, Cailing
AU	5	870	870	Li, RQ	Li, Ruiqing
AU	6	871	871	Che, L	Che, Long
AU	7	872	872	Li, EK	Li, Enkai

Record 7 | WOS:001598251800001

Exploration of oxidative stress-mediated genetic toxicology modes of action using a pathway analysis, Connectivity Mapping, and transcriptional benchmark dosing-based framework

Field	Position	Original line	Normalised line	Original value	Normalised value
AU	1	1043	1043	De Abrew, KN	de_Abrew, K_Nadira
AU	2	1044	1044	Selman, BG	Selman, Bastian_G
AU	3	1045	1045	Shobair, M	Shobair, Mahmoud
AU	4	1046	1046	Zhang, XL	Zhang, Xiaoling
AU	5	1047	1047	Allemang, AJ	Allemang, Ashley_J
AU	6	1048	1048	Pfuhler, S	Pfuhler, Stefan
AF	1	1049	1049	De Abrew, K. Nadira	de_Abrew, K_Nadira
AF	2	1050	1050	Selman, Bastian G.	Selman, Bastian_G
AF	5	1053	1053	Allemang, Ashley J.	Allemang, Ashley_J

Record 8 | WOS:001591221900001

Prevention and detoxification of mycotoxins in food and feed: A review

Field	Position	Original line	Normalised line	Original value	Normalised value
AU	1	1212	1212	Deliklitas, K	Deliklitas, Kubra
AU	2	1213	1213	Gokbulut, C	Gokbulut, Cengiz

Record 9 | WOS:001603917600001

Astaxanthin Alleviates Ochratoxin A (OTA)-Induced Spleen Dysfunction and Apoptosis in Broiler Chickens by Modulating the PTEN/PI3K/AKT Signaling Pathway

Field	Position	Original line	Normalised line	Original value	Normalised value
AU	1	1554	1554	Cheng, ZB	Cheng, Zhibi
AU	2	1555	1555	Sang, WL	Sang, Weilun
AU	3	1556	1556	Li, P	Li, Peng
AU	4	1557	1557	Yang, SH	Yang, Shuhua

Record 10 | WOS:001593782400001

Mycotoxin Contamination: Occurrence, Biotransformation, Pathogenic Mechanisms, and Strategies for Nutritional Intervention

Field	Position	Original line	Normalised line	Original value	Normalised value
AU	1	1678	1678	Yao, CY	Yao, Chenyu
AU	2	1679	1679	Ye, MY	Ye, Mengyu
AU	3	1680	1680	Wang, C	Wang, Cong
AU	4	1681	1681	Zou, L	Zou, Lin
AU	5	1682	1682	Zhang, XM	Zhang, Ximeng
AU	6	1683	1683	Chai, X	Chai, Xin

AU	7	1684	1684	Yu, HJ	Yu, Huijuan
AU	8	1685	1685	Zhang, CY	Zhang, Chengyu
AU	9	1686	1686	Wang, YF	Wang, Yuefei

Record 11 | WOS:001579727300001

Chlorogenic Acid and VX765 Alleviate Deoxynivalenol-Induced Enterohepatic Injury and Lipid Metabolism Disorders by Improving Intestinal Microecology

Field	Position	Original line	Normalised line	Original value	Normalised value
AU	1	1956	1956	Wen, T	Wen, Tao
AU	2	1957	1957	Li, SR	Li, Sirui
AU	3	1958	1958	Guo, HJ	Guo, Huijun
AU	4	1959	1959	Wang, JB	Wang, Jinbo
AU	5	1960	1960	Zhang, XR	Zhang, Xinru
AU	6	1961	1961	Wang, CY	Wang, Chunyang

Record 12 | WOS:001573742900001

Fungal metabolite Ochratoxin A inhibits MrkD1P of multidrug-resistant Klebsiella pneumoniae: Integrated computational and in vitro validation

Field	Position	Original line	Normalised line	Original value	Normalised value
AU	1	2075	2075	Roqunuzzaman, M	Roqunuzzaman, Md
AU	2	2076	2076	Islam, A	Islam, Ariful
AU	3	2077	2077	Supti, SJ	Supti, Sumaiya_Jahan
AU	4	2078	2078	Rifat, MH	Rifat, Mahbub_Hasan
AU	5	2079	2079	Islam, MS	Islam, Mohammad_Saiful
AU	6	2080	2080	Ananna, UH	Ananna, Ummay_Habiba
AU	7	2081	2081	Tusher, KS	Tusher, Khalid_Saifullah
AU	8	2082	2082	Al-Mutairi, AA	Al-Mutairi, Aamal_A
AU	9	2083	2083	Zaki, MEA	Zaki, Magdi_E_A
AU	10	2084	2084	Sarker, S	Sarker, Subir
AU	11	2085	2085	Hosen, ME	Hosen, Md_Eram
AF	3	2088	2088	Supti, Sumaiya Jahan	Supti, Sumaiya_Jahan
AF	4	2089	2089	Rifat, Mahbub Hasan	Rifat, Mahbub_Hasan
AF	5	2090	2090	Islam, Mohammad Saiful	Islam, Mohammad_Saiful
AF	6	2091	2091	Ananna, Ummay Habiba	Ananna, Ummay_Habiba
AF	7	2092	2092	Tusher, Khalid Saifullah	Tusher, Khalid_Saifullah
AF	8	2093	2093	Al-Mutairi, Aamal A.	Al-Mutairi, Aamal_A
AF	9	2094	2094	Zaki, Magdi E. A.	Zaki, Magdi_E_A
AF	11	2096	2096	Hosen, Md. Eram	Hosen, Md_Eram

Record 13 | WOS:001581342800001

Biocatalytic Detoxification of Ochratoxins A/B by a Fungal Dye-Decolorizing Peroxidase: Mechanistic Insights and Toxicity Assessment

Field	Position	Original line	Normalised line	Original value	Normalised value
AU	1	2253	2253	Xia, WJ	Xia, Wenjing
AU	2	2254	2254	Zhu, NQ	Zhu, Nianqing
AU	3	2255	2255	Mei, J	Mei, Jie

AU	4	2256	2256	Peng, YQ	Peng, Yueqin
AU	5	2257	2257	Song, FL	Song, Fanglin
AU	6	2258	2258	Ding, S	Ding, Shuai
AU	7	2259	2259	Li, F	Li, Fei
AU	8	2260	2260	Zhou, X	Zhou, Xue

Record 14 | WOS:001556476900001

Identification of an M20 Family Metallopeptidase with Amidohydrolase Activity from Alcaligenes faecalis HNGD-HTN06 and Simultaneous Degradation of Ochratoxin A, Aflatoxin B1, and Zearalenone

Field	Position	Original line	Normalised line	Original value	Normalised value
AU	1	2357	2357	Hou, MJ	Hou, Minjie
AU	2	2358	2358	Mei, XD	Mei, Xuedong
AU	3	2359	2359	Mwabulili, F	Mwabulili, Fred
AU	4	2360	2360	Li, Q	Li, Qian
AU	5	2361	2361	Jia, H	Jia, Hang
AU	6	2362	2362	Huang, QR	Huang, Qingrong
AU	7	2363	2363	Xie, YL	Xie, Yanli

Record 15 | WOS:001590628300001

Roles of microRNAs in multiple organs under mycotoxin stress by bioinformatic analysis microRNA regulation during mycotoxin exposure

Field	Position	Original line	Normalised line	Original value	Normalised value
AU	1	2499	2499	Sohn, EJ	Sohn, Eun_Jung
AU	2	2500	2500	Park, KT	Park, Kun-Taek
AF	1	2501	2501	Sohn, Eun Jung	Sohn, Eun_Jung

Record 16 | WOS:001556296100001

The protective effect of Gentiana lutea flower against mycotoxin toxicity in a gastrointestinal barrier in vitro model

Field	Position	Original line	Normalised line	Original value	Normalised value
AU	1	2719	2719	Di Matteo, G	di_Matteo, Giacomo
AU	2	2720	2720	Frangiamone, M	Frangiamone, Massimo
AU	3	2721	2721	Vila-Donat, P	Vila-Donat, Pilar
AU	4	2722	2722	Di Cecco, V	di_Cecco, Valter
AU	5	2723	2723	Di Martino, L	di_Martino, Luciano
AU	6	2724	2724	Manyes, L	Manyes, Lara
AU	7	2725	2725	Mannina, L	Mannina, Luisa
AF	1	2726	2726	Di Matteo, Giacomo	di_Matteo, Giacomo
AF	4	2729	2729	Di Cecco, Valter	di_Cecco, Valter
AF	5	2730	2730	Di Martino, Luciano	di_Martino, Luciano

Record 17 | WOS:001546906900003

Mitochondrial dynamics imbalance and energy dyshomeostasis mediated developmental toxicity of ochratoxin a in zebrafish (Danio rerio)

Field	Position	Original line	Normalised line	Original value	Normalised value
AU	1	2855	2855	Li, M	Li, Meng
AU	2	2856	2856	He, MG	He, Mengai
AU	3	2857	2857	Liao, ZF	Liao, Zhenfeng
AU	4	2858	2858	Li, CX	Li, Chenxi
AU	5	2859	2859	Mi, YQ	Mi, Yingqiu
AU	6	2860	2860	Pan, NN	Pan, Ningning
AU	7	2861	2861	Xie, XY	Xie, Xinyi
AU	8	2862	2862	Liu, ZY	Liu, Zhiyu
AU	9	2863	2863	Hou, JY	Hou, Jiayin
AU	10	2864	2864	Yu, XL	Yu, Xueli
AU	11	2865	2865	Chen, LZ	Chen, Liezhong

Record 18 | WOS:001582665400002

L-epicatechin partially alleviates Ochratoxin A-induced oxidative and endoplasmic reticulum stress and improves developmental competence of porcine embryos during in vitro culture

Field	Position	Original line	Normalised line	Original value	Normalised value
AU	1	3007	3007	Wang, X	Wang, Xu
AU	2	3008	3008	Li, MY	Li, Mingyu
AU	3	3009	3009	Jiang, N	Jiang, Nan
AU	4	3010	3010	Jin, L	Jin, Long
AU	5	3011	3011	Li, C	Li, Chuang

Record 19 | WOS:001539646900001

Emerging Mycotoxins in Aquaculture: Current Insights on Toxicity, Biocontrol Strategies, and Occurrence in Aquafeed and Fish

Field	Position	Original line	Normalised line	Original value	Normalised value
AU	1	3115	3115	Lorusso, P	Lorusso, Patrizio
AU	2	3116	3116	Rusco, G	Rusco, Giusy
AU	3	3117	3117	Manfredi, A	Manfredi, Alessio
AU	4	3118	3118	Iaffaldano, N	Iaffaldano, Nicolaia
AU	5	3119	3119	Di Pinto, A	di_Pinto, Angela
AU	6	3120	3120	Bonerba, E	Bonerba, Elisabetta
AF	5	3125	3125	Di Pinto, Angela	di_Pinto, Angela

Record 20 | WOS:001529843100001

Decontamination of Ochratoxin A in Food: Emerging Strategies and Safety Perspectives

Field	Position	Original line	Normalised line	Original value	Normalised value
AU	1	3304	3304	Khan, R	Khan, Rahim
AU	2	3305	3305	Anwar, F	Anwar, Farooq
AU	3	3306	3306	Ghazali, FM	Ghazali, Farinazleen_Mohamad
AU	4	3307	3307	Mahyudin, NA	Mahyudin, Nor_Ainy
AU	5	3308	3308	Iqbal, A	Iqbal, Arshad
AF	3	3311	3311	Ghazali, Farinazleen Mohamad	Ghazali, Farinazleen_Mohamad
AF	4	3312	3312	Mahyudin, Nor Ainy	Mahyudin, Nor_Ainy

Record 21 | WOS:001527764700005

Through its genoprotective, mitochondrial bioenergetic modulation, and antioxidant effects, Fucoxanthin and its metabolite minimize Ochratoxin A-induced nephrotoxicity in HK-2 human kidney cells

Field	Position	Original line	Normalised line	Original value	Normalised value
AU	1	3518	3518	Elmorsy, EM	Elmorsy, Ekramy_M
AU	2	3519	3519	Al Doghaither, HA	al_Doghaither, Huda_A
AU	3	3520	3520	Al-Ghafari, AB	Al-Ghafari, Ayat_B
AU	4	3521	3521	Alyamani, SA	Alyamani, Shaza_A
AU	5	3522	3522	Mohammed, ZMS	Mohammed, Zakariya_M_S
AU	6	3523	3523	Ebrahim, NA	Ebrahim, Neven_A
AU	7	3524	3524	Elshopakey, GE	Elshopakey, Gehad_E
AU	8	3525	3525	Shabana, SM	Shabana, Sameh_M
AF	1	3526	3526	Elmorsy, Ekramy M.	Elmorsy, Ekramy_M
AF	2	3527	3527	Al Doghaither, Huda A.	al_Doghaither, Huda_A
AF	3	3528	3528	Al-Ghafari, Ayat B.	Al-Ghafari, Ayat_B
AF	4	3529	3529	Alyamani, Shaza A.	Alyamani, Shaza_A
AF	5	3530	3530	Mohammed, Zakariya M. S.	Mohammed, Zakariya_M_S
AF	6	3531	3531	Ebrahim, Neven A.	Ebrahim, Neven_A
AF	7	3532	3532	Elshopakey, Gehad E.	Elshopakey, Gehad_E
AF	8	3533	3533	Shabana, Sameh M.	Shabana, Sameh_M

Record 22 | WOS:001539670300001

Oral Exposure to Chlorella sorokiniana Detoxifies Deoxynivalenol, Ochratoxin A, and Fumonisin B1 In Vitro and In Vivo

Field	Position	Original line	Normalised line	Original value	Normalised value
AU	1	3677	3677	Yamaguchi, H	Yamaguchi, Hiroki
AU	2	3678	3678	Ando, M	Ando, Mana
AU	3	3679	3679	Ohira, C	Ohira, Chiharu
AU	4	3680	3680	Magami, T	Magami, Tensei
AU	5	3681	3681	Kaneki, M	Kaneki, Mao
AU	6	3682	3682	Sugita, K	Sugita, Kazutoshi
AU	7	3683	3683	Ogawa, T	Ogawa, Taro
AU	8	3684	3684	Nakashima, A	Nakashima, Ayaka
AU	9	3685	3685	Fukuyama, T	Fukuyama, Tomoki

Record 23 | WOS:001513772100001

Schisandrin B mitigates oxidative damage in early porcine embryos exposed to Ochratoxin A via NRF2 pathway activation

Field	Position	Original line	Normalised line	Original value	Normalised value
AU	1	3772	3772	Li, C	Li, Chuang
AU	2	3773	3773	Bin Ji, K	bin_Ji, Kuk
AU	3	3774	3774	Kim, EY	Kim, Eun_Young
AU	4	3775	3775	Choi, HY	Choi, Ho_Yong
AU	5	3776	3776	Yoon, JH	Yoon, Jae_Hyeok
AU	6	3777	3777	Zhang, X	Zhang, Xiang

AU	7	3778	3778	Kim, RE	Kim, Ryoung_Eun
AU	8	3779	3779	Park, KS	Park, Kang_Sun
AU	9	3780	3780	Liu, HX	Liu, Haixing
AU	10	3781	3781	Kim, MK	Kim, Min_Kyu
AF	2	3783	3783	Bin Ji, Kuk	bin_Ji, Kuk
AF	3	3784	3784	Kim, Eun Young	Kim, Eun_Young
AF	4	3785	3785	Choi, Ho Yong	Choi, Ho_Yong
AF	5	3786	3786	Yoon, Jae Hyeok	Yoon, Jae_Hyeok
AF	7	3788	3788	Kim, Ryoung Eun	Kim, Ryoung_Eun
AF	8	3789	3789	Park, Kang Sun	Park, Kang_Sun
AF	10	3791	3791	Kim, Min Kyu	Kim, Min_Kyu

Record 24 | WOS:001505840900001

Biocontrol Agents and Natural Feed Supplements as a Safe and Cost-Effective Way for Preventing Health Ailments Provoked by Mycotoxins

Field	Position	Original line	Normalised line	Original value	Normalised value
AU	1	3890	3890	Stoev, SD	Stoev, Stoycho_D
AF	1	3891	3891	Stoev, Stoycho D.	Stoev, Stoycho_D

Record 25 | WOS:001514636100001

Modulation of the Antioxidant System of Caco-2 Cells in the Presence of Aflatoxin B1, Ochratoxin A, and Ferulic Acid

Field	Position	Original line	Normalised line	Original value	Normalised value
AU	1	4251	4251	Radulescu, AL	Radulescu, Andreea-Luminita
AU	2	4252	4252	Popescu, RG	Popescu, Roua_Gabriela
AU	3	4253	4253	Balas, M	Balas, Mihaela
AU	4	4254	4254	Marinescu, GC	Marinescu, George_Catalin
AU	5	4255	4255	Dinischiotu, A	Dinischiotu, Anca
AF	2	4257	4257	Popescu, Roua Gabriela	Popescu, Roua_Gabriela
AF	4	4259	4259	Marinescu, George Catalin	Marinescu, George_Catalin

Record 26 | WOS:001496132800001

Evaluation of Dietary Bioactive Agents Against Aflatoxin B1 and Ochratoxin A-Induced Duodenal Toxicity in Rats

Field	Position	Original line	Normalised line	Original value	Normalised value
AU	1	4406	4406	Rafai, S	Rafai, Sarra
AU	2	4407	4407	Cimbalo, A	Cimbalo, Alessandra
AU	3	4408	4408	Manyes, L	Manyes, Lara

Record 27 | WOS:001488085400001

Bi2WO6-TiO2 carbon nanoparticles controlled postharvest blue mold of table grapes caused by Talaromyces rugulosus and the possible action mechanisms

Field	Position	Original line	Normalised line	Original value	Normalised value
AU	1	4509	4509	Li, JZ	Li, Jinzhu
AU	2	4510	4510	Xi, JH	Xi, Jihui

AU	3	4511	4511	Wang, RX	Wang, Ruoxing
AU	4	4512	4512	Zhang, QR	Zhang, Qingru
AU	5	4513	4513	Fan, HY	Fan, Haiyan
AU	6	4514	4514	Lyu, L	Lyu, Liang
AU	7	4515	4515	Xue, HL	Xue, Huali
AU	8	4516	4516	Bi, Y	Bi, Yang

Record 28 | WOS:001486309200001

Immunotoxic effect and mechanisms of Fusarium mycotoxins on human immune cells: A focus on T cells and macrophages

Field	Position	Original line	Normalised line	Original value	Normalised value
AU	1	4610	4610	Yang, J	Yang, Jing
AU	2	4611	4611	Wang, XH	Wang, Xiaohong
AU	3	4612	4612	Zhang, QN	Zhang, Qiannan
AU	4	4613	4613	Cui, HY	Cui, Hongyang
AU	5	4614	4614	You, LJ	You, Lijuan
AU	6	4615	4615	Zhuang, ZS	Zhuang, Zhisen
AU	7	4616	4616	Tian, YR	Tian, Yaru
AU	8	4617	4617	Han, XM	Han, Xiaomin
AU	9	4618	4618	Shi, MY	Shi, Miaoying
AU	10	4619	4619	Bai, L	Bai, Li
AU	11	4620	4620	Jia, XD	Jia, Xudong
AU	12	4621	4621	Yang, H	Yang, Hui

Record 29 | WOS:001484254000002

Quantification and metabolic variations of mycotoxins in raw milk: Implications for dairy cow health and human safety

Field	Position	Original line	Normalised line	Original value	Normalised value
AU	1	4741	4741	Chen, YY	Chen, Yuanyuan
AU	2	4742	4742	Cheng, JX	Cheng, Jiaxin
AU	3	4743	4743	Wang, ZS	Wang, Zhuangshu
AU	4	4744	4744	Liu, HL	Liu, Honglin
AU	5	4745	4745	Xia, C	Xia, Cheng
AU	6	4746	4746	Zhang, HF	Zhang, Haifeng
AU	7	4747	4747	Zou, SQ	Zou, Siqi
AU	8	4748	4748	Chang, QC	Chang, Qiaocheng
AU	9	4749	4749	Xu, C	Xu, Chuang

Record 30 | WOS:001484241000001

Oxidative stress promotes post-translational down-regulation of MRP2 in Caco-2 cells: Involvement of proteasomal degradation and toxicological implications

Field	Position	Original line	Normalised line	Original value	Normalised value
AU	1	4889	4889	Ricardi, LL	Ricardi, Laura_Lis
AU	2	4890	4890	Zecchinati, F	Zecchinati, Felipe
AU	3	4891	4891	Perdomo, VG	Perdomo, Virginia_Gabriela
AU	4	4892	4892	Basiglio, CL	Basiglio, Cecilia_Lorena

AU	5	4893	4893	Garcia, F	Garcia, Fabiana
AU	6	4894	4894	Arana, MR	Arana, Maite_Rocio
AU	7	4895	4895	Villanueva, SSM	Villanueva, Silvina_Stella_Maris
AF	1	4896	4896	Ricardi, Laura Lis	Ricardi, Laura_Lis
AF	3	4898	4898	Perdomo, Virginia Gabriela	Perdomo, Virginia_Gabriela
AF	4	4899	4899	Basiglio, Cecilia Lorena	Basiglio, Cecilia_Lorena
AF	6	4901	4901	Arana, Maite Rocio	Arana, Maite_Rocio
AF	7	4902	4902	Villanueva, Silvina Stella Maris	Villanueva, Silvina_Stella_Maris

Record 31 | WOS:001471942000001

Ginsenoside Rg1 alleviates ochratoxin A-induced liver inflammation in ducklings: Involvement of intestinal microbiota modulation and the TLR4/ NF-κB pathway inhibition

Field	Position	Original line	Normalised line	Original value	Normalised value
AU	1	5029	5029	Zhai, SS	Zhai, Shuangshuang
AU	2	5030	5030	Peng, X	Peng, Xin
AU	3	5031	5031	Liu, C	Liu, Cheng
AU	4	5032	5032	Zhang, R	Zhang, Ran
AU	5	5033	5033	Jin, CL	Jin, Chunlong
AU	6	5034	5034	Jiang, XY	Jiang, Xiayu
AU	7	5035	5035	Feng, PS	Feng, Peishi
AU	8	5036	5036	Liang, YT	Liang, Yuting
AU	9	5037	5037	Yuan, X	Yuan, Xin
AU	10	5038	5038	Zhang, JQ	Zhang, Jinqiu
AU	11	5039	5039	Yang, Y	Yang, Ye
AF	9	5048	5048	Yuan, Xi	Yuan, Xin

Record 32 | WOS:001471582100001

Mitigating AFB1 and OTA hepatic residues using pumpkin and milk fermented whey as functional ingredients

Field	Position	Original line	Normalised line	Original value	Normalised value
AU	1	5155	5155	Cimbalo, A	Cimbalo, Alessandra
AU	2	5156	5156	da Silva, JL	da_Silva, Juliane_Lima
AU	3	5157	5157	Izzo, L	Izzo, Luana
AU	4	5158	5158	Ritieni, A	Ritieni, Alberto
AU	5	5159	5159	Manyes, L	Manyes, Lara
AF	2	5161	5161	da Silva, Juliane Lima	da_Silva, Juliane_Lima

Record 33 | WOS:001456981800001

Ochratoxin A-induced mitochondrial pathway apoptosis and ferroptosis by promoting glycolysis

Field	Position	Original line	Normalised line	Original value	Normalised value
AU	1	5274	5274	Zhou, Y	Zhou, Yao
AU	2	5275	5275	Chen, WY	Chen, Wenying
AU	3	5276	5276	Feng, SY	Feng, Shiyu
AU	4	5277	5277	Liu, SC	Liu, Shuangchao
AU	5	5278	5278	Chen, C	Chen, Cheng

AU	6	5279	5279	Yao, BX	Yao, Bingxu
AU	7	5280	5280	Shen, XL	Shen, Xiao_Li
AF	7	5287	5287	Shen, Xiao Li	Shen, Xiao_Li

Record 34 | WOS:001463798400001

Combined Cytotoxic Effects of the Fungicide Azoxystrobin and Common Food-Contaminating Mycotoxins

Field	Position	Original line	Normalised line	Original value	Normalised value
AU	1	5422	5422	Fuentes, C	Fuentes, Cristina
AU	2	5423	5423	Zingales, V	Zingales, Veronica
AU	3	5424	5424	Barat, JM	Barat, Jose_Manuel
AU	4	5425	5425	Ruiz, MJ	Ruiz, Maria-Jose
AF	3	5428	5428	Barat, Jose Manuel	Barat, Jose_Manuel

Record 35 | WOS:001475234400001

Mycotoxins in Feed: Hazards, Toxicology, and Plant Extract-Based Remedies

Field	Position	Original line	Normalised line	Original value	Normalised value
AU	1	5555	5555	Zhang, XN	Zhang, Xiangnan
AU	2	5556	5556	Chen, JS	Chen, Jiashun
AU	3	5557	5557	Ma, XK	Ma, Xiaokang
AU	4	5558	5558	Tang, XZ	Tang, Xiongzhuo
AU	5	5559	5559	Tan, B	Tan, Bie
AU	6	5560	5560	Liao, P	Liao, Peng
AU	7	5561	5561	Yao, K	Yao, Kang
AU	8	5562	5562	Jiang, Q	Jiang, Qian

Record 36 | WOS:001437492200001

Potential role of ochratoxin A in Parkinson's disease: a systematic review of current evidence

Field	Position	Original line	Normalised line	Original value	Normalised value
AU	3	5776	5776	alvarez-Erviti, L	Alvarez-Erviti, Lydia
AU	4	5777	5777	de Cerain, AL	de_Cerain, A_Lopez
AU	5	5778	5778	Vettorazzi, A	Vettorazzi, Ariane
AF	1	5779	5779	Serrano-Civantos, M.	Serrano-Civantos, M
AF	2	5780	5780	Beraza, E.	Beraza, E
AF	3	5781	5781	alvarez-Erviti, L.	Alvarez-Erviti, Lydia
AF	4	5782	5782	de Cerain, A. Lopez	de_Cerain, A_Lopez
AF	5	5783	5783	Vettorazzi, A.	Vettorazzi, Ariane

Record 37 | WOS:001427487800001

Neurotoxic implications of gliotoxin and ochratoxin A in SH-SY5Y cells: ROS-induced apoptosis and genotoxicity

Field	Position	Original line	Normalised line	Original value	Normalised value
AU	1	5924	5924	Penalva-Olcina, R	Penalva-Olcina, Raquel

AU	2	5925	5925	Juan, CSA	Juan, Cristina
AU	3	5926	5926	Fernández-Franzón, M	Fernandez-Franzon, Monica
AU	4	5927	5927	Juan-García, A	Juan-Garcia, Ana

Record 38 | WOS:001426276300001

Ochratoxin A induces lung cell PANoptosis through activation of the AIM 2 inflammasome

Field	Position	Original line	Normalised line	Original value	Normalised value
AU	1	6036	6036	Xie, JL	Xie, Jianlong
AU	2	6037	6037	Fu, QJ	Fu, Qiujuan
AU	3	6038	6038	Qin, LL	Qin, Lingling
AU	4	6039	6039	Lin, LY	Lin, Liyao
AU	5	6040	6040	Wu, Q	Wu, Qin
AU	6	6041	6041	Zeng, KQ	Zeng, Kaiqi
AU	7	6042	6042	Wu, JH	Wu, Jiahuan
AU	8	6043	6043	Cao, ZY	Cao, Ziyi
AU	9	6044	6044	Ou, YF	Ou, Yongfang

Record 39 | WOS:001425801400001

Probiotics mitigate reproductive toxicity in male mice induced by co-exposure of ochratoxin A and alcohol

Field	Position	Original line	Normalised line	Original value	Normalised value
AU	1	6137	6137	Ning, X	Ning, Xiao
AU	2	6138	6138	Cao, J	Cao, Jin
AU	3	6139	6139	Li, JY	Li, Jingyun
AU	4	6140	6140	Ye, YL	Ye, Yongli
AU	5	6141	6141	Jin, SM	Jin, Shaoming
AU	6	6142	6142	Zhang, YZ	Zhang, Yinzhi
AU	7	6143	6143	Cui, SH	Cui, Shenghui
AU	8	6144	6144	Sun, XL	Sun, Xiulan

Record 40 | WOS:001416011800001

Metabolic disruption by mycotoxins: focus on metabolic endpoints steatosis, adipogenesis and glucose metabolism in vivo and in vitro

Field	Position	Original line	Normalised line	Original value	Normalised value
AU	1	6253	6253	Heikkinen, T	Heikkinen, Tia
AU	2	6254	6254	Küblbeck, J	Kublbeck, Jenni
AU	3	6255	6255	Rysä, J	Rysa, Jaana

Record 41 | WOS:001403208800001

Curcumin: A Potential Detoxifier Against Chemical and Natural Toxicants

Field	Position	Original line	Normalised line	Original value	Normalised value
AU	1	6367	6367	Kumar, H	Kumar, Harsh
AU	2	6368	6368	Dhalaria, R	Dhalaria, Rajni

AU	3	6369	6369	Kimta, N	Kimta, Neetika
AU	4	6370	6370	Guleria, S	Guleria, Shivani
AU	5	6371	6371	Upadhyay, NK	Upadhyay, Navneet_Kumar
AU	6	6372	6372	Nepovimova, E	Nepovimova, Eugenie
AU	7	6373	6373	Dhanjal, DS	Dhanjal, Daljeet_Singh
AU	8	6374	6374	Sethi, N	Sethi, Nidhi
AU	9	6375	6375	Manickam, S	Manickam, Sivakumar
AF	5	6380	6380	Upadhyay, Navneet Kumar	Upadhyay, Navneet_Kumar
AF	7	6382	6382	Dhanjal, Daljeet Singh	Dhanjal, Daljeet_Singh

Record 42 | WOS:001416156300001

Ochratoxin A induces immunotoxicity by targeting Annexin A1 mediated neutrophil apoptosis in zebrafish

Field	Position	Original line	Normalised line	Original value	Normalised value
AU	1	6845	6845	Zheng, YH	Zheng, Yihong
AU	2	6846	6846	Liu, YN	Liu, Yinuo
AU	3	6847	6847	Tian, J	Tian, Jin
AU	4	6848	6848	Liu, SH	Liu, Shuhong
AU	5	6849	6849	Ma, GW	Ma, Gaowei
AU	6	6850	6850	Xie, YP	Xie, Yupeng
AU	7	6851	6851	Zheng, CH	Zheng, Chenhua
AU	8	6852	6852	Wu, ZK	Wu, Zekai

Record 43 | WOS:001393437600001

Chemical Composition, In Vivo, and In Silico Molecular Docking Studies of the Effect of Syzygium aromaticum (Clove) Essential Oil on Ochratoxin A-Induced Acute Neurotoxicity

Field	Position	Original line	Normalised line	Original value	Normalised value
AU	1	6948	6948	Brahmi, M	Brahmi, Mostapha
AU	2	6949	6949	Adli, DEH	Adli, Djallal_Eddine_H
AU	3	6950	6950	Kaoudj, I	Kaoudj, Imane
AU	4	6951	6951	Alkholifi, FK	Alkholifi, Faisal_K
AU	5	6952	6952	Arabi, W	Arabi, Wafaa
AU	6	6953	6953	Sohbi, S	Sohbi, Soumia
AU	7	6954	6954	Ziani, K	Ziani, Kaddour
AU	8	6955	6955	Kahloula, K	Kahloula, Khaled
AU	9	6956	6956	Slimani, M	Slimani, Miloud
AU	10	6957	6957	Sweilam, SH	Sweilam, Sherouk_Hussein
AF	2	6959	6959	Adli, Djallal Eddine H.	Adli, Djallal_Eddine_H
AF	4	6961	6961	Alkholifi, Faisal K.	Alkholifi, Faisal_K
AF	10	6967	6967	Sweilam, Sherouk Hussein	Sweilam, Sherouk_Hussein

Record 44 | WOS:001404966700001

Proteomic Analysis of the Murine Liver Response to Oral Exposure to Aflatoxin B1 and Ochratoxin A: The Protective Role to Bioactive Compounds

Field	Position	Original line	Normalised line	Original value	Normalised value
AU	1	7110	7110	Trombetti, S	Trombetti, Silvia

AU	2	7111	7111	Cimbalo, A	Cimbalo, Alessandra
AU	3	7112	7112	Grosso, M	Grosso, Michela
AU	4	7113	7113	Vila-Donat, P	Vila-Donat, Pilar
AU	5	7114	7114	Mañes, J	Manes, Jordi
AU	6	7115	7115	Manyes, L	Manyes, Lara

Record 45 | WOS:001370729500001

Alterations in the proteomes of HepG2 and IHKE cells inflicted by six selected mycotoxins

Field	Position	Original line	Normalised line	Original value	Normalised value
AU	1	7231	7231	Keuter, L	Keuter, Lucas
AU	2	7232	7232	Fortmann, M	Fortmann, Marco
AU	3	7233	7233	Behrens, M	Behrens, Matthias
AU	4	7234	7234	Humpf, HU	Humpf, Hans-Ulrich

Record 46 | WOS:001375296600001

Bioavailability study of OTA, ZEN, and AFB1 along with bioactive compounds from tiger nut beverage and its by-products

Field	Position	Original line	Normalised line	Original value	Normalised value
AU	1	7372	7372	Llorens, P	Llorens, Paula
AU	2	7373	7373	Juan-García, A	Juan-Garcia, Ana
AU	5	7376	7376	Martí-Quijal, FJ	Marti-Quijal, F_J
AU	6	7377	7377	Esteve-Turrillas, FA	Esteve-Turrillas, F_A
AU	7	7378	7378	Barba, FJ	Barba, F_J
AU	8	7379	7379	Chiacchio, MF	Chiacchio, M_F
AU	10	7381	7381	Moltó, JC	Molto, Juan_Carlos
AU	11	7382	7382	Juan, C	Juan, Cristina
AF	1	7383	7383	Llorens, P.	Llorens, Paula
AF	2	7384	7384	Juan-Garcia, A.	Juan-Garcia, Ana
AF	3	7385	7385	Pardo, O.	Pardo, O
AF	4	7386	7386	Arjona-Mudarra, P.	Arjona-Mudarra, P
AF	5	7387	7387	Marti-Quijal, F. J.	Marti-Quijal, F_J
AF	6	7388	7388	Esteve-Turrillas, F. A.	Esteve-Turrillas, F_A
AF	7	7389	7389	Barba, F. J.	Barba, F_J
AF	8	7390	7390	Chiacchio, M. F.	Chiacchio, M_F
AF	9	7391	7391	Vitaglione, P.	Vitaglione, P
AF	10	7392	7392	Molto, J. C.	Molto, Juan_Carlos
AF	11	7393	7393	Juan, C.	Juan, Cristina

Record 47 | WOS:001384323500001

Development of an Easy-To-Use Microfluidic System to Assess Dynamic Exposure to Mycotoxins in 3D Culture Models: Evaluation of Ochratoxin A and Patulin Cytotoxicity

Field	Position	Original line	Normalised line	Original value	Normalised value
AU	1	7538	7538	Zingales, V	Zingales, Veronica
AU	2	7539	7539	Piunti, C	Piunti, Caterina
AU	3	7540	7540	Micheli, S	Micheli, Sara

AU	4	7541	7541	Cimetta, E	Cimetta, Elisa
AU	5	7542	7542	Ruiz, MJ	Ruiz, Maria-Jose

Record 48 | WOS:001356112700001

DNA ligase III mediates deoxynivalenol exposure-induced DNA damage in intestinal epithelial cells by regulating oxidative stress and interaction with PCNA

Field	Position	Original line	Normalised line	Original value	Normalised value
AU	1	7672	7672	Zhu, XY	Zhu, Xiaoyang
AU	2	7673	7673	Wu, JY	Wu, Jiayun
AU	3	7674	7674	Chen, XL	Chen, Xiaolei
AU	4	7675	7675	Shi, DF	Shi, Dongfeng
AU	5	7676	7676	Hui, P	Hui, Peng
AU	6	7677	7677	Wang, HF	Wang, Haifei
AU	7	7678	7678	Wu, ZC	Wu, Zhengchang
AU	8	7679	7679	Wu, SL	Wu, Shenglong
AU	9	7680	7680	Bao, WB	Bao, Wenbin
AU	10	7681	7681	Fan, HR	Fan, Hairui

Record 49 | WOS:001366569000001

Chlorophyll-Amended Organoclays for the Detoxification of Ochratoxin A

Field	Position	Original line	Normalised line	Original value	Normalised value
AU	1	7812	7812	Oladele, JO	Oladele, Johnson_O
AU	2	7813	7813	Wang, MC	Wang, Meichen
AU	3	7814	7814	Xenophontos, X	Xenophontos, Xenophon
AU	4	7815	7815	Lilly, K	Lilly, Kendall
AU	5	7816	7816	Tamamis, P	Tamamis, Phanourios
AU	6	7817	7817	Phillips, TD	Phillips, Timothy_D
AF	1	7818	7818	Oladele, Johnson O.	Oladele, Johnson_O
AF	6	7823	7823	Phillips, Timothy D.	Phillips, Timothy_D

Record 50 | WOS:001351279800001

Protective Effects of Carotenoid-Loaded Nanostructured Lipid Carriers Against Ochratoxin-A-Induced Cytotoxicity

Field	Position	Original line	Normalised line	Original value	Normalised value
AU	1	7955	7955	Pinna, N	Pinna, Nicola
AU	2	7956	7956	Vila-Donat, P	Vila-Donat, Pilar
AU	3	7957	7957	Pasca, D	Pasca, Denisia
AU	4	7958	7958	Blasi, F	Blasi, Francesca
AU	5	7959	7959	Schoubben, A	Schoubben, Aurelie
AU	6	7960	7960	Manyes, L	Manyes, Lara

Record 51 | WOS:001329697100001

Unveiling the hub genes associated with ochratoxin A-induced hepatotoxicity in broiler chickens

Field	Position	Original line	Normalised line	Original value	Normalised value
AU	1	8073	8073	Li, XF	Li, Xiaofeng
AU	2	8074	8074	Wang, ZY	Wang, Zhongyuan
AU	3	8075	8075	Wang, SJ	Wang, Shujuan
AU	4	8076	8076	Yang, B	Yang, Bing

Record 52 | WOS:001314852000005

Investigating the protective properties of Panax ginseng and its constituents against biotoxins and metal toxicity: a mechanistic review

Field	Position	Original line	Normalised line	Original value	Normalised value
AU	1	8174	8174	Rameshrad, M	Rameshrad, Maryam
AU	2	8175	8175	Memariani, Z	Memariani, Zahra
AU	3	8176	8176	Naraki, K	Naraki, Karim
AU	4	8177	8177	Hosseinzadeh, H	Hosseinzadeh, Hossein

Record 53 | WOS:001323506900001

Phytochemical Characterization of Bilberries and Their Potential as a Functional Ingredient to Mitigate Ochratoxin A Toxicity in Cereal-Based Products

Field	Position	Original line	Normalised line	Original value	Normalised value
AU	1	8379	8379	Pasca, D	Pasca, Denisia
AU	2	8380	8380	Frangiamone, M	Frangiamone, Massimo
AU	3	8381	8381	Mangiapelo, L	Mangiapelo, Luciano
AU	4	8382	8382	Vila-Donat, P	Vila-Donat, Pilar
AU	5	8383	8383	Mirza, O	Mirza, Oana
AU	6	8384	8384	Vlase, AM	Vlase, Ana-Maria
AU	7	8385	8385	Miere, D	Miere, Doina
AU	8	8386	8386	Filip, L	Filip, Lorena
AU	9	8387	8387	Mañes, J	Manes, Jordi
AU	10	8388	8388	Loghin, F	Loghin, Felicia
AU	11	8389	8389	Manyes, L	Manyes, Lara

Record 54 | WOS:001345720100005

Review: Strategies and technologies in preventing regulated and emerging mycotoxin co-contamination in forage for safeguarding ruminant health

Field	Position	Original line	Normalised line	Original value	Normalised value
AU	1	8524	8524	Gallo, A	Gallo, Antonio
AF	1	8529	8529	Gallo, A.	Gallo, Antonio
AF	2	8530	8530	Catellani, A.	Catellani, A
AF	3	8531	8531	Ghilardelli, F.	Ghilardelli, F
AF	4	8532	8532	Lapris, M.	Lapris, M
AF	5	8533	8533	Mastroeni, C.	Mastroeni, C

Record 55 | WOS:001296248000001

Effects of isochlorogenic acid A on mitochondrial dynamics imbalance and RLR damage in PAM cells induced by combined mycotoxins

Field	Position	Original line	Normalised line	Original value	Normalised value
AU	1	8769	8769	Lai, XN	Lai, Xinuo
AU	2	8770	8770	Fan, P	Fan, Peng
AU	3	8771	8771	Deng, HD	Deng, Huidan
AU	4	8772	8772	Jia, GL	Jia, Guilin
AU	5	8773	8773	Zuo, ZC	Zuo, Zhicai
AU	6	8774	8774	Hu, YC	Hu, Yanchun
AU	7	8775	8775	Wang, Y	Wang, Ya
AU	8	8776	8776	Cai, DJ	Cai, Dongjie
AU	9	8777	8777	Gou, LP	Gou, Liping
AU	10	8778	8778	Wen, YP	Wen, Yiping
AU	11	8779	8779	Yu, SM	Yu, Shumin
AU	12	8780	8780	Cao, SZ	Cao, Suizhong
AU	13	8781	8781	Shen, LH	Shen, Liuhong
AU	14	8782	8782	Deng, JL	Deng, Junliang
AU	15	8783	8783	Ren, ZH	Ren, Zhihua

Record 56 | WOS:001291367000001

Ochratoxin A-induced DNA damage triggers G2 phase arrest via hMLH1-p53-p21 signaling pathway in human gastric epithelium immortalized cells in vitro

Field	Position	Original line	Normalised line	Original value	Normalised value
AU	1	8904	8904	Jia, X	Jia, Xin
AU	2	8905	8905	Wang, Y	Wang, Yuan
AU	3	8906	8906	Cui, JF	Cui, Jinfeng
AU	4	8907	8907	Li, YH	Li, Yuehong
AU	5	8908	8908	Wu, WX	Wu, Wenxin
AU	6	8909	8909	Zhang, XH	Zhang, Xianghong
AU	7	8910	8910	Wang, J	Wang, Juan

Record 57 | WOS:001325349600001

Evaluating the combined and individual cytotoxic effect of beauvericin, enniatin B and ochratoxin a on breast cancer cells, leukemia cells, and fresh peripheral blood mononuclear cells

Field	Position	Original line	Normalised line	Original value	Normalised value
AU	1	9008	9008	Juan-García, A	Juan-Garcia, Ana
AU	2	9009	9009	Ilie, AM	Ilie, Ana-Maria
AU	3	9010	9010	Juan, CSA	Juan, Cristina
AU	4	9011	9011	Martínez, L	Martinez, Lola

Record 58 | WOS:001277668700001

The Novel Role of the NLRP3 Inflammasome in Mycotoxin-Induced Toxicological Mechanisms

Field	Position	Original line	Normalised line	Original value	Normalised value
AU	1	9126	9126	Liao, CS	Liao, Chengshui
AU	2	9127	9127	Xu, FR	Xu, Fengru
AU	3	9128	9128	Yu, ZH	Yu, Zuhua
AU	4	9129	9129	Ding, K	Ding, Ke

AU	5	9130	9130	Jia, YY	Jia, Yanyan

Record 59 | WOS:001263565100001

Reduction of aflatoxins and ochratoxin A by addition of commercial Koji during fermentation of the Korean traditional soybean paste, Doenjang

Field	Position	Original line	Normalised line	Original value	Normalised value
AU	1	9326	9326	Woo, SY	Woo, So_Young
AU	2	9327	9327	Tian, F	Tian, Fei
AU	3	9328	9328	Lee, SY	Lee, Sang_Yoo
AU	4	9329	9329	Park, SB	Park, Su_Been
AU	5	9330	9330	Han, KH	Han, Kap-Hoon
AU	6	9331	9331	Kim, HY	Kim, Hye-Young
AU	7	9332	9332	Chun, HS	Chun, Hyang_Sook
AF	1	9333	9333	Woo, So Young	Woo, So_Young
AF	3	9335	9335	Lee, Sang Yoo	Lee, Sang_Yoo
AF	4	9336	9336	Park, Su Been	Park, Su_Been
AF	7	9339	9339	Chun, Hyang Sook	Chun, Hyang_Sook

Record 60 | WOS:001257266000001

Zearalenone modulates the function of goat endometrial cells via the mitochondrial quality control system

Field	Position	Original line	Normalised line	Original value	Normalised value
AU	1	9438	9438	Zhang, GM	Zhang, Guomin
AU	2	9439	9439	Zeng, C	Zeng, Cheng
AU	3	9440	9440	Sun, X	Sun, Xuan
AU	4	9441	9441	Zhang, Q	Zhang, Qi
AU	5	9442	9442	Wang, YF	Wang, Yifei
AU	6	9443	9443	Xia, RX	Xia, Rongxin
AU	7	9444	9444	Mai, QY	Mai, Qingyang
AU	8	9445	9445	Xue, G	Xue, Gang
AU	9	9446	9446	Huang, H	Huang, Han
AU	10	9447	9447	Wang, F	Wang, Feng

Record 61 | WOS:001250935500001

Central role of Sigma-1 receptor in ochratoxin A-induced ferroptosis

Field	Position	Original line	Normalised line	Original value	Normalised value
AU	1	9583	9583	Chen, WY	Chen, Wenying
AU	2	9584	9584	Han, LY	Han, Lingyun
AU	3	9585	9585	Yang, RR	Yang, Ruiran
AU	4	9586	9586	Wang, HW	Wang, Hongwei
AU	5	9587	9587	Yao, S	Yao, Song
AU	6	9588	9588	Deng, HQ	Deng, Huiqiong
AU	7	9589	9589	Liu, SC	Liu, Shuangchao
AU	8	9590	9590	Zhou, Y	Zhou, Yao
AU	9	9591	9591	Shen, XL	Shen, Xiao_Li

AF	9	9600	9600	Shen, Xiao Li	Shen, Xiao_Li

Record 62 | WOS:001263872600001

Occurrence of the two major regulated mycotoxins, ochratoxin A and fumonisin B1, in cereal and cereal-based products in Europe and toxicological effects: A review

Field	Position	Original line	Normalised line	Original value	Normalised value
AU	1	9715	9715	Arce-López, B	Arce-Lopez, Beatriz
AU	2	9716	9716	Coton, M	Coton, Monika
AU	3	9717	9717	Coton, E	Coton, Emmanuel
AU	4	9718	9718	Hymery, N	Hymery, Nolwenn

Record 63 | WOS:001256451400001

High-Coverage UHPLC-MS/MS Analysis of 67 Mycotoxins in Plasma for Male Infertility Exposure Studies

Field	Position	Original line	Normalised line	Original value	Normalised value
AU	1	10002	10002	Ning, X	Ning, Xiao
AU	2	10003	10003	Wang, LL	Wang, Lulu
AU	3	10004	10004	Wang, JS	Wang, Jia-Sheng
AU	4	10005	10005	Ji, J	Ji, Jian
AU	5	10006	10006	Jin, SM	Jin, Shaoming
AU	6	10007	10007	Sun, JD	Sun, Jiadi
AU	7	10008	10008	Ye, YL	Ye, Yongli
AU	8	10009	10009	Mei, SH	Mei, Shenghui
AU	9	10010	10010	Zhang, YZ	Zhang, Yinzhi
AU	10	10011	10011	Cao, J	Cao, Jin
AU	11	10012	10012	Sun, XL	Sun, Xiulan

Record 64 | WOS:001234877500001

Investigation of the mechanisms behind ochratoxin A-induced cytotoxicity in human astrocytes and the protective effects of N-acetylcysteine

Field	Position	Original line	Normalised line	Original value	Normalised value
AU	1	10154	10154	Chu, CS	Chu, Che-Sheng
AU	2	10155	10155	Chen, YT	Chen, Ying-Tso
AU	3	10156	10156	Liang, WZ	Liang, Wei-Zhe

Record 65 | WOS:001229022000001

The efficiency of mycotoxin binding by sorbents in the in vitro model using a naturally contaminated animal feed

Field	Position	Original line	Normalised line	Original value	Normalised value
AU	1	10272	10272	Zybura, A	Zybura, Adrianna
AU	2	10273	10273	Jedziniak, P	Jedziniak, Piotr

Record 66 | WOS:001228887800001

In-vivo evaluation of the adverse effects of ochratoxin A on broiler chicken health and adsorption efficacy of indigenous and commercial clay of Balochistan, Pakistan

Field	Position	Original line	Normalised line	Original value	Normalised value
AU	1	10357	10357	Abro, MR	Abro, Mustafa_Rahim
AU	2	10358	10358	Rashid, N	Rashid, Nadeem
AU	4	10360	10360	Siddique, Z	Siddique, Zainab
AF	1	10361	10361	Abro, Mustafa Rahim	Abro, Mustafa_Rahim

Record 67 | WOS:001222378900001

Role of albumin in the metabolism and excretion of ochratoxin A

Field	Position	Original line	Normalised line	Original value	Normalised value
AU	1	10489	10489	Kuhn, M	Kuhn, Michael
AU	2	10490	10490	Hassan, R	Hassan, Reham
AU	3	10491	10491	González, D	Gonzalez, Daniela
AU	4	10492	10492	Myllys, M	Myllys, Maiju
AU	5	10493	10493	Hobloss, Z	Hobloss, Zaynab
AU	6	10494	10494	Degen, GH	Degen, Gisela_H
AU	7	10495	10495	Humpf, HU	Humpf, Hans-Ulrich
AU	8	10496	10496	Hengstler, JG	Hengstler, Jan_G
AU	9	10497	10497	Cramer, B	Cramer, Benedikt
AU	10	10498	10498	Ghallab, A	Ghallab, Ahmed
AF	6	10504	10504	Degen, Gisela H.	Degen, Gisela_H
AF	8	10506	10506	Hengstler, Jan G.	Hengstler, Jan_G

Record 68 | WOS:001220328900001

Unveiling the emerging role of curcumin to alleviate ochratoxin A-induced muscle toxicity in grass carp (Ctenopharyngodon idella): in vitro and in vivo studies

Field	Position	Original line	Normalised line	Original value	Normalised value
AU	1	10616	10616	Zhao, P	Zhao, Piao
AU	2	10617	10617	Feng, L	Feng, Lin
AU	3	10618	10618	Jiang, WD	Jiang, Weidan
AU	4	10619	10619	Wu, P	Wu, Pei
AU	5	10620	10620	Liu, Y	Liu, Yang
AU	6	10621	10621	Ren, HM	Ren, Hongmei
AU	7	10622	10622	Jin, XW	Jin, Xiaowan
AU	8	10623	10623	Zhang, L	Zhang, Lu
AU	9	10624	10624	Mi, HF	Mi, Haifeng
AU	10	10625	10625	Zhou, XQ	Zhou, Xiaoqiu

Record 69 | WOS:001215577500001

Protective Effect of Boric Acid Against Ochratoxin A-Induced Toxic Effects in Human Embryonal Kidney Cells (HEK293): A Study on Cytotoxic, Genotoxic, Oxidative, and Apoptotic Effects

Field	Position	Original line	Normalised line	Original value	Normalised value
AU	1	10779	10779	Tekin, A	Tekin, Askin
AU	2	10780	10780	Güner, A	Guner, Adem
AU	3	10781	10781	Akkan, T	Akkan, Tamer

Record 70 | WOS:001231234500001

Alternariol Monomethyl-Ether Induces Toxicity via Cell Death and Oxidative Stress in Swine Intestinal Epithelial Cells

Field	Position	Original line	Normalised line	Original value	Normalised value
AU	1	10911	10911	Marin, DE	Marin, Daniela_Eliza
AU	2	10912	10912	Bulgaru, VC	Bulgaru, Valeria_Cristina
AU	3	10913	10913	Pertea, A	Pertea, Anamaria
AU	4	10914	10914	Grosu, IA	Grosu, Iulian_Alexandru
AU	5	10915	10915	Pistol, GC	Pistol, Gina_Cecilia
AU	6	10916	10916	Taranu, I	Taranu, Ionelia
AF	1	10917	10917	Marin, Daniela Eliza	Marin, Daniela_Eliza
AF	2	10918	10918	Bulgaru, Valeria Cristina	Bulgaru, Valeria_Cristina
AF	4	10920	10920	Grosu, Iulian Alexandru	Grosu, Iulian_Alexandru
AF	5	10921	10921	Pistol, Gina Cecilia	Pistol, Gina_Cecilia

Record 71 | WOS:001227638700001

Development and application of the physiologically-based toxicokinetic (PBTK) model for ochratoxin A (OTA) in rats and humans

Field	Position	Original line	Normalised line	Original value	Normalised value
AU	1	11029	11029	Su, BD	Su, Bu-Da
AU	2	11030	11030	Li, XM	Li, Xiao-Meng
AU	3	11031	11031	Huang, ZW	Huang, Zhi-Wei
AU	4	11032	11032	Wang, Y	Wang, Yue
AU	5	11033	11033	Shao, J	Shao, Jia
AU	6	11034	11034	Xu, YY	Xu, Yan-Yan
AU	7	11035	11035	Shu, LX	Shu, Le-Xin
AU	8	11036	11036	Li, YB	Li, Yu-Bo

Record 72 | WOS:001209938800001

Comprehensive Insights into Ochratoxin A: Occurrence, Analysis, and Control Strategies

Field	Position	Original line	Normalised line	Original value	Normalised value
AU	1	11195	11195	Ben Miri, Y	Ben_Miri, Yamina
AU	2	11196	11196	Benabdallah, A	Benabdallah, Amina
AU	3	11197	11197	Chentir, I	Chentir, Imene
AU	4	11198	11198	Djenane, D	Djenane, Djamel
AU	5	11199	11199	Luvisi, A	Luvisi, Andrea
AU	6	11200	11200	De Bellis, L	de_Bellis, Luigi
AF	1	11201	11201	Ben Miri, Yamina	Ben_Miri, Yamina
AF	6	11206	11206	De Bellis, Luigi	de_Bellis, Luigi

Record 73 | WOS:001221312500001

In vitro and in vivo assessment of AFB1 and OTA toxic effects and the beneficial role of bioactive compounds. A systematic review

Field	Position	Original line	Normalised line	Original value	Normalised value

AU	1	11592	11592	Frangiamone, M	Frangiamone, Massimo
AU	2	11593	11593	Cimbalo, A	Cimbalo, Alessandra
AU	3	11594	11594	Font, G	Font, Guillermina
AU	4	11595	11595	Manyes, L	Manyes, Lara

Record 74 | WOS:001219077100001

Disruption of intestinal epithelial permeability in the Co-culture system of Caco-2/HT29-MTX cells exposed individually or simultaneously to acrylamide and ochratoxin A

Field	Position	Original line	Normalised line	Original value	Normalised value
AU	1	11749	11749	Lu, JW	Lu, Jiawen
AU	2	11750	11750	Su, D	Su, Dan
AU	3	11751	11751	Yang, Y	Yang, Ying
AU	4	11752	11752	Shu, MN	Shu, Mengni
AU	5	11753	11753	Wang, YT	Wang, Yuting
AU	6	11754	11754	Zhou, XT	Zhou, Xingtao
AU	7	11755	11755	Yu, Q	Yu, Qiang
AU	8	11756	11756	Li, C	Li, Chang
AU	9	11757	11757	Xie, JH	Xie, Jianhua
AU	10	11758	11758	Chen, Y	Chen, Yi

Record 75 | WOS:001185505600042

A high-throughput gut-on-chip platform to study the epithelial responses to enterotoxins

Field	Position	Original line	Normalised line	Original value	Normalised value
AU	1	11899	11899	Morelli, M	Morelli, Moran
AU	2	11900	11900	Rodríguez, MC	Rodriguez, Marta_Cabezuelo
AU	3	11901	11901	Queiroz, K	Queiroz, Karla
AF	2	11903	11903	Rodriguez, Marta Cabezuelo	Rodriguez, Marta_Cabezuelo

Record 76 | WOS:001187481100001

Efficacy of a multicomponent binding agent against combined exposure to zearalenone and ochratoxin A in weaned pigs

Field	Position	Original line	Normalised line	Original value	Normalised value
AU	1	12012	12012	Tassis, P	Tassis, Panagiotis
AU	2	12013	12013	Raj, J	Raj, Jog
AU	3	12014	12014	Floros, D	Floros, Dimitrios
AU	4	12015	12015	Mittas, N	Mittas, Nikolaos
AU	5	12016	12016	Ntarampa, N	Ntarampa, Niki
AU	6	12017	12017	Farkas, H	Farkas, Hunor
AU	7	12018	12018	Polizopoulou, Z	Polizopoulou, Zoe
AU	8	12019	12019	Vasilievic, M	Vasilievic, Marko

Record 77 | WOS:001192691800001

Red Orange and Lemon Extract Ameliorates the Renal Oxidative Stress and Inflammation Induced by Ochratoxin A through the Modulation of Nrf2

Field	Position	Original line	Normalised line	Original value	Normalised value
AU	1	12133	12133	Longobardi, C	Longobardi, Consiglia
AU	2	12134	12134	Damiano, S	Damiano, Sara
AU	3	12135	12135	Fabroni, S	Fabroni, Simona
AU	4	12136	12136	Montagnaro, S	Montagnaro, Serena
AU	5	12137	12137	Russo, V	Russo, Valeria
AU	6	12138	12138	Vaccaro, E	Vaccaro, Emanuela
AU	7	12139	12139	Giordano, A	Giordano, Antonio
AU	8	12140	12140	Florio, S	Florio, Salvatore
AU	9	12141	12141	Ciarcia, R	Ciarcia, Roberto

Record 78 | WOS:001176181400001

Natural feed additives and bioactive supplements versus chemical additives as a safe and practical approach to combat foodborne mycotoxicoses

Field	Position	Original line	Normalised line	Original value	Normalised value
AU	1	12259	12259	Stoev, SD	Stoev, Stoycho_D
AF	1	12260	12260	Stoev, Stoycho D.	Stoev, Stoycho_D

Record 79 | WOS:001179683700001

In vitro and in vivo induction of ochratoxin A exposure-related micronucleus formation in rat proximal tubular epithelial cells and expression profiling of chromosomal instability-related genes

Field	Position	Original line	Normalised line	Original value	Normalised value
AU	1	12613	12613	Ozawa, S	Ozawa, Shunsuke
AU	2	12614	12614	Ojiro, R	Ojiro, Ryota
AU	3	12615	12615	Tang, Q	Tang, Qian
AU	4	12616	12616	Zou, XY	Zou, Xinyu
AU	5	12617	12617	Jin, ML	Jin, Meilan
AU	6	12618	12618	Yoshida, T	Yoshida, Toshinori
AU	7	12619	12619	Shibutani, M	Shibutani, Makoto

Record 80 | WOS:001172173100001

Comparative Study of Spheroids (3D) and Monolayer Cultures (2D) for the In Vitro Assessment of Cytotoxicity Induced by the Mycotoxins Sterigmatocystin, Ochratoxin A and Patulin

Field	Position	Original line	Normalised line	Original value	Normalised value
AU	1	12761	12761	Zingales, V	Zingales, Veronica
AU	2	12762	12762	Esposito, MR	Esposito, Maria_Rosaria
AU	3	12763	12763	Quagliata, M	Quagliata, Martina
AU	4	12764	12764	Cimetta, E	Cimetta, Elisa
AU	5	12765	12765	Ruiz, MJ	Ruiz, Maria-Jose
AF	2	12767	12767	Esposito, Maria Rosaria	Esposito, Maria_Rosaria

Record 81 | WOS:001175293000001

Involvement of pro-inflammatory mediators and cell cycle disruption in neuronal cells induced by gliotoxin and ochratoxin A after individual and combined exposure

Field	Position	Original line	Normalised line	Original value	Normalised value

AU	1	12895	12895	Penalva-Olcina, R	Penalva-Olcina, Raquel
AU	2	12896	12896	Juan, CSA	Juan, Cristina
AU	3	12897	12897	Fernández-Franzón, M	Fernandez-Franzon, Monica
AU	4	12898	12898	Juan-García, A	Juan-Garcia, Ana

Record 82 | WOS:001154746400001

Recent advances in the degradation efficacy and mechanisms of mycotoxins in food by atmospheric cold plasma

Field	Position	Original line	Normalised line	Original value	Normalised value
AU	1	13007	13007	Liu, MJ	Liu, Mengjie
AU	2	13008	13008	Feng, JX	Feng, Junxia
AU	3	13009	13009	Yang, XD	Yang, Xudong
AU	4	13010	13010	Yu, B	Yu, Bo
AU	5	13011	13011	Zhuang, J	Zhuang, Jie
AU	6	13012	13012	Xu, HB	Xu, Hangbo
AU	7	13013	13013	Xiang, QS	Xiang, Qisen
AU	8	13014	13014	Ma, RA	Ma, Ruonan
AU	9	13015	13015	Jiao, Z	Jiao, Zhen

Record 83 | WOS:001201732900001

Comparative Assessment of Thermal Treatment for Mycotoxins Adsorption Potential of Indigenous Clay from Balochistan, Pakistan

Field	Position	Original line	Normalised line	Original value	Normalised value
AU	1	13176	13176	Abro, MR	Abro, Mustafa_Rahim
AU	2	13177	13177	Rashid, N	Rashid, Nadeem
AU	4	13179	13179	Soomro, RN	Soomro, Rab_Nawaz
AU	5	13180	13180	Siddique, Z	Siddique, Zainab
AF	1	13181	13181	Abro, Mustafa Rahim	Abro, Mustafa_Rahim
AF	4	13184	13184	Soomro, Rab Nawaz	Soomro, Rab_Nawaz

Record 84 | WOS:001151094300001

Practical Strategies to Reduce Ochratoxin A in Foods

Field	Position	Original line	Normalised line	Original value	Normalised value
AU	1	13273	13273	Lee, HJ	Lee, Hyun_Jung
AU	2	13274	13274	Kim, HD	Kim, Hae_Dun
AU	3	13275	13275	Ryu, D	Ryu, Dojin
AF	1	13276	13276	Lee, Hyun Jung	Lee, Hyun_Jung
AF	2	13277	13277	Kim, Hae Dun	Kim, Hae_Dun

Record 85 | WOS:001262485700001

Grape pomace as a novel functional ingredient: Mitigating ochratoxin A bioaccessibility and unraveling cytoprotective mechanisms in vitro

Field	Position	Original line	Normalised line	Original value	Normalised value
AU	1	13497	13497	Mangiapelo, L	Mangiapelo, Luciano

AU	2	13498	13498	Frangiamone, M	Frangiamone, Massimo
AU	3	13499	13499	Vila-Donat, P	Vila-Donat, Pilar
AU	4	13500	13500	Pasca, D	Pasca, Denisia
AU	5	13501	13501	Ianni, F	Ianni, Federica
AU	6	13502	13502	Cossignani, L	Cossignani, Lina
AU	7	13503	13503	Manyes, L	Manyes, Lara

Record 86 | WOS:001145669600001

Ochratoxin A-enhanced glycolysis induces inflammatory responses in human gastric epithelium cells through mTOR/HIF-1α signaling pathway

Field	Position	Original line	Normalised line	Original value	Normalised value
AU	1	13629	13629	Wang, Y	Wang, Yuan
AU	2	13630	13630	Zhao, M	Zhao, Man
AU	3	13631	13631	Cui, JF	Cui, Jinfeng
AU	4	13632	13632	Lian, HG	Lian, Hongguang
AU	5	13633	13633	Hao, ZF	Hao, Zengfang
AU	6	13634	13634	Lou, L	Lou, Lei
AU	7	13635	13635	Jia, X	Jia, Xin
AU	8	13636	13636	Zhao, W	Zhao, Wei
AU	9	13637	13637	Shen, HT	Shen, Haitao
AU	10	13638	13638	Xing, LX	Xing, Lingxiao
AU	11	13639	13639	Zhang, XH	Zhang, Xianghong

Record 87 | WOS:001119207400001

Elucidating the mechanism of action of mycotoxins through machine learning-driven QSAR models: Focus on lipid peroxidation

Field	Position	Original line	Normalised line	Original value	Normalised value
AU	1	13742	13742	Galvez-Llompart, M	Galvez-Llompart, Maria
AU	2	13743	13743	Zanni, R	Zanni, Riccardo
AU	3	13744	13744	Manyes, L	Manyes, Lara
AU	4	13745	13745	Meca, G	Meca, Giuseppe

Record 88 | WOS:001099952500001

Risks for animal health related to the presence of ochratoxin A (OTA) in feed

Field	Position	Original line	Normalised line	Original value	Normalised value
AU	1	13896	13896	Schrenk, D	Schrenk, Dieter
AU	2	13897	13897	Bignami, M	Bignami, Margherita
AU	3	13898	13898	Bodin, L	Bodin, Laurent
AU	4	13899	13899	Chipman, JK	Chipman, James_Kevin
AU	5	13900	13900	del Mazo, J	del_Mazo, Jesus
AU	6	13901	13901	Grasl-Kraupp, B	Grasl-Kraupp, Bettina
AU	7	13902	13902	Hogstrand, C	Hogstrand, Christer
AU	8	13903	13903	Hoogenboom, L	Hoogenboom, Laurentius_(ron)
AU	9	13904	13904	Leblanc, JC	Leblanc, Jean-Charles
AU	10	13905	13905	Nielsen, E	Nielsen, Elsa

AU	11	13906	13906	Ntzani, E	Ntzani, Evangelia
AU	12	13907	13907	Sand, S	Sand, Salomon
AU	13	13908	13908	Schwerdtle, T	Schwerdtle, Tanja
AU	14	13909	13909	Vleminckx, C	Vleminckx, Christiane
AU	15	13910	13910	Wallace, H	Wallace, Heather
AU	16	13911	13911	Gropp, J	Gropp, Jurgen
AU	17	13912	13912	Antonissen, G	Antonissen, Gunther
AU	18	13913	13913	Rychen, G	Rychen, Guido
AU	19	13914	13914	Ruiz, JAG	Ruiz, Jose_Angel_Gomez
AU	20	13915	13915	Innocenti, ML	Innocenti, Matteo_Lorenzo
AU	21	13916	13916	Rovesti, E	Rovesti, Elena
AU	22	13917	13917	Petersen, A	Petersen, Annette
AF	4	13921	13921	Chipman, James Kevin	Chipman, James_Kevin
AF	5	13922	13922	del Mazo, Jesus	del_Mazo, Jesus
AF	8	13925	13925	Hoogenboom, Laurentius (Ron)	Hoogenboom, Laurentius_(ron)
AF	19	13936	13936	Ruiz, Jose Angel Gomez	Ruiz, Jose_Angel_Gomez
AF	20	13937	13937	Innocenti, Matteo Lorenzo	Innocenti, Matteo_Lorenzo

Record 89 | WOS:001072761100001

Farnesoid X Receptor Plays a Key Role in Ochratoxin A-Induced Nephrotoxicity by Targeting Ferroptosis In Vivo and In Vitro

Field	Position	Original line	Normalised line	Original value	Normalised value
AU	1	14168	14168	Tang, JY	Tang, Jiangyu
AU	2	14169	14169	Zeng, JY	Zeng, Junya
AU	3	14170	14170	Chen, L	Chen, Li
AU	4	14171	14171	Wang, MM	Wang, Mengmeng
AU	5	14172	14172	He, SB	He, Suibin
AU	6	14173	14173	Muhmood, A	Muhmood, Azhar
AU	7	14174	14174	Chen, XX	Chen, Xingxiang
AU	8	14175	14175	Huang, KH	Huang, Kehe
AU	9	14176	14176	Gan, F	Gan, Fang

Record 90 | WOS:001079796800001

Neurotoxicity of ochratoxin A: Molecular mechanisms and neurotherapeutic strategies

Field	Position	Original line	Normalised line	Original value	Normalised value
AU	1	14296	14296	Obafemi, BA	Obafemi, Blessing_A
AU	2	14297	14297	Adedara, IA	Adedara, Isaac_A
AU	3	14298	14298	Rocha, JBT	Rocha, Joao_B_T
AF	1	14299	14299	Obafemi, Blessing A.	Obafemi, Blessing_A
AF	2	14300	14300	Adedara, Isaac A.	Adedara, Isaac_A
AF	3	14301	14301	Rocha, Joao B. T.	Rocha, Joao_B_T

Record 91 | WOS:001078658300001

The coexistence of aflatoxin M1 and ochratoxin A induced intestinal barrier disruption via the regulation of key differentially expressed microRNAs and long non-coding RNAs in BALB/c mice

Field	Position	Original line	Normalised line	Original value	Normalised value
AU	1	14506	14506	Gao, YN	Gao, Ya-Nan
AU	2	14507	14507	Min, L	Min, Li
AU	3	14508	14508	Yang, X	Yang, Xue
AU	4	14509	14509	Wang, JQ	Wang, Jia-Qi
AU	5	14510	14510	Zheng, N	Zheng, Nan

Record 92 | WOS:001077853000001

Ochratoxin A: Overview of Prevention, Removal, and Detoxification Methods

Field	Position	Original line	Normalised line	Original value	Normalised value
AU	1	14625	14625	Ding, LJ	Ding, Lijuan
AU	2	14626	14626	Han, MH	Han, Meihua
AU	3	14627	14627	Wang, XT	Wang, Xiangtao
AU	4	14628	14628	Guo, YF	Guo, Yifei

Record 93 | WOS:001060917800001

Exploring the Toxic Effects of ZEA on IPEC-J2 Cells from the Inflammatory Response and Apoptosis

Field	Position	Original line	Normalised line	Original value	Normalised value
AU	1	14795	14795	Guan, HY	Guan, Haoyue
AU	2	14796	14796	Ma, WX	Ma, Wenxue
AU	3	14797	14797	Wu, Q	Wu, Qiong
AU	4	14798	14798	Cai, JZ	Cai, Jingzeng
AU	5	14799	14799	Zhang, ZW	Zhang, Ziwei

Record 94 | WOS:001074097100001

The Effect of Mycotoxins and Their Mixtures on Bovine Spermatozoa Characteristics

Field	Position	Original line	Normalised line	Original value	Normalised value
AU	1	14904	14904	Kalo, D	Kalo, Dorit
AU	2	14905	14905	Mendelson, P	Mendelson, Paz
AU	3	14906	14906	Komsky-Elbaz, A	Komsky-Elbaz, Alisa
AU	4	14907	14907	Voet, H	Voet, Hillary
AU	5	14908	14908	Roth, Z	Roth, Zvi

Record 95 | WOS:001070923600001

Cell cycle and enzymatic activity alterations induced by ROS production in human neuroblastoma cells SH-SY5Y exposed to Fumonisin B1, Ochratoxin A and their combination

Field	Position	Original line	Normalised line	Original value	Normalised value
AU	1	15063	15063	Penalva-Olcina, R	Penalva-Olcina, Raquel
AU	2	15064	15064	Juan, CSA	Juan, Cristina
AU	3	15065	15065	Fernández-Franzón, M	Fernandez-Franzon, Monica
AU	4	15066	15066	Juan-García, A	Juan-Garcia, Ana

Record 96 | WOS:001054307000001

An integrated approach for evaluating the interactive effects between azoxystrobin and ochratoxin A: Pathway-based in vivo analyses

Field	Position	Original line	Normalised line	Original value	Normalised value
AU	1	15170	15170	Wang, RK	Wang, Ruike
AU	2	15171	15171	Huang, N	Huang, Nan
AU	3	15172	15172	Ji, J	Ji, Jing
AU	4	15173	15173	Chen, C	Chen, Chen

Record 97 | WOS:001041623900001

The Protective Effect of Pumpkin and Fermented Whey Mixture against AFB1 and OTA Immune Toxicity In Vitro. A Transcriptomic Approach

Field	Position	Original line	Normalised line	Original value	Normalised value
AU	1	15268	15268	Frangiamone, M	Frangiamone, Massimo
AU	2	15269	15269	Lozano, M	Lozano, Manuel
AU	3	15270	15270	Cimbalo, A	Cimbalo, Alessandra
AU	4	15271	15271	Lazaro, A	Lazaro, Alvaro
AU	5	15272	15272	Font, G	Font, Guillermina
AU	6	15273	15273	Manyes, L	Manyes, Lara

Record 98 | WOS:001055768800001

Mycotoxins in Cereal-Based Products and Their Impacts on the Health of Humans, Livestock Animals and Pets

Field	Position	Original line	Normalised line	Original value	Normalised value
AU	1	15432	15432	Yu, JM	Yu, Jianmei
AU	2	15433	15433	Pedroso, IR	Pedroso, Ivana_Ramos
AF	2	15435	15435	Pedroso, Ivana Ramos	Pedroso, Ivana_Ramos

Record 99 | WOS:001036576500001

Pathological Role of Oxidative Stress in Aflatoxin-Induced Toxicity in Different Experimental Models and Protective Effect of Phytochemicals: A Review

Field	Position	Original line	Normalised line	Original value	Normalised value
AU	1	15747	15747	Jobe, MC	Jobe, Martha_Cebile
AU	2	15748	15748	Mthiyane, DMN	Mthiyane, Doctor_M_N
AU	3	15749	15749	Dludla, PV	Dludla, Phiwayinkosi_V
AU	4	15750	15750	Mazibuko-Mbeje, SE	Mazibuko-Mbeje, Sithandiwe_E
AU	5	15751	15751	Onwudiwe, DC	Onwudiwe, Damian_C
AU	6	15752	15752	Mwanza, M	Mwanza, Mulunda
AF	1	15753	15753	Jobe, Martha Cebile	Jobe, Martha_Cebile
AF	2	15754	15754	Mthiyane, Doctor M. N.	Mthiyane, Doctor_M_N
AF	3	15755	15755	Dludla, Phiwayinkosi V.	Dludla, Phiwayinkosi_V
AF	4	15756	15756	Mazibuko-Mbeje, Sithandiwe E.	Mazibuko-Mbeje, Sithandiwe_E
AF	5	15757	15757	Onwudiwe, Damian C.	Onwudiwe, Damian_C

Record 100 | WOS:001040619100001

MiR-155-5p Elevated by Ochratoxin A Induces Intestinal Fibrosis and Epithelial-to-Mesenchymal Transition through TGF-β Regulated Signaling Pathway In Vitro and In Vivo

Field	Position	Original line	Normalised line	Original value	Normalised value
AU	1	15953	15953	Rhee, KH	Rhee, Kyu_Hyun
AU	2	15954	15954	Yang, SA	Yang, Seon_Ah
AU	3	15955	15955	Pyo, MC	Pyo, Min_Cheol
AU	4	15956	15956	Lim, JM	Lim, Jae-Min
AU	5	15957	15957	Lee, KW	Lee, Kwang-Won
AF	1	15958	15958	Rhee, Kyu Hyun	Rhee, Kyu_Hyun
AF	2	15959	15959	Yang, Seon Ah	Yang, Seon_Ah
AF	3	15960	15960	Pyo, Min Cheol	Pyo, Min_Cheol

Record 101 | WOS:001013318000001

Enzymatic degradation of ochratoxin A in the gastrointestinal tract of piglets

Field	Position	Original line	Normalised line	Original value	Normalised value
AU	1	16058	16058	Prasad, S	Prasad, Shreenath
AU	2	16059	16059	Streit, B	Streit, Barbara
AU	3	16060	16060	Gruber, C	Gruber, Christina
AU	4	16061	16061	Gonaus, C	Gonaus, Christoph

Record 102 | WOS:001011146700001

A Chemically Defined Medium That Supports Mycotoxin Production by Stachybotrys chartarum Enabled Analysis of the Impact of Nitrogen and Carbon Sources on the Biosynthesis of Macrocyclic Trichothecenes and Stachybotrylactam

Field	Position	Original line	Normalised line	Original value	Normalised value
AU	1	16180	16180	Tribelhorn, K	Tribelhorn, Katharina
AU	2	16181	16181	Twaruzek, M	Twaruzek, Magdalena
AU	3	16182	16182	Kosicki, R	Kosicki, Robert
AU	4	16183	16183	Straubinger, RK	Straubinger, Reinhard_K
AU	5	16184	16184	Ebel, F	Ebel, Frank
AU	6	16185	16185	Ulrich, S	Ulrich, Sebastian
AF	4	16189	16189	Straubinger, Reinhard K.	Straubinger, Reinhard_K

Record 103 | WOS:001013506800001

Citrinin as a potential anti-cancer therapy: A comprehensive review

Field	Position	Original line	Normalised line	Original value	Normalised value
AU	1	16347	16347	Menezes, AAPMD	Menezes, Ag-Anne_P_M_de
AU	2	16348	16348	Aguiar, RPS	Aguiar, Rai_P_S
AU	3	16349	16349	Santos, JVO	Santos, Jose_V_O
AU	4	16350	16350	Sarkar, C	Sarkar, Chandan
AU	5	16351	16351	Islam, MT	Islam, Muhammad_T
AU	6	16352	16352	Braga, AL	Braga, Antonio_L

AU	7	16353	16353	Hasan, MM	Hasan, Mohammad_M
AU	8	16354	16354	Silva, FCCD	Silva, Felipe_C_C_da
AU	9	16355	16355	Sharifi-Rad, J	Sharifi-Rad, Javad
AU	10	16356	16356	Dey, A	Dey, Abhijit
AU	11	16357	16357	Calina, D	Calina, Daniela
AU	12	16358	16358	Melo-Cavalcante, AAC	Melo-Cavalcante, Ana_A_C
AU	13	16359	16359	Sousa, JMC	Sousa, Joao_M_C
AF	1	16360	16360	Menezes, Ag-Anne P. M. de	Menezes, Ag-Anne_P_M_de
AF	2	16361	16361	Aguiar, Rai P. S.	Aguiar, Rai_P_S
AF	3	16362	16362	Santos, Jose V. O.	Santos, Jose_V_O
AF	5	16364	16364	Islam, Muhammad T.	Islam, Muhammad_T
AF	6	16365	16365	Braga, Antonio L.	Braga, Antonio_L
AF	7	16366	16366	Hasan, Mohammad M.	Hasan, Mohammad_M
AF	8	16367	16367	Silva, Felipe C. C. da	Silva, Felipe_C_C_da
AF	12	16371	16371	Melo-Cavalcante, Ana A. C.	Melo-Cavalcante, Ana_A_C
AF	13	16372	16372	Sousa, Joao M. C.	Sousa, Joao_M_C

Record 104 | WOS:001141751900001

Determination of Ochratoxin-A Detoxification and Antibiotic Resistance Potential of Acinetobacter calcoaceticus

Field	Position	Original line	Normalised line	Original value	Normalised value
AU	1	16578	16578	Al-Nussairawi, M	Al-Nussairawi, Mohammed
AU	2	16579	16579	Al-Tulaibawi, NAJ	Al-Tulaibawi, Nooralden_Abdulkarem_Jasim
AU	3	16580	16580	Aal-Aaboda, M	Aal-Aaboda, Munaf
AF	2	16582	16582	Al-Tulaibawi, Nooralden Abdulkarem Jasim	Al-Tulaibawi, Nooralden_Abdulkarem_Jasim

Record 105 | WOS:000988737300001

Natural mycotoxin contamination in dog food: A review on toxicity and detoxification methods

Field	Position	Original line	Normalised line	Original value	Normalised value
AU	1	16660	16660	Yang, L	Yang, Ling
AU	2	16661	16661	Yang, LH	Yang, Lihan
AU	3	16662	16662	Cai, YQ	Cai, Yuqing
AU	4	16663	16663	Luo, YF	Luo, Yifei
AU	5	16664	16664	Wang, H	Wang, Hui
AU	6	16665	16665	Wang, L	Wang, Li
AU	7	16666	16666	Chen, JQ	Chen, Jingqing
AU	8	16667	16667	Liu, XM	Liu, Xiaoming
AU	9	16668	16668	Wu, YJ	Wu, Yingjie
AU	10	16669	16669	Qin, YH	Qin, Yinghe
AU	11	16670	16670	Wu, ZL	Wu, Zhenlong
AU	12	16671	16671	Liu, N	Liu, Ning

Record 106 | WOS:000983760800001

Excessive ER-phagy contributes to ochratoxin A-induced apoptosis

Field	Position	Original line	Normalised line	Original value	Normalised value
AU	1	16957	16957	Deng, HQ	Deng, Huiqiong
AU	2	16958	16958	Chen, WY	Chen, Wenying
AU	3	16959	16959	Zhang, BY	Zhang, Boyang
AU	4	16960	16960	Zhang, YW	Zhang, Yiwen
AU	5	16961	16961	Han, LY	Han, Lingyun
AU	6	16962	16962	Zhang, QP	Zhang, Qipeng
AU	7	16963	16963	Yao, S	Yao, Song
AU	8	16964	16964	Wang, HW	Wang, Hongwei
AU	9	16965	16965	Shen, XL	Shen, Xiao_Li
AF	9	16974	16974	Shen, Xiao Li	Shen, Xiao_Li

Record 107 | WOS:000977904100001

New perspectives in application of kidney biomarkers in mycotoxin induced nephrotoxicity, with a particular focus on domestic pigs

Field	Position	Original line	Normalised line	Original value	Normalised value
AU	1	17090	17090	Ráduly, Z	Raduly, Zsolt
AU	2	17091	17091	Szabó, A	Szabo, Andras
AU	3	17092	17092	Mézes, M	Mezes, Miklos
AU	4	17093	17093	Balatoni, I	Balatoni, Ildiko
AU	5	17094	17094	Price, RG	Price, Robert_G
AU	6	17095	17095	Dockrell, ME	Dockrell, Mark_E
AU	7	17096	17096	Pócsi, I	Pocsi, Istvan
AU	8	17097	17097	Csernoch, L	Csernoch, Laszlo
AF	5	17102	17102	Price, Robert G.	Price, Robert_G
AF	6	17103	17103	Dockrell, Mark E.	Dockrell, Mark_E

Record 108 | WOS:000968395600001

Fermented whey modulated AFB1 and OTA-induced hepatotoxicity and nephrotoxicity in vivo. A relative and absolute quantification about sex differences

Field	Position	Original line	Normalised line	Original value	Normalised value
AU	1	17318	17318	Frangiamone, M	Frangiamone, Massimo
AU	2	17319	17319	Yemelin, A	Yemelin, Alexander
AU	3	17320	17320	Cimbalo, A	Cimbalo, Alessandra
AU	4	17321	17321	Font, G	Font, Guillermina
AU	5	17322	17322	Thines, E	Thines, Eckhard
AU	6	17323	17323	Manyes, L	Manyes, Lara

Record 109 | WOS:000982062800001

Protective Effect of alpha-Tocopherol Against Ochratoxin A in Kidney Cell Line HK-2

Field	Position	Original line	Normalised line	Original value	Normalised value

AU	1	17473	17473	Lee, HJ	Lee, Hyun_Jung
AU	2	17474	17474	Kim, HD	Kim, Hae_Dun
AU	3	17475	17475	Ryu, D	Ryu, Dojin
AF	1	17476	17476	Lee, Hyun Jung	Lee, Hyun_Jung
AF	2	17477	17477	Kim, Hae Dun	Kim, Hae_Dun

Record 110 | WOS:001094910400007

INITIAL PHASE ESTABLISHMENT OF AN IN VITRO METHOD FOR DEVELOP-MENTAL NEUROTOXICITY TEST USING KI-67 IN HUMAN NEURAL PROGENITOR CELLS

Field	Position	Original line	Normalised line	Original value	Normalised value
AU	1	17579	17579	Go, SM	Go, S_M
AU	4	17582	17582	Jeong, SH	Jeong, S_H
AU	5	17583	17583	Jo, NR	Jo, N_R
AU	6	17584	17584	Park, SM	Park, S_M
AU	8	17586	17586	Tran, DN	Tran, D_N
AU	9	17587	17587	Jung, EM	Jung, E_-M
AU	10	17588	17588	Lee, SD	Lee, S_D
AU	11	17589	17589	Jeung, EB	Jeung, E_-B
AF	1	17590	17590	Go, S. M.	Go, S_M
AF	2	17591	17591	Lee, B.	Lee, B
AF	3	17592	17592	Ahn, C.	Ahn, C
AF	4	17593	17593	Jeong, S. H.	Jeong, S_H
AF	5	17594	17594	Jo, N. R.	Jo, N_R
AF	6	17595	17595	Park, S. M.	Park, S_M
AF	7	17596	17596	Lee, M.	Lee, M
AF	8	17597	17597	Tran, D. N.	Tran, D_N
AF	9	17598	17598	Jung, E. -M.	Jung, E_-M
AF	10	17599	17599	Lee, S. D.	Lee, S_D
AF	11	17600	17600	Jeung, E. -B.	Jeung, E_-B

Record 111 | WOS:000984555200005

Microcapsules loaded with date seed extract and its inhibitory potential to modulate the toxic effects of mycotoxins in mice received mold-contaminated diet

Field	Position	Original line	Normalised line	Original value	Normalised value
AU	1	17708	17708	Sanei, S	Sanei, Sogand
AU	2	17709	17709	Kasgari, MB	Kasgari, Mohammadamin_Bozorgi
AU	3	17710	17710	Abedinzadeh, F	Abedinzadeh, Fatemeh
AU	4	17711	17711	Sasan, AP	Sasan, Atena_Poor
AU	5	17712	17712	Hassani, S	Hassani, Saeid
AU	6	17713	17713	Karimi, E	Karimi, Ehsan
AU	7	17714	17714	Oskoueian, E	Oskoueian, Ehsan
AU	8	17715	17715	Jahromi, MF	Jahromi, Mohammad_Faseleh
AF	2	17717	17717	Kasgari, Mohammadamin Bozorgi	Kasgari, Mohammadamin_Bozorgi
AF	4	17719	17719	Sasan, Atena Poor	Sasan, Atena_Poor
AF	8	17723	17723	Jahromi, Mohammad Faseleh	Jahromi, Mohammad_Faseleh

Record 112 | WOS:000951983500001

Hazard characterisation for significant mycotoxins in food

Field	Position	Original line	Normalised line	Original value	Normalised value
AU	1	17815	17815	Malir, F	Malir, Frantisek
AU	2	17816	17816	Pickova, D	Pickova, Darina
AU	3	17817	17817	Toman, J	Toman, Jakub
AU	4	17818	17818	Grosse, Y	Grosse, Yann
AU	5	17819	17819	Ostry, V	Ostry, Vladimir

Record 113 | WOS:000958249600001

The Effect of Mushroom Culture Filtrates on the Inhibition of Mycotoxins Produced by Aspergillus flavus and Aspergillus carbonarius

Field	Position	Original line	Normalised line	Original value	Normalised value
AU	1	18032	18032	Loncar, J	Loncar, Jelena
AU	2	18033	18033	Bellich, B	Bellich, Barbara
AU	3	18034	18034	Cescutti, P	Cescutti, Paola
AU	4	18035	18035	Motola, A	Motola, Alice
AU	5	18036	18036	Beccaccioli, M	Beccaccioli, Marzia
AU	6	18037	18037	Zjalic, S	Zjalic, Slaven
AU	7	18038	18038	Reverberi, M	Reverberi, Massimo

Record 114 | WOS:000955634100001

Combined Effects of Acrylamide and Ochratoxin A on the Intestinal Barrier in Caco-2 Cells

Field	Position	Original line	Normalised line	Original value	Normalised value
AU	1	18142	18142	Su, D	Su, Dan
AU	2	18143	18143	Lu, JW	Lu, Jiawen
AU	3	18144	18144	Nie, CC	Nie, Chunchao
AU	4	18145	18145	Guo, ZY	Guo, Ziyan
AU	5	18146	18146	Li, C	Li, Chang
AU	6	18147	18147	Yu, Q	Yu, Qiang
AU	7	18148	18148	Xie, JH	Xie, Jianhua
AU	8	18149	18149	Chen, Y	Chen, Yi

Record 115 | WOS:000960233300001

Immunotoxicity of Three Environmental Mycotoxins and Their Risks of Increasing Pathogen Infections

Field	Position	Original line	Normalised line	Original value	Normalised value
AU	1	18263	18263	Sun, YH	Sun, Yuhang
AU	2	18264	18264	Song, YQ	Song, Yuqi
AU	3	18265	18265	Long, M	Long, Miao
AU	4	18266	18266	Yang, SH	Yang, Shuhua

Record 116 | WOS:000944193800011

Dietary Ochratoxin A Contamination Modulates Oxidative Stress, Inflammation Processes and Causes Fibrosis in in vitro and in vivo Lung Models

Field	Position	Original line	Normalised line	Original value	Normalised value
AU	1	18388	18388	Ou, YF	Ou, Yongfang
AU	2	18389	18389	Fu, QJ	Fu, Qiujuan
AU	3	18390	18390	Chen, YH	Chen, Yonghua
AU	4	18391	18391	Lin, LY	Lin, Liyao
AU	5	18392	18392	Wang, JF	Wang, Junfeng
AU	6	18393	18393	Wu, D	Wu, Dong
AU	7	18394	18394	Wu, Q	Wu, Qin
AU	8	18395	18395	Xie, JL	Xie, Jianlong

Record 117 | WOS:000942033800001

Comparative Assessment of Different Yeast Cell Wall-Based Mycotoxin Adsorbents Using a Model- and Bioassay-Based In Vitro Approach

Field	Position	Original line	Normalised line	Original value	Normalised value
AU	1	18495	18495	Xu, R	Xu, Ran
AU	2	18496	18496	Yiannikouris, A	Yiannikouris, Alexandros
AU	3	18497	18497	Shandilya, UK	Shandilya, Umesh_K_K
AU	4	18498	18498	Karrow, NA	Karrow, Niel_A_A
AF	3	18501	18501	Shandilya, Umesh K. K.	Shandilya, Umesh_K_K
AF	4	18502	18502	Karrow, Niel A. A.	Karrow, Niel_A_A

Record 118 | WOS:000929015600001

Potential application of probiotics in mycotoxicosis reduction in mammals and poultry

Field	Position	Original line	Normalised line	Original value	Normalised value
AU	1	18627	18627	Zoghi, A	Zoghi, Alaleh
AU	2	18628	18628	Todorov, SD	Todorov, Svetoslav_Dimitrov
AU	3	18629	18629	Khosravi-Darani, K	Khosravi-Darani, Kianoush
AF	2	18631	18631	Todorov, Svetoslav Dimitrov	Todorov, Svetoslav_Dimitrov

Record 119 | WOS:000918850400001

Lonp1 and Sig-1R contribute to the counteraction of ursolic acid against ochratoxin A-induced mitochondrial apoptosis

Field	Position	Original line	Normalised line	Original value	Normalised value
AU	1	18756	18756	Zhang, QP	Zhang, Qipeng
AU	2	18757	18757	Chen, WY	Chen, Wenying
AU	3	18758	18758	Zhang, BY	Zhang, Boyang
AU	4	18759	18759	Zhang, YW	Zhang, Yiwen
AU	5	18760	18760	Xiao, YQ	Xiao, Yuqing
AU	6	18761	18761	An, YC	An, Yichen
AU	7	18762	18762	Han, LY	Han, Lingyun
AU	8	18763	18763	Deng, HQ	Deng, Huiqiong

AU	9	18764	18764	Yao, S	Yao, Song
AU	10	18765	18765	Wang, HW	Wang, Hongwei
AU	11	18766	18766	Shen, XL	Shen, Xiao_Li
AF	11	18777	18777	Shen, Xiao Li	Shen, Xiao_Li

Record 120 | WOS:001000273300006

Protective role of fermented whey and pumpkin extract against aflatoxin B1 and ochratoxin A toxicity in Jurkat T-cells

Field	Position	Original line	Normalised line	Original value	Normalised value
AU	1	18846	18846	Cimbalo, A	Cimbalo, Alessandra
AU	2	18847	18847	Frangiamone, M	Frangiamone, Massimo
AU	3	18848	18848	Lozano, M	Lozano, Manuel
AU	4	18849	18849	Escrivá, L	Escriva, L
AU	5	18850	18850	Vila-Donat, P	Vila-Donat, Pilar
AU	6	18851	18851	Manyes, L	Manyes, Lara
AF	1	18852	18852	Cimbalo, A.	Cimbalo, Alessandra
AF	2	18853	18853	Frangiamone, M.	Frangiamone, Massimo
AF	3	18854	18854	Lozano, M.	Lozano, Manuel
AF	4	18855	18855	Escriva, L.	Escriva, L
AF	5	18856	18856	Vila-Donat, P.	Vila-Donat, Pilar
AF	6	18857	18857	Manyes, L.	Manyes, Lara

Record 121 | WOS:000915148500001

AFB1 and OTA Promote Immune Toxicity in Human LymphoBlastic T Cells at Transcriptomic Level

Field	Position	Original line	Normalised line	Original value	Normalised value
AU	1	18957	18957	Frangiamone, M	Frangiamone, Massimo
AU	2	18958	18958	Lozano, M	Lozano, Manuel
AU	3	18959	18959	Cimbalo, A	Cimbalo, Alessandra
AU	4	18960	18960	Font, G	Font, Guillermina
AU	5	18961	18961	Manyes, L	Manyes, Lara

Record 122 | WOS:000912325600001

Toxico-pathological effects of ochratoxin A and its diastereoisomer under in ovo conditions and in vitro evaluation of the toxicity of these toxins against the embryo Gallus gallus fibroblast cell line

Field	Position	Original line	Normalised line	Original value	Normalised value
AU	1	19140	19140	Bryla, M	Bryla, Marcin
AU	2	19141	19141	Damaziak, K	Damaziak, Krzysztof
AU	3	19142	19142	Twaruzek, M	Twaruzek, Magdalena
AU	4	19143	19143	Waskiewicz, A	Waskiewicz, Agnieszka
AU	5	19144	19144	Stepien, L	Stepien, Lukasz
AU	6	19145	19145	Roszko, M	Roszko, Marek
AU	7	19146	19146	Pierzgalski, A	Pierzgalski, Adam
AU	8	19147	19147	Soszczynska, E	Soszczynska, Ewelina

AU	9	19148	19148	Lukasiewicz-Mierzejewska, M	Lukasiewicz-Mierzejewska, Monika
AU	10	19149	19149	Chmiel, M	Chmiel, Marta
AU	11	19150	19150	Wojcik, W	Wojcik, Wojciech

Record 123 | WOS:000900483500001

Modified Mycotoxins, a Still Unresolved Issue

Field	Position	Original line	Normalised line	Original value	Normalised value
AU	1	19267	19267	Angioni, A	Angioni, Alberto
AU	2	19268	19268	Russo, M	Russo, Mariateresa
AU	3	19269	19269	La Rocca, C	la_Rocca, Cinzia
AU	4	19270	19270	Pinto, O	Pinto, Ornella
AU	5	19271	19271	Mantovani, A	Mantovani, Alberto
AF	3	19274	19274	La Rocca, Cinzia	la_Rocca, Cinzia

Record 124 | WOS:000898846200001

Innovative in vitro approaches to toxicological investigations of mycotoxins effects

Field	Position	Original line	Normalised line	Original value	Normalised value
AU	1	19473	19473	Beatriz, AL	Beatriz, Arce_Lopez
AU	2	19474	19474	Nolwenn, H	Nolwenn, Hymery
AF	1	19475	19475	Beatriz, Arce Lopez	Beatriz, Arce_Lopez

Record 125 | WOS:000903192900001

Possible Mechanisms of the Interplay between Drugs and Mycotoxins-Is There a Possible Impact?

Field	Position	Original line	Normalised line	Original value	Normalised value
AU	1	19567	19567	Lootens, O	Lootens, Orphelie
AU	2	19568	19568	Vermeulen, A	Vermeulen, An
AU	3	19569	19569	Croubels, S	Croubels, Siska
AU	4	19570	19570	De Saeger, S	de_Saeger, Sarah
AU	5	19571	19571	Van Bocxlaer, J	van_Bocxlaer, Jan
AU	6	19572	19572	De Boevre, M	de_Boevre, Marthe
AF	4	19576	19576	De Saeger, Sarah	de_Saeger, Sarah
AF	5	19577	19577	Van Bocxlaer, Jan	van_Bocxlaer, Jan
AF	6	19578	19578	De Boevre, Marthe	de_Boevre, Marthe

Record 126 | WOS:000888695100001

Mycotoxins and cellular senescence: the impact of oxidative stress, hypoxia, and immunosuppression

Field	Position	Original line	Normalised line	Original value	Normalised value
AU	1	19735	19735	You, L	You, Lijuan
AU	2	19736	19736	Nepovimova, E	Nepovimova, Eugenie
AU	3	19737	19737	Valko, M	Valko, Marian

AU	4	19738	19738	Wu, QH	Wu, Qinghua
AU	5	19739	19739	Kuca, K	Kuca, Kamil
AF	1	19740	19740	You, Li	You, Lijuan

Record 127 | WOS:000892528400007

The efficacy of mycotoxin binders to control mycotoxins in feeds and the potential risk of interactions with nutrient: a review

Field	Position	Original line	Normalised line	Original value	Normalised value
AU	1	19896	19896	Kihal, A	Kihal, Abdelhacib
AU	2	19897	19897	Rodríguez-Prado, M	Rodriguez-Prado, Maria
AU	3	19898	19898	Calsamiglia, S	Calsamiglia, Sergio

Record 128 | WOS:000886894200008

Protective effect of selenomethionine on kidney injury induced by ochratoxin A in rabbits

Field	Position	Original line	Normalised line	Original value	Normalised value
AU	1	20044	20044	Zhang, ZQ	Zhang, Ziqiang
AU	2	20045	20045	Sun, YY	Sun, Yingying
AU	3	20046	20046	Xie, H	Xie, Hui
AU	4	20047	20047	Wang, JJ	Wang, Jiajia
AU	5	20048	20048	Zhang, X	Zhang, Xin
AU	6	20049	20049	Shi, ZY	Shi, Zhangyu
AU	7	20050	20050	Liu, YM	Liu, Yumei

Record 129 | WOS:000896944500005

In vitro study of ochratoxin A in the expression of genes associated with neuron survival and viability

Field	Position	Original line	Normalised line	Original value	Normalised value
AU	1	20184	20184	Sharma, R	Sharma, Riddhi
AU	2	20185	20185	Gettings, SM	Gettings, Sean_M
AU	3	20186	20186	Hazell, G	Hazell, Gareth
AU	4	20187	20187	Bourbia, N	Bourbia, Nora
AF	2	20189	20189	Gettings, Sean M.	Gettings, Sean_M

Record 130 | WOS:000974547800001

Low doses of fumonisin B1 exacerbate ochratoxin A-induced renal injury in mice and the protective roles of heat shock protein 70

Field	Position	Original line	Normalised line	Original value	Normalised value
AU	1	20310	20310	Li, HL	Li, Haolei
AU	2	20311	20311	He, WM	He, Wenmiao
AU	3	20312	20312	Yue, DM	Yue, Dongmei
AU	4	20313	20313	Wang, MM	Wang, Mengmeng
AU	5	20314	20314	Yuan, X	Yuan, Xin
AU	6	20315	20315	Huang, KH	Huang, Kehe

Record 131 | WOS:000887299000001

Mycotoxins and Essential Oils-From a Meat Industry Hazard to a Possible Solution: A Brief Review

Field	Position	Original line	Normalised line	Original value	Normalised value
AU	1	20454	20454	Gheorghe-Irimia, RA	Gheorghe-Irimia, Raluca_Aniela
AU	2	20455	20455	Tapaloaga, D	Tapaloaga, Dana
AU	3	20456	20456	Tapaloaga, PR	Tapaloaga, Paul_Rodian
AU	4	20457	20457	Ilie, LI	Ilie, Lucian_Ionel
AU	5	20458	20458	Sonea, C	Sonea, Cosmin
AU	6	20459	20459	Serban, AI	Serban, Andreea_Iren
AF	1	20460	20460	Gheorghe-Irimia, Raluca Aniela	Gheorghe-Irimia, Raluca_Aniela
AF	3	20462	20462	Tapaloaga, Paul Rodian	Tapaloaga, Paul_Rodian
AF	4	20463	20463	Ilie, Lucian Ionel	Ilie, Lucian_Ionel
AF	6	20465	20465	Serban, Andreea Iren	Serban, Andreea_Iren

Record 132 | WOS:000894684300001

Co-Contamination of Food and Feed with Mycotoxin and Bacteria and Possible Implications for Health

Field	Position	Original line	Normalised line	Original value	Normalised value
AU	1	20669	20669	Marin, DE	Marin, Daniela_Eliza
AU	2	20670	20670	Pistol, GC	Pistol, Gina_Cecilia
AU	3	20671	20671	Procudin, CV	Procudin, Cristina_Valeria
AU	4	20672	20672	Taranu, I	Taranu, Ionelia
AF	1	20673	20673	Marin, Daniela Eliza	Marin, Daniela_Eliza
AF	2	20674	20674	Pistol, Gina Cecilia	Pistol, Gina_Cecilia
AF	3	20675	20675	Procudin, Cristina Valeria	Procudin, Cristina_Valeria

Record 133 | WOS:000883142900001

Current Review of Mycotoxin Biodegradation and Bioadsorption: Microorganisms, Mechanisms, and Main Important Applications

Field	Position	Original line	Normalised line	Original value	Normalised value
AU	1	20780	20780	Ndiaye, S	Ndiaye, Seyni
AU	2	20781	20781	Zhang, MH	Zhang, Minhui
AU	3	20782	20782	Fall, M	Fall, Mouhamed
AU	4	20783	20783	Ayessou, NM	Ayessou, Nicolas_M
AU	5	20784	20784	Zhang, Q	Zhang, Qi
AU	6	20785	20785	Li, PW	Li, Peiwu
AF	4	20789	20789	Ayessou, Nicolas M.	Ayessou, Nicolas_M

Record 134 | WOS:000867775600001

The Effect of Aflatoxin B1 on Tumor-Related Genes and Phenotypic Characters of MCF7 and MCF10A Cells

Field	Position	Original line	Normalised line	Original value	Normalised value
AU	1	21029	21029	Adam, MAA	Adam, Mowaffaq_Adam_Ahmed
AU	2	21030	21030	Kamal, LZM	Kamal, Laina_Zarisa_Muhd

AU	3	21031	21031	Kanakal, M	Kanakal, Mahibub
AU	4	21032	21032	Babu, D	Babu, Dinesh
AU	5	21033	21033	Dahham, SS	Dahham, Saad_Sabbar
AU	6	21034	21034	Tabana, Y	Tabana, Yasser
AU	7	21035	21035	Lok, B	Lok, Bronwyn
AU	8	21036	21036	Bermoy, BM	Bermoy, Brittany_M
AU	9	21037	21037	Yunus, MA	Yunus, Muhammad_Amir
AU	10	21038	21038	Than, LTL	Than, Leslie_Thian_Lung
AU	11	21039	21039	Barakat, K	Barakat, Khaled
AU	12	21040	21040	Sandai, D	Sandai, Doblin
AF	1	21041	21041	Adam, Mowaffaq Adam Ahmed	Adam, Mowaffaq_Adam_Ahmed
AF	2	21042	21042	Kamal, Laina Zarisa Muhd	Kamal, Laina_Zarisa_Muhd
AF	5	21045	21045	Dahham, Saad Sabbar	Dahham, Saad_Sabbar
AF	8	21048	21048	Bermoy, Brittany M.	Bermoy, Brittany_M
AF	9	21049	21049	Yunus, Muhammad Amir	Yunus, Muhammad_Amir
AF	10	21050	21050	Than, Leslie Thian Lung	Than, Leslie_Thian_Lung

Record 135 | WOS:000911765600013

Ochratoxin A induced differentiation nephrotoxicity in renal tubule and glomeruli via autophagy differential regulation

Field	Position	Original line	Normalised line	Original value	Normalised value
AU	1	21213	21213	Le, GN	le, Guannan
AU	2	21214	21214	Du, H	du, Heng
AU	3	21215	21215	Sylia, A	Sylia, Ardache
AU	4	21216	21216	Hou, LL	Hou, Lili
AU	5	21217	21217	Muhmood, A	Muhmood, Azhar
AU	6	21218	21218	Wei, W	Wei, Wei
AU	7	21219	21219	Huang, KH	Huang, Kehe
AF	1	21220	21220	Le, Guannan	le, Guannan
AF	2	21221	21221	Du, Heng	du, Heng

Record 136 | WOS:000866918500001

Quercetin: Its Antioxidant Mechanism, Antibacterial Properties and Potential Application in Prevention and Control of Toxipathy

Field	Position	Original line	Normalised line	Original value	Normalised value
AU	1	21324	21324	Qi, WD	Qi, Weidong
AU	2	21325	21325	Qi, WX	Qi, Wanxiang
AU	3	21326	21326	Xiong, DW	Xiong, Dongwei
AU	4	21327	21327	Long, M	Long, Miao

Record 137 | WOS:000872026500001

Luteolin and Chrysin Could Prevent E. coli Lipopolysaccharide-Ochratoxin A Combination-Caused Inflammation and Oxidative Stress in In Vitro Porcine Intestinal Model

Field	Position	Original line	Normalised line	Original value	Normalised value
AU	1	21534	21534	Wohlert, A	Wohlert, Annelie
AU	2	21535	21535	Pézsa, NP	Pezsa, Nikolett_Palkovicsne

AU	3	21536	21536	Móritz, AV	Moritz, Alma_Virag
AU	4	21537	21537	Jerzsele, A	Jerzsele, Akos
AU	5	21538	21538	Farkas, O	Farkas, Orsolya
AU	6	21539	21539	Pászti-Gere, E	Paszti-Gere, Erzsebet
AF	2	21541	21541	Pezsa, Nikolett Palkovicsne	Pezsa, Nikolett_Palkovicsne
AF	3	21542	21542	Moritz, Alma Virag	Moritz, Alma_Virag

Record 138 | WOS:000864053100001

In-vitro alleviation of ochratoxin a mediated cytotoxicity using N-acetyl-L-tryptophan glucoside pretreatment

Field	Position	Original line	Normalised line	Original value	Normalised value
AU	1	21646	21646	Agarwal, P	Agarwal, Prerna
AU	2	21647	21647	Khodlan, P	Khodlan, Pallavi
AU	3	21648	21648	Kalra, N	Kalra, Namita
AU	4	21649	21649	Raisuddin, S	Raisuddin, Sheikh
AU	5	21650	21650	Kumar, R	Kumar, Raj

Record 139 | WOS:000884231200001

Aflatoxin M1 and ochratoxin A induce a competitive endogenous RNA regulatory network of intestinal immunosuppression by whole-transcriptome analysis

Field	Position	Original line	Normalised line	Original value	Normalised value
AU	1	21743	21743	Gao, YN	Gao, Ya-Nan
AU	2	21744	21744	Wang, ZW	Wang, Zi-Wei
AU	3	21745	21745	Yang, X	Yang, Xue
AU	4	21746	21746	Wang, JQ	Wang, Jia-Qi
AU	5	21747	21747	Zheng, N	Zheng, Nan

Record 140 | WOS:000876186700006

The importance of transcriptomics and proteomics for studying molecular mechanisms of mycotoxin exposure: A review

Field	Position	Original line	Normalised line	Original value	Normalised value
AU	1	21876	21876	Cimbalo, A	Cimbalo, Alessandra
AU	2	21877	21877	Frangiamone, M	Frangiamone, Massimo
AU	3	21878	21878	Font, G	Font, Guillermina
AU	4	21879	21879	Manyes, L	Manyes, Lara
AF	1	21880	21880	Cimbalo, A.	Cimbalo, Alessandra
AF	2	21881	21881	Frangiamone, M.	Frangiamone, Massimo
AF	3	21882	21882	Font, G.	Font, Guillermina
AF	4	21883	21883	Manyes, L.	Manyes, Lara

Record 141 | WOS:000851604100001

Mycotoxins in soybean-based foods fermented with filamentous fungi: Occurrence and preventive strategies

Field	Position	Original line	Normalised line	Original value	Normalised value
AU	1	21998	21998	Tian, F	Tian, Fei

AU	2	21999	21999	Woo, SY	Woo, So_Young
AU	3	22000	22000	Lee, SY	Lee, Sang_Yoo
AU	4	22001	22001	Park, SB	Park, Su_Been
AU	5	22002	22002	Im, JH	Im, Ju_Hee
AU	6	22003	22003	Chun, HS	Chun, Hyang_Sook
AF	2	22005	22005	Woo, So Young	Woo, So_Young
AF	3	22006	22006	Lee, Sang Yoo	Lee, Sang_Yoo
AF	4	22007	22007	Park, Su Been	Park, Su_Been
AF	5	22008	22008	Im, Ju Hee	Im, Ju_Hee
AF	6	22009	22009	Chun, Hyang Sook	Chun, Hyang_Sook

Record 142 | WOS:000851281600001

Mechanisms underlying citrinin-induced toxicity via oxidative stress and apoptosis-mediated by mitochondrial-dependent pathway in SH-SY5Y cells

Field	Position	Original line	Normalised line	Original value	Normalised value
AU	1	22202	22202	Abudayyak, M	Abudayyak, Mahmoud
AU	2	22203	22203	Karaman, EF	Karaman, Ecem_Fatma
AU	3	22204	22204	Ozden, S	Ozden, Sibel
AF	2	22206	22206	Karaman, Ecem Fatma	Karaman, Ecem_Fatma

Record 143 | WOS:000862185500005

Co-occurrence of mycotoxins: A review on bioanalytical methods for simultaneous analysis in human biological samples, mixture toxicity and risk assessment strategies

Field	Position	Original line	Normalised line	Original value	Normalised value
AU	1	22335	22335	Karsauliya, K	Karsauliya, Kajal
AU	3	22337	22337	Pandey, A	Pandey, Anushka
AU	4	22338	22338	Bhateria, M	Bhateria, Manisha
AU	5	22339	22339	Sonker, AK	Sonker, Ashish_Kumar
AU	6	22340	22340	Pandey, H	Pandey, Harshita
AU	7	22341	22341	Sharma, M	Sharma, Manu
AU	8	22342	22342	Singh, SP	Singh, Sheelendra_Pratap
AF	2	22344	22344	Yahavi, C.	Yahavi, C
AF	5	22347	22347	Sonker, Ashish Kumar	Sonker, Ashish_Kumar
AF	8	22350	22350	Singh, Sheelendra Pratap	Singh, Sheelendra_Pratap

Record 144 | WOS:000857038900001

The Neurotoxic Effect of Ochratoxin-A on the Hippocampal Neurogenic Niche of Adult Mouse Brain

Field	Position	Original line	Normalised line	Original value	Normalised value
AU	1	22528	22528	Mateo, E	Mateo, Eva
AU	2	22529	22529	Tonino, RPB	Bernardus_Tonino, Rik_Paulus
AU	3	22530	22530	Canto, A	Canto, Antolin
AU	4	22531	22531	Noyola, AM	Monroy_Noyola, Antonio
AU	5	22532	22532	Miranda, M	Miranda, Maria
AU	6	22533	22533	Soria, JM	Miguel_Soria, Jose
AU	7	22534	22534	Esparza, MAG	Garcia_Esparza, Maria_Angeles

AF	2	22536	22536	Bernardus Tonino, Rik Paulus	Bernardus_Tonino, Rik_Paulus
AF	4	22538	22538	Monroy Noyola, Antonio	Monroy_Noyola, Antonio
AF	6	22540	22540	Miguel Soria, Jose	Miguel_Soria, Jose
AF	7	22541	22541	Garcia Esparza, Maria Angeles	Garcia_Esparza, Maria_Angeles

Record 145 | WOS:000859602700001

Ochratoxin A and Citrinin Differentially Modulate Bovine Mammary Epithelial Cell Permeability and Innate Immune Function

Field	Position	Original line	Normalised line	Original value	Normalised value
AU	1	22655	22655	Xu, R	Xu, Ran
AU	2	22656	22656	Shandilya, UK	Shandilya, Umesh_K
AU	3	22657	22657	Yiannikouris, A	Yiannikouris, Alexandros
AU	4	22658	22658	Karrow, NA	Karrow, Niel_A
AF	2	22660	22660	Shandilya, Umesh K.	Shandilya, Umesh_K
AF	4	22662	22662	Karrow, Niel A.	Karrow, Niel_A

Record 146 | WOS:000858998000001

Protective activities of ellagic acid and urolithins against kidney toxicity of environmental pollutants: A review

Field	Position	Original line	Normalised line	Original value	Normalised value
AU	1	22829	22829	García-Niño, WR	Ramses_Garcia-Nino, Wylly
AU	2	22830	22830	Ibarra-Lara, L	Ibarra-Lara, Luz
AU	3	22831	22831	Cuevas-Magaña, MY	Yael_Cuevas-Magana, Mayra
AU	4	22832	22832	Sánchez-Mendoza, A	Sanchez-Mendoza, Alicia
AU	5	22833	22833	Armada, E	Armada, Elisabeth
AF	1	22834	22834	Ramses Garcia-Nino, Wylly	Ramses_Garcia-Nino, Wylly
AF	3	22836	22836	Yael Cuevas-Magana, Mayra	Yael_Cuevas-Magana, Mayra

Record 147 | WOS:000836768600001

Metabolic profiling as a powerful tool for the analysis of cellular alterations caused by 20 mycotoxins in HepG2 cells

Field	Position	Original line	Normalised line	Original value	Normalised value
AU	1	23119	23119	Gerdemann, A	Gerdemann, Andrea
AU	2	23120	23120	Behrens, M	Behrens, Matthias
AU	3	23121	23121	Esselen, M	Esselen, Melanie
AU	4	23122	23122	Humpf, HU	Humpf, Hans-Ulrich

Record 148 | WOS:000836539000001

Combined effects of cadmium and ochratoxin A on intestinal barrier dysfunction in human Caco-2 cells and pig small intestinal epithelial cells

Field	Position	Original line	Normalised line	Original value	Normalised value
AU	1	23227	23227	Kim, SH	Kim, So-Hee
AU	2	23228	23228	Jeong, YJ	Jeong, Yu-Jin
AU	3	23229	23229	Pyo, MC	Pyo, Min_Cheol
AU	4	23230	23230	Lee, KW	Lee, Kwang-Won

AF	3	23233	23233	Pyo, Min Cheol	Pyo, Min_Cheol

Record 149 | WOS:000827733300003

Antifungal and antimycotoxic activities of 3 essential oils against 3 mycotoxinogenic fungi

Field	Position	Original line	Normalised line	Original value	Normalised value
AU	1	23336	23336	Chelaghema, A	Chelaghema, Asma
AU	2	23337	23337	Durand, N	Durand, Noel
AU	3	23338	23338	Servent, A	Servent, Adrien
AU	4	23339	23339	Mamouni, M	Mamouni, Myriam
AU	5	23340	23340	Poucheret, P	Poucheret, Patrick
AU	6	23341	23341	Schorr-Galindo, S	Schorr-Galindo, Sabine
AU	7	23342	23342	Fontana, A	Fontana, Angelique
AU	8	23343	23343	Strub, C	Strub, Caroline

Record 150 | WOS:000845596200001

Evaluation of the Safety and Ochratoxin A Degradation Capacity of Pediococcus pentosaceus as a Dietary Probiotic with Molecular Docking Approach and Pharmacokinetic Toxicity Assessment

Field	Position	Original line	Normalised line	Original value	Normalised value
AU	1	23464	23464	Park, S	Park, Sungkwon
AU	2	23465	23465	Koo, J	Koo, Jinsu
AU	3	23466	23466	Kim, B	Kim, Bosung
AU	4	23467	23467	Pushparaj, K	Pushparaj, Karthika
AU	5	23468	23468	Malaisamy, A	Malaisamy, Arunkumar
AU	6	23469	23469	Liu, WC	Liu, Wen-Chao
AU	7	23470	23470	Balasubramanian, B	Balasubramanian, Balamuralikrishnan

Record 151 | WOS:000810935900001

Paper The multiple biotoxicity integrated study in grass carp (Ctenopharyngodon idella) caused by Ochratoxin A: Oxidative damage, apoptosis and immunosuppression

Field	Position	Original line	Normalised line	Original value	Normalised value
AU	1	23579	23579	Zhao, P	Zhao, Piao
AU	2	23580	23580	Liu, X	Liu, Xin
AU	3	23581	23581	Jiang, WD	Jiang, Wei-Dan
AU	4	23582	23582	Wu, P	Wu, Pei
AU	5	23583	23583	Liu, Y	Liu, Yang
AU	6	23584	23584	Jiang, J	Jiang, Jun
AU	7	23585	23585	Zhang, L	Zhang, Lu
AU	8	23586	23586	Mi, HF	Mi, Hai-Feng
AU	9	23587	23587	Kuang, SY	Kuang, Sheng-Yao
AU	10	23588	23588	Tang, L	Tang, Ling
AU	11	23589	23589	Zhou, XQ	Zhou, Xiao-Qiu
AU	12	23590	23590	Feng, L	Feng, Lin

Record 152 | WOS:000807511200002

Nutritional impact of mycotoxins in food animal production and strategies for mitigation

Field	Position	Original line	Normalised line	Original value	Normalised value
AU	1	23790	23790	Xu, R	Xu, Ran
AU	2	23791	23791	Kiarie, EG	Kiarie, Elijah_G
AU	3	23792	23792	Yiannikouris, A	Yiannikouris, Alexandros
AU	4	23793	23793	Sun, L	Sun, Lvhui
AU	5	23794	23794	Karrow, NA	Karrow, Niel_A
AF	2	23796	23796	Kiarie, Elijah G.	Kiarie, Elijah_G
AF	5	23799	23799	Karrow, Niel A.	Karrow, Niel_A

Record 153 | WOS:000818403700001

Genotoxicity of 12 Mycotoxins by the SOS/umu Test: Comparison of Liver and Kidney S9 Fraction

Field	Position	Original line	Normalised line	Original value	Normalised value
AU	1	24058	24058	Alonso-Jauregui, M	Alonso-Jauregui, Maria
AU	2	24059	24059	Gonzalez-Peñas, E	Gonzalez-Penas, Elena
AU	3	24060	24060	de Cerain, AL	Lopez_de_Cerain, Adela
AU	4	24061	24061	Vettorazzi, A	Vettorazzi, Ariane
AF	3	24064	24064	Lopez de Cerain, Adela	Lopez_de_Cerain, Adela

Record 154 | WOS:000819631900001

Ochratoxin A and Kidney Oxidative Stress: The Role of Nutraceuticals in Veterinary Medicine-A Review

Field	Position	Original line	Normalised line	Original value	Normalised value
AU	1	24186	24186	Longobardi, C	Longobardi, Consiglia
AU	2	24187	24187	Ferrara, G	Ferrara, Gianmarco
AU	3	24188	24188	Andretta, E	Andretta, Emanuela
AU	4	24189	24189	Montagnaro, S	Montagnaro, Serena
AU	5	24190	24190	Damiano, S	Damiano, Sara
AU	6	24191	24191	Ciarcia, R	Ciarcia, Roberto

Record 155 | WOS:000806405700007

In vitro mycotoxin binding capacities of clays, glucomannan and their combinations

Field	Position	Original line	Normalised line	Original value	Normalised value
AU	1	24357	24357	Oguz, H	Oguz, Halis
AU	2	24358	24358	Bahcivan, E	Bahcivan, Emre
AU	3	24359	24359	Erdogan, T	Erdogan, Teslime
AU	4	24360	24360	Yalcin, NF	Yalcin, Nihayet_Fadime
AU	5	24361	24361	Ozdas, A	Ozdas, Ayse
AU	6	24362	24362	Isik, MK	Isik, Mehmet_Kursat
AU	7	24363	24363	Altunbas, O	Altunbas, Osman
AF	4	24367	24367	Yalcin, Nihayet Fadime	Yalcin, Nihayet_Fadime

AF	6	24369	24369	Isik, Mehmet Kursat	Isik, Mehmet_Kursat

Record 156 | WOS:000832788700002

Effectiveness of beetroot extract in SH-SY5Y neuronal cell protection against Fumonisin B1, Ochratoxin A and its combination

Field	Position	Original line	Normalised line	Original value	Normalised value
AU	1	24496	24496	Penalva-Olcina, R	Penalva-Olcina, Raquel
AU	2	24497	24497	Juan, C	Juan, Cristina
AU	3	24498	24498	Fernández-Franzón, M	Fernandez-Franzon, Monica
AU	4	24499	24499	Juan-García, A	Juan-Garcia, Ana

Record 157 | WOS:000793099900001

Ochratoxin a induces hepatic fibrosis through TGF-β receptor I/Smad2/3 signaling pathway

Field	Position	Original line	Normalised line	Original value	Normalised value
AU	1	24606	24606	Chae, SA	Chae, Seung_A
AU	2	24607	24607	Pyo, MC	Pyo, Min_Cheol
AU	3	24608	24608	Yoo, HJ	Yoo, Hee_Joon
AU	4	24609	24609	Lee, KW	Lee, Kwang-Won
AF	1	24610	24610	Chae, Seung A.	Chae, Seung_A
AF	2	24611	24611	Pyo, Min Cheol	Pyo, Min_Cheol
AF	3	24612	24612	Yoo, Hee Joon	Yoo, Hee_Joon

Record 158 | WOS:000801766500001

Degradation of Ochratoxin A by a UV-Mutated Aspergillus niger Strain

Field	Position	Original line	Normalised line	Original value	Normalised value
AU	1	24712	24712	Zou, D	Zou, Dong
AU	2	24713	24713	Ji, J	Ji, Jian
AU	3	24714	24714	Ye, YL	Ye, Yongli
AU	4	24715	24715	Yang, Y	Yang, Yang
AU	5	24716	24716	Yu, J	Yu, Jian
AU	6	24717	24717	Wang, M	Wang, Meng
AU	7	24718	24718	Zheng, Y	Zheng, Yihong
AU	8	24719	24719	Sun, XL	Sun, Xiulan
AF	7	24726	24726	Zheng, Yi	Zheng, Yihong

Record 159 | WOS:000802213700006

Pumpkin extract and fermented whey individually and in combination alleviated AFB1-and OTA-induced alterations on neuronal differentiation in vitro

Field	Position	Original line	Normalised line	Original value	Normalised value
AU	1	24843	24843	Frangiamone, M	Frangiamone, Massimo
AU	2	24844	24844	Alonso-Garrido, M	Alonso-Garrido, Manuel
AU	3	24845	24845	Font, G	Font, Guillermina
AU	4	24846	24846	Cimbalo, A	Cimbalo, Alessandra

AU	5	24847	24847	Manyes, L	Manyes, Lara

Record 160 | WOS:000793768800008

Ochratoxin A as an alarming health threat for livestock and human: A review on molecular interactions, mechanism of toxicity, detection, detoxification, and dietary prophylaxis

Field	Position	Original line	Normalised line	Original value	Normalised value
AU	1	24987	24987	Liu, WC	Liu, Wen-Chao
AU	2	24988	24988	Pushparaj, K	Pushparaj, Karthika
AU	3	24989	24989	Meyyazhagan, A	Meyyazhagan, Arun
AU	4	24990	24990	Arumugam, VA	Arumugam, Vijaya_Anand
AU	5	24991	24991	Pappuswamy, M	Pappuswamy, Manikantan
AU	6	24992	24992	Bhotla, HK	Bhotla, Haripriya_Kuchi
AU	7	24993	24993	Baskaran, R	Baskaran, Rathinasamy
AU	8	24994	24994	Issara, U	Issara, Utthapon
AU	9	24995	24995	Balasubramanian, B	Balasubramanian, Balamuralikrishnan
AU	10	24996	24996	Khaneghah, AM	Khaneghah, Amin_Mousavi
AF	4	25000	25000	Arumugam, Vijaya Anand	Arumugam, Vijaya_Anand
AF	6	25002	25002	Bhotla, Haripriya Kuchi	Bhotla, Haripriya_Kuchi
AF	10	25006	25006	Khaneghah, Amin Mousavi	Khaneghah, Amin_Mousavi

Record 161 | WOS:000786151200001

Cytotoxicity of Mycotoxins and Their Combinations on Different Cell Lines: A Review

Field	Position	Original line	Normalised line	Original value	Normalised value
AU	1	25263	25263	Skrzydlewski, P	Skrzydlewski, Pawel
AU	2	25264	25264	Twaruzek, M	Twaruzek, Magdalena
AU	3	25265	25265	Grajewski, J	Grajewski, Jan

Record 162 | WOS:000784471500001

Mycotoxins in food and feed: toxicity, preventive challenges, and advanced detection techniques for associated diseases

Field	Position	Original line	Normalised line	Original value	Normalised value
AU	1	25417	25417	Dey, DK	Dey, Debasish_Kumar
AU	2	25418	25418	Kang, JI	Kang, Ji_In
AU	3	25419	25419	Bajpai, VK	Bajpai, Vivek_K
AU	4	25420	25420	Kim, K	Kim, Kwanwoo
AU	5	25421	25421	Lee, H	Lee, Hoomin
AU	6	25422	25422	Sonwal, S	Sonwal, Sonam
AU	7	25423	25423	Simal-Gandara, J	Simal-Gandara, Jesus
AU	8	25424	25424	Xiao, JB	Xiao, Jianbo
AU	9	25425	25425	Ali, S	Ali, Sajad
AU	10	25426	25426	Huh, YS	Huh, Yun_Suk
AU	11	25427	25427	Han, YK	Han, Yong-Kyu
AU	12	25428	25428	Shukla, S	Shukla, Shruti
AF	1	25429	25429	Dey, Debasish Kumar	Dey, Debasish_Kumar

AF	2	25430	25430	Kang, Ji In	Kang, Ji_In
AF	3	25431	25431	Bajpai, Vivek K.	Bajpai, Vivek_K
AF	10	25438	25438	Huh, Yun Suk	Huh, Yun_Suk

Record 163 | WOS:000793342700006

PCV2 infection aggravates OTA-induced immunotoxicity in vivo and in vitro

Field	Position	Original line	Normalised line	Original value	Normalised value
AU	1	25703	25703	Gan, F	Gan, Fang
AU	2	25704	25704	Hou, LL	Hou, Lili
AU	3	25705	25705	Xu, HB	Xu, Haibin
AU	4	25706	25706	Liu, YH	Liu, Yunhuan
AU	5	25707	25707	Chen, XX	Chen, Xingxiang
AU	6	25708	25708	Huang, KH	Huang, Kehe

Record 164 | WOS:000805486800007

An update on immunotoxicity and mechanisms of action of six environmental mycotoxins

Field	Position	Original line	Normalised line	Original value	Normalised value
AU	1	25831	25831	Sun, YH	Sun, Yuhang
AU	2	25832	25832	Huang, KH	Huang, Kehe
AU	3	25833	25833	Long, M	Long, Miao
AU	4	25834	25834	Yang, SH	Yang, Shuhua
AU	5	25835	25835	Zhang, Y	Zhang, Ying

Record 165 | WOS:000774405900001

Ex Vivo and In Vitro Studies Revealed Underlying Mechanisms of Immature Intestinal Inflammatory Responses Caused by Aflatoxin M1 Together with Ochratoxin A

Field	Position	Original line	Normalised line	Original value	Normalised value
AU	1	25967	25967	Wang, ZW	Wang, Zi-Wei
AU	2	25968	25968	Gao, YN	Gao, Ya-Nan
AU	3	25969	25969	Huang, SN	Huang, Sheng-Nan
AU	4	25970	25970	Wang, JQ	Wang, Jia-Qi
AU	5	25971	25971	Zheng, N	Zheng, Nan

Record 166 | WOS:000798517400010

Detoxification of ochratoxin A and zearalenone by Pleurotus ostreatus during in vitro gastrointestinal digestion

Field	Position	Original line	Normalised line	Original value	Normalised value
AU	1	26125	26125	Nobre, C	Nobre, Clarisse
AU	2	26126	26126	González, A	Gonzalez, Alondra
AU	3	26127	26127	Losoya, C	Losoya, Carolina
AU	4	26128	26128	Teixeira, JA	Teixeira, Jose_A
AU	5	26129	26129	Belmares, R	Belmares, Ruth
AU	6	26130	26130	Abrunhosa, L	Abrunhosa, Luis

AF	4	26134	26134	Teixeira, Jose A.	Teixeira, Jose_A

Record 167 | WOS:000760242900001

MicroRNA regulates the toxicological mechanism of four mycotoxins in vivo and in vitro

Field	Position	Original line	Normalised line	Original value	Normalised value
AU	1	26246	26246	Chen, J	Chen, Jia
AU	2	26247	26247	Yang, SH	Yang, Shuhua
AU	3	26248	26248	Li, P	Li, Peng
AU	4	26249	26249	Wu, AB	Wu, Aibo
AU	5	26250	26250	Nepovimova, E	Nepovimova, Eugenie
AU	6	26251	26251	Long, M	Long, Miao
AU	7	26252	26252	Wu, WD	Wu, Wenda
AU	8	26253	26253	Kuca, K	Kuca, Kamil

Record 168 | WOS:000796935500003

Studies on teratogenic effect of ochratoxin A given via mouldy diet in mice in various sensitive periods of the pregnancy and the putative protection of phenylalanine

Field	Position	Original line	Normalised line	Original value	Normalised value
AU	1	26445	26445	Stoev, SD	Stoev, Stoycho_D
AF	1	26446	26446	Stoev, Stoycho D.	Stoev, Stoycho_D

Record 169 | WOS:000762109100001

Spent Coffee Grounds Valorization as Bioactive Phenolic Source Acquired Antifungal, Anti-Mycotoxigenic, and Anti-Cytotoxic Activities

Field	Position	Original line	Normalised line	Original value	Normalised value
AU	1	26546	26546	Badr, AN	Badr, Ahmed_Noah
AU	2	26547	26547	El-Attar, MM	El-Attar, Marwa_M
AU	3	26548	26548	Ali, HS	Ali, Hatem_S
AU	4	26549	26549	Elkhadragy, MF	Elkhadragy, Manal_F
AU	5	26550	26550	Yehia, HM	Yehia, Hany_M
AU	6	26551	26551	Farouk, A	Farouk, Amr
AF	1	26552	26552	Badr, Ahmed Noah	Badr, Ahmed_Noah
AF	2	26553	26553	El-Attar, Marwa M.	El-Attar, Marwa_M
AF	3	26554	26554	Ali, Hatem S.	Ali, Hatem_S
AF	4	26555	26555	Elkhadragy, Manal F.	Elkhadragy, Manal_F
AF	5	26556	26556	Yehia, Hany M.	Yehia, Hany_M

Record 170 | WOS:000762022600001

Metabolomic Analysis Reveals the Mechanisms of Hepatotoxicity Induced by Aflatoxin M1 and Ochratoxin A

Field	Position	Original line	Normalised line	Original value	Normalised value
AU	1	26670	26670	Gao, YN	Gao, Ya-Nan
AU	2	26671	26671	Wu, CQ	Wu, Chen-Qing
AU	3	26672	26672	Wang, JQ	Wang, Jia-Qi

AU	4	26673	26673	Zheng, N	Zheng, Nan

Record 171 | WOS:000789897500004

A synergism of in silico and statistical approaches to discover new potential endocrine disruptor mycotoxins

Field	Position	Original line	Normalised line	Original value	Normalised value
AU	1	26792	26792	Spaggiari, G	Spaggiari, Giulia
AU	2	26793	26793	Morelli, G	Morelli, Gianluca
AU	3	26794	26794	Riani, M	Riani, Marco
AU	4	26795	26795	Cozzini, P	Cozzini, Pietro

Record 172 | WOS:000738826400005

Combined protective effects of icariin and selenomethionine on novel chronic tubulointerstitial nephropathy models in vivo and in vitro

Field	Position	Original line	Normalised line	Original value	Normalised value
AU	1	26891	26891	Hou, LL	Hou, Lili
AU	2	26892	26892	Lin, ZM	Lin, Ziman
AU	3	26893	26893	Xu, AY	Xu, Anyuan
AU	4	26894	26894	Le, GN	le, Guannan
AU	5	26895	26895	Ge, L	Ge, Lei
AU	6	26896	26896	Liu, SP	Liu, Shuiping
AU	7	26897	26897	Muhmood, A	Muhmood, Azhar
AU	8	26898	26898	Gan, F	Gan, Fang
AU	9	26899	26899	Huang, KH	Huang, Kehe
AF	4	26903	26903	Le, Guannan	le, Guannan

Record 173 | WOS:000742259000001

Subchronic exposure of individual and combined ochratoxin A and citrinin selectively affects the expression of rat renal organic cation transporters

Field	Position	Original line	Normalised line	Original value	Normalised value
AU	1	27019	27019	Karaica, D	Karaica, Dean
AU	2	27020	27020	Micek, V	Micek, Vedran
AU	3	27021	27021	Rasic, D	Rasic, Dubravka
AU	4	27022	27022	Peraica, M	Peraica, Maja
AU	5	27023	27023	Klaric, MS	Klaric, Maja_Segvic
AU	6	27024	27024	Breljak, D	Breljak, Davorka
AF	5	27029	27029	Klaric, Maja Segvic	Klaric, Maja_Segvic

Record 174 | WOS:000750801500002

In vitro and in vivo evaluation of AFB1 and OTA-toxicity through immunofluorescence and flow cytometry techniques: A systematic review

Field	Position	Original line	Normalised line	Original value	Normalised value
AU	1	27132	27132	Frangiamone, M	Frangiamone, Massimo
AU	2	27133	27133	Cimbalo, A	Cimbalo, Alessandra
AU	3	27134	27134	Alonso-Garrido, M	Alonso-Garrido, Manuel

AU	4	27135	27135	Vila-Donat, P	Vila-Donat, Pilar
AU	5	27136	27136	Manyes, L	Manyes, Lara

Record 175 | WOS:000767939000005

An overview of mycotoxins, their pathogenic effects, foods where they are found and their diagnostic biomarkers

Field	Position	Original line	Normalised line	Original value	Normalised value
AU	1	27341	27341	da Silva, JVB	Batista_da_Silva, Joao_Victor
AU	2	27342	27342	de Oliveira, CAF	Fernandes_de_Oliveira, Carlos_Augusto
AU	3	27343	27343	Ramalho, LNZ	Zambelli_Ramalho, Leandra_Naira
AF	1	27344	27344	Batista da Silva, Joao Victor	Batista_da_Silva, Joao_Victor
AF	2	27345	27345	Fernandes de Oliveira, Carlos Augusto	Fernandes_de_Oliveira, Carlos_Augusto
AF	3	27346	27346	Zambelli Ramalho, Leandra Naira	Zambelli_Ramalho, Leandra_Naira

Record 176 | WOS:000912776700001

Mycotoxin Interactions along the Gastrointestinal Tract: In Vitro Semi-Dynamic Digestion and Static Colonic Fermentation of a Contaminated Meal

Field	Position	Original line	Normalised line	Original value	Normalised value
AU	1	27502	27502	Sobral, MMC	Costa_Sobral, Maria_Madalena
AU	2	27503	27503	Gonçalves, T	Goncalves, Tiago
AU	3	27504	27504	Martins, ZE	Martins, Zita_E
AU	4	27505	27505	Bäuerl, C	Bauerl, Christine
AU	5	27506	27506	Cortés-Macías, E	Cortes-Macias, Erika
AU	6	27507	27507	Collado, MC	Collado, Maria_Carmen
AU	7	27508	27508	Ferreira, IMPLVO	Ferreira, Isabel_M_P_L_V_O
AF	1	27509	27509	Costa Sobral, Maria Madalena	Costa_Sobral, Maria_Madalena
AF	3	27511	27511	Martins, Zita E.	Martins, Zita_E
AF	6	27514	27514	Collado, Maria Carmen	Collado, Maria_Carmen
AF	7	27515	27515	Ferreira, Isabel M. P. L. V. O.	Ferreira, Isabel_M_P_L_V_O

Record 177 | WOS:000786544000007

Adsorption efficiency of sodium & calcium bentonite for ochratoxin A in some Egyptian cheeses: an innovative fortification model, in vitro and in vivo experiments

Field	Position	Original line	Normalised line	Original value	Normalised value
AU	1	27629	27629	Hamad, GM	Hamad, G_M
AU	2	27630	27630	El-Makarem, HA	El-Makarem, H_A
AU	3	27631	27631	Abd Elaziz, A	Abd_Elaziz, A_I
AU	4	27632	27632	Amer, AA	Amer, A_A
AU	5	27633	27633	El-Nogoumy, BA	El-Nogoumy, B_A
AU	6	27634	27634	Abou-Alella, SA	Abou-Alella, S_A
AF	1	27635	27635	Hamad, G. M.	Hamad, G_M
AF	2	27636	27636	El-Makarem, H. A.	El-Makarem, H_A
AF	3	27637	27637	Abd Elaziz, A., I	Abd_Elaziz, A_I
AF	4	27638	27638	Amer, A. A.	Amer, A_A

AF	5	27639	27639	El-Nogoumy, B. A.	El-Nogoumy, B_A
AF	6	27640	27640	Abou-Alella, S. A.	Abou-Alella, S_A

Record 178 | WOS:000746960900001

Development of an Extraction Method of Aflatoxins and Ochratoxin A from Oral, Gastric and Intestinal Phases of Digested Bread by In Vitro Model

Field	Position	Original line	Normalised line	Original value	Normalised value
AU	1	27753	27753	Llorens, P	Llorens, Paula
AU	2	27754	27754	Pietrzak-Fiecko, R	Pietrzak-Fiecko, Renata
AU	3	27755	27755	Molto, JC	Molto, Juan_Carlos
AU	4	27756	27756	Manes, J	Manes, Jordi
AU	5	27757	27757	Juan, CSA	Juan, Cristina
AF	3	27760	27760	Molto, Juan Carlos	Molto, Juan_Carlos

Record 179 | WOS:000891197500003

Gene expression profiling after ochratoxin A treatment in small intestinal epithelial cells from pigs

Field	Position	Original line	Normalised line	Original value	Normalised value
AU	1	27856	27856	Yoon, JW	Yoon, Jung_Woong
AU	2	27857	27857	Lee, SI	Lee, Sang_In
AF	1	27858	27858	Yoon, Jung Woong	Yoon, Jung_Woong
AF	2	27859	27859	Lee, Sang In	Lee, Sang_In

Record 180 | WOS:000730029700001

Agro-industrial Waste Products as Mycotoxin Biosorbents: A Review of in Vitro and in Vivo Studies

Field	Position	Original line	Normalised line	Original value	Normalised value
AU	1	27949	27949	Aguilar-Zuniga, K	Aguilar-Zuniga, Kateryn
AU	2	27950	27950	Laurie, VF	Laurie, V_Felipe
AU	3	27951	27951	Moore-Carrasco, R	Moore-Carrasco, Rodrigo
AU	4	27952	27952	Ortiz-Villeda, B	Ortiz-Villeda, Bryan
AU	5	27953	27953	Carrasco-Sánchez, V	Carrasco-Sanchez, Veronica
AF	2	27955	27955	Laurie, V. Felipe	Laurie, V_Felipe

Record 181 | WOS:000740093400002

Ochratoxin A induces NLRP3 inflammasome-mediated pyroptosis via activation of ROS modulated by autophagy in vitro

Field	Position	Original line	Normalised line	Original value	Normalised value
AU	1	28084	28084	Liu, K	Liu, Kai
AU	2	28085	28085	Yue, DM	Yue, Dongmei
AU	3	28086	28086	Mao, XR	Mao, Xinru
AU	4	28087	28087	Li, H	Li, Hu
AU	5	28088	28088	Qu, J	Qu, Jie
AU	6	28089	28089	Liu, SP	Liu, Shuiping
AU	7	28090	28090	Korolchuk, V	Korolchuk, Viktor_I

AU	8	28091	28091	Liu, YH	Liu, Yunhuan
AU	9	28092	28092	Gan, F	Gan, Fang
AU	10	28093	28093	Pan, CL	Pan, Cuiling
AU	11	28094	28094	Huang, KH	Huang, Kehe
AU	12	28095	28095	Chen, XX	Chen, Xingxiang
AF	7	28102	28102	Korolchuk, Viktor, I	Korolchuk, Viktor_I

Record 182 | WOS:000737483600001

Urinary Biomarkers of Mycotoxin Induced Nephrotoxicity-Current Status and Expected Future Trends

Field	Position	Original line	Normalised line	Original value	Normalised value
AU	1	28219	28219	Raduly, Z	Raduly, Zsolt
AU	2	28220	28220	Price, RG	Price, Robert_G
AU	3	28221	28221	Dockrell, MEC	Dockrell, Mark_E_C
AU	4	28222	28222	Csernoch, L	Csernoch, Laszlo
AU	5	28223	28223	Pocsi, I	Pocsi, Istvan
AF	2	28225	28225	Price, Robert G.	Price, Robert_G
AF	3	28226	28226	Dockrell, Mark E. C.	Dockrell, Mark_E_C

Record 183 | WOS:000734651000014

Non-embryotoxic dosage of alternariol aggravates ochratoxin A-triggered deleterious effects on embryonic development through ROS-dependent apoptotic processes

Field	Position	Original line	Normalised line	Original value	Normalised value
AU	1	28432	28432	Huang, CH	Huang, Chien-Hsun
AU	2	28433	28433	Wang, FT	Wang, Fu-Ting
AU	3	28434	28434	Hsuuw, YD	Hsuuw, Yan-Der
AU	4	28435	28435	Huang, FJ	Huang, Fu-Jen
AU	5	28436	28436	Chan, WH	Chan, Wen-Hsiung

Record 184 | WOS:000721173800002

The individual and combined effects of ochratoxin A with citrinin and their metabolites (ochratoxin B, ochratoxin C, and dihydrocitrinone) on 2D/3D cell cultures, and zebrafish embryo models

Field	Position	Original line	Normalised line	Original value	Normalised value
AU	1	28561	28561	Csenki, Z	Csenki, Zsolt
AU	2	28562	28562	Garai, E	Garai, Edina
AU	3	28563	28563	Faisal, Z	Faisal, Zelma
AU	4	28564	28564	Csepregi, R	Csepregi, Rita
AU	5	28565	28565	Garai, K	Garai, Kitti
AU	6	28566	28566	Sipos, DK	Sipos, Dora_Kanaine
AU	7	28567	28567	Szabó, I	Szabo, Istvan
AU	8	28568	28568	Koszegi, T	Koszegi, Tamas
AU	9	28569	28569	Czéh, A	Czeh, Arpad
AU	10	28570	28570	Czömpöly, T	Czompoly, Tamas
AU	11	28571	28571	Kvell, K	Kvell, Krisztian
AU	12	28572	28572	Poór, M	Poor, Miklos
AF	6	28578	28578	Sipos, Dora Kanaine	Sipos, Dora_Kanaine

Record 185 | WOS:000874698700001

Food Mycotoxins: Dietary Interventions Implicated in the Prevention of Mycotoxicosis

Field	Position	Original line	Normalised line	Original value	Normalised value
AU	1	28730	28730	Sharma, V	Sharma, Vinesh
AU	2	28731	28731	Patial, V	Patial, Vikram

Record 186 | WOS:000724718300001

Human Proximal Tubule Epithelial Cells (HK-2) as a Sensitive In Vitro System for Ochratoxin A Induced Oxidative Stress

Field	Position	Original line	Normalised line	Original value	Normalised value
AU	1	28974	28974	Garcia-Perez, E	Garcia-Perez, Enrique
AU	2	28975	28975	Ryu, D	Ryu, Dojin
AU	3	28976	28976	Kim, HY	Kim, Hwa-Young
AU	4	28977	28977	Kim, HD	Kim, Hae_Dun
AU	5	28978	28978	Lee, HJ	Lee, Hyun_Jung
AF	4	28982	28982	Kim, Hae Dun	Kim, Hae_Dun
AF	5	28983	28983	Lee, Hyun Jung	Lee, Hyun_Jung

Record 187 | WOS:000724371000001

Microbiological Decontamination of Mycotoxins: Opportunities and Limitations

Field	Position	Original line	Normalised line	Original value	Normalised value
AU	1	29134	29134	Piotrowska, M	Piotrowska, Malgorzata

Record 188 | WOS:000723720700001

Anti-Apoptotic Effect of Flavokawain A on Ochratoxin-A-Induced Endothelial Cell Injury by Attenuation of Oxidative Stress via PI3K/AKT-Mediated Nrf2 Signaling Cascade

Field	Position	Original line	Normalised line	Original value	Normalised value
AU	1	29333	29333	Rajendran, P	Rajendran, Peramaiyan
AU	2	29334	29334	Alzahrani, AM	Alzahrani, Abdullah_M
AU	3	29335	29335	Veeraraghavan, VP	Veeraraghavan, Vishnu_Priya
AU	4	29336	29336	Ahmed, EA	Ahmed, Emad_A
AF	2	29338	29338	Alzahrani, Abdullah M.	Alzahrani, Abdullah_M
AF	3	29339	29339	Veeraraghavan, Vishnu Priya	Veeraraghavan, Vishnu_Priya
AF	4	29340	29340	Ahmed, Emad A.	Ahmed, Emad_A

Record 189 | WOS:000807169400001

Ochratoxin A Induces Steatosis via PPARγ-CD36 Axis

Field	Position	Original line	Normalised line	Original value	Normalised value
AU	1	29437	29437	Zheng, QW	Zheng, Qian-Wen
AU	2	29438	29438	Ding, XF	Ding, Xu-Fen
AU	3	29439	29439	Cao, HJ	Cao, Hui-Jun

AU	4	29440	29440	Ni, QZ	Ni, Qian-Zhi
AU	5	29441	29441	Zhu, B	Zhu, Bing
AU	6	29442	29442	Ma, N	Ma, Ning
AU	7	29443	29443	Zhang, FK	Zhang, Feng-Kun
AU	8	29444	29444	Wang, YK	Wang, Yi-Kang
AU	9	29445	29445	Xu, S	Xu, Sheng
AU	10	29446	29446	Chen, TW	Chen, Tian-Wei
AU	11	29447	29447	Xia, J	Xia, Ji
AU	12	29448	29448	Qiu, XS	Qiu, Xiao-Song
AU	13	29449	29449	Yu, DZ	Yu, Dian-Zhen
AU	14	29450	29450	Xie, D	Xie, Dong
AU	15	29451	29451	Li, JJ	Li, Jing-Jing

Record 190 | WOS:000718125600004

Combination of zinc and selenium alleviates ochratoxin A-induced fibrosis via blocking ROS-dependent autophagy in HK-2 cells

Field	Position	Original line	Normalised line	Original value	Normalised value
AU	1	29570	29570	Le, GN	le, Guannan
AU	2	29571	29571	Yang, LL	Yang, Lulu
AU	3	29572	29572	Du, H	du, Heng
AU	4	29573	29573	Hou, LL	Hou, Lili
AU	5	29574	29574	Ge, L	Ge, Lei
AU	6	29575	29575	Sylia, A	Sylia, Ardache
AU	7	29576	29576	Muhmood, A	Muhmood, Azhar
AU	8	29577	29577	Chen, XX	Chen, Xinxiang
AU	9	29578	29578	Han, B	Han, Bo
AU	10	29579	29579	Huang, KH	Huang, Kehe
AF	1	29580	29580	Le, Guannan	le, Guannan
AF	3	29582	29582	Du, Heng	du, Heng

Record 191 | WOS:000709727500001

Selenomethionine alleviated Ochratoxin A induced pyroptosis and renal fibrotic factors expressions in MDCK cells

Field	Position	Original line	Normalised line	Original value	Normalised value
AU	1	29686	29686	Mao, XR	Mao, Xinru
AU	2	29687	29687	Li, H	Li, Hu
AU	3	29688	29688	Ge, L	Ge, Lei
AU	4	29689	29689	Liu, SP	Liu, Shuiping
AU	5	29690	29690	Hou, LL	Hou, Lili
AU	6	29691	29691	Yue, DM	Yue, Dongmei
AU	7	29692	29692	Du, H	du, Heng
AU	8	29693	29693	Pan, CL	Pan, Cuiling
AU	9	29694	29694	Gan, F	Gan, Fang
AU	10	29695	29695	Liu, YH	Liu, Yunhuan
AU	11	29696	29696	Huang, KH	Huang, Kehe
AU	12	29697	29697	Chen, XX	Chen, Xingxiang
AF	7	29704	29704	Du, Heng	du, Heng

Record 192 | WOS:000715414400001

Prioritization of Mycotoxins Based on Their Genotoxic Potential with an In Silico-In Vitro Strategy

Field	Position	Original line	Normalised line	Original value	Normalised value
AU	1	29810	29810	Alonso-Jauregui, M	Alonso-Jauregui, Maria
AU	2	29811	29811	Font, M	Font, Maria
AU	3	29812	29812	Gonzalez-Penas, E	Gonzalez-Penas, Elena
AU	4	29813	29813	de Cerain, AL	Lopez_de_Cerain, Adela
AU	5	29814	29814	Vettorazzi, A	Vettorazzi, Ariane
AF	4	29818	29818	Lopez de Cerain, Adela	Lopez_de_Cerain, Adela

Record 193 | WOS:000714429400001

Ochratoxin A-Induced Nephrotoxicity: Up-to-Date Evidence

Field	Position	Original line	Normalised line	Original value	Normalised value
AU	1	29967	29967	Khoi, CS	Khoi, Chong-Sun
AU	2	29968	29968	Chen, JH	Chen, Jia-Huang
AU	3	29969	29969	Lin, TY	Lin, Tzu-Yu
AU	4	29970	29970	Chiang, CK	Chiang, Chih-Kang
AU	5	29971	29971	Hung, KY	Hung, Kuan-Yu

Record 194 | WOS:000726706400001

The role of pumpkin pulp extract carotenoids against mycotoxin damage in the blood brain barrier in vitro

Field	Position	Original line	Normalised line	Original value	Normalised value
AU	1	30153	30153	Alonso-Garrido, M	Alonso-Garrido, Manuel
AU	2	30154	30154	Pallarés, N	Pallares, Noelia
AU	3	30155	30155	Font, G	Font, Guillermina
AU	4	30156	30156	Tedeschi, P	Tedeschi, Paola
AU	5	30157	30157	Manyes, L	Manyes, Lara
AU	6	30158	30158	Lozano, M	Lozano, Manuel

Record 195 | WOS:000714098000254

Evaluation of larval zebrafish as an alternative whole-animal model for nephrotoxicity testing

Field	Position	Original line	Normalised line	Original value	Normalised value
AU	1	30261	30261	Bauer, B	Bauer, Benedikt
AU	2	30262	30262	Liedtke, D	Liedtke, Daniel
AU	3	30263	30263	Jarzina, S	Jarzina, Sebastian
AU	4	30264	30264	Stammler, E	Stammler, Emilia
AU	5	30265	30265	Kreisel, K	Kreisel, Katrin
AU	6	30266	30266	Lalomia, V	Lalomia, Viola
AU	8	30268	30268	Diefenbacher, M	Diefenbacher, Markus
AU	9	30269	30269	Klopocki, E	Klopocki, Eva
AU	10	30270	30270	Mally, A	Mally, Angela

AF	1	30271	30271	Bauer, B.	Bauer, Benedikt
AF	2	30272	30272	Liedtke, D.	Liedtke, Daniel
AF	3	30273	30273	Jarzina, S.	Jarzina, Sebastian
AF	4	30274	30274	Stammler, E.	Stammler, Emilia
AF	5	30275	30275	Kreisel, K.	Kreisel, Katrin
AF	6	30276	30276	Lalomia, V.	Lalomia, Viola
AF	7	30277	30277	Kruse, M.	Kruse, M
AF	8	30278	30278	Diefenbacher, M.	Diefenbacher, Markus
AF	9	30279	30279	Klopocki, E.	Klopocki, Eva
AF	10	30280	30280	Mally, A.	Mally, Angela

Record 196 | WOS:000701125500001

The Reduction of the Combined Effects of Aflatoxin and Ochratoxin A in Piglet Livers and Kidneys by Dietary Antioxidants

Field	Position	Original line	Normalised line	Original value	Normalised value
AU	1	30327	30327	Popescu, RG	Popescu, Roua_Gabriela
AU	2	30328	30328	Avramescu, S	Avramescu, Sorin
AU	3	30329	30329	Marin, DE	Marin, Daniela_Eliza
AU	4	30330	30330	Taranu, I	Taranu, Ionelia
AU	5	30331	30331	Georgescu, SE	Georgescu, Sergiu_Emil
AU	6	30332	30332	Dinischiotu, A	Dinischiotu, Anca
AF	1	30333	30333	Popescu, Roua Gabriela	Popescu, Roua_Gabriela
AF	3	30335	30335	Marin, Daniela Eliza	Marin, Daniela_Eliza
AF	5	30337	30337	Georgescu, Sergiu Emil	Georgescu, Sergiu_Emil

Record 197 | WOS:000689864300001

Cytotoxicity of Mycotoxins Frequently Present in Aquafeeds to the Fish Cell Line RTGill-W1

Field	Position	Original line	Normalised line	Original value	Normalised value
AU	1	30514	30514	Bernal-Algaba, E	Bernal-Algaba, Elena
AU	2	30515	30515	Pulgarín-Alfaro, M	Pulgarin-Alfaro, Marta
AU	3	30516	30516	Fernández-Cruz, ML	Fernandez-Cruz, Maria_Luisa
AF	3	30519	30519	Fernandez-Cruz, Maria Luisa	Fernandez-Cruz, Maria_Luisa

Record 198 | WOS:000688617200001

Curcumin Modulates Nitrosative Stress, Inflammation, and DNA Damage and Protects against Ochratoxin A-Induced Hepatotoxicity and Nephrotoxicity in Rats

Field	Position	Original line	Normalised line	Original value	Normalised value
AU	1	30633	30633	Longobardi, C	Longobardi, Consiglia
AU	2	30634	30634	Damiano, S	Damiano, Sara
AU	3	30635	30635	Andretta, E	Andretta, Emanuela
AU	4	30636	30636	Prisco, F	Prisco, Francesco
AU	5	30637	30637	Russo, V	Russo, Valeria
AU	6	30638	30638	Pagnini, F	Pagnini, Francesco
AU	7	30639	30639	Florio, S	Florio, Salvatore
AU	8	30640	30640	Ciarcia, R	Ciarcia, Roberto

Record 199 | WOS:000681272600001

Hepatotoxicity of food-borne mycotoxins: molecular mechanism, anti-hepatotoxic medicines and target prediction

Field	Position	Original line	Normalised line	Original value	Normalised value
AU	1	30777	30777	Ruan, HN	Ruan, Haonan
AU	2	30778	30778	Lu, Q	Lu, Qian
AU	3	30779	30779	Wu, JS	Wu, Jiashuo
AU	4	30780	30780	Qin, JA	Qin, Jiaan
AU	5	30781	30781	Sui, M	Sui, Ming
AU	6	30782	30782	Sun, XQ	Sun, Xinqi
AU	7	30783	30783	Shi, Y	Shi, Yue
AU	8	30784	30784	Luo, JY	Luo, Jiaoyang
AU	9	30785	30785	Yang, MH	Yang, Meihua

Record 200 | WOS:000674669100002

Metabolomics analysis underlay mechanisms in the renal impairment of mice caused by combination of aflatoxin M1 and ochratoxin A

Field	Position	Original line	Normalised line	Original value	Normalised value
AU	1	31044	31044	Wang, ZW	Wang, Ziwei
AU	2	31045	31045	Gao, YA	Gao, Yanan
AU	3	31046	31046	Huang, X	Huang, Xin
AU	4	31047	31047	Huang, SN	Huang, Shengnan
AU	5	31048	31048	Yang, X	Yang, Xue
AU	6	31049	31049	Wang, JQ	Wang, Jiaqi
AU	7	31050	31050	Zheng, N	Zheng, Nan

Record 201 | WOS:000675540600002

Ochratoxin A induces ERK1/2 phosphorylation-dependent apoptosis through NF-KB/ERK axis in human proximal tubule HK-2 cell line

Field	Position	Original line	Normalised line	Original value	Normalised value
AU	1	31196	31196	Darbuka, E	Darbuka, Elif
AU	2	31197	31197	Gürkaslar, C	Gurkaslar, Can
AU	3	31198	31198	Yaman, I	Yaman, Ibrahim

Record 202 | WOS:000656878400004

Mycotoxins in human food: A challenge for research

Field	Position	Original line	Normalised line	Original value	Normalised value
AU	1	31299	31299	Payros, D	Payros, Delphine
AU	2	31300	31300	Garofalo, M	Garofalo, Marion
AU	3	31301	31301	Pierron, A	Pierron, Alix
AU	4	31302	31302	Soler-Vasco, L	Soler-Vasco, Laura
AU	5	31303	31303	Al-Ayoubi, C	Al-Ayoubi, Carine
AU	6	31304	31304	Maruo, VM	Maruo, Viviane_M
AU	7	31305	31305	Alassane-Kpembi, I	Alassane-Kpembi, Imourana

AU	8	31306	31306	Pinton, P	Pinton, Philippe
AU	9	31307	31307	Oswald, IP	Oswald, Isabelle_P
AF	6	31313	31313	Maruo, Viviane M.	Maruo, Viviane_M
AF	9	31316	31316	Oswald, Isabelle P.	Oswald, Isabelle_P

Record 203 | WOS:000661314200027

In vitro blood brain barrier exposure to mycotoxins and carotenoids pumpkin extract alters mitochondrial gene expression and oxidative stress

Field	Position	Original line	Normalised line	Original value	Normalised value
AU	1	31496	31496	Alonso-Garrido, M	Alonso-Garrido, Manuel
AU	2	31497	31497	Frangiamone, M	Frangiamone, Massimo
AU	3	31498	31498	Font, G	Font, Guillermina
AU	4	31499	31499	Cimbalo, A	Cimbalo, Alessandra
AU	5	31500	31500	Manyes, L	Manyes, Lara
AF	1	31501	31501	Alonso-Garrido, M.	Alonso-Garrido, Manuel
AF	2	31502	31502	Frangiamone, M.	Frangiamone, Massimo
AF	3	31503	31503	Font, G.	Font, Guillermina
AF	4	31504	31504	Cimbalo, A.	Cimbalo, Alessandra
AF	5	31505	31505	Manyes, L.	Manyes, Lara

Record 204 | WOS:000661314200019

Multidimensional analysis of the epigenetic alterations in toxicities induced by mycotoxins

Field	Position	Original line	Normalised line	Original value	Normalised value
AU	1	31605	31605	Zhu, LY	Zhu, Liye
AU	2	31606	31606	Yuhan, JY	Yuhan, Jieyu
AU	3	31607	31607	Huang, KL	Huang, Kunlun
AU	4	31608	31608	He, XY	He, Xiaoyun
AU	5	31609	31609	Liang, ZH	Liang, Zhihong
AU	6	31610	31610	Xu, WT	Xu, Wentao

Record 205 | WOS:000659169000010

The effect of ochratoxin A on cytotoxicity and glucose metabolism in human esophageal epithelium Het-1A cells

Field	Position	Original line	Normalised line	Original value	Normalised value
AU	1	31786	31786	Zhao, M	Zhao, Man
AU	2	31787	31787	Wang, Y	Wang, Yuan
AU	3	31788	31788	Jia, X	Jia, Xin
AU	4	31789	31789	Liu, WN	Liu, Weina
AU	5	31790	31790	Zhang, XH	Zhang, Xianghong
AU	6	31791	31791	Cui, JF	Cui, Jinfeng

Record 206 | WOS:000664290000006

Non-cytotoxic dosage of fumonisin B1 aggravates ochratoxin A-induced nephrocytotoxicity and apoptosis via ROS-dependent JNK/MAPK signaling pathway

Field	Position	Original line	Normalised line	Original value	Normalised value
AU	1	31886	31886	Li, HL	Li, Haolei
AU	2	31887	31887	Wang, MM	Wang, Mengmeng
AU	3	31888	31888	Kang, WL	Kang, Weili
AU	4	31889	31889	Lin, ZM	Lin, Ziman
AU	5	31890	31890	Gan, F	Gan, Fang
AU	6	31891	31891	Huang, KH	Huang, Kehe

Record 207 | WOS:000654061000009

Oral subchronic exposure to the mycotoxin ochratoxin A induces key pathological features of Parkinson's disease in mice six months after the end of the treatment

Field	Position	Original line	Normalised line	Original value	Normalised value
AU	1	32002	32002	Izco, M	Izco, Maria
AU	2	32003	32003	Vettorazzi, A	Vettorazzi, Ariane
AU	3	32004	32004	Forcen, R	Forcen, Raquel
AU	4	32005	32005	Blesa, J	Blesa, Javier
AU	5	32006	32006	de Toro, M	de_Toro, Maria
AU	6	32007	32007	Alvarez-Herrera, N	Alvarez-Herrera, Natalia
AU	7	32008	32008	Cooper, JM	Cooper, J_Mark
AU	8	32009	32009	Gonzalez-Peñas, E	Gonzalez-Penas, Elena
AU	9	32010	32010	de Cerain, AL	de_Cerain, Adela_Lopez
AU	10	32011	32011	Alvarez-Erviti, L	Alvarez-Erviti, Lydia
AF	5	32016	32016	de Toro, Maria	de_Toro, Maria
AF	7	32018	32018	Cooper, J. Mark	Cooper, J_Mark
AF	9	32020	32020	de Cerain, Adela Lopez	de_Cerain, Adela_Lopez

Record 208 | WOS:000643671100001

Ochratoxin A Induces Oxidative Stress in HepG2 Cells by Impairing the Gene Expression of Antioxidant Enzymes

Field	Position	Original line	Normalised line	Original value	Normalised value
AU	1	32179	32179	García-Pérez, E	Garcia-Perez, Enrique
AU	2	32180	32180	Ryu, D	Ryu, Dojin
AU	3	32181	32181	Lee, C	Lee, Chan
AU	4	32182	32182	Lee, HJ	Lee, Hyun_Jung
AF	4	32186	32186	Lee, Hyun Jung	Lee, Hyun_Jung

Record 209 | WOS:000637397900008

Exploration of zebrafish larvae as an alternative whole-animal model for nephrotoxicity testing

Field	Position	Original line	Normalised line	Original value	Normalised value
AU	1	32301	32301	Bauer, B	Bauer, Benedikt
AU	2	32302	32302	Liedtke, D	Liedtke, Daniel
AU	3	32303	32303	Jarzina, S	Jarzina, Sebastian
AU	4	32304	32304	Stammler, E	Stammler, Emilia
AU	5	32305	32305	Kreisel, K	Kreisel, Katrin
AU	6	32306	32306	Lalomia, V	Lalomia, Viola

AU	7	32307	32307	Diefenbacher, M	Diefenbacher, Markus
AU	8	32308	32308	Klopocki, E	Klopocki, Eva
AU	9	32309	32309	Mally, A	Mally, Angela

Record 210 | WOS:000634193100001

Rat Tumour Histopathology Associated with Experimental Chronic Dietary Exposure to Ochratoxin A in Prediction of the Mycotoxin's Risk for Human Cancers

Field	Position	Original line	Normalised line	Original value	Normalised value
AU	1	32463	32463	Herman, D	Herman, Diana
AU	2	32464	32464	Mantle, P	Mantle, Peter

Record 211 | WOS:000634190600001

The Impact of the Nephrotoxin Ochratoxin A on Human Renal Cells Studied by a Novel Co-Culture Model Is Influenced by the Presence of Fibroblasts

Field	Position	Original line	Normalised line	Original value	Normalised value
AU	1	32537	32537	Schwerdt, G	Schwerdt, Gerald
AU	2	32538	32538	Kopf, M	Kopf, Michael
AU	3	32539	32539	Gekle, M	Gekle, Michael

Record 212 | WOS:000637502500004

Screening of Macrofungi Isolates for Aflatoxin B1 and Ochratoxin A Degradation

Field	Position	Original line	Normalised line	Original value	Normalised value
AU	1	32625	32625	Söylemez, T	Soylemez, Tuncay
AU	2	32626	32626	Yamaç, M	Yamac, Mustafa

Record 213 | WOS:000614680800001

Ochratoxin A induces nephrotoxicity in vitro and in vivo via pyroptosis

Field	Position	Original line	Normalised line	Original value	Normalised value
AU	1	32713	32713	Li, H	Li, Hu
AU	2	32714	32714	Mao, XR	Mao, Xinru
AU	3	32715	32715	Liu, K	Liu, Kai
AU	4	32716	32716	Sun, JH	Sun, Jiahao
AU	5	32717	32717	Li, BR	Li, Benrui
AU	6	32718	32718	Malyar, RM	Malyar, Rahmani_Mohammad
AU	7	32719	32719	Liu, DD	Liu, Dandan
AU	8	32720	32720	Pan, CL	Pan, Cuiling
AU	9	32721	32721	Gan, F	Gan, Fang
AU	10	32722	32722	Liu, YH	Liu, Yunhuan
AU	11	32723	32723	Huang, KH	Huang, Kehe
AU	12	32724	32724	Chen, XX	Chen, Xingxiang
AF	6	32730	32730	Malyar, Rahmani Mohammad	Malyar, Rahmani_Mohammad

Record 214 | WOS:000623049400013

Ochratoxin A and its reaction products affected by sugars during heat processing

Field	Position	Original line	Normalised line	Original value	Normalised value
AU	1	32853	32853	Gu, KJ	Gu, Kejia
AU	2	32854	32854	Ryu, D	Ryu, Dojin
AU	3	32855	32855	Lee, HJ	Lee, Hyun_Jung
AF	3	32858	32858	Lee, Hyun Jung	Lee, Hyun_Jung

Record 215 | WOS:000615712900001

Editorial overview of the special issue on application of tissue chips in toxicology

Field	Position	Original line	Normalised line	Original value	Normalised value
AU	1	32952	32952	Rusyn, I	Rusyn, Ivan
AU	2	32953	32953	Roth, A	Roth, Adrian

Record 216 | WOS:000615712900006

Central role of TRAP1 in the ameliorative effect of oleanolic acid on the mitochondrial-mediated and endoplasmic reticulum stress-excitated apoptosis induced by ochratoxin A

Field	Position	Original line	Normalised line	Original value	Normalised value
AU	1	33036	33036	Zhang, QP	Zhang, Qipeng
AU	2	33037	33037	Chen, WY	Chen, Wenying
AU	3	33038	33038	Zhang, BY	Zhang, Boyang
AU	4	33039	33039	Li, C	Li, Chen
AU	5	33040	33040	Zhang, XY	Zhang, Xunyao
AU	6	33041	33041	Wang, Q	Wang, Qian
AU	7	33042	33042	Wang, Y	Wang, Yan
AU	8	33043	33043	Zhou, Q	Zhou, Qian
AU	9	33044	33044	Li, XH	Li, Xiaohong
AU	10	33045	33045	Shen, XL	Shen, Xiao_Li
AF	10	33055	33055	Shen, Xiao Li	Shen, Xiao_Li

Record 217 | WOS:000606891900001

Mycotoxins: Impact on Health and Strategies for Prevention and Detoxification in the Food Chain

Field	Position	Original line	Normalised line	Original value	Normalised value
AU	1	33180	33180	Peivasteh-Roudsari, L	Peivasteh-Roudsari, Leila
AU	2	33181	33181	Pirhadi, M	Pirhadi, Mohadeseh
AU	3	33182	33182	Shahbazi, R	Shahbazi, Razieh
AU	4	33183	33183	Eghbaljoo-Gharehgheshlaghi, H	Eghbaljoo-Gharehgheshlaghi, Hadi
AU	5	33184	33184	Sepahi, M	Sepahi, Mahtab
AU	6	33185	33185	Alizadeh, AM	Mirza_Alizadeh, Adel
AU	7	33186	33186	Tajdar-oranj, B	Tajdar-Oranj, Behrouz
AU	8	33187	33187	Jazaeri, S	Jazaeri, Sahar
AF	6	33193	33193	Mirza Alizadeh, Adel	Mirza_Alizadeh, Adel

AF	7	33194	33194	Tajdar-oranj, Behrouz	Tajdar-Oranj, Behrouz

Record 218 | WOS:000638197700008

Comet assay: a versatile but complex tool in genotoxicity testing

Field	Position	Original line	Normalised line	Original value	Normalised value
AU	1	33494	33494	Cordelli, E	Cordelli, Eugenia
AU	2	33495	33495	Bignami, M	Bignami, Margherita
AU	3	33496	33496	Pacchierotti, F	Pacchierotti, Francesca

Record 219 | WOS:000701996600001

Aspergillus derived mycotoxins in food and the environment: Prevalence, detection, and toxicity

Field	Position	Original line	Normalised line	Original value	Normalised value
AU	1	33648	33648	Navale, V	Navale, Vishwambar
AU	2	33649	33649	Vamkudoth, KR	Vamkudoth, Koteswara_Rao
AU	3	33650	33650	Ajmera, S	Ajmera, Shanthipriya
AU	4	33651	33651	Dhuri, V	Dhuri, Vaibhavi
AF	2	33653	33653	Vamkudoth, Koteswara Rao	Vamkudoth, Koteswara_Rao

Record 220 | WOS:000670072600004

Neurotoxicity mechanism of Ochratoxin A

Field	Position	Original line	Normalised line	Original value	Normalised value
AU	1	34012	34012	Nourbakhsh, F	Nourbakhsh, Fahimeh
AU	2	34013	34013	Tajbakhsh, E	Tajbakhsh, Elahe

Record 221 | WOS:000603090600001

Mycotoxin and Gut Microbiota Interactions

Field	Position	Original line	Normalised line	Original value	Normalised value
AU	1	34149	34149	Guerre, P	Guerre, Philippe

Record 222 | WOS:000597367400001

Ameliorative role of dietary activated carbon against ochratoxin-A induced oxidative damage, suppressed performance and toxicological effects

Field	Position	Original line	Normalised line	Original value	Normalised value
AU	1	34409	34409	Bhatti, SA	Bhatti, Sheraz_Ahmed
AU	2	34410	34410	Khan, MZ	Khan, Muhammad_Zargham
AU	3	34411	34411	Saleemi, MK	Saleemi, Muhammad_Kashif
AU	4	34412	34412	Ul Hassan, Z	Ul_Hassan, Zahoor
AU	5	34413	34413	Khan, A	Khan, Ahrar
AF	1	34414	34414	Bhatti, Sheraz Ahmed	Bhatti, Sheraz_Ahmed
AF	2	34415	34415	Khan, Muhammad Zargham	Khan, Muhammad_Zargham

AF	3	34416	34416	Saleemi, Muhammad Kashif	Saleemi, Muhammad_Kashif
AF	4	34417	34417	Ul Hassan, Zahoor	Ul_Hassan, Zahoor

Record 223 | WOS:000584401800010

Transcriptomic alterations induced by aflatoxin B1 and ochratoxin A in LMH cell line

Field	Position	Original line	Normalised line	Original value	Normalised value
AU	1	34532	34532	Choi, SY	Choi, So-Young
AU	2	34533	34533	Kim, TH	Kim, Tae_Hyun
AU	3	34534	34534	Hong, MW	Hong, Min-Wook
AU	4	34535	34535	Park, TS	Park, Tae_Sub
AU	5	34536	34536	Lee, H	Lee, Hyojeong
AU	6	34537	34537	Lee, SJ	Lee, Sung-Jin
AF	2	34539	34539	Kim, Tae Hyun	Kim, Tae_Hyun
AF	4	34541	34541	Park, Tae Sub	Park, Tae_Sub

Record 224 | WOS:000594353000001

Effects of Single and Repeated Oral Doses of Ochratoxin A on the Lipid Peroxidation and Antioxidant Defense Systems in Mouse Kidneys

Field	Position	Original line	Normalised line	Original value	Normalised value
AU	1	34649	34649	Ferenczi, S	Ferenczi, Szilamer
AU	2	34650	34650	Kuti, D	Kuti, Daniel
AU	3	34651	34651	Cserháti, M	Cserhati, Matyas
AU	4	34652	34652	Krifaton, C	Krifaton, Csilla
AU	5	34653	34653	Szoboszlay, S	Szoboszlay, Sandor
AU	6	34654	34654	Kukolya, J	Kukolya, Jozsef
AU	7	34655	34655	Szoke, Z	Szoke, Zsuzsanna
AU	8	34656	34656	Albert, M	Albert, Mihaly
AU	9	34657	34657	Kriszt, B	Kriszt, Balazs
AU	10	34658	34658	Kovács, KJ	Kovacs, Krisztina_J
AU	11	34659	34659	Mézes, M	Mezes, Miklos
AU	12	34660	34660	Balogh, K	Balogh, Krisztian
AF	10	34670	34670	Kovacs, Krisztina J.	Kovacs, Krisztina_J

Record 225 | WOS:000580526300009

Microphysiological system modeling of ochratoxin A-associated nephrotoxicity

Field	Position	Original line	Normalised line	Original value	Normalised value
AU	1	34796	34796	Imaoka, T	Imaoka, Tomoki
AU	2	34797	34797	Yang, J	Yang, Jade
AU	3	34798	34798	Wang, L	Wang, Lu
AU	4	34799	34799	McDonald, MG	Mcdonald, Matthew_G
AU	5	34800	34800	Afsharinejad, Z	Afsharinejad, Zahra
AU	6	34801	34801	Bammler, TK	Bammler, Theo_K
AU	7	34802	34802	Van Ness, K	van_Ness, Kirk
AU	8	34803	34803	Yeung, CK	Yeung, Catherine_K

AU	9	34804	34804	Rettie, AE	Rettie, Allan_E
AU	10	34805	34805	Himmelfarb, J	Himmelfarb, Jonathan
AU	11	34806	34806	Kelly, EJ	Kelly, Edward_J
AF	4	34810	34810	McDonald, Matthew G.	Mcdonald, Matthew_G
AF	6	34812	34812	Bammler, Theo K.	Bammler, Theo_K
AF	7	34813	34813	Van Ness, Kirk	van_Ness, Kirk
AF	8	34814	34814	Yeung, Catherine K.	Yeung, Catherine_K
AF	9	34815	34815	Rettie, Allan E.	Rettie, Allan_E
AF	11	34817	34817	Kelly, Edward J.	Kelly, Edward_J

Record 226 | WOS:000582386000020

Ochratoxins in food and feed: Occurrence and its impact on human health and management strategies

Field	Position	Original line	Normalised line	Original value	Normalised value
AU	1	34945	34945	Kumar, P	Kumar, Pradeep
AU	2	34946	34946	Mahato, DK	Mahato, Dipendra_Kumar
AU	3	34947	34947	Sharma, B	Sharma, Bharti
AU	4	34948	34948	Borah, R	Borah, Rituraj
AU	5	34949	34949	Haque, S	Haque, Shafiul
AU	6	34950	34950	Mahmud, MMC	Mahmud, M_M_Chayan
AU	7	34951	34951	Shah, AK	Shah, Ajay_Kumar
AU	8	34952	34952	Rawal, D	Rawal, Deepaksshi
AU	9	34953	34953	Bora, H	Bora, Himashree
AU	10	34954	34954	Bui, S	Bui, Sukni
AF	2	34956	34956	Mahato, Dipendra Kumar	Mahato, Dipendra_Kumar
AF	6	34960	34960	Mahmud, M. M. Chayan	Mahmud, M_M_Chayan
AF	7	34961	34961	Shah, Ajay Kumar	Shah, Ajay_Kumar

Record 227 | WOS:000584874600001

The protective effect of stilbenes resveratrol and pterostilbene individually and combined with mycotoxin citrinin in human adenocarcinoma HT-29 cell line in vitro

Field	Position	Original line	Normalised line	Original value	Normalised value
AU	1	35254	35254	Spevakova, I	Spevakova, Ivana
AU	2	35255	35255	Fernandez-Cruz, ML	Fernandez-Cruz, Maria_Luisa
AU	3	35256	35256	Tokarova, K	Tokarova, Katarina
AU	4	35257	35257	Greifova, H	Greifova, Hana
AU	5	35258	35258	Capcarova, M	Capcarova, Marcela
AF	2	35260	35260	Fernandez-Cruz, Maria-Luisa	Fernandez-Cruz, Maria_Luisa

Record 228 | WOS:000563527100012

Cytoprotective effects of curcumin and silymarin on PK-15 cells exposed to ochratoxin A, fumonisin B1 and deoxynivalenol

Field	Position	Original line	Normalised line	Original value	Normalised value
AU	1	35394	35394	Ledur, PC	Ledur, Pauline_Christ
AU	2	35395	35395	Santurio, JM	Santurio, Janio_M
AF	1	35396	35396	Ledur, Pauline Christ	Ledur, Pauline_Christ

AF	2	35397	35397	Santurio, Janio M.	Santurio, Janio_M

Record 229 | WOS:000563527100013

Genotoxic effects of mycotoxins

Field	Position	Original line	Normalised line	Original value	Normalised value
AU	1	35497	35497	Ülger, TG	Ulger, Taha_Gokmen
AU	2	35498	35498	Uçar, A	Ucar, Asli
AU	3	35499	35499	Çakiroglu, FP	Cakiroglu, Funda_Pinar
AU	4	35500	35500	Yilmaz, S	Yilmaz, Serkan
AF	1	35501	35501	Ulger, Taha Gokmen	Ulger, Taha_Gokmen
AF	3	35503	35503	Cakiroglu, Funda Pinar	Cakiroglu, Funda_Pinar

Record 230 | WOS:000579907000025

Ochratoxin A induces reprogramming of glucose metabolism by switching energy metabolism from oxidative phosphorylation to glycolysis in human gastric epithelium GES-1 cells in vitro

Field	Position	Original line	Normalised line	Original value	Normalised value
AU	1	35683	35683	Wang, Y	Wang, Yuan
AU	2	35684	35684	Zhao, M	Zhao, Man
AU	3	35685	35685	Cui, JF	Cui, Jinfeng
AU	4	35686	35686	Wu, X	Wu, Xin
AU	5	35687	35687	Li, YH	Li, Yuehong
AU	6	35688	35688	Wu, WX	Wu, Wenxin
AU	7	35689	35689	Zhang, XH	Zhang, Xianghong

Record 231 | WOS:000566774200008

Dechlorination and demethylation of ochratoxin A enhance blocking activity of PXR activation, suppress PXR expression and reduce cytotoxicity

Field	Position	Original line	Normalised line	Original value	Normalised value
AU	1	35798	35798	Shen, YJ	Shen, Yuanjun
AU	2	35799	35799	Shi, ZQ	Shi, Zhanquan
AU	3	35800	35800	Fan, JT	Fan, Jun_Ting
AU	4	35801	35801	Yan, BF	Yan, Bingfang
AF	3	35804	35804	Fan, Jun Ting	Fan, Jun_Ting

Record 232 | WOS:000585534200001

The Compromised Intestinal Barrier Induced by Mycotoxins

Field	Position	Original line	Normalised line	Original value	Normalised value
AU	1	35914	35914	Gao, YA	Gao, Yanan
AU	2	35915	35915	Meng, L	Meng, Lu
AU	3	35916	35916	Liu, HM	Liu, Huimin
AU	4	35917	35917	Wang, JQ	Wang, Jiaqi
AU	5	35918	35918	Zheng, N	Zheng, Nan

Record 233 | WOS:000564335100008

Larval zebrafish as an in vitro model for evaluating toxicological effects of mycotoxins

Field	Position	Original line	Normalised line	Original value	Normalised value
AU	1	36210	36210	Juan-García, A	Juan-Garcia, Ana
AU	2	36211	36211	Bind, MA	Bind, Marie-Abele
AU	3	36212	36212	Engert, F	Engert, Florian

Record 234 | WOS:000585500900001

Toxin Degradation by Rumen Microorganisms: A Review

Field	Position	Original line	Normalised line	Original value	Normalised value
AU	1	36360	36360	Loh, ZH	Loh, Zhi_Hung
AU	2	36361	36361	Ouwerkerk, D	Ouwerkerk, Diane
AU	3	36362	36362	Klieve, AV	Klieve, Athol_V
AU	4	36363	36363	Hungerford, NL	Hungerford, Natasha_L
AU	5	36364	36364	Fletcher, MT	Fletcher, Mary_T
AF	1	36365	36365	Loh, Zhi Hung	Loh, Zhi_Hung
AF	3	36367	36367	Klieve, Athol V.	Klieve, Athol_V
AF	4	36368	36368	Hungerford, Natasha L.	Hungerford, Natasha_L
AF	5	36369	36369	Fletcher, Mary T.	Fletcher, Mary_T

Record 235 | WOS:000585462600001

Mycotoxins: Biotransformation and Bioavailability Assessment Using Caco-2 Cell Monolayer

Field	Position	Original line	Normalised line	Original value	Normalised value
AU	1	36690	36690	Tran, VN	Tran, van_Nguyen
AU	2	36691	36691	Viktorová, J	Viktorova, Jitka
AU	3	36692	36692	Ruml, T	Ruml, Tomas
AF	1	36693	36693	Tran, Van Nguyen	Tran, van_Nguyen

Record 236 | WOS:000573807900002

Ochratoxin A affects oocyte maturation and subsequent embryo developmental dynamics in the juvenile sheep model

Field	Position	Original line	Normalised line	Original value	Normalised value
AU	1	36997	36997	Dell'Aquila, ME	Dell'Aquila, Maria_Elena
AU	2	36998	36998	Asif, S	Asif, Shafaq
AU	3	36999	36999	Temerario, L	Temerario, Letizia
AU	4	37000	37000	Mastrorocco, A	Mastrorocco, Antonella
AU	5	37001	37001	Marzano, G	Marzano, Giuseppina
AU	6	37002	37002	Martino, NA	Martino, Nicola_Antonio
AU	7	37003	37003	Lacalandra, GM	Lacalandra, Giovanni_Michele
AU	8	37004	37004	Roelen, BAJ	Roelen, Bernard_A_J
AU	9	37005	37005	Carluccio, A	Carluccio, Augusto
AU	10	37006	37006	Robbe, D	Robbe, Domenico

AU	11	37007	37007	Minervini, F	Minervini, Fiorenza
AF	1	37008	37008	Dell'Aquila, Maria Elena	Dell'Aquila, Maria_Elena
AF	6	37013	37013	Martino, Nicola Antonio	Martino, Nicola_Antonio
AF	7	37014	37014	Lacalandra, Giovanni Michele	Lacalandra, Giovanni_Michele
AF	8	37015	37015	Roelen, Bernard A. J.	Roelen, Bernard_A_J

Record 237 | WOS:000545982700021

E ffects of Ochratoxin A exposure on DNA damage in porcine granulosa cells in vitro

Field	Position	Original line	Normalised line	Original value	Normalised value
AU	1	37144	37144	Zhang, TY	Zhang, Tian-Yu
AU	2	37145	37145	Kong, L	Kong, Li
AU	3	37146	37146	Hao, JX	Hao, Jia-Xing
AU	4	37147	37147	Wang, H	Wang, Han
AU	5	37148	37148	Yan, ZH	Yan, Zi-Hui
AU	6	37149	37149	Sun, XF	Sun, Xiao-Feng
AU	7	37150	37150	Shen, W	Shen, Wei

Record 238 | WOS:000568157900004

Multiple-endpoint in vitro carcinogenicity test in human cell line TK6 distinguishes carcinogens from non-carcinogens and highlights mechanisms of action

Field	Position	Original line	Normalised line	Original value	Normalised value
AU	1	37259	37259	Chapman, KE	Chapman, Katherine_E
AU	2	37260	37260	Wilde, EC	Wilde, Eleanor_C
AU	3	37261	37261	Chapman, FM	Chapman, Fiona_M
AU	4	37262	37262	Verma, JR	Verma, Jatin_R
AU	5	37263	37263	Shah, UK	Shah, Ume-Kulsoom
AU	6	37264	37264	Stannard, LM	Stannard, Leanne_M
AU	7	37265	37265	Seager, AL	Seager, Anna_L
AU	8	37266	37266	Tonkin, JA	Tonkin, James_A
AU	9	37267	37267	Brown, MR	Brown, M_Rowan
AU	10	37268	37268	Doherty, AT	Doherty, Ann_T
AU	11	37269	37269	Johnson, GE	Johnson, George_E
AU	12	37270	37270	Doak, SH	Doak, Shareen_H
AU	13	37271	37271	Jenkins, GJS	Jenkins, Gareth_J_S
AF	1	37272	37272	Chapman, Katherine E.	Chapman, Katherine_E
AF	2	37273	37273	Wilde, Eleanor C.	Wilde, Eleanor_C
AF	3	37274	37274	Chapman, Fiona M.	Chapman, Fiona_M
AF	4	37275	37275	Verma, Jatin R.	Verma, Jatin_R
AF	6	37277	37277	Stannard, Leanne M.	Stannard, Leanne_M
AF	7	37278	37278	Seager, Anna L.	Seager, Anna_L
AF	8	37279	37279	Tonkin, James A.	Tonkin, James_A
AF	9	37280	37280	Brown, M. Rowan	Brown, M_Rowan
AF	10	37281	37281	Doherty, Ann T.	Doherty, Ann_T
AF	11	37282	37282	Johnson, George E.	Johnson, George_E
AF	12	37283	37283	Doak, Shareen H.	Doak, Shareen_H
AF	13	37284	37284	Jenkins, Gareth J. S.	Jenkins, Gareth_J_S

Record 239 | WOS:000567625500001

Protective role of lactic acid bacteria and yeasts as dietary carcinogen-binding agents - a review

Field	Position	Original line	Normalised line	Original value	Normalised value
AU	1	37409	37409	Cuevas-González, PF	Cuevas-Gonzalez, P_F
AU	2	37410	37410	González-Córdova, AF	Gonzalez-Cordova, A_F
AU	4	37412	37412	Aguilar-Toalá, JE	Aguilar-Toala, J_E
AU	5	37413	37413	Hall, FG	Hall, F_G
AU	6	37414	37414	Urbizo-Reyes, UC	Urbizo-Reyes, U_C
AU	7	37415	37415	Liceaga, AM	Liceaga, A_M
AU	9	37417	37417	García, HS	Garcia, H_S
AF	1	37418	37418	Cuevas-Gonzalez, P. F.	Cuevas-Gonzalez, P_F
AF	2	37419	37419	Gonzalez-Cordova, A. F.	Gonzalez-Cordova, A_F
AF	3	37420	37420	Vallejo-Cordoba, B.	Vallejo-Cordoba, B
AF	4	37421	37421	Aguilar-Toala, J. E.	Aguilar-Toala, J_E
AF	5	37422	37422	Hall, F. G.	Hall, F_G
AF	6	37423	37423	Urbizo-Reyes, U. C.	Urbizo-Reyes, U_C
AF	7	37424	37424	Liceaga, A. M.	Liceaga, A_M
AF	8	37425	37425	Hernandez-Mendoza, A.	Hernandez-Mendoza, A
AF	9	37426	37426	Garcia, H. S.	Garcia, H_S

Record 240 | WOS:000562731900014

Ochratoxin A induces glomerular injury through activating the ERK/NF-κB signaling pathway

Field	Position	Original line	Normalised line	Original value	Normalised value
AU	1	37685	37685	Le, GN	le, Guannan
AU	2	37686	37686	Yuan, X	Yuan, Xin
AU	3	37687	37687	Hou, LL	Hou, Lili
AU	4	37688	37688	Ge, L	Ge, Lei
AU	5	37689	37689	Liu, SP	Liu, Shuiping
AU	6	37690	37690	Muhmood, A	Muhmood, Azhar
AU	7	37691	37691	Liu, K	Liu, Kai
AU	8	37692	37692	Lin, ZM	Lin, Ziman
AU	9	37693	37693	Liu, DD	Liu, Dandan
AU	10	37694	37694	Gan, F	Gan, Fang
AU	11	37695	37695	Song, SQ	Song, Suquan
AU	12	37696	37696	Pan, CL	Pan, Cuilin
AU	13	37697	37697	Chen, XX	Chen, Xingxiang
AU	14	37698	37698	Huang, KH	Huang, Kehe
AF	1	37699	37699	Le, Guannan	le, Guannan

Record 241 | WOS:000585802200008

Synergistic Interaction of Ochratoxin A and Acrylamide Toxins in Human Kidney and Liver Cells

Field	Position	Original line	Normalised line	Original value	Normalised value
AU	1	37810	37810	Pyo, MC	Pyo, Min_Cheol

AU	2	37811	37811	Shin, HS	Shin, Hye_Soo
AU	3	37812	37812	Jeon, GY	Jeon, Gyeong_Yun
AU	4	37813	37813	Lee, KW	Lee, Kwang-Won
AF	1	37814	37814	Pyo, Min Cheol	Pyo, Min_Cheol
AF	2	37815	37815	Shin, Hye Soo	Shin, Hye_Soo
AF	3	37816	37816	Jeon, Gyeong Yun	Jeon, Gyeong_Yun

Record 242 | WOS:000560097800001

Ochratoxin A presence in Cabernet Sauvignon wine changes antioxidant activityin vitroand oxidative stress markersin vivo

Field	Position	Original line	Normalised line	Original value	Normalised value
AU	1	37942	37942	Schmidt, L	Schmidt, Luana
AU	2	37943	37943	Heck, ND	Heck, Natalia_de_Vargas
AU	3	37944	37944	Ferreira, I	Ferreira, Ingrid
AU	4	37945	37945	Göethel, G	Goethel, Gabriela
AU	5	37946	37946	Somacal, S	Somacal, Sabrina
AU	6	37947	37947	Emanuelli, T	Emanuelli, Tatiana
AU	7	37948	37948	Rodrigues, E	Rodrigues, Eliseu
AU	8	37949	37949	Garcia, SC	Garcia, Solange_Cristina
AU	9	37950	37950	Welke, JE	Welke, Juliane_Elisa
AU	10	37951	37951	Augusti, PR	Augusti, Paula_Rossini
AF	2	37953	37953	Heck, Natalia de Vargas	Heck, Natalia_de_Vargas
AF	8	37959	37959	Garcia, Solange Cristina	Garcia, Solange_Cristina
AF	9	37960	37960	Welke, Juliane Elisa	Welke, Juliane_Elisa
AF	10	37961	37961	Augusti, Paula Rossini	Augusti, Paula_Rossini

Record 243 | WOS:000539161100006

Mycotoxins, invisible danger of feedstuff with toxic effect on animals

Field	Position	Original line	Normalised line	Original value	Normalised value
AU	1	38075	38075	Buszewska-Forajta, M	Buszewska-Forajta, Magdalena

Record 244 | WOS:000547359800001

Subchronic exposure to individual and combined ochratoxin A and citrinin affects the expression of rat renal organic anion transporters

Field	Position	Original line	Normalised line	Original value	Normalised value
AU	1	38249	38249	Karaica, D	Karaica, Dean
AU	2	38250	38250	Micek, V	Micek, Vedran
AU	3	38251	38251	Rasic, D	Rasic, Dubravka
AU	4	38252	38252	Peraica, M	Peraica, Maja
AU	5	38253	38253	Klaric, MS	Klaric, Maja_Segvic
AU	6	38254	38254	Breljak, D	Breljak, Davorka
AF	5	38259	38259	Klaric, Maja Segvic	Klaric, Maja_Segvic

Record 245 | WOS:000545059200001

Ochratoxin A induces epithelial-to-mesenchymal transition and renal fibrosis through TGF-β/Smad2/3 and Wnt1/β-catenin signaling pathways in vitro and in vivo

Field	Position	Original line	Normalised line	Original value	Normalised value
AU	1	38382	38382	Pyo, MC	Pyo, Min_Cheol
AU	2	38383	38383	Chae, SA	Chae, Seung_A
AU	3	38384	38384	Yoo, HJ	Yoo, Hee_Joon
AU	4	38385	38385	Lee, KW	Lee, Kwang-Won
AF	1	38386	38386	Pyo, Min Cheol	Pyo, Min_Cheol
AF	2	38387	38387	Chae, Seung A.	Chae, Seung_A
AF	3	38388	38388	Yoo, Hee Joon	Yoo, Hee_Joon

Record 246 | WOS:000541110800046

Luteolin alleviates ochratoxin A induced oxidative stress by regulating Nrf2 and HIF-1α pathways in NRK-52E rat kidney cells

Field	Position	Original line	Normalised line	Original value	Normalised value
AU	1	38512	38512	Liu, M	Liu, Man
AU	2	38513	38513	Cheng, C	Cheng, Chao
AU	3	38514	38514	Li, XZ	Li, Xuezhi
AU	4	38515	38515	Zhou, SH	Zhou, Sihan
AU	5	38516	38516	Hua, JL	Hua, Jiali
AU	6	38517	38517	Huang, J	Huang, Jie
AU	7	38518	38518	Li, YX	Li, Yongxin
AU	8	38519	38519	Yang, KL	Yang, Kunlong
AU	9	38520	38520	Zhang, P	Zhang, Peng
AU	10	38521	38521	Zhang, Y	Zhang, Yan
AU	11	38522	38522	Tian, J	Tian, Jun

Record 247 | WOS:000556892100001

Reducing Ochratoxin A Content in Grape Pomace by Different Methods

Field	Position	Original line	Normalised line	Original value	Normalised value
AU	1	38646	38646	Yu, JM	Yu, Jianmei
AU	2	38647	38647	Smith, IN	Smith, Ivy_N
AU	3	38648	38648	Mikiashvili, N	Mikiashvili, Nona
AF	2	38650	38650	Smith, Ivy N.	Smith, Ivy_N

Record 248 | WOS:000530851100002

Ochratoxin A causes cell cycle arrest in G1 and G1/S phases through p53 in HK-2 cells

Field	Position	Original line	Normalised line	Original value	Normalised value
AU	1	38741	38741	Çelik, DA	Celik, Dilek_Asci
AU	2	38742	38742	Gurbuz, N	Gurbuz, Nilgun
AU	3	38743	38743	Togay, VA	Togay, Vehbi_Atahan
AU	4	38744	38744	Özçelik, N	Ozcelik, Nurten

AF	1	38745	38745	Celik, Dilek Asci	Celik, Dilek_Asci
AF	3	38747	38747	Togay, Vehbi Atahan	Togay, Vehbi_Atahan

Record 249 | WOS:000541267600001

Cytogenotoxic evaluation of the acetonitrile extract, citrinin and dicitrinin-A fromPenicillium citrinum

Field	Position	Original line	Normalised line	Original value	Normalised value
AU	1	38836	38836	de Oliveira, JWG	Gomes_de_Oliveira_Filho, Jose_Williams
AU	2	38837	38837	Andrade, TDAD	Aguiar_dos_Santos_Andrade, Teresinha_de_Jesus
AU	3	38838	38838	de Lima, RMT	Torres_de_Lima, Rosalia_Maria
AU	4	38839	38839	Silva, DHS	Siqueira_Silva, Dulce_Helena
AU	5	38840	38840	dos Reis, AC	dos_Reis, Antonielly_Campinho
AU	6	38841	38841	Santos, JVD	de_Oliveira_Santos, Jose_Victor
AU	7	38842	38842	de Meneses, AAPM	Melo_de_Meneses, Ag-Anne_Pereira
AU	8	38843	38843	de Carvalho, RM	de_Carvalho, Ricardo_Melo
AU	9	38844	38844	da Mata, AMO	Oliveira_da_Mata, Ana_Maria
AU	10	38845	38845	de Alencar, MVOB	Oliveira_Barros_de_Alencar, Marcus_Vinicius
AU	11	38846	38846	Dias, ACS	Soares_Dias, Ana_Carolina
AU	12	38847	38847	da Silva, FCC	Carneiro_da_Silva, Felipe_Cavalcanti
AU	13	38848	38848	Islam, MT	Islam, Muhammad_Torequl
AU	14	38849	38849	Clark, CCT	Clark, Cain_C_T
AU	15	38850	38850	Sousa, JMDE	de_Castro_E_Sousa, Joao_Marcelo
AU	16	38851	38851	Melo-Cavalcante, AAD	de_Carvalho_Melo-Cavalcante, Ana_Amelia
AF	1	38852	38852	Gomes de Oliveira Filho, Jose Williams	Gomes_de_Oliveira_Filho, Jose_Williams
AF	2	38853	38853	Aguiar dos Santos Andrade, Teresinha de Jesus	Aguiar_dos_Santos_Andrade, Teresinha_de_Jesus
AF	3	38854	38854	Torres de Lima, Rosalia Maria	Torres_de_Lima, Rosalia_Maria
AF	4	38855	38855	Siqueira Silva, Dulce Helena	Siqueira_Silva, Dulce_Helena
AF	5	38856	38856	dos Reis, Antonielly Campinho	dos_Reis, Antonielly_Campinho
AF	6	38857	38857	de Oliveira Santos, Jose Victor	de_Oliveira_Santos, Jose_Victor
AF	7	38858	38858	Melo de Meneses, Ag-Anne Pereira	Melo_de_Meneses, Ag-Anne_Pereira
AF	8	38859	38859	de Carvalho, Ricardo Melo	de_Carvalho, Ricardo_Melo
AF	9	38860	38860	Oliveira da Mata, Ana Maria	Oliveira_da_Mata, Ana_Maria
AF	10	38861	38861	Oliveira Barros de Alencar, Marcus Vinicius	Oliveira_Barros_de_Alencar, Marcus_Vinicius
AF	11	38862	38862	Soares Dias, Ana Carolina	Soares_Dias, Ana_Carolina
AF	12	38863	38863	Carneiro da Silva, Felipe Cavalcanti	Carneiro_da_Silva, Felipe_Cavalcanti
AF	13	38864	38864	Islam, Muhammad Torequl	Islam, Muhammad_Torequl
AF	14	38865	38865	Clark, Cain C. T.	Clark, Cain_C_T
AF	15	38866	38866	de Castro e Sousa, Joao Marcelo	de_Castro_E_Sousa, Joao_Marcelo

AF	16	38867	38867	de Carvalho Melo-Cavalcante, Ana Amelia	de_Carvalho_Melo-Cavalcante, Ana_Amelia

Record 250 | WOS:000533844900008

Ochratoxin A-induced genotoxic and epigenetic mechanisms lead to Alzheimer disease: its modulation with strategies

Field	Position	Original line	Normalised line	Original value	Normalised value
AU	1	39023	39023	Niaz, K	Niaz, Kamal
AU	2	39024	39024	Shah, SZA	Shah, Syed_Zahid_Ali
AU	3	39025	39025	Khan, F	Khan, Fazlullah
AU	4	39026	39026	Bule, M	Bule, Mohammed
AF	2	39028	39028	Shah, Syed Zahid Ali	Shah, Syed_Zahid_Ali

Record 251 | WOS:000542374900003

Milk thistle (Silybum Marianum) as an antidote or a protective agent against natural or chemical toxicities: a review

Field	Position	Original line	Normalised line	Original value	Normalised value
AU	1	39343	39343	Fanoudi, S	Fanoudi, Sahar
AU	2	39344	39344	Alavi, MS	Alavi, Mohaddeseh_Sadat
AU	3	39345	39345	Karimi, G	Karimi, Gholamreza
AU	4	39346	39346	Hosseinzadeh, H	Hosseinzadeh, Hossein
AF	2	39348	39348	Alavi, Mohaddeseh Sadat	Alavi, Mohaddeseh_Sadat

Record 252 | WOS:000527365800027

Ochratoxin A exposure causes meiotic failure and oocyte deterioration in mice

Field	Position	Original line	Normalised line	Original value	Normalised value
AU	1	39584	39584	Jia, HQ	Jia, Huiqun
AU	2	39585	39585	Jia, CQ	Jia, Chenqi
AU	3	39586	39586	An, QL	An, Quanli
AU	4	39587	39587	Cheng, YY	Cheng, Yuyao
AU	5	39588	39588	Jiang, XL	Jiang, Xianlei
AU	6	39589	39589	Xu, Y	Xu, Yue
AU	7	39590	39590	Zhao, RL	Zhao, Ruolin
AU	8	39591	39591	Peng, W	Peng, Wei
AU	9	39592	39592	Zhang, Y	Zhang, Yong
AU	10	39593	39593	Su, JM	Su, Jianmin

Record 253 | WOS:000526412300005

Beauvericin and ochratoxin A mycotoxins individually and combined in HepG2 cells alter lipid peroxidation, levels of reactive oxygen species and glutathione

Field	Position	Original line	Normalised line	Original value	Normalised value
AU	1	39730	39730	Juan-García, A	Juan-Garcia, Ana
AU	2	39731	39731	Carbone, S	Carbone, Saverio
AU	3	39732	39732	Ben-Mahmoud, M	Ben-Mahmoud, Maissa

AU	4	39733	39733	Sagratini, G	Sagratini, Gianni
AU	5	39734	39734	Mañes, J	Manes, Jordi

Record 254 | WOS:000537553700018

Risk assessment of ochratoxin A in food

Field	Position	Original line	Normalised line	Original value	Normalised value
AU	1	39848	39848	Schrenk, D	Schrenk, Dieter
AU	2	39849	39849	Bodin, L	Bodin, Laurent
AU	3	39850	39850	Chipman, JK	Chipman, James_Kevin
AU	4	39851	39851	del Mazo, J	del_Mazo, Jesus
AU	5	39852	39852	Grasl-Kraupp, B	Grasl-Kraupp, Bettina
AU	6	39853	39853	Hogstrand, C	Hogstrand, Christer
AU	7	39854	39854	Hoogenboom, L	Hoogenboom, Laurentius_(ron)
AU	8	39855	39855	Leblanc, JC	Leblanc, Jean-Charles
AU	9	39856	39856	Nebbia, CS	Nebbia, Carlo_Stefano
AU	10	39857	39857	Nielsen, E	Nielsen, Elsa
AU	11	39858	39858	Ntzani, E	Ntzani, Evangelia
AU	12	39859	39859	Petersen, A	Petersen, Annette
AU	13	39860	39860	Sand, S	Sand, Salomon
AU	14	39861	39861	Schwerdtle, T	Schwerdtle, Tanja
AU	15	39862	39862	Vleminckx, C	Vleminckx, Christiane
AU	16	39863	39863	Wallace, H	Wallace, Heather
AU	17	39864	39864	Alexander, J	Alexander, Jan
AU	18	39865	39865	Dall'Asta, C	Dall'Asta, Chiara
AU	19	39866	39866	Mally, A	Mally, Angela
AU	20	39867	39867	Metzler, M	Metzler, Manfred
AU	21	39868	39868	Binaglia, M	Binaglia, Marco
AU	22	39869	39869	Horváth, Z	Horvath, Zsuzsanna
AU	23	39870	39870	Steinkellner, H	Steinkellner, Hans
AU	24	39871	39871	Bignami, M	Bignami, Margherita
AF	3	39874	39874	Chipman, James Kevin	Chipman, James_Kevin
AF	4	39875	39875	del Mazo, Jesus	del_Mazo, Jesus
AF	7	39878	39878	Hoogenboom, Laurentius (Ron)	Hoogenboom, Laurentius_(ron)
AF	9	39880	39880	Nebbia, Carlo Stefano	Nebbia, Carlo_Stefano

Record 255 | WOS:000505957800037

Aptamer assisted ultrafiltration cleanup with high performance liquid chromatography-fluorescence detector for the determination of OTA in green coffee

Field	Position	Original line	Normalised line	Original value	Normalised value
AU	1	40405	40405	Moez, E	Moez, Elsaadani
AU	2	40406	40406	Noel, D	Noel, Durand
AU	3	40407	40407	Brice, S	Brice, Sorli
AU	4	40408	40408	Benjamin, G	Benjamin, Guibert
AU	5	40409	40409	Pascaline, A	Pascaline, Atelier
AU	6	40410	40410	Didier, M	Didier, Montet

Record 256 | WOS:000526746500001

Protective Effect of Hydroxytyrosol Against Oxidative Stress Induced by the Ochratoxin in Kidney Cells: in vitro and in vivo Study

Field	Position	Original line	Normalised line	Original value	Normalised value
AU	1	40510	40510	Crupi, R	Crupi, Rosalia
AU	2	40511	40511	Palma, E	Palma, Ernesto
AU	3	40512	40512	Siracusa, R	Siracusa, Rosalba
AU	4	40513	40513	Fusco, R	Fusco, Roberta
AU	5	40514	40514	Gugliandolo, E	Gugliandolo, Enrico
AU	6	40515	40515	Cordaro, M	Cordaro, Marika
AU	7	40516	40516	Impellizzeri, D	Impellizzeri, Daniela
AU	8	40517	40517	De Caro, C	de_Caro, Carmen
AU	9	40518	40518	Calzetta, L	Calzetta, Luigino
AU	10	40519	40519	Cuzzocrea, S	Cuzzocrea, Salvatore
AU	11	40520	40520	Di Paola, R	di_Paola, Rosanna
AF	8	40528	40528	De Caro, Carmen	de_Caro, Carmen
AF	11	40531	40531	Di Paola, Rosanna	di_Paola, Rosanna

Record 257 | WOS:000524713000001

Development of ELISA and Lateral Flow Immunoassays for Ochratoxins (OTA and OTB) Detection Based on Monoclonal Antibody

Field	Position	Original line	Normalised line	Original value	Normalised value
AU	1	40628	40628	Fadlalla, MH	Fadlalla, Mohamed_Hassan
AU	2	40629	40629	Ling, SM	Ling, Sumei
AU	3	40630	40630	Wang, RZ	Wang, Rongzhi
AU	4	40631	40631	Li, XL	Li, Xiulan
AU	5	40632	40632	Yuan, J	Yuan, Jun
AU	6	40633	40633	Xiao, SW	Xiao, Shiwei
AU	7	40634	40634	Wang, K	Wang, Ke
AU	8	40635	40635	Tang, SQ	Tang, Shuqin
AU	9	40636	40636	Elsir, H	Elsir, Hoyda
AU	10	40637	40637	Wang, SH	Wang, Shihua
AF	1	40638	40638	Fadlalla, Mohamed Hassan	Fadlalla, Mohamed_Hassan

Record 258 | WOS:000530248700129

Protective Effect of Astaxanthin on Ochratoxin A-Induced Kidney Injury to Mice by Regulating Oxidative Stress-Related NRF2/KEAP1 Pathway

Field	Position	Original line	Normalised line	Original value	Normalised value
AU	1	40751	40751	Li, L	Li, Lin
AU	2	40752	40752	Chen, YL	Chen, Yueli
AU	3	40753	40753	Jiao, DY	Jiao, Danyang
AU	4	40754	40754	Yang, SH	Yang, Shuhua
AU	5	40755	40755	Li, L	Li, Lin
AU	6	40756	40756	Li, P	Li, Peng

Record 259 | WOS:000517659300003

Toxicity of mycotoxins in vivo on vertebrate organisms: A review

Field	Position	Original line	Normalised line	Original value	Normalised value
AU	1	40870	40870	Cimbalo, A	Cimbalo, Alessandra
AU	2	40871	40871	Alonso-Garrido, M	Alonso-Garrido, Manuel
AU	3	40872	40872	Font, G	Font, Guillermina
AU	4	40873	40873	Manyes, L	Manyes, Lara
AF	1	40874	40874	Cimbalo, A.	Cimbalo, Alessandra
AF	2	40875	40875	Alonso-Garrido, M.	Alonso-Garrido, Manuel
AF	3	40876	40876	Font, G.	Font, Guillermina
AF	4	40877	40877	Manyes, L.	Manyes, Lara

Record 260 | WOS:000526529800024

Influences of Electron Beam Irradiation on the Physical and Chemical Properties of Zearalenone- and Ochratoxin A-Contaminated Corn and In Vivo Toxicity Assessment

Field	Position	Original line	Normalised line	Original value	Normalised value
AU	1	41104	41104	Luo, XH	Luo, Xiaohu
AU	2	41105	41105	Zhai, YH	Zhai, Yuheng
AU	3	41106	41106	Qi, LJ	Qi, Lijun
AU	4	41107	41107	Pan, LH	Pan, Lihong
AU	5	41108	41108	Wang, J	Wang, Jing
AU	6	41109	41109	Xing, JL	Xing, Jiali
AU	7	41110	41110	Wang, R	Wang, Ren
AU	8	41111	41111	Wang, L	Wang, Li
AU	9	41112	41112	Zhang, QC	Zhang, Qingchuan
AU	10	41113	41113	Yang, K	Yang, Kai
AU	11	41114	41114	Chen, ZX	Chen, Zhengxing

Record 261 | WOS:000525009500057

In-Vitro Cell Culture for Efficient Assessment of Mycotoxin Exposure, Toxicity and Risk Mitigation

Field	Position	Original line	Normalised line	Original value	Normalised value
AU	1	41237	41237	Xu, R	Xu, Ran
AU	2	41238	41238	Karrow, NA	Karrow, Niel_A
AU	3	41239	41239	Shandilya, UK	Shandilya, Umesh_K
AU	4	41240	41240	Sun, LH	Sun, Lv-Hui
AU	5	41241	41241	Kitazawa, H	Kitazawa, Haruki
AF	2	41243	41243	Karrow, Niel A.	Karrow, Niel_A
AF	3	41244	41244	Shandilya, Umesh K.	Shandilya, Umesh_K
AF	4	41245	41245	Sun, Lv-hui	Sun, Lv-Hui

Record 262 | WOS:000543783800001

Nontoxic concentration of ochratoxin A decreases the dosage of cyclosporine A to induce chronic nephropathy model via autophagy mediated by toll-like receptor 4

Field	Position	Original line	Normalised line	Original value	Normalised value
AU	1	41570	41570	Hou, LL	Hou, Lili
AU	2	41571	41571	Le, GN	le, Guannan
AU	3	41572	41572	Lin, ZM	Lin, Ziman
AU	4	41573	41573	Qian, G	Qian, Gang
AU	5	41574	41574	Gan, F	Gan, Fang
AU	6	41575	41575	Gu, C	Gu, Cong
AU	7	41576	41576	Jiang, S	Jiang, Shuai
AU	8	41577	41577	Mu, JX	Mu, Jiaxin
AU	9	41578	41578	Ge, L	Ge, Lei
AU	10	41579	41579	Huang, KH	Huang, Kehe
AF	2	41581	41581	Le, Guannan	le, Guannan

Record 263 | WOS:000512092100007

Ochratoxin A induces global DNA hypomethylation and oxidative stress in neuronal cells in vitro

Field	Position	Original line	Normalised line	Original value	Normalised value
AU	1	41682	41682	Babayan, N	Babayan, Nelly
AU	2	41683	41683	Tadevosyan, G	Tadevosyan, Gohar
AU	3	41684	41684	Khondkaryan, L	Khondkaryan, Lusine
AU	4	41685	41685	Grigoryan, R	Grigoryan, Ruzanna
AU	5	41686	41686	Sarkisyan, N	Sarkisyan, Natalya
AU	6	41687	41687	Haroutiounian, R	Haroutiounian, Rouben
AU	7	41688	41688	Stopper, H	Stopper, Helga

Record 264 | WOS:000506899900001

Toxicity effects of mycotoxins and autophagy: a mechanistic view

Field	Position	Original line	Normalised line	Original value	Normalised value
AU	1	41787	41787	Ariafar, S	Ariafar, Saba
AU	2	41788	41788	Harsin, AO	Oftadeh_Harsin, Akram
AU	3	41789	41789	Fadaiie, A	Fadaiie, Ahmad
AU	4	41790	41790	Mahboobian, MM	Mahboobian, Mohammad_Mehdi
AU	5	41791	41791	Mohammadi, M	Mohammadi, Mojdeh
AF	2	41793	41793	Oftadeh Harsin, Akram	Oftadeh_Harsin, Akram
AF	4	41795	41795	Mahboobian, Mohammad Mehdi	Mahboobian, Mohammad_Mehdi

Record 265 | WOS:000505295600001

Red orange and lemon extract prevents the renal toxicity induced by ochratoxin A in rats

Field	Position	Original line	Normalised line	Original value	Normalised value
AU	1	41936	41936	Damiano, S	Damiano, Sara
AU	2	41937	41937	Iovane, V	Iovane, Valentina
AU	3	41938	41938	Squillacioti, C	Squillacioti, Caterina
AU	4	41939	41939	Mirabella, N	Mirabella, Nicola
AU	5	41940	41940	Prisco, F	Prisco, Francesco

AU	6	41941	41941	Ariano, A	Ariano, Andrea
AU	7	41942	41942	Amenta, M	Amenta, Margherita
AU	8	41943	41943	Giordano, A	Giordano, Antonio
AU	9	41944	41944	Florio, S	Florio, Salvatore
AU	10	41945	41945	Ciarcia, R	Ciarcia, Roberto

Record 266 | WOS:000488786200007

Mycotoxin-induced toxicities and diseases

Field	Position	Original line	Normalised line	Original value	Normalised value
AU	1	42064	42064	Gacem, MA	Gacem, Mohamed_Amine
AU	2	42065	42065	Gacem, H	Gacem, Hiba
AU	3	42066	42066	Telli, A	Telli, Alia
AU	4	42067	42067	Khelil, AOE	Khelil, Aminata_Ould_el_Hadj
AF	1	42068	42068	Gacem, Mohamed Amine	Gacem, Mohamed_Amine
AF	4	42071	42071	Khelil, Aminata Ould El Hadj	Khelil, Aminata_Ould_el_Hadj

Record 267 | WOS:000680216400001

Mycotoxins Occurrence, Toxicity and Detection Methods

Field	Position	Original line	Normalised line	Original value	Normalised value
AU	1	42354	42354	Gacem, MA	Gacem, Mohamed_Amine
AU	2	42355	42355	El Hadj-Khelil, AO	el_Hadj-Khelil, Aminata_Ould
AU	3	42356	42356	Boudjemaa, B	Boudjemaa, Badreddine
AU	4	42357	42357	Gacem, H	Gacem, Hiba
AF	1	42358	42358	Gacem, Mohamed Amine	Gacem, Mohamed_Amine
AF	2	42359	42359	El Hadj-Khelil, Aminata Ould	el_Hadj-Khelil, Aminata_Ould

Record 268 | WOS:000504757200032

Melatonin ameliorates ochratoxin A-induced oxidative stress and apoptosis in porcine oocytes

Field	Position	Original line	Normalised line	Original value	Normalised value
AU	1	42713	42713	Lan, M	Lan, Mei
AU	2	42714	42714	Zhang, Y	Zhang, Yu
AU	3	42715	42715	Wan, X	Wan, Xiang
AU	4	42716	42716	Pan, MH	Pan, Meng-Hao
AU	5	42717	42717	Xu, Y	Xu, Yao
AU	6	42718	42718	Sun, SC	Sun, Shao-Chen

Record 269 | WOS:000514650700067

Modelling Fungal Growth, Mycotoxin Production and Release in Grana Cheese

Field	Position	Original line	Normalised line	Original value	Normalised value
AU	1	42815	42815	Leggieri, MC	Leggieri, Marco_Camardo
AU	2	42816	42816	Pietri, A	Pietri, Amedeo

AU	3	42817	42817	Battilani, P	Battilani, Paola
AF	1	42818	42818	Leggieri, Marco Camardo	Leggieri, Marco_Camardo

Record 270 | WOS:000545943400001

Penicillium spp. mycotoxins found in food and feed and their health effects

Field	Position	Original line	Normalised line	Original value	Normalised value
AU	3	42920	42920	Echeverría-Vega, A	Echeverria-Vega, A
AF	1	42922	42922	Otero, C.	Otero, C
AF	2	42923	42923	Arredondo, C.	Arredondo, C
AF	3	42924	42924	Echeverria-Vega, A.	Echeverria-Vega, A
AF	4	42925	42925	Gordillo-Fuenzalida, F.	Gordillo-Fuenzalida, F

Record 271 | WOS:000514630000034

Comprehensive Evaluation of the Efficiency of Yeast Cell Wall Extract to Adsorb Ochratoxin A and Mitigate Accumulation of the Toxin in Broiler Chickens

Field	Position	Original line	Normalised line	Original value	Normalised value
AU	1	43142	43142	Vartiainen, S	Vartiainen, Suvi
AU	2	43143	43143	Yiannikouris, A	Yiannikouris, Alexandros
AU	3	43144	43144	Apajalahti, J	Apajalahti, Juha
AU	4	43145	43145	Moran, CA	Moran, Colm_A
AF	4	43149	43149	Moran, Colm A.	Moran, Colm_A

Record 272 | WOS:000514630000026

Transcriptome Analysis of Ochratoxin A-Induced Apoptosis in Differentiated Caco-2 Cells

Field	Position	Original line	Normalised line	Original value	Normalised value
AU	1	43247	43247	Yang, X	Yang, Xue
AU	2	43248	43248	Gao, YN	Gao, Yanan
AU	3	43249	43249	Yan, QY	Yan, Qiaoyan
AU	4	43250	43250	Bao, XY	Bao, Xiaoyu
AU	5	43251	43251	Zhao, SG	Zhao, Shengguo
AU	6	43252	43252	Wang, JQ	Wang, Jiaqi
AU	7	43253	43253	Zheng, N	Zheng, Nan

Record 273 | WOS:000498748700011

Epithelial-fibroblast cross talk aggravates the impact of the nephrotoxin ochratoxin A

Field	Position	Original line	Normalised line	Original value	Normalised value
AU	1	43414	43414	Schulz, MC	Schulz, Marie-Christin
AU	2	43415	43415	Gekle, M	Gekle, Michael
AU	3	43416	43416	Schwerdt, G	Schwerdt, Gerald

Record 274 | WOS:000498194100002

Capsaicin, an inhibitor of Ochratoxin A production by Aspergillus section Nigri strains in grapes (Vitis vinifera L.)

Field	Position	Original line	Normalised line	Original value	Normalised value
AU	1	43524	43524	Kollia, E	Kollia, Eleni
AU	2	43525	43525	Proestos, C	Proestos, Charalampos
AU	3	43526	43526	Zoumpoulakis, P	Zoumpoulakis, Panagiotis
AU	4	43527	43527	Markaki, P	Markaki, Panagiota

Record 275 | WOS:000501604700005

Ochratoxin A Sequentially Activates Autophagy and the Ubiquitin-Proteasome System

Field	Position	Original line	Normalised line	Original value	Normalised value
AU	1	43641	43641	Akpinar, HA	Akpinar, Hafize_Aysin
AU	2	43642	43642	Kahraman, H	Kahraman, Hilal
AU	3	43643	43643	Yaman, I	Yaman, Ibrahim
AF	1	43644	43644	Akpinar, Hafize Aysin	Akpinar, Hafize_Aysin

Record 276 | WOS:000501604700006

Comparative In Vitro Assessment of a Range of Commercial Feed Additives with Multiple Mycotoxin Binding Claims

Field	Position	Original line	Normalised line	Original value	Normalised value
AU	1	43766	43766	Kolawole, O	Kolawole, Oluwatobi
AU	2	43767	43767	Meneely, J	Meneely, Julie
AU	3	43768	43768	Greer, B	Greer, Brett
AU	4	43769	43769	Chevallier, O	Chevallier, Olivier
AU	5	43770	43770	Jones, DS	Jones, David_S
AU	6	43771	43771	Connolly, L	Connolly, Lisa
AU	7	43772	43772	Elliott, C	Elliott, Christopher
AF	5	43777	43777	Jones, David S.	Jones, David_S

Record 277 | WOS:000477691300004

Reduction of ochratoxin A in oats and rice by twin-screw extrusion processing with baking soda

Field	Position	Original line	Normalised line	Original value	Normalised value
AU	1	43889	43889	Ryu, D	Ryu, Dojin
AU	2	43890	43890	Kowalski, RJ	Kowalski, Ryan_Joseph
AU	3	43891	43891	Ganjyal, G	Ganjyal, Girish
AU	4	43892	43892	Lee, HJ	Lee, Hyun_Jung
AF	2	43894	43894	Kowalski, Ryan Joseph	Kowalski, Ryan_Joseph
AF	4	43896	43896	Lee, Hyun Jung	Lee, Hyun_Jung

Record 278 | WOS:000475915000013

A multi-endpoint approach to the combined toxic effects of patulin and ochratoxin a in human intestinal cells

Field	Position	Original line	Normalised line	Original value	Normalised value
AU	1	43998	43998	Assunçao, R	Assuncao, Ricardo
AU	2	43999	43999	Pinhao, M	Pinhao, Mariana
AU	3	44000	44000	Loureiro, S	Loureiro, Susana
AU	4	44001	44001	Alvito, P	Alvito, Paula
AU	5	44002	44002	Silva, MJ	Silva, Maria_Joao
AF	5	44007	44007	Silva, Maria Joao	Silva, Maria_Joao

Record 279 | WOS:000484647100041

Impact of dietary Trichosporon mycotoxinivorans on ochratoxin A induced immunotoxicity; In vivo study

Field	Position	Original line	Normalised line	Original value	Normalised value
AU	1	44157	44157	Bhatti, SA	Bhatti, Sheraz_Ahmed
AU	2	44158	44158	Khan, MZ	Khan, Muhammad_Zargham
AU	3	44159	44159	Saleemi, MK	Saleemi, Muhammad_Kashif
AU	4	44160	44160	Ul Hassan, Z	Ul_Hassan, Zahoor
AF	1	44161	44161	Bhatti, Sheraz Ahmed	Bhatti, Sheraz_Ahmed
AF	2	44162	44162	Khan, Muhammad Zargham	Khan, Muhammad_Zargham
AF	3	44163	44163	Saleemi, Muhammad Kashif	Saleemi, Muhammad_Kashif
AF	4	44164	44164	Ul Hassan, Zahoor	Ul_Hassan, Zahoor

Record 280 | WOS:000486105900030

In vitro effects of single and binary mixtures of regulated mycotoxins and persistent organochloride pesticides on steroid hormone production in MA-10 Leydig cell line

Field	Position	Original line	Normalised line	Original value	Normalised value
AU	1	44265	44265	Eze, UA	Eze, Ukpai_A
AU	2	44266	44266	Huntriss, JD	Huntriss, John_D
AU	3	44267	44267	Routledge, MN	Routledge, Michael_N
AU	4	44268	44268	Gong, YY	Gong, Yun_Yun
AF	1	44269	44269	Eze, Ukpai A.	Eze, Ukpai_A
AF	2	44270	44270	Huntriss, John D.	Huntriss, John_D
AF	3	44271	44271	Routledge, Michael N.	Routledge, Michael_N
AF	4	44272	44272	Gong, Yun Yun	Gong, Yun_Yun

Record 281 | WOS:000498064500026

Detecting Neurodevelopmental Toxicity of Domoic Acid and Ochratoxin A Using Rat Fetal Neural Stem Cells

Field	Position	Original line	Normalised line	Original value	Normalised value
AU	1	44386	44386	Gill, S	Gill, Santokh
AU	2	44387	44387	Kumara, VMR	Kumara, V_M_Ruvin
AF	2	44389	44389	Kumara, V. M. Ruvin	Kumara, V_M_Ruvin

Record 282 | WOS:000487934500032

Zinc supplementation alleviates OTA-induced oxidative stress and apoptosis in MDCK cells by up-regulating metallothioneins

Field	Position	Original line	Normalised line	Original value	Normalised value
AU	1	44498	44498	Li, H	Li, Hu
AU	2	44499	44499	Malyar, RM	Malyar, Rahmani_Mohammad
AU	3	44500	44500	Zhai, NH	Zhai, Nianhui
AU	4	44501	44501	Wang, H	Wang, Hong
AU	5	44502	44502	Liu, K	Liu, Kai
AU	6	44503	44503	Liu, DD	Liu, Dandan
AU	7	44504	44504	Pan, CL	Pan, Cuiling
AU	8	44505	44505	Can, F	Can, Fang
AU	9	44506	44506	Huang, KH	Huang, Kehe
AU	10	44507	44507	Miao, JF	Miao, Jinfeng
AU	11	44508	44508	Chen, XX	Chen, Xingxiang
AF	2	44510	44510	Malyar, Rahmani Mohammad	Malyar, Rahmani_Mohammad

Record 283 | WOS:000505648100002

Roles of microRNAs and prospective view of competing endogenous RNAs in mycotoxicosis

Field	Position	Original line	Normalised line	Original value	Normalised value
AU	1	44628	44628	Liao, YX	Liao, Yuxiao
AU	2	44629	44629	Peng, Z	Peng, Zhao
AU	3	44630	44630	Chen, LK	Chen, Liangkai
AU	4	44631	44631	Liu, LG	Liu, Liegang
AU	5	44632	44632	Wu, QH	Wu, Qinghua
AU	6	44633	44633	Yang, W	Yang, Wei

Record 284 | WOS:000487079300009

Antifungal activity of selected essential oils against Fusarium culmorum and F. graminearum and their secondary metabolites in wheat seeds

Field	Position	Original line	Normalised line	Original value	Normalised value
AU	1	44810	44810	Perczak, A	Perczak, Adam
AU	2	44811	44811	Gwiazdowska, D	Gwiazdowska, Daniela
AU	3	44812	44812	Marchwinska, K	Marchwinska, Katarzyna
AU	4	44813	44813	Jus, K	Jus, Krzysztof
AU	5	44814	44814	Gwiazdowski, R	Gwiazdowski, Romuald
AU	6	44815	44815	Waskiewicz, A	Waskiewicz, Agnieszka

Record 285 | WOS:000475915000016

Annexin A3 may play an important role in ochratoxin-induced malignant transformation of human gastric epithelium cells

Field	Position	Original line	Normalised line	Original value	Normalised value
AU	1	44955	44955	Wang, J	Wang, Juan
AU	2	44956	44956	Jia, X	Jia, Xin
AU	3	44957	44957	Meng, XX	Meng, Xinxing
AU	4	44958	44958	Li, YH	Li, Yuehong
AU	5	44959	44959	Wu, WX	Wu, Wenxin
AU	6	44960	44960	Zhang, XH	Zhang, Xianghong

AU	7	44961	44961	Xu, H	Xu, Hong
AU	8	44962	44962	Cui, JF	Cui, Jinfeng

Record 286 | WOS:000468622000013

Nontoxic concentrations of OTA aggravate DON-induced intestinal barrier dysfunction in IPEC-J2 cells via activation of NF-κB signaling pathway

Field	Position	Original line	Normalised line	Original value	Normalised value
AU	1	45067	45067	Chen, Y	Chen, Ying
AU	2	45068	45068	Wang, H	Wang, Hong
AU	3	45069	45069	Zhai, NH	Zhai, Nianhui
AU	4	45070	45070	Wang, CL	Wang, Chunlei
AU	5	45071	45071	Huang, KH	Huang, Kehe
AU	6	45072	45072	Pan, CL	Pan, Cuiling

Record 287 | WOS:000482246600031

Transport of mycotoxins across human gastric NCI-N87 and intestinal Caco-2 cell models

Field	Position	Original line	Normalised line	Original value	Normalised value
AU	1	45185	45185	Sobral, MMC	Sobral, M_Madalena_C
AU	2	45186	45186	Faria, MA	Faria, Miguel_A
AU	3	45187	45187	Cunha, SC	Cunha, Sara_C
AU	4	45188	45188	Miladinovic, B	Miladinovic, Bojana
AU	5	45189	45189	Ferreira, IMPLVO	Ferreira, Isabel_M_P_L_V_O
AF	1	45190	45190	Sobral, M. Madalena C.	Sobral, M_Madalena_C
AF	2	45191	45191	Faria, Miguel A.	Faria, Miguel_A
AF	3	45192	45192	Cunha, Sara C.	Cunha, Sara_C
AF	5	45194	45194	Ferreira, Isabel M. P. L. V. O.	Ferreira, Isabel_M_P_L_V_O

Record 288 | WOS:000487968300046

Astaxanthin Protects OTA-Induced Lung Injury in Mice through the Nrf2/NF-κB Pathway

Field	Position	Original line	Normalised line	Original value	Normalised value
AU	1	45318	45318	Xu, WX	Xu, Weixiang
AU	2	45319	45319	Wang, MY	Wang, Mingyang
AU	3	45320	45320	Cui, GY	Cui, Gengyuan
AU	4	45321	45321	Li, L	Li, Lin
AU	5	45322	45322	Jiao, DY	Jiao, Danyang
AU	6	45323	45323	Yao, BB	Yao, Beibei
AU	7	45324	45324	Xu, KT	Xu, Ketao
AU	8	45325	45325	Chen, YL	Chen, Yueli
AU	9	45326	45326	Long, M	Long, Miao
AU	10	45327	45327	Yang, SH	Yang, Shuhua
AU	11	45328	45328	He, JB	He, Jianbin

Record 289 | WOS:000487968300014

Heterologous Expression and Characterization of A Novel Ochratoxin A Degrading Enzyme, N-acyl-L-amino Acid Amidohydrolase, from Alcaligenes faecalis

Field	Position	Original line	Normalised line	Original value	Normalised value
AU	1	45449	45449	Zhang, HH	Zhang, Honghai
AU	2	45450	45450	Zhang, YP	Zhang, Yunpeng
AU	3	45451	45451	Yin, T	Yin, Tie
AU	4	45452	45452	Wang, J	Wang, Jing
AU	5	45453	45453	Zhang, XL	Zhang, Xiaolin

Record 290 | WOS:000483690200021

Accuracy, discriminative properties and reliability of a human ESC-based in vitro toxicity assay to distinguish teratogens responsible for neural tube defects

Field	Position	Original line	Normalised line	Original value	Normalised value
AU	1	45542	45542	Feutz, AC	Feutz, Anne-Catherine
AU	2	45543	45543	De Geyter, C	de_Geyter, Christian
AF	2	45545	45545	De Geyter, Christian	de_Geyter, Christian

Record 291 | WOS:000477777500023

Effects of Gamma Irradiation on Ochratoxin A Stability and Cytotoxicity in Methanolic Solutions and Potential Application in Tunisian Millet Samples

Field	Position	Original line	Normalised line	Original value	Normalised value
AU	1	45637	45637	Maatouk, I	Maatouk, Imed
AU	2	45638	45638	Mehrez, A	Mehrez, Amel
AU	3	45639	45639	Ben Amara, A	Ben_Amara, Aya
AU	4	45640	45640	Chayma, R	Chayma, Ragoubi
AU	5	45641	45641	Abid, S	Abid, Salwa
AU	6	45642	45642	Jerbi, T	Jerbi, Taieb
AU	7	45643	45643	Landoulsi, A	Landoulsi, Ahmed
AF	3	45646	45646	Ben Amara, Aya	Ben_Amara, Aya

Record 292 | WOS:000478525600001

An in vitro attempt at precision toxicology reveals the involvement of DNA methylation alteration in ochratoxin A-induced G0/G1 phase arrest

Field	Position	Original line	Normalised line	Original value	Normalised value
AU	1	45760	45760	Zhang, BY	Zhang, Boyang
AU	2	45761	45761	Zhu, LY	Zhu, Liye
AU	3	45762	45762	Dai, YQ	Dai, Yaqi
AU	4	45763	45763	Li, HY	Li, Hongyu
AU	5	45764	45764	Huang, KL	Huang, Kunlun
AU	6	45765	45765	Luo, YB	Luo, Yunbo
AU	7	45766	45766	Xu, WT	Xu, Wentao

Record 293 | WOS:000474669600029

Long-Time Instead of Short-Time Exposure in Vitro and Administration in Vivo of Ochratoxin A Is Consistent in Immunosuppression

Field	Position	Original line	Normalised line	Original value	Normalised value
AU	1	45890	45890	Su, JR	Su, Jiarui
AU	2	45891	45891	Liu, DD	Liu, Dandan
AU	3	45892	45892	Wang, Q	Wang, Qing
AU	4	45893	45893	Lin, JS	Lin, Jiashan
AU	5	45894	45894	Song, SQ	Song, Suquan
AU	6	45895	45895	Huang, KH	Huang, Kehe

Record 294 | WOS:000482110000051

Ochratoxin A-Induced Hepatotoxicity through Phase I and Phase II Reactions Regulated by AhR in Liver Cells

Field	Position	Original line	Normalised line	Original value	Normalised value
AU	1	46008	46008	Shin, HS	Shin, Hye_Soo
AU	2	46009	46009	Lee, HJ	Lee, Hyun_Jung
AU	3	46010	46010	Pyo, MC	Pyo, Min_Cheol
AU	4	46011	46011	Ryu, D	Ryu, Dojin
AU	5	46012	46012	Lee, KW	Lee, Kwang-Won
AF	1	46013	46013	Shin, Hye Soo	Shin, Hye_Soo
AF	2	46014	46014	Lee, Hyun Jung	Lee, Hyun_Jung
AF	3	46015	46015	Pyo, Min Cheol	Pyo, Min_Cheol

Record 295 | WOS:000475328000001

Assorted Methods for Decontamination of Aflatoxin M1 in Milk Using Microbial Adsorbents

Field	Position	Original line	Normalised line	Original value	Normalised value
AU	1	46121	46121	Assaf, JC	Assaf, Jean_Claude
AU	2	46122	46122	Nahle, S	Nahle, Sahar
AU	3	46123	46123	Chokr, A	Chokr, Ali
AU	4	46124	46124	Louka, N	Louka, Nicolas
AU	5	46125	46125	Atoui, A	Atoui, Ali
AU	6	46126	46126	El Khoury, A	el_Khoury, Andre
AF	1	46127	46127	Assaf, Jean Claude	Assaf, Jean_Claude
AF	6	46132	46132	El Khoury, Andre	el_Khoury, Andre

Record 296 | WOS:000475328000038

Cytotoxicity, Genotoxicity and Disturbance of Cell Cycle in HepG2 Cells Exposed to OTA and BEA: Single and Combined Actions

Field	Position	Original line	Normalised line	Original value	Normalised value
AU	1	46338	46338	Juan-García, A	Juan-Garcia, Ana
AU	2	46339	46339	Tolosa, J	Tolosa, Josefa
AU	3	46340	46340	Juan, C	Juan, Cristina
AU	4	46341	46341	Ruiz, MJ	Ruiz, Maria-Jose

Record 297 | WOS:000475288200030

Long-Term Effects of Ochratoxin A on the Glutathione Redox System and Its Regulation in Chicken

Field	Position	Original line	Normalised line	Original value	Normalised value
AU	1	46446	46446	Kövesi, B	Kovesi, Benjamin
AU	2	46447	46447	Cserháti, M	Cserhati, Matyas
AU	3	46448	46448	Erdélyi, M	Erdelyi, Marta
AU	4	46449	46449	Zándoki, E	Zandoki, Erika
AU	5	46450	46450	Mézes, M	Mezes, Miklos
AU	6	46451	46451	Balogh, K	Balogh, Krisztian

Record 298 | WOS:000461534700004

Effects of medicinal herb and Brazilian traditional plant extracts on in vitro mycotoxin decontamination

Field	Position	Original line	Normalised line	Original value	Normalised value
AU	2	46569	46569	Rottinghaus, GE	Rottinghaus, G_E
AU	3	46570	46570	Landers, BR	Landers, B_R
AU	4	46571	46571	Oliveira, CAF	Oliveira, C_A_F
AF	1	46572	46572	Ponzilacqua, B.	Ponzilacqua, B
AF	2	46573	46573	Rottinghaus, G. E.	Rottinghaus, G_E
AF	3	46574	46574	Landers, B. R.	Landers, B_R
AF	4	46575	46575	Oliveira, C. A. F.	Oliveira, C_A_F

Record 299 | WOS:000465050300026

Ochratoxin A upregulates biomarkers associated with hypoxia and transformation in human kidney cells

Field	Position	Original line	Normalised line	Original value	Normalised value
AU	1	46656	46656	Raghubeer, S	Raghubeer, Shanel
AU	2	46657	46657	Nagiah, S	Nagiah, Savania
AU	3	46658	46658	Chuturgoon, A	Chuturgoon, Anil

Record 300 | WOS:000475328000057

The In Vivo and In Vitro Toxicokinetics of Citreoviridin Extracted from Penicillium citreonigrum

Field	Position	Original line	Normalised line	Original value	Normalised value
AU	1	46751	46751	Uchiyama, Y	Uchiyama, Yosuke
AU	2	46752	46752	Takino, M	Takino, Masahiko
AU	3	46753	46753	Noguchi, M	Noguchi, Michiko
AU	4	46754	46754	Shiratori, N	Shiratori, Nozomi
AU	5	46755	46755	Kobayashi, N	Kobayashi, Naoki
AU	6	46756	46756	Sugita-Konishi, Y	Sugita-Konishi, Yoshiko

Record 301 | WOS:000463851800006

Biotransformation of plantain pseudostem fibres using local enzyme sources; analysis of their potential as commercial poultry feed

Field	Position	Original line	Normalised line	Original value	Normalised value
AU	1	46862	46862	Amadi, PU	Amadi, Peter_Uchenna
AU	2	46863	46863	Nnoka, CO	Nnoka, Charity_Ogunka
AU	3	46864	46864	Abbey, BW	Abbey, Bene_Willie
AF	1	46865	46865	Amadi, Peter Uchenna	Amadi, Peter_Uchenna
AF	2	46866	46866	Nnoka, Charity Ogunka	Nnoka, Charity_Ogunka
AF	3	46867	46867	Abbey, Bene Willie	Abbey, Bene_Willie

Record 302 | WOS:000472672300064

Degrading Ochratoxin A and Zearalenone Mycotoxins Using a Multifunctional Recombinant Enzyme

Field	Position	Original line	Normalised line	Original value	Normalised value
AU	1	46973	46973	Azam, MS	Azam, Md_Shofiul
AU	2	46974	46974	Yu, DZ	Yu, Dianzhen
AU	3	46975	46975	Liu, N	Liu, Ning
AU	4	46976	46976	Wu, AB	Wu, Aibo
AF	1	46977	46977	Azam, Md Shofiul	Azam, Md_Shofiul
AF	3	46979	46979	Liu, Na	Liu, Ning

Record 303 | WOS:000469753500299

Interaction of Mycotoxin Alternariol with Serum Albumin

Field	Position	Original line	Normalised line	Original value	Normalised value
AU	1	47078	47078	Fliszár-Nyúl, E	Fliszar-Nyul, Eszter
AU	2	47079	47079	Lemli, B	Lemli, Beata
AU	3	47080	47080	Kunsági-Máté, S	Kunsagi-Mate, Sandor
AU	4	47081	47081	Dellafiora, L	Dellafiora, Luca
AU	5	47082	47082	Dall'Asta, C	Dall'Asta, Chiara
AU	6	47083	47083	Cruciani, G	Cruciani, Gabriele
AU	7	47084	47084	Petho, G	Petho, Gabor
AU	8	47085	47085	Poór, M	Poor, Miklos

Record 304 | WOS:000467512200032

Cytotoxicity against fish and mammalian cell lines and endocrine activity of the mycotoxins beauvericin, deoxynivalenol and ochratoxin-A

Field	Position	Original line	Normalised line	Original value	Normalised value
AU	3	47219	47219	Navas, JM	Navas, J_M
AU	4	47220	47220	Fernández-Cruz, ML	Fernandez-Cruz, Maria_Luisa
AF	2	47222	47222	Valdehita, A.	Valdehita, A
AF	3	47223	47223	Navas, J. M.	Navas, J_M
AF	4	47224	47224	Fernandez-Cruz, M. L.	Fernandez-Cruz, Maria_Luisa

Record 305 | WOS:000468044200002

Prevention of ochratoxin A-induced oxidative stress-mediated apoptotic processes and impairment of embryonic development in mouse blastocysts by liquiritigenin

Field	Position	Original line	Normalised line	Original value	Normalised value
AU	1	47353	47353	Huang, CH	Huang, Chien-Hsun
AU	2	47354	47354	Wang, FT	Wang, Fu-Ting
AU	3	47355	47355	Chan, WH	Chan, Wen-Hsiung

Record 306 | WOS:000467299600035

Assessment of Toxic Effects of Ochratoxin A in Human Embryonic Stem Cells

Field	Position	Original line	Normalised line	Original value	Normalised value
AU	1	47483	47483	Erceg, S	Erceg, Slaven
AU	2	47484	47484	Mateo, EM	Maria_Mateo, Eva
AU	3	47485	47485	Zipancic, I	Zipancic, Ivan
AU	4	47486	47486	Jiménez, FJR	Rodriguez_Jimenez, Francisco_Javier
AU	5	47487	47487	Aragó, MAP	Perez_Arago, Maria_Amparo
AU	6	47488	47488	Jiménez, M	Jimenez, Misericordia
AU	7	47489	47489	Soria, JM	Miguel_Soria, Jose
AU	8	47490	47490	Garcia-Esparza, MA	Angeles_Garcia-Esparza, M
AF	2	47492	47492	Maria Mateo, Eva	Maria_Mateo, Eva
AF	4	47494	47494	Rodriguez Jimenez, Francisco Javier	Rodriguez_Jimenez, Francisco_Javier
AF	5	47495	47495	Perez Arago, Maria Amparo	Perez_Arago, Maria_Amparo
AF	7	47497	47497	Miguel Soria, Jose	Miguel_Soria, Jose
AF	8	47498	47498	Angeles Garcia-Esparza, M.	Angeles_Garcia-Esparza, M

Record 307 | WOS:000467645200017

Nephrotoxicity instead of immunotoxicity of OTA is induced through DNMT1-dependent activation of JAK2/STAT3 signaling pathway by targeting SOCS3

Field	Position	Original line	Normalised line	Original value	Normalised value
AU	1	47626	47626	Gan, F	Gan, Fang
AU	2	47627	47627	Zhou, X	Zhou, Xuan
AU	3	47628	47628	Zhou, YJ	Zhou, Yajiao
AU	4	47629	47629	Hou, LL	Hou, Lili
AU	5	47630	47630	Chen, XX	Chen, Xingxiang
AU	6	47631	47631	Pan, CL	Pan, Cuiling
AU	7	47632	47632	Huang, KH	Huang, Kehe

Record 308 | WOS:000467299600005

Biomarkers for Exposure as a Tool for Efficacy Testing of a Mycotoxin Detoxifier in Broiler Chickens and Pigs

Field	Position	Original line	Normalised line	Original value	Normalised value
AU	1	47752	47752	Lauwers, M	Lauwers, Marianne
AU	2	47753	47753	Croubels, S	Croubels, Siska
AU	3	47754	47754	Letor, B	Letor, Ben
AU	4	47755	47755	Gougoulias, C	Gougoulias, Christos
AU	5	47756	47756	Devreese, M	Devreese, Mathias

Record 309 | WOS:000470119800012

Antifungal and antimycotoxigenic effect of Lactobacillus plantarum CRL 778 at different water activity values

Field	Position	Original line	Normalised line	Original value	Normalised value
AU	1	47875	47875	Dallagnol, AM	Micaela_Dallagnol, Andrea
AU	2	47876	47876	Bustos, AY	Yanina_Bustos, Ana
AU	3	47877	47877	Martos, GI	Irma_Martos, Gladys
AU	4	47878	47878	de Valdez, GF	Font_de_Valdez, Graciela
AU	5	47879	47879	Gereza, CL	Luciana_Gereza, Carla
AF	1	47880	47880	Micaela Dallagnol, Andrea	Micaela_Dallagnol, Andrea
AF	2	47881	47881	Yanina Bustos, Ana	Yanina_Bustos, Ana
AF	3	47882	47882	Irma Martos, Gladys	Irma_Martos, Gladys
AF	4	47883	47883	Font de Valdez, Graciela	Font_de_Valdez, Graciela
AF	5	47884	47884	Luciana Gereza, Carla	Luciana_Gereza, Carla

Record 310 | WOS:000462049400006

Oxidative stress as a mechanism of combined OTA and CTN toxicity in rat plasma, liver and kidney

Field	Position	Original line	Normalised line	Original value	Normalised value
AU	1	47973	47973	Rasic, D	Rasic, Dubravka
AU	2	47974	47974	Micek, V	Micek, Vedran
AU	3	47975	47975	Klaric, MS	Klaric, M_S
AU	4	47976	47976	Peraica, M	Peraica, Maja
AF	1	47977	47977	Rasic, D.	Rasic, Dubravka
AF	2	47978	47978	Micek, V.	Micek, Vedran
AF	3	47979	47979	Klaric, M. S.	Klaric, M_S
AF	4	47980	47980	Peraica, M.	Peraica, Maja

Record 311 | WOS:000467299600032

Fluorescence Spectroscopic Investigation of Competitive Interactions between Quercetin and Aflatoxin B1 for Binding to Human Serum Albumin

Field	Position	Original line	Normalised line	Original value	Normalised value
AU	1	48083	48083	Tan, HX	Tan, Hongxia
AU	2	48084	48084	Chen, L	Chen, Lu
AU	3	48085	48085	Ma, L	Ma, Liang
AU	4	48086	48086	Liu, S	Liu, Shuang
AU	5	48087	48087	Zhou, HY	Zhou, Hongyuan
AU	6	48088	48088	Zhang, YH	Zhang, Yuhao
AU	7	48089	48089	Guo, T	Guo, Ting
AU	8	48090	48090	Liu, W	Liu, Wei
AU	9	48091	48091	Dai, HJ	Dai, Hongjie
AU	10	48092	48092	Yu, Y	Yu, Yong

Record 312 | WOS:000462420300003

Review: Biotechnology of mycotoxins detoxification using microorganisms and enzymes

Field	Position	Original line	Normalised line	Original value	Normalised value
AU	1	48217	48217	Ben Taheur, F	Ben_Taheur, Fadia
AU	2	48218	48218	Kouidhi, B	Kouidhi, Bochra
AU	3	48219	48219	Al Qurashi, YMA	al_Qurashi, Yasir_Mohammed_A
AU	4	48220	48220	Ben Salah-Abbès, J	Ben_Salah-Abbes, Jalila
AU	5	48221	48221	Chaieb, K	Chaieb, Kamel
AF	1	48222	48222	Ben Taheur, Fadia	Ben_Taheur, Fadia
AF	3	48224	48224	Al Qurashi, Yasir Mohammed A.	al_Qurashi, Yasir_Mohammed_A
AF	4	48225	48225	Ben Salah-Abbes, Jalila	Ben_Salah-Abbes, Jalila

Record 313 | WOS:000460996800027

Ochratoxin A Exposure Impairs Porcine Granulosa Cell Growth via the PI3K/AKT Signaling Pathway

Field	Position	Original line	Normalised line	Original value	Normalised value
AU	1	48488	48488	Zhang, TY	Zhang, Tian-Yu
AU	2	48489	48489	Sun, XF	Sun, Xiao-Feng
AU	3	48490	48490	Li, L	Li, Lan
AU	4	48491	48491	Ma, JM	Ma, Jin-Mei
AU	5	48492	48492	Zhang, RQ	Zhang, Rui-Qjan
AU	6	48493	48493	Li, N	Li, Na
AU	7	48494	48494	Liu, XL	Liu, Xue-Lian
AU	8	48495	48495	Dyce, PW	Dyce, Paul_W
AU	9	48496	48496	Shen, W	Shen, Wei
AF	8	48504	48504	Dyce, Paul W.	Dyce, Paul_W

Record 314 | WOS:000465449900001

Cytotoxicity of gamma irradiated aflatoxin B1 and ochratoxin A

Field	Position	Original line	Normalised line	Original value	Normalised value
AU	1	48610	48610	Domijan, AM	Domijan, Ana-Marija
AU	2	48611	48611	Cermak, AMM	Cermak, Ana_Marija_Marjanovic
AU	3	48612	48612	Vulic, A	Vulic, Ana
AU	4	48613	48613	Bujak, IT	Bujak, Ivana_Tartaro
AU	5	48614	48614	Pavicic, I	Pavicic, Ivan
AU	6	48615	48615	Pleadin, J	Pleadin, Jelka
AU	7	48616	48616	Markov, K	Markov, Ksenija
AU	8	48617	48617	Mihaljevic, B	Mihaljevic, Branka
AF	2	48619	48619	Cermak, Ana Marija Marjanovic	Cermak, Ana_Marija_Marjanovic
AF	4	48621	48621	Bujak, Ivana Tartaro	Bujak, Ivana_Tartaro

Record 315 | WOS:000481430100007

Inhibitory effect of ochratoxin A on DNMT-mediated flocculation of yeast

Field	Position	Original line	Normalised line	Original value	Normalised value
AU	1	48723	48723	Sugiyama, K	Sugiyama, Kei-Ichi
AU	2	48724	48724	Furusawa, H	Furusawa, Hiroko

AU	3	48725	48725	Gruz, P	Gruz, Petr
AU	4	48726	48726	Kinoshita, M	Kinoshita, Mawo
AU	5	48727	48727	Honma, M	Honma, Masamitsu
AF	1	48728	48728	Sugiyama, Kei-ichi	Sugiyama, Kei-Ichi

Record 316 | WOS:000460801500074

Modulation of Mucin (MUC2, MUC5AC and MUC5B) mRNA Expression and Protein Production and Secretion in Caco-2/HT29-MTX Co-Cultures Following Exposure to Individual and Combined Aflatoxin M1 and Ochratoxin A

Field	Position	Original line	Normalised line	Original value	Normalised value
AU	1	48805	48805	Huang, X	Huang, Xin
AU	2	48806	48806	Gao, YN	Gao, Yanan
AU	3	48807	48807	Li, SL	Li, Songli
AU	4	48808	48808	Wu, CQ	Wu, Chenqing
AU	5	48809	48809	Wang, JQ	Wang, Jiaqi
AU	6	48810	48810	Zheng, N	Zheng, Nan

Record 317 | WOS:000457296600055

Precision toxicology shows that troxerutin alleviates ochratoxin A-induced renal lipotoxicity

Field	Position	Original line	Normalised line	Original value	Normalised value
AU	1	48939	48939	Yang, X	Yang, Xuan
AU	2	48940	48940	Xu, WT	Xu, Wentao
AU	3	48941	48941	Huang, KL	Huang, Kunlun
AU	4	48942	48942	Zhang, BY	Zhang, Boyang
AU	5	48943	48943	Wang, HM	Wang, Haomiao
AU	6	48944	48944	Zhang, XQ	Zhang, Xueqin
AU	7	48945	48945	Gong, LJ	Gong, Lijing
AU	8	48946	48946	Luo, YB	Luo, Yunbo
AU	9	48947	48947	He, XY	He, Xiaoyun

Record 318 | WOS:000566844400029

Potential Mycotoxin Effects on Coffee Consumers' Health

Field	Position	Original line	Normalised line	Original value	Normalised value
AU	1	49128	49128	Cruz, R	Cruz, Rebeca
AU	2	49129	49129	Casal, S	Casal, Susana

Record 319 | WOS:000533508400001

Protective activity of melatonin against mycotoxins-induced toxicity: a review

Field	Position	Original line	Normalised line	Original value	Normalised value
AU	1	49217	49217	Iranshahy, M	Iranshahy, Milad
AU	2	49218	49218	Etemad, L	Etemad, Leila
AU	3	49219	49219	Shakeri, A	Shakeri, Abolfazl

AU	4	49220	49220	Badibostan, H	Badibostan, Hasan
AU	5	49221	49221	Karimi, G	Karimi, Gholamreza

Record 320 | WOS:000467038800009

OCRATOXINS AND THEIR NEPHROTOXIC POTENTIAL

Field	Position	Original line	Normalised line	Original value	Normalised value
AU	1	49334	49334	Torres, CCM	Martinez_Torres, Cristhian_Camilo
AU	2	49335	49335	Silva, DCC	Chaves_Silva, Diana_Carolina
AF	1	49336	49336	Martinez Torres, Cristhian Camilo	Martinez_Torres, Cristhian_Camilo
AF	2	49337	49337	Chaves Silva, Diana Carolina	Chaves_Silva, Diana_Carolina

Record 321 | WOS:000576729800009

Mycotoxins in food and feed

Field	Position	Original line	Normalised line	Original value	Normalised value
AU	1	49438	49438	Pleadin, J	Pleadin, Jelka
AU	2	49439	49439	Frece, J	Frece, Jadranka
AU	3	49440	49440	Markov, K	Markov, Ksenija

Record 322 | WOS:000463362400007

DNA damage in rat kidneys and liver upon subchronic exposure to single and combined ochratoxin A and citrinin

Field	Position	Original line	Normalised line	Original value	Normalised value
AU	1	49619	49619	Rasic, D	Rasic, Dubravka
AU	2	49620	49620	Zeljezic, D	Zeljezic, Davor
AU	3	49621	49621	Kopjar, N	Kopjar, Nevenka
AU	5	49623	49623	Klaric, MS	Klaric, M_Segvic
AU	6	49624	49624	Peraica, M	Peraica, Maja
AF	1	49625	49625	Rasic, D.	Rasic, Dubravka
AF	2	49626	49626	Zeljezic, D.	Zeljezic, Davor
AF	3	49627	49627	Kopjar, N.	Kopjar, Nevenka
AF	4	49628	49628	Kifer, D.	Kifer, D
AF	5	49629	49629	Klaric, M. Segvic	Klaric, M_Segvic
AF	6	49630	49630	Peraica, M.	Peraica, Maja

Record 323 | WOS:000456754300029

Sex-dependent gene expression after ochratoxin A insult in F344 rat kidney

Field	Position	Original line	Normalised line	Original value	Normalised value
AU	1	49743	49743	Vettorazzi, A	Vettorazzi, Ariane
AU	2	49744	49744	Pastor, L	Pastor, Laura
AU	3	49745	49745	Guruceaga, E	Guruceaga, Elizabeth
AU	4	49746	49746	de Cerain, AL	Lopez_de_Cerain, Adela
AF	4	49750	49750	Lopez de Cerain, Adela	Lopez_de_Cerain, Adela

Record 324 | WOS:000694027800516

Modulation of intestinal epithelial permeability and mucin mRNA (MUC2, MUC5AC, and MUC5B) expression and protein secretion in Caco-2/HT29-MTX co-cultures exposed to aflatoxin M1, ochratoxin A, and zearalenone individually or collectively.

Field	Position	Original line	Normalised line	Original value	Normalised value
AU	1	49872	49872	Wu, C	Wu, Chenqing
AU	2	49873	49873	Zheng, N	Zheng, Nan
AU	3	49874	49874	Gao, Y	Gao, Yanan
AF	1	49876	49876	Wu, C.	Wu, Chenqing
AF	2	49877	49877	Zheng, N.	Zheng, Nan
AF	3	49878	49878	Gao, Y.	Gao, Yanan
AF	4	49879	49879	Wang, J.	Wang, J

Record 325 | WOS:000507379000008

The in vivo efficacy of highly porous carbon nanocomposites prepared from sugar beet waste for the Ochratoxin A detoxification

Field	Position	Original line	Normalised line	Original value	Normalised value
AU	1	49924	49924	Zahoor, M	Zahoor, Muhammad
AU	2	49925	49925	Gulfam, N	Gulfam, Naila
AU	3	49926	49926	Khisroon, M	Khisroon, Muhammad
AU	4	49927	49927	Khan, FA	Khan, Farhat_Ali
AF	4	49931	49931	Khan, Farhat Ali	Khan, Farhat_Ali

Record 326 | WOS:000452098600001

Persistence of Epigenomic Effects After Recovery From Repeated Treatment With Two Nephrocarcinogens

Field	Position	Original line	Normalised line	Original value	Normalised value
AU	1	50010	50010	Limonciel, A	Limonciel, Alice
AU	2	50011	50011	van Breda, SG	van_Breda, Simone_G
AU	3	50012	50012	Jiang, XQ	Jiang, Xiaoqi
AU	4	50013	50013	Tredwell, GD	Tredwell, Gregory_D
AU	5	50014	50014	Wilmes, A	Wilmes, Anja
AU	6	50015	50015	Aschauer, L	Aschauer, Lydia
AU	7	50016	50016	Siskos, AP	Siskos, Alexandros_P
AU	8	50017	50017	Sachinidis, A	Sachinidis, Agapios
AU	9	50018	50018	Keun, HC	Keun, Hector_C
AU	10	50019	50019	Kopp-Schneider, A	Kopp-Schneider, Annette
AU	11	50020	50020	de Kok, TM	de_Kok, Theo_M
AU	12	50021	50021	Kleinjans, JCS	Kleinjans, Jos_C_S
AU	13	50022	50022	Jennings, P	Jennings, Paul
AF	2	50024	50024	van Breda, Simone G.	van_Breda, Simone_G
AF	4	50026	50026	Tredwell, Gregory D.	Tredwell, Gregory_D
AF	7	50029	50029	Siskos, Alexandros P.	Siskos, Alexandros_P
AF	9	50031	50031	Keun, Hector C.	Keun, Hector_C
AF	11	50033	50033	de Kok, Theo M.	de_Kok, Theo_M
AF	12	50034	50034	Kleinjans, Jos C. S.	Kleinjans, Jos_C_S

Record 327 | WOS:000447547900018

Fumonisin production and toxic capacity in airborne black Aspergilli

Field	Position	Original line	Normalised line	Original value	Normalised value
AU	1	50157	50157	Jaksic, D	Jaksic, Daniela
AU	2	50158	50158	Kocsubé, S	Kocsube, Sandor
AU	3	50159	50159	Bencsik, O	Bencsik, Otto
AU	4	50160	50160	Kecskeméti, A	Kecskemeti, Anita
AU	5	50161	50161	Szekeres, A	Szekeres, Andras
AU	6	50162	50162	Jelic, D	Jelic, Dubravko
AU	7	50163	50163	Kopjar, N	Kopjar, Nevenka
AU	8	50164	50164	Vágvölgyi, C	Vagvolgyi, Csaba
AU	9	50165	50165	Varga, J	Varga, Janos
AU	10	50166	50166	Klaric, MS	Klaric, Maja_Segvic
AF	10	50176	50176	Klaric, Maja Segvic	Klaric, Maja_Segvic

Record 328 | WOS:000452587500077

Activation of AMPK-dependent SIRT-1 by astragalus polysaccharide protects against ochratoxin A-induced immune stress in vitro and in vivo

Field	Position	Original line	Normalised line	Original value	Normalised value
AU	1	50311	50311	Liu, DD	Liu, Dandan
AU	2	50312	50312	Su, JR	Su, Jiarui
AU	3	50313	50313	Lin, JS	Lin, Jiashan
AU	4	50314	50314	Qian, G	Qian, Gang
AU	5	50315	50315	Chen, XX	Chen, Xingxiang
AU	6	50316	50316	Song, SQ	Song, Suquan
AU	7	50317	50317	Huang, KH	Huang, Kehe

Record 329 | WOS:000449893700013

Ochratoxin A induces cytoprotective autophagy via blocking AKT/mTOR signaling pathway in PK-15 cells

Field	Position	Original line	Normalised line	Original value	Normalised value
AU	1	50419	50419	Qian, G	Qian, Gang
AU	2	50420	50420	Liu, DD	Liu, Dandan
AU	3	50421	50421	Hou, LL	Hou, Lili
AU	4	50422	50422	Hamid, M	Hamid, Mohammed
AU	5	50423	50423	Chen, XX	Chen, Xingxiang
AU	6	50424	50424	Gan, F	Gan, Fang
AU	7	50425	50425	Song, SQ	Song, Suquan
AU	8	50426	50426	Huang, KH	Huang, Kehe

Record 330 | WOS:000451238100005

ERK1/2-mediated autophagy is essential for cell survival under Ochratoxin A exposure in IPEC-J2 cells

Field	Position	Original line	Normalised line	Original value	Normalised value

AU	1	50545	50545	Wang, H	Wang, Hong
AU	2	50546	50546	Li, H	Li, Hu
AU	3	50547	50547	Chen, XX	Chen, Xingxiang
AU	4	50548	50548	Huang, KH	Huang, Kehe

Record 331 | WOS:000449242800003

Mitigation of cell apoptosis induced by ochratoxin A (OTA) is possibly through organic cation transport 2 (OCT2) knockout

Field	Position	Original line	Normalised line	Original value	Normalised value
AU	1	50639	50639	Qi, XZ	Qi, Xiaozhe
AU	2	50640	50640	Zhu, LY	Zhu, Liye
AU	3	50641	50641	Yang, B	Yang, Bing
AU	4	50642	50642	Luo, HS	Luo, Haoshu
AU	5	50643	50643	Xu, WT	Xu, Wentao
AU	6	50644	50644	He, XY	He, Xiaoyun
AU	7	50645	50645	Huang, KL	Huang, Kunlun
AF	3	50648	50648	Yang, Bo	Yang, Bing

Record 332 | WOS:000446150300012

OTA induces intestinal epithelial barrier dysfunction and tight junction disruption in IPEC-J2 cells through ROS/Ca2+-mediated MLCK activation

Field	Position	Original line	Normalised line	Original value	Normalised value
AU	1	50741	50741	Wang, H	Wang, Hong
AU	2	50742	50742	Zhai, NH	Zhai, Nianhui
AU	3	50743	50743	Chen, Y	Chen, Ying
AU	4	50744	50744	Fu, CY	Fu, Chongyang
AU	5	50745	50745	Huang, KH	Huang, Kehe

Record 333 | WOS:000440879500026

Mechanism and effects of Zearalenone on mouse T lymphocytes activation in vitro

Field	Position	Original line	Normalised line	Original value	Normalised value
AU	1	50855	50855	Cai, GD	Cai, Guodong
AU	2	50856	50856	Sun, K	Sun, Kai
AU	3	50857	50857	Wang, T	Wang, Tao
AU	4	50858	50858	Zou, H	Zou, Hui
AU	5	50859	50859	Gu, JH	Gu, Jianhong
AU	6	50860	50860	Yuan, Y	Yuan, Yan
AU	7	50861	50861	Liu, XZ	Liu, Xuezhong
AU	8	50862	50862	Liu, ZP	Liu, Zongping
AU	9	50863	50863	Bian, JC	Bian, Jianchun

Record 334 | WOS:000448628600032

The registration of aptamer-ligand (ochratoxin A) interactions based on ligand fluorescence changes

Field	Position	Original line	Normalised line	Original value	Normalised value
AU	1	50970	50970	Samokhvalov, AV	Samokhvalov, Alexey_V
AU	2	50971	50971	Safenkova, IV	Safenkova, Irina_V
AU	3	50972	50972	Zherdev, AV	Zherdev, Anatoly_V
AU	4	50973	50973	Dzantiev, BB	Dzantiev, Boris_B
AF	1	50974	50974	Samokhvalov, Alexey, V	Samokhvalov, Alexey_V
AF	2	50975	50975	Safenkova, Irina, V	Safenkova, Irina_V
AF	3	50976	50976	Zherdev, Anatoly, V	Zherdev, Anatoly_V
AF	4	50977	50977	Dzantiev, Boris B.	Dzantiev, Boris_B

Record 335 | WOS:000447114600010

Dietary L-carnitine and vitamin-E; a strategy to combat ochratoxin-A induced immunosuppression

Field	Position	Original line	Normalised line	Original value	Normalised value
AU	1	51067	51067	Bhatti, SA	Bhatti, Sheraz_Ahmed
AU	2	51068	51068	Khan, MZ	Khan, Muhammad_Zargham
AU	3	51069	51069	Hassan, ZU	Hassan, Zahoor_Ul
AU	4	51070	51070	Saleemi, MK	Saleemi, Muhammad_Kashif
AU	5	51071	51071	Khatoon, A	Khatoon, Aisha
AU	6	51072	51072	Abidin, ZU	Abidin, Zain_Ul
AU	7	51073	51073	Hameed, MR	Hameed, Muhammad_Raza
AF	1	51074	51074	Bhatti, Sheraz Ahmed	Bhatti, Sheraz_Ahmed
AF	2	51075	51075	Khan, Muhammad Zargham	Khan, Muhammad_Zargham
AF	3	51076	51076	Hassan, Zahoor Ul	Hassan, Zahoor_Ul
AF	4	51077	51077	Saleemi, Muhammad Kashif	Saleemi, Muhammad_Kashif
AF	6	51079	51079	Abidin, Zain ul	Abidin, Zain_Ul
AF	7	51080	51080	Hameed, Muhammad Raza	Hameed, Muhammad_Raza

Record 336 | WOS:000453415000007

In Vivo Detoxification of Ochratoxin A by Highly Porous Magnetic Nanocomposites Prepared from Coconut Shell

Field	Position	Original line	Normalised line	Original value	Normalised value
AU	1	51175	51175	Gulfam, N	Gulfam, Naila
AU	2	51176	51176	Zahoor, M	Zahoor, Muhammad
AU	4	51178	51178	Khan, FA	Khan, Farhat_Ali
AF	1	51179	51179	Gulfam, N.	Gulfam, Naila
AF	2	51180	51180	Zahoor, M.	Zahoor, Muhammad
AF	3	51181	51181	Khisroom, M.	Khisroom, M
AF	4	51182	51182	Khan, F. A.	Khan, Farhat_Ali

Record 337 | WOS:000442067900032

Research progress on the raw and modified montmorillonites as adsorbents for mycotoxins: A review

Field	Position	Original line	Normalised line	Original value	Normalised value
AU	1	51272	51272	Li, Y	Li, Yan
AU	2	51273	51273	Tian, GY	Tian, Guangyan

AU	3	51274	51274	Dong, GY	Dong, Gongyue
AU	4	51275	51275	Bai, SS	Bai, Shansong
AU	5	51276	51276	Han, XY	Han, Xiaoyu
AU	6	51277	51277	Liang, JS	Liang, Jinsheng
AU	7	51278	51278	Meng, JP	Meng, Junping
AU	8	51279	51279	Zhang, H	Zhang, Hong

Record 338 | WOS:000439674200027

Restorative effect of L-Dopa treatment against Ochratoxin A induced neurotoxicity

Field	Position	Original line	Normalised line	Original value	Normalised value
AU	1	51481	51481	Bhat, PV	Bhat, Pratiksha_V
AU	3	51483	51483	Manu, TM	Manu, Mohan_T
AU	4	51484	51484	Khanum, F	Khanum, Farhath
AF	1	51485	51485	Bhat, Pratiksha V.	Bhat, Pratiksha_V
AF	2	51486	51486	Anand, T.	Anand, T
AF	3	51487	51487	Manu, Mohan T.	Manu, Mohan_T

Record 339 | WOS:000448339000017

Interaction of 2R-ochratoxin A with Serum Albumins: Binding Site, Effects of Site Markers, Thermodynamics, Species Differences of Albumin-binding, and Influence of Albumin on Its Toxicity in MDCK Cells

Field	Position	Original line	Normalised line	Original value	Normalised value
AU	1	51626	51626	Faisal, Z	Faisal, Zelma
AU	2	51627	51627	Derdák, D	Derdak, Diana
AU	3	51628	51628	Lemli, B	Lemli, Beata
AU	4	51629	51629	Kunsági-Máté, S	Kunsagi-Mate, Sandor
AU	5	51630	51630	Bálint, M	Balint, Monika
AU	6	51631	51631	Hetényi, C	Hetenyi, Csaba
AU	7	51632	51632	Csepregi, R	Csepregi, Rita
AU	8	51633	51633	Koszegi, T	Koszegi, Tamas
AU	9	51634	51634	Sueck, F	Sueck, Franziska
AU	10	51635	51635	Cramer, B	Cramer, Benedikt
AU	11	51636	51636	Humpf, HU	Humpf, Hans-Ulrich
AU	12	51637	51637	Poór, M	Poor, Miklos

Record 340 | WOS:000442890400004

Protective effects of compound ammonium glycyrrhizin, L-arginine, silymarin and glucurolactone against liver damage induced by ochratoxin A in primary chicken hepatocytes

Field	Position	Original line	Normalised line	Original value	Normalised value
AU	1	51767	51767	Yu, ZG	Yu, Zugong
AU	2	51768	51768	Wu, F	Wu, Feng
AU	3	51769	51769	Tian, J	Tian, Jing
AU	4	51770	51770	Guo, XW	Guo, Xuewen
AU	5	51771	51771	An, R	An, Ran

Record 341 | WOS:000442714300074

Micronucleus induction and cell cycle alterations produced by deoxynivalenol and its acetylated derivatives in individual and combined exposure on HepG2 cells

Field	Position	Original line	Normalised line	Original value	Normalised value
AU	1	51883	51883	Juan-García, A	Juan-Garcia, Ana
AU	2	51884	51884	Taroncher, M	Taroncher, Mercedes
AU	3	51885	51885	Font, G	Font, Guillermina
AU	4	51886	51886	Ruiz, MJ	Ruiz, Maria-Jose

Record 342 | WOS:000442489000011

Molecular Dynamics Simulations of Mismatched DNA Duplexes Associated with the Major C8-Linked 2′-Deoxyguanosine Adduct of the Food Mutagen Ochratoxin A: Influence of Opposing Base, Adduct Ionization State, and Sequence on the Structure of Damaged DNA

Field	Position	Original line	Normalised line	Original value	Normalised value
AU	1	51979	51979	Kathuria, P	Kathuria, Preetleen
AU	2	51980	51980	Sharma, P	Sharma, Purshotam
AU	3	51981	51981	Manderville, RA	Manderville, Richard_A
AU	4	51982	51982	Wetmore, SD	Wetmore, Stacey_D
AF	3	51985	51985	Manderville, Richard A.	Manderville, Richard_A
AF	4	51986	51986	Wetmore, Stacey D.	Wetmore, Stacey_D

Record 343 | WOS:000440099900007

Comparison of base-line and chemical-induced transcriptomic responses in HepaRG and RPTEC/TERT1 cells using TempO-Seq

Field	Position	Original line	Normalised line	Original value	Normalised value
AU	1	52105	52105	Limonciel, A	Limonciel, Alice
AU	2	52106	52106	Ates, G	Ates, Gamze
AU	3	52107	52107	Carta, G	Carta, Giada
AU	4	52108	52108	Wilmes, A	Wilmes, Anja
AU	5	52109	52109	Watzele, M	Watzele, Manfred
AU	6	52110	52110	Shepard, PJ	Shepard, Peter_J
AU	7	52111	52111	VanSteenhouse, HC	Vansteenhouse, Harper_C
AU	8	52112	52112	Seligmann, B	Seligmann, Bruce
AU	9	52113	52113	Yeakley, JM	Yeakley, Joanne_M
AU	10	52114	52114	van de Water, B	van_de_Water, Bob
AU	11	52115	52115	Vinken, M	Vinken, Mathieu
AU	12	52116	52116	Jennings, P	Jennings, Paul
AF	6	52122	52122	Shepard, Peter J.	Shepard, Peter_J
AF	7	52123	52123	VanSteenhouse, Harper C.	Vansteenhouse, Harper_C
AF	9	52125	52125	Yeakley, Joanne M.	Yeakley, Joanne_M
AF	10	52126	52126	van de Water, Bob	van_de_Water, Bob

Record 344 | WOS:000438651800008

Occurrence of ochratoxin A in Astragalus propinquus root and its transfer to decoction

Field	Position	Original line	Normalised line	Original value	Normalised value
AU	1	52250	52250	Toman, J	Toman, Jakub
AU	2	52251	52251	Ostry, V	Ostry, Vladimir
AU	3	52252	52252	Grosse, Y	Grosse, Yann
AU	4	52253	52253	Roubal, T	Roubal, Tomas
AU	5	52254	52254	Malir, F	Malir, Frantisek

Record 345 | WOS:000434754600071

PCV2 infection aggravates ochratoxin A-induced nephrotoxicity via autophagy involving p38 signaling pathway in vivo and in vitro

Field	Position	Original line	Normalised line	Original value	Normalised value
AU	1	52357	52357	Gan, F	Gan, Fang
AU	2	52358	52358	Zhou, YJ	Zhou, Yajiao
AU	3	52359	52359	Qian, G	Qian, Gang
AU	4	52360	52360	Huang, D	Huang, da
AU	5	52361	52361	Hou, LL	Hou, Lili
AU	6	52362	52362	Liu, DD	Liu, Dandan
AU	7	52363	52363	Chen, XX	Chen, Xingxiang
AU	8	52364	52364	Wang, T	Wang, Tian
AU	9	52365	52365	Jiang, P	Jiang, Ping
AU	10	52366	52366	Lei, XG	Lei, Xingen
AU	11	52367	52367	Huang, KH	Huang, Kehe
AF	4	52371	52371	Huang, Da	Huang, da

Record 346 | WOS:000442594000032

Toxicity Assay for Citrinin, Zearalenone and Zearalenone-14-Sulfate Using the Nematode Caenorhabditis elegans as Model Organism

Field	Position	Original line	Normalised line	Original value	Normalised value
AU	1	52485	52485	Keller, J	Keller, Julia
AU	2	52486	52486	Borzekowski, A	Borzekowski, Antje
AU	3	52487	52487	Haase, H	Haase, Hajo
AU	4	52488	52488	Menzel, R	Menzel, Ralph
AU	5	52489	52489	Ruess, L	Ruess, Liliane
AU	6	52490	52490	Koch, M	Koch, Matthias

Record 347 | WOS:000430882700061

Toxicological interactions between mycotoxins from ubiquitous fungi: Impact on hepatic and intestinal human epithelial cells

Field	Position	Original line	Normalised line	Original value	Normalised value
AU	3	52597	52597	Faria, MA	Faria, Miguel_A
AU	4	52598	52598	Cunha, SC	Cunha, Sara_C
AU	5	52599	52599	Ferreira, IMPLVO	Ferreira, Isabel_M_P_L_V_O
AF	1	52600	52600	Madalena, M.	Madalena, M
AF	2	52601	52601	Sobral, C.	Sobral, C
AF	3	52602	52602	Faria, Miguel A.	Faria, Miguel_A
AF	4	52603	52603	Cunha, Sara C.	Cunha, Sara_C

AF	5	52604	52604	Ferreira, Isabel M. P. L. V. O.	Ferreira, Isabel_M_P_L_V_O

Record 348 | WOS:000432602600016

Synergistic action of the nephrotoxic mycotoxins ochratoxin A and citrinin at nanomolar concentrations in human proximal tubule-derived cells

Field	Position	Original line	Normalised line	Original value	Normalised value
AU	1	52737	52737	Schulz, MC	Schulz, Marie-Christin
AU	2	52738	52738	Schumann, L	Schumann, Luise
AU	3	52739	52739	Rottkord, U	Rottkord, Ulrike
AU	4	52740	52740	Humpf, HU	Humpf, Hans-Ulrich
AU	5	52741	52741	Gekle, M	Gekle, Michael
AU	6	52742	52742	Schwerdt, G	Schwerdt, Gerald

Record 349 | WOS:000437960800009

Risk assessment of exposure to aflatoxin B1 and ochratoxin A through consumption of different Pistachio (Pistacia vera L.) cultivars collected from four geographical regions of Iran

Field	Position	Original line	Normalised line	Original value	Normalised value
AU	1	52840	52840	Taghizadeh, SF	Taghizadeh, Seyedeh_Faezeh
AU	2	52841	52841	Rezaee, R	Rezaee, Ramin
AU	3	52842	52842	Davarynejad, G	Davarynejad, Gholamhossein
AU	4	52843	52843	Asili, J	Asili, Javad
AU	5	52844	52844	Nemati, SH	Nemati, Seyed_Hossein
AU	6	52845	52845	Goumenou, M	Goumenou, Marina
AU	7	52846	52846	Tsakiris, I	Tsakiris, Ioannis
AU	8	52847	52847	Tsatsakis, AM	Tsatsakis, Aristides_M
AU	9	52848	52848	Shirani, K	Shirani, Kobra
AU	10	52849	52849	Karimi, G	Karimi, Gholamreza
AF	1	52850	52850	Taghizadeh, Seyedeh Faezeh	Taghizadeh, Seyedeh_Faezeh
AF	5	52854	52854	Nemati, Seyed Hossein	Nemati, Seyed_Hossein
AF	8	52857	52857	Tsatsakis, Aristides M.	Tsatsakis, Aristides_M

Record 350 | WOS:000435525800022

Combination of Selenomethionine and N-Acetylcysteine Alleviates the Joint Toxicities of Aflatoxin B1 and Ochratoxin A by ERK MAPK Signal Pathway in Porcine Alveolar Macrophages

Field	Position	Original line	Normalised line	Original value	Normalised value
AU	1	52961	52961	Hou, LL	Hou, Lili
AU	2	52962	52962	Zhou, X	Zhou, Xuan
AU	3	52963	52963	Gan, F	Gan, Fang
AU	4	52964	52964	Liu, ZX	Liu, Zixuan
AU	5	52965	52965	Zhou, YJ	Zhou, Yajiao
AU	6	52966	52966	Qjan, G	Qjan, Gang
AU	7	52967	52967	Huang, KH	Huang, Kehe

Record 351 | WOS:000433656100042

Is oxidative stress involved in the sex-dependent response to ochratoxin A renal toxicity?

Field	Position	Original line	Normalised line	Original value	Normalised value
AU	1	53086	53086	Enciso, JM	Manuel_Enciso, Jose
AU	2	53087	53087	de Cerain, AL	Lopez_de_Cerain, Adela
AU	3	53088	53088	Pastor, L	Pastor, Laura
AU	4	53089	53089	Azqueta, A	Azqueta, Amaya
AU	5	53090	53090	Vettorazzi, A	Vettorazzi, Ariane
AF	1	53091	53091	Manuel Enciso, Jose	Manuel_Enciso, Jose
AF	2	53092	53092	Lopez de Cerain, Adela	Lopez_de_Cerain, Adela

Record 352 | WOS:000433016800013

Effects of combined treatment with ochratoxin A and citrinin on oxidative damage in kidneys and liver of rats

Field	Position	Original line	Normalised line	Original value	Normalised value
AU	1	53228	53228	Rasic, D	Rasic, Dubravka
AU	2	53229	53229	Mladinic, M	Mladinic, Marin
AU	3	53230	53230	Zeljezic, D	Zeljezic, Davor
AU	4	53231	53231	Pizent, A	Pizent, Alica
AU	5	53232	53232	Stefanovic, S	Stefanovic, Srdan
AU	6	53233	53233	Milicevic, D	Milicevic, Dragan
AU	7	53234	53234	Konjevoda, P	Konjevoda, Pasko
AU	8	53235	53235	Peraica, M	Peraica, Maja

Record 353 | WOS:000432498000011

The importance of accounting for sex in the search of proteomic signatures of mycotoxin exposure

Field	Position	Original line	Normalised line	Original value	Normalised value
AU	2	53357	53357	Oswald, IP	Oswald, I_P
AF	1	53358	53358	Soler, L.	Soler, L
AF	2	53359	53359	Oswald, I. P.	Oswald, I_P

Record 354 | WOS:000428605400010

Toxicological effects of regulated mycotoxins and persistent organochloride pesticides: In vitro cytotoxic assessment of single and defined mixtures on MA-10 murine Leydig cell line

Field	Position	Original line	Normalised line	Original value	Normalised value
AU	1	53557	53557	Eze, UA	Eze, Ukpai_A
AU	2	53558	53558	Huntriss, J	Huntriss, John
AU	3	53559	53559	Routledge, MN	Routledge, Michael_N
AU	4	53560	53560	Gong, YY	Gong, Yun_Yun
AF	1	53561	53561	Eze, Ukpai A.	Eze, Ukpai_A
AF	3	53563	53563	Routledge, Michael N.	Routledge, Michael_N
AF	4	53564	53564	Gong, Yun Yun	Gong, Yun_Yun

Record 355 | WOS:000434061300005

Ochratoxin A, citrinin and deoxynivalenol decrease claudin-2 expression in mouse rectum CMT93-II cells

Field	Position	Original line	Normalised line	Original value	Normalised value
AU	1	53724	53724	Nakayama, H	Nakayama, Hideaki
AU	2	53725	53725	Kitagawa, N	Kitagawa, Norio
AU	3	53726	53726	Otani, T	Otani, Takahito
AU	4	53727	53727	Iida, H	Iida, Hiroshi
AU	5	53728	53728	Anan, H	Anan, Hisashi
AU	6	53729	53729	Inai, T	Inai, Tetsuichiro

Record 356 | WOS:000429758100013

Toxicity assessment of mycotoxins extracted from contaminated commercial dog pelleted feed on canine blood mononuclear cells

Field	Position	Original line	Normalised line	Original value	Normalised value
AU	1	53861	53861	Singh, SD	Singh, Sanil_D
AU	2	53862	53862	Abdul, NS	Abdul, Naeem_Sheik
AU	3	53863	53863	Phulukdaree, A	Phulukdaree, Alisa
AU	4	53864	53864	Tiloke, C	Tiloke, Charlette
AU	5	53865	53865	Nagiah, S	Nagiah, Savania
AU	6	53866	53866	Baijnath, S	Baijnath, Sooraj
AU	7	53867	53867	Chuturgoon, AA	Chuturgoon, Anil_A
AF	1	53868	53868	Singh, Sanil D.	Singh, Sanil_D
AF	2	53869	53869	Abdul, Naeem Sheik	Abdul, Naeem_Sheik
AF	7	53874	53874	Chuturgoon, Anil A.	Chuturgoon, Anil_A

Record 357 | WOS:000428799300003

The efficiency of lactic acid bacteria against pathogenic fungi and mycotoxins

Field	Position	Original line	Normalised line	Original value	Normalised value
AU	1	53979	53979	Perczak, A	Perczak, Adam
AU	2	53980	53980	Golinski, P	Golinski, Piotr
AU	3	53981	53981	Bryla, M	Bryla, Marcin
AU	4	53982	53982	Waskiewicz, A	Waskiewicz, Agnieszka

Record 358 | WOS:000422811300020

Metabolism-dependent cytotoxicity of citrinin and ochratoxin A alone and in combination as assessed adopting integrated discrete multiple organ co-culture (IdMOC)

Field	Position	Original line	Normalised line	Original value	Normalised value
AU	1	54209	54209	Gayathri, L	Gayathri, Loganathan
AU	2	54210	54210	Karthikeyan, BS	Karthikeyan, Bagavathy_Shanmugam
AU	3	54211	54211	Rajalakshmi, M	Rajalakshmi, Manikkam
AU	4	54212	54212	Dhanasekaran, D	Dhanasekaran, Dharumadurai
AU	5	54213	54213	Li, AP	Li, Albert_P
AU	6	54214	54214	Akbarsha, MA	Akbarsha, Mohammad_A
AF	2	54216	54216	Karthikeyan, Bagavathy Shanmugam	Karthikeyan, Bagavathy_Shanmugam
AF	5	54219	54219	Li, Albert P.	Li, Albert_P

AF	6	54220	54220	Akbarsha, Mohammad A.	Akbarsha, Mohammad_A

Record 359 | WOS:000425526000033

Linking site-specific loss of histone acetylation to repression of gene expression by the mycotoxin ochratoxin A

Field	Position	Original line	Normalised line	Original value	Normalised value
AU	1	54344	54344	Limbeck, E	Limbeck, Elisabeth
AU	2	54345	54345	Vanselow, JT	Vanselow, Jens_T
AU	3	54346	54346	Hofmann, J	Hofmann, Julian
AU	4	54347	54347	Schlosser, A	Schlosser, Andreas
AU	5	54348	54348	Mally, A	Mally, Angela
AF	2	54350	54350	Vanselow, Jens T.	Vanselow, Jens_T

Record 360 | WOS:000425568900007

Toxicity reduction of ochratoxin A by lactic acid bacteria

Field	Position	Original line	Normalised line	Original value	Normalised value
AU	3	54474	54474	Mañes, J	Manes, Jordi
AU	4	54475	54475	Meca, G	Meca, Giuseppe
AF	1	54476	54476	Luz, C.	Luz, C
AF	2	54477	54477	Ferrer, J.	Ferrer, J
AF	3	54478	54478	Manes, J.	Manes, Jordi
AF	4	54479	54479	Meca, G.	Meca, Giuseppe

Record 361 | WOS:000425568900035

Ochratoxin A: Toxicity, oxidative stress and metabolism

Field	Position	Original line	Normalised line	Original value	Normalised value
AU	1	54557	54557	Tao, YF	Tao, Yanfei
AU	2	54558	54558	Xie, SY	Xie, Shuyu
AU	3	54559	54559	Xu, FF	Xu, Fanfan
AU	4	54560	54560	Liu, AM	Liu, Aimei
AU	5	54561	54561	Wang, YX	Wang, Yanxin
AU	6	54562	54562	Chen, DM	Chen, Dongmei
AU	7	54563	54563	Pan, YH	Pan, Yuanhu
AU	8	54564	54564	Huang, LL	Huang, Lingli
AU	9	54565	54565	Peng, DP	Peng, Dapeng
AU	10	54566	54566	Wang, X	Wang, Xu
AU	11	54567	54567	Yuan, ZH	Yuan, Zonghui

Record 362 | WOS:000424317600001

Disruption of liver development and coagulation pathway by ochratoxin A in embryonic zebrafish

Field	Position	Original line	Normalised line	Original value	Normalised value
AU	1	54762	54762	Wu, TS	Wu, Ting-Shuan

AU	2	54763	54763	Lin, YT	Lin, Yu-Ting
AU	3	54764	54764	Huang, YT	Huang, Ying-Tzu
AU	4	54765	54765	Cheng, YC	Cheng, Ya-Chih
AU	5	54766	54766	Yu, FY	Yu, Feng-Yih
AU	6	54767	54767	Liu, BH	Liu, Biing-Hui

Record 363 | WOS:000424317600006

Ochratoxin A exposure decreased sperm motility via the AMPK and PTEN signaling pathways

Field	Position	Original line	Normalised line	Original value	Normalised value
AU	1	54874	54874	Zhang, TY	Zhang, Tian-Yu
AU	2	54875	54875	Wu, RY	Wu, Rui-Ying
AU	3	54876	54876	Zhao, Y	Zhao, Yong
AU	4	54877	54877	Xu, CS	Xu, Chun-Shuang
AU	5	54878	54878	Zhang, WD	Zhang, Wei-Dong
AU	6	54879	54879	Ge, W	Ge, Wei
AU	7	54880	54880	Liu, J	Liu, Jing
AU	8	54881	54881	Sun, ZY	Sun, Zhong-Yi
AU	9	54882	54882	Zou, SH	Zou, Shu-Hua
AU	10	54883	54883	Shen, W	Shen, Wei

Record 364 | WOS:000423566000030

Individual and combined cytotoxicity assessment of zearalenone with ochratoxin A or α-zearalenol by full factorial design

Field	Position	Original line	Normalised line	Original value	Normalised value
AU	1	54999	54999	Zheng, N	Zheng, Nan
AU	2	55000	55000	Gao, YN	Gao, Ya-Nan
AU	3	55001	55001	Liu, J	Liu, Jing
AU	4	55002	55002	Wang, HW	Wang, H_W
AU	5	55003	55003	Wang, JQ	Wang, Jia-Qi
AF	1	55004	55004	Zheng, N.	Zheng, Nan
AF	2	55005	55005	Gao, Y. N.	Gao, Ya-Nan
AF	3	55006	55006	Liu, J.	Liu, Jing
AF	4	55007	55007	Wang, H. W.	Wang, H_W
AF	5	55008	55008	Wang, J. Q.	Wang, Jia-Qi

Record 365 | WOS:000416024300043

Effect of rMnSOD on Sodium Reabsorption in Renal Proximal Tubule in Ochratoxin ATreated Rats

Field	Position	Original line	Normalised line	Original value	Normalised value
AU	1	55102	55102	Damiano, S	Damiano, Sara
AU	2	55103	55103	Puzio, MV	Puzio, Maria_V
AU	3	55104	55104	Squillacioti, C	Squillacioti, Caterina
AU	4	55105	55105	Mirabella, N	Mirabella, Nicola
AU	5	55106	55106	Zona, E	Zona, Enrica
AU	6	55107	55107	Mancini, A	Mancini, Aldo

AU	7	55108	55108	Borrelli, A	Borrelli, Antonella
AU	8	55109	55109	Astarita, C	Astarita, Carlo
AU	9	55110	55110	Boffo, S	Boffo, Silvia
AU	10	55111	55111	Giordano, A	Giordano, Antonio
AU	11	55112	55112	Avallone, L	Avallone, Luigi
AU	12	55113	55113	Florio, S	Florio, Salvatore
AU	13	55114	55114	Ciarcia, R	Ciarcia, Roberto
AF	2	55116	55116	Puzio, Maria V.	Puzio, Maria_V

Record 366 | WOS:000435675100011

Cinnamon (Cinnamomum zeylanicum) as an antidote or a protective agent against natural or chemical toxicities: a review

Field	Position	Original line	Normalised line	Original value	Normalised value
AU	1	55221	55221	Dorri, M	Dorri, Mahyar
AU	2	55222	55222	Hashemitabar, S	Hashemitabar, Shirin
AU	3	55223	55223	Hosseinzadeh, H	Hosseinzadeh, Hossein

Record 367 | WOS:000423248100019

Modified mycotoxins: An updated review on their formation, detection, occurrence, and toxic effects

Field	Position	Original line	Normalised line	Original value	Normalised value
AU	1	55362	55362	Freire, L	Freire, Luisa
AU	2	55363	55363	Sant'Ana, AS	Sant'Ana, Anderson_S
AF	2	55365	55365	Sant'Ana, Anderson S.	Sant'Ana, Anderson_S

Record 368 | WOS:000423248100048

In vitro mechanisms of Beauvericin toxicity: A review

Field	Position	Original line	Normalised line	Original value	Normalised value
AU	1	55617	55617	Mallebrera, B	Mallebrera, Beatriz
AU	2	55618	55618	Prosperini, A	Prosperini, Alessandra
AU	3	55619	55619	Font, G	Font, Guillermina
AU	4	55620	55620	Ruiz, MJ	Jose_Ruiz, Maria
AF	4	55624	55624	Jose Ruiz, Maria	Jose_Ruiz, Maria

Record 369 | WOS:000418302000021

Modulations of DNMT1 and HDAC1 are involved in the OTA-induced cytotoxicity and apoptosis in vitro

Field	Position	Original line	Normalised line	Original value	Normalised value
AU	1	55774	55774	Zhou, YJ	Zhou, Yajiao
AU	2	55775	55775	Gan, F	Gan, Fang
AU	3	55776	55776	Hou, LL	Hou, Lili
AU	4	55777	55777	Zhou, X	Zhou, Xuan
AU	5	55778	55778	Ibrahim, YAA	Ibrahim, Yassin_Abdulrahim_Adam

AU	6	55779	55779	Huang, K	Huang, Kehe
AF	5	55784	55784	Ibrahim, Yassin Abdulrahim Adam	Ibrahim, Yassin_Abdulrahim_Adam

Record 370 | WOS:000417670500029

Ochratoxin A-Induced Apoptosis of IPEC-J2 Cells through ROS-Mediated Mitochondrial Permeability Transition Pore Opening Pathway

Field	Position	Original line	Normalised line	Original value	Normalised value
AU	1	55881	55881	Wang, H	Wang, Hong
AU	2	55882	55882	Chen, Y	Chen, Ying
AU	3	55883	55883	Zhai, NH	Zhai, Nianhui
AU	4	55884	55884	Chen, XX	Chen, Xingxiang
AU	5	55885	55885	Gan, F	Gan, Fang
AU	6	55886	55886	Li, H	Li, Hu
AU	7	55887	55887	Huang, KH	Huang, Kehe

Record 371 | WOS:000418211700013

A comprehensive review on biological properties of citrinin

Field	Position	Original line	Normalised line	Original value	Normalised value
AU	1	55996	55996	de Oliveira, JW	Gomes_de_Oliveira_Filho, Jose_Williams
AU	2	55997	55997	Islam, MT	Islam, Muhammad_Torequl
AU	3	55998	55998	Ali, ES	Ali, Eunus_S
AU	4	55999	55999	Uddin, SJ	Uddin, Shaikh_Jamal
AU	5	56000	56000	Santos, JVD	de_Oliveira_Santos, Jose_Victor
AU	6	56001	56001	de Alencar, MVOB	Oliveira_Barros_de_Alencar, Marcus_Vinicius
AU	7	56002	56002	Gomes, AL	Gomes_Junior, Antonio_Luiz
AU	8	56003	56003	Paz, MFCJ	Correia_Jardim_Paz, Marcia_Fernanda
AU	9	56004	56004	de Brito, MDM	Mendes_de_Brito, Maria_dos_Remedios
AU	10	56005	56005	Sousa, JMDE	de_Castro_E_Sousa, Joao_Marcelo
AU	11	56006	56006	Shaw, S	Shaw, Subrata
AU	12	56007	56007	de Medeiros, MDF	Freire_de_Medeiros, Maria_das_Gracas
AU	13	56008	56008	Dantas, SMMD	Mendes_de_Moura_Dantas, Sandra_Maria
AU	14	56009	56009	Rolim, HML	Lins_Rolim, Hercilia_Maria
AU	15	56010	56010	Ferreira, PMP	Pinheiro_Ferreira, Paulo_Michel
AU	16	56011	56011	Kamal, MA	Kamal, Mohammad_Amjad
AU	17	56012	56012	Pieczynska, MD	Pieczynska, Magdalena_D
AU	18	56013	56013	Das, N	das, Niranjan
AU	19	56014	56014	Gupta, VK	Gupta, Vijai_K
AU	20	56015	56015	Mocan, A	Mocan, Andrei
AU	21	56016	56016	Andrade, TDAD	Aguiar_dos_Santos_Andrade, Teresinha_de_Jesus

AU	22	56017	56017	Singh, BN	Singh, Brahma_Nand
AU	23	56018	56018	Mishra, SK	Mishra, Siddhartha_K
AU	24	56019	56019	Atanasov, AG	Atanasov, Atanas_G
AU	25	56020	56020	Melo-Cavalcante, AAD	Melo-Cavalcante, Ana_Amelia_de_Carvalho
AF	1	56021	56021	Gomes de Oliveira Filho, Jose Williams	Gomes_de_Oliveira_Filho, Jose_Williams
AF	2	56022	56022	Islam, Muhammad Torequl	Islam, Muhammad_Torequl
AF	3	56023	56023	Ali, Eunus S.	Ali, Eunus_S
AF	4	56024	56024	Uddin, Shaikh Jamal	Uddin, Shaikh_Jamal
AF	5	56025	56025	de Oliveira Santos, Jose Victor	de_Oliveira_Santos, Jose_Victor
AF	6	56026	56026	Oliveira Barros de Alencar, Marcus Vinicius	Oliveira_Barros_de_Alencar, Marcus_Vinicius
AF	7	56027	56027	Gomes Junior, Antonio Luiz	Gomes_Junior, Antonio_Luiz
AF	8	56028	56028	Correia Jardim Paz, Marcia Fernanda	Correia_Jardim_Paz, Marcia_Fernanda
AF	9	56029	56029	Mendes de Brito, Maria dos Remedios	Mendes_de_Brito, Maria_dos_Remedios
AF	10	56030	56030	de Castro e Sousa, Joao Marcelo	de_Castro_E_Sousa, Joao_Marcelo
AF	12	56032	56032	Freire de Medeiros, Maria das Gracas	Freire_de_Medeiros, Maria_das_Gracas
AF	13	56033	56033	Mendes de Moura Dantas, Sandra Maria	Mendes_de_Moura_Dantas, Sandra_Maria
AF	14	56034	56034	Lins Rolim, Hercilia Maria	Lins_Rolim, Hercilia_Maria
AF	15	56035	56035	Pinheiro Ferreira, Paulo Michel	Pinheiro_Ferreira, Paulo_Michel
AF	16	56036	56036	Kamal, Mohammad Amjad	Kamal, Mohammad_Amjad
AF	17	56037	56037	Pieczynska, Magdalena D.	Pieczynska, Magdalena_D
AF	18	56038	56038	Das, Niranjan	das, Niranjan
AF	19	56039	56039	Gupta, Vijai K.	Gupta, Vijai_K
AF	21	56041	56041	Aguiar dos Santos Andrade, Teresinha de Jesus	Aguiar_dos_Santos_Andrade, Teresinha_de_Jesus
AF	22	56042	56042	Singh, Brahma Nand	Singh, Brahma_Nand
AF	23	56043	56043	Mishra, Siddhartha K.	Mishra, Siddhartha_K
AF	24	56044	56044	Atanasov, Atanas G.	Atanasov, Atanas_G
AF	25	56045	56045	Melo-Cavalcante, Ana Amelia de Carvalho	Melo-Cavalcante, Ana_Amelia_de_Carvalho

Record 372 | WOS:000416589800031

Differential Gene Expression Analysis of Bovine Macrophages after Exposure to the Penicillium Mycotoxins Citrinin and/or Ochratoxin A

Field	Position	Original line	Normalised line	Original value	Normalised value
AU	1	56260	56260	Brennan, KM	Brennan, Kristen_M
AU	2	56261	56261	Oh, SY	Oh, Se-Young
AU	3	56262	56262	Yiannikouris, A	Yiannikouris, Alexandros
AU	4	56263	56263	Graugnard, DE	Graugnard, Daniel_E
AU	5	56264	56264	Karrow, NA	Karrow, Niel_A
AF	1	56265	56265	Brennan, Kristen M.	Brennan, Kristen_M
AF	4	56268	56268	Graugnard, Daniel E.	Graugnard, Daniel_E
AF	5	56269	56269	Karrow, Niel A.	Karrow, Niel_A

Record 373 | WOS:000415911400030

UPLC-MS/MS analysis of ochratoxin A metabolites produced by Caco-2 and HepG2 cells in a co-culture system

Field	Position	Original line	Normalised line	Original value	Normalised value
AU	1	56392	56392	González-Arias, CA	Gonzalez-Arias, Cyndia_A
AU	2	56393	56393	Marín, S	Marin, Sonia
AU	3	56394	56394	Rojas-García, AE	Rojas-Garcia, Aurora_E
AU	4	56395	56395	Sanchis, V	Sanchis, Vicente
AU	5	56396	56396	Ramos, AJ	Ramos, Antonio_J
AF	1	56397	56397	Gonzalez-Arias, Cyndia A.	Gonzalez-Arias, Cyndia_A
AF	3	56399	56399	Rojas-Garcia, Aurora E.	Rojas-Garcia, Aurora_E
AF	5	56401	56401	Ramos, Antonio J.	Ramos, Antonio_J

Record 374 | WOS:000410635200006

Effects of ochratoxin A on ER stress, MAPK signaling pathway and autophagy of kidney and spleen in pigs

Field	Position	Original line	Normalised line	Original value	Normalised value
AU	1	56522	56522	Gan, F	Gan, Fang
AU	2	56523	56523	Hou, LL	Hou, Lili
AU	3	56524	56524	Zhou, YJ	Zhou, Yajiao
AU	4	56525	56525	Liu, YH	Liu, Yunhuan
AU	5	56526	56526	Huang, D	Huang, da
AU	6	56527	56527	Chen, XX	Chen, Xingxiang
AU	7	56528	56528	Huang, KH	Huang, Kehe
AF	5	56533	56533	Huang, Da	Huang, da

Record 375 | WOS:000414913800067

Evaluation of Chitosan and Cellulosic Polymers as Binding Adsorbent Materials to Prevent Aflatoxin B1, Fumonisin B1, Ochratoxin, Trichothecene, Deoxynivalenol, and Zearalenone Mycotoxicoses Through an In Vitro Gastrointestinal Model for Poultry

Field	Position	Original line	Normalised line	Original value	Normalised value
AU	1	56636	56636	Solís-Cruz, B	Solis-Cruz, Bruno
AU	2	56637	56637	Hernández-Patlán, D	Hernandez-Patlan, Daniel
AU	3	56638	56638	Beyssac, E	Beyssac, Eric
AU	4	56639	56639	Latorre, JD	Latorre, Juan_D
AU	5	56640	56640	Hernandez-Velasco, X	Hernandez-Velasco, Xochitl
AU	6	56641	56641	Merino-Guzman, R	Merino-Guzman, Ruben
AU	7	56642	56642	Tellez, G	Tellez, Guillermo
AU	8	56643	56643	López-Arellano, R	Lopez-Arellano, Raquel
AF	4	56647	56647	Latorre, Juan D.	Latorre, Juan_D

Record 376 | WOS:000408520200003

Worldwide Occurrence of Mycotoxins in Cereals and Cereal-Derived Food Products: Public Health Perspectives of Their Co-occurrence

Field	Position	Original line	Normalised line	Original value	Normalised value

AU	1	56761	56761	Lee, HJ	Lee, Hyun_Jung
AU	2	56762	56762	Ryu, D	Ryu, Dojin
AF	1	56763	56763	Lee, Hyun Jung	Lee, Hyun_Jung

Record 377 | WOS:000408520200008

Mutagenicity of Ochratoxin A: Role for a Carbon-Linked C8-Deoxyguanosine Adduct?

Field	Position	Original line	Normalised line	Original value	Normalised value
AU	1	57050	57050	Manderville, RA	Manderville, Richard_A
AU	2	57051	57051	Wetmore, SD	Wetmore, Stacey_D
AF	1	57052	57052	Manderville, Richard A.	Manderville, Richard_A
AF	2	57053	57053	Wetmore, Stacey D.	Wetmore, Stacey_D

Record 378 | WOS:000408074600028

Overexpression and Low Expression of Selenoprotein S Impact Ochratoxin A-Induced Porcine Cytotoxicity and Apoptosis in Vitro

Field	Position	Original line	Normalised line	Original value	Normalised value
AU	1	57170	57170	Gan, F	Gan, Fang
AU	2	57171	57171	Hu, ZH	Hu, Zhihua
AU	3	57172	57172	Zhou, YJ	Zhou, Yajiao
AU	4	57173	57173	Huang, KH	Huang, Kehe

Record 379 | WOS:000411538100005

Nrf2 deficiency exacerbates ochratoxin A-induced toxicity in vitro and in vivo

Field	Position	Original line	Normalised line	Original value	Normalised value
AU	1	57274	57274	Loboda, A	Loboda, Agnieszka
AU	2	57275	57275	Stachurska, A	Stachurska, Anna
AU	3	57276	57276	Sobczak, M	Sobczak, Mateusz
AU	4	57277	57277	Podkalicka, P	Podkalicka, Paulina
AU	5	57278	57278	Mucha, O	Mucha, Olga
AU	6	57279	57279	Jozkowicz, A	Jozkowicz, Alicja
AU	7	57280	57280	Dulak, J	Dulak, Jozef

Record 380 | WOS:000406735700003

Ochratoxin A transport by the human breast cancer resistance protein (BCRP), multidrug resistance protein 2 (MRP2), and organic anion-transporting polypeptides 1A2, 1B1 and 2B1

Field	Position	Original line	Normalised line	Original value	Normalised value
AU	1	57395	57395	Qi, XZ	Qi, Xiaozhe
AU	2	57396	57396	Wagenaar, E	Wagenaar, Els
AU	3	57397	57397	Xu, WT	Xu, Wentao
AU	4	57398	57398	Huang, KL	Huang, Kunlun
AU	5	57399	57399	Schinkel, AH	Schinkel, Alfred_H
AF	5	57404	57404	Schinkel, Alfred H.	Schinkel, Alfred_H

Record 381 | WOS:000407370700051

Health Risks Associated with Exposure to Filamentous Fungi

Field	Position	Original line	Normalised line	Original value	Normalised value
AU	1	57474	57474	Egbuta, MA	Egbuta, Mary_Augustina
AU	2	57475	57475	Mwanza, M	Mwanza, Mulunda
AU	3	57476	57476	Babalola, OO	Babalola, Olubukola_Oluranti
AF	1	57477	57477	Egbuta, Mary Augustina	Egbuta, Mary_Augustina
AF	3	57479	57479	Babalola, Olubukola Oluranti	Babalola, Olubukola_Oluranti

Record 382 | WOS:000404237400013

Evaluation of the automated MicroFlow® and Metafer™ platforms for high-throughput micronucleus scoring and dose response analysis in human lymphoblastoid TK6 cells

Field	Position	Original line	Normalised line	Original value	Normalised value
AU	1	57669	57669	Verma, JR	Verma, Jatin_R
AU	2	57670	57670	Rees, BJ	Rees, Benjamin_J
AU	3	57671	57671	Wilde, EC	Wilde, Eleanor_C
AU	4	57672	57672	Thornton, CA	Thornton, Catherine_A
AU	5	57673	57673	Jenkins, GJS	Jenkins, Gareth_J_S
AU	6	57674	57674	Doak, SH	Doak, Shareen_H
AU	7	57675	57675	Johnson, GE	Johnson, George_E
AF	1	57676	57676	Verma, Jatin R.	Verma, Jatin_R
AF	2	57677	57677	Rees, Benjamin J.	Rees, Benjamin_J
AF	3	57678	57678	Wilde, Eleanor C.	Wilde, Eleanor_C
AF	4	57679	57679	Thornton, Catherine A.	Thornton, Catherine_A
AF	5	57680	57680	Jenkins, Gareth J. S.	Jenkins, Gareth_J_S
AF	6	57681	57681	Doak, Shareen H.	Doak, Shareen_H
AF	7	57682	57682	Johnson, George E.	Johnson, George_E

Record 383 | WOS:000404198300012

In vitro immune toxicity of ochratoxin A in porcine alveolar macrophages: A role for the ROS-relative TLR4/MyD88 signaling pathway

Field	Position	Original line	Normalised line	Original value	Normalised value
AU	1	57763	57763	Xu, HB	Xu, Haibin
AU	2	57764	57764	Hao, S	Hao, Shu
AU	3	57765	57765	Gan, F	Gan, Fang
AU	4	57766	57766	Wang, H	Wang, Hong
AU	5	57767	57767	Xu, J	Xu, Jing
AU	6	57768	57768	Liu, DD	Liu, Dandan
AU	7	57769	57769	Huang, KH	Huang, Kehe

Record 384 | WOS:000401600400001

Adsorption of aflatoxin B1, zearalenone and ochratoxin A by microorganisms isolated from Kefir grains

Field	Position	Original line	Normalised line	Original value	Normalised value

AU	1	57875	57875	Ben Taheur, F	Ben_Taheur, Fadia
AU	2	57876	57876	Fedhila, K	Fedhila, Kais
AU	3	57877	57877	Chaieb, K	Chaieb, Kamel
AU	4	57878	57878	Kouidhi, B	Kouidhi, Bochra
AU	5	57879	57879	Bakhrouf, A	Bakhrouf, Amina
AU	6	57880	57880	Abrunhosa, L	Abrunhosa, Luis
AF	1	57881	57881	Ben Taheur, Fadia	Ben_Taheur, Fadia

Record 385 | WOS:000408731500007

Lycopene restores trace element levels in ochratoxin A-treated rats

Field	Position	Original line	Normalised line	Original value	Normalised value
AU	1	57998	57998	Palabiyik, SS	Palabiyik, Saziye_Sezin
AU	2	57999	57999	Erkekoglu, P	Erkekoglu, Pinar
AU	3	58000	58000	Kizilgun, M	Kizilgun, Murat
AU	4	58001	58001	Sahin, G	Sahin, Gonul
AU	5	58002	58002	Kocer-Gumusel, B	Kocer-Gumusel, Belma
AF	1	58003	58003	Palabiyik, Saziye Sezin	Palabiyik, Saziye_Sezin

Record 386 | WOS:000403637300009

Ochratoxin A induced premature senescence in human renal proximal tubular cells

Field	Position	Original line	Normalised line	Original value	Normalised value
AU	1	58098	58098	Yang, XA	Yang, Xuan
AU	2	58099	58099	Liu, S	Liu, Sheng
AU	3	58100	58100	Huang, CC	Huang, Chuchu
AU	4	58101	58101	Wang, HM	Wang, Haomiao
AU	5	58102	58102	Luo, YB	Luo, Yunbo
AU	6	58103	58103	Xu, WT	Xu, Wentao
AU	7	58104	58104	Huang, KL	Huang, Kunlun

Record 387 | WOS:000394982800001

The intestinal barrier as an emerging target in the toxicological assessment of mycotoxins

Field	Position	Original line	Normalised line	Original value	Normalised value
AU	1	58228	58228	Akbari, P	Akbari, Peyman
AU	2	58229	58229	Braber, S	Braber, Saskia
AU	3	58230	58230	Varasteh, S	Varasteh, Soheil
AU	4	58231	58231	Alizadeh, A	Alizadeh, Arash
AU	5	58232	58232	Garssen, J	Garssen, Johan
AU	6	58233	58233	Fink-Gremmels, J	Fink-Gremmels, Johanna

Record 388 | WOS:000394558400005

Assessing interactions of binary mixtures of Penicillium mycotoxins (PMs) by using a bovine macrophage cell line (BoMacs)

Field	Position	Original line	Normalised line	Original value	Normalised value
AU	1	58500	58500	Oh, SY	Oh, Se-Young
AU	2	58501	58501	Cedergreen, N	Cedergreen, Nina
AU	3	58502	58502	Yiannikouris, A	Yiannikouris, Alexandros
AU	4	58503	58503	Swamy, HVLN	Swamy, H_V_L_N
AU	5	58504	58504	Karrow, NA	Karrow, Niel_A
AF	4	58508	58508	Swamy, H. V. L. N.	Swamy, H_V_L_N
AF	5	58509	58509	Karrow, Niel A.	Karrow, Niel_A

Record 389 | WOS:000392347800007

Mycotoxins as human carcinogens-the IARC Monographs classification

Field	Position	Original line	Normalised line	Original value	Normalised value
AU	1	58592	58592	Ostry, V	Ostry, Vladimir
AU	2	58593	58593	Malir, F	Malir, Frantisek
AU	3	58594	58594	Toman, J	Toman, Jakub
AU	4	58595	58595	Grosse, Y	Grosse, Yann

Record 390 | WOS:000389167600013

Mycotoxin detoxifiers detoxifiers attenuate deoxynivalenol-induced pro-inflammatory barrier insult in porcine enterocytes as an in vitro evaluation model of feed mycotoxin reduction

Field	Position	Original line	Normalised line	Original value	Normalised value
AU	1	58753	58753	Park, SH	Park, Seong-Hwan
AU	2	58754	58754	Kim, J	Kim, Juil
AU	3	58755	58755	Kim, D	Kim, Dongwook
AU	4	58756	58756	Moon, Y	Moon, Yuseok

Record 391 | WOS:000392789800020

Chitosan nanoparticles plus quercetin suppress the oxidative stress, modulate DNA fragmentation and gene expression in the kidney of rats fed ochratoxin A-contaminated diet

Field	Position	Original line	Normalised line	Original value	Normalised value
AU	1	58870	58870	Abdel-Wahhab, MA	Abdel-Wahhab, Mosaad_A
AU	2	58871	58871	Aljawish, A	Aljawish, Abdulhadi
AU	3	58872	58872	El-Nekeety, AA	El-Nekeety, Aziza_A
AU	4	58873	58873	Abdel-Aziem, SH	Abdel-Aziem, Sekena_H
AU	5	58874	58874	Hassan, NS	Hassan, Nabila_S
AF	1	58875	58875	Abdel-Wahhab, Mosaad A.	Abdel-Wahhab, Mosaad_A
AF	3	58877	58877	El-Nekeety, Aziza A.	El-Nekeety, Aziza_A
AF	4	58878	58878	Abdel-Aziem, Sekena H.	Abdel-Aziem, Sekena_H
AF	5	58879	58879	Hassan, Nabila S.	Hassan, Nabila_S

Record 392 | WOS:000402081300011

Mycotoxins co-contamination: Methodological aspects and biological relevance of combined toxicity studies

Field	Position	Original line	Normalised line	Original value	Normalised value

AU	1	59040	59040	Alassane-Kpembi, I	Alassane-Kpembi, Imourana
AU	2	59041	59041	Schatzmayr, G	Schatzmayr, Gerd
AU	3	59042	59042	Taranu, I	Taranu, Ionelia
AU	4	59043	59043	Marin, D	Marin, Daniela
AU	5	59044	59044	Puel, O	Puel, Olivier
AU	6	59045	59045	Oswald, IP	Oswald, Isabelle_Paule
AF	6	59051	59051	Oswald, Isabelle Paule	Oswald, Isabelle_Paule

Record 393 | WOS:000399597700009

Protective role of bentonite against aflatoxin B1- and ochratoxin A-induced immunotoxicity in broilers

Field	Position	Original line	Normalised line	Original value	Normalised value
AU	1	59248	59248	Bhatti, SA	Bhatti, Sheraz_Ahmed
AU	2	59249	59249	Khan, MZ	Khan, Muhammad_Zargham
AU	3	59250	59250	Saleemi, MK	Saleemi, Muhammad_Kashif
AU	4	59251	59251	Saqib, M	Saqib, Muhammad
AU	5	59252	59252	Khan, A	Khan, Ahrar
AU	6	59253	59253	ul-Hassan, Z	Ul_Hassan, Zahoor
AF	1	59254	59254	Bhatti, Sheraz Ahmed	Bhatti, Sheraz_Ahmed
AF	2	59255	59255	Khan, Muhammad Zargham	Khan, Muhammad_Zargham
AF	3	59256	59256	Saleemi, Muhammad Kashif	Saleemi, Muhammad_Kashif
AF	6	59259	59259	ul-Hassan, Zahoor	Ul_Hassan, Zahoor

Record 394 | WOS:000448708300012

Aflatoxin in Rice Crop: Prevalence and Assessment of Daily Exposure

Field	Position	Original line	Normalised line	Original value	Normalised value
AU	1	59373	59373	Elzupir, AO	Elzupir, Amin_O
AU	2	59374	59374	Alamer, AS	Alamer, Abdulaziz_S
AU	3	59375	59375	Dutton, MF	Dutton, Michael_F
AF	1	59376	59376	Elzupir, Amin O.	Elzupir, Amin_O
AF	2	59377	59377	Alamer, Abdulaziz S.	Alamer, Abdulaziz_S
AF	3	59378	59378	Dutton, Michael F.	Dutton, Michael_F

Record 395 | WOS:000416384800008

Effect of PR toxin on THP1 and Caco-2 cells: an in vitro study

Field	Position	Original line	Normalised line	Original value	Normalised value
AU	1	59486	59486	Hymery, N	Hymery, Nolwenn
AU	2	59487	59487	Puel, O	Puel, Olivier
AU	5	59490	59490	Le Scouarnec, H	le_Scouarnec, H
AU	6	59491	59491	Coton, E	Coton, Emmanuel
AU	7	59492	59492	Coton, M	Coton, Monika
AF	1	59493	59493	Hymery, N.	Hymery, Nolwenn
AF	2	59494	59494	Puel, O.	Puel, Olivier
AF	3	59495	59495	Tadrist, S.	Tadrist, S

AF	4	59496	59496	Canlet, C.	Canlet, C
AF	5	59497	59497	Le Scouarnec, H.	le_Scouarnec, H
AF	6	59498	59498	Coton, E.	Coton, Emmanuel
AF	7	59499	59499	Coton, M.	Coton, Monika

Record 396 | WOS:000413016300007

Efficacy of mixed adsorbent in ameliorating ochratoxicosis in broilers fed ochratoxin A contaminated diets

Field	Position	Original line	Normalised line	Original value	Normalised value
AU	1	59610	59610	Qu, DF	Qu, Daofeng
AU	2	59611	59611	Huang, XL	Huang, Xiaolin
AU	3	59612	59612	Han, JZ	Han, Jianzhong
AU	4	59613	59613	Man, NN	Man, Nana

Record 397 | WOS:000417403600001

Citrinin induces apoptosis in human HCT116 colon cancer cells through endoplasmic reticulum stress

Field	Position	Original line	Normalised line	Original value	Normalised value
AU	1	59691	59691	Salah, A	Salah, Amal
AU	2	59692	59692	Bouaziz, C	Bouaziz, Chayma
AU	3	59693	59693	Prola, A	Prola, Alexandre
AU	4	59694	59694	Da Silva, JP	da_Silva, Julie_Pires
AU	5	59695	59695	Bacha, H	Bacha, Hassen
AU	6	59696	59696	Abid-Essefi, S	Abid-Essefi, Salwa
AU	7	59697	59697	Lemaire, C	Lemaire, Christophe
AF	4	59701	59701	Da Silva, Julie Pires	da_Silva, Julie_Pires

Record 398 | WOS:000424458100023

Mycotoxins

Field	Position	Original line	Normalised line	Original value	Normalised value
AU	1	59829	59829	Stein, RA	Stein, R_A
AU	2	59830	59830	Bulboaca, AE	Bulboaca, A_E
AF	1	59831	59831	Stein, R. A.	Stein, R_A
AF	2	59832	59832	Bulboaca, A. E.	Bulboaca, A_E

Record 399 | WOS:000448708300031

Application of Fungal Metabolites Against Mycotoxins Production

Field	Position	Original line	Normalised line	Original value	Normalised value
AU	1	60107	60107	Verheecke, C	Verheecke, Carol
AU	2	60108	60108	Choque, E	Choque, Elodie
AU	3	60109	60109	Mathieu, F	Mathieu, Florence

Record 400 | WOS:000391769900002

Toxicity study of ochratoxin A using HEK293 and HepG2 cell lines based on microRNA profiling

Field	Position	Original line	Normalised line	Original value	Normalised value
AU	2	60331	60331	Qi, X	Qi, Xiaozhe
AU	6	60335	60335	Guo, M	Guo, Mingzhang
AU	9	60338	60338	Luo, H	Luo, Haoshu
AF	1	60341	60341	Zhao, J.	Zhao, J
AF	2	60342	60342	Qi, X.	Qi, Xiaozhe
AF	3	60343	60343	Dai, Q.	Dai, Q
AF	4	60344	60344	He, X.	He, X
AF	5	60345	60345	Dweep, H.	Dweep, H
AF	6	60346	60346	Guo, M.	Guo, Mingzhang
AF	7	60347	60347	Luo, Y.	Luo, Y
AF	8	60348	60348	Gretz, N.	Gretz, N
AF	9	60349	60349	Luo, H.	Luo, Haoshu
AF	10	60350	60350	Huang, K.	Huang, K
AF	11	60351	60351	Xu, W.	Xu, W

Record 401 | WOS:000393580000007

Limited Link between Oxidative Stress and Ochratoxin A-Induced Renal Injury in an Acute Toxicity Rat Model

Field	Position	Original line	Normalised line	Original value	Normalised value
AU	1	60460	60460	Zhu, LY	Zhu, Liye
AU	2	60461	60461	Yu, T	Yu, Tao
AU	3	60462	60462	Qi, XZ	Qi, Xiaozhe
AU	4	60463	60463	Gao, J	Gao, Jing
AU	5	60464	60464	Huang, KL	Huang, Kunlun
AU	6	60465	60465	He, XY	He, Xiaoyun
AU	7	60466	60466	Luo, HS	Luo, Haoshu
AU	8	60467	60467	Xu, WT	Xu, Wentao

Record 402 | WOS:000387780500027

TOXICOPATHOLOGICAL AND SERUM BIOCHEMICAL ALTERATIONS INDUCED BY OCHRATOXIN A IN BROILER CHICKS AND THEIR AMELIORATION BY LOCALLY AVAILABLE BENTONITE CLAY

Field	Position	Original line	Normalised line	Original value	Normalised value
AU	1	60592	60592	Khatoon, A	Khatoon, Aisha
AU	2	60593	60593	Khan, MZ	Khan, Muhammad_Zargham
AU	3	60594	60594	Khan, A	Khan, Ahrar
AU	4	60595	60595	Javed, I	Javed, Ijaz
AF	2	60597	60597	Khan, Muhammad Zargham	Khan, Muhammad_Zargham

Record 403 | WOS:000383825300009

Aflatoxin M1 cytotoxicity against human intestinal Caco-2 cells is enhanced in the presence of other mycotoxins

Field	Position	Original line	Normalised line	Original value	Normalised value
AU	1	60696	60696	Gao, YN	Gao, Ya-Nan
AU	2	60697	60697	Wang, JQ	Wang, Jia-Qi
AU	3	60698	60698	Li, SL	Li, S_L
AU	4	60699	60699	Zhang, YD	Zhang, Y_D
AU	5	60700	60700	Zheng, N	Zheng, Nan
AF	1	60701	60701	Gao, Y. N.	Gao, Ya-Nan
AF	2	60702	60702	Wang, J. Q.	Wang, Jia-Qi
AF	3	60703	60703	Li, S. L.	Li, S_L
AF	4	60704	60704	Zhang, Y. D.	Zhang, Y_D
AF	5	60705	60705	Zheng, N.	Zheng, Nan

Record 404 | WOS:000387347300005

Different Toxicity Mechanisms for Citrinin and Ochratoxin A Revealed by Transcriptomic Analysis in Yeast

Field	Position	Original line	Normalised line	Original value	Normalised value
AU	1	60826	60826	Vanacloig-Pedros, E	Vanacloig-Pedros, Elena
AU	2	60827	60827	Proft, M	Proft, Markus
AU	3	60828	60828	Pascual-Ahuir, A	Pascual-Ahuir, Amparo

Record 405 | WOS:000383733300020

Quantitation of the DNA Adduct of Semicarbazide in Organs of Semicarbazide-Treated Rats by Isotope-Dilution Liquid Chromatography-Tandem Mass Spectrometry: A Comparative Study with the RNA Adduct

Field	Position	Original line	Normalised line	Original value	Normalised value
AU	1	60953	60953	Wang, YA	Wang, Yinan
AU	2	60954	60954	Wong, TY	Wong, Tin-Yan
AU	3	60955	60955	Chan, W	Chan, Wan

Record 406 | WOS:000387199300010

Ochratoxin A activates neutrophils and kills these cells through necrosis, an effect eliminated through its conversion into ochratoxin α

Field	Position	Original line	Normalised line	Original value	Normalised value
AU	1	61039	61039	Kupski, L	Kupski, Larine
AU	2	61040	61040	Freitas, M	Freitas, Marisa
AU	3	61041	61041	Ribeiro, D	Ribeiro, Daniela
AU	4	61042	61042	Furlong, EB	Furlong, Eliana_Badiale
AU	5	61043	61043	Fernandes, E	Fernandes, Eduarda
AF	4	61047	61047	Furlong, Eliana Badiale	Furlong, Eliana_Badiale

Record 407 | WOS:000380647600001

Cytotoxic Effects of Ochratoxin A in Neuro-2a Cells: Role of Oxidative Stress Evidenced by N-acetylcysteine

Field	Position	Original line	Normalised line	Original value	Normalised value
AU	1	61171	61171	Bhat, PV	Bhat, Pratiksha_V

AU	2	61172	61172	Pandareesh, MD	Pandareesh, M_D
AU	3	61173	61173	Khanum, F	Khanum, Farhath
AU	4	61174	61174	Tamatam, A	Tamatam, Anand
AF	1	61175	61175	Bhat, Pratiksha V.	Bhat, Pratiksha_V
AF	2	61176	61176	Pandareesh, M. D.	Pandareesh, M_D

Record 408 | WOS:000380763800002

Ochratoxin A: 50 Years of Research

Field	Position	Original line	Normalised line	Original value	Normalised value
AU	1	61298	61298	Malir, F	Malir, Frantisek
AU	2	61299	61299	Ostry, V	Ostry, Vladimir
AU	3	61300	61300	Pfohl-Leszkowicz, A	Pfohl-Leszkowicz, Annie
AU	4	61301	61301	Malir, J	Malir, Jan
AU	5	61302	61302	Toman, J	Toman, Jakub

Record 409 | WOS:000377720700011

Inhibition of CXCL12-mediated chemotaxis of Jurkat cells by direct immunotoxicants

Field	Position	Original line	Normalised line	Original value	Normalised value
AU	1	61746	61746	Shao, J	Shao, Jia
AU	2	61747	61747	Stout, I	Stout, Inge
AU	3	61748	61748	Volger, OL	Volger, Oscar_L
AU	4	61749	61749	Hendriksen, PJM	Hendriksen, Peter_J_M
AU	5	61750	61750	van Loveren, H	van_Loveren, Henk
AU	6	61751	61751	Peijnenburg, AACM	Peijnenburg, Ad_A_C_M
AF	3	61754	61754	Volger, Oscar L.	Volger, Oscar_L
AF	4	61755	61755	Hendriksen, Peter J. M.	Hendriksen, Peter_J_M
AF	5	61756	61756	van Loveren, Henk	van_Loveren, Henk
AF	6	61757	61757	Peijnenburg, Ad A. C. M.	Peijnenburg, Ad_A_C_M

Record 410 | WOS:000376990200009

Cytotoxicity, apoptosis, DNA damage and methylation in mammary and kidney epithelial cell lines exposed to ochratoxin A

Field	Position	Original line	Normalised line	Original value	Normalised value
AU	1	61864	61864	Giromini, C	Giromini, Carlotta
AU	2	61865	61865	Rebucci, R	Rebucci, Raffaella
AU	3	61866	61866	Fusi, E	Fusi, Eleonora
AU	4	61867	61867	Rossi, L	Rossi, Luciana
AU	5	61868	61868	Saccone, F	Saccone, Francesca
AU	6	61869	61869	Baldi, A	Baldi, Antonella

Record 411 | WOS:000378297500009

Effects of Ochratoxin A on Mouse Oocyte Maturation and Fertilization, and Apoptosis During Fetal Development

Field	Position	Original line	Normalised line	Original value	Normalised value
AU	1	61957	61957	Huang, FJ	Huang, Fu-Jen
AU	2	61958	61958	Chan, WH	Chan, Wen-Hsiung

Record 412 | WOS:000375813200002

Innovative technologies for the mitigation of mycotoxins in animal feed and ingredients-A review of recent patents

Field	Position	Original line	Normalised line	Original value	Normalised value
AU	1	62067	62067	Zhu, Y	Zhu, Yan
AU	2	62068	62068	Hassan, YI	Hassan, Yousef_I
AU	3	62069	62069	Watts, C	Watts, Christena
AU	4	62070	62070	Zhou, T	Zhou, Ting
AF	2	62072	62072	Hassan, Yousef I.	Hassan, Yousef_I

Record 413 | WOS:000373624800006

Multiparametric luminescent cell viability assay in toxicology models: A critical evaluation

Field	Position	Original line	Normalised line	Original value	Normalised value
AU	1	62201	62201	Sali, N	Sali, Nikolett
AU	2	62202	62202	Nagy, S	Nagy, Sandor
AU	3	62203	62203	Poór, M	Poor, Miklos
AU	4	62204	62204	Koszegi, T	Koszegi, Tamas

Record 414 | WOS:000377731900003

Mycotoxins modify the barrier function of Caco-2 cells through differential gene expression of specific claudin isoforms: Protective effect of illite mineral clay

Field	Position	Original line	Normalised line	Original value	Normalised value
AU	1	62312	62312	Romero, A	Romero, Alejandro
AU	2	62313	62313	Ares, I	Ares, Irma
AU	3	62314	62314	Ramos, E	Ramos, Eva
AU	4	62315	62315	Castellano, V	Castellano, Victor
AU	5	62316	62316	Martínez, M	Martinez, Marta
AU	6	62317	62317	Martínez-Larrañaga, MR	Martinez-Larranaga, Maria-Rosa
AU	7	62318	62318	Anadón, A	Anadon, Arturo
AU	8	62319	62319	Martínez, MA	Martinez, Maria-Aranzazu

Record 415 | WOS:000372000700010

Alleviative effect of myricetin on ochratoxin A-induced oxidative stress in rat renal cortex: histological and biochemical study

Field	Position	Original line	Normalised line	Original value	Normalised value
AU	1	62475	62475	Abd El-Haleem, MR	Abd_El-Haleem, Manal_R
AU	2	62476	62476	Kattaia, AAA	Kattaia, Asmaa_A_A
AU	3	62477	62477	Abd El-Baset, SA	Abd_El-Baset, Samia_A
AU	4	62478	62478	Mostafa, HE	Mostafa, Heba_el_Sayed
AF	1	62479	62479	Abd El-Haleem, Manal R.	Abd_El-Haleem, Manal_R

AF	2	62480	62480	Kattaia, Asmaa A. A.	Kattaia, Asmaa_A_A
AF	3	62481	62481	Abd El-Baset, Samia A.	Abd_El-Baset, Samia_A
AF	4	62482	62482	Mostafa, Heba El Sayed	Mostafa, Heba_el_Sayed

Record 416 | WOS:000375861700010

Natural Co-Occurrence of Mycotoxins in Foods and Feeds and Their in vitro Combined Toxicological Effects

Field	Position	Original line	Normalised line	Original value	Normalised value
AU	1	62605	62605	Smith, MC	Smith, Marie-Caroline
AU	2	62606	62606	Madec, S	Madec, Stephanie
AU	3	62607	62607	Coton, E	Coton, Emmanuel
AU	4	62608	62608	Hymery, N	Hymery, Nolwenn

Record 417 | WOS:000431903800102

Mycotoxins at the blood-brain barrier: Metabolism, toxicity, barrier integrity and transfer to the brain

Field	Position	Original line	Normalised line	Original value	Normalised value
AU	1	62873	62873	Behrens, M	Behrens, Matthias
AU	2	62874	62874	Hüwel, S	Huwel, Sabine
AU	3	62875	62875	Galla, HJ	Galla, Hans-Joachim
AU	4	62876	62876	Humpf, HU	Humpf, Hans-Ulrich

Record 418 | WOS:000372963300003

Ochratoxin A and T-2 Toxin Induce Clonogenicity and Cell Migration in Human Colon Carcinoma and Fetal Lung Fibroblast Cell Lines

Field	Position	Original line	Normalised line	Original value	Normalised value
AU	1	62916	62916	Abassi, H	Abassi, Haila
AU	2	62917	62917	Ayed-Boussema, I	Ayed-Boussema, Imen
AU	3	62918	62918	Shirley, S	Shirley, Sarah
AU	4	62919	62919	Abid, S	Abid, Salwa
AU	5	62920	62920	Bacha, H	Bacha, Hassen

Record 419 | WOS:000370832600004

Malignant transformation of human gastric epithelium cells via reactive oxygen species production and Wnt/β-catenin pathway activation following 40-week exposure to ochratoxin A

Field	Position	Original line	Normalised line	Original value	Normalised value
AU	1	63031	63031	Jia, X	Jia, Xin
AU	2	63032	63032	Cui, JF	Cui, Jinfeng
AU	3	63033	63033	Meng, XX	Meng, Xinxing
AU	4	63034	63034	Xing, LX	Xing, Lingxiao
AU	5	63035	63035	Shen, HT	Shen, Haitao
AU	6	63036	63036	Wang, J	Wang, Juan
AU	7	63037	63037	Liu, J	Liu, Jing
AU	8	63038	63038	Wang, Y	Wang, Yuan
AU	9	63039	63039	Lian, W	Lian, Weiguang

AU	10	63040	63040	Zhang, XH	Zhang, Xianghong

Record 420 | WOS:000369179000011

Effects of aflatoxin B1 on oxidative stress markers and apoptosis of spleens in broilers

Field	Position	Original line	Normalised line	Original value	Normalised value
AU	1	63147	63147	Chen, J	Chen, Jin
AU	2	63148	63148	Chen, KJ	Chen, Kejie
AU	3	63149	63149	Yuan, SB	Yuan, Shibin
AU	4	63150	63150	Peng, X	Peng, Xin
AU	5	63151	63151	Fang, J	Fang, Jing
AU	6	63152	63152	Wang, FY	Wang, Fengyuan
AU	7	63153	63153	Cui, HM	Cui, Hengmin
AU	8	63154	63154	Chen, ZL	Chen, Zhengli
AU	9	63155	63155	Yuan, JX	Yuan, Jingxin
AU	10	63156	63156	Geng, Y	Geng, Yi
AF	4	63160	63160	Peng, Xi	Peng, Xin

Record 421 | WOS:000370094500037

Grafting of gallic acid onto chitosan nano particles enhances antioxidant activities in vitro and protects against ochratoxin A toxicity in catfish (Clarias gariepinus)

Field	Position	Original line	Normalised line	Original value	Normalised value
AU	1	63265	63265	Abdel-Wahhab, MA	Abdel-Wahhab, Mosaad_A
AU	2	63266	63266	Aljawish, A	Aljawish, Abdulhadi
AU	3	63267	63267	Kenawy, AM	Kenawy, Amany_M
AU	4	63268	63268	El-Nekeety, AA	El-Nekeety, Aziza_A
AU	5	63269	63269	Hamed, HS	Hamed, Heba_S
AU	6	63270	63270	Abdel-Aziem, SH	Abdel-Aziem, Sekena_H
AF	1	63271	63271	Abdel-Wahhab, Mosaad A.	Abdel-Wahhab, Mosaad_A
AF	3	63273	63273	Kenawy, Amany M.	Kenawy, Amany_M
AF	4	63274	63274	El-Nekeety, Aziza A.	El-Nekeety, Aziza_A
AF	5	63275	63275	Hamed, Heba S.	Hamed, Heba_S
AF	6	63276	63276	Abdel-Aziem, Sekena H.	Abdel-Aziem, Sekena_H

Record 422 | WOS:000388323700013

Mycotoxin Outbreak in Animal Feed

Field	Position	Original line	Normalised line	Original value	Normalised value
AU	1	63400	63400	Cheat, S	Cheat, Sophal
AU	2	63401	63401	Oswald, IP	Oswald, Isabelle_P
AU	3	63402	63402	Kolf-Clauw, M	Kolf-Clauw, Martine
AF	2	63404	63404	Oswald, Isabelle P.	Oswald, Isabelle_P

Record 423 | WOS:000368966200007

Ochratoxin A-induced cytotoxicity, genotoxicity and reactive oxygen species in kidney cells: An integrative approach of complementary endpoints

Field	Position	Original line	Normalised line	Original value	Normalised value
AU	1	63619	63619	Costa, JG	Costa, Joao_G
AU	2	63620	63620	Saraiva, N	Saraiva, Nuno
AU	3	63621	63621	Guerreiro, PS	Guerreiro, Patricia_S
AU	4	63622	63622	Louro, H	Louro, Henriqueta
AU	5	63623	63623	Silva, MJ	Silva, Maria_J
AU	6	63624	63624	Miranda, JP	Miranda, Joana_P
AU	7	63625	63625	Castro, M	Castro, Matilde
AU	8	63626	63626	Batinic-Haberle, I	Batinic-Haberle, Ines
AU	9	63627	63627	Fernandes, AS	Fernandes, Ana_S
AU	10	63628	63628	Oliveira, NG	Oliveira, Nuno_G
AF	1	63629	63629	Costa, Joao G.	Costa, Joao_G
AF	3	63631	63631	Guerreiro, Patricia S.	Guerreiro, Patricia_S
AF	5	63633	63633	Silva, Maria J.	Silva, Maria_J
AF	6	63634	63634	Miranda, Joana P.	Miranda, Joana_P
AF	9	63637	63637	Fernandes, Ana S.	Fernandes, Ana_S
AF	10	63638	63638	Oliveira, Nuno G.	Oliveira, Nuno_G

Record 424 | WOS:000371816100005

Individual and combined effects of roquefortine C and mycophenolic acid on human monocytic and intestinal cells

Field	Position	Original line	Normalised line	Original value	Normalised value
AU	3	63783	63783	Coton, E	Coton, Emmanuel
AU	4	63784	63784	Hymery, N	Hymery, Nolwenn
AF	1	63785	63785	Fontaine, K.	Fontaine, K
AF	2	63786	63786	Mounier, J.	Mounier, J
AF	3	63787	63787	Coton, E.	Coton, Emmanuel
AF	4	63788	63788	Hymery, N.	Hymery, Nolwenn

Record 425 | WOS:000376604100010

Evaluation of renal in vitro models used in ochratoxin research

Field	Position	Original line	Normalised line	Original value	Normalised value
AU	1	63881	63881	Heussner, AH	Heussner, Alexandra_H
AF	1	63883	63883	Heussner, A. H.	Heussner, Alexandra_H
AF	2	63884	63884	Paget, T.	Paget, T

Record 426 | WOS:000389199300011

Diet contaminated with ochratoxin A at the highest level allowed by EU recommendation disturbs liver metabolism in weaned piglets

Field	Position	Original line	Normalised line	Original value	Normalised value
AU	1	64172	64172	Marin, DE	Marin, D_E
AU	2	64173	64173	Motiu, M	Motiu, Monica
AU	3	64174	64174	Pistol, GC	Pistol, Gina_Cecilia
AU	4	64175	64175	Gras, MA	Gras, Mihail_Alexandru
AU	5	64176	64176	Israel-Roming, F	Israel-Roming, Florentina

AU	6	64177	64177	Calin, L	Calin, Loredana
AU	7	64178	64178	Stancu, M	Stancu, Mariana
AU	8	64179	64179	Taranu, I	Taranu, Ionelia
AF	1	64180	64180	Marin, D. E.	Marin, D_E
AF	2	64181	64181	Motiu, M.	Motiu, Monica
AF	3	64182	64182	Pistol, G. C.	Pistol, Gina_Cecilia
AF	4	64183	64183	Gras, M. A.	Gras, Mihail_Alexandru
AF	5	64184	64184	Israel-Roming, F.	Israel-Roming, Florentina
AF	6	64185	64185	Calin, L.	Calin, Loredana
AF	7	64186	64186	Stancu, M.	Stancu, Mariana
AF	8	64187	64187	Taranu, I.	Taranu, Ionelia

Record 427 | WOS:000368260800005

Ochratoxin A as possible factor trigging autism and its male prevalence via epigenetic mechanism

Field	Position	Original line	Normalised line	Original value	Normalised value
AU	2	64295	64295	Raggi, ME	Raggi, M_E
AF	1	64298	64298	Mezzelani, A.	Mezzelani, A
AF	2	64299	64299	Raggi, M. E.	Raggi, M_E
AF	3	64300	64300	Marabotti, A.	Marabotti, A
AF	4	64301	64301	Milanesi, L.	Milanesi, L

Record 428 | WOS:000387395900007

In Vitro Assays to Estimate the Toxicological Effects of Masked Mycotoxins

Field	Position	Original line	Normalised line	Original value	Normalised value
AU	1	64374	64374	Nathanail, AV	Nathanail, Alexis_V
AU	2	64375	64375	Jestoi, M	Jestoi, Marika
AU	3	64376	64376	Jonsson, M	Jonsson, Martina
AU	4	64377	64377	Peltonen, K	Peltonen, Kimmo
AF	1	64378	64378	Nathanail, Alexis V.	Nathanail, Alexis_V

Record 429 | WOS:000384724200015

Probiotic preparation reduces faecal water genotoxicity and cytotoxicity in chickens fed ochratoxin A contaminated feed (in vivo study)

Field	Position	Original line	Normalised line	Original value	Normalised value
AU	1	64553	64553	Slizewska, K	Slizewska, Katarzyna
AU	2	64554	64554	Nowak, A	Nowak, Adriana
AU	3	64555	64555	Smulikowska, S	Smulikowska, Stefania

Record 430 | WOS:000366832700013

miR-122 plays an important role in ochratoxin A-induced hepatocyte apoptosis in vitro and in vivo

Field	Position	Original line	Normalised line	Original value	Normalised value
AU	1	64655	64655	Zhu, LY	Zhu, Liye

AU	2	64656	64656	Yu, T	Yu, Tao
AU	3	64657	64657	Qi, XZ	Qi, Xiaozhe
AU	4	64658	64658	Yang, B	Yang, Bing
AU	5	64659	64659	Shi, L	Shi, Lei
AU	6	64660	64660	Luo, HS	Luo, Haoshu
AU	7	64661	64661	He, XY	He, Xiaoyun
AU	8	64662	64662	Huang, KL	Huang, Kunlun
AU	9	64663	64663	Xu, WT	Xu, Wentao
AF	4	64667	64667	Yang, Bo	Yang, Bing

Record 431 | WOS:000367635500005

MiR-122 partly mediates the ochratoxin A-induced GC-2 cell apoptosis

Field	Position	Original line	Normalised line	Original value	Normalised value
AU	1	64770	64770	Chen, R	Chen, Ran
AU	2	64771	64771	Deng, L	Deng, Liang
AU	3	64772	64772	Yu, XX	Yu, Xinxin
AU	4	64773	64773	Wang, XX	Wang, Xiuxiu
AU	5	64774	64774	Zhu, LY	Zhu, Liye
AU	6	64775	64775	Yu, T	Yu, Tao
AU	7	64776	64776	Zhang, YH	Zhang, Yiheng
AU	8	64777	64777	Zhou, B	Zhou, Bo
AU	9	64778	64778	Xu, WT	Xu, Wentao
AU	10	64779	64779	Chen, L	Chen, Liang
AU	11	64780	64780	Luo, HS	Luo, Haoshu

Record 432 | WOS:000367013500020

Interaction of Citrinin with Human Serum Albumin

Field	Position	Original line	Normalised line	Original value	Normalised value
AU	1	64893	64893	Poór, M	Poor, Miklos
AU	2	64894	64894	Lemli, B	Lemli, Beata
AU	3	64895	64895	Bálint, M	Balint, Monika
AU	4	64896	64896	Hetényi, C	Hetenyi, Csaba
AU	5	64897	64897	Sali, N	Sali, Nikolett
AU	6	64898	64898	Koszegi, T	Koszegi, Tamas
AU	7	64899	64899	Kunsági-Máté, S	Kunsagi-Mate, Sandor

Record 433 | WOS:000362726700025

The Phytoalexin Resveratrol Ameliorates Ochratoxin A Toxicity in Human Embryonic Kidney (HEK293) Cells

Field	Position	Original line	Normalised line	Original value	Normalised value
AU	1	65017	65017	Raghubeer, S	Raghubeer, Shanel
AU	2	65018	65018	Nagiah, S	Nagiah, Savania
AU	3	65019	65019	Phulukdaree, A	Phulukdaree, Alisa
AU	4	65020	65020	Chuturgoon, A	Chuturgoon, Anil

Record 434 | WOS:000363821500008

A food contaminant ochratoxin A suppresses pregnane X receptor (PXR)-mediated CYP3A4 induction in primary cultures of human hepatocytes

Field	Position	Original line	Normalised line	Original value	Normalised value
AU	1	65109	65109	Doricakova, A	Doricakova, Aneta
AU	2	65110	65110	Vrzal, R	Vrzal, Radim

Record 435 | WOS:000363259300002

In vitro exposure of Penicillium mycotoxins with or without a modified yeast cell wall extract (mYCW) on bovine macrophages (BoMacs)

Field	Position	Original line	Normalised line	Original value	Normalised value
AU	1	65195	65195	Oh, SY	Oh, Se-Young
AU	2	65196	65196	Quinton, VM	Quinton, V_Margaret
AU	3	65197	65197	Boermans, HJ	Boermans, Herman_J
AU	4	65198	65198	Swamy, HVLN	Swamy, H_V_L_N
AU	5	65199	65199	Karrow, NA	Karrow, Niel_A
AF	2	65201	65201	Quinton, V. Margaret	Quinton, V_Margaret
AF	3	65202	65202	Boermans, Herman J.	Boermans, Herman_J
AF	4	65203	65203	Swamy, H. V. L. N.	Swamy, H_V_L_N
AF	5	65204	65204	Karrow, Niel A.	Karrow, Niel_A

Record 436 | WOS:000360322300014

Ochratoxin A at low concentrations inhibits in vitro growth of canine umbilical cord matrix mesenchymal stem cells through oxidative chromatin and DNA damage

Field	Position	Original line	Normalised line	Original value	Normalised value
AU	1	65301	65301	Rutigliano, L	Rutigliano, Lucia
AU	2	65302	65302	Valentini, L	Valentini, Luisa
AU	3	65303	65303	Martino, NA	Martino, Nicola_Antonio
AU	4	65304	65304	Pizzi, F	Pizzi, Flavia
AU	5	65305	65305	Zanghì, A	Zanghi, Antonina
AU	6	65306	65306	Dell'Aquila, ME	Dell'Aquila, Maria_Elena
AU	7	65307	65307	Minervini, F	Minervini, Fiorenza
AF	3	65310	65310	Martino, Nicola Antonio	Martino, Nicola_Antonio
AF	6	65313	65313	Dell'Aquila, Maria Elena	Dell'Aquila, Maria_Elena

Record 437 | WOS:000365417800020

The influence of ochratoxin A on DNA adduct formation by the carcinogen aristolochic acid in rats

Field	Position	Original line	Normalised line	Original value	Normalised value
AU	1	65464	65464	Stiborová, M	Stiborova, Marie
AU	2	65465	65465	Bárta, F	Barta, Frantisek
AU	3	65466	65466	Levová, K	Levova, Katerina
AU	4	65467	65467	Hodek, P	Hodek, Petr
AU	5	65468	65468	Frei, E	Frei, Eva
AU	6	65469	65469	Arlt, VM	Arlt, Volker_M

AU	7	65470	65470	Schmeiser, HH	Schmeiser, Heinz_H
AF	6	65476	65476	Arlt, Volker M.	Arlt, Volker_M
AF	7	65477	65477	Schmeiser, Heinz H.	Schmeiser, Heinz_H

Record 438 | WOS:000357839100054

Cross-linked chitosan polymers as generic adsorbents for simultaneous adsorption of multiple mycotoxins

Field	Position	Original line	Normalised line	Original value	Normalised value
AU	1	65648	65648	Zhao, ZY	Zhao, Zhiyong
AU	2	65649	65649	Liu, N	Liu, Ning
AU	3	65650	65650	Yang, LC	Yang, Lingchen
AU	4	65651	65651	Wang, JH	Wang, Jianhua
AU	5	65652	65652	Song, SQ	Song, Suquan
AU	6	65653	65653	Nie, DX	Nie, Dongxia
AU	7	65654	65654	Yang, XL	Yang, Xianli
AU	8	65655	65655	Hou, JF	Hou, Jiafa
AU	9	65656	65656	Wu, AB	Wu, Aibo
AF	2	65658	65658	Liu, Na	Liu, Ning

Record 439 | WOS:000370693801841

A contribution to hazard assessment of combined exposure to mycotoxins using in vitro toxicity testing

Field	Position	Original line	Normalised line	Original value	Normalised value
AU	2	65757	65757	Mendonça, I	Mendonca, I
AU	3	65758	65758	Louro, H	Louro, Henriqueta
AU	4	65759	65759	Pinhao, M	Pinhao, Mariana
AU	5	65760	65760	Loureiro, S	Loureiro, Susana
AU	6	65761	65761	Alvito, P	Alvito, Paula
AU	7	65762	65762	Silva, MJ	Silva, M_J
AF	1	65763	65763	Tavares, A.	Tavares, A
AF	2	65764	65764	Mendonca, I.	Mendonca, I
AF	3	65765	65765	Louro, H.	Louro, Henriqueta
AF	4	65766	65766	Pinhao, M.	Pinhao, Mariana
AF	5	65767	65767	Loureiro, S.	Loureiro, Susana
AF	6	65768	65768	Alvito, P.	Alvito, Paula
AF	7	65769	65769	Silva, M. J.	Silva, M_J

Record 440 | WOS:000361263500036

Cytotoxicity of the mycotoxins deoxynivalenol and ochratoxin A on Caco-2 cell line in presence of resveratrol

Field	Position	Original line	Normalised line	Original value	Normalised value
AU	2	65825	65825	González-Arias, CA	Gonzalez-Arias, Cyndia_A
AU	3	65826	65826	Ramos, AJ	Ramos, Antonio_J
AU	4	65827	65827	Sanchis, V	Sanchis, Vicente
AU	5	65828	65828	Fernández-Cruz, ML	Fernandez-Cruz, Maria_Luisa
AF	1	65829	65829	Cano-Sancho, G.	Cano-Sancho, G

AF	2	65830	65830	Gonzalez-Arias, C. A.	Gonzalez-Arias, Cyndia_A
AF	3	65831	65831	Ramos, A. J.	Ramos, Antonio_J
AF	4	65832	65832	Sanchis, V.	Sanchis, Vicente
AF	5	65833	65833	Fernandez-Cruz, M. L.	Fernandez-Cruz, Maria_Luisa

Record 441 | WOS:000367407400003

Dietary mycotoxins, co-exposure, and carcinogenesis in humans: Short review

Field	Position	Original line	Normalised line	Original value	Normalised value
AU	1	65942	65942	De Ruyck, K	de_Ruyck, Karl
AU	2	65943	65943	De Boevre, M	de_Boevre, Marthe
AU	3	65944	65944	Huybrechts, I	Huybrechts, Inge
AU	4	65945	65945	De Saeger, S	de_Saeger, Sarah
AF	1	65946	65946	De Ruyck, Karl	de_Ruyck, Karl
AF	2	65947	65947	De Boevre, Marthe	de_Boevre, Marthe
AF	4	65949	65949	De Saeger, Sarah	de_Saeger, Sarah

Record 442 | WOS:000364960400028

Comparative Ochratoxin Toxicity: A Review of the Available Data

Field	Position	Original line	Normalised line	Original value	Normalised value
AU	1	66117	66117	Heussner, AH	Heussner, Alexandra_H
AU	2	66118	66118	Bingle, LEH	Bingle, Lewis_E_H
AF	1	66119	66119	Heussner, Alexandra H.	Heussner, Alexandra_H
AF	2	66120	66120	Bingle, Lewis E. H.	Bingle, Lewis_E_H

Record 443 | WOS:000355886400028

Development of an aptasensor for the fast detection of Versicolorin A

Field	Position	Original line	Normalised line	Original value	Normalised value
AU	1	66382	66382	Jiang, HL	Jiang, Hai-Lan
AU	2	66383	66383	Liu, XY	Liu, Xiao-Yun
AU	3	66384	66384	Qiu, YX	Qiu, Yu-Xin
AU	4	66385	66385	Yao, DS	Yao, Dong-Sheng
AU	5	66386	66386	Xie, CF	Xie, Chun-Fang
AU	6	66387	66387	Liu, DL	Liu, Da-Ling
AF	3	66390	66390	Qiu, Yu-xin	Qiu, Yu-Xin

Record 444 | WOS:000359029000029

Cytotoxic and genotoxic potencies of single and combined spore extracts of airborne OTA-producing and OTA-non-producing Aspergilli in Human lung A549 cells

Field	Position	Original line	Normalised line	Original value	Normalised value
AU	1	66496	66496	Klaric, MS	Klaric, Maja_Segvic
AU	2	66497	66497	Despot, DJ	Despot, Daniela_Jaksic
AU	3	66498	66498	Kopjar, N	Kopjar, Nevenka

AU	4	66499	66499	Rasic, D	Rasic, Dubravka
AU	5	66500	66500	Kocsubé, S	Kocsube, Sandor
AU	6	66501	66501	Varga, J	Varga, Janos
AU	7	66502	66502	Peraica, M	Peraica, Maja
AF	1	66503	66503	Klaric, Maja Segvic	Klaric, Maja_Segvic
AF	2	66504	66504	Despot, Daniela Jaksic	Despot, Daniela_Jaksic

Record 445 | WOS:000362622500013

Ochratoxin A induces DNA damage and G2 phase arrest in human esophageal epithelium Het-1A cells in vitro

Field	Position	Original line	Normalised line	Original value	Normalised value
AU	1	66633	66633	Liu, J	Liu, Jing
AU	2	66634	66634	Wu, S	Wu, Sha
AU	3	66635	66635	Shen, HT	Shen, Haitao
AU	4	66636	66636	Cui, JF	Cui, Jinfeng
AU	5	66637	66637	Wang, Y	Wang, Yuan
AU	6	66638	66638	Xing, LX	Xing, Lingxiao
AU	7	66639	66639	Wang, JL	Wang, Junling
AU	8	66640	66640	Yan, X	Yan, Xia
AU	9	66641	66641	Zhang, XH	Zhang, Xianghong

Record 446 | WOS:000362860200019

Risk analysis of main mycotoxins occurring in food for children: An overview

Field	Position	Original line	Normalised line	Original value	Normalised value
AU	1	66755	66755	Raiola, A	Raiola, Assunta
AU	2	66756	66756	Tenore, GC	Tenore, Gian_Carlo
AU	3	66757	66757	Manyes, L	Manyes, Lara
AU	4	66758	66758	Meca, G	Meca, Giuseppe
AU	5	66759	66759	Ritieni, A	Ritieni, Alberto
AF	2	66761	66761	Tenore, Gian Carlo	Tenore, Gian_Carlo

Record 447 | WOS:000362551400018

Biomonitoring using dried blood spots: Detection of ochratoxin A and its degradation product 2'R-ochratoxin A in blood from coffee drinkers

Field	Position	Original line	Normalised line	Original value	Normalised value
AU	1	66997	66997	Cramer, B	Cramer, Benedikt
AU	2	66998	66998	Osteresch, B	Osteresch, Bernd
AU	3	66999	66999	Muñoz, KA	Munoz, Katherine_A
AU	4	67000	67000	Hillmann, H	Hillmann, Hartmut
AU	5	67001	67001	Sibrowski, W	Sibrowski, Walter
AU	6	67002	67002	Humpf, HU	Humpf, Hans-Ulrich
AF	3	67005	67005	Munoz, Katherine A.	Munoz, Katherine_A

Record 448 | WOS:000361078800019

Hepatotoxic effect of ochratoxin A and citrinin, alone and in combination, and protective effect of vitamin E: In vitro study in HepG2 cell

Field	Position	Original line	Normalised line	Original value	Normalised value
AU	1	67104	67104	Gayathri, L	Gayathri, Loganathan
AU	2	67105	67105	Dhivya, R	Dhivya, Rajakumar
AU	3	67106	67106	Dhanasekaran, D	Dhanasekaran, Dharumadurai
AU	4	67107	67107	Periasamy, VS	Periasamy, Vaiyapuri_S
AU	5	67108	67108	Alshatwi, AA	Alshatwi, Ali_A
AU	6	67109	67109	Akbarsha, MA	Akbarsha, Mohammad_A
AF	4	67113	67113	Periasamy, Vaiyapuri S.	Periasamy, Vaiyapuri_S
AF	5	67114	67114	Alshatwi, Ali A.	Alshatwi, Ali_A
AF	6	67115	67115	Akbarsha, Mohammad A.	Akbarsha, Mohammad_A

Record 449 | WOS:000361762200007

Effects of Dietary Exposure to Zearalenone (ZEN) on Carp (Cyprinus carpio L.)

Field	Position	Original line	Normalised line	Original value	Normalised value
AU	1	67258	67258	Pietsch, C	Pietsch, Constanze
AU	2	67259	67259	Kersten, S	Kersten, Susanne
AU	3	67260	67260	Valenta, H	Valenta, Hana
AU	4	67261	67261	Dänicke, S	Daenicke, Sven
AU	5	67262	67262	Schulz, C	Schulz, Carsten
AU	6	67263	67263	Burkhardt-Holm, P	Burkhardt-Holm, Patricia
AU	7	67264	67264	Junge, R	Junge, Ranka

Record 450 | WOS:000360343000001

Cytotoxic effects of oosporein isolated from endophytic fungus Cochliobolus kusanoi

Field	Position	Original line	Normalised line	Original value	Normalised value
AU	1	67404	67404	Ramesha, A	Ramesha, Alurappa
AU	3	67406	67406	Nirmaladevi, D	Nirmaladevi, Dhamodaran
AU	4	67407	67407	Gupta, VK	Gupta, Vijai_K
AU	6	67409	67409	Srinivas, C	Srinivas, Chowdappa
AF	2	67411	67411	Venkataramana, M.	Venkataramana, M
AF	4	67413	67413	Gupta, Vijai K.	Gupta, Vijai_K
AF	5	67414	67414	Chandranayaka, S.	Chandranayaka, S

Record 451 | WOS:000360202700022

Review on Mycotoxin Issues in Ruminants: Occurrence in Forages, Effects of Mycotoxin Ingestion on Health Status and Animal Performance and Practical Strategies to Counteract Their Negative Effects

Field	Position	Original line	Normalised line	Original value	Normalised value
AU	1	67509	67509	Gallo, A	Gallo, Antonio
AU	2	67510	67510	Giuberti, G	Giuberti, Gianluca

AU	3	67511	67511	Frisvad, JC	Frisvad, Jens_C
AU	4	67512	67512	Bertuzzi, T	Bertuzzi, Terenzio
AU	5	67513	67513	Nielsen, KF	Nielsen, Kristian_F
AF	3	67516	67516	Frisvad, Jens C.	Frisvad, Jens_C
AF	5	67518	67518	Nielsen, Kristian F.	Nielsen, Kristian_F

Record 452 | WOS:000358238100012

Ochratoxin A activates opposing c-MET/PI3K/Akt and MAPK/ERK 1-2 pathways in human proximal tubule HK-2 cells

Field	Position	Original line	Normalised line	Original value	Normalised value
AU	1	67892	67892	Özcan, Z	Ozcan, Zeynep
AU	2	67893	67893	Gül, G	Gul, Gizem
AU	3	67894	67894	Yaman, I	Yaman, Ibrahim

Record 453 | WOS:000357537400012

Effects of Chronic Ochratoxin A Exposure on p53 Heterozygous and p53 Homozygous Mice

Field	Position	Original line	Normalised line	Original value	Normalised value
AU	1	68032	68032	Bondy, GS	Bondy, Genevieve_S
AU	2	68033	68033	Caldwell, DS	Caldwell, Donald_S
AU	3	68034	68034	Aziz, SA	Aziz, Syed_A
AU	4	68035	68035	Coady, LC	Coady, Laurie_C
AU	5	68036	68036	Armstrong, CL	Armstrong, Cheryl_L
AU	6	68037	68037	Curran, IHA	Curran, Ivan_H_A
AU	7	68038	68038	Koffman, RL	Koffman, Robyn_L
AU	8	68039	68039	Kapal, K	Kapal, Kamla
AU	9	68040	68040	Lefebvre, DE	Lefebvre, David_E
AU	10	68041	68041	Mehta, R	Mehta, Rekha
AF	1	68042	68042	Bondy, Genevieve S.	Bondy, Genevieve_S
AF	2	68043	68043	Caldwell, Donald S.	Caldwell, Donald_S
AF	3	68044	68044	Aziz, Syed A.	Aziz, Syed_A
AF	4	68045	68045	Coady, Laurie C.	Coady, Laurie_C
AF	5	68046	68046	Armstrong, Cheryl L.	Armstrong, Cheryl_L
AF	6	68047	68047	Curran, Ivan H. A.	Curran, Ivan_H_A
AF	7	68048	68048	Koffman, Robyn L.	Koffman, Robyn_L
AF	9	68050	68050	Lefebvre, David E.	Lefebvre, David_E

Record 454 | WOS:000355330300006

Neurotoxic effects of ochratoxin A on the subventricular zone of adult mouse brain

Field	Position	Original line	Normalised line	Original value	Normalised value
AU	1	68174	68174	Paradells, S	Paradells, Sara
AU	2	68175	68175	Rocamonde, B	Rocamonde, Brenda
AU	3	68176	68176	Llinares, C	Llinares, Cristina
AU	4	68177	68177	Herranz-Pérez, V	Herranz-Perez, Vicente
AU	5	68178	68178	Jimenez, M	Jimenez, Misericordia

AU	6	68179	68179	Garcia-Verdugo, JM	Manuel_Garcia-Verdugo, Jose
AU	7	68180	68180	Zipancic, I	Zipancic, Ivan
AU	8	68181	68181	Soria, JM	Miguel_Soria, Jose
AU	9	68182	68182	Garcia-Esparza, MA	Angeles_Garcia-Esparza, Ma
AF	6	68188	68188	Manuel Garcia-Verdugo, Jose	Manuel_Garcia-Verdugo, Jose
AF	8	68190	68190	Miguel Soria, Jose	Miguel_Soria, Jose
AF	9	68191	68191	Angeles Garcia-Esparza, Ma	Angeles_Garcia-Esparza, Ma

Record 455 | WOS:000357490500001

Mycotoxins - prevention and decontamination by yeasts

Field	Position	Original line	Normalised line	Original value	Normalised value
AU	1	68357	68357	Pfliegler, WP	Pfliegler, Walter_P
AU	2	68358	68358	Pusztahelyi, T	Pusztahelyi, Tuende
AU	3	68359	68359	Pócsi, I	Pocsi, Istvan
AF	1	68360	68360	Pfliegler, Walter P.	Pfliegler, Walter_P

Record 456 | WOS:000362080300002

Structure elucidation and in vitro cytotoxicity of ochratoxin α amide, a new degradation product of ochratoxin A

Field	Position	Original line	Normalised line	Original value	Normalised value
AU	1	68542	68542	Bittner, A	Bittner, Andrea
AU	2	68543	68543	Cramer, B	Cramer, Benedikt
AU	3	68544	68544	Harrer, H	Harrer, Henning
AU	4	68545	68545	Humpf, HU	Humpf, Hans-Ulrich

Record 457 | WOS:000357242500002

Polyphenols as possible bioprotectors against cytotoxicity and DNA damage induced by ochratoxin A

Field	Position	Original line	Normalised line	Original value	Normalised value
AU	1	68623	68623	Cariddi, LN	Cariddi, L_N
AU	2	68624	68624	Sabini, MC	Sabini, M_C
AU	3	68625	68625	Escobar, FM	Escobar, F_M
AU	5	68627	68627	Mañas, F	Manas, F
AU	7	68629	68629	Comini, LR	Comini, L_R
AU	8	68630	68630	Sabini, LI	Sabini, L_I
AU	9	68631	68631	Dalcero, AM	Dalcero, A_M
AF	1	68632	68632	Cariddi, L. N.	Cariddi, L_N
AF	2	68633	68633	Sabini, M. C.	Sabini, M_C
AF	3	68634	68634	Escobar, F. M.	Escobar, F_M
AF	4	68635	68635	Montironi, I.	Montironi, I
AF	5	68636	68636	Manas, F.	Manas, F
AF	6	68637	68637	Iglesias, D.	Iglesias, D
AF	7	68638	68638	Comini, L. R.	Comini, L_R
AF	8	68639	68639	Sabini, L. I.	Sabini, L_I
AF	9	68640	68640	Dalcero, A. M.	Dalcero, A_M

Record 458 | WOS:000353230400005

In vitro and in vivo metabolism of ochratoxin A: a comparative study using ultra-performance liquid chromatography-quadrupole/time-of-flight hybrid mass spectrometry

Field	Position	Original line	Normalised line	Original value	Normalised value
AU	1	68748	68748	Yang, S	Yang, Shupeng
AU	2	68749	68749	Zhang, H	Zhang, Huiyan
AU	3	68750	68750	De Saeger, S	de_Saeger, Sarah
AU	4	68751	68751	De Boevre, M	de_Boevre, Marthe
AU	5	68752	68752	Sun, FF	Sun, Feifei
AU	6	68753	68753	Zhang, SX	Zhang, Suxia
AU	7	68754	68754	Cao, XY	Cao, Xingyuan
AU	8	68755	68755	Wang, ZH	Wang, Zhanhui
AF	3	68758	68758	De Saeger, Sarah	de_Saeger, Sarah
AF	4	68759	68759	De Boevre, Marthe	de_Boevre, Marthe

Record 459 | WOS:000351834600019

Assessment and characterisation of yeast-based products intended to mitigate ochratoxin exposure using in vitro and in vivo models

Field	Position	Original line	Normalised line	Original value	Normalised value
AU	1	68857	68857	Pfohl-Leszkowicz, A	Pfohl-Leszkowicz, Annie
AU	2	68858	68858	Hadjeba-Medjdoub, K	Hadjeba-Medjdoub, Kheira
AU	3	68859	68859	Ballet, N	Ballet, Nathalie
AU	5	68861	68861	Fink-Gremmels, J	Fink-Gremmels, Johanna
AF	1	68862	68862	Pfohl-Leszkowicz, A.	Pfohl-Leszkowicz, Annie
AF	2	68863	68863	Hadjeba-Medjdoub, K.	Hadjeba-Medjdoub, Kheira
AF	3	68864	68864	Ballet, N.	Ballet, Nathalie
AF	4	68865	68865	Schrickx, J.	Schrickx, J
AF	5	68866	68866	Fink-Gremmels, J.	Fink-Gremmels, Johanna

Record 460 | WOS:000353889600047

Selenium Alleviates Porcine Nephrotoxicity of Ochratoxin A by Improving Selenoenzyme Expression In Vitro

Field	Position	Original line	Normalised line	Original value	Normalised value
AU	1	68993	68993	Gan, F	Gan, Fang
AU	2	68994	68994	Xue, HX	Xue, Hongxia
AU	3	68995	68995	Huang, Y	Huang, Yu
AU	4	68996	68996	Pan, CL	Pan, Cuiling
AU	5	68997	68997	Huang, KH	Huang, Kehe

Record 461 | WOS:000349553900004

Determination of Aflatoxins and Ochratoxins in Sicilian Sweet Wines by High-Performance Liquid Chromatography with Fluorometric Detection and Immunoaffinity Cleanup

Field	Position	Original line	Normalised line	Original value	Normalised value
AU	1	69104	69104	Di Stefano, V	di_Stefano, Vita
AU	2	69105	69105	Pitonzo, R	Pitonzo, Rosa

AU	3	69106	69106	Avellone, G	Avellone, Giuseppe
AU	4	69107	69107	Di Fiore, A	di_Fiore, Antonina
AU	5	69108	69108	Monte, L	Monte, Lucio
AU	6	69109	69109	Ogorka, AZT	Ogorka, Agata_Zofia_Tabis
AF	1	69110	69110	Di Stefano, Vita	di_Stefano, Vita
AF	4	69113	69113	Di Fiore, Antonina	di_Fiore, Antonina
AF	6	69115	69115	Ogorka, Agata Zofia Tabis	Ogorka, Agata_Zofia_Tabis

Record 462 | WOS:000343780400062

In vitro genotoxicity of mycotoxins ochratoxin A and fumonisin B1 could be prevented by sodium copper chlorophyllin - Implication to their genotoxic mechanism

Field	Position	Original line	Normalised line	Original value	Normalised value
AU	1	69205	69205	Domijan, AM	Domijan, Ana-Marija
AU	2	69206	69206	Gajski, G	Gajski, Goran
AU	3	69207	69207	Jovanovic, IN	Jovanovic, Ivana_Novak
AU	4	69208	69208	Genic, M	Genic, Marko
AU	5	69209	69209	Garaj-Vrhovac, V	Garaj-Vrhovac, Vera
AF	3	69212	69212	Jovanovic, Ivana Novak	Jovanovic, Ivana_Novak

Record 463 | WOS:000351485000004

Ochratoxin A promotes porcine circovirus type 2 replication in vitro and in vivo

Field	Position	Original line	Normalised line	Original value	Normalised value
AU	1	69325	69325	Gan, F	Gan, Fang
AU	2	69326	69326	Zhang, ZQ	Zhang, Zheqian
AU	3	69327	69327	Hu, ZH	Hu, Zhihua
AU	4	69328	69328	Hesketh, J	Hesketh, John
AU	5	69329	69329	Xue, HX	Xue, Hongxia
AU	6	69330	69330	Chen, XX	Chen, Xingxiang
AU	7	69331	69331	Hao, S	Hao, Shu
AU	8	69332	69332	Huang, Y	Huang, Yu
AU	9	69333	69333	Ezea, PC	Ezea, Patience_Cole
AU	10	69334	69334	Parveen, F	Parveen, Fahmida
AU	11	69335	69335	Huang, KH	Huang, Kehe
AF	9	69344	69344	Ezea, Patience Cole	Ezea, Patience_Cole

Record 464 | WOS:000350023000003

Ochratoxin A and its effects on immunity

Field	Position	Original line	Normalised line	Original value	Normalised value
AU	1	69471	69471	Marin, DE	Marin, Daniela_E
AU	2	69472	69472	Taranu, I	Taranu, Ionelia
AF	1	69473	69473	Marin, Daniela E.	Marin, Daniela_E

Record 465 | WOS:000350094000016

Genotoxicity of Aflatoxin B1 and Ochratoxin A after simultaneous application of the in vivo micronucleus and comet assay

Field	Position	Original line	Normalised line	Original value	Normalised value
AU	1	69628	69628	Corcuera, LA	Corcuera, Laura-Ana
AU	2	69629	69629	Vettorazzi, A	Vettorazzi, Ariane
AU	3	69630	69630	Arbillaga, L	Arbillaga, Leire
AU	4	69631	69631	Pérez, N	Perez, Noemi
AU	5	69632	69632	Gil, AG	Gloria_Gil, Ana
AU	6	69633	69633	Azqueta, A	Azqueta, Amaya
AU	7	69634	69634	González-Peñas, E	Gonzalez-Penas, Elena
AU	8	69635	69635	García-Jalón, JA	Antonio_Garcia-Jalon, Jose
AU	9	69636	69636	de Cerain, AL	Lopez_de_Cerain, Adela
AF	5	69641	69641	Gloria Gil, Ana	Gloria_Gil, Ana
AF	8	69644	69644	Antonio Garcia-Jalon, Jose	Antonio_Garcia-Jalon, Jose
AF	9	69645	69645	Lopez de Cerain, Adela	Lopez_de_Cerain, Adela

Record 466 | WOS:000362800100005

Mycotoxin mechanisms of action and health impact: 'in vitro' or 'in vivo' tests, that is the question

Field	Position	Original line	Normalised line	Original value	Normalised value
AU	2	69772	69772	Giromini, C	Giromini, Carlotta
AU	3	69773	69773	Baldi, A	Baldi, Antonella
AF	1	69774	69774	Cheli, F.	Cheli, F
AF	2	69775	69775	Giromini, C.	Giromini, Carlotta
AF	3	69776	69776	Baldi, A.	Baldi, Antonella

Record 467 | WOS:000351213600013

Toxic Effects of Aflatoxin B1 on Embryonic Development of Zebrafish (Danio rerio): Potential Activity of Piceatannol Encapsulated Chitosan/poly (Lactic Acid) Nanoparticles

Field	Position	Original line	Normalised line	Original value	Normalised value
AU	1	69975	69975	Dhanapal, J	Dhanapal, Jeevitha
AU	2	69976	69976	Ravindrran, MB	Ravindrran, Malathy_Balaraman
AU	3	69977	69977	Baskar, SK	Baskar, Santhosh_K
AF	2	69979	69979	Ravindrran, Malathy Balaraman	Ravindrran, Malathy_Balaraman
AF	3	69980	69980	Baskar, Santhosh K.	Baskar, Santhosh_K

Record 468 | WOS:000349133200010

Bioaccessibility of ochratoxin A from red wine in an in vitro dynamic gastrointestinal model

Field	Position	Original line	Normalised line	Original value	Normalised value
AU	1	70108	70108	González-Arias, CA	Gonzalez-Arias, Cyndia_A
AU	3	70110	70110	Marin, S	Marin, Sonia
AU	4	70111	70111	Sanchis, V	Sanchis, Vicente
AU	5	70112	70112	Ramos, AJ	Ramos, Antonio_J

AF	1	70113	70113	Gonzalez-Arias, C. A.	Gonzalez-Arias, Cyndia_A
AF	2	70114	70114	Piquer-Garcia, I.	Piquer-Garcia, I
AF	3	70115	70115	Marin, S.	Marin, Sonia
AF	4	70116	70116	Sanchis, V.	Sanchis, Vicente
AF	5	70117	70117	Ramos, A. J.	Ramos, Antonio_J

Record 469 | WOS:000415214500006

Current Insights into Mycotoxins

Field	Position	Original line	Normalised line	Original value	Normalised value
AU	1	70204	70204	Karimi, G	Karimi, Gholamreza
AU	2	70205	70205	Mehri, S	Mehri, Soghra

Record 470 | WOS:000360184800009

Dynamic changes of global DNA methylation and hypermethylation of cell adhesion-related genes in rat kidneys in response to ochratoxin A

Field	Position	Original line	Normalised line	Original value	Normalised value
AU	2	70307	70307	Gao, J	Gao, Jing
AU	4	70309	70309	Qi, X	Qi, Xiaozhe
AF	1	70313	70313	Li, X.	Li, X
AF	2	70314	70314	Gao, J.	Gao, Jing
AF	3	70315	70315	Huang, K.	Huang, K
AF	4	70316	70316	Qi, X.	Qi, Xiaozhe
AF	5	70317	70317	Dai, Q.	Dai, Q
AF	6	70318	70318	Mei, X.	Mei, X
AF	7	70319	70319	Xu, W.	Xu, W

Record 471 | WOS:000367698600008

POTENTIAL GENOTOXICITY OF ACETAMIPRID AND PROPINEB SINGLY OR IN COMBINATION IN CULTURED HUMAN PERIPHERAL BLOOD LYMPHOCYTES BY USING MN ASSAY

Field	Position	Original line	Normalised line	Original value	Normalised value
AU	1	70429	70429	Muranli, FDG	Muranli, Fulya_Dilek_Gokalp
AU	2	70430	70430	Rasgele, PG	Rasgele, Pinar_Goc
AU	3	70431	70431	Kekecoglu, M	Kekecoglu, Meral
AU	4	70432	70432	Kanev, M	Kanev, Martin
AU	5	70433	70433	Ozdemir, K	Ozdemir, Kezban
AF	1	70434	70434	Muranli, Fulya Dilek Gokalp	Muranli, Fulya_Dilek_Gokalp
AF	2	70435	70435	Rasgele, Pinar Goc	Rasgele, Pinar_Goc

Record 472 | WOS:000347274600033

Low doses of ochratoxin A induce micronucleus formation and delay DNA repair in human lymphocytes

Field	Position	Original line	Normalised line	Original value	Normalised value
AU	1	70553	70553	González-Arias, CA	Gonzalez-Arias, Cyndia_A
AU	2	70554	70554	Benitez-Trinidad, AB	Benitez-Trinidad, Alma_B

AU	3	70555	70555	Sordo, M	Sordo, Monserrat
AU	4	70556	70556	Robledo-Marenco, L	Robledo-Marenco, Lourdes
AU	5	70557	70557	Medina-Díaz, IM	Medina-Diaz, Irma_M
AU	6	70558	70558	Barrón-Vivanco, BS	Barron-Vivanco, Briscia_S
AU	7	70559	70559	Marín, S	Marin, Sonia
AU	8	70560	70560	Sanchis, V	Sanchis, Vicente
AU	9	70561	70561	Ramos, AJ	Ramos, Antonio_J
AU	10	70562	70562	Rojas-García, AE	Rojas-Garcia, Aurora_E
AF	1	70563	70563	Gonzalez-Arias, Cyndia A.	Gonzalez-Arias, Cyndia_A
AF	2	70564	70564	Benitez-Trinidad, Alma B.	Benitez-Trinidad, Alma_B
AF	5	70567	70567	Medina-Diaz, Irma M.	Medina-Diaz, Irma_M
AF	6	70568	70568	Barron-Vivanco, Briscia S.	Barron-Vivanco, Briscia_S
AF	9	70571	70571	Ramos, Antonio J.	Ramos, Antonio_J
AF	10	70572	70572	Rojas-Garcia, Aurora E.	Rojas-Garcia, Aurora_E

Record 473 | WOS:000349038800005

In vitro effects of ochratoxin A, deoxynivalenol and zearalenone on cell viability and E-ADA activity in broiler chickens lymphocytes

Field	Position	Original line	Normalised line	Original value	Normalised value
AU	1	70698	70698	Lautert, C	Lautert, Claudia
AU	2	70699	70699	Ferreiro, L	Ferreiro, Laerte
AU	3	70700	70700	Zimmermann, CEP	Zimmermann, Carine_E_P
AU	4	70701	70701	Castilhos, LG	Castilhos, Livia_G
AU	5	70702	70702	de Jesus, FPK	de_Jesus, Francielli_P_K
AU	6	70703	70703	Zanette, RA	Zanette, Regis_A
AU	7	70704	70704	Leal, DBR	Leal, Daniela_B_R
AU	8	70705	70705	Santurio, JM	Santurio, Janio_M
AF	3	70708	70708	Zimmermann, Carine E. P.	Zimmermann, Carine_E_P
AF	4	70709	70709	Castilhos, Livia G.	Castilhos, Livia_G
AF	5	70710	70710	de Jesus, Francielli P. K.	de_Jesus, Francielli_P_K
AF	6	70711	70711	Zanette, Regis A.	Zanette, Regis_A
AF	7	70712	70712	Leal, Daniela B. R.	Leal, Daniela_B_R
AF	8	70713	70713	Santurio, Janio M.	Santurio, Janio_M

Record 474 | WOS:000344696100002

The mycotoxin beauvericin induces apoptotic cell death in H4IIE hepatoma cells accompanied by an inhibition of NF-κB-activity and modulation of MAP-kinases

Field	Position	Original line	Normalised line	Original value	Normalised value
AU	1	70812	70812	Wätjen, W	Waetjen, Wim
AU	2	70813	70813	Debbab, A	Debbab, Abdessamab
AU	3	70814	70814	Hohlfeld, A	Hohlfeld, Anke
AU	4	70815	70815	Chovolou, Y	Chovolou, Yvonni
AU	5	70816	70816	Proksch, P	Proksch, Peter

Record 475 | WOS:000344980400016

Ochratoxin A induces rat renal carcinogenicity with limited induction of oxidative stress responses

Field	Position	Original line	Normalised line	Original value	Normalised value
AU	1	70905	70905	Qi, XZ	Qi, Xiaozhe
AU	2	70906	70906	Yu, T	Yu, Tao
AU	3	70907	70907	Zhu, LY	Zhu, Liye
AU	4	70908	70908	Gao, J	Gao, Jing
AU	5	70909	70909	He, XY	He, Xiaoyun
AU	6	70910	70910	Huang, KL	Huang, Kunlun
AU	7	70911	70911	Luo, YB	Luo, Yunbo
AU	8	70912	70912	Xu, WT	Xu, Wentao

Record 476 | WOS:000343730400082

A New Ochratoxin A Biodegradation Strategy Using Cupriavidus basilensis Or16 Strain

Field	Position	Original line	Normalised line	Original value	Normalised value
AU	1	71025	71025	Ferenczi, S	Ferenczi, Szilamer
AU	2	71026	71026	Cserháti, M	Cserhati, Matyas
AU	3	71027	71027	Krifaton, C	Krifaton, Csilla
AU	4	71028	71028	Szoboszlay, S	Szoboszlay, Sandor
AU	5	71029	71029	Kukolya, J	Kukolya, Jozsef
AU	6	71030	71030	Szoke, Z	Szoke, Zsuzsanna
AU	7	71031	71031	Koszegi, B	Koszegi, Balazs
AU	8	71032	71032	Albert, M	Albert, Mihaly
AU	9	71033	71033	Barna, T	Barna, Terez
AU	10	71034	71034	Mézes, M	Mezes, Miklos
AU	11	71035	71035	Kovács, KJ	Kovacs, Krisztina_J
AU	12	71036	71036	Kriszt, B	Kriszt, Balazs
AF	11	71047	71047	Kovacs, Krisztina J.	Kovacs, Krisztina_J

Record 477 | WOS:000341677200007

Biodegradation of ochratoxin A by Pediococcus parvulus isolated from Douro wines

Field	Position	Original line	Normalised line	Original value	Normalised value
AU	1	71167	71167	Abrunhosa, L	Abrunhosa, Luis
AU	2	71168	71168	Inês, A	Ines, Antonio
AU	3	71169	71169	Rodrigues, AI	Rodrigues, Ana_I
AU	4	71170	71170	Guimaraes, A	Guimaraes, Ana
AU	5	71171	71171	Pereira, VL	Pereira, Vania_L
AU	6	71172	71172	Parpot, P	Parpot, Pier
AU	7	71173	71173	Mendes-Faia, A	Mendes-Faia, Arlete
AU	8	71174	71174	Venâncio, A	Venancio, Armando
AF	3	71177	71177	Rodrigues, Ana I.	Rodrigues, Ana_I
AF	5	71179	71179	Pereira, Vania L.	Pereira, Vania_L

Record 478 | WOS:000343630800010

Biological detoxification of zearalenone by Aspergillus niger strain FS10

Field	Position	Original line	Normalised line	Original value	Normalised value
AU	1	71310	71310	Sun, XL	Sun, Xiulan
AU	2	71311	71311	He, XX	He, Xingxing
AU	3	71312	71312	Xue, KS	Xue, Kathy_Siyu
AU	4	71313	71313	Li, Y	Li, Yun
AU	5	71314	71314	Xu, D	Xu, Dan
AU	6	71315	71315	Qian, H	Qian, He
AF	3	71318	71318	Xue, Kathy Siyu	Xue, Kathy_Siyu

Record 479 | WOS:000343630800034

Ochratoxin A kinetics: A review of analytical methods and studies in rat model

Field	Position	Original line	Normalised line	Original value	Normalised value
AU	1	71425	71425	Vettorazzi, A	Vettorazzi, Ariane
AU	2	71426	71426	González-Peñas, E	Gonzalez-Penas, Elena
AU	3	71427	71427	de Cerain, AL	Lopez_de_Cerain, Adela
AF	1	71428	71428	Vettorazzi, A.	Vettorazzi, Ariane
AF	2	71429	71429	Gonzalez-Penas, E.	Gonzalez-Penas, Elena
AF	3	71430	71430	Lopez de Cerain, A.	Lopez_de_Cerain, Adela

Record 480 | WOS:000340827200008

Synergism of ochratoxin B and calcium-channel antagonist verapamil caused mitochondrial dysfunction

Field	Position	Original line	Normalised line	Original value	Normalised value
AU	1	71582	71582	Chatopadhyay, P	Chatopadhyay, Pronobesh
AU	2	71583	71583	Tariang, B	Tariang, Banlumlang
AU	3	71584	71584	Agnihotri, A	Agnihotri, Amit
AU	4	71585	71585	Veer, V	Veer, Vijay

Record 481 | WOS:000340837600026

Development of an in vitro method for the prediction of mycotoxin binding on yeast-based products: case of aflatoxin B1, zearalenone and ochratoxin A

Field	Position	Original line	Normalised line	Original value	Normalised value
AU	1	71668	71668	Faucet-Marquis, V	Faucet-Marquis, Virginie
AU	2	71669	71669	Joannis-Cassan, C	Joannis-Cassan, Claire
AU	3	71670	71670	Hadjeba-Medjdoub, K	Hadjeba-Medjdoub, Kheira
AU	4	71671	71671	Ballet, N	Ballet, Nathalie
AU	5	71672	71672	Pfohl-Leszkowicz, A	Pfohl-Leszkowicz, Annie

Record 482 | WOS:000342990200034

Combination of Metagenomics and Culture-Based Methods to Study the Interaction Between Ochratoxin A and Gut Microbiota

Field	Position	Original line	Normalised line	Original value	Normalised value
AU	1	71793	71793	Guo, MZ	Guo, Mingzhang
AU	2	71794	71794	Huang, KL	Huang, Kunlun

AU	3	71795	71795	Chen, SY	Chen, Siyuan
AU	4	71796	71796	Qi, XZ	Qi, Xiaozhe
AU	5	71797	71797	He, XY	He, Xiaoyun
AU	6	71798	71798	Cheng, WH	Cheng, Wen-Hsing
AU	7	71799	71799	Luo, YB	Luo, Yunbo
AU	8	71800	71800	Xia, K	Xia, Kai
AU	9	71801	71801	Xu, WT	Xu, Wentao

Record 483 | WOS:000340087200003

Biological detoxification of mycotoxins: a review

Field	Position	Original line	Normalised line	Original value	Normalised value
AU	1	71904	71904	Hathout, AS	Hathout, Amal_S
AU	2	71905	71905	Aly, SE	Aly, Soher_E
AF	1	71906	71906	Hathout, Amal S.	Hathout, Amal_S
AF	2	71907	71907	Aly, Soher E.	Aly, Soher_E

Record 484 | WOS:000341466700016

Hepatotoxicity and genotoxicity of patulin in mice, and its modulation by green tea polyphenols administration

Field	Position	Original line	Normalised line	Original value	Normalised value
AU	1	72140	72140	Song, EQ	Song, Erqun
AU	2	72141	72141	Xia, XM	Xia, Xiaomin
AU	3	72142	72142	Su, CY	Su, Chuanyang
AU	4	72143	72143	Dong, WJ	Dong, Wenjing
AU	5	72144	72144	Xian, YP	Xian, Yaping
AU	6	72145	72145	Wang, W	Wang, Wei
AU	7	72146	72146	Song, Y	Song, Yang

Record 485 | WOS:000341466700027

Cytotoxicity induced by ochratoxin A, zearalenone, and α-zearalenol: Effects of individual and combined treatment

Field	Position	Original line	Normalised line	Original value	Normalised value
AU	1	72268	72268	Wang, HW	Wang, H_W
AU	2	72269	72269	Wang, JQ	Wang, Jia-Qi
AU	3	72270	72270	Zheng, BQ	Zheng, B_Q
AU	4	72271	72271	Li, SL	Li, S_L
AU	5	72272	72272	Zhang, YD	Zhang, Y_D
AU	6	72273	72273	Li, FD	Li, F_D
AU	7	72274	72274	Zheng, N	Zheng, Nan
AF	1	72275	72275	Wang, H. W.	Wang, H_W
AF	2	72276	72276	Wang, J. Q.	Wang, Jia-Qi
AF	3	72277	72277	Zheng, B. Q.	Zheng, B_Q
AF	4	72278	72278	Li, S. L.	Li, S_L
AF	5	72279	72279	Zhang, Y. D.	Zhang, Y_D
AF	6	72280	72280	Li, F. D.	Li, F_D

AF	7	72281	72281	Zheng, N.	Zheng, Nan

Record 486 | WOS:000338616800026

Cytotoxicity and immunotoxicity of cyclopiazonic acid on human cells

Field	Position	Original line	Normalised line	Original value	Normalised value
AU	1	72376	72376	Hymery, N	Hymery, Nolwenn
AU	2	72377	72377	Masson, F	Masson, Floriane
AU	3	72378	72378	Barbier, G	Barbier, Georges
AU	4	72379	72379	Coton, E	Coton, Emmanuel

Record 487 | WOS:000338817900023

Central role of Nix in the autophagic response to ochratoxin A

Field	Position	Original line	Normalised line	Original value	Normalised value
AU	1	72492	72492	Shen, XL	Shen, Xiao_Li
AU	2	72493	72493	Zhang, BY	Zhang, Boyang
AU	3	72494	72494	Liang, R	Liang, Rui
AU	4	72495	72495	Cheng, WH	Cheng, Wen-Hsing
AU	5	72496	72496	Xu, WT	Xu, Wentao
AU	6	72497	72497	Luo, YB	Luo, Yunbo
AU	7	72498	72498	Zhao, CH	Zhao, Changhui
AU	8	72499	72499	Huang, KL	Huang, Kunlun
AF	1	72500	72500	Shen, Xiao Li	Shen, Xiao_Li
AF	6	72505	72505	Luo, YunBo	Luo, Yunbo

Record 488 | WOS:000336730800004

Toxicity of the mycotoxin citrinin and its metabolite dihydrocitrinone and of mixtures of citrinin and ochratoxin A in vitro

Field	Position	Original line	Normalised line	Original value	Normalised value
AU	1	72609	72609	Föllmann, W	Foellmann, Wolfram
AU	2	72610	72610	Behm, C	Behm, Claudia
AU	3	72611	72611	Degen, GH	Degen, Gisela_H
AF	3	72614	72614	Degen, Gisela H.	Degen, Gisela_H

Record 489 | WOS:000362078100003

Modulation of innate and antigen-specific immune functions directed against Listeria monocytogenes by fungal toxins in vitro

Field	Position	Original line	Normalised line	Original value	Normalised value
AF	1	72714	72714	Herter, I.	Herter, I
AF	2	72715	72715	Geginat, G.	Geginat, G
AF	3	72716	72716	Hof, H.	Hof, H
AF	4	72717	72717	Kupfahl, C.	Kupfahl, C

Record 490 | WOS:000333247100013

Downregulation of Rad51 participates in OTA-induced DNA double-strand breaks in GES-1 cells in vitro

Field	Position	Original line	Normalised line	Original value	Normalised value
AU	1	72809	72809	Lian, HG	Lian, Hongguang
AU	2	72810	72810	Cui, JF	Cui, Jinfeng
AU	3	72811	72811	Wang, Y	Wang, Yuan
AU	4	72812	72812	Liu, J	Liu, Jing
AU	5	72813	72813	Wang, J	Wang, Juan
AU	6	72814	72814	Shen, HT	Shen, Haitao
AU	7	72815	72815	Xing, LX	Xing, Lingxiao
AU	8	72816	72816	Wang, JL	Wang, Junling
AU	9	72817	72817	Yan, X	Yan, Xia
AU	10	72818	72818	Zhang, XH	Zhang, Xianghong

Record 491 | WOS:000335104500012

Protective role of the mitochondrial Lon protease 1 in ochratoxin A-induced cytotoxicity in HEK293 cells

Field	Position	Original line	Normalised line	Original value	Normalised value
AU	1	72927	72927	Zhang, BY	Zhang, Boyang
AU	2	72928	72928	Shen, XL	Shen, Xiao_Li
AU	3	72929	72929	Liang, R	Liang, Rui
AU	4	72930	72930	Li, YZ	Li, Yuzhe
AU	5	72931	72931	Huang, KL	Huang, Kunlun
AU	6	72932	72932	Zhao, CH	Zhao, Changhui
AU	7	72933	72933	Luo, YB	Luo, Yunbo
AU	8	72934	72934	Xu, WT	Xu, Wentao
AF	2	72936	72936	Shen, Xiao Li	Shen, Xiao_Li

Record 492 | WOS:000332034900015

Mutant frequency in comparison to oxidative DNA damage induced by ochratoxin A in L5178Y tk+/- (3.7.2C) mouse lymphoma cells

Field	Position	Original line	Normalised line	Original value	Normalised value
AU	1	73085	73085	Ali, R	Ali, Rahat
AU	2	73086	73086	Guo, XQ	Guo, Xiaoqing
AU	3	73087	73087	Lin, HX	Lin, Haixia
AU	4	73088	73088	Khan, QM	Khan, Qaiser_M
AU	5	73089	73089	Ismail, M	Ismail, Muhammad
AU	6	73090	73090	Waheed, U	Waheed, Usman
AU	7	73091	73091	Ali, T	Ali, Tayyaba
AU	8	73092	73092	Bhalli, JA	Bhalli, Javed_A
AF	4	73096	73096	Khan, Qaiser M.	Khan, Qaiser_M
AF	8	73100	73100	Bhalli, Javed A.	Bhalli, Javed_A

Record 493 | WOS:000335760200003

Analysis of Individual and Combined Effects of Ochratoxin A and Zearalenone on HepG2 and KK-1 Cells with Mathematical Models

Field	Position	Original line	Normalised line	Original value	Normalised value
AU	1	73205	73205	Li, YZ	Li, Yuzhe
AU	2	73206	73206	Zhang, BY	Zhang, Boyang
AU	3	73207	73207	He, XY	He, Xiaoyun
AU	4	73208	73208	Cheng, WH	Cheng, Wen-Hsing
AU	5	73209	73209	Xu, WT	Xu, Wentao
AU	6	73210	73210	Luo, YB	Luo, Yunbo
AU	7	73211	73211	Liang, R	Liang, Rui
AU	8	73212	73212	Luo, HS	Luo, Haoshu
AU	9	73213	73213	Huang, KL	Huang, Kunlun

Record 494 | WOS:000338818900001

Flavonoid diosmetin increases ATP levels in kidney cells and relieves ATP depleting effect of ochratoxin A

Field	Position	Original line	Normalised line	Original value	Normalised value
AU	1	73331	73331	Poór, M	Poor, Miklos
AU	2	73332	73332	Veres, B	Veres, Balazs
AU	3	73333	73333	Jakus, PB	Jakus, Peter_B
AU	4	73334	73334	Antus, C	Antus, Csenge
AU	5	73335	73335	Montskó, G	Montsko, Gergely
AU	6	73336	73336	Zrínyi, Z	Zrinyi, Zita
AU	7	73337	73337	Vladimir-Knezevic, S	Vladimir-Knezevic, Sanda
AU	8	73338	73338	Petrik, J	Petrik, Jozsef
AU	9	73339	73339	Koszegi, T	Koszegi, Tumas
AF	3	73342	73342	Jakus, Peter B.	Jakus, Peter_B

Record 495 | WOS:000332451700009

Natural feed contaminant zearalenone decreases the expressions of important pro- and anti-inflammatory mediators and mitogen-activated protein kinase/NF-kB signalling molecules in pigs

Field	Position	Original line	Normalised line	Original value	Normalised value
AU	1	73459	73459	Pistol, GC	Pistol, Gina_Cecilia
AU	2	73460	73460	Gras, MA	Gras, Mihail_Alexandru
AU	3	73461	73461	Marin, DE	Marin, Daniela_Eliza
AU	4	73462	73462	Israel-Roming, F	Israel-Roming, Florentina
AU	5	73463	73463	Stancu, M	Stancu, Mariana
AU	6	73464	73464	Taranu, I	Taranu, Ionelia
AF	1	73465	73465	Pistol, Gina Cecilia	Pistol, Gina_Cecilia
AF	2	73466	73466	Gras, Mihail Alexandru	Gras, Mihail_Alexandru
AF	3	73467	73467	Marin, Daniela Eliza	Marin, Daniela_Eliza

Record 496 | WOS:000334436600041

Mycotoxins' Activity at Toxic and Sub-Toxic Concentrations: Differential Cytotoxic and Genotoxic Effects of Single and Combined Administration of Sterigmatocystin, Ochratoxin A and Citrinin on the Hepatocellular Cancer Cell Line Hep3B

Field	Position	Original line	Normalised line	Original value	Normalised value
AU	1	73607	73607	Anninou, N	Anninou, Nikolia
AU	2	73608	73608	Chatzaki, E	Chatzaki, Ekaterini
AU	3	73609	73609	Papachristou, F	Papachristou, Fotini
AU	4	73610	73610	Pitiakoudis, M	Pitiakoudis, Michail
AU	5	73611	73611	Simopoulos, C	Simopoulos, Constantinos

Record 497 | WOS:000335746400013

Deficient Glutathione in the Pathophysiology of Mycotoxin-Related Illness

Field	Position	Original line	Normalised line	Original value	Normalised value
AU	1	73734	73734	Guilford, FT	Guilford, Frederick_T
AU	2	73735	73735	Hope, J	Hope, Janette
AF	1	73736	73736	Guilford, Frederick T.	Guilford, Frederick_T

Record 498 | WOS:000329417100014

Interaction effects of Fusarium enniatins (A, A1, B and B1) combinations on in vitro cytotoxicity of Caco-2 cells

Field	Position	Original line	Normalised line	Original value	Normalised value
AU	1	73880	73880	Prosperini, A	Prosperini, Alessandra
AU	2	73881	73881	Font, G	Font, Guillermina
AU	3	73882	73882	Ruiz, MJ	Ruiz, Maria-Jose
AF	1	73883	73883	Prosperini, A.	Prosperini, Alessandra
AF	2	73884	73884	Font, G.	Font, Guillermina
AF	3	73885	73885	Ruiz, M. J.	Ruiz, Maria-Jose

Record 499 | WOS:000334568500006

Cell-based models for mycotoxin screening and toxicity evaluation: an update

Field	Position	Original line	Normalised line	Original value	Normalised value
AU	2	73968	73968	Fusi, E	Fusi, Eleonora
AU	3	73969	73969	Baldi, A	Baldi, Antonella
AF	1	73970	73970	Cheli, F.	Cheli, F
AF	2	73971	73971	Fusi, E.	Fusi, Eleonora
AF	3	73972	73972	Baldi, A.	Baldi, Antonella

Record 500 | WOS:000339460900017

Ochratoxin A induces cytotoxicity, DNA damage and apoptosis in rat hepatocyte primary cell culture at nanomolar concentration

Field	Position	Original line	Normalised line	Original value	Normalised value
AU	6	74127	74127	Veer, V	Veer, Vijay
AF	1	74128	74128	Goyary, D.	Goyary, D
AF	2	74129	74129	Chattopadhyay, P.	Chattopadhyay, P
AF	3	74130	74130	Giri, S.	Giri, S
AF	4	74131	74131	Aher, V.	Aher, V
AF	5	74132	74132	Upadhyay, A.	Upadhyay, A
AF	6	74133	74133	Veer, V.	Veer, Vijay

Record 501 | WOS:000329058600004

Ochratoxin A induces DNA double-strand breaks and large deletion mutations in the carcinogenic target site of gpt delta rats

Field	Position	Original line	Normalised line	Original value	Normalised value
AU	1	74244	74244	Kuroda, K	Kuroda, Ken
AU	2	74245	74245	Hibi, D	Hibi, Daisuke
AU	3	74246	74246	Ishii, Y	Ishii, Yuji
AU	4	74247	74247	Takasu, S	Takasu, Shinji
AU	5	74248	74248	Kijima, A	Kijima, Aki
AU	6	74249	74249	Matsushita, K	Matsushita, Kohei
AU	7	74250	74250	Masumura, K	Masumura, Ken-Ichi
AU	8	74251	74251	Watanabe, M	Watanabe, Maiko
AU	9	74252	74252	Sugita-Konishi, Y	Sugita-Konishi, Yoshiko
AU	10	74253	74253	Sakai, H	Sakai, Hiroki
AU	11	74254	74254	Yanai, T	Yanai, Tokuma
AU	12	74255	74255	Nohmi, T	Nohmi, Takehiko
AU	13	74256	74256	Ogawa, K	Ogawa, Kumiko
AU	14	74257	74257	Umemura, T	Umemura, Takashi
AF	7	74264	74264	Masumura, Ken-ichi	Masumura, Ken-Ichi

Record 502 | WOS:000336092900022

A Review of the Evidence that Ochratoxin A Is an Nrf2 Inhibitor: Implications for Nephrotoxicity and Renal Carcinogenicity

Field	Position	Original line	Normalised line	Original value	Normalised value
AU	1	74387	74387	Limonciel, A	Limonciel, Alice
AU	2	74388	74388	Jennings, P	Jennings, Paul

Record 503 | WOS:000339460900010

Effects of lactic acid bacteria and Saccharomyces cerevisiae on growth of Aspergillus westerdijkiae and ochratoxin A production and toxicity

Field	Position	Original line	Normalised line	Original value	Normalised value
AU	1	74480	74480	Piotrowska, M	Piotrowska, Malgorzata
AF	1	74486	74486	Piotrowska, M.	Piotrowska, Malgorzata
AF	2	74487	74487	Roszak, J.	Roszak, J
AF	3	74488	74488	Stanczyk, M.	Stanczyk, M
AF	4	74489	74489	Palus, J.	Palus, J
AF	5	74490	74490	Dziubaltowska, E.	Dziubaltowska, E

AF	6	74491	74491	Stepnik, M.	Stepnik, M

Record 504 | WOS:000329960400028

Ochratoxin-induced toxicity, oxidative stress and apoptosis ameliorated by quercetin - Modulation by Nrf2

Field	Position	Original line	Normalised line	Original value	Normalised value
AU	1	74592	74592	Ramyaa, P	Ramyaa, Periasamy
AU	2	74593	74593	Padma, VV	Padma, Viswanadha_Vijaya
AF	2	74595	74595	Padma, Viswanadha Vijaya	Padma, Viswanadha_Vijaya

Record 505 | WOS:000328628900003

Deleterious Effects of Mycotoxin Combinations Involving Ochratoxin A

Field	Position	Original line	Normalised line	Original value	Normalised value
AU	1	74709	74709	Klaric, MS	Klaric, Maja_Segvic
AU	2	74710	74710	Rasic, D	Rasic, Dubravka
AU	3	74711	74711	Peraica, M	Peraica, Maja
AF	1	74712	74712	Klaric, Maja Segvic	Klaric, Maja_Segvic

Record 506 | WOS:000327686300025

In vitro investigation of individual and combined cytotoxic effects of aflatoxin B1 and other selected mycotoxins on the cell line porcine kidney 15

Field	Position	Original line	Normalised line	Original value	Normalised value
AU	1	74895	74895	Lei, MY	Lei, Mingyan
AU	2	74896	74896	Zhang, NY	Zhang, Niya
AU	3	74897	74897	Qi, DS	Qi, Desheng

Record 507 | WOS:000326211800003

Multi-mycotoxin determination in baby foods and in vitro combined cytotoxic effects of aflatoxin M1 and ochratoxin A

Field	Position	Original line	Normalised line	Original value	Normalised value
AU	1	74986	74986	Tavares, AM	Tavares, A_M
AU	2	74987	74987	Alvito, P	Alvito, Paula
AU	3	74988	74988	Loureiro, S	Loureiro, Susana
AU	4	74989	74989	Louro, H	Louro, Henriqueta
AU	5	74990	74990	Silva, MJ	Silva, M_J
AF	1	74991	74991	Tavares, A. M.	Tavares, A_M
AF	2	74992	74992	Alvito, P.	Alvito, Paula
AF	3	74993	74993	Loureiro, S.	Loureiro, Susana
AF	4	74994	74994	Louro, H.	Louro, Henriqueta
AF	5	74995	74995	Silva, M. J.	Silva, M_J

Record 508 | WOS:000324301900014

Beauvericin-induced cytotoxicity via ROS production and mitochondrial damage in Caco-2 cells

Field	Position	Original line	Normalised line	Original value	Normalised value
AU	1	75106	75106	Prosperini, A	Prosperini, Alessandra
AU	2	75107	75107	Juan-García, A	Juan-Garcia, Ana
AU	3	75108	75108	Font, G	Font, Guillermina
AU	4	75109	75109	Ruiz, MJ	Ruiz, Maria-Jose
AF	1	75110	75110	Prosperini, A.	Prosperini, Alessandra
AF	2	75111	75111	Juan-Garcia, A.	Juan-Garcia, Ana
AF	3	75112	75112	Font, G.	Font, Guillermina
AF	4	75113	75113	Ruiz, M. J.	Ruiz, Maria-Jose

Record 509 | WOS:000324773300006

Oxidative DNA damage is involved in ochratoxin A-induced G2 arrest through ataxia telangiectasia-mutated (ATM) pathways in human gastric epithelium GES-1 cells in vitro

Field	Position	Original line	Normalised line	Original value	Normalised value
AU	1	75214	75214	Cui, JF	Cui, Jinfeng
AU	2	75215	75215	Liu, J	Liu, Jing
AU	3	75216	75216	Wu, S	Wu, Sha
AU	4	75217	75217	Wang, Y	Wang, Yuan
AU	5	75218	75218	Shen, HT	Shen, Haitao
AU	6	75219	75219	Xing, LX	Xing, Lingxiao
AU	7	75220	75220	Wang, JL	Wang, Junling
AU	8	75221	75221	Yan, X	Yan, Xia
AU	9	75222	75222	Zhang, XH	Zhang, Xianghong

Record 510 | WOS:000326384200009

Toxicogenomics-Based Identification of Mechanisms for Direct Immunotoxicity

Field	Position	Original line	Normalised line	Original value	Normalised value
AU	1	75346	75346	Shao, J	Shao, Jia
AU	2	75347	75347	Katika, MR	Katika, Madhumohan_R
AU	3	75348	75348	Schmeits, PCJ	Schmeits, Peter_C_J
AU	4	75349	75349	Hendriksen, PJM	Hendriksen, Peter_J_M
AU	5	75350	75350	van Loveren, H	van_Loveren, Henk
AU	6	75351	75351	Peijnenburg, AACM	Peijnenburg, Ad_A_C_M
AU	7	75352	75352	Volger, OL	Volger, Oscar_L
AF	2	75354	75354	Katika, Madhumohan R.	Katika, Madhumohan_R
AF	3	75355	75355	Schmeits, Peter C. J.	Schmeits, Peter_C_J
AF	4	75356	75356	Hendriksen, Peter J. M.	Hendriksen, Peter_J_M
AF	5	75357	75357	van Loveren, Henk	van_Loveren, Henk
AF	6	75358	75358	Peijnenburg, Ad A. C. M.	Peijnenburg, Ad_A_C_M
AF	7	75359	75359	Volger, Oscar L.	Volger, Oscar_L

Record 511 | WOS:000332372600004

Toxicity of Ochratoxin A and Its Modulation by Antioxidants: A Review

Field	Position	Original line	Normalised line	Original value	Normalised value

AU	1	75563	75563	Sorrenti, V	Sorrenti, Valeria
AU	2	75564	75564	Di Giacomo, C	di_Giacomo, Claudia
AU	3	75565	75565	Acquaviva, R	Acquaviva, Rosaria
AU	4	75566	75566	Barbagallo, I	Barbagallo, Ignazio
AU	5	75567	75567	Bognanno, M	Bognanno, Matteo
AU	6	75568	75568	Galvano, F	Galvano, Fabio
AF	2	75570	75570	Di Giacomo, Claudia	di_Giacomo, Claudia

Record 512 | WOS:000322929800012

Identification of proteins related to early changes observed in Human hepatocellular carcinoma cells after treatment with the mycotoxin Zearalenone

Field	Position	Original line	Normalised line	Original value	Normalised value
AU	1	75795	75795	Gazzah, AC	Gazzah, Amel_Chatti
AU	2	75796	75796	Camoin, L	Camoin, Luc
AU	3	75797	75797	Abid, S	Abid, Salwa
AU	4	75798	75798	Bouaziz, C	Bouaziz, Chayma
AU	5	75799	75799	Ladjimi, M	Ladjimi, Moncef
AU	6	75800	75800	Bacha, H	Bacha, Hassen
AF	1	75801	75801	Gazzah, Amel Chatti	Gazzah, Amel_Chatti

Record 513 | WOS:000324847800043

Individual and combined effects of mycotoxins from typical indoor moulds

Field	Position	Original line	Normalised line	Original value	Normalised value
AU	6	75930	75930	Schüürmann, G	Schueuermann, G
AF	1	75932	75932	Mueller, A.	Mueller, A
AF	2	75933	75933	Schlink, U.	Schlink, U
AF	3	75934	75934	Wichmann, G.	Wichmann, G
AF	4	75935	75935	Bauer, M.	Bauer, M
AF	5	75936	75936	Graebsch, C.	Graebsch, C
AF	6	75937	75937	Schueuermann, G.	Schueuermann, G
AF	7	75938	75938	Herbarth, O.	Herbarth, O

Record 514 | WOS:000328628700005

Producers and Important Dietary Sources of Ochratoxin A and Citrinin

Field	Position	Original line	Normalised line	Original value	Normalised value
AU	1	76076	76076	Ostry, V	Ostry, Vladimir
AU	2	76077	76077	Malir, F	Malir, Frantisek
AU	3	76078	76078	Ruprich, J	Ruprich, Jiri

Record 515 | WOS:000328416400017

Fluorescence spectroscopic investigation of competitive interactions between ochratoxin A and 13 drug molecules for binding to human serum albumin

Field	Position	Original line	Normalised line	Original value	Normalised value

AU	1	76219	76219	Poór, M	Poor, Miklos
AU	2	76220	76220	Kunsági-Máté, S	Kunsagi-Mate, Sandor
AU	3	76221	76221	Czibulya, Z	Czibulya, Zsuzsanna
AU	4	76222	76222	Li, Y	Li, Yin
AU	5	76223	76223	Peles-Lemli, B	Peles-Lemli, Beata
AU	6	76224	76224	Petrik, J	Petrik, Jozsef
AU	7	76225	76225	Vladimir-Knezevic, S	Vladimir-Knezevic, Sanda
AU	8	76226	76226	Koszegi, T	Koszegi, Tamas

Record 516 | WOS:000324280000095

A review on ochratoxin A transcriptomic studies

Field	Position	Original line	Normalised line	Original value	Normalised value
AU	1	76344	76344	Vettorazzi, A	Vettorazzi, Ariane
AU	2	76345	76345	van Delft, J	van_Delft, Joost
AU	3	76346	76346	de Cerain, AL	Lopez_de_Cerain, Adela
AF	2	76348	76348	van Delft, Joost	van_Delft, Joost
AF	3	76349	76349	Lopez de Cerain, Adela	Lopez_de_Cerain, Adela

Record 517 | WOS:000320498300035

Individual and combined cytotoxic effects of Fusarium toxins (deoxynivalenol, nivalenol, zearalenone and fumonisins B1) on swine jejunal epithelial cells

Field	Position	Original line	Normalised line	Original value	Normalised value
AU	1	76508	76508	Wan, LYM	Wan, Lam_Yim_Murphy
AU	2	76509	76509	Turner, PC	Turner, Paul_C
AU	3	76510	76510	El-Nezami, H	El-Nezami, Hani
AF	1	76511	76511	Wan, Lam Yim Murphy	Wan, Lam_Yim_Murphy
AF	2	76512	76512	Turner, Paul C.	Turner, Paul_C

Record 518 | WOS:000320770400009

Computational Design of Peptide Ligands for Ochratoxin A

Field	Position	Original line	Normalised line	Original value	Normalised value
AU	1	76655	76655	Heurich, M	Heurich, Meike
AU	2	76656	76656	Altintas, Z	Altintas, Zeynep
AU	3	76657	76657	Tothill, IE	Tothill, Ibtisam_E
AF	3	76660	76660	Tothill, Ibtisam E.	Tothill, Ibtisam_E

Record 519 | WOS:000320571000011

Effect of quercetin on the toxicokinetics of ochratoxin A in rats

Field	Position	Original line	Normalised line	Original value	Normalised value
AU	1	76756	76756	Abbas, Z	Abbas, Zein
AU	2	76757	76757	Blank, R	Blank, Ralf
AU	3	76758	76758	Wein, S	Wein, Silvia

AU	4	76759	76759	Wolffram, S	Wolffram, Siegfried

Record 520 | WOS:000316978700004

Zinc protects HepG2 cells against the oxidative damage and DNA damage induced by ochratoxin A

Field	Position	Original line	Normalised line	Original value	Normalised value
AU	1	76844	76844	Zheng, JJ	Zheng, Juanjuan
AU	2	76845	76845	Zhang, Y	Zhang, Yu
AU	3	76846	76846	Xu, WT	Xu, Wentao
AU	4	76847	76847	Luo, YB	Luo, Yunbo
AU	5	76848	76848	Mao, JR	Mao, Junran
AU	6	76849	76849	Shen, XL	Shen, Xiao_Li
AU	7	76850	76850	Yang, X	Yang, Xuan
AU	8	76851	76851	Li, XH	Li, Xiaohong
AU	9	76852	76852	Huang, KL	Huang, Kunlun
AF	4	76856	76856	Luo, YunBo	Luo, Yunbo
AF	6	76858	76858	Shen, Xiao Li	Shen, Xiao_Li

Record 521 | WOS:000315160400007

Role of hMLH1 in sterigmatocystin-induced G2 phase arrest in human esophageal epithelial Het-1A cells in vitro

Field	Position	Original line	Normalised line	Original value	Normalised value
AU	1	76962	76962	Wang, J	Wang, Juan
AU	2	76963	76963	Huang, SJ	Huang, Shujuan
AU	3	76964	76964	Xing, LX	Xing, Lingxiao
AU	4	76965	76965	Shen, HT	Shen, Haitao
AU	5	76966	76966	Yan, X	Yan, Xia
AU	6	76967	76967	Wang, JL	Wang, Junling
AU	7	76968	76968	Zhang, XH	Zhang, Xianghong

Record 522 | WOS:000316229200014

Cross-talk between microRNAs, nuclear factor E2-related factor 2, and heme oxygenase-1 in ochratoxin A-induced toxic effects in renal proximal tubular epithelial cells

Field	Position	Original line	Normalised line	Original value	Normalised value
AU	1	77084	77084	Stachurska, A	Stachurska, Anna
AU	2	77085	77085	Ciesla, M	Ciesla, Maciej
AU	3	77086	77086	Kozakowska, M	Kozakowska, Magdalena
AU	4	77087	77087	Wolffram, S	Wolffram, Siegfried
AU	5	77088	77088	Boesch-Saadatmandi, C	Boesch-Saadatmandi, Christine
AU	6	77089	77089	Rimbach, G	Rimbach, Gerald
AU	7	77090	77090	Jozkowicz, A	Jozkowicz, Alicja
AU	8	77091	77091	Dulak, J	Dulak, Jozef
AU	9	77092	77092	Loboda, A	Loboda, Agnieszka

Record 523 | WOS:000316522300011

Effects of p53 knockout on ochratoxin A-induced genotoxicity in p53-deficient gpt delta mice

Field	Position	Original line	Normalised line	Original value	Normalised value
AU	1	77235	77235	Hibi, D	Hibi, Daisuke
AU	2	77236	77236	Kijima, A	Kijima, Aki
AU	3	77237	77237	Suzuki, Y	Suzuki, Yuta
AU	4	77238	77238	Ishii, Y	Ishii, Yuji
AU	5	77239	77239	Jin, M	Jin, Meilan
AU	6	77240	77240	Sugita-Konishi, Y	Sugita-Konishi, Yoshiko
AU	7	77241	77241	Yanai, T	Yanai, Tokuma
AU	8	77242	77242	Nishikawa, A	Nishikawa, Akiyoshi
AU	9	77243	77243	Umemura, T	Umemura, Takashi

Record 524 | WOS:000315408000011

Modulation of Intestinal Functions Following Mycotoxin Ingestion: Meta-Analysis of Published Experiments in Animals

Field	Position	Original line	Normalised line	Original value	Normalised value
AU	1	77349	77349	Grenier, B	Grenier, Bertrand
AU	2	77350	77350	Applegate, TJ	Applegate, Todd_J
AF	2	77352	77352	Applegate, Todd J.	Applegate, Todd_J

Record 525 | WOS:000314671300007

Molecular mechanisms underlying ochratoxin A-induced genotoxicity: global gene expression analysis suggests induction of DNA double-strand breaks and cell cycle progression

Field	Position	Original line	Normalised line	Original value	Normalised value
AU	1	77508	77508	Hibi, D	Hibi, Daisuke
AU	2	77509	77509	Kijima, A	Kijima, Aki
AU	3	77510	77510	Kuroda, K	Kuroda, Ken
AU	4	77511	77511	Suzuki, Y	Suzuki, Yuta
AU	5	77512	77512	Ishii, Y	Ishii, Yuji
AU	6	77513	77513	Jin, ML	Jin, Meilan
AU	7	77514	77514	Nakajima, M	Nakajima, Masahiro
AU	8	77515	77515	Sugita-Konishi, Y	Sugita-Konishi, Yoshiko
AU	9	77516	77516	Yanai, T	Yanai, Tokuma
AU	10	77517	77517	Nohmi, T	Nohmi, Takehiko
AU	11	77518	77518	Nishikawa, A	Nishikawa, Akiyoshi
AU	12	77519	77519	Umemura, T	Umemura, Takashi

Record 526 | WOS:000316706800031

An iTRAQ-based mitoproteomics approach for profiling the nephrotoxicity mechanisms of ochratoxin A in HEK 293 cells

Field	Position	Original line	Normalised line	Original value	Normalised value
AU	1	77645	77645	Shen, XL	Shen, Xiao_Li
AU	2	77646	77646	Zhang, Y	Zhang, Yu

AU	3	77647	77647	Xu, WT	Xu, Wentao
AU	4	77648	77648	Liang, R	Liang, Rui
AU	5	77649	77649	Zheng, JJ	Zheng, Juanjuan
AU	6	77650	77650	Luo, YB	Luo, Yunbo
AU	7	77651	77651	Wang, Y	Wang, Yan
AU	8	77652	77652	Huang, KL	Huang, Kunlun
AF	1	77653	77653	Shen, Xiao Li	Shen, Xiao_Li
AF	6	77658	77658	Luo, YunBo	Luo, Yunbo

Record 527 | WOS:000318093700001

A Review of the Mechanism of Injury and Treatment Approaches for Illness Resulting from Exposure to Water-Damaged Buildings, Mold, and Mycotoxins

Field	Position	Original line	Normalised line	Original value	Normalised value
AU	1	77793	77793	Hope, J	Hope, Janette

Record 528 | WOS:000314048800053

Ochratoxin A Inhibits Mouse Embryonic Development by Activating a Mitochondrion-Dependent Apoptotic Signaling Pathway

Field	Position	Original line	Normalised line	Original value	Normalised value
AU	1	78048	78048	Hsuuw, YD	Hsuuw, Yan-Der
AU	2	78049	78049	Chan, WH	Chan, Wen-Hsiung
AU	3	78050	78050	Yu, JS	Yu, Jau-Song

Record 529 | WOS:000313124600008

Role of intestinal microflora in xenobiotic-induced toxicity

Field	Position	Original line	Normalised line	Original value	Normalised value
AU	1	78170	78170	Jeong, HG	Jeong, Hye_Gwang
AU	2	78171	78171	Kang, MJ	Kang, Mi_Jeong
AU	3	78172	78172	Kim, HG	Kim, Hyung_Gyun
AU	4	78173	78173	Oh, DG	Oh, do_Gyeong
AU	5	78174	78174	Kim, JS	Kim, Jin_Sung
AU	6	78175	78175	Lee, SK	Lee, Sang_Kyu
AU	7	78176	78176	Jeong, TC	Jeong, Tae_Cheon
AF	1	78177	78177	Jeong, Hye Gwang	Jeong, Hye_Gwang
AF	2	78178	78178	Kang, Mi Jeong	Kang, Mi_Jeong
AF	3	78179	78179	Kim, Hyung Gyun	Kim, Hyung_Gyun
AF	4	78180	78180	Oh, Do Gyeong	Oh, do_Gyeong
AF	5	78181	78181	Kim, Jin Sung	Kim, Jin_Sung
AF	6	78182	78182	Lee, Sang Kyu	Lee, Sang_Kyu
AF	7	78183	78183	Jeong, Tae Cheon	Jeong, Tae_Cheon

Record 530 | WOS:000315356800004

Amelioration of Ochratoxin A-induced immunotoxic effects by silymarin and vitamin E in White Leghorn cockerels

Field	Position	Original line	Normalised line	Original value	Normalised value
AU	1	78313	78313	Khatoon, A	Khatoon, Aisha
AU	2	78314	78314	Khan, MZ	Khan, Muhammad_Zargham
AU	3	78315	78315	Khan, A	Khan, Ahrar
AU	4	78316	78316	Saleemi, MK	Saleemi, Muhammad_Kashif
AU	5	78317	78317	Javed, I	Javed, Ijaz
AF	2	78319	78319	Khan, Muhammad Zargham	Khan, Muhammad_Zargham
AF	4	78321	78321	Saleemi, Muhammad Kashif	Saleemi, Muhammad_Kashif

Record 531 | WOS:000312386800135

Nivalenol and Deoxynivalenol Affect Rat Intestinal Epithelial Cells: A Concentration Related Study

Field	Position	Original line	Normalised line	Original value	Normalised value
AU	1	78421	78421	Bianco, G	Bianco, Giuseppe
AU	2	78422	78422	Fontanella, B	Fontanella, Bianca
AU	3	78423	78423	Severino, L	Severino, Lorella
AU	4	78424	78424	Quaroni, A	Quaroni, Andrea
AU	5	78425	78425	Autore, G	Autore, Giuseppina
AU	6	78426	78426	Marzocco, S	Marzocco, Stefania

Record 532 | WOS:000312288000010

Lactobacillus reuteri CRL 1098 and Lactobacillus acidophilus CRL 1014 differently reduce in vitro immunotoxic effect induced by Ochratoxin A

Field	Position	Original line	Normalised line	Original value	Normalised value
AU	1	78541	78541	Mechoud, MA	Mechoud, Monica_A
AU	2	78542	78542	Juarez, GE	Juarez, Guillermo_E
AU	3	78543	78543	de Valdez, GF	Font_de_Valdez, Graciela
AU	4	78544	78544	Rodriguez, AV	Rodriguez, Ana_V
AF	1	78545	78545	Mechoud, Monica A.	Mechoud, Monica_A
AF	2	78546	78546	Juarez, Guillermo E.	Juarez, Guillermo_E
AF	3	78547	78547	Font de Valdez, Graciela	Font_de_Valdez, Graciela
AF	4	78548	78548	Rodriguez, Ana V.	Rodriguez, Ana_V

Record 533 | WOS:000310867400006

Increase in Mitochondrial DNA Copy Number in Response to Ochratoxin A and Methanol-Induced Mitochondrial DNA Damage in Drosophila

Field	Position	Original line	Normalised line	Original value	Normalised value
AU	1	78654	78654	Mutlu, AG	Mutlu, Ayse_Gul
AF	1	78655	78655	Mutlu, Ayse Gul	Mutlu, Ayse_Gul

Record 534 | WOS:000312288000021

Evaluation of nephrotoxic effects of mycotoxins, citrinin and patulin, on zebrafish (Danio rerio) embryos

Field	Position	Original line	Normalised line	Original value	Normalised value
AU	1	78724	78724	Wu, TS	Wu, Ting-Shuan

AU	2	78725	78725	Yang, JJ	Yang, Jiann-Jou
AU	3	78726	78726	Yu, FY	Yu, Feng-Yih
AU	4	78727	78727	Liu, BH	Liu, Biing-Hui

Record 535 | WOS:000308629100003

Human skin penetration of selected model mycotoxins

Field	Position	Original line	Normalised line	Original value	Normalised value
AU	1	78851	78851	Boonen, J	Boonen, Jente
AU	2	78852	78852	Malysheva, SV	Malysheva, Svetlana_V
AU	3	78853	78853	Taevernier, L	Taevernier, Lien
AU	4	78854	78854	Di Mavungu, JD	di_Mavungu, Jose_Diana
AU	5	78855	78855	De Saeger, S	de_Saeger, Sarah
AU	6	78856	78856	De Spiegeleer, B	de_Spiegeleer, Bart
AF	2	78858	78858	Malysheva, Svetlana V.	Malysheva, Svetlana_V
AF	4	78860	78860	Di Mavungu, Jose Diana	di_Mavungu, Jose_Diana
AF	5	78861	78861	De Saeger, Sarah	de_Saeger, Sarah
AF	6	78862	78862	De Spiegeleer, Bart	de_Spiegeleer, Bart

Record 536 | WOS:000315406500009

Apoptosis Induction by OTA and TNF-α in Cultured Primary Rat Hepatocytes and Prevention by Silibinin

Field	Position	Original line	Normalised line	Original value	Normalised value
AU	1	79033	79033	Essid, E	Essid, Ebtisam
AU	2	79034	79034	Dernawi, Y	Dernawi, Yousef
AU	3	79035	79035	Petzinger, E	Petzinger, Ernst

Record 537 | WOS:000309849700023

Co-exposure of Fusarium mycotoxins: In vitro myelotoxicity assessment on human hematopoietic progenitors

Field	Position	Original line	Normalised line	Original value	Normalised value
AU	1	79141	79141	Ficheux, AS	Ficheux, A_S
AF	1	79144	79144	Ficheux, A. S.	Ficheux, A_S
AF	2	79145	79145	Sibiril, Y.	Sibiril, Y
AF	3	79146	79146	Parent-Massin, D.	Parent-Massin, D

Record 538 | WOS:000309897100023

Immune modulatory effects of the foodborne contaminant citrinin in mice

Field	Position	Original line	Normalised line	Original value	Normalised value
AU	1	79238	79238	Islam, MR	Islam, Mohammad_Rafiqul
AU	2	79239	79239	Roh, YS	Roh, Yoon-Seok
AU	3	79240	79240	Cho, A	Cho, Ara
AU	4	79241	79241	Kim, J	Kim, Jinho
AU	5	79242	79242	Kim, JH	Kim, Jong-Hoon

AU	6	79243	79243	Eo, SK	Eo, Seong-Kug
AU	7	79244	79244	Lim, CW	Lim, Chae-Woong
AU	8	79245	79245	Kim, B	Kim, Bumseok
AF	1	79246	79246	Islam, Mohammad Rafiqul	Islam, Mohammad_Rafiqul
AF	6	79251	79251	Eo, Seong-kug	Eo, Seong-Kug

Record 539 | WOS:000309897100058

Fumonisin B1 and the kidney: Modes of action for renal tumor formation by fumonisin B1 in rodents

Field	Position	Original line	Normalised line	Original value	Normalised value
AU	1	79350	79350	Müller, S	Mueller, Stephanie
AU	2	79351	79351	Dekant, W	Dekant, Wolfgang
AU	3	79352	79352	Mally, A	Mally, Angela

Record 540 | WOS:000308289000002

The food contaminant and nephrotoxin ochratoxin A enhances Wnt1 inducible signaling protein 1 and tumor necrosis factor-α expression in human primary proximal tubule cells

Field	Position	Original line	Normalised line	Original value	Normalised value
AU	1	79543	79543	Hennemeier, I	Hennemeier, Isabell
AU	2	79544	79544	Humpf, HU	Humpf, Hans-Ulrich
AU	3	79545	79545	Gekle, M	Gekle, Michael
AU	4	79546	79546	Schwerdt, G	Schwerdt, Gerald

Record 541 | WOS:000307323900012

Citrinin reduces testosterone secretion by inducing apoptosis in rat Leydig cells

Field	Position	Original line	Normalised line	Original value	Normalised value
AU	1	79641	79641	Liu, SQ	Liu, Shuqiang
AU	2	79642	79642	Wang, D	Wang, Dan
AU	3	79643	79643	Zhang, JW	Zhang, Junwen
AU	4	79644	79644	Zhang, DZ	Zhang, Dongzhi
AU	5	79645	79645	Gong, M	Gong, Meng
AU	6	79646	79646	Wang, C	Wang, Chao
AU	7	79647	79647	Wei, N	Wei, Ning
AU	8	79648	79648	Liu, WH	Liu, Wenhua
AU	9	79649	79649	Wang, YQ	Wang, Yongqi
AU	10	79650	79650	Zhao, CX	Zhao, Chongxue
AU	11	79651	79651	Cui, YX	Cui, Yaxiong
AU	12	79652	79652	Hu, DF	Hu, Defu

Record 542 | WOS:000307766500014

Cellular distribution of cell cycle-related molecules in the renal tubules of rats treated with renal carcinogens for 28 days: relationship between cell cycle aberration and carcinogenesis

Field	Position	Original line	Normalised line	Original value	Normalised value
AU	1	79750	79750	Taniai, E	Taniai, Eriko

AU	2	79751	79751	Hayashi, H	Hayashi, Hitomi
AU	3	79752	79752	Yafune, A	Yafune, Atsunori
AU	4	79753	79753	Watanabe, M	Watanabe, Maiko
AU	5	79754	79754	Akane, H	Akane, Hirotoshi
AU	6	79755	79755	Suzuki, K	Suzuki, Kazuhiko
AU	7	79756	79756	Mitsumori, K	Mitsumori, Kunitoshi
AU	8	79757	79757	Shibutani, M	Shibutani, Makoto

Record 543 | WOS:000307487100016

Protective Effect of Deferricoprogen Isolated from Monascus purpureus NTU 568 on Citrinin-Induced Apoptosis in HEK-293 Cells

Field	Position	Original line	Normalised line	Original value	Normalised value
AU	1	79878	79878	Hsu, LC	Hsu, Li-Chuan
AU	2	79879	79879	Hsu, YW	Hsu, Ya-Wen
AU	3	79880	79880	Liang, YH	Liang, Yu-Han
AU	4	79881	79881	Lin, ZH	Lin, Zhi-Hu
AU	5	79882	79882	Kuo, YH	Kuo, Yao-Haur
AU	6	79883	79883	Pan, TM	Pan, Tzu-Ming

Record 544 | WOS:000304513300011

Toxic effects of citrinin on the male reproductive system in mice

Field	Position	Original line	Normalised line	Original value	Normalised value
AU	1	79984	79984	Han, QQ	Han_Qingqing
AU	2	79985	79985	Yu, LB	Yu_Linbo
AU	3	79986	79986	Guo, YQ	Guo_Yunqian
AU	4	79987	79987	Liu, SQ	Liu_Shuqiang
AF	1	79988	79988	Han Qingqing	Han_Qingqing
AF	2	79989	79989	Yu Linbo	Yu_Linbo
AF	3	79990	79990	Guo Yunqian	Guo_Yunqian
AF	4	79991	79991	Liu Shuqiang	Liu_Shuqiang

Record 545 | WOS:000304401100023

Collaborative study for the detection of toxic compounds in shellfish extracts using cell-based assays. Part II: application to shellfish extracts spiked with lipophilic marine toxins

Field	Position	Original line	Normalised line	Original value	Normalised value
AU	1	80079	80079	Ledreux, A	Ledreux, Aurelie
AU	2	80080	80080	Sérandour, AL	Serandour, Anne-Laure
AU	3	80081	80081	Morin, B	Morin, Benedicte
AU	4	80082	80082	Derick, S	Derick, Sylvain
AU	5	80083	80083	Lanceleur, R	Lanceleur, Rachelle
AU	6	80084	80084	Hamlaoui, S	Hamlaoui, Sahima
AU	7	80085	80085	Furger, C	Furger, Christophe
AU	8	80086	80086	Biré, R	Bire, Ronel
AU	9	80087	80087	Krys, S	Krys, Sophie
AU	10	80088	80088	Fessard, V	Fessard, Valerie

AU	11	80089	80089	Troussellier, M	Troussellier, Marc
AU	12	80090	80090	Bernard, C	Bernard, Cecile

Record 546 | WOS:000304744700011

Ochratoxin A induces oxidative DNA damage and G1 phase arrest in human peripheral blood mononuclear cells in vitro

Field	Position	Original line	Normalised line	Original value	Normalised value
AU	1	80213	80213	Liu, J	Liu, Jing
AU	2	80214	80214	Wang, Y	Wang, Yuan
AU	3	80215	80215	Cui, JF	Cui, Jinfeng
AU	4	80216	80216	Xing, LX	Xing, Lingxiao
AU	5	80217	80217	Shen, HT	Shen, Haitao
AU	6	80218	80218	Wu, S	Wu, Sha
AU	7	80219	80219	Lian, HG	Lian, Hongguang
AU	8	80220	80220	Wang, JL	Wang, Junling
AU	9	80221	80221	Yan, X	Yan, Xia
AU	10	80222	80222	Zhang, XH	Zhang, Xianghong

Record 547 | WOS:000304198400001

Ochratoxin A and Mitotic Disruption: Mode of Action Analysis of Renal Tumor Formation by Ochratoxin A

Field	Position	Original line	Normalised line	Original value	Normalised value
AU	1	80339	80339	Mally, A	Mally, Angela

Record 548 | WOS:000304401100022

Collaborative study for the detection of toxic compounds in shellfish extracts using cell-based assays. Part I: screening strategy and pre-validation study with lipophilic marine toxins

Field	Position	Original line	Normalised line	Original value	Normalised value
AU	1	80548	80548	Sérandour, AL	Serandour, Anne-Laure
AU	2	80549	80549	Ledreux, A	Ledreux, Aurelie
AU	3	80550	80550	Morin, B	Morin, Benedicte
AU	4	80551	80551	Derick, S	Derick, Sylvain
AU	5	80552	80552	Augier, E	Augier, Elie
AU	6	80553	80553	Lanceleur, R	Lanceleur, Rachelle
AU	7	80554	80554	Hamlaoui, S	Hamlaoui, Sahima
AU	8	80555	80555	Moukha, S	Moukha, Serge
AU	9	80556	80556	Furger, C	Furger, Christophe
AU	10	80557	80557	Biré, R	Bire, Ronel
AU	11	80558	80558	Krys, S	Krys, Sophie
AU	12	80559	80559	Fessard, V	Fessard, Valerie
AU	13	80560	80560	Troussellier, M	Troussellier, Marc
AU	14	80561	80561	Bernard, C	Bernard, Cecile

Record 549 | WOS:000302950000003

Assaying embryotoxicity in the test tube: Current limitations of the embryonic stem cell test (EST) challenging its applicability domain

Field	Position	Original line	Normalised line	Original value	Normalised value
AU	1	80691	80691	Riebeling, C	Riebeling, Christian
AU	2	80692	80692	Hayess, K	Hayess, Katrin
AU	3	80693	80693	Peters, AK	Peters, Annelieke_K
AU	4	80694	80694	Steemans, M	Steemans, Margino
AU	5	80695	80695	Spielmann, H	Spielmann, Horst
AU	6	80696	80696	Luch, A	Luch, Andreas
AU	7	80697	80697	Seiler, AEM	Seiler, Andrea_E_M
AF	3	80700	80700	Peters, Annelieke K.	Peters, Annelieke_K
AF	7	80704	80704	Seiler, Andrea E. M.	Seiler, Andrea_E_M

Record 550 | WOS:000307982300003

Mutagenicity of Ochratoxin A and Its Hydroquinone Metabolite in the SupF Gene of the Mutation Reporter Plasmid Ps189

Field	Position	Original line	Normalised line	Original value	Normalised value
AU	1	80999	80999	Akman, SA	Akman, Steven_A
AU	2	81000	81000	Adams, M	Adams, Marissa
AU	3	81001	81001	Case, D	Case, Doug
AU	4	81002	81002	Park, G	Park, Gyungse
AU	5	81003	81003	Manderville, RA	Manderville, Richard_A
AF	1	81004	81004	Akman, Steven A.	Akman, Steven_A
AF	5	81008	81008	Manderville, Richard A.	Manderville, Richard_A

Record 551 | WOS:000301792400008

Transcriptomic alterations induced by Ochratoxin A in rat and human renal proximal tubular in vitro models and comparison to a rat in vivo model

Field	Position	Original line	Normalised line	Original value	Normalised value
AU	1	81126	81126	Jennings, P	Jennings, Paul
AU	2	81127	81127	Weiland, C	Weiland, Christina
AU	3	81128	81128	Limonciel, A	Limonciel, Alice
AU	4	81129	81129	Bloch, KM	Bloch, Katarzyna_M
AU	5	81130	81130	Radford, R	Radford, Robert
AU	6	81131	81131	Aschauer, L	Aschauer, Lydia
AU	7	81132	81132	McMorrow, T	Mcmorrow, Tara
AU	8	81133	81133	Wilmes, A	Wilmes, Anja
AU	9	81134	81134	Pfaller, W	Pfaller, Walter
AU	10	81135	81135	Ahr, HJ	Ahr, Hans_J
AU	11	81136	81136	Slattery, C	Slattery, Craig
AU	12	81137	81137	Lock, EA	Lock, Edward_A
AU	13	81138	81138	Ryan, MP	Ryan, Michael_P
AU	14	81139	81139	Ellinger-Ziegelbauer, H	Ellinger-Ziegelbauer, Heidrun
AF	4	81143	81143	Bloch, Katarzyna M.	Bloch, Katarzyna_M
AF	7	81146	81146	McMorrow, Tara	Mcmorrow, Tara
AF	10	81149	81149	Ahr, Hans J.	Ahr, Hans_J
AF	12	81151	81151	Lock, Edward A.	Lock, Edward_A
AF	13	81152	81152	Ryan, Michael P.	Ryan, Michael_P

Record 552 | WOS:000302917900001

Carcinogens induce loss of the primary cilium in human renal proximal tubular epithelial cells independently of effects on the cell cycle

Field	Position	Original line	Normalised line	Original value	Normalised value
AU	1	81246	81246	Radford, R	Radford, Robert
AU	2	81247	81247	Slattery, C	Slattery, Craig
AU	3	81248	81248	Jennings, P	Jennings, Paul
AU	4	81249	81249	Blaque, O	Blaque, Oliver
AU	5	81250	81250	Pfaller, W	Pfaller, Walter
AU	6	81251	81251	Gmuender, H	Gmuender, Hans
AU	7	81252	81252	Van Delft, J	van_Delft, Joost
AU	8	81253	81253	Ryan, MP	Ryan, Michael_P
AU	9	81254	81254	McMorrow, T	Mcmorrow, Tara
AF	7	81261	81261	Van Delft, Joost	van_Delft, Joost
AF	8	81262	81262	Ryan, Michael P.	Ryan, Michael_P
AF	9	81263	81263	McMorrow, Tara	Mcmorrow, Tara

Record 553 | WOS:000301277200012

ERK and p38 MAPK signaling pathways are involved in ochratoxin A-induced G2 phase arrest in human gastric epithelium cells

Field	Position	Original line	Normalised line	Original value	Normalised value
AU	1	81373	81373	Wang, Y	Wang, Yuan
AU	2	81374	81374	Liu, J	Liu, Jing
AU	3	81375	81375	Cui, JF	Cui, Jinfeng
AU	4	81376	81376	Xing, LX	Xing, Lingxiao
AU	5	81377	81377	Wang, JL	Wang, Junling
AU	6	81378	81378	Yan, X	Yan, Xia
AU	7	81379	81379	Zhang, XH	Zhang, Xianghong

Record 554 | WOS:000302911300002

Assessment on pollution of ochratoxin a in grain in China and its apoptosis effect on vitro-cultured human tubular kidney cells

Field	Position	Original line	Normalised line	Original value	Normalised value
AU	1	81466	81466	Li, ZN	Li, Zengning
AU	2	81467	81467	Zhang, XH	Zhang, Xianghong
AU	3	81468	81468	Cui, JF	Cui, Jinfeng
AU	4	81469	81469	Kang, WJ	Kang, Weijun

Record 555 | WOS:000300700100002

Inactivation of A. ochraceus Spores and Detoxification of Ochratoxin A in Coffee Beans by Gamma Irradiation

Field	Position	Original line	Normalised line	Original value	Normalised value
AU	1	81561	81561	Kumar, S	Kumar, Sanjeev
AU	2	81562	81562	Kunwar, A	Kunwar, Amit
AU	3	81563	81563	Gautam, S	Gautam, Satyendra

AU	4	81564	81564	Sharma, A	Sharma, Arun

Record 556 | WOS:000309118000007

CYTOTOXIC POTENCY OF MYCOTOXINS IN CULTURES OF V79 LUNG FIBROBLAST CELLS

Field	Position	Original line	Normalised line	Original value	Normalised value
AU	1	81652	81652	Behm, C	Behm, Claudia
AU	2	81653	81653	Föllmann, W	Foellmann, Wolfram
AU	3	81654	81654	Degen, GH	Degen, Gisela_H
AF	3	81657	81657	Degen, Gisela H.	Degen, Gisela_H

Record 557 | WOS:000209177700107

Transport of aflatoxin M1 in human intestinal Caco-2/TC7 cells

Field	Position	Original line	Normalised line	Original value	Normalised value
AU	1	81730	81730	Caloni, F	Caloni, Francesca
AU	2	81731	81731	Cortinovis, C	Cortinovis, Cristina
AU	3	81732	81732	Pizzo, F	Pizzo, Fabiola
AU	4	81733	81733	De Angelis, I	de_Angelis, Isabella
AF	4	81737	81737	De Angelis, Isabella	de_Angelis, Isabella

Record 558 | WOS:000300137300023

In vitro myelotoxicity assessment of the emerging mycotoxins Beauvericin, Enniatin b and Moniliformin on human hematopoietic progenitors

Field	Position	Original line	Normalised line	Original value	Normalised value
AU	1	81843	81843	Ficheux, AS	Ficheux, A_S
AU	3	81845	81845	Le Garrec, R	le_Garrec, R
AF	1	81847	81847	Ficheux, A. S.	Ficheux, A_S
AF	2	81848	81848	Sibiril, Y.	Sibiril, Y
AF	3	81849	81849	Le Garrec, R.	le_Garrec, R
AF	4	81850	81850	Parent-Massin, D.	Parent-Massin, D

Record 559 | WOS:000299028700020

Structure-Activity Relationships Imply Different Mechanisms of Action for Ochratoxin A-Mediated Cytotoxicity and Genotoxicity

Field	Position	Original line	Normalised line	Original value	Normalised value
AU	1	81944	81944	Hadjeba-Medjdoub, K	Hadjeba-Medjdoub, Kheira
AU	2	81945	81945	Tozlovanu, M	Tozlovanu, Mariana
AU	3	81946	81946	Pfohl-Leszkowicz, A	Pfohl-Leszkowicz, Annie
AU	4	81947	81947	Frenette, C	Frenette, Christine
AU	5	81948	81948	Paugh, RJ	Paugh, Robert_J
AU	6	81949	81949	Manderville, RA	Manderville, Richard_A
AF	5	81954	81954	Paugh, Robert J.	Paugh, Robert_J
AF	6	81955	81955	Manderville, Richard A.	Manderville, Richard_A

Record 560 | WOS:000299902300007

In vivo and ex vivo phagocytic potential of macrophages from progeny of breeder hens kept on ochratoxin A (OTA)-contaminated diet

Field	Position	Original line	Normalised line	Original value	Normalised value
AU	1	82085	82085	Zahoor-ul-Hassan	Zahoor-Ul-Hassan
AU	2	82086	82086	Khan, MZ	Khan, Muhammad_Zargham
AU	3	82087	82087	Khan, A	Khan, Ahrar
AU	4	82088	82088	Javed, I	Javed, Ijaz
AU	5	82089	82089	Noreen, M	Noreen, Mnaza
AF	1	82090	82090	Zahoor-ul-Hassan	Zahoor-Ul-Hassan
AF	2	82091	82091	Khan, Muhammad Zargham	Khan, Muhammad_Zargham

Record 561 | WOS:000297778200001

Ochratoxin A in feed of food-producing animals: An undesirable mycotoxin with health and performance effects

Field	Position	Original line	Normalised line	Original value	Normalised value
AU	1	82202	82202	Duarte, SC	Duarte, Sofia_C
AU	2	82203	82203	Lino, CM	Lino, Celeste_M
AU	3	82204	82204	Pena, A	Pena, Angelina
AF	1	82205	82205	Duarte, Sofia C.	Duarte, Sofia_C
AF	2	82206	82206	Lino, Celeste M.	Lino, Celeste_M

Record 562 | WOS:000298362500059

Effects of zearalenone and its derivatives on porcine immune response

Field	Position	Original line	Normalised line	Original value	Normalised value
AU	1	82390	82390	Marin, DE	Marin, Daniela_E
AU	2	82391	82391	Taranu, I	Taranu, Ionelia
AU	3	82392	82392	Burlacu, R	Burlacu, Radu
AU	4	82393	82393	Manda, G	Manda, Gina
AU	5	82394	82394	Motiu, M	Motiu, Monica
AU	6	82395	82395	Neagoe, I	Neagoe, Ionela
AU	7	82396	82396	Dragomir, C	Dragomir, Catalin
AU	8	82397	82397	Stancu, M	Stancu, Mariana
AU	9	82398	82398	Calin, L	Calin, Loredana
AF	1	82399	82399	Marin, Daniela E.	Marin, Daniela_E

Record 563 | WOS:000297102800002

Molecular Events Involved in Ochratoxin A Induced Mitochondrial Pathway of Apoptosis, Modulation by Bcl-2 Family Members

Field	Position	Original line	Normalised line	Original value	Normalised value
AU	1	82524	82524	Bouaziz, C	Bouaziz, Chayma
AU	2	82525	82525	el Dein, OS	el_Dein, Ossama_Sharaf
AU	3	82526	82526	Martel, C	Martel, Cecile
AU	4	82527	82527	El Golli, E	el_Golli, Emna

AU	5	82528	82528	Abid-Essefi, S	Abid-Essefi, Salwa
AU	6	82529	82529	Brenner, C	Brenner, Catherine
AU	7	82530	82530	Lemaire, C	Lemaire, Christophe
AU	8	82531	82531	Bacha, H	Bacha, Hassen
AF	2	82533	82533	el Dein, Ossama Sharaf	el_Dein, Ossama_Sharaf
AF	4	82535	82535	El Golli, Emna	el_Golli, Emna

Record 564 | WOS:000296681600021

Ochratoxin A reduces aflatoxin B1 induced DNA damage detected by the comet assay in Hep G2 cells

Field	Position	Original line	Normalised line	Original value	Normalised value
AU	1	82645	82645	Corcuera, LA	Corcuera, Laura-Ana
AU	2	82646	82646	Arbillaga, L	Arbillaga, Leire
AU	3	82647	82647	Vettorazzi, A	Vettorazzi, Ariane
AU	5	82649	82649	de Cerain, AL	Lopez_de_Cerain, Adela
AF	1	82650	82650	Corcuera, L. A.	Corcuera, Laura-Ana
AF	2	82651	82651	Arbillaga, L.	Arbillaga, Leire
AF	3	82652	82652	Vettorazzi, A.	Vettorazzi, Ariane
AF	4	82653	82653	Azqueta, A.	Azqueta, A
AF	5	82654	82654	Lopez de Cerain, A.	Lopez_de_Cerain, Adela

Record 565 | WOS:000295241700033

Cytotoxic effects of mycotoxin combinations in mammalian kidney cells

Field	Position	Original line	Normalised line	Original value	Normalised value
AU	1	82764	82764	Ruiz, MJ	Ruiz, Maria-Jose
AU	2	82765	82765	Macáková, P	Macakova, Petra
AU	3	82766	82766	Juan-García, A	Juan-Garcia, Ana
AU	4	82767	82767	Font, G	Font, Guillermina

Record 566 | WOS:000298538400030

Mycotoxins profiling of the culture material of Fusarium verticillioides (Sacc.) Nirenberg culture (CABI-IMI 392668) isolated from rice in Niger State, Nigeria and its cytotoxic effects on human lymphocytes comparatively to those of some mycotoxin standards

Field	Position	Original line	Normalised line	Original value	Normalised value
AU	1	82849	82849	Makun, HA	Makun, Hussaini_Anthony
AU	2	82850	82850	Dutton, MF	Dutton, Michael_Francis
AU	3	82851	82851	Mwanza, M	Mwanza, Mulunda
AU	4	82852	82852	Njobeh, PB	Njobeh, Patrick_Berka
AF	1	82853	82853	Makun, Hussaini Anthony	Makun, Hussaini_Anthony
AF	2	82854	82854	Dutton, Michael Francis	Dutton, Michael_Francis
AF	4	82856	82856	Njobeh, Patrick Berka	Njobeh, Patrick_Berka

Record 567 | WOS:000295307400004

Toxicological interactions between the mycotoxins beauvericin, deoxynivalenol and T-2 toxin in CHO-K1 cells in vitro

Field	Position	Original line	Normalised line	Original value	Normalised value
AU	1	82953	82953	Ruiz, MJ	Ruiz, Maria-Jose
AU	3	82955	82955	Juan-García, A	Juan-Garcia, Ana
AU	4	82956	82956	Font, G	Font, Guillermina
AF	1	82957	82957	Ruiz, M. J.	Ruiz, Maria-Jose
AF	2	82958	82958	Franzova, P.	Franzova, P
AF	3	82959	82959	Juan-Garcia, A.	Juan-Garcia, Ana
AF	4	82960	82960	Font, G.	Font, Guillermina

Record 568 | WOS:000296746400005

Comparative cytotoxicity of the possibly occurring mycotoxins in wheat on the basis of a mycological analysis

Field	Position	Original line	Normalised line	Original value	Normalised value
AU	1	83042	83042	Bensassi, F	Bensassi, Fatma
AU	2	83043	83043	Bouslimi, A	Bouslimi, Amel
AU	3	83044	83044	El Golli-Bennour, E	el_Golli-Bennour, Emna
AU	4	83045	83045	Gargouri, S	Gargouri, Samia
AU	5	83046	83046	Hajlaoui, MR	Hajlaoui, Mohamed_Rabeh
AU	6	83047	83047	Bacha, H	Bacha, Hassen
AF	3	83050	83050	El Golli-Bennour, Emna	el_Golli-Bennour, Emna
AF	5	83052	83052	Hajlaoui, Mohamed Rabeh	Hajlaoui, Mohamed_Rabeh

Record 569 | WOS:000295697800004

Herbal hepatotoxicity by kava: Update on pipermethystine, flavokavain B, and mould hepatotoxins as primarily assumed culprits

Field	Position	Original line	Normalised line	Original value	Normalised value
AU	1	83196	83196	Teschke, R	Teschke, Rolf
AU	2	83197	83197	Qiu, SX	Qiu, Samuel_X
AU	3	83198	83198	Lebot, V	Lebot, Vincent
AF	2	83200	83200	Qiu, Samuel X.	Qiu, Samuel_X

Record 570 | WOS:000294029600014

Ochratoxin A at nanomolar concentration perturbs the homeostasis of neural stem cells in highly differentiated but not in immature three-dimensional brain cell cultures

Field	Position	Original line	Normalised line	Original value	Normalised value
AU	1	83308	83308	Zurich, MG	Zurich, Marie-Gabrielle
AU	2	83309	83309	Honegger, P	Honegger, Paul

Record 571 | WOS:000293914500008

Perturbation of Mitosis through Inhibition of Histone Acetyltransferases: The Key to Ochratoxin A Toxicity and Carcinogenicity?

Field	Position	Original line	Normalised line	Original value	Normalised value
AU	1	83429	83429	Czakai, K	Czakai, Kristin
AU	2	83430	83430	Müller, K	Mueller, Katja
AU	3	83431	83431	Mosesso, P	Mosesso, Pasquale

AU	4	83432	83432	Pepe, G	Pepe, Gaetano
AU	5	83433	83433	Schulze, M	Schulze, Markus
AU	6	83434	83434	Gohla, A	Gohla, Antje
AU	7	83435	83435	Patnaik, D	Patnaik, Debasis
AU	8	83436	83436	Dekant, W	Dekant, Wolfgang
AU	9	83437	83437	Higgins, JMG	Higgins, Jonathan_M_G
AU	10	83438	83438	Mally, A	Mally, Angela
AF	9	83447	83447	Higgins, Jonathan M. G.	Higgins, Jonathan_M_G

Record 572 | WOS:000293914500016

Site-Specific In Vivo Mutagenicity in the Kidney of gpt Delta Rats Given a Carcinogenic Dose of Ochratoxin A

Field	Position	Original line	Normalised line	Original value	Normalised value
AU	1	83567	83567	Hibi, D	Hibi, Daisuke
AU	2	83568	83568	Suzuki, Y	Suzuki, Yuta
AU	3	83569	83569	Ishii, Y	Ishii, Yuji
AU	4	83570	83570	Jin, ML	Jin, Meilan
AU	5	83571	83571	Watanabe, M	Watanabe, Maiko
AU	6	83572	83572	Sugita-Konishi, Y	Sugita-Konishi, Yoshiko
AU	7	83573	83573	Yanai, T	Yanai, Tokuma
AU	8	83574	83574	Nohmi, T	Nohmi, Takehiko
AU	9	83575	83575	Nishikawa, A	Nishikawa, Akiyoshi
AU	10	83576	83576	Umemura, T	Umemura, Takashi

Record 573 | WOS:000293914500030

Citrinin-Generated Reactive Oxygen Species Cause Cell Cycle Arrest Leading to Apoptosis via the Intrinsic Mitochondrial Pathway in Mouse Skin

Field	Position	Original line	Normalised line	Original value	Normalised value
AU	1	83680	83680	Kumar, R	Kumar, Rahul
AU	2	83681	83681	Dwivedi, PD	Dwivedi, Premendra_D
AU	3	83682	83682	Dhawan, A	Dhawan, Alok
AU	4	83683	83683	Das, M	das, Mukul
AU	5	83684	83684	Ansari, KM	Ansari, Kausar_M
AF	2	83686	83686	Dwivedi, Premendra D.	Dwivedi, Premendra_D
AF	4	83688	83688	Das, Mukul	das, Mukul
AF	5	83689	83689	Ansari, Kausar M.	Ansari, Kausar_M

Record 574 | WOS:000292672700008

Comparative analysis of micronuclei and DNA damage induced by Ochratoxin A in two mammalian cell lines

Field	Position	Original line	Normalised line	Original value	Normalised value
AU	1	83791	83791	Ali, R	Ali, Rahat
AU	2	83792	83792	Mittelstaedt, RA	Mittelstaedt, Roberta_A
AU	3	83793	83793	Shaddock, JG	Shaddock, Joseph_G
AU	4	83794	83794	Ding, W	Ding, Wei
AU	5	83795	83795	Bhalli, JA	Bhalli, Javed_A

AU	6	83796	83796	Khan, QM	Khan, Qaiser_M
AU	7	83797	83797	Heflich, RH	Heflich, Robert_H
AF	2	83799	83799	Mittelstaedt, Roberta A.	Mittelstaedt, Roberta_A
AF	3	83800	83800	Shaddock, Joseph G.	Shaddock, Joseph_G
AF	5	83802	83802	Bhalli, Javed A.	Bhalli, Javed_A
AF	6	83803	83803	Khan, Qaiser M.	Khan, Qaiser_M
AF	7	83804	83804	Heflich, Robert H.	Heflich, Robert_H

Record 575 | WOS:000286856200015

Enzyme-Linked Aptamer Assays (ELAAs), based on a competition format for a rapid and sensitive detection of Ochratoxin A in wine

Field	Position	Original line	Normalised line	Original value	Normalised value
AU	1	83920	83920	Barthelmebs, L	Barthelmebs, Lise
AU	2	83921	83921	Jonca, J	Jonca, Justyna
AU	3	83922	83922	Hayat, A	Hayat, Akhtar
AU	4	83923	83923	Prieto-Simon, B	Prieto-Simon, Beatriz
AU	5	83924	83924	Marty, JL	Marty, Jean-Louis

Record 576 | WOS:000288933100012

Aflatoxin B1 and fumonisin B1 affect the oxidative status of bovine peripheral blood mononuclear cells

Field	Position	Original line	Normalised line	Original value	Normalised value
AU	1	84023	84023	Bernabucci, U	Bernabucci, Umberto
AU	2	84024	84024	Colavecchia, L	Colavecchia, Luciana
AU	3	84025	84025	Danieli, PP	Danieli, Pier_Paolo
AU	4	84026	84026	Basiricò, L	Basirico, Loredana
AU	5	84027	84027	Lacetera, N	Lacetera, Nicola
AU	6	84028	84028	Nardone, A	Nardone, Alessandro
AU	7	84029	84029	Ronchi, B	Ronchi, Bruno
AF	3	84032	84032	Danieli, Pier Paolo	Danieli, Pier_Paolo

Record 577 | WOS:000289042100014

Lymphocyte blastic transformation test in the evaluation of ochratoxin A influence on the immune system of hens and their progeny

Field	Position	Original line	Normalised line	Original value	Normalised value
AU	1	84160	84160	Zaleska, M	Zaleska, Magdalena
AU	2	84161	84161	Zbikowski, A	Zbikowski, Artur
AU	3	84162	84162	Niemiec, J	Niemiec, Jan
AU	4	84163	84163	Kosowska, G	Kosowska, Grazyna

Record 578 | WOS:000288341900004

Zebrafish (Danio rerio) embryos as a model for testing proteratogens

Field	Position	Original line	Normalised line	Original value	Normalised value
AU	1	84229	84229	Weigt, S	Weigt, Stefan

AU	2	84230	84230	Huebler, N	Huebler, Nicole
AU	3	84231	84231	Strecker, R	Strecker, Ruben
AU	4	84232	84232	Braunbeck, T	Braunbeck, Thomas
AU	5	84233	84233	Broschard, TH	Broschard, Thomas_H
AF	5	84238	84238	Broschard, Thomas H.	Broschard, Thomas_H

Record 579 | WOS:000286679100003

Protective effects of melatonin and Glycyrrhiza glabra extract on ochratoxin A-induced damages on testes in mature rats

Field	Position	Original line	Normalised line	Original value	Normalised value
AU	1	84430	84430	Malekinejad, H	Malekinejad, Hassan
AU	2	84431	84431	Mirzakhani, N	Mirzakhani, Navideh
AU	3	84432	84432	Razi, M	Razi, Mazdak
AU	4	84433	84433	Cheraghi, H	Cheraghi, Hadi
AU	5	84434	84434	Alizadeh, A	Alizadeh, Arash
AU	6	84435	84435	Dardmeh, F	Dardmeh, Fereshteh

Record 580 | WOS:000285845400008

Mycotoxin Citrinin Induced Cell Cycle G2/M Arrest and Numerical Chromosomal Aberration Associated with Disruption of Microtubule Formation in Human Cells

Field	Position	Original line	Normalised line	Original value	Normalised value
AU	1	84524	84524	Chang, CH	Chang, Chia-Hao
AU	2	84525	84525	Yu, FY	Yu, Feng-Yih
AU	3	84526	84526	Wu, TS	Wu, Ting-Shun
AU	4	84527	84527	Wang, LT	Wang, Li-Ting
AU	5	84528	84528	Liu, BH	Liu, Biing-Hui

Record 581 | WOS:000285100300026

Metabolic response to low-level toxicant exposure in a novel renal tubule epithelial cell system

Field	Position	Original line	Normalised line	Original value	Normalised value
AU	1	84630	84630	Ellis, JK	Ellis, James_Keith
AU	2	84631	84631	Athersuch, TJ	Athersuch, Toby_James
AU	3	84632	84632	Cavill, R	Cavill, Rachel
AU	4	84633	84633	Radford, R	Radford, Robert
AU	5	84634	84634	Slattery, C	Slattery, Craig
AU	6	84635	84635	Jennings, P	Jennings, Paul
AU	7	84636	84636	McMorrow, T	Mcmorrow, Tara
AU	8	84637	84637	Ryan, MP	Ryan, Michael_P
AU	9	84638	84638	Ebbels, TMD	Ebbels, Timothy_Mark_David
AU	10	84639	84639	Keun, HC	Keun, Hector_Charles
AF	1	84640	84640	Ellis, James Keith	Ellis, James_Keith
AF	2	84641	84641	Athersuch, Toby James	Athersuch, Toby_James
AF	7	84646	84646	McMorrow, Tara	Mcmorrow, Tara
AF	8	84647	84647	Ryan, Michael P.	Ryan, Michael_P
AF	9	84648	84648	Ebbels, Timothy Mark David	Ebbels, Timothy_Mark_David

AF	10	84649	84649	Keun, Hector Charles	Keun, Hector_Charles

Record 582 | WOS:000297106700013

Review of mycotoxin reduction in food and feed: from prevention in the field to detoxification by adsorption or transformation

Field	Position	Original line	Normalised line	Original value	Normalised value
AU	3	84766	84766	Mathieu, F	Mathieu, Florence
AU	4	84767	84767	Guyonvarc'h, A	Guyonvarc'H, A
AF	1	84769	84769	Jard, G.	Jard, G
AF	2	84770	84770	Liboz, T.	Liboz, T
AF	3	84771	84771	Mathieu, F.	Mathieu, Florence
AF	4	84772	84772	Guyonvarc'h, A.	Guyonvarc'H, A
AF	5	84773	84773	Lebrihi, A.	Lebrihi, A

Record 583 | WOS:000298090700025

Experimentally Induced Toxicity of Ochratoxin A and Endosulfan in Male Wistar Rats: A Hormonal Disorder

Field	Position	Original line	Normalised line	Original value	Normalised value
AU	1	85066	85066	Kumar, SN	Kumar, Shashi_Nandar
AU	2	85067	85067	Telang, AG	Telang, Avinash_Gopal
AU	3	85068	85068	Singh, KP	Singh, Karan_Pal
AU	4	85069	85069	Jain, AK	Jain, Arun_Kumar
AU	5	85070	85070	Afroz, M	Afroz, Masarrat
AU	6	85071	85071	Patil, RD	Patil, Rajendra_Damu
AF	1	85072	85072	Kumar, Shashi Nandar	Kumar, Shashi_Nandar
AF	2	85073	85073	Telang, Avinash Gopal	Telang, Avinash_Gopal
AF	3	85074	85074	Singh, Karan Pal	Singh, Karan_Pal
AF	4	85075	85075	Jain, Arun Kumar	Jain, Arun_Kumar
AF	6	85077	85077	Patil, Rajendra Damu	Patil, Rajendra_Damu

Record 584 | WOS:000295693800002

Potential of an in vitro toolbox combined with exposure data as a first step for the risk assessment of dietary chemical contaminants

Field	Position	Original line	Normalised line	Original value	Normalised value
AU	1	85171	85171	Ribonnet, L	Ribonnet, Laurence
AU	2	85172	85172	van der Heiden, E	van_der_Heiden, Edwige
AU	5	85175	85175	Remacle, AS	Remacle, A_-S
AU	6	85176	85176	De Saeger, S	de_Saeger, Sarah
AU	7	85177	85177	Schneider, YJ	Schneider, Yves-Jacques
AU	8	85178	85178	Scippo, ML	Scippo, Marie-Louise
AU	10	85180	85180	Pussemier, L	Pussemier, Luc
AU	11	85181	85181	Larondelle, Y	Larondelle, Yvan
AF	1	85182	85182	Ribonnet, L.	Ribonnet, Laurence
AF	2	85183	85183	van der Heiden, E.	van_der_Heiden, Edwige
AF	3	85184	85184	Nobels, I.	Nobels, I
AF	4	85185	85185	Chaumont, A.	Chaumont, A

AF	5	85186	85186	Remacle, A. -S.	Remacle, A_-S
AF	6	85187	85187	De Saeger, S.	de_Saeger, Sarah
AF	7	85188	85188	Schneider, Y. -J.	Schneider, Yves-Jacques
AF	8	85189	85189	Scippo, M. -L.	Scippo, Marie-Louise
AF	9	85190	85190	Blust, R.	Blust, R
AF	10	85191	85191	Pussemier, L.	Pussemier, Luc
AF	11	85192	85192	Larondelle, Y.	Larondelle, Yvan

Record 585 | WOS:000292496700010

Developing mechanism-based and exposure biomarkers for mycotoxins in animals

Field	Position	Original line	Normalised line	Original value	Normalised value
AU	1	85377	85377	Riley, RT	Riley, R_T
AU	2	85378	85378	Voss, KA	Voss, K_A
AU	3	85379	85379	Coulombe, RA	Coulombe, R_A
AU	4	85380	85380	Pestka, JJ	Pestka, J_J
AU	5	85381	85381	Williams, DE	Williams, D_E
AF	1	85382	85382	Riley, R. T.	Riley, R_T
AF	2	85383	85383	Voss, K. A.	Voss, K_A
AF	3	85384	85384	Coulombe, R. A.	Coulombe, R_A
AF	4	85385	85385	Pestka, J. J.	Pestka, J_J
AF	5	85386	85386	Williams, D. E.	Williams, D_E

Record 586 | WOS:000297106700012

Isotherm modeling of organic activated bentonite and humic acid polymer used as mycotoxin adsorbents

Field	Position	Original line	Normalised line	Original value	Normalised value
AU	1	85595	85595	Santos, RR	Santos, R_R
AU	4	85598	85598	Fink-Gremmels, J	Fink-Gremmels, Johanna
AF	1	85599	85599	Santos, R. R.	Santos, R_R
AF	2	85600	85600	Vermeulen, S.	Vermeulen, S
AF	3	85601	85601	Haritova, A.	Haritova, A
AF	4	85602	85602	Fink-Gremmels, J.	Fink-Gremmels, Johanna

Record 587 | WOS:000290627500016

Aptamer-functionalized magnetic nanoparticle-based bioassay for the detection of ochratoxin a using upconversion nanoparticles as labels

Field	Position	Original line	Normalised line	Original value	Normalised value
AU	1	85698	85698	Wu, SJ	Wu, Shijia
AU	2	85699	85699	Duan, N	Duan, Nuo
AU	3	85700	85700	Wang, ZP	Wang, Zhouping
AU	4	85701	85701	Wang, HX	Wang, Hongxin

Record 588 | WOS:000283743900015

Immunotoxicity of zearalenone in Balb/c mice in a high subchronic dosing study counteracted by Raphanus sativus extract

Field	Position	Original line	Normalised line	Original value	Normalised value
AU	1	85812	85812	Ben Salah-Abbès, J	Ben_Salah-Abbes, Jalila
AU	2	85813	85813	Abbès, S	Abbes, Samir
AU	3	85814	85814	Abdel-Wahhab, MA	Abdel-Wahhab, Mosaad_A
AU	4	85815	85815	Oueslati, R	Oueslati, Ridha
AF	1	85816	85816	Ben Salah-Abbes, Jalila	Ben_Salah-Abbes, Jalila
AF	3	85818	85818	Abdel-Wahhab, M. A.	Abdel-Wahhab, Mosaad_A

Record 589 | WOS:000284436000007

Evaluation of a urinary kidney biomarker panel in rat models of acute and subchronic nephrotoxicity

Field	Position	Original line	Normalised line	Original value	Normalised value
AU	1	85921	85921	Hoffmann, D	Hoffmann, Dana
AU	2	85922	85922	Fuchs, TC	Fuchs, Tobias_C
AU	3	85923	85923	Henzler, T	Henzler, Tanja
AU	4	85924	85924	Matheis, KA	Matheis, Katja_A
AU	5	85925	85925	Herget, T	Herget, Thomas
AU	6	85926	85926	Dekant, W	Dekant, Wolfgang
AU	7	85927	85927	Hewitt, P	Hewitt, Philip
AU	8	85928	85928	Mally, A	Mally, Angela
AF	2	85930	85930	Fuchs, Tobias C.	Fuchs, Tobias_C
AF	4	85932	85932	Matheis, Katja A.	Matheis, Katja_A

Record 590 | WOS:000283810500022

Protein Biomarkers for in Vitro Testing of Embryotoxicity

Field	Position	Original line	Normalised line	Original value	Normalised value
AU	1	86053	86053	Groebe, K	Groebe, Karlfried
AU	2	86054	86054	Hayess, K	Hayess, Katrin
AU	3	86055	86055	Klemm-Manns, M	Klemm-Manns, Martina
AU	4	86056	86056	Schwall, G	Schwall, Gerhard
AU	5	86057	86057	Wozny, W	Wozny, Woijciech
AU	6	86058	86058	Steemans, M	Steemans, Margino
AU	7	86059	86059	Peters, AK	Peters, Annelieke_K
AU	8	86060	86060	Sastri, C	Sastri, Chaturvedala
AU	9	86061	86061	Jaeckel, P	Jaeckel, Petra
AU	10	86062	86062	Stegmann, W	Stegmann, Werner
AU	11	86063	86063	Zengerling, H	Zengerling, Helmut
AU	12	86064	86064	Schöpf, R	Schoepf, Rainer
AU	13	86065	86065	Poznanovic, S	Poznanovic, Slobodan
AU	14	86066	86066	Stummann, TC	Stummann, Tina_C
AU	15	86067	86067	Seiler, A	Seiler, Andrea
AU	16	86068	86068	Spielmann, H	Spielmann, Horst
AU	17	86069	86069	Schrattenholz, A	Schrattenholz, Andre
AF	7	86076	86076	Peters, Annelieke K.	Peters, Annelieke_K
AF	14	86083	86083	Stummann, Tina C.	Stummann, Tina_C

Record 591 | WOS:000280344000003

Some food-associated mycotoxins as potential risk factors in humans predisposed to chronic intestinal inflammatory diseases

Field	Position	Original line	Normalised line	Original value	Normalised value
AU	1	86205	86205	Maresca, M	Maresca, Marc
AU	2	86206	86206	Fantini, J	Fantini, Jacques

Record 592 | WOS:000281740900017

Mycotoxins and Their Biotransformation in the Rumen: A Review

Field	Position	Original line	Normalised line	Original value	Normalised value
AU	1	86421	86421	Upadhaya, SD	Upadhaya, Santi_Devi
AU	2	86422	86422	Park, MA	Park, M_A
AU	3	86423	86423	Ha, JK	Ha, Jong_K
AF	1	86424	86424	Upadhaya, Santi Devi	Upadhaya, Santi_Devi
AF	2	86425	86425	Park, M. A.	Park, M_A
AF	3	86426	86426	Ha, Jong K.	Ha, Jong_K

Record 593 | WOS:000282996200006

Free radicals and antioxidants in two oxidative-stress cell models exposed to ochratoxin A and amyloid β: unexpected results

Field	Position	Original line	Normalised line	Original value	Normalised value
AU	4	86603	86603	Guerra, MC	Guerra, M_C
AF	1	86605	86605	Costa, S.	Costa, S
AF	2	86606	86606	Cervellati, R.	Cervellati, R
AF	3	86607	86607	Speroni, E.	Speroni, E
AF	4	86608	86608	Guerra, M. C.	Guerra, M_C
AF	5	86609	86609	Greco, E.	Greco, E

Record 594 | WOS:000280991100075

Safety studies on products from whole coffee fruit

Field	Position	Original line	Normalised line	Original value	Normalised value
AU	1	86684	86684	Heimbach, JT	Heimbach, J_T
AU	2	86685	86685	Marone, PA	Marone, P_A
AU	3	86686	86686	Hunter, JM	Hunter, J_M
AU	4	86687	86687	Nemzer, BV	Nemzer, B_V
AU	5	86688	86688	Stanley, SM	Stanley, S_M
AF	1	86690	86690	Heimbach, J. T.	Heimbach, J_T
AF	2	86691	86691	Marone, P. A.	Marone, P_A
AF	3	86692	86692	Hunter, J. M.	Hunter, J_M
AF	4	86693	86693	Nemzer, B. V.	Nemzer, B_V
AF	5	86694	86694	Stanley, S. M.	Stanley, S_M
AF	6	86695	86695	Kennepohl, E.	Kennepohl, E

Record 595 | WOS:000280256800007

Beauvericin and ochratoxin A genotoxicity evaluated using the alkaline comet assay: single and combined genotoxic action

Field	Position	Original line	Normalised line	Original value	Normalised value
AU	1	86783	86783	Klaric, MS	Klaric, Maja_Segvic
AU	2	86784	86784	Darabos, D	Darabos, Dina
AU	3	86785	86785	Rozgaj, R	Rozgaj, Ruzica
AU	4	86786	86786	Kasuba, V	Kasuba, Vilena
AU	5	86787	86787	Pepeljnjak, S	Pepeljnjak, Stjepan
AF	1	86788	86788	Klaric, Maja Segvic	Klaric, Maja_Segvic

Record 596 | WOS:000282996200009

Mycotoxins in grapes and wine in Europe: occurrence, factors affecting the occurrence and related toxicological effects

Field	Position	Original line	Normalised line	Original value	Normalised value
AU	2	86888	86888	van der Fels-Klerx, HJ	van_der_Fels-Klerx, H_J
AF	1	86889	86889	Stratakou, I.	Stratakou, I
AF	2	86890	86890	van der Fels-Klerx, H. J.	van_der_Fels-Klerx, H_J

Record 597 | WOS:000278803100015

Allium cepa as a biomonitor of ochratoxin A toxicity and genotoxicity

Field	Position	Original line	Normalised line	Original value	Normalised value
AU	2	87023	87023	Bistoni, MB	Bistoni, M_Biagi
AF	1	87026	87026	Lerda, D.	Lerda, D
AF	2	87027	87027	Bistoni, M. Biagi	Bistoni, M_Biagi
AF	3	87028	87028	Pelliccioni, P.	Pelliccioni, P
AF	4	87029	87029	Litterio, N.	Litterio, N

Record 598 | WOS:000277103900009

Neuronal models for evaluation of proliferation in vitro using high content screening

Field	Position	Original line	Normalised line	Original value	Normalised value
AU	1	87102	87102	Mundy, WR	Mundy, William_R
AU	2	87103	87103	Radio, NM	Radio, Nicholas_M
AU	3	87104	87104	Freudenrich, TM	Freudenrich, Theresa_M
AF	1	87105	87105	Mundy, William R.	Mundy, William_R
AF	2	87106	87106	Radio, Nicholas M.	Radio, Nicholas_M
AF	3	87107	87107	Freudenrich, Theresa M.	Freudenrich, Theresa_M

Record 599 | WOS:000208434800018

Antigenotoxic Studies of Different Substances to Reduce the DNA Damage Induced by Aflatoxin B1 and Ochratoxin A

Field	Position	Original line	Normalised line	Original value	Normalised value

AU	1	87196	87196	Madrigal-Santillán, E	Madrigal-Santillan, Eduardo
AU	2	87197	87197	Morales-González, JA	Morales-Gonzalez, Jose_A
AU	3	87198	87198	Vargas-Mendoza, N	Vargas-Mendoza, Nancy
AU	4	87199	87199	Reyes-Ramírez, P	Reyes-Ramirez, Patricia
AU	5	87200	87200	Cruz-Jaime, S	Cruz-Jaime, Sandra
AU	6	87201	87201	Sumaya-Martínez, T	Sumaya-Martinez, Teresa
AU	7	87202	87202	Pérez-Pastén, R	Perez-Pasten, Ricardo
AU	8	87203	87203	Madrigal-Bujaidar, E	Madrigal-Bujaidar, Eduardo
AF	2	87205	87205	Morales-Gonzalez, Jose A.	Morales-Gonzalez, Jose_A

Record 600 | WOS:000276175200004

Ochratoxin A induces G2 phase arrest in human gastric epithelium GES-1 cells in vitro

Field	Position	Original line	Normalised line	Original value	Normalised value
AU	1	87356	87356	Cui, JF	Cui, Jinfeng
AU	2	87357	87357	Xing, LX	Xing, Lingxiao
AU	3	87358	87358	Li, ZN	Li, Zengning
AU	4	87359	87359	Wu, S	Wu, Sha
AU	5	87360	87360	Wang, J	Wang, Juan
AU	6	87361	87361	Liu, J	Liu, Jing
AU	7	87362	87362	Wang, JL	Wang, Junling
AU	8	87363	87363	Yan, X	Yan, Xia
AU	9	87364	87364	Zhang, XH	Zhang, Xianghong

Record 601 | WOS:000274582400001

PREVENTIVE AND THERAPEUTIC METHODS AGAINST THE TOXIC EFFECTS OF MYCOTOXINS - A REVIEW

Field	Position	Original line	Normalised line	Original value	Normalised value
AU	1	87457	87457	Mézes, M	Mezes, Miklos
AU	2	87458	87458	Balogh, K	Balogh, Krisztian
AU	3	87459	87459	Tóth, K	Toth, Katalin

Record 602 | WOS:000276819800011

Decontamination and detoxification strategies for the Fusarium mycotoxin deoxynivalenol in animal feed and the effectiveness of microbial biodegradation

Field	Position	Original line	Normalised line	Original value	Normalised value
AU	1	87635	87635	Awad, WA	Awad, Wageha_A
AU	2	87636	87636	Ghareeb, K	Ghareeb, Khaled
AU	3	87637	87637	Böhm, J	Boehm, Josef
AU	4	87638	87638	Zentek, J	Zentek, Juergen
AF	1	87639	87639	Awad, Wageha A.	Awad, Wageha_A

Record 603 | WOS:000279331700013

Modification of aflatoxin B1 and ochratoxin A toxicokinetics in rats administered a yeast cell wall preparation

Field	Position	Original line	Normalised line	Original value	Normalised value
AU	3	87777	87777	Morgavi, DP	Morgavi, D_P
AU	5	87779	87779	Jouany, JP	Jouany, J_P
AF	1	87782	87782	Firmin, S.	Firmin, S
AF	2	87783	87783	Gandia, P.	Gandia, P
AF	3	87784	87784	Morgavi, D. P.	Morgavi, D_P
AF	4	87785	87785	Houin, G.	Houin, G
AF	5	87786	87786	Jouany, J. P.	Jouany, J_P
AF	6	87787	87787	Bertin, G.	Bertin, G
AF	7	87788	87788	Boudra, H.	Boudra, H

Record 604 | WOS:000273528200008

Zearalenone and its Derivatives: Known Toxins in New Aspects

Field	Position	Original line	Normalised line	Original value	Normalised value
AU	1	87895	87895	Golinski, P	Golinski, Piotr
AU	2	87896	87896	Waskiewicz, A	Waskiewicz, Agnieszka
AU	3	87897	87897	Gromadzka, K	Gromadzka, Karolina

Record 605 | WOS:000281731700007

Lifetime, low-dose ochratoxin A dietary study on renal carcinogenesis in male Fischer rats

Field	Position	Original line	Normalised line	Original value	Normalised value
AU	1	88034	88034	Mantle, P	Mantle, Peter
AU	2	88035	88035	Kulinskaya, E	Kulinskaya, Elena

Record 606 | WOS:000273618500012

Structures of Covalent Adducts between DNA and Ochratoxin A: A New Factor in Debate about Genotoxicity and Human Risk Assessment

Field	Position	Original line	Normalised line	Original value	Normalised value
AU	1	88111	88111	Mantle, PG	Mantle, Peter_G
AU	2	88112	88112	Faucet-Marquis, V	Faucet-Marquis, Virginie
AU	3	88113	88113	Manderville, RA	Manderville, Richard_A
AU	4	88114	88114	Squillaci, B	Squillaci, Bianca
AU	5	88115	88115	Pfohl-Leszkowicz, A	Pfohl-Leszkowicz, Annie
AF	1	88116	88116	Mantle, Peter G.	Mantle, Peter_G
AF	3	88118	88118	Manderville, Richard A.	Manderville, Richard_A

Record 607 | WOS:000286528700008

Ochratoxin A-induced serum biochemical alterations in New Zealand white rabbits (Oryctolagus cuniculus)

Field	Position	Original line	Normalised line	Original value	Normalised value
AU	1	88229	88229	Mir, MS	Mir, Masood_S
AU	2	88230	88230	Dwivedi, P	Dwivedi, Prabakar
AF	1	88231	88231	Mir, Masood S.	Mir, Masood_S

Record 608 | WOS:000274143800002

Toxicology of mycotoxins

Field	Position	Original line	Normalised line	Original value	Normalised value
AU	1	88322	88322	Paterson, RRM	Paterson, Robert_R_M
AU	2	88323	88323	Lima, N	Lima, Nelson
AF	1	88324	88324	Paterson, Robert R. M.	Paterson, Robert_R_M

Record 609 | WOS:000272760200016

CYP1A1 and CYP3A4 modulation by dietary flavonoids in human intestinal Caco-2 cells

Field	Position	Original line	Normalised line	Original value	Normalised value
AU	1	88487	88487	Sergent, T	Sergent, Therese
AU	2	88488	88488	Dupont, I	Dupont, Isabelle
AU	3	88489	88489	Van der Heiden, E	van_der_Heiden, Edwige
AU	4	88490	88490	Scippo, ML	Scippo, Marie-Louise
AU	5	88491	88491	Pussemier, L	Pussemier, Luc
AU	6	88492	88492	Larondelle, Y	Larondelle, Yvan
AU	7	88493	88493	Schneider, YJ	Schneider, Yves-Jacques
AF	3	88496	88496	Van der Heiden, Edwige	van_der_Heiden, Edwige

Record 610 | WOS:000271971100003

Comparative Mechanisms of Zearalenone and Ochratoxin A Toxicities on Cultured HepG2 cells: Is Oxidative Stress a Common Process?

Field	Position	Original line	Normalised line	Original value	Normalised value
AU	1	88623	88623	Bennour, EEG	Bennour, Emna_el_Golli
AU	2	88624	88624	Bouaziz, C	Bouaziz, Chayma
AU	3	88625	88625	Ladjimi, M	Ladjimi, Moncef
AU	4	88626	88626	Renaud, F	Renaud, Flore
AU	5	88627	88627	Bacha, H	Bacha, Hassen
AF	1	88628	88628	Bennour, Emna El Golli	Bennour, Emna_el_Golli

Record 611 | WOS:000208434400002

Cyclopiazonic Acid Biosynthesis of Aspergillus flavus and Aspergillus oryzae

Field	Position	Original line	Normalised line	Original value	Normalised value
AU	1	88736	88736	Chang, PK	Chang, Perng-Kuang
AU	2	88737	88737	Ehrlich, KC	Ehrlich, Kenneth_C
AU	3	88738	88738	Fujii, I	Fujii, Isao
AF	2	88740	88740	Ehrlich, Kenneth C.	Ehrlich, Kenneth_C

Record 612 | WOS:000272276600011

Reactive oxygen species induced by beauvericin, patulin and zearalenone in CHO-K1 cells

Field	Position	Original line	Normalised line	Original value	Normalised value
AU	2	88916	88916	Juan-García, A	Juan-Garcia, Ana
AU	3	88917	88917	Font, G	Font, Guillermina
AU	4	88918	88918	Ruiz, MJ	Ruiz, Maria-Jose
AF	1	88919	88919	Ferrer, E.	Ferrer, E
AF	2	88920	88920	Juan-Garcia, A.	Juan-Garcia, Ana
AF	3	88921	88921	Font, G.	Font, Guillermina
AF	4	88922	88922	Ruiz, M. J.	Ruiz, Maria-Jose

Record 613 | WOS:000272276600013

Intracellular zinc stores protect the intestinal epithelium from Ochratoxin A toxicity

Field	Position	Original line	Normalised line	Original value	Normalised value
AF	1	89004	89004	Ranaldi, G.	Ranaldi, G
AF	2	89005	89005	Caprini, V.	Caprini, V
AF	3	89006	89006	Sambuy, Y.	Sambuy, Y
AF	4	89007	89007	Perozzi, G.	Perozzi, G
AF	5	89008	89008	Murgia, C.	Murgia, C

Record 614 | WOS:000270527000004

Minimum tolerable exposure period and maximum threshold dietary intake of ochratoxin A for causing renal cancer in male Dark Agouti rats

Field	Position	Original line	Normalised line	Original value	Normalised value
AU	1	89098	89098	Mantle, PG	Mantle, Peter_G
AF	1	89099	89099	Mantle, Peter G.	Mantle, Peter_G

Record 615 | WOS:000270503100034

Molecular Characterization of Preneoplastic Lesions Provides Insight on the Development of Renal Tumors

Field	Position	Original line	Normalised line	Original value	Normalised value
AU	1	89165	89165	Stemmer, K	Stemmer, Kerstin
AU	2	89166	89166	Ellinger-Ziegelbauer, H	Ellinger-Ziegelbauer, Heidrun
AU	3	89167	89167	Ahr, HJ	Ahr, Hans-Juergen
AU	4	89168	89168	Dietrich, DR	Dietrich, Daniel_R
AF	4	89172	89172	Dietrich, Daniel R.	Dietrich, Daniel_R

Record 616 | WOS:000269677000009

Low doses of ochratoxin A upregulate the protein expression of organic anion transporters Oat1, Oat2, Oat3 and Oat5 in rat kidney cortex

Field	Position	Original line	Normalised line	Original value	Normalised value
AU	1	89280	89280	Zlender, V	Zlender, Vilim
AU	2	89281	89281	Breljak, D	Breljak, Davorka
AU	3	89282	89282	Ljubojevic, M	Ljubojevic, Marija
AU	4	89283	89283	Flajs, D	Flajs, Dubravka
AU	5	89284	89284	Balen, D	Balen, Daniela

AU	6	89285	89285	Brzica, H	Brzica, Hrvoje
AU	7	89286	89286	Domijan, AM	Domijan, Ana-Marija
AU	8	89287	89287	Peraica, M	Peraica, Maja
AU	9	89288	89288	Fuchs, R	Fuchs, Radovan
AU	10	89289	89289	Anzai, N	Anzai, Naohiko
AU	11	89290	89290	Sabolic, I	Sabolic, Ivan

Record 617 | WOS:000269551500021

The mycotoxin ochratoxin A inhibits DNA topoisomerase II and induces polyploidy in cultured CHO cells

Field	Position	Original line	Normalised line	Original value	Normalised value
AU	1	89428	89428	Cosimi, S	Cosimi, Simona
AU	2	89429	89429	Orta, L	Orta, Luis
AU	3	89430	89430	Mateos, S	Mateos, Santiago
AU	4	89431	89431	Cortés, F	Cortes, Felipe

Record 618 | WOS:000269551500029

Multiplexed assay panel of cytotoxicity in HK-2 cells for detection of renal proximal tubule injury potential of compounds

Field	Position	Original line	Normalised line	Original value	Normalised value
AU	1	89539	89539	Wu, Y	Wu, Yang
AU	2	89540	89540	Connors, D	Connors, David
AU	3	89541	89541	Barber, L	Barber, Lauren
AU	4	89542	89542	Jayachandra, S	Jayachandra, Sukhanya
AU	5	89543	89543	Hanumegowda, UM	Hanumegowda, Umesh_M
AU	6	89544	89544	Adams, SP	Adams, Stephen_P
AF	5	89549	89549	Hanumegowda, Umesh M.	Hanumegowda, Umesh_M
AF	6	89550	89550	Adams, Stephen P.	Adams, Stephen_P

Record 619 | WOS:000268006900004

Pro-apoptotic effects of nivalenol and deoxynivalenol trichothecenes in J774A.1 murine macrophages

Field	Position	Original line	Normalised line	Original value	Normalised value
AU	1	89646	89646	Marzocco, S	Marzocco, Stefania
AU	2	89647	89647	Russo, R	Russo, Rosario
AU	3	89648	89648	Bianco, G	Bianco, Giuseppe
AU	4	89649	89649	Autore, G	Autore, Giuseppina
AU	5	89650	89650	Severino, L	Severino, Lorella

Record 620 | WOS:000282995500010

Occurrence of ochratoxin A and heavy metals in tissues associated with porcine nephropathy in Serbia

Field	Position	Original line	Normalised line	Original value	Normalised value
AU	1	89748	89748	Milicevic, D	Milicevic, Dragan
AU	3	89750	89750	Stefanovic, S	Stefanovic, Srdan

AF	1	89754	89754	Milicevic, D.	Milicevic, Dragan
AF	2	89755	89755	Juric, V.	Juric, V
AF	3	89756	89756	Stefanovic, S.	Stefanovic, Srdan
AF	4	89757	89757	Jovanovic, M.	Jovanovic, M
AF	5	89758	89758	Petrovic, Z.	Petrovic, Z
AF	6	89759	89759	Vukovic, D.	Vukovic, D

Record 621 | WOS:000267227800008

Ochratoxin A-Mediated DNA and Protein Damage: Roles of Nitrosative and Oxidative Stresses

Field	Position	Original line	Normalised line	Original value	Normalised value
AU	1	89871	89871	Cavin, C	Cavin, Christophe
AU	2	89872	89872	Delatour, T	Delatour, Thierry
AU	3	89873	89873	Marin-Kuan, M	Marin-Kuan, Maricel
AU	4	89874	89874	Fenaille, F	Fenaille, Francois
AU	5	89875	89875	Holzhäuser, D	Holzhaeuser, Daisy
AU	6	89876	89876	Guignard, G	Guignard, Gabriela
AU	7	89877	89877	Bezençon, C	Bezencon, Claudine
AU	8	89878	89878	Piguet, D	Piguet, Dominique
AU	9	89879	89879	Parisod, V	Parisod, Veronique
AU	10	89880	89880	Richoz-Payot, J	Richoz-Payot, Janique
AU	11	89881	89881	Schilter, B	Schilter, Benoit

Record 622 | WOS:000268796500023

A Review of the Implementation of the Embryonic Stem Cell Test (EST) The report and recommendations of an ECVAM/ReProTect Workshop

Field	Position	Original line	Normalised line	Original value	Normalised value
AU	1	90011	90011	Marx-Stoelting, P	Marx-Stoelting, Philip
AU	2	90012	90012	Adriaens, E	Adriaens, Els
AU	3	90013	90013	Ahr, HJ	Ahr, Hans-Juergen
AU	4	90014	90014	Bremer, S	Bremer, Susanne
AU	5	90015	90015	Garthoff, B	Garthoff, Bernward
AU	6	90016	90016	Gelbke, HP	Gelbke, Heinz-Peter
AU	7	90017	90017	Piersma, A	Piersma, Aldert
AU	8	90018	90018	Pellizzer, C	Pellizzer, Cristian
AU	9	90019	90019	Reuter, U	Reuter, Ulrike
AU	10	90020	90020	Rogiers, V	Rogiers, Vera
AU	11	90021	90021	Schenk, B	Schenk, Barbara
AU	12	90022	90022	Schwengberg, S	Schwengberg, Silke
AU	13	90023	90023	Seiler, A	Seiler, Andrea
AU	14	90024	90024	Spielmann, H	Spielmann, Horst
AU	15	90025	90025	Steemans, M	Steemans, Margino
AU	16	90026	90026	Stedman, DB	Stedman, Donald_B
AU	17	90027	90027	Vanparys, P	Vanparys, Philippe
AU	18	90028	90028	Vericat, JA	Vericat, Joan_Albert
AU	19	90029	90029	Verwei, M	Verwei, Miriam
AU	20	90030	90030	Van De Water, F	van_de_Water, Femke

AU	21	90031	90031	Weimer, M	Weimer, Marc
AU	22	90032	90032	Schwarz, M	Schwarz, Michael
AF	16	90048	90048	Stedman, Donald B.	Stedman, Donald_B
AF	18	90050	90050	Vericat, Joan Albert	Vericat, Joan_Albert
AF	20	90052	90052	van de Water, Femke	van_de_Water, Femke

Record 623 | WOS:000265685000003

Oxidative stress and apoptotic changes in primary cultures of rat proximal tubular cells exposed to lead

Field	Position	Original line	Normalised line	Original value	Normalised value
AU	1	90150	90150	Wang, L	Wang, Lin
AU	2	90151	90151	Wang, H	Wang, Heng
AU	3	90152	90152	Hu, MZ	Hu, Maozhi
AU	4	90153	90153	Cao, J	Cao, Jin
AU	5	90154	90154	Chen, DW	Chen, Dawei
AU	6	90155	90155	Liu, ZP	Liu, Zongping

Record 624 | WOS:000265474500006

Silibinin protects OTA-mediated TNF-α release from perfused rat livers and isolated rat Kupffer cells

Field	Position	Original line	Normalised line	Original value	Normalised value
AU	1	90253	90253	Al-Anati, L	Al-Anati, Lauy
AU	2	90254	90254	Essid, E	Essid, Ebtisam
AU	3	90255	90255	Reinehr, R	Reinehr, Roland
AU	4	90256	90256	Petzinger, E	Petzinger, Ernst

Record 625 | WOS:000265474500007

Mycotoxins and the kidney: Modes of action for renal tumor formation by ochratoxin A in rodents

Field	Position	Original line	Normalised line	Original value	Normalised value
AU	1	90352	90352	Mally, A	Mally, Angela
AU	2	90353	90353	Dekant, W	Dekant, Wolfgang

Record 626 | WOS:000263803400010

Effects of the mycotoxin fumonisin B1 on cell death in human kidney cells and human lung fibroblasts in primary culture

Field	Position	Original line	Normalised line	Original value	Normalised value
AU	1	90495	90495	Schwerdt, G	Schwerdt, Gerald
AU	2	90496	90496	Königs, M	Koenigs, Maika
AU	3	90497	90497	Holzinger, H	Holzinger, Hildegard
AU	4	90498	90498	Humpf, HU	Humpf, Hans-Ulrich
AU	5	90499	90499	Gekle, M	Gekle, Michael
AF	1	90500	90500	Schwerdt, G.	Schwerdt, Gerald
AF	2	90501	90501	Koenigs, M.	Koenigs, Maika
AF	3	90502	90502	Holzinger, H.	Holzinger, Hildegard

AF	4	90503	90503	Humpf, H. U.	Humpf, Hans-Ulrich
AF	5	90504	90504	Gekle, M.	Gekle, Michael

Record 627 | WOS:000266663300004

Ochratoxin A induces apoptosis in neuronal cells

Field	Position	Original line	Normalised line	Original value	Normalised value
AU	1	90608	90608	Zhang, XN	Zhang, Xiangnan
AU	2	90609	90609	Boesch-Saadatmandi, C	Boesch-Saadatmandi, Christine
AU	3	90610	90610	Lou, YJ	Lou, Yijia
AU	4	90611	90611	Wolffram, S	Wolffram, Siegfried
AU	5	90612	90612	Huebbe, P	Huebbe, Patricia
AU	6	90613	90613	Rimbach, G	Rimbach, Gerald

Record 628 | WOS:000263382500003

CYP1A1 induction and CYP3A4 inhibition by the fungicide imazalil in the human intestinal Caco-2 cells-Comparison with other conazole pesticides

Field	Position	Original line	Normalised line	Original value	Normalised value
AU	1	90705	90705	Sergent, T	Sergent, Therese
AU	2	90706	90706	Dupont, I	Dupont, Isabelle
AU	3	90707	90707	Jassogne, C	Jassogne, Coralie
AU	4	90708	90708	Ribonnet, L	Ribonnet, Laurence
AU	5	90709	90709	van der Heiden, E	van_der_Heiden, Edwige
AU	6	90710	90710	Scippo, ML	Scippo, Marie-Louise
AU	7	90711	90711	Muller, M	Muller, Marc
AU	8	90712	90712	McAlister, D	Mcalister, Dan
AU	9	90713	90713	Pussemier, L	Pussemier, Luc
AU	10	90714	90714	Larondelle, Y	Larondelle, Yvan
AU	11	90715	90715	Schneider, YJ	Schneider, Yves-Jacques
AF	5	90720	90720	van der Heiden, Edwige	van_der_Heiden, Edwige
AF	8	90723	90723	McAlister, Dan	Mcalister, Dan

Record 629 | WOS:000262717200017

Effects of live yeast cell supplementation to high concentrate diets on the toxicokinetics of ochratoxin A in sheep

Field	Position	Original line	Normalised line	Original value	Normalised value
AU	1	90857	90857	Blank, R	Blank, Ralf
AU	2	90858	90858	Wolffram, S	Wolffram, Siegfried
AF	1	90859	90859	Blank, R.	Blank, Ralf
AF	2	90860	90860	Wolffram, S.	Wolffram, Siegfried

Record 630 | WOS:000267272900021

The biotransformation of chosen mycotoxins

Field	Position	Original line	Normalised line	Original value	Normalised value

AF	1	90952	90952	Gajecka, M.	Gajecka, M
AF	2	90953	90953	Jakimiuk, E.	Jakimiuk, E
AF	3	90954	90954	Zielonka, L.	Zielonka, L
AF	4	90955	90955	Obremski, K.	Obremski, K
AF	5	90956	90956	Gajecki, M.	Gajecki, M

Record 631 | WOS:000273001000009

Effects of probiotic bacteria on the bioaccessibility of aflatoxin B1 and ochratoxin A using an in vitro digestion model under fed conditions

Field	Position	Original line	Normalised line	Original value	Normalised value
AU	1	91056	91056	Kabak, B	Kabak, Bulent
AU	2	91057	91057	Brandon, EFA	Brandon, Esther_F_A
AU	3	91058	91058	Var, I	Var, Isil
AU	4	91059	91059	Blokland, M	Blokland, Marco
AU	5	91060	91060	Sips, AJAM	Sips, Adrienne_J_A_M
AF	2	91062	91062	Brandon, Esther F. A.	Brandon, Esther_F_A
AF	5	91065	91065	Sips, Adrienne J. A. M.	Sips, Adrienne_J_A_M

Record 632 | WOS:000292954300008

Microorganisms and their enzymes for detoxifying mycotoxins posing a risk to livestock animals

Field	Position	Original line	Normalised line	Original value	Normalised value
AU	2	91162	91162	Binder, EM	Binder, Eva_Maria
AU	3	91163	91163	Schatzmayr, G	Schatzmayr, Gerd
AF	1	91164	91164	Rodrigues, I.	Rodrigues, I
AF	2	91165	91165	Binder, E. M.	Binder, Eva_Maria
AF	3	91166	91166	Schatzmayr, G.	Schatzmayr, Gerd

Record 633 | WOS:000262775900032

Effect of ochratoxin A on cell survival and collagen homeostasis in human mesangial cells in primary culture

Field	Position	Original line	Normalised line	Original value	Normalised value
AU	1	91260	91260	Schwerdt, G	Schwerdt, Gerald
AU	2	91261	91261	Holzinger, H	Holzinger, Hildegard
AU	3	91262	91262	Königs, M	Koenigs, Maika
AU	4	91263	91263	Humpf, HU	Humpf, Hans-Ulrich
AU	5	91264	91264	Gekle, M	Gekle, Michael

Record 634 | WOS:000264793900023

EFFECTS OF AFLATOXIN B1, OCHRATOXIN A, PATULIN, CITRININ, AND ZEARALENONE ON THE IN VITRO PROLIFERATION OF PIG BLOOD LYMPHOCYTES

Field	Position	Original line	Normalised line	Original value	Normalised value
AU	1	91349	91349	Stec, J	Stec, Jan
AU	2	91350	91350	Zmudzki, J	Zmudzki, Jacek
AU	3	91351	91351	Rachubik, J	Rachubik, Jaroslaw

AU	4	91352	91352	Szczotka, M	Szczotka, Maria

Record 635 | WOS:000262790100004

Developmental Toxicity of Ochratoxin A in Rat Embryo Midbrain Micromass Cultures

Field	Position	Original line	Normalised line	Original value	Normalised value
AU	1	91437	91437	Wilk-Zasadna, I	Wilk-Zasadna, Iwona
AU	2	91438	91438	Minta, M	Minta, Maria

Record 636 | WOS:000261056700090

Safety and Mutagenicity Evaluation of Nanoparticulate Red Mold Rice

Field	Position	Original line	Normalised line	Original value	Normalised value
AU	1	91532	91532	Yu, CC	Yu, Chiun-Chieh
AU	2	91533	91533	Wang, JJ	Wang, Jyh-Jye
AU	3	91534	91534	Lee, CL	Lee, Chun-Lin
AU	4	91535	91535	Lee, SH	Lee, Shu-Hui
AU	5	91536	91536	Pan, TM	Pan, Tzu-Ming

Record 637 | WOS:000207684500010

Oxidative stress induced by ochratoxin A in LLC-PK1 cell line and the chemoprotective effects of carnosic acid

Field	Position	Original line	Normalised line	Original value	Normalised value
AU	7	91652	91652	Guerra, MC	Guerra, M_C
AF	1	91653	91653	Costa, S.	Costa, S
AF	2	91654	91654	Utan, A.	Utan, A
AF	3	91655	91655	Speroni, E.	Speroni, E
AF	4	91656	91656	Cervellati, R.	Cervellati, R
AF	5	91657	91657	Piva, G.	Piva, G
AF	6	91658	91658	Prandini, A.	Prandini, A
AF	7	91659	91659	Guerra, M. C.	Guerra, M_C

Record 638 | WOS:000260367000001

Individual and combined effects of ochratoxin A and citrinin on viability and DNA fragmentation in cultured Vero cells and on chromosome aberrations in mice bone marrow cells

Field	Position	Original line	Normalised line	Original value	Normalised value
AU	1	91730	91730	Bouslimi, A	Bouslimi, Amel
AU	2	91731	91731	Bouaziz, C	Bouaziz, Chayma
AU	3	91732	91732	Ayed-Boussema, I	Ayed-Boussema, Imen
AU	4	91733	91733	Hassen, W	Hassen, Wafa
AU	5	91734	91734	Bacha, H	Bacha, Hassen

Record 639 | WOS:000259798900005

Inula crithmoides extract protects against ochratoxin A-induced oxidative stress, clastogenic and mutagenic alterations in male rats

Field	Position	Original line	Normalised line	Original value	Normalised value
AU	1	91863	91863	Abdel-Wahhab, MA	Abdel-Wahhab, Mosaad_A
AU	2	91864	91864	Abdel-Azim, SH	Abdel-Azim, Sekena_H
AU	3	91865	91865	El-Nekeety, AA	El-Nekeety, Aziza_A
AF	1	91866	91866	Abdel-Wahhab, Mosaad A.	Abdel-Wahhab, Mosaad_A
AF	2	91867	91867	Abdel-Azim, Sekena H.	Abdel-Azim, Sekena_H
AF	3	91868	91868	El-Nekeety, Aziza A.	El-Nekeety, Aziza_A

Record 640 | WOS:000260145000006

Differential modification of inflammatory enzymes in J774A.1 macrophages by ochratoxin A alone or in combination with lipopolysaccharide

Field	Position	Original line	Normalised line	Original value	Normalised value
AU	1	91991	91991	Ferrante, MC	Ferrante, Maria_Carmela
AU	2	91992	91992	Raso, GM	Raso, G_Mattace
AU	3	91993	91993	Bilancione, M	Bilancione, Marcella
AU	4	91994	91994	Esposito, E	Esposito, Emanuela
AU	5	91995	91995	Iacono, A	Iacono, Anna
AU	6	91996	91996	Meli, R	Meli, Rosaria
AF	1	91997	91997	Ferrante, M. C.	Ferrante, Maria_Carmela
AF	2	91998	91998	Raso, G. Mattace	Raso, G_Mattace
AF	3	91999	91999	Bilancione, M.	Bilancione, Marcella
AF	4	92000	92000	Esposito, E.	Esposito, Emanuela
AF	5	92001	92001	Iacono, A.	Iacono, Anna
AF	6	92002	92002	Meli, R.	Meli, Rosaria

Record 641 | WOS:000258440100010

Effect of ochratoxin A on redox-regulated transcription factors, antioxidant enzymes and glutathione-S-transferase in cultured kidney tubulus cells

Field	Position	Original line	Normalised line	Original value	Normalised value
AU	1	92110	92110	Boesch-Saadatmandi, C	Boesch-Saadatmandi, Christine
AU	2	92111	92111	Loboda, A	Loboda, Agnieszka
AU	3	92112	92112	Jozkowicz, A	Jozkowicz, Alicja
AU	4	92113	92113	Huebbe, P	Huebbe, Patricia
AU	5	92114	92114	Blank, R	Blank, Ralf
AU	6	92115	92115	Wolffram, S	Wolffram, Siegfried
AU	7	92116	92116	Dulak, J	Dulak, Jozef
AU	8	92117	92117	Rimbach, G	Rimbach, Gerald
AF	1	92118	92118	Boesch-Saadatmandi, C.	Boesch-Saadatmandi, Christine
AF	2	92119	92119	Loboda, A.	Loboda, Agnieszka
AF	3	92120	92120	Jozkowicz, A.	Jozkowicz, Alicja
AF	4	92121	92121	Huebbe, P.	Huebbe, Patricia
AF	5	92122	92122	Blank, R.	Blank, Ralf
AF	6	92123	92123	Wolffram, S.	Wolffram, Siegfried
AF	7	92124	92124	Dulak, J.	Dulak, Jozef
AF	8	92125	92125	Rimbach, G.	Rimbach, Gerald

Record 642 | WOS:000257645600013

Species-specific toxicity of aristolochic acid (AA) in vitro

Field	Position	Original line	Normalised line	Original value	Normalised value
AU	2	92237	92237	Bruske, EI	Bruske, E_I
AU	4	92239	92239	O'Brien, E	O'Brien, Evelyn
AU	5	92240	92240	Dietrich, DR	Dietrich, Daniel_R
AF	1	92241	92241	Huljic, S.	Huljic, S
AF	2	92242	92242	Bruske, E. I.	Bruske, E_I
AF	3	92243	92243	Pfitzenmaier, N.	Pfitzenmaier, N
AF	4	92244	92244	O'Brien, E.	O'Brien, Evelyn
AF	5	92245	92245	Dietrich, D. R.	Dietrich, Daniel_R

Record 643 | WOS:000257721400033

Identification and in vitro cytotoxicity of ochratoxin a degradation products formed during coffee roasting

Field	Position	Original line	Normalised line	Original value	Normalised value
AU	1	92334	92334	Cramer, B	Cramer, Benedikt
AU	2	92335	92335	Königs, M	Koenigs, Maika
AU	3	92336	92336	Humpf, HU	Humpf, Hans-Ulrich

Record 644 | WOS:000257577500008

Gene expression changes induced by ochratoxin A in renal and hepatic tissues of male F344 rat after oral repeated administration

Field	Position	Original line	Normalised line	Original value	Normalised value
AU	1	92424	92424	Arbillaga, L	Arbillaga, Leire
AU	2	92425	92425	Vettorazzi, A	Vettorazzi, Ariane
AU	3	92426	92426	Gil, AG	Gil, Ana_G
AU	4	92427	92427	van Delft, JHM	van_Delft, Joost_H_M
AU	5	92428	92428	García-Jalón, JA	Garcia-Jalon, Jose_Antonio
AU	6	92429	92429	de Cerain, AL	de_Cerain, Adela_Lopez
AF	3	92432	92432	Gil, Ana G.	Gil, Ana_G
AF	4	92433	92433	van Delft, Joost H. M.	van_Delft, Joost_H_M
AF	5	92434	92434	Garcia-Jalon, Jose Antonio	Garcia-Jalon, Jose_Antonio
AF	6	92435	92435	de Cerain, Adela Lopez	de_Cerain, Adela_Lopez

Record 645 | WOS:000256601000019

Structure-activity relationships for the fluorescence of ochratoxin A: Insight for detection of ochratoxin A metabolites

Field	Position	Original line	Normalised line	Original value	Normalised value
AU	1	92531	92531	Frenette, C	Frenette, Christine
AU	2	92532	92532	Paugh, RJ	Paugh, Robert_J
AU	3	92533	92533	Tozlovanu, M	Tozlovanu, Mariana
AU	4	92534	92534	Maud, JB	Maud, Juzio_B
AU	5	92535	92535	Pfohl-Leszkowicz, A	Pfohl-Leszkowicz, Annie

AU	6	92536	92536	Manderville, RA	Manderville, Richard_A
AF	2	92538	92538	Paugh, Robert J.	Paugh, Robert_J
AF	4	92540	92540	Maud, Juzio B.	Maud, Juzio_B
AF	6	92542	92542	Manderville, Richard. A.	Manderville, Richard_A

Record 646 | WOS:000256806600009

In vitro cytogenetic results supporting a DNA nonreactive mechanism for Ochratoxin A, potentially relevant for its carcinogenicity

Field	Position	Original line	Normalised line	Original value	Normalised value
AU	1	92633	92633	Mosesso, P	Mosesso, Pasquale
AU	2	92634	92634	Cinelli, S	Cinelli, Serena
AU	3	92635	92635	Piñero, J	Pinero, Joaquin
AU	4	92636	92636	Bellacima, R	Bellacima, Raffaela
AU	5	92637	92637	Pepe, G	Pepe, Gaetano

Record 647 | WOS:000256206000004

Modulating effects of fumonisin B1 and ochratoxin A on leukocytes and messenger cytokines of the human immune system

Field	Position	Original line	Normalised line	Original value	Normalised value
AU	1	92761	92761	Odhav, B	Odhav, Bhard
AU	2	92762	92762	Adam, JK	Adam, Jamila_K
AU	3	92763	92763	Bhoola, KD	Bhoola, Kand_D
AF	2	92765	92765	Adam, Jamila K.	Adam, Jamila_K
AF	3	92766	92766	Bhoola, Kand D.	Bhoola, Kand_D

Record 648 | WOS:000255760800015

Evaluation of putative biomarkers of nephrotoxicity after exposure to ochratoxin a in vivo and in vitro

Field	Position	Original line	Normalised line	Original value	Normalised value
AU	1	92843	92843	Rached, E	Rached, Eva
AU	2	92844	92844	Hoffmann, D	Hoffmann, Dana
AU	3	92845	92845	Blumbach, K	Blumbach, Kai
AU	4	92846	92846	Weber, K	Weber, Klaus
AU	5	92847	92847	Dekant, W	Dekant, Wolfgang
AU	6	92848	92848	Mally, A	Mally, Angela

Record 649 | WOS:000256713600009

Ochratoxin A inhibits the production of tissue factor and plasminogen activator inhibitor-2 by human blood mononuclear cells: Another potential mechanism of immune-suppression

Field	Position	Original line	Normalised line	Original value	Normalised value
AU	1	92956	92956	Rossiello, MR	Rossiello, Maria_R
AU	2	92957	92957	Rotunno, C	Rotunno, Crescenzia
AU	3	92958	92958	Coluccia, A	Coluccia, Addolorata
AU	4	92959	92959	Carratu, MR	Carratu, Maria_R
AU	5	92960	92960	Di Santo, A	di_Santo, Angelomaria

AU	6	92961	92961	Evangelista, V	Evangelista, Virgilio
AU	7	92962	92962	Semeraro, N	Semeraro, Nicola
AU	8	92963	92963	Colucci, M	Colucci, Mario
AF	1	92964	92964	Rossiello, Maria R.	Rossiello, Maria_R
AF	4	92967	92967	Carratu, Maria R.	Carratu, Maria_R
AF	5	92968	92968	Di Santo, Angelomaria	di_Santo, Angelomaria

Record 650 | WOS:000256381400006

Medical relevance of mycotoxins

Field	Position	Original line	Normalised line	Original value	Normalised value
AF	1	93058	93058	Hof, H.	Hof, H

Record 651 | WOS:000254689700022

Detoxification of patulin and ochratoxin A, two abundant mycotoxins, by lactic acid bacteria

Field	Position	Original line	Normalised line	Original value	Normalised value
AU	6	93122	93122	Knasmüller, S	Knasmueller, S
AF	1	93123	93123	Fuchs, S.	Fuchs, S
AF	2	93124	93124	Sontag, G.	Sontag, G
AF	3	93125	93125	Stidl, R.	Stidl, R
AF	4	93126	93126	Ehrlich, V.	Ehrlich, V
AF	5	93127	93127	Kundi, M.	Kundi, M
AF	6	93128	93128	Knasmueller, S.	Knasmueller, S

Record 652 | WOS:000254544300011

Both direct and indirect effects account for the pro-inflammatory activity of enteropathogenic mycotoxins on the human intestinal epithelium:: Stimulation of interleukin-8 secretion, potentiation of interleukin-1β effect and increase in the transepithelial passage of commensal bacteria

Field	Position	Original line	Normalised line	Original value	Normalised value
AU	1	93260	93260	Maresca, M	Maresca, Marc
AU	2	93261	93261	Yahi, N	Yahi, Nouara
AU	3	93262	93262	Younes-Sakr, L	Younes-Sakr, Lama
AU	4	93263	93263	Boyron, M	Boyron, Marilyn
AU	5	93264	93264	Caporiccio, B	Caporiccio, Bertrand
AU	6	93265	93265	Fantini, J	Fantini, Jacques

Record 653 | WOS:000257197500001

Risk-benefit considerations of mitigation measures on acrylamide content of foods - A case study on potatoes, cereals and coffee

Field	Position	Original line	Normalised line	Original value	Normalised value
AU	1	93387	93387	Seal, CJ	Seal, C_J
AU	2	93388	93388	de Mul, A	de_Mul, A
AU	4	93390	93390	Haverkort, AJ	Haverkort, A_J
AU	6	93392	93392	Lalljie, SPD	Lalljie, S_P_D

AU	12	93398	93398	van Boekel, M	van_Boekel, M
AU	13	93399	93399	van Klaveren, J	van_Klaveren, J
AU	14	93400	93400	Wilcockson, SJ	Wilcockson, S_J
AF	1	93402	93402	Seal, C. J.	Seal, C_J
AF	2	93403	93403	de Mul, A.	de_Mul, A
AF	3	93404	93404	Eisenbrand, G.	Eisenbrand, G
AF	4	93405	93405	Haverkort, A. J.	Haverkort, A_J
AF	5	93406	93406	Franke, K.	Franke, K
AF	6	93407	93407	Lalljie, S. P. D.	Lalljie, S_P_D
AF	7	93408	93408	Mykkaenen, H.	Mykkaenen, H
AF	8	93409	93409	Reimerdes, E.	Reimerdes, E
AF	9	93410	93410	Scholz, G.	Scholz, G
AF	10	93411	93411	Somoza, V.	Somoza, V
AF	11	93412	93412	Tuijtelaars, S.	Tuijtelaars, S
AF	12	93413	93413	van Boekel, M.	van_Boekel, M
AF	13	93414	93414	van Klaveren, J.	van_Klaveren, J
AF	14	93415	93415	Wilcockson, S. J.	Wilcockson, S_J
AF	15	93416	93416	Wilms, L.	Wilms, L

Record 654 | WOS:000258542600115

In vitro investigation of individual and combined cytotoxicity of ochratoxin a and arsenic in Chinese hamster lung fibroblasts and human urothelial cells

Field	Position	Original line	Normalised line	Original value	Normalised value
AU	2	93771	93771	Behm, C	Behm, Claudia
AU	3	93772	93772	Foellmann, W	Foellmann, Wolfram
AU	4	93773	93773	Malik, JK	Malik, J_K
AU	5	93774	93774	Degen, GH	Degen, Gisela_H
AF	1	93775	93775	Aggarwal, M.	Aggarwal, M
AF	2	93776	93776	Behm, C.	Behm, Claudia
AF	3	93777	93777	Foellmann, W.	Foellmann, Wolfram
AF	4	93778	93778	Malik, J. K.	Malik, J_K
AF	5	93779	93779	Degen, G. H.	Degen, Gisela_H

Record 655 | WOS:000252706500006

Ameliorative effect of artichoke (Cynara scolymus L.) extracts on creatine monohydrate-induced renal dysfunction in male rats

Field	Position	Original line	Normalised line	Original value	Normalised value
AU	1	93822	93822	Ahmed, HH	Ahmed, Hanaa_H
AU	2	93823	93823	Mannaa, F	Mannaa, Fathia
AU	3	93824	93824	El-Sayed, EM	El-Sayed, Eman_M
AF	1	93825	93825	Ahmed, Hanaa H.	Ahmed, Hanaa_H
AF	3	93827	93827	El-Sayed, Eman M.	El-Sayed, Eman_M

Record 656 | WOS:000255988000006

Cytotoxicity and oxidative damage in kidney cells exposed to the mycotoxins ochratoxin A and citrinin: Individual and combined effects

Field	Position	Original line	Normalised line	Original value	Normalised value
AU	1	93948	93948	Bouslimi, A	Bouslimi, Amel
AU	2	93949	93949	Ouannes, Z	Ouannes, Zouhour
AU	3	93950	93950	El Golli, E	el_Golli, Emna
AU	4	93951	93951	Bouaziz, C	Bouaziz, Chayma
AU	5	93952	93952	Hassen, W	Hassen, Wafa
AU	6	93953	93953	Bacha, H	Bacha, Hassen
AF	3	93956	93956	El Golli, Emna	el_Golli, Emna

Record 657 | WOS:000253659900006

Mycotoxins in cattle feeds and carry-over to dairy milk: A review

Field	Position	Original line	Normalised line	Original value	Normalised value
AU	1	94060	94060	Fink-Gremmels, J	Fink-Gremmels, Johanna

Record 658 | WOS:000268886600003

Combined Toxic Effects of Ochratoxin A and Citrinin, In Vivo and In Vitro

Field	Position	Original line	Normalised line	Original value	Normalised value
AU	1	94179	94179	Pfohl-Leszkowicz, A	Pfohl-Leszkowicz, Annie
AU	2	94180	94180	Molinié, A	Molinie, A
AU	3	94181	94181	Tozlovanu, M	Tozlovanu, Mariana
AU	4	94182	94182	Manderville, RA	Manderville, Richard_A
AF	1	94183	94183	Pfohl-Leszkowicz, A.	Pfohl-Leszkowicz, Annie
AF	2	94184	94184	Molinie, A.	Molinie, A
AF	3	94185	94185	Tozlovanu, M.	Tozlovanu, Mariana
AF	4	94186	94186	Manderville, R. A.	Manderville, Richard_A

Record 659 | WOS:000251052700012

Toxigenic fungi and mycotoxins in mature corn silage

Field	Position	Original line	Normalised line	Original value	Normalised value
AF	1	94294	94294	Richard, E.	Richard, E
AF	2	94295	94295	Heutte, N.	Heutte, N
AF	3	94296	94296	Sage, L.	Sage, L
AF	4	94297	94297	Pottier, D.	Pottier, D
AF	5	94298	94298	Bouchart, V.	Bouchart, V
AF	6	94299	94299	Lebailly, P.	Lebailly, P
AF	7	94300	94300	Garon, D.	Garon, D

Record 660 | WOS:000249598500015

Catechins: natural free-radical scavengers against ochratoxin A-induced cell damage in a pig kidney cell line (LLC-PK1)

Field	Position	Original line	Normalised line	Original value	Normalised value
AU	5	94385	94385	Guerra, MC	Guerra, M_C

AF	1	94386	94386	Costa, S.	Costa, S
AF	2	94387	94387	Utan, A.	Utan, A
AF	3	94388	94388	Cervellati, R.	Cervellati, R
AF	4	94389	94389	Speroni, E.	Speroni, E
AF	5	94390	94390	Guerra, M. C.	Guerra, M_C

Record 661 | WOS:000249983300013

New molecular and field evidences for the implication of mycotoxins but not aristolochic acid in human nephropathy and urinary tract tumor

Field	Position	Original line	Normalised line	Original value	Normalised value
AU	1	94487	94487	Pfohl-Leszkowicz, A	Pfohl-Leszkowicz, Annie
AU	2	94488	94488	Tozlovanu, M	Tozlovanu, Mariana
AU	3	94489	94489	Manderville, R	Manderville, Richard
AU	4	94490	94490	Peraica, M	Peraica, Maja
AU	5	94491	94491	Castegnaro, M	Castegnaro, Marcel
AU	6	94492	94492	Stefanovic, V	Stefanovic, Vladislav

Record 662 | WOS:000248014300007

Ochratoxin A-induced mutagenesis in mammalian cells is consistent with the production of oxidative stress

Field	Position	Original line	Normalised line	Original value	Normalised value
AU	1	94613	94613	Palma, N	Palma, Nieves
AU	2	94614	94614	Cinelli, S	Cinelli, Serena
AU	3	94615	94615	Sapora, O	Sapora, Orazio
AU	4	94616	94616	Wilson, SH	Wilson, Samuel_H
AU	5	94617	94617	Dogliotti, E	Dogliotti, Eugenia
AF	4	94621	94621	Wilson, Samuel H.	Wilson, Samuel_H

Record 663 | WOS:000247601800018

Adult hippocampal neural stem/progenitor cells in vitro are vulnerable to the mycotoxin ochratoxin-A

Field	Position	Original line	Normalised line	Original value	Normalised value
AU	1	94755	94755	Sava, V	Sava, Vasyl
AU	2	94756	94756	Velasquez, A	Velasquez, Adriana
AU	3	94757	94757	Song, S	Song, Shijie
AU	4	94758	94758	Sanchez-Ramos, J	Sanchez-Ramos, Juan

Record 664 | WOS:000246927300041

Ochratoxin A displaces claudins from detergent resistant membrane microdomains

Field	Position	Original line	Normalised line	Original value	Normalised value
AU	1	94868	94868	Lambert, D	Lambert, Daniel
AU	2	94869	94869	Padfield, PJ	Padfield, Philip_J
AU	3	94870	94870	McLaughlin, J	Mclaughlin, John
AU	4	94871	94871	Cannell, S	Cannell, Stephanie

AU	5	94872	94872	O'Neill, CA	O'Neill, Catherine_A
AF	2	94874	94874	Padfield, Philip J.	Padfield, Philip_J
AF	3	94875	94875	McLaughlin, John	Mclaughlin, John
AF	5	94877	94877	O'Neill, Catherine A.	O'Neill, Catherine_A

Record 665 | WOS:000247178200008

Ochratoxin A:: 13-week oral toxicity and cell proliferation in male F344/N rats

Field	Position	Original line	Normalised line	Original value	Normalised value
AU	1	94945	94945	Rached, E	Rached, Eva
AU	2	94946	94946	Hard, GC	Hard, Gordon_C
AU	3	94947	94947	Blumbach, K	Blumbach, Kai
AU	4	94948	94948	Weber, K	Weber, Klaus
AU	5	94949	94949	Draheim, R	Draheim, Regina
AU	6	94950	94950	Lutz, WK	Lutz, Werner_K
AU	7	94951	94951	Özden, S	Ozden, Sibel
AU	8	94952	94952	Steger, U	Steger, Ulrich
AU	9	94953	94953	Dekant, W	Dekant, Wolfgang
AU	10	94954	94954	Mally, A	Mally, Angela
AF	2	94956	94956	Hard, Gordon C.	Hard, Gordon_C
AF	6	94960	94960	Lutz, Werner K.	Lutz, Werner_K

Record 666 | WOS:000247092600009

Transepithelial transport of fusariotoxin nivalenol: Mediation of secretion by ABC transporters

Field	Position	Original line	Normalised line	Original value	Normalised value
AU	1	95055	95055	Tep, J	Tep, Jonathan
AU	2	95056	95056	Videmann, B	Videmann, Bernadette
AU	3	95057	95057	Mazallon, M	Mazallon, Michele
AU	4	95058	95058	Balleydier, S	Balleydier, Sabine
AU	5	95059	95059	Cavret, S	Cavret, Severine
AU	6	95060	95060	Lecoeur, S	Lecoeur, Sylvaine

Record 667 | WOS:000246213800014

Production and characterization of monoclonal antibodies against ochratoxin B

Field	Position	Original line	Normalised line	Original value	Normalised value
AU	1	95159	95159	Heussner, AH	Heussner, Alexandra_H
AU	2	95160	95160	Moeller, I	Moeller, Ines
AU	3	95161	95161	Day, BW	Day, Billy_W
AU	4	95162	95162	Dietrich, DR	Dietrich, Daniel_R
AU	5	95163	95163	O'Brien, E	O'Brien, Evelyn
AF	1	95164	95164	Heussner, Alexandra H.	Heussner, Alexandra_H
AF	3	95166	95166	Day, Billy W.	Day, Billy_W
AF	4	95167	95167	Dietrich, Daniel R.	Dietrich, Daniel_R

Record 668 | WOS:000246875200005

Combined effects of mycotoxin mixtures on human T cell function

Field	Position	Original line	Normalised line	Original value	Normalised value
AU	1	95253	95253	Tammer, B	Tammer, Beate
AU	2	95254	95254	Lehmann, I	Lehmann, Irina
AU	3	95255	95255	Nieber, K	Nieber, Karen
AU	4	95256	95256	Altenburger, R	Altenburger, Rolf

Record 669 | WOS:000245707900011

In vitro gene expression data supporting a DNA non-reactive genotoxic mechanism for ochratoxin A

Field	Position	Original line	Normalised line	Original value	Normalised value
AU	1	95356	95356	Arbillaga, L	Arbillaga, Leire
AU	2	95357	95357	Azqueta, A	Azqueta, Amaia
AU	3	95358	95358	van Delft, JHM	van_Delft, Joost_H_M
AU	4	95359	95359	de Cerain, AL	Lopez_de_Cerain, Adela
AF	3	95362	95362	van Delft, Joost H. M.	van_Delft, Joost_H_M
AF	4	95363	95363	Lopez de Cerain, Adela	Lopez_de_Cerain, Adela

Record 670 | WOS:000244262900005

Reduction in antioxidant defenses may contribute to ochratoxin a toxicity and carcinogenicity

Field	Position	Original line	Normalised line	Original value	Normalised value
AU	1	95461	95461	Cavin, C	Cavin, Christophe
AU	2	95462	95462	Delatour, T	Delatour, Thierry
AU	3	95463	95463	Marin-Kuan, M	Marin-Kuan, Maricel
AU	4	95464	95464	Holzhäuser, D	Holzhauser, Daisy
AU	5	95465	95465	Higgins, L	Higgins, Larry
AU	6	95466	95466	Bezençon, C	Bezencon, Claudine
AU	7	95467	95467	Guignard, G	Guignard, Gabriela
AU	8	95468	95468	Junod, S	Junod, Sylviane
AU	9	95469	95469	Richoz-Payot, J	Richoz-Payot, Janique
AU	10	95470	95470	Gremaud, E	Gremaud, Eric
AU	11	95471	95471	Hayes, JD	Hayes, John_D
AU	12	95472	95472	Nestler, S	Nestler, Sandra
AU	13	95473	95473	Mantle, P	Mantle, Peter
AU	14	95474	95474	Schilter, B	Schilter, Benoit
AF	11	95485	95485	Hayes, John D.	Hayes, John_D

Record 671 | WOS:000244244100003

Lipid peroxidation and glutathione levels in porcine kidney PK15 cells after individual and combined treatment with fumonisin B1, beauvericin and ochratoxin A

Field	Position	Original line	Normalised line	Original value	Normalised value
AU	1	95593	95593	Klaric, MS	Klaric, Maja_Segvic

AU	2	95594	95594	Pepeljnjak, S	Pepeljnjak, Stjepan
AU	3	95595	95595	Domijan, AM	Domijan, Ana-Marija
AU	4	95596	95596	Petrik, J	Petrik, Jozsef
AF	1	95597	95597	Klaric, Maja Segvic	Klaric, Maja_Segvic

Record 672 | WOS:000244169500009

Effects of repeated ochratoxin exposure on renal cells in vitro

Field	Position	Original line	Normalised line	Original value	Normalised value
AU	1	95693	95693	Heussner, AH	Heussner, Alexandra_H
AU	2	95694	95694	O'Brien, E	O'Brien, Evelyn
AU	3	95695	95695	Dietrich, DR	Dietrich, Daniel_R
AF	1	95696	95696	Heussner, A. H.	Heussner, Alexandra_H
AF	2	95697	95697	O'Brien, E.	O'Brien, Evelyn
AF	3	95698	95698	Dietrich, D. R.	Dietrich, Daniel_R

Record 673 | WOS:000243327000004

Oxidative DNA damage induced by Ochratoxin A in the HK-2 human kidney cell line: evidence of the relationship with cytotoxicity

Field	Position	Original line	Normalised line	Original value	Normalised value
AU	1	95771	95771	Arbillaga, L	Arbillaga, Leire
AU	2	95772	95772	Azqueta, A	Azqueta, Amaia
AU	3	95773	95773	Ezpeleta, O	Ezpeleta, Olga
AU	4	95774	95774	de Cerain, AL	de_Cerain, Adela_Lopez
AF	4	95778	95778	de Cerain, Adela Lopez	de_Cerain, Adela_Lopez

Record 674 | WOS:000244790500003

Ochratoxin A: An overview on toxicity and carcinogenicity in animals and humans

Field	Position	Original line	Normalised line	Original value	Normalised value
AU	1	95884	95884	Pfohl-Leszkowicz, A	Pfohl-Leszkowicz, Annie
AU	2	95885	95885	Manderville, RA	Manderville, Richard_A
AF	2	95887	95887	Manderville, Richard A.	Manderville, Richard_A

Record 675 | WOS:000254907600043

Cytotoxicity of feed-borne mycotoxins to animal cell lines in vitro using the MTT assay

Field	Position	Original line	Normalised line	Original value	Normalised value
AU	1	96435	96435	Stec, J	Stec, Jan
AU	2	96436	96436	Szczotka, M	Szczotka, Maria
AU	3	96437	96437	Kuzmak, J	Kuzmak, Jacek

Record 676 | WOS:000248276300289

Effect of Saccharomyces cerevisiae and esterified glucomannan on residues of Ochratoxin A in kidney, muscle and blood of laying hens

Field	Position	Original line	Normalised line	Original value	Normalised value
AF	1	96507	96507	Zaghini, A.	Zaghini, A
AF	2	96508	96508	Simioli, M.	Simioli, M
AF	3	96509	96509	Roncada, R.	Roncada, R
AF	4	96510	96510	Rizzi, L.	Rizzi, L

Record 677 | WOS:000243441300001

Review on the toxicity, occurrence, metabolism, detoxification, regulations and intake of zearalenone:: An oestrogenic mycotoxin

Field	Position	Original line	Normalised line	Original value	Normalised value
AU	1	96573	96573	Zinedine, A	Zinedine, Abdellah
AU	2	96574	96574	Soriano, JM	Soriano, Jose_Miguel
AU	3	96575	96575	Moltó, JC	Molto, Juan_Carlos
AU	4	96576	96576	Mañes, J	Manes, Jordi
AF	2	96578	96578	Soriano, Jose Miguel	Soriano, Jose_Miguel
AF	3	96579	96579	Molto, Juan Carlos	Molto, Juan_Carlos

Record 678 | WOS:000242136100016

Interactive effects of fumonisin B1 and α-zearalenol on proliferation and cytokine expression in Jurkat T cells

Field	Position	Original line	Normalised line	Original value	Normalised value
AU	2	96819	96819	Severino, L	Severino, Lorella
AU	4	96821	96821	De Luna, R	de_Luna, R
AF	1	96824	96824	Luongo, D.	Luongo, D
AF	2	96825	96825	Severino, L.	Severino, Lorella
AF	3	96826	96826	Bergamo, P.	Bergamo, P
AF	4	96827	96827	De Luna, R.	de_Luna, R
AF	5	96828	96828	Lucisano, A.	Lucisano, A
AF	6	96829	96829	Rossi, M.	Rossi, M

Record 679 | WOS:000243461200014

Ochratoxin A-induced cytotoxicity in liver (HepG2) cells:: Impact of serum concentration, dietary antioxidants and glutathione-modulating compounds

Field	Position	Original line	Normalised line	Original value	Normalised value
AU	1	96928	96928	Bösch-Saadatmandi, C	Boesch-Saadatmandi, Christine
AU	2	96929	96929	Hundhausen, C	Hundhausen, Christoph
AU	3	96930	96930	Jofre-Monseny, L	Jofre-Monseny, Laia
AU	4	96931	96931	Blank, R	Blank, Ralf
AU	5	96932	96932	Wolffram, S	Wolffram, Siegfried
AU	6	96933	96933	Rimbach, G	Rimbach, Gerald

Record 680 | WOS:000241622800019

Critical period and minimum single oral dose of ochratoxin A for inducing developmental toxicity in pregnant Wistar rats

Field	Position	Original line	Normalised line	Original value	Normalised value

AU	1	97045	97045	Patil, RD	Patil, Rajendra_D
AU	2	97046	97046	Dwivedi, P	Dwivedi, Prabhaker
AU	3	97047	97047	Sharma, AK	Sharma, Anil_K
AF	1	97048	97048	Patil, Rajendra D.	Patil, Rajendra_D
AF	3	97050	97050	Sharma, Anil K.	Sharma, Anil_K

Record 681 | WOS:000239496200009

A study of zearalenone cytotoxicity on human peripheral blood mononuclear cells

Field	Position	Original line	Normalised line	Original value	Normalised value
AU	1	97132	97132	Vlata, Z	Vlata, Zaxarenia
AU	2	97133	97133	Porichis, F	Porichis, Filippos
AU	3	97134	97134	Tzanakakis, G	Tzanakakis, George
AU	4	97135	97135	Tsatsakis, A	Tsatsakis, Aristidis
AU	5	97136	97136	Krambovitis, E	Krambovitis, Elias

Record 682 | WOS:000239973600004

Expression of COX-2 and hsp72 in peritoneal macrophages after an acute ochratoxin A treatment in mice

Field	Position	Original line	Normalised line	Original value	Normalised value
AU	1	97230	97230	Ferrante, MC	Ferrante, Maria_Carmela
AU	2	97231	97231	Bilancione, M	Bilancione, Marcella
AU	3	97232	97232	Raso, GM	Raso, Giuseppina_Mattace
AU	4	97233	97233	Esposito, E	Esposito, Emanuela
AU	5	97234	97234	Iacono, A	Iacono, Anna
AU	6	97235	97235	Zaccaroni, A	Zaccaroni, Annalisa
AU	7	97236	97236	Meli, R	Meli, Rosaria
AF	1	97237	97237	Ferrante, Maria Carmela	Ferrante, Maria_Carmela
AF	3	97239	97239	Raso, Giuseppina Mattace	Raso, Giuseppina_Mattace

Record 683 | WOS:000240051000015

Early cytotoxic effects of ochratoxin A in rat liver: A morphological, biochemical and molecular study

Field	Position	Original line	Normalised line	Original value	Normalised value
AU	1	97347	97347	Gagliano, N	Gagliano, Nicoletta
AU	2	97348	97348	Donne, ID	Donne, Isabella_Dalle
AU	3	97349	97349	Torri, C	Torri, Carlo
AU	4	97350	97350	Migliori, M	Migliori, Massimiliano
AU	5	97351	97351	Grizzi, F	Grizzi, Fabio
AU	6	97352	97352	Milzani, A	Milzani, Aldo
AU	7	97353	97353	Filippi, C	Filippi, Cristina
AU	8	97354	97354	Annoni, G	Annoni, Giorgio
AU	9	97355	97355	Colombo, P	Colombo, Piergiuseppe
AU	10	97356	97356	Costa, F	Costa, Francesco
AU	11	97357	97357	Ceva-Grimaldi, G	Ceva-Grimaldi, Giorgia
AU	12	97358	97358	Bertelli, AAE	Bertelli, Alberto_A_E

AU	13	97359	97359	Giovannini, L	Giovannini, Luca
AU	14	97360	97360	Gioia, M	Gioia, Magda
AF	2	97362	97362	Donne, Isabella Dalle	Donne, Isabella_Dalle
AF	12	97372	97372	Bertelli, Alberto A. E.	Bertelli, Alberto_A_E

Record 684 | WOS:000240051000002

Adverse effects of T-2 toxin on chicken lymphocytes blastogenesis and its protection with Vitamin E

Field	Position	Original line	Normalised line	Original value	Normalised value
AU	1	97482	97482	Jaradat, ZW	Jaradat, Ziad_W
AU	2	97483	97483	Vila, B	Vila, Borja
AU	3	97484	97484	Marquardt, RR	Marquardt, Ronal_R
AF	1	97485	97485	Jaradat, Ziad W.	Jaradat, Ziad_W
AF	3	97487	97487	Marquardt, Ronal R.	Marquardt, Ronal_R

Record 685 | WOS:000239031400009

Glutathione S-transferase polymorphisms and ochratoxin A toxicity in primary human urothelial cells

Field	Position	Original line	Normalised line	Original value	Normalised value
AU	1	97578	97578	Lebrun, S	Lebrun, Stefan
AU	2	97579	97579	Golka, K	Golka, Klaus
AU	3	97580	97580	Schulze, H	Schulze, Harald
AU	4	97581	97581	Foellmann, W	Foellmann, Wolfram

Record 686 | WOS:000238650400009

Ochratoxin A: Apoptosis and aberrant exit from mitosis due to perturbation of microtubule dynamics?

Field	Position	Original line	Normalised line	Original value	Normalised value
AU	1	97679	97679	Rached, E	Rached, Eva
AU	2	97680	97680	Pfeiffer, E	Pfeiffer, Erika
AU	3	97681	97681	Dekant, W	Dekant, Wolfgang
AU	4	97682	97682	Mally, A	Mally, Angela

Record 687 | WOS:000237912300008

Deoxynivalenol transport across human intestinal Caco-2 cells and its effects on cellular metabolism at realistic intestinal concentrations

Field	Position	Original line	Normalised line	Original value	Normalised value
AU	1	97786	97786	Sergent, T	Sergent, Therese
AU	2	97787	97787	Parys, M	Parys, Marie
AU	3	97788	97788	Garsou, S	Garsou, Serge
AU	4	97789	97789	Pussemier, L	Pussemier, Luc
AU	5	97790	97790	Schneider, YJ	Schneider, Yves-Jacques
AU	6	97791	97791	Larondelle, Y	Larondelle, Yvan

Record 688 | WOS:000238344300009

Balkan endemic nephropathy: Role of ochratoxins A through biomarkers

Field	Position	Original line	Normalised line	Original value	Normalised value
AU	1	97894	97894	Castegnaro, M	Castegnaro, Marcel
AU	2	97895	97895	Canadas, D	Canadas, Delphine
AU	3	97896	97896	Vrabcheva, T	Vrabcheva, Terry
AU	4	97897	97897	Petkova-Bocharova, T	Petkova-Bocharova, Theodora
AU	5	97898	97898	Chernozemsky, IN	Chernozemsky, Ivan_N
AU	6	97899	97899	Pfohl-Leszkowicz, A	Pfohl-Leszkowicz, Annie
AF	5	97904	97904	Chernozemsky, Ivan N.	Chernozemsky, Ivan_N

Record 689 | WOS:000238344300010

Evidence of a new dechlorinated ochratoxin A derivative formed in opossum kidney cell cultures after pretreatment by modulators of glutathione pathways: Correlation with DNA-adduct formation

Field	Position	Original line	Normalised line	Original value	Normalised value
AU	1	97996	97996	Faucet-Marquis, V	Faucet-Marquis, Virginie
AU	2	97997	97997	Pont, F	Pont, Frederic
AU	3	97998	97998	Stormer, FC	Stormer, Frederik_C
AU	4	97999	97999	Rizk, T	Rizk, Toufik
AU	5	98000	98000	Castegnaro, M	Castegnaro, Marcel
AU	6	98001	98001	Pfohl-Leszkowicz, A	Pfohl-Leszkowicz, Annie
AF	3	98004	98004	Stormer, Frederik C.	Stormer, Frederik_C

Record 690 | WOS:000238344300011

Microbiologicals for deactivating mycotoxins

Field	Position	Original line	Normalised line	Original value	Normalised value
AU	1	98139	98139	Schatzmayr, G	Schatzmayr, Gerd
AU	2	98140	98140	Zehner, F	Zehner, Florian
AU	3	98141	98141	Täubel, M	Taubel, Martin
AU	4	98142	98142	Schatzmayr, D	Schatzmayr, Dian
AU	5	98143	98143	Klimitsch, A	Klimitsch, Alfred
AU	6	98144	98144	Loibner, AP	Loibner, Andreas_Paul
AU	7	98145	98145	Binder, EM	Binder, Eva_Maria
AF	6	98151	98151	Loibner, Andreas Paul	Loibner, Andreas_Paul
AF	7	98152	98152	Binder, Eva Maria	Binder, Eva_Maria

Record 691 | WOS:000236846900001

Ochratoxin A secretion by ATP-dependent membrane transporters in Caco-2 cells

Field	Position	Original line	Normalised line	Original value	Normalised value
AU	3	98238	98238	Fink-Gremmels, J	Fink-Gremmels, Johanna
AF	3	98241	98241	Fink-Gremmels, J	Fink-Gremmels, Johanna

Record 692 | WOS:000236987100010

In vitro investigation of individual and combined cytotoxic effects of ochratoxin A and other selected mycotoxins on renal cells

Field	Position	Original line	Normalised line	Original value	Normalised value
AU	1	98334	98334	Heussner, AH	Heussner, Ah
AU	2	98335	98335	Dietrich, DR	Dietrich, Dr
AU	3	98336	98336	O'Brien, E	O'Brien, Evelyn
AF	1	98337	98337	Heussner, AH	Heussner, Ah
AF	2	98338	98338	Dietrich, DR	Dietrich, Dr
AF	3	98339	98339	O'Brien, E	O'Brien, Evelyn

Record 693 | WOS:000236605600004

Ochratoxin A: Its cancer risk and potential for exposure

Field	Position	Original line	Normalised line	Original value	Normalised value
AU	1	98421	98421	Clark, HA	Clark, Ha
AU	2	98422	98422	Snedeker, SM	Snedeker, Sm
AF	1	98423	98423	Clark, HA	Clark, Ha
AF	2	98424	98424	Snedeker, SM	Snedeker, Sm

Record 694 | WOS:000234793300002

Toxicokinetics and toxicodynamics of ochratoxin A, an update

Field	Position	Original line	Normalised line	Original value	Normalised value
AU	3	98661	98661	Schneider, YJ	Schneider, Yj
AU	4	98662	98662	Larondelle, Y	Larondelle, Yvan
AF	3	98665	98665	Schneider, YJ	Schneider, Yj
AF	4	98666	98666	Larondelle, Y	Larondelle, Yvan

Record 695 | WOS:000234140400042

The impact of plasma protein binding on the renal transport of organic anions

Field	Position	Original line	Normalised line	Original value	Normalised value
AU	1	98951	98951	Bow, DAJ	Bow, Daj
AU	2	98952	98952	Perry, JL	Perry, Jl
AU	3	98953	98953	Simon, JD	Simon, Jd
AU	4	98954	98954	Pritchard, JB	Pritchard, Jb
AF	1	98955	98955	Bow, DAJ	Bow, Daj
AF	2	98956	98956	Perry, JL	Perry, Jl
AF	3	98957	98957	Simon, JD	Simon, Jd
AF	4	98958	98958	Pritchard, JB	Pritchard, Jb

Record 696 | WOS:000235953600006

Mycoflora, cytotoxicity, and DNA interaction of polyherbal products from Malaysia

Field	Position	Original line	Normalised line	Original value	Normalised value
AU	1	99048	99048	Fuat, ARM	Fuat, Arm
AU	2	99049	99049	Aidoo, KE	Aidoo, Ke
AU	3	99050	99050	Calvert, TW	Calvert, Tw
AU	4	99051	99051	Candlish, AAG	Candlish, Aag
AF	1	99052	99052	Fuat, ARM	Fuat, Arm
AF	2	99053	99053	Aidoo, KE	Aidoo, Ke
AF	3	99054	99054	Calvert, TW	Calvert, Tw
AF	4	99055	99055	Candlish, AAG	Candlish, Aag

Record 697 | WOS:000233991000012

A toxicogenomics approach to identify new plausible epigenetic mechanisms of ochratoxin a carcinogenicity in rat

Field	Position	Original line	Normalised line	Original value	Normalised value
AU	1	99142	99142	Marin-Kuan, M	Marin-Kuan, Maricel
AU	2	99143	99143	Nestler, S	Nestler, Sandra
AU	4	99145	99145	Bezençon, C	Bezencon, Claudine
AU	5	99146	99146	Piguet, D	Piguet, Dominique
AU	9	99150	99150	Delatour, T	Delatour, Thierry
AU	10	99151	99151	Mantle, P	Mantle, Peter
AU	11	99152	99152	Cavin, C	Cavin, Christophe
AU	12	99153	99153	Schilter, B	Schilter, Benoit
AF	1	99154	99154	Marin-Kuan, M	Marin-Kuan, Maricel
AF	2	99155	99155	Nestler, S	Nestler, Sandra
AF	4	99157	99157	Bezençon, C	Bezencon, Claudine
AF	5	99158	99158	Piguet, D	Piguet, Dominique
AF	9	99162	99162	Delatour, T	Delatour, Thierry
AF	10	99163	99163	Mantle, P	Mantle, Peter
AF	11	99164	99164	Cavin, C	Cavin, Christophe
AF	12	99165	99165	Schilter, B	Schilter, Benoit

Record 698 | WOS:000234194400007

An in vitro approach to detect metabolite toxicity due to CYP3A4-dependent bioactivation of xenobiotics

Field	Position	Original line	Normalised line	Original value	Normalised value
AU	5	99299	99299	de Kanter, R	de_Kanter, R
AF	5	99305	99305	de Kanter, R	de_Kanter, R

Record 699 | WOS:000234094800009

Ochratoxin A induces oxidative DNA damage in liver and kidney after oral dosing to rats

Field	Position	Original line	Normalised line	Original value	Normalised value
AU	1	99404	99404	Kamp, HG	Kamp, Hg
AU	5	99408	99408	Latendresse, JR	Latendresse, Jr
AU	7	99410	99410	Turesky, RJ	Turesky, Rj
AF	1	99411	99411	Kamp, HG	Kamp, Hg

AF	5	99415	99415	Latendresse, JR	Latendresse, Jr
AF	7	99417	99417	Turesky, RJ	Turesky, Rj

Record 700 | WOS:000233125300003

Teratogenic effects in rabbits of simultaneous exposure to ochratoxin A and aflatoxin B1 with special reference to microscopic effects

Field	Position	Original line	Normalised line	Original value	Normalised value
AU	1	99530	99530	Wangikar, PB	Wangikar, Pb
AU	4	99533	99533	Sharma, AK	Sharma, Arun
AU	5	99534	99534	Telang, AG	Telang, Ag
AF	1	99535	99535	Wangikar, PB	Wangikar, Pb
AF	4	99538	99538	Sharma, AK	Sharma, Arun
AF	5	99539	99539	Telang, AG	Telang, Ag

Record 701 | WOS:000232435300026

The effects of mycotoxins, fungal food contaminants, on the intestinal epithelial cell-derived innate immune response

Field	Position	Original line	Normalised line	Original value	Normalised value
AU	2	99662	99662	Oswald, IP	Oswald, Ip
AF	2	99664	99664	Oswald, IP	Oswald, Ip

Record 702 | WOS:000233069800005

Investigation of the teratogenic potential of ochratoxin A and B using the FETAX system

Field	Position	Original line	Normalised line	Original value	Normalised value
AU	1	99790	99790	O'Brien, E	O'Brien, Evelyn
AU	3	99792	99792	Dietrich, DR	Dietrich, Dr
AF	1	99793	99793	O'Brien, E	O'Brien, Evelyn
AF	3	99795	99795	Dietrich, DR	Dietrich, Dr

Record 703 | WOS:000232409600014

Fate of ochratoxin A during cooking of naturally contaminated polished rice

Field	Position	Original line	Normalised line	Original value	Normalised value
AU	1	99895	99895	Park, JW	Park, Jw
AU	2	99896	99896	Chung, SH	Chung, Sh
AU	3	99897	99897	Lee, C	Lee, Chan
AU	4	99898	99898	Kim, YB	Kim, Yb
AF	1	99899	99899	Park, JW	Park, Jw
AF	2	99900	99900	Chung, SH	Chung, Sh
AF	3	99901	99901	Lee, C	Lee, Chan
AF	4	99902	99902	Kim, YB	Kim, Yb

Record 704 | WOS:000232524600005

Alterations induced in vitro by ochratoxin A in rat lymphoid cells

Field	Position	Original line	Normalised line	Original value	Normalised value
AU	1	99973	99973	Alvarez-Erviti, L	Alvarez-Erviti, Lydia
AU	3	99975	99975	González-Peñas, E	Gonzalez-Penas, Elena
AU	4	99976	99976	de Cerain, AL	de_Cerain, al
AF	1	99977	99977	Alvarez-Erviti, L	Alvarez-Erviti, Lydia
AF	3	99979	99979	González-Peñas, E	Gonzalez-Penas, Elena
AF	4	99980	99980	de Cerain, AL	de_Cerain, al

Record 705 | WOS:000231364400010

Cyanidin-3-O-β-glucopyranoside, a natural free-radical scavenger against aflatoxin B1-and ochratoxin A-induced cell damage in a human hepatoma cell line (Hep G2) and a human colonic adenocarcinoma cell line (CaCo-2)

Field	Position	Original line	Normalised line	Original value	Normalised value
AU	1	100071	100071	Guerra, MC	Guerra, Mc
AU	2	100072	100072	Galvano, F	Galvano, Fabio
AF	1	100078	100078	Guerra, MC	Guerra, Mc
AF	2	100079	100079	Galvano, F	Galvano, Fabio

Record 706 | WOS:000231235000007

Functional, biochemical, and pathological effects of repeated oral administration of ochratoxin A to rats

Field	Position	Original line	Normalised line	Original value	Normalised value
AU	1	100181	100181	Mally, A	Mally, Angela
AU	2	100182	100182	Völkel, W	Volkel, W
AU	8	100188	100188	Dekant, W	Dekant, Wolfgang
AF	1	100189	100189	Mally, A	Mally, Angela
AF	2	100190	100190	Völkel, W	Volkel, W
AF	8	100196	100196	Dekant, W	Dekant, Wolfgang

Record 707 | WOS:000231235000008

Ochratoxin A causes DNA damage and cytogenetic effects but no DNA adducts in rats

Field	Position	Original line	Normalised line	Original value	Normalised value
AU	1	100316	100316	Mally, A	Mally, Angela
AU	2	100317	100317	Pepe, G	Pepe, Gaetano
AU	5	100320	100320	Gupta, RC	Gupta, Rc
AU	6	100321	100321	Dekant, W	Dekant, Wolfgang
AU	7	100322	100322	Mosesso, P	Mosesso, Pasquale
AF	1	100323	100323	Mally, A	Mally, Angela
AF	2	100324	100324	Pepe, G	Pepe, Gaetano
AF	5	100327	100327	Gupta, RC	Gupta, Rc
AF	6	100328	100328	Dekant, W	Dekant, Wolfgang

AF	7	100329	100329	Mosesso, P	Mosesso, Pasquale

Record 708 | WOS:000230120200005

Biotransformation and nephrotoxicity of ochratoxin B in rats

Field	Position	Original line	Normalised line	Original value	Normalised value
AU	1	100434	100434	Mally, A	Mally, Angela
AU	6	100439	100439	Mantle, P	Mantle, Peter
AU	7	100440	100440	Völkel, W	Volkel, W
AU	8	100441	100441	Hard, GC	Hard, Gc
AU	9	100442	100442	Dekant, W	Dekant, Wolfgang
AF	1	100443	100443	Mally, A	Mally, Angela
AF	6	100448	100448	Mantle, P	Mantle, Peter
AF	7	100449	100449	Völkel, W	Volkel, W
AF	8	100450	100450	Hard, GC	Hard, Gc
AF	9	100451	100451	Dekant, W	Dekant, Wolfgang

Record 709 | WOS:000228398000034

Cisplatin-induced apoptosis is enhanced by hypoxia and by inhibition of mitochondria in renal collecting duct cells

Field	Position	Original line	Normalised line	Original value	Normalised value
AU	1	100529	100529	Schwerdt, G	Schwerdt, Gerald
AU	6	100534	100534	Gekle, M	Gekle, Michael
AF	1	100535	100535	Schwerdt, G	Schwerdt, Gerald
AF	6	100540	100540	Gekle, M	Gekle, Michael

Record 710 | WOS:000229004100015

Comparison of in vitro cytotoxicity of Fusarium mycotoxins, deoxynivalenol, T-2 toxin and zearalenone on selected human epithelial cell lines

Field	Position	Original line	Normalised line	Original value	Normalised value
AU	1	100624	100624	Calvert, TW	Calvert, Tw
AU	2	100625	100625	Aidoo, KE	Aidoo, Ke
AU	3	100626	100626	Candlish, AGG	Candlish, Agg
AU	4	100627	100627	Fuat, ARM	Fuat, Arm
AF	1	100628	100628	Calvert, TW	Calvert, Tw
AF	2	100629	100629	Aidoo, KE	Aidoo, Ke
AF	3	100630	100630	Candlish, AGG	Candlish, Agg
AF	4	100631	100631	Fuat, ARM	Fuat, Arm

Record 711 | WOS:000227733000027

Proximal tubular toxicity of ochratoxin A is amplified by simultaneous inhibition of the extracellular signal-regulated kinases 1/2

Field	Position	Original line	Normalised line	Original value	Normalised value
AU	2	100720	100720	Holzinger, H	Holzinger, Hildegard
AU	3	100721	100721	Gekle, M	Gekle, Michael

AF	2	100723	100723	Holzinger, H	Holzinger, Hildegard
AF	3	100724	100724	Gekle, M	Gekle, Michael

Record 712 | WOS:000227112000002

Interference of arachidonic acid and its metabolites with TNF-α release by ochratoxin A from rat liver

Field	Position	Original line	Normalised line	Original value	Normalised value
AU	1	100810	100810	Al-Anati, L	Al-Anati, Lauy
AU	3	100812	100812	Petzinger, E	Petzinger, Ernst
AF	1	100813	100813	Al-Anati, L	Al-Anati, Lauy
AF	3	100815	100815	Petzinger, E	Petzinger, Ernst

Record 713 | WOS:000227242200003

Ochratoxin A at nanomolar concentrations: A signal modulator in renal cells

Field	Position	Original line	Normalised line	Original value	Normalised value
AU	1	100934	100934	Gekle, M	Gekle, Michael
AU	3	100936	100936	Schwerdt, G	Schwerdt, Gerald
AF	1	100937	100937	Gekle, M	Gekle, Michael
AF	3	100939	100939	Schwerdt, G	Schwerdt, Gerald

Record 714 | WOS:000234123000008

Ochratoxin A: Comparative pharmacokinetics and toxicological implications (experimental and domestic animals and humans)

Field	Position	Original line	Normalised line	Original value	Normalised value
AU	1	101094	101094	Dietrich, DR	Dietrich, Dr
AU	2	101095	101095	Heussner, AH	Heussner, Ah
AU	3	101096	101096	O'Brien, E	O'Brien, Evelyn
AF	1	101097	101097	Dietrich, DR	Dietrich, Dr
AF	2	101098	101098	Heussner, AH	Heussner, Ah
AF	3	101099	101099	O'Brien, E	O'Brien, Evelyn

Record 715 | WOS:000234123000011

DNA adduct formation by ochratoxin A: Review of the available evidence

Field	Position	Original line	Normalised line	Original value	Normalised value
AU	1	101194	101194	Mally, A	Mally, Angela
AU	2	101195	101195	Dekant, W	Dekant, Wolfgang
AF	1	101196	101196	Mally, A	Mally, Angela
AF	2	101197	101197	Dekant, W	Dekant, Wolfgang

Record 716 | WOS:000226837900002

Ochratoxin A: The continuing enigma

Field	Position	Original line	Normalised line	Original value	Normalised value
AU	1	101307	101307	O'Brien, E	O'Brien, Evelyn
AU	2	101308	101308	Dietrich, DR	Dietrich, Dr
AF	1	101309	101309	O'Brien, E	O'Brien, Evelyn
AF	2	101310	101310	Dietrich, DR	Dietrich, Dr

Record 717 | WOS:000234123000012

Further arguments in favour of direct covalent binding of Ochratoxin A (OTA) after metabolic biotransformation

Field	Position	Original line	Normalised line	Original value	Normalised value
AU	1	101575	101575	Pfohl-Leszkowicz, A	Pfohl-Leszkowicz, Annie
AU	2	101576	101576	Castegnaro, M	Castegnaro, Marcel
AF	1	101577	101577	Pfohl-Leszkowicz, A	Pfohl-Leszkowicz, Annie
AF	2	101578	101578	Castegnaro, M	Castegnaro, Marcel

Record 719 | WOS:000226875200005

Ochratoxin A-induced DNA damage in human fibroblast:: protective effect of cyanidin 3-O-β-D-glucoside

Field	Position	Original line	Normalised line	Original value	Normalised value
AU	2	101777	101777	La Fauci, L	la_Fauci, L
AU	3	101778	101778	Acquaviva, R	Acquaviva, Rosaria
AU	10	101785	101785	Galvano, F	Galvano, Fabio
AF	2	101787	101787	La Fauci, L	la_Fauci, L
AF	3	101788	101788	Acquaviva, R	Acquaviva, Rosaria
AF	10	101795	101795	Galvano, F	Galvano, Fabio

Record 720 | WOS:000226469400004

Exposure to nephrotoxic Ochratoxin A enhances collagen secretion in human renal proximal tubular cells

Field	Position	Original line	Normalised line	Original value	Normalised value
AU	2	101899	101899	Holzinger, H	Holzinger, Hildegard
AU	4	101901	101901	Gekle, M	Gekle, Michael
AF	2	101903	101903	Holzinger, H	Holzinger, Hildegard
AF	4	101905	101905	Gekle, M	Gekle, Michael

Record 721 | WOS:000226446300003

Applicability of an in vitro digestion model in assessing the bioaccessibility of mycotoxins from food

Field	Position	Original line	Normalised line	Original value	Normalised value
AU	1	101993	101993	Versantvoort, CHM	Versantvoort, Chm
AU	2	101994	101994	Oomen, AG	Oomen, Ag
AU	3	101995	101995	Van de Kamp, E	van_de_Kamp, E
AU	4	101996	101996	Rompelberg, CJM	Rompelberg, Cjm
AU	5	101997	101997	Sips, AJAM	Sips, Ajam
AF	1	101998	101998	Versantvoort, CHM	Versantvoort, Chm

AF	2	101999	101999	Oomen, AG	Oomen, Ag
AF	3	102000	102000	Van de Kamp, E	van_de_Kamp, E
AF	4	102001	102001	Rompelberg, CJM	Rompelberg, Cjm
AF	5	102002	102002	Sips, AJAM	Sips, Ajam

Record 722 | WOS:000234123000002

Ochratoxin A: Previous risk assessments and issues arising

Field	Position	Original line	Normalised line	Original value	Normalised value
AU	2	102086	102086	Larsen, JC	Larsen, Jc
AF	2	102088	102088	Larsen, JC	Larsen, Jc

Record 723 | WOS:000231491200006

Unusual astrocyte reactivity caused by the food mycotoxin ochratoxin A in aggregating rat brain cell cultures

Field	Position	Original line	Normalised line	Original value	Normalised value
AU	1	102143	102143	Zurich, MG	Zurich, Mg
AU	6	102148	102148	Honegger, P	Honegger, Paul
AF	1	102149	102149	Zurich, MG	Zurich, Mg
AF	6	102154	102154	Honegger, P	Honegger, Paul

Record 724 | WOS:000225312300008

Effects of deoxynivalenol on general performance and electrophysiological properties of intestinal mucosa of broiler chickens

Field	Position	Original line	Normalised line	Original value	Normalised value
AU	1	102280	102280	Awad, WA	Awad, Wa
AU	2	102281	102281	Böhm, J	Bohm, J
AU	4	102283	102283	Hulan, HW	Hulan, Hw
AU	5	102284	102284	Zentek, J	Zentek, Juergen
AF	1	102285	102285	Awad, WA	Awad, Wa
AF	2	102286	102286	Böhm, J	Bohm, J
AF	4	102288	102288	Hulan, HW	Hulan, Hw
AF	5	102289	102289	Zentek, J	Zentek, Juergen

Record 725 | WOS:000226727000007

Combined effects of selected Penicillium mycotoxins on in vitro proliferation of porcine lymphocytes

Field	Position	Original line	Normalised line	Original value	Normalised value
AU	4	102415	102415	Larsen, HJS	Larsen, Hjs
AU	5	102416	102416	Flåoyen, A	Flaoyen, A
AF	4	102420	102420	Larsen, HJS	Larsen, Hjs
AF	5	102421	102421	Flåoyen, A	Flaoyen, A

Record 726 | WOS:000226361400006

Molecular aspects of the transport and toxicity of ochratoxin A

Field	Position	Original line	Normalised line	Original value	Normalised value
AU	2	102480	102480	Park, G	Park, Gyungse
AU	3	102481	102481	Perry, JL	Perry, Jl
AU	4	102482	102482	Il'Ichev, YV	Il'Ichev, Yv
AU	5	102483	102483	Bow, DAJ	Bow, Daj
AU	6	102484	102484	Pritchard, JB	Pritchard, Jb
AU	8	102486	102486	Pfohl-Leszkowicz, A	Pfohl-Leszkowicz, Annie
AU	9	102487	102487	Manderville, RA	Manderville, Richard
AU	10	102488	102488	Simon, JD	Simon, Jd
AF	2	102490	102490	Park, G	Park, Gyungse
AF	3	102491	102491	Perry, JL	Perry, Jl
AF	4	102492	102492	Il'Ichev, YV	Il'Ichev, Yv
AF	5	102493	102493	Bow, DAJ	Bow, Daj
AF	6	102494	102494	Pritchard, JB	Pritchard, Jb
AF	8	102496	102496	Pfohl-Leszkowicz, A	Pfohl-Leszkowicz, Annie
AF	9	102497	102497	Manderville, RA	Manderville, Richard
AF	10	102498	102498	Simon, JD	Simon, Jd

Record 727 | WOS:000224725300008

Experimental mycotoxicosis in chickens induced by ochratoxin A and penicillic acid and intervention with natural plant extracts

Field	Position	Original line	Normalised line	Original value	Normalised value
AU	1	102600	102600	Stoev, SD	Stoev, Sd
AU	5	102604	102604	Domijan, AM	Domijan, Am
AU	6	102605	102605	Peraica, M	Peraica, Maja
AF	1	102606	102606	Stoev, SD	Stoev, Sd
AF	5	102610	102610	Domijan, AM	Domijan, Am
AF	6	102611	102611	Peraica, M	Peraica, Maja

Record 728 | WOS:000226417300009

The effects of the Penicillium mycotoxins citrinin, cyclopiazonic acid, ochratoxin A, patulin, penicillic acid, and roquefortine C on in vitro proliferation of porcine lymphocytes

Field	Position	Original line	Normalised line	Original value	Normalised value
AU	3	102714	102714	Höfer, CC	Hofer, Cc
AU	5	102716	102716	Larsen, HJS	Larsen, Hjs
AU	6	102717	102717	Flåoyen, A	Flaoyen, A
AF	3	102720	102720	Höfer, CC	Hofer, Cc
AF	5	102722	102722	Larsen, HJS	Larsen, Hjs
AF	6	102723	102723	Flåoyen, A	Flaoyen, A

Record 730 | WOS:000223753400030

Alkalinization of urinary pH accelerates renal excretion of ochratoxin A in pigs

Field	Position	Original line	Normalised line	Original value	Normalised value
AU	1	102896	102896	Blank, R	Blank, Ralf
AU	2	102897	102897	Wolffram, S	Wolffram, Siegfried
AF	1	102898	102898	Blank, R	Blank, Ralf
AF	2	102899	102899	Wolffram, S	Wolffram, Siegfried

Record 731 | WOS:000224015700013

Synergistic effects of fumonisin B1 and ochratoxin A: are in vitro cytotoxicity data predictive of in vivo acute toxicity?

Field	Position	Original line	Normalised line	Original value	Normalised value
AU	1	102979	102979	Creppy, EE	Creppy, Ee
AU	4	102982	102982	Moukha, S	Moukha, Serge
AU	5	102983	102983	Carratù, MR	Carratu, Mr
AF	1	102984	102984	Creppy, EE	Creppy, Ee
AF	4	102987	102987	Moukha, S	Moukha, Serge
AF	5	102988	102988	Carratù, MR	Carratu, Mr

Record 732 | WOS:000224048300016

Evidence for covalent DNA adduction by ochratoxin A following chronic exposure to rat and subacute exposure to pig

Field	Position	Original line	Normalised line	Original value	Normalised value
AU	2	103103	103103	Pfohl-Leszkowicz, A	Pfohl-Leszkowicz, Annie
AU	4	103105	103105	Castegnaro, M	Castegnaro, Marcel
AU	5	103106	103106	Manderville, RA	Manderville, Richard
AF	2	103108	103108	Pfohl-Leszkowicz, A	Pfohl-Leszkowicz, Annie
AF	4	103110	103110	Castegnaro, M	Castegnaro, Marcel
AF	5	103111	103111	Manderville, RA	Manderville, Richard

Record 733 | WOS:000225210900008

Levels of mycotoxins and sample cytotoxicity of selected organic and conventional grain-based products purchased from Finnish and Italian markets

Field	Position	Original line	Normalised line	Original value	Normalised value
AU	1	103215	103215	Jestoi, M	Jestoi, Marika
AU	2	103216	103216	Somma, MC	Somma, Mc
AU	6	103220	103220	Ritieni, A	Ritieni, Alberto
AU	7	103221	103221	Peltonen, K	Peltonen, Kimmo
AF	1	103222	103222	Jestoi, M	Jestoi, Marika
AF	2	103223	103223	Somma, MC	Somma, Mc
AF	6	103227	103227	Ritieni, A	Ritieni, Alberto
AF	7	103228	103228	Peltonen, K	Peltonen, Kimmo

Record 734 | WOS:000223297700006

Effects of rosmarinic acid against aflatoxin B1 and ochratoxin-A-induced cell damage in a human hepatoma cell line (Hep G2)

Field	Position	Original line	Normalised line	Original value	Normalised value

AU	2	103307	103307	Galvano, F	Galvano, Fabio
AU	5	103310	103310	Guerra, MC	Guerra, Mc
AF	2	103312	103312	Galvano, F	Galvano, Fabio
AF	5	103315	103315	Guerra, MC	Guerra, Mc

Record 736 | WOS:000221555600005

Metabolism-mediated cytotoxicity of ochratoxin A

Field	Position	Original line	Normalised line	Original value	Normalised value
AU	1	103502	103502	Doorten, AYS	Doorten, Ays
AU	3	103504	103504	Van der Doelen, MAM	van_der_Doelen, Mam
AU	4	103505	103505	Fink-Gremmels, J	Fink-Gremmels, Johanna
AF	1	103506	103506	Doorten, AYS	Doorten, Ays
AF	3	103508	103508	Van der Doelen, MAM	van_der_Doelen, Mam
AF	4	103509	103509	Fink-Gremmels, J	Fink-Gremmels, Johanna

Record 737 | WOS:000220837300016

Immunotoxic effects of Ochratoxin A in wistar rats after oral administration

Field	Position	Original line	Normalised line	Original value	Normalised value
AU	2	103598	103598	Gil, AG	Gil, Ag
AU	3	103599	103599	Ezpeleta, O	Ezpeleta, Olga
AU	4	103600	103600	García-Jalón, JA	Garcia-Jalon, Ja
AU	5	103601	103601	de Cerain, AL	de_Cerain, al
AF	2	103603	103603	Gil, AG	Gil, Ag
AF	3	103604	103604	Ezpeleta, O	Ezpeleta, Olga
AF	4	103605	103605	García-Jalón, JA	Garcia-Jalon, Ja
AF	5	103606	103606	de Cerain, AL	de_Cerain, al

Record 738 | WOS:000221514400030

Synergistic interaction in simultaneous exposure to Streptomyces californicus and Stachybotrys chartarum

Field	Position	Original line	Normalised line	Original value	Normalised value
AU	3	103712	103712	Nielsen, KF	Nielsen, Kf
AU	6	103715	103715	Hirvonen, MM	Hirvonen, Mm
AF	3	103718	103718	Nielsen, KF	Nielsen, Kf
AF	6	103721	103721	Hirvonen, MM	Hirvonen, Mm

Record 739 | WOS:000220235700005

Inhibitory effects of ochratoxin A on nerve growth factor-induced neurite extension through downregulation of p38 map kinase and AP-1 activation in cultured pheochromocytoma cells

Field	Position	Original line	Normalised line	Original value	Normalised value
AU	1	103820	103820	Jae, HH	Jae, Hh
AU	2	103821	103821	Jung, HK	Jung, Hk
AU	3	103822	103822	Park, YJ	Park, Yj

AU	4	103823	103823	Kim, CK	Kim, Ck
AU	5	103824	103824	Chun, SY	Chun, Sy
AU	6	103825	103825	Park, NG	Park, Ng
AU	7	103826	103826	Yun, YW	Yun, Yw
AU	8	103827	103827	Kim, DJ	Kim, Dongwook
AU	9	103828	103828	Ha, TY	Ha, Ty
AU	10	103829	103829	Song, YS	Song, Ys
AU	11	103830	103830	Lee, YM	Lee, Ym
AU	12	103831	103831	Oh, KW	Oh, Kw
AU	13	103832	103832	Hong, JT	Hong, Jt
AF	1	103833	103833	Jae, HH	Jae, Hh
AF	2	103834	103834	Jung, HK	Jung, Hk
AF	3	103835	103835	Park, YJ	Park, Yj
AF	4	103836	103836	Kim, CK	Kim, Ck
AF	5	103837	103837	Chun, SY	Chun, Sy
AF	6	103838	103838	Park, NG	Park, Ng
AF	7	103839	103839	Yun, YW	Yun, Yw
AF	8	103840	103840	Kim, DJ	Kim, Dongwook
AF	9	103841	103841	Ha, TY	Ha, Ty
AF	10	103842	103842	Song, YS	Song, Ys
AF	11	103843	103843	Lee, YM	Lee, Ym
AF	12	103844	103844	Oh, KW	Oh, Kw
AF	13	103845	103845	Hong, JT	Hong, Jt

Record 740 | WOS:000189087700013

Ochratoxin A: Lack of formation of covalent DNA adducts

Field	Position	Original line	Normalised line	Original value	Normalised value
AU	1	103932	103932	Mally, A	Mally, Angela
AU	7	103938	103938	Vökel, W	Vokel, W
AU	8	103939	103939	Dekant, W	Dekant, Wolfgang
AF	1	103940	103940	Mally, A	Mally, Angela
AF	7	103946	103946	Vökel, W	Vokel, W
AF	8	103947	103947	Dekant, W	Dekant, Wolfgang

Record 741 | WOS:000188530700003

Ochratoxin A and zearalenone: a comparative study on genotoxic effects and cell death induced in bovine lymphocytes

Field	Position	Original line	Normalised line	Original value	Normalised value
AU	1	104023	104023	Lioi, MB	Lioi, Mb
AU	3	104025	104025	Barbieri, RB	Barbieri, Rb
AU	4	104026	104026	Salzano, SS	Salzano, Ss
AU	5	104027	104027	Ursini, MV	Ursini, Mv
AF	1	104028	104028	Lioi, MB	Lioi, Mb
AF	3	104030	104030	Barbieri, RB	Barbieri, Rb
AF	4	104031	104031	Salzano, SS	Salzano, Ss
AF	5	104032	104032	Ursini, MV	Ursini, Mv

Record 743 | WOS:000189041600006

Inhibition of mitochondria and extracellular acidification enhance ochratoxin A-induced apoptosis in renal collecting duct-derived MDCK-C7 cells

Field	Position	Original line	Normalised line	Original value	Normalised value
AU	1	104225	104225	Schwerdt, G	Schwerdt, Gerald
AU	5	104229	104229	Gekle, M	Gekle, Michael
AF	1	104230	104230	Schwerdt, G	Schwerdt, Gerald
AF	5	104234	104234	Gekle, M	Gekle, Michael

Record 744 | WOS:000231756500075

Natural antioxidants and mycotoxins: Theoretical considerations and practical applications

Field	Position	Original line	Normalised line	Original value	Normalised value
AU	1	104315	104315	Surai, PF	Surai, Pf
AU	2	104316	104316	Dvorska, JE	Dvorska, Je
AU	3	104317	104317	Sparks, NHC	Sparks, Nhc
AF	1	104318	104318	Surai, PF	Surai, Pf
AF	2	104319	104319	Dvorska, JE	Dvorska, Je
AF	3	104320	104320	Sparks, NHC	Sparks, Nhc

Record 745 | WOS:000186953000004

Apoptosis and oxidative stress induced by ochratoxin A in rat kidney

Field	Position	Original line	Normalised line	Original value	Normalised value
AU	1	104413	104413	Petrik, J	Petrik, Jozsef
AU	4	104416	104416	Pepeljnjak, S	Pepeljnjak, Stjepan
AF	1	104420	104420	Petrik, J	Petrik, Jozsef
AF	4	104423	104423	Pepeljnjak, S	Pepeljnjak, Stjepan

Record 746 | WOS:000186282400042

Effects of chronic ingestion of ochratoxin A on blood levels and excretion of the mycotoxin in sheep

Field	Position	Original line	Normalised line	Original value	Normalised value
AU	1	104539	104539	Blank, R	Blank, Ralf
AU	2	104540	104540	Rolfs, JP	Rolfs, Jp
AU	3	104541	104541	Südekum, KH	Sudekum, Kh
AU	4	104542	104542	Frohlich, AA	Frohlich, Aa
AU	5	104543	104543	Marquardt, RR	Marquardt, Rr
AF	1	104545	104545	Blank, R	Blank, Ralf
AF	2	104546	104546	Rolfs, JP	Rolfs, Jp
AF	3	104547	104547	Südekum, KH	Sudekum, Kh
AF	4	104548	104548	Frohlich, AA	Frohlich, Aa
AF	5	104549	104549	Marquardt, RR	Marquardt, Rr

Record 747 | WOS:000184259400007

Effects of the mycotoxin ochratoxin A and some of its metabolites on human kidney cell lines

Field	Position	Original line	Normalised line	Original value	Normalised value
AU	1	104634	104634	Müller, G	Muller, G
AU	4	104637	104637	Köhler, H	Kohler, H
AF	1	104638	104638	Müller, G	Muller, G
AF	4	104641	104641	Köhler, H	Kohler, H

Record 748 | WOS:000184243200002

An oxygen-bonded C8-deoxyguanosine nucleoside adduct of pentachlorophenol by peroxidase activation: Evidence for ambident C8 reactivity by phenoxyl radicals

Field	Position	Original line	Normalised line	Original value	Normalised value
AU	2	104736	104736	Wright, MW	Wright, Mw
AU	3	104737	104737	Manderville, RA	Manderville, Richard
AF	2	104739	104739	Wright, MW	Wright, Mw
AF	3	104740	104740	Manderville, RA	Manderville, Richard

Record 749 | WOS:000184363500006

Effect of Ochratoxin A on human haematopoietic progenitors proliferation and differentiation: an in vitro study

Field	Position	Original line	Normalised line	Original value	Normalised value
AU	2	104806	104806	Le Dréan, G	le_Drean, G
AF	2	104809	104809	Le Dréan, G	le_Drean, G

Record 750 | WOS:000185415100022

Evaluation of two mycotoxin binders to reduce toxicity of broiler diets containing ochratoxin A and T-2 toxin contaminated grain

Field	Position	Original line	Normalised line	Original value	Normalised value
AU	1	104905	104905	García, AR	Garcia, Ar
AU	4	104908	104908	Petrone, VM	Petrone, Vm
AF	1	104909	104909	García, AR	Garcia, Ar
AF	4	104912	104912	Petrone, VM	Petrone, Vm

Record 751 | WOS:000183040200012

A new approach to studying ochratoxin A (OTA)-induced nephrotoxicity: Expression profiling in vivo and in vitro employing cDNA microarrays

Field	Position	Original line	Normalised line	Original value	Normalised value
AU	1	105005	105005	Lühe, A	Luhe, A
AU	5	105009	105009	Ahr, HJ	Ahr, Hj
AF	1	105010	105010	Lühe, A	Luhe, A
AF	5	105014	105014	Ahr, HJ	Ahr, Hj

Record 752 | WOS:000183875100009

Effects of the mycotoxin ochratoxin A in a bacterial and a mammalian in vitro mutagenicity test system

Field	Position	Original line	Normalised line	Original value	Normalised value
AU	1	105126	105126	Föllmann, W	Follmann, W
AF	1	105128	105128	Föllmann, W	Follmann, W

Record 753 | WOS:000182307900052

Interaction of ochratoxin A with human intestinal Caco-2 cells: possible implication of a multidrug resistance-associated protein (MRP2)

Field	Position	Original line	Normalised line	Original value	Normalised value
AU	2	105212	105212	Gabriel, AF	Gabriel, Af
AU	3	105213	105213	Sergent, T	Sergent, Therese
AU	5	105215	105215	Larondelle, Y	Larondelle, Yvan
AU	6	105216	105216	Schneider, YJ	Schneider, Yj
AF	2	105218	105218	Gabriel, AF	Gabriel, Af
AF	3	105219	105219	Sergent, T	Sergent, Therese
AF	5	105221	105221	Larondelle, Y	Larondelle, Yvan
AF	6	105222	105222	Schneider, YJ	Schneider, Yj

Record 755 | WOS:000180037400011

Photochemically catalyzed reaction of ochratoxin A with D- and L-cysteine

Field	Position	Original line	Normalised line	Original value	Normalised value
AU	1	105359	105359	Brow, ME	Brow, Me
AU	3	105361	105361	Park, G	Park, Gyungse
AU	4	105362	105362	Wright, MW	Wright, Mw
AU	5	105363	105363	Gillman, IG	Gillman, Ig
AU	6	105364	105364	Manderville, RA	Manderville, Richard
AF	1	105365	105365	Brow, ME	Brow, Me
AF	3	105367	105367	Park, G	Park, Gyungse
AF	4	105368	105368	Wright, MW	Wright, Mw
AF	5	105369	105369	Gillman, IG	Gillman, Ig
AF	6	105370	105370	Manderville, RA	Manderville, Richard

Record 756 | WOS:000179850500004

Prevention and control of mycotoxins in the poultry production chain:: a European view

Field	Position	Original line	Normalised line	Original value	Normalised value
AU	1	105456	105456	Dänicke, S	Danicke, S
AF	1	105457	105457	Dänicke, S	Danicke, S

Record 758 | WOS:000177393700006

Genotoxic effects of ochratoxin A in human-derived hepatoma (HepG2) cells

Field	Position	Original line	Normalised line	Original value	Normalised value
AU	4	105845	105845	Steinkellner, H	Steinkellner, Hans
AU	6	105847	105847	Majer, BJ	Majer, Bj
AU	8	105849	105849	Knasmüller, S	Knasmuller, S
AF	4	105853	105853	Steinkellner, H	Steinkellner, Hans
AF	6	105855	105855	Majer, BJ	Majer, Bj
AF	8	105857	105857	Knasmüller, S	Knasmuller, S

Record 759 | WOS:000177631900009

Species- and sex-specific variations in binding of ochratoxin A by renal proteins in vitro

Field	Position	Original line	Normalised line	Original value	Normalised value
AU	1	105947	105947	Heussner, AH	Heussner, Ah
AU	2	105948	105948	O'Brien, E	O'Brien, Evelyn
AU	3	105949	105949	Dietrich, DR	Dietrich, Dr
AF	1	105950	105950	Heussner, AH	Heussner, Ah
AF	2	105951	105951	O'Brien, E	O'Brien, Evelyn
AF	3	105952	105952	Dietrich, DR	Dietrich, Dr

Record 760 | WOS:000176910000001

Aristolochic acid as a probable human cancer hazard in herbal remedies: a review

Field	Position	Original line	Normalised line	Original value	Normalised value
AU	1	106043	106043	Arlt, VM	Arlt, Vm
AU	2	106044	106044	Stiborova, M	Stiborova, Marie
AU	3	106045	106045	Schmeiser, HH	Schmeiser, Hh
AF	1	106046	106046	Arlt, VM	Arlt, Vm
AF	2	106047	106047	Stiborova, M	Stiborova, Marie
AF	3	106048	106048	Schmeiser, HH	Schmeiser, Hh

Record 761 | WOS:000175922300023

Esterified-glucomannan in broiler chicken diets-contaminated with aflatoxin, ochratoxin and T-2 toxin:: Evaluation of its binding ability (in vitro) and efficacy as immunomodulator

Field	Position	Original line	Normalised line	Original value	Normalised value
AU	1	106224	106224	Raju, MVLN	Raju, Mvln
AF	1	106226	106226	Raju, MVLN	Raju, Mvln

Record 762 | WOS:000176518700006

Role of human organic anion transporter 4 in the transport of ochratoxin A

Field	Position	Original line	Normalised line	Original value	Normalised value
AU	7	106299	106299	Cha, SH	Cha, Sh
AF	7	106309	106309	Cha, SH	Cha, Sh

Record 763 | WOS:000176216800005

Expression of Hsp70 in kidney cells exposed to ochratoxin A

Field	Position	Original line	Normalised line	Original value	Normalised value
AU	2	106414	106414	Petrik, J	Petrik, Jozsef
AU	5	106417	106417	Grubisic, TZ	Grubisic, Tz
AF	2	106419	106419	Petrik, J	Petrik, Jozsef
AF	5	106422	106422	Grubisic, TZ	Grubisic, Tz

Record 764 | WOS:000175152300005

Effect of T-2 toxin on in vivo lipid peroxidation and vitamin E status in mice

Field	Position	Original line	Normalised line	Original value	Normalised value
AU	1	106519	106519	Vilà, B	Vila, Borja
AU	2	106520	106520	Jaradat, ZW	Jaradat, Zw
AU	3	106521	106521	Marquardt, RR	Marquardt, Rr
AU	4	106522	106522	Frohlich, AA	Frohlich, Aa
AF	1	106523	106523	Vilà, B	Vila, Borja
AF	2	106524	106524	Jaradat, ZW	Jaradat, Zw
AF	3	106525	106525	Marquardt, RR	Marquardt, Rr
AF	4	106526	106526	Frohlich, AA	Frohlich, Aa

Record 765 | WOS:000173055800005

Determination of the epigenetic effects of ochratoxin in a human kidney and a rat liver epithelial cell line

Field	Position	Original line	Normalised line	Original value	Normalised value
AU	2	106605	106605	Upham, BL	Upham, Bl
AU	4	106607	106607	Trosko, JE	Trosko, Je
AF	2	106609	106609	Upham, BL	Upham, Bl
AF	4	106611	106611	Trosko, JE	Trosko, Je

Record 766 | WOS:000176962600010

Toxins of filamentous fungi

Field	Position	Original line	Normalised line	Original value	Normalised value
AU	2	106737	106737	Yu, JJ	Yu, Jj
AU	3	106738	106738	Ehrlich, KC	Ehrlich, Kc
AF	2	106740	106740	Yu, JJ	Yu, Jj
AF	3	106741	106741	Ehrlich, KC	Ehrlich, Kc

Record 767 | WOS:000174243300013

Detection of ochratoxin A-induced DNA damage in MDCK cells by alkaline single cell gel electrophoresis (comet assay)

Field	Position	Original line	Normalised line	Original value	Normalised value

AU	1	107007	107007	Lebrun, S	Lebrun, Stefan
AU	2	107008	107008	Föllmann, W	Follmann, W
AF	1	107009	107009	Lebrun, S	Lebrun, Stefan
AF	2	107010	107010	Föllmann, W	Follmann, W

Record 770 | WOS:000171478500001

Toxicity, metabolism, and impact of mycotoxins on humans and animals

Field	Position	Original line	Normalised line	Original value	Normalised value
AU	1	107302	107302	Hussein, HS	Hussein, Hs
AU	2	107303	107303	Brasel, JM	Brasel, Jm
AF	1	107304	107304	Hussein, HS	Hussein, Hs
AF	2	107305	107305	Brasel, JM	Brasel, Jm

Record 771 | WOS:000171571300006

The mycotoxin ochratoxin A alters intestinal barrier and absorption functions but has no effect on chloride secretion

Field	Position	Original line	Normalised line	Original value	Normalised value
AU	1	107619	107619	Maresca, M	Maresca, Marc
AU	3	107621	107621	Pfohl-Leszkowicz, A	Pfohl-Leszkowicz, Annie
AU	4	107622	107622	Fantini, J	Fantini, Jacques
AF	1	107623	107623	Maresca, M	Maresca, Marc
AF	3	107625	107625	Pfohl-Leszkowicz, A	Pfohl-Leszkowicz, Annie
AF	4	107626	107626	Fantini, J	Fantini, Jacques

Record 772 | WOS:000171310400014

Species-, sex-, and cell type-specific effects of ochratoxin A and B

Field	Position	Original line	Normalised line	Original value	Normalised value
AU	1	107731	107731	O'Brien, E	O'Brien, Evelyn
AU	2	107732	107732	Heussner, AH	Heussner, Ah
AU	3	107733	107733	Dietrich, DR	Dietrich, Dr
AF	1	107734	107734	O'Brien, E	O'Brien, Evelyn
AF	2	107735	107735	Heussner, AH	Heussner, Ah
AF	3	107736	107736	Dietrich, DR	Dietrich, Dr

Record 773 | WOS:000171274400005

Characterization of ochratoxin A transport by human organic anion transporters

Field	Position	Original line	Normalised line	Original value	Normalised value
AU	1	107851	107851	Jung, KY	Jung, Ky
AU	3	107853	107853	Kim, DK	Kim, Dongwook
AU	6	107856	107856	Yoo, BS	Yoo, Bs
AU	8	107858	107858	Cha, SH	Cha, Sh
AF	1	107861	107861	Jung, KY	Jung, Ky

AF	3	107863	107863	Kim, DK	Kim, Dongwook
AF	6	107866	107866	Yoo, BS	Yoo, Bs
AF	8	107868	107868	Cha, SH	Cha, Sh

Record 774 | WOS:000170039000017

Species- and sex-specific renal cytotoxicity of Ochratoxin A and B in vitro

Field	Position	Original line	Normalised line	Original value	Normalised value
AU	1	107951	107951	Dietrich, DR	Dietrich, Dr
AU	2	107952	107952	O'Brien, E	O'Brien, Evelyn
AU	3	107953	107953	Stack, ME	Stack, Me
AU	4	107954	107954	Heussner, AH	Heussner, Ah
AF	1	107955	107955	Dietrich, DR	Dietrich, Dr
AF	2	107956	107956	O'Brien, E	O'Brien, Evelyn
AF	3	107957	107957	Stack, ME	Stack, Me
AF	4	107958	107958	Heussner, AH	Heussner, Ah

Record 775 | WOS:000169038800006

Aspartame prevents the karyomegaly induced by ochratoxin A in rat kidney

Field	Position	Original line	Normalised line	Original value	Normalised value
AU	4	108048	108048	Betbeder, AM	Betbeder, Am
AU	7	108051	108051	Steyn, PS	Steyn, Ps
AU	8	108052	108052	Creppy, EE	Creppy, Ee
AF	4	108056	108056	Betbeder, AM	Betbeder, Am
AF	7	108059	108059	Steyn, PS	Steyn, Ps
AF	8	108060	108060	Creppy, EE	Creppy, Ee

Record 776 | WOS:000167652300006

Age-related differences in the toxicity of ochratoxin A in female rats

Field	Position	Original line	Normalised line	Original value	Normalised value
AU	1	108150	108150	Dortant, PM	Dortant, Pm
AU	2	108151	108151	Peters-Volleberg, GWM	Peters-Volleberg, Gwm
AU	3	108152	108152	Van Loveren, H	van_Loveren, Henk
AU	4	108153	108153	Marquardt, RR	Marquardt, Rr
AU	5	108154	108154	Speijers, GJA	Speijers, Gja
AF	1	108155	108155	Dortant, PM	Dortant, Pm
AF	2	108156	108156	Peters-Volleberg, GWM	Peters-Volleberg, Gwm
AF	3	108157	108157	Van Loveren, H	van_Loveren, Henk
AF	4	108158	108158	Marquardt, RR	Marquardt, Rr
AF	5	108159	108159	Speijers, GJA	Speijers, Gja

Record 777 | WOS:000166500400005

Metabolism of ochratoxin A: Absence of formation of genotoxic derivatives by human and rat enzymes

Field	Position	Original line	Normalised line	Original value	Normalised value
AU	1	108257	108257	Gautier, JC	Gautier, Jc
AU	3	108259	108259	Welti, DH	Welti, Dh
AU	5	108261	108261	Gremaud, E	Gremaud, Eric
AU	6	108262	108262	Guengerich, FP	Guengerich, Fp
AU	7	108263	108263	Turesky, RJ	Turesky, Rj
AF	1	108264	108264	Gautier, JC	Gautier, Jc
AF	3	108266	108266	Welti, DH	Welti, Dh
AF	5	108268	108268	Gremaud, E	Gremaud, Eric
AF	6	108269	108269	Guengerich, FP	Guengerich, Fp
AF	7	108270	108270	Turesky, RJ	Turesky, Rj

Record 778 | WOS:000165378500005

Effects of ochratoxin A on cytotoxicity and cell differentiation in cultured rat embryonic cells

Field	Position	Original line	Normalised line	Original value	Normalised value
AU	1	108384	108384	Hong, JT	Hong, Jt
AU	2	108385	108385	Park, KL	Park, Kl
AU	3	108386	108386	Han, SY	Han, Sy
AU	4	108387	108387	Park, KS	Park, Ks
AU	5	108388	108388	Kim, HS	Kim, Hs
AU	6	108389	108389	Oh, SD	Oh, Sd
AU	7	108390	108390	Lee, RD	Lee, Rd
AU	8	108391	108391	Jang, SJ	Jang, Sj
AF	1	108392	108392	Hong, JT	Hong, Jt
AF	2	108393	108393	Park, KL	Park, Kl
AF	3	108394	108394	Han, SY	Han, Sy
AF	4	108395	108395	Park, KS	Park, Ks
AF	5	108396	108396	Kim, HS	Kim, Hs
AF	6	108397	108397	Oh, SD	Oh, Sd
AF	7	108398	108398	Lee, RD	Lee, Rd
AF	8	108399	108399	Jang, SJ	Jang, Sj

Record 779 | WOS:000089269500007

Studies of ochratoxin A-induced inhibition of phenylalanine hydroxylase and its reversal by phenylalanine

Field	Position	Original line	Normalised line	Original value	Normalised value
AU	4	108480	108480	Petrik, J	Petrik, Jozsef
AU	6	108482	108482	Pepeljnjak, S	Pepeljnjak, Stjepan
AF	4	108486	108486	Petrik, J	Petrik, Jozsef
AF	6	108488	108488	Pepeljnjak, S	Pepeljnjak, Stjepan

Record 781 | WOS:000087555100005

Ochratoxin A from a toxicological perspective

Field	Position	Original line	Normalised line	Original value	Normalised value

AU	1	108695	108695	Petzinger, E	Petzinger, Ernst
AF	1	108697	108697	Petzinger, E	Petzinger, Ernst

Record 782 | WOS:000085306200013

Induction of unscheduled DNA synthesis in primary human urothelial cells by the mycotoxin ochratoxin A

Field	Position	Original line	Normalised line	Original value	Normalised value
AU	1	108832	108832	Dörrenhaus, A	Dorrenhaus, A
AU	3	108834	108834	Golka, K	Golka, Klaus
AU	4	108835	108835	Schulze, H	Schulze, Harald
AU	6	108837	108837	Degen, GH	Degen, Gh
AU	7	108838	108838	Föllmann, W	Follmann, W
AF	1	108839	108839	Dörrenhaus, A	Dorrenhaus, A
AF	3	108841	108841	Golka, K	Golka, Klaus
AF	4	108842	108842	Schulze, H	Schulze, Harald
AF	6	108844	108844	Degen, GH	Degen, Gh
AF	7	108845	108845	Föllmann, W	Follmann, W

Record 783 | WOS:000084146300015

Cholestyramine protection against ochratoxin A toxicity: Role of ochratoxin A sorption by the resin and bile enterohepatic circulation

Field	Position	Original line	Normalised line	Original value	Normalised value
AU	3	108945	108945	Marquardt, RR	Marquardt, Rr
AU	4	108946	108946	Frohlich, AA	Frohlich, Aa
AU	5	108947	108947	Yousef, IM	Yousef, Im
AU	6	108948	108948	Zhu, XX	Zhu, Xiaoyang
AF	3	108952	108952	Marquardt, RR	Marquardt, Rr
AF	4	108953	108953	Frohlich, AA	Frohlich, Aa
AF	5	108954	108954	Yousef, IM	Yousef, Im
AF	6	108955	108955	Zhu, XX	Zhu, Xiaoyang

Record 784 | WOS:000165381000007

Increased cytotoxicity of food-borne mycotoxins toward human cell lines in vitro via enhanced cytochrome p450 expression using the MTT bioassay

Field	Position	Original line	Normalised line	Original value	Normalised value
AU	1	109037	109037	Lewis, CW	Lewis, Cw
AU	2	109038	109038	Smith, JE	Smith, Je
AU	3	109039	109039	Anderson, JG	Anderson, Jg
AU	4	109040	109040	Freshney, RI	Freshney, Ri
AF	1	109041	109041	Lewis, CW	Lewis, Cw
AF	2	109042	109042	Smith, JE	Smith, Je
AF	3	109043	109043	Anderson, JG	Anderson, Jg
AF	4	109044	109044	Freshney, RI	Freshney, Ri

Record 786 | WOS:000082038700010

Ochratoxin binding to phenylalanyl-tRNA synthetase: Computational approach to the mechanism of ochratoxicosis and its antagonism

Field	Position	Original line	Normalised line	Original value	Normalised value
AU	1	109235	109235	McMasters, DR	Mcmasters, Dr
AF	1	109237	109237	McMasters, DR	Mcmasters, Dr

Record 787 | WOS:000081907800002

Mutagenicity of commercial Monascus fermentation products and the role of citrinin contamination

Field	Position	Original line	Normalised line	Original value	Normalised value
AU	2	109329	109329	Maas, RFM	Maas, Rfm
AU	3	109330	109330	Fink-Gremmels, J	Fink-Gremmels, Johanna
AF	2	109332	109332	Maas, RFM	Maas, Rfm
AF	3	109333	109333	Fink-Gremmels, J	Fink-Gremmels, Johanna

Record 788 | WOS:000081759900001

Ochratoxin A-binding proteins in rat organs and plasma and in different cell lines of the kidney

Field	Position	Original line	Normalised line	Original value	Normalised value
AU	1	109421	109421	Schwerdt, G	Schwerdt, Gerald
AU	4	109424	109424	Gekle, M	Gekle, Michael
AF	1	109425	109425	Schwerdt, G	Schwerdt, Gerald
AF	4	109428	109428	Gekle, M	Gekle, Michael

Record 789 | WOS:000080471300017

Transport of ochratoxin A by renal multispecific organic anion transporter 1

Field	Position	Original line	Normalised line	Original value	Normalised value
AU	4	109524	109524	Cha, SH	Cha, Sh
AF	4	109531	109531	Cha, SH	Cha, Sh

Record 790 | WOS:000080762800002

Roles of cyclooxygenase and lipoxygenases in ochratoxin A genotoxicity in human epithelial lung cells

Field	Position	Original line	Normalised line	Original value	Normalised value
AU	2	109606	109606	El Adlouni, C	el_Adlouni, C
AU	5	109609	109609	Pfohl-Leszkowicz, A	Pfohl-Leszkowicz, Annie
AF	2	109611	109611	El Adlouni, C	el_Adlouni, C
AF	5	109614	109614	Pfohl-Leszkowicz, A	Pfohl-Leszkowicz, Annie

Record 791 | WOS:000080209100001

Inactivation of ochratoxin in ruminal fluid with variation of pH-value and fermentation parameters in an in vitro system

Field	Position	Original line	Normalised line	Original value	Normalised value
AU	1	109729	109729	Özpinar, H	Ozpinar, H
AF	1	109732	109732	Özpinar, H	Ozpinar, H

Record 792 | WOS:000083320300013

Cytotoxic and genotoxic effects of zearalenone:: Prevention by vitamin E

Field	Position	Original line	Normalised line	Original value	Normalised value
AU	1	109803	109803	Ghédira-Chékir, L	Ghedira-Chekir, L
AU	3	109805	109805	Creppy, EE	Creppy, Ee
AU	4	109806	109806	Bacha, H	Bacha, Hassen
AF	1	109807	109807	Ghédira-Chékir, L	Ghedira-Chekir, L
AF	3	109809	109809	Creppy, EE	Creppy, Ee
AF	4	109810	109810	Bacha, H	Bacha, Hassen

Record 793 | WOS:000087386900028

Biotransformation and lack of mutagenicity of ochratoxin A using combinations of mammalian biotransformation enzymes

Field	Position	Original line	Normalised line	Original value	Normalised value
AU	2	109889	109889	Pähler, A	Pahler, A
AU	4	109891	109891	Dekant, W	Dekant, Wolfgang
AF	2	109893	109893	Pähler, A	Pahler, A
AF	4	109895	109895	Dekant, W	Dekant, Wolfgang

Record 794 | WOS:000078111000012

Apical uptake of radiolabelled ochratoxin A into Madin-Darby canine kidney cells

Field	Position	Original line	Normalised line	Original value	Normalised value
AU	1	109972	109972	Schwerdt, G	Schwerdt, Gerald
AU	4	109975	109975	Gekle, M	Gekle, Michael
AF	1	109976	109976	Schwerdt, G	Schwerdt, Gerald
AF	4	109979	109979	Gekle, M	Gekle, Michael

Record 795 | WOS:000077419100015

Apoptosis in the kidney

Field	Position	Original line	Normalised line	Original value	Normalised value
AU	1	110080	110080	Davis, MA	Davis, Ma
AU	2	110081	110081	Ryan, DH	Ryan, Dh
AF	1	110082	110082	Davis, MA	Davis, Ma
AF	2	110083	110083	Ryan, DH	Ryan, Dh

Record 797 | WOS:000076443800002

Cytotoxicity of nephrotoxic fungal toxins to kidney-derived LLC-PK1 and OK cell lines

Field	Position	Original line	Normalised line	Original value	Normalised value
AU	1	110357	110357	Bondy, GS	Bondy, Gs
AU	2	110358	110358	Armstrong, CL	Armstrong, Cl
AF	1	110359	110359	Bondy, GS	Bondy, Gs
AF	2	110360	110360	Armstrong, CL	Armstrong, Cl

Record 798 | WOS:000076413600003

Exposure to ochratoxin A impairs organic anion transport in proximal-tubule-derived opossum kidney cells

Field	Position	Original line	Normalised line	Original value	Normalised value
AU	3	110444	110444	Gekle, M	Gekle, Michael
AF	3	110447	110447	Gekle, M	Gekle, Michael

Record 799 | WOS:000074665700021

Detailed mapping of ochratoxin a reabsorption along the rat nephron in vivo: The nephrotoxin can be reabsorbed in all nephron segments by different mechanisms

Field	Position	Original line	Normalised line	Original value	Normalised value
AU	2	110533	110533	Dantzler, WH	Dantzler, Wh
AU	4	110535	110535	Gekle, M	Gekle, Michael
AF	2	110537	110537	Dantzler, WH	Dantzler, Wh
AF	4	110539	110539	Gekle, M	Gekle, Michael

Record 800 | WOS:000075078200010

Haematotoxicity of mycotoxins

Field	Position	Original line	Normalised line	Original value	Normalised value
AU	2	110612	110612	Parchment, RE	Parchment, Re
AF	2	110614	110614	Parchment, RE	Parchment, Re

Record 801 | WOS:000073042900016

Activated carbons: In vitro affinity for ochratoxin A and deoxynivalenol and relation of adsorption ability to physicochemical parameters

Field	Position	Original line	Normalised line	Original value	Normalised value
AU	1	110747	110747	Galvano, F	Galvano, Fabio
AU	2	110748	110748	Pietri, A	Pietri, Amedeo
AU	3	110749	110749	Bertuzzi, T	Bertuzzi, Terenzio
AF	1	110753	110753	Galvano, F	Galvano, Fabio
AF	2	110754	110754	Pietri, A	Pietri, Amedeo
AF	3	110755	110755	Bertuzzi, T	Bertuzzi, Terenzio

Record 803 | WOS:000079327200007

The effects of four mycotoxins on the mitogen stimulated proliferation of bovine peripheral blood mononuclear cells in vitro

Field	Position	Original line	Normalised line	Original value	Normalised value

AU	2	110942	110942	Fitzpatrick, JL	Fitzpatrick, Jl
AU	3	110943	110943	Smith, JE	Smith, Je
AF	2	110945	110945	Fitzpatrick, JL	Fitzpatrick, Jl
AF	3	110946	110946	Smith, JE	Smith, Je

Record 804 | WOS:000081940900027

Mycotoxins: Mechanisms of action at the molecular level

Field	Position	Original line	Normalised line	Original value	Normalised value
AU	1	111046	111046	Fink-Gremmels, J	Fink-Gremmels, Johanna
AF	1	111047	111047	Fink-Gremmels, J	Fink-Gremmels, Johanna

Record 805 | WOS:000075950800023

Tubulotoxic mechanisms of ochratoxin a

Field	Position	Original line	Normalised line	Original value	Normalised value
AU	1	111167	111167	Gekle, M	Gekle, Michael
AU	3	111169	111169	Schwerdt, G	Schwerdt, Gerald
AF	1	111171	111171	Gekle, M	Gekle, Michael
AF	3	111173	111173	Schwerdt, G	Schwerdt, Gerald

Record 806 | WOS:000080521300009

Ochratoxins: Molecular strategies for developing an antidote

Field	Position	Original line	Normalised line	Original value	Normalised value
AU	1	111257	111257	McMasters, DR	Mcmasters, Dr
AF	1	111259	111259	McMasters, DR	Mcmasters, Dr

Record 807 | WOS:000071510600005

Acute toxicity of the mycotoxin roridin E on liver and kidney of rats

Field	Position	Original line	Normalised line	Original value	Normalised value
AU	1	111322	111322	Omar, HEM	Omar, Hem
AU	2	111323	111323	El Sawi, NM	el_Sawi, Nm
AU	3	111324	111324	Meki, ARMA	Meki, Arma
AF	1	111325	111325	Omar, HEM	Omar, Hem
AF	2	111326	111326	El Sawi, NM	el_Sawi, Nm
AF	3	111327	111327	Meki, ARMA	Meki, Arma

Record 809 | WOS:A1997XM61400017

Effects of inorganic adsorbents and cyclopiazonic acid in broiler chickens

Field	Position	Original line	Normalised line	Original value	Normalised value
AU	1	111471	111471	Dwyer, MR	Dwyer, Mr

AU	2	111472	111472	Kubena, LF	Kubena, Lf
AU	3	111473	111473	Harvey, RB	Harvey, Rb
AU	5	111475	111475	Sarr, AB	Sarr, Ab
AU	7	111477	111477	Bailey, RH	Bailey, Rh
AU	8	111478	111478	Phillips, TD	Phillips, Td
AF	1	111479	111479	Dwyer, MR	Dwyer, Mr
AF	2	111480	111480	Kubena, LF	Kubena, Lf
AF	3	111481	111481	Harvey, RB	Harvey, Rb
AF	5	111483	111483	Sarr, AB	Sarr, Ab
AF	7	111485	111485	Bailey, RH	Bailey, Rh
AF	8	111486	111486	Phillips, TD	Phillips, Td

Record 810 | WOS:A1997XN68000014

Lipid peroxidation as one mode of action in ochratoxin A toxicity in rats and chicks

Field	Position	Original line	Normalised line	Original value	Normalised value
AU	2	111588	111588	Marquardt, RR	Marquardt, Rr
AU	3	111589	111589	Frohlich, AA	Frohlich, Aa
AF	2	111591	111591	Marquardt, RR	Marquardt, Rr
AF	3	111592	111592	Frohlich, AA	Frohlich, Aa

Record 811 | WOS:A1997XA91600004

Induction of micronuclei with ochratoxin A in ovine seminal vesicle cell cultures

Field	Position	Original line	Normalised line	Original value	Normalised value
AU	1	111657	111657	Degen, GH	Degen, Gh
AU	2	111658	111658	Gerber, MM	Gerber, Mm
AU	3	111659	111659	ObrechtPflumio, S	Obrechtpflumio, S
AF	1	111661	111661	Degen, GH	Degen, Gh
AF	2	111662	111662	Gerber, MM	Gerber, Mm
AF	3	111663	111663	ObrechtPflumio, S	Obrechtpflumio, S

Record 813 | WOS:A1997WW09700003

Reduction of the ochratoxin A-induced cytotoxicity in Vero cells by aspartame

Field	Position	Original line	Normalised line	Original value	Normalised value
AU	2	111855	111855	Betbeder, AM	Betbeder, Am
AU	3	111856	111856	Creppy, EE	Creppy, Ee
AF	2	111858	111858	Betbeder, AM	Betbeder, Am
AF	3	111859	111859	Creppy, EE	Creppy, Ee

Record 814 | WOS:A1997XZ55900019

Effects of the naturally occurring food mycotoxin ochratoxin A on brain cells in culture

Field	Position	Original line	Normalised line	Original value	Normalised value

AU	1	111949	111949	MonnetTschudi, F	Monnettschudi, F
AU	3	111951	111951	Honegger, P	Honegger, Paul
AU	4	111952	111952	Zurich, MG	Zurich, Mg
AU	5	111953	111953	Huggett, AC	Huggett, Ac
AU	6	111954	111954	Schilter, B	Schilter, Benoit
AF	1	111955	111955	MonnetTschudi, F	Monnettschudi, F
AF	3	111957	111957	Honegger, P	Honegger, Paul
AF	4	111958	111958	Zurich, MG	Zurich, Mg
AF	5	111959	111959	Huggett, AC	Huggett, Ac
AF	6	111960	111960	Schilter, B	Schilter, Benoit

Record 815 | WOS:A1997WC04100026

Reabsorption of the nephrotoxin ochratoxin A along the rat nephron in vivo

Field	Position	Original line	Normalised line	Original value	Normalised value
AU	3	112058	112058	Gekle, M	Gekle, Michael
AF	3	112061	112061	Gekle, M	Gekle, Michael

Record 816 | WOS:A1996WB42500013

Influence of vitamins E and C on the toxic effects of ochratoxin A and T-2 toxin in chicks

Field	Position	Original line	Normalised line	Original value	Normalised value
AU	2	112125	112125	Marquardt, RR	Marquardt, Rr
AF	2	112127	112127	Marquardt, RR	Marquardt, Rr

Record 817 | WOS:A1996UZ36400005

Effects of mycotoxins on cytokine production and proliferation in EL-4 thymoma cells

Field	Position	Original line	Normalised line	Original value	Normalised value
AU	1	112202	112202	Marin, ML	Marin, Ml
AU	3	112204	112204	Dong, WM	Dong, Wenjing
AU	4	112205	112205	Pestka, JJ	Pestka, Jj
AF	1	112206	112206	Marin, ML	Marin, Ml
AF	3	112208	112208	Dong, WM	Dong, Wenjing
AF	4	112209	112209	Pestka, JJ	Pestka, Jj

Record 818 | WOS:A1996UT94000012

Prevention of toxic effects of mycotoxins by means of nonnutritive adsorbent compounds

Field	Position	Original line	Normalised line	Original value	Normalised value
AU	1	112312	112312	Ramos, AJ	Ramos, Aj
AU	2	112313	112313	FinkGremmels, J	Fink-Gremmels, Johanna
AF	1	112315	112315	Ramos, AJ	Ramos, Aj
AF	2	112316	112316	FinkGremmels, J	Fink-Gremmels, Johanna

Record 819 | WOS:A1996VJ28200003

Aspartame as a preventive agent of chronic toxic effects of ochratoxin A in experimental animals

Field	Position	Original line	Normalised line	Original value	Normalised value
AU	1	112474	112474	Creppy, EE	Creppy, Ee
AU	4	112477	112477	Betbeder, AM	Betbeder, Am
AF	1	112478	112478	Creppy, EE	Creppy, Ee
AF	4	112481	112481	Betbeder, AM	Betbeder, Am

Record 820 | WOS:A1996VW20400002

Renal toxicodynamics of ochratoxin A: A pathophysiological approach

Field	Position	Original line	Normalised line	Original value	Normalised value
AU	1	112593	112593	Gekle, M	Gekle, Michael
AF	1	112595	112595	Gekle, M	Gekle, Michael

Record 821 | WOS:A1995TU51400124

Prevention of nephrotoxicity of ochratoxin A, a food contaminant

Field	Position	Original line	Normalised line	Original value	Normalised value
AU	1	112712	112712	Creppy, EE	Creppy, Ee
AU	3	112714	112714	Betbeder, AM	Betbeder, Am
AF	1	112715	112715	Creppy, EE	Creppy, Ee
AF	3	112717	112717	Betbeder, AM	Betbeder, Am

Record 822 | WOS:A1995TN07400005

Activation markers and cell proliferation as indicators of toxicity: A flow cytometric approach

Field	Position	Original line	Normalised line	Original value	Normalised value
AU	3	112817	112817	Gadhasson, IL	Gadhasson, Il
AF	3	112820	112820	Gadhasson, IL	Gadhasson, Il

Record 823 | WOS:A1995TC07600004

OCHRATOXINES AND TOXICOLOGICAL CONSEQUENCES

Field	Position	Original line	Normalised line	Original value	Normalised value
AU	1	112896	112896	CREPPY, EE	Creppy, Ee
AU	2	112897	112897	BAUDRIMONT, I	Baudrimont, I
AU	3	112898	112898	BETBEDER, AM	Betbeder, Am
AF	1	112899	112899	CREPPY, EE	Creppy, Ee
AF	2	112900	112900	BAUDRIMONT, I	Baudrimont, I
AF	3	112901	112901	BETBEDER, AM	Betbeder, Am

Record 824 | WOS:A1995QJ43400010

SISTER-CHROMATID EXCHANGE FREQUENCY IN CULTURED ISOLATED PORCINE URINARY-BLADDER EPITHELIAL-CELLS (PUBEC) TREATED WITH OCHRATOXIN-A AND OCHRATOXIN-ALPHA

Field	Position	Original line	Normalised line	Original value	Normalised value
AU	1	112934	112934	FOLLMANN, W	Follmann, W
AU	2	112935	112935	HILLEBRAND, IE	Hillebrand, Ie
AU	3	112936	112936	CREPPY, EE	Creppy, Ee
AU	4	112937	112937	BOLT, HM	Bolt, Hm
AF	1	112938	112938	FOLLMANN, W	Follmann, W
AF	2	112939	112939	HILLEBRAND, IE	Hillebrand, Ie
AF	3	112940	112940	CREPPY, EE	Creppy, Ee
AF	4	112941	112941	BOLT, HM	Bolt, Hm

Record 825 | WOS:A1995QC67100017

EFFECT OF PIROXICAM ON THE NEPHROTOXICITY INDUCED BY OCHRATOXIN-A IN RATS

Field	Position	Original line	Normalised line	Original value	Normalised value
AU	1	113031	113031	BAUDRIMONT, I	Baudrimont, I
AU	2	113032	113032	MURN, M	Murn, M
AU	3	113033	113033	BETBEDER, AM	Betbeder, Am
AU	4	113034	113034	GUILCHER, J	Guilcher, J
AU	5	113035	113035	CREPPY, EE	Creppy, Ee
AF	1	113036	113036	BAUDRIMONT, I	Baudrimont, I
AF	2	113037	113037	MURN, M	Murn, M
AF	3	113038	113038	BETBEDER, AM	Betbeder, Am
AF	4	113039	113039	GUILCHER, J	Guilcher, J
AF	5	113040	113040	CREPPY, EE	Creppy, Ee

Record 826 | WOS:A1994NN32900003

EFFECT OF SUPEROXIDE-DISMUTASE AND CATALASE ON THE NEPHROTOXICITY INDUCED BY SUBCHRONICAL ADMINISTRATION OF OCHRATOXIN-A IN RATS

Field	Position	Original line	Normalised line	Original value	Normalised value
AU	1	113133	113133	BAUDRIMONT, I	Baudrimont, I
AU	2	113134	113134	BETBEDER, AM	Betbeder, Am
AU	3	113135	113135	GHARBI, A	Gharbi, A
AU	4	113136	113136	PFOHLLESZKOWICZ, A	Pfohl-Leszkowicz, Annie
AU	5	113137	113137	DIRHEIMER, G	Dirheimer, G
AU	6	113138	113138	CREPPY, EE	Creppy, Ee
AF	1	113139	113139	BAUDRIMONT, I	Baudrimont, I
AF	2	113140	113140	BETBEDER, AM	Betbeder, Am
AF	3	113141	113141	GHARBI, A	Gharbi, A
AF	4	113142	113142	PFOHLLESZKOWICZ, A	Pfohl-Leszkowicz, Annie
AF	5	113143	113143	DIRHEIMER, G	Dirheimer, G
AF	6	113144	113144	CREPPY, EE	Creppy, Ee

Record 827 | WOS:A1994MM85700005

THE ROLE OF THE PROXIMAL TUBULE IN OCHRATOXIN-A NEPHROTOXICITY IN-VIVO - TOXODYNAMIC AND TOXOKINETIC ASPECTS

Field	Position	Original line	Normalised line	Original value	Normalised value
AU	1	113229	113229	GEKLE, M	Gekle, Michael
AU	2	113230	113230	SILBERNAGL, S	Silbernagl, S
AF	1	113231	113231	GEKLE, M	Gekle, Michael
AF	2	113232	113232	SILBERNAGL, S	Silbernagl, S

Record 828 | WOS:A1994NV49900013

EFFECT OF MYCOTOXINS ON IN-VITRO MOVEMENT OF TRACHEAL CILIA FROM ONE-DAY-OLD CHICKS

Field	Position	Original line	Normalised line	Original value	Normalised value
AU	1	113307	113307	JESENSKA, Z	Jesenska, Z
AU	2	113308	113308	BERNAT, D	Bernat, D
AF	1	113309	113309	JESENSKA, Z	Jesenska, Z
AF	2	113310	113310	BERNAT, D	Bernat, D

Record 829 | WOS:A1993MU47600008

EVALUATION OF THE DEVELOPMENTAL TOXICITY OF CITRININ USING HYDRA-ATTENUATA AND POSTIMPLANTATION RAT WHOLE-EMBRYO CULTURE

Field	Position	Original line	Normalised line	Original value	Normalised value
AU	1	113392	113392	YANG, YG	Yang, Yg
AU	2	113393	113393	MAYURA, K	Mayura, K
AU	3	113394	113394	SPAINHOUR, CB	Spainhour, Cb
AU	4	113395	113395	EDWARDS, JF	Edwards, Jf
AU	5	113396	113396	PHILLIPS, TD	Phillips, Td
AF	1	113397	113397	YANG, YG	Yang, Yg
AF	2	113398	113398	MAYURA, K	Mayura, K
AF	3	113399	113399	SPAINHOUR, CB	Spainhour, Cb
AF	4	113400	113400	EDWARDS, JF	Edwards, Jf
AF	5	113401	113401	PHILLIPS, TD	Phillips, Td

Record 830 | WOS:A1993MM43500024

POSSIBLE ROLE OF AN IRON-OXYGEN COMPLEX IN 4(S)-4-HYDROXYOCHRATOXIN-A FORMATION BY RAT-LIVER MICROSOMES

Field	Position	Original line	Normalised line	Original value	Normalised value
AU	1	113501	113501	OMAR, RF	Omar, Rf
AU	2	113502	113502	RAHIMTULA, AD	Rahimtula, Ad
AF	1	113503	113503	OMAR, RF	Omar, Rf
AF	2	113504	113504	RAHIMTULA, AD	Rahimtula, Ad

Record 831 | WOS:A1993MQ49500006

OCHRATOXIN-A IMPAIRS POSTPROXIMAL NEPHRON FUNCTION IN-VIVO AND BLOCKS PLASMA-MEMBRANE ANION CONDUCTANCE IN MADIN-DARBY CANINE KIDNEY-CELLS IN-VITRO

Field	Position	Original line	Normalised line	Original value	Normalised value
AU	1	113586	113586	GEKLE, M	Gekle, Michael
AU	2	113587	113587	OBERLEITHNER, H	Oberleithner, H
AU	3	113588	113588	SILBERNAGL, S	Silbernagl, S
AF	1	113589	113589	GEKLE, M	Gekle, Michael
AF	2	113590	113590	OBERLEITHNER, H	Oberleithner, H
AF	3	113591	113591	SILBERNAGL, S	Silbernagl, S

Record 832 | WOS:A1993LR82100028

EVALUATION AND APPLICATION OF A BIOLUMINESCENT BACTERIAL GENOTOXICITY TEST

Field	Position	Original line	Normalised line	Original value	Normalised value
AU	1	113667	113667	SUN, TSC	Sun, Tsc
AU	2	113668	113668	STAHR, HM	Stahr, Hm
AF	1	113669	113669	SUN, TSC	Sun, Tsc
AF	2	113670	113670	STAHR, HM	Stahr, Hm

Record 833 | WOS:A1993LN86000012

COMPARISON OF IN-VITRO AND IN-VIVO BIOLOGICAL-ACTIVITY OF MYCOTOXINS

Field	Position	Original line	Normalised line	Original value	Normalised value
AU	1	113733	113733	TERSE, PS	Terse, Ps
AU	2	113734	113734	MADHYASTHA, MS	Madhyastha, Ms
AU	3	113735	113735	ZUROVAC, O	Zurovac, O
AU	4	113736	113736	STRINGFELLOW, D	Stringfellow, D
AU	5	113737	113737	MARQUARDT, RR	Marquardt, Rr
AU	6	113738	113738	KEMPPAINEN, BW	Kemppainen, Bw
AF	1	113739	113739	TERSE, PS	Terse, Ps
AF	2	113740	113740	MADHYASTHA, MS	Madhyastha, Ms
AF	3	113741	113741	ZUROVAC, O	Zurovac, O
AF	4	113742	113742	STRINGFELLOW, D	Stringfellow, D
AF	5	113743	113743	MARQUARDT, RR	Marquardt, Rr
AF	6	113744	113744	KEMPPAINEN, BW	Kemppainen, Bw

Record 834 | WOS:A1993LJ01500043

EXTRACELLULAR ACIDOSIS AMELIORATES METABOLIC-INHIBITOR-INDUCED AND POTENTIATES OXIDANT-INDUCED CELL-DEATH IN RENAL PROXIMAL TUBULES

Field	Position	Original line	Normalised line	Original value	Normalised value
AU	1	113825	113825	RODEHEAVER, DP	Rodeheaver, Dp
AU	2	113826	113826	SCHNELLMANN, RG	Schnellmann, Rg
AF	1	113827	113827	RODEHEAVER, DP	Rodeheaver, Dp

AF	2	113828	113828	SCHNELLMANN, RG	Schnellmann, Rg

Record 835 | WOS:A1993KQ30600025

IMMUNOTOXICITY OF MYCOTOXINS

Field	Position	Original line	Normalised line	Original value	Normalised value
AU	1	113903	113903	SHARMA, RP	Sharma, Riddhi
AF	1	113904	113904	SHARMA, RP	Sharma, Riddhi

Record 836 | WOS:A1992JU40900011

HYDROLYSIS OF OCHRATOXIN-A BY THE MICROBIAL ACTIVITY OF DIGESTA IN THE GASTROINTESTINAL-TRACT OF RATS

Field	Position	Original line	Normalised line	Original value	Normalised value
AU	1	113994	113994	MADHYASTHA, MS	Madhyastha, Ms
AU	2	113995	113995	MARQUARDT, RR	Marquardt, Rr
AU	3	113996	113996	FROHLICH, AA	Frohlich, Aa
AF	1	113997	113997	MADHYASTHA, MS	Madhyastha, Ms
AF	2	113998	113998	MARQUARDT, RR	Marquardt, Rr
AF	3	113999	113999	FROHLICH, AA	Frohlich, Aa

Record 837 | WOS:A1992KT90400023

ALTERATIONS IN ATP-DEPENDENT CALCIUM-UPTAKE BY RAT RENAL-CORTEX MICROSOMES FOLLOWING OCHRATOXIN A ADMINISTRATION INVIVO OR ADDITION INVITRO

Field	Position	Original line	Normalised line	Original value	Normalised value
AU	1	114065	114065	CHONG, X	Chong, X
AU	2	114066	114066	RAHIMTULA, AD	Rahimtula, Ad
AF	1	114067	114067	CHONG, X	Chong, X
AF	2	114068	114068	RAHIMTULA, AD	Rahimtula, Ad

Record 838 | WOS:A1992HP59700018

INVITRO EFFECTS OF THE NEPHROTOXINS OCHRATOXIN-A AND CITRININ UPON BIOCHEMICAL FUNCTION OF PORCINE KIDNEY

Field	Position	Original line	Normalised line	Original value	Normalised value
AU	1	114160	114160	BRAUNBERG, RC	Braunberg, Rc
AU	2	114161	114161	GANTT, O	Gantt, O
AU	3	114162	114162	BARTON, C	Barton, C
AU	4	114163	114163	FRIEDMAN, L	Friedman, L
AF	1	114164	114164	BRAUNBERG, RC	Braunberg, Rc
AF	2	114165	114165	GANTT, O	Gantt, O
AF	3	114166	114166	BARTON, C	Barton, C
AF	4	114167	114167	FRIEDMAN, L	Friedman, L

Record 839 | WOS:A1991GJ33400005

ROLE OF CYTOCHROME-P-450 IN OCHRATOXIN-A STIMULATED LIPID-PEROXIDATION

Field	Position	Original line	Normalised line	Original value	Normalised value
AU	1	114264	114264	OMAR, RF	Omar, Rf
AU	2	114265	114265	RAHIMTULA, AD	Rahimtula, Ad
AU	3	114266	114266	BARTSCH, H	Bartsch, H
AF	1	114267	114267	OMAR, RF	Omar, Rf
AF	2	114268	114268	RAHIMTULA, AD	Rahimtula, Ad
AF	3	114269	114269	BARTSCH, H	Bartsch, H

Record 840 | WOS:A1991GJ33400006

INVITRO INHIBITION OF RAT PLATELET-AGGREGATION BY OCHRATOXIN-A

Field	Position	Original line	Normalised line	Original value	Normalised value
AU	1	114337	114337	OMAR, RF	Omar, Rf
AU	2	114338	114338	RANDELL, E	Randell, E
AU	3	114339	114339	RAHIMTULA, AD	Rahimtula, Ad
AF	1	114340	114340	OMAR, RF	Omar, Rf
AF	2	114341	114341	RANDELL, E	Randell, E
AF	3	114342	114342	RAHIMTULA, AD	Rahimtula, Ad

Record 841 | WOS:A1991GA29400009

CHARACTERIZATION OF PIG-LIVER PURIFIED CYTOCHROME-P-450 ISOENZYMES FOR OCHRATOXIN-A METABOLISM STUDIES

Field	Position	Original line	Normalised line	Original value	Normalised value
AU	1	114406	114406	OSTER, T	Oster, T
AU	2	114407	114407	JAYYOSI, Z	Jayyosi, Z
AU	3	114408	114408	CREPPY, EE	Creppy, Ee
AU	4	114409	114409	ELAMRI, HS	Elamri, Hs
AU	5	114410	114410	BATT, AM	Batt, Am
AF	1	114411	114411	OSTER, T	Oster, T
AF	2	114412	114412	JAYYOSI, Z	Jayyosi, Z
AF	3	114413	114413	CREPPY, EE	Creppy, Ee
AF	4	114414	114414	ELAMRI, HS	Elamri, Hs
AF	5	114415	114415	BATT, AM	Batt, Am

Record 842 | WOS:A1991FC20700661

STIMULATION OF RENAL ENDOPLASMIC-RETICULUM CALCIUM-UPTAKE BY OCHRATOXIN-A

Field	Position	Original line	Normalised line	Original value	Normalised value
AU	1	114496	114496	CHONG, X	Chong, X
AU	2	114497	114497	RAHIMTULA, AD	Rahimtula, Ad
AF	1	114498	114498	CHONG, X	Chong, X
AF	2	114499	114499	RAHIMTULA, AD	Rahimtula, Ad

Record 843 | WOS:A1991FR33200006

INVIVO ABSORPTION OF C-14 GLUCOSE AND C-14 GLYCINE BY THE RAT INTESTINE DURING OCHRATOXIN A TOXICOSIS

Field	Position	Original line	Normalised line	Original value	Normalised value
AU	1	114536	114536	SUBRAMANIAN, S	Subramanian, S
AU	2	114537	114537	BALASUBRAMANIAN, N	Balasubramanian, N
AU	3	114538	114538	WILLIAM, S	William, S
AU	4	114539	114539	GOVINDASAMY, S	Govindasamy, S
AF	1	114540	114540	SUBRAMANIAN, S	Subramanian, S
AF	2	114541	114541	BALASUBRAMANIAN, N	Balasubramanian, N
AF	3	114542	114542	WILLIAM, S	William, S
AF	4	114543	114543	GOVINDASAMY, S	Govindasamy, S

Record 844 | WOS:A1991BW81R00026

MECHANISM OF ACTION OF OCHRATOXIN-A

Field	Position	Original line	Normalised line	Original value	Normalised value
AU	1	114603	114603	DIRHEIMER, G	Dirheimer, G
AU	2	114604	114604	CREPPY, EE	Creppy, Ee
AF	1	114605	114605	DIRHEIMER, G	Dirheimer, G
AF	2	114606	114606	CREPPY, EE	Creppy, Ee

Record 845 | WOS:A1991BW81R00032

DNA ADDUCT FORMATION IN MICE TREATED WITH OCHRATOXIN-A

Field	Position	Original line	Normalised line	Original value	Normalised value
AU	1	114648	114648	PFOHLLESZKOWICZ, A	Pfohl-Leszkowicz, Annie
AU	2	114649	114649	CHAKOR, K	Chakor, K
AU	3	114650	114650	CREPPY, EE	Creppy, Ee
AU	4	114651	114651	DIRHEIMER, G	Dirheimer, G
AF	1	114652	114652	PFOHLLESZKOWICZ, A	Pfohl-Leszkowicz, Annie
AF	2	114653	114653	CHAKOR, K	Chakor, K
AF	3	114654	114654	CREPPY, EE	Creppy, Ee
AF	4	114655	114655	DIRHEIMER, G	Dirheimer, G

Record 846 | WOS:A1990EP18900002

EFFECTS OF OCHRATOXIN-A AND OCHRATOXIN-B ON PRECHONDROGENIC MESENCHYMAL CELLS FROM CHICK-EMBRYO LIMB BUDS

Field	Position	Original line	Normalised line	Original value	Normalised value
AU	1	114696	114696	WIGER, R	Wiger, R
AU	2	114697	114697	STORMER, FC	Stormer, Fc
AF	1	114698	114698	WIGER, R	Wiger, R
AF	2	114699	114699	STORMER, FC	Stormer, Fc

Record 847 | WOS:A1990EF19400012

NADPH-CYTOCHROME-P-450 REDUCTASE PROMOTED HYDROXYL RADICAL PRODUCTION BY THE IRON(III)-OCHRATOXIN-A COMPLEX

Field	Position	Original line	Normalised line	Original value	Normalised value

AU	1	114760	114760	HASINOFF, BB	Hasinoff, Bb
AU	2	114761	114761	RAHIMTULA, AD	Rahimtula, Ad
AU	3	114762	114762	OMAR, RF	Omar, Rf
AF	1	114763	114763	HASINOFF, BB	Hasinoff, Bb
AF	2	114764	114764	RAHIMTULA, AD	Rahimtula, Ad
AF	3	114765	114765	OMAR, RF	Omar, Rf

Record 848 | WOS:A1990DV94600004

INDUCTION OF CHARACTERISTIC CHROMOSOMAL-ABERRATIONS, PARTICULARLY X-TRISOMY, IN CULTURED HUMAN-LYMPHOCYTES TREATED BY OCHRATOXIN-A, A MYCOTOXIN IMPLICATED IN BALKAN ENDEMIC NEPHROPATHY

Field	Position	Original line	Normalised line	Original value	Normalised value
AU	1	114825	114825	MANOLOVA, Y	Manolova, Y
AU	2	114826	114826	MANOLOV, G	Manolov, G
AU	3	114827	114827	PARVANOVA, L	Parvanova, L
AU	4	114828	114828	PETKOVABOCHAROVA, T	Petkova-Bocharova, Theodora
AU	5	114829	114829	CASTEGNARO, M	Castegnaro, Marcel
AU	6	114830	114830	CHERNOZEMSKY, IN	Chernozemsky, In
AF	1	114831	114831	MANOLOVA, Y	Manolova, Y
AF	2	114832	114832	MANOLOV, G	Manolov, G
AF	3	114833	114833	PARVANOVA, L	Parvanova, L
AF	4	114834	114834	PETKOVABOCHAROVA, T	Petkova-Bocharova, Theodora
AF	5	114835	114835	CASTEGNARO, M	Castegnaro, Marcel
AF	6	114836	114836	CHERNOZEMSKY, IN	Chernozemsky, In

Record 849 | WOS:A1990CF01500012

EFFECTS OF FUNGAL METABOLITES ON TESTOSTERONE SECRETION INVITRO

Field	Position	Original line	Normalised line	Original value	Normalised value
AU	1	114897	114897	FENSKE, M	Fenske, M
AU	2	114898	114898	FINKGREMMELS, J	Fink-Gremmels, Johanna
AF	1	114899	114899	FENSKE, M	Fenske, M
AF	2	114900	114900	FINKGREMMELS, J	Fink-Gremmels, Johanna

Record 850 | WOS:A1990CJ12700002

ENSILING AS A MEANS OF REDUCING OCHRATOXIN-A CONCENTRATIONS IN CONTAMINATED BARLEY

Field	Position	Original line	Normalised line	Original value	Normalised value
AU	1	114961	114961	ROTTER, RG	Rotter, Rg
AU	2	114962	114962	MARQUARDT, RR	Marquardt, Rr
AU	3	114963	114963	FROHLICH, AA	Frohlich, Aa
AU	4	114964	114964	ABRAMSON, D	Abramson, D
AF	1	114965	114965	ROTTER, RG	Rotter, Rg
AF	2	114966	114966	MARQUARDT, RR	Marquardt, Rr
AF	3	114967	114967	FROHLICH, AA	Frohlich, Aa
AF	4	114968	114968	ABRAMSON, D	Abramson, D

Record 851 | WOS:A1989CA57100015

INFLUENCE OF DIETARY CHARCOAL ON OCHRATOXIN-A TOXICITY IN LEGHORN CHICKS

Field	Position	Original line	Normalised line	Original value	Normalised value
AU	1	115043	115043	ROTTER, RG	Rotter, Rg
AU	2	115044	115044	FROHLICH, AA	Frohlich, Aa
AU	3	115045	115045	MARQUARDT, RR	Marquardt, Rr
AF	1	115046	115046	ROTTER, RG	Rotter, Rg
AF	2	115047	115047	FROHLICH, AA	Frohlich, Aa
AF	3	115048	115048	MARQUARDT, RR	Marquardt, Rr

Record 852 | WOS:A1989AT97300002

NEPHROTOXICITY ASSESSMENT BY MEASURING CELLULAR ATP CONTENT .2. INTRANEPHRON SITE OF OCHRATOXIN-A NEPHROTOXICITY

Field	Position	Original line	Normalised line	Original value	Normalised value
AU	1	115110	115110	JUNG, KY	Jung, Ky
AU	2	115111	115111	ENDOU, H	Endou, H
AF	1	115112	115112	JUNG, KY	Jung, Ky
AF	2	115113	115113	ENDOU, H	Endou, H

Record 853 | WOS:A1989CB27000014

MECHANISM OF OCHRATOXIN A-INDUCED IMMUNOSUPPRESSION

Field	Position	Original line	Normalised line	Original value	Normalised value
AU	1	115181	115181	LEA, T	Lea, T
AU	2	115182	115182	STEIEN, K	Steien, K
AU	3	115183	115183	STORMER, FC	Stormer, Fc
AF	1	115184	115184	LEA, T	Lea, T
AF	2	115185	115185	STEIEN, K	Steien, K
AF	3	115186	115186	STORMER, FC	Stormer, Fc

Record 854 | WOS:A1989T676500015

THE EFFECTS OF OCHRATOXIN-A ON POSTIMPLANTATION RAT EMBRYOS IN CULTURE

Field	Position	Original line	Normalised line	Original value	Normalised value
AU	1	115246	115246	MAYURA, K	Mayura, K
AU	2	115247	115247	EDWARDS, JF	Edwards, Jf
AU	3	115248	115248	MAULL, EA	Maull, Ea
AU	4	115249	115249	PHILLIPS, TD	Phillips, Td
AF	1	115250	115250	MAYURA, K	Mayura, K
AF	2	115251	115251	EDWARDS, JF	Edwards, Jf
AF	3	115252	115252	MAULL, EA	Maull, Ea
AF	4	115253	115253	PHILLIPS, TD	Phillips, Td

Record 855 | WOS:A1989T320900022

INFLUENCE OF OCHRATOXIN B ON THE OCHRATOXIN-A INHIBITION OF PHENYLALANYL-TRANSFER RNA FORMATION INVITRO AND PROTEIN-SYNTHESIS IN HEPATOMA TISSUE-CULTURE CELLS

Field	Position	Original line	Normalised line	Original value	Normalised value
AU	1	115318	115318	ROTH, A	Roth, Adrian
AU	2	115319	115319	CREPPY, EE	Creppy, Ee
AU	3	115320	115320	KANE, A	Kane, A
AU	4	115321	115321	BACHA, H	Bacha, Hassen
AU	5	115322	115322	STEYN, PS	Steyn, Ps
AU	6	115323	115323	ROSCHENTHALER, R	Roschenthaler, R
AU	7	115324	115324	DIRHEIMER, G	Dirheimer, G
AF	1	115325	115325	ROTH, A	Roth, Adrian
AF	2	115326	115326	CREPPY, EE	Creppy, Ee
AF	3	115327	115327	KANE, A	Kane, A
AF	4	115328	115328	BACHA, H	Bacha, Hassen
AF	5	115329	115329	STEYN, PS	Steyn, Ps
AF	6	115330	115330	ROSCHENTHALER, R	Roschenthaler, R
AF	7	115331	115331	DIRHEIMER, G	Dirheimer, G

Record 856 | WOS:A1989R524600010

PERTURBATION OF LIVER MICROSOMAL CALCIUM HOMEOSTASIS BY OCHRATOXIN-A

Field	Position	Original line	Normalised line	Original value	Normalised value
AU	1	115393	115393	KHAN, S	Khan, S
AU	2	115394	115394	MARTIN, M	Martin, M
AU	3	115395	115395	BARTSCH, H	Bartsch, H
AU	4	115396	115396	RAHIMTULA, AD	Rahimtula, Ad
AF	1	115397	115397	KHAN, S	Khan, S
AF	2	115398	115398	MARTIN, M	Martin, M
AF	3	115399	115399	BARTSCH, H	Bartsch, H
AF	4	115400	115400	RAHIMTULA, AD	Rahimtula, Ad

Record 857 | WOS:A1988R353500008

LIPID-PEROXIDATION AS A POSSIBLE CAUSE OF OCHRATOXIN A TOXICITY

Field	Position	Original line	Normalised line	Original value	Normalised value
AU	1	115482	115482	RAHIMTULA, AD	Rahimtula, Ad
AU	2	115483	115483	BEREZIAT, JC	Bereziat, Jc
AU	3	115484	115484	BUSSACCHINIGRIOT, V	Bussacchinigriot, V
AU	4	115485	115485	BARTSCH, H	Bartsch, H
AF	1	115486	115486	RAHIMTULA, AD	Rahimtula, Ad
AF	2	115487	115487	BEREZIAT, JC	Bereziat, Jc
AF	3	115488	115488	BUSSACCHINIGRIOT, V	Bussacchinigriot, V
AF	4	115489	115489	BARTSCH, H	Bartsch, H

Record 858 | WOS:A1988P563200002

INDUCTION OF SOS FUNCTION IN ESCHERICHIA-COLI BY SOME MYCOTOXINS

Field	Position	Original line	Normalised line	Original value	Normalised value
AU	1	115573	115573	AUFFRAY, Y	Auffray, Y
AU	2	115574	115574	BOUTIBONNES, P	Boutibonnes, P
AF	1	115575	115575	AUFFRAY, Y	Auffray, Y
AF	2	115576	115576	BOUTIBONNES, P	Boutibonnes, P

Record 859 | WOS:A1988R574700010

OCHRATOXIN-A AS A SUPPRESSOR OF MITOGEN-INDUCED BLASTOGENESIS OF PORCINE BLOOD-LYMPHOCYTES

Field	Position	Original line	Normalised line	Original value	Normalised value
AU	1	115609	115609	HOLMBERG, T	Holmberg, T
AU	2	115610	115610	THUVANDER, A	Thuvander, A
AU	3	115611	115611	HULT, K	Hult, K
AF	1	115612	115612	HOLMBERG, T	Holmberg, T
AF	2	115613	115613	THUVANDER, A	Thuvander, A
AF	3	115614	115614	HULT, K	Hult, K

Record 860 | WOS:A1987L417900004

PROTEIN-BINDING POTENTIAL OF OCHRATOXIN-A INVITRO AND ITS FLUORESCENCE ENHANCEMENT

Field	Position	Original line	Normalised line	Original value	Normalised value
AU	1	115669	115669	UCHIYAMA, S	Uchiyama, S
AU	2	115670	115670	SAITO, Y	Saito, Y
AF	1	115671	115671	UCHIYAMA, S	Uchiyama, S
AF	2	115672	115672	SAITO, Y	Saito, Y

Record 861 | WOS:A1987K378200008

GENOTOXIC ACTIVITY OF SOME MYCOTOXINS USING THE SOS CHROMOTEST

Field	Position	Original line	Normalised line	Original value	Normalised value
AU	1	115707	115707	AUFFRAY, Y	Auffray, Y
AU	2	115708	115708	BOUTIBONNES, P	Boutibonnes, P
AF	1	115709	115709	AUFFRAY, Y	Auffray, Y
AF	2	115710	115710	BOUTIBONNES, P	Boutibonnes, P

Record 862 | WOS:A1987G499300004

CARCINOGENICITY AND GENOTOXICITY OF MYCOTOXINS

Field	Position	Original line	Normalised line	Original value	Normalised value
AU	1	115777	115777	MULLER, T	Muller, T
AF	1	115778	115778	MULLER, T	Muller, T

Record 863 | WOS:A1986AYL5800018

INTERSTRAIN COMPARISON OF HEPATIC AND RENAL MICROSOMAL CARCINOGEN METABOLISM AND LIVER S9-MEDIATED MUTAGENICITY IN DA AND LEWIS RATS PHENOTYPED AS POOR AND EXTENSIVE METABOLIZERS OF DEBRISOQUINE

Field	Position	Original line	Normalised line	Original value	Normalised value
AU	1	115866	115866	HIETANEN, E	Hietanen, E
AU	2	115867	115867	MALAVEILLE, C	Malaveille, C
AU	3	115868	115868	CAMUS, AM	Camus, Am
AU	4	115869	115869	BEREZIAT, JC	Bereziat, Jc
AU	5	115870	115870	BRUN, G	Brun, G
AU	6	115871	115871	CASTEGNARO, M	Castegnaro, Marcel
AU	7	115872	115872	MICHELON, J	Michelon, J
AU	8	115873	115873	IDLE, JR	Idle, Jr
AU	9	115874	115874	BARTSCH, H	Bartsch, H
AF	1	115875	115875	HIETANEN, E	Hietanen, E
AF	2	115876	115876	MALAVEILLE, C	Malaveille, C
AF	3	115877	115877	CAMUS, AM	Camus, Am
AF	4	115878	115878	BEREZIAT, JC	Bereziat, Jc
AF	5	115879	115879	BRUN, G	Brun, G
AF	6	115880	115880	CASTEGNARO, M	Castegnaro, Marcel
AF	7	115881	115881	MICHELON, J	Michelon, J
AF	8	115882	115882	IDLE, JR	Idle, Jr
AF	9	115883	115883	BARTSCH, H	Bartsch, H

Record 864 | WOS:A1985AWD1800005

EVALUATION OF OCHRATOXIN-A FOR MUTAGENICITY IN A BATTERY OF BACTERIAL AND MAMMALIAN-CELL ASSAYS

Field	Position	Original line	Normalised line	Original value	Normalised value
AU	1	115974	115974	BENDELE, AM	Bendele, Am
AU	2	115975	115975	NEAL, SB	Neal, Sb
AU	3	115976	115976	OBERLY, TJ	Oberly, Tj
AU	4	115977	115977	THOMPSON, CZ	Thompson, Cz
AU	5	115978	115978	BEWSEY, BJ	Bewsey, Bj
AU	6	115979	115979	HILL, LE	Hill, le
AU	7	115980	115980	REXROAT, MA	Rexroat, Ma
AU	8	115981	115981	CARLTON, WW	Carlton, Ww
AU	9	115982	115982	PROBST, GS	Probst, Gs
AF	1	115983	115983	BENDELE, AM	Bendele, Am
AF	2	115984	115984	NEAL, SB	Neal, Sb
AF	3	115985	115985	OBERLY, TJ	Oberly, Tj
AF	4	115986	115986	THOMPSON, CZ	Thompson, Cz
AF	5	115987	115987	BEWSEY, BJ	Bewsey, Bj
AF	6	115988	115988	HILL, LE	Hill, le
AF	7	115989	115989	REXROAT, MA	Rexroat, Ma
AF	8	115990	115990	CARLTON, WW	Carlton, Ww
AF	9	115991	115991	PROBST, GS	Probst, Gs

Record 865 | WOS:A1985A019900016

GENOTOXICITY OF OCHRATOXIN-A IN MICE - DNA SINGLE-STRAND BREAKS EVALUATION IN SPLEEN, KIDNEY AND LIVER

Field	Position	Original line	Normalised line	Original value	Normalised value
AU	1	116063	116063	CREPPY, EE	Creppy, Ee
AU	2	116064	116064	KANE, A	Kane, A
AU	3	116065	116065	DIRHEIMER, G	Dirheimer, G
AU	4	116066	116066	LAFARGEFRAYSSINET, C	Lafargefrayssinet, C
AU	5	116067	116067	MOUSSET, S	Mousset, S
AU	6	116068	116068	FRAYSSINET, C	Frayssinet, C
AF	1	116069	116069	CREPPY, EE	Creppy, Ee
AF	2	116070	116070	KANE, A	Kane, A
AF	3	116071	116071	DIRHEIMER, G	Dirheimer, G
AF	4	116072	116072	LAFARGEFRAYSSINET, C	Lafargefrayssinet, C
AF	5	116073	116073	MOUSSET, S	Mousset, S
AF	6	116074	116074	FRAYSSINET, C	Frayssinet, C

Record 866 | WOS:A1985AUK4000005

GENOTOXICITY OF OCHRATOXIN-A IN MICE - DNA SINGLE-STRAND BREAK EVALUATION IN SPLEEN, LIVER AND KIDNEY

Field	Position	Original line	Normalised line	Original value	Normalised value
AU	1	116138	116138	CREPPY, EE	Creppy, Ee
AU	2	116139	116139	KANE, A	Kane, A
AU	3	116140	116140	DIRHEIMER, G	Dirheimer, G
AU	4	116141	116141	LAFARGEFRAYSSINET, C	Lafargefrayssinet, C
AU	5	116142	116142	MOUSSET, S	Mousset, S
AU	6	116143	116143	FRAYSSINET, C	Frayssinet, C
AF	1	116144	116144	CREPPY, EE	Creppy, Ee
AF	2	116145	116145	KANE, A	Kane, A
AF	3	116146	116146	DIRHEIMER, G	Dirheimer, G
AF	4	116147	116147	LAFARGEFRAYSSINET, C	Lafargefrayssinet, C
AF	5	116148	116148	MOUSSET, S	Mousset, S
AF	6	116149	116149	FRAYSSINET, C	Frayssinet, C

Record 867 | WOS:A1985ASM7700004

EFFECT OF OCHRATOXIN A ON RAT-LIVER MITOCHONDRIAL RESPIRATION AND OXIDATIVE-PHOSPHORYLATION

Field	Position	Original line	Normalised line	Original value	Normalised value
AU	1	116221	116221	WEI, YH	Wei, Yh
AU	2	116222	116222	LU, CY	Lu, Cy
AU	3	116223	116223	LIN, TN	Lin, Tn
AU	4	116224	116224	WEI, RD	Wei, Rd
AF	1	116225	116225	WEI, YH	Wei, Yh
AF	2	116226	116226	LU, CY	Lu, Cy
AF	3	116227	116227	LIN, TN	Lin, Tn
AF	4	116228	116228	WEI, RD	Wei, Rd

Record 868 | WOS:A1984SW21300010

EFFECTS OF FUNGAL TOXINS ON RENAL SLICE CALCIUM BALANCE

Field	Position	Original line	Normalised line	Original value	Normalised value
AU	1	116295	116295	BERNDT, WO	Berndt, Wo
AU	2	116296	116296	HAYES, AW	Hayes, Aw
AU	3	116297	116297	BAGGETT, JM	Baggett, Jm
AF	1	116298	116298	BERNDT, WO	Berndt, Wo
AF	2	116299	116299	HAYES, AW	Hayes, Aw
AF	3	116300	116300	BAGGETT, JM	Baggett, Jm

Record 869 | WOS:A1984TF49500004

EFFECTS OF SOME MYCOTOXINS ON MITOGEN-INDUCED BLASTOGENESIS AND SCE FREQUENCY IN HUMAN-LYMPHOCYTES

Field	Position	Original line	Normalised line	Original value	Normalised value
AU	1	116367	116367	COORAY, R	Cooray, R
AF	1	116368	116368	COORAY, R	Cooray, R

Record 870 | WOS:A1984SZ35800008

CONVERSION OF OCHRATOXIN-C INTO OCHRATOXIN-A INVIVO

Field	Position	Original line	Normalised line	Original value	Normalised value
AU	1	116439	116439	FUCHS, R	Fuchs, Radovan
AU	2	116440	116440	HULT, K	Hult, K
AU	3	116441	116441	PERAICA, M	Peraica, Maja
AU	4	116442	116442	RADIC, B	Radic, B
AU	5	116443	116443	PLESTINA, R	Plestina, R
AF	1	116444	116444	FUCHS, R	Fuchs, Radovan
AF	2	116445	116445	HULT, K	Hult, K
AF	3	116446	116446	PERAICA, M	Peraica, Maja
AF	4	116447	116447	RADIC, B	Radic, B
AF	5	116448	116448	PLESTINA, R	Plestina, R

Record 871 | WOS:A1984SX17000033

GENOTOXICITY OF A VARIETY OF MYCOTOXINS IN THE HEPATOCYTE PRIMARY CULTURE DNA-REPAIR TEST USING RAT AND MOUSE HEPATOCYTES

Field	Position	Original line	Normalised line	Original value	Normalised value
AU	1	116496	116496	MORI, H	Mori, H
AU	2	116497	116497	KAWAI, K	Kawai, K
AU	3	116498	116498	OHBAYASHI, F	Ohbayashi, F
AU	4	116499	116499	KUNIYASU, T	Kuniyasu, T
AU	5	116500	116500	YAMAZAKI, M	Yamazaki, M
AU	6	116501	116501	HAMASAKI, T	Hamasaki, T
AU	7	116502	116502	WILLIAMS, GM	Williams, Gm
AF	1	116503	116503	MORI, H	Mori, H

AF	2	116504	116504	KAWAI, K	Kawai, K
AF	3	116505	116505	OHBAYASHI, F	Ohbayashi, F
AF	4	116506	116506	KUNIYASU, T	Kuniyasu, T
AF	5	116507	116507	YAMAZAKI, M	Yamazaki, M
AF	6	116508	116508	HAMASAKI, T	Hamasaki, T
AF	7	116509	116509	WILLIAMS, GM	Williams, Gm

Record 872 | WOS:A1983RZ78000009

EFFECTS OF OCHRATOXIN A METABOLITES ON YEAST PHENYLALANYL-TRNA SYNTHETASE AND ON THE GROWTH AND INVIVO PROTEIN-SYNTHESIS OF HEPATOMA-CELLS

Field	Position	Original line	Normalised line	Original value	Normalised value
AU	1	116593	116593	CREPPY, EE	Creppy, Ee
AU	2	116594	116594	STORMER, FC	Stormer, Fc
AU	3	116595	116595	KERN, D	Kern, D
AU	4	116596	116596	ROSCHENTHALER, R	Roschenthaler, R
AU	5	116597	116597	DIRHEIMER, G	Dirheimer, G
AF	1	116598	116598	CREPPY, EE	Creppy, Ee
AF	2	116599	116599	STORMER, FC	Stormer, Fc
AF	3	116600	116600	KERN, D	Kern, D
AF	4	116601	116601	ROSCHENTHALER, R	Roschenthaler, R
AF	5	116602	116602	DIRHEIMER, G	Dirheimer, G

Record 873 | WOS:A1982NN76100006

PREVENTION OF MOLD GROWTH AND TOXIN PRODUCTION THROUGH CONTROL OF ENVIRONMENTAL-CONDITIONS

Field	Position	Original line	Normalised line	Original value	Normalised value
AU	1	116667	116667	NORTHOLT, MD	Northolt, Md
AU	2	116668	116668	BULLERMAN, LB	Bullerman, Lb
AF	1	116669	116669	NORTHOLT, MD	Northolt, Md
AF	2	116670	116670	BULLERMAN, LB	Bullerman, Lb

Record 874 | WOS:A1980KP16100001

MEASURING OF A DOUBLE CONTAMINATION BY PATULIN AND OCHRATOXIN

Field	Position	Original line	Normalised line	Original value	Normalised value
AU	1	116762	116762	ESCOULA, L	Escoula, L
AU	2	116763	116763	LARRIEU, G	Larrieu, G
AF	1	116764	116764	ESCOULA, L	Escoula, L
AF	2	116765	116765	LARRIEU, G	Larrieu, G

Record 875 | WOS:A1980KR30600008

EVIDENCE FOR INVITRO AND INVIVO INTERACTION BETWEEN OCHRATOXIN-A AND 3 ACIDIC DRUGS

Field	Position	Original line	Normalised line	Original value	Normalised value
AU	1	116805	116805	GALTIER, P	Galtier, P

AU	2	116806	116806	CAMGUILHEM, R	Camguilhem, R
AU	3	116807	116807	BODIN, G	Bodin, G
AF	1	116808	116808	GALTIER, P	Galtier, P
AF	2	116809	116809	CAMGUILHEM, R	Camguilhem, R
AF	3	116810	116810	BODIN, G	Bodin, G

Record 876 | WOS:A1979HH25500002

INVIVO AND INVITRO CHANGES IN RENAL-FUNCTION CAUSED BY OCHRATOXIN A IN THE RAT

Field	Position	Original line	Normalised line	Original value	Normalised value
AU	1	116860	116860	BERNDT, WO	Berndt, Wo
AU	2	116861	116861	HAYES, AW	Hayes, Aw
AF	1	116862	116862	BERNDT, WO	Berndt, Wo
AF	2	116863	116863	HAYES, AW	Hayes, Aw

Record 877 | WOS:A1978GE26800006

OCHRATOXIN-A AND PROTEIN-UTILIZATION INVIVO

Field	Position	Original line	Normalised line	Original value	Normalised value
AU	1	116922	116922	KIESSLING, KH	Kiessling, Kh
AU	2	116923	116923	PETTERSSON, H	Pettersson, H
AF	1	116924	116924	KIESSLING, KH	Kiessling, Kh
AF	2	116925	116925	PETTERSSON, H	Pettersson, H

Record 878 | WOS:A1977DR04200006

FATE OF OCHRATOXIN A IN RATS

Field	Position	Original line	Normalised line	Original value	Normalised value
AU	1	116959	116959	CHANG, FC	Chang, Fc
AU	2	116960	116960	CHU, FS	Chu, Fs
AF	1	116961	116961	CHANG, FC	Chang, Fc
AF	2	116962	116962	CHU, FS	Chu, Fs

Record 879 | WOS:A1975AC00900024

INVITRO TOXICITY OF ANALOGS OF OCHRATOXIN-A IN MONKEY KIDNEY EPITHELIAL-CELLS

Field	Position	Original line	Normalised line	Original value	Normalised value
AU	1	117022	117022	STEYN, PS	Steyn, Ps
AU	2	117023	117023	VLEGGAAR, R	Vleggaar, R
AU	3	117024	117024	DUPREEZ, NP	Dupreez, Np
AU	4	117025	117025	BLYTH, AA	Blyth, Aa
AU	5	117026	117026	SEEGERS, JC	Seegers, Jc
AF	1	117027	117027	STEYN, PS	Steyn, Ps
AF	2	117028	117028	VLEGGAAR, R	Vleggaar, R
AF	3	117029	117029	DUPREEZ, NP	Dupreez, Np

AF	4	117030	117030	BLYTH, AA	Blyth, Aa
AF	5	117031	117031	SEEGERS, JC	Seegers, Jc

Record 880 | WOS:A1975BC07400010

STUDIES ON NEPHROTOXICITY OF OCHRATOXIN A IN RATS

Field	Position	Original line	Normalised line	Original value	Normalised value
AU	1	117081	117081	SUZUKI, S	Suzuki, S
AU	2	117082	117082	KOZUKA, Y	Kozuka, Y
AU	3	117083	117083	SATOH, T	Satoh, T
AU	4	117084	117084	YAMAZAKI, M	Yamazaki, M
AF	1	117085	117085	SUZUKI, S	Suzuki, S
AF	2	117086	117086	KOZUKA, Y	Kozuka, Y
AF	3	117087	117087	SATOH, T	Satoh, T
AF	4	117088	117088	YAMAZAKI, M	Yamazaki, M

Record 881 | WOS:A1972M753500008

COMPARATIVE RATES OF HYDROLYSIS OF OCHRATOXINS A AND B IN-VITRO

Field	Position	Original line	Normalised line	Original value	Normalised value
AU	1	117159	117159	DOSTER, RC	Doster, Rc
AU	2	117160	117160	SINNHUBER, RO	Sinnhuber, Ro
AF	1	117161	117161	DOSTER, RC	Doster, Rc
AF	2	117162	117162	SINNHUBER, RO	Sinnhuber, Ro

Record 882 | WOS:A1971K640300003

TOXICITY OF 5-CHLORO-8-HYDROXY-3, 4-DIHYDRO-3-METHYL-ISOCOUMARIN-7-CARBOXYLIC ACID, A HYDROLYZATE OF OCHRATOXIN-A

Field	Position	Original line	Normalised line	Original value	Normalised value
AU	1	117206	117206	YAMAZAKI, M	Yamazaki, M
AU	2	117207	117207	SUZUKI, S	Suzuki, S
AU	3	117208	117208	SAKAKIBARA, Y	Sakakibara, Y
AU	4	117209	117209	MIYAKI, K	Miyaki, K
AF	1	117210	117210	YAMAZAKI, M	Yamazaki, M
AF	2	117211	117211	SUZUKI, S	Suzuki, S
AF	3	117212	117212	SAKAKIBARA, Y	Sakakibara, Y
AF	4	117213	117213	MIYAKI, K	Miyaki, K

Record 883 | WOS:A19656230000050

OCHRATOXIN A A TOXIC METABOLITE PRODUCED BY ASPERGILLUS OCHRACEUS WILH

Field	Position	Original line	Normalised line	Original value	Normalised value
AU	1	117258	117258	VANDERME.KJ	Vanderme, kj
AU	2	117259	117259	STEYN, PS	Steyn, Ps
AU	3	117260	117260	FOURIE, L	Fourie, L

AU	4	117261	117261	SCOTT, DB	Scott, Db
AU	5	117262	117262	THERON, JJ	Theron, Jj
AF	1	117263	117263	VANDERME.KJ	Vandermekj
AF	2	117264	117264	STEYN, PS	Steyn, Ps
AF	3	117265	117265	FOURIE, L	Fourie, L
AF	4	117266	117266	SCOTT, DB	Scott, Db
AF	5	117267	117267	THERON, JJ	Theron, Jj
