# Supplementary material for: Ochratoxin A Toxicity Research in In Vitro and In Vivo Experimental Settings: A Cross-Software Bibliometric and Science-Mapping Study
Source: Toxins (Basel). 2026 Aug 11;18(8):346. doi: 10.3390/toxins18080346 (PMC13517462; doi:10.3390/toxins18080346)
Supplement: Supplementary file 1 [file toxins-18-00346-s001.zip › Supplementary File S6.pdf]

Journal normalisation audit – ALL RECORDS, article-by-article

Compared files: WoS 1-883_Original.txt and WoS_1-883_Journals_Normalized.txt. This corrected Word version includes every matched record, including records with no journal-related changes.

Metric	Value
records_before	883
records_after	883
matched	883
records_with_changes	417
records_without_changes	466

Record 1 | WOS:001628370900001

Dietary Quercetin Mitigates DON-Induced Intestinal Injury via Inhibiting MAPK/NF-Î°B-Mediated Pyroptosis and Tight Junction Disruption in Chicken

SO original: JOURNAL OF AGRICULTURAL AND FOOD CHEMISTRY | SO normalised: JOURNAL OF AGRICULTURAL AND FOOD CHEMISTRY | CR count: 58 → 58

No journal-related changes detected in SO, J9, JI or CR for this record.

Record 2 | WOS:001646793400001

Silent Saboteurs: Decoding Mycotoxins-From Chemistry and Prevalence to Health Risks, Detection, Management and Emerging Frontiers

SO original: JOURNAL OF FUNGI | SO normalised: JOURNAL OF FUNGI | CR count: 246 → 246

Field	Status	Orig. pos	Norm. pos	Orig. line	Norm. line	Original cited/source value	Normalised cited/source value
CR	replaced	8	8	192	192	Mycotoxins in Food<br>Alexander N.J., 2018, Mycotoxins in Food, Feed and Bioweapons, P75	Mycotoxins in food<br>Alexander N.J., 2018, Mycotoxins in food, Feed and Bioweapons, P75
CR	replaced	14	14	198	198	Food Funct<br>Assuncao R., 2016, Food Funct, V7, P2056	FOOD FUNCT<br>Assuncao R., 2016, FOOD FUNCT, V7, P2056
CR	replaced	25	25	209	209	Mycotoxins: Detection Methods<br>Bhatnagar D., 2006, Mycotoxins: Detection Methods, Management, Public Health and Agricultural Trade, P171	Mycotoxins: detection methods<br>Bhatnagar D., 2006, Mycotoxins: detection methods, Management, Public Health and Agricultural Trade, P171
CR	replaced	28	28	212	212	Food Chem. Toxicol<br>Bourdiol F., 1990, Food Chem. Toxicol, V28, P723	FOOD CHEM TOXICOL<br>Bourdiol F., 1990, FOOD CHEM TOXICOL, V28, P723
CR	replaced	31	31	215	215	Curr. Neuropharmacol<br>Chaudhary N., 2010, Curr. Neuropharmacol, V8, P174	CURR NEUROPHARMACOL<br>Chaudhary N., 2010, CURR NEUROPHARMACOL, V8, P174
CR	replaced	32	32	216	216	Food Control<br>Cheli F., 2013, Food Control, V34, P429	FOOD CONTROL<br>Cheli F., 2013, FOOD CONTROL, V34, P429

CR	replaced	37	37	221	221	Mycotoxin Res<br>Cotty P J, 2006, Mycotoxin Res, V22, P110, DOI 10.1007/BF02956774	MYCOTOXIN RES<br>Cotty P J, 2006, MYCOTOXIN RES, V22, P110, DOI 10.1007/BF02956774
CR	replaced	39	39	223	223	Food Chem. Toxicol<br>Creppy E.E., 1983, Food Chem. Toxicol, V21, P587	FOOD CHEM TOXICOL<br>Creppy E.E., 1983, FOOD CHEM TOXICOL, V21, P587
CR	replaced	42	42	226	226	Aflatoxin Toxicity<br>Dhakal A., 2020, Aflatoxin Toxicity	AFLATOXIN TOXICITY<br>Dhakal A., 2020, AFLATOXIN TOXICITY
CR	replaced	43	43	227	227	Environ. Toxicol. Pharmacol<br>Ding L., 2006, Environ. Toxicol. Pharmacol, V22, P234	ENVIRON TOXICOL PHAR<br>Ding L., 2006, ENVIRON TOXICOL PHAR, V22, P234
CR	replaced	51	51	235	235	Off. J. Eur. Union<br>European Commission, 2024, Off. J. Eur. Union, VL, P1	OFF J EUR UNION<br>European Commission, 2024, OFF J EUR UNION, VL, P1
CR	replaced	63	63	247	247	Toxins<br>Frizzell C., 2011, Toxins, V3, P1234	TOXINS<br>Frizzell C., 2011, TOXINS, V3, P1234
CR	replaced	68	68	252	252	J. Pharmacol. Exp. Ther<br>Gekle M., 1994, J. Pharmacol. Exp. Ther, V268, P138	J PHARMACOL EXP THER<br>Gekle M., 1994, J PHARMACOL EXP THER, V268, P138
CR	replaced	74	74	258	258	Food Control<br>Gonzalez M.A., 2023, Food Control, V132, P108503	FOOD CONTROL<br>Gonzalez M.A., 2023, FOOD CONTROL, V132, P108503
CR	replaced	84	84	268	268	Adv Appl Microbiol<br>Hidy P H, 1977, Adv Appl Microbiol, V22, P59, DOI 10.1016/S0065-2164(08)70160-6	ADV APPL MICROBIOL<br>Hidy P H, 1977, ADV APPL MICROBIOL, V22, P59, DOI 10.1016/S0065-2164(08)70160-6
CR	replaced	95	95	279	279	Toxicology<br>Islam Z., 2006, Toxicology, V219, P56	TOXICOLOGY<br>Islam Z., 2006, TOXICOLOGY, V219, P56
CR	replaced	101	101	285	285	Chem. Res. Toxicol<br>Johnson W.W., 1997, Chem. Res. Toxicol, V10, P362	CHEM RES TOXICOL<br>Johnson W.W., 1997, CHEM RES TOXICOL, V10, P362
CR	replaced	110	110	294	294	Fungal Genet. Biol<br>Kim H.K., 2005, Fungal Genet. Biol, V42, P44	FUNGAL GENET BIOL<br>Kim H.K., 2005, FUNGAL GENET BIOL, V42, P44
CR	replaced	126	126	310	310	Toxicol. Lett<br>Liu B.H., 2003, Toxicol. Lett, V143, P243	TOXICOL LETT<br>Liu B.H., 2003, TOXICOL LETT, V143, P243
CR	replaced	145	145	329	329	Environ. Health Perspect<br>Merrill A.H., 2001, Environ. Health Perspect, V109, P283	ENVIRON HEALTH PERSP<br>Merrill A.H., 2001, ENVIRON HEALTH PERSP, V109, P283
CR	replaced	156	156	340	340	Mycoses<br>Muller G., 1999, Mycoses, V42, P495, DOI [10.1046/j.1439-0507.1999.00504.x, DOI 10.1046/J.1439-0507.1999.00504.X]	MYCOSES<br>Muller G., 1999, MYCOSES, V42, P495, DOI [10.1046/j.1439-0507.1999.00504.x, DOI 10.1046/J.1439-0507.1999.00504.X]
CR	replaced	165	165	349	349	Toxicol Rep<br>Nji Queenta Ngum, 2024, Toxicol Rep, V13, P101711, DOI	TOXICOL REP<br>Nji Queenta Ngum, 2024, TOXICOL REP, V13, P101711, DOI

						[10.1016/j.toxrep.2024.101711, 10.1016/j.toxrep.2024.101711]	[10.1016/j.toxrep.2024.101711, 10.1016/j.toxrep.2024.101711]
CR	replaced	174	174	358	358	Toxicol. Appl. Pharmacol<br>Osuchowski M.F., 2005, Toxicol. Appl. Pharmacol, V205, P198	TOXICOL APPL PHARM<br>Osuchowski M.F., 2005, TOXICOL APPL PHARM, V205, P198
CR	replaced	180	180	364	364	Toxicol. Appl. Pharmacol<br>Phillips T.D., 1990, Toxicol. Appl. Pharmacol, V104, P94	TOXICOL APPL PHARM<br>Phillips T.D., 1990, TOXICOL APPL PHARM, V104, P94
CR	replaced	186	186	370	370	Toxins<br>Polak-Sliwinska M., 2021, Toxins, V13, P876	TOXINS<br>Polak-Sliwinska M., 2021, TOXINS, V13, P876
CR	replaced	202	202	386	386	Toxicol Rep<br>Shekhar Ravikant, 2025, Toxicol Rep, V14, P101952, DOI [10.1016/j.toxrep.2025.101952, 10.1016/j.toxrep.2025.101952]	TOXICOL REP<br>Shekhar Ravikant, 2025, TOXICOL REP, V14, P101952, DOI [10.1016/j.toxrep.2025.101952, 10.1016/j.toxrep.2025.101952]
CR	replaced	226	226	410	410	Toxins<br>Wang X., 2019, Toxins, V11, P466	TOXINS<br>Wang X., 2019, TOXINS, V11, P466
CR	replaced	229	229	413	413	FEMS Microbiol. Lett<br>White T.C., 2006, FEMS Microbiol. Lett, V255, P17, DOI [10.1111/j.1574-6968.2005.00051.x, DOI 10.1111/J.1574-6968.2005.00051.X]	FEMS MICROBIOL LETT<br>White T.C., 2006, FEMS MICROBIOL LETT, V255, P17, DOI [10.1111/j.1574-6968.2005.00051.x, DOI 10.1111/J.1574-6968.2005.00051.X]
CR	replaced	244	244	428	428	Toxicol. Sci<br>Zhou H.R., 2003, Toxicol. Sci, V74, P297	TOXICOL SCI<br>Zhou H.R., 2003, TOXICOL SCI, V74, P297

Record 3 | WOS:001648209500001

Dose-Dependent but Non-Interactive Effects of Ochratoxin A and Selenomethionine on Hepatic Lipid Metabolism and Oxidative Stress in Broiler Chickens

SO original: TOXINS | SO normalised: TOXINS | CR count: 60 → 60

No journal-related changes detected in SO, J9, JI or CR for this record.

Record 4 | WOS:001635022200001

Selective Cytotoxicity of Ochratoxin A: Pro-Apoptotic Effects on Healthy Immune Cells Compared to Leukemia Cells

SO original: MOLECULES | SO normalised: MOLECULES | CR count: 68 → 68

Field	Status	Orig. pos	Norm. pos	Orig. line	Norm. line	Original cited/source value	Normalised cited/source value
CR	replaced	48	48	708	708	Braz. J. Poult. Sci.<br>Moura MA, 2004, Braz. J. Poult. Sci., V6, P187, DOI 10.1590/S1516-635X2004000300010	BRAZ J POULTRY SCI<br>Moura MA, 2004, BRAZ J POULTRY SCI, V6, P187, DOI 10.1590/S1516-635X2004000300010

CR	replaced	49	49	709	709	IARC Sci Publ<br>Petkova-Bocharova T, 1991, IARC Sci Publ, P135	IARC SCI PUBL<br>Petkova-Bocharova T, 1991, IARC SCI PUBL, P135
CR	replaced	61	61	721	721	Mycotoxin Res<br>Weidenbach A, 2000, Mycotoxin Res, V16 Suppl 2, P189, DOI 10.1007/BF02940035	MYCOTOXIN RES<br>Weidenbach A, 2000, MYCOTOXIN RES, V16 Suppl 2, P189, DOI 10.1007/BF02940035

Record 5 | WOS:001625561500001

Cytotoxic Effects Induced by Combined Exposure of the Patulin, Ochratoxin A, and Acetamiprid to HK-2 and SK-N-SH Cell Lines

SO original: TOXINS | SO normalised: TOXINS | CR count: 49 → 49

No journal-related changes detected in SO, J9, JI or CR for this record.

Record 6 | WOS:001623564900001

Mycotoxins and the Intestinal Epithelium: From Barrier Injury to Stem Cell Dysfunction

SO original: TOXINS | SO normalised: TOXINS | CR count: 113 → 113

Field	Status	Orig. pos	Norm. pos	Orig. line	Norm. line	Original cited/source value	Normalised cited/source value
CR	replaced	76	76	976	976	arXiv<br>Tong R., 2025, arXiv, DOI [10.48550/arXiv.2510.16677, DOI 10.48550/ARXIV.2510.16677]	Arxiv<br>Tong R., 2025, Arxiv, DOI [10.48550/arXiv.2510.16677, DOI 10.48550/ARXIV.2510.16677]

Record 7 | WOS:001598251800001

Exploration of oxidative stress-mediated genetic toxicology modes of action using a pathway analysis, Connectivity Mapping, and transcriptional benchmark dosing-based framework

SO original: TOXICOLOGICAL SCIENCES | SO normalised: TOXICOLOGICAL SCIENCES | CR count: 106 → 106

No journal-related changes detected in SO, J9, JI or CR for this record.

Record 8 | WOS:001591221900001

Prevention and detoxification of mycotoxins in food and feed: A review

SO original: FOOD BIOSCIENCE | SO normalised: FOOD BIOSCIENCE | CR count: 291 → 291

Field	Status	Orig. pos	Norm. pos	Orig. line	Norm. line	Original cited/source value	Normalised cited/source value
CR	replaced	19	19	1253	1253	Braz. J. Biol.<br>Ali E. M., 2024, Braz. J. Biol., V84, pe263814, DOI [10.1590/1519-6984.263814, 10.1590/1519-6984.263814]	BRAZ J BIOL<br>Ali E. M., 2024, BRAZ J BIOL, V84, pe263814, DOI [10.1590/1519-6984.263814, 10.1590/1519-6984.263814]

CR	replaced	233	233	1467	1467	Toxicol Rep<br>Shekhar Ravikant, 2025, Toxicol Rep, V14, P101952, DOI [10.1016/j.toxrep.2025.101952, 10.1016/j.toxrep.2025.101952]	TOXICOL REP<br>Shekhar Ravikant, 2025, TOXICOL REP, V14, P101952, DOI [10.1016/j.toxrep.2025.101952, 10.1016/j.toxrep.2025.101952]

Record 9 | WOS:001603917600001

Astaxanthin Alleviates Ochratoxin A (OTA)-Induced Spleen Dysfunction and Apoptosis in Broiler Chickens by Modulating the PTEN/PI3K/AKT Signaling Pathway

SO original: ANTIOXIDANTS | SO normalised: ANTIOXIDANTS | CR count: 65 → 65

Field	Status	Orig. pos	Norm. pos	Orig. line	Norm. line	Original cited/source value	Normalised cited/source value
CR	replaced	48	48	1629	1629	OXIDATIVE STRESS<br>Sies H., 1985, OXIDATIVE STRESS, DOI [10.1016/B978%2D0%2D12%2D642760%2D8.50005%2D3, DOI 10.1016/B978-0-12-64272D8.50005-3, 10.1016/B978-0-12-642760-8.50005-3, DOI 10.1016/C2009-0-02957-2]	Oxidative Stress<br>Sies H., 1985, Oxidative Stress, DOI [10.1016/B978%2D0%2D12%2D642760%2D8.50005%2D3, DOI 10.1016/B978-0-12-64272D8.50005-3, 10.1016/B978-0-12-642760-8.50005-3, DOI 10.1016/C2009-0-02957-2]

Record 10 | WOS:001593782400001

Mycotoxin Contamination: Occurrence, Biotransformation, Pathogenic Mechanisms, and Strategies for Nutritional Intervention

SO original: MOLECULES | SO normalised: MOLECULES | CR count: 196 → 196

Field	Status	Orig. pos	Norm. pos	Orig. line	Norm. line	Original cited/source value	Normalised cited/source value
CR	replaced	43	43	1773	1773	Chemosphere<br>Feng Chi, 2023, Chemosphere, V340, P139940, DOI [10.1016/j.chemosphere.2023.139940, 10.1016/j.chemosphere.2023.139940]	CHEMOSPHERE<br>Feng Chi, 2023, CHEMOSPHERE, V340, P139940, DOI [10.1016/j.chemosphere.2023.139940, 10.1016/j.chemosphere.2023.139940]
CR	replaced	52	52	1782	1782	Mycotoxin Res<br>Geisen R, 2006, Mycotoxin Res, V22, P134, DOI 10.1007/BF02956777	MYCOTOXIN RES<br>Geisen R, 2006, MYCOTOXIN RES, V22, P134, DOI 10.1007/BF02956777

Record 11 | WOS:001579727300001

Chlorogenic Acid and VX765 Alleviate Deoxynivalenol-Induced Enterohepatic Injury and Lipid Metabolism Disorders by Improving Intestinal Microecology

SO original: TOXINS | SO normalised: TOXINS | CR count: 54 → 54

Field	Status	Orig. pos	Norm. pos	Orig. line	Norm. line	Original cited/source value	Normalised cited/source value
CR	replaced	21	21	2012	2012	Int J Environ Res Public Health<br>Gonya Susan, 2024, Int J Environ Res Public Health, V21, DOI [10.3390/ijerph21060808, 10.3390/ijerph21060808]	INT J ENV RES PUB HE<br>Gonya Susan, 2024, INT J ENV RES PUB HE, V21, DOI [10.3390/ijerph21060808, 10.3390/ijerph21060808]

Record 12 | WOS:001573742900001

Fungal metabolite Ochratoxin A inhibits MrkD1P of multidrug-resistant Klebsiella pneumoniae: Integrated computational and in vitro validation

SO original: JOURNAL OF COMPUTER-AIDED MOLECULAR DESIGN | SO normalised: JOURNAL OF COMPUTER-AIDED MOLECULAR DESIGN | CR count: 93 → 93

Field	Status	Orig. pos	Norm. pos	Orig. line	Norm. line	Original cited/source value	Normalised cited/source value
CR	replaced	12	12	2140	2140	MBio<br>B. M. D, 2017, MBio, V8	MBIO<br>B. M. D, 2017, MBIO, V8
CR	replaced	79	79	2207	2207	Heliyon<br>Rojmala JV, 2024, Heliyon, V10, DOI [10.1016/j.heliyon.2024.e40211.inKlebsiellapneumoniaeusinginsilicoapproach, DOI 10.1016/J.HELIYON.2024.E40211.INKLEBSIELLAPNEUMONIAEUSINGINSILICOAPPROACH]	HELIYON<br>Rojmala JV, 2024, HELIYON, V10, DOI [10.1016/j.heliyon.2024.e40211.inKlebsiellapneumoniaeusinginsilicoapproach, DOI 10.1016/J.HELIYON.2024.E40211.INKLEBSIELLAPNEUMONIAEUSINGINSILICOAPPROACH]

Record 13 | WOS:001581342800001

Biocatalytic Detoxification of Ochratoxins A/B by a Fungal Dye-Decolorizing Peroxidase: Mechanistic Insights and Toxicity Assessment

SO original: TOXINS | SO normalised: TOXINS | CR count: 35 → 35

Field	Status	Orig. pos	Norm. pos	Orig. line	Norm. line	Original cited/source value	Normalised cited/source value
CR	replaced	3	3	2295	2295	Heliyon<br>Banahene Joel Cox Menka, 2024, Heliyon, V10, pe39313, DOI [10.1016/j.heliyon.2024.e39313, 10.1016/j.heliyon.2024.e39313]	HELIYON<br>Banahene Joel Cox Menka, 2024, HELIYON, V10, pe39313, DOI [10.1016/j.heliyon.2024.e39313, 10.1016/j.heliyon.2024.e39313]

Record 14 | WOS:001556476900001

Identification of an M20 Family Metallopeptidase with Amidohydrolase Activity from Alcaligenes faecalis HNGD-HTN06 and Simultaneous Degradation of Ochratoxin A, Aflatoxin B1, and Zearalenone

SO original: JOURNAL OF AGRICULTURAL AND FOOD CHEMISTRY | SO normalised: JOURNAL OF AGRICULTURAL AND FOOD CHEMISTRY | CR count: 69 → 69

No journal-related changes detected in SO, J9, JI or CR for this record.

Record 15 | WOS:001590628300001

Roles of microRNAs in multiple organs under mycotoxin stress by bioinformatic analysis microRNA regulation during mycotoxin exposure

SO original: POULTRY SCIENCE | SO normalised: POULTRY SCIENCE | CR count: 166 → 166

No journal-related changes detected in SO, J9, JI or CR for this record.

Record 16 | WOS:001556296100001

The protective effect of Gentiana lutea flower against mycotoxin toxicity in a gastrointestinal barrier in vitro model

SO original: FRONTIERS IN NUTRITION | SO normalised: FRONTIERS IN NUTRITION | CR count: 65 → 65

No journal-related changes detected in SO, J9, JI or CR for this record.

Record 17 | WOS:001546906900003

Mitochondrial dynamics imbalance and energy dyshomeostasis mediated developmental toxicity of ochratoxin a in zebrafish (Danio rerio)

SO original: FOOD AND CHEMICAL TOXICOLOGY | SO normalised: FOOD AND CHEMICAL TOXICOLOGY | CR count: 72 → 72

Field	Status	Orig. pos	Norm. pos	Orig. line	Norm. line	Original cited/source value	Normalised cited/source value
CR	replaced	55	55	2960	2960	Chemosphere<br>Wang Yanhua, 2023, Chemosphere, V326, P138429, DOI [10.1016/j.chemosphere.2023.138429, 10.1016/j.chemosphere.2023.138429]	CHEMOSPHERE<br>Wang Yanhua, 2023, CHEMOSPHERE, V326, P138429, DOI [10.1016/j.chemosphere.2023.138429, 10.1016/j.chemosphere.2023.138429]

Record 18 | WOS:001582665400002

L-epicatechin partially alleviates Ochratoxin A-induced oxidative and endoplasmic reticulum stress and improves developmental competence of porcine embryos during in vitro culture

SO original: THERIOGENOLOGY | SO normalised: THERIOGENOLOGY | CR count: 50 → 50

No journal-related changes detected in SO, J9, JI or CR for this record.

Record 19 | WOS:001539646900001

Emerging Mycotoxins in Aquaculture: Current Insights on Toxicity, Biocontrol Strategies, and Occurrence in Aquafeed and Fish

SO original: TOXINS | SO normalised: TOXINS | CR count: 115 → 115

Field	Status	Orig. pos	Norm. pos	Orig. line	Norm. line	Original cited/source value	Normalised cited/source value
CR	replaced	27	27	3188	3188	Off J Eur Union<br>European Commission, 2017, Off J Eur Union, V138, P92	OFF J EUR UNION<br>European Commission, 2017, OFF J EUR UNION, V138, P92
CR	replaced	115	115	3276	3276	2011<br>,, 2011, EFSA Journal, V9, P2407	2011<br>,, 2011, EFSA J, V9, P2407

Record 20 | WOS:001529843100001

Decontamination of Ochratoxin A in Food: Emerging Strategies and Safety Perspectives

SO original: INTERNATIONAL JOURNAL OF FOOD SCIENCE AND TECHNOLOGY | SO normalised: INTERNATIONAL JOURNAL OF FOOD SCIENCE AND TECHNOLOGY | CR count: 153 → 153

Field	Status	Orig. pos	Norm. pos	Orig. line	Norm. line	Original cited/source value	Normalised cited/source value

CR	replaced	9	9	3347	3347	Heliyon<br>Banahene Joel Cox Menka, 2024, Heliyon, V10, pe39313, DOI [10.1016/j.heliyon.2024.e39313, 10.1016/j.heliyon.2024.e39313]	HELIYON<br>Banahene Joel Cox Menka, 2024, HELIYON, V10, pe39313, DOI [10.1016/j.heliyon.2024.e39313, 10.1016/j.heliyon.2024.e39313]

Record 21 | WOS:001527764700005

Through its genoprotective, mitochondrial bioenergetic modulation, and antioxidant effects, Fucoxanthin and its metabolite minimize Ochratoxin A-induced nephrotoxicity in HK-2 human kidney cells

SO original: BMC NEPHROLOGY | SO normalised: BMC NEPHROLOGY | CR count: 73 → 73

Field	Status	Orig. pos	Norm. pos	Orig. line	Norm. line	Original cited/source value	Normalised cited/source value
CR	replaced	5	5	3580	3580	Heliyon<br>Banahene JCM, 2024, Heliyon	HELIYON<br>Banahene JCM, 2024, HELIYON

Record 22 | WOS:001539670300001

Oral Exposure to Chlorella sorokiniana Detoxifies Deoxynivalenol, Ochratoxin A, and Fumonisin B1 In Vitro and In Vivo

SO original: TOXINS | SO normalised: TOXINS | CR count: 30 → 30

No journal-related changes detected in SO, J9, JI or CR for this record.

Record 23 | WOS:001513772100001

Schisandrin B mitigates oxidative damage in early porcine embryos exposed to Ochratoxin A via NRF2 pathway activation

SO original: ANIMAL REPRODUCTION SCIENCE | SO normalised: ANIMAL REPRODUCTION SCIENCE | CR count: 42 → 42

No journal-related changes detected in SO, J9, JI or CR for this record.

Record 24 | WOS:001505840900001

Biocontrol Agents and Natural Feed Supplements as a Safe and Cost-Effective Way for Preventing Health Ailments Provoked by Mycotoxins

SO original: FOODS | SO normalised: FOODS | CR count: 301 → 301

Field	Status	Orig. pos	Norm. pos	Orig. line	Norm. line	Original cited/source value	Normalised cited/source value
CR	replaced	33	33	3954	3954	J Toxicol Sci<br>Bishayi Biswadev, 2002, J Toxicol Sci, V27, P139, DOI 10.2131/jts.27.139	J TOXICOL SCI<br>Bishayi Biswadev, 2002, J TOXICOL SCI, V27, P139, DOI 10.2131/jts.27.139
CR	replaced	64	64	3985	3985	Mycotoxin Res<br>Deberghes P, 1995, Mycotoxin Res, V11, P37, DOI 10.1007/BF03192060	MYCOTOXIN RES<br>Deberghes P, 1995, MYCOTOXIN RES, V11, P37, DOI 10.1007/BF03192060

CR	replaced	71	71	3992	3992	Mycotoxin Res<br>Dorner J W, 2006, Mycotoxin Res, V22, P33, DOI 10.1007/BF02954555	MYCOTOXIN RES<br>Dorner J W, 2006, MYCOTOXIN RES, V22, P33, DOI 10.1007/BF02954555
CR	replaced	79	79	4000	4000	Mycotoxin Res<br>Engelhardt G, 2002, Mycotoxin Res, V18, P37, DOI 10.1007/BF02946138	MYCOTOXIN RES<br>Engelhardt G, 2002, MYCOTOXIN RES, V18, P37, DOI 10.1007/BF02946138
CR	replaced	212	212	4133	4133	Veterinarski Arhiv<br>Sakhare Pramodkumar Shrirarn, 2007, Veterinarski Arhiv, V77, P129	VET ARHIV<br>Sakhare Pramodkumar Shrirarn, 2007, VET ARHIV, V77, P129
CR	replaced	214	214	4135	4135	Mycotoxin Res<br>Schatzmayr G, 2003, Mycotoxin Res, V19, P124, DOI 10.1007/BF02942950	MYCOTOXIN RES<br>Schatzmayr G, 2003, MYCOTOXIN RES, V19, P124, DOI 10.1007/BF02942950
CR	replaced	289	289	4210	4210	Food Sci. Technol<br>YU Meizhen, 2023, Food Sci. Technol, V43, pe114622, DOI [10.1590/fst.114622, 10.1590/fst.114622]	FOOD SCI TECH-BRAZIL<br>YU Meizhen, 2023, FOOD SCI TECH-BRAZIL, V43, pe114622, DOI [10.1590/fst.114622, 10.1590/fst.114622]

Record 25 | WOS:001514636100001

Modulation of the Antioxidant System of Caco-2 Cells in the Presence of Aflatoxin B1, Ochratoxin A, and Ferulic Acid

SO original: TOXINS | SO normalised: TOXINS | CR count: 87 → 87

No journal-related changes detected in SO, J9, JI or CR for this record.

Record 26 | WOS:001496132800001

Evaluation of Dietary Bioactive Agents Against Aflatoxin B1 and Ochratoxin A-Induced Duodenal Toxicity in Rats

SO original: FOODS | SO normalised: FOODS | CR count: 46 → 46

No journal-related changes detected in SO, J9, JI or CR for this record.

Record 27 | WOS:001488085400001

Bi2WO6-TiO2 carbon nanoparticles controlled postharvest blue mold of table grapes caused by Talaromyces rugulosus and the possible action mechanisms

SO original: NEW ZEALAND JOURNAL OF CROP AND HORTICULTURAL SCIENCE | SO normalised: NEW ZEALAND JOURNAL OF CROP AND HORTICULTURAL SCIENCE | CR count: 33 → 33

No journal-related changes detected in SO, J9, JI or CR for this record.

Record 28 | WOS:001486309200001

Immunotoxic effect and mechanisms of Fusarium mycotoxins on human immune cells: A focus on T cells and macrophages

SO original: TOXICOLOGY | SO normalised: TOXICOLOGY | CR count: 51 → 51

No journal-related changes detected in SO, J9, JI or CR for this record.

Record 29 | WOS:001484254000002

Quantification and metabolic variations of mycotoxins in raw milk: Implications for dairy cow health and human safety

SO original: JOURNAL OF DAIRY SCIENCE | SO normalised: JOURNAL OF DAIRY SCIENCE | CR count: 62 → 62

No journal-related changes detected in SO, J9, JI or CR for this record.

Record 30 | WOS:001484241000001

Oxidative stress promotes post-translational down-regulation of MRP2 in Caco-2 cells: Involvement of proteasomal degradation and toxicological implications

SO original: FOOD AND CHEMICAL TOXICOLOGY | SO normalised: FOOD AND CHEMICAL TOXICOLOGY | CR count: 64 → 64

No journal-related changes detected in SO, J9, JI or CR for this record.

Record 31 | WOS:001471942000001

Ginsenoside Rg1 alleviates ochratoxin A-induced liver inflammation in ducklings: Involvement of intestinal microbiota modulation and the TLR4/ NF-Î°B pathway inhibition

SO original: ECOTOXICOLOGY AND ENVIRONMENTAL SAFETY | SO normalised: ECOTOXICOLOGY AND ENVIRONMENTAL SAFETY | CR count: 54 → 54

No journal-related changes detected in SO, J9, JI or CR for this record.

Record 32 | WOS:001471582100001

Mitigating AFB1 and OTA hepatic residues using pumpkin and milk fermented whey as functional ingredients

SO original: FOOD BIOSCIENCE | SO normalised: FOOD BIOSCIENCE | CR count: 45 → 45

**Field**	**Status**	**Orig. pos**	**Norm. pos**	**Orig. line**	**Norm. line**	**Original cited/source value**	**Normalised cited/source value**
CR	replaced	36	36	5237	5237	Mycotoxin Res<br>Thieu N Q, 2009, Mycotoxin Res, V25, P59, DOI 10.1007/s12550-009-0009-z	MYCOTOXIN RES<br>Thieu N Q, 2009, MYCOTOXIN RES, V25, P59, DOI 10.1007/s12550-009-0009-z

Record 33 | WOS:001456981800001

Ochratoxin A-induced mitochondrial pathway apoptosis and ferroptosis by promoting glycolysis

SO original: APOPTOSIS | SO normalised: APOPTOSIS | CR count: 68 → 68

No journal-related changes detected in SO, J9, JI or CR for this record.

Record 34 | WOS:001463798400001

Combined Cytotoxic Effects of the Fungicide Azoxystrobin and Common Food-Contaminating Mycotoxins

SO original: FOODS | SO normalised: FOODS | CR count: 63 → 63

Field	Status	Orig. pos	Norm. pos	Orig. line	Norm. line	Original cited/source value	Normalised cited/source value
CR	replaced	19	19	5482	5482	Off. J. Eur. Union European Parliament and Council, 2005, Off. J. Eur. Union, VL 70, P1	OFF J EUR UNION European Parliament and Council, 2005, OFF J EUR UNION, VL 70, P1
CR	replaced	29	29	5492	5492	Biomarkers in Toxicology Gupta P.K., 2014, Biomarkers in Toxicology, V409, P431	BIOMARKERS IN TOXICOLOGY Gupta P.K., 2014, BIOMARKERS IN TOXICOLOGY, V409, P431
CR	replaced	51	51	5514	5514	Assay Guidance Manual Riss T.L., 2013, Assay Guidance Manual	Assay guidance manual Riss T.L., 2013, Assay guidance manual

Record 35 | WOS:001475234400001

Mycotoxins in Feed: Hazards, Toxicology, and Plant Extract-Based Remedies

SO original: METABOLITES | SO normalised: METABOLITES | CR count: 138 → 138

Field	Status	Orig. pos	Norm. pos	Orig. line	Norm. line	Original cited/source value	Normalised cited/source value
CR	replaced	6	6	5613	5613	Heliyon Banahene Joel Cox Menka, 2024, Heliyon, V10, pe39313, DOI [10.1016/j.heliyon.2024.e39313, 10.1016/j.heliyon.2024.e39313]	HELIYON Banahene Joel Cox Menka, 2024, HELIYON, V10, pe39313, DOI [10.1016/j.heliyon.2024.e39313, 10.1016/j.heliyon.2024.e39313]
CR	replaced	92	92	5699	5699	Mycotoxin Res Raters M, 2008, Mycotoxin Res, V24, P130, DOI 10.1007/BF03032339	MYCOTOXIN RES Raters M, 2008, MYCOTOXIN RES, V24, P130, DOI 10.1007/BF03032339

Record 36 | WOS:001437492200001

Potential role of ochratoxin A in Parkinson's disease: a systematic review of current evidence

SO original: ARCHIVES OF TOXICOLOGY | SO normalised: ARCHIVES OF TOXICOLOGY | CR count: 71 → 71

Field	Status	Orig. pos	Norm. pos	Orig. line	Norm. line	Original cited/source value	Normalised cited/source value
CR	replaced	53	53	5874	5874	Mycotoxin Res Raters M, 2008, Mycotoxin Res, V24, P130, DOI 10.1007/BF03032339	MYCOTOXIN RES Raters M, 2008, MYCOTOXIN RES, V24,

							P130, DOI 10.1007/BF03032339

Record 37 | WOS:001427487800001

Neurotoxic implications of gliotoxin and ochratoxin A in SH-SY5Y cells: ROS-induced apoptosis and genotoxicity

SO original: TOXICOLOGY LETTERS | SO normalised: TOXICOLOGY LETTERS | CR count: 49 → 49

Field	Status	Orig. pos	Norm. pos	Orig. line	Norm. line	Original cited/source value	Normalised cited/source value
CR	replaced	3	3	5959	5959	OECD Guideline for the Testing of Chemicals<br>[Anonymous], 2016, OECD Guideline for the Testing of Chemicals, DOI [DOI 10.1787/9789264264861-EN, https://doi.org/10.1787/9789264264861-en]	OECD Guideline for the testing of chemicals<br>[Anonymous], 2016, OECD Guideline for the testing of chemicals, DOI [DOI 10.1787/9789264264861-EN, https://doi.org/10.1787/9789264264861-en]

Record 38 | WOS:001426276300001

Ochratoxin A induces lung cell PANoptosis through activation of the AIM 2 inflammasome

SO original: INTERNATIONAL IMMUNOPHARMACOLOGY | SO normalised: INTERNATIONAL IMMUNOPHARMACOLOGY | CR count: 27 → 27

No journal-related changes detected in SO, J9, JI or CR for this record.

Record 39 | WOS:001425801400001

Probiotics mitigate reproductive toxicity in male mice induced by co-exposure of ochratoxin A and alcohol

SO original: FOOD BIOSCIENCE | SO normalised: FOOD BIOSCIENCE | CR count: 52 → 52

Field	Status	Orig. pos	Norm. pos	Orig. line	Norm. line	Original cited/source value	Normalised cited/source value
CR	replaced	44	44	6217	6217	Alcohol<br>World Health Organization, 2024, Alcohol	ALCOHOL<br>World Health Organization, 2024, ALCOHOL

Record 40 | WOS:001416011800001

Metabolic disruption by mycotoxins: focus on metabolic endpoints steatosis, adipogenesis and glucose metabolism in vivo and in vitro

SO original: ARCHIVES OF TOXICOLOGY | SO normalised: ARCHIVES OF TOXICOLOGY | CR count: 55 → 55

Field	Status	Orig. pos	Norm. pos	Orig. line	Norm. line	Original cited/source value	Normalised cited/source value
CR	replaced	18	18	6298	6298	Biomed Pharmacother<br>Fang Jia-You, 2022, Biomed Pharmacother, V146, P112497, DOI [10.1016/j.biopha.2021.112497, 10.1016/j.biopha.2021.112497]	BIOMED PHARMACOTHER<br>Fang Jia-You, 2022, BIOMED PHARMACOTHER, V146, P112497, DOI [10.1016/j.biopha.2021.112497, 10.1016/j.biopha.2021.112497]

Record 41 | WOS:001403208800001

Curcumin: A Potential Detoxifier Against Chemical and Natural Toxicants

SO original: PHYTOTHERAPY RESEARCH | SO normalised: PHYTOTHERAPY RESEARCH | CR count: 394 → 394

Field	Status	Orig. pos	Norm. pos	Orig. line	Norm. line	Original cited/source value	Normalised cited/source value
CR	replaced	167	167	6587	6587	Saudi J Biol Sci<br>Jarrar Bashir M, 2012, Saudi J Biol Sci, V19, P203, DOI 10.1016/j.sjbs.2011.12.005	SAUDI J BIOL SCI<br>Jarrar Bashir M, 2012, SAUDI J BIOL SCI, V19, P203, DOI 10.1016/j.sjbs.2011.12.005
CR	replaced	200	200	6620	6620	Mycotoxin Res<br>Leong Yin-Hui, 2012, Mycotoxin Res, V28, P79, DOI 10.1007/s12550-012-0129-8	MYCOTOXIN RES<br>Leong Yin-Hui, 2012, MYCOTOXIN RES, V28, P79, DOI 10.1007/s12550-012-0129-8

Record 42 | WOS:001416156300001

Ochratoxin A induces immunotoxicity by targeting Annexin A1 mediated neutrophil apoptosis in zebrafish

SO original: FRONTIERS IN IMMUNOLOGY | SO normalised: FRONTIERS IN IMMUNOLOGY | CR count: 37 → 37

No journal-related changes detected in SO, J9, JI or CR for this record.

Record 43 | WOS:001393437600001

Chemical Composition, In Vivo, and In Silico Molecular Docking Studies of the Effect of Syzygium aromaticum (Clove) Essential Oil on Ochratoxin A-Induced Acute Neurotoxicity

SO original: PLANTS-BASEL | SO normalised: PLANTS-BASEL | CR count: 75 → 75

Field	Status	Orig. pos	Norm. pos	Orig. line	Norm. line	Original cited/source value	Normalised cited/source value
CR	replaced	70	70	7076	7076	Int J Pharm Sci Res<br>Taroq A, 2021, Int J Pharm Sci Res, V12, P776, DOI [10.13040/IJPSR.0975-8232.12(2).776-82, DOI 10.13040/IJPSR.0975-8232.12(2).776-82]	INT J PHARM SCI RES<br>Taroq A, 2021, INT J PHARM SCI RES, V12, P776, DOI [10.13040/IJPSR.0975-8232.12(2).776-82, DOI 10.13040/IJPSR.0975-8232.12(2).776-82]

Record 44 | WOS:001404966700001

Proteomic Analysis of the Murine Liver Response to Oral Exposure to Aflatoxin B1 and Ochratoxin A: The Protective Role to Bioactive Compounds

SO original: TOXINS | SO normalised: TOXINS | CR count: 47 → 47

Field	Status	Orig. pos	Norm. pos	Orig. line	Norm. line	Original cited/source value	Normalised cited/source value

CR	replaced	38	38	7193	7193	Drug Safety<br>Temple R, 2009, Drug Safety	DRUG SAFETY<br>Temple R, 2009, DRUG SAFETY

Record 45 | WOS:001370729500001

Alterations in the proteomes of HepG2 and IHKE cells inflicted by six selected mycotoxins

SO original: ARCHIVES OF TOXICOLOGY | SO normalised: ARCHIVES OF TOXICOLOGY | CR count: 82 → 82

Field	Status	Orig. pos	Norm. pos	Orig. line	Norm. line	Original cited/source value	Normalised cited/source value
CR	replaced	38	38	7296	7296	Mycotoxin Res<br>Knecht A, 2005, Mycotoxin Res, V21, P176, DOI 10.1007/BF02959258	MYCOTOXIN RES<br>Knecht A, 2005, MYCOTOXIN RES, V21, P176, DOI 10.1007/BF02959258

Record 46 | WOS:001375296600001

Bioavailability study of OTA, ZEN, and AFB1 along with bioactive compounds from tiger nut beverage and its by-products

SO original: FOOD RESEARCH INTERNATIONAL | SO normalised: FOOD RESEARCH INTERNATIONAL | CR count: 75 → 75

No journal-related changes detected in SO, J9, JI or CR for this record.

Record 47 | WOS:001384323500001

Development of an Easy-To-Use Microfluidic System to Assess Dynamic Exposure to Mycotoxins in 3D Culture Models: Evaluation of Ochratoxin A and Patulin Cytotoxicity

SO original: FOODS | SO normalised: FOODS | CR count: 62 → 62

No journal-related changes detected in SO, J9, JI or CR for this record.

Record 48 | WOS:001356112700001

DNA ligase III mediates deoxynivalenol exposure-induced DNA damage in intestinal epithelial cells by regulating oxidative stress and interaction with PCNA

SO original: INTERNATIONAL JOURNAL OF BIOLOGICAL MACROMOLECULES | SO normalised: INTERNATIONAL JOURNAL OF BIOLOGICAL MACROMOLECULES | CR count: 64 → 64

No journal-related changes detected in SO, J9, JI or CR for this record.

Record 49 | WOS:001366569000001

Chlorophyll-Amended Organoclays for the Detoxification of Ochratoxin A

SO original: TOXINS | SO normalised: TOXINS | CR count: 76 → 76

Field	Status	Orig. pos	Norm. pos	Orig. line	Norm. line	Original cited/source value	Normalised cited/source value
CR	replaced	3	3	7853	7853	Hypertension [Anonymous], 2021, Hypertension	HYPERTENSION [Anonymous], 2021, HYPERTENSION
CR	replaced	11	11	7861	7861	Methods Mol Biol Boyd Windy A, 2012, Methods Mol Biol, V889, P15, DOI 10.1007/978-1-61779-867-2_3	METHODS MOL BIOL Boyd Windy A, 2012, METHODS MOL BIOL, V889, P15, DOI 10.1007/978-1-61779-867-2_3

Record 50 | WOS:001351279800001

Protective Effects of Carotenoid-Loaded Nanostructured Lipid Carriers Against Ochratoxin-A-Induced Cytotoxicity

SO original: FOODS | SO normalised: FOODS | CR count: 41 → 41

No journal-related changes detected in SO, J9, JI or CR for this record.

Record 51 | WOS:001329697100001

Unveiling the hub genes associated with ochratoxin A-induced hepatotoxicity in broiler chickens

SO original: ANIMAL SCIENCE JOURNAL | SO normalised: ANIMAL SCIENCE JOURNAL | CR count: 30 → 30

No journal-related changes detected in SO, J9, JI or CR for this record.

Record 52 | WOS:001314852000005

Investigating the protective properties of Panax ginseng and its constituents against biotoxins and metal toxicity: a mechanistic review

SO original: NAUNYN-SCHMIEDEBERGS ARCHIVES OF PHARMACOLOGY | SO normalised: NAUNYN-SCHMIEDEBERGS ARCHIVES OF PHARMACOLOGY | CR count: 145 → 145

Field	Status	Orig. pos	Norm. pos	Orig. line	Norm. line	Original cited/source value	Normalised cited/source value
CR	replaced	94	94	8297	8297	ALTERN MED REV Patrick L, 2006, ALTERN MED REV, V11, P2	Altern Med Rev Patrick L, 2006, Altern Med Rev, V11, P2

Record 53 | WOS:001323506900001

Phytochemical Characterization of Bilberries and Their Potential as a Functional Ingredient to Mitigate Ochratoxin A Toxicity in Cereal-Based Products

SO original: NUTRIENTS | SO normalised: NUTRIENTS | CR count: 53 → 53

No journal-related changes detected in SO, J9, JI or CR for this record.

Record 54 | WOS:001345720100005

Review: Strategies and technologies in preventing regulated and emerging mycotoxin co-contamination in forage for safeguarding ruminant health

SO original: ANIMAL | SO normalised: ANIMAL | CR count: 178 → 178

Field	Status	Orig. pos	Norm. pos	Orig. line	Norm. line	Original cited/source value	Normalised cited/source value
CR	replaced	5	5	8565	8565	Off J Eur Union<br>[Anonymous], 2006, Off J Eur Union, V229, P7, DOI DOI 10.3390/ECMC2021-11474	OFF J EUR UNION<br>[Anonymous], 2006, OFF J EUR UNION, V229, P7, DOI DOI 10.3390/ECMC2021-11474
CR	replaced	67	67	8627	8627	Adv Appl Microbiol<br>Hidy P H, 1977, Adv Appl Microbiol, V22, P59, DOI 10.1016/S0065-2164(08)70160-6	ADV APPL MICROBIOL<br>Hidy P H, 1977, ADV APPL MICROBIOL, V22, P59, DOI 10.1016/S0065-2164(08)70160-6
CR	replaced	88	88	8648	8648	Mycotoxin reduction in grain chains<br>Leslie J.F., 2006, Mycotoxin reduction in grain chains	MYCOTOXIN REDUCTION IN GRAIN CHAINS<br>Leslie J.F., 2006, MYCOTOXIN REDUCTION IN GRAIN CHAINS
CR	replaced	113	113	8673	8673	Corn: chemistry and technology<br>Munkvold G. P., 2003, Corn: chemistry and technology, P811	CORN: CHEMISTRY AND TECHNOLOGY<br>Munkvold G. P., 2003, CORN: CHEMISTRY AND TECHNOLOGY, P811
CR	replaced	122	122	8682	8682	Mycotoxin Res<br>Oldenburg E, 2005, Mycotoxin Res, V21, P105, DOI 10.1007/BF02954430	MYCOTOXIN RES<br>Oldenburg E, 2005, MYCOTOXIN RES, V21, P105, DOI 10.1007/BF02954430

Record 55 | WOS:001296248000001

Effects of isochlorogenic acid A on mitochondrial dynamics imbalance and RLR damage in PAM cells induced by combined mycotoxins

SO original: TOXICOLOGY | SO normalised: TOXICOLOGY | CR count: 57 → 57

Field	Status	Orig. pos	Norm. pos	Orig. line	Norm. line	Original cited/source value	Normalised cited/source value
CR	replaced	35	35	8852	8852	ADV PHARMACOL<br>Miao MS, 2020, ADV PHARMACOL, V87, P71, DOI 10.1016/bs.apha.2019.12.002	Adv Pharmacol<br>Miao MS, 2020, Adv Pharmacol, V87, P71, DOI 10.1016/bs.apha.2019.12.002

Record 56 | WOS:001291367000001

Ochratoxin A-induced DNA damage triggers G2 phase arrest via hMLH1-p53-p21 signaling pathway in human gastric epithelium immortalized cells in vitro

SO original: TOXICOLOGY LETTERS | SO normalised: TOXICOLOGY LETTERS | CR count: 32 → 32

No journal-related changes detected in SO, J9, JI or CR for this record.

Record 57 | WOS:001325349600001

Evaluating the combined and individual cytotoxic effect of beauvericin, enniatin B and ochratoxin a on breast cancer cells, leukemia cells, and fresh peripheral blood mononuclear cells

SO original: TOXICOLOGY IN VITRO | SO normalised: TOXICOLOGY IN VITRO | CR count: 51 → 51

No journal-related changes detected in SO, J9, JI or CR for this record.

Record 58 | WOS:001277668700001

The Novel Role of the NLRP3 Inflammasome in Mycotoxin-Induced Toxicological Mechanisms

SO original: VETERINARY SCIENCES | SO normalised: VETERINARY SCIENCES | CR count: 133 → 133

No journal-related changes detected in SO, J9, JI or CR for this record.

Record 59 | WOS:001263565100001

Reduction of aflatoxins and ochratoxin A by addition of commercial Koji during fermentation of the Korean traditional soybean paste, Doenjang

SO original: FOOD CONTROL | SO normalised: FOOD CONTROL | CR count: 49 → 49

Field	Status	Orig. pos	Norm. pos	Orig. line	Norm. line	Original cited/source value	Normalised cited/source value
CR	replaced	28	28	9389	9389	Korean Journal of Food Science & Technology<br>Park K. Y., 1989, Korean Journal of Food Science & Technology, V21, P419	Korean Journal of Food Science and Technology<br>Park K. Y., 1989, Korean Journal of Food Science and Technology, V21, P419

Record 60 | WOS:001257266000001

Zearalenone modulates the function of goat endometrial cells via the mitochondrial quality control system

SO original: FASEB JOURNAL | SO normalised: FASEB JOURNAL | CR count: 58 → 58

Field	Status	Orig. pos	Norm. pos	Orig. line	Norm. line	Original cited/source value	Normalised cited/source value
CR	replaced	34	34	9529	9529	Endocrinology<br>Meresman G., 2010, Endocrinology, V8, P1	ENDOCRINOLOGY<br>Meresman G., 2010, ENDOCRINOLOGY, V8, P1

Record 61 | WOS:001250935500001

Central role of Sigma-1 receptor in ochratoxin A-induced ferroptosis

SO original: ARCHIVES OF TOXICOLOGY | SO normalised: ARCHIVES OF TOXICOLOGY | CR count: 48 → 48

No journal-related changes detected in SO, J9, JI or CR for this record.

Record 62 | WOS:001263872600001

Occurrence of the two major regulated mycotoxins, ochratoxin A and fumonisin B1, in cereal and cereal-based products in Europe and toxicological effects: A review

SO original: ENVIRONMENTAL TOXICOLOGY AND PHARMACOLOGY | SO normalised: ENVIRONMENTAL TOXICOLOGY AND PHARMACOLOGY | CR count: 221 → 221

Field	Status	Orig. pos	Norm. pos	Orig. line	Norm. line	Original cited/source value	Normalised cited/source value
CR	replaced	66	66	9817	9817	Off. J. Eur. Union<br>European Commission, 2022, Off. J. Eur. Union, V206, P11	OFF J EUR UNION<br>European Commission, 2022, OFF J EUR UNION, V206, P11
CR	replaced	151	151	9902	9902	Ser. Test. Assess.<br>OECD, 2018, Ser. Test. Assess., V119	Ser. Test. Assess<br>OECD, 2018, Ser. Test. Assess, V119

Record 63 | WOS:001256451400001

High-Coverage UHPLC-MS/MS Analysis of 67 Mycotoxins in Plasma for Male Infertility Exposure Studies

SO original: TOXICS | SO normalised: TOXICS | CR count: 67 → 67

Field	Status	Orig. pos	Norm. pos	Orig. line	Norm. line	Original cited/source value	Normalised cited/source value
CR	replaced	67	67	10125	10125	2011<br>,, 2011, EFSA Journal, V9, P2407	2011<br>,, 2011, EFSA J, V9, P2407

Record 64 | WOS:001234877500001

Investigation of the mechanisms behind ochratoxin A-induced cytotoxicity in human astrocytes and the protective effects of N-acetylcysteine

SO original: JOURNAL OF APPLIED TOXICOLOGY | SO normalised: JOURNAL OF APPLIED TOXICOLOGY | CR count: 51 → 51

Field	Status	Orig. pos	Norm. pos	Orig. line	Norm. line	Original cited/source value	Normalised cited/source value
CR	replaced	23	23	10212	10212	Springerplus<br>Lautert Claudia, 2014, Springerplus, V3, P506, DOI 10.1186/2193-1801-3-506	SPRINGERPLUS<br>Lautert Claudia, 2014, SPRINGERPLUS, V3, P506, DOI 10.1186/2193-1801-3-506

Record 65 | WOS:001229022000001

The efficiency of mycotoxin binding by sorbents in the in vitro model using a naturally contaminated animal feed

SO original: JOURNAL OF VETERINARY RESEARCH | SO normalised: JOURNAL OF VETERINARY RESEARCH | CR count: 32 → 32

Field	Status	Orig. pos	Norm. pos	Orig. line	Norm. line	Original cited/source value	Normalised cited/source value
CR	replaced	25	25	10318	10318	Mycotoxin Res<br>Thieu N Q, 2008, Mycotoxin	MYCOTOXIN RES<br>Thieu N Q, 2008, MYCOTOXIN RES, V24,

						Res, V24, P124, DOI 10.1007/BF03032338	P124, DOI 10.1007/BF03032338

Record 66 | WOS:001228887800001

In-vivo evaluation of the adverse effects of ochratoxin A on broiler chicken health and adsorption efficacy of indigenous and commercial clay of Balochistan, Pakistan

SO original: FOOD ADDITIVES AND CONTAMINANTS PART A-CHEMISTRY ANALYSIS CONTROL ; EXPOSURE & RISK ASSESSMENT | SO normalised: FOOD ADDITIVES AND CONTAMINANTS PART A-CHEMISTRY ANALYSIS CONTROL ; EXPOSURE & RISK ASSESSMENT | CR count: 76 → 76

No journal-related changes detected in SO, J9, JI or CR for this record.

Record 67 | WOS:001222378900001

Role of albumin in the metabolism and excretion of ochratoxin A

SO original: MYCOTOXIN RESEARCH | SO normalised: MYCOTOXIN RESEARCH | CR count: 56 → 56

No journal-related changes detected in SO, J9, JI or CR for this record.

Record 68 | WOS:001220328900001

Unveiling the emerging role of curcumin to alleviate ochratoxin A-induced muscle toxicity in grass carp (Ctenopharyngodon idella): in vitro and in vivo studies

SO original: JOURNAL OF ANIMAL SCIENCE AND BIOTECHNOLOGY | SO normalised: JOURNAL OF ANIMAL SCIENCE AND BIOTECHNOLOGY | CR count: 85 → 85

No journal-related changes detected in SO, J9, JI or CR for this record.

Record 69 | WOS:001215577500001

Protective Effect of Boric Acid Against Ochratoxin A-Induced Toxic Effects in Human Embryonal Kidney Cells (HEK293): A Study on Cytotoxic, Genotoxic, Oxidative, and Apoptotic Effects

SO original: BIOLOGICAL TRACE ELEMENT RESEARCH | SO normalised: BIOLOGICAL TRACE ELEMENT RESEARCH | CR count: 72 → 72

Field	Status	Orig. pos	Norm. pos	Orig. line	Norm. line	Original cited/source value	Normalised cited/source value
CR	replaced	40	40	10847	10847	Int J Toxicol<br>Kishore A S, 2008, Int J Toxicol, V27, P449, DOI 10.1080/10915810802656996	INT J TOXICOL<br>Kishore A S, 2008, INT J TOXICOL, V27, P449, DOI 10.1080/10915810802656996

Record 70 | WOS:001231234500001

Alternariol Monomethyl-Ether Induces Toxicity via Cell Death and Oxidative Stress in Swine Intestinal Epithelial Cells

SO original: TOXINS | SO normalised: TOXINS | CR count: 56 → 56

Field	Status	Orig. pos	Norm. pos	Orig. line	Norm. line	Original cited/source value	Normalised cited/source value

CR	replaced	9	9	10953	10953	Mycotoxin Res<br>Burkhardt Britta, 2009, Mycotoxin Res, V25, P149, DOI 10.1007/s12550-009-0022-2	MYCOTOXIN RES<br>Burkhardt Britta, 2009, MYCOTOXIN RES, V25, P149, DOI 10.1007/s12550-009-0022-2
CR	replaced	40	40	10984	10984	Mycotoxin Res<br>Pfeiffer E, 2007, Mycotoxin Res, V23, P152, DOI 10.1007/BF02951512	MYCOTOXIN RES<br>Pfeiffer E, 2007, MYCOTOXIN RES, V23, P152, DOI 10.1007/BF02951512
CR	replaced	56	56	11000	11000	2011<br>,, 2011, EFSA Journal, V9, P2407	2011<br>,, 2011, EFSA J, V9, P2407

Record 71 | WOS:001227638700001

Development and application of the physiologically-based toxicokinetic (PBTK) model for ochratoxin A (OTA) in rats and humans

SO original: ECOTOXICOLOGY AND ENVIRONMENTAL SAFETY | SO normalised: ECOTOXICOLOGY AND ENVIRONMENTAL SAFETY | CR count: 97 → 97

Field	Status	Orig. pos	Norm. pos	Orig. line	Norm. line	Original cited/source value	Normalised cited/source value
CR	replaced	21	21	11089	11089	EFSA Journal<br>EFSA (European Food Safety Authority), 2020, EFSA Journal, V18, P6113	EFSA J<br>EFSA (European Food Safety Authority), 2020, EFSA J, V18, P6113

Record 72 | WOS:001209938800001

Comprehensive Insights into Ochratoxin A: Occurrence, Analysis, and Control Strategies

SO original: FOODS | SO normalised: FOODS | CR count: 329 → 329

Field	Status	Orig. pos	Norm. pos	Orig. line	Norm. line	Original cited/source value	Normalised cited/source value
CR	replaced	93	93	11327	11327	EFSA Journal<br>EFSA (European Food Safety Authority), 2020, EFSA Journal, V18, P6113	EFSA J<br>EFSA (European Food Safety Authority), 2020, EFSA J, V18, P6113
CR	replaced	133	133	11367	11367	Int J Environ Res Public Health<br>Hassan Hussein F, 2023, Int J Environ Res Public Health, V20, DOI [10.3390/ijerph20166556, 10.3390/ijerph20166556]	INT J ENV RES PUB HE<br>Hassan Hussein F, 2023, INT J ENV RES PUB HE, V20, DOI [10.3390/ijerph20166556, 10.3390/ijerph20166556]

Record 73 | WOS:001221312500001

In vitro and in vivo assessment of AFB1 and OTA toxic effects and the beneficial role of bioactive compounds. A systematic review

SO original: FOOD CHEMISTRY | SO normalised: FOOD CHEMISTRY | CR count: 97 → 97

Field	Status	Orig. pos	Norm. pos	Orig. line	Norm. line	Original cited/source value	Normalised cited/source value
CR	replaced	5	5	11627	11627	IARC Sci Publ<br>[Anonymous], 2012, IARC Sci Publ, P105	IARC SCI PUBL<br>[Anonymous], 2012, IARC SCI PUBL, P105

Record 74 | WOS:001219077100001

Disruption of intestinal epithelial permeability in the Co-culture system of Caco-2/HT29-MTX cells exposed individually or simultaneously to acrylamide and ochratoxin A

SO original: FOOD AND CHEMICAL TOXICOLOGY | SO normalised: FOOD AND CHEMICAL TOXICOLOGY | CR count: 75 → 75

Field	Status	Orig. pos	Norm. pos	Orig. line	Norm. line	Original cited/source value	Normalised cited/source value
CR	replaced	31	31	11826	11826	Int. J. Biol. Sci.<br>Khoi C.-S., 2021, Int. J. Biol. Sci., V22	INT J BIOL SCI<br>Khoi C.-S., 2021, INT J BIOL SCI, V22

Record 75 | WOS:001185505600042

A high-throughput gut-on-chip platform to study the epithelial responses to enterotoxins

SO original: SCIENTIFIC REPORTS | SO normalised: SCIENTIFIC REPORTS | CR count: 63 → 63

Field	Status	Orig. pos	Norm. pos	Orig. line	Norm. line	Original cited/source value	Normalised cited/source value
CR	replaced	54	54	11974	11974	bioRxiv<br>Tejeda-Mora H, 2019, bioRxiv, DOI [10.1101/761627, 10.1101/761627, DOI 10.1101/761627]	BIORXIV<br>Tejeda-Mora H, 2019, BIORXIV, DOI [10.1101/761627, 10.1101/761627, DOI 10.1101/761627]

Record 76 | WOS:001187481100001

Efficacy of a multicomponent binding agent against combined exposure to zearalenone and ochratoxin A in weaned pigs

SO original: FRONTIERS IN VETERINARY SCIENCE | SO normalised: FRONTIERS IN VETERINARY SCIENCE | CR count: 50 → 50

Field	Status	Orig. pos	Norm. pos	Orig. line	Norm. line	Original cited/source value	Normalised cited/source value
CR	replaced	7	7	12062	12062	Off J Eur Union<br>[Anonymous], 2006, Off J Eur Union, V229, P7, DOI DOI 10.3390/ECMC2021-11474	OFF J EUR UNION<br>[Anonymous], 2006, OFF J EUR UNION, V229, P7, DOI DOI 10.3390/ECMC2021-11474
CR	replaced	29	29	12084	12084	Mycotoxin Res<br>Lusky K, 2000, Mycotoxin Res, V16 Suppl 2, P158, DOI 10.1007/BF02940026	MYCOTOXIN RES<br>Lusky K, 2000, MYCOTOXIN RES, V16 Suppl 2, P158, DOI 10.1007/BF02940026

Record 77 | WOS:001192691800001

Red Orange and Lemon Extract Ameliorates the Renal Oxidative Stress and Inflammation Induced by Ochratoxin A through the Modulation of Nrf2

SO original: TOXINS | SO normalised: TOXINS | CR count: 51 → 51

Field	Status	Orig. pos	Norm. pos	Orig. line	Norm. line	Original cited/source value	Normalised cited/source value
CR	replaced	18	18	12197	12197	Oxid Med Cell Longev<br>Hussain Tarique, 2016, Oxid Med Cell Longev, V2016, P7432797	OXID MED CELL LONGEV<br>Hussain Tarique, 2016, OXID MED CELL LONGEV, V2016, P7432797

Record 78 | WOS:001176181400001

Natural feed additives and bioactive supplements versus chemical additives as a safe and practical approach to combat foodborne mycotoxicoses

SO original: FRONTIERS IN NUTRITION | SO normalised: FRONTIERS IN NUTRITION | CR count: 294 → 294

Field	Status	Orig. pos	Norm. pos	Orig. line	Norm. line	Original cited/source value	Normalised cited/source value
CR	replaced	21	21	12312	12312	Off J Eur Union<br>[Anonymous], 2006, Off J Eur Union, V229, P7, DOI DOI 10.3390/ECMC2021-11474	OFF J EUR UNION<br>[Anonymous], 2006, OFF J EUR UNION, V229, P7, DOI DOI 10.3390/ECMC2021-11474
CR	replaced	36	36	12327	12327	J Toxicol Sci<br>Bishayi Biswadev, 2002, J Toxicol Sci, V27, P139, DOI 10.2131/jts.27.139	J TOXICOL SCI<br>Bishayi Biswadev, 2002, J TOXICOL SCI, V27, P139, DOI 10.2131/jts.27.139
CR	replaced	64	64	12355	12355	Mycotoxin Res<br>Deberghes P, 1995, Mycotoxin Res, V11, P37, DOI 10.1007/BF03192060	MYCOTOXIN RES<br>Deberghes P, 1995, MYCOTOXIN RES, V11, P37, DOI 10.1007/BF03192060
CR	replaced	75	75	12366	12366	Mycotoxin Res<br>Engelhardt G, 2002, Mycotoxin Res, V18, P37, DOI 10.1007/BF02946138	MYCOTOXIN RES<br>Engelhardt G, 2002, MYCOTOXIN RES, V18, P37, DOI 10.1007/BF02946138
CR	replaced	130	130	12421	12421	Revue de Medecine Veterinaire<br>Laciakova A., 1998, Revue de Medecine Veterinaire, V149, P567	REV MED VET-TOULOUSE<br>Laciakova A., 1998, REV MED VET-TOULOUSE, V149, P567
CR	replaced	212	212	12503	12503	Mycotoxin Res<br>Schatzmayr G, 2003, Mycotoxin Res, V19, P124, DOI 10.1007/BF02942950	MYCOTOXIN RES<br>Schatzmayr G, 2003, MYCOTOXIN RES, V19, P124, DOI 10.1007/BF02942950
CR	replaced	254	254	12545	12545	Mycotoxin Res<br>Stoev Stoycho D, 2010, Mycotoxin Res, V26, P31, DOI 10.1007/s12550-009-0038-7	MYCOTOXIN RES<br>Stoev Stoycho D, 2010, MYCOTOXIN RES, V26, P31, DOI 10.1007/s12550-009-0038-7
CR	replaced	283	283	12574	12574	Food Sci. Technol<br>YU Meizhen, 2023, Food Sci. Technol, V43, pe114622, DOI	FOOD SCI TECH-BRAZIL<br>YU Meizhen, 2023, FOOD SCI TECH-BRAZIL, V43,

						[10.1590/fst.114622, 10.1590/fst.114622]	pe114622, DOI [10.1590/fst.114622, 10.1590/fst.114622]

Record 79 | WOS:001179683700001

In vitro and in vivo induction of ochratoxin A exposure-related micronucleus formation in rat proximal tubular epithelial cells and expression profiling of chromosomal instability-related genes

SO original: FOOD AND CHEMICAL TOXICOLOGY | SO normalised: FOOD AND CHEMICAL TOXICOLOGY | CR count: 74 → 74

Field	Status	Orig. pos	Norm. pos	Orig. line	Norm. line	Original cited/source value	Normalised cited/source value
CR	replaced	39	39	12696	12696	Frontiers in Genetics Luzhna Lidiya, 2013, Frontiers in Genetics, V4, P131, DOI 10.3389/fgene.2013.00131	FRONT GENET Luzhna Lidiya, 2013, FRONT GENET, V4, P131, DOI 10.3389/fgene.2013.00131

Record 80 | WOS:001172173100001

Comparative Study of Spheroids (3D) and Monolayer Cultures (2D) for the In Vitro Assessment of Cytotoxicity Induced by the Mycotoxins Sterigmatocystin, Ochratoxin A and Patulin

SO original: FOODS | SO normalised: FOODS | CR count: 69 → 69

Field	Status	Orig. pos	Norm. pos	Orig. line	Norm. line	Original cited/source value	Normalised cited/source value
CR	replaced	6	6	12803	12803	Off. J. Eur. Union [Anonymous], 2003, Off. J. Eur. Union, VL203, P1	OFF J EUR UNION [Anonymous], 2003, OFF J EUR UNION, VL203, P1
CR	replaced	7	7	12804	12804	NIH Publ [Anonymous], 2006, NIH Publ	NIH PUBL [Anonymous], 2006, NIH PUBL
CR	replaced	30	30	12827	12827	IARC Monogr IARC, 1987, IARC Monogr, V1	IARC MONOGR IARC, 1987, IARC MONOGR, V1

Record 81 | WOS:001175293000001

Involvement of pro-inflammatory mediators and cell cycle disruption in neuronal cells induced by gliotoxin and ochratoxin A after individual and combined exposure

SO original: TOXICOLOGY LETTERS | SO normalised: TOXICOLOGY LETTERS | CR count: 49 → 49

Field	Status	Orig. pos	Norm. pos	Orig. line	Norm. line	Original cited/source value	Normalised cited/source value
CR	replaced	41	41	12967	12967	Methods Mol Biol Tabatabaei Mahdis Sadat, 2022, Methods Mol Biol, V2508, P115, DOI 10.1007/978-1-0716-2376-3_10	METHODS MOL BIOL Tabatabaei Mahdis Sadat, 2022, METHODS MOL BIOL, V2508, P115, DOI 10.1007/978-1-0716-2376-3_10

Record 82 | WOS:001154746400001

Recent advances in the degradation efficacy and mechanisms of mycotoxins in food by atmospheric cold plasma

SO original: ECOTOXICOLOGY AND ENVIRONMENTAL SAFETY | SO normalised: ECOTOXICOLOGY AND ENVIRONMENTAL SAFETY | CR count: 84 → 84

No journal-related changes detected in SO, J9, JI or CR for this record.

Record 83 | WOS:001201732900001

Comparative Assessment of Thermal Treatment for Mycotoxins Adsorption Potential of Indigenous Clay from Balochistan, Pakistan

SO original: PAKISTAN JOURNAL OF ANALYTICAL & ENVIRONMENTAL CHEMISTRY | SO normalised: PAKISTAN JOURNAL OF ANALYTICAL & ENVIRONMENTAL CHEMISTRY | CR count: 43 → 43

No journal-related changes detected in SO, J9, JI or CR for this record.

Record 84 | WOS:001151094300001

Practical Strategies to Reduce Ochratoxin A in Foods

SO original: TOXINS | SO normalised: TOXINS | CR count: 171 → 171

Field	Status	Orig. pos	Norm. pos	Orig. line	Norm. line	Original cited/source value	Normalised cited/source value
CR	replaced	113	113	13410	13410	Mycotoxin Res<br>Munoz Katherine, 2010, Mycotoxin Res, V26, P59, DOI 10.1007/s12550-009-0040-0	MYCOTOXIN RES<br>Munoz Katherine, 2010, MYCOTOXIN RES, V26, P59, DOI 10.1007/s12550-009-0040-0

Record 85 | WOS:001262485700001

Grape pomace as a novel functional ingredient: Mitigating ochratoxin A bioaccessibility and unraveling cytoprotective mechanisms in vitro

SO original: CURRENT RESEARCH IN FOOD SCIENCE | SO normalised: CURRENT RESEARCH IN FOOD SCIENCE | CR count: 55 → 55

No journal-related changes detected in SO, J9, JI or CR for this record.

Record 86 | WOS:001145669600001

Ochratoxin A-enhanced glycolysis induces inflammatory responses in human gastric epithelium cells through mTOR/HIF-1Î± signaling pathway

SO original: ECOTOXICOLOGY AND ENVIRONMENTAL SAFETY | SO normalised: ECOTOXICOLOGY AND ENVIRONMENTAL SAFETY | CR count: 37 → 37

No journal-related changes detected in SO, J9, JI or CR for this record.

Record 87 | WOS:001119207400001

Elucidating the mechanism of action of mycotoxins through machine learning-driven QSAR models: Focus on lipid peroxidation

SO original: FOOD AND CHEMICAL TOXICOLOGY | SO normalised: FOOD AND CHEMICAL TOXICOLOGY | CR count: 92 → 92

Field	Status	Orig. pos	Norm. pos	Orig. line	Norm. line	Original cited/source value	Normalised cited/source value
CR	replaced	17	17	13791	13791	Aflatoxin toxicity Dhakal A., 2023, Aflatoxin toxicity	AFLATOXIN TOXICITY Dhakal A., 2023, AFLATOXIN TOXICITY

Record 88 | WOS:001099952500001

Risks for animal health related to the presence of ochratoxin A (OTA) in feed

SO original: EFSA JOURNAL | SO normalised: EFSA JOURNAL | CR count: 180 → 180

Field	Status	Orig. pos	Norm. pos	Orig. line	Norm. line	Original cited/source value	Normalised cited/source value
CR	replaced	4	4	13963	13963	Mycotoxin Res Almeida Ines, 2011, Mycotoxin Res, V27, P177, DOI 10.1007/s12550-011-0093-8	MYCOTOXIN RES Almeida Ines, 2011, MYCOTOXIN RES, V27, P177, DOI 10.1007/s12550-011-0093-8
CR	replaced	22	22	13981	13981	Mycotoxin Res Blank R, 2005, Mycotoxin Res, V21, P147, DOI 10.1007/BF02954441	MYCOTOXIN RES Blank R, 2005, MYCOTOXIN RES, V21, P147, DOI 10.1007/BF02954441
CR	replaced	25	25	13984	13984	Mycotoxin Res Bohm Josef, 2010, Mycotoxin Res, V26, P147, DOI 10.1007/s12550-010-0049-4	MYCOTOXIN RES Bohm Josef, 2010, MYCOTOXIN RES, V26, P147, DOI 10.1007/s12550-010-0049-4
CR	replaced	40	40	13999	13999	EFSA Journal EFSA (European Food Safety Authority), 2020, EFSA Journal, V18, P6113	EFSA J EFSA (European Food Safety Authority), 2020, EFSA J, V18, P6113
CR	replaced	83	83	14042	14042	Veterinarski Arhiv Koynarsky Vencislav, 2007, Veterinarski Arhiv, V77, P113	VET ARHIV Koynarsky Vencislav, 2007, VET ARHIV, V77, P113
CR	replaced	90	90	14049	14049	Mycotoxin Res Liesener Kristina, 2010, Mycotoxin Res, V26, P23, DOI 10.1007/s12550-009-0037-8	MYCOTOXIN RES Liesener Kristina, 2010, MYCOTOXIN RES, V26, P23, DOI 10.1007/s12550-009-0037-8
CR	replaced	102	102	14061	14061	Mycotoxin Res Martins H M, 2008, Mycotoxin Res, V24, P19, DOI 10.1007/BF02985266	MYCOTOXIN RES Martins H M, 2008, MYCOTOXIN RES, V24, P19, DOI 10.1007/BF02985266
CR	replaced	103	103	14062	14062	Mycotoxin Res Martins H Marina, 2012, Mycotoxin Res, V28, P107, DOI 10.1007/s12550-011-0118-3	MYCOTOXIN RES Martins H Marina, 2012, MYCOTOXIN RES, V28, P107, DOI 10.1007/s12550-011-0118-3

Record 89 | WOS:001072761100001

Farnesoid X Receptor Plays a Key Role in Ochratoxin A-Induced Nephrotoxicity by Targeting Ferroptosis In Vivo and In Vitro

SO original: JOURNAL OF AGRICULTURAL AND FOOD CHEMISTRY | SO normalised: JOURNAL OF AGRICULTURAL AND FOOD CHEMISTRY | CR count: 55 → 55

No journal-related changes detected in SO, J9, JI or CR for this record.

Record 90 | WOS:001079796800001

Neurotoxicity of ochratoxin A: Molecular mechanisms and neurotherapeutic strategies

SO original: TOXICOLOGY | SO normalised: TOXICOLOGY | CR count: 148 → 148

Field	Status	Orig. pos	Norm. pos	Orig. line	Norm. line	Original cited/source value	Normalised cited/source value
CR	replaced	65	65	14393	14393	Essays Biochem<br>Latham R.L., 2023, Essays Biochem, V14	ESSAYS BIOCHEM<br>Latham R.L., 2023, ESSAYS BIOCHEM, V14

Record 91 | WOS:001078658300001

The coexistence of aflatoxin M1 and ochratoxin A induced intestinal barrier disruption via the regulation of key differentially expressed microRNAs and long non-coding RNAs in BALB/c mice

SO original: ECOTOXICOLOGY AND ENVIRONMENTAL SAFETY | SO normalised: ECOTOXICOLOGY AND ENVIRONMENTAL SAFETY | CR count: 52 → 52

No journal-related changes detected in SO, J9, JI or CR for this record.

Record 92 | WOS:001077853000001

Ochratoxin A: Overview of Prevention, Removal, and Detoxification Methods

SO original: TOXINS | SO normalised: TOXINS | CR count: 112 → 112

No journal-related changes detected in SO, J9, JI or CR for this record.

Record 93 | WOS:001060917800001

Exploring the Toxic Effects of ZEA on IPEC-J2 Cells from the Inflammatory Response and Apoptosis

SO original: ANIMALS | SO normalised: ANIMALS | CR count: 45 → 45

No journal-related changes detected in SO, J9, JI or CR for this record.

Record 94 | WOS:001074097100001

The Effect of Mycotoxins and Their Mixtures on Bovine Spermatozoa Characteristics

SO original: TOXINS | SO normalised: TOXINS | CR count: 97 → 97

Field	Status	Orig. pos	Norm. pos	Orig. line	Norm. line	Original cited/source value	Normalised cited/source value
CR	replaced	36	36	14973	14973	Mycotoxin Res<br>Hassanane M, 2000,	MYCOTOXIN RES<br>Hassanane M, 2000,

						Mycotoxin Res, V16, P53, DOI 10.1007/BF02946105	MYCOTOXIN RES, V16, P53, DOI 10.1007/BF02946105

Record 95 | WOS:001070923600001

Cell cycle and enzymatic activity alterations induced by ROS production in human neuroblastoma cells SH-SY5Y exposed to Fumonisin B1, Ochratoxin A and their combination

SO original: TOXICOLOGY IN VITRO | SO normalised: TOXICOLOGY IN VITRO | CR count: 47 → 47

No journal-related changes detected in SO, J9, JI or CR for this record.

Record 96 | WOS:001054307000001

An integrated approach for evaluating the interactive effects between azoxystrobin and ochratoxin A: Pathway-based in vivo analyses

SO original: PESTICIDE BIOCHEMISTRY AND PHYSIOLOGY | SO normalised: PESTICIDE BIOCHEMISTRY AND PHYSIOLOGY | CR count: 43 → 43

No journal-related changes detected in SO, J9, JI or CR for this record.

Record 97 | WOS:001041623900001

The Protective Effect of Pumpkin and Fermented Whey Mixture against AFB1 and OTA Immune Toxicity In Vitro. A Transcriptomic Approach

SO original: MOLECULAR NUTRITION & FOOD RESEARCH | SO normalised: MOLECULAR NUTRITION & FOOD RESEARCH | CR count: 100 → 100

No journal-related changes detected in SO, J9, JI or CR for this record.

Record 98 | WOS:001055768800001

Mycotoxins in Cereal-Based Products and Their Impacts on the Health of Humans, Livestock Animals and Pets

SO original: TOXINS | SO normalised: TOXINS | CR count: 258 → 258

Field	Status	Orig. pos	Norm. pos	Orig. line	Norm. line	Original cited/source value	Normalised cited/source value
CR	replaced	67	67	15527	15527	Diseases of Swine<br>Ensley S. M., 2019, Diseases of Swine, V11th, P1055, DOI DOI 10.1002/9781119350927.CH69	Diseases of swine<br>Ensley S. M., 2019, Diseases of swine, V11th, P1055, DOI DOI 10.1002/9781119350927.CH69
CR	replaced	137	137	15597	15597	Mycotoxin Res<br>Makun Hussaini Anthony, 2011, Mycotoxin Res, V27, P97, DOI 10.1007/s12550-010-0080-5	MYCOTOXIN RES<br>Makun Hussaini Anthony, 2011, MYCOTOXIN RES, V27, P97, DOI 10.1007/s12550-010-0080-5

Record 99 | WOS:001036576500001

Pathological Role of Oxidative Stress in Aflatoxin-Induced Toxicity in Different Experimental Models and Protective Effect of Phytochemicals: A Review

SO original: MOLECULES | SO normalised: MOLECULES | CR count: 138 → 138

Field	Status	Orig. pos	Norm. pos	Orig. line	Norm. line	Original cited/source value	Normalised cited/source value
CR	replaced	105	105	15891	15891	Natural Antioxidants in Avian Nutrition and Reproduction<br>Surai P.F., 2002, Natural Antioxidants in Avian Nutrition and Reproduction, P5	Natural antioxidants in avian nutrition and reproduction<br>Surai P.F., 2002, Natural antioxidants in avian nutrition and reproduction, P5

Record 100 | WOS:001040619100001

MiR-155-5p Elevated by Ochratoxin A Induces Intestinal Fibrosis and Epithelial-to-Mesenchymal Transition through TGF-Î² Regulated Signaling Pathway In Vitro and In Vivo

SO original: TOXINS | SO normalised: TOXINS | CR count: 45 → 45

Field	Status	Orig. pos	Norm. pos	Orig. line	Norm. line	Original cited/source value	Normalised cited/source value
CR	replaced	39	39	16023	16023	Toxicol Rep<br>Yang Seon Ah, 2023, Toxicol Rep, V10, P133, DOI [10.1016/j.toxrep.2023.01.006, 10.1016/j.toxrep.2023.01.006]	TOXICOL REP<br>Yang Seon Ah, 2023, TOXICOL REP, V10, P133, DOI [10.1016/j.toxrep.2023.01.006, 10.1016/j.toxrep.2023.01.006]

Record 101 | WOS:001013318000001

Enzymatic degradation of ochratoxin A in the gastrointestinal tract of piglets

SO original: JOURNAL OF ANIMAL SCIENCE | SO normalised: JOURNAL OF ANIMAL SCIENCE | CR count: 64 → 64

Field	Status	Orig. pos	Norm. pos	Orig. line	Norm. line	Original cited/source value	Normalised cited/source value
CR	replaced	3	3	16089	16089	Off J Eur Union<br>[Anonymous], 2006, Off J Eur Union, V229, P7, DOI DOI 10.3390/ECMC2021-11474	OFF J EUR UNION<br>[Anonymous], 2006, OFF J EUR UNION, V229, P7, DOI DOI 10.3390/ECMC2021-11474
CR	replaced	5	5	16091	16091	EFSA Journal<br>Authority E. F. S., 2006, EFSA Journal, V4, P365	EFSA J<br>Authority E. F. S., 2006, EFSA J, V4, P365

Record 102 | WOS:001011146700001

A Chemically Defined Medium That Supports Mycotoxin Production by Stachybotrys chartarum Enabled Analysis of the Impact of Nitrogen and Carbon Sources on the Biosynthesis of Macrocyclic Trichothecenes and Stachybotrylactam

SO original: APPLIED AND ENVIRONMENTAL MICROBIOLOGY | SO normalised: APPLIED AND ENVIRONMENTAL MICROBIOLOGY | CR count: 87 → 87

Field	Status	Orig. pos	Norm. pos	Orig. line	Norm. line	Original cited/source value	Normalised cited/source value

CR	replaced	38	38	16268	16268	Mycotoxin Res<br>Jarvis B, 2000, Mycotoxin Res, V16 Suppl 1, P105, DOI 10.1007/BF02942994	MYCOTOXIN RES<br>Jarvis B, 2000, MYCOTOXIN RES, V16 Suppl 1, P105, DOI 10.1007/BF02942994
CR	replaced	53	53	16283	16283	Mycotoxins in Human and Animal Health<br>McLaughlin C.S., 1977, Mycotoxins in Human and Animal Health, Park Forest South Pathotox, P263	Mycotoxins in human and animal health<br>McLaughlin C.S., 1977, Mycotoxins in human and animal health, Park Forest South Pathotox, P263

Record 103 | WOS:001013506800001

Citrinin as a potential anti-cancer therapy: A comprehensive review

SO original: CHEMICO-BIOLOGICAL INTERACTIONS | SO normalised: CHEMICO-BIOLOGICAL INTERACTIONS | CR count: 137 → 137

Field	Status	Orig. pos	Norm. pos	Orig. line	Norm. line	Original cited/source value	Normalised cited/source value
CR	replaced	39	39	16450	16450	Cell Physiol Biochem<br>Escribano-Lopez Irene, 2019, Cell Physiol Biochem, V52, P186, DOI [10.33594/000000013, 10.33594/000000013]	CELL PHYSIOL BIOCHEM<br>Escribano-Lopez Irene, 2019, CELL PHYSIOL BIOCHEM, V52, P186, DOI [10.33594/000000013, 10.33594/000000013]
CR	replaced	77	77	16488	16488	Mycotoxin Res<br>Knecht A, 2005, Mycotoxin Res, V21, P176, DOI 10.1007/BF02959258	MYCOTOXIN RES<br>Knecht A, 2005, MYCOTOXIN RES, V21, P176, DOI 10.1007/BF02959258
CR	replaced	137	137	16548	16548	2012<br>,, 2012, EFSA Journal, V10, P2605	2012<br>,, 2012, EFSA J, V10, P2605

Record 104 | WOS:001141751900001

Determination of Ochratoxin-A Detoxification and Antibiotic Resistance Potential of Acinetobacter calcoaceticus

SO original: JOURNAL OF PURE AND APPLIED MICROBIOLOGY | SO normalised: JOURNAL OF PURE AND APPLIED MICROBIOLOGY | CR count: 28 → 28

No journal-related changes detected in SO, J9, JI or CR for this record.

Record 105 | WOS:000988737300001

Natural mycotoxin contamination in dog food: A review on toxicity and detoxification methods

SO original: ECOTOXICOLOGY AND ENVIRONMENTAL SAFETY | SO normalised: ECOTOXICOLOGY AND ENVIRONMENTAL SAFETY | CR count: 205 → 205

Field	Status	Orig. pos	Norm. pos	Orig. line	Norm. line	Original cited/source value	Normalised cited/source value
CR	replaced	26	26	16748	16748	Mycotoxin Res<br>Bohm J, 2000, Mycotoxin Res, V16 Suppl 1, P70, DOI 10.1007/BF02942985	MYCOTOXIN RES<br>Bohm J, 2000, MYCOTOXIN RES, V16 Suppl 1, P70, DOI 10.1007/BF02942985

CR	replaced	28	28	16750	16750	Mycotoxin Res<br>Bohm Josef, 2010, Mycotoxin Res, V26, P147, DOI 10.1007/s12550-010-0049-4	MYCOTOXIN RES<br>Bohm Josef, 2010, MYCOTOXIN RES, V26, P147, DOI 10.1007/s12550-010-0049-4
CR	replaced	59	59	16781	16781	Polish Journal of Veterinary Sciences<br>Gajecka M., 2004, Polish Journal of Veterinary Sciences, V7, P175	POL J VET SCI<br>Gajecka M., 2004, POL J VET SCI, V7, P175
CR	replaced	60	60	16782	16782	Polish Journal of Veterinary Sciences<br>Gajecka M., 2004, Polish Journal of Veterinary Sciences, V7, P59	POL J VET SCI<br>Gajecka M., 2004, POL J VET SCI, V7, P59
CR	replaced	82	82	16804	16804	Adv Appl Microbiol<br>Hidy P H, 1977, Adv Appl Microbiol, V22, P59, DOI 10.1016/S0065-2164(08)70160-6	ADV APPL MICROBIOL<br>Hidy P H, 1977, ADV APPL MICROBIOL, V22, P59, DOI 10.1016/S0065-2164(08)70160-6
CR	replaced	166	166	16888	16888	Mycotoxin Res<br>Songsermsakul P, 2007, Mycotoxin Res, V23, P65, DOI 10.1007/BF02946027	MYCOTOXIN RES<br>Songsermsakul P, 2007, MYCOTOXIN RES, V23, P65, DOI 10.1007/BF02946027
CR	replaced	205	205	16927	16927	Polish Journal of Veterinary Sciences<br>Zwierzchowski W., 2004, Polish Journal of Veterinary Sciences, V7, P289	POL J VET SCI<br>Zwierzchowski W., 2004, POL J VET SCI, V7, P289

Record 106 | WOS:000983760800001

Excessive ER-phagy contributes to ochratoxin A-induced apoptosis

SO original: FOOD AND CHEMICAL TOXICOLOGY | SO normalised: FOOD AND CHEMICAL TOXICOLOGY | CR count: 52 → 52

No journal-related changes detected in SO, J9, JI or CR for this record.

Record 107 | WOS:000977904100001

New perspectives in application of kidney biomarkers in mycotoxin induced nephrotoxicity, with a particular focus on domestic pigs

SO original: FRONTIERS IN MICROBIOLOGY | SO normalised: FRONTIERS IN MICROBIOLOGY | CR count: 133 → 133

**Field**	**Status**	**Orig. pos**	**Norm. pos**	**Orig. line**	**Norm. line**	**Original cited/source value**	**Normalised cited/source value**
CR	replaced	6	6	17163	17163	Off J Eur Union<br>[Anonymous], 2006, Off J Eur Union, V229, P7, DOI DOI 10.3390/ECMC2021-11474	OFF J EUR UNION<br>[Anonymous], 2006, OFF J EUR UNION, V229, P7, DOI DOI 10.3390/ECMC2021-11474
CR	replaced	105	105	17262	17262	Mycotoxin Res<br>Seeling K, 2005, Mycotoxin Res, V21, P132, DOI 10.1007/BF02954437	MYCOTOXIN RES<br>Seeling K, 2005, MYCOTOXIN RES, V21,

							P132, DOI 10.1007/BF02954437

Record 108 | WOS:000968395600001

Fermented whey modulated AFB1 and OTA-induced hepatotoxicity and nephrotoxicity in vivo. A relative and absolute quantification about sex differences

SO original: TOXICOLOGY MECHANISMS AND METHODS | SO normalised: TOXICOLOGY MECHANISMS AND METHODS | CR count: 87 → 87

Field	Status	Orig. pos	Norm. pos	Orig. line	Norm. line	Original cited/source value	Normalised cited/source value
CR	replaced	17	17	17372	17372	BIOMED ENVIRON SCI<br>Deng ZJ, 2020, BIOMED ENVIRON SCI, V33, P238, DOI 10.3967/bes2020.033	Biomed Environ Sci<br>Deng ZJ, 2020, Biomed Environ Sci, V33, P238, DOI 10.3967/bes2020.033

Record 109 | WOS:000982062800001

Protective Effect of alpha-Tocopherol Against Ochratoxin A in Kidney Cell Line HK-2

SO original: JOURNAL OF FOOD PROTECTION | SO normalised: JOURNAL OF FOOD PROTECTION | CR count: 48 → 48

No journal-related changes detected in SO, J9, JI or CR for this record.

Record 110 | WOS:001094910400007

INITIAL PHASE ESTABLISHMENT OF AN IN VITRO METHOD FOR DEVELOP-MENTAL NEUROTOXICITY TEST USING KI-67 IN HUMAN NEURAL PROGENITOR CELLS

SO original: JOURNAL OF PHYSIOLOGY AND PHARMACOLOGY | SO normalised: JOURNAL OF PHYSIOLOGY AND PHARMACOLOGY | CR count: 46 → 46

Field	Status	Orig. pos	Norm. pos	Orig. line	Norm. line	Original cited/source value	Normalised cited/source value
CR	replaced	18	18	17650	17650	Reprod Toxicol<br>Kang Hee Young, 2017, Reprod Toxicol, V72, P74, DOI [10.1016/j.reprotox.2017.06.185, 10.1016/j.reprotox.2017.06.185]	REPROD TOXICOL<br>Kang Hee Young, 2017, REPROD TOXICOL, V72, P74, DOI [10.1016/j.reprotox.2017.06.185, 10.1016/j.reprotox.2017.06.185]
CR	replaced	38	38	17670	17670	Curr Protoc Cytom<br>Rothaeusler Kristina, 2007, Curr Protoc Cytom, VChapter 7, DOI 10.1002/0471142956.cy0731s40	CURR PROTOC CYTOM<br>Rothaeusler Kristina, 2007, CURR PROTOC CYTOM, VChapter 7, DOI 10.1002/0471142956.cy0731s40

Record 111 | WOS:000984555200005

Microcapsules loaded with date seed extract and its inhibitory potential to modulate the toxic effects of mycotoxins in mice received mold-contaminated diet

SO original: ENVIRONMENTAL SCIENCE AND POLLUTION RESEARCH | SO normalised: ENVIRONMENTAL SCIENCE AND POLLUTION RESEARCH | CR count: 40 → 40

No journal-related changes detected in SO, J9, JI or CR for this record.

Record 112 | WOS:000951983500001

Hazard characterisation for significant mycotoxins in food

SO original: MYCOTOXIN RESEARCH | SO normalised: MYCOTOXIN RESEARCH | CR count: 147 → 147

Field	Status	Orig. pos	Norm. pos	Orig. line	Norm. line	Original cited/source value	Normalised cited/source value
CR	replaced	13	13	17867	17867	J Appl Toxicol<br>Becci P J, 1981, J Appl Toxicol, V1, P256, DOI 10.1002/jat.2550010504	J APPL TOXICOL<br>Becci P J, 1981, J APPL TOXICOL, V1, P256, DOI 10.1002/jat.2550010504
CR	replaced	41	41	17895	17895	EFSA Support. Publ.<br>EFSA (European Food Safety Authority), 2016, EFSA Support. Publ., DOI [10.2903/sp.efsa.2016.EN-1094, DOI 10.2903/SP.EFSA.2016.EN-1006]	EFSA SUPPORT PUBL<br>EFSA (European Food Safety Authority), 2016, EFSA SUPPORT PUBL, DOI [10.2903/sp.efsa.2016.EN-1094, DOI 10.2903/SP.EFSA.2016.EN-1006]

Record 113 | WOS:000958249600001

The Effect of Mushroom Culture Filtrates on the Inhibition of Mycotoxins Produced by Aspergillus flavus and Aspergillus carbonarius

SO original: TOXINS | SO normalised: TOXINS | CR count: 46 → 46

No journal-related changes detected in SO, J9, JI or CR for this record.

Record 114 | WOS:000955634100001

Combined Effects of Acrylamide and Ochratoxin A on the Intestinal Barrier in Caco-2 Cells

SO original: FOODS | SO normalised: FOODS | CR count: 55 → 55

Field	Status	Orig. pos	Norm. pos	Orig. line	Norm. line	Original cited/source value	Normalised cited/source value
CR	replaced	52	52	18231	18231	MUTAT RES GEN TOX EN<br>Yma B., 2021, MUTAT RES GEN TOX EN, V873, P503420	MUTAT RES-GEN TOX EN<br>Yma B., 2021, MUTAT RES-GEN TOX EN, V873, P503420

Record 115 | WOS:000960233300001

Immunotoxicity of Three Environmental Mycotoxins and Their Risks of Increasing Pathogen Infections

SO original: TOXINS | SO normalised: TOXINS | CR count: 65 → 65

No journal-related changes detected in SO, J9, JI or CR for this record.

Record 116 | WOS:000944193800011

Dietary Ochratoxin A Contamination Modulates Oxidative Stress, Inflammation Processes and Causes Fibrosis in in vitro and in vivo Lung Models

SO original: FRONTIERS IN BIOSCIENCE-LANDMARK | SO normalised: FRONTIERS IN BIOSCIENCE-LANDMARK | CR count: 35 → 35

No journal-related changes detected in SO, J9, JI or CR for this record.

Record 117 | WOS:000942033800001

Comparative Assessment of Different Yeast Cell Wall-Based Mycotoxin Adsorbents Using a Model- and Bioassay-Based In Vitro Approach

SO original: TOXINS | SO normalised: TOXINS | CR count: 73 → 73

No journal-related changes detected in SO, J9, JI or CR for this record.

Record 118 | WOS:000929015600001

Potential application of probiotics in mycotoxicosis reduction in mammals and poultry

SO original: CRITICAL REVIEWS IN TOXICOLOGY | SO normalised: CRITICAL REVIEWS IN TOXICOLOGY | CR count: 69 → 69

No journal-related changes detected in SO, J9, JI or CR for this record.

Record 119 | WOS:000918850400001

Lonp1 and Sig-1R contribute to the counteraction of ursolic acid against ochratoxin A-induced mitochondrial apoptosis

SO original: FOOD AND CHEMICAL TOXICOLOGY | SO normalised: FOOD AND CHEMICAL TOXICOLOGY | CR count: 6 → 6

No journal-related changes detected in SO, J9, JI or CR for this record.

Record 120 | WOS:001000273300006

Protective role of fermented whey and pumpkin extract against aflatoxin B1 and ochratoxin A toxicity in Jurkat T-cells

SO original: WORLD MYCOTOXIN JOURNAL | SO normalised: WORLD MYCOTOXIN JOURNAL | CR count: 47 → 47

No journal-related changes detected in SO, J9, JI or CR for this record.

Record 121 | WOS:000915148500001

AFB1 and OTA Promote Immune Toxicity in Human LymphoBlastic T Cells at Transcriptomic Level

SO original: FOODS | SO normalised: FOODS | CR count: 121 → 121

Field	Status	Orig. pos	Norm. pos	Orig. line	Norm. line	Original cited/source value	Normalised cited/source value
CR	replaced	60	60	19050	19050	Mycotoxin Res<br>Leong Yin-Hui, 2012, Mycotoxin Res, V28, P79, DOI 10.1007/s12550-012-0129-8	MYCOTOXIN RES<br>Leong Yin-Hui, 2012, MYCOTOXIN RES, V28, P79, DOI 10.1007/s12550-012-0129-8
CR	replaced	94	94	19084	19084	Frontiers in Genetics<br>Stracker Travis H., 2013, Frontiers in Genetics, V4, P37, DOI 10.3389/fgene.2013.00037	FRONT GENET<br>Stracker Travis H., 2013, FRONT GENET, V4, P37, DOI 10.3389/fgene.2013.00037

Record 122 | WOS:000912325600001

Toxico-pathological effects of ochratoxin A and its diastereoisomer under in ovo conditions and in vitro evaluation of the toxicity of these toxins against the embryo Gallus gallus fibroblast cell line

SO original: POULTRY SCIENCE | SO normalised: POULTRY SCIENCE | CR count: 48 → 48

No journal-related changes detected in SO, J9, JI or CR for this record.

Record 123 | WOS:000900483500001

Modified Mycotoxins, a Still Unresolved Issue

SO original: CHEMISTRY-SWITZERLAND | SO normalised: CHEMISTRY-SWITZERLAND | CR count: 146 → 146

Field	Status	Orig. pos	Norm. pos	Orig. line	Norm. line	Original cited/source value	Normalised cited/source value
CR	replaced	51	51	19349	19349	EFSA Journal<br>EFSA (European Food Safety Authority), 2020, EFSA Journal, V18, P6113	EFSA J<br>EFSA (European Food Safety Authority), 2020, EFSA J, V18, P6113
CR	replaced	112	112	19410	19410	Mycotoxin Res<br>Ruhland M, 1997, Mycotoxin Res, V13, P54, DOI 10.1007/BF02945066	MYCOTOXIN RES<br>Ruhland M, 1997, MYCOTOXIN RES, V13, P54, DOI 10.1007/BF02945066

Record 124 | WOS:000898846200001

Innovative in vitro approaches to toxicological investigations of mycotoxins effects

SO original: EFSA JOURNAL | SO normalised: EFSA JOURNAL | CR count: 39 → 39

Field	Status	Orig. pos	Norm. pos	Orig. line	Norm. line	Original cited/source value	Normalised cited/source value
CR	replaced	6	6	19504	19504	Toxicity Testing in the 21st Century: A Vision and a Strategy<br>[Anonymous], 2007, Toxicity Testing in the 21st Century: A Vision and a Strategy	Toxicity testing in the 21st century: A vision and a strategy<br>[Anonymous], 2007, Toxicity testing in the 21st century: A vision and a strategy
CR	replaced	7	7	19505	19505	Mycopathologia<br>[Anonymous], 2003, Mycopathologia, V65, P43	MYCOPATHOLOGIA<br>[Anonymous], 2003,

							MYCOPATHOLOGIA, V65, P43

Record 125 | WOS:000903192900001

Possible Mechanisms of the Interplay between Drugs and Mycotoxins-Is There a Possible Impact?

SO original: TOXINS | SO normalised: TOXINS | CR count: 103 → 103

Field	Status	Orig. pos	Norm. pos	Orig. line	Norm. line	Original cited/source value	Normalised cited/source value
CR	replaced	14	14	19617	19617	Health<br>Bbosa G.S., 2013, Health, V5, P21, DOI [10.4236/health.2013.510A1003, DOI 10.4236/HEALTH.2013.510A1003]	HEALTH<br>Bbosa G.S., 2013, HEALTH, V5, P21, DOI [10.4236/health.2013.510A1003, DOI 10.4236/HEALTH.2013.510A1003]
CR	replaced	43	43	19646	19646	Aflatoxins-Biochemistry and Molecular Biology<br>Guevara-Gonzalez R.G., 2011, Aflatoxins-Biochemistry and Molecular Biology	AFLATOXINS - BIOCHEMISTRY AND MOLECULAR BIOLOGY<br>Guevara-Gonzalez R.G., 2011, AFLATOXINS - BIOCHEMISTRY AND MOLECULAR BIOLOGY

Record 126 | WOS:000888695100001

Mycotoxins and cellular senescence: the impact of oxidative stress, hypoxia, and immunosuppression

SO original: ARCHIVES OF TOXICOLOGY | SO normalised: ARCHIVES OF TOXICOLOGY | CR count: 90 → 90

No journal-related changes detected in SO, J9, JI or CR for this record.

Record 127 | WOS:000892528400007

The efficacy of mycotoxin binders to control mycotoxins in feeds and the potential risk of interactions with nutrient: a review

SO original: JOURNAL OF ANIMAL SCIENCE | SO normalised: JOURNAL OF ANIMAL SCIENCE | CR count: 89 → 89

Field	Status	Orig. pos	Norm. pos	Orig. line	Norm. line	Original cited/source value	Normalised cited/source value
CR	replaced	79	79	20004	20004	Mycotoxin Res<br>Thieu N Q, 2008, Mycotoxin Res, V24, P124, DOI 10.1007/BF03032338	MYCOTOXIN RES<br>Thieu N Q, 2008, MYCOTOXIN RES, V24, P124, DOI 10.1007/BF03032338
CR	replaced	83	83	20008	20008	Mycotoxin Res<br>Vekiru E, 2007, Mycotoxin Res, V23, P27, DOI 10.1007/BF02946021	MYCOTOXIN RES<br>Vekiru E, 2007, MYCOTOXIN RES, V23, P27, DOI 10.1007/BF02946021

Record 128 | WOS:000886894200008

Protective effect of selenomethionine on kidney injury induced by ochratoxin A in rabbits

SO original: ENVIRONMENTAL SCIENCE AND POLLUTION RESEARCH | SO normalised: ENVIRONMENTAL SCIENCE AND POLLUTION RESEARCH | CR count: 70 → 70

No journal-related changes detected in SO, J9, JI or CR for this record.

Record 129 | WOS:000896944500005

In vitro study of ochratoxin A in the expression of genes associated with neuron survival and viability

SO original: TOXICOLOGY | SO normalised: TOXICOLOGY | CR count: 63 → 63

No journal-related changes detected in SO, J9, JI or CR for this record.

Record 130 | WOS:000974547800001

Low doses of fumonisin B1 exacerbate ochratoxin A-induced renal injury in mice and the protective roles of heat shock protein 70

SO original: CHEMICO-BIOLOGICAL INTERACTIONS | SO normalised: CHEMICO-BIOLOGICAL INTERACTIONS | CR count: 77 → 77

No journal-related changes detected in SO, J9, JI or CR for this record.

Record 131 | WOS:000887299000001

Mycotoxins and Essential Oils-From a Meat Industry Hazard to a Possible Solution: A Brief Review

SO original: FOODS | SO normalised: FOODS | CR count: 150 → 150

**Field**	**Status**	**Orig. pos**	**Norm. pos**	**Orig. line**	**Norm. line**	**Original cited/source value**	**Normalised cited/source value**
CR	replaced	1	1	20491	20491	Mycotoxin Res<br>Abd Alla el-S A, 1996, Mycotoxin Res, V12, P41, DOI 10.1007/BF03192079	MYCOTOXIN RES<br>Abd Alla el-S A, 1996, MYCOTOXIN RES, V12, P41, DOI 10.1007/BF03192079
CR	replaced	2	2	20492	20492	J. Am. Sci.<br>Abd-El Fattah S., 2010, J. Am. Sci., V6, P582, DOI 10.13140/RG.2.1.1899.2729	J AM SCI<br>Abd-El Fattah S., 2010, J AM SCI, V6, P582, DOI 10.13140/RG.2.1.1899.2729

Record 132 | WOS:000894684300001

Co-Contamination of Food and Feed with Mycotoxin and Bacteria and Possible Implications for Health

SO original: AGRICULTURE-BASEL | SO normalised: AGRICULTURE-BASEL | CR count: 54 → 54

**Field**	**Status**	**Orig. pos**	**Norm. pos**	**Orig. line**	**Norm. line**	**Original cited/source value**	**Normalised cited/source value**
CR	replaced	11	11	20709	20709	Oncotarget<br>Chen Linlin, 2018, Oncotarget, V9, P7204, DOI 10.18632/oncotarget.23208	ONCOTARGET<br>Chen Linlin, 2018, ONCOTARGET, V9, P7204, DOI 10.18632/oncotarget.23208

Record 133 | WOS:000883142900001

Current Review of Mycotoxin Biodegradation and Bioadsorption: Microorganisms, Mechanisms, and Main Important Applications

SO original: TOXINS | SO normalised: TOXINS | CR count: 179 → 179

Field	Status	Orig. pos	Norm. pos	Orig. line	Norm. line	Original cited/source value	Normalised cited/source value
CR	replaced	24	24	20845	20845	Nat Toxins<br>Boswald C, 1995, Nat Toxins, V3, P138, DOI 10.1002/nt.2620030304	NAT TOXINS<br>Boswald C, 1995, NAT TOXINS, V3, P138, DOI 10.1002/nt.2620030304

Record 134 | WOS:000867775600001

The Effect of Aflatoxin B1 on Tumor-Related Genes and Phenotypic Characters of MCF7 and MCF10A Cells

SO original: INTERNATIONAL JOURNAL OF MOLECULAR SCIENCES | SO normalised: INTERNATIONAL JOURNAL OF MOLECULAR SCIENCES | CR count: 97 → 97

Field	Status	Orig. pos	Norm. pos	Orig. line	Norm. line	Original cited/source value	Normalised cited/source value
CR	replaced	39	39	21125	21125	J Biochem Mol Toxicol<br>Golli-Bennour Emna El, 2010, J Biochem Mol Toxicol, V24, P42, DOI 10.1002/jbt.20310	J BIOCHEM MOL TOXIC<br>Golli-Bennour Emna El, 2010, J BIOCHEM MOL TOXIC, V24, P42, DOI 10.1002/jbt.20310

Record 135 | WOS:000911765600013

Ochratoxin A induced differentiation nephrotoxicity in renal tubule and glomeruli via autophagy differential regulation

SO original: ENVIRONMENTAL TOXICOLOGY AND PHARMACOLOGY | SO normalised: ENVIRONMENTAL TOXICOLOGY AND PHARMACOLOGY | CR count: 47 → 47

No journal-related changes detected in SO, J9, JI or CR for this record.

Record 136 | WOS:000866918500001

Quercetin: Its Antioxidant Mechanism, Antibacterial Properties and Potential Application in Prevention and Control of Toxipathy

SO original: MOLECULES | SO normalised: MOLECULES | CR count: 150 → 150

Field	Status	Orig. pos	Norm. pos	Orig. line	Norm. line	Original cited/source value	Normalised cited/source value
CR	replaced	91	91	21446	21446	Biomed J<br>Patil Shrikant L, 2014, Biomed J, V37, P305, DOI 10.4103/2319-4170.132880	BIOMED J<br>Patil Shrikant L, 2014, BIOMED J, V37, P305, DOI 10.4103/2319-4170.132880
CR	replaced	108	108	21463	21463	Mycotoxin Res<br>Romero Stella Maris, 2009, Mycotoxin Res, V25, P165, DOI 10.1007/s12550-009-0026-y	MYCOTOXIN RES<br>Romero Stella Maris, 2009, MYCOTOXIN RES, V25, P165, DOI 10.1007/s12550-009-0026-y

Record 137 | WOS:000872026500001

Luteolin and Chrysin Could Prevent E. coli Lipopolysaccharide-Ochratoxin A Combination-Caused Inflammation and Oxidative Stress in In Vitro Porcine Intestinal Model

SO original: ANIMALS | SO normalised: ANIMALS | CR count: 43 → 43

No journal-related changes detected in SO, J9, JI or CR for this record.

Record 138 | WOS:000864053100001

In-vitro alleviation of ochratoxin a mediated cytotoxicity using N-acetyl-L-tryptophan glucoside pretreatment

SO original: LIFE SCIENCES | SO normalised: LIFE SCIENCES | CR count: 33 → 33

Field	Status	Orig. pos	Norm. pos	Orig. line	Norm. line	Original cited/source value	Normalised cited/source value
CR	replaced	9	9	21690	21690	Journal of Toxicological Sciences<br>Creppy Edmond Ekue, 1998, Journal of Toxicological Sciences, V23, P165	J TOXICOL SCI<br>Creppy Edmond Ekue, 1998, J TOXICOL SCI, V23, P165
CR	replaced	21	21	21702	21702	Curr Issues Mol Biol<br>Nunez R, 2001, Curr Issues Mol Biol, V3, P67	CURR ISSUES MOL BIOL<br>Nunez R, 2001, CURR ISSUES MOL BIOL, V3, P67

Record 139 | WOS:000884231200001

Aflatoxin M1 and ochratoxin A induce a competitive endogenous RNA regulatory network of intestinal immunosuppression by whole-transcriptome analysis

SO original: SCIENCE OF THE TOTAL ENVIRONMENT | SO normalised: SCIENCE OF THE TOTAL ENVIRONMENT | CR count: 63 → 63

No journal-related changes detected in SO, J9, JI or CR for this record.

Record 140 | WOS:000876186700006

The importance of transcriptomics and proteomics for studying molecular mechanisms of mycotoxin exposure: A review

SO original: FOOD AND CHEMICAL TOXICOLOGY | SO normalised: FOOD AND CHEMICAL TOXICOLOGY | CR count: 66 → 66

No journal-related changes detected in SO, J9, JI or CR for this record.

Record 141 | WOS:000851604100001

Mycotoxins in soybean-based foods fermented with filamentous fungi: Occurrence and preventive strategies

SO original: COMPREHENSIVE REVIEWS IN FOOD SCIENCE AND FOOD SAFETY | SO normalised: COMPREHENSIVE REVIEWS IN FOOD SCIENCE AND FOOD SAFETY | CR count: 141 → 141

Field	Status	Orig. pos	Norm. pos	Orig. line	Norm. line	Original cited/source value	Normalised cited/source value

CR	replaced	36	36	22066	22066	Mycotoxin Res<br>Ellner F M, 2005, Mycotoxin Res, V21, P112, DOI 10.1007/BF02954432	MYCOTOXIN RES<br>Ellner F M, 2005, MYCOTOXIN RES, V21, P112, DOI 10.1007/BF02954432
CR	replaced	61	61	22091	22091	[Korean Journal of Food Science and Technology<br>ë° ì ±êµ-, 2004, [Korean Journal of Food Science and Technology,<br>í êµ-ì í ê³¼í í ì§], V36, P158	Korean Journal of Food Science and Technology<br>ë° ì ±êµ-, 2004, Korean Journal of Food Science and Technology,<br>í êµ-ì í ê³¼í í ì§], V36, P158
CR	replaced	86	86	22116	22116	Mycotoxin Res<br>Oviedo M S, 2012, Mycotoxin Res, V28, P169, DOI 10.1007/s12550-012-0132-0	MYCOTOXIN RES<br>Oviedo M S, 2012, MYCOTOXIN RES, V28, P169, DOI 10.1007/s12550-012-0132-0
CR	replaced	115	115	22145	22145	Food Science and Technology<br>Sun Ting-Ting, 2010, Food Science and Technology, V35, P296	FOOD SCI TECH-BRAZIL<br>Sun Ting-Ting, 2010, FOOD SCI TECH-BRAZIL, V35, P296
CR	replaced	119	119	22149	22149	Mycotoxin Res<br>Tanaka K, 2006, Mycotoxin Res, V22, P153, DOI 10.1007/BF02959268	MYCOTOXIN RES<br>Tanaka K, 2006, MYCOTOXIN RES, V22, P153, DOI 10.1007/BF02959268

Record 142 | WOS:000851281600001

Mechanisms underlying citrinin-induced toxicity via oxidative stress and apoptosis-mediated by mitochondrial-dependent pathway in SH-SY5Y cells

SO original: DRUG AND CHEMICAL TOXICOLOGY | SO normalised: DRUG AND CHEMICAL TOXICOLOGY | CR count: 72 → 72

Field	Status	Orig. pos	Norm. pos	Orig. line	Norm. line	Original cited/source value	Normalised cited/source value
CR	replaced	1	1	22233	22233	J Biomed Biotechnol<br>Abdel Wahab Siddig Ibrahim, 2009, J Biomed Biotechnol, V2009, P769568, DOI 10.1155/2009/769568	J BIOMED BIOTECHNOL<br>Abdel Wahab Siddig Ibrahim, 2009, J BIOMED BIOTECHNOL, V2009, P769568, DOI 10.1155/2009/769568
CR	replaced	72	72	22304	22304	2012<br>,, 2012, EFSA Journal, V10, P2605	2012<br>,, 2012, EFSA J, V10, P2605

Record 143 | WOS:000862185500005

Co-occurrence of mycotoxins: A review on bioanalytical methods for simultaneous analysis in human biological samples, mixture toxicity and risk assessment strategies

SO original: TOXICON | SO normalised: TOXICON | CR count: 116 → 116

Field	Status	Orig. pos	Norm. pos	Orig. line	Norm. line	Original cited/source value	Normalised cited/source value
CR	replaced	55	55	22437	22437	Mycotoxin Res<br>Knecht A, 2005, Mycotoxin	MYCOTOXIN RES<br>Knecht A, 2005, MYCOTOXIN RES, V21,

						Res, V21, P176, DOI 10.1007/BF02959258	P176, DOI 10.1007/BF02959258

Record 144 | WOS:000857038900001

The Neurotoxic Effect of Ochratoxin-A on the Hippocampal Neurogenic Niche of Adult Mouse Brain

SO original: TOXINS | SO normalised: TOXINS | CR count: 45 → 45

Field	Status	Orig. pos	Norm. pos	Orig. line	Norm. line	Original cited/source value	Normalised cited/source value
CR	replaced	28	28	22609	22609	Oxid Med Cell Longev Pei Xingyao, 2021, Oxid Med Cell Longev, V2021, P9967334, DOI [10.1155/2021/9967334, 10.1155/2021/9967334]	OXID MED CELL LONGEV Pei Xingyao, 2021, OXID MED CELL LONGEV, V2021, P9967334, DOI [10.1155/2021/9967334, 10.1155/2021/9967334]

Record 145 | WOS:000859602700001

Ochratoxin A and Citrinin Differentially Modulate Bovine Mammary Epithelial Cell Permeability and Innate Immune Function

SO original: TOXINS | SO normalised: TOXINS | CR count: 115 → 115

Field	Status	Orig. pos	Norm. pos	Orig. line	Norm. line	Original cited/source value	Normalised cited/source value
CR	replaced	61	61	22746	22746	Mycotoxin Res Knecht A, 2005, Mycotoxin Res, V21, P176, DOI 10.1007/BF02959258	MYCOTOXIN RES Knecht A, 2005, MYCOTOXIN RES, V21, P176, DOI 10.1007/BF02959258

Record 146 | WOS:000858998000001

Protective activities of ellagic acid and urolithins against kidney toxicity of environmental pollutants: A review

SO original: ENVIRONMENTAL TOXICOLOGY AND PHARMACOLOGY | SO normalised: ENVIRONMENTAL TOXICOLOGY AND PHARMACOLOGY | CR count: 230 → 230

Field	Status	Orig. pos	Norm. pos	Orig. line	Norm. line	Original cited/source value	Normalised cited/source value
CR	replaced	27	27	22887	22887	Nephron Physiol Barbier O., 2005, Nephron Physiol, V99, P105, DOI [10.1159/000083981, DOI 10.1159/000083981]	NEPHRON PHYSIOL Barbier O., 2005, NEPHRON PHYSIOL, V99, P105, DOI [10.1159/000083981, DOI 10.1159/000083981]
CR	replaced	43	43	22903	22903	Oncotarget Chen Linlin, 2018, Oncotarget, V9, P7204, DOI 10.18632/oncotarget.23208	ONCOTARGET Chen Linlin, 2018, ONCOTARGET, V9, P7204, DOI 10.18632/oncotarget.23208

Record 147 | WOS:000836768600001

Metabolic profiling as a powerful tool for the analysis of cellular alterations caused by 20 mycotoxins in HepG2 cells

SO original: ARCHIVES OF TOXICOLOGY | SO normalised: ARCHIVES OF TOXICOLOGY | CR count: 48 → 48

No journal-related changes detected in SO, J9, JI or CR for this record.

Record 148 | WOS:000836539000001

Combined effects of cadmium and ochratoxin A on intestinal barrier dysfunction in human Caco-2 cells and pig small intestinal epithelial cells

SO original: MYCOTOXIN RESEARCH | SO normalised: MYCOTOXIN RESEARCH | CR count: 50 → 50

No journal-related changes detected in SO, J9, JI or CR for this record.

Record 149 | WOS:000827733300003

Antifungal and antimycotoxic activities of 3 essential oils against 3 mycotoxinogenic fungi

SO original: ARCHIVES OF MICROBIOLOGY | SO normalised: ARCHIVES OF MICROBIOLOGY | CR count: 56 → 56

No journal-related changes detected in SO, J9, JI or CR for this record.

Record 150 | WOS:000845596200001

Evaluation of the Safety and Ochratoxin A Degradation Capacity of Pediococcus pentosaceus as a Dietary Probiotic with Molecular Docking Approach and Pharmacokinetic Toxicity Assessment

SO original: INTERNATIONAL JOURNAL OF MOLECULAR SCIENCES | SO normalised: INTERNATIONAL JOURNAL OF MOLECULAR SCIENCES | CR count: 39 → 39

**Field**	**Status**	**Orig. pos**	**Norm. pos**	**Orig. line**	**Norm. line**	**Original cited/source value**	**Normalised cited/source value**
CR	replaced	12	12	23522	23522	EFSA Journal<br>European Food Safety Authority, 2020, EFSA Journal, V18	EFSA J<br>European Food Safety Authority, 2020, EFSA J, V18

Record 151 | WOS:000810935900001

Paper The multiple biotoxicity integrated study in grass carp (Ctenopharyngodon idella) caused by Ochratoxin A: Oxidative damage, apoptosis and immunosuppression

SO original: JOURNAL OF HAZARDOUS MATERIALS | SO normalised: JOURNAL OF HAZARDOUS MATERIALS | CR count: 123 → 123

No journal-related changes detected in SO, J9, JI or CR for this record.

Record 152 | WOS:000807511200002

Nutritional impact of mycotoxins in food animal production and strategies for mitigation

SO original: JOURNAL OF ANIMAL SCIENCE AND BIOTECHNOLOGY | SO normalised: JOURNAL OF ANIMAL SCIENCE AND BIOTECHNOLOGY | CR count: 203 → 203

Field	Status	Orig. pos	Norm. pos	Orig. line	Norm. line	Original cited/source value	Normalised cited/source value
CR	replaced	44	44	23869	23869	EFSA Journal<br>European Food Safety Authority (EFSA), 2010, EFSA Journal, V8, P1693, DOI DOI 10.2903/J.EFSA.2010.1693	EFSA J<br>European Food Safety Authority (EFSA), 2010, EFSA J, V8, P1693, DOI DOI 10.2903/J.EFSA.2010.1693

Record 153 | WOS:000818403700001

Genotoxicity of 12 Mycotoxins by the SOS/umu Test: Comparison of Liver and Kidney S9 Fraction

SO original: TOXINS | SO normalised: TOXINS | CR count: 63 → 63

No journal-related changes detected in SO, J9, JI or CR for this record.

Record 154 | WOS:000819631900001

Ochratoxin A and Kidney Oxidative Stress: The Role of Nutraceuticals in Veterinary Medicine-A Review

SO original: TOXINS | SO normalised: TOXINS | CR count: 107 → 107

Field	Status	Orig. pos	Norm. pos	Orig. line	Norm. line	Original cited/source value	Normalised cited/source value
CR	replaced	70	70	24291	24291	FORUM NUTR<br>Miyashita K, 2009, FORUM NUTR, V61, P136, DOI 10.1159/000212746	Forum Nutr<br>Miyashita K, 2009, Forum Nutr, V61, P136, DOI 10.1159/000212746
CR	replaced	85	85	24306	24306	Mycotoxin Res<br>Raters M, 2008, Mycotoxin Res, V24, P130, DOI 10.1007/BF03032339	MYCOTOXIN RES<br>Raters M, 2008, MYCOTOXIN RES, V24, P130, DOI 10.1007/BF03032339

Record 155 | WOS:000806405700007

In vitro mycotoxin binding capacities of clays, glucomannan and their combinations

SO original: TOXICON | SO normalised: TOXICON | CR count: 68 → 68

Field	Status	Orig. pos	Norm. pos	Orig. line	Norm. line	Original cited/source value	Normalised cited/source value
CR	replaced	13	13	24411	24411	Cienc. Rural<br>Batina Patricia Neves, 2005, Cienc. Rural, V35, P826, DOI 10.1590/S0103-84782005000400012	CIENC RURAL<br>Batina Patricia Neves, 2005, CIENC RURAL, V35, P826, DOI 10.1590/S0103-84782005000400012
CR	replaced	21	21	24419	24419	EFSA Journal<br>European Food Safety Authority (EFSA), 2010, EFSA	EFSA J<br>European Food Safety Authority (EFSA), 2010, EFSA

						Journal, V8, P1693, DOI DOI 10.2903/J.EFSA.2010.1693	J, V8, P1693, DOI DOI 10.2903/J.EFSA.2010.1693

Record 156 | WOS:000832788700002

Effectiveness of beetroot extract in SH-SY5Y neuronal cell protection against Fumonisin B1, Ochratoxin A and its combination

SO original: FOOD AND CHEMICAL TOXICOLOGY | SO normalised: FOOD AND CHEMICAL TOXICOLOGY | CR count: 52 → 52

No journal-related changes detected in SO, J9, JI or CR for this record.

Record 157 | WOS:000793099900001

Ochratoxin a induces hepatic fibrosis through TGF-Î² receptor I/Smad2/3 signaling pathway

SO original: ENVIRONMENTAL TOXICOLOGY | SO normalised: ENVIRONMENTAL TOXICOLOGY | CR count: 45 → 45

No journal-related changes detected in SO, J9, JI or CR for this record.

Record 158 | WOS:000801766500001

Degradation of Ochratoxin A by a UV-Mutated Aspergillus niger Strain

SO original: TOXINS | SO normalised: TOXINS | CR count: 52 → 52

No journal-related changes detected in SO, J9, JI or CR for this record.

Record 159 | WOS:000802213700006

Pumpkin extract and fermented whey individually and in combination alleviated AFB1-and OTA-induced alterations on neuronal differentiation in vitro

SO original: FOOD AND CHEMICAL TOXICOLOGY | SO normalised: FOOD AND CHEMICAL TOXICOLOGY | CR count: 87 → 87

No journal-related changes detected in SO, J9, JI or CR for this record.

Record 160 | WOS:000793768800008

Ochratoxin A as an alarming health threat for livestock and human: A review on molecular interactions, mechanism of toxicity, detection, detoxification, and dietary prophylaxis

SO original: TOXICON | SO normalised: TOXICON | CR count: 186 → 186

Field	Status	Orig. pos	Norm. pos	Orig. line	Norm. line	Original cited/source value	Normalised cited/source value
CR	replaced	141	141	25188	25188	Mycotoxin Res Schatzmayr G, 2003, Mycotoxin Res, V19, P124, DOI 10.1007/BF02942950	MYCOTOXIN RES Schatzmayr G, 2003, MYCOTOXIN RES, V19, P124, DOI 10.1007/BF02942950

Record 161 | WOS:000786151200001

Cytotoxicity of Mycotoxins and Their Combinations on Different Cell Lines: A Review

SO original: TOXINS | SO normalised: TOXINS | CR count: 99 → 99

Field	Status	Orig. pos	Norm. pos	Orig. line	Norm. line	Original cited/source value	Normalised cited/source value
CR	replaced	41	41	25330	25330	Mycotoxin Res<br>Knecht A, 2005, Mycotoxin Res, V21, P176, DOI 10.1007/BF02959258	MYCOTOXIN RES<br>Knecht A, 2005, MYCOTOXIN RES, V21, P176, DOI 10.1007/BF02959258
CR	replaced	70	70	25359	25359	Mycotoxin Res<br>Reubel G H, 1987, Mycotoxin Res, V3, P85, DOI 10.1007/BF03191994	MYCOTOXIN RES<br>Reubel G H, 1987, MYCOTOXIN RES, V3, P85, DOI 10.1007/BF03191994

Record 162 | WOS:000784471500001

Mycotoxins in food and feed: toxicity, preventive challenges, and advanced detection techniques for associated diseases

SO original: CRITICAL REVIEWS IN FOOD SCIENCE AND NUTRITION | SO normalised: CRITICAL REVIEWS IN FOOD SCIENCE AND NUTRITION | CR count: 194 → 194

No journal-related changes detected in SO, J9, JI or CR for this record.

Record 163 | WOS:000793342700006

PCV2 infection aggravates OTA-induced immunotoxicity in vivo and in vitro

SO original: ECOTOXICOLOGY AND ENVIRONMENTAL SAFETY | SO normalised: ECOTOXICOLOGY AND ENVIRONMENTAL SAFETY | CR count: 60 → 60

Field	Status	Orig. pos	Norm. pos	Orig. line	Norm. line	Original cited/source value	Normalised cited/source value
CR	replaced	41	41	25782	25782	Mycotoxin Res<br>Martins H Marina, 2012, Mycotoxin Res, V28, P107, DOI 10.1007/s12550-011-0118-3	MYCOTOXIN RES<br>Martins H Marina, 2012, MYCOTOXIN RES, V28, P107, DOI 10.1007/s12550-011-0118-3

Record 164 | WOS:000805486800007

An update on immunotoxicity and mechanisms of action of six environmental mycotoxins

SO original: FOOD AND CHEMICAL TOXICOLOGY | SO normalised: FOOD AND CHEMICAL TOXICOLOGY | CR count: 74 → 74

Field	Status	Orig. pos	Norm. pos	Orig. line	Norm. line	Original cited/source value	Normalised cited/source value
CR	replaced	59	59	25923	25923	Mycotoxin Res<br>Taylor M J, 1985, Mycotoxin Res, V1, P57, DOI 10.1007/BF03192004	MYCOTOXIN RES<br>Taylor M J, 1985, MYCOTOXIN RES, V1, P57, DOI 10.1007/BF03192004

Record 165 | WOS:000774405900001

Ex Vivo and In Vitro Studies Revealed Underlying Mechanisms of Immature Intestinal Inflammatory Responses Caused by Aflatoxin M1 Together with Ochratoxin A

SO original: TOXINS | SO normalised: TOXINS | CR count: 88 → 88

No journal-related changes detected in SO, J9, JI or CR for this record.

Record 166 | WOS:000798517400010

Detoxification of ochratoxin A and zearalenone by Pleurotus ostreatus during in vitro gastrointestinal digestion

SO original: FOOD CHEMISTRY | SO normalised: FOOD CHEMISTRY | CR count: 40 → 40

Field	Status	Orig. pos	Norm. pos	Orig. line	Norm. line	Original cited/source value	Normalised cited/source value
CR	replaced	12	12	26188	26188	Mycotoxin Res<br>Engelhardt G, 2002, Mycotoxin Res, V18, P37, DOI 10.1007/BF02946138	MYCOTOXIN RES<br>Engelhardt G, 2002, MYCOTOXIN RES, V18, P37, DOI 10.1007/BF02946138

Record 167 | WOS:000760242900001

MicroRNA regulates the toxicological mechanism of four mycotoxins in vivo and in vitro

SO original: JOURNAL OF ANIMAL SCIENCE AND BIOTECHNOLOGY | SO normalised: JOURNAL OF ANIMAL SCIENCE AND BIOTECHNOLOGY | CR count: 118 → 118

Field	Status	Orig. pos	Norm. pos	Orig. line	Norm. line	Original cited/source value	Normalised cited/source value
CR	replaced	28	28	26325	26325	Polish Journal of Veterinary Sciences<br>Gajecki M., 2004, Polish Journal of Veterinary Sciences, V7, P153	POL J VET SCI<br>Gajecki M., 2004, POL J VET SCI, V7, P153

Record 168 | WOS:000796935500003

Studies on teratogenic effect of ochratoxin A given via mouldy diet in mice in various sensitive periods of the pregnancy and the putative protection of phenylalanine

SO original: TOXICON | SO normalised: TOXICON | CR count: 41 → 41

No journal-related changes detected in SO, J9, JI or CR for this record.

Record 169 | WOS:000762109100001

Spent Coffee Grounds Valorization as Bioactive Phenolic Source Acquired Antifungal, Anti-Mycotoxigenic, and Anti-Cytotoxic Activities

SO original: TOXINS | SO normalised: TOXINS | CR count: 47 → 47

No journal-related changes detected in SO, J9, JI or CR for this record.

Record 170 | WOS:000762022600001

Metabolomic Analysis Reveals the Mechanisms of Hepatotoxicity Induced by Aflatoxin M1 and Ochratoxin A

SO original: TOXINS | SO normalised: TOXINS | CR count: 63 → 63

Field	Status	Orig. pos	Norm. pos	Orig. line	Norm. line	Original cited/source value	Normalised cited/source value
CR	replaced	4	4	26704	26704	BIOMED ENVIRON SCI Deng ZJ, 2020, BIOMED ENVIRON SCI, V33, P238, DOI 10.3967/bes2020.033	Biomed Environ Sci Deng ZJ, 2020, Biomed Environ Sci, V33, P238, DOI 10.3967/bes2020.033

Record 171 | WOS:000789897500004

A synergism of in silico and statistical approaches to discover new potential endocrine disruptor mycotoxins

SO original: TOXICOLOGY AND APPLIED PHARMACOLOGY | SO normalised: TOXICOLOGY AND APPLIED PHARMACOLOGY | CR count: 42 → 42

Field	Status	Orig. pos	Norm. pos	Orig. line	Norm. line	Original cited/source value	Normalised cited/source value
CR	replaced	6	6	26827	26827	Some Traditional Herbal Medicines [Anonymous], 2002, Some Traditional Herbal Medicines, Some Mycotoxins, Naphthalene and Styrene	Some traditional herbal medicines [Anonymous], 2002, Some traditional herbal medicines, Some Mycotoxins, Naphthalene and Styrene
CR	replaced	34	34	26855	26855	Mycotoxin Res Pfeiffer E, 2007, Mycotoxin Res, V23, P152, DOI 10.1007/BF02951512	MYCOTOXIN RES Pfeiffer E, 2007, MYCOTOXIN RES, V23, P152, DOI 10.1007/BF02951512

Record 172 | WOS:000738826400005

Combined protective effects of icariin and selenomethionine on novel chronic tubulointerstitial nephropathy models in vivo and in vitro

SO original: BRITISH JOURNAL OF NUTRITION | SO normalised: BRITISH JOURNAL OF NUTRITION | CR count: 50 → 50

No journal-related changes detected in SO, J9, JI or CR for this record.

Record 173 | WOS:000742259000001

Subchronic exposure of individual and combined ochratoxin A and citrinin selectively affects the expression of rat renal organic cation transporters

SO original: MYCOTOXIN RESEARCH | SO normalised: MYCOTOXIN RESEARCH | CR count: 43 → 43

Field	Status	Orig. pos	Norm. pos	Orig. line	Norm. line	Original cited/source value	Normalised cited/source value
CR	replaced	17	17	27075	27075	Mycotoxin Res Knecht A, 2005, Mycotoxin	MYCOTOXIN RES Knecht A, 2005, MYCOTOXIN RES, V21,

						Res, V21, P176, DOI 10.1007/BF02959258	P176, DOI 10.1007/BF02959258

Record 174 | WOS:000750801500002

In vitro and in vivo evaluation of AFB1 and OTA-toxicity through immunofluorescence and flow cytometry techniques: A systematic review

SO original: FOOD AND CHEMICAL TOXICOLOGY | SO normalised: FOOD AND CHEMICAL TOXICOLOGY | CR count: 142 → 142

No journal-related changes detected in SO, J9, JI or CR for this record.

Record 175 | WOS:000767939000005

An overview of mycotoxins, their pathogenic effects, foods where they are found and their diagnostic biomarkers

SO original: FOOD SCIENCE AND TECHNOLOGY | SO normalised: FOOD SCIENCE AND TECHNOLOGY | CR count: 107 → 107

Field	Status	Orig. pos	Norm. pos	Orig. line	Norm. line	Original cited/source value	Normalised cited/source value
CR	replaced	47	47	27414	27414	J Vet Diagn Invest Harrison L R, 1990, J Vet Diagn Invest, V2, P217	J VET DIAGN INVEST Harrison L R, 1990, J VET DIAGN INVEST, V2, P217

Record 176 | WOS:000912776700001

Mycotoxin Interactions along the Gastrointestinal Tract: In Vitro Semi-Dynamic Digestion and Static Colonic Fermentation of a Contaminated Meal

SO original: TOXINS | SO normalised: TOXINS | CR count: 57 → 57

Field	Status	Orig. pos	Norm. pos	Orig. line	Norm. line	Original cited/source value	Normalised cited/source value
CR	replaced	44	44	27587	27587	Mycotoxin Res Raters M, 2008, Mycotoxin Res, V24, P130, DOI 10.1007/BF03032339	MYCOTOXIN RES Raters M, 2008, MYCOTOXIN RES, V24, P130, DOI 10.1007/BF03032339

Record 177 | WOS:000786544000007

Adsorption efficiency of sodium & calcium bentonite for ochratoxin A in some Egyptian cheeses: an innovative fortification model, in vitro and in vivo experiments

SO original: WORLD MYCOTOXIN JOURNAL | SO normalised: WORLD MYCOTOXIN JOURNAL | CR count: 62 → 62

Field	Status	Orig. pos	Norm. pos	Orig. line	Norm. line	Original cited/source value	Normalised cited/source value
CR	replaced	5	5	27668	27668	Off J Eur Union [Anonymous], 2006, Off J Eur Union, V229, P7, DOI DOI 10.3390/ECMC2021-11474	OFF J EUR UNION [Anonymous], 2006, OFF J EUR UNION, V229, P7, DOI DOI 10.3390/ECMC2021-11474

Record 178 | WOS:000746960900001

Development of an Extraction Method of Aflatoxins and Ochratoxin A from Oral, Gastric and Intestinal Phases of Digested Bread by In Vitro Model

SO original: TOXINS | SO normalised: TOXINS | CR count: 35 → 35

No journal-related changes detected in SO, J9, JI or CR for this record.

Record 179 | WOS:000891197500003

Gene expression profiling after ochratoxin A treatment in small intestinal epithelial cells from pigs

SO original: JOURNAL OF ANIMAL SCIENCE AND TECHNOLOGY | SO normalised: JOURNAL OF ANIMAL SCIENCE AND TECHNOLOGY | CR count: 39 → 39

No journal-related changes detected in SO, J9, JI or CR for this record.

Record 180 | WOS:000730029700001

Agro-industrial Waste Products as Mycotoxin Biosorbents: A Review of in Vitro and in Vivo Studies

SO original: FOOD REVIEWS INTERNATIONAL | SO normalised: FOOD REVIEWS INTERNATIONAL | CR count: 75 → 75

No journal-related changes detected in SO, J9, JI or CR for this record.

Record 181 | WOS:000740093400002

Ochratoxin A induces NLRP3 inflammasome-mediated pyroptosis via activation of ROS modulated by autophagy in vitro

SO original: ECOTOXICOLOGY AND ENVIRONMENTAL SAFETY | SO normalised: ECOTOXICOLOGY AND ENVIRONMENTAL SAFETY | CR count: 53 → 53

No journal-related changes detected in SO, J9, JI or CR for this record.

Record 182 | WOS:000737483600001

Urinary Biomarkers of Mycotoxin Induced Nephrotoxicity-Current Status and Expected Future Trends

SO original: TOXINS | SO normalised: TOXINS | CR count: 126 → 126

No journal-related changes detected in SO, J9, JI or CR for this record.

Record 183 | WOS:000734651000014

Non-embryotoxic dosage of alternariol aggravates ochratoxin A-triggered deleterious effects on embryonic development through ROS-dependent apoptotic processes

SO original: TOXICOLOGY RESEARCH | SO normalised: TOXICOLOGY RESEARCH | CR count: 56 → 56

No journal-related changes detected in SO, J9, JI or CR for this record.

Record 184 | WOS:000721173800002

The individual and combined effects of ochratoxin A with citrinin and their metabolites (ochratoxin B, ochratoxin C, and dihydrocitrinone) on 2D/3D cell cultures, and zebrafish embryo models

SO original: FOOD AND CHEMICAL TOXICOLOGY | SO normalised: FOOD AND CHEMICAL TOXICOLOGY | CR count: 71 → 71

Field	Status	Orig. pos	Norm. pos	Orig. line	Norm. line	Original cited/source value	Normalised cited/source value
CR	replaced	28	28	28657	28657	Mycotoxin Res<br>Follmann W, 2000, Mycotoxin Res, V16 Suppl 1, P123, DOI 10.1007/BF02942998	MYCOTOXIN RES<br>Follmann W, 2000, MYCOTOXIN RES, V16 Suppl 1, P123, DOI 10.1007/BF02942998
CR	replaced	40	40	28669	28669	Mycotoxin Res<br>Knecht A, 2005, Mycotoxin Res, V21, P176, DOI 10.1007/BF02959258	MYCOTOXIN RES<br>Knecht A, 2005, MYCOTOXIN RES, V21, P176, DOI 10.1007/BF02959258
CR	replaced	42	42	28671	28671	Mycotoxin Res<br>Kohler H, 2002, Mycotoxin Res, V18 Suppl 2, P169, DOI 10.1007/BF02946089	MYCOTOXIN RES<br>Kohler H, 2002, MYCOTOXIN RES, V18 Suppl 2, P169, DOI 10.1007/BF02946089

Record 185 | WOS:000874698700001

Food Mycotoxins: Dietary Interventions Implicated in the Prevention of Mycotoxicosis

SO original: ACS FOOD SCIENCE & TECHNOLOGY | SO normalised: ACS FOOD SCIENCE & TECHNOLOGY | CR count: 194 → 194

Field	Status	Orig. pos	Norm. pos	Orig. line	Norm. line	Original cited/source value	Normalised cited/source value
CR	replaced	158	158	28910	28910	Mycotoxin Res<br>Sugiyama Kei-ichi, 2011, Mycotoxin Res, V27, P281, DOI 10.1007/s12550-011-0105-8	MYCOTOXIN RES<br>Sugiyama Kei-ichi, 2011, MYCOTOXIN RES, V27, P281, DOI 10.1007/s12550-011-0105-8
CR	replaced	165	165	28917	28917	Mycotoxin Res<br>Vekiru E, 2007, Mycotoxin Res, V23, P27, DOI 10.1007/BF02946021	MYCOTOXIN RES<br>Vekiru E, 2007, MYCOTOXIN RES, V23, P27, DOI 10.1007/BF02946021

Record 186 | WOS:000724718300001

Human Proximal Tubule Epithelial Cells (HK-2) as a Sensitive In Vitro System for Ochratoxin A Induced Oxidative Stress

SO original: TOXINS | SO normalised: TOXINS | CR count: 93 → 93

Field	Status	Orig. pos	Norm. pos	Orig. line	Norm. line	Original cited/source value	Normalised cited/source value
CR	replaced	12	12	29024	29024	Biochimica et Biophysica Acta<br>CHANG S E, 1986, Biochimica et Biophysica	BIOCHIM BIOPHYS ACTA<br>CHANG S E, 1986, BIOCHIM BIOPHYS ACTA, V823, P161,

						Acta, V823, P161, DOI 10.1016/0304-419X(86)90001-6	DOI 10.1016/0304-419X(86)90001-6

Record 187 | WOS:000724371000001

Microbiological Decontamination of Mycotoxins: Opportunities and Limitations

SO original: TOXINS | SO normalised: TOXINS | CR count: 150 → 150

Field	Status	Orig. pos	Norm. pos	Orig. line	Norm. line	Original cited/source value	Normalised cited/source value
CR	replaced	13	13	29167	29167	Off. J. Eur. Union [Anonymous], 2012, Off. J. Eur. Union	OFF J EUR UNION [Anonymous], 2012, OFF J EUR UNION
CR	replaced	41	41	29195	29195	Off. J. Eur. Union European Comission, 2015, Off. J. Eur. Union, VL185, P11	OFF J EUR UNION European Comission, 2015, OFF J EUR UNION, VL185, P11
CR	replaced	44	44	29198	29198	Off. J. Eur. Union European Commission, 2019, Off. J. Eur. Union, V293, P2	OFF J EUR UNION European Commission, 2019, OFF J EUR UNION, V293, P2
CR	replaced	108	108	29262	29262	Mycotoxin Res Styriak I, 2001, Mycotoxin Res, V17 Suppl 1, P24, DOI 10.1007/BF03036705	MYCOTOXIN RES Styriak I, 2001, MYCOTOXIN RES, V17 Suppl 1, P24, DOI 10.1007/BF03036705

Record 188 | WOS:000723720700001

Anti-Apoptotic Effect of Flavokawain A on Ochratoxin-A-Induced Endothelial Cell Injury by Attenuation of Oxidative Stress via PI3K/AKT-Mediated Nrf2 Signaling Cascade

SO original: TOXINS | SO normalised: TOXINS | CR count: 40 → 40

No journal-related changes detected in SO, J9, JI or CR for this record.

Record 189 | WOS:000807169400001

Ochratoxin A Induces Steatosis via PPARÎ³-CD36 Axis

SO original: TOXINS | SO normalised: TOXINS | CR count: 39 → 39

No journal-related changes detected in SO, J9, JI or CR for this record.

Record 190 | WOS:000718125600004

Combination of zinc and selenium alleviates ochratoxin A-induced fibrosis via blocking ROS-dependent autophagy in HK-2 cells

SO original: JOURNAL OF TRACE ELEMENTS IN MEDICINE AND BIOLOGY | SO normalised: JOURNAL OF TRACE ELEMENTS IN MEDICINE AND BIOLOGY | CR count: 41 → 41

Field	Status	Orig. pos	Norm. pos	Orig. line	Norm. line	Original cited/source value	Normalised cited/source value

CR	replaced	16	16	29632	29632	INFLAMMOPHARMACOLOGY<br>Latronico T, 2021, INFLAMMOPHARMACOLOGY, V29, P561, DOI 10.1007/s10787-020-00772-w	Inflammopharmacology<br>Latronico T, 2021, Inflammopharmacology, V29, P561, DOI 10.1007/s10787-020-00772-w
CR	replaced	22	22	29638	29638	Mycotoxin Res<br>Makun Hussaini Anthony, 2011, Mycotoxin Res, V27, P97, DOI 10.1007/s12550-010-0080-5	MYCOTOXIN RES<br>Makun Hussaini Anthony, 2011, MYCOTOXIN RES, V27, P97, DOI 10.1007/s12550-010-0080-5

Record 191 | WOS:000709727500001

Selenomethionine alleviated Ochratoxin A induced pyroptosis and renal fibrotic factors expressions in MDCK cells

SO original: JOURNAL OF BIOCHEMICAL AND MOLECULAR TOXICOLOGY | SO normalised: JOURNAL OF BIOCHEMICAL AND MOLECULAR TOXICOLOGY | CR count: 48 → 48

Field	Status	Orig. pos	Norm. pos	Orig. line	Norm. line	Original cited/source value	Normalised cited/source value
CR	replaced	2	2	29734	29734	Nephron Physiol<br>Aukema H M, 2004, Nephron Physiol, V96, pP19, DOI 10.1159/000075576	NEPHRON PHYSIOL<br>Aukema H M, 2004, NEPHRON PHYSIOL, V96, pP19, DOI 10.1159/000075576

Record 192 | WOS:000715414400001

Prioritization of Mycotoxins Based on Their Genotoxic Potential with an In Silico-In Vitro Strategy

SO original: TOXINS | SO normalised: TOXINS | CR count: 93 → 93

No journal-related changes detected in SO, J9, JI or CR for this record.

Record 193 | WOS:000714429400001

Ochratoxin A-Induced Nephrotoxicity: Up-to-Date Evidence

SO original: INTERNATIONAL JOURNAL OF MOLECULAR SCIENCES | SO normalised: INTERNATIONAL JOURNAL OF MOLECULAR SCIENCES | CR count: 115 → 115

Field	Status	Orig. pos	Norm. pos	Orig. line	Norm. line	Original cited/source value	Normalised cited/source value
CR	replaced	75	75	30083	30083	Mycotoxin Res<br>Ostry V, 2005, Mycotoxin Res, V21, P49, DOI 10.1007/BF02954817	MYCOTOXIN RES<br>Ostry V, 2005, MYCOTOXIN RES, V21, P49, DOI 10.1007/BF02954817

Record 194 | WOS:000726706400001

The role of pumpkin pulp extract carotenoids against mycotoxin damage in the blood brain barrier in vitro

SO original: ARHIV ZA HIGIJENU RADA I TOKSIKOLOGIJU-ARCHIVES OF INDUSTRIAL HYGIENE ; AND TOXICOLOGY | SO normalised: ARHIV ZA HIGIJENU RADA I TOKSIKOLOGIJU-ARCHIVES OF INDUSTRIAL HYGIENE ; AND TOXICOLOGY | CR count: 34 → 34

No journal-related changes detected in SO, J9, JI or CR for this record.

Record 195 | WOS:000714098000254

Evaluation of larval zebrafish as an alternative whole-animal model for nephrotoxicity testing

SO original: TOXICOLOGY LETTERS | SO normalised: TOXICOLOGY LETTERS | CR count: 0 → 0

No journal-related changes detected in SO, J9, JI or CR for this record.

Record 196 | WOS:000701125500001

The Reduction of the Combined Effects of Aflatoxin and Ochratoxin A in Piglet Livers and Kidneys by Dietary Antioxidants

SO original: TOXINS | SO normalised: TOXINS | CR count: 115 → 115

Field	Status	Orig. pos	Norm. pos	Orig. line	Norm. line	Original cited/source value	Normalised cited/source value
CR	replaced	19	19	30389	30389	Methods Mol Biol<br>Chang Thomas K H, 2006, Methods Mol Biol, V320, P127	METHODS MOL BIOL<br>Chang Thomas K H, 2006, METHODS MOL BIOL, V320, P127

Record 197 | WOS:000689864300001

Cytotoxicity of Mycotoxins Frequently Present in Aquafeeds to the Fish Cell Line RTGill-W1

SO original: TOXINS | SO normalised: TOXINS | CR count: 57 → 57

No journal-related changes detected in SO, J9, JI or CR for this record.

Record 198 | WOS:000688617200001

Curcumin Modulates Nitrosative Stress, Inflammation, and DNA Damage and Protects against Ochratoxin A-Induced Hepatotoxicity and Nephrotoxicity in Rats

SO original: ANTIOXIDANTS | SO normalised: ANTIOXIDANTS | CR count: 70 → 70

No journal-related changes detected in SO, J9, JI or CR for this record.

Record 199 | WOS:000681272600001

Hepatotoxicity of food-borne mycotoxins: molecular mechanism, anti-hepatotoxic medicines and target prediction

SO original: CRITICAL REVIEWS IN FOOD SCIENCE AND NUTRITION | SO normalised: CRITICAL REVIEWS IN FOOD SCIENCE AND NUTRITION | CR count: 192 → 192

Field	Status	Orig. pos	Norm. pos	Orig. line	Norm. line	Original cited/source value	Normalised cited/source value
CR	replaced	137	137	30958	30958	J Appl Toxicol<br>Salah-Abbes Jalila Ben, 2008, J	J APPL TOXICOL<br>Salah-Abbes Jalila Ben, 2008, J

						Appl Toxicol, V28, P6, DOI 10.1002/jat.1240	APPL TOXICOL, V28, P6, DOI 10.1002/jat.1240

Record 200 | WOS:000674669100002

Metabolomics analysis underlay mechanisms in the renal impairment of mice caused by combination of aflatoxin M1 and ochratoxin A

SO original: TOXICOLOGY | SO normalised: TOXICOLOGY | CR count: 75 → 75

No journal-related changes detected in SO, J9, JI or CR for this record.

Record 201 | WOS:000675540600002

Ochratoxin A induces ERK1/2 phosphorylation-dependent apoptosis through NF-KB/ERK axis in human proximal tubule HK-2 cell line

SO original: TOXICON | SO normalised: TOXICON | CR count: 44 → 44

Field	Status	Orig. pos	Norm. pos	Orig. line	Norm. line	Original cited/source value	Normalised cited/source value
CR	replaced	4	4	31229	31229	Arch Tierernahr Bohm J, 1992, Arch Tierernahr, V42, P95	ARCH TIERERNAHR Bohm J, 1992, ARCH TIERERNAHR, V42, P95

Record 202 | WOS:000656878400004

Mycotoxins in human food: A challenge for research

SO original: CAHIERS DE NUTRITION ET DE DIETETIQUE | SO normalised: CAHIERS DE NUTRITION ET DE DIETETIQUE | CR count: 125 → 125

No journal-related changes detected in SO, J9, JI or CR for this record.

Record 203 | WOS:000661314200027

In vitro blood brain barrier exposure to mycotoxins and carotenoids pumpkin extract alters mitochondrial gene expression and oxidative stress

SO original: FOOD AND CHEMICAL TOXICOLOGY | SO normalised: FOOD AND CHEMICAL TOXICOLOGY | CR count: 46 → 46

Field	Status	Orig. pos	Norm. pos	Orig. line	Norm. line	Original cited/source value	Normalised cited/source value
CR	replaced	18	18	31548	31548	Revista de ToxicologÃ-a Gil L, 2016, Revista de ToxicologÃ-a, V33, P50	Revista de toxicologÃ-a Gil L, 2016, Revista de toxicologÃ-a, V33, P50

Record 204 | WOS:000661314200019

Multidimensional analysis of the epigenetic alterations in toxicities induced by mycotoxins

SO original: FOOD AND CHEMICAL TOXICOLOGY | SO normalised: FOOD AND CHEMICAL TOXICOLOGY | CR count: 115 → 115

No journal-related changes detected in SO, J9, JI or CR for this record.

Record 205 | WOS:000659169000010

The effect of ochratoxin A on cytotoxicity and glucose metabolism in human esophageal epithelium Het-1A cells

SO original: TOXICON | SO normalised: TOXICON | CR count: 33 → 33

No journal-related changes detected in SO, J9, JI or CR for this record.

Record 206 | WOS:000664290000006

Non-cytotoxic dosage of fumonisin B1 aggravates ochratoxin A-induced nephrocytotoxicity and apoptosis via ROS-dependent JNK/MAPK signaling pathway

SO original: TOXICOLOGY | SO normalised: TOXICOLOGY | CR count: 48 → 48

Field	Status	Orig. pos	Norm. pos	Orig. line	Norm. line	Original cited/source value	Normalised cited/source value
CR	replaced	33	33	31957	31957	Mycotoxin Res<br>Seefelder W, 2001, Mycotoxin Res, V17 Suppl 1, P107, DOI 10.1007/BF03036723	MYCOTOXIN RES<br>Seefelder W, 2001, MYCOTOXIN RES, V17 Suppl 1, P107, DOI 10.1007/BF03036723

Record 207 | WOS:000654061000009

Oral subchronic exposure to the mycotoxin ochratoxin A induces key pathological features of Parkinson's disease in mice six months after the end of the treatment

SO original: FOOD AND CHEMICAL TOXICOLOGY | SO normalised: FOOD AND CHEMICAL TOXICOLOGY | CR count: 78 → 78

No journal-related changes detected in SO, J9, JI or CR for this record.

Record 208 | WOS:000643671100001

Ochratoxin A Induces Oxidative Stress in HepG2 Cells by Impairing the Gene Expression of Antioxidant Enzymes

SO original: TOXINS | SO normalised: TOXINS | CR count: 57 → 57

No journal-related changes detected in SO, J9, JI or CR for this record.

Record 209 | WOS:000637397900008

Exploration of zebrafish larvae as an alternative whole-animal model for nephrotoxicity testing

SO original: TOXICOLOGY LETTERS | SO normalised: TOXICOLOGY LETTERS | CR count: 85 → 85

Field	Status	Orig. pos	Norm. pos	Orig. line	Norm. line	Original cited/source value	Normalised cited/source value
CR	replaced	4	4	32351	32351	EFSA Journal<br>[Anonymous], 2005, EFSA Journal, V292, P1, DOI DOI 10.2903/J.EFSA.2005.292	EFSA J<br>[Anonymous], 2005, EFSA J, V292, P1, DOI DOI 10.2903/J.EFSA.2005.292

CR	replaced	7	7	32354	32354	Journal of Pharmacological and Toxicological Methods Berghmans Stephane, 2008, Journal of Pharmacological and Toxicological Methods, V58, P59, DOI 10.1016/j.vascn.2008.05.130	J PHARMACOL TOX MET Berghmans Stephane, 2008, J PHARMACOL TOX MET, V58, P59, DOI 10.1016/j.vascn.2008.05.130

Record 210 | WOS:000634193100001

Rat Tumour Histopathology Associated with Experimental Chronic Dietary Exposure to Ochratoxin A in Prediction of the Mycotoxin's Risk for Human Cancers

SO original: TOXINS | SO normalised: TOXINS | CR count: 26 → 26

No journal-related changes detected in SO, J9, JI or CR for this record.

Record 211 | WOS:000634190600001

The Impact of the Nephrotoxin Ochratoxin A on Human Renal Cells Studied by a Novel Co-Culture Model Is Influenced by the Presence of Fibroblasts

SO original: TOXINS | SO normalised: TOXINS | CR count: 39 → 39

No journal-related changes detected in SO, J9, JI or CR for this record.

Record 212 | WOS:000637502500004

Screening of Macrofungi Isolates for Aflatoxin B1 and Ochratoxin A Degradation

SO original: BIOLOGY BULLETIN | SO normalised: BIOLOGY BULLETIN | CR count: 40 → 40

No journal-related changes detected in SO, J9, JI or CR for this record.

Record 213 | WOS:000614680800001

Ochratoxin A induces nephrotoxicity in vitro and in vivo via pyroptosis

SO original: ARCHIVES OF TOXICOLOGY | SO normalised: ARCHIVES OF TOXICOLOGY | CR count: 59 → 59

Field	Status	Orig. pos	Norm. pos	Orig. line	Norm. line	Original cited/source value	Normalised cited/source value
CR	replaced	49	49	32812	32812	Mycotoxin Res Songsermsakul P, 2007, Mycotoxin Res, V23, P65, DOI 10.1007/BF02946027	MYCOTOXIN RES Songsermsakul P, 2007, MYCOTOXIN RES, V23, P65, DOI 10.1007/BF02946027

Record 214 | WOS:000623049400013

Ochratoxin A and its reaction products affected by sugars during heat processing

SO original: FOOD CHEMISTRY | SO normalised: FOOD CHEMISTRY | CR count: 39 → 39

No journal-related changes detected in SO, J9, JI or CR for this record.

Record 215 | WOS:000615712900001

Editorial overview of the special issue on application of tissue chips in toxicology

SO original: TOXICOLOGY | SO normalised: TOXICOLOGY | CR count: 29 → 29

No journal-related changes detected in SO, J9, JI or CR for this record.

Record 216 | WOS:000615712900006

Central role of TRAP1 in the ameliorative effect of oleanolic acid on the mitochondrial-mediated and endoplasmic reticulum stress-excitated apoptosis induced by ochratoxin A

SO original: TOXICOLOGY | SO normalised: TOXICOLOGY | CR count: 57 → 57

No journal-related changes detected in SO, J9, JI or CR for this record.

Record 217 | WOS:000606891900001

Mycotoxins: Impact on Health and Strategies for Prevention and Detoxification in the Food Chain

SO original: FOOD REVIEWS INTERNATIONAL | SO normalised: FOOD REVIEWS INTERNATIONAL | CR count: 239 → 239

Field	Status	Orig. pos	Norm. pos	Orig. line	Norm. line	Original cited/source value	Normalised cited/source value
CR	replaced	3	3	33228	33228	J FOOD PROT<br>Afolabi C. G, 2006, J FOOD PROT, V69	J FOOD PROTECT<br>Afolabi C. G, 2006, J FOOD PROTECT, V69
CR	replaced	6	6	33231	33231	J FOOD PROT<br>Amezqueta S, 2008, J FOOD PROT, V71	J FOOD PROTECT<br>Amezqueta S, 2008, J FOOD PROTECT, V71
CR	replaced	14	14	33239	33239	Cancer Biol Ther<br>[Anonymous], 2005, Cancer Biol Ther, DOI DOI 10.4161/CBT.4.8.2034	CANCER BIOL THER<br>[Anonymous], 2005, CANCER BIOL THER, DOI DOI 10.4161/CBT.4.8.2034
CR	replaced	28	28	33253	33253	Food Research International<br>Bajpai V. K., 2012, Food Research International, V45, P722, DOI 10.1016/j.foodres.2011.04.052	FOOD RES INT<br>Bajpai V. K., 2012, FOOD RES INT, V45, P722, DOI 10.1016/j.foodres.2011.04.052
CR	replaced	41	41	33266	33266	J FOOD PROT<br>Bueno D. J, 2007, J FOOD PROT, V70, P2148	J FOOD PROTECT<br>Bueno D. J, 2007, J FOOD PROTECT, V70, P2148
CR	replaced	94	94	33319	33319	Mycotoxin Factbook: Food & Feed Topics<br>Hammond B, 2006, Mycotoxin Factbook: Food & Feed Topics, P199	MYCOTOXIN FACTBOOK: FOOD & FEED TOPICS<br>Hammond B, 2006, MYCOTOXIN FACTBOOK: FOOD & FEED TOPICS, P199
CR	replaced	162	162	33387	33387	Phytopathology<br>Paul P.A., 2008, Phytopathology, V98	PHYTOPATHOLOGY<br>Paul P.A., 2008, PHYTOPATHOLOGY, V98

CR	replaced	227	227	33452	33452	Plant Dis<br>Willyerd K.T., 2012, Plant Dis, V96	PLANT DIS<br>Willyerd K.T., 2012, PLANT DIS, V96

Record 218 | WOS:000638197700008

Comet assay: a versatile but complex tool in genotoxicity testing

SO original: TOXICOLOGY RESEARCH | SO normalised: TOXICOLOGY RESEARCH | CR count: 100 → 100

Field	Status	Orig. pos	Norm. pos	Orig. line	Norm. line	Original cited/source value	Normalised cited/source value
CR	replaced	32	32	33548	33548	EFSA Journal<br>EFSA (European Food Safety Authority), 2020, EFSA Journal, V18, P6113	EFSA J<br>EFSA (European Food Safety Authority), 2020, EFSA J, V18, P6113
CR	replaced	47	47	33563	33563	Exp Suppl<br>Henkler Frank, 2012, Exp Suppl, V101, P107, DOI 10.1007/978-3-7643-8340-4_5	EXPERIENTIA SUPPL<br>Henkler Frank, 2012, EXPERIENTIA SUPPL, V101, P107, DOI 10.1007/978-3-7643-8340-4_5
CR	replaced	100	100	33616	33616	2012<br>,, 2012, EFSA Journal, V10, P2578	2012<br>,, 2012, EFSA J, V10, P2578

Record 219 | WOS:000701996600001

Aspergillus derived mycotoxins in food and the environment: Prevalence, detection, and toxicity

SO original: TOXICOLOGY REPORTS | SO normalised: TOXICOLOGY REPORTS | CR count: 296 → 296

No journal-related changes detected in SO, J9, JI or CR for this record.

Record 220 | WOS:000670072600004

Neurotoxicity mechanism of Ochratoxin A

SO original: QUALITY ASSURANCE AND SAFETY OF CROPS & FOODS | SO normalised: QUALITY ASSURANCE AND SAFETY OF CROPS & FOODS | CR count: 89 → 89

Field	Status	Orig. pos	Norm. pos	Orig. line	Norm. line	Original cited/source value	Normalised cited/source value
CR	replaced	20	20	34051	34051	Mycotoxin Res<br>Czerwiecki L, 2001, Mycotoxin Res, V17 Suppl 2, P125, DOI 10.1007/BF03036419	MYCOTOXIN RES<br>Czerwiecki L, 2001, MYCOTOXIN RES, V17 Suppl 2, P125, DOI 10.1007/BF03036419
CR	replaced	29	29	34060	34060	Mycotoxin Res<br>Fuchs E, 2001, Mycotoxin Res, V17 Suppl 2, P142, DOI 10.1007/BF03036423	MYCOTOXIN RES<br>Fuchs E, 2001, MYCOTOXIN RES, V17 Suppl 2, P142, DOI 10.1007/BF03036423
CR	replaced	43	43	34074	34074	Mycotoxin Res<br>Kohler H, 2002, Mycotoxin Res, V18 Suppl 2, P169, DOI 10.1007/BF02946089	MYCOTOXIN RES<br>Kohler H, 2002, MYCOTOXIN RES, V18

							Suppl 2, P169, DOI 10.1007/BF02946089
CR	replaced	57	57	34088	34088	Mycotoxin Res Otteneder H, 2000, Mycotoxin Res, V16 Suppl 1, P131, DOI 10.1007/BF02943000	MYCOTOXIN RES Otteneder H, 2000, MYCOTOXIN RES, V16 Suppl 1, P131, DOI 10.1007/BF02943000
CR	replaced	83	83	34114	34114	Mycotoxin Res Weidenbach A, 2000, Mycotoxin Res, V16 Suppl 2, P189, DOI 10.1007/BF02940035	MYCOTOXIN RES Weidenbach A, 2000, MYCOTOXIN RES, V16 Suppl 2, P189, DOI 10.1007/BF02940035

Record 221 | WOS:000603090600001

Mycotoxin and Gut Microbiota Interactions

SO original: TOXINS | SO normalised: TOXINS | CR count: 214 → 214

No journal-related changes detected in SO, J9, JI or CR for this record.

Record 222 | WOS:000597367400001

Ameliorative role of dietary activated carbon against ochratoxin-A induced oxidative damage, suppressed performance and toxicological effects

SO original: TOXIN REVIEWS | SO normalised: TOXIN REVIEWS | CR count: 55 → 55

No journal-related changes detected in SO, J9, JI or CR for this record.

Record 223 | WOS:000584401800010

Transcriptomic alterations induced by aflatoxin B1 and ochratoxin A in LMH cell line

SO original: POULTRY SCIENCE | SO normalised: POULTRY SCIENCE | CR count: 53 → 53

Field	Status	Orig. pos	Norm. pos	Orig. line	Norm. line	Original cited/source value	Normalised cited/source value
CR	replaced	13	13	34579	34579	J Biochem Mol Toxicol Golli-Bennour Emna El, 2010, J Biochem Mol Toxicol, V24, P42, DOI 10.1002/jbt.20310	J BIOCHEM MOL TOXIC Golli-Bennour Emna El, 2010, J BIOCHEM MOL TOXIC, V24, P42, DOI 10.1002/jbt.20310
CR	replaced	49	49	34615	34615	POULT SCI Zeferino C. P., 2016, POULT SCI, V96	POULTRY SCI Zeferino C. P., 2016, POULTRY SCI, V96

Record 224 | WOS:000594353000001

Effects of Single and Repeated Oral Doses of Ochratoxin A on the Lipid Peroxidation and Antioxidant Defense Systems in Mouse Kidneys

SO original: TOXINS | SO normalised: TOXINS | CR count: 56 → 56

No journal-related changes detected in SO, J9, JI or CR for this record.

Record 225 | WOS:000580526300009

Microphysiological system modeling of ochratoxin A-associated nephrotoxicity

SO original: TOXICOLOGY | SO normalised: TOXICOLOGY | CR count: 44 → 44

Field	Status	Orig. pos	Norm. pos	Orig. line	Norm. line	Original cited/source value	Normalised cited/source value
CR	replaced	27	27	34898	34898	Mycotoxin Res<br>Lebrun Stefan, 2002, Mycotoxin Res, V18 Suppl 2, P173, DOI 10.1007/BF02946090	MYCOTOXIN RES<br>Lebrun Stefan, 2002, MYCOTOXIN RES, V18 Suppl 2, P173, DOI 10.1007/BF02946090

Record 226 | WOS:000582386000020

Ochratoxins in food and feed: Occurrence and its impact on human health and management strategies

SO original: TOXICON | SO normalised: TOXICON | CR count: 228 → 228

Field	Status	Orig. pos	Norm. pos	Orig. line	Norm. line	Original cited/source value	Normalised cited/source value
CR	replaced	17	17	35014	35014	African Journal of Biotechnology<br>Bankole S. A., 2003, African Journal of Biotechnology, V2, P254	AFR J BIOTECHNOL<br>Bankole S. A., 2003, AFR J BIOTECHNOL, V2, P254
CR	replaced	35	35	35032	35032	Nat Toxins<br>Boswald C, 1995, Nat Toxins, V3, P138, DOI 10.1002/nt.2620030304	NAT TOXINS<br>Boswald C, 1995, NAT TOXINS, V3, P138, DOI 10.1002/nt.2620030304
CR	replaced	74	74	35071	35071	Mycotoxin Res<br>Geisen R, 2006, Mycotoxin Res, V22, P134, DOI 10.1007/BF02956777	MYCOTOXIN RES<br>Geisen R, 2006, MYCOTOXIN RES, V22, P134, DOI 10.1007/BF02956777
CR	replaced	101	101	35098	35098	Mycotoxin Res<br>Kotowski K, 2000, Mycotoxin Res, V16, P66, DOI 10.1007/BF02946106	MYCOTOXIN RES<br>Kotowski K, 2000, MYCOTOXIN RES, V16, P66, DOI 10.1007/BF02946106
CR	replaced	132	132	35129	35129	Nat Toxins<br>Muller H M, 1998, Nat Toxins, V6, P251, DOI 10.1002/(SICI)1522-7189(199811/12)6:6<251::AID-NT35>3.0.CO;2-P	NAT TOXINS<br>Muller H M, 1998, NAT TOXINS, V6, P251, DOI 10.1002/(SICI)1522-7189(199811/12)6:6<251::AID-NT35>3.0.CO;2-P
CR	replaced	157	157	35154	35154	Mycotoxin Res<br>Ruhland M, 1997, Mycotoxin Res, V13, P54, DOI 10.1007/BF02945066	MYCOTOXIN RES<br>Ruhland M, 1997, MYCOTOXIN RES, V13, P54, DOI 10.1007/BF02945066
CR	replaced	158	158	35155	35155	Mycotoxin Res<br>Ruhland M, 1996, Mycotoxin	MYCOTOXIN RES<br>Ruhland M, 1996,

						Res, V12, P7, DOI 10.1007/BF03192075	MYCOTOXIN RES, V12, P7, DOI 10.1007/BF03192075

Record 227 | WOS:000584874600001

The protective effect of stilbenes resveratrol and pterostilbene individually and combined with mycotoxin citrinin in human adenocarcinoma HT-29 cell line in vitro

SO original: JOURNAL OF ENVIRONMENTAL SCIENCE AND HEALTH PART A-TOXIC/HAZARDOUS ; SUBSTANCES & ENVIRONMENTAL ENGINEERING | SO normalised: JOURNAL OF ENVIRONMENTAL SCIENCE AND HEALTH PART A-TOXIC/HAZARDOUS ; SUBSTANCES & ENVIRONMENTAL ENGINEERING | CR count: 70 → 70

Field	Status	Orig. pos	Norm. pos	Orig. line	Norm. line	Original cited/source value	Normalised cited/source value
CR	replaced	25	25	35317	35317	Int J Microbiol<br>Jeswal Punam, 2015, Int J Microbiol, V2015, P242486, DOI [10.1155/2015/242486, 10.1155/2015/242486]	INT J MICROBIOL<br>Jeswal Punam, 2015, INT J MICROBIOL, V2015, P242486, DOI [10.1155/2015/242486, 10.1155/2015/242486]
CR	replaced	42	42	35334	35334	The Impact of Food Bioactives on Health: In Vitro and Ex Vivo Models<br>Martinez-Maqueda D., 2015, The Impact of Food Bioactives on Health: In Vitro and Ex Vivo Models, DOI [DOI 10.1007/978-3-319-16104-4, 10.1007/978-3-319-16104-4, DOI 10.1007/978-3-319-16104-4_11]	The Impact of Food Bioactives on Health: in vitro and ex vivo models<br>Martinez-Maqueda D., 2015, The Impact of Food Bioactives on Health: in vitro and ex vivo models, DOI [DOI 10.1007/978-3-319-16104-4, 10.1007/978-3-319-16104-4, DOI 10.1007/978-3-319-16104-4_11]

Record 228 | WOS:000563527100012

Cytoprotective effects of curcumin and silymarin on PK-15 cells exposed to ochratoxin A, fumonisin B1 and deoxynivalenol

SO original: TOXICON | SO normalised: TOXICON | CR count: 48 → 48

No journal-related changes detected in SO, J9, JI or CR for this record.

Record 229 | WOS:000563527100013

Genotoxic effects of mycotoxins

SO original: TOXICON | SO normalised: TOXICON | CR count: 129 → 129

Field	Status	Orig. pos	Norm. pos	Orig. line	Norm. line	Original cited/source value	Normalised cited/source value
CR	replaced	36	36	35561	35561	Mycotoxin Res<br>Dutton Michael F, 2009, Mycotoxin Res, V25, P29, DOI 10.1007/s12550-008-0005-8	MYCOTOXIN RES<br>Dutton Michael F, 2009, MYCOTOXIN RES, V25, P29, DOI 10.1007/s12550-008-0005-8

Record 230 | WOS:000579907000025

Ochratoxin A induces reprogramming of glucose metabolism by switching energy metabolism from oxidative phosphorylation to glycolysis in human gastric epithelium GES-1 cells in vitro

SO original: TOXICOLOGY LETTERS | SO normalised: TOXICOLOGY LETTERS | CR count: 44 → 44

No journal-related changes detected in SO, J9, JI or CR for this record.

Record 231 | WOS:000566774200008

Dechlorination and demethylation of ochratoxin A enhance blocking activity of PXR activation, suppress PXR expression and reduce cytotoxicity

SO original: TOXICOLOGY LETTERS | SO normalised: TOXICOLOGY LETTERS | CR count: 57 → 57

No journal-related changes detected in SO, J9, JI or CR for this record.

Record 232 | WOS:000585534200001

The Compromised Intestinal Barrier Induced by Mycotoxins

SO original: TOXINS | SO normalised: TOXINS | CR count: 220 → 220

No journal-related changes detected in SO, J9, JI or CR for this record.

Record 233 | WOS:000564335100008

Larval zebrafish as an in vitro model for evaluating toxicological effects of mycotoxins

SO original: ECOTOXICOLOGY AND ENVIRONMENTAL SAFETY | SO normalised: ECOTOXICOLOGY AND ENVIRONMENTAL SAFETY | CR count: 85 → 85

No journal-related changes detected in SO, J9, JI or CR for this record.

Record 234 | WOS:000585500900001

Toxin Degradation by Rumen Microorganisms: A Review

SO original: TOXINS | SO normalised: TOXINS | CR count: 267 → 267

Field	Status	Orig. pos	Norm. pos	Orig. line	Norm. line	Original cited/source value	Normalised cited/source value
CR	replaced	68	68	36462	36462	Annales de Recherches Veterinaires<br>GALTIER P, 1976, Annales de Recherches Veterinaires, V7, P91	ANN RECH VET<br>GALTIER P, 1976, ANN RECH VET, V7, P91
CR	replaced	113	113	36507	36507	J. Crit. Rev<br>Kaur R., 2015, J. Crit. Rev, V2, P1, DOI DOI 10.31838/JCR.02.01.01	J CRIT REV<br>Kaur R., 2015, J CRIT REV, V2, P1, DOI DOI 10.31838/JCR.02.01.01

CR	replaced	241	241	36635	36635	Asian-Australasian Journal of Animal Sciences Wang R. J., 2005, Asian-Australasian Journal of Animal Sciences, V19, P72	ASIAN AUSTRAL J ANIM Wang R. J., 2005, ASIAN AUSTRAL J ANIM, V19, P72

Record 235 | WOS:000585462600001

Mycotoxins: Biotransformation and Bioavailability Assessment Using Caco-2 Cell Monolayer

SO original: TOXINS | SO normalised: TOXINS | CR count: 247 → 247

Field	Status	Orig. pos	Norm. pos	Orig. line	Norm. line	Original cited/source value	Normalised cited/source value
CR	replaced	29	29	36750	36750	Mycotoxin Res Burkhardt Britta, 2009, Mycotoxin Res, V25, P149, DOI 10.1007/s12550-009-0022-2	MYCOTOXIN RES Burkhardt Britta, 2009, MYCOTOXIN RES, V25, P149, DOI 10.1007/s12550-009-0022-2
CR	replaced	154	154	36875	36875	Mycotoxin Res Pfeiffer E, 2009, Mycotoxin Res, V25, P3, DOI 10.1007/s12550-008-0001-z	MYCOTOXIN RES Pfeiffer E, 2009, MYCOTOXIN RES, V25, P3, DOI 10.1007/s12550-008-0001-z
CR	replaced	155	155	36876	36876	Mycotoxin Res Pfeiffer E, 2008, Mycotoxin Res, V24, P117, DOI 10.1007/BF03032337	MYCOTOXIN RES Pfeiffer E, 2008, MYCOTOXIN RES, V24, P117, DOI 10.1007/BF03032337

Record 236 | WOS:000573807900002

Ochratoxin A affects oocyte maturation and subsequent embryo developmental dynamics in the juvenile sheep model

SO original: MYCOTOXIN RESEARCH | SO normalised: MYCOTOXIN RESEARCH | CR count: 52 → 52

Field	Status	Orig. pos	Norm. pos	Orig. line	Norm. line	Original cited/source value	Normalised cited/source value
CR	replaced	38	38	37098	37098	Mycotoxin Res Munoz Katherine, 2010, Mycotoxin Res, V26, P59, DOI 10.1007/s12550-009-0040-0	MYCOTOXIN RES Munoz Katherine, 2010, MYCOTOXIN RES, V26, P59, DOI 10.1007/s12550-009-0040-0

Record 237 | WOS:000545982700021

E ffects of Ochratoxin A exposure on DNA damage in porcine granulosa cells in vitro

SO original: TOXICOLOGY LETTERS | SO normalised: TOXICOLOGY LETTERS | CR count: 49 → 49

Field	Status	Orig. pos	Norm. pos	Orig. line	Norm. line	Original cited/source value	Normalised cited/source value
CR	replaced	13	13	37193	37193	Mycotoxin Res Degen G H, 2007, Mycotoxin	MYCOTOXIN RES Degen G H, 2007,

						Res, V23, P88, DOI 10.1007/BF02946032	MYCOTOXIN RES, V23, P88, DOI 10.1007/BF02946032

Record 238 | WOS:000568157900004

Multiple-endpoint in vitro carcinogenicity test in human cell line TK6 distinguishes carcinogens from non-carcinogens and highlights mechanisms of action

SO original: ARCHIVES OF TOXICOLOGY | SO normalised: ARCHIVES OF TOXICOLOGY | CR count: 63 → 63

No journal-related changes detected in SO, J9, JI or CR for this record.

Record 239 | WOS:000567625500001

Protective role of lactic acid bacteria and yeasts as dietary carcinogen-binding agents - a review

SO original: CRITICAL REVIEWS IN FOOD SCIENCE AND NUTRITION | SO normalised: CRITICAL REVIEWS IN FOOD SCIENCE AND NUTRITION | CR count: 199 → 199

Field	Status	Orig. pos	Norm. pos	Orig. line	Norm. line	Original cited/source value	Normalised cited/source value
CR	replaced	28	28	37483	37483	Mycotoxin Res<br>Bohm J, 2000, Mycotoxin Res, V16 Suppl 1, P70, DOI 10.1007/BF02942985	MYCOTOXIN RES<br>Bohm J, 2000, MYCOTOXIN RES, V16 Suppl 1, P70, DOI 10.1007/BF02942985

Record 240 | WOS:000562731900014

Ochratoxin A induces glomerular injury through activating the ERK/NF-ÎºB signaling pathway

SO original: FOOD AND CHEMICAL TOXICOLOGY | SO normalised: FOOD AND CHEMICAL TOXICOLOGY | CR count: 40 → 40

No journal-related changes detected in SO, J9, JI or CR for this record.

Record 241 | WOS:000585802200008

Synergistic Interaction of Ochratoxin A and Acrylamide Toxins in Human Kidney and Liver Cells

SO original: BIOLOGICAL & PHARMACEUTICAL BULLETIN | SO normalised: BIOLOGICAL & PHARMACEUTICAL BULLETIN | CR count: 68 → 68

No journal-related changes detected in SO, J9, JI or CR for this record.

Record 242 | WOS:000560097800001

Ochratoxin A presence in Cabernet Sauvignon wine changes antioxidant activityin vitroand oxidative stress markersin vivo

SO original: FOOD ADDITIVES AND CONTAMINANTS PART A-CHEMISTRY ANALYSIS CONTROL ; EXPOSURE & RISK ASSESSMENT | SO normalised: FOOD ADDITIVES AND CONTAMINANTS PART A-CHEMISTRY ANALYSIS CONTROL ; EXPOSURE & RISK ASSESSMENT | CR count: 43 → 43

No journal-related changes detected in SO, J9, JI or CR for this record.

Record 243 | WOS:000539161100006

Mycotoxins, invisible danger of feedstuff with toxic effect on animals

SO original: TOXICON | SO normalised: TOXICON | CR count: 126 → 126

Field	Status	Orig. pos	Norm. pos	Orig. line	Norm. line	Original cited/source value	Normalised cited/source value
CR	replaced	2	2	38096	38096	Arch Tierernahr<br>Abdelhamid A M, 1992, Arch Tierernahr, V42, P63	ARCH TIERERNAHR<br>Abdelhamid A M, 1992, ARCH TIERERNAHR, V42, P63
CR	replaced	74	74	38168	38168	Nat Toxins<br>Muller H M, 1997, Nat Toxins, V5, P24, DOI 10.1002/(SICI)(1997)5:1<24::AID-NT4>3.0.CO;2-#	NAT TOXINS<br>Muller H M, 1997, NAT TOXINS, V5, P24, DOI 10.1002/(SICI)(1997)5:1<24::AID-NT4>3.0.CO;2-#
CR	replaced	78	78	38172	38172	Mycotoxin Res<br>Oldenburg E, 2005, Mycotoxin Res, V21, P105, DOI 10.1007/BF02954430	MYCOTOXIN RES<br>Oldenburg E, 2005, MYCOTOXIN RES, V21, P105, DOI 10.1007/BF02954430
CR	replaced	86	86	38180	38180	Mycotoxin Res<br>Perkowski J, 1990, Mycotoxin Res, V6, P7, DOI 10.1007/BF03192133	MYCOTOXIN RES<br>Perkowski J, 1990, MYCOTOXIN RES, V6, P7, DOI 10.1007/BF03192133
CR	replaced	113	113	38207	38207	Toxicological and Environmental Chemistry<br>Tulayakul Phitsanu, 2006, Toxicological and Environmental Chemistry, V88, P479, DOI 10.1080/02772240600662203	TOXICOL ENVIRON CHEM<br>Tulayakul Phitsanu, 2006, TOXICOL ENVIRON CHEM, V88, P479, DOI 10.1080/02772240600662203
CR	replaced	126	126	38220	38220	Polish Journal of Veterinary Sciences<br>Zwierzchowski W., 2005, Polish Journal of Veterinary Sciences, V8, P209	POL J VET SCI<br>Zwierzchowski W., 2005, POL J VET SCI, V8, P209

Record 244 | WOS:000547359800001

Subchronic exposure to individual and combined ochratoxin A and citrinin affects the expression of rat renal organic anion transporters

SO original: MYCOTOXIN RESEARCH | SO normalised: MYCOTOXIN RESEARCH | CR count: 61 → 61

Field	Status	Orig. pos	Norm. pos	Orig. line	Norm. line	Original cited/source value	Normalised cited/source value
CR	replaced	30	30	38320	38320	Mycotoxin Res<br>Knecht A, 2005, Mycotoxin Res, V21, P176, DOI 10.1007/BF02959258	MYCOTOXIN RES<br>Knecht A, 2005, MYCOTOXIN RES, V21, P176, DOI 10.1007/BF02959258

Record 245 | WOS:000545059200001

Ochratoxin A induces epithelial-to-mesenchymal transition and renal fibrosis through TGF-Î²/Smad2/3 and Wnt1/Î²-catenin signaling pathways in vitro and in vivo

SO original: ARCHIVES OF TOXICOLOGY | SO normalised: ARCHIVES OF TOXICOLOGY | CR count: 61 → 61

No journal-related changes detected in SO, J9, JI or CR for this record.

Record 246 | WOS:000541110800046

Luteolin alleviates ochratoxin A induced oxidative stress by regulating Nrf2 and HIF-1Î± pathways in NRK-52E rat kidney cells

SO original: FOOD AND CHEMICAL TOXICOLOGY | SO normalised: FOOD AND CHEMICAL TOXICOLOGY | CR count: 49 → 49

No journal-related changes detected in SO, J9, JI or CR for this record.

Record 247 | WOS:000556892100001

Reducing Ochratoxin A Content in Grape Pomace by Different Methods

SO original: TOXINS | SO normalised: TOXINS | CR count: 44 → 44

Field	Status	Orig. pos	Norm. pos	Orig. line	Norm. line	Original cited/source value	Normalised cited/source value
CR	replaced	15	15	38683	38683	Mycotoxin Res Deberghes P, 1995, Mycotoxin Res, V11, P37, DOI 10.1007/BF03192060	MYCOTOXIN RES Deberghes P, 1995, MYCOTOXIN RES, V11, P37, DOI 10.1007/BF03192060

Record 248 | WOS:000530851100002

Ochratoxin A causes cell cycle arrest in G1 and G1/S phases through p53 in HK-2 cells

SO original: TOXICON | SO normalised: TOXICON | CR count: 38 → 38

No journal-related changes detected in SO, J9, JI or CR for this record.

Record 249 | WOS:000541267600001

Cytogenotoxic evaluation of the acetonitrile extract, citrinin and dicitrinin-A fromPenicillium citrinum

SO original: DRUG AND CHEMICAL TOXICOLOGY | SO normalised: DRUG AND CHEMICAL TOXICOLOGY | CR count: 85 → 85

No journal-related changes detected in SO, J9, JI or CR for this record.

Record 250 | WOS:000533844900008

Ochratoxin A-induced genotoxic and epigenetic mechanisms lead to Alzheimer disease: its modulation with strategies

SO original: ENVIRONMENTAL SCIENCE AND POLLUTION RESEARCH | SO normalised: ENVIRONMENTAL SCIENCE AND POLLUTION RESEARCH | CR count: 258 → 258

Field	Status	Orig. pos	Norm. pos	Orig. line	Norm. line	Original cited/source value	Normalised cited/source value
CR	replaced	41	41	39095	39095	Mycotoxin Res Bohm J, 2000, Mycotoxin Res,	MYCOTOXIN RES Bohm J, 2000, MYCOTOXIN

						V16 Suppl 1, P70, DOI 10.1007/BF02942985	RES, V16 Suppl 1, P70, DOI 10.1007/BF02942985
CR	replaced	53	53	39107	39107	Int J Environ Res Public Health<br>Creppy Edmond E, 2005, Int J Environ Res Public Health, V2, P186, DOI 10.3390/ijerph2005010186	INT J ENV RES PUB HE<br>Creppy Edmond E, 2005, INT J ENV RES PUB HE, V2, P186, DOI 10.3390/ijerph2005010186
CR	replaced	54	54	39108	39108	Journal of Toxicological Sciences<br>Creppy Edmond Ekue, 1998, Journal of Toxicological Sciences, V23, P165	J TOXICOL SCI<br>Creppy Edmond Ekue, 1998, J TOXICOL SCI, V23, P165
CR	replaced	75	75	39129	39129	Mycotoxin Res<br>Engelhardt G, 2002, Mycotoxin Res, V18, P37, DOI 10.1007/BF02946138	MYCOTOXIN RES<br>Engelhardt G, 2002, MYCOTOXIN RES, V18, P37, DOI 10.1007/BF02946138
CR	replaced	76	76	39130	39130	Mycotoxin Res<br>Essid E, 2011, Mycotoxin Res, V27, P167, DOI 10.1007/s12550-011-0092-9	MYCOTOXIN RES<br>Essid E, 2011, MYCOTOXIN RES, V27, P167, DOI 10.1007/s12550-011-0092-9
CR	replaced	203	203	39257	39257	Mycotoxin Res<br>Schatzmayr G, 2002, Mycotoxin Res, V18 Suppl 2, P183, DOI 10.1007/BF02946092	MYCOTOXIN RES<br>Schatzmayr G, 2002, MYCOTOXIN RES, V18 Suppl 2, P183, DOI 10.1007/BF02946092
CR	replaced	204	204	39258	39258	Mycotoxin Res<br>Schatzmayr G, 2003, Mycotoxin Res, V19, P124, DOI 10.1007/BF02942950	MYCOTOXIN RES<br>Schatzmayr G, 2003, MYCOTOXIN RES, V19, P124, DOI 10.1007/BF02942950
CR	replaced	212	212	39266	39266	Mycotoxin Res<br>Songsermsakul P, 2007, Mycotoxin Res, V23, P65, DOI 10.1007/BF02946027	MYCOTOXIN RES<br>Songsermsakul P, 2007, MYCOTOXIN RES, V23, P65, DOI 10.1007/BF02946027

Record 251 | WOS:000542374900003

Milk thistle (Silybum Marianum) as an antidote or a protective agent against natural or chemical toxicities: a review

SO original: DRUG AND CHEMICAL TOXICOLOGY | SO normalised: DRUG AND CHEMICAL TOXICOLOGY | CR count: 181 → 181

Field	Status	Orig. pos	Norm. pos	Orig. line	Norm. line	Original cited/source value	Normalised cited/source value
CR	replaced	44	44	39417	39417	Mycotoxin Res<br>Essid E, 2011, Mycotoxin Res, V27, P167, DOI 10.1007/s12550-011-0092-9	MYCOTOXIN RES<br>Essid E, 2011, MYCOTOXIN RES, V27, P167, DOI 10.1007/s12550-011-0092-9

Record 252 | WOS:000527365800027

Ochratoxin A exposure causes meiotic failure and oocyte deterioration in mice

SO original: THERIOGENOLOGY | SO normalised: THERIOGENOLOGY | CR count: 75 → 75

No journal-related changes detected in SO, J9, JI or CR for this record.

Record 253 | WOS:000526412300005

Beauvericin and ochratoxin A mycotoxins individually and combined in HepG2 cells alter lipid peroxidation, levels of reactive oxygen species and glutathione

SO original: FOOD AND CHEMICAL TOXICOLOGY | SO normalised: FOOD AND CHEMICAL TOXICOLOGY | CR count: 57 → 57

No journal-related changes detected in SO, J9, JI or CR for this record.

Record 254 | WOS:000537553700018

Risk assessment of ochratoxin A in food

SO original: EFSA JOURNAL | SO normalised: EFSA JOURNAL | CR count: 456 → 456

Field	Status	Orig. pos	Norm. pos	Orig. line	Norm. line	Original cited/source value	Normalised cited/source value
CR	replaced	9	9	39929	39929	Mycotoxin Res<br>Ahn Jongsung, 2010, Mycotoxin Res, V26, P279, DOI 10.1007/s12550-010-0067-2	MYCOTOXIN RES<br>Ahn Jongsung, 2010, MYCOTOXIN RES, V26, P279, DOI 10.1007/s12550-010-0067-2
CR	replaced	37	37	39957	39957	EFSA Journal<br>[Anonymous], 2005, EFSA Journal, P1	EFSA J<br>[Anonymous], 2005, EFSA J, P1
CR	replaced	144	144	40064	40064	Mycotoxin Res<br>Essid E, 2011, Mycotoxin Res, V27, P167, DOI 10.1007/s12550-011-0092-9	MYCOTOXIN RES<br>Essid E, 2011, MYCOTOXIN RES, V27, P167, DOI 10.1007/s12550-011-0092-9
CR	replaced	152	152	40072	40072	Mycotoxin Res<br>Follmann W, 2007, Mycotoxin Res, V23, P101, DOI 10.1007/BF02946034	MYCOTOXIN RES<br>Follmann W, 2007, MYCOTOXIN RES, V23, P101, DOI 10.1007/BF02946034
CR	replaced	308	308	40228	40228	Mycotoxin Res<br>Munoz Katherine, 2010, Mycotoxin Res, V26, P59, DOI 10.1007/s12550-009-0040-0	MYCOTOXIN RES<br>Munoz Katherine, 2010, MYCOTOXIN RES, V26, P59, DOI 10.1007/s12550-009-0040-0
CR	replaced	363	363	40283	40283	Mycotoxin Res<br>Ruhland M, 1997, Mycotoxin Res, V13, P54, DOI 10.1007/BF02945066	MYCOTOXIN RES<br>Ruhland M, 1997, MYCOTOXIN RES, V13, P54, DOI 10.1007/BF02945066

Record 255 | WOS:000505957800037

Aptamer assisted ultrafiltration cleanup with high performance liquid chromatography-fluorescence detector for the determination of OTA in green coffee

SO original: FOOD CHEMISTRY | SO normalised: FOOD CHEMISTRY | CR count: 40 → 40

No journal-related changes detected in SO, J9, JI or CR for this record.

Record 256 | WOS:000526746500001

Protective Effect of Hydroxytyrosol Against Oxidative Stress Induced by the Ochratoxin in Kidney Cells: in vitro and in vivo Study

SO original: FRONTIERS IN VETERINARY SCIENCE | SO normalised: FRONTIERS IN VETERINARY SCIENCE | CR count: 40 → 40

No journal-related changes detected in SO, J9, JI or CR for this record.

Record 257 | WOS:000524713000001

Development of ELISA and Lateral Flow Immunoassays for Ochratoxins (OTA and OTB) Detection Based on Monoclonal Antibody

SO original: FRONTIERS IN CELLULAR AND INFECTION MICROBIOLOGY | SO normalised: FRONTIERS IN CELLULAR AND INFECTION MICROBIOLOGY | CR count: 49 → 49

No journal-related changes detected in SO, J9, JI or CR for this record.

Record 258 | WOS:000530248700129

Protective Effect of Astaxanthin on Ochratoxin A-Induced Kidney Injury to Mice by Regulating Oxidative Stress-Related NRF2/KEAP1 Pathway

SO original: MOLECULES | SO normalised: MOLECULES | CR count: 61 → 61

No journal-related changes detected in SO, J9, JI or CR for this record.

Record 259 | WOS:000517659300003

Toxicity of mycotoxins in vivo on vertebrate organisms: A review

SO original: FOOD AND CHEMICAL TOXICOLOGY | SO normalised: FOOD AND CHEMICAL TOXICOLOGY | CR count: 175 → 175

Field	Status	Orig. pos	Norm. pos	Orig. line	Norm. line	Original cited/source value	Normalised cited/source value
CR	replaced	52	52	40953	40953	Crit. Rev. Food Sci. Nutr.<br>Eskola M., 2019, Crit. Rev. Food Sci. Nutr., P1	CRIT REV FOOD SCI<br>Eskola M., 2019, CRIT REV FOOD SCI, P1

Record 260 | WOS:000526529800024

Influences of Electron Beam Irradiation on the Physical and Chemical Properties of Zearalenone- and Ochratoxin A-Contaminated Corn and In Vivo Toxicity Assessment

SO original: FOODS | SO normalised: FOODS | CR count: 43 → 43

Field	Status	Orig. pos	Norm. pos	Orig. line	Norm. line	Original cited/source value	Normalised cited/source value
CR	replaced	16	16	41182	41182	[æ ¸å å-¦æ ¥<br>Li Chao], 2011, [æ ¸å å-¦æ ¥, Acta Agriculturae Nucleatae Sinica], V25, P959	[é£ å ç§ å-¦<br>Li Chao], 2011, [é£ å ç§ å-¦, Acta Agriculturae Nucleatae Sinica], V25, P959
CR	replaced	25	25	41191	41191	Food Chemistry<br>Oneh Abu J., 2006, Food	FOOD CHEM<br>Oneh Abu J., 2006, FOOD

						Chemistry, V95, P386, DOI 10.1016/j.foodchem.2005.01.008	CHEM, V95, P386, DOI 10.1016/j.foodchem.2005.01.008
CR	replaced	40	40	41206	41206	[é£ å ä¸ æ ºæ¢°<br>[å¼ è ¹ Zhang Ying], 2013, [é£ å ä¸ æ ºæ¢°, Food and Machinery], V29, P236	[é£ å ç§ å-¦<br>[å¼ è ¹ Zhang Ying], 2013, [é£ å ç§ å-¦, Food and Machinery], V29, P236

Record 261 | WOS:000525009500057

In-Vitro Cell Culture for Efficient Assessment of Mycotoxin Exposure, Toxicity and Risk Mitigation

SO original: TOXINS | SO normalised: TOXINS | CR count: 261 → 261

Field	Status	Orig. pos	Norm. pos	Orig. line	Norm. line	Original cited/source value	Normalised cited/source value
CR	replaced	62	62	41343	41343	Mycotoxin Res<br>Dzidic Anamarija, 2010, Mycotoxin Res, V26, P235, DOI 10.1007/s12550-010-0061-8	MYCOTOXIN RES<br>Dzidic Anamarija, 2010, MYCOTOXIN RES, V26, P235, DOI 10.1007/s12550-010-0061-8
CR	replaced	110	110	41391	41391	The Impact of Food Bioactives on Health: In Vitro and Ex Vivo Models<br>Kleiveland CR, 2015, The Impact of Food Bioactives on Health: In Vitro and Ex Vivo Models, P197, DOI [10.1007/978-3-319-16104-4_18, DOI 10.1007/978-3-319-16104-4_18]	The Impact of Food Bioactives on Health: in vitro and ex vivo models<br>Kleiveland CR, 2015, The Impact of Food Bioactives on Health: in vitro and ex vivo models, P197, DOI [10.1007/978-3-319-16104-4_18, DOI 10.1007/978-3-319-16104-4_18]

Record 262 | WOS:000543783800001

Nontoxic concentration of ochratoxin A decreases the dosage of cyclosporine A to induce chronic nephropathy model via autophagy mediated by toll-like receptor 4

SO original: CELL DEATH & DISEASE | SO normalised: CELL DEATH & DISEASE | CR count: 39 → 39

No journal-related changes detected in SO, J9, JI or CR for this record.

Record 263 | WOS:000512092100007

Ochratoxin A induces global DNA hypomethylation and oxidative stress in neuronal cells in vitro

SO original: MYCOTOXIN RESEARCH | SO normalised: MYCOTOXIN RESEARCH | CR count: 43 → 43

No journal-related changes detected in SO, J9, JI or CR for this record.

Record 264 | WOS:000506899900001

Toxicity effects of mycotoxins and autophagy: a mechanistic view

SO original: TOXIN REVIEWS | SO normalised: TOXIN REVIEWS | CR count: 93 → 93

No journal-related changes detected in SO, J9, JI or CR for this record.

Record 265 | WOS:000505295600001

Red orange and lemon extract prevents the renal toxicity induced by ochratoxin A in rats

SO original: JOURNAL OF CELLULAR PHYSIOLOGY | SO normalised: JOURNAL OF CELLULAR PHYSIOLOGY | CR count: 50 → 50

No journal-related changes detected in SO, J9, JI or CR for this record.

Record 266 | WOS:000488786200007

Mycotoxin-induced toxicities and diseases

SO original: NANOMYCOTOXICOLOGY: TREATING MYCOTOXINS IN THE NANO WAY | SO normalised: NANOMYCOTOXICOLOGY: TREATING MYCOTOXINS IN THE NANO WAY | CR count: 238 → 238

Field	Status	Orig. pos	Norm. pos	Orig. line	Norm. line	Original cited/source value	Normalised cited/source value
CR	replaced	76	76	42169	42169	J Biochem Mol Toxicol Golli-Bennour Emna El, 2010, J Biochem Mol Toxicol, V24, P42, DOI 10.1002/jbt.20310	J BIOCHEM MOL TOXIC Golli-Bennour Emna El, 2010, J BIOCHEM MOL TOXIC, V24, P42, DOI 10.1002/jbt.20310

Record 267 | WOS:000680216400001

Mycotoxins Occurrence, Toxicity and Detection Methods

SO original: SUSTAINABLE AGRICULTURE REVIEWS 40 | SO normalised: SUSTAINABLE AGRICULTURE REVIEWS 40 | CR count: 303 → 303

Field	Status	Orig. pos	Norm. pos	Orig. line	Norm. line	Original cited/source value	Normalised cited/source value
CR	replaced	13	13	42395	42395	Braz. J. Microbiol. Almeida-Ferreira Giovanna Caputo, 2013, Braz. J. Microbiol., V44, P371, DOI 10.1590/S1517-83822013005000037	BRAZ J MICROBIOL Almeida-Ferreira Giovanna Caputo, 2013, BRAZ J MICROBIOL, V44, P371, DOI 10.1590/S1517-83822013005000037
CR	replaced	160	160	42542	42542	Mycotoxin Res Makun Hussaini Anthony, 2011, Mycotoxin Res, V27, P97, DOI 10.1007/s12550-010-0080-5	MYCOTOXIN RES Makun Hussaini Anthony, 2011, MYCOTOXIN RES, V27, P97, DOI 10.1007/s12550-010-0080-5

Record 268 | WOS:000504757200032

Melatonin ameliorates ochratoxin A-induced oxidative stress and apoptosis in porcine oocytes

SO original: ENVIRONMENTAL POLLUTION | SO normalised: ENVIRONMENTAL POLLUTION | CR count: 41 → 41

No journal-related changes detected in SO, J9, JI or CR for this record.

Record 269 | WOS:000514650700067

Modelling Fungal Growth, Mycotoxin Production and Release in Grana Cheese

SO original: MICROORGANISMS | SO normalised: MICROORGANISMS | CR count: 40 → 40

No journal-related changes detected in SO, J9, JI or CR for this record.

Record 270 | WOS:000545943400001

Penicillium spp. mycotoxins found in food and feed and their health effects

SO original: WORLD MYCOTOXIN JOURNAL | SO normalised: WORLD MYCOTOXIN JOURNAL | CR count: 167 → 167

Field	Status	Orig. pos	Norm. pos	Orig. line	Norm. line	Original cited/source value	Normalised cited/source value
CR	replaced	80	80	43028	43028	JSM MYCOTOXINS<br>Kawai K., 1983, JSM MYCOTOXINS, V1983, P50, DOI [10.2520/myco1975.1983.50, DOI 10.2520/MYCO1975.1983.50]	JSM Mycotoxins<br>Kawai K., 1983, JSM Mycotoxins, V1983, P50, DOI [10.2520/myco1975.1983.50, DOI 10.2520/MYCO1975.1983.50]
CR	replaced	122	122	43070	43070	JSM MYCOTOXINS<br>Pitt J.I., 2014, JSM MYCOTOXINS, V1988, P159, DOI [10.1007/978-1-4615-0629-4_4, DOI 10.1007/978-1-4615-0629-4_4]	JSM Mycotoxins<br>Pitt J.I., 2014, JSM Mycotoxins, V1988, P159, DOI [10.1007/978-1-4615-0629-4_4, DOI 10.1007/978-1-4615-0629-4_4]
CR	replaced	149	149	43097	43097	Journal of Toxicological Sciences<br>UENO Y, 1978, Journal of Toxicological Sciences, V3, P11, DOI 10.2131/jts.3.11	J TOXICOL SCI<br>UENO Y, 1978, J TOXICOL SCI, V3, P11, DOI 10.2131/jts.3.11
CR	replaced	150	150	43098	43098	Journal of Toxicological Sciences<br>UENO Y, 1980, Journal of Toxicological Sciences, V5, P295, DOI 10.2131/jts.5.295	J TOXICOL SCI<br>UENO Y, 1980, J TOXICOL SCI, V5, P295, DOI 10.2131/jts.5.295

Record 271 | WOS:000514630000034

Comprehensive Evaluation of the Efficiency of Yeast Cell Wall Extract to Adsorb Ochratoxin A and Mitigate Accumulation of the Toxin in Broiler Chickens

SO original: TOXINS | SO normalised: TOXINS | CR count: 45 → 45

Field	Status	Orig. pos	Norm. pos	Orig. line	Norm. line	Original cited/source value	Normalised cited/source value
CR	replaced	2	2	43176	43176	Off J Eur Union<br>[Anonymous], 2006, Off J Eur Union, VL229, P7	OFF J EUR UNION<br>[Anonymous], 2006, OFF J EUR UNION, VL229, P7

CR	replaced	35	35	43209	43209	Braz. J. Poult. Sci.<br>Santin E, 2006, Braz. J. Poult. Sci., V8, P221, DOI 10.1590/S1516-635X2006000400004	BRAZ J POULTRY SCI<br>Santin E, 2006, BRAZ J POULTRY SCI, V8, P221, DOI 10.1590/S1516-635X2006000400004

Record 272 | WOS:000514630000026

Transcriptome Analysis of Ochratoxin A-Induced Apoptosis in Differentiated Caco-2 Cells

SO original: TOXINS | SO normalised: TOXINS | CR count: 87 → 87

No journal-related changes detected in SO, J9, JI or CR for this record.

Record 273 | WOS:000498748700011

Epithelial-fibroblast cross talk aggravates the impact of the nephrotoxin ochratoxin A

SO original: BIOCHIMICA ET BIOPHYSICA ACTA-MOLECULAR CELL RESEARCH | SO normalised: BIOCHIMICA ET BIOPHYSICA ACTA-MOLECULAR CELL RESEARCH | CR count: 52 → 52

No journal-related changes detected in SO, J9, JI or CR for this record.

Record 274 | WOS:000498194100002

Capsaicin, an inhibitor of Ochratoxin A production by Aspergillus section Nigri strains in grapes (Vitis vinifera L.)

SO original: FOOD ADDITIVES AND CONTAMINANTS PART A-CHEMISTRY ANALYSIS CONTROL ; EXPOSURE & RISK ASSESSMENT | SO normalised: FOOD ADDITIVES AND CONTAMINANTS PART A-CHEMISTRY ANALYSIS CONTROL ; EXPOSURE & RISK ASSESSMENT | CR count: 50 → 50

Field	Status	Orig. pos	Norm. pos	Orig. line	Norm. line	Original cited/source value	Normalised cited/source value
CR	replaced	31	31	43593	43593	Int J Microbiol<br>Meimaroglou Dido Maria, 2009, Int J Microbiol, V2009, P842626, DOI 10.1155/2009/842626	INT J MICROBIOL<br>Meimaroglou Dido Maria, 2009, INT J MICROBIOL, V2009, P842626, DOI 10.1155/2009/842626
CR	replaced	39	39	43601	43601	Mycotoxin Res<br>Romero Stella Maris, 2009, Mycotoxin Res, V25, P165, DOI 10.1007/s12550-009-0026-y	MYCOTOXIN RES<br>Romero Stella Maris, 2009, MYCOTOXIN RES, V25, P165, DOI 10.1007/s12550-009-0026-y

Record 275 | WOS:000501604700005

Ochratoxin A Sequentially Activates Autophagy and the Ubiquitin-Proteasome System

SO original: TOXINS | SO normalised: TOXINS | CR count: 65 → 65

No journal-related changes detected in SO, J9, JI or CR for this record.

Record 276 | WOS:000501604700006

Comparative In Vitro Assessment of a Range of Commercial Feed Additives with Multiple Mycotoxin Binding Claims

SO original: TOXINS | SO normalised: TOXINS | CR count: 56 → 56

Field	Status	Orig. pos	Norm. pos	Orig. line	Norm. line	Original cited/source value	Normalised cited/source value
CR	replaced	20	20	43825	43825	Mycotoxin Res<br>Gomaa M N, 1997, Mycotoxin Res, V13, P23, DOI 10.1007/BF02945059	MYCOTOXIN RES<br>Gomaa M N, 1997, MYCOTOXIN RES, V13, P23, DOI 10.1007/BF02945059
CR	replaced	45	45	43850	43850	Mycotoxin Res<br>Raters M, 2008, Mycotoxin Res, V24, P130, DOI 10.1007/BF03032339	MYCOTOXIN RES<br>Raters M, 2008, MYCOTOXIN RES, V24, P130, DOI 10.1007/BF03032339
CR	replaced	51	51	43856	43856	MYCOTOXINS<br>Tengjaroenkul U., 2006, MYCOTOXINS, V2006, P240, DOI [10.2520/myco1975.2006.Suppl4_240, DOI 10.2520/MYCO1975.2006.SUPPL4_240]	Mycotoxins<br>Tengjaroenkul U., 2006, Mycotoxins, V2006, P240, DOI [10.2520/myco1975.2006.Suppl4_240, DOI 10.2520/MYCO1975.2006.SUPPL4_240]
CR	replaced	54	54	43859	43859	Mycotoxin Res<br>Vekiru E, 2007, Mycotoxin Res, V23, P27, DOI 10.1007/BF02946021	MYCOTOXIN RES<br>Vekiru E, 2007, MYCOTOXIN RES, V23, P27, DOI 10.1007/BF02946021

Record 277 | WOS:000477691300004

Reduction of ochratoxin A in oats and rice by twin-screw extrusion processing with baking soda

SO original: FOOD CONTROL | SO normalised: FOOD CONTROL | CR count: 45 → 45

No journal-related changes detected in SO, J9, JI or CR for this record.

Record 278 | WOS:000475915000013

A multi-endpoint approach to the combined toxic effects of patulin and ochratoxin a in human intestinal cells

SO original: TOXICOLOGY LETTERS | SO normalised: TOXICOLOGY LETTERS | CR count: 73 → 73

No journal-related changes detected in SO, J9, JI or CR for this record.

Record 279 | WOS:000484647100041

Impact of dietary Trichosporon mycotoxinivorans on ochratoxin A induced immunotoxicity; In vivo study

SO original: FOOD AND CHEMICAL TOXICOLOGY | SO normalised: FOOD AND CHEMICAL TOXICOLOGY | CR count: 48 → 48

Field	Status	Orig. pos	Norm. pos	Orig. line	Norm. line	Original cited/source value	Normalised cited/source value
CR	replaced	39	39	44229	44229	Veterinarski Arhiv<br>Sakhare Pramodkumar Shrirarn, 2007, Veterinarski Arhiv, V77, P129	VET ARHIV<br>Sakhare Pramodkumar Shrirarn, 2007, VET ARHIV, V77, P129

Record 280 | WOS:000486105900030

In vitro effects of single and binary mixtures of regulated mycotoxins and persistent organochloride pesticides on steroid hormone production in MA-10 Leydig cell line

SO original: TOXICOLOGY IN VITRO | SO normalised: TOXICOLOGY IN VITRO | CR count: 60 → 60

No journal-related changes detected in SO, J9, JI or CR for this record.

Record 281 | WOS:000498064500026

Detecting Neurodevelopmental Toxicity of Domoic Acid and Ochratoxin A Using Rat Fetal Neural Stem Cells

SO original: MARINE DRUGS | SO normalised: MARINE DRUGS | CR count: 64 → 64

Field	Status	Orig. pos	Norm. pos	Orig. line	Norm. line	Original cited/source value	Normalised cited/source value
CR	replaced	55	55	44461	44461	J Toxicol Sci<br>Tohyama Chiharu, 2016, J Toxicol Sci, V41, pSP69, DOI [10.2131/jts.41.sp69, 10.2131/jts.41.SP69]	J TOXICOL SCI<br>Tohyama Chiharu, 2016, J TOXICOL SCI, V41, pSP69, DOI [10.2131/jts.41.sp69, 10.2131/jts.41.SP69]

Record 282 | WOS:000487934500032

Zinc supplementation alleviates OTA-induced oxidative stress and apoptosis in MDCK cells by up-regulating metallothioneins

SO original: LIFE SCIENCES | SO normalised: LIFE SCIENCES | CR count: 45 → 45

Field	Status	Orig. pos	Norm. pos	Orig. line	Norm. line	Original cited/source value	Normalised cited/source value
CR	replaced	3	3	44559	44559	Mycotoxin Res<br>Bohm Josef, 2010, Mycotoxin Res, V26, P147, DOI 10.1007/s12550-010-0049-4	MYCOTOXIN RES<br>Bohm Josef, 2010, MYCOTOXIN RES, V26, P147, DOI 10.1007/s12550-010-0049-4
CR	replaced	36	36	44592	44592	Mycotoxin Res<br>Songsermsakul P, 2007, Mycotoxin Res, V23, P65, DOI 10.1007/BF02946027	MYCOTOXIN RES<br>Songsermsakul P, 2007, MYCOTOXIN RES, V23, P65, DOI 10.1007/BF02946027

Record 283 | WOS:000505648100002

Roles of microRNAs and prospective view of competing endogenous RNAs in mycotoxicosis

SO original: MUTATION RESEARCH-REVIEWS IN MUTATION RESEARCH | SO normalised: MUTATION RESEARCH-REVIEWS IN MUTATION RESEARCH | CR count: 110 → 110

No journal-related changes detected in SO, J9, JI or CR for this record.

Record 284 | WOS:000487079300009

Antifungal activity of selected essential oils against Fusarium culmorum and F. graminearum and their secondary metabolites in wheat seeds

SO original: ARCHIVES OF MICROBIOLOGY | SO normalised: ARCHIVES OF MICROBIOLOGY | CR count: 78 → 78

No journal-related changes detected in SO, J9, JI or CR for this record.

Record 285 | WOS:000475915000016

Annexin A3 may play an important role in ochratoxin-induced malignant transformation of human gastric epithelium cells

SO original: TOXICOLOGY LETTERS | SO normalised: TOXICOLOGY LETTERS | CR count: 40 → 40

No journal-related changes detected in SO, J9, JI or CR for this record.

Record 286 | WOS:000468622000013

Nontoxic concentrations of OTA aggravate DON-induced intestinal barrier dysfunction in IPEC-J2 cells via activation of NF-Îº B signaling pathway

SO original: TOXICOLOGY LETTERS | SO normalised: TOXICOLOGY LETTERS | CR count: 56 → 56

Field	Status	Orig. pos	Norm. pos	Orig. line	Norm. line	Original cited/source value	Normalised cited/source value
CR	replaced	45	45	45145	45145	Mycotoxin Res<br>Stoev Stoycho D, 2010, Mycotoxin Res, V26, P31, DOI 10.1007/s12550-009-0038-7	MYCOTOXIN RES<br>Stoev Stoycho D, 2010, MYCOTOXIN RES, V26, P31, DOI 10.1007/s12550-009-0038-7

Record 287 | WOS:000482246600031

Transport of mycotoxins across human gastric NCI-N87 and intestinal Caco-2 cell models

SO original: FOOD AND CHEMICAL TOXICOLOGY | SO normalised: FOOD AND CHEMICAL TOXICOLOGY | CR count: 60 → 60

Field	Status	Orig. pos	Norm. pos	Orig. line	Norm. line	Original cited/source value	Normalised cited/source value
CR	replaced	54	54	45285	45285	Methods Mol Biol<br>Tavelin Staffan, 2002, Methods Mol Biol, V188, P233	METHODS MOL BIOL<br>Tavelin Staffan, 2002, METHODS MOL BIOL, V188, P233

Record 288 | WOS:000487968300046

Astaxanthin Protects OTA-Induced Lung Injury in Mice through the Nrf2/NF-Îº B Pathway

SO original: TOXINS | SO normalised: TOXINS | CR count: 52 → 52

No journal-related changes detected in SO, J9, JI or CR for this record.

Record 289 | WOS:000487968300014

Heterologous Expression and Characterization of A Novel Ochratoxin A Degrading Enzyme, N-acyl-L-amino Acid Amidohydrolase, from Alcaligenes faecalis

SO original: TOXINS | SO normalised: TOXINS | CR count: 32 → 32

No journal-related changes detected in SO, J9, JI or CR for this record.

Record 290 | WOS:000483690200021

Accuracy, discriminative properties and reliability of a human ESC-based in vitro toxicity assay to distinguish teratogens responsible for neural tube defects

SO original: ARCHIVES OF TOXICOLOGY | SO normalised: ARCHIVES OF TOXICOLOGY | CR count: 37 → 37

No journal-related changes detected in SO, J9, JI or CR for this record.

Record 291 | WOS:000477777500023

Effects of Gamma Irradiation on Ochratoxin A Stability and Cytotoxicity in Methanolic Solutions and Potential Application in Tunisian Millet Samples

SO original: JOURNAL OF FOOD PROTECTION | SO normalised: JOURNAL OF FOOD PROTECTION | CR count: 55 → 55

No journal-related changes detected in SO, J9, JI or CR for this record.

Record 292 | WOS:000478525600001

An in vitro attempt at precision toxicology reveals the involvement of DNA methylation alteration in ochratoxin A-induced G0/G1 phase arrest

SO original: EPIGENETICS | SO normalised: EPIGENETICS | CR count: 62 → 62

No journal-related changes detected in SO, J9, JI or CR for this record.

Record 293 | WOS:000474669600029

Long-Time Instead of Short-Time Exposure in Vitro and Administration in Vivo of Ochratoxin A Is Consistent in Immunosuppression

SO original: JOURNAL OF AGRICULTURAL AND FOOD CHEMISTRY | SO normalised: JOURNAL OF AGRICULTURAL AND FOOD CHEMISTRY | CR count: 48 → 48

No journal-related changes detected in SO, J9, JI or CR for this record.

Record 294 | WOS:000482110000051

Ochratoxin A-Induced Hepatotoxicity through Phase I and Phase II Reactions Regulated by AhR in Liver Cells

SO original: TOXINS | SO normalised: TOXINS | CR count: 51 → 51

Field	Status	Orig. pos	Norm. pos	Orig. line	Norm. line	Original cited/source value	Normalised cited/source value
CR	replaced	30	30	46072	46072	IARC Sci Publ Petkova-Bocharova T, 1991, IARC Sci Publ, P83	IARC SCI PUBL Petkova-Bocharova T, 1991, IARC SCI PUBL, P83

Record 295 | WOS:000475328000001

Assorted Methods for Decontamination of Aflatoxin M1 in Milk Using Microbial Adsorbents

SO original: TOXINS | SO normalised: TOXINS | CR count: 149 → 149

Field	Status	Orig. pos	Norm. pos	Orig. line	Norm. line	Original cited/source value	Normalised cited/source value
CR	replaced	80	80	46242	46242	FOOD SCI TECHNOL Magan N., 2005, FOOD SCI TECHNOL, V40, P337	FOOD SCI TECH-BRAZIL Magan N., 2005, FOOD SCI TECH-BRAZIL, V40, P337

Record 296 | WOS:000475328000038

Cytotoxicity, Genotoxicity and Disturbance of Cell Cycle in HepG2 Cells Exposed to OTA and BEA: Single and Combined Actions

SO original: TOXINS | SO normalised: TOXINS | CR count: 46 → 46

Field	Status	Orig. pos	Norm. pos	Orig. line	Norm. line	Original cited/source value	Normalised cited/source value
CR	replaced	39	39	46412	46412	Toxicological and Environmental Chemistry Ruiz Maria Jose, 2006, Toxicological and Environmental Chemistry, V88, P345, DOI 10.1080/02772240600630622	TOXICOL ENVIRON CHEM Ruiz Maria Jose, 2006, TOXICOL ENVIRON CHEM, V88, P345, DOI 10.1080/02772240600630622

Record 297 | WOS:000475288200030

Long-Term Effects of Ochratoxin A on the Glutathione Redox System and Its Regulation in Chicken

SO original: ANTIOXIDANTS | SO normalised: ANTIOXIDANTS | CR count: 50 → 50

Field	Status	Orig. pos	Norm. pos	Orig. line	Norm. line	Original cited/source value	Normalised cited/source value
CR	replaced	47	47	46536	46536	Am J Clin Pathol WEICHSELBAUM T E, 1946, Am J Clin Pathol, V10, P40	AM J CLIN PATHOL WEICHSELBAUM T E, 1946, AM J CLIN PATHOL, V10, P40

Record 298 | WOS:000461534700004

Effects of medicinal herb and Brazilian traditional plant extracts on in vitro mycotoxin decontamination

SO original: FOOD CONTROL | SO normalised: FOOD CONTROL | CR count: 30 → 30

No journal-related changes detected in SO, J9, JI or CR for this record.

Record 299 | WOS:000465050300026

Ochratoxin A upregulates biomarkers associated with hypoxia and transformation in human kidney cells

SO original: TOXICOLOGY IN VITRO | SO normalised: TOXICOLOGY IN VITRO | CR count: 41 → 41

No journal-related changes detected in SO, J9, JI or CR for this record.

Record 300 | WOS:000475328000057

The In Vivo and In Vitro Toxicokinetics of Citreoviridin Extracted from Penicillium citreonigrum

SO original: TOXINS | SO normalised: TOXINS | CR count: 50 → 50

No journal-related changes detected in SO, J9, JI or CR for this record.

Record 301 | WOS:000463851800006

Biotransformation of plantain pseudostem fibres using local enzyme sources; analysis of their potential as commercial poultry feed

SO original: BIOCATALYSIS AND BIOTRANSFORMATION | SO normalised: BIOCATALYSIS AND BIOTRANSFORMATION | CR count: 60 → 60

Field	Status	Orig. pos	Norm. pos	Orig. line	Norm. line	Original cited/source value	Normalised cited/source value
CR	replaced	5	5	46890	46890	Saudi J Biol Sci<br>Al-Hazmi N A, 2010, Saudi J Biol Sci, V17, P353, DOI 10.1016/j.sjbs.2010.06.006	SAUDI J BIOL SCI<br>Al-Hazmi N A, 2010, SAUDI J BIOL SCI, V17, P353, DOI 10.1016/j.sjbs.2010.06.006

Record 302 | WOS:000472672300064

Degrading Ochratoxin A and Zearalenone Mycotoxins Using a Multifunctional Recombinant Enzyme

SO original: TOXINS | SO normalised: TOXINS | CR count: 42 → 42

Field	Status	Orig. pos	Norm. pos	Orig. line	Norm. line	Original cited/source value	Normalised cited/source value
CR	replaced	4	4	47012	47012	Saudi J Biol Sci<br>Al-Hazmi N A, 2010, Saudi J Biol Sci, V17, P353, DOI 10.1016/j.sjbs.2010.06.006	SAUDI J BIOL SCI<br>Al-Hazmi N A, 2010, SAUDI J BIOL SCI, V17, P353, DOI 10.1016/j.sjbs.2010.06.006

Record 303 | WOS:000469753500299

Interaction of Mycotoxin Alternariol with Serum Albumin

SO original: INTERNATIONAL JOURNAL OF MOLECULAR SCIENCES | SO normalised: INTERNATIONAL JOURNAL OF MOLECULAR SCIENCES | CR count: 60 → 60

Field	Status	Orig. pos	Norm. pos	Orig. line	Norm. line	Original cited/source value	Normalised cited/source value

CR	replaced	60	60	47189	47189	2011<br>,, 2011, EFSA Journal, V9, P2407	2011<br>,, 2011, EFSA J, V9, P2407

Record 304 | WOS:000467512200032

Cytotoxicity against fish and mammalian cell lines and endocrine activity of the mycotoxins beauvericin, deoxynivalenol and ochratoxin-A

SO original: FOOD AND CHEMICAL TOXICOLOGY | SO normalised: FOOD AND CHEMICAL TOXICOLOGY | CR count: 77 → 77

No journal-related changes detected in SO, J9, JI or CR for this record.

Record 305 | WOS:000468044200002

Prevention of ochratoxin A-induced oxidative stress-mediated apoptotic processes and impairment of embryonic development in mouse blastocysts by liquiritigenin

SO original: ENVIRONMENTAL TOXICOLOGY | SO normalised: ENVIRONMENTAL TOXICOLOGY | CR count: 71 → 71

No journal-related changes detected in SO, J9, JI or CR for this record.

Record 306 | WOS:000467299600035

Assessment of Toxic Effects of Ochratoxin A in Human Embryonic Stem Cells

SO original: TOXINS | SO normalised: TOXINS | CR count: 56 → 56

Field	Status	Orig. pos	Norm. pos	Orig. line	Norm. line	Original cited/source value	Normalised cited/source value
CR	replaced	36	36	47578	47578	IARC Sci Publ<br>Petkova-Bocharova T, 1991, IARC Sci Publ, P83	IARC SCI PUBL<br>Petkova-Bocharova T, 1991, IARC SCI PUBL, P83

Record 307 | WOS:000467645200017

Nephrotoxicity instead of immunotoxicity of OTA is induced through DNMT1-dependent activation of JAK2/STAT3 signaling pathway by targeting SOCS3

SO original: ARCHIVES OF TOXICOLOGY | SO normalised: ARCHIVES OF TOXICOLOGY | CR count: 55 → 55

Field	Status	Orig. pos	Norm. pos	Orig. line	Norm. line	Original cited/source value	Normalised cited/source value
CR	replaced	36	36	47704	47704	Mycotoxin Res<br>Martins H Marina, 2012, Mycotoxin Res, V28, P107, DOI 10.1007/s12550-011-0118-3	MYCOTOXIN RES<br>Martins H Marina, 2012, MYCOTOXIN RES, V28, P107, DOI 10.1007/s12550-011-0118-3

Record 308 | WOS:000467299600005

Biomarkers for Exposure as a Tool for Efficacy Testing of a Mycotoxin Detoxifier in Broiler Chickens and Pigs

SO original: TOXINS | SO normalised: TOXINS | CR count: 61 → 61

Field	Status	Orig. pos	Norm. pos	Orig. line	Norm. line	Original cited/source value	Normalised cited/source value
CR	replaced	37	37	47823	47823	Mycotoxin Res<br>Leong Yin-Hui, 2012, Mycotoxin Res, V28, P79, DOI 10.1007/s12550-012-0129-8	MYCOTOXIN RES<br>Leong Yin-Hui, 2012, MYCOTOXIN RES, V28, P79, DOI 10.1007/s12550-012-0129-8
CR	replaced	56	56	47842	47842	Mycotoxin Res<br>Shehata S, 2004, Mycotoxin Res, V20, P42, DOI 10.1007/BF02946709	MYCOTOXIN RES<br>Shehata S, 2004, MYCOTOXIN RES, V20, P42, DOI 10.1007/BF02946709

Record 309 | WOS:000470119800012

Antifungal and antimycotoxigenic effect of Lactobacillus plantarum CRL 778 at different water activity values

SO original: REVISTA ARGENTINA DE MICROBIOLOGIA | SO normalised: REVISTA ARGENTINA DE MICROBIOLOGIA | CR count: 33 → 33

Field	Status	Orig. pos	Norm. pos	Orig. line	Norm. line	Original cited/source value	Normalised cited/source value
CR	replaced	31	31	47941	47941	Mycotoxin Res<br>Stoev Stoycho D, 2010, Mycotoxin Res, V26, P31, DOI 10.1007/s12550-009-0038-7	MYCOTOXIN RES<br>Stoev Stoycho D, 2010, MYCOTOXIN RES, V26, P31, DOI 10.1007/s12550-009-0038-7

Record 310 | WOS:000462049400006

Oxidative stress as a mechanism of combined OTA and CTN toxicity in rat plasma, liver and kidney

SO original: HUMAN & EXPERIMENTAL TOXICOLOGY | SO normalised: HUMAN & EXPERIMENTAL TOXICOLOGY | CR count: 48 → 48

Field	Status	Orig. pos	Norm. pos	Orig. line	Norm. line	Original cited/source value	Normalised cited/source value
CR	replaced	22	22	48028	48028	Mycotoxin Res<br>Knecht A, 2005, Mycotoxin Res, V21, P176, DOI 10.1007/BF02959258	MYCOTOXIN RES<br>Knecht A, 2005, MYCOTOXIN RES, V21, P176, DOI 10.1007/BF02959258

Record 311 | WOS:000467299600032

Fluorescence Spectroscopic Investigation of Competitive Interactions between Quercetin and Aflatoxin B1 for Binding to Human Serum Albumin

SO original: TOXINS | SO normalised: TOXINS | CR count: 58 → 58

No journal-related changes detected in SO, J9, JI or CR for this record.

Record 312 | WOS:000462420300003

Review: Biotechnology of mycotoxins detoxification using microorganisms and enzymes

SO original: TOXICON | SO normalised: TOXICON | CR count: 211 → 211

Field	Status	Orig. pos	Norm. pos	Orig. line	Norm. line	Original cited/source value	Normalised cited/source value
CR	replaced	30	30	48280	48280	Mycotoxin Res<br>Bohm J, 2000, Mycotoxin Res, V16 Suppl 1, P70, DOI 10.1007/BF02942985	MYCOTOXIN RES<br>Bohm J, 2000, MYCOTOXIN RES, V16 Suppl 1, P70, DOI 10.1007/BF02942985
CR	replaced	41	41	48291	48291	Mycotoxin Res<br>Cotty P J, 2006, Mycotoxin Res, V22, P110, DOI 10.1007/BF02956774	MYCOTOXIN RES<br>Cotty P J, 2006, MYCOTOXIN RES, V22, P110, DOI 10.1007/BF02956774
CR	replaced	79	79	48329	48329	J Vet Diagn Invest<br>Harrison L R, 1990, J Vet Diagn Invest, V2, P217	J VET DIAGN INVEST<br>Harrison L R, 1990, J VET DIAGN INVEST, V2, P217

Record 313 | WOS:000460996800027

Ochratoxin A Exposure Impairs Porcine Granulosa Cell Growth via the PI3K/AKT Signaling Pathway

SO original: JOURNAL OF AGRICULTURAL AND FOOD CHEMISTRY | SO normalised: JOURNAL OF AGRICULTURAL AND FOOD CHEMISTRY | CR count: 50 → 50

No journal-related changes detected in SO, J9, JI or CR for this record.

Record 314 | WOS:000465449900001

Cytotoxicity of gamma irradiated aflatoxin B1 and ochratoxin A

SO original: JOURNAL OF ENVIRONMENTAL SCIENCE AND HEALTH PART B-PESTICIDES FOOD ; CONTAMINANTS AND AGRICULTURAL WASTES | SO normalised: JOURNAL OF ENVIRONMENTAL SCIENCE AND HEALTH PART B-PESTICIDES FOOD ; CONTAMINANTS AND AGRICULTURAL WASTES | CR count: 40 → 40

No journal-related changes detected in SO, J9, JI or CR for this record.

Record 315 | WOS:000481430100007

Inhibitory effect of ochratoxin A on DNMT-mediated flocculation of yeast

SO original: MUTAGENESIS | SO normalised: MUTAGENESIS | CR count: 27 → 27

No journal-related changes detected in SO, J9, JI or CR for this record.

Record 316 | WOS:000460801500074

Modulation of Mucin (MUC2, MUC5AC and MUC5B) mRNA Expression and Protein Production and Secretion in Caco-2/HT29-MTX Co-Cultures Following Exposure to Individual and Combined Aflatoxin M1 and Ochratoxin A

SO original: TOXINS | SO normalised: TOXINS | CR count: 55 → 55

No journal-related changes detected in SO, J9, JI or CR for this record.

Record 317 | WOS:000457296600055

Precision toxicology shows that troxerutin alleviates ochratoxin A-induced renal lipotoxicity

SO original: FASEB JOURNAL | SO normalised: FASEB JOURNAL | CR count: 111 → 111

Field	Status	Orig. pos	Norm. pos	Orig. line	Norm. line	Original cited/source value	Normalised cited/source value
CR	replaced	48	48	49035	49035	Nat Rev Nephrol<br>Kirk Rebecca, 2013, Nat Rev Nephrol, V9, P627, DOI 10.1038/nrneph.2013.194	NAT REV NEPHROL<br>Kirk Rebecca, 2013, NAT REV NEPHROL, V9, P627, DOI 10.1038/nrneph.2013.194
CR	replaced	58	58	49045	49045	Lancet<br>Levin Adeera, 2017, Lancet, V390, P1888, DOI 10.1016/S0140-6736(17)30788-2	LANCET<br>Levin Adeera, 2017, LANCET, V390, P1888, DOI 10.1016/S0140-6736(17)30788-2
CR	replaced	83	83	49070	49070	Mycotoxin Res<br>Sauvant C, 2003, Mycotoxin Res, V19, P118, DOI 10.1007/BF02942949	MYCOTOXIN RES<br>Sauvant C, 2003, MYCOTOXIN RES, V19, P118, DOI 10.1007/BF02942949

Record 318 | WOS:000566844400029

Potential Mycotoxin Effects on Coffee Consumers' Health

SO original: COFFEE: CONSUMPTION AND HEALTH IMPLICATIONS | SO normalised: COFFEE: CONSUMPTION AND HEALTH IMPLICATIONS | CR count: 43 → 43

No journal-related changes detected in SO, J9, JI or CR for this record.

Record 319 | WOS:000533508400001

Protective activity of melatonin against mycotoxins-induced toxicity: a review

SO original: TOXICOLOGICAL AND ENVIRONMENTAL CHEMISTRY | SO normalised: TOXICOLOGICAL AND ENVIRONMENTAL CHEMISTRY | CR count: 56 → 56

Field	Status	Orig. pos	Norm. pos	Orig. line	Norm. line	Original cited/source value	Normalised cited/source value
CR	replaced	20	20	49271	49271	Afr J Reprod Health<br>Ibeh I N, 1997, Afr J Reprod Health, V1, P85, DOI 10.2307/3583379	AFR J REPROD HEALTH<br>Ibeh I N, 1997, AFR J REPROD HEALTH, V1, P85, DOI 10.2307/3583379

Record 320 | WOS:000467038800009

OCRATOXINS AND THEIR NEPHROTOXIC POTENTIAL

SO original: REVISTA DE NEFROLOGIA DIALISIS Y TRASPLANTE | SO normalised: REVISTA DE NEFROLOGIA DIALISIS Y TRASPLANTE | CR count: 58 → 58

No journal-related changes detected in SO, J9, JI or CR for this record.

Record 321 | WOS:000576729800009

Mycotoxins in food and feed

SO original: ADVANCES IN FOOD AND NUTRITION RESEARCH, VOL 89 | SO normalised: ADVANCES IN FOOD AND NUTRITION RESEARCH, VOL 89 | CR count: 125 → 125

Field	Status	Orig. pos	Norm. pos	Orig. line	Norm. line	Original cited/source value	Normalised cited/source value
CR	replaced	46	46	49513	49513	Adv Appl Microbiol Hidy P H, 1977, Adv Appl Microbiol, V22, P59, DOI 10.1016/S0065-2164(08)70160-6	ADV APPL MICROBIOL Hidy P H, 1977, ADV APPL MICROBIOL, V22, P59, DOI 10.1016/S0065-2164(08)70160-6

Record 322 | WOS:000463362400007

DNA damage in rat kidneys and liver upon subchronic exposure to single and combined ochratoxin A and citrinin

SO original: WORLD MYCOTOXIN JOURNAL | SO normalised: WORLD MYCOTOXIN JOURNAL | CR count: 56 → 56

Field	Status	Orig. pos	Norm. pos	Orig. line	Norm. line	Original cited/source value	Normalised cited/source value
CR	replaced	5	5	49665	49665	DNA Repair Azqueta A., 2011, DNA Repair, P615, DOI DOI 10.5772/22504	DNA REPAIR Azqueta A., 2011, DNA REPAIR, P615, DOI DOI 10.5772/22504
CR	replaced	13	13	49673	49673	Mol Biotechnol Collins Andrew R, 2004, Mol Biotechnol, V26, P249	MOL BIOTECHNOL Collins Andrew R, 2004, MOL BIOTECHNOL, V26, P249
CR	replaced	56	56	49716	49716	2012 ,, 2012, EFSA Journal, V10, P2605	2012 ,, 2012, EFSA J, V10, P2605

Record 323 | WOS:000456754300029

Sex-dependent gene expression after ochratoxin A insult in F344 rat kidney

SO original: FOOD AND CHEMICAL TOXICOLOGY | SO normalised: FOOD AND CHEMICAL TOXICOLOGY | CR count: 64 → 64

No journal-related changes detected in SO, J9, JI or CR for this record.

Record 324 | WOS:000694027800516

Modulation of intestinal epithelial permeability and mucin mRNA (MUC2, MUC5AC, and MUC5B) expression and protein secretion in Caco-2/HT29-MTX co-cultures exposed to aflatoxin M1, ochratoxin A, and zearalenone individually or collectively.

SO original: JOURNAL OF DAIRY SCIENCE | SO normalised: JOURNAL OF DAIRY SCIENCE | CR count: 0 → 0

No journal-related changes detected in SO, J9, JI or CR for this record.

Record 325 | WOS:000507379000008

The in vivo efficacy of highly porous carbon nanocomposites prepared from sugar beet waste for the Ochratoxin A detoxification

SO original: VETERINARSKI ARHIV | SO normalised: VETERINARSKI ARHIV | CR count: 33 → 33

No journal-related changes detected in SO, J9, JI or CR for this record.

Record 326 | WOS:000452098600001

Persistence of Epigenomic Effects After Recovery From Repeated Treatment With Two Nephrocarcinogens

SO original: FRONTIERS IN GENETICS | SO normalised: FRONTIERS IN GENETICS | CR count: 52 → 52

Field	Status	Orig. pos	Norm. pos	Orig. line	Norm. line	Original cited/source value	Normalised cited/source value
CR	replaced	20	20	50098	50098	IARC Monographs on the Evaluation of Carcinogenic Risks to Humans International Agency for Research on Cancer Weltgesundheitsorganisation IARC, 2018, IARC Monographs on the Evaluation of Carcinogenic Risks to Humans, V114	IARC Monographs on the evaluation of carcinogenic risks to humans International Agency for Research on Cancer Weltgesundheitsorganisation IARC, 2018, IARC Monographs on the evaluation of carcinogenic risks to humans, V114

Record 327 | WOS:000447547900018

Fumonisin production and toxic capacity in airborne black Aspergilli

SO original: TOXICOLOGY IN VITRO | SO normalised: TOXICOLOGY IN VITRO | CR count: 72 → 72

No journal-related changes detected in SO, J9, JI or CR for this record.

Record 328 | WOS:000452587500077

Activation of AMPK-dependent SIRT-1 by astragalus polysaccharide protects against ochratoxin A-induced immune stress in vitro and in vivo

SO original: INTERNATIONAL JOURNAL OF BIOLOGICAL MACROMOLECULES | SO normalised: INTERNATIONAL JOURNAL OF BIOLOGICAL MACROMOLECULES | CR count: 39 → 39

No journal-related changes detected in SO, J9, JI or CR for this record.

Record 329 | WOS:000449893700013

Ochratoxin A induces cytoprotective autophagy via blocking AKT/mTOR signaling pathway in PK-15 cells

SO original: FOOD AND CHEMICAL TOXICOLOGY | SO normalised: FOOD AND CHEMICAL TOXICOLOGY | CR count: 54 → 54

No journal-related changes detected in SO, J9, JI or CR for this record.

Record 330 | WOS:000451238100005

ERK1/2-mediated autophagy is essential for cell survival under Ochratoxin A exposure in IPEC-J2 cells

SO original: TOXICOLOGY AND APPLIED PHARMACOLOGY | SO normalised: TOXICOLOGY AND APPLIED PHARMACOLOGY | CR count: 31 → 31

No journal-related changes detected in SO, J9, JI or CR for this record.

Record 331 | WOS:000449242800003

Mitigation of cell apoptosis induced by ochratoxin A (OTA) is possibly through organic cation transport 2 (OCT2) knockout

SO original: FOOD AND CHEMICAL TOXICOLOGY | SO normalised: FOOD AND CHEMICAL TOXICOLOGY | CR count: 34 → 34

Field	Status	Orig. pos	Norm. pos	Orig. line	Norm. line	Original cited/source value	Normalised cited/source value
CR	replaced	11	11	50690	50690	J Biochem Mol Toxicol Golli-Bennour Emna El, 2010, J Biochem Mol Toxicol, V24, P42, DOI 10.1002/jbt.20310	J BIOCHEM MOL TOXIC Golli-Bennour Emna El, 2010, J BIOCHEM MOL TOXIC, V24, P42, DOI 10.1002/jbt.20310

Record 332 | WOS:000446150300012

OTA induces intestinal epithelial barrier dysfunction and tight junction disruption in IPEC-J2 cells through ROS/Ca2+-mediated MLCK activation

SO original: ENVIRONMENTAL POLLUTION | SO normalised: ENVIRONMENTAL POLLUTION | CR count: 46 → 46

No journal-related changes detected in SO, J9, JI or CR for this record.

Record 333 | WOS:000440879500026

Mechanism and effects of Zearalenone on mouse T lymphocytes activation in vitro

SO original: ECOTOXICOLOGY AND ENVIRONMENTAL SAFETY | SO normalised: ECOTOXICOLOGY AND ENVIRONMENTAL SAFETY | CR count: 43 → 43

No journal-related changes detected in SO, J9, JI or CR for this record.

Record 334 | WOS:000448628600032

The registration of aptamer-ligand (ochratoxin A) interactions based on ligand fluorescence changes

SO original: BIOCHEMICAL AND BIOPHYSICAL RESEARCH COMMUNICATIONS | SO normalised: BIOCHEMICAL AND BIOPHYSICAL RESEARCH COMMUNICATIONS | CR count: 35 → 35

No journal-related changes detected in SO, J9, JI or CR for this record.

Record 335 | WOS:000447114600010

Dietary L-carnitine and vitamin-E; a strategy to combat ochratoxin-A induced immunosuppression

SO original: TOXICON | SO normalised: TOXICON | CR count: 43 → 43

No journal-related changes detected in SO, J9, JI or CR for this record.

Record 336 | WOS:000453415000007

In Vivo Detoxification of Ochratoxin A by Highly Porous Magnetic Nanocomposites Prepared from Coconut Shell

SO original: BRAZILIAN JOURNAL OF POULTRY SCIENCE | SO normalised: BRAZILIAN JOURNAL OF POULTRY SCIENCE | CR count: 35 → 35

No journal-related changes detected in SO, J9, JI or CR for this record.

Record 337 | WOS:000442067900032

Research progress on the raw and modified montmorillonites as adsorbents for mycotoxins: A review

SO original: APPLIED CLAY SCIENCE | SO normalised: APPLIED CLAY SCIENCE | CR count: 143 → 142

Field	Status	Orig. pos	Norm. pos	Orig. line	Norm. line	Original cited/source value	Normalised cited/source value
CR	replaced	22	22	51334	51334	Mycotoxin Res<br>Broggi L E, 2007, Mycotoxin Res, V23, P59, DOI 10.1007/BF02946026	MYCOTOXIN RES<br>Broggi L E, 2007, MYCOTOXIN RES, V23, P59, DOI 10.1007/BF02946026
CR	replaced	36	36	51348	51348	Mycopathologia<br>Diaz Duarte E, 2002, Mycopathologia, V156, P223, DOI 10.1023/A:1023388321713	MYCOPATHOLOGIA<br>Diaz Duarte E, 2002, MYCOPATHOLOGIA, V156, P223, DOI 10.1023/A:1023388321713
CR	replaced	42	42	51354	51354	[å è ½æ æ<br>[æ ¹å £ç ¼ Fang Shengqiong], 2016, [å è ½æ æ , Journal of Functional Materials], V47, P8143	[é£ å ç§ å-¦<br>[æ ¹å £ç ¼ Fang Shengqiong], 2016, [é£ å ç§ å-¦, Journal of Functional Materials], V47, P8143
CR	replaced	59	59	51371	51371	[é é å± ç ¿<br>[è æ ç§ Jiang Yuexiu], 2004, [é é å± ç ¿, Non-metallic Mines], V27, P19	[é£ å ç§ å-¦<br>[è æ ç§ Jiang Yuexiu], 2004, [é£ å ç§ å-¦, Non-metallic Mines], V27, P19
CR	replaced	74	74	51386	51386	FEED IND<br>Luo Y., 2014, FEED IND, V1, P114	Feed. Ind<br>Luo Y., 2014, Feed. Ind, V1, P114
CR	replaced	108	108	51420	51420	[ç ç §å<br>[é½ å¾·ç Qi Desheng], 2003, [ç ç §å	[é£ å ç§ å-¦<br>[é½ å¾·ç Qi Desheng], 2003, [é£ å ç§ å-¦, Acta Veterinaria et Zootechnica Sinica], V34, P620

CR	deleted	109		51421		V34<br>½å »å-¦æ ¥, Acta Veterinaria et Zootechnica Sinica], V34, P620	
CR	replaced	124	123	51436	51435	Applied Clay Science<br>Stockmeyer M.R., 1991, Applied Clay Science, V6, P39, DOI DOI 10.1016/0169-1317(91)90011-4	APPL CLAY SCI<br>Stockmeyer M.R., 1991, APPL CLAY SCI, V6, P39, DOI DOI 10.1016/0169-1317(91)90011-4
CR	replaced	131	130	51443	51442	Mycotoxin Res<br>Vekiru E, 2007, Mycotoxin Res, V23, P27, DOI 10.1007/BF02946021	MYCOTOXIN RES<br>Vekiru E, 2007, MYCOTOXIN RES, V23, P27, DOI 10.1007/BF02946021

Record 338 | WOS:000439674200027

Restorative effect of L-Dopa treatment against Ochratoxin A induced neurotoxicity

SO original: NEUROCHEMISTRY INTERNATIONAL | SO normalised: NEUROCHEMISTRY INTERNATIONAL | CR count: 89 → 89

Field	Status	Orig. pos	Norm. pos	Orig. line	Norm. line	Original cited/source value	Normalised cited/source value
CR	replaced	25	25	51535	51534	Nutr. Neurosci.<br>De Santis B., 2017, Nutr. Neurosci., P1	NUTR NEUROSCI<br>De Santis B., 2017, NUTR NEUROSCI, P1

Record 339 | WOS:000448339000017

Interaction of 2R-ochratoxin A with Serum Albumins: Binding Site, Effects of Site Markers, Thermodynamics, Species Differences of Albumin-binding, and Influence of Albumin on Its Toxicity in MDCK Cells

SO original: TOXINS | SO normalised: TOXINS | CR count: 47 → 47

No journal-related changes detected in SO, J9, JI or CR for this record.

Record 340 | WOS:000442890400004

Protective effects of compound ammonium glycyrrhizin, L-arginine, silymarin and glucurolactone against liver damage induced by ochratoxin A in primary chicken hepatocytes

SO original: MOLECULAR MEDICINE REPORTS | SO normalised: MOLECULAR MEDICINE REPORTS | CR count: 55 → 55

Field	Status	Orig. pos	Norm. pos	Orig. line	Norm. line	Original cited/source value	Normalised cited/source value
CR	replaced	11	11	51810	51809	J Clin Diagn Res<br>Dr Shweta, 2014, J Clin Diagn Res, V8, pOC10, DOI 10.7860/JCDR/2014/8343.4966	J CLIN DIAGN RES<br>Dr Shweta, 2014, J CLIN DIAGN RES, V8, pOC10, DOI 10.7860/JCDR/2014/8343.4966
CR	replaced	46	46	51845	51844	BIOMED ENVIRON SCI<br>Turkey MJ, 2015, BIOMED ENVIRON SCI, V28, P36, DOI 10.3967/bes2015.004	Biomed Environ Sci<br>Tuorkey MJ, 2015, Biomed Environ Sci, V28, P36, DOI 10.3967/bes2015.004

Record 341 | WOS:000442714300074

Micronucleus induction and cell cycle alterations produced by deoxynivalenol and its acetylated derivatives in individual and combined exposure on HepG2 cells

SO original: FOOD AND CHEMICAL TOXICOLOGY | SO normalised: FOOD AND CHEMICAL TOXICOLOGY | CR count: 37 → 37

No journal-related changes detected in SO, J9, JI or CR for this record.

Record 342 | WOS:000442489000011

Molecular Dynamics Simulations of Mismatched DNA Duplexes Associated with the Major C8-Linked 2â ²-Deoxyguanosine Adduct of the Food Mutagen Ochratoxin A: Influence of Opposing Base, Adduct Ionization State, and Sequence on the Structure of Damaged DNA

SO original: CHEMICAL RESEARCH IN TOXICOLOGY | SO normalised: CHEMICAL RESEARCH IN TOXICOLOGY | CR count: 55 → 55

No journal-related changes detected in SO, J9, JI or CR for this record.

Record 343 | WOS:000440099900007

Comparison of base-line and chemical-induced transcriptomic responses in HepaRG and RPTEC/TERT1 cells using TempO-Seq

SO original: ARCHIVES OF TOXICOLOGY | SO normalised: ARCHIVES OF TOXICOLOGY | CR count: 50 → 50

No journal-related changes detected in SO, J9, JI or CR for this record.

Record 344 | WOS:000438651800008

Occurrence of ochratoxin A in Astragalus propinquus root and its transfer to decoction

SO original: MYCOTOXIN RESEARCH | SO normalised: MYCOTOXIN RESEARCH | CR count: 38 → 38

Field	Status	Orig. pos	Norm. pos	Orig. line	Norm. line	Original cited/source value	Normalised cited/source value
CR	replaced	30	30	52321	52320	ALTERN MED REV<br>Roxas M, 2007, ALTERN MED REV, V12, P25	Altern Med Rev<br>Roxas M, 2007, Altern Med Rev, V12, P25
CR	replaced	35	35	52326	52325	í êµ-ì í ì ì ¥ì í µí í ì§<br>ìµ í ì , 2007,<br>í êµ-ì í ì ì ¥ì í µí í ì§ , V14, P356	[é£ å ç§ å-¦<br>ìµ í ì , 2007,<br>[é£ å ç§ å-¦, V14, P356

Record 345 | WOS:000434754600071

PCV2 infection aggravates ochratoxin A-induced nephrotoxicity via autophagy involving p38 signaling pathway in vivo and in vitro

SO original: ENVIRONMENTAL POLLUTION | SO normalised: ENVIRONMENTAL POLLUTION | CR count: 48 → 48

No journal-related changes detected in SO, J9, JI or CR for this record.

Record 346 | WOS:000442594000032

Toxicity Assay for Citrinin, Zearalenone and Zearalenone-14-Sulfate Using the Nematode Caenorhabditis elegans as Model Organism

SO original: TOXINS | SO normalised: TOXINS | CR count: 47 → 47

Field	Status	Orig. pos	Norm. pos	Orig. line	Norm. line	Original cited/source value	Normalised cited/source value
CR	replaced	25	25	52546	52545	Mycotoxin Res<br>Hildebrand Andreas A, 2012, Mycotoxin Res, V28, P1, DOI 10.1007/s12550-011-0109-4	MYCOTOXIN RES<br>Hildebrand Andreas A, 2012, MYCOTOXIN RES, V28, P1, DOI 10.1007/s12550-011-0109-4

Record 347 | WOS:000430882700061

Toxicological interactions between mycotoxins from ubiquitous fungi: Impact on hepatic and intestinal human epithelial cells

SO original: CHEMOSPHERE | SO normalised: CHEMOSPHERE | CR count: 70 → 70

Field	Status	Orig. pos	Norm. pos	Orig. line	Norm. line	Original cited/source value	Normalised cited/source value
CR	replaced	9	9	52649	52648	Mycotoxin Res<br>Becker Christiane, 2011, Mycotoxin Res, V27, P287, DOI 10.1007/s12550-011-0106-7	MYCOTOXIN RES<br>Becker Christiane, 2011, MYCOTOXIN RES, V27, P287, DOI 10.1007/s12550-011-0106-7

Record 348 | WOS:000432602600016

Synergistic action of the nephrotoxic mycotoxins ochratoxin A and citrinin at nanomolar concentrations in human proximal tubule-derived cells

SO original: TOXICOLOGY LETTERS | SO normalised: TOXICOLOGY LETTERS | CR count: 38 → 38

Field	Status	Orig. pos	Norm. pos	Orig. line	Norm. line	Original cited/source value	Normalised cited/source value
CR	replaced	16	16	52789	52788	Int J Microbiol<br>Jeswal Punam, 2015, Int J Microbiol, V2015, P242486, DOI [10.1155/2015/242486, 10.1155/2015/242486]	INT J MICROBIOL<br>Jeswal Punam, 2015, INT J MICROBIOL, V2015, P242486, DOI [10.1155/2015/242486, 10.1155/2015/242486]
CR	replaced	21	21	52794	52793	Mycotoxin Res<br>Knecht A, 2005, Mycotoxin Res, V21, P176, DOI 10.1007/BF02959258	MYCOTOXIN RES<br>Knecht A, 2005, MYCOTOXIN RES, V21, P176, DOI 10.1007/BF02959258
CR	replaced	25	25	52798	52797	Mycotoxin Res<br>Martlbauer Erwin, 2009, Mycotoxin Res, V25, P175, DOI 10.1007/s12550-009-0025-z	MYCOTOXIN RES<br>Martlbauer Erwin, 2009, MYCOTOXIN RES, V25, P175, DOI 10.1007/s12550-009-0025-z

Record 349 | WOS:000437960800009

Risk assessment of exposure to aflatoxin B1 and ochratoxin A through consumption of different Pistachio (Pistacia vera L.) cultivars collected from four geographical regions of Iran

SO original: ENVIRONMENTAL TOXICOLOGY AND PHARMACOLOGY | SO normalised: ENVIRONMENTAL TOXICOLOGY AND PHARMACOLOGY | CR count: 37 → 37

Field	Status	Orig. pos	Norm. pos	Orig. line	Norm. line	Original cited/source value	Normalised cited/source value
CR	replaced	6	6	52903	52902	Mycotoxin Res Bensassi Fatma, 2010, Mycotoxin Res, V26, P199, DOI 10.1007/s12550-010-0054-7	MYCOTOXIN RES Bensassi Fatma, 2010, MYCOTOXIN RES, V26, P199, DOI 10.1007/s12550-010-0054-7

Record 350 | WOS:000435525800022

Combination of Selenomethionine and N-Acetylcysteine Alleviates the Joint Toxicities of Aflatoxin B1 and Ochratoxin A by ERK MAPK Signal Pathway in Porcine Alveolar Macrophages

SO original: JOURNAL OF AGRICULTURAL AND FOOD CHEMISTRY | SO normalised: JOURNAL OF AGRICULTURAL AND FOOD CHEMISTRY | CR count: 53 → 53

Field	Status	Orig. pos	Norm. pos	Orig. line	Norm. line	Original cited/source value	Normalised cited/source value
CR	replaced	8	8	53012	53011	[ä¸-å æ£ éª å »å-¦æ å¿ [å ¯æ°¸ä¸ Feng Yongdong], 2004, [ä¸-å æ£ éª å »å-¦æ å¿ , Chinese Journal of Laboratory Medicine], V27, P582	[é£ å ç§ å-¦ [å ¯æ°¸ä¸ Feng Yongdong], 2004, [é£ å ç§ å-¦, Chinese Journal of Laboratory Medicine], V27, P582
CR	replaced	12	12	53016	53015	J Biochem Mol Toxicol Golli-Bennour Emna El, 2010, J Biochem Mol Toxicol, V24, P42, DOI 10.1002/jbt.20310	J BIOCHEM MOL TOXIC Golli-Bennour Emna El, 2010, J BIOCHEM MOL TOXIC, V24, P42, DOI 10.1002/jbt.20310

Record 351 | WOS:000433656100042

Is oxidative stress involved in the sex-dependent response to ochratoxin A renal toxicity?

SO original: FOOD AND CHEMICAL TOXICOLOGY | SO normalised: FOOD AND CHEMICAL TOXICOLOGY | CR count: 69 → 69

No journal-related changes detected in SO, J9, JI or CR for this record.

Record 352 | WOS:000433016800013

Effects of combined treatment with ochratoxin A and citrinin on oxidative damage in kidneys and liver of rats

SO original: TOXICON | SO normalised: TOXICON | CR count: 53 → 53

Field	Status	Orig. pos	Norm. pos	Orig. line	Norm. line	Original cited/source value	Normalised cited/source value

CR	replaced	44	44	53321	53320	Essential statistics for the pharmaceutical sciences<br>Rowe P., 2016, Essential statistics for the pharmaceutical sciences, V2nd	ESSENTIAL STATISTICS FOR THE PHARMACEUTICAL SCIENCES<br>Rowe P., 2016, ESSENTIAL STATISTICS FOR THE PHARMACEUTICAL SCIENCES, V2nd

Record 353 | WOS:000432498000011

The importance of accounting for sex in the search of proteomic signatures of mycotoxin exposure

SO original: JOURNAL OF PROTEOMICS | SO normalised: JOURNAL OF PROTEOMICS | CR count: 137 → 137

No journal-related changes detected in SO, J9, JI or CR for this record.

Record 354 | WOS:000428605400010

Toxicological effects of regulated mycotoxins and persistent organochloride pesticides: In vitro cytotoxic assessment of single and defined mixtures on MA-10 murine Leydig cell line

SO original: TOXICOLOGY IN VITRO | SO normalised: TOXICOLOGY IN VITRO | CR count: 106 → 106

Field	Status	Orig. pos	Norm. pos	Orig. line	Norm. line	Original cited/source value	Normalised cited/source value
CR	replaced	38	38	53630	53629	J Biochem Mol Toxicol<br>Golli-Bennour Emna El, 2010, J Biochem Mol Toxicol, V24, P42, DOI 10.1002/jbt.20310	J BIOCHEM MOL TOXIC<br>Golli-Bennour Emna El, 2010, J BIOCHEM MOL TOXIC, V24, P42, DOI 10.1002/jbt.20310

Record 355 | WOS:000434061300005

Ochratoxin A, citrinin and deoxynivalenol decrease claudin-2 expression in mouse rectum CMT93-II cells

SO original: MICROSCOPY | SO normalised: MICROSCOPY | CR count: 73 → 73

No journal-related changes detected in SO, J9, JI or CR for this record.

Record 356 | WOS:000429758100013

Toxicity assessment of mycotoxins extracted from contaminated commercial dog pelleted feed on canine blood mononuclear cells

SO original: FOOD AND CHEMICAL TOXICOLOGY | SO normalised: FOOD AND CHEMICAL TOXICOLOGY | CR count: 53 → 53

Field	Status	Orig. pos	Norm. pos	Orig. line	Norm. line	Original cited/source value	Normalised cited/source value
CR	replaced	22	22	53921	53920	Mycotoxin Res<br>Kohler H, 2002, Mycotoxin Res, V18 Suppl 2, P169, DOI 10.1007/BF02946089	MYCOTOXIN RES<br>Kohler H, 2002, MYCOTOXIN RES, V18 Suppl 2, P169, DOI 10.1007/BF02946089

CR	replaced	37	37	53936	53935	Mycotoxin Res Mwanza Mulunda, 2009, Mycotoxin Res, V25, P233, DOI 10.1007/s12550-009-0033-z	MYCOTOXIN RES Mwanza Mulunda, 2009, MYCOTOXIN RES, V25, P233, DOI 10.1007/s12550-009-0033-z

Record 357 | WOS:000428799300003

The efficiency of lactic acid bacteria against pathogenic fungi and mycotoxins

SO original: ARHIV ZA HIGIJENU RADA I TOKSIKOLOGIJU-ARCHIVES OF INDUSTRIAL HYGIENE ; AND TOXICOLOGY | SO normalised: ARHIV ZA HIGIJENU RADA I TOKSIKOLOGIJU-ARCHIVES OF INDUSTRIAL HYGIENE ; AND TOXICOLOGY | CR count: 171 → 171

Field	Status	Orig. pos	Norm. pos	Orig. line	Norm. line	Original cited/source value	Normalised cited/source value
CR	replaced	5	5	54014	54013	Cogent Food and Agriculture Adeyeye S.A. O., 2016, Cogent Food and Agriculture, V2, DOI [DOI 10.1080/23311932.2016.1213127, 10.1080/ 23311932.2016.1213127, 10.1080/23311932.2016.1213127]	Cogent Food & Agriculture Adeyeye S.A. O., 2016, Cogent Food & Agriculture, V2, DOI [DOI 10.1080/23311932.2016.1213127, 10.1080/ 23311932.2016.1213127, 10.1080/23311932.2016.1213127]
CR	replaced	15	15	54024	54023	Mycotoxin Res Bohm J, 2000, Mycotoxin Res, V16 Suppl 1, P70, DOI 10.1007/BF02942985	MYCOTOXIN RES Bohm J, 2000, MYCOTOXIN RES, V16 Suppl 1, P70, DOI 10.1007/BF02942985
CR	replaced	44	44	54053	54052	Mycotoxin Res Fernandez-Juri Maria Guillermina, 2011, Mycotoxin Res, V27, P273, DOI 10.1007/s12550-011-0104-9	MYCOTOXIN RES Fernandez-Juri Maria Guillermina, 2011, MYCOTOXIN RES, V27, P273, DOI 10.1007/s12550-011-0104-9
CR	replaced	114	114	54123	54122	Mycotoxin Res Ostry V, 2001, Mycotoxin Res, V17 Suppl 2, P188, DOI 10.1007/BF03036433	MYCOTOXIN RES Ostry V, 2001, MYCOTOXIN RES, V17 Suppl 2, P188, DOI 10.1007/BF03036433
CR	replaced	171	171	54180	54179	Polish Journal of Veterinary Sciences Zwierzchowski W., 2005, Polish Journal of Veterinary Sciences, V8, P209	POL J VET SCI Zwierzchowski W., 2005, POL J VET SCI, V8, P209

Record 358 | WOS:000422811300020

Metabolism-dependent cytotoxicity of citrinin and ochratoxin A alone and in combination as assessed adopting integrated discrete multiple organ co-culture (IdMOC)

SO original: TOXICOLOGY IN VITRO | SO normalised: TOXICOLOGY IN VITRO | CR count: 59 → 59

Field	Status	Orig. pos	Norm. pos	Orig. line	Norm. line	Original cited/source value	Normalised cited/source value
CR	replaced	20	20	54279	54278	Mycotoxin Res Follmann W, 2007, Mycotoxin Res, V23, P101, DOI 10.1007/BF02946034	MYCOTOXIN RES Follmann W, 2007, MYCOTOXIN RES, V23, P101, DOI 10.1007/BF02946034

CR	replaced	23	23	54282	54281	J Biochem Mol Toxicol<br>Golli-Bennour Emna El, 2010, J Biochem Mol Toxicol, V24, P42, DOI 10.1002/jbt.20310	J BIOCHEM MOL TOXIC<br>Golli-Bennour Emna El, 2010, J BIOCHEM MOL TOXIC, V24, P42, DOI 10.1002/jbt.20310
CR	replaced	30	30	54289	54288	Mycotoxin Res<br>Knecht A, 2005, Mycotoxin Res, V21, P176, DOI 10.1007/BF02959258	MYCOTOXIN RES<br>Knecht A, 2005, MYCOTOXIN RES, V21, P176, DOI 10.1007/BF02959258
CR	replaced	51	51	54310	54309	Med. J. Australia<br>Russell WMS., 1960, Med. J. Australia, DOI DOI 10.1038/1841675B0	MED J AUSTRALIA<br>Russell WMS., 1960, MED J AUSTRALIA, DOI DOI 10.1038/1841675B0

Record 359 | WOS:000425526000033

Linking site-specific loss of histone acetylation to repression of gene expression by the mycotoxin ochratoxin A

SO original: ARCHIVES OF TOXICOLOGY | SO normalised: ARCHIVES OF TOXICOLOGY | CR count: 67 → 67

No journal-related changes detected in SO, J9, JI or CR for this record.

Record 360 | WOS:000425568900007

Toxicity reduction of ochratoxin A by lactic acid bacteria

SO original: FOOD AND CHEMICAL TOXICOLOGY | SO normalised: FOOD AND CHEMICAL TOXICOLOGY | CR count: 25 → 25

No journal-related changes detected in SO, J9, JI or CR for this record.

Record 361 | WOS:000425568900035

Ochratoxin A: Toxicity, oxidative stress and metabolism

SO original: FOOD AND CHEMICAL TOXICOLOGY | SO normalised: FOOD AND CHEMICAL TOXICOLOGY | CR count: 129 → 129

Field	Status	Orig. pos	Norm. pos	Orig. line	Norm. line	Original cited/source value	Normalised cited/source value
CR	replaced	38	38	54644	54643	Annales de Recherches Veterinaires<br>GALTIER P, 1976, Annales de Recherches Veterinaires, V7, P91	ANN RECH VET<br>GALTIER P, 1976, ANN RECH VET, V7, P91

Record 362 | WOS:000424317600001

Disruption of liver development and coagulation pathway by ochratoxin A in embryonic zebrafish

SO original: TOXICOLOGY AND APPLIED PHARMACOLOGY | SO normalised: TOXICOLOGY AND APPLIED PHARMACOLOGY | CR count: 49 → 49

No journal-related changes detected in SO, J9, JI or CR for this record.

Record 363 | WOS:000424317600006

Ochratoxin A exposure decreased sperm motility via the AMPK and PTEN signaling pathways

SO original: TOXICOLOGY AND APPLIED PHARMACOLOGY | SO normalised: TOXICOLOGY AND APPLIED PHARMACOLOGY | CR count: 49 → 49

Field	Status	Orig. pos	Norm. pos	Orig. line	Norm. line	Original cited/source value	Normalised cited/source value
CR	replaced	3	3	54926	54925	SCI REP<br>[Anonymous], 2016, SCI REP	SCI REP-UK<br>[Anonymous], 2016, SCI REP-UK

Record 364 | WOS:000423566000030

Individual and combined cytotoxicity assessment of zearalenone with ochratoxin A or Î±-zearalenol by full factorial design

SO original: FOOD SCIENCE AND BIOTECHNOLOGY | SO normalised: FOOD SCIENCE AND BIOTECHNOLOGY | CR count: 33 → 33

No journal-related changes detected in SO, J9, JI or CR for this record.

Record 365 | WOS:000416024300043

Effect of rMnSOD on Sodium Reabsorption in Renal Proximal Tubule in Ochratoxin ATreated Rats

SO original: JOURNAL OF CELLULAR BIOCHEMISTRY | SO normalised: JOURNAL OF CELLULAR BIOCHEMISTRY | CR count: 38 → 38

Field	Status	Orig. pos	Norm. pos	Orig. line	Norm. line	Original cited/source value	Normalised cited/source value
CR	replaced	10	10	55165	55164	Mycotoxin Res<br>Degen G H, 2007, Mycotoxin Res, V23, P88, DOI 10.1007/BF02946032	MYCOTOXIN RES<br>Degen G H, 2007, MYCOTOXIN RES, V23, P88, DOI 10.1007/BF02946032

Record 366 | WOS:000435675100011

Cinnamon (Cinnamomum zeylanicum) as an antidote or a protective agent against natural or chemical toxicities: a review

SO original: DRUG AND CHEMICAL TOXICOLOGY | SO normalised: DRUG AND CHEMICAL TOXICOLOGY | CR count: 86 → 86

No journal-related changes detected in SO, J9, JI or CR for this record.

Record 367 | WOS:000423248100019

Modified mycotoxins: An updated review on their formation, detection, occurrence, and toxic effects

SO original: FOOD AND CHEMICAL TOXICOLOGY | SO normalised: FOOD AND CHEMICAL TOXICOLOGY | CR count: 191 → 191

Field	Status	Orig. pos	Norm. pos	Orig. line	Norm. line	Original cited/source value	Normalised cited/source value
CR	replaced	135	135	55534	55533	Feedstuffs<br>Pedrosa K., 2011, Feedstuffs, V83, P1	FEEDSTUFFS<br>Pedrosa K., 2011, FEEDSTUFFS, V83, P1

Record 368 | WOS:000423248100048

In vitro mechanisms of Beauvericin toxicity: A review

SO original: FOOD AND CHEMICAL TOXICOLOGY | SO normalised: FOOD AND CHEMICAL TOXICOLOGY | CR count: 99 → 99

Field	Status	Orig. pos	Norm. pos	Orig. line	Norm. line	Original cited/source value	Normalised cited/source value
CR	replaced	13	13	55661	55660	EFSA J.<br>EFSA, 2014, EFSA J., V12, P2902	EFSA J<br>EFSA, 2014, EFSA J, V12, P2902

Record 369 | WOS:000418302000021

Modulations of DNMT1 and HDAC1 are involved in the OTA-induced cytotoxicity and apoptosis in vitro

SO original: CHEMICO-BIOLOGICAL INTERACTIONS | SO normalised: CHEMICO-BIOLOGICAL INTERACTIONS | CR count: 40 → 40

No journal-related changes detected in SO, J9, JI or CR for this record.

Record 370 | WOS:000417670500029

Ochratoxin A-Induced Apoptosis of IPEC-J2 Cells through ROS-Mediated Mitochondrial Permeability Transition Pore Opening Pathway

SO original: JOURNAL OF AGRICULTURAL AND FOOD CHEMISTRY | SO normalised: JOURNAL OF AGRICULTURAL AND FOOD CHEMISTRY | CR count: 46 → 46

No journal-related changes detected in SO, J9, JI or CR for this record.

Record 371 | WOS:000418211700013

A comprehensive review on biological properties of citrinin

SO original: FOOD AND CHEMICAL TOXICOLOGY | SO normalised: FOOD AND CHEMICAL TOXICOLOGY | CR count: 111 → 111

Field	Status	Orig. pos	Norm. pos	Orig. line	Norm. line	Original cited/source value	Normalised cited/source value
CR	replaced	51	51	56173	56172	Int J Microbiol<br>Jeswal Punam, 2015, Int J Microbiol, V2015, P242486, DOI [10.1155/2015/242486, 10.1155/2015/242486]	INT J MICROBIOL<br>Jeswal Punam, 2015, INT J MICROBIOL, V2015, P242486, DOI [10.1155/2015/242486, 10.1155/2015/242486]
CR	replaced	61	61	56183	56182	Mycotoxin Res<br>Knecht A, 2005, Mycotoxin Res, V21, P176, DOI 10.1007/BF02959258	MYCOTOXIN RES<br>Knecht A, 2005, MYCOTOXIN RES, V21,

							P176, DOI 10.1007/BF02959258

Record 372 | WOS:000416589800031

Differential Gene Expression Analysis of Bovine Macrophages after Exposure to the Penicillium Mycotoxins Citrinin and/or Ochratoxin A

SO original: TOXINS | SO normalised: TOXINS | CR count: 72 → 72

No journal-related changes detected in SO, J9, JI or CR for this record.

Record 373 | WOS:000415911400030

UPLC-MS/MS analysis of ochratoxin A metabolites produced by Caco-2 and HepG2 cells in a co-culture system

SO original: FOOD AND CHEMICAL TOXICOLOGY | SO normalised: FOOD AND CHEMICAL TOXICOLOGY | CR count: 61 → 61

No journal-related changes detected in SO, J9, JI or CR for this record.

Record 374 | WOS:000410635200006

Effects of ochratoxin A on ER stress, MAPK signaling pathway and autophagy of kidney and spleen in pigs

SO original: ENVIRONMENTAL TOXICOLOGY | SO normalised: ENVIRONMENTAL TOXICOLOGY | CR count: 46 → 46

**Field**	**Status**	**Orig. pos**	**Norm. pos**	**Orig. line**	**Norm. line**	**Original cited/source value**	**Normalised cited/source value**
CR	replaced	4	4	56566	56565	SCI REP UK [Anonymous], 2016, SCI REP UK	SCI REP-UK [Anonymous], 2016, SCI REP-UK
CR	replaced	30	30	56592	56591	Mycotoxin Res Martins H Marina, 2012, Mycotoxin Res, V28, P107, DOI 10.1007/s12550-011-0118-3	MYCOTOXIN RES Martins H Marina, 2012, MYCOTOXIN RES, V28, P107, DOI 10.1007/s12550-011-0118-3

Record 375 | WOS:000414913800067

Evaluation of Chitosan and Cellulosic Polymers as Binding Adsorbent Materials to Prevent Aflatoxin B1, Fumonisin B1, Ochratoxin, Trichothecene, Deoxynivalenol, and Zearalenone Mycotoxicoses Through an In Vitro Gastrointestinal Model for Poultry

SO original: POLYMERS | SO normalised: POLYMERS | CR count: 45 → 45

No journal-related changes detected in SO, J9, JI or CR for this record.

Record 376 | WOS:000408520200003

Worldwide Occurrence of Mycotoxins in Cereals and Cereal-Derived Food Products: Public Health Perspectives of Their Co-occurrence

SO original: JOURNAL OF AGRICULTURAL AND FOOD CHEMISTRY | SO normalised: JOURNAL OF AGRICULTURAL AND FOOD CHEMISTRY | CR count: 234 → 234

Field	Status	Orig. pos	Norm. pos	Orig. line	Norm. line	Original cited/source value	Normalised cited/source value
CR	replaced	11	11	56798	56797	FUNGAL METABOLITES [Anonymous], 1983, FUNGAL METABOLITES	REF SER PHYTOCHEM [Anonymous], 1983, REF SER PHYTOCHEM
CR	replaced	33	33	56820	56819	Mycotoxin Res Broggi L E, 2007, Mycotoxin Res, V23, P59, DOI 10.1007/BF02946026	MYCOTOXIN RES Broggi L E, 2007, MYCOTOXIN RES, V23, P59, DOI 10.1007/BF02946026
CR	replaced	39	39	56826	56825	J FOOD PROT Cappozzo J., 2017, J FOOD PROT, V80	J FOOD PROTECT Cappozzo J., 2017, J FOOD PROTECT, V80
CR	replaced	61	61	56848	56847	Mycotoxin Res Dutton Michael F, 2009, Mycotoxin Res, V25, P29, DOI 10.1007/s12550-008-0005-8	MYCOTOXIN RES Dutton Michael F, 2009, MYCOTOXIN RES, V25, P29, DOI 10.1007/s12550-008-0005-8
CR	replaced	81	81	56868	56867	J Biochem Mol Toxicol Golli-Bennour Emna El, 2010, J Biochem Mol Toxicol, V24, P42, DOI 10.1002/jbt.20310	J BIOCHEM MOL TOXIC Golli-Bennour Emna El, 2010, J BIOCHEM MOL TOXIC, V24, P42, DOI 10.1002/jbt.20310
CR	replaced	132	132	56919	56918	Mycotoxin Res Makun Hussaini Anthony, 2011, Mycotoxin Res, V27, P97, DOI 10.1007/s12550-010-0080-5	MYCOTOXIN RES Makun Hussaini Anthony, 2011, MYCOTOXIN RES, V27, P97, DOI 10.1007/s12550-010-0080-5
CR	replaced	154	154	56941	56940	Mycotoxin Res Mwanza Mulunda, 2009, Mycotoxin Res, V25, P233, DOI 10.1007/s12550-009-0033-z	MYCOTOXIN RES Mwanza Mulunda, 2009, MYCOTOXIN RES, V25, P233, DOI 10.1007/s12550-009-0033-z
CR	replaced	157	157	56944	56943	Mycotoxin Res Njobeh Patrick B, 2010, Mycotoxin Res, V26, P47, DOI 10.1007/s12550-009-0039-6	MYCOTOXIN RES Njobeh Patrick B, 2010, MYCOTOXIN RES, V26, P47, DOI 10.1007/s12550-009-0039-6
CR	replaced	214	214	57001	57000	FUNGAL METABOLITES Turner W.B., 1971, FUNGAL METABOLITES	REF SER PHYTOCHEM Turner W.B., 1971, REF SER PHYTOCHEM

Record 377 | WOS:000408520200008

Mutagenicity of Ochratoxin A: Role for a Carbon-Linked C8-Deoxyguanosine Adduct?

SO original: JOURNAL OF AGRICULTURAL AND FOOD CHEMISTRY | SO normalised: JOURNAL OF AGRICULTURAL AND FOOD CHEMISTRY | CR count: 59 → 59

No journal-related changes detected in SO, J9, JI or CR for this record.

Record 378 | WOS:000408074600028

Overexpression and Low Expression of Selenoprotein S Impact Ochratoxin A-Induced Porcine Cytotoxicity and Apoptosis in Vitro

SO original: JOURNAL OF AGRICULTURAL AND FOOD CHEMISTRY | SO normalised: JOURNAL OF AGRICULTURAL AND FOOD CHEMISTRY | CR count: 38 → 38

No journal-related changes detected in SO, J9, JI or CR for this record.

Record 379 | WOS:000411538100005

Nrf2 deficiency exacerbates ochratoxin A-induced toxicity in vitro and in vivo

SO original: TOXICOLOGY | SO normalised: TOXICOLOGY | CR count: 45 → 45

No journal-related changes detected in SO, J9, JI or CR for this record.

Record 380 | WOS:000406735700003

Ochratoxin A transport by the human breast cancer resistance protein (BCRP), multidrug resistance protein 2 (MRP2), and organic anion-transporting polypeptides 1A2, 1B1 and 2B1

SO original: TOXICOLOGY AND APPLIED PHARMACOLOGY | SO normalised: TOXICOLOGY AND APPLIED PHARMACOLOGY | CR count: 23 → 23

No journal-related changes detected in SO, J9, JI or CR for this record.

Record 381 | WOS:000407370700051

Health Risks Associated with Exposure to Filamentous Fungi

SO original: INTERNATIONAL JOURNAL OF ENVIRONMENTAL RESEARCH AND PUBLIC HEALTH | SO normalised: INTERNATIONAL JOURNAL OF ENVIRONMENTAL RESEARCH AND PUBLIC HEALTH | CR count: 132 → 132

Field	Status	Orig. pos	Norm. pos	Orig. line	Norm. line	Original cited/source value	Normalised cited/source value
CR	replaced	5	5	57514	57513	Some Traditional Herbal Medicines<br>[Anonymous], 2002, Some Traditional Herbal Medicines, Some Mycotoxins, Naphthalene and Styrene	Some traditional herbal medicines<br>[Anonymous], 2002, Some traditional herbal medicines, Some Mycotoxins, Naphthalene and Styrene
CR	replaced	23	23	57532	57531	Eur J Cancer Prev<br>Craddock V M, 1992, Eur J Cancer Prev, V1, P89, DOI 10.1097/00008469-199202000-00002	EUR J CANCER PREV<br>Craddock V M, 1992, EUR J CANCER PREV, V1, P89, DOI 10.1097/00008469-199202000-00002
CR	replaced	33	33	57542	57541	Mycotoxin Res<br>Fehr Markus, 2010, Mycotoxin Res, V26, P247, DOI 10.1007/s12550-010-0063-6	MYCOTOXIN RES<br>Fehr Markus, 2010, MYCOTOXIN RES, V26, P247, DOI 10.1007/s12550-010-0063-6
CR	replaced	90	90	57599	57598	Mycotoxin Res<br>Mwanza Mulunda, 2009, Mycotoxin Res, V25, P233, DOI 10.1007/s12550-009-0033-z	MYCOTOXIN RES<br>Mwanza Mulunda, 2009, MYCOTOXIN RES, V25, P233, DOI 10.1007/s12550-009-0033-z

Record 382 | WOS:000404237400013

Evaluation of the automated MicroFlowÂ® and Metaferâ ¢ platforms for high-throughput micronucleus scoring and dose response analysis in human lymphoblastoid TK6 cells

SO original: ARCHIVES OF TOXICOLOGY | SO normalised: ARCHIVES OF TOXICOLOGY | CR count: 22 → 22

No journal-related changes detected in SO, J9, JI or CR for this record.

Record 383 | WOS:000404198300012

In vitro immune toxicity of ochratoxin A in porcine alveolar macrophages: A role for the ROS-relative TLR4/MyD88 signaling pathway

SO original: CHEMICO-BIOLOGICAL INTERACTIONS | SO normalised: CHEMICO-BIOLOGICAL INTERACTIONS | CR count: 43 → 43

Field	Status	Orig. pos	Norm. pos	Orig. line	Norm. line	Original cited/source value	Normalised cited/source value
CR	replaced	32	32	57836	57835	Mycotoxin Res<br>Martins H Marina, 2012, Mycotoxin Res, V28, P107, DOI 10.1007/s12550-011-0118-3	MYCOTOXIN RES<br>Martins H Marina, 2012, MYCOTOXIN RES, V28, P107, DOI 10.1007/s12550-011-0118-3

Record 384 | WOS:000401600400001

Adsorption of aflatoxin B1, zearalenone and ochratoxin A by microorganisms isolated from Kefir grains

SO original: INTERNATIONAL JOURNAL OF FOOD MICROBIOLOGY | SO normalised: INTERNATIONAL JOURNAL OF FOOD MICROBIOLOGY | CR count: 44 → 44

No journal-related changes detected in SO, J9, JI or CR for this record.

Record 385 | WOS:000408731500007

Lycopene restores trace element levels in ochratoxin A-treated rats

SO original: ARHIV ZA HIGIJENU RADA I TOKSIKOLOGIJU-ARCHIVES OF INDUSTRIAL HYGIENE ; AND TOXICOLOGY | SO normalised: ARHIV ZA HIGIJENU RADA I TOKSIKOLOGIJU-ARCHIVES OF INDUSTRIAL HYGIENE ; AND TOXICOLOGY | CR count: 37 → 37

No journal-related changes detected in SO, J9, JI or CR for this record.

Record 386 | WOS:000403637300009

Ochratoxin A induced premature senescence in human renal proximal tubular cells

SO original: TOXICOLOGY | SO normalised: TOXICOLOGY | CR count: 68 → 68

Field	Status	Orig. pos	Norm. pos	Orig. line	Norm. line	Original cited/source value	Normalised cited/source value
CR	replaced	4	4	58135	58134	Mycotoxin Res<br>Aslam M, 2005, Mycotoxin	MYCOTOXIN RES<br>Aslam M, 2005,

						Res, V21, P164, DOI 10.1007/BF02959255	MYCOTOXIN RES, V21, P164, DOI 10.1007/BF02959255

Record 387 | WOS:000394982800001

The intestinal barrier as an emerging target in the toxicological assessment of mycotoxins

SO original: ARCHIVES OF TOXICOLOGY | SO normalised: ARCHIVES OF TOXICOLOGY | CR count: 210 → 210

Field	Status	Orig. pos	Norm. pos	Orig. line	Norm. line	Original cited/source value	Normalised cited/source value
CR	replaced	90	90	58351	58350	Mycotoxin Res<br>Leong Yin-Hui, 2012, Mycotoxin Res, V28, P79, DOI 10.1007/s12550-012-0129-8	MYCOTOXIN RES<br>Leong Yin-Hui, 2012, MYCOTOXIN RES, V28, P79, DOI 10.1007/s12550-012-0129-8

Record 388 | WOS:000394558400005

Assessing interactions of binary mixtures of Penicillium mycotoxins (PMs) by using a bovine macrophage cell line (BoMacs)

SO original: TOXICOLOGY AND APPLIED PHARMACOLOGY | SO normalised: TOXICOLOGY AND APPLIED PHARMACOLOGY | CR count: 31 → 31

No journal-related changes detected in SO, J9, JI or CR for this record.

Record 389 | WOS:000392347800007

Mycotoxins as human carcinogens-the IARC Monographs classification

SO original: MYCOTOXIN RESEARCH | SO normalised: MYCOTOXIN RESEARCH | CR count: 95 → 95

Field	Status	Orig. pos	Norm. pos	Orig. line	Norm. line	Original cited/source value	Normalised cited/source value
CR	replaced	63	63	58693	58692	Environmental Health Perspectives<br>Newberne P. M., 1974, Environmental Health Perspectives, V9, P1, DOI 10.2307/3428255	ENVIRON HEALTH PERSP<br>Newberne P. M., 1974, ENVIRON HEALTH PERSP, V9, P1, DOI 10.2307/3428255

Record 390 | WOS:000389167600013

Mycotoxin detoxifiers detoxifiers attenuate deoxynivalenol-induced pro-inflammatory barrier insult in porcine enterocytes as an in vitro evaluation model of feed mycotoxin reduction

SO original: TOXICOLOGY IN VITRO | SO normalised: TOXICOLOGY IN VITRO | CR count: 53 → 53

Field	Status	Orig. pos	Norm. pos	Orig. line	Norm. line	Original cited/source value	Normalised cited/source value
CR	replaced	21	21	58811	58810	Mycotoxin Res<br>Goyarts T, 2005, Mycotoxin	MYCOTOXIN RES<br>Goyarts T, 2005, MYCOTOXIN RES, V21,

						Res, V21, P139, DOI 10.1007/BF02954439	P139, DOI 10.1007/BF02954439
CR	replaced	46	46	58836	58835	Mycotoxin Res Shehata S, 2004, Mycotoxin Res, V20, P42, DOI 10.1007/BF02946709	MYCOTOXIN RES Shehata S, 2004, MYCOTOXIN RES, V20, P42, DOI 10.1007/BF02946709

Record 391 | WOS:000392789800020

Chitosan nanoparticles plus quercetin suppress the oxidative stress, modulate DNA fragmentation and gene expression in the kidney of rats fed ochratoxin A-contaminated diet

SO original: FOOD AND CHEMICAL TOXICOLOGY | SO normalised: FOOD AND CHEMICAL TOXICOLOGY | CR count: 103 → 103

No journal-related changes detected in SO, J9, JI or CR for this record.

Record 392 | WOS:000402081300011

Mycotoxins co-contamination: Methodological aspects and biological relevance of combined toxicity studies

SO original: CRITICAL REVIEWS IN FOOD SCIENCE AND NUTRITION | SO normalised: CRITICAL REVIEWS IN FOOD SCIENCE AND NUTRITION | CR count: 135 → 135

Field	Status	Orig. pos	Norm. pos	Orig. line	Norm. line	Original cited/source value	Normalised cited/source value
CR	replaced	64	64	59149	59148	Mycotoxin Res Knecht A, 2005, Mycotoxin Res, V21, P176, DOI 10.1007/BF02959258	MYCOTOXIN RES Knecht A, 2005, MYCOTOXIN RES, V21, P176, DOI 10.1007/BF02959258
CR	replaced	84	84	59169	59168	Mycotoxin Res Mwanza Mulunda, 2009, Mycotoxin Res, V25, P233, DOI 10.1007/s12550-009-0033-z	MYCOTOXIN RES Mwanza Mulunda, 2009, MYCOTOXIN RES, V25, P233, DOI 10.1007/s12550-009-0033-z

Record 393 | WOS:000399597700009

Protective role of bentonite against aflatoxin B1- and ochratoxin A-induced immunotoxicity in broilers

SO original: JOURNAL OF IMMUNOTOXICOLOGY | SO normalised: JOURNAL OF IMMUNOTOXICOLOGY | CR count: 57 → 57

No journal-related changes detected in SO, J9, JI or CR for this record.

Record 394 | WOS:000448708300012

Aflatoxin in Rice Crop: Prevalence and Assessment of Daily Exposure

SO original: FUNGAL METABOLITES | SO normalised: FUNGAL METABOLITES | CR count: 63 → 63

Field	Status	Orig. pos	Norm. pos	Orig. line	Norm. line	Original cited/source value	Normalised cited/source value

CR	replaced	10	10	59410	59409	Foodborne diseases<br>Chu F. S., 2002, Foodborne diseases, P271	FOODBORNE DISEASES<br>Chu F. S., 2002, FOODBORNE DISEASES, P271
CR	replaced	13	13	59413	59412	Mycotoxin Res<br>Elzupir Amin O, 2012, Mycotoxin Res, V28, P131, DOI 10.1007/s12550-012-0127-x	MYCOTOXIN RES<br>Elzupir Amin O, 2012, MYCOTOXIN RES, V28, P131, DOI 10.1007/s12550-012-0127-x
CR	replaced	14	14	59414	59413	Mycotoxin Res<br>Elzupir Amin O, 2010, Mycotoxin Res, V26, P69, DOI 10.1007/s12550-010-0041-z	MYCOTOXIN RES<br>Elzupir Amin O, 2010, MYCOTOXIN RES, V26, P69, DOI 10.1007/s12550-010-0041-z
CR	replaced	17	17	59417	59416	Mycotoxin Res<br>Elzupir Amin Osman, 2011, Mycotoxin Res, V27, P183, DOI 10.1007/s12550-011-0094-7	MYCOTOXIN RES<br>Elzupir Amin Osman, 2011, MYCOTOXIN RES, V27, P183, DOI 10.1007/s12550-011-0094-7
CR	replaced	24	24	59424	59423	Mycotoxin Res<br>Iqbal Shahzad Z, 2012, Mycotoxin Res, V28, P175, DOI 10.1007/s12550-012-0131-1	MYCOTOXIN RES<br>Iqbal Shahzad Z, 2012, MYCOTOXIN RES, V28, P175, DOI 10.1007/s12550-012-0131-1
CR	replaced	27	27	59427	59426	Mycotoxin Res<br>Leong Yin-Hui, 2012, Mycotoxin Res, V28, P79, DOI 10.1007/s12550-012-0129-8	MYCOTOXIN RES<br>Leong Yin-Hui, 2012, MYCOTOXIN RES, V28, P79, DOI 10.1007/s12550-012-0129-8
CR	replaced	30	30	59430	59429	Mycotoxin Res<br>Makun Hussaini Anthony, 2011, Mycotoxin Res, V27, P97, DOI 10.1007/s12550-010-0080-5	MYCOTOXIN RES<br>Makun Hussaini Anthony, 2011, MYCOTOXIN RES, V27, P97, DOI 10.1007/s12550-010-0080-5
CR	replaced	59	59	59459	59458	BIOMED ENVIRON SCI<br>Wang J, 2007, BIOMED ENVIRON SCI, V20, P483	Biomed Environ Sci<br>Wang J, 2007, Biomed Environ Sci, V20, P483

Record 395 | WOS:000416384800008

Effect of PR toxin on THP1 and Caco-2 cells: an in vitro study

SO original: WORLD MYCOTOXIN JOURNAL | SO normalised: WORLD MYCOTOXIN JOURNAL | CR count: 61 → 61

No journal-related changes detected in SO, J9, JI or CR for this record.

Record 396 | WOS:000413016300007

Efficacy of mixed adsorbent in ameliorating ochratoxicosis in broilers fed ochratoxin A contaminated diets

SO original: ITALIAN JOURNAL OF ANIMAL SCIENCE | SO normalised: ITALIAN JOURNAL OF ANIMAL SCIENCE | CR count: 25 → 25

**Field**	**Status**	**Orig. pos**	**Norm. pos**	**Orig. line**	**Norm. line**	**Original cited/source value**	**Normalised cited/source value**

CR	replaced	16	16	59654	59653	VET MED INT<br>Sakthivelan SM, 2010, VET MED INT, V1, P1, DOI DOI 10.4061/2010/590432	Vet Med Int<br>Sakthivelan SM, 2010, Vet Med Int, V1, P1, DOI DOI 10.4061/2010/590432

Record 397 | WOS:000417403600001

Citrinin induces apoptosis in human HCT116 colon cancer cells through endoplasmic reticulum stress

SO original: JOURNAL OF TOXICOLOGY AND ENVIRONMENTAL HEALTH-PART A-CURRENT ISSUES | SO normalised: JOURNAL OF TOXICOLOGY AND ENVIRONMENTAL HEALTH-PART A-CURRENT ISSUES | CR count: 64 → 64

No journal-related changes detected in SO, J9, JI or CR for this record.

Record 398 | WOS:000424458100023

Mycotoxins

SO original: FOODBORNE DISEASES, 3RD EDITION | SO normalised: FOODBORNE DISEASES, 3RD EDITION | CR count: 232 → 232

Field	Status	Orig. pos	Norm. pos	Orig. line	Norm. line	Original cited/source value	Normalised cited/source value
CR	replaced	137	137	59991	59990	Mycotoxin Res<br>Mori S, 1996, Mycotoxin Res, V12, P91, DOI 10.1007/BF03192267	MYCOTOXIN RES<br>Mori S, 1996, MYCOTOXIN RES, V12, P91, DOI 10.1007/BF03192267
CR	replaced	164	164	60018	60017	J Vet Diagn Invest<br>Ross P F, 1991, J Vet Diagn Invest, V3, P238	J VET DIAGN INVEST<br>Ross P F, 1991, J VET DIAGN INVEST, V3, P238
CR	replaced	194	194	60048	60047	Journal of Toxicological Sciences<br>UENO Y, 1980, Journal of Toxicological Sciences, V5, P295, DOI 10.2131/jts.5.295	J TOXICOL SCI<br>UENO Y, 1980, J TOXICOL SCI, V5, P295, DOI 10.2131/jts.5.295

Record 399 | WOS:000448708300031

Application of Fungal Metabolites Against Mycotoxins Production

SO original: FUNGAL METABOLITES | SO normalised: FUNGAL METABOLITES | CR count: 169 → 169

Field	Status	Orig. pos	Norm. pos	Orig. line	Norm. line	Original cited/source value	Normalised cited/source value
CR	replaced	34	34	60172	60171	Mycotoxin Res<br>Cole R J, 1986, Mycotoxin Res, V2, P3, DOI 10.1007/BF03191956	MYCOTOXIN RES<br>Cole R J, 1986, MYCOTOXIN RES, V2, P3, DOI 10.1007/BF03191956
CR	replaced	137	137	60275	60274	Mycotoxin Res<br>Schmidt-Heydt Markus, 2009, Mycotoxin Res, V25, P141, DOI 10.1007/s12550-009-0019-x	MYCOTOXIN RES<br>Schmidt-Heydt Markus, 2009, MYCOTOXIN RES, V25, P141, DOI 10.1007/s12550-009-0019-x

Record 400 | WOS:000391769900002

Toxicity study of ochratoxin A using HEK293 and HepG2 cell lines based on microRNA profiling

SO original: HUMAN & EXPERIMENTAL TOXICOLOGY | SO normalised: HUMAN & EXPERIMENTAL TOXICOLOGY | CR count: 54 → 54

No journal-related changes detected in SO, J9, JI or CR for this record.

Record 401 | WOS:000393580000007

Limited Link between Oxidative Stress and Ochratoxin A-Induced Renal Injury in an Acute Toxicity Rat Model

SO original: TOXINS | SO normalised: TOXINS | CR count: 60 → 60

Field	Status	Orig. pos	Norm. pos	Orig. line	Norm. line	Original cited/source value	Normalised cited/source value
CR	replaced	28	28	60533	60532	Springerplus<br>Lautert Claudia, 2014, Springerplus, V3, P506, DOI 10.1186/2193-1801-3-506	SPRINGERPLUS<br>Lautert Claudia, 2014, SPRINGERPLUS, V3, P506, DOI 10.1186/2193-1801-3-506
CR	replaced	45	45	60550	60549	SCI REP<br>Qi X, 2014, SCI REP	SCI REP-UK<br>Qi X, 2014, SCI REP-UK

Record 402 | WOS:000387780500027

TOXICOPATHOLOGICAL AND SERUM BIOCHEMICAL ALTERATIONS INDUCED BY OCHRATOXIN A IN BROILER CHICKS AND THEIR AMELIORATION BY LOCALLY AVAILABLE BENTONITE CLAY

SO original: PAKISTAN JOURNAL OF AGRICULTURAL SCIENCES | SO normalised: PAKISTAN JOURNAL OF AGRICULTURAL SCIENCES | CR count: 47 → 47

No journal-related changes detected in SO, J9, JI or CR for this record.

Record 403 | WOS:000383825300009

Aflatoxin M1 cytotoxicity against human intestinal Caco-2 cells is enhanced in the presence of other mycotoxins

SO original: FOOD AND CHEMICAL TOXICOLOGY | SO normalised: FOOD AND CHEMICAL TOXICOLOGY | CR count: 63 → 63

No journal-related changes detected in SO, J9, JI or CR for this record.

Record 404 | WOS:000387347300005

Different Toxicity Mechanisms for Citrinin and Ochratoxin A Revealed by Transcriptomic Analysis in Yeast

SO original: TOXINS | SO normalised: TOXINS | CR count: 61 → 61

No journal-related changes detected in SO, J9, JI or CR for this record.

Record 405 | WOS:000383733300020

Quantitation of the DNA Adduct of Semicarbazide in Organs of Semicarbazide-Treated Rats by Isotope-Dilution Liquid Chromatography-Tandem Mass Spectrometry: A Comparative Study with the RNA Adduct

SO original: CHEMICAL RESEARCH IN TOXICOLOGY | SO normalised: CHEMICAL RESEARCH IN TOXICOLOGY | CR count: 29 → 29

No journal-related changes detected in SO, J9, JI or CR for this record.

Record 406 | WOS:000387199300010

Ochratoxin A activates neutrophils and kills these cells through necrosis, an effect eliminated through its conversion into ochratoxin Î±

SO original: TOXICOLOGY | SO normalised: TOXICOLOGY | CR count: 56 → 56

No journal-related changes detected in SO, J9, JI or CR for this record.

Record 407 | WOS:000380647600001

Cytotoxic Effects of Ochratoxin A in Neuro-2a Cells: Role of Oxidative Stress Evidenced by N-acetylcysteine

SO original: FRONTIERS IN MICROBIOLOGY | SO normalised: FRONTIERS IN MICROBIOLOGY | CR count: 73 → 73

Field	Status	Orig. pos	Norm. pos	Orig. line	Norm. line	Original cited/source value	Normalised cited/source value
CR	replaced	34	34	61233	61232	Springerplus<br>Lautert Claudia, 2014, Springerplus, V3, P506, DOI 10.1186/2193-1801-3-506	SPRINGERPLUS<br>Lautert Claudia, 2014, SPRINGERPLUS, V3, P506, DOI 10.1186/2193-1801-3-506
CR	replaced	67	67	61266	61265	[å °å-¦å ç¼<br>[ç æ°´ç ¯ Wang Shuijiong], 2014, [å °å-¦å ç¼ , Earth Science Frontiers], V21, P21	[é£ å ç§ å-¦<br>[ç æ°´ç ¯ Wang Shuijiong], 2014, [é£ å ç§ å-¦, Earth Science Frontiers], V21, P21

Record 408 | WOS:000380763800002

Ochratoxin A: 50 Years of Research

SO original: TOXINS | SO normalised: TOXINS | CR count: 374 → 374

Field	Status	Orig. pos	Norm. pos	Orig. line	Norm. line	Original cited/source value	Normalised cited/source value
CR	replaced	62	62	61407	61406	Mutation Research<br>Azemar Brigitte, 1997, Mutation Research, V379, pS157, DOI 10.1016/S0027-5107(97)83165-X	MUTAT RES<br>Azemar Brigitte, 1997, MUTAT RES, V379, pS157, DOI 10.1016/S0027-5107(97)83165-X
CR	replaced	103	103	61448	61447	Mycotoxin Res<br>Degen G H, 2007, Mycotoxin Res, V23, P88, DOI 10.1007/BF02946032	MYCOTOXIN RES<br>Degen G H, 2007, MYCOTOXIN RES, V23, P88, DOI 10.1007/BF02946032

CR	replaced	126	126	61471	61470	Mycotoxin Res<br>El-Sayed Am Abdalla, 2002, Mycotoxin Res, V18, P23, DOI 10.1007/BF02946136	MYCOTOXIN RES<br>El-Sayed Am Abdalla, 2002, MYCOTOXIN RES, V18, P23, DOI 10.1007/BF02946136
CR	replaced	140	140	61485	61484	Annales de Recherches Veterinaires<br>GALTIER P, 1976, Annales de Recherches Veterinaires, V7, P91	ANN RECH VET<br>GALTIER P, 1976, ANN RECH VET, V7, P91
CR	replaced	156	156	61501	61500	Mycotoxin Res<br>Grajewski J, 2007, Mycotoxin Res, V23, P22, DOI 10.1007/BF02946020	MYCOTOXIN RES<br>Grajewski J, 2007, MYCOTOXIN RES, V23, P22, DOI 10.1007/BF02946020
CR	replaced	186	186	61531	61530	Journal of Nutritional and Environmental Medicine (Abingdon)<br>Jonsyn Felixtina E., 1999, Journal of Nutritional and Environmental Medicine (Abingdon), V9, P15, DOI 10.1080/13590849762763	Journal of Nutritional & Environmental Medicine (Abingdon)<br>Jonsyn Felixtina E., 1999, Journal of Nutritional & Environmental Medicine (Abingdon), V9, P15, DOI 10.1080/13590849762763
CR	replaced	201	201	61546	61545	Endemic nephropathy<br>Krogh P., 1972, Endemic nephropathy, P266	ENDEMIC NEPHROPATHY<br>Krogh P., 1972, ENDEMIC NEPHROPATHY, P266
CR	replaced	215	215	61560	61559	Mycotoxin Research<br>Malir F., 2001, Mycotoxin Research, V17, P39	MYCOTOXIN RES<br>Malir F., 2001, MYCOTOXIN RES, V17, P39
CR	replaced	247	247	61592	61591	Mycotoxin Res<br>Munoz Katherine, 2010, Mycotoxin Res, V26, P59, DOI 10.1007/s12550-009-0040-0	MYCOTOXIN RES<br>Munoz Katherine, 2010, MYCOTOXIN RES, V26, P59, DOI 10.1007/s12550-009-0040-0
CR	replaced	255	255	61600	61599	Mycotoxin Res<br>Ostry V, 2005, Mycotoxin Res, V21, P49, DOI 10.1007/BF02954817	MYCOTOXIN RES<br>Ostry V, 2005, MYCOTOXIN RES, V21, P49, DOI 10.1007/BF02954817
CR	replaced	287	287	61632	61631	IARC Sci Publ<br>Plestina S, 1991, IARC Sci Publ, P43	IARC SCI PUBL<br>Plestina S, 1991, IARC SCI PUBL, P43

Record 409 | WOS:000377720700011

Inhibition of CXCL12-mediated chemotaxis of Jurkat cells by direct immunotoxicants

SO original: ARCHIVES OF TOXICOLOGY | SO normalised: ARCHIVES OF TOXICOLOGY | CR count: 50 → 50

No journal-related changes detected in SO, J9, JI or CR for this record.

Record 410 | WOS:000376990200009

Cytotoxicity, apoptosis, DNA damage and methylation in mammary and kidney epithelial cell lines exposed to ochratoxin A

SO original: CELL BIOLOGY AND TOXICOLOGY | SO normalised: CELL BIOLOGY AND TOXICOLOGY | CR count: 34 → 34

No journal-related changes detected in SO, J9, JI or CR for this record.

Record 411 | WOS:000378297500009

Effects of Ochratoxin A on Mouse Oocyte Maturation and Fertilization, and Apoptosis During Fetal Development

SO original: ENVIRONMENTAL TOXICOLOGY | SO normalised: ENVIRONMENTAL TOXICOLOGY | CR count: 52 → 52

No journal-related changes detected in SO, J9, JI or CR for this record.

Record 412 | WOS:000375813200002

Innovative technologies for the mitigation of mycotoxins in animal feed and ingredients-A review of recent patents

SO original: ANIMAL FEED SCIENCE AND TECHNOLOGY | SO normalised: ANIMAL FEED SCIENCE AND TECHNOLOGY | CR count: 83 → 83

No journal-related changes detected in SO, J9, JI or CR for this record.

Record 413 | WOS:000373624800006

Multiparametric luminescent cell viability assay in toxicology models: A critical evaluation

SO original: JOURNAL OF PHARMACOLOGICAL AND TOXICOLOGICAL METHODS | SO normalised: JOURNAL OF PHARMACOLOGICAL AND TOXICOLOGICAL METHODS | CR count: 46 → 46

No journal-related changes detected in SO, J9, JI or CR for this record.

Record 414 | WOS:000377731900003

Mycotoxins modify the barrier function of Caco-2 cells through differential gene expression of specific claudin isoforms: Protective effect of illite mineral clay

SO original: TOXICOLOGY | SO normalised: TOXICOLOGY | CR count: 87 → 87

No journal-related changes detected in SO, J9, JI or CR for this record.

Record 415 | WOS:000372000700010

Alleviative effect of myricetin on ochratoxin A-induced oxidative stress in rat renal cortex: histological and biochemical study

SO original: HISTOLOGY AND HISTOPATHOLOGY | SO normalised: HISTOLOGY AND HISTOPATHOLOGY | CR count: 76 → 76

Field	Status	Orig. pos	Norm. pos	Orig. line	Norm. line	Original cited/source value	Normalised cited/source value
CR	replaced	42	42	62544	62543	Mycotoxin Res<br>Martins H Marina, 2012, Mycotoxin Res, V28, P107, DOI 10.1007/s12550-011-0118-3	MYCOTOXIN RES<br>Martins H Marina, 2012, MYCOTOXIN RES, V28, P107, DOI 10.1007/s12550-011-0118-3

Record 416 | WOS:000375861700010

Natural Co-Occurrence of Mycotoxins in Foods and Feeds and Their in vitro Combined Toxicological Effects

SO original: TOXINS | SO normalised: TOXINS | CR count: 212 → 212

Field	Status	Orig. pos	Norm. pos	Orig. line	Norm. line	Original cited/source value	Normalised cited/source value
CR	replaced	7	7	62641	62640	Mycotoxin Res<br>Almeida Ines, 2011, Mycotoxin Res, V27, P177, DOI 10.1007/s12550-011-0093-8	MYCOTOXIN RES<br>Almeida Ines, 2011, MYCOTOXIN RES, V27, P177, DOI 10.1007/s12550-011-0093-8
CR	replaced	24	24	62658	62657	Food Industrial Processes - Methods and Equipment<br>Bosco F., 2012, Food Industrial Processes - Methods and Equipment	FOOD INDUSTRIAL PROCESSES - METHODS AND EQUIPMENT<br>Bosco F., 2012, FOOD INDUSTRIAL PROCESSES - METHODS AND EQUIPMENT
CR	replaced	64	64	62698	62697	Mycotoxin Res<br>Follmann W, 2000, Mycotoxin Res, V16 Suppl 1, P123, DOI 10.1007/BF02942998	MYCOTOXIN RES<br>Follmann W, 2000, MYCOTOXIN RES, V16 Suppl 1, P123, DOI 10.1007/BF02942998
CR	replaced	73	73	62707	62706	J Biochem Mol Toxicol<br>Golli-Bennour Emna El, 2010, J Biochem Mol Toxicol, V24, P42, DOI 10.1002/jbt.20310	J BIOCHEM MOL TOXIC<br>Golli-Bennour Emna El, 2010, J BIOCHEM MOL TOXIC, V24, P42, DOI 10.1002/jbt.20310
CR	replaced	112	112	62746	62745	Mycotoxin Res<br>Knecht A, 2005, Mycotoxin Res, V21, P176, DOI 10.1007/BF02959258	MYCOTOXIN RES<br>Knecht A, 2005, MYCOTOXIN RES, V21, P176, DOI 10.1007/BF02959258
CR	replaced	130	130	62764	62763	Mycotoxin Res<br>Makun Hussaini Anthony, 2011, Mycotoxin Res, V27, P97, DOI 10.1007/s12550-010-0080-5	MYCOTOXIN RES<br>Makun Hussaini Anthony, 2011, MYCOTOXIN RES, V27, P97, DOI 10.1007/s12550-010-0080-5
CR	replaced	134	134	62768	62767	Mycotoxin Res<br>Martins H M, 2006, Mycotoxin Res, V22, P206, DOI 10.1007/BF02946743	MYCOTOXIN RES<br>Martins H M, 2006, MYCOTOXIN RES, V22, P206, DOI 10.1007/BF02946743

Record 417 | WOS:000431903800102

Mycotoxins at the blood-brain barrier: Metabolism, toxicity, barrier integrity and transfer to the brain

SO original: ABSTRACTS OF PAPERS OF THE AMERICAN CHEMICAL SOCIETY | SO normalised: ABSTRACTS OF PAPERS OF THE AMERICAN CHEMICAL SOCIETY | CR count: 0 → 0

No journal-related changes detected in SO, J9, JI or CR for this record.

Record 418 | WOS:000372963300003

Ochratoxin A and T-2 Toxin Induce Clonogenicity and Cell Migration in Human Colon Carcinoma and Fetal Lung Fibroblast Cell Lines

SO original: JOURNAL OF BIOCHEMICAL AND MOLECULAR TOXICOLOGY | SO normalised: JOURNAL OF BIOCHEMICAL AND MOLECULAR TOXICOLOGY | CR count: 50 → 50

No journal-related changes detected in SO, J9, JI or CR for this record.

Record 419 | WOS:000370832600004

Malignant transformation of human gastric epithelium cells via reactive oxygen species production and Wnt/Î²-catenin pathway activation following 40-week exposure to ochratoxin A

SO original: CANCER LETTERS | SO normalised: CANCER LETTERS | CR count: 43 → 43

No journal-related changes detected in SO, J9, JI or CR for this record.

Record 420 | WOS:000369179000011

Effects of aflatoxin B1 on oxidative stress markers and apoptosis of spleens in broilers

SO original: TOXICOLOGY AND INDUSTRIAL HEALTH | SO normalised: TOXICOLOGY AND INDUSTRIAL HEALTH | CR count: 47 → 47

Field	Status	Orig. pos	Norm. pos	Orig. line	Norm. line	Original cited/source value	Normalised cited/source value
CR	replaced	33	33	63223	63222	J Immunotoxicol<br>Sabourin Patrick J, 2006, J Immunotoxicol, V3, P11, DOI 10.1080/15476910500468635	J IMMUNOTOXICOL<br>Sabourin Patrick J, 2006, J IMMUNOTOXICOL, V3, P11, DOI 10.1080/15476910500468635

Record 421 | WOS:000370094500037

Grafting of gallic acid onto chitosan nano particles enhances antioxidant activities in vitro and protects against ochratoxin A toxicity in catfish (Clarias gariepinus)

SO original: ENVIRONMENTAL TOXICOLOGY AND PHARMACOLOGY | SO normalised: ENVIRONMENTAL TOXICOLOGY AND PHARMACOLOGY | CR count: 64 → 64

No journal-related changes detected in SO, J9, JI or CR for this record.

Record 422 | WOS:000388323700013

Mycotoxin Outbreak in Animal Feed

SO original: FOODBORNE DISEASES: CASE STUDIES OF OUTBREAKS IN THE AGRI-FOOD ; INDUSTRIES | SO normalised: FOODBORNE DISEASES: CASE STUDIES OF OUTBREAKS IN THE AGRI-FOOD ; INDUSTRIES | CR count: 167 → 167

Field	Status	Orig. pos	Norm. pos	Orig. line	Norm. line	Original cited/source value	Normalised cited/source value
CR	replaced	53	53	63482	63481	Polish Journal of Veterinary Sciences<br>Gajecki M., 2002, Polish Journal of Veterinary Sciences, V5, P117	POL J VET SCI<br>Gajecki M., 2002, POL J VET SCI, V5, P117
CR	replaced	54	54	63483	63482	Annales de Recherches Veterinaires<br>GALTIER P, 1976, Annales de Recherches Veterinaires, V7, P91	ANN RECH VET<br>GALTIER P, 1976, ANN RECH VET, V7, P91

CR	replaced	70	70	63499	63498	MYCOTOXINS IN FOOD<br>Ingle A, 2010, MYCOTOXINS IN FOOD, FEED AND BIOWEAPONS, P291, DOI 10.1007/978-3-642-00725-5_17	Mycotoxins in food<br>Ingle A, 2010, Mycotoxins in food, FEED AND BIOWEAPONS, P291, DOI 10.1007/978-3-642-00725-5_17
CR	replaced	125	125	63554	63553	FUNGI AND FOOD SPOILAGE<br>Pitt JI, 2009, FUNGI AND FOOD SPOILAGE, THIRD EDITION, P3, DOI 10.1007/978-0-387-92207-2_2	Fungi and food spoilage<br>Pitt JI, 2009, Fungi and food spoilage, THIRD EDITION, P3, DOI 10.1007/978-0-387-92207-2_2
CR	replaced	145	145	63574	63573	MYCOTOXINS IN FOOD<br>Sugita-Konishi Y, 2010, MYCOTOXINS IN FOOD, FEED AND BIOWEAPONS, P253, DOI 10.1007/978-3-642-00725-5_15	Mycotoxins in food<br>Sugita-Konishi Y, 2010, Mycotoxins in food, FEED AND BIOWEAPONS, P253, DOI 10.1007/978-3-642-00725-5_15
CR	replaced	166	166	63595	63594	2014<br>,, 2014, EFSA Journal, V12, P3667	2014<br>,, 2014, EFSA J, V12, P3667
CR	replaced	167	167	63596	63595	2012<br>,, 2012, EFSA Journal, V10, P2557	2012<br>,, 2012, EFSA J, V10, P2557

Record 423 | WOS:000368966200007

Ochratoxin A-induced cytotoxicity, genotoxicity and reactive oxygen species in kidney cells: An integrative approach of complementary endpoints

SO original: FOOD AND CHEMICAL TOXICOLOGY | SO normalised: FOOD AND CHEMICAL TOXICOLOGY | CR count: 71 → 71

No journal-related changes detected in SO, J9, JI or CR for this record.

Record 424 | WOS:000371816100005

Individual and combined effects of roquefortine C and mycophenolic acid on human monocytic and intestinal cells

SO original: WORLD MYCOTOXIN JOURNAL | SO normalised: WORLD MYCOTOXIN JOURNAL | CR count: 37 → 37

No journal-related changes detected in SO, J9, JI or CR for this record.

Record 425 | WOS:000376604100010

Evaluation of renal in vitro models used in ochratoxin research

SO original: WORLD MYCOTOXIN JOURNAL | SO normalised: WORLD MYCOTOXIN JOURNAL | CR count: 243 → 243

Field	Status	Orig. pos	Norm. pos	Orig. line	Norm. line	Original cited/source value	Normalised cited/source value
CR	replaced	63	63	63966	63965	Mycotoxin Res<br>Follmann W, 2005, Mycotoxin Res, V21, P53, DOI 10.1007/BF02954818	MYCOTOXIN RES<br>Follmann W, 2005, MYCOTOXIN RES, V21, P53, DOI 10.1007/BF02954818

CR	replaced	75	75	63978	63977	J Biochem Mol Toxicol<br>Golli-Bennour Emna El, 2010, J Biochem Mol Toxicol, V24, P42, DOI 10.1002/jbt.20310	J BIOCHEM MOL TOXIC<br>Golli-Bennour Emna El, 2010, J BIOCHEM MOL TOXIC, V24, P42, DOI 10.1002/jbt.20310
CR	replaced	133	133	64036	64035	Mycotoxin Res<br>Lebrun S, 2001, Mycotoxin Res, V17 Suppl 2, P132, DOI 10.1007/BF03036421	MYCOTOXIN RES<br>Lebrun S, 2001, MYCOTOXIN RES, V17 Suppl 2, P132, DOI 10.1007/BF03036421
CR	replaced	197	197	64100	64099	Mycotoxin Res<br>Sauvant C, 2003, Mycotoxin Res, V19, P118, DOI 10.1007/BF02942949	MYCOTOXIN RES<br>Sauvant C, 2003, MYCOTOXIN RES, V19, P118, DOI 10.1007/BF02942949
CR	replaced	208	208	64111	64110	Mycotoxin Res<br>Schwerdt G, 2000, Mycotoxin Res, V16 Suppl 2, P154, DOI 10.1007/BF02940025	MYCOTOXIN RES<br>Schwerdt G, 2000, MYCOTOXIN RES, V16 Suppl 2, P154, DOI 10.1007/BF02940025
CR	replaced	225	225	64128	64127	Mycotoxin Res<br>Stoev Stoycho D, 2010, Mycotoxin Res, V26, P31, DOI 10.1007/s12550-009-0038-7	MYCOTOXIN RES<br>Stoev Stoycho D, 2010, MYCOTOXIN RES, V26, P31, DOI 10.1007/s12550-009-0038-7
CR	replaced	229	229	64132	64131	Methods Mol Biol<br>Taub M, 1997, Methods Mol Biol, V75, P153	METHODS MOL BIOL<br>Taub M, 1997, METHODS MOL BIOL, V75, P153
CR	replaced	235	235	64138	64137	Mycotoxin Res<br>Weber F, 2003, Mycotoxin Res, V19, P20, DOI 10.1007/BF02940085	MYCOTOXIN RES<br>Weber F, 2003, MYCOTOXIN RES, V19, P20, DOI 10.1007/BF02940085

Record 426 | WOS:000389199300011

Diet contaminated with ochratoxin A at the highest level allowed by EU recommendation disturbs liver metabolism in weaned piglets

SO original: WORLD MYCOTOXIN JOURNAL | SO normalised: WORLD MYCOTOXIN JOURNAL | CR count: 56 → 56

Field	Status	Orig. pos	Norm. pos	Orig. line	Norm. line	Original cited/source value	Normalised cited/source value
CR	replaced	5	5	64217	64216	Off J Eur Union<br>[Anonymous], 2006, Off J Eur Union, V229, P7, DOI DOI 10.3390/ECMC2021-11474	OFF J EUR UNION<br>[Anonymous], 2006, OFF J EUR UNION, V229, P7, DOI DOI 10.3390/ECMC2021-11474
CR	replaced	34	34	64246	64245	Mycotoxin Res<br>Martins H Marina, 2012, Mycotoxin Res, V28, P107, DOI 10.1007/s12550-011-0118-3	MYCOTOXIN RES<br>Martins H Marina, 2012, MYCOTOXIN RES, V28, P107, DOI 10.1007/s12550-011-0118-3

Record 427 | WOS:000368260800005

Ochratoxin A as possible factor trigging autism and its male prevalence via epigenetic mechanism

SO original: NUTRITIONAL NEUROSCIENCE | SO normalised: NUTRITIONAL NEUROSCIENCE | CR count: 17 → 17

No journal-related changes detected in SO, J9, JI or CR for this record.

Record 428 | WOS:000387395900007

In Vitro Assays to Estimate the Toxicological Effects of Masked Mycotoxins

SO original: MASKED MYCOTOXINS IN FOOD: FORMATION, OCCURRENCE AND TOXICOLOGICAL ; RELEVANCE | SO normalised: MASKED MYCOTOXINS IN FOOD: FORMATION, OCCURRENCE AND TOXICOLOGICAL ; RELEVANCE | CR count: 126 → 126

Field	Status	Orig. pos	Norm. pos	Orig. line	Norm. line	Original cited/source value	Normalised cited/source value
CR	replaced	39	39	64442	64441	MYCOTOXINS IN FOOD<br>Dall'Asta C, 2010, MYCOTOXINS IN FOOD, FEED AND BIOWEAPONS, P385, DOI 10.1007/978-3-642-00725-5_22	Mycotoxins in food<br>Dall'Asta C, 2010, Mycotoxins in food, FEED AND BIOWEAPONS, P385, DOI 10.1007/978-3-642-00725-5_22
CR	replaced	69	69	64472	64471	Environmental Toxicology<br>Kennedy C.J., 2013, Environmental Toxicology, P689	ENVIRON TOXICOL<br>Kennedy C.J., 2013, ENVIRON TOXICOL, P689
CR	replaced	87	87	64490	64489	Mycotoxin Res<br>Motta E L, 2009, Mycotoxin Res, V25, P229, DOI 10.1007/s12550-009-0032-0	MYCOTOXIN RES<br>Motta E L, 2009, MYCOTOXIN RES, V25, P229, DOI 10.1007/s12550-009-0032-0
CR	replaced	104	104	64507	64506	Mycotoxin Res<br>Ruhland M, 1997, Mycotoxin Res, V13, P54, DOI 10.1007/BF02945066	MYCOTOXIN RES<br>Ruhland M, 1997, MYCOTOXIN RES, V13, P54, DOI 10.1007/BF02945066
CR	replaced	126	126	64529	64528	2011<br>,, 2011, EFSA Journal, V9, P2197	2011<br>,, 2011, EFSA J, V9, P2197

Record 429 | WOS:000384724200015

Probiotic preparation reduces faecal water genotoxicity and cytotoxicity in chickens fed ochratoxin A contaminated feed (in vivo study)

SO original: ACTA BIOCHIMICA POLONICA | SO normalised: ACTA BIOCHIMICA POLONICA | CR count: 44 → 44

No journal-related changes detected in SO, J9, JI or CR for this record.

Record 430 | WOS:000366832700013

miR-122 plays an important role in ochratoxin A-induced hepatocyte apoptosis in vitro and in vivo

SO original: TOXICOLOGY RESEARCH | SO normalised: TOXICOLOGY RESEARCH | CR count: 42 → 42

Field	Status	Orig. pos	Norm. pos	Orig. line	Norm. line	Original cited/source value	Normalised cited/source value

CR	replaced	12	12	64711	64710	Mycotoxin Res<br>Essid E, 2011, Mycotoxin Res, V27, P167, DOI 10.1007/s12550-011-0092-9	MYCOTOXIN RES<br>Essid E, 2011, MYCOTOXIN RES, V27, P167, DOI 10.1007/s12550-011-0092-9

Record 431 | WOS:000367635500005

MiR-122 partly mediates the ochratoxin A-induced GC-2 cell apoptosis

SO original: TOXICOLOGY IN VITRO | SO normalised: TOXICOLOGY IN VITRO | CR count: 50 → 50

No journal-related changes detected in SO, J9, JI or CR for this record.

Record 432 | WOS:000367013500020

Interaction of Citrinin with Human Serum Albumin

SO original: TOXINS | SO normalised: TOXINS | CR count: 37 → 37

No journal-related changes detected in SO, J9, JI or CR for this record.

Record 433 | WOS:000362726700025

The Phytoalexin Resveratrol Ameliorates Ochratoxin A Toxicity in Human Embryonic Kidney (HEK293) Cells

SO original: JOURNAL OF CELLULAR BIOCHEMISTRY | SO normalised: JOURNAL OF CELLULAR BIOCHEMISTRY | CR count: 33 → 33

**Field**	**Status**	**Orig. pos**	**Norm. pos**	**Orig. line**	**Norm. line**	**Original cited/source value**	**Normalised cited/source value**
CR	replaced	3	3	65051	65050	Mycotoxin Res<br>Bankole S, 2006, Mycotoxin Res, V22, P163, DOI 10.1007/BF02959270	MYCOTOXIN RES<br>Bankole S, 2006, MYCOTOXIN RES, V22, P163, DOI 10.1007/BF02959270

Record 434 | WOS:000363821500008

A food contaminant ochratoxin A suppresses pregnane X receptor (PXR)-mediated CYP3A4 induction in primary cultures of human hepatocytes

SO original: TOXICOLOGY | SO normalised: TOXICOLOGY | CR count: 32 → 32

No journal-related changes detected in SO, J9, JI or CR for this record.

Record 435 | WOS:000363259300002

In vitro exposure of Penicillium mycotoxins with or without a modified yeast cell wall extract (mYCW) on bovine macrophages (BoMacs)

SO original: MYCOTOXIN RESEARCH | SO normalised: MYCOTOXIN RESEARCH | CR count: 46 → 46

Field	Status	Orig. pos	Norm. pos	Orig. line	Norm. line	Original cited/source value	Normalised cited/source value
CR	replaced	16	16	65243	65242	Feedstuffs<br>Forgacs J., 1962, Feedstuffs, V34, P124	FEEDSTUFFS<br>Forgacs J., 1962, FEEDSTUFFS, V34, P124
CR	replaced	26	26	65253	65252	Mycotoxin Res<br>Martins H Marina, 2012, Mycotoxin Res, V28, P107, DOI 10.1007/s12550-011-0118-3	MYCOTOXIN RES<br>Martins H Marina, 2012, MYCOTOXIN RES, V28, P107, DOI 10.1007/s12550-011-0118-3

Record 436 | WOS:000360322300014

Ochratoxin A at low concentrations inhibits in vitro growth of canine umbilical cord matrix mesenchymal stem cells through oxidative chromatin and DNA damage

SO original: REPRODUCTIVE TOXICOLOGY | SO normalised: REPRODUCTIVE TOXICOLOGY | CR count: 76 → 76

No journal-related changes detected in SO, J9, JI or CR for this record.

Record 437 | WOS:000365417800020

The influence of ochratoxin A on DNA adduct formation by the carcinogen aristolochic acid in rats

SO original: ARCHIVES OF TOXICOLOGY | SO normalised: ARCHIVES OF TOXICOLOGY | CR count: 107 → 107

Field	Status	Orig. pos	Norm. pos	Orig. line	Norm. line	Original cited/source value	Normalised cited/source value
CR	replaced	52	52	65565	65564	IARC Sci Publ<br>Pfohl-Leszkowicz A, 1993, IARC Sci Publ, P141	IARC SCI PUBL<br>Pfohl-Leszkowicz A, 1993, IARC SCI PUBL, P141

Record 438 | WOS:000357839100054

Cross-linked chitosan polymers as generic adsorbents for simultaneous adsorption of multiple mycotoxins

SO original: FOOD CONTROL | SO normalised: FOOD CONTROL | CR count: 38 → 38

No journal-related changes detected in SO, J9, JI or CR for this record.

Record 439 | WOS:000370693801841

A contribution to hazard assessment of combined exposure to mycotoxins using in vitro toxicity testing

SO original: TOXICOLOGY LETTERS | SO normalised: TOXICOLOGY LETTERS | CR count: 0 → 0

No journal-related changes detected in SO, J9, JI or CR for this record.

Record 440 | WOS:000361263500036

Cytotoxicity of the mycotoxins deoxynivalenol and ochratoxin A on Caco-2 cell line in presence of resveratrol

SO original: TOXICOLOGY IN VITRO | SO normalised: TOXICOLOGY IN VITRO | CR count: 46 → 46

No journal-related changes detected in SO, J9, JI or CR for this record.

Record 441 | WOS:000367407400003

Dietary mycotoxins, co-exposure, and carcinogenesis in humans: Short review

SO original: MUTATION RESEARCH-REVIEWS IN MUTATION RESEARCH | SO normalised: MUTATION RESEARCH-REVIEWS IN MUTATION RESEARCH | CR count: 113 → 113

No journal-related changes detected in SO, J9, JI or CR for this record.

Record 442 | WOS:000364960400028

Comparative Ochratoxin Toxicity: A Review of the Available Data

SO original: TOXINS | SO normalised: TOXINS | CR count: 215 → 215

Field	Status	Orig. pos	Norm. pos	Orig. line	Norm. line	Original cited/source value	Normalised cited/source value
CR	replaced	70	70	66209	66208	Mycotoxin Res<br>Geisen R, 2006, Mycotoxin Res, V22, P134, DOI 10.1007/BF02956777	MYCOTOXIN RES<br>Geisen R, 2006, MYCOTOXIN RES, V22, P134, DOI 10.1007/BF02956777
CR	replaced	111	111	66250	66249	Mycotoxin Res<br>Kohler H, 2003, Mycotoxin Res, V19, P108, DOI 10.1007/BF02942947	MYCOTOXIN RES<br>Kohler H, 2003, MYCOTOXIN RES, V19, P108, DOI 10.1007/BF02942947
CR	replaced	112	112	66251	66250	Mycotoxin Res<br>Kohler H, 2002, Mycotoxin Res, V18 Suppl 2, P169, DOI 10.1007/BF02946089	MYCOTOXIN RES<br>Kohler H, 2002, MYCOTOXIN RES, V18 Suppl 2, P169, DOI 10.1007/BF02946089
CR	replaced	171	171	66310	66309	Mycotoxin Res<br>Scheuer Rainer, 2002, Mycotoxin Res, V18, P62, DOI 10.1007/BF02946698	MYCOTOXIN RES<br>Scheuer Rainer, 2002, MYCOTOXIN RES, V18, P62, DOI 10.1007/BF02946698
CR	replaced	188	188	66327	66326	Mycotoxin Res<br>Stoev Stoycho D, 2010, Mycotoxin Res, V26, P31, DOI 10.1007/s12550-009-0038-7	MYCOTOXIN RES<br>Stoev Stoycho D, 2010, MYCOTOXIN RES, V26, P31, DOI 10.1007/s12550-009-0038-7

Record 443 | WOS:000355886400028

Development of an aptasensor for the fast detection of Versicolorin A

SO original: FOOD CONTROL | SO normalised: FOOD CONTROL | CR count: 57 → 57

Field	Status	Orig. pos	Norm. pos	Orig. line	Norm. line	Original cited/source value	Normalised cited/source value

CR	replaced	27	27	66440	66439	Mycotoxin Res<br>Leong Yin-Hui, 2011, Mycotoxin Res, V27, P207, DOI 10.1007/s12550-011-0097-4	MYCOTOXIN RES<br>Leong Yin-Hui, 2011, MYCOTOXIN RES, V27, P207, DOI 10.1007/s12550-011-0097-4

Record 444 | WOS:000359029000029

Cytotoxic and genotoxic potencies of single and combined spore extracts of airborne OTA-producing and OTA-non-producing Aspergilli in Human lung A549 cells

SO original: ECOTOXICOLOGY AND ENVIRONMENTAL SAFETY | SO normalised: ECOTOXICOLOGY AND ENVIRONMENTAL SAFETY | CR count: 62 → 62

Field	Status	Orig. pos	Norm. pos	Orig. line	Norm. line	Original cited/source value	Normalised cited/source value
CR	replaced	46	46	66589	66588	FUNGI AND FOOD SPOILAGE<br>Pitt JI, 2009, FUNGI AND FOOD SPOILAGE, THIRD EDITION, P275, DOI 10.1007/978-0-387-92207-2_8	Fungi and food spoilage<br>Pitt JI, 2009, Fungi and food spoilage, THIRD EDITION, P275, DOI 10.1007/978-0-387-92207-2_8

Record 445 | WOS:000362622500013

Ochratoxin A induces DNA damage and G2 phase arrest in human esophageal epithelium Het-1A cells in vitro

SO original: JOURNAL OF TOXICOLOGICAL SCIENCES | SO normalised: JOURNAL OF TOXICOLOGICAL SCIENCES | CR count: 53 → 53

No journal-related changes detected in SO, J9, JI or CR for this record.

Record 446 | WOS:000362860200019

Risk analysis of main mycotoxins occurring in food for children: An overview

SO original: FOOD AND CHEMICAL TOXICOLOGY | SO normalised: FOOD AND CHEMICAL TOXICOLOGY | CR count: 172 → 172

Field	Status	Orig. pos	Norm. pos	Orig. line	Norm. line	Original cited/source value	Normalised cited/source value
CR	replaced	33	33	66831	66830	Mycotoxins: Detection methods<br>Bhatnagar D., 2008, Mycotoxins: Detection methods, management, public health and agricultural trade	Mycotoxins: detection methods<br>Bhatnagar D., 2008, Mycotoxins: detection methods, management, public health and agricultural trade
CR	replaced	38	38	66836	66835	African Journal of Biotechnology<br>Brown Robert L., 2003, African Journal of Biotechnology, V2, P557	AFR J BIOTECHNOL<br>Brown Robert L., 2003, AFR J BIOTECHNOL, V2, P557
CR	replaced	51	51	66849	66848	Mycotoxin Res<br>Daenicke Sven, 2011, Mycotoxin Res, V27, P49, DOI 10.1007/s12550-010-0074-3	MYCOTOXIN RES<br>Daenicke Sven, 2011, MYCOTOXIN RES, V27, P49, DOI 10.1007/s12550-010-0074-3

CR	replaced	54	54	66852	66851	FUSARIUM MYCOTOXINS<br>Desjardins A. E, 2006, FUSARIUM MYCOTOXINS, P53	Fusarium: mycotoxins<br>Desjardins A. E, 2006, Fusarium: mycotoxins, P53
CR	replaced	55	55	66853	66852	FOOD SCI TECHNOL<br>Dors G.C., 2009, FOOD SCI TECHNOL, V42, P433	FOOD SCI TECH-BRAZIL<br>Dors G.C., 2009, FOOD SCI TECH-BRAZIL, V42, P433
CR	replaced	76	76	66874	66873	Mycotoxins: Detection Methods<br>Hell K., 2008, Mycotoxins: Detection Methods, Management, Public Health and Agricultural Trade, P210	Mycotoxins: detection methods<br>Hell K., 2008, Mycotoxins: detection methods, Management, Public Health and Agricultural Trade, P210

Record 447 | WOS:000362551400018

Biomonitoring using dried blood spots: Detection of ochratoxin A and its degradation product 2'R-ochratoxin A in blood from coffee drinkers

SO original: MOLECULAR NUTRITION & FOOD RESEARCH | SO normalised: MOLECULAR NUTRITION & FOOD RESEARCH | CR count: 42 → 42

Field	Status	Orig. pos	Norm. pos	Orig. line	Norm. line	Original cited/source value	Normalised cited/source value
CR	replaced	12	12	67045	67044	Mycotoxin Res<br>Degen G H, 2007, Mycotoxin Res, V23, P88, DOI 10.1007/BF02946032	MYCOTOXIN RES<br>Degen G H, 2007, MYCOTOXIN RES, V23, P88, DOI 10.1007/BF02946032
CR	replaced	29	29	67062	67061	Mycotoxin Res<br>Martlbauer Erwin, 2009, Mycotoxin Res, V25, P175, DOI 10.1007/s12550-009-0025-z	MYCOTOXIN RES<br>Martlbauer Erwin, 2009, MYCOTOXIN RES, V25, P175, DOI 10.1007/s12550-009-0025-z
CR	replaced	32	32	67065	67064	Mycotoxin Res<br>Munoz Katherine, 2011, Mycotoxin Res, V27, P239, DOI 10.1007/s12550-011-0100-0	MYCOTOXIN RES<br>Munoz Katherine, 2011, MYCOTOXIN RES, V27, P239, DOI 10.1007/s12550-011-0100-0

Record 448 | WOS:000361078800019

Hepatotoxic effect of ochratoxin A and citrinin, alone and in combination, and protective effect of vitamin E: In vitro study in HepG2 cell

SO original: FOOD AND CHEMICAL TOXICOLOGY | SO normalised: FOOD AND CHEMICAL TOXICOLOGY | CR count: 83 → 83

Field	Status	Orig. pos	Norm. pos	Orig. line	Norm. line	Original cited/source value	Normalised cited/source value
CR	replaced	29	29	67177	67176	Mycotoxin Res<br>Follmann W, 2007, Mycotoxin Res, V23, P101, DOI 10.1007/BF02946034	MYCOTOXIN RES<br>Follmann W, 2007, MYCOTOXIN RES, V23, P101, DOI 10.1007/BF02946034
CR	replaced	30	30	67178	67177	Mycotoxin Res<br>Follmann W, 2000, Mycotoxin Res, V16 Suppl 1, P123, DOI 10.1007/BF02942998	MYCOTOXIN RES<br>Follmann W, 2000, MYCOTOXIN RES, V16 Suppl 1, P123, DOI 10.1007/BF02942998

CR	replaced	31	31	67179	67178	J Biochem Mol Toxicol Golli-Bennour Emna El, 2010, J Biochem Mol Toxicol, V24, P42, DOI 10.1002/jbt.20310	J BIOCHEM MOL TOXIC Golli-Bennour Emna El, 2010, J BIOCHEM MOL TOXIC, V24, P42, DOI 10.1002/jbt.20310
CR	replaced	43	43	67191	67190	Mycotoxin Res Knecht A, 2005, Mycotoxin Res, V21, P176, DOI 10.1007/BF02959258	MYCOTOXIN RES Knecht A, 2005, MYCOTOXIN RES, V21, P176, DOI 10.1007/BF02959258
CR	replaced	61	61	67209	67208	IARC Sci Publ Petkova-Bocharova T, 1991, IARC Sci Publ, P83	IARC SCI PUBL Petkova-Bocharova T, 1991, IARC SCI PUBL, P83

Record 449 | WOS:000361762200007

Effects of Dietary Exposure to Zearalenone (ZEN) on Carp (Cyprinus carpio L.)

SO original: TOXINS | SO normalised: TOXINS | CR count: 74 → 74

Field	Status	Orig. pos	Norm. pos	Orig. line	Norm. line	Original cited/source value	Normalised cited/source value
CR	replaced	66	66	67368	67367	Mycotoxin Res Valenta H, 2003, Mycotoxin Res, V19, P51, DOI 10.1007/BF02940093	MYCOTOXIN RES Valenta H, 2003, MYCOTOXIN RES, V19, P51, DOI 10.1007/BF02940093

Record 450 | WOS:000360343000001

Cytotoxic effects of oosporein isolated from endophytic fungus Cochliobolus kusanoi

SO original: FRONTIERS IN MICROBIOLOGY | SO normalised: FRONTIERS IN MICROBIOLOGY | CR count: 44 → 44

Field	Status	Orig. pos	Norm. pos	Orig. line	Norm. line	Original cited/source value	Normalised cited/source value
CR	replaced	32	32	67471	67470	J Vet Diagn Invest Ross P F, 1989, J Vet Diagn Invest, V1, P271	J VET DIAGN INVEST Ross P F, 1989, J VET DIAGN INVEST, V1, P271

Record 451 | WOS:000360202700022

Review on Mycotoxin Issues in Ruminants: Occurrence in Forages, Effects of Mycotoxin Ingestion on Health Status and Animal Performance and Practical Strategies to Counteract Their Negative Effects

SO original: TOXINS | SO normalised: TOXINS | CR count: 314 → 314

Field	Status	Orig. pos	Norm. pos	Orig. line	Norm. line	Original cited/source value	Normalised cited/source value
CR	replaced	15	15	67565	67564	Mycopathologia [Anonymous], 2003, Mycopathologia, V65, P43	MYCOPATHOLOGIA [Anonymous], 2003, MYCOPATHOLOGIA, V65, P43
CR	replaced	29	29	67579	67578	Off. J. Eur. Union [Anonymous], 2013, Off. J. Eur. Union	OFF J EUR UNION [Anonymous], 2013, OFF J EUR UNION

CR	replaced	31	31	67581	67580	The Mycotoxin Blue Book<br>[Anonymous], 2005, The Mycotoxin Blue Book	The mycotoxin blue book<br>[Anonymous], 2005, The mycotoxin blue book
CR	replaced	73	73	67623	67622	Fusarium: Mycotoxins<br>Chelkowski J., 2014, Fusarium: Mycotoxins, Taxonomy, Pathogenicity	Fusarium: mycotoxins<br>Chelkowski J., 2014, Fusarium: mycotoxins, Taxonomy, Pathogenicity
CR	replaced	93	93	67643	67642	Mycotoxin Res<br>Dzidic Anamarija, 2010, Mycotoxin Res, V26, P235, DOI 10.1007/s12550-010-0061-8	MYCOTOXIN RES<br>Dzidic Anamarija, 2010, MYCOTOXIN RES, V26, P235, DOI 10.1007/s12550-010-0061-8
CR	replaced	110	110	67660	67659	Introduction to food- and airborne fungi<br>Frisvad J. C., 2004, Introduction to food- and airborne fungi, P321	Introduction to Food and Airborne Fungi<br>Frisvad J. C., 2004, Introduction to Food and Airborne Fungi, P321
CR	replaced	211	211	67761	67760	Nat Toxins<br>Muller H M, 1998, Nat Toxins, V6, P251, DOI 10.1002/(SICI)1522-7189(199811/12)6:6<251::AID-NT35>3.0.CO;2-P	NAT TOXINS<br>Muller H M, 1998, NAT TOXINS, V6, P251, DOI 10.1002/(SICI)1522-7189(199811/12)6:6<251::AID-NT35>3.0.CO;2-P
CR	replaced	220	220	67770	67769	Mycotoxin Res<br>Oldenburg E, 2005, Mycotoxin Res, V21, P105, DOI 10.1007/BF02954430	MYCOTOXIN RES<br>Oldenburg E, 2005, MYCOTOXIN RES, V21, P105, DOI 10.1007/BF02954430
CR	replaced	226	226	67776	67775	Feedstuffs<br>Pedrosa K., 2011, Feedstuffs, V83, P1	FEEDSTUFFS<br>Pedrosa K., 2011, FEEDSTUFFS, V83, P1

Record 452 | WOS:000358238100012

Ochratoxin A activates opposing c-MET/PI3K/Akt and MAPK/ERK 1-2 pathways in human proximal tubule HK-2 cells

SO original: ARCHIVES OF TOXICOLOGY | SO normalised: ARCHIVES OF TOXICOLOGY | CR count: 86 → 86

No journal-related changes detected in SO, J9, JI or CR for this record.

Record 453 | WOS:000357537400012

Effects of Chronic Ochratoxin A Exposure on p53 Heterozygous and p53 Homozygous Mice

SO original: TOXICOLOGIC PATHOLOGY | SO normalised: TOXICOLOGIC PATHOLOGY | CR count: 75 → 75

Field	Status	Orig. pos	Norm. pos	Orig. line	Norm. line	Original cited/source value	Normalised cited/source value
CR	replaced	1	1	68072	68071	Toxicology<br>[Anonymous], 1998, Toxicology, V125, P183	TOXICOLOGY<br>[Anonymous], 1998, TOXICOLOGY, V125, P183

Record 454 | WOS:000355330300006

Neurotoxic effects of ochratoxin A on the subventricular zone of adult mouse brain

SO original: JOURNAL OF APPLIED TOXICOLOGY | SO normalised: JOURNAL OF APPLIED TOXICOLOGY | CR count: 84 → 84

No journal-related changes detected in SO, J9, JI or CR for this record.

Record 455 | WOS:000357490500001

Mycotoxins - prevention and decontamination by yeasts

SO original: JOURNAL OF BASIC MICROBIOLOGY | SO normalised: JOURNAL OF BASIC MICROBIOLOGY | CR count: 129 → 129

Field	Status	Orig. pos	Norm. pos	Orig. line	Norm. line	Original cited/source value	Normalised cited/source value
CR	replaced	104	104	68489	68488	Mycotoxin Res Schatzmayr G, 2003, Mycotoxin Res, V19, P124, DOI 10.1007/BF02942950	MYCOTOXIN RES Schatzmayr G, 2003, MYCOTOXIN RES, V19, P124, DOI 10.1007/BF02942950
CR	replaced	114	114	68499	68498	Mycotoxin Res Styriak I, 2001, Mycotoxin Res, V17 Suppl 1, P24, DOI 10.1007/BF03036705	MYCOTOXIN RES Styriak I, 2001, MYCOTOXIN RES, V17 Suppl 1, P24, DOI 10.1007/BF03036705

Record 456 | WOS:000362080300002

Structure elucidation and in vitro cytotoxicity of ochratoxin Î± amide, a new degradation product of ochratoxin A

SO original: MYCOTOXIN RESEARCH | SO normalised: MYCOTOXIN RESEARCH | CR count: 24 → 24

Field	Status	Orig. pos	Norm. pos	Orig. line	Norm. line	Original cited/source value	Normalised cited/source value
CR	replaced	4	4	68575	68574	Mycotoxin Res Beyer Marita, 2010, Mycotoxin Res, V26, P109, DOI 10.1007/s12550-010-0046-7	MYCOTOXIN RES Beyer Marita, 2010, MYCOTOXIN RES, V26, P109, DOI 10.1007/s12550-010-0046-7

Record 457 | WOS:000357242500002

Polyphenols as possible bioprotectors against cytotoxicity and DNA damage induced by ochratoxin A

SO original: ENVIRONMENTAL TOXICOLOGY AND PHARMACOLOGY | SO normalised: ENVIRONMENTAL TOXICOLOGY AND PHARMACOLOGY | CR count: 42 → 42

No journal-related changes detected in SO, J9, JI or CR for this record.

Record 458 | WOS:000353230400005

In vitro and in vivo metabolism of ochratoxin A: a comparative study using ultra-performance liquid chromatography-quadrupole/time-of-flight hybrid mass spectrometry

SO original: ANALYTICAL AND BIOANALYTICAL CHEMISTRY | SO normalised: ANALYTICAL AND BIOANALYTICAL CHEMISTRY | CR count: 36 → 36

Field	Status	Orig. pos	Norm. pos	Orig. line	Norm. line	Original cited/source value	Normalised cited/source value
CR	replaced	35	35	68828	68827	Mycotoxin Res<br>Zepnik H, 2003, Mycotoxin Res, V19, P102, DOI 10.1007/BF02942946	MYCOTOXIN RES<br>Zepnik H, 2003, MYCOTOXIN RES, V19, P102, DOI 10.1007/BF02942946

Record 459 | WOS:000351834600019

Assessment and characterisation of yeast-based products intended to mitigate ochratoxin exposure using in vitro and in vivo models

SO original: FOOD ADDITIVES AND CONTAMINANTS PART A-CHEMISTRY ANALYSIS CONTROL ; EXPOSURE & RISK ASSESSMENT | SO normalised: FOOD ADDITIVES AND CONTAMINANTS PART A-CHEMISTRY ANALYSIS CONTROL ; EXPOSURE & RISK ASSESSMENT | CR count: 61 → 61

Field	Status	Orig. pos	Norm. pos	Orig. line	Norm. line	Original cited/source value	Normalised cited/source value
CR	replaced	18	18	68921	68920	EFSA Journal<br>European Food Safety Authority (EFSA), 2010, EFSA Journal, V8, P1693, DOI DOI 10.2903/J.EFSA.2010.1693	EFSA J<br>European Food Safety Authority (EFSA), 2010, EFSA J, V8, P1693, DOI DOI 10.2903/J.EFSA.2010.1693

Record 460 | WOS:000353889600047

Selenium Alleviates Porcine Nephrotoxicity of Ochratoxin A by Improving Selenoenzyme Expression In Vitro

SO original: PLOS ONE | SO normalised: PLOS ONE | CR count: 50 → 50

No journal-related changes detected in SO, J9, JI or CR for this record.

Record 461 | WOS:000349553900004

Determination of Aflatoxins and Ochratoxins in Sicilian Sweet Wines by High-Performance Liquid Chromatography with Fluorometric Detection and Immunoaffinity Cleanup

SO original: FOOD ANALYTICAL METHODS | SO normalised: FOOD ANALYTICAL METHODS | CR count: 33 → 33

No journal-related changes detected in SO, J9, JI or CR for this record.

Record 462 | WOS:000343780400062

In vitro genotoxicity of mycotoxins ochratoxin A and fumonisin B1 could be prevented by sodium copper chlorophyllin - Implication to their genotoxic mechanism

SO original: FOOD CHEMISTRY | SO normalised: FOOD CHEMISTRY | CR count: 53 → 53

No journal-related changes detected in SO, J9, JI or CR for this record.

Record 463 | WOS:000351485000004

Ochratoxin A promotes porcine circovirus type 2 replication in vitro and in vivo

SO original: FREE RADICAL BIOLOGY AND MEDICINE | SO normalised: FREE RADICAL BIOLOGY AND MEDICINE | CR count: 65 → 65

Field	Status	Orig. pos	Norm. pos	Orig. line	Norm. line	Original cited/source value	Normalised cited/source value
CR	replaced	39	39	69417	69416	Mycotoxin Res<br>Martins H Marina, 2012, Mycotoxin Res, V28, P107, DOI 10.1007/s12550-011-0118-3	MYCOTOXIN RES<br>Martins H Marina, 2012, MYCOTOXIN RES, V28, P107, DOI 10.1007/s12550-011-0118-3

Record 464 | WOS:000350023000003

Ochratoxin A and its effects on immunity

SO original: TOXIN REVIEWS | SO normalised: TOXIN REVIEWS | CR count: 108 → 108

Field	Status	Orig. pos	Norm. pos	Orig. line	Norm. line	Original cited/source value	Normalised cited/source value
CR	replaced	27	27	69520	69519	Mycotoxin Res<br>Essid E, 2011, Mycotoxin Res, V27, P167, DOI 10.1007/s12550-011-0092-9	MYCOTOXIN RES<br>Essid E, 2011, MYCOTOXIN RES, V27, P167, DOI 10.1007/s12550-011-0092-9
CR	replaced	84	84	69577	69576	Mycotoxin Res<br>Reinhold Lilli, 2011, Mycotoxin Res, V27, P137, DOI 10.1007/s12550-011-0086-7	MYCOTOXIN RES<br>Reinhold Lilli, 2011, MYCOTOXIN RES, V27, P137, DOI 10.1007/s12550-011-0086-7
CR	replaced	104	104	69597	69596	Mycotoxin Res<br>Weidenbach A, 2000, Mycotoxin Res, V16 Suppl 2, P189, DOI 10.1007/BF02940035	MYCOTOXIN RES<br>Weidenbach A, 2000, MYCOTOXIN RES, V16 Suppl 2, P189, DOI 10.1007/BF02940035

Record 465 | WOS:000350094000016

Genotoxicity of Aflatoxin B1 and Ochratoxin A after simultaneous application of the in vivo micronucleus and comet assay

SO original: FOOD AND CHEMICAL TOXICOLOGY | SO normalised: FOOD AND CHEMICAL TOXICOLOGY | CR count: 65 → 65

Field	Status	Orig. pos	Norm. pos	Orig. line	Norm. line	Original cited/source value	Normalised cited/source value
CR	replaced	14	14	69693	69692	Methods Mol Biol<br>Collins Andrew R, 2002, Methods Mol Biol, V186, P147	METHODS MOL BIOL<br>Collins Andrew R, 2002, METHODS MOL BIOL, V186, P147

Record 466 | WOS:000362800100005

Mycotoxin mechanisms of action and health impact: 'in vitro' or 'in vivo' tests, that is the question

SO original: WORLD MYCOTOXIN JOURNAL | SO normalised: WORLD MYCOTOXIN JOURNAL | CR count: 153 → 153

Field	Status	Orig. pos	Norm. pos	Orig. line	Norm. line	Original cited/source value	Normalised cited/source value
CR	replaced	27	27	69823	69822	Mycotoxin Res<br>Burkhardt Britta, 2009, Mycotoxin Res, V25, P149, DOI 10.1007/s12550-009-0022-2	MYCOTOXIN RES<br>Burkhardt Britta, 2009, MYCOTOXIN RES, V25, P149, DOI 10.1007/s12550-009-0022-2
CR	replaced	53	53	69849	69848	EFSA Journal<br>European Food Safety Authority (EFSA), 2010, EFSA Journal, V8, P1693, DOI DOI 10.2903/J.EFSA.2010.1693	EFSA J<br>European Food Safety Authority (EFSA), 2010, EFSA J, V8, P1693, DOI DOI 10.2903/J.EFSA.2010.1693

Record 467 | WOS:000351213600013

Toxic Effects of Aflatoxin B1 on Embryonic Development of Zebrafish (Danio rerio): Potential Activity of Piceatannol Encapsulated Chitosan/poly (Lactic Acid) Nanoparticles

SO original: ANTI-CANCER AGENTS IN MEDICINAL CHEMISTRY | SO normalised: ANTI-CANCER AGENTS IN MEDICINAL CHEMISTRY | CR count: 76 → 76

No journal-related changes detected in SO, J9, JI or CR for this record.

Record 468 | WOS:000349133200010

Bioaccessibility of ochratoxin A from red wine in an in vitro dynamic gastrointestinal model

SO original: WORLD MYCOTOXIN JOURNAL | SO normalised: WORLD MYCOTOXIN JOURNAL | CR count: 32 → 32

No journal-related changes detected in SO, J9, JI or CR for this record.

Record 469 | WOS:000415214500006

Current Insights into Mycotoxins

SO original: BIOLOGICAL TOXINS AND BIOTERRORISM | SO normalised: BIOLOGICAL TOXINS AND BIOTERRORISM | CR count: 50 → 50

No journal-related changes detected in SO, J9, JI or CR for this record.

Record 470 | WOS:000360184800009

Dynamic changes of global DNA methylation and hypermethylation of cell adhesion-related genes in rat kidneys in response to ochratoxin A

SO original: WORLD MYCOTOXIN JOURNAL | SO normalised: WORLD MYCOTOXIN JOURNAL | CR count: 63 → 63

No journal-related changes detected in SO, J9, JI or CR for this record.

Record 471 | WOS:000367698600008

POTENTIAL GENOTOXICITY OF ACETAMIPRID AND PROPINEB SINGLY OR IN COMBINATION IN CULTURED HUMAN PERIPHERAL BLOOD LYMPHOCYTES BY USING MN ASSAY

SO original: FRESENIUS ENVIRONMENTAL BULLETIN | SO normalised: FRESENIUS ENVIRONMENTAL BULLETIN | CR count: 65 → 65

Field	Status	Orig. pos	Norm. pos	Orig. line	Norm. line	Original cited/source value	Normalised cited/source value
CR	replaced	64	64	70527	70526	BIOMED ENVIRON SCI Yao XH, 2006, BIOMED ENVIRON SCI, V19, P309	Biomed Environ Sci Yao XH, 2006, Biomed Environ Sci, V19, P309

Record 472 | WOS:000347274600033

Low doses of ochratoxin A induce micronucleus formation and delay DNA repair in human lymphocytes

SO original: FOOD AND CHEMICAL TOXICOLOGY | SO normalised: FOOD AND CHEMICAL TOXICOLOGY | CR count: 62 → 62

Field	Status	Orig. pos	Norm. pos	Orig. line	Norm. line	Original cited/source value	Normalised cited/source value
CR	replaced	27	27	70635	70634	J Biochem Mol Toxicol Golli-Bennour Emna El, 2010, J Biochem Mol Toxicol, V24, P42, DOI 10.1002/jbt.20310	J BIOCHEM MOL TOXIC Golli-Bennour Emna El, 2010, J BIOCHEM MOL TOXIC, V24, P42, DOI 10.1002/jbt.20310

Record 473 | WOS:000349038800005

In vitro effects of ochratoxin A, deoxynivalenol and zearalenone on cell viability and E-ADA activity in broiler chickens lymphocytes

SO original: PESQUISA VETERINARIA BRASILEIRA | SO normalised: PESQUISA VETERINARIA BRASILEIRA | CR count: 48 → 48

Field	Status	Orig. pos	Norm. pos	Orig. line	Norm. line	Original cited/source value	Normalised cited/source value
CR	replaced	20	20	70756	70755	MYCOTOXINS IN FOOD Gajecki M, 2010, MYCOTOXINS IN FOOD, FEED AND BIOWEAPONS, P131, DOI 10.1007/978-3-642-00725-5_9	Mycotoxins in food Gajecki M, 2010, Mycotoxins in food, FEED AND BIOWEAPONS, P131, DOI 10.1007/978-3-642-00725-5_9
CR	replaced	24	24	70760	70759	Methods of enzymatic analysis Guisti, 1984, Methods of enzymatic analysis, V2nd, P315	Methods of Enzymatic Analysis Guisti, 1984, Methods of Enzymatic Analysis, V2nd, P315

Record 474 | WOS:000344696100002

The mycotoxin beauvericin induces apoptotic cell death in H4IIE hepatoma cells accompanied by an inhibition of NF-ÎºB-activity and modulation of MAP-kinases

SO original: TOXICOLOGY LETTERS | SO normalised: TOXICOLOGY LETTERS | CR count: 26 → 26

No journal-related changes detected in SO, J9, JI or CR for this record.

Record 475 | WOS:000344980400016

Ochratoxin A induces rat renal carcinogenicity with limited induction of oxidative stress responses

SO original: TOXICOLOGY AND APPLIED PHARMACOLOGY | SO normalised: TOXICOLOGY AND APPLIED PHARMACOLOGY | CR count: 52 → 52

No journal-related changes detected in SO, J9, JI or CR for this record.

Record 476 | WOS:000343730400082

A New Ochratoxin A Biodegradation Strategy Using Cupriavidus basilensis Or16 Strain

SO original: PLOS ONE | SO normalised: PLOS ONE | CR count: 51 → 51

Field	Status	Orig. pos	Norm. pos	Orig. line	Norm. line	Original cited/source value	Normalised cited/source value
CR	replaced	35	35	71124	71123	Mycotoxin Res<br>Raters M, 2005, Mycotoxin Res, V21, P182, DOI 10.1007/BF02959259	MYCOTOXIN RES<br>Raters M, 2005, MYCOTOXIN RES, V21, P182, DOI 10.1007/BF02959259
CR	replaced	39	39	71128	71127	Mycotoxin Res<br>Schatzmayr G, 2003, Mycotoxin Res, V19, P124, DOI 10.1007/BF02942950	MYCOTOXIN RES<br>Schatzmayr G, 2003, MYCOTOXIN RES, V19, P124, DOI 10.1007/BF02942950

Record 477 | WOS:000341677200007

Biodegradation of ochratoxin A by Pediococcus parvulus isolated from Douro wines

SO original: INTERNATIONAL JOURNAL OF FOOD MICROBIOLOGY | SO normalised: INTERNATIONAL JOURNAL OF FOOD MICROBIOLOGY | CR count: 54 → 54

Field	Status	Orig. pos	Norm. pos	Orig. line	Norm. line	Original cited/source value	Normalised cited/source value
CR	replaced	6	6	71234	71233	Mycotoxin Res<br>Bohm J, 2000, Mycotoxin Res, V16 Suppl 1, P70, DOI 10.1007/BF02942985	MYCOTOXIN RES<br>Bohm J, 2000, MYCOTOXIN RES, V16 Suppl 1, P70, DOI 10.1007/BF02942985

Record 478 | WOS:000343630800010

Biological detoxification of zearalenone by Aspergillus niger strain FS10

SO original: FOOD AND CHEMICAL TOXICOLOGY | SO normalised: FOOD AND CHEMICAL TOXICOLOGY | CR count: 42 → 42

Field	Status	Orig. pos	Norm. pos	Orig. line	Norm. line	Original cited/source value	Normalised cited/source value
CR	replaced	25	25	71381	71380	Rev Iberoam Micol<br>Robledo M de L, 2001, Rev Iberoam Micol, V18, P141	REV IBEROAM MICOL<br>Robledo M de L, 2001, REV IBEROAM MICOL, V18, P141

Record 479 | WOS:000343630800034

Ochratoxin A kinetics: A review of analytical methods and studies in rat model

SO original: FOOD AND CHEMICAL TOXICOLOGY | SO normalised: FOOD AND CHEMICAL TOXICOLOGY | CR count: 102 → 102

No journal-related changes detected in SO, J9, JI or CR for this record.

Record 480 | WOS:000340827200008

Synergism of ochratoxin B and calcium-channel antagonist verapamil caused mitochondrial dysfunction

SO original: TOXICOLOGY MECHANISMS AND METHODS | SO normalised: TOXICOLOGY MECHANISMS AND METHODS | CR count: 29 → 29

No journal-related changes detected in SO, J9, JI or CR for this record.

Record 481 | WOS:000340837600026

Development of an in vitro method for the prediction of mycotoxin binding on yeast-based products: case of aflatoxin B1, zearalenone and ochratoxin A

SO original: APPLIED MICROBIOLOGY AND BIOTECHNOLOGY | SO normalised: APPLIED MICROBIOLOGY AND BIOTECHNOLOGY | CR count: 54 → 54

No journal-related changes detected in SO, J9, JI or CR for this record.

Record 482 | WOS:000342990200034

Combination of Metagenomics and Culture-Based Methods to Study the Interaction Between Ochratoxin A and Gut Microbiota

SO original: TOXICOLOGICAL SCIENCES | SO normalised: TOXICOLOGICAL SCIENCES | CR count: 44 → 44

No journal-related changes detected in SO, J9, JI or CR for this record.

Record 483 | WOS:000340087200003

Biological detoxification of mycotoxins: a review

SO original: ANNALS OF MICROBIOLOGY | SO normalised: ANNALS OF MICROBIOLOGY | CR count: 187 → 187

Field	Status	Orig. pos	Norm. pos	Orig. line	Norm. line	Original cited/source value	Normalised cited/source value
CR	replaced	20	20	71945	71944	Mycotoxin Res<br>Bohm J, 2000, Mycotoxin Res, V16 Suppl 1, P70, DOI 10.1007/BF02942985	MYCOTOXIN RES<br>Bohm J, 2000, MYCOTOXIN RES, V16 Suppl 1, P70, DOI 10.1007/BF02942985
CR	replaced	23	23	71948	71947	Nat Toxins<br>Boswald C, 1995, Nat Toxins, V3, P138, DOI 10.1002/nt.2620030304	NAT TOXINS<br>Boswald C, 1995, NAT TOXINS, V3, P138, DOI 10.1002/nt.2620030304

CR	replaced	64	64	71989	71988	Mycotoxin Res<br>Fuchs E, 2000, Mycotoxin Res, V16 Suppl 1, P66, DOI 10.1007/BF02942984	MYCOTOXIN RES<br>Fuchs E, 2000, MYCOTOXIN RES, V16 Suppl 1, P66, DOI 10.1007/BF02942984

Record 484 | WOS:000341466700016

Hepatotoxicity and genotoxicity of patulin in mice, and its modulation by green tea polyphenols administration

SO original: FOOD AND CHEMICAL TOXICOLOGY | SO normalised: FOOD AND CHEMICAL TOXICOLOGY | CR count: 55 → 55

Field	Status	Orig. pos	Norm. pos	Orig. line	Norm. line	Original cited/source value	Normalised cited/source value
CR	replaced	4	4	72190	72189	J Appl Toxicol<br>Becci P J, 1981, J Appl Toxicol, V1, P256, DOI 10.1002/jat.2550010504	J APPL TOXICOL<br>Becci P J, 1981, J APPL TOXICOL, V1, P256, DOI 10.1002/jat.2550010504
CR	replaced	5	5	72191	72190	Integr Cancer Ther<br>Borek Carmia, 2004, Integr Cancer Ther, V3, P333, DOI 10.1177/1534735404270578	INTEGR CANCER THER<br>Borek Carmia, 2004, INTEGR CANCER THER, V3, P333, DOI 10.1177/1534735404270578

Record 485 | WOS:000341466700027

Cytotoxicity induced by ochratoxin A, zearalenone, and Î±-zearalenol: Effects of individual and combined treatment

SO original: FOOD AND CHEMICAL TOXICOLOGY | SO normalised: FOOD AND CHEMICAL TOXICOLOGY | CR count: 39 → 39

No journal-related changes detected in SO, J9, JI or CR for this record.

Record 486 | WOS:000338616800026

Cytotoxicity and immunotoxicity of cyclopiazonic acid on human cells

SO original: TOXICOLOGY IN VITRO | SO normalised: TOXICOLOGY IN VITRO | CR count: 63 → 63

Field	Status	Orig. pos	Norm. pos	Orig. line	Norm. line	Original cited/source value	Normalised cited/source value
CR	replaced	11	11	72413	72412	Mycotoxin Res<br>Cole R J, 1986, Mycotoxin Res, V2, P3, DOI 10.1007/BF03191956	MYCOTOXIN RES<br>Cole R J, 1986, MYCOTOXIN RES, V2, P3, DOI 10.1007/BF03191956

Record 487 | WOS:000338817900023

Central role of Nix in the autophagic response to ochratoxin A

SO original: FOOD AND CHEMICAL TOXICOLOGY | SO normalised: FOOD AND CHEMICAL TOXICOLOGY | CR count: 44 → 44

No journal-related changes detected in SO, J9, JI or CR for this record.

Record 488 | WOS:000336730800004

Toxicity of the mycotoxin citrinin and its metabolite dihydrocitrinone and of mixtures of citrinin and ochratoxin A in vitro

SO original: ARCHIVES OF TOXICOLOGY | SO normalised: ARCHIVES OF TOXICOLOGY | CR count: 49 → 49

Field	Status	Orig. pos	Norm. pos	Orig. line	Norm. line	Original cited/source value	Normalised cited/source value
CR	replaced	26	26	72659	72658	Mycotoxin Res<br>Follmann W, 2007, Mycotoxin Res, V23, P101, DOI 10.1007/BF02946034	MYCOTOXIN RES<br>Follmann W, 2007, MYCOTOXIN RES, V23, P101, DOI 10.1007/BF02946034
CR	replaced	27	27	72660	72659	Mycotoxin Res<br>Follmann W, 2000, Mycotoxin Res, V16 Suppl 1, P123, DOI 10.1007/BF02942998	MYCOTOXIN RES<br>Follmann W, 2000, MYCOTOXIN RES, V16 Suppl 1, P123, DOI 10.1007/BF02942998
CR	replaced	28	28	72661	72660	Mycotoxin Res<br>Follmann Wolfram, 2009, Mycotoxin Res, V25, P11, DOI 10.1007/s12550-008-0002-y	MYCOTOXIN RES<br>Follmann Wolfram, 2009, MYCOTOXIN RES, V25, P11, DOI 10.1007/s12550-008-0002-y
CR	replaced	38	38	72671	72670	Mycotoxin Res<br>Knecht A, 2005, Mycotoxin Res, V21, P176, DOI 10.1007/BF02959258	MYCOTOXIN RES<br>Knecht A, 2005, MYCOTOXIN RES, V21, P176, DOI 10.1007/BF02959258
CR	replaced	42	42	72675	72674	IARC Sci Publ<br>Petkova-Bocharova T, 1991, IARC Sci Publ, P83	IARC SCI PUBL<br>Petkova-Bocharova T, 1991, IARC SCI PUBL, P83

Record 489 | WOS:000362078100003

Modulation of innate and antigen-specific immune functions directed against Listeria monocytogenes by fungal toxins in vitro

SO original: MYCOTOXIN RESEARCH | SO normalised: MYCOTOXIN RESEARCH | CR count: 46 → 46

No journal-related changes detected in SO, J9, JI or CR for this record.

Record 490 | WOS:000333247100013

Downregulation of Rad51 participates in OTA-induced DNA double-strand breaks in GES-1 cells in vitro

SO original: TOXICOLOGY LETTERS | SO normalised: TOXICOLOGY LETTERS | CR count: 39 → 39

No journal-related changes detected in SO, J9, JI or CR for this record.

Record 491 | WOS:000335104500012

Protective role of the mitochondrial Lon protease 1 in ochratoxin A-induced cytotoxicity in HEK293 cells

SO original: JOURNAL OF PROTEOMICS | SO normalised: JOURNAL OF PROTEOMICS | CR count: 83 → 83

No journal-related changes detected in SO, J9, JI or CR for this record.

Record 492 | WOS:000332034900015

Mutant frequency in comparison to oxidative DNA damage induced by ochratoxin A in L5178Y tk+/- (3.7.2C) mouse lymphoma cells

SO original: DRUG AND CHEMICAL TOXICOLOGY | SO normalised: DRUG AND CHEMICAL TOXICOLOGY | CR count: 42 → 42

Field	Status	Orig. pos	Norm. pos	Orig. line	Norm. line	Original cited/source value	Normalised cited/source value
CR	replaced	4	4	73139	73138	Mycotoxin Res<br>Aslam M, 2005, Mycotoxin Res, V21, P164, DOI 10.1007/BF02959255	MYCOTOXIN RES<br>Aslam M, 2005, MYCOTOXIN RES, V21, P164, DOI 10.1007/BF02959255

Record 493 | WOS:000335760200003

Analysis of Individual and Combined Effects of Ochratoxin A and Zearalenone on HepG2 and KK-1 Cells with Mathematical Models

SO original: TOXINS | SO normalised: TOXINS | CR count: 52 → 52

No journal-related changes detected in SO, J9, JI or CR for this record.

Record 494 | WOS:000338818900001

Flavonoid diosmetin increases ATP levels in kidney cells and relieves ATP depleting effect of ochratoxin A

SO original: JOURNAL OF PHOTOCHEMISTRY AND PHOTOBIOLOGY B-BIOLOGY | SO normalised: JOURNAL OF PHOTOCHEMISTRY AND PHOTOBIOLOGY B-BIOLOGY | CR count: 54 → 54

No journal-related changes detected in SO, J9, JI or CR for this record.

Record 495 | WOS:000332451700009

Natural feed contaminant zearalenone decreases the expressions of important pro- and anti-inflammatory mediators and mitogen-activated protein kinase/NF-kB signalling molecules in pigs

SO original: BRITISH JOURNAL OF NUTRITION | SO normalised: BRITISH JOURNAL OF NUTRITION | CR count: 78 → 78

Field	Status	Orig. pos	Norm. pos	Orig. line	Norm. line	Original cited/source value	Normalised cited/source value
CR	replaced	12	12	73512	73511	BMC Genomics<br>Chowdhury Shankar R, 2007, BMC Genomics, V8, P215, DOI 10.1186/1471-2164-8-215	BMC GENOMICS<br>Chowdhury Shankar R, 2007, BMC GENOMICS, V8, P215, DOI 10.1186/1471-2164-8-215
CR	replaced	21	21	73521	73520	Mycotoxin Res<br>Gazzah Amel Chatti, 2010, Mycotoxin Res, V26, P187, DOI 10.1007/s12550-010-0053-8	MYCOTOXIN RES<br>Gazzah Amel Chatti, 2010, MYCOTOXIN RES, V26, P187, DOI 10.1007/s12550-010-0053-8

Record 496 | WOS:000334436600041

Mycotoxins' Activity at Toxic and Sub-Toxic Concentrations: Differential Cytotoxic and Genotoxic Effects of Single and Combined Administration of Sterigmatocystin, Ochratoxin A and Citrinin on the Hepatocellular Cancer Cell Line Hep3B

SO original: INTERNATIONAL JOURNAL OF ENVIRONMENTAL RESEARCH AND PUBLIC HEALTH | SO normalised: INTERNATIONAL JOURNAL OF ENVIRONMENTAL RESEARCH AND PUBLIC HEALTH | CR count: 63 → 63

Field	Status	Orig. pos	Norm. pos	Orig. line	Norm. line	Original cited/source value	Normalised cited/source value
CR	replaced	18	18	73660	73659	Mycotoxin Res Follmann W, 2000, Mycotoxin Res, V16 Suppl 1, P123, DOI 10.1007/BF02942998	MYCOTOXIN RES Follmann W, 2000, MYCOTOXIN RES, V16 Suppl 1, P123, DOI 10.1007/BF02942998

Record 497 | WOS:000335746400013

Deficient Glutathione in the Pathophysiology of Mycotoxin-Related Illness

SO original: TOXINS | SO normalised: TOXINS | CR count: 98 → 98

No journal-related changes detected in SO, J9, JI or CR for this record.

Record 498 | WOS:000329417100014

Interaction effects of Fusarium enniatins (A, A1, B and B1) combinations on in vitro cytotoxicity of Caco-2 cells

SO original: TOXICOLOGY IN VITRO | SO normalised: TOXICOLOGY IN VITRO | CR count: 29 → 29

Field	Status	Orig. pos	Norm. pos	Orig. line	Norm. line	Original cited/source value	Normalised cited/source value
CR	replaced	23	23	73933	73932	Toxicological and Environmental Chemistry Ruiz Maria Jose, 2006, Toxicological and Environmental Chemistry, V88, P345, DOI 10.1080/02772240600630622	TOXICOL ENVIRON CHEM Ruiz Maria Jose, 2006, TOXICOL ENVIRON CHEM, V88, P345, DOI 10.1080/02772240600630622

Record 499 | WOS:000334568500006

Cell-based models for mycotoxin screening and toxicity evaluation: an update

SO original: WORLD MYCOTOXIN JOURNAL | SO normalised: WORLD MYCOTOXIN JOURNAL | CR count: 105 → 105

Field	Status	Orig. pos	Norm. pos	Orig. line	Norm. line	Original cited/source value	Normalised cited/source value
CR	replaced	57	57	74048	74047	Mycotoxin Res Kohler H, 2003, Mycotoxin	MYCOTOXIN RES Kohler H, 2003, MYCOTOXIN RES, V19,

						Res, V19, P108, DOI 10.1007/BF02942947	P108, DOI 10.1007/BF02942947

Record 500 | WOS:000339460900017

Ochratoxin A induces cytotoxicity, DNA damage and apoptosis in rat hepatocyte primary cell culture at nanomolar concentration

SO original: WORLD MYCOTOXIN JOURNAL | SO normalised: WORLD MYCOTOXIN JOURNAL | CR count: 62 → 62

No journal-related changes detected in SO, J9, JI or CR for this record.

Record 501 | WOS:000329058600004

Ochratoxin A induces DNA double-strand breaks and large deletion mutations in the carcinogenic target site of gpt delta rats

SO original: MUTAGENESIS | SO normalised: MUTAGENESIS | CR count: 60 → 60

No journal-related changes detected in SO, J9, JI or CR for this record.

Record 502 | WOS:000336092900022

A Review of the Evidence that Ochratoxin A Is an Nrf2 Inhibitor: Implications for Nephrotoxicity and Renal Carcinogenicity

SO original: TOXINS | SO normalised: TOXINS | CR count: 40 → 40

No journal-related changes detected in SO, J9, JI or CR for this record.

Record 503 | WOS:000339460900010

Effects of lactic acid bacteria and Saccharomyces cerevisiae on growth of Aspergillus westerdijkiae and ochratoxin A production and toxicity

SO original: WORLD MYCOTOXIN JOURNAL | SO normalised: WORLD MYCOTOXIN JOURNAL | CR count: 52 → 52

No journal-related changes detected in SO, J9, JI or CR for this record.

Record 504 | WOS:000329960400028

Ochratoxin-induced toxicity, oxidative stress and apoptosis ameliorated by quercetin - Modulation by Nrf2

SO original: FOOD AND CHEMICAL TOXICOLOGY | SO normalised: FOOD AND CHEMICAL TOXICOLOGY | CR count: 66 → 66

No journal-related changes detected in SO, J9, JI or CR for this record.

Record 505 | WOS:000328628900003

Deleterious Effects of Mycotoxin Combinations Involving Ochratoxin A

SO original: TOXINS | SO normalised: TOXINS | CR count: 132 → 132

Field	Status	Orig. pos	Norm. pos	Orig. line	Norm. line	Original cited/source value	Normalised cited/source value
CR	replaced	24	24	74759	74758	Microbial Toxins Ciegler A., 1971, Microbial Toxins, V6, P409	MICROBIAL TOXINS Ciegler A., 1971, MICROBIAL TOXINS, V6, P409
CR	replaced	38	38	74773	74772	Mycotoxin Res Follmann W, 2000, Mycotoxin Res, V16 Suppl 1, P123, DOI 10.1007/BF02942998	MYCOTOXIN RES Follmann W, 2000, MYCOTOXIN RES, V16 Suppl 1, P123, DOI 10.1007/BF02942998
CR	replaced	45	45	74780	74779	J Biochem Mol Toxicol Golli-Bennour Emna El, 2010, J Biochem Mol Toxicol, V24, P42, DOI 10.1002/jbt.20310	J BIOCHEM MOL TOXIC Golli-Bennour Emna El, 2010, J BIOCHEM MOL TOXIC, V24, P42, DOI 10.1002/jbt.20310
CR	replaced	60	60	74795	74794	Mycotoxin Res Jurjevic Z, 1999, Mycotoxin Res, V15, P67, DOI 10.1007/BF02945217	MYCOTOXIN RES Jurjevic Z, 1999, MYCOTOXIN RES, V15, P67, DOI 10.1007/BF02945217
CR	replaced	72	72	74807	74806	Mycotoxin Res Knecht A, 2005, Mycotoxin Res, V21, P176, DOI 10.1007/BF02959258	MYCOTOXIN RES Knecht A, 2005, MYCOTOXIN RES, V21, P176, DOI 10.1007/BF02959258
CR	replaced	105	105	74840	74839	Veterinarski Arhiv Sakhare Pramodkumar Shrirarn, 2007, Veterinarski Arhiv, V77, P129	VET ARHIV Sakhare Pramodkumar Shrirarn, 2007, VET ARHIV, V77, P129
CR	replaced	118	118	74853	74852	Mycotoxin Res Stoev Stoycho D, 2010, Mycotoxin Res, V26, P31, DOI 10.1007/s12550-009-0038-7	MYCOTOXIN RES Stoev Stoycho D, 2010, MYCOTOXIN RES, V26, P31, DOI 10.1007/s12550-009-0038-7

Record 506 | WOS:000327686300025

In vitro investigation of individual and combined cytotoxic effects of aflatoxin B1 and other selected mycotoxins on the cell line porcine kidney 15

SO original: EXPERIMENTAL AND TOXICOLOGIC PATHOLOGY | SO normalised: EXPERIMENTAL AND TOXICOLOGIC PATHOLOGY | CR count: 36 → 36

Field	Status	Orig. pos	Norm. pos	Orig. line	Norm. line	Original cited/source value	Normalised cited/source value
CR	replaced	9	9	74931	74930	J Biochem Mol Toxicol Golli-Bennour Emna El, 2010, J Biochem Mol Toxicol, V24, P42, DOI 10.1002/jbt.20310	J BIOCHEM MOL TOXIC Golli-Bennour Emna El, 2010, J BIOCHEM MOL TOXIC, V24, P42, DOI 10.1002/jbt.20310
CR	replaced	10	10	74932	74931	J Vet Diagn Invest Harrison L R, 1990, J Vet Diagn Invest, V2, P217	J VET DIAGN INVEST Harrison L R, 1990, J VET DIAGN INVEST, V2, P217
CR	replaced	27	27	74949	74948	Toxicological and Environmental Chemistry Ruiz Maria Jose, 2006, Toxicological and Environmental Chemistry,	TOXICOL ENVIRON CHEM Ruiz Maria Jose, 2006, TOXICOL ENVIRON CHEM, V88, P345, DOI 10.1080/02772240600630622

						V88, P345, DOI 10.1080/02772240600630622	

Record 507 | WOS:000326211800003

Multi-mycotoxin determination in baby foods and in vitro combined cytotoxic effects of aflatoxin M1 and ochratoxin A

SO original: WORLD MYCOTOXIN JOURNAL | SO normalised: WORLD MYCOTOXIN JOURNAL | CR count: 52 → 52

No journal-related changes detected in SO, J9, JI or CR for this record.

Record 508 | WOS:000324301900014

Beauvericin-induced cytotoxicity via ROS production and mitochondrial damage in Caco-2 cells

SO original: TOXICOLOGY LETTERS | SO normalised: TOXICOLOGY LETTERS | CR count: 43 → 43

Field	Status	Orig. pos	Norm. pos	Orig. line	Norm. line	Original cited/source value	Normalised cited/source value
CR	replaced	33	33	75176	75175	Toxicological and Environmental Chemistry Ruiz Maria Jose, 2006, Toxicological and Environmental Chemistry, V88, P345, DOI 10.1080/02772240600630622	TOXICOL ENVIRON CHEM Ruiz Maria Jose, 2006, TOXICOL ENVIRON CHEM, V88, P345, DOI 10.1080/02772240600630622

Record 509 | WOS:000324773300006

Oxidative DNA damage is involved in ochratoxin A-induced G2 arrest through ataxia telangiectasia-mutated (ATM) pathways in human gastric epithelium GES-1 cells in vitro

SO original: ARCHIVES OF TOXICOLOGY | SO normalised: ARCHIVES OF TOXICOLOGY | CR count: 59 → 59

No journal-related changes detected in SO, J9, JI or CR for this record.

Record 510 | WOS:000326384200009

Toxicogenomics-Based Identification of Mechanisms for Direct Immunotoxicity

SO original: TOXICOLOGICAL SCIENCES | SO normalised: TOXICOLOGICAL SCIENCES | CR count: 144 → 144

No journal-related changes detected in SO, J9, JI or CR for this record.

Record 511 | WOS:000332372600004

Toxicity of Ochratoxin A and Its Modulation by Antioxidants: A Review

SO original: TOXINS | SO normalised: TOXINS | CR count: 169 → 169

Field	Status	Orig. pos	Norm. pos	Orig. line	Norm. line	Original cited/source value	Normalised cited/source value
CR	replaced	84	84	75682	75681	CYTOBIOS<br>Jeswal P, 1998, CYTOBIOS, V93, P123	Cytobios<br>Jeswal P, 1998, Cytobios, V93, P123

Record 512 | WOS:000322929800012

Identification of proteins related to early changes observed in Human hepatocellular carcinoma cells after treatment with the mycotoxin Zearalenone

SO original: EXPERIMENTAL AND TOXICOLOGIC PATHOLOGY | SO normalised: EXPERIMENTAL AND TOXICOLOGIC PATHOLOGY | CR count: 58 → 58

Field	Status	Orig. pos	Norm. pos	Orig. line	Norm. line	Original cited/source value	Normalised cited/source value
CR	replaced	19	19	75858	75857	Mycotoxin Res<br>Gazzah Amel Chatti, 2010, Mycotoxin Res, V26, P187, DOI 10.1007/s12550-010-0053-8	MYCOTOXIN RES<br>Gazzah Amel Chatti, 2010, MYCOTOXIN RES, V26, P187, DOI 10.1007/s12550-010-0053-8

Record 513 | WOS:000324847800043

Individual and combined effects of mycotoxins from typical indoor moulds

SO original: TOXICOLOGY IN VITRO | SO normalised: TOXICOLOGY IN VITRO | CR count: 70 → 70

Field	Status	Orig. pos	Norm. pos	Orig. line	Norm. line	Original cited/source value	Normalised cited/source value
CR	replaced	59	59	76037	76036	BIOMED ENVIRON SCI<br>Sun XM, 2002, BIOMED ENVIRON SCI, V15, P145	Biomed Environ Sci<br>Sun XM, 2002, Biomed Environ Sci, V15, P145

Record 514 | WOS:000328628700005

Producers and Important Dietary Sources of Ochratoxin A and Citrinin

SO original: TOXINS | SO normalised: TOXINS | CR count: 88 → 88

Field	Status	Orig. pos	Norm. pos	Orig. line	Norm. line	Original cited/source value	Normalised cited/source value
CR	replaced	20	20	76123	76122	Mycotoxin Res<br>Degen G H, 2007, Mycotoxin Res, V23, P88, DOI 10.1007/BF02946032	MYCOTOXIN RES<br>Degen G H, 2007, MYCOTOXIN RES, V23, P88, DOI 10.1007/BF02946032
CR	replaced	21	21	76124	76123	Mycotoxin Res<br>Dietrich R, 2001, Mycotoxin Res, V17 Suppl 2, P156, DOI 10.1007/BF03036426	MYCOTOXIN RES<br>Dietrich R, 2001, MYCOTOXIN RES, V17 Suppl 2, P156, DOI 10.1007/BF03036426
CR	replaced	63	63	76166	76165	Studies in Mycology<br>Samson R. A., 2004, Studies in Mycology	Studies in mycology<br>Samson R. A., 2004, Studies in mycology

Record 515 | WOS:000328416400017

Fluorescence spectroscopic investigation of competitive interactions between ochratoxin A and 13 drug molecules for binding to human serum albumin

SO original: LUMINESCENCE | SO normalised: LUMINESCENCE | CR count: 48 → 48

Field	Status	Orig. pos	Norm. pos	Orig. line	Norm. line	Original cited/source value	Normalised cited/source value
CR	replaced	8	8	76276	76275	IARC Sci Publ Chernozemsky I N, 1991, IARC Sci Publ, P3	IARC SCI PUBL Chernozemsky I N, 1991, IARC SCI PUBL, P3

Record 516 | WOS:000324280000095

A review on ochratoxin A transcriptomic studies

SO original: FOOD AND CHEMICAL TOXICOLOGY | SO normalised: FOOD AND CHEMICAL TOXICOLOGY | CR count: 111 → 111

No journal-related changes detected in SO, J9, JI or CR for this record.

Record 517 | WOS:000320498300035

Individual and combined cytotoxic effects of Fusarium toxins (deoxynivalenol, nivalenol, zearalenone and fumonisins B1) on swine jejunal epithelial cells

SO original: FOOD AND CHEMICAL TOXICOLOGY | SO normalised: FOOD AND CHEMICAL TOXICOLOGY | CR count: 83 → 83

Field	Status	Orig. pos	Norm. pos	Orig. line	Norm. line	Original cited/source value	Normalised cited/source value
CR	replaced	54	54	76599	76598	Mycotoxin Res Mwanza Mulunda, 2009, Mycotoxin Res, V25, P233, DOI 10.1007/s12550-009-0033-z	MYCOTOXIN RES Mwanza Mulunda, 2009, MYCOTOXIN RES, V25, P233, DOI 10.1007/s12550-009-0033-z
CR	replaced	60	60	76605	76604	Mycotoxin Res Reubel G H, 1987, Mycotoxin Res, V3, P85, DOI 10.1007/BF03191994	MYCOTOXIN RES Reubel G H, 1987, MYCOTOXIN RES, V3, P85, DOI 10.1007/BF03191994

Record 518 | WOS:000320770400009

Computational Design of Peptide Ligands for Ochratoxin A

SO original: TOXINS | SO normalised: TOXINS | CR count: 46 → 46

Field	Status	Orig. pos	Norm. pos	Orig. line	Norm. line	Original cited/source value	Normalised cited/source value
CR	replaced	32	32	76714	76713	Mycotoxin Res Rolle-Kampczyk U, 2000, Mycotoxin Res, V16 Suppl 1, P100, DOI 10.1007/BF02942993	MYCOTOXIN RES Rolle-Kampczyk U, 2000, MYCOTOXIN RES, V16 Suppl 1, P100, DOI 10.1007/BF02942993

Record 519 | WOS:000320571000011

Effect of quercetin on the toxicokinetics of ochratoxin A in rats

SO original: FOOD ADDITIVES AND CONTAMINANTS PART A-CHEMISTRY ANALYSIS CONTROL ; EXPOSURE & RISK ASSESSMENT | SO normalised: FOOD ADDITIVES AND CONTAMINANTS PART A-CHEMISTRY ANALYSIS CONTROL ; EXPOSURE & RISK ASSESSMENT | CR count: 35 → 35

No journal-related changes detected in SO, J9, JI or CR for this record.

Record 520 | WOS:000316978700004

Zinc protects HepG2 cells against the oxidative damage and DNA damage induced by ochratoxin A

SO original: TOXICOLOGY AND APPLIED PHARMACOLOGY | SO normalised: TOXICOLOGY AND APPLIED PHARMACOLOGY | CR count: 54 → 54

No journal-related changes detected in SO, J9, JI or CR for this record.

Record 521 | WOS:000315160400007

Role of hMLH1 in sterigmatocystin-induced G2 phase arrest in human esophageal epithelial Het-1A cells in vitro

SO original: TOXICOLOGY LETTERS | SO normalised: TOXICOLOGY LETTERS | CR count: 54 → 54

Field	Status	Orig. pos	Norm. pos	Orig. line	Norm. line	Original cited/source value	Normalised cited/source value
CR	replaced	44	44	77045	77044	BIOMED ENVIRON SCI Sun XM, 2002, BIOMED ENVIRON SCI, V15, P145	Biomed Environ Sci Sun XM, 2002, Biomed Environ Sci, V15, P145

Record 522 | WOS:000316229200014

Cross-talk between microRNAs, nuclear factor E2-related factor 2, and heme oxygenase-1 in ochratoxin A-induced toxic effects in renal proximal tubular epithelial cells

SO original: MOLECULAR NUTRITION & FOOD RESEARCH | SO normalised: MOLECULAR NUTRITION & FOOD RESEARCH | CR count: 58 → 58

Field	Status	Orig. pos	Norm. pos	Orig. line	Norm. line	Original cited/source value	Normalised cited/source value
CR	replaced	33	33	77181	77180	Am J Nephrol Lee Seung Hun, 2005, Am J Nephrol, V25, P64, DOI 10.1159/000084275	AM J NEPHROL Lee Seung Hun, 2005, AM J NEPHROL, V25, P64, DOI 10.1159/000084275

Record 523 | WOS:000316522300011

Effects of p53 knockout on ochratoxin A-induced genotoxicity in p53-deficient gpt delta mice

SO original: TOXICOLOGY | SO normalised: TOXICOLOGY | CR count: 42 → 42

Field	Status	Orig. pos	Norm. pos	Orig. line	Norm. line	Original cited/source value	Normalised cited/source value

CR	replaced	3	3	77283	77282	WHO Food additives series [Anonymous], 2008, WHO Food additives series	WHO Food Additives Series [Anonymous], 2008, WHO Food Additives Series

Record 524 | WOS:000315408000011

Modulation of Intestinal Functions Following Mycotoxin Ingestion: Meta-Analysis of Published Experiments in Animals

SO original: TOXINS | SO normalised: TOXINS | CR count: 105 → 105

Field	Status	Orig. pos	Norm. pos	Orig. line	Norm. line	Original cited/source value	Normalised cited/source value
CR	replaced	57	57	77432	77431	Veterinarski Arhiv Koynarsky Vencislav, 2007, Veterinarski Arhiv, V77, P113	VET ARHIV Koynarsky Vencislav, 2007, VET ARHIV, V77, P113

Record 525 | WOS:000314671300007

Molecular mechanisms underlying ochratoxin A-induced genotoxicity: global gene expression analysis suggests induction of DNA double-strand breaks and cell cycle progression

SO original: JOURNAL OF TOXICOLOGICAL SCIENCES | SO normalised: JOURNAL OF TOXICOLOGICAL SCIENCES | CR count: 52 → 52

No journal-related changes detected in SO, J9, JI or CR for this record.

Record 526 | WOS:000316706800031

An iTRAQ-based mitoproteomics approach for profiling the nephrotoxicity mechanisms of ochratoxin A in HEK 293 cells

SO original: JOURNAL OF PROTEOMICS | SO normalised: JOURNAL OF PROTEOMICS | CR count: 81 → 81

No journal-related changes detected in SO, J9, JI or CR for this record.

Record 527 | WOS:000318093700001

A Review of the Mechanism of Injury and Treatment Approaches for Illness Resulting from Exposure to Water-Damaged Buildings, Mold, and Mycotoxins

SO original: SCIENTIFIC WORLD JOURNAL | SO normalised: SCIENTIFIC WORLD JOURNAL | CR count: 213 → 213

Field	Status	Orig. pos	Norm. pos	Orig. line	Norm. line	Original cited/source value	Normalised cited/source value
CR	replaced	6	6	77817	77816	J Vet Sci Agawane S B, 2004, J Vet Sci, V5, P359	J VET SCI Agawane S B, 2004, J VET SCI, V5, P359
CR	replaced	16	16	77827	77826	Appl Neuropsychol Baldo Juliana V, 2002, Appl Neuropsychol, V9, P193, DOI 10.1207/S15324826AN0904_1	APPL NEUROPSYCHOL Baldo Juliana V, 2002, APPL NEUROPSYCHOL, V9, P193, DOI 10.1207/S15324826AN0904_1
CR	replaced	46	46	77857	77856	ALTERN MED REV Crinnion WJ, 2010, ALTERN MED REV, V15, P101	Altern Med Rev Crinnion WJ, 2010, Altern Med Rev, V15, P101

CR	replaced	47	47	77858	77857	ALTERN MED REV Crinnion WJ, 2011, ALTERN MED REV, V16, P215	Altern Med Rev Crinnion WJ, 2011, Altern Med Rev, V16, P215

Record 528 | WOS:000314048800053

Ochratoxin A Inhibits Mouse Embryonic Development by Activating a Mitochondrion-Dependent Apoptotic Signaling Pathway

SO original: INTERNATIONAL JOURNAL OF MOLECULAR SCIENCES | SO normalised: INTERNATIONAL JOURNAL OF MOLECULAR SCIENCES | CR count: 62 → 62

Field	Status	Orig. pos	Norm. pos	Orig. line	Norm. line	Original cited/source value	Normalised cited/source value
CR	replaced	23	23	78103	78102	J Biochem Mol Toxicol Golli-Bennour Emna El, 2010, J Biochem Mol Toxicol, V24, P42, DOI 10.1002/jbt.20310	J BIOCHEM MOL TOXIC Golli-Bennour Emna El, 2010, J BIOCHEM MOL TOXIC, V24, P42, DOI 10.1002/jbt.20310

Record 529 | WOS:000313124600008

Role of intestinal microflora in xenobiotic-induced toxicity

SO original: MOLECULAR NUTRITION & FOOD RESEARCH | SO normalised: MOLECULAR NUTRITION & FOOD RESEARCH | CR count: 78 → 78

No journal-related changes detected in SO, J9, JI or CR for this record.

Record 530 | WOS:000315356800004

Amelioration of Ochratoxin A-induced immunotoxic effects by silymarin and vitamin E in White Leghorn cockerels

SO original: JOURNAL OF IMMUNOTOXICOLOGY | SO normalised: JOURNAL OF IMMUNOTOXICOLOGY | CR count: 48 → 48

No journal-related changes detected in SO, J9, JI or CR for this record.

Record 531 | WOS:000312386800135

Nivalenol and Deoxynivalenol Affect Rat Intestinal Epithelial Cells: A Concentration Related Study

SO original: PLOS ONE | SO normalised: PLOS ONE | CR count: 55 → 55

No journal-related changes detected in SO, J9, JI or CR for this record.

Record 532 | WOS:000312288000010

Lactobacillus reuteri CRL 1098 and Lactobacillus acidophilus CRL 1014 differently reduce in vitro immunotoxic effect induced by Ochratoxin A

SO original: FOOD AND CHEMICAL TOXICOLOGY | SO normalised: FOOD AND CHEMICAL TOXICOLOGY | CR count: 52 → 52

No journal-related changes detected in SO, J9, JI or CR for this record.

Record 533 | WOS:000310867400006

Increase in Mitochondrial DNA Copy Number in Response to Ochratoxin A and Methanol-Induced Mitochondrial DNA Damage in Drosophila

SO original: BULLETIN OF ENVIRONMENTAL CONTAMINATION AND TOXICOLOGY | SO normalised: BULLETIN OF ENVIRONMENTAL CONTAMINATION AND TOXICOLOGY | CR count: 26 → 26

Field	Status	Orig. pos	Norm. pos	Orig. line	Norm. line	Original cited/source value	Normalised cited/source value
CR	replaced	20	20	78690	78689	Methods Mol Biol<br>Santos Janine H, 2002, Methods Mol Biol, V197, P159, DOI 10.1385/1-59259-284-8:159	METHODS MOL BIOL<br>Santos Janine H, 2002, METHODS MOL BIOL, V197, P159, DOI 10.1385/1-59259-284-8:159

Record 534 | WOS:000312288000021

Evaluation of nephrotoxic effects of mycotoxins, citrinin and patulin, on zebrafish (Danio rerio) embryos

SO original: FOOD AND CHEMICAL TOXICOLOGY | SO normalised: FOOD AND CHEMICAL TOXICOLOGY | CR count: 56 → 56

No journal-related changes detected in SO, J9, JI or CR for this record.

Record 535 | WOS:000308629100003

Human skin penetration of selected model mycotoxins

SO original: TOXICOLOGY | SO normalised: TOXICOLOGY | CR count: 115 → 115

No journal-related changes detected in SO, J9, JI or CR for this record.

Record 536 | WOS:000315406500009

Apoptosis Induction by OTA and TNF-Î± in Cultured Primary Rat Hepatocytes and Prevention by Silibinin

SO original: TOXINS | SO normalised: TOXINS | CR count: 55 → 55

Field	Status	Orig. pos	Norm. pos	Orig. line	Norm. line	Original cited/source value	Normalised cited/source value
CR	replaced	16	16	79074	79073	Mycotoxin Res<br>Essid E, 2011, Mycotoxin Res, V27, P167, DOI 10.1007/s12550-011-0092-9	MYCOTOXIN RES<br>Essid E, 2011, MYCOTOXIN RES, V27, P167, DOI 10.1007/s12550-011-0092-9

Record 537 | WOS:000309849700023

Co-exposure of Fusarium mycotoxins: In vitro myelotoxicity assessment on human hematopoietic progenitors

SO original: TOXICON | SO normalised: TOXICON | CR count: 39 → 39

Field	Status	Orig. pos	Norm. pos	Orig. line	Norm. line	Original cited/source value	Normalised cited/source value
CR	replaced	21	21	79193	79192	Mycopathologia<br>Lautraite Sophie, 1997, Mycopathologia, V140, P59, DOI 10.1023/A:1006833606209	MYCOPATHOLOGIA<br>Lautraite Sophie, 1997, MYCOPATHOLOGIA, V140, P59, DOI 10.1023/A:1006833606209

Record 538 | WOS:000309897100023

Immune modulatory effects of the foodborne contaminant citrinin in mice

SO original: FOOD AND CHEMICAL TOXICOLOGY | SO normalised: FOOD AND CHEMICAL TOXICOLOGY | CR count: 42 → 42

No journal-related changes detected in SO, J9, JI or CR for this record.

Record 539 | WOS:000309897100058

Fumonisin B1 and the kidney: Modes of action for renal tumor formation by fumonisin B1 in rodents

SO original: FOOD AND CHEMICAL TOXICOLOGY | SO normalised: FOOD AND CHEMICAL TOXICOLOGY | CR count: 141 → 141

Field	Status	Orig. pos	Norm. pos	Orig. line	Norm. line	Original cited/source value	Normalised cited/source value
CR	replaced	27	27	79401	79400	Mycotoxin Res<br>Dutton Michael F, 2009, Mycotoxin Res, V25, P29, DOI 10.1007/s12550-008-0005-8	MYCOTOXIN RES<br>Dutton Michael F, 2009, MYCOTOXIN RES, V25, P29, DOI 10.1007/s12550-008-0005-8
CR	replaced	31	31	79405	79404	Mycotoxin Res<br>Gelderblom W C, 1991, Mycotoxin Res, V7, P46, DOI 10.1007/BF03192165	MYCOTOXIN RES<br>Gelderblom W C, 1991, MYCOTOXIN RES, V7, P46, DOI 10.1007/BF03192165
CR	replaced	46	46	79420	79419	J Vet Diagn Invest<br>Harrison L R, 1990, J Vet Diagn Invest, V2, P217	J VET DIAGN INVEST<br>Harrison L R, 1990, J VET DIAGN INVEST, V2, P217
CR	replaced	139	139	79513	79512	Mycotoxin Res<br>Zimmer I, 2008, Mycotoxin Res, V24, P40, DOI 10.1007/BF02985269	MYCOTOXIN RES<br>Zimmer I, 2008, MYCOTOXIN RES, V24, P40, DOI 10.1007/BF02985269

Record 540 | WOS:000308289000002

The food contaminant and nephrotoxin ochratoxin A enhances Wnt1 inducible signaling protein 1 and tumor necrosis factor-Î± expression in human primary proximal tubule cells

SO original: MOLECULAR NUTRITION & FOOD RESEARCH | SO normalised: MOLECULAR NUTRITION & FOOD RESEARCH | CR count: 38 → 38

No journal-related changes detected in SO, J9, JI or CR for this record.

Record 541 | WOS:000307323900012

Citrinin reduces testosterone secretion by inducing apoptosis in rat Leydig cells

SO original: TOXICOLOGY IN VITRO | SO normalised: TOXICOLOGY IN VITRO | CR count: 33 → 33

Field	Status	Orig. pos	Norm. pos	Orig. line	Norm. line	Original cited/source value	Normalised cited/source value
CR	replaced	14	14	79704	79703	Cell<br>LI P., 2004, Cell, V116, P57	CELL<br>LI P., 2004, CELL, V116, P57

Record 542 | WOS:000307766500014

Cellular distribution of cell cycle-related molecules in the renal tubules of rats treated with renal carcinogens for 28 days: relationship between cell cycle aberration and carcinogenesis

SO original: ARCHIVES OF TOXICOLOGY | SO normalised: ARCHIVES OF TOXICOLOGY | CR count: 53 → 53

Field	Status	Orig. pos	Norm. pos	Orig. line	Norm. line	Original cited/source value	Normalised cited/source value
CR	replaced	4	4	79801	79800	IARC Sci Publ<br>Ashby J, 1992, IARC Sci Publ, P135	IARC SCI PUBL<br>Ashby J, 1992, IARC SCI PUBL, P135

Record 543 | WOS:000307487100016

Protective Effect of Deferricoprogen Isolated from Monascus purpureus NTU 568 on Citrinin-Induced Apoptosis in HEK-293 Cells

SO original: JOURNAL OF AGRICULTURAL AND FOOD CHEMISTRY | SO normalised: JOURNAL OF AGRICULTURAL AND FOOD CHEMISTRY | CR count: 39 → 39

Field	Status	Orig. pos	Norm. pos	Orig. line	Norm. line	Original cited/source value	Normalised cited/source value
CR	replaced	30	30	79946	79945	Methods Mol Biol<br>Morgan D M, 1998, Methods Mol Biol, V79, P179	METHODS MOL BIOL<br>Morgan D M, 1998, METHODS MOL BIOL, V79, P179

Record 544 | WOS:000304513300011

Toxic effects of citrinin on the male reproductive system in mice

SO original: EXPERIMENTAL AND TOXICOLOGIC PATHOLOGY | SO normalised: EXPERIMENTAL AND TOXICOLOGIC PATHOLOGY | CR count: 33 → 33

No journal-related changes detected in SO, J9, JI or CR for this record.

Record 545 | WOS:000304401100023

Collaborative study for the detection of toxic compounds in shellfish extracts using cell-based assays. Part II: application to shellfish extracts spiked with lipophilic marine toxins

SO original: ANALYTICAL AND BIOANALYTICAL CHEMISTRY | SO normalised: ANALYTICAL AND BIOANALYTICAL CHEMISTRY | CR count: 36 → 36

No journal-related changes detected in SO, J9, JI or CR for this record.

Record 546 | WOS:000304744700011

Ochratoxin A induces oxidative DNA damage and G1 phase arrest in human peripheral blood mononuclear cells in vitro

SO original: TOXICOLOGY LETTERS | SO normalised: TOXICOLOGY LETTERS | CR count: 56 → 56

No journal-related changes detected in SO, J9, JI or CR for this record.

Record 547 | WOS:000304198400001

Ochratoxin A and Mitotic Disruption: Mode of Action Analysis of Renal Tumor Formation by Ochratoxin A

SO original: TOXICOLOGICAL SCIENCES | SO normalised: TOXICOLOGICAL SCIENCES | CR count: 155 → 155

No journal-related changes detected in SO, J9, JI or CR for this record.

Record 548 | WOS:000304401100022

Collaborative study for the detection of toxic compounds in shellfish extracts using cell-based assays. Part I: screening strategy and pre-validation study with lipophilic marine toxins

SO original: ANALYTICAL AND BIOANALYTICAL CHEMISTRY | SO normalised: ANALYTICAL AND BIOANALYTICAL CHEMISTRY | CR count: 40 → 40

No journal-related changes detected in SO, J9, JI or CR for this record.

Record 549 | WOS:000302950000003

Assaying embryotoxicity in the test tube: Current limitations of the embryonic stem cell test (EST) challenging its applicability domain

SO original: CRITICAL REVIEWS IN TOXICOLOGY | SO normalised: CRITICAL REVIEWS IN TOXICOLOGY | CR count: 239 → 239

No journal-related changes detected in SO, J9, JI or CR for this record.

Record 550 | WOS:000307982300003

Mutagenicity of Ochratoxin A and Its Hydroquinone Metabolite in the SupF Gene of the Mutation Reporter Plasmid Ps189

SO original: TOXINS | SO normalised: TOXINS | CR count: 64 → 64

No journal-related changes detected in SO, J9, JI or CR for this record.

Record 551 | WOS:000301792400008

Transcriptomic alterations induced by Ochratoxin A in rat and human renal proximal tubular in vitro models and comparison to a rat in vivo model

SO original: ARCHIVES OF TOXICOLOGY | SO normalised: ARCHIVES OF TOXICOLOGY | CR count: 37 → 37

No journal-related changes detected in SO, J9, JI or CR for this record.

Record 552 | WOS:000302917900001

Carcinogens induce loss of the primary cilium in human renal proximal tubular epithelial cells independently of effects on the cell cycle

SO original: AMERICAN JOURNAL OF PHYSIOLOGY-RENAL PHYSIOLOGY | SO normalised: AMERICAN JOURNAL OF PHYSIOLOGY-RENAL PHYSIOLOGY | CR count: 50 → 50

No journal-related changes detected in SO, J9, JI or CR for this record.

Record 553 | WOS:000301277200012

ERK and p38 MAPK signaling pathways are involved in ochratoxin A-induced G2 phase arrest in human gastric epithelium cells

SO original: TOXICOLOGY LETTERS | SO normalised: TOXICOLOGY LETTERS | CR count: 26 → 26

No journal-related changes detected in SO, J9, JI or CR for this record.

Record 554 | WOS:000302911300002

Assessment on pollution of ochratoxin a in grain in China and its apoptosis effect on vitro-cultured human tubular kidney cells

SO original: JOURNAL OF BIOCHEMICAL AND MOLECULAR TOXICOLOGY | SO normalised: JOURNAL OF BIOCHEMICAL AND MOLECULAR TOXICOLOGY | CR count: 34 → 34

No journal-related changes detected in SO, J9, JI or CR for this record.

Record 555 | WOS:000300700100002

Inactivation of A. ochraceus Spores and Detoxification of Ochratoxin A in Coffee Beans by Gamma Irradiation

SO original: JOURNAL OF FOOD SCIENCE | SO normalised: JOURNAL OF FOOD SCIENCE | CR count: 35 → 35

No journal-related changes detected in SO, J9, JI or CR for this record.

Record 556 | WOS:000309118000007

CYTOTOXIC POTENCY OF MYCOTOXINS IN CULTURES OF V79 LUNG FIBROBLAST CELLS

SO original: JOURNAL OF TOXICOLOGY AND ENVIRONMENTAL HEALTH-PART A-CURRENT ISSUES | SO normalised: JOURNAL OF TOXICOLOGY AND ENVIRONMENTAL HEALTH-PART A-CURRENT ISSUES | CR count: 21 → 21

Field	Status	Orig. pos	Norm. pos	Orig. line	Norm. line	Original cited/source value	Normalised cited/source value
CR	replaced	10	10	81689	81688	Mycotoxin Res Follmann W, 2007, Mycotoxin Res, V23, P101, DOI 10.1007/BF02946034	MYCOTOXIN RES Follmann W, 2007, MYCOTOXIN RES, V23, P101, DOI 10.1007/BF02946034
CR	replaced	11	11	81690	81689	Mycotoxin Res Follmann Wolfram, 2009, Mycotoxin Res, V25, P11,	MYCOTOXIN RES Follmann Wolfram, 2009, MYCOTOXIN RES, V25, P11,

						DOI 10.1007/s12550-008-0002-y	DOI 10.1007/s12550-008-0002-y
CR	replaced	16	16	81695	81694	Mycotoxin Res<br>Mayer S, 2008, Mycotoxin Res, V24, P151, DOI 10.1007/BF03032342	MYCOTOXIN RES<br>Mayer S, 2008, MYCOTOXIN RES, V24, P151, DOI 10.1007/BF03032342

Record 557 | WOS:000209177700107

Transport of aflatoxin M1 in human intestinal Caco-2/TC7 cells

SO original: FRONTIERS IN PHARMACOLOGY | SO normalised: FRONTIERS IN PHARMACOLOGY | CR count: 60 → 60

No journal-related changes detected in SO, J9, JI or CR for this record.

Record 558 | WOS:000300137300023

In vitro myelotoxicity assessment of the emerging mycotoxins Beauvericin, Enniatin b and Moniliformin on human hematopoietic progenitors

SO original: TOXICON | SO normalised: TOXICON | CR count: 46 → 46

No journal-related changes detected in SO, J9, JI or CR for this record.

Record 559 | WOS:000299028700020

Structure-Activity Relationships Imply Different Mechanisms of Action for Ochratoxin A-Mediated Cytotoxicity and Genotoxicity

SO original: CHEMICAL RESEARCH IN TOXICOLOGY | SO normalised: CHEMICAL RESEARCH IN TOXICOLOGY | CR count: 69 → 69

No journal-related changes detected in SO, J9, JI or CR for this record.

Record 560 | WOS:000299902300007

In vivo and ex vivo phagocytic potential of macrophages from progeny of breeder hens kept on ochratoxin A (OTA)-contaminated diet

SO original: JOURNAL OF IMMUNOTOXICOLOGY | SO normalised: JOURNAL OF IMMUNOTOXICOLOGY | CR count: 52 → 52

No journal-related changes detected in SO, J9, JI or CR for this record.

Record 561 | WOS:000297778200001

Ochratoxin A in feed of food-producing animals: An undesirable mycotoxin with health and performance effects

SO original: VETERINARY MICROBIOLOGY | SO normalised: VETERINARY MICROBIOLOGY | CR count: 126 → 126

Field	Status	Orig. pos	Norm. pos	Orig. line	Norm. line	Original cited/source value	Normalised cited/source value
CR	replaced	3	3	82239	82238	Off J Eur Union<br>[Anonymous], 2006, Off J Eur	OFF J EUR UNION<br>[Anonymous], 2006, OFF J

						Union, V229, P7, DOI DOI 10.3390/ECMC2021-11474	EUR UNION, V229, P7, DOI DOI 10.3390/ECMC2021-11474
CR	replaced	15	15	82251	82250	Rev Bras Med Vet<br>Cruz LCH., 1984, Rev Bras Med Vet, V6, P17	REV BRAS MED VET<br>Cruz LCH., 1984, REV BRAS MED VET, V6, P17
CR	replaced	59	59	82295	82294	Mycotoxin Res<br>Kotowski K, 2000, Mycotoxin Res, V16, P66, DOI 10.1007/BF02946106	MYCOTOXIN RES<br>Kotowski K, 2000, MYCOTOXIN RES, V16, P66, DOI 10.1007/BF02946106
CR	replaced	69	69	82305	82304	Indian Vet J<br>Kunjamma CRL., 1997, Indian Vet J, V74, P22	INDIAN VET J<br>Kunjamma CRL., 1997, INDIAN VET J, V74, P22
CR	replaced	83	83	82319	82318	VET MED INT<br>PEREYRA CM, 2010, VET MED INT, P1	Vet Med Int<br>PEREYRA CM, 2010, Vet Med Int, P1
CR	replaced	93	93	82329	82328	VET MED INT<br>SAKTHIVELAN SM, 2010, VET MED INT, P1	Vet Med Int<br>SAKTHIVELAN SM, 2010, Vet Med Int, P1
CR	replaced	110	110	82346	82345	Mycotoxin Res<br>Stoev Stoycho D, 2010, Mycotoxin Res, V26, P31, DOI 10.1007/s12550-009-0038-7	MYCOTOXIN RES<br>Stoev Stoycho D, 2010, MYCOTOXIN RES, V26, P31, DOI 10.1007/s12550-009-0038-7
CR	replaced	126	126	82362	82361	Pak J Pharm Sci<br>Zafar F, 2001, Pak J Pharm Sci, V14, P5	PAK J PHARM SCI<br>Zafar F, 2001, PAK J PHARM SCI, V14, P5

Record 562 | WOS:000298362500059

Effects of zearalenone and its derivatives on porcine immune response

SO original: TOXICOLOGY IN VITRO | SO normalised: TOXICOLOGY IN VITRO | CR count: 55 → 55

No journal-related changes detected in SO, J9, JI or CR for this record.

Record 563 | WOS:000297102800002

Molecular Events Involved in Ochratoxin A Induced Mitochondrial Pathway of Apoptosis, Modulation by Bcl-2 Family Members

SO original: ENVIRONMENTAL TOXICOLOGY | SO normalised: ENVIRONMENTAL TOXICOLOGY | CR count: 44 → 44

Field	Status	Orig. pos	Norm. pos	Orig. line	Norm. line	Original cited/source value	Normalised cited/source value
CR	replaced	9	9	82582	82581	Int J Environ Res Public Health<br>Creppy Edmond E, 2005, Int J Environ Res Public Health, V2, P186, DOI 10.3390/ijerph2005010186	INT J ENV RES PUB HE<br>Creppy Edmond E, 2005, INT J ENV RES PUB HE, V2, P186, DOI 10.3390/ijerph2005010186

Record 564 | WOS:000296681600021

Ochratoxin A reduces aflatoxin B1 induced DNA damage detected by the comet assay in Hep G2 cells

SO original: FOOD AND CHEMICAL TOXICOLOGY | SO normalised: FOOD AND CHEMICAL TOXICOLOGY | CR count: 48 → 48

Field	Status	Orig. pos	Norm. pos	Orig. line	Norm. line	Original cited/source value	Normalised cited/source value
CR	replaced	11	11	82699	82698	Methods Mol Biol Collins Andrew R, 2002, Methods Mol Biol, V186, P147	METHODS MOL BIOL Collins Andrew R, 2002, METHODS MOL BIOL, V186, P147

Record 565 | WOS:000295241700033

Cytotoxic effects of mycotoxin combinations in mammalian kidney cells

SO original: FOOD AND CHEMICAL TOXICOLOGY | SO normalised: FOOD AND CHEMICAL TOXICOLOGY | CR count: 28 → 28

Field	Status	Orig. pos	Norm. pos	Orig. line	Norm. line	Original cited/source value	Normalised cited/source value
CR	replaced	10	10	82804	82803	J Biochem Mol Toxicol Golli-Bennour Emna El, 2010, J Biochem Mol Toxicol, V24, P42, DOI 10.1002/jbt.20310	J BIOCHEM MOL TOXIC Golli-Bennour Emna El, 2010, J BIOCHEM MOL TOXIC, V24, P42, DOI 10.1002/jbt.20310
CR	replaced	23	23	82817	82816	Toxicological and Environmental Chemistry Ruiz Maria Jose, 2006, Toxicological and Environmental Chemistry, V88, P345, DOI 10.1080/02772240600630622	TOXICOL ENVIRON CHEM Ruiz Maria Jose, 2006, TOXICOL ENVIRON CHEM, V88, P345, DOI 10.1080/02772240600630622

Record 566 | WOS:000298538400030

Mycotoxins profiling of the culture material of Fusarium verticillioides (Sacc.) Nirenberg culture (CABI-IMI 392668) isolated from rice in Niger State, Nigeria and its cytotoxic effects on human lymphocytes comparatively to those of some mycotoxin standards

SO original: AFRICAN JOURNAL OF BIOTECHNOLOGY | SO normalised: AFRICAN JOURNAL OF BIOTECHNOLOGY | CR count: 45 → 45

Field	Status	Orig. pos	Norm. pos	Orig. line	Norm. line	Original cited/source value	Normalised cited/source value
CR	replaced	19	19	82902	82901	J Biochem Mol Toxicol Golli-Bennour Emna El, 2010, J Biochem Mol Toxicol, V24, P42, DOI 10.1002/jbt.20310	J BIOCHEM MOL TOXIC Golli-Bennour Emna El, 2010, J BIOCHEM MOL TOXIC, V24, P42, DOI 10.1002/jbt.20310

Record 567 | WOS:000295307400004

Toxicological interactions between the mycotoxins beauvericin, deoxynivalenol and T-2 toxin in CHO-K1 cells in vitro

SO original: TOXICON | SO normalised: TOXICON | CR count: 31 → 31

Field	Status	Orig. pos	Norm. pos	Orig. line	Norm. line	Original cited/source value	Normalised cited/source value

CR	replaced	11	11	82995	82994	Mycotoxin Res<br>Follmann Wolfram, 2009, Mycotoxin Res, V25, P11, DOI 10.1007/s12550-008-0002-y	MYCOTOXIN RES<br>Follmann Wolfram, 2009, MYCOTOXIN RES, V25, P11, DOI 10.1007/s12550-008-0002-y
CR	replaced	12	12	82996	82995	J Biochem Mol Toxicol<br>Golli-Bennour Emna El, 2010, J Biochem Mol Toxicol, V24, P42, DOI 10.1002/jbt.20310	J BIOCHEM MOL TOXIC<br>Golli-Bennour Emna El, 2010, J BIOCHEM MOL TOXIC, V24, P42, DOI 10.1002/jbt.20310
CR	replaced	27	27	83011	83010	Mycotoxin Res<br>Nielsen Carina, 2009, Mycotoxin Res, V25, P77, DOI 10.1007/s12550-009-0011-5	MYCOTOXIN RES<br>Nielsen Carina, 2009, MYCOTOXIN RES, V25, P77, DOI 10.1007/s12550-009-0011-5
CR	replaced	29	29	83013	83012	Toxicological and Environmental Chemistry<br>Ruiz Maria Jose, 2006, Toxicological and Environmental Chemistry, V88, P345, DOI 10.1080/02772240600630622	TOXICOL ENVIRON CHEM<br>Ruiz Maria Jose, 2006, TOXICOL ENVIRON CHEM, V88, P345, DOI 10.1080/02772240600630622

Record 568 | WOS:000296746400005

Comparative cytotoxicity of the possibly occurring mycotoxins in wheat on the basis of a mycological analysis

SO original: CRYPTOGAMIE MYCOLOGIE | SO normalised: CRYPTOGAMIE MYCOLOGIE | CR count: 90 → 90

**Field**	**Status**	**Orig. pos**	**Norm. pos**	**Orig. line**	**Norm. line**	**Original cited/source value**	**Normalised cited/source value**
CR	replaced	11	11	83090	83089	Mycotoxins in Agriculture and Food Safety<br>Bottalico A., 1998, Mycotoxins in Agriculture and Food Safety, P65	Mycotoxins in agriculture and food safety<br>Bottalico A., 1998, Mycotoxins in agriculture and food safety, P65
CR	replaced	19	19	83098	83097	FUSARIUM MYCOTOXINS<br>Desjardins A.E., 2006, FUSARIUM MYCOTOXINS	Fusarium: mycotoxins<br>Desjardins A.E., 2006, Fusarium: mycotoxins
CR	replaced	26	26	83105	83104	Introduction to Food- and Airborne Fungi<br>Frisvad J.C., 2000, Introduction to Food- and Airborne Fungi, V6th, P321	Introduction to Food and Airborne Fungi<br>Frisvad J.C., 2000, Introduction to Food and Airborne Fungi, V6th, P321
CR	replaced	31	31	83110	83109	Mycopathologia<br>Gonzalez H. H. L., 1999, Mycopathologia, V144, P97, DOI 10.1023/A:1007020822134	MYCOPATHOLOGIA<br>Gonzalez H. H. L., 1999, MYCOPATHOLOGIA, V144, P97, DOI 10.1023/A:1007020822134
CR	replaced	65	65	83144	83143	Mycotoxin Res<br>Pfeiffer E, 2007, Mycotoxin Res, V23, P152, DOI 10.1007/BF02951512	MYCOTOXIN RES<br>Pfeiffer E, 2007, MYCOTOXIN RES, V23, P152, DOI 10.1007/BF02951512

Record 569 | WOS:000295697800004

Herbal hepatotoxicity by kava: Update on pipermethystine, flavokavain B, and mould hepatotoxins as primarily assumed culprits

SO original: DIGESTIVE AND LIVER DISEASE | SO normalised: DIGESTIVE AND LIVER DISEASE | CR count: 60 → 60

Field	Status	Orig. pos	Norm. pos	Orig. line	Norm. line	Original cited/source value	Normalised cited/source value
CR	replaced	12	12	83232	83231	HerbalGram<br>Lebot V., 2006, HerbalGram, V71, P34	HERBALGRAM<br>Lebot V., 2006, HERBALGRAM, V71, P34

Record 570 | WOS:000294029600014

Ochratoxin A at nanomolar concentration perturbs the homeostasis of neural stem cells in highly differentiated but not in immature three-dimensional brain cell cultures

SO original: TOXICOLOGY LETTERS | SO normalised: TOXICOLOGY LETTERS | CR count: 61 → 61

Field	Status	Orig. pos	Norm. pos	Orig. line	Norm. line	Original cited/source value	Normalised cited/source value
CR	replaced	30	30	83369	83368	Neuroscience<br>Liu Y., 2009, Neuroscience	NEUROSCIENCE<br>Liu Y., 2009, NEUROSCIENCE

Record 571 | WOS:000293914500008

Perturbation of Mitosis through Inhibition of Histone Acetyltransferases: The Key to Ochratoxin A Toxicity and Carcinogenicity?

SO original: TOXICOLOGICAL SCIENCES | SO normalised: TOXICOLOGICAL SCIENCES | CR count: 57 → 57

No journal-related changes detected in SO, J9, JI or CR for this record.

Record 572 | WOS:000293914500016

Site-Specific In Vivo Mutagenicity in the Kidney of gpt Delta Rats Given a Carcinogenic Dose of Ochratoxin A

SO original: TOXICOLOGICAL SCIENCES | SO normalised: TOXICOLOGICAL SCIENCES | CR count: 39 → 39

Field	Status	Orig. pos	Norm. pos	Orig. line	Norm. line	Original cited/source value	Normalised cited/source value
CR	replaced	5	5	83617	83616	WHO Food additives series<br>[Anonymous], 2008, WHO Food additives series	WHO Food Additives Series<br>[Anonymous], 2008, WHO Food Additives Series
CR	replaced	29	29	83641	83640	IARC Sci Publ<br>Pfohl-Leszkowicz A, 1993, IARC Sci Publ, P141	IARC SCI PUBL<br>Pfohl-Leszkowicz A, 1993, IARC SCI PUBL, P141

Record 573 | WOS:000293914500030

Citrinin-Generated Reactive Oxygen Species Cause Cell Cycle Arrest Leading to Apoptosis via the Intrinsic Mitochondrial Pathway in Mouse Skin

SO original: TOXICOLOGICAL SCIENCES | SO normalised: TOXICOLOGICAL SCIENCES | CR count: 40 → 40

No journal-related changes detected in SO, J9, JI or CR for this record.

Record 574 | WOS:000292672700008

Comparative analysis of micronuclei and DNA damage induced by Ochratoxin A in two mammalian cell lines

SO original: MUTATION RESEARCH-GENETIC TOXICOLOGY AND ENVIRONMENTAL MUTAGENESIS | SO normalised: MUTATION RESEARCH-GENETIC TOXICOLOGY AND ENVIRONMENTAL MUTAGENESIS | CR count: 54 → 54

Field	Status	Orig. pos	Norm. pos	Orig. line	Norm. line	Original cited/source value	Normalised cited/source value
CR	replaced	17	17	83855	83854	Mycotoxin Res<br>Degen G H, 2005, Mycotoxin Res, V21, P57, DOI 10.1007/BF02954819	MYCOTOXIN RES<br>Degen G H, 2005, MYCOTOXIN RES, V21, P57, DOI 10.1007/BF02954819
CR	replaced	24	24	83862	83861	Mycotoxin Res<br>Follmann W, 2007, Mycotoxin Res, V23, P101, DOI 10.1007/BF02946034	MYCOTOXIN RES<br>Follmann W, 2007, MYCOTOXIN RES, V23, P101, DOI 10.1007/BF02946034

Record 575 | WOS:000286856200015

Enzyme-Linked Aptamer Assays (ELAAs), based on a competition format for a rapid and sensitive detection of Ochratoxin A in wine

SO original: FOOD CONTROL | SO normalised: FOOD CONTROL | CR count: 32 → 32

Field	Status	Orig. pos	Norm. pos	Orig. line	Norm. line	Original cited/source value	Normalised cited/source value
CR	replaced	5	5	83970	83969	J Biotechnol<br>Brody E N, 2000, J Biotechnol, V74, P5, DOI 10.1016/S1389-0352(99)00004-5	J BIOTECHNOL<br>Brody E N, 2000, J BIOTECHNOL, V74, P5, DOI 10.1016/S1389-0352(99)00004-5

Record 576 | WOS:000288933100012

Aflatoxin B1 and fumonisin B1 affect the oxidative status of bovine peripheral blood mononuclear cells

SO original: TOXICOLOGY IN VITRO | SO normalised: TOXICOLOGY IN VITRO | CR count: 70 → 70

Field	Status	Orig. pos	Norm. pos	Orig. line	Norm. line	Original cited/source value	Normalised cited/source value
CR	replaced	34	34	84097	84096	J Vet Diagn Invest<br>Harrison L R, 1990, J Vet Diagn Invest, V2, P217	J VET DIAGN INVEST<br>Harrison L R, 1990, J VET DIAGN INVEST, V2, P217
CR	replaced	36	36	84099	84098	J Biochem Toxicol<br>Kang Y J, 1996, J Biochem Toxicol, V11, P121, DOI 10.1002/(SICI)1522-	J BIOCHEM TOXICOL<br>Kang Y J, 1996, J BIOCHEM TOXICOL, V11, P121, DOI 10.1002/(SICI)1522-

						7146(1996)11:3<121::AID-JBT3>3.3.CO;2-Y	7146(1996)11:3<121::AID-JBT3>3.3.CO;2-Y

Record 577 | WOS:000289042100014

Lymphocyte blastic transformation test in the evaluation of ochratoxin A influence on the immune system of hens and their progeny

SO original: MEDYCYNA WETERYNARYJNA-VETERINARY MEDICINE-SCIENCE AND PRACTICE | SO normalised: MEDYCYNA WETERYNARYJNA-VETERINARY MEDICINE-SCIENCE AND PRACTICE | CR count: 17 → 17

No journal-related changes detected in SO, J9, JI or CR for this record.

Record 578 | WOS:000288341900004

Zebrafish (Danio rerio) embryos as a model for testing proteratogens

SO original: TOXICOLOGY | SO normalised: TOXICOLOGY | CR count: 141 → 141

Field	Status	Orig. pos	Norm. pos	Orig. line	Norm. line	Original cited/source value	Normalised cited/source value
CR	replaced	17	17	84277	84276	Int J Med Sci Banhidy Ferenc, 2005, Int J Med Sci, V2, P100	INT J MED SCI Banhidy Ferenc, 2005, INT J MED SCI, V2, P100
CR	replaced	45	45	84305	84304	Obstet Gynecol Surv Gililland J, 1983, Obstet Gynecol Surv, V38, P6	OBSTET GYNECOL SURV Gililland J, 1983, OBSTET GYNECOL SURV, V38, P6
CR	replaced	98	98	84358	84357	Journal of Pharmacological and Toxicological Methods Pugsley Michael K., 2008, Journal of Pharmacological and Toxicological Methods, V58, P69, DOI 10.1016/j.vascn.2008.07.002	J PHARMACOL TOX MET Pugsley Michael K., 2008, J PHARMACOL TOX MET, V58, P69, DOI 10.1016/j.vascn.2008.07.002
CR	replaced	101	101	84361	84360	Journal of Pharmacological and Toxicological Methods Redfern William S., 2008, Journal of Pharmacological and Toxicological Methods, V58, P110, DOI 10.1016/j.vascn.2008.05.006	J PHARMACOL TOX MET Redfern William S., 2008, J PHARMACOL TOX MET, V58, P110, DOI 10.1016/j.vascn.2008.05.006
CR	replaced	112	112	84372	84371	Prog Clin Biol Res Shepard T H, 1983, Prog Clin Biol Res, V135, P147	PROG CLIN BIOL RES Shepard T H, 1983, PROG CLIN BIOL RES, V135, P147
CR	replaced	126	126	84386	84385	Journal of Pharmacological and Toxicological Methods Valentin Jean-Pierre, 2009, Journal of Pharmacological and Toxicological Methods, V60, P152, DOI 10.1016/j.vascn.2009.05.011	J PHARMACOL TOX MET Valentin Jean-Pierre, 2009, J PHARMACOL TOX MET, V60, P152, DOI 10.1016/j.vascn.2009.05.011
CR	replaced	134	134	84394	84393	Toxicol Appl Pharmacol Wells Peter G, 2005, Toxicol Appl Pharmacol, V207, P354, DOI 10.1016/j.taap.2005.01.061	TOXICOL APPL PHARM Wells Peter G, 2005, TOXICOL APPL PHARM, V207, P354, DOI 10.1016/j.taap.2005.01.061

CR	replaced	140	140	84400	84399	Journal of Toxicological Sciences<br>Yukiyama Shiro, 1996, Journal of Toxicological Sciences, V21, P603	J TOXICOL SCI<br>Yukiyama Shiro, 1996, J TOXICOL SCI, V21, P603

Record 579 | WOS:000286679100003

Protective effects of melatonin and Glycyrrhiza glabra extract on ochratoxin A-induced damages on testes in mature rats

SO original: HUMAN & EXPERIMENTAL TOXICOLOGY | SO normalised: HUMAN & EXPERIMENTAL TOXICOLOGY | CR count: 31 → 31

No journal-related changes detected in SO, J9, JI or CR for this record.

Record 580 | WOS:000285845400008

Mycotoxin Citrinin Induced Cell Cycle G2/M Arrest and Numerical Chromosomal Aberration Associated with Disruption of Microtubule Formation in Human Cells

SO original: TOXICOLOGICAL SCIENCES | SO normalised: TOXICOLOGICAL SCIENCES | CR count: 41 → 41

No journal-related changes detected in SO, J9, JI or CR for this record.

Record 581 | WOS:000285100300026

Metabolic response to low-level toxicant exposure in a novel renal tubule epithelial cell system

SO original: MOLECULAR BIOSYSTEMS | SO normalised: MOLECULAR BIOSYSTEMS | CR count: 51 → 51

Field	Status	Orig. pos	Norm. pos	Orig. line	Norm. line	Original cited/source value	Normalised cited/source value
CR	replaced	19	19	84703	84702	Reprod Toxicol<br>Gibb Steven, 2008, Reprod Toxicol, V25, P136, DOI 10.1016/j.reprotox.2007.10.013	REPROD TOXICOL<br>Gibb Steven, 2008, REPROD TOXICOL, V25, P136, DOI 10.1016/j.reprotox.2007.10.013

Record 582 | WOS:000297106700013

Review of mycotoxin reduction in food and feed: from prevention in the field to detoxification by adsorption or transformation

SO original: FOOD ADDITIVES AND CONTAMINANTS PART A-CHEMISTRY ANALYSIS CONTROL ; EXPOSURE & RISK ASSESSMENT | SO normalised: FOOD ADDITIVES AND CONTAMINANTS PART A-CHEMISTRY ANALYSIS CONTROL ; EXPOSURE & RISK ASSESSMENT | CR count: 238 → 238

Field	Status	Orig. pos	Norm. pos	Orig. line	Norm. line	Original cited/source value	Normalised cited/source value
CR	replaced	33	33	84833	84832	Nat Toxins<br>Boswald C, 1995, Nat Toxins, V3, P138, DOI 10.1002/nt.2620030304	NAT TOXINS<br>Boswald C, 1995, NAT TOXINS, V3, P138, DOI 10.1002/nt.2620030304
CR	replaced	82	82	84882	84881	Mycotoxin Res<br>Engelhardt G, 2002,	MYCOTOXIN RES<br>Engelhardt G, 2002,

						Mycotoxin Res, V18, P37, DOI 10.1007/BF02946138	MYCOTOXIN RES, V18, P37, DOI 10.1007/BF02946138
CR	replaced	94	94	84894	84893	Annales de Recherches Veterinaires<br>GALTIER P, 1976, Annales de Recherches Veterinaires, V7, P91	ANN RECH VET<br>GALTIER P, 1976, ANN RECH VET, V7, P91
CR	replaced	127	127	84927	84926	US patent<br>Karlovsky P, 2003, US patent, Patent No. 20030073239	US Patent<br>Karlovsky P, 2003, US Patent, Patent No. 20030073239
CR	replaced	135	135	84935	84934	US patent<br>Kubo K, 1996, US patent, Patent No. [5 549 890, 5549890]	US Patent<br>Kubo K, 1996, US Patent, Patent No. [5 549 890, 5549890]
CR	replaced	189	189	84989	84988	Toxicologist<br>Sarr A.B., 1990, Toxicologist, V10, P163	TOXICOLOGIST<br>Sarr A.B., 1990, TOXICOLOGIST, V10, P163
CR	replaced	191	191	84991	84990	Mycotoxin Res<br>Schatzmayr G, 2003, Mycotoxin Res, V19, P124, DOI 10.1007/BF02942950	MYCOTOXIN RES<br>Schatzmayr G, 2003, MYCOTOXIN RES, V19, P124, DOI 10.1007/BF02942950
CR	replaced	218	218	85018	85017	US patent<br>Tangni EK, 2006, US patent, Patent No. [2006/ 0263410 A1, 20060263410]	US Patent<br>Tangni EK, 2006, US Patent, Patent No. [2006/ 0263410 A1, 20060263410]

Record 583 | WOS:000298090700025

Experimentally Induced Toxicity of Ochratoxin A and Endosulfan in Male Wistar Rats: A Hormonal Disorder

SO original: JOURNAL OF ANIMAL AND VETERINARY ADVANCES | SO normalised: JOURNAL OF ANIMAL AND VETERINARY ADVANCES | CR count: 44 → 44

No journal-related changes detected in SO, J9, JI or CR for this record.

Record 584 | WOS:000295693800002

Potential of an in vitro toolbox combined with exposure data as a first step for the risk assessment of dietary chemical contaminants

SO original: FOOD ADDITIVES AND CONTAMINANTS PART A-CHEMISTRY ANALYSIS CONTROL ; EXPOSURE & RISK ASSESSMENT | SO normalised: FOOD ADDITIVES AND CONTAMINANTS PART A-CHEMISTRY ANALYSIS CONTROL ; EXPOSURE & RISK ASSESSMENT | CR count: 128 → 128

Field	Status	Orig. pos	Norm. pos	Orig. line	Norm. line	Original cited/source value	Normalised cited/source value
CR	replaced	9	9	85230	85229	In vitro Toxicol<br>[Anonymous], 1995, In vitro Toxicol	IN VITRO TOXICOL<br>[Anonymous], 1995, IN VITRO TOXICOL
CR	replaced	12	12	85233	85232	OJ.L<br>[Anonymous], 2005, OJ.L, V70, P1	OJ L<br>[Anonymous], 2005, OJ L, V70, P1

Record 585 | WOS:000292496700010

Developing mechanism-based and exposure biomarkers for mycotoxins in animals

SO original: DETERMINING MYCOTOXINS AND MYCOTOXIGENIC FUNGI IN FOOD AND FEED | SO normalised: DETERMINING MYCOTOXINS AND MYCOTOXIGENIC FUNGI IN FOOD AND FEED | CR count: 158 → 158

No journal-related changes detected in SO, J9, JI or CR for this record.

Record 586 | WOS:000297106700012

Isotherm modeling of organic activated bentonite and humic acid polymer used as mycotoxin adsorbents

SO original: FOOD ADDITIVES AND CONTAMINANTS PART A-CHEMISTRY ANALYSIS CONTROL ; EXPOSURE & RISK ASSESSMENT | SO normalised: FOOD ADDITIVES AND CONTAMINANTS PART A-CHEMISTRY ANALYSIS CONTROL ; EXPOSURE & RISK ASSESSMENT | CR count: 48 → 48

Field	Status	Orig. pos	Norm. pos	Orig. line	Norm. line	Original cited/source value	Normalised cited/source value
CR	replaced	1	1	85623	85622	Mycopathologia [Anonymous], 2003, Mycopathologia, V65, P43	MYCOPATHOLOGIA [Anonymous], 2003, MYCOPATHOLOGIA, V65, P43

Record 587 | WOS:000290627500016

Aptamer-functionalized magnetic nanoparticle-based bioassay for the detection of ochratoxin a using upconversion nanoparticles as labels

SO original: ANALYST | SO normalised: ANALYST | CR count: 56 → 56

No journal-related changes detected in SO, J9, JI or CR for this record.

Record 588 | WOS:000283743900015

Immunotoxicity of zearalenone in Balb/c mice in a high subchronic dosing study counteracted by Raphanus sativus extract

SO original: IMMUNOPHARMACOLOGY AND IMMUNOTOXICOLOGY | SO normalised: IMMUNOPHARMACOLOGY AND IMMUNOTOXICOLOGY | CR count: 40 → 40

Field	Status	Orig. pos	Norm. pos	Orig. line	Norm. line	Original cited/source value	Normalised cited/source value
CR	replaced	5	5	85858	85857	Off J Eur Union [Anonymous], 2006, Off J Eur Union, V229, P7, DOI DOI 10.3390/ECMC2021-11474	OFF J EUR UNION [Anonymous], 2006, OFF J EUR UNION, V229, P7, DOI DOI 10.3390/ECMC2021-11474

Record 589 | WOS:000284436000007

Evaluation of a urinary kidney biomarker panel in rat models of acute and subchronic nephrotoxicity

SO original: TOXICOLOGY | SO normalised: TOXICOLOGY | CR count: 62 → 62

No journal-related changes detected in SO, J9, JI or CR for this record.

Record 590 | WOS:000283810500022

Protein Biomarkers for in Vitro Testing of Embryotoxicity

SO original: JOURNAL OF PROTEOME RESEARCH | SO normalised: JOURNAL OF PROTEOME RESEARCH | CR count: 63 → 63

No journal-related changes detected in SO, J9, JI or CR for this record.

Record 591 | WOS:000280344000003

Some food-associated mycotoxins as potential risk factors in humans predisposed to chronic intestinal inflammatory diseases

SO original: TOXICON | SO normalised: TOXICON | CR count: 163 → 163

Field	Status	Orig. pos	Norm. pos	Orig. line	Norm. line	Original cited/source value	Normalised cited/source value
CR	replaced	59	59	86290	86289	J Physiol Pharmacol<br>Hollander D, 2003, J Physiol Pharmacol, V54 Suppl 4, P183	J PHYSIOL PHARMACOL<br>Hollander D, 2003, J PHYSIOL PHARMACOL, V54 Suppl 4, P183
CR	replaced	62	62	86293	86292	Toxicol Appl Pharmacol<br>Jeffrey A M, 2005, Toxicol Appl Pharmacol, V207, P628, DOI 10.1016/j.taap.2005.03.024	TOXICOL APPL PHARM<br>Jeffrey A M, 2005, TOXICOL APPL PHARM, V207, P628, DOI 10.1016/j.taap.2005.03.024
CR	replaced	108	108	86339	86338	Polish Journal of Veterinary Sciences<br>Obremski K., 2005, Polish Journal of Veterinary Sciences, V8, P301	POL J VET SCI<br>Obremski K., 2005, POL J VET SCI, V8, P301

Record 592 | WOS:000281740900017

Mycotoxins and Their Biotransformation in the Rumen: A Review

SO original: ASIAN-AUSTRALASIAN JOURNAL OF ANIMAL SCIENCES | SO normalised: ASIAN-AUSTRALASIAN JOURNAL OF ANIMAL SCIENCES | CR count: 123 → 123

Field	Status	Orig. pos	Norm. pos	Orig. line	Norm. line	Original cited/source value	Normalised cited/source value
CR	replaced	44	44	86492	86491	J Vet Diagn Invest<br>Harrison L R, 1990, J Vet Diagn Invest, V2, P217	J VET DIAGN INVEST<br>Harrison L R, 1990, J VET DIAGN INVEST, V2, P217
CR	replaced	92	92	86540	86539	Mycotoxin Res<br>Schatzmayr G, 2002, Mycotoxin Res, V18 Suppl 2, P183, DOI 10.1007/BF02946092	MYCOTOXIN RES<br>Schatzmayr G, 2002, MYCOTOXIN RES, V18 Suppl 2, P183, DOI 10.1007/BF02946092
CR	replaced	123	123	86571	86570	Polish Journal of Veterinary Sciences<br>Zwierzchowski W., 2005, Polish Journal of Veterinary Sciences, V8, P209	POL J VET SCI<br>Zwierzchowski W., 2005, POL J VET SCI, V8, P209

Record 593 | WOS:000282996200006

Free radicals and antioxidants in two oxidative-stress cell models exposed to ochratoxin A and amyloid Î²: unexpected results

SO original: WORLD MYCOTOXIN JOURNAL | SO normalised: WORLD MYCOTOXIN JOURNAL | CR count: 24 → 24

No journal-related changes detected in SO, J9, JI or CR for this record.

Record 594 | WOS:000280991100075

Safety studies on products from whole coffee fruit

SO original: FOOD AND CHEMICAL TOXICOLOGY | SO normalised: FOOD AND CHEMICAL TOXICOLOGY | CR count: 38 → 38

No journal-related changes detected in SO, J9, JI or CR for this record.

Record 595 | WOS:000280256800007

Beauvericin and ochratoxin A genotoxicity evaluated using the alkaline comet assay: single and combined genotoxic action

SO original: ARCHIVES OF TOXICOLOGY | SO normalised: ARCHIVES OF TOXICOLOGY | CR count: 39 → 39

Field	Status	Orig. pos	Norm. pos	Orig. line	Norm. line	Original cited/source value	Normalised cited/source value
CR	replaced	18	18	86838	86837	Mycotoxin Res<br>Jurjevic Z, 1999, Mycotoxin Res, V15, P67, DOI 10.1007/BF02945217	MYCOTOXIN RES<br>Jurjevic Z, 1999, MYCOTOXIN RES, V15, P67, DOI 10.1007/BF02945217

Record 596 | WOS:000282996200009

Mycotoxins in grapes and wine in Europe: occurrence, factors affecting the occurrence and related toxicological effects

SO original: WORLD MYCOTOXIN JOURNAL | SO normalised: WORLD MYCOTOXIN JOURNAL | CR count: 86 → 86

Field	Status	Orig. pos	Norm. pos	Orig. line	Norm. line	Original cited/source value	Normalised cited/source value
CR	replaced	51	51	86959	86958	Mycotoxin Res<br>Majerus P, 2008, Mycotoxin Res, V24, P135, DOI 10.1007/BF03032340	MYCOTOXIN RES<br>Majerus P, 2008, MYCOTOXIN RES, V24, P135, DOI 10.1007/BF03032340
CR	replaced	63	63	86971	86970	Mycotoxin Res<br>Pfeiffer E, 2008, Mycotoxin Res, V24, P117, DOI 10.1007/BF03032337	MYCOTOXIN RES<br>Pfeiffer E, 2008, MYCOTOXIN RES, V24, P117, DOI 10.1007/BF03032337

Record 597 | WOS:000278803100015

Allium cepa as a biomonitor of ochratoxin A toxicity and genotoxicity

SO original: PLANT BIOLOGY | SO normalised: PLANT BIOLOGY | CR count: 22 → 22

No journal-related changes detected in SO, J9, JI or CR for this record.

Record 598 | WOS:000277103900009

Neuronal models for evaluation of proliferation in vitro using high content screening

SO original: TOXICOLOGY | SO normalised: TOXICOLOGY | CR count: 44 → 44

No journal-related changes detected in SO, J9, JI or CR for this record.

Record 599 | WOS:000208434800018

Antigenotoxic Studies of Different Substances to Reduce the DNA Damage Induced by Aflatoxin B1 and Ochratoxin A

SO original: TOXINS | SO normalised: TOXINS | CR count: 90 → 90

Field	Status	Orig. pos	Norm. pos	Orig. line	Norm. line	Original cited/source value	Normalised cited/source value
CR	replaced	22	22	87260	87259	Journal of Toxicological Sciences<br>Creppy Edmond Ekue, 1998, Journal of Toxicological Sciences, V23, P165	J TOXICOL SCI<br>Creppy Edmond Ekue, 1998, J TOXICOL SCI, V23, P165
CR	replaced	25	25	87263	87262	Mycotoxin Res<br>Diaz G, 2001, Mycotoxin Res, V17, P15, DOI 10.1007/BF02946113	MYCOTOXIN RES<br>Diaz G, 2001, MYCOTOXIN RES, V17, P15, DOI 10.1007/BF02946113
CR	replaced	36	36	87274	87273	Journal of Dairy Science<br>Girard I. D., 1994, Journal of Dairy Science, V77, P300	J DAIRY SCI<br>Girard I. D., 1994, J DAIRY SCI, V77, P300
CR	replaced	88	88	87326	87325	BIOMED ENVIRON SCI<br>Wang J, 2007, BIOMED ENVIRON SCI, V20, P483	Biomed Environ Sci<br>Wang J, 2007, Biomed Environ Sci, V20, P483

Record 600 | WOS:000276175200004

Ochratoxin A induces G2 phase arrest in human gastric epithelium GES-1 cells in vitro

SO original: TOXICOLOGY LETTERS | SO normalised: TOXICOLOGY LETTERS | CR count: 38 → 38

No journal-related changes detected in SO, J9, JI or CR for this record.

Record 601 | WOS:000274582400001

PREVENTIVE AND THERAPEUTIC METHODS AGAINST THE TOXIC EFFECTS OF MYCOTOXINS - A REVIEW

SO original: ACTA VETERINARIA HUNGARICA | SO normalised: ACTA VETERINARIA HUNGARICA | CR count: 123 → 123

Field	Status	Orig. pos	Norm. pos	Orig. line	Norm. line	Original cited/source value	Normalised cited/source value
CR	replaced	38	38	87522	87521	Feedstuffs<br>Devegowda G.M., 1998, Feedstuffs, V70, P12	FEEDSTUFFS<br>Devegowda G.M., 1998, FEEDSTUFFS, V70, P12
CR	replaced	39	39	87523	87522	Journal of Dairy Science<br>Diaz D.E., 1997, Journal of Dairy Science, V80, P261	J DAIRY SCI<br>Diaz D.E., 1997, J DAIRY SCI, V80, P261
CR	replaced	92	92	87576	87575	Asian-Australasian Journal of Animal Sciences<br>Raju M. V. L. N., 2002, Asian-Australasian Journal of Animal Sciences, V15, P1051	ASIAN AUSTRAL J ANIM<br>Raju M. V. L. N., 2002, ASIAN AUSTRAL J ANIM, V15, P1051

Record 602 | WOS:000276819800011

Decontamination and detoxification strategies for the Fusarium mycotoxin deoxynivalenol in animal feed and the effectiveness of microbial biodegradation

SO original: FOOD ADDITIVES AND CONTAMINANTS PART A-CHEMISTRY ANALYSIS CONTROL ; EXPOSURE & RISK ASSESSMENT | SO normalised: FOOD ADDITIVES AND CONTAMINANTS PART A-CHEMISTRY ANALYSIS CONTROL ; EXPOSURE & RISK ASSESSMENT | CR count: 80 → 80

Field	Status	Orig. pos	Norm. pos	Orig. line	Norm. line	Original cited/source value	Normalised cited/source value
CR	replaced	57	57	87724	87723	Food Addit Contam<br>Scott P M, 1984, Food Addit Contam, V1, P313, DOI 10.1080/02652038409385862	FOOD ADDIT CONTAM<br>Scott P M, 1984, FOOD ADDIT CONTAM, V1, P313, DOI 10.1080/02652038409385862
CR	replaced	67	67	87734	87733	FUNDAM APPL TOXICOL<br>UENO Y, 1984, FUNDAM APPL TOXICOL, V4, P124	Fundam Appl Toxicol<br>UENO Y, 1984, Fundam Appl Toxicol, V4, P124

Record 603 | WOS:000279331700013

Modification of aflatoxin B1 and ochratoxin A toxicokinetics in rats administered a yeast cell wall preparation

SO original: FOOD ADDITIVES AND CONTAMINANTS PART A-CHEMISTRY ANALYSIS CONTROL ; EXPOSURE & RISK ASSESSMENT | SO normalised: FOOD ADDITIVES AND CONTAMINANTS PART A-CHEMISTRY ANALYSIS CONTROL ; EXPOSURE & RISK ASSESSMENT | CR count: 45 → 45

No journal-related changes detected in SO, J9, JI or CR for this record.

Record 604 | WOS:000273528200008

Zearalenone and its Derivatives: Known Toxins in New Aspects

SO original: MYCOTOXINS IN FOOD, FEED AND BIOWEAPONS | SO normalised: MYCOTOXINS IN FOOD, FEED AND BIOWEAPONS | CR count: 94 → 94

Field	Status	Orig. pos	Norm. pos	Orig. line	Norm. line	Original cited/source value	Normalised cited/source value
CR	replaced	11	11	87929	87928	Mycotoxin Res<br>Berthiller F, 2007, Mycotoxin Res, V23, P68, DOI 10.1007/BF02946028	MYCOTOXIN RES<br>Berthiller F, 2007, MYCOTOXIN RES, V23, P68, DOI 10.1007/BF02946028

CR	replaced	33	33	87951	87950	Polish Journal of Veterinary Sciences<br>Gajecki M., 2004, Polish Journal of Veterinary Sciences, V7, P153	POL J VET SCI<br>Gajecki M., 2004, POL J VET SCI, V7, P153
CR	replaced	39	39	87957	87956	Polish Journal of Veterinary Sciences<br>Gora M., 2004, Polish Journal of Veterinary Sciences, V7, P181	POL J VET SCI<br>Gora M., 2004, POL J VET SCI, V7, P181
CR	replaced	42	42	87960	87959	Adv Appl Microbiol<br>Hidy P H, 1977, Adv Appl Microbiol, V22, P59, DOI 10.1016/S0065-2164(08)70160-6	ADV APPL MICROBIOL<br>Hidy P H, 1977, ADV APPL MICROBIOL, V22, P59, DOI 10.1016/S0065-2164(08)70160-6
CR	replaced	64	64	87982	87981	Polish Journal of Veterinary Sciences<br>Popiel J., 2004, Polish Journal of Veterinary Sciences, V7, P149	POL J VET SCI<br>Popiel J., 2004, POL J VET SCI, V7, P149
CR	replaced	75	75	87993	87992	Polish Journal of Veterinary Sciences<br>Skorska-Wyszynska E., 2004, Polish Journal of Veterinary Sciences, V7, P305	POL J VET SCI<br>Skorska-Wyszynska E., 2004, POL J VET SCI, V7, P305
CR	replaced	94	94	88012	88011	Polish Journal of Veterinary Sciences<br>Zwierzchowski W., 2005, Polish Journal of Veterinary Sciences, V8, P209	POL J VET SCI<br>Zwierzchowski W., 2005, POL J VET SCI, V8, P209

Record 605 | WOS:000281731700007

Lifetime, low-dose ochratoxin A dietary study on renal carcinogenesis in male Fischer rats

SO original: FOOD ADDITIVES AND CONTAMINANTS PART A-CHEMISTRY ANALYSIS CONTROL ; EXPOSURE & RISK ASSESSMENT | SO normalised: FOOD ADDITIVES AND CONTAMINANTS PART A-CHEMISTRY ANALYSIS CONTROL ; EXPOSURE & RISK ASSESSMENT | CR count: 22 → 22

No journal-related changes detected in SO, J9, JI or CR for this record.

Record 606 | WOS:000273618500012

Structures of Covalent Adducts between DNA and Ochratoxin A: A New Factor in Debate about Genotoxicity and Human Risk Assessment

SO original: CHEMICAL RESEARCH IN TOXICOLOGY | SO normalised: CHEMICAL RESEARCH IN TOXICOLOGY | CR count: 43 → 43

No journal-related changes detected in SO, J9, JI or CR for this record.

Record 607 | WOS:000286528700008

Ochratoxin A-induced serum biochemical alterations in New Zealand white rabbits (Oryctolagus cuniculus)

SO original: TURKISH JOURNAL OF VETERINARY & ANIMAL SCIENCES | SO normalised: TURKISH JOURNAL OF VETERINARY & ANIMAL SCIENCES | CR count: 42 → 42

Field	Status	Orig. pos	Norm. pos	Orig. line	Norm. line	Original cited/source value	Normalised cited/source value
CR	replaced	1	1	88255	88254	J Vet Sci<br>Agawane S B, 2004, J Vet Sci, V5, P359	J VET SCI<br>Agawane S B, 2004, J VET SCI, V5, P359
CR	replaced	23	23	88277	88276	SKUAST J. Res<br>Mir M.S., 2008, SKUAST J. Res, V10, P99	SKUAST J RES<br>Mir M.S., 2008, SKUAST J RES, V10, P99

Record 608 | WOS:000274143800002

Toxicology of mycotoxins

SO original: MOLECULAR, CLINICAL AND ENVIRONMENTAL TOXICOLOGY VOL 2: CLINICAL ; TOXICOLOGY | SO normalised: MOLECULAR, CLINICAL AND ENVIRONMENTAL TOXICOLOGY VOL 2: CLINICAL ; TOXICOLOGY | CR count: 116 → 116

Field	Status	Orig. pos	Norm. pos	Orig. line	Norm. line	Original cited/source value	Normalised cited/source value
CR	replaced	1	1	88346	88345	Mycopathologia<br>[Anonymous], 2003, Mycopathologia, V65, P43	MYCOPATHOLOGIA<br>[Anonymous], 2003, MYCOPATHOLOGIA, V65, P43
CR	replaced	25	25	88370	88369	Microbial toxins<br>Forgacs J., 1972, Microbial toxins, VVIII., P95	MICROBIAL TOXINS<br>Forgacs J., 1972, MICROBIAL TOXINS, VVIII., P95
CR	replaced	96	96	88441	88440	BIOMED ENVIRON SCI<br>Sun XM, 2002, BIOMED ENVIRON SCI, V15, P145	Biomed Environ Sci<br>Sun XM, 2002, Biomed Environ Sci, V15, P145

Record 609 | WOS:000272760200016

CYP1A1 and CYP3A4 modulation by dietary flavonoids in human intestinal Caco-2 cells

SO original: TOXICOLOGY LETTERS | SO normalised: TOXICOLOGY LETTERS | CR count: 66 → 66

Field	Status	Orig. pos	Norm. pos	Orig. line	Norm. line	Original cited/source value	Normalised cited/source value
CR	replaced	48	48	88576	88575	BR J NUTR<br>ROMIER B, 2008, BR J NUTR, V100, P42	BRIT J NUTR<br>ROMIER B, 2008, BRIT J NUTR, V100, P42

Record 610 | WOS:000271971100003

Comparative Mechanisms of Zearalenone and Ochratoxin A Toxicities on Cultured HepG2 cells: Is Oxidative Stress a Common Process?

SO original: ENVIRONMENTAL TOXICOLOGY | SO normalised: ENVIRONMENTAL TOXICOLOGY | CR count: 56 → 56

No journal-related changes detected in SO, J9, JI or CR for this record.

Record 611 | WOS:000208434400002

Cyclopiazonic Acid Biosynthesis of Aspergillus flavus and Aspergillus oryzae

SO original: TOXINS | SO normalised: TOXINS | CR count: 126 → 126

Field	Status	Orig. pos	Norm. pos	Orig. line	Norm. line	Original cited/source value	Normalised cited/source value
CR	replaced	19	19	88780	88779	Mycotoxin Res<br>Cole R J, 1986, Mycotoxin Res, V2, P3, DOI 10.1007/BF03191956	MYCOTOXIN RES<br>Cole R J, 1986, MYCOTOXIN RES, V2, P3, DOI 10.1007/BF03191956

Record 612 | WOS:000272276600011

Reactive oxygen species induced by beauvericin, patulin and zearalenone in CHO-K1 cells

SO original: TOXICOLOGY IN VITRO | SO normalised: TOXICOLOGY IN VITRO | CR count: 21 → 21

Field	Status	Orig. pos	Norm. pos	Orig. line	Norm. line	Original cited/source value	Normalised cited/source value
CR	replaced	18	18	88968	88967	Toxicological and Environmental Chemistry<br>Ruiz Maria Jose, 2006, Toxicological and Environmental Chemistry, V88, P345, DOI 10.1080/02772240600630622	TOXICOL ENVIRON CHEM<br>Ruiz Maria Jose, 2006, TOXICOL ENVIRON CHEM, V88, P345, DOI 10.1080/02772240600630622

Record 613 | WOS:000272276600013

Intracellular zinc stores protect the intestinal epithelium from Ochratoxin A toxicity

SO original: TOXICOLOGY IN VITRO | SO normalised: TOXICOLOGY IN VITRO | CR count: 36 → 36

No journal-related changes detected in SO, J9, JI or CR for this record.

Record 614 | WOS:000270527000004

Minimum tolerable exposure period and maximum threshold dietary intake of ochratoxin A for causing renal cancer in male Dark Agouti rats

SO original: FOOD AND CHEMICAL TOXICOLOGY | SO normalised: FOOD AND CHEMICAL TOXICOLOGY | CR count: 17 → 17

No journal-related changes detected in SO, J9, JI or CR for this record.

Record 615 | WOS:000270503100034

Molecular Characterization of Preneoplastic Lesions Provides Insight on the Development of Renal Tumors

SO original: AMERICAN JOURNAL OF PATHOLOGY | SO normalised: AMERICAN JOURNAL OF PATHOLOGY | CR count: 58 → 58

Field	Status	Orig. pos	Norm. pos	Orig. line	Norm. line	Original cited/source value	Normalised cited/source value

CR	replaced	3	3	89196	89195	Contrib Nephrol Bannasch P, 1999, Contrib Nephrol, V128, P99	CONTRIB NEPHROL Bannasch P, 1999, CONTRIB NEPHROL, V128, P99
CR	replaced	19	19	89212	89211	Adv Cancer Res Hamilton J M, 1975, Adv Cancer Res, V22, P1	ADV CANCER RES Hamilton J M, 1975, ADV CANCER RES, V22, P1

Record 616 | WOS:000269677000009

Low doses of ochratoxin A upregulate the protein expression of organic anion transporters Oat1, Oat2, Oat3 and Oat5 in rat kidney cortex

SO original: TOXICOLOGY AND APPLIED PHARMACOLOGY | SO normalised: TOXICOLOGY AND APPLIED PHARMACOLOGY | CR count: 68 → 68

No journal-related changes detected in SO, J9, JI or CR for this record.

Record 617 | WOS:000269551500021

The mycotoxin ochratoxin A inhibits DNA topoisomerase II and induces polyploidy in cultured CHO cells

SO original: TOXICOLOGY IN VITRO | SO normalised: TOXICOLOGY IN VITRO | CR count: 51 → 51

No journal-related changes detected in SO, J9, JI or CR for this record.

Record 618 | WOS:000269551500029

Multiplexed assay panel of cytotoxicity in HK-2 cells for detection of renal proximal tubule injury potential of compounds

SO original: TOXICOLOGY IN VITRO | SO normalised: TOXICOLOGY IN VITRO | CR count: 50 → 50

No journal-related changes detected in SO, J9, JI or CR for this record.

Record 619 | WOS:000268006900004

Pro-apoptotic effects of nivalenol and deoxynivalenol trichothecenes in J774A.1 murine macrophages

SO original: TOXICOLOGY LETTERS | SO normalised: TOXICOLOGY LETTERS | CR count: 42 → 42

No journal-related changes detected in SO, J9, JI or CR for this record.

Record 620 | WOS:000282995500010

Occurrence of ochratoxin A and heavy metals in tissues associated with porcine nephropathy in Serbia

SO original: WORLD MYCOTOXIN JOURNAL | SO normalised: WORLD MYCOTOXIN JOURNAL | CR count: 57 → 57

No journal-related changes detected in SO, J9, JI or CR for this record.

Record 621 | WOS:000267227800008

Ochratoxin A-Mediated DNA and Protein Damage: Roles of Nitrosative and Oxidative Stresses

SO original: TOXICOLOGICAL SCIENCES | SO normalised: TOXICOLOGICAL SCIENCES | CR count: 69 → 69

No journal-related changes detected in SO, J9, JI or CR for this record.

Record 622 | WOS:000268796500023

A Review of the Implementation of the Embryonic Stem Cell Test (EST) The report and recommendations of an ECVAM/ReProTect Workshop

SO original: ATLA-ALTERNATIVES TO LABORATORY ANIMALS | SO normalised: ATLA-ALTERNATIVES TO LABORATORY ANIMALS | CR count: 23 → 23

No journal-related changes detected in SO, J9, JI or CR for this record.

Record 623 | WOS:000265685000003

Oxidative stress and apoptotic changes in primary cultures of rat proximal tubular cells exposed to lead

SO original: ARCHIVES OF TOXICOLOGY | SO normalised: ARCHIVES OF TOXICOLOGY | CR count: 42 → 42

No journal-related changes detected in SO, J9, JI or CR for this record.

Record 624 | WOS:000265474500006

Silibinin protects OTA-mediated TNF-Î± release from perfused rat livers and isolated rat Kupffer cells

SO original: MOLECULAR NUTRITION & FOOD RESEARCH | SO normalised: MOLECULAR NUTRITION & FOOD RESEARCH | CR count: 43 → 43

Field	Status	Orig. pos	Norm. pos	Orig. line	Norm. line	Original cited/source value	Normalised cited/source value
CR	replaced	38	38	90319	90318	Mycotoxin Res Weidenbach A, 2000, Mycotoxin Res, V16 Suppl 2, P189, DOI 10.1007/BF02940035	MYCOTOXIN RES Weidenbach A, 2000, MYCOTOXIN RES, V16 Suppl 2, P189, DOI 10.1007/BF02940035

Record 625 | WOS:000265474500007

Mycotoxins and the kidney: Modes of action for renal tumor formation by ochratoxin A in rodents

SO original: MOLECULAR NUTRITION & FOOD RESEARCH | SO normalised: MOLECULAR NUTRITION & FOOD RESEARCH | CR count: 94 → 94

No journal-related changes detected in SO, J9, JI or CR for this record.

Record 626 | WOS:000263803400010

Effects of the mycotoxin fumonisin B1 on cell death in human kidney cells and human lung fibroblasts in primary culture

SO original: JOURNAL OF APPLIED TOXICOLOGY | SO normalised: JOURNAL OF APPLIED TOXICOLOGY | CR count: 50 → 50

Field	Status	Orig. pos	Norm. pos	Orig. line	Norm. line	Original cited/source value	Normalised cited/source value
CR	replaced	15	15	90545	90544	J Vet Diagn Invest<br>Harrison L R, 1990, J Vet Diagn Invest, V2, P217	J VET DIAGN INVEST<br>Harrison L R, 1990, J VET DIAGN INVEST, V2, P217

Record 627 | WOS:000266663300004

Ochratoxin A induces apoptosis in neuronal cells

SO original: GENES AND NUTRITION | SO normalised: GENES AND NUTRITION | CR count: 35 → 35

No journal-related changes detected in SO, J9, JI or CR for this record.

Record 628 | WOS:000263382500003

CYP1A1 induction and CYP3A4 inhibition by the fungicide imazalil in the human intestinal Caco-2 cells-Comparison with other conazole pesticides

SO original: TOXICOLOGY LETTERS | SO normalised: TOXICOLOGY LETTERS | CR count: 72 → 72

No journal-related changes detected in SO, J9, JI or CR for this record.

Record 629 | WOS:000262717200017

Effects of live yeast cell supplementation to high concentrate diets on the toxicokinetics of ochratoxin A in sheep

SO original: FOOD ADDITIVES AND CONTAMINANTS PART A-CHEMISTRY ANALYSIS CONTROL ; EXPOSURE & RISK ASSESSMENT | SO normalised: FOOD ADDITIVES AND CONTAMINANTS PART A-CHEMISTRY ANALYSIS CONTROL ; EXPOSURE & RISK ASSESSMENT | CR count: 40 → 40

Field	Status	Orig. pos	Norm. pos	Orig. line	Norm. line	Original cited/source value	Normalised cited/source value
CR	replaced	12	12	90891	90890	Biochemical Pharmacology<br>CHU F S, 1974, Biochemical Pharmacology, V23, P1105, DOI 10.1016/0006-2952(74)90011-2	BIOCHEM PHARMACOL<br>CHU F S, 1974, BIOCHEM PHARMACOL, V23, P1105, DOI 10.1016/0006-2952(74)90011-2
CR	replaced	29	29	90908	90907	Nat Toxins<br>Muller H M, 1998, Nat Toxins, V6, P251, DOI 10.1002/(SICI)1522-7189(199811/12)6:6<251::AID-NT35>3.0.CO;2-P	NAT TOXINS<br>Muller H M, 1998, NAT TOXINS, V6, P251, DOI 10.1002/(SICI)1522-7189(199811/12)6:6<251::AID-NT35>3.0.CO;2-P

Record 630 | WOS:000267272900021

The biotransformation of chosen mycotoxins

SO original: POLISH JOURNAL OF VETERINARY SCIENCES | SO normalised: POLISH JOURNAL OF VETERINARY SCIENCES | CR count: 54 → 54

No journal-related changes detected in SO, J9, JI or CR for this record.

Record 631 | WOS:000273001000009

Effects of probiotic bacteria on the bioaccessibility of aflatoxin B1 and ochratoxin A using an in vitro digestion model under fed conditions

SO original: JOURNAL OF ENVIRONMENTAL SCIENCE AND HEALTH PART B-PESTICIDES FOOD ; CONTAMINANTS AND AGRICULTURAL WASTES | SO normalised: JOURNAL OF ENVIRONMENTAL SCIENCE AND HEALTH PART B-PESTICIDES FOOD ; CONTAMINANTS AND AGRICULTURAL WASTES | CR count: 29 → 29

No journal-related changes detected in SO, J9, JI or CR for this record.

Record 632 | WOS:000292954300008

Microorganisms and their enzymes for detoxifying mycotoxins posing a risk to livestock animals

SO original: MYCOTOXIN PREVENTION AND CONTROL IN AGRICULTURE | SO normalised: MYCOTOXIN PREVENTION AND CONTROL IN AGRICULTURE | CR count: 44 → 44

Field	Status	Orig. pos	Norm. pos	Orig. line	Norm. line	Original cited/source value	Normalised cited/source value
CR	replaced	35	35	91224	91223	Mycotoxin Res Schatzmayr G, 2003, Mycotoxin Res, V19, P124, DOI 10.1007/BF02942950	MYCOTOXIN RES Schatzmayr G, 2003, MYCOTOXIN RES, V19, P124, DOI 10.1007/BF02942950

Record 633 | WOS:000262775900032

Effect of ochratoxin A on cell survival and collagen homeostasis in human mesangial cells in primary culture

SO original: FOOD AND CHEMICAL TOXICOLOGY | SO normalised: FOOD AND CHEMICAL TOXICOLOGY | CR count: 28 → 28

No journal-related changes detected in SO, J9, JI or CR for this record.

Record 634 | WOS:000264793900023

EFFECTS OF AFLATOXIN B1, OCHRATOXIN A, PATULIN, CITRININ, AND ZEARALENONE ON THE IN VITRO PROLIFERATION OF PIG BLOOD LYMPHOCYTES

SO original: BULLETIN OF THE VETERINARY INSTITUTE IN PULAWY | SO normalised: BULLETIN OF THE VETERINARY INSTITUTE IN PULAWY | CR count: 37 → 37

No journal-related changes detected in SO, J9, JI or CR for this record.

Record 635 | WOS:000262790100004

Developmental Toxicity of Ochratoxin A in Rat Embryo Midbrain Micromass Cultures

SO original: INTERNATIONAL JOURNAL OF MOLECULAR SCIENCES | SO normalised: INTERNATIONAL JOURNAL OF MOLECULAR SCIENCES | CR count: 47 → 47

No journal-related changes detected in SO, J9, JI or CR for this record.

Record 636 | WOS:000261056700090

Safety and Mutagenicity Evaluation of Nanoparticulate Red Mold Rice

SO original: JOURNAL OF AGRICULTURAL AND FOOD CHEMISTRY | SO normalised: JOURNAL OF AGRICULTURAL AND FOOD CHEMISTRY | CR count: 56 → 56

Field	Status	Orig. pos	Norm. pos	Orig. line	Norm. line	Original cited/source value	Normalised cited/source value
CR	replaced	56	56	91617	91616	Journal of Nutrition<br>Bieri J. G., 1977, Journal of Nutrition, V107, P1394	J NUTR<br>Bieri J. G., 1977, J NUTR, V107, P1394

Record 637 | WOS:000207684500010

Oxidative stress induced by ochratoxin A in LLC-PK1 cell line and the chemoprotective effects of carnosic acid

SO original: WORLD MYCOTOXIN JOURNAL | SO normalised: WORLD MYCOTOXIN JOURNAL | CR count: 27 → 27

No journal-related changes detected in SO, J9, JI or CR for this record.

Record 638 | WOS:000260367000001

Individual and combined effects of ochratoxin A and citrinin on viability and DNA fragmentation in cultured Vero cells and on chromosome aberrations in mice bone marrow cells

SO original: TOXICOLOGY | SO normalised: TOXICOLOGY | CR count: 72 → 72

No journal-related changes detected in SO, J9, JI or CR for this record.

Record 639 | WOS:000259798900005

Inula crithmoides extract protects against ochratoxin A-induced oxidative stress, clastogenic and mutagenic alterations in male rats

SO original: TOXICON | SO normalised: TOXICON | CR count: 75 → 75

No journal-related changes detected in SO, J9, JI or CR for this record.

Record 640 | WOS:000260145000006

Differential modification of inflammatory enzymes in J774A.1 macrophages by ochratoxin A alone or in combination with lipopolysaccharide

SO original: TOXICOLOGY LETTERS | SO normalised: TOXICOLOGY LETTERS | CR count: 53 → 53

No journal-related changes detected in SO, J9, JI or CR for this record.

Record 641 | WOS:000258440100010

Effect of ochratoxin A on redox-regulated transcription factors, antioxidant enzymes and glutathione-S-transferase in cultured kidney tubulus cells

SO original: FOOD AND CHEMICAL TOXICOLOGY | SO normalised: FOOD AND CHEMICAL TOXICOLOGY | CR count: 50 → 50

No journal-related changes detected in SO, J9, JI or CR for this record.

Record 642 | WOS:000257645600013

Species-specific toxicity of aristolochic acid (AA) in vitro

SO original: TOXICOLOGY IN VITRO | SO normalised: TOXICOLOGY IN VITRO | CR count: 43 → 43

No journal-related changes detected in SO, J9, JI or CR for this record.

Record 643 | WOS:000257721400033

Identification and in vitro cytotoxicity of ochratoxin a degradation products formed during coffee roasting

SO original: JOURNAL OF AGRICULTURAL AND FOOD CHEMISTRY | SO normalised: JOURNAL OF AGRICULTURAL AND FOOD CHEMISTRY | CR count: 35 → 35

Field	Status	Orig. pos	Norm. pos	Orig. line	Norm. line	Original cited/source value	Normalised cited/source value
CR	replaced	18	18	92378	92377	IARC Sci Publ Petkova-Bocharova T, 1991, IARC Sci Publ, P135	IARC SCI PUBL Petkova-Bocharova T, 1991, IARC SCI PUBL, P135

Record 644 | WOS:000257577500008

Gene expression changes induced by ochratoxin A in renal and hepatic tissues of male F344 rat after oral repeated administration

SO original: TOXICOLOGY AND APPLIED PHARMACOLOGY | SO normalised: TOXICOLOGY AND APPLIED PHARMACOLOGY | CR count: 44 → 44

No journal-related changes detected in SO, J9, JI or CR for this record.

Record 645 | WOS:000256601000019

Structure-activity relationships for the fluorescence of ochratoxin A: Insight for detection of ochratoxin A metabolites

SO original: ANALYTICA CHIMICA ACTA | SO normalised: ANALYTICA CHIMICA ACTA | CR count: 38 → 38

No journal-related changes detected in SO, J9, JI or CR for this record.

Record 646 | WOS:000256806600009

In vitro cytogenetic results supporting a DNA nonreactive mechanism for Ochratoxin A, potentially relevant for its carcinogenicity

SO original: CHEMICAL RESEARCH IN TOXICOLOGY | SO normalised: CHEMICAL RESEARCH IN TOXICOLOGY | CR count: 70 → 70

Field	Status	Orig. pos	Norm. pos	Orig. line	Norm. line	Original cited/source value	Normalised cited/source value

CR	replaced	8	8	92670	92669	Int Rev Cytol<br>Brodsky W Y, 1977, Int Rev Cytol, V50, P275	INT REV CYTOL<br>Brodsky W Y, 1977, INT REV CYTOL, V50, P275

Record 647 | WOS:000256206000004

Modulating effects of fumonisin B1 and ochratoxin A on leukocytes and messenger cytokines of the human immune system

SO original: INTERNATIONAL IMMUNOPHARMACOLOGY | SO normalised: INTERNATIONAL IMMUNOPHARMACOLOGY | CR count: 29 → 29

No journal-related changes detected in SO, J9, JI or CR for this record.

Record 648 | WOS:000255760800015

Evaluation of putative biomarkers of nephrotoxicity after exposure to ochratoxin a in vivo and in vitro

SO original: TOXICOLOGICAL SCIENCES | SO normalised: TOXICOLOGICAL SCIENCES | CR count: 52 → 52

Field	Status	Orig. pos	Norm. pos	Orig. line	Norm. line	Original cited/source value	Normalised cited/source value
CR	replaced	42	42	92917	92916	Journal of Toxicological Sciences<br>Tamura Kotaro, 2006, Journal of Toxicological Sciences, V31, P471, DOI 10.2131/jts.31.471	J TOXICOL SCI<br>Tamura Kotaro, 2006, J TOXICOL SCI, V31, P471, DOI 10.2131/jts.31.471

Record 649 | WOS:000256713600009

Ochratoxin A inhibits the production of tissue factor and plasminogen activator inhibitor-2 by human blood mononuclear cells: Another potential mechanism of immune-suppression

SO original: TOXICOLOGY AND APPLIED PHARMACOLOGY | SO normalised: TOXICOLOGY AND APPLIED PHARMACOLOGY | CR count: 37 → 37

No journal-related changes detected in SO, J9, JI or CR for this record.

Record 650 | WOS:000256381400006

Medical relevance of mycotoxins

SO original: DEUTSCHE MEDIZINISCHE WOCHENSCHRIFT | SO normalised: DEUTSCHE MEDIZINISCHE WOCHENSCHRIFT | CR count: 18 → 18

Field	Status	Orig. pos	Norm. pos	Orig. line	Norm. line	Original cited/source value	Normalised cited/source value
CR	replaced	7	7	93079	93078	Mycotoxin Res<br>Degen G H, 2007, Mycotoxin Res, V23, P88, DOI 10.1007/BF02946032	MYCOTOXIN RES<br>Degen G H, 2007, MYCOTOXIN RES, V23, P88, DOI 10.1007/BF02946032
CR	replaced	11	11	93083	93082	Mycotoxin Res<br>Grajewski J, 2007, Mycotoxin Res, V23, P22, DOI 10.1007/BF02946020	MYCOTOXIN RES<br>Grajewski J, 2007, MYCOTOXIN RES, V23, P22, DOI 10.1007/BF02946020

Record 651 | WOS:000254689700022

Detoxification of patulin and ochratoxin A, two abundant mycotoxins, by lactic acid bacteria

SO original: FOOD AND CHEMICAL TOXICOLOGY | SO normalised: FOOD AND CHEMICAL TOXICOLOGY | CR count: 77 → 77

No journal-related changes detected in SO, J9, JI or CR for this record.

Record 652 | WOS:000254544300011

Both direct and indirect effects account for the pro-inflammatory activity of enteropathogenic mycotoxins on the human intestinal epithelium:: Stimulation of interleukin-8 secretion, potentiation of interleukin-1Î² effect and increase in the transepithelial passage of commensal bacteria

SO original: TOXICOLOGY AND APPLIED PHARMACOLOGY | SO normalised: TOXICOLOGY AND APPLIED PHARMACOLOGY | CR count: 64 → 64

No journal-related changes detected in SO, J9, JI or CR for this record.

Record 653 | WOS:000257197500001

Risk-benefit considerations of mitigation measures on acrylamide content of foods - A case study on potatoes, cereals and coffee

SO original: BRITISH JOURNAL OF NUTRITION | SO normalised: BRITISH JOURNAL OF NUTRITION | CR count: 289 → 289

Field	Status	Orig. pos	Norm. pos	Orig. line	Norm. line	Original cited/source value	Normalised cited/source value
CR	replaced	253	253	93705	93704	FOOD SCI TECHNOL<br>SUMMA CA, 2007, FOOD SCI TECHNOL	FOOD SCI TECH-BRAZIL<br>SUMMA CA, 2007, FOOD SCI TECH-BRAZIL
CR	replaced	286	286	93738	93737	Potato Res.<br>Wustman R, 2005, Potato Res., V48, P117, DOI [10.1007/BF02742371, DOI 10.1007/BF02742371]	POTATO RES<br>Wustman R, 2005, POTATO RES, V48, P117, DOI [10.1007/BF02742371, DOI 10.1007/BF02742371]

Record 654 | WOS:000258542600115

In vitro investigation of individual and combined cytotoxicity of ochratoxin a and arsenic in Chinese hamster lung fibroblasts and human urothelial cells

SO original: IN VITRO CELLULAR & DEVELOPMENTAL BIOLOGY-ANIMAL | SO normalised: IN VITRO CELLULAR & DEVELOPMENTAL BIOLOGY-ANIMAL | CR count: 0 → 0

No journal-related changes detected in SO, J9, JI or CR for this record.

Record 655 | WOS:000252706500006

Ameliorative effect of artichoke (Cynara scolymus L.) extracts on creatine monohydrate-induced renal dysfunction in male rats

SO original: DEUTSCHE LEBENSMITTEL-RUNDSCHAU | SO normalised: DEUTSCHE LEBENSMITTEL-RUNDSCHAU | CR count: 73 → 73

No journal-related changes detected in SO, J9, JI or CR for this record.

Record 656 | WOS:000255988000006

Cytotoxicity and oxidative damage in kidney cells exposed to the mycotoxins ochratoxin A and citrinin: Individual and combined effects

SO original: TOXICOLOGY MECHANISMS AND METHODS | SO normalised: TOXICOLOGY MECHANISMS AND METHODS | CR count: 54 → 54

Field	Status	Orig. pos	Norm. pos	Orig. line	Norm. line	Original cited/source value	Normalised cited/source value
CR	replaced	35	35	94014	94013	IARC Sci Publ<br>Petkova-Bocharova T, 1991, IARC Sci Publ, P135	IARC SCI PUBL<br>Petkova-Bocharova T, 1991, IARC SCI PUBL, P135

Record 657 | WOS:000253659900006

Mycotoxins in cattle feeds and carry-over to dairy milk: A review

SO original: FOOD ADDITIVES AND CONTAMINANTS PART A-CHEMISTRY ANALYSIS CONTROL ; EXPOSURE & RISK ASSESSMENT | SO normalised: FOOD ADDITIVES AND CONTAMINANTS PART A-CHEMISTRY ANALYSIS CONTROL ; EXPOSURE & RISK ASSESSMENT | CR count: 67 → 67

Field	Status	Orig. pos	Norm. pos	Orig. line	Norm. line	Original cited/source value	Normalised cited/source value
CR	replaced	1	1	94085	94084	REV MED VET<br>AUERBACH H, 1998, REV MED VET, P573	REV MED VET-TOULOUSE<br>AUERBACH H, 1998, REV MED VET-TOULOUSE, P573
CR	replaced	14	14	94098	94097	Annales de Recherches Veterinaires<br>ESCOULA L, 1975, Annales de Recherches Veterinaires, V6, P303	ANN RECH VET<br>ESCOULA L, 1975, ANN RECH VET, V6, P303
CR	replaced	19	19	94103	94102	The Toxicology of Aflatoxins: Human Health<br>Hendricks J.D., 1994, The Toxicology of Aflatoxins: Human Health, Veterinary and Agricultural Significance, P103	The toxicology of aflatoxins: human health<br>Hendricks J.D., 1994, The toxicology of aflatoxins: human health, Veterinary and Agricultural Significance, P103

Record 658 | WOS:000268886600003

Combined Toxic Effects of Ochratoxin A and Citrinin, In Vivo and In Vitro

SO original: FOOD CONTAMINANTS: MYCOTOXINS AND FOOD ALLERGENS | SO normalised: FOOD CONTAMINANTS: MYCOTOXINS AND FOOD ALLERGENS | CR count: 49 → 49

Field	Status	Orig. pos	Norm. pos	Orig. line	Norm. line	Original cited/source value	Normalised cited/source value
CR	replaced	33	33	94245	94244	IARC Sci Publ<br>Petkova-Bocharova T, 1991, IARC Sci Publ, P83	IARC SCI PUBL<br>Petkova-Bocharova T, 1991, IARC SCI PUBL, P83

Record 659 | WOS:000251052700012

Toxigenic fungi and mycotoxins in mature corn silage

SO original: FOOD AND CHEMICAL TOXICOLOGY | SO normalised: FOOD AND CHEMICAL TOXICOLOGY | CR count: 31 → 31

Field	Status	Orig. pos	Norm. pos	Orig. line	Norm. line	Original cited/source value	Normalised cited/source value
CR	replaced	1	1	94323	94322	BMC Nephrol<br>Abbott Kevin C, 2003, BMC Nephrol, V4, P1, DOI 10.1186/1471-2369-4-1	BMC NEPHROL<br>Abbott Kevin C, 2003, BMC NEPHROL, V4, P1, DOI 10.1186/1471-2369-4-1
CR	replaced	18	18	94340	94339	Mycotoxin Res<br>Oldenburg E, 1993, Mycotoxin Res, V9, P72, DOI 10.1007/BF03192237	MYCOTOXIN RES<br>Oldenburg E, 1993, MYCOTOXIN RES, V9, P72, DOI 10.1007/BF03192237

Record 660 | WOS:000249598500015

Catechins: natural free-radical scavengers against ochratoxin A-induced cell damage in a pig kidney cell line (LLC-PK1)

SO original: FOOD AND CHEMICAL TOXICOLOGY | SO normalised: FOOD AND CHEMICAL TOXICOLOGY | CR count: 48 → 48

No journal-related changes detected in SO, J9, JI or CR for this record.

Record 661 | WOS:000249983300013

New molecular and field evidences for the implication of mycotoxins but not aristolochic acid in human nephropathy and urinary tract tumor

SO original: MOLECULAR NUTRITION & FOOD RESEARCH | SO normalised: MOLECULAR NUTRITION & FOOD RESEARCH | CR count: 56 → 56

Field	Status	Orig. pos	Norm. pos	Orig. line	Norm. line	Original cited/source value	Normalised cited/source value
CR	replaced	27	27	94556	94555	IARC Sci Publ<br>Petkova-Bocharova T, 1991, IARC Sci Publ, P83	IARC SCI PUBL<br>Petkova-Bocharova T, 1991, IARC SCI PUBL, P83
CR	replaced	32	32	94561	94560	IARC Sci Publ<br>Pfohl-Leszkowicz A, 1993, IARC Sci Publ, P141	IARC SCI PUBL<br>Pfohl-Leszkowicz A, 1993, IARC SCI PUBL, P141
CR	replaced	45	45	94574	94573	IARC Sci Publ<br>Swenberg James A, 2004, IARC Sci Publ, P237	IARC SCI PUBL<br>Swenberg James A, 2004, IARC SCI PUBL, P237

Record 662 | WOS:000248014300007

Ochratoxin A-induced mutagenesis in mammalian cells is consistent with the production of oxidative stress

SO original: CHEMICAL RESEARCH IN TOXICOLOGY | SO normalised: CHEMICAL RESEARCH IN TOXICOLOGY | CR count: 81 → 81

No journal-related changes detected in SO, J9, JI or CR for this record.

Record 663 | WOS:000247601800018

Adult hippocampal neural stem/progenitor cells in vitro are vulnerable to the mycotoxin ochratoxin-A

SO original: TOXICOLOGICAL SCIENCES | SO normalised: TOXICOLOGICAL SCIENCES | CR count: 57 → 57

Field	Status	Orig. pos	Norm. pos	Orig. line	Norm. line	Original cited/source value	Normalised cited/source value
CR	replaced	12	12	94795	94794	Neurobiol Aging<br>Chen H., 2006, Neurobiol Aging	NEUROBIOL AGING<br>Chen H., 2006, NEUROBIOL AGING
CR	replaced	43	43	94826	94825	IARC Sci Publ<br>Pfohl-Leszkowicz A, 1993, IARC Sci Publ, P141	IARC SCI PUBL<br>Pfohl-Leszkowicz A, 1993, IARC SCI PUBL, P141

Record 664 | WOS:000246927300041

Ochratoxin A displaces claudins from detergent resistant membrane microdomains

SO original: BIOCHEMICAL AND BIOPHYSICAL RESEARCH COMMUNICATIONS | SO normalised: BIOCHEMICAL AND BIOPHYSICAL RESEARCH COMMUNICATIONS | CR count: 19 → 19

No journal-related changes detected in SO, J9, JI or CR for this record.

Record 665 | WOS:000247178200008

Ochratoxin A:: 13-week oral toxicity and cell proliferation in male F344/N rats

SO original: TOXICOLOGICAL SCIENCES | SO normalised: TOXICOLOGICAL SCIENCES | CR count: 40 → 40

No journal-related changes detected in SO, J9, JI or CR for this record.

Record 666 | WOS:000247092600009

Transepithelial transport of fusariotoxin nivalenol: Mediation of secretion by ABC transporters

SO original: TOXICOLOGY LETTERS | SO normalised: TOXICOLOGY LETTERS | CR count: 45 → 45

No journal-related changes detected in SO, J9, JI or CR for this record.

Record 667 | WOS:000246213800014

Production and characterization of monoclonal antibodies against ochratoxin B

SO original: FOOD AND CHEMICAL TOXICOLOGY | SO normalised: FOOD AND CHEMICAL TOXICOLOGY | CR count: 37 → 37

No journal-related changes detected in SO, J9, JI or CR for this record.

Record 668 | WOS:000246875200005

Combined effects of mycotoxin mixtures on human T cell function

SO original: TOXICOLOGY LETTERS | SO normalised: TOXICOLOGY LETTERS | CR count: 44 → 44

No journal-related changes detected in SO, J9, JI or CR for this record.

Record 669 | WOS:000245707900011

In vitro gene expression data supporting a DNA non-reactive genotoxic mechanism for ochratoxin A

SO original: TOXICOLOGY AND APPLIED PHARMACOLOGY | SO normalised: TOXICOLOGY AND APPLIED PHARMACOLOGY | CR count: 48 → 48

Field	Status	Orig. pos	Norm. pos	Orig. line	Norm. line	Original cited/source value	Normalised cited/source value
CR	replaced	41	41	95426	95425	Journal of Toxicological Sciences<br>Tamura Kotaro, 2006, Journal of Toxicological Sciences, V31, P471, DOI 10.2131/jts.31.471	J TOXICOL SCI<br>Tamura Kotaro, 2006, J TOXICOL SCI, V31, P471, DOI 10.2131/jts.31.471

Record 670 | WOS:000244262900005

Reduction in antioxidant defenses may contribute to ochratoxin a toxicity and carcinogenicity

SO original: TOXICOLOGICAL SCIENCES | SO normalised: TOXICOLOGICAL SCIENCES | CR count: 52 → 52

No journal-related changes detected in SO, J9, JI or CR for this record.

Record 671 | WOS:000244244100003

Lipid peroxidation and glutathione levels in porcine kidney PK15 cells after individual and combined treatment with fumonisin B1, beauvericin and ochratoxin A

SO original: BASIC & CLINICAL PHARMACOLOGY & TOXICOLOGY | SO normalised: BASIC & CLINICAL PHARMACOLOGY & TOXICOLOGY | CR count: 44 → 44

Field	Status	Orig. pos	Norm. pos	Orig. line	Norm. line	Original cited/source value	Normalised cited/source value
CR	replaced	14	14	95634	95633	J Vet Diagn Invest<br>Harrison L R, 1990, J Vet Diagn Invest, V2, P217	J VET DIAGN INVEST<br>Harrison L R, 1990, J VET DIAGN INVEST, V2, P217
CR	replaced	17	17	95637	95636	Mycotoxin Res<br>Jurjevic Z, 1999, Mycotoxin Res, V15, P67, DOI 10.1007/BF02945217	MYCOTOXIN RES<br>Jurjevic Z, 1999, MYCOTOXIN RES, V15, P67, DOI 10.1007/BF02945217
CR	replaced	19	19	95639	95638	J Biochem Toxicol<br>Kang Y J, 1996, J Biochem Toxicol, V11, P121, DOI 10.1002/(SICI)1522-7146(1996)11:3<121::AID-JBT3>3.3.CO;2-Y	J BIOCHEM TOXICOL<br>Kang Y J, 1996, J BIOCHEM TOXICOL, V11, P121, DOI 10.1002/(SICI)1522-7146(1996)11:3<121::AID-JBT3>3.3.CO;2-Y

Record 672 | WOS:000244169500009

Effects of repeated ochratoxin exposure on renal cells in vitro

SO original: TOXICOLOGY IN VITRO | SO normalised: TOXICOLOGY IN VITRO | CR count: 27 → 27

No journal-related changes detected in SO, J9, JI or CR for this record.

Record 673 | WOS:000243327000004

Oxidative DNA damage induced by Ochratoxin A in the HK-2 human kidney cell line: evidence of the relationship with cytotoxicity

SO original: MUTAGENESIS | SO normalised: MUTAGENESIS | CR count: 59 → 59

No journal-related changes detected in SO, J9, JI or CR for this record.

Record 674 | WOS:000244790500003

Ochratoxin A: An overview on toxicity and carcinogenicity in animals and humans

SO original: MOLECULAR NUTRITION & FOOD RESEARCH | SO normalised: MOLECULAR NUTRITION & FOOD RESEARCH | CR count: 497 → 497

Field	Status	Orig. pos	Norm. pos	Orig. line	Norm. line	Original cited/source value	Normalised cited/source value
CR	replaced	24	24	95934	95933	Mutation Research<br>Azemar Brigitte, 1997, Mutation Research, V379, pS157, DOI 10.1016/S0027-5107(97)83165-X	MUTAT RES<br>Azemar Brigitte, 1997, MUTAT RES, V379, pS157, DOI 10.1016/S0027-5107(97)83165-X
CR	replaced	75	75	95985	95984	Biochemical Pharmacology<br>CHU F S, 1974, Biochemical Pharmacology, V23, P1105, DOI 10.1016/0006-2952(74)90011-2	BIOCHEM PHARMACOL<br>CHU F S, 1974, BIOCHEM PHARMACOL, V23, P1105, DOI 10.1016/0006-2952(74)90011-2
CR	replaced	90	90	96000	95999	Mycotoxin Res<br>Degen G H, 2003, Mycotoxin Res, V19, P3, DOI 10.1007/BF02940082	MYCOTOXIN RES<br>Degen G H, 2003, MYCOTOXIN RES, V19, P3, DOI 10.1007/BF02940082
CR	replaced	97	97	96007	96006	Facta Univ Ser Med Biol<br>Dimitrov Tsvetan., 2002, Facta Univ Ser Med Biol, V9, P7	Facta Univ. Ser. Med. Biol<br>Dimitrov Tsvetan., 2002, Facta Univ. Ser. Med. Biol, V9, P7
CR	replaced	194	194	96104	96103	Teratology<br>HOOD R D, 1975, Teratology, V11, p23A	TERATOLOGY<br>HOOD R D, 1975, TERATOLOGY, V11, p23A
CR	replaced	211	211	96121	96120	CYTOBIOS<br>JESWAL P, 1986, CYTOBIOS, V86, P329	Cytobios<br>JESWAL P, 1986, Cytobios, V86, P329
CR	replaced	215	215	96125	96124	Journal of Nutritional and Environmental Medicine (Abingdon)<br>Jonsyn Felixtina E., 1999, Journal of Nutritional and Environmental Medicine (Abingdon), V9, P15, DOI 10.1080/13590849762763	Journal of Nutritional & Environmental Medicine (Abingdon)<br>Jonsyn Felixtina E., 1999, Journal of Nutritional & Environmental Medicine (Abingdon), V9, P15, DOI 10.1080/13590849762763
CR	replaced	223	223	96133	96132	Mycotoxin Res<br>Jurjevic Z, 1999, Mycotoxin Res, V15, P67, DOI 10.1007/BF02945217	MYCOTOXIN RES<br>Jurjevic Z, 1999, MYCOTOXIN RES, V15, P67, DOI 10.1007/BF02945217

CR	replaced	272	272	96182	96181	REV MED VET MALIR F, 1998, REV MED VET, V149, P710	REV MED VET-TOULOUSE MALIR F, 1998, REV MED VET-TOULOUSE, V149, P710
CR	replaced	332	332	96242	96241	IARC Sci Publ Petkova-Bocharova T, 1991, IARC Sci Publ, P135	IARC SCI PUBL Petkova-Bocharova T, 1991, IARC SCI PUBL, P135
CR	replaced	345	345	96255	96254	IARC Sci Publ Pfohl-Leszkowicz A, 1993, IARC Sci Publ, P141	IARC SCI PUBL Pfohl-Leszkowicz A, 1993, IARC SCI PUBL, P141
CR	replaced	360	360	96270	96269	IARC Sci Publ Plestina S, 1991, IARC Sci Publ, P43	IARC SCI PUBL Plestina S, 1991, IARC SCI PUBL, P43
CR	replaced	480	480	96390	96389	Mycotoxin Res Weidenbach A, 2000, Mycotoxin Res, V16 Suppl 2, P189, DOI 10.1007/BF02940035	MYCOTOXIN RES Weidenbach A, 2000, MYCOTOXIN RES, V16 Suppl 2, P189, DOI 10.1007/BF02940035

Record 675 | WOS:000254907600043

Cytotoxicity of feed-borne mycotoxins to animal cell lines in vitro using the MTT assay

SO original: BULLETIN OF THE VETERINARY INSTITUTE IN PULAWY | SO normalised: BULLETIN OF THE VETERINARY INSTITUTE IN PULAWY | CR count: 24 → 24

Field	Status	Orig. pos	Norm. pos	Orig. line	Norm. line	Original cited/source value	Normalised cited/source value
CR	replaced	7	7	96462	96461	BIOMED ENVIRON SCI KNIPERGOODMAN T, 1989, BIOMED ENVIRON SCI, V2, P179	Biomed Environ Sci KNIPERGOODMAN T, 1989, Biomed Environ Sci, V2, P179
CR	replaced	17	17	96472	96471	Mycotoxin Res Reubel G H, 1987, Mycotoxin Res, V3, P85, DOI 10.1007/BF03191994	MYCOTOXIN RES Reubel G H, 1987, MYCOTOXIN RES, V3, P85, DOI 10.1007/BF03191994

Record 676 | WOS:000248276300289

Effect of Saccharomyces cerevisiae and esterified glucomannan on residues of Ochratoxin A in kidney, muscle and blood of laying hens

SO original: ITALIAN JOURNAL OF ANIMAL SCIENCE | SO normalised: ITALIAN JOURNAL OF ANIMAL SCIENCE | CR count: 15 → 15

No journal-related changes detected in SO, J9, JI or CR for this record.

Record 677 | WOS:000243441300001

Review on the toxicity, occurrence, metabolism, detoxification, regulations and intake of zearalenone:: An oestrogenic mycotoxin

SO original: FOOD AND CHEMICAL TOXICOLOGY | SO normalised: FOOD AND CHEMICAL TOXICOLOGY | CR count: 186 → 186

Field	Status	Orig. pos	Norm. pos	Orig. line	Norm. line	Original cited/source value	Normalised cited/source value
CR	replaced	4	4	96608	96607	Arch Tierernahr Abdelhamid A M, 1992, Arch Tierernahr, V42, P63	ARCH TIERERNAHR Abdelhamid A M, 1992, ARCH TIERERNAHR, V42, P63

CR	replaced	15	15	96619	96618	IARC Monographs<br>[Anonymous], 1999, IARC Monographs, V1, P1	IARC MONOGRAPHS<br>[Anonymous], 1999, IARC MONOGRAPHS, V1, P1
CR	replaced	52	52	96656	96655	Mycotoxin Res<br>Doll S, 2003, Mycotoxin Res, V19, P73, DOI 10.1007/BF02940098	MYCOTOXIN RES<br>Doll S, 2003, MYCOTOXIN RES, V19, P73, DOI 10.1007/BF02940098
CR	replaced	76	76	96680	96679	Adv Appl Microbiol<br>Hidy P H, 1977, Adv Appl Microbiol, V22, P59, DOI 10.1016/S0065-2164(08)70160-6	ADV APPL MICROBIOL<br>Hidy P H, 1977, ADV APPL MICROBIOL, V22, P59, DOI 10.1016/S0065-2164(08)70160-6
CR	replaced	104	104	96708	96707	Revista Argentina de Microbiologia<br>LOPEZ T A, 1980, Revista Argentina de Microbiologia, V12, P29	REV ARGENT MICROBIOL<br>LOPEZ T A, 1980, REV ARGENT MICROBIOL, V12, P29
CR	replaced	118	118	96722	96721	Nat Toxins<br>Muller H M, 1997, Nat Toxins, V5, P24, DOI 10.1002/(SICI)(1997)5:1<24::AID-NT4>3.0.CO;2-#	NAT TOXINS<br>Muller H M, 1997, NAT TOXINS, V5, P24, DOI 10.1002/(SICI)(1997)5:1<24::AID-NT4>3.0.CO;2-#
CR	replaced	134	134	96738	96737	Mycotoxin Res<br>Perkowski J, 1990, Mycotoxin Res, V6, P7, DOI 10.1007/BF03192133	MYCOTOXIN RES<br>Perkowski J, 1990, MYCOTOXIN RES, V6, P7, DOI 10.1007/BF03192133
CR	replaced	186	186	96790	96789	Polish Journal of Veterinary Sciences<br>Zwierzchowski W., 2005, Polish Journal of Veterinary Sciences, V8, P209	POL J VET SCI<br>Zwierzchowski W., 2005, POL J VET SCI, V8, P209

Record 678 | WOS:000242136100016

Interactive effects of fumonisin B1 and Î±-zearalenol on proliferation and cytokine expression in Jurkat T cells

SO original: TOXICOLOGY IN VITRO | SO normalised: TOXICOLOGY IN VITRO | CR count: 47 → 47

No journal-related changes detected in SO, J9, JI or CR for this record.

Record 679 | WOS:000243461200014

Ochratoxin A-induced cytotoxicity in liver (HepG2) cells:: Impact of serum concentration, dietary antioxidants and glutathione-modulating compounds

SO original: JOURNAL OF APPLIED BOTANY AND FOOD QUALITY | SO normalised: JOURNAL OF APPLIED BOTANY AND FOOD QUALITY | CR count: 59 → 59

Field	Status	Orig. pos	Norm. pos	Orig. line	Norm. line	Original cited/source value	Normalised cited/source value
CR	replaced	9	9	96970	96969	Biochemical Pharmacology<br>CHU F S, 1974, Biochemical Pharmacology, V23, P1105, DOI 10.1016/0006-2952(74)90011-2	BIOCHEM PHARMACOL<br>CHU F S, 1974, BIOCHEM PHARMACOL, V23, P1105, DOI 10.1016/0006-2952(74)90011-2
CR	replaced	11	11	96972	96971	Journal of Toxicological Sciences<br>Creppy Edmond Ekue, 1998,	J TOXICOL SCI<br>Creppy Edmond Ekue, 1998, J TOXICOL SCI, V23, P165

						Journal of Toxicological Sciences, V23, P165	

Record 680 | WOS:000241622800019

Critical period and minimum single oral dose of ochratoxin A for inducing developmental toxicity in pregnant Wistar rats

SO original: REPRODUCTIVE TOXICOLOGY | SO normalised: REPRODUCTIVE TOXICOLOGY | CR count: 37 → 37

Field	Status	Orig. pos	Norm. pos	Orig. line	Norm. line	Original cited/source value	Normalised cited/source value
CR	replaced	23	23	97091	97090	Indian Vet J Kunjamma CRL., 1997, Indian Vet J, V74, P22	INDIAN VET J Kunjamma CRL., 1997, INDIAN VET J, V74, P22

Record 681 | WOS:000239496200009

A study of zearalenone cytotoxicity on human peripheral blood mononuclear cells

SO original: TOXICOLOGY LETTERS | SO normalised: TOXICOLOGY LETTERS | CR count: 39 → 39

Field	Status	Orig. pos	Norm. pos	Orig. line	Norm. line	Original cited/source value	Normalised cited/source value
CR	replaced	1	1	97164	97163	Arch Tierernahr Abdelhamid A M, 1992, Arch Tierernahr, V42, P63	ARCH TIERERNAHR Abdelhamid A M, 1992, ARCH TIERERNAHR, V42, P63

Record 682 | WOS:000239973600004

Expression of COX-2 and hsp72 in peritoneal macrophages after an acute ochratoxin A treatment in mice

SO original: LIFE SCIENCES | SO normalised: LIFE SCIENCES | CR count: 50 → 50

Field	Status	Orig. pos	Norm. pos	Orig. line	Norm. line	Original cited/source value	Normalised cited/source value
CR	replaced	4	4	97273	97272	Nephron Physiol Aukema H M, 2004, Nephron Physiol, V96, pP19, DOI 10.1159/000075576	NEPHRON PHYSIOL Aukema H M, 2004, NEPHRON PHYSIOL, V96, pP19, DOI 10.1159/000075576
CR	replaced	49	49	97318	97317	Mycotoxin Res Weidenbach A, 2000, Mycotoxin Res, V16 Suppl 2, P189, DOI 10.1007/BF02940035	MYCOTOXIN RES Weidenbach A, 2000, MYCOTOXIN RES, V16 Suppl 2, P189, DOI 10.1007/BF02940035

Record 683 | WOS:000240051000015

Early cytotoxic effects of ochratoxin A in rat liver: A morphological, biochemical and molecular study

SO original: TOXICOLOGY | SO normalised: TOXICOLOGY | CR count: 43 → 43

No journal-related changes detected in SO, J9, JI or CR for this record.

Record 684 | WOS:000240051000002

Adverse effects of T-2 toxin on chicken lymphocytes blastogenesis and its protection with Vitamin E

SO original: TOXICOLOGY | SO normalised: TOXICOLOGY | CR count: 44 → 44

Field	Status	Orig. pos	Norm. pos	Orig. line	Norm. line	Original cited/source value	Normalised cited/source value
CR	replaced	8	8	97514	97513	Feed Mix<br>Brake J., 1997, Feed Mix, V5, P21	FEED MIX<br>Brake J., 1997, FEED MIX, V5, P21

Record 685 | WOS:000239031400009

Glutathione S-transferase polymorphisms and ochratoxin A toxicity in primary human urothelial cells

SO original: TOXICOLOGY | SO normalised: TOXICOLOGY | CR count: 48 → 48

Field	Status	Orig. pos	Norm. pos	Orig. line	Norm. line	Original cited/source value	Normalised cited/source value
CR	replaced	2	2	97605	97604	IARC Sci Publ<br>Chernozemsky I N, 1991, IARC Sci Publ, P3	IARC SCI PUBL<br>Chernozemsky I N, 1991, IARC SCI PUBL, P3
CR	replaced	32	32	97635	97634	IARC Sci Publ<br>Petkova-Bocharova T, 1991, IARC Sci Publ, P135	IARC SCI PUBL<br>Petkova-Bocharova T, 1991, IARC SCI PUBL, P135

Record 686 | WOS:000238650400009

Ochratoxin A: Apoptosis and aberrant exit from mitosis due to perturbation of microtubule dynamics?

SO original: TOXICOLOGICAL SCIENCES | SO normalised: TOXICOLOGICAL SCIENCES | CR count: 50 → 50

No journal-related changes detected in SO, J9, JI or CR for this record.

Record 687 | WOS:000237912300008

Deoxynivalenol transport across human intestinal Caco-2 cells and its effects on cellular metabolism at realistic intestinal concentrations

SO original: TOXICOLOGY LETTERS | SO normalised: TOXICOLOGY LETTERS | CR count: 47 → 47

No journal-related changes detected in SO, J9, JI or CR for this record.

Record 688 | WOS:000238344300009

Balkan endemic nephropathy: Role of ochratoxins A through biomarkers

SO original: MOLECULAR NUTRITION & FOOD RESEARCH | SO normalised: MOLECULAR NUTRITION & FOOD RESEARCH | CR count: 34 → 34

Field	Status	Orig. pos	Norm. pos	Orig. line	Norm. line	Original cited/source value	Normalised cited/source value
CR	replaced	19	19	97952	97951	Endemic nephropathy Krogh P., 1972, Endemic nephropathy, P266	ENDEMIC NEPHROPATHY Krogh P., 1972, ENDEMIC NEPHROPATHY, P266
CR	replaced	22	22	97955	97954	IARC Sci Publ Petkova-Bocharova T, 1991, IARC Sci Publ, P83	IARC SCI PUBL Petkova-Bocharova T, 1991, IARC SCI PUBL, P83

Record 689 | WOS:000238344300010

Evidence of a new dechlorinated ochratoxin A derivative formed in opossum kidney cell cultures after pretreatment by modulators of glutathione pathways: Correlation with DNA-adduct formation

SO original: MOLECULAR NUTRITION & FOOD RESEARCH | SO normalised: MOLECULAR NUTRITION & FOOD RESEARCH | CR count: 66 → 66

Field	Status	Orig. pos	Norm. pos	Orig. line	Norm. line	Original cited/source value	Normalised cited/source value
CR	replaced	49	49	98093	98092	IARC Sci Publ Pfohl-Leszkowicz A, 1993, IARC Sci Publ, P141	IARC SCI PUBL Pfohl-Leszkowicz A, 1993, IARC SCI PUBL, P141

Record 690 | WOS:000238344300011

Microbiologicals for deactivating mycotoxins

SO original: MOLECULAR NUTRITION & FOOD RESEARCH | SO normalised: MOLECULAR NUTRITION & FOOD RESEARCH | CR count: 29 → 29

Field	Status	Orig. pos	Norm. pos	Orig. line	Norm. line	Original cited/source value	Normalised cited/source value
CR	replaced	22	22	98200	98199	Mycotoxin Res Schatzmayr G, 2003, Mycotoxin Res, V19, P124, DOI 10.1007/BF02942950	MYCOTOXIN RES Schatzmayr G, 2003, MYCOTOXIN RES, V19, P124, DOI 10.1007/BF02942950

Record 691 | WOS:000236846900001

Ochratoxin A secretion by ATP-dependent membrane transporters in Caco-2 cells

SO original: ARCHIVES OF TOXICOLOGY | SO normalised: ARCHIVES OF TOXICOLOGY | CR count: 47 → 47

No journal-related changes detected in SO, J9, JI or CR for this record.

Record 692 | WOS:000236987100010

In vitro investigation of individual and combined cytotoxic effects of ochratoxin A and other selected mycotoxins on renal cells

SO original: TOXICOLOGY IN VITRO | SO normalised: TOXICOLOGY IN VITRO | CR count: 34 → 34

No journal-related changes detected in SO, J9, JI or CR for this record.

Record 693 | WOS:000236605600004

Ochratoxin A: Its cancer risk and potential for exposure

SO original: JOURNAL OF TOXICOLOGY AND ENVIRONMENTAL HEALTH-PART B-CRITICAL REVIEWS | SO normalised: JOURNAL OF TOXICOLOGY AND ENVIRONMENTAL HEALTH-PART B-CRITICAL REVIEWS | CR count: 190 → 190

Field	Status	Orig. pos	Norm. pos	Orig. line	Norm. line	Original cited/source value	Normalised cited/source value
CR	replaced	29	29	98468	98467	Alimentaria<br>Burdaspal P. A., 1999, Alimentaria, V36, P107	ALIMENTARIA<br>Burdaspal P. A., 1999, ALIMENTARIA, V36, P107
CR	replaced	37	37	98476	98475	Journal of Toxicological Sciences<br>Creppy Edmond Ekue, 1998, Journal of Toxicological Sciences, V23, P165	J TOXICOL SCI<br>Creppy Edmond Ekue, 1998, J TOXICOL SCI, V23, P165
CR	replaced	87	87	98526	98525	CYTOBIOS<br>Jeswal P, 1998, CYTOBIOS, V93, P123	Cytobios<br>Jeswal P, 1998, Cytobios, V93, P123
CR	replaced	132	132	98571	98570	IARC Sci Publ<br>Petkova-Bocharova T, 1991, IARC Sci Publ, P135	IARC SCI PUBL<br>Petkova-Bocharova T, 1991, IARC SCI PUBL, P135
CR	replaced	133	133	98572	98571	IARC Sci Publ<br>Petkova-Bocharova T, 1991, IARC Sci Publ, P83	IARC SCI PUBL<br>Petkova-Bocharova T, 1991, IARC SCI PUBL, P83

Record 694 | WOS:000234793300002

Toxicokinetics and toxicodynamics of ochratoxin A, an update

SO original: CHEMICO-BIOLOGICAL INTERACTIONS | SO normalised: CHEMICO-BIOLOGICAL INTERACTIONS | CR count: 236 → 236

Field	Status	Orig. pos	Norm. pos	Orig. line	Norm. line	Original cited/source value	Normalised cited/source value
CR	replaced	34	34	98720	98719	Biochemical Pharmacology<br>CHU F S, 1974, Biochemical Pharmacology, V23, P1105, DOI 10.1016/0006-2952(74)90011-2	BIOCHEM PHARMACOL<br>CHU F S, 1974, BIOCHEM PHARMACOL, V23, P1105, DOI 10.1016/0006-2952(74)90011-2
CR	replaced	59	59	98745	98744	Annales de Recherches Veterinaires<br>GALTIER P, 1976, Annales de Recherches Veterinaires, V7, P91	ANN RECH VET<br>GALTIER P, 1976, ANN RECH VET, V7, P91
CR	replaced	149	149	98835	98834	Nat Toxins<br>Muller H M, 1998, Nat Toxins, V6, P251, DOI 10.1002/(SICI)1522-7189(199811/12)6:6<251::AID-NT35>3.0.CO;2-P	NAT TOXINS<br>Muller H M, 1998, NAT TOXINS, V6, P251, DOI 10.1002/(SICI)1522-7189(199811/12)6:6<251::AID-NT35>3.0.CO;2-P
CR	replaced	224	224	98910	98909	Mycotoxin Res<br>Weidenbach A, 2000, Mycotoxin Res, V16 Suppl 2,	MYCOTOXIN RES<br>Weidenbach A, 2000, MYCOTOXIN RES, V16

						P189, DOI 10.1007/BF02940035	Suppl 2, P189, DOI 10.1007/BF02940035

Record 695 | WOS:000234140400042

The impact of plasma protein binding on the renal transport of organic anions

SO original: JOURNAL OF PHARMACOLOGY AND EXPERIMENTAL THERAPEUTICS | SO normalised: JOURNAL OF PHARMACOLOGY AND EXPERIMENTAL THERAPEUTICS | CR count: 43 → 43

No journal-related changes detected in SO, J9, JI or CR for this record.

Record 696 | WOS:000235953600006

Mycoflora, cytotoxicity, and DNA interaction of polyherbal products from Malaysia

SO original: PHARMACEUTICAL BIOLOGY | SO normalised: PHARMACEUTICAL BIOLOGY | CR count: 43 → 43

No journal-related changes detected in SO, J9, JI or CR for this record.

Record 697 | WOS:000233991000012

A toxicogenomics approach to identify new plausible epigenetic mechanisms of ochratoxin a carcinogenicity in rat

SO original: TOXICOLOGICAL SCIENCES | SO normalised: TOXICOLOGICAL SCIENCES | CR count: 80 → 80

Field	Status	Orig. pos	Norm. pos	Orig. line	Norm. line	Original cited/source value	Normalised cited/source value
CR	replaced	2	2	99188	99187	Front Biosci<br>Amati B., 1998, Front Biosci, V3, pd250, DOI [10.2741/A239, DOI 10.2741/A239]	FRONT BIOSCI-LANDMRK<br>Amati B., 1998, FRONT BIOSCI-LANDMRK, V3, pd250, DOI [10.2741/A239, DOI 10.2741/A239]

Record 698 | WOS:000234194400007

An in vitro approach to detect metabolite toxicity due to CYP3A4-dependent bioactivation of xenobiotics

SO original: TOXICOLOGY | SO normalised: TOXICOLOGY | CR count: 50 → 50

Field	Status	Orig. pos	Norm. pos	Orig. line	Norm. line	Original cited/source value	Normalised cited/source value
CR	replaced	6	6	99332	99331	Journal of Pharmacological and Toxicological Methods<br>Day S. H., 2005, Journal of Pharmacological and Toxicological Methods, V52, P278, DOI 10.1016/j.vascn.2004.11.006	J PHARMACOL TOX MET<br>Day S. H., 2005, J PHARMACOL TOX MET, V52, P278, DOI 10.1016/j.vascn.2004.11.006

Record 699 | WOS:000234094800009

Ochratoxin A induces oxidative DNA damage in liver and kidney after oral dosing to rats

SO original: MOLECULAR NUTRITION & FOOD RESEARCH | SO normalised: MOLECULAR NUTRITION & FOOD RESEARCH | CR count: 64 → 64

No journal-related changes detected in SO, J9, JI or CR for this record.

Record 700 | WOS:000233125300003

Teratogenic effects in rabbits of simultaneous exposure to ochratoxin A and aflatoxin B1 with special reference to microscopic effects

SO original: TOXICOLOGY | SO normalised: TOXICOLOGY | CR count: 70 → 70

Field	Status	Orig. pos	Norm. pos	Orig. line	Norm. line	Original cited/source value	Normalised cited/source value
CR	replaced	33	33	99595	99594	Indian Vet J<br>Kunjamma CRL., 1997, Indian Vet J, V74, P22	INDIAN VET J<br>Kunjamma CRL., 1997, INDIAN VET J, V74, P22
CR	replaced	34	34	99596	99595	Food Cosmet Toxicol<br>LEBRETON E, 1964, Food Cosmet Toxicol, V2, P675	FOOD COSMET TOXICOL<br>LEBRETON E, 1964, FOOD COSMET TOXICOL, V2, P675
CR	replaced	35	35	99597	99596	Journal of Toxicological Sciences<br>Makarananda Kittima, 1998, Journal of Toxicological Sciences, V23, P155	J TOXICOL SCI<br>Makarananda Kittima, 1998, J TOXICOL SCI, V23, P155

Record 701 | WOS:000232435300026

The effects of mycotoxins, fungal food contaminants, on the intestinal epithelial cell-derived innate immune response

SO original: VETERINARY IMMUNOLOGY AND IMMUNOPATHOLOGY | SO normalised: VETERINARY IMMUNOLOGY AND IMMUNOPATHOLOGY | CR count: 73 → 73

No journal-related changes detected in SO, J9, JI or CR for this record.

Record 702 | WOS:000233069800005

Investigation of the teratogenic potential of ochratoxin A and B using the FETAX system

SO original: BIRTH DEFECTS RESEARCH PART B-DEVELOPMENTAL AND REPRODUCTIVE TOXICOLOGY | SO normalised: BIRTH DEFECTS RESEARCH PART B-DEVELOPMENTAL AND REPRODUCTIVE TOXICOLOGY | CR count: 54 → 54

No journal-related changes detected in SO, J9, JI or CR for this record.

Record 703 | WOS:000232409600014

Fate of ochratoxin A during cooking of naturally contaminated polished rice

SO original: JOURNAL OF FOOD PROTECTION | SO normalised: JOURNAL OF FOOD PROTECTION | CR count: 27 → 27

No journal-related changes detected in SO, J9, JI or CR for this record.

Record 704 | WOS:000232524600005

Alterations induced in vitro by ochratoxin A in rat lymphoid cells

SO original: HUMAN & EXPERIMENTAL TOXICOLOGY | SO normalised: HUMAN & EXPERIMENTAL TOXICOLOGY | CR count: 43 → 43

Field	Status	Orig. pos	Norm. pos	Orig. line	Norm. line	Original cited/source value	Normalised cited/source value
CR	replaced	2	2	100002	100001	Off J Eur Union [Anonymous], 2002, Off J Eur Union, VL220, P18	OFF J EUR UNION [Anonymous], 2002, OFF J EUR UNION, VL220, P18

Record 705 | WOS:000231364400010

Cyanidin-3-O-Î²-glucopyranoside, a natural free-radical scavenger against aflatoxin B1-and ochratoxin A-induced cell damage in a human hepatoma cell line (Hep G2) and a human colonic adenocarcinoma cell line (CaCo-2)

SO original: BRITISH JOURNAL OF NUTRITION | SO normalised: BRITISH JOURNAL OF NUTRITION | CR count: 42 → 42

No journal-related changes detected in SO, J9, JI or CR for this record.

Record 706 | WOS:000231235000007

Functional, biochemical, and pathological effects of repeated oral administration of ochratoxin A to rats

SO original: CHEMICAL RESEARCH IN TOXICOLOGY | SO normalised: CHEMICAL RESEARCH IN TOXICOLOGY | CR count: 70 → 70

No journal-related changes detected in SO, J9, JI or CR for this record.

Record 707 | WOS:000231235000008

Ochratoxin A causes DNA damage and cytogenetic effects but no DNA adducts in rats

SO original: CHEMICAL RESEARCH IN TOXICOLOGY | SO normalised: CHEMICAL RESEARCH IN TOXICOLOGY | CR count: 55 → 55

Field	Status	Orig. pos	Norm. pos	Orig. line	Norm. line	Original cited/source value	Normalised cited/source value
CR	replaced	23	23	100373	100372	IARC Sci Publ Gupta R C, 1993, IARC Sci Publ, P11	IARC SCI PUBL Gupta R C, 1993, IARC SCI PUBL, P11

Record 708 | WOS:000230120200005

Biotransformation and nephrotoxicity of ochratoxin B in rats

SO original: TOXICOLOGY AND APPLIED PHARMACOLOGY | SO normalised: TOXICOLOGY AND APPLIED PHARMACOLOGY | CR count: 28 → 28

No journal-related changes detected in SO, J9, JI or CR for this record.

Record 709 | WOS:000228398000034

Cisplatin-induced apoptosis is enhanced by hypoxia and by inhibition of mitochondria in renal collecting duct cells

SO original: TOXICOLOGICAL SCIENCES | SO normalised: TOXICOLOGICAL SCIENCES | CR count: 37 → 37

Field	Status	Orig. pos	Norm. pos	Orig. line	Norm. line	Original cited/source value	Normalised cited/source value
CR	replaced	24	24	100583	100582	Biochimica et Biophysica Acta<br>PINTO A L, 1985, Biochimica et Biophysica Acta, V780, P167, DOI 10.1016/0304-419X(85)90001-0	BIOCHIM BIOPHYS ACTA<br>PINTO A L, 1985, BIOCHIM BIOPHYS ACTA, V780, P167, DOI 10.1016/0304-419X(85)90001-0

Record 710 | WOS:000229004100015

Comparison of in vitro cytotoxicity of Fusarium mycotoxins, deoxynivalenol, T-2 toxin and zearalenone on selected human epithelial cell lines

SO original: MYCOPATHOLOGIA | SO normalised: MYCOPATHOLOGIA | CR count: 42 → 42

Field	Status	Orig. pos	Norm. pos	Orig. line	Norm. line	Original cited/source value	Normalised cited/source value
CR	replaced	32	32	100681	100680	BIOMED ENVIRON SCI<br>Sun XM, 2002, BIOMED ENVIRON SCI, V15, P145	Biomed Environ Sci<br>Sun XM, 2002, Biomed Environ Sci, V15, P145

Record 711 | WOS:000227733000027

Proximal tubular toxicity of ochratoxin A is amplified by simultaneous inhibition of the extracellular signal-regulated kinases 1/2

SO original: JOURNAL OF PHARMACOLOGY AND EXPERIMENTAL THERAPEUTICS | SO normalised: JOURNAL OF PHARMACOLOGY AND EXPERIMENTAL THERAPEUTICS | CR count: 40 → 40

Field	Status	Orig. pos	Norm. pos	Orig. line	Norm. line	Original cited/source value	Normalised cited/source value
CR	replaced	2	2	100744	100743	Nephron Physiol<br>Aukema H M, 2004, Nephron Physiol, V96, pP19, DOI 10.1159/000075576	NEPHRON PHYSIOL<br>Aukema H M, 2004, NEPHRON PHYSIOL, V96, pP19, DOI 10.1159/000075576

Record 712 | WOS:000227112000002

Interference of arachidonic acid and its metabolites with TNF-Î± release by ochratoxin A from rat liver

SO original: TOXICOLOGY | SO normalised: TOXICOLOGY | CR count: 73 → 73

Field	Status	Orig. pos	Norm. pos	Orig. line	Norm. line	Original cited/source value	Normalised cited/source value
CR	replaced	70	70	100902	100901	Mycotoxin Res Weidenbach A, 2000, Mycotoxin Res, V16 Suppl 2, P189, DOI 10.1007/BF02940035	MYCOTOXIN RES Weidenbach A, 2000, MYCOTOXIN RES, V16 Suppl 2, P189, DOI 10.1007/BF02940035

Record 713 | WOS:000227242200003

Ochratoxin A at nanomolar concentrations: A signal modulator in renal cells

SO original: MOLECULAR NUTRITION & FOOD RESEARCH | SO normalised: MOLECULAR NUTRITION & FOOD RESEARCH | CR count: 108 → 108

Field	Status	Orig. pos	Norm. pos	Orig. line	Norm. line	Original cited/source value	Normalised cited/source value
CR	replaced	19	19	100977	100976	PFLUGERS ARCH EDER S, 2000, PFLUGERS ARCH, V40, P521	PFLUG ARCH EUR J PHY EDER S, 2000, PFLUG ARCH EUR J PHY, V40, P521

Record 714 | WOS:000234123000008

Ochratoxin A: Comparative pharmacokinetics and toxicological implications (experimental and domestic animals and humans)

SO original: FOOD ADDITIVES AND CONTAMINANTS PART A-CHEMISTRY ANALYSIS CONTROL ; EXPOSURE & RISK ASSESSMENT | SO normalised: FOOD ADDITIVES AND CONTAMINANTS PART A-CHEMISTRY ANALYSIS CONTROL ; EXPOSURE & RISK ASSESSMENT | CR count: 44 → 44

No journal-related changes detected in SO, J9, JI or CR for this record.

Record 715 | WOS:000234123000011

DNA adduct formation by ochratoxin A: Review of the available evidence

SO original: FOOD ADDITIVES AND CONTAMINANTS PART A-CHEMISTRY ANALYSIS CONTROL ; EXPOSURE & RISK ASSESSMENT | SO normalised: FOOD ADDITIVES AND CONTAMINANTS PART A-CHEMISTRY ANALYSIS CONTROL ; EXPOSURE & RISK ASSESSMENT | CR count: 60 → 60

Field	Status	Orig. pos	Norm. pos	Orig. line	Norm. line	Original cited/source value	Normalised cited/source value
CR	replaced	47	47	101266	101265	IARC Sci Publ Randerath K, 1993, IARC Sci Publ, P157	IARC SCI PUBL Randerath K, 1993, IARC SCI PUBL, P157

Record 716 | WOS:000226837900002

Ochratoxin A: The continuing enigma

SO original: CRITICAL REVIEWS IN TOXICOLOGY | SO normalised: CRITICAL REVIEWS IN TOXICOLOGY | CR count: 219 → 219

Field	Status	Orig. pos	Norm. pos	Orig. line	Norm. line	Original cited/source value	Normalised cited/source value

CR	replaced	88	88	101415	101414	Mycotoxin Res Heller M, 2000, Mycotoxin Res, V16 Suppl 2, P194, DOI 10.1007/BF02940036	MYCOTOXIN RES Heller M, 2000, MYCOTOXIN RES, V16 Suppl 2, P194, DOI 10.1007/BF02940036

Record 717 | WOS:000234123000012

Further arguments in favour of direct covalent binding of Ochratoxin A (OTA) after metabolic biotransformation

SO original: FOOD ADDITIVES AND CONTAMINANTS PART A-CHEMISTRY ANALYSIS CONTROL ; EXPOSURE & RISK ASSESSMENT | SO normalised: FOOD ADDITIVES AND CONTAMINANTS PART A-CHEMISTRY ANALYSIS CONTROL ; EXPOSURE & RISK ASSESSMENT | CR count: 62 → 62

Field	Status	Orig. pos	Norm. pos	Orig. line	Norm. line	Original cited/source value	Normalised cited/source value
CR	replaced	47	47	101651	101650	IARC Sci Publ Pfohl-Leszkowicz A, 1993, IARC Sci Publ, P141	IARC SCI PUBL Pfohl-Leszkowicz A, 1993, IARC SCI PUBL, P141

Record 718 | WOS:000232477800006

Effect of mycotoxins on some activities of isolated human neutrophils

SO original: IMMUNOPHARMACOLOGY AND IMMUNOTOXICOLOGY | SO normalised: IMMUNOPHARMACOLOGY AND IMMUNOTOXICOLOGY | CR count: 31 → 31

No journal-related changes detected in SO, J9, JI or CR for this record.

Record 719 | WOS:000226875200005

Ochratoxin A-induced DNA damage in human fibroblast:: protective effect of cyanidin 3-O-Î²-D-glucoside

SO original: JOURNAL OF NUTRITIONAL BIOCHEMISTRY | SO normalised: JOURNAL OF NUTRITIONAL BIOCHEMISTRY | CR count: 48 → 48

No journal-related changes detected in SO, J9, JI or CR for this record.

Record 720 | WOS:000226469400004

Exposure to nephrotoxic Ochratoxin A enhances collagen secretion in human renal proximal tubular cells

SO original: MOLECULAR NUTRITION & FOOD RESEARCH | SO normalised: MOLECULAR NUTRITION & FOOD RESEARCH | CR count: 42 → 42

Field	Status	Orig. pos	Norm. pos	Orig. line	Norm. line	Original cited/source value	Normalised cited/source value
CR	replaced	1	1	101925	101924	Nephron Physiol Aukema H M, 2004, Nephron Physiol, V96, pP19, DOI 10.1159/000075576	NEPHRON PHYSIOL Aukema H M, 2004, NEPHRON PHYSIOL, V96, pP19, DOI 10.1159/000075576

Record 721 | WOS:000226446300003

Applicability of an in vitro digestion model in assessing the bioaccessibility of mycotoxins from food

SO original: FOOD AND CHEMICAL TOXICOLOGY | SO normalised: FOOD AND CHEMICAL TOXICOLOGY | CR count: 35 → 35

No journal-related changes detected in SO, J9, JI or CR for this record.

Record 722 | WOS:000234123000002

Ochratoxin A: Previous risk assessments and issues arising

SO original: FOOD ADDITIVES AND CONTAMINANTS PART A-CHEMISTRY ANALYSIS CONTROL ; EXPOSURE & RISK ASSESSMENT | SO normalised: FOOD ADDITIVES AND CONTAMINANTS PART A-CHEMISTRY ANALYSIS CONTROL ; EXPOSURE & RISK ASSESSMENT | CR count: 8 → 8

No journal-related changes detected in SO, J9, JI or CR for this record.

Record 723 | WOS:000231491200006

Unusual astrocyte reactivity caused by the food mycotoxin ochratoxin A in aggregating rat brain cell cultures

SO original: NEUROSCIENCE | SO normalised: NEUROSCIENCE | CR count: 75 → 75

No journal-related changes detected in SO, J9, JI or CR for this record.

Record 724 | WOS:000225312300008

Effects of deoxynivalenol on general performance and electrophysiological properties of intestinal mucosa of broiler chickens

SO original: POULTRY SCIENCE | SO normalised: POULTRY SCIENCE | CR count: 71 → 71

No journal-related changes detected in SO, J9, JI or CR for this record.

Record 725 | WOS:000226727000007

Combined effects of selected Penicillium mycotoxins on in vitro proliferation of porcine lymphocytes

SO original: MYCOPATHOLOGIA | SO normalised: MYCOPATHOLOGIA | CR count: 13 → 13

No journal-related changes detected in SO, J9, JI or CR for this record.

Record 726 | WOS:000226361400006

Molecular aspects of the transport and toxicity of ochratoxin A

SO original: ACCOUNTS OF CHEMICAL RESEARCH | SO normalised: ACCOUNTS OF CHEMICAL RESEARCH | CR count: 50 → 50

No journal-related changes detected in SO, J9, JI or CR for this record.

Record 727 | WOS:000224725300008

Experimental mycotoxicosis in chickens induced by ochratoxin A and penicillic acid and intervention with natural plant extracts

SO original: VETERINARY RESEARCH COMMUNICATIONS | SO normalised: VETERINARY RESEARCH COMMUNICATIONS | CR count: 49 → 49

Field	Status	Orig. pos	Norm. pos	Orig. line	Norm. line	Original cited/source value	Normalised cited/source value
CR	replaced	21	21	102657	102656	Endemic nephropathy Krogh P., 1972, Endemic nephropathy, P266	ENDEMIC NEPHROPATHY Krogh P., 1972, ENDEMIC NEPHROPATHY, P266

Record 728 | WOS:000226417300009

The effects of the Penicillium mycotoxins citrinin, cyclopiazonic acid, ochratoxin A, patulin, penicillic acid, and roquefortine C on in vitro proliferation of porcine lymphocytes

SO original: MYCOPATHOLOGIA | SO normalised: MYCOPATHOLOGIA | CR count: 36 → 36

No journal-related changes detected in SO, J9, JI or CR for this record.

Record 729 | WOS:000223666000030

Fungal microflora and ochratoxin a risk in French vineyards

SO original: JOURNAL OF AGRICULTURAL AND FOOD CHEMISTRY | SO normalised: JOURNAL OF AGRICULTURAL AND FOOD CHEMISTRY | CR count: 36 → 36

No journal-related changes detected in SO, J9, JI or CR for this record.

Record 730 | WOS:000223753400030

Alkalinization of urinary pH accelerates renal excretion of ochratoxin A in pigs

SO original: JOURNAL OF NUTRITION | SO normalised: JOURNAL OF NUTRITION | CR count: 29 → 29

Field	Status	Orig. pos	Norm. pos	Orig. line	Norm. line	Original cited/source value	Normalised cited/source value
CR	replaced	19	19	102940	102939	Pharmacology Rang HP., 2003, Pharmacology, V5th	PHARMACOLOGY Rang HP., 2003, PHARMACOLOGY, V5th

Record 731 | WOS:000224015700013

Synergistic effects of fumonisin B1 and ochratoxin A: are in vitro cytotoxicity data predictive of in vivo acute toxicity?

SO original: TOXICOLOGY | SO normalised: TOXICOLOGY | CR count: 60 → 60

Field	Status	Orig. pos	Norm. pos	Orig. line	Norm. line	Original cited/source value	Normalised cited/source value
CR	replaced	28	28	103041	103040	J Vet Diagn Invest Harrison L R, 1990, J Vet Diagn Invest, V2, P217	J VET DIAGN INVEST Harrison L R, 1990, J VET DIAGN INVEST, V2, P217

Record 732 | WOS:000224048300016

Evidence for covalent DNA adduction by ochratoxin A following chronic exposure to rat and subacute exposure to pig

SO original: CHEMICAL RESEARCH IN TOXICOLOGY | SO normalised: CHEMICAL RESEARCH IN TOXICOLOGY | CR count: 52 → 52

No journal-related changes detected in SO, J9, JI or CR for this record.

Record 733 | WOS:000225210900008

Levels of mycotoxins and sample cytotoxicity of selected organic and conventional grain-based products purchased from Finnish and Italian markets

SO original: MOLECULAR NUTRITION & FOOD RESEARCH | SO normalised: MOLECULAR NUTRITION & FOOD RESEARCH | CR count: 30 → 30

Field	Status	Orig. pos	Norm. pos	Orig. line	Norm. line	Original cited/source value	Normalised cited/source value
CR	replaced	25	25	103274	103273	Food Addit Contam Scott P M, 1984, Food Addit Contam, V1, P313, DOI 10.1080/02652038409385862	FOOD ADDIT CONTAM Scott P M, 1984, FOOD ADDIT CONTAM, V1, P313, DOI 10.1080/02652038409385862

Record 734 | WOS:000223297700006

Effects of rosmarinic acid against aflatoxin B1 and ochratoxin-A-induced cell damage in a human hepatoma cell line (Hep G2)

SO original: JOURNAL OF APPLIED TOXICOLOGY | SO normalised: JOURNAL OF APPLIED TOXICOLOGY | CR count: 26 → 26

No journal-related changes detected in SO, J9, JI or CR for this record.

Record 735 | WOS:000221551200016

Ochratoxin A induces apoptosis in human lymphocytes through down regulation of Bcl-xL

SO original: TOXICOLOGICAL SCIENCES | SO normalised: TOXICOLOGICAL SCIENCES | CR count: 57 → 57

No journal-related changes detected in SO, J9, JI or CR for this record.

Record 736 | WOS:000221555600005

Metabolism-mediated cytotoxicity of ochratoxin A

SO original: TOXICOLOGY IN VITRO | SO normalised: TOXICOLOGY IN VITRO | CR count: 43 → 43

No journal-related changes detected in SO, J9, JI or CR for this record.

Record 737 | WOS:000220837300016

Immunotoxic effects of Ochratoxin A in wistar rats after oral administration

SO original: FOOD AND CHEMICAL TOXICOLOGY | SO normalised: FOOD AND CHEMICAL TOXICOLOGY | CR count: 54 → 54

No journal-related changes detected in SO, J9, JI or CR for this record.

Record 738 | WOS:000221514400030

Synergistic interaction in simultaneous exposure to Streptomyces californicus and Stachybotrys chartarum

SO original: ENVIRONMENTAL HEALTH PERSPECTIVES | SO normalised: ENVIRONMENTAL HEALTH PERSPECTIVES | CR count: 42 → 42

No journal-related changes detected in SO, J9, JI or CR for this record.

Record 739 | WOS:000220235700005

Inhibitory effects of ochratoxin A on nerve growth factor-induced neurite extension through downregulation of p38 map kinase and AP-1 activation in cultured pheochromocytoma cells

SO original: JOURNAL OF TOXICOLOGY AND ENVIRONMENTAL HEALTH-PART A-CURRENT ISSUES | SO normalised: JOURNAL OF TOXICOLOGY AND ENVIRONMENTAL HEALTH-PART A-CURRENT ISSUES | CR count: 35 → 35

No journal-related changes detected in SO, J9, JI or CR for this record.

Record 740 | WOS:000189087700013

Ochratoxin A: Lack of formation of covalent DNA adducts

SO original: CHEMICAL RESEARCH IN TOXICOLOGY | SO normalised: CHEMICAL RESEARCH IN TOXICOLOGY | CR count: 30 → 30

Field	Status	Orig. pos	Norm. pos	Orig. line	Norm. line	Original cited/source value	Normalised cited/source value
CR	replaced	26	26	103991	103990	IARC Sci Publ Turteltaub K W, 1993, IARC Sci Publ, P293	IARC SCI PUBL Turteltaub K W, 1993, IARC SCI PUBL, P293

Record 741 | WOS:000188530700003

Ochratoxin A and zearalenone: a comparative study on genotoxic effects and cell death induced in bovine lymphocytes

SO original: MUTATION RESEARCH-GENETIC TOXICOLOGY AND ENVIRONMENTAL MUTAGENESIS | SO normalised: MUTATION RESEARCH-GENETIC TOXICOLOGY AND ENVIRONMENTAL MUTAGENESIS | CR count: 47 → 47

No journal-related changes detected in SO, J9, JI or CR for this record.

Record 742 | WOS:000189121300007

New insights into the mechanisms involved in renal proximal tubular damage induced in vitro by ochratoxin A

SO original: JOURNAL OF BIOCHEMICAL AND MOLECULAR TOXICOLOGY | SO normalised: JOURNAL OF BIOCHEMICAL AND MOLECULAR TOXICOLOGY | CR count: 33 → 33

No journal-related changes detected in SO, J9, JI or CR for this record.

Record 743 | WOS:000189041600006

Inhibition of mitochondria and extracellular acidification enhance ochratoxin A-induced apoptosis in renal collecting duct-derived MDCK-C7 cells

SO original: CELLULAR PHYSIOLOGY AND BIOCHEMISTRY | SO normalised: CELLULAR PHYSIOLOGY AND BIOCHEMISTRY | CR count: 35 → 35

No journal-related changes detected in SO, J9, JI or CR for this record.

Record 744 | WOS:000231756500075

Natural antioxidants and mycotoxins: Theoretical considerations and practical applications

SO original: POISONOUS PLANTS AND RELATED TOXINS | SO normalised: POISONOUS PLANTS AND RELATED TOXINS | CR count: 50 → 50

No journal-related changes detected in SO, J9, JI or CR for this record.

Record 745 | WOS:000186953000004

Apoptosis and oxidative stress induced by ochratoxin A in rat kidney

SO original: ARCHIVES OF TOXICOLOGY | SO normalised: ARCHIVES OF TOXICOLOGY | CR count: 61 → 61

Field	Status	Orig. pos	Norm. pos	Orig. line	Norm. line	Original cited/source value	Normalised cited/source value
CR	replaced	40	40	104490	104489	IARC Sci Publ Pfohl-Leszkowicz A, 1993, IARC Sci Publ, P141	IARC SCI PUBL Pfohl-Leszkowicz A, 1993, IARC SCI PUBL, P141

Record 746 | WOS:000186282400042

Effects of chronic ingestion of ochratoxin A on blood levels and excretion of the mycotoxin in sheep

SO original: JOURNAL OF AGRICULTURAL AND FOOD CHEMISTRY | SO normalised: JOURNAL OF AGRICULTURAL AND FOOD CHEMISTRY | CR count: 37 → 37

Field	Status	Orig. pos	Norm. pos	Orig. line	Norm. line	Original cited/source value	Normalised cited/source value
CR	replaced	6	6	104575	104574	Biochemical Pharmacology CHU F S, 1974, Biochemical Pharmacology, V23, P1105, DOI 10.1016/0006-2952(74)90011-2	BIOCHEM PHARMACOL CHU F S, 1974, BIOCHEM PHARMACOL, V23, P1105, DOI 10.1016/0006-2952(74)90011-2

CR	replaced	25	25	104594	104593	Nat Toxins Muller H M, 1998, Nat Toxins, V6, P251, DOI 10.1002/(SICI)1522-7189(199811/12)6:6<251::AID-NT35>3.0.CO;2-P	NAT TOXINS Muller H M, 1998, NAT TOXINS, V6, P251, DOI 10.1002/(SICI)1522-7189(199811/12)6:6<251::AID-NT35>3.0.CO;2-P

Record 747 | WOS:000184259400007

Effects of the mycotoxin ochratoxin A and some of its metabolites on human kidney cell lines

SO original: TOXICOLOGY IN VITRO | SO normalised: TOXICOLOGY IN VITRO | CR count: 52 → 52

No journal-related changes detected in SO, J9, JI or CR for this record.

Record 748 | WOS:000184243200002

An oxygen-bonded C8-deoxyguanosine nucleoside adduct of pentachlorophenol by peroxidase activation: Evidence for ambident C8 reactivity by phenoxyl radicals

SO original: CHEMICAL RESEARCH IN TOXICOLOGY | SO normalised: CHEMICAL RESEARCH IN TOXICOLOGY | CR count: 21 → 21

No journal-related changes detected in SO, J9, JI or CR for this record.

Record 749 | WOS:000184363500006

Effect of Ochratoxin A on human haematopoietic progenitors proliferation and differentiation: an in vitro study

SO original: HUMAN & EXPERIMENTAL TOXICOLOGY | SO normalised: HUMAN & EXPERIMENTAL TOXICOLOGY | CR count: 51 → 51

No journal-related changes detected in SO, J9, JI or CR for this record.

Record 750 | WOS:000185415100022

Evaluation of two mycotoxin binders to reduce toxicity of broiler diets containing ochratoxin A and T-2 toxin contaminated grain

SO original: AVIAN DISEASES | SO normalised: AVIAN DISEASES | CR count: 45 → 45

No journal-related changes detected in SO, J9, JI or CR for this record.

Record 751 | WOS:000183040200012

A new approach to studying ochratoxin A (OTA)-induced nephrotoxicity: Expression profiling in vivo and in vitro employing cDNA microarrays

SO original: TOXICOLOGICAL SCIENCES | SO normalised: TOXICOLOGICAL SCIENCES | CR count: 61 → 61

No journal-related changes detected in SO, J9, JI or CR for this record.

Record 752 | WOS:000183875100009

Effects of the mycotoxin ochratoxin A in a bacterial and a mammalian in vitro mutagenicity test system

SO original: ARCHIVES OF TOXICOLOGY | SO normalised: ARCHIVES OF TOXICOLOGY | CR count: 36 → 36

Field	Status	Orig. pos	Norm. pos	Orig. line	Norm. line	Original cited/source value	Normalised cited/source value
CR	replaced	23	23	105170	105169	IARC Sci Publ<br>Petkova-Bocharova T, 1991, IARC Sci Publ, P135	IARC SCI PUBL<br>Petkova-Bocharova T, 1991, IARC SCI PUBL, P135

Record 753 | WOS:000182307900052

Interaction of ochratoxin A with human intestinal Caco-2 cells: possible implication of a multidrug resistance-associated protein (MRP2)

SO original: TOXICOLOGY LETTERS | SO normalised: TOXICOLOGY LETTERS | CR count: 33 → 33

No journal-related changes detected in SO, J9, JI or CR for this record.

Record 754 | WOS:000181518501697

Cellular and molecular mechanisms underlying ochratoxin A-induced in vitro nephrotoxicity.

SO original: TOXICOLOGICAL SCIENCES | SO normalised: TOXICOLOGICAL SCIENCES | CR count: 0 → 0

No journal-related changes detected in SO, J9, JI or CR for this record.

Record 755 | WOS:000180037400011

Photochemically catalyzed reaction of ochratoxin A with D- and L-cysteine

SO original: PHOTOCHEMISTRY AND PHOTOBIOLOGY | SO normalised: PHOTOCHEMISTRY AND PHOTOBIOLOGY | CR count: 40 → 40

No journal-related changes detected in SO, J9, JI or CR for this record.

Record 756 | WOS:000179850500004

Prevention and control of mycotoxins in the poultry production chain:: a European view

SO original: WORLDS POULTRY SCIENCE JOURNAL | SO normalised: WORLDS POULTRY SCIENCE JOURNAL | CR count: 128 → 128

Field	Status	Orig. pos	Norm. pos	Orig. line	Norm. line	Original cited/source value	Normalised cited/source value
CR	replaced	18	18	105498	105497	Arch Tierernahr<br>Bohm J, 1992, Arch Tierernahr, V42, P95	ARCH TIERERNAHR<br>Bohm J, 1992, ARCH TIERERNAHR, V42, P95
CR	replaced	31	31	105511	105510	Mycotoxin Res<br>Curtui V G, 2000, Mycotoxin Res, V16, P43, DOI 10.1007/BF02946104	MYCOTOXIN RES<br>Curtui V G, 2000, MYCOTOXIN RES, V16, P43, DOI 10.1007/BF02946104

CR	replaced	39	39	105519	105518	Mycotoxin Res<br>Doll S, 2000, Mycotoxin Res, V16 Suppl 1, P38, DOI 10.1007/BF02942977	MYCOTOXIN RES<br>Doll S, 2000, MYCOTOXIN RES, V16 Suppl 1, P38, DOI 10.1007/BF02942977
CR	replaced	42	42	105522	105521	Mycotoxins in Agriculture and Food Safety<br>Dowd PF., 1998, Mycotoxins in Agriculture and Food Safety, P307	Mycotoxins in agriculture and food safety<br>Dowd PF., 1998, Mycotoxins in agriculture and food safety, P307
CR	replaced	49	49	105529	105528	Mycotoxin Res<br>Fuchs E, 2000, Mycotoxin Res, V16 Suppl 1, P66, DOI 10.1007/BF02942984	MYCOTOXIN RES<br>Fuchs E, 2000, MYCOTOXIN RES, V16 Suppl 1, P66, DOI 10.1007/BF02942984
CR	replaced	88	88	105568	105567	Mycotoxin Res<br>Meier A, 2000, Mycotoxin Res, V16 Suppl 1, P12, DOI 10.1007/BF02942971	MYCOTOXIN RES<br>Meier A, 2000, MYCOTOXIN RES, V16 Suppl 1, P12, DOI 10.1007/BF02942971
CR	replaced	95	95	105575	105574	Nat Toxins<br>Muller H M, 1997, Nat Toxins, V5, P24, DOI 10.1002/(SICI)(1997)5:1<24::AID-NT4>3.0.CO;2-#	NAT TOXINS<br>Muller H M, 1997, NAT TOXINS, V5, P24, DOI 10.1002/(SICI)(1997)5:1<24::AID-NT4>3.0.CO;2-#
CR	replaced	99	99	105579	105578	Mycotoxin Res<br>Obst A, 2000, Mycotoxin Res, V16 Suppl 1, P16, DOI 10.1007/BF02942972	MYCOTOXIN RES<br>Obst A, 2000, MYCOTOXIN RES, V16 Suppl 1, P16, DOI 10.1007/BF02942972
CR	replaced	117	117	105597	105596	Poultry Science<br>SQUIRES E J, 1992, Poultry Science, V71, P42	POULTRY SCI<br>SQUIRES E J, 1992, POULTRY SCI, V71, P42

Record 757 | WOS:000178857500004

RETRACTED: Antioxidant nutrients and mycotoxins (Retracted Article)

SO original: TOXICOLOGY | SO normalised: TOXICOLOGY | CR count: 151 → 151

Field	Status	Orig. pos	Norm. pos	Orig. line	Norm. line	Original cited/source value	Normalised cited/source value
CR	replaced	3	3	105664	105663	In Vivo<br>Aboobaker V S, 1994, In Vivo, V8, P1095	IN VIVO<br>Aboobaker V S, 1994, IN VIVO, V8, P1095
CR	replaced	34	34	105695	105694	Feedstuffs<br>Coelho M., 1996, Feedstuffs, V68	FEEDSTUFFS<br>Coelho M., 1996, FEEDSTUFFS, V68
CR	replaced	72	72	105733	105732	CYTOBIOS<br>JESVAL P, 1998, CYTOBIOS, V93, P123	Cytobios<br>JESVAL P, 1998, Cytobios, V93, P123
CR	replaced	105	105	105766	105765	Lipids<br>Nawar W.W., 1996, Lipids, Food chemistry, V3th, P225	LIPIDS<br>Nawar W.W., 1996, LIPIDS, Food chemistry, V3th, P225

Record 758 | WOS:000177393700006

Genotoxic effects of ochratoxin A in human-derived hepatoma (HepG2) cells

SO original: FOOD AND CHEMICAL TOXICOLOGY | SO normalised: FOOD AND CHEMICAL TOXICOLOGY | CR count: 38 → 38

No journal-related changes detected in SO, J9, JI or CR for this record.

Record 759 | WOS:000177631900009

Species- and sex-specific variations in binding of ochratoxin A by renal proteins in vitro

SO original: EXPERIMENTAL AND TOXICOLOGIC PATHOLOGY | SO normalised: EXPERIMENTAL AND TOXICOLOGIC PATHOLOGY | CR count: 42 → 42

No journal-related changes detected in SO, J9, JI or CR for this record.

Record 760 | WOS:000176910000001

Aristolochic acid as a probable human cancer hazard in herbal remedies: a review

SO original: MUTAGENESIS | SO normalised: MUTAGENESIS | CR count: 124 → 124

Field	Status	Orig. pos	Norm. pos	Orig. line	Norm. line	Original cited/source value	Normalised cited/source value
CR	replaced	27	27	106099	106098	IARC Sci Publ<br>Fernando R C, 1993, IARC Sci Publ, P167	IARC SCI PUBL<br>Fernando R C, 1993, IARC SCI PUBL, P167
CR	replaced	76	76	106148	106147	IARC Sci Publ<br>Pfohl-Leszkowicz A, 1993, IARC Sci Publ, P141	IARC SCI PUBL<br>Pfohl-Leszkowicz A, 1993, IARC SCI PUBL, P141

Record 761 | WOS:000175922300023

Esterified-glucomannan in broiler chicken diets-contaminated with aflatoxin, ochratoxin and T-2 toxin:: Evaluation of its binding ability (in vitro) and efficacy as immunomodulator

SO original: ASIAN-AUSTRALASIAN JOURNAL OF ANIMAL SCIENCES | SO normalised: ASIAN-AUSTRALASIAN JOURNAL OF ANIMAL SCIENCES | CR count: 23 → 23

No journal-related changes detected in SO, J9, JI or CR for this record.

Record 762 | WOS:000176518700006

Role of human organic anion transporter 4 in the transport of ochratoxin A

SO original: BIOCHIMICA ET BIOPHYSICA ACTA-MOLECULAR CELL RESEARCH | SO normalised: BIOCHIMICA ET BIOPHYSICA ACTA-MOLECULAR CELL RESEARCH | CR count: 50 → 50

Field	Status	Orig. pos	Norm. pos	Orig. line	Norm. line	Original cited/source value	Normalised cited/source value
CR	replaced	9	9	106343	106342	Journal of Toxicological Sciences<br>Creppy Edmond Ekue, 1998, Journal of Toxicological Sciences, V23, P165	J TOXICOL SCI<br>Creppy Edmond Ekue, 1998, J TOXICOL SCI, V23, P165

Record 763 | WOS:000176216800005

Expression of Hsp70 in kidney cells exposed to ochratoxin A

SO original: ARCHIVES OF TOXICOLOGY | SO normalised: ARCHIVES OF TOXICOLOGY | CR count: 52 → 52

No journal-related changes detected in SO, J9, JI or CR for this record.

Record 764 | WOS:000175152300005

Effect of T-2 toxin on in vivo lipid peroxidation and vitamin E status in mice

SO original: FOOD AND CHEMICAL TOXICOLOGY | SO normalised: FOOD AND CHEMICAL TOXICOLOGY | CR count: 33 → 33

No journal-related changes detected in SO, J9, JI or CR for this record.

Record 765 | WOS:000173055800005

Determination of the epigenetic effects of ochratoxin in a human kidney and a rat liver epithelial cell line

SO original: TOXICON | SO normalised: TOXICON | CR count: 69 → 69

No journal-related changes detected in SO, J9, JI or CR for this record.

Record 766 | WOS:000176962600010

Toxins of filamentous fungi

SO original: FUNGAL ALLERGY AND PATHOGENICITY | SO normalised: FUNGAL ALLERGY AND PATHOGENICITY | CR count: 222 → 222

Field	Status	Orig. pos	Norm. pos	Orig. line	Norm. line	Original cited/source value	Normalised cited/source value
CR	replaced	6	6	106766	106765	FUNGAL METABOLITES [Anonymous], 1983, FUNGAL METABOLITES	REF SER PHYTOCHEM [Anonymous], 1983, REF SER PHYTOCHEM
CR	replaced	24	24	106784	106783	Mycotoxins in Agriculture and Food Safety Bottalico A., 1998, Mycotoxins in Agriculture and Food Safety, P65	Mycotoxins in agriculture and food safety Bottalico A., 1998, Mycotoxins in agriculture and food safety, P65
CR	replaced	100	100	106860	106859	Mycotoxin Res Huang X, 1994, Mycotoxin Res, V10, P101, DOI 10.1007/BF03192259	MYCOTOXIN RES Huang X, 1994, MYCOTOXIN RES, V10, P101, DOI 10.1007/BF03192259
CR	replaced	171	171	106931	106930	MYCOTOXINS SCOTT PM, 1975, MYCOTOXINS, P383	Mycotoxins SCOTT PM, 1975, Mycotoxins, P383
CR	replaced	194	194	106954	106953	MYCOTOXINS VANDERWATT JJ, 1977, MYCOTOXINS, P368	Mycotoxins VANDERWATT JJ, 1977, Mycotoxins, P368

Record 767 | WOS:000174243300013

Detection of ochratoxin A-induced DNA damage in MDCK cells by alkaline single cell gel electrophoresis (comet assay)

SO original: ARCHIVES OF TOXICOLOGY | SO normalised: ARCHIVES OF TOXICOLOGY | CR count: 42 → 42

Field	Status	Orig. pos	Norm. pos	Orig. line	Norm. line	Original cited/source value	Normalised cited/source value
CR	replaced	13	13	107041	107040	Mycotoxin Res<br>Follmann W, 2000, Mycotoxin Res, V16 Suppl 1, P123, DOI 10.1007/BF02942998	MYCOTOXIN RES<br>Follmann W, 2000, MYCOTOXIN RES, V16 Suppl 1, P123, DOI 10.1007/BF02942998
CR	replaced	28	28	107056	107055	IARC Sci Publ<br>Petkova-Bocharova T, 1991, IARC Sci Publ, P135	IARC SCI PUBL<br>Petkova-Bocharova T, 1991, IARC SCI PUBL, P135

Record 768 | WOS:000172508300013

Improvement of megakaryocytic progenitor culture for toxicological investigations

SO original: TOXICOLOGY IN VITRO | SO normalised: TOXICOLOGY IN VITRO | CR count: 35 → 35

Field	Status	Orig. pos	Norm. pos	Orig. line	Norm. line	Original cited/source value	Normalised cited/source value
CR	replaced	19	19	107138	107137	Journal of Toxicological Sciences<br>Ohtani Katsumi, 1993, Journal of Toxicological Sciences, V18, P155	J TOXICOL SCI<br>Ohtani Katsumi, 1993, J TOXICOL SCI, V18, P155
CR	replaced	31	31	107150	107149	Journal of Toxicological Sciences<br>RYU J-C, 1987, Journal of Toxicological Sciences, V12, P11	J TOXICOL SCI<br>RYU J-C, 1987, J TOXICOL SCI, V12, P11

Record 769 | WOS:000173171400003

Horseradish peroxidase mediates DNA and deoxyguanosine 3â ²-monophosphate adduct formation in the presence of ochratoxin A

SO original: ARCHIVES OF TOXICOLOGY | SO normalised: ARCHIVES OF TOXICOLOGY | CR count: 67 → 67

Field	Status	Orig. pos	Norm. pos	Orig. line	Norm. line	Original cited/source value	Normalised cited/source value
CR	replaced	36	36	107243	107242	Mutation Research<br>Obrecht-Pflumio Sophie, 1997, Mutation Research, V379, pS156, DOI 10.1016/S0027-5107(97)83159-4	MUTAT RES<br>Obrecht-Pflumio Sophie, 1997, MUTAT RES, V379, pS156, DOI 10.1016/S0027-5107(97)83159-4
CR	replaced	41	41	107248	107247	IARC Sci Publ<br>Pfohl-Leszkowicz A, 1993, IARC Sci Publ, P141	IARC SCI PUBL<br>Pfohl-Leszkowicz A, 1993, IARC SCI PUBL, P141

Record 770 | WOS:000171478500001

Toxicity, metabolism, and impact of mycotoxins on humans and animals

SO original: TOXICOLOGY | SO normalised: TOXICOLOGY | CR count: 269 → 269

Field	Status	Orig. pos	Norm. pos	Orig. line	Norm. line	Original cited/source value	Normalised cited/source value
CR	replaced	45	45	107366	107365	J. Anim. Sci<br>Cantley D.C., 1982, J. Anim. Sci, V55, p104S	J ANIM SCI<br>Cantley D.C., 1982, J ANIM SCI, V55, p104S
CR	replaced	54	54	107375	107374	Biochemical Pharmacology<br>CHU F S, 1974, Biochemical Pharmacology, V23, P1105, DOI 10.1016/0006-2952(74)90011-2	BIOCHEM PHARMACOL<br>CHU F S, 1974, BIOCHEM PHARMACOL, V23, P1105, DOI 10.1016/0006-2952(74)90011-2
CR	replaced	91	91	107412	107411	Mycotoxin Res<br>Gelderblom W C, 1991, Mycotoxin Res, V7, P46, DOI 10.1007/BF03192165	MYCOTOXIN RES<br>Gelderblom W C, 1991, MYCOTOXIN RES, V7, P46, DOI 10.1007/BF03192165
CR	replaced	100	100	107421	107420	J Vet Diagn Invest<br>Harrison L R, 1990, J Vet Diagn Invest, V2, P217	J VET DIAGN INVEST<br>Harrison L R, 1990, J VET DIAGN INVEST, V2, P217
CR	replaced	216	216	107537	107536	The toxicology of aflatoxins: Human health<br>Shane S.H., 1994, The toxicology of aflatoxins: Human health, veterinary, and agricultural significance, P513, DOI DOI 10.1016/B978-0-12-228255-3.50028-3	The toxicology of aflatoxins: human health<br>Shane S.H., 1994, The toxicology of aflatoxins: human health, veterinary, and agricultural significance, P513, DOI DOI 10.1016/B978-0-12-228255-3.50028-3

Record 771 | WOS:000171571300006

The mycotoxin ochratoxin A alters intestinal barrier and absorption functions but has no effect on chloride secretion

SO original: TOXICOLOGY AND APPLIED PHARMACOLOGY | SO normalised: TOXICOLOGY AND APPLIED PHARMACOLOGY | CR count: 56 → 56

No journal-related changes detected in SO, J9, JI or CR for this record.

Record 772 | WOS:000171310400014

Species-, sex-, and cell type-specific effects of ochratoxin A and B

SO original: TOXICOLOGICAL SCIENCES | SO normalised: TOXICOLOGICAL SCIENCES | CR count: 68 → 68

No journal-related changes detected in SO, J9, JI or CR for this record.

Record 773 | WOS:000171274400005

Characterization of ochratoxin A transport by human organic anion transporters

SO original: LIFE SCIENCES | SO normalised: LIFE SCIENCES | CR count: 34 → 34

Field	Status	Orig. pos	Norm. pos	Orig. line	Norm. line	Original cited/source value	Normalised cited/source value

CR	replaced	9	9	107898	107897	Journal of Toxicological Sciences<br>Creppy Edmond Ekue, 1998, Journal of Toxicological Sciences, V23, P165	J TOXICOL SCI<br>Creppy Edmond Ekue, 1998, J TOXICOL SCI, V23, P165

Record 774 | WOS:000170039000017

Species- and sex-specific renal cytotoxicity of Ochratoxin A and B in vitro

SO original: EXPERIMENTAL AND TOXICOLOGIC PATHOLOGY | SO normalised: EXPERIMENTAL AND TOXICOLOGIC PATHOLOGY | CR count: 41 → 41

Field	Status	Orig. pos	Norm. pos	Orig. line	Norm. line	Original cited/source value	Normalised cited/source value
CR	replaced	3	3	107979	107978	PFLUGERS ARCH<br>BENESIC A, 1999, PFLUGERS ARCH, V439, P278	PFLUG ARCH EUR J PHY<br>BENESIC A, 1999, PFLUG ARCH EUR J PHY, V439, P278

Record 775 | WOS:000169038800006

Aspartame prevents the karyomegaly induced by ochratoxin A in rat kidney

SO original: ARCHIVES OF TOXICOLOGY | SO normalised: ARCHIVES OF TOXICOLOGY | CR count: 42 → 42

Field	Status	Orig. pos	Norm. pos	Orig. line	Norm. line	Original cited/source value	Normalised cited/source value
CR	replaced	28	28	108109	108108	PFLUGERS ARCH<br>KUROMACHI G, 1997, PFLUGERS ARCH, V434, P392	PFLUG ARCH EUR J PHY<br>KUROMACHI G, 1997, PFLUG ARCH EUR J PHY, V434, P392
CR	replaced	38	38	108119	108118	IARC Sci Publ<br>Pfohl-Leszkowicz A, 1993, IARC Sci Publ, P141	IARC SCI PUBL<br>Pfohl-Leszkowicz A, 1993, IARC SCI PUBL, P141

Record 776 | WOS:000167652300006

Age-related differences in the toxicity of ochratoxin A in female rats

SO original: FOOD AND CHEMICAL TOXICOLOGY | SO normalised: FOOD AND CHEMICAL TOXICOLOGY | CR count: 47 → 47

Field	Status	Orig. pos	Norm. pos	Orig. line	Norm. line	Original cited/source value	Normalised cited/source value
CR	replaced	35	35	108217	108216	IARC Sci Publ<br>Petkova-Bocharova T, 1991, IARC Sci Publ, P83	IARC SCI PUBL<br>Petkova-Bocharova T, 1991, IARC SCI PUBL, P83

Record 777 | WOS:000166500400005

Metabolism of ochratoxin A: Absence of formation of genotoxic derivatives by human and rat enzymes

SO original: CHEMICAL RESEARCH IN TOXICOLOGY | SO normalised: CHEMICAL RESEARCH IN TOXICOLOGY | CR count: 67 → 67

No journal-related changes detected in SO, J9, JI or CR for this record.

Record 778 | WOS:000165378500005

Effects of ochratoxin A on cytotoxicity and cell differentiation in cultured rat embryonic cells

SO original: JOURNAL OF TOXICOLOGY AND ENVIRONMENTAL HEALTH-PART A-CURRENT ISSUES | SO normalised: JOURNAL OF TOXICOLOGY AND ENVIRONMENTAL HEALTH-PART A-CURRENT ISSUES | CR count: 34 → 34

No journal-related changes detected in SO, J9, JI or CR for this record.

Record 779 | WOS:000089269500007

Studies of ochratoxin A-induced inhibition of phenylalanine hydroxylase and its reversal by phenylalanine

SO original: TOXICOLOGY AND APPLIED PHARMACOLOGY | SO normalised: TOXICOLOGY AND APPLIED PHARMACOLOGY | CR count: 47 → 47

No journal-related changes detected in SO, J9, JI or CR for this record.

Record 780 | WOS:000087816200003

In vitro DNA and dGMP adducts formation caused by ochratoxin A

SO original: CHEMICO-BIOLOGICAL INTERACTIONS | SO normalised: CHEMICO-BIOLOGICAL INTERACTIONS | CR count: 60 → 60

Field	Status	Orig. pos	Norm. pos	Orig. line	Norm. line	Original cited/source value	Normalised cited/source value
CR	replaced	12	12	108618	108617	Food and Chemical Toxicology Dharmshilal Kumari, 1994, Food and Chemical Toxicology, V32, P471, DOI 10.1016/0278-6915(94)90044-2	FOOD CHEM TOXICOL Dharmshilal Kumari, 1994, FOOD CHEM TOXICOL, V32, P471, DOI 10.1016/0278-6915(94)90044-2
CR	replaced	37	37	108643	108642	Mutation Research Obrecht-Pflumio Sophie, 1997, Mutation Research, V379, pS156, DOI 10.1016/S0027-5107(97)83159-4	MUTAT RES Obrecht-Pflumio Sophie, 1997, MUTAT RES, V379, pS156, DOI 10.1016/S0027-5107(97)83159-4

Record 781 | WOS:000087555100005

Ochratoxin A from a toxicological perspective

SO original: JOURNAL OF VETERINARY PHARMACOLOGY AND THERAPEUTICS | SO normalised: JOURNAL OF VETERINARY PHARMACOLOGY AND THERAPEUTICS | CR count: 93 → 93

Field	Status	Orig. pos	Norm. pos	Orig. line	Norm. line	Original cited/source value	Normalised cited/source value
CR	replaced	38	38	108751	108750	REV MED VET HUBNER M, 1998, REV MED VET, V149, P507	REV MED VET-TOULOUSE HUBNER M, 1998, REV MED VET-TOULOUSE, V149, P507

Record 782 | WOS:000085306200013

Induction of unscheduled DNA synthesis in primary human urothelial cells by the mycotoxin ochratoxin A

SO original: TOXICOLOGICAL SCIENCES | SO normalised: TOXICOLOGICAL SCIENCES | CR count: 47 → 47

Field	Status	Orig. pos	Norm. pos	Orig. line	Norm. line	Original cited/source value	Normalised cited/source value
CR	replaced	35	35	108902	108901	IARC Sci Publ Petkova-Bocharova T, 1991, IARC Sci Publ, P135	IARC SCI PUBL Petkova-Bocharova T, 1991, IARC SCI PUBL, P135

Record 783 | WOS:000084146300015

Cholestyramine protection against ochratoxin A toxicity: Role of ochratoxin A sorption by the resin and bile enterohepatic circulation

SO original: JOURNAL OF FOOD PROTECTION | SO normalised: JOURNAL OF FOOD PROTECTION | CR count: 34 → 34

No journal-related changes detected in SO, J9, JI or CR for this record.

Record 784 | WOS:000165381000007

Increased cytotoxicity of food-borne mycotoxins toward human cell lines in vitro via enhanced cytochrome p450 expression using the MTT bioassay

SO original: MYCOPATHOLOGIA | SO normalised: MYCOPATHOLOGIA | CR count: 27 → 27

Field	Status	Orig. pos	Norm. pos	Orig. line	Norm. line	Original cited/source value	Normalised cited/source value
CR	replaced	21	21	109085	109084	Mycotoxin Res Reubel G H, 1987, Mycotoxin Res, V3, P85, DOI 10.1007/BF03191994	MYCOTOXIN RES Reubel G H, 1987, MYCOTOXIN RES, V3, P85, DOI 10.1007/BF03191994

Record 785 | WOS:000083648200009

Genotoxicity of ochratoxin A by Salmonella mutagenicity test after bioactivation by mouse kidney microsomes

SO original: MUTATION RESEARCH-GENETIC TOXICOLOGY AND ENVIRONMENTAL MUTAGENESIS | SO normalised: MUTATION RESEARCH-GENETIC TOXICOLOGY AND ENVIRONMENTAL MUTAGENESIS | CR count: 59 → 59

Field	Status	Orig. pos	Norm. pos	Orig. line	Norm. line	Original cited/source value	Normalised cited/source value
CR	replaced	18	18	109167	109166	Food and Chemical Toxicology Dharmshilal Kumari, 1994, Food and Chemical Toxicology, V32, P471, DOI 10.1016/0278-6915(94)90044-2	FOOD CHEM TOXICOL Dharmshilal Kumari, 1994, FOOD CHEM TOXICOL, V32, P471, DOI 10.1016/0278-6915(94)90044-2
CR	replaced	40	40	109189	109188	Mutation Research Obrecht-Pflumio Sophie, 1997, Mutation Research, V379, pS156, DOI	MUTAT RES Obrecht-Pflumio Sophie, 1997, MUTAT RES, V379,

						10.1016/S0027-5107(97)83159-4	pS156, DOI 10.1016/S0027-5107(97)83159-4
CR	replaced	44	44	109193	109192	IARC Sci Publ<br>Pfohl-Leszkowicz A, 1993, IARC Sci Publ, P141	IARC SCI PUBL<br>Pfohl-Leszkowicz A, 1993, IARC SCI PUBL, P141
CR	replaced	50	50	109199	109198	Cell Biology and Toxicology<br>Pinelli E., 1995, Cell Biology and Toxicology, V11, P211	CELL BIOL TOXICOL<br>Pinelli E., 1995, CELL BIOL TOXICOL, V11, P211

Record 786 | WOS:000082038700010

Ochratoxin binding to phenylalanyl-tRNA synthetase: Computational approach to the mechanism of ochratoxicosis and its antagonism

SO original: JOURNAL OF MEDICINAL CHEMISTRY | SO normalised: JOURNAL OF MEDICINAL CHEMISTRY | CR count: 48 → 48

No journal-related changes detected in SO, J9, JI or CR for this record.

Record 787 | WOS:000081907800002

Mutagenicity of commercial Monascus fermentation products and the role of citrinin contamination

SO original: MUTATION RESEARCH-GENETIC TOXICOLOGY AND ENVIRONMENTAL MUTAGENESIS | SO normalised: MUTATION RESEARCH-GENETIC TOXICOLOGY AND ENVIRONMENTAL MUTAGENESIS | CR count: 44 → 44

Field	Status	Orig. pos	Norm. pos	Orig. line	Norm. line	Original cited/source value	Normalised cited/source value
CR	replaced	12	12	109362	109361	Journal of Veterinary Pharmacology and Therapeutics<br>De Groene E. M., 1997, Journal of Veterinary Pharmacology and Therapeutics, V20, P275	J VET PHARMACOL THER<br>De Groene E. M., 1997, J VET PHARMACOL THER, V20, P275

Record 788 | WOS:000081759900001

Ochratoxin A-binding proteins in rat organs and plasma and in different cell lines of the kidney

SO original: TOXICOLOGY | SO normalised: TOXICOLOGY | CR count: 46 → 46

No journal-related changes detected in SO, J9, JI or CR for this record.

Record 789 | WOS:000080471300017

Transport of ochratoxin A by renal multispecific organic anion transporter 1

SO original: JOURNAL OF PHARMACOLOGY AND EXPERIMENTAL THERAPEUTICS | SO normalised: JOURNAL OF PHARMACOLOGY AND EXPERIMENTAL THERAPEUTICS | CR count: 28 → 28

No journal-related changes detected in SO, J9, JI or CR for this record.

Record 790 | WOS:000080762800002

Roles of cyclooxygenase and lipoxygenases in ochratoxin A genotoxicity in human epithelial lung cells

SO original: ENVIRONMENTAL TOXICOLOGY AND PHARMACOLOGY | SO normalised: ENVIRONMENTAL TOXICOLOGY AND PHARMACOLOGY | CR count: 64 → 64

Field	Status	Orig. pos	Norm. pos	Orig. line	Norm. line	Original cited/source value	Normalised cited/source value
CR	replaced	44	44	109681	109680	IARC Sci Publ Pfohl-Leszkowicz A, 1993, IARC Sci Publ, P141	IARC SCI PUBL Pfohl-Leszkowicz A, 1993, IARC SCI PUBL, P141

Record 791 | WOS:000080209100001

Inactivation of ochratoxin in ruminal fluid with variation of pH-value and fermentation parameters in an in vitro system

SO original: ENVIRONMENTAL TOXICOLOGY AND PHARMACOLOGY | SO normalised: ENVIRONMENTAL TOXICOLOGY AND PHARMACOLOGY | CR count: 26 → 26

Field	Status	Orig. pos	Norm. pos	Orig. line	Norm. line	Original cited/source value	Normalised cited/source value
CR	replaced	10	10	109760	109759	Annales de Recherches Veterinaires GALTIER P, 1976, Annales de Recherches Veterinaires, V7, P91	ANN RECH VET GALTIER P, 1976, ANN RECH VET, V7, P91

Record 792 | WOS:000083320300013

Cytotoxic and genotoxic effects of zearalenone:: Prevention by vitamin E

SO original: JOURNAL OF TOXICOLOGY-TOXIN REVIEWS | SO normalised: JOURNAL OF TOXICOLOGY-TOXIN REVIEWS | CR count: 34 → 34

Field	Status	Orig. pos	Norm. pos	Orig. line	Norm. line	Original cited/source value	Normalised cited/source value
CR	replaced	11	11	109840	109839	Adv Appl Microbiol Hidy P H, 1977, Adv Appl Microbiol, V22, P59, DOI 10.1016/S0065-2164(08)70160-6	ADV APPL MICROBIOL Hidy P H, 1977, ADV APPL MICROBIOL, V22, P59, DOI 10.1016/S0065-2164(08)70160-6

Record 793 | WOS:000087386900028

Biotransformation and lack of mutagenicity of ochratoxin A using combinations of mammalian biotransformation enzymes

SO original: 18TH INTERNATIONAL SCIENTIFIC COLLOQUIUM ON COFFEE | SO normalised: 18TH INTERNATIONAL SCIENTIFIC COLLOQUIUM ON COFFEE | CR count: 38 → 38

No journal-related changes detected in SO, J9, JI or CR for this record.

Record 794 | WOS:000078111000012

Apical uptake of radiolabelled ochratoxin A into Madin-Darby canine kidney cells

SO original: TOXICOLOGY | SO normalised: TOXICOLOGY | CR count: 51 → 51

Field	Status	Orig. pos	Norm. pos	Orig. line	Norm. line	Original cited/source value	Normalised cited/source value
CR	replaced	33	33	110033	110032	Comptes Rendus des Seances de la Societe de Biologie et de ses Filiales<br>Pfohl-Leszkowicz Anne, 1994, Comptes Rendus des Seances de la Societe de Biologie et de ses Filiales, V188, P335	CR SOC BIOL<br>Pfohl-Leszkowicz Anne, 1994, CR SOC BIOL, V188, P335

Record 795 | WOS:000077419100015

Apoptosis in the kidney

SO original: TOXICOLOGIC PATHOLOGY | SO normalised: TOXICOLOGIC PATHOLOGY | CR count: 164 → 164

No journal-related changes detected in SO, J9, JI or CR for this record.

Record 796 | WOS:000076860300002

Measurement of antibacterial activities of T-2 toxin, deoxynivalenol, ochratoxin A, aflatoxin B1 and fumonisin B1 using microtitration tray-based turbidimetric techniques

SO original: JOURNAL OF VETERINARY MEDICINE SERIES A-PHYSIOLOGY PATHOLOGY CLINICAL ; MEDICINE | SO normalised: JOURNAL OF VETERINARY MEDICINE SERIES A-PHYSIOLOGY PATHOLOGY CLINICAL ; MEDICINE | CR count: 13 → 13

No journal-related changes detected in SO, J9, JI or CR for this record.

Record 797 | WOS:000076443800002

Cytotoxicity of nephrotoxic fungal toxins to kidney-derived LLC-PK1 and OK cell lines

SO original: CELL BIOLOGY AND TOXICOLOGY | SO normalised: CELL BIOLOGY AND TOXICOLOGY | CR count: 39 → 39

Field	Status	Orig. pos	Norm. pos	Orig. line	Norm. line	Original cited/source value	Normalised cited/source value
CR	replaced	28	28	110404	110403	Mycotoxin Res<br>Reubel G H, 1987, Mycotoxin Res, V3, P85, DOI 10.1007/BF03191994	MYCOTOXIN RES<br>Reubel G H, 1987, MYCOTOXIN RES, V3, P85, DOI 10.1007/BF03191994

Record 798 | WOS:000076413600003

Exposure to ochratoxin A impairs organic anion transport in proximal-tubule-derived opossum kidney cells

SO original: JOURNAL OF PHARMACOLOGY AND EXPERIMENTAL THERAPEUTICS | SO normalised: JOURNAL OF PHARMACOLOGY AND EXPERIMENTAL THERAPEUTICS | CR count: 42 → 42

Field	Status	Orig. pos	Norm. pos	Orig. line	Norm. line	Original cited/source value	Normalised cited/source value
CR	replaced	31	31	110494	110493	IARC Sci Publ<br>Petkova-Bocharova T, 1991, IARC Sci Publ, P135	IARC SCI PUBL<br>Petkova-Bocharova T, 1991, IARC SCI PUBL, P135

Record 799 | WOS:000074665700021

Detailed mapping of ochratoxin a reabsorption along the rat nephron in vivo: The nephrotoxin can be reabsorbed in all nephron segments by different mechanisms

SO original: JOURNAL OF PHARMACOLOGY AND EXPERIMENTAL THERAPEUTICS | SO normalised: JOURNAL OF PHARMACOLOGY AND EXPERIMENTAL THERAPEUTICS | CR count: 27 → 27

Field	Status	Orig. pos	Norm. pos	Orig. line	Norm. line	Original cited/source value	Normalised cited/source value
CR	replaced	3	3	110560	110559	Biochemical Pharmacology<br>CHU F S, 1974, Biochemical Pharmacology, V23, P1105, DOI 10.1016/0006-2952(74)90011-2	BIOCHEM PHARMACOL<br>CHU F S, 1974, BIOCHEM PHARMACOL, V23, P1105, DOI 10.1016/0006-2952(74)90011-2

Record 800 | WOS:000075078200010

Haematotoxicity of mycotoxins

SO original: REVUE DE MEDECINE VETERINAIRE | SO normalised: REVUE DE MEDECINE VETERINAIRE | CR count: 84 → 84

Field	Status	Orig. pos	Norm. pos	Orig. line	Norm. line	Original cited/source value	Normalised cited/source value
CR	replaced	3	3	110641	110640	World Rev Nutr Diet<br>Bababunmi E A, 1980, World Rev Nutr Diet, V34, P161	WORLD REV NUTR DIET<br>Bababunmi E A, 1980, WORLD REV NUTR DIET, V34, P161
CR	replaced	70	70	110708	110707	Journal of Toxicological Sciences<br>RYU J-C, 1987, Journal of Toxicological Sciences, V12, P11	J TOXICOL SCI<br>RYU J-C, 1987, J TOXICOL SCI, V12, P11
CR	replaced	73	73	110711	110710	Journal of Toxicological Sciences<br>SATO N, 1978, Journal of Toxicological Sciences, V3, P335	J TOXICOL SCI<br>SATO N, 1978, J TOXICOL SCI, V3, P335

Record 801 | WOS:000073042900016

Activated carbons: In vitro affinity for ochratoxin A and deoxynivalenol and relation of adsorption ability to physicochemical parameters

SO original: JOURNAL OF FOOD PROTECTION | SO normalised: JOURNAL OF FOOD PROTECTION | CR count: 19 → 19

Field	Status	Orig. pos	Norm. pos	Orig. line	Norm. line	Original cited/source value	Normalised cited/source value
CR	replaced	12	12	110793	110792	Mycotoxin Res<br>Niessen L, 1993, Mycotoxin	MYCOTOXIN RES<br>Niessen L, 1993,

						Res, V9, P99, DOI 10.1007/BF03192241	MYCOTOXIN RES, V9, P99, DOI 10.1007/BF03192241

Record 802 | WOS:000072874100001

Ochratoxin A in food and feed: occurrence, legislation and mode of action

SO original: ZEITSCHRIFT FUR ERNAHRUNGSWISSENSCHAFT | SO normalised: ZEITSCHRIFT FUR ERNAHRUNGSWISSENSCHAFT | CR count: 70 → 70

Field	Status	Orig. pos	Norm. pos	Orig. line	Norm. line	Original cited/source value	Normalised cited/source value
CR	replaced	30	30	110875	110874	OXIDATIVE STRESS<br>Kappus H., 1985, OXIDATIVE STRESS	Oxidative Stress<br>Kappus H., 1985, Oxidative Stress

Record 803 | WOS:000079327200007

The effects of four mycotoxins on the mitogen stimulated proliferation of bovine peripheral blood mononuclear cells in vitro

SO original: MYCOPATHOLOGIA | SO normalised: MYCOPATHOLOGIA | CR count: 54 → 54

Field	Status	Orig. pos	Norm. pos	Orig. line	Norm. line	Original cited/source value	Normalised cited/source value
CR	replaced	5	5	110970	110969	Int Rev Cytol<br>Brown J C, 1978, Int Rev Cytol, V52, P277, DOI 10.1016/S0074-7696(08)60758-5	INT REV CYTOL<br>Brown J C, 1978, INT REV CYTOL, V52, P277, DOI 10.1016/S0074-7696(08)60758-5
CR	replaced	40	40	111005	111004	Mycotoxin Res<br>Reubel G H, 1987, Mycotoxin Res, V3, P85, DOI 10.1007/BF03191994	MYCOTOXIN RES<br>Reubel G H, 1987, MYCOTOXIN RES, V3, P85, DOI 10.1007/BF03191994

Record 804 | WOS:000081940900027

Mycotoxins: Mechanisms of action at the molecular level

SO original: MYCOTOXINS AND PHYCOTOXINS - DEVELOPMENTS IN CHEMISTRY, TOXICOLOGY AND ; FOOD SAFETY | SO normalised: MYCOTOXINS AND PHYCOTOXINS - DEVELOPMENTS IN CHEMISTRY, TOXICOLOGY AND ; FOOD SAFETY | CR count: 72 → 72

Field	Status	Orig. pos	Norm. pos	Orig. line	Norm. line	Original cited/source value	Normalised cited/source value
CR	replaced	23	23	111096	111095	Mycotoxin Res<br>Gelderblom W C, 1991, Mycotoxin Res, V7, P46, DOI 10.1007/BF03192165	MYCOTOXIN RES<br>Gelderblom W C, 1991, MYCOTOXIN RES, V7, P46, DOI 10.1007/BF03192165

Record 805 | WOS:000075950800023

Tubulotoxic mechanisms of ochratoxin a

SO original: KIDNEY & BLOOD PRESSURE RESEARCH | SO normalised: KIDNEY & BLOOD PRESSURE RESEARCH | CR count: 33 → 33

No journal-related changes detected in SO, J9, JI or CR for this record.

Record 806 | WOS:000080521300009

Ochratoxins: Molecular strategies for developing an antidote

SO original: ALTEX-ALTERNATIVEN ZU TIEREXPERIMENTEN | SO normalised: ALTEX-ALTERNATIVEN ZU TIEREXPERIMENTEN | CR count: 21 → 21

No journal-related changes detected in SO, J9, JI or CR for this record.

Record 807 | WOS:000071510600005

Acute toxicity of the mycotoxin roridin E on liver and kidney of rats

SO original: JOURNAL OF APPLIED ANIMAL RESEARCH | SO normalised: JOURNAL OF APPLIED ANIMAL RESEARCH | CR count: 22 → 22

No journal-related changes detected in SO, J9, JI or CR for this record.

Record 808 | WOS:A1997YD92300001

The neurotoxic effects of ochratoxin-A are reduced by protein binding but are not affected by I-phenylalanine

SO original: TOXICOLOGY AND APPLIED PHARMACOLOGY | SO normalised: TOXICOLOGY AND APPLIED PHARMACOLOGY | CR count: 35 → 35

No journal-related changes detected in SO, J9, JI or CR for this record.

Record 809 | WOS:A1997XM61400017

Effects of inorganic adsorbents and cyclopiazonic acid in broiler chickens

SO original: POULTRY SCIENCE | SO normalised: POULTRY SCIENCE | CR count: 56 → 56

Field	Status	Orig. pos	Norm. pos	Orig. line	Norm. line	Original cited/source value	Normalised cited/source value
CR	replaced	2	2	111505	111504	Mycopathologia Balachandran C., 1996, Mycopathologia, V132, P161, DOI 10.1007/BF01103982	MYCOPATHOLOGIA Balachandran C., 1996, MYCOPATHOLOGIA, V132, P161, DOI 10.1007/BF01103982
CR	replaced	5	5	111508	111507	Mycotoxin Res Cole R J, 1986, Mycotoxin Res, V2, P3, DOI 10.1007/BF03191956	MYCOTOXIN RES Cole R J, 1986, MYCOTOXIN RES, V2, P3, DOI 10.1007/BF03191956
CR	replaced	50	50	111553	111552	Statistical methods Snedecor G., 1967, Statistical methods, P258	STATISTICAL METHODS Snedecor G., 1967, STATISTICAL METHODS, P258
CR	replaced	56	56	111559	111558	Poultry Science WILSON M E, 1986, Poultry Science, V65, P145	POULTRY SCI WILSON M E, 1986, POULTRY SCI, V65, P145

Record 810 | WOS:A1997XN68000014

Lipid peroxidation as one mode of action in ochratoxin A toxicity in rats and chicks

SO original: CANADIAN JOURNAL OF ANIMAL SCIENCE | SO normalised: CANADIAN JOURNAL OF ANIMAL SCIENCE | CR count: 26 → 26

No journal-related changes detected in SO, J9, JI or CR for this record.

Record 811 | WOS:A1997XA91600004

Induction of micronuclei with ochratoxin A in ovine seminal vesicle cell cultures

SO original: ARCHIVES OF TOXICOLOGY | SO normalised: ARCHIVES OF TOXICOLOGY | CR count: 66 → 66

No journal-related changes detected in SO, J9, JI or CR for this record.

Record 812 | WOS:A1997WT39200011

Reduction of ochratoxin A toxicity by heat-induced epimerization. In vitro effects of ochratoxins on embryonic chick meningeal and other cell cultures

SO original: TOXICOLOGY | SO normalised: TOXICOLOGY | CR count: 23 → 23

No journal-related changes detected in SO, J9, JI or CR for this record.

Record 813 | WOS:A1997WW09700003

Reduction of the ochratoxin A-induced cytotoxicity in Vero cells by aspartame

SO original: ARCHIVES OF TOXICOLOGY | SO normalised: ARCHIVES OF TOXICOLOGY | CR count: 46 → 46

Field	Status	Orig. pos	Norm. pos	Orig. line	Norm. line	Original cited/source value	Normalised cited/source value
CR	replaced	22	22	111897	111896	Annales de Recherches Veterinaires<br>Galtier P., 1974, Annales de Recherches Veterinaires, V5, P311	ANN RECH VET<br>Galtier P., 1974, ANN RECH VET, V5, P311

Record 814 | WOS:A1997XZ55900019

Effects of the naturally occurring food mycotoxin ochratoxin A on brain cells in culture

SO original: NEUROTOXICOLOGY | SO normalised: NEUROTOXICOLOGY | CR count: 51 → 51

Field	Status	Orig. pos	Norm. pos	Orig. line	Norm. line	Original cited/source value	Normalised cited/source value
CR	replaced	40	40	112019	112018	Comptes Rendus des Seances de la Societe de Biologie et de ses Filiales<br>Pfohl-Leszkowicz Anne,	CR SOC BIOL<br>Pfohl-Leszkowicz Anne, 1994, CR SOC BIOL, V188, P335

						1994, Comptes Rendus des Seances de la Societe de Biologie et de ses Filiales, V188, P335	

Record 815 | WOS:A1997WC04100026

Reabsorption of the nephrotoxin ochratoxin A along the rat nephron in vivo

SO original: JOURNAL OF PHARMACOLOGY AND EXPERIMENTAL THERAPEUTICS | SO normalised: JOURNAL OF PHARMACOLOGY AND EXPERIMENTAL THERAPEUTICS | CR count: 22 → 22

Field	Status	Orig. pos	Norm. pos	Orig. line	Norm. line	Original cited/source value	Normalised cited/source value
CR	replaced	4	4	112079	112078	Biochemical Pharmacology CHU F S, 1974, Biochemical Pharmacology, V23, P1105, DOI 10.1016/0006-2952(74)90011-2	BIOCHEM PHARMACOL CHU F S, 1974, BIOCHEM PHARMACOL, V23, P1105, DOI 10.1016/0006-2952(74)90011-2
CR	replaced	11	11	112086	112085	PFLUGERS ARCH GERLE M, 1993, PFLUGERS ARCH, V425, P401	PFLUG ARCH EUR J PHY GERLE M, 1993, PFLUG ARCH EUR J PHY, V425, P401
CR	replaced	16	16	112091	112090	PFLUGERS ARCH SCHWERDT G, 1996, PFLUGERS ARCH, V431, pR109	PFLUG ARCH EUR J PHY SCHWERDT G, 1996, PFLUG ARCH EUR J PHY, V431, pR109

Record 816 | WOS:A1996WB42500013

Influence of vitamins E and C on the toxic effects of ochratoxin A and T-2 toxin in chicks

SO original: POULTRY SCIENCE | SO normalised: POULTRY SCIENCE | CR count: 32 → 32

Field	Status	Orig. pos	Norm. pos	Orig. line	Norm. line	Original cited/source value	Normalised cited/source value
CR	replaced	13	13	112155	112154	Journal of Animal Science Hoehler D., 1995, Journal of Animal Science, V73, P208	J ANIM SCI Hoehler D., 1995, J ANIM SCI, V73, P208

Record 817 | WOS:A1996UZ36400005

Effects of mycotoxins on cytokine production and proliferation in EL-4 thymoma cells

SO original: JOURNAL OF TOXICOLOGY AND ENVIRONMENTAL HEALTH | SO normalised: JOURNAL OF TOXICOLOGY AND ENVIRONMENTAL HEALTH | CR count: 58 → 58

Field	Status	Orig. pos	Norm. pos	Orig. line	Norm. line	Original cited/source value	Normalised cited/source value
CR	replaced	47	47	112272	112271	Mycotoxin Res Reubel G H, 1987, Mycotoxin Res, V3, P85, DOI 10.1007/BF03191994	MYCOTOXIN RES Reubel G H, 1987, MYCOTOXIN RES, V3, P85, DOI 10.1007/BF03191994

Record 818 | WOS:A1996UT94000012

Prevention of toxic effects of mycotoxins by means of nonnutritive adsorbent compounds

SO original: JOURNAL OF FOOD PROTECTION | SO normalised: JOURNAL OF FOOD PROTECTION | CR count: 111 → 111

Field	Status	Orig. pos	Norm. pos	Orig. line	Norm. line	Original cited/source value	Normalised cited/source value
CR	replaced	7	7	112343	112342	Poultry Science<br>ARABA M, 1991, Poultry Science, V70, P6	POULTRY SCI<br>ARABA M, 1991, POULTRY SCI, V70, P6
CR	replaced	25	25	112361	112360	Poultry Science<br>Davidson J. N., 1987, Poultry Science, V66, P89	POULTRY SCI<br>Davidson J. N., 1987, POULTRY SCI, V66, P89
CR	replaced	29	29	112365	112364	Poultry Science<br>DOERR J A, 1989, Poultry Science, V68, P45	POULTRY SCI<br>DOERR J A, 1989, POULTRY SCI, V68, P45
CR	replaced	32	32	112368	112367	Mycotoxins in dairy products.<br>Egmond H. P. van, 1989, Mycotoxins in dairy products., P11	Mycotoxins in Dairy Products<br>Egmond H. P. van, 1989, Mycotoxins in Dairy Products, P11
CR	replaced	49	49	112385	112384	Poultry Science<br>KUBENA L F, 1988, Poultry Science, V67, P106	POULTRY SCI<br>KUBENA L F, 1988, POULTRY SCI, V67, P106
CR	replaced	57	57	112393	112392	Journal of Animal Science<br>LINDEMANN M D, 1989, Journal of Animal Science, V67, P36	J ANIM SCI<br>LINDEMANN M D, 1989, J ANIM SCI, V67, P36
CR	replaced	63	63	112399	112398	LEBENSM WISS TECHNOL<br>MILLER N, 1985, LEBENSM WISS TECHNOL, V18, P201	LEBENSM-WISS TECHNOL<br>MILLER N, 1985, LEBENSM-WISS TECHNOL, V18, P201
CR	replaced	92	92	112428	112427	Toxicologist<br>Sarr A.B., 1990, Toxicologist, V10, P163	TOXICOLOGIST<br>Sarr A.B., 1990, TOXICOLOGIST, V10, P163
CR	replaced	93	93	112429	112428	Poultry Science<br>SCHEIDELER S E, 1989, Poultry Science, V68, P203	POULTRY SCI<br>SCHEIDELER S E, 1989, POULTRY SCI, V68, P203

Record 819 | WOS:A1996VJ28200003

Aspartame as a preventive agent of chronic toxic effects of ochratoxin A in experimental animals

SO original: JOURNAL OF TOXICOLOGY-TOXIN REVIEWS | SO normalised: JOURNAL OF TOXICOLOGY-TOXIN REVIEWS | CR count: 62 → 62

Field	Status	Orig. pos	Norm. pos	Orig. line	Norm. line	Original cited/source value	Normalised cited/source value
CR	replaced	12	12	112518	112517	Archives of Toxicology<br>Chakor K., 1988, Archives of Toxicology, P201	ARCH TOXICOL<br>Chakor K., 1988, ARCH TOXICOL, P201

Record 820 | WOS:A1996VW20400002

Renal toxicodynamics of ochratoxin A: A pathophysiological approach

SO original: KIDNEY & BLOOD PRESSURE RESEARCH | SO normalised: KIDNEY & BLOOD PRESSURE RESEARCH | CR count: 74 → 74

Field	Status	Orig. pos	Norm. pos	Orig. line	Norm. line	Original cited/source value	Normalised cited/source value
CR	replaced	10	10	112621	112620	Biochemical Pharmacology CHU F S, 1974, Biochemical Pharmacology, V23, P1105, DOI 10.1016/0006-2952(74)90011-2	BIOCHEM PHARMACOL CHU F S, 1974, BIOCHEM PHARMACOL, V23, P1105, DOI 10.1016/0006-2952(74)90011-2
CR	replaced	46	46	112657	112656	Cellular Physiology and Biochemistry OBERLEITHNER H, 1992, Cellular Physiology and Biochemistry, V2, P180, DOI 10.1159/000154639	CELL PHYSIOL BIOCHEM OBERLEITHNER H, 1992, CELL PHYSIOL BIOCHEM, V2, P180, DOI 10.1159/000154639
CR	replaced	56	56	112667	112666	PFLUGERS ARCH SCHWERDT G, 1995, PFLUGERS ARCH, V429, pR122	PFLUG ARCH EUR J PHY SCHWERDT G, 1995, PFLUG ARCH EUR J PHY, V429, pR122

Record 821 | WOS:A1995TU51400124

Prevention of nephrotoxicity of ochratoxin A, a food contaminant

SO original: TOXICOLOGY LETTERS | SO normalised: TOXICOLOGY LETTERS | CR count: 54 → 54

No journal-related changes detected in SO, J9, JI or CR for this record.

Record 822 | WOS:A1995TN07400005

Activation markers and cell proliferation as indicators of toxicity: A flow cytometric approach

SO original: CELL BIOLOGY AND TOXICOLOGY | SO normalised: CELL BIOLOGY AND TOXICOLOGY | CR count: 31 → 31

Field	Status	Orig. pos	Norm. pos	Orig. line	Norm. line	Original cited/source value	Normalised cited/source value
CR	replaced	16	16	112854	112853	FUNDAM APPL TOXICOL LANG D, 1993, FUNDAM APPL TOXICOL, V18, P200	Fundam Appl Toxicol LANG D, 1993, Fundam Appl Toxicol, V18, P200

Record 823 | WOS:A1995TC07600004

OCHRATOXINES AND TOXICOLOGICAL CONSEQUENCES

SO original: CRYPTOGAMIE MYCOLOGIE | SO normalised: CRYPTOGAMIE MYCOLOGIE | CR count: 0 → 0

No journal-related changes detected in SO, J9, JI or CR for this record.

Record 824 | WOS:A1995QJ43400010

SISTER-CHROMATID EXCHANGE FREQUENCY IN CULTURED ISOLATED PORCINE URINARY-BLADDER EPITHELIAL-CELLS (PUBEC) TREATED WITH OCHRATOXIN-A AND OCHRATOXIN-ALPHA

SO original: ARCHIVES OF TOXICOLOGY | SO normalised: ARCHIVES OF TOXICOLOGY | CR count: 46 → 46

No journal-related changes detected in SO, J9, JI or CR for this record.

Record 825 | WOS:A1995QC67100017

EFFECT OF PIROXICAM ON THE NEPHROTOXICITY INDUCED BY OCHRATOXIN-A IN RATS

SO original: TOXICOLOGY | SO normalised: TOXICOLOGY | CR count: 50 → 50

No journal-related changes detected in SO, J9, JI or CR for this record.

Record 826 | WOS:A1994NN32900003

EFFECT OF SUPEROXIDE-DISMUTASE AND CATALASE ON THE NEPHROTOXICITY INDUCED BY SUBCHRONICAL ADMINISTRATION OF OCHRATOXIN-A IN RATS

SO original: TOXICOLOGY | SO normalised: TOXICOLOGY | CR count: 38 → 38

No journal-related changes detected in SO, J9, JI or CR for this record.

Record 827 | WOS:A1994MM85700005

THE ROLE OF THE PROXIMAL TUBULE IN OCHRATOXIN-A NEPHROTOXICITY IN-VIVO - TOXODYNAMIC AND TOXOKINETIC ASPECTS

SO original: RENAL PHYSIOLOGY AND BIOCHEMISTRY | SO normalised: RENAL PHYSIOLOGY AND BIOCHEMISTRY | CR count: 34 → 34

Field	Status	Orig. pos	Norm. pos	Orig. line	Norm. line	Original cited/source value	Normalised cited/source value
CR	replaced	6	6	113253	113252	Biochemical Pharmacology CHU F S, 1974, Biochemical Pharmacology, V23, P1105, DOI 10.1016/0006-2952(74)90011-2	BIOCHEM PHARMACOL CHU F S, 1974, BIOCHEM PHARMACOL, V23, P1105, DOI 10.1016/0006-2952(74)90011-2
CR	replaced	10	10	113257	113256	PFLUGERS ARCH GEKL M, 1993, PFLUGERS ARCH, V422, pR126	PFLUG ARCH EUR J PHY GEKL M, 1993, PFLUG ARCH EUR J PHY, V422, pR126

Record 828 | WOS:A1994NV49900013

EFFECT OF MYCOTOXINS ON IN-VITRO MOVEMENT OF TRACHEAL CILIA FROM ONE-DAY-OLD CHICKS

SO original: FOLIA MICROBIOLOGICA | SO normalised: FOLIA MICROBIOLOGICA | CR count: 42 → 42

No journal-related changes detected in SO, J9, JI or CR for this record.

Record 829 | WOS:A1993MU47600008

EVALUATION OF THE DEVELOPMENTAL TOXICITY OF CITRININ USING HYDRA-ATTENUATA AND POSTIMPLANTATION RAT WHOLE-EMBRYO CULTURE

SO original: TOXICOLOGY | SO normalised: TOXICOLOGY | CR count: 56 → 56

Field	Status	Orig. pos	Norm. pos	Orig. line	Norm. line	Original cited/source value	Normalised cited/source value
CR	replaced	42	42	113460	113459	IARC Sci Publ<br>Petkova-Bocharova T, 1991, IARC Sci Publ, P83	IARC SCI PUBL<br>Petkova-Bocharova T, 1991, IARC SCI PUBL, P83
CR	replaced	53	53	113471	113470	Teratology<br>WANG Y-H, 1991, Teratology, V43, P463	TERATOLOGY<br>WANG Y-H, 1991, TERATOLOGY, V43, P463

Record 830 | WOS:A1993MM43500024

POSSIBLE ROLE OF AN IRON-OXYGEN COMPLEX IN 4(S)-4-HYDROXYOCHRATOXIN-A FORMATION BY RAT-LIVER MICROSOMES

SO original: BIOCHEMICAL PHARMACOLOGY | SO normalised: BIOCHEMICAL PHARMACOLOGY | CR count: 39 → 39

No journal-related changes detected in SO, J9, JI or CR for this record.

Record 831 | WOS:A1993MQ49500006

OCHRATOXIN-A IMPAIRS POSTPROXIMAL NEPHRON FUNCTION IN-VIVO AND BLOCKS PLASMA-MEMBRANE ANION CONDUCTANCE IN MADIN-DARBY CANINE KIDNEY-CELLS IN-VITRO

SO original: PFLUGERS ARCHIV-EUROPEAN JOURNAL OF PHYSIOLOGY | SO normalised: PFLUGERS ARCHIV-EUROPEAN JOURNAL OF PHYSIOLOGY | CR count: 32 → 32

Field	Status	Orig. pos	Norm. pos	Orig. line	Norm. line	Original cited/source value	Normalised cited/source value
CR	replaced	7	7	113615	113614	PFLUGERS ARCH<br>GEKL M, 1993, PFLUGERS ARCH, V422, pR126	PFLUG ARCH EUR J PHY<br>GEKL M, 1993, PFLUG ARCH EUR J PHY, V422, pR126

Record 832 | WOS:A1993LR82100028

EVALUATION AND APPLICATION OF A BIOLUMINESCENT BACTERIAL GENOTOXICITY TEST

SO original: JOURNAL OF AOAC INTERNATIONAL | SO normalised: JOURNAL OF AOAC INTERNATIONAL | CR count: 24 → 24

No journal-related changes detected in SO, J9, JI or CR for this record.

Record 833 | WOS:A1993LN86000012

COMPARISON OF IN-VITRO AND IN-VIVO BIOLOGICAL-ACTIVITY OF MYCOTOXINS

SO original: TOXICON | SO normalised: TOXICON | CR count: 40 → 40

Field	Status	Orig. pos	Norm. pos	Orig. line	Norm. line	Original cited/source value	Normalised cited/source value
CR	replaced	16	16	113774	113773	Adv Appl Microbiol<br>Hidy P H, 1977, Adv Appl Microbiol, V22, P59, DOI 10.1016/S0065-2164(08)70160-6	ADV APPL MICROBIOL<br>Hidy P H, 1977, ADV APPL MICROBIOL, V22, P59, DOI 10.1016/S0065-2164(08)70160-6
CR	replaced	17	17	113775	113774	S Afr Med J<br>Holzapfel C W, 1966, S Afr Med J, V40, P1100	S AFR MED J<br>Holzapfel C W, 1966, S AFR MED J, V40, P1100
CR	replaced	23	23	113781	113780	Annales de Recherches Veterinaires<br>More J., 1974, Annales de Recherches Veterinaires, V5, P167	ANN RECH VET<br>More J., 1974, ANN RECH VET, V5, P167
CR	replaced	30	30	113788	113787	Theriogenology<br>STRINGFELLOW D A, 1991, Theriogenology, V35, P275, DOI 10.1016/0093-691X(91)90251-8	THERIOGENOLOGY<br>STRINGFELLOW D A, 1991, THERIOGENOLOGY, V35, P275, DOI 10.1016/0093-691X(91)90251-8

Record 834 | WOS:A1993LJ01500043

EXTRACELLULAR ACIDOSIS AMELIORATES METABOLIC-INHIBITOR-INDUCED AND POTENTIATES OXIDANT-INDUCED CELL-DEATH IN RENAL PROXIMAL TUBULES

SO original: JOURNAL OF PHARMACOLOGY AND EXPERIMENTAL THERAPEUTICS | SO normalised: JOURNAL OF PHARMACOLOGY AND EXPERIMENTAL THERAPEUTICS | CR count: 32 → 32

No journal-related changes detected in SO, J9, JI or CR for this record.

Record 835 | WOS:A1993KQ30600025

IMMUNOTOXICITY OF MYCOTOXINS

SO original: JOURNAL OF DAIRY SCIENCE | SO normalised: JOURNAL OF DAIRY SCIENCE | CR count: 50 → 50

Field	Status	Orig. pos	Norm. pos	Orig. line	Norm. line	Original cited/source value	Normalised cited/source value
CR	replaced	29	29	113945	113944	Avian Dis<br>Pier A C, 1970, Avian Dis, V14, P797, DOI 10.2307/1588651	AVIAN DIS<br>Pier A C, 1970, AVIAN DIS, V14, P797, DOI 10.2307/1588651
CR	replaced	44	44	113960	113959	Mycotoxin Res<br>Taylor M J, 1985, Mycotoxin Res, V1, P57, DOI 10.1007/BF03192004	MYCOTOXIN RES<br>Taylor M J, 1985, MYCOTOXIN RES, V1, P57, DOI 10.1007/BF03192004

Record 836 | WOS:A1992JU40900011

HYDROLYSIS OF OCHRATOXIN-A BY THE MICROBIAL ACTIVITY OF DIGESTA IN THE GASTROINTESTINAL-TRACT OF RATS

SO original: ARCHIVES OF ENVIRONMENTAL CONTAMINATION AND TOXICOLOGY | SO normalised: ARCHIVES OF ENVIRONMENTAL CONTAMINATION AND TOXICOLOGY | CR count: 28 → 28

No journal-related changes detected in SO, J9, JI or CR for this record.

Record 837 | WOS:A1992KT90400023

ALTERATIONS IN ATP-DEPENDENT CALCIUM-UPTAKE BY RAT RENAL-CORTEX MICROSOMES FOLLOWING OCHRATOXIN A ADMINISTRATION INVIVO OR ADDITION INVITRO

SO original: BIOCHEMICAL PHARMACOLOGY | SO normalised: BIOCHEMICAL PHARMACOLOGY | CR count: 50 → 50

No journal-related changes detected in SO, J9, JI or CR for this record.

Record 838 | WOS:A1992HP59700018

INVITRO EFFECTS OF THE NEPHROTOXINS OCHRATOXIN-A AND CITRININ UPON BIOCHEMICAL FUNCTION OF PORCINE KIDNEY

SO original: ARCHIVES OF ENVIRONMENTAL CONTAMINATION AND TOXICOLOGY | SO normalised: ARCHIVES OF ENVIRONMENTAL CONTAMINATION AND TOXICOLOGY | CR count: 58 → 58

No journal-related changes detected in SO, J9, JI or CR for this record.

Record 839 | WOS:A1991GJ33400005

ROLE OF CYTOCHROME-P-450 IN OCHRATOXIN-A STIMULATED LIPID-PEROXIDATION

SO original: JOURNAL OF BIOCHEMICAL TOXICOLOGY | SO normalised: JOURNAL OF BIOCHEMICAL TOXICOLOGY | CR count: 23 → 23

No journal-related changes detected in SO, J9, JI or CR for this record.

Record 840 | WOS:A1991GJ33400006

INVITRO INHIBITION OF RAT PLATELET-AGGREGATION BY OCHRATOXIN-A

SO original: JOURNAL OF BIOCHEMICAL TOXICOLOGY | SO normalised: JOURNAL OF BIOCHEMICAL TOXICOLOGY | CR count: 23 → 23

No journal-related changes detected in SO, J9, JI or CR for this record.

Record 841 | WOS:A1991GA29400009

CHARACTERIZATION OF PIG-LIVER PURIFIED CYTOCHROME-P-450 ISOENZYMES FOR OCHRATOXIN-A METABOLISM STUDIES

SO original: TOXICOLOGY LETTERS | SO normalised: TOXICOLOGY LETTERS | CR count: 37 → 37

No journal-related changes detected in SO, J9, JI or CR for this record.

Record 842 | WOS:A1991FC20700661

STIMULATION OF RENAL ENDOPLASMIC-RETICULUM CALCIUM-UPTAKE BY OCHRATOXIN-A

SO original: FASEB JOURNAL | SO normalised: FASEB JOURNAL | CR count: 0 → 0

No journal-related changes detected in SO, J9, JI or CR for this record.

Record 843 | WOS:A1991FR33200006

INVIVO ABSORPTION OF C-14 GLUCOSE AND C-14 GLYCINE BY THE RAT INTESTINE DURING OCHRATOXIN A TOXICOSIS

SO original: BIOCHEMISTRY INTERNATIONAL | SO normalised: BIOCHEMISTRY INTERNATIONAL | CR count: 25 → 25

No journal-related changes detected in SO, J9, JI or CR for this record.

Record 844 | WOS:A1991BW81R00026

MECHANISM OF ACTION OF OCHRATOXIN-A

SO original: MYCOTOXINS, ENDEMIC NEPHROPATHY AND URINARY TRACT TUMOURS | SO normalised: MYCOTOXINS, ENDEMIC NEPHROPATHY AND URINARY TRACT TUMOURS | CR count: 0 → 0

No journal-related changes detected in SO, J9, JI or CR for this record.

Record 845 | WOS:A1991BW81R00032

DNA ADDUCT FORMATION IN MICE TREATED WITH OCHRATOXIN-A

SO original: MYCOTOXINS, ENDEMIC NEPHROPATHY AND URINARY TRACT TUMOURS | SO normalised: MYCOTOXINS, ENDEMIC NEPHROPATHY AND URINARY TRACT TUMOURS | CR count: 0 → 0

No journal-related changes detected in SO, J9, JI or CR for this record.

Record 846 | WOS:A1990EP18900002

EFFECTS OF OCHRATOXIN-A AND OCHRATOXIN-B ON PRECHONDROGENIC MESENCHYMAL CELLS FROM CHICK-EMBRYO LIMB BUDS

SO original: TOXICOLOGY LETTERS | SO normalised: TOXICOLOGY LETTERS | CR count: 19 → 19

No journal-related changes detected in SO, J9, JI or CR for this record.

Record 847 | WOS:A1990EF19400012

NADPH-CYTOCHROME-P-450 REDUCTASE PROMOTED HYDROXYL RADICAL PRODUCTION BY THE IRON(III)-OCHRATOXIN-A COMPLEX

SO original: BIOCHIMICA ET BIOPHYSICA ACTA | SO normalised: BIOCHIMICA ET BIOPHYSICA ACTA | CR count: 18 → 18

No journal-related changes detected in SO, J9, JI or CR for this record.

Record 848 | WOS:A1990DV94600004

INDUCTION OF CHARACTERISTIC CHROMOSOMAL-ABERRATIONS, PARTICULARLY X-TRISOMY, IN CULTURED HUMAN-LYMPHOCYTES TREATED BY OCHRATOXIN-A, A MYCOTOXIN IMPLICATED IN BALKAN ENDEMIC NEPHROPATHY

SO original: MUTATION RESEARCH | SO normalised: MUTATION RESEARCH | CR count: 18 → 18

No journal-related changes detected in SO, J9, JI or CR for this record.

Record 849 | WOS:A1990CF01500012

EFFECTS OF FUNGAL METABOLITES ON TESTOSTERONE SECRETION INVITRO

SO original: ARCHIVES OF TOXICOLOGY | SO normalised: ARCHIVES OF TOXICOLOGY | CR count: 22 → 22

Field	Status	Orig. pos	Norm. pos	Orig. line	Norm. line	Original cited/source value	Normalised cited/source value
CR	replaced	19	19	114931	114930	Mycotoxins in Human and Animal Health McLaughlin C.S., 1977, Mycotoxins in Human and Animal Health, Park Forest South Pathotox, P263	Mycotoxins in human and animal health McLaughlin C.S., 1977, Mycotoxins in human and animal health, Park Forest South Pathotox, P263

Record 850 | WOS:A1990CJ12700002

ENSILING AS A MEANS OF REDUCING OCHRATOXIN-A CONCENTRATIONS IN CONTAMINATED BARLEY

SO original: JOURNAL OF THE SCIENCE OF FOOD AND AGRICULTURE | SO normalised: JOURNAL OF THE SCIENCE OF FOOD AND AGRICULTURE | CR count: 36 → 36

No journal-related changes detected in SO, J9, JI or CR for this record.

Record 851 | WOS:A1989CA57100015

INFLUENCE OF DIETARY CHARCOAL ON OCHRATOXIN-A TOXICITY IN LEGHORN CHICKS

SO original: CANADIAN JOURNAL OF VETERINARY RESEARCH-REVUE CANADIENNE DE RECHERCHE ; VETERINAIRE | SO normalised: CANADIAN JOURNAL OF VETERINARY RESEARCH-REVUE CANADIENNE DE RECHERCHE ; VETERINAIRE | CR count: 26 → 26

No journal-related changes detected in SO, J9, JI or CR for this record.

Record 852 | WOS:A1989AT97300002

NEPHROTOXICITY ASSESSMENT BY MEASURING CELLULAR ATP CONTENT .2. INTRANEPHRON SITE OF OCHRATOXIN-A NEPHROTOXICITY

SO original: TOXICOLOGY AND APPLIED PHARMACOLOGY | SO normalised: TOXICOLOGY AND APPLIED PHARMACOLOGY | CR count: 30 → 30

No journal-related changes detected in SO, J9, JI or CR for this record.

Record 853 | WOS:A1989CB27000014

MECHANISM OF OCHRATOXIN A-INDUCED IMMUNOSUPPRESSION

SO original: MYCOPATHOLOGIA | SO normalised: MYCOPATHOLOGIA | CR count: 21 → 21

No journal-related changes detected in SO, J9, JI or CR for this record.

Record 854 | WOS:A1989T676500015

THE EFFECTS OF OCHRATOXIN-A ON POSTIMPLANTATION RAT EMBRYOS IN CULTURE

SO original: ARCHIVES OF ENVIRONMENTAL CONTAMINATION AND TOXICOLOGY | SO normalised: ARCHIVES OF ENVIRONMENTAL CONTAMINATION AND TOXICOLOGY | CR count: 25 → 25

Field	Status	Orig. pos	Norm. pos	Orig. line	Norm. line	Original cited/source value	Normalised cited/source value
CR	replaced	6	6	115271	115270	Teratology<br>GILANI S H, 1975, Teratology, V11, p18A	TERATOLOGY<br>GILANI S H, 1975, TERATOLOGY, V11, p18A
CR	replaced	20	20	115285	115284	Annales de Recherches Veterinaires<br>More J., 1974, Annales de Recherches Veterinaires, V5, P167	ANN RECH VET<br>More J., 1974, ANN RECH VET, V5, P167

Record 855 | WOS:A1989T320900022

INFLUENCE OF OCHRATOXIN B ON THE OCHRATOXIN-A INHIBITION OF PHENYLALANYL-TRANSFER RNA FORMATION INVITRO AND PROTEIN-SYNTHESIS IN HEPATOMA TISSUE-CULTURE CELLS

SO original: TOXICOLOGY LETTERS | SO normalised: TOXICOLOGY LETTERS | CR count: 14 → 14

No journal-related changes detected in SO, J9, JI or CR for this record.

Record 856 | WOS:A1989R524600010

PERTURBATION OF LIVER MICROSOMAL CALCIUM HOMEOSTASIS BY OCHRATOXIN-A

SO original: BIOCHEMICAL PHARMACOLOGY | SO normalised: BIOCHEMICAL PHARMACOLOGY | CR count: 42 → 42

Field	Status	Orig. pos	Norm. pos	Orig. line	Norm. line	Original cited/source value	Normalised cited/source value
CR	replaced	17	17	115429	115428	Journal of Biochemical Toxicology<br>KHAN S, 1986, Journal of Biochemical Toxicology, V1,	J BIOCHEM TOXICOL<br>KHAN S, 1986, J BIOCHEM TOXICOL, V1, P31, DOI 10.1002/jbt.2570010404

						P31, DOI 10.1002/jbt.2570010404	

Record 857 | WOS:A1988R353500008

LIPID-PEROXIDATION AS A POSSIBLE CAUSE OF OCHRATOXIN A TOXICITY

SO original: BIOCHEMICAL PHARMACOLOGY | SO normalised: BIOCHEMICAL PHARMACOLOGY | CR count: 45 → 45

No journal-related changes detected in SO, J9, JI or CR for this record.

Record 858 | WOS:A1988P563200002

INDUCTION OF SOS FUNCTION IN ESCHERICHIA-COLI BY SOME MYCOTOXINS

SO original: TOXICITY ASSESSMENT | SO normalised: TOXICITY ASSESSMENT | CR count: 0 → 0

No journal-related changes detected in SO, J9, JI or CR for this record.

Record 859 | WOS:A1988R574700010

OCHRATOXIN-A AS A SUPPRESSOR OF MITOGEN-INDUCED BLASTOGENESIS OF PORCINE BLOOD-LYMPHOCYTES

SO original: ACTA VETERINARIA SCANDINAVICA | SO normalised: ACTA VETERINARIA SCANDINAVICA | CR count: 17 → 17

No journal-related changes detected in SO, J9, JI or CR for this record.

Record 860 | WOS:A1987L417900004

PROTEIN-BINDING POTENTIAL OF OCHRATOXIN-A INVITRO AND ITS FLUORESCENCE ENHANCEMENT

SO original: JOURNAL OF THE FOOD HYGIENIC SOCIETY OF JAPAN | SO normalised: JOURNAL OF THE FOOD HYGIENIC SOCIETY OF JAPAN | CR count: 0 → 0

No journal-related changes detected in SO, J9, JI or CR for this record.

Record 861 | WOS:A1987K378200008

GENOTOXIC ACTIVITY OF SOME MYCOTOXINS USING THE SOS CHROMOTEST

SO original: MYCOPATHOLOGIA | SO normalised: MYCOPATHOLOGIA | CR count: 30 → 30

No journal-related changes detected in SO, J9, JI or CR for this record.

Record 862 | WOS:A1987G499300004

CARCINOGENICITY AND GENOTOXICITY OF MYCOTOXINS

SO original: NAHRUNG-FOOD | SO normalised: NAHRUNG-FOOD | CR count: 55 → 55

Field	Status	Orig. pos	Norm. pos	Orig. line	Norm. line	Original cited/source value	Normalised cited/source value
CR	replaced	5	5	115791	115790	J Appl Toxicol<br>Becci P J, 1981, J Appl Toxicol, V1, P256, DOI 10.1002/jat.2550010504	J APPL TOXICOL<br>Becci P J, 1981, J APPL TOXICOL, V1, P256, DOI 10.1002/jat.2550010504
CR	replaced	8	8	115794	115793	Adv Appl Microbiol<br>Chu F S, 1977, Adv Appl Microbiol, V22, P83, DOI 10.1016/S0065-2164(08)70161-8	ADV APPL MICROBIOL<br>Chu F S, 1977, ADV APPL MICROBIOL, V22, P83, DOI 10.1016/S0065-2164(08)70161-8

Record 863 | WOS:A1986AYL5800018

INTERSTRAIN COMPARISON OF HEPATIC AND RENAL MICROSOMAL CARCINOGEN METABOLISM AND LIVER S9-MEDIATED MUTAGENICITY IN DA AND LEWIS RATS PHENOTYPED AS POOR AND EXTENSIVE METABOLIZERS OF DEBRISOQUINE

SO original: DRUG METABOLISM AND DISPOSITION | SO normalised: DRUG METABOLISM AND DISPOSITION | CR count: 48 → 48

No journal-related changes detected in SO, J9, JI or CR for this record.

Record 864 | WOS:A1985AWD1800005

EVALUATION OF OCHRATOXIN-A FOR MUTAGENICITY IN A BATTERY OF BACTERIAL AND MAMMALIAN-CELL ASSAYS

SO original: FOOD AND CHEMICAL TOXICOLOGY | SO normalised: FOOD AND CHEMICAL TOXICOLOGY | CR count: 33 → 33

No journal-related changes detected in SO, J9, JI or CR for this record.

Record 865 | WOS:A1985A019900016

GENOTOXICITY OF OCHRATOXIN-A IN MICE - DNA SINGLE-STRAND BREAKS EVALUATION IN SPLEEN, KIDNEY AND LIVER

SO original: COMPTES RENDUS DES SEANCES DE LA SOCIETE DE BIOLOGIE ET DE SES FILIALES | SO normalised: COMPTES RENDUS DES SEANCES DE LA SOCIETE DE BIOLOGIE ET DE SES FILIALES | CR count: 25 → 25

Field	Status	Orig. pos	Norm. pos	Orig. line	Norm. line	Original cited/source value	Normalised cited/source value
CR	replaced	6	6	116094	116093	Annales de Recherches Veterinaires<br>Galtier P., 1974, Annales de Recherches Veterinaires, V5, P319	ANN RECH VET<br>Galtier P., 1974, ANN RECH VET, V5, P319
CR	replaced	7	7	116095	116094	MYCOTOXINS<br>HARVIG J, 1974, MYCOTOXINS, P345	Mycotoxins<br>HARVIG J, 1974, Mycotoxins, P345

Record 866 | WOS:A1985AUK4000005

GENOTOXICITY OF OCHRATOXIN-A IN MICE - DNA SINGLE-STRAND BREAK EVALUATION IN SPLEEN, LIVER AND KIDNEY

SO original: TOXICOLOGY LETTERS | SO normalised: TOXICOLOGY LETTERS | CR count: 28 → 28

Field	Status	Orig. pos	Norm. pos	Orig. line	Norm. line	Original cited/source value	Normalised cited/source value
CR	replaced	7	7	116172	116171	Annales de Recherches Veterinaires<br>Galtier P., 1974, Annales de Recherches Veterinaires, V5, P319	ANN RECH VET<br>Galtier P., 1974, ANN RECH VET, V5, P319
CR	replaced	8	8	116173	116172	MYCOTOXINS<br>HARVIG J, 1974, MYCOTOXINS, P345	Mycotoxins<br>HARVIG J, 1974, Mycotoxins, P345

Record 867 | WOS:A1985ASM7700004

EFFECT OF OCHRATOXIN A ON RAT-LIVER MITOCHONDRIAL RESPIRATION AND OXIDATIVE-PHOSPHORYLATION

SO original: TOXICOLOGY | SO normalised: TOXICOLOGY | CR count: 26 → 26

Field	Status	Orig. pos	Norm. pos	Orig. line	Norm. line	Original cited/source value	Normalised cited/source value
CR	replaced	8	8	116249	116248	METHODS ENZYMOL<br>Johnson D., 1967, METHODS ENZYMOL, V10, P94, DOI DOI 10.1016/0076-6879(67)10018-9	Methods Enzymol<br>Johnson D., 1967, Methods Enzymol, V10, P94, DOI DOI 10.1016/0076-6879(67)10018-9
CR	replaced	9	9	116250	116249	METHODS ENZYMOL<br>King T.E., 1967, METHODS ENZYMOL, V10, P216	Methods Enzymol<br>King T.E., 1967, Methods Enzymol, V10, P216
CR	replaced	18	18	116259	116258	METHODS ENZYMOL<br>Slater E.C., 1967, METHODS ENZYMOL, V10, P48	Methods Enzymol<br>Slater E.C., 1967, Methods Enzymol, V10, P48

Record 868 | WOS:A1984SW21300010

EFFECTS OF FUNGAL TOXINS ON RENAL SLICE CALCIUM BALANCE

SO original: TOXICOLOGY AND APPLIED PHARMACOLOGY | SO normalised: TOXICOLOGY AND APPLIED PHARMACOLOGY | CR count: 27 → 27

No journal-related changes detected in SO, J9, JI or CR for this record.

Record 869 | WOS:A1984TF49500004

EFFECTS OF SOME MYCOTOXINS ON MITOGEN-INDUCED BLASTOGENESIS AND SCE FREQUENCY IN HUMAN-LYMPHOCYTES

SO original: FOOD AND CHEMICAL TOXICOLOGY | SO normalised: FOOD AND CHEMICAL TOXICOLOGY | CR count: 32 → 32

Field	Status	Orig. pos	Norm. pos	Orig. line	Norm. line	Original cited/source value	Normalised cited/source value
CR	replaced	17	17	116397	116396	Toxicon<br>PRIOR M G, 1979, Toxicon, V17, P146	TOXICON<br>PRIOR M G, 1979, TOXICON, V17, P146

Record 870 | WOS:A1984SZ35800008

CONVERSION OF OCHRATOXIN-C INTO OCHRATOXIN-A INVIVO

SO original: APPLIED AND ENVIRONMENTAL MICROBIOLOGY | SO normalised: APPLIED AND ENVIRONMENTAL MICROBIOLOGY | CR count: 9 → 9

Field	Status	Orig. pos	Norm. pos	Orig. line	Norm. line	Original cited/source value	Normalised cited/source value
CR	replaced	3	3	116462	116461	Annales de Recherches Veterinaires<br>GALTIER P, 1976, Annales de Recherches Veterinaires, V7, P91	ANN RECH VET<br>GALTIER P, 1976, ANN RECH VET, V7, P91

Record 871 | WOS:A1984SX17000033

GENOTOXICITY OF A VARIETY OF MYCOTOXINS IN THE HEPATOCYTE PRIMARY CULTURE DNA-REPAIR TEST USING RAT AND MOUSE HEPATOCYTES

SO original: CANCER RESEARCH | SO normalised: CANCER RESEARCH | CR count: 42 → 42

Field	Status	Orig. pos	Norm. pos	Orig. line	Norm. line	Original cited/source value	Normalised cited/source value
CR	replaced	28	28	116553	116552	Journal of Toxicological Sciences<br>UENO Y, 1980, Journal of Toxicological Sciences, V5, P295, DOI 10.2131/jts.5.295	J TOXICOL SCI<br>UENO Y, 1980, J TOXICOL SCI, V5, P295, DOI 10.2131/jts.5.295

Record 872 | WOS:A1983RZ78000009

EFFECTS OF OCHRATOXIN A METABOLITES ON YEAST PHENYLALANYL-TRNA SYNTHETASE AND ON THE GROWTH AND INVIVO PROTEIN-SYNTHESIS OF HEPATOMA-CELLS

SO original: CHEMICO-BIOLOGICAL INTERACTIONS | SO normalised: CHEMICO-BIOLOGICAL INTERACTIONS | CR count: 19 → 19

No journal-related changes detected in SO, J9, JI or CR for this record.

Record 873 | WOS:A1982NN76100006

PREVENTION OF MOLD GROWTH AND TOXIN PRODUCTION THROUGH CONTROL OF ENVIRONMENTAL-CONDITIONS

SO original: JOURNAL OF FOOD PROTECTION | SO normalised: JOURNAL OF FOOD PROTECTION | CR count: 55 → 55

Field	Status	Orig. pos	Norm. pos	Orig. line	Norm. line	Original cited/source value	Normalised cited/source value
CR	replaced	20	20	116701	116700	Mycopathologia<br>Hitokoto H., 1978,	MYCOPATHOLOGIA<br>Hitokoto H., 1978,

						Mycopathologia, V66, P161, DOI 10.1007/BF00683965	MYCOPATHOLOGIA, V66, P161, DOI 10.1007/BF00683965
CR	replaced	21	21	116702	116701	Fleischwirtschaft Incze K., 1976, Fleischwirtschaft, V56, P219	FLEISCHWIRTSCHAFT Incze K., 1976, FLEISCHWIRTSCHAFT, V56, P219
CR	replaced	54	54	116735	116734	Feedstuffs Young J. C., 1979, Feedstuffs, V51, P23	FEEDSTUFFS Young J. C., 1979, FEEDSTUFFS, V51, P23

Record 874 | WOS:A1980KP16100001

MEASURING OF A DOUBLE CONTAMINATION BY PATULIN AND OCHRATOXIN

SO original: ANNALES DE RECHERCHES VETERINAIRES | SO normalised: ANNALES DE RECHERCHES VETERINAIRES | CR count: 7 → 7

Field	Status	Orig. pos	Norm. pos	Orig. line	Norm. line	Original cited/source value	Normalised cited/source value
CR	replaced	2	2	116776	116775	Annales de Recherches Veterinaires ESCOULA L, 1975, Annales de Recherches Veterinaires, V6, P155	ANN RECH VET ESCOULA L, 1975, ANN RECH VET, V6, P155
CR	replaced	3	3	116777	116776	Annales de Recherches Veterinaires ESCOULA L, 1973, Annales de Recherches Veterinaires, V4, P253	ANN RECH VET ESCOULA L, 1973, ANN RECH VET, V4, P253
CR	replaced	5	5	116779	116778	Annales de Recherches Veterinaires Galtier P., 1974, Annales de Recherches Veterinaires, V5, P155	ANN RECH VET Galtier P., 1974, ANN RECH VET, V5, P155

Record 875 | WOS:A1980KR30600008

EVIDENCE FOR INVITRO AND INVIVO INTERACTION BETWEEN OCHRATOXIN-A AND 3 ACIDIC DRUGS

SO original: FOOD AND COSMETICS TOXICOLOGY | SO normalised: FOOD AND COSMETICS TOXICOLOGY | CR count: 13 → 13

Field	Status	Orig. pos	Norm. pos	Orig. line	Norm. line	Original cited/source value	Normalised cited/source value
CR	replaced	4	4	116826	116825	Annales de Recherches Veterinaires GALTIER P, 1975, Annales de Recherches Veterinaires, V6, P207	ANN RECH VET GALTIER P, 1975, ANN RECH VET, V6, P207
CR	replaced	5	5	116827	116826	Annales de Recherches Veterinaires Galtier P., 1974, Annales de Recherches Veterinaires, V5, P155	ANN RECH VET Galtier P., 1974, ANN RECH VET, V5, P155
CR	replaced	6	6	116828	116827	Annales de Recherches Veterinaires Galtier P., 1974, Annales de	ANN RECH VET Galtier P., 1974, ANN RECH VET, V5, P233

						Recherches Veterinaires, V5, P233	
CR	replaced	7	7	116829	116828	Annales de Recherches Veterinaires<br>Galtier P., 1974, Annales de Recherches Veterinaires, V5, P311	ANN RECH VET<br>Galtier P., 1974, ANN RECH VET, V5, P311

Record 876 | WOS:A1979HH25500002

INVIVO AND INVITRO CHANGES IN RENAL-FUNCTION CAUSED BY OCHRATOXIN A IN THE RAT

SO original: TOXICOLOGY | SO normalised: TOXICOLOGY | CR count: 21 → 21

No journal-related changes detected in SO, J9, JI or CR for this record.

Record 877 | WOS:A1978GE26800006

OCHRATOXIN-A AND PROTEIN-UTILIZATION INVIVO

SO original: SWEDISH JOURNAL OF AGRICULTURAL RESEARCH | SO normalised: SWEDISH JOURNAL OF AGRICULTURAL RESEARCH | CR count: 0 → 0

No journal-related changes detected in SO, J9, JI or CR for this record.

Record 878 | WOS:A1977DR04200006

FATE OF OCHRATOXIN A IN RATS

SO original: FOOD AND COSMETICS TOXICOLOGY | SO normalised: FOOD AND COSMETICS TOXICOLOGY | CR count: 23 → 23

**Field**	**Status**	**Orig. pos**	**Norm. pos**	**Orig. line**	**Norm. line**	**Original cited/source value**	**Normalised cited/source value**
CR	replaced	1	1	116975	116974	Adv Appl Microbiol<br>Applegate K L, 1973, Adv Appl Microbiol, V16, P97, DOI 10.1016/S0065-2164(08)70024-8	ADV APPL MICROBIOL<br>Applegate K L, 1973, ADV APPL MICROBIOL, V16, P97, DOI 10.1016/S0065-2164(08)70024-8
CR	replaced	7	7	116981	116980	Biochemical Pharmacology<br>CHU F S, 1974, Biochemical Pharmacology, V23, P1105, DOI 10.1016/0006-2952(74)90011-2	BIOCHEM PHARMACOL<br>CHU F S, 1974, BIOCHEM PHARMACOL, V23, P1105, DOI 10.1016/0006-2952(74)90011-2

Record 879 | WOS:A1975AC00900024

INVITRO TOXICITY OF ANALOGS OF OCHRATOXIN-A IN MONKEY KIDNEY EPITHELIAL-CELLS

SO original: TOXICOLOGY AND APPLIED PHARMACOLOGY | SO normalised: TOXICOLOGY AND APPLIED PHARMACOLOGY | CR count: 13 → 13

**Field**	**Status**	**Orig. pos**	**Norm. pos**	**Orig. line**	**Norm. line**	**Original cited/source value**	**Normalised cited/source value**

CR	replaced	3	3	117045	117044	S Afr Med J Engelbrecht J C, 1969, S Afr Med J, V43, P524	S AFR MED J Engelbrecht J C, 1969, S AFR MED J, V43, P524

Record 880 | WOS:A1975BC07400010

STUDIES ON NEPHROTOXICITY OF OCHRATOXIN A IN RATS

SO original: TOXICOLOGY AND APPLIED PHARMACOLOGY | SO normalised: TOXICOLOGY AND APPLIED PHARMACOLOGY | CR count: 36 → 36

Field	Status	Orig. pos	Norm. pos	Orig. line	Norm. line	Original cited/source value	Normalised cited/source value
CR	replaced	13	13	117110	117109	S Afr Med J Engelbrecht J C, 1969, S Afr Med J, V43, P524	S AFR MED J Engelbrecht J C, 1969, S AFR MED J, V43, P524

Record 881 | WOS:A1972M753500008

COMPARATIVE RATES OF HYDROLYSIS OF OCHRATOXINS A AND B IN-VITRO

SO original: FOOD AND COSMETICS TOXICOLOGY | SO normalised: FOOD AND COSMETICS TOXICOLOGY | CR count: 12 → 12

No journal-related changes detected in SO, J9, JI or CR for this record.

Record 882 | WOS:A1971K640300003

TOXICITY OF 5-CHLORO-8-HYDROXY-3, 4-DIHYDRO-3-METHYL-ISOCOUMARIN-7-CARBOXYLIC ACID, A HYDROLYZATE OF OCHRATOXIN-A

SO original: JAPANESE JOURNAL OF MEDICAL SCIENCE & BIOLOGY | SO normalised: JAPANESE JOURNAL OF MEDICAL SCIENCE & BIOLOGY | CR count: 8 → 8

No journal-related changes detected in SO, J9, JI or CR for this record.

Record 883 | WOS:A19656230000050

OCHRATOXIN A A TOXIC METABOLITE PRODUCED BY ASPERGILLUS OCHRACEUS WILH

SO original: NATURE | SO normalised: NATURE | CR count: 4 → 4

No journal-related changes detected in SO, J9, JI or CR for this record.
