# Supplementary material for: Ochratoxin A Toxicity Research in In Vitro and In Vivo Experimental Settings: A Cross-Software Bibliometric and Science-Mapping Study
Source: Toxins (Basel). 2026 Aug 11;18(8):346. doi: 10.3390/toxins18080346 (PMC13517462; doi:10.3390/toxins18080346)
Supplement: Supplementary file 1 [file toxins-18-00346-s001.zip › Supplementary File S8.pdf]

Countries and institutions normalisation audit – line-by-line and article-by-article

Files compared: WoS 1-883_Original.txt vs WoS_1-883_Countries and Instititutions_Normalized.txt

Corrected country extraction: postal/state fragments are not treated as countries (e.g., “OH 45217 USA” → “USA”), and China variants such as “Peoples R China” are reported as “China”. Records are ordered from 1 to 883.

Field	Entries before	Entries after	Changed mapping pairs	Records with count mismatch
C1	2102	2102	1363	0
C3	2567	2567	12	0
RP	1044	1044	532	0

Record 1 | WOS:001628370900001

Dietary Quercetin Mitigates DON-Induced Intestinal Injury via Inhibiting MAPK/NF-Î°B-Mediated Pyroptosis and Tight Junction Disruption in Chicken

Field	Position	Original line	Normalised line	Original value	Normalised value	Original country / institution	Normalised country / institution
C1	1	21	21	[Guan, Chunwei; Mei, Xueyi; Sun, Shilong; Ding, Yuyang; Cai, Jingzeng] Northeast Agr Univ, Coll Vet Med, Harbin 150030, Peoples R China.	[Guan, Chunwei; Mei, Xueyi; Sun, Shilong; Ding, Yuyang; Cai, Jingzeng] Northeast Agricultural University, Coll Vet Med, Harbin 150030, China.	Country: China Institution: Northeast Agr Univ	Country: China Institution: Northeast Agricultural University
C1	2	22	22	[Cai, Jingzeng] Northeast Agr Univ, Key Lab Prov Educ Dept Heilongjiang Common Anim Di, Harbin 150030, Peoples R China.	[Cai, Jingzeng] Northeast Agricultural University, Key Lab Prov Educ Dept Heilongjiang Common Anim Di, Harbin 150030, China.	Country: China Institution: Northeast Agr Univ	Country: China Institution: Northeast Agricultural University
RP	1	25	25	Cai, JZ (corresponding author), Northeast Agr Univ, Coll Vet Med, Harbin 150030, Peoples R China	Cai, JZ (corresponding author), Northeast Agricultural University, Coll Vet Med, Harbin 150030, Peoples R China	Country: China Institution: Northeast Agr Univ	Country: China Institution: Northeast Agricultural University
RP	2	25	25	Cai, JZ (corresponding author), Northeast Agr Univ, Key Lab Prov Educ Dept Heilongjiang Common Anim Di, Harbin 150030, Peoples R China	Cai, JZ (corresponding author), Northeast Agr Univ, Key Lab Prov Educ Dept Heilongjiang Common Anim Di, Harbin 150030, China	Country: China Institution: Northeast Agr Univ	Country: China Institution: Northeast Agr Univ

Record 2 | WOS:001646793400001

Silent Saboteurs: Decoding Mycotoxins-From Chemistry and Prevalence to Health Risks, Detection, Management and Emerging Frontiers

Field	Position	Original line	Normalised line	Original value	Normalised value	Original country / institution	Normalised country / institution
C1	1	160	160	[Thambugala, Kasun M.; Dayananda, Dilakshini; Tennakoon, Samawansha; Harischandra, Hiruni; Jayatunga, Pamoda; de Silva, Nissanka; Dhanusha, Asanthi; Madusanka, Sahan] Univ Sri Jayewardenepura, Fac Appl Sci, Genet & Mol Biol Unit, Nugegoda 10250, Sri Lanka.	[Thambugala, Kasun M.; Dayananda, Dilakshini; Tennakoon, Samawansha; Harischandra, Hiruni; Jayatunga, Pamoda; de Silva, Nissanka; Dhanusha, Asanthi; Madusanka, Sahan] University Sri Jayewardenepura, Fac Appl Sci, Genet & Mol Biol Unit, Nugegoda 10250, Sri Lanka.	Country: Sri Lanka Institution: Univ Sri Jayewardenepura	Country: Sri Lanka Institution: University Sri Jayewardenepura
C1	2	161	161	[Thambugala, Kasun M.] Univ Sri Jayewardenepura, Ctr Plant	[Thambugala, Kasun M.] University Sri Jayewardenepura, Ctr Plant Mat	Country: Sri Lanka Institution: Univ Sri Jayewardenepura	Country: Sri Lanka Institution: University Sri Jayewardenepura

				Mat & Herbal Prod Res, Nugegoda 10250, Sri Lanka.	& Herbal Prod Res, Nugegoda 10250, Sri Lanka.		
C1	3	162	162	[Thambugala, Kasun M.; de Silva, Nissanka] Univ Sri Jayewardenepura, Ctr Biotechnol, Dept Zool, Nugegoda 10250, Sri Lanka.	[Thambugala, Kasun M.; de Silva, Nissanka] University Sri Jayewardenepura, Ctr Biotechnol, Dept Zool, Nugegoda 10250, Sri Lanka.	Country: Sri Lanka Institution: Univ Sri Jayewardenepura	Country: Sri Lanka Institution: University Sri Jayewardenepura
C1	4	163	163	[Daranagama, Dinushani A.] Univ Kelaniya, Fac Sci, Dept Plant & Mol Biol, Kelaniya 11300, Sri Lanka.	[Daranagama, Dinushani A.] University Kelaniya, Fac Sci, Dept Plant & Mol Biol, Kelaniya 11300, Sri Lanka.	Country: Sri Lanka Institution: Univ Kelaniya	Country: Sri Lanka Institution: University Kelaniya
C1	5	164	164	[Gonapaladeniya, Madhusha] Univ Sri Jayewardenepura, Fac Allied Hlth Sci, Dept Med Lab Sci, Nugegoda 10250, Sri Lanka.	[Gonapaladeniya, Madhusha] University Sri Jayewardenepura, Fac Allied Hlth Sci, Dept Med Lab Sci, Nugegoda 10250, Sri Lanka.	Country: Sri Lanka Institution: Univ Sri Jayewardenepura	Country: Sri Lanka Institution: University Sri Jayewardenepura
C1	6	165	165	[Haituk, Sukanya] Chiang Mai Univ, Off Res Adm, Chiang Mai 50200, Thailand.	[Haituk, Sukanya] Chiang Mai University, Off Res Adm, Chiang Mai 50200, Thailand.	Country: Thailand Institution: Chiang Mai Univ	Country: Thailand Institution: Chiang Mai University
C1	7	166	166	[Haituk, Sukanya; Cheewangkoon, Ratchadawan] Chiang Mai Univ, Fac Agr, Dept Entomol & Plant Pathol, Chiang Mai 50200, Thailand.	[Haituk, Sukanya; Cheewangkoon, Ratchadawan] Chiang Mai University, Fac Agr, Dept Entomol & Plant Pathol, Chiang Mai 50200, Thailand.	Country: Thailand Institution: Chiang Mai Univ	Country: Thailand Institution: Chiang Mai University
RP	1	170	170	Thambugala, KM (corresponding author), Univ Sri Jayewardenepura, Fac Appl Sci, Genet & Mol Biol Unit, Nugegoda 10250, Sri Lanka	Thambugala, KM (corresponding author), University Sri Jayewardenepura, Fac Appl Sci, Genet & Mol Biol Unit, Nugegoda 10250, Sri Lanka	Country: Sri Lanka Institution: Univ Sri Jayewardenepura	Country: Sri Lanka Institution: University Sri Jayewardenepura

Record 3 | WOS:001648209500001

Dose-Dependent but Non-Interactive Effects of Ochratoxin A and Selenomethionine on Hepatic Lipid Metabolism and Oxidative Stress in Broiler Chickens

No C1/C3/RP normalisation changes detected for this record.

Record 4 | WOS:001635022200001

Selective Cytotoxicity of Ochratoxin A: Pro-Apoptotic Effects on Healthy Immune Cells Compared to Leukemia Cells

Field	Position	Original line	Normalised line	Original value	Normalised value	Original country / institution	Normalised country / institution
C1	1	640	640	[Wieckowska, Magdalena; Janik-Karpinska, Edyta; Cichon, Natalia; Szelenberger, Rafal; Stela, Maksymilian; Podogrocki, Marcin; Gorniak, Leslaw; Bijak, Michal] Univ Lodz, Fac Biol & Environm Protect, Biohazard Prevent Ctr, Pomorska 141-143, PL-90236 Lodz, Poland.	[Wieckowska, Magdalena; Janik-Karpinska, Edyta; Cichon, Natalia; Szelenberger, Rafal; Stela, Maksymilian; Podogrocki, Marcin; Gorniak, Leslaw; Bijak, Michal] University of Lodz, Fac Biol & Environm Protect, Biohazard Prevent Ctr, Pomorska 141-143, PL-90236 Lodz, Poland.	Country: Poland Institution: Univ Lodz	Country: Poland Institution: University of Lodz
C1	2	641	641	[Synowiec, Ewelina; Sliwinski, Tomasz] Univ Lodz, Fac Biol & Environm Protect, Dept Mol Genet, Pomorska 141-143, PL-90236 Lodz, Poland.	[Synowiec, Ewelina; Sliwinski, Tomasz] University of Lodz, Fac Biol & Environm Protect, Dept Mol Genet, Pomorska 141-143, PL-90236 Lodz, Poland.	Country: Poland Institution: Univ Lodz	Country: Poland Institution: University of Lodz
C1	3	642	642	[Poplawski, Tomasz] Med Univ Lodz, Dept Microbiol & Pharmaceut Biochem,	[Poplawski, Tomasz] Medical University Lodz, Dept Microbiol & Pharmaceut Biochem,	Country: Poland Institution: Med Univ Lodz	Country: Poland Institution: Medical University Lodz

				Mazowiecka 5, PL-92215 Lodz, Poland.	Mazowiecka 5, PL-92215 Lodz, Poland.		
RP	1	646	646	Bijak, M (corresponding author), Univ Lodz, Fac Biol & Environm Protect, Biohazard Prevent Ctr, Pomorska 141-143, PL-90236 Lodz, Poland	Bijak, M (corresponding author), University of Lodz, Fac Biol & Environm Protect, Biohazard Prevent Ctr, Pomorska 141-143, PL-90236 Lodz, Poland	Country: Poland<br>Institution: Univ Lodz	Country: Poland<br>Institution: University of Lodz

Record 5 | WOS:001625561500001

Cytotoxic Effects Induced by Combined Exposure of the Patulin, Ochratoxin A, and Acetamiprid to HK-2 and SK-N-SH Cell Lines

Field	Position	Original line	Normalised line	Original value	Normalised value	Original country / institution	Normalised country / institution
C1	1	779	779	[Zhu, Zuoyin; Zhao, Hanke; Yang, Xianli; Nie, Dongxia; Yang, Junhua; Han, Zheng] Shanghai Acad Agr Sci, Inst Agrofood Stand & Testing Technol, Shanghai 201403, Peoples R China.	[Zhu, Zuoyin; Zhao, Hanke; Yang, Xianli; Nie, Dongxia; Yang, Junhua; Han, Zheng] Shanghai Academy of Agricultural Sciences, Inst Agrofood Stand & Testing Technol, Shanghai 201403, China.	Country: China<br>Institution: Shanghai Acad Agr Sci	Country: China<br>Institution: Shanghai Academy of Agricultural Sciences
RP	2	781	781	Han, Z (corresponding author), Shanghai Acad Agr Sci, Inst Agrofood Stand & Testing Technol, Shanghai 201403, Peoples R China	Han, Z (corresponding author), Shanghai Academy of Agricultural Sciences, Inst Agrofood Stand & Testing Technol, Shanghai 201403, China	Country: China<br>Institution: Shanghai Acad Agr Sci	Country: China<br>Institution: Shanghai Academy of Agricultural Sciences

Record 6 | WOS:001623564900001

Mycotoxins and the Intestinal Epithelium: From Barrier Injury to Stem Cell Dysfunction

Field	Position	Original line	Normalised line	Original value	Normalised value	Original country / institution	Normalised country / institution
C1	1	890	890	[Huo, Wenying; Qiao, Yingying; Wang, Cailing; Li, Ruiqing; Che, Long] Henan Univ Anim Husb & Econ, Coll Anim Sci & Technol, Zhengzhou 450046, Peoples R China.	[Huo, Wenying; Qiao, Yingying; Wang, Cailing; Li, Ruiqing; Che, Long] Henan Univ Anim Husb & Econ, Coll Anim Sci & Technol, Zhengzhou 450046, China.	Country: China<br>Institution: Henan Univ Anim Husb & Econ	Country: China<br>Institution: Henan Univ Anim Husb & Econ
C1	2	891	891	[He, Xiangru] Columbia Univ, Grad Sch Arts & Sci, Dept Stat, New York, NY 10032 USA.	[He, Xiangru] Columbia University, Grad Sch Arts & Sci, Dept Stat, New York, NY 10032 USA.	Country: USA<br>Institution: Columbia Univ	Country: USA<br>Institution: Columbia University
C1	3	892	892	[Li, Enkai] Washington Univ, Sch Med, Dept Mol Microbiol, St Louis, MO 63110 USA.	[Li, Enkai] University of Washington, Sch Med, Dept Mol Microbiol, St Louis, MO 63110 USA.	Country: USA<br>Institution: Washington Univ	Country: USA<br>Institution: University of Washington

Record 7 | WOS:001598251800001

Exploration of oxidative stress-mediated genetic toxicology modes of action using a pathway analysis, Connectivity Mapping, and transcriptional benchmark dosing-based framework

Field	Position	Original line	Normalised line	Original value	Normalised value	Original country / institution	Normalised country / institution
C1	1	1069	1069	[De Abrew, K. Nadira] Procter & Gamble Co, Ivorydale Innovat Ctr, Cincinnati, OH 45217 USA.	[De Abrew, K. Nadira] Procter & Gamble, Ivorydale Innovat Ctr, Cincinnati, OH 45217 USA.	Country: USA<br>Institution: Procter & Gamble Co	Country: USA<br>Institution: Procter & Gamble

C1	2	1070	1070	[Selman, Bastian G.; Shobair, Mahmoud; Zhang, Xiaoling; Allemang, Ashley J.; Pfuhler, Stefan] Procter & Gamble Co, Mason Business & Innovat Ctr, Mason, OH 45040 USA.	[Selman, Bastian G.; Shobair, Mahmoud; Zhang, Xiaoling; Allemang, Ashley J.; Pfuhler, Stefan] Procter & Gamble, Mason Business & Innovat Ctr, Mason, OH 45040 USA.	Country: USA Institution: Procter & Gamble Co	Country: USA Institution: Procter & Gamble

Record 8 | WOS:001591221900001

Prevention and detoxification of mycotoxins in food and feed: A review

Field	Position	Original line	Normalised line	Original value	Normalised value	Original country / institution	Normalised country / institution
C1	1	1226	1226	[Deliklitas, Kubra] Balikesir Univ, Inst Hlth Sci, Dept Vet Pharmacol & Toxicol, CoHE 100-2000 Scholarship Holder, Balikesir, Turkiye.	[Deliklitas, Kubra] Balikesir University, Inst Hlth Sci, Dept Vet Pharmacol & Toxicol, CoHE 100-2000 Scholarship Holder, Balikesir, Turkiye.	Country: Turkey Institution: Balikesir Univ	Country: Turkey Institution: Balikesir University
C1	2	1227	1227	[Gokbulut, Cengiz] Balikesir Univ, Fac Med, Dept Med Pharmacol, Balikesir, Turkiye.	[Gokbulut, Cengiz] Balikesir University, Fac Med, Dept Med Pharmacol, Balikesir, Turkey.	Country: Turkey Institution: Balikesir Univ	Country: Turkey Institution: Balikesir University
C1	3	1228	1228	[Gokbulut, Cengiz] Inst Hlth Sci, Dept Vet Pharmacol & Toxicol, Balikesir, Turkiye.	[Gokbulut, Cengiz] Inst Hlth Sci, Dept Vet Pharmacol & Toxicol, Balikesir, Turkey.	Country: Turkey Institution: Inst Hlth Sci	Country: Turkey Institution: Inst Hlth Sci
RP	1	1230	1230	Gokbulut, C (corresponding author), Balikesir Univ, Fac Med, Dept Med Pharmacol, Balikesir, Turkiye	Gokbulut, C (corresponding author), Balikesir University, Fac Med, Dept Med Pharmacol, Balikesir, Turkiye	Country: Turkey Institution: Balikesir Univ	Country: Turkey Institution: Balikesir University
RP	2	1230	1230	Gokbulut, C (corresponding author), Inst Hlth Sci, Dept Vet Pharmacol & Toxicol, Balikesir, Turkiye	Gokbulut, C (corresponding author), Inst Hlth Sci, Dept Vet Pharmacol & Toxicol, Balikesir, Turkey	Country: Turkey Institution: Inst Hlth Sci	Country: Turkey Institution: Inst Hlth Sci

Record 9 | WOS:001603917600001

Astaxanthin Alleviates Ochratoxin A (OTA)-Induced Spleen Dysfunction and Apoptosis in Broiler Chickens by Modulating the PTEN/PI3K/AKT Signaling Pathway

Field	Position	Original line	Normalised line	Original value	Normalised value	Original country / institution	Normalised country / institution
C1	1	1572	1572	[Cheng, Zhibi; Sang, Weilun; Li, Peng; Yang, Shuhua] Shenyang Agr Univ, Coll Anim Sci & Vet Med, Shenyang 110866, Peoples R China.	[Cheng, Zhibi; Sang, Weilun; Li, Peng; Yang, Shuhua] Shenyang Agricultural University, Coll Anim Sci & Vet Med, Shenyang 110866, China.	Country: China Institution: Shenyang Agr Univ	Country: China Institution: Shenyang Agricultural University
RP	2	1574	1574	Yang, SH (corresponding author), Shenyang Agr Univ, Coll Anim Sci & Vet Med, Shenyang 110866, Peoples R China	Yang, SH (corresponding author), Shenyang Agricultural University, Coll Anim Sci & Vet Med, Shenyang 110866, China	Country: China Institution: Shenyang Agr Univ	Country: China Institution: Shenyang Agricultural University

Record 10 | WOS:001593782400001

Mycotoxin Contamination: Occurrence, Biotransformation, Pathogenic Mechanisms, and Strategies for Nutritional Intervention

Field	Position	Original line	Normalised line	Original value	Normalised value	Original country / institution	Normalised country / institution
C1	1	1707	1707	[Yao, Chenyu; Ye, Mengyu; Wang, Cong; Zou, Lin; Zhang, Ximeng; Chai, Xin; Yu, Huijuan; Zhang, Chengyu; Wang, Yuefei]	[Yao, Chenyu; Ye, Mengyu; Wang, Cong; Zou, Lin; Zhang, Ximeng; Chai, Xin; Yu, Huijuan; Zhang, Chengyu; Wang, Yuefei]	Country: China Institution: Tianjin Univ Tradit Chinese Med	Country: China Institution: Tianjin University of Traditional Chinese Medicine

				Tianjin Univ Tradit Chinese Med, State Key Lab Chinese Med Modernizat, Tianjin Key Lab TCM Chem & Anal, Tianjin 301617, Peoples R China.	Tianjin University of Traditional Chinese Medicine, State Key Lab Chinese Med Modernizat, Tianjin Key Lab TCM Chem & Anal, Tianjin 301617, China.		
C1	2	1708	1708	[Chai, Xin; Yu, Huijuan; Zhang, Chengyu; Wang, Yuefei] Haihe Lab Modern Chinese Med, Tianjin 301617, Peoples R China.	[Chai, Xin; Yu, Huijuan; Zhang, Chengyu; Wang, Yuefei] Haihe Lab Modern Chinese Med, Tianjin 301617, China.	Country: China Institution: Haihe Lab Modern Chinese Med	Country: China Institution: Haihe Lab Modern Chinese Med
RP	2	1710	1710	Wang, YF (corresponding author), Tianjin Univ Tradit Chinese Med, State Key Lab Chinese Med Modernizat, Tianjin Key Lab TCM Chem & Anal, Tianjin 301617, Peoples R China	Wang, YF (corresponding author), Tianjin University of Traditional Chinese Medicine, State Key Lab Chinese Med Modernizat, Tianjin Key Lab TCM Chem & Anal, Tianjin 301617, Peoples R China	Country: China Institution: Tianjin Univ Tradit Chinese Med	Country: China Institution: Tianjin University of Traditional Chinese Medicine
RP	4	1710	1710	Wang, YF (corresponding author), Haihe Lab Modern Chinese Med, Tianjin 301617, Peoples R China	Wang, YF (corresponding author), Haihe Lab Modern Chinese Med, Tianjin 301617, China	Country: China Institution: Haihe Lab Modern Chinese Med	Country: China Institution: Haihe Lab Modern Chinese Med

Record 11 | WOS:001579727300001

Chlorogenic Acid and VX765 Alleviate Deoxynivalenol-Induced Enterohepatic Injury and Lipid Metabolism Disorders by Improving Intestinal Microecology

Field	Position	Original line	Normalised line	Original value	Normalised value	Original country / institution	Normalised country / institution
C1	1	1978	1978	[Wen, Tao; Li, Sirui; Guo, Huijun; Wang, Jinbo; Zhang, Xinru; Wang, Chunyang] Shandong Agr Univ, Coll Anim Vet Med, Shandong Prov Key Lab Zoonoses, Tai An 271018, Peoples R China.	[Wen, Tao; Li, Sirui; Guo, Huijun; Wang, Jinbo; Zhang, Xinru; Wang, Chunyang] Shandong Agricultural University, Coll Anim Vet Med, Shandong Prov Key Lab Zoonoses, Tai An 271018, China.	Country: China Institution: Shandong Agr Univ	Country: China Institution: Shandong Agricultural University
RP	1	1980	1980	Wang, CY (corresponding author), Shandong Agr Univ, Coll Anim Vet Med, Shandong Prov Key Lab Zoonoses, Tai An 271018, Peoples R China	Wang, CY (corresponding author), Shandong Agricultural University, Coll Anim Vet Med, Shandong Prov Key Lab Zoonoses, Tai An 271018, China	Country: China Institution: Shandong Agr Univ	Country: China Institution: Shandong Agricultural University

Record 12 | WOS:001573742900001

Fungal metabolite Ochratoxin A inhibits MrkD1P of multidrug-resistant Klebsiella pneumoniae: Integrated computational and in vitro validation

Field	Position	Original line	Normalised line	Original value	Normalised value	Original country / institution	Normalised country / institution
C1	1	2110	2110	[Roqunuzzaman, Md; Islam, Ariful; Supti, Sumaiya Jahan; Rifat, Mahbub Hasan; Islam, Mohammad Saiful; Ananna, Ummay Habiba; Tusher, Khalid Saifullah] Univ Rajshahi, Dept Genet Engn & Biotechnol, Rajshahi, Bangladesh.	[Roqunuzzaman, Md; Islam, Ariful; Supti, Sumaiya Jahan; Rifat, Mahbub Hasan; Islam, Mohammad Saiful; Ananna, Ummay Habiba; Tusher, Khalid Saifullah] University of Rajshahi, Dept Genet Engn & Biotechnol, Rajshahi, Bangladesh.	Country: Bangladesh Institution: Univ Rajshahi	Country: Bangladesh Institution: University of Rajshahi
C1	2	2111	2111	[Al-Mutairi, Aamal A.; Zaki, Magdi E. A.] Imam Mohammad Ibn Saud Islamic Univ IMSIU, Coll Sci, Dept Chem, Riyadh 11623, Saudi Arabia.	[Al-Mutairi, Aamal A.; Zaki, Magdi E. A.] Imam Mohammad Ibn Saud Islamic University (IMSIU), Coll Sci, Dept Chem, Riyadh 11623, Saudi Arabia.	Country: Saudi Arabia Institution: Imam Mohammad Ibn Saud Islamic Univ IMSIU	Country: Saudi Arabia Institution: Imam Mohammad Ibn Saud Islamic University (IMSIU)

C1	3	2112	2112	[Sarker, Subir; Hosen, Md. Eram] James Cook Univ, Coll Med & Dent, Biomed Sci & Mol Biol, Townsville, Qld 4811, Australia.	[Sarker, Subir; Hosen, Md. Eram] James Cook University, Coll Med & Dent, Biomed Sci & Mol Biol, Townsville, Qld 4811, Australia.	Country: Australia<br>Institution: James Cook Univ	Country: Australia<br>Institution: James Cook University
C1	4	2113	2113	[Hosen, Md. Eram] Univ Rajshahi, Shaheed Shamsuzzoha Inst Biosci, Dept Microbiol, Rajshahi, Bangladesh.	[Hosen, Md. Eram] University of Rajshahi, Shaheed Shamsuzzoha Inst Biosci, Dept Microbiol, Rajshahi, Bangladesh.	Country: Bangladesh<br>Institution: Univ Rajshahi	Country: Bangladesh<br>Institution: University of Rajshahi
RP	2	2116	2116	Hosen, ME (corresponding author), James Cook Univ, Coll Med & Dent, Biomed Sci & Mol Biol, Townsville, Qld 4811, Australia	Hosen, ME (corresponding author), James Cook University, Coll Med & Dent, Biomed Sci & Mol Biol, Townsville, Qld 4811, Australia	Country: Australia<br>Institution: James Cook Univ	Country: Australia<br>Institution: James Cook University

Record 13 | WOS:001581342800001

Biocatalytic Detoxification of Ochratoxins A/B by a Fungal Dye-Decolorizing Peroxidase: Mechanistic Insights and Toxicity Assessment

Field	Position	Original line	Normalised line	Original value	Normalised value	Original country / institution	Normalised country / institution
C1	1	2278	2278	[Xia, Wenjing; Zhu, Nianqing; Mei, Jie; Peng, Yueqin; Song, Fanglin; Zhou, Xue] Nanjing Normal Univ, Coll Chem & Biol Engn, Taizhou Coll, Taizhou 225300, Peoples R China.	[Xia, Wenjing; Zhu, Nianqing; Mei, Jie; Peng, Yueqin; Song, Fanglin; Zhou, Xue] Nanjing Normal University, Coll Chem & Biol Engn, Taizhou Coll, Taizhou 225300, China.	Country: China<br>Institution: Nanjing Normal Univ	Country: China<br>Institution: Nanjing Normal University
C1	2	2279	2279	[Zhu, Nianqing] Taizhou Univ, Coll Pharm & Chem & Chem Engn, Jiangsu Key Lab Chiral Pharmaceut Biosynth, Taizhou 225300, Peoples R China.	[Zhu, Nianqing] Taizhou Univ, Coll Pharm & Chem & Chem Engn, Jiangsu Key Lab Chiral Pharmaceut Biosynth, Taizhou 225300, China.	Country: China<br>Institution: Taizhou Univ	Country: China<br>Institution: Taizhou Univ
C1	3	2280	2280	[Ding, Shuai; Li, Fei] Wuhan Univ Sci & Technol, Sch Chem & Chem Engn, Dept Bioengn, Wuhan 430081, Peoples R China.	[Ding, Shuai; Li, Fei] Wuhan University of Science & Technology, Sch Chem & Chem Engn, Dept Bioengn, Wuhan 430081, China.	Country: China<br>Institution: Wuhan Univ Sci & Technol	Country: China<br>Institution: Wuhan University of Science & Technology
C3	2	2281	2281	Taizhou University - Jiangsu	Taizhou University	Institution: Taizhou University - Jiangsu	Institution: Taizhou University
RP	1	2283	2283	Zhou, X (corresponding author), Nanjing Normal Univ, Coll Chem & Biol Engn, Taizhou Coll, Taizhou 225300, Peoples R China	Zhou, X (corresponding author), Nanjing Normal University, Coll Chem & Biol Engn, Taizhou Coll, Taizhou 225300, China	Country: China<br>Institution: Nanjing Normal Univ	Country: China<br>Institution: Nanjing Normal University

Record 14 | WOS:001556476900001

Identification of an M20 Family Metallopeptidase with Amidohydrolase Activity from Alcaligenes faecalis HNGD-HTN06 and Simultaneous Degradation of Ochratoxin A, Aflatoxin B1, and Zearalenone

Field	Position	Original line	Normalised line	Original value	Normalised value	Original country / institution	Normalised country / institution
C1	1	2382	2382	[Hou, Minjie; Mei, Xuedong; Mwabulili, Fred; Li, Qian; Jia, Hang; Xie, Yanli] Henan Univ Technol, Coll Food Sci & Engn, Zhengzhou 450001, Peoples R China.	[Hou, Minjie; Mei, Xuedong; Mwabulili, Fred; Li, Qian; Jia, Hang; Xie, Yanli] Henan University of Science & Technology, Coll Food Sci & Engn, Zhengzhou 450001, China.	Country: China<br>Institution: Henan Univ Technol	Country: China<br>Institution: Henan University of Science & Technology

C1	2	2383	2383	[Hou, Minjie; Mei, Xuedong; Mwabulili, Fred; Li, Qian; Jia, Hang; Xie, Yanli] Henan Key Lab Cereal & Oil Food Safety & Nutr, Zhengzhou 450001, Peoples R China.	[Hou, Minjie; Mei, Xuedong; Mwabulili, Fred; Li, Qian; Jia, Hang; Xie, Yanli] Henan Key Lab Cereal & Oil Food Safety & Nutr, Zhengzhou 450001, China.	Country: China<br>Institution: Henan Key Lab Cereal & Oil Food Safety & Nutr	Country: China<br>Institution: Henan Key Lab Cereal & Oil Food Safety & Nutr
RP	1	2387	2387	Xie, YL (corresponding author), Henan Univ Technol, Coll Food Sci & Engn, Zhengzhou 450001, Peoples R China	Xie, YL (corresponding author), Henan University of Science & Technology, Coll Food Sci & Engn, Zhengzhou 450001, Peoples R China	Country: China<br>Institution: Henan Univ Technol	Country: China<br>Institution: Henan University of Science & Technology
RP	2	2387	2387	Xie, YL (corresponding author), Henan Key Lab Cereal & Oil Food Safety & Nutr, Zhengzhou 450001, Peoples R China	Xie, YL (corresponding author), Henan Key Lab Cereal & Oil Food Safety & Nutr, Zhengzhou 450001, China	Country: China<br>Institution: Henan Key Lab Cereal & Oil Food Safety & Nutr	Country: China<br>Institution: Henan Key Lab Cereal & Oil Food Safety & Nutr

Record 15 | WOS:001590628300001

Roles of microRNAs in multiple organs under mycotoxin stress by bioinformatic analysis microRNA regulation during mycotoxin exposure

Field	Position	Original line	Normalised line	Original value	Normalised value	Original country / institution	Normalised country / institution
C1	1	2514	2514	[Sohn, Eun Jung; Park, Kun-Taek] Inje Univ, Dept Biotechnol, 197 Inje Ro, Gimhae 50834, South Korea.	[Sohn, Eun Jung; Park, Kun-Taek] Inje University, Dept Biotechnol, 197 Inje Ro, Gimhae 50834, South Korea.	Country: South Korea<br>Institution: Inje Univ	Country: South Korea<br>Institution: Inje University
C1	2	2515	2515	[Sohn, Eun Jung] Kyungsung Univ, Dept SmartBio, Busan 48434, South Korea.	[Sohn, Eun Jung] Kyungsung University, Dept SmartBio, Busan 48434, South Korea.	Country: South Korea<br>Institution: Kyungsung Univ	Country: South Korea<br>Institution: Kyungsung University
C1	3	2516	2516	[Sohn, Eun Jung] Dongguk Univ, Dept Biopharmaceut Engn, WISE, Gyeongju 38066, South Korea.	[Sohn, Eun Jung] Dongguk University, Dept Biopharmaceut Engn, WISE, Gyeongju 38066, South Korea.	Country: South Korea<br>Institution: Dongguk Univ	Country: South Korea<br>Institution: Dongguk University
RP	1	2518	2518	Park, KT (corresponding author), Inje Univ, Dept Biotechnol, 197 Inje Ro, Gimhae 50834, South Korea	Park, KT (corresponding author), Inje University, Dept Biotechnol, 197 Inje Ro, Gimhae 50834, South Korea	Country: South Korea<br>Institution: Inje Univ	Country: South Korea<br>Institution: Inje University

Record 16 | WOS:001556296100001

The protective effect of Gentiana lutea flower against mycotoxin toxicity in a gastrointestinal barrier in vitro model

Field	Position	Original line	Normalised line	Original value	Normalised value	Original country / institution	Normalised country / institution
C1	1	2743	2743	[Di Matteo, Giacomo; Mannina, Luisa] Sapienza Univ Rome, Dept Chem & Technol Drugs, Food Chem Lab, Rome, Italy.	[Di Matteo, Giacomo; Mannina, Luisa] Sapienza University Rome, Dept Chem & Technol Drugs, Food Chem Lab, Rome, Italy.	Country: Italy<br>Institution: Sapienza Univ Rome	Country: Italy<br>Institution: Sapienza University Rome
C1	2	2744	2744	[Di Matteo, Giacomo; Mannina, Luisa] Sapienza Univ Rome, NMR Based Metabol Lab Sapienza NMLab, Rome, Italy.	[Di Matteo, Giacomo; Mannina, Luisa] Sapienza University Rome, NMR Based Metabol Lab Sapienza NMLab, Rome, Italy.	Country: Italy<br>Institution: Sapienza Univ Rome	Country: Italy<br>Institution: Sapienza University Rome
C1	4	2746	2746	[Frangiamone, Massimo] Univ Lausanne, Dept Biomed Sci, Lausanne, Switzerland.	[Frangiamone, Massimo] University of Lausanne, Dept Biomed Sci, Lausanne, Switzerland.	Country: Switzerland<br>Institution: Univ Lausanne	Country: Switzerland<br>Institution: University of Lausanne
C1	5	2747	2747	[Vila-Donat, Pilar; Manyes, Lara] Univ Valencia, Fac Pharm, Lab Food Chem & Toxicol, Valencia, Spain.	[Vila-Donat, Pilar; Manyes, Lara] University of Valencia, Fac Pharm, Lab Food Chem & Toxicol, Valencia, Spain.	Country: Spain<br>Institution: Univ Valencia	Country: Spain<br>Institution: University of Valencia

RP	1	2751	2751	Manyes, L (corresponding author), Univ Valencia, Fac Pharm, Lab Food Chem & Toxicol, Valencia, Spain	Manyes, L (corresponding author), University of Valencia, Fac Pharm, Lab Food Chem & Toxicol, Valencia, Spain	Country: Spain<br>Institution: Univ Valencia	Country: Spain<br>Institution: University of Valencia

Record 17 | WOS:001546906900003

Mitochondrial dynamics imbalance and energy dyshomeostasis mediated developmental toxicity of ochratoxin a in zebrafish (Danio rerio)

Field	Position	Original line	Normalised line	Original value	Normalised value	Original country / institution	Normalised country / institution
C1	1	2887	2887	[Li, Meng; He, Mengai; Liao, Zhenfeng; Li, Chenxi; Mi, Yingqiu; Pan, Ningning; Xie, Xinyi; Liu, Zhiyu; Hou, Jiayin; Chen, Liezhong] Zhejiang Acad Agr Sci, State Key Lab Qual & Safety Agroprod, Hangzhou 310021, Zhejiang, Peoples R China.	[Li, Meng; He, Mengai; Liao, Zhenfeng; Li, Chenxi; Mi, Yingqiu; Pan, Ningning; Xie, Xinyi; Liu, Zhiyu; Hou, Jiayin; Chen, Liezhong] Zhejiang Academy of Agricultural Sciences, State Key Lab Qual & Safety Agroprod, Hangzhou 310021, Zhejiang, China.	Country: China<br>Institution: Zhejiang Acad Agr Sci	Country: China<br>Institution: Zhejiang Academy of Agricultural Sciences
C1	2	2888	2888	[He, Mengai; Mi, Yingqiu; Yu, Xueli] Shenyang Agr Univ, Coll Food Sci, Shenyang 110866, Liaoning, Peoples R China.	[He, Mengai; Mi, Yingqiu; Yu, Xueli] Shenyang Agricultural University, Coll Food Sci, Shenyang 110866, Liaoning, China.	Country: China<br>Institution: Shenyang Agr Univ	Country: China<br>Institution: Shenyang Agricultural University
RP	1	2891	2891	Chen, LZ (corresponding author), Zhejiang Acad Agr Sci, State Key Lab Qual & Safety Agroprod, Hangzhou 310021, Zhejiang, Peoples R China	Chen, LZ (corresponding author), Zhejiang Academy of Agricultural Sciences, State Key Lab Qual & Safety Agroprod, Hangzhou 310021, Zhejiang, China	Country: China<br>Institution: Zhejiang Acad Agr Sci	Country: China<br>Institution: Zhejiang Academy of Agricultural Sciences

Record 18 | WOS:001582665400002

L-epicatechin partially alleviates Ochratoxin A-induced oxidative and endoplasmic reticulum stress and improves developmental competence of porcine embryos during in vitro culture

Field	Position	Original line	Normalised line	Original value	Normalised value	Original country / institution	Normalised country / institution
C1	1	3027	3027	[Wang, Xu; Li, Mingyu; Jiang, Nan; Jin, Long] Second Hosp Jilin Univ, Dept Urol, Changchun 130033, Peoples R China.	[Wang, Xu; Li, Mingyu; Jiang, Nan; Jin, Long] Second Hosp Jilin Univ, Dept Urol, Changchun 130033, China.	Country: China<br>Institution: Second Hosp Jilin Univ	Country: China<br>Institution: Second Hosp Jilin Univ
C1	2	3028	3028	[Li, Chuang] Changchun Polytech Univ, Coll Modern Agr, Changchun 130022, Peoples R China.	[Li, Chuang] Changchun Polytech Univ, Coll Modern Agr, Changchun 130022, China.	Country: China<br>Institution: Changchun Polytech Univ	Country: China<br>Institution: Changchun Polytech Univ
C1	3	3029	3029	[Li, Chuang] Chungnam Natl Univ, Coll Agr & Life Sci, Daejeon 34314, South Korea.	[Li, Chuang] Chungnam National University, Coll Agr & Life Sci, Daejeon 34314, South Korea.	Country: South Korea<br>Institution: Chungnam Natl Univ	Country: South Korea<br>Institution: Chungnam National University
RP	2	3032	3032	Li, C (corresponding author), Changchun Polytech Univ, Coll Modern Agr, Changchun 130022, Peoples R China	Li, C (corresponding author), Changchun Polytech Univ, Coll Modern Agr, Changchun 130022, China	Country: China<br>Institution: Changchun Polytech Univ	Country: China<br>Institution: Changchun Polytech Univ

Record 19 | WOS:001539646900001

Emerging Mycotoxins in Aquaculture: Current Insights on Toxicity, Biocontrol Strategies, and Occurrence in Aquafeed and Fish

Field	Position	Original line	Normalised line	Original value	Normalised value	Original country / institution	Normalised country / institution
C1	2	3138	3138	[Rusco, Giusy; Iaffaldano, Nicolaia] Univ Molise, Dept Agr Environm & Food Sci, I-86100 Campobasso, Italy.	[Rusco, Giusy; Iaffaldano, Nicolaia] University of Molise, Dept Agr Environm & Food Sci, I-86100 Campobasso, Italy.	Country: Italy<br>Institution: Univ Molise	Country: Italy<br>Institution: University of Molise

Record 20 | WOS:001529843100001

Decontamination of Ochratoxin A in Food: Emerging Strategies and Safety Perspectives

Field	Position	Original line	Normalised line	Original value	Normalised value	Original country / institution	Normalised country / institution
C1	1	3325	3325	[Khan, Rahim; Anwar, Farooq; Ghazali, Farinazleen Mohamad] Univ Putra Malaysia, Fac Food Sci & Technol, Dept Food Sci, Serdang 43400, Selangor, Malaysia.	[Khan, Rahim; Anwar, Farooq; Ghazali, Farinazleen Mohamad] Universiti Putra Malaysia, Fac Food Sci & Technol, Dept Food Sci, Serdang 43400, Selangor, Malaysia.	Country: Malaysia<br>Institution: Univ Putra Malaysia	Country: Malaysia<br>Institution: Universiti Putra Malaysia
C1	2	3326	3326	[Anwar, Farooq] Univ Sargodha, Inst Chem, Sargodha 40100, Pakistan.	[Anwar, Farooq] University of Sargodha, Inst Chem, Sargodha 40100, Pakistan.	Country: Pakistan<br>Institution: Univ Sargodha	Country: Pakistan<br>Institution: University of Sargodha
C1	4	3328	3328	[Mahyudin, Nor Ainy] Univ Putra Malaysia, Fac Food Sci & Technol, Dept Food Serv & Management, Serdang 43300, Selangor, Malaysia.	[Mahyudin, Nor Ainy] Universiti Putra Malaysia, Fac Food Sci & Technol, Dept Food Serv & Management, Serdang 43300, Selangor, Malaysia.	Country: Malaysia<br>Institution: Univ Putra Malaysia	Country: Malaysia<br>Institution: Universiti Putra Malaysia
C1	5	3329	3329	[Mahyudin, Nor Ainy] Univ Putra Malaysia, Halal Prod Res Inst, Lab Halal Sci Res, Serdang 43300, Selangor, Malaysia.	[Mahyudin, Nor Ainy] Universiti Putra Malaysia, Halal Prod Res Inst, Lab Halal Sci Res, Serdang 43300, Selangor, Malaysia.	Country: Malaysia<br>Institution: Univ Putra Malaysia	Country: Malaysia<br>Institution: Universiti Putra Malaysia
RP	1	3334	3334	Khan, R (corresponding author), Univ Putra Malaysia, Fac Food Sci & Technol, Dept Food Sci, Serdang 43400, Selangor, Malaysia	Khan, R (corresponding author), Universiti Putra Malaysia, Fac Food Sci & Technol, Dept Food Sci, Serdang 43400, Selangor, Malaysia	Country: Malaysia<br>Institution: Univ Putra Malaysia	Country: Malaysia<br>Institution: Universiti Putra Malaysia

Record 21 | WOS:001527764700005

Through its genoprotective, mitochondrial bioenergetic modulation, and antioxidant effects, Fucoxanthin and its metabolite minimize Ochratoxin A-induced nephrotoxicity in HK-2 human kidney cells

Field	Position	Original line	Normalised line	Original value	Normalised value	Original country / institution	Normalised country / institution
C1	1	3545	3545	[Elmorsy, Ekramy M.] Northern Border Univ, Ctr Hlth Res, Ar Ar 91431, Saudi Arabia.	[Elmorsy, Ekramy M.] Northern Border University, Ctr Hlth Res, Ar Ar 91431, Saudi Arabia.	Country: Saudi Arabia<br>Institution: Northern Border Univ	Country: Saudi Arabia<br>Institution: Northern Border University
C1	2	3546	3546	[Al Doghaither, Huda A.; Al-Ghafari, Ayat B.] King Abdulaziz Univ, Fac Sci, Dept Biochem, Jeddah 21589, Saudi Arabia.	[Al Doghaither, Huda A.; Al-Ghafari, Ayat B.] King Abdulaziz University, Fac Sci, Dept Biochem, Jeddah 21589, Saudi Arabia.	Country: Saudi Arabia<br>Institution: King Abdulaziz Univ	Country: Saudi Arabia<br>Institution: King Abdulaziz University
C1	3	3547	3547	[Al-Ghafari, Ayat B.] King Abdulaziz Univ, King Fahd Med Res Ctr, Expt Biochem Unit, Jeddah 21589, Saudi Arabia.	[Al-Ghafari, Ayat B.] King Abdulaziz University, King Fahd Med Res Ctr, Expt Biochem Unit, Jeddah 21589, Saudi Arabia.	Country: Saudi Arabia<br>Institution: King Abdulaziz Univ	Country: Saudi Arabia<br>Institution: King Abdulaziz University
C1	4	3548	3548	[Alyamani, Shaza A.] Batterjee Med Coll, Dept Biol, POB 6231, Jeddah 21442, Saudi Arabia.	[Alyamani, Shaza A.] Batterjee Medical College, Dept Biol, POB 6231, Jeddah 21442, Saudi Arabia.	Country: Saudi Arabia<br>Institution: Batterjee Med Coll	Country: Saudi Arabia<br>Institution: Batterjee Medical College

C1	5	3549	3549	[Mohammed, Zakariya M. S.] Northern Border Univ, Ctr Sci Res & Entrepreneurship, Ar Ar, Saudi Arabia.	[Mohammed, Zakariya M. S.] Northern Border University, Ctr Sci Res & Entrepreneurship, Ar Ar, Saudi Arabia.	Country: Saudi Arabia Institution: Northern Border Univ	Country: Saudi Arabia Institution: Northern Border University
C1	6	3550	3550	[Mohammed, Zakariya M. S.] Northern Border Univ, Coll Sci, Dept Math, POB 1321, Ar Ar 91431, Saudi Arabia.	[Mohammed, Zakariya M. S.] Northern Border University, Coll Sci, Dept Math, POB 1321, Ar Ar 91431, Saudi Arabia.	Country: Saudi Arabia Institution: Northern Border Univ	Country: Saudi Arabia Institution: Northern Border University
C1	7	3551	3551	[Ebrahim, Neven A.] Taibah Univ, Coll Med, Dept Basic Med Sci, Madinah, Saudi Arabia.	[Ebrahim, Neven A.] Taibah University, Coll Med, Dept Basic Med Sci, Madinah, Saudi Arabia.	Country: Saudi Arabia Institution: Taibah Univ	Country: Saudi Arabia Institution: Taibah University
C1	8	3552	3552	[Ebrahim, Neven A.] Mansoura Univ, Fac Med, Dept Human Anat & Embryol, Mansoura, Egypt.	[Ebrahim, Neven A.] Mansoura University, Fac Med, Dept Human Anat & Embryol, Mansoura, Egypt.	Country: Egypt Institution: Mansoura Univ	Country: Egypt Institution: Mansoura University
C1	9	3553	3553	[Elshopakey, Gehad E.] Mansoura Univ, Fac Vet Med, Dept Clin Pathol, Mansoura 35516, Egypt.	[Elshopakey, Gehad E.] Mansoura University, Fac Vet Med, Dept Clin Pathol, Mansoura 35516, Egypt.	Country: Egypt Institution: Mansoura Univ	Country: Egypt Institution: Mansoura University
C1	10	3554	3554	[Elshopakey, Gehad E.] Delta Univ Sci & Technol, Fac Vet Med, Dept Vet Dis, Gamasa 35712, Egypt.	[Elshopakey, Gehad E.] Delta University for Science & Technology, Fac Vet Med, Dept Vet Dis, Gamasa 35712, Egypt.	Country: Egypt Institution: Delta Univ Sci & Technol	Country: Egypt Institution: Delta University for Science & Technology
C1	11	3555	3555	[Shabana, Sameh M.] Mansoura Univ, Fac Sci, Dept Zool, Mansoura 35516, Egypt.	[Shabana, Sameh M.] Mansoura University, Fac Sci, Dept Zool, Mansoura 35516, Egypt.	Country: Egypt Institution: Mansoura Univ	Country: Egypt Institution: Mansoura University
RP	1	3562	3562	Elmorsy, EM (corresponding author), Northern Border Univ, Ctr Hlth Res, Ar Ar 91431, Saudi Arabia	Elmorsy, EM (corresponding author), Northern Border University, Ctr Hlth Res, Ar Ar 91431, Saudi Arabia	Country: Saudi Arabia Institution: Northern Border Univ	Country: Saudi Arabia Institution: Northern Border University

Record 22 | WOS:001539670300001

Oral Exposure to Chlorella sorokiniana Detoxifies Deoxynivalenol, Ochratoxin A, and Fumonisin B1 In Vitro and In Vivo

Field	Position	Original line	Normalised line	Original value	Normalised value	Original country / institution	Normalised country / institution
C1	1	3703	3703	[Yamaguchi, Hiroki; Ando, Mana; Ohira, Chiharu; Magami, Tensei; Kaneki, Mao; Sugita, Kazutoshi; Fukuyama, Tomoki] Azabu Univ, Sch Vet Med, 1-17-71 Fuchinobe,Chuo Ku, Sagamihara, Kanagawa 2525201, Japan.	[Yamaguchi, Hiroki; Ando, Mana; Ohira, Chiharu; Magami, Tensei; Kaneki, Mao; Sugita, Kazutoshi; Fukuyama, Tomoki] Azabu University, Sch Vet Med, 1-17-71 Fuchinobe,Chuo Ku, Sagamihara, Kanagawa 2525201, Japan.	Country: Japan Institution: Azabu Univ	Country: Japan Institution: Azabu University
C1	3	3705	3705	[Fukuyama, Tomoki] Azabu Univ, Ctr Human & Anim Symbiosis Sci, 1-17-71 Fuchinobe,Chuo Ku, Sagamihara, Kanagawa 2525201, Japan.	[Fukuyama, Tomoki] Azabu University, Ctr Human & Anim Symbiosis Sci, 1-17-71 Fuchinobe,Chuo Ku, Sagamihara, Kanagawa 2525201, Japan.	Country: Japan Institution: Azabu Univ	Country: Japan Institution: Azabu University
RP	1	3707	3707	Fukuyama, T (corresponding author), Azabu Univ, Sch Vet Med, 1-17-71 Fuchinobe,Chuo Ku, Sagamihara, Kanagawa 2525201, Japan	Fukuyama, T (corresponding author), Azabu University, Sch Vet Med, 1-17-71 Fuchinobe,Chuo Ku, Sagamihara, Kanagawa 2525201, Japan	Country: Japan Institution: Azabu Univ	Country: Japan Institution: Azabu University

Record 23 | WOS:001513772100001

Schisandrin B mitigates oxidative damage in early porcine embryos exposed to Ochratoxin A via NRF2 pathway activation

Field	Position	Original line	Normalised line	Original value	Normalised value	Original country / institution	Normalised country / institution
C1	1	3802	3802	[Li, Chuang; Bin Ji, Kuk; Choi, Ho Yong; Yoon, Jae Hyeok; Zhang, Xiang; Kim, Min Kyu] Chungnam Natl Univ, Coll Agr & Life Sci, Div Anim & Dairy Sci, Daejeon, South Korea.	[Li, Chuang; Bin Ji, Kuk; Choi, Ho Yong; Yoon, Jae Hyeok; Zhang, Xiang; Kim, Min Kyu] Chungnam National University, Coll Agr & Life Sci, Div Anim & Dairy Sci, Daejeon, South Korea.	Country: South Korea Institution: Chungnam Natl Univ	Country: South Korea Institution: Chungnam National University
C1	3	3804	3804	[Liu, Haixing] Yanbian Univ, Coll Agr, Dept Anim Sci, Yanji, Jilin, Peoples R China.	[Liu, Haixing] Yanbian University, Coll Agr, Dept Anim Sci, Yanji, Jilin, China.	Country: China Institution: Yanbian Univ	Country: China Institution: Yanbian University
RP	1	3806	3806	Kim, MK (corresponding author), Chungnam Natl Univ, Coll Agr & Life Sci, Div Anim & Dairy Sci, Daejeon, South Korea	Kim, MK (corresponding author), Chungnam National University, Coll Agr & Life Sci, Div Anim & Dairy Sci, Daejeon, South Korea	Country: South Korea Institution: Chungnam Natl Univ	Country: South Korea Institution: Chungnam National University

Record 24 | WOS:001505840900001

Biocontrol Agents and Natural Feed Supplements as a Safe and Cost-Effective Way for Preventing Health Ailments Provoked by Mycotoxins

Field	Position	Original line	Normalised line	Original value	Normalised value	Original country / institution	Normalised country / institution
C1	1	3904	3904	[Stoev, Stoycho D.] Trakia Univ, Fac Vet Med, Dept Gen & Clin Pathol, Stara Zagora 6000, Bulgaria.	[Stoev, Stoycho D.] Trakia University, Fac Vet Med, Dept Gen & Clin Pathol, Stara Zagora 6000, Bulgaria.	Country: Bulgaria Institution: Trakia Univ	Country: Bulgaria Institution: Trakia University
RP	1	3906	3906	Stoev, SD (corresponding author), Trakia Univ, Fac Vet Med, Dept Gen & Clin Pathol, Stara Zagora 6000, Bulgaria	Stoev, SD (corresponding author), Trakia University, Fac Vet Med, Dept Gen & Clin Pathol, Stara Zagora 6000, Bulgaria	Country: Bulgaria Institution: Trakia Univ	Country: Bulgaria Institution: Trakia University

Record 25 | WOS:001514636100001

Modulation of the Antioxidant System of Caco-2 Cells in the Presence of Aflatoxin B1, Ochratoxin A, and Ferulic Acid

Field	Position	Original line	Normalised line	Original value	Normalised value	Original country / institution	Normalised country / institution
C1	1	4270	4270	[Radulescu, Andreea-Luminita; Balas, Mihaela; Dinischiotu, Anca] Univ Bucharest, Fac Biol, Dept Biochem & Mol Biol, Bucharest 050095, Romania.	[Radulescu, Andreea-Luminita; Balas, Mihaela; Dinischiotu, Anca] University of Bucharest, Fac Biol, Dept Biochem & Mol Biol, Bucharest 050095, Romania.	Country: Romania Institution: Univ Bucharest	Country: Romania Institution: University of Bucharest
RP	1	4273	4273	Dinischiotu, A (corresponding author), Univ Bucharest, Fac Biol, Dept Biochem & Mol Biol, Bucharest 050095, Romania	Dinischiotu, A (corresponding author), University of Bucharest, Fac Biol, Dept Biochem & Mol Biol, Bucharest 050095, Romania	Country: Romania Institution: Univ Bucharest	Country: Romania Institution: University of Bucharest

Record 26 | WOS:001496132800001

Evaluation of Dietary Bioactive Agents Against Aflatoxin B1 and Ochratoxin A-Induced Duodenal Toxicity in Rats

Field	Position	Original line	Normalised line	Original value	Normalised value	Original country / institution	Normalised country / institution
C1	1	4420	4420	[Rafai, Sarra; Cimbalo, Alessandra; Manyes, Lara] Univ Valencia, Fac Pharm & Food Sci, Biotech Agrifood, Avda Vicent	[Rafai, Sarra; Cimbalo, Alessandra; Manyes, Lara] University of Valencia, Fac Pharm & Food Sci, Biotech	Country: Spain Institution: Univ Valencia	Country: Spain Institution: University of Valencia

				Andres Estelles S-N, Burjassot 46100, Spain.	Agrifood, Avda Vicent Andres Estelles S-N, Burjassot 46100, Spain.		
RP	1	4422	4422	Cimbalo, A (corresponding author), Univ Valencia, Fac Pharm & Food Sci, Biotech Agrifood, Avda Vicent Andres Estelles S-N, Burjassot 46100, Spain	Cimbalo, A (corresponding author), University of Valencia, Fac Pharm & Food Sci, Biotech Agrifood, Avda Vicent Andres Estelles S-N, Burjassot 46100, Spain	Country: Spain<br>Institution: Univ Valencia	Country: Spain<br>Institution: University of Valencia

Record 27 | WOS:001488085400001

Bi2WO6-TiO2 carbon nanoparticles controlled postharvest blue mold of table grapes caused by Talaromyces rugulosus and the possible action mechanisms

Field	Position	Original line	Normalised line	Original value	Normalised value	Original country / institution	Normalised country / institution
C1	1	4534	4534	[Li, Jinzhu; Xi, Jihui; Wang, Ruoxing; Zhang, Qingru; Xue, Huali] Gansu Agr Univ, Coll Sci, Lanzhou, Peoples R China.	[Li, Jinzhu; Xi, Jihui; Wang, Ruoxing; Zhang, Qingru; Xue, Huali] Gansu Agricultural University, Coll Sci, Lanzhou, China.	Country: China<br>Institution: Gansu Agr Univ	Country: China<br>Institution: Gansu Agricultural University
C1	2	4535	4535	[Fan, Haiyan] Nazarbayev Univ, Coll Sci & Humanities, Astana, Kazakhstan.	[Fan, Haiyan] Nazarbayev University, Coll Sci & Humanities, Astana, Kazakhstan.	Country: Kazakhstan<br>Institution: Nazarbayev Univ	Country: Kazakhstan<br>Institution: Nazarbayev University
C1	3	4536	4536	[Bi, Yang] Gansu Agr Univ, Coll Food Sci & Engn, Lanzhou, Peoples R China.	[Bi, Yang] Gansu Agricultural University, Coll Food Sci & Engn, Lanzhou, China.	Country: China<br>Institution: Gansu Agr Univ	Country: China<br>Institution: Gansu Agricultural University
C1	4	4537	4537	[Lyu, Liang] Lanzhou Jiaotong Univ, Coll Biol & Pharmaceut Engn, Lanzhou, Peoples R China.	[Lyu, Liang] Lanzhou Jiaotong University, Coll Biol & Pharmaceut Engn, Lanzhou, China.	Country: China<br>Institution: Lanzhou Jiaotong Univ	Country: China<br>Institution: Lanzhou Jiaotong University
RP	1	4540	4540	Xue, HL (corresponding author), Gansu Agr Univ, Coll Sci, Lanzhou, Peoples R China	Xue, HL (corresponding author), Gansu Agricultural University, Coll Sci, Lanzhou, Peoples R China	Country: China<br>Institution: Gansu Agr Univ	Country: China<br>Institution: Gansu Agricultural University
RP	2	4540	4540	Bi, Y (corresponding author), Gansu Agr Univ, Coll Food Sci & Engn, Lanzhou, Peoples R China	Bi, Y (corresponding author), Gansu Agr Univ, Coll Food Sci & Engn, Lanzhou, China	Country: China<br>Institution: Gansu Agr Univ	Country: China<br>Institution: Gansu Agr University

Record 28 | WOS:001486309200001

Immunotoxic effect and mechanisms of Fusarium mycotoxins on human immune cells: A focus on T cells and macrophages

Field	Position	Original line	Normalised line	Original value	Normalised value	Original country / institution	Normalised country / institution
C1	1	4644	4644	[Yang, Jing; You, Lijuan; Bai, Li; Yang, Hui] Shandong Second Med Univ, Sch Publ Hlth, Weifang 261053, Shandong, Peoples R China.	[Yang, Jing; You, Lijuan; Bai, Li; Yang, Hui] Shandong Second Medical University, Sch Publ Hlth, Weifang 261053, Shandong, China.	Country: China<br>Institution: Shandong Second Med Univ	Country: China<br>Institution: Shandong Second Medical University
C1	2	4645	4645	[Wang, Xiaohong; Zhang, Qiannan; Tian, Yaru; Han, Xiaomin; Shi, Miaoying; Bai, Li; Jia, Xudong; Yang, Hui] China Natl Ctr Food Safety Risk Assessment, NHC Key Lab Food Safety Risk Assessment, Beijing 100021, Peoples R China.	[Wang, Xiaohong; Zhang, Qiannan; Tian, Yaru; Han, Xiaomin; Shi, Miaoying; Bai, Li; Jia, Xudong; Yang, Hui] China Natl Ctr Food Safety Risk Assessment, NHC Key Lab Food Safety Risk Assessment, Beijing 100021, China.	Country: China<br>Institution: China Natl Ctr Food Safety Risk Assessment	Country: China<br>Institution: China Natl Ctr Food Safety Risk Assessment

C1	3	4646	4646	[Cui, Hongyang] Chinese Ctr Dis Control & Prevent, Natl Inst Environm Hlth, China CDC Key Lab Environm & Populat Hlth, Beijing 100021, Peoples R China.	[Cui, Hongyang] Chinese Ctr Dis Control & Prevent, Natl Inst Environm Hlth, China CDC Key Lab Environm & Populat Hlth, Beijing 100021, China.	Country: China Institution: Chinese Ctr Dis Control & Prevent	Country: China Institution: Chinese Ctr Dis Control & Prevent
C1	4	4647	4647	[Zhuang, Zhisen; Bai, Li; Yang, Hui] Southern Med Univ, Food Safety & Hlth Res Ctr, Sch Publ Hlth, Guangdong Key Lab Trop Dis Res, Guangzhou 510515, Peoples R China.	[Zhuang, Zhisen; Bai, Li; Yang, Hui] Southern Medical University - China, Food Safety & Hlth Res Ctr, Sch Publ Hlth, Guangdong Key Lab Trop Dis Res, Guangzhou 510515, China.	Country: China Institution: Southern Med Univ	Country: China Institution: Southern Medical University - China
C3	4	4648	4648	Southern Medical University - China	Southern Medical University	Institution: Southern Medical University - China	Institution: Southern Medical University
RP	1	4651	4651	Yang, H (corresponding author), China Natl Ctr Food Safety Risk Assessment CFSA, Lab Toxicol, 7 Panjiayuan Nanli, Beijing 100021, Peoples R China	Yang, H (corresponding author), China Natl Ctr Food Safety Risk Assessment CFSA, Lab Toxicol, 7 Panjiayuan Nanli, Beijing 100021, China	Country: China Institution: China Natl Ctr Food Safety Risk Assessment CFSA	Country: China Institution: China Natl Ctr Food Safety Risk Assessment CFSA

Record 29 | WOS:001484254000002

Quantification and metabolic variations of mycotoxins in raw milk: Implications for dairy cow health and human safety

Field	Position	Original line	Normalised line	Original value	Normalised value	Original country / institution	Normalised country / institution
C1	1	4768	4768	[Chen, Yuanyuan; Cheng, Jiaxin; Liu, Honglin; Xia, Cheng; Zhang, Haifeng; Zou, Siqi] Heilongjiang Bayi Agr Univ, Coll Anim Sci & Vet Med, Heilongjiang Prov Key Lab Prevent & Control Bovine, Daqing 163319, Heilongjiang, Peoples R China.	[Chen, Yuanyuan; Cheng, Jiaxin; Liu, Honglin; Xia, Cheng; Zhang, Haifeng; Zou, Siqi] Heilongjiang Bayi Agricultural University, Coll Anim Sci & Vet Med, Heilongjiang Prov Key Lab Prevent & Control Bovine, Daqing 163319, Heilongjiang, China.	Country: China Institution: Heilongjiang Bayi Agr Univ	Country: China Institution: Heilongjiang Bayi Agricultural University
C1	2	4769	4769	[Wang, Zhuangshu] Dis Prevent & Control Ctr Huiyang Dist, Huizhou 516003, Guangdong, Peoples R China.	[Wang, Zhuangshu] Dis Prevent & Control Ctr Huiyang Dist, Huizhou 516003, Guangdong, China.	Country: China Institution: Dis Prevent & Control Ctr Huiyang Dist	Country: China Institution: Dis Prevent & Control Ctr Huiyang Dist
C1	3	4770	4770	[Chang, Qiaocheng] Shantou Univ, Sch Publ Hlth, Shantou 515063, Guangdong, Peoples R China.	[Chang, Qiaocheng] Shantou University, Sch Publ Hlth, Shantou 515063, Guangdong, China.	Country: China Institution: Shantou Univ	Country: China Institution: Shantou University
C1	4	4771	4771	[Xu, Chuang] China Agr Univ, Coll Anim Sci & Technol, Beijing 100193, Peoples R China.	[Xu, Chuang] China Agricultural University, Coll Anim Sci & Technol, Beijing 100193, China.	Country: China Institution: China Agr Univ	Country: China Institution: China Agricultural University
RP	1	4774	4774	Chang, QC (corresponding author), Shantou Univ, Sch Publ Hlth, Shantou 515063, Guangdong, Peoples R China	Chang, QC (corresponding author), Shantou University, Sch Publ Hlth, Shantou 515063, Guangdong, China	Country: China Institution: Shantou Univ	Country: China Institution: Shantou University

Record 30 | WOS:001484241000001

Oxidative stress promotes post-translational down-regulation of MRP2 in Caco-2 cells: Involvement of proteasomal degradation and toxicological implications

No C1/C3/RP normalisation changes detected for this record.

Record 31 | WOS:001471942000001

Ginsenoside Rg1 alleviates ochratoxin A-induced liver inflammation in ducklings: Involvement of intestinal microbiota modulation and the TLR4/ NF-Î°B pathway inhibition

Field	Position	Original line	Normalised line	Original value	Normalised value	Original country / institution	Normalised country / institution
C1	1	5062	5062	[Zhai, Shuangshuang; Peng, Xin; Liu, Cheng; Jiang, Xiayu; Liang, Yuting; Yuan, Xi; Zhang, Jinqiu; Yang, Ye] Yangtze Univ, Coll Anim Sci & Technol, Jingzhou 434023, Hubei, Peoples R China.	[Zhai, Shuangshuang; Peng, Xin; Liu, Cheng; Jiang, Xiayu; Liang, Yuting; Yuan, Xi; Zhang, Jinqiu; Yang, Ye] Yangtze University, Coll Anim Sci & Technol, Jingzhou 434023, Hubei, China.	Country: China<br>Institution: Yangtze Univ	Country: China<br>Institution: Yangtze University
C1	2	5063	5063	[Feng, Peishi] Zhejiang Univ Technol, Coll Pharm, Hangzhou 310014, Zhejiang, Peoples R China.	[Feng, Peishi] Zhejiang University of Technology, Coll Pharm, Hangzhou 310014, Zhejiang, China.	Country: China<br>Institution: Zhejiang Univ Technol	Country: China<br>Institution: Zhejiang University of Technology
C1	3	5064	5064	[Zhang, Ran; Jin, Chunlong] Shenzhen Customs Dist, Shenzhen Int Travel Hlth Care Ctr, Dept Endocrinol, Shenzhen Customs Dist Port Outpatient Clin, Shenzhen 518000, Peoples R China.	[Zhang, Ran; Jin, Chunlong] Shenzhen Customs Dist, Shenzhen Int Travel Hlth Care Ctr, Dept Endocrinol, Shenzhen Customs Dist Port Outpatient Clin, Shenzhen 518000, China.	Country: China<br>Institution: Shenzhen Customs Dist	Country: China<br>Institution: Shenzhen Customs Dist
RP	1	5066	5066	Zhai, SS (corresponding author), Yangtze Univ, Coll Anim Sci & Technol, Jingzhou 434023, Hubei, Peoples R China	Zhai, SS (corresponding author), Yangtze University, Coll Anim Sci & Technol, Jingzhou 434023, Hubei, China	Country: China<br>Institution: Yangtze Univ	Country: China<br>Institution: Yangtze University

Record 32 | WOS:001471582100001

Mitigating AFB1 and OTA hepatic residues using pumpkin and milk fermented whey as functional ingredients

Field	Position	Original line	Normalised line	Original value	Normalised value	Original country / institution	Normalised country / institution
C1	1	5174	5174	[Cimbalo, Alessandra; Manyes, Lara] Univ Valencia, Fac Pharm & Food Sci, Biotech Agrifood, Avda Vicent Andres Estelles S-N, Burjassot 46100, Spain.	[Cimbalo, Alessandra; Manyes, Lara] University of Valencia, Fac Pharm & Food Sci, Biotech Agrifood, Avda Vicent Andres Estelles S-N, Burjassot 46100, Spain.	Country: Spain<br>Institution: Univ Valencia	Country: Spain<br>Institution: University of Valencia
C1	3	5176	5176	[Izzo, Luana; Ritieni, Alberto] Univ Naples Federico II, Dept Pharm, FoodLab, Via Domen Montesano 49, I-80131 Naples, Italy.	[Izzo, Luana; Ritieni, Alberto] University of Naples Federico II, Dept Pharm, FoodLab, Via Domen Montesano 49, I-80131 Naples, Italy.	Country: Italy<br>Institution: Univ Naples Federico II	Country: Italy<br>Institution: University of Naples Federico II
RP	1	5179	5179	Izzo, L (corresponding author), Univ Naples Federico II, Dept Pharm, FoodLab, Via Domen Montesano 49, I-80131 Naples, Italy	Izzo, L (corresponding author), University of Naples Federico II, Dept Pharm, FoodLab, Via Domen Montesano 49, I-80131 Naples, Italy	Country: Italy<br>Institution: Univ Naples Federico II	Country: Italy<br>Institution: University of Naples Federico II

Record 33 | WOS:001456981800001

Ochratoxin A-induced mitochondrial pathway apoptosis and ferroptosis by promoting glycolysis

Field	Position	Original line	Normalised line	Original value	Normalised value	Original country / institution	Normalised country / institution
C1	1	5298	5298	[Zhou, Yao; Chen, Wenying; Feng, Shiyu; Liu, Shuangchao; Chen, Cheng; Yao, Bingxu; Shen, Xiao Li] Zunyi Med Univ, Sch Publ Hlth, 1 Campus Rd, Zunyi 563000, Guizhou, Peoples R China.	[Zhou, Yao; Chen, Wenying; Feng, Shiyu; Liu, Shuangchao; Chen, Cheng; Yao, Bingxu; Shen, Xiao Li] Zunyi Medical University, Sch Publ Hlth, 1 Campus Rd, Zunyi 563000, Guizhou, China.	Country: China<br>Institution: Zunyi Med Univ	Country: China<br>Institution: Zunyi Medical University

C1	2	5299	5299	[Chen, Wenying; Shen, Xiao Li] Guizhou Prov Dept Educ, Key Lab Maternal & Child Hlth & Exposure Sci, Zunyi 563000, Guizhou, Peoples R China.	[Chen, Wenying; Shen, Xiao Li] Guizhou Prov Dept Educ, Key Lab Maternal & Child Hlth & Exposure Sci, Zunyi 563000, Guizhou, China.	Country: China Institution: Guizhou Prov Dept Educ	Country: China Institution: Guizhou Prov Dept Educ
RP	1	5301	5301	Shen, XL (corresponding author), Zunyi Med Univ, Sch Publ Hlth, 1 Campus Rd, Zunyi 563000, Guizhou, Peoples R China	Shen, XL (corresponding author), Zunyi Medical University, Sch Publ Hlth, 1 Campus Rd, Zunyi 563000, Guizhou, Peoples R China	Country: China Institution: Zunyi Med Univ	Country: China Institution: Zunyi Medical University
RP	2	5301	5301	Shen, XL (corresponding author), Guizhou Prov Dept Educ, Key Lab Maternal & Child Hlth & Exposure Sci, Zunyi 563000, Guizhou, Peoples R China	Shen, XL (corresponding author), Guizhou Prov Dept Educ, Key Lab Maternal & Child Hlth & Exposure Sci, Zunyi 563000, Guizhou, China	Country: China Institution: Guizhou Prov Dept Educ	Country: China Institution: Guizhou Prov Dept Educ

Record 34 | WOS:001463798400001

Combined Cytotoxic Effects of the Fungicide Azoxystrobin and Common Food-Contaminating Mycotoxins

Field	Position	Original line	Normalised line	Original value	Normalised value	Original country / institution	Normalised country / institution
C1	2	5439	5439	[Fuentes, Cristina; Zingales, Veronica; Ruiz, Maria-Jose] Univ Valencia, Res Grp Alternat Methods Determining Tox Effects &, Ave Vicent Andres Estelles s-n, Burjassot 46100, Spain.	[Fuentes, Cristina; Zingales, Veronica; Ruiz, Maria-Jose] University of Valencia, Res Grp Alternat Methods Determining Tox Effects &, Ave Vicent Andres Estelles s-n, Burjassot 46100, Spain.	Country: Spain Institution: Univ Valencia	Country: Spain Institution: University of Valencia
C1	3	5440	5440	[Zingales, Veronica; Ruiz, Maria-Jose] Univ Valencia, Fac Pharm, Lab Food Chem & Toxicol, Ave Vicent Andres Estelles S-N, Burjassot 46100, Spain.	[Zingales, Veronica; Ruiz, Maria-Jose] University of Valencia, Fac Pharm, Lab Food Chem & Toxicol, Ave Vicent Andres Estelles S-N, Burjassot 46100, Spain.	Country: Spain Institution: Univ Valencia	Country: Spain Institution: University of Valencia

Record 35 | WOS:001475234400001

Mycotoxins in Feed: Hazards, Toxicology, and Plant Extract-Based Remedies

Field	Position	Original line	Normalised line	Original value	Normalised value	Original country / institution	Normalised country / institution
C1	1	5582	5582	[Zhang, Xiangnan; Chen, Jiashun; Ma, Xiaokang; Tang, Xiongzhuo; Tan, Bie; Jiang, Qian] Hunan Agr Univ, Coll Anim Sci & Technol, Anim Nutr Genome & Germplasm Innovat Res Ctr, Changsha 410128, Peoples R China.	[Zhang, Xiangnan; Chen, Jiashun; Ma, Xiaokang; Tang, Xiongzhuo; Tan, Bie; Jiang, Qian] Hunan Agricultural University, Coll Anim Sci & Technol, Anim Nutr Genome & Germplasm Innovat Res Ctr, Changsha 410128, China.	Country: China Institution: Hunan Agr Univ	Country: China Institution: Hunan Agricultural University
C1	2	5583	5583	[Zhang, Xiangnan; Chen, Jiashun; Ma, Xiaokang; Tang, Xiongzhuo; Tan, Bie; Jiang, Qian] Yuelushan Lab, Changsha 410128, Peoples R China.	[Zhang, Xiangnan; Chen, Jiashun; Ma, Xiaokang; Tang, Xiongzhuo; Tan, Bie; Jiang, Qian] Yuelushan Lab, Changsha 410128, China.	Country: China Institution: Yuelushan Lab	Country: China Institution: Yuelushan Lab
C1	3	5584	5584	[Zhang, Xiangnan; Liao, Peng; Yao, Kang; Jiang, Qian] Chinese Acad Sci, Inst Subtrop Agr, Changsha 410125, Peoples R China.	[Zhang, Xiangnan; Liao, Peng; Yao, Kang; Jiang, Qian] Chinese Academy of Sciences, Inst Subtrop Agr, Changsha 410125, China.	Country: China Institution: Chinese Acad Sci	Country: China Institution: Chinese Academy of Sciences

RP	1	5587	5587	Jiang, Q (corresponding author), Hunan Agr Univ, Coll Anim Sci & Technol, Anim Nutr Genome & Germplasm Innovat Res Ctr, Changsha 410128, Peoples R China	Jiang, Q (corresponding author), Hunan Agricultural University, Coll Anim Sci & Technol, Anim Nutr Genome & Germplasm Innovat Res Ctr, Changsha 410128, Peoples R China	Country: China<br>Institution: Hunan Agr Univ	Country: China<br>Institution: Hunan Agricultural University
RP	4	5587	5587	Jiang, Q (corresponding author), Chinese Acad Sci, Inst Subtrop Agr, Changsha 410125, Peoples R China	Jiang, Q (corresponding author), Chinese Acad Sci, Inst Subtrop Agr, Changsha 410125, China	Country: China<br>Institution: Chinese Acad Sci	Country: China<br>Institution: Chinese Acad Sci

Record 36 | WOS:001437492200001

Potential role of ochratoxin A in Parkinson's disease: a systematic review of current evidence

Field	Position	Original line	Normalised line	Original value	Normalised value	Original country / institution	Normalised country / institution
C1	1	5793	5793	[Serrano-Civantos, M.; Beraza, E.; de Cerain, A. Lopez; Vettorazzi, A.] Univ Navarra, Sch Pharm & Nutr, Dept Pharmaceut Sci, MITOX Res Grp, Pamplona, Spain.	[Serrano-Civantos, M.; Beraza, E.; de Cerain, A. Lopez; Vettorazzi, A.] University of Navarra, Sch Pharm & Nutr, Dept Pharmaceut Sci, MITOX Res Grp, Pamplona, Spain.	Country: Spain<br>Institution: Univ Navarra	Country: Spain<br>Institution: University of Navarra
RP	1	5796	5796	Vettorazzi, A (corresponding author), Univ Navarra, Sch Pharm & Nutr, Dept Pharmaceut Sci, MITOX Res Grp, Pamplona, Spain	Vettorazzi, A (corresponding author), University of Navarra, Sch Pharm & Nutr, Dept Pharmaceut Sci, MITOX Res Grp, Pamplona, Spain	Country: Spain<br>Institution: Univ Navarra	Country: Spain<br>Institution: University of Navarra

Record 37 | WOS:001427487800001

Neurotoxic implications of gliotoxin and ochratoxin A in SH-SY5Y cells: ROS-induced apoptosis and genotoxicity

Field	Position	Original line	Normalised line	Original value	Normalised value	Original country / institution	Normalised country / institution
C1	1	5942	5942	[Penalva-Olcina, Raquel; Juan, Cristina; Fernandez-Franzon, Monica; Juan-Garcia, Ana] Univ Valencia, Fac Pharm & Food Sci, Lab Food Chem & Toxicol, Av Vicent Andres Estelles S-N, Valencia 46100, Spain.	[Penalva-Olcina, Raquel; Juan, Cristina; Fernandez-Franzon, Monica; Juan-Garcia, Ana] University of Valencia, Fac Pharm & Food Sci, Lab Food Chem & Toxicol, Av Vicent Andres Estelles S-N, Valencia 46100, Spain.	Country: Spain<br>Institution: Univ Valencia	Country: Spain<br>Institution: University of Valencia
RP	1	5944	5944	Juan-GarcÃ-a, A (corresponding author), Univ Valencia, Fac Pharm & Food Sci, Lab Food Chem & Toxicol, Av Vicent Andres Estelles S-N, Valencia 46100, Spain	Juan-GarcÃ-a, A (corresponding author), University of Valencia, Fac Pharm & Food Sci, Lab Food Chem & Toxicol, Av Vicent Andres Estelles S-N, Valencia 46100, Spain	Country: Spain<br>Institution: Univ Valencia	Country: Spain<br>Institution: University of Valencia

Record 38 | WOS:001426276300001

Ochratoxin A induces lung cell PANoptosis through activation of the AIM 2 inflammasome

Field	Position	Original line	Normalised line	Original value	Normalised value	Original country / institution	Normalised country / institution
C1	1	6062	6062	[Xie, Jianlong; Lin, Liyao; Wu, Qin] Guangdong Med Univ, Affiliated Hosp, Dept Thorac	[Xie, Jianlong; Lin, Liyao; Wu, Qin] Guangdong Medical University, Affiliated Hosp,	Country: China<br>Institution: Guangdong Med Univ	Country: China<br>Institution: Guangdong Medical University

				Surg, Zhanjiang 524000, Guangdong, Peoples R China.	Dept Thorac Surg, Zhanjiang 524000, Guangdong, China.		
C1	2	6063	6063	[Fu, Qiujuan; Qin, Lingling; Zeng, Kaiqi; Wu, Jiahuan; Cao, Ziyi; Ou, Yongfang] Guangdong Med Univ, Affiliated Hosp, Pathol Diag & Res Ctr, Zhanjiang 524000, Guangdong, Peoples R China.	[Fu, Qiujuan; Qin, Lingling; Zeng, Kaiqi; Wu, Jiahuan; Cao, Ziyi; Ou, Yongfang] Guangdong Medical University, Affiliated Hosp, Pathol Diag & Res Ctr, Zhanjiang 524000, Guangdong, China.	Country: China Institution: Guangdong Med Univ	Country: China Institution: Guangdong Medical University
C1	3	6064	6064	[Qin, Lingling; Zeng, Kaiqi; Wu, Jiahuan; Cao, Ziyi] Guangdong Med Univ, Clin Coll 1, Zhanjiang 524000, Guangdong, Peoples R China.	[Qin, Lingling; Zeng, Kaiqi; Wu, Jiahuan; Cao, Ziyi] Guangdong Medical University, Clin Coll 1, Zhanjiang 524000, Guangdong, China.	Country: China Institution: Guangdong Med Univ	Country: China Institution: Guangdong Medical University
RP	1	6067	6067	Ou, YF (corresponding author), Guangdong Med Univ, Affiliated Hosp, Pathol Diag & Res Ctr, Zhanjiang 524000, Guangdong, Peoples R China	Ou, YF (corresponding author), Guangdong Medical University, Affiliated Hosp, Pathol Diag & Res Ctr, Zhanjiang 524000, Guangdong, China	Country: China Institution: Guangdong Med Univ	Country: China Institution: Guangdong Medical University

Record 39 | WOS:001425801400001

Probiotics mitigate reproductive toxicity in male mice induced by co-exposure of ochratoxin A and alcohol

Field	Position	Original line	Normalised line	Original value	Normalised value	Original country / institution	Normalised country / institution
C1	1	6161	6161	[Ning, Xiao; Ye, Yongli; Zhang, Yinzhi; Sun, Xiulan] Jiangnan Univ, Synerget Innovat Ctr Food Safety & Qual Control, Sch Food Sci & Technol, Int Joint Lab Food Safety, Wuxi 214122, Jiangsu, Peoples R China.	[Ning, Xiao; Ye, Yongli; Zhang, Yinzhi; Sun, Xiulan] Jiangnan University, Synerget Innovat Ctr Food Safety & Qual Control, Sch Food Sci & Technol, Int Joint Lab Food Safety, Wuxi 214122, Jiangsu, China.	Country: China Institution: Jiangnan Univ	Country: China Institution: Jiangnan University
C1	2	6162	6162	[Ning, Xiao; Cao, Jin; Li, Jingyun; Jin, Shaoming; Cui, Shenghui] Natl Inst Food & Drug Control, Key Lab Food Qual & Safety State Market Regulat, Beijing 100050, Peoples R China.	[Ning, Xiao; Cao, Jin; Li, Jingyun; Jin, Shaoming; Cui, Shenghui] National Institute of Food & Drug Control - China, Key Lab Food Qual & Safety State Market Regulat, Beijing 100050, China.	Country: China Institution: Natl Inst Food & Drug Control	Country: China Institution: National Institute of Food & Drug Control - China
C1	3	6163	6163	[Ye, Yongli; Sun, Xiulan] State Adm Market Regulat, Key Lab Food Qual & Safety, Beijing 100176, Peoples R China.	[Ye, Yongli; Sun, Xiulan] State Adm Market Regulat, Key Lab Food Qual & Safety, Beijing 100176, China.	Country: China Institution: State Adm Market Regulat	Country: China Institution: State Adm Market Regulat
C3	2	6164	6164	National Institute of Food & Drug Control - China	National Institute of Food & Drug Control	Institution: National Institute of Food & Drug Control - China	Institution: National Institute of Food & Drug Control
RP	1	6165	6165	Sun, XL (corresponding author), Jiangnan Univ, Synerget Innovat Ctr Food Safety & Qual Control, Sch Food Sci & Technol, Int Joint Lab Food Safety, Wuxi 214122, Jiangsu, Peoples R China	Sun, XL (corresponding author), Jiangnan University, Synerget Innovat Ctr Food Safety & Qual Control, Sch Food Sci & Technol, Int Joint Lab Food Safety, Wuxi 214122, Jiangsu, Peoples R China	Country: China Institution: Jiangnan Univ	Country: China Institution: Jiangnan University
RP	2	6165	6165	Cui, SH (corresponding author), Natl Inst Food & Drug Control, Key Lab Food Qual & Safety State Market Regulat, Beijing 100050, Peoples R China	Cui, SH (corresponding author), Natl Inst Food & Drug Control, Key Lab Food Qual & Safety State Market Regulat, Beijing 100050, China	Country: China Institution: Natl Inst Food & Drug Control	Country: China Institution: Natl Inst Food & Drug Control

Record 40 | WOS:001416011800001

Metabolic disruption by mycotoxins: focus on metabolic endpoints steatosis, adipogenesis and glucose metabolism in vivo and in vitro

Field	Position	Original line	Normalised line	Original value	Normalised value	Original country / institution	Normalised country / institution
C1	1	6269	6269	[Heikkinen, Tia; Rysa, Jaana] Univ Eastern Finland, Fac Hlth Sci, Sch Pharm, POB 1627, Kuopio 70211, Finland.	[Heikkinen, Tia; Rysa, Jaana] University of Eastern Finland, Fac Hlth Sci, Sch Pharm, POB 1627, Kuopio 70211, Finland.	Country: Finland Institution: Univ Eastern Finland	Country: Finland Institution: University of Eastern Finland
C1	2	6270	6270	[Kublbeck, Jenni] Univ Eastern Finland, AI Virtanen Inst Mol Sci, Kuopio, Finland.	[Kublbeck, Jenni] University of Eastern Finland, AI Virtanen Inst Mol Sci, Kuopio, Finland.	Country: Finland Institution: Univ Eastern Finland	Country: Finland Institution: University of Eastern Finland
RP	1	6272	6272	RysÃ¤, J (corresponding author), Univ Eastern Finland, Fac Hlth Sci, Sch Pharm, POB 1627, Kuopio 70211, Finland	RysÃ¤, J (corresponding author), University of Eastern Finland, Fac Hlth Sci, Sch Pharm, POB 1627, Kuopio 70211, Finland	Country: Finland Institution: Univ Eastern Finland	Country: Finland Institution: University of Eastern Finland

Record 41 | WOS:001403208800001

Curcumin: A Potential Detoxifier Against Chemical and Natural Toxicants

Field	Position	Original line	Normalised line	Original value	Normalised value	Original country / institution	Normalised country / institution
C1	1	6395	6395	[Kumar, Harsh] Univ Hradec Kralove, Fac Sci, Ctr Adv Technol, Hradec Kralove, Czech Republic.	[Kumar, Harsh] University of Hradec Kralove, Fac Sci, Ctr Adv Technol, Hradec Kralove, Czech Republic.	Country: Czech Republic Institution: Univ Hradec Kralove	Country: Czech Republic Institution: University of Hradec Kralove
C1	5	6399	6399	[Nepovimova, Eugenie] Univ Hradec Kralove, Fac Sci, Dept Chem, Hradec Kralove, Czech Republic.	[Nepovimova, Eugenie] University of Hradec Kralove, Fac Sci, Dept Chem, Hradec Kralove, Czech Republic.	Country: Czech Republic Institution: Univ Hradec Kralove	Country: Czech Republic Institution: University of Hradec Kralove
C1	8	6402	6402	[Sethi, Nidhi] Guru Nanak Dev Univ, Dept Pharmaceut Sci, Amritsar, India.	[Sethi, Nidhi] Guru Nanak Dev University, Dept Pharmaceut Sci, Amritsar, India.	Country: India Institution: Guru Nanak Dev Univ	Country: India Institution: Guru Nanak Dev University
RP	1	6408	6408	Nepovimova, E (corresponding author), Univ Hradec Kralove, Fac Sci, Dept Chem, Hradec Kralove, Czech Republic	Nepovimova, E (corresponding author), University of Hradec Kralove, Fac Sci, Dept Chem, Hradec Kralove, Czech Republic	Country: Czech Republic Institution: Univ Hradec Kralove	Country: Czech Republic Institution: University of Hradec Kralove

Record 42 | WOS:001416156300001

Ochratoxin A induces immunotoxicity by targeting Annexin A1 mediated neutrophil apoptosis in zebrafish

Field	Position	Original line	Normalised line	Original value	Normalised value	Original country / institution	Normalised country / institution
C1	1	6869	6869	[Zheng, Yihong; Liu, Yinuo; Tian, Jin; Liu, Shuhong; Ma, Gaowei; Xie, Yupeng; Wu, Zekai] Fujian Med Univ, Sch Basic Med Sci, Key Lab Gastrointestinal Canc, Minist Educ, Fuzhou, Peoples R China.	[Zheng, Yihong; Liu, Yinuo; Tian, Jin; Liu, Shuhong; Ma, Gaowei; Xie, Yupeng; Wu, Zekai] Fujian Medical University, Sch Basic Med Sci, Key Lab Gastrointestinal Canc, Minist Educ, Fuzhou, China.	Country: China Institution: Fujian Med Univ	Country: China Institution: Fujian Medical University
C1	2	6870	6870	[Zheng, Chenhua] Fujian Med Univ, Expt Teaching Ctr Basic Med Sci, Sch Basic Med Sci, Fuzhou, Peoples R China.	[Zheng, Chenhua] Fujian Medical University, Expt Teaching Ctr Basic Med Sci, Sch Basic Med Sci, Fuzhou, China.	Country: China Institution: Fujian Med Univ	Country: China Institution: Fujian Medical University
RP	1	6872	6872	Wu, ZK (corresponding author), Fujian Med Univ, Sch Basic Med Sci, Key Lab Gastrointestinal Canc, Minist Educ, Fuzhou, Peoples R China	Wu, ZK (corresponding author), Fujian Medical University, Sch Basic Med Sci, Key Lab Gastrointestinal Canc, Minist Educ, Fuzhou, Peoples R China	Country: China Institution: Fujian Med Univ	Country: China Institution: Fujian Medical University
RP	2	6872	6872	Zheng, CH (corresponding author), Fujian Med Univ, Expt	Zheng, CH (corresponding author), Fujian Med Univ, Expt	Country: China Institution: Fujian Med Univ	Country: China Institution: Fujian Med Univ

				Teaching Ctr Basic Med Sci, Sch Basic Med Sci, Fuzhou, Peoples R China	Teaching Ctr Basic Med Sci, Sch Basic Med Sci, Fuzhou, China		

Record 43 | WOS:001393437600001

Chemical Composition, In Vivo, and In Silico Molecular Docking Studies of the Effect of Syzygium aromaticum (Clove) Essential Oil on Ochratoxin A-Induced Acute Neurotoxicity

Field	Position	Original line	Normalised line	Original value	Normalised value	Original country / institution	Normalised country / institution
C1	1	6979	6979	[Brahmi, Mostapha] Univ Ahmed Zabana, Fac Nat & Life Sci, Dept Biol Sci, Relizane 48000, Algeria.	[Brahmi, Mostapha] University Ahmed Zabana of Relizane, Fac Nat & Life Sci, Dept Biol Sci, Relizane 48000, Algeria.	Country: Algeria Institution: Univ Ahmed Zabana	Country: Algeria Institution: University Ahmed Zabana of Relizane
C1	3	6981	6981	[Alkholifi, Faisal K.] Prince Sattam Bin Abdulaziz Univ, Coll Pharm, Dept Pharmacol, Alkharj 11942, Saudi Arabia.	[Alkholifi, Faisal K.] Prince Sattam Bin Abdulaziz University, Coll Pharm, Dept Pharmacol, Alkharj 11942, Saudi Arabia.	Country: Saudi Arabia Institution: Prince Sattam Bin Abdulaziz Univ	Country: Saudi Arabia Institution: Prince Sattam Bin Abdulaziz University
C1	4	6982	6982	[Sweilam, Sherouk Hussein] Prince Sattam Bin Abdulaziz Univ, Coll Pharm, Dept Pharmacognosy, Alkharj 11942, Saudi Arabia.	[Sweilam, Sherouk Hussein] Prince Sattam Bin Abdulaziz University, Coll Pharm, Dept Pharmacognosy, Alkharj 11942, Saudi Arabia.	Country: Saudi Arabia Institution: Prince Sattam Bin Abdulaziz Univ	Country: Saudi Arabia Institution: Prince Sattam Bin Abdulaziz University
C1	5	6983	6983	[Sweilam, Sherouk Hussein] Egyptian Russian Univ, Fac Pharm, Dept Pharmacognosy, Cairo Suez Rd, Badr City 11829, Cairo, Egypt.	[Sweilam, Sherouk Hussein] Egyptian Russian University, Fac Pharm, Dept Pharmacognosy, Cairo Suez Rd, Badr City 11829, Cairo, Egypt.	Country: Egypt Institution: Egyptian Russian Univ	Country: Egypt Institution: Egyptian Russian University
RP	1	6987	6987	Brahmi, M (corresponding author), Univ Ahmed Zabana, Fac Nat & Life Sci, Dept Biol Sci, Relizane 48000, Algeria	Brahmi, M (corresponding author), University Ahmed Zabana of Relizane, Fac Nat & Life Sci, Dept Biol Sci, Relizane 48000, Algeria	Country: Algeria Institution: Univ Ahmed Zabana	Country: Algeria Institution: University Ahmed Zabana of Relizane

Record 44 | WOS:001404966700001

Proteomic Analysis of the Murine Liver Response to Oral Exposure to Aflatoxin B1 and Ochratoxin A: The Protective Role to Bioactive Compounds

Field	Position	Original line	Normalised line	Original value	Normalised value	Original country / institution	Normalised country / institution
C1	1	7131	7131	[Trombetti, Silvia; Grosso, Michela] Univ Naples Federico II, Dept Mol Med & Med Biotechnol, Via Pansini 5, I-80131 Naples, Italy.	[Trombetti, Silvia; Grosso, Michela] University of Naples Federico II, Dept Mol Med & Med Biotechnol, Via Pansini 5, I-80131 Naples, Italy.	Country: Italy Institution: Univ Naples Federico II	Country: Italy Institution: University of Naples Federico II
C1	2	7132	7132	[Cimbalo, Alessandra; Vila-Donat, Pilar; Manes, Jordi; Manyes, Lara] Univ Valencia, Fac Pharm & Food Sci, Biotech Agrifood, Avda Vicent Andres Estelles S-N, Burjassot 46100, Spain.	[Cimbalo, Alessandra; Vila-Donat, Pilar; Manes, Jordi; Manyes, Lara] University of Valencia, Fac Pharm & Food Sci, Biotech Agrifood, Avda Vicent Andres Estelles S-N, Burjassot 46100, Spain.	Country: Spain Institution: Univ Valencia	Country: Spain Institution: University of Valencia
RP	1	7134	7134	Vila-Donat, P (corresponding author), Univ Valencia, Fac Pharm & Food Sci, Biotech Agrifood, Avda Vicent Andres Estelles S-N, Burjassot 46100, Spain	Vila-Donat, P (corresponding author), University of Valencia, Fac Pharm & Food Sci, Biotech Agrifood, Avda Vicent Andres Estelles S-N, Burjassot 46100, Spain	Country: Spain Institution: Univ Valencia	Country: Spain Institution: University of Valencia

Record 45 | WOS:001370729500001

Alterations in the proteomes of HepG2 and IHKE cells inflicted by six selected mycotoxins

Field	Position	Original line	Normalised line	Original value	Normalised value	Original country / institution	Normalised country / institution
C1	1	7249	7249	[Keuter, Lucas; Fortmann, Marco; Behrens, Matthias; Humpf, Hans-Ulrich] Univ Munster, Inst Food Chem, Corrensstr 45, D-48149 Munster, Germany.	[Keuter, Lucas; Fortmann, Marco; Behrens, Matthias; Humpf, Hans-Ulrich] University of Munster, Inst Food Chem, Corrensstr 45, D-48149 Munster, Germany.	Country: Germany Institution: Univ Munster	Country: Germany Institution: University of Munster
RP	1	7251	7251	Humpf, HU (corresponding author), Univ Munster, Inst Food Chem, Corrensstr 45, D-48149 Munster, Germany	Humpf, HU (corresponding author), University of Munster, Inst Food Chem, Corrensstr 45, D-48149 Munster, Germany	Country: Germany Institution: Univ Munster	Country: Germany Institution: University of Munster

Record 46 | WOS:001375296600001

Bioavailability study of OTA, ZEN, and AFB1 along with bioactive compounds from tiger nut beverage and its by-products

Field	Position	Original line	Normalised line	Original value	Normalised value	Original country / institution	Normalised country / institution
C1	1	7404	7404	[Llorens, P.; Juan-Garcia, A.; Molto, J. C.; Juan, C.] Univ Valencia, Fac Pharm, Dept Prevent Med & Publ Hlth Food Sci Toxicol & Fo, Lab Food Chem & Toxicol, Avda Vicent Andres Estelles S-N, Burjassot 46100, Spain.	[Llorens, P.; Juan-Garcia, A.; Molto, J. C.; Juan, C.] University of Valencia, Fac Pharm, Dept Prevent Med & Publ Hlth Food Sci Toxicol & Fo, Lab Food Chem & Toxicol, Avda Vicent Andres Estelles S-N, Burjassot 46100, Spain.	Country: Spain Institution: Univ Valencia	Country: Spain Institution: University of Valencia
C1	2	7405	7405	[Pardo, O.; Arjona-Mudarra, P.; Esteve-Turrillas, F. A.] Univ Valencia, Dept Analyt Chem, Doctor Moliner 50, Burjassot 46100, Spain.	[Pardo, O.; Arjona-Mudarra, P.; Esteve-Turrillas, F. A.] University of Valencia, Dept Analyt Chem, Doctor Moliner 50, Burjassot 46100, Spain.	Country: Spain Institution: Univ Valencia	Country: Spain Institution: University of Valencia
C1	3	7406	7406	[Marti-Quijal, F. J.; Barba, F. J.] Univ Valencia, Fac Pharm, Dept Prevent Med & Publ Hlth Food Sci Toxicol & Fo, Res Grp Innovat Technol Sustainable Food ALISOST, Avda Vicent Andres Estelles S-N, Burjassot 46100, Valencia, Spain.	[Marti-Quijal, F. J.; Barba, F. J.] University of Valencia, Fac Pharm, Dept Prevent Med & Publ Hlth Food Sci Toxicol & Fo, Res Grp Innovat Technol Sustainable Food ALISOST, Avda Vicent Andres Estelles S-N, Burjassot 46100, Valencia, Spain.	Country: Spain Institution: Univ Valencia	Country: Spain Institution: University of Valencia
C1	4	7407	7407	[Chiacchio, M. F.] Univ Naples Federico II, Dept Agr Sci, I-80055 Portici, Italy.	[Chiacchio, M. F.] University of Naples Federico II, Dept Agr Sci, I-80055 Portici, Italy.	Country: Italy Institution: Univ Naples Federico II	Country: Italy Institution: University of Naples Federico II
RP	1	7410	7410	Juan-GarcÃ-a, A (corresponding author), Univ Valencia, Fac Pharm, Dept Prevent Med & Publ Hlth Food Sci Toxicol & Fo, Lab Food Chem & Toxicol, Avda Vicent Andres Estelles S-N, Burjassot 46100, Spain	Juan-GarcÃ-a, A (corresponding author), University of Valencia, Fac Pharm, Dept Prevent Med & Publ Hlth Food Sci Toxicol & Fo, Lab Food Chem & Toxicol, Avda Vicent Andres Estelles S-N, Burjassot 46100, Spain	Country: Spain Institution: Univ Valencia	Country: Spain Institution: University of Valencia

Record 47 | WOS:001384323500001

Development of an Easy-To-Use Microfluidic System to Assess Dynamic Exposure to Mycotoxins in 3D Culture Models: Evaluation of Ochratoxin A and Patulin Cytotoxicity

Field	Position	Original line	Normalised line	Original value	Normalised value	Original country / institution	Normalised country / institution
C1	2	7559	7559	[Zingales, Veronica; Ruiz, Maria-Jose] Univ Valencia, Fac Pharm, Lab Toxicol, Ave Vicent Andres Estelles S-N, Valencia 46100, Spain.	[Zingales, Veronica; Ruiz, Maria-Jose] University of Valencia, Fac Pharm, Lab Toxicol, Ave Vicent Andres Estelles S-N, Valencia 46100, Spain.	Country: Spain Institution: Univ Valencia	Country: Spain Institution: University of Valencia
C1	3	7560	7560	[Zingales, Veronica; Piunti, Caterina; Micheli, Sara; Cimetta, Elisa] Univ Padua, Dept Ind Engn DII, Via Marzolo 9, I-35131 Padua, Italy.	[Zingales, Veronica; Piunti, Caterina; Micheli, Sara; Cimetta, Elisa] University of Padua, Dept Ind Engn DII, Via Marzolo 9, I-35131 Padua, Italy.	Country: Italy Institution: Univ Padua	Country: Italy Institution: University of Padua

Record 48 | WOS:001356112700001

DNA ligase III mediates deoxynivalenol exposure-induced DNA damage in intestinal epithelial cells by regulating oxidative stress and interaction with PCNA

Field	Position	Original line	Normalised line	Original value	Normalised value	Original country / institution	Normalised country / institution
C1	1	7702	7702	[Zhu, Xiaoyang; Chen, Xiaolei; Shi, Dongfeng; Hui, Peng; Wang, Haifei; Wu, Zhengchang; Wu, Shenglong; Bao, Wenbin; Fan, Hairui] Yangzhou Univ, Coll Anim Sci & Technol, Key Lab Anim Genet Breeding Reprod & Mol Design, Yangzhou 225009, Peoples R China.	[Zhu, Xiaoyang; Chen, Xiaolei; Shi, Dongfeng; Hui, Peng; Wang, Haifei; Wu, Zhengchang; Wu, Shenglong; Bao, Wenbin; Fan, Hairui] Yangzhou University, Coll Anim Sci & Technol, Key Lab Anim Genet Breeding Reprod & Mol Design, Yangzhou 225009, China.	Country: China Institution: Yangzhou Univ	Country: China Institution: Yangzhou University
C1	2	7703	7703	[Wu, Jiayun] Jiangsu Agrianim Husb Vocat Coll, Taizhou 225300, Peoples R China.	[Wu, Jiayun] Jiangsu Agrianim Husb Vocat Coll, Taizhou 225300, China.	Country: China Institution: Jiangsu Agrianim Husb Vocat Coll	Country: China Institution: Jiangsu Agrianim Husb Vocat Coll
C1	3	7704	7704	[Wang, Haifei; Wu, Shenglong; Bao, Wenbin] Yangzhou Univ, Joint Int Res Lab Agr & Agriprod Safety, Minist Educ China, Yangzhou 225009, Jiangsu, Peoples R China.	[Wang, Haifei; Wu, Shenglong; Bao, Wenbin] Yangzhou University, Joint Int Res Lab Agr & Agriprod Safety, Minist Educ China, Yangzhou 225009, Jiangsu, China.	Country: China Institution: Yangzhou Univ	Country: China Institution: Yangzhou University
C1	4	7705	7705	[Fan, Hairui] Yangzhou Univ, Inst Comparat Med, Coll Vet Med, Yangzhou 225009, Jiangsu, Peoples R China.	[Fan, Hairui] Yangzhou University, Inst Comparat Med, Coll Vet Med, Yangzhou 225009, Jiangsu, China.	Country: China Institution: Yangzhou Univ	Country: China Institution: Yangzhou University
RP	1	7708	7708	Fan, HR (corresponding author), Yangzhou Univ, Coll Anim Sci & Technol, Key Lab Anim Genet Breeding Reprod & Mol Design, Yangzhou 225009, Peoples R China	Fan, HR (corresponding author), Yangzhou University, Coll Anim Sci & Technol, Key Lab Anim Genet Breeding Reprod & Mol Design, Yangzhou 225009, Peoples R China	Country: China Institution: Yangzhou Univ	Country: China Institution: Yangzhou University
RP	2	7708	7708	Fan, HR (corresponding author), Yangzhou Univ, Inst Comparat Med, Coll Vet Med, Yangzhou 225009, Jiangsu, Peoples R China	Fan, HR (corresponding author), Yangzhou Univ, Inst Comparat Med, Coll Vet Med, Yangzhou 225009, Jiangsu, China	Country: China Institution: Yangzhou Univ	Country: China Institution: Yangzhou Univ

Record 49 | WOS:001366569000001

Chlorophyll-Amended Organoclays for the Detoxification of Ochratoxin A

Field	Position	Original line	Normalised line	Original value	Normalised value	Original country / institution	Normalised country / institution

C1	1	7833	7833	[Oladele, Johnson O.; Wang, Meichen; Phillips, Timothy D.] Texas A&M Univ, Interdisciplinary Fac Toxicol, College Stn, TX 77843 USA.	[Oladele, Johnson O.; Wang, Meichen; Phillips, Timothy D.] Texas A&M University College Station, Interdisciplinary Fac Toxicol, College Stn, TX 77843 USA.	Country: USA<br>Institution: Texas A&M Univ	Country: USA<br>Institution: Texas A&M University College Station
C1	2	7834	7834	[Oladele, Johnson O.; Wang, Meichen; Phillips, Timothy D.] Texas A&M Univ, Coll Vet Med & Biomed Sci, Dept Vet Physiol & Pharmacol, College Stn, TX 77843 USA.	[Oladele, Johnson O.; Wang, Meichen; Phillips, Timothy D.] Texas A&M University College Station, Coll Vet Med & Biomed Sci, Dept Vet Physiol & Pharmacol, College Stn, TX 77843 USA.	Country: USA<br>Institution: Texas A&M Univ	Country: USA<br>Institution: Texas A&M University College Station
C1	3	7835	7835	[Xenophontos, Xenophon; Tamamis, Phanourios] Texas A&M Univ, Coll Engn, Artie McFerrin Dept Chem Engn, College Stn, TX 77843 USA.	[Xenophontos, Xenophon; Tamamis, Phanourios] Texas A&M University College Station, Coll Engn, Artie McFerrin Dept Chem Engn, College Stn, TX 77843 USA.	Country: USA<br>Institution: Texas A&M Univ	Country: USA<br>Institution: Texas A&M University College Station
C1	4	7836	7836	[Lilly, Kendall; Tamamis, Phanourios] Texas A&M Univ, Coll Engn, Dept Mat Sci & Engn, College Stn, TX 77843 USA.	[Lilly, Kendall; Tamamis, Phanourios] Texas A&M University College Station, Coll Engn, Dept Mat Sci & Engn, College Stn, TX 77843 USA.	Country: USA<br>Institution: Texas A&M Univ	Country: USA<br>Institution: Texas A&M University College Station
RP	1	7841	7841	Phillips, TD (corresponding author), Texas A&M Univ, Interdisciplinary Fac Toxicol, College Stn, TX 77843 USA	Phillips, TD (corresponding author), Texas A&M University College Station, Interdisciplinary Fac Toxicol, College Stn, TX 77843 USA	Country: USA<br>Institution: Texas A&M Univ	Country: USA<br>Institution: Texas A&M University College Station

Record 50 | WOS:001351279800001

Protective Effects of Carotenoid-Loaded Nanostructured Lipid Carriers Against Ochratoxin-A-Induced Cytotoxicity

Field	Position	Original line	Normalised line	Original value	Normalised value	Original country / institution	Normalised country / institution
C1	1	7976	7976	[Pinna, Nicola; Blasi, Francesca] Univ Perugia, Dept Pharmaceut Sci, Sect Food Sci & Nutr, I-06123 Perugia, Italy.	[Pinna, Nicola; Blasi, Francesca] University of Perugia, Dept Pharmaceut Sci, Sect Food Sci & Nutr, I-06123 Perugia, Italy.	Country: Italy<br>Institution: Univ Perugia	Country: Italy<br>Institution: University of Perugia
C1	2	7977	7977	[Pinna, Nicola; Schoubben, Aurelie] Univ Perugia, Dept Pharmaceut Sci, Sect Pharmaceut Technol, I-06123 Perugia, Italy.	[Pinna, Nicola; Schoubben, Aurelie] University of Perugia, Dept Pharmaceut Sci, Sect Pharmaceut Technol, I-06123 Perugia, Italy.	Country: Italy<br>Institution: Univ Perugia	Country: Italy<br>Institution: University of Perugia
C1	3	7978	7978	[Vila-Donat, Pilar; Pasca, Denisia; Manyes, Lara] Univ Valencia, Biotech Agrifood, Fac Farm & Ciencies Alimentacio, Burjassot 46100, Spain.	[Vila-Donat, Pilar; Pasca, Denisia; Manyes, Lara] University of Valencia, Biotech Agrifood, Fac Farm & Ciencies Alimentacio, Burjassot 46100, Spain.	Country: Spain<br>Institution: Univ Valencia	Country: Spain<br>Institution: University of Valencia
RP	1	7982	7982	Vila-Donat, P (corresponding author), Univ Valencia, Biotech Agrifood, Fac Farm & Ciencies Alimentacio, Burjassot 46100, Spain	Vila-Donat, P (corresponding author), University of Valencia, Biotech Agrifood, Fac Farm & Ciencies Alimentacio, Burjassot 46100, Spain	Country: Spain<br>Institution: Univ Valencia	Country: Spain<br>Institution: University of Valencia

Record 51 | WOS:001329697100001

Unveiling the hub genes associated with ochratoxin A-induced hepatotoxicity in broiler chickens

Field	Position	Original line	Normalised line	Original value	Normalised value	Original country / institution	Normalised country / institution
C1	1	8089	8089	[Li, Xiaofeng; Wang, Zhongyuan; Wang, Shujuan; Yang, Bing] Anhui Sci & Technol Univ, Coll Anim Sci, Fengyang 233100, Peoples R China.	[Li, Xiaofeng; Wang, Zhongyuan; Wang, Shujuan; Yang, Bing] Anhui Science & Technology University, Coll Anim Sci, Fengyang 233100, China.	Country: China Institution: Anhui Sci & Technol Univ	Country: China Institution: Anhui Science & Technology University
RP	1	8091	8091	Yang, B (corresponding author), Anhui Sci & Technol Univ, Coll Anim Sci, Fengyang 233100, Peoples R China	Yang, B (corresponding author), Anhui Science & Technology University, Coll Anim Sci, Fengyang 233100, China	Country: China Institution: Anhui Sci & Technol Univ	Country: China Institution: Anhui Science & Technology University

Record 52 | WOS:001314852000005

Investigating the protective properties of Panax ginseng and its constituents against biotoxins and metal toxicity: a mechanistic review

Field	Position	Original line	Normalised line	Original value	Normalised value	Original country / institution	Normalised country / institution
C1	1	8192	8192	[Rameshrad, Maryam; Naraki, Karim; Hosseinzadeh, Hossein] Mashhad Univ Med Sci, Sch Pharm, Dept Pharmacodynam & Toxicol, Mashhad, Iran.	[Rameshrad, Maryam; Naraki, Karim; Hosseinzadeh, Hossein] Mashhad University of Medical Sciences, Sch Pharm, Dept Pharmacodynam & Toxicol, Mashhad, Iran.	Country: Iran Institution: Mashhad Univ Med Sci	Country: Iran Institution: Mashhad University of Medical Sciences
C1	2	8193	8193	[Rameshrad, Maryam; Hosseinzadeh, Hossein] Mashhad Univ Med Sci, Pharmaceut Technol Inst, Pharmaceut Res Ctr, Mashhad, Iran.	[Rameshrad, Maryam; Hosseinzadeh, Hossein] Mashhad University of Medical Sciences, Pharmaceut Technol Inst, Pharmaceut Res Ctr, Mashhad, Iran.	Country: Iran Institution: Mashhad Univ Med Sci	Country: Iran Institution: Mashhad University of Medical Sciences
C1	3	8194	8194	[Memariani, Zahra] Babol Univ Med Sci, Hlth Res Inst, Tradit Med & Hist Med Sci Res Ctr, Babol, Iran.	[Memariani, Zahra] Babol University of Medical Sciences, Hlth Res Inst, Tradit Med & Hist Med Sci Res Ctr, Babol, Iran.	Country: Iran Institution: Babol Univ Med Sci	Country: Iran Institution: Babol University of Medical Sciences
C1	4	8195	8195	[Naraki, Karim] Mashhad Univ Med Sci, Student Res Comm, Mashhad, Iran.	[Naraki, Karim] Mashhad University of Medical Sciences, Student Res Comm, Mashhad, Iran.	Country: Iran Institution: Mashhad Univ Med Sci	Country: Iran Institution: Mashhad University of Medical Sciences
RP	1	8199	8199	Hosseinzadeh, H (corresponding author), Mashhad Univ Med Sci, Sch Pharm, Dept Pharmacodynam & Toxicol, Mashhad, Iran	Hosseinzadeh, H (corresponding author), Mashhad University of Medical Sciences, Sch Pharm, Dept Pharmacodynam & Toxicol, Mashhad, Iran	Country: Iran Institution: Mashhad Univ Med Sci	Country: Iran Institution: Mashhad University of Medical Sciences

Record 53 | WOS:001323506900001

Phytochemical Characterization of Bilberries and Their Potential as a Functional Ingredient to Mitigate Ochratoxin A Toxicity in Cereal-Based Products

Field	Position	Original line	Normalised line	Original value	Normalised value	Original country / institution	Normalised country / institution
C1	1	8410	8410	[Pasca, Denisia; Frangiamone, Massimo; Vila-Donat, Pilar; Manes, Jordi; Manyes, Lara] Univ Valencia, Fac Pharm & Food Sci, Biotech AgriFood Lab, Burjassot 46100, Valencia, Spain.	[Pasca, Denisia; Frangiamone, Massimo; Vila-Donat, Pilar; Manes, Jordi; Manyes, Lara] University of Valencia, Fac Pharm & Food Sci, Biotech AgriFood Lab, Burjassot 46100, Valencia, Spain.	Country: Spain Institution: Univ Valencia	Country: Spain Institution: University of Valencia

C1	4	8413	8413	[Mangiapelo, Luciano] Univ Perugia, Dept Pharmaceut Sci, I-06123 Perugia, Italy.	[Mangiapelo, Luciano] University of Perugia, Dept Pharmaceut Sci, I-06123 Perugia, Italy.	Country: Italy<br>Institution: Univ Perugia	Country: Italy<br>Institution: University of Perugia
C1	6	8415	8415	[Filip, Lorena] Acad Romanian Scientists AOSR, 3 Ilfov St, Bucharest 050044, Romania.	[Filip, Lorena] Academy of Romanian Scientists (AOSR), 3 Ilfov St, Bucharest 050044, Romania.	Country: Romania<br>Institution: Acad Romanian Scientists AOSR	Country: Romania<br>Institution: Academy of Romanian Scientists (AOSR)
RP	1	8420	8420	Vila-Donat, P (corresponding author), Univ Valencia, Fac Pharm & Food Sci, Biotech AgriFood Lab, Burjassot 46100, Valencia, Spain	Vila-Donat, P (corresponding author), University of Valencia, Fac Pharm & Food Sci, Biotech AgriFood Lab, Burjassot 46100, Valencia, Spain	Country: Spain<br>Institution: Univ Valencia	Country: Spain<br>Institution: University of Valencia

Record 54 | WOS:001345720100005

Review: Strategies and technologies in preventing regulated and emerging mycotoxin co-contamination in forage for safeguarding ruminant health

No C1/C3/RP normalisation changes detected for this record.

Record 55 | WOS:001296248000001

Effects of isochlorogenic acid A on mitochondrial dynamics imbalance and RLR damage in PAM cells induced by combined mycotoxins

Field	Position	Original line	Normalised line	Original value	Normalised value	Original country / institution	Normalised country / institution
C1	1	8808	8808	[Lai, Xinuo; Fan, Peng; Deng, Huidan; Jia, Guilin; Zuo, Zhicai; Hu, Yanchun; Wang, Ya; Cai, Dongjie; Gou, Liping; Wen, Yiping; Yu, Shumin; Cao, Suizhong; Shen, Liuhong; Deng, Junliang; Ren, Zhihua] Sichuan Agr Univ, Coll Vet Med, Key Lab Anim Dis & Human Hlth Sichuan Prov, Chengdu 611130, Peoples R China.	[Lai, Xinuo; Fan, Peng; Deng, Huidan; Jia, Guilin; Zuo, Zhicai; Hu, Yanchun; Wang, Ya; Cai, Dongjie; Gou, Liping; Wen, Yiping; Yu, Shumin; Cao, Suizhong; Shen, Liuhong; Deng, Junliang; Ren, Zhihua] Sichuan Agricultural University, Coll Vet Med, Key Lab Anim Dis & Human Hlth Sichuan Prov, Chengdu 611130, China.	Country: China<br>Institution: Sichuan Agr Univ	Country: China<br>Institution: Sichuan Agricultural University
RP	2	8811	8811	Ren, ZH (corresponding author), Sichuan Agr Univ, Coll Vet Med, Key Lab Anim Dis & Human Hlth Sichuan Prov, Chengdu 611130, Peoples R China	Ren, ZH (corresponding author), Sichuan Agricultural University, Coll Vet Med, Key Lab Anim Dis & Human Hlth Sichuan Prov, Chengdu 611130, China	Country: China<br>Institution: Sichuan Agr Univ	Country: China<br>Institution: Sichuan Agricultural University

Record 56 | WOS:001291367000001

Ochratoxin A-induced DNA damage triggers G2 phase arrest via hMLH1-p53-p21 signaling pathway in human gastric epithelium immortalized cells in vitro

Field	Position	Original line	Normalised line	Original value	Normalised value	Original country / institution	Normalised country / institution
C1	1	8929	8929	[Jia, Xin; Wang, Yuan; Cui, Jinfeng; Li, Yuehong; Wu, Wenxin; Zhang, Xianghong; Wang, Juan] Hebei Med Univ, Hosp 2, Dept Pathol, 215 Heping Western Rd, Shijiazhuang, Peoples R China.	[Jia, Xin; Wang, Yuan; Cui, Jinfeng; Li, Yuehong; Wu, Wenxin; Zhang, Xianghong; Wang, Juan] Hebei Medical University, Hosp 2, Dept Pathol, 215 Heping Western Rd, Shijiazhuang, China.	Country: China<br>Institution: Hebei Med Univ	Country: China<br>Institution: Hebei Medical University

C1	2	8930	8930	[Zhang, Xianghong] Hebei Med Univ, Lab Pathol, Shijiazhuang, Peoples R China.	[Zhang, Xianghong] Hebei Medical University, Lab Pathol, Shijiazhuang, China.	Country: China Institution: Hebei Med Univ	Country: China Institution: Hebei Medical University
RP	1	8932	8932	Wang, J (corresponding author), Hebei Med Univ, Hosp 2, Dept Pathol, 215 Heping Western Rd, Shijiazhuang, Peoples R China	Wang, J (corresponding author), Hebei Medical University, Hosp 2, Dept Pathol, 215 Heping Western Rd, Shijiazhuang, China	Country: China Institution: Hebei Med Univ	Country: China Institution: Hebei Medical University

Record 57 | WOS:001325349600001

Evaluating the combined and individual cytotoxic effect of beauvericin, enniatin B and ochratoxin a on breast cancer cells, leukemia cells, and fresh peripheral blood mononuclear cells

Field	Position	Original line	Normalised line	Original value	Normalised value	Original country / institution	Normalised country / institution
C1	1	9027	9027	[Juan-Garcia, Ana; Juan, Cristina] Univ Valencia, Fac Pharm, Lab Food Chem & Toxicol, Ave Vicent Andres Estelles s-n, Burjassot 46100, Valencia, Spain.	[Juan-Garcia, Ana; Juan, Cristina] University of Valencia, Fac Pharm, Lab Food Chem & Toxicol, Ave Vicent Andres Estelles s-n, Burjassot 46100, Valencia, Spain.	Country: Spain Institution: Univ Valencia	Country: Spain Institution: University of Valencia
RP	1	9031	9031	Juan-GarcÃ-a, A (corresponding author), Univ Valencia, Fac Pharm, Lab Food Chem & Toxicol, Ave Vicent Andres Estelles s-n, Burjassot 46100, Valencia, Spain	Juan-GarcÃ-a, A (corresponding author), University of Valencia, Fac Pharm, Lab Food Chem & Toxicol, Ave Vicent Andres Estelles s-n, Burjassot 46100, Valencia, Spain	Country: Spain Institution: Univ Valencia	Country: Spain Institution: University of Valencia

Record 58 | WOS:001277668700001

The Novel Role of the NLRP3 Inflammasome in Mycotoxin-Induced Toxicological Mechanisms

Field	Position	Original line	Normalised line	Original value	Normalised value	Original country / institution	Normalised country / institution
C1	1	9146	9146	[Liao, Chengshui; Xu, Fengru; Yu, Zuhua; Ding, Ke; Jia, Yanyan] Henan Univ Sci & Technol, Coll Anim Sci & Technol, Lab Funct Microbiol & Anim Hlth, Luoyang 471003, Peoples R China.	[Liao, Chengshui; Xu, Fengru; Yu, Zuhua; Ding, Ke; Jia, Yanyan] Henan University of Science & Technology, Coll Anim Sci & Technol, Lab Funct Microbiol & Anim Hlth, Luoyang 471003, China.	Country: China Institution: Henan Univ Sci & Technol	Country: China Institution: Henan University of Science & Technology
C1	2	9147	9147	[Liao, Chengshui; Xu, Fengru; Yu, Zuhua; Ding, Ke; Jia, Yanyan] Luoyang Key Lab Live Carrier Biomat & Anim Dis Pre, Luoyang 471023, Peoples R China.	[Liao, Chengshui; Xu, Fengru; Yu, Zuhua; Ding, Ke; Jia, Yanyan] Luoyang Key Lab Live Carrier Biomat & Anim Dis Pre, Luoyang 471023, China.	Country: China Institution: Luoyang Key Lab Live Carrier Biomat & Anim Dis Pre	Country: China Institution: Luoyang Key Lab Live Carrier Biomat & Anim Dis Pre
C1	3	9148	9148	[Liao, Chengshui; Xu, Fengru; Yu, Zuhua; Ding, Ke; Jia, Yanyan] Henan Univ Sci & Technol, Key Lab Anim Dis & Publ Hlth, Luoyang 471023, Peoples R China.	[Liao, Chengshui; Xu, Fengru; Yu, Zuhua; Ding, Ke; Jia, Yanyan] Henan University of Science & Technology, Key Lab Anim Dis & Publ Hlth, Luoyang 471023, China.	Country: China Institution: Henan Univ Sci & Technol	Country: China Institution: Henan University of Science & Technology
RP	1	9151	9151	Jia, YY (corresponding author), Henan Univ Sci & Technol, Coll Anim Sci & Technol, Lab Funct Microbiol & Anim Hlth, Luoyang 471003, Peoples R China	Jia, YY (corresponding author), Henan University of Science & Technology, Coll Anim Sci & Technol, Lab Funct Microbiol & Anim Hlth, Luoyang 471003, Peoples R China	Country: China Institution: Henan Univ Sci & Technol	Country: China Institution: Henan University of Science & Technology
RP	3	9151	9151	Jia, YY (corresponding author), Henan Univ Sci & Technol, Key Lab Anim Dis & Publ Hlth,	Jia, YY (corresponding author), Henan Univ Sci & Technol, Key	Country: China Institution: Henan Univ Sci & Technol	Country: China Institution: Henan Univ Sci & Technol

				Luoyang 471023, Peoples R China	Lab Anim Dis & Publ Hlth, Luoyang 471023, China		

Record 59 | WOS:001263565100001

Reduction of aflatoxins and ochratoxin A by addition of commercial Koji during fermentation of the Korean traditional soybean paste, Doenjang

Field	Position	Original line	Normalised line	Original value	Normalised value	Original country / institution	Normalised country / institution
C1	1	9348	9348	[Woo, So Young; Tian, Fei; Lee, Sang Yoo; Park, Su Been; Chun, Hyang Sook] Chung Ang Univ, Sch Food Sci & Technol, GreenTech based Food Safety Res Grp, BK21 Four, Anseong, South Korea.	[Woo, So Young; Tian, Fei; Lee, Sang Yoo; Park, Su Been; Chun, Hyang Sook] Chung Ang University, Sch Food Sci & Technol, GreenTech based Food Safety Res Grp, BK21 Four, Anseong, South Korea.	Country: South Korea Institution: Chung Ang Univ	Country: South Korea Institution: Chung Ang University
C1	2	9349	9349	[Tian, Fei] Chinese Acad Sci, Shenzhen Inst Adv Technol, Shenzhen, Peoples R China.	[Tian, Fei] Chinese Academy of Sciences, Shenzhen Inst Adv Technol, Shenzhen, China.	Country: China Institution: Chinese Acad Sci	Country: China Institution: Chinese Academy of Sciences
C1	3	9350	9350	[Han, Kap-Hoon] Woosuk Univ, Dept Pharmaceut Engn, Wonju, South Korea.	[Han, Kap-Hoon] Woosuk University, Dept Pharmaceut Engn, Wonju, South Korea.	Country: South Korea Institution: Woosuk Univ	Country: South Korea Institution: Woosuk University
C1	4	9351	9351	[Kim, Hye-Young] Yongin Univ, Dept Foods & Nutr, Yongin, South Korea.	[Kim, Hye-Young] Yongin University, Dept Foods & Nutr, Yongin, South Korea.	Country: South Korea Institution: Yongin Univ	Country: South Korea Institution: Yongin University
RP	1	9354	9354	Chun, HS (corresponding author), Chung Ang Univ, Sch Food Sci & Technol, GreenTech based Food Safety Res Grp, BK21 Four, Anseong, South Korea	Chun, HS (corresponding author), Chung Ang University, Sch Food Sci & Technol, GreenTech based Food Safety Res Grp, BK21 Four, Anseong, South Korea	Country: South Korea Institution: Chung Ang Univ	Country: South Korea Institution: Chung Ang University

Record 60 | WOS:001257266000001

Zearalenone modulates the function of goat endometrial cells via the mitochondrial quality control system

Field	Position	Original line	Normalised line	Original value	Normalised value	Original country / institution	Normalised country / institution
C1	1	9470	9470	[Zhang, Guomin; Zeng, Cheng; Zhang, Qi; Wang, Yifei; Xia, Rongxin; Mai, Qingyang] Nanjing Agr Univ, Coll Vet Med, Nanjing 210095, Peoples R China.	[Zhang, Guomin; Zeng, Cheng; Zhang, Qi; Wang, Yifei; Xia, Rongxin; Mai, Qingyang] Nanjing Agricultural University, Coll Vet Med, Nanjing 210095, China.	Country: China Institution: Nanjing Agr Univ	Country: China Institution: Nanjing Agricultural University
C1	2	9471	9471	[Zhang, Guomin; Zeng, Cheng; Sun, Xuan; Zhang, Qi; Wang, Yifei; Xia, Rongxin; Wang, Feng] Nanjing Agr Univ, Jiangsu Livestock Embryo Engn Lab, Nanjing, Peoples R China.	[Zhang, Guomin; Zeng, Cheng; Sun, Xuan; Zhang, Qi; Wang, Yifei; Xia, Rongxin; Wang, Feng] Nanjing Agricultural University, Jiangsu Livestock Embryo Engn Lab, Nanjing, China.	Country: China Institution: Nanjing Agr Univ	Country: China Institution: Nanjing Agricultural University
C1	3	9472	9472	[Xue, Gang] Yangtze River Delta White Goat Breeding Res Inst, Nantong, Peoples R China.	[Xue, Gang] Yangtze River Delta White Goat Breeding Res Inst, Nantong, China.	Country: China Institution: Yangtze River Delta White Goat Breeding Res Inst	Country: China Institution: Yangtze River Delta White Goat Breeding Res Inst
C1	4	9473	9473	[Huang, Han] Mashan Cty Ctr Anim Dis Control & Prevent, Nanning, Peoples R China.	[Huang, Han] Mashan Cty Ctr Anim Dis Control & Prevent, Nanning, China.	Country: China Institution: Mashan Cty Ctr Anim Dis Control & Prevent	Country: China Institution: Mashan Cty Ctr Anim Dis Control & Prevent
RP	1	9475	9475	Zhang, GM (corresponding author), Nanjing Agr Univ, Coll Vet Med, Nanjing 210095, Peoples R China	Zhang, GM (corresponding author), Nanjing Agricultural University, Coll Vet Med, Nanjing 210095, China	Country: China Institution: Nanjing Agr Univ	Country: China Institution: Nanjing Agricultural University

Record 61 | WOS:001250935500001

Central role of Sigma-1 receptor in ochratoxin A-induced ferroptosis

Field	Position	Original line	Normalised line	Original value	Normalised value	Original country / institution	Normalised country / institution
C1	1	9608	9608	[Chen, Wenying; Han, Lingyun; Yang, Ruiran; Wang, Hongwei; Yao, Song; Deng, Huiqiong; Liu, Shuangchao; Zhou, Yao; Shen, Xiao Li] Zunyi Med Univ, Sch Publ Hlth, 1 Campus Rd, Zunyi 563000, Guizhou, Peoples R China.	[Chen, Wenying; Han, Lingyun; Yang, Ruiran; Wang, Hongwei; Yao, Song; Deng, Huiqiong; Liu, Shuangchao; Zhou, Yao; Shen, Xiao Li] Zunyi Medical University, Sch Publ Hlth, 1 Campus Rd, Zunyi 563000, Guizhou, China.	Country: China<br>Institution: Zunyi Med Univ	Country: China<br>Institution: Zunyi Medical University
C1	2	9609	9609	[Chen, Wenying; Shen, Xiao Li] Exposure Sci Guizhou Higher Educ Inst, Key Lab Maternal & Child Hlth, Zunyi 563000, Guizhou, Peoples R China.	[Chen, Wenying; Shen, Xiao Li] Exposure Sci Guizhou Higher Educ Inst, Key Lab Maternal & Child Hlth, Zunyi 563000, Guizhou, China.	Country: China<br>Institution: Exposure Sci Guizhou Higher Educ Inst	Country: China<br>Institution: Exposure Sci Guizhou Higher Educ Inst
C1	3	9610	9610	[Deng, Huiqiong] Fuling Dist Ctr Dis Control & Prevent, Chongqing 408000, Peoples R China.	[Deng, Huiqiong] Fuling Dist Ctr Dis Control & Prevent, Chongqing 408000, China.	Country: China<br>Institution: Fuling Dist Ctr Dis Control & Prevent	Country: China<br>Institution: Fuling Dist Ctr Dis Control & Prevent
RP	1	9612	9612	Shen, XL (corresponding author), Zunyi Med Univ, Sch Publ Hlth, 1 Campus Rd, Zunyi 563000, Guizhou, Peoples R China	Shen, XL (corresponding author), Zunyi Medical University, Sch Publ Hlth, 1 Campus Rd, Zunyi 563000, Guizhou, Peoples R China	Country: China<br>Institution: Zunyi Med Univ	Country: China<br>Institution: Zunyi Medical University
RP	2	9612	9612	Shen, XL (corresponding author), Exposure Sci Guizhou Higher Educ Inst, Key Lab Maternal & Child Hlth, Zunyi 563000, Guizhou, Peoples R China	Shen, XL (corresponding author), Exposure Sci Guizhou Higher Educ Inst, Key Lab Maternal & Child Hlth, Zunyi 563000, Guizhou, China	Country: China<br>Institution: Exposure Sci Guizhou Higher Educ Inst	Country: China<br>Institution: Exposure Sci Guizhou Higher Educ Inst

Record 62 | WOS:001263872600001

Occurrence of the two major regulated mycotoxins, ochratoxin A and fumonisin B1, in cereal and cereal-based products in Europe and toxicological effects: A review

No C1/C3/RP normalisation changes detected for this record.

Record 63 | WOS:001256451400001

High-Coverage UHPLC-MS/MS Analysis of 67 Mycotoxins in Plasma for Male Infertility Exposure Studies

Field	Position	Original line	Normalised line	Original value	Normalised value	Original country / institution	Normalised country / institution
C1	1	10034	10034	[Ning, Xiao; Ji, Jian; Sun, Jiadi; Ye, Yongli; Zhang, Yinzhi; Sun, Xiulan] Jiangnan Univ, Synerget Innovat Ctr Food Safety & Qual Control, Sch Food Sci & Technol, Int Joint Lab Food Safety, Wuxi 214122, Peoples R China.	[Ning, Xiao; Ji, Jian; Sun, Jiadi; Ye, Yongli; Zhang, Yinzhi; Sun, Xiulan] Jiangnan University, Synerget Innovat Ctr Food Safety & Qual Control, Sch Food Sci & Technol, Int Joint Lab Food Safety, Wuxi 214122, China.	Country: China<br>Institution: Jiangnan Univ	Country: China<br>Institution: Jiangnan University
C1	2	10035	10035	[Ning, Xiao; Wang, Lulu; Jin, Shaoming; Cao, Jin] Natl Inst Food & Drug Control, Key Lab Food Qual & Safety State	[Ning, Xiao; Wang, Lulu; Jin, Shaoming; Cao, Jin] National Institute of Food & Drug Control - China, Key Lab Food	Country: China<br>Institution: Natl Inst Food & Drug Control	Country: China<br>Institution: National Institute of Food & Drug Control - China

				Market Regulat, Beijing 100050, Peoples R China.	Qual & Safety State Market Regulat, Beijing 100050, China.		
C1	3	10036	10036	[Wang, Jia-Sheng] Univ Georgia, Coll Publ Hlth, Dept Environm Hlth Sci, Athens, GA 30602 USA.	[Wang, Jia-Sheng] University of Georgia, Coll Publ Hlth, Dept Environm Hlth Sci, Athens, GA 30602 USA.	Country: USA<br>Institution: Univ Georgia	Country: USA<br>Institution: University of Georgia
C1	4	10037	10037	[Mei, Shenghui] Capital Med Univ, Beijing Tiantan Hosp, Dept Pharm, Beijing 100070, Peoples R China.	[Mei, Shenghui] Capital Medical University, Beijing Tiantan Hosp, Dept Pharm, Beijing 100070, China.	Country: China<br>Institution: Capital Med Univ	Country: China<br>Institution: Capital Medical University
C1	5	10038	10038	[Mei, Shenghui] Capital Med Univ, Sch Pharmaceut Sci, Dept Clin Pharmacol, Beijing 100069, Peoples R China.	[Mei, Shenghui] Capital Medical University, Sch Pharmaceut Sci, Dept Clin Pharmacol, Beijing 100069, China.	Country: China<br>Institution: Capital Med Univ	Country: China<br>Institution: Capital Medical University
C3	2	10039	10039	National Institute of Food & Drug Control - China	National Institute of Food & Drug Control	Institution: National Institute of Food & Drug Control - China	Institution: National Institute of Food & Drug Control
RP	1	10042	10042	Sun, XL (corresponding author), Jiangnan Univ, Synerget Innovat Ctr Food Safety & Qual Control, Sch Food Sci & Technol, Int Joint Lab Food Safety, Wuxi 214122, Peoples R China	Sun, XL (corresponding author), Jiangnan University, Synerget Innovat Ctr Food Safety & Qual Control, Sch Food Sci & Technol, Int Joint Lab Food Safety, Wuxi 214122, Peoples R China	Country: China<br>Institution: Jiangnan Univ	Country: China<br>Institution: Jiangnan University
RP	2	10042	10042	Cao, J (corresponding author), Natl Inst Food & Drug Control, Key Lab Food Qual & Safety State Market Regulat, Beijing 100050, Peoples R China	Cao, J (corresponding author), Natl Inst Food & Drug Control, Key Lab Food Qual & Safety State Market Regulat, Beijing 100050, China	Country: China<br>Institution: Natl Inst Food & Drug Control	Country: China<br>Institution: Natl Inst Food & Drug Control

Record 64 | WOS:001234877500001

Investigation of the mechanisms behind ochratoxin A-induced cytotoxicity in human astrocytes and the protective effects of N-acetylcysteine

No C1/C3/RP normalisation changes detected for this record.

Record 65 | WOS:001229022000001

The efficiency of mycotoxin binding by sorbents in the in vitro model using a naturally contaminated animal feed

Field	Position	Original line	Normalised line	Original value	Normalised value	Original country / institution	Normalised country / institution
C1	1	10284	10284	[Zybura, Adrianna; Jedziniak, Piotr] Natl Vet Res Inst, Dept Pharmacol & Toxicol, PL-24100 Pulawy, Poland.	[Zybura, Adrianna; Jedziniak, Piotr] National Veterinary Institute - National Research Institute, Dept Pharmacol & Toxicol, PL-24100 Pulawy, Poland.	Country: Poland<br>Institution: Natl Vet Res Inst	Country: Poland<br>Institution: National Veterinary Institute - National Research Institute
RP	1	10286	10286	Zybura, A (corresponding author), Natl Vet Res Inst, Dept Pharmacol & Toxicol, PL-24100 Pulawy, Poland	Zybura, A (corresponding author), National Veterinary Institute - National Research Institute, Dept Pharmacol & Toxicol, PL-24100 Pulawy, Poland	Country: Poland<br>Institution: Natl Vet Res Inst	Country: Poland<br>Institution: National Veterinary Institute - National Research Institute

Record 66 | WOS:001228887800001

In-vivo evaluation of the adverse effects of ochratoxin A on broiler chicken health and adsorption efficacy of indigenous and commercial clay of Balochistan, Pakistan

Field	Position	Original line	Normalised line	Original value	Normalised value	Original country / institution	Normalised country / institution
C1	1	10376	10376	[Abro, Mustafa Rahim; Rashid, Nadeem] Univ Balochistan, Ctr Adv Studies Vaccinol & Biotechnol, Dept Nutr & Toxicol, Quetta, Pakistan.	[Abro, Mustafa Rahim; Rashid, Nadeem] University of Balochistan, Ctr Adv Studies Vaccinol & Biotechnol, Dept Nutr & Toxicol, Quetta, Pakistan.	Country: Pakistan Institution: Univ Balochistan	Country: Pakistan Institution: University of Balochistan

Record 67 | WOS:001222378900001

Role of albumin in the metabolism and excretion of ochratoxin A

Field	Position	Original line	Normalised line	Original value	Normalised value	Original country / institution	Normalised country / institution
C1	1	10517	10517	[Kuhn, Michael; Humpf, Hans-Ulrich; Cramer, Benedikt] Univ Munster, Inst Food Chem, Corrensstr 45, D-48149 Munster, Germany.	[Kuhn, Michael; Humpf, Hans-Ulrich; Cramer, Benedikt] University of Munster, Inst Food Chem, Corrensstr 45, D-48149 Munster, Germany.	Country: Germany Institution: Univ Munster	Country: Germany Institution: University of Munster
RP	1	10523	10523	Cramer, B (corresponding author), Univ Munster, Inst Food Chem, Corrensstr 45, D-48149 Munster, Germany	Cramer, B (corresponding author), University of Munster, Inst Food Chem, Corrensstr 45, D-48149 Munster, Germany	Country: Germany Institution: Univ Munster	Country: Germany Institution: University of Munster

Record 68 | WOS:001220328900001

Unveiling the emerging role of curcumin to alleviate ochratoxin A-induced muscle toxicity in grass carp (Ctenopharyngodon idella): in vitro and in vivo studies

Field	Position	Original line	Normalised line	Original value	Normalised value	Original country / institution	Normalised country / institution
C1	1	10646	10646	[Zhao, Piao; Feng, Lin; Jiang, Weidan; Wu, Pei; Liu, Yang; Ren, Hongmei; Jin, Xiaowan; Zhou, Xiaoqiu] Sichuan Agr Univ, Anim Nutr Inst, Chengdu 611130, Sichuan, Peoples R China.	[Zhao, Piao; Feng, Lin; Jiang, Weidan; Wu, Pei; Liu, Yang; Ren, Hongmei; Jin, Xiaowan; Zhou, Xiaoqiu] Sichuan Agricultural University, Anim Nutr Inst, Chengdu 611130, Sichuan, China.	Country: China Institution: Sichuan Agr Univ	Country: China Institution: Sichuan Agricultural University
C1	2	10647	10647	[Feng, Lin; Jiang, Weidan; Wu, Pei; Liu, Yang; Ren, Hongmei; Jin, Xiaowan; Zhou, Xiaoqiu] Sichuan Agr Univ, Fish Nutr & Safety Prod Univ Key Lab Sichuan Prov, Chengdu 611130, Sichuan, Peoples R China.	[Feng, Lin; Jiang, Weidan; Wu, Pei; Liu, Yang; Ren, Hongmei; Jin, Xiaowan; Zhou, Xiaoqiu] Sichuan Agricultural University, Fish Nutr & Safety Prod Univ Key Lab Sichuan Prov, Chengdu 611130, Sichuan, China.	Country: China Institution: Sichuan Agr Univ	Country: China Institution: Sichuan Agricultural University
C1	3	10648	10648	[Feng, Lin; Jiang, Weidan; Wu, Pei; Liu, Yang; Ren, Hongmei; Jin, Xiaowan; Zhou, Xiaoqiu] Minist Educ, Minist Agr & Rural Affairs, Key Lab Anim Dis Resistance Nutr, Key Lab Sichuan Prov, Chengdu 611130, Sichuan, Peoples R China.	[Feng, Lin; Jiang, Weidan; Wu, Pei; Liu, Yang; Ren, Hongmei; Jin, Xiaowan; Zhou, Xiaoqiu] Minist Educ, Minist Agr & Rural Affairs, Key Lab Anim Dis Resistance Nutr, Key Lab Sichuan Prov, Chengdu 611130, Sichuan, China.	Country: China Institution: Minist Educ	Country: China Institution: Minist Educ
C1	4	10649	10649	[Zhang, Lu; Mi, Haifeng] Tongwei Co Ltd, Hlth Aquaculture Key Lab Sichuan Prov, Chengdu 610041, Sichuan, Peoples R China.	[Zhang, Lu; Mi, Haifeng] Tongwei Co Ltd, Hlth Aquaculture Key Lab Sichuan Prov, Chengdu 610041, Sichuan, China.	Country: China Institution: Tongwei Co Ltd	Country: China Institution: Tongwei Co Ltd
RP	1	10651	10651	Zhou, XQ (corresponding author), Sichuan Agr Univ, Anim Nutr Inst, Chengdu	Zhou, XQ (corresponding author), Sichuan Agricultural University, Anim Nutr Inst,	Country: China Institution: Sichuan Agr Univ	Country: China Institution: Sichuan Agricultural University

				611130, Sichuan, Peoples R China	Chengdu 611130, Sichuan, Peoples R China		
RP	3	10651	10651	Zhou, XQ (corresponding author), Minist Educ, Minist Agr & Rural Affairs, Key Lab Anim Dis Resistance Nutr, Key Lab Sichuan Prov, Chengdu 611130, Sichuan, Peoples R China	Zhou, XQ (corresponding author), Minist Educ, Minist Agr & Rural Affairs, Key Lab Anim Dis Resistance Nutr, Key Lab Sichuan Prov, Chengdu 611130, Sichuan, China	Country: China<br>Institution: Minist Educ	Country: China<br>Institution: Minist Educ

Record 69 | WOS:001215577500001

Protective Effect of Boric Acid Against Ochratoxin A-Induced Toxic Effects in Human Embryonal Kidney Cells (HEK293): A Study on Cytotoxic, Genotoxic, Oxidative, and Apoptotic Effects

Field	Position	Original line	Normalised line	Original value	Normalised value	Original country / institution	Normalised country / institution
C1	1	10795	10795	[Tekin, Askin] Sinop Univ, Fac Hlth Sci, Dept Occupat Hlth & Safety, Sinop, Turkiye.	[Tekin, Askin] Sinop University, Fac Hlth Sci, Dept Occupat Hlth & Safety, Sinop, Turkey.	Country: Turkey<br>Institution: Sinop Univ	Country: Turkey<br>Institution: Sinop University
C1	2	10796	10796	[Guner, Adem] Giresun Univ, Sebinkarahisar Vocat Sch Hlth Serv, Giresun, Turkiye.	[Guner, Adem] Giresun University, Sebinkarahisar Vocat Sch Hlth Serv, Giresun, Turkey.	Country: Turkey<br>Institution: Giresun Univ	Country: Turkey<br>Institution: Giresun University
C1	3	10797	10797	[Akkan, Tamer] Giresun Univ, Fac Arts & Sci, Biol Dept, Giresun, Turkiye.	[Akkan, Tamer] Giresun University, Fac Arts & Sci, Biol Dept, Giresun, Turkey.	Country: Turkey<br>Institution: Giresun Univ	Country: Turkey<br>Institution: Giresun University
RP	1	10799	10799	Tekin, A (corresponding author), Sinop Univ, Fac Hlth Sci, Dept Occupat Hlth & Safety, Sinop, Turkiye	Tekin, A (corresponding author), Sinop University, Fac Hlth Sci, Dept Occupat Hlth & Safety, Sinop, Turkey	Country: Turkey<br>Institution: Sinop Univ	Country: Turkey<br>Institution: Sinop University

Record 70 | WOS:001231234500001

Alternariol Monomethyl-Ether Induces Toxicity via Cell Death and Oxidative Stress in Swine Intestinal Epithelial Cells

No C1/C3/RP normalisation changes detected for this record.

Record 71 | WOS:001227638700001

Development and application of the physiologically-based toxicokinetic (PBTK) model for ochratoxin A (OTA) in rats and humans

Field	Position	Original line	Normalised line	Original value	Normalised value	Original country / institution	Normalised country / institution
C1	1	11055	11055	[Su, Bu-Da; Li, Xiao-Meng; Wang, Yue; Xu, Yan-Yan; Shu, Le-Xin; Li, Yu-Bo] Tianjin Univ Tradit Chinese Med, Sch Chinese Mat Med, Tianjin State Key Lab Modern Chinese Med, Tianjin 301617, Peoples R China.	[Su, Bu-Da; Li, Xiao-Meng; Wang, Yue; Xu, Yan-Yan; Shu, Le-Xin; Li, Yu-Bo] Tianjin University of Traditional Chinese Medicine, Sch Chinese Mat Med, Tianjin State Key Lab Modern Chinese Med, Tianjin 301617, China.	Country: China<br>Institution: Tianjin Univ Tradit Chinese Med	Country: China<br>Institution: Tianjin University of Traditional Chinese Medicine
C1	2	11056	11056	[Huang, Zhi-Wei] Fudan Univ, Huashan Hosp, Phase I Clin Res Ctr, Shanghai 200040, Peoples R China.	[Huang, Zhi-Wei] Fudan University, Huashan Hosp, Phase I Clin Res Ctr, Shanghai 200040, China.	Country: China<br>Institution: Fudan Univ	Country: China<br>Institution: Fudan University
C1	3	11057	11057	[Shao, Jia] Tianjin First Cent Hosp, Dept Pharm, Tianjin 300192, Peoples R China.	[Shao, Jia] Tianjin First Cent Hosp, Dept Pharm, Tianjin 300192, China.	Country: China<br>Institution: Tianjin First Cent Hosp	Country: China<br>Institution: Tianjin First Cent Hosp

C1	4	11058	11058	[Xu, Yan-Yan; Shu, Le-Xin; Li, Yu-Bo] Tianjin Univ Tradit Chinese Med, 10 Poyanghu Rd, Tianjin, Peoples R China.	[Xu, Yan-Yan; Shu, Le-Xin; Li, Yu-Bo] Tianjin University of Traditional Chinese Medicine, 10 Poyanghu Rd, Tianjin, China.	Country: China<br>Institution: Tianjin Univ Tradit Chinese Med	Country: China<br>Institution: Tianjin University of Traditional Chinese Medicine
RP	3	11062	11062	Li, YB (corresponding author), Tianjin Univ Tradit Chinese Med, 10 Poyanghu Rd, Tianjin, Peoples R China	Li, YB (corresponding author), Tianjin University of Traditional Chinese Medicine, 10 Poyanghu Rd, Tianjin, China	Country: China<br>Institution: Tianjin Univ Tradit Chinese Med	Country: China<br>Institution: Tianjin University of Traditional Chinese Medicine

Record 72 | WOS:001209938800001

Comprehensive Insights into Ochratoxin A: Occurrence, Analysis, and Control Strategies

Field	Position	Original line	Normalised line	Original value	Normalised value	Original country / institution	Normalised country / institution
C1	5	11222	11222	[Luvisi, Andrea; De Bellis, Luigi] Univ Salento, Dipartimento Sci & Tecnol Biol & Ambientali, Palazzina Acentro Ecotekne Via Prov, I-73100 Lecce, Italy.	[Luvisi, Andrea; De Bellis, Luigi] University of Salento, Dipartimento Sci & Tecnol Biol & Ambientali, Palazzina Acentro Ecotekne Via Prov, I-73100 Lecce, Italy.	Country: Italy<br>Institution: Univ Salento	Country: Italy<br>Institution: University of Salento
RP	1	11225	11225	De Bellis, L (corresponding author), Univ Salento, Dipartimento Sci & Tecnol Biol & Ambientali, Palazzina Acentro Ecotekne Via Prov, I-73100 Lecce, Italy	De Bellis, L (corresponding author), University of Salento, Dipartimento Sci & Tecnol Biol & Ambientali, Palazzina Acentro Ecotekne Via Prov, I-73100 Lecce, Italy	Country: Italy<br>Institution: Univ Salento	Country: Italy<br>Institution: University of Salento

Record 73 | WOS:001221312500001

In vitro and in vivo assessment of AFB1 and OTA toxic effects and the beneficial role of bioactive compounds. A systematic review

Field	Position	Original line	Normalised line	Original value	Normalised value	Original country / institution	Normalised country / institution
C1	1	11609	11609	[Frangiamone, Massimo; Cimbalo, Alessandra; Font, Guillermina; Manyes, Lara] Univ Valencia, Fac Pharm, Lab Food Chem & Toxicol, Av Carrer Vicent Andres Estelles S-N, Burjassot 46100, Spain.	[Frangiamone, Massimo; Cimbalo, Alessandra; Font, Guillermina; Manyes, Lara] University of Valencia, Fac Pharm, Lab Food Chem & Toxicol, Av Carrer Vicent Andres Estelles S-N, Burjassot 46100, Spain.	Country: Spain<br>Institution: Univ Valencia	Country: Spain<br>Institution: University of Valencia
RP	1	11611	11611	Cimbalo, A (corresponding author), Univ Valencia, Fac Pharm, Lab Food Chem & Toxicol, Av Carrer Vicent Andres Estelles S-N, Burjassot 46100, Spain	Cimbalo, A (corresponding author), University of Valencia, Fac Pharm, Lab Food Chem & Toxicol, Av Carrer Vicent Andres Estelles S-N, Burjassot 46100, Spain	Country: Spain<br>Institution: Univ Valencia	Country: Spain<br>Institution: University of Valencia

Record 74 | WOS:001219077100001

Disruption of intestinal epithelial permeability in the Co-culture system of Caco-2/HT29-MTX cells exposed individually or simultaneously to acrylamide and ochratoxin A

Field	Position	Original line	Normalised line	Original value	Normalised value	Original country / institution	Normalised country / institution
C1	1	11780	11780	[Lu, Jiawen; Su, Dan; Yang, Ying; Shu, Mengni; Wang, Yuting; Zhou, Xingtao; Yu, Qiang; Li, Chang; Xie, Jianhua; Chen, Yi] Nanchang Univ, State	[Lu, Jiawen; Su, Dan; Yang, Ying; Shu, Mengni; Wang, Yuting; Zhou, Xingtao; Yu, Qiang; Li, Chang; Xie, Jianhua; Chen, Yi] Nanchang University,	Country: China<br>Institution: Nanchang Univ	Country: China<br>Institution: Nanchang University

				Key Lab Food Sci & Resources, 235 Nanjing East Rd, Nanchang 330047, Jiangxi, Peoples R China.	State Key Lab Food Sci & Resources, 235 Nanjing East Rd, Nanchang 330047, Jiangxi, China.		
RP	1	11782	11782	Chen, Y (corresponding author), Nanchang Univ, State Key Lab Food Sci & Resources, 235 Nanjing East Rd, Nanchang 330047, Jiangxi, Peoples R China	Chen, Y (corresponding author), Nanchang University, State Key Lab Food Sci & Resources, 235 Nanjing East Rd, Nanchang 330047, Jiangxi, China	Country: China Institution: Nanchang Univ	Country: China Institution: Nanchang University

Record 75 | WOS:001185505600042

A high-throughput gut-on-chip platform to study the epithelial responses to enterotoxins

No C1/C3/RP normalisation changes detected for this record.

Record 76 | WOS:001187481100001

Efficacy of a multicomponent binding agent against combined exposure to zearalenone and ochratoxin A in weaned pigs

Field	Position	Original line	Normalised line	Original value	Normalised value	Original country / institution	Normalised country / institution
C1	1	12038	12038	[Tassis, Panagiotis; Floros, Dimitrios; Ntarampa, Niki] Aristotle Univ Thessaloniki, Sch Vet Med, Farm Anim Clin, Thessaloniki, Greece.	[Tassis, Panagiotis; Floros, Dimitrios; Ntarampa, Niki] Aristotle University of Thessaloniki, Sch Vet Med, Farm Anim Clin, Thessaloniki, Greece.	Country: Greece Institution: Aristotle Univ Thessaloniki	Country: Greece Institution: Aristotle University of Thessaloniki
C1	4	12041	12041	[Polizopoulou, Zoe] Aristotle Univ Thessaloniki, Sch Vet Med, Diagnost Lab, Thessaloniki, Greece.	[Polizopoulou, Zoe] Aristotle University of Thessaloniki, Sch Vet Med, Diagnost Lab, Thessaloniki, Greece.	Country: Greece Institution: Aristotle Univ Thessaloniki	Country: Greece Institution: Aristotle University of Thessaloniki
RP	1	12044	12044	Tassis, P (corresponding author), Aristotle Univ Thessaloniki, Sch Vet Med, Farm Anim Clin, Thessaloniki, Greece	Tassis, P (corresponding author), Aristotle University of Thessaloniki, Sch Vet Med, Farm Anim Clin, Thessaloniki, Greece	Country: Greece Institution: Aristotle Univ Thessaloniki	Country: Greece Institution: Aristotle University of Thessaloniki

Record 77 | WOS:001192691800001

Red Orange and Lemon Extract Ameliorates the Renal Oxidative Stress and Inflammation Induced by Ochratoxin A through the Modulation of Nrf2

Field	Position	Original line	Normalised line	Original value	Normalised value	Original country / institution	Normalised country / institution
C1	1	12159	12159	[Longobardi, Consiglia; Damiano, Sara; Montagnaro, Serena; Russo, Valeria; Vaccaro, Emanuela; Florio, Salvatore; Ciarcia, Roberto] Univ Naples Federico II, Dept Vet Med & Anim Prod, Via F Delpino 1, I-80137 Naples, Italy.	[Longobardi, Consiglia; Damiano, Sara; Montagnaro, Serena; Russo, Valeria; Vaccaro, Emanuela; Florio, Salvatore; Ciarcia, Roberto] University of Naples Federico II, Dept Vet Med & Anim Prod, Via F Delpino 1, I-80137 Naples, Italy.	Country: Italy Institution: Univ Naples Federico II	Country: Italy Institution: University of Naples Federico II
C1	3	12161	12161	[Giordano, Antonio] Temple Univ, Sbarro Inst Canc Res & Mol Med, Coll Sci & Technol, Ctr Biotechnol, Philadelphia, PA 19122 USA.	[Giordano, Antonio] Temple University, Sbarro Inst Canc Res & Mol Med, Coll Sci & Technol, Ctr Biotechnol, Philadelphia, PA 19122 USA.	Country: USA Institution: Temple Univ	Country: USA Institution: Temple University

C1	4	12162	12162	[Giordano, Antonio] Univ Siena, Dept Med Biotechnol, I-53100 Siena, Italy.	[Giordano, Antonio] University of Siena, Dept Med Biotechnol, I-53100 Siena, Italy.	Country: Italy<br>Institution: Univ Siena	Country: Italy<br>Institution: University of Siena
RP	2	12167	12167	Ciarcia, R (corresponding author), Univ Naples Federico II, Dept Vet Med & Anim Prod, Via F Delpino 1, I-80137 Naples, Italy	Ciarcia, R (corresponding author), University of Naples Federico II, Dept Vet Med & Anim Prod, Via F Delpino 1, I-80137 Naples, Italy	Country: Italy<br>Institution: Univ Naples Federico II	Country: Italy<br>Institution: University of Naples Federico II

Record 78 | WOS:001176181400001

Natural feed additives and bioactive supplements versus chemical additives as a safe and practical approach to combat foodborne mycotoxicoses

Field	Position	Original line	Normalised line	Original value	Normalised value	Original country / institution	Normalised country / institution
C1	1	12273	12273	[Stoev, Stoycho D.] Trakia Univ, Fac Vet Med, Dept Gen & Clin Pathol, Stara Zagora, Bulgaria.	[Stoev, Stoycho D.] Trakia University, Fac Vet Med, Dept Gen & Clin Pathol, Stara Zagora, Bulgaria.	Country: Bulgaria<br>Institution: Trakia Univ	Country: Bulgaria<br>Institution: Trakia University
RP	1	12275	12275	Stoev, SD (corresponding author), Trakia Univ, Fac Vet Med, Dept Gen & Clin Pathol, Stara Zagora, Bulgaria	Stoev, SD (corresponding author), Trakia University, Fac Vet Med, Dept Gen & Clin Pathol, Stara Zagora, Bulgaria	Country: Bulgaria<br>Institution: Trakia Univ	Country: Bulgaria<br>Institution: Trakia University

Record 79 | WOS:001179683700001

In vitro and in vivo induction of ochratoxin A exposure-related micronucleus formation in rat proximal tubular epithelial cells and expression profiling of chromosomal instability-related genes

Field	Position	Original line	Normalised line	Original value	Normalised value	Original country / institution	Normalised country / institution
C1	3	12642	12642	[Jin, Meilan] Southwest Univ, Coll Vet Med, Lab Vet Pathol, 2 Tiansheng Rd, Chongqing 400715, Peoples R China.	[Jin, Meilan] Southwest Univ, Coll Vet Med, Lab Vet Pathol, 2 Tiansheng Rd, Chongqing 400715, China.	Country: China<br>Institution: Southwest Univ	Country: China<br>Institution: Southwest Univ
C3	3	12644	12644	Southwest University - China	Southwest University	Institution: Southwest University - China	Institution: Southwest University

Record 80 | WOS:001172173100001

Comparative Study of Spheroids (3D) and Monolayer Cultures (2D) for the In Vitro Assessment of Cytotoxicity Induced by the Mycotoxins Sterigmatocystin, Ochratoxin A and Patulin

Field	Position	Original line	Normalised line	Original value	Normalised value	Original country / institution	Normalised country / institution
C1	2	12783	12783	[Zingales, Veronica; Ruiz, Maria-Jose] Univ Valencia, Fac Pharm, Lab Toxicol, Av Vicent Andres Estelles S-N, Valencia 46100, Spain.	[Zingales, Veronica; Ruiz, Maria-Jose] University of Valencia, Fac Pharm, Lab Toxicol, Av Vicent Andres Estelles S-N, Valencia 46100, Spain.	Country: Spain<br>Institution: Univ Valencia	Country: Spain<br>Institution: University of Valencia
C1	3	12784	12784	[Zingales, Veronica; Esposito, Maria Rosaria; Quagliata, Martina; Cimetta, Elisa] Univ Padua, Dept Ind Engn DII, Via Marzolo 9, I-35131 Padua, Italy.	[Zingales, Veronica; Esposito, Maria Rosaria; Quagliata, Martina; Cimetta, Elisa] University of Padua, Dept Ind Engn DII, Via Marzolo 9, I-35131 Padua, Italy.	Country: Italy<br>Institution: Univ Padua	Country: Italy<br>Institution: University of Padua

Record 81 | WOS:001175293000001

Involvement of pro-inflammatory mediators and cell cycle disruption in neuronal cells induced by gliotoxin and ochratoxin A after individual and combined exposure

Field	Position	Original line	Normalised line	Original value	Normalised value	Original country / institution	Normalised country / institution
C1	1	12912	12912	[Penalva-Olcina, Raquel; Juan, Cristina; Fernandez-Franzon, Monica; Juan-Garcia, Ana] Univ Valencia, Fac Pharm & Food Sci, Lab Food Chem & Toxicol, Ave Vicent Andres Estelles s-n, Burjassot 46100, Valencia, Spain.	[Penalva-Olcina, Raquel; Juan, Cristina; Fernandez-Franzon, Monica; Juan-Garcia, Ana] University of Valencia, Fac Pharm & Food Sci, Lab Food Chem & Toxicol, Ave Vicent Andres Estelles s-n, Burjassot 46100, Valencia, Spain.	Country: Spain Institution: Univ Valencia	Country: Spain Institution: University of Valencia
RP	1	12914	12914	Juan-GarcÃ-a, A (corresponding author), Univ Valencia, Fac Pharm & Food Sci, Lab Food Chem & Toxicol, Ave Vicent Andres Estelles s-n, Burjassot 46100, Valencia, Spain	Juan-GarcÃ-a, A (corresponding author), University of Valencia, Fac Pharm & Food Sci, Lab Food Chem & Toxicol, Ave Vicent Andres Estelles s-n, Burjassot 46100, Valencia, Spain	Country: Spain Institution: Univ Valencia	Country: Spain Institution: University of Valencia

Record 82 | WOS:001154746400001

Recent advances in the degradation efficacy and mechanisms of mycotoxins in food by atmospheric cold plasma

Field	Position	Original line	Normalised line	Original value	Normalised value	Original country / institution	Normalised country / institution
C1	1	13034	13034	[Liu, Mengjie; Yang, Xudong; Yu, Bo; Xu, Hangbo; Ma, Ruonan; Jiao, Zhen] Zhengzhou Univ, Sch Agr Sci, State Key Lab Cotton Biol, Zhengzhou Res Base, Zhengzhou 450001, Peoples R China.	[Liu, Mengjie; Yang, Xudong; Yu, Bo; Xu, Hangbo; Ma, Ruonan; Jiao, Zhen] Zhengzhou University, Sch Agr Sci, State Key Lab Cotton Biol, Zhengzhou Res Base, Zhengzhou 450001, China.	Country: China Institution: Zhengzhou Univ	Country: China Institution: Zhengzhou University
C1	2	13035	13035	[Liu, Mengjie; Yang, Xudong; Xu, Hangbo; Ma, Ruonan; Jiao, Zhen] Zhengzhou Univ, Henan Key Lab Ion beam Bioengn, Zhengzhou 450052, Peoples R China.	[Liu, Mengjie; Yang, Xudong; Xu, Hangbo; Ma, Ruonan; Jiao, Zhen] Zhengzhou University, Henan Key Lab Ion beam Bioengn, Zhengzhou 450052, China.	Country: China Institution: Zhengzhou Univ	Country: China Institution: Zhengzhou University
C1	3	13036	13036	[Xiang, Qisen] Zhengzhou Univ Light Ind, Coll Food & Bioengn, Zhengzhou 450001, Peoples R China.	[Xiang, Qisen] Zhengzhou Univ Light Ind, Coll Food & Bioengn, Zhengzhou 450001, China.	Country: China Institution: Zhengzhou Univ Light Ind	Country: China Institution: Zhengzhou Univ Light Ind
C1	4	13037	13037	[Feng, Junxia] Huadu Dist Peoples Hosp Guangzhou, Dept Urol, Guangzhou 510800, Peoples R China.	[Feng, Junxia] Huadu Dist Peoples Hosp Guangzhou, Dept Urol, Guangzhou 510800, China.	Country: China Institution: Huadu Dist Peoples Hosp Guangzhou	Country: China Institution: Huadu Dist Peoples Hosp Guangzhou
C1	5	13038	13038	[Zhuang, Jie] Chinese Acad Sci, Suzhou Inst Biomed Engn & Technol, Suzhou 215163, Peoples R China.	[Zhuang, Jie] Chinese Academy of Sciences, Suzhou Inst Biomed Engn & Technol, Suzhou 215163, China.	Country: China Institution: Chinese Acad Sci	Country: China Institution: Chinese Academy of Sciences
RP	1	13042	13042	Ma, RA (corresponding author), Zhengzhou Univ, Sch Agr Sci, State Key Lab Cotton Biol, Zhengzhou Res Base, Zhengzhou 450001, Peoples R China	Ma, RA (corresponding author), Zhengzhou University, Sch Agr Sci, State Key Lab Cotton Biol, Zhengzhou Res Base, Zhengzhou 450001, Peoples R China	Country: China Institution: Zhengzhou Univ	Country: China Institution: Zhengzhou University
RP	2	13042	13042	Xiang, QS (corresponding author), Zhengzhou Univ Light Ind, Coll Food & Bioengn, Zhengzhou 450001, Peoples R China	Xiang, QS (corresponding author), Zhengzhou Univ Light Ind, Coll Food & Bioengn, Zhengzhou 450001, China	Country: China Institution: Zhengzhou Univ Light Ind	Country: China Institution: Zhengzhou Univ Light Ind

Record 83 | WOS:001201732900001

Comparative Assessment of Thermal Treatment for Mycotoxins Adsorption Potential of Indigenous Clay from Balochistan, Pakistan

Field	Position	Original line	Normalised line	Original value	Normalised value	Original country / institution	Normalised country / institution
C1	1	13195	13195	[Abro, Mustafa Rahim; Rashid, Nadeem] Univ Balochistan, Ctr Adv Studies Vaccinol & Biotechnol, Quetta 873000, Pakistan.	[Abro, Mustafa Rahim; Rashid, Nadeem] University of Balochistan, Ctr Adv Studies Vaccinol & Biotechnol, Quetta 873000, Pakistan.	Country: Pakistan Institution: Univ Balochistan	Country: Pakistan Institution: University of Balochistan
RP	1	13199	13199	Abro, MR (corresponding author), Univ Balochistan, Ctr Adv Studies Vaccinol & Biotechnol, Quetta 873000, Pakistan	Abro, MR (corresponding author), University of Balochistan, Ctr Adv Studies Vaccinol & Biotechnol, Quetta 873000, Pakistan	Country: Pakistan Institution: Univ Balochistan	Country: Pakistan Institution: University of Balochistan

Record 84 | WOS:001151094300001

Practical Strategies to Reduce Ochratoxin A in Foods

Field	Position	Original line	Normalised line	Original value	Normalised value	Original country / institution	Normalised country / institution
C1	1	13288	13288	[Lee, Hyun Jung; Kim, Hae Dun] Univ Idaho, Dept Anim Vet & Food Sci, Moscow, ID 83844 USA.	[Lee, Hyun Jung; Kim, Hae Dun] University of Idaho, Dept Anim Vet & Food Sci, Moscow, ID 83844 USA.	Country: USA Institution: Univ Idaho	Country: USA Institution: University of Idaho
RP	1	13292	13292	Lee, HJ (corresponding author), Univ Idaho, Dept Anim Vet & Food Sci, Moscow, ID 83844 USA	Lee, HJ (corresponding author), University of Idaho, Dept Anim Vet & Food Sci, Moscow, ID 83844 USA	Country: USA Institution: Univ Idaho	Country: USA Institution: University of Idaho

Record 85 | WOS:001262485700001

Grape pomace as a novel functional ingredient: Mitigating ochratoxin A bioaccessibility and unraveling cytoprotective mechanisms in vitro

Field	Position	Original line	Normalised line	Original value	Normalised value	Original country / institution	Normalised country / institution
C1	1	13519	13519	[Mangiapelo, Luciano; Ianni, Federica; Cossignani, Lina] Univ Perugia, Dept Pharmaceut Sci, Sect Food Sci & Nutr, I-06123 Perugia, Italy.	[Mangiapelo, Luciano; Ianni, Federica; Cossignani, Lina] University of Perugia, Dept Pharmaceut Sci, Sect Food Sci & Nutr, I-06123 Perugia, Italy.	Country: Italy Institution: Univ Perugia	Country: Italy Institution: University of Perugia
C1	2	13520	13520	[Frangiamone, Massimo] Univ Lausanne, Dept Biomed Sci, Rue Bugnon, CH-1005 Lausanne, Switzerland.	[Frangiamone, Massimo] University of Lausanne, Dept Biomed Sci, Rue Bugnon, CH-1005 Lausanne, Switzerland.	Country: Switzerland Institution: Univ Lausanne	Country: Switzerland Institution: University of Lausanne
C1	3	13521	13521	[Vila-Donat, Pilar; Pasca, Denisia; Manyes, Lara] Univ Valencia, Fac Farm & Ciencies Alimentacio, Lab Food Chem & Toxicol, Burjassot 46100, Spain.	[Vila-Donat, Pilar; Pasca, Denisia; Manyes, Lara] University of Valencia, Fac Farm & Ciencies Alimentacio, Lab Food Chem & Toxicol, Burjassot 46100, Spain.	Country: Spain Institution: Univ Valencia	Country: Spain Institution: University of Valencia
RP	1	13525	13525	Cossignani, L (corresponding author), Univ Perugia, Dept Pharmaceut Sci, Sect Food Sci & Nutr, I-06123 Perugia, Italy	Cossignani, L (corresponding author), University of Perugia, Dept Pharmaceut Sci, Sect Food Sci & Nutr, I-06123 Perugia, Italy	Country: Italy Institution: Univ Perugia	Country: Italy Institution: University of Perugia

Record 86 | WOS:001145669600001

Ochratoxin A-enhanced glycolysis induces inflammatory responses in human gastric epithelium cells through mTOR/HIF-1Î± signaling pathway

Field	Position	Original line	Normalised line	Original value	Normalised value	Original country / institution	Normalised country / institution
C1	1	13661	13661	[Wang, Yuan; Cui, Jinfeng; Lian, Hongguang; Hao, Zengfang; Lou, Lei; Jia, Xin; Zhao, Wei; Zhang, Xianghong] Hebei Med Univ, Hosp 2, Dept Pathol, Shijiazhuang, Peoples R China.	[Wang, Yuan; Cui, Jinfeng; Lian, Hongguang; Hao, Zengfang; Lou, Lei; Jia, Xin; Zhao, Wei; Zhang, Xianghong] Hebei Medical University, Hosp 2, Dept Pathol, Shijiazhuang, China.	Country: China<br>Institution: Hebei Med Univ	Country: China<br>Institution: Hebei Medical University
C1	2	13662	13662	[Zhao, Man; Shen, Haitao; Xing, Lingxiao; Zhang, Xianghong] Hebei Med Univ, Dept Pathol, Shijiazhuang, Peoples R China.	[Zhao, Man; Shen, Haitao; Xing, Lingxiao; Zhang, Xianghong] Hebei Medical University, Dept Pathol, Shijiazhuang, China.	Country: China<br>Institution: Hebei Med Univ	Country: China<br>Institution: Hebei Medical University
C1	3	13663	13663	[Zhang, Xianghong] Hebei Med Univ, Hosp 2, Dept Pathol, 215 Heping Western Rd, Shijiazhuang, Peoples R China.	[Zhang, Xianghong] Hebei Medical University, Hosp 2, Dept Pathol, 215 Heping Western Rd, Shijiazhuang, China.	Country: China<br>Institution: Hebei Med Univ	Country: China<br>Institution: Hebei Medical University
RP	1	13666	13666	Zhang, XH (corresponding author), Hebei Med Univ, Hosp 2, Dept Pathol, 215 Heping Western Rd, Shijiazhuang, Peoples R China	Zhang, XH (corresponding author), Hebei Medical University, Hosp 2, Dept Pathol, 215 Heping Western Rd, Shijiazhuang, China	Country: China<br>Institution: Hebei Med Univ	Country: China<br>Institution: Hebei Medical University

Record 87 | WOS:001119207400001

Elucidating the mechanism of action of mycotoxins through machine learning-driven QSAR models: Focus on lipid peroxidation

Field	Position	Original line	Normalised line	Original value	Normalised value	Original country / institution	Normalised country / institution
C1	1	13760	13760	[Galvez-Llompart, Maria; Manyes, Lara; Meca, Giuseppe] Univ Valencia, Fac Pharm, Dept Prevent Med & Publ Hlth, Food Sci Toxicol & Forens Med, Valencia 46100, Spain.	[Galvez-Llompart, Maria; Manyes, Lara; Meca, Giuseppe] University of Valencia, Fac Pharm, Dept Prevent Med & Publ Hlth, Food Sci Toxicol & Forens Med, Valencia 46100, Spain.	Country: Spain<br>Institution: Univ Valencia	Country: Spain<br>Institution: University of Valencia
C1	2	13761	13761	[Galvez-Llompart, Maria; Zanni, Riccardo] Univ Valencia, Dept Phys Chem, E-46100 Burjassot, Valencia, Spain.	[Galvez-Llompart, Maria; Zanni, Riccardo] University of Valencia, Dept Phys Chem, E-46100 Burjassot, Valencia, Spain.	Country: Spain<br>Institution: Univ Valencia	Country: Spain<br>Institution: University of Valencia
RP	1	13763	13763	Galvez-Llompart, M (corresponding author), Univ Valencia, Fac Pharm, Dept Prevent Med & Publ Hlth, Food Sci Toxicol & Forens Med, Valencia 46100, Spain	Galvez-Llompart, M (corresponding author), University of Valencia, Fac Pharm, Dept Prevent Med & Publ Hlth, Food Sci Toxicol & Forens Med, Valencia 46100, Spain	Country: Spain<br>Institution: Univ Valencia	Country: Spain<br>Institution: University of Valencia

Record 88 | WOS:001099952500001

Risks for animal health related to the presence of ochratoxin A (OTA) in feed

No C1/C3/RP normalisation changes detected for this record.

Record 89 | WOS:001072761100001

Farnesoid X Receptor Plays a Key Role in Ochratoxin A-Induced Nephrotoxicity by Targeting Ferroptosis In Vivo and In Vitro

Field	Position	Original line	Normalised line	Original value	Normalised value	Original country / institution	Normalised country / institution
C1	1	14194	14194	[Tang, Jiangyu; Zeng, Junya; Chen, Li; Wang, Mengmeng; He, Suibin; Muhmood, Azhar; Chen, Xingxiang; Huang, Kehe; Gan, Fang] Nanjing Agr Univ, Coll Vet Med, Nanjing 210095, Jiangsu, Peoples R China.	[Tang, Jiangyu; Zeng, Junya; Chen, Li; Wang, Mengmeng; He, Suibin; Muhmood, Azhar; Chen, Xingxiang; Huang, Kehe; Gan, Fang] Nanjing Agricultural University, Coll Vet Med, Nanjing 210095, Jiangsu, China.	Country: China<br>Institution: Nanjing Agr Univ	Country: China<br>Institution: Nanjing Agricultural University
C1	2	14195	14195	[Tang, Jiangyu; Zeng, Junya; Chen, Li; Wang, Mengmeng; Muhmood, Azhar; Chen, Xingxiang; Huang, Kehe; Gan, Fang] Nanjing Agr Univ, Inst Anim Nutr Hlth, Nanjing 210095, Jiangsu, Peoples R China.	[Tang, Jiangyu; Zeng, Junya; Chen, Li; Wang, Mengmeng; Muhmood, Azhar; Chen, Xingxiang; Huang, Kehe; Gan, Fang] Nanjing Agricultural University, Inst Anim Nutr Hlth, Nanjing 210095, Jiangsu, China.	Country: China<br>Institution: Nanjing Agr Univ	Country: China<br>Institution: Nanjing Agricultural University
C1	3	14196	14196	[Tang, Jiangyu; Zeng, Junya; Chen, Li; Wang, Mengmeng; Muhmood, Azhar; Chen, Xingxiang; Huang, Kehe; Gan, Fang] Nanjing Agr Univ, Coll Vet Med, MOE Joint Int Res Lab Anim Hlth & Food Safety, Nanjing 210095, Jiangsu, Peoples R China.	[Tang, Jiangyu; Zeng, Junya; Chen, Li; Wang, Mengmeng; Muhmood, Azhar; Chen, Xingxiang; Huang, Kehe; Gan, Fang] Nanjing Agricultural University, Coll Vet Med, MOE Joint Int Res Lab Anim Hlth & Food Safety, Nanjing 210095, Jiangsu, China.	Country: China<br>Institution: Nanjing Agr Univ	Country: China<br>Institution: Nanjing Agricultural University
C1	4	14197	14197	[He, Suibin] Anim Husb & Aquat Prod Technol Promot Ctr Pudong, Shanghai 201299, Peoples R China.	[He, Suibin] Anim Husb & Aquat Prod Technol Promot Ctr Pudong, Shanghai 201299, China.	Country: China<br>Institution: Anim Husb & Aquat Prod Technol Promot Ctr Pudong	Country: China<br>Institution: Anim Husb & Aquat Prod Technol Promot Ctr Pudong
RP	1	14200	14200	Gan, F (corresponding author), Nanjing Agr Univ, Coll Vet Med, Nanjing 210095, Jiangsu, Peoples R China	Gan, F (corresponding author), Nanjing Agricultural University, Coll Vet Med, Nanjing 210095, Jiangsu, Peoples R China	Country: China<br>Institution: Nanjing Agr Univ	Country: China<br>Institution: Nanjing Agricultural University
RP	3	14200	14200	Gan, F (corresponding author), Nanjing Agr Univ, Coll Vet Med, MOE Joint Int Res Lab Anim Hlth & Food Safety, Nanjing 210095, Jiangsu, Peoples R China	Gan, F (corresponding author), Nanjing Agr Univ, Coll Vet Med, MOE Joint Int Res Lab Anim Hlth & Food Safety, Nanjing 210095, Jiangsu, China	Country: China<br>Institution: Nanjing Agr Univ	Country: China<br>Institution: Nanjing Agr Univ

Record 90 | WOS:001079796800001

Neurotoxicity of ochratoxin A: Molecular mechanisms and neurotherapeutic strategies

No C1/C3/RP normalisation changes detected for this record.

Record 91 | WOS:001078658300001

The coexistence of aflatoxin M1 and ochratoxin A induced intestinal barrier disruption via the regulation of key differentially expressed microRNAs and long non-coding RNAs in BALB/c mice

Field	Position	Original line	Normalised line	Original value	Normalised value	Original country / institution	Normalised country / institution

C1	1	14526	14526	[Gao, Ya-Nan; Yang, Xue; Wang, Jia-Qi; Zheng, Nan] Chinese Acad Agr Sci, Inst Anim Sci, Key Lab Qual & Safety Control Milk & Dairy Prod, Minist Agr & Rural Affairs,Lab Qual & Safety Risk, Beijing 100193, Peoples R China.	[Gao, Ya-Nan; Yang, Xue; Wang, Jia-Qi; Zheng, Nan] Chinese Academy of Agricultural Sciences, Inst Anim Sci, Key Lab Qual & Safety Control Milk & Dairy Prod, Minist Agr & Rural Affairs,Lab Qual & Safety Risk, Beijing 100193, China.	Country: China<br>Institution: Chinese Acad Agr Sci	Country: China<br>Institution: Chinese Academy of Agricultural Sciences
C1	2	14527	14527	[Min, Li] Guangdong Acad Agr Sci, Inst Anim Sci, Key Lab Anim Nutr & Feed Sci South China, Minist Agr,Guangdong Publ Lab Anim Breeding & Nutr, Guangzhou 510640, Peoples R China.	[Min, Li] Guangdong Academy of Agricultural Sciences, Inst Anim Sci, Key Lab Anim Nutr & Feed Sci South China, Minist Agr,Guangdong Publ Lab Anim Breeding & Nutr, Guangzhou 510640, China.	Country: China<br>Institution: Guangdong Acad Agr Sci	Country: China<br>Institution: Guangdong Academy of Agricultural Sciences
C1	3	14528	14528	[Zheng, Nan] Chinese Acad Agr Sci, Inst Anim Sci, 2 Yuanmingyuan West Rd, Beijing 100193, Peoples R China.	[Zheng, Nan] Chinese Academy of Agricultural Sciences, Inst Anim Sci, 2 Yuanmingyuan West Rd, Beijing 100193, China.	Country: China<br>Institution: Chinese Acad Agr Sci	Country: China<br>Institution: Chinese Academy of Agricultural Sciences
RP	1	14532	14532	Zheng, N (corresponding author), Chinese Acad Agr Sci, Inst Anim Sci, 2 Yuanmingyuan West Rd, Beijing 100193, Peoples R China	Zheng, N (corresponding author), Chinese Academy of Agricultural Sciences, Inst Anim Sci, 2 Yuanmingyuan West Rd, Beijing 100193, China	Country: China<br>Institution: Chinese Acad Agr Sci	Country: China<br>Institution: Chinese Academy of Agricultural Sciences

Record 92 | WOS:001077853000001

Ochratoxin A: Overview of Prevention, Removal, and Detoxification Methods

Field	Position	Original line	Normalised line	Original value	Normalised value	Original country / institution	Normalised country / institution
C1	1	14642	14642	[Ding, Lijuan; Han, Meihua; Wang, Xiangtao; Guo, Yifei] Chinese Acad Med Sci & Peking Union Med Coll, Inst Med Plant Dev, 151 Malianwa North Rd, Beijing 100193, Peoples R China.	[Ding, Lijuan; Han, Meihua; Wang, Xiangtao; Guo, Yifei] Chinese Academy of Medical Sciences - Peking Union Medical College, Inst Med Plant Dev, 151 Malianwa North Rd, Beijing 100193, China.	Country: China<br>Institution: Chinese Acad Med Sci & Peking Union Med Coll	Country: China<br>Institution: Chinese Academy of Medical Sciences - Peking Union Medical College
RP	1	14646	14646	Guo, YF (corresponding author), Chinese Acad Med Sci & Peking Union Med Coll, Inst Med Plant Dev, 151 Malianwa North Rd, Beijing 100193, Peoples R China	Guo, YF (corresponding author), Chinese Academy of Medical Sciences - Peking Union Medical College, Inst Med Plant Dev, 151 Malianwa North Rd, Beijing 100193, China	Country: China<br>Institution: Chinese Acad Med Sci & Peking Union Med Coll	Country: China<br>Institution: Chinese Academy of Medical Sciences - Peking Union Medical College

Record 93 | WOS:001060917800001

Exploring the Toxic Effects of ZEA on IPEC-J2 Cells from the Inflammatory Response and Apoptosis

Field	Position	Original line	Normalised line	Original value	Normalised value	Original country / institution	Normalised country / institution
C1	1	14815	14815	[Guan, Haoyue; Ma, Wenxue; Cai, Jingzeng; Zhang, Ziwei] Northeast Agr Univ, Coll Vet Med, Harbin 150030, Peoples R China.	[Guan, Haoyue; Ma, Wenxue; Cai, Jingzeng; Zhang, Ziwei] Northeast Agricultural University, Coll Vet Med, Harbin 150030, China.	Country: China<br>Institution: Northeast Agr Univ	Country: China<br>Institution: Northeast Agricultural University
C1	2	14816	14816	[Guan, Haoyue] Sichuan Agr Univ, Coll Anim Sci & Vet Med, Chengdu 611130, Peoples R China.	[Guan, Haoyue] Sichuan Agricultural University, Coll Anim Sci & Vet Med, Chengdu 611130, China.	Country: China<br>Institution: Sichuan Agr Univ	Country: China<br>Institution: Sichuan Agricultural University

C1	3	14817	14817	[Wu, Qiong] Beijing Univ Agr, Anim Sci & Technol Coll, Beijing 100096, Peoples R China.	[Wu, Qiong] Beijing Univ Agr, Anim Sci & Technol Coll, Beijing 100096, China.	Country: China Institution: Beijing Univ Agr	Country: China Institution: Beijing Univ Agr
C3	1	14818	14818	Northeast Agricultural University - China	Northeast Agricultural University	Institution: Northeast Agricultural University - China	Institution: Northeast Agricultural University
RP	1	14820	14820	Zhang, ZW (corresponding author), Northeast Agr Univ, Coll Vet Med, Harbin 150030, Peoples R China	Zhang, ZW (corresponding author), Northeast Agricultural University, Coll Vet Med, Harbin 150030, Peoples R China	Country: China Institution: Northeast Agr Univ	Country: China Institution: Northeast Agricultural University
RP	2	14820	14820	Wu, Q (corresponding author), Beijing Univ Agr, Anim Sci & Technol Coll, Beijing 100096, Peoples R China	Wu, Q (corresponding author), Beijing Univ Agr, Anim Sci & Technol Coll, Beijing 100096, China	Country: China Institution: Beijing Univ Agr	Country: China Institution: Beijing Univ Agr

Record 94 | WOS:001074097100001

The Effect of Mycotoxins and Their Mixtures on Bovine Spermatozoa Characteristics

Field	Position	Original line	Normalised line	Original value	Normalised value	Original country / institution	Normalised country / institution
C1	1	14924	14924	[Kalo, Dorit; Mendelson, Paz; Komsky-Elbaz, Alisa; Roth, Zvi] Hebrew Univ Jerusalem, Dept Anim Sci, Robert H Smith Fac Agr Food & Environm, IL-7610001 Rehovot, Israel.	[Kalo, Dorit; Mendelson, Paz; Komsky-Elbaz, Alisa; Roth, Zvi] Hebrew University of Jerusalem, Dept Anim Sci, Robert H Smith Fac Agr Food & Environm, IL-7610001 Rehovot, Israel.	Country: Israel Institution: Hebrew Univ Jerusalem	Country: Israel Institution: Hebrew University of Jerusalem
C1	2	14925	14925	[Voet, Hillary] Hebrew Univ Jerusalem, Robert H Smith Fac Agr Food & Environm, Dept Agr Econ & Management, IL-7610001 Rehovot, Israel.	[Voet, Hillary] Hebrew University of Jerusalem, Robert H Smith Fac Agr Food & Environm, Dept Agr Econ & Management, IL-7610001 Rehovot, Israel.	Country: Israel Institution: Hebrew Univ Jerusalem	Country: Israel Institution: Hebrew University of Jerusalem
RP	1	14927	14927	Roth, Z (corresponding author), Hebrew Univ Jerusalem, Dept Anim Sci, Robert H Smith Fac Agr Food & Environm, IL-7610001 Rehovot, Israel	Roth, Z (corresponding author), Hebrew University of Jerusalem, Dept Anim Sci, Robert H Smith Fac Agr Food & Environm, IL-7610001 Rehovot, Israel	Country: Israel Institution: Hebrew Univ Jerusalem	Country: Israel Institution: Hebrew University of Jerusalem

Record 95 | WOS:001070923600001

Cell cycle and enzymatic activity alterations induced by ROS production in human neuroblastoma cells SH-SY5Y exposed to Fumonisin B1, Ochratoxin A and their combination

Field	Position	Original line	Normalised line	Original value	Normalised value	Original country / institution	Normalised country / institution
C1	1	15081	15081	[Juan, Cristina; Fernandez-Franzon, Monica; Juan-Garcia, Ana] Univ Valencia, Fac Pharm, Lab Food Chem & Toxicol, Av Vicent Andres Estelles S-N, Burjassot 46100, Spain.	[Juan, Cristina; Fernandez-Franzon, Monica; Juan-Garcia, Ana] University of Valencia, Fac Pharm, Lab Food Chem & Toxicol, Av Vicent Andres Estelles S-N, Burjassot 46100, Spain.	Country: Spain Institution: Univ Valencia	Country: Spain Institution: University of Valencia
RP	1	15083	15083	Juan-GarcÃ-a, A (corresponding author), Univ Valencia, Fac Pharm, Lab Food Chem & Toxicol, Av Vicent Andres Estelles S-N, Burjassot 46100, Spain	Juan-GarcÃ-a, A (corresponding author), University of Valencia, Fac Pharm, Lab Food Chem & Toxicol, Av Vicent Andres Estelles S-N, Burjassot 46100, Spain	Country: Spain Institution: Univ Valencia	Country: Spain Institution: University of Valencia

Record 96 | WOS:001054307000001

An integrated approach for evaluating the interactive effects between azoxystrobin and ochratoxin A: Pathway-based in vivo analyses

Field	Position	Original line	Normalised line	Original value	Normalised value	Original country / institution	Normalised country / institution
C1	1	15188	15188	[Wang, Ruike; Huang, Nan; Ji, Jing; Chen, Chen] Shandong Univ, Cheeloo Coll Med, Sch Publ Hlth, Jinan 250012, Shandong, Peoples R China.	[Wang, Ruike; Huang, Nan; Ji, Jing; Chen, Chen] Shandong University, Cheeloo Coll Med, Sch Publ Hlth, Jinan 250012, Shandong, China.	Country: China<br>Institution: Shandong Univ	Country: China<br>Institution: Shandong University
RP	1	15190	15190	Chen, C (corresponding author), Shandong Univ, Cheeloo Coll Med, Sch Publ Hlth, Jinan 250012, Shandong, Peoples R China	Chen, C (corresponding author), Shandong University, Cheeloo Coll Med, Sch Publ Hlth, Jinan 250012, Shandong, China	Country: China<br>Institution: Shandong Univ	Country: China<br>Institution: Shandong University

Record 97 | WOS:001041623900001

The Protective Effect of Pumpkin and Fermented Whey Mixture against AFB1 and OTA Immune Toxicity In Vitro. A Transcriptomic Approach

Field	Position	Original line	Normalised line	Original value	Normalised value	Original country / institution	Normalised country / institution
C1	1	15290	15290	[Frangiamone, Massimo; Lozano, Manuel; Cimbalo, Alessandra; Lazaro, Alvaro; Font, Guillermina; Manyes, Lara] Univ Valencia, Fac Pharm, Lab Food Chem & Toxicol, Av Vicent Andres Estelles S-N, Burjassot 46100, Spain.	[Frangiamone, Massimo; Lozano, Manuel; Cimbalo, Alessandra; Lazaro, Alvaro; Font, Guillermina; Manyes, Lara] University of Valencia, Fac Pharm, Lab Food Chem & Toxicol, Av Vicent Andres Estelles S-N, Burjassot 46100, Spain.	Country: Spain<br>Institution: Univ Valencia	Country: Spain<br>Institution: University of Valencia
RP	1	15292	15292	Cimbalo, A (corresponding author), Univ Valencia, Fac Pharm, Lab Food Chem & Toxicol, Av Vicent Andres Estelles S-N, Burjassot 46100, Spain	Cimbalo, A (corresponding author), University of Valencia, Fac Pharm, Lab Food Chem & Toxicol, Av Vicent Andres Estelles S-N, Burjassot 46100, Spain	Country: Spain<br>Institution: Univ Valencia	Country: Spain<br>Institution: University of Valencia

Record 98 | WOS:001055768800001

Mycotoxins in Cereal-Based Products and Their Impacts on the Health of Humans, Livestock Animals and Pets

No C1/C3/RP normalisation changes detected for this record.

Record 99 | WOS:001036576500001

Pathological Role of Oxidative Stress in Aflatoxin-Induced Toxicity in Different Experimental Models and Protective Effect of Phytochemicals: A Review

Field	Position	Original line	Normalised line	Original value	Normalised value	Original country / institution	Normalised country / institution
C1	3	15771	15771	[Dludla, Phiwayinkosi V.] South African Med Res Council, Cochrane South Africa, ZA-7505 Tygerberg, South Africa.	[Dludla, Phiwayinkosi V.] South African Medical Research Council, Cochrane South Africa, ZA-7505 Tygerberg, South Africa.	Country: South Africa<br>Institution: South African Med Res Council	Country: South Africa<br>Institution: South African Medical Research Council

C1	4	15772	15772	[Dludla, Phiwayinkosi V.] Univ Zululand, Dept Biochem & Microbiol, ZA-3886 Kwa Dlangezwa, South Africa.	[Dludla, Phiwayinkosi V.] University of Zululand, Dept Biochem & Microbiol, ZA-3886 Kwa Dlangezwa, South Africa.	Country: South Africa Institution: Univ Zululand	Country: South Africa Institution: University of Zululand

Record 100 | WOS:001040619100001

MiR-155-5p Elevated by Ochratoxin A Induces Intestinal Fibrosis and Epithelial-to-Mesenchymal Transition through TGF-Î² Regulated Signaling Pathway In Vitro and In Vivo

Field	Position	Original line	Normalised line	Original value	Normalised value	Original country / institution	Normalised country / institution
C1	1	15972	15972	[Rhee, Kyu Hyun; Yang, Seon Ah; Pyo, Min Cheol; Lim, Jae-Min; Lee, Kwang-Won] Korea Univ, Coll Life Sci & Biotechnol, Dept Biotechnol, Seoul 02841, South Korea.	[Rhee, Kyu Hyun; Yang, Seon Ah; Pyo, Min Cheol; Lim, Jae-Min; Lee, Kwang-Won] Korea University, Coll Life Sci & Biotechnol, Dept Biotechnol, Seoul 02841, South Korea.	Country: South Korea Institution: Korea Univ	Country: South Korea Institution: Korea University
RP	1	15974	15974	Lee, KW (corresponding author), Korea Univ, Coll Life Sci & Biotechnol, Dept Biotechnol, Seoul 02841, South Korea	Lee, KW (corresponding author), Korea University, Coll Life Sci & Biotechnol, Dept Biotechnol, Seoul 02841, South Korea	Country: South Korea Institution: Korea Univ	Country: South Korea Institution: Korea University

Record 101 | WOS:001013318000001

Enzymatic degradation of ochratoxin A in the gastrointestinal tract of piglets

No C1/C3/RP normalisation changes detected for this record.

Record 102 | WOS:001011146700001

A Chemically Defined Medium That Supports Mycotoxin Production by Stachybotrys chartarum Enabled Analysis of the Impact of Nitrogen and Carbon Sources on the Biosynthesis of Macrocyclic Trichothecenes and Stachybotrylactam

Field	Position	Original line	Normalised line	Original value	Normalised value	Original country / institution	Normalised country / institution
C1	2	16207	16207	[Twaruzek, Magdalena; Kosicki, Robert] Kazimierz Wielki Univ, Fac Biol Sci, Dept Physiol & Toxicol, Bydgoszcz, Poland.	[Twaruzek, Magdalena; Kosicki, Robert] Kazimierz Wielki University, Fac Biol Sci, Dept Physiol & Toxicol, Bydgoszcz, Poland.	Country: Poland Institution: Kazimierz Wielki Univ	Country: Poland Institution: Kazimierz Wielki University

Record 103 | WOS:001013506800001

Citrinin as a potential anti-cancer therapy: A comprehensive review

Field	Position	Original line	Normalised line	Original value	Normalised value	Original country / institution	Normalised country / institution
C1	4	16387	16387	[Hasan, Mohammad M.] Mawlana Bhashani Sci & Technol Univ, Fac Life Sci, Dept Biochem & Mol Biol, Tangail, Bangladesh.	[Hasan, Mohammad M.] Mawlana Bhashani Science & Technology University, Fac Life Sci, Dept Biochem & Mol Biol, Tangail, Bangladesh.	Country: Bangladesh Institution: Mawlana Bhashani Sci & Technol Univ	Country: Bangladesh Institution: Mawlana Bhashani Science & Technology University

Record 104 | WOS:001141751900001

Determination of Ochratoxin-A Detoxification and Antibiotic Resistance Potential of Acinetobacter calcoaceticus

Field	Position	Original line	Normalised line	Original value	Normalised value	Original country / institution	Normalised country / institution
C1	1	16593	16593	[Al-Nussairawi, Mohammed; Al-Tulaibawi, Nooralden Abdulkarem Jasim] Univ Misan, Dept Clin & Lab Sci, Coll Pharm, Misan, Iraq.	[Al-Nussairawi, Mohammed; Al-Tulaibawi, Nooralden Abdulkarem Jasim] Misan University, Dept Clin & Lab Sci, Coll Pharm, Misan, Iraq.	Country: Iraq<br>Institution: Univ Misan	Country: Iraq<br>Institution: Misan University
C1	2	16594	16594	[Aal-Aaboda, Munaf] Univ Misan, Dept Pharmacol, Coll Pharm, Misan, Iraq.	[Aal-Aaboda, Munaf] Misan University, Dept Pharmacol, Coll Pharm, Misan, Iraq.	Country: Iraq<br>Institution: Univ Misan	Country: Iraq<br>Institution: Misan University
RP	1	16596	16596	Al-Nussairawi, M (corresponding author), Univ Misan, Dept Clin & Lab Sci, Coll Pharm, Misan, Iraq	Al-Nussairawi, M (corresponding author), Misan University, Dept Clin & Lab Sci, Coll Pharm, Misan, Iraq	Country: Iraq<br>Institution: Univ Misan	Country: Iraq<br>Institution: Misan University

Record 105 | WOS:000988737300001

Natural mycotoxin contamination in dog food: A review on toxicity and detoxification methods

Field	Position	Original line	Normalised line	Original value	Normalised value	Original country / institution	Normalised country / institution
C1	1	16695	16695	[Yang, Ling; Wang, Hui; Wang, Li] Beijing Vocat Coll Agr, Dept Food & Bioengn, Beijing 102442, Peoples R China.	[Yang, Ling; Wang, Hui; Wang, Li] Beijing Vocat Coll Agr, Dept Food & Bioengn, Beijing 102442, China.	Country: China<br>Institution: Beijing Vocat Coll Agr	Country: China<br>Institution: Beijing Vocat Coll Agr
C1	2	16696	16696	[Yang, Lihan; Cai, Yuqing; Luo, Yifei; Wu, Yingjie; Qin, Yinghe; Wu, Zhenlong; Liu, Ning] China Agr Univ, Coll Anim Sci & Technol, Beijing 100193, Peoples R China.	[Yang, Lihan; Cai, Yuqing; Luo, Yifei; Wu, Yingjie; Qin, Yinghe; Wu, Zhenlong; Liu, Ning] China Agricultural University, Coll Anim Sci & Technol, Beijing 100193, China.	Country: China<br>Institution: China Agr Univ	Country: China<br>Institution: China Agricultural University
C1	3	16697	16697	[Chen, Jingqing] Acad Mil Med Sci, Lab Anim Ctr, Beijing 100071, Peoples R China.	[Chen, Jingqing] Acad Mil Med Sci, Lab Anim Ctr, Beijing 100071, China.	Country: China<br>Institution: Acad Mil Med Sci	Country: China<br>Institution: Acad Mil Med Sci
C1	4	16698	16698	[Liu, Xiaoming] Shandong Agr Univ, Coll Anim Sci & Technol, Tai An, Peoples R China.	[Liu, Xiaoming] Shandong Agricultural University, Coll Anim Sci & Technol, Tai An, China.	Country: China<br>Institution: Shandong Agr Univ	Country: China<br>Institution: Shandong Agricultural University
RP	2	16701	16701	Liu, N (corresponding author), China Agr Univ, Coll Anim Sci & Technol, Beijing 100193, Peoples R China	Liu, N (corresponding author), China Agricultural University, Coll Anim Sci & Technol, Beijing 100193, China	Country: China<br>Institution: China Agr Univ	Country: China<br>Institution: China Agricultural University

Record 106 | WOS:000983760800001

Excessive ER-phagy contributes to ochratoxin A-induced apoptosis

Field	Position	Original line	Normalised line	Original value	Normalised value	Original country / institution	Normalised country / institution
C1	1	16984	16984	[Deng, Huiqiong; Chen, Wenying; Zhang, Yiwen; Han, Lingyun; Zhang, Qipeng; Yao, Song; Wang, Hongwei; Shen, Xiao Li] Zunyi Med Univ, Sch Publ Hlth, Univ West Rd 6,	[Deng, Huiqiong; Chen, Wenying; Zhang, Yiwen; Han, Lingyun; Zhang, Qipeng; Yao, Song; Wang, Hongwei; Shen, Xiao Li] Zunyi Medical University, Sch Publ Hlth, Univ	Country: China<br>Institution: Zunyi Med Univ	Country: China<br>Institution: Zunyi Medical University

				Zunyi 563000, Guizhou, Peoples R China.	West Rd 6, Zunyi 563000, Guizhou, China.		
C1	2	16985	16985	[Zhang, Qipeng] Zunyi Med Univ, Affiliated Hosp, Dept Hosp Infect Control, Zunyi 563000, Guizhou, Peoples R China.	[Zhang, Qipeng] Zunyi Medical University, Affiliated Hosp, Dept Hosp Infect Control, Zunyi 563000, Guizhou, China.	Country: China<br>Institution: Zunyi Med Univ	Country: China<br>Institution: Zunyi Medical University
C1	3	16986	16986	[Zhang, Boyang] China Agr Univ, Dept Nutr & Hlth, Key Lab Precis Nutr & Food Qual, Beijing 100083, Peoples R China.	[Zhang, Boyang] China Agricultural University, Dept Nutr & Hlth, Key Lab Precis Nutr & Food Qual, Beijing 100083, China.	Country: China<br>Institution: China Agr Univ	Country: China<br>Institution: China Agricultural University
RP	1	16989	16989	Shen, XL (corresponding author), Zunyi Med Univ, Sch Publ Hlth, Univ West Rd 6, Zunyi 563000, Guizhou, Peoples R China	Shen, XL (corresponding author), Zunyi Medical University, Sch Publ Hlth, Univ West Rd 6, Zunyi 563000, Guizhou, China	Country: China<br>Institution: Zunyi Med Univ	Country: China<br>Institution: Zunyi Medical University

Record 107 | WOS:000977904100001

New perspectives in application of kidney biomarkers in mycotoxin induced nephrotoxicity, with a particular focus on domestic pigs

Field	Position	Original line	Normalised line	Original value	Normalised value	Original country / institution	Normalised country / institution
C1	1	17117	17117	[Raduly, Zsolt; Csernoch, Laszlo] Univ Debrecen, Fac Med, Dept Physiol, Debrecen, Hungary.	[Raduly, Zsolt; Csernoch, Laszlo] University of Debrecen, Fac Med, Dept Physiol, Debrecen, Hungary.	Country: Hungary<br>Institution: Univ Debrecen	Country: Hungary<br>Institution: University of Debrecen
C1	2	17118	17118	[Raduly, Zsolt] Univ Debrecen, ELKH DE, Cell Physiol Res Grp, Debrecen, Hungary.	[Raduly, Zsolt] University of Debrecen, ELKH DE, Cell Physiol Res Grp, Debrecen, Hungary.	Country: Hungary<br>Institution: Univ Debrecen	Country: Hungary<br>Institution: University of Debrecen
C1	3	17119	17119	[Raduly, Zsolt] Univ Debrecen, Doctoral Sch Mol Med, Debrecen, Hungary.	[Raduly, Zsolt] University of Debrecen, Doctoral Sch Mol Med, Debrecen, Hungary.	Country: Hungary<br>Institution: Univ Debrecen	Country: Hungary<br>Institution: University of Debrecen
C1	7	17123	17123	[Balatoni, Ildiko] Univ Debrecen, Clin Ctr, Debrecen, Hungary.	[Balatoni, Ildiko] University of Debrecen, Clin Ctr, Debrecen, Hungary.	Country: Hungary<br>Institution: Univ Debrecen	Country: Hungary<br>Institution: University of Debrecen
C1	8	17124	17124	[Price, Robert G.] Kings Coll London, Dept Nutr, Franklin Wilkins Bldg, London, England.	[Price, Robert G.] Kings Coll London, Dept Nutr, Franklin Wilkins Bldg, London, United Kingdom.	Country: England<br>Institution: Kings Coll London	Country: United Kingdom<br>Institution: Kings Coll London
C1	9	17125	17125	[Dockrell, Mark E.] SWT Inst Renal Res, London, England.	[Dockrell, Mark E.] SWT Inst Renal Res, London, United Kingdom.	Country: England<br>Institution: SWT Inst Renal Res	Country: United Kingdom<br>Institution: SWT Inst Renal Res
C1	10	17126	17126	[Dockrell, Mark E.] St Georges Univ, Dept Mol & Clin Sci, London, England.	[Dockrell, Mark E.] St Georges Univ, Dept Mol & Clin Sci, London, United Kingdom.	Country: England<br>Institution: St Georges Univ	Country: United Kingdom<br>Institution: St Georges Univ
C1	11	17127	17127	[Pocsi, Istvan] Univ Debrecen, Inst Biotechnol, Fac Sci & Technol, Dept Mol Biotechnol & Microbiol, Debrecen, Hungary.	[Pocsi, Istvan] University of Debrecen, Inst Biotechnol, Fac Sci & Technol, Dept Mol Biotechnol & Microbiol, Debrecen, Hungary.	Country: Hungary<br>Institution: Univ Debrecen	Country: Hungary<br>Institution: University of Debrecen
RP	1	17134	17134	RÃ¡duly, Z (corresponding author), Univ Debrecen, Fac Med, Dept Physiol, Debrecen, Hungary	RÃ¡duly, Z (corresponding author), University of Debrecen, Fac Med, Dept Physiol, Debrecen, Hungary	Country: Hungary<br>Institution: Univ Debrecen	Country: Hungary<br>Institution: University of Debrecen

Record 108 | WOS:000968395600001

Fermented whey modulated AFB1 and OTA-induced hepatotoxicity and nephrotoxicity in vivo. A relative and absolute quantification about sex differences

Field	Position	Original line	Normalised line	Original value	Normalised value	Original country / institution	Normalised country / institution
C1	1	17341	17341	[Frangiamone, Massimo; Cimbalo, Alessandra; Font, Guillermina; Manyes, Lara] Univ Valencia, Fac Pharm, Lab Food Chem & Toxicol, Burjassot, Spain.	[Frangiamone, Massimo; Cimbalo, Alessandra; Font, Guillermina; Manyes, Lara] University of Valencia, Fac Pharm, Lab Food Chem & Toxicol, Burjassot, Spain.	Country: Spain Institution: Univ Valencia	Country: Spain Institution: University of Valencia
C1	3	17343	17343	[Thines, Eckhard] Johannes Gutenberg Univ Mainz, Mikrobiol & Biotechnol, Mainz, Germany.	[Thines, Eckhard] Johannes Gutenberg University of Mainz, Mikrobiol & Biotechnol, Mainz, Germany.	Country: Germany Institution: Johannes Gutenberg Univ Mainz	Country: Germany Institution: Johannes Gutenberg University of Mainz
C1	4	17344	17344	[Cimbalo, Alessandra] Univ Valencia, Fac Pharm, Lab Food Chem & Toxicol, Ave Vicent A Estelles s-n, Burjassot 46100, Valencia, Spain.	[Cimbalo, Alessandra] University of Valencia, Fac Pharm, Lab Food Chem & Toxicol, Ave Vicent A Estelles s-n, Burjassot 46100, Valencia, Spain.	Country: Spain Institution: Univ Valencia	Country: Spain Institution: University of Valencia
RP	1	17347	17347	Cimbalo, A (corresponding author), Univ Valencia, Fac Pharm, Lab Food Chem & Toxicol, Ave Vicent A Estelles s-n, Burjassot 46100, Valencia, Spain	Cimbalo, A (corresponding author), University of Valencia, Fac Pharm, Lab Food Chem & Toxicol, Ave Vicent A Estelles s-n, Burjassot 46100, Valencia, Spain	Country: Spain Institution: Univ Valencia	Country: Spain Institution: University of Valencia

Record 109 | WOS:000982062800001

Protective Effect of alpha-Tocopherol Against Ochratoxin A in Kidney Cell Line HK-2

Field	Position	Original line	Normalised line	Original value	Normalised value	Original country / institution	Normalised country / institution
C1	1	17489	17489	[Lee, Hyun Jung; Kim, Hae Dun; Ryu, Dojin] Univ Idaho, Dept Anim Vet & Food Sci, 875 Perimeter Dr MS 2330, Moscow, ID 83844 USA.	[Lee, Hyun Jung; Kim, Hae Dun; Ryu, Dojin] University of Idaho, Dept Anim Vet & Food Sci, 875 Perimeter Dr MS 2330, Moscow, ID 83844 USA.	Country: USA Institution: Univ Idaho	Country: USA Institution: University of Idaho
RP	1	17491	17491	Lee, HJ (corresponding author), Univ Idaho, Dept Anim Vet & Food Sci, 875 Perimeter Dr MS 2330, Moscow, ID 83844 USA	Lee, HJ (corresponding author), University of Idaho, Dept Anim Vet & Food Sci, 875 Perimeter Dr MS 2330, Moscow, ID 83844 USA	Country: USA Institution: Univ Idaho	Country: USA Institution: University of Idaho

Record 110 | WOS:001094910400007

INITIAL PHASE ESTABLISHMENT OF AN IN VITRO METHOD FOR DEVELOP-MENTAL NEUROTOXICITY TEST USING KI-67 IN HUMAN NEURAL PROGENITOR CELLS

Field	Position	Original line	Normalised line	Original value	Normalised value	Original country / institution	Normalised country / institution
C1	1	17615	17615	[Go, S. M.; Lee, B.; Jeong, S. H.; Park, S. M.; Lee, M.; Tran, D. N.; Jeung, E. -B.] Chungbuk Natl Univ, Coll Vet Med, Lab Vet Biochem & Mol Biol, Cheongju 28644, Chungbuk, South Korea.	[Go, S. M.; Lee, B.; Jeong, S. H.; Park, S. M.; Lee, M.; Tran, D. N.; Jeung, E. -B.] Chungbuk National University, Coll Vet Med, Lab Vet Biochem & Mol Biol, Cheongju 28644, Chungbuk, South Korea.	Country: South Korea Institution: Chungbuk Natl Univ	Country: South Korea Institution: Chungbuk National University
C1	2	17616	17616	[Ahn, C.] Jeju Natl Univ, Coll Vet Med, Lab Vet Physiol, Jeju 63243, South Korea.	[Ahn, C.] Jeju National University, Coll Vet Med, Lab Vet Physiol, Jeju 63243, South Korea.	Country: South Korea Institution: Jeju Natl Univ	Country: South Korea Institution: Jeju National University

C1	3	17617	17617	[Jo, N. R.; Lee, S. D.] Chungbuk Natl Univ, Coll Nat Sci, Dept Informat & Stat, Cheongju 28644, Chungbuk, South Korea.	[Jo, N. R.; Lee, S. D.] Chungbuk National University, Coll Nat Sci, Dept Informat & Stat, Cheongju 28644, Chungbuk, South Korea.	Country: South Korea Institution: Chungbuk Natl Univ	Country: South Korea Institution: Chungbuk National University
C1	4	17618	17618	[Jung, E. -M.] Pusan Natl Univ, Coll Nat Sci, Dept Mol Biol, Busan 46241, South Korea.	[Jung, E. -M.] Pusan National University, Coll Nat Sci, Dept Mol Biol, Busan 46241, South Korea.	Country: South Korea Institution: Pusan Natl Univ	Country: South Korea Institution: Pusan National University
C1	5	17619	17619	[Jeung, E. -B.] Chungbuk Natl Univ, Coll Vet Med, Lab Vet Biochem & Mol Biol, Cheongju 28644, Chungbuk, South Korea.	[Jeung, E. -B.] Chungbuk National University, Coll Vet Med, Lab Vet Biochem & Mol Biol, Cheongju 28644, Chungbuk, South Korea.	Country: South Korea Institution: Chungbuk Natl Univ	Country: South Korea Institution: Chungbuk National University
RP	1	17623	17623	Jeung, EB (corresponding author), Chungbuk Natl Univ, Coll Vet Med, Lab Vet Biochem & Mol Biol, Cheongju 28644, Chungbuk, South Korea	Jeung, EB (corresponding author), Chungbuk National University, Coll Vet Med, Lab Vet Biochem & Mol Biol, Cheongju 28644, Chungbuk, South Korea	Country: South Korea Institution: Chungbuk Natl Univ	Country: South Korea Institution: Chungbuk National University

Record 111 | WOS:000984555200005

Microcapsules loaded with date seed extract and its inhibitory potential to modulate the toxic effects of mycotoxins in mice received mold-contaminated diet

Field	Position	Original line	Normalised line	Original value	Normalised value	Original country / institution	Normalised country / institution
C1	1	17735	17735	[Sanei, Sogand; Kasgari, Mohammadamin Bozorgi; Abedinzadeh, Fatemeh; Sasan, Atena Poor; Hassani, Saeid; Karimi, Ehsan; Oskoueian, Ehsan] Islamic Azad Univ, Dept Biol, Mashhad Branch, Mashhad, Iran.	[Sanei, Sogand; Kasgari, Mohammadamin Bozorgi; Abedinzadeh, Fatemeh; Sasan, Atena Poor; Hassani, Saeid; Karimi, Ehsan; Oskoueian, Ehsan] Islamic Azad University, Dept Biol, Mashhad Branch, Mashhad, Iran.	Country: Iran Institution: Islamic Azad Univ	Country: Iran Institution: Islamic Azad University
RP	1	17738	17738	Karimi, E (corresponding author), Islamic Azad Univ, Dept Biol, Mashhad Branch, Mashhad, Iran	Karimi, E (corresponding author), Islamic Azad University, Dept Biol, Mashhad Branch, Mashhad, Iran	Country: Iran Institution: Islamic Azad Univ	Country: Iran Institution: Islamic Azad University

Record 112 | WOS:000951983500001

Hazard characterisation for significant mycotoxins in food

Field	Position	Original line	Normalised line	Original value	Normalised value	Original country / institution	Normalised country / institution
C1	1	17835	17835	[Malir, Frantisek; Pickova, Darina; Toman, Jakub] Univ Hradec Kralove, Dept Biol, Fac Sci, Rokitanskeho 62, CZ-50003 Hradec Kralove, Czech Republic.	[Malir, Frantisek; Pickova, Darina; Toman, Jakub] University of Hradec Kralove, Dept Biol, Fac Sci, Rokitanskeho 62, CZ-50003 Hradec Kralove, Czech Republic.	Country: Czech Republic Institution: Univ Hradec Kralove	Country: Czech Republic Institution: University of Hradec Kralove
RP	1	17841	17841	Malir, F (corresponding author), Univ Hradec Kralove, Dept Biol, Fac Sci, Rokitanskeho 62, CZ-50003 Hradec Kralove, Czech Republic	Malir, F (corresponding author), University of Hradec Kralove, Dept Biol, Fac Sci, Rokitanskeho 62, CZ-50003 Hradec Kralove, Czech Republic	Country: Czech Republic Institution: Univ Hradec Kralove	Country: Czech Republic Institution: University of Hradec Kralove

Record 113 | WOS:000958249600001

The Effect of Mushroom Culture Filtrates on the Inhibition of Mycotoxins Produced by Aspergillus flavus and Aspergillus carbonarius

Field	Position	Original line	Normalised line	Original value	Normalised value	Original country / institution	Normalised country / institution
C1	1	18055	18055	[Loncar, Jelena; Zjalic, Slaven] Univ Zadar, Dept Ecol Aquaculture & Agr, Zadar 23000, Croatia.	[Loncar, Jelena; Zjalic, Slaven] University of Zadar, Dept Ecol Aquaculture & Agr, Zadar 23000, Croatia.	Country: Croatia<br>Institution: Univ Zadar	Country: Croatia<br>Institution: University of Zadar
C1	2	18056	18056	[Loncar, Jelena; Motola, Alice; Beccaccioli, Marzia; Reverberi, Massimo] Sapienza Univ Rome, Dept Environm Biol, I-00185 Rome, Italy.	[Loncar, Jelena; Motola, Alice; Beccaccioli, Marzia; Reverberi, Massimo] Sapienza University Rome, Dept Environm Biol, I-00185 Rome, Italy.	Country: Italy<br>Institution: Sapienza Univ Rome	Country: Italy<br>Institution: Sapienza University Rome
C1	3	18057	18057	[Bellich, Barbara; Cescutti, Paola] Univ Trieste, Dept Life Sci, I-34127 Trieste, Italy.	[Bellich, Barbara; Cescutti, Paola] University of Trieste, Dept Life Sci, I-34127 Trieste, Italy.	Country: Italy<br>Institution: Univ Trieste	Country: Italy<br>Institution: University of Trieste
RP	1	18059	18059	Loncar, J (corresponding author), Univ Zadar, Dept Ecol Aquaculture & Agr, Zadar 23000, Croatia	Loncar, J (corresponding author), University of Zadar, Dept Ecol Aquaculture & Agr, Zadar 23000, Croatia	Country: Croatia<br>Institution: Univ Zadar	Country: Croatia<br>Institution: University of Zadar

Record 114 | WOS:000955634100001

Combined Effects of Acrylamide and Ochratoxin A on the Intestinal Barrier in Caco-2 Cells

Field	Position	Original line	Normalised line	Original value	Normalised value	Original country / institution	Normalised country / institution
C1	1	18167	18167	[Su, Dan; Lu, Jiawen; Nie, Chunchao; Guo, Ziyan; Li, Chang; Yu, Qiang; Xie, Jianhua; Chen, Yi] Nanchang Univ, State Key Lab Food Sci & Technol, 235 Nanjing East Rd, Nanchang 330047, Peoples R China.	[Su, Dan; Lu, Jiawen; Nie, Chunchao; Guo, Ziyan; Li, Chang; Yu, Qiang; Xie, Jianhua; Chen, Yi] Nanchang University, State Key Lab Food Sci & Technol, 235 Nanjing East Rd, Nanchang 330047, China.	Country: China<br>Institution: Nanchang Univ	Country: China<br>Institution: Nanchang University
RP	1	18169	18169	Chen, Y (corresponding author), Nanchang Univ, State Key Lab Food Sci & Technol, 235 Nanjing East Rd, Nanchang 330047, Peoples R China	Chen, Y (corresponding author), Nanchang University, State Key Lab Food Sci & Technol, 235 Nanjing East Rd, Nanchang 330047, China	Country: China<br>Institution: Nanchang Univ	Country: China<br>Institution: Nanchang University

Record 115 | WOS:000960233300001

Immunotoxicity of Three Environmental Mycotoxins and Their Risks of Increasing Pathogen Infections

Field	Position	Original line	Normalised line	Original value	Normalised value	Original country / institution	Normalised country / institution
C1	1	18282	18282	[Sun, Yuhang; Song, Yuqi; Long, Miao; Yang, Shuhua] Shenyang Agr Univ, Coll Anim Sci & Vet Med, Shenyang 110866, Peoples R China.	[Sun, Yuhang; Song, Yuqi; Long, Miao; Yang, Shuhua] Shenyang Agricultural University, Coll Anim Sci & Vet Med, Shenyang 110866, China.	Country: China<br>Institution: Shenyang Agr Univ	Country: China<br>Institution: Shenyang Agricultural University
RP	1	18284	18284	Yang, SH (corresponding author), Shenyang Agr Univ, Coll Anim Sci & Vet Med, Shenyang 110866, Peoples R China	Yang, SH (corresponding author), Shenyang Agricultural University, Coll Anim Sci & Vet Med, Shenyang 110866, China	Country: China<br>Institution: Shenyang Agr Univ	Country: China<br>Institution: Shenyang Agricultural University

Record 116 | WOS:000944193800011

Dietary Ochratoxin A Contamination Modulates Oxidative Stress, Inflammation Processes and Causes Fibrosis in in vitro and in vivo Lung Models

Field	Position	Original line	Normalised line	Original value	Normalised value	Original country / institution	Normalised country / institution
C1	1	18413	18413	[Ou, Yongfang; Fu, Qiujuan; Chen, Yonghua] Guangdong Med Univ, Affiliated Hosp, Pathol Diag & Res Ctr, Zhanjiang 524000, Guangdong, Peoples R China.	[Ou, Yongfang; Fu, Qiujuan; Chen, Yonghua] Guangdong Medical University, Affiliated Hosp, Pathol Diag & Res Ctr, Zhanjiang 524000, Guangdong, China.	Country: China<br>Institution: Guangdong Med Univ	Country: China<br>Institution: Guangdong Medical University
C1	2	18414	18414	[Lin, Liyao; Wang, Junfeng; Wu, Dong; Wu, Qin; Xie, Jianlong] Guangdong Med Univ, Affiliated Hosp, Dept Cardiothorac Surg Ctr, Zhanjiang 524000, Guangdong, Peoples R China.	[Lin, Liyao; Wang, Junfeng; Wu, Dong; Wu, Qin; Xie, Jianlong] Guangdong Medical University, Affiliated Hosp, Dept Cardiothorac Surg Ctr, Zhanjiang 524000, Guangdong, China.	Country: China<br>Institution: Guangdong Med Univ	Country: China<br>Institution: Guangdong Medical University
RP	1	18416	18416	Xie, JL (corresponding author), Guangdong Med Univ, Affiliated Hosp, Dept Cardiothorac Surg Ctr, Zhanjiang 524000, Guangdong, Peoples R China	Xie, JL (corresponding author), Guangdong Medical University, Affiliated Hosp, Dept Cardiothorac Surg Ctr, Zhanjiang 524000, Guangdong, China	Country: China<br>Institution: Guangdong Med Univ	Country: China<br>Institution: Guangdong Medical University

Record 117 | WOS:000942033800001

Comparative Assessment of Different Yeast Cell Wall-Based Mycotoxin Adsorbents Using a Model- and Bioassay-Based In Vitro Approach

Field	Position	Original line	Normalised line	Original value	Normalised value	Original country / institution	Normalised country / institution
C1	1	18514	18514	[Xu, Ran; Shandilya, Umesh K. K.; Karrow, Niel A. A.] Univ Guelph, Dept Anim Biosci, Guelph, ON N1G 2W1, Canada.	[Xu, Ran; Shandilya, Umesh K. K.; Karrow, Niel A. A.] University of Guelph, Dept Anim Biosci, Guelph, ON N1G 2W1, Canada.	Country: Canada<br>Institution: Univ Guelph	Country: Canada<br>Institution: University of Guelph
RP	1	18517	18517	Karrow, NA (corresponding author), Univ Guelph, Dept Anim Biosci, Guelph, ON N1G 2W1, Canada	Karrow, NA (corresponding author), University of Guelph, Dept Anim Biosci, Guelph, ON N1G 2W1, Canada	Country: Canada<br>Institution: Univ Guelph	Country: Canada<br>Institution: University of Guelph

Record 118 | WOS:000929015600001

Potential application of probiotics in mycotoxicosis reduction in mammals and poultry

Field	Position	Original line	Normalised line	Original value	Normalised value	Original country / institution	Normalised country / institution
C1	1	18644	18644	[Zoghi, Alaleh; Khosravi-Darani, Kianoush] Shahid Beheshti Univ Med Sci, Natl Nutr & Food Technol Res Inst, Fac Nutr Sci & Food Technol, Dept Food Sci & Technol, Tehran, Iran.	[Zoghi, Alaleh; Khosravi-Darani, Kianoush] Shahid Beheshti University Medical Sciences, Natl Nutr & Food Technol Res Inst, Fac Nutr Sci & Food Technol, Dept Food Sci & Technol, Tehran, Iran.	Country: Iran<br>Institution: Shahid Beheshti Univ Med Sci	Country: Iran<br>Institution: Shahid Beheshti University Medical Sciences
C1	2	18645	18645	[Todorov, Svetoslav Dimitrov] Handong Global Univ, Dept Adv Convergence, ProBacLab, Pohang, Gyeongbuk, South Korea.	[Todorov, Svetoslav Dimitrov] Handong Global University, Dept Adv Convergence, ProBacLab, Pohang, Gyeongbuk, South Korea.	Country: South Korea<br>Institution: Handong Global Univ	Country: South Korea<br>Institution: Handong Global University

C1	3	18646	18646	[Khosravi-Darani, Kianoush] Shahid Beheshti Univ Med Sci, Natl Nutr & food Technol Res Inst, Fac Food & Nutr Sci, Res Dept Food Technol, POB 19395-4741, Tehran, Iran.	[Khosravi-Darani, Kianoush] Shahid Beheshti University Medical Sciences, Natl Nutr & food Technol Res Inst, Fac Food & Nutr Sci, Res Dept Food Technol, POB 19395-4741, Tehran, Iran.	Country: Iran<br>Institution: Shahid Beheshti Univ Med Sci	Country: Iran<br>Institution: Shahid Beheshti University Medical Sciences
RP	1	18649	18649	Khosravi-Darani, K (corresponding author), Shahid Beheshti Univ Med Sci, Natl Nutr & food Technol Res Inst, Fac Food & Nutr Sci, Res Dept Food Technol, POB 19395-4741, Tehran, Iran	Khosravi-Darani, K (corresponding author), Shahid Beheshti University Medical Sciences, Natl Nutr & food Technol Res Inst, Fac Food & Nutr Sci, Res Dept Food Technol, POB 19395-4741, Tehran, Iran	Country: Iran<br>Institution: Shahid Beheshti Univ Med Sci	Country: Iran<br>Institution: Shahid Beheshti University Medical Sciences

Record 119 | WOS:000918850400001

Lonp1 and Sig-1R contribute to the counteraction of ursolic acid against ochratoxin A-induced mitochondrial apoptosis

Field	Position	Original line	Normalised line	Original value	Normalised value	Original country / institution	Normalised country / institution
C1	1	18786	18786	[Zhang, Qipeng; Chen, Wenying; Zhang, Yiwen; Xiao, Yuqing; An, Yichen; Han, Lingyun; Deng, Huiqiong; Yao, Song; Wang, Hongwei; Shen, Xiao Li] Zunyi Med Univ, Sch Publ Hlth, Univ West Rd 6, Zunyi 563000, Guizhou, Peoples R China.	[Zhang, Qipeng; Chen, Wenying; Zhang, Yiwen; Xiao, Yuqing; An, Yichen; Han, Lingyun; Deng, Huiqiong; Yao, Song; Wang, Hongwei; Shen, Xiao Li] Zunyi Medical University, Sch Publ Hlth, Univ West Rd 6, Zunyi 563000, Guizhou, China.	Country: China<br>Institution: Zunyi Med Univ	Country: China<br>Institution: Zunyi Medical University
C1	2	18787	18787	[Zhang, Qipeng] Zunyi Med Univ, Depatment Hosp Infect Control, Affiliated Hosp, Zunyi 563000, Guizhou, Peoples R China.	[Zhang, Qipeng] Zunyi Medical University, Depatment Hosp Infect Control, Affiliated Hosp, Zunyi 563000, Guizhou, China.	Country: China<br>Institution: Zunyi Med Univ	Country: China<br>Institution: Zunyi Medical University
C1	3	18788	18788	[Zhang, Boyang] China Agr Univ, Dept Nutr & Hlth, Key Lab Precis Nutr & Food Qual, Beijing 100083, Peoples R China.	[Zhang, Boyang] China Agricultural University, Dept Nutr & Hlth, Key Lab Precis Nutr & Food Qual, Beijing 100083, China.	Country: China<br>Institution: China Agr Univ	Country: China<br>Institution: China Agricultural University
RP	1	18791	18791	Shen, XL (corresponding author), Zunyi Med Univ, Sch Publ Hlth, Univ West Rd 6, Zunyi 563000, Guizhou, Peoples R China	Shen, XL (corresponding author), Zunyi Medical University, Sch Publ Hlth, Univ West Rd 6, Zunyi 563000, Guizhou, China	Country: China<br>Institution: Zunyi Med Univ	Country: China<br>Institution: Zunyi Medical University

Record 120 | WOS:001000273300006

Protective role of fermented whey and pumpkin extract against aflatoxin B1 and ochratoxin A toxicity in Jurkat T-cells

Field	Position	Original line	Normalised line	Original value	Normalised value	Original country / institution	Normalised country / institution
C1	1	18866	18866	[Cimbalo, A.; Frangiamone, M.; Lozano, M.; Escriva, L.; Vila-Donat, P.; Manyes, L.] Univ Valencia, Lab Food Chem & Toxicol, Fac Pharm, Ave Vicent Andres Estelles s-n, Burjassot 46100, Spain.	[Cimbalo, A.; Frangiamone, M.; Lozano, M.; Escriva, L.; Vila-Donat, P.; Manyes, L.] University of Valencia, Lab Food Chem & Toxicol, Fac Pharm, Ave Vicent Andres Estelles s-n, Burjassot 46100, Spain.	Country: Spain<br>Institution: Univ Valencia	Country: Spain<br>Institution: University of Valencia

RP	1	18868	18868	Vila-Donat, P (corresponding author), Univ Valencia, Lab Food Chem & Toxicol, Fac Pharm, Ave Vicent Andres Estelles s-n, Burjassot 46100, Spain	Vila-Donat, P (corresponding author), University of Valencia, Lab Food Chem & Toxicol, Fac Pharm, Ave Vicent Andres Estelles s-n, Burjassot 46100, Spain	Country: Spain Institution: Univ Valencia	Country: Spain Institution: University of Valencia

Record 121 | WOS:000915148500001

AFB1 and OTA Promote Immune Toxicity in Human LymphoBlastic T Cells at Transcriptomic Level

Field	Position	Original line	Normalised line	Original value	Normalised value	Original country / institution	Normalised country / institution
C1	1	18978	18978	[Frangiamone, Massimo; Lozano, Manuel; Cimbalo, Alessandra; Font, Guillermina; Manyes, Lara] Univ Valencia, Fac Pharm, Lab Food Chem & Toxicol, Burjassot 46100, Spain.	[Frangiamone, Massimo; Lozano, Manuel; Cimbalo, Alessandra; Font, Guillermina; Manyes, Lara] University of Valencia, Fac Pharm, Lab Food Chem & Toxicol, Burjassot 46100, Spain.	Country: Spain Institution: Univ Valencia	Country: Spain Institution: University of Valencia
RP	1	18980	18980	Cimbalo, A (corresponding author), Univ Valencia, Fac Pharm, Lab Food Chem & Toxicol, Burjassot 46100, Spain	Cimbalo, A (corresponding author), University of Valencia, Fac Pharm, Lab Food Chem & Toxicol, Burjassot 46100, Spain	Country: Spain Institution: Univ Valencia	Country: Spain Institution: University of Valencia

Record 122 | WOS:000912325600001

Toxico-pathological effects of ochratoxin A and its diastereoisomer under in ovo conditions and in vitro evaluation of the toxicity of these toxins against the embryo Gallus gallus fibroblast cell line

Field	Position	Original line	Normalised line	Original value	Normalised value	Original country / institution	Normalised country / institution
C1	3	19173	19173	[Twaruzek, Magdalena; Soszczynska, Ewelina] Kazimierz Wielki Univ, Fac Biol Sci, Dept Physiol & Toxicol, PL-85064 Bydgoszcz, Poland.	[Twaruzek, Magdalena; Soszczynska, Ewelina] Kazimierz Wielki University, Fac Biol Sci, Dept Physiol & Toxicol, PL-85064 Bydgoszcz, Poland.	Country: Poland Institution: Kazimierz Wielki Univ	Country: Poland Institution: Kazimierz Wielki University
C1	4	19174	19174	[Waskiewicz, Agnieszka] Poznan Univ Life Sci, Fac Forestry & Wood Technol, Dept Chem, PL-60625 Poznan, Poland.	[Waskiewicz, Agnieszka] Poznan University of Life Sciences, Fac Forestry & Wood Technol, Dept Chem, PL-60625 Poznan, Poland.	Country: Poland Institution: Poznan Univ Life Sci	Country: Poland Institution: Poznan University of Life Sciences
C1	5	19175	19175	[Stepien, Lukasz] Polish Acad Sci, Inst Plant Genet, Dept Plant Pathogen Interact, PL-60479 Poznan, Poland.	[Stepien, Lukasz] Polish Academy of Sciences, Inst Plant Genet, Dept Plant Pathogen Interact, PL-60479 Poznan, Poland.	Country: Poland Institution: Polish Acad Sci	Country: Poland Institution: Polish Academy of Sciences

Record 123 | WOS:000900483500001

Modified Mycotoxins, a Still Unresolved Issue

Field	Position	Original line	Normalised line	Original value	Normalised value	Original country / institution	Normalised country / institution
C1	1	19287	19287	[Angioni, Alberto] Univ Cagliari, Dept Life & Environm Sci, I-09126 Cagliari, Italy.	[Angioni, Alberto] University of Cagliari, Dept Life & Environm Sci, I-09126 Cagliari, Italy.	Country: Italy Institution: Univ Cagliari	Country: Italy Institution: University of Cagliari
RP	1	19292	19292	Angioni, A (corresponding author), Univ Cagliari, Dept Life	Angioni, A (corresponding author), University of Cagliari,	Country: Italy Institution: Univ Cagliari	Country: Italy Institution: University of Cagliari

				& Environm Sci, I-09126 Cagliari, Italy	Dept Life & Environm Sci, I-09126 Cagliari, Italy		

Record 124 | WOS:000898846200001

Innovative in vitro approaches to toxicological investigations of mycotoxins effects

Field	Position	Original line	Normalised line	Original value	Normalised value	Original country / institution	Normalised country / institution
C1	1	19486	19486	[Beatriz, Arce Lopez; Nolwenn, Hymery] Univ Bretagne Occidentale, Lab Univ Biodivers & Ecol Microbienne, LUBEM, UBO, Brest, France.	[Beatriz, Arce Lopez; Nolwenn, Hymery] Universite de Bretagne Occidentale, Lab Univ Biodivers & Ecol Microbienne, LUBEM, UBO, Brest, France.	Country: France Institution: Univ Bretagne Occidentale	Country: France Institution: Universite de Bretagne Occidentale
RP	1	19488	19488	Beatriz, AL (corresponding author), Univ Bretagne Occidentale, Lab Univ Biodivers & Ecol Microbienne, LUBEM, UBO, Brest, France	Beatriz, AL (corresponding author), Universite de Bretagne Occidentale, Lab Univ Biodivers & Ecol Microbienne, LUBEM, UBO, Brest, France	Country: France Institution: Univ Bretagne Occidentale	Country: France Institution: Universite de Bretagne Occidentale

Record 125 | WOS:000903192900001

Possible Mechanisms of the Interplay between Drugs and Mycotoxins-Is There a Possible Impact?

Field	Position	Original line	Normalised line	Original value	Normalised value	Original country / institution	Normalised country / institution
C1	1	19591	19591	[Lootens, Orphelie; De Saeger, Sarah; De Boevre, Marthe] Univ Ghent, Fac Pharmaceut Sci, Ctr Excellence Mycotoxicol & Publ Hlth, Dept Bioanal, Ottergemsesteenweg 460, B-9000 Ghent, Belgium.	[Lootens, Orphelie; De Saeger, Sarah; De Boevre, Marthe] Ghent University, Fac Pharmaceut Sci, Ctr Excellence Mycotoxicol & Publ Hlth, Dept Bioanal, Ottergemsesteenweg 460, B-9000 Ghent, Belgium.	Country: Belgium Institution: Univ Ghent	Country: Belgium Institution: Ghent University
C1	2	19592	19592	[Lootens, Orphelie; Vermeulen, An; Van Bocxlaer, Jan] Univ Ghent, Dept Bioanal, Lab Med Biochem & Clin Anal, Ottergemsesteenweg 460, B-9000 Ghent, Belgium.	[Lootens, Orphelie; Vermeulen, An; Van Bocxlaer, Jan] Ghent University, Dept Bioanal, Lab Med Biochem & Clin Anal, Ottergemsesteenweg 460, B-9000 Ghent, Belgium.	Country: Belgium Institution: Univ Ghent	Country: Belgium Institution: Ghent University
C1	3	19593	19593	[Lootens, Orphelie; Croubels, Siska; De Saeger, Sarah; De Boevre, Marthe] Univ Ghent, MYTOX SOUTH, Int Themat Network, Ottergemsesteenweg 460, B-9000 Ghent, Belgium.	[Lootens, Orphelie; Croubels, Siska; De Saeger, Sarah; De Boevre, Marthe] Ghent University, MYTOX SOUTH, Int Themat Network, Ottergemsesteenweg 460, B-9000 Ghent, Belgium.	Country: Belgium Institution: Univ Ghent	Country: Belgium Institution: Ghent University
C1	4	19594	19594	[Croubels, Siska] Univ Ghent, Fac Vet Med, Dept Pathobiol Pharmacol & Zool Med, Lab Pharmacol & Toxicol, Salisburylaan 133, B-9820 Merelbeke, Belgium.	[Croubels, Siska] Ghent University, Fac Vet Med, Dept Pathobiol Pharmacol & Zool Med, Lab Pharmacol & Toxicol, Salisburylaan 133, B-9820 Merelbeke, Belgium.	Country: Belgium Institution: Univ Ghent	Country: Belgium Institution: Ghent University
C1	5	19595	19595	[De Saeger, Sarah] Univ Johannesburg, Fac Sci, Dept Food Technol, Doornfontein Campus,POB 17011, ZA-2028 Gauteng, South Africa.	[De Saeger, Sarah] University of Johannesburg, Fac Sci, Dept Food Technol, Doornfontein Campus,POB 17011, ZA-2028 Gauteng, South Africa.	Country: South Africa Institution: Univ Johannesburg	Country: South Africa Institution: University of Johannesburg
RP	2	19598	19598	De Boevre, M (corresponding author), Univ Ghent, Fac Pharmaceut Sci, Ctr Excellence Mycotoxicol & Publ Hlth, Dept	De Boevre, M (corresponding author), Ghent University, Fac Pharmaceut Sci, Ctr Excellence Mycotoxicol & Publ Hlth, Dept	Country: Belgium Institution: Univ Ghent	Country: Belgium Institution: Ghent University

				Bioanal, Ottergemsesteenweg 460, B-9000 Ghent, Belgium	Bioanal, Ottergemsesteenweg 460, B-9000 Ghent, Belgium		

Record 126 | WOS:000888695100001

Mycotoxins and cellular senescence: the impact of oxidative stress, hypoxia, and immunosuppression

Field	Position	Original line	Normalised line	Original value	Normalised value	Original country / institution	Normalised country / institution
C1	1	19755	19755	[You, Li] Chongqing Coll Int Business & Econ, Coll Phys Educ & Hlth, Chongqing 401520, Peoples R China.	[You, Li] Chongqing Coll Int Business & Econ, Coll Phys Educ & Hlth, Chongqing 401520, China.	Country: China<br>Institution: Chongqing Coll Int Business & Econ	Country: China<br>Institution: Chongqing Coll Int Business & Econ
C1	2	19756	19756	[You, Li; Wu, Qinghua] Yangtze Univ, Coll Life Sci, Jingzhou 434025, Peoples R China.	[You, Li; Wu, Qinghua] Yangtze University, Coll Life Sci, Jingzhou 434025, China.	Country: China<br>Institution: Yangtze Univ	Country: China<br>Institution: Yangtze University
C1	3	19757	19757	[Nepovimova, Eugenie; Wu, Qinghua; Kuca, Kamil] Univ Hradec Kralove, Fac Sci, Dept Chem, Hradec Kralove 50003, Czech Republic.	[Nepovimova, Eugenie; Wu, Qinghua; Kuca, Kamil] University of Hradec Kralove, Fac Sci, Dept Chem, Hradec Kralove 50003, Czech Republic.	Country: Czech Republic<br>Institution: Univ Hradec Kralove	Country: Czech Republic<br>Institution: University of Hradec Kralove
C1	4	19758	19758	[Valko, Marian] Slovak Univ Technol Bratislava, Fac Chem & Food Technol, Bratislava 81237, Slovakia.	[Valko, Marian] Slovak University of Technology Bratislava, Fac Chem & Food Technol, Bratislava 81237, Slovakia.	Country: Slovakia<br>Institution: Slovak Univ Technol Bratislava	Country: Slovakia<br>Institution: Slovak University of Technology Bratislava
C1	5	19759	19759	[Kuca, Kamil] Univ Granada, Andalusian Res Inst Data Sci & Computat Intellige, Granada, Spain.	[Kuca, Kamil] University of Granada, Andalusian Res Inst Data Sci & Computat Intellige, Granada, Spain.	Country: Spain<br>Institution: Univ Granada	Country: Spain<br>Institution: University of Granada
RP	1	19762	19762	Wu, QH (corresponding author), Yangtze Univ, Coll Life Sci, Jingzhou 434025, Peoples R China	Wu, QH (corresponding author), Yangtze University, Coll Life Sci, Jingzhou 434025, Peoples R China	Country: China<br>Institution: Yangtze Univ	Country: China<br>Institution: Yangtze University

Record 127 | WOS:000892528400007

The efficacy of mycotoxin binders to control mycotoxins in feeds and the potential risk of interactions with nutrient: a review

No C1/C3/RP normalisation changes detected for this record.

Record 128 | WOS:000886894200008

Protective effect of selenomethionine on kidney injury induced by ochratoxin A in rabbits

Field	Position	Original line	Normalised line	Original value	Normalised value	Original country / institution	Normalised country / institution
C1	1	20069	20069	[Zhang, Ziqiang; Sun, Yingying; Xie, Hui; Wang, Jiajia; Zhang, Xin; Shi, Zhangyu; Liu, Yumei] Henan Univ Sci & Technol, Coll Anim Sci & Technol, Luoyang 471000, Henan, Peoples R China.	[Zhang, Ziqiang; Sun, Yingying; Xie, Hui; Wang, Jiajia; Zhang, Xin; Shi, Zhangyu; Liu, Yumei] Henan University of Science & Technology, Coll Anim Sci & Technol, Luoyang 471000, Henan, China.	Country: China<br>Institution: Henan Univ Sci & Technol	Country: China<br>Institution: Henan University of Science & Technology
RP	1	20071	20071	Liu, YM (corresponding author), Henan Univ Sci & Technol, Coll Anim Sci & Technol, Luoyang 471000, Henan, Peoples R China	Liu, YM (corresponding author), Henan University of Science & Technology, Coll Anim Sci & Technol, Luoyang 471000, Henan, China	Country: China<br>Institution: Henan Univ Sci & Technol	Country: China<br>Institution: Henan University of Science & Technology

Record 129 | WOS:000896944500005

In vitro study of ochratoxin A in the expression of genes associated with neuron survival and viability

Field	Position	Original line	Normalised line	Original value	Normalised value	Original country / institution	Normalised country / institution
C1	1	20201	20201	[Sharma, Riddhi] Univ Bath, Dept Pharm & Pharmacol, Bath BA2, Somerset, England.	[Sharma, Riddhi] University of Bath, Dept Pharm & Pharmacol, Bath BA2, Somerset, United Kingdom.	Country: England Institution: Univ Bath	Country: United Kingdom Institution: University of Bath
C1	2	20202	20202	[Gettings, Sean M.; Hazell, Gareth; Bourbia, Nora] UK Hlth Secur Agcy, Radiat Effects Dept, Radiat Protect Sci Div, Harwell Sci Campus, Didcot OX110RQ, Oxfordshire, England.	[Gettings, Sean M.; Hazell, Gareth; Bourbia, Nora] UK Hlth Secur Agcy, Radiat Effects Dept, Radiat Protect Sci Div, Harwell Sci Campus, Didcot OX110RQ, Oxfordshire, United Kingdom.	Country: England Institution: UK Hlth Secur Agcy	Country: United Kingdom Institution: UK Hlth Secur Agcy
C3	2	20203	20203	UK Health Security Agency (UKHSA)	United Kingdom Health Security Agency (UKHSA)	Institution: UK Health Security Agency (UKHSA)	Institution: United Kingdom Health Security Agency (UKHSA)
RP	1	20204	20204	Bourbia, N (corresponding author), UK Hlth Secur Agcy, Radiat Effects Dept, Radiat Protect Sci Div, Harwell Sci Campus, Didcot OX110RQ, Oxfordshire, England	Bourbia, N (corresponding author), UK Hlth Secur Agcy, Radiat Effects Dept, Radiat Protect Sci Div, Harwell Sci Campus, Didcot OX110RQ, Oxfordshire, United Kingdom	Country: England Institution: UK Hlth Secur Agcy	Country: United Kingdom Institution: UK Hlth Secur Agcy

Record 130 | WOS:000974547800001

Low doses of fumonisin B1 exacerbate ochratoxin A-induced renal injury in mice and the protective roles of heat shock protein 70

Field	Position	Original line	Normalised line	Original value	Normalised value	Original country / institution	Normalised country / institution
C1	1	20332	20332	[Li, Haolei; He, Wenmiao; Yue, Dongmei; Wang, Mengmeng; Yuan, Xin; Huang, Kehe] Nanjing Agr Univ, Coll Vet Med, Nanjing 210095, Jiangsu, Peoples R China.	[Li, Haolei; He, Wenmiao; Yue, Dongmei; Wang, Mengmeng; Yuan, Xin; Huang, Kehe] Nanjing Agricultural University, Coll Vet Med, Nanjing 210095, Jiangsu, China.	Country: China Institution: Nanjing Agr Univ	Country: China Institution: Nanjing Agricultural University
C1	2	20333	20333	[Li, Haolei; He, Wenmiao; Yue, Dongmei; Wang, Mengmeng; Yuan, Xin; Huang, Kehe] Nanjing Agr Univ, Inst Anim Nutr Hlth, Nanjing 210095, Jiangsu, Peoples R China.	[Li, Haolei; He, Wenmiao; Yue, Dongmei; Wang, Mengmeng; Yuan, Xin; Huang, Kehe] Nanjing Agricultural University, Inst Anim Nutr Hlth, Nanjing 210095, Jiangsu, China.	Country: China Institution: Nanjing Agr Univ	Country: China Institution: Nanjing Agricultural University
C1	3	20334	20334	[Li, Haolei; He, Wenmiao; Yue, Dongmei; Wang, Mengmeng; Yuan, Xin; Huang, Kehe] Nanjing Agr Univ, Coll Vet Med, MOE Joint Int Res Lab Anim Hlth & Food Safety, Nanjing 210095, Jiangsu, Peoples R China.	[Li, Haolei; He, Wenmiao; Yue, Dongmei; Wang, Mengmeng; Yuan, Xin; Huang, Kehe] Nanjing Agricultural University, Coll Vet Med, MOE Joint Int Res Lab Anim Hlth & Food Safety, Nanjing 210095, Jiangsu, China.	Country: China Institution: Nanjing Agr Univ	Country: China Institution: Nanjing Agricultural University
C1	4	20335	20335	[Huang, Kehe] Nanjing Agr Univ, Nanjing 210095, Jiangsu, Peoples R China.	[Huang, Kehe] Nanjing Agricultural University, Nanjing 210095, Jiangsu, China.	Country: China Institution: Nanjing Agr Univ	Country: China Institution: Nanjing Agricultural University
RP	1	20338	20338	Huang, KH (corresponding author), Nanjing Agr Univ, Nanjing 210095, Jiangsu, Peoples R China	Huang, KH (corresponding author), Nanjing Agricultural University, Nanjing 210095, Jiangsu, China	Country: China Institution: Nanjing Agr Univ	Country: China Institution: Nanjing Agricultural University

Record 131 | WOS:000887299000001

Mycotoxins and Essential Oils-From a Meat Industry Hazard to a Possible Solution: A Brief Review

Field	Position	Original line	Normalised line	Original value	Normalised value	Original country / institution	Normalised country / institution
C1	3	20479	20479	[Serban, Andreea Iren] Univ Bucharest, Fac Biol, 91-95 Blvd, Bucharest 050095, Romania.	[Serban, Andreea Iren] University of Bucharest, Fac Biol, 91-95 Blvd, Bucharest 050095, Romania.	Country: Romania<br>Institution: Univ Bucharest	Country: Romania<br>Institution: University of Bucharest

Record 132 | WOS:000894684300001

Co-Contamination of Food and Feed with Mycotoxin and Bacteria and Possible Implications for Health

No C1/C3/RP normalisation changes detected for this record.

Record 133 | WOS:000883142900001

Current Review of Mycotoxin Biodegradation and Bioadsorption: Microorganisms, Mechanisms, and Main Important Applications

Field	Position	Original line	Normalised line	Original value	Normalised value	Original country / institution	Normalised country / institution
C1	1	20803	20803	[Ndiaye, Seyni; Zhang, Minhui; Zhang, Qi; Li, Peiwu] Minist Agr & Rural Affairs, Key Lab Biol & Genet Improvement Oil Crops, Wuhan 430062, Hubei, Peoples R China.	[Ndiaye, Seyni; Zhang, Minhui; Zhang, Qi; Li, Peiwu] Minist Agr & Rural Affairs, Key Lab Biol & Genet Improvement Oil Crops, Wuhan 430062, Hubei, China.	Country: China<br>Institution: Minist Agr & Rural Affairs	Country: China<br>Institution: Minist Agr & Rural Affairs
C1	2	20804	20804	[Ndiaye, Seyni; Zhang, Minhui; Zhang, Qi; Li, Peiwu] Chinese Acad Agr Sci, Oil Crops Res Inst, Wuhan 430062, Hubei, Peoples R China.	[Ndiaye, Seyni; Zhang, Minhui; Zhang, Qi; Li, Peiwu] Chinese Academy of Agricultural Sciences, Oil Crops Res Inst, Wuhan 430062, Hubei, China.	Country: China<br>Institution: Chinese Acad Agr Sci	Country: China<br>Institution: Chinese Academy of Agricultural Sciences
C1	3	20805	20805	[Ndiaye, Seyni; Zhang, Minhui; Zhang, Qi; Li, Peiwu] Minist Agr & Rural Affairs, Key Lab Detect Mycotoxins, Wuhan 430062, Hubei, Peoples R China.	[Ndiaye, Seyni; Zhang, Minhui; Zhang, Qi; Li, Peiwu] Minist Agr & Rural Affairs, Key Lab Detect Mycotoxins, Wuhan 430062, Hubei, China.	Country: China<br>Institution: Minist Agr & Rural Affairs	Country: China<br>Institution: Minist Agr & Rural Affairs
C1	4	20806	20806	[Ndiaye, Seyni; Zhang, Minhui; Zhang, Qi; Li, Peiwu] Minist Agr & Rural Affairs, Lab Risk Assessment Oilseeds Prod Wuhan, Wuhan 430062, Hubei, Peoples R China.	[Ndiaye, Seyni; Zhang, Minhui; Zhang, Qi; Li, Peiwu] Minist Agr & Rural Affairs, Lab Risk Assessment Oilseeds Prod Wuhan, Wuhan 430062, Hubei, China.	Country: China<br>Institution: Minist Agr & Rural Affairs	Country: China<br>Institution: Minist Agr & Rural Affairs
C1	5	20807	20807	[Ndiaye, Seyni; Ayessou, Nicolas M.] Univ Cheikh Anta Diop, Lab Anal & Essai, Ecole Super Polytech, Fann Dakar 5085, Senegal.	[Ndiaye, Seyni; Ayessou, Nicolas M.] University Cheikh Anta Diop Dakar, Lab Anal & Essai, Ecole Super Polytech, Fann Dakar 5085, Senegal.	Country: Senegal<br>Institution: Univ Cheikh Anta Diop	Country: Senegal<br>Institution: University Cheikh Anta Diop Dakar
C1	6	20808	20808	[Fall, Mouhamed] Chinese Acad Agr Sci, Key Lab Agroprod Proc, Inst Agroprod Proc Sci & Technol, Minist Agr, Beijing 100193, Peoples R China.	[Fall, Mouhamed] Chinese Academy of Agricultural Sciences, Key Lab Agroprod Proc, Inst Agroprod Proc Sci & Technol, Minist Agr, Beijing 100193, China.	Country: China<br>Institution: Chinese Acad Agr Sci	Country: China<br>Institution: Chinese Academy of Agricultural Sciences
C1	7	20809	20809	[Zhang, Qi; Li, Peiwu] Hubei Hongshan Lab, Wuhan 430070, Hubei, Peoples R China.	[Zhang, Qi; Li, Peiwu] Hubei Hongshan Lab, Wuhan 430070, Hubei, China.	Country: China<br>Institution: Hubei Hongshan Lab	Country: China<br>Institution: Hubei Hongshan Lab

RP	5	20813	20813	Zhang, Q (corresponding author), Hubei Hongshan Lab, Wuhan 430070, Hubei, Peoples R China	Zhang, Q (corresponding author), Hubei Hongshan Lab, Wuhan 430070, Hubei, China	Country: China Institution: Hubei Hongshan Lab	Country: China Institution: Hubei Hongshan Lab

Record 134 | WOS:000867775600001

The Effect of Aflatoxin B1 on Tumor-Related Genes and Phenotypic Characters of MCF7 and MCF10A Cells

Field	Position	Original line	Normalised line	Original value	Normalised value	Original country / institution	Normalised country / institution
C1	1	21063	21063	[Adam, Mowaffaq Adam Ahmed; Kamal, Laina Zarisa Muhd; Lok, Bronwyn; Yunus, Muhammad Amir; Sandai, Doblin] Univ Sains Malaysia, Adv Med & Dent Inst, Dept Biomed Sci, George Town 13200, Malaysia.	[Adam, Mowaffaq Adam Ahmed; Kamal, Laina Zarisa Muhd; Lok, Bronwyn; Yunus, Muhammad Amir; Sandai, Doblin] Universiti Sains Malaysia, Adv Med & Dent Inst, Dept Biomed Sci, George Town 13200, Malaysia.	Country: Malaysia Institution: Univ Sains Malaysia	Country: Malaysia Institution: Universiti Sains Malaysia
C1	2	21064	21064	[Adam, Mowaffaq Adam Ahmed; Bermoy, Brittany M.] San Diego State Univ, Dept Chem & Biochem, San Diego, CA 92182 USA.	[Adam, Mowaffaq Adam Ahmed; Bermoy, Brittany M.] San Diego State University, Dept Chem & Biochem, San Diego, CA 92182 USA.	Country: USA Institution: San Diego State Univ	Country: USA Institution: San Diego State University
C1	4	21066	21066	[Babu, Dinesh; Tabana, Yasser; Barakat, Khaled] Univ Alberta, Fac Pharm & Pharmaceut Sci, Edmonton, AB T6G 2E1, Canada.	[Babu, Dinesh; Tabana, Yasser; Barakat, Khaled] University of Alberta, Fac Pharm & Pharmaceut Sci, Edmonton, AB T6G 2E1, Canada.	Country: Canada Institution: Univ Alberta	Country: Canada Institution: University of Alberta
C1	6	21068	21068	[Than, Leslie Thian Lung] Univ Putra Malaysia, Fac Med & Hlth Sci, Dept Med Microbiol & Parasitol, Serdang 43400, Malaysia.	[Than, Leslie Thian Lung] Universiti Putra Malaysia, Fac Med & Hlth Sci, Dept Med Microbiol & Parasitol, Serdang 43400, Malaysia.	Country: Malaysia Institution: Univ Putra Malaysia	Country: Malaysia Institution: Universiti Putra Malaysia
RP	1	21071	21071	Sandai, D (corresponding author), Univ Sains Malaysia, Adv Med & Dent Inst, Dept Biomed Sci, George Town 13200, Malaysia	Sandai, D (corresponding author), Universiti Sains Malaysia, Adv Med & Dent Inst, Dept Biomed Sci, George Town 13200, Malaysia	Country: Malaysia Institution: Univ Sains Malaysia	Country: Malaysia Institution: Universiti Sains Malaysia

Record 135 | WOS:000911765600013

Ochratoxin A induced differentiation nephrotoxicity in renal tubule and glomeruli via autophagy differential regulation

Field	Position	Original line	Normalised line	Original value	Normalised value	Original country / institution	Normalised country / institution
C1	1	21236	21236	[Le, Guannan; Wei, Wei] Southeast Univ, Nanjing 211189, Peoples R China.	[Le, Guannan; Wei, Wei] Southeast Univ, Nanjing 211189, China.	Country: China Institution: Southeast Univ	Country: China Institution: Southeast Univ
C1	2	21237	21237	[Le, Guannan; Du, Heng; Sylia, Ardache; Hou, Lili; Muhmood, Azhar; Huang, Kehe] Nanjing Agr Univ, Coll Vet Med, Nanjing 210095, Jiangsu, Peoples R China.	[Le, Guannan; Du, Heng; Sylia, Ardache; Hou, Lili; Muhmood, Azhar; Huang, Kehe] Nanjing Agricultural University, Coll Vet Med, Nanjing 210095, Jiangsu, China.	Country: China Institution: Nanjing Agr Univ	Country: China Institution: Nanjing Agricultural University
C1	3	21238	21238	[Le, Guannan] Univ Chinese Acad Sci, Engn Res Ctr Clin Funct Mat & Diag & Treatment De, Wenzhou Inst, Wenzhou 325001, Peoples R China.	[Le, Guannan] Chinese Academy of Sciences, Engn Res Ctr Clin Funct Mat & Diag & Treatment De, Wenzhou Inst, Wenzhou 325001, China.	Country: China Institution: Univ Chinese Acad Sci	Country: China Institution: Chinese Academy of Sciences

C3	1	21239	21239	Southeast University - China	Southeast University	Institution: Southeast University - China	Institution: Southeast University
RP	1	21241	21241	Huang, KH (corresponding author), Nanjing Agr Univ, Coll Vet Med, Nanjing 210095, Jiangsu, Peoples R China	Huang, KH (corresponding author), Nanjing Agricultural University, Coll Vet Med, Nanjing 210095, Jiangsu, China	Country: China<br>Institution: Nanjing Agr Univ	Country: China<br>Institution: Nanjing Agricultural University

Record 136 | WOS:000866918500001

Quercetin: Its Antioxidant Mechanism, Antibacterial Properties and Potential Application in Prevention and Control of Toxipathy

Field	Position	Original line	Normalised line	Original value	Normalised value	Original country / institution	Normalised country / institution
C1	1	21342	21342	[Qi, Weidong; Qi, Wanxiang; Xiong, Dongwei; Long, Miao] Shenyang Agr Univ, Coll Anim Sci & Vet Med, Key Lab Livestock Infect Dis, Minist Educ, Shenyang 110866, Peoples R China.	[Qi, Weidong; Qi, Wanxiang; Xiong, Dongwei; Long, Miao] Shenyang Agricultural University, Coll Anim Sci & Vet Med, Key Lab Livestock Infect Dis, Minist Educ, Shenyang 110866, China.	Country: China<br>Institution: Shenyang Agr Univ	Country: China<br>Institution: Shenyang Agricultural University
RP	1	21344	21344	Long, M (corresponding author), Shenyang Agr Univ, Coll Anim Sci & Vet Med, Key Lab Livestock Infect Dis, Minist Educ, Shenyang 110866, Peoples R China	Long, M (corresponding author), Shenyang Agricultural University, Coll Anim Sci & Vet Med, Key Lab Livestock Infect Dis, Minist Educ, Shenyang 110866, China	Country: China<br>Institution: Shenyang Agr Univ	Country: China<br>Institution: Shenyang Agricultural University

Record 137 | WOS:000872026500001

Luteolin and Chrysin Could Prevent E. coli Lipopolysaccharide-Ochratoxin A Combination-Caused Inflammation and Oxidative Stress in In Vitro Porcine Intestinal Model

No C1/C3/RP normalisation changes detected for this record.

Record 138 | WOS:000864053100001

In-vitro alleviation of ochratoxin a mediated cytotoxicity using N-acetyl-L-tryptophan glucoside pretreatment

Field	Position	Original line	Normalised line	Original value	Normalised value	Original country / institution	Normalised country / institution
C1	2	21667	21667	[Agarwal, Prerna; Raisuddin, Sheikh] Jamia Hamdard, Fac Sci, Dept Med Elementol & Toxicol, Delhi 110062, India.	[Agarwal, Prerna; Raisuddin, Sheikh] Jamia Hamdard University, Fac Sci, Dept Med Elementol & Toxicol, Delhi 110062, India.	Country: India<br>Institution: Jamia Hamdard	Country: India<br>Institution: Jamia Hamdard University

Record 139 | WOS:000884231200001

Aflatoxin M1 and ochratoxin A induce a competitive endogenous RNA regulatory network of intestinal immunosuppression by whole-transcriptome analysis

Field	Position	Original line	Normalised line	Original value	Normalised value	Original country / institution	Normalised country / institution
C1	1	21763	21763	Chinese Acad Agr Sci, Inst Anim Sci, Key Lab Qual Safety Control Milk & Dairy Prod, Minist Agr & Rural Affairs, Beijing 100193, Peoples R China. Chinese Acad Agr Sci, Inst Anim Sci, Lab Qual & Safety	Chinese Academy of Agricultural Sciences, Inst Anim Sci, Key Lab Qual Safety Control Milk & Dairy Prod, Minist Agr & Rural Affairs, Beijing 100193, China. Chinese Academy of Agricultural	Country: China<br>Institution: Chinese Acad Agr Sci	Country: China<br>Institution: Chinese Academy of Agricultural Sciences

				Risk Assessment Dairy Prod, Minist Agr & Rural Affairs, Beijing 100193, Peoples R China. Chinese Acad Agr Sci, Inst Anim Sci, Minist Agr & Rural Affairs, Milk & Milk Prod Inspect Ctr, Beijing 100193, Peoples R China. Chinese Acad Agr Sci, Inst Anim Sci, State Key Lab Anim Nutr, Beijing 100193, Peoples R China.	Sciences, Inst Anim Sci, Lab Qual & Safety Risk Assessment Dairy Prod, Minist Agr & Rural Affairs, Beijing 100193, China. Chinese Academy of Agricultural Sciences, Inst Anim Sci, Minist Agr & Rural Affairs, Milk & Milk Prod Inspect Ctr, Beijing 100193, China. Chinese Academy of Agricultural Sciences, Inst Anim Sci, State Key Lab Anim Nutr, Beijing 100193, China.		
RP	1	21772	21772	Zheng, N (corresponding author), Chinese Acad Agr Sci, Inst Anim Sci, 2 Yuanmingyuan West Rd, Beijing 100193, Peoples R China	Zheng, N (corresponding author), Chinese Academy of Agricultural Sciences, Inst Anim Sci, 2 Yuanmingyuan West Rd, Beijing 100193, China	Country: China Institution: Chinese Acad Agr Sci	Country: China Institution: Chinese Academy of Agricultural Sciences

Record 140 | WOS:000876186700006

The importance of transcriptomics and proteomics for studying molecular mechanisms of mycotoxin exposure: A review

Field	Position	Original line	Normalised line	Original value	Normalised value	Original country / institution	Normalised country / institution
C1	1	21892	21892	[Cimbalo, A.; Frangiamone, M.; Font, G.; Manyes, L.] Univ Valencia, Fac Pharm, Lab Food Chem & Toxicol, Ave Vicent Andres Estelles S-N, Burjassot 46100, Spain.	[Cimbalo, A.; Frangiamone, M.; Font, G.; Manyes, L.] University of Valencia, Fac Pharm, Lab Food Chem & Toxicol, Ave Vicent Andres Estelles S-N, Burjassot 46100, Spain.	Country: Spain Institution: Univ Valencia	Country: Spain Institution: University of Valencia
RP	1	21894	21894	Frangiamone, M (corresponding author), Univ Valencia, Fac Pharm, Lab Food Chem & Toxicol, Ave Vicent Andres Estelles S-N, Burjassot 46100, Spain	Frangiamone, M (corresponding author), University of Valencia, Fac Pharm, Lab Food Chem & Toxicol, Ave Vicent Andres Estelles S-N, Burjassot 46100, Spain	Country: Spain Institution: Univ Valencia	Country: Spain Institution: University of Valencia

Record 141 | WOS:000851604100001

Mycotoxins in soybean-based foods fermented with filamentous fungi: Occurrence and preventive strategies

Field	Position	Original line	Normalised line	Original value	Normalised value	Original country / institution	Normalised country / institution
C1	1	22021	22021	[Tian, Fei; Woo, So Young; Lee, Sang Yoo; Park, Su Been; Im, Ju Hee; Chun, Hyang Sook] Chung Ang Univ, Sch Food Sci & Technol, Food Toxicol Lab, Anseong, South Korea.	[Tian, Fei; Woo, So Young; Lee, Sang Yoo; Park, Su Been; Im, Ju Hee; Chun, Hyang Sook] Chung Ang University, Sch Food Sci & Technol, Food Toxicol Lab, Anseong, South Korea.	Country: South Korea Institution: Chung Ang Univ	Country: South Korea Institution: Chung Ang University
RP	1	22023	22023	Chun, HS (corresponding author), Chung Ang Univ, Sch Food Sci & Technol, Anseong 17546, South Korea	Chun, HS (corresponding author), Chung Ang University, Sch Food Sci & Technol, Anseong 17546, South Korea	Country: South Korea Institution: Chung Ang Univ	Country: South Korea Institution: Chung Ang University

Record 142 | WOS:000851281600001

Mechanisms underlying citrinin-induced toxicity via oxidative stress and apoptosis-mediated by mitochondrial-dependent pathway in SH-SY5Y cells

Field	Position	Original line	Normalised line	Original value	Normalised value	Original country / institution	Normalised country / institution
C1	1	22218	22218	[Abudayyak, Mahmoud; Ozden, Sibel] Istanbul Univ, Fac Pharm, Dept Pharmaceut Toxicol, TR-34116 Istanbul, Turkey.	[Abudayyak, Mahmoud; Ozden, Sibel] Istanbul University, Fac Pharm, Dept Pharmaceut Toxicol, TR-34116 Istanbul, Turkey.	Country: Turkey<br>Institution: Istanbul Univ	Country: Turkey<br>Institution: Istanbul University
C1	2	22219	22219	[Karaman, Ecem Fatma] Biruni Univ, Fac Pharm, Dept Pharmaceut Toxicol, Istanbul, Turkey.	[Karaman, Ecem Fatma] Biruni University, Fac Pharm, Dept Pharmaceut Toxicol, Istanbul, Turkey.	Country: Turkey<br>Institution: Biruni Univ	Country: Turkey<br>Institution: Biruni University
RP	1	22221	22221	Ozden, S (corresponding author), Istanbul Univ, Fac Pharm, Dept Pharmaceut Toxicol, TR-34116 Istanbul, Turkey	Ozden, S (corresponding author), Istanbul University, Fac Pharm, Dept Pharmaceut Toxicol, TR-34116 Istanbul, Turkey	Country: Turkey<br>Institution: Istanbul Univ	Country: Turkey<br>Institution: Istanbul University

Record 143 | WOS:000862185500005

Co-occurrence of mycotoxins: A review on bioanalytical methods for simultaneous analysis in human biological samples, mixture toxicity and risk assessment strategies

No C1/C3/RP normalisation changes detected for this record.

Record 144 | WOS:000857038900001

The Neurotoxic Effect of Ochratoxin-A on the Hippocampal Neurogenic Niche of Adult Mouse Brain

Field	Position	Original line	Normalised line	Original value	Normalised value	Original country / institution	Normalised country / institution
C1	1	22552	22552	[Mateo, Eva] Univ Valencia, Sch Med & Dent, Dept Microbiol & Ecol, Valencia 46001, Spain.	[Mateo, Eva] University of Valencia, Sch Med & Dent, Dept Microbiol & Ecol, Valencia 46001, Spain.	Country: Spain<br>Institution: Univ Valencia	Country: Spain<br>Institution: University of Valencia
C1	2	22553	22553	[Bernardus Tonino, Rik Paulus] Leiden Univ, Haematol Dept, NL-1043 AJ Leiden, Netherlands.	[Bernardus Tonino, Rik Paulus] Leiden University, Haematol Dept, NL-1043 AJ Leiden, Netherlands.	Country: Netherlands<br>Institution: Leiden Univ	Country: Netherlands<br>Institution: Leiden University
C1	3	22554	22554	[Canto, Antolin; Miranda, Maria; Miguel Soria, Jose] Cardenal Herrera Univ CEU Univ, Dept Biomed Sci, Valencia 46001, Spain.	[Canto, Antolin; Miranda, Maria; Miguel Soria, Jose] Universidad CEU Cardenal Herrera, Dept Biomed Sci, Valencia 46001, Spain.	Country: Spain<br>Institution: Cardenal Herrera Univ CEU Univ	Country: Spain<br>Institution: Universidad CEU Cardenal Herrera
C1	5	22556	22556	[Garcia Esparza, Maria Angeles] Cardenal Herrera Univ CEU Univ, Dept Pharm, Valencia 46001, Spain.	[Garcia Esparza, Maria Angeles] Universidad CEU Cardenal Herrera, Dept Pharm, Valencia 46001, Spain.	Country: Spain<br>Institution: Cardenal Herrera Univ CEU Univ	Country: Spain<br>Institution: Universidad CEU Cardenal Herrera
RP	1	22559	22559	Soria, JM (corresponding author), Cardenal Herrera Univ CEU Univ, Dept Biomed Sci, Valencia 46001, Spain	Soria, JM (corresponding author), Universidad CEU Cardenal Herrera, Dept Biomed Sci, Valencia 46001, Spain	Country: Spain<br>Institution: Cardenal Herrera Univ CEU Univ	Country: Spain<br>Institution: Universidad CEU Cardenal Herrera

Record 145 | WOS:000859602700001

Ochratoxin A and Citrinin Differentially Modulate Bovine Mammary Epithelial Cell Permeability and Innate Immune Function

Field	Position	Original line	Normalised line	Original value	Normalised value	Original country / institution	Normalised country / institution

C1	1	22674	22674	[Xu, Ran; Shandilya, Umesh K.; Karrow, Niel A.] Univ Guelph, Dept Anim Biosci, Guelph, ON N1G 2W1, Canada.	[Xu, Ran; Shandilya, Umesh K.; Karrow, Niel A.] University of Guelph, Dept Anim Biosci, Guelph, ON N1G 2W1, Canada.	Country: Canada<br>Institution: Univ Guelph	Country: Canada<br>Institution: University of Guelph
RP	1	22677	22677	Karrow, NA (corresponding author), Univ Guelph, Dept Anim Biosci, Guelph, ON N1G 2W1, Canada	Karrow, NA (corresponding author), University of Guelph, Dept Anim Biosci, Guelph, ON N1G 2W1, Canada	Country: Canada<br>Institution: Univ Guelph	Country: Canada<br>Institution: University of Guelph

Record 146 | WOS:000858998000001

Protective activities of ellagic acid and urolithins against kidney toxicity of environmental pollutants: A review

No C1/C3/RP normalisation changes detected for this record.

Record 147 | WOS:000836768600001

Metabolic profiling as a powerful tool for the analysis of cellular alterations caused by 20 mycotoxins in HepG2 cells

Field	Position	Original line	Normalised line	Original value	Normalised value	Original country / institution	Normalised country / institution
C1	1	23137	23137	[Gerdemann, Andrea; Behrens, Matthias; Esselen, Melanie; Humpf, Hans-Ulrich] Univ Munster, Inst Food Chem, Corrensstr 45, D-48149 Munster, Germany.	[Gerdemann, Andrea; Behrens, Matthias; Esselen, Melanie; Humpf, Hans-Ulrich] University of Munster, Inst Food Chem, Corrensstr 45, D-48149 Munster, Germany.	Country: Germany<br>Institution: Univ Munster	Country: Germany<br>Institution: University of Munster
RP	1	23139	23139	Humpf, HU (corresponding author), Univ Munster, Inst Food Chem, Corrensstr 45, D-48149 Munster, Germany	Humpf, HU (corresponding author), University of Munster, Inst Food Chem, Corrensstr 45, D-48149 Munster, Germany	Country: Germany<br>Institution: Univ Munster	Country: Germany<br>Institution: University of Munster

Record 148 | WOS:000836539000001

Combined effects of cadmium and ochratoxin A on intestinal barrier dysfunction in human Caco-2 cells and pig small intestinal epithelial cells

Field	Position	Original line	Normalised line	Original value	Normalised value	Original country / institution	Normalised country / institution
C1	1	23246	23246	[Kim, So-Hee; Jeong, Yu-Jin; Pyo, Min Cheol; Lee, Kwang-Won] Korea Univ, Coll Life Sci & Biotechnol, Dept Biotechnol, Seoul 02841, South Korea.	[Kim, So-Hee; Jeong, Yu-Jin; Pyo, Min Cheol; Lee, Kwang-Won] Korea University, Coll Life Sci & Biotechnol, Dept Biotechnol, Seoul 02841, South Korea.	Country: South Korea<br>Institution: Korea Univ	Country: South Korea<br>Institution: Korea University
C1	2	23247	23247	[Lee, Kwang-Won] Korea Univ, Coll Life Sci & Biotechnol, Dept Food Biosci & Technol, Seoul 02841, South Korea.	[Lee, Kwang-Won] Korea University, Coll Life Sci & Biotechnol, Dept Food Biosci & Technol, Seoul 02841, South Korea.	Country: South Korea<br>Institution: Korea Univ	Country: South Korea<br>Institution: Korea University
RP	1	23249	23249	Lee, KW (corresponding author), Korea Univ, Coll Life Sci & Biotechnol, Dept Biotechnol, Seoul 02841, South Korea	Lee, KW (corresponding author), Korea University, Coll Life Sci & Biotechnol, Dept Biotechnol, Seoul 02841, South Korea	Country: South Korea<br>Institution: Korea Univ	Country: South Korea<br>Institution: Korea University

Record 149 | WOS:000827733300003

Antifungal and antimycotoxic activities of 3 essential oils against 3 mycotoxinogenic fungi

Field	Position	Original line	Normalised line	Original value	Normalised value	Original country / institution	Normalised country / institution
C1	1	23361	23361	[Chelaghema, Asma; Durand, Noel; Servent, Adrien; Mamouni, Myriam; Poucheret, Patrick; Schorr-Galindo, Sabine; Fontana, Angelique; Strub, Caroline] Univ La Reunion, Qualisud, Univ Montpellier, CIRAD,Inst Agro,IRD,Avignon Univ, Montpellier, France.	[Chelaghema, Asma; Durand, Noel; Servent, Adrien; Mamouni, Myriam; Poucheret, Patrick; Schorr-Galindo, Sabine; Fontana, Angelique; Strub, Caroline] University of La Reunion, Qualisud, Univ Montpellier, CIRAD,Inst Agro,IRD,Avignon Univ, Montpellier, France.	Country: France Institution: Univ La Reunion	Country: France Institution: University of La Reunion
RP	1	23366	23366	Strub, C (corresponding author), Univ La Reunion, Qualisud, Univ Montpellier, CIRAD,Inst Agro,IRD,Avignon Univ, Montpellier, France	Strub, C (corresponding author), University of La Reunion, Qualisud, Univ Montpellier, CIRAD,Inst Agro,IRD,Avignon Univ, Montpellier, France	Country: France Institution: Univ La Reunion	Country: France Institution: University of La Reunion

Record 150 | WOS:000845596200001

Evaluation of the Safety and Ochratoxin A Degradation Capacity of Pediococcus pentosaceus as a Dietary Probiotic with Molecular Docking Approach and Pharmacokinetic Toxicity Assessment

Field	Position	Original line	Normalised line	Original value	Normalised value	Original country / institution	Normalised country / institution
C1	1	23488	23488	[Park, Sungkwon; Koo, Jinsu; Kim, Bosung; Balasubramanian, Balamuralikrishnan] Sejong Univ, Coll Life Sci, Dept Food Sci & Biotechnol, Seoul 05006, South Korea.	[Park, Sungkwon; Koo, Jinsu; Kim, Bosung; Balasubramanian, Balamuralikrishnan] Sejong University, Coll Life Sci, Dept Food Sci & Biotechnol, Seoul 05006, South Korea.	Country: South Korea Institution: Sejong Univ	Country: South Korea Institution: Sejong University
C1	4	23491	23491	[Liu, Wen-Chao] Guangdong Ocean Univ, Coll Coastal Agr Sci, Dept Anim Sci, Zhanjiang 524088, Peoples R China.	[Liu, Wen-Chao] Guangdong Ocean University, Coll Coastal Agr Sci, Dept Anim Sci, Zhanjiang 524088, China.	Country: China Institution: Guangdong Ocean Univ	Country: China Institution: Guangdong Ocean University
RP	1	23496	23496	Balasubramanian, B (corresponding author), Sejong Univ, Coll Life Sci, Dept Food Sci & Biotechnol, Seoul 05006, South Korea	Balasubramanian, B (corresponding author), Sejong University, Coll Life Sci, Dept Food Sci & Biotechnol, Seoul 05006, South Korea	Country: South Korea Institution: Sejong Univ	Country: South Korea Institution: Sejong University
RP	2	23496	23496	Liu, WC (corresponding author), Guangdong Ocean Univ, Coll Coastal Agr Sci, Dept Anim Sci, Zhanjiang 524088, Peoples R China	Liu, WC (corresponding author), Guangdong Ocean Univ, Coll Coastal Agr Sci, Dept Anim Sci, Zhanjiang 524088, China	Country: China Institution: Guangdong Ocean Univ	Country: China Institution: Guangdong Ocean Univ

Record 151 | WOS:000810935900001

Paper The multiple biotoxicity integrated study in grass carp (Ctenopharyngodon idella) caused by Ochratoxin A: Oxidative damage, apoptosis and immunosuppression

Field	Position	Original line	Normalised line	Original value	Normalised value	Original country / institution	Normalised country / institution
C1	1	23613	23613	[Zhao, Piao; Liu, Xin; Jiang, Wei-Dan; Wu, Pei; Liu, Yang; Jiang, Jun; Zhou, Xiao-Qiu; Feng, Lin] Sichuan Agr Univ, Anim Nutr Inst, Chengdu 611130, Sichuan, Peoples R China.	[Zhao, Piao; Liu, Xin; Jiang, Wei-Dan; Wu, Pei; Liu, Yang; Jiang, Jun; Zhou, Xiao-Qiu; Feng, Lin] Sichuan Agricultural University, Anim Nutr Inst, Chengdu 611130, Sichuan, China.	Country: China Institution: Sichuan Agr Univ	Country: China Institution: Sichuan Agricultural University
C1	2	23614	23614	[Jiang, Wei-Dan; Wu, Pei; Liu, Yang; Zhou, Xiao-Qiu; Feng, Lin] Sichuan Agr Univ, Fish Nutr & Safety Prod Univ, Key	[Jiang, Wei-Dan; Wu, Pei; Liu, Yang; Zhou, Xiao-Qiu; Feng, Lin] Sichuan Agricultural University, Fish Nutr & Safety	Country: China Institution: Sichuan Agr Univ	Country: China Institution: Sichuan Agricultural University

				Lab Sichuan Prov, Chengdu 611130, Sichuan, Peoples R China.	Prod Univ, Key Lab Sichuan Prov, Chengdu 611130, Sichuan, China.		
C1	3	23615	23615	[Jiang, Wei-Dan; Wu, Pei; Liu, Yang; Zhou, Xiao-Qiu; Feng, Lin] Minist Agr & Rural Affairs, Minist Educ, Key Lab Anim Dis Resistance Nutr, Key Lab Sichuan Prov, Sichuan 611130, Peoples R China.	[Jiang, Wei-Dan; Wu, Pei; Liu, Yang; Zhou, Xiao-Qiu; Feng, Lin] Minist Agr & Rural Affairs, Minist Educ, Key Lab Anim Dis Resistance Nutr, Key Lab Sichuan Prov, Sichuan 611130, China.	Country: China<br>Institution: Minist Agr & Rural Affairs	Country: China<br>Institution: Minist Agr & Rural Affairs
C1	4	23616	23616	[Zhang, Lu; Mi, Hai-Feng] Tongwei Co Ltd, Hlth Aquaculture Key Lab Sichuan Prov, Chengdu 610041, Sichuan, Peoples R China.	[Zhang, Lu; Mi, Hai-Feng] Tongwei Co Ltd, Hlth Aquaculture Key Lab Sichuan Prov, Chengdu 610041, Sichuan, China.	Country: China<br>Institution: Tongwei Co Ltd	Country: China<br>Institution: Tongwei Co Ltd
C1	5	23617	23617	[Kuang, Sheng-Yao; Tang, Ling] Sichuan Acad Anim Sci, Anim Nutr Inst, Chengdu 610066, Peoples R China.	[Kuang, Sheng-Yao; Tang, Ling] Sichuan Acad Anim Sci, Anim Nutr Inst, Chengdu 610066, China.	Country: China<br>Institution: Sichuan Acad Anim Sci	Country: China<br>Institution: Sichuan Acad Anim Sci
RP	2	23620	23620	Feng, L (corresponding author), Sichuan Agr Univ, Anim Nutr Inst, Chengdu 611130, Sichuan, Peoples R China	Feng, L (corresponding author), Sichuan Agricultural University, Anim Nutr Inst, Chengdu 611130, Sichuan, China	Country: China<br>Institution: Sichuan Agr Univ	Country: China<br>Institution: Sichuan Agricultural University

Record 152 | WOS:000807511200002

Nutritional impact of mycotoxins in food animal production and strategies for mitigation

Field	Position	Original line	Normalised line	Original value	Normalised value	Original country / institution	Normalised country / institution
C1	1	23811	23811	[Xu, Ran; Kiarie, Elijah G.; Karrow, Niel A.] Univ Guelph, Dept Anim Biosci, Guelph, ON N1G 2W1, Canada.	[Xu, Ran; Kiarie, Elijah G.; Karrow, Niel A.] University of Guelph, Dept Anim Biosci, Guelph, ON N1G 2W1, Canada.	Country: Canada<br>Institution: Univ Guelph	Country: Canada<br>Institution: University of Guelph
C1	3	23813	23813	[Sun, Lvhui] Huazhong Agr Univ, Coll Anim Sci & Technol, Hubei Hongshan Lab, Wuhan 430070, Hubei, Peoples R China.	[Sun, Lvhui] Huazhong Agr Univ, Coll Anim Sci & Technol, Hubei Hongshan Lab, Wuhan 430070, Hubei, China.	Country: China<br>Institution: Huazhong Agr Univ	Country: China<br>Institution: Huazhong Agr Univ
RP	1	23816	23816	Karrow, NA (corresponding author), Univ Guelph, Dept Anim Biosci, Guelph, ON N1G 2W1, Canada	Karrow, NA (corresponding author), University of Guelph, Dept Anim Biosci, Guelph, ON N1G 2W1, Canada	Country: Canada<br>Institution: Univ Guelph	Country: Canada<br>Institution: University of Guelph

Record 153 | WOS:000818403700001

Genotoxicity of 12 Mycotoxins by the SOS/umu Test: Comparison of Liver and Kidney S9 Fraction

Field	Position	Original line	Normalised line	Original value	Normalised value	Original country / institution	Normalised country / institution
C1	1	24076	24076	[Alonso-Jauregui, Maria; Lopez de Cerain, Adela; Vettorazzi, Ariane] Univ Navarra, Sch Pharm & Nutr, Dept Pharmacol & Toxicol, MITOX Res Grp, Pamplona 31008, Spain.	[Alonso-Jauregui, Maria; Lopez de Cerain, Adela; Vettorazzi, Ariane] University of Navarra, Sch Pharm & Nutr, Dept Pharmacol & Toxicol, MITOX Res Grp, Pamplona 31008, Spain.	Country: Spain<br>Institution: Univ Navarra	Country: Spain<br>Institution: University of Navarra
C1	2	24077	24077	[Gonzalez-Penas, Elena] Univ Navarra, Sch Pharm & Nutr, Dept Pharmaceut Technol & Chem, MITOX Res Grp, Pamplona 31008, Spain.	[Gonzalez-Penas, Elena] University of Navarra, Sch Pharm & Nutr, Dept Pharmaceut Technol & Chem,	Country: Spain<br>Institution: Univ Navarra	Country: Spain<br>Institution: University of Navarra

					MITOX Res Grp, Pamplona 31008, Spain.		
RP	1	24079	24079	Vettorazzi, A (corresponding author), Univ Navarra, Sch Pharm & Nutr, Dept Pharmacol & Toxicol, MITOX Res Grp, Pamplona 31008, Spain	Vettorazzi, A (corresponding author), University of Navarra, Sch Pharm & Nutr, Dept Pharmacol & Toxicol, MITOX Res Grp, Pamplona 31008, Spain	Country: Spain<br>Institution: Univ Navarra	Country: Spain<br>Institution: University of Navarra

Record 154 | WOS:000819631900001

Ochratoxin A and Kidney Oxidative Stress: The Role of Nutraceuticals in Veterinary Medicine-A Review

Field	Position	Original line	Normalised line	Original value	Normalised value	Original country / institution	Normalised country / institution
C1	2	24210	24210	[Ferrara, Gianmarco; Andretta, Emanuela; Montagnaro, Serena; Damiano, Sara; Ciarcia, Roberto] Univ Naples Federico II, Dept Vet Med & Anim Prod, Via Federico Delpino 1, I-80137 Naples, Italy.	[Ferrara, Gianmarco; Andretta, Emanuela; Montagnaro, Serena; Damiano, Sara; Ciarcia, Roberto] University of Naples Federico II, Dept Vet Med & Anim Prod, Via Federico Delpino 1, I-80137 Naples, Italy.	Country: Italy<br>Institution: Univ Naples Federico II	Country: Italy<br>Institution: University of Naples Federico II
RP	1	24212	24212	Damiano, S (corresponding author), Univ Naples Federico II, Dept Vet Med & Anim Prod, Via Federico Delpino 1, I-80137 Naples, Italy	Damiano, S (corresponding author), University of Naples Federico II, Dept Vet Med & Anim Prod, Via Federico Delpino 1, I-80137 Naples, Italy	Country: Italy<br>Institution: Univ Naples Federico II	Country: Italy<br>Institution: University of Naples Federico II

Record 155 | WOS:000806405700007

In vitro mycotoxin binding capacities of clays, glucomannan and their combinations

Field	Position	Original line	Normalised line	Original value	Normalised value	Original country / institution	Normalised country / institution
C1	1	24381	24381	[Oguz, Halis] Selcuk Univ, Fac Vet Med, Dept Pharmacol & Toxicol, TR-42130 Konya, Turkey.	[Oguz, Halis] Selcuk University, Fac Vet Med, Dept Pharmacol & Toxicol, TR-42130 Konya, Turkey.	Country: Turkey<br>Institution: Selcuk Univ	Country: Turkey<br>Institution: Selcuk University
C1	2	24382	24382	[Bahcivan, Emre] Kafkas Univ, Fac Vet Med, Dept Pharmacol & Toxicol, Pasacayiri Kampusu, TR-36100 Kars, Turkey.	[Bahcivan, Emre] Kafkas University, Fac Vet Med, Dept Pharmacol & Toxicol, Pasacayiri Kampusu, TR-36100 Kars, Turkey.	Country: Turkey<br>Institution: Kafkas Univ	Country: Turkey<br>Institution: Kafkas University
RP	1	24387	24387	Oguz, H (corresponding author), Selcuk Univ, Fac Vet Med, Dept Pharmacol & Toxicol, TR-42130 Konya, Turkey	Oguz, H (corresponding author), Selcuk University, Fac Vet Med, Dept Pharmacol & Toxicol, TR-42130 Konya, Turkey	Country: Turkey<br>Institution: Selcuk Univ	Country: Turkey<br>Institution: Selcuk University

Record 156 | WOS:000832788700002

Effectiveness of beetroot extract in SH-SY5Y neuronal cell protection against Fumonisin B1, Ochratoxin A and its combination

Field	Position	Original line	Normalised line	Original value	Normalised value	Original country / institution	Normalised country / institution
C1	1	24514	24514	[Penalva-Olcina, Raquel; Juan, Cristina; Fernandez-Franzon, Monica; Juan-Garcia, Ana] Univ Valencia, Fac Pharm, Lab Food Chem & Toxicol, Av Vicent	[Penalva-Olcina, Raquel; Juan, Cristina; Fernandez-Franzon, Monica; Juan-Garcia, Ana] University of Valencia, Fac Pharm, Lab Food Chem & Toxicol, Av Vicent Andres	Country: Spain<br>Institution: Univ Valencia	Country: Spain<br>Institution: University of Valencia

				Andres Estelles S-N, Valencia 46100, Spain.	Estelles S-N, Valencia 46100, Spain.		
RP	1	24516	24516	Juan-GarcÃ-a, A (corresponding author), Univ Valencia, Fac Pharm, Lab Food Chem & Toxicol, Av Vicent Andres Estelles S-N, Valencia 46100, Spain	Juan-GarcÃ-a, A (corresponding author), University of Valencia, Fac Pharm, Lab Food Chem & Toxicol, Av Vicent Andres Estelles S-N, Valencia 46100, Spain	Country: Spain<br>Institution: Univ Valencia	Country: Spain<br>Institution: University of Valencia

Record 157 | WOS:000793099900001

Ochratoxin a induces hepatic fibrosis through TGF-Î² receptor I/Smad2/3 signaling pathway

Field	Position	Original line	Normalised line	Original value	Normalised value	Original country / institution	Normalised country / institution
C1	1	24623	24623	[Chae, Seung A.; Pyo, Min Cheol; Yoo, Hee Joon; Lee, Kwang-Won] Korea Univ, Dept Biotechnol, Coll Life Sci & Biotechnol, Seoul 02841, South Korea.	[Chae, Seung A.; Pyo, Min Cheol; Yoo, Hee Joon; Lee, Kwang-Won] Korea University, Dept Biotechnol, Coll Life Sci & Biotechnol, Seoul 02841, South Korea.	Country: South Korea<br>Institution: Korea Univ	Country: South Korea<br>Institution: Korea University
C1	2	24624	24624	[Lee, Kwang-Won] Korea Univ, Dept Food Biosci & Technol, Coll Life Sci & Biotechnol, Seoul, South Korea.	[Lee, Kwang-Won] Korea University, Dept Food Biosci & Technol, Coll Life Sci & Biotechnol, Seoul, South Korea.	Country: South Korea<br>Institution: Korea Univ	Country: South Korea<br>Institution: Korea University
RP	2	24626	24626	Lee, KW (corresponding author), Korea Univ, Dept Biotechnol, Coll Life Sci & Biotechnol, Seoul 02841, South Korea	Lee, KW (corresponding author), Korea University, Dept Biotechnol, Coll Life Sci & Biotechnol, Seoul 02841, South Korea	Country: South Korea<br>Institution: Korea Univ	Country: South Korea<br>Institution: Korea University

Record 158 | WOS:000801766500001

Degradation of Ochratoxin A by a UV-Mutated Aspergillus niger Strain

Field	Position	Original line	Normalised line	Original value	Normalised value	Original country / institution	Normalised country / institution
C1	1	24738	24738	[Zou, Dong; Ji, Jian; Ye, Yongli; Yang, Yang; Yu, Jian; Sun, Xiulan] Jiangnan Univ, Synerget Innovat Ctr Food Safety & Qual Control, Sch Food Sci & Technol, State Key Lab Food Sci & Technol, Wuxi 214122, Jiangsu, Peoples R China.	[Zou, Dong; Ji, Jian; Ye, Yongli; Yang, Yang; Yu, Jian; Sun, Xiulan] Jiangnan University, Synerget Innovat Ctr Food Safety & Qual Control, Sch Food Sci & Technol, State Key Lab Food Sci & Technol, Wuxi 214122, Jiangsu, China.	Country: China<br>Institution: Jiangnan Univ	Country: China<br>Institution: Jiangnan University
C1	2	24739	24739	[Ji, Jian] Xinjiang Agr Univ, Coll Food Sci & Pharm, Urumqi 830052, Peoples R China.	[Ji, Jian] Xinjiang Agricultural University, Coll Food Sci & Pharm, Urumqi 830052, China.	Country: China<br>Institution: Xinjiang Agr Univ	Country: China<br>Institution: Xinjiang Agricultural University
C1	3	24740	24740	[Wang, Meng] Beijing Acad Agr & Forestry Sci, Inst Qual Stand & Testing Technol, Beijing 100097, Peoples R China.	[Wang, Meng] Beijing Acad Agr & Forestry Sci, Inst Qual Stand & Testing Technol, Beijing 100097, China.	Country: China<br>Institution: Beijing Acad Agr & Forestry Sci	Country: China<br>Institution: Beijing Acad Agr & Forestry Sci
C1	4	24741	24741	[Zheng, Yi] Jiangsu Agrianim Husb Vocat Coll, Key Lab High Tech Res & Dev Vet Biopharmaceut, Taizhou 214122, Peoples R China.	[Zheng, Yi] Jiangsu Agrianim Husb Vocat Coll, Key Lab High Tech Res & Dev Vet Biopharmaceut, Taizhou 214122, China.	Country: China<br>Institution: Jiangsu Agrianim Husb Vocat Coll	Country: China<br>Institution: Jiangsu Agrianim Husb Vocat Coll
RP	1	24744	24744	Sun, XL (corresponding author), Jiangnan Univ, Synerget Innovat Ctr Food Safety & Qual Control, Sch Food Sci &	Sun, XL (corresponding author), Jiangnan University, Synerget Innovat Ctr Food Safety & Qual Control, Sch Food Sci &	Country: China<br>Institution: Jiangnan Univ	Country: China<br>Institution: Jiangnan University

				Technol, State Key Lab Food Sci & Technol, Wuxi 214122, Jiangsu, Peoples R China	Technol, State Key Lab Food Sci & Technol, Wuxi 214122, Jiangsu, China		

Record 159 | WOS:000802213700006

Pumpkin extract and fermented whey individually and in combination alleviated AFB1-and OTA-induced alterations on neuronal differentiation in vitro

Field	Position	Original line	Normalised line	Original value	Normalised value	Original country / institution	Normalised country / institution
C1	1	24863	24863	[Frangiamone, Massimo; Alonso-Garrido, Manuel; Font, Guillermina; Cimbalo, Alessandra; Manyes, Lara] Univ Valencia, Fac Pharm, Lab Food Chem & Toxicol, Vicent Andres Estelles s n, Burjassot 46100, Spain.	[Frangiamone, Massimo; Alonso-Garrido, Manuel; Font, Guillermina; Cimbalo, Alessandra; Manyes, Lara] University of Valencia, Fac Pharm, Lab Food Chem & Toxicol, Vicent Andres Estelles s n, Burjassot 46100, Spain.	Country: Spain Institution: Univ Valencia	Country: Spain Institution: University of Valencia
RP	1	24865	24865	Cimbalo, A (corresponding author), Univ Valencia, Fac Pharm, Lab Food Chem & Toxicol, Vicent Andres Estelles s n, Burjassot 46100, Spain	Cimbalo, A (corresponding author), University of Valencia, Fac Pharm, Lab Food Chem & Toxicol, Vicent Andres Estelles s n, Burjassot 46100, Spain	Country: Spain Institution: Univ Valencia	Country: Spain Institution: University of Valencia

Record 160 | WOS:000793768800008

Ochratoxin A as an alarming health threat for livestock and human: A review on molecular interactions, mechanism of toxicity, detection, detoxification, and dietary prophylaxis

Field	Position	Original line	Normalised line	Original value	Normalised value	Original country / institution	Normalised country / institution
C1	1	25019	25019	[Liu, Wen-Chao; Bhotla, Haripriya Kuchi] Guangdong Ocean Univ, Coll Coastal Agr Sci, Dept Anim Sci, Zhanjiang 524088, Peoples R China.	[Liu, Wen-Chao; Bhotla, Haripriya Kuchi] Guangdong Ocean University, Coll Coastal Agr Sci, Dept Anim Sci, Zhanjiang 524088, China.	Country: China Institution: Guangdong Ocean Univ	Country: China Institution: Guangdong Ocean University
C1	4	25022	25022	[Arumugam, Vijaya Anand] Bharathiar Univ, Dept Human Genet & Mol Biol, Coimbatore 641046, Tamil Nadu, India.	[Arumugam, Vijaya Anand] Bharathiar University, Dept Human Genet & Mol Biol, Coimbatore 641046, Tamil Nadu, India.	Country: India Institution: Bharathiar Univ	Country: India Institution: Bharathiar University
C1	6	25024	25024	[Issara, Utthapon] Rajamangala Univ Technol Thanyaburi, Fac Sci & Technol, Div Food Sci & Technol Management, Pathum Thani 12110, Thailand.	[Issara, Utthapon] Rajamangala University of Technology Thanyaburi, Fac Sci & Technol, Div Food Sci & Technol Management, Pathum Thani 12110, Thailand.	Country: Thailand Institution: Rajamangala Univ Technol Thanyaburi	Country: Thailand Institution: Rajamangala University of Technology Thanyaburi
C1	7	25025	25025	[Balasubramanian, Balamuralikrishnan] Sejong Univ, Coll Life Sci, Dept Food Sci & Biotechnol, Seoul 05006, South Korea.	[Balasubramanian, Balamuralikrishnan] Sejong University, Coll Life Sci, Dept Food Sci & Biotechnol, Seoul 05006, South Korea.	Country: South Korea Institution: Sejong Univ	Country: South Korea Institution: Sejong University

Record 161 | WOS:000786151200001

Cytotoxicity of Mycotoxins and Their Combinations on Different Cell Lines: A Review

Field	Position	Original line	Normalised line	Original value	Normalised value	Original country / institution	Normalised country / institution

C1	1	25279	25279	[Skrzydlewski, Pawel; Twaruzek, Magdalena; Grajewski, Jan] Kazimierz Wielki Univ, Fac Biol Sci, Dept Physiol & Toxicol, PL-85064 Bydgoszcz, Poland.	[Skrzydlewski, Pawel; Twaruzek, Magdalena; Grajewski, Jan] Kazimierz Wielki University, Fac Biol Sci, Dept Physiol & Toxicol, PL-85064 Bydgoszcz, Poland.	Country: Poland<br>Institution: Kazimierz Wielki Univ	Country: Poland<br>Institution: Kazimierz Wielki University
RP	1	25281	25281	Twaruzek, M (corresponding author), Kazimierz Wielki Univ, Fac Biol Sci, Dept Physiol & Toxicol, PL-85064 Bydgoszcz, Poland	Twaruzek, M (corresponding author), Kazimierz Wielki University, Fac Biol Sci, Dept Physiol & Toxicol, PL-85064 Bydgoszcz, Poland	Country: Poland<br>Institution: Kazimierz Wielki Univ	Country: Poland<br>Institution: Kazimierz Wielki University

Record 162 | WOS:000784471500001

Mycotoxins in food and feed: toxicity, preventive challenges, and advanced detection techniques for associated diseases

Field	Position	Original line	Normalised line	Original value	Normalised value	Original country / institution	Normalised country / institution
C1	1	25451	25451	[Dey, Debasish Kumar] Daegu Univ, Dept Biotechnol, Gyongsan, South Korea.	[Dey, Debasish Kumar] Daegu University, Dept Biotechnol, Gyongsan, South Korea.	Country: South Korea<br>Institution: Daegu Univ	Country: South Korea<br>Institution: Daegu University
C1	4	25454	25454	[Bajpai, Vivek K.; Han, Yong-Kyu] Dongguk Univ, Dept Energy & Materials Engn, Seoul, South Korea.	[Bajpai, Vivek K.; Han, Yong-Kyu] Dongguk University, Dept Energy & Materials Engn, Seoul, South Korea.	Country: South Korea<br>Institution: Dongguk Univ	Country: South Korea<br>Institution: Dongguk University
C1	5	25455	25455	[Kim, Kwanwoo; Lee, Hoomin; Sonwal, Sonam; Huh, Yun Suk] Inha Univ, Biohybrid Syst Res Ctr BSRC, Dept Biol Engn, Incheon, South Korea.	[Kim, Kwanwoo; Lee, Hoomin; Sonwal, Sonam; Huh, Yun Suk] Inha University, Biohybrid Syst Res Ctr BSRC, Dept Biol Engn, Incheon, South Korea.	Country: South Korea<br>Institution: Inha Univ	Country: South Korea<br>Institution: Inha University
C1	7	25457	25457	[Ali, Sajad] Yeungnam Univ, Dept Biotechnol, Gyongsan, South Korea.	[Ali, Sajad] Yeungnam University, Dept Biotechnol, Gyongsan, South Korea.	Country: South Korea<br>Institution: Yeungnam Univ	Country: South Korea<br>Institution: Yeungnam University
RP	1	25463	25463	Han, YK (corresponding author), Dongguk Univ, Dept Energy & Materials Engn, Seoul, South Korea	Han, YK (corresponding author), Dongguk University, Dept Energy & Materials Engn, Seoul, South Korea	Country: South Korea<br>Institution: Dongguk Univ	Country: South Korea<br>Institution: Dongguk University

Record 163 | WOS:000793342700006

PCV2 infection aggravates OTA-induced immunotoxicity in vivo and in vitro

Field	Position	Original line	Normalised line	Original value	Normalised value	Original country / institution	Normalised country / institution
C1	1	25726	25726	[Gan, Fang; Hou, Lili; Xu, Haibin; Liu, Yunhuan; Chen, Xingxiang; Huang, Kehe] Nanjing Agr Univ, Coll Vet Med, Nanjing 210095, Jiangsu, Peoples R China.	[Gan, Fang; Hou, Lili; Xu, Haibin; Liu, Yunhuan; Chen, Xingxiang; Huang, Kehe] Nanjing Agricultural University, Coll Vet Med, Nanjing 210095, Jiangsu, China.	Country: China<br>Institution: Nanjing Agr Univ	Country: China<br>Institution: Nanjing Agricultural University
C1	2	25727	25727	[Gan, Fang; Hou, Lili; Xu, Haibin; Liu, Yunhuan; Chen, Xingxiang; Huang, Kehe] Nanjing Agr Univ, Inst Anim Nutr Hlth, Nanjing 210095, Jiangsu, Peoples R China.	[Gan, Fang; Hou, Lili; Xu, Haibin; Liu, Yunhuan; Chen, Xingxiang; Huang, Kehe] Nanjing Agricultural University, Inst Anim Nutr Hlth, Nanjing 210095, Jiangsu, China.	Country: China<br>Institution: Nanjing Agr Univ	Country: China<br>Institution: Nanjing Agricultural University
C1	3	25728	25728	[Gan, Fang; Hou, Lili; Xu, Haibin; Liu, Yunhuan; Chen, Xingxiang; Huang, Kehe] Nanjing Agr Univ, Coll Vet	[Gan, Fang; Hou, Lili; Xu, Haibin; Liu, Yunhuan; Chen, Xingxiang; Huang, Kehe] Nanjing Agricultural	Country: China<br>Institution: Nanjing Agr Univ	Country: China<br>Institution: Nanjing Agricultural University

				Med, MOE Joint Int Res Lab Anim Hlth & Food Safety, Nanjing 210095, Jiangsu, Peoples R China.	University, Coll Vet Med, MOE Joint Int Res Lab Anim Hlth & Food Safety, Nanjing 210095, Jiangsu, China.		
RP	1	25731	25731	Huang, KH (corresponding author), Nanjing Agr Univ, Coll Vet Med, Nanjing 210095, Jiangsu, Peoples R China	Huang, KH (corresponding author), Nanjing Agricultural University, Coll Vet Med, Nanjing 210095, Jiangsu, China	Country: China<br>Institution: Nanjing Agr Univ	Country: China<br>Institution: Nanjing Agricultural University

Record 164 | WOS:000805486800007

An update on immunotoxicity and mechanisms of action of six environmental mycotoxins

Field	Position	Original line	Normalised line	Original value	Normalised value	Original country / institution	Normalised country / institution
C1	1	25851	25851	[Sun, Yuhang; Long, Miao; Yang, Shuhua; Zhang, Ying] Shenyang Agr Univ, Coll Anim Sci & Vet Med, Shenyang 110866, Peoples R China.	[Sun, Yuhang; Long, Miao; Yang, Shuhua; Zhang, Ying] Shenyang Agricultural University, Coll Anim Sci & Vet Med, Shenyang 110866, China.	Country: China<br>Institution: Shenyang Agr Univ	Country: China<br>Institution: Shenyang Agricultural University
C1	2	25852	25852	[Huang, Kehe] Nanjing Agr Univ, Coll Vet Med, Nanjing 210095, Peoples R China.	[Huang, Kehe] Nanjing Agricultural University, Coll Vet Med, Nanjing 210095, China.	Country: China<br>Institution: Nanjing Agr Univ	Country: China<br>Institution: Nanjing Agricultural University
RP	2	25854	25854	Zhang, Y (corresponding author), Shenyang Agr Univ, Coll Anim Sci & Vet Med, Shenyang 110866, Peoples R China	Zhang, Y (corresponding author), Shenyang Agricultural University, Coll Anim Sci & Vet Med, Shenyang 110866, China	Country: China<br>Institution: Shenyang Agr Univ	Country: China<br>Institution: Shenyang Agricultural University

Record 165 | WOS:000774405900001

Ex Vivo and In Vitro Studies Revealed Underlying Mechanisms of Immature Intestinal Inflammatory Responses Caused by Aflatoxin M1 Together with Ochratoxin A

Field	Position	Original line	Normalised line	Original value	Normalised value	Original country / institution	Normalised country / institution
C1	1	25987	25987	[Wang, Zi-Wei; Gao, Ya-Nan; Huang, Sheng-Nan; Wang, Jia-Qi; Zheng, Nan] Chinese Acad Agr Sci, Inst Anim Sci, Key Lab Qual & Safety Control Milk & Dairy Prod, Minist Agr & Rural Affairs, Beijing 100193, Peoples R China.	[Wang, Zi-Wei; Gao, Ya-Nan; Huang, Sheng-Nan; Wang, Jia-Qi; Zheng, Nan] Chinese Academy of Agricultural Sciences, Inst Anim Sci, Key Lab Qual & Safety Control Milk & Dairy Prod, Minist Agr & Rural Affairs, Beijing 100193, China.	Country: China<br>Institution: Chinese Acad Agr Sci	Country: China<br>Institution: Chinese Academy of Agricultural Sciences
C1	2	25988	25988	[Wang, Zi-Wei; Gao, Ya-Nan; Huang, Sheng-Nan; Wang, Jia-Qi; Zheng, Nan] Chinese Acad Agr Sci, Inst Anim Sci, Lab Qual & Safety Risk Assessment Dairy Prod, Minist Agr & Rural Affairs, Beijing 100193, Peoples R China.	[Wang, Zi-Wei; Gao, Ya-Nan; Huang, Sheng-Nan; Wang, Jia-Qi; Zheng, Nan] Chinese Academy of Agricultural Sciences, Inst Anim Sci, Lab Qual & Safety Risk Assessment Dairy Prod, Minist Agr & Rural Affairs, Beijing 100193, China.	Country: China<br>Institution: Chinese Acad Agr Sci	Country: China<br>Institution: Chinese Academy of Agricultural Sciences
C1	3	25989	25989	[Wang, Zi-Wei; Gao, Ya-Nan; Huang, Sheng-Nan; Wang, Jia-Qi; Zheng, Nan] Chinese Acad Agr Sci, Milk & Milk Prod Inspect Ctr, Inst Anim Sci, Minist Agr & Rural Affairs, Beijing 100193, Peoples R China.	[Wang, Zi-Wei; Gao, Ya-Nan; Huang, Sheng-Nan; Wang, Jia-Qi; Zheng, Nan] Chinese Academy of Agricultural Sciences, Milk & Milk Prod Inspect Ctr, Inst Anim Sci, Minist Agr & Rural Affairs, Beijing 100193, China.	Country: China<br>Institution: Chinese Acad Agr Sci	Country: China<br>Institution: Chinese Academy of Agricultural Sciences

C1	4	25990	25990	[Wang, Zi-Wei; Gao, Ya-Nan; Huang, Sheng-Nan; Wang, Jia-Qi; Zheng, Nan] Chinese Acad Agr Sci, Inst Anim Sci, State Key Lab Anim Nutr, Beijing 100193, Peoples R China.	[Wang, Zi-Wei; Gao, Ya-Nan; Huang, Sheng-Nan; Wang, Jia-Qi; Zheng, Nan] Chinese Academy of Agricultural Sciences, Inst Anim Sci, State Key Lab Anim Nutr, Beijing 100193, China.	Country: China<br>Institution: Chinese Acad Agr Sci	Country: China<br>Institution: Chinese Academy of Agricultural Sciences
RP	1	25996	25996	Zheng, N (corresponding author), Chinese Acad Agr Sci, Inst Anim Sci, Key Lab Qual & Safety Control Milk & Dairy Prod, Minist Agr & Rural Affairs, Beijing 100193, Peoples R China	Zheng, N (corresponding author), Chinese Academy of Agricultural Sciences, Inst Anim Sci, Key Lab Qual & Safety Control Milk & Dairy Prod, Minist Agr & Rural Affairs, Beijing 100193, Peoples R China	Country: China<br>Institution: Chinese Acad Agr Sci	Country: China<br>Institution: Chinese Academy of Agricultural Sciences
RP	4	25996	25996	Zheng, N (corresponding author), Chinese Acad Agr Sci, Inst Anim Sci, State Key Lab Anim Nutr, Beijing 100193, Peoples R China	Zheng, N (corresponding author), Chinese Acad Agr Sci, Inst Anim Sci, State Key Lab Anim Nutr, Beijing 100193, China	Country: China<br>Institution: Chinese Acad Agr Sci	Country: China<br>Institution: Chinese Acad Agr Sci

Record 166 | WOS:000798517400010

Detoxification of ochratoxin A and zearalenone by Pleurotus ostreatus during in vitro gastrointestinal digestion

No C1/C3/RP normalisation changes detected for this record.

Record 167 | WOS:000760242900001

MicroRNA regulates the toxicological mechanism of four mycotoxins in vivo and in vitro

Field	Position	Original line	Normalised line	Original value	Normalised value	Original country / institution	Normalised country / institution
C1	1	26271	26271	[Chen, Jia; Yang, Shuhua; Li, Peng; Long, Miao] Shenyang Agr Univ, Coll Anim Sci & Vet Med, Shenyang 110866, Peoples R China.	[Chen, Jia; Yang, Shuhua; Li, Peng; Long, Miao] Shenyang Agricultural University, Coll Anim Sci & Vet Med, Shenyang 110866, China.	Country: China<br>Institution: Shenyang Agr Univ	Country: China<br>Institution: Shenyang Agricultural University
C1	2	26272	26272	[Wu, Aibo] Chinese Acad Sci, Univ Chinese Acad Sci, SIBS UGENT SJTU Joint Lab Mycotoxin Res, CAS Key Lab Nutr Metab & Food Safety,Shanghai Ins, Shanghai 200031, Peoples R China.	[Wu, Aibo] Chinese Academy of Sciences, Univ Chinese Acad Sci, SIBS UGENT SJTU Joint Lab Mycotoxin Res, CAS Key Lab Nutr Metab & Food Safety,Shanghai Ins, Shanghai 200031, China.	Country: China<br>Institution: Chinese Acad Sci	Country: China<br>Institution: Chinese Academy of Sciences
C1	3	26273	26273	[Nepovimova, Eugenie; Wu, Wenda; Kuca, Kamil] Univ Hradec Kralove, Fac Sci, Dept Chem, Hradec Kralove 50003, Czech Republic.	[Nepovimova, Eugenie; Wu, Wenda; Kuca, Kamil] University of Hradec Kralove, Fac Sci, Dept Chem, Hradec Kralove 50003, Czech Republic.	Country: Czech Republic<br>Institution: Univ Hradec Kralove	Country: Czech Republic<br>Institution: University of Hradec Kralove
C1	4	26274	26274	[Wu, Wenda] Nanjing Agr Univ, Coll Vet Med, MOE Joint Int Res Lab Anim Hlth & Food Safety, Nanjing 210095, Peoples R China.	[Wu, Wenda] Nanjing Agricultural University, Coll Vet Med, MOE Joint Int Res Lab Anim Hlth & Food Safety, Nanjing 210095, China.	Country: China<br>Institution: Nanjing Agr Univ	Country: China<br>Institution: Nanjing Agricultural University
C1	5	26275	26275	[Kuca, Kamil] Univ Hosp Hradec Kralove, Biomed Res Ctr, Hradec Kralove 50003, Czech Republic.	[Kuca, Kamil] University of Hradec Kralove, Biomed Res Ctr, Hradec Kralove 50003, Czech Republic.	Country: Czech Republic<br>Institution: Univ Hosp Hradec Kralove	Country: Czech Republic<br>Institution: University of Hradec Kralove
RP	1	26280	26280	Long, M (corresponding author), Shenyang Agr Univ, Coll Anim Sci & Vet Med,	Long, M (corresponding author), Shenyang Agricultural University, Coll Anim Sci & Vet	Country: China<br>Institution: Shenyang Agr Univ	Country: China<br>Institution: Shenyang Agricultural University

				Shenyang 110866, Peoples R China	Med, Shenyang 110866, Peoples R China		

Record 168 | WOS:000796935500003

Studies on teratogenic effect of ochratoxin A given via mouldy diet in mice in various sensitive periods of the pregnancy and the putative protection of phenylalanine

Field	Position	Original line	Normalised line	Original value	Normalised value	Original country / institution	Normalised country / institution
C1	1	26459	26459	[Stoev, Stoycho D.] Trakia Univ, Fac Vet Med, Dept Gen & Clin Pathol, Students Campus, Stara Zagora 6000, Bulgaria.	[Stoev, Stoycho D.] Trakia University, Fac Vet Med, Dept Gen & Clin Pathol, Students Campus, Stara Zagora 6000, Bulgaria.	Country: Bulgaria<br>Institution: Trakia Univ	Country: Bulgaria<br>Institution: Trakia University
RP	1	26461	26461	Stoev, SD (corresponding author), Trakia Univ, Fac Vet Med, Dept Gen & Clin Pathol, Students Campus, Stara Zagora 6000, Bulgaria	Stoev, SD (corresponding author), Trakia University, Fac Vet Med, Dept Gen & Clin Pathol, Students Campus, Stara Zagora 6000, Bulgaria	Country: Bulgaria<br>Institution: Trakia Univ	Country: Bulgaria<br>Institution: Trakia University

Record 169 | WOS:000762109100001

Spent Coffee Grounds Valorization as Bioactive Phenolic Source Acquired Antifungal, Anti-Mycotoxigenic, and Anti-Cytotoxic Activities

Field	Position	Original line	Normalised line	Original value	Normalised value	Original country / institution	Normalised country / institution
C1	4	26571	26571	[Elkhadragy, Manal F.] Princess Nourah bint Abdulrahman Univ, Coll Sci, Dept Biol, POB 84428, Riyadh 11671, Saudi Arabia.	[Elkhadragy, Manal F.] Princess Nourah bint Abdulrahman University, Coll Sci, Dept Biol, POB 84428, Riyadh 11671, Saudi Arabia.	Country: Saudi Arabia<br>Institution: Princess Nourah bint Abdulrahman Univ	Country: Saudi Arabia<br>Institution: Princess Nourah bint Abdulrahman University
C1	5	26572	26572	[Yehia, Hany M.] King Saud Univ, Coll Food & Agr Sci, Food Sci & Nutr Dept, POB 2460, Riyadh 11451, Saudi Arabia.	[Yehia, Hany M.] King Saud University, Coll Food & Agr Sci, Food Sci & Nutr Dept, POB 2460, Riyadh 11451, Saudi Arabia.	Country: Saudi Arabia<br>Institution: King Saud Univ	Country: Saudi Arabia<br>Institution: King Saud University

Record 170 | WOS:000762022600001

Metabolomic Analysis Reveals the Mechanisms of Hepatotoxicity Induced by Aflatoxin M1 and Ochratoxin A

Field	Position	Original line	Normalised line	Original value	Normalised value	Original country / institution	Normalised country / institution
C1	1	26687	26687	[Gao, Ya-Nan; Wu, Chen-Qing; Wang, Jia-Qi; Zheng, Nan] Chinese Acad Agr Sci, Inst Anim Sci, Minist Agr & Rural Affairs, Key Lab Qual & Safety Control Milk & Dairy Prod, Beijing 100193, Peoples R China.	[Gao, Ya-Nan; Wu, Chen-Qing; Wang, Jia-Qi; Zheng, Nan] Chinese Academy of Agricultural Sciences, Inst Anim Sci, Minist Agr & Rural Affairs, Key Lab Qual & Safety Control Milk & Dairy Prod, Beijing 100193, China.	Country: China<br>Institution: Chinese Acad Agr Sci	Country: China<br>Institution: Chinese Academy of Agricultural Sciences
C1	2	26688	26688	[Gao, Ya-Nan; Wu, Chen-Qing; Wang, Jia-Qi; Zheng, Nan] Chinese Acad Agr Sci, Inst Anim Sci, Minist Agr & Rural Affairs, Lab Qual & Safety Risk Assessment Dairy Prod, Beijing 100193, Peoples R China.	[Gao, Ya-Nan; Wu, Chen-Qing; Wang, Jia-Qi; Zheng, Nan] Chinese Academy of Agricultural Sciences, Inst Anim Sci, Minist Agr & Rural Affairs, Lab Qual & Safety Risk Assessment Dairy Prod, Beijing 100193, China.	Country: China<br>Institution: Chinese Acad Agr Sci	Country: China<br>Institution: Chinese Academy of Agricultural Sciences

C1	3	26689	26689	[Gao, Ya-Nan; Wu, Chen-Qing; Wang, Jia-Qi; Zheng, Nan] Chinese Acad Agr Sci, Inst Anim Sci, Minist Agr & Rural Affairs, Milk & Milk Prod Inspect Ctr, Beijing 100193, Peoples R China.	[Gao, Ya-Nan; Wu, Chen-Qing; Wang, Jia-Qi; Zheng, Nan] Chinese Academy of Agricultural Sciences, Inst Anim Sci, Minist Agr & Rural Affairs, Milk & Milk Prod Inspect Ctr, Beijing 100193, China.	Country: China<br>Institution: Chinese Acad Agr Sci	Country: China<br>Institution: Chinese Academy of Agricultural Sciences
C1	4	26690	26690	[Gao, Ya-Nan; Wu, Chen-Qing; Wang, Jia-Qi; Zheng, Nan] Chinese Acad Agr Sci, Inst Anim Sci, State Key Lab Anim Nutr, Beijing 100193, Peoples R China.	[Gao, Ya-Nan; Wu, Chen-Qing; Wang, Jia-Qi; Zheng, Nan] Chinese Academy of Agricultural Sciences, Inst Anim Sci, State Key Lab Anim Nutr, Beijing 100193, China.	Country: China<br>Institution: Chinese Acad Agr Sci	Country: China<br>Institution: Chinese Academy of Agricultural Sciences
RP	1	26696	26696	Zheng, N (corresponding author), Chinese Acad Agr Sci, Inst Anim Sci, Minist Agr & Rural Affairs, Key Lab Qual & Safety Control Milk & Dairy Prod, Beijing 100193, Peoples R China	Zheng, N (corresponding author), Chinese Academy of Agricultural Sciences, Inst Anim Sci, Minist Agr & Rural Affairs, Key Lab Qual & Safety Control Milk & Dairy Prod, Beijing 100193, Peoples R China	Country: China<br>Institution: Chinese Acad Agr Sci	Country: China<br>Institution: Chinese Academy of Agricultural Sciences
RP	4	26696	26696	Zheng, N (corresponding author), Chinese Acad Agr Sci, Inst Anim Sci, State Key Lab Anim Nutr, Beijing 100193, Peoples R China	Zheng, N (corresponding author), Chinese Acad Agr Sci, Inst Anim Sci, State Key Lab Anim Nutr, Beijing 100193, China	Country: China<br>Institution: Chinese Acad Agr Sci	Country: China<br>Institution: Chinese Acad Agr Sci

Record 171 | WOS:000789897500004

A synergism of in silico and statistical approaches to discover new potential endocrine disruptor mycotoxins

Field	Position	Original line	Normalised line	Original value	Normalised value	Original country / institution	Normalised country / institution
C1	1	26810	26810	[Spaggiari, Giulia; Cozzini, Pietro] Univ Parma, Dept Food & Drug, Mol Modeling Lab, Parco Area Sci 17-A, I-43124 Parma, Italy.	[Spaggiari, Giulia; Cozzini, Pietro] University of Parma, Dept Food & Drug, Mol Modeling Lab, Parco Area Sci 17-A, I-43124 Parma, Italy.	Country: Italy<br>Institution: Univ Parma	Country: Italy<br>Institution: University of Parma
C1	2	26811	26811	[Morelli, Gianluca; Riani, Marco] Univ Parma, Interdept Ctr Robust Stat, Dept Econ & Management, Via J F Kennedy 6, I-43100 Parma, Italy.	[Morelli, Gianluca; Riani, Marco] University of Parma, Interdept Ctr Robust Stat, Dept Econ & Management, Via J F Kennedy 6, I-43100 Parma, Italy.	Country: Italy<br>Institution: Univ Parma	Country: Italy<br>Institution: University of Parma
RP	1	26813	26813	Cozzini, P (corresponding author), Univ Parma, Dept Food & Drug, Mol Modeling Lab, Parco Area Sci 17-A, I-43124 Parma, Italy	Cozzini, P (corresponding author), University of Parma, Dept Food & Drug, Mol Modeling Lab, Parco Area Sci 17-A, I-43124 Parma, Italy	Country: Italy<br>Institution: Univ Parma	Country: Italy<br>Institution: University of Parma

Record 172 | WOS:000738826400005

Combined protective effects of icariin and selenomethionine on novel chronic tubulointerstitial nephropathy models in vivo and in vitro

Field	Position	Original line	Normalised line	Original value	Normalised value	Original country / institution	Normalised country / institution
C1	1	26921	26921	[Hou, Lili; Lin, Ziman; Xu, Anyuan; Le, Guannan; Ge, Lei; Liu, Shuiping; Muhmood, Azhar; Gan, Fang; Huang, Kehe] Nanjing Agr Univ, Coll Vet Med, Nanjing 210095, Jiangsu, Peoples R China.	[Hou, Lili; Lin, Ziman; Xu, Anyuan; Le, Guannan; Ge, Lei; Liu, Shuiping; Muhmood, Azhar; Gan, Fang; Huang, Kehe] Nanjing Agricultural University, Coll Vet Med, Nanjing 210095, Jiangsu, China.	Country: China<br>Institution: Nanjing Agr Univ	Country: China<br>Institution: Nanjing Agricultural University

C1	2	26922	26922	[Hou, Lili; Lin, Ziman; Le, Guannan; Ge, Lei; Liu, Shuiping; Muhmood, Azhar; Gan, Fang; Huang, Kehe] Nanjing Agr Univ, Inst Nutr & Metab Disorders Domest Anim & Fowls, Nanjing 210095, Jiangsu, Peoples R China.	[Hou, Lili; Lin, Ziman; Le, Guannan; Ge, Lei; Liu, Shuiping; Muhmood, Azhar; Gan, Fang; Huang, Kehe] Nanjing Agricultural University, Inst Nutr & Metab Disorders Domest Anim & Fowls, Nanjing 210095, Jiangsu, China.	Country: China<br>Institution: Nanjing Agr Univ	Country: China<br>Institution: Nanjing Agricultural University
C1	3	26923	26923	[Hou, Lili; Lin, Ziman; Le, Guannan; Ge, Lei; Liu, Shuiping; Muhmood, Azhar; Gan, Fang; Huang, Kehe] Nanjing Agr Univ, Coll Vet Med, MOE Joint Int Res Lab Anim Hlth & Food Safety, Nanjing 210095, Jiangsu, Peoples R China.	[Hou, Lili; Lin, Ziman; Le, Guannan; Ge, Lei; Liu, Shuiping; Muhmood, Azhar; Gan, Fang; Huang, Kehe] Nanjing Agricultural University, Coll Vet Med, MOE Joint Int Res Lab Anim Hlth & Food Safety, Nanjing 210095, Jiangsu, China.	Country: China<br>Institution: Nanjing Agr Univ	Country: China<br>Institution: Nanjing Agricultural University
RP	1	26926	26926	Huang, KH (corresponding author), Nanjing Agr Univ, Coll Vet Med, Nanjing 210095, Jiangsu, Peoples R China	Huang, KH (corresponding author), Nanjing Agricultural University, Coll Vet Med, Nanjing 210095, Jiangsu, Peoples R China	Country: China<br>Institution: Nanjing Agr Univ	Country: China<br>Institution: Nanjing Agricultural University
RP	3	26926	26926	Huang, KH (corresponding author), Nanjing Agr Univ, Coll Vet Med, MOE Joint Int Res Lab Anim Hlth & Food Safety, Nanjing 210095, Jiangsu, Peoples R China	Huang, KH (corresponding author), Nanjing Agr Univ, Coll Vet Med, MOE Joint Int Res Lab Anim Hlth & Food Safety, Nanjing 210095, Jiangsu, China	Country: China<br>Institution: Nanjing Agr Univ	Country: China<br>Institution: Nanjing Agr Univ

Record 173 | WOS:000742259000001

Subchronic exposure of individual and combined ochratoxin A and citrinin selectively affects the expression of rat renal organic cation transporters

Field	Position	Original line	Normalised line	Original value	Normalised value	Original country / institution	Normalised country / institution
C1	1	27041	27041	[Karaica, Dean; Breljak, Davorka] Inst Med Res & Occupat Hlth, Mol Toxicol Unit, Zagreb 10000, Croatia.	[Karaica, Dean; Breljak, Davorka] Institute for Medical Research & Occupational Health (IMROH), Mol Toxicol Unit, Zagreb 10000, Croatia.	Country: Croatia<br>Institution: Inst Med Res & Occupat Hlth	Country: Croatia<br>Institution: Institute for Medical Research & Occupational Health (IMROH)
C1	2	27042	27042	[Micek, Vedran] Inst Med Res & Occupat Hlth, Anim Breeding Unit, Zagreb 10000, Croatia.	[Micek, Vedran] Institute for Medical Research & Occupational Health (IMROH), Anim Breeding Unit, Zagreb 10000, Croatia.	Country: Croatia<br>Institution: Inst Med Res & Occupat Hlth	Country: Croatia<br>Institution: Institute for Medical Research & Occupational Health (IMROH)
C1	3	27043	27043	[Rasic, Dubravka; Peraica, Maja] Inst Med Res & Occupat Hlth, Toxicol Unit, Zagreb 10000, Croatia.	[Rasic, Dubravka; Peraica, Maja] Institute for Medical Research & Occupational Health (IMROH), Toxicol Unit, Zagreb 10000, Croatia.	Country: Croatia<br>Institution: Inst Med Res & Occupat Hlth	Country: Croatia<br>Institution: Institute for Medical Research & Occupational Health (IMROH)
C1	4	27044	27044	[Klaric, Maja Segvic] Univ Zagreb, Fac Pharm & Biochem, Dept Microbiol, Zagreb, Croatia.	[Klaric, Maja Segvic] University of Zagreb, Fac Pharm & Biochem, Dept Microbiol, Zagreb, Croatia.	Country: Croatia<br>Institution: Univ Zagreb	Country: Croatia<br>Institution: University of Zagreb
RP	1	27048	27048	Karaica, D (corresponding author), Inst Med Res & Occupat Hlth, Mol Toxicol Unit, Zagreb 10000, Croatia	Karaica, D (corresponding author), Institute for Medical Research & Occupational Health (IMROH), Mol Toxicol Unit, Zagreb 10000, Croatia	Country: Croatia<br>Institution: Inst Med Res & Occupat Hlth	Country: Croatia<br>Institution: Institute for Medical Research & Occupational Health (IMROH)

Record 174 | WOS:000750801500002

In vitro and in vivo evaluation of AFB1 and OTA-toxicity through immunofluorescence and flow cytometry techniques: A systematic review

Field	Position	Original line	Normalised line	Original value	Normalised value	Original country / institution	Normalised country / institution
C1	1	27154	27154	[Frangiamone, Massimo; Cimbalo, Alessandra; Alonso-Garrido, Manuel; Vila-Donat, Pilar; Manyes, Lara] Univ Valencia, Fac Pharm, Lab Food Chem & Toxicol, Av Vicent Andres Estelles S-N, Burjassot 46100, Spain.	[Frangiamone, Massimo; Cimbalo, Alessandra; Alonso-Garrido, Manuel; Vila-Donat, Pilar; Manyes, Lara] University of Valencia, Fac Pharm, Lab Food Chem & Toxicol, Av Vicent Andres Estelles S-N, Burjassot 46100, Spain.	Country: Spain<br>Institution: Univ Valencia	Country: Spain<br>Institution: University of Valencia
RP	1	27156	27156	Vila-Donat, P (corresponding author), Univ Valencia, Fac Pharm, Lab Food Chem & Toxicol, Av Vicent Andres Estelles S-N, Burjassot 46100, Spain	Vila-Donat, P (corresponding author), University of Valencia, Fac Pharm, Lab Food Chem & Toxicol, Av Vicent Andres Estelles S-N, Burjassot 46100, Spain	Country: Spain<br>Institution: Univ Valencia	Country: Spain<br>Institution: University of Valencia

Record 175 | WOS:000767939000005

An overview of mycotoxins, their pathogenic effects, foods where they are found and their diagnostic biomarkers

Field	Position	Original line	Normalised line	Original value	Normalised value	Original country / institution	Normalised country / institution
C1	1	27357	27357	[Batista da Silva, Joao Victor; Zambelli Ramalho, Leandra Naira] Univ Sao Paulo, Fac Med Ribeirao Preto, Dept Patol & Med Legal, USP, Ribeirao Preto, SP, Brazil.	[Batista da Silva, Joao Victor; Zambelli Ramalho, Leandra Naira] Universidade de Sao Paulo, Fac Med Ribeirao Preto, Dept Patol & Med Legal, USP, Ribeirao Preto, SP, Brazil.	Country: Brazil<br>Institution: Univ Sao Paulo	Country: Brazil<br>Institution: Universidade de Sao Paulo
C1	2	27358	27358	[Fernandes de Oliveira, Carlos Augusto] Univ Sao Paulo, Fac Zootecnia & Engn Alimentos, Dept Engn Alimentos, USP, Pirassununga, SP, Brazil.	[Fernandes de Oliveira, Carlos Augusto] Universidade de Sao Paulo, Fac Zootecnia & Engn Alimentos, Dept Engn Alimentos, USP, Pirassununga, SP, Brazil.	Country: Brazil<br>Institution: Univ Sao Paulo	Country: Brazil<br>Institution: Universidade de Sao Paulo
RP	1	27360	27360	Ramalho, LNZ (corresponding author), Univ Sao Paulo, Fac Med Ribeirao Preto, Dept Patol & Med Legal, USP, Ribeirao Preto, SP, Brazil	Ramalho, LNZ (corresponding author), Universidade de Sao Paulo, Fac Med Ribeirao Preto, Dept Patol & Med Legal, USP, Ribeirao Preto, SP, Brazil	Country: Brazil<br>Institution: Univ Sao Paulo	Country: Brazil<br>Institution: Universidade de Sao Paulo

Record 176 | WOS:000912776700001

Mycotoxin Interactions along the Gastrointestinal Tract: In Vitro Semi-Dynamic Digestion and Static Colonic Fermentation of a Contaminated Meal

No C1/C3/RP normalisation changes detected for this record.

Record 177 | WOS:000786544000007

Adsorption efficiency of sodium & calcium bentonite for ochratoxin A in some Egyptian cheeses: an innovative fortification model, in vitro and in vivo experiments

Field	Position	Original line	Normalised line	Original value	Normalised value	Original country / institution	Normalised country / institution

C1	2	27651	27651	[El-Makarem, H. A.; Amer, A. A.; Abou-Alella, S. A.] Alexandria Univ, Fac Vet Med, Dept Food Hyg & Control, Alexandria, Egypt.	[El-Makarem, H. A.; Amer, A. A.; Abou-Alella, S. A.] Alexandria University, Fac Vet Med, Dept Food Hyg & Control, Alexandria, Egypt.	Country: Egypt<br>Institution: Alexandria Univ	Country: Egypt<br>Institution: Alexandria University
C1	3	27652	27652	[Abd Elaziz, A., I] Al Azhar Univ, Fac Med, Dept Pharmacol, Cairo, Egypt.	[Abd Elaziz, A., I] Al Azhar University, Fac Med, Dept Pharmacol, Cairo, Egypt.	Country: Egypt<br>Institution: Al Azhar Univ	Country: Egypt<br>Institution: Al Azhar University
C1	4	27653	27653	[El-Nogoumy, B. A.] Kafrelsheikh Univ, Fac Sci, Dept Bot & Microbiol, Kafrelsheikh, Egypt.	[El-Nogoumy, B. A.] Kafrelsheikh University, Fac Sci, Dept Bot & Microbiol, Kafrelsheikh, Egypt.	Country: Egypt<br>Institution: Kafrelsheikh Univ	Country: Egypt<br>Institution: Kafrelsheikh University

Record 178 | WOS:000746960900001

Development of an Extraction Method of Aflatoxins and Ochratoxin A from Oral, Gastric and Intestinal Phases of Digested Bread by In Vitro Model

Field	Position	Original line	Normalised line	Original value	Normalised value	Original country / institution	Normalised country / institution
C1	1	27773	27773	[Llorens, Paula; Molto, Juan Carlos; Manes, Jordi; Juan, Cristina] Univ Valencia, Fac Pharm, Lab Food Chem & Toxicol, Burjassot 46100, Spain.	[Llorens, Paula; Molto, Juan Carlos; Manes, Jordi; Juan, Cristina] University of Valencia, Fac Pharm, Lab Food Chem & Toxicol, Burjassot 46100, Spain.	Country: Spain<br>Institution: Univ Valencia	Country: Spain<br>Institution: University of Valencia
C1	2	27774	27774	[Pietrzak-Fiecko, Renata] Univ Warmia & Mazury, Fac Food Sci, Dept Commod & Food Anal, PL-10719 Olsztyn, Poland.	[Pietrzak-Fiecko, Renata] University of Warmia & Mazury, Fac Food Sci, Dept Commod & Food Anal, PL-10719 Olsztyn, Poland.	Country: Poland<br>Institution: Univ Warmia & Mazury	Country: Poland<br>Institution: University of Warmia & Mazury
RP	1	27776	27776	Juan, CSA (corresponding author), Univ Valencia, Fac Pharm, Lab Food Chem & Toxicol, Burjassot 46100, Spain	Juan, CSA (corresponding author), University of Valencia, Fac Pharm, Lab Food Chem & Toxicol, Burjassot 46100, Spain	Country: Spain<br>Institution: Univ Valencia	Country: Spain<br>Institution: University of Valencia

Record 179 | WOS:000891197500003

Gene expression profiling after ochratoxin A treatment in small intestinal epithelial cells from pigs

Field	Position	Original line	Normalised line	Original value	Normalised value	Original country / institution	Normalised country / institution
C1	1	27868	27868	[Yoon, Jung Woong; Lee, Sang In] Kyungpook Natl Univ, Dept Anim Sci & Biotechnol, Sangju 37224, South Korea.	[Yoon, Jung Woong; Lee, Sang In] Kyungpook National University (KNU), Dept Anim Sci & Biotechnol, Sangju 37224, South Korea.	Country: South Korea<br>Institution: Kyungpook Natl Univ	Country: South Korea<br>Institution: Kyungpook National University (KNU)
RP	1	27870	27870	Lee, SI (corresponding author), Kyungpook Natl Univ, Dept Anim Sci & Biotechnol, Sangju 37224, South Korea	Lee, SI (corresponding author), Kyungpook National University (KNU), Dept Anim Sci & Biotechnol, Sangju 37224, South Korea	Country: South Korea<br>Institution: Kyungpook Natl Univ	Country: South Korea<br>Institution: Kyungpook National University (KNU)

Record 180 | WOS:000730029700001

Agro-industrial Waste Products as Mycotoxin Biosorbents: A Review of in Vitro and in Vivo Studies

No C1/C3/RP normalisation changes detected for this record.

Record 181 | WOS:000740093400002

Ochratoxin A induces NLRP3 inflammasome-mediated pyroptosis via activation of ROS modulated by autophagy in vitro

Field	Position	Original line	Normalised line	Original value	Normalised value	Original country / institution	Normalised country / institution
C1	1	28118	28118	[Liu, Kai; Yue, Dongmei; Mao, Xinru; Li, Hu; Qu, Jie; Liu, Shuiping; Liu, Yunhuan; Gan, Fang; Pan, Cuiling; Huang, Kehe; Chen, Xingxiang] Nanjing Agr Univ, Coll Vet Med, Nanjing 210095, Jiangsu, Peoples R China.	[Liu, Kai; Yue, Dongmei; Mao, Xinru; Li, Hu; Qu, Jie; Liu, Shuiping; Liu, Yunhuan; Gan, Fang; Pan, Cuiling; Huang, Kehe; Chen, Xingxiang] Nanjing Agricultural University, Coll Vet Med, Nanjing 210095, Jiangsu, China.	Country: China<br>Institution: Nanjing Agr Univ	Country: China<br>Institution: Nanjing Agricultural University
C1	2	28119	28119	[Liu, Kai; Yue, Dongmei; Mao, Xinru; Li, Hu; Qu, Jie; Liu, Shuiping; Liu, Yunhuan; Gan, Fang; Pan, Cuiling; Huang, Kehe; Chen, Xingxiang] Nanjing Agr Univ, Inst Nutr & Metab Disorders Domest Anim & Fowls, Nanjing 210095, Jiangsu, Peoples R China.	[Liu, Kai; Yue, Dongmei; Mao, Xinru; Li, Hu; Qu, Jie; Liu, Shuiping; Liu, Yunhuan; Gan, Fang; Pan, Cuiling; Huang, Kehe; Chen, Xingxiang] Nanjing Agricultural University, Inst Nutr & Metab Disorders Domest Anim & Fowls, Nanjing 210095, Jiangsu, China.	Country: China<br>Institution: Nanjing Agr Univ	Country: China<br>Institution: Nanjing Agricultural University
C1	3	28120	28120	[Korolchuk, Viktor, I] Newcastle Univ, Inst Cell & Mol Biosci, Newcastle Upon Tyne NE4 5PL, Tyne & Wear, England.	[Korolchuk, Viktor, I] Newcastle Univ, Inst Cell & Mol Biosci, Newcastle Upon Tyne NE4 5PL, Tyne & Wear, United Kingdom.	Country: England<br>Institution: Newcastle Univ	Country: United Kingdom<br>Institution: Newcastle Univ
C3	3	28121	28121	Newcastle University - UK	Newcastle University - United Kingdom	Institution: Newcastle University - UK	Institution: Newcastle University - United Kingdom
RP	1	28123	28123	Chen, XX (corresponding author), Nanjing Agr Univ, Coll Vet Med, Nanjing 210095, Jiangsu, Peoples R China	Chen, XX (corresponding author), Nanjing Agricultural University, Coll Vet Med, Nanjing 210095, Jiangsu, China	Country: China<br>Institution: Nanjing Agr Univ	Country: China<br>Institution: Nanjing Agricultural University

Record 182 | WOS:000737483600001

Urinary Biomarkers of Mycotoxin Induced Nephrotoxicity-Current Status and Expected Future Trends

Field	Position	Original line	Normalised line	Original value	Normalised value	Original country / institution	Normalised country / institution
C1	1	28240	28240	[Raduly, Zsolt] Univ Debrecen, Doctoral Sch Mol Med, H-4032 Debrecen, Hungary.	[Raduly, Zsolt] University of Debrecen, Doctoral Sch Mol Med, H-4032 Debrecen, Hungary.	Country: Hungary<br>Institution: Univ Debrecen	Country: Hungary<br>Institution: University of Debrecen
C1	2	28241	28241	[Raduly, Zsolt; Csernoch, Laszlo] Univ Debrecen, Dept Physiol, Fac Med, H-4032 Debrecen, Hungary.	[Raduly, Zsolt; Csernoch, Laszlo] University of Debrecen, Dept Physiol, Fac Med, H-4032 Debrecen, Hungary.	Country: Hungary<br>Institution: Univ Debrecen	Country: Hungary<br>Institution: University of Debrecen
C1	3	28242	28242	[Price, Robert G.] Kings Coll London, Dept Nutr, Franklin Wilkins Bldg,150 Stamford St, London SE1 9NH, England.	[Price, Robert G.] Kings Coll London, Dept Nutr, Franklin Wilkins Bldg,150 Stamford St, London SE1 9NH, United Kingdom.	Country: England<br>Institution: Kings Coll London	Country: United Kingdom<br>Institution: Kings Coll London
C1	4	28243	28243	[Dockrell, Mark E. C.] South West Thames Inst Renal Res, Carshalton SM5 1AA, Surrey, England.	[Dockrell, Mark E. C.] South West Thames Inst Renal Res, Carshalton SM5 1AA, Surrey, United Kingdom.	Country: England<br>Institution: South West Thames Inst Renal Res	Country: United Kingdom<br>Institution: South West Thames Inst Renal Res
C1	5	28244	28244	[Pocsi, Istvan] Univ Debrecen, Fac Sci & Technol, Dept Mol Biotechnol & Microbiol, H-4032 Debrecen, Hungary.	[Pocsi, Istvan] University of Debrecen, Fac Sci & Technol, Dept Mol Biotechnol & Microbiol, H-4032 Debrecen, Hungary.	Country: Hungary<br>Institution: Univ Debrecen	Country: Hungary<br>Institution: University of Debrecen

RP	1	28247	28247	Raduly, Z (corresponding author), Univ Debrecen, Doctoral Sch Mol Med, H-4032 Debrecen, Hungary	Raduly, Z (corresponding author), University of Debrecen, Doctoral Sch Mol Med, H-4032 Debrecen, Hungary	Country: Hungary<br>Institution: Univ Debrecen	Country: Hungary<br>Institution: University of Debrecen

Record 183 | WOS:000734651000014

Non-embryotoxic dosage of alternariol aggravates ochratoxin A-triggered deleterious effects on embryonic development through ROS-dependent apoptotic processes

Field	Position	Original line	Normalised line	Original value	Normalised value	Original country / institution	Normalised country / institution
C1	3	28458	28458	[Hsuuw, Yan-Der; Huang, Fu-Jen] Natl Pingtung Univ Sci & Technol, Dept Trop Agr & Int Cooperat, Pingtung 91201, Taiwan.	[Hsuuw, Yan-Der; Huang, Fu-Jen] National Pingtung University Science & Technology, Dept Trop Agr & Int Cooperat, Pingtung 91201, Taiwan.	Country: Taiwan<br>Institution: Natl Pingtung Univ Sci & Technol	Country: Taiwan<br>Institution: National Pingtung University Science & Technology
C1	6	28461	28461	[Chan, Wen-Hsiung] Chung Yuan Christian Univ, Dept Biosci Technol, Taoyuan 32023, Taiwan.	[Chan, Wen-Hsiung] Chung Yuan Christian University, Dept Biosci Technol, Taoyuan 32023, Taiwan.	Country: Taiwan<br>Institution: Chung Yuan Christian Univ	Country: Taiwan<br>Institution: Chung Yuan Christian University
C1	7	28462	28462	[Chan, Wen-Hsiung] Chung Yuan Christian Univ, Ctr Nanotechnol, Taoyuan 32023, Taiwan.	[Chan, Wen-Hsiung] Chung Yuan Christian University, Ctr Nanotechnol, Taoyuan 32023, Taiwan.	Country: Taiwan<br>Institution: Chung Yuan Christian Univ	Country: Taiwan<br>Institution: Chung Yuan Christian University
RP	1	28466	28466	Chan, WH (corresponding author), Chung Yuan Christian Univ, Dept Biosci Technol, Taoyuan 32023, Taiwan	Chan, WH (corresponding author), Chung Yuan Christian University, Dept Biosci Technol, Taoyuan 32023, Taiwan	Country: Taiwan<br>Institution: Chung Yuan Christian Univ	Country: Taiwan<br>Institution: Chung Yuan Christian University

Record 184 | WOS:000721173800002

The individual and combined effects of ochratoxin A with citrinin and their metabolites (ochratoxin B, ochratoxin C, and dihydrocitrinone) on 2D/3D cell cultures, and zebrafish embryo models

Field	Position	Original line	Normalised line	Original value	Normalised value	Original country / institution	Normalised country / institution
C1	2	28597	28597	[Faisal, Zelma; Poor, Miklos] Univ Pecs, Dept Pharmacol, Fac Pharm, Rokus U 2, H-7624 Pecs, Hungary.	[Faisal, Zelma; Poor, Miklos] University of Pecs, Dept Pharmacol, Fac Pharm, Rokus U 2, H-7624 Pecs, Hungary.	Country: Hungary<br>Institution: Univ Pecs	Country: Hungary<br>Institution: University of Pecs
C1	3	28598	28598	[Faisal, Zelma; Garai, Kitti; Czeh, Arpad; Czompoly, Tamas; Kvell, Krisztian; Poor, Miklos] Univ Pecs, Food Biotechnol Res Grp, Janos Szentagothai Res Ctr, Ifjusag Utja 20, H-7624 Pecs, Hungary.	[Faisal, Zelma; Garai, Kitti; Czeh, Arpad; Czompoly, Tamas; Kvell, Krisztian; Poor, Miklos] University of Pecs, Food Biotechnol Res Grp, Janos Szentagothai Res Ctr, Ifjusag Utja 20, H-7624 Pecs, Hungary.	Country: Hungary<br>Institution: Univ Pecs	Country: Hungary<br>Institution: University of Pecs
C1	4	28599	28599	[Csepregi, Rita; Koszegi, Tamas] Univ Pecs, Lab Chip Res Grp, Janos Szentagothai Res Ctr, Ifjusag Utja 20, H-7624 Pecs, Hungary.	[Csepregi, Rita; Koszegi, Tamas] University of Pecs, Lab Chip Res Grp, Janos Szentagothai Res Ctr, Ifjusag Utja 20, H-7624 Pecs, Hungary.	Country: Hungary<br>Institution: Univ Pecs	Country: Hungary<br>Institution: University of Pecs
C1	5	28600	28600	[Csepregi, Rita; Koszegi, Tamas] Univ Pecs, Med Sch, Dept Lab Med, Ifjusag Ut 13, H-7624 Pecs, Hungary.	[Csepregi, Rita; Koszegi, Tamas] University of Pecs, Med Sch, Dept Lab Med, Ifjusag Ut 13, H-7624 Pecs, Hungary.	Country: Hungary<br>Institution: Univ Pecs	Country: Hungary<br>Institution: University of Pecs
C1	6	28601	28601	[Garai, Kitti; Kvell, Krisztian] Univ Pecs, Dept Pharmaceut Biotechnol, Fac Pharm, Rokus U 2, H-7624 Pecs, Hungary.	[Garai, Kitti; Kvell, Krisztian] University of Pecs, Dept Pharmaceut Biotechnol, Fac Pharm, Rokus U 2, H-7624 Pecs, Hungary.	Country: Hungary<br>Institution: Univ Pecs	Country: Hungary<br>Institution: University of Pecs

RP	1	28606	28606	PoÃ³r, M (corresponding author), Univ Pecs, Dept Pharmacol, Fac Pharm, Rokus U 2, H-7624 Pecs, Hungary	PoÃ³r, M (corresponding author), University of Pecs, Dept Pharmacol, Fac Pharm, Rokus U 2, H-7624 Pecs, Hungary	Country: Hungary<br>Institution: Univ Pecs	Country: Hungary<br>Institution: University of Pecs

Record 185 | WOS:000874698700001

Food Mycotoxins: Dietary Interventions Implicated in the Prevention of Mycotoxicosis

Field	Position	Original line	Normalised line	Original value	Normalised value	Original country / institution	Normalised country / institution
C1	1	28744	28744	[Sharma, Vinesh; Patial, Vikram] CSIR Inst Himalayan Bioresource Technol, Dietet & Nutr Technol Div, Pharmacol & Toxicol Lab, Palampur 176061, Himachal Prades, India.	[Sharma, Vinesh; Patial, Vikram] CSIR - Institute of Himalayan Bioresource Technology (IHBT), Dietet & Nutr Technol Div, Pharmacol & Toxicol Lab, Palampur 176061, Himachal Prades, India.	Country: India<br>Institution: CSIR Inst Himalayan Bioresource Technol	Country: India<br>Institution: CSIR - Institute of Himalayan Bioresource Technology (IHBT)
RP	1	28749	28749	Patial, V (corresponding author), CSIR Inst Himalayan Bioresource Technol, Dietet & Nutr Technol Div, Pharmacol & Toxicol Lab, Palampur 176061, Himachal Prades, India	Patial, V (corresponding author), CSIR - Institute of Himalayan Bioresource Technology (IHBT), Dietet & Nutr Technol Div, Pharmacol & Toxicol Lab, Palampur 176061, Himachal Prades, India	Country: India<br>Institution: CSIR Inst Himalayan Bioresource Technol	Country: India<br>Institution: CSIR - Institute of Himalayan Bioresource Technology (IHBT)

Record 186 | WOS:000724718300001

Human Proximal Tubule Epithelial Cells (HK-2) as a Sensitive In Vitro System for Ochratoxin A Induced Oxidative Stress

Field	Position	Original line	Normalised line	Original value	Normalised value	Original country / institution	Normalised country / institution
C1	1	28995	28995	[Garcia-Perez, Enrique] Washington State Univ, Sch Food Sci, POB 646376, Pullman, WA 99164 USA.	[Garcia-Perez, Enrique] Washington State University, Sch Food Sci, POB 646376, Pullman, WA 99164 USA.	Country: USA<br>Institution: Washington State Univ	Country: USA<br>Institution: Washington State University
C1	2	28996	28996	[Ryu, Dojin; Kim, Hae Dun; Lee, Hyun Jung] Univ Idaho, Dept Anim Vet & Food Sci, 875 Perimeter Dr MS 2330, Moscow, ID 83844 USA.	[Ryu, Dojin; Kim, Hae Dun; Lee, Hyun Jung] University of Idaho, Dept Anim Vet & Food Sci, 875 Perimeter Dr MS 2330, Moscow, ID 83844 USA.	Country: USA<br>Institution: Univ Idaho	Country: USA<br>Institution: University of Idaho
C1	3	28997	28997	[Kim, Hwa-Young] Yeungnam Univ, Dept Biochem & Mol Biol, Coll Med, Daegu 42415, South Korea.	[Kim, Hwa-Young] Yeungnam University, Dept Biochem & Mol Biol, Coll Med, Daegu 42415, South Korea.	Country: South Korea<br>Institution: Yeungnam Univ	Country: South Korea<br>Institution: Yeungnam University
RP	1	28999	28999	Lee, HJ (corresponding author), Univ Idaho, Dept Anim Vet & Food Sci, 875 Perimeter Dr MS 2330, Moscow, ID 83844 USA	Lee, HJ (corresponding author), University of Idaho, Dept Anim Vet & Food Sci, 875 Perimeter Dr MS 2330, Moscow, ID 83844 USA	Country: USA<br>Institution: Univ Idaho	Country: USA<br>Institution: University of Idaho

Record 187 | WOS:000724371000001

Microbiological Decontamination of Mycotoxins: Opportunities and Limitations

Field	Position	Original line	Normalised line	Original value	Normalised value	Original country / institution	Normalised country / institution

C1	1	29148	29148	[Piotrowska, Malgorzata] Lodz Univ Technol, Fac Biotechnol & Food Sci, Inst Fermentat Technol & Microbiol, Wolczanska 171-173, PL-90530 Lodz, Poland.	[Piotrowska, Malgorzata] Lodz University of Technology, Fac Biotechnol & Food Sci, Inst Fermentat Technol & Microbiol, Wolczanska 171-173, PL-90530 Lodz, Poland.	Country: Poland<br>Institution: Lodz Univ Technol	Country: Poland<br>Institution: Lodz University of Technology
RP	1	29150	29150	Piotrowska, M (corresponding author), Lodz Univ Technol, Fac Biotechnol & Food Sci, Inst Fermentat Technol & Microbiol, Wolczanska 171-173, PL-90530 Lodz, Poland	Piotrowska, M (corresponding author), Lodz University of Technology, Fac Biotechnol & Food Sci, Inst Fermentat Technol & Microbiol, Wolczanska 171-173, PL-90530 Lodz, Poland	Country: Poland<br>Institution: Lodz Univ Technol	Country: Poland<br>Institution: Lodz University of Technology

Record 188 | WOS:000723720700001

Anti-Apoptotic Effect of Flavokawain A on Ochratoxin-A-Induced Endothelial Cell Injury by Attenuation of Oxidative Stress via PI3K/AKT-Mediated Nrf2 Signaling Cascade

Field	Position	Original line	Normalised line	Original value	Normalised value	Original country / institution	Normalised country / institution
C1	1	29351	29351	[Rajendran, Peramaiyan; Alzahrani, Abdullah M.; Ahmed, Emad A.] King Faisal Univ, Coll Sci, Dept Biol Sci, Al Hasa 31982, Saudi Arabia.	[Rajendran, Peramaiyan; Alzahrani, Abdullah M.; Ahmed, Emad A.] King Faisal University, Coll Sci, Dept Biol Sci, Al Hasa 31982, Saudi Arabia.	Country: Saudi Arabia<br>Institution: King Faisal Univ	Country: Saudi Arabia<br>Institution: King Faisal University
C1	3	29353	29353	[Ahmed, Emad A.] Assiut Univ, Fac Sci, Zool Dept, Lab Mol Physiol, Assiut 71515, Egypt.	[Ahmed, Emad A.] Assiut University, Fac Sci, Zool Dept, Lab Mol Physiol, Assiut 71515, Egypt.	Country: Egypt<br>Institution: Assiut Univ	Country: Egypt<br>Institution: Assiut University
RP	1	29357	29357	Rajendran, P (corresponding author), King Faisal Univ, Coll Sci, Dept Biol Sci, Al Hasa 31982, Saudi Arabia	Rajendran, P (corresponding author), King Faisal University, Coll Sci, Dept Biol Sci, Al Hasa 31982, Saudi Arabia	Country: Saudi Arabia<br>Institution: King Faisal Univ	Country: Saudi Arabia<br>Institution: King Faisal University

Record 189 | WOS:000807169400001

Ochratoxin A Induces Steatosis via PPARÎ³-CD36 Axis

Field	Position	Original line	Normalised line	Original value	Normalised value	Original country / institution	Normalised country / institution
C1	1	29475	29475	[Zheng, Qian-Wen; Ding, Xu-Fen; Cao, Hui-Jun; Ni, Qian-Zhi; Zhu, Bing; Ma, Ning; Zhang, Feng-Kun; Wang, Yi-Kang; Xu, Sheng; Chen, Tian-Wei; Xia, Ji; Qiu, Xiao-Song; Yu, Dian-Zhen; Xie, Dong; Li, Jing-Jing] Chinese Acad Sci, Univ Chinese Acad Sci, Shanghai Inst Nutr & Hlth, CAS Key Lab Nutr Metab & Food Safety, Shanghai 200031, Peoples R China.	[Zheng, Qian-Wen; Ding, Xu-Fen; Cao, Hui-Jun; Ni, Qian-Zhi; Zhu, Bing; Ma, Ning; Zhang, Feng-Kun; Wang, Yi-Kang; Xu, Sheng; Chen, Tian-Wei; Xia, Ji; Qiu, Xiao-Song; Yu, Dian-Zhen; Xie, Dong; Li, Jing-Jing] Chinese Academy of Sciences, Univ Chinese Acad Sci, Shanghai Inst Nutr & Hlth, CAS Key Lab Nutr Metab & Food Safety, Shanghai 200031, China.	Country: China<br>Institution: Chinese Acad Sci	Country: China<br>Institution: Chinese Academy of Sciences
C1	2	29476	29476	[Zheng, Qian-Wen; Qiu, Xiao-Song; Xie, Dong] ShanghaiTech Univ, Sch Life Sci & Technol, Shanghai 201210, Peoples R China.	[Zheng, Qian-Wen; Qiu, Xiao-Song; Xie, Dong] ShanghaiTech University, Sch Life Sci & Technol, Shanghai 201210, China.	Country: China<br>Institution: ShanghaiTech Univ	Country: China<br>Institution: ShanghaiTech University
C1	3	29477	29477	[Xie, Dong] China Natl Ctr Food Safety Risk Assessment, NHC Key Lab Food Safety Risk	[Xie, Dong] China Natl Ctr Food Safety Risk Assessment, NHC Key Lab Food Safety Risk	Country: China<br>Institution: China Natl Ctr Food Safety Risk Assessment	Country: China<br>Institution: China Natl Ctr Food Safety Risk Assessment

				Assessment, Beijing 100022, Peoples R China.	Assessment, Beijing 100022, China.		
RP	2	29481	29481	Li, JJ (corresponding author), Chinese Acad Sci, Univ Chinese Acad Sci, Shanghai Inst Nutr & Hlth, CAS Key Lab Nutr Metab & Food Safety, Shanghai 200031, Peoples R China	Li, JJ (corresponding author), Chinese Academy of Sciences, Univ Chinese Acad Sci, Shanghai Inst Nutr & Hlth, CAS Key Lab Nutr Metab & Food Safety, Shanghai 200031, Peoples R China	Country: China Institution: Chinese Acad Sci	Country: China Institution: Chinese Academy of Sciences
RP	4	29481	29481	Xie, D (corresponding author), China Natl Ctr Food Safety Risk Assessment, NHC Key Lab Food Safety Risk Assessment, Beijing 100022, Peoples R China	Xie, D (corresponding author), China Natl Ctr Food Safety Risk Assessment, NHC Key Lab Food Safety Risk Assessment, Beijing 100022, China	Country: China Institution: China Natl Ctr Food Safety Risk Assessment	Country: China Institution: China Natl Ctr Food Safety Risk Assessment

Record 190 | WOS:000718125600004

Combination of zinc and selenium alleviates ochratoxin A-induced fibrosis via blocking ROS-dependent autophagy in HK-2 cells

Field	Position	Original line	Normalised line	Original value	Normalised value	Original country / institution	Normalised country / institution
C1	1	29601	29601	[Le, Guannan; Du, Heng; Hou, Lili; Ge, Lei; Sylia, Ardache; Muhmood, Azhar; Chen, Xinxiang; Huang, Kehe] Nanjing Agr Univ, Coll Vet Med, Nanjing 210095, Jiangsu, Peoples R China.	[Le, Guannan; Du, Heng; Hou, Lili; Ge, Lei; Sylia, Ardache; Muhmood, Azhar; Chen, Xinxiang; Huang, Kehe] Nanjing Agricultural University, Coll Vet Med, Nanjing 210095, Jiangsu, China.	Country: China Institution: Nanjing Agr Univ	Country: China Institution: Nanjing Agricultural University
C1	2	29602	29602	[Yang, Lulu] Northwest A&F Univ, Coll Vet Med, Yangling, Shaanxi, Peoples R China.	[Yang, Lulu] Northwest A&F Univ, Coll Vet Med, Yangling, Shaanxi, China.	Country: China Institution: Northwest A&F Univ	Country: China Institution: Northwest A&F Univ
C1	3	29603	29603	[Han, Bo] China Agr Univ, Coll Vet Med, Dept Clin Vet Med, Beijing 100193, Peoples R China.	[Han, Bo] China Agricultural University, Coll Vet Med, Dept Clin Vet Med, Beijing 100193, China.	Country: China Institution: China Agr Univ	Country: China Institution: China Agricultural University
C3	2	29604	29604	Northwest A&F University - China	Northwest A&F University	Institution: Northwest A&F University - China	Institution: Northwest A&F University
RP	1	29606	29606	Huang, KH (corresponding author), Nanjing Agr Univ, Coll Vet Med, Nanjing 210095, Jiangsu, Peoples R China	Huang, KH (corresponding author), Nanjing Agricultural University, Coll Vet Med, Nanjing 210095, Jiangsu, China	Country: China Institution: Nanjing Agr Univ	Country: China Institution: Nanjing Agricultural University

Record 191 | WOS:000709727500001

Selenomethionine alleviated Ochratoxin A induced pyroptosis and renal fibrotic factors expressions in MDCK cells

Field	Position	Original line	Normalised line	Original value	Normalised value	Original country / institution	Normalised country / institution
C1	1	29720	29720	[Mao, Xinru; Li, Hu; Ge, Lei; Liu, Shuiping; Hou, Lili; Yue, Dongmei; Du, Heng; Pan, Cuiling; Gan, Fang; Liu, Yunhuan; Huang, Kehe; Chen, Xingxiang] Nanjing Agr Univ, Coll Vet Med, Nanjing 210095, Jiangsu, Peoples R China.	[Mao, Xinru; Li, Hu; Ge, Lei; Liu, Shuiping; Hou, Lili; Yue, Dongmei; Du, Heng; Pan, Cuiling; Gan, Fang; Liu, Yunhuan; Huang, Kehe; Chen, Xingxiang] Nanjing Agricultural University, Coll Vet Med, Nanjing 210095, Jiangsu, China.	Country: China Institution: Nanjing Agr Univ	Country: China Institution: Nanjing Agricultural University
C1	2	29721	29721	[Mao, Xinru; Li, Hu; Ge, Lei; Liu, Shuiping; Hou, Lili; Yue, Dongmei; Du, Heng; Pan, Cuiling; Gan, Fang; Liu, Yunhuan; Huang, Kehe; Chen,	[Mao, Xinru; Li, Hu; Ge, Lei; Liu, Shuiping; Hou, Lili; Yue, Dongmei; Du, Heng; Pan, Cuiling; Gan, Fang; Liu, Yunhuan; Huang, Kehe; Chen,	Country: China Institution: Nanjing Agr Univ	Country: China Institution: Nanjing Agricultural University

				Xingxiang] Nanjing Agr Univ, Inst Anim Nutr & Hlth, Nanjing, Jiangsu, Peoples R China.	Xingxiang] Nanjing Agricultural University, Inst Anim Nutr & Hlth, Nanjing, Jiangsu, China.		
RP	1	29723	29723	Chen, XX (corresponding author), Nanjing Agr Univ, Coll Vet Med, Nanjing 210095, Jiangsu, Peoples R China	Chen, XX (corresponding author), Nanjing Agricultural University, Coll Vet Med, Nanjing 210095, Jiangsu, China	Country: China<br>Institution: Nanjing Agr Univ	Country: China<br>Institution: Nanjing Agricultural University

Record 192 | WOS:000715414400001

Prioritization of Mycotoxins Based on Their Genotoxic Potential with an In Silico-In Vitro Strategy

Field	Position	Original line	Normalised line	Original value	Normalised value	Original country / institution	Normalised country / institution
C1	1	29830	29830	[Alonso-Jauregui, Maria; Lopez de Cerain, Adela; Vettorazzi, Ariane] Univ Navarra, Dept Pharmacol & Toxicol, Res Grp MITOX, Sch Pharm & Nutr, Pamplona 31008, Spain.	[Alonso-Jauregui, Maria; Lopez de Cerain, Adela; Vettorazzi, Ariane] University of Navarra, Dept Pharmacol & Toxicol, Res Grp MITOX, Sch Pharm & Nutr, Pamplona 31008, Spain.	Country: Spain<br>Institution: Univ Navarra	Country: Spain<br>Institution: University of Navarra
C1	2	29831	29831	[Font, Maria; Gonzalez-Penas, Elena] Univ Navarra, Dept Pharmaceut Technol & Chem, Res Grp MITOX, Sch Pharm & Nutr, Pamplona 31008, Spain.	[Font, Maria; Gonzalez-Penas, Elena] University of Navarra, Dept Pharmaceut Technol & Chem, Res Grp MITOX, Sch Pharm & Nutr, Pamplona 31008, Spain.	Country: Spain<br>Institution: Univ Navarra	Country: Spain<br>Institution: University of Navarra
RP	1	29834	29834	Vettorazzi, A (corresponding author), Univ Navarra, Dept Pharmacol & Toxicol, Res Grp MITOX, Sch Pharm & Nutr, Pamplona 31008, Spain	Vettorazzi, A (corresponding author), University of Navarra, Dept Pharmacol & Toxicol, Res Grp MITOX, Sch Pharm & Nutr, Pamplona 31008, Spain	Country: Spain<br>Institution: Univ Navarra	Country: Spain<br>Institution: University of Navarra

Record 193 | WOS:000714429400001

Ochratoxin A-Induced Nephrotoxicity: Up-to-Date Evidence

Field	Position	Original line	Normalised line	Original value	Normalised value	Original country / institution	Normalised country / institution
C1	1	29986	29986	[Khoi, Chong-Sun; Chen, Jia-Huang; Chiang, Chih-Kang] Natl Taiwan Univ, Grad Inst Toxicol, Coll Med, Taipei 106, Taiwan.	[Khoi, Chong-Sun; Chen, Jia-Huang; Chiang, Chih-Kang] National Taiwan University, Grad Inst Toxicol, Coll Med, Taipei 106, Taiwan.	Country: Taiwan<br>Institution: Natl Taiwan Univ	Country: Taiwan<br>Institution: National Taiwan University
C1	3	29988	29988	[Chiang, Chih-Kang; Hung, Kuan-Yu] Natl Taiwan Univ, Dept Internal Med, Coll Med & Hosp, Taipei 106, Taiwan.	[Chiang, Chih-Kang; Hung, Kuan-Yu] National Taiwan University, Dept Internal Med, Coll Med & Hosp, Taipei 106, Taiwan.	Country: Taiwan<br>Institution: Natl Taiwan Univ	Country: Taiwan<br>Institution: National Taiwan University
RP	1	29993	29993	Chiang, CK (corresponding author), Natl Taiwan Univ, Grad Inst Toxicol, Coll Med, Taipei 106, Taiwan	Chiang, CK (corresponding author), National Taiwan University, Grad Inst Toxicol, Coll Med, Taipei 106, Taiwan	Country: Taiwan<br>Institution: Natl Taiwan Univ	Country: Taiwan<br>Institution: National Taiwan University

Record 194 | WOS:000726706400001

The role of pumpkin pulp extract carotenoids against mycotoxin damage in the blood brain barrier in vitro

Field	Position	Original line	Normalised line	Original value	Normalised value	Original country / institution	Normalised country / institution

C1	1	30175	30175	[Alonso-Garrido, Manuel; Pallares, Noelia; Font, Guillermina; Manyes, Lara; Lozano, Manuel] Univ Valencia, Lab Food Chem & Toxicol, Fac Pharm, Ave Vicent Andres Estelles S-N, Burjassot 46100, Spain.	[Alonso-Garrido, Manuel; Pallares, Noelia; Font, Guillermina; Manyes, Lara; Lozano, Manuel] University of Valencia, Lab Food Chem & Toxicol, Fac Pharm, Ave Vicent Andres Estelles S-N, Burjassot 46100, Spain.	Country: Spain<br>Institution: Univ Valencia	Country: Spain<br>Institution: University of Valencia
C1	2	30176	30176	[Tedeschi, Paola] Univ Ferrara, Dept Chem & Pharmaceut Sci, Ferrara, Italy.	[Tedeschi, Paola] University of Ferrara, Dept Chem & Pharmaceut Sci, Ferrara, Italy.	Country: Italy<br>Institution: Univ Ferrara	Country: Italy<br>Institution: University of Ferrara
C1	4	30178	30178	[Lozano, Manuel] Univ Valencia, Fdn Promot Hlth & Biomed Res Valencian Reg FISABI, Epidemiol & Environm Hlth Joint Res Unit, Valencia, Spain.	[Lozano, Manuel] University of Valencia, Fdn Promot Hlth & Biomed Res Valencian Reg FISABI, Epidemiol & Environm Hlth Joint Res Unit, Valencia, Spain.	Country: Spain<br>Institution: Univ Valencia	Country: Spain<br>Institution: University of Valencia
RP	1	30181	30181	Manyes, L (corresponding author), Univ Valencia, Lab Food Chem & Toxicol, Fac Pharm, Ave Vicent Andres Estelles S-N, Burjassot 46100, Spain	Manyes, L (corresponding author), University of Valencia, Lab Food Chem & Toxicol, Fac Pharm, Ave Vicent Andres Estelles S-N, Burjassot 46100, Spain	Country: Spain<br>Institution: Univ Valencia	Country: Spain<br>Institution: University of Valencia

Record 195 | WOS:000714098000254

Evaluation of larval zebrafish as an alternative whole-animal model for nephrotoxicity testing

No C1/C3/RP normalisation changes detected for this record.

Record 196 | WOS:000701125500001

The Reduction of the Combined Effects of Aflatoxin and Ochratoxin A in Piglet Livers and Kidneys by Dietary Antioxidants

Field	Position	Original line	Normalised line	Original value	Normalised value	Original country / institution	Normalised country / institution
C1	1	30350	30350	[Popescu, Roua Gabriela; Georgescu, Sergiu Emil; Dinischiotu, Anca] Univ Bucharest, Fac Biol, Dept Biochem & Mol Biol, Splaiul Independentei 91-95, Bucharest 050095, Romania.	[Popescu, Roua Gabriela; Georgescu, Sergiu Emil; Dinischiotu, Anca] University of Bucharest, Fac Biol, Dept Biochem & Mol Biol, Splaiul Independentei 91-95, Bucharest 050095, Romania.	Country: Romania<br>Institution: Univ Bucharest	Country: Romania<br>Institution: University of Bucharest
C1	2	30351	30351	[Avramescu, Sorin] Univ Bucharest, Fac Chem, Dept Organ Chem Biochem & Catalysis, Soseaua Panduri 90-92, Bucharest 050663, Romania.	[Avramescu, Sorin] University of Bucharest, Fac Chem, Dept Organ Chem Biochem & Catalysis, Soseaua Panduri 90-92, Bucharest 050663, Romania.	Country: Romania<br>Institution: Univ Bucharest	Country: Romania<br>Institution: University of Bucharest
RP	1	30354	30354	Georgescu, SE (corresponding author), Univ Bucharest, Fac Biol, Dept Biochem & Mol Biol, Splaiul Independentei 91-95, Bucharest 050095, Romania	Georgescu, SE (corresponding author), University of Bucharest, Fac Biol, Dept Biochem & Mol Biol, Splaiul Independentei 91-95, Bucharest 050095, Romania	Country: Romania<br>Institution: Univ Bucharest	Country: Romania<br>Institution: University of Bucharest

Record 197 | WOS:000689864300001

Cytotoxicity of Mycotoxins Frequently Present in Aquafeeds to the Fish Cell Line RTGill-W1

Field	Position	Original line	Normalised line	Original value	Normalised value	Original country / institution	Normalised country / institution
C1	2	30531	30531	[Pulgarin-Alfaro, Marta] Inst Salud Carlos III, Ctr Nacl Invest Cardiovasc Carlos III, Madrid 28029, Spain.	[Pulgarin-Alfaro, Marta] Instituto de Salud Carlos III, Ctr Nacl Invest Cardiovasc Carlos III, Madrid 28029, Spain.	Country: Spain<br>Institution: Inst Salud Carlos III	Country: Spain<br>Institution: Instituto de Salud Carlos III

Record 198 | WOS:000688617200001

Curcumin Modulates Nitrosative Stress, Inflammation, and DNA Damage and Protects against Ochratoxin A-Induced Hepatotoxicity and Nephrotoxicity in Rats

Field	Position	Original line	Normalised line	Original value	Normalised value	Original country / institution	Normalised country / institution
C1	2	30661	30661	[Damiano, Sara; Andretta, Emanuela; Prisco, Francesco; Russo, Valeria; Florio, Salvatore; Ciarcia, Roberto] Univ Naples Federico II, Dept Vet Med & Anim Prod, Via Delpino 1, I-80137 Naples, Italy.	[Damiano, Sara; Andretta, Emanuela; Prisco, Francesco; Russo, Valeria; Florio, Salvatore; Ciarcia, Roberto] University of Naples Federico II, Dept Vet Med & Anim Prod, Via Delpino 1, I-80137 Naples, Italy.	Country: Italy<br>Institution: Univ Naples Federico II	Country: Italy<br>Institution: University of Naples Federico II
C1	3	30662	30662	[Pagnini, Francesco] Univ Parma, Dept Med & Surg, Unit Radiol, Via Gramsci 14, I-43126 Parma, Italy.	[Pagnini, Francesco] University of Parma, Dept Med & Surg, Unit Radiol, Via Gramsci 14, I-43126 Parma, Italy.	Country: Italy<br>Institution: Univ Parma	Country: Italy<br>Institution: University of Parma
RP	2	30665	30665	Ciarcia, R (corresponding author), Univ Naples Federico II, Dept Vet Med & Anim Prod, Via Delpino 1, I-80137 Naples, Italy	Ciarcia, R (corresponding author), University of Naples Federico II, Dept Vet Med & Anim Prod, Via Delpino 1, I-80137 Naples, Italy	Country: Italy<br>Institution: Univ Naples Federico II	Country: Italy<br>Institution: University of Naples Federico II

Record 199 | WOS:000681272600001

Hepatotoxicity of food-borne mycotoxins: molecular mechanism, anti-hepatotoxic medicines and target prediction

Field	Position	Original line	Normalised line	Original value	Normalised value	Original country / institution	Normalised country / institution
C1	1	30806	30806	[Ruan, Haonan; Lu, Qian; Wu, Jiashuo; Qin, Jiaan; Sui, Ming; Sun, Xinqi; Shi, Yue; Luo, Jiaoyang; Yang, Meihua] Chinese Acad Med Sci & Peking Union Med Coll, Inst Med Plant Dev, Minist Educ, Key Lab Bioact Subst & Resources Utilizat Chinese, Beijing, Peoples R China.	[Ruan, Haonan; Lu, Qian; Wu, Jiashuo; Qin, Jiaan; Sui, Ming; Sun, Xinqi; Shi, Yue; Luo, Jiaoyang; Yang, Meihua] Chinese Academy of Medical Sciences - Peking Union Medical College, Inst Med Plant Dev, Minist Educ, Key Lab Bioact Subst & Resources Utilizat Chinese, Beijing, China.	Country: China<br>Institution: Chinese Acad Med Sci & Peking Union Med Coll	Country: China<br>Institution: Chinese Academy of Medical Sciences - Peking Union Medical College
RP	3	30810	30810	Yang, MH (corresponding author), Chinese Acad Med Sci & Peking Union Med Coll, Inst Med Plant Dev, Minist Educ, Key Lab Bioact Subst & Resources Utilizat Chinese, Beijing, Peoples R China	Yang, MH (corresponding author), Chinese Academy of Medical Sciences - Peking Union Medical College, Inst Med Plant Dev, Minist Educ, Key Lab Bioact Subst & Resources Utilizat Chinese, Beijing, China	Country: China<br>Institution: Chinese Acad Med Sci & Peking Union Med Coll	Country: China<br>Institution: Chinese Academy of Medical Sciences - Peking Union Medical College

Record 200 | WOS:000674669100002

Metabolomics analysis underlay mechanisms in the renal impairment of mice caused by combination of aflatoxin M1 and ochratoxin A

Field	Position	Original line	Normalised line	Original value	Normalised value	Original country / institution	Normalised country / institution

C1	1	31067	31067	[Wang, Ziwei; Gao, Yanan; Huang, Xin; Huang, Shengnan; Yang, Xue; Wang, Jiaqi; Zheng, Nan] Chinese Acad Agr Sci, Inst Anim Sci, Minist Agr & Rural Affairs, Key Lab Qual & Safety Control Milk & Dairy Prod, Beijing 100193, Peoples R China.	[Wang, Ziwei; Gao, Yanan; Huang, Xin; Huang, Shengnan; Yang, Xue; Wang, Jiaqi; Zheng, Nan] Chinese Academy of Agricultural Sciences, Inst Anim Sci, Minist Agr & Rural Affairs, Key Lab Qual & Safety Control Milk & Dairy Prod, Beijing 100193, China.	Country: China Institution: Chinese Acad Agr Sci	Country: China Institution: Chinese Academy of Agricultural Sciences
C1	2	31068	31068	[Wang, Ziwei; Gao, Yanan; Huang, Xin; Huang, Shengnan; Yang, Xue; Wang, Jiaqi; Zheng, Nan] Chinese Acad Agr Sci, Inst Anim Sci, Minist Agr & Rural Affairs, Lab Qual & Safety Risk Assessment Dairy Prod, Beijing 100193, Peoples R China.	[Wang, Ziwei; Gao, Yanan; Huang, Xin; Huang, Shengnan; Yang, Xue; Wang, Jiaqi; Zheng, Nan] Chinese Academy of Agricultural Sciences, Inst Anim Sci, Minist Agr & Rural Affairs, Lab Qual & Safety Risk Assessment Dairy Prod, Beijing 100193, China.	Country: China Institution: Chinese Acad Agr Sci	Country: China Institution: Chinese Academy of Agricultural Sciences
C1	3	31069	31069	[Wang, Ziwei; Gao, Yanan; Huang, Xin; Huang, Shengnan; Yang, Xue; Wang, Jiaqi; Zheng, Nan] Chinese Acad Agr Sci, Inst Anim Sci, Minist Agr & Rural Affairs, Milk & Milk Prod Inspect Ctr, Beijing 100193, Peoples R China.	[Wang, Ziwei; Gao, Yanan; Huang, Xin; Huang, Shengnan; Yang, Xue; Wang, Jiaqi; Zheng, Nan] Chinese Academy of Agricultural Sciences, Inst Anim Sci, Minist Agr & Rural Affairs, Milk & Milk Prod Inspect Ctr, Beijing 100193, China.	Country: China Institution: Chinese Acad Agr Sci	Country: China Institution: Chinese Academy of Agricultural Sciences
C1	4	31070	31070	[Wang, Ziwei; Gao, Yanan; Huang, Xin; Huang, Shengnan; Yang, Xue; Wang, Jiaqi; Zheng, Nan] Chinese Acad Agr Sci, Inst Anim Sci, State Key Lab Anim Nutr, Beijing 100193, Peoples R China.	[Wang, Ziwei; Gao, Yanan; Huang, Xin; Huang, Shengnan; Yang, Xue; Wang, Jiaqi; Zheng, Nan] Chinese Academy of Agricultural Sciences, Inst Anim Sci, State Key Lab Anim Nutr, Beijing 100193, China.	Country: China Institution: Chinese Acad Agr Sci	Country: China Institution: Chinese Academy of Agricultural Sciences
RP	1	31076	31076	Zheng, N (corresponding author), Chinese Acad Agr Sci, Inst Anim Sci, 2 Yuanmingyuan West Rd, Beijing 100193, Peoples R China	Zheng, N (corresponding author), Chinese Academy of Agricultural Sciences, Inst Anim Sci, 2 Yuanmingyuan West Rd, Beijing 100193, China	Country: China Institution: Chinese Acad Agr Sci	Country: China Institution: Chinese Academy of Agricultural Sciences

Record 201 | WOS:000675540600002

Ochratoxin A induces ERK1/2 phosphorylation-dependent apoptosis through NF-KB/ERK axis in human proximal tubule HK-2 cell line

Field	Position	Original line	Normalised line	Original value	Normalised value	Original country / institution	Normalised country / institution
C1	1	31211	31211	[Darbuka, Elif; Gurkaslar, Can; Yaman, Ibrahim] Bogazici Univ, Dept Mol Biol & Genet, Mol Toxicol & Canc Res Lab, TR-34342 Istanbul, Turkey.	[Darbuka, Elif; Gurkaslar, Can; Yaman, Ibrahim] Bogazici University, Dept Mol Biol & Genet, Mol Toxicol & Canc Res Lab, TR-34342 Istanbul, Turkey.	Country: Turkey Institution: Bogazici Univ	Country: Turkey Institution: Bogazici University
C1	2	31212	31212	[Yaman, Ibrahim] Bogazici Univ, Ctr Life Sci & Technol, TR-34342 Istanbul, Turkey.	[Yaman, Ibrahim] Bogazici University, Ctr Life Sci & Technol, TR-34342 Istanbul, Turkey.	Country: Turkey Institution: Bogazici Univ	Country: Turkey Institution: Bogazici University
RP	1	31214	31214	Yaman, I (corresponding author), Bogazici Univ, Ctr Life Sci & Technol, TR-34342 Istanbul, Turkey	Yaman, I (corresponding author), Bogazici University, Ctr Life Sci & Technol, TR-34342 Istanbul, Turkey	Country: Turkey Institution: Bogazici Univ	Country: Turkey Institution: Bogazici University

Record 202 | WOS:000656878400004

Mycotoxins in human food: A challenge for research

Field	Position	Original line	Normalised line	Original value	Normalised value	Original country / institution	Normalised country / institution
C1	1	31325	31325	[Payros, Delphine; Garofalo, Marion] Univ Toulouse, IRSD, INSERM, INRAE,ENVT,UPS, Toulouse, France.	[Payros, Delphine; Garofalo, Marion] Universite de Toulouse, IRSD, INSERM, INRAE,ENVT,UPS, Toulouse, France.	Country: France Institution: Univ Toulouse	Country: France Institution: Universite de Toulouse
C1	2	31326	31326	[Garofalo, Marion; Pierron, Alix; Soler-Vasco, Laura; Al-Ayoubi, Carine; Maruo, Viviane M.; Pinton, Philippe; Oswald, Isabelle P.] Univ Toulouse, INRA, ENVT, Toxalim,Res Ctr Food Toxicol,INP PURPAN,UPS, Toulouse, France.	[Garofalo, Marion; Pierron, Alix; Soler-Vasco, Laura; Al-Ayoubi, Carine; Maruo, Viviane M.; Pinton, Philippe; Oswald, Isabelle P.] Universite de Toulouse, INRA, ENVT, Toxalim,Res Ctr Food Toxicol,INP PURPAN,UPS, Toulouse, France.	Country: France Institution: Univ Toulouse	Country: France Institution: Universite de Toulouse
RP	1	31335	31335	Payros, D (corresponding author), Univ Toulouse, IRSD, INSERM, INRAE,ENVT,UPS, Toulouse, France	Payros, D (corresponding author), Universite de Toulouse, IRSD, INSERM, INRAE,ENVT,UPS, Toulouse, France	Country: France Institution: Univ Toulouse	Country: France Institution: Universite de Toulouse

Record 203 | WOS:000661314200027

In vitro blood brain barrier exposure to mycotoxins and carotenoids pumpkin extract alters mitochondrial gene expression and oxidative stress

Field	Position	Original line	Normalised line	Original value	Normalised value	Original country / institution	Normalised country / institution
C1	1	31516	31516	[Alonso-Garrido, M.; Frangiamone, M.; Font, G.; Cimbalo, A.; Manyes, L.] Univ Valencia, Fac Pharm, Lab Food Chem & Toxicol, Ave Vicent Andres Estelles S-N, Burjassot 46100, Spain.	[Alonso-Garrido, M.; Frangiamone, M.; Font, G.; Cimbalo, A.; Manyes, L.] University of Valencia, Fac Pharm, Lab Food Chem & Toxicol, Ave Vicent Andres Estelles S-N, Burjassot 46100, Spain.	Country: Spain Institution: Univ Valencia	Country: Spain Institution: University of Valencia
RP	1	31518	31518	Cimbalo, A (corresponding author), Univ Valencia, Fac Pharm, Lab Food Chem & Toxicol, Ave Vicent Andres Estelles S-N, Burjassot 46100, Spain	Cimbalo, A (corresponding author), University of Valencia, Fac Pharm, Lab Food Chem & Toxicol, Ave Vicent Andres Estelles S-N, Burjassot 46100, Spain	Country: Spain Institution: Univ Valencia	Country: Spain Institution: University of Valencia

Record 204 | WOS:000661314200019

Multidimensional analysis of the epigenetic alterations in toxicities induced by mycotoxins

Field	Position	Original line	Normalised line	Original value	Normalised value	Original country / institution	Normalised country / institution
C1	1	31628	31628	[Zhu, Liye; Xu, Wentao] China Agr Univ, Dept Nutr & Hlth, Key Lab Precis Nutr & Food Qual, Beijing 100083, Peoples R China.	[Zhu, Liye; Xu, Wentao] China Agricultural University, Dept Nutr & Hlth, Key Lab Precis Nutr & Food Qual, Beijing 100083, China.	Country: China Institution: China Agr Univ	Country: China Institution: China Agricultural University
C1	2	31629	31629	[Yuhan, Jieyu; Huang, Kunlun; He, Xiaoyun; Liang, Zhihong] China Agr Univ, Coll Food Sci & Nutr Engn, Beijing 100083, Peoples R China.	[Yuhan, Jieyu; Huang, Kunlun; He, Xiaoyun; Liang, Zhihong] China Agricultural University, Coll Food Sci & Nutr Engn, Beijing 100083, China.	Country: China Institution: China Agr Univ	Country: China Institution: China Agricultural University
RP	1	31631	31631	Xu, WT (corresponding author), China Agr Univ, 17 Qinghua	Xu, WT (corresponding author), China Agricultural University,	Country: China Institution: China Agr Univ	Country: China Institution: China Agricultural University

				Donglu, Beijing 100083, Peoples R China	17 Qinghua Donglu, Beijing 100083, China		

Record 205 | WOS:000659169000010

The effect of ochratoxin A on cytotoxicity and glucose metabolism in human esophageal epithelium Het-1A cells

Field	Position	Original line	Normalised line	Original value	Normalised value	Original country / institution	Normalised country / institution
C1	1	31808	31808	[Zhao, Man; Zhang, Xianghong] Hebei Med Univ, Metab Dis & Canc Res Ctr, Lab Pathol, Shijiazhuang, Hebei, Peoples R China.	[Zhao, Man; Zhang, Xianghong] Hebei Medical University, Metab Dis & Canc Res Ctr, Lab Pathol, Shijiazhuang, Hebei, China.	Country: China Institution: Hebei Med Univ	Country: China Institution: Hebei Medical University
C1	2	31809	31809	[Wang, Yuan; Jia, Xin; Liu, Weina; Zhang, Xianghong; Cui, Jinfeng] Hebei Med Univ, Hosp 2, Dept Pathol, Shijiazhuang, Hebei, Peoples R China.	[Wang, Yuan; Jia, Xin; Liu, Weina; Zhang, Xianghong; Cui, Jinfeng] Hebei Medical University, Hosp 2, Dept Pathol, Shijiazhuang, Hebei, China.	Country: China Institution: Hebei Med Univ	Country: China Institution: Hebei Medical University
RP	1	31811	31811	Cui, JF (corresponding author), Hebei Med Univ, Hosp 2, Dept Pathol, Shijiazhuang, Hebei, Peoples R China	Cui, JF (corresponding author), Hebei Medical University, Hosp 2, Dept Pathol, Shijiazhuang, Hebei, China	Country: China Institution: Hebei Med Univ	Country: China Institution: Hebei Medical University

Record 206 | WOS:000664290000006

Non-cytotoxic dosage of fumonisin B1 aggravates ochratoxin A-induced nephrocytotoxicity and apoptosis via ROS-dependent JNK/MAPK signaling pathway

Field	Position	Original line	Normalised line	Original value	Normalised value	Original country / institution	Normalised country / institution
C1	1	31909	31909	[Li, Haolei; Wang, Mengmeng; Kang, Weili; Lin, Ziman; Gan, Fang; Huang, Kehe] Nanjing Agr Univ, Coll Vet Med, Nanjing 210095, Jiangsu, Peoples R China.	[Li, Haolei; Wang, Mengmeng; Kang, Weili; Lin, Ziman; Gan, Fang; Huang, Kehe] Nanjing Agricultural University, Coll Vet Med, Nanjing 210095, Jiangsu, China.	Country: China Institution: Nanjing Agr Univ	Country: China Institution: Nanjing Agricultural University
C1	2	31910	31910	[Li, Haolei; Wang, Mengmeng; Kang, Weili; Lin, Ziman; Gan, Fang; Huang, Kehe] Nanjing Agr Univ, Inst Nutr & Metab Disorders Domest Anim & Fowls, Nanjing 210095, Jiangsu, Peoples R China.	[Li, Haolei; Wang, Mengmeng; Kang, Weili; Lin, Ziman; Gan, Fang; Huang, Kehe] Nanjing Agricultural University, Inst Nutr & Metab Disorders Domest Anim & Fowls, Nanjing 210095, Jiangsu, China.	Country: China Institution: Nanjing Agr Univ	Country: China Institution: Nanjing Agricultural University
C1	3	31911	31911	[Li, Haolei; Wang, Mengmeng; Kang, Weili; Lin, Ziman; Gan, Fang; Huang, Kehe] Nanjing Agr Univ, Coll Vet Med, MOE Joint Int Res Lab Anim Hlth & Food Safety, Nanjing 210095, Jiangsu, Peoples R China.	[Li, Haolei; Wang, Mengmeng; Kang, Weili; Lin, Ziman; Gan, Fang; Huang, Kehe] Nanjing Agricultural University, Coll Vet Med, MOE Joint Int Res Lab Anim Hlth & Food Safety, Nanjing 210095, Jiangsu, China.	Country: China Institution: Nanjing Agr Univ	Country: China Institution: Nanjing Agricultural University
RP	1	31914	31914	Huang, KH (corresponding author), Nanjing Agr Univ, Nanjing 210095, Jiangsu, Peoples R China	Huang, KH (corresponding author), Nanjing Agricultural University, Nanjing 210095, Jiangsu, China	Country: China Institution: Nanjing Agr Univ	Country: China Institution: Nanjing Agricultural University

Record 207 | WOS:000654061000009

Oral subchronic exposure to the mycotoxin ochratoxin A induces key pathological features of Parkinson's disease in mice six months after the end of the treatment

Field	Position	Original line	Normalised line	Original value	Normalised value	Original country / institution	Normalised country / institution
C1	2	32035	32035	[Vettorazzi, Ariane; de Cerain, Adela Lopez] Univ Navarra, Dept Pharmacol & Toxicol, MITOX Res Grp, Pamplona 31008, Spain.	[Vettorazzi, Ariane; de Cerain, Adela Lopez] University of Navarra, Dept Pharmacol & Toxicol, MITOX Res Grp, Pamplona 31008, Spain.	Country: Spain Institution: Univ Navarra	Country: Spain Institution: University of Navarra
C1	6	32039	32039	[Cooper, J. Mark] UCL, Dept Clin & Movement Neurosci, Inst Neurol, Gower St, London, England.	[Cooper, J. Mark] UCL, Dept Clin & Movement Neurosci, Inst Neurol, Gower St, London, United Kingdom.	Country: England Institution: UCL	Country: United Kingdom Institution: UCL
C1	7	32040	32040	[Gonzalez-Penas, Elena] Univ Navarra, Dept Pharmaceut Technol & Chem, Pamplona 31008, Spain.	[Gonzalez-Penas, Elena] University of Navarra, Dept Pharmaceut Technol & Chem, Pamplona 31008, Spain.	Country: Spain Institution: Univ Navarra	Country: Spain Institution: University of Navarra

Record 208 | WOS:000643671100001

Ochratoxin A Induces Oxidative Stress in HepG2 Cells by Impairing the Gene Expression of Antioxidant Enzymes

Field	Position	Original line	Normalised line	Original value	Normalised value	Original country / institution	Normalised country / institution
C1	1	32197	32197	[Garcia-Perez, Enrique] Washington State Univ, Sch Food Sci, POB 646376, Pullman, WA 99164 USA.	[Garcia-Perez, Enrique] Washington State University, Sch Food Sci, POB 646376, Pullman, WA 99164 USA.	Country: USA Institution: Washington State Univ	Country: USA Institution: Washington State University
C1	2	32198	32198	[Ryu, Dojin; Lee, Hyun Jung] Univ Idaho, Dept Anim Vet & Food Sci, 875 Perimeter Dr MS 2330, Moscow, ID 83844 USA.	[Ryu, Dojin; Lee, Hyun Jung] University of Idaho, Dept Anim Vet & Food Sci, 875 Perimeter Dr MS 2330, Moscow, ID 83844 USA.	Country: USA Institution: Univ Idaho	Country: USA Institution: University of Idaho
C1	3	32199	32199	[Lee, Chan] Chung Ang Univ, Dept Food Sci & Technol, Anseong, Gyeonggi, South Korea.	[Lee, Chan] Chung Ang University, Dept Food Sci & Technol, Anseong, Gyeonggi, South Korea.	Country: South Korea Institution: Chung Ang Univ	Country: South Korea Institution: Chung Ang University
RP	1	32201	32201	Lee, HJ (corresponding author), Univ Idaho, Dept Anim Vet & Food Sci, 875 Perimeter Dr MS 2330, Moscow, ID 83844 USA	Lee, HJ (corresponding author), University of Idaho, Dept Anim Vet & Food Sci, 875 Perimeter Dr MS 2330, Moscow, ID 83844 USA	Country: USA Institution: Univ Idaho	Country: USA Institution: University of Idaho

Record 209 | WOS:000637397900008

Exploration of zebrafish larvae as an alternative whole-animal model for nephrotoxicity testing

Field	Position	Original line	Normalised line	Original value	Normalised value	Original country / institution	Normalised country / institution
C1	1	32327	32327	[Bauer, Benedikt; Jarzina, Sebastian; Stammler, Emilia; Kreisel, Katrin; Lalomia, Viola; Mally, Angela] Univ Wurzburg, Dept Toxicol, Versbacher Str 9, D-97078 Wurzburg, Germany.	[Bauer, Benedikt; Jarzina, Sebastian; Stammler, Emilia; Kreisel, Katrin; Lalomia, Viola; Mally, Angela] University of Wurzburg, Dept Toxicol, Versbacher Str 9, D-97078 Wurzburg, Germany.	Country: Germany Institution: Univ Wurzburg	Country: Germany Institution: University of Wurzburg
C1	2	32328	32328	[Liedtke, Daniel; Klopocki, Eva] Univ Wurzburg, Inst Human Genet, D-97074 Wurzburg, Germany.	[Liedtke, Daniel; Klopocki, Eva] University of Wurzburg, Inst Human Genet, D-97074 Wurzburg, Germany.	Country: Germany Institution: Univ Wurzburg	Country: Germany Institution: University of Wurzburg
C1	3	32329	32329	[Diefenbacher, Markus] Univ Wurzburg, Chair Biochem &	[Diefenbacher, Markus] University of Wurzburg, Chair	Country: Germany Institution: Univ Wurzburg	Country: Germany Institution: University of Wurzburg

				Mol Biol, D-97074 Wurzburg, Germany.	Biochem & Mol Biol, D-97074 Wurzburg, Germany.		

Record 210 | WOS:000634193100001

Rat Tumour Histopathology Associated with Experimental Chronic Dietary Exposure to Ochratoxin A in Prediction of the Mycotoxin's Risk for Human Cancers

Field	Position	Original line	Normalised line	Original value	Normalised value	Original country / institution	Normalised country / institution
C1	2	32477	32477	[Mantle, Peter] Imperial Coll London, Ctr Environm Policy, London SW7 2AZ, England.	[Mantle, Peter] Imperial College London, Ctr Environm Policy, London SW7 2AZ, United Kingdom.	Country: England Institution: Imperial Coll London	Country: United Kingdom Institution: Imperial College London
RP	1	32479	32479	Mantle, P (corresponding author), Imperial Coll London, Ctr Environm Policy, London SW7 2AZ, England	Mantle, P (corresponding author), Imperial College London, Ctr Environm Policy, London SW7 2AZ, United Kingdom	Country: England Institution: Imperial Coll London	Country: United Kingdom Institution: Imperial College London

Record 211 | WOS:000634190600001

The Impact of the Nephrotoxin Ochratoxin A on Human Renal Cells Studied by a Novel Co-Culture Model Is Influenced by the Presence of Fibroblasts

No C1/C3/RP normalisation changes detected for this record.

Record 212 | WOS:000637502500004

Screening of Macrofungi Isolates for Aflatoxin B1 and Ochratoxin A Degradation

Field	Position	Original line	Normalised line	Original value	Normalised value	Original country / institution	Normalised country / institution
C1	2	32638	32638	[Yamac, Mustafa] Eskisehir Osmangazi Univ, Fac Arts & Sci, Dept Biol, TR-26480 Eskisehir, Turkey.	[Yamac, Mustafa] Eskisehir Osmangazi University, Fac Arts & Sci, Dept Biol, TR-26480 Eskisehir, Turkey.	Country: Turkey Institution: Eskisehir Osmangazi Univ	Country: Turkey Institution: Eskisehir Osmangazi University

Record 213 | WOS:000614680800001

Ochratoxin A induces nephrotoxicity in vitro and in vivo via pyroptosis

Field	Position	Original line	Normalised line	Original value	Normalised value	Original country / institution	Normalised country / institution
C1	1	32743	32743	[Li, Hu; Mao, Xinru; Liu, Kai; Sun, Jiahao; Li, Benrui; Malyar, Rahmani Mohammad; Liu, Dandan; Pan, Cuiling; Gan, Fang; Liu, Yunhuan; Huang, Kehe; Chen, Xingxiang] Nanjing Agr Univ, Coll Vet Med, Nanjing 210095, Peoples R China.	[Li, Hu; Mao, Xinru; Liu, Kai; Sun, Jiahao; Li, Benrui; Malyar, Rahmani Mohammad; Liu, Dandan; Pan, Cuiling; Gan, Fang; Liu, Yunhuan; Huang, Kehe; Chen, Xingxiang] Nanjing Agricultural University, Coll Vet Med, Nanjing 210095, China.	Country: China Institution: Nanjing Agr Univ	Country: China Institution: Nanjing Agricultural University
C1	2	32744	32744	[Li, Hu; Mao, Xinru; Liu, Kai; Sun, Jiahao; Li, Benrui; Malyar, Rahmani Mohammad; Liu, Dandan; Pan, Cuiling; Gan, Fang; Liu, Yunhuan; Huang, Kehe; Chen, Xingxiang] Nanjing Agr Univ, Inst Nutr & Metab	[Li, Hu; Mao, Xinru; Liu, Kai; Sun, Jiahao; Li, Benrui; Malyar, Rahmani Mohammad; Liu, Dandan; Pan, Cuiling; Gan, Fang; Liu, Yunhuan; Huang, Kehe; Chen, Xingxiang] Nanjing Agricultural University, Inst	Country: China Institution: Nanjing Agr Univ	Country: China Institution: Nanjing Agricultural University

				Disorders Domest Anim & Fowls, Nanjing 210095, Peoples R China.	Nutr & Metab Disorders Domest Anim & Fowls, Nanjing 210095, China.		
RP	1	32746	32746	Chen, XX (corresponding author), Nanjing Agr Univ, Coll Vet Med, Nanjing 210095, Peoples R China	Chen, XX (corresponding author), Nanjing Agricultural University, Coll Vet Med, Nanjing 210095, Peoples R China	Country: China<br>Institution: Nanjing Agr Univ	Country: China<br>Institution: Nanjing Agricultural University
RP	2	32746	32746	Chen, XX (corresponding author), Nanjing Agr Univ, Inst Nutr & Metab Disorders Domest Anim & Fowls, Nanjing 210095, Peoples R China	Chen, XX (corresponding author), Nanjing Agr Univ, Inst Nutr & Metab Disorders Domest Anim & Fowls, Nanjing 210095, China	Country: China<br>Institution: Nanjing Agr Univ	Country: China<br>Institution: Nanjing Agr Univ

Record 214 | WOS:000623049400013

Ochratoxin A and its reaction products affected by sugars during heat processing

Field	Position	Original line	Normalised line	Original value	Normalised value	Original country / institution	Normalised country / institution
C1	1	32869	32869	[Gu, Kejia] Washington State Univ, Sch Food Sci, POB 646376, Pullman, WA 99164 USA.	[Gu, Kejia] Washington State University, Sch Food Sci, POB 646376, Pullman, WA 99164 USA.	Country: USA<br>Institution: Washington State Univ	Country: USA<br>Institution: Washington State University
C1	2	32870	32870	[Ryu, Dojin; Lee, Hyun Jung] Univ Idaho, Dept Anim Vet & Food Sci, 875 Perimeter Dr MS 2330, Moscow, ID 83844 USA.	[Ryu, Dojin; Lee, Hyun Jung] University of Idaho, Dept Anim Vet & Food Sci, 875 Perimeter Dr MS 2330, Moscow, ID 83844 USA.	Country: USA<br>Institution: Univ Idaho	Country: USA<br>Institution: University of Idaho
RP	1	32872	32872	Lee, HJ (corresponding author), Univ Idaho, Dept Anim Vet & Food Sci, 875 Perimeter Dr MS 2330, Moscow, ID 83844 USA	Lee, HJ (corresponding author), University of Idaho, Dept Anim Vet & Food Sci, 875 Perimeter Dr MS 2330, Moscow, ID 83844 USA	Country: USA<br>Institution: Univ Idaho	Country: USA<br>Institution: University of Idaho

Record 215 | WOS:000615712900001

Editorial overview of the special issue on application of tissue chips in toxicology

Field	Position	Original line	Normalised line	Original value	Normalised value	Original country / institution	Normalised country / institution
C1	1	32962	32962	[Rusyn, Ivan] Texas A&M Univ, Dept Vet Integrat Biosci, Coll Vet Med & Biomed Sci, College Stn, TX 77843 USA.	[Rusyn, Ivan] Texas A&M University College Station, Dept Vet Integrat Biosci, Coll Vet Med & Biomed Sci, College Stn, TX 77843 USA.	Country: USA<br>Institution: Texas A&M Univ	Country: USA<br>Institution: Texas A&M University College Station
RP	1	32966	32966	Rusyn, I (corresponding author), Texas A&M Univ, Dept Vet Integrat Biosci, Coll Vet Med & Biomed Sci, College Stn, TX 77843 USA	Rusyn, I (corresponding author), Texas A&M University College Station, Dept Vet Integrat Biosci, Coll Vet Med & Biomed Sci, College Stn, TX 77843 USA	Country: USA<br>Institution: Texas A&M Univ	Country: USA<br>Institution: Texas A&M University College Station

Record 216 | WOS:000615712900006

Central role of TRAP1 in the ameliorative effect of oleanolic acid on the mitochondrial-mediated and endoplasmic reticulum stress-excitated apoptosis induced by ochratoxin A

Field	Position	Original line	Normalised line	Original value	Normalised value	Original country / institution	Normalised country / institution

C1	1	33067	33067	[Zhang, Qipeng; Chen, Wenying; Li, Chen; Zhang, Xunyao; Wang, Qian; Shen, Xiao Li] Zunyi Med Univ, Sch Publ Hlth, Zunyi 563000, Guizhou, Peoples R China.	[Zhang, Qipeng; Chen, Wenying; Li, Chen; Zhang, Xunyao; Wang, Qian; Shen, Xiao Li] Zunyi Medical University, Sch Publ Hlth, Zunyi 563000, Guizhou, China.	Country: China Institution: Zunyi Med Univ	Country: China Institution: Zunyi Medical University
C1	2	33068	33068	[Zhou, Qian] Hebei Agr Univ, Coll Food Sci & Technol, Baoding 071000, Hebei, Peoples R China.	[Zhou, Qian] Hebei Agricultural University, Coll Food Sci & Technol, Baoding 071000, Hebei, China.	Country: China Institution: Hebei Agr Univ	Country: China Institution: Hebei Agricultural University
C1	3	33069	33069	[Zhang, Boyang] China Agr Univ, Dept Nutr & Hlth, Key Lab Precis Nutr & Food Qual, Beijing 100083, Peoples R China.	[Zhang, Boyang] China Agricultural University, Dept Nutr & Hlth, Key Lab Precis Nutr & Food Qual, Beijing 100083, China.	Country: China Institution: China Agr Univ	Country: China Institution: China Agricultural University
C1	4	33070	33070	[Wang, Yan] Zhejiang Univ Technol, Dept Food Sci & Technol, Hangzhou 310014, Zhejiang, Peoples R China.	[Wang, Yan] Zhejiang University of Technology, Dept Food Sci & Technol, Hangzhou 310014, Zhejiang, China.	Country: China Institution: Zhejiang Univ Technol	Country: China Institution: Zhejiang University of Technology
C1	5	33071	33071	[Li, Xiaohong] Beijing Agr Vocat Coll, Dept Food & Bioengn, Beijing 102442, Peoples R China.	[Li, Xiaohong] Beijing Agr Vocat Coll, Dept Food & Bioengn, Beijing 102442, China.	Country: China Institution: Beijing Agr Vocat Coll	Country: China Institution: Beijing Agr Vocat Coll
RP	1	33074	33074	Shen, XL (corresponding author), Zunyi Med Univ, Sch Publ Hlth, Dept Food Safety, Univ West Rd 6, Zunyi 563000, Guizhou, Peoples R China	Shen, XL (corresponding author), Zunyi Medical University, Sch Publ Hlth, Dept Food Safety, Univ West Rd 6, Zunyi 563000, Guizhou, China	Country: China Institution: Zunyi Med Univ	Country: China Institution: Zunyi Medical University

Record 217 | WOS:000606891900001

Mycotoxins: Impact on Health and Strategies for Prevention and Detoxification in the Food Chain

Field	Position	Original line	Normalised line	Original value	Normalised value	Original country / institution	Normalised country / institution
C1	2	33207	33207	[Peivasteh-Roudsari, Leila; Pirhadi, Mohadeseh; Shahbazi, Razieh; Eghbaljoo-Gharehgheshlaghi, Hadi] Univ Tehran Med Sci, Food Safety & Hyg Div, Dept Environm Hlth Engn, Tehran, Iran.	[Peivasteh-Roudsari, Leila; Pirhadi, Mohadeseh; Shahbazi, Razieh; Eghbaljoo-Gharehgheshlaghi, Hadi] Tehran University of Medical Sciences, Food Safety & Hyg Div, Dept Environm Hlth Engn, Tehran, Iran.	Country: Iran Institution: Univ Tehran Med Sci	Country: Iran Institution: Tehran University of Medical Sciences
C1	3	33208	33208	[Eghbaljoo-Gharehgheshlaghi, Hadi] Univ Tehran Med Sci, Students Sci Res Ctr, Tehran, Iran.	[Eghbaljoo-Gharehgheshlaghi, Hadi] Tehran University of Medical Sciences, Students Sci Res Ctr, Tehran, Iran.	Country: Iran Institution: Univ Tehran Med Sci	Country: Iran Institution: Tehran University of Medical Sciences
C1	5	33210	33210	[Mirza Alizadeh, Adel; Tajdar-oranj, Behrouz] Shahid Beheshti Univ Med Sci, Natl Nutr & Food Technol Res Inst, Fac Nutr Sci & Food Technol, Student Res Comm,Dept Food Technol, Tehran, Iran.	[Mirza Alizadeh, Adel; Tajdar-oranj, Behrouz] Shahid Beheshti University Medical Sciences, Natl Nutr & Food Technol Res Inst, Fac Nutr Sci & Food Technol, Student Res Comm,Dept Food Technol, Tehran, Iran.	Country: Iran Institution: Shahid Beheshti Univ Med Sci	Country: Iran Institution: Shahid Beheshti University Medical Sciences
C1	6	33211	33211	[Jazaeri, Sahar] Shahid Beheshti Univ Med Sci, Natl Nutr & Food Technol Res Inst, Fac Nutr Sci Food Sci & Technol, Dept Food Sci & Technol, Tehran, Iran.	[Jazaeri, Sahar] Shahid Beheshti University Medical Sciences, Natl Nutr & Food Technol Res Inst, Fac Nutr Sci Food Sci & Technol, Dept Food Sci & Technol, Tehran, Iran.	Country: Iran Institution: Shahid Beheshti Univ Med Sci	Country: Iran Institution: Shahid Beheshti University Medical Sciences
RP	1	33215	33215	Tajdar-oranj, B (corresponding author), Shahid Beheshti Univ Med Sci, Natl Nutr & Food	Tajdar-oranj, B (corresponding author), Shahid Beheshti University Medical Sciences,	Country: Iran Institution: Shahid Beheshti Univ Med Sci	Country: Iran Institution: Shahid Beheshti University Medical Sciences

				Technol Res Inst, Fac Nutr Sci & Food Technol, Student Res Comm,Dept Food Technol, Tehran, Iran	Natl Nutr & Food Technol Res Inst, Fac Nutr Sci & Food Technol, Student Res Comm,Dept Food Technol, Tehran, Iran		

Record 218 | WOS:000638197700008

Comet assay: a versatile but complex tool in genotoxicity testing

No C1/C3/RP normalisation changes detected for this record.

Record 219 | WOS:000701996600001

Aspergillus derived mycotoxins in food and the environment: Prevalence, detection, and toxicity

No C1/C3/RP normalisation changes detected for this record.

Record 220 | WOS:000670072600004

Neurotoxicity mechanism of Ochratoxin A

Field	Position	Original line	Normalised line	Original value	Normalised value	Original country / institution	Normalised country / institution
C1	1	34024	34024	[Nourbakhsh, Fahimeh] Mashhad Univ Med Sci, Fac Med, Med Toxicol Res Ctr, Mashhad, Razavi Khorasan, Iran.	[Nourbakhsh, Fahimeh] Mashhad University of Medical Sciences, Fac Med, Med Toxicol Res Ctr, Mashhad, Razavi Khorasan, Iran.	Country: Iran<br>Institution: Mashhad Univ Med Sci	Country: Iran<br>Institution: Mashhad University of Medical Sciences
C1	2	34025	34025	[Tajbakhsh, Elahe] Islamic Azad Univ, Fac Basic Sci, Dept Microbiol, Shahrekord Branch, Shahrekord, Iran.	[Tajbakhsh, Elahe] Islamic Azad University, Fac Basic Sci, Dept Microbiol, Shahrekord Branch, Shahrekord, Iran.	Country: Iran<br>Institution: Islamic Azad Univ	Country: Iran<br>Institution: Islamic Azad University
RP	1	34027	34027	Nourbakhsh, F (corresponding author), Mashhad Univ Med Sci, Fac Med, Med Toxicol Res Ctr, Mashhad, Razavi Khorasan, Iran	Nourbakhsh, F (corresponding author), Mashhad University of Medical Sciences, Fac Med, Med Toxicol Res Ctr, Mashhad, Razavi Khorasan, Iran	Country: Iran<br>Institution: Mashhad Univ Med Sci	Country: Iran<br>Institution: Mashhad University of Medical Sciences

Record 221 | WOS:000603090600001

Mycotoxin and Gut Microbiota Interactions

Field	Position	Original line	Normalised line	Original value	Normalised value	Original country / institution	Normalised country / institution
C1	1	34160	34160	[Guerre, Philippe] Univ Toulouse, ENVT, Ecole Natl Vet Toulouse, F-31076 Toulouse, France.	[Guerre, Philippe] Universite de Toulouse, ENVT, Ecole Natl Vet Toulouse, F-31076 Toulouse, France.	Country: France<br>Institution: Univ Toulouse	Country: France<br>Institution: Universite de Toulouse
RP	1	34162	34162	Guerre, P (corresponding author), Univ Toulouse, ENVT, Ecole Natl Vet Toulouse, F-31076 Toulouse, France	Guerre, P (corresponding author), Universite de Toulouse, ENVT, Ecole Natl Vet Toulouse, F-31076 Toulouse, France	Country: France<br>Institution: Univ Toulouse	Country: France<br>Institution: Universite de Toulouse

Record 222 | WOS:000597367400001

Ameliorative role of dietary activated carbon against ochratoxin-A induced oxidative damage, suppressed performance and toxicological effects

Field	Position	Original line	Normalised line	Original value	Normalised value	Original country / institution	Normalised country / institution
C1	1	34430	34430	[Bhatti, Sheraz Ahmed] Bahauddin Zakariya Univ, Fac Vet Sci, Dept Pathobiol, Multan 60800, Pakistan.	[Bhatti, Sheraz Ahmed] Bahauddin Zakariya University, Fac Vet Sci, Dept Pathobiol, Multan 60800, Pakistan.	Country: Pakistan Institution: Bahauddin Zakariya Univ	Country: Pakistan Institution: Bahauddin Zakariya University
C1	3	34432	34432	[Ul Hassan, Zahoor] Qatar Univ, Coll Arts & Sci, Dept Biol & Environm Sci, Doha, Qatar.	[Ul Hassan, Zahoor] Qatar University, Coll Arts & Sci, Dept Biol & Environm Sci, Doha, Qatar.	Country: Qatar Institution: Qatar Univ	Country: Qatar Institution: Qatar University
C1	4	34433	34433	[Khan, Ahrar] Shandong Vocat Anim Sci & Vet Coll, Weifang, Peoples R China.	[Khan, Ahrar] Shandong Vocat Anim Sci & Vet Coll, Weifang, China.	Country: China Institution: Shandong Vocat Anim Sci & Vet Coll	Country: China Institution: Shandong Vocat Anim Sci & Vet Coll
RP	1	34436	34436	Bhatti, SA (corresponding author), Bahauddin Zakariya Univ, Fac Vet Sci, Dept Pathobiol, Multan 60800, Pakistan	Bhatti, SA (corresponding author), Bahauddin Zakariya University, Fac Vet Sci, Dept Pathobiol, Multan 60800, Pakistan	Country: Pakistan Institution: Bahauddin Zakariya Univ	Country: Pakistan Institution: Bahauddin Zakariya University

Record 223 | WOS:000584401800010

Transcriptomic alterations induced by aflatoxin B1 and ochratoxin A in LMH cell line

Field	Position	Original line	Normalised line	Original value	Normalised value	Original country / institution	Normalised country / institution
C1	1	34553	34553	[Choi, So-Young; Hong, Min-Wook; Lee, Hyojeong; Lee, Sung-Jin] Kangwon Natl Univ, Dept Anim Life Sci, Chuncheon Si 24341, Gangwon Do, South Korea.	[Choi, So-Young; Hong, Min-Wook; Lee, Hyojeong; Lee, Sung-Jin] Kangwon National University, Dept Anim Life Sci, Chuncheon Si 24341, Gangwon Do, South Korea.	Country: South Korea Institution: Kangwon Natl Univ	Country: South Korea Institution: Kangwon National University
C1	3	34555	34555	[Park, Tae Sub] Seoul Natl Univ, Grad Sch Int Agr Technol, Pyeongchang Gun 25354, Gangwon Do, South Korea.	[Park, Tae Sub] Seoul National University (SNU), Grad Sch Int Agr Technol, Pyeongchang Gun 25354, Gangwon Do, South Korea.	Country: South Korea Institution: Seoul Natl Univ	Country: South Korea Institution: Seoul National University (SNU)
C1	4	34556	34556	[Park, Tae Sub] Seoul Natl Univ, Inst Greenbio Sci & Technol, Pyeongchang Gun 25354, Gangwon Do, South Korea.	[Park, Tae Sub] Seoul National University (SNU), Inst Greenbio Sci & Technol, Pyeongchang Gun 25354, Gangwon Do, South Korea.	Country: South Korea Institution: Seoul Natl Univ	Country: South Korea Institution: Seoul National University (SNU)
RP	1	34560	34560	Lee, SJ (corresponding author), Kangwon Natl Univ, Dept Anim Life Sci, Chuncheon Si 24341, Gangwon Do, South Korea	Lee, SJ (corresponding author), Kangwon National University, Dept Anim Life Sci, Chuncheon Si 24341, Gangwon Do, South Korea	Country: South Korea Institution: Kangwon Natl Univ	Country: South Korea Institution: Kangwon National University

Record 224 | WOS:000594353000001

Effects of Single and Repeated Oral Doses of Ochratoxin A on the Lipid Peroxidation and Antioxidant Defense Systems in Mouse Kidneys

Field	Position	Original line	Normalised line	Original value	Normalised value	Original country / institution	Normalised country / institution
C1	7	34689	34689	[Mezes, Miklos; Balogh, Krisztian] Hungarian Acad Sci, MTA KE SZIE Mycotoxins Food	[Mezes, Miklos; Balogh, Krisztian] Hungarian Academy of Sciences, MTA KE SZIE	Country: Hungary Institution: Hungarian Acad Sci	Country: Hungary Institution: Hungarian Academy of Sciences

				Chain Res Grp, 40 Guba Sandor St, H-7400 Kaposvar, Hungary.	Mycotoxins Food Chain Res Grp, 40 Guba Sandor St, H-7400 Kaposvar, Hungary.		

Record 225 | WOS:000580526300009

Microphysiological system modeling of ochratoxin A-associated nephrotoxicity

Field	Position	Original line	Normalised line	Original value	Normalised value	Original country / institution	Normalised country / institution
C1	1	34828	34828	[Imaoka, Tomoki; Yang, Jade; Van Ness, Kirk; Kelly, Edward J.] Univ Washington, Sch Pharm, Dept Pharmaceut, Seattle, WA 98195 USA.	[Imaoka, Tomoki; Yang, Jade; Van Ness, Kirk; Kelly, Edward J.] University of Washington, Sch Pharm, Dept Pharmaceut, Seattle, WA 98195 USA.	Country: USA<br>Institution: Univ Washington	Country: USA<br>Institution: University of Washington
C1	2	34829	34829	[McDonald, Matthew G.; Rettie, Allan E.] Univ Washington, Sch Pharm, Dept Med Chem, Seattle, WA 98195 USA.	[McDonald, Matthew G.; Rettie, Allan E.] University of Washington, Sch Pharm, Dept Med Chem, Seattle, WA 98195 USA.	Country: USA<br>Institution: Univ Washington	Country: USA<br>Institution: University of Washington
C1	3	34830	34830	[Wang, Lu; Afsharinejad, Zahra; Bammler, Theo K.] Univ Washington, Dept Environm & Occupat Hlth Sci, Seattle, WA 98195 USA.	[Wang, Lu; Afsharinejad, Zahra; Bammler, Theo K.] University of Washington, Dept Environm & Occupat Hlth Sci, Seattle, WA 98195 USA.	Country: USA<br>Institution: Univ Washington	Country: USA<br>Institution: University of Washington
C1	4	34831	34831	[Yeung, Catherine K.] Univ Washington, Sch Pharm, Dept Pharm, Seattle, WA 98195 USA.	[Yeung, Catherine K.] University of Washington, Sch Pharm, Dept Pharm, Seattle, WA 98195 USA.	Country: USA<br>Institution: Univ Washington	Country: USA<br>Institution: University of Washington
C1	5	34832	34832	[Yeung, Catherine K.; Himmelfarb, Jonathan; Kelly, Edward J.] Univ Washington, Kidney Res Inst, Dept Med, Div Nephrol, Seattle, WA 98104 USA.	[Yeung, Catherine K.; Himmelfarb, Jonathan; Kelly, Edward J.] University of Washington, Kidney Res Inst, Dept Med, Div Nephrol, Seattle, WA 98104 USA.	Country: USA<br>Institution: Univ Washington	Country: USA<br>Institution: University of Washington

Record 226 | WOS:000582386000020

Ochratoxins in food and feed: Occurrence and its impact on human health and management strategies

Field	Position	Original line	Normalised line	Original value	Normalised value	Original country / institution	Normalised country / institution
C1	2	34978	34978	[Mahato, Dipendra Kumar; Mahmud, M. M. Chayan; Rawal, Deepaksshi] Deakin Univ, Sch Exercise & Nutr Sci, 221 Burwood Hwy, Burwood, Vic 3125, Australia.	[Mahato, Dipendra Kumar; Mahmud, M. M. Chayan; Rawal, Deepaksshi] Deakin University, Sch Exercise & Nutr Sci, 221 Burwood Hwy, Burwood, Vic 3125, Australia.	Country: Australia<br>Institution: Deakin Univ	Country: Australia<br>Institution: Deakin University
C1	3	34979	34979	[Sharma, Bharti] Banaras Hindu Univ, Ctr Food Sci & Technol, Varanasi 221005, Uttar Pradesh, India.	[Sharma, Bharti] Banaras Hindu University (BHU), Ctr Food Sci & Technol, Varanasi 221005, Uttar Pradesh, India.	Country: India<br>Institution: Banaras Hindu Univ	Country: India<br>Institution: Banaras Hindu University (BHU)
C1	4	34980	34980	[Haque, Shafiul] Jazan Univ, Coll Nursing & Allied Hlth Sci, Res & Sci Studies Unit, Jazan 45142, Saudi Arabia.	[Haque, Shafiul] Jazan University, Coll Nursing & Allied Hlth Sci, Res & Sci Studies Unit, Jazan 45142, Saudi Arabia.	Country: Saudi Arabia<br>Institution: Jazan Univ	Country: Saudi Arabia<br>Institution: Jazan University

Record 227 | WOS:000584874600001

The protective effect of stilbenes resveratrol and pterostilbene individually and combined with mycotoxin citrinin in human adenocarcinoma HT-29 cell line in vitro

No C1/C3/RP normalisation changes detected for this record.

Record 228 | WOS:000563527100012

Cytoprotective effects of curcumin and silymarin on PK-15 cells exposed to ochratoxin A, fumonisin B1 and deoxynivalenol

No C1/C3/RP normalisation changes detected for this record.

Record 229 | WOS:000563527100013

Genotoxic effects of mycotoxins

Field	Position	Original line	Normalised line	Original value	Normalised value	Original country / institution	Normalised country / institution
C1	1	35515	35515	[Ulger, Taha Gokmen] Abant Izzet Baysal Univ, Fac Hlth Sci, Golkoy Campus, TR-14030 Bolu, Turkey.	[Ulger, Taha Gokmen] Abant Izzet Baysal University, Fac Hlth Sci, Golkoy Campus, TR-14030 Bolu, Turkey.	Country: Turkey<br>Institution: Abant Izzet Baysal Univ	Country: Turkey<br>Institution: Abant Izzet Baysal University
C1	2	35516	35516	[Ucar, Asli; Cakiroglu, Funda Pinar] Univ Ankara, Fac Hlth Sci, Dept Nutr & Dietet, Fatih St,197-A, TR-06290 Ankara, Turkey.	[Ucar, Asli; Cakiroglu, Funda Pinar] Ankara University, Fac Hlth Sci, Dept Nutr & Dietet, Fatih St,197-A, TR-06290 Ankara, Turkey.	Country: Turkey<br>Institution: Univ Ankara	Country: Turkey<br>Institution: Ankara University
C1	3	35517	35517	[Yilmaz, Serkan] Univ Ankara, Fac Nursing, Dept Midwifery, Plevne St 5, TR-06230 Ankara, Turkey.	[Yilmaz, Serkan] Ankara University, Fac Nursing, Dept Midwifery, Plevne St 5, TR-06230 Ankara, Turkey.	Country: Turkey<br>Institution: Univ Ankara	Country: Turkey<br>Institution: Ankara University
C1	4	35518	35518	[Yilmaz, Serkan] Univ Ankara, Fac Hlth Sci, Fatih St 197-A, TR-06290 Kecioren Ankara, Turkey.	[Yilmaz, Serkan] Ankara University, Fac Hlth Sci, Fatih St 197-A, TR-06290 Kecioren Ankara, Turkey.	Country: Turkey<br>Institution: Univ Ankara	Country: Turkey<br>Institution: Ankara University
RP	1	35520	35520	Yilmaz, S (corresponding author), Univ Ankara, Fac Nursing, Dept Midwifery, Plevne St 5, TR-06230 Ankara, Turkey	Yilmaz, S (corresponding author), Ankara University, Fac Nursing, Dept Midwifery, Plevne St 5, TR-06230 Ankara, Turkey	Country: Turkey<br>Institution: Univ Ankara	Country: Turkey<br>Institution: Ankara University

Record 230 | WOS:000579907000025

Ochratoxin A induces reprogramming of glucose metabolism by switching energy metabolism from oxidative phosphorylation to glycolysis in human gastric epithelium GES-1 cells in vitro

Field	Position	Original line	Normalised line	Original value	Normalised value	Original country / institution	Normalised country / institution
C1	1	35709	35709	[Wang, Yuan; Cui, Jinfeng; Li, Yuehong; Wu, Wenxin; Zhang, Xianghong] Hebei Med Univ, Hosp 2, Dept Pathol, 215 Heping Western Rd, Shijiazhuang, Hebei, Peoples R China.	[Wang, Yuan; Cui, Jinfeng; Li, Yuehong; Wu, Wenxin; Zhang, Xianghong] Hebei Medical University, Hosp 2, Dept Pathol, 215 Heping Western Rd, Shijiazhuang, Hebei, China.	Country: China<br>Institution: Hebei Med Univ	Country: China<br>Institution: Hebei Medical University
C1	2	35710	35710	[Zhao, Man; Zhang, Xianghong] Hebei Med Univ, Metab Dis & Canc Res Ctr, Lab Pathol,	[Zhao, Man; Zhang, Xianghong] Hebei Medical University, Metab Dis & Canc Res Ctr, Lab	Country: China<br>Institution: Hebei Med Univ	Country: China<br>Institution: Hebei Medical University

				Shijiazhuang, Hebei, Peoples R China.	Pathol, Shijiazhuang, Hebei, China.		
C1	3	35711	35711	[Wu, Xin] Hebei North Univ, Dept Pathol, Zhangjiakou, Peoples R China.	[Wu, Xin] Hebei North University, Dept Pathol, Zhangjiakou, China.	Country: China Institution: Hebei North Univ	Country: China Institution: Hebei North University
RP	1	35714	35714	Zhang, XH (corresponding author), Hebei Med Univ, Hosp 2, Dept Pathol, 215 Heping Western Rd, Shijiazhuang, Hebei, Peoples R China	Zhang, XH (corresponding author), Hebei Medical University, Hosp 2, Dept Pathol, 215 Heping Western Rd, Shijiazhuang, Hebei, China	Country: China Institution: Hebei Med Univ	Country: China Institution: Hebei Medical University

Record 231 | WOS:000566774200008

Dechlorination and demethylation of ochratoxin A enhance blocking activity of PXR activation, suppress PXR expression and reduce cytotoxicity

Field	Position	Original line	Normalised line	Original value	Normalised value	Original country / institution	Normalised country / institution
C1	1	35818	35818	[Shen, Yuanjun; Shi, Zhanquan; Fan, Jun Ting; Yan, Bingfang] Univ Cincinnati, James L Winkle Coll Pharm, Div Pharmaceut Sci, Cincinnati, OH 45229 USA.	[Shen, Yuanjun; Shi, Zhanquan; Fan, Jun Ting; Yan, Bingfang] University of Cincinnati, James L Winkle Coll Pharm, Div Pharmaceut Sci, Cincinnati, OH 45229 USA.	Country: USA Institution: Univ Cincinnati	Country: USA Institution: University of Cincinnati
RP	1	35820	35820	Yan, BF (corresponding author), Univ Cincinnati, James L Winkle Coll Pharm, Div Pharmaceut Sci, Cincinnati, OH 45229 USA	Yan, BF (corresponding author), University of Cincinnati, James L Winkle Coll Pharm, Div Pharmaceut Sci, Cincinnati, OH 45229 USA	Country: USA Institution: Univ Cincinnati	Country: USA Institution: University of Cincinnati

Record 232 | WOS:000585534200001

The Compromised Intestinal Barrier Induced by Mycotoxins

Field	Position	Original line	Normalised line	Original value	Normalised value	Original country / institution	Normalised country / institution
C1	1	35934	35934	[Gao, Yanan; Meng, Lu; Liu, Huimin; Wang, Jiaqi; Zheng, Nan] Chinese Acad Agr Sci, Inst Anim Sci, Key Lab Qual & Safety Control Milk & Dairy Prod, Minist Agr & Rural Affairs, Beijing 100193, Peoples R China.	[Gao, Yanan; Meng, Lu; Liu, Huimin; Wang, Jiaqi; Zheng, Nan] Chinese Academy of Agricultural Sciences, Inst Anim Sci, Key Lab Qual & Safety Control Milk & Dairy Prod, Minist Agr & Rural Affairs, Beijing 100193, China.	Country: China Institution: Chinese Acad Agr Sci	Country: China Institution: Chinese Academy of Agricultural Sciences
C1	2	35935	35935	[Gao, Yanan; Meng, Lu; Liu, Huimin; Wang, Jiaqi; Zheng, Nan] Chinese Acad Agr Sci, Inst Anim Sci, Minist Agr & Rural Affairs, Lab Qual & Safety Risk Assessment Dairy Prod, Beijing 100193, Peoples R China.	[Gao, Yanan; Meng, Lu; Liu, Huimin; Wang, Jiaqi; Zheng, Nan] Chinese Academy of Agricultural Sciences, Inst Anim Sci, Minist Agr & Rural Affairs, Lab Qual & Safety Risk Assessment Dairy Prod, Beijing 100193, China.	Country: China Institution: Chinese Acad Agr Sci	Country: China Institution: Chinese Academy of Agricultural Sciences
C1	3	35936	35936	[Gao, Yanan; Meng, Lu; Liu, Huimin; Wang, Jiaqi; Zheng, Nan] Chinese Acad Agr Sci, Inst Anim Sci, Milk & Milk Prod Inspect Ctr, Minist Agr & Rural Affairs, Beijing 100193, Peoples R China.	[Gao, Yanan; Meng, Lu; Liu, Huimin; Wang, Jiaqi; Zheng, Nan] Chinese Academy of Agricultural Sciences, Inst Anim Sci, Milk & Milk Prod Inspect Ctr, Minist Agr & Rural Affairs, Beijing 100193, China.	Country: China Institution: Chinese Acad Agr Sci	Country: China Institution: Chinese Academy of Agricultural Sciences
C1	4	35937	35937	[Gao, Yanan; Meng, Lu; Liu, Huimin; Wang, Jiaqi; Zheng, Nan] Chinese Acad Agr Sci, State Key Lab Anim Nutr, Inst	[Gao, Yanan; Meng, Lu; Liu, Huimin; Wang, Jiaqi; Zheng, Nan] Chinese Academy of Agricultural Sciences, State Key	Country: China Institution: Chinese Acad Agr Sci	Country: China Institution: Chinese Academy of Agricultural Sciences

				Anim Sci, Beijing 100193, Peoples R China.	Lab Anim Nutr, Inst Anim Sci, Beijing 100193, China.		
RP	2	35943	35943	Zheng, N (corresponding author), Chinese Acad Agr Sci, Inst Anim Sci, Key Lab Qual & Safety Control Milk & Dairy Prod, Minist Agr & Rural Affairs, Beijing 100193, Peoples R China	Zheng, N (corresponding author), Chinese Academy of Agricultural Sciences, Inst Anim Sci, Key Lab Qual & Safety Control Milk & Dairy Prod, Minist Agr & Rural Affairs, Beijing 100193, Peoples R China	Country: China<br>Institution: Chinese Acad Agr Sci	Country: China<br>Institution: Chinese Academy of Agricultural Sciences
RP	8	35943	35943	Zheng, N (corresponding author), Chinese Acad Agr Sci, State Key Lab Anim Nutr, Inst Anim Sci, Beijing 100193, Peoples R China	Zheng, N (corresponding author), Chinese Acad Agr Sci, State Key Lab Anim Nutr, Inst Anim Sci, Beijing 100193, China	Country: China<br>Institution: Chinese Acad Agr Sci	Country: China<br>Institution: Chinese Acad Agr Sci

Record 233 | WOS:000564335100008

Larval zebrafish as an in vitro model for evaluating toxicological effects of mycotoxins

Field	Position	Original line	Normalised line	Original value	Normalised value	Original country / institution	Normalised country / institution
C1	1	36226	36226	[Juan-Garcia, Ana] Univ Valencia, Lab Food Chem & Toxicol, Fac Pharm, Ave Vicent Andres Estelles S-N, Valencia 46100, Spain.	[Juan-Garcia, Ana] University of Valencia, Lab Food Chem & Toxicol, Fac Pharm, Ave Vicent Andres Estelles S-N, Valencia 46100, Spain.	Country: Spain<br>Institution: Univ Valencia	Country: Spain<br>Institution: University of Valencia
C1	2	36227	36227	[Bind, Marie-Abele] Harvard Univ, Dept Stat, Fac Arts & Sci, Cambridge, MA 02138 USA.	[Bind, Marie-Abele] Harvard University, Dept Stat, Fac Arts & Sci, Cambridge, MA 02138 USA.	Country: USA<br>Institution: Harvard Univ	Country: USA<br>Institution: Harvard University
C1	3	36228	36228	[Juan-Garcia, Ana; Engert, Florian] Harvard Univ, Dept Mol & Cellular Biol, 16 Divin Ave, Cambridge, MA 02138 USA.	[Juan-Garcia, Ana; Engert, Florian] Harvard University, Dept Mol & Cellular Biol, 16 Divin Ave, Cambridge, MA 02138 USA.	Country: USA<br>Institution: Harvard Univ	Country: USA<br>Institution: Harvard University
RP	1	36230	36230	Juan-GarcÃ-a, A (corresponding author), Univ Valencia, Lab Food Chem & Toxicol, Fac Pharm, Ave Vicent Andres Estelles S-N, Valencia 46100, Spain	Juan-GarcÃ-a, A (corresponding author), University of Valencia, Lab Food Chem & Toxicol, Fac Pharm, Ave Vicent Andres Estelles S-N, Valencia 46100, Spain	Country: Spain<br>Institution: Univ Valencia	Country: Spain<br>Institution: University of Valencia

Record 234 | WOS:000585500900001

Toxin Degradation by Rumen Microorganisms: A Review

Field	Position	Original line	Normalised line	Original value	Normalised value	Original country / institution	Normalised country / institution
C1	1	36380	36380	[Loh, Zhi Hung; Ouwerkerk, Diane; Klieve, Athol V.; Hungerford, Natasha L.; Fletcher, Mary T.] Univ Queensland, Hlth & Food Sci Precinct, Queensland Alliance Agr & Food Innovat QAAFI, Coopers Plains, Qld 4108, Australia.	[Loh, Zhi Hung; Ouwerkerk, Diane; Klieve, Athol V.; Hungerford, Natasha L.; Fletcher, Mary T.] University of Queensland, Hlth & Food Sci Precinct, Queensland Alliance Agr & Food Innovat QAAFI, Coopers Plains, Qld 4108, Australia.	Country: Australia<br>Institution: Univ Queensland	Country: Australia<br>Institution: University of Queensland
RP	1	36383	36383	Fletcher, MT (corresponding author), Univ Queensland, Hlth & Food Sci Precinct, Queensland Alliance Agr & Food Innovat	Fletcher, MT (corresponding author), University of Queensland, Hlth & Food Sci Precinct, Queensland Alliance Agr & Food Innovat QAAFI,	Country: Australia<br>Institution: Univ Queensland	Country: Australia<br>Institution: University of Queensland

				QAAFI, Coopers Plains, Qld 4108, Australia	Coopers Plains, Qld 4108, Australia		

Record 235 | WOS:000585462600001

Mycotoxins: Biotransformation and Bioavailability Assessment Using Caco-2 Cell Monolayer

No C1/C3/RP normalisation changes detected for this record.

Record 236 | WOS:000573807900002

Ochratoxin A affects oocyte maturation and subsequent embryo developmental dynamics in the juvenile sheep model

Field	Position	Original line	Normalised line	Original value	Normalised value	Original country / institution	Normalised country / institution
C1	2	37032	37032	[Asif, Shafaq; Mastrorocco, Antonella; Carluccio, Augusto; Robbe, Domenico] Univ Teramo, Fac Vet Med, SP18, I-64100 Teramo, Italy.	[Asif, Shafaq; Mastrorocco, Antonella; Carluccio, Augusto; Robbe, Domenico] University of Teramo, Fac Vet Med, SP18, I-64100 Teramo, Italy.	Country: Italy<br>Institution: Univ Teramo	Country: Italy<br>Institution: University of Teramo
C1	3	37033	37033	[Marzano, Giuseppina] Univ Salento, Dept Math & Phys E Giorgi, Via Arnesano, I-73100 Lecce, Italy.	[Marzano, Giuseppina] University of Salento, Dept Math & Phys E Giorgi, Via Arnesano, I-73100 Lecce, Italy.	Country: Italy<br>Institution: Univ Salento	Country: Italy<br>Institution: University of Salento
C1	6	37036	37036	[Roelen, Bernard A. J.] Univ Utrecht, Fac Vet Med, Dept Clin Sci, Uppsalalaan 8, NL-3584 CT Utrecht, Netherlands.	[Roelen, Bernard A. J.] Utrecht University, Fac Vet Med, Dept Clin Sci, Uppsalalaan 8, NL-3584 CT Utrecht, Netherlands.	Country: Netherlands<br>Institution: Univ Utrecht	Country: Netherlands<br>Institution: Utrecht University

Record 237 | WOS:000545982700021

E ffects of Ochratoxin A exposure on DNA damage in porcine granulosa cells in vitro

Field	Position	Original line	Normalised line	Original value	Normalised value	Original country / institution	Normalised country / institution
C1	1	37168	37168	[Zhang, Tian-Yu; Kong, Li; Wang, Han; Yan, Zi-Hui; Sun, Xiao-Feng; Shen, Wei] Qingdao Agr Univ, Key Lab Anim Reprod & Germplasm Enhancement Univ, Coll Life Sci, Qingdao 266109, Peoples R China.	[Zhang, Tian-Yu; Kong, Li; Wang, Han; Yan, Zi-Hui; Sun, Xiao-Feng; Shen, Wei] Qingdao Agricultural University, Key Lab Anim Reprod & Germplasm Enhancement Univ, Coll Life Sci, Qingdao 266109, China.	Country: China<br>Institution: Qingdao Agr Univ	Country: China<br>Institution: Qingdao Agricultural University
C1	2	37169	37169	[Zhang, Tian-Yu] Qingdao Univ, Sch Publ Hlth, Qingdao 266034, Peoples R China.	[Zhang, Tian-Yu] Qingdao University, Sch Publ Hlth, Qingdao 266034, China.	Country: China<br>Institution: Qingdao Univ	Country: China<br>Institution: Qingdao University
C1	3	37170	37170	[Hao, Jia-Xing] Qingdao Univ, Qingdao Womens & Childrens Hosp, Ctr Reprod Med, Qingdao 266034, Peoples R China.	[Hao, Jia-Xing] Qingdao University, Qingdao Womens & Childrens Hosp, Ctr Reprod Med, Qingdao 266034, China.	Country: China<br>Institution: Qingdao Univ	Country: China<br>Institution: Qingdao University
RP	1	37172	37172	Shen, W (corresponding author), Qingdao Agr Univ, Key Lab Anim Reprod & Germplasm Enhancement Univ, Coll Life Sci, Qingdao 266109, Peoples R China	Shen, W (corresponding author), Qingdao Agricultural University, Key Lab Anim Reprod & Germplasm Enhancement Univ, Coll Life Sci, Qingdao 266109, China	Country: China<br>Institution: Qingdao Agr Univ	Country: China<br>Institution: Qingdao Agricultural University

Record 238 | WOS:000568157900004

Multiple-endpoint in vitro carcinogenicity test in human cell line TK6 distinguishes carcinogens from non-carcinogens and highlights mechanisms of action

Field	Position	Original line	Normalised line	Original value	Normalised value	Original country / institution	Normalised country / institution
C1	1	37296	37296	[Chapman, Katherine E.; Wilde, Eleanor C.; Chapman, Fiona M.; Verma, Jatin R.; Shah, Ume-Kulsoom; Stannard, Leanne M.; Seager, Anna L.; Johnson, George E.; Doak, Shareen H.; Jenkins, Gareth J. S.] Swansea Univ, Med Sch, Inst Life Sci 1, In Vitro Toxicol Grp, Singleton Campus, Swansea SA2 8PP, W Glam, Wales.	[Chapman, Katherine E.; Wilde, Eleanor C.; Chapman, Fiona M.; Verma, Jatin R.; Shah, Ume-Kulsoom; Stannard, Leanne M.; Seager, Anna L.; Johnson, George E.; Doak, Shareen H.; Jenkins, Gareth J. S.] Swansea University, Med Sch, Inst Life Sci 1, In Vitro Toxicol Grp, Singleton Campus, Swansea SA2 8PP, W Glam, United Kingdom.	Country: Wales<br>Institution: Swansea Univ	Country: United Kingdom<br>Institution: Swansea University
C1	2	37297	37297	[Tonkin, James A.; Brown, M. Rowan] Swansea Univ, Coll Engn, Bay Campus, Swansea SA1 8EN, W Glam, Wales.	[Tonkin, James A.; Brown, M. Rowan] Swansea University, Coll Engn, Bay Campus, Swansea SA1 8EN, W Glam, United Kingdom.	Country: Wales<br>Institution: Swansea Univ	Country: United Kingdom<br>Institution: Swansea University
C1	3	37298	37298	[Doherty, Ann T.] AstraZeneca, DSM, Discovery Safety, Darwin Bldg,Cambridge Sci Pk,Milton Rd, Cambridge CB4 0WG, England.	[Doherty, Ann T.] AstraZeneca, DSM, Discovery Safety, Darwin Bldg,Cambridge Sci Pk,Milton Rd, Cambridge CB4 0WG, United Kingdom.	Country: England<br>Institution: AstraZeneca	Country: United Kingdom<br>Institution: AstraZeneca
RP	1	37300	37300	Chapman, KE (corresponding author), Swansea Univ, Med Sch, Inst Life Sci 1, In Vitro Toxicol Grp, Singleton Campus, Swansea SA2 8PP, W Glam, Wales	Chapman, KE (corresponding author), Swansea University, Med Sch, Inst Life Sci 1, In Vitro Toxicol Grp, Singleton Campus, Swansea SA2 8PP, W Glam, United Kingdom	Country: Wales<br>Institution: Swansea Univ	Country: United Kingdom<br>Institution: Swansea University

Record 239 | WOS:000567625500001

Protective role of lactic acid bacteria and yeasts as dietary carcinogen-binding agents - a review

Field	Position	Original line	Normalised line	Original value	Normalised value	Original country / institution	Normalised country / institution
C1	2	37439	37439	[Hall, F. G.; Urbizo-Reyes, U. C.; Liceaga, A. M.] Purdue Univ, Dept Food Sci, Smith Hall, W Lafayette, IN 47907 USA.	[Hall, F. G.; Urbizo-Reyes, U. C.; Liceaga, A. M.] Purdue University, Dept Food Sci, Smith Hall, W Lafayette, IN 47907 USA.	Country: USA<br>Institution: Purdue Univ	Country: USA<br>Institution: Purdue University

Record 240 | WOS:000562731900014

Ochratoxin A induces glomerular injury through activating the ERK/NF-ÎºB signaling pathway

Field	Position	Original line	Normalised line	Original value	Normalised value	Original country / institution	Normalised country / institution
C1	1	37723	37723	[Le, Guannan; Yuan, Xin; Hou, Lili; Ge, Lei; Liu, Shuiping; Muhmood, Azhar; Liu, Kai; Lin, Ziman; Liu, Dandan; Gan, Fang; Song, Suquan; Pan, Cuilin; Chen, Xingxiang; Huang, Kehe] Nanjing Agr Univ, Coll Vet	[Le, Guannan; Yuan, Xin; Hou, Lili; Ge, Lei; Liu, Shuiping; Muhmood, Azhar; Liu, Kai; Lin, Ziman; Liu, Dandan; Gan, Fang; Song, Suquan; Pan, Cuilin; Chen, Xingxiang; Huang, Kehe] Nanjing Agricultural	Country: China<br>Institution: Nanjing Agr Univ	Country: China<br>Institution: Nanjing Agricultural University

				Med, Nanjing 210095, Jiangsu, Peoples R China.	University, Coll Vet Med, Nanjing 210095, Jiangsu, China.		
C1	2	37724	37724	[Le, Guannan; Yuan, Xin; Hou, Lili; Ge, Lei; Liu, Shuiping; Muhmood, Azhar; Liu, Kai; Lin, Ziman; Liu, Dandan; Gan, Fang; Song, Suquan; Pan, Cuilin; Chen, Xingxiang; Huang, Kehe] Nanjing Agr Univ, Inst Nutr & Metab Disorders Domest Anim & Fowls, Nanjing 210095, Jiangsu, Peoples R China.	[Le, Guannan; Yuan, Xin; Hou, Lili; Ge, Lei; Liu, Shuiping; Muhmood, Azhar; Liu, Kai; Lin, Ziman; Liu, Dandan; Gan, Fang; Song, Suquan; Pan, Cuilin; Chen, Xingxiang; Huang, Kehe] Nanjing Agricultural University, Inst Nutr & Metab Disorders Domest Anim & Fowls, Nanjing 210095, Jiangsu, China.	Country: China<br>Institution: Nanjing Agr Univ	Country: China<br>Institution: Nanjing Agricultural University
C1	3	37725	37725	[Le, Guannan; Yuan, Xin; Hou, Lili; Ge, Lei; Liu, Shuiping; Muhmood, Azhar; Liu, Kai; Lin, Ziman; Liu, Dandan; Gan, Fang; Song, Suquan; Pan, Cuilin; Chen, Xingxiang; Huang, Kehe] Nanjing Agr Univ, Coll Vet Med, MOE Joint Int Res Lab Anim Hlth & Food Safety, Nanjing 210095, Jiangsu, Peoples R China.	[Le, Guannan; Yuan, Xin; Hou, Lili; Ge, Lei; Liu, Shuiping; Muhmood, Azhar; Liu, Kai; Lin, Ziman; Liu, Dandan; Gan, Fang; Song, Suquan; Pan, Cuilin; Chen, Xingxiang; Huang, Kehe] Nanjing Agricultural University, Coll Vet Med, MOE Joint Int Res Lab Anim Hlth & Food Safety, Nanjing 210095, Jiangsu, China.	Country: China<br>Institution: Nanjing Agr Univ	Country: China<br>Institution: Nanjing Agricultural University
RP	1	37728	37728	Huang, KH (corresponding author), Nanjing Agr Univ, Coll Vet Med, Nanjing 210095, Jiangsu, Peoples R China	Huang, KH (corresponding author), Nanjing Agricultural University, Coll Vet Med, Nanjing 210095, Jiangsu, China	Country: China<br>Institution: Nanjing Agr Univ	Country: China<br>Institution: Nanjing Agricultural University

Record 241 | WOS:000585802200008

Synergistic Interaction of Ochratoxin A and Acrylamide Toxins in Human Kidney and Liver Cells

Field	Position	Original line	Normalised line	Original value	Normalised value	Original country / institution	Normalised country / institution
C1	1	37828	37828	[Pyo, Min Cheol; Shin, Hye Soo; Jeon, Gyeong Yun; Lee, Kwang-Won] Korea Univ, Coll Life Sci & Biotechnol, Dept Biotechnol, Seoul 02841, South Korea.	[Pyo, Min Cheol; Shin, Hye Soo; Jeon, Gyeong Yun; Lee, Kwang-Won] Korea University, Coll Life Sci & Biotechnol, Dept Biotechnol, Seoul 02841, South Korea.	Country: South Korea<br>Institution: Korea Univ	Country: South Korea<br>Institution: Korea University
RP	1	37830	37830	Lee, KW (corresponding author), Korea Univ, Coll Life Sci & Biotechnol, Dept Biotechnol, Seoul 02841, South Korea	Lee, KW (corresponding author), Korea University, Coll Life Sci & Biotechnol, Dept Biotechnol, Seoul 02841, South Korea	Country: South Korea<br>Institution: Korea Univ	Country: South Korea<br>Institution: Korea University

Record 242 | WOS:000560097800001

Ochratoxin A presence in Cabernet Sauvignon wine changes antioxidant activityin vitroand oxidative stress markersin vivo

No C1/C3/RP normalisation changes detected for this record.

Record 243 | WOS:000539161100006

Mycotoxins, invisible danger of feedstuff with toxic effect on animals

Field	Position	Original line	Normalised line	Original value	Normalised value	Original country / institution	Normalised country / institution

C1	1	38090	38090	[Buszewska-Forajta, Magdalena] Nicolaus Copernicus Univ Torun, Vet Ctr, Torun, Poland.	[Buszewska-Forajta, Magdalena] Nicolaus Copernicus University, Vet Ctr, Torun, Poland.	Country: Poland Institution: Nicolaus Copernicus Univ Torun	Country: Poland Institution: Nicolaus Copernicus University
RP	1	38092	38092	Buszewska-Forajta, M (corresponding author), Nicolaus Copernicus Univ Torun, Vet Ctr, Torun, Poland	Buszewska-Forajta, M (corresponding author), Nicolaus Copernicus University, Vet Ctr, Torun, Poland	Country: Poland Institution: Nicolaus Copernicus Univ Torun	Country: Poland Institution: Nicolaus Copernicus University

Record 244 | WOS:000547359800001

Subchronic exposure to individual and combined ochratoxin A and citrinin affects the expression of rat renal organic anion transporters

Field	Position	Original line	Normalised line	Original value	Normalised value	Original country / institution	Normalised country / institution
C1	1	38271	38271	[Karaica, Dean; Breljak, Davorka] Inst Med Res & Occupat Hlth, Mol Toxicol Unit, Ksaverska Cesta 2, Zagreb 10000, Croatia.	[Karaica, Dean; Breljak, Davorka] Institute for Medical Research & Occupational Health (IMROH), Mol Toxicol Unit, Ksaverska Cesta 2, Zagreb 10000, Croatia.	Country: Croatia Institution: Inst Med Res & Occupat Hlth	Country: Croatia Institution: Institute for Medical Research & Occupational Health (IMROH)
C1	2	38272	38272	[Micek, Vedran] Inst Med Res & Occupat Hlth, Anim Breeding Unit, Ksaverska Cesta 2, Zagreb 10000, Croatia.	[Micek, Vedran] Institute for Medical Research & Occupational Health (IMROH), Anim Breeding Unit, Ksaverska Cesta 2, Zagreb 10000, Croatia.	Country: Croatia Institution: Inst Med Res & Occupat Hlth	Country: Croatia Institution: Institute for Medical Research & Occupational Health (IMROH)
C1	3	38273	38273	[Rasic, Dubravka; Peraica, Maja] Inst Med Res & Occupat Hlth, Toxicol Unit, Ksaverska Cesta 2, Zagreb 10000, Croatia.	[Rasic, Dubravka; Peraica, Maja] Institute for Medical Research & Occupational Health (IMROH), Toxicol Unit, Ksaverska Cesta 2, Zagreb 10000, Croatia.	Country: Croatia Institution: Inst Med Res & Occupat Hlth	Country: Croatia Institution: Institute for Medical Research & Occupational Health (IMROH)
C1	4	38274	38274	[Klaric, Maja Segvic] Univ Zagreb, Fac Pharm & Biochem, Dept Microbiol, Schrottova 39,1 Kat, Zagreb 10000, Croatia.	[Klaric, Maja Segvic] University of Zagreb, Fac Pharm & Biochem, Dept Microbiol, Schrottova 39,1 Kat, Zagreb 10000, Croatia.	Country: Croatia Institution: Univ Zagreb	Country: Croatia Institution: University of Zagreb
RP	1	38278	38278	Karaica, D (corresponding author), Inst Med Res & Occupat Hlth, Mol Toxicol Unit, Ksaverska Cesta 2, Zagreb 10000, Croatia	Karaica, D (corresponding author), Institute for Medical Research & Occupational Health (IMROH), Mol Toxicol Unit, Ksaverska Cesta 2, Zagreb 10000, Croatia	Country: Croatia Institution: Inst Med Res & Occupat Hlth	Country: Croatia Institution: Institute for Medical Research & Occupational Health (IMROH)

Record 245 | WOS:000545059200001

Ochratoxin A induces epithelial-to-mesenchymal transition and renal fibrosis through TGF-Î²/Smad2/3 and Wnt1/Î²-catenin signaling pathways in vitro and in vivo

Field	Position	Original line	Normalised line	Original value	Normalised value	Original country / institution	Normalised country / institution
C1	1	38401	38401	[Pyo, Min Cheol; Chae, Seung A.; Yoo, Hee Joon; Lee, Kwang-Won] Korea Univ, Coll Life Sci & Biotechnol, Dept Biotechnol, Seoul 02841, South Korea.	[Pyo, Min Cheol; Chae, Seung A.; Yoo, Hee Joon; Lee, Kwang-Won] Korea University, Coll Life Sci & Biotechnol, Dept Biotechnol, Seoul 02841, South Korea.	Country: South Korea Institution: Korea Univ	Country: South Korea Institution: Korea University
RP	1	38403	38403	Lee, KW (corresponding author), Korea Univ, Coll Life Sci & Biotechnol, Dept Biotechnol, Seoul 02841, South Korea	Lee, KW (corresponding author), Korea University, Coll Life Sci & Biotechnol, Dept Biotechnol, Seoul 02841, South Korea	Country: South Korea Institution: Korea Univ	Country: South Korea Institution: Korea University

Record 246 | WOS:000541110800046

Luteolin alleviates ochratoxin A induced oxidative stress by regulating Nrf2 and HIF-1Î± pathways in NRK-52E rat kidney cells

Field	Position	Original line	Normalised line	Original value	Normalised value	Original country / institution	Normalised country / institution
C1	1	38544	38544	[Liu, Man; Cheng, Chao; Li, Xuezhi; Zhou, Sihan; Hua, Jiali; Huang, Jie; Li, Yongxin; Yang, Kunlong; Zhang, Peng; Tian, Jun] Jiangsu Normal Univ, Sch Life Sci, Xuzhou 221116, Jiangsu, Peoples R China.	[Liu, Man; Cheng, Chao; Li, Xuezhi; Zhou, Sihan; Hua, Jiali; Huang, Jie; Li, Yongxin; Yang, Kunlong; Zhang, Peng; Tian, Jun] Jiangsu Normal University, Sch Life Sci, Xuzhou 221116, Jiangsu, China.	Country: China<br>Institution: Jiangsu Normal Univ	Country: China<br>Institution: Jiangsu Normal University
C1	2	38545	38545	[Liu, Man; Tian, Jun] Beijing Technol & Business Univ, Beijing Adv Innovat Ctr Food Nutr & Human Hlth, Beijing 10048, Peoples R China.	[Liu, Man; Tian, Jun] Beijing Technology & Business University, Beijing Adv Innovat Ctr Food Nutr & Human Hlth, Beijing 10048, China.	Country: China<br>Institution: Beijing Technol & Business Univ	Country: China<br>Institution: Beijing Technology & Business University
C1	3	38546	38546	[Zhang, Yan] Xuzhou Canc Hosp, Dept Osteol, Xuzhou 221005, Jiangsu, Peoples R China.	[Zhang, Yan] Xuzhou Canc Hosp, Dept Osteol, Xuzhou 221005, Jiangsu, China.	Country: China<br>Institution: Xuzhou Canc Hosp	Country: China<br>Institution: Xuzhou Canc Hosp
RP	2	38548	38548	Tian, J (corresponding author), Jiangsu Normal Univ, Sch Life Sci, Xuzhou 221116, Jiangsu, Peoples R China	Tian, J (corresponding author), Jiangsu Normal University, Sch Life Sci, Xuzhou 221116, Jiangsu, Peoples R China	Country: China<br>Institution: Jiangsu Normal Univ	Country: China<br>Institution: Jiangsu Normal University
RP	3	38548	38548	Zhang, Y (corresponding author), Xuzhou Canc Hosp, Dept Osteol, Xuzhou 221005, Jiangsu, Peoples R China	Zhang, Y (corresponding author), Xuzhou Canc Hosp, Dept Osteol, Xuzhou 221005, Jiangsu, China	Country: China<br>Institution: Xuzhou Canc Hosp	Country: China<br>Institution: Xuzhou Canc Hosp

Record 247 | WOS:000556892100001

Reducing Ochratoxin A Content in Grape Pomace by Different Methods

No C1/C3/RP normalisation changes detected for this record.

Record 248 | WOS:000530851100002

Ochratoxin A causes cell cycle arrest in G1 and G1/S phases through p53 in HK-2 cells

Field	Position	Original line	Normalised line	Original value	Normalised value	Original country / institution	Normalised country / institution
C1	1	38758	38758	[Celik, Dilek Asci; Gurbuz, Nilgun; Togay, Vehbi Atahan; Ozcelik, Nurten] Suleyman Demirel Univ, Fac Med, Dept Med Biol, Isparta, Turkey.	[Celik, Dilek Asci; Gurbuz, Nilgun; Togay, Vehbi Atahan; Ozcelik, Nurten] Suleyman Demirel University, Fac Med, Dept Med Biol, Isparta, Turkey.	Country: Turkey<br>Institution: Suleyman Demirel Univ	Country: Turkey<br>Institution: Suleyman Demirel University
RP	1	38760	38760	Ã elik, DA (corresponding author), Suleyman Demirel Univ, Fac Med, Dept Med Biol, Isparta, Turkey	Ã elik, DA (corresponding author), Suleyman Demirel University, Fac Med, Dept Med Biol, Isparta, Turkey	Country: Turkey<br>Institution: Suleyman Demirel Univ	Country: Turkey<br>Institution: Suleyman Demirel University

Record 249 | WOS:000541267600001

Cytogenotoxic evaluation of the acetonitrile extract, citrinin and dicitrinin-A fromPenicillium citrinum

Field	Position	Original line	Normalised line	Original value	Normalised value	Original country / institution	Normalised country / institution
C1	7	38885	38885	[Islam, Muhammad Torequl] Ton Duc Thang Univ, Lab Theoret & Computat Biophys, Ho Chi Minh City 700000, Vietnam.	[Islam, Muhammad Torequl] Ton Duc Thang University, Lab Theoret & Computat Biophys, Ho Chi Minh City 700000, Vietnam.	Country: Vietnam Institution: Ton Duc Thang Univ	Country: Vietnam Institution: Ton Duc Thang University
C1	8	38886	38886	[Islam, Muhammad Torequl] Ton Duc Thang Univ, Fac Pharm, Ho Chi Minh City, Vietnam.	[Islam, Muhammad Torequl] Ton Duc Thang University, Fac Pharm, Ho Chi Minh City, Vietnam.	Country: Vietnam Institution: Ton Duc Thang Univ	Country: Vietnam Institution: Ton Duc Thang University
C1	9	38887	38887	[Clark, Cain C. T.] Coventry Univ, Ctr Sport Exercise & Life Sci, Coventry, W Midlands, England.	[Clark, Cain C. T.] Coventry University, Ctr Sport Exercise & Life Sci, Coventry, W Midlands, United Kingdom.	Country: England Institution: Coventry Univ	Country: United Kingdom Institution: Coventry University
RP	1	38892	38892	Islam, MT (corresponding author), Ton Duc Thang Univ, Lab Theoret & Computat Biophys, Ho Chi Minh City 700000, Vietnam	Islam, MT (corresponding author), Ton Duc Thang University, Lab Theoret & Computat Biophys, Ho Chi Minh City 700000, Vietnam	Country: Vietnam Institution: Ton Duc Thang Univ	Country: Vietnam Institution: Ton Duc Thang University

Record 250 | WOS:000533844900008

Ochratoxin A-induced genotoxic and epigenetic mechanisms lead to Alzheimer disease: its modulation with strategies

Field	Position	Original line	Normalised line	Original value	Normalised value	Original country / institution	Normalised country / institution
C1	4	39045	39045	[Khan, Fazlullah] Univ Tehran Med Sci, Fac Pharm, Dept Pharmacol & Toxicol, Tehran 1417614411, Iran.	[Khan, Fazlullah] Tehran University of Medical Sciences, Fac Pharm, Dept Pharmacol & Toxicol, Tehran 1417614411, Iran.	Country: Iran Institution: Univ Tehran Med Sci	Country: Iran Institution: Tehran University of Medical Sciences
C1	5	39046	39046	[Bule, Mohammed] Ambo Univ, Coll Med & Hlth Sci, Dept Pharm, Ambo, Oromia, Ethiopia.	[Bule, Mohammed] Ambo University, Coll Med & Hlth Sci, Dept Pharm, Ambo, Oromia, Ethiopia.	Country: Ethiopia Institution: Ambo Univ	Country: Ethiopia Institution: Ambo University

Record 251 | WOS:000542374900003

Milk thistle (Silybum Marianum) as an antidote or a protective agent against natural or chemical toxicities: a review

Field	Position	Original line	Normalised line	Original value	Normalised value	Original country / institution	Normalised country / institution
C1	1	39362	39362	[Fanoudi, Sahar; Alavi, Mohaddeseh Sadat] Mashhad Univ Med Sci, Dept Pharmacol, Fac Med, Mashhad, Razavi Khorasan, Iran.	[Fanoudi, Sahar; Alavi, Mohaddeseh Sadat] Mashhad University of Medical Sciences, Dept Pharmacol, Fac Med, Mashhad, Razavi Khorasan, Iran.	Country: Iran Institution: Mashhad Univ Med Sci	Country: Iran Institution: Mashhad University of Medical Sciences
C1	2	39363	39363	[Karimi, Gholamreza; Hosseinzadeh, Hossein] Mashhad Univ Med Sci, Pharmaceut Technol Inst, Pharmaceut Res Ctr, Mashhad, Razavi Khorasan, Iran.	[Karimi, Gholamreza; Hosseinzadeh, Hossein] Mashhad University of Medical Sciences, Pharmaceut Technol Inst, Pharmaceut Res Ctr, Mashhad, Razavi Khorasan, Iran.	Country: Iran Institution: Mashhad Univ Med Sci	Country: Iran Institution: Mashhad University of Medical Sciences
C1	3	39364	39364	[Karimi, Gholamreza; Hosseinzadeh, Hossein] Mashhad Univ Med Sci, Sch Pharm, Dept Pharmacodynam	[Karimi, Gholamreza; Hosseinzadeh, Hossein] Mashhad University of Medical Sciences, Sch Pharm, Dept Pharmacodynam & Toxicol,	Country: Iran Institution: Mashhad Univ Med Sci	Country: Iran Institution: Mashhad University of Medical Sciences

				& Toxicol, Mashhad, Razavi Khorasan, Iran.	Mashhad, Razavi Khorasan, Iran.		
RP	1	39367	39367	Hosseinzadeh, H (corresponding author), Mashhad Univ Med Sci, Pharmaceut Technol Inst, Pharmaceut Res Ctr, Mashhad, Razavi Khorasan, Iran	Hosseinzadeh, H (corresponding author), Mashhad University of Medical Sciences, Pharmaceut Technol Inst, Pharmaceut Res Ctr, Mashhad, Razavi Khorasan, Iran	Country: Iran<br>Institution: Mashhad Univ Med Sci	Country: Iran<br>Institution: Mashhad University of Medical Sciences

Record 252 | WOS:000527365800027

Ochratoxin A exposure causes meiotic failure and oocyte deterioration in mice

Field	Position	Original line	Normalised line	Original value	Normalised value	Original country / institution	Normalised country / institution
C1	1	39614	39614	[Jia, Huiqun; Jia, Chenqi; An, Quanli; Cheng, Yuyao; Jiang, Xianlei; Xu, Yue; Zhao, Ruolin; Zhang, Yong; Su, Jianmin] Northwest A&F Univ, Coll Vet Med, Yangling 712100, Shaanxi, Peoples R China.	[Jia, Huiqun; Jia, Chenqi; An, Quanli; Cheng, Yuyao; Jiang, Xianlei; Xu, Yue; Zhao, Ruolin; Zhang, Yong; Su, Jianmin] Northwest A&F Univ, Coll Vet Med, Yangling 712100, Shaanxi, China.	Country: China<br>Institution: Northwest A&F Univ	Country: China<br>Institution: Northwest A&F Univ
C1	2	39615	39615	[Peng, Wei] Qinghai Univ, Acad Anim Sci & Vet Med, Xining 810003, Qinghai, Peoples R China.	[Peng, Wei] Qinghai University, Acad Anim Sci & Vet Med, Xining 810003, Qinghai, China.	Country: China<br>Institution: Qinghai Univ	Country: China<br>Institution: Qinghai University
C3	1	39616	39616	Northwest A&F University - China	Northwest A&F University	Institution: Northwest A&F University - China	Institution: Northwest A&F University
RP	1	39617	39617	Su, JM (corresponding author), 3 Tai Cheng Rd, Xianyang 712100, Shaanxi, Peoples R China	Su, JM (corresponding author), 3 Tai Cheng Rd, Xianyang 712100, Shaanxi, China	Country: China<br>Institution: 3 Tai Cheng Rd	Country: China<br>Institution: 3 Tai Cheng Rd

Record 253 | WOS:000526412300005

Beauvericin and ochratoxin A mycotoxins individually and combined in HepG2 cells alter lipid peroxidation, levels of reactive oxygen species and glutathione

Field	Position	Original line	Normalised line	Original value	Normalised value	Original country / institution	Normalised country / institution
C1	1	39751	39751	[Juan-Garcia, Ana; Manes, Jordi] Univ Valencia, Fac Pharm, Lab Food Chem & Toxicol, Av Vicent Andres Estelles S-N, E-46100 Valencia, Spain.	[Juan-Garcia, Ana; Manes, Jordi] University of Valencia, Fac Pharm, Lab Food Chem & Toxicol, Av Vicent Andres Estelles S-N, E-46100 Valencia, Spain.	Country: Spain<br>Institution: Univ Valencia	Country: Spain<br>Institution: University of Valencia
C1	2	39752	39752	[Carbone, Saverio; Sagratini, Gianni] Univ Camerino, Sch Pharm, Lab Food Chem, Via S Agostino 1, I-62032 Camerino, Italy.	[Carbone, Saverio; Sagratini, Gianni] University of Camerino, Sch Pharm, Lab Food Chem, Via S Agostino 1, I-62032 Camerino, Italy.	Country: Italy<br>Institution: Univ Camerino	Country: Italy<br>Institution: University of Camerino
RP	1	39755	39755	Juan-GarcÃ-a, A (corresponding author), Univ Valencia, Fac Pharm, Lab Food Chem & Toxicol, Av Vicent Andres Estelles S-N, E-46100 Valencia, Spain	Juan-GarcÃ-a, A (corresponding author), University of Valencia, Fac Pharm, Lab Food Chem & Toxicol, Av Vicent Andres Estelles S-N, E-46100 Valencia, Spain	Country: Spain<br>Institution: Univ Valencia	Country: Spain<br>Institution: University of Valencia

Record 254 | WOS:000537553700018

Risk assessment of ochratoxin A in food

No C1/C3/RP normalisation changes detected for this record.

Record 255 | WOS:000505957800037

Aptamer assisted ultrafiltration cleanup with high performance liquid chromatography-fluorescence detector for the determination of OTA in green coffee

Field	Position	Original line	Normalised line	Original value	Normalised value	Original country / institution	Normalised country / institution
C1	2	40428	40428	[Moez, Elsaadani; Noel, Durand; Didier, Montet] Univ Reunion, Univ Avignon, Univ Montpellier, Montpellier SupAgro,UMR 95 QualiSud,CIRAD, Montpellier, France.	[Moez, Elsaadani; Noel, Durand; Didier, Montet] University of La Reunion, Univ Avignon, Univ Montpellier, Montpellier SupAgro,UMR 95 QualiSud,CIRAD, Montpellier, France.	Country: France<br>Institution: Univ Reunion	Country: France<br>Institution: University of La Reunion
C1	3	40429	40429	[Moez, Elsaadani] Misr Univ Sci & Technol, Fac Biotechnol, 6th 13 October City, Egypt.	[Moez, Elsaadani] Misr University for Science & Technology, Fac Biotechnol, 6th 13 October City, Egypt.	Country: Egypt<br>Institution: Misr Univ Sci & Technol	Country: Egypt<br>Institution: Misr University for Science & Technology

Record 256 | WOS:000526746500001

Protective Effect of Hydroxytyrosol Against Oxidative Stress Induced by the Ochratoxin in Kidney Cells: in vitro and in vivo Study

Field	Position	Original line	Normalised line	Original value	Normalised value	Original country / institution	Normalised country / institution
C1	1	40543	40543	[Crupi, Rosalia] Univ Messina, Dept Vet Sci, Messina, Italy.	[Crupi, Rosalia] University of Messina, Dept Vet Sci, Messina, Italy.	Country: Italy<br>Institution: Univ Messina	Country: Italy<br>Institution: University of Messina
C1	2	40544	40544	[Palma, Ernesto; De Caro, Carmen; Calzetta, Luigino] Univ Catanzaro Magna Graecia, Dept Hlth Sci, Catanzaro, Italy.	[Palma, Ernesto; De Caro, Carmen; Calzetta, Luigino] Magna Graecia University of Catanzaro, Dept Hlth Sci, Catanzaro, Italy.	Country: Italy<br>Institution: Univ Catanzaro Magna Graecia	Country: Italy<br>Institution: Magna Graecia University of Catanzaro
C1	3	40545	40545	[Siracusa, Rosalba; Fusco, Roberta; Gugliandolo, Enrico; Cordaro, Marika; Impellizzeri, Daniela; Cuzzocrea, Salvatore; Di Paola, Rosanna] Univ Messina, Dept Chem Biol Pharmaceut & Environm Sci, Messina, Italy.	[Siracusa, Rosalba; Fusco, Roberta; Gugliandolo, Enrico; Cordaro, Marika; Impellizzeri, Daniela; Cuzzocrea, Salvatore; Di Paola, Rosanna] University of Messina, Dept Chem Biol Pharmaceut & Environm Sci, Messina, Italy.	Country: Italy<br>Institution: Univ Messina	Country: Italy<br>Institution: University of Messina
RP	1	40550	40550	Cuzzocrea, S (corresponding author), Univ Messina, Dept Chem Biol Pharmaceut & Environm Sci, Messina, Italy	Cuzzocrea, S (corresponding author), University of Messina, Dept Chem Biol Pharmaceut & Environm Sci, Messina, Italy	Country: Italy<br>Institution: Univ Messina	Country: Italy<br>Institution: University of Messina

Record 257 | WOS:000524713000001

Development of ELISA and Lateral Flow Immunoassays for Ochratoxins (OTA and OTB) Detection Based on Monoclonal Antibody

Field	Position	Original line	Normalised line	Original value	Normalised value	Original country / institution	Normalised country / institution

C1	1	40659	40659	[Fadlalla, Mohamed Hassan; Ling, Sumei; Wang, Rongzhi; Li, Xiulan; Yuan, Jun; Xiao, Shiwei; Wang, Ke; Tang, Shuqin; Elsir, Hoyda; Wang, Shihua] Fujian Agr & Forestry Univ, Fujian Key Lab Pathogen Fungi & Mycotoxins, Fuzhou, Peoples R China.	[Fadlalla, Mohamed Hassan; Ling, Sumei; Wang, Rongzhi; Li, Xiulan; Yuan, Jun; Xiao, Shiwei; Wang, Ke; Tang, Shuqin; Elsir, Hoyda; Wang, Shihua] Fujian Agr & Forestry Univ, Fujian Key Lab Pathogen Fungi & Mycotoxins, Fuzhou, China.	Country: China Institution: Fujian Agr & Forestry Univ	Country: China Institution: Fujian Agr & Forestry Univ
C1	2	40660	40660	[Fadlalla, Mohamed Hassan; Ling, Sumei; Wang, Rongzhi; Li, Xiulan; Yuan, Jun; Wang, Ke; Tang, Shuqin; Elsir, Hoyda; Wang, Shihua] Fujian Agr & Forestry Univ, Sch Life Sci, Fuzhou, Peoples R China.	[Fadlalla, Mohamed Hassan; Ling, Sumei; Wang, Rongzhi; Li, Xiulan; Yuan, Jun; Wang, Ke; Tang, Shuqin; Elsir, Hoyda; Wang, Shihua] Fujian Agr & Forestry Univ, Sch Life Sci, Fuzhou, China.	Country: China Institution: Fujian Agr & Forestry Univ	Country: China Institution: Fujian Agr & Forestry Univ
C1	3	40661	40661	[Ling, Sumei; Wang, Rongzhi; Xiao, Shiwei; Wang, Shihua] Fujian Agr & Forestry Univ, Key Lab Biopesticide & Chem Biol, Educ Minist, Fuzhou, Peoples R China.	[Ling, Sumei; Wang, Rongzhi; Xiao, Shiwei; Wang, Shihua] Fujian Agr & Forestry Univ, Key Lab Biopesticide & Chem Biol, Educ Minist, Fuzhou, China.	Country: China Institution: Fujian Agr & Forestry Univ	Country: China Institution: Fujian Agr & Forestry Univ
RP	3	40664	40664	Wang, SH (corresponding author), Fujian Agr & Forestry Univ, Key Lab Biopesticide & Chem Biol, Educ Minist, Fuzhou, Peoples R China	Wang, SH (corresponding author), Fujian Agr & Forestry Univ, Key Lab Biopesticide & Chem Biol, Educ Minist, Fuzhou, China	Country: China Institution: Fujian Agr & Forestry Univ	Country: China Institution: Fujian Agr & Forestry Univ

Record 258 | WOS:000530248700129

Protective Effect of Astaxanthin on Ochratoxin A-Induced Kidney Injury to Mice by Regulating Oxidative Stress-Related NRF2/KEAP1 Pathway

Field	Position	Original line	Normalised line	Original value	Normalised value	Original country / institution	Normalised country / institution
C1	1	40771	40771	[Li, Lin; Chen, Yueli; Jiao, Danyang; Yang, Shuhua; Li, Lin; Li, Peng] Shenyang Agr Univ, Coll Anim Sci & Vet Med, Key Lab Zoonosis Liaoning Prov, Shenyang 110866, Peoples R China.	[Li, Lin; Chen, Yueli; Jiao, Danyang; Yang, Shuhua; Li, Lin; Li, Peng] Shenyang Agricultural University, Coll Anim Sci & Vet Med, Key Lab Zoonosis Liaoning Prov, Shenyang 110866, China.	Country: China Institution: Shenyang Agr Univ	Country: China Institution: Shenyang Agricultural University
RP	3	40773	40773	Li, P (corresponding author), Shenyang Agr Univ, Coll Anim Sci & Vet Med, Key Lab Zoonosis Liaoning Prov, Shenyang 110866, Peoples R China	Li, P (corresponding author), Shenyang Agricultural University, Coll Anim Sci & Vet Med, Key Lab Zoonosis Liaoning Prov, Shenyang 110866, China	Country: China Institution: Shenyang Agr Univ	Country: China Institution: Shenyang Agricultural University

Record 259 | WOS:000517659300003

Toxicity of mycotoxins in vivo on vertebrate organisms: A review

Field	Position	Original line	Normalised line	Original value	Normalised value	Original country / institution	Normalised country / institution
C1	1	40888	40888	[Cimbalo, A.; Alonso-Garrido, M.; Font, G.; Manyes, L.] Univ Valencia, Fac Pharm, Lab Food Chem & Toxicol, Avinguda Vicent Andres Estelles S-N, E-46100 Burjassot, Spain.	[Cimbalo, A.; Alonso-Garrido, M.; Font, G.; Manyes, L.] University of Valencia, Fac Pharm, Lab Food Chem & Toxicol, Avinguda Vicent Andres Estelles S-N, E-46100 Burjassot, Spain.	Country: Spain Institution: Univ Valencia	Country: Spain Institution: University of Valencia

RP	1	40890	40890	Cimbalo, A (corresponding author), Univ Valencia, Fac Pharm, Lab Food Chem & Toxicol, Avinguda Vicent Andres Estelles S-N, E-46100 Burjassot, Spain	Cimbalo, A (corresponding author), University of Valencia, Fac Pharm, Lab Food Chem & Toxicol, Avinguda Vicent Andres Estelles S-N, E-46100 Burjassot, Spain	Country: Spain<br>Institution: Univ Valencia	Country: Spain<br>Institution: University of Valencia

Record 260 | WOS:000526529800024

Influences of Electron Beam Irradiation on the Physical and Chemical Properties of Zearalenone- and Ochratoxin A-Contaminated Corn and In Vivo Toxicity Assessment

Field	Position	Original line	Normalised line	Original value	Normalised value	Original country / institution	Normalised country / institution
C1	1	41137	41137	[Luo, Xiaohu; Wang, Jing] BTBU, Beijing Adv Innovat Ctr Food Nutr & Human Hlth, Beijing 100048, Peoples R China.	[Luo, Xiaohu; Wang, Jing] BTBU, Beijing Adv Innovat Ctr Food Nutr & Human Hlth, Beijing 100048, China.	Country: China<br>Institution: BTBU	Country: China<br>Institution: BTBU
C1	2	41138	41138	[Luo, Xiaohu; Zhai, Yuheng; Qi, Lijun; Pan, Lihong; Wang, Ren; Wang, Li; Yang, Kai; Chen, Zhengxing] Jiangnan Univ, Natl Engn Lab Cereal Fermentat Technol, Wuxi 214122, Jiangsu, Peoples R China.	[Luo, Xiaohu; Zhai, Yuheng; Qi, Lijun; Pan, Lihong; Wang, Ren; Wang, Li; Yang, Kai; Chen, Zhengxing] Jiangnan University, Natl Engn Lab Cereal Fermentat Technol, Wuxi 214122, Jiangsu, China.	Country: China<br>Institution: Jiangnan Univ	Country: China<br>Institution: Jiangnan University
C1	3	41139	41139	[Luo, Xiaohu; Zhang, Qingchuan] Beijing Technol & Business Univ, Natl Engn Lab Agri Prod Qual Traceabil, Beijing 100048, Peoples R China.	[Luo, Xiaohu; Zhang, Qingchuan] Beijing Technology & Business University, Natl Engn Lab Agri Prod Qual Traceabil, Beijing 100048, China.	Country: China<br>Institution: Beijing Technol & Business Univ	Country: China<br>Institution: Beijing Technology & Business University
C1	4	41140	41140	[Xing, Jiali] Ningbo Inst Food Control, Ningbo 315048, Peoples R China.	[Xing, Jiali] Ningbo Inst Food Control, Ningbo 315048, China.	Country: China<br>Institution: Ningbo Inst Food Control	Country: China<br>Institution: Ningbo Inst Food Control
RP	3	41143	41143	Luo, XH (corresponding author), Beijing Technol & Business Univ, Natl Engn Lab Agri Prod Qual Traceabil, Beijing 100048, Peoples R China	Luo, XH (corresponding author), Beijing Technol & Business Univ, Natl Engn Lab Agri Prod Qual Traceabil, Beijing 100048, China	Country: China<br>Institution: Beijing Technol & Business Univ	Country: China<br>Institution: Beijing Technol & Business Univ

Record 261 | WOS:000525009500057

In-Vitro Cell Culture for Efficient Assessment of Mycotoxin Exposure, Toxicity and Risk Mitigation

Field	Position	Original line	Normalised line	Original value	Normalised value	Original country / institution	Normalised country / institution
C1	1	41258	41258	[Xu, Ran; Karrow, Niel A.; Shandilya, Umesh K.] Univ Guelph, Dept Anim Biosci, Guelph, ON N1G 2W1, Canada.	[Xu, Ran; Karrow, Niel A.; Shandilya, Umesh K.] University of Guelph, Dept Anim Biosci, Guelph, ON N1G 2W1, Canada.	Country: Canada<br>Institution: Univ Guelph	Country: Canada<br>Institution: University of Guelph
C1	2	41259	41259	[Sun, Lv-hui] Huazhong Agr Univ, Coll Anim Sci & Technol, Dept Anim Nutr & Feed Sci, Wuhan 430070, Peoples R China.	[Sun, Lv-hui] Huazhong Agr Univ, Coll Anim Sci & Technol, Dept Anim Nutr & Feed Sci, Wuhan 430070, China.	Country: China<br>Institution: Huazhong Agr Univ	Country: China<br>Institution: Huazhong Agr Univ
C1	3	41260	41260	[Kitazawa, Haruki] Tohoku Univ, Food & Feed Immunol Grp, Lab Anim Prod Chem, Grad Sch Agr Sci, Sendai, Miyagi 9808572, Japan.	[Kitazawa, Haruki] Tohoku University, Food & Feed Immunol Grp, Lab Anim Prod Chem, Grad Sch Agr Sci, Sendai, Miyagi 9808572, Japan.	Country: Japan<br>Institution: Tohoku Univ	Country: Japan<br>Institution: Tohoku University
C1	4	41261	41261	[Kitazawa, Haruki] Tohoku Univ, Grad Sch Agr Sci,	[Kitazawa, Haruki] Tohoku University, Grad Sch Agr Sci,	Country: Japan<br>Institution: Tohoku Univ	Country: Japan<br>Institution: Tohoku University

				Livestock Immunol Unit, Int Educ & Res Ctr Food & Agr Immunol CFAI, Sendai, Miyagi 9808572, Japan.	Livestock Immunol Unit, Int Educ & Res Ctr Food & Agr Immunol CFAI, Sendai, Miyagi 9808572, Japan.		
RP	1	41264	41264	Karrow, NA (corresponding author), Univ Guelph, Dept Anim Biosci, Guelph, ON N1G 2W1, Canada	Karrow, NA (corresponding author), University of Guelph, Dept Anim Biosci, Guelph, ON N1G 2W1, Canada	Country: Canada<br>Institution: Univ Guelph	Country: Canada<br>Institution: University of Guelph

Record 262 | WOS:000543783800001

Nontoxic concentration of ochratoxin A decreases the dosage of cyclosporine A to induce chronic nephropathy model via autophagy mediated by toll-like receptor 4

Field	Position	Original line	Normalised line	Original value	Normalised value	Original country / institution	Normalised country / institution
C1	1	41599	41599	[Hou, Lili; Le, Guannan; Lin, Ziman; Qian, Gang; Gan, Fang; Gu, Cong; Jiang, Shuai; Mu, Jiaxin; Ge, Lei; Huang, Kehe] Nanjing Agr Univ, Coll Vet Med, Nanjing 210095, Jiangsu, Peoples R China.	[Hou, Lili; Le, Guannan; Lin, Ziman; Qian, Gang; Gan, Fang; Gu, Cong; Jiang, Shuai; Mu, Jiaxin; Ge, Lei; Huang, Kehe] Nanjing Agricultural University, Coll Vet Med, Nanjing 210095, Jiangsu, China.	Country: China<br>Institution: Nanjing Agr Univ	Country: China<br>Institution: Nanjing Agricultural University
C1	2	41600	41600	[Hou, Lili; Le, Guannan; Lin, Ziman; Qian, Gang; Gan, Fang; Gu, Cong; Jiang, Shuai; Mu, Jiaxin; Ge, Lei; Huang, Kehe] Nanjing Agr Univ, Inst Nutr & Metab Disorders Domest Anim & Fowls, Nanjing 210095, Jiangsu, Peoples R China.	[Hou, Lili; Le, Guannan; Lin, Ziman; Qian, Gang; Gan, Fang; Gu, Cong; Jiang, Shuai; Mu, Jiaxin; Ge, Lei; Huang, Kehe] Nanjing Agricultural University, Inst Nutr & Metab Disorders Domest Anim & Fowls, Nanjing 210095, Jiangsu, China.	Country: China<br>Institution: Nanjing Agr Univ	Country: China<br>Institution: Nanjing Agricultural University
C1	3	41601	41601	[Hou, Lili; Le, Guannan; Lin, Ziman; Qian, Gang; Gan, Fang; Gu, Cong; Jiang, Shuai; Mu, Jiaxin; Ge, Lei; Huang, Kehe] Nanjing Agr Univ, Coll Vet Med, MOE Joint Int Res Lab Anim Hlth & Food Safety, Nanjing 210095, Jiangsu, Peoples R China.	[Hou, Lili; Le, Guannan; Lin, Ziman; Qian, Gang; Gan, Fang; Gu, Cong; Jiang, Shuai; Mu, Jiaxin; Ge, Lei; Huang, Kehe] Nanjing Agricultural University, Coll Vet Med, MOE Joint Int Res Lab Anim Hlth & Food Safety, Nanjing 210095, Jiangsu, China.	Country: China<br>Institution: Nanjing Agr Univ	Country: China<br>Institution: Nanjing Agricultural University
RP	1	41604	41604	Huang, KH (corresponding author), Nanjing Agr Univ, Coll Vet Med, Nanjing 210095, Jiangsu, Peoples R China	Huang, KH (corresponding author), Nanjing Agricultural University, Coll Vet Med, Nanjing 210095, Jiangsu, Peoples R China	Country: China<br>Institution: Nanjing Agr Univ	Country: China<br>Institution: Nanjing Agricultural University
RP	2	41604	41604	Huang, KH (corresponding author), Nanjing Agr Univ, Inst Nutr & Metab Disorders Domest Anim & Fowls, Nanjing 210095, Jiangsu, Peoples R China	Huang, KH (corresponding author), Nanjing Agr Univ, Inst Nutr & Metab Disorders Domest Anim & Fowls, Nanjing 210095, Jiangsu, China	Country: China<br>Institution: Nanjing Agr Univ	Country: China<br>Institution: Nanjing Agr Univ

Record 263 | WOS:000512092100007

Ochratoxin A induces global DNA hypomethylation and oxidative stress in neuronal cells in vitro

Field	Position	Original line	Normalised line	Original value	Normalised value	Original country / institution	Normalised country / institution
C1	1	41706	41706	[Babayan, Nelly; Tadevosyan, Gohar; Khondkaryan, Lusine; Grigoryan, Ruzanna; Sarkisyan, Natalya] Natl Acad Sci	[Babayan, Nelly; Tadevosyan, Gohar; Khondkaryan, Lusine; Grigoryan, Ruzanna; Sarkisyan, Natalya] National Academy of	Country: Armenia<br>Institution: Natl Acad Sci Armenia	Country: Armenia<br>Institution: National Academy of Sciences of Armenia

				Armenia, Inst Mol Biol, Hasratyan 7, Yerevan 0014, Armenia.	Sciences of Armenia, Inst Mol Biol, Hasratyan 7, Yerevan 0014, Armenia.		
C1	2	41707	41707	[Babayan, Nelly; Haroutiounian, Rouben] Yerevan State Univ, A Manoogian 1, Yerevan 0025, Armenia.	[Babayan, Nelly; Haroutiounian, Rouben] Yerevan State University, A Manoogian 1, Yerevan 0025, Armenia.	Country: Armenia<br>Institution: Yerevan State Univ	Country: Armenia<br>Institution: Yerevan State University
C1	3	41708	41708	[Stopper, Helga] Univ Wurzburg, Inst Pharmacol & Toxicol, Versbacher 9, D-997078 Wurzburg, Germany.	[Stopper, Helga] University of Wurzburg, Inst Pharmacol & Toxicol, Versbacher 9, D-997078 Wurzburg, Germany.	Country: Germany<br>Institution: Univ Wurzburg	Country: Germany<br>Institution: University of Wurzburg
RP	1	41711	41711	Babayan, N (corresponding author), Natl Acad Sci Armenia, Inst Mol Biol, Hasratyan 7, Yerevan 0014, Armenia	Babayan, N (corresponding author), National Academy of Sciences of Armenia, Inst Mol Biol, Hasratyan 7, Yerevan 0014, Armenia	Country: Armenia<br>Institution: Natl Acad Sci Armenia	Country: Armenia<br>Institution: National Academy of Sciences of Armenia

Record 264 | WOS:000506899900001

Toxicity effects of mycotoxins and autophagy: a mechanistic view

Field	Position	Original line	Normalised line	Original value	Normalised value	Original country / institution	Normalised country / institution
C1	1	41805	41805	[Ariafar, Saba; Oftadeh Harsin, Akram; Fadaiie, Ahmad; Mohammadi, Mojdeh] Hamadan Univ Med Sci, Sch Pharm, Dept Pharmacol & Toxicol, Hamadan, Iran.	[Ariafar, Saba; Oftadeh Harsin, Akram; Fadaiie, Ahmad; Mohammadi, Mojdeh] Hamadan University of Medical Sciences, Sch Pharm, Dept Pharmacol & Toxicol, Hamadan, Iran.	Country: Iran<br>Institution: Hamadan Univ Med Sci	Country: Iran<br>Institution: Hamadan University of Medical Sciences
C1	2	41806	41806	[Mahboobian, Mohammad Mehdi] Hamadan Univ Med Sci, Sch Pharm, Dept Pharmaceut, Hamadan, Iran.	[Mahboobian, Mohammad Mehdi] Hamadan University of Medical Sciences, Sch Pharm, Dept Pharmaceut, Hamadan, Iran.	Country: Iran<br>Institution: Hamadan Univ Med Sci	Country: Iran<br>Institution: Hamadan University of Medical Sciences
RP	1	41809	41809	Mohammadi, M (corresponding author), Hamadan Univ Med Sci, Sch Pharm, Dept Pharmacol & Toxicol, Hamadan, Iran	Mohammadi, M (corresponding author), Hamadan University of Medical Sciences, Sch Pharm, Dept Pharmacol & Toxicol, Hamadan, Iran	Country: Iran<br>Institution: Hamadan Univ Med Sci	Country: Iran<br>Institution: Hamadan University of Medical Sciences

Record 265 | WOS:000505295600001

Red orange and lemon extract prevents the renal toxicity induced by ochratoxin A in rats

Field	Position	Original line	Normalised line	Original value	Normalised value	Original country / institution	Normalised country / institution
C1	1	41966	41966	[Damiano, Sara; Squillacioti, Caterina; Mirabella, Nicola; Prisco, Francesco; Ariano, Andrea; Florio, Salvatore; Ciarcia, Roberto] Univ Naples Federico II, Dept Vet Med & Anim Prod, Naples, Italy.	[Damiano, Sara; Squillacioti, Caterina; Mirabella, Nicola; Prisco, Francesco; Ariano, Andrea; Florio, Salvatore; Ciarcia, Roberto] University of Naples Federico II, Dept Vet Med & Anim Prod, Naples, Italy.	Country: Italy<br>Institution: Univ Naples Federico II	Country: Italy<br>Institution: University of Naples Federico II
C1	2	41967	41967	[Iovane, Valentina] Univ Salerno, Dept Pharm, Fisciano, Italy.	[Iovane, Valentina] University of Salerno, Dept Pharm, Fisciano, Italy.	Country: Italy<br>Institution: Univ Salerno	Country: Italy<br>Institution: University of Salerno
C1	4	41969	41969	[Giordano, Antonio] Univ Siena, Dept Med Biotechnol, Siena, Italy.	[Giordano, Antonio] University of Siena, Dept Med Biotechnol, Siena, Italy.	Country: Italy<br>Institution: Univ Siena	Country: Italy<br>Institution: University of Siena

C1	5	41970	41970	[Giordano, Antonio] Temple Univ, Coll Sci & Technol, Ctr Biotechnol, Sbarro Inst Canc Res & Mol Med, Philadelphia, PA 19122 USA.	[Giordano, Antonio] Temple University, Coll Sci & Technol, Ctr Biotechnol, Sbarro Inst Canc Res & Mol Med, Philadelphia, PA 19122 USA.	Country: USA<br>Institution: Temple Univ	Country: USA<br>Institution: Temple University
RP	1	41975	41975	Ciarcia, R (corresponding author), Univ Naples Federico II, Dept Vet Med & Anim Prod, Via Delpino 1, I-80137 Naples, Italy	Ciarcia, R (corresponding author), University of Naples Federico II, Dept Vet Med & Anim Prod, Via Delpino 1, I-80137 Naples, Italy	Country: Italy<br>Institution: Univ Naples Federico II	Country: Italy<br>Institution: University of Naples Federico II

Record 266 | WOS:000488786200007

Mycotoxin-induced toxicities and diseases

Field	Position	Original line	Normalised line	Original value	Normalised value	Original country / institution	Normalised country / institution
C1	3	42083	42083	[Gacem, Hiba] Univ Djillali Liabes Sidi Bel Abbes, Hassani Abdelkader Univ Hosp Ctr, Fac Med, Epidemiol Serv, Sidi Bel Abbes, Algeria.	[Gacem, Hiba] University Djillali Liabes Sidi Bel Abbes, Hassani Abdelkader Univ Hosp Ctr, Fac Med, Epidemiol Serv, Sidi Bel Abbes, Algeria.	Country: Algeria<br>Institution: Univ Djillali Liabes Sidi Bel Abbes	Country: Algeria<br>Institution: University Djillali Liabes Sidi Bel Abbes
C1	4	42084	42084	[Gacem, Hiba] Univ Djillali Liabes Sidi Bel Abbes, Hassani Abdelkader Univ Hosp Ctr, Fac Med, Prevent Med, Sidi Bel Abbes, Algeria.	[Gacem, Hiba] University Djillali Liabes Sidi Bel Abbes, Hassani Abdelkader Univ Hosp Ctr, Fac Med, Prevent Med, Sidi Bel Abbes, Algeria.	Country: Algeria<br>Institution: Univ Djillali Liabes Sidi Bel Abbes	Country: Algeria<br>Institution: University Djillali Liabes Sidi Bel Abbes

Record 267 | WOS:000680216400001

Mycotoxins Occurrence, Toxicity and Detection Methods

Field	Position	Original line	Normalised line	Original value	Normalised value	Original country / institution	Normalised country / institution
C1	3	42375	42375	[Gacem, Hiba] Univ Djillali Liabes Sidi Bel Abbes, Fac Med, Epidemiol Serv & Prevent Med, Sidi Bel Abbes, Algeria.	[Gacem, Hiba] University Djillali Liabes Sidi Bel Abbes, Fac Med, Epidemiol Serv & Prevent Med, Sidi Bel Abbes, Algeria.	Country: Algeria<br>Institution: Univ Djillali Liabes Sidi Bel Abbes	Country: Algeria<br>Institution: University Djillali Liabes Sidi Bel Abbes

Record 268 | WOS:000504757200032

Melatonin ameliorates ochratoxin A-induced oxidative stress and apoptosis in porcine oocytes

Field	Position	Original line	Normalised line	Original value	Normalised value	Original country / institution	Normalised country / institution
C1	1	42734	42734	[Lan, Mei; Zhang, Yu; Wan, Xiang; Pan, Meng-Hao; Xu, Yao; Sun, Shao-Chen] Nanjing Agr Univ, Coll Anim Sci & Technol, Nanjing 210095, Jiangsu, Peoples R China.	[Lan, Mei; Zhang, Yu; Wan, Xiang; Pan, Meng-Hao; Xu, Yao; Sun, Shao-Chen] Nanjing Agricultural University, Coll Anim Sci & Technol, Nanjing 210095, Jiangsu, China.	Country: China<br>Institution: Nanjing Agr Univ	Country: China<br>Institution: Nanjing Agricultural University
RP	1	42736	42736	Sun, SC (corresponding author), Nanjing Agr Univ, Coll Anim Sci & Technol, Nanjing 210095, Jiangsu, Peoples R China	Sun, SC (corresponding author), Nanjing Agricultural University, Coll Anim Sci & Technol, Nanjing 210095, Jiangsu, China	Country: China<br>Institution: Nanjing Agr Univ	Country: China<br>Institution: Nanjing Agricultural University

Record 269 | WOS:000514650700067

Modelling Fungal Growth, Mycotoxin Production and Release in Grana Cheese

No C1/C3/RP normalisation changes detected for this record.

Record 270 | WOS:000545943400001

Penicillium spp. mycotoxins found in food and feed and their health effects

Field	Position	Original line	Normalised line	Original value	Normalised value	Original country / institution	Normalised country / institution
C1	1	42936	42936	[Otero, C.] Univ Andres Bello, Fac Med, Escuela Quim & Farm, Republ 252, Santiago, Chile.	[Otero, C.] Universidad Andres Bello, Fac Med, Escuela Quim & Farm, Republ 252, Santiago, Chile.	Country: Chile Institution: Univ Andres Bello	Country: Chile Institution: Universidad Andres Bello
C1	2	42937	42937	[Arredondo, C.] Univ Andres Bello, Fac Med, Lab Neuroepigenet, Inst Ciencias Biomed ICB, Republ 330, Santiago, Chile.	[Arredondo, C.] Universidad Andres Bello, Fac Med, Lab Neuroepigenet, Inst Ciencias Biomed ICB, Republ 330, Santiago, Chile.	Country: Chile Institution: Univ Andres Bello	Country: Chile Institution: Universidad Andres Bello
C1	3	42938	42938	[Arredondo, C.] Univ Andres Bello, Fac Ciencias Vida, Republ 330, Santiago, Chile.	[Arredondo, C.] Universidad Andres Bello, Fac Ciencias Vida, Republ 330, Santiago, Chile.	Country: Chile Institution: Univ Andres Bello	Country: Chile Institution: Universidad Andres Bello
C1	4	42939	42939	[Echeverria-Vega, A.] Univ Catolica Maule, Ctr Invest Estudios Avanzados Maule CIEAM, Vicerrectoria Invest & Postgrad, Talca, Chile.	[Echeverria-Vega, A.] Universidad Catolica del Maule, Ctr Invest Estudios Avanzados Maule CIEAM, Vicerrectoria Invest & Postgrad, Talca, Chile.	Country: Chile Institution: Univ Catolica Maule	Country: Chile Institution: Universidad Catolica del Maule
C1	5	42940	42940	[Gordillo-Fuenzalida, F.] Univ Catolica Maule, Fac Ciencias Agr & Forestales, Ctr Biotecnol Recursos Nat CENBIO, Lab Microbiol Aplicada, Avda San Miguel 3605, Talca, Chile.	[Gordillo-Fuenzalida, F.] Universidad Catolica del Maule, Fac Ciencias Agr & Forestales, Ctr Biotecnol Recursos Nat CENBIO, Lab Microbiol Aplicada, Avda San Miguel 3605, Talca, Chile.	Country: Chile Institution: Univ Catolica Maule	Country: Chile Institution: Universidad Catolica del Maule
RP	1	42943	42943	Gordillo-Fuenzalida, F (corresponding author), Univ Catolica Maule, Fac Ciencias Agr & Forestales, Ctr Biotecnol Recursos Nat CENBIO, Lab Microbiol Aplicada, Avda San Miguel 3605, Talca, Chile	Gordillo-Fuenzalida, F (corresponding author), Universidad Catolica del Maule, Fac Ciencias Agr & Forestales, Ctr Biotecnol Recursos Nat CENBIO, Lab Microbiol Aplicada, Avda San Miguel 3605, Talca, Chile	Country: Chile Institution: Univ Catolica Maule	Country: Chile Institution: Universidad Catolica del Maule

Record 271 | WOS:000514630000034

Comprehensive Evaluation of the Efficiency of Yeast Cell Wall Extract to Adsorb Ochratoxin A and Mitigate Accumulation of the Toxin in Broiler Chickens

No C1/C3/RP normalisation changes detected for this record.

Record 272 | WOS:000514630000026

Transcriptome Analysis of Ochratoxin A-Induced Apoptosis in Differentiated Caco-2 Cells

Field	Position	Original line	Normalised line	Original value	Normalised value	Original country / institution	Normalised country / institution

C1	1	43272	43272	[Yang, Xue; Gao, Yanan; Yan, Qiaoyan; Bao, Xiaoyu; Zhao, Shengguo; Wang, Jiaqi; Zheng, Nan] Chinese Acad Agr Sci, Inst Anim Sci, Key Lab Qual & Safety Control Milk & Dairy Prod, Minist Agr & Rural Affairs, Beijing 100193, Peoples R China.	[Yang, Xue; Gao, Yanan; Yan, Qiaoyan; Bao, Xiaoyu; Zhao, Shengguo; Wang, Jiaqi; Zheng, Nan] Chinese Academy of Agricultural Sciences, Inst Anim Sci, Key Lab Qual & Safety Control Milk & Dairy Prod, Minist Agr & Rural Affairs, Beijing 100193, China.	Country: China<br>Institution: Chinese Acad Agr Sci	Country: China<br>Institution: Chinese Academy of Agricultural Sciences
C1	2	43273	43273	[Yang, Xue; Gao, Yanan; Yan, Qiaoyan; Bao, Xiaoyu; Zhao, Shengguo; Wang, Jiaqi; Zheng, Nan] Chinese Acad Agr Sci, Inst Anim Sci, Lab Qual & Safety Risk Assessment Dairy Prod, Minist Agr & Rural Affairs, Beijing 100193, Peoples R China.	[Yang, Xue; Gao, Yanan; Yan, Qiaoyan; Bao, Xiaoyu; Zhao, Shengguo; Wang, Jiaqi; Zheng, Nan] Chinese Academy of Agricultural Sciences, Inst Anim Sci, Lab Qual & Safety Risk Assessment Dairy Prod, Minist Agr & Rural Affairs, Beijing 100193, China.	Country: China<br>Institution: Chinese Acad Agr Sci	Country: China<br>Institution: Chinese Academy of Agricultural Sciences
C1	3	43274	43274	[Yang, Xue; Gao, Yanan; Yan, Qiaoyan; Bao, Xiaoyu; Zhao, Shengguo; Wang, Jiaqi; Zheng, Nan] Chinese Acad Agr Sci, Milk & Dairy Prod Inspect Ctr, Inst Anim Sci, Minist Agr & Rural Affairs, Beijing 100193, Peoples R China.	[Yang, Xue; Gao, Yanan; Yan, Qiaoyan; Bao, Xiaoyu; Zhao, Shengguo; Wang, Jiaqi; Zheng, Nan] Chinese Academy of Agricultural Sciences, Milk & Dairy Prod Inspect Ctr, Inst Anim Sci, Minist Agr & Rural Affairs, Beijing 100193, China.	Country: China<br>Institution: Chinese Acad Agr Sci	Country: China<br>Institution: Chinese Academy of Agricultural Sciences
C1	4	43275	43275	[Yang, Xue; Gao, Yanan; Yan, Qiaoyan; Bao, Xiaoyu; Zhao, Shengguo; Wang, Jiaqi; Zheng, Nan] Chinese Acad Agr Sci, Inst Anim Sci, State Key Lab Anim Nutr, Beijing 100193, Peoples R China.	[Yang, Xue; Gao, Yanan; Yan, Qiaoyan; Bao, Xiaoyu; Zhao, Shengguo; Wang, Jiaqi; Zheng, Nan] Chinese Academy of Agricultural Sciences, Inst Anim Sci, State Key Lab Anim Nutr, Beijing 100193, China.	Country: China<br>Institution: Chinese Acad Agr Sci	Country: China<br>Institution: Chinese Academy of Agricultural Sciences
RP	1	43281	43281	Zheng, N (corresponding author), Chinese Acad Agr Sci, Inst Anim Sci, Key Lab Qual & Safety Control Milk & Dairy Prod, Minist Agr & Rural Affairs, Beijing 100193, Peoples R China	Zheng, N (corresponding author), Chinese Academy of Agricultural Sciences, Inst Anim Sci, Key Lab Qual & Safety Control Milk & Dairy Prod, Minist Agr & Rural Affairs, Beijing 100193, Peoples R China	Country: China<br>Institution: Chinese Acad Agr Sci	Country: China<br>Institution: Chinese Academy of Agricultural Sciences
RP	4	43281	43281	Zheng, N (corresponding author), Chinese Acad Agr Sci, Inst Anim Sci, State Key Lab Anim Nutr, Beijing 100193, Peoples R China	Zheng, N (corresponding author), Chinese Acad Agr Sci, Inst Anim Sci, State Key Lab Anim Nutr, Beijing 100193, China	Country: China<br>Institution: Chinese Acad Agr Sci	Country: China<br>Institution: Chinese Acad Agr Sci

Record 273 | WOS:000498748700011

Epithelial-fibroblast cross talk aggravates the impact of the nephrotoxin ochratoxin A

Field	Position	Original line	Normalised line	Original value	Normalised value	Original country / institution	Normalised country / institution
C1	1	43433	43433	[Schulz, Marie-Christin; Gekle, Michael; Schwerdt, Gerald] Martin Luther Univ Halle Wittenberg, Julius Bernstein Inst Physiol, Magdeburger Str 6, D-06112 Halle, Saale, Germany.	[Schulz, Marie-Christin; Gekle, Michael; Schwerdt, Gerald] Martin Luther University Halle Wittenberg, Julius Bernstein Inst Physiol, Magdeburger Str 6, D-06112 Halle, Saale, Germany.	Country: Germany<br>Institution: Martin Luther Univ Halle Wittenberg	Country: Germany<br>Institution: Martin Luther University Halle Wittenberg
RP	1	43435	43435	Schulz, MC (corresponding author), Martin Luther Univ Halle Wittenberg, Julius Bernstein Inst Physiol,	Schulz, MC (corresponding author), Martin Luther University Halle Wittenberg, Julius Bernstein Inst Physiol,	Country: Germany<br>Institution: Martin Luther Univ Halle Wittenberg	Country: Germany<br>Institution: Martin Luther University Halle Wittenberg

				Magdeburger Str 6, D-06112 Halle, Saale, Germany	Magdeburger Str 6, D-06112 Halle, Saale, Germany		

Record 274 | WOS:000498194100002

Capsaicin, an inhibitor of Ochratoxin A production by Aspergillus section Nigri strains in grapes (Vitis vinifera L.)

No C1/C3/RP normalisation changes detected for this record.

Record 275 | WOS:000501604700005

Ochratoxin A Sequentially Activates Autophagy and the Ubiquitin-Proteasome System

Field	Position	Original line	Normalised line	Original value	Normalised value	Original country / institution	Normalised country / institution
C1	1	43658	43658	[Akpinar, Hafize Aysin; Kahraman, Hilal; Yaman, Ibrahim] Bogazici Univ, Dept Mol Biol & Genet, Mol Toxicol & Canc Res Lab, TR-34342 Bebek, Turkey.	[Akpinar, Hafize Aysin; Kahraman, Hilal; Yaman, Ibrahim] Bogazici University, Dept Mol Biol & Genet, Mol Toxicol & Canc Res Lab, TR-34342 Bebek, Turkey.	Country: Turkey Institution: Bogazici Univ	Country: Turkey Institution: Bogazici University
C1	2	43659	43659	[Yaman, Ibrahim] Bogazici Univ, Ctr Life Sci & Technol, TR-34342 Bebek, Turkey.	[Yaman, Ibrahim] Bogazici University, Ctr Life Sci & Technol, TR-34342 Bebek, Turkey.	Country: Turkey Institution: Bogazici Univ	Country: Turkey Institution: Bogazici University
RP	1	43661	43661	Yaman, I (corresponding author), Bogazici Univ, Dept Mol Biol & Genet, Mol Toxicol & Canc Res Lab, TR-34342 Bebek, Turkey	Yaman, I (corresponding author), Bogazici University, Dept Mol Biol & Genet, Mol Toxicol & Canc Res Lab, TR-34342 Bebek, Turkey	Country: Turkey Institution: Bogazici Univ	Country: Turkey Institution: Bogazici University

Record 276 | WOS:000501604700006

Comparative In Vitro Assessment of a Range of Commercial Feed Additives with Multiple Mycotoxin Binding Claims

Field	Position	Original line	Normalised line	Original value	Normalised value	Original country / institution	Normalised country / institution
C1	1	43789	43789	[Kolawole, Oluwatobi; Meneely, Julie; Greer, Brett; Chevallier, Olivier; Connolly, Lisa; Elliott, Christopher] Queens Univ Belfast, Inst Global Food Secur, Belfast BT9 SDL, Antrim, North Ireland.	[Kolawole, Oluwatobi; Meneely, Julie; Greer, Brett; Chevallier, Olivier; Connolly, Lisa; Elliott, Christopher] Queens University Belfast, Inst Global Food Secur, Belfast BT9 SDL, Antrim, United Kingdom.	Country: North Ireland Institution: Queens Univ Belfast	Country: United Kingdom Institution: Queens University Belfast
C1	2	43790	43790	[Jones, David S.] Queens Univ Belfast, Sch Pharm, Belfast BT9 7BL, Antrim, North Ireland.	[Jones, David S.] Queens University Belfast, Sch Pharm, Belfast BT9 7BL, Antrim, United Kingdom.	Country: North Ireland Institution: Queens Univ Belfast	Country: United Kingdom Institution: Queens University Belfast
RP	1	43792	43792	Elliott, C (corresponding author), Queens Univ Belfast, Inst Global Food Secur, Belfast BT9 SDL, Antrim, North Ireland	Elliott, C (corresponding author), Queens University Belfast, Inst Global Food Secur, Belfast BT9 SDL, Antrim, United Kingdom	Country: North Ireland Institution: Queens Univ Belfast	Country: United Kingdom Institution: Queens University Belfast

Record 277 | WOS:000477691300004

Reduction of ochratoxin A in oats and rice by twin-screw extrusion processing with baking soda

Field	Position	Original line	Normalised line	Original value	Normalised value	Original country / institution	Normalised country / institution
C1	1	43907	43907	[Ryu, Dojin; Lee, Hyun Jung] Univ Idaho, Sch Food Sci, 875 Perimeter Dr,MS 2312, Moscow, ID 83844 USA.	[Ryu, Dojin; Lee, Hyun Jung] University of Idaho, Sch Food Sci, 875 Perimeter Dr,MS 2312, Moscow, ID 83844 USA.	Country: USA<br>Institution: Univ Idaho	Country: USA<br>Institution: University of Idaho
C1	2	43908	43908	[Kowalski, Ryan Joseph; Ganjyal, Girish] Washington State Univ, Sch Food Sci, POB 646376, Pullman, WA 99164 USA.	[Kowalski, Ryan Joseph; Ganjyal, Girish] Washington State University, Sch Food Sci, POB 646376, Pullman, WA 99164 USA.	Country: USA<br>Institution: Washington State Univ	Country: USA<br>Institution: Washington State University
RP	1	43910	43910	Lee, HJ (corresponding author), Univ Idaho, Sch Food Sci, 875 Perimeter Dr,MS 2312, Moscow, ID 83844 USA	Lee, HJ (corresponding author), University of Idaho, Sch Food Sci, 875 Perimeter Dr,MS 2312, Moscow, ID 83844 USA	Country: USA<br>Institution: Univ Idaho	Country: USA<br>Institution: University of Idaho

Record 278 | WOS:000475915000013

A multi-endpoint approach to the combined toxic effects of patulin and ochratoxin a in human intestinal cells

Field	Position	Original line	Normalised line	Original value	Normalised value	Original country / institution	Normalised country / institution
C1	2	44020	44020	[Assuncao, Ricardo] Univ Evora, IIFA, Apartado 94, P-7002554 Evora, Portugal.	[Assuncao, Ricardo] University of Evora, IIFA, Apartado 94, P-7002554 Evora, Portugal.	Country: Portugal<br>Institution: Univ Evora	Country: Portugal<br>Institution: University of Evora
C1	6	44024	44024	[Silva, Maria Joao] Univ Nova Lisboa, FCM, Nova Med Sch, Ctr Toxicogen & Human Hlth ToxOm, Lisbon, Portugal.	[Silva, Maria Joao] Universidade Nova de Lisboa, FCM, Nova Med Sch, Ctr Toxicogen & Human Hlth ToxOm, Lisbon, Portugal.	Country: Portugal<br>Institution: Univ Nova Lisboa	Country: Portugal<br>Institution: Universidade Nova de Lisboa

Record 279 | WOS:000484647100041

Impact of dietary Trichosporon mycotoxinivorans on ochratoxin A induced immunotoxicity; In vivo study

Field	Position	Original line	Normalised line	Original value	Normalised value	Original country / institution	Normalised country / institution
C1	2	44176	44176	[Ul Hassan, Zahoor] Qatar Univ, Dept Biol & Environm Sci, Coll Arts & Sci, Doha 2713, Qatar.	[Ul Hassan, Zahoor] Qatar University, Dept Biol & Environm Sci, Coll Arts & Sci, Doha 2713, Qatar.	Country: Qatar<br>Institution: Qatar Univ	Country: Qatar<br>Institution: Qatar University
C1	3	44177	44177	[Bhatti, Sheraz Ahmed] Bahauddin Zakariya Univ, Dept Pathobiol, Fac Vet Sci, Multan, Pakistan.	[Bhatti, Sheraz Ahmed] Bahauddin Zakariya University, Dept Pathobiol, Fac Vet Sci, Multan, Pakistan.	Country: Pakistan<br>Institution: Bahauddin Zakariya Univ	Country: Pakistan<br>Institution: Bahauddin Zakariya University

Record 280 | WOS:000486105900030

In vitro effects of single and binary mixtures of regulated mycotoxins and persistent organochloride pesticides on steroid hormone production in MA-10 Leydig cell line

Field	Position	Original line	Normalised line	Original value	Normalised value	Original country / institution	Normalised country / institution
C1	1	44285	44285	[Eze, Ukpai A.; Gong, Yun Yun] Univ Leeds, Sch Food Sci & Nutr, Food Sci Bldg, Leeds LS2 9JT, W Yorkshire, England.	[Eze, Ukpai A.; Gong, Yun Yun] University of Leeds, Sch Food Sci & Nutr, Food Sci Bldg, Leeds LS2 9JT, W Yorkshire, United Kingdom.	Country: England<br>Institution: Univ Leeds	Country: United Kingdom<br>Institution: University of Leeds
C1	3	44287	44287	[Huntriss, John D.; Routledge, Michael N.] Univ Leeds, Sch Med, Leeds Inst Cardiovasc &	[Huntriss, John D.; Routledge, Michael N.] University of Leeds, Sch Med, Leeds Inst Cardiovasc	Country: England<br>Institution: Univ Leeds	Country: United Kingdom<br>Institution: University of Leeds

				Metab Med, Leeds LS2 9JT, W Yorkshire, England.	& Metab Med, Leeds LS2 9JT, W Yorkshire, United Kingdom.		
C1	4	44288	44288	[Gong, Yun Yun] Minist Hlth, China Natl Ctr Food Safety Risk Assessment, Dept Food Safety Risk Assessment, Beijing 100021, Peoples R China.	[Gong, Yun Yun] Minist Hlth, China Natl Ctr Food Safety Risk Assessment, Dept Food Safety Risk Assessment, Beijing 100021, China.	Country: China<br>Institution: Minist Hlth	Country: China<br>Institution: Minist Hlth
C1	5	44289	44289	[Eze, Ukpai A.] Coventry Univ, Fac Hlth & Life Sci, Sch Life Sci, Coventry CV1 2DS, W Midlands, England.	[Eze, Ukpai A.] Coventry University, Fac Hlth & Life Sci, Sch Life Sci, Coventry CV1 2DS, W Midlands, United Kingdom.	Country: England<br>Institution: Coventry Univ	Country: United Kingdom<br>Institution: Coventry University
RP	1	44291	44291	Routledge, MN (corresponding author), Univ Leeds, Sch Med, Leeds Inst Cardiovasc & Metab Med, Leeds LS2 9JT, W Yorkshire, England	Routledge, MN (corresponding author), University of Leeds, Sch Med, Leeds Inst Cardiovasc & Metab Med, Leeds LS2 9JT, W Yorkshire, United Kingdom	Country: England<br>Institution: Univ Leeds	Country: United Kingdom<br>Institution: University of Leeds

Record 281 | WOS:000498064500026

Detecting Neurodevelopmental Toxicity of Domoic Acid and Ochratoxin A Using Rat Fetal Neural Stem Cells

Field	Position	Original line	Normalised line	Original value	Normalised value	Original country / institution	Normalised country / institution
C1	1	44401	44401	[Gill, Santokh; Kumara, V. M. Ruvin] Hlth Canada, Regulatory Toxicol Res Div, Hlth Prod & Food Branch, Tunneys Pasture, 251 Sir Frederick Banting Driveway, Ottawa, ON K1A 0K9, Canada.	[Gill, Santokh; Kumara, V. M. Ruvin] Health Canada, Regulatory Toxicol Res Div, Hlth Prod & Food Branch, Tunneys Pasture, 251 Sir Frederick Banting Driveway, Ottawa, ON K1A 0K9, Canada.	Country: Canada<br>Institution: Hlth Canada	Country: Canada<br>Institution: Health Canada
RP	1	44403	44403	Gill, S (corresponding author), Hlth Canada, Regulatory Toxicol Res Div, Hlth Prod & Food Branch, Tunneys Pasture, 251 Sir Frederick Banting Driveway, Ottawa, ON K1A 0K9, Canada	Gill, S (corresponding author), Health Canada, Regulatory Toxicol Res Div, Hlth Prod & Food Branch, Tunneys Pasture, 251 Sir Frederick Banting Driveway, Ottawa, ON K1A 0K9, Canada	Country: Canada<br>Institution: Hlth Canada	Country: Canada<br>Institution: Health Canada

Record 282 | WOS:000487934500032

Zinc supplementation alleviates OTA-induced oxidative stress and apoptosis in MDCK cells by up-regulating metallothioneins

Field	Position	Original line	Normalised line	Original value	Normalised value	Original country / institution	Normalised country / institution
C1	1	44532	44532	[Li, Hu; Malyar, Rahmani Mohammad; Zhai, Nianhui; Wang, Hong; Liu, Kai; Liu, Dandan; Pan, Cuiling; Can, Fang; Huang, Kehe; Miao, Jinfeng; Chen, Xingxiang] Nanjing Agr Univ, Coll Vet Med, Nanjing 210095, Jiangsu, Peoples R China.	[Li, Hu; Malyar, Rahmani Mohammad; Zhai, Nianhui; Wang, Hong; Liu, Kai; Liu, Dandan; Pan, Cuiling; Can, Fang; Huang, Kehe; Miao, Jinfeng; Chen, Xingxiang] Nanjing Agricultural University, Coll Vet Med, Nanjing 210095, Jiangsu, China.	Country: China<br>Institution: Nanjing Agr Univ	Country: China<br>Institution: Nanjing Agricultural University
C1	2	44533	44533	[Li, Hu; Malyar, Rahmani Mohammad; Zhai, Nianhui; Wang, Hong; Liu, Kai; Liu, Dandan; Pan, Cuiling; Can, Fang; Huang, Kehe; Miao, Jinfeng; Chen, Xingxiang] Nanjing Agr Univ, Inst Nutr & Metab Disorders Domest Anim	[Li, Hu; Malyar, Rahmani Mohammad; Zhai, Nianhui; Wang, Hong; Liu, Kai; Liu, Dandan; Pan, Cuiling; Can, Fang; Huang, Kehe; Miao, Jinfeng; Chen, Xingxiang] Nanjing Agricultural University, Inst Nutr & Metab Disorders Domest Anim &	Country: China<br>Institution: Nanjing Agr Univ	Country: China<br>Institution: Nanjing Agricultural University

				& Fowls, Nanjing 210095, Jiangsu, Peoples R China.	Fowls, Nanjing 210095, Jiangsu, China.		
C1	3	44534	44534	[Li, Hu; Malyar, Rahmani Mohammad; Zhai, Nianhui; Wang, Hong; Liu, Kai; Liu, Dandan; Pan, Cuiling; Can, Fang; Huang, Kehe; Miao, Jinfeng; Chen, Xingxiang] Nanjing Agr Univ, Coll Vet Med, MOE Joint Int Res Lab Anim Hlth & Food Safety, Nanjing, Jiangsu, Peoples R China.	[Li, Hu; Malyar, Rahmani Mohammad; Zhai, Nianhui; Wang, Hong; Liu, Kai; Liu, Dandan; Pan, Cuiling; Can, Fang; Huang, Kehe; Miao, Jinfeng; Chen, Xingxiang] Nanjing Agricultural University, Coll Vet Med, MOE Joint Int Res Lab Anim Hlth & Food Safety, Nanjing, Jiangsu, China.	Country: China<br>Institution: Nanjing Agr Univ	Country: China<br>Institution: Nanjing Agricultural University
RP	1	44537	44537	Chen, XX (corresponding author), Nanjing Agr Univ, Coll Vet Med, Nanjing 210095, Jiangsu, Peoples R China	Chen, XX (corresponding author), Nanjing Agricultural University, Coll Vet Med, Nanjing 210095, Jiangsu, China	Country: China<br>Institution: Nanjing Agr Univ	Country: China<br>Institution: Nanjing Agricultural University

Record 283 | WOS:000505648100002

Roles of microRNAs and prospective view of competing endogenous RNAs in mycotoxicosis

Field	Position	Original line	Normalised line	Original value	Normalised value	Original country / institution	Normalised country / institution
C1	1	44651	44651	[Liao, Yuxiao; Peng, Zhao; Chen, Liangkai; Liu, Liegang; Yang, Wei] Huazhong Univ Sci & Technol, Tongji Med Coll, Hubei Key Lab Food Nutr & Safety, Dept Nutr & Food Hyg, Hongkong Rd 13, Wuhan 430030, Hubei, Peoples R China.	[Liao, Yuxiao; Peng, Zhao; Chen, Liangkai; Liu, Liegang; Yang, Wei] Huazhong University of Science & Technology, Tongji Med Coll, Hubei Key Lab Food Nutr & Safety, Dept Nutr & Food Hyg, Hongkong Rd 13, Wuhan 430030, Hubei, China.	Country: China<br>Institution: Huazhong Univ Sci & Technol	Country: China<br>Institution: Huazhong University of Science & Technology
C1	2	44652	44652	[Liao, Yuxiao; Peng, Zhao; Chen, Liangkai; Liu, Liegang; Yang, Wei] Huazhong Univ Sci & Technol, Dept Nutr & Food Hyg, Tongji Med Coll, Sch Publ Hlth, Hangkong Rd 13, Wuhan 430030, Hubei, Peoples R China.	[Liao, Yuxiao; Peng, Zhao; Chen, Liangkai; Liu, Liegang; Yang, Wei] Huazhong University of Science & Technology, Dept Nutr & Food Hyg, Tongji Med Coll, Sch Publ Hlth, Hangkong Rd 13, Wuhan 430030, Hubei, China.	Country: China<br>Institution: Huazhong Univ Sci & Technol	Country: China<br>Institution: Huazhong University of Science & Technology
C1	3	44653	44653	[Liao, Yuxiao; Peng, Zhao; Chen, Liangkai; Liu, Liegang; Yang, Wei] Huazhong Univ Sci & Technol, MOE Key Lab Environm & Hlth, Tongji Med Coll, Sch Publ Hlth, Hongkong Rd 13, Wuhan 430030, Hubei, Peoples R China.	[Liao, Yuxiao; Peng, Zhao; Chen, Liangkai; Liu, Liegang; Yang, Wei] Huazhong University of Science & Technology, MOE Key Lab Environm & Hlth, Tongji Med Coll, Sch Publ Hlth, Hongkong Rd 13, Wuhan 430030, Hubei, China.	Country: China<br>Institution: Huazhong Univ Sci & Technol	Country: China<br>Institution: Huazhong University of Science & Technology
C1	4	44654	44654	[Wu, Qinghua] Yangtze Univ, Inst Biomed, Coll Life Sci, Jingzhou, Peoples R China.	[Wu, Qinghua] Yangtze University, Inst Biomed, Coll Life Sci, Jingzhou, China.	Country: China<br>Institution: Yangtze Univ	Country: China<br>Institution: Yangtze University
C1	5	44655	44655	[Wu, Qinghua] Univ Hradec Kralove, Fac Sci, Dept Chem, Hradec Kralove, Czech Republic.	[Wu, Qinghua] University of Hradec Kralove, Fac Sci, Dept Chem, Hradec Kralove, Czech Republic.	Country: Czech Republic<br>Institution: Univ Hradec Kralove	Country: Czech Republic<br>Institution: University of Hradec Kralove
RP	1	44659	44659	Yang, W (corresponding author), Huazhong Univ Sci & Technol, Dept Nutr & Food Hyg, Tongji Med Coll, Sch Publ Hlth, Hangkong Rd 13, Wuhan 430030, Hubei, Peoples R China	Yang, W (corresponding author), Huazhong University of Science & Technology, Dept Nutr & Food Hyg, Tongji Med Coll, Sch Publ Hlth, Hangkong	Country: China<br>Institution: Huazhong Univ Sci & Technol	Country: China<br>Institution: Huazhong University of Science & Technology

					Rd 13, Wuhan 430030, Hubei, Peoples R China		
RP	2	44659	44659	Yang, W (corresponding author), Huazhong Univ Sci & Technol, MOE Key Lab Environm & Hlth, Tongji Med Coll, Sch Publ Hlth, Hongkong Rd 13, Wuhan 430030, Hubei, Peoples R China	Yang, W (corresponding author), Huazhong Univ Sci & Technol, MOE Key Lab Environm & Hlth, Tongji Med Coll, Sch Publ Hlth, Hongkong Rd 13, Wuhan 430030, Hubei, China	Country: China<br>Institution: Huazhong Univ Sci & Technol	Country: China<br>Institution: Huazhong Univ Sci & Technol

Record 284 | WOS:000487079300009

Antifungal activity of selected essential oils against Fusarium culmorum and F. graminearum and their secondary metabolites in wheat seeds

Field	Position	Original line	Normalised line	Original value	Normalised value	Original country / institution	Normalised country / institution
C1	1	44834	44834	[Perczak, Adam; Waskiewicz, Agnieszka] Poznan Univ Life Sci, Dept Chem, Wojska Polskiego St 75, PL-60625 Poznan, Poland.	[Perczak, Adam; Waskiewicz, Agnieszka] Poznan University of Life Sciences, Dept Chem, Wojska Polskiego St 75, PL-60625 Poznan, Poland.	Country: Poland<br>Institution: Poznan Univ Life Sci	Country: Poland<br>Institution: Poznan University of Life Sciences
RP	1	44839	44839	Perczak, A (corresponding author), Poznan Univ Life Sci, Dept Chem, Wojska Polskiego St 75, PL-60625 Poznan, Poland	Perczak, A (corresponding author), Poznan University of Life Sciences, Dept Chem, Wojska Polskiego St 75, PL-60625 Poznan, Poland	Country: Poland<br>Institution: Poznan Univ Life Sci	Country: Poland<br>Institution: Poznan University of Life Sciences

Record 285 | WOS:000475915000016

Annexin A3 may play an important role in ochratoxin-induced malignant transformation of human gastric epithelium cells

Field	Position	Original line	Normalised line	Original value	Normalised value	Original country / institution	Normalised country / institution
C1	1	44981	44981	[Wang, Juan; Jia, Xin; Meng, Xinxing; Li, Yuehong; Wu, Wenxin; Zhang, Xianghong; Cui, Jinfeng] Hebei Med Univ, Hosp 2, Dept Pathol, 215 Heping Western Rd, Shijiazhuang, Hebei, Peoples R China.	[Wang, Juan; Jia, Xin; Meng, Xinxing; Li, Yuehong; Wu, Wenxin; Zhang, Xianghong; Cui, Jinfeng] Hebei Medical University, Hosp 2, Dept Pathol, 215 Heping Western Rd, Shijiazhuang, Hebei, China.	Country: China<br>Institution: Hebei Med Univ	Country: China<br>Institution: Hebei Medical University
C1	2	44982	44982	[Xu, Hong] North China Univ Sci & Technol, Med Res Ctr, Tangshan, Peoples R China.	[Xu, Hong] North China University of Science & Technology, Med Res Ctr, Tangshan, China.	Country: China<br>Institution: North China Univ Sci & Technol	Country: China<br>Institution: North China University of Science & Technology
RP	1	44984	44984	Cui, JF (corresponding author), Hebei Med Univ, Hosp 2, Dept Pathol, 215 Heping Western Rd, Shijiazhuang, Hebei, Peoples R China	Cui, JF (corresponding author), Hebei Medical University, Hosp 2, Dept Pathol, 215 Heping Western Rd, Shijiazhuang, Hebei, China	Country: China<br>Institution: Hebei Med Univ	Country: China<br>Institution: Hebei Medical University

Record 286 | WOS:000468622000013

Nontoxic concentrations of OTA aggravate DON-induced intestinal barrier dysfunction in IPEC-J2 cells via activation of NF-Î°B signaling pathway

Field	Position	Original line	Normalised line	Original value	Normalised value	Original country / institution	Normalised country / institution
C1	1	45090	45090	[Chen, Ying; Wang, Hong; Zhai, Nianhui; Wang, Chunlei; Huang, Kehe; Pan, Cuiling]	[Chen, Ying; Wang, Hong; Zhai, Nianhui; Wang, Chunlei; Huang, Kehe; Pan, Cuiling]	Country: China<br>Institution: Nanjing Agr Univ	Country: China<br>Institution: Nanjing Agricultural University

				Nanjing Agr Univ, Coll Vet Med, Nanjing 210095, Jiangsu, Peoples R China.	Nanjing Agricultural University, Coll Vet Med, Nanjing 210095, Jiangsu, China.		
C1	2	45091	45091	[Chen, Ying; Wang, Hong; Zhai, Nianhui; Wang, Chunlei; Huang, Kehe; Pan, Cuiling] Nanjing Agr Univ, Inst Nutr & Metab Disorders Domest Anim & Fowls, Nanjing 210095, Jiangsu, Peoples R China.	[Chen, Ying; Wang, Hong; Zhai, Nianhui; Wang, Chunlei; Huang, Kehe; Pan, Cuiling] Nanjing Agricultural University, Inst Nutr & Metab Disorders Domest Anim & Fowls, Nanjing 210095, Jiangsu, China.	Country: China<br>Institution: Nanjing Agr Univ	Country: China<br>Institution: Nanjing Agricultural University
RP	2	45093	45093	Pan, CL (corresponding author), Nanjing Agr Univ, Coll Vet Med, Nanjing 210095, Jiangsu, Peoples R China	Pan, CL (corresponding author), Nanjing Agricultural University, Coll Vet Med, Nanjing 210095, Jiangsu, China	Country: China<br>Institution: Nanjing Agr Univ	Country: China<br>Institution: Nanjing Agricultural University

Record 287 | WOS:000482246600031

Transport of mycotoxins across human gastric NCI-N87 and intestinal Caco-2 cell models

Field	Position	Original line	Normalised line	Original value	Normalised value	Original country / institution	Normalised country / institution
C1	2	45207	45207	[Miladinovic, Bojana] Univ Nis, Fac Med, Dept Pharm, Nish, Serbia.	[Miladinovic, Bojana] University of Nis, Fac Med, Dept Pharm, Nish, Serbia.	Country: Serbia<br>Institution: Univ Nis	Country: Serbia<br>Institution: University of Nis

Record 288 | WOS:000487968300046

Astaxanthin Protects OTA-Induced Lung Injury in Mice through the Nrf2/NF-ÎºB Pathway

Field	Position	Original line	Normalised line	Original value	Normalised value	Original country / institution	Normalised country / institution
C1	1	45351	45351	[Xu, Weixiang; Wang, Mingyang; Cui, Gengyuan; Li, Lin; Jiao, Danyang; Yao, Beibei; Xu, Ketao; Chen, Yueli; Long, Miao; Yang, Shuhua; He, Jianbin] Shenyang Agr Univ, Coll Anim Sci & Vet Med, Key Lab Zoonosis Liaoning Prov, Shenyang 110866, Liaoning, Peoples R China.	[Xu, Weixiang; Wang, Mingyang; Cui, Gengyuan; Li, Lin; Jiao, Danyang; Yao, Beibei; Xu, Ketao; Chen, Yueli; Long, Miao; Yang, Shuhua; He, Jianbin] Shenyang Agricultural University, Coll Anim Sci & Vet Med, Key Lab Zoonosis Liaoning Prov, Shenyang 110866, Liaoning, China.	Country: China<br>Institution: Shenyang Agr Univ	Country: China<br>Institution: Shenyang Agricultural University
C1	2	45352	45352	[Li, Lin] Dept Epidem Control Fushun Modern Agr & Poverty A, Fushun 113006, Peoples R China.	[Li, Lin] Dept Epidem Control Fushun Modern Agr & Poverty A, Fushun 113006, China.	Country: China<br>Institution: Dept Epidem Control Fushun Modern Agr & Poverty A	Country: China<br>Institution: Dept Epidem Control Fushun Modern Agr & Poverty A
RP	3	45354	45354	He, JB (corresponding author), Shenyang Agr Univ, Coll Anim Sci & Vet Med, Key Lab Zoonosis Liaoning Prov, Shenyang 110866, Liaoning, Peoples R China	He, JB (corresponding author), Shenyang Agricultural University, Coll Anim Sci & Vet Med, Key Lab Zoonosis Liaoning Prov, Shenyang 110866, Liaoning, China	Country: China<br>Institution: Shenyang Agr Univ	Country: China<br>Institution: Shenyang Agricultural University

Record 289 | WOS:000487968300014

Heterologous Expression and Characterization of A Novel Ochratoxin A Degrading Enzyme, N-acyl-L-amino Acid Amidohydrolase, from Alcaligenes faecalis

Field	Position	Original line	Normalised line	Original value	Normalised value	Original country / institution	Normalised country / institution

C1	1	45469	45469	[Zhang, Honghai; Wang, Jing] Gansu Agr Univ, Coll Food Sci & Engn, Gansu Key Lab Viticulture & Enol, Lanzhou 730070, Gansu, Peoples R China.	[Zhang, Honghai; Wang, Jing] Gansu Agricultural University, Coll Food Sci & Engn, Gansu Key Lab Viticulture & Enol, Lanzhou 730070, Gansu, China.	Country: China Institution: Gansu Agr Univ	Country: China Institution: Gansu Agricultural University
C1	2	45470	45470	[Zhang, Yunpeng; Yin, Tie] Acad State Adm Grain, Beijing 100032, Peoples R China.	[Zhang, Yunpeng; Yin, Tie] Acad State Adm Grain, Beijing 100032, China.	Country: China Institution: Acad State Adm Grain	Country: China Institution: Acad State Adm Grain
C1	3	45471	45471	[Zhang, Xiaolin] COFCO Nutr & Hlth Inst, Beijing Key Lab Nutr & Hlth & Food Safety, Beijing 102209, Peoples R China.	[Zhang, Xiaolin] COFCO Nutr & Hlth Inst, Beijing Key Lab Nutr & Hlth & Food Safety, Beijing 102209, China.	Country: China Institution: COFCO Nutr & Hlth Inst	Country: China Institution: COFCO Nutr & Hlth Inst
RP	1	45473	45473	Wang, J (corresponding author), Gansu Agr Univ, Coll Food Sci & Engn, Gansu Key Lab Viticulture & Enol, Lanzhou 730070, Gansu, Peoples R China	Wang, J (corresponding author), Gansu Agricultural University, Coll Food Sci & Engn, Gansu Key Lab Viticulture & Enol, Lanzhou 730070, Gansu, Peoples R China	Country: China Institution: Gansu Agr Univ	Country: China Institution: Gansu Agricultural University
RP	2	45473	45473	Zhang, XL (corresponding author), COFCO Nutr & Hlth Inst, Beijing Key Lab Nutr & Hlth & Food Safety, Beijing 102209, Peoples R China	Zhang, XL (corresponding author), COFCO Nutr & Hlth Inst, Beijing Key Lab Nutr & Hlth & Food Safety, Beijing 102209, China	Country: China Institution: COFCO Nutr & Hlth Inst	Country: China Institution: COFCO Nutr & Hlth Inst

Record 290 | WOS:000483690200021

Accuracy, discriminative properties and reliability of a human ESC-based in vitro toxicity assay to distinguish teratogens responsible for neural tube defects

Field	Position	Original line	Normalised line	Original value	Normalised value	Original country / institution	Normalised country / institution
C1	1	45557	45557	[Feutz, Anne-Catherine; De Geyter, Christian] Univ Basel, Univ Hosp, Reprod Med & Gynaecol Endocrinol RME, Vogesenstr 134, CH-4031 Basel, Switzerland.	[Feutz, Anne-Catherine; De Geyter, Christian] University of Basel, Univ Hosp, Reprod Med & Gynaecol Endocrinol RME, Vogesenstr 134, CH-4031 Basel, Switzerland.	Country: Switzerland Institution: Univ Basel	Country: Switzerland Institution: University of Basel
C1	2	45558	45558	[Feutz, Anne-Catherine] Univ Basel, Univ Hosp, Dept Biomed DBM, Hebelstr 20, CH-4031 Basel, Switzerland.	[Feutz, Anne-Catherine] University of Basel, Univ Hosp, Dept Biomed DBM, Hebelstr 20, CH-4031 Basel, Switzerland.	Country: Switzerland Institution: Univ Basel	Country: Switzerland Institution: University of Basel
C1	3	45559	45559	[Feutz, Anne-Catherine; De Geyter, Christian] Univ Basel, SCAHT, Missionsstr 64, CH-4055 Basel, Switzerland.	[Feutz, Anne-Catherine; De Geyter, Christian] University of Basel, SCAHT, Missionsstr 64, CH-4055 Basel, Switzerland.	Country: Switzerland Institution: Univ Basel	Country: Switzerland Institution: University of Basel
RP	1	45561	45561	De Geyter, C (corresponding author), Univ Basel, Univ Hosp, Reprod Med & Gynaecol Endocrinol RME, Vogesenstr 134, CH-4031 Basel, Switzerland	De Geyter, C (corresponding author), University of Basel, Univ Hosp, Reprod Med & Gynaecol Endocrinol RME, Vogesenstr 134, CH-4031 Basel, Switzerland	Country: Switzerland Institution: Univ Basel	Country: Switzerland Institution: University of Basel

Record 291 | WOS:000477777500023

Effects of Gamma Irradiation on Ochratoxin A Stability and Cytotoxicity in Methanolic Solutions and Potential Application in Tunisian Millet Samples

No C1/C3/RP normalisation changes detected for this record.

Record 292 | WOS:000478525600001

An in vitro attempt at precision toxicology reveals the involvement of DNA methylation alteration in ochratoxin A-induced G0/G1 phase arrest

Field	Position	Original line	Normalised line	Original value	Normalised value	Original country / institution	Normalised country / institution
C1	1	45784	45784	[Zhang, Boyang; Zhu, Liye; Dai, Yaqi; Li, Hongyu; Huang, Kunlun; Luo, Yunbo; Xu, Wentao] China Agr Univ, Coll Food Sci & Nutr Engn, Beijing Adv Innovat Ctr Food Nutr & Human Hlth, Beijing, Peoples R China.	[Zhang, Boyang; Zhu, Liye; Dai, Yaqi; Li, Hongyu; Huang, Kunlun; Luo, Yunbo; Xu, Wentao] China Agricultural University, Coll Food Sci & Nutr Engn, Beijing Adv Innovat Ctr Food Nutr & Human Hlth, Beijing, China.	Country: China Institution: China Agr Univ	Country: China Institution: China Agricultural University
C1	3	45786	45786	[Huang, Kunlun; Luo, Yunbo; Xu, Wentao] Minist Agr, Key Lab Safety Assessment Genetically Modified Or, Beijing, Peoples R China.	[Huang, Kunlun; Luo, Yunbo; Xu, Wentao] Minist Agr, Key Lab Safety Assessment Genetically Modified Or, Beijing, China.	Country: China Institution: Minist Agr	Country: China Institution: Minist Agr
RP	2	45788	45788	Xu, WT (corresponding author), China Agr Univ, Coll Food Sci & Nutr Engn, 17 Tsinghua East Rd, Beijing 100083, Peoples R China	Xu, WT (corresponding author), China Agricultural University, Coll Food Sci & Nutr Engn, 17 Tsinghua East Rd, Beijing 100083, China	Country: China Institution: China Agr Univ	Country: China Institution: China Agricultural University

Record 293 | WOS:000474669600029

Long-Time Instead of Short-Time Exposure in Vitro and Administration in Vivo of Ochratoxin A Is Consistent in Immunosuppression

Field	Position	Original line	Normalised line	Original value	Normalised value	Original country / institution	Normalised country / institution
C1	1	45913	45913	[Su, Jiarui; Liu, Dandan; Wang, Qing; Lin, Jiashan; Song, Suquan; Huang, Kehe] Nanjing Agr Univ, Coll Vet Med, Nanjing 210095, Jiangsu, Peoples R China.	[Su, Jiarui; Liu, Dandan; Wang, Qing; Lin, Jiashan; Song, Suquan; Huang, Kehe] Nanjing Agricultural University, Coll Vet Med, Nanjing 210095, Jiangsu, China.	Country: China Institution: Nanjing Agr Univ	Country: China Institution: Nanjing Agricultural University
C1	2	45914	45914	[Su, Jiarui; Liu, Dandan; Wang, Qing; Lin, Jiashan; Song, Suquan; Huang, Kehe] Nanjing Agr Univ, Coll Vet Med, Inst Nutr & Metab Disorders Domest Anim & Fowls, Nanjing 210095, Jiangsu, Peoples R China.	[Su, Jiarui; Liu, Dandan; Wang, Qing; Lin, Jiashan; Song, Suquan; Huang, Kehe] Nanjing Agricultural University, Coll Vet Med, Inst Nutr & Metab Disorders Domest Anim & Fowls, Nanjing 210095, Jiangsu, China.	Country: China Institution: Nanjing Agr Univ	Country: China Institution: Nanjing Agricultural University
C1	3	45915	45915	[Su, Jiarui; Liu, Dandan; Wang, Qing; Lin, Jiashan; Song, Suquan; Huang, Kehe] Nanjing Agr Univ, Coll Vet Med, Minist Educ, Joint Int Res Lab Anim Hlth & Food Safety, Nanjing 210095, Jiangsu, Peoples R China.	[Su, Jiarui; Liu, Dandan; Wang, Qing; Lin, Jiashan; Song, Suquan; Huang, Kehe] Nanjing Agricultural University, Coll Vet Med, Minist Educ, Joint Int Res Lab Anim Hlth & Food Safety, Nanjing 210095, Jiangsu, China.	Country: China Institution: Nanjing Agr Univ	Country: China Institution: Nanjing Agricultural University
RP	1	45918	45918	Huang, KH (corresponding author), Nanjing Agr Univ, Coll Vet Med, Nanjing 210095, Jiangsu, Peoples R China	Huang, KH (corresponding author), Nanjing Agricultural University, Coll Vet Med, Nanjing 210095, Jiangsu, Peoples R China	Country: China Institution: Nanjing Agr Univ	Country: China Institution: Nanjing Agricultural University
RP	3	45918	45918	Huang, KH (corresponding author), Nanjing Agr Univ, Coll Vet Med, Minist Educ, Joint Int Res Lab Anim Hlth & Food Safety, Nanjing 210095, Jiangsu, Peoples R China	Huang, KH (corresponding author), Nanjing Agr Univ, Coll Vet Med, Minist Educ, Joint Int Res Lab Anim Hlth & Food Safety, Nanjing 210095, Jiangsu, China	Country: China Institution: Nanjing Agr Univ	Country: China Institution: Nanjing Agr Univ

Record 294 | WOS:000482110000051

Ochratoxin A-Induced Hepatotoxicity through Phase I and Phase II Reactions Regulated by AhR in Liver Cells

Field	Position	Original line	Normalised line	Original value	Normalised value	Original country / institution	Normalised country / institution
C1	1	46029	46029	[Shin, Hye Soo; Pyo, Min Cheol; Lee, Kwang-Won] Korea Univ, Dept Biotechnol, Coll Life Sci & Biotechnol, Seoul 02841, South Korea.	[Shin, Hye Soo; Pyo, Min Cheol; Lee, Kwang-Won] Korea University, Dept Biotechnol, Coll Life Sci & Biotechnol, Seoul 02841, South Korea.	Country: South Korea<br>Institution: Korea Univ	Country: South Korea<br>Institution: Korea University
C1	2	46030	46030	[Lee, Hyun Jung; Ryu, Dojin] Univ Idaho, Sch Food Sci, 875 Perimeter Dr, Moscow, MS USA.	[Lee, Hyun Jung; Ryu, Dojin] University of Idaho, Sch Food Sci, 875 Perimeter Dr, Moscow, MS USA.	Country: USA<br>Institution: Univ Idaho	Country: USA<br>Institution: University of Idaho
RP	1	46032	46032	Lee, KW (corresponding author), Korea Univ, Dept Biotechnol, Coll Life Sci & Biotechnol, Seoul 02841, South Korea	Lee, KW (corresponding author), Korea University, Dept Biotechnol, Coll Life Sci & Biotechnol, Seoul 02841, South Korea	Country: South Korea<br>Institution: Korea Univ	Country: South Korea<br>Institution: Korea University

Record 295 | WOS:000475328000001

Assorted Methods for Decontamination of Aflatoxin M1 in Milk Using Microbial Adsorbents

Field	Position	Original line	Normalised line	Original value	Normalised value	Original country / institution	Normalised country / institution
C1	2	46144	46144	[Assaf, Jean Claude; Nahle, Sahar; Chokr, Ali; Atoui, Ali] Lebanese Univ, Fac Sci 1, Dept Life & Earth Sci, Res Lab Microbiol, Hadat Campus,POB 5, Beirut, Lebanon.	[Assaf, Jean Claude; Nahle, Sahar; Chokr, Ali; Atoui, Ali] Lebanese University, Fac Sci 1, Dept Life & Earth Sci, Res Lab Microbiol, Hadat Campus,POB 5, Beirut, Lebanon.	Country: Lebanon<br>Institution: Lebanese Univ	Country: Lebanon<br>Institution: Lebanese University
C1	3	46145	46145	[Assaf, Jean Claude; Nahle, Sahar; Chokr, Ali] Lebanese Univ, Platform Res & Anal Environm Sci PRASE, Doctoral Sch Sci & Technol, Hadat Campus,POB 6573-14, Beirut, Lebanon.	[Assaf, Jean Claude; Nahle, Sahar; Chokr, Ali] Lebanese University, Platform Res & Anal Environm Sci PRASE, Doctoral Sch Sci & Technol, Hadat Campus,POB 6573-14, Beirut, Lebanon.	Country: Lebanon<br>Institution: Lebanese Univ	Country: Lebanon<br>Institution: Lebanese University

Record 296 | WOS:000475328000038

Cytotoxicity, Genotoxicity and Disturbance of Cell Cycle in HepG2 Cells Exposed to OTA and BEA: Single and Combined Actions

Field	Position	Original line	Normalised line	Original value	Normalised value	Original country / institution	Normalised country / institution
C1	1	46357	46357	[Juan-Garcia, Ana; Tolosa, Josefa; Juan, Cristina; Ruiz, Maria-Jose] Univ Valencia, Fac Pharm, Lab Food Chem & Toxicol, Av Vicent Andres Estelles S-N, E-46100 Valencia, Spain.	[Juan-Garcia, Ana; Tolosa, Josefa; Juan, Cristina; Ruiz, Maria-Jose] University of Valencia, Fac Pharm, Lab Food Chem & Toxicol, Av Vicent Andres Estelles S-N, E-46100 Valencia, Spain.	Country: Spain<br>Institution: Univ Valencia	Country: Spain<br>Institution: University of Valencia
RP	1	46360	46360	Juan-GarcÃ-a, A (corresponding author), Univ Valencia, Fac Pharm, Lab Food Chem & Toxicol, Av Vicent Andres Estelles S-N, E-46100 Valencia, Spain	Juan-GarcÃ-a, A (corresponding author), University of Valencia, Fac Pharm, Lab Food Chem & Toxicol, Av Vicent Andres Estelles S-N, E-46100 Valencia, Spain	Country: Spain<br>Institution: Univ Valencia	Country: Spain<br>Institution: University of Valencia

Record 297 | WOS:000475288200030

Long-Term Effects of Ochratoxin A on the Glutathione Redox System and Its Regulation in Chicken

Field	Position	Original line	Normalised line	Original value	Normalised value	Original country / institution	Normalised country / institution
C1	3	46470	46470	[Zandoki, Erika; Mezes, Miklos; Balogh, Krisztian] Kaposvar Univ, MTA KE SZIE Mycotoxins Food Chain Res Grp, H-7400 Kaposvar, Hungary.	[Zandoki, Erika; Mezes, Miklos; Balogh, Krisztian] Kaposvar University, MTA KE SZIE Mycotoxins Food Chain Res Grp, H-7400 Kaposvar, Hungary.	Country: Hungary<br>Institution: Kaposvar Univ	Country: Hungary<br>Institution: Kaposvar University

Record 298 | WOS:000461534700004

Effects of medicinal herb and Brazilian traditional plant extracts on in vitro mycotoxin decontamination

Field	Position	Original line	Normalised line	Original value	Normalised value	Original country / institution	Normalised country / institution
C1	1	46585	46585	[Ponzilacqua, B.; Oliveira, C. A. F.] Univ Sao Paulo, Sch Anim Sci & Food Engn, Dept Food Engn, Av Duque de Caxias Norte 225, BR-13635900 Pirassununga, SP, Brazil.	[Ponzilacqua, B.; Oliveira, C. A. F.] Universidade de Sao Paulo, Sch Anim Sci & Food Engn, Dept Food Engn, Av Duque de Caxias Norte 225, BR-13635900 Pirassununga, SP, Brazil.	Country: Brazil<br>Institution: Univ Sao Paulo	Country: Brazil<br>Institution: Universidade de Sao Paulo
RP	1	46589	46589	Oliveira, CAF (corresponding author), Univ Sao Paulo, Sch Anim Sci & Food Engn, Dept Food Engn, Av Duque de Caxias Norte 225, BR-13635900 Pirassununga, SP, Brazil	Oliveira, CAF (corresponding author), Universidade de Sao Paulo, Sch Anim Sci & Food Engn, Dept Food Engn, Av Duque de Caxias Norte 225, BR-13635900 Pirassununga, SP, Brazil	Country: Brazil<br>Institution: Univ Sao Paulo	Country: Brazil<br>Institution: Universidade de Sao Paulo

Record 299 | WOS:000465050300026

Ochratoxin A upregulates biomarkers associated with hypoxia and transformation in human kidney cells

Field	Position	Original line	Normalised line	Original value	Normalised value	Original country / institution	Normalised country / institution
C1	1	46671	46671	[Raghubeer, Shanel; Nagiah, Savania; Chuturgoon, Anil] Univ KwaZulu Natal, Discipline Med Biochem, Sch Lab Med & Med Sci, Coll Hlth Sci, ZA-4041 Durban, South Africa.	[Raghubeer, Shanel; Nagiah, Savania; Chuturgoon, Anil] University of Kwazulu Natal, Discipline Med Biochem, Sch Lab Med & Med Sci, Coll Hlth Sci, ZA-4041 Durban, South Africa.	Country: South Africa<br>Institution: Univ KwaZulu Natal	Country: South Africa<br>Institution: University of Kwazulu Natal
RP	1	46673	46673	Chuturgoon, A (corresponding author), Univ KwaZulu Natal, Discipline Med Biochem, Sch Lab Med & Med Sci, Coll Hlth Sci, ZA-4041 Durban, South Africa	Chuturgoon, A (corresponding author), University of Kwazulu Natal, Discipline Med Biochem, Sch Lab Med & Med Sci, Coll Hlth Sci, ZA-4041 Durban, South Africa	Country: South Africa<br>Institution: Univ KwaZulu Natal	Country: South Africa<br>Institution: University of Kwazulu Natal

Record 300 | WOS:000475328000057

The In Vivo and In Vitro Toxicokinetics of Citreoviridin Extracted from Penicillium citreonigrum

Field	Position	Original line	Normalised line	Original value	Normalised value	Original country / institution	Normalised country / institution

C1	1	46772	46772	[Uchiyama, Yosuke; Shiratori, Nozomi; Kobayashi, Naoki; Sugita-Konishi, Yoshiko] Azabu Univ, Dept Food & Life Sci, Grad Sch Life & Environm Sci, Chuo Ku, 1-17-71 Fuchinobe, Sagamihara, Kanagawa 2525201, Japan.	[Uchiyama, Yosuke; Shiratori, Nozomi; Kobayashi, Naoki; Sugita-Konishi, Yoshiko] Azabu University, Dept Food & Life Sci, Grad Sch Life & Environm Sci, Chuo Ku, 1-17-71 Fuchinobe, Sagamihara, Kanagawa 2525201, Japan.	Country: Japan<br>Institution: Azabu Univ	Country: Japan<br>Institution: Azabu University
C1	3	46774	46774	[Noguchi, Michiko] Azabu Univ, Dept Vet Med, Lab Theriogenol, Chuo Ku, 1-17-71 Fuchinobe, Sagamihara, Kanagawa 2525201, Japan.	[Noguchi, Michiko] Azabu University, Dept Vet Med, Lab Theriogenol, Chuo Ku, 1-17-71 Fuchinobe, Sagamihara, Kanagawa 2525201, Japan.	Country: Japan<br>Institution: Azabu Univ	Country: Japan<br>Institution: Azabu University
RP	1	46776	46776	Sugita-Konishi, Y (corresponding author), Azabu Univ, Dept Food & Life Sci, Grad Sch Life & Environm Sci, Chuo Ku, 1-17-71 Fuchinobe, Sagamihara, Kanagawa 2525201, Japan	Sugita-Konishi, Y (corresponding author), Azabu University, Dept Food & Life Sci, Grad Sch Life & Environm Sci, Chuo Ku, 1-17-71 Fuchinobe, Sagamihara, Kanagawa 2525201, Japan	Country: Japan<br>Institution: Azabu Univ	Country: Japan<br>Institution: Azabu University

Record 301 | WOS:000463851800006

Biotransformation of plantain pseudostem fibres using local enzyme sources; analysis of their potential as commercial poultry feed

No C1/C3/RP normalisation changes detected for this record.

Record 302 | WOS:000472672300064

Degrading Ochratoxin A and Zearalenone Mycotoxins Using a Multifunctional Recombinant Enzyme

Field	Position	Original line	Normalised line	Original value	Normalised value	Original country / institution	Normalised country / institution
C1	1	46991	46991	[Azam, Md Shofiul; Yu, Dianzhen; Liu, Na; Wu, Aibo] Univ Chinese Acad Sci, SIBS UGENT SJTU Joint Lab Mycotoxin Res, CAS Key Lab Nutr Metab & Food Safety,Chinese Acad, Shanghai Inst Nutr & Hlth,Shanghai Inst Biol Sci, Shanghai 200031, Peoples R China.	[Azam, Md Shofiul; Yu, Dianzhen; Liu, Na; Wu, Aibo] Chinese Academy of Sciences, SIBS UGENT SJTU Joint Lab Mycotoxin Res, CAS Key Lab Nutr Metab & Food Safety,Chinese Acad, Shanghai Inst Nutr & Hlth,Shanghai Inst Biol Sci, Shanghai 200031, China.	Country: China<br>Institution: Univ Chinese Acad Sci	Country: China<br>Institution: Chinese Academy of Sciences
RP	1	46994	46994	Wu, AB (corresponding author), Univ Chinese Acad Sci, SIBS UGENT SJTU Joint Lab Mycotoxin Res, CAS Key Lab Nutr Metab & Food Safety,Chinese Acad, Shanghai Inst Nutr & Hlth,Shanghai Inst Biol Sci, Shanghai 200031, Peoples R China	Wu, AB (corresponding author), Chinese Academy of Sciences, SIBS UGENT SJTU Joint Lab Mycotoxin Res, CAS Key Lab Nutr Metab & Food Safety,Chinese Acad, Shanghai Inst Nutr & Hlth,Shanghai Inst Biol Sci, Shanghai 200031, China	Country: China<br>Institution: Univ Chinese Acad Sci	Country: China<br>Institution: Chinese Academy of Sciences

Record 303 | WOS:000469753500299

Interaction of Mycotoxin Alternariol with Serum Albumin

Field	Position	Original line	Normalised line	Original value	Normalised value	Original country / institution	Normalised country / institution

C1	1	47104	47104	[Fliszar-Nyul, Eszter; Petho, Gabor; Poor, Miklos] Univ Pecs, Dept Pharmacol, Fac Pharm, Szigeti Ut 12, H-7642 Pecs, Hungary.	[Fliszar-Nyul, Eszter; Petho, Gabor; Poor, Miklos] University of Pecs, Dept Pharmacol, Fac Pharm, Szigeti Ut 12, H-7642 Pecs, Hungary.	Country: Hungary Institution: Univ Pecs	Country: Hungary Institution: University of Pecs
C1	2	47105	47105	[Fliszar-Nyul, Eszter; Lemli, Beata; Kunsagi-Mate, Sandor; Poor, Miklos] Univ Pecs, Janos Szentagothai Res Ctr, Ifjusag Utja 20, H-7642 Pecs, Hungary.	[Fliszar-Nyul, Eszter; Lemli, Beata; Kunsagi-Mate, Sandor; Poor, Miklos] University of Pecs, Janos Szentagothai Res Ctr, Ifjusag Utja 20, H-7642 Pecs, Hungary.	Country: Hungary Institution: Univ Pecs	Country: Hungary Institution: University of Pecs
C1	3	47106	47106	[Lemli, Beata] Univ Pecs, Inst Organ & Med Chem, Med Sch, Szigeti Ut 12, H-7624 Pecs, Hungary.	[Lemli, Beata] University of Pecs, Inst Organ & Med Chem, Med Sch, Szigeti Ut 12, H-7624 Pecs, Hungary.	Country: Hungary Institution: Univ Pecs	Country: Hungary Institution: University of Pecs
C1	4	47107	47107	[Kunsagi-Mate, Sandor] Univ Pecs, Dept Pharmaceut Chem, Fac Pharm, Rokus Utca 2, H-7642 Pecs, Hungary.	[Kunsagi-Mate, Sandor] University of Pecs, Dept Pharmaceut Chem, Fac Pharm, Rokus Utca 2, H-7642 Pecs, Hungary.	Country: Hungary Institution: Univ Pecs	Country: Hungary Institution: University of Pecs
C1	5	47108	47108	[Dellafiora, Luca; Dall'Asta, Chiara] Univ Parma, Dept Food & Drug, Via GP 7 Usberti 17-A, I-43124 Parma, Italy.	[Dellafiora, Luca; Dall'Asta, Chiara] University of Parma, Dept Food & Drug, Via GP 7 Usberti 17-A, I-43124 Parma, Italy.	Country: Italy Institution: Univ Parma	Country: Italy Institution: University of Parma
C1	6	47109	47109	[Cruciani, Gabriele] Univ Perugia, Dept Chem Biol & Biotechnol, Via Elce Sotto 8, I-06123 Perugia, Italy.	[Cruciani, Gabriele] University of Perugia, Dept Chem Biol & Biotechnol, Via Elce Sotto 8, I-06123 Perugia, Italy.	Country: Italy Institution: Univ Perugia	Country: Italy Institution: University of Perugia
C1	7	47110	47110	[Petho, Gabor] Univ Pecs, Med Sch, Dept Pharmacol & Pharmacotherapy, Szigeti Ut 12, H-7624 Pecs, Hungary.	[Petho, Gabor] University of Pecs, Med Sch, Dept Pharmacol & Pharmacotherapy, Szigeti Ut 12, H-7624 Pecs, Hungary.	Country: Hungary Institution: Univ Pecs	Country: Hungary Institution: University of Pecs
RP	1	47113	47113	PoÃ³r, M (corresponding author), Univ Pecs, Dept Pharmacol, Fac Pharm, Szigeti Ut 12, H-7642 Pecs, Hungary	PoÃ³r, M (corresponding author), University of Pecs, Dept Pharmacol, Fac Pharm, Szigeti Ut 12, H-7642 Pecs, Hungary	Country: Hungary Institution: Univ Pecs	Country: Hungary Institution: University of Pecs

Record 304 | WOS:000467512200032

Cytotoxicity against fish and mammalian cell lines and endocrine activity of the mycotoxins beauvericin, deoxynivalenol and ochratoxin-A

No C1/C3/RP normalisation changes detected for this record.

Record 305 | WOS:000468044200002

Prevention of ochratoxin A-induced oxidative stress-mediated apoptotic processes and impairment of embryonic development in mouse blastocysts by liquiritigenin

Field	Position	Original line	Normalised line	Original value	Normalised value	Original country / institution	Normalised country / institution
C1	3	47372	47372	[Chan, Wen-Hsiung] Chung Yuan Christian Univ, Dept Biosci Technol, Taoyuan 32023, Taiwan.	[Chan, Wen-Hsiung] Chung Yuan Christian University, Dept Biosci Technol, Taoyuan 32023, Taiwan.	Country: Taiwan Institution: Chung Yuan Christian Univ	Country: Taiwan Institution: Chung Yuan Christian University
C1	4	47373	47373	[Chan, Wen-Hsiung] Chung Yuan Christian Univ, Ctr Nanotechnol, Taoyuan 32023, Taiwan.	[Chan, Wen-Hsiung] Chung Yuan Christian University, Ctr Nanotechnol, Taoyuan 32023, Taiwan.	Country: Taiwan Institution: Chung Yuan Christian Univ	Country: Taiwan Institution: Chung Yuan Christian University

RP	1	47376	47376	Chan, WH (corresponding author), Chung Yuan Christian Univ, Dept Biosci Technol, Taoyuan 32023, Taiwan	Chan, WH (corresponding author), Chung Yuan Christian University, Dept Biosci Technol, Taoyuan 32023, Taiwan	Country: Taiwan<br>Institution: Chung Yuan Christian Univ	Country: Taiwan<br>Institution: Chung Yuan Christian University

Record 306 | WOS:000467299600035

Assessment of Toxic Effects of Ochratoxin A in Human Embryonic Stem Cells

Field	Position	Original line	Normalised line	Original value	Normalised value	Original country / institution	Normalised country / institution
C1	2	47510	47510	[Maria Mateo, Eva; Jimenez, Misericordia] Univ Valencia, Dept Microbiol & Ecol, E-46100 Valencia, Spain.	[Maria Mateo, Eva; Jimenez, Misericordia] University of Valencia, Dept Microbiol & Ecol, E-46100 Valencia, Spain.	Country: Spain<br>Institution: Univ Valencia	Country: Spain<br>Institution: University of Valencia
C1	3	47511	47511	[Zipancic, Ivan] Univ Cardenal Herrera CEU, CEU Univ, Sch Hlth Sci, Dept Biomed Sci, Valencia 46115, Spain.	[Zipancic, Ivan] Universidad CEU Cardenal Herrera, CEU Univ, Sch Hlth Sci, Dept Biomed Sci, Valencia 46115, Spain.	Country: Spain<br>Institution: Univ Cardenal Herrera CEU	Country: Spain<br>Institution: Universidad CEU Cardenal Herrera
C1	4	47512	47512	[Miguel Soria, Jose] Univ Cardenal Herrera CEU, CEU Univ, Sch Hlth Sci, Dept Biomed Sci, Alicante 03204, Spain.	[Miguel Soria, Jose] Universidad CEU Cardenal Herrera, CEU Univ, Sch Hlth Sci, Dept Biomed Sci, Alicante 03204, Spain.	Country: Spain<br>Institution: Univ Cardenal Herrera CEU	Country: Spain<br>Institution: Universidad CEU Cardenal Herrera
C1	5	47513	47513	[Angeles Garcia-Esparza, M.] Univ Cardenal Herrera CEU, CEU Univ, Sch Hlth Sci, Dept Pharm, Alicante 03204, Spain.	[Angeles Garcia-Esparza, M.] Universidad CEU Cardenal Herrera, CEU Univ, Sch Hlth Sci, Dept Pharm, Alicante 03204, Spain.	Country: Spain<br>Institution: Univ Cardenal Herrera CEU	Country: Spain<br>Institution: Universidad CEU Cardenal Herrera

Record 307 | WOS:000467645200017

Nephrotoxicity instead of immunotoxicity of OTA is induced through DNMT1-dependent activation of JAK2/STAT3 signaling pathway by targeting SOCS3

Field	Position	Original line	Normalised line	Original value	Normalised value	Original country / institution	Normalised country / institution
C1	1	47652	47652	[Gan, Fang; Zhou, Xuan; Zhou, Yajiao; Hou, Lili; Chen, Xingxiang; Pan, Cuiling; Huang, Kehe] Nanjing Agr Univ, Coll Vet Med, Nanjing 210095, Jiangsu, Peoples R China.	[Gan, Fang; Zhou, Xuan; Zhou, Yajiao; Hou, Lili; Chen, Xingxiang; Pan, Cuiling; Huang, Kehe] Nanjing Agricultural University, Coll Vet Med, Nanjing 210095, Jiangsu, China.	Country: China<br>Institution: Nanjing Agr Univ	Country: China<br>Institution: Nanjing Agricultural University
C1	2	47653	47653	[Gan, Fang; Zhou, Xuan; Zhou, Yajiao; Hou, Lili; Chen, Xingxiang; Pan, Cuiling; Huang, Kehe] Nanjing Agr Univ, Inst Nutr & Metab Disorders Domest Anim & Fowls, Nanjing 210095, Jiangsu, Peoples R China.	[Gan, Fang; Zhou, Xuan; Zhou, Yajiao; Hou, Lili; Chen, Xingxiang; Pan, Cuiling; Huang, Kehe] Nanjing Agricultural University, Inst Nutr & Metab Disorders Domest Anim & Fowls, Nanjing 210095, Jiangsu, China.	Country: China<br>Institution: Nanjing Agr Univ	Country: China<br>Institution: Nanjing Agricultural University
C1	3	47654	47654	[Gan, Fang; Zhou, Xuan; Zhou, Yajiao; Hou, Lili; Chen, Xingxiang; Pan, Cuiling; Huang, Kehe] Nanjing Agr Univ, Coll Vet Med, MOE Joint Int Res Lab Anim Hlth & Food Safety, Nanjing 210095, Jiangsu, Peoples R China.	[Gan, Fang; Zhou, Xuan; Zhou, Yajiao; Hou, Lili; Chen, Xingxiang; Pan, Cuiling; Huang, Kehe] Nanjing Agricultural University, Coll Vet Med, MOE Joint Int Res Lab Anim Hlth & Food Safety, Nanjing 210095, Jiangsu, China.	Country: China<br>Institution: Nanjing Agr Univ	Country: China<br>Institution: Nanjing Agricultural University
RP	1	47657	47657	Huang, KH (corresponding author), Nanjing Agr Univ, Coll Vet Med, Nanjing 210095, Jiangsu, Peoples R China	Huang, KH (corresponding author), Nanjing Agricultural University, Coll Vet Med, Nanjing 210095, Jiangsu, Peoples R China	Country: China<br>Institution: Nanjing Agr Univ	Country: China<br>Institution: Nanjing Agricultural University

RP	3	47657	47657	Huang, KH (corresponding author), Nanjing Agr Univ, Coll Vet Med, MOE Joint Int Res Lab Anim Hlth & Food Safety, Nanjing 210095, Jiangsu, Peoples R China	Huang, KH (corresponding author), Nanjing Agr Univ, Coll Vet Med, MOE Joint Int Res Lab Anim Hlth & Food Safety, Nanjing 210095, Jiangsu, China	Country: China<br>Institution: Nanjing Agr Univ	Country: China<br>Institution: Nanjing Agr Univ

Record 308 | WOS:000467299600005

Biomarkers for Exposure as a Tool for Efficacy Testing of a Mycotoxin Detoxifier in Broiler Chickens and Pigs

Field	Position	Original line	Normalised line	Original value	Normalised value	Original country / institution	Normalised country / institution
C1	1	47773	47773	[Lauwers, Marianne; Croubels, Siska; Devreese, Mathias] Univ Ghent, Dept Pharmacol Toxicol & Biochem, Fac Vet Med, B-9820 Merelbeke, Belgium.	[Lauwers, Marianne; Croubels, Siska; Devreese, Mathias] Ghent University, Dept Pharmacol Toxicol & Biochem, Fac Vet Med, B-9820 Merelbeke, Belgium.	Country: Belgium<br>Institution: Univ Ghent	Country: Belgium<br>Institution: Ghent University
RP	1	47776	47776	Croubels, S (corresponding author), Univ Ghent, Dept Pharmacol Toxicol & Biochem, Fac Vet Med, B-9820 Merelbeke, Belgium	Croubels, S (corresponding author), Ghent University, Dept Pharmacol Toxicol & Biochem, Fac Vet Med, B-9820 Merelbeke, Belgium	Country: Belgium<br>Institution: Univ Ghent	Country: Belgium<br>Institution: Ghent University

Record 309 | WOS:000470119800012

Antifungal and antimycotoxigenic effect of Lactobacillus plantarum CRL 778 at different water activity values

No C1/C3/RP normalisation changes detected for this record.

Record 310 | WOS:000462049400006

Oxidative stress as a mechanism of combined OTA and CTN toxicity in rat plasma, liver and kidney

Field	Position	Original line	Normalised line	Original value	Normalised value	Original country / institution	Normalised country / institution
C1	1	47991	47991	[Rasic, D.; Peraica, M.] Inst Med Res & Occupat Hlth, Toxicol Unit, Ksaverska Cesta 2, Zagreb 10001, Croatia.	[Rasic, D.; Peraica, M.] Institute for Medical Research & Occupational Health (IMROH), Toxicol Unit, Ksaverska Cesta 2, Zagreb 10001, Croatia.	Country: Croatia<br>Institution: Inst Med Res & Occupat Hlth	Country: Croatia<br>Institution: Institute for Medical Research & Occupational Health (IMROH)
C1	2	47992	47992	[Micek, V.] Inst Med Res & Occupat Hlth, Lab Anim Unit, Zagreb, Croatia.	[Micek, V.] Institute for Medical Research & Occupational Health (IMROH), Lab Anim Unit, Zagreb, Croatia.	Country: Croatia<br>Institution: Inst Med Res & Occupat Hlth	Country: Croatia<br>Institution: Institute for Medical Research & Occupational Health (IMROH)
C1	3	47993	47993	[Klaric, M. S.] Univ Zagreb, Dept Microbiol, Zagreb, Croatia.	[Klaric, M. S.] University of Zagreb, Dept Microbiol, Zagreb, Croatia.	Country: Croatia<br>Institution: Univ Zagreb	Country: Croatia<br>Institution: University of Zagreb
RP	1	47996	47996	Rasic, D (corresponding author), Inst Med Res & Occupat Hlth, Toxicol Unit, Ksaverska Cesta 2, Zagreb 10001, Croatia	Rasic, D (corresponding author), Institute for Medical Research & Occupational Health (IMROH), Toxicol Unit, Ksaverska Cesta 2, Zagreb 10001, Croatia	Country: Croatia<br>Institution: Inst Med Res & Occupat Hlth	Country: Croatia<br>Institution: Institute for Medical Research & Occupational Health (IMROH)

Record 311 | WOS:000467299600032

Fluorescence Spectroscopic Investigation of Competitive Interactions between Quercetin and Aflatoxin B1 for Binding to Human Serum Albumin

Field	Position	Original line	Normalised line	Original value	Normalised value	Original country / institution	Normalised country / institution
C1	1	48114	48114	[Tan, Hongxia; Chen, Lu; Ma, Liang; Liu, Shuang; Zhou, Hongyuan; Zhang, Yuhao; Guo, Ting; Liu, Wei; Dai, Hongjie; Yu, Yong] Southwest Univ, Coll Food Sci, Chongqing 400715, Peoples R China.	[Tan, Hongxia; Chen, Lu; Ma, Liang; Liu, Shuang; Zhou, Hongyuan; Zhang, Yuhao; Guo, Ting; Liu, Wei; Dai, Hongjie; Yu, Yong] Southwest Univ, Coll Food Sci, Chongqing 400715, China.	Country: China<br>Institution: Southwest Univ	Country: China<br>Institution: Southwest Univ
C1	2	48115	48115	[Ma, Liang; Zhang, Yuhao] Southwest Univ, Biol Sci Res Ctr, Chongqing 400715, Peoples R China.	[Ma, Liang; Zhang, Yuhao] Southwest Univ, Biol Sci Res Ctr, Chongqing 400715, China.	Country: China<br>Institution: Southwest Univ	Country: China<br>Institution: Southwest Univ
C3	1	48116	48116	Southwest University - China	Southwest University	Institution: Southwest University - China	Institution: Southwest University
C3	2	48116	48116	Southwest University - China	Southwest University	Institution: Southwest University - China	Institution: Southwest University
RP	2	48117	48117	Ma, L (corresponding author), Southwest Univ, Biol Sci Res Ctr, Chongqing 400715, Peoples R China	Ma, L (corresponding author), Southwest Univ, Biol Sci Res Ctr, Chongqing 400715, China	Country: China<br>Institution: Southwest Univ	Country: China<br>Institution: Southwest Univ

Record 312 | WOS:000462420300003

Review: Biotechnology of mycotoxins detoxification using microorganisms and enzymes

Field	Position	Original line	Normalised line	Original value	Normalised value	Original country / institution	Normalised country / institution
C1	2	48239	48239	[Kouidhi, Bochra; Al Qurashi, Yasir Mohammed A.] Taibah Univ, Coll Appl Med Sci, Med Lab Dept, Yanbu El Bahr, Saudi Arabia.	[Kouidhi, Bochra; Al Qurashi, Yasir Mohammed A.] Taibah University, Coll Appl Med Sci, Med Lab Dept, Yanbu El Bahr, Saudi Arabia.	Country: Saudi Arabia<br>Institution: Taibah Univ	Country: Saudi Arabia<br>Institution: Taibah University
RP	1	48242	48242	Kouidhi, B (corresponding author), Taibah Univ, Fac Appl Med Sci, Med Lab Technol Dept, Yanbu, Saudi Arabia	Kouidhi, B (corresponding author), Taibah University, Fac Appl Med Sci, Med Lab Technol Dept, Yanbu, Saudi Arabia	Country: Saudi Arabia<br>Institution: Taibah Univ	Country: Saudi Arabia<br>Institution: Taibah University

Record 313 | WOS:000460996800027

Ochratoxin A Exposure Impairs Porcine Granulosa Cell Growth via the PI3K/AKT Signaling Pathway

Field	Position	Original line	Normalised line	Original value	Normalised value	Original country / institution	Normalised country / institution
C1	1	48515	48515	[Zhang, Tian-Yu; Zhang, Rui-Qjan; Li, Na; Liu, Xue-Lian] Qingdao Agr Univ, Coll Anim Sci & Technol, Qingdao 266109, Peoples R China.	[Zhang, Tian-Yu; Zhang, Rui-Qjan; Li, Na; Liu, Xue-Lian] Qingdao Agricultural University, Coll Anim Sci & Technol, Qingdao 266109, China.	Country: China<br>Institution: Qingdao Agr Univ	Country: China<br>Institution: Qingdao Agricultural University
C1	2	48516	48516	[Sun, Xiao-Feng; Li, Lan; Shen, Wei] Qingdao Agr Univ, Inst Reprod Sci, Coll Life Sci, Qingdao 266109, Peoples R China.	[Sun, Xiao-Feng; Li, Lan; Shen, Wei] Qingdao Agricultural University, Inst Reprod Sci, Coll Life Sci, Qingdao 266109, China.	Country: China<br>Institution: Qingdao Agr Univ	Country: China<br>Institution: Qingdao Agricultural University
C1	3	48517	48517	[Ma, Jin-Mei] Anim Husb & Vet Stn Penglai City, Yantai 265600, Peoples R China.	[Ma, Jin-Mei] Anim Husb & Vet Stn Penglai City, Yantai 265600, China.	Country: China<br>Institution: Anim Husb & Vet Stn Penglai City	Country: China<br>Institution: Anim Husb & Vet Stn Penglai City
RP	1	48521	48521	Shen, W (corresponding author), Qingdao Agr Univ, Inst Reprod Sci, Coll Life Sci,	Shen, W (corresponding author), Qingdao Agricultural	Country: China<br>Institution: Qingdao Agr Univ	Country: China<br>Institution: Qingdao Agricultural University

				Qingdao 266109, Peoples R China	University, Inst Reprod Sci, Coll Life Sci, Qingdao 266109, China		

Record 314 | WOS:000465449900001

Cytotoxicity of gamma irradiated aflatoxin B1 and ochratoxin A

Field	Position	Original line	Normalised line	Original value	Normalised value	Original country / institution	Normalised country / institution
C1	1	48636	48636	[Domijan, Ana-Marija] Univ Zagreb, Fac Pharm & Biochem, Zagreb, Croatia.	[Domijan, Ana-Marija] University of Zagreb, Fac Pharm & Biochem, Zagreb, Croatia.	Country: Croatia<br>Institution: Univ Zagreb	Country: Croatia<br>Institution: University of Zagreb
C1	2	48637	48637	[Cermak, Ana Marija Marjanovic; Pavicic, Ivan] Inst Med Res & Occupat Hlth, Zagreb, Croatia.	[Cermak, Ana Marija Marjanovic; Pavicic, Ivan] Institute for Medical Research & Occupational Health (IMROH), Zagreb, Croatia.	Country: Croatia<br>Institution: Inst Med Res & Occupat Hlth	Country: Croatia<br>Institution: Institute for Medical Research & Occupational Health (IMROH)
C1	3	48638	48638	[Vulic, Ana; Pleadin, Jelka] Croatian Vet Inst, Zagreb, Croatia.	[Vulic, Ana; Pleadin, Jelka] Croatian Veterinary Institute Zagreb, Zagreb, Croatia.	Country: Croatia<br>Institution: Croatian Vet Inst	Country: Croatia<br>Institution: Croatian Veterinary Institute Zagreb
C1	4	48639	48639	[Bujak, Ivana Tartaro; Mihaljevic, Branka] Rudjer Boskovic Inst, Zagreb, Croatia.	[Bujak, Ivana Tartaro; Mihaljevic, Branka] Rudjer Boskovic Institute, Zagreb, Croatia.	Country: Croatia<br>Institution: Rudjer Boskovic Inst	Country: Croatia<br>Institution: Rudjer Boskovic Institute
C1	5	48640	48640	[Markov, Ksenija] Univ Zagreb, Fac Food Technol & Biotechnol, Zagreb, Croatia.	[Markov, Ksenija] University of Zagreb, Fac Food Technol & Biotechnol, Zagreb, Croatia.	Country: Croatia<br>Institution: Univ Zagreb	Country: Croatia<br>Institution: University of Zagreb
RP	1	48644	48644	Domijan, AM (corresponding author), Univ Zagreb, Fac Pharm & Biochem, Zagreb, Croatia	Domijan, AM (corresponding author), University of Zagreb, Fac Pharm & Biochem, Zagreb, Croatia	Country: Croatia<br>Institution: Univ Zagreb	Country: Croatia<br>Institution: University of Zagreb

Record 315 | WOS:000481430100007

Inhibitory effect of ochratoxin A on DNMT-mediated flocculation of yeast

No C1/C3/RP normalisation changes detected for this record.

Record 316 | WOS:000460801500074

Modulation of Mucin (MUC2, MUC5AC and MUC5B) mRNA Expression and Protein Production and Secretion in Caco-2/HT29-MTX Co-Cultures Following Exposure to Individual and Combined Aflatoxin M1 and Ochratoxin A

Field	Position	Original line	Normalised line	Original value	Normalised value	Original country / institution	Normalised country / institution
C1	1	48829	48829	[Huang, Xin; Gao, Yanan; Li, Songli; Wu, Chenqing; Wang, Jiaqi; Zheng, Nan] Chinese Acad Agr Sci, Inst Anim Sci, Minist Agr & Rural Affairs, Key Lab Qual & Safety Control Milk & Dairy Prod, Beijing 100193, Peoples R China.	[Huang, Xin; Gao, Yanan; Li, Songli; Wu, Chenqing; Wang, Jiaqi; Zheng, Nan] Chinese Academy of Agricultural Sciences, Inst Anim Sci, Minist Agr & Rural Affairs, Key Lab Qual & Safety Control Milk & Dairy Prod, Beijing 100193, China.	Country: China<br>Institution: Chinese Acad Agr Sci	Country: China<br>Institution: Chinese Academy of Agricultural Sciences
C1	2	48830	48830	[Huang, Xin; Gao, Yanan; Li, Songli; Wu, Chenqing; Wang, Jiaqi; Zheng, Nan] Chinese Acad Agr Sci, Inst Anim Sci, Minist Agr & Rural Affairs, Lab Qual &	[Huang, Xin; Gao, Yanan; Li, Songli; Wu, Chenqing; Wang, Jiaqi; Zheng, Nan] Chinese Academy of Agricultural Sciences, Inst Anim Sci, Minist	Country: China<br>Institution: Chinese Acad Agr Sci	Country: China<br>Institution: Chinese Academy of Agricultural Sciences

				Safety Risk Assessment Dairy Prod, Beijing 100193, Peoples R China.	Agr & Rural Affairs, Lab Qual & Safety Risk Assessment Dairy Prod, Beijing 100193, China.		
C1	3	48831	48831	[Huang, Xin; Gao, Yanan; Li, Songli; Wu, Chenqing; Wang, Jiaqi; Zheng, Nan] Chinese Acad Agr Sci, Inst Anim Sci, Minist Agr & Rural Affairs, Milk & Milk Prod Inspect Ctr, Beijing 100193, Peoples R China.	[Huang, Xin; Gao, Yanan; Li, Songli; Wu, Chenqing; Wang, Jiaqi; Zheng, Nan] Chinese Academy of Agricultural Sciences, Inst Anim Sci, Minist Agr & Rural Affairs, Milk & Milk Prod Inspect Ctr, Beijing 100193, China.	Country: China Institution: Chinese Acad Agr Sci	Country: China Institution: Chinese Academy of Agricultural Sciences
C1	4	48832	48832	[Huang, Xin; Gao, Yanan; Li, Songli; Wu, Chenqing; Wang, Jiaqi; Zheng, Nan] Chinese Acad Agr Sci, Inst Anim Sci, State Key Lab Anim Nutr, Beijing 100193, Peoples R China.	[Huang, Xin; Gao, Yanan; Li, Songli; Wu, Chenqing; Wang, Jiaqi; Zheng, Nan] Chinese Academy of Agricultural Sciences, Inst Anim Sci, State Key Lab Anim Nutr, Beijing 100193, China.	Country: China Institution: Chinese Acad Agr Sci	Country: China Institution: Chinese Academy of Agricultural Sciences
RP	1	48838	48838	Zheng, N (corresponding author), Chinese Acad Agr Sci, Inst Anim Sci, Minist Agr & Rural Affairs, Key Lab Qual & Safety Control Milk & Dairy Prod, Beijing 100193, Peoples R China	Zheng, N (corresponding author), Chinese Academy of Agricultural Sciences, Inst Anim Sci, Minist Agr & Rural Affairs, Key Lab Qual & Safety Control Milk & Dairy Prod, Beijing 100193, Peoples R China	Country: China Institution: Chinese Acad Agr Sci	Country: China Institution: Chinese Academy of Agricultural Sciences
RP	4	48838	48838	Zheng, N (corresponding author), Chinese Acad Agr Sci, Inst Anim Sci, State Key Lab Anim Nutr, Beijing 100193, Peoples R China	Zheng, N (corresponding author), Chinese Acad Agr Sci, Inst Anim Sci, State Key Lab Anim Nutr, Beijing 100193, China	Country: China Institution: Chinese Acad Agr Sci	Country: China Institution: Chinese Acad Agr Sci

Record 317 | WOS:000457296600055

Precision toxicology shows that troxerutin alleviates ochratoxin A-induced renal lipotoxicity

Field	Position	Original line	Normalised line	Original value	Normalised value	Original country / institution	Normalised country / institution
C1	1	48968	48968	[Yang, Xuan; Xu, Wentao; Huang, Kunlun; Zhang, Boyang; Wang, Haomiao; Zhang, Xueqin; Luo, Yunbo; He, Xiaoyun] China Agr Univ, Coll Food Sci & Nutr Engn, Beijing Adv Innovat Ctr Food Nutr & Human Hlth, 17 Qinghua Donglu, Beijing 100083, Peoples R China.	[Yang, Xuan; Xu, Wentao; Huang, Kunlun; Zhang, Boyang; Wang, Haomiao; Zhang, Xueqin; Luo, Yunbo; He, Xiaoyun] China Agricultural University, Coll Food Sci & Nutr Engn, Beijing Adv Innovat Ctr Food Nutr & Human Hlth, 17 Qinghua Donglu, Beijing 100083, China.	Country: China Institution: China Agr Univ	Country: China Institution: China Agricultural University
C1	2	48969	48969	[Xu, Wentao; Huang, Kunlun] Beijing Lab Food Qual & Safety, Beijing, Peoples R China.	[Xu, Wentao; Huang, Kunlun] Beijing Lab Food Qual & Safety, Beijing, China.	Country: China Institution: Beijing Lab Food Qual & Safety	Country: China Institution: Beijing Lab Food Qual & Safety
C1	3	48970	48970	[Gong, Lijing] Beijing Sport Univ, China Acad Sport & Hlth Sci, Beijing, Peoples R China.	[Gong, Lijing] Beijing Sport University, China Acad Sport & Hlth Sci, Beijing, China.	Country: China Institution: Beijing Sport Univ	Country: China Institution: Beijing Sport University
C1	4	48971	48971	[Luo, Yunbo; He, Xiaoyun] Minist Agr, Key Lab Safety Assessment Genetically Modified Or, Beijing, Peoples R China.	[Luo, Yunbo; He, Xiaoyun] Minist Agr, Key Lab Safety Assessment Genetically Modified Or, Beijing, China.	Country: China Institution: Minist Agr	Country: China Institution: Minist Agr
RP	1	48973	48973	He, XY (corresponding author), China Agr Univ, Coll Food Sci & Nutr Engn, Beijing Adv Innovat Ctr Food Nutr & Human Hlth, 17 Qinghua	He, XY (corresponding author), China Agricultural University, Coll Food Sci & Nutr Engn, Beijing Adv Innovat Ctr Food Nutr & Human Hlth, 17	Country: China Institution: China Agr Univ	Country: China Institution: China Agricultural University

				Donglu, Beijing 100083, Peoples R China	Qinghua Donglu, Beijing 100083, China		

Record 318 | WOS:000566844400029

Potential Mycotoxin Effects on Coffee Consumers' Health

No C1/C3/RP normalisation changes detected for this record.

Record 319 | WOS:000533508400001

Protective activity of melatonin against mycotoxins-induced toxicity: a review

Field	Position	Original line	Normalised line	Original value	Normalised value	Original country / institution	Normalised country / institution
C1	1	49237	49237	[Iranshahy, Milad; Shakeri, Abolfazl] Mashhad Univ Med Sci, Sch Pharm, Dept Pharmacognosy, Mashhad, Razavi Khorasan, Iran.	[Iranshahy, Milad; Shakeri, Abolfazl] Mashhad University of Medical Sciences, Sch Pharm, Dept Pharmacognosy, Mashhad, Razavi Khorasan, Iran.	Country: Iran<br>Institution: Mashhad Univ Med Sci	Country: Iran<br>Institution: Mashhad University of Medical Sciences
C1	2	49238	49238	[Etemad, Leila; Badibostan, Hasan; Karimi, Gholamreza] Mashhad Univ Med Sci, Pharmaceut Res Ctr, Pharmaceut Technol Inst, Mashhad, Razavi Khorasan, Iran.	[Etemad, Leila; Badibostan, Hasan; Karimi, Gholamreza] Mashhad University of Medical Sciences, Pharmaceut Res Ctr, Pharmaceut Technol Inst, Mashhad, Razavi Khorasan, Iran.	Country: Iran<br>Institution: Mashhad Univ Med Sci	Country: Iran<br>Institution: Mashhad University of Medical Sciences
C1	3	49239	49239	[Karimi, Gholamreza] Mashhad Univ Med Sci, Dept Pharmacodynam & Toxicol, Sch Pharm, Mashhad, Razavi Khorasan, Iran.	[Karimi, Gholamreza] Mashhad University of Medical Sciences, Dept Pharmacodynam & Toxicol, Sch Pharm, Mashhad, Razavi Khorasan, Iran.	Country: Iran<br>Institution: Mashhad Univ Med Sci	Country: Iran<br>Institution: Mashhad University of Medical Sciences
RP	1	49242	49242	Karimi, G (corresponding author), Mashhad Univ Med Sci, Pharmaceut Res Ctr, Pharmaceut Technol Inst, Mashhad, Razavi Khorasan, Iran	Karimi, G (corresponding author), Mashhad University of Medical Sciences, Pharmaceut Res Ctr, Pharmaceut Technol Inst, Mashhad, Razavi Khorasan, Iran	Country: Iran<br>Institution: Mashhad Univ Med Sci	Country: Iran<br>Institution: Mashhad University of Medical Sciences

Record 320 | WOS:000467038800009

OCRATOXINS AND THEIR NEPHROTOXIC POTENTIAL

No C1/C3/RP normalisation changes detected for this record.

Record 321 | WOS:000576729800009

Mycotoxins in food and feed

Field	Position	Original line	Normalised line	Original value	Normalised value	Original country / institution	Normalised country / institution
C1	1	49454	49454	[Pleadin, Jelka] Croatian Vet Inst, Analyt Chem Lab, Zagreb, Croatia.	[Pleadin, Jelka] Croatian Veterinary Institute Zagreb, Analyt Chem Lab, Zagreb, Croatia.	Country: Croatia<br>Institution: Croatian Vet Inst	Country: Croatia<br>Institution: Croatian Veterinary Institute Zagreb
C1	2	49455	49455	[Frece, Jadranka; Markov, Ksenija] Univ Zagreb, Fac Food	[Frece, Jadranka; Markov, Ksenija] University of Zagreb,	Country: Croatia<br>Institution: Univ Zagreb	Country: Croatia<br>Institution: University of Zagreb

				Technol & Biotechnol, Zagreb, Croatia.	Fac Food Technol & Biotechnol, Zagreb, Croatia.		
RP	1	49457	49457	Pleadin, J (corresponding author), Croatian Vet Inst, Analyt Chem Lab, Zagreb, Croatia	Pleadin, J (corresponding author), Croatian Veterinary Institute Zagreb, Analyt Chem Lab, Zagreb, Croatia	Country: Croatia Institution: Croatian Vet Inst	Country: Croatia Institution: Croatian Veterinary Institute Zagreb

Record 322 | WOS:000463362400007

DNA damage in rat kidneys and liver upon subchronic exposure to single and combined ochratoxin A and citrinin

Field	Position	Original line	Normalised line	Original value	Normalised value	Original country / institution	Normalised country / institution
C1	1	49641	49641	[Rasic, D.; Peraica, M.] Inst Med Res & Occupat Hlth, Toxicol Unit, Ksaverska Cesta 2, Zagreb 10000, Croatia.	[Rasic, D.; Peraica, M.] Institute for Medical Research & Occupational Health (IMROH), Toxicol Unit, Ksaverska Cesta 2, Zagreb 10000, Croatia.	Country: Croatia Institution: Inst Med Res & Occupat Hlth	Country: Croatia Institution: Institute for Medical Research & Occupational Health (IMROH)
C1	2	49642	49642	[Zeljezic, D.; Kopjar, N.] Inst Med Res & Occupat Hlth, Mutagenesis Unit, Ksaverska Cesta 2, Zagreb 10000, Croatia.	[Zeljezic, D.; Kopjar, N.] Institute for Medical Research & Occupational Health (IMROH), Mutagenesis Unit, Ksaverska Cesta 2, Zagreb 10000, Croatia.	Country: Croatia Institution: Inst Med Res & Occupat Hlth	Country: Croatia Institution: Institute for Medical Research & Occupational Health (IMROH)
C1	3	49643	49643	[Kifer, D.] Univ Zagreb, Dept Biophys, Fac Pharm & Biochem, A Kovacica 1, Zagreb 10000, Croatia.	[Kifer, D.] University of Zagreb, Dept Biophys, Fac Pharm & Biochem, A Kovacica 1, Zagreb 10000, Croatia.	Country: Croatia Institution: Univ Zagreb	Country: Croatia Institution: University of Zagreb
C1	4	49644	49644	[Klaric, M. Segvic] Univ Zagreb, Fac Pharm & Biochem, Dept Microbiol, Schrottova 39, Zagreb 10000, Croatia.	[Klaric, M. Segvic] University of Zagreb, Fac Pharm & Biochem, Dept Microbiol, Schrottova 39, Zagreb 10000, Croatia.	Country: Croatia Institution: Univ Zagreb	Country: Croatia Institution: University of Zagreb
RP	1	49648	49648	Peraica, M (corresponding author), Inst Med Res & Occupat Hlth, Toxicol Unit, Ksaverska Cesta 2, Zagreb 10000, Croatia	Peraica, M (corresponding author), Institute for Medical Research & Occupational Health (IMROH), Toxicol Unit, Ksaverska Cesta 2, Zagreb 10000, Croatia	Country: Croatia Institution: Inst Med Res & Occupat Hlth	Country: Croatia Institution: Institute for Medical Research & Occupational Health (IMROH)

Record 323 | WOS:000456754300029

Sex-dependent gene expression after ochratoxin A insult in F344 rat kidney

Field	Position	Original line	Normalised line	Original value	Normalised value	Original country / institution	Normalised country / institution
C1	1	49761	49761	[Vettorazzi, Ariane; Pastor, Laura; Lopez de Cerain, Adela] Univ Navarra, Dept Pharmacol & Toxicol, Fac Pharm & Nutr, CIFA Bldg,C Irunlarrea 1, E-31008 Pamplona, Spain.	[Vettorazzi, Ariane; Pastor, Laura; Lopez de Cerain, Adela] University of Navarra, Dept Pharmacol & Toxicol, Fac Pharm & Nutr, CIFA Bldg,C Irunlarrea 1, E-31008 Pamplona, Spain.	Country: Spain Institution: Univ Navarra	Country: Spain Institution: University of Navarra
C1	3	49763	49763	[Guruceaga, Elizabeth] Univ Navarra, Ctr Appl Med Res CIMA, Bioinformat Platform, E-31008 Pamplona, Spain.	[Guruceaga, Elizabeth] University of Navarra, Ctr Appl Med Res CIMA, Bioinformat Platform, E-31008 Pamplona, Spain.	Country: Spain Institution: Univ Navarra	Country: Spain Institution: University of Navarra
RP	1	49765	49765	Vettorazzi, A (corresponding author), Univ Navarra, Dept Pharmacol & Toxicol, Fac Pharm & Nutr, CIFA Bldg,C	Vettorazzi, A (corresponding author), University of Navarra, Dept Pharmacol & Toxicol, Fac Pharm & Nutr, CIFA Bldg,C	Country: Spain Institution: Univ Navarra	Country: Spain Institution: University of Navarra

				Irunlarrea 1, E-31008 Pamplona, Spain	Irunlarrea 1, E-31008 Pamplona, Spain		

Record 324 | WOS:000694027800516

Modulation of intestinal epithelial permeability and mucin mRNA (MUC2, MUC5AC, and MUC5B) expression and protein secretion in Caco-2/HT29-MTX co-cultures exposed to aflatoxin M1, ochratoxin A, and zearalenone individually or collectively.

Field	Position	Original line	Normalised line	Original value	Normalised value	Original country / institution	Normalised country / institution
C1	1	49889	49889	[Wu, C.; Zheng, N.; Gao, Y.; Wang, J.] Chinese Acad Agr Sci, Inst Anim Sci, State Key Lab Anim Nutr, Beijing, Peoples R China.	[Wu, C.; Zheng, N.; Gao, Y.; Wang, J.] Chinese Academy of Agricultural Sciences, Inst Anim Sci, State Key Lab Anim Nutr, Beijing, China.	Country: China Institution: Chinese Acad Agr Sci	Country: China Institution: Chinese Academy of Agricultural Sciences
C1	2	49890	49890	[Wu, C.; Zheng, N.; Gao, Y.; Wang, J.] Chinese Acad Agr Sci, Inst Anim Sci, Minist Agr & Rural Affairs, Key Lab Qual & Safety Control Milk & Dairy Prod, Beijing, Peoples R China.	[Wu, C.; Zheng, N.; Gao, Y.; Wang, J.] Chinese Academy of Agricultural Sciences, Inst Anim Sci, Minist Agr & Rural Affairs, Key Lab Qual & Safety Control Milk & Dairy Prod, Beijing, China.	Country: China Institution: Chinese Acad Agr Sci	Country: China Institution: Chinese Academy of Agricultural Sciences

Record 325 | WOS:000507379000008

The in vivo efficacy of highly porous carbon nanocomposites prepared from sugar beet waste for the Ochratoxin A detoxification

Field	Position	Original line	Normalised line	Original value	Normalised value	Original country / institution	Normalised country / institution
C1	1	49941	49941	[Zahoor, Muhammad] Univ Malakand, Dept Chem, Dir Lower 18800, Kpk, Pakistan.	[Zahoor, Muhammad] University of Malakand, Dept Chem, Dir Lower 18800, Kpk, Pakistan.	Country: Pakistan Institution: Univ Malakand	Country: Pakistan Institution: University of Malakand
C1	2	49942	49942	[Gulfam, Naila; Khisroon, Muhammad] Univ Peshawar, Dept Zool, Khyber Pakhtunkhwa, Pakistan.	[Gulfam, Naila; Khisroon, Muhammad] University of Peshawar, Dept Zool, Khyber Pakhtunkhwa, Pakistan.	Country: Pakistan Institution: Univ Peshawar	Country: Pakistan Institution: University of Peshawar
RP	1	49945	49945	Zahoor, M (corresponding author), Univ Malakand, Dept Chem, Dir Lower 18800, Kpk, Pakistan	Zahoor, M (corresponding author), University of Malakand, Dept Chem, Dir Lower 18800, Kpk, Pakistan	Country: Pakistan Institution: Univ Malakand	Country: Pakistan Institution: University of Malakand

Record 326 | WOS:000452098600001

Persistence of Epigenomic Effects After Recovery From Repeated Treatment With Two Nephrocarcinogens

Field	Position	Original line	Normalised line	Original value	Normalised value	Original country / institution	Normalised country / institution
C1	1	50046	50046	[Limonciel, Alice; Wilmes, Anja; Jennings, Paul] Vrije Univ Amsterdam, Amsterdam Inst Mol Med & Syst, Div Mol & Computat Toxicol, Amsterdam, Netherlands.	[Limonciel, Alice; Wilmes, Anja; Jennings, Paul] Vrije Universiteit Amsterdam, Amsterdam Inst Mol Med & Syst, Div Mol & Computat Toxicol, Amsterdam, Netherlands.	Country: Netherlands Institution: Vrije Univ Amsterdam	Country: Netherlands Institution: Vrije Universiteit Amsterdam
C1	2	50047	50047	[Limonciel, Alice; Wilmes, Anja; Aschauer, Lydia; Jennings, Paul] Med Univ Innsbruck, Dept	[Limonciel, Alice; Wilmes, Anja; Aschauer, Lydia; Jennings, Paul] Medical University of Innsbruck, Dept Physiol & Med	Country: Austria Institution: Med Univ Innsbruck	Country: Austria Institution: Medical University of Innsbruck

				Physiol & Med Phys, Div Physiol, Innsbruck, Austria.	Phys, Div Physiol, Innsbruck, Austria.		
C1	3	50048	50048	[van Breda, Simone G.; de Kok, Theo M.; Kleinjans, Jos C. S.] Maastricht Univ, Med Ctr, GROW Sch Oncol & Dev Biol, Dept Toxicogen, Maastricht, Netherlands.	[van Breda, Simone G.; de Kok, Theo M.; Kleinjans, Jos C. S.] Maastricht University, Med Ctr, GROW Sch Oncol & Dev Biol, Dept Toxicogen, Maastricht, Netherlands.	Country: Netherlands Institution: Maastricht Univ	Country: Netherlands Institution: Maastricht University
C1	5	50050	50050	[Tredwell, Gregory D.; Siskos, Alexandros P.; Keun, Hector C.] Imperial Coll London, Hammersmith Hosp, Fac Med, Div Canc,Dept Surg & Canc, London, England.	[Tredwell, Gregory D.; Siskos, Alexandros P.; Keun, Hector C.] Imperial College London, Hammersmith Hosp, Fac Med, Div Canc,Dept Surg & Canc, London, United Kingdom.	Country: England Institution: Imperial Coll London	Country: United Kingdom Institution: Imperial College London
C1	6	50051	50051	[Tredwell, Gregory D.] Australian Natl Univ, Res Sch Phys & Engn, Dept Appl Math, Canberra, ACT, Australia.	[Tredwell, Gregory D.] Australian National University, Res Sch Phys & Engn, Dept Appl Math, Canberra, ACT, Australia.	Country: Australia Institution: Australian Natl Univ	Country: Australia Institution: Australian National University
C1	7	50052	50052	[Aschauer, Lydia] Orbit Discovery, Brookes Innovat Hub, Oxford, England.	[Aschauer, Lydia] Orbit Discovery, Brookes Innovat Hub, Oxford, United Kingdom.	Country: England Institution: Orbit Discovery	Country: United Kingdom Institution: Orbit Discovery
RP	2	50059	50059	Jennings, P (corresponding author), Vrije Univ Amsterdam, Amsterdam Inst Mol Med & Syst, Div Mol & Computat Toxicol, Amsterdam, Netherlands	Jennings, P (corresponding author), Vrije Universiteit Amsterdam, Amsterdam Inst Mol Med & Syst, Div Mol & Computat Toxicol, Amsterdam, Netherlands	Country: Netherlands Institution: Vrije Univ Amsterdam	Country: Netherlands Institution: Vrije Universiteit Amsterdam

Record 327 | WOS:000447547900018

Fumonisin production and toxic capacity in airborne black Aspergilli

Field	Position	Original line	Normalised line	Original value	Normalised value	Original country / institution	Normalised country / institution
C1	1	50186	50186	[Jaksic, Daniela; Klaric, Maja Segvic] Univ Zagreb, Fac Pharm & Biochem, Dept Microbiol, Schrottova 39, Zagreb 10000, Croatia.	[Jaksic, Daniela; Klaric, Maja Segvic] University of Zagreb, Fac Pharm & Biochem, Dept Microbiol, Schrottova 39, Zagreb 10000, Croatia.	Country: Croatia Institution: Univ Zagreb	Country: Croatia Institution: University of Zagreb
C1	2	50187	50187	[Kocsube, Sandor; Bencsik, Otto; Kecskemeti, Anita; Szekeres, Andras; Vagvolgyi, Csaba; Varga, Janos] Univ Szeged, Fac Sci & Informat, Dept Microbiol, Kozep Fasor 52, H-6726 Szeged, Hungary.	[Kocsube, Sandor; Bencsik, Otto; Kecskemeti, Anita; Szekeres, Andras; Vagvolgyi, Csaba; Varga, Janos] Szeged University, Fac Sci & Informat, Dept Microbiol, Kozep Fasor 52, H-6726 Szeged, Hungary.	Country: Hungary Institution: Univ Szeged	Country: Hungary Institution: Szeged University
C1	4	50189	50189	[Kopjar, Nevenka] Inst Med Res & Occupat Hlth, Mutagenesis Unit, Ksaverska Cesta 2, Zagreb 10000, Croatia.	[Kopjar, Nevenka] Institute for Medical Research & Occupational Health (IMROH), Mutagenesis Unit, Ksaverska Cesta 2, Zagreb 10000, Croatia.	Country: Croatia Institution: Inst Med Res & Occupat Hlth	Country: Croatia Institution: Institute for Medical Research & Occupational Health (IMROH)
RP	1	50192	50192	Klaric, MS (corresponding author), Univ Zagreb, Fac Pharm & Biochem, Dept Microbiol, Schrottova 39, Zagreb 10000, Croatia	Klaric, MS (corresponding author), University of Zagreb, Fac Pharm & Biochem, Dept Microbiol, Schrottova 39, Zagreb 10000, Croatia	Country: Croatia Institution: Univ Zagreb	Country: Croatia Institution: University of Zagreb

Record 328 | WOS:000452587500077

Activation of AMPK-dependent SIRT-1 by astragalus polysaccharide protects against ochratoxin A-induced immune stress in vitro and in vivo

Field	Position	Original line	Normalised line	Original value	Normalised value	Original country / institution	Normalised country / institution
C1	1	50335	50335	[Huang, Kehe] Nanjing Agr Univ, Coll Vet Med, Nanjing 210095, Jiangsu, Peoples R China. Nanjing Agr Univ, Inst Nutr & Metab Disorders Domest Anim & Fowls, Nanjing 210095, Jiangsu, Peoples R China.	[Huang, Kehe] Nanjing Agricultural University, Coll Vet Med, Nanjing 210095, Jiangsu, China. Nanjing Agricultural University, Inst Nutr & Metab Disorders Domest Anim & Fowls, Nanjing 210095, Jiangsu, China.	Country: China Institution: Nanjing Agr Univ	Country: China Institution: Nanjing Agricultural University
RP	1	50338	50338	Huang, KH (corresponding author), Nanjing Agr Univ, Coll Vet Med, Nanjing 210095, Jiangsu, Peoples R China	Huang, KH (corresponding author), Nanjing Agricultural University, Coll Vet Med, Nanjing 210095, Jiangsu, China	Country: China Institution: Nanjing Agr Univ	Country: China Institution: Nanjing Agricultural University

Record 329 | WOS:000449893700013

Ochratoxin A induces cytoprotective autophagy via blocking AKT/mTOR signaling pathway in PK-15 cells

Field	Position	Original line	Normalised line	Original value	Normalised value	Original country / institution	Normalised country / institution
C1	1	50444	50444	[Qian, Gang; Liu, Dandan; Hou, Lili; Hamid, Mohammed; Chen, Xingxiang; Gan, Fang; Song, Suquan; Huang, Kehe] Nanjing Agr Univ, Coll Vet Med, Nanjing 210095, Jiangsu, Peoples R China.	[Qian, Gang; Liu, Dandan; Hou, Lili; Hamid, Mohammed; Chen, Xingxiang; Gan, Fang; Song, Suquan; Huang, Kehe] Nanjing Agricultural University, Coll Vet Med, Nanjing 210095, Jiangsu, China.	Country: China Institution: Nanjing Agr Univ	Country: China Institution: Nanjing Agricultural University
C1	2	50445	50445	[Qian, Gang; Liu, Dandan; Hou, Lili; Hamid, Mohammed; Chen, Xingxiang; Gan, Fang; Huang, Kehe] Nanjing Agr Univ, Inst Nutr & Metab Disorders Domest Anim & Fowls, Nanjing 210095, Jiangsu, Peoples R China.	[Qian, Gang; Liu, Dandan; Hou, Lili; Hamid, Mohammed; Chen, Xingxiang; Gan, Fang; Huang, Kehe] Nanjing Agricultural University, Inst Nutr & Metab Disorders Domest Anim & Fowls, Nanjing 210095, Jiangsu, China.	Country: China Institution: Nanjing Agr Univ	Country: China Institution: Nanjing Agricultural University
C1	3	50446	50446	[Qian, Gang] Jinling Inst Technol, Dept Anim Sci & Technol, Nanjing 210095, Jiangsu, Peoples R China.	[Qian, Gang] Jinling Institute of Technology, Dept Anim Sci & Technol, Nanjing 210095, Jiangsu, China.	Country: China Institution: Jinling Inst Technol	Country: China Institution: Jinling Institute of Technology
RP	1	50449	50449	Huang, KH (corresponding author), Nanjing Agr Univ, Coll Vet Med, Nanjing 210095, Jiangsu, Peoples R China	Huang, KH (corresponding author), Nanjing Agricultural University, Coll Vet Med, Nanjing 210095, Jiangsu, China	Country: China Institution: Nanjing Agr Univ	Country: China Institution: Nanjing Agricultural University

Record 330 | WOS:000451238100005

ERK1/2-mediated autophagy is essential for cell survival under Ochratoxin A exposure in IPEC-J2 cells

Field	Position	Original line	Normalised line	Original value	Normalised value	Original country / institution	Normalised country / institution
C1	1	50564	50564	[Wang, Hong; Li, Hu; Chen, Xingxiang; Huang, Kehe] Nanjing Agr Univ, Coll Vet Med, Nanjing 210095, Jiangsu, Peoples R China.	[Wang, Hong; Li, Hu; Chen, Xingxiang; Huang, Kehe] Nanjing Agricultural University, Coll Vet Med, Nanjing 210095, Jiangsu, China.	Country: China Institution: Nanjing Agr Univ	Country: China Institution: Nanjing Agricultural University
C1	2	50565	50565	[Wang, Hong; Li, Hu; Chen, Xingxiang; Huang, Kehe] Nanjing Agr Univ, Inst Nutr & Metab Disorders Domest Animals & Fowl, Nanjing	[Wang, Hong; Li, Hu; Chen, Xingxiang; Huang, Kehe] Nanjing Agricultural University, Inst Nutr & Metab Disorders Domest Animals &	Country: China Institution: Nanjing Agr Univ	Country: China Institution: Nanjing Agricultural University

				210095, Jiangsu, Peoples R China.	Fowl, Nanjing 210095, Jiangsu, China.		
RP	1	50567	50567	Huang, KH (corresponding author), Nanjing Agr Univ, Coll Vet Med, Nanjing 210095, Jiangsu, Peoples R China	Huang, KH (corresponding author), Nanjing Agricultural University, Coll Vet Med, Nanjing 210095, Jiangsu, Peoples R China	Country: China Institution: Nanjing Agr Univ	Country: China Institution: Nanjing Agricultural University
RP	2	50567	50567	Huang, KH (corresponding author), Nanjing Agr Univ, Inst Nutr & Metab Disorders Domest Animals & Fowl, Nanjing 210095, Jiangsu, Peoples R China	Huang, KH (corresponding author), Nanjing Agr Univ, Inst Nutr & Metab Disorders Domest Animals & Fowl, Nanjing 210095, Jiangsu, China	Country: China Institution: Nanjing Agr Univ	Country: China Institution: Nanjing Agr Univ

Record 331 | WOS:000449242800003

Mitigation of cell apoptosis induced by ochratoxin A (OTA) is possibly through organic cation transport 2 (OCT2) knockout

Field	Position	Original line	Normalised line	Original value	Normalised value	Original country / institution	Normalised country / institution
C1	1	50663	50663	[Qi, Xiaozhe; Zhu, Liye; Xu, Wentao; He, Xiaoyun; Huang, Kunlun] China Agr Univ, Beijing Adv Innovat Ctr Food Nutr & Human Hlth, Coll Food Sci & Nutr Engn, Beijing 100083, Peoples R China.	[Qi, Xiaozhe; Zhu, Liye; Xu, Wentao; He, Xiaoyun; Huang, Kunlun] China Agricultural University, Beijing Adv Innovat Ctr Food Nutr & Human Hlth, Coll Food Sci & Nutr Engn, Beijing 100083, China.	Country: China Institution: China Agr Univ	Country: China Institution: China Agricultural University
C1	2	50664	50664	[Qi, Xiaozhe] State Adm Grain, Stand & Qual Ctr, Beijing 100037, Peoples R China.	[Qi, Xiaozhe] State Adm Grain, Stand & Qual Ctr, Beijing 100037, China.	Country: China Institution: State Adm Grain	Country: China Institution: State Adm Grain
C1	3	50665	50665	[Yang, Bo; Xu, Wentao; He, Xiaoyun; Huang, Kunlun] China Agr Univ, Beijing Lab Food Qual & Safety, Coll Food Sci & Nutr Engn, Beijing 100083, Peoples R China.	[Yang, Bo; Xu, Wentao; He, Xiaoyun; Huang, Kunlun] China Agricultural University, Beijing Lab Food Qual & Safety, Coll Food Sci & Nutr Engn, Beijing 100083, China.	Country: China Institution: China Agr Univ	Country: China Institution: China Agricultural University
C1	4	50666	50666	[Luo, Haoshu] China Agr Univ, State Key Lab Agrobiotechnol, Coll Biol Sci, Beijing 100193, Peoples R China.	[Luo, Haoshu] China Agricultural University, State Key Lab Agrobiotechnol, Coll Biol Sci, Beijing 100193, China.	Country: China Institution: China Agr Univ	Country: China Institution: China Agricultural University
RP	2	50669	50669	Huang, KL (corresponding author), China Agr Univ, Beijing Adv Innovat Ctr Food Nutr & Human Hlth, Coll Food Sci & Nutr Engn, Beijing 100083, Peoples R China	Huang, KL (corresponding author), China Agricultural University, Beijing Adv Innovat Ctr Food Nutr & Human Hlth, Coll Food Sci & Nutr Engn, Beijing 100083, China	Country: China Institution: China Agr Univ	Country: China Institution: China Agricultural University

Record 332 | WOS:000446150300012

OTA induces intestinal epithelial barrier dysfunction and tight junction disruption in IPEC-J2 cells through ROS/Ca2+-mediated MLCK activation

Field	Position	Original line	Normalised line	Original value	Normalised value	Original country / institution	Normalised country / institution
C1	1	50763	50763	[Wang, Hong; Zhai, Nianhui; Chen, Ying; Fu, Chongyang; Huang, Kehe] Nanjing Agr Univ, Coll Vet Med, Nanjing 210095, Jiangsu, Peoples R China.	[Wang, Hong; Zhai, Nianhui; Chen, Ying; Fu, Chongyang; Huang, Kehe] Nanjing Agricultural University, Coll Vet Med, Nanjing 210095, Jiangsu, China.	Country: China Institution: Nanjing Agr Univ	Country: China Institution: Nanjing Agricultural University

C1	2	50764	50764	[Wang, Hong; Zhai, Nianhui; Chen, Ying; Fu, Chongyang; Huang, Kehe] Nanjing Agr Univ, Inst Nutr & Metab Disorders Domest Anim & Fowls, Nanjing 210095, Jiangsu, Peoples R China.	[Wang, Hong; Zhai, Nianhui; Chen, Ying; Fu, Chongyang; Huang, Kehe] Nanjing Agricultural University, Inst Nutr & Metab Disorders Domest Anim & Fowls, Nanjing 210095, Jiangsu, China.	Country: China<br>Institution: Nanjing Agr Univ	Country: China<br>Institution: Nanjing Agricultural University
RP	1	50766	50766	Huang, KH (corresponding author), Nanjing Agr Univ, Coll Vet Med, Nanjing 210095, Jiangsu, Peoples R China	Huang, KH (corresponding author), Nanjing Agricultural University, Coll Vet Med, Nanjing 210095, Jiangsu, China	Country: China<br>Institution: Nanjing Agr Univ	Country: China<br>Institution: Nanjing Agricultural University

Record 333 | WOS:000440879500026

Mechanism and effects of Zearalenone on mouse T lymphocytes activation in vitro

Field	Position	Original line	Normalised line	Original value	Normalised value	Original country / institution	Normalised country / institution
C1	1	50884	50884	[Cai, Guodong; Sun, Kai; Wang, Tao; Zou, Hui; Gu, Jianhong; Yuan, Yan; Liu, Xuezhong; Liu, Zongping; Bian, Jianchun] Yangzhou Univ, Coll Vet Med, 12 Wenhui East Rd, Yangzhou 225009, Jiangsu, Peoples R China.	[Cai, Guodong; Sun, Kai; Wang, Tao; Zou, Hui; Gu, Jianhong; Yuan, Yan; Liu, Xuezhong; Liu, Zongping; Bian, Jianchun] Yangzhou University, Coll Vet Med, 12 Wenhui East Rd, Yangzhou 225009, Jiangsu, China.	Country: China<br>Institution: Yangzhou Univ	Country: China<br>Institution: Yangzhou University
C1	2	50885	50885	[Cai, Guodong; Sun, Kai; Wang, Tao; Zou, Hui; Gu, Jianhong; Yuan, Yan; Liu, Xuezhong; Liu, Zongping; Bian, Jianchun] Jiangsu Coinnovat Ctr Prevent & Control Important, Yangzhou 225009, Jiangsu, Peoples R China.	[Cai, Guodong; Sun, Kai; Wang, Tao; Zou, Hui; Gu, Jianhong; Yuan, Yan; Liu, Xuezhong; Liu, Zongping; Bian, Jianchun] Jiangsu Coinnovat Ctr Prevent & Control Important, Yangzhou 225009, Jiangsu, China.	Country: China<br>Institution: Jiangsu Coinnovat Ctr Prevent & Control Important	Country: China<br>Institution: Jiangsu Coinnovat Ctr Prevent & Control Important
C1	3	50886	50886	[Cai, Guodong; Liu, Zongping; Bian, Jianchun] Yangzhou Univ, Minist Educ China, Joint Int Res Lab Agr & Agri Prod Safety, Yangzhou 225009, Jiangsu, Peoples R China.	[Cai, Guodong; Liu, Zongping; Bian, Jianchun] Yangzhou University, Minist Educ China, Joint Int Res Lab Agr & Agri Prod Safety, Yangzhou 225009, Jiangsu, China.	Country: China<br>Institution: Yangzhou Univ	Country: China<br>Institution: Yangzhou University
RP	1	50888	50888	Bian, JC (corresponding author), Yangzhou Univ, Coll Vet Med, 12 Wenhui East Rd, Yangzhou 225009, Jiangsu, Peoples R China	Bian, JC (corresponding author), Yangzhou University, Coll Vet Med, 12 Wenhui East Rd, Yangzhou 225009, Jiangsu, China	Country: China<br>Institution: Yangzhou Univ	Country: China<br>Institution: Yangzhou University

Record 334 | WOS:000448628600032

The registration of aptamer-ligand (ochratoxin A) interactions based on ligand fluorescence changes

Field	Position	Original line	Normalised line	Original value	Normalised value	Original country / institution	Normalised country / institution
C1	1	50989	50989	[Samokhvalov, Alexey, V; Safenkova, Irina, V; Zherdev, Anatoly, V; Dzantiev, Boris B.] Russian Acad Sci, AN Bach Inst Biochem, Res Ctr Biotechnol, Leninsky Prospect 33, Moscow 119071, Russia.	[Samokhvalov, Alexey, V; Safenkova, Irina, V; Zherdev, Anatoly, V; Dzantiev, Boris B.] Russian Academy of Sciences, AN Bach Inst Biochem, Res Ctr Biotechnol, Leninsky Prospect 33, Moscow 119071, Russia.	Country: Russia<br>Institution: Russian Acad Sci	Country: Russia<br>Institution: Russian Academy of Sciences
RP	1	50991	50991	Dzantiev, BB (corresponding author), Russian Acad Sci, AN Bach Inst Biochem, Res Ctr	Dzantiev, BB (corresponding author), Russian Academy of Sciences, AN Bach Inst Biochem,	Country: Russia<br>Institution: Russian Acad Sci	Country: Russia<br>Institution: Russian Academy of Sciences

				Biotechnol, Leninsky Prospect 33, Moscow 119071, Russia	Res Ctr Biotechnol, Leninsky Prospect 33, Moscow 119071, Russia		

Record 335 | WOS:000447114600010

Dietary L-carnitine and vitamin-E; a strategy to combat ochratoxin-A induced immunosuppression

Field	Position	Original line	Normalised line	Original value	Normalised value	Original country / institution	Normalised country / institution
C1	2	51092	51092	[Hassan, Zahoor Ul] Qatar Univ, Coll Arts & Sci, Dept Biol & Environm Sci, Doha 2713, Qatar.	[Hassan, Zahoor Ul] Qatar University, Coll Arts & Sci, Dept Biol & Environm Sci, Doha 2713, Qatar.	Country: Qatar<br>Institution: Qatar Univ	Country: Qatar<br>Institution: Qatar University
C1	3	51093	51093	[Abidin, Zain ul] Vet Res Inst, Zarrar Shaheed Rd, Lahore Cantt 13, Pakistan.	[Abidin, Zain ul] National Veterinary Institute - National Research Institute, Zarrar Shaheed Rd, Lahore Cantt 13, Pakistan.	Country: Pakistan<br>Institution: Vet Res Inst	Country: Pakistan<br>Institution: National Veterinary Institute - National Research Institute
C1	4	51094	51094	[Bhatti, Sheraz Ahmed; Hameed, Muhammad Raza] Bahauddin Zakariya Univ, Fac Vet Sci, Dept Pathobiol, Multan, Pakistan.	[Bhatti, Sheraz Ahmed; Hameed, Muhammad Raza] Bahauddin Zakariya University, Fac Vet Sci, Dept Pathobiol, Multan, Pakistan.	Country: Pakistan<br>Institution: Bahauddin Zakariya Univ	Country: Pakistan<br>Institution: Bahauddin Zakariya University

Record 336 | WOS:000453415000007

In Vivo Detoxification of Ochratoxin A by Highly Porous Magnetic Nanocomposites Prepared from Coconut Shell

Field	Position	Original line	Normalised line	Original value	Normalised value	Original country / institution	Normalised country / institution
C1	1	51195	51195	[Gulfam, N.; Khisroom, M.] Peshawar Univ, Dept Zool, Khyber Pakhtunkhwa, Pakistan.	[Gulfam, N.; Khisroom, M.] University of Peshawar, Dept Zool, Khyber Pakhtunkhwa, Pakistan.	Country: Pakistan<br>Institution: Peshawar Univ	Country: Pakistan<br>Institution: University of Peshawar
C1	2	51196	51196	[Zahoor, M.] Univ Malakand, Dept Chem, Dir Lower 18800, Kpk, Pakistan.	[Zahoor, M.] University of Malakand, Dept Chem, Dir Lower 18800, Kpk, Pakistan.	Country: Pakistan<br>Institution: Univ Malakand	Country: Pakistan<br>Institution: University of Malakand
RP	1	51199	51199	Zahoor, M (corresponding author), Univ Malakand, Dept Chem, Dir Lower 18800, Kpk, Pakistan	Zahoor, M (corresponding author), University of Malakand, Dept Chem, Dir Lower 18800, Kpk, Pakistan	Country: Pakistan<br>Institution: Univ Malakand	Country: Pakistan<br>Institution: University of Malakand

Record 337 | WOS:000442067900032

Research progress on the raw and modified montmorillonites as adsorbents for mycotoxins: A review

Field	Position	Original line	Normalised line	Original value	Normalised value	Original country / institution	Normalised country / institution
C1	1	51298	51298	[Li, Yan; Tian, Guangyan; Dong, Gongyue; Bai, Shansong; Han, Xiaoyu; Liang, Jinsheng; Meng, Junping; Zhang, Hong] Hebei Univ Technol, Minist Educ, Key Lab Special Funct Mat Ecol Environm & Informa, Tianjin 300130, Peoples R China.	[Li, Yan; Tian, Guangyan; Dong, Gongyue; Bai, Shansong; Han, Xiaoyu; Liang, Jinsheng; Meng, Junping; Zhang, Hong] Hebei University of Technology, Minist Educ, Key Lab Special Funct Mat Ecol Environm & Informa, Tianjin 300130, China.	Country: China<br>Institution: Hebei Univ Technol	Country: China<br>Institution: Hebei University of Technology
C1	2	51299	51299	[Li, Yan; Tian, Guangyan; Dong, Gongyue; Bai, Shansong; Han, Xiaoyu; Liang, Jinsheng; Meng,	[Li, Yan; Tian, Guangyan; Dong, Gongyue; Bai, Shansong; Han, Xiaoyu; Liang, Jinsheng; Meng,	Country: China<br>Institution: Hebei Univ Technol	Country: China<br>Institution: Hebei University of Technology

				Junping; Zhang, Hong] Hebei Univ Technol, Inst Power Source & Ecomat Sci, Tianjin 300130, Peoples R China.	Junping; Zhang, Hong] Hebei University of Technology, Inst Power Source & Ecomat Sci, Tianjin 300130, China.		
RP	2	51301	51301	Liang, JS (corresponding author), Hebei Univ Technol, Minist Educ, Key Lab Special Funct Mat Ecol Environm & Informa, Tianjin 300130, Peoples R China	Liang, JS (corresponding author), Hebei University of Technology, Minist Educ, Key Lab Special Funct Mat Ecol Environm & Informa, Tianjin 300130, China	Country: China<br>Institution: Hebei Univ Technol	Country: China<br>Institution: Hebei University of Technology

Record 338 | WOS:000439674200027

Restorative effect of L-Dopa treatment against Ochratoxin A induced neurotoxicity

No C1/C3/RP normalisation changes detected for this record.

Record 339 | WOS:000448339000017

Interaction of 2R-ochratoxin A with Serum Albumins: Binding Site, Effects of Site Markers, Thermodynamics, Species Differences of Albumin-binding, and Influence of Albumin on Its Toxicity in MDCK Cells

Field	Position	Original line	Normalised line	Original value	Normalised value	Original country / institution	Normalised country / institution
C1	1	51661	51661	[Faisal, Zelma; Poor, Miklos] Univ Pecs, Fac Pharm, Dept Pharmacol, Szigeti Ut 12, H-7624 Pecs, Hungary.	[Faisal, Zelma; Poor, Miklos] University of Pecs, Fac Pharm, Dept Pharmacol, Szigeti Ut 12, H-7624 Pecs, Hungary.	Country: Hungary<br>Institution: Univ Pecs	Country: Hungary<br>Institution: University of Pecs
C1	2	51662	51662	[Faisal, Zelma; Derdak, Diana; Lemli, Beata; Kunsagi-Mate, Sandor; Csepregi, Rita; Koszegi, Tamas; Poor, Miklos] Univ Pecs, Janos Szentagothai Res Ctr, Ifjusag Utja 20, H-7624 Pecs, Hungary.	[Faisal, Zelma; Derdak, Diana; Lemli, Beata; Kunsagi-Mate, Sandor; Csepregi, Rita; Koszegi, Tamas; Poor, Miklos] University of Pecs, Janos Szentagothai Res Ctr, Ifjusag Utja 20, H-7624 Pecs, Hungary.	Country: Hungary<br>Institution: Univ Pecs	Country: Hungary<br>Institution: University of Pecs
C1	3	51663	51663	[Derdak, Diana; Lemli, Beata; Kunsagi-Mate, Sandor] Univ Pecs, Dept Gen & Phys Chem, Ifjusag Utja 6, H-7624 Pecs, Hungary.	[Derdak, Diana; Lemli, Beata; Kunsagi-Mate, Sandor] University of Pecs, Dept Gen & Phys Chem, Ifjusag Utja 6, H-7624 Pecs, Hungary.	Country: Hungary<br>Institution: Univ Pecs	Country: Hungary<br>Institution: University of Pecs
C1	4	51664	51664	[Derdak, Diana; Lemli, Beata; Kunsagi-Mate, Sandor] Univ Pecs, Dept Pharmaceut Chem, Fac Pharm, Rokus U 2, H-7624 Pecs, Hungary.	[Derdak, Diana; Lemli, Beata; Kunsagi-Mate, Sandor] University of Pecs, Dept Pharmaceut Chem, Fac Pharm, Rokus U 2, H-7624 Pecs, Hungary.	Country: Hungary<br>Institution: Univ Pecs	Country: Hungary<br>Institution: University of Pecs
C1	5	51665	51665	[Balint, Monika; Hetenyi, Csaba] Univ Pecs, Med Sch, Dept Pharmacol & Pharmacotherapy, Szigeti Ut 12, H-7624 Pecs, Hungary.	[Balint, Monika; Hetenyi, Csaba] University of Pecs, Med Sch, Dept Pharmacol & Pharmacotherapy, Szigeti Ut 12, H-7624 Pecs, Hungary.	Country: Hungary<br>Institution: Univ Pecs	Country: Hungary<br>Institution: University of Pecs
C1	6	51666	51666	[Csepregi, Rita; Koszegi, Tamas] Univ Pecs, Med Sch, Dept Lab Med, Ifjusag Utja 13, H-7624 Pecs, Hungary.	[Csepregi, Rita; Koszegi, Tamas] University of Pecs, Med Sch, Dept Lab Med, Ifjusag Utja 13, H-7624 Pecs, Hungary.	Country: Hungary<br>Institution: Univ Pecs	Country: Hungary<br>Institution: University of Pecs
RP	1	51670	51670	PoÃ³r, M (corresponding author), Univ Pecs, Fac Pharm, Dept Pharmacol, Szigeti Ut 12, H-7624 Pecs, Hungary	PoÃ³r, M (corresponding author), University of Pecs, Fac Pharm, Dept Pharmacol, Szigeti Ut 12, H-7624 Pecs, Hungary	Country: Hungary<br>Institution: Univ Pecs	Country: Hungary<br>Institution: University of Pecs

Record 340 | WOS:000442890400004

Protective effects of compound ammonium glycyrrhizin, L-arginine, silymarin and glucurolactone against liver damage induced by ochratoxin A in primary chicken hepatocytes

Field	Position	Original line	Normalised line	Original value	Normalised value	Original country / institution	Normalised country / institution
C1	1	51789	51789	[Yu, Zugong; Wu, Feng; Tian, Jing; Guo, Xuewen; An, Ran] Nanjing Agr Univ, Coll Vet Med, Lab Vet Pharmacol & Toxicol, 1 Weigang St, Nanjing 210095, Jiangsu, Peoples R China.	[Yu, Zugong; Wu, Feng; Tian, Jing; Guo, Xuewen; An, Ran] Nanjing Agricultural University, Coll Vet Med, Lab Vet Pharmacol & Toxicol, 1 Weigang St, Nanjing 210095, Jiangsu, China.	Country: China<br>Institution: Nanjing Agr Univ	Country: China<br>Institution: Nanjing Agricultural University
RP	1	51791	51791	Yu, ZG (corresponding author), Nanjing Agr Univ, Coll Vet Med, Lab Vet Pharmacol & Toxicol, 1 Weigang St, Nanjing 210095, Jiangsu, Peoples R China	Yu, ZG (corresponding author), Nanjing Agricultural University, Coll Vet Med, Lab Vet Pharmacol & Toxicol, 1 Weigang St, Nanjing 210095, Jiangsu, China	Country: China<br>Institution: Nanjing Agr Univ	Country: China<br>Institution: Nanjing Agricultural University

Record 341 | WOS:000442714300074

Micronucleus induction and cell cycle alterations produced by deoxynivalenol and its acetylated derivatives in individual and combined exposure on HepG2 cells

Field	Position	Original line	Normalised line	Original value	Normalised value	Original country / institution	Normalised country / institution
C1	1	51902	51902	[Juan-Garcia, Ana; Taroncher, Mercedes; Font, Guillermina; Ruiz, Maria-Jose] Univ Valencia, Fac Pharm, Lab Food Chem & Toxicol, Av Vicent Andres Estelles S-N, E-46100 Valencia, Spain.	[Juan-Garcia, Ana; Taroncher, Mercedes; Font, Guillermina; Ruiz, Maria-Jose] University of Valencia, Fac Pharm, Lab Food Chem & Toxicol, Av Vicent Andres Estelles S-N, E-46100 Valencia, Spain.	Country: Spain<br>Institution: Univ Valencia	Country: Spain<br>Institution: University of Valencia
RP	1	51904	51904	Juan-GarcÃ-a, A (corresponding author), Univ Valencia, Fac Pharm, Lab Food Chem & Toxicol, Av Vicent Andres Estelles S-N, E-46100 Valencia, Spain	Juan-GarcÃ-a, A (corresponding author), University of Valencia, Fac Pharm, Lab Food Chem & Toxicol, Av Vicent Andres Estelles S-N, E-46100 Valencia, Spain	Country: Spain<br>Institution: Univ Valencia	Country: Spain<br>Institution: University of Valencia

Record 342 | WOS:000442489000011

Molecular Dynamics Simulations of Mismatched DNA Duplexes Associated with the Major C8-Linked 2â ²-Deoxyguanosine Adduct of the Food Mutagen Ochratoxin A: Influence of Opposing Base, Adduct Ionization State, and Sequence on the Structure of Damaged DNA

Field	Position	Original line	Normalised line	Original value	Normalised value	Original country / institution	Normalised country / institution
C1	1	51998	51998	[Kathuria, Preetleen; Sharma, Purshotam] Panjab Univ, Dept Chem, Computat Biochem Lab, Chandigarh 160014, India.	[Kathuria, Preetleen; Sharma, Purshotam] Panjab University, Dept Chem, Computat Biochem Lab, Chandigarh 160014, India.	Country: India<br>Institution: Panjab Univ	Country: India<br>Institution: Panjab University
C1	2	51999	51999	[Kathuria, Preetleen; Sharma, Purshotam] Panjab Univ, Ctr Adv Studies Chem, Chandigarh 160014, India.	[Kathuria, Preetleen; Sharma, Purshotam] Panjab University, Ctr Adv Studies Chem, Chandigarh 160014, India.	Country: India<br>Institution: Panjab Univ	Country: India<br>Institution: Panjab University
C1	3	52000	52000	[Manderville, Richard A.] Univ Guelph, Dept Chem, Guelph, ON N1G 2W1, Canada.	[Manderville, Richard A.] University of Guelph, Dept Chem, Guelph, ON N1G 2W1, Canada.	Country: Canada<br>Institution: Univ Guelph	Country: Canada<br>Institution: University of Guelph

C1	4	52001	52001	[Manderville, Richard A.] Univ Guelph, Dept Toxicol, Guelph, ON N1G 2W1, Canada.	[Manderville, Richard A.] University of Guelph, Dept Toxicol, Guelph, ON N1G 2W1, Canada.	Country: Canada Institution: Univ Guelph	Country: Canada Institution: University of Guelph
C1	5	52002	52002	[Wetmore, Stacey D.] Univ Lethbridge, Dept Chem & Biochem, Lethbridge, AB T1K 3M4, Canada.	[Wetmore, Stacey D.] University of Lethbridge, Dept Chem & Biochem, Lethbridge, AB T1K 3M4, Canada.	Country: Canada Institution: Univ Lethbridge	Country: Canada Institution: University of Lethbridge
RP	1	52005	52005	Wetmore, SD (corresponding author), Univ Lethbridge, Dept Chem & Biochem, Lethbridge, AB T1K 3M4, Canada	Wetmore, SD (corresponding author), University of Lethbridge, Dept Chem & Biochem, Lethbridge, AB T1K 3M4, Canada	Country: Canada Institution: Univ Lethbridge	Country: Canada Institution: University of Lethbridge

Record 343 | WOS:000440099900007

Comparison of base-line and chemical-induced transcriptomic responses in HepaRG and RPTEC/TERT1 cells using TempO-Seq

Field	Position	Original line	Normalised line	Original value	Normalised value	Original country / institution	Normalised country / institution
C1	1	52138	52138	[Limonciel, Alice; Carta, Giada; Wilmes, Anja; Jennings, Paul] Vrije Univ Amsterdam, Amsterdam Inst Mol Med & Syst, Div Mol & Computat Toxicol, De Boelelaan 1108, NL-1081 HZ Amsterdam, Netherlands.	[Limonciel, Alice; Carta, Giada; Wilmes, Anja; Jennings, Paul] Vrije Universiteit Amsterdam, Amsterdam Inst Mol Med & Syst, Div Mol & Computat Toxicol, De Boelelaan 1108, NL-1081 HZ Amsterdam, Netherlands.	Country: Netherlands Institution: Vrije Univ Amsterdam	Country: Netherlands Institution: Vrije Universiteit Amsterdam
C1	2	52139	52139	[Ates, Gamze; Vinken, Mathieu] Vrije Univ Brussel, Dept In Vitro Toxicol & Dermatocosmetol, Laarbeeklaan 103, B-1090 Brussels, Belgium.	[Ates, Gamze; Vinken, Mathieu] Vrije Universiteit Brussel, Dept In Vitro Toxicol & Dermatocosmetol, Laarbeeklaan 103, B-1090 Brussels, Belgium.	Country: Belgium Institution: Vrije Univ Brussel	Country: Belgium Institution: Vrije Universiteit Brussel
C1	3	52140	52140	[Limonciel, Alice; Carta, Giada; Wilmes, Anja; Jennings, Paul] Med Univ Innsbruck, Div Physiol, Dept Physiol & Med Phys, A-6020 Innsbruck, Austria.	[Limonciel, Alice; Carta, Giada; Wilmes, Anja; Jennings, Paul] Medical University of Innsbruck, Div Physiol, Dept Physiol & Med Phys, A-6020 Innsbruck, Austria.	Country: Austria Institution: Med Univ Innsbruck	Country: Austria Institution: Medical University of Innsbruck
C1	6	52143	52143	[van de Water, Bob] Leiden Univ, Leiden Acad Ctr Drug Res, Div Toxicol, POB 9503, NL-2300 RA Leiden, Netherlands.	[van de Water, Bob] Leiden University, Leiden Acad Ctr Drug Res, Div Toxicol, POB 9503, NL-2300 RA Leiden, Netherlands.	Country: Netherlands Institution: Leiden Univ	Country: Netherlands Institution: Leiden University
RP	1	52147	52147	Jennings, P (corresponding author), Vrije Univ Amsterdam, Amsterdam Inst Mol Med & Syst, Div Mol & Computat Toxicol, De Boelelaan 1108, NL-1081 HZ Amsterdam, Netherlands	Jennings, P (corresponding author), Vrije Universiteit Amsterdam, Amsterdam Inst Mol Med & Syst, Div Mol & Computat Toxicol, De Boelelaan 1108, NL-1081 HZ Amsterdam, Netherlands	Country: Netherlands Institution: Vrije Univ Amsterdam	Country: Netherlands Institution: Vrije Universiteit Amsterdam

Record 344 | WOS:000438651800008

Occurrence of ochratoxin A in Astragalus propinquus root and its transfer to decoction

Field	Position	Original line	Normalised line	Original value	Normalised value	Original country / institution	Normalised country / institution
C1	1	52270	52270	[Toman, Jakub; Ostry, Vladimir; Malir, Frantisek] Univ Hradec Kralove, Dept Biol, Fac Sci,	[Toman, Jakub; Ostry, Vladimir; Malir, Frantisek] University of Hradec Kralove, Dept Biol, Fac	Country: Czech Republic Institution: Univ Hradec Kralove	Country: Czech Republic Institution: University of Hradec Kralove

				Rokitanskeho 62, Hradec Kralove 50003, Czech Republic.	Sci, Rokitanskeho 62, Hradec Kralove 50003, Czech Republic.		
RP	1	52277	52277	Toman, J (corresponding author), Univ Hradec Kralove, Dept Biol, Fac Sci, Rokitanskeho 62, Hradec Kralove 50003, Czech Republic	Toman, J (corresponding author), University of Hradec Kralove, Dept Biol, Fac Sci, Rokitanskeho 62, Hradec Kralove 50003, Czech Republic	Country: Czech Republic<br>Institution: Univ Hradec Kralove	Country: Czech Republic<br>Institution: University of Hradec Kralove

Record 345 | WOS:000434754600071

PCV2 infection aggravates ochratoxin A-induced nephrotoxicity via autophagy involving p38 signaling pathway in vivo and in vitro

Field	Position	Original line	Normalised line	Original value	Normalised value	Original country / institution	Normalised country / institution
C1	1	52390	52390	[Gan, Fang; Zhou, Yajiao; Qian, Gang; Huang, Da; Hou, Lili; Liu, Dandan; Chen, Xingxiang; Jiang, Ping; Huang, Kehe] Nanjing Agr Univ, Coll Vet Med, Nanjing 210095, Jiangsu, Peoples R China.	[Gan, Fang; Zhou, Yajiao; Qian, Gang; Huang, Da; Hou, Lili; Liu, Dandan; Chen, Xingxiang; Jiang, Ping; Huang, Kehe] Nanjing Agricultural University, Coll Vet Med, Nanjing 210095, Jiangsu, China.	Country: China<br>Institution: Nanjing Agr Univ	Country: China<br>Institution: Nanjing Agricultural University
C1	2	52391	52391	[Gan, Fang; Zhou, Yajiao; Qian, Gang; Huang, Da; Hou, Lili; Liu, Dandan; Chen, Xingxiang; Jiang, Ping; Huang, Kehe] Nanjing Agr Univ, Inst Nutrit & Metab Disorders Domest Animals & Fo, Nanjing 210095, Jiangsu, Peoples R China.	[Gan, Fang; Zhou, Yajiao; Qian, Gang; Huang, Da; Hou, Lili; Liu, Dandan; Chen, Xingxiang; Jiang, Ping; Huang, Kehe] Nanjing Agricultural University, Inst Nutrit & Metab Disorders Domest Animals & Fo, Nanjing 210095, Jiangsu, China.	Country: China<br>Institution: Nanjing Agr Univ	Country: China<br>Institution: Nanjing Agricultural University
C1	3	52392	52392	[Wang, Tian] Nanjing Agr Univ, Coll Anim Sci & Technol, Nanjing 210095, Jiangsu, Peoples R China.	[Wang, Tian] Nanjing Agricultural University, Coll Anim Sci & Technol, Nanjing 210095, Jiangsu, China.	Country: China<br>Institution: Nanjing Agr Univ	Country: China<br>Institution: Nanjing Agricultural University
C1	4	52393	52393	[Lei, Xingen] Cornell Univ, Dept Anim Sci, Ithaca, NY 14853 USA.	[Lei, Xingen] Cornell University, Dept Anim Sci, Ithaca, NY 14853 USA.	Country: USA<br>Institution: Cornell Univ	Country: USA<br>Institution: Cornell University
RP	1	52396	52396	Huang, KH (corresponding author), Nanjing Agr Univ, Coll Vet Med, Nanjing 210095, Jiangsu, Peoples R China	Huang, KH (corresponding author), Nanjing Agricultural University, Coll Vet Med, Nanjing 210095, Jiangsu, Peoples R China	Country: China<br>Institution: Nanjing Agr Univ	Country: China<br>Institution: Nanjing Agricultural University

Record 346 | WOS:000442594000032

Toxicity Assay for Citrinin, Zearalenone and Zearalenone-14-Sulfate Using the Nematode Caenorhabditis elegans as Model Organism

No C1/C3/RP normalisation changes detected for this record.

Record 347 | WOS:000430882700061

Toxicological interactions between mycotoxins from ubiquitous fungi: Impact on hepatic and intestinal human epithelial cells

No C1/C3/RP normalisation changes detected for this record.

Record 348 | WOS:000432602600016

Synergistic action of the nephrotoxic mycotoxins ochratoxin A and citrinin at nanomolar concentrations in human proximal tubule-derived cells

Field	Position	Original line	Normalised line	Original value	Normalised value	Original country / institution	Normalised country / institution
C1	1	52761	52761	[Schulz, Marie-Christin; Schumann, Luise; Gekle, Michael; Schwerdt, Gerald] Martin Luther Univ Halle Wittenberg, Julius Bernstein Inst Physiol, Magdeburger Str 6, D-06112 Halle, Germany.	[Schulz, Marie-Christin; Schumann, Luise; Gekle, Michael; Schwerdt, Gerald] Martin Luther University Halle Wittenberg, Julius Bernstein Inst Physiol, Magdeburger Str 6, D-06112 Halle, Germany.	Country: Germany Institution: Martin Luther Univ Halle Wittenberg	Country: Germany Institution: Martin Luther University Halle Wittenberg
RP	1	52764	52764	Schwerdt, G (corresponding author), Martin Luther Univ Halle Wittenberg, Julius Bernstein Inst Physiol, Magdeburger Str 6, D-06112 Halle, Germany	Schwerdt, G (corresponding author), Martin Luther University Halle Wittenberg, Julius Bernstein Inst Physiol, Magdeburger Str 6, D-06112 Halle, Germany	Country: Germany Institution: Martin Luther Univ Halle Wittenberg	Country: Germany Institution: Martin Luther University Halle Wittenberg

Record 349 | WOS:000437960800009

Risk assessment of exposure to aflatoxin B1 and ochratoxin A through consumption of different Pistachio (Pistacia vera L.) cultivars collected from four geographical regions of Iran

Field	Position	Original line	Normalised line	Original value	Normalised value	Original country / institution	Normalised country / institution
C1	1	52871	52871	[Taghizadeh, Seyedeh Faezeh; Davarynejad, Gholamhossein; Nemati, Seyed Hossein] Ferdowsi Univ Mashhad, Dept Hort & Landscape Engn, Fac Agr, Mashhad, Iran.	[Taghizadeh, Seyedeh Faezeh; Davarynejad, Gholamhossein; Nemati, Seyed Hossein] Ferdowsi University Mashhad, Dept Hort & Landscape Engn, Fac Agr, Mashhad, Iran.	Country: Iran Institution: Ferdowsi Univ Mashhad	Country: Iran Institution: Ferdowsi University Mashhad
C1	2	52872	52872	[Rezaee, Ramin] Mashhad Univ Med Sci, Fac Med, Clin Res Unit, Mashhad, Iran.	[Rezaee, Ramin] Mashhad University of Medical Sciences, Fac Med, Clin Res Unit, Mashhad, Iran.	Country: Iran Institution: Mashhad Univ Med Sci	Country: Iran Institution: Mashhad University of Medical Sciences
C1	3	52873	52873	[Asili, Javad] Mashhad Univ Med Sci, Fac Pharm, Dept Pharmacognosy, Mashhad, Iran.	[Asili, Javad] Mashhad University of Medical Sciences, Fac Pharm, Dept Pharmacognosy, Mashhad, Iran.	Country: Iran Institution: Mashhad Univ Med Sci	Country: Iran Institution: Mashhad University of Medical Sciences
C1	6	52876	52876	[Tsatsakis, Aristides M.] Univ Crete, Med Sch, Ctr Toxicol Sci & Res, Iraklion, Crete, Greece.	[Tsatsakis, Aristides M.] University of Crete, Med Sch, Ctr Toxicol Sci & Res, Iraklion, Crete, Greece.	Country: Greece Institution: Univ Crete	Country: Greece Institution: University of Crete
C1	7	52877	52877	[Shirani, Kobra] Mashhad Univ Med Sci, Dept Pharmacodynamy & Toxicol, Fac Pharm, Mashhad, Iran.	[Shirani, Kobra] Mashhad University of Medical Sciences, Dept Pharmacodynamy & Toxicol, Fac Pharm, Mashhad, Iran.	Country: Iran Institution: Mashhad Univ Med Sci	Country: Iran Institution: Mashhad University of Medical Sciences
C1	8	52878	52878	[Karimi, Gholamreza] Mashhad Univ Med Sci, Inst Pharmaceut Technol, Pharmaceut Res Ctr, POB 1365-91775, Mashhad, Iran.	[Karimi, Gholamreza] Mashhad University of Medical Sciences, Inst Pharmaceut Technol, Pharmaceut Res Ctr, POB 1365-91775, Mashhad, Iran.	Country: Iran Institution: Mashhad Univ Med Sci	Country: Iran Institution: Mashhad University of Medical Sciences
C1	9	52879	52879	[Karimi, Gholamreza] Mashhad Univ Med Sci, Sch Pharm, Mashhad, Iran.	[Karimi, Gholamreza] Mashhad University of Medical Sciences, Sch Pharm, Mashhad, Iran.	Country: Iran Institution: Mashhad Univ Med Sci	Country: Iran Institution: Mashhad University of Medical Sciences
RP	1	52884	52884	Karimi, G (corresponding author), Mashhad Univ Med Sci, Inst Pharmaceut Technol, Pharmaceut Res Ctr, POB 1365-91775, Mashhad, Iran	Karimi, G (corresponding author), Mashhad University of Medical Sciences, Inst Pharmaceut Technol, Pharmaceut Res Ctr, POB 1365-91775, Mashhad, Iran	Country: Iran Institution: Mashhad Univ Med Sci	Country: Iran Institution: Mashhad University of Medical Sciences

Record 350 | WOS:000435525800022

Combination of Selenomethionine and N-Acetylcysteine Alleviates the Joint Toxicities of Aflatoxin B1 and Ochratoxin A by ERK MAPK Signal Pathway in Porcine Alveolar Macrophages

Field	Position	Original line	Normalised line	Original value	Normalised value	Original country / institution	Normalised country / institution
C1	1	52987	52987	[Hou, Lili; Zhou, Xuan; Gan, Fang; Liu, Zixuan; Zhou, Yajiao; Qjan, Gang; Huang, Kehe] Nanjing Agr Univ, Coll Vet Med, Nanjing 210095, Jiangsu, Peoples R China.	[Hou, Lili; Zhou, Xuan; Gan, Fang; Liu, Zixuan; Zhou, Yajiao; Qjan, Gang; Huang, Kehe] Nanjing Agricultural University, Coll Vet Med, Nanjing 210095, Jiangsu, China.	Country: China<br>Institution: Nanjing Agr Univ	Country: China<br>Institution: Nanjing Agricultural University
C1	2	52988	52988	[Hou, Lili; Zhou, Xuan; Gan, Fang; Liu, Zixuan; Zhou, Yajiao; Qjan, Gang; Huang, Kehe] Nanjing Agr Univ, Inst Nutr & Metab Disorders Domest Anim & Fowls, Nanjing 210095, Jiangsu, Peoples R China.	[Hou, Lili; Zhou, Xuan; Gan, Fang; Liu, Zixuan; Zhou, Yajiao; Qjan, Gang; Huang, Kehe] Nanjing Agricultural University, Inst Nutr & Metab Disorders Domest Anim & Fowls, Nanjing 210095, Jiangsu, China.	Country: China<br>Institution: Nanjing Agr Univ	Country: China<br>Institution: Nanjing Agricultural University
RP	1	52990	52990	Huang, KH (corresponding author), Nanjing Agr Univ, Coll Vet Med, Nanjing 210095, Jiangsu, Peoples R China	Huang, KH (corresponding author), Nanjing Agricultural University, Coll Vet Med, Nanjing 210095, Jiangsu, Peoples R China	Country: China<br>Institution: Nanjing Agr Univ	Country: China<br>Institution: Nanjing Agricultural University
RP	2	52990	52990	Huang, KH (corresponding author), Nanjing Agr Univ, Inst Nutr & Metab Disorders Domest Anim & Fowls, Nanjing 210095, Jiangsu, Peoples R China	Huang, KH (corresponding author), Nanjing Agr Univ, Inst Nutr & Metab Disorders Domest Anim & Fowls, Nanjing 210095, Jiangsu, China	Country: China<br>Institution: Nanjing Agr Univ	Country: China<br>Institution: Nanjing Agr Univ

Record 351 | WOS:000433656100042

Is oxidative stress involved in the sex-dependent response to ochratoxin A renal toxicity?

Field	Position	Original line	Normalised line	Original value	Normalised value	Original country / institution	Normalised country / institution
C1	1	53106	53106	[Manuel Enciso, Jose; Lopez de Cerain, Adela; Pastor, Laura; Azqueta, Amaya; Vettorazzi, Ariane] Univ Navarra, Fac Pharm & Nutr, Dept Pharmacol & Toxicol, C Irunlarrea 1, Pamplona 31008, Spain.	[Manuel Enciso, Jose; Lopez de Cerain, Adela; Pastor, Laura; Azqueta, Amaya; Vettorazzi, Ariane] University of Navarra, Fac Pharm & Nutr, Dept Pharmacol & Toxicol, C Irunlarrea 1, Pamplona 31008, Spain.	Country: Spain<br>Institution: Univ Navarra	Country: Spain<br>Institution: University of Navarra
RP	1	53109	53109	Azqueta, A (corresponding author), Univ Navarra, Fac Pharm & Nutr, Dept Pharmacol & Toxicol, C Irunlarrea 1, Pamplona 31008, Spain	Azqueta, A (corresponding author), University of Navarra, Fac Pharm & Nutr, Dept Pharmacol & Toxicol, C Irunlarrea 1, Pamplona 31008, Spain	Country: Spain<br>Institution: Univ Navarra	Country: Spain<br>Institution: University of Navarra

Record 352 | WOS:000433016800013

Effects of combined treatment with ochratoxin A and citrinin on oxidative damage in kidneys and liver of rats

Field	Position	Original line	Normalised line	Original value	Normalised value	Original country / institution	Normalised country / institution

C1	1	53253	53253	[Rasic, Dubravka; Peraica, Maja] Inst Med Res & Occupat Hlth, Toxicol Unit, Ksaverska Cesta 2, Zagreb, Croatia.	[Rasic, Dubravka; Peraica, Maja] Institute for Medical Research & Occupational Health (IMROH), Toxicol Unit, Ksaverska Cesta 2, Zagreb, Croatia.	Country: Croatia<br>Institution: Inst Med Res & Occupat Hlth	Country: Croatia<br>Institution: Institute for Medical Research & Occupational Health (IMROH)
C1	2	53254	53254	[Mladinic, Marin; Zeljezic, Davor] Inst Med Res & Occupat Hlth, Mutagenesis Unit, Zagreb, Croatia.	[Mladinic, Marin; Zeljezic, Davor] Institute for Medical Research & Occupational Health (IMROH), Mutagenesis Unit, Zagreb, Croatia.	Country: Croatia<br>Institution: Inst Med Res & Occupat Hlth	Country: Croatia<br>Institution: Institute for Medical Research & Occupational Health (IMROH)
C1	4	53256	53256	[Pizent, Alica] Inst Med Res & Occupat Hlth, Analyt Toxicol & Mineral Metab Unit, Zagreb, Croatia.	[Pizent, Alica] Institute for Medical Research & Occupational Health (IMROH), Analyt Toxicol & Mineral Metab Unit, Zagreb, Croatia.	Country: Croatia<br>Institution: Inst Med Res & Occupat Hlth	Country: Croatia<br>Institution: Institute for Medical Research & Occupational Health (IMROH)
C1	6	53258	53258	[Konjevoda, Pasko] Rudjer Boskovic Inst, Div Mol Med, Lab Epigen, Zagreb, Croatia.	[Konjevoda, Pasko] Rudjer Boskovic Institute, Div Mol Med, Lab Epigen, Zagreb, Croatia.	Country: Croatia<br>Institution: Rudjer Boskovic Inst	Country: Croatia<br>Institution: Rudjer Boskovic Institute
RP	1	53263	53263	Rasic, D (corresponding author), Inst Med Res & Occupat Hlth, Toxicol Unit, Ksaverska Cesta 2, Zagreb, Croatia	Rasic, D (corresponding author), Institute for Medical Research & Occupational Health (IMROH), Toxicol Unit, Ksaverska Cesta 2, Zagreb, Croatia	Country: Croatia<br>Institution: Inst Med Res & Occupat Hlth	Country: Croatia<br>Institution: Institute for Medical Research & Occupational Health (IMROH)

Record 353 | WOS:000432498000011

The importance of accounting for sex in the search of proteomic signatures of mycotoxin exposure

Field	Position	Original line	Normalised line	Original value	Normalised value	Original country / institution	Normalised country / institution
C1	1	53372	53372	[Soler, L.; Oswald, I. P.] Univ Toulouse, Toxalim Res Ctr Food Toxicol, INRA, ENVT,INP Purpan,UPS, Toulouse, France.	[Soler, L.; Oswald, I. P.] Universite de Toulouse, Toxalim Res Ctr Food Toxicol, INRA, ENVT,INP Purpan,UPS, Toulouse, France.	Country: France<br>Institution: Univ Toulouse	Country: France<br>Institution: Universite de Toulouse
RP	1	53375	53375	Oswald, IP (corresponding author), Univ Toulouse, Toxalim Res Ctr Food Toxicol, INRA, ENVT,INP Purpan,UPS, Toulouse, France	Oswald, IP (corresponding author), Universite de Toulouse, Toxalim Res Ctr Food Toxicol, INRA, ENVT,INP Purpan,UPS, Toulouse, France	Country: France<br>Institution: Univ Toulouse	Country: France<br>Institution: Universite de Toulouse

Record 354 | WOS:000428605400010

Toxicological effects of regulated mycotoxins and persistent organochloride pesticides: In vitro cytotoxic assessment of single and defined mixtures on MA-10 murine Leydig cell line

Field	Position	Original line	Normalised line	Original value	Normalised value	Original country / institution	Normalised country / institution
C1	1	53577	53577	[Eze, Ukpai A.; Gong, Yun Yun] Univ Leeds, Sch Food Sci & Nutr, Food Sci Bldg, Leeds LS2 9JT, W Yorkshire, England.	[Eze, Ukpai A.; Gong, Yun Yun] University of Leeds, Sch Food Sci & Nutr, Food Sci Bldg, Leeds LS2 9JT, W Yorkshire, United Kingdom.	Country: England<br>Institution: Univ Leeds	Country: United Kingdom<br>Institution: University of Leeds
C1	3	53579	53579	[Huntriss, John] Univ Leeds, Sch Med, Leeds Inst Cardiovasc & Metab Med, Div Reprod & Early Dev, Leeds LS2 9JT, W Yorkshire, England.	[Huntriss, John] University of Leeds, Sch Med, Leeds Inst Cardiovasc & Metab Med, Div Reprod & Early Dev, Leeds LS2 9JT, W Yorkshire, United Kingdom.	Country: England<br>Institution: Univ Leeds	Country: United Kingdom<br>Institution: University of Leeds
C1	4	53580	53580	[Routledge, Michael N.] Univ Leeds, Sch Med, Leeds Inst	[Routledge, Michael N.] University of Leeds, Sch Med,	Country: England<br>Institution: Univ Leeds	Country: United Kingdom<br>Institution: University of Leeds

				Cardiovasc & Metab Med, Leeds LS2 9JT, W Yorkshire, England.	Leeds Inst Cardiovasc & Metab Med, Leeds LS2 9JT, W Yorkshire, United Kingdom.		
RP	1	53582	53582	Eze, UA (corresponding author), Univ Leeds, Sch Food Sci & Nutr, Food Sci Bldg, Leeds LS2 9JT, W Yorkshire, England	Eze, UA (corresponding author), University of Leeds, Sch Food Sci & Nutr, Food Sci Bldg, Leeds LS2 9JT, W Yorkshire, United Kingdom	Country: England<br>Institution: Univ Leeds	Country: United Kingdom<br>Institution: University of Leeds

Record 355 | WOS:000434061300005

Ochratoxin A, citrinin and deoxynivalenol decrease claudin-2 expression in mouse rectum CMT93-II cells

Field	Position	Original line	Normalised line	Original value	Normalised value	Original country / institution	Normalised country / institution
C1	3	53749	53749	[Iida, Hiroshi] Kyushu Univ, Grad Sch Agr, Lab Zool, Higashi Ku, 6-10-1 Hakozaki, Fukuoka, Fukuoka 8128581, Japan.	[Iida, Hiroshi] Kyushu University, Grad Sch Agr, Lab Zool, Higashi Ku, 6-10-1 Hakozaki, Fukuoka, Fukuoka 8128581, Japan.	Country: Japan<br>Institution: Kyushu Univ	Country: Japan<br>Institution: Kyushu University

Record 356 | WOS:000429758100013

Toxicity assessment of mycotoxins extracted from contaminated commercial dog pelleted feed on canine blood mononuclear cells

Field	Position	Original line	Normalised line	Original value	Normalised value	Original country / institution	Normalised country / institution
C1	1	53885	53885	[Singh, Sanil D.; Abdul, Naeem Sheik; Phulukdaree, Alisa; Tiloke, Charlette; Nagiah, Savania; Baijnath, Sooraj; Chuturgoon, Anil A.] Univ KwaZulu Natal, Sch Lab Med & Med Sci, Discipline Med Biochem, Durban, South Africa.	[Singh, Sanil D.; Abdul, Naeem Sheik; Phulukdaree, Alisa; Tiloke, Charlette; Nagiah, Savania; Baijnath, Sooraj; Chuturgoon, Anil A.] University of Kwazulu Natal, Sch Lab Med & Med Sci, Discipline Med Biochem, Durban, South Africa.	Country: South Africa<br>Institution: Univ KwaZulu Natal	Country: South Africa<br>Institution: University of Kwazulu Natal
C1	2	53886	53886	[Phulukdaree, Alisa] Univ Pretoria, Dept Physiol, Fac Hlth Sci, Pretoria, South Africa.	[Phulukdaree, Alisa] University of Pretoria, Dept Physiol, Fac Hlth Sci, Pretoria, South Africa.	Country: South Africa<br>Institution: Univ Pretoria	Country: South Africa<br>Institution: University of Pretoria
RP	1	53888	53888	Chuturgoon, AA (corresponding author), Univ KwaZulu Natal, Sch Lab Med & Med Sci, Med Biochem, Private Bag 7, ZA-4013 Durban, South Africa	Chuturgoon, AA (corresponding author), University of Kwazulu Natal, Sch Lab Med & Med Sci, Med Biochem, Private Bag 7, ZA-4013 Durban, South Africa	Country: South Africa<br>Institution: Univ KwaZulu Natal	Country: South Africa<br>Institution: University of Kwazulu Natal

Record 357 | WOS:000428799300003

The efficiency of lactic acid bacteria against pathogenic fungi and mycotoxins

Field	Position	Original line	Normalised line	Original value	Normalised value	Original country / institution	Normalised country / institution
C1	1	53999	53999	[Perczak, Adam; Golinski, Piotr; Waskiewicz, Agnieszka] Poznan Univ Life Sci, Dept Chem, Wojska Polskiego 75, Poznan, Poland.	[Perczak, Adam; Golinski, Piotr; Waskiewicz, Agnieszka] Poznan University of Life Sciences, Dept Chem, Wojska Polskiego 75, Poznan, Poland.	Country: Poland<br>Institution: Poznan Univ Life Sci	Country: Poland<br>Institution: Poznan University of Life Sciences
RP	1	54003	54003	Waskiewicz, A (corresponding author), Poznan Univ Life Sci, Dept Chem, Wojska Polskiego 75, Poznan, Poland	Waskiewicz, A (corresponding author), Poznan University of Life Sciences, Dept Chem,	Country: Poland<br>Institution: Poznan Univ Life Sci	Country: Poland<br>Institution: Poznan University of Life Sciences

					Wojska Polskiego 75, Poznan, Poland		

Record 358 | WOS:000422811300020

Metabolism-dependent cytotoxicity of citrinin and ochratoxin A alone and in combination as assessed adopting integrated discrete multiple organ co-culture (IdMOC)

Field	Position	Original line	Normalised line	Original value	Normalised value	Original country / institution	Normalised country / institution
C1	1	54233	54233	[Gayathri, Loganathan; Dhanasekaran, Dharumadurai] Bharathidasan Univ, Dept Microbiol, Tiruchirappalli 620024, Tamil Nadu, India.	[Gayathri, Loganathan; Dhanasekaran, Dharumadurai] Bharathidasan University, Dept Microbiol, Tiruchirappalli 620024, Tamil Nadu, India.	Country: India<br>Institution: Bharathidasan Univ	Country: India<br>Institution: Bharathidasan University
C1	2	54234	54234	[Gayathri, Loganathan; Karthikeyan, Bagavathy Shanmugam; Akbarsha, Mohammad A.] Bharathidasan Univ, Mahatma Gandhi Doerenkamp Ctr, Tiruchirappalli 620024, Tamil Nadu, India.	[Gayathri, Loganathan; Karthikeyan, Bagavathy Shanmugam; Akbarsha, Mohammad A.] Bharathidasan University, Mahatma Gandhi Doerenkamp Ctr, Tiruchirappalli 620024, Tamil Nadu, India.	Country: India<br>Institution: Bharathidasan Univ	Country: India<br>Institution: Bharathidasan University
C1	5	54237	54237	[Akbarsha, Mohammad A.] King Saud Univ, Dept Food Sci & Nutr, Coll Food Sci & Agr, Nanobiotechnol & Mol Biol Res Lab, Riyadh 11451, Saudi Arabia.	[Akbarsha, Mohammad A.] King Saud University, Dept Food Sci & Nutr, Coll Food Sci & Agr, Nanobiotechnol & Mol Biol Res Lab, Riyadh 11451, Saudi Arabia.	Country: Saudi Arabia<br>Institution: King Saud Univ	Country: Saudi Arabia<br>Institution: King Saud University
RP	1	54240	54240	Akbarsha, MA (corresponding author), Bharathidasan Univ, Mahatma Gandhi Doerenkamp Ctr, Tiruchirappalli 620024, Tamil Nadu, India	Akbarsha, MA (corresponding author), Bharathidasan University, Mahatma Gandhi Doerenkamp Ctr, Tiruchirappalli 620024, Tamil Nadu, India	Country: India<br>Institution: Bharathidasan Univ	Country: India<br>Institution: Bharathidasan University

Record 359 | WOS:000425526000033

Linking site-specific loss of histone acetylation to repression of gene expression by the mycotoxin ochratoxin A

Field	Position	Original line	Normalised line	Original value	Normalised value	Original country / institution	Normalised country / institution
C1	1	54364	54364	[Limbeck, Elisabeth; Hofmann, Julian; Mally, Angela] Univ Wurzburg, Dept Toxicol, Versbacher Str 9, D-97078 Wurzburg, Germany.	[Limbeck, Elisabeth; Hofmann, Julian; Mally, Angela] University of Wurzburg, Dept Toxicol, Versbacher Str 9, D-97078 Wurzburg, Germany.	Country: Germany<br>Institution: Univ Wurzburg	Country: Germany<br>Institution: University of Wurzburg
C1	2	54365	54365	[Vanselow, Jens T.; Schlosser, Andreas] Univ Wurzburg, Rudolf Virchow Ctr Expt Biomed, D-97078 Wurzburg, Germany.	[Vanselow, Jens T.; Schlosser, Andreas] University of Wurzburg, Rudolf Virchow Ctr Expt Biomed, D-97078 Wurzburg, Germany.	Country: Germany<br>Institution: Univ Wurzburg	Country: Germany<br>Institution: University of Wurzburg
RP	1	54367	54367	Mally, A (corresponding author), Univ Wurzburg, Dept Toxicol, Versbacher Str 9, D-97078 Wurzburg, Germany	Mally, A (corresponding author), University of Wurzburg, Dept Toxicol, Versbacher Str 9, D-97078 Wurzburg, Germany	Country: Germany<br>Institution: Univ Wurzburg	Country: Germany<br>Institution: University of Wurzburg

Record 360 | WOS:000425568900007

Toxicity reduction of ochratoxin A by lactic acid bacteria

Field	Position	Original line	Normalised line	Original value	Normalised value	Original country / institution	Normalised country / institution
C1	1	54489	54489	[Luz, C.; Ferrer, J.; Manes, J.; Meca, G.] Univ Valencia, Fac Pharm, Lab Food Chem & Toxicol, Av Vicent Andres Estelles S-N, E-46100 Burjassot, Spain.	[Luz, C.; Ferrer, J.; Manes, J.; Meca, G.] University of Valencia, Fac Pharm, Lab Food Chem & Toxicol, Av Vicent Andres Estelles S-N, E-46100 Burjassot, Spain.	Country: Spain<br>Institution: Univ Valencia	Country: Spain<br>Institution: University of Valencia
RP	1	54491	54491	Meca, G (corresponding author), Univ Valencia, Fac Pharm, Lab Food Chem & Toxicol, Av Vicent Andres Estelles S-N, E-46100 Burjassot, Spain	Meca, G (corresponding author), University of Valencia, Fac Pharm, Lab Food Chem & Toxicol, Av Vicent Andres Estelles S-N, E-46100 Burjassot, Spain	Country: Spain<br>Institution: Univ Valencia	Country: Spain<br>Institution: University of Valencia

Record 361 | WOS:000425568900035

Ochratoxin A: Toxicity, oxidative stress and metabolism

Field	Position	Original line	Normalised line	Original value	Normalised value	Original country / institution	Normalised country / institution
C1	1	54588	54588	[Xie, Shuyu; Xu, Fanfan; Pan, Yuanhu; Peng, Dapeng; Wang, Xu; Yuan, Zonghui] Natl Reference Lab Vet Drug Residues HZAU, Wuhan 430070, Hubei, Peoples R China.	[Xie, Shuyu; Xu, Fanfan; Pan, Yuanhu; Peng, Dapeng; Wang, Xu; Yuan, Zonghui] Natl Reference Lab Vet Drug Residues HZAU, Wuhan 430070, Hubei, China.	Country: China<br>Institution: Natl Reference Lab Vet Drug Residues HZAU	Country: China<br>Institution: Natl Reference Lab Vet Drug Residues HZAU
C1	2	54589	54589	[Xie, Shuyu; Xu, Fanfan; Pan, Yuanhu; Peng, Dapeng; Wang, Xu; Yuan, Zonghui] MAO Key Lab Detect Vet Drug Residues, Wuhan 430070, Hubei, Peoples R China.	[Xie, Shuyu; Xu, Fanfan; Pan, Yuanhu; Peng, Dapeng; Wang, Xu; Yuan, Zonghui] MAO Key Lab Detect Vet Drug Residues, Wuhan 430070, Hubei, China.	Country: China<br>Institution: MAO Key Lab Detect Vet Drug Residues	Country: China<br>Institution: MAO Key Lab Detect Vet Drug Residues
C1	3	54590	54590	[Tao, Yanfei; Liu, Aimei; Wang, Yanxin; Chen, Dongmei; Huang, Lingli; Yuan, Zonghui] MOA Lab Risk Assessment Qual & Safety Livestock &, Wuhan 430070, Hubei, Peoples R China.	[Tao, Yanfei; Liu, Aimei; Wang, Yanxin; Chen, Dongmei; Huang, Lingli; Yuan, Zonghui] MOA Lab Risk Assessment Qual & Safety Livestock &, Wuhan 430070, Hubei, China.	Country: China<br>Institution: MOA Lab Risk Assessment Qual & Safety Livestock &	Country: China<br>Institution: MOA Lab Risk Assessment Qual & Safety Livestock &
C1	4	54591	54591	[Tao, Yanfei; Xie, Shuyu; Xu, Fanfan; Wang, Yanxin; Chen, Dongmei; Huang, Lingli; Yuan, Zonghui] Huazhong Agr Univ, Hubei Collaborat Innovat Ctr Anim Nutr & Feed Saf, Wuhan 430070, Hubei, Peoples R China.	[Tao, Yanfei; Xie, Shuyu; Xu, Fanfan; Wang, Yanxin; Chen, Dongmei; Huang, Lingli; Yuan, Zonghui] Huazhong Agr Univ, Hubei Collaborat Innovat Ctr Anim Nutr & Feed Saf, Wuhan 430070, Hubei, China.	Country: China<br>Institution: Huazhong Agr Univ	Country: China<br>Institution: Huazhong Agr Univ
RP	2	54593	54593	Wang, X (corresponding author), MAO Key Lab Detect Vet Drug Residues, Wuhan 430070, Hubei, Peoples R China	Wang, X (corresponding author), MAO Key Lab Detect Vet Drug Residues, Wuhan 430070, Hubei, China	Country: China<br>Institution: MAO Key Lab Detect Vet Drug Residues	Country: China<br>Institution: MAO Key Lab Detect Vet Drug Residues

Record 362 | WOS:000424317600001

Disruption of liver development and coagulation pathway by ochratoxin A in embryonic zebrafish

Field	Position	Original line	Normalised line	Original value	Normalised value	Original country / institution	Normalised country / institution
C1	1	54782	54782	[Wu, Ting-Shuan; Lin, Yu-Ting; Huang, Ying-Tzu; Cheng, Ya-Chih; Liu, Biing-Hui] Natl Taiwan Univ, Grad Inst Toxicol,	[Wu, Ting-Shuan; Lin, Yu-Ting; Huang, Ying-Tzu; Cheng, Ya-Chih; Liu, Biing-Hui] National Taiwan University, Grad Inst	Country: Taiwan<br>Institution: Natl Taiwan Univ	Country: Taiwan<br>Institution: National Taiwan University

				Coll Med, 1,Sec 1,Jen Ai Rd, Taipei 10043, Taiwan.	Toxicol, Coll Med, 1,Sec 1,Jen Ai Rd, Taipei 10043, Taiwan.		
C1	2	54783	54783	[Yu, Feng-Yih] Chung Shan Med Univ, Dept Biomed Sci, Taichung, Taiwan.	[Yu, Feng-Yih] Chung Shan Medical University, Dept Biomed Sci, Taichung, Taiwan.	Country: Taiwan<br>Institution: Chung Shan Med Univ	Country: Taiwan<br>Institution: Chung Shan Medical University
C1	3	54784	54784	[Yu, Feng-Yih] Chung Shan Med Univ Hosp, Dept Med Res, Taichung, Taiwan.	[Yu, Feng-Yih] Chung Shan Medical University, Dept Med Res, Taichung, Taiwan.	Country: Taiwan<br>Institution: Chung Shan Med Univ Hosp	Country: Taiwan<br>Institution: Chung Shan Medical University
RP	1	54787	54787	Liu, BH (corresponding author), Natl Taiwan Univ, Grad Inst Toxicol, Coll Med, 1,Sec 1,Jen Ai Rd, Taipei 10043, Taiwan	Liu, BH (corresponding author), National Taiwan University, Grad Inst Toxicol, Coll Med, 1,Sec 1,Jen Ai Rd, Taipei 10043, Taiwan	Country: Taiwan<br>Institution: Natl Taiwan Univ	Country: Taiwan<br>Institution: National Taiwan University

Record 363 | WOS:000424317600006

Ochratoxin A exposure decreased sperm motility via the AMPK and PTEN signaling pathways

Field	Position	Original line	Normalised line	Original value	Normalised value	Original country / institution	Normalised country / institution
C1	1	54903	54903	[Zhang, Tian-Yu; Zhang, Wei-Dong; Shen, Wei] Qingdao Agr Univ, Coll Anim Sci & Technol, Qingdao 266109, Peoples R China.	[Zhang, Tian-Yu; Zhang, Wei-Dong; Shen, Wei] Qingdao Agricultural University, Coll Anim Sci & Technol, Qingdao 266109, China.	Country: China<br>Institution: Qingdao Agr Univ	Country: China<br>Institution: Qingdao Agricultural University
C1	2	54904	54904	[Wu, Rui-Ying; Xu, Chun-Shuang; Zou, Shu-Hua] Qingdao Univ, Qingdao Womens & Childrens Hosp, Ctr Reprod Med, Qingdao 266034, Peoples R China.	[Wu, Rui-Ying; Xu, Chun-Shuang; Zou, Shu-Hua] Qingdao University, Qingdao Womens & Childrens Hosp, Ctr Reprod Med, Qingdao 266034, China.	Country: China<br>Institution: Qingdao Univ	Country: China<br>Institution: Qingdao University
C1	3	54905	54905	[Zhao, Yong; Ge, Wei; Shen, Wei] Qingdao Agr Univ, Inst Reprod Sci, Coll Life Sci, Qingdao 266109, Peoples R China.	[Zhao, Yong; Ge, Wei; Shen, Wei] Qingdao Agricultural University, Inst Reprod Sci, Coll Life Sci, Qingdao 266109, China.	Country: China<br>Institution: Qingdao Agr Univ	Country: China<br>Institution: Qingdao Agricultural University
C1	4	54906	54906	[Liu, Jing] Qingdao Agr Univ, Core Labs, Qingdao 266109, Peoples R China.	[Liu, Jing] Qingdao Agricultural University, Core Labs, Qingdao 266109, China.	Country: China<br>Institution: Qingdao Agr Univ	Country: China<br>Institution: Qingdao Agricultural University
C1	5	54907	54907	[Sun, Zhong-Yi] Third Mil Med Univ, Daping Hosp, Inst Surg Res, Dept Urol, Chongqing 400042, Peoples R China.	[Sun, Zhong-Yi] Third Mil Med Univ, Daping Hosp, Inst Surg Res, Dept Urol, Chongqing 400042, China.	Country: China<br>Institution: Third Mil Med Univ	Country: China<br>Institution: Third Mil Med Univ
RP	1	54911	54911	Shen, W (corresponding author), Qingdao Agr Univ, Coll Anim Sci & Technol, Qingdao 266109, Peoples R China	Shen, W (corresponding author), Qingdao Agricultural University, Coll Anim Sci & Technol, Qingdao 266109, Peoples R China	Country: China<br>Institution: Qingdao Agr Univ	Country: China<br>Institution: Qingdao Agricultural University
RP	2	54911	54911	Shen, W (corresponding author), Qingdao Agr Univ, Inst Reprod Sci, Coll Life Sci, Qingdao 266109, Peoples R China	Shen, W (corresponding author), Qingdao Agr Univ, Inst Reprod Sci, Coll Life Sci, Qingdao 266109, China	Country: China<br>Institution: Qingdao Agr Univ	Country: China<br>Institution: Qingdao Agr Univ

Record 364 | WOS:000423566000030

Individual and combined cytotoxicity assessment of zearalenone with ochratoxin A or Î±-zearalenol by full factorial design

Field	Position	Original line	Normalised line	Original value	Normalised value	Original country / institution	Normalised country / institution

C1	1	55019	55019	[Zheng, N.; Gao, Y. N.; Wang, H. W.; Wang, J. Q.] Chinese Acad Agr Sci, Inst Anim Sci, Minist Agr, Lab Qual & Safety Risk Assessment Dairy Prod Beij, Beijing 100193, Peoples R China.	[Zheng, N.; Gao, Y. N.; Wang, H. W.; Wang, J. Q.] Chinese Academy of Agricultural Sciences, Inst Anim Sci, Minist Agr, Lab Qual & Safety Risk Assessment Dairy Prod Beij, Beijing 100193, China.	Country: China<br>Institution: Chinese Acad Agr Sci	Country: China<br>Institution: Chinese Academy of Agricultural Sciences
C1	2	55020	55020	[Zheng, N.; Gao, Y. N.; Wang, J. Q.] Chinese Acad Agr Sci, Inst Anim Sci, State Key Lab Anim Nutr, 2 Yuanmingyuan West Rd, Beijing 100193, Peoples R China.	[Zheng, N.; Gao, Y. N.; Wang, J. Q.] Chinese Academy of Agricultural Sciences, Inst Anim Sci, State Key Lab Anim Nutr, 2 Yuanmingyuan West Rd, Beijing 100193, China.	Country: China<br>Institution: Chinese Acad Agr Sci	Country: China<br>Institution: Chinese Academy of Agricultural Sciences
C1	3	55021	55021	[Wang, H. W.] Gansu Agr Univ, Coll Anim Sci & Technol, Lanzhou 730070, Gansu, Peoples R China.	[Wang, H. W.] Gansu Agricultural University, Coll Anim Sci & Technol, Lanzhou 730070, Gansu, China.	Country: China<br>Institution: Gansu Agr Univ	Country: China<br>Institution: Gansu Agricultural University
C1	4	55022	55022	[Liu, J.] China Natl Res Inst Food & Fermentat Ind, Beijing 100027, Peoples R China.	[Liu, J.] China Natl Res Inst Food & Fermentat Ind, Beijing 100027, China.	Country: China<br>Institution: China Natl Res Inst Food & Fermentat Ind	Country: China<br>Institution: China Natl Res Inst Food & Fermentat Ind
RP	1	55026	55026	Wang, JQ (corresponding author), Chinese Acad Agr Sci, Inst Anim Sci, Minist Agr, Lab Qual & Safety Risk Assessment Dairy Prod Beij, Beijing 100193, Peoples R China	Wang, JQ (corresponding author), Chinese Academy of Agricultural Sciences, Inst Anim Sci, Minist Agr, Lab Qual & Safety Risk Assessment Dairy Prod Beij, Beijing 100193, Peoples R China	Country: China<br>Institution: Chinese Acad Agr Sci	Country: China<br>Institution: Chinese Academy of Agricultural Sciences
RP	2	55026	55026	Wang, JQ (corresponding author), Chinese Acad Agr Sci, Inst Anim Sci, State Key Lab Anim Nutr, 2 Yuanmingyuan West Rd, Beijing 100193, Peoples R China	Wang, JQ (corresponding author), Chinese Acad Agr Sci, Inst Anim Sci, State Key Lab Anim Nutr, 2 Yuanmingyuan West Rd, Beijing 100193, China	Country: China<br>Institution: Chinese Acad Agr Sci	Country: China<br>Institution: Chinese Acad Agr Sci

Record 365 | WOS:000416024300043

Effect of rMnSOD on Sodium Reabsorption in Renal Proximal Tubule in Ochratoxin ATreated Rats

Field	Position	Original line	Normalised line	Original value	Normalised value	Original country / institution	Normalised country / institution
C1	1	55137	55137	[Damiano, Sara; Puzio, Maria V.; Squillacioti, Caterina; Mirabella, Nicola; Avallone, Luigi; Florio, Salvatore; Ciarcia, Roberto] Univ Naples Federico II, Dept Vet Med, Anim Prod, I-80137 Naples, Italy.	[Damiano, Sara; Puzio, Maria V.; Squillacioti, Caterina; Mirabella, Nicola; Avallone, Luigi; Florio, Salvatore; Ciarcia, Roberto] University of Naples Federico II, Dept Vet Med, Anim Prod, I-80137 Naples, Italy.	Country: Italy<br>Institution: Univ Naples Federico II	Country: Italy<br>Institution: University of Naples Federico II
C1	5	55141	55141	[Astarita, Carlo] Univ Siena, Dept Med Surg & Neurosci, Siena, Italy.	[Astarita, Carlo] University of Siena, Dept Med Surg & Neurosci, Siena, Italy.	Country: Italy<br>Institution: Univ Siena	Country: Italy<br>Institution: University of Siena
C1	6	55142	55142	[Astarita, Carlo; Boffo, Silvia; Giordano, Antonio] Temple Univ, Coll Sci & Technol, Ctr Biotechnol, Sbarro Inst Canc Res & Mol Med, Philadelphia, PA 19122 USA.	[Astarita, Carlo; Boffo, Silvia; Giordano, Antonio] Temple University, Coll Sci & Technol, Ctr Biotechnol, Sbarro Inst Canc Res & Mol Med, Philadelphia, PA 19122 USA.	Country: USA<br>Institution: Temple Univ	Country: USA<br>Institution: Temple University
RP	1	55146	55146	Ciarcia, R (corresponding author), Univ Naples Federico II, Dept Vet Med & Anim Prod, Via Delpino 1, I-80137 Naples, Italy	Ciarcia, R (corresponding author), University of Naples Federico II, Dept Vet Med & Anim Prod, Via Delpino 1, I-80137 Naples, Italy	Country: Italy<br>Institution: Univ Naples Federico II	Country: Italy<br>Institution: University of Naples Federico II

Record 366 | WOS:000435675100011

Cinnamon (Cinnamomum zeylanicum) as an antidote or a protective agent against natural or chemical toxicities: a review

Field	Position	Original line	Normalised line	Original value	Normalised value	Original country / institution	Normalised country / institution
C1	1	55237	55237	[Dorri, Mahyar] Mashhad Univ Med Sci, Dept Pharmacognosy, Sch Pharm, Mashhad, Iran.	[Dorri, Mahyar] Mashhad University of Medical Sciences, Dept Pharmacognosy, Sch Pharm, Mashhad, Iran.	Country: Iran Institution: Mashhad Univ Med Sci	Country: Iran Institution: Mashhad University of Medical Sciences
C1	2	55238	55238	[Hashemitabar, Shirin] Mashhad Univ Med Sci, Biotechnol Res Ctr, Mashhad, Iran.	[Hashemitabar, Shirin] Mashhad University of Medical Sciences, Biotechnol Res Ctr, Mashhad, Iran.	Country: Iran Institution: Mashhad Univ Med Sci	Country: Iran Institution: Mashhad University of Medical Sciences
C1	3	55239	55239	[Hosseinzadeh, Hossein] Mashhad Univ Med Sci, Dept Pharmacodynamy & Toxicol, Sch Pharm, Mashhad, Iran.	[Hosseinzadeh, Hossein] Mashhad University of Medical Sciences, Dept Pharmacodynamy & Toxicol, Sch Pharm, Mashhad, Iran.	Country: Iran Institution: Mashhad Univ Med Sci	Country: Iran Institution: Mashhad University of Medical Sciences
RP	1	55242	55242	Hosseinzadeh, H (corresponding author), Mashhad Univ Med Sci, Pharmaceut Res Ctr, Inst Pharmaceut Technol, Mashhad, Iran	Hosseinzadeh, H (corresponding author), Mashhad University of Medical Sciences, Pharmaceut Res Ctr, Inst Pharmaceut Technol, Mashhad, Iran	Country: Iran Institution: Mashhad Univ Med Sci	Country: Iran Institution: Mashhad University of Medical Sciences

Record 367 | WOS:000423248100019

Modified mycotoxins: An updated review on their formation, detection, occurrence, and toxic effects

Field	Position	Original line	Normalised line	Original value	Normalised value	Original country / institution	Normalised country / institution
C1	1	55377	55377	[Freire, Luisa; Sant'Ana, Anderson S.] Univ Estadual Campinas, Fac Food Engn FEA, Dept Food Sci, Campinas, SP, Brazil.	[Freire, Luisa; Sant'Ana, Anderson S.] Universidade Estadual de Campinas, Fac Food Engn FEA, Dept Food Sci, Campinas, SP, Brazil.	Country: Brazil Institution: Univ Estadual Campinas	Country: Brazil Institution: Universidade Estadual de Campinas
RP	1	55379	55379	Sant'Ana, AS (corresponding author), Univ Estadual Campinas, Fac Food Engn FEA, Dept Food Sci, Campinas, SP, Brazil	Sant'Ana, AS (corresponding author), Universidade Estadual de Campinas, Fac Food Engn FEA, Dept Food Sci, Campinas, SP, Brazil	Country: Brazil Institution: Univ Estadual Campinas	Country: Brazil Institution: Universidade Estadual de Campinas

Record 368 | WOS:000423248100048

In vitro mechanisms of Beauvericin toxicity: A review

Field	Position	Original line	Normalised line	Original value	Normalised value	Original country / institution	Normalised country / institution
C1	1	55636	55636	[Mallebrera, Beatriz; Prosperini, Alessandra; Font, Guillermina; Jose Ruiz, Maria] Univ Valencia, Lab Toxicol, Fac Pharm, Av Vicent Andres Estelles S-N, E-46100 Valencia, Spain.	[Mallebrera, Beatriz; Prosperini, Alessandra; Font, Guillermina; Jose Ruiz, Maria] University of Valencia, Lab Toxicol, Fac Pharm, Av Vicent Andres Estelles S-N, E-46100 Valencia, Spain.	Country: Spain Institution: Univ Valencia	Country: Spain Institution: University of Valencia
RP	1	55638	55638	Prosperini, A (corresponding author), Univ Valencia, Lab Toxicol, Fac Pharm, Av Vicent	Prosperini, A (corresponding author), University of Valencia, Lab Toxicol, Fac Pharm, Av	Country: Spain Institution: Univ Valencia	Country: Spain Institution: University of Valencia

				Andres Estelles S-N, E-46100 Valencia, Spain	Vicent Andres Estelles S-N, E-46100 Valencia, Spain		

Record 369 | WOS:000418302000021

Modulations of DNMT1 and HDAC1 are involved in the OTA-induced cytotoxicity and apoptosis in vitro

Field	Position	Original line	Normalised line	Original value	Normalised value	Original country / institution	Normalised country / institution
C1	1	55795	55795	[Zhou, Yajiao; Gan, Fang; Hou, Lili; Zhou, Xuan; Ibrahim, Yassin Abdulrahim Adam; Huang, Kehe] Nanjing Agr Univ, Coll Vet Med, Nanjing 210095, Jiangsu, Peoples R China.	[Zhou, Yajiao; Gan, Fang; Hou, Lili; Zhou, Xuan; Ibrahim, Yassin Abdulrahim Adam; Huang, Kehe] Nanjing Agricultural University, Coll Vet Med, Nanjing 210095, Jiangsu, China.	Country: China<br>Institution: Nanjing Agr Univ	Country: China<br>Institution: Nanjing Agricultural University
C1	2	55796	55796	[Zhou, Yajiao; Gan, Fang; Hou, Lili; Zhou, Xuan; Ibrahim, Yassin Abdulrahim Adam; Huang, Kehe] Nanjing Agr Univ, Inst Nutr & Metab Disorders Domest Anim & Fowls, Nanjing 210095, Jiangsu, Peoples R China.	[Zhou, Yajiao; Gan, Fang; Hou, Lili; Zhou, Xuan; Ibrahim, Yassin Abdulrahim Adam; Huang, Kehe] Nanjing Agricultural University, Inst Nutr & Metab Disorders Domest Anim & Fowls, Nanjing 210095, Jiangsu, China.	Country: China<br>Institution: Nanjing Agr Univ	Country: China<br>Institution: Nanjing Agricultural University
RP	1	55798	55798	Huang, KH (corresponding author), Nanjing Agr Univ, Coll Vet Med, Nanjing 210095, Jiangsu, Peoples R China	Huang, KH (corresponding author), Nanjing Agricultural University, Coll Vet Med, Nanjing 210095, Jiangsu, China	Country: China<br>Institution: Nanjing Agr Univ	Country: China<br>Institution: Nanjing Agricultural University

Record 370 | WOS:000417670500029

Ochratoxin A-Induced Apoptosis of IPEC-J2 Cells through ROS-Mediated Mitochondrial Permeability Transition Pore Opening Pathway

Field	Position	Original line	Normalised line	Original value	Normalised value	Original country / institution	Normalised country / institution
C1	1	55906	55906	[Wang, Hong; Chen, Ying; Zhai, Nianhui; Chen, Xingxiang; Gan, Fang; Li, Hu; Huang, Kehe] Nanjing Agr Univ, Coll Vet Med, Nanjing 210095, Jiangsu, Peoples R China.	[Wang, Hong; Chen, Ying; Zhai, Nianhui; Chen, Xingxiang; Gan, Fang; Li, Hu; Huang, Kehe] Nanjing Agricultural University, Coll Vet Med, Nanjing 210095, Jiangsu, China.	Country: China<br>Institution: Nanjing Agr Univ	Country: China<br>Institution: Nanjing Agricultural University
C1	2	55907	55907	[Wang, Hong; Chen, Ying; Zhai, Nianhui; Chen, Xingxiang; Gan, Fang; Li, Hu; Huang, Kehe] Nanjing Agr Univ, Inst Nutr & Metab Disorders Domest Anim & Fowls, Nanjing 210095, Jiangsu, Peoples R China.	[Wang, Hong; Chen, Ying; Zhai, Nianhui; Chen, Xingxiang; Gan, Fang; Li, Hu; Huang, Kehe] Nanjing Agricultural University, Inst Nutr & Metab Disorders Domest Anim & Fowls, Nanjing 210095, Jiangsu, China.	Country: China<br>Institution: Nanjing Agr Univ	Country: China<br>Institution: Nanjing Agricultural University
RP	1	55909	55909	Huang, KH (corresponding author), Nanjing Agr Univ, Coll Vet Med, Nanjing 210095, Jiangsu, Peoples R China	Huang, KH (corresponding author), Nanjing Agricultural University, Coll Vet Med, Nanjing 210095, Jiangsu, Peoples R China	Country: China<br>Institution: Nanjing Agr Univ	Country: China<br>Institution: Nanjing Agricultural University
RP	2	55909	55909	Huang, KH (corresponding author), Nanjing Agr Univ, Inst Nutr & Metab Disorders Domest Anim & Fowls, Nanjing 210095, Jiangsu, Peoples R China	Huang, KH (corresponding author), Nanjing Agr Univ, Inst Nutr & Metab Disorders Domest Anim & Fowls, Nanjing 210095, Jiangsu, China	Country: China<br>Institution: Nanjing Agr Univ	Country: China<br>Institution: Nanjing Agr Univ

Record 371 | WOS:000418211700013

A comprehensive review on biological properties of citrinin

Field	Position	Original line	Normalised line	Original value	Normalised value	Original country / institution	Normalised country / institution
C1	6	56060	56060	[Uddin, Shaikh Jamal] Khulna Univ, Life Sci Sch, Pharm Discipline, Khulna 9208, Bangladesh.	[Uddin, Shaikh Jamal] Khulna University, Life Sci Sch, Pharm Discipline, Khulna 9208, Bangladesh.	Country: Bangladesh Institution: Khulna Univ	Country: Bangladesh Institution: Khulna University
C1	10	56064	56064	[Kamal, Mohammad Amjad] King Abdulaziz Univ, King Fand Med Res Ctr, Jeddah, Saudi Arabia.	[Kamal, Mohammad Amjad] King Abdulaziz University, King Fand Med Res Ctr, Jeddah, Saudi Arabia.	Country: Saudi Arabia Institution: King Abdulaziz Univ	Country: Saudi Arabia Institution: King Abdulaziz University
C1	13	56067	56067	[Pieczynska, Magdalena D.; Atanasov, Atanas G.] Polish Acad Sci, Inst Genet & Anim Breeding, Jastrzebiec, Poland.	[Pieczynska, Magdalena D.; Atanasov, Atanas G.] Polish Academy of Sciences, Inst Genet & Anim Breeding, Jastrzebiec, Poland.	Country: Poland Institution: Polish Acad Sci	Country: Poland Institution: Polish Academy of Sciences
C1	17	56071	56071	[Atanasov, Atanas G.] Univ Vienna, Fac Life Sci, Dept Pharmacognosy, Althan Str 14, A-1090 Vienna, Austria.	[Atanasov, Atanas G.] University of Vienna, Fac Life Sci, Dept Pharmacognosy, Althan Str 14, A-1090 Vienna, Austria.	Country: Austria Institution: Univ Vienna	Country: Austria Institution: University of Vienna
C1	19	56073	56073	[Gupta, Vijai K.] Tallinn Univ Technol, Sch Sci, ERA Chair Green Chem, Dept Chem & Biotechnol, EE-12618 Tallinn, Estonia.	[Gupta, Vijai K.] Tallinn University of Technology, Sch Sci, ERA Chair Green Chem, Dept Chem & Biotechnol, EE-12618 Tallinn, Estonia.	Country: Estonia Institution: Tallinn Univ Technol	Country: Estonia Institution: Tallinn University of Technology
RP	1	56087	56087	Atanasov, AG (corresponding author), Polish Acad Sci, Inst Genet & Anim Breeding, Jastrzebiec, Poland	Atanasov, AG (corresponding author), Polish Academy of Sciences, Inst Genet & Anim Breeding, Jastrzebiec, Poland	Country: Poland Institution: Polish Acad Sci	Country: Poland Institution: Polish Academy of Sciences

Record 372 | WOS:000416589800031

Differential Gene Expression Analysis of Bovine Macrophages after Exposure to the Penicillium Mycotoxins Citrinin and/or Ochratoxin A

Field	Position	Original line	Normalised line	Original value	Normalised value	Original country / institution	Normalised country / institution
C1	2	56282	56282	[Oh, Se-Young; Karrow, Niel A.] Univ Guelph, Dept Anim Biosci, Guelph, ON N1G 2W1, Canada.	[Oh, Se-Young; Karrow, Niel A.] University of Guelph, Dept Anim Biosci, Guelph, ON N1G 2W1, Canada.	Country: Canada Institution: Univ Guelph	Country: Canada Institution: University of Guelph

Record 373 | WOS:000415911400030

UPLC-MS/MS analysis of ochratoxin A metabolites produced by Caco-2 and HepG2 cells in a co-culture system

No C1/C3/RP normalisation changes detected for this record.

Record 374 | WOS:000410635200006

Effects of ochratoxin A on ER stress, MAPK signaling pathway and autophagy of kidney and spleen in pigs

Field	Position	Original line	Normalised line	Original value	Normalised value	Original country / institution	Normalised country / institution

C1	1	56545	56545	[Gan, Fang; Hou, Lili; Zhou, Yajiao; Liu, Yunhuan; Huang, Da; Chen, Xingxiang; Huang, Kehe] Nanjing Agr Univ, Coll Vet Med, Nanjing 210095, Jiangsu, Peoples R China.	[Gan, Fang; Hou, Lili; Zhou, Yajiao; Liu, Yunhuan; Huang, Da; Chen, Xingxiang; Huang, Kehe] Nanjing Agricultural University, Coll Vet Med, Nanjing 210095, Jiangsu, China.	Country: China<br>Institution: Nanjing Agr Univ	Country: China<br>Institution: Nanjing Agricultural University
C1	2	56546	56546	[Gan, Fang; Hou, Lili; Zhou, Yajiao; Liu, Yunhuan; Huang, Da; Chen, Xingxiang; Huang, Kehe] Nanjing Agr Univ, Inst Nutr & Metab Disorders Domest Anim & Fowls, Nanjing 210095, Jiangsu, Peoples R China.	[Gan, Fang; Hou, Lili; Zhou, Yajiao; Liu, Yunhuan; Huang, Da; Chen, Xingxiang; Huang, Kehe] Nanjing Agricultural University, Inst Nutr & Metab Disorders Domest Anim & Fowls, Nanjing 210095, Jiangsu, China.	Country: China<br>Institution: Nanjing Agr Univ	Country: China<br>Institution: Nanjing Agricultural University
RP	1	56548	56548	Huang, KH (corresponding author), Nanjing Agr Univ, Coll Vet Med, Nanjing 210095, Jiangsu, Peoples R China	Huang, KH (corresponding author), Nanjing Agricultural University, Coll Vet Med, Nanjing 210095, Jiangsu, China	Country: China<br>Institution: Nanjing Agr Univ	Country: China<br>Institution: Nanjing Agricultural University

Record 375 | WOS:000414913800067

Evaluation of Chitosan and Cellulosic Polymers as Binding Adsorbent Materials to Prevent Aflatoxin B1, Fumonisin B1, Ochratoxin, Trichothecene, Deoxynivalenol, and Zearalenone Mycotoxicoses Through an In Vitro Gastrointestinal Model for Poultry

No C1/C3/RP normalisation changes detected for this record.

Record 376 | WOS:000408520200003

Worldwide Occurrence of Mycotoxins in Cereals and Cereal-Derived Food Products: Public Health Perspectives of Their Co-occurrence

Field	Position	Original line	Normalised line	Original value	Normalised value	Original country / institution	Normalised country / institution
C1	1	56775	56775	[Lee, Hyun Jung; Ryu, Dojin] Univ Idaho, Sch Food Sci, 875 Perimeter Dr MS 2312, Moscow, ID 83844 USA.	[Lee, Hyun Jung; Ryu, Dojin] University of Idaho, Sch Food Sci, 875 Perimeter Dr MS 2312, Moscow, ID 83844 USA.	Country: USA<br>Institution: Univ Idaho	Country: USA<br>Institution: University of Idaho
RP	1	56777	56777	Ryu, D (corresponding author), Univ Idaho, Sch Food Sci, 875 Perimeter Dr MS 2312, Moscow, ID 83844 USA	Ryu, D (corresponding author), University of Idaho, Sch Food Sci, 875 Perimeter Dr MS 2312, Moscow, ID 83844 USA	Country: USA<br>Institution: Univ Idaho	Country: USA<br>Institution: University of Idaho

Record 377 | WOS:000408520200008

Mutagenicity of Ochratoxin A: Role for a Carbon-Linked C8-Deoxyguanosine Adduct?

Field	Position	Original line	Normalised line	Original value	Normalised value	Original country / institution	Normalised country / institution
C1	1	57065	57065	[Manderville, Richard A.] Univ Guelph, Dept Chem, Guelph, ON N1G 2W1, Canada.	[Manderville, Richard A.] University of Guelph, Dept Chem, Guelph, ON N1G 2W1, Canada.	Country: Canada<br>Institution: Univ Guelph	Country: Canada<br>Institution: University of Guelph
C1	2	57066	57066	[Manderville, Richard A.] Univ Guelph, Dept Toxicol, Guelph, ON N1G 2W1, Canada.	[Manderville, Richard A.] University of Guelph, Dept Toxicol, Guelph, ON N1G 2W1, Canada.	Country: Canada<br>Institution: Univ Guelph	Country: Canada<br>Institution: University of Guelph
C1	3	57067	57067	[Wetmore, Stacey D.] Univ Lethbridge, Dept Chem & Biochem, Lethbridge, AB T1K 3M4, Canada.	[Wetmore, Stacey D.] University of Lethbridge, Dept Chem & Biochem, Lethbridge, AB T1K 3M4, Canada.	Country: Canada<br>Institution: Univ Lethbridge	Country: Canada<br>Institution: University of Lethbridge

RP	1	57069	57069	Manderville, RA (corresponding author), Univ Guelph, Dept Chem, Guelph, ON N1G 2W1, Canada	Manderville, RA (corresponding author), University of Guelph, Dept Chem, Guelph, ON N1G 2W1, Canada	Country: Canada Institution: Univ Guelph	Country: Canada Institution: University of Guelph

Record 378 | WOS:000408074600028

Overexpression and Low Expression of Selenoprotein S Impact Ochratoxin A-Induced Porcine Cytotoxicity and Apoptosis in Vitro

Field	Position	Original line	Normalised line	Original value	Normalised value	Original country / institution	Normalised country / institution
C1	1	57189	57189	[Gan, Fang; Hu, Zhihua; Zhou, Yajiao; Huang, Kehe] Nanjing Agr Univ, Coll Vet Med, Nanjing 210095, Jiangsu, Peoples R China.	[Gan, Fang; Hu, Zhihua; Zhou, Yajiao; Huang, Kehe] Nanjing Agricultural University, Coll Vet Med, Nanjing 210095, Jiangsu, China.	Country: China Institution: Nanjing Agr Univ	Country: China Institution: Nanjing Agricultural University
RP	1	57191	57191	Huang, KH (corresponding author), Nanjing Agr Univ, Coll Vet Med, Nanjing 210095, Jiangsu, Peoples R China	Huang, KH (corresponding author), Nanjing Agricultural University, Coll Vet Med, Nanjing 210095, Jiangsu, China	Country: China Institution: Nanjing Agr Univ	Country: China Institution: Nanjing Agricultural University

Record 379 | WOS:000411538100005

Nrf2 deficiency exacerbates ochratoxin A-induced toxicity in vitro and in vivo

Field	Position	Original line	Normalised line	Original value	Normalised value	Original country / institution	Normalised country / institution
C1	1	57301	57301	[Loboda, Agnieszka; Stachurska, Anna; Sobczak, Mateusz; Podkalicka, Paulina; Mucha, Olga; Jozkowicz, Alicja; Dulak, Jozef] Jagiellonian Univ, Fac Biochem Biophys & Biotechnol, Dept Med Biotechnol, Gronostajowa 7, PL-30387 Krakow, Poland.	[Loboda, Agnieszka; Stachurska, Anna; Sobczak, Mateusz; Podkalicka, Paulina; Mucha, Olga; Jozkowicz, Alicja; Dulak, Jozef] Jagiellonian University, Fac Biochem Biophys & Biotechnol, Dept Med Biotechnol, Gronostajowa 7, PL-30387 Krakow, Poland.	Country: Poland Institution: Jagiellonian Univ	Country: Poland Institution: Jagiellonian University
RP	1	57303	57303	Loboda, A (corresponding author), Jagiellonian Univ, Fac Biochem Biophys & Biotechnol, Dept Med Biotechnol, Gronostajowa 7, PL-30387 Krakow, Poland	Loboda, A (corresponding author), Jagiellonian University, Fac Biochem Biophys & Biotechnol, Dept Med Biotechnol, Gronostajowa 7, PL-30387 Krakow, Poland	Country: Poland Institution: Jagiellonian Univ	Country: Poland Institution: Jagiellonian University

Record 380 | WOS:000406735700003

Ochratoxin A transport by the human breast cancer resistance protein (BCRP), multidrug resistance protein 2 (MRP2), and organic anion-transporting polypeptides 1A2, 1B1 and 2B1

Field	Position	Original line	Normalised line	Original value	Normalised value	Original country / institution	Normalised country / institution
C1	1	57414	57414	[Qi, Xiaozhe; Xu, Wentao; Huang, Kunlun] China Agr Univ, Coll Food Sci & Nutr Engn, Beijing Adv Innovat Ctr Food Nutr & Human Hlth, Beijing 100083, Peoples R China.	[Qi, Xiaozhe; Xu, Wentao; Huang, Kunlun] China Agricultural University, Coll Food Sci & Nutr Engn, Beijing Adv Innovat Ctr Food Nutr & Human Hlth, Beijing 100083, China.	Country: China Institution: China Agr Univ	Country: China Institution: China Agricultural University
C1	3	57416	57416	[Xu, Wentao; Huang, Kunlun] China Agr Univ, Beijing Lab Food Qual & Safety, Coll Food	[Xu, Wentao; Huang, Kunlun] China Agricultural University, Beijing Lab Food Qual & Safety,	Country: China Institution: China Agr Univ	Country: China Institution: China Agricultural University

				Sci & Nutr Engn, Beijing 100083, Peoples R China.	Coll Food Sci & Nutr Engn, Beijing 100083, China.		
RP	1	57419	57419	Huang, KL (corresponding author), China Agr Univ, Coll Food Sci & Nutr Engn, Beijing Adv Innovat Ctr Food Nutr & Human Hlth, Beijing 100083, Peoples R China	Huang, KL (corresponding author), China Agricultural University, Coll Food Sci & Nutr Engn, Beijing Adv Innovat Ctr Food Nutr & Human Hlth, Beijing 100083, Peoples R China	Country: China<br>Institution: China Agr Univ	Country: China<br>Institution: China Agricultural University

Record 381 | WOS:000407370700051

Health Risks Associated with Exposure to Filamentous Fungi

Field	Position	Original line	Normalised line	Original value	Normalised value	Original country / institution	Normalised country / institution
C1	2	57490	57490	[Egbuta, Mary Augustina] Southern Cross Univ, Southern Cross Plant Sci, Lismore, NSW 2480, Australia.	[Egbuta, Mary Augustina] Southern Cross University, Southern Cross Plant Sci, Lismore, NSW 2480, Australia.	Country: Australia<br>Institution: Southern Cross Univ	Country: Australia<br>Institution: Southern Cross University

Record 382 | WOS:000404237400013

Evaluation of the automated MicroFlowÂ® and Metaferâ ¢ platforms for high-throughput micronucleus scoring and dose response analysis in human lymphoblastoid TK6 cells

Field	Position	Original line	Normalised line	Original value	Normalised value	Original country / institution	Normalised country / institution
C1	1	57693	57693	[Verma, Jatin R.; Rees, Benjamin J.; Wilde, Eleanor C.; Thornton, Catherine A.; Jenkins, Gareth J. S.; Doak, Shareen H.; Johnson, George E.] Swansea Univ, Inst Life Sci, Sch Med, Swansea SA2 8PP, W Glam, Wales.	[Verma, Jatin R.; Rees, Benjamin J.; Wilde, Eleanor C.; Thornton, Catherine A.; Jenkins, Gareth J. S.; Doak, Shareen H.; Johnson, George E.] Swansea University, Inst Life Sci, Sch Med, Swansea SA2 8PP, W Glam, United Kingdom.	Country: Wales<br>Institution: Swansea Univ	Country: United Kingdom<br>Institution: Swansea University
RP	1	57695	57695	Verma, JR (corresponding author), Swansea Univ, Inst Life Sci, Sch Med, Swansea SA2 8PP, W Glam, Wales	Verma, JR (corresponding author), Swansea University, Inst Life Sci, Sch Med, Swansea SA2 8PP, W Glam, United Kingdom	Country: Wales<br>Institution: Swansea Univ	Country: United Kingdom<br>Institution: Swansea University

Record 383 | WOS:000404198300012

In vitro immune toxicity of ochratoxin A in porcine alveolar macrophages: A role for the ROS-relative TLR4/MyD88 signaling pathway

Field	Position	Original line	Normalised line	Original value	Normalised value	Original country / institution	Normalised country / institution
C1	1	57787	57787	[Xu, Haibin; Hao, Shu; Gan, Fang; Wang, Hong; Xu, Jing; Liu, Dandan; Huang, Kehe] Nanjing Agr Univ, Coll Vet Med, Nanjing 210095, Jiangsu, Peoples R China.	[Xu, Haibin; Hao, Shu; Gan, Fang; Wang, Hong; Xu, Jing; Liu, Dandan; Huang, Kehe] Nanjing Agricultural University, Coll Vet Med, Nanjing 210095, Jiangsu, China.	Country: China<br>Institution: Nanjing Agr Univ	Country: China<br>Institution: Nanjing Agricultural University
C1	2	57788	57788	[Xu, Haibin; Hao, Shu; Gan, Fang; Wang, Hong; Xu, Jing; Liu, Dandan; Huang, Kehe] Nanjing Agr Univ, Inst Nutr & Metab Disorders Domest Anim & Fowls, Nanjing 210095, Jiangsu, Peoples R China.	[Xu, Haibin; Hao, Shu; Gan, Fang; Wang, Hong; Xu, Jing; Liu, Dandan; Huang, Kehe] Nanjing Agricultural University, Inst Nutr & Metab Disorders Domest Anim & Fowls, Nanjing 210095, Jiangsu, China.	Country: China<br>Institution: Nanjing Agr Univ	Country: China<br>Institution: Nanjing Agricultural University

RP	1	57790	57790	Huang, KH (corresponding author), Nanjing Agr Univ, Coll Vet Med, Nanjing 210095, Jiangsu, Peoples R China	Huang, KH (corresponding author), Nanjing Agricultural University, Coll Vet Med, Nanjing 210095, Jiangsu, China	Country: China<br>Institution: Nanjing Agr Univ	Country: China<br>Institution: Nanjing Agricultural University

Record 384 | WOS:000401600400001

Adsorption of aflatoxin B1, zearalenone and ochratoxin A by microorganisms isolated from Kefir grains

Field	Position	Original line	Normalised line	Original value	Normalised value	Original country / institution	Normalised country / institution
C1	2	57898	57898	[Chaieb, Kamel] Taibah Univ, Biol Dept, Coll Sci, Al Madinah Al Monawarah, Saudi Arabia.	[Chaieb, Kamel] Taibah University, Biol Dept, Coll Sci, Al Madinah Al Monawarah, Saudi Arabia.	Country: Saudi Arabia<br>Institution: Taibah Univ	Country: Saudi Arabia<br>Institution: Taibah University
C1	3	57899	57899	[Kouidhi, Bochra] Taibah Univ, Med Lab Dept, Coll Appl Med Sci, Al Madinah Al Monawarah, Saudi Arabia.	[Kouidhi, Bochra] Taibah University, Med Lab Dept, Coll Appl Med Sci, Al Madinah Al Monawarah, Saudi Arabia.	Country: Saudi Arabia<br>Institution: Taibah Univ	Country: Saudi Arabia<br>Institution: Taibah University
RP	1	57903	57903	Chaieb, K (corresponding author), Taibah Univ, Biol Dept, Coll Sci, Al Madinah Al Monawarah, Saudi Arabia	Chaieb, K (corresponding author), Taibah University, Biol Dept, Coll Sci, Al Madinah Al Monawarah, Saudi Arabia	Country: Saudi Arabia<br>Institution: Taibah Univ	Country: Saudi Arabia<br>Institution: Taibah University

Record 385 | WOS:000408731500007

Lycopene restores trace element levels in ochratoxin A-treated rats

Field	Position	Original line	Normalised line	Original value	Normalised value	Original country / institution	Normalised country / institution
C1	1	58016	58016	[Palabiyik, Saziye Sezin] Ataturk Univ, Fac Pharm, Dept Toxicol, Erzurum, Turkey.	[Palabiyik, Saziye Sezin] Ataturk University, Fac Pharm, Dept Toxicol, Erzurum, Turkey.	Country: Turkey<br>Institution: Ataturk Univ	Country: Turkey<br>Institution: Ataturk University
C1	2	58017	58017	[Erkekoglu, Pinar; Kocer-Gumusel, Belma] Hacettepe Univ, Fac Pharm, Dept Toxicol, TR-06100 Ankara, Turkey.	[Erkekoglu, Pinar; Kocer-Gumusel, Belma] Hacettepe University, Fac Pharm, Dept Toxicol, TR-06100 Ankara, Turkey.	Country: Turkey<br>Institution: Hacettepe Univ	Country: Turkey<br>Institution: Hacettepe University
C1	4	58019	58019	[Sahin, Gonul] Eastern Mediterranean Univ, Fac Pharm, Dept Toxicol, Mersin, Turkey.	[Sahin, Gonul] Eastern Mediterranean University, Fac Pharm, Dept Toxicol, Mersin, Turkey.	Country: Turkey<br>Institution: Eastern Mediterranean Univ	Country: Turkey<br>Institution: Eastern Mediterranean University
RP	1	58023	58023	Kocer-Gumusel, B (corresponding author), Hacettepe Univ, Fac Pharm, Dept Toxicol, TR-06100 Ankara, Turkey	Kocer-Gumusel, B (corresponding author), Hacettepe University, Fac Pharm, Dept Toxicol, TR-06100 Ankara, Turkey	Country: Turkey<br>Institution: Hacettepe Univ	Country: Turkey<br>Institution: Hacettepe University

Record 386 | WOS:000403637300009

Ochratoxin A induced premature senescence in human renal proximal tubular cells

Field	Position	Original line	Normalised line	Original value	Normalised value	Original country / institution	Normalised country / institution
C1	1	58122	58122	[Yang, Xuan; Liu, Sheng; Huang, Chuchu; Wang, Haomiao; Luo, Yunbo; Xu, Wentao; Huang, Kunlun] China Agr Univ, Coll Food Sci & Nutr Engn, Beijing Adv Innovat Ctr	[Yang, Xuan; Liu, Sheng; Huang, Chuchu; Wang, Haomiao; Luo, Yunbo; Xu, Wentao; Huang, Kunlun] China Agricultural University, Coll Food Sci & Nutr Engn, Beijing	Country: China<br>Institution: China Agr Univ	Country: China<br>Institution: China Agricultural University

				Food Nutr & Human Hlth, Beijing 100083, Peoples R China.	Adv Innovat Ctr Food Nutr & Human Hlth, Beijing 100083, China.		
C1	2	58123	58123	[Xu, Wentao; Huang, Kunlun] Beijing Lab Food Qual & Safety, Beijing 100083, Peoples R China.	[Xu, Wentao; Huang, Kunlun] Beijing Lab Food Qual & Safety, Beijing 100083, China.	Country: China<br>Institution: Beijing Lab Food Qual & Safety	Country: China<br>Institution: Beijing Lab Food Qual & Safety
RP	1	58125	58125	Huang, KL (corresponding author), China Agr Univ, Coll Food Sci & Nutr Engn, 17 Qinghua Donglu, Beijing 100083, Peoples R China	Huang, KL (corresponding author), China Agricultural University, Coll Food Sci & Nutr Engn, 17 Qinghua Donglu, Beijing 100083, China	Country: China<br>Institution: China Agr Univ	Country: China<br>Institution: China Agricultural University

Record 387 | WOS:000394982800001

The intestinal barrier as an emerging target in the toxicological assessment of mycotoxins

Field	Position	Original line	Normalised line	Original value	Normalised value	Original country / institution	Normalised country / institution
C1	1	58251	58251	[Akbari, Peyman; Braber, Saskia; Varasteh, Soheil; Alizadeh, Arash; Fink-Gremmels, Johanna] Univ Utrecht, Inst Risk Assessment Sci, Div Vet Pharmacol Pharmacotherapy & Toxicol, Yalelaan 104, NL-3584 CM Utrecht, Netherlands.	[Akbari, Peyman; Braber, Saskia; Varasteh, Soheil; Alizadeh, Arash; Fink-Gremmels, Johanna] Utrecht University, Inst Risk Assessment Sci, Div Vet Pharmacol Pharmacotherapy & Toxicol, Yalelaan 104, NL-3584 CM Utrecht, Netherlands.	Country: Netherlands<br>Institution: Univ Utrecht	Country: Netherlands<br>Institution: Utrecht University
C1	2	58252	58252	[Akbari, Peyman; Varasteh, Soheil; Alizadeh, Arash; Garssen, Johan] Univ Utrecht, Utrecht Inst Pharmaceut Sci, Div Pharmacol, Fac Sci, NL-3584 CG Utrecht, Netherlands.	[Akbari, Peyman; Varasteh, Soheil; Alizadeh, Arash; Garssen, Johan] Utrecht University, Utrecht Inst Pharmaceut Sci, Div Pharmacol, Fac Sci, NL-3584 CG Utrecht, Netherlands.	Country: Netherlands<br>Institution: Univ Utrecht	Country: Netherlands<br>Institution: Utrecht University
RP	1	58255	58255	Braber, S (corresponding author), Univ Utrecht, Inst Risk Assessment Sci, Div Vet Pharmacol Pharmacotherapy & Toxicol, Yalelaan 104, NL-3584 CM Utrecht, Netherlands	Braber, S (corresponding author), Utrecht University, Inst Risk Assessment Sci, Div Vet Pharmacol Pharmacotherapy & Toxicol, Yalelaan 104, NL-3584 CM Utrecht, Netherlands	Country: Netherlands<br>Institution: Univ Utrecht	Country: Netherlands<br>Institution: Utrecht University

Record 388 | WOS:000394558400005

Assessing interactions of binary mixtures of Penicillium mycotoxins (PMs) by using a bovine macrophage cell line (BoMacs)

Field	Position	Original line	Normalised line	Original value	Normalised value	Original country / institution	Normalised country / institution
C1	1	58519	58519	[Oh, Se-Young; Karrow, Niel A.] Univ Guelph, Dept Anim Biosci, OAC, Guelph, ON N1G 2W1, Canada.	[Oh, Se-Young; Karrow, Niel A.] University of Guelph, Dept Anim Biosci, OAC, Guelph, ON N1G 2W1, Canada.	Country: Canada<br>Institution: Univ Guelph	Country: Canada<br>Institution: University of Guelph
C1	2	58520	58520	[Cedergreen, Nina] Univ Copenhagen, Dept Life Sci, Frederiksberg, Denmark.	[Cedergreen, Nina] University of Copenhagen, Dept Life Sci, Frederiksberg, Denmark.	Country: Denmark<br>Institution: Univ Copenhagen	Country: Denmark<br>Institution: University of Copenhagen
RP	2	58524	58524	Karrow, NA (corresponding author), Univ Guelph, Dept Toxicol, Dept Anim Biosci, CGIL, Guelph, ON N1G 2W1, Canada	Karrow, NA (corresponding author), University of Guelph, Dept Toxicol, Dept Anim Biosci, CGIL, Guelph, ON N1G 2W1, Canada	Country: Canada<br>Institution: Univ Guelph	Country: Canada<br>Institution: University of Guelph

Record 389 | WOS:000392347800007

Mycotoxins as human carcinogens-the IARC Monographs classification

Field	Position	Original line	Normalised line	Original value	Normalised value	Original country / institution	Normalised country / institution
C1	2	58611	58611	[Malir, Frantisek; Toman, Jakub] Univ Hradec Kralove, Fac Sci, Dept Biol, Rokitanskeho 62, Hradec Kralove 50003, Czech Republic.	[Malir, Frantisek; Toman, Jakub] University of Hradec Kralove, Fac Sci, Dept Biol, Rokitanskeho 62, Hradec Kralove 50003, Czech Republic.	Country: Czech Republic Institution: Univ Hradec Kralove	Country: Czech Republic Institution: University of Hradec Kralove

Record 390 | WOS:000389167600013

Mycotoxin detoxifiers detoxifiers attenuate deoxynivalenol-induced pro-inflammatory barrier insult in porcine enterocytes as an in vitro evaluation model of feed mycotoxin reduction

Field	Position	Original line	Normalised line	Original value	Normalised value	Original country / institution	Normalised country / institution
C1	1	58772	58772	[Park, Seong-Hwan; Kim, Juil; Moon, Yuseok] Pusan Natl Univ, Sch Med, Dept Biomed Sci, Lab Mucosal Exposome & Biomodulat, Yangsan, South Korea.	[Park, Seong-Hwan; Kim, Juil; Moon, Yuseok] Pusan National University, Sch Med, Dept Biomed Sci, Lab Mucosal Exposome & Biomodulat, Yangsan, South Korea.	Country: South Korea Institution: Pusan Natl Univ	Country: South Korea Institution: Pusan National University
C1	2	58773	58773	[Park, Seong-Hwan; Moon, Yuseok] Pusan Natl Univ, Res Inst Basic Sci, Busan, South Korea.	[Park, Seong-Hwan; Moon, Yuseok] Pusan National University, Res Inst Basic Sci, Busan, South Korea.	Country: South Korea Institution: Pusan Natl Univ	Country: South Korea Institution: Pusan National University
C1	3	58774	58774	[Park, Seong-Hwan; Moon, Yuseok] Pusan Natl Univ, Med Res Inst, Busan, South Korea.	[Park, Seong-Hwan; Moon, Yuseok] Pusan National University, Med Res Inst, Busan, South Korea.	Country: South Korea Institution: Pusan Natl Univ	Country: South Korea Institution: Pusan National University
RP	1	58781	58781	Moon, Y (corresponding author), Pusan Natl Univ, Sch Med, Dept Biomed Sci, Yangsan 50612, South Korea	Moon, Y (corresponding author), Pusan National University, Sch Med, Dept Biomed Sci, Yangsan 50612, South Korea	Country: South Korea Institution: Pusan Natl Univ	Country: South Korea Institution: Pusan National University

Record 391 | WOS:000392789800020

Chitosan nanoparticles plus quercetin suppress the oxidative stress, modulate DNA fragmentation and gene expression in the kidney of rats fed ochratoxin A-contaminated diet

No C1/C3/RP normalisation changes detected for this record.

Record 392 | WOS:000402081300011

Mycotoxins co-contamination: Methodological aspects and biological relevance of combined toxicity studies

Field	Position	Original line	Normalised line	Original value	Normalised value	Original country / institution	Normalised country / institution
C1	1	59064	59064	[Alassane-Kpembi, Imourana; Puel, Olivier; Oswald, Isabelle Paule] Univ Toulouse, Toxalim, Res Ctr Food Toxicol, INRA,ENVT,INP PURPAN,UPS, Toulouse, France.	[Alassane-Kpembi, Imourana; Puel, Olivier; Oswald, Isabelle Paule] Universite de Toulouse, Toxalim, Res Ctr Food Toxicol, INRA,ENVT,INP PURPAN,UPS, Toulouse, France.	Country: France Institution: Univ Toulouse	Country: France Institution: Universite de Toulouse

RP	1	59071	59071	Oswald, IP (corresponding author), INRA, UMR Toxalim 1331, Res Ctr Food Toxicol, 180 Chem Tournefeuille,BP93173, F-31027 Toulouse 03, France	Oswald, IP (corresponding author), INRAE, UMR Toxalim 1331, Res Ctr Food Toxicol, 180 Chem Tournefeuille,BP93173, F-31027 Toulouse 03, France	Country: France<br>Institution: INRA	Country: France<br>Institution: INRAE

Record 393 | WOS:000399597700009

Protective role of bentonite against aflatoxin B1- and ochratoxin A-induced immunotoxicity in broilers

Field	Position	Original line	Normalised line	Original value	Normalised value	Original country / institution	Normalised country / institution
C1	1	59269	59269	[Bhatti, Sheraz Ahmed] Bahauddin Zakariya Univ, Fac Vet Sci, Multan, Pakistan.	[Bhatti, Sheraz Ahmed] Bahauddin Zakariya University, Fac Vet Sci, Multan, Pakistan.	Country: Pakistan<br>Institution: Bahauddin Zakariya Univ	Country: Pakistan<br>Institution: Bahauddin Zakariya University

Record 394 | WOS:000448708300012

Aflatoxin in Rice Crop: Prevalence and Assessment of Daily Exposure

Field	Position	Original line	Normalised line	Original value	Normalised value	Original country / institution	Normalised country / institution
C1	1	59390	59390	[Elzupir, Amin O.; Alamer, Abdulaziz S.] Al Imam Mohammad Ibn Saud Islamic Univ IMSIU, Comm Radiat & Environm Pollut Protect, Coll Sci, Riyadh, Saudi Arabia.	[Elzupir, Amin O.; Alamer, Abdulaziz S.] Imam Mohammad Ibn Saud Islamic University (IMSIU), Comm Radiat & Environm Pollut Protect, Coll Sci, Riyadh, Saudi Arabia.	Country: Saudi Arabia<br>Institution: Al Imam Mohammad Ibn Saud Islamic Univ IMSIU	Country: Saudi Arabia<br>Institution: Imam Mohammad Ibn Saud Islamic University (IMSIU)
C1	3	59392	59392	[Dutton, Michael F.] Univ Johannesburg, Fac Hlth Sci, Food Environm & Hlth Res Grp, Johannesburg, South Africa.	[Dutton, Michael F.] University of Johannesburg, Fac Hlth Sci, Food Environm & Hlth Res Grp, Johannesburg, South Africa.	Country: South Africa<br>Institution: Univ Johannesburg	Country: South Africa<br>Institution: University of Johannesburg
RP	1	59395	59395	Elzupir, AO (corresponding author), Al Imam Mohammad Ibn Saud Islamic Univ IMSIU, Comm Radiat & Environm Pollut Protect, Coll Sci, Riyadh, Saudi Arabia	Elzupir, AO (corresponding author), Imam Mohammad Ibn Saud Islamic University (IMSIU), Comm Radiat & Environm Pollut Protect, Coll Sci, Riyadh, Saudi Arabia	Country: Saudi Arabia<br>Institution: Al Imam Mohammad Ibn Saud Islamic Univ IMSIU	Country: Saudi Arabia<br>Institution: Imam Mohammad Ibn Saud Islamic University (IMSIU)

Record 395 | WOS:000416384800008

Effect of PR toxin on THP1 and Caco-2 cells: an in vitro study

Field	Position	Original line	Normalised line	Original value	Normalised value	Original country / institution	Normalised country / institution
C1	2	59511	59511	[Puel, O.; Tadrist, S.; Canlet, C.] Univ Toulouse, INRA, ENVT, INP Purpan,UPS,Toxalim Res Ctr Food Toxicol, Toulouse, France.	[Puel, O.; Tadrist, S.; Canlet, C.] Universite de Toulouse, INRA, ENVT, INP Purpan,UPS,Toxalim Res Ctr Food Toxicol, Toulouse, France.	Country: France<br>Institution: Univ Toulouse	Country: France<br>Institution: Universite de Toulouse

Record 396 | WOS:000413016300007

Efficacy of mixed adsorbent in ameliorating ochratoxicosis in broilers fed ochratoxin A contaminated diets

Field	Position	Original line	Normalised line	Original value	Normalised value	Original country / institution	Normalised country / institution

C1	1	59627	59627	[Qu, Daofeng; Huang, Xiaolin; Han, Jianzhong] Zhejiang Gongshang Univ, Food Safety Key Lab Zhejiang Prov, Hangzhou, Zhejiang, Peoples R China.	[Qu, Daofeng; Huang, Xiaolin; Han, Jianzhong] Zhejiang Gongshang University, Food Safety Key Lab Zhejiang Prov, Hangzhou, Zhejiang, China.	Country: China<br>Institution: Zhejiang Gongshang Univ	Country: China<br>Institution: Zhejiang Gongshang University
C1	2	59628	59628	[Man, Nana] Hangzhou Seed Stn, Hangzhou, Zhejiang, Peoples R China.	[Man, Nana] Hangzhou Seed Stn, Hangzhou, Zhejiang, China.	Country: China<br>Institution: Hangzhou Seed Stn	Country: China<br>Institution: Hangzhou Seed Stn
RP	1	59630	59630	Han, JZ (corresponding author), Zhejiang Gongshang Univ, Sch Food Sci & Biotechnol, Food Safety Key Lab Zhejiang Prov, Hangzhou 310035, Zhejiang, Peoples R China	Han, JZ (corresponding author), Zhejiang Gongshang University, Sch Food Sci & Biotechnol, Food Safety Key Lab Zhejiang Prov, Hangzhou 310035, Zhejiang, China	Country: China<br>Institution: Zhejiang Gongshang Univ	Country: China<br>Institution: Zhejiang Gongshang University

Record 397 | WOS:000417403600001

Citrinin induces apoptosis in human HCT116 colon cancer cells through endoplasmic reticulum stress

Field	Position	Original line	Normalised line	Original value	Normalised value	Original country / institution	Normalised country / institution
C1	3	59716	59716	[Prola, Alexandre; Da Silva, Julie Pires] Univ Paris Saclay, Univ Paris Sud, INSERM, UMR S 1180, Chatenay Malabry, France.	[Prola, Alexandre; Da Silva, Julie Pires] Universite Paris Saclay, Univ Paris Sud, INSERM, UMR S 1180, Chatenay Malabry, France.	Country: France<br>Institution: Univ Paris Saclay	Country: France<br>Institution: Universite Paris Saclay
C1	4	59717	59717	[Lemaire, Christophe] Univ Paris Saclay, Univ Paris Sud, INSERM, UMR S 1180,Univ Versailles St Quentin, Chatenay Malabry, France.	[Lemaire, Christophe] Universite Paris Saclay, Univ Paris Sud, INSERM, UMR S 1180,Univ Versailles St Quentin, Chatenay Malabry, France.	Country: France<br>Institution: Univ Paris Saclay	Country: France<br>Institution: Universite Paris Saclay

Record 398 | WOS:000424458100023

Mycotoxins

No C1/C3/RP normalisation changes detected for this record.

Record 399 | WOS:000448708300031

Application of Fungal Metabolites Against Mycotoxins Production

Field	Position	Original line	Normalised line	Original value	Normalised value	Original country / institution	Normalised country / institution
C1	1	60127	60127	[Verheecke, Carol; Choque, Elodie; Mathieu, Florence] Univ Toulouse, Lab Genie Chim, CNRS, INPT,UPS, Toulouse, France.	[Verheecke, Carol; Choque, Elodie; Mathieu, Florence] Universite de Toulouse, Lab Genie Chim, CNRS, INPT,UPS, Toulouse, France.	Country: France<br>Institution: Univ Toulouse	Country: France<br>Institution: Universite de Toulouse
RP	1	60132	60132	Mathieu, F (corresponding author), Univ Toulouse, Lab Genie Chim, CNRS, INPT,UPS, Toulouse, France	Mathieu, F (corresponding author), Universite de Toulouse, Lab Genie Chim, CNRS, INPT,UPS, Toulouse, France	Country: France<br>Institution: Univ Toulouse	Country: France<br>Institution: Universite de Toulouse

Record 400 | WOS:000391769900002

Toxicity study of ochratoxin A using HEK293 and HepG2 cell lines based on microRNA profiling

Field	Position	Original line	Normalised line	Original value	Normalised value	Original country / institution	Normalised country / institution
C1	1	60361	60361	[Zhao, J.; Qi, X.; Dai, Q.; He, X.; Guo, M.; Luo, Y.; Huang, K.; Xu, W.] China Agr Univ, Coll Food Sci & Nutr Engn, Beijing Adv Innovat Ctr Food Nutr & Human Hlth, 17 Qinghua Donglu, Beijing, Peoples R China.	[Zhao, J.; Qi, X.; Dai, Q.; He, X.; Guo, M.; Luo, Y.; Huang, K.; Xu, W.] China Agricultural University, Coll Food Sci & Nutr Engn, Beijing Adv Innovat Ctr Food Nutr & Human Hlth, 17 Qinghua Donglu, Beijing, China.	Country: China Institution: China Agr Univ	Country: China Institution: China Agricultural University
C1	3	60363	60363	[Luo, H.] China Agr Univ, Coll Biol Sci, State Key Lab Agrobiotechnol, Beijing, Peoples R China.	[Luo, H.] China Agricultural University, Coll Biol Sci, State Key Lab Agrobiotechnol, Beijing, China.	Country: China Institution: China Agr Univ	Country: China Institution: China Agricultural University
C1	4	60364	60364	[Xu, W.] China Agr Univ, Coll Food Sci & Nutr Engn, Beijing Lab Food Qual & Safety, Beijing, Peoples R China.	[Xu, W.] China Agricultural University, Coll Food Sci & Nutr Engn, Beijing Lab Food Qual & Safety, Beijing, China.	Country: China Institution: China Agr Univ	Country: China Institution: China Agricultural University
RP	1	60367	60367	Xu, W (corresponding author), China Agr Univ, Coll Food Sci & Nutr Engn, Beijing Adv Innovat Ctr Food Nutr & Human Hlth, 17 Qinghua Donglu, Beijing, Peoples R China	Xu, W (corresponding author), China Agricultural University, Coll Food Sci & Nutr Engn, Beijing Adv Innovat Ctr Food Nutr & Human Hlth, 17 Qinghua Donglu, Beijing, China	Country: China Institution: China Agr Univ	Country: China Institution: China Agricultural University

Record 401 | WOS:000393580000007

Limited Link between Oxidative Stress and Ochratoxin A-Induced Renal Injury in an Acute Toxicity Rat Model

Field	Position	Original line	Normalised line	Original value	Normalised value	Original country / institution	Normalised country / institution
C1	1	60487	60487	[Zhu, Liye; Yu, Tao; Qi, Xiaozhe; Gao, Jing; Huang, Kunlun; He, Xiaoyun; Xu, Wentao] China Agr Univ, Beijing Adv Innovat Ctr Food Nutr & Human Hlth, Coll Food Sci & Nutr Engn, 17 Tsinghua Donglu, Beijing 100083, Peoples R China.	[Zhu, Liye; Yu, Tao; Qi, Xiaozhe; Gao, Jing; Huang, Kunlun; He, Xiaoyun; Xu, Wentao] China Agricultural University, Beijing Adv Innovat Ctr Food Nutr & Human Hlth, Coll Food Sci & Nutr Engn, 17 Tsinghua Donglu, Beijing 100083, China.	Country: China Institution: China Agr Univ	Country: China Institution: China Agricultural University
C1	2	60488	60488	[Luo, Haoshu] China Agr Univ, State Key Lab Agrobiotechnol, Dept Anim Physiol, Coll Biol Sci, Beijing 100193, Peoples R China.	[Luo, Haoshu] China Agricultural University, State Key Lab Agrobiotechnol, Dept Anim Physiol, Coll Biol Sci, Beijing 100193, China.	Country: China Institution: China Agr Univ	Country: China Institution: China Agricultural University
C1	3	60489	60489	[Zhu, Liye; Huang, Kunlun; He, Xiaoyun; Xu, Wentao] Minist Agr, Supervis Inspect & Testing Ctr Genet Modified Org, Beijing 100083, Peoples R China.	[Zhu, Liye; Huang, Kunlun; He, Xiaoyun; Xu, Wentao] Minist Agr, Supervis Inspect & Testing Ctr Genet Modified Org, Beijing 100083, China.	Country: China Institution: Minist Agr	Country: China Institution: Minist Agr
C1	4	60490	60490	[Huang, Kunlun; He, Xiaoyun; Xu, Wentao] Beijing Lab Food Qual & Safety, Beijing 100083, Peoples R China.	[Huang, Kunlun; He, Xiaoyun; Xu, Wentao] Beijing Lab Food Qual & Safety, Beijing 100083, China.	Country: China Institution: Beijing Lab Food Qual & Safety	Country: China Institution: Beijing Lab Food Qual & Safety
RP	1	60492	60492	Xu, WT (corresponding author), China Agr Univ, Beijing Adv Innovat Ctr Food Nutr & Human Hlth, Coll Food Sci & Nutr Engn, 17 Tsinghua Donglu, Beijing 100083, Peoples R China	Xu, WT (corresponding author), China Agricultural University, Beijing Adv Innovat Ctr Food Nutr & Human Hlth, Coll Food Sci & Nutr Engn, 17 Tsinghua Donglu, Beijing 100083, Peoples R China	Country: China Institution: China Agr Univ	Country: China Institution: China Agricultural University

RP	4	60492	60492	Xu, WT (corresponding author), Beijing Lab Food Qual & Safety, Beijing 100083, Peoples R China	Xu, WT (corresponding author), Beijing Lab Food Qual & Safety, Beijing 100083, China	Country: China Institution: Beijing Lab Food Qual & Safety	Country: China Institution: Beijing Lab Food Qual & Safety

Record 402 | WOS:000387780500027

TOXICOPATHOLOGICAL AND SERUM BIOCHEMICAL ALTERATIONS INDUCED BY OCHRATOXIN A IN BROILER CHICKS AND THEIR AMELIORATION BY LOCALLY AVAILABLE BENTONITE CLAY

No C1/C3/RP normalisation changes detected for this record.

Record 403 | WOS:000383825300009

Aflatoxin M1 cytotoxicity against human intestinal Caco-2 cells is enhanced in the presence of other mycotoxins

Field	Position	Original line	Normalised line	Original value	Normalised value	Original country / institution	Normalised country / institution
C1	1	60717	60717	[Gao, Y. N.; Wang, J. Q.; Li, S. L.; Zhang, Y. D.; Zheng, N.] Chinese Acad Agr Sci, Inst Anim Sci, Minist Agr, Lab Qual & Safety Risk Assessment Daily Prod Beij, Beijing 100193, Peoples R China.	[Gao, Y. N.; Wang, J. Q.; Li, S. L.; Zhang, Y. D.; Zheng, N.] Chinese Academy of Agricultural Sciences, Inst Anim Sci, Minist Agr, Lab Qual & Safety Risk Assessment Daily Prod Beij, Beijing 100193, China.	Country: China Institution: Chinese Acad Agr Sci	Country: China Institution: Chinese Academy of Agricultural Sciences
C1	2	60718	60718	[Gao, Y. N.; Wang, J. Q.; Li, S. L.; Zhang, Y. D.; Zheng, N.] Chinese Acad Agr Sci, Inst Anim Sci, State Key Lab Anim Nutr, Beijing 100193, Peoples R China.	[Gao, Y. N.; Wang, J. Q.; Li, S. L.; Zhang, Y. D.; Zheng, N.] Chinese Academy of Agricultural Sciences, Inst Anim Sci, State Key Lab Anim Nutr, Beijing 100193, China.	Country: China Institution: Chinese Acad Agr Sci	Country: China Institution: Chinese Academy of Agricultural Sciences
RP	1	60722	60722	Zheng, N (corresponding author), Chinese Acad Agr Sci, Inst Anim Sci, 2 Yuanmingyuan West Rd, Beijing 100193, Peoples R China	Zheng, N (corresponding author), Chinese Academy of Agricultural Sciences, Inst Anim Sci, 2 Yuanmingyuan West Rd, Beijing 100193, China	Country: China Institution: Chinese Acad Agr Sci	Country: China Institution: Chinese Academy of Agricultural Sciences

Record 404 | WOS:000387347300005

Different Toxicity Mechanisms for Citrinin and Ochratoxin A Revealed by Transcriptomic Analysis in Yeast

No C1/C3/RP normalisation changes detected for this record.

Record 405 | WOS:000383733300020

Quantitation of the DNA Adduct of Semicarbazide in Organs of Semicarbazide-Treated Rats by Isotope-Dilution Liquid Chromatography-Tandem Mass Spectrometry: A Comparative Study with the RNA Adduct

Field	Position	Original line	Normalised line	Original value	Normalised value	Original country / institution	Normalised country / institution
C1	1	60970	60970	[Wang, Yinan; Wong, Tin-Yan; Chan, Wan] Hong Kong Univ Sci & Technol, Dept Chem, Room 4520,Acad Bldg, Kowloon, Hong Kong, Peoples R China.	[Wang, Yinan; Wong, Tin-Yan; Chan, Wan] Hong Kong University of Science & Technology, Dept Chem, Room 4520,Acad Bldg, Kowloon, Hong Kong, China.	Country: China Institution: Hong Kong Univ Sci & Technol	Country: China Institution: Hong Kong University of Science & Technology

RP	1	60972	60972	Chan, W (corresponding author), Hong Kong Univ Sci & Technol, Dept Chem, Room 4520,Acad Bldg, Kowloon, Hong Kong, Peoples R China	Chan, W (corresponding author), Hong Kong University of Science & Technology, Dept Chem, Room 4520,Acad Bldg, Kowloon, Hong Kong, China	Country: China<br>Institution: Hong Kong Univ Sci & Technol	Country: China<br>Institution: Hong Kong University of Science & Technology

Record 406 | WOS:000387199300010

Ochratoxin A activates neutrophils and kills these cells through necrosis, an effect eliminated through its conversion into ochratoxin Î±

No C1/C3/RP normalisation changes detected for this record.

Record 407 | WOS:000380647600001

Cytotoxic Effects of Ochratoxin A in Neuro-2a Cells: Role of Oxidative Stress Evidenced by N-acetylcysteine

No C1/C3/RP normalisation changes detected for this record.

Record 408 | WOS:000380763800002

Ochratoxin A: 50 Years of Research

Field	Position	Original line	Normalised line	Original value	Normalised value	Original country / institution	Normalised country / institution
C1	1	61320	61320	[Malir, Frantisek; Toman, Jakub] Univ Hradec Kralove, Dept Biol, Fac Sci, Hradec Kralove 50003, Czech Republic.	[Malir, Frantisek; Toman, Jakub] University of Hradec Kralove, Dept Biol, Fac Sci, Hradec Kralove 50003, Czech Republic.	Country: Czech Republic<br>Institution: Univ Hradec Kralove	Country: Czech Republic<br>Institution: University of Hradec Kralove
C1	3	61322	61322	[Pfohl-Leszkowicz, Annie] Univ Toulouse, Dept Bioproc & Microbial Syst, Chem Engn Lab, INP ENSA Toulouse,UMR CNRS INPT UPS 5503, F-31320 Auzeville Tolosane, France.	[Pfohl-Leszkowicz, Annie] Universite de Toulouse, Dept Bioproc & Microbial Syst, Chem Engn Lab, INP ENSA Toulouse,UMR CNRS INPT UPS 5503, F-31320 Auzeville Tolosane, France.	Country: France<br>Institution: Univ Toulouse	Country: France<br>Institution: Universite de Toulouse
C1	4	61323	61323	[Malir, Jan] Czech Acad Sci, Inst State & Law, Narodni 18, Prague 11600, Czech Republic.	[Malir, Jan] Czech Academy of Sciences, Inst State & Law, Narodni 18, Prague 11600, Czech Republic.	Country: Czech Republic<br>Institution: Czech Acad Sci	Country: Czech Republic<br>Institution: Czech Academy of Sciences
RP	1	61330	61330	Malir, F (corresponding author), Univ Hradec Kralove, Dept Biol, Fac Sci, Hradec Kralove 50003, Czech Republic	Malir, F (corresponding author), University of Hradec Kralove, Dept Biol, Fac Sci, Hradec Kralove 50003, Czech Republic	Country: Czech Republic<br>Institution: Univ Hradec Kralove	Country: Czech Republic<br>Institution: University of Hradec Kralove

Record 409 | WOS:000377720700011

Inhibition of CXCL12-mediated chemotaxis of Jurkat cells by direct immunotoxicants

Field	Position	Original line	Normalised line	Original value	Normalised value	Original country / institution	Normalised country / institution
C1	1	61768	61768	[Shao, Jia; Stout, Inge; Volger, Oscar L.; Hendriksen, Peter J. M.; Peijnenburg, Ad A. C. M.] Univ Wageningen & Res Ctr, RIKILT Inst Food Safety, POB 230, NL-6700 AE Wageningen, Netherlands.	[Shao, Jia; Stout, Inge; Volger, Oscar L.; Hendriksen, Peter J. M.; Peijnenburg, Ad A. C. M.] Wageningen University & Research, RIKILT Inst Food Safety, POB 230, NL-6700 AE Wageningen, Netherlands.	Country: Netherlands<br>Institution: Univ Wageningen & Res Ctr	Country: Netherlands<br>Institution: Wageningen University & Research

C1	3	61770	61770	[Shao, Jia; van Loveren, Henk] Maastricht Univ, Dept Toxicogen, POB 616, NL-6200 MD Maastricht, Netherlands.	[Shao, Jia; van Loveren, Henk] Maastricht Univ, Dept Toxicogen, POB 616, NL-6200 MD Maastricht, Netherlands.	Country: Netherlands Institution: Maastricht Univ	Country: Netherlands Institution: Maastricht University
C1	4	61771	61771	[Shao, Jia; Volger, Oscar L.; Hendriksen, Peter J. M.; van Loveren, Henk; Peijnenburg, Ad A. C. M.] Maastricht Univ, Netherlands Toxicogen Ctr, POB 616, NL-6200 MD Maastricht, Netherlands.	[Shao, Jia; Volger, Oscar L.; Hendriksen, Peter J. M.; van Loveren, Henk; Peijnenburg, Ad A. C. M.] Maastricht University, Netherlands Toxicogen Ctr, POB 616, NL-6200 MD Maastricht, Netherlands.	Country: Netherlands Institution: Maastricht Univ	Country: Netherlands Institution: Maastricht University
RP	1	61775	61775	Peijnenburg, AACM (corresponding author), Univ Wageningen & Res Ctr, RIKILT Inst Food Safety, POB 230, NL-6700 AE Wageningen, Netherlands	Peijnenburg, AACM (corresponding author), Wageningen University & Research, RIKILT Inst Food Safety, POB 230, NL-6700 AE Wageningen, Netherlands	Country: Netherlands Institution: Univ Wageningen & Res Ctr	Country: Netherlands Institution: Wageningen University & Research

Record 410 | WOS:000376990200009

Cytotoxicity, apoptosis, DNA damage and methylation in mammary and kidney epithelial cell lines exposed to ochratoxin A

Field	Position	Original line	Normalised line	Original value	Normalised value	Original country / institution	Normalised country / institution
C1	1	61885	61885	[Giromini, Carlotta; Rebucci, Raffaella; Fusi, Eleonora; Rossi, Luciana; Saccone, Francesca; Baldi, Antonella] Univ Milan, Dept Hlth Anim Sci & Food Safety, Via D Trentacoste 2, I-20134 Milan, Italy.	[Giromini, Carlotta; Rebucci, Raffaella; Fusi, Eleonora; Rossi, Luciana; Saccone, Francesca; Baldi, Antonella] University of Milan, Dept Hlth Anim Sci & Food Safety, Via D Trentacoste 2, I-20134 Milan, Italy.	Country: Italy Institution: Univ Milan	Country: Italy Institution: University of Milan
RP	1	61887	61887	Giromini, C (corresponding author), Univ Milan, Dept Hlth Anim Sci & Food Safety, Via D Trentacoste 2, I-20134 Milan, Italy	Giromini, C (corresponding author), University of Milan, Dept Hlth Anim Sci & Food Safety, Via D Trentacoste 2, I-20134 Milan, Italy	Country: Italy Institution: Univ Milan	Country: Italy Institution: University of Milan

Record 411 | WOS:000378297500009

Effects of Ochratoxin A on Mouse Oocyte Maturation and Fertilization, and Apoptosis During Fetal Development

Field	Position	Original line	Normalised line	Original value	Normalised value	Original country / institution	Normalised country / institution
C1	2	61971	61971	[Huang, Fu-Jen] Chang Gung Univ, Sch Med, Kaohsiung, Taiwan.	[Huang, Fu-Jen] Chang Gung University, Sch Med, Kaohsiung, Taiwan.	Country: Taiwan Institution: Chang Gung Univ	Country: Taiwan Institution: Chang Gung University
C1	3	61972	61972	[Chan, Wen-Hsiung] Chung Yuan Christian Univ, Dept Biosci Technol, Chungli, Taiwan.	[Chan, Wen-Hsiung] Chung Yuan Christian University, Dept Biosci Technol, Chungli, Taiwan.	Country: Taiwan Institution: Chung Yuan Christian Univ	Country: Taiwan Institution: Chung Yuan Christian University
C1	4	61973	61973	[Chan, Wen-Hsiung] Chung Yuan Christian Univ, Ctr Nanotechnol, Chungli, Taiwan.	[Chan, Wen-Hsiung] Chung Yuan Christian University, Ctr Nanotechnol, Chungli, Taiwan.	Country: Taiwan Institution: Chung Yuan Christian Univ	Country: Taiwan Institution: Chung Yuan Christian University
C1	5	61974	61974	[Chan, Wen-Hsiung] Chung Yuan Christian Univ, Ctr Biomed Technol, Chungli, Taiwan.	[Chan, Wen-Hsiung] Chung Yuan Christian University, Ctr Biomed Technol, Chungli, Taiwan.	Country: Taiwan Institution: Chung Yuan Christian Univ	Country: Taiwan Institution: Chung Yuan Christian University
RP	1	61978	61978	Chan, WH (corresponding author), Chung Yuan Christian Univ, Dept Biosci Technol, Chungli, Taiwan	Chan, WH (corresponding author), Chung Yuan Christian University, Dept Biosci Technol, Chungli, Taiwan	Country: Taiwan Institution: Chung Yuan Christian Univ	Country: Taiwan Institution: Chung Yuan Christian University

Record 412 | WOS:000375813200002

Innovative technologies for the mitigation of mycotoxins in animal feed and ingredients-A review of recent patents

Field	Position	Original line	Normalised line	Original value	Normalised value	Original country / institution	Normalised country / institution
C1	1	62085	62085	[Zhu, Yan; Hassan, Yousef I.; Watts, Christena; Zhou, Ting] Agr & Agri Food Canada, Guelph Food Res Ctr, 93 Stone Rd West, Guelph, ON N1G 5C9, Canada.	[Zhu, Yan; Hassan, Yousef I.; Watts, Christena; Zhou, Ting] Agriculture & Agri Food Canada, Guelph Food Res Ctr, 93 Stone Rd West, Guelph, ON N1G 5C9, Canada.	Country: Canada Institution: Agr & Agri Food Canada	Country: Canada Institution: Agriculture & Agri Food Canada
RP	1	62087	62087	Zhou, T (corresponding author), Agr & Agri Food Canada, Guelph Food Res Ctr, 93 Stone Rd West, Guelph, ON N1G 5C9, Canada	Zhou, T (corresponding author), Agriculture & Agri Food Canada, Guelph Food Res Ctr, 93 Stone Rd West, Guelph, ON N1G 5C9, Canada	Country: Canada Institution: Agr & Agri Food Canada	Country: Canada Institution: Agriculture & Agri Food Canada

Record 413 | WOS:000373624800006

Multiparametric luminescent cell viability assay in toxicology models: A critical evaluation

Field	Position	Original line	Normalised line	Original value	Normalised value	Original country / institution	Normalised country / institution
C1	1	62222	62222	[Sali, Nikolett; Poor, Miklos; Koszegi, Tamas] Univ Pecs, Dept Lab Med, Ifjusag U 13, H-7624 Pecs, Hungary.	[Sali, Nikolett; Poor, Miklos; Koszegi, Tamas] University of Pecs, Dept Lab Med, Ifjusag U 13, H-7624 Pecs, Hungary.	Country: Hungary Institution: Univ Pecs	Country: Hungary Institution: University of Pecs
C1	2	62223	62223	[Nagy, Sandor] Univ Pecs, Inst Pharmaceut Technol & Biopharm, Rokus U 2, H-7624 Pecs, Hungary.	[Nagy, Sandor] University of Pecs, Inst Pharmaceut Technol & Biopharm, Rokus U 2, H-7624 Pecs, Hungary.	Country: Hungary Institution: Univ Pecs	Country: Hungary Institution: University of Pecs
C1	5	62226	62226	[Poor, Miklos] Univ Pecs, Dept Pharmacol & Pharmacotherapy, Toxicol Sect, H-7624 Pecs, Hungary.	[Poor, Miklos] University of Pecs, Dept Pharmacol & Pharmacotherapy, Toxicol Sect, H-7624 Pecs, Hungary.	Country: Hungary Institution: Univ Pecs	Country: Hungary Institution: University of Pecs
RP	1	62228	62228	Koszegi, T (corresponding author), Univ Pecs, Dept Lab Med, Ifjusag U 13, H-7624 Pecs, Hungary	Koszegi, T (corresponding author), University of Pecs, Dept Lab Med, Ifjusag U 13, H-7624 Pecs, Hungary	Country: Hungary Institution: Univ Pecs	Country: Hungary Institution: University of Pecs

Record 414 | WOS:000377731900003

Mycotoxins modify the barrier function of Caco-2 cells through differential gene expression of specific claudin isoforms: Protective effect of illite mineral clay

Field	Position	Original line	Normalised line	Original value	Normalised value	Original country / institution	Normalised country / institution
C1	1	62340	62340	[Romero, Alejandro; Ares, Irma; Ramos, Eva; Castellano, Victor; Martinez, Marta; Martinez-Larranaga, Maria-Rosa; Anadon, Arturo; Martinez, Maria-Aranzazu] Univ Complutense Madrid, Fac Vet Med, Dept Pharmacol & Toxicol, E-28040 Madrid, Spain.	[Romero, Alejandro; Ares, Irma; Ramos, Eva; Castellano, Victor; Martinez, Marta; Martinez-Larranaga, Maria-Rosa; Anadon, Arturo; Martinez, Maria-Aranzazu] Complutense University of Madrid, Fac Vet Med, Dept Pharmacol & Toxicol, E-28040 Madrid, Spain.	Country: Spain Institution: Univ Complutense Madrid	Country: Spain Institution: Complutense University of Madrid
RP	1	62342	62342	AnadÃ³n, A (corresponding author), Univ Complutense Madrid, Fac Vet Med, Dept	AnadÃ³n, A (corresponding author), Complutense University of Madrid, Fac Vet	Country: Spain Institution: Univ Complutense Madrid	Country: Spain Institution: Complutense University of Madrid

				Pharmacol & Toxicol, E-28040 Madrid, Spain	Med, Dept Pharmacol & Toxicol, E-28040 Madrid, Spain		

Record 415 | WOS:000372000700010

Alleviative effect of myricetin on ochratoxin A-induced oxidative stress in rat renal cortex: histological and biochemical study

Field	Position	Original line	Normalised line	Original value	Normalised value	Original country / institution	Normalised country / institution
C1	1	62492	62492	[Abd El-Haleem, Manal R.; Kattaia, Asmaa A. A.; Abd El-Baset, Samia A.] Zagazig Univ, Fac Med, Dept Histol & Cell Biol, Zagazig 44519, Asharquia, Egypt.	[Abd El-Haleem, Manal R.; Kattaia, Asmaa A. A.; Abd El-Baset, Samia A.] Zagazig University, Fac Med, Dept Histol & Cell Biol, Zagazig 44519, Asharquia, Egypt.	Country: Egypt<br>Institution: Zagazig Univ	Country: Egypt<br>Institution: Zagazig University
C1	2	62493	62493	[Mostafa, Heba El Sayed] Zagazig Univ, Fac Med, Dept Forens Med & Clin Toxicol, Zagazig 44519, Asharquia, Egypt.	[Mostafa, Heba El Sayed] Zagazig University, Fac Med, Dept Forens Med & Clin Toxicol, Zagazig 44519, Asharquia, Egypt.	Country: Egypt<br>Institution: Zagazig Univ	Country: Egypt<br>Institution: Zagazig University
RP	1	62496	62496	Kattaia, AAA (corresponding author), Zagazig Univ, Fac Med, Zagazig 44519, Asharquia, Egypt	Kattaia, AAA (corresponding author), Zagazig University, Fac Med, Zagazig 44519, Asharquia, Egypt	Country: Egypt<br>Institution: Zagazig Univ	Country: Egypt<br>Institution: Zagazig University

Record 416 | WOS:000375861700010

Natural Co-Occurrence of Mycotoxins in Foods and Feeds and Their in vitro Combined Toxicological Effects

No C1/C3/RP normalisation changes detected for this record.

Record 417 | WOS:000431903800102

Mycotoxins at the blood-brain barrier: Metabolism, toxicity, barrier integrity and transfer to the brain

No C1/C3/RP normalisation changes detected for this record.

Record 418 | WOS:000372963300003

Ochratoxin A and T-2 Toxin Induce Clonogenicity and Cell Migration in Human Colon Carcinoma and Fetal Lung Fibroblast Cell Lines

No C1/C3/RP normalisation changes detected for this record.

Record 419 | WOS:000370832600004

Malignant transformation of human gastric epithelium cells via reactive oxygen species production and Wnt/Î²-catenin pathway activation following 40-week exposure to ochratoxin A

Field	Position	Original line	Normalised line	Original value	Normalised value	Original country / institution	Normalised country / institution
C1	1	63062	63062	[Jia, Xin; Meng, Xinxing; Xing, Lingxiao; Shen, Haitao; Zhang, Xianghong] Hebei Med Univ, Pathol Lab, Shijiazhuang, Peoples R China.	[Jia, Xin; Meng, Xinxing; Xing, Lingxiao; Shen, Haitao; Zhang, Xianghong] Hebei Medical University, Pathol Lab, Shijiazhuang, China.	Country: China<br>Institution: Hebei Med Univ	Country: China<br>Institution: Hebei Medical University

C1	2	63063	63063	[Cui, Jinfeng; Wang, Juan; Liu, Jing; Wang, Yuan; Lian, Weiguang; Zhang, Xianghong] Hebei Med Univ, Hosp 2, Dept Pathol, Shijiazhuang, Peoples R China.	[Cui, Jinfeng; Wang, Juan; Liu, Jing; Wang, Yuan; Lian, Weiguang; Zhang, Xianghong] Hebei Medical University, Hosp 2, Dept Pathol, Shijiazhuang, China.	Country: China<br>Institution: Hebei Med Univ	Country: China<br>Institution: Hebei Medical University
RP	1	63065	63065	Zhang, XH (corresponding author), Hebei Med Univ, Pathol Lab, Shijiazhuang, Peoples R China	Zhang, XH (corresponding author), Hebei Medical University, Pathol Lab, Shijiazhuang, Peoples R China	Country: China<br>Institution: Hebei Med Univ	Country: China<br>Institution: Hebei Medical University
RP	2	63065	63065	Zhang, XH (corresponding author), Hebei Med Univ, Hosp 2, Dept Pathol, Shijiazhuang, Peoples R China	Zhang, XH (corresponding author), Hebei Med Univ, Hosp 2, Dept Pathol, Shijiazhuang, China	Country: China<br>Institution: Hebei Med Univ	Country: China<br>Institution: Hebei Med Univ

Record 420 | WOS:000369179000011

Effects of aflatoxin B1 on oxidative stress markers and apoptosis of spleens in broilers

Field	Position	Original line	Normalised line	Original value	Normalised value	Original country / institution	Normalised country / institution
C1	1	63177	63177	[Chen, Jin; Chen, Kejie; Peng, Xi; Fang, Jing; Wang, Fengyuan; Cui, Hengmin; Chen, Zhengli; Yuan, Jingxin; Geng, Yi] Sichuan Agr Univ, Dept Vet Med, Yaan 625014, Sichuan, Peoples R China.	[Chen, Jin; Chen, Kejie; Peng, Xi; Fang, Jing; Wang, Fengyuan; Cui, Hengmin; Chen, Zhengli; Yuan, Jingxin; Geng, Yi] Sichuan Agricultural University, Dept Vet Med, Yaan 625014, Sichuan, China.	Country: China<br>Institution: Sichuan Agr Univ	Country: China<br>Institution: Sichuan Agricultural University
C1	2	63178	63178	[Yuan, Shibin] China West Normal Univ, Inst Rare Anim & Plants, Coll Life Sci, Nanchong, Sichuan, Peoples R China.	[Yuan, Shibin] China West Normal University, Inst Rare Anim & Plants, Coll Life Sci, Nanchong, Sichuan, China.	Country: China<br>Institution: China West Normal Univ	Country: China<br>Institution: China West Normal University
RP	1	63180	63180	Peng, X (corresponding author), Sichuan Agr Univ, Dept Vet Med, Yaan 625014, Sichuan, Peoples R China	Peng, X (corresponding author), Sichuan Agricultural University, Dept Vet Med, Yaan 625014, Sichuan, China	Country: China<br>Institution: Sichuan Agr Univ	Country: China<br>Institution: Sichuan Agricultural University

Record 421 | WOS:000370094500037

Grafting of gallic acid onto chitosan nano particles enhances antioxidant activities in vitro and protects against ochratoxin A toxicity in catfish (Clarias gariepinus)

Field	Position	Original line	Normalised line	Original value	Normalised value	Original country / institution	Normalised country / institution
C1	4	63292	63292	[Hamed, Heba S.] Ain Shams Univ, Dept Zool, Fac Women Arts Sci & Educ, Cairo, Egypt.	[Hamed, Heba S.] Ain Shams University, Dept Zool, Fac Women Arts Sci & Educ, Cairo, Egypt.	Country: Egypt<br>Institution: Ain Shams Univ	Country: Egypt<br>Institution: Ain Shams University

Record 422 | WOS:000388323700013

Mycotoxin Outbreak in Animal Feed

No C1/C3/RP normalisation changes detected for this record.

Record 423 | WOS:000368966200007

Ochratoxin A-induced cytotoxicity, genotoxicity and reactive oxygen species in kidney cells: An integrative approach of complementary endpoints

Field	Position	Original line	Normalised line	Original value	Normalised value	Original country / institution	Normalised country / institution
C1	1	63651	63651	[Costa, Joao G.; Saraiva, Nuno; Fernandes, Ana S.] Univ Lusofona, CBIOS, Res Ctr Biosci & Hlth Technol, Campo Grande 376, P-1749024 Lisbon, Portugal.	[Costa, Joao G.; Saraiva, Nuno; Fernandes, Ana S.] Lusofona University, CBIOS, Res Ctr Biosci & Hlth Technol, Campo Grande 376, P-1749024 Lisbon, Portugal.	Country: Portugal Institution: Univ Lusofona	Country: Portugal Institution: Lusofona University
C1	5	63655	63655	[Louro, Henriqueta; Silva, Maria J.] Univ Nova Lisboa, FCM, NMS, Ctr Toxicogen & Human Hlth Tox, Lisbon, Portugal.	[Louro, Henriqueta; Silva, Maria J.] Universidade Nova de Lisboa, FCM, NMS, Ctr Toxicogen & Human Hlth Tox, Lisbon, Portugal.	Country: Portugal Institution: Univ Nova Lisboa	Country: Portugal Institution: Universidade Nova de Lisboa
C1	6	63656	63656	[Batinic-Haberle, Ines] Duke Univ, Sch Med, Dept Radiat Oncol, Durham, NC USA.	[Batinic-Haberle, Ines] Duke University, Sch Med, Dept Radiat Oncol, Durham, NC USA.	Country: USA Institution: Duke Univ	Country: USA Institution: Duke University
RP	1	63660	63660	Fernandes, AS (corresponding author), Univ Lusofona, CBIOS, Res Ctr Biosci & Hlth Technol, Campo Grande 376, P-1749024 Lisbon, Portugal	Fernandes, AS (corresponding author), Lusofona University, CBIOS, Res Ctr Biosci & Hlth Technol, Campo Grande 376, P-1749024 Lisbon, Portugal	Country: Portugal Institution: Univ Lusofona	Country: Portugal Institution: Lusofona University

Record 424 | WOS:000371816100005

Individual and combined effects of roquefortine C and mycophenolic acid on human monocytic and intestinal cells

No C1/C3/RP normalisation changes detected for this record.

Record 425 | WOS:000376604100010

Evaluation of renal in vitro models used in ochratoxin research

Field	Position	Original line	Normalised line	Original value	Normalised value	Original country / institution	Normalised country / institution
C1	1	63895	63895	[Heussner, A. H.] Univ Konstanz, Human & Environm Toxicol, Univ Str 10, D-78457 Constance, Germany.	[Heussner, A. H.] University of Konstanz, Human & Environm Toxicol, Univ Str 10, D-78457 Constance, Germany.	Country: Germany Institution: Univ Konstanz	Country: Germany Institution: University of Konstanz
C1	2	63896	63896	[Heussner, A. H.; Paget, T.] Univ Sunderland, Pharm Hlth & Well Being, Sci Complex,Wharncliffe St, Sunderland SR1 3SD, Tyne & Wear, England.	[Heussner, A. H.; Paget, T.] University of Sunderland, Pharm Hlth & Well Being, Sci Complex,Wharncliffe St, Sunderland SR1 3SD, Tyne & Wear, United Kingdom.	Country: England Institution: Univ Sunderland	Country: United Kingdom Institution: University of Sunderland
RP	1	63898	63898	Heussner, AH (corresponding author), Univ Konstanz, Human & Environm Toxicol, Univ Str 10, D-78457 Constance, Germany	Heussner, AH (corresponding author), University of Konstanz, Human & Environm Toxicol, Univ Str 10, D-78457 Constance, Germany	Country: Germany Institution: Univ Konstanz	Country: Germany Institution: University of Konstanz
RP	2	63898	63898	Heussner, AH (corresponding author), Univ Sunderland, Pharm Hlth & Well Being, Sci Complex,Wharncliffe St, Sunderland SR1 3SD, Tyne & Wear, England	Heussner, AH (corresponding author), Univ Sunderland, Pharm Hlth & Well Being, Sci Complex,Wharncliffe St, Sunderland SR1 3SD, Tyne & Wear, United Kingdom	Country: England Institution: Univ Sunderland	Country: United Kingdom Institution: Univ Sunderland

Record 426 | WOS:000389199300011

Diet contaminated with ochratoxin A at the highest level allowed by EU recommendation disturbs liver metabolism in weaned piglets

No C1/C3/RP normalisation changes detected for this record.

Record 427 | WOS:000368260800005

Ochratoxin A as possible factor trigging autism and its male prevalence via epigenetic mechanism

Field	Position	Original line	Normalised line	Original value	Normalised value	Original country / institution	Normalised country / institution
C1	3	64313	64313	[Marabotti, A.] Univ Salerno, Dept Chem & Biol, I-84084 Fisciano, SA, Italy.	[Marabotti, A.] University of Salerno, Dept Chem & Biol, I-84084 Fisciano, SA, Italy.	Country: Italy<br>Institution: Univ Salerno	Country: Italy<br>Institution: University of Salerno

Record 428 | WOS:000387395900007

In Vitro Assays to Estimate the Toxicological Effects of Masked Mycotoxins

No C1/C3/RP normalisation changes detected for this record.

Record 429 | WOS:000384724200015

Probiotic preparation reduces faecal water genotoxicity and cytotoxicity in chickens fed ochratoxin A contaminated feed (in vivo study)

Field	Position	Original line	Normalised line	Original value	Normalised value	Original country / institution	Normalised country / institution
C1	1	64568	64568	[Slizewska, Katarzyna; Nowak, Adriana] Lodz Univ Technol, Inst Fermentat Technol & Microbiol, Lodz, Poland.	[Slizewska, Katarzyna; Nowak, Adriana] Lodz University of Technology, Inst Fermentat Technol & Microbiol, Lodz, Poland.	Country: Poland<br>Institution: Lodz Univ Technol	Country: Poland<br>Institution: Lodz University of Technology
C1	2	64569	64569	[Smulikowska, Stefania] Polish Acad Sci, Kielanowski Inst Anim Psysiol & Nutr, Jablonna, Poland.	[Smulikowska, Stefania] Polish Academy of Sciences, Kielanowski Inst Anim Psysiol & Nutr, Jablonna, Poland.	Country: Poland<br>Institution: Polish Acad Sci	Country: Poland<br>Institution: Polish Academy of Sciences
RP	1	64571	64571	Slizewska, K (corresponding author), Lodz Univ Technol, Inst Fermentat Technol & Microbiol, Lodz, Poland	Slizewska, K (corresponding author), Lodz University of Technology, Inst Fermentat Technol & Microbiol, Lodz, Poland	Country: Poland<br>Institution: Lodz Univ Technol	Country: Poland<br>Institution: Lodz University of Technology

Record 430 | WOS:000366832700013

miR-122 plays an important role in ochratoxin A-induced hepatocyte apoptosis in vitro and in vivo

Field	Position	Original line	Normalised line	Original value	Normalised value	Original country / institution	Normalised country / institution
C1	1	64681	64681	[Zhu, Liye; Yu, Tao; Qi, Xiaozhe; Yang, Bo; Shi, Lei; He, Xiaoyun; Huang, Kunlun; Xu, Wentao] China Agr Univ, Coll Food Sci & Nutr Engn, Beijing 100083, Peoples R China.	[Zhu, Liye; Yu, Tao; Qi, Xiaozhe; Yang, Bo; Shi, Lei; He, Xiaoyun; Huang, Kunlun; Xu, Wentao] China Agricultural University, Coll Food Sci & Nutr Engn, Beijing 100083, China.	Country: China<br>Institution: China Agr Univ	Country: China<br>Institution: China Agricultural University
C1	2	64682	64682	[Luo, Haoshu] China Agr Univ, Coll Biol Sci, Beijing 100083, Peoples R China.	[Luo, Haoshu] China Agricultural University, Coll Biol Sci, Beijing 100083, China.	Country: China<br>Institution: China Agr Univ	Country: China<br>Institution: China Agricultural University
C1	3	64683	64683	[He, Xiaoyun; Huang, Kunlun; Xu, Wentao] Minist Agr, Supervis Inspect & Testing Ctr	[He, Xiaoyun; Huang, Kunlun; Xu, Wentao] Minist Agr, Supervis Inspect & Testing Ctr	Country: China<br>Institution: Minist Agr	Country: China<br>Institution: Minist Agr

				Genet Modified Org, Beijing 100083, Peoples R China.	Genet Modified Org, Beijing 100083, China.		
RP	1	64685	64685	Zhu, LY (corresponding author), China Agr Univ, Coll Food Sci & Nutr Engn, Beijing 100083, Peoples R China	Zhu, LY (corresponding author), China Agricultural University, Coll Food Sci & Nutr Engn, Beijing 100083, China	Country: China<br>Institution: China Agr Univ	Country: China<br>Institution: China Agricultural University

Record 431 | WOS:000367635500005

MiR-122 partly mediates the ochratoxin A-induced GC-2 cell apoptosis

Field	Position	Original line	Normalised line	Original value	Normalised value	Original country / institution	Normalised country / institution
C1	1	64800	64800	[Chen, Ran; Yu, Xinxin; Wang, Xiuxiu; Zhang, Yiheng; Zhou, Bo; Luo, Haoshu] China Agr Univ, Coll Biol Sci, State Key Lab Agrobiotechnol, Beijing, Peoples R China.	[Chen, Ran; Yu, Xinxin; Wang, Xiuxiu; Zhang, Yiheng; Zhou, Bo; Luo, Haoshu] China Agricultural University, Coll Biol Sci, State Key Lab Agrobiotechnol, Beijing, China.	Country: China<br>Institution: China Agr Univ	Country: China<br>Institution: China Agricultural University
C1	2	64801	64801	[Zhu, Liye; Yu, Tao; Xu, Wentao] China Agr Univ, Coll Food Sci & Nutr Engn, Lab Food Safety & Mol Biol, Beijing, Peoples R China.	[Zhu, Liye; Yu, Tao; Xu, Wentao] China Agricultural University, Coll Food Sci & Nutr Engn, Lab Food Safety & Mol Biol, Beijing, China.	Country: China<br>Institution: China Agr Univ	Country: China<br>Institution: China Agricultural University
C1	3	64802	64802	[Chen, Liang] Peking Univ, Hosp 1, Med Ctr Reprod & Genet, Beijing, Peoples R China.	[Chen, Liang] Peking University, Hosp 1, Med Ctr Reprod & Genet, Beijing, China.	Country: China<br>Institution: Peking Univ	Country: China<br>Institution: Peking University
C1	4	64803	64803	[Deng, Liang] Shenyang Agr Univ, Coll Anim Sci & Vet Med, Dept Anim Genet & Reprod, Shenyang, Peoples R China.	[Deng, Liang] Shenyang Agricultural University, Coll Anim Sci & Vet Med, Dept Anim Genet & Reprod, Shenyang, China.	Country: China<br>Institution: Shenyang Agr Univ	Country: China<br>Institution: Shenyang Agricultural University
RP	1	64806	64806	Chen, L (corresponding author), Peking Univ, Hosp 1, Med Ctr Reprod & Genet, Beijing, Peoples R China	Chen, L (corresponding author), Peking University, Hosp 1, Med Ctr Reprod & Genet, Beijing, China	Country: China<br>Institution: Peking Univ	Country: China<br>Institution: Peking University

Record 432 | WOS:000367013500020

Interaction of Citrinin with Human Serum Albumin

Field	Position	Original line	Normalised line	Original value	Normalised value	Original country / institution	Normalised country / institution
C1	1	64917	64917	[Poor, Miklos] Univ Pecs, Toxicol Sect, Dept Pharmacol & Pharmacotherapy, H-7624 Pecs, Hungary.	[Poor, Miklos] University of Pecs, Toxicol Sect, Dept Pharmacol & Pharmacotherapy, H-7624 Pecs, Hungary.	Country: Hungary<br>Institution: Univ Pecs	Country: Hungary<br>Institution: University of Pecs
C1	2	64918	64918	[Lemli, Beata; Kunsagi-Mate, Sandor] Univ Pecs, Dept Gen & Phys Chem, H-7624 Pecs, Hungary.	[Lemli, Beata; Kunsagi-Mate, Sandor] University of Pecs, Dept Gen & Phys Chem, H-7624 Pecs, Hungary.	Country: Hungary<br>Institution: Univ Pecs	Country: Hungary<br>Institution: University of Pecs
C1	4	64920	64920	[Balint, Monika] Eotvos Lorand Univ, Dept Biochem, H-1117 Budapest, Hungary.	[Balint, Monika] Eotvos Lorand University, Dept Biochem, H-1117 Budapest, Hungary.	Country: Hungary<br>Institution: Eotvos Lorand Univ	Country: Hungary<br>Institution: Eotvos Lorand University
C1	5	64921	64921	[Hetenyi, Csaba] Hungarian Acad Sci, MTA ELTE Mol Biophys Res Grp, H-1117 Budapest, Hungary.	[Hetenyi, Csaba] Hungarian Academy of Sciences, MTA ELTE Mol Biophys Res Grp, H-1117 Budapest, Hungary.	Country: Hungary<br>Institution: Hungarian Acad Sci	Country: Hungary<br>Institution: Hungarian Academy of Sciences
C1	6	64922	64922	[Sali, Nikolett; Koszegi, Tamas] Univ Pecs, Dept Lab Med, H-7624 Pecs, Hungary.	[Sali, Nikolett; Koszegi, Tamas] University of Pecs, Dept Lab Med, H-7624 Pecs, Hungary.	Country: Hungary<br>Institution: Univ Pecs	Country: Hungary<br>Institution: University of Pecs

RP	1	64925	64925	PoÃ³r, M (corresponding author), Univ Pecs, Toxicol Sect, Dept Pharmacol & Pharmacotherapy, Szigeti Ut 12, H-7624 Pecs, Hungary	PoÃ³r, M (corresponding author), University of Pecs, Toxicol Sect, Dept Pharmacol & Pharmacotherapy, Szigeti Ut 12, H-7624 Pecs, Hungary	Country: Hungary Institution: Univ Pecs	Country: Hungary Institution: University of Pecs

Record 433 | WOS:000362726700025

The Phytoalexin Resveratrol Ameliorates Ochratoxin A Toxicity in Human Embryonic Kidney (HEK293) Cells

Field	Position	Original line	Normalised line	Original value	Normalised value	Original country / institution	Normalised country / institution
C1	1	65034	65034	[Raghubeer, Shanel; Nagiah, Savania; Chuturgoon, Anil] Univ KwaZulu Natal, Sch Lab Med & Med Sci, Coll Hlth Sci, Discipline Med Biochem, ZA-4041 Durban, South Africa.	[Raghubeer, Shanel; Nagiah, Savania; Chuturgoon, Anil] University of Kwazulu Natal, Sch Lab Med & Med Sci, Coll Hlth Sci, Discipline Med Biochem, ZA-4041 Durban, South Africa.	Country: South Africa Institution: Univ KwaZulu Natal	Country: South Africa Institution: University of Kwazulu Natal
C1	2	65035	65035	[Phulukdaree, Alisa] Univ Pretoria, Sch Med, Fac Hlth Sci, Dept Physiol, ZA-0002 Pretoria, South Africa.	[Phulukdaree, Alisa] University of Pretoria, Sch Med, Fac Hlth Sci, Dept Physiol, ZA-0002 Pretoria, South Africa.	Country: South Africa Institution: Univ Pretoria	Country: South Africa Institution: University of Pretoria
RP	1	65037	65037	Chuturgoon, A (corresponding author), Univ KwaZulu Natal, Discipline Med Biochem & Chem Pathol, Fac Hlth Sci, Howard Coll, George Campbell Bldg, ZA-4041 Durban, South Africa	Chuturgoon, A (corresponding author), University of Kwazulu Natal, Discipline Med Biochem & Chem Pathol, Fac Hlth Sci, Howard Coll, George Campbell Bldg, ZA-4041 Durban, South Africa	Country: South Africa Institution: Univ KwaZulu Natal	Country: South Africa Institution: University of Kwazulu Natal

Record 434 | WOS:000363821500008

A food contaminant ochratoxin A suppresses pregnane X receptor (PXR)-mediated CYP3A4 induction in primary cultures of human hepatocytes

No C1/C3/RP normalisation changes detected for this record.

Record 435 | WOS:000363259300002

In vitro exposure of Penicillium mycotoxins with or without a modified yeast cell wall extract (mYCW) on bovine macrophages (BoMacs)

Field	Position	Original line	Normalised line	Original value	Normalised value	Original country / institution	Normalised country / institution
C1	1	65216	65216	[Oh, Se-Young; Quinton, V. Margaret; Karrow, Niel A.] Univ Guelph, OAC, Dept Anim & Poultry Sci APS, Guelph, ON N1G 2W1, Canada.	[Oh, Se-Young; Quinton, V. Margaret; Karrow, Niel A.] University of Guelph, OAC, Dept Anim & Poultry Sci APS, Guelph, ON N1G 2W1, Canada.	Country: Canada Institution: Univ Guelph	Country: Canada Institution: University of Guelph
C1	2	65217	65217	[Boermans, Herman J.] Univ Guelph, OVC, Dept Biomed Sci, Guelph, ON N1G 2W1, Canada.	[Boermans, Herman J.] University of Guelph, OVC, Dept Biomed Sci, Guelph, ON N1G 2W1, Canada.	Country: Canada Institution: Univ Guelph	Country: Canada Institution: University of Guelph
C1	4	65219	65219	[Oh, Se-Young; Karrow, Niel A.] Univ Guelph, CGIL, Dept Anim & Poultry Sci, Dept Toxicol, Guelph, ON N1G 2W1, Canada.	[Oh, Se-Young; Karrow, Niel A.] University of Guelph, CGIL, Dept Anim & Poultry Sci, Dept Toxicol, Guelph, ON N1G 2W1, Canada.	Country: Canada Institution: Univ Guelph	Country: Canada Institution: University of Guelph

RP	1	65221	65221	Oh, SY (corresponding author), Univ Guelph, OAC, Dept Anim & Poultry Sci APS, Guelph, ON N1G 2W1, Canada	Oh, SY (corresponding author), University of Guelph, OAC, Dept Anim & Poultry Sci APS, Guelph, ON N1G 2W1, Canada	Country: Canada<br>Institution: Univ Guelph	Country: Canada<br>Institution: University of Guelph

Record 436 | WOS:000360322300014

Ochratoxin A at low concentrations inhibits in vitro growth of canine umbilical cord matrix mesenchymal stem cells through oxidative chromatin and DNA damage

Field	Position	Original line	Normalised line	Original value	Normalised value	Original country / institution	Normalised country / institution
C1	6	65331	65331	[Zanghi, Antonina] Univ Messina, Dept Vet Sci, Polo Univ SS Annunziata, I-98168 Messina, Italy.	[Zanghi, Antonina] University of Messina, Dept Vet Sci, Polo Univ SS Annunziata, I-98168 Messina, Italy.	Country: Italy<br>Institution: Univ Messina	Country: Italy<br>Institution: University of Messina

Record 437 | WOS:000365417800020

The influence of ochratoxin A on DNA adduct formation by the carcinogen aristolochic acid in rats

Field	Position	Original line	Normalised line	Original value	Normalised value	Original country / institution	Normalised country / institution
C1	1	65490	65490	[Stiborova, Marie; Barta, Frantisek; Levova, Katerina; Hodek, Petr] Charles Univ Prague, Fac Sci, Dept Biochem, Prague 12840 2, Czech Republic.	[Stiborova, Marie; Barta, Frantisek; Levova, Katerina; Hodek, Petr] Charles University Prague, Fac Sci, Dept Biochem, Prague 12840 2, Czech Republic.	Country: Czech Republic<br>Institution: Charles Univ Prague	Country: Czech Republic<br>Institution: Charles University Prague
C1	3	65492	65492	[Arlt, Volker M.] Kings Coll London, MRC PHE Ctr Environm & Hlth, Analyt & Environm Sci Div, London SE1 9NH, England.	[Arlt, Volker M.] Kings Coll London, MRC PHE Ctr Environm & Hlth, Analyt & Environm Sci Div, London SE1 9NH, United Kingdom.	Country: England<br>Institution: Kings Coll London	Country: United Kingdom<br>Institution: Kings Coll London
RP	1	65498	65498	StiborovÃ¡, M (corresponding author), Charles Univ Prague, Fac Sci, Dept Biochem, Albertov 2030, Prague 12840 2, Czech Republic	StiborovÃ¡, M (corresponding author), Charles University Prague, Fac Sci, Dept Biochem, Albertov 2030, Prague 12840 2, Czech Republic	Country: Czech Republic<br>Institution: Charles Univ Prague	Country: Czech Republic<br>Institution: Charles University Prague

Record 438 | WOS:000357839100054

Cross-linked chitosan polymers as generic adsorbents for simultaneous adsorption of multiple mycotoxins

Field	Position	Original line	Normalised line	Original value	Normalised value	Original country / institution	Normalised country / institution
C1	1	65676	65676	[Zhao, Zhiyong; Wu, Aibo] Chinese Acad Sci, Shanghai Inst Biol Sci, Inst Nutr Sci, Key Lab Food Safety Res, Shanghai 200031, Peoples R China.	[Zhao, Zhiyong; Wu, Aibo] Chinese Academy of Sciences, Shanghai Inst Biol Sci, Inst Nutr Sci, Key Lab Food Safety Res, Shanghai 200031, China.	Country: China<br>Institution: Chinese Acad Sci	Country: China<br>Institution: Chinese Academy of Sciences
C1	2	65677	65677	[Zhao, Zhiyong; Yang, Lingchen; Song, Suquan; Hou, Jiafa] Nanjing Agr Univ, Coll Vet Med, Nanjing 210095, Jiangsu, Peoples R China.	[Zhao, Zhiyong; Yang, Lingchen; Song, Suquan; Hou, Jiafa] Nanjing Agricultural University, Coll Vet Med, Nanjing 210095, Jiangsu, China.	Country: China<br>Institution: Nanjing Agr Univ	Country: China<br>Institution: Nanjing Agricultural University
C1	3	65678	65678	[Zhao, Zhiyong; Liu, Na; Wang, Jianhua; Nie, Dongxia; Yang, Xianli] Shanghai Acad Agr Sci, Inst Agrofood Stand & Testing Technol, Lab Qual & Safety Risk Assessment Agroprod Shangh,	[Zhao, Zhiyong; Liu, Na; Wang, Jianhua; Nie, Dongxia; Yang, Xianli] Shanghai Academy of Agricultural Sciences, Inst Agrofood Stand & Testing Technol, Lab Qual & Safety Risk	Country: China<br>Institution: Shanghai Acad Agr Sci	Country: China<br>Institution: Shanghai Academy of Agricultural Sciences

				Minist Agr, Shanghai 201403, Peoples R China.	Assessment Agroprod Shangh, Minist Agr, Shanghai 201403, China.		
RP	1	65681	65681	Wu, AB (corresponding author), Chinese Acad Sci, Shanghai Inst Biol Sci, Inst Nutr Sci, Key Lab Food Safety Res, 294 Taiyuan Rd, Shanghai 200031, Peoples R China	Wu, AB (corresponding author), Chinese Academy of Sciences, Shanghai Inst Biol Sci, Inst Nutr Sci, Key Lab Food Safety Res, 294 Taiyuan Rd, Shanghai 200031, China	Country: China<br>Institution: Chinese Acad Sci	Country: China<br>Institution: Chinese Academy of Sciences

Record 439 | WOS:000370693801841

A contribution to hazard assessment of combined exposure to mycotoxins using in vitro toxicity testing

No C1/C3/RP normalisation changes detected for this record.

Record 440 | WOS:000361263500036

Cytotoxicity of the mycotoxins deoxynivalenol and ochratoxin A on Caco-2 cell line in presence of resveratrol

No C1/C3/RP normalisation changes detected for this record.

Record 441 | WOS:000367407400003

Dietary mycotoxins, co-exposure, and carcinogenesis in humans: Short review

Field	Position	Original line	Normalised line	Original value	Normalised value	Original country / institution	Normalised country / institution
C1	1	65961	65961	[De Ruyck, Karl; De Boevre, Marthe; De Saeger, Sarah] Univ Ghent, Fac Pharmaceut Sci, Lab Food Anal, B-9000 Ghent, Belgium.	[De Ruyck, Karl; De Boevre, Marthe; De Saeger, Sarah] Ghent University, Fac Pharmaceut Sci, Lab Food Anal, B-9000 Ghent, Belgium.	Country: Belgium<br>Institution: Univ Ghent	Country: Belgium<br>Institution: Ghent University
RP	1	65965	65965	De Ruyck, K (corresponding author), Univ Ghent, Fac Pharmaceut Sci, Lab Food Anal, Ottergemsesteenweg 460, B-9000 Ghent, Belgium	De Ruyck, K (corresponding author), Ghent University, Fac Pharmaceut Sci, Lab Food Anal, Ottergemsesteenweg 460, B-9000 Ghent, Belgium	Country: Belgium<br>Institution: Univ Ghent	Country: Belgium<br>Institution: Ghent University

Record 442 | WOS:000364960400028

Comparative Ochratoxin Toxicity: A Review of the Available Data

Field	Position	Original line	Normalised line	Original value	Normalised value	Original country / institution	Normalised country / institution
C1	1	66130	66130	[Heussner, Alexandra H.] Univ Konstanz, Human & Environm Toxicol, D-78457 Constance, Germany.	[Heussner, Alexandra H.] University of Konstanz, Human & Environm Toxicol, D-78457 Constance, Germany.	Country: Germany<br>Institution: Univ Konstanz	Country: Germany<br>Institution: University of Konstanz
C1	2	66131	66131	[Heussner, Alexandra H.; Bingle, Lewis E. H.] Univ Sunderland, Dept Pharm Hlth & Well Being, Sunderland SR1 3SD, Durham, England.	[Heussner, Alexandra H.; Bingle, Lewis E. H.] University of Sunderland, Dept Pharm Hlth & Well Being, Sunderland SR1 3SD, Durham, United Kingdom.	Country: England<br>Institution: Univ Sunderland	Country: United Kingdom<br>Institution: University of Sunderland

RP	1	66133	66133	Heussner, AH (corresponding author), Univ Konstanz, Human & Environm Toxicol, Univ Str 10, D-78457 Constance, Germany	Heussner, AH (corresponding author), University of Konstanz, Human & Environm Toxicol, Univ Str 10, D-78457 Constance, Germany	Country: Germany<br>Institution: Univ Konstanz	Country: Germany<br>Institution: University of Konstanz

Record 443 | WOS:000355886400028

Development of an aptasensor for the fast detection of Versicolorin A

Field	Position	Original line	Normalised line	Original value	Normalised value	Original country / institution	Normalised country / institution
C1	1	66404	66404	[Jiang, Hai-Lan; Liu, Xiao-Yun; Qiu, Yu-xin; Xie, Chun-Fang; Liu, Da-Ling] Ji Nan Univ, Dept Biotechnol, Guangzhou 510632, Guangdong, Peoples R China.	[Jiang, Hai-Lan; Liu, Xiao-Yun; Qiu, Yu-xin; Xie, Chun-Fang; Liu, Da-Ling] Ji Nan Univ, Dept Biotechnol, Guangzhou 510632, Guangdong, China.	Country: China<br>Institution: Ji Nan Univ	Country: China<br>Institution: Ji Nan Univ
C1	2	66405	66405	[Yao, Dong-Sheng] Jinan Univ, Natl Engn Res Ctr Genet Med, Guangzhou 510632, Guangdong, Peoples R China.	[Yao, Dong-Sheng] Jinan University, Natl Engn Res Ctr Genet Med, Guangzhou 510632, Guangdong, China.	Country: China<br>Institution: Jinan Univ	Country: China<br>Institution: Jinan University
RP	1	66407	66407	Liu, DL (corresponding author), Ji Nan Univ, Dept Biotechnol, Guangzhou 510632, Guangdong, Peoples R China	Liu, DL (corresponding author), Ji Nan Univ, Dept Biotechnol, Guangzhou 510632, Guangdong, China	Country: China<br>Institution: Ji Nan Univ	Country: China<br>Institution: Ji Nan Univ

Record 444 | WOS:000359029000029

Cytotoxic and genotoxic potencies of single and combined spore extracts of airborne OTA-producing and OTA-non-producing Aspergilli in Human lung A549 cells

Field	Position	Original line	Normalised line	Original value	Normalised value	Original country / institution	Normalised country / institution
C1	1	66522	66522	[Klaric, Maja Segvic; Despot, Daniela Jaksic] Univ Zagreb, Fac Pharm & Biochem, Dept Microbiol, Zagreb 10000, Croatia.	[Klaric, Maja Segvic; Despot, Daniela Jaksic] University of Zagreb, Fac Pharm & Biochem, Dept Microbiol, Zagreb 10000, Croatia.	Country: Croatia<br>Institution: Univ Zagreb	Country: Croatia<br>Institution: University of Zagreb
C1	2	66523	66523	[Kopjar, Nevenka] Inst Med Res & Occupat Hlth, Mutagenesis Unit, Zagreb 41000, Croatia.	[Kopjar, Nevenka] Institute for Medical Research & Occupational Health (IMROH), Mutagenesis Unit, Zagreb 41000, Croatia.	Country: Croatia<br>Institution: Inst Med Res & Occupat Hlth	Country: Croatia<br>Institution: Institute for Medical Research & Occupational Health (IMROH)
C1	3	66524	66524	[Rasic, Dubravka; Peraica, Maja] Inst Med Res & Occupat Hlth, Toxicol Unit, Zagreb 41000, Croatia.	[Rasic, Dubravka; Peraica, Maja] Institute for Medical Research & Occupational Health (IMROH), Toxicol Unit, Zagreb 41000, Croatia.	Country: Croatia<br>Institution: Inst Med Res & Occupat Hlth	Country: Croatia<br>Institution: Institute for Medical Research & Occupational Health (IMROH)
C1	4	66525	66525	[Kocsube, Sandor; Varga, Janos] Univ Szeged, Fac Sci & Informat, Dept Microbiol, Szeged, Hungary.	[Kocsube, Sandor; Varga, Janos] Szeged University, Fac Sci & Informat, Dept Microbiol, Szeged, Hungary.	Country: Hungary<br>Institution: Univ Szeged	Country: Hungary<br>Institution: Szeged University
RP	1	66529	66529	Klaric, MS (corresponding author), Univ Zagreb, Fac Pharm & Biochem, Dept Microbiol, Schmtwva 39, Zagreb 10000, Croatia	Klaric, MS (corresponding author), University of Zagreb, Fac Pharm & Biochem, Dept Microbiol, Schmtwva 39, Zagreb 10000, Croatia	Country: Croatia<br>Institution: Univ Zagreb	Country: Croatia<br>Institution: University of Zagreb

Record 445 | WOS:000362622500013

Ochratoxin A induces DNA damage and G2 phase arrest in human esophageal epithelium Het-1A cells in vitro

Field	Position	Original line	Normalised line	Original value	Normalised value	Original country / institution	Normalised country / institution
C1	1	66661	66661	[Liu, Jing] Chinese Peoples Armed Police Force, Gen Hosp, Dept Pathol, Beijing, Peoples R China.	[Liu, Jing] Chinese Peoples Armed Police Force, Gen Hosp, Dept Pathol, Beijing, China.	Country: China Institution: Chinese Peoples Armed Police Force	Country: China Institution: Chinese Peoples Armed Police Force
C1	2	66662	66662	[Liu, Jing; Wu, Sha; Shen, Haitao; Xing, Lingxiao; Wang, Junling; Yan, Xia; Zhang, Xianghong] Hebei Med Univ, Pathol Lab, Shijiazhuang, Peoples R China.	[Liu, Jing; Wu, Sha; Shen, Haitao; Xing, Lingxiao; Wang, Junling; Yan, Xia; Zhang, Xianghong] Hebei Medical University, Pathol Lab, Shijiazhuang, China.	Country: China Institution: Hebei Med Univ	Country: China Institution: Hebei Medical University
C1	3	66663	66663	[Cui, Jinfeng; Wang, Yuan; Zhang, Xianghong] Hebei Med Univ, Hosp 2, Dept Pathol, Shijiazhuang, Peoples R China.	[Cui, Jinfeng; Wang, Yuan; Zhang, Xianghong] Hebei Medical University, Hosp 2, Dept Pathol, Shijiazhuang, China.	Country: China Institution: Hebei Med Univ	Country: China Institution: Hebei Medical University
RP	1	66665	66665	Zhang, XH (corresponding author), Hebei Med Univ, Pathol Lab, Shijiazhuang, Peoples R China	Zhang, XH (corresponding author), Hebei Medical University, Pathol Lab, Shijiazhuang, China	Country: China Institution: Hebei Med Univ	Country: China Institution: Hebei Medical University

Record 446 | WOS:000362860200019

Risk analysis of main mycotoxins occurring in food for children: An overview

Field	Position	Original line	Normalised line	Original value	Normalised value	Original country / institution	Normalised country / institution
C1	1	66777	66777	[Raiola, Assunta] Univ Naples Federico II, Fac Agr, Dept Agr, I-80055 Naples, Italy.	[Raiola, Assunta] University of Naples Federico II, Fac Agr, Dept Agr, I-80055 Naples, Italy.	Country: Italy Institution: Univ Naples Federico II	Country: Italy Institution: University of Naples Federico II
C1	2	66778	66778	[Tenore, Gian Carlo; Ritieni, Alberto] Univ Naples Federico II, Fac Pharm, Dept Pharm, I-80131 Naples, Italy.	[Tenore, Gian Carlo; Ritieni, Alberto] University of Naples Federico II, Fac Pharm, Dept Pharm, I-80131 Naples, Italy.	Country: Italy Institution: Univ Naples Federico II	Country: Italy Institution: University of Naples Federico II
C1	3	66779	66779	[Manyes, Lara; Meca, Giuseppe] Univ Valencia, Fac Pharm, Lab Food Chem & Toxicol, E-46100 Burjassot, Spain.	[Manyes, Lara; Meca, Giuseppe] University of Valencia, Fac Pharm, Lab Food Chem & Toxicol, E-46100 Burjassot, Spain.	Country: Spain Institution: Univ Valencia	Country: Spain Institution: University of Valencia
RP	1	66782	66782	Raiola, A (corresponding author), Univ Naples Federico II, Fac Agr, Dept Agr, Via Univ 100, I-80055 Naples, Italy	Raiola, A (corresponding author), University of Naples Federico II, Fac Agr, Dept Agr, Via Univ 100, I-80055 Naples, Italy	Country: Italy Institution: Univ Naples Federico II	Country: Italy Institution: University of Naples Federico II

Record 447 | WOS:000362551400018

Biomonitoring using dried blood spots: Detection of ochratoxin A and its degradation product 2'R-ochratoxin A in blood from coffee drinkers

Field	Position	Original line	Normalised line	Original value	Normalised value	Original country / institution	Normalised country / institution
C1	1	67021	67021	[Cramer, Benedikt; Osteresch, Bernd; Humpf, Hans-Ulrich] Univ Munster, Inst Food Chem, D-48149 Munster, Germany.	[Cramer, Benedikt; Osteresch, Bernd; Humpf, Hans-Ulrich] University of Munster, Inst Food Chem, D-48149 Munster, Germany.	Country: Germany Institution: Univ Munster	Country: Germany Institution: University of Munster
RP	1	67026	67026	Humpf, HU (corresponding author), Univ Munster, Inst Food Chem, D-48149 Munster, Germany	Humpf, HU (corresponding author), University of Munster, Inst Food Chem, D-48149 Munster, Germany	Country: Germany Institution: Univ Munster	Country: Germany Institution: University of Munster

Record 448 | WOS:000361078800019

Hepatotoxic effect of ochratoxin A and citrinin, alone and in combination, and protective effect of vitamin E: In vitro study in HepG2 cell

Field	Position	Original line	Normalised line	Original value	Normalised value	Original country / institution	Normalised country / institution
C1	1	67128	67128	[Gayathri, Loganathan; Dhanasekaran, Dharumadurai] Bharathidasan Univ, Dept Microbiol, Tiruchirappalli 620024, India.	[Gayathri, Loganathan; Dhanasekaran, Dharumadurai] Bharathidasan University, Dept Microbiol, Tiruchirappalli 620024, India.	Country: India Institution: Bharathidasan Univ	Country: India Institution: Bharathidasan University
C1	2	67129	67129	[Gayathri, Loganathan; Dhivya, Rajakumar; Akbarsha, Mohammad A.] Bharathidasan Univ, Mahatma Gandhi Doerenkamp Ctr, Tiruchirappalli 620024, India.	[Gayathri, Loganathan; Dhivya, Rajakumar; Akbarsha, Mohammad A.] Bharathidasan University, Mahatma Gandhi Doerenkamp Ctr, Tiruchirappalli 620024, India.	Country: India Institution: Bharathidasan Univ	Country: India Institution: Bharathidasan University
C1	3	67130	67130	[Periasamy, Vaiyapuri S.; Alshatwi, Ali A.; Akbarsha, Mohammad A.] King Saud Univ, Dept Food Sci & Nutr, Coll Food Sci & Agr, Nanobiotechnol & Mol Biol Res Lab, Riyadh 11451, Saudi Arabia.	[Periasamy, Vaiyapuri S.; Alshatwi, Ali A.; Akbarsha, Mohammad A.] King Saud University, Dept Food Sci & Nutr, Coll Food Sci & Agr, Nanobiotechnol & Mol Biol Res Lab, Riyadh 11451, Saudi Arabia.	Country: Saudi Arabia Institution: King Saud Univ	Country: Saudi Arabia Institution: King Saud University
RP	1	67132	67132	Akbarsha, MA (corresponding author), Bharathidasan Univ, Mahatma Gandhi Doerenkamp Ctr, Tiruchirappalli 620024, India	Akbarsha, MA (corresponding author), Bharathidasan University, Mahatma Gandhi Doerenkamp Ctr, Tiruchirappalli 620024, India	Country: India Institution: Bharathidasan Univ	Country: India Institution: Bharathidasan University

Record 449 | WOS:000361762200007

Effects of Dietary Exposure to Zearalenone (ZEN) on Carp (Cyprinus carpio L.)

Field	Position	Original line	Normalised line	Original value	Normalised value	Original country / institution	Normalised country / institution
C1	2	67284	67284	[Pietsch, Constanze; Burkhardt-Holm, Patricia] Univ Basel, Dept Environm Sci, Man Soc Environm, CH-4051 Basel, Switzerland.	[Pietsch, Constanze; Burkhardt-Holm, Patricia] University of Basel, Dept Environm Sci, Man Soc Environm, CH-4051 Basel, Switzerland.	Country: Switzerland Institution: Univ Basel	Country: Switzerland Institution: University of Basel
C1	4	67286	67286	[Schulz, Carsten] Univ Kiel, Inst Tierzucht & Tierhaltung, D-24098 Kiel, Germany.	[Schulz, Carsten] University of Kiel, Inst Tierzucht & Tierhaltung, D-24098 Kiel, Germany.	Country: Germany Institution: Univ Kiel	Country: Germany Institution: University of Kiel
C1	6	67288	67288	[Burkhardt-Holm, Patricia] Univ Alberta, Dept Biol Sci, Edmonton, AB T6G 2E9, Canada.	[Burkhardt-Holm, Patricia] University of Alberta, Dept Biol Sci, Edmonton, AB T6G 2E9, Canada.	Country: Canada Institution: Univ Alberta	Country: Canada Institution: University of Alberta

Record 450 | WOS:000360343000001

Cytotoxic effects of oosporein isolated from endophytic fungus Cochliobolus kusanoi

Field	Position	Original line	Normalised line	Original value	Normalised value	Original country / institution	Normalised country / institution
C1	1	67425	67425	[Ramesha, Alurappa; Nirmaladevi, Dhamodaran; Srinivas, Chowdappa]	[Ramesha, Alurappa; Nirmaladevi, Dhamodaran; Srinivas, Chowdappa]	Country: India Institution: Bangalore Univ	Country: India Institution: Bangalore University

				Bangalore Univ, Dept Microbiol & Biotechnol, Bangalore 560056, Karnataka, India.	Bangalore University, Dept Microbiol & Biotechnol, Bangalore 560056, Karnataka, India.		
C1	2	67426	67426	[Venkataramana, M.] Bharathiar Univ, Toxicol & Immunol Div, DRDO BU Ctr Life Sci, Coimbatore, Tamil Nadu, India.	[Venkataramana, M.] Bharathiar University, Toxicol & Immunol Div, DRDO BU Ctr Life Sci, Coimbatore, Tamil Nadu, India.	Country: India Institution: Bharathiar Univ	Country: India Institution: Bharathiar University
C1	4	67428	67428	[Chandranayaka, S.] Univ Mysore, Dept Studies Biotechnol, Mysore 570006, Karnataka, India.	[Chandranayaka, S.] University of Mysore, Dept Studies Biotechnol, Mysore 570006, Karnataka, India.	Country: India Institution: Univ Mysore	Country: India Institution: University of Mysore
RP	1	67432	67432	Chandranayaka, S (corresponding author), Univ Mysore, Dept Studies Biotechnol, Mysore 570006, Karnataka, India	Chandranayaka, S (corresponding author), University of Mysore, Dept Studies Biotechnol, Mysore 570006, Karnataka, India	Country: India Institution: Univ Mysore	Country: India Institution: University of Mysore

Record 451 | WOS:000360202700022

Review on Mycotoxin Issues in Ruminants: Occurrence in Forages, Effects of Mycotoxin Ingestion on Health Status and Animal Performance and Practical Strategies to Counteract Their Negative Effects

No C1/C3/RP normalisation changes detected for this record.

Record 452 | WOS:000358238100012

Ochratoxin A activates opposing c-MET/PI3K/Akt and MAPK/ERK 1-2 pathways in human proximal tubule HK-2 cells

Field	Position	Original line	Normalised line	Original value	Normalised value	Original country / institution	Normalised country / institution
C1	1	67908	67908	[Ozcan, Zeynep; Gul, Gizem; Yaman, Ibrahim] Bogazici Univ, Dept Mol Biol & Genet, Mol Toxicol & Canc Res Lab, TR-34342 Bebek, Turkey.	[Ozcan, Zeynep; Gul, Gizem; Yaman, Ibrahim] Bogazici University, Dept Mol Biol & Genet, Mol Toxicol & Canc Res Lab, TR-34342 Bebek, Turkey.	Country: Turkey Institution: Bogazici Univ	Country: Turkey Institution: Bogazici University
C1	3	67910	67910	[Yaman, Ibrahim] Bogazici Univ, Ctr Life Sci & Technol, TR-34342 Istanbul, Turkey.	[Yaman, Ibrahim] Bogazici University, Ctr Life Sci & Technol, TR-34342 Istanbul, Turkey.	Country: Turkey Institution: Bogazici Univ	Country: Turkey Institution: Bogazici University
RP	1	67912	67912	Yaman, I (corresponding author), Bogazici Univ, Dept Mol Biol & Genet, Mol Toxicol & Canc Res Lab, TR-34342 Bebek, Turkey	Yaman, I (corresponding author), Bogazici University, Dept Mol Biol & Genet, Mol Toxicol & Canc Res Lab, TR-34342 Bebek, Turkey	Country: Turkey Institution: Bogazici Univ	Country: Turkey Institution: Bogazici University

Record 453 | WOS:000357537400012

Effects of Chronic Ochratoxin A Exposure on p53 Heterozygous and p53 Homozygous Mice

Field	Position	Original line	Normalised line	Original value	Normalised value	Original country / institution	Normalised country / institution
C1	1	68062	68062	[Bondy, Genevieve S.; Caldwell, Donald S.; Aziz, Syed A.; Coady, Laurie C.; Armstrong, Cheryl L.; Curran, Ivan H. A.; Kapal, Kamla; Lefebvre, David E.; Mehta, Rekha] Hlth Canada, Food Directorate, Hlth Prod &	[Bondy, Genevieve S.; Caldwell, Donald S.; Aziz, Syed A.; Coady, Laurie C.; Armstrong, Cheryl L.; Curran, Ivan H. A.; Kapal, Kamla; Lefebvre, David E.; Mehta, Rekha] Health Canada, Food Directorate, Hlth	Country: Canada Institution: Hlth Canada	Country: Canada Institution: Health Canada

				Food Branch, Ottawa, ON K1A 0K9, Canada.	Prod & Food Branch, Ottawa, ON K1A 0K9, Canada.		
C1	2	68063	68063	[Koffman, Robyn L.] Hlth Canada, Regions & Programs Bur, Burnaby, BC, Canada.	[Koffman, Robyn L.] Health Canada, Regions & Programs Bur, Burnaby, BC, Canada.	Country: Canada<br>Institution: Hlth Canada	Country: Canada<br>Institution: Health Canada
RP	1	68065	68065	Bondy, GS (corresponding author), Hlth Canada, Toxicol Res Div 2202D, Hlth Prod & Food Branch, Bur Chem Safety,Food Directorate, Ottawa, ON K1A 0K9, Canada	Bondy, GS (corresponding author), Health Canada, Toxicol Res Div 2202D, Hlth Prod & Food Branch, Bur Chem Safety,Food Directorate, Ottawa, ON K1A 0K9, Canada	Country: Canada<br>Institution: Hlth Canada	Country: Canada<br>Institution: Health Canada

Record 454 | WOS:000355330300006

Neurotoxic effects of ochratoxin A on the subventricular zone of adult mouse brain

Field	Position	Original line	Normalised line	Original value	Normalised value	Original country / institution	Normalised country / institution
C1	1	68202	68202	[Paradells, Sara; Rocamonde, Brenda; Llinares, Cristina; Zipancic, Ivan; Miguel Soria, Jose; Angeles Garcia-Esparza, Ma] Univ CEU Cardenal Herrera, Fac Ciencias Salud, Valencia 46113, Spain.	[Paradells, Sara; Rocamonde, Brenda; Llinares, Cristina; Zipancic, Ivan; Miguel Soria, Jose; Angeles Garcia-Esparza, Ma] Universidad CEU Cardenal Herrera, Fac Ciencias Salud, Valencia 46113, Spain.	Country: Spain<br>Institution: Univ CEU Cardenal Herrera	Country: Spain<br>Institution: Universidad CEU Cardenal Herrera
C1	2	68203	68203	[Herranz-Perez, Vicente; Manuel Garcia-Verdugo, Jose] Univ Valencia, Inst Cavanilles Biodiversidad & Biol Evolut, Lab Neurobiol Comparada, CIBERNED, Valencia 46980, Spain.	[Herranz-Perez, Vicente; Manuel Garcia-Verdugo, Jose] University of Valencia, Inst Cavanilles Biodiversidad & Biol Evolut, Lab Neurobiol Comparada, CIBERNED, Valencia 46980, Spain.	Country: Spain<br>Institution: Univ Valencia	Country: Spain<br>Institution: University of Valencia
C1	4	68205	68205	[Jimenez, Misericordia] Univ Valencia, Dept Microbiol & Ecol, E-46100 Burjassot, Spain.	[Jimenez, Misericordia] University of Valencia, Dept Microbiol & Ecol, E-46100 Burjassot, Spain.	Country: Spain<br>Institution: Univ Valencia	Country: Spain<br>Institution: University of Valencia
C1	5	68206	68206	[Zipancic, Ivan; Miguel Soria, Jose; Angeles Garcia-Esparza, Ma] Univ CEU Cardenal Herrera, Inst Ciencias Biomed, Moncada 46113, Spain.	[Zipancic, Ivan; Miguel Soria, Jose; Angeles Garcia-Esparza, Ma] Universidad CEU Cardenal Herrera, Inst Ciencias Biomed, Moncada 46113, Spain.	Country: Spain<br>Institution: Univ CEU Cardenal Herrera	Country: Spain<br>Institution: Universidad CEU Cardenal Herrera
RP	1	68209	68209	Garcia-Esparza, MA (corresponding author), Univ CEU Cardenal Herrera, Fac Ciencias Salud, Dept Farm, Ave Seminario S-N, Valencia 46313, Spain	Garcia-Esparza, MA (corresponding author), Universidad CEU Cardenal Herrera, Fac Ciencias Salud, Dept Farm, Ave Seminario S-N, Valencia 46313, Spain	Country: Spain<br>Institution: Univ CEU Cardenal Herrera	Country: Spain<br>Institution: Universidad CEU Cardenal Herrera

Record 455 | WOS:000357490500001

Mycotoxins - prevention and decontamination by yeasts

Field	Position	Original line	Normalised line	Original value	Normalised value	Original country / institution	Normalised country / institution
C1	1	68372	68372	[Pfliegler, Walter P.] Univ Debrecen, Dept Genet & Appl Microbiol, Fac Sci & Technol, H-4032 Debrecen, Hungary.	[Pfliegler, Walter P.] University of Debrecen, Dept Genet & Appl Microbiol, Fac Sci & Technol, H-4032 Debrecen, Hungary.	Country: Hungary<br>Institution: Univ Debrecen	Country: Hungary<br>Institution: University of Debrecen
C1	2	68373	68373	[Pfliegler, Walter P.; Pocsi, Istvan] Univ Debrecen, Dept Biotechnol & Microbiol, Fac Sci	[Pfliegler, Walter P.; Pocsi, Istvan] University of Debrecen, Dept Biotechnol & Microbiol,	Country: Hungary<br>Institution: Univ Debrecen	Country: Hungary<br>Institution: University of Debrecen

				& Technol, H-4032 Debrecen, Hungary.	Fac Sci & Technol, H-4032 Debrecen, Hungary.		
C1	4	68375	68375	[Pusztahelyi, Tuende] Univ Debrecen, Fac Agr & Food Sci & Environm Management, Cent Lab, H-4032 Debrecen, Hungary.	[Pusztahelyi, Tuende] University of Debrecen, Fac Agr & Food Sci & Environm Management, Cent Lab, H-4032 Debrecen, Hungary.	Country: Hungary Institution: Univ Debrecen	Country: Hungary Institution: University of Debrecen
RP	1	68378	68378	Pfliegler, WP (corresponding author), Univ Debrecen, Dept Biotechnol & Microbiol, Fac Sci & Technol, Egyet Ter 1, H-4032 Debrecen, Hungary	Pfliegler, WP (corresponding author), University of Debrecen, Dept Biotechnol & Microbiol, Fac Sci & Technol, Egyet Ter 1, H-4032 Debrecen, Hungary	Country: Hungary Institution: Univ Debrecen	Country: Hungary Institution: University of Debrecen

Record 456 | WOS:000362080300002

Structure elucidation and in vitro cytotoxicity of ochratoxin Î± amide, a new degradation product of ochratoxin A

Field	Position	Original line	Normalised line	Original value	Normalised value	Original country / institution	Normalised country / institution
C1	1	68559	68559	[Bittner, Andrea; Cramer, Benedikt; Harrer, Henning; Humpf, Hans-Ulrich] Univ Munster, Inst Food Chem, D-48149 Munster, Germany.	[Bittner, Andrea; Cramer, Benedikt; Harrer, Henning; Humpf, Hans-Ulrich] University of Munster, Inst Food Chem, D-48149 Munster, Germany.	Country: Germany Institution: Univ Munster	Country: Germany Institution: University of Munster
RP	1	68561	68561	Humpf, HU (corresponding author), Univ Munster, Inst Food Chem, Corrensstr 45, D-48149 Munster, Germany	Humpf, HU (corresponding author), University of Munster, Inst Food Chem, Corrensstr 45, D-48149 Munster, Germany	Country: Germany Institution: Univ Munster	Country: Germany Institution: University of Munster

Record 457 | WOS:000357242500002

Polyphenols as possible bioprotectors against cytotoxicity and DNA damage induced by ochratoxin A

No C1/C3/RP normalisation changes detected for this record.

Record 458 | WOS:000353230400005

In vitro and in vivo metabolism of ochratoxin A: a comparative study using ultra-performance liquid chromatography-quadrupole/time-of-flight hybrid mass spectrometry

Field	Position	Original line	Normalised line	Original value	Normalised value	Original country / institution	Normalised country / institution
C1	1	68775	68775	[Yang, Shupeng; Zhang, Huiyan; Sun, Feifei; Zhang, Suxia; Cao, Xingyuan; Wang, Zhanhui] China Agr Univ, Beijing Key Lab Detect Technol Anim Derived Food, Natl Reference Lab Vet Drug Residues, Coll Vet Med,Beijing Lab Food Qual & Safety, Beijing 100193, Peoples R China.	[Yang, Shupeng; Zhang, Huiyan; Sun, Feifei; Zhang, Suxia; Cao, Xingyuan; Wang, Zhanhui] China Agricultural University, Beijing Key Lab Detect Technol Anim Derived Food, Natl Reference Lab Vet Drug Residues, Coll Vet Med,Beijing Lab Food Qual & Safety, Beijing 100193, China.	Country: China Institution: China Agr Univ	Country: China Institution: China Agricultural University
C1	2	68776	68776	[De Saeger, Sarah; De Boevre, Marthe] Univ Ghent, Fac Pharmaceut Sci, Lab Food Anal, B-9000 Ghent, Belgium.	[De Saeger, Sarah; De Boevre, Marthe] Ghent University, Fac Pharmaceut Sci, Lab Food Anal, B-9000 Ghent, Belgium.	Country: Belgium Institution: Univ Ghent	Country: Belgium Institution: Ghent University
RP	1	68778	68778	Wang, ZH (corresponding author), China Agr Univ, Beijing Key Lab Detect Technol Anim	Wang, ZH (corresponding author), China Agricultural University, Beijing Key Lab	Country: China Institution: China Agr Univ	Country: China Institution: China Agricultural University

				Derived Food, Natl Reference Lab Vet Drug Residues, Coll Vet Med,Beijing Lab Food Qual & Safety, Beijing 100193, Peoples R China	Detect Technol Anim Derived Food, Natl Reference Lab Vet Drug Residues, Coll Vet Med,Beijing Lab Food Qual & Safety, Beijing 100193, China		

Record 459 | WOS:000351834600019

Assessment and characterisation of yeast-based products intended to mitigate ochratoxin exposure using in vitro and in vivo models

Field	Position	Original line	Normalised line	Original value	Normalised value	Original country / institution	Normalised country / institution
C1	1	68884	68884	[Pfohl-Leszkowicz, A.; Hadjeba-Medjdoub, K.] Univ Toulouse, Dept Bioproc & Microbial Syst, Chem Engn Lab, INPT,ENSAT,UMR CNRS 5503, Auzeville Tolosane, France.	[Pfohl-Leszkowicz, A.; Hadjeba-Medjdoub, K.] Universite de Toulouse, Dept Bioproc & Microbial Syst, Chem Engn Lab, INPT,ENSAT,UMR CNRS 5503, Auzeville Tolosane, France.	Country: France<br>Institution: Univ Toulouse	Country: France<br>Institution: Universite de Toulouse
C1	3	68886	68886	[Schrickx, J.; Fink-Gremmels, J.] Univ Utrecht, Fac Vet Med, Inst Risk Assessment Sci, Utrecht, Netherlands.	[Schrickx, J.; Fink-Gremmels, J.] Utrecht University, Fac Vet Med, Inst Risk Assessment Sci, Utrecht, Netherlands.	Country: Netherlands<br>Institution: Univ Utrecht	Country: Netherlands<br>Institution: Utrecht University
RP	1	68891	68891	Pfohl-Leszkowicz, A (corresponding author), Univ Toulouse, Dept Bioproc & Microbial Syst, Chem Engn Lab, INPT,ENSAT,UMR CNRS 5503, Auzeville Tolosane, France	Pfohl-Leszkowicz, A (corresponding author), Universite de Toulouse, Dept Bioproc & Microbial Syst, Chem Engn Lab, INPT,ENSAT,UMR CNRS 5503, Auzeville Tolosane, France	Country: France<br>Institution: Univ Toulouse	Country: France<br>Institution: Universite de Toulouse

Record 460 | WOS:000353889600047

Selenium Alleviates Porcine Nephrotoxicity of Ochratoxin A by Improving Selenoenzyme Expression In Vitro

Field	Position	Original line	Normalised line	Original value	Normalised value	Original country / institution	Normalised country / institution
C1	1	69012	69012	[Gan, Fang; Xue, Hongxia; Huang, Yu; Pan, Cuiling; Huang, Kehe] Nanjing Agr Univ, Coll Vet Med, Nanjing, Jiangsu, Peoples R China.	[Gan, Fang; Xue, Hongxia; Huang, Yu; Pan, Cuiling; Huang, Kehe] Nanjing Agricultural University, Coll Vet Med, Nanjing, Jiangsu, China.	Country: China<br>Institution: Nanjing Agr Univ	Country: China<br>Institution: Nanjing Agricultural University
RP	1	69014	69014	Huang, KH (corresponding author), Nanjing Agr Univ, Coll Vet Med, Nanjing, Jiangsu, Peoples R China	Huang, KH (corresponding author), Nanjing Agricultural University, Coll Vet Med, Nanjing, Jiangsu, China	Country: China<br>Institution: Nanjing Agr Univ	Country: China<br>Institution: Nanjing Agricultural University

Record 461 | WOS:000349553900004

Determination of Aflatoxins and Ochratoxins in Sicilian Sweet Wines by High-Performance Liquid Chromatography with Fluorometric Detection and Immunoaffinity Cleanup

Field	Position	Original line	Normalised line	Original value	Normalised value	Original country / institution	Normalised country / institution
C1	1	69127	69127	[Di Stefano, Vita; Avellone, Giuseppe; Di Fiore, Antonina] Univ Palermo, Dept Biol Chem & Pharmaceut Sci & Technol STEBICE, I-90123 Palermo, Italy.	[Di Stefano, Vita; Avellone, Giuseppe; Di Fiore, Antonina] University of Palermo, Dept Biol Chem & Pharmaceut Sci & Technol STEBICE, I-90123 Palermo, Italy.	Country: Italy<br>Institution: Univ Palermo	Country: Italy<br>Institution: University of Palermo

C1	2	69128	69128	[Pitonzo, Rosa] Univ Palermo, CGA, UniNetLab, I-90123 Palermo, Italy.	[Pitonzo, Rosa] University of Palermo, CGA, UniNetLab, I-90123 Palermo, Italy.	Country: Italy<br>Institution: Univ Palermo	Country: Italy<br>Institution: University of Palermo
C1	4	69130	69130	[Ogorka, Agata Zofia Tabis] Jagiellonian Univ, Fac Pharm Toxicol, Krakow, Poland.	[Ogorka, Agata Zofia Tabis] Jagiellonian University, Fac Pharm Toxicol, Krakow, Poland.	Country: Poland<br>Institution: Jagiellonian Univ	Country: Poland<br>Institution: Jagiellonian University
C1	5	69131	69131	[Di Stefano, Vita] Univ Palermo, Dipartimento Sci & Tecnol Biol Chim & Farmaceut S, I-90123 Palermo, Italy.	[Di Stefano, Vita] University of Palermo, Dipartimento Sci & Tecnol Biol Chim & Farmaceut S, I-90123 Palermo, Italy.	Country: Italy<br>Institution: Univ Palermo	Country: Italy<br>Institution: University of Palermo
RP	1	69134	69134	Di Stefano, V (corresponding author), Univ Palermo, Dipartimento Sci & Tecnol Biol Chim & Farmaceut S, Via Archirafi 32, I-90123 Palermo, Italy	Di Stefano, V (corresponding author), University of Palermo, Dipartimento Sci & Tecnol Biol Chim & Farmaceut S, Via Archirafi 32, I-90123 Palermo, Italy	Country: Italy<br>Institution: Univ Palermo	Country: Italy<br>Institution: University of Palermo

Record 462 | WOS:000343780400062

In vitro genotoxicity of mycotoxins ochratoxin A and fumonisin B1 could be prevented by sodium copper chlorophyllin - Implication to their genotoxic mechanism

Field	Position	Original line	Normalised line	Original value	Normalised value	Original country / institution	Normalised country / institution
C1	1	69227	69227	[Domijan, Ana-Marija] Univ Zagreb, Fac Pharm & Biochem, Dept Pharmaceut Bot, Zagreb 41000, Croatia.	[Domijan, Ana-Marija] University of Zagreb, Fac Pharm & Biochem, Dept Pharmaceut Bot, Zagreb 41000, Croatia.	Country: Croatia<br>Institution: Univ Zagreb	Country: Croatia<br>Institution: University of Zagreb
C1	2	69228	69228	[Gajski, Goran; Genic, Marko; Garaj-Vrhovac, Vera] Inst Med Res & Occupat Hlth, Mutagenesis Unit, Zagreb 41000, Croatia.	[Gajski, Goran; Genic, Marko; Garaj-Vrhovac, Vera] Institute for Medical Research & Occupational Health (IMROH), Mutagenesis Unit, Zagreb 41000, Croatia.	Country: Croatia<br>Institution: Inst Med Res & Occupat Hlth	Country: Croatia<br>Institution: Institute for Medical Research & Occupational Health (IMROH)
C1	3	69229	69229	[Jovanovic, Ivana Novak] Inst Med Res & Occupat Hlth, Toxicol Unit, Zagreb 41000, Croatia.	[Jovanovic, Ivana Novak] Institute for Medical Research & Occupational Health (IMROH), Toxicol Unit, Zagreb 41000, Croatia.	Country: Croatia<br>Institution: Inst Med Res & Occupat Hlth	Country: Croatia<br>Institution: Institute for Medical Research & Occupational Health (IMROH)
RP	1	69233	69233	Domijan, AM (corresponding author), Univ Zagreb, Fac Pharm & Biochem, Dept Pharmaceut Bot, A Kovacica 1, Zagreb 41000, Croatia	Domijan, AM (corresponding author), University of Zagreb, Fac Pharm & Biochem, Dept Pharmaceut Bot, A Kovacica 1, Zagreb 41000, Croatia	Country: Croatia<br>Institution: Univ Zagreb	Country: Croatia<br>Institution: University of Zagreb

Record 463 | WOS:000351485000004

Ochratoxin A promotes porcine circovirus type 2 replication in vitro and in vivo

Field	Position	Original line	Normalised line	Original value	Normalised value	Original country / institution	Normalised country / institution
C1	1	69358	69358	[Gan, Fang; Zhang, Zheqian; Hu, Zhihua; Xue, Hongxia; Chen, Xingxiang; Hao, Shu; Huang, Yu; Parveen, Fahmida; Huang, Kehe] Nanjing Agr Univ, Coll Vet Med, Nanjing 210095, Jiangsu, Peoples R China.	[Gan, Fang; Zhang, Zheqian; Hu, Zhihua; Xue, Hongxia; Chen, Xingxiang; Hao, Shu; Huang, Yu; Parveen, Fahmida; Huang, Kehe] Nanjing Agricultural University, Coll Vet Med, Nanjing 210095, Jiangsu, China.	Country: China<br>Institution: Nanjing Agr Univ	Country: China<br>Institution: Nanjing Agricultural University
C1	2	69359	69359	[Hesketh, John; Ezea, Patience Cole] Newcastle Univ, Sch Med, Inst Cell & Mol Biosci,	[Hesketh, John; Ezea, Patience Cole] Newcastle Univ, Sch Med, Inst Cell & Mol Biosci,	Country: England<br>Institution: Newcastle Univ	Country: United Kingdom<br>Institution: Newcastle Univ

				Newcastle Upon Tyne NE2 4HH, Tyne & Wear, England.	Newcastle Upon Tyne NE2 4HH, Tyne & Wear, United Kingdom.		
C3	2	69360	69360	Newcastle University - UK	Newcastle University - United Kingdom	Institution: Newcastle University - UK	Institution: Newcastle University - United Kingdom
RP	1	69361	69361	Huang, KH (corresponding author), Nanjing Agr Univ, Coll Vet Med, Nanjing 210095, Jiangsu, Peoples R China	Huang, KH (corresponding author), Nanjing Agricultural University, Coll Vet Med, Nanjing 210095, Jiangsu, China	Country: China<br>Institution: Nanjing Agr Univ	Country: China<br>Institution: Nanjing Agricultural University

Record 464 | WOS:000350023000003

Ochratoxin A and its effects on immunity

No C1/C3/RP normalisation changes detected for this record.

Record 465 | WOS:000350094000016

Genotoxicity of Aflatoxin B1 and Ochratoxin A after simultaneous application of the in vivo micronucleus and comet assay

Field	Position	Original line	Normalised line	Original value	Normalised value	Original country / institution	Normalised country / institution
C1	1	69656	69656	[Corcuera, Laura-Ana; Vettorazzi, Ariane; Arbillaga, Leire; Perez, Noemi; Gloria Gil, Ana; Azqueta, Amaya; Lopez de Cerain, Adela] Univ Navarra, Fac Pharm, Dept Pharmacol & Toxicol, Pamplona 31008, Spain.	[Corcuera, Laura-Ana; Vettorazzi, Ariane; Arbillaga, Leire; Perez, Noemi; Gloria Gil, Ana; Azqueta, Amaya; Lopez de Cerain, Adela] University of Navarra, Fac Pharm, Dept Pharmacol & Toxicol, Pamplona 31008, Spain.	Country: Spain<br>Institution: Univ Navarra	Country: Spain<br>Institution: University of Navarra
C1	2	69657	69657	[Antonio Garcia-Jalon, Jose] Univ Zaragoza, Fac Vet, Dept Anim Pathol, E-50013 Zaragoza, Spain.	[Antonio Garcia-Jalon, Jose] University of Zaragoza, Fac Vet, Dept Anim Pathol, E-50013 Zaragoza, Spain.	Country: Spain<br>Institution: Univ Zaragoza	Country: Spain<br>Institution: University of Zaragoza
C1	3	69658	69658	[Gonzalez-Penas, Elena] Univ Navarra, Fac Pharm, Dept Organ & Pharmaceut Chem, Pamplona 31008, Spain.	[Gonzalez-Penas, Elena] University of Navarra, Fac Pharm, Dept Organ & Pharmaceut Chem, Pamplona 31008, Spain.	Country: Spain<br>Institution: Univ Navarra	Country: Spain<br>Institution: University of Navarra
RP	1	69660	69660	de Cerain, AL (corresponding author), Univ Navarra, Fac Pharm, Dept Pharmacol & Toxicol, Irunlarrea 1, Pamplona 31008, Spain	de Cerain, AL (corresponding author), University of Navarra, Fac Pharm, Dept Pharmacol & Toxicol, Irunlarrea 1, Pamplona 31008, Spain	Country: Spain<br>Institution: Univ Navarra	Country: Spain<br>Institution: University of Navarra

Record 466 | WOS:000362800100005

Mycotoxin mechanisms of action and health impact: 'in vitro' or 'in vivo' tests, that is the question

Field	Position	Original line	Normalised line	Original value	Normalised value	Original country / institution	Normalised country / institution
C1	1	69787	69787	[Cheli, F.; Giromini, C.; Baldi, A.] Univ Milan, Dept Hlth Anim Sci & Food Safety, Via Trentacoste 2, I-20134 Milan, Italy.	[Cheli, F.; Giromini, C.; Baldi, A.] University of Milan, Dept Hlth Anim Sci & Food Safety, Via Trentacoste 2, I-20134 Milan, Italy.	Country: Italy<br>Institution: Univ Milan	Country: Italy<br>Institution: University of Milan
RP	1	69789	69789	Cheli, F (corresponding author), Univ Milan, Dept Hlth Anim Sci	Cheli, F (corresponding author), University of Milan, Dept Hlth Anim Sci & Food Safety, Via	Country: Italy<br>Institution: Univ Milan	Country: Italy<br>Institution: University of Milan

				& Food Safety, Via Trentacoste 2, I-20134 Milan, Italy	Trentacoste 2, I-20134 Milan, Italy		

Record 467 | WOS:000351213600013

Toxic Effects of Aflatoxin B1 on Embryonic Development of Zebrafish (Danio rerio): Potential Activity of Piceatannol Encapsulated Chitosan/poly (Lactic Acid) Nanoparticles

No C1/C3/RP normalisation changes detected for this record.

Record 468 | WOS:000349133200010

Bioaccessibility of ochratoxin A from red wine in an in vitro dynamic gastrointestinal model

No C1/C3/RP normalisation changes detected for this record.

Record 469 | WOS:000415214500006

Current Insights into Mycotoxins

Field	Position	Original line	Normalised line	Original value	Normalised value	Original country / institution	Normalised country / institution
C1	1	70220	70220	[Karimi, Gholamreza] Mashhad Univ Med Sci, Med Toxicol Res Ctr, Mashhad, Iran.	[Karimi, Gholamreza] Mashhad University of Medical Sciences, Med Toxicol Res Ctr, Mashhad, Iran.	Country: Iran<br>Institution: Mashhad Univ Med Sci	Country: Iran<br>Institution: Mashhad University of Medical Sciences
C1	2	70221	70221	[Karimi, Gholamreza] Mashhad Univ Med Sci, Sch Pharm, Mashhad, Iran.	[Karimi, Gholamreza] Mashhad University of Medical Sciences, Sch Pharm, Mashhad, Iran.	Country: Iran<br>Institution: Mashhad Univ Med Sci	Country: Iran<br>Institution: Mashhad University of Medical Sciences
C1	3	70222	70222	[Mehri, Soghra] Mashhad Univ Med Sci, Pharmaceut Res Ctr, Mashhad, Iran.	[Mehri, Soghra] Mashhad University of Medical Sciences, Pharmaceut Res Ctr, Mashhad, Iran.	Country: Iran<br>Institution: Mashhad Univ Med Sci	Country: Iran<br>Institution: Mashhad University of Medical Sciences
C1	4	70223	70223	[Mehri, Soghra] Mashhad Univ Med Sci, Sch Pharm, Dept Pharmacodynamy & Toxicol, Mashhad, Iran.	[Mehri, Soghra] Mashhad University of Medical Sciences, Sch Pharm, Dept Pharmacodynamy & Toxicol, Mashhad, Iran.	Country: Iran<br>Institution: Mashhad Univ Med Sci	Country: Iran<br>Institution: Mashhad University of Medical Sciences
RP	1	70227	70227	Karimi, G (corresponding author), Mashhad Univ Med Sci, Med Toxicol Res Ctr, Mashhad, Iran	Karimi, G (corresponding author), Mashhad University of Medical Sciences, Med Toxicol Res Ctr, Mashhad, Iran	Country: Iran<br>Institution: Mashhad Univ Med Sci	Country: Iran<br>Institution: Mashhad University of Medical Sciences

Record 470 | WOS:000360184800009

Dynamic changes of global DNA methylation and hypermethylation of cell adhesion-related genes in rat kidneys in response to ochratoxin A

Field	Position	Original line	Normalised line	Original value	Normalised value	Original country / institution	Normalised country / institution
C1	1	70329	70329	[Li, X.; Gao, J.; Huang, K.; Qi, X.; Dai, Q.; Mei, X.; Xu, W.] China Agr Univ, Coll Food Sci & Nutr Engn, Lab Food Safety & Mol Biol, Beijing 100083, Peoples R China.	[Li, X.; Gao, J.; Huang, K.; Qi, X.; Dai, Q.; Mei, X.; Xu, W.] China Agricultural University, Coll Food Sci & Nutr Engn, Lab Food Safety & Mol Biol, Beijing 100083, China.	Country: China<br>Institution: China Agr Univ	Country: China<br>Institution: China Agricultural University
C1	2	70330	70330	[Huang, K.] Lab Food Qual & Safety, Beijing 100083, Peoples R China.	[Huang, K.] Lab Food Qual & Safety, Beijing 100083, China.	Country: China<br>Institution: Lab Food Qual & Safety	Country: China<br>Institution: Lab Food Qual & Safety

RP	1	70332	70332	Li, X (corresponding author), China Agr Univ, Coll Food Sci & Nutr Engn, Lab Food Safety & Mol Biol, Beijing 100083, Peoples R China	Li, X (corresponding author), China Agricultural University, Coll Food Sci & Nutr Engn, Lab Food Safety & Mol Biol, Beijing 100083, China	Country: China<br>Institution: China Agr Univ	Country: China<br>Institution: China Agricultural University

Record 471 | WOS:000367698600008

POTENTIAL GENOTOXICITY OF ACETAMIPRID AND PROPINEB SINGLY OR IN COMBINATION IN CULTURED HUMAN PERIPHERAL BLOOD LYMPHOCYTES BY USING MN ASSAY

Field	Position	Original line	Normalised line	Original value	Normalised value	Original country / institution	Normalised country / institution
C1	1	70450	70450	[Muranli, Fulya Dilek Gokalp; Kanev, Martin; Ozdemir, Kezban] Trakya Univ, Fac Sci, Dept Biol, TR-22030 Edirne, Turkey.	[Muranli, Fulya Dilek Gokalp; Kanev, Martin; Ozdemir, Kezban] Trakya University, Fac Sci, Dept Biol, TR-22030 Edirne, Turkey.	Country: Turkey<br>Institution: Trakya Univ	Country: Turkey<br>Institution: Trakya University
C1	2	70451	70451	[Rasgele, Pinar Goc] Duzce Univ, Agr & Nat Sci Fac, Dept Biosyst Engn, TR-81620 Duzce, Turkey.	[Rasgele, Pinar Goc] Duzce University, Agr & Nat Sci Fac, Dept Biosyst Engn, TR-81620 Duzce, Turkey.	Country: Turkey<br>Institution: Duzce Univ	Country: Turkey<br>Institution: Duzce University
C1	3	70452	70452	[Kekecoglu, Meral] Duzce Univ, Fac Sci & Letters, Dept Biol, TR-81620 Duzce, Turkey.	[Kekecoglu, Meral] Duzce University, Fac Sci & Letters, Dept Biol, TR-81620 Duzce, Turkey.	Country: Turkey<br>Institution: Duzce Univ	Country: Turkey<br>Institution: Duzce University
RP	1	70454	70454	Muranli, FDG (corresponding author), Trakya Univ, Fac Sci, Dept Biol, TR-22030 Edirne, Turkey	Muranli, FDG (corresponding author), Trakya University, Fac Sci, Dept Biol, TR-22030 Edirne, Turkey	Country: Turkey<br>Institution: Trakya Univ	Country: Turkey<br>Institution: Trakya University

Record 472 | WOS:000347274600033

Low doses of ochratoxin A induce micronucleus formation and delay DNA repair in human lymphocytes

No C1/C3/RP normalisation changes detected for this record.

Record 473 | WOS:000349038800005

In vitro effects of ochratoxin A, deoxynivalenol and zearalenone on cell viability and E-ADA activity in broiler chickens lymphocytes

No C1/C3/RP normalisation changes detected for this record.

Record 474 | WOS:000344696100002

The mycotoxin beauvericin induces apoptotic cell death in H4IIE hepatoma cells accompanied by an inhibition of NF-ÎºB-activity and modulation of MAP-kinases

No C1/C3/RP normalisation changes detected for this record.

Record 475 | WOS:000344980400016

Ochratoxin A induces rat renal carcinogenicity with limited induction of oxidative stress responses

Field	Position	Original line	Normalised line	Original value	Normalised value	Original country / institution	Normalised country / institution

C1	1	70931	70931	[Qi, Xiaozhe; Yu, Tao; Zhu, Liye; Gao, Jing; He, Xiaoyun; Huang, Kunlun; Luo, Yunbo; Xu, Wentao] China Agr Univ, Coll Food Sci & Nutr Engn, Beijing 100083, Peoples R China.	[Qi, Xiaozhe; Yu, Tao; Zhu, Liye; Gao, Jing; He, Xiaoyun; Huang, Kunlun; Luo, Yunbo; Xu, Wentao] China Agricultural University, Coll Food Sci & Nutr Engn, Beijing 100083, China.	Country: China<br>Institution: China Agr Univ	Country: China<br>Institution: China Agricultural University
C1	2	70932	70932	[He, Xiaoyun; Huang, Kunlun; Luo, Yunbo; Xu, Wentao] Minist Agr, Inspect & Testing Ctr Genet Modified Organisms, Beijing 100083, Peoples R China.	[He, Xiaoyun; Huang, Kunlun; Luo, Yunbo; Xu, Wentao] Minist Agr, Inspect & Testing Ctr Genet Modified Organisms, Beijing 100083, China.	Country: China<br>Institution: Minist Agr	Country: China<br>Institution: Minist Agr
RP	1	70934	70934	Xu, WT (corresponding author), China Agr Univ, Coll Food Sci & Nutr Engn, 17 Tsinghua Donglu, Beijing 100083, Peoples R China	Xu, WT (corresponding author), China Agricultural University, Coll Food Sci & Nutr Engn, 17 Tsinghua Donglu, Beijing 100083, China	Country: China<br>Institution: China Agr Univ	Country: China<br>Institution: China Agricultural University

Record 476 | WOS:000343730400082

A New Ochratoxin A Biodegradation Strategy Using Cupriavidus basilensis Or16 Strain

Field	Position	Original line	Normalised line	Original value	Normalised value	Original country / institution	Normalised country / institution
C1	1	71058	71058	[Ferenczi, Szilamer; Kovacs, Krisztina J.] Hungarian Acad Sci, Inst Expt Med, Lab Mol Neuroendocrinol, Budapest, Hungary.	[Ferenczi, Szilamer; Kovacs, Krisztina J.] Hungarian Academy of Sciences, Inst Expt Med, Lab Mol Neuroendocrinol, Budapest, Hungary.	Country: Hungary<br>Institution: Hungarian Acad Sci	Country: Hungary<br>Institution: Hungarian Academy of Sciences
C1	6	71063	71063	[Barna, Terez] Univ Debrecen, Dept Genet & Appl Microbiol, H-4012 Debrecen, Hungary.	[Barna, Terez] University of Debrecen, Dept Genet & Appl Microbiol, H-4012 Debrecen, Hungary.	Country: Hungary<br>Institution: Univ Debrecen	Country: Hungary<br>Institution: University of Debrecen
RP	1	71069	71069	Ferenczi, S (corresponding author), Hungarian Acad Sci, Inst Expt Med, Lab Mol Neuroendocrinol, Budapest, Hungary	Ferenczi, S (corresponding author), Hungarian Academy of Sciences, Inst Expt Med, Lab Mol Neuroendocrinol, Budapest, Hungary	Country: Hungary<br>Institution: Hungarian Acad Sci	Country: Hungary<br>Institution: Hungarian Academy of Sciences

Record 477 | WOS:000341677200007

Biodegradation of ochratoxin A by Pediococcus parvulus isolated from Douro wines

Field	Position	Original line	Normalised line	Original value	Normalised value	Original country / institution	Normalised country / institution
C1	2	71193	71193	[Ines, Antonio; Pereira, Vania L.; Mendes-Faia, Arlete] Univ Tras Os Montes & Alto Douro, Inst Biotechnol & Bioengn, Ctr Genom & Biotechnol, Sch Life Sci & Environm, P-5001801 Vila Real, Portugal.	[Ines, Antonio; Pereira, Vania L.; Mendes-Faia, Arlete] University of Tras-os-Montes & Alto Douro, Inst Biotechnol & Bioengn, Ctr Genom & Biotechnol, Sch Life Sci & Environm, P-5001801 Vila Real, Portugal.	Country: Portugal<br>Institution: Univ Tras Os Montes & Alto Douro	Country: Portugal<br>Institution: University of Tras-os-Montes & Alto Douro

Record 478 | WOS:000343630800010

Biological detoxification of zearalenone by Aspergillus niger strain FS10

Field	Position	Original line	Normalised line	Original value	Normalised value	Original country / institution	Normalised country / institution

C1	1	71333	71333	[Sun, Xiulan; He, Xingxing; Li, Yun; Qian, He] Jiangnan Univ, Synerget Innovat Ctr Food Safety & Nutr, Sch Food Sci & Technol, State Key Lab Food Sci & Technol, Wuxi 214122, Jiangsu, Peoples R China.	[Sun, Xiulan; He, Xingxing; Li, Yun; Qian, He] Jiangnan University, Synerget Innovat Ctr Food Safety & Nutr, Sch Food Sci & Technol, State Key Lab Food Sci & Technol, Wuxi 214122, Jiangsu, China.	Country: China<br>Institution: Jiangnan Univ	Country: China<br>Institution: Jiangnan University
C1	2	71334	71334	[Li, Yun] Chinese Acad Agr Sci, Inst Qual Stand & Testing Technol Agroprod, Beijing 100081, Peoples R China.	[Li, Yun] Chinese Academy of Agricultural Sciences, Inst Qual Stand & Testing Technol Agroprod, Beijing 100081, China.	Country: China<br>Institution: Chinese Acad Agr Sci	Country: China<br>Institution: Chinese Academy of Agricultural Sciences
C1	3	71335	71335	[Xu, Dan] Shaanxi Univ Sci Technol, Dept Food Sci, Xian 710021, Peoples R China.	[Xu, Dan] Shaanxi University of Science & Technology, Dept Food Sci, Xian 710021, China.	Country: China<br>Institution: Shaanxi Univ Sci Technol	Country: China<br>Institution: Shaanxi University of Science & Technology
C1	4	71336	71336	[Xue, Kathy Siyu] Univ Georgia, Dept Environm Hlth Sci, Athens, GA 30602 USA.	[Xue, Kathy Siyu] University of Georgia, Dept Environm Hlth Sci, Athens, GA 30602 USA.	Country: USA<br>Institution: Univ Georgia	Country: USA<br>Institution: University of Georgia
RP	1	71341	71341	Sun, XL (corresponding author), Jiangnan Univ, Synerget Innovat Ctr Food Safety & Nutr, Sch Food Sci & Technol, State Key Lab Food Sci & Technol, Wuxi 214122, Jiangsu, Peoples R China	Sun, XL (corresponding author), Jiangnan University, Synerget Innovat Ctr Food Safety & Nutr, Sch Food Sci & Technol, State Key Lab Food Sci & Technol, Wuxi 214122, Jiangsu, China	Country: China<br>Institution: Jiangnan Univ	Country: China<br>Institution: Jiangnan University

Record 479 | WOS:000343630800034

Ochratoxin A kinetics: A review of analytical methods and studies in rat model

Field	Position	Original line	Normalised line	Original value	Normalised value	Original country / institution	Normalised country / institution
C1	1	71441	71441	[Vettorazzi, A.; Lopez de Cerain, A.] Univ Navarra, Sch Pharm, Dept Pharmacol & Toxicol, E-31080 Pamplona, Spain.	[Vettorazzi, A.; Lopez de Cerain, A.] University of Navarra, Sch Pharm, Dept Pharmacol & Toxicol, E-31080 Pamplona, Spain.	Country: Spain<br>Institution: Univ Navarra	Country: Spain<br>Institution: University of Navarra
C1	2	71442	71442	[Gonzalez-Penas, E.] Univ Navarra, Sch Pharm, Dept Organ & Pharmaceut Chem, E-31080 Pamplona, Spain.	[Gonzalez-Penas, E.] University of Navarra, Sch Pharm, Dept Organ & Pharmaceut Chem, E-31080 Pamplona, Spain.	Country: Spain<br>Institution: Univ Navarra	Country: Spain<br>Institution: University of Navarra
RP	1	71444	71444	Vettorazzi, A (corresponding author), Univ Navarra, Sch Pharm, Dept Pharmacol & Toxicol, Irunlarrea 1, E-31080 Pamplona, Spain	Vettorazzi, A (corresponding author), University of Navarra, Sch Pharm, Dept Pharmacol & Toxicol, Irunlarrea 1, E-31080 Pamplona, Spain	Country: Spain<br>Institution: Univ Navarra	Country: Spain<br>Institution: University of Navarra

Record 480 | WOS:000340827200008

Synergism of ochratoxin B and calcium-channel antagonist verapamil caused mitochondrial dysfunction

No C1/C3/RP normalisation changes detected for this record.

Record 481 | WOS:000340837600026

Development of an in vitro method for the prediction of mycotoxin binding on yeast-based products: case of aflatoxin B1, zearalenone and ochratoxin A

Field	Position	Original line	Normalised line	Original value	Normalised value	Original country / institution	Normalised country / institution

Field	Position	Original line	Normalised line	Original value	Normalised value	Original country / institution	Normalised country / institution
C1	1	71690	71690	[Faucet-Marquis, Virginie; Joannis-Cassan, Claire; Hadjeba-Medjdoub, Kheira; Pfohl-Leszkowicz, Annie] Univ Toulouse, UMR CNRS INPT UPS 5503, Chem Engn Lab, F-31320 Auzeville Tolosane, France.	[Faucet-Marquis, Virginie; Joannis-Cassan, Claire; Hadjeba-Medjdoub, Kheira; Pfohl-Leszkowicz, Annie] Universite de Toulouse, UMR CNRS INPT UPS 5503, Chem Engn Lab, F-31320 Auzeville Tolosane, France.	Country: France<br>Institution: Univ Toulouse	Country: France<br>Institution: Universite de Toulouse
RP	1	71699	71699	Pfohl-Leszkowicz, A (corresponding author), Univ Toulouse, UMR CNRS INPT UPS 5503, Chem Engn Lab, 1 Ave Agrobiople, F-31320 Auzeville Tolosane, France	Pfohl-Leszkowicz, A (corresponding author), Universite de Toulouse, UMR CNRS INPT UPS 5503, Chem Engn Lab, 1 Ave Agrobiople, F-31320 Auzeville Tolosane, France	Country: France<br>Institution: Univ Toulouse	Country: France<br>Institution: Universite de Toulouse

Record 482 | WOS:000342990200034

Combination of Metagenomics and Culture-Based Methods to Study the Interaction Between Ochratoxin A and Gut Microbiota

Field	Position	Original line	Normalised line	Original value	Normalised value	Original country / institution	Normalised country / institution
C1	1	71820	71820	[Guo, Mingzhang; Huang, Kunlun; Chen, Siyuan; Qi, Xiaozhe; He, Xiaoyun; Luo, Yunbo; Xia, Kai; Xu, Wentao] China Agr Univ, Coll Food Sci & Nutr Engn, Lab Food Safety & Mol Biol, Beijing 100083, Peoples R China.	[Guo, Mingzhang; Huang, Kunlun; Chen, Siyuan; Qi, Xiaozhe; He, Xiaoyun; Luo, Yunbo; Xia, Kai; Xu, Wentao] China Agricultural University, Coll Food Sci & Nutr Engn, Lab Food Safety & Mol Biol, Beijing 100083, China.	Country: China<br>Institution: China Agr Univ	Country: China<br>Institution: China Agricultural University
C1	2	71821	71821	[Cheng, Wen-Hsing] Mississippi State Univ, Dept Food Sci Nutr & Hlth Promot, Mississippi State, MS 39762 USA.	[Cheng, Wen-Hsing] Mississippi State University, Dept Food Sci Nutr & Hlth Promot, Mississippi State, MS 39762 USA.	Country: USA<br>Institution: Mississippi State Univ	Country: USA<br>Institution: Mississippi State University
RP	1	71823	71823	Xu, WT (corresponding author), China Agr Univ, Coll Food Sci & Nutr Engn, Lab Food Safety & Mol Biol, Beijing 100083, Peoples R China	Xu, WT (corresponding author), China Agricultural University, Coll Food Sci & Nutr Engn, Lab Food Safety & Mol Biol, Beijing 100083, China	Country: China<br>Institution: China Agr Univ	Country: China<br>Institution: China Agricultural University

Record 483 | WOS:000340087200003

Biological detoxification of mycotoxins: a review

No C1/C3/RP normalisation changes detected for this record.

Record 484 | WOS:000341466700016

Hepatotoxicity and genotoxicity of patulin in mice, and its modulation by green tea polyphenols administration

Field	Position	Original line	Normalised line	Original value	Normalised value	Original country / institution	Normalised country / institution
C1	1	72165	72165	[Song, Erqun; Xia, Xiaomin; Su, Chuanyang; Dong, Wenjing; Xian, Yaping; Wang, Wei; Song, Yang] Southwest Univ, Coll Pharmaceut Sci, Key Lab Luminescence & Real Time Analyt Chem, Minist Educ,	[Song, Erqun; Xia, Xiaomin; Su, Chuanyang; Dong, Wenjing; Xian, Yaping; Wang, Wei; Song, Yang] Southwest Univ, Coll Pharmaceut Sci, Key Lab Luminescence & Real Time	Country: China<br>Institution: Southwest Univ	Country: China<br>Institution: Southwest Univ

				Chongqing 400715, Peoples R China.	Analyt Chem, Minist Educ, Chongqing 400715, China.		
C3	1	72166	72166	Southwest University - China	Southwest University	Institution: Southwest University - China	Institution: Southwest University
RP	1	72167	72167	Song, Y (corresponding author), Southwest Univ, Coll Pharmaceut Sci, Chongqing 400715, Peoples R China	Song, Y (corresponding author), Southwest Univ, Coll Pharmaceut Sci, Chongqing 400715, China	Country: China<br>Institution: Southwest Univ	Country: China<br>Institution: Southwest Univ

Record 485 | WOS:000341466700027

Cytotoxicity induced by ochratoxin A, zearalenone, and Î±-zearalenol: Effects of individual and combined treatment

Field	Position	Original line	Normalised line	Original value	Normalised value	Original country / institution	Normalised country / institution
C1	1	72293	72293	[Wang, H. W.; Wang, J. Q.; Li, S. L.; Zhang, Y. D.; Zheng, N.] Chinese Acad Agr Sci, Inst Anim Sci, Minist Agr, Lab Qual & Safety Risk Assessment Dairy Prod Beij, Beijing 100193, Peoples R China.	[Wang, H. W.; Wang, J. Q.; Li, S. L.; Zhang, Y. D.; Zheng, N.] Chinese Academy of Agricultural Sciences, Inst Anim Sci, Minist Agr, Lab Qual & Safety Risk Assessment Dairy Prod Beij, Beijing 100193, China.	Country: China<br>Institution: Chinese Acad Agr Sci	Country: China<br>Institution: Chinese Academy of Agricultural Sciences
C1	2	72294	72294	[Wang, H. W.; Li, F. D.] Gansu Agr Univ, Coll Anim Sci & Technol, Lanzhou 730070, Peoples R China.	[Wang, H. W.; Li, F. D.] Gansu Agricultural University, Coll Anim Sci & Technol, Lanzhou 730070, China.	Country: China<br>Institution: Gansu Agr Univ	Country: China<br>Institution: Gansu Agricultural University
C1	3	72295	72295	[Wang, H. W.; Wang, J. Q.; Li, S. L.; Zhang, Y. D.; Zheng, N.] Chinese Acad Agr Sci, Inst Anim Sci, State Key Lab Anim Nutr, Beijing 100193, Peoples R China.	[Wang, H. W.; Wang, J. Q.; Li, S. L.; Zhang, Y. D.; Zheng, N.] Chinese Academy of Agricultural Sciences, Inst Anim Sci, State Key Lab Anim Nutr, Beijing 100193, China.	Country: China<br>Institution: Chinese Acad Agr Sci	Country: China<br>Institution: Chinese Academy of Agricultural Sciences
C1	4	72296	72296	[Zheng, B. Q.] Tangshan Livestock & Aquat Prod Qual Monitoring C, Tangshan 06300, Peoples R China.	[Zheng, B. Q.] Tangshan Livestock & Aquat Prod Qual Monitoring C, Tangshan 06300, China.	Country: China<br>Institution: Tangshan Livestock & Aquat Prod Qual Monitoring C	Country: China<br>Institution: Tangshan Livestock & Aquat Prod Qual Monitoring C
RP	1	72300	72300	Zheng, N (corresponding author), Chinese Acad Agr Sci, Inst Anim Sci, State Key Lab Anim Nutr, 2 Yuanmingyuan West Rd, Beijing 100193, Peoples R China	Zheng, N (corresponding author), Chinese Academy of Agricultural Sciences, Inst Anim Sci, State Key Lab Anim Nutr, 2 Yuanmingyuan West Rd, Beijing 100193, China	Country: China<br>Institution: Chinese Acad Agr Sci	Country: China<br>Institution: Chinese Academy of Agricultural Sciences

Record 486 | WOS:000338616800026

Cytotoxicity and immunotoxicity of cyclopiazonic acid on human cells

No C1/C3/RP normalisation changes detected for this record.

Record 487 | WOS:000338817900023

Central role of Nix in the autophagic response to ochratoxin A

Field	Position	Original line	Normalised line	Original value	Normalised value	Original country / institution	Normalised country / institution
C1	1	72516	72516	[Shen, Xiao Li; Zhang, Boyang; Liang, Rui; Xu, Wentao; Luo, YunBo; Huang, Kunlun] China Agr Univ, Coll Food Sci & Nutr	[Shen, Xiao Li; Zhang, Boyang; Liang, Rui; Xu, Wentao; Luo, YunBo; Huang, Kunlun] China Agricultural University, Coll	Country: China<br>Institution: China Agr Univ	Country: China<br>Institution: China Agricultural University

				Engn, Lab Food Safety & Mol Biol, Beijing 100083, Peoples R China.	Food Sci & Nutr Engn, Lab Food Safety & Mol Biol, Beijing 100083, China.		
C1	2	72517	72517	[Shen, Xiao Li] Zunyi Med Univ, Sch Publ Hlth, Zunyi 563003, Guizhou, Peoples R China.	[Shen, Xiao Li] Zunyi Medical University, Sch Publ Hlth, Zunyi 563003, Guizhou, China.	Country: China<br>Institution: Zunyi Med Univ	Country: China<br>Institution: Zunyi Medical University
C1	3	72518	72518	[Cheng, Wen-Hsing] Mississippi State Univ, Dept Food Sci Nutr & Hlth Promot, Mississippi State, MS 39762 USA.	[Cheng, Wen-Hsing] Mississippi State University, Dept Food Sci Nutr & Hlth Promot, Mississippi State, MS 39762 USA.	Country: USA<br>Institution: Mississippi State Univ	Country: USA<br>Institution: Mississippi State University
RP	1	72523	72523	Xu, WT (corresponding author), China Agr Univ, Coll Food Sci & Nutr Engn, Lab Food Safety & Mol Biol, Beijing 100083, Peoples R China	Xu, WT (corresponding author), China Agricultural University, Coll Food Sci & Nutr Engn, Lab Food Safety & Mol Biol, Beijing 100083, China	Country: China<br>Institution: China Agr Univ	Country: China<br>Institution: China Agricultural University

Record 488 | WOS:000336730800004

Toxicity of the mycotoxin citrinin and its metabolite dihydrocitrinone and of mixtures of citrinin and ochratoxin A in vitro

No C1/C3/RP normalisation changes detected for this record.

Record 489 | WOS:000362078100003

Modulation of innate and antigen-specific immune functions directed against Listeria monocytogenes by fungal toxins in vitro

No C1/C3/RP normalisation changes detected for this record.

Record 490 | WOS:000333247100013

Downregulation of Rad51 participates in OTA-induced DNA double-strand breaks in GES-1 cells in vitro

Field	Position	Original line	Normalised line	Original value	Normalised value	Original country / institution	Normalised country / institution
C1	1	72841	72841	[Lian, Hongguang; Liu, Jing; Shen, Haitao; Xing, Lingxiao; Wang, Junling; Yan, Xia; Zhang, Xianghong] Hebei Med Univ, Pathol Lab, Shijiazhuang, Peoples R China.	[Lian, Hongguang; Liu, Jing; Shen, Haitao; Xing, Lingxiao; Wang, Junling; Yan, Xia; Zhang, Xianghong] Hebei Medical University, Pathol Lab, Shijiazhuang, China.	Country: China<br>Institution: Hebei Med Univ	Country: China<br>Institution: Hebei Medical University
C1	2	72842	72842	[Cui, Jinfeng; Wang, Yuan; Wang, Juan] Hebei Med Univ, Dept Pathol, Hosp 2, Shijiazhuang, Peoples R China.	[Cui, Jinfeng; Wang, Yuan; Wang, Juan] Hebei Medical University, Dept Pathol, Hosp 2, Shijiazhuang, China.	Country: China<br>Institution: Hebei Med Univ	Country: China<br>Institution: Hebei Medical University
RP	1	72844	72844	Zhang, XH (corresponding author), Hebei Med Univ, Pathol Lab, 361 East Zhongshan Rd, Shijiazhuang, Peoples R China	Zhang, XH (corresponding author), Hebei Medical University, Pathol Lab, 361 East Zhongshan Rd, Shijiazhuang, China	Country: China<br>Institution: Hebei Med Univ	Country: China<br>Institution: Hebei Medical University

Record 491 | WOS:000335104500012

Protective role of the mitochondrial Lon protease 1 in ochratoxin A-induced cytotoxicity in HEK293 cells

Field	Position	Original line	Normalised line	Original value	Normalised value	Original country / institution	Normalised country / institution

C1	1	72954	72954	[Zhang, Boyang; Shen, Xiao Li; Liang, Rui; Li, Yuzhe; Huang, Kunlun; Luo, Yunbo; Xu, Wentao] China Agr Univ, Coll Food Sci & Nutr Engn, Lab Food Safety & Mol Biol, Beijing 100083, Peoples R China.	[Zhang, Boyang; Shen, Xiao Li; Liang, Rui; Li, Yuzhe; Huang, Kunlun; Luo, Yunbo; Xu, Wentao] China Agricultural University, Coll Food Sci & Nutr Engn, Lab Food Safety & Mol Biol, Beijing 100083, China.	Country: China<br>Institution: China Agr Univ	Country: China<br>Institution: China Agricultural University
C1	2	72955	72955	[Shen, Xiao Li] Zunyi Med Univ, Sch Publ Hlth, Zunyi 563003, Guizhou, Peoples R China.	[Shen, Xiao Li] Zunyi Medical University, Sch Publ Hlth, Zunyi 563003, Guizhou, China.	Country: China<br>Institution: Zunyi Med Univ	Country: China<br>Institution: Zunyi Medical University
RP	1	72959	72959	Xu, WT (corresponding author), China Agr Univ, Coll Food Sci & Nutr Engn, Lab Food Safety & Mol Biol, Beijing 100083, Peoples R China	Xu, WT (corresponding author), China Agricultural University, Coll Food Sci & Nutr Engn, Lab Food Safety & Mol Biol, Beijing 100083, China	Country: China<br>Institution: China Agr Univ	Country: China<br>Institution: China Agricultural University

Record 492 | WOS:000332034900015

Mutant frequency in comparison to oxidative DNA damage induced by ochratoxin A in L5178Y tk+/- (3.7.2C) mouse lymphoma cells

Field	Position	Original line	Normalised line	Original value	Normalised value	Original country / institution	Normalised country / institution
C1	1	73112	73112	[Ali, Rahat; Guo, Xiaoqing; Lin, Haixia; Bhalli, Javed A.] US FDA, Natl Ctr Toxicol Res, Jefferson, AR 72079 USA.	[Ali, Rahat; Guo, Xiaoqing; Lin, Haixia; Bhalli, Javed A.] US Food & Drug Administration (FDA), Natl Ctr Toxicol Res, Jefferson, AR 72079 USA.	Country: USA<br>Institution: US FDA	Country: USA<br>Institution: US Food & Drug Administration (FDA)

Record 493 | WOS:000335760200003

Analysis of Individual and Combined Effects of Ochratoxin A and Zearalenone on HepG2 and KK-1 Cells with Mathematical Models

Field	Position	Original line	Normalised line	Original value	Normalised value	Original country / institution	Normalised country / institution
C1	1	73233	73233	[Li, Yuzhe; Zhang, Boyang; He, Xiaoyun; Xu, Wentao; Luo, Yunbo; Liang, Rui; Luo, Haoshu; Huang, Kunlun] China Agr Univ, Coll Food Sci & Nutr Engn, Lab Food Safety & Mol Biol, Beijing 100083, Peoples R China.	[Li, Yuzhe; Zhang, Boyang; He, Xiaoyun; Xu, Wentao; Luo, Yunbo; Liang, Rui; Luo, Haoshu; Huang, Kunlun] China Agricultural University, Coll Food Sci & Nutr Engn, Lab Food Safety & Mol Biol, Beijing 100083, China.	Country: China<br>Institution: China Agr Univ	Country: China<br>Institution: China Agricultural University
C1	2	73234	73234	[Cheng, Wen-Hsing] Mississippi State Univ, Dept Food Sci Nutr & Hlth Promot, Mississippi State, MS 39762 USA.	[Cheng, Wen-Hsing] Mississippi State University, Dept Food Sci Nutr & Hlth Promot, Mississippi State, MS 39762 USA.	Country: USA<br>Institution: Mississippi State Univ	Country: USA<br>Institution: Mississippi State University
RP	1	73236	73236	Xu, WT (corresponding author), China Agr Univ, Coll Food Sci & Nutr Engn, Lab Food Safety & Mol Biol, Beijing 100083, Peoples R China	Xu, WT (corresponding author), China Agricultural University, Coll Food Sci & Nutr Engn, Lab Food Safety & Mol Biol, Beijing 100083, China	Country: China<br>Institution: China Agr Univ	Country: China<br>Institution: China Agricultural University

Record 494 | WOS:000338818900001

Flavonoid diosmetin increases ATP levels in kidney cells and relieves ATP depleting effect of ochratoxin A

Field	Position	Original line	Normalised line	Original value	Normalised value	Original country / institution	Normalised country / institution

C1	1	73359	73359	[Poor, Miklos; Montsko, Gergely; Zrinyi, Zita; Koszegi, Tumas] Univ Pecs, Inst Lab Med, H-7624 Pecs, Hungary.	[Poor, Miklos; Montsko, Gergely; Zrinyi, Zita; Koszegi, Tumas] University of Pecs, Inst Lab Med, H-7624 Pecs, Hungary.	Country: Hungary Institution: Univ Pecs	Country: Hungary Institution: University of Pecs
C1	2	73360	73360	[Veres, Balazs; Jakus, Peter B.; Antus, Csenge] Univ Pecs, Dept Biochem & Med Chem, H-7624 Pecs, Hungary.	[Veres, Balazs; Jakus, Peter B.; Antus, Csenge] University of Pecs, Dept Biochem & Med Chem, H-7624 Pecs, Hungary.	Country: Hungary Institution: Univ Pecs	Country: Hungary Institution: University of Pecs
C1	3	73361	73361	[Vladimir-Knezevic, Sanda] Univ Zagreb, Fac Pharm & Biochem, Dept Pharmacognosy, HR-10000 Zagreb, Croatia.	[Vladimir-Knezevic, Sanda] University of Zagreb, Fac Pharm & Biochem, Dept Pharmacognosy, HR-10000 Zagreb, Croatia.	Country: Croatia Institution: Univ Zagreb	Country: Croatia Institution: University of Zagreb
C1	4	73362	73362	[Petrik, Jozsef] Univ Zagreb, Fac Pharm & Biochem, Dept Med Biochem & Haematol, HR-10000 Zagreb, Croatia.	[Petrik, Jozsef] University of Zagreb, Fac Pharm & Biochem, Dept Med Biochem & Haematol, HR-10000 Zagreb, Croatia.	Country: Croatia Institution: Univ Zagreb	Country: Croatia Institution: University of Zagreb
RP	1	73365	73365	Koszegi, T (corresponding author), Univ Pecs, Inst Lab Med, Ifjusag U 13, H-7624 Pecs, Hungary	Koszegi, T (corresponding author), University of Pecs, Inst Lab Med, Ifjusag U 13, H-7624 Pecs, Hungary	Country: Hungary Institution: Univ Pecs	Country: Hungary Institution: University of Pecs

Record 495 | WOS:000332451700009

Natural feed contaminant zearalenone decreases the expressions of important pro- and anti-inflammatory mediators and mitogen-activated protein kinase/NF-kB signalling molecules in pigs

No C1/C3/RP normalisation changes detected for this record.

Record 496 | WOS:000344366600041

Mycotoxins' Activity at Toxic and Sub-Toxic Concentrations: Differential Cytotoxic and Genotoxic Effects of Single and Combined Administration of Sterigmatocystin, Ochratoxin A and Citrinin on the Hepatocellular Cancer Cell Line Hep3B

Field	Position	Original line	Normalised line	Original value	Normalised value	Original country / institution	Normalised country / institution
C1	1	73630	73630	[Anninou, Nikolia; Papachristou, Fotini; Simopoulos, Constantinos] Democritus Univ Thrace, Sch Med, Lab Expt Surg & Surg Res, Cell Culture Unit, Alexandroupolis 68100, Greece.	[Anninou, Nikolia; Papachristou, Fotini; Simopoulos, Constantinos] Democritus University of Thrace, Sch Med, Lab Expt Surg & Surg Res, Cell Culture Unit, Alexandroupolis 68100, Greece.	Country: Greece Institution: Democritus Univ Thrace	Country: Greece Institution: Democritus University of Thrace
C1	2	73631	73631	[Chatzaki, Ekaterini] Democritus Univ Thrace, Sch Med, Pharmacol Lab, Alexandroupolis 68100, Greece.	[Chatzaki, Ekaterini] Democritus University of Thrace, Sch Med, Pharmacol Lab, Alexandroupolis 68100, Greece.	Country: Greece Institution: Democritus Univ Thrace	Country: Greece Institution: Democritus University of Thrace
C1	3	73632	73632	[Pitiakoudis, Michail; Simopoulos, Constantinos] Democritus Univ Thrace, Sch Med, Univ Gen Hosp Alexandroupolis, Dept Surg 2, Alexandroupolis 68100, Greece.	[Pitiakoudis, Michail; Simopoulos, Constantinos] Democritus University of Thrace, Sch Med, Univ Gen Hosp Alexandroupolis, Dept Surg 2, Alexandroupolis 68100, Greece.	Country: Greece Institution: Democritus Univ Thrace	Country: Greece Institution: Democritus University of Thrace
RP	1	73635	73635	Anninou, N (corresponding author), Democritus Univ Thrace, Sch Med, Lab Expt Surg & Surg Res, Cell Culture Unit, Alexandroupolis 68100, Greece	Anninou, N (corresponding author), Democritus University of Thrace, Sch Med, Lab Expt Surg & Surg Res, Cell Culture	Country: Greece Institution: Democritus Univ Thrace	Country: Greece Institution: Democritus University of Thrace

					Unit, Alexandroupolis 68100, Greece		

Record 497 | WOS:000335746400013

Deficient Glutathione in the Pathophysiology of Mycotoxin-Related Illness

No C1/C3/RP normalisation changes detected for this record.

Record 498 | WOS:000329417100014

Interaction effects of Fusarium enniatins (A, A1, B and B1) combinations on in vitro cytotoxicity of Caco-2 cells

Field	Position	Original line	Normalised line	Original value	Normalised value	Original country / institution	Normalised country / institution
C1	1	73896	73896	[Prosperini, A.; Font, G.; Ruiz, M. J.] Univ Valencia, Fac Pharm, Lab Food Chem & Toxicol, E-46100 Valencia, Spain.	[Prosperini, A.; Font, G.; Ruiz, M. J.] University of Valencia, Fac Pharm, Lab Food Chem & Toxicol, E-46100 Valencia, Spain.	Country: Spain<br>Institution: Univ Valencia	Country: Spain<br>Institution: University of Valencia

Record 499 | WOS:000334568500006

Cell-based models for mycotoxin screening and toxicity evaluation: an update

Field	Position	Original line	Normalised line	Original value	Normalised value	Original country / institution	Normalised country / institution
C1	1	73983	73983	[Cheli, F.; Fusi, E.; Baldi, A.] Univ Milan, Dept Hlth Anim Sci & Food Safety, I-20134 Milan, Italy.	[Cheli, F.; Fusi, E.; Baldi, A.] University of Milan, Dept Hlth Anim Sci & Food Safety, I-20134 Milan, Italy.	Country: Italy<br>Institution: Univ Milan	Country: Italy<br>Institution: University of Milan
RP	1	73985	73985	Cheli, F (corresponding author), Univ Milan, Dept Hlth Anim Sci & Food Safety, Via Trentacoste 2, I-20134 Milan, Italy	Cheli, F (corresponding author), University of Milan, Dept Hlth Anim Sci & Food Safety, Via Trentacoste 2, I-20134 Milan, Italy	Country: Italy<br>Institution: Univ Milan	Country: Italy<br>Institution: University of Milan

Record 500 | WOS:000339460900017

Ochratoxin A induces cytotoxicity, DNA damage and apoptosis in rat hepatocyte primary cell culture at nanomolar concentration

No C1/C3/RP normalisation changes detected for this record.

Record 501 | WOS:000329058600004

Ochratoxin A induces DNA double-strand breaks and large deletion mutations in the carcinogenic target site of gpt delta rats

Field	Position	Original line	Normalised line	Original value	Normalised value	Original country / institution	Normalised country / institution
C1	4	74284	74284	[Sugita-Konishi, Yoshiko] Azabu Univ, Sch Life & Environm Sci, Dept Food Life Sci, Chuo Ku, Sagamihara, Kanagawa 2525201, Japan.	[Sugita-Konishi, Yoshiko] Azabu University, Sch Life & Environm Sci, Dept Food Life Sci, Chuo Ku, Sagamihara, Kanagawa 2525201, Japan.	Country: Japan<br>Institution: Azabu Univ	Country: Japan<br>Institution: Azabu University
C1	5	74285	74285	[Sakai, Hiroki; Yanai, Tokuma] Gifu Univ, Fac Appl Biol Sci,	[Sakai, Hiroki; Yanai, Tokuma] Gifu University, Fac Appl Biol	Country: Japan<br>Institution: Gifu Univ	Country: Japan<br>Institution: Gifu University

				Dept Vet Med, Gifu 5011193, Japan.	Sci, Dept Vet Med, Gifu 5011193, Japan.		

Record 502 | WOS:000336092900022

A Review of the Evidence that Ochratoxin A Is an Nrf2 Inhibitor: Implications for Nephrotoxicity and Renal Carcinogenicity

Field	Position	Original line	Normalised line	Original value	Normalised value	Original country / institution	Normalised country / institution
C1	1	74401	74401	[Limonciel, Alice; Jennings, Paul] Med Univ Innsbruck, Div Physiol, Dept Physiol & Med Phys, A-6020 Innsbruck, Austria.	[Limonciel, Alice; Jennings, Paul] Medical University of Innsbruck, Div Physiol, Dept Physiol & Med Phys, A-6020 Innsbruck, Austria.	Country: Austria<br>Institution: Med Univ Innsbruck	Country: Austria<br>Institution: Medical University of Innsbruck
RP	1	74403	74403	Jennings, P (corresponding author), Med Univ Innsbruck, Div Physiol, Dept Physiol & Med Phys, A-6020 Innsbruck, Austria	Jennings, P (corresponding author), Medical University of Innsbruck, Div Physiol, Dept Physiol & Med Phys, A-6020 Innsbruck, Austria	Country: Austria<br>Institution: Med Univ Innsbruck	Country: Austria<br>Institution: Medical University of Innsbruck

Record 503 | WOS:000339460900010

Effects of lactic acid bacteria and Saccharomyces cerevisiae on growth of Aspergillus westerdijkiae and ochratoxin A production and toxicity

No C1/C3/RP normalisation changes detected for this record.

Record 504 | WOS:000329960400028

Ochratoxin-induced toxicity, oxidative stress and apoptosis ameliorated by quercetin - Modulation by Nrf2

Field	Position	Original line	Normalised line	Original value	Normalised value	Original country / institution	Normalised country / institution
C1	1	74605	74605	[Ramyaa, Periasamy; Padma, Viswanadha Vijaya] Bharathiar Univ, Sch Biotechnol & Genet Engn, Dept Biotechnol, Anim Tissue Culture & Mol Genet Lab, Coimbatore 641046, Tamil Nadu, India.	[Ramyaa, Periasamy; Padma, Viswanadha Vijaya] Bharathiar University, Sch Biotechnol & Genet Engn, Dept Biotechnol, Anim Tissue Culture & Mol Genet Lab, Coimbatore 641046, Tamil Nadu, India.	Country: India<br>Institution: Bharathiar Univ	Country: India<br>Institution: Bharathiar University
RP	1	74607	74607	Padma, VV (corresponding author), Bharathiar Univ, Sch Biotechnol & Genet Engn, Dept Biotechnol, Anim Tissue Culture & Mol Genet Lab, Coimbatore 641046, Tamil Nadu, India	Padma, VV (corresponding author), Bharathiar University, Sch Biotechnol & Genet Engn, Dept Biotechnol, Anim Tissue Culture & Mol Genet Lab, Coimbatore 641046, Tamil Nadu, India	Country: India<br>Institution: Bharathiar Univ	Country: India<br>Institution: Bharathiar University

Record 505 | WOS:000328628900003

Deleterious Effects of Mycotoxin Combinations Involving Ochratoxin A

Field	Position	Original line	Normalised line	Original value	Normalised value	Original country / institution	Normalised country / institution
C1	1	74725	74725	[Klaric, Maja Segvic] Univ Zagreb, Fac Pharm & Biochem, Dept Microbiol, HR-10000 Zagreb, Croatia.	[Klaric, Maja Segvic] University of Zagreb, Fac Pharm & Biochem, Dept Microbiol, HR-10000 Zagreb, Croatia.	Country: Croatia<br>Institution: Univ Zagreb	Country: Croatia<br>Institution: University of Zagreb

C1	2	74726	74726	[Rasic, Dubravka; Peraica, Maja] Inst Med Res & Occupat Hlth, Toxicol Unit, HR-10000 Zagreb, Croatia.	[Rasic, Dubravka; Peraica, Maja] Institute for Medical Research & Occupational Health (IMROH), Toxicol Unit, HR-10000 Zagreb, Croatia.	Country: Croatia<br>Institution: Inst Med Res & Occupat Hlth	Country: Croatia<br>Institution: Institute for Medical Research & Occupational Health (IMROH)
RP	1	74729	74729	Klaric, MS (corresponding author), Univ Zagreb, Fac Pharm & Biochem, Dept Microbiol, Schrottova 39, HR-10000 Zagreb, Croatia	Klaric, MS (corresponding author), University of Zagreb, Fac Pharm & Biochem, Dept Microbiol, Schrottova 39, HR-10000 Zagreb, Croatia	Country: Croatia<br>Institution: Univ Zagreb	Country: Croatia<br>Institution: University of Zagreb

Record 506 | WOS:000327686300025

In vitro investigation of individual and combined cytotoxic effects of aflatoxin B1 and other selected mycotoxins on the cell line porcine kidney 15

Field	Position	Original line	Normalised line	Original value	Normalised value	Original country / institution	Normalised country / institution
C1	1	74912	74912	[Lei, Mingyan; Zhang, Niya; Qi, Desheng] HuaZhong Agr Univ, Coll Anim Sci & Technol, Dept Anim Nutr & Feed Sci, Wuhan 430070, Peoples R China.	[Lei, Mingyan; Zhang, Niya; Qi, Desheng] Huazhong Agricultural University, Coll Anim Sci & Technol, Dept Anim Nutr & Feed Sci, Wuhan 430070, China.	Country: China<br>Institution: HuaZhong Agr Univ	Country: China<br>Institution: Huazhong Agricultural University
RP	1	74914	74914	Qi, DS (corresponding author), HuaZhong Agr Univ, Coll Anim Sci & Technol, Dept Anim Nutr & Feed Sci, Wuhan 430070, Peoples R China	Qi, DS (corresponding author), Huazhong Agricultural University, Coll Anim Sci & Technol, Dept Anim Nutr & Feed Sci, Wuhan 430070, China	Country: China<br>Institution: HuaZhong Agr Univ	Country: China<br>Institution: Huazhong Agricultural University

Record 507 | WOS:000326211800003

Multi-mycotoxin determination in baby foods and in vitro combined cytotoxic effects of aflatoxin M1 and ochratoxin A

No C1/C3/RP normalisation changes detected for this record.

Record 508 | WOS:000324301900014

Beauvericin-induced cytotoxicity via ROS production and mitochondrial damage in Caco-2 cells

Field	Position	Original line	Normalised line	Original value	Normalised value	Original country / institution	Normalised country / institution
C1	1	75125	75125	[Prosperini, A.; Juan-Garcia, A.; Font, G.; Ruiz, M. J.] Univ Valencia, Fac Pharm, Lab Food Chem & Toxicol, E-46100 Valencia, Spain.	[Prosperini, A.; Juan-Garcia, A.; Font, G.; Ruiz, M. J.] University of Valencia, Fac Pharm, Lab Food Chem & Toxicol, E-46100 Valencia, Spain.	Country: Spain<br>Institution: Univ Valencia	Country: Spain<br>Institution: University of Valencia
RP	1	75127	75127	Prosperini, A (corresponding author), Univ Valencia, Fac Pharm, Lab Food Chem & Toxicol, Av Vicent Andres Estelles S-N, E-46100 Valencia, Spain	Prosperini, A (corresponding author), University of Valencia, Fac Pharm, Lab Food Chem & Toxicol, Av Vicent Andres Estelles S-N, E-46100 Valencia, Spain	Country: Spain<br>Institution: Univ Valencia	Country: Spain<br>Institution: University of Valencia

Record 509 | WOS:000324773300006

Oxidative DNA damage is involved in ochratoxin A-induced G2 arrest through ataxia telangiectasia-mutated (ATM) pathways in human gastric epithelium GES-1 cells in vitro

Field	Position	Original line	Normalised line	Original value	Normalised value	Original country / institution	Normalised country / institution
C1	1	75243	75243	[Cui, Jinfeng; Zhang, Xianghong] Hebei Med Univ, Hosp 2, Dept Pathol, Shijiazhuang, Hebei, Peoples R China.	[Cui, Jinfeng; Zhang, Xianghong] Hebei Medical University, Hosp 2, Dept Pathol, Shijiazhuang, Hebei, China.	Country: China<br>Institution: Hebei Med Univ	Country: China<br>Institution: Hebei Medical University
C1	2	75244	75244	[Liu, Jing; Wu, Sha; Wang, Yuan; Shen, Haitao; Xing, Lingxiao; Wang, Junling; Yan, Xia] Hebei Med Univ, Pathol Lab, Shijiazhuang, Hebei, Peoples R China.	[Liu, Jing; Wu, Sha; Wang, Yuan; Shen, Haitao; Xing, Lingxiao; Wang, Junling; Yan, Xia] Hebei Medical University, Pathol Lab, Shijiazhuang, Hebei, China.	Country: China<br>Institution: Hebei Med Univ	Country: China<br>Institution: Hebei Medical University
RP	1	75246	75246	Zhang, XH (corresponding author), Hebei Med Univ, Hosp 2, Dept Pathol, 215 Heping Western Rd, Shijiazhuang, Hebei, Peoples R China	Zhang, XH (corresponding author), Hebei Medical University, Hosp 2, Dept Pathol, 215 Heping Western Rd, Shijiazhuang, Hebei, China	Country: China<br>Institution: Hebei Med Univ	Country: China<br>Institution: Hebei Medical University

Record 510 | WOS:000326384200009

Toxicogenomics-Based Identification of Mechanisms for Direct Immunotoxicity

Field	Position	Original line	Normalised line	Original value	Normalised value	Original country / institution	Normalised country / institution
C1	1	75371	75371	[Shao, Jia; Katika, Madhumohan R.; Schmeits, Peter C. J.; Hendriksen, Peter J. M.; Peijnenburg, Ad A. C. M.; Volger, Oscar L.] Univ Wageningen & Res Ctr, RIKILT Inst Food Safety, NL-6700 AE Wageningen, Netherlands.	[Shao, Jia; Katika, Madhumohan R.; Schmeits, Peter C. J.; Hendriksen, Peter J. M.; Peijnenburg, Ad A. C. M.; Volger, Oscar L.] Wageningen University & Research, RIKILT Inst Food Safety, NL-6700 AE Wageningen, Netherlands.	Country: Netherlands<br>Institution: Univ Wageningen & Res Ctr	Country: Netherlands<br>Institution: Wageningen University & Research
C1	2	75372	75372	[Shao, Jia; Katika, Madhumohan R.; Schmeits, Peter C. J.; van Loveren, Henk] Maastricht Univ, Dept Toxicogen, NL-6200 MD Maastricht, Netherlands.	[Shao, Jia; Katika, Madhumohan R.; Schmeits, Peter C. J.; van Loveren, Henk] Maastricht University, Dept Toxicogen, NL-6200 MD Maastricht, Netherlands.	Country: Netherlands<br>Institution: Maastricht Univ	Country: Netherlands<br>Institution: Maastricht University
C1	3	75373	75373	[Shao, Jia; Katika, Madhumohan R.; Schmeits, Peter C. J.; Hendriksen, Peter J. M.; van Loveren, Henk; Peijnenburg, Ad A. C. M.; Volger, Oscar L.] Maastricht Univ, Netherlands Toxicogen Ctr, NL-6200 MD Maastricht, Netherlands.	[Shao, Jia; Katika, Madhumohan R.; Schmeits, Peter C. J.; Hendriksen, Peter J. M.; van Loveren, Henk; Peijnenburg, Ad A. C. M.; Volger, Oscar L.] Maastricht University, Netherlands Toxicogen Ctr, NL-6200 MD Maastricht, Netherlands.	Country: Netherlands<br>Institution: Maastricht Univ	Country: Netherlands<br>Institution: Maastricht University

Record 511 | WOS:000332372600004

Toxicity of Ochratoxin A and Its Modulation by Antioxidants: A Review

Field	Position	Original line	Normalised line	Original value	Normalised value	Original country / institution	Normalised country / institution
C1	1	75585	75585	[Sorrenti, Valeria; Di Giacomo, Claudia; Acquaviva, Rosaria; Barbagallo, Ignazio; Galvano, Fabio] Univ Catania, Biochem Sect, Dept Drug Sci, I-95125 Catania, Italy.	[Sorrenti, Valeria; Di Giacomo, Claudia; Acquaviva, Rosaria; Barbagallo, Ignazio; Galvano, Fabio] University of Catania, Biochem Sect, Dept Drug Sci, I-95125 Catania, Italy.	Country: Italy<br>Institution: Univ Catania	Country: Italy<br>Institution: University of Catania

RP	1	75588	75588	Sorrenti, V (corresponding author), Univ Catania, Biochem Sect, Dept Drug Sci, I-95125 Catania, Italy	Sorrenti, V (corresponding author), University of Catania, Biochem Sect, Dept Drug Sci, I-95125 Catania, Italy	Country: Italy<br>Institution: Univ Catania	Country: Italy<br>Institution: University of Catania

Record 512 | WOS:000322929800012

Identification of proteins related to early changes observed in Human hepatocellular carcinoma cells after treatment with the mycotoxin Zearalenone

Field	Position	Original line	Normalised line	Original value	Normalised value	Original country / institution	Normalised country / institution
C1	3	75821	75821	[Camoin, Luc] INSERM, CRCM, U1068, F-13009 Marseille, France.	[Camoin, Luc] Institut National de la Sante et de la Recherche Medicale (Inserm), CRCM, U1068, F-13009 Marseille, France.	Country: France<br>Institution: INSERM	Country: France<br>Institution: Institut National de la Sante et de la Recherche Medicale (Inserm)
C1	5	75823	75823	[Camoin, Luc] Aix Marseille Univ, F-13284 Marseille, France.	[Camoin, Luc] Aix-Marseille Universite, F-13284 Marseille, France.	Country: France<br>Institution: Aix Marseille Univ	Country: France<br>Institution: Aix-Marseille Universite
C1	6	75824	75824	[Camoin, Luc] CNRS, CRCM, UMR7258, F-13009 Marseille, France.	[Camoin, Luc] Centre National de la Recherche Scientifique (CNRS), CRCM, UMR7258, F-13009 Marseille, France.	Country: France<br>Institution: CNRS	Country: France<br>Institution: Centre National de la Recherche Scientifique (CNRS)

Record 513 | WOS:000324847800043

Individual and combined effects of mycotoxins from typical indoor moulds

Field	Position	Original line	Normalised line	Original value	Normalised value	Original country / institution	Normalised country / institution
C1	2	75951	75951	[Wichmann, G.] Univ Leipzig, Fac Med, Dept Otorhinolaryngol, D-04103 Leipzig, Germany.	[Wichmann, G.] Leipzig University, Fac Med, Dept Otorhinolaryngol, D-04103 Leipzig, Germany.	Country: Germany<br>Institution: Univ Leipzig	Country: Germany<br>Institution: Leipzig University
C1	5	75954	75954	[Herbarth, O.] Univ Leipzig, Fac Med Environm Med & Hyg, D-04103 Leipzig, Germany.	[Herbarth, O.] Leipzig University, Fac Med Environm Med & Hyg, D-04103 Leipzig, Germany.	Country: Germany<br>Institution: Univ Leipzig	Country: Germany<br>Institution: Leipzig University

Record 514 | WOS:000328628700005

Producers and Important Dietary Sources of Ochratoxin A and Citrinin

Field	Position	Original line	Normalised line	Original value	Normalised value	Original country / institution	Normalised country / institution
C1	2	76092	76092	[Malir, Frantisek] Univ Hradec Kralove, Fac Sci, Dept Biol, Hradec Kralove 50003, Czech Republic.	[Malir, Frantisek] University of Hradec Kralove, Fac Sci, Dept Biol, Hradec Kralove 50003, Czech Republic.	Country: Czech Republic<br>Institution: Univ Hradec Kralove	Country: Czech Republic<br>Institution: University of Hradec Kralove

Record 515 | WOS:000328416400017

Fluorescence spectroscopic investigation of competitive interactions between ochratoxin A and 13 drug molecules for binding to human serum albumin

Field	Position	Original line	Normalised line	Original value	Normalised value	Original country / institution	Normalised country / institution

C1	1	76246	76246	[Poor, Miklos; Koszegi, Tamas] Univ Pecs, Inst Lab Med, H-7624 Pecs, Hungary.	[Poor, Miklos; Koszegi, Tamas] University of Pecs, Inst Lab Med, H-7624 Pecs, Hungary.	Country: Hungary<br>Institution: Univ Pecs	Country: Hungary<br>Institution: University of Pecs
C1	2	76247	76247	[Kunsagi-Mate, Sandor; Czibulya, Zsuzsanna; Li, Yin; Peles-Lemli, Beata] Univ Pecs, Dept Gen & Phys Chem, H-7624 Pecs, Hungary.	[Kunsagi-Mate, Sandor; Czibulya, Zsuzsanna; Li, Yin; Peles-Lemli, Beata] University of Pecs, Dept Gen & Phys Chem, H-7624 Pecs, Hungary.	Country: Hungary<br>Institution: Univ Pecs	Country: Hungary<br>Institution: University of Pecs
C1	4	76249	76249	[Petrik, Jozsef] Univ Zagreb, Fac Pharm & Biochem, Dept Med Biochem & Haematol, HR-10000 Zagreb, Croatia.	[Petrik, Jozsef] University of Zagreb, Fac Pharm & Biochem, Dept Med Biochem & Haematol, HR-10000 Zagreb, Croatia.	Country: Croatia<br>Institution: Univ Zagreb	Country: Croatia<br>Institution: University of Zagreb
C1	5	76250	76250	[Vladimir-Knezevic, Sanda] Univ Zagreb, Fac Pharm & Biochem, Dept Pharmacognosy, HR-10000 Zagreb, Croatia.	[Vladimir-Knezevic, Sanda] University of Zagreb, Fac Pharm & Biochem, Dept Pharmacognosy, HR-10000 Zagreb, Croatia.	Country: Croatia<br>Institution: Univ Zagreb	Country: Croatia<br>Institution: University of Zagreb
RP	1	76253	76253	Koszegi, T (corresponding author), Univ Pecs, Inst Lab Med, Ifjusag U 13, H-7624 Pecs, Hungary	Koszegi, T (corresponding author), University of Pecs, Inst Lab Med, Ifjusag U 13, H-7624 Pecs, Hungary	Country: Hungary<br>Institution: Univ Pecs	Country: Hungary<br>Institution: University of Pecs

Record 516 | WOS:000324280000095

A review on ochratoxin A transcriptomic studies

Field	Position	Original line	Normalised line	Original value	Normalised value	Original country / institution	Normalised country / institution
C1	1	76360	76360	[Vettorazzi, Ariane; Lopez de Cerain, Adela] Univ Navarra, Dept Pharmacol & Toxicol, Pamplona 31008, Spain.	[Vettorazzi, Ariane; Lopez de Cerain, Adela] University of Navarra, Dept Pharmacol & Toxicol, Pamplona 31008, Spain.	Country: Spain<br>Institution: Univ Navarra	Country: Spain<br>Institution: University of Navarra
C1	2	76361	76361	[van Delft, Joost] Maastricht Univ, Dept Toxicogen, NL-6229ER Maastricht, Netherlands.	[van Delft, Joost] Maastricht University, Dept Toxicogen, NL-6229ER Maastricht, Netherlands.	Country: Netherlands<br>Institution: Maastricht Univ	Country: Netherlands<br>Institution: Maastricht University
RP	1	76363	76363	de Cerain, AL (corresponding author), Univ Navarra, Dept Pharmacol & Toxicol, Pamplona 31008, Spain	de Cerain, AL (corresponding author), University of Navarra, Dept Pharmacol & Toxicol, Pamplona 31008, Spain	Country: Spain<br>Institution: Univ Navarra	Country: Spain<br>Institution: University of Navarra

Record 517 | WOS:000320498300035

Individual and combined cytotoxic effects of Fusarium toxins (deoxynivalenol, nivalenol, zearalenone and fumonisins B1) on swine jejunal epithelial cells

Field	Position	Original line	Normalised line	Original value	Normalised value	Original country / institution	Normalised country / institution
C1	1	76525	76525	[Wan, Lam Yim Murphy; El-Nezami, Hani] Univ Hong Kong, Sch Biol Sci, Fac Sci, Pokfulam, Hong Kong, Peoples R China.	[Wan, Lam Yim Murphy; El-Nezami, Hani] University of Hong Kong, Sch Biol Sci, Fac Sci, Pokfulam, Hong Kong, China.	Country: China<br>Institution: Univ Hong Kong	Country: China<br>Institution: University of Hong Kong
C1	3	76527	76527	[El-Nezami, Hani] Univ Eastern Finland, Inst Publ Hlth & Clin Nutr, Kuopio, Finland.	[El-Nezami, Hani] University of Eastern Finland, Inst Publ Hlth & Clin Nutr, Kuopio, Finland.	Country: Finland<br>Institution: Univ Eastern Finland	Country: Finland<br>Institution: University of Eastern Finland

Record 518 | WOS:000320770400009

Computational Design of Peptide Ligands for Ochratoxin A

Field	Position	Original line	Normalised line	Original value	Normalised value	Original country / institution	Normalised country / institution
C1	1	76670	76670	[Heurich, Meike; Altintas, Zeynep; Tothill, Ibtisam E.] Cranfield Univ, Cranfield Hlth, Cranfield MK43 0AL, Beds, England.	[Heurich, Meike; Altintas, Zeynep; Tothill, Ibtisam E.] Cranfield University, Cranfield Hlth, Cranfield MK43 0AL, Beds, United Kingdom.	Country: England Institution: Cranfield Univ	Country: United Kingdom Institution: Cranfield University
RP	1	76672	76672	Tothill, IE (corresponding author), Cranfield Univ, Cranfield Hlth, Cranfield MK43 0AL, Beds, England	Tothill, IE (corresponding author), Cranfield University, Cranfield Hlth, Cranfield MK43 0AL, Beds, United Kingdom	Country: England Institution: Cranfield Univ	Country: United Kingdom Institution: Cranfield University

Record 519 | WOS:000320571000011

Effect of quercetin on the toxicokinetics of ochratoxin A in rats

Field	Position	Original line	Normalised line	Original value	Normalised value	Original country / institution	Normalised country / institution
C1	1	76774	76774	[Abbas, Zein; Blank, Ralf; Wein, Silvia; Wolffram, Siegfried] Univ Kiel, Inst Anim Nutr & Physiol, Kiel, Germany.	[Abbas, Zein; Blank, Ralf; Wein, Silvia; Wolffram, Siegfried] University of Kiel, Inst Anim Nutr & Physiol, Kiel, Germany.	Country: Germany Institution: Univ Kiel	Country: Germany Institution: University of Kiel
RP	1	76776	76776	Blank, R (corresponding author), Univ Kiel, Inst Anim Nutr & Physiol, Kiel, Germany	Blank, R (corresponding author), University of Kiel, Inst Anim Nutr & Physiol, Kiel, Germany	Country: Germany Institution: Univ Kiel	Country: Germany Institution: University of Kiel

Record 520 | WOS:000316978700004

Zinc protects HepG2 cells against the oxidative damage and DNA damage induced by ochratoxin A

Field	Position	Original line	Normalised line	Original value	Normalised value	Original country / institution	Normalised country / institution
C1	1	76871	76871	[Zheng, Juanjuan; Zhang, Yu; Xu, Wentao; Luo, YunBo; Mao, Junran; Yang, Xuan; Huang, Kunlun] China Agr Univ, Coll Food Sci & Nutr Engn, Lab Food Safety & Mol Biol, Beijing 100083, Peoples R China.	[Zheng, Juanjuan; Zhang, Yu; Xu, Wentao; Luo, YunBo; Mao, Junran; Yang, Xuan; Huang, Kunlun] China Agricultural University, Coll Food Sci & Nutr Engn, Lab Food Safety & Mol Biol, Beijing 100083, China.	Country: China Institution: China Agr Univ	Country: China Institution: China Agricultural University
C1	2	76872	76872	[Xu, Wentao; Luo, YunBo; Shen, Xiao Li; Li, Xiaohong; Huang, Kunlun] Minist Agr, Supervis Inspect & Testing Ctr Genetically Modifi, Beijing 100083, Peoples R China.	[Xu, Wentao; Luo, YunBo; Shen, Xiao Li; Li, Xiaohong; Huang, Kunlun] Minist Agr, Supervis Inspect & Testing Ctr Genetically Modifi, Beijing 100083, China.	Country: China Institution: Minist Agr	Country: China Institution: Minist Agr
RP	1	76874	76874	Xu, WT (corresponding author), China Agr Univ, Coll Food Sci & Nutr Engn, Lab Food Safety & Mol Biol, Beijing 100083, Peoples R China	Xu, WT (corresponding author), China Agricultural University, Coll Food Sci & Nutr Engn, Lab Food Safety & Mol Biol, Beijing 100083, China	Country: China Institution: China Agr Univ	Country: China Institution: China Agricultural University

Record 521 | WOS:000315160400007

Role of hMLH1 in sterigmatocystin-induced G2 phase arrest in human esophageal epithelial Het-1A cells in vitro

Field	Position	Original line	Normalised line	Original value	Normalised value	Original country / institution	Normalised country / institution

C1	1	76985	76985	[Wang, Juan; Huang, Shujuan; Xing, Lingxiao; Shen, Haitao; Zhang, Xianghong] Hebei Med Univ, Dept Pathol, Hosp 2, Shijiazhuang, Peoples R China.	[Wang, Juan; Huang, Shujuan; Xing, Lingxiao; Shen, Haitao; Zhang, Xianghong] Hebei Medical University, Dept Pathol, Hosp 2, Shijiazhuang, China.	Country: China<br>Institution: Hebei Med Univ	Country: China<br>Institution: Hebei Medical University
C1	2	76986	76986	[Xing, Lingxiao; Shen, Haitao; Yan, Xia; Wang, Junling; Zhang, Xianghong] Hebei Med Univ, Pathol Lab, Shijiazhuang, Peoples R China.	[Xing, Lingxiao; Shen, Haitao; Yan, Xia; Wang, Junling; Zhang, Xianghong] Hebei Medical University, Pathol Lab, Shijiazhuang, China.	Country: China<br>Institution: Hebei Med Univ	Country: China<br>Institution: Hebei Medical University
RP	1	76988	76988	Zhang, XH (corresponding author), Hebei Med Univ, Pathol Lab, 361,Zhongshan Eastern Rd, Shijiazhuang, Peoples R China	Zhang, XH (corresponding author), Hebei Medical University, Pathol Lab, 361,Zhongshan Eastern Rd, Shijiazhuang, China	Country: China<br>Institution: Hebei Med Univ	Country: China<br>Institution: Hebei Medical University

Record 522 | WOS:000316229200014

Cross-talk between microRNAs, nuclear factor E2-related factor 2, and heme oxygenase-1 in ochratoxin A-induced toxic effects in renal proximal tubular epithelial cells

Field	Position	Original line	Normalised line	Original value	Normalised value	Original country / institution	Normalised country / institution
C1	1	77113	77113	[Stachurska, Anna; Ciesla, Maciej; Kozakowska, Magdalena; Jozkowicz, Alicja; Dulak, Jozef; Loboda, Agnieszka] Jagiellonian Univ, Fac Biochem Biophys & Biotechnol, Dept Med Biotechnol, PL-30387 Krakow, Poland.	[Stachurska, Anna; Ciesla, Maciej; Kozakowska, Magdalena; Jozkowicz, Alicja; Dulak, Jozef; Loboda, Agnieszka] Jagiellonian University, Fac Biochem Biophys & Biotechnol, Dept Med Biotechnol, PL-30387 Krakow, Poland.	Country: Poland<br>Institution: Jagiellonian Univ	Country: Poland<br>Institution: Jagiellonian University
C1	2	77114	77114	[Wolffram, Siegfried] Univ Kiel, Inst Anim Nutr & Physiol, Kiel, Germany.	[Wolffram, Siegfried] University of Kiel, Inst Anim Nutr & Physiol, Kiel, Germany.	Country: Germany<br>Institution: Univ Kiel	Country: Germany<br>Institution: University of Kiel
C1	3	77115	77115	[Boesch-Saadatmandi, Christine; Rimbach, Gerald] Univ Kiel, Inst Human Nutr & Food Sci, Kiel, Germany.	[Boesch-Saadatmandi, Christine; Rimbach, Gerald] University of Kiel, Inst Human Nutr & Food Sci, Kiel, Germany.	Country: Germany<br>Institution: Univ Kiel	Country: Germany<br>Institution: University of Kiel
RP	1	77117	77117	Loboda, A (corresponding author), Jagiellonian Univ, Fac Biochem Biophys & Biotechnol, Dept Med Biotechnol, Gronostajowa 7, PL-30387 Krakow, Poland	Loboda, A (corresponding author), Jagiellonian University, Fac Biochem Biophys & Biotechnol, Dept Med Biotechnol, Gronostajowa 7, PL-30387 Krakow, Poland	Country: Poland<br>Institution: Jagiellonian Univ	Country: Poland<br>Institution: Jagiellonian University

Record 523 | WOS:000316522300011

Effects of p53 knockout on ochratoxin A-induced genotoxicity in p53-deficient gpt delta mice

Field	Position	Original line	Normalised line	Original value	Normalised value	Original country / institution	Normalised country / institution
C1	4	77266	77266	[Yanai, Tokuma] Gifu Univ, Fac Appl Biol Sci, Dept Vet Med, Lab Vet Pathol, Gifu 5011193, Japan.	[Yanai, Tokuma] Gifu University, Fac Appl Biol Sci, Dept Vet Med, Lab Vet Pathol, Gifu 5011193, Japan.	Country: Japan<br>Institution: Gifu Univ	Country: Japan<br>Institution: Gifu University

Record 524 | WOS:000315408000011

Modulation of Intestinal Functions Following Mycotoxin Ingestion: Meta-Analysis of Published Experiments in Animals

Field	Position	Original line	Normalised line	Original value	Normalised value	Original country / institution	Normalised country / institution
C1	1	77364	77364	[Grenier, Bertrand; Applegate, Todd J.] Purdue Univ, Dept Anim Sci, W Lafayette, IN 47907 USA.	[Grenier, Bertrand; Applegate, Todd J.] Purdue University, Dept Anim Sci, W Lafayette, IN 47907 USA.	Country: USA<br>Institution: Purdue Univ	Country: USA<br>Institution: Purdue University
RP	1	77367	77367	Applegate, TJ (corresponding author), Purdue Univ, Dept Anim Sci, W Lafayette, IN 47907 USA	Applegate, TJ (corresponding author), Purdue University, Dept Anim Sci, W Lafayette, IN 47907 USA	Country: USA<br>Institution: Purdue Univ	Country: USA<br>Institution: Purdue University

Record 525 | WOS:000314671300007

Molecular mechanisms underlying ochratoxin A-induced genotoxicity: global gene expression analysis suggests induction of DNA double-strand breaks and cell cycle progression

Field	Position	Original line	Normalised line	Original value	Normalised value	Original country / institution	Normalised country / institution
C1	4	77545	77545	[Yanai, Tokuma] Gifu Univ, Fac Appl Biol Sci, Dept Vet Med, Lab Vet Pathol, Gifu 5011193, Japan.	[Yanai, Tokuma] Gifu University, Fac Appl Biol Sci, Dept Vet Med, Lab Vet Pathol, Gifu 5011193, Japan.	Country: Japan<br>Institution: Gifu Univ	Country: Japan<br>Institution: Gifu University

Record 526 | WOS:000316706800031

An iTRAQ-based mitoproteomics approach for profiling the nephrotoxicity mechanisms of ochratoxin A in HEK 293 cells

Field	Position	Original line	Normalised line	Original value	Normalised value	Original country / institution	Normalised country / institution
C1	1	77672	77672	[Shen, Xiao Li; Xu, Wentao; Liang, Rui; Zheng, Juanjuan; Luo, YunBo; Wang, Yan; Huang, Kunlun] China Agr Univ, Coll Food Sci & Nutr Engn, Lab Food Safety & Mol Biol, Beijing 100083, Peoples R China.	[Shen, Xiao Li; Xu, Wentao; Liang, Rui; Zheng, Juanjuan; Luo, YunBo; Wang, Yan; Huang, Kunlun] China Agricultural University, Coll Food Sci & Nutr Engn, Lab Food Safety & Mol Biol, Beijing 100083, China.	Country: China<br>Institution: China Agr Univ	Country: China<br>Institution: China Agricultural University
C1	2	77673	77673	[Zhang, Yu; Xu, Wentao; Luo, YunBo; Huang, Kunlun] Minist Agr, Supervis Inspect & Testing Ctr Genet Modified Org, Beijing 100083, Peoples R China.	[Zhang, Yu; Xu, Wentao; Luo, YunBo; Huang, Kunlun] Minist Agr, Supervis Inspect & Testing Ctr Genet Modified Org, Beijing 100083, China.	Country: China<br>Institution: Minist Agr	Country: China<br>Institution: Minist Agr
RP	1	77675	77675	Xu, WT (corresponding author), China Agr Univ, Coll Food Sci & Nutr Engn, Lab Food Safety & Mol Biol, Beijing 100083, Peoples R China	Xu, WT (corresponding author), China Agricultural University, Coll Food Sci & Nutr Engn, Lab Food Safety & Mol Biol, Beijing 100083, China	Country: China<br>Institution: China Agr Univ	Country: China<br>Institution: China Agricultural University

Record 527 | WOS:000318093700001

A Review of the Mechanism of Injury and Treatment Approaches for Illness Resulting from Exposure to Water-Damaged Buildings, Mold, and Mycotoxins

No C1/C3/RP normalisation changes detected for this record.

Record 528 | WOS:000314048800053

Ochratoxin A Inhibits Mouse Embryonic Development by Activating a Mitochondrion-Dependent Apoptotic Signaling Pathway

Field	Position	Original line	Normalised line	Original value	Normalised value	Original country / institution	Normalised country / institution
C1	1	78064	78064	[Hsuuw, Yan-Der] Natl Pingtung Univ Sci & Technol, Dept Life Sci, Pingtung 912, Taiwan.	[Hsuuw, Yan-Der] National Pingtung University Science & Technology, Dept Life Sci, Pingtung 912, Taiwan.	Country: Taiwan<br>Institution: Natl Pingtung Univ Sci & Technol	Country: Taiwan<br>Institution: National Pingtung University Science & Technology
C1	2	78065	78065	[Chan, Wen-Hsiung] Chung Yuan Christian Univ, Dept Biosci Technol, Chungli 320, Taiwan.	[Chan, Wen-Hsiung] Chung Yuan Christian University, Dept Biosci Technol, Chungli 320, Taiwan.	Country: Taiwan<br>Institution: Chung Yuan Christian Univ	Country: Taiwan<br>Institution: Chung Yuan Christian University
C1	3	78066	78066	[Chan, Wen-Hsiung] Chung Yuan Christian Univ, Ctr Nanotechnol, Chungli 320, Taiwan.	[Chan, Wen-Hsiung] Chung Yuan Christian University, Ctr Nanotechnol, Chungli 320, Taiwan.	Country: Taiwan<br>Institution: Chung Yuan Christian Univ	Country: Taiwan<br>Institution: Chung Yuan Christian University
C1	4	78067	78067	[Yu, Jau-Song] Chang Gung Univ, Coll Med, Inst Biomed Sci, Dept Cell & Mol Biol, Tao Yuan 333, Taiwan.	[Yu, Jau-Song] Chang Gung University, Coll Med, Inst Biomed Sci, Dept Cell & Mol Biol, Tao Yuan 333, Taiwan.	Country: Taiwan<br>Institution: Chang Gung Univ	Country: Taiwan<br>Institution: Chang Gung University
RP	1	78070	78070	Chan, WH (corresponding author), Chung Yuan Christian Univ, Dept Biosci Technol, Chungli 320, Taiwan	Chan, WH (corresponding author), Chung Yuan Christian University, Dept Biosci Technol, Chungli 320, Taiwan	Country: Taiwan<br>Institution: Chung Yuan Christian Univ	Country: Taiwan<br>Institution: Chung Yuan Christian University

Record 529 | WOS:000313124600008

Role of intestinal microflora in xenobiotic-induced toxicity

Field	Position	Original line	Normalised line	Original value	Normalised value	Original country / institution	Normalised country / institution
C1	1	78194	78194	[Jeong, Hye Gwang; Kim, Hyung Gyun] Chungnam Natl Univ, Coll Pharm, Taejon, South Korea.	[Jeong, Hye Gwang; Kim, Hyung Gyun] Chungnam National University, Coll Pharm, Taejon, South Korea.	Country: South Korea<br>Institution: Chungnam Natl Univ	Country: South Korea<br>Institution: Chungnam National University
C1	2	78195	78195	[Kang, Mi Jeong; Oh, Do Gyeong; Kim, Jin Sung; Jeong, Tae Cheon] Yeungnam Univ, Coll Pharm, Kyongsan 712749, South Korea.	[Kang, Mi Jeong; Oh, Do Gyeong; Kim, Jin Sung; Jeong, Tae Cheon] Yeungnam University, Coll Pharm, Kyongsan 712749, South Korea.	Country: South Korea<br>Institution: Yeungnam Univ	Country: South Korea<br>Institution: Yeungnam University
C1	3	78196	78196	[Lee, Sang Kyu] Kyungpook Natl Univ, Coll Pharm, Taegu, South Korea.	[Lee, Sang Kyu] Kyungpook National University (KNU), Coll Pharm, Taegu, South Korea.	Country: South Korea<br>Institution: Kyungpook Natl Univ	Country: South Korea<br>Institution: Kyungpook National University (KNU)
RP	1	78199	78199	Jeong, TC (corresponding author), Yeungnam Univ, Coll Pharm, 214-1 Dae Dong, Kyongsan 712749, South Korea	Jeong, TC (corresponding author), Yeungnam University, Coll Pharm, 214-1 Dae Dong, Kyongsan 712749, South Korea	Country: South Korea<br>Institution: Yeungnam Univ	Country: South Korea<br>Institution: Yeungnam University

Record 530 | WOS:000315356800004

Amelioration of Ochratoxin A-induced immunotoxic effects by silymarin and vitamin E in White Leghorn cockerels

No C1/C3/RP normalisation changes detected for this record.

Record 531 | WOS:000312386800135

Nivalenol and Deoxynivalenol Affect Rat Intestinal Epithelial Cells: A Concentration Related Study

Field	Position	Original line	Normalised line	Original value	Normalised value	Original country / institution	Normalised country / institution

C1	1	78441	78441	[Bianco, Giuseppe; Autore, Giuseppina; Marzocco, Stefania] Univ Salerno, Sch Pharm, Dept Pharmaceut & Biomed Sci, Fisciano, Italy.	[Bianco, Giuseppe; Autore, Giuseppina; Marzocco, Stefania] University of Salerno, Sch Pharm, Dept Pharmaceut & Biomed Sci, Fisciano, Italy.	Country: Italy<br>Institution: Univ Salerno	Country: Italy<br>Institution: University of Salerno
C1	3	78443	78443	[Severino, Lorella] Univ Naples Federico II, Sch Vet Med, Div Toxicol, Dept Pathol & Anim Hlth, Naples, Italy.	[Severino, Lorella] University of Naples Federico II, Sch Vet Med, Div Toxicol, Dept Pathol & Anim Hlth, Naples, Italy.	Country: Italy<br>Institution: Univ Naples Federico II	Country: Italy<br>Institution: University of Naples Federico II
C1	4	78444	78444	[Quaroni, Andrea] Cornell Univ, Dept Biomed Sci, Ithaca, NY USA.	[Quaroni, Andrea] Cornell University, Dept Biomed Sci, Ithaca, NY USA.	Country: USA<br>Institution: Cornell Univ	Country: USA<br>Institution: Cornell University
RP	1	78448	78448	Marzocco, S (corresponding author), Univ Salerno, Sch Pharm, Dept Pharmaceut & Biomed Sci, Fisciano, Italy	Marzocco, S (corresponding author), University of Salerno, Sch Pharm, Dept Pharmaceut & Biomed Sci, Fisciano, Italy	Country: Italy<br>Institution: Univ Salerno	Country: Italy<br>Institution: University of Salerno

Record 532 | WOS:000312288000010

Lactobacillus reuteri CRL 1098 and Lactobacillus acidophilus CRL 1014 differently reduce in vitro immunotoxic effect induced by Ochratoxin A

No C1/C3/RP normalisation changes detected for this record.

Record 533 | WOS:000310867400006

Increase in Mitochondrial DNA Copy Number in Response to Ochratoxin A and Methanol-Induced Mitochondrial DNA Damage in Drosophila

Field	Position	Original line	Normalised line	Original value	Normalised value	Original country / institution	Normalised country / institution
C1	1	78664	78664	Mehmet Akif Ersoy Univ, Dept Biol, Burdur, Turkey.	Mehmet Akif Ersoy University, Dept Biol, Burdur, Turkey.	Country: Turkey<br>Institution: Mehmet Akif Ersoy Univ	Country: Turkey<br>Institution: Mehmet Akif Ersoy University
RP	1	78666	78666	Mutlu, AG (corresponding author), Mehmet Akif Ersoy Univ, Dept Biol, Burdur, Turkey	Mutlu, AG (corresponding author), Mehmet Akif Ersoy University, Dept Biol, Burdur, Turkey	Country: Turkey<br>Institution: Mehmet Akif Ersoy Univ	Country: Turkey<br>Institution: Mehmet Akif Ersoy University

Record 534 | WOS:000312288000021

Evaluation of nephrotoxic effects of mycotoxins, citrinin and patulin, on zebrafish (Danio rerio) embryos

Field	Position	Original line	Normalised line	Original value	Normalised value	Original country / institution	Normalised country / institution
C1	1	78741	78741	[Yang, Jiann-Jou; Yu, Feng-Yih; Liu, Biing-Hui] Chung Shan Med Univ, Dept Biomed Sci, Taichung 402, Taiwan.	[Yang, Jiann-Jou; Yu, Feng-Yih; Liu, Biing-Hui] Chung Shan Medical University, Dept Biomed Sci, Taichung 402, Taiwan.	Country: Taiwan<br>Institution: Chung Shan Med Univ	Country: Taiwan<br>Institution: Chung Shan Medical University
C1	2	78742	78742	[Wu, Ting-Shuan] Chung Shan Med Univ, Inst Med, Taichung 402, Taiwan.	[Wu, Ting-Shuan] Chung Shan Medical University, Inst Med, Taichung 402, Taiwan.	Country: Taiwan<br>Institution: Chung Shan Med Univ	Country: Taiwan<br>Institution: Chung Shan Medical University
C1	3	78743	78743	[Yang, Jiann-Jou; Yu, Feng-Yih] Chung Shan Med Univ Hosp, Dept Med Res, Taichung, Taiwan.	[Yang, Jiann-Jou; Yu, Feng-Yih] Chung Shan Medical University, Dept Med Res, Taichung, Taiwan.	Country: Taiwan<br>Institution: Chung Shan Med Univ Hosp	Country: Taiwan<br>Institution: Chung Shan Medical University
C1	4	78744	78744	[Liu, Biing-Hui] Natl Taiwan Univ, Grad Inst Toxicol, Coll Med, Taipei 10764, Taiwan.	[Liu, Biing-Hui] National Taiwan University, Grad Inst Toxicol, Coll Med, Taipei 10764, Taiwan.	Country: Taiwan<br>Institution: Natl Taiwan Univ	Country: Taiwan<br>Institution: National Taiwan University

RP	1	78748	78748	Yu, FY (corresponding author), Chung Shan Med Univ, Dept Biomed Sci, 110,Sec 1,Chien Kuo N Rd, Taichung 402, Taiwan	Yu, FY (corresponding author), Chung Shan Medical University, Dept Biomed Sci, 110,Sec 1,Chien Kuo N Rd, Taichung 402, Taiwan	Country: Taiwan<br>Institution: Chung Shan Med Univ	Country: Taiwan<br>Institution: Chung Shan Medical University

Record 535 | WOS:000308629100003

Human skin penetration of selected model mycotoxins

Field	Position	Original line	Normalised line	Original value	Normalised value	Original country / institution	Normalised country / institution
C1	1	78873	78873	[Boonen, Jente; Taevernier, Lien; De Spiegeleer, Bart] Univ Ghent, Fac Pharmaceut Sci, Drug Qual & Registrat DruQuaR Grp, B-9000 Ghent, Belgium.	[Boonen, Jente; Taevernier, Lien; De Spiegeleer, Bart] Ghent University, Fac Pharmaceut Sci, Drug Qual & Registrat DruQuaR Grp, B-9000 Ghent, Belgium.	Country: Belgium<br>Institution: Univ Ghent	Country: Belgium<br>Institution: Ghent University
C1	2	78874	78874	[Malysheva, Svetlana V.; Di Mavungu, Jose Diana; De Saeger, Sarah] Univ Ghent, Fac Pharmaceut Sci, Lab Food Anal, B-9000 Ghent, Belgium.	[Malysheva, Svetlana V.; Di Mavungu, Jose Diana; De Saeger, Sarah] Ghent University, Fac Pharmaceut Sci, Lab Food Anal, B-9000 Ghent, Belgium.	Country: Belgium<br>Institution: Univ Ghent	Country: Belgium<br>Institution: Ghent University
RP	1	78876	78876	De Spiegeleer, B (corresponding author), Univ Ghent, Fac Pharmaceut Sci, Drug Qual & Registrat DruQuaR Grp, Harelbekestr 72, B-9000 Ghent, Belgium	De Spiegeleer, B (corresponding author), Ghent University, Fac Pharmaceut Sci, Drug Qual & Registrat DruQuaR Grp, Harelbekestr 72, B-9000 Ghent, Belgium	Country: Belgium<br>Institution: Univ Ghent	Country: Belgium<br>Institution: Ghent University

Record 536 | WOS:000315406500009

Apoptosis Induction by OTA and TNF-Î± in Cultured Primary Rat Hepatocytes and Prevention by Silibinin

No C1/C3/RP normalisation changes detected for this record.

Record 537 | WOS:000309849700023

Co-exposure of Fusarium mycotoxins: In vitro myelotoxicity assessment on human hematopoietic progenitors

No C1/C3/RP normalisation changes detected for this record.

Record 538 | WOS:000309897100023

Immune modulatory effects of the foodborne contaminant citrinin in mice

No C1/C3/RP normalisation changes detected for this record.

Record 539 | WOS:000309897100058

Fumonisin B1 and the kidney: Modes of action for renal tumor formation by fumonisin B1 in rodents

Field	Position	Original line	Normalised line	Original value	Normalised value	Original country / institution	Normalised country / institution

C1	1	79367	79367	[Mueller, Stephanie; Dekant, Wolfgang; Mally, Angela] Univ Wurzburg, Dept Toxicol, D-97078 Wurzburg, Germany.	[Mueller, Stephanie; Dekant, Wolfgang; Mally, Angela] University of Wurzburg, Dept Toxicol, D-97078 Wurzburg, Germany.	Country: Germany Institution: Univ Wurzburg	Country: Germany Institution: University of Wurzburg
RP	1	79369	79369	Mally, A (corresponding author), Univ Wurzburg, Dept Toxicol, Versbacher Str 9, D-97078 Wurzburg, Germany	Mally, A (corresponding author), University of Wurzburg, Dept Toxicol, Versbacher Str 9, D-97078 Wurzburg, Germany	Country: Germany Institution: Univ Wurzburg	Country: Germany Institution: University of Wurzburg

Record 540 | WOS:000308289000002

The food contaminant and nephrotoxin ochratoxin A enhances Wnt1 inducible signaling protein 1 and tumor necrosis factor-Î± expression in human primary proximal tubule cells

Field	Position	Original line	Normalised line	Original value	Normalised value	Original country / institution	Normalised country / institution
C1	2	79564	79564	[Humpf, Hans-Ulrich] Univ Munster, Inst Lebensmittelchem, D-4400 Munster, Germany.	[Humpf, Hans-Ulrich] University of Munster, Inst Lebensmittelchem, D-4400 Munster, Germany.	Country: Germany Institution: Univ Munster	Country: Germany Institution: University of Munster

Record 541 | WOS:000307323900012

Citrinin reduces testosterone secretion by inducing apoptosis in rat Leydig cells

Field	Position	Original line	Normalised line	Original value	Normalised value	Original country / institution	Normalised country / institution
C1	1	79674	79674	[Hu, Defu] Beijing Forestry Univ, Physiol Anim Lab, Coll Biol Sci & Technol, Beijing 100083, Peoples R China.	[Hu, Defu] Beijing Forestry University, Physiol Anim Lab, Coll Biol Sci & Technol, Beijing 100083, China.	Country: China Institution: Beijing Forestry Univ	Country: China Institution: Beijing Forestry University
C1	2	79675	79675	[Wang, Dan] Qingdao Entry Exit Inspect & Quarantine Bur, Food Serv, Qingdao 266000, Peoples R China.	[Wang, Dan] Qingdao Entry Exit Inspect & Quarantine Bur, Food Serv, Qingdao 266000, China.	Country: China Institution: Qingdao Entry Exit Inspect & Quarantine Bur	Country: China Institution: Qingdao Entry Exit Inspect & Quarantine Bur
C1	3	79676	79676	[Liu, Wenhua; Wang, Yongqi] Shanxi Inst Zool, Xian 710032, Peoples R China.	[Liu, Wenhua; Wang, Yongqi] Shanxi Inst Zool, Xian 710032, China.	Country: China Institution: Shanxi Inst Zool	Country: China Institution: Shanxi Inst Zool
C1	4	79677	79677	[Zhao, Chongxue; Cui, Yaxiong] Endangered Anim Rescue Ctr Wuwei, Wuwei 733300, Peoples R China.	[Zhao, Chongxue; Cui, Yaxiong] Endangered Anim Rescue Ctr Wuwei, Wuwei 733300, China.	Country: China Institution: Endangered Anim Rescue Ctr Wuwei	Country: China Institution: Endangered Anim Rescue Ctr Wuwei
RP	1	79679	79679	Hu, DF (corresponding author), Beijing Forestry Univ, Physiol Anim Lab, Coll Biol Sci & Technol, Beijing 100083, Peoples R China	Hu, DF (corresponding author), Beijing Forestry University, Physiol Anim Lab, Coll Biol Sci & Technol, Beijing 100083, China	Country: China Institution: Beijing Forestry Univ	Country: China Institution: Beijing Forestry University

Record 542 | WOS:000307766500014

Cellular distribution of cell cycle-related molecules in the renal tubules of rats treated with renal carcinogens for 28 days: relationship between cell cycle aberration and carcinogenesis

Field	Position	Original line	Normalised line	Original value	Normalised value	Original country / institution	Normalised country / institution
C1	2	79779	79779	[Taniai, Eriko; Hayashi, Hitomi; Yafune, Atsunori] Gifu Univ, United Grad Sch Vet Sci, Gifu 5011193, Japan.	[Taniai, Eriko; Hayashi, Hitomi; Yafune, Atsunori] Gifu University, United Grad Sch Vet Sci, Gifu 5011193, Japan.	Country: Japan Institution: Gifu Univ	Country: Japan Institution: Gifu University

Record 543 | WOS:000307487100016

Protective Effect of Deferricoprogen Isolated from Monascus purpureus NTU 568 on Citrinin-Induced Apoptosis in HEK-293 Cells

Field	Position	Original line	Normalised line	Original value	Normalised value	Original country / institution	Normalised country / institution
C1	2	79901	79901	[Hsu, Li-Chuan; Hsu, Ya-Wen; Liang, Yu-Han; Pan, Tzu-Ming] Natl Taiwan Univ, Dept Biochem Sci & Technol, Coll Life Sci, Taipei 106, Taiwan.	[Hsu, Li-Chuan; Hsu, Ya-Wen; Liang, Yu-Han; Pan, Tzu-Ming] National Taiwan University, Dept Biochem Sci & Technol, Coll Life Sci, Taipei 106, Taiwan.	Country: Taiwan Institution: Natl Taiwan Univ	Country: Taiwan Institution: National Taiwan University
C1	3	79902	79902	[Kuo, Yao-Haur] China Med Univ, Grad Inst Integrated Med, Taichung 404, Taiwan.	[Kuo, Yao-Haur] China Medical University Taiwan, Grad Inst Integrated Med, Taichung 404, Taiwan.	Country: Taiwan Institution: China Med Univ	Country: Taiwan Institution: China Medical University Taiwan

Record 544 | WOS:000304513300011

Toxic effects of citrinin on the male reproductive system in mice

Field	Position	Original line	Normalised line	Original value	Normalised value	Original country / institution	Normalised country / institution
C1	1	80001	80001	[Liu Shuqiang] Beijing Forestry Univ, Coll Biol Sci & Technol, Physiol Anim Lab, Beijing 100083, Peoples R China.	[Liu Shuqiang] Beijing Forestry University, Coll Biol Sci & Technol, Physiol Anim Lab, Beijing 100083, China.	Country: China Institution: Beijing Forestry Univ	Country: China Institution: Beijing Forestry University
RP	1	80003	80003	Liu, SQ (corresponding author), Beijing Forestry Univ, Coll Biol Sci & Technol, Physiol Anim Lab, Beijing 100083, Peoples R China	Liu, SQ (corresponding author), Beijing Forestry University, Coll Biol Sci & Technol, Physiol Anim Lab, Beijing 100083, China	Country: China Institution: Beijing Forestry Univ	Country: China Institution: Beijing Forestry University

Record 545 | WOS:000304401100023

Collaborative study for the detection of toxic compounds in shellfish extracts using cell-based assays. Part II: application to shellfish extracts spiked with lipophilic marine toxins

No C1/C3/RP normalisation changes detected for this record.

Record 546 | WOS:000304744700011

Ochratoxin A induces oxidative DNA damage and G1 phase arrest in human peripheral blood mononuclear cells in vitro

Field	Position	Original line	Normalised line	Original value	Normalised value	Original country / institution	Normalised country / institution
C1	1	80244	80244	[Liu, Jing; Wang, Yuan; Xing, Lingxiao; Shen, Haitao; Wu, Sha; Lian, Hongguang; Wang, Junling; Yan, Xia; Zhang, Xianghong] Hebei Med Univ, Pathol Lab, Shijiazhuang, Peoples R China.	[Liu, Jing; Wang, Yuan; Xing, Lingxiao; Shen, Haitao; Wu, Sha; Lian, Hongguang; Wang, Junling; Yan, Xia; Zhang, Xianghong] Hebei Medical University, Pathol Lab, Shijiazhuang, China.	Country: China Institution: Hebei Med Univ	Country: China Institution: Hebei Medical University
C1	2	80245	80245	[Cui, Jinfeng; Zhang, Xianghong] Hebei Med Univ, Hosp 2, Dept Pathol, Shijiazhuang, Peoples R China.	[Cui, Jinfeng; Zhang, Xianghong] Hebei Medical University, Hosp 2, Dept Pathol, Shijiazhuang, China.	Country: China Institution: Hebei Med Univ	Country: China Institution: Hebei Medical University
RP	1	80247	80247	Zhang, XH (corresponding author), Hebei Med Univ, Pathol Lab, 361 Zhongshan	Zhang, XH (corresponding author), Hebei Medical University, Pathol Lab, 361	Country: China Institution: Hebei Med Univ	Country: China Institution: Hebei Medical University

				Eastern Rd, Shijiazhuang, Peoples R China	Zhongshan Eastern Rd, Shijiazhuang, China		

Record 547 | WOS:000304198400001

Ochratoxin A and Mitotic Disruption: Mode of Action Analysis of Renal Tumor Formation by Ochratoxin A

Field	Position	Original line	Normalised line	Original value	Normalised value	Original country / institution	Normalised country / institution
C1	1	80352	80352	Univ Wurzburg, Dept Toxicol, D-97078 Wurzburg, Germany.	University of Wurzburg, Dept Toxicol, D-97078 Wurzburg, Germany.	Country: Germany Institution: Univ Wurzburg	Country: Germany Institution: University of Wurzburg
RP	1	80354	80354	Mally, A (corresponding author), Univ Wurzburg, Dept Toxicol, Versbacher Str 9, D-97078 Wurzburg, Germany	Mally, A (corresponding author), University of Wurzburg, Dept Toxicol, Versbacher Str 9, D-97078 Wurzburg, Germany	Country: Germany Institution: Univ Wurzburg	Country: Germany Institution: University of Wurzburg

Record 548 | WOS:000304401100022

Collaborative study for the detection of toxic compounds in shellfish extracts using cell-based assays. Part I: screening strategy and pre-validation study with lipophilic marine toxins

No C1/C3/RP normalisation changes detected for this record.

Record 549 | WOS:000302950000003

Assaying embryotoxicity in the test tube: Current limitations of the embryonic stem cell test (EST) challenging its applicability domain

No C1/C3/RP normalisation changes detected for this record.

Record 550 | WOS:000307982300003

Mutagenicity of Ochratoxin A and Its Hydroquinone Metabolite in the SupF Gene of the Mutation Reporter Plasmid Ps189

Field	Position	Original line	Normalised line	Original value	Normalised value	Original country / institution	Normalised country / institution
C1	1	81019	81019	[Akman, Steven A.; Adams, Marissa] Wake Forest Univ Hlth Sci, Dept Canc Biol, Winston Salem, NC 27106 USA.	[Akman, Steven A.; Adams, Marissa] Wake Forest University, Dept Canc Biol, Winston Salem, NC 27106 USA.	Country: USA Institution: Wake Forest Univ Hlth Sci	Country: USA Institution: Wake Forest University
C1	2	81020	81020	[Case, Doug] Wake Forest Univ Hlth Sci, Dept Publ Hlth Sci, Winston Salem, NC USA.	[Case, Doug] Wake Forest University, Dept Publ Hlth Sci, Winston Salem, NC USA.	Country: USA Institution: Wake Forest Univ Hlth Sci	Country: USA Institution: Wake Forest University
C1	3	81021	81021	[Park, Gyungse] Kunsan Natl Univ, Coll Sci & Technol, Dept Chem, Kusan, South Korea.	[Park, Gyungse] Kunsan National University, Coll Sci & Technol, Dept Chem, Kusan, South Korea.	Country: South Korea Institution: Kunsan Natl Univ	Country: South Korea Institution: Kunsan National University
C1	4	81022	81022	[Manderville, Richard A.] Univ Guelph, Dept Chem & Toxicol, Guelph, ON N1G 2W1, Canada.	[Manderville, Richard A.] University of Guelph, Dept Chem & Toxicol, Guelph, ON N1G 2W1, Canada.	Country: Canada Institution: Univ Guelph	Country: Canada Institution: University of Guelph
RP	1	81026	81026	Akman, SA (corresponding author), Wake Forest Univ Hlth Sci, Dept Canc Biol, Winston Salem, NC 27106 USA	Akman, SA (corresponding author), Wake Forest University, Dept Canc Biol, Winston Salem, NC 27106 USA	Country: USA Institution: Wake Forest Univ Hlth Sci	Country: USA Institution: Wake Forest University

Record 551 | WOS:000301792400008

Transcriptomic alterations induced by Ochratoxin A in rat and human renal proximal tubular in vitro models and comparison to a rat in vivo model

Field	Position	Original line	Normalised line	Original value	Normalised value	Original country / institution	Normalised country / institution
C1	1	81165	81165	[Jennings, Paul; Limonciel, Alice; Aschauer, Lydia; Wilmes, Anja; Pfaller, Walter] Innsbruck Med Univ, Dept Physiol & Med Phys, Div Physiol, A-6020 Innsbruck, Austria.	[Jennings, Paul; Limonciel, Alice; Aschauer, Lydia; Wilmes, Anja; Pfaller, Walter] Medical University of Innsbruck, Dept Physiol & Med Phys, Div Physiol, A-6020 Innsbruck, Austria.	Country: Austria<br>Institution: Innsbruck Med Univ	Country: Austria<br>Institution: Medical University of Innsbruck
C1	2	81166	81166	[Weiland, Christina; Ahr, Hans J.; Ellinger-Ziegelbauer, Heidrun] Bayer Pharma AG, Toxicol, D-42096 Wuppertal, Germany.	[Weiland, Christina; Ahr, Hans J.; Ellinger-Ziegelbauer, Heidrun] Bayer AG, Toxicol, D-42096 Wuppertal, Germany.	Country: Germany<br>Institution: Bayer Pharma AG	Country: Germany<br>Institution: Bayer AG
C1	3	81167	81167	[Bloch, Katarzyna M.; Lock, Edward A.] Liverpool John Moores Univ, Sch Pharm & Biomol Sci, Liverpool L3 5UX, Merseyside, England.	[Bloch, Katarzyna M.; Lock, Edward A.] Liverpool John Moores University, Sch Pharm & Biomol Sci, Liverpool L3 5UX, Merseyside, United Kingdom.	Country: England<br>Institution: Liverpool John Moores Univ	Country: United Kingdom<br>Institution: Liverpool John Moores University
C1	4	81168	81168	[Radford, Robert; McMorrow, Tara; Slattery, Craig; Ryan, Michael P.] Univ Coll Dublin, UCD Conway Inst, Renal Dis Res Grp, Sch Biomol & Biomed Sci, Dublin 2, Ireland.	[Radford, Robert; McMorrow, Tara; Slattery, Craig; Ryan, Michael P.] University College Dublin, UCD Conway Inst, Renal Dis Res Grp, Sch Biomol & Biomed Sci, Dublin 2, Ireland.	Country: Ireland<br>Institution: Univ Coll Dublin	Country: Ireland<br>Institution: University College Dublin
RP	1	81172	81172	Jennings, P (corresponding author), Innsbruck Med Univ, Dept Physiol & Med Phys, Div Physiol, Fritz Pregl Str 3, A-6020 Innsbruck, Austria	Jennings, P (corresponding author), Medical University of Innsbruck, Dept Physiol & Med Phys, Div Physiol, Fritz Pregl Str 3, A-6020 Innsbruck, Austria	Country: Austria<br>Institution: Innsbruck Med Univ	Country: Austria<br>Institution: Medical University of Innsbruck

Record 552 | WOS:000302917900001

Carcinogens induce loss of the primary cilium in human renal proximal tubular epithelial cells independently of effects on the cell cycle

Field	Position	Original line	Normalised line	Original value	Normalised value	Original country / institution	Normalised country / institution
C1	1	81273	81273	[Radford, Robert; Slattery, Craig; Blaque, Oliver; Ryan, Michael P.; McMorrow, Tara] Univ Coll Dublin, UCD Sch Biomol & Biomed Res, UCD Conway Inst, Dublin 4, Ireland.	[Radford, Robert; Slattery, Craig; Blaque, Oliver; Ryan, Michael P.; McMorrow, Tara] University College Dublin, UCD Sch Biomol & Biomed Res, UCD Conway Inst, Dublin 4, Ireland.	Country: Ireland<br>Institution: Univ Coll Dublin	Country: Ireland<br>Institution: University College Dublin
C1	2	81274	81274	[Jennings, Paul; Pfaller, Walter] Innsbruck Med Univ, Div Physiol, Dept Physiol & Med Phys, Innsbruck, Austria.	[Jennings, Paul; Pfaller, Walter] Medical University of Innsbruck, Div Physiol, Dept Physiol & Med Phys, Innsbruck, Austria.	Country: Austria<br>Institution: Innsbruck Med Univ	Country: Austria<br>Institution: Medical University of Innsbruck
C1	4	81276	81276	[Van Delft, Joost] Maastricht Univ, NTC, Dept Human Biol, Dept Toxicogen, Maastricht, Netherlands.	[Van Delft, Joost] Maastricht University, NTC, Dept Human Biol, Dept Toxicogen, Maastricht, Netherlands.	Country: Netherlands<br>Institution: Maastricht Univ	Country: Netherlands<br>Institution: Maastricht University
RP	1	81279	81279	McMorrow, T (corresponding author), Univ Coll Dublin, Renal Dis Res Grp, Sch Biomol & Biomed Sci, UCD Conway Inst, Dublin 4, Ireland	McMorrow, T (corresponding author), University College Dublin, Renal Dis Res Grp, Sch Biomol & Biomed Sci, UCD Conway Inst, Dublin 4, Ireland	Country: Ireland<br>Institution: Univ Coll Dublin	Country: Ireland<br>Institution: University College Dublin

Record 553 | WOS:000301277200012

ERK and p38 MAPK signaling pathways are involved in ochratoxin A-induced G2 phase arrest in human gastric epithelium cells

Field	Position	Original line	Normalised line	Original value	Normalised value	Original country / institution	Normalised country / institution
C1	1	81396	81396	[Wang, Yuan; Liu, Jing; Xing, Lingxiao; Wang, Junling; Yan, Xia; Zhang, Xianghong] Hebei Med Univ, Pathol Lab, Shijiazhuang, Peoples R China.	[Wang, Yuan; Liu, Jing; Xing, Lingxiao; Wang, Junling; Yan, Xia; Zhang, Xianghong] Hebei Medical University, Pathol Lab, Shijiazhuang, China.	Country: China<br>Institution: Hebei Med Univ	Country: China<br>Institution: Hebei Medical University
C1	2	81397	81397	[Cui, Jinfeng; Zhang, Xianghong] Hebei Med Univ, Hosp 2, Dept Pathol, Shijiazhuang, Peoples R China.	[Cui, Jinfeng; Zhang, Xianghong] Hebei Medical University, Hosp 2, Dept Pathol, Shijiazhuang, China.	Country: China<br>Institution: Hebei Med Univ	Country: China<br>Institution: Hebei Medical University
RP	1	81399	81399	Zhang, XH (corresponding author), Hebei Med Univ, Pathol Lab, 361 Zhongshan Eastern Rd, Shijiazhuang, Peoples R China	Zhang, XH (corresponding author), Hebei Medical University, Pathol Lab, 361 Zhongshan Eastern Rd, Shijiazhuang, China	Country: China<br>Institution: Hebei Med Univ	Country: China<br>Institution: Hebei Medical University

Record 554 | WOS:000302911300002

Assessment on pollution of ochratoxin a in grain in China and its apoptosis effect on vitro-cultured human tubular kidney cells

Field	Position	Original line	Normalised line	Original value	Normalised value	Original country / institution	Normalised country / institution
C1	1	81484	81484	[Li, Zengning] Hebei Med Univ, Attached Hosp 1, Dept Nutr, Shijiazhuang, Peoples R China.	[Li, Zengning] Hebei Medical University, Attached Hosp 1, Dept Nutr, Shijiazhuang, China.	Country: China<br>Institution: Hebei Med Univ	Country: China<br>Institution: Hebei Medical University
C1	2	81485	81485	[Zhang, Xianghong; Cui, Jinfeng] Hebei Med Univ, Dept Pathol, Shijiazhuang, Peoples R China.	[Zhang, Xianghong; Cui, Jinfeng] Hebei Medical University, Dept Pathol, Shijiazhuang, China.	Country: China<br>Institution: Hebei Med Univ	Country: China<br>Institution: Hebei Medical University
C1	3	81486	81486	[Kang, Weijun] Hebei Med Univ, Sch Publ Hlth, Shijiazhuang, Peoples R China.	[Kang, Weijun] Hebei Medical University, Sch Publ Hlth, Shijiazhuang, China.	Country: China<br>Institution: Hebei Med Univ	Country: China<br>Institution: Hebei Medical University
RP	1	81489	81489	Li, ZN (corresponding author), Hebei Med Univ, Attached Hosp 1, Dept Nutr, Shijiazhuang, Peoples R China	Li, ZN (corresponding author), Hebei Medical University, Attached Hosp 1, Dept Nutr, Shijiazhuang, China	Country: China<br>Institution: Hebei Med Univ	Country: China<br>Institution: Hebei Medical University

Record 555 | WOS:000300700100002

Inactivation of A. ochraceus Spores and Detoxification of Ochratoxin A in Coffee Beans by Gamma Irradiation

No C1/C3/RP normalisation changes detected for this record.

Record 556 | WOS:000309118000007

CYTOTOXIC POTENCY OF MYCOTOXINS IN CULTURES OF V79 LUNG FIBROBLAST CELLS

No C1/C3/RP normalisation changes detected for this record.

Record 557 | WOS:000209177700107

Transport of aflatoxin M1 in human intestinal Caco-2/TC7 cells

Field	Position	Original line	Normalised line	Original value	Normalised value	Original country / institution	Normalised country / institution
C1	1	81748	81748	[Caloni, Francesca; Cortinovis, Cristina; Pizzo, Fabiola] Univ Milan, Dept Hlth Anim Sci & Food Safety, I-20133 Milan, Italy.	[Caloni, Francesca; Cortinovis, Cristina; Pizzo, Fabiola] University of Milan, Dept Hlth Anim Sci & Food Safety, I-20133 Milan, Italy.	Country: Italy<br>Institution: Univ Milan	Country: Italy<br>Institution: University of Milan
RP	1	81751	81751	Caloni, F (corresponding author), Univ Milan, Dept Hlth Anim Sci & Food Safety, Via Celoria 10, I-20133 Milan, Italy	Caloni, F (corresponding author), University of Milan, Dept Hlth Anim Sci & Food Safety, Via Celoria 10, I-20133 Milan, Italy	Country: Italy<br>Institution: Univ Milan	Country: Italy<br>Institution: University of Milan

Record 558 | WOS:000300137300023

In vitro myelotoxicity assessment of the emerging mycotoxins Beauvericin, Enniatin b and Moniliformin on human hematopoietic progenitors

No C1/C3/RP normalisation changes detected for this record.

Record 559 | WOS:000299028700020

Structure-Activity Relationships Imply Different Mechanisms of Action for Ochratoxin A-Mediated Cytotoxicity and Genotoxicity

Field	Position	Original line	Normalised line	Original value	Normalised value	Original country / institution	Normalised country / institution
C1	2	81966	81966	[Frenette, Christine; Paugh, Robert J.; Manderville, Richard A.] Univ Guelph, Dept Chem, Guelph, ON N1G 2W1, Canada.	[Frenette, Christine; Paugh, Robert J.; Manderville, Richard A.] University of Guelph, Dept Chem, Guelph, ON N1G 2W1, Canada.	Country: Canada<br>Institution: Univ Guelph	Country: Canada<br>Institution: University of Guelph
C1	3	81967	81967	[Frenette, Christine; Paugh, Robert J.; Manderville, Richard A.] Univ Guelph, Dept Toxicol, Guelph, ON N1G 2W1, Canada.	[Frenette, Christine; Paugh, Robert J.; Manderville, Richard A.] University of Guelph, Dept Toxicol, Guelph, ON N1G 2W1, Canada.	Country: Canada<br>Institution: Univ Guelph	Country: Canada<br>Institution: University of Guelph

Record 560 | WOS:000299902300007

In vivo and ex vivo phagocytic potential of macrophages from progeny of breeder hens kept on ochratoxin A (OTA)-contaminated diet

No C1/C3/RP normalisation changes detected for this record.

Record 561 | WOS:000297778200001

Ochratoxin A in feed of food-producing animals: An undesirable mycotoxin with health and performance effects

No C1/C3/RP normalisation changes detected for this record.

Record 562 | WOS:000298362500059

Effects of zearalenone and its derivatives on porcine immune response

No C1/C3/RP normalisation changes detected for this record.

Record 563 | WOS:000297102800002

Molecular Events Involved in Ochratoxin A Induced Mitochondrial Pathway of Apoptosis, Modulation by Bcl-2 Family Members

No C1/C3/RP normalisation changes detected for this record.

Record 564 | WOS:000296681600021

Ochratoxin A reduces aflatoxin B1 induced DNA damage detected by the comet assay in Hep G2 cells

Field	Position	Original line	Normalised line	Original value	Normalised value	Original country / institution	Normalised country / institution
C1	1	82665	82665	[Corcuera, L. A.; Arbillaga, L.; Vettorazzi, A.; Azqueta, A.; Lopez de Cerain, A.] Univ Navarra, Dept Nutr Food Sci Physiol & Toxicol, Fac Pharm, E-31008 Pamplona, Spain.	[Corcuera, L. A.; Arbillaga, L.; Vettorazzi, A.; Azqueta, A.; Lopez de Cerain, A.] University of Navarra, Dept Nutr Food Sci Physiol & Toxicol, Fac Pharm, E-31008 Pamplona, Spain.	Country: Spain<br>Institution: Univ Navarra	Country: Spain<br>Institution: University of Navarra
RP	1	82667	82667	de Cerain, AL (corresponding author), Univ Navarra, Dept Nutr Food Sci Physiol & Toxicol, Fac Pharm, Irunlarrea 1, E-31008 Pamplona, Spain	de Cerain, AL (corresponding author), University of Navarra, Dept Nutr Food Sci Physiol & Toxicol, Fac Pharm, Irunlarrea 1, E-31008 Pamplona, Spain	Country: Spain<br>Institution: Univ Navarra	Country: Spain<br>Institution: University of Navarra

Record 565 | WOS:000295241700033

Cytotoxic effects of mycotoxin combinations in mammalian kidney cells

Field	Position	Original line	Normalised line	Original value	Normalised value	Original country / institution	Normalised country / institution
C1	1	82781	82781	[Ruiz, Maria-Jose; Macakova, Petra; Juan-Garcia, Ana; Font, Guillermina] Univ Valencia, Toxicol Lab, Fac Pharm, E-46100 Burjassot, Spain.	[Ruiz, Maria-Jose; Macakova, Petra; Juan-Garcia, Ana; Font, Guillermina] University of Valencia, Toxicol Lab, Fac Pharm, E-46100 Burjassot, Spain.	Country: Spain<br>Institution: Univ Valencia	Country: Spain<br>Institution: University of Valencia
RP	1	82783	82783	Ruiz, MJ (corresponding author), Univ Valencia, Toxicol Lab, Fac Pharm, C Vicent Andres Estelles S-N, E-46100 Burjassot, Spain	Ruiz, MJ (corresponding author), University of Valencia, Toxicol Lab, Fac Pharm, C Vicent Andres Estelles S-N, E-46100 Burjassot, Spain	Country: Spain<br>Institution: Univ Valencia	Country: Spain<br>Institution: University of Valencia

Record 566 | WOS:000298538400030

Mycotoxins profiling of the culture material of Fusarium verticillioides (Sacc.) Nirenberg culture (CABI-IMI 392668) isolated from rice in Niger State, Nigeria and its cytotoxic effects on human lymphocytes comparatively to those of some mycotoxin standards

Field	Position	Original line	Normalised line	Original value	Normalised value	Original country / institution	Normalised country / institution

C1	2	82869	82869	[Dutton, Michael Francis; Mwanza, Mulunda; Njobeh, Patrick Berka] Univ Johannesburg, Fac Hlth Sci, Food Environm & Hlth Res Grp, ZA-2028 Gauteng, South Africa.	[Dutton, Michael Francis; Mwanza, Mulunda; Njobeh, Patrick Berka] University of Johannesburg, Fac Hlth Sci, Food Environm & Hlth Res Grp, ZA-2028 Gauteng, South Africa.	Country: South Africa<br>Institution: Univ Johannesburg	Country: South Africa<br>Institution: University of Johannesburg

Record 567 | WOS:000295307400004

Toxicological interactions between the mycotoxins beauvericin, deoxynivalenol and T-2 toxin in CHO-K1 cells in vitro

Field	Position	Original line	Normalised line	Original value	Normalised value	Original country / institution	Normalised country / institution
C1	1	82971	82971	[Ruiz, M. J.; Franzova, P.; Juan-Garcia, A.; Font, G.] Univ Valencia, Fac Pharm, Lab Toxicol, E-46100 Valencia, Spain.	[Ruiz, M. J.; Franzova, P.; Juan-Garcia, A.; Font, G.] University of Valencia, Fac Pharm, Lab Toxicol, E-46100 Valencia, Spain.	Country: Spain<br>Institution: Univ Valencia	Country: Spain<br>Institution: University of Valencia
RP	1	82973	82973	Ruiz, MJ (corresponding author), Univ Valencia, Fac Pharm, Lab Toxicol, C Vicent Andres Estelles S-N, E-46100 Valencia, Spain	Ruiz, MJ (corresponding author), University of Valencia, Fac Pharm, Lab Toxicol, C Vicent Andres Estelles S-N, E-46100 Valencia, Spain	Country: Spain<br>Institution: Univ Valencia	Country: Spain<br>Institution: University of Valencia

Record 568 | WOS:000296746400005

Comparative cytotoxicity of the possibly occurring mycotoxins in wheat on the basis of a mycological analysis

No C1/C3/RP normalisation changes detected for this record.

Record 569 | WOS:000295697800004

Herbal hepatotoxicity by kava: Update on pipermethystine, flavokavain B, and mould hepatotoxins as primarily assumed culprits

Field	Position	Original line	Normalised line	Original value	Normalised value	Original country / institution	Normalised country / institution
C1	1	83212	83212	[Teschke, Rolf] Goethe Univ Frankfurt Main, Dept Internal Med 2, Klinikum Hanau, Teaching Hosp,Div Gastroenterol & Hepatol, D-63450 Hanau, Germany.	[Teschke, Rolf] Goethe University Frankfurt, Dept Internal Med 2, Klinikum Hanau, Teaching Hosp,Div Gastroenterol & Hepatol, D-63450 Hanau, Germany.	Country: Germany<br>Institution: Goethe Univ Frankfurt Main	Country: Germany<br>Institution: Goethe University Frankfurt
C1	2	83213	83213	[Qiu, Samuel X.] Chinese Acad Sci, S China Bot Garden, Key Lab Plant Resources Conservat & Sustainable U, Guangzhou, Guangdong, Peoples R China.	[Qiu, Samuel X.] Chinese Academy of Sciences, S China Bot Garden, Key Lab Plant Resources Conservat & Sustainable U, Guangzhou, Guangdong, China.	Country: China<br>Institution: Chinese Acad Sci	Country: China<br>Institution: Chinese Academy of Sciences
RP	1	83216	83216	Teschke, R (corresponding author), Goethe Univ Frankfurt Main, Dept Internal Med 2, Klinikum Hanau, Teaching Hosp,Div Gastroenterol & Hepatol, Leimenstr 20, D-63450 Hanau, Germany	Teschke, R (corresponding author), Goethe University Frankfurt, Dept Internal Med 2, Klinikum Hanau, Teaching Hosp,Div Gastroenterol & Hepatol, Leimenstr 20, D-63450 Hanau, Germany	Country: Germany<br>Institution: Goethe Univ Frankfurt Main	Country: Germany<br>Institution: Goethe University Frankfurt

Record 570 | WOS:000294029600014

Ochratoxin A at nanomolar concentration perturbs the homeostasis of neural stem cells in highly differentiated but not in immature three-dimensional brain cell cultures

Field	Position	Original line	Normalised line	Original value	Normalised value	Original country / institution	Normalised country / institution
C1	1	83324	83324	[Zurich, Marie-Gabrielle] Univ Lausanne, Fac Biol & Med, Dept Physiol, CH-1005 Lausanne, Switzerland.	[Zurich, Marie-Gabrielle] University of Lausanne, Fac Biol & Med, Dept Physiol, CH-1005 Lausanne, Switzerland.	Country: Switzerland Institution: Univ Lausanne	Country: Switzerland Institution: University of Lausanne
C1	2	83325	83325	[Zurich, Marie-Gabrielle; Honegger, Paul] Univ Lausanne, SCAHT, CH-1005 Lausanne, Switzerland.	[Zurich, Marie-Gabrielle; Honegger, Paul] University of Lausanne, SCAHT, CH-1005 Lausanne, Switzerland.	Country: Switzerland Institution: Univ Lausanne	Country: Switzerland Institution: University of Lausanne
RP	1	83327	83327	Zurich, MG (corresponding author), Univ Lausanne, Fac Biol & Med, Dept Physiol, Rue Bugnon 7, CH-1005 Lausanne, Switzerland	Zurich, MG (corresponding author), University of Lausanne, Fac Biol & Med, Dept Physiol, Rue Bugnon 7, CH-1005 Lausanne, Switzerland	Country: Switzerland Institution: Univ Lausanne	Country: Switzerland Institution: University of Lausanne

Record 571 | WOS:000293914500008

Perturbation of Mitosis through Inhibition of Histone Acetyltransferases: The Key to Ochratoxin A Toxicity and Carcinogenicity?

Field	Position	Original line	Normalised line	Original value	Normalised value	Original country / institution	Normalised country / institution
C1	1	83461	83461	[Czakai, Kristin; Mueller, Katja; Dekant, Wolfgang; Mally, Angela] Univ Wurzburg, Inst Pharmacol & Toxicol, Dept Toxicol, D-97078 Wurzburg, Germany.	[Czakai, Kristin; Mueller, Katja; Dekant, Wolfgang; Mally, Angela] University of Wurzburg, Inst Pharmacol & Toxicol, Dept Toxicol, D-97078 Wurzburg, Germany.	Country: Germany Institution: Univ Wurzburg	Country: Germany Institution: University of Wurzburg
C1	2	83462	83462	[Mosesso, Pasquale; Pepe, Gaetano] Univ Tuscia, Dipartimento Agrobiol & Agrochim, I-01100 Viterbo, Italy.	[Mosesso, Pasquale; Pepe, Gaetano] Tuscia University, Dipartimento Agrobiol & Agrochim, I-01100 Viterbo, Italy.	Country: Italy Institution: Univ Tuscia	Country: Italy Institution: Tuscia University
C1	3	83463	83463	[Schulze, Markus; Gohla, Antje] Univ Wurzburg, Inst Pharmacol & Toxicol, Dept Pharmacol, D-97078 Wurzburg, Germany.	[Schulze, Markus; Gohla, Antje] University of Wurzburg, Inst Pharmacol & Toxicol, Dept Pharmacol, D-97078 Wurzburg, Germany.	Country: Germany Institution: Univ Wurzburg	Country: Germany Institution: University of Wurzburg
C1	4	83464	83464	[Schulze, Markus; Gohla, Antje] Univ Wurzburg, Rudolf Virchow Ctr Expt Biomed, D-97078 Wurzburg, Germany.	[Schulze, Markus; Gohla, Antje] University of Wurzburg, Rudolf Virchow Ctr Expt Biomed, D-97078 Wurzburg, Germany.	Country: Germany Institution: Univ Wurzburg	Country: Germany Institution: University of Wurzburg
C1	5	83465	83465	[Patnaik, Debasis; Higgins, Jonathan M. G.] Harvard Univ, Brigham & Womens Hosp, Sch Med, Div Rheumatol Immunol & Allergy, Boston, MA 02115 USA.	[Patnaik, Debasis; Higgins, Jonathan M. G.] Harvard University, Brigham & Womens Hosp, Sch Med, Div Rheumatol Immunol & Allergy, Boston, MA 02115 USA.	Country: USA Institution: Harvard Univ	Country: USA Institution: Harvard University
RP	1	83469	83469	Mally, A (corresponding author), Univ Wurzburg, Inst Pharmacol & Toxicol, Dept Toxicol, Versbacher Str 9, D-97078 Wurzburg, Germany	Mally, A (corresponding author), University of Wurzburg, Inst Pharmacol & Toxicol, Dept Toxicol, Versbacher Str 9, D-97078 Wurzburg, Germany	Country: Germany Institution: Univ Wurzburg	Country: Germany Institution: University of Wurzburg

Record 572 | WOS:000293914500016

Site-Specific In Vivo Mutagenicity in the Kidney of gpt Delta Rats Given a Carcinogenic Dose of Ochratoxin A

Field	Position	Original line	Normalised line	Original value	Normalised value	Original country / institution	Normalised country / institution
C1	3	83597	83597	[Yanai, Tokuma] Gifu Univ, Fac Appl Biol Sci, Dept Vet Med, Lab Vet Pathol, Gifu 5011193, Japan.	[Yanai, Tokuma] Gifu University, Fac Appl Biol Sci, Dept Vet Med, Lab Vet Pathol, Gifu 5011193, Japan.	Country: Japan<br>Institution: Gifu Univ	Country: Japan<br>Institution: Gifu University

Record 573 | WOS:000293914500030

Citrinin-Generated Reactive Oxygen Species Cause Cell Cycle Arrest Leading to Apoptosis via the Intrinsic Mitochondrial Pathway in Mouse Skin

No C1/C3/RP normalisation changes detected for this record.

Record 574 | WOS:000292672700008

Comparative analysis of micronuclei and DNA damage induced by Ochratoxin A in two mammalian cell lines

Field	Position	Original line	Normalised line	Original value	Normalised value	Original country / institution	Normalised country / institution
C1	1	83815	83815	[Heflich, Robert H.] US FDA, Div Genet & Mol Toxicol, Natl Ctr Toxicol Res, Jefferson, AR USA.	[Heflich, Robert H.] US Food & Drug Administration (FDA), Div Genet & Mol Toxicol, Natl Ctr Toxicol Res, Jefferson, AR USA.	Country: USA<br>Institution: US FDA	Country: USA<br>Institution: US Food & Drug Administration (FDA)
RP	1	83819	83819	Heflich, RH (corresponding author), US FDA, Div Genet & Mol Toxicol, Natl Ctr Toxicol Res, 3900 NCTR Rd, Jefferson, AR USA	Heflich, RH (corresponding author), US Food & Drug Administration (FDA), Div Genet & Mol Toxicol, Natl Ctr Toxicol Res, 3900 NCTR Rd, Jefferson, AR USA	Country: USA<br>Institution: US FDA	Country: USA<br>Institution: US Food & Drug Administration (FDA)

Record 575 | WOS:000286856200015

Enzyme-Linked Aptamer Assays (ELAAs), based on a competition format for a rapid and sensitive detection of Ochratoxin A in wine

No C1/C3/RP normalisation changes detected for this record.

Record 576 | WOS:000288933100012

Aflatoxin B1 and fumonisin B1 affect the oxidative status of bovine peripheral blood mononuclear cells

Field	Position	Original line	Normalised line	Original value	Normalised value	Original country / institution	Normalised country / institution
C1	1	84047	84047	[Bernabucci, Umberto; Colavecchia, Luciana; Danieli, Pier Paolo; Basirico, Loredana; Lacetera, Nicola; Nardone, Alessandro; Ronchi, Bruno] Univ Tuscia, Dept Anim Sci, I-01100 Viterbo, Italy.	[Bernabucci, Umberto; Colavecchia, Luciana; Danieli, Pier Paolo; Basirico, Loredana; Lacetera, Nicola; Nardone, Alessandro; Ronchi, Bruno] Tuscia University, Dept Anim Sci, I-01100 Viterbo, Italy.	Country: Italy<br>Institution: Univ Tuscia	Country: Italy<br>Institution: Tuscia University
RP	1	84049	84049	Bernabucci, U (corresponding author), Univ Tuscia, Dept	Bernabucci, U (corresponding author), Tuscia University, Dept	Country: Italy<br>Institution: Univ Tuscia	Country: Italy<br>Institution: Tuscia University

				Anim Sci, Via SC De Lellis, I-01100 Viterbo, Italy	Anim Sci, Via SC De Lellis, I-01100 Viterbo, Italy		

Record 577 | WOS:000289042100014

Lymphocyte blastic transformation test in the evaluation of ochratoxin A influence on the immune system of hens and their progeny

No C1/C3/RP normalisation changes detected for this record.

Record 578 | WOS:000288341900004

Zebrafish (Danio rerio) embryos as a model for testing proteratogens

No C1/C3/RP normalisation changes detected for this record.

Record 579 | WOS:000286679100003

Protective effects of melatonin and Glycyrrhiza glabra extract on ochratoxin A-induced damages on testes in mature rats

Field	Position	Original line	Normalised line	Original value	Normalised value	Original country / institution	Normalised country / institution
C1	1	84450	84450	[Malekinejad, Hassan; Mirzakhani, Navideh; Cheraghi, Hadi; Alizadeh, Arash; Dardmeh, Fereshteh] Urmia Univ, Dept Pharmacol & Toxicol, Fac Vet Med, Orumiyeh, Iran.	[Malekinejad, Hassan; Mirzakhani, Navideh; Cheraghi, Hadi; Alizadeh, Arash; Dardmeh, Fereshteh] Urmia University, Dept Pharmacol & Toxicol, Fac Vet Med, Orumiyeh, Iran.	Country: Iran<br>Institution: Urmia Univ	Country: Iran<br>Institution: Urmia University
C1	2	84451	84451	[Razi, Mazdak] Urmia Univ, Dept Histol & Embryol, Fac Vet Med, Orumiyeh, Iran.	[Razi, Mazdak] Urmia University, Dept Histol & Embryol, Fac Vet Med, Orumiyeh, Iran.	Country: Iran<br>Institution: Urmia Univ	Country: Iran<br>Institution: Urmia University
RP	1	84453	84453	Malekinejad, H (corresponding author), Urmia Univ, Dept Pharmacol & Toxicol, Fac Vet Med, POB 1177, Orumiyeh, Iran	Malekinejad, H (corresponding author), Urmia University, Dept Pharmacol & Toxicol, Fac Vet Med, POB 1177, Orumiyeh, Iran	Country: Iran<br>Institution: Urmia Univ	Country: Iran<br>Institution: Urmia University

Record 580 | WOS:000285845400008

Mycotoxin Citrinin Induced Cell Cycle G2/M Arrest and Numerical Chromosomal Aberration Associated with Disruption of Microtubule Formation in Human Cells

Field	Position	Original line	Normalised line	Original value	Normalised value	Original country / institution	Normalised country / institution
C1	1	84546	84546	[Chang, Chia-Hao; Yu, Feng-Yih; Wu, Ting-Shun; Liu, Biing-Hui] Chung Shan Med Univ Hosp, Dept Biomed Sci, Taichung 402, Taiwan.	[Chang, Chia-Hao; Yu, Feng-Yih; Wu, Ting-Shun; Liu, Biing-Hui] Chung Shan Medical University, Dept Biomed Sci, Taichung 402, Taiwan.	Country: Taiwan<br>Institution: Chung Shan Med Univ Hosp	Country: Taiwan<br>Institution: Chung Shan Medical University
C1	2	84547	84547	[Liu, Biing-Hui] Chung Shan Med Univ Hosp, Dept Med Res, Taichung 402, Taiwan.	[Liu, Biing-Hui] Chung Shan Medical University, Dept Med Res, Taichung 402, Taiwan.	Country: Taiwan<br>Institution: Chung Shan Med Univ Hosp	Country: Taiwan<br>Institution: Chung Shan Medical University
RP	1	84550	84550	Liu, BH (corresponding author), Chung Shan Med Univ, Dept Biomed Sci, 110,Sect 1,Chien Kuo N Rd, Taichung 402, Taiwan	Liu, BH (corresponding author), Chung Shan Medical University, Dept Biomed Sci, 110,Sect 1,Chien Kuo N Rd, Taichung 402, Taiwan	Country: Taiwan<br>Institution: Chung Shan Med Univ	Country: Taiwan<br>Institution: Chung Shan Medical University

Record 581 | WOS:000285100300026

Metabolic response to low-level toxicant exposure in a novel renal tubule epithelial cell system

Field	Position	Original line	Normalised line	Original value	Normalised value	Original country / institution	Normalised country / institution
C1	1	84658	84658	[Ellis, James Keith; Athersuch, Toby James; Cavill, Rachel; Ebbels, Timothy Mark David; Keun, Hector Charles] Univ London Imperial Coll Sci Technol & Med, Dept Surg & Canc, Fac Med, London SW7 2AZ, England.	[Ellis, James Keith; Athersuch, Toby James; Cavill, Rachel; Ebbels, Timothy Mark David; Keun, Hector Charles] Univ London Imperial Coll Sci Technol & Med, Dept Surg & Canc, Fac Med, London SW7 2AZ, United Kingdom.	Country: England Institution: Univ London Imperial Coll Sci Technol & Med	Country: United Kingdom Institution: Univ London Imperial Coll Sci Technol & Med
C1	2	84659	84659	[Radford, Robert; Slattery, Craig; McMorrow, Tara; Ryan, Michael P.] Univ Coll Dublin, UCD Sch Biomol & Biomed Sci, UCD Conway Inst, Dublin 2, Ireland.	[Radford, Robert; Slattery, Craig; McMorrow, Tara; Ryan, Michael P.] University College Dublin, UCD Sch Biomol & Biomed Sci, UCD Conway Inst, Dublin 2, Ireland.	Country: Ireland Institution: Univ Coll Dublin	Country: Ireland Institution: University College Dublin
C1	3	84660	84660	[Athersuch, Toby James] Univ London Imperial Coll Sci Technol & Med, MRC HPA Ctr Environm & Hlth, Dept Epidemiol & Biostat, Sch Publ Hlth,Fac Med, London SW7 2AZ, England.	[Athersuch, Toby James] Univ London Imperial Coll Sci Technol & Med, MRC HPA Ctr Environm & Hlth, Dept Epidemiol & Biostat, Sch Publ Hlth,Fac Med, London SW7 2AZ, United Kingdom.	Country: England Institution: Univ London Imperial Coll Sci Technol & Med	Country: United Kingdom Institution: Univ London Imperial Coll Sci Technol & Med
C1	4	84661	84661	[Jennings, Paul] Innsbruck Med Univ, Div Physiol, Dept Physiol & Med Phys, Innsbruck, Austria.	[Jennings, Paul] Medical University of Innsbruck, Div Physiol, Dept Physiol & Med Phys, Innsbruck, Austria.	Country: Austria Institution: Innsbruck Med Univ	Country: Austria Institution: Medical University of Innsbruck
RP	1	84664	84664	Ebbels, TMD (corresponding author), Univ London Imperial Coll Sci Technol & Med, Dept Surg & Canc, Fac Med, Sir Alexander Fleming Bldg, London SW7 2AZ, England	Ebbels, TMD (corresponding author), Univ London Imperial Coll Sci Technol & Med, Dept Surg & Canc, Fac Med, Sir Alexander Fleming Bldg, London SW7 2AZ, United Kingdom	Country: England Institution: Univ London Imperial Coll Sci Technol & Med	Country: United Kingdom Institution: Univ London Imperial Coll Sci Technol & Med

Record 582 | WOS:000297106700013

Review of mycotoxin reduction in food and feed: from prevention in the field to detoxification by adsorption or transformation

Field	Position	Original line	Normalised line	Original value	Normalised value	Original country / institution	Normalised country / institution
C1	1	84785	84785	[Jard, G.; Liboz, T.; Mathieu, F.; Lebrihi, A.] Univ Toulouse, LGC, INP ENSAT, Castanet Tolosan, France.	[Jard, G.; Liboz, T.; Mathieu, F.; Lebrihi, A.] Universite de Toulouse, LGC, INP ENSAT, Castanet Tolosan, France.	Country: France Institution: Univ Toulouse	Country: France Institution: Universite de Toulouse
RP	1	84789	84789	Liboz, T (corresponding author), Univ Toulouse, LGC, INP ENSAT, Castanet Tolosan, France	Liboz, T (corresponding author), Universite de Toulouse, LGC, INP ENSAT, Castanet Tolosan, France	Country: France Institution: Univ Toulouse	Country: France Institution: Universite de Toulouse

Record 583 | WOS:000298090700025

Experimentally Induced Toxicity of Ochratoxin A and Endosulfan in Male Wistar Rats: A Hormonal Disorder

Field	Position	Original line	Normalised line	Original value	Normalised value	Original country / institution	Normalised country / institution

C1	3	85089	85089	[Telang, Avinash Gopal; Singh, Karan Pal] Indian Vet Res Inst, Ctr Anim Dis Res & Diag, Izatnagar 243122, Uttar Pradesh, India.	[Telang, Avinash Gopal; Singh, Karan Pal] ICAR - Indian Veterinary Research Institute, Ctr Anim Dis Res & Diag, Izatnagar 243122, Uttar Pradesh, India.	Country: India<br>Institution: Indian Vet Res Inst	Country: India<br>Institution: ICAR - Indian Veterinary Research Institute

Record 584 | WOS:000295693800002

Potential of an in vitro toolbox combined with exposure data as a first step for the risk assessment of dietary chemical contaminants

Field	Position	Original line	Normalised line	Original value	Normalised value	Original country / institution	Normalised country / institution
C1	2	85206	85206	[van der Heiden, E.; Remacle, A. -S.; Scippo, M. -L.] Univ Liege, CART, Fac Vet Med, Lab Food Anal,Dept Food Sci, B-4000 Liege, Belgium.	[van der Heiden, E.; Remacle, A. -S.; Scippo, M. -L.] University of Liege, CART, Fac Vet Med, Lab Food Anal,Dept Food Sci, B-4000 Liege, Belgium.	Country: Belgium<br>Institution: Univ Liege	Country: Belgium<br>Institution: University of Liege
C1	3	85207	85207	[Nobels, I.; Blust, R.] Univ Antwerp, Lab Ecophysiol Biochem & Toxicol, B-2020 Antwerp, Belgium.	[Nobels, I.; Blust, R.] University of Antwerp, Lab Ecophysiol Biochem & Toxicol, B-2020 Antwerp, Belgium.	Country: Belgium<br>Institution: Univ Antwerp	Country: Belgium<br>Institution: University of Antwerp
C1	4	85208	85208	[De Saeger, S.] Univ Ghent, Fac Pharmaceut Sci, Lab Food Anal, B-9000 Ghent, Belgium.	[De Saeger, S.] Ghent University, Fac Pharmaceut Sci, Lab Food Anal, B-9000 Ghent, Belgium.	Country: Belgium<br>Institution: Univ Ghent	Country: Belgium<br>Institution: Ghent University

Record 585 | WOS:000292496700010

Developing mechanism-based and exposure biomarkers for mycotoxins in animals

Field	Position	Original line	Normalised line	Original value	Normalised value	Original country / institution	Normalised country / institution
C1	2	85401	85401	[Coulombe, R. A.] Utah State Univ, Dept Vet Sci, Logan, UT 84322 USA.	[Coulombe, R. A.] Utah State University, Dept Vet Sci, Logan, UT 84322 USA.	Country: USA<br>Institution: Utah State Univ	Country: USA<br>Institution: Utah State University
C1	3	85402	85402	[Pestka, J. J.] Michigan State Univ, Dept Food Sci & Human Nutr, E Lansing, MI 48824 USA.	[Pestka, J. J.] Michigan State University, Dept Food Sci & Human Nutr, E Lansing, MI 48824 USA.	Country: USA<br>Institution: Michigan State Univ	Country: USA<br>Institution: Michigan State University
C1	4	85403	85403	[Williams, D. E.] Oregon State Univ, Dept Environm & Mol Toxicol, Corvallis, OR 97331 USA.	[Williams, D. E.] Oregon State University, Dept Environm & Mol Toxicol, Corvallis, OR 97331 USA.	Country: USA<br>Institution: Oregon State Univ	Country: USA<br>Institution: Oregon State University

Record 586 | WOS:000297106700012

Isotherm modeling of organic activated bentonite and humic acid polymer used as mycotoxin adsorbents

Field	Position	Original line	Normalised line	Original value	Normalised value	Original country / institution	Normalised country / institution
C1	1	85613	85613	[Santos, R. R.; Vermeulen, S.; Fink-Gremmels, J.] Univ Utrecht, Fac Vet Med, Div Pharmacol & Toxicol, Dept Equine Sci, Utrecht, Netherlands.	[Santos, R. R.; Vermeulen, S.; Fink-Gremmels, J.] Utrecht University, Fac Vet Med, Div Pharmacol & Toxicol, Dept Equine Sci, Utrecht, Netherlands.	Country: Netherlands<br>Institution: Univ Utrecht	Country: Netherlands<br>Institution: Utrecht University
C1	2	85614	85614	[Haritova, A.] Trakia Univ, Dept Pharmacol Physiol Anim &	[Haritova, A.] Trakia University, Dept Pharmacol Physiol Anim	Country: Bulgaria<br>Institution: Trakia Univ	Country: Bulgaria<br>Institution: Trakia University

				Physiol Chem, Fac Vet Med, Stara Zagora, Bulgaria.	& Physiol Chem, Fac Vet Med, Stara Zagora, Bulgaria.		
RP	1	85616	85616	Santos, RR (corresponding author), Univ Utrecht, Fac Vet Med, Div Pharmacol & Toxicol, Dept Equine Sci, Utrecht, Netherlands	Santos, RR (corresponding author), Utrecht University, Fac Vet Med, Div Pharmacol & Toxicol, Dept Equine Sci, Utrecht, Netherlands	Country: Netherlands<br>Institution: Univ Utrecht	Country: Netherlands<br>Institution: Utrecht University

Record 587 | WOS:000290627500016

Aptamer-functionalized magnetic nanoparticle-based bioassay for the detection of ochratoxin a using upconversion nanoparticles as labels

Field	Position	Original line	Normalised line	Original value	Normalised value	Original country / institution	Normalised country / institution
C1	1	85714	85714	[Wu, Shijia; Duan, Nuo; Wang, Zhouping; Wang, Hongxin] Jiangnan Univ, Sch Food Sci & Technol, State Key Lab Food Sci & Technol, Wuxi 214122, Peoples R China.	[Wu, Shijia; Duan, Nuo; Wang, Zhouping; Wang, Hongxin] Jiangnan University, Sch Food Sci & Technol, State Key Lab Food Sci & Technol, Wuxi 214122, China.	Country: China<br>Institution: Jiangnan Univ	Country: China<br>Institution: Jiangnan University
RP	1	85716	85716	Wang, ZP (corresponding author), Jiangnan Univ, Sch Food Sci & Technol, State Key Lab Food Sci & Technol, Wuxi 214122, Peoples R China	Wang, ZP (corresponding author), Jiangnan University, Sch Food Sci & Technol, State Key Lab Food Sci & Technol, Wuxi 214122, China	Country: China<br>Institution: Jiangnan Univ	Country: China<br>Institution: Jiangnan University

Record 588 | WOS:000283743900015

Immunotoxicity of zearalenone in Balb/c mice in a high subchronic dosing study counteracted by Raphanus sativus extract

No C1/C3/RP normalisation changes detected for this record.

Record 589 | WOS:000284436000007

Evaluation of a urinary kidney biomarker panel in rat models of acute and subchronic nephrotoxicity

Field	Position	Original line	Normalised line	Original value	Normalised value	Original country / institution	Normalised country / institution
C1	1	85949	85949	[Hoffmann, Dana; Dekant, Wolfgang; Mally, Angela] Univ Wurzburg, Dept Toxicol, D-97078 Wurzburg, Germany.	[Hoffmann, Dana; Dekant, Wolfgang; Mally, Angela] University of Wurzburg, Dept Toxicol, D-97078 Wurzburg, Germany.	Country: Germany<br>Institution: Univ Wurzburg	Country: Germany<br>Institution: University of Wurzburg
RP	1	85953	85953	Mally, A (corresponding author), Univ Wurzburg, Dept Toxicol, Versbacher Str 9, D-97078 Wurzburg, Germany	Mally, A (corresponding author), University of Wurzburg, Dept Toxicol, Versbacher Str 9, D-97078 Wurzburg, Germany	Country: Germany<br>Institution: Univ Wurzburg	Country: Germany<br>Institution: University of Wurzburg

Record 590 | WOS:000283810500022

Protein Biomarkers for in Vitro Testing of Embryotoxicity

No C1/C3/RP normalisation changes detected for this record.

Record 591 | WOS:000280344000003

Some food-associated mycotoxins as potential risk factors in humans predisposed to chronic intestinal inflammatory diseases

Field	Position	Original line	Normalised line	Original value	Normalised value	Original country / institution	Normalised country / institution
C1	1	86220	86220	[Maresca, Marc] Univ Aix Marseille 2, CRN2M, CNRS,Fac Sci St Jerome, UMR 6231,INRA,USC 2027,Lab Interact Mol & Syst Me, F-13397 Marseille 20, France. Univ Aix Marseille 3, CRN2M, CNRS,Fac Sci St Jerome, UMR 6231,INRA,USC 2027,Lab Interact Mol & Syst Me, F-13397 Marseille 20, France.	[Maresca, Marc] Aix-Marseille Universite, CRN2M, CNRS,Fac Sci St Jerome, UMR 6231,INRA,USC 2027,Lab Interact Mol & Syst Me, F-13397 Marseille 20, France. Aix-Marseille Universite, CRN2M, CNRS,Fac Sci St Jerome, UMR 6231,INRA,USC 2027,Lab Interact Mol & Syst Me, F-13397 Marseille 20, France.	Country: France<br>Institution: Univ Aix Marseille 2	Country: France<br>Institution: Aix-Marseille Universite
RP	1	86225	86225	Maresca, M (corresponding author), Univ Aix Marseille 2, CRN2M, CNRS,Fac Sci St Jerome, UMR 6231,INRA,USC 2027,Lab Interact Mol & Syst Me, F-13397 Marseille 20, France	Maresca, M (corresponding author), Aix-Marseille Universite, CRN2M, CNRS,Fac Sci St Jerome, UMR 6231,INRA,USC 2027,Lab Interact Mol & Syst Me, F-13397 Marseille 20, France	Country: France<br>Institution: Univ Aix Marseille 2	Country: France<br>Institution: Aix-Marseille Universite

Record 592 | WOS:000281740900017

Mycotoxins and Their Biotransformation in the Rumen: A Review

Field	Position	Original line	Normalised line	Original value	Normalised value	Original country / institution	Normalised country / institution
C1	1	86436	86436	[Upadhaya, Santi Devi; Park, M. A.; Ha, Jong K.] Seoul Natl Univ, Dept Agr Biotechnol, Res Inst Agr & Life Sci, Coll Agr & Life Sci, Seoul 151, South Korea.	[Upadhaya, Santi Devi; Park, M. A.; Ha, Jong K.] Seoul National University (SNU), Dept Agr Biotechnol, Res Inst Agr & Life Sci, Coll Agr & Life Sci, Seoul 151, South Korea.	Country: South Korea<br>Institution: Seoul Natl Univ	Country: South Korea<br>Institution: Seoul National University (SNU)
RP	1	86438	86438	Ha, JK (corresponding author), Seoul Natl Univ, Dept Agr Biotechnol, Res Inst Agr & Life Sci, Coll Agr & Life Sci, Seoul 151, South Korea	Ha, JK (corresponding author), Seoul National University (SNU), Dept Agr Biotechnol, Res Inst Agr & Life Sci, Coll Agr & Life Sci, Seoul 151, South Korea	Country: South Korea<br>Institution: Seoul Natl Univ	Country: South Korea<br>Institution: Seoul National University (SNU)

Record 593 | WOS:000282996200006

Free radicals and antioxidants in two oxidative-stress cell models exposed to ochratoxin A and amyloid Î²: unexpected results

Field	Position	Original line	Normalised line	Original value	Normalised value	Original country / institution	Normalised country / institution
C1	1	86619	86619	[Costa, S.; Speroni, E.; Guerra, M. C.] Univ Bologna, Dept Pharmacol, I-40126 Bologna, Italy.	[Costa, S.; Speroni, E.; Guerra, M. C.] University of Bologna, Dept Pharmacol, I-40126 Bologna, Italy.	Country: Italy<br>Institution: Univ Bologna	Country: Italy<br>Institution: University of Bologna
RP	1	86622	86622	Costa, S (corresponding author), Univ Bologna, Dept Pharmacol, Via Irnerio 48, I-40126 Bologna, Italy	Costa, S (corresponding author), University of Bologna, Dept Pharmacol, Via Irnerio 48, I-40126 Bologna, Italy	Country: Italy<br>Institution: Univ Bologna	Country: Italy<br>Institution: University of Bologna

Record 594 | WOS:000280991100075

Safety studies on products from whole coffee fruit

No C1/C3/RP normalisation changes detected for this record.

Record 595 | WOS:000280256800007

Beauvericin and ochratoxin A genotoxicity evaluated using the alkaline comet assay: single and combined genotoxic action

Field	Position	Original line	Normalised line	Original value	Normalised value	Original country / institution	Normalised country / institution
C1	1	86802	86802	[Klaric, Maja Segvic; Darabos, Dina; Pepeljnjak, Stjepan] Univ Zagreb, Fac Pharm & Biochem, Dept Microbiol, Zagreb 10000, Croatia.	[Klaric, Maja Segvic; Darabos, Dina; Pepeljnjak, Stjepan] University of Zagreb, Fac Pharm & Biochem, Dept Microbiol, Zagreb 10000, Croatia.	Country: Croatia<br>Institution: Univ Zagreb	Country: Croatia<br>Institution: University of Zagreb
C1	2	86803	86803	[Rozgaj, Ruzica; Kasuba, Vilena] Inst Med Res & Occupat Hlth, Mutagenesis Unit, Zagreb 41000, Croatia.	[Rozgaj, Ruzica; Kasuba, Vilena] Institute for Medical Research & Occupational Health (IMROH), Mutagenesis Unit, Zagreb 41000, Croatia.	Country: Croatia<br>Institution: Inst Med Res & Occupat Hlth	Country: Croatia<br>Institution: Institute for Medical Research & Occupational Health (IMROH)
RP	1	86806	86806	Klaric, MS (corresponding author), Univ Zagreb, Fac Pharm & Biochem, Dept Microbiol, Schrottova 39, Zagreb 10000, Croatia	Klaric, MS (corresponding author), University of Zagreb, Fac Pharm & Biochem, Dept Microbiol, Schrottova 39, Zagreb 10000, Croatia	Country: Croatia<br>Institution: Univ Zagreb	Country: Croatia<br>Institution: University of Zagreb

Record 596 | WOS:000282996200009

Mycotoxins in grapes and wine in Europe: occurrence, factors affecting the occurrence and related toxicological effects

No C1/C3/RP normalisation changes detected for this record.

Record 597 | WOS:000278803100015

Allium cepa as a biomonitor of ochratoxin A toxicity and genotoxicity

Field	Position	Original line	Normalised line	Original value	Normalised value	Original country / institution	Normalised country / institution
C1	1	87040	87040	[Lerda, D.; Bistoni, M. Biagi; Pelliccioni, P.] Cordoba Catholic Univ, Genet Lab, Sch Med, Cordoba, Argentina.	[Lerda, D.; Bistoni, M. Biagi; Pelliccioni, P.] Catholic University of Cordoba, Genet Lab, Sch Med, Cordoba, Argentina.	Country: Argentina<br>Institution: Cordoba Catholic Univ	Country: Argentina<br>Institution: Catholic University of Cordoba
C1	2	87041	87041	[Litterio, N.] Cordoba Catholic Univ, Agr Sci Sch, Cordoba, Argentina.	[Litterio, N.] Catholic University of Cordoba, Agr Sci Sch, Cordoba, Argentina.	Country: Argentina<br>Institution: Cordoba Catholic Univ	Country: Argentina<br>Institution: Catholic University of Cordoba
RP	1	87043	87043	Lerda, D (corresponding author), Cordoba Catholic Univ, Genet Lab, Sch Med, Cordoba, Argentina	Lerda, D (corresponding author), Catholic University of Cordoba, Genet Lab, Sch Med, Cordoba, Argentina	Country: Argentina<br>Institution: Cordoba Catholic Univ	Country: Argentina<br>Institution: Catholic University of Cordoba

Record 598 | WOS:000277103900009

Neuronal models for evaluation of proliferation in vitro using high content screening

Field	Position	Original line	Normalised line	Original value	Normalised value	Original country / institution	Normalised country / institution
C1	1	87118	87118	[Mundy, William R.; Radio, Nicholas M.; Freudenrich, Theresa M.] US EPA, Integrated Syst Toxicol Div, Natl Hlth & Environm Effects Res Lab, Off Res & Dev, Res Triangle Pk, NC 27711 USA.	[Mundy, William R.; Radio, Nicholas M.; Freudenrich, Theresa M.] United States Environmental Protection Agency, Integrated Syst Toxicol Div, Natl Hlth & Environm Effects Res Lab, Off Res & Dev, Res Triangle Pk, NC 27711 USA.	Country: USA<br>Institution: US EPA	Country: USA<br>Institution: United States Environmental Protection Agency
RP	1	87120	87120	Mundy, WR (corresponding author), US EPA, Integrated Syst Toxicol Div, Natl Hlth & Environm Effects Res Lab, Off Res & Dev, B105-06, Res Triangle Pk, NC 27711 USA	Mundy, WR (corresponding author), United States Environmental Protection Agency, Integrated Syst Toxicol Div, Natl Hlth & Environm Effects Res Lab, Off Res & Dev, B105-06, Res Triangle Pk, NC 27711 USA	Country: USA<br>Institution: US EPA	Country: USA<br>Institution: United States Environmental Protection Agency

Record 599 | WOS:000208434800018

Antigenotoxic Studies of Different Substances to Reduce the DNA Damage Induced by Aflatoxin B1 and Ochratoxin A

Field	Position	Original line	Normalised line	Original value	Normalised value	Original country / institution	Normalised country / institution
C1	1	87222	87222	[Madrigal-Santillan, Eduardo; Morales-Gonzalez, Jose A.; Vargas-Mendoza, Nancy; Reyes-Ramirez, Patricia; Cruz-Jaime, Sandra; Sumaya-Martinez, Teresa] Univ Autonoma Estado Hidalgo, Inst Ciencias Salud, Pachuca 42080, Hidalgo, Mexico.	[Madrigal-Santillan, Eduardo; Morales-Gonzalez, Jose A.; Vargas-Mendoza, Nancy; Reyes-Ramirez, Patricia; Cruz-Jaime, Sandra; Sumaya-Martinez, Teresa] Universidad Autonoma del Estado de Hidalgo, Inst Ciencias Salud, Pachuca 42080, Hidalgo, Mexico.	Country: Mexico<br>Institution: Univ Autonoma Estado Hidalgo	Country: Mexico<br>Institution: Universidad Autonoma del Estado de Hidalgo
RP	1	87227	87227	Madrigal-SantillÃ¡n, E (corresponding author), Univ Autonoma Estado Hidalgo, Inst Ciencias Salud, Pachuca 42080, Hidalgo, Mexico	Madrigal-SantillÃ¡n, E (corresponding author), Universidad Autonoma del Estado de Hidalgo, Inst Ciencias Salud, Pachuca 42080, Hidalgo, Mexico	Country: Mexico<br>Institution: Univ Autonoma Estado Hidalgo	Country: Mexico<br>Institution: Universidad Autonoma del Estado de Hidalgo

Record 600 | WOS:000276175200004

Ochratoxin A induces G2 phase arrest in human gastric epithelium GES-1 cells in vitro

Field	Position	Original line	Normalised line	Original value	Normalised value	Original country / institution	Normalised country / institution
C1	1	87383	87383	[Cui, Jinfeng; Xing, Lingxiao; Li, Zengning; Wu, Sha; Wang, Juan; Liu, Jing; Wang, Junling; Yan, Xia; Zhang, Xianghong] Hebei Med Univ, Pathol Lab, Shijiazhuang, Peoples R China.	[Cui, Jinfeng; Xing, Lingxiao; Li, Zengning; Wu, Sha; Wang, Juan; Liu, Jing; Wang, Junling; Yan, Xia; Zhang, Xianghong] Hebei Medical University, Pathol Lab, Shijiazhuang, China.	Country: China<br>Institution: Hebei Med Univ	Country: China<br>Institution: Hebei Medical University
C1	2	87384	87384	[Cui, Jinfeng; Zhang, Xianghong] Hebei Med Univ, Hosp 2, Dept Pathol, Shijiazhuang, Peoples R China.	[Cui, Jinfeng; Zhang, Xianghong] Hebei Medical University, Hosp 2, Dept Pathol, Shijiazhuang, China.	Country: China<br>Institution: Hebei Med Univ	Country: China<br>Institution: Hebei Medical University
RP	1	87386	87386	Zhang, XH (corresponding author), Hebei Med Univ, Pathol Lab, 361 Zhongshan Eastern R, Shijiazhuang, Peoples R China	Zhang, XH (corresponding author), Hebei Medical University, Pathol Lab, 361 Zhongshan Eastern R, Shijiazhuang, China	Country: China<br>Institution: Hebei Med Univ	Country: China<br>Institution: Hebei Medical University

Record 601 | WOS:000274582400001

PREVENTIVE AND THERAPEUTIC METHODS AGAINST THE TOXIC EFFECTS OF MYCOTOXINS - A REVIEW

Field	Position	Original line	Normalised line	Original value	Normalised value	Original country / institution	Normalised country / institution
C1	2	87475	87475	[Balogh, Krisztian] Univ Kaposvar, Fac Anim Sci, Res Grp Anim Breeding & Hyg, Kaposvar, Hungary.	[Balogh, Krisztian] Kaposvar University, Fac Anim Sci, Res Grp Anim Breeding & Hyg, Kaposvar, Hungary.	Country: Hungary Institution: Univ Kaposvar	Country: Hungary Institution: Kaposvar University

Record 602 | WOS:000276819800011

Decontamination and detoxification strategies for the Fusarium mycotoxin deoxynivalenol in animal feed and the effectiveness of microbial biodegradation

Field	Position	Original line	Normalised line	Original value	Normalised value	Original country / institution	Normalised country / institution
C1	1	87656	87656	[Awad, Wageha A.; Zentek, Juergen] Free Univ Berlin, Fachbereich Vet Med, Inst Tierernahrung, D-14195 Berlin, Germany.	[Awad, Wageha A.; Zentek, Juergen] Free University of Berlin, Fachbereich Vet Med, Inst Tierernahrung, D-14195 Berlin, Germany.	Country: Germany Institution: Free Univ Berlin	Country: Germany Institution: Free University of Berlin
RP	1	87661	87661	Zentek, J (corresponding author), Free Univ Berlin, Fachbereich Vet Med, Inst Tierernahrung, Brummerstr 34, D-14195 Berlin, Germany	Zentek, J (corresponding author), Free University of Berlin, Fachbereich Vet Med, Inst Tierernahrung, Brummerstr 34, D-14195 Berlin, Germany	Country: Germany Institution: Free Univ Berlin	Country: Germany Institution: Free University of Berlin

Record 603 | WOS:000279331700013

Modification of aflatoxin B1 and ochratoxin A toxicokinetics in rats administered a yeast cell wall preparation

Field	Position	Original line	Normalised line	Original value	Normalised value	Original country / institution	Normalised country / institution
C1	1	87800	87800	[Firmin, S.; Morgavi, D. P.; Jouany, J. P.; Boudra, H.] INRA, Herbivores UR1213, F-63122 St Genes Champanelle, France.	[Firmin, S.; Morgavi, D. P.; Jouany, J. P.; Boudra, H.] INRAE, Herbivores UR1213, F-63122 St Genes Champanelle, France.	Country: France Institution: INRA	Country: France Institution: INRAE
RP	1	87805	87805	Boudra, H (corresponding author), INRA, Herbivores UR1213, Site Theix, F-63122 St Genes Champanelle, France	Boudra, H (corresponding author), INRAE, Herbivores UR1213, Site Theix, F-63122 St Genes Champanelle, France	Country: France Institution: INRA	Country: France Institution: INRAE

Record 604 | WOS:000273528200008

Zearalenone and its Derivatives: Known Toxins in New Aspects

No C1/C3/RP normalisation changes detected for this record.

Record 605 | WOS:000281731700007

Lifetime, low-dose ochratoxin A dietary study on renal carcinogenesis in male Fischer rats

Field	Position	Original line	Normalised line	Original value	Normalised value	Original country / institution	Normalised country / institution

C1	1	88047	88047	[Mantle, Peter] Univ London Imperial Coll Sci Technol & Med, Ctr Environm Policy, London SW7 2AZ, England.	[Mantle, Peter] Univ London Imperial Coll Sci Technol & Med, Ctr Environm Policy, London SW7 2AZ, United Kingdom.	Country: England Institution: Univ London Imperial Coll Sci Technol & Med	Country: United Kingdom Institution: Univ London Imperial Coll Sci Technol & Med
C1	2	88048	88048	[Kulinskaya, Elena] Univ E Anglia, Sch Comp Sci, Norwich NR4 7TJ, Norfolk, England.	[Kulinskaya, Elena] Univ E Anglia, Sch Comp Sci, Norwich NR4 7TJ, Norfolk, United Kingdom.	Country: England Institution: Univ E Anglia	Country: United Kingdom Institution: Univ E Anglia
RP	1	88050	88050	Mantle, P (corresponding author), Univ London Imperial Coll Sci Technol & Med, Ctr Environm Policy, London SW7 2AZ, England	Mantle, P (corresponding author), Univ London Imperial Coll Sci Technol & Med, Ctr Environm Policy, London SW7 2AZ, United Kingdom	Country: England Institution: Univ London Imperial Coll Sci Technol & Med	Country: United Kingdom Institution: Univ London Imperial Coll Sci Technol & Med

Record 606 | WOS:000273618500012

Structures of Covalent Adducts between DNA and Ochratoxin A: A New Factor in Debate about Genotoxicity and Human Risk Assessment

Field	Position	Original line	Normalised line	Original value	Normalised value	Original country / institution	Normalised country / institution
C1	1	88130	88130	[Mantle, Peter G.] Univ London Imperial Coll Sci Technol & Med, Ctr Environm Policy, London SW7 2AZ, England.	[Mantle, Peter G.] Univ London Imperial Coll Sci Technol & Med, Ctr Environm Policy, London SW7 2AZ, United Kingdom.	Country: England Institution: Univ London Imperial Coll Sci Technol & Med	Country: United Kingdom Institution: Univ London Imperial Coll Sci Technol & Med
C1	3	88132	88132	[Manderville, Richard A.] Univ Guelph, Dept Chem, Guelph, ON N1G 2W1, Canada.	[Manderville, Richard A.] University of Guelph, Dept Chem, Guelph, ON N1G 2W1, Canada.	Country: Canada Institution: Univ Guelph	Country: Canada Institution: University of Guelph
C1	4	88133	88133	[Manderville, Richard A.] Univ Guelph, Dept Toxicol, Guelph, ON N1G 2W1, Canada.	[Manderville, Richard A.] University of Guelph, Dept Toxicol, Guelph, ON N1G 2W1, Canada.	Country: Canada Institution: Univ Guelph	Country: Canada Institution: University of Guelph
C1	5	88134	88134	[Squillaci, Bianca] GlaxoSmithKline, Drug Metab & Pharmacokinet Dept, Ware SG12 0DP, Herts, England.	[Squillaci, Bianca] GlaxoSmithKline, Drug Metab & Pharmacokinet Dept, Ware SG12 0DP, Herts, United Kingdom.	Country: England Institution: GlaxoSmithKline	Country: United Kingdom Institution: GlaxoSmithKline
RP	1	88140	88140	Mantle, PG (corresponding author), Univ London Imperial Coll Sci Technol & Med, Ctr Environm Policy, London SW7 2AZ, England	Mantle, PG (corresponding author), Univ London Imperial Coll Sci Technol & Med, Ctr Environm Policy, London SW7 2AZ, United Kingdom	Country: England Institution: Univ London Imperial Coll Sci Technol & Med	Country: United Kingdom Institution: Univ London Imperial Coll Sci Technol & Med

Record 607 | WOS:000286528700008

Ochratoxin A-induced serum biochemical alterations in New Zealand white rabbits (Oryctolagus cuniculus)

Field	Position	Original line	Normalised line	Original value	Normalised value	Original country / institution	Normalised country / institution
C1	1	88244	88244	[Mir, Masood S.; Dwivedi, Prabakar] Indian Vet Res Inst, Div Pathol, Bareilly 243122, Uttar Pradesh, India.	[Mir, Masood S.; Dwivedi, Prabakar] ICAR - Indian Veterinary Research Institute, Div Pathol, Bareilly 243122, Uttar Pradesh, India.	Country: India Institution: Indian Vet Res Inst	Country: India Institution: ICAR - Indian Veterinary Research Institute
RP	1	88249	88249	Mir, MS (corresponding author), Indian Vet Res Inst, Div Pathol, Bareilly 243122, Uttar Pradesh, India	Mir, MS (corresponding author), ICAR - Indian Veterinary Research Institute, Div Pathol, Bareilly 243122, Uttar Pradesh, India	Country: India Institution: Indian Vet Res Inst	Country: India Institution: ICAR - Indian Veterinary Research Institute

Record 608 | WOS:000274143800002

Toxicology of mycotoxins

No C1/C3/RP normalisation changes detected for this record.

Record 609 | WOS:000272760200016

CYP1A1 and CYP3A4 modulation by dietary flavonoids in human intestinal Caco-2 cells

Field	Position	Original line	Normalised line	Original value	Normalised value	Original country / institution	Normalised country / institution
C1	2	88513	88513	[Van der Heiden, Edwige; Scippo, Marie-Louise] Univ Liege, CART, Fac Vet Med, Dept Food Sci,Lab Food Anal, B-4000 Liege, Belgium.	[Van der Heiden, Edwige; Scippo, Marie-Louise] University of Liege, CART, Fac Vet Med, Dept Food Sci,Lab Food Anal, B-4000 Liege, Belgium.	Country: Belgium Institution: Univ Liege	Country: Belgium Institution: University of Liege

Record 610 | WOS:000271971100003

Comparative Mechanisms of Zearalenone and Ochratoxin A Toxicities on Cultured HepG2 cells: Is Oxidative Stress a Common Process?

No C1/C3/RP normalisation changes detected for this record.

Record 611 | WOS:000208434400002

Cyclopiazonic Acid Biosynthesis of Aspergillus flavus and Aspergillus oryzae

Field	Position	Original line	Normalised line	Original value	Normalised value	Original country / institution	Normalised country / institution
C1	1	88753	88753	[Chang, Perng-Kuang; Ehrlich, Kenneth C.] USDA ARS, So Reg Res Ctr, New Orleans, LA 70124 USA.	[Chang, Perng-Kuang; Ehrlich, Kenneth C.] United States Department of Agriculture (USDA), So Reg Res Ctr, New Orleans, LA 70124 USA.	Country: USA Institution: USDA ARS	Country: USA Institution: United States Department of Agriculture (USDA)
C1	2	88754	88754	[Fujii, Isao] Iwate Med Univ, Sch Pharm, Yahaba, Iwate 0283694, Japan.	[Fujii, Isao] Iwate Medical University, Sch Pharm, Yahaba, Iwate 0283694, Japan.	Country: Japan Institution: Iwate Med Univ	Country: Japan Institution: Iwate Medical University
RP	1	88756	88756	Chang, PK (corresponding author), USDA ARS, So Reg Res Ctr, 1100 Robert E Lee Blvd, New Orleans, LA 70124 USA	Chang, PK (corresponding author), United States Department of Agriculture (USDA), So Reg Res Ctr, 1100 Robert E Lee Blvd, New Orleans, LA 70124 USA	Country: USA Institution: USDA ARS	Country: USA Institution: United States Department of Agriculture (USDA)

Record 612 | WOS:000272276600011

Reactive oxygen species induced by beauvericin, patulin and zearalenone in CHO-K1 cells

Field	Position	Original line	Normalised line	Original value	Normalised value	Original country / institution	Normalised country / institution
C1	1	88939	88939	[Ferrer, E.; Juan-Garcia, A.; Font, G.; Ruiz, M. J.] Univ Valencia, Toxicol Lab, Fac Farm, E-46100 Valencia, Spain.	[Ferrer, E.; Juan-Garcia, A.; Font, G.; Ruiz, M. J.] University of Valencia, Toxicol Lab, Fac Farm, E-46100 Valencia, Spain.	Country: Spain Institution: Univ Valencia	Country: Spain Institution: University of Valencia

RP	1	88941	88941	Ruiz, MJ (corresponding author), Univ Valencia, Toxicol Lab, Fac Farm, Av Vicent Andres Estelles, E-46100 Valencia, Spain	Ruiz, MJ (corresponding author), University of Valencia, Toxicol Lab, Fac Farm, Av Vicent Andres Estelles, E-46100 Valencia, Spain	Country: Spain<br>Institution: Univ Valencia	Country: Spain<br>Institution: University of Valencia

Record 613 | WOS:000272276600013

Intracellular zinc stores protect the intestinal epithelium from Ochratoxin A toxicity

No C1/C3/RP normalisation changes detected for this record.

Record 614 | WOS:000270527000004

Minimum tolerable exposure period and maximum threshold dietary intake of ochratoxin A for causing renal cancer in male Dark Agouti rats

Field	Position	Original line	Normalised line	Original value	Normalised value	Original country / institution	Normalised country / institution
C1	1	89109	89109	Univ London Imperial Coll Sci Technol & Med, Ctr Environm Policy, London SW7 2AZ, England.	Univ London Imperial Coll Sci Technol & Med, Ctr Environm Policy, London SW7 2AZ, United Kingdom.	Country: England<br>Institution: Univ London Imperial Coll Sci Technol & Med	Country: United Kingdom<br>Institution: Univ London Imperial Coll Sci Technol & Med
RP	1	89111	89111	Mantle, PG (corresponding author), Univ London Imperial Coll Sci Technol & Med, Ctr Environm Policy, London SW7 2AZ, England	Mantle, PG (corresponding author), Univ London Imperial Coll Sci Technol & Med, Ctr Environm Policy, London SW7 2AZ, United Kingdom	Country: England<br>Institution: Univ London Imperial Coll Sci Technol & Med	Country: United Kingdom<br>Institution: Univ London Imperial Coll Sci Technol & Med

Record 615 | WOS:000270503100034

Molecular Characterization of Preneoplastic Lesions Provides Insight on the Development of Renal Tumors

Field	Position	Original line	Normalised line	Original value	Normalised value	Original country / institution	Normalised country / institution
C1	1	89182	89182	[Dietrich, Daniel R.] Univ Konstanz, Fac Biol, Dept Human & Ervironm Toxicol, D-78457 Constance, Germany.	[Dietrich, Daniel R.] University of Konstanz, Fac Biol, Dept Human & Ervironm Toxicol, D-78457 Constance, Germany.	Country: Germany<br>Institution: Univ Konstanz	Country: Germany<br>Institution: University of Konstanz
C1	2	89183	89183	[Ellinger-Ziegelbauer, Heidrun; Ahr, Hans-Juergen] Bayer HealthCare, Dept Special Toxicol, Wuppertal, Germany.	[Ellinger-Ziegelbauer, Heidrun; Ahr, Hans-Juergen] Bayer Healthcare Pharmaceuticals, Dept Special Toxicol, Wuppertal, Germany.	Country: Germany<br>Institution: Bayer HealthCare	Country: Germany<br>Institution: Bayer Healthcare Pharmaceuticals
RP	1	89185	89185	Dietrich, DR (corresponding author), Univ Konstanz, Fac Biol, Dept Human & Ervironm Toxicol, Jacob Burckhardstr 25, D-78457 Constance, Germany	Dietrich, DR (corresponding author), University of Konstanz, Fac Biol, Dept Human & Ervironm Toxicol, Jacob Burckhardstr 25, D-78457 Constance, Germany	Country: Germany<br>Institution: Univ Konstanz	Country: Germany<br>Institution: University of Konstanz

Record 616 | WOS:000269677000009

Low doses of ochratoxin A upregulate the protein expression of organic anion transporters Oat1, Oat2, Oat3 and Oat5 in rat kidney cortex

Field	Position	Original line	Normalised line	Original value	Normalised value	Original country / institution	Normalised country / institution

C1	1	89313	89313	[Zlender, Vilim; Flajs, Dubravka; Domijan, Ana-Marija; Peraica, Maja; Sabolic, Ivan] Inst Med Res & Occupat Hlth, Toxicol Unit, HR-10001 Zagreb, Croatia.	[Zlender, Vilim; Flajs, Dubravka; Domijan, Ana-Marija; Peraica, Maja; Sabolic, Ivan] Institute for Medical Research & Occupational Health (IMROH), Toxicol Unit, HR-10001 Zagreb, Croatia.	Country: Croatia<br>Institution: Inst Med Res & Occupat Hlth	Country: Croatia<br>Institution: Institute for Medical Research & Occupational Health (IMROH)
C1	2	89314	89314	[Anzai, Naohiko] Kyorin Univ, Sch Med, Dept Pharmacol & Toxicol, Tokyo, Japan.	[Anzai, Naohiko] Kyorin University, Sch Med, Dept Pharmacol & Toxicol, Tokyo, Japan.	Country: Japan<br>Institution: Kyorin Univ	Country: Japan<br>Institution: Kyorin University
RP	1	89317	89317	Sabolic, I (corresponding author), Inst Med Res & Occupat Hlth, Toxicol Unit, Ksaverska Cesta 2, HR-10001 Zagreb, Croatia	Sabolic, I (corresponding author), Institute for Medical Research & Occupational Health (IMROH), Toxicol Unit, Ksaverska Cesta 2, HR-10001 Zagreb, Croatia	Country: Croatia<br>Institution: Inst Med Res & Occupat Hlth	Country: Croatia<br>Institution: Institute for Medical Research & Occupational Health (IMROH)

Record 617 | WOS:000269551500021

The mycotoxin ochratoxin A inhibits DNA topoisomerase II and induces polyploidy in cultured CHO cells

No C1/C3/RP normalisation changes detected for this record.

Record 618 | WOS:000269551500029

Multiplexed assay panel of cytotoxicity in HK-2 cells for detection of renal proximal tubule injury potential of compounds

Field	Position	Original line	Normalised line	Original value	Normalised value	Original country / institution	Normalised country / institution
C1	1	89561	89561	[Wu, Yang; Hanumegowda, Umesh M.; Adams, Stephen P.] Bristol Myers Squibb Co, Discovery Toxicol Res & Dev, Wallingford, CT 06492 USA.	[Wu, Yang; Hanumegowda, Umesh M.; Adams, Stephen P.] Bristol-Myers Squibb, Discovery Toxicol Res & Dev, Wallingford, CT 06492 USA.	Country: USA<br>Institution: Bristol Myers Squibb Co	Country: USA<br>Institution: Bristol-Myers Squibb
C1	2	89562	89562	[Connors, David; Barber, Lauren; Jayachandra, Sukhanya] Bristol Myers Squibb Co, Lead Discovery Res & Dev, Wallingford, CT 06492 USA.	[Connors, David; Barber, Lauren; Jayachandra, Sukhanya] Bristol-Myers Squibb, Lead Discovery Res & Dev, Wallingford, CT 06492 USA.	Country: USA<br>Institution: Bristol Myers Squibb Co	Country: USA<br>Institution: Bristol-Myers Squibb
RP	1	89564	89564	Wu, Y (corresponding author), Bristol Myers Squibb Co, Discovery Toxicol Res & Dev, 5 Res Pkwy, Wallingford, CT 06492 USA	Wu, Y (corresponding author), Bristol-Myers Squibb, Discovery Toxicol Res & Dev, 5 Res Pkwy, Wallingford, CT 06492 USA	Country: USA<br>Institution: Bristol Myers Squibb Co	Country: USA<br>Institution: Bristol-Myers Squibb

Record 619 | WOS:000268006900004

Pro-apoptotic effects of nivalenol and deoxynivalenol trichothecenes in J774A.1 murine macrophages

Field	Position	Original line	Normalised line	Original value	Normalised value	Original country / institution	Normalised country / institution
C1	1	89667	89667	[Marzocco, Stefania] Univ Salerno, Dept Pharmaceut Sci, Fac Pharm, Sch Pharm, I-84084 Fisciano, SA, Italy.	[Marzocco, Stefania] University of Salerno, Dept Pharmaceut Sci, Fac Pharm, Sch Pharm, I-84084 Fisciano, SA, Italy.	Country: Italy<br>Institution: Univ Salerno	Country: Italy<br>Institution: University of Salerno
C1	2	89668	89668	[Russo, Rosario; Severino, Lorella] Univ Naples Federico II, Dept Pathol & Anim Hlth,	[Russo, Rosario; Severino, Lorella] University of Naples Federico II, Dept Pathol & Anim	Country: Italy<br>Institution: Univ Naples Federico II	Country: Italy<br>Institution: University of Naples Federico II

				Div Toxicol, Sch Vet Med, Naples, Italy.	Hlth, Div Toxicol, Sch Vet Med, Naples, Italy.		
RP	1	89670	89670	Marzocco, S (corresponding author), Univ Salerno, Dept Pharmaceut Sci, Fac Pharm, Sch Pharm, Via Ponte Don Melillo 11-C, I-84084 Fisciano, SA, Italy	Marzocco, S (corresponding author), University of Salerno, Dept Pharmaceut Sci, Fac Pharm, Sch Pharm, Via Ponte Don Melillo 11-C, I-84084 Fisciano, SA, Italy	Country: Italy<br>Institution: Univ Salerno	Country: Italy<br>Institution: University of Salerno

Record 620 | WOS:000282995500010

Occurrence of ochratoxin A and heavy metals in tissues associated with porcine nephropathy in Serbia

Field	Position	Original line	Normalised line	Original value	Normalised value	Original country / institution	Normalised country / institution
C1	2	89770	89770	[Juric, V.] Univ Novi Sad, Dept Anim Sci, Fac Agr, Novi Sad 21000, Serbia.	[Juric, V.] University of Novi Sad, Dept Anim Sci, Fac Agr, Novi Sad 21000, Serbia.	Country: Serbia<br>Institution: Univ Novi Sad	Country: Serbia<br>Institution: University of Novi Sad
C1	3	89771	89771	[Jovanovic, M.] Univ Belgrade, Dept Pathomorphol, Fac Vet Med, Belgrade 11000, Serbia.	[Jovanovic, M.] University of Belgrade, Dept Pathomorphol, Fac Vet Med, Belgrade 11000, Serbia.	Country: Serbia<br>Institution: Univ Belgrade	Country: Serbia<br>Institution: University of Belgrade

Record 621 | WOS:000267227800008

Ochratoxin A-Mediated DNA and Protein Damage: Roles of Nitrosative and Oxidative Stresses

Field	Position	Original line	Normalised line	Original value	Normalised value	Original country / institution	Normalised country / institution
C1	1	89905	89905	[Cavin, Christophe; Delatour, Thierry; Marin-Kuan, Maricel; Holzhaeuser, Daisy; Guignard, Gabriela; Bezencon, Claudine; Piguet, Dominique; Parisod, Veronique; Richoz-Payot, Janique; Schilter, Benoit] Nestle Res Ctr, Qual & Safety Dept, CH-1000 Lausanne 26, Switzerland.	[Cavin, Christophe; Delatour, Thierry; Marin-Kuan, Maricel; Holzhaeuser, Daisy; Guignard, Gabriela; Bezencon, Claudine; Piguet, Dominique; Parisod, Veronique; Richoz-Payot, Janique; Schilter, Benoit] Nestle Research Center, Qual & Safety Dept, CH-1000 Lausanne 26, Switzerland.	Country: Switzerland<br>Institution: Nestle Res Ctr	Country: Switzerland<br>Institution: Nestle Research Center
RP	1	89908	89908	Cavin, C (corresponding author), Nestle Res Ctr, Qual & Safety Dept, POB 44,Vers Chez Les Blanc, CH-1000 Lausanne 26, Switzerland	Cavin, C (corresponding author), Nestle Research Center, Qual & Safety Dept, POB 44,Vers Chez Les Blanc, CH-1000 Lausanne 26, Switzerland	Country: Switzerland<br>Institution: Nestle Res Ctr	Country: Switzerland<br>Institution: Nestle Research Center

Record 622 | WOS:000268796500023

A Review of the Implementation of the Embryonic Stem Cell Test (EST) The report and recommendations of an ECVAM/ReProTect Workshop

Field	Position	Original line	Normalised line	Original value	Normalised value	Original country / institution	Normalised country / institution
C1	5	90071	90071	[Garthoff, Bernward] Bayer Crop Sci AG, Monheim, Germany.	[Garthoff, Bernward] Bayer AG, Monheim, Germany.	Country: Germany<br>Institution: Bayer Crop Sci AG	Country: Germany<br>Institution: Bayer AG
C1	9	90075	90075	[Rogiers, Vera] Vrije Univ Brussel, Brussels, Belgium.	[Rogiers, Vera] Vrije Universiteit Brussel, Brussels, Belgium.	Country: Belgium<br>Institution: Vrije Univ Brussel	Country: Belgium<br>Institution: Vrije Universiteit Brussel
C3	15	90085	90085	Pfizer USA	Pfizer United States	Institution: Pfizer USA	Institution: Pfizer United States

Record 623 | WOS:000265685000003

Oxidative stress and apoptotic changes in primary cultures of rat proximal tubular cells exposed to lead

Field	Position	Original line	Normalised line	Original value	Normalised value	Original country / institution	Normalised country / institution
C1	1	90173	90173	[Wang, Lin; Wang, Heng; Cao, Jin; Chen, Dawei; Liu, Zongping] Yangzhou Univ, Coll Vet Med, Yangzhou 225009, Peoples R China.	[Wang, Lin; Wang, Heng; Cao, Jin; Chen, Dawei; Liu, Zongping] Yangzhou University, Coll Vet Med, Yangzhou 225009, China.	Country: China Institution: Yangzhou Univ	Country: China Institution: Yangzhou University
C1	2	90174	90174	[Hu, Maozhi] Yangzhou Univ, Testing Ctr, Yangzhou 225009, Peoples R China.	[Hu, Maozhi] Yangzhou University, Testing Ctr, Yangzhou 225009, China.	Country: China Institution: Yangzhou Univ	Country: China Institution: Yangzhou University
RP	1	90176	90176	Liu, ZP (corresponding author), Yangzhou Univ, Coll Vet Med, 12 E Wenhui Rd, Yangzhou 225009, Peoples R China	Liu, ZP (corresponding author), Yangzhou University, Coll Vet Med, 12 E Wenhui Rd, Yangzhou 225009, China	Country: China Institution: Yangzhou Univ	Country: China Institution: Yangzhou University

Record 624 | WOS:000265474500006

Silibinin protects OTA-mediated TNF-Î± release from perfused rat livers and isolated rat Kupffer cells

No C1/C3/RP normalisation changes detected for this record.

Record 625 | WOS:000265474500007

Mycotoxins and the kidney: Modes of action for renal tumor formation by ochratoxin A in rodents

Field	Position	Original line	Normalised line	Original value	Normalised value	Original country / institution	Normalised country / institution
C1	1	90366	90366	[Mally, Angela; Dekant, Wolfgang] Univ Wurzburg, Dept Toxicol, D-997078 Wurzburg, Germany.	[Mally, Angela; Dekant, Wolfgang] University of Wurzburg, Dept Toxicol, D-997078 Wurzburg, Germany.	Country: Germany Institution: Univ Wurzburg	Country: Germany Institution: University of Wurzburg
RP	1	90368	90368	Mally, A (corresponding author), Univ Wurzburg, Dept Toxicol, Versbacher Str, D-997078 Wurzburg, Germany	Mally, A (corresponding author), University of Wurzburg, Dept Toxicol, Versbacher Str, D-997078 Wurzburg, Germany	Country: Germany Institution: Univ Wurzburg	Country: Germany Institution: University of Wurzburg

Record 626 | WOS:000263803400010

Effects of the mycotoxin fumonisin B1 on cell death in human kidney cells and human lung fibroblasts in primary culture

Field	Position	Original line	Normalised line	Original value	Normalised value	Original country / institution	Normalised country / institution
C1	1	90515	90515	[Koenigs, M.; Humpf, H. U.] Univ Munster, Inst Lebensmittelchem, D-4400 Munster, Germany.	[Koenigs, M.; Humpf, H. U.] University of Munster, Inst Lebensmittelchem, D-4400 Munster, Germany.	Country: Germany Institution: Univ Munster	Country: Germany Institution: University of Munster
C1	2	90516	90516	[Holzinger, H.] Univ Wurzburg, Inst Physiol, D-8700 Wurzburg, Germany.	[Holzinger, H.] University of Wurzburg, Inst Physiol, D-8700 Wurzburg, Germany.	Country: Germany Institution: Univ Wurzburg	Country: Germany Institution: University of Wurzburg

Record 627 | WOS:000266663300004

Ochratoxin A induces apoptosis in neuronal cells

Field	Position	Original line	Normalised line	Original value	Normalised value	Original country / institution	Normalised country / institution
C1	1	90630	90630	[Boesch-Saadatmandi, Christine; Huebbe, Patricia; Rimbach, Gerald] Univ Kiel, Inst Human Nutr & Food Sci, D-24098 Kiel, Germany.	[Boesch-Saadatmandi, Christine; Huebbe, Patricia; Rimbach, Gerald] University of Kiel, Inst Human Nutr & Food Sci, D-24098 Kiel, Germany.	Country: Germany<br>Institution: Univ Kiel	Country: Germany<br>Institution: University of Kiel
C1	2	90631	90631	[Zhang, Xiangnan; Lou, Yijia] Zhejiang Univ, Coll Pharmaceut Sci, Inst Pharmacol Toxicol & Biochem Pharmaceut, Hangzhou 310058, Zhejiang, Peoples R China.	[Zhang, Xiangnan; Lou, Yijia] Zhejiang University, Coll Pharmaceut Sci, Inst Pharmacol Toxicol & Biochem Pharmaceut, Hangzhou 310058, Zhejiang, China.	Country: China<br>Institution: Zhejiang Univ	Country: China<br>Institution: Zhejiang University
C1	3	90632	90632	[Wolffram, Siegfried] Univ Kiel, Inst Anim Nutr & Physiol, D-24098 Kiel, Germany.	[Wolffram, Siegfried] University of Kiel, Inst Anim Nutr & Physiol, D-24098 Kiel, Germany.	Country: Germany<br>Institution: Univ Kiel	Country: Germany<br>Institution: University of Kiel
RP	1	90634	90634	Rimbach, G (corresponding author), Univ Kiel, Inst Human Nutr & Food Sci, Hermann Rodewald Str 6, D-24098 Kiel, Germany	Rimbach, G (corresponding author), University of Kiel, Inst Human Nutr & Food Sci, Hermann Rodewald Str 6, D-24098 Kiel, Germany	Country: Germany<br>Institution: Univ Kiel	Country: Germany<br>Institution: University of Kiel

Record 628 | WOS:000263382500003

CYP1A1 induction and CYP3A4 inhibition by the fungicide imazalil in the human intestinal Caco-2 cells-Comparison with other conazole pesticides

Field	Position	Original line	Normalised line	Original value	Normalised value	Original country / institution	Normalised country / institution
C1	3	90740	90740	[van der Heiden, Edwige; Scippo, Marie-Louise] Univ Liege, Dept Food Sci, Lab Food Anal, B-4000 Liege, Belgium.	[van der Heiden, Edwige; Scippo, Marie-Louise] University of Liege, Dept Food Sci, Lab Food Anal, B-4000 Liege, Belgium.	Country: Belgium<br>Institution: Univ Liege	Country: Belgium<br>Institution: University of Liege
C1	4	90741	90741	[Muller, Marc] Univ Liege, Dept Life Sci, GIGA R, Lab Mol Biol & Genet Engn, B-4000 Liege, Belgium.	[Muller, Marc] University of Liege, Dept Life Sci, GIGA R, Lab Mol Biol & Genet Engn, B-4000 Liege, Belgium.	Country: Belgium<br>Institution: Univ Liege	Country: Belgium<br>Institution: University of Liege

Record 629 | WOS:000262717200017

Effects of live yeast cell supplementation to high concentrate diets on the toxicokinetics of ochratoxin A in sheep

Field	Position	Original line	Normalised line	Original value	Normalised value	Original country / institution	Normalised country / institution
C1	1	90871	90871	[Blank, R.; Wolffram, S.] Univ Kiel, Inst Anim Nutr & Physiol, D-24098 Kiel, Germany.	[Blank, R.; Wolffram, S.] University of Kiel, Inst Anim Nutr & Physiol, D-24098 Kiel, Germany.	Country: Germany<br>Institution: Univ Kiel	Country: Germany<br>Institution: University of Kiel
RP	1	90873	90873	Blank, R (corresponding author), Univ Kiel, Inst Anim Nutr & Physiol, Olshausenstr 40, D-24098 Kiel, Germany	Blank, R (corresponding author), University of Kiel, Inst Anim Nutr & Physiol, Olshausenstr 40, D-24098 Kiel, Germany	Country: Germany<br>Institution: Univ Kiel	Country: Germany<br>Institution: University of Kiel

Record 630 | WOS:000267272900021

The biotransformation of chosen mycotoxins

Field	Position	Original line	Normalised line	Original value	Normalised value	Original country / institution	Normalised country / institution
C1	1	90967	90967	[Gajecka, M.; Jakimiuk, E.; Zielonka, L.; Obremski, K.; Gajecki, M.] Univ Warmia & Mazury, Dept Vet Prevent & Feed Hyg, Fac Vet Med, PL-10718 Olsztyn, Poland.	[Gajecka, M.; Jakimiuk, E.; Zielonka, L.; Obremski, K.; Gajecki, M.] University of Warmia & Mazury, Dept Vet Prevent & Feed Hyg, Fac Vet Med, PL-10718 Olsztyn, Poland.	Country: Poland Institution: Univ Warmia & Mazury	Country: Poland Institution: University of Warmia & Mazury
RP	1	90969	90969	Gajecka, M (corresponding author), Univ Warmia & Mazury, Dept Vet Prevent & Feed Hyg, Fac Vet Med, Oczapowskiego 13, PL-10718 Olsztyn, Poland	Gajecka, M (corresponding author), University of Warmia & Mazury, Dept Vet Prevent & Feed Hyg, Fac Vet Med, Oczapowskiego 13, PL-10718 Olsztyn, Poland	Country: Poland Institution: Univ Warmia & Mazury	Country: Poland Institution: University of Warmia & Mazury

Record 631 | WOS:000273001000009

Effects of probiotic bacteria on the bioaccessibility of aflatoxin B1 and ochratoxin A using an in vitro digestion model under fed conditions

Field	Position	Original line	Normalised line	Original value	Normalised value	Original country / institution	Normalised country / institution
C1	1	91079	91079	[Kabak, Bulent] Hitit Univ, Dept Food Engn, Corum, Turkey.	[Kabak, Bulent] Hitit University, Dept Food Engn, Corum, Turkey.	Country: Turkey Institution: Hitit Univ	Country: Turkey Institution: Hitit University
C1	3	91081	91081	[Var, Isil] Cukurova Univ, Dept Food Engn, Adana, Turkey.	[Var, Isil] Cukurova University, Dept Food Engn, Adana, Turkey.	Country: Turkey Institution: Cukurova Univ	Country: Turkey Institution: Cukurova University
RP	1	91084	91084	Kabak, B (corresponding author), Hitit Univ, Dept Food Engn, Corum, Turkey	Kabak, B (corresponding author), Hitit University, Dept Food Engn, Corum, Turkey	Country: Turkey Institution: Hitit Univ	Country: Turkey Institution: Hitit University

Record 632 | WOS:000292954300008

Microorganisms and their enzymes for detoxifying mycotoxins posing a risk to livestock animals

Field	Position	Original line	Normalised line	Original value	Normalised value	Original country / institution	Normalised country / institution
C1	1	91183	91183	[Rodrigues, I.] BIOMIN Holding GmbH, A-3130 Herzogenburg, Austria.	[Rodrigues, I.] BIOMIN GmbH, A-3130 Herzogenburg, Austria.	Country: Austria Institution: BIOMIN Holding GmbH	Country: Austria Institution: BIOMIN GmbH
RP	1	91185	91185	Rodrigues, I (corresponding author), BIOMIN Holding GmbH, Ind Str 21, A-3130 Herzogenburg, Austria	Rodrigues, I (corresponding author), BIOMIN GmbH, Ind Str 21, A-3130 Herzogenburg, Austria	Country: Austria Institution: BIOMIN Holding GmbH	Country: Austria Institution: BIOMIN GmbH

Record 633 | WOS:000262775900032

Effect of ochratoxin A on cell survival and collagen homeostasis in human mesangial cells in primary culture

Field	Position	Original line	Normalised line	Original value	Normalised value	Original country / institution	Normalised country / institution
C1	2	91280	91280	[Holzinger, Hildegard] Univ Wurzburg, Inst Physiol, D-97070 Wurzburg, Germany.	[Holzinger, Hildegard] University of Wurzburg, Inst	Country: Germany Institution: Univ Wurzburg	Country: Germany Institution: University of Wurzburg

					Physiol, D-97070 Wurzburg, Germany.		
C1	3	91281	91281	[Koenigs, Maika; Humpf, Hans-Ulrich] Univ Munster, Inst Lebensmittelchem, D-4400 Munster, Germany.	[Koenigs, Maika; Humpf, Hans-Ulrich] University of Munster, Inst Lebensmittelchem, D-4400 Munster, Germany.	Country: Germany<br>Institution: Univ Munster	Country: Germany<br>Institution: University of Munster

Record 634 | WOS:000264793900023

EFFECTS OF AFLATOXIN B1, OCHRATOXIN A, PATULIN, CITRININ, AND ZEARALENONE ON THE IN VITRO PROLIFERATION OF PIG BLOOD LYMPHOCYTES

Field	Position	Original line	Normalised line	Original value	Normalised value	Original country / institution	Normalised country / institution
C1	1	91366	91366	[Stec, Jan; Szczotka, Maria] Natl Inst Vet Res, Dept Biochem, PL-24100 Pulawy, Poland.	[Stec, Jan; Szczotka, Maria] National Veterinary Institute - National Research Institute, Dept Biochem, PL-24100 Pulawy, Poland.	Country: Poland<br>Institution: Natl Inst Vet Res	Country: Poland<br>Institution: National Veterinary Institute - National Research Institute
C1	2	91367	91367	[Zmudzki, Jacek] Natl Inst Vet Res, Dept Swine Dis, PL-24100 Pulawy, Poland.	[Zmudzki, Jacek] National Veterinary Institute - National Research Institute, Dept Swine Dis, PL-24100 Pulawy, Poland.	Country: Poland<br>Institution: Natl Inst Vet Res	Country: Poland<br>Institution: National Veterinary Institute - National Research Institute
C1	3	91368	91368	[Stec, Jan; Szczotka, Maria] Natl Inst Vet Res, Dept Radiobiol, PL-24100 Pulawy, Poland.	[Stec, Jan; Szczotka, Maria] National Veterinary Institute - National Research Institute, Dept Radiobiol, PL-24100 Pulawy, Poland.	Country: Poland<br>Institution: Natl Inst Vet Res	Country: Poland<br>Institution: National Veterinary Institute - National Research Institute
RP	1	91372	91372	Stec, J (corresponding author), Natl Inst Vet Res, Dept Biochem, PL-24100 Pulawy, Poland	Stec, J (corresponding author), National Veterinary Institute - National Research Institute, Dept Biochem, PL-24100 Pulawy, Poland	Country: Poland<br>Institution: Natl Inst Vet Res	Country: Poland<br>Institution: National Veterinary Institute - National Research Institute

Record 635 | WOS:000262790100004

Developmental Toxicity of Ochratoxin A in Rat Embryo Midbrain Micromass Cultures

Field	Position	Original line	Normalised line	Original value	Normalised value	Original country / institution	Normalised country / institution
C1	1	91451	91451	[Wilk-Zasadna, Iwona; Minta, Maria] Natl Inst Vet Res, Dept Pharmacol & Toxicol, PL-24100 Pulawy, Poland.	[Wilk-Zasadna, Iwona; Minta, Maria] National Veterinary Institute - National Research Institute, Dept Pharmacol & Toxicol, PL-24100 Pulawy, Poland.	Country: Poland<br>Institution: Natl Inst Vet Res	Country: Poland<br>Institution: National Veterinary Institute - National Research Institute
RP	1	91453	91453	Minta, M (corresponding author), Natl Inst Vet Res, Dept Pharmacol & Toxicol, Al Partyzantow 57, PL-24100 Pulawy, Poland	Minta, M (corresponding author), National Veterinary Institute - National Research Institute, Dept Pharmacol & Toxicol, Al Partyzantow 57, PL-24100 Pulawy, Poland	Country: Poland<br>Institution: Natl Inst Vet Res	Country: Poland<br>Institution: National Veterinary Institute - National Research Institute

Record 636 | WOS:000261056700090

Safety and Mutagenicity Evaluation of Nanoparticulate Red Mold Rice

Field	Position	Original line	Normalised line	Original value	Normalised value	Original country / institution	Normalised country / institution

C1	1	91551	91551	[Yu, Chiun-Chieh; Lee, Chun-Lin; Pan, Tzu-Ming] Natl Taiwan Univ, Inst Microbiol & Biochem, Taipei 10764, Taiwan.	[Yu, Chiun-Chieh; Lee, Chun-Lin; Pan, Tzu-Ming] National Taiwan University, Inst Microbiol & Biochem, Taipei 10764, Taiwan.	Country: Taiwan<br>Institution: Natl Taiwan Univ	Country: Taiwan<br>Institution: National Taiwan University
RP	1	91555	91555	Pan, TM (corresponding author), Natl Taiwan Univ, Inst Microbiol & Biochem, Taipei 10764, Taiwan	Pan, TM (corresponding author), National Taiwan University, Inst Microbiol & Biochem, Taipei 10764, Taiwan	Country: Taiwan<br>Institution: Natl Taiwan Univ	Country: Taiwan<br>Institution: National Taiwan University

Record 637 | WOS:000207684500010

Oxidative stress induced by ochratoxin A in LLC-PK1 cell line and the chemoprotective effects of carnosic acid

Field	Position	Original line	Normalised line	Original value	Normalised value	Original country / institution	Normalised country / institution
C1	1	91667	91667	[Costa, S.; Utan, A.; Speroni, E.; Guerra, M. C.] Univ Bologna, Dept Pharmacol, I-40126 Bologna, Italy.	[Costa, S.; Utan, A.; Speroni, E.; Guerra, M. C.] University of Bologna, Dept Pharmacol, I-40126 Bologna, Italy.	Country: Italy<br>Institution: Univ Bologna	Country: Italy<br>Institution: University of Bologna
C1	2	91668	91668	[Cervellati, R.] Univ Bologna, Dept Chem, I-40126 Bologna, Italy.	[Cervellati, R.] University of Bologna, Dept Chem, I-40126 Bologna, Italy.	Country: Italy<br>Institution: Univ Bologna	Country: Italy<br>Institution: University of Bologna
RP	1	91672	91672	Costa, S (corresponding author), Univ Bologna, Dept Pharmacol, Via Irnerio 48, I-40126 Bologna, Italy	Costa, S (corresponding author), University of Bologna, Dept Pharmacol, Via Irnerio 48, I-40126 Bologna, Italy	Country: Italy<br>Institution: Univ Bologna	Country: Italy<br>Institution: University of Bologna

Record 638 | WOS:000260367000001

Individual and combined effects of ochratoxin A and citrinin on viability and DNA fragmentation in cultured Vero cells and on chromosome aberrations in mice bone marrow cells

No C1/C3/RP normalisation changes detected for this record.

Record 639 | WOS:000259798900005

Inula crithmoides extract protects against ochratoxin A-induced oxidative stress, clastogenic and mutagenic alterations in male rats

No C1/C3/RP normalisation changes detected for this record.

Record 640 | WOS:000260145000006

Differential modification of inflammatory enzymes in J774A.1 macrophages by ochratoxin A alone or in combination with lipopolysaccharide

Field	Position	Original line	Normalised line	Original value	Normalised value	Original country / institution	Normalised country / institution
C1	1	92018	92018	[Raso, G. Mattace; Esposito, E.; Iacono, A.; Meli, R.] Univ Naples Federico 2, Dept Expt Pharmacol, I-80131 Naples, Italy.	[Raso, G. Mattace; Esposito, E.; Iacono, A.; Meli, R.] University of Naples Federico II, Dept Expt Pharmacol, I-80131 Naples, Italy.	Country: Italy<br>Institution: Univ Naples Federico 2	Country: Italy<br>Institution: University of Naples Federico II
C1	2	92019	92019	[Bilancione, M.] Univ Naples Federico 2, Dept Pathol & Anim Hlth, I-80137 Naples, Italy.	[Bilancione, M.] University of Naples Federico II, Dept Pathol & Anim Hlth, I-80137 Naples, Italy.	Country: Italy<br>Institution: Univ Naples Federico 2	Country: Italy<br>Institution: University of Naples Federico II

RP	1	92021	92021	Meli, R (corresponding author), Univ Naples Federico 2, Dept Expt Pharmacol, Via D Montesano 49, I-80131 Naples, Italy	Meli, R (corresponding author), University of Naples Federico II, Dept Expt Pharmacol, Via D Montesano 49, I-80131 Naples, Italy	Country: Italy Institution: Univ Naples Federico 2	Country: Italy Institution: University of Naples Federico II

Record 641 | WOS:000258440100010

Effect of ochratoxin A on redox-regulated transcription factors, antioxidant enzymes and glutathione-S-transferase in cultured kidney tubulus cells

Field	Position	Original line	Normalised line	Original value	Normalised value	Original country / institution	Normalised country / institution
C1	1	92137	92137	[Boesch-Saadatmandi, C.; Huebbe, P.; Rimbach, G.] Univ Kiel, Inst Human Nutr & Food Sci, D-24118 Kiel, Germany.	[Boesch-Saadatmandi, C.; Huebbe, P.; Rimbach, G.] University of Kiel, Inst Human Nutr & Food Sci, D-24118 Kiel, Germany.	Country: Germany Institution: Univ Kiel	Country: Germany Institution: University of Kiel
C1	2	92138	92138	[Loboda, A.; Jozkowicz, A.; Dulak, J.] Jagiellonian Univ, Fac Biochem Biophys & Biotechnol, Dept Med Biotechnol, Krakow, Poland.	[Loboda, A.; Jozkowicz, A.; Dulak, J.] Jagiellonian University, Fac Biochem Biophys & Biotechnol, Dept Med Biotechnol, Krakow, Poland.	Country: Poland Institution: Jagiellonian Univ	Country: Poland Institution: Jagiellonian University
C1	3	92139	92139	[Blank, R.; Wolffram, S.] Univ Kiel, Inst Anim Nutr & Physiol, D-24118 Kiel, Germany.	[Blank, R.; Wolffram, S.] University of Kiel, Inst Anim Nutr & Physiol, D-24118 Kiel, Germany.	Country: Germany Institution: Univ Kiel	Country: Germany Institution: University of Kiel
RP	1	92141	92141	Rimbach, G (corresponding author), Univ Kiel, Inst Human Nutr & Food Sci, Herrman Rodewald Str 6, D-24118 Kiel, Germany	Rimbach, G (corresponding author), University of Kiel, Inst Human Nutr & Food Sci, Herrman Rodewald Str 6, D-24118 Kiel, Germany	Country: Germany Institution: Univ Kiel	Country: Germany Institution: University of Kiel

Record 642 | WOS:000257645600013

Species-specific toxicity of aristolochic acid (AA) in vitro

Field	Position	Original line	Normalised line	Original value	Normalised value	Original country / institution	Normalised country / institution
C1	1	92256	92256	[Huljic, S.; Bruske, E. I.; Pfitzenmaier, N.; O'Brien, E.; Dietrich, D. R.] Univ Konstanz, Fac Biol, D-78457 Constance, Germany.	[Huljic, S.; Bruske, E. I.; Pfitzenmaier, N.; O'Brien, E.; Dietrich, D. R.] University of Konstanz, Fac Biol, D-78457 Constance, Germany.	Country: Germany Institution: Univ Konstanz	Country: Germany Institution: University of Konstanz
RP	1	92258	92258	Dietrich, DR (corresponding author), Univ Konstanz, Fac Biol, Jacob Burckhardtstr 25, D-78457 Constance, Germany	Dietrich, DR (corresponding author), University of Konstanz, Fac Biol, Jacob Burckhardtstr 25, D-78457 Constance, Germany	Country: Germany Institution: Univ Konstanz	Country: Germany Institution: University of Konstanz

Record 643 | WOS:000257721400033

Identification and in vitro cytotoxicity of ochratoxin a degradation products formed during coffee roasting

Field	Position	Original line	Normalised line	Original value	Normalised value	Original country / institution	Normalised country / institution
C1	1	92351	92351	[Cramer, Benedikt; Koenigs, Maika; Humpf, Hans-Ulrich] Univ Munster, Inst Lebensmittelchem, D-48149 Munster, Germany.	[Cramer, Benedikt; Koenigs, Maika; Humpf, Hans-Ulrich] University of Munster, Inst Lebensmittelchem, D-48149 Munster, Germany.	Country: Germany Institution: Univ Munster	Country: Germany Institution: University of Munster

RP	1	92353	92353	Humpf, HU (corresponding author), Univ Munster, Inst Lebensmittelchem, Corrensstr 45, D-48149 Munster, Germany	Humpf, HU (corresponding author), University of Munster, Inst Lebensmittelchem, Corrensstr 45, D-48149 Munster, Germany	Country: Germany Institution: Univ Munster	Country: Germany Institution: University of Munster

Record 644 | WOS:000257577500008

Gene expression changes induced by ochratoxin A in renal and hepatic tissues of male F344 rat after oral repeated administration

Field	Position	Original line	Normalised line	Original value	Normalised value	Original country / institution	Normalised country / institution
C1	1	92447	92447	[Arbillaga, Leire; Vettorazzi, Ariane; Gil, Ana G.; de Cerain, Adela Lopez] Univ Navarra, Dept Nutr & Food Sci, Fac Pharm, Pamplona 31008, Spain.	[Arbillaga, Leire; Vettorazzi, Ariane; Gil, Ana G.; de Cerain, Adela Lopez] University of Navarra, Dept Nutr & Food Sci, Fac Pharm, Pamplona 31008, Spain.	Country: Spain Institution: Univ Navarra	Country: Spain Institution: University of Navarra
C1	2	92448	92448	[van Delft, Joost H. M.] Maastricht Univ, Dept Hlth Risk Anal & Toxicol, Fac Hlth Sci, NL-6229 ER Maastricht, Netherlands.	[van Delft, Joost H. M.] Maastricht University, Dept Hlth Risk Anal & Toxicol, Fac Hlth Sci, NL-6229 ER Maastricht, Netherlands.	Country: Netherlands Institution: Maastricht Univ	Country: Netherlands Institution: Maastricht University
C1	3	92449	92449	[Garcia-Jalon, Jose Antonio] Univ Zaragoza, Dept Anim Pathol, Fac Vet, Zaragoza, Spain.	[Garcia-Jalon, Jose Antonio] University of Zaragoza, Dept Anim Pathol, Fac Vet, Zaragoza, Spain.	Country: Spain Institution: Univ Zaragoza	Country: Spain Institution: University of Zaragoza
RP	1	92451	92451	de Cerain, AL (corresponding author), Univ Navarra, Dept Nutr & Food Sci, Fac Pharm, C Irunlarrea 1, Pamplona 31008, Spain	de Cerain, AL (corresponding author), University of Navarra, Dept Nutr & Food Sci, Fac Pharm, C Irunlarrea 1, Pamplona 31008, Spain	Country: Spain Institution: Univ Navarra	Country: Spain Institution: University of Navarra

Record 645 | WOS:000256601000019

Structure-activity relationships for the fluorescence of ochratoxin A: Insight for detection of ochratoxin A metabolites

Field	Position	Original line	Normalised line	Original value	Normalised value	Original country / institution	Normalised country / institution
C1	1	92556	92556	[Tozlovanu, Mariana; Maud, Juzio B.; Pfohl-Leszkowicz, Annie] CNRS, UMR 5503, ENSAT, F-31326 Auzeville Tolosane, France.	[Tozlovanu, Mariana; Maud, Juzio B.; Pfohl-Leszkowicz, Annie] Centre National de la Recherche Scientifique (CNRS), UMR 5503, ENSAT, F-31326 Auzeville Tolosane, France.	Country: France Institution: CNRS	Country: France Institution: Centre National de la Recherche Scientifique (CNRS)
C1	2	92557	92557	[Frenette, Christine; Paugh, Robert J.; Manderville, Richard. A.] Univ Guelph, Dept Chem & Toxicol, Guelph, ON N1G 2W1, Canada.	[Frenette, Christine; Paugh, Robert J.; Manderville, Richard. A.] University of Guelph, Dept Chem & Toxicol, Guelph, ON N1G 2W1, Canada.	Country: Canada Institution: Univ Guelph	Country: Canada Institution: University of Guelph
RP	1	92563	92563	Pfohl-Leszkowicz, A (corresponding author), CNRS, UMR 5503, ENSAT, 1 Ave Agrobiopole, F-31326 Auzeville Tolosane, France	Pfohl-Leszkowicz, A (corresponding author), Centre National de la Recherche Scientifique (CNRS), UMR 5503, ENSAT, 1 Ave Agrobiopole, F-31326 Auzeville Tolosane, France	Country: France Institution: CNRS	Country: France Institution: Centre National de la Recherche Scientifique (CNRS)

Record 646 | WOS:000256806600009

In vitro cytogenetic results supporting a DNA nonreactive mechanism for Ochratoxin A, potentially relevant for its carcinogenicity

Field	Position	Original line	Normalised line	Original value	Normalised value	Original country / institution	Normalised country / institution
C1	1	92652	92652	[Mosesso, Pasquale] Univ Tuscia, Dipartimento Agrobiol & Agrochim, I-01100 Viterbo, Italy.	[Mosesso, Pasquale] Tuscia University, Dipartimento Agrobiol & Agrochim, I-01100 Viterbo, Italy.	Country: Italy<br>Institution: Univ Tuscia	Country: Italy<br>Institution: Tuscia University
RP	1	92656	92656	Mosesso, P (corresponding author), Univ Tuscia, Dipartimento Agrobiol & Agrochim, Via San Camillo de Lellis Snc, I-01100 Viterbo, Italy	Mosesso, P (corresponding author), Tuscia University, Dipartimento Agrobiol & Agrochim, Via San Camillo de Lellis Snc, I-01100 Viterbo, Italy	Country: Italy<br>Institution: Univ Tuscia	Country: Italy<br>Institution: Tuscia University

Record 647 | WOS:000256206000004

Modulating effects of fumonisin B1 and ochratoxin A on leukocytes and messenger cytokines of the human immune system

Field	Position	Original line	Normalised line	Original value	Normalised value	Original country / institution	Normalised country / institution
C1	1	92777	92777	[Bhoola, Kand D.] Univ Western Australia, Lung Inst Western Australia, Perth, WA 6009, Australia.	[Bhoola, Kand D.] University of Western Australia, Lung Inst Western Australia, Perth, WA 6009, Australia.	Country: Australia<br>Institution: Univ Western Australia	Country: Australia<br>Institution: University of Western Australia
C1	2	92778	92778	[Bhoola, Kand D.] Univ Western Australia, Ctr Asthma Allergy & Resp Res, Perth, WA 6009, Australia.	[Bhoola, Kand D.] University of Western Australia, Ctr Asthma Allergy & Resp Res, Perth, WA 6009, Australia.	Country: Australia<br>Institution: Univ Western Australia	Country: Australia<br>Institution: University of Western Australia
C1	3	92779	92779	[Odhav, Bhard] Durban Univ Technol, Dept Biotechnol, Durban, South Africa.	[Odhav, Bhard] Durban University of Technology, Dept Biotechnol, Durban, South Africa.	Country: South Africa<br>Institution: Durban Univ Technol	Country: South Africa<br>Institution: Durban University of Technology
C1	4	92780	92780	[Adam, Jamila K.] Durban Univ Technol, Dept Clin Technol, Durban, South Africa.	[Adam, Jamila K.] Durban University of Technology, Dept Clin Technol, Durban, South Africa.	Country: South Africa<br>Institution: Durban Univ Technol	Country: South Africa<br>Institution: Durban University of Technology
RP	1	92783	92783	Bhoola, KD (corresponding author), Univ Western Australia, Lung Inst Western Australia, Perth, WA 6009, Australia	Bhoola, KD (corresponding author), University of Western Australia, Lung Inst Western Australia, Perth, WA 6009, Australia	Country: Australia<br>Institution: Univ Western Australia	Country: Australia<br>Institution: University of Western Australia

Record 648 | WOS:000255760800015

Evaluation of putative biomarkers of nephrotoxicity after exposure to ochratoxin a in vivo and in vitro

Field	Position	Original line	Normalised line	Original value	Normalised value	Original country / institution	Normalised country / institution
C1	1	92866	92866	[Rached, Eva; Hoffmann, Dana; Dekant, Wolfgang; Mally, Angela] Univ Wurzburg, Dept Toxicol, D-97078 Wurzburg, Germany.	[Rached, Eva; Hoffmann, Dana; Dekant, Wolfgang; Mally, Angela] University of Wurzburg, Dept Toxicol, D-97078 Wurzburg, Germany.	Country: Germany<br>Institution: Univ Wurzburg	Country: Germany<br>Institution: University of Wurzburg
RP	1	92870	92870	Mally, A (corresponding author), Univ Wurzburg, Dept Toxicol, Versbacher Str 9, D-97078 Wurzburg, Germany	Mally, A (corresponding author), University of Wurzburg, Dept Toxicol, Versbacher Str 9, D-97078 Wurzburg, Germany	Country: Germany<br>Institution: Univ Wurzburg	Country: Germany<br>Institution: University of Wurzburg

Record 649 | WOS:000256713600009

Ochratoxin A inhibits the production of tissue factor and plasminogen activator inhibitor-2 by human blood mononuclear cells: Another potential mechanism of immune-suppression

No C1/C3/RP normalisation changes detected for this record.

Record 650 | WOS:000256381400006

Medical relevance of mycotoxins

No C1/C3/RP normalisation changes detected for this record.

Record 651 | WOS:000254689700022

Detoxification of patulin and ochratoxin A, two abundant mycotoxins, by lactic acid bacteria

Field	Position	Original line	Normalised line	Original value	Normalised value	Original country / institution	Normalised country / institution
C1	1	93144	93144	[Fuchs, S.; Sontag, G.; Stidl, R.] Univ Vienna, Fac Chem, Dept Analyt & Food Chem, Vienna, Austria.	[Fuchs, S.; Sontag, G.; Stidl, R.] University of Vienna, Fac Chem, Dept Analyt & Food Chem, Vienna, Austria.	Country: Austria<br>Institution: Univ Vienna	Country: Austria<br>Institution: University of Vienna
C1	2	93145	93145	[Ehrlich, V.; Knasmueller, S.] Med Univ Vienna, Dept Med 1, Inst Canc Res, Vienna, Austria.	[Ehrlich, V.; Knasmueller, S.] Medical University of Vienna, Dept Med 1, Inst Canc Res, Vienna, Austria.	Country: Austria<br>Institution: Med Univ Vienna	Country: Austria<br>Institution: Medical University of Vienna
C1	3	93146	93146	[Kundi, M.] Med Univ Vienna, Inst Environm Ctr Publ Hlth, Vienna, Austria.	[Kundi, M.] Medical University of Vienna, Inst Environm Ctr Publ Hlth, Vienna, Austria.	Country: Austria<br>Institution: Med Univ Vienna	Country: Austria<br>Institution: Medical University of Vienna
RP	1	93149	93149	Ehrlich, V (corresponding author), Univ Vienna, Fac Chem, Dept Analyt & Food Chem, Vienna, Austria	Ehrlich, V (corresponding author), University of Vienna, Fac Chem, Dept Analyt & Food Chem, Vienna, Austria	Country: Austria<br>Institution: Univ Vienna	Country: Austria<br>Institution: University of Vienna

Record 652 | WOS:000254544300011

Both direct and indirect effects account for the pro-inflammatory activity of enteropathogenic mycotoxins on the human intestinal epithelium:: Stimulation of interleukin-8 secretion, potentiation of interleukin-1Î² effect and increase in the transepithelial passage of commensal bacteria

No C1/C3/RP normalisation changes detected for this record.

Record 653 | WOS:000257197500001

Risk-benefit considerations of mitigation measures on acrylamide content of foods - A case study on potatoes, cereals and coffee

Field	Position	Original line	Normalised line	Original value	Normalised value	Original country / institution	Normalised country / institution
C1	1	93427	93427	[Seal, C. J.; Wilcockson, S. J.] Newcastle Univ, Sch Agr Food & Rural Dev, Newcastle Upon Tyne NE1 7RU, Tyne & Wear, England.	[Seal, C. J.; Wilcockson, S. J.] Newcastle Univ, Sch Agr Food & Rural Dev, Newcastle Upon Tyne NE1 7RU, Tyne & Wear, United Kingdom.	Country: England<br>Institution: Newcastle Univ	Country: United Kingdom<br>Institution: Newcastle Univ
C1	6	93432	93432	[Lalljie, S. P. D.] Unilever Res Labs Colworth, Colworth	[Lalljie, S. P. D.] Unilever Res Labs Colworth, Colworth	Country: England<br>Institution: Unilever Res Labs Colworth	Country: United Kingdom<br>Institution: Unilever Res Labs Colworth

				House, Sharnbrook MK44 1LQ, Beds, England.	House, Sharnbrook MK44 1LQ, Beds, United Kingdom.		
C1	8	93434	93434	[Scholz, G.] Nestle Res Ctr, CH-1000 Lausanne 26, Switzerland.	[Scholz, G.] Nestle Research Center, CH-1000 Lausanne 26, Switzerland.	Country: Switzerland<br>Institution: Nestle Res Ctr	Country: Switzerland<br>Institution: Nestle Research Center
C1	11	93437	93437	[van Boekel, M.] Wageningen Univ, Dept Agrotechnol & Food Sci, NL-6700 EV Wageningen, Netherlands.	[van Boekel, M.] Wageningen University & Research, Dept Agrotechnol & Food Sci, NL-6700 EV Wageningen, Netherlands.	Country: Netherlands<br>Institution: Wageningen Univ	Country: Netherlands<br>Institution: Wageningen University & Research
C3	1	93439	93439	Newcastle University - UK	Newcastle University - United Kingdom	Institution: Newcastle University - UK	Institution: Newcastle University - United Kingdom
RP	1	93444	93444	Seal, CJ (corresponding author), Newcastle Univ, Sch Agr Food & Rural Dev, Newcastle Upon Tyne NE1 7RU, Tyne & Wear, England	Seal, CJ (corresponding author), Newcastle Univ, Sch Agr Food & Rural Dev, Newcastle Upon Tyne NE1 7RU, Tyne & Wear, United Kingdom	Country: England<br>Institution: Newcastle Univ	Country: United Kingdom<br>Institution: Newcastle Univ

Record 654 | WOS:000258542600115

In vitro investigation of individual and combined cytotoxicity of ochratoxin a and arsenic in Chinese hamster lung fibroblasts and human urothelial cells

Field	Position	Original line	Normalised line	Original value	Normalised value	Original country / institution	Normalised country / institution
C1	2	93787	93787	[Aggarwal, M.; Malik, J. K.] Indian Vet Res Inst, Div Pharmacol & Toxicol, Izatnagar 243122, Uttar Pradesh, India.	[Aggarwal, M.; Malik, J. K.] ICAR - Indian Veterinary Research Institute, Div Pharmacol & Toxicol, Izatnagar 243122, Uttar Pradesh, India.	Country: India<br>Institution: Indian Vet Res Inst	Country: India<br>Institution: ICAR - Indian Veterinary Research Institute

Record 655 | WOS:000252706500006

Ameliorative effect of artichoke (Cynara scolymus L.) extracts on creatine monohydrate-induced renal dysfunction in male rats

No C1/C3/RP normalisation changes detected for this record.

Record 656 | WOS:000255988000006

Cytotoxicity and oxidative damage in kidney cells exposed to the mycotoxins ochratoxin A and citrinin: Individual and combined effects

No C1/C3/RP normalisation changes detected for this record.

Record 657 | WOS:000253659900006

Mycotoxins in cattle feeds and carry-over to dairy milk: A review

Field	Position	Original line	Normalised line	Original value	Normalised value	Original country / institution	Normalised country / institution
C1	1	94077	94077	Univ Utrecht, Div Vet Pharmacol Pharm & Toxicol, Fac Vet Med, Utrecht, Netherlands.	Utrecht University, Div Vet Pharmacol Pharm & Toxicol, Fac Vet Med, Utrecht, Netherlands.	Country: Netherlands<br>Institution: Univ Utrecht	Country: Netherlands<br>Institution: Utrecht University
RP	1	94079	94079	Fink-Gremmels, J (corresponding author), Univ Utrecht, Div Vet Pharmacol Pharm & Toxicol, Fac Vet Med, Utrecht, Netherlands	Fink-Gremmels, J (corresponding author), Utrecht University, Div Vet Pharmacol Pharm & Toxicol, Fac Vet Med, Utrecht, Netherlands	Country: Netherlands<br>Institution: Univ Utrecht	Country: Netherlands<br>Institution: Utrecht University

Record 658 | WOS:000268886600003

Combined Toxic Effects of Ochratoxin A and Citrinin, In Vivo and In Vitro

No C1/C3/RP normalisation changes detected for this record.

Record 659 | WOS:000251052700012

Toxigenic fungi and mycotoxins in mature corn silage

No C1/C3/RP normalisation changes detected for this record.

Record 660 | WOS:000249598500015

Catechins: natural free-radical scavengers against ochratoxin A-induced cell damage in a pig kidney cell line (LLC-PK1)

Field	Position	Original line	Normalised line	Original value	Normalised value	Original country / institution	Normalised country / institution
C1	1	94404	94404	Univ Bologna, Dept Pharmacol, I-40126 Bologna, Italy. Univ Bologna, Dept Chem G Ciamician, I-40126 Bologna, Italy.	University of Bologna, Dept Pharmacol, I-40126 Bologna, Italy. University of Bologna, Dept Chem G Ciamician, I-40126 Bologna, Italy.	Country: Italy<br>Institution: Univ Bologna	Country: Italy<br>Institution: University of Bologna
RP	1	94407	94407	Costa, S (corresponding author), Univ Bologna, Dept Pharmacol, Via Irnerio 48, I-40126 Bologna, Italy	Costa, S (corresponding author), University of Bologna, Dept Pharmacol, Via Irnerio 48, I-40126 Bologna, Italy	Country: Italy<br>Institution: Univ Bologna	Country: Italy<br>Institution: University of Bologna

Record 661 | WOS:000249983300013

New molecular and field evidences for the implication of mycotoxins but not aristolochic acid in human nephropathy and urinary tract tumor

Field	Position	Original line	Normalised line	Original value	Normalised value	Original country / institution	Normalised country / institution
C1	1	94510	94510	ENSAT, CNRS 5503, UMR, F-31326 Castanet Tolosan, France. ENSA, INP, CNRS, UPS 5503,Lab Genie Chim,UMR, Toulouse, France. Univ Guelph, Dept Chem, Guelph, ON N1G 2W1, Canada. Inst Med Res & Occupat Hlth, Zagreb, Croatia. Les Collanges, St Jean Chambre, France. Fac Med, Inst Nephrol, Nish, Serbia.	ENSAT, CNRS 5503, UMR, F-31326 Castanet Tolosan, France. ENSA, INP, CNRS, UPS 5503,Lab Genie Chim,UMR, Toulouse, France. University of Guelph, Dept Chem, Guelph, ON N1G 2W1, Canada. Institute for Medical Research & Occupational Health (IMROH), Zagreb, Croatia. Les Collanges, St Jean Chambre, France. Fac Med, Inst Nephrol, Nish, Serbia.	Country: Serbia<br>Institution: ENSAT	Country: Serbia<br>Institution: ENSAT

Record 662 | WOS:000248014300007

Ochratoxin A-induced mutagenesis in mammalian cells is consistent with the production of oxidative stress

Field	Position	Original line	Normalised line	Original value	Normalised value	Original country / institution	Normalised country / institution
C3	2	94636	94636	National Institutes of Health (NIH) - USA	National Institutes of Health (NIH)	Institution: National Institutes of Health (NIH) - USA	Institution: National Institutes of Health (NIH)

Record 663 | WOS:000247601800018

Adult hippocampal neural stem/progenitor cells in vitro are vulnerable to the mycotoxin ochratoxin-A

No C1/C3/RP normalisation changes detected for this record.

Record 664 | WOS:000246927300041

Ochratoxin A displaces claudins from detergent resistant membrane microdomains

Field	Position	Original line	Normalised line	Original value	Normalised value	Original country / institution	Normalised country / institution
C1	1	94887	94887	Univ Manchester, Sch Med, Sect Gastrointestinal Sect, Manchester, Lancs, England.	University of Manchester, Sch Med, Sect Gastrointestinal Sect, Manchester, Lancs, United Kingdom.	Country: England<br>Institution: Univ Manchester	Country: United Kingdom<br>Institution: University of Manchester
RP	1	94889	94889	O'Neill, CA (corresponding author), Univ Manchester, Sch Med, Sect Gastrointestinal Sect, Stopford Bldg,Oxfors Rd, Manchester, Lancs, England	O'Neill, CA (corresponding author), University of Manchester, Sch Med, Sect Gastrointestinal Sect, Stopford Bldg,Oxfors Rd, Manchester, Lancs, United Kingdom	Country: England<br>Institution: Univ Manchester	Country: United Kingdom<br>Institution: University of Manchester

Record 665 | WOS:000247178200008

Ochratoxin A:: 13-week oral toxicity and cell proliferation in male F344/N rats

Field	Position	Original line	Normalised line	Original value	Normalised value	Original country / institution	Normalised country / institution
C1	1	94975	94975	Univ Wurzburg, Dept Toxicol, D-97078 Wurzburg, Germany. RCC Ltd, CH-4414 Fullinsdorf, Switzerland. RCC Ltd, CH-4452 Itingen, Switzerland. Univ Wurzburg, Dept Surg, D-97078 Wurzburg, Germany.	University of Wurzburg, Dept Toxicol, D-97078 Wurzburg, Germany. RCC Ltd, CH-4414 Fullinsdorf, Switzerland. RCC Ltd, CH-4452 Itingen, Switzerland. University of Wurzburg, Dept Surg, D-97078 Wurzburg, Germany.	Country: Germany<br>Institution: Univ Wurzburg	Country: Germany<br>Institution: University of Wurzburg
RP	1	94980	94980	Mally, A (corresponding author), Univ Wurzburg, Dept Toxicol, Versbacher Str 9, D-97078 Wurzburg, Germany	Mally, A (corresponding author), University of Wurzburg, Dept Toxicol, Versbacher Str 9, D-97078 Wurzburg, Germany	Country: Germany<br>Institution: Univ Wurzburg	Country: Germany<br>Institution: University of Wurzburg

Record 666 | WOS:000247092600009

Transepithelial transport of fusariotoxin nivalenol: Mediation of secretion by ABC transporters

No C1/C3/RP normalisation changes detected for this record.

Record 667 | WOS:000246213800014

Production and characterization of monoclonal antibodies against ochratoxin B

Field	Position	Original line	Normalised line	Original value	Normalised value	Original country / institution	Normalised country / institution

C1	1	95179	95179	Univ Konstanz, D-78457 Constance, Germany. Univ Pittsburgh, Pittsburgh, PA 15261 USA.	University of Konstanz, D-78457 Constance, Germany. University of Pittsburgh, Pittsburgh, PA 15261 USA.	Country: USA<br>Institution: Univ Konstanz	Country: USA<br>Institution: University of Konstanz
RP	1	95183	95183	O'Brien, E (corresponding author), Univ Konstanz, POB X-918, D-78457 Constance, Germany	O'Brien, E (corresponding author), University of Konstanz, POB X-918, D-78457 Constance, Germany	Country: Germany<br>Institution: Univ Konstanz	Country: Germany<br>Institution: University of Konstanz

Record 668 | WOS:000246875200005

Combined effects of mycotoxin mixtures on human T cell function

Field	Position	Original line	Normalised line	Original value	Normalised value	Original country / institution	Normalised country / institution
C1	1	95271	95271	UFZ Helmholtz Ctr Environm Res, Helmholtz Ctr Environm Res, Dept Environm Immunol, D-04318 Leipzig, Germany. Univ Leipzig, Inst Pharm, D-7010 Leipzig, Germany. UFZ Helmholtz Ctr Environm Res, Helmholtz Ctr Environm Res, Dept Bioanalyt Ecotoxicol, D-04318 Leipzig, Germany.	UFZ Helmholtz Ctr Environm Res, Helmholtz Ctr Environm Res, Dept Environm Immunol, D-04318 Leipzig, Germany. Leipzig University, Inst Pharm, D-7010 Leipzig, Germany. UFZ Helmholtz Ctr Environm Res, Helmholtz Ctr Environm Res, Dept Bioanalyt Ecotoxicol, D-04318 Leipzig, Germany.	Country: Germany<br>Institution: UFZ Helmholtz Ctr Environm Res	Country: Germany<br>Institution: UFZ Helmholtz Ctr Environm Res

Record 669 | WOS:000245707900011

In vitro gene expression data supporting a DNA non-reactive genotoxic mechanism for ochratoxin A

Field	Position	Original line	Normalised line	Original value	Normalised value	Original country / institution	Normalised country / institution
C1	1	95374	95374	Univ Navarra, Fac Pharm, Dept Food Sci & Toxicol, Pamplona 31008, Spain. Maastricht Univ, Fac Hlth Sci, Dept Hlth Risk Anal & Toxicol, NL-6229 ER Maastricht, Netherlands.	University of Navarra, Fac Pharm, Dept Food Sci & Toxicol, Pamplona 31008, Spain. Maastricht University, Fac Hlth Sci, Dept Hlth Risk Anal & Toxicol, NL-6229 ER Maastricht, Netherlands.	Country: Netherlands<br>Institution: Univ Navarra	Country: Netherlands<br>Institution: University of Navarra
RP	1	95377	95377	de Cerain, AL (corresponding author), Univ Navarra, Fac Pharm, Dept Food Sci & Toxicol, C-Irunlarrea 1, Pamplona 31008, Spain	de Cerain, AL (corresponding author), University of Navarra, Fac Pharm, Dept Food Sci & Toxicol, C-Irunlarrea 1, Pamplona 31008, Spain	Country: Spain<br>Institution: Univ Navarra	Country: Spain<br>Institution: University of Navarra

Record 670 | WOS:000244262900005

Reduction in antioxidant defenses may contribute to ochratoxin a toxicity and carcinogenicity

Field	Position	Original line	Normalised line	Original value	Normalised value	Original country / institution	Normalised country / institution
C1	1	95500	95500	Nestle Res Ctr, Qual & Safety Dept, CH-1000 Lausanne 26, Switzerland. Univ Dundee, Biomed Res Ctr, Dundee DD1 9SY, Scotland. UCL, Nephrourol Unit, Inst Child Hlth, London WC1E 1EH, England. Univ London Imperial Coll Sci Technol & Med, Dept Environm	Nestle Research Center, Qual & Safety Dept, CH-1000 Lausanne 26, Switzerland. University of Dundee, Biomed Res Ctr, Dundee DD1 9SY, United Kingdom. UCL, Nephrourol Unit, Inst Child Hlth, London WC1E 1EH, United Kingdom. Univ London Imperial Coll Sci	Country: England<br>Institution: Nestle Res Ctr	Country: United Kingdom<br>Institution: Nestle Research Center

				Sci & Technol, London SW7 2AZ, England.	Technol & Med, Dept Environm Sci & Technol, London SW7 2AZ, United Kingdom.		
RP	1	95506	95506	Cavin, C (corresponding author), Nestle Res Ctr, Qual & Safety Dept, POB 44, CH-1000 Lausanne 26, Switzerland	Cavin, C (corresponding author), Nestle Research Center, Qual & Safety Dept, POB 44, CH-1000 Lausanne 26, Switzerland	Country: Switzerland<br>Institution: Nestle Res Ctr	Country: Switzerland<br>Institution: Nestle Research Center

Record 671 | WOS:000244244100003

Lipid peroxidation and glutathione levels in porcine kidney PK15 cells after individual and combined treatment with fumonisin B1, beauvericin and ochratoxin A

Field	Position	Original line	Normalised line	Original value	Normalised value	Original country / institution	Normalised country / institution
C1	1	95610	95610	Univ Zagreb, Fac Pharm & Biochem, Dept Microbiol, HR-1000 Zagreb, Croatia. Inst Med Res & Occupat Hlth, Toxicol Unit, Zagreb, Croatia.	University of Zagreb, Fac Pharm & Biochem, Dept Microbiol, HR-1000 Zagreb, Croatia. Institute for Medical Research & Occupational Health (IMROH), Toxicol Unit, Zagreb, Croatia.	Country: Croatia<br>Institution: Univ Zagreb	Country: Croatia<br>Institution: University of Zagreb
RP	1	95614	95614	Klaric, MS (corresponding author), Univ Zagreb, Fac Pharm & Biochem, Dept Microbiol, Schrottova 39, HR-1000 Zagreb, Croatia	Klaric, MS (corresponding author), University of Zagreb, Fac Pharm & Biochem, Dept Microbiol, Schrottova 39, HR-1000 Zagreb, Croatia	Country: Croatia<br>Institution: Univ Zagreb	Country: Croatia<br>Institution: University of Zagreb

Record 672 | WOS:000244169500009

Effects of repeated ochratoxin exposure on renal cells in vitro

Field	Position	Original line	Normalised line	Original value	Normalised value	Original country / institution	Normalised country / institution
C1	1	95708	95708	Univ Konstanz, D-78457 Constance, Germany.	University of Konstanz, D-78457 Constance, Germany.	Country: Germany<br>Institution: Univ Konstanz	Country: Germany<br>Institution: University of Konstanz
RP	1	95710	95710	Dietrich, DR (corresponding author), Univ Konstanz, POB X-918, D-78457 Constance, Germany	Dietrich, DR (corresponding author), University of Konstanz, POB X-918, D-78457 Constance, Germany	Country: Germany<br>Institution: Univ Konstanz	Country: Germany<br>Institution: University of Konstanz

Record 673 | WOS:000243327000004

Oxidative DNA damage induced by Ochratoxin A in the HK-2 human kidney cell line: evidence of the relationship with cytotoxicity

Field	Position	Original line	Normalised line	Original value	Normalised value	Original country / institution	Normalised country / institution
C1	1	95787	95787	Univ Navarra, Fac Pharm, Dept Food Sci & Toxicol, Pamplona 31008, Spain.	University of Navarra, Fac Pharm, Dept Food Sci & Toxicol, Pamplona 31008, Spain.	Country: Spain<br>Institution: Univ Navarra	Country: Spain<br>Institution: University of Navarra
RP	1	95789	95789	de Cerain, AL (corresponding author), Univ Navarra, Fac Pharm, Dept Food Sci & Toxicol, C Irunlarrea 1, Pamplona 31008, Spain	de Cerain, AL (corresponding author), University of Navarra, Fac Pharm, Dept Food Sci & Toxicol, C Irunlarrea 1, Pamplona 31008, Spain	Country: Spain<br>Institution: Univ Navarra	Country: Spain<br>Institution: University of Navarra

Record 674 | WOS:000244790500003

Ochratoxin A: An overview on toxicity and carcinogenicity in animals and humans

Field	Position	Original line	Normalised line	Original value	Normalised value	Original country / institution	Normalised country / institution
C1	1	95899	95899	CNRS, Lab Genie Chim, UMR 5503, INPT,UPS,INP,ENSA Toulouse, F-31326 Auzeville Tolosane, France. Univ Guelph, Dept Chem, Guelph, ON N1G 2W1, Canada.	Centre National de la Recherche Scientifique (CNRS), Lab Genie Chim, UMR 5503, INPT,UPS,INP,ENSA Toulouse, F-31326 Auzeville Tolosane, France. University of Guelph, Dept Chem, Guelph, ON N1G 2W1, Canada.	Country: Canada<br>Institution: CNRS	Country: Canada<br>Institution: Centre National de la Recherche Scientifique (CNRS)
RP	1	95906	95906	Manderville, RA (corresponding author), CNRS, Lab Genie Chim, UMR 5503, INPT,UPS,INP,ENSA Toulouse, F-31326 Auzeville Tolosane, France	Manderville, RA (corresponding author), Centre National de la Recherche Scientifique (CNRS), Lab Genie Chim, UMR 5503, INPT,UPS,INP,ENSA Toulouse, F-31326 Auzeville Tolosane, France	Country: France<br>Institution: CNRS	Country: France<br>Institution: Centre National de la Recherche Scientifique (CNRS)

Record 675 | WOS:000254907600043

Cytotoxicity of feed-borne mycotoxins to animal cell lines in vitro using the MTT assay

Field	Position	Original line	Normalised line	Original value	Normalised value	Original country / institution	Normalised country / institution
C1	1	96449	96449	[Stec, Jan; Szczotka, Maria; Kuzmak, Jacek] Natl Inst Vet Res, PL-24100 Pulawy, Poland.	[Stec, Jan; Szczotka, Maria; Kuzmak, Jacek] National Veterinary Institute - National Research Institute, PL-24100 Pulawy, Poland.	Country: Poland<br>Institution: Natl Inst Vet Res	Country: Poland<br>Institution: National Veterinary Institute - National Research Institute
RP	1	96451	96451	Stec, J (corresponding author), Natl Inst Vet Res, PL-24100 Pulawy, Poland	Stec, J (corresponding author), National Veterinary Institute - National Research Institute, PL-24100 Pulawy, Poland	Country: Poland<br>Institution: Natl Inst Vet Res	Country: Poland<br>Institution: National Veterinary Institute - National Research Institute

Record 676 | WOS:000248276300289

Effect of Saccharomyces cerevisiae and esterified glucomannan on residues of Ochratoxin A in kidney, muscle and blood of laying hens

Field	Position	Original line	Normalised line	Original value	Normalised value	Original country / institution	Normalised country / institution
C1	1	96522	96522	Univ Bologna, Dipartimento Sanita Pubbl Vet & Patol Anim, I-40064 Ozzano Dell Emilia, Italy. Univ Bologna, Dipartimento Morfofisiol Vet & Prod Anim, I-40126 Bologna, Italy.	University of Bologna, Dipartimento Sanita Pubbl Vet & Patol Anim, I-40064 Ozzano Dell Emilia, Italy. University of Bologna, Dipartimento Morfofisiol Vet & Prod Anim, I-40126 Bologna, Italy.	Country: Italy<br>Institution: Univ Bologna	Country: Italy<br>Institution: University of Bologna
RP	1	96525	96525	Zaghini, A (corresponding author), Univ Bologna, Dipartimento Sanita Pubbl Vet & Patol Anim, Via Tolara Sopra 50, I-40064 Ozzano Dell Emilia, Italy	Zaghini, A (corresponding author), University of Bologna, Dipartimento Sanita Pubbl Vet & Patol Anim, Via Tolara Sopra 50, I-40064 Ozzano Dell Emilia, Italy	Country: Italy<br>Institution: Univ Bologna	Country: Italy<br>Institution: University of Bologna

Record 677 | WOS:000243441300001

Review on the toxicity, occurrence, metabolism, detoxification, regulations and intake of zearalenone:: An oestrogenic mycotoxin

Field	Position	Original line	Normalised line	Original value	Normalised value	Original country / institution	Normalised country / institution

C1	1	96592	96592	Univ Valencia, Fac Pharm, Lab Food Chem & Toxicol, E-46100 Valencia, Spain. Natl Inst Hlth, Lab Food Toxicol, Rabat, Morocco.	University of Valencia, Fac Pharm, Lab Food Chem & Toxicol, E-46100 Valencia, Spain. Natl Inst Hlth, Lab Food Toxicol, Rabat, Morocco.	Country: Morocco<br>Institution: Univ Valencia	Country: Morocco<br>Institution: University of Valencia
RP	1	96595	96595	Soriano, JM (corresponding author), Univ Valencia, Fac Pharm, Lab Food Chem & Toxicol, Av Vicent Andres Estelles S-N, E-46100 Valencia, Spain	Soriano, JM (corresponding author), University of Valencia, Fac Pharm, Lab Food Chem & Toxicol, Av Vicent Andres Estelles S-N, E-46100 Valencia, Spain	Country: Spain<br>Institution: Univ Valencia	Country: Spain<br>Institution: University of Valencia

Record 678 | WOS:000242136100016

Interactive effects of fumonisin B1 and Î±-zearalenol on proliferation and cytokine expression in Jurkat T cells

Field	Position	Original line	Normalised line	Original value	Normalised value	Original country / institution	Normalised country / institution
C1	1	96840	96840	CNR, Ist Sci Alimentaz, I-83100 Avellino, Italy. Univ Naples Federico II, Dept Pathol & Anim Hlth, Div Toxicol, I-80137 Naples, Italy. Univ Naples Federico II, Dept Vet Clin Sci, I-80137 Naples, Italy.	CNR, Ist Sci Alimentaz, I-83100 Avellino, Italy. University of Naples Federico II, Dept Pathol & Anim Hlth, Div Toxicol, I-80137 Naples, Italy. University of Naples Federico II, Dept Vet Clin Sci, I-80137 Naples, Italy.	Country: Italy<br>Institution: CNR	Country: Italy<br>Institution: CNR

Record 679 | WOS:000243461200014

Ochratoxin A-induced cytotoxicity in liver (HepG2) cells:: Impact of serum concentration, dietary antioxidants and glutathione-modulating compounds

Field	Position	Original line	Normalised line	Original value	Normalised value	Original country / institution	Normalised country / institution
C1	1	96950	96950	Univ Kiel, Inst Human Nutr & Food Sci, D-24118 Kiel, Germany. Univ Kiel, Inst Anim Nutr & Physiol, D-24118 Kiel, Germany.	University of Kiel, Inst Human Nutr & Food Sci, D-24118 Kiel, Germany. University of Kiel, Inst Anim Nutr & Physiol, D-24118 Kiel, Germany.	Country: Germany<br>Institution: Univ Kiel	Country: Germany<br>Institution: University of Kiel
RP	1	96953	96953	Rimbach, G (corresponding author), Univ Kiel, Inst Human Nutr & Food Sci, Hermann Rodewald Str 6, D-24118 Kiel, Germany	Rimbach, G (corresponding author), University of Kiel, Inst Human Nutr & Food Sci, Hermann Rodewald Str 6, D-24118 Kiel, Germany	Country: Germany<br>Institution: Univ Kiel	Country: Germany<br>Institution: University of Kiel

Record 680 | WOS:000241622800019

Critical period and minimum single oral dose of ochratoxin A for inducing developmental toxicity in pregnant Wistar rats

Field	Position	Original line	Normalised line	Original value	Normalised value	Original country / institution	Normalised country / institution
C1	1	97060	97060	Indian Vet Res Inst, Div Pathol, Izatnagar 243122, Uttar Pradesh, India.	ICAR - Indian Veterinary Research Institute, Div Pathol, Izatnagar 243122, Uttar Pradesh, India.	Country: India<br>Institution: Indian Vet Res Inst	Country: India<br>Institution: ICAR - Indian Veterinary Research Institute
RP	1	97063	97063	Dwivedi, P (corresponding author), Indian Vet Res Inst, Div Pathol, Izatnagar 243122, Uttar Pradesh, India	Dwivedi, P (corresponding author), ICAR - Indian Veterinary Research Institute, Div Pathol, Izatnagar 243122, Uttar Pradesh, India	Country: India<br>Institution: Indian Vet Res Inst	Country: India<br>Institution: ICAR - Indian Veterinary Research Institute

Record 681 | WOS:000239496200009

A study of zearalenone cytotoxicity on human peripheral blood mononuclear cells

Field	Position	Original line	Normalised line	Original value	Normalised value	Original country / institution	Normalised country / institution
C1	1	97152	97152	Inst Mol Biol & Biotechnol, Dept Appl Biochem & Immunol, Iraklion, Crete, Greece. Univ Thessaly, Dept Vet Med, Thessaly, Greece. Univ Crete, Dept Med, Toxicol Lab, Iraklion, Crete, Greece.	Inst Mol Biol & Biotechnol, Dept Appl Biochem & Immunol, Iraklion, Crete, Greece. University of Thessaly, Dept Vet Med, Thessaly, Greece. University of Crete, Dept Med, Toxicol Lab, Iraklion, Crete, Greece.	Country: Greece<br>Institution: Inst Mol Biol & Biotechnol	Country: Greece<br>Institution: Inst Mol Biol & Biotechnol

Record 682 | WOS:000239973600004

Expression of COX-2 and hsp72 in peritoneal macrophages after an acute ochratoxin A treatment in mice

Field	Position	Original line	Normalised line	Original value	Normalised value	Original country / institution	Normalised country / institution
C1	1	97255	97255	Univ Naples Federico II, Dept Expt Pharmacol, I-80131 Naples, Italy. Univ Naples Federico II, Dept Pathol & Anim Hlth, I-80131 Naples, Italy. Univ Bologna, Dept Vet Publ Hlth & Anim Pathol, Bologna, Italy.	University of Naples Federico II, Dept Expt Pharmacol, I-80131 Naples, Italy. University of Naples Federico II, Dept Pathol & Anim Hlth, I-80131 Naples, Italy. University of Bologna, Dept Vet Publ Hlth & Anim Pathol, Bologna, Italy.	Country: Italy<br>Institution: Univ Naples Federico II	Country: Italy<br>Institution: University of Naples Federico II
RP	1	97260	97260	Meli, R (corresponding author), Univ Naples Federico II, Dept Expt Pharmacol, Via D Montesano 49, I-80131 Naples, Italy	Meli, R (corresponding author), University of Naples Federico II, Dept Expt Pharmacol, Via D Montesano 49, I-80131 Naples, Italy	Country: Italy<br>Institution: Univ Naples Federico II	Country: Italy<br>Institution: University of Naples Federico II

Record 683 | WOS:000240051000015

Early cytotoxic effects of ochratoxin A in rat liver: A morphological, biochemical and molecular study

Field	Position	Original line	Normalised line	Original value	Normalised value	Original country / institution	Normalised country / institution
C1	1	97386	97386	LITA Segrate, Dept Human Morphol, I-20090 Milan, Italy. Univ Milan, Dept Biol, Milan, Italy. Univ Pisa, Dept Neurosci, Pharmacol Sect, I-56100 Pisa, Italy. Univ Pisa, Dept Internal Med, Pharmacol Sect, I-56100 Pisa, Italy. IRCCS, Ist Clin Humanitas, Labs Quantitat Med, Milan, Italy. Univ Milano Bicocca, Dept Clin Med Prevent & Med Biotechnol, Milan, Italy. Univ Milano Bicocca, S Gerardo Hosp, Milan, Italy. IRCCS, Ist Clin Humanitas, Dept Pathol, Milan, Italy.	LITA Segrate, Dept Human Morphol, I-20090 Milan, Italy. University of Milan, Dept Biol, Milan, Italy. University of Pisa, Dept Neurosci, Pharmacol Sect, I-56100 Pisa, Italy. University of Pisa, Dept Internal Med, Pharmacol Sect, I-56100 Pisa, Italy. IRCCS, Ist Clin Humanitas, Labs Quantitat Med, Milan, Italy. University of Milano-Bicocca, Dept Clin Med Prevent & Med Biotechnol, Milan, Italy. University of Milano-Bicocca, S Gerardo Hosp, Milan, Italy. IRCCS, Ist Clin Humanitas, Dept Pathol, Milan, Italy.	Country: Italy<br>Institution: LITA Segrate	Country: Italy<br>Institution: LITA Segrate

Record 684 | WOS:000240051000002

Adverse effects of T-2 toxin on chicken lymphocytes blastogenesis and its protection with Vitamin E

Field	Position	Original line	Normalised line	Original value	Normalised value	Original country / institution	Normalised country / institution
C1	1	97498	97498	Jordan Univ Sci & Technol, Dept Biotechnol & Genet Engn, Irbid 22110, Jordan. Univ Manitoba, Dept Anim Sci, Winnipeg, MB R3T 2N2, Canada.	Jordan University of Science & Technology, Dept Biotechnol & Genet Engn, Irbid 22110, Jordan. Univ Manitoba, Dept Anim Sci, Winnipeg, MB R3T 2N2, Canada.	Country: Canada<br>Institution: Jordan Univ Sci & Technol	Country: Canada<br>Institution: Jordan University of Science & Technology
RP	1	97501	97501	Jaradat, ZW (corresponding author), Jordan Univ Sci & Technol, Dept Biotechnol & Genet Engn, POB 3030, Irbid 22110, Jordan	Jaradat, ZW (corresponding author), Jordan University of Science & Technology, Dept Biotechnol & Genet Engn, POB 3030, Irbid 22110, Jordan	Country: Jordan<br>Institution: Jordan Univ Sci & Technol	Country: Jordan<br>Institution: Jordan University of Science & Technology

Record 685 | WOS:000239031400009

Glutathione S-transferase polymorphisms and ochratoxin A toxicity in primary human urothelial cells

Field	Position	Original line	Normalised line	Original value	Normalised value	Original country / institution	Normalised country / institution
C1	1	97596	97596	Univ Dortmund, Inst Arbeitsphysiol, D-44139 Dortmund, Germany. Klinikum Dortmund gGmbH, Urol Klin, D-44145 Dortmund, Germany.	Univ Dortmund, Inst Arbeitsphysiol, D-44139 Dortmund, Germany. Klinikum Dortmund, Urol Klin, D-44145 Dortmund, Germany.	Country: Germany<br>Institution: Univ Dortmund	Country: Germany<br>Institution: Univ Dortmund

Record 686 | WOS:000238650400009

Ochratoxin A: Apoptosis and aberrant exit from mitosis due to perturbation of microtubule dynamics?

Field	Position	Original line	Normalised line	Original value	Normalised value	Original country / institution	Normalised country / institution
C1	1	97698	97698	Univ Wurzburg, Dept Toxicol, D-97078 Wurzburg, Germany. Univ Karlsruhe, Inst Appl Biosci, Sect Food Chem & Toxicol, D-76128 Karlsruhe, Germany.	University of Wurzburg, Dept Toxicol, D-97078 Wurzburg, Germany. Univ Karlsruhe, Inst Appl Biosci, Sect Food Chem & Toxicol, D-76128 Karlsruhe, Germany.	Country: Germany<br>Institution: Univ Wurzburg	Country: Germany<br>Institution: University of Wurzburg
RP	1	97702	97702	Dekant, W (corresponding author), Univ Wurzburg, Dept Toxicol, Versbacher Str 9, D-97078 Wurzburg, Germany	Dekant, W (corresponding author), University of Wurzburg, Dept Toxicol, Versbacher Str 9, D-97078 Wurzburg, Germany	Country: Germany<br>Institution: Univ Wurzburg	Country: Germany<br>Institution: University of Wurzburg

Record 687 | WOS:000237912300008

Deoxynivalenol transport across human intestinal Caco-2 cells and its effects on cellular metabolism at realistic intestinal concentrations

No C1/C3/RP normalisation changes detected for this record.

Record 688 | WOS:000238344300009

Balkan endemic nephropathy: Role of ochratoxins A through biomarkers

Field	Position	Original line	Normalised line	Original value	Normalised value	Original country / institution	Normalised country / institution
C1	1	97919	97919	Int Agcy Res Canc, F-69372 Lyon, France. CNRS, INPT, UMR 5503, UPS,Lab Genie Chim,Dept Bioprocedes & Syst Microb, Auzeville Tolosane, France. Natl Ctr Hyg, Mycotoxicol Lab, Sofia, Bulgaria. Inst Oncol, Sofia, Bulgaria.	Int Agcy Res Canc, F-69372 Lyon, France. Centre National de la Recherche Scientifique (CNRS), INPT, UMR 5503, UPS,Lab Genie Chim,Dept Bioprocedes & Syst Microb, Auzeville Tolosane, France. Natl Ctr Hyg, Mycotoxicol Lab, Sofia, Bulgaria. Inst Oncol, Sofia, Bulgaria.	Country: Bulgaria<br>Institution: Int Agcy Res Canc	Country: Bulgaria<br>Institution: Int Agcy Res Canc

Record 689 | WOS:000238344300010

Evidence of a new dechlorinated ochratoxin A derivative formed in opossum kidney cell cultures after pretreatment by modulators of glutathione pathways: Correlation with DNA-adduct formation

Field	Position	Original line	Normalised line	Original value	Normalised value	Original country / institution	Normalised country / institution
C1	1	98025	98025	CNRS, UMR 5503, INPT, Dept BioSyM,Lab Genie Chim, Auzeville Tolosane, France. CHU Purpan, INSERM, U563, Dept Mass Spectrometry,IFR 30, Toulouse, France. Norwegian Inst Publ Hlth, Dept Environm Med, Oslo, Norway. Univ St Joseph, Beirut, Lebanon. Collanges, St Jean Chambre, France.	Centre National de la Recherche Scientifique (CNRS), UMR 5503, INPT, Dept BioSyM,Lab Genie Chim, Auzeville Tolosane, France. CHU Purpan, INSERM, U563, Dept Mass Spectrometry,IFR 30, Toulouse, France. Norwegian Inst Publ Hlth, Dept Environm Med, Oslo, Norway. Univ St Joseph, Beirut, Lebanon. Collanges, St Jean Chambre, France.	Country: France<br>Institution: CNRS	Country: France<br>Institution: Centre National de la Recherche Scientifique (CNRS)

Record 690 | WOS:000238344300011

Microbiologicals for deactivating mycotoxins

No C1/C3/RP normalisation changes detected for this record.

Record 691 | WOS:000236846900001

Ochratoxin A secretion by ATP-dependent membrane transporters in Caco-2 cells

Field	Position	Original line	Normalised line	Original value	Normalised value	Original country / institution	Normalised country / institution
C1	1	98252	98252	Univ Utrecht, Fac Vet Med, Dept Vet Pharmacol Pharm & Toxicol, NL-3584 CM Utrecht, Netherlands. Univ Dortmund, Inst Occupat Physiol, D-44139 Dortmund, Germany.	Utrecht University, Fac Vet Med, Dept Vet Pharmacol Pharm & Toxicol, NL-3584 CM Utrecht, Netherlands. Univ Dortmund, Inst Occupat Physiol, D-44139 Dortmund, Germany.	Country: Germany<br>Institution: Univ Utrecht	Country: Germany<br>Institution: Utrecht University
RP	1	98255	98255	Schrickx, J (corresponding author), Univ Utrecht, Fac Vet Med, Dept Vet Pharmacol Pharm & Toxicol, Yalelaan 16, NL-3584 CM Utrecht, Netherlands	Schrickx, J (corresponding author), Utrecht University, Fac Vet Med, Dept Vet Pharmacol Pharm & Toxicol, Yalelaan 16, NL-3584 CM Utrecht, Netherlands	Country: Netherlands<br>Institution: Univ Utrecht	Country: Netherlands<br>Institution: Utrecht University

Record 692 | WOS:000236987100010

In vitro investigation of individual and combined cytotoxic effects of ochratoxin A and other selected mycotoxins on renal cells

Field	Position	Original line	Normalised line	Original value	Normalised value	Original country / institution	Normalised country / institution
C1	1	98350	98350	Univ Konstanz, Dept Expt Toxicol, D-78457 Constance, Germany.	University of Konstanz, Dept Expt Toxicol, D-78457 Constance, Germany.	Country: Germany Institution: Univ Konstanz	Country: Germany Institution: University of Konstanz
RP	1	98352	98352	O'Brien, E (corresponding author), Univ Konstanz, Dept Expt Toxicol, Jakob Burkhardt St 25,POB X-918, D-78457 Constance, Germany	O'Brien, E (corresponding author), University of Konstanz, Dept Expt Toxicol, Jakob Burkhardt St 25,POB X-918, D-78457 Constance, Germany	Country: Germany Institution: Univ Konstanz	Country: Germany Institution: University of Konstanz

Record 693 | WOS:000236605600004

Ochratoxin A: Its cancer risk and potential for exposure

Field	Position	Original line	Normalised line	Original value	Normalised value	Original country / institution	Normalised country / institution
C1	1	98433	98433	Cornell Univ, Coll Vet Med, Program BCERF, Sprecher Inst Comparat Canc Res, Ithaca, NY 14853 USA.	Cornell University, Coll Vet Med, Program BCERF, Sprecher Inst Comparat Canc Res, Ithaca, NY 14853 USA.	Country: USA Institution: Cornell Univ	Country: USA Institution: Cornell University
RP	1	98435	98435	Clark, HA (corresponding author), Cornell Univ, Coll Vet Med, Program BCERF, Sprecher Inst Comparat Canc Res, Vet Box 31, Ithaca, NY 14853 USA	Clark, HA (corresponding author), Cornell University, Coll Vet Med, Program BCERF, Sprecher Inst Comparat Canc Res, Vet Box 31, Ithaca, NY 14853 USA	Country: USA Institution: Cornell Univ	Country: USA Institution: Cornell University

Record 694 | WOS:000234793300002

Toxicokinetics and toxicodynamics of ochratoxin A, an update

No C1/C3/RP normalisation changes detected for this record.

Record 695 | WOS:000234140400042

The impact of plasma protein binding on the renal transport of organic anions

Field	Position	Original line	Normalised line	Original value	Normalised value	Original country / institution	Normalised country / institution
C1	1	98968	98968	Natl Inst Environm Hlth Sci, Lab Pharmacol & Chem, NIH, Res Triangle Pk, NC 27709 USA. Duke Univ, Dept Chem & Biochem, Durham, NC 27706 USA.	Natl Inst Environm Hlth Sci, Lab Pharmacol & Chem, NIH, Res Triangle Pk, NC 27709 USA. Duke University, Dept Chem & Biochem, Durham, NC 27706 USA.	Country: USA Institution: Natl Inst Environm Hlth Sci	Country: USA Institution: Natl Inst Environm Hlth Sci
C3	1	98970	98970	National Institutes of Health (NIH) - USA	National Institutes of Health (NIH)	Institution: National Institutes of Health (NIH) - USA	Institution: National Institutes of Health (NIH)

Record 696 | WOS:000235953600006

Mycoflora, cytotoxicity, and DNA interaction of polyherbal products from Malaysia

Field	Position	Original line	Normalised line	Original value	Normalised value	Original country / institution	Normalised country / institution
C1	1	99066	99066	Glasgow Caledonian Univ, Dept Biol & Biomed Sci, Glasgow G4 0BA, Lanark, Scotland.	Glasgow Caledonian University, Dept Biol & Biomed Sci, Glasgow G4 0BA, Lanark, United Kingdom.	Country: Scotland Institution: Glasgow Caledonian Univ	Country: United Kingdom Institution: Glasgow Caledonian University
RP	1	99068	99068	Aidoo, KE (corresponding author), Glasgow Caledonian Univ, Dept Biol & Biomed Sci, Cowcaddens Rd, Glasgow G4 0BA, Lanark, Scotland	Aidoo, KE (corresponding author), Glasgow Caledonian University, Dept Biol & Biomed Sci, Cowcaddens Rd, Glasgow G4 0BA, Lanark, United Kingdom	Country: Scotland Institution: Glasgow Caledonian Univ	Country: United Kingdom Institution: Glasgow Caledonian University

Record 697 | WOS:000233991000012

A toxicogenomics approach to identify new plausible epigenetic mechanisms of ochratoxin a carcinogenicity in rat

Field	Position	Original line	Normalised line	Original value	Normalised value	Original country / institution	Normalised country / institution
C1	1	99176	99176	Nestle Res Ctr, CH-1000 Lausanne, Switzerland. UCL, Inst Child Hlth, NephroUrol Unit, London WC1E 1EH, England. Univ London Imperial Coll Sci Technol & Med, Dept Environm Sci & Technol, London SW7 2AZ, England.	Nestle Research Center, CH-1000 Lausanne, Switzerland. UCL, Inst Child Hlth, NephroUrol Unit, London WC1E 1EH, United Kingdom. Univ London Imperial Coll Sci Technol & Med, Dept Environm Sci & Technol, London SW7 2AZ, United Kingdom.	Country: England Institution: Nestle Res Ctr	Country: United Kingdom Institution: Nestle Research Center
RP	1	99181	99181	Marin-Kuan, M (corresponding author), Nestle Res Ctr, POB 44, CH-1000 Lausanne, Switzerland	Marin-Kuan, M (corresponding author), Nestle Research Center, POB 44, CH-1000 Lausanne, Switzerland	Country: Switzerland Institution: Nestle Res Ctr	Country: Switzerland Institution: Nestle Research Center

Record 698 | WOS:000234194400007

An in vitro approach to detect metabolite toxicity due to CYP3A4-dependent bioactivation of xenobiotics

Field	Position	Original line	Normalised line	Original value	Normalised value	Original country / institution	Normalised country / institution
C1	1	99319	99319	Nerviano Med Sci, Dept Pre Clin Dev, I-20014 Nerviano, MI, Italy.	Nerviano Medical Sciences S.r.l, Dept Pre Clin Dev, I-20014 Nerviano, MI, Italy.	Country: Italy Institution: Nerviano Med Sci	Country: Italy Institution: Nerviano Medical Sciences S.r.l
RP	1	99321	99321	Vignati, L (corresponding author), Nerviano Med Sci, Dept Pre Clin Dev, V Pasteur 10, I-20014 Nerviano, MI, Italy	Vignati, L (corresponding author), Nerviano Medical Sciences S.r.l, Dept Pre Clin Dev, V Pasteur 10, I-20014 Nerviano, MI, Italy	Country: Italy Institution: Nerviano Med Sci	Country: Italy Institution: Nerviano Medical Sciences S.r.l

Record 699 | WOS:000234094800009

Ochratoxin A induces oxidative DNA damage in liver and kidney after oral dosing to rats

Field	Position	Original line	Normalised line	Original value	Normalised value	Original country / institution	Normalised country / institution
C1	1	99428	99428	Univ Kaiserslautern, Dept Chem, Div Food Chem & Environm Toxicol, D-67663 Kaiserslautern, Germany. US FDA, Natl Ctr Toxicol Res, Jefferson, AR 72079 USA.	Univ Kaiserslautern, Dept Chem, Div Food Chem & Environm Toxicol, D-67663 Kaiserslautern, Germany. US Food & Drug Administration (FDA), Natl Ctr Toxicol Res,	Country: Switzerland Institution: Univ Kaiserslautern	Country: Switzerland Institution: Univ Kaiserslautern

				Toxicol Pathol Associates, Jefferson, AR USA. Swiss Fed Off Publ Hlth, Food Toxicol Sect, Zurich, Switzerland.	Jefferson, AR 72079 USA. Toxicol Pathol Associates, Jefferson, AR USA. Swiss Fed Off Publ Hlth, Food Toxicol Sect, Zurich, Switzerland.		

Record 700 | WOS:000233125300003

Teratogenic effects in rabbits of simultaneous exposure to ochratoxin A and aflatoxin B1 with special reference to microscopic effects

Field	Position	Original line	Normalised line	Original value	Normalised value	Original country / institution	Normalised country / institution
C1	1	99551	99551	Indian Vet Res Inst, Div Pathol, Izatnagar 243122, Uttar Pradesh, India. Cent Drug Res Inst, Div Toxicol, Lucknow, Uttar Pradesh, India.	ICAR - Indian Veterinary Research Institute, Div Pathol, Izatnagar 243122, Uttar Pradesh, India. Cent Drug Res Inst, Div Toxicol, Lucknow, Uttar Pradesh, India.	Country: India<br>Institution: Indian Vet Res Inst	Country: India<br>Institution: ICAR - Indian Veterinary Research Institute
RP	1	99556	99556	Dwivedi, P (corresponding author), Indian Vet Res Inst, Div Pathol, Izatnagar 243122, Uttar Pradesh, India	Dwivedi, P (corresponding author), ICAR - Indian Veterinary Research Institute, Div Pathol, Izatnagar 243122, Uttar Pradesh, India	Country: India<br>Institution: Indian Vet Res Inst	Country: India<br>Institution: ICAR - Indian Veterinary Research Institute

Record 701 | WOS:000232435300026

The effects of mycotoxins, fungal food contaminants, on the intestinal epithelial cell-derived innate immune response

Field	Position	Original line	Normalised line	Original value	Normalised value	Original country / institution	Normalised country / institution
C1	1	99680	99680	INRA, Lab Pharmacol Toxicol, F-31931 Toulouse, France.	INRAE, Lab Pharmacol Toxicol, F-31931 Toulouse, France.	Country: France<br>Institution: INRA	Country: France<br>Institution: INRAE
RP	1	99682	99682	Oswald, IP (corresponding author), INRA, Lab Pharmacol Toxicol, 180 Chemin Tournefeuille,BP 3, F-31931 Toulouse, France	Oswald, IP (corresponding author), INRAE, Lab Pharmacol Toxicol, 180 Chemin Tournefeuille,BP 3, F-31931 Toulouse, France	Country: France<br>Institution: INRA	Country: France<br>Institution: INRAE

Record 702 | WOS:000233069800005

Investigation of the teratogenic potential of ochratoxin A and B using the FETAX system

Field	Position	Original line	Normalised line	Original value	Normalised value	Original country / institution	Normalised country / institution
C1	1	99805	99805	Univ Konstanz, D-78457 Constance, Germany.	University of Konstanz, D-78457 Constance, Germany.	Country: Germany<br>Institution: Univ Konstanz	Country: Germany<br>Institution: University of Konstanz
RP	1	99807	99807	O'Brien, E (corresponding author), Univ Konstanz, Jacob Burckhardstr 25,POB X918, D-78457 Constance, Germany	O'Brien, E (corresponding author), University of Konstanz, Jacob Burckhardstr 25,POB X918, D-78457 Constance, Germany	Country: Germany<br>Institution: Univ Konstanz	Country: Germany<br>Institution: University of Konstanz

Record 703 | WOS:000232409600014

Fate of ochratoxin A during cooking of naturally contaminated polished rice

Field	Position	Original line	Normalised line	Original value	Normalised value	Original country / institution	Normalised country / institution
C1	1	99911	99911	Korea Univ, Grad Sch Biotechnol, Seoul 136701, South Korea. Korea Univ, Coll Hlth Sci, Seoul 136703, South Korea. Chung Ang Univ, Coll Ind Sci, Gyeonggi 456756, South Korea.	Korea University, Grad Sch Biotechnol, Seoul 136701, South Korea. Korea University, Coll Hlth Sci, Seoul 136703, South Korea. Chung Ang University, Coll Ind Sci, Gyeonggi 456756, South Korea.	Country: South Korea Institution: Korea Univ	Country: South Korea Institution: Korea University

Record 704 | WOS:000232524600005

Alterations induced in vitro by ochratoxin A in rat lymphoid cells

Field	Position	Original line	Normalised line	Original value	Normalised value	Original country / institution	Normalised country / institution
C1	1	99991	99991	Univ Navarra, Fac Pharm, Food Sci & Toxicol Dept, E-31080 Pamplona, Spain. Univ Navarra, Fac Pharm, Organ & Pharmaceut Chem Dept, E-31080 Pamplona, Spain.	University of Navarra, Fac Pharm, Food Sci & Toxicol Dept, E-31080 Pamplona, Spain. University of Navarra, Fac Pharm, Organ & Pharmaceut Chem Dept, E-31080 Pamplona, Spain.	Country: Spain Institution: Univ Navarra	Country: Spain Institution: University of Navarra
RP	1	99994	99994	de Cerain, AL (corresponding author), Univ Navarra, Fac Pharm, Food Sci & Toxicol Dept, Irunlarrea 1, E-31080 Pamplona, Spain	de Cerain, AL (corresponding author), University of Navarra, Fac Pharm, Food Sci & Toxicol Dept, Irunlarrea 1, E-31080 Pamplona, Spain	Country: Spain Institution: Univ Navarra	Country: Spain Institution: University of Navarra

Record 705 | WOS:000231364400010

Cyanidin-3-O-Î²-glucopyranoside, a natural free-radical scavenger against aflatoxin B1-and ochratoxin A-induced cell damage in a human hepatoma cell line (Hep G2) and a human colonic adenocarcinoma cell line (CaCo-2)

Field	Position	Original line	Normalised line	Original value	Normalised value	Original country / institution	Normalised country / institution
C1	1	100098	100098	Univ Bologna, Dept Pharmacol, I-40126 Bologna, Italy. Univ Reggio Calabria, Dept Agroforestry Environm Sci & Technol, I-89100 Reggio Di Calabria, Italy. Univ Bologna, Inst Histol & Gen Embryol, I-40126 Bologna, Italy. Univ Bologna, Dept Chem G Ciamician, I-40126 Bologna, Italy.	University of Bologna, Dept Pharmacol, I-40126 Bologna, Italy. Univ Reggio Calabria, Dept Agroforestry Environm Sci & Technol, I-89100 Reggio Di Calabria, Italy. University of Bologna, Inst Histol & Gen Embryol, I-40126 Bologna, Italy. University of Bologna, Dept Chem G Ciamician, I-40126 Bologna, Italy.	Country: Italy Institution: Univ Bologna	Country: Italy Institution: University of Bologna
RP	1	100104	100104	Guerra, MC (corresponding author), Univ Bologna, Dept Pharmacol, Via Irnerio 48, I-40126 Bologna, Italy	Guerra, MC (corresponding author), University of Bologna, Dept Pharmacol, Via Irnerio 48, I-40126 Bologna, Italy	Country: Italy Institution: Univ Bologna	Country: Italy Institution: University of Bologna

Record 706 | WOS:000231235000007

Functional, biochemical, and pathological effects of repeated oral administration of ochratoxin A to rats

Field	Position	Original line	Normalised line	Original value	Normalised value	Original country / institution	Normalised country / institution

C1	1	100206	100206	Univ Wurzburg, Dept Toxicol, D-97078 Wurzburg, Germany. Aventis Pharma, Drug Safety Evaluat, Hattersheim, Germany. Aventis Pharma, Frankfurt, Germany. NTP Archives, Res Triangle Pk, NC USA.	University of Wurzburg, Dept Toxicol, D-97078 Wurzburg, Germany. Aventis Pharma, Drug Safety Evaluat, Hattersheim, Germany. Aventis Pharma, Frankfurt, Germany. NTP Archives, Res Triangle Pk, NC USA.	Country: USA Institution: Univ Wurzburg	Country: USA Institution: University of Wurzburg
RP	1	100212	100212	Dekant, W (corresponding author), Univ Wurzburg, Dept Toxicol, Versbacher Str 9, D-97078 Wurzburg, Germany	Dekant, W (corresponding author), University of Wurzburg, Dept Toxicol, Versbacher Str 9, D-97078 Wurzburg, Germany	Country: Germany Institution: Univ Wurzburg	Country: Germany Institution: University of Wurzburg

Record 707 | WOS:000231235000008

Ochratoxin A causes DNA damage and cytogenetic effects but no DNA adducts in rats

Field	Position	Original line	Normalised line	Original value	Normalised value	Original country / institution	Normalised country / institution
C1	1	100339	100339	Univ Wurzburg, Dept Toxicol, D-97078 Wurzburg, Germany. Univ Tuscia, Dipartimento Agrobiol & Agrochim, I-01100 Viterbo, Italy. Univ Louisville, Brown Canc Ctr, Louisville, KY 40292 USA.	University of Wurzburg, Dept Toxicol, D-97078 Wurzburg, Germany. Tuscia University, Dipartimento Agrobiol & Agrochim, I-01100 Viterbo, Italy. University of Louisville, Brown Canc Ctr, Louisville, KY 40292 USA.	Country: USA Institution: Univ Wurzburg	Country: USA Institution: University of Wurzburg
RP	1	100343	100343	Dekant, W (corresponding author), Univ Wurzburg, Dept Toxicol, Versbacher Str 9, D-97078 Wurzburg, Germany	Dekant, W (corresponding author), University of Wurzburg, Dept Toxicol, Versbacher Str 9, D-97078 Wurzburg, Germany	Country: Germany Institution: Univ Wurzburg	Country: Germany Institution: University of Wurzburg

Record 708 | WOS:000230120200005

Biotransformation and nephrotoxicity of ochratoxin B in rats

Field	Position	Original line	Normalised line	Original value	Normalised value	Original country / institution	Normalised country / institution
C1	1	100460	100460	Univ Wurzburg, Inst Toxikol, D-97078 Wurzburg, Germany. Aventis Pharma, Drug Safety Evaluat, Hattersheim, Germany. Aventis Pharma, Chem, Frankfurt, Germany. Univ London Imperial Coll Sci Technol & Med, Dept Environm Sci & Technol, London, England. NTP Archives, Res Triangle Pk, NC 27709 USA.	University of Wurzburg, Inst Toxikol, D-97078 Wurzburg, Germany. Aventis Pharma, Drug Safety Evaluat, Hattersheim, Germany. Aventis Pharma, Chem, Frankfurt, Germany. Univ London Imperial Coll Sci Technol & Med, Dept Environm Sci & Technol, London, United Kingdom. NTP Archives, Res Triangle Pk, NC 27709 USA.	Country: USA Institution: Univ Wurzburg	Country: USA Institution: University of Wurzburg
RP	1	100467	100467	Dekant, W (corresponding author), Univ Wurzburg, Dept Toxicol, Versbacher Str 9, D-97078 Wurzburg, Germany	Dekant, W (corresponding author), University of Wurzburg, Dept Toxicol, Versbacher Str 9, D-97078 Wurzburg, Germany	Country: Germany Institution: Univ Wurzburg	Country: Germany Institution: University of Wurzburg

Record 709 | WOS:000228398000034

Cisplatin-induced apoptosis is enhanced by hypoxia and by inhibition of mitochondria in renal collecting duct cells

Field	Position	Original line	Normalised line	Original value	Normalised value	Original country / institution	Normalised country / institution
C1	1	100551	100551	Univ Wurzburg, Inst Physiol, D-97070 Wurzburg, Germany.	University of Wurzburg, Inst Physiol, D-97070 Wurzburg, Germany.	Country: Germany Institution: Univ Wurzburg	Country: Germany Institution: University of Wurzburg
RP	1	100553	100553	Schwerdt, G (corresponding author), Univ Wurzburg, Inst Physiol, Rontgenring 9, D-97070 Wurzburg, Germany	Schwerdt, G (corresponding author), University of Wurzburg, Inst Physiol, Rontgenring 9, D-97070 Wurzburg, Germany	Country: Germany Institution: Univ Wurzburg	Country: Germany Institution: University of Wurzburg

Record 710 | WOS:000229004100015

Comparison of in vitro cytotoxicity of Fusarium mycotoxins, deoxynivalenol, T-2 toxin and zearalenone on selected human epithelial cell lines

Field	Position	Original line	Normalised line	Original value	Normalised value	Original country / institution	Normalised country / institution
C1	1	100643	100643	Glasgow Caledonian Univ, Sch Biol & Biomed Sci, Glasgow G4 0BA, Lanark, Scotland.	Glasgow Caledonian University, Sch Biol & Biomed Sci, Glasgow G4 0BA, Lanark, United Kingdom.	Country: Scotland Institution: Glasgow Caledonian Univ	Country: United Kingdom Institution: Glasgow Caledonian University
RP	1	100645	100645	Aidoo, KE (corresponding author), Glasgow Caledonian Univ, Sch Biol & Biomed Sci, Cowcaddens Rd, Glasgow G4 0BA, Lanark, Scotland	Aidoo, KE (corresponding author), Glasgow Caledonian University, Sch Biol & Biomed Sci, Cowcaddens Rd, Glasgow G4 0BA, Lanark, United Kingdom	Country: Scotland Institution: Glasgow Caledonian Univ	Country: United Kingdom Institution: Glasgow Caledonian University

Record 711 | WOS:000227733000027

Proximal tubular toxicity of ochratoxin A is amplified by simultaneous inhibition of the extracellular signal-regulated kinases 1/2

Field	Position	Original line	Normalised line	Original value	Normalised value	Original country / institution	Normalised country / institution
C1	1	100734	100734	Univ Wurzburg, Inst Physiol, D-97070 Wurzburg, Germany.	University of Wurzburg, Inst Physiol, D-97070 Wurzburg, Germany.	Country: Germany Institution: Univ Wurzburg	Country: Germany Institution: University of Wurzburg
RP	1	100736	100736	Sauvant, C (corresponding author), Univ Wurzburg, Inst Physiol, Rontgenring 9, D-97070 Wurzburg, Germany	Sauvant, C (corresponding author), University of Wurzburg, Inst Physiol, Rontgenring 9, D-97070 Wurzburg, Germany	Country: Germany Institution: Univ Wurzburg	Country: Germany Institution: University of Wurzburg

Record 712 | WOS:000227112000002

Interference of arachidonic acid and its metabolites with TNF-Î± release by ochratoxin A from rat liver

No C1/C3/RP normalisation changes detected for this record.

Record 713 | WOS:000227242200003

Ochratoxin A at nanomolar concentrations: A signal modulator in renal cells

Field	Position	Original line	Normalised line	Original value	Normalised value	Original country / institution	Normalised country / institution
C1	1	100950	100950	Univ Wurzburg, Physiol Inst, D-97070 Wurzburg, Germany.	University of Wurzburg, Physiol Inst, D-97070 Wurzburg, Germany.	Country: Germany Institution: Univ Wurzburg	Country: Germany Institution: University of Wurzburg

RP	1	100952	100952	Gekle, M (corresponding author), Univ Wurzburg, Physiol Inst, Rontgenring 9, D-97070 Wurzburg, Germany	Gekle, M (corresponding author), University of Wurzburg, Physiol Inst, Rontgenring 9, D-97070 Wurzburg, Germany	Country: Germany Institution: Univ Wurzburg	Country: Germany Institution: University of Wurzburg

Record 714 | WOS:000234123000008

Ochratoxin A: Comparative pharmacokinetics and toxicological implications (experimental and domestic animals and humans)

Field	Position	Original line	Normalised line	Original value	Normalised value	Original country / institution	Normalised country / institution
C1	1	101114	101114	Univ Konstanz, Dept Environm & Human Toxicol, D-7750 Constance, Germany.	University of Konstanz, Dept Environm & Human Toxicol, D-7750 Constance, Germany.	Country: Germany Institution: Univ Konstanz	Country: Germany Institution: University of Konstanz
RP	1	101116	101116	Dietrich, DR (corresponding author), Univ Konstanz, Dept Environm & Human Toxicol, D-7750 Constance, Germany	Dietrich, DR (corresponding author), University of Konstanz, Dept Environm & Human Toxicol, D-7750 Constance, Germany	Country: Germany Institution: Univ Konstanz	Country: Germany Institution: University of Konstanz

Record 715 | WOS:000234123000011

DNA adduct formation by ochratoxin A: Review of the available evidence

Field	Position	Original line	Normalised line	Original value	Normalised value	Original country / institution	Normalised country / institution
C1	1	101212	101212	Univ Wurzburg, Dept Toxicol, Wurzburg, Germany.	University of Wurzburg, Dept Toxicol, Wurzburg, Germany.	Country: Germany Institution: Univ Wurzburg	Country: Germany Institution: University of Wurzburg
RP	1	101214	101214	Dekant, W (corresponding author), Univ Wurzburg, Dept Toxicol, Wurzburg, Germany	Dekant, W (corresponding author), University of Wurzburg, Dept Toxicol, Wurzburg, Germany	Country: Germany Institution: Univ Wurzburg	Country: Germany Institution: University of Wurzburg

Record 716 | WOS:000226837900002

Ochratoxin A: The continuing enigma

Field	Position	Original line	Normalised line	Original value	Normalised value	Original country / institution	Normalised country / institution
C1	1	101320	101320	Univ Konstanz, D-78457 Constance, Germany.	University of Konstanz, D-78457 Constance, Germany.	Country: Germany Institution: Univ Konstanz	Country: Germany Institution: University of Konstanz
RP	1	101322	101322	O'Brien, E (corresponding author), Univ Konstanz, Jacob Burckhardt Str 25, D-78457 Constance, Germany	O'Brien, E (corresponding author), University of Konstanz, Jacob Burckhardt Str 25, D-78457 Constance, Germany	Country: Germany Institution: Univ Konstanz	Country: Germany Institution: University of Konstanz

Record 717 | WOS:000234123000012

Further arguments in favour of direct covalent binding of Ochratoxin A (OTA) after metabolic biotransformation

No C1/C3/RP normalisation changes detected for this record.

Record 718 | WOS:000232477800006

Effect of mycotoxins on some activities of isolated human neutrophils

Field	Position	Original line	Normalised line	Original value	Normalised value	Original country / institution	Normalised country / institution
C1	1	101710	101710	Univ Messina, Dept Expt Pathol & Microbiol, I-98100 Messina, Italy. Univ Messina, Dept Vet Publ Hlth, I-98100 Messina, Italy. Univ Messina, Dept Environm Sanitary Social & Ind Protectionist, I-98100 Messina, Italy.	University of Messina, Dept Expt Pathol & Microbiol, I-98100 Messina, Italy. University of Messina, Dept Vet Publ Hlth, I-98100 Messina, Italy. University of Messina, Dept Environm Sanitary Social & Ind Protectionist, I-98100 Messina, Italy.	Country: Italy<br>Institution: Univ Messina	Country: Italy<br>Institution: University of Messina
RP	1	101714	101714	Fimiani, V (corresponding author), Univ Messina, Dept Expt Pathol & Microbiol, I-98100 Messina, Italy	Fimiani, V (corresponding author), University of Messina, Dept Expt Pathol & Microbiol, I-98100 Messina, Italy	Country: Italy<br>Institution: Univ Messina	Country: Italy<br>Institution: University of Messina

Record 719 | WOS:000226875200005

Ochratoxin A-induced DNA damage in human fibroblast:: protective effect of cyanidin 3-O-Î²-D-glucoside

Field	Position	Original line	Normalised line	Original value	Normalised value	Original country / institution	Normalised country / institution
C1	1	101807	101807	Mediterannean Univ Reggio Calabria, Dept Agroforestry Environm Sci & Technol, I-89061 Gallina, Italy. Catania Univ, Dept Biochem, I-95100 Catania, Italy. Univ Catania, Dept Agron, I-95123 Catania, Italy.	Mediterannean Univ Reggio Calabria, Dept Agroforestry Environm Sci & Technol, I-89061 Gallina, Italy. University of Catania, Dept Biochem, I-95100 Catania, Italy. University of Catania, Dept Agron, I-95123 Catania, Italy.	Country: Italy<br>Institution: Mediterannean Univ Reggio Calabria	Country: Italy<br>Institution: Mediterannean Univ Reggio Calabria

Record 720 | WOS:000226469400004

Exposure to nephrotoxic Ochratoxin A enhances collagen secretion in human renal proximal tubular cells

Field	Position	Original line	Normalised line	Original value	Normalised value	Original country / institution	Normalised country / institution
C1	1	101916	101916	Univ Wurzburg, Inst Physiol, D-97070 Wurzburg, Germany.	University of Wurzburg, Inst Physiol, D-97070 Wurzburg, Germany.	Country: Germany<br>Institution: Univ Wurzburg	Country: Germany<br>Institution: University of Wurzburg
RP	1	101918	101918	Sauvant, C (corresponding author), Univ Wurzburg, Inst Physiol, Rontgenring 9, D-97070 Wurzburg, Germany	Sauvant, C (corresponding author), University of Wurzburg, Inst Physiol, Rontgenring 9, D-97070 Wurzburg, Germany	Country: Germany<br>Institution: Univ Wurzburg	Country: Germany<br>Institution: University of Wurzburg

Record 721 | WOS:000226446300003

Applicability of an in vitro digestion model in assessing the bioaccessibility of mycotoxins from food

No C1/C3/RP normalisation changes detected for this record.

Record 722 | WOS:000234123000002

Ochratoxin A: Previous risk assessments and issues arising

Field	Position	Original line	Normalised line	Original value	Normalised value	Original country / institution	Normalised country / institution
C1	1	102100	102100	Univ Surrey, Sch Biomed & Mol Sci, Guildford GU2 5XH, Surrey, England. Danish Inst Food & Vet Res, Soborg, Denmark.	University of Surrey, Sch Biomed & Mol Sci, Guildford GU2 5XH, Surrey, United Kingdom. Danish Inst Food & Vet Res, Soborg, Denmark.	Country: Denmark<br>Institution: Univ Surrey	Country: Denmark<br>Institution: University of Surrey
RP	1	102103	102103	Walker, R (corresponding author), Univ Surrey, Sch Biomed & Mol Sci, Guildford GU2 5XH, Surrey, England	Walker, R (corresponding author), University of Surrey, Sch Biomed & Mol Sci, Guildford GU2 5XH, Surrey, United Kingdom	Country: England<br>Institution: Univ Surrey	Country: United Kingdom<br>Institution: University of Surrey

Record 723 | WOS:000231491200006

Unusual astrocyte reactivity caused by the food mycotoxin ochratoxin A in aggregating rat brain cell cultures

Field	Position	Original line	Normalised line	Original value	Normalised value	Original country / institution	Normalised country / institution
C1	1	102166	102166	Univ Lausanne, Dept Physiol, CH-1005 Lausanne, Switzerland. Univ Lausanne Hosp, Clin Chem Lab, CH-1011 Lausanne, Switzerland.	University of Lausanne, Dept Physiol, CH-1005 Lausanne, Switzerland. Univ Lausanne Hosp, Clin Chem Lab, CH-1011 Lausanne, Switzerland.	Country: Switzerland<br>Institution: Univ Lausanne	Country: Switzerland<br>Institution: University of Lausanne
RP	1	102170	102170	Zurich, MG (corresponding author), Univ Lausanne, Dept Physiol, Rue Bugnon 7, CH-1005 Lausanne, Switzerland	Zurich, MG (corresponding author), University of Lausanne, Dept Physiol, Rue Bugnon 7, CH-1005 Lausanne, Switzerland	Country: Switzerland<br>Institution: Univ Lausanne	Country: Switzerland<br>Institution: University of Lausanne

Record 724 | WOS:000225312300008

Effects of deoxynivalenol on general performance and electrophysiological properties of intestinal mucosa of broiler chickens

No C1/C3/RP normalisation changes detected for this record.

Record 725 | WOS:000226727000007

Combined effects of selected Penicillium mycotoxins on in vitro proliferation of porcine lymphocytes

Field	Position	Original line	Normalised line	Original value	Normalised value	Original country / institution	Normalised country / institution
C1	1	102431	102431	Natl Vet Inst, N-0033 Oslo, Norway. Norwegian Sch Vet Med, Dept Food Saftey & Infect Biol, N-0033 Oslo, Norway.	National Veterinary Institute - National Research Institute, N-0033 Oslo, Norway. Norwegian Veterinary Institute, Dept Food Saftey & Infect Biol, N-0033 Oslo, Norway.	Country: Norway<br>Institution: Natl Vet Inst	Country: Norway<br>Institution: National Veterinary Institute - National Research Institute
RP	1	102434	102434	Bernhoft, A (corresponding author), Natl Vet Inst, POB 8156 DEP, N-0033 Oslo, Norway	Bernhoft, A (corresponding author), National Veterinary Institute - National Research Institute, POB 8156 DEP, N-0033 Oslo, Norway	Country: Norway<br>Institution: Natl Vet Inst	Country: Norway<br>Institution: National Veterinary Institute - National Research Institute

Record 726 | WOS:000226361400006

Molecular aspects of the transport and toxicity of ochratoxin A

Field	Position	Original line	Normalised line	Original value	Normalised value	Original country / institution	Normalised country / institution
C1	1	102507	102507	Wake Forest Univ, Dept Chem, Winston Salem, NC 27106 USA. Duke Univ, Dept Chem, Durham, NC 27708 USA. NIEHS, Lab Pharmacol & Chem, NIH, Res Triangle Pk, NC 27709 USA. Ecole Natl Super Agron Toulouse, UMR CNRS 5503, Lab Genie Chim, Dept Toxicol & Food Safety, F-31326 Castanet Tolosan, France.	Wake Forest University, Dept Chem, Winston Salem, NC 27106 USA. Duke University, Dept Chem, Durham, NC 27708 USA. NIEHS, Lab Pharmacol & Chem, NIH, Res Triangle Pk, NC 27709 USA. Ecole Natl Super Agron Toulouse, UMR CNRS 5503, Lab Genie Chim, Dept Toxicol & Food Safety, F-31326 Castanet Tolosan, France.	Country: France<br>Institution: Wake Forest Univ	Country: France<br>Institution: Wake Forest University
C3	3	102511	102511	National Institutes of Health (NIH) - USA	National Institutes of Health (NIH)	Institution: National Institutes of Health (NIH) - USA	Institution: National Institutes of Health (NIH)
RP	1	102517	102517	Manderville, RA (corresponding author), Wake Forest Univ, Dept Chem, Winston Salem, NC 27106 USA	Manderville, RA (corresponding author), Wake Forest University, Dept Chem, Winston Salem, NC 27106 USA	Country: USA<br>Institution: Wake Forest Univ	Country: USA<br>Institution: Wake Forest University

Record 727 | WOS:000224725300008

Experimental mycotoxicosis in chickens induced by ochratoxin A and penicillic acid and intervention with natural plant extracts

Field	Position	Original line	Normalised line	Original value	Normalised value	Original country / institution	Normalised country / institution
C1	1	102622	102622	Trakia Univ, Fac Vet Med, Dept Gen & Clin Pathol, Stara Zagora 6000, Bulgaria. Trakia Univ, Fac Agr, Dept Morphol Anim, Stara Zagora 6000, Bulgaria. Trakia Univ, Fac Agr, Dept Microbiol, Stara Zagora 6000, Bulgaria. Univ Zagreb, Inst Med Res & Occupat Hlth, Zagreb 41000, Croatia.	Trakia University, Fac Vet Med, Dept Gen & Clin Pathol, Stara Zagora 6000, Bulgaria. Trakia University, Fac Agr, Dept Morphol Anim, Stara Zagora 6000, Bulgaria. Trakia University, Fac Agr, Dept Microbiol, Stara Zagora 6000, Bulgaria. University of Zagreb, Inst Med Res & Occupat Hlth, Zagreb 41000, Croatia.	Country: Croatia<br>Institution: Trakia Univ	Country: Croatia<br>Institution: Trakia University
RP	1	102628	102628	Stoev, SD (corresponding author), Trakia Univ, Fac Vet Med, Dept Gen & Clin Pathol, Stara Zagora 6000, Bulgaria	Stoev, SD (corresponding author), Trakia University, Fac Vet Med, Dept Gen & Clin Pathol, Stara Zagora 6000, Bulgaria	Country: Bulgaria<br>Institution: Trakia Univ	Country: Bulgaria<br>Institution: Trakia University

Record 728 | WOS:000226417300009

The effects of the Penicillium mycotoxins citrinin, cyclopiazonic acid, ochratoxin A, patulin, penicillic acid, and roquefortine C on in vitro proliferation of porcine lymphocytes

Field	Position	Original line	Normalised line	Original value	Normalised value	Original country / institution	Normalised country / institution
C1	1	102734	102734	Natl Vet Inst, N-0033 Oslo, Norway. Norwegian Sch Vet Sci, Dept Food Safety & Infect Biol, N-0033 Oslo, Norway.	National Veterinary Institute - National Research Institute, N-0033 Oslo, Norway. Norwegian Veterinary Institute, Dept Food Safety & Infect Biol, N-0033 Oslo, Norway.	Country: Norway<br>Institution: Natl Vet Inst	Country: Norway<br>Institution: National Veterinary Institute - National Research Institute

RP	1	102737	102737	Bernhoft, A (corresponding author), Natl Vet Inst, POB 8156 DEP, N-0033 Oslo, Norway	Bernhoft, A (corresponding author), National Veterinary Institute - National Research Institute, POB 8156 DEP, N-0033 Oslo, Norway	Country: Norway<br>Institution: Natl Vet Inst	Country: Norway<br>Institution: National Veterinary Institute - National Research Institute

Record 729 | WOS:000223666000030

Fungal microflora and ochratoxin a risk in French vineyards

No C1/C3/RP normalisation changes detected for this record.

Record 730 | WOS:000223753400030

Alkalinization of urinary pH accelerates renal excretion of ochratoxin A in pigs

Field	Position	Original line	Normalised line	Original value	Normalised value	Original country / institution	Normalised country / institution
C1	1	102913	102913	Univ Kiel, Inst Anim Nutr Physiol & Metab, D-24098 Kiel, Germany.	University of Kiel, Inst Anim Nutr Physiol & Metab, D-24098 Kiel, Germany.	Country: Germany<br>Institution: Univ Kiel	Country: Germany<br>Institution: University of Kiel
RP	1	102915	102915	Blank, R (corresponding author), Univ Kiel, Inst Anim Nutr Physiol & Metab, D-24098 Kiel, Germany	Blank, R (corresponding author), University of Kiel, Inst Anim Nutr Physiol & Metab, D-24098 Kiel, Germany	Country: Germany<br>Institution: Univ Kiel	Country: Germany<br>Institution: University of Kiel

Record 731 | WOS:000224015700013

Synergistic effects of fumonisin B1 and ochratoxin A: are in vitro cytotoxicity data predictive of in vivo acute toxicity?

Field	Position	Original line	Normalised line	Original value	Normalised value	Original country / institution	Normalised country / institution
C1	1	103004	103004	Univ Bordeaux 2, Dept Toxicol, 146 Rue Leo Saignat, F-33076 Bordeaux, France. Univ Bari, Policlin, Sch Med, Dept Pharmacol & Human Physiol, I-70124 Bari, Italy. INRA, Ctr Rech Bordeaux Aquitaine, Unite Biol & Technol Apres Recolte, F-33883 Villenave Dornon, France.	Univ Bordeaux 2, Dept Toxicol, 146 Rue Leo Saignat, F-33076 Bordeaux, France. Univ Bari, Policlin, Sch Med, Dept Pharmacol & Human Physiol, I-70124 Bari, Italy. INRAE, Ctr Rech Bordeaux Aquitaine, Unite Biol & Technol Apres Recolte, F-33883 Villenave Dornon, France.	Country: France<br>Institution: Univ Bordeaux 2	Country: France<br>Institution: Univ Bordeaux 2

Record 732 | WOS:000224048300016

Evidence for covalent DNA adduction by ochratoxin A following chronic exposure to rat and subacute exposure to pig

Field	Position	Original line	Normalised line	Original value	Normalised value	Original country / institution	Normalised country / institution
C1	1	103120	103120	Univ Guelph, Dept Chem, Guelph, ON N1G 2W1, Canada. Ecole Natl Super Agron Toulouse, Dept Toxicol & Food Safety, Lab Genie Chim, CNRS,UMR 5503, F-31326 Castanet Tolosan, France. Wake Forest Univ, Dept Chem, Winston Salem, NC 27109 USA. IARC, F-69000 Lyon, France.	University of Guelph, Dept Chem, Guelph, ON N1G 2W1, Canada. Ecole Natl Super Agron Toulouse, Dept Toxicol & Food Safety, Lab Genie Chim, CNRS,UMR 5503, F-31326 Castanet Tolosan, France. Wake Forest University, Dept Chem, Winston Salem, NC 27109 USA. International Agency for	Country: France<br>Institution: Univ Guelph	Country: France<br>Institution: University of Guelph

					Research on Cancer (IARC), F-69000 Lyon, France.		
RP	1	103130	103130	Pfohl-Leszkowicz, A (corresponding author), Univ Guelph, Dept Chem, Guelph, ON N1G 2W1, Canada	Pfohl-Leszkowicz, A (corresponding author), University of Guelph, Dept Chem, Guelph, ON N1G 2W1, Canada	Country: Canada<br>Institution: Univ Guelph	Country: Canada<br>Institution: University of Guelph

Record 733 | WOS:000225210900008

Levels of mycotoxins and sample cytotoxicity of selected organic and conventional grain-based products purchased from Finnish and Italian markets

Field	Position	Original line	Normalised line	Original value	Normalised value	Original country / institution	Normalised country / institution
C1	1	103239	103239	Nat Vet & Food Res Inst, EELA, Dept Chem, FIN-00581 Helsinki, Finland. Univ Naples Federico II, Dept Food Sci, Portici, Italy. Nat Vet & Food Res Inst, EELA, Dept Virol, Helsinki, Finland.	Nat Vet & Food Res Inst, EELA, Dept Chem, FIN-00581 Helsinki, Finland. University of Naples Federico II, Dept Food Sci, Portici, Italy. Nat Vet & Food Res Inst, EELA, Dept Virol, Helsinki, Finland.	Country: Finland<br>Institution: Nat Vet & Food Res Inst	Country: Finland<br>Institution: Nat Vet & Food Res Inst

Record 734 | WOS:000223297700006

Effects of rosmarinic acid against aflatoxin B1 and ochratoxin-A-induced cell damage in a human hepatoma cell line (Hep G2)

Field	Position	Original line	Normalised line	Original value	Normalised value	Original country / institution	Normalised country / institution
C1	1	103326	103326	Univ Bologna, Dept Pharmacol, I-40126 Bologna, Italy. Univ Reggio Calabria, Dept Agroforestry Environm Sci & Technol, Reggio Di Calabria, Italy. Univ Bologna, Inst Histol & Gen Embryol, Bologna, Italy.	University of Bologna, Dept Pharmacol, I-40126 Bologna, Italy. Univ Reggio Calabria, Dept Agroforestry Environm Sci & Technol, Reggio Di Calabria, Italy. University of Bologna, Inst Histol & Gen Embryol, Bologna, Italy.	Country: Italy<br>Institution: Univ Bologna	Country: Italy<br>Institution: University of Bologna
RP	1	103331	103331	Guerra, MC (corresponding author), Univ Bologna, Dept Pharmacol, Via Irnerio 48, I-40126 Bologna, Italy	Guerra, MC (corresponding author), University of Bologna, Dept Pharmacol, Via Irnerio 48, I-40126 Bologna, Italy	Country: Italy<br>Institution: Univ Bologna	Country: Italy<br>Institution: University of Bologna

Record 735 | WOS:000221551200016

Ochratoxin A induces apoptosis in human lymphocytes through down regulation of Bcl-xL

No C1/C3/RP normalisation changes detected for this record.

Record 736 | WOS:000221555600005

Metabolism-mediated cytotoxicity of ochratoxin A

Field	Position	Original line	Normalised line	Original value	Normalised value	Original country / institution	Normalised country / institution
C1	1	103519	103519	Univ Utrecht, Dept Vet Pharm Pharmacol & Toxicol, Fac Vet Med, NL-3584 CM Utrecht, Netherlands.	Utrecht University, Dept Vet Pharm Pharmacol & Toxicol, Fac Vet Med, NL-3584 CM Utrecht, Netherlands.	Country: Netherlands<br>Institution: Univ Utrecht	Country: Netherlands<br>Institution: Utrecht University

RP	1	103521	103521	Fink-Gremmels, J (corresponding author), Univ Utrecht, Dept Vet Pharm Pharmacol & Toxicol, Fac Vet Med, Yalelaan 16, NL-3584 CM Utrecht, Netherlands	Fink-Gremmels, J (corresponding author), Utrecht University, Dept Vet Pharm Pharmacol & Toxicol, Fac Vet Med, Yalelaan 16, NL-3584 CM Utrecht, Netherlands	Country: Netherlands Institution: Univ Utrecht	Country: Netherlands Institution: Utrecht University

Record 737 | WOS:000220837300016

Immunotoxic effects of Ochratoxin A in wistar rats after oral administration

Field	Position	Original line	Normalised line	Original value	Normalised value	Original country / institution	Normalised country / institution
C1	1	103617	103617	Univ Navarra, Dept Food Sci & Toxicol, Fac Pharm, E-31080 Pamplona, Spain. Univ Zaragoza, Dept Anim Pathol, Fac Vet, Zaragoza, Spain.	University of Navarra, Dept Food Sci & Toxicol, Fac Pharm, E-31080 Pamplona, Spain. University of Zaragoza, Dept Anim Pathol, Fac Vet, Zaragoza, Spain.	Country: Spain Institution: Univ Navarra	Country: Spain Institution: University of Navarra
RP	1	103620	103620	de Cerain, AL (corresponding author), Univ Navarra, Dept Food Sci & Toxicol, Fac Pharm, E-31080 Pamplona, Spain	de Cerain, AL (corresponding author), University of Navarra, Dept Food Sci & Toxicol, Fac Pharm, E-31080 Pamplona, Spain	Country: Spain Institution: Univ Navarra	Country: Spain Institution: University of Navarra

Record 738 | WOS:000221514400030

Synergistic interaction in simultaneous exposure to Streptomyces californicus and Stachybotrys chartarum

No C1/C3/RP normalisation changes detected for this record.

Record 739 | WOS:000220235700005

Inhibitory effects of ochratoxin A on nerve growth factor-induced neurite extension through downregulation of p38 map kinase and AP-1 activation in cultured pheochromocytoma cells

Field	Position	Original line	Normalised line	Original value	Normalised value	Original country / institution	Normalised country / institution
C1	1	103856	103856	Chungbuk Natl Univ, Coll Pharm, Cheongju 361763, Chungbuk, South Korea. Korea Food & Drug Adm, Seoul, South Korea. Pukyong Natl Univ, Dept Biotechnol & Bioengn, Pusan, South Korea. Korea Food Res Inst, Keongki Do, South Korea. Coll Vet Med, Seoul, South Korea.	Chungbuk National University, Coll Pharm, Cheongju 361763, Chungbuk, South Korea. Korea Food & Drug Adm, Seoul, South Korea. Pukyong National University, Dept Biotechnol & Bioengn, Pusan, South Korea. Korea Food Research Institute (KFRI), Keongki Do, South Korea. Coll Vet Med, Seoul, South Korea.	Country: South Korea Institution: Chungbuk Natl Univ	Country: South Korea Institution: Chungbuk National University
RP	1	103863	103863	Hong, JT (corresponding author), Chungbuk Natl Univ, Coll Pharm, 48 Gaesin Dong, Cheongju 361763, Chungbuk, South Korea	Hong, JT (corresponding author), Chungbuk National University, Coll Pharm, 48 Gaesin Dong, Cheongju 361763, Chungbuk, South Korea	Country: South Korea Institution: Chungbuk Natl Univ	Country: South Korea Institution: Chungbuk National University

Record 740 | WOS:000189087700013

Ochratoxin A: Lack of formation of covalent DNA adducts

Field	Position	Original line	Normalised line	Original value	Normalised value	Original country / institution	Normalised country / institution
C1	1	103955	103955	Univ Wurzburg, Inst Toxikol, D-97078 Wurzburg, Germany. Univ Wurzburg, Inst Organ Chem, D-97074 Wurzburg, Germany. Lawrence Livermore Natl Lab, Biol & Biotechnol Res Program, Livermore, CA 94550 USA.	University of Wurzburg, Inst Toxikol, D-97078 Wurzburg, Germany. University of Wurzburg, Inst Organ Chem, D-97074 Wurzburg, Germany. Lawrence Livermore Natl Lab, Biol & Biotechnol Res Program, Livermore, CA 94550 USA.	Country: USA Institution: Univ Wurzburg	Country: USA Institution: University of Wurzburg
RP	1	103960	103960	Dekant, W (corresponding author), Univ Wurzburg, Inst Toxikol, Versbacher Str 9, D-97078 Wurzburg, Germany	Dekant, W (corresponding author), University of Wurzburg, Inst Toxikol, Versbacher Str 9, D-97078 Wurzburg, Germany	Country: Germany Institution: Univ Wurzburg	Country: Germany Institution: University of Wurzburg

Record 741 | WOS:000188530700003

Ochratoxin A and zearalenone: a comparative study on genotoxic effects and cell death induced in bovine lymphocytes

Field	Position	Original line	Normalised line	Original value	Normalised value	Original country / institution	Normalised country / institution
C1	1	104044	104044	Univ Basilicata, Dipartimento Sci Prod Anim, I-85100 Potenza, Italy. CNR, Ist Genet & Biofis, A Buzzati Traverso, I-80125 Naples, Italy. CNR, Ist Endocrinol & Oncol Sperimentale, I-80125 Naples, Italy.	University of Basilicata, Dipartimento Sci Prod Anim, I-85100 Potenza, Italy. CNR, Ist Genet & Biofis, A Buzzati Traverso, I-80125 Naples, Italy. CNR, Ist Endocrinol & Oncol Sperimentale, I-80125 Naples, Italy.	Country: Italy Institution: Univ Basilicata	Country: Italy Institution: University of Basilicata
RP	1	104051	104051	Lioi, MB (corresponding author), Univ Basilicata, Dipartimento Sci Prod Anim, Vi N Sauro 85, I-85100 Potenza, Italy	Lioi, MB (corresponding author), University of Basilicata, Dipartimento Sci Prod Anim, Vi N Sauro 85, I-85100 Potenza, Italy	Country: Italy Institution: Univ Basilicata	Country: Italy Institution: University of Basilicata

Record 742 | WOS:000189121300007

New insights into the mechanisms involved in renal proximal tubular damage induced in vitro by ochratoxin A

Field	Position	Original line	Normalised line	Original value	Normalised value	Original country / institution	Normalised country / institution
C1	1	104155	104155	ECVAM, Inst Hlth & Consumer Protect, I-21020 Ispra, VA, Italy. Univ Milan, Dept Pharmacol Sci, I-20133 Milan, Italy.	ECVAM, Inst Hlth & Consumer Protect, I-21020 Ispra, VA, Italy. University of Milan, Dept Pharmacol Sci, I-20133 Milan, Italy.	Country: Italy Institution: ECVAM	Country: Italy Institution: ECVAM

Record 743 | WOS:000189041600006

Inhibition of mitochondria and extracellular acidification enhance ochratoxin A-induced apoptosis in renal collecting duct-derived MDCK-C7 cells

Field	Position	Original line	Normalised line	Original value	Normalised value	Original country / institution	Normalised country / institution
C1	1	104246	104246	Univ Wurzburg, Inst Physiol, D-97070 Wurzburg, Germany.	University of Wurzburg, Inst Physiol, D-97070 Wurzburg, Germany.	Country: Germany Institution: Univ Wurzburg	Country: Germany Institution: University of Wurzburg
RP	1	104248	104248	Schwerdt, G (corresponding author), Univ Wurzburg, Inst	Schwerdt, G (corresponding author), University of Wurzburg, Inst Physiol,	Country: Germany Institution: Univ Wurzburg	Country: Germany Institution: University of Wurzburg

				Physiol, Rontgenring 9, D-97070 Wurzburg, Germany	Rontgenring 9, D-97070 Wurzburg, Germany		

Record 744 | WOS:000231756500075

Natural antioxidants and mycotoxins: Theoretical considerations and practical applications

Field	Position	Original line	Normalised line	Original value	Normalised value	Original country / institution	Normalised country / institution
C1	1	104336	104336	Scottish Agr Coll, Avian Sci Res Ctr, Auchincruive KA6 5HW, Ayr, Scotland.	Scottish Agricultural College, Avian Sci Res Ctr, Auchincruive KA6 5HW, Ayr, United Kingdom.	Country: Scotland Institution: Scottish Agr Coll	Country: United Kingdom Institution: Scottish Agricultural College
RP	1	104338	104338	Surai, PF (corresponding author), Scottish Agr Coll, Avian Sci Res Ctr, Auchincruive KA6 5HW, Ayr, Scotland	Surai, PF (corresponding author), Scottish Agricultural College, Avian Sci Res Ctr, Auchincruive KA6 5HW, Ayr, United Kingdom	Country: Scotland Institution: Scottish Agr Coll	Country: United Kingdom Institution: Scottish Agricultural College

Record 745 | WOS:000186953000004

Apoptosis and oxidative stress induced by ochratoxin A in rat kidney

Field	Position	Original line	Normalised line	Original value	Normalised value	Original country / institution	Normalised country / institution
C1	1	104437	104437	Univ Zagreb, Fac Pharm & Biochem, Dept Med Biochem & Haematol, Zagreb 10000, Croatia. Fac Pharm & Biochem, Dept Microbiol, Zagreb 10000, Croatia. Inst Med Res & Occupat Hlth, Zagreb 10000, Croatia. PLIVA Res Inst, Lab Toxicol & Pathol, Zagreb 10000, Croatia.	University of Zagreb, Fac Pharm & Biochem, Dept Med Biochem & Haematol, Zagreb 10000, Croatia. Fac Pharm & Biochem, Dept Microbiol, Zagreb 10000, Croatia. Institute for Medical Research & Occupational Health (IMROH), Zagreb 10000, Croatia. Pliva, Lab Toxicol & Pathol, Zagreb 10000, Croatia.	Country: Croatia Institution: Univ Zagreb	Country: Croatia Institution: University of Zagreb
RP	1	104444	104444	Petrik, J (corresponding author), Univ Zagreb, Fac Pharm & Biochem, Dept Med Biochem & Haematol, Ante Kovacica 1,POB 156, Zagreb 10000, Croatia	Petrik, J (corresponding author), University of Zagreb, Fac Pharm & Biochem, Dept Med Biochem & Haematol, Ante Kovacica 1,POB 156, Zagreb 10000, Croatia	Country: Croatia Institution: Univ Zagreb	Country: Croatia Institution: University of Zagreb

Record 746 | WOS:000186282400042

Effects of chronic ingestion of ochratoxin A on blood levels and excretion of the mycotoxin in sheep

Field	Position	Original line	Normalised line	Original value	Normalised value	Original country / institution	Normalised country / institution
C1	1	104561	104561	Univ Kiel, Inst Anim Nutr Physiol & Metab, D-24098 Kiel, Germany. Univ Manitoba, Dept Anim Sci, Winnipeg, MB R3T 2N2, Canada.	University of Kiel, Inst Anim Nutr Physiol & Metab, D-24098 Kiel, Germany. Univ Manitoba, Dept Anim Sci, Winnipeg, MB R3T 2N2, Canada.	Country: Canada Institution: Univ Kiel	Country: Canada Institution: University of Kiel
RP	1	104564	104564	Blank, R (corresponding author), Univ Kiel, Inst Anim Nutr Physiol & Metab, D-24098 Kiel, Germany	Blank, R (corresponding author), University of Kiel, Inst Anim Nutr Physiol & Metab, D-24098 Kiel, Germany	Country: Germany Institution: Univ Kiel	Country: Germany Institution: University of Kiel

Record 747 | WOS:000184259400007

Effects of the mycotoxin ochratoxin A and some of its metabolites on human kidney cell lines

No C1/C3/RP normalisation changes detected for this record.

Record 748 | WOS:000184243200002

An oxygen-bonded C8-deoxyguanosine nucleoside adduct of pentachlorophenol by peroxidase activation: Evidence for ambident C8 reactivity by phenoxyl radicals

Field	Position	Original line	Normalised line	Original value	Normalised value	Original country / institution	Normalised country / institution
C1	1	104749	104749	Wake Forest Univ, Dept Chem, Winston Salem, NC 27109 USA.	Wake Forest University, Dept Chem, Winston Salem, NC 27109 USA.	Country: USA<br>Institution: Wake Forest Univ	Country: USA<br>Institution: Wake Forest University
RP	1	104751	104751	Manderville, RA (corresponding author), Wake Forest Univ, Dept Chem, Winston Salem, NC 27109 USA	Manderville, RA (corresponding author), Wake Forest University, Dept Chem, Winston Salem, NC 27109 USA	Country: USA<br>Institution: Wake Forest Univ	Country: USA<br>Institution: Wake Forest University

Record 749 | WOS:000184363500006

Effect of Ochratoxin A on human haematopoietic progenitors proliferation and differentiation: an in vitro study

No C1/C3/RP normalisation changes detected for this record.

Record 750 | WOS:000185415100022

Evaluation of two mycotoxin binders to reduce toxicity of broiler diets containing ochratoxin A and T-2 toxin contaminated grain

Field	Position	Original line	Normalised line	Original value	Normalised value	Original country / institution	Normalised country / institution
RP	1	104927	104927	GarcÃ-a, AR (corresponding author), Univ Georgia, 301 Poultry Sci Bldg, Athens, GA 30602 USA	GarcÃ-a, AR (corresponding author), University of Georgia, 301 Poultry Sci Bldg, Athens, GA 30602 USA	Country: USA<br>Institution: Univ Georgia	Country: USA<br>Institution: University of Georgia

Record 751 | WOS:000183040200012

A new approach to studying ochratoxin A (OTA)-induced nephrotoxicity: Expression profiling in vivo and in vitro employing cDNA microarrays

Field	Position	Original line	Normalised line	Original value	Normalised value	Original country / institution	Normalised country / institution
C1	1	105026	105026	Bayer AG, Dept Mol & Genet Toxicol, D-42096 Wuppertal, Germany. Bayer AG, Dept Pathol, D-42096 Wuppertal, Germany. Goethe Univ Frankfurt, Inst Pharmaceut Biol, D-60439 Frankfurt, Germany.	Bayer AG, Dept Mol & Genet Toxicol, D-42096 Wuppertal, Germany. Bayer AG, Dept Pathol, D-42096 Wuppertal, Germany. Goethe University Frankfurt, Inst Pharmaceut Biol, D-60439 Frankfurt, Germany.	Country: Germany<br>Institution: Bayer AG	Country: Germany<br>Institution: Bayer AG

Record 752 | WOS:000183875100009

Effects of the mycotoxin ochratoxin A in a bacterial and a mammalian in vitro mutagenicity test system

No C1/C3/RP normalisation changes detected for this record.

Record 753 | WOS:000182307900052

Interaction of ochratoxin A with human intestinal Caco-2 cells: possible implication of a multidrug resistance-associated protein (MRP2)

No C1/C3/RP normalisation changes detected for this record.

Record 754 | WOS:000181518501697

Cellular and molecular mechanisms underlying ochratoxin A-induced in vitro nephrotoxicity.

No C1/C3/RP normalisation changes detected for this record.

Record 755 | WOS:000180037400011

Photochemically catalyzed reaction of ochratoxin A with D- and L-cysteine

Field	Position	Original line	Normalised line	Original value	Normalised value	Original country / institution	Normalised country / institution
C1	1	105379	105379	Wake Forest Univ, Dept Chem, Winston Salem, NC 27109 USA.	Wake Forest University, Dept Chem, Winston Salem, NC 27109 USA.	Country: USA<br>Institution: Wake Forest Univ	Country: USA<br>Institution: Wake Forest University
RP	1	105381	105381	Manderville, RA (corresponding author), Wake Forest Univ, Dept Chem, Winston Salem, NC 27109 USA	Manderville, RA (corresponding author), Wake Forest University, Dept Chem, Winston Salem, NC 27109 USA	Country: USA<br>Institution: Wake Forest Univ	Country: USA<br>Institution: Wake Forest University

Record 756 | WOS:000179850500004

Prevention and control of mycotoxins in the poultry production chain:: a European view

No C1/C3/RP normalisation changes detected for this record.

Record 757 | WOS:000178857500004

RETRACTED: Antioxidant nutrients and mycotoxins (Retracted Article)

Field	Position	Original line	Normalised line	Original value	Normalised value	Original country / institution	Normalised country / institution
C1	1	105653	105653	Univ Helsinki, Fac Med Vet, Dept Clin Sci Pharmacol & Toxicol, FIN-00014 Helsinki, Finland. Natl Vet & Food Res Inst, Dept Chem, Helsinki, Finland. Univ Helsinki, Fac Med Vet, Dept Microbiol & Epidemiol, FIN-00014 Helsinki, Finland.	University of Helsinki, Fac Med Vet, Dept Clin Sci Pharmacol & Toxicol, FIN-00014 Helsinki, Finland. Natl Vet & Food Res Inst, Dept Chem, Helsinki, Finland. University of Helsinki, Fac Med Vet, Dept Microbiol & Epidemiol, FIN-00014 Helsinki, Finland.	Country: Finland<br>Institution: Univ Helsinki	Country: Finland<br>Institution: University of Helsinki
RP	1	105657	105657	Atroshi, F (corresponding author), Univ Helsinki, Fac Med Vet, Dept Clin Sci Pharmacol & Toxicol, POB 57, FIN-00014 Helsinki, Finland	Atroshi, F (corresponding author), University of Helsinki, Fac Med Vet, Dept Clin Sci Pharmacol & Toxicol, POB 57, FIN-00014 Helsinki, Finland	Country: Finland<br>Institution: Univ Helsinki	Country: Finland<br>Institution: University of Helsinki

Record 758 | WOS:000177393700006

Genotoxic effects of ochratoxin A in human-derived hepatoma (HepG2) cells

Field	Position	Original line	Normalised line	Original value	Normalised value	Original country / institution	Normalised country / institution
C1	1	105870	105870	Univ Vienna, Inst Canc Res, A-1090 Vienna, Austria. Leiden State Univ, Med Ctr, Dept Radiat Genet & Chem Mutagenesis, NL-2333 AL Leiden, Netherlands. Inst Bldg Biol & Ecol, A-1090 Vienna, Austria.	University of Vienna, Inst Canc Res, A-1090 Vienna, Austria. Leiden State Univ, Med Ctr, Dept Radiat Genet & Chem Mutagenesis, NL-2333 AL Leiden, Netherlands. Inst Bldg Biol & Ecol, A-1090 Vienna, Austria.	Country: Austria Institution: Univ Vienna	Country: Austria Institution: University of Vienna
RP	1	105874	105874	Ehrlich, V (corresponding author), Univ Vienna, Inst Canc Res, Borschkegasse 8A, A-1090 Vienna, Austria	Ehrlich, V (corresponding author), University of Vienna, Inst Canc Res, Borschkegasse 8A, A-1090 Vienna, Austria	Country: Austria Institution: Univ Vienna	Country: Austria Institution: University of Vienna

Record 759 | WOS:000177631900009

Species- and sex-specific variations in binding of ochratoxin A by renal proteins in vitro

Field	Position	Original line	Normalised line	Original value	Normalised value	Original country / institution	Normalised country / institution
C1	1	105964	105964	Univ Konstanz, D-78457 Constance, Germany.	University of Konstanz, D-78457 Constance, Germany.	Country: Germany Institution: Univ Konstanz	Country: Germany Institution: University of Konstanz
RP	1	105966	105966	Dietrich, DR (corresponding author), Univ Konstanz, Jacob Burckhardstr 25,POB X918, D-78457 Constance, Germany	Dietrich, DR (corresponding author), University of Konstanz, Jacob Burckhardstr 25,POB X918, D-78457 Constance, Germany	Country: Germany Institution: Univ Konstanz	Country: Germany Institution: University of Konstanz

Record 760 | WOS:000176910000001

Aristolochic acid as a probable human cancer hazard in herbal remedies: a review

Field	Position	Original line	Normalised line	Original value	Normalised value	Original country / institution	Normalised country / institution
C1	1	106059	106059	Inst Canc Res, Sutton SM2 5NG, Surrey, England. Charles Univ Prague, Fac Sci, Dept Biochem, Prague 12840 2, Czech Republic. German Canc Res Ctr, Div Mol Toxicol, D-69120 Heidelberg, Germany.	Inst Canc Res, Sutton SM2 5NG, Surrey, United Kingdom. Charles University Prague, Fac Sci, Dept Biochem, Prague 12840 2, Czech Republic. German Canc Res Ctr, Div Mol Toxicol, D-69120 Heidelberg, Germany.	Country: Germany Institution: Inst Canc Res	Country: Germany Institution: Inst Canc Res
C3	2	106062	106062	Institute of Cancer Research - UK	Institute of Cancer Research - United Kingdom	Institution: Institute of Cancer Research - UK	Institution: Institute of Cancer Research - United Kingdom
RP	1	106065	106065	Arlt, VM (corresponding author), Inst Canc Res, Cotswold Rd, Sutton SM2 5NG, Surrey, England	Arlt, VM (corresponding author), Inst Canc Res, Cotswold Rd, Sutton SM2 5NG, Surrey, United Kingdom	Country: England Institution: Inst Canc Res	Country: United Kingdom Institution: Inst Canc Res

Record 761 | WOS:000175922300023

Esterified-glucomannan in broiler chicken diets-contaminated with aflatoxin, ochratoxin and T-2 toxin:: Evaluation of its binding ability (in vitro) and efficacy as immunomodulator

Field	Position	Original line	Normalised line	Original value	Normalised value	Original country / institution	Normalised country / institution
C1	1	106236	106236	Univ Agr Sci Bangalore, Dept Poultry Sci, Bangalore 560024, Karnataka, India.	University of Agricultural Sciences Bangalore, Dept Poultry Sci, Bangalore 560024, Karnataka, India.	Country: India Institution: Univ Agr Sci Bangalore	Country: India Institution: University of Agricultural Sciences Bangalore

Record 762 | WOS:000176518700006

Role of human organic anion transporter 4 in the transport of ochratoxin A

Field	Position	Original line	Normalised line	Original value	Normalised value	Original country / institution	Normalised country / institution
C1	1	106324	106324	Kyorin Univ, Sch Med, Dept Pharmacol & Toxicol, Mitaka, Tokyo 1818611, Japan. Univ Madras, Dept Med Biochem, Chennai, India. Univ Tokyo, Dept Internal Med, Div Nephrol, Tokyo, Japan.	Kyorin University, Sch Med, Dept Pharmacol & Toxicol, Mitaka, Tokyo 1818611, Japan. University of Madras, Dept Med Biochem, Chennai, India. University of Tokyo, Dept Internal Med, Div Nephrol, Tokyo, Japan.	Country: Japan Institution: Kyorin Univ	Country: Japan Institution: Kyorin University
RP	1	106328	106328	Endou, H (corresponding author), Kyorin Univ, Sch Med, Dept Pharmacol & Toxicol, 6-20-2 Shinkawa, Mitaka, Tokyo 1818611, Japan	Endou, H (corresponding author), Kyorin University, Sch Med, Dept Pharmacol & Toxicol, 6-20-2 Shinkawa, Mitaka, Tokyo 1818611, Japan	Country: Japan Institution: Kyorin Univ	Country: Japan Institution: Kyorin University

Record 763 | WOS:000176216800005

Expression of Hsp70 in kidney cells exposed to ochratoxin A

Field	Position	Original line	Normalised line	Original value	Normalised value	Original country / institution	Normalised country / institution
C1	1	106433	106433	Univ Zagreb, Fac Pharm & Biochem, Dept Med Biochem & Hematol, Zagreb 10000, Croatia.	University of Zagreb, Fac Pharm & Biochem, Dept Med Biochem & Hematol, Zagreb 10000, Croatia.	Country: Croatia Institution: Univ Zagreb	Country: Croatia Institution: University of Zagreb
RP	1	106435	106435	Barisic, K (corresponding author), Univ Zagreb, Fac Pharm & Biochem, Dept Med Biochem & Hematol, A Kovacica 1, Zagreb 10000, Croatia	Barisic, K (corresponding author), University of Zagreb, Fac Pharm & Biochem, Dept Med Biochem & Hematol, A Kovacica 1, Zagreb 10000, Croatia	Country: Croatia Institution: Univ Zagreb	Country: Croatia Institution: University of Zagreb

Record 764 | WOS:000175152300005

Effect of T-2 toxin on in vivo lipid peroxidation and vitamin E status in mice

Field	Position	Original line	Normalised line	Original value	Normalised value	Original country / institution	Normalised country / institution
C1	1	106536	106536	Purdue Univ, Dept Food Sci, W Lafayette, IN 47907 USA. Univ Manitoba, Dept Anim Sci, Winnipeg, MB R3T 2N2, Canada.	Purdue University, Dept Food Sci, W Lafayette, IN 47907 USA. Univ Manitoba, Dept Anim Sci, Winnipeg, MB R3T 2N2, Canada.	Country: Canada Institution: Purdue Univ	Country: Canada Institution: Purdue University
RP	1	106539	106539	Jaradat, ZW (corresponding author), Purdue Univ, Dept Food Sci, W Lafayette, IN 47907 USA	Jaradat, ZW (corresponding author), Purdue University, Dept Food Sci, W Lafayette, IN 47907 USA	Country: USA Institution: Purdue Univ	Country: USA Institution: Purdue University

Record 765 | WOS:000173055800005

Determination of the epigenetic effects of ochratoxin in a human kidney and a rat liver epithelial cell line

Field	Position	Original line	Normalised line	Original value	Normalised value	Original country / institution	Normalised country / institution
C1	1	106624	106624	Michigan State Univ, Dept Pediat & Human Dev, E Lansing, MI 48824 USA. Michigan State Univ, Natl Food Safety & Toxicol Ctr, E Lansing, MI 48824 USA. Michigan State Univ, Inst Int Hlth, E Lansing, MI 48824 USA. Med Univ Sofia, Dept Chem & Biochem, Sofia, Bulgaria.	Michigan State University, Dept Pediat & Human Dev, E Lansing, MI 48824 USA. Michigan State University, Natl Food Safety & Toxicol Ctr, E Lansing, MI 48824 USA. Michigan State University, Inst Int Hlth, E Lansing, MI 48824 USA. Medical University Sofia, Dept Chem & Biochem, Sofia, Bulgaria.	Country: Bulgaria<br>Institution: Michigan State Univ	Country: Bulgaria<br>Institution: Michigan State University
RP	1	106630	106630	Upham, BL (corresponding author), Michigan State Univ, Dept Pediat & Human Dev, E Lansing, MI 48824 USA	Upham, BL (corresponding author), Michigan State University, Dept Pediat & Human Dev, E Lansing, MI 48824 USA	Country: USA<br>Institution: Michigan State Univ	Country: USA<br>Institution: Michigan State University

Record 766 | WOS:000176962600010

Toxins of filamentous fungi

Field	Position	Original line	Normalised line	Original value	Normalised value	Original country / institution	Normalised country / institution
C1	1	106754	106754	USDA ARS, So Reg Res Ctr, New Orleans, LA 70124 USA.	United States Department of Agriculture (USDA), So Reg Res Ctr, New Orleans, LA 70124 USA.	Country: USA<br>Institution: USDA ARS	Country: USA<br>Institution: United States Department of Agriculture (USDA)
RP	1	106756	106756	Bhatnagar, D (corresponding author), USDA ARS, So Reg Res Ctr, 1100 Robert E Lee Blvd, New Orleans, LA 70124 USA	Bhatnagar, D (corresponding author), United States Department of Agriculture (USDA), So Reg Res Ctr, 1100 Robert E Lee Blvd, New Orleans, LA 70124 USA	Country: USA<br>Institution: USDA ARS	Country: USA<br>Institution: United States Department of Agriculture (USDA)

Record 767 | WOS:000174243300013

Detection of ochratoxin A-induced DNA damage in MDCK cells by alkaline single cell gel electrophoresis (comet assay)

No C1/C3/RP normalisation changes detected for this record.

Record 768 | WOS:000172508300013

Improvement of megakaryocytic progenitor culture for toxicological investigations

No C1/C3/RP normalisation changes detected for this record.

Record 769 | WOS:000173171400003

Horseradish peroxidase mediates DNA and deoxyguanosine 3â ²-monophosphate adduct formation in the presence of ochratoxin A

No C1/C3/RP normalisation changes detected for this record.

Record 770 | WOS:000171478500001

Toxicity, metabolism, and impact of mycotoxins on humans and animals

No C1/C3/RP normalisation changes detected for this record.

Record 771 | WOS:000171571300006

The mycotoxin ochratoxin A alters intestinal barrier and absorption functions but has no effect on chloride secretion

No C1/C3/RP normalisation changes detected for this record.

Record 772 | WOS:000171310400014

Species-, sex-, and cell type-specific effects of ochratoxin A and B

Field	Position	Original line	Normalised line	Original value	Normalised value	Original country / institution	Normalised country / institution
C1	1	107746	107746	Univ Konstanz, D-78457 Constance, Germany.	University of Konstanz, D-78457 Constance, Germany.	Country: Germany Institution: Univ Konstanz	Country: Germany Institution: University of Konstanz
RP	1	107748	107748	Dietrich, DR (corresponding author), Univ Konstanz, Jacob Burckhardtstr 25,POB 5560 X918, D-78457 Constance, Germany	Dietrich, DR (corresponding author), University of Konstanz, Jacob Burckhardtstr 25,POB 5560 X918, D-78457 Constance, Germany	Country: Germany Institution: Univ Konstanz	Country: Germany Institution: University of Konstanz

Record 773 | WOS:000171274400005

Characterization of ochratoxin A transport by human organic anion transporters

Field	Position	Original line	Normalised line	Original value	Normalised value	Original country / institution	Normalised country / institution
C1	1	107881	107881	Kyorin Univ, Sch Med, Dept Pharmacol & Toxicol, Mitaka, Tokyo 1818611, Japan. Wonkwang Univ, Sch Med, Dept Pharmacol & Toxicol, Chonju, South Korea. Univ Tokyo, Fac Med, Dept Internal Med 2, Tokyo 113, Japan.	Kyorin University, Sch Med, Dept Pharmacol & Toxicol, Mitaka, Tokyo 1818611, Japan. Wonkwang University, Sch Med, Dept Pharmacol & Toxicol, Chonju, South Korea. University of Tokyo, Fac Med, Dept Internal Med 2, Tokyo 113, Japan.	Country: Japan Institution: Kyorin Univ	Country: Japan Institution: Kyorin University
RP	1	107885	107885	Endou, H (corresponding author), Kyorin Univ, Sch Med, Dept Pharmacol & Toxicol, 6-20-2 Shinkawa, Mitaka, Tokyo 1818611, Japan	Endou, H (corresponding author), Kyorin University, Sch Med, Dept Pharmacol & Toxicol, 6-20-2 Shinkawa, Mitaka, Tokyo 1818611, Japan	Country: Japan Institution: Kyorin Univ	Country: Japan Institution: Kyorin University

Record 774 | WOS:000170039000017

Species- and sex-specific renal cytotoxicity of Ochratoxin A and B in vitro

Field	Position	Original line	Normalised line	Original value	Normalised value	Original country / institution	Normalised country / institution
C1	1	107967	107967	Univ Konstanz, D-78464 Constance, Germany. US FDA, Washington, DC 20204 USA.	University of Konstanz, D-78464 Constance, Germany. US Food & Drug Administration (FDA), Washington, DC 20204 USA.	Country: USA Institution: Univ Konstanz	Country: USA Institution: University of Konstanz
RP	1	107970	107970	Dietrich, DR (corresponding author), Univ Konstanz, Jacob Burckhardt Str 25, D-78464 Constance, Germany	Dietrich, DR (corresponding author), University of Konstanz, Jacob Burckhardt Str 25, D-78464 Constance, Germany	Country: Germany Institution: Univ Konstanz	Country: Germany Institution: University of Konstanz

Record 775 | WOS:000169038800006

Aspartame prevents the karyomegaly induced by ochratoxin A in rat kidney

Field	Position	Original line	Normalised line	Original value	Normalised value	Original country / institution	Normalised country / institution
C1	1	108069	108069	Univ Bordeaux 2, UFR Sci Pharmaceut, Lab Toxikol & Hyg Appliquee, F-33076 Bordeaux, France. Inst Med Res & Occupat Hlth, Zagreb 41001, Croatia. Univ Abidjan, Fac Pharm, Abidjan, Cote Ivoire. Univ Natl Benin, FAST, Dept Biochim & Biol Cellulaire, Cotonou, Benin. Univ Stellenbosch, Div Res Dev, ZA-7600 Stellenbosch, South Africa.	Univ Bordeaux 2, UFR Sci Pharmaceut, Lab Toxikol & Hyg Appliquee, F-33076 Bordeaux, France. Institute for Medical Research & Occupational Health (IMROH), Zagreb 41001, Croatia. Univ Abidjan, Fac Pharm, Abidjan, Cote d'Ivoire. Univ Natl Benin, FAST, Dept Biochim & Biol Cellulaire, Cotonou, Benin. Stellenbosch University, Div Res Dev, ZA-7600 Stellenbosch, South Africa.	Country: South Africa Institution: Univ Bordeaux 2	Country: South Africa Institution: Univ Bordeaux 2

Record 776 | WOS:000167652300006

Age-related differences in the toxicity of ochratoxin A in female rats

No C1/C3/RP normalisation changes detected for this record.

Record 777 | WOS:000166500400005

Metabolism of ochratoxin A: Absence of formation of genotoxic derivatives by human and rat enzymes

Field	Position	Original line	Normalised line	Original value	Normalised value	Original country / institution	Normalised country / institution
C1	1	108280	108280	Vanderbilt Univ, Sch Med, Ctr Mol Toxicol, Nashville, TN 37232 USA. Vanderbilt Univ, Sch Med, Dept Biochem, Nashville, TN 37232 USA. Nestec Ltd, Nestle Res Ctr, CH-1000 Lausanne 26, Switzerland.	Vanderbilt University, Sch Med, Ctr Mol Toxicol, Nashville, TN 37232 USA. Vanderbilt University, Sch Med, Dept Biochem, Nashville, TN 37232 USA. Nestec Ltd, Nestle Res Ctr, CH-1000 Lausanne 26, Switzerland.	Country: Switzerland Institution: Vanderbilt Univ	Country: Switzerland Institution: Vanderbilt University

Record 778 | WOS:000165378500005

Effects of ochratoxin A on cytotoxicity and cell differentiation in cultured rat embryonic cells

No C1/C3/RP normalisation changes detected for this record.

Record 779 | WOS:000089269500007

Studies of ochratoxin A-induced inhibition of phenylalanine hydroxylase and its reversal by phenylalanine

Field	Position	Original line	Normalised line	Original value	Normalised value	Original country / institution	Normalised country / institution
C1	1	108500	108500	Univ Zagreb, Dept Med Biochem & Hematol, Zagreb 10000, Croatia. Univ Zagreb, Dept Microbiol, Fac Pharm & Biochem, Zagreb 10000, Croatia. Inst Med Res & Occupat Hlth, Zagreb 10000, Croatia.	University of Zagreb, Dept Med Biochem & Hematol, Zagreb 10000, Croatia. University of Zagreb, Dept Microbiol, Fac Pharm & Biochem, Zagreb 10000, Croatia. Institute for Medical Research & Occupational Health (IMROH), Zagreb 10000, Croatia.	Country: Croatia<br>Institution: Univ Zagreb	Country: Croatia<br>Institution: University of Zagreb
RP	1	108505	108505	Zanic-Grubisic, T (corresponding author), Univ Zagreb, Dept Med Biochem & Hematol, Zagreb 10000, Croatia	Zanic-Grubisic, T (corresponding author), University of Zagreb, Dept Med Biochem & Hematol, Zagreb 10000, Croatia	Country: Croatia<br>Institution: Univ Zagreb	Country: Croatia<br>Institution: University of Zagreb

Record 780 | WOS:000087816200003

In vitro DNA and dGMP adducts formation caused by ochratoxin A

Field	Position	Original line	Normalised line	Original value	Normalised value	Original country / institution	Normalised country / institution
C1	1	108595	108595	CNRS, Inst Biol Mol & Cellulaire, UPR 9002, F-67084 Strasbourg, France. IUT Louis Pasteur, Lab Biol Vegetale Appl, F-67300 Schiltigheim, France.	Centre National de la Recherche Scientifique (CNRS), Inst Biol Mol & Cellulaire, UPR 9002, F-67084 Strasbourg, France. IUT Louis Pasteur, Lab Biol Vegetale Appl, F-67300 Schiltigheim, France.	Country: France<br>Institution: CNRS	Country: France<br>Institution: Centre National de la Recherche Scientifique (CNRS)
RP	1	108602	108602	Dirheimer, G (corresponding author), CNRS, Inst Biol Mol & Cellulaire, UPR 9002, 15 Rue Rene Descartes, F-67084 Strasbourg, France	Dirheimer, G (corresponding author), Centre National de la Recherche Scientifique (CNRS), Inst Biol Mol & Cellulaire, UPR 9002, 15 Rue Rene Descartes, F-67084 Strasbourg, France	Country: France<br>Institution: CNRS	Country: France<br>Institution: Centre National de la Recherche Scientifique (CNRS)

Record 781 | WOS:000087555100005

Ochratoxin A from a toxicological perspective

No C1/C3/RP normalisation changes detected for this record.

Record 782 | WOS:000085306200013

Induction of unscheduled DNA synthesis in primary human urothelial cells by the mycotoxin ochratoxin A

Field	Position	Original line	Normalised line	Original value	Normalised value	Original country / institution	Normalised country / institution
C1	1	108856	108856	Ruhr Univ Bochum, Inst Hyg Sozial & Umweltmed, D-44801 Bochum, Germany. Univ Dortmund, Inst Arbeitsphysiol, D-4600 Dortmund, Germany.	Ruhr University Bochum, Inst Hyg Sozial & Umweltmed, D-44801 Bochum, Germany. Univ Dortmund, Inst Arbeitsphysiol, D-4600 Dortmund, Germany.	Country: Germany<br>Institution: Ruhr Univ Bochum	Country: Germany<br>Institution: Ruhr University Bochum

				Stadt Kliniken Dortmund, Urol Klin, Dortmund, Germany. Stadt Kliniken Dortmund, Kinderchirurg Klin, Dortmund, Germany.	Stadt Kliniken Dortmund, Urol Klin, Dortmund, Germany. Stadt Kliniken Dortmund, Kinderchirurg Klin, Dortmund, Germany.		
RP	1	108862	108862	Flieger, A (corresponding author), Ruhr Univ Bochum, Inst Hyg Sozial & Umweltmed, Univ Str 150, D-44801 Bochum, Germany	Flieger, A (corresponding author), Ruhr University Bochum, Inst Hyg Sozial & Umweltmed, Univ Str 150, D-44801 Bochum, Germany	Country: Germany Institution: Ruhr Univ Bochum	Country: Germany Institution: Ruhr University Bochum

Record 783 | WOS:000084146300015

Cholestyramine protection against ochratoxin A toxicity: Role of ochratoxin A sorption by the resin and bile enterohepatic circulation

No C1/C3/RP normalisation changes detected for this record.

Record 784 | WOS:000165381000007

Increased cytotoxicity of food-borne mycotoxins toward human cell lines in vitro via enhanced cytochrome p450 expression using the MTT bioassay

Field	Position	Original line	Normalised line	Original value	Normalised value	Original country / institution	Normalised country / institution
C1	1	109055	109055	Univ Strathclyde, Dept Biosci & Biotechnol, Glasgow G1 1XW, Lanark, Scotland. Univ Glasgow, CRC, Dept Med Oncol, Glasgow G61 1BD, Lanark, Scotland.	University of Strathclyde, Dept Biosci & Biotechnol, Glasgow G1 1XW, Lanark, United Kingdom. University of Glasgow, CRC, Dept Med Oncol, Glasgow G61 1BD, Lanark, United Kingdom.	Country: Scotland Institution: Univ Strathclyde	Country: United Kingdom Institution: University of Strathclyde
RP	1	109058	109058	Lewis, CW (corresponding author), 38 Crarae Ave, Bearsden G61 1HY, E Dunbartonshir, Scotland	Lewis, CW (corresponding author), 38 Crarae Ave, Bearsden G61 1HY, E Dunbartonshir, United Kingdom	Country: Scotland Institution: 38 Crarae Ave	Country: United Kingdom Institution: 38 Crarae Ave

Record 785 | WOS:000083648200009

Genotoxicity of ochratoxin A by Salmonella mutagenicity test after bioactivation by mouse kidney microsomes

Field	Position	Original line	Normalised line	Original value	Normalised value	Original country / institution	Normalised country / institution
C1	1	109137	109137	Inst Pasteur, Toxicol Lab, F-59019 Lille, France. IUT Louis Pasteur, Lab Biol Vegetale Appl, F-67300 Schiltigheim, France. CNRS, Inst Biol Mol & Cellulaire, UPR 9002, F-67084 Strasbourg, France.	Inst Pasteur, Toxicol Lab, F-59019 Lille, France. IUT Louis Pasteur, Lab Biol Vegetale Appl, F-67300 Schiltigheim, France. Centre National de la Recherche Scientifique (CNRS), Inst Biol Mol & Cellulaire, UPR 9002, F-67084 Strasbourg, France.	Country: France Institution: Inst Pasteur	Country: France Institution: Inst Pasteur

Record 786 | WOS:000082038700010

Ochratoxin binding to phenylalanyl-tRNA synthetase: Computational approach to the mechanism of ochratoxicosis and its antagonism

No C1/C3/RP normalisation changes detected for this record.

Record 787 | WOS:000081907800002

Mutagenicity of commercial Monascus fermentation products and the role of citrinin contamination

Field	Position	Original line	Normalised line	Original value	Normalised value	Original country / institution	Normalised country / institution
C1	1	109344	109344	Univ Utrecht, Dept Vet Pharmacol Pharm & Toxicol, NL-3508 TD Utrecht, Netherlands.	Utrecht University, Dept Vet Pharmacol Pharm & Toxicol, NL-3508 TD Utrecht, Netherlands.	Country: Netherlands Institution: Univ Utrecht	Country: Netherlands Institution: Utrecht University
RP	1	109346	109346	Sabater-Vilar, M (corresponding author), Univ Utrecht, Dept Vet Pharmacol Pharm & Toxicol, POB 80152, NL-3508 TD Utrecht, Netherlands	Sabater-Vilar, M (corresponding author), Utrecht University, Dept Vet Pharmacol Pharm & Toxicol, POB 80152, NL-3508 TD Utrecht, Netherlands	Country: Netherlands Institution: Univ Utrecht	Country: Netherlands Institution: Utrecht University

Record 788 | WOS:000081759900001

Ochratoxin A-binding proteins in rat organs and plasma and in different cell lines of the kidney

Field	Position	Original line	Normalised line	Original value	Normalised value	Original country / institution	Normalised country / institution
C1	1	109438	109438	Univ Wurzburg, Inst Physiol, D-97070 Wurzburg, Germany.	University of Wurzburg, Inst Physiol, D-97070 Wurzburg, Germany.	Country: Germany Institution: Univ Wurzburg	Country: Germany Institution: University of Wurzburg
RP	1	109440	109440	Schwerdt, G (corresponding author), Univ Wurzburg, Inst Physiol, Rontgenring 9, D-97070 Wurzburg, Germany	Schwerdt, G (corresponding author), University of Wurzburg, Inst Physiol, Rontgenring 9, D-97070 Wurzburg, Germany	Country: Germany Institution: Univ Wurzburg	Country: Germany Institution: University of Wurzburg

Record 789 | WOS:000080471300017

Transport of ochratoxin A by renal multispecific organic anion transporter 1

Field	Position	Original line	Normalised line	Original value	Normalised value	Original country / institution	Normalised country / institution
C1	1	109543	109543	Kyorin Univ, Sch Med, Dept Pharmacol & Toxicol, Tokyo 1818611, Japan. Kyoritsu Coll Pharmaceut Sci, Dept Toxicol, Minato Ku, Tokyo, Japan.	Kyorin University, Sch Med, Dept Pharmacol & Toxicol, Tokyo 1818611, Japan. Kyoritsu Coll Pharmaceut Sci, Dept Toxicol, Minato Ku, Tokyo, Japan.	Country: Japan Institution: Kyorin Univ	Country: Japan Institution: Kyorin University
RP	1	109546	109546	Endou, H (corresponding author), Kyorin Univ, Sch Med, Dept Pharmacol & Toxicol, 6-20-2 Shinkawa, Tokyo 1818611, Japan	Endou, H (corresponding author), Kyorin University, Sch Med, Dept Pharmacol & Toxicol, 6-20-2 Shinkawa, Tokyo 1818611, Japan	Country: Japan Institution: Kyorin Univ	Country: Japan Institution: Kyorin University

Record 790 | WOS:000080762800002

Roles of cyclooxygenase and lipoxygenases in ochratoxin A genotoxicity in human epithelial lung cells

No C1/C3/RP normalisation changes detected for this record.

Record 791 | WOS:000080209100001

Inactivation of ochratoxin in ruminal fluid with variation of pH-value and fermentation parameters in an in vitro system

Field	Position	Original line	Normalised line	Original value	Normalised value	Original country / institution	Normalised country / institution
C1	1	109743	109743	Univ Istanbul, Fac Med Vet, Dept Anim Nutr & Nutrit Dis, Istanbul, Turkey. Acad Vet Med, Dept Anim Nutr, Kaunas, Lithuania. Univ Hohenheim, Dept Anim Nutr, D-7000 Stuttgart, Germany.	Istanbul University, Fac Med Vet, Dept Anim Nutr & Nutrit Dis, Istanbul, Turkey. Acad Vet Med, Dept Anim Nutr, Kaunas, Lithuania. University Hohenheim, Dept Anim Nutr, D-7000 Stuttgart, Germany.	Country: Germany<br>Institution: Univ Istanbul	Country: Germany<br>Institution: Istanbul University
RP	1	109747	109747	Ã zpinar, H (corresponding author), Univ Istanbul, Fac Med Vet, Dept Anim Nutr & Nutrit Dis, Istanbul, Turkey	Ã zpinar, H (corresponding author), Istanbul University, Fac Med Vet, Dept Anim Nutr & Nutrit Dis, Istanbul, Turkey	Country: Turkey<br>Institution: Univ Istanbul	Country: Turkey<br>Institution: Istanbul University

Record 792 | WOS:000083320300013

Cytotoxic and genotoxic effects of zearalenone:: Prevention by vitamin E

No C1/C3/RP normalisation changes detected for this record.

Record 793 | WOS:000087386900028

Biotransformation and lack of mutagenicity of ochratoxin A using combinations of mammalian biotransformation enzymes

Field	Position	Original line	Normalised line	Original value	Normalised value	Original country / institution	Normalised country / institution
C1	1	109909	109909	Univ Wurzburg, Dept Toxicol, D-97078 Wurzburg, Germany.	University of Wurzburg, Dept Toxicol, D-97078 Wurzburg, Germany.	Country: Germany<br>Institution: Univ Wurzburg	Country: Germany<br>Institution: University of Wurzburg
RP	1	109911	109911	Dekant, W (corresponding author), Univ Wurzburg, Dept Toxicol, Versbacher Str 9, D-97078 Wurzburg, Germany	Dekant, W (corresponding author), University of Wurzburg, Dept Toxicol, Versbacher Str 9, D-97078 Wurzburg, Germany	Country: Germany<br>Institution: Univ Wurzburg	Country: Germany<br>Institution: University of Wurzburg

Record 794 | WOS:000078111000012

Apical uptake of radiolabelled ochratoxin A into Madin-Darby canine kidney cells

Field	Position	Original line	Normalised line	Original value	Normalised value	Original country / institution	Normalised country / institution
C1	1	109992	109992	Univ Wurzburg, Inst Physiol, D-97070 Wurzburg, Germany.	University of Wurzburg, Inst Physiol, D-97070 Wurzburg, Germany.	Country: Germany<br>Institution: Univ Wurzburg	Country: Germany<br>Institution: University of Wurzburg
RP	1	109994	109994	Schwerdt, G (corresponding author), Univ Wurzburg, Inst Physiol, Rontgenring 9, D-97070 Wurzburg, Germany	Schwerdt, G (corresponding author), University of Wurzburg, Inst Physiol, Rontgenring 9, D-97070 Wurzburg, Germany	Country: Germany<br>Institution: Univ Wurzburg	Country: Germany<br>Institution: University of Wurzburg

Record 795 | WOS:000077419100015

Apoptosis in the kidney

No C1/C3/RP normalisation changes detected for this record.

Record 796 | WOS:000076860300002

Measurement of antibacterial activities of T-2 toxin, deoxynivalenol, ochratoxin A, aflatoxin B1 and fumonisin B1 using microtitration tray-based turbidimetric techniques

Field	Position	Original line	Normalised line	Original value	Normalised value	Original country / institution	Normalised country / institution
C1	1	110311	110311	Univ Helsinki, Fac Vet Med, Dept Clin Sci, Helsinki, Finland. Natl Vet & Food Res Inst, Helsinki, Finland.	University of Helsinki, Fac Vet Med, Dept Clin Sci, Helsinki, Finland. Natl Vet & Food Res Inst, Helsinki, Finland.	Country: Finland<br>Institution: Univ Helsinki	Country: Finland<br>Institution: University of Helsinki
RP	1	110314	110314	Atroshi, F (corresponding author), Univ Helsinki, Fac Vet Med, Dept Clin Sci, Helsinki, Finland	Atroshi, F (corresponding author), University of Helsinki, Fac Vet Med, Dept Clin Sci, Helsinki, Finland	Country: Finland<br>Institution: Univ Helsinki	Country: Finland<br>Institution: University of Helsinki

Record 797 | WOS:000076443800002

Cytotoxicity of nephrotoxic fungal toxins to kidney-derived LLC-PK1 and OK cell lines

Field	Position	Original line	Normalised line	Original value	Normalised value	Original country / institution	Normalised country / institution
C1	1	110371	110371	Hlth Canada, Toxicol Res Div, Bur Chem Safety, Food Directorate,Hlth Protect Branch, Ottawa, ON K1A 0L2, Canada.	Health Canada, Toxicol Res Div, Bur Chem Safety, Food Directorate,Hlth Protect Branch, Ottawa, ON K1A 0L2, Canada.	Country: Canada<br>Institution: Hlth Canada	Country: Canada<br>Institution: Health Canada
RP	1	110373	110373	Bondy, GS (corresponding author), Hlth Canada, Toxicol Res Div, Bur Chem Safety, Food Directorate,Hlth Protect Branch, Ottawa, ON K1A 0L2, Canada	Bondy, GS (corresponding author), Health Canada, Toxicol Res Div, Bur Chem Safety, Food Directorate,Hlth Protect Branch, Ottawa, ON K1A 0L2, Canada	Country: Canada<br>Institution: Hlth Canada	Country: Canada<br>Institution: Health Canada

Record 798 | WOS:000076413600003

Exposure to ochratoxin A impairs organic anion transport in proximal-tubule-derived opossum kidney cells

Field	Position	Original line	Normalised line	Original value	Normalised value	Original country / institution	Normalised country / institution
C1	1	110456	110456	Univ Wurzburg, Inst Physiol, D-97070 Wurzburg, Germany.	University of Wurzburg, Inst Physiol, D-97070 Wurzburg, Germany.	Country: Germany<br>Institution: Univ Wurzburg	Country: Germany<br>Institution: University of Wurzburg
RP	1	110458	110458	Sauvant, C (corresponding author), Univ Wurzburg, Inst Physiol, Rontgenring 9, D-97070 Wurzburg, Germany	Sauvant, C (corresponding author), University of Wurzburg, Inst Physiol, Rontgenring 9, D-97070 Wurzburg, Germany	Country: Germany<br>Institution: Univ Wurzburg	Country: Germany<br>Institution: University of Wurzburg

Record 799 | WOS:000074665700021

Detailed mapping of ochratoxin a reabsorption along the rat nephron in vivo: The nephrotoxin can be reabsorbed in all nephron segments by different mechanisms

Field	Position	Original line	Normalised line	Original value	Normalised value	Original country / institution	Normalised country / institution
C1	1	110548	110548	Univ Wurzburg, Inst Physiol, D-97070 Wurzburg, Germany. Univ Arizona, Coll Med, Dept Physiol, Tucson, AZ 85724 USA.	University of Wurzburg, Inst Physiol, D-97070 Wurzburg, Germany. University of	Country: USA<br>Institution: Univ Wurzburg	Country: USA<br>Institution: University of Wurzburg

					Arizona, Coll Med, Dept Physiol, Tucson, AZ 85724 USA.		
RP	1	110551	110551	Gekle, M (corresponding author), Univ Wurzburg, Inst Physiol, Rontgenring 9, D-97070 Wurzburg, Germany	Gekle, M (corresponding author), University of Wurzburg, Inst Physiol, Rontgenring 9, D-97070 Wurzburg, Germany	Country: Germany<br>Institution: Univ Wurzburg	Country: Germany<br>Institution: University of Wurzburg

Record 800 | WOS:000075078200010

Haematotoxicity of mycotoxins

Field	Position	Original line	Normalised line	Original value	Normalised value	Original country / institution	Normalised country / institution
C1	1	110631	110631	Univ Bretagne Occidentale, Technopole Brest Iroise, ISAMOR, ESMISA,Lab Microbiol & Secur Alimentaire, F-29280 Plouzane, France. Wayne State Univ, Harper Hosp, Barbara Ann Karmanos Canc Inst, Div Hematol Oncol, Detroit, MI 48201 USA.	Universite de Bretagne Occidentale, Technopole Brest Iroise, ISAMOR, ESMISA,Lab Microbiol & Secur Alimentaire, F-29280 Plouzane, France. Wayne State University, Harper Hosp, Barbara Ann Karmanos Canc Inst, Div Hematol Oncol, Detroit, MI 48201 USA.	Country: USA<br>Institution: Univ Bretagne Occidentale	Country: USA<br>Institution: Universite de Bretagne Occidentale
RP	1	110635	110635	Parent-Massin, D (corresponding author), Univ Bretagne Occidentale, Technopole Brest Iroise, ISAMOR, ESMISA,Lab Microbiol & Secur Alimentaire, F-29280 Plouzane, France	Parent-Massin, D (corresponding author), Universite de Bretagne Occidentale, Technopole Brest Iroise, ISAMOR, ESMISA,Lab Microbiol & Secur Alimentaire, F-29280 Plouzane, France	Country: France<br>Institution: Univ Bretagne Occidentale	Country: France<br>Institution: Universite de Bretagne Occidentale

Record 801 | WOS:000073042900016

Activated carbons: In vitro affinity for ochratoxin A and deoxynivalenol and relation of adsorption ability to physicochemical parameters

Field	Position	Original line	Normalised line	Original value	Normalised value	Original country / institution	Normalised country / institution
C1	1	110767	110767	Univ Reggio Calabria, Dipartimento Sci & Tecnol Agroforestali & Ambient, I-89061 Reggio Di Calabria, Italy. Univ Cattolica Sacro Cuore, Fac Agr, Ist Sci Alimenti & Nutr, I-29100 Piacenza, Italy. Univ Bologna, Dipartimento Morfofisiol Vet & Prod Anim, I-40064 Ozzano Dell Emilia, Bologna, Italy. Univ Catania, Fac Biol Sci, Ist Sci Biochim & Farmacol, I-95125 Catania, Italy.	Univ Reggio Calabria, Dipartimento Sci & Tecnol Agroforestali & Ambient, I-89061 Reggio Di Calabria, Italy. Univ Cattolica Sacro Cuore, Fac Agr, Ist Sci Alimenti & Nutr, I-29100 Piacenza, Italy. University of Bologna, Dipartimento Morfofisiol Vet & Prod Anim, I-40064 Ozzano Dell Emilia, Bologna, Italy. University of Catania, Fac Biol Sci, Ist Sci Biochim & Farmacol, I-95125 Catania, Italy.	Country: Italy<br>Institution: Univ Reggio Calabria	Country: Italy<br>Institution: Univ Reggio Calabria

Record 802 | WOS:000072874100001

Ochratoxin A in food and feed: occurrence, legislation and mode of action

Field	Position	Original line	Normalised line	Original value	Normalised value	Original country / institution	Normalised country / institution

C1	1	110839	110839	Univ Kiel, Inst Tierernahrung & Stoffwechselphysiol, D-24098 Kiel, Germany.	University of Kiel, Inst Tierernahrung & Stoffwechselphysiol, D-24098 Kiel, Germany.	Country: Germany Institution: Univ Kiel	Country: Germany Institution: University of Kiel
RP	1	110841	110841	Hohler, D (corresponding author), Univ Kiel, Inst Tierernahrung & Stoffwechselphysiol, Olshausenstr 40, D-24098 Kiel, Germany	Hohler, D (corresponding author), University of Kiel, Inst Tierernahrung & Stoffwechselphysiol, Olshausenstr 40, D-24098 Kiel, Germany	Country: Germany Institution: Univ Kiel	Country: Germany Institution: University of Kiel

Record 803 | WOS:000079327200007

The effects of four mycotoxins on the mitogen stimulated proliferation of bovine peripheral blood mononuclear cells in vitro

Field	Position	Original line	Normalised line	Original value	Normalised value	Original country / institution	Normalised country / institution
C1	1	110959	110959	Univ Strathclyde, Dept Biosci & Biotechnol, Glasgow G1 1XW, Lanark, Scotland. Univ Glasgow, Sch Vet, Dept Vet Clin Studies, Glasgow G61 1QH, Lanark, Scotland.	University of Strathclyde, Dept Biosci & Biotechnol, Glasgow G1 1XW, Lanark, United Kingdom. University of Glasgow, Sch Vet, Dept Vet Clin Studies, Glasgow G61 1QH, Lanark, United Kingdom.	Country: Scotland Institution: Univ Strathclyde	Country: United Kingdom Institution: University of Strathclyde
RP	1	110962	110962	Smith, JE (corresponding author), Univ Strathclyde, Dept Biosci & Biotechnol, Glasgow G1 1XW, Lanark, Scotland	Smith, JE (corresponding author), University of Strathclyde, Dept Biosci & Biotechnol, Glasgow G1 1XW, Lanark, United Kingdom	Country: Scotland Institution: Univ Strathclyde	Country: United Kingdom Institution: University of Strathclyde

Record 804 | WOS:000081940900027

Mycotoxins: Mechanisms of action at the molecular level

No C1/C3/RP normalisation changes detected for this record.

Record 805 | WOS:000075950800023

Tubulotoxic mechanisms of ochratoxin a

Field	Position	Original line	Normalised line	Original value	Normalised value	Original country / institution	Normalised country / institution
C1	1	111188	111188	Univ Wurzburg, Inst Physiol, D-97070 Wurzburg, Germany.	University of Wurzburg, Inst Physiol, D-97070 Wurzburg, Germany.	Country: Germany Institution: Univ Wurzburg	Country: Germany Institution: University of Wurzburg
RP	1	111190	111190	Gekle, M (corresponding author), Univ Wurzburg, Inst Physiol, Rontgenring 9, D-97070 Wurzburg, Germany	Gekle, M (corresponding author), University of Wurzburg, Inst Physiol, Rontgenring 9, D-97070 Wurzburg, Germany	Country: Germany Institution: Univ Wurzburg	Country: Germany Institution: University of Wurzburg

Record 806 | WOS:000080521300009

Ochratoxins: Molecular strategies for developing an antidote

No C1/C3/RP normalisation changes detected for this record.

Record 807 | WOS:000071510600005

Acute toxicity of the mycotoxin roridin E on liver and kidney of rats

Field	Position	Original line	Normalised line	Original value	Normalised value	Original country / institution	Normalised country / institution
C1	1	111336	111336	Univ Assiut, Fac Sci, Dept Zool, Assiut, Egypt.	Assiut University, Fac Sci, Dept Zool, Assiut, Egypt.	Country: Egypt<br>Institution: Univ Assiut	Country: Egypt<br>Institution: Assiut University

Record 808 | WOS:A1997YD92300001

The neurotoxic effects of ochratoxin-A are reduced by protein binding but are not affected by l-phenylalanine

Field	Position	Original line	Normalised line	Original value	Normalised value	Original country / institution	Normalised country / institution
C1	1	111402	111402	ETH ZURICH, INST TOXICOL, CH-8603 SCHWERZENBACH, SWITZERLAND. UNIV ZURICH, CH-8603 SCHWERZENBACH, SWITZERLAND.	ETH ZURICH, INST TOXICOL, CH-8603 SCHWERZENBACH, Switzerland. University of Zurich, CH-8603 SCHWERZENBACH, Switzerland.	Country: Switzerland<br>Institution: ETH ZURICH	Country: Switzerland<br>Institution: ETH ZURICH

Record 809 | WOS:A1997XM61400017

Effects of inorganic adsorbents and cyclopiazonic acid in broiler chickens

Field	Position	Original line	Normalised line	Original value	Normalised value	Original country / institution	Normalised country / institution
C1	1	111496	111496	TEXAS A&M UNIV,COLL VET MED,DEPT VET ANAT & PUBL HLTH,COLLEGE STN,TX 77843. ARS,USDA,FOOD ANIM PROTECT RES LAB,COLLEGE STN,TX 77845.	Texas A&M University College Station,COLL VET MED,DEPT VET ANAT & PUBL HLTH,COLLEGE STN,TX 77843. ARS,USDA,FOOD ANIM PROTECT RES LAB,COLLEGE STN,TX 77845.	Country: TX 77845<br>Institution: TEXAS A&M UNIV	Country: TX 77845<br>Institution: Texas A&M University College Station

Record 810 | WOS:A1997XN68000014

Lipid peroxidation as one mode of action in ochratoxin A toxicity in rats and chicks

Field	Position	Original line	Normalised line	Original value	Normalised value	Original country / institution	Normalised country / institution
C1	1	111601	111601	UNIV MANITOBA,DEPT ANIM SCI,WINNIPEG,MB R3T 2N2,CANADA.	University of Manitoba,DEPT ANIM SCI,WINNIPEG,MB R3T 2N2,Canada.	Country: Canada<br>Institution: UNIV MANITOBA	Country: Canada<br>Institution: University of Manitoba

Record 811 | WOS:A1997XA91600004

Induction of micronuclei with ochratoxin A in ovine seminal vesicle cell cultures

Field	Position	Original line	Normalised line	Original value	Normalised value	Original country / institution	Normalised country / institution
C1	1	111676	111676	INST BIOL MOL & CELLULAIRE, CNRS, F-67084 STRASBOURG, FRANCE. UNIV STRASBOURG 1, F-67084 STRASBOURG, FRANCE.	INST BIOL MOL & CELLULAIRE, CNRS, F-67084 STRASBOURG, France. UNIV STRASBOURG 1, F-67084 STRASBOURG, France.	Country: France<br>Institution: INST BIOL MOL & CELLULAIRE	Country: France<br>Institution: INST BIOL MOL & CELLULAIRE

RP	1	111683	111683	Degen, GH (corresponding author), UNIV DORTMUND, INST ARBEITSPHYSIOL, ARDEYSTR 67, D-44139 DORTMUND, GERMANY	Degen, GH (corresponding author), UNIV DORTMUND, INST ARBEITSPHYSIOL, ARDEYSTR 67, D-44139 DORTMUND, Germany	Country: Germany Institution: UNIV DORTMUND	Country: Germany Institution: UNIV DORTMUND

Record 812 | WOS:A1997WT39200011

Reduction of ochratoxin A toxicity by heat-induced epimerization. In vitro effects of ochratoxins on embryonic chick meningeal and other cell cultures

Field	Position	Original line	Normalised line	Original value	Normalised value	Original country / institution	Normalised country / institution
C1	1	111797	111797	ETH ZURICH,INST TOXICOL,CH-8603 SCHWERZENBACH,SWITZERLAND. UNIV ZURICH,CH-8603 SCHWERZENBACH,SWITZERLAND.	ETH ZURICH,INST TOXICOL,CH-8603 SCHWERZENBACH,Switzerland. University of Zurich,CH-8603 SCHWERZENBACH,Switzerland.	Country: Switzerland Institution: ETH ZURICH	Country: Switzerland Institution: ETH ZURICH

Record 813 | WOS:A1997WW09700003

Reduction of the ochratoxin A-induced cytotoxicity in Vero cells by aspartame

Field	Position	Original line	Normalised line	Original value	Normalised value	Original country / institution	Normalised country / institution
C1	1	111871	111871	UNIV BORDEAUX 2, UNITE FORMAT & RECH SCI PHARMACEUT, LAB TOXICOL & HYG APPL, F-33076 BORDEAUX, FRANCE.	UNIV BORDEAUX 2, UNITE FORMAT & RECH SCI PHARMACEUT, LAB TOXICOL & HYG APPL, F-33076 BORDEAUX, France.	Country: France Institution: UNIV BORDEAUX 2	Country: France Institution: UNIV BORDEAUX 2

Record 814 | WOS:A1997XZ55900019

Effects of the naturally occurring food mycotoxin ochratoxin A on brain cells in culture

Field	Position	Original line	Normalised line	Original value	Normalised value	Original country / institution	Normalised country / institution
C1	1	111971	111971	NESTEC LTD, NESTLE RES CTR, CH-1000 LAUSANNE, SWITZERLAND.	NESTEC LTD, NESTLE RES CTR, CH-1000 LAUSANNE, Switzerland.	Country: Switzerland Institution: NESTEC LTD	Country: Switzerland Institution: NESTEC LTD
RP	1	111973	111973	MonnetTschudi, F (corresponding author), UNIV LAUSANNE, INST PHYSIOL, 7 RUE BUGNON, CH-1005 LAUSANNE, SWITZERLAND	MonnetTschudi, F (corresponding author), University of Lausanne, INST PHYSIOL, 7 RUE BUGNON, CH-1005 LAUSANNE, Switzerland	Country: Switzerland Institution: UNIV LAUSANNE	Country: Switzerland Institution: University of Lausanne

Record 815 | WOS:A1997WC04100026

Reabsorption of the nephrotoxin ochratoxin A along the rat nephron in vivo

Field	Position	Original line	Normalised line	Original value	Normalised value	Original country / institution	Normalised country / institution
C1	1	112069	112069	UNIV WURZBURG, INST PHYSIOL, D-97070 WURZBURG, GERMANY.	University of Wurzburg, INST PHYSIOL, D-97070 WURZBURG, Germany.	Country: Germany Institution: UNIV WURZBURG	Country: Germany Institution: University of Wurzburg

Record 816 | WOS:A1996WB42500013

Influence of vitamins E and C on the toxic effects of ochratoxin A and T-2 toxin in chicks

Field	Position	Original line	Normalised line	Original value	Normalised value	Original country / institution	Normalised country / institution
C1	1	112138	112138	UNIV MANITOBA,DEPT ANIM SCI,WINNIPEG,MB R3T 2N2,CANADA.	University of Manitoba,DEPT ANIM SCI,WINNIPEG,MB R3T 2N2,Canada.	Country: Canada<br>Institution: UNIV MANITOBA	Country: Canada<br>Institution: University of Manitoba

Record 817 | WOS:A1996UZ36400005

Effects of mycotoxins on cytokine production and proliferation in EL-4 thymoma cells

No C1/C3/RP normalisation changes detected for this record.

Record 818 | WOS:A1996UT94000012

Prevention of toxic effects of mycotoxins by means of nonnutritive adsorbent compounds

Field	Position	Original line	Normalised line	Original value	Normalised value	Original country / institution	Normalised country / institution
C1	1	112328	112328	UNIV UTRECHT,DEPT VET PHARMACOL PHARM & TOXICOL,3508 TD UTRECHT,NETHERLANDS. UNIV POLITECN VALENCIA,DEPT BIOTECHNOL,E-46071 VALENCIA,SPAIN.	UNIV UTRECHT,DEPT VET PHARMACOL PHARM & TOXICOL,3508 TD UTRECHT,Netherlands. UNIV POLITECN VALENCIA,DEPT BIOTECHNOL,E-46071 VALENCIA,Spain.	Country: Spain<br>Institution: UNIV UTRECHT	Country: Spain<br>Institution: UNIV UTRECHT
RP	1	112331	112331	Ramos, AJ (corresponding author), UNIV LLEIDA,DEPT FOOD TECHNOL,ALCALDE ROVIRA ROURE 177,E-25198 LLEIDA,SPAIN	Ramos, AJ (corresponding author), UNIV LLEIDA,DEPT FOOD TECHNOL,ALCALDE ROVIRA ROURE 177,E-25198 LLEIDA,Spain	Country: Spain<br>Institution: UNIV LLEIDA	Country: Spain<br>Institution: UNIV LLEIDA

Record 819 | WOS:A1996VJ28200003

Aspartame as a preventive agent of chronic toxic effects of ochratoxin A in experimental animals

Field	Position	Original line	Normalised line	Original value	Normalised value	Original country / institution	Normalised country / institution
RP	1	112503	112503	Creppy, EE (corresponding author), UNIV BORDEAUX 2, DEPT TOXICOL, 146 RUE LEO SAIGNAT, F-33076 BORDEAUX, FRANCE	Creppy, EE (corresponding author), UNIV BORDEAUX 2, DEPT TOXICOL, 146 RUE LEO SAIGNAT, F-33076 BORDEAUX, France	Country: France<br>Institution: UNIV BORDEAUX 2	Country: France<br>Institution: UNIV BORDEAUX 2

Record 820 | WOS:A1996VW20400002

Renal toxicodynamics of ochratoxin A: A pathophysiological approach

Field	Position	Original line	Normalised line	Original value	Normalised value	Original country / institution	Normalised country / institution

RP	1	112606	112606	Gekle, M (corresponding author), UNIV WURZBURG, INST PHYSIOL, RONTGENRING 9, D-97070 WURZBURG, GERMANY	Gekle, M (corresponding author), University of Wurzburg, INST PHYSIOL, RONTGENRING 9, D-97070 WURZBURG, Germany	Country: Germany Institution: UNIV WURZBURG	Country: Germany Institution: University of Wurzburg

Record 821 | WOS:A1995TU51400124

Prevention of nephrotoxicity of ochratoxin A, a food contaminant

Field	Position	Original line	Normalised line	Original value	Normalised value	Original country / institution	Normalised country / institution
RP	1	112730	112730	Creppy, EE (corresponding author), UNIV BORDEAUX 2, DEPT TOXICOL, 3TER PL VICTOIRE, F-33000 BORDEAUX, FRANCE	Creppy, EE (corresponding author), UNIV BORDEAUX 2, DEPT TOXICOL, 3TER PL VICTOIRE, F-33000 BORDEAUX, France	Country: France Institution: UNIV BORDEAUX 2	Country: France Institution: UNIV BORDEAUX 2

Record 822 | WOS:A1995TN07400005

Activation markers and cell proliferation as indicators of toxicity: A flow cytometric approach

Field	Position	Original line	Normalised line	Original value	Normalised value	Original country / institution	Normalised country / institution
C1	1	112832	112832	NATL FOOD ADM TOXICOL LAB,DIV TOXICOL,UPPSALA,SWEDEN.	NATL FOOD ADM TOXICOL LAB,DIV TOXICOL,UPPSALA,Sweden.	Country: Sweden Institution: NATL FOOD ADM TOXICOL LAB	Country: Sweden Institution: NATL FOOD ADM TOXICOL LAB
RP	1	112833	112833	Johannisson, A (corresponding author), SWEDISH UNIV AGR SCI,FAC VET MED,DEPT PATHOL,BOX 7028,S-75007 UPPSALA,SWEDEN	Johannisson, A (corresponding author), Swedish University of Agricultural Sciences,FAC VET MED,DEPT PATHOL,BOX 7028,S-75007 UPPSALA,Sweden	Country: Sweden Institution: SWEDISH UNIV AGR SCI	Country: Sweden Institution: Swedish University of Agricultural Sciences

Record 823 | WOS:A1995TC07600004

OCHRATOXINES AND TOXICOLOGICAL CONSEQUENCES

Field	Position	Original line	Normalised line	Original value	Normalised value	Original country / institution	Normalised country / institution
RP	1	112906	112906	CREPPY, EE (corresponding author), UNIV BORDEAUX 2, TOXICOL & HYG APPL LAB, 3 TER PL VICTOIRE, F-33000 BORDEAUX, FRANCE	CREPPY, EE (corresponding author), UNIV BORDEAUX 2, TOXICOL & HYG APPL LAB, 3 TER PL VICTOIRE, F-33000 BORDEAUX, France	Country: France Institution: UNIV BORDEAUX 2	Country: France Institution: UNIV BORDEAUX 2

Record 824 | WOS:A1995QJ43400010

SISTER-CHROMATID EXCHANGE FREQUENCY IN CULTURED ISOLATED PORCINE URINARY-BLADDER EPITHELIAL-CELLS (PUBEC) TREATED WITH OCHRATOXIN-A AND OCHRATOXIN-ALPHA

Field	Position	Original line	Normalised line	Original value	Normalised value	Original country / institution	Normalised country / institution
C1	1	112953	112953	UNIV BORDEAUX 2,UFR SCI PHARMACEUT,TOXICOL & HYG APPL LAB,F-33076 BORDEAUX,FRANCE.	UNIV BORDEAUX 2,UFR SCI PHARMACEUT,TOXICOL & HYG APPL LAB,F-33076 BORDEAUX,France.	Country: France Institution: UNIV BORDEAUX 2	Country: France Institution: UNIV BORDEAUX 2
RP	1	112955	112955	FOLLMANN, W (corresponding author), UNIV	FOLLMANN, W (corresponding author), UNIV	Country: Germany Institution: UNIV DORTMUND	Country: Germany Institution: UNIV DORTMUND

				DORTMUND,INST ARBEITSPHYSIOL,ARDEYSTR 67,D-44139 DORTMUND,GERMANY	DORTMUND,INST ARBEITSPHYSIOL,ARDEYSTR 67,D-44139 DORTMUND,Germany		

Record 825 | WOS:A1995QC67100017

EFFECT OF PIROXICAM ON THE NEPHROTOXICITY INDUCED BY OCHRATOXIN-A IN RATS

Field	Position	Original line	Normalised line	Original value	Normalised value	Original country / institution	Normalised country / institution
C1	1	113050	113050	UNIV BORDEAUX 2, TOXICOL LAB, F-33076 BORDEAUX, FRANCE. KRKA PO NOVO MESTO, DEPT TOXICOL, 68000 NOVO MESTO, SLOVENIA.	UNIV BORDEAUX 2, TOXICOL LAB, F-33076 BORDEAUX, France. KRKA PO NOVO MESTO, DEPT TOXICOL, 68000 NOVO MESTO, Slovenia.	Country: SLOVENIA Institution: UNIV BORDEAUX 2	Country: Slovenia Institution: UNIV BORDEAUX 2

Record 826 | WOS:A1994NN32900003

EFFECT OF SUPEROXIDE-DISMUTASE AND CATALASE ON THE NEPHROTOXICITY INDUCED BY SUBCHRONICAL ADMINISTRATION OF OCHRATOXIN-A IN RATS

Field	Position	Original line	Normalised line	Original value	Normalised value	Original country / institution	Normalised country / institution
C1	1	113153	113153	UNIV BORDEAUX 2, TOXICOL & HYG APPL LAB, F-33076 BORDEAUX, FRANCE. CNRS, INST BIOL MOLEC & CELLULAIRE, F-67084 STRASBOURG, FRANCE. UNIV STRASBOURG 1, F-67084 STRASBOURG, FRANCE.	UNIV BORDEAUX 2, TOXICOL & HYG APPL LAB, F-33076 BORDEAUX, France. Centre National de la Recherche Scientifique (CNRS), INST BIOL MOLEC & CELLULAIRE, F-67084 STRASBOURG, France. UNIV STRASBOURG 1, F-67084 STRASBOURG, France.	Country: France Institution: UNIV BORDEAUX 2	Country: France Institution: UNIV BORDEAUX 2

Record 827 | WOS:A1994MM85700005

THE ROLE OF THE PROXIMAL TUBULE IN OCHRATOXIN-A NEPHROTOXICITY IN-VIVO - TOXODYNAMIC AND TOXOKINETIC ASPECTS

Field	Position	Original line	Normalised line	Original value	Normalised value	Original country / institution	Normalised country / institution
RP	1	113242	113242	GEKLE, M (corresponding author), UNIV WURZBURG, INST PHYSIOL, RONTGENRING 9, D-97070 WURZBURG, GERMANY	GEKLE, M (corresponding author), University of Wurzburg, INST PHYSIOL, RONTGENRING 9, D-97070 WURZBURG, Germany	Country: Germany Institution: UNIV WURZBURG	Country: Germany Institution: University of Wurzburg

Record 828 | WOS:A1994NV49900013

EFFECT OF MYCOTOXINS ON IN-VITRO MOVEMENT OF TRACHEAL CILIA FROM ONE-DAY-OLD CHICKS

Field	Position	Original line	Normalised line	Original value	Normalised value	Original country / institution	Normalised country / institution
RP	1	113320	113320	JESENSKA, Z (corresponding author), INST PREVENT & CLIN MED, BRATISLAVA 83301, SLOVAKIA	JESENSKA, Z (corresponding author), INST PREVENT & CLIN MED, BRATISLAVA 83301, Slovakia	Country: Slovakia Institution: INST PREVENT & CLIN MED	Country: Slovakia Institution: INST PREVENT & CLIN MED

Record 829 | WOS:A1993MU47600008

EVALUATION OF THE DEVELOPMENTAL TOXICITY OF CITRININ USING HYDRA-ATTENUATA AND POSTIMPLANTATION RAT WHOLE-EMBRYO CULTURE

Field	Position	Original line	Normalised line	Original value	Normalised value	Original country / institution	Normalised country / institution
C1	1	113411	113411	TEXAS A&M UNIV, COLL VET MED, DEPT VET ANAT & PUBL HLTH, COLL STN, TX 77843 USA. TEXAS A&M UNIV, COLL VET MED, DEPT VET PATHOBIOL, COLL STN, TX 77843 USA.	Texas A&M University College Station, COLL VET MED, DEPT VET ANAT & PUBL HLTH, COLL STN, TX 77843 USA. Texas A&M University College Station, COLL VET MED, DEPT VET PATHOBIOL, COLL STN, TX 77843 USA.	Country: USA<br>Institution: TEXAS A&M UNIV	Country: USA<br>Institution: Texas A&M University College Station

Record 830 | WOS:A1993MM43500024

POSSIBLE ROLE OF AN IRON-OXYGEN COMPLEX IN 4(S)-4-HYDROXYOCHRATOXIN-A FORMATION BY RAT-LIVER MICROSOMES

Field	Position	Original line	Normalised line	Original value	Normalised value	Original country / institution	Normalised country / institution
C1	1	113513	113513	MEM UNIV NEWFOUNDLAND, DEPT BIOCHEM, St John A1B 3X9, NF, CANADA.	MEM UNIV NEWFOUNDLAND, DEPT BIOCHEM, St John A1B 3X9, NF, Canada.	Country: Canada<br>Institution: MEM UNIV NEWFOUNDLAND	Country: Canada<br>Institution: MEM UNIV NEWFOUNDLAND

Record 831 | WOS:A1993MQ49500006

OCHRATOXIN-A IMPAIRS POSTPROXIMAL NEPHRON FUNCTION IN-VIVO AND BLOCKS PLASMA-MEMBRANE ANION CONDUCTANCE IN MADIN-DARBY CANINE KIDNEY-CELLS IN-VITRO

Field	Position	Original line	Normalised line	Original value	Normalised value	Original country / institution	Normalised country / institution
RP	1	113603	113603	GEKLE, M (corresponding author), UNIV WURZBURG,DEPT PHYSIOL,RONTGENRING 9,D-97070 WURZBURG,GERMANY	GEKLE, M (corresponding author), University of Wurzburg,DEPT PHYSIOL,RONTGENRING 9,D-97070 WURZBURG,Germany	Country: Germany<br>Institution: UNIV WURZBURG	Country: Germany<br>Institution: University of Wurzburg

Record 832 | WOS:A1993LR82100028

EVALUATION AND APPLICATION OF A BIOLUMINESCENT BACTERIAL GENOTOXICITY TEST

Field	Position	Original line	Normalised line	Original value	Normalised value	Original country / institution	Normalised country / institution
C1	1	113679	113679	IOWA STATE UNIV SCI & TECHNOL,VET DIAGNOST LAB,AMES,IA 50011.	Iowa State University,VET DIAGNOST LAB,AMES,IA 50011.	Country: IA 50011<br>Institution: IOWA STATE UNIV SCI & TECHNOL	Country: IA 50011<br>Institution: Iowa State University

Record 833 | WOS:A1993LN86000012

COMPARISON OF IN-VITRO AND IN-VIVO BIOLOGICAL-ACTIVITY OF MYCOTOXINS

Field	Position	Original line	Normalised line	Original value	Normalised value	Original country / institution	Normalised country / institution

C1	1	113751	113751	AUBURN UNIV,COLL VET MED,DEPT PHYSIOL & PHARMACOL,AUBURN,AL 36849. UNIV MANITOBA,DEPT ANIM SCI,WINNIPEG R3T 2N2,MANITOBA,CANADA. AUBURN UNIV,COLL VET MED,DEPT PATHOBIOL,AUBURN,AL 36849.	Auburn University,COLL VET MED,DEPT PHYSIOL & PHARMACOL,AUBURN,AL 36849. University of Manitoba,DEPT ANIM SCI,WINNIPEG R3T 2N2,MANITOBA,Canada. Auburn University,COLL VET MED,DEPT PATHOBIOL,AUBURN,AL 36849.	Country: AL 36849<br>Institution: AUBURN UNIV	Country: AL 36849<br>Institution: Auburn University

Record 834 | WOS:A1993LJ01500043

EXTRACELLULAR ACIDOSIS AMELIORATES METABOLIC-INHIBITOR-INDUCED AND POTENTIATES OXIDANT-INDUCED CELL-DEATH IN RENAL PROXIMAL TUBULES

No C1/C3/RP normalisation changes detected for this record.

Record 835 | WOS:A1993KQ30600025

IMMUNOTOXICITY OF MYCOTOXINS

Field	Position	Original line	Normalised line	Original value	Normalised value	Original country / institution	Normalised country / institution
RP	1	113913	113913	SHARMA, RP (corresponding author), UTAH STATE UNIV,DEPT ANIM DAIRY & VET SCI,PROGRAM TOXICOL,LOGAN,UT 84322, USA	SHARMA, RP (corresponding author), Utah State University,DEPT ANIM DAIRY & VET SCI,PROGRAM TOXICOL,LOGAN,UT 84322, United States	Country: USA<br>Institution: UTAH STATE UNIV	Country: USA<br>Institution: Utah State University

Record 836 | WOS:A1992JU40900011

HYDROLYSIS OF OCHRATOXIN-A BY THE MICROBIAL ACTIVITY OF DIGESTA IN THE GASTROINTESTINAL-TRACT OF RATS

Field	Position	Original line	Normalised line	Original value	Normalised value	Original country / institution	Normalised country / institution
C1	1	114007	114007	UNIV MANITOBA,DEPT ANIM SCI,WINNIPEG R3T 2N2,MANITOBA,CANADA.	University of Manitoba,DEPT ANIM SCI,WINNIPEG R3T 2N2,MANITOBA,Canada.	Country: Canada<br>Institution: UNIV MANITOBA	Country: Canada<br>Institution: University of Manitoba

Record 837 | WOS:A1992KT90400023

ALTERATIONS IN ATP-DEPENDENT CALCIUM-UPTAKE BY RAT RENAL-CORTEX MICROSOMES FOLLOWING OCHRATOXIN A ADMINISTRATION INVIVO OR ADDITION INVITRO

Field	Position	Original line	Normalised line	Original value	Normalised value	Original country / institution	Normalised country / institution
C1	1	114078	114078	MEM UNIV NEWFOUNDLAND, DEPT BIOCHEM, St John A1B 3X9, NEWFOUNDLAND, CANADA.	MEM UNIV NEWFOUNDLAND, DEPT BIOCHEM, St John A1B 3X9, NEWFOUNDLAND, Canada.	Country: Canada<br>Institution: MEM UNIV NEWFOUNDLAND	Country: Canada<br>Institution: MEM UNIV NEWFOUNDLAND

Record 838 | WOS:A1992HP59700018

INVITRO EFFECTS OF THE NEPHROTOXINS OCHRATOXIN-A AND CITRININ UPON BIOCHEMICAL FUNCTION OF PORCINE KIDNEY

Field	Position	Original line	Normalised line	Original value	Normalised value	Original country / institution	Normalised country / institution
RP	1	114176	114176	BRAUNBERG, RC (corresponding author), US FDA,CTR FOOD SAFETY & APPL NUTR,BELTSVILLE,MD 20708, USA	BRAUNBERG, RC (corresponding author), US Food & Drug Administration (FDA),CTR FOOD SAFETY & APPL NUTR,BELTSVILLE,MD 20708, United States	Country: USA<br>Institution: US FDA	Country: USA<br>Institution: US Food & Drug Administration (FDA)

Record 839 | WOS:A1991GJ33400005

ROLE OF CYTOCHROME-P-450 IN OCHRATOXIN-A STIMULATED LIPID-PEROXIDATION

Field	Position	Original line	Normalised line	Original value	Normalised value	Original country / institution	Normalised country / institution
C1	1	114279	114279	MEM UNIV NEWFOUNDLAND,DEPT BIOCHEM,ST JOHNS A1B 3X9,NEWFOUNDLAND,CANADA. INT AGCY RES CANC,ENVIRONM CARCINOGENS & HOST FACTORS UNIT,F-69372 LYONS,FRANCE.	MEM UNIV NEWFOUNDLAND,DEPT BIOCHEM,ST JOHNS A1B 3X9,NEWFOUNDLAND,Canada. International Agency for Research on Cancer (IARC),ENVIRONM CARCINOGENS & HOST FACTORS UNIT,F-69372 LYONS,France.	Country: France<br>Institution: MEM UNIV NEWFOUNDLAND	Country: France<br>Institution: MEM UNIV NEWFOUNDLAND

Record 840 | WOS:A1991GJ33400006

INVITRO INHIBITION OF RAT PLATELET-AGGREGATION BY OCHRATOXIN-A

Field	Position	Original line	Normalised line	Original value	Normalised value	Original country / institution	Normalised country / institution
C1	1	114350	114350	MEM UNIV NEWFOUNDLAND, DEPT BIOCHEM, St John A1B 3X9, NEWFOUNDLAND, CANADA.	MEM UNIV NEWFOUNDLAND, DEPT BIOCHEM, St John A1B 3X9, NEWFOUNDLAND, Canada.	Country: Canada<br>Institution: MEM UNIV NEWFOUNDLAND	Country: Canada<br>Institution: MEM UNIV NEWFOUNDLAND

Record 841 | WOS:A1991GA29400009

CHARACTERIZATION OF PIG-LIVER PURIFIED CYTOCHROME-P-450 ISOENZYMES FOR OCHRATOXIN-A METABOLISM STUDIES

Field	Position	Original line	Normalised line	Original value	Normalised value	Original country / institution	Normalised country / institution
C1	1	114425	114425	UNIV NANCY 1,FAC SCI PHARMACEUT & BIOL,CTR MED,CNRS,URA 597,30 RUE LIONNOIS,BP 403,F-54001 NANCY,FRANCE. INST BIOL MOLEC & CELLULAIRE,F-67084 STRASBOURG,FRANCE.	UNIV NANCY 1,FAC SCI PHARMACEUT & BIOL,CTR MED,CNRS,URA 597,30 RUE LIONNOIS,BP 403,F-54001 NANCY,France. INST BIOL MOLEC & CELLULAIRE,F-67084 STRASBOURG,France.	Country: France<br>Institution: UNIV NANCY 1	Country: France<br>Institution: UNIV NANCY 1

Record 842 | WOS:A1991FC20700661

STIMULATION OF RENAL ENDOPLASMIC-RETICULUM CALCIUM-UPTAKE BY OCHRATOXIN-A

Field	Position	Original line	Normalised line	Original value	Normalised value	Original country / institution	Normalised country / institution

C1	1	114505	114505	MEM UNIV NEWFOUNDLAND,DEPT BIOCHEM,ST JOHNS A1B 3X9,NEWFOUNDLAND,CANADA.	MEM UNIV NEWFOUNDLAND,DEPT BIOCHEM,ST JOHNS A1B 3X9,NEWFOUNDLAND,Canada.	Country: Canada Institution: MEM UNIV NEWFOUNDLAND	Country: Canada Institution: MEM UNIV NEWFOUNDLAND

Record 843 | WOS:A1991FR33200006

INVIVO ABSORPTION OF C-14 GLUCOSE AND C-14 GLYCINE BY THE RAT INTESTINE DURING OCHRATOXIN A TOXICOSIS

Field	Position	Original line	Normalised line	Original value	Normalised value	Original country / institution	Normalised country / institution
RP	1	114550	114550	SUBRAMANIAN, S (corresponding author), UNIV MADRAS, DEPT BIOCHEM, GUINDY CAMPUS, MADRAS 600025, TAMIL NADU, INDIA	SUBRAMANIAN, S (corresponding author), University of Madras, DEPT BIOCHEM, GUINDY CAMPUS, MADRAS 600025, TAMIL NADU, India	Country: India Institution: UNIV MADRAS	Country: India Institution: University of Madras

Record 844 | WOS:A1991BW81R00026

MECHANISM OF ACTION OF OCHRATOXIN-A

Field	Position	Original line	Normalised line	Original value	Normalised value	Original country / institution	Normalised country / institution
RP	1	114622	114622	DIRHEIMER, G (corresponding author), CNRS,INST BIOL MOLEC & CELLULAIRE,15 RUE RENE DESCARTES,F-67084 STRASBOURG,FRANCE	DIRHEIMER, G (corresponding author), Centre National de la Recherche Scientifique (CNRS),INST BIOL MOLEC & CELLULAIRE,15 RUE RENE DESCARTES,F-67084 STRASBOURG,France	Country: France Institution: CNRS	Country: France Institution: Centre National de la Recherche Scientifique (CNRS)

Record 845 | WOS:A1991BW81R00032

DNA ADDUCT FORMATION IN MICE TREATED WITH OCHRATOXIN-A

No C1/C3/RP normalisation changes detected for this record.

Record 846 | WOS:A1990EP18900002

EFFECTS OF OCHRATOXIN-A AND OCHRATOXIN-B ON PRECHONDROGENIC MESENCHYMAL CELLS FROM CHICK-EMBRYO LIMB BUDS

Field	Position	Original line	Normalised line	Original value	Normalised value	Original country / institution	Normalised country / institution
C1	1	114710	114710	NATL INST PUBL HLTH,DEPT ENVIRONM MED,GEITMYRSVEIEN 75,N-0462 OSLO 4,NORWAY.	NATL INST PUBL HLTH,DEPT ENVIRONM MED,GEITMYRSVEIEN 75,N-0462 OSLO 4,Norway.	Country: Norway Institution: NATL INST PUBL HLTH	Country: Norway Institution: NATL INST PUBL HLTH

Record 847 | WOS:A1990EF19400012

NADPH-CYTOCHROME-P-450 REDUCTASE PROMOTED HYDROXYL RADICAL PRODUCTION BY THE IRON(III)-OCHRATOXIN-A COMPLEX

Field	Position	Original line	Normalised line	Original value	Normalised value	Original country / institution	Normalised country / institution

C1	1	114772	114772	MEM UNIV NEWFOUNDLAND, FAC MED, ST JOHNS A1B 3X7, NEWFOUNDLAND, CANADA. MEM UNIV NEWFOUNDLAND, DEPT BIOCHEM, ST JOHNS A1B 3X7, NEWFOUNDLAND, CANADA.	MEM UNIV NEWFOUNDLAND, FAC MED, ST JOHNS A1B 3X7, NEWFOUNDLAND, Canada. MEM UNIV NEWFOUNDLAND, DEPT BIOCHEM, ST JOHNS A1B 3X7, NEWFOUNDLAND, Canada.	Country: Canada Institution: MEM UNIV NEWFOUNDLAND	Country: Canada Institution: MEM UNIV NEWFOUNDLAND
RP	1	114775	114775	HASINOFF, BB (corresponding author), MEM UNIV NEWFOUNDLAND, DEPT CHEM, ST JOHNS A1B 3X7, NEWFOUNDLAND, CANADA	HASINOFF, BB (corresponding author), MEM UNIV NEWFOUNDLAND, DEPT CHEM, ST JOHNS A1B 3X7, NEWFOUNDLAND, Canada	Country: Canada Institution: MEM UNIV NEWFOUNDLAND	Country: Canada Institution: MEM UNIV NEWFOUNDLAND

Record 848 | WOS:A1990DV94600004

INDUCTION OF CHARACTERISTIC CHROMOSOMAL-ABERRATIONS, PARTICULARLY X-TRISOMY, IN CULTURED HUMAN-LYMPHOCYTES TREATED BY OCHRATOXIN-A, A MYCOTOXIN IMPLICATED IN BALKAN ENDEMIC NEPHROPATHY

Field	Position	Original line	Normalised line	Original value	Normalised value	Original country / institution	Normalised country / institution
C1	1	114844	114844	INT AGCY RES CANC, ENVIRONM CARCINOGENS & HOST FACTORS UNIT, 150 COURS ALBERT THOMAS, F-69372 Lyon, FRANCE. BULGARIAN ACAD SCI, INST CELL BIOL & MORPHOL, BU-1040 SOFIA, BULGARIA. BULGARIAN ACAD MED, NATL ONCOL CTR, BU-1156 SOFIA, BULGARIA.	International Agency for Research on Cancer (IARC), ENVIRONM CARCINOGENS & HOST FACTORS UNIT, 150 COURS ALBERT THOMAS, F-69372 Lyon, France. Bulgarian Academy of Sciences, INST CELL BIOL & MORPHOL, BU-1040 SOFIA, Bulgaria. BULGARIAN ACAD MED, NATL ONCOL CTR, BU-1156 SOFIA, Bulgaria.	Country: BULGARIA Institution: INT AGCY RES CANC	Country: Bulgaria Institution: International Agency for Research on Cancer (IARC)

Record 849 | WOS:A1990CF01500012

EFFECTS OF FUNGAL METABOLITES ON TESTOSTERONE SECRETION INVITRO

Field	Position	Original line	Normalised line	Original value	Normalised value	Original country / institution	Normalised country / institution
C1	1	114906	114906	UNIV BAYREUTH, DEPT ANIM PHYSIOL, W-8580 BAYREUTH, GERMANY. FED CTR MEAT RES, INST MICROBIOL TOXICOL & HISTOL, W-8650 KULMBACH, GERMANY.	University of Bayreuth, DEPT ANIM PHYSIOL, W-8580 BAYREUTH, Germany. FED CTR MEAT RES, INST MICROBIOL TOXICOL & HISTOL, W-8650 KULMBACH, Germany.	Country: Germany Institution: UNIV BAYREUTH	Country: Germany Institution: University of Bayreuth

Record 850 | WOS:A1990CJ12700002

ENSILING AS A MEANS OF REDUCING OCHRATOXIN-A CONCENTRATIONS IN CONTAMINATED BARLEY

Field	Position	Original line	Normalised line	Original value	Normalised value	Original country / institution	Normalised country / institution
C1	1	114975	114975	AGR CANADA, RES STN, WINNIPEG R3T 2M9, MANITOBA, CANADA.	AGR CANADA, RES STN, WINNIPEG R3T 2M9, MANITOBA, Canada.	Country: Canada Institution: AGR CANADA	Country: Canada Institution: AGR CANADA

RP	1	114977	114977	ROTTER, RG (corresponding author), UNIV MANITOBA, DEPT ANIM SCI, WINNIPEG R3T 2N2, MANITOBA, CANADA	ROTTER, RG (corresponding author), University of Manitoba, DEPT ANIM SCI, WINNIPEG R3T 2N2, MANITOBA, Canada	Country: Canada Institution: UNIV MANITOBA	Country: Canada Institution: University of Manitoba

Record 851 | WOS:A1989CA57100015

INFLUENCE OF DIETARY CHARCOAL ON OCHRATOXIN-A TOXICITY IN LEGHORN CHICKS

Field	Position	Original line	Normalised line	Original value	Normalised value	Original country / institution	Normalised country / institution
RP	1	115055	115055	ROTTER, RG (corresponding author), UNIV MANITOBA, DEPT ANIM SCI, WINNIPEG R3T 2N2, MANITOBA, CANADA	ROTTER, RG (corresponding author), University of Manitoba, DEPT ANIM SCI, WINNIPEG R3T 2N2, MANITOBA, Canada	Country: Canada Institution: UNIV MANITOBA	Country: Canada Institution: University of Manitoba

Record 852 | WOS:A1989AT97300002

NEPHROTOXICITY ASSESSMENT BY MEASURING CELLULAR ATP CONTENT .2. INTRANEPHRON SITE OF OCHRATOXIN-A NEPHROTOXICITY

Field	Position	Original line	Normalised line	Original value	Normalised value	Original country / institution	Normalised country / institution
C1	1	115120	115120	UNIV TOKYO, FAC MED, DEPT PHARMACOL, BUNKYO KU, TOKYO 113, JAPAN.	UNIV TOKYO, FAC MED, DEPT PHARMACOL, BUNKYO KU, TOKYO 113, Japan.	Country: Japan Institution: UNIV TOKYO	Country: Japan Institution: UNIV TOKYO

Record 853 | WOS:A1989CB27000014

MECHANISM OF OCHRATOXIN A-INDUCED IMMUNOSUPPRESSION

Field	Position	Original line	Normalised line	Original value	Normalised value	Original country / institution	Normalised country / institution
C1	1	115192	115192	NATL INST PUBL HLTH, DEPT TOXICOL, N-0462 OSLO 4, NORWAY. NATL HOSP NORWAY, INST IMMUNOL & RHEUMATOL, OSLO 1, NORWAY.	NATL INST PUBL HLTH, DEPT TOXICOL, N-0462 OSLO 4, Norway. NATL HOSP NORWAY, INST IMMUNOL & RHEUMATOL, OSLO 1, Norway.	Country: Norway Institution: NATL INST PUBL HLTH	Country: Norway Institution: NATL INST PUBL HLTH

Record 854 | WOS:A1989T676500015

THE EFFECTS OF OCHRATOXIN-A ON POSTIMPLANTATION RAT EMBRYOS IN CULTURE

Field	Position	Original line	Normalised line	Original value	Normalised value	Original country / institution	Normalised country / institution
C1	1	115259	115259	TEXAS A&M UNIV, COLL VET MED, DEPT VET PUBL HLTH, COLLEGE STN, TX 77843 USA. TEXAS A&M UNIV, COLL VET MED, DEPT VET PATHOL, COLLEGE STN, TX 77843 USA.	Texas A&M University College Station, COLL VET MED, DEPT VET PUBL HLTH, COLLEGE STN, TX 77843 USA. Texas A&M University College Station, COLL VET MED, DEPT VET PATHOL, COLLEGE STN, TX 77843 USA.	Country: USA Institution: TEXAS A&M UNIV	Country: USA Institution: Texas A&M University College Station

Record 855 | WOS:A1989T320900022

INFLUENCE OF OCHRATOXIN B ON THE OCHRATOXIN-A INHIBITION OF PHENYLALANYL-TRANSFER RNA FORMATION INVITRO AND PROTEIN-SYNTHESIS IN HEPATOMA TISSUE-CULTURE CELLS

Field	Position	Original line	Normalised line	Original value	Normalised value	Original country / institution	Normalised country / institution
C1	1	115339	115339	UNIV STRASBOURG 1, CNRS, INST BIOL MOLEC & CELLULAIRE, 15 RUE DESCARTES, F-67084 STRASBOURG, FRANCE. UNIV STRASBOURG 1, FAC PHARM, F-67084 STRASBOURG, FRANCE. UNIV MUNSTER, INST MICROBIOL, D-4400 MUNSTER, GERMANY. NATL FOOD RES INST, PRETORIA, SOUTH AFRICA.	UNIV STRASBOURG 1, CNRS, INST BIOL MOLEC & CELLULAIRE, 15 RUE DESCARTES, F-67084 STRASBOURG, France. UNIV STRASBOURG 1, FAC PHARM, F-67084 STRASBOURG, France. UNIV MUNSTER, INST MICROBIOL, D-4400 MUNSTER, Germany. NATL FOOD RES INST, PRETORIA, South Africa.	Country: South Africa Institution: UNIV STRASBOURG 1	Country: South Africa Institution: UNIV STRASBOURG 1

Record 856 | WOS:A1989R524600010

PERTURBATION OF LIVER MICROSOMAL CALCIUM HOMEOSTASIS BY OCHRATOXIN-A

Field	Position	Original line	Normalised line	Original value	Normalised value	Original country / institution	Normalised country / institution
C1	1	115406	115406	MEM UNIV NEWFOUNDLAND, DEPT BIOCHEM, ST JOHNS A1B 3X9, NEWFOUNDLAND, CANADA. INT AGCY RES CANC, ENVIRONM CARCINOGENS & HOST FACTORS, F-69372 LYON 08, FRANCE.	MEM UNIV NEWFOUNDLAND, DEPT BIOCHEM, ST JOHNS A1B 3X9, NEWFOUNDLAND, Canada. International Agency for Research on Cancer (IARC), ENVIRONM CARCINOGENS & HOST FACTORS, F-69372 LYON 08, France.	Country: France Institution: MEM UNIV NEWFOUNDLAND	Country: France Institution: MEM UNIV NEWFOUNDLAND

Record 857 | WOS:A1988R353500008

LIPID-PEROXIDATION AS A POSSIBLE CAUSE OF OCHRATOXIN A TOXICITY

Field	Position	Original line	Normalised line	Original value	Normalised value	Original country / institution	Normalised country / institution
C1	1	115495	115495	INT AGCY RES CANC, 150 COURS ALBERT THOMAS, F-69372 LYON 08, FRANCE.	International Agency for Research on Cancer (IARC), 150 COURS ALBERT THOMAS, F-69372 LYON 08, France.	Country: France Institution: INT AGCY RES CANC	Country: France Institution: International Agency for Research on Cancer (IARC)

Record 858 | WOS:A1988P563200002

INDUCTION OF SOS FUNCTION IN ESCHERICHIA-COLI BY SOME MYCOTOXINS

Field	Position	Original line	Normalised line	Original value	Normalised value	Original country / institution	Normalised country / institution
RP	1	115581	115581	AUFFRAY, Y (corresponding author), UNIV CAEN,UNITE TOXICOL GENET,GENET	AUFFRAY, Y (corresponding author), UNIV CAEN,UNITE TOXICOL GENET,GENET	Country: France Institution: UNIV CAEN	Country: France Institution: UNIV CAEN

				MICROBIENNE LAB,F-14032 CAEN,FRANCE	MICROBIENNE LAB,F-14032 CAEN,France		

Record 859 | WOS:A1988R574700010

OCHRATOXIN-A AS A SUPPRESSOR OF MITOGEN-INDUCED BLASTOGENESIS OF PORCINE BLOOD-LYMPHOCYTES

Field	Position	Original line	Normalised line	Original value	Normalised value	Original country / institution	Normalised country / institution
C1	1	115621	115621	ROYAL INST TECHNOL, DEPT BIOCHEM & BIOTECHNOL, S-10044 STOCKHOLM 70, SWEDEN.	Royal Institute of Technology, DEPT BIOCHEM & BIOTECHNOL, S-10044 STOCKHOLM 70, Sweden.	Country: Sweden<br>Institution: ROYAL INST TECHNOL	Country: Sweden<br>Institution: Royal Institute of Technology
RP	1	115623	115623	HOLMBERG, T (corresponding author), SWEDISH UNIV AGR SCI, COLL VET, NATL VET INST, DEPT PATHOL, POB 7073, S-75007 UPPSALA, SWEDEN	HOLMBERG, T (corresponding author), Swedish University of Agricultural Sciences, COLL VET, NATL VET INST, DEPT PATHOL, POB 7073, S-75007 UPPSALA, Sweden	Country: Sweden<br>Institution: SWEDISH UNIV AGR SCI	Country: Sweden<br>Institution: Swedish University of Agricultural Sciences

Record 860 | WOS:A1987L417900004

PROTEIN-BINDING POTENTIAL OF OCHRATOXIN-A INVITRO AND ITS FLUORESCENCE ENHANCEMENT

Field	Position	Original line	Normalised line	Original value	Normalised value	Original country / institution	Normalised country / institution
RP	1	115679	115679	UCHIYAMA, S (corresponding author), NATL INST HYG SCI, 1-18-1 KAMIYOGA, SETAGAYA KU, TOKYO 158, JAPAN	UCHIYAMA, S (corresponding author), NATL INST HYG SCI, 1-18-1 KAMIYOGA, SETAGAYA KU, TOKYO 158, Japan	Country: Japan<br>Institution: NATL INST HYG SCI	Country: Japan<br>Institution: NATL INST HYG SCI

Record 861 | WOS:A1987K378200008

GENOTOXIC ACTIVITY OF SOME MYCOTOXINS USING THE SOS CHROMOTEST

Field	Position	Original line	Normalised line	Original value	Normalised value	Original country / institution	Normalised country / institution
RP	1	115716	115716	AUFFRAY, Y (corresponding author), UNIV CAEN, INST RECH BIOL AGR, UNITE TOXICOL GENET, GENET MICROBIENNE LAB, F-14032 CAEN, FRANCE	AUFFRAY, Y (corresponding author), UNIV CAEN, INST RECH BIOL AGR, UNITE TOXICOL GENET, GENET MICROBIENNE LAB, F-14032 CAEN, France	Country: France<br>Institution: UNIV CAEN	Country: France<br>Institution: UNIV CAEN

Record 862 | WOS:A1987G499300004

CARCINOGENICITY AND GENOTOXICITY OF MYCOTOXINS

Field	Position	Original line	Normalised line	Original value	Normalised value	Original country / institution	Normalised country / institution
RP	1	115783	115783	MULLER, T (corresponding author), AKAD LANDWIRTSCHAFTSWISSENSCH DDR,INST FUTTERPROD,DDR-1551 PAULINENAUE,GER DEM REP	MULLER, T (corresponding author), AKAD LANDWIRTSCHAFTSWISSENSCH DDR,INST FUTTERPROD,DDR-1551 PAULINENAUE,Germany	Country: GER DEM REP<br>Institution: AKAD LANDWIRTSCHAFTSWISSENSCH DDR	Country: Germany<br>Institution: AKAD LANDWIRTSCHAFTSWISSENSCH DDR

Record 863 | WOS:A1986AYL5800018

INTERSTRAIN COMPARISON OF HEPATIC AND RENAL MICROSOMAL CARCINOGEN METABOLISM AND LIVER S9-MEDIATED MUTAGENICITY IN DA AND LEWIS RATS PHENOTYPED AS POOR AND EXTENSIVE METABOLIZERS OF DEBRISOQUINE

Field	Position	Original line	Normalised line	Original value	Normalised value	Original country / institution	Normalised country / institution
C1	1	115891	115891	INT AGCY RES CANC, 150 COURS ALBERT THOMAS, F-69372 LYON 8, FRANCE. ST MARYS HOSP, SCH MED, DEPT PHARMACOL, F-69372 LYON 8, FRANCE.	International Agency for Research on Cancer (IARC), 150 COURS ALBERT THOMAS, F-69372 LYON 8, France. ST MARYS HOSP, SCH MED, DEPT PHARMACOL, F-69372 LYON 8, France.	Country: France<br>Institution: INT AGCY RES CANC	Country: France<br>Institution: International Agency for Research on Cancer (IARC)

Record 864 | WOS:A1985AWD1800005

EVALUATION OF OCHRATOXIN-A FOR MUTAGENICITY IN A BATTERY OF BACTERIAL AND MAMMALIAN-CELL ASSAYS

Field	Position	Original line	Normalised line	Original value	Normalised value	Original country / institution	Normalised country / institution
C1	1	115998	115998	PURDUE UNIV, SCH VET MED, W LAFAYETTE, IN 47907 USA. ELI LILLY & CO, GREENFIELD, IN 46140 USA.	PURDUE UNIV, SCH VET MED, W LAFAYETTE, IN 47907 USA. Eli Lilly, GREENFIELD, IN 46140 USA.	Country: USA<br>Institution: PURDUE UNIV	Country: USA<br>Institution: PURDUE UNIV

Record 865 | WOS:A1985A019900016

GENOTOXICITY OF OCHRATOXIN-A IN MICE - DNA SINGLE-STRAND BREAKS EVALUATION IN SPLEEN, KIDNEY AND LIVER

Field	Position	Original line	Normalised line	Original value	Normalised value	Original country / institution	Normalised country / institution
C1	1	116081	116081	FAC PHARM STRASBOURG, F-67084 STRASBOURG, FRANCE. INST RECH SCI CANC, F-94802 VILLEJUIF, FRANCE.	FAC PHARM STRASBOURG, F-67084 STRASBOURG, France. INST RECH SCI CANC, F-94802 VILLEJUIF, France.	Country: France<br>Institution: FAC PHARM STRASBOURG	Country: France<br>Institution: FAC PHARM STRASBOURG
RP	1	116085	116085	CREPPY, EE (corresponding author), CNRS, INST BIOL MOLEC & CELLULAIRE, TOXICOL & BIOL MOLEC LAB, 15 RUE DESCARTES, F-67084 STRASBOURG, FRANCE	CREPPY, EE (corresponding author), Centre National de la Recherche Scientifique (CNRS), INST BIOL MOLEC & CELLULAIRE, TOXICOL & BIOL MOLEC LAB, 15 RUE DESCARTES, F-67084 STRASBOURG, France	Country: France<br>Institution: CNRS	Country: France<br>Institution: Centre National de la Recherche Scientifique (CNRS)

Record 866 | WOS:A1985AUK4000005

GENOTOXICITY OF OCHRATOXIN-A IN MICE - DNA SINGLE-STRAND BREAK EVALUATION IN SPLEEN, LIVER AND KIDNEY

Field	Position	Original line	Normalised line	Original value	Normalised value	Original country / institution	Normalised country / institution
C1	1	116156	116156	FAC PHARM STRASBOURG, 15 RUE DESCARTES, STRASBOURG, FRANCE. CNRS, INST BIOL MOLEC & CELLULAIRE, TOXICOL & BIOL MOLEC LAB,	FAC PHARM STRASBOURG, 15 RUE DESCARTES, STRASBOURG, France. Centre National de la Recherche Scientifique (CNRS), INST BIOL MOLEC & CELLULAIRE,	Country: France<br>Institution: FAC PHARM STRASBOURG	Country: France<br>Institution: FAC PHARM STRASBOURG

				STRASBOURG, FRANCE. INST RECH SCI CANC, F-94802 VILLEJUIF, FRANCE.	TOXICOL & BIOL MOLEC LAB, STRASBOURG, France. INST RECH SCI CANC, F-94802 VILLEJUIF, France.		

Record 867 | WOS:A1985ASM7700004

EFFECT OF OCHRATOXIN A ON RAT-LIVER MITOCHONDRIAL RESPIRATION AND OXIDATIVE-PHOSPHORYLATION

Field	Position	Original line	Normalised line	Original value	Normalised value	Original country / institution	Normalised country / institution
C1	1	116235	116235	NATL YANG MING MED COLL, DEPT BIOCHEM, TAIPEI, TAIWAN.	NATL YANG MING MED COLL, DEPT BIOCHEM, TAIPEI, Taiwan.	Country: TAIWAN Institution: NATL YANG MING MED COLL	Country: Taiwan Institution: NATL YANG MING MED COLL

Record 868 | WOS:A1984SW21300010

EFFECTS OF FUNGAL TOXINS ON RENAL SLICE CALCIUM BALANCE

Field	Position	Original line	Normalised line	Original value	Normalised value	Original country / institution	Normalised country / institution
C1	1	116306	116306	UNIV NEBRASKA, MED CTR, COLL MED, DEPT PHARMACOL, OMAHA, NE 68105 USA. UNIV MISSISSIPPI, MED CTR, DEPT PHARMACOL & TOXICOL, JACKSON, MS 39216 USA.	UNIV NEBRASKA, MED CTR, COLL MED, DEPT PHARMACOL, OMAHA, NE 68105 USA. University of Mississippi, MED CTR, DEPT PHARMACOL & TOXICOL, JACKSON, MS 39216 USA.	Country: USA Institution: UNIV NEBRASKA	Country: USA Institution: UNIV NEBRASKA

Record 869 | WOS:A1984TF49500004

EFFECTS OF SOME MYCOTOXINS ON MITOGEN-INDUCED BLASTOGENESIS AND SCE FREQUENCY IN HUMAN-LYMPHOCYTES

Field	Position	Original line	Normalised line	Original value	Normalised value	Original country / institution	Normalised country / institution
C1	1	116375	116375	SWEDISH UNIV AGR SCI, DEPT ANIM NUTR, S-75007 UPPSALA, SWEDEN.	Swedish University of Agricultural Sciences, DEPT ANIM NUTR, S-75007 UPPSALA, Sweden.	Country: Sweden Institution: SWEDISH UNIV AGR SCI	Country: Sweden Institution: Swedish University of Agricultural Sciences
RP	1	116377	116377	COORAY, R (corresponding author), UNIV UPPSALA, INST ZOOPHYSIOL, S-75122 UPPSALA, SWEDEN	COORAY, R (corresponding author), UNIV UPPSALA, INST ZOOPHYSIOL, S-75122 UPPSALA, Sweden	Country: Sweden Institution: UNIV UPPSALA	Country: Sweden Institution: UNIV UPPSALA

Record 870 | WOS:A1984SZ35800008

CONVERSION OF OCHRATOXIN-C INTO OCHRATOXIN-A INVIVO

Field	Position	Original line	Normalised line	Original value	Normalised value	Original country / institution	Normalised country / institution
C1	1	116454	116454	ROYAL INST TECHNOL, DEPT BIOCHEM & BIOTECHNOL, S-10044 STOCKHOLM 70, SWEDEN. INST MED RES & OCCUPAT HLTH, POB 291, YU-41001 Zagreb, YUGOSLAVIA.	Royal Institute of Technology, DEPT BIOCHEM & BIOTECHNOL, S-10044 STOCKHOLM 70, Sweden. Institute for Medical Research & Occupational Health (IMROH), POB 291, YU-41001 Zagreb, Croatia.	Country: YUGOSLAVIA Institution: ROYAL INST TECHNOL	Country: Croatia Institution: Royal Institute of Technology

Record 871 | WOS:A1984SX17000033

GENOTOXICITY OF A VARIETY OF MYCOTOXINS IN THE HEPATOCYTE PRIMARY CULTURE DNA-REPAIR TEST USING RAT AND MOUSE HEPATOCYTES

Field	Position	Original line	Normalised line	Original value	Normalised value	Original country / institution	Normalised country / institution
C1	1	116516	116516	GIFU UNIV, SCH MED, DEPT PATHOL, GIFU 500, JAPAN. GIFU UNIV, SCH MED, DEPT BIOCHEM, GIFU 500, JAPAN. TOTTORI UNIV, FAC AGR, TOTTORI 680, JAPAN. AMER HLTH FDN, NAYLOR DANA INST DIS PREVENT, VALHALLA, NY 10595 USA. CHIBA UNIV, CHEMOBIODYNAM RES INST, CHIBA 280, JAPAN.	GIFU UNIV, SCH MED, DEPT PATHOL, GIFU 500, Japan. GIFU UNIV, SCH MED, DEPT BIOCHEM, GIFU 500, Japan. Tottori University, FAC AGR, TOTTORI 680, Japan. AMER HLTH FDN, NAYLOR DANA INST DIS PREVENT, VALHALLA, NY 10595 USA. Chiba University, CHEMOBIODYNAM RES INST, CHIBA 280, Japan.	Country: Japan<br>Institution: GIFU UNIV	Country: Japan<br>Institution: GIFU UNIV

Record 872 | WOS:A1983RZ78000009

EFFECTS OF OCHRATOXIN A METABOLITES ON YEAST PHENYLALANYL-TRNA SYNTHETASE AND ON THE GROWTH AND INVIVO PROTEIN-SYNTHESIS OF HEPATOMA-CELLS

Field	Position	Original line	Normalised line	Original value	Normalised value	Original country / institution	Normalised country / institution
C1	1	116610	116610	CNRS, INST BIOL MOLEC & CELLULAIRE, 15 RUE RENE DESCARTES, F-67084 STRASBOURG, FRANCE. UNIV MUENSTER, INST MICROBIOL, D-4400 MUENSTER, FED REP GER. UNIV STRASBOURG 1, FAC PHARM, F-67048 STRASBOURG, FRANCE. NATL INST PUBL HLTH, DEPT TOXICOL, OSLO, NORWAY.	Centre National de la Recherche Scientifique (CNRS), INST BIOL MOLEC & CELLULAIRE, 15 RUE RENE DESCARTES, F-67084 STRASBOURG, France. UNIV MUENSTER, INST MICROBIOL, D-4400 MUENSTER, Germany. UNIV STRASBOURG 1, FAC PHARM, F-67048 STRASBOURG, France. NATL INST PUBL HLTH, DEPT TOXICOL, OSLO, Norway.	Country: Norway<br>Institution: CNRS	Country: Norway<br>Institution: Centre National de la Recherche Scientifique (CNRS)

Record 873 | WOS:A1982NN76100006

PREVENTION OF MOLD GROWTH AND TOXIN PRODUCTION THROUGH CONTROL OF ENVIRONMENTAL-CONDITIONS

Field	Position	Original line	Normalised line	Original value	Normalised value	Original country / institution	Normalised country / institution
C1	1	116676	116676	UNIV NEBRASKA,INST AGR & NAT RESOURCES,DEPT FOOD SCI & TECHNOL,LINCOLN,NE 68583. NATL INST PUBL HLTH,ZOONOSES & FOOD MICROBIOL LAB,3720 BA BILTHOVEN,NETHERLANDS.	UNIV NEBRASKA,INST AGR & NAT RESOURCES,DEPT FOOD SCI & TECHNOL,LINCOLN,NE 68583. NATL INST PUBL HLTH,ZOONOSES & FOOD MICROBIOL LAB,3720 BA BILTHOVEN,Netherlands.	Country: Netherlands<br>Institution: UNIV NEBRASKA	Country: Netherlands<br>Institution: UNIV NEBRASKA

Record 874 | WOS:A1980KP16100001

MEASURING OF A DOUBLE CONTAMINATION BY PATULIN AND OCHRATOXIN

Field	Position	Original line	Normalised line	Original value	Normalised value	Original country / institution	Normalised country / institution
C1	1	116771	116771	INRA, PHARMACOL TOXICOL STN, 180 CHEMIN TOURNEFEUILLE, F-31300 TOULOUSE, FRANCE.	INRAE, PHARMACOL TOXICOL STN, 180 CHEMIN TOURNEFEUILLE, F-31300 TOULOUSE, France.	Country: France<br>Institution: INRA	Country: France<br>Institution: INRAE

Record 875 | WOS:A1980KR30600008

EVIDENCE FOR INVITRO AND INVIVO INTERACTION BETWEEN OCHRATOXIN-A AND 3 ACIDIC DRUGS

Field	Position	Original line	Normalised line	Original value	Normalised value	Original country / institution	Normalised country / institution
C1	1	116817	116817	ECOLE NATL VET, F-31300 TOULOUSE, FRANCE.	ECOLE NATL VET, F-31300 TOULOUSE, France.	Country: France<br>Institution: ECOLE NATL VET	Country: France<br>Institution: ECOLE NATL VET
RP	1	116819	116819	GALTIER, P (corresponding author), INRA, PHARMACOL TOXICOL LAB, 180 CHEMIN DE TOURNEFEUILL, F-31300 TOULOUSE, FRANCE	GALTIER, P (corresponding author), INRAE, PHARMACOL TOXICOL LAB, 180 CHEMIN DE TOURNEFEUILL, F-31300 TOULOUSE, France	Country: France<br>Institution: INRA	Country: France<br>Institution: INRAE

Record 876 | WOS:A1979HH25500002

INVIVO AND INVITRO CHANGES IN RENAL-FUNCTION CAUSED BY OCHRATOXIN A IN THE RAT

Field	Position	Original line	Normalised line	Original value	Normalised value	Original country / institution	Normalised country / institution
RP	1	116870	116870	BERNDT, WO (corresponding author), UNIV MISSISSIPPI, MED CTR, DEPT PHARMACOL & TOXICOL, JACKSON, MS 39216 USA	BERNDT, WO (corresponding author), University of Mississippi, MED CTR, DEPT PHARMACOL & TOXICOL, JACKSON, MS 39216 USA	Country: USA<br>Institution: UNIV MISSISSIPPI	Country: USA<br>Institution: University of Mississippi

Record 877 | WOS:A1978GE26800006

OCHRATOXIN-A AND PROTEIN-UTILIZATION INVIVO

Field	Position	Original line	Normalised line	Original value	Normalised value	Original country / institution	Normalised country / institution
RP	1	116931	116931	KIESSLING, KH (corresponding author), SWEDISH UNIV AGR SCI, DEPT ANIM PHYSIOL, S-75007 UPPSALA, SWEDEN	KIESSLING, KH (corresponding author), Swedish University of Agricultural Sciences, DEPT ANIM PHYSIOL, S-75007 UPPSALA, Sweden	Country: Sweden<br>Institution: SWEDISH UNIV AGR SCI	Country: Sweden<br>Institution: Swedish University of Agricultural Sciences

Record 878 | WOS:A1977DR04200006

FATE OF OCHRATOXIN A IN RATS

No C1/C3/RP normalisation changes detected for this record.

Record 879 | WOS:A1975AC00900024

INVITRO TOXICITY OF ANALOGS OF OCHRATOXIN-A IN MONKEY KIDNEY EPITHELIAL-CELLS

Field	Position	Original line	Normalised line	Original value	Normalised value	Original country / institution	Normalised country / institution
C1	1	117037	117037	CSIR,NATL CHEM RES LAB,PRETORIA 0001,SOUTH AFRICA. NATL RES INST NUTR DIS,PRETORIA 0001,SOUTH AFRICA.	CSIR,NATL CHEM RES LAB,PRETORIA 0001,South Africa. NATL RES INST NUTR DIS,PRETORIA 0001,South Africa.	Country: South Africa<br>Institution: CSIR	Country: South Africa<br>Institution: CSIR

Record 880 | WOS:A1975BC07400010

STUDIES ON NEPHROTOXICITY OF OCHRATOXIN A IN RATS

Field	Position	Original line	Normalised line	Original value	Normalised value	Original country / institution	Normalised country / institution
C1	1	117093	117093	CHIBA UNIV,RES INST CHEMOBIODYNAM,NARASHINO 275,CHIBA,JAPAN.	Chiba University,RES INST CHEMOBIODYNAM,NARASHINO 275,CHIBA,Japan.	Country: Japan<br>Institution: CHIBA UNIV	Country: Japan<br>Institution: Chiba University

Record 881 | WOS:A1972M753500008

COMPARATIVE RATES OF HYDROLYSIS OF OCHRATOXINS A AND B IN-VITRO

No C1/C3/RP normalisation changes detected for this record.

Record 882 | WOS:A1971K640300003

TOXICITY OF 5-CHLORO-8-HYDROXY-3, 4-DIHYDRO-3-METHYL-ISOCOUMARIN-7-CARBOXYLIC ACID, A HYDROLYZATE OF OCHRATOXIN-A

No C1/C3/RP normalisation changes detected for this record.

Record 883 | WOS:A19656230000050

OCHRATOXIN A A TOXIC METABOLITE PRODUCED BY ASPERGILLUS OCHRACEUS WILH

No C1/C3/RP normalisation changes detected for this record.
